# Identification of long non-coding RNAs in the immature and mature rat anterior pituitary

**DOI:** 10.1038/s41598-017-17996-6

**Published:** 2017-12-19

**Authors:** Dong-Xu Han, Xu-Lei Sun, Yao Fu, Chang-Jiang Wang, Jian-Bo Liu, Hao Jiang, Yan Gao, Cheng-Zhen Chen, Bao Yuan, Jia-Bao Zhang

**Affiliations:** Department of Laboratory Animals, College of Animal Sciences, Jilin University, Changchun, 130062 Jilin, P.R. China

## Abstract

Many long non-coding RNAs (lncRNAs) have been identified in several types of human pituitary adenomas and normal anterior pituitary, some of which are involved in the pathogenesis of pituitary adenomas. However, a systematic analysis of lncRNAs expressed at different developmental stages of normal pituitary, particularly in rats, has not been performed. Therefore, we contrasted two cDNA libraries of immature (D15) and mature (D120) anterior pituitary in rat that were sequenced on an Illumina HiSeq Xten platform, and a total of 29,568,806,352 clean reads were identified. Notably, 7039 lncRNA transcripts corresponded to 4442 lncRNA genes, and 1181 lncRNA transcripts were significantly differentially expressed in D15 and D120. In addition, 6839 protein-coding genes (<100 kb upstream and downstream) were the nearest neighbors of 4074 lncRNA genes. An interaction network of lncRNAs and the follicle-stimulating hormone beta-subunit (FSHb) gene was constructed using the lncRNATargets platform, and three novel lncRNAs were obtained. Furthermore, we detected the expression of the novel lncRNAs and ten highly expressed lncRNAs that were randomly selected through quantitative PCR (qPCR). The rat anterior pituitary lncRNA content identified in this study provides a more in-depth understanding of the roles of these lncRNAs in hormone and reproduction development and regulation in mammals.

## Introduction

Recent genome sequencing studies have shown that less than 2% of the genome represents protein-coding genes, whereas more than 90% is considered non-coding RNAs (ncRNAs)^1,2^. In general, ncRNAs can be divided into two classes by length. ncRNAs shorter than 200 nucleotides are called short ncRNAs, such as small interfering RNAs (siRNAs) and micro-RNAs (miRNAs), and this category also includes classical ncRNAs, such as small nucleolar RNAs (snoRNAs), whereas long non-coding RNAs (lncRNAs) are ncRNAs longer than 200 nucleotides^3^. lncRNAs have some general features: they are mostly smaller than mRNAs (with a length of more than 200 nt and a median size of ~500 nt), and 80% have two to four exons^4^. In recent years, lncRNAs have been discovered in several types of mammals, such as *Homo sapiens* ^5,6^, *Mus musculus* ^7,8^, *Rattus norvegicus* ^9,10^ and *Bos taurus* ^11,12^. An increasing amount of evidence indicates that lncRNAs play a key role in diverse biological processes through distinct mechanisms^4,13^.

As an important experimental animal, rats are widely used worldwide in life science studies and play a key role in many types of functional studies, particularly in the field of reproduction development. Therefore, the relevant molecular mechanisms in reproduction development should be more of a research focus. Reproduction development contains extensive content and is regulated by many factors, such as hormones, essential micronutrients, and temperature^14–18^. Several recent studies have reported that lncRNAs perform an important role in reproduction. In 2015, Wei Jiang *et al*. indicated that lncRNA *DEANR1* is involved in human endoderm differentiation^19^. Furthermore, lncRNAs also play a role in sex-chromosome dosage compensation, stem cell maintenance and differentiation, and sex determination, among other functions^20–23^. However, information on the role of lncRNAs in reproductive hormones, particularly in rats, is limited.

In this study, we systematically investigated the lncRNA contents in the immature (D15) and mature (D120) rat anterior pituitary using an Illumina HiSeq Xten platform. A total of 7039 lncRNA transcripts were identified, and 1181 of these transcripts were significantly differentially expressed between two libraries, D15 and D120. To the best of our knowledge, only a few reports are available on normal pituitary lncRNAs, and no reports have been published on their biological functions in rat. Therefore, our results will provide a powerful resource for a more in-depth understanding of the regulatory functions of lncRNAs in rat and will contribute to a better comprehension of reproduction and development in mammals.

## Results

### Morphology of immature and mature pituitary tissues

**Figure 1:**
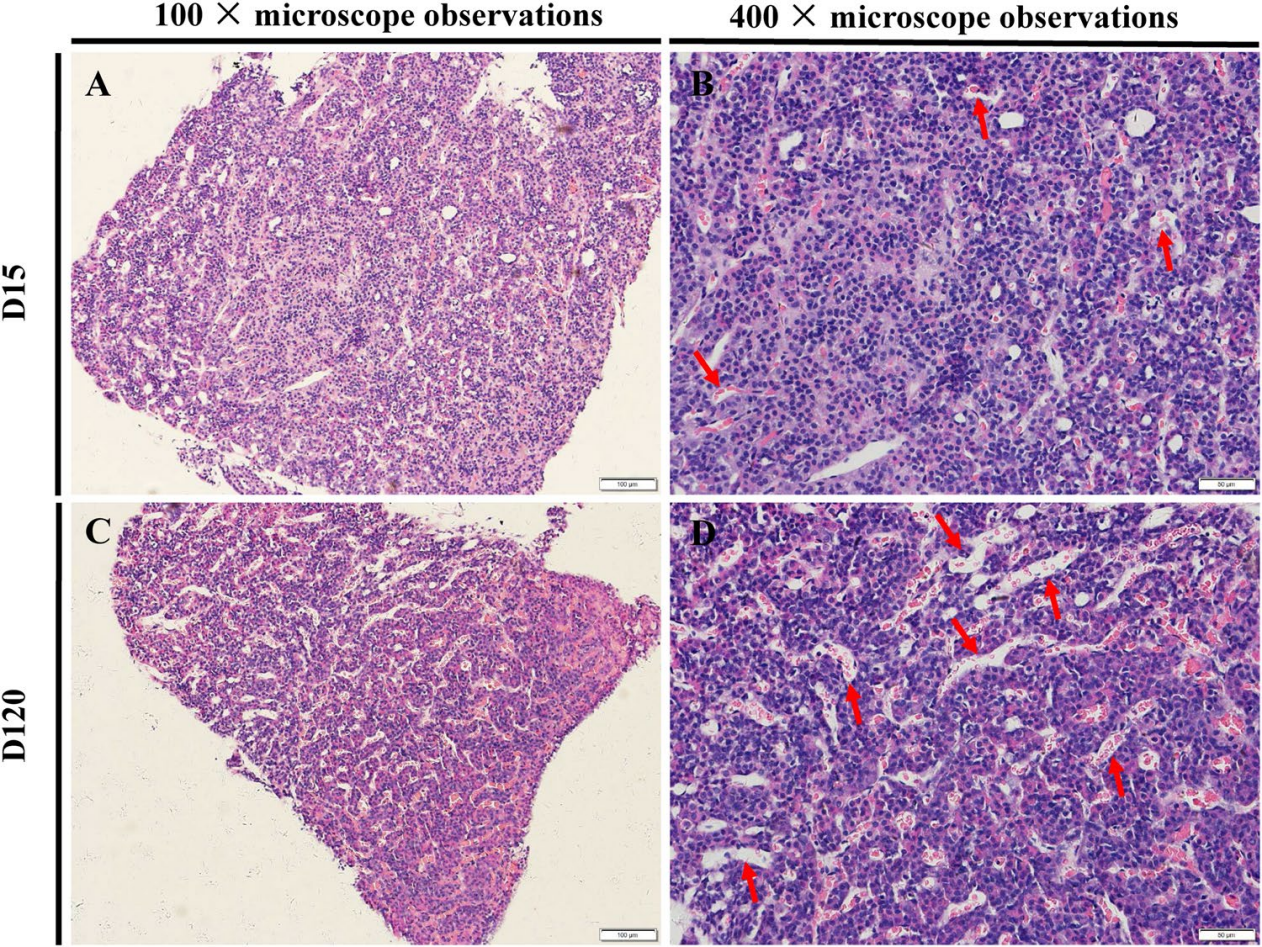
Morphology of immature and mature pituitary tissues. (**A** and **B**) Morphology of immature anterior pituitary tissue under 100× and 400× magnification, respectively. (**C** and **D**) Morphology of the mature anterior pituitary tissue under 100× and 400× magnification, respectively. The red arrows point to capillaries.

The morphology of immature and mature anterior pituitary tissues was different. The anterior pituitary was divided into distal, middle and nodule sections. Under 100 × magnification, the boundaries of the mature anterior pituitary were clearer than those of the immature tissue (Fig. 1A,C), and under 400 × magnification, a more sinusoidal capillary network was detected in the mature anterior pituitary than in the immature anterior pituitary (Fig. 1B,D). In general, capillaries contained red blood cells. Moreover, the sinusoidal capillary network tended to gradually develop with the growth and development of the pituitary.

### Overview of RNA sequencing

We identified the lncRNAs expressed in the immature and mature rat anterior pituitary by constructing two cDNA libraries, denoted D15 and D120, and each of these libraries was obtained from three rat anterior pituitary RNA samples. The libraries were sequenced using an Illumina HiSeq Xten platform. A total of 14,374,898,087 and 15,310,557,636 raw reads were identified from D15 and D120, respectively. After discarding the reads that contained adaptor sequences and were of low quality, 14,308,773,556 (99.54%) and 15,260,032,796 (99.67%) clean reads were identified from D15 and D120, respectively. Moreover, the GC content of the D15 and D120 libraries were 48.53% and 51.28%, respectively. In addition, 95.06% to 95.15% of the clean data were mapped to the rat reference genome sequence (Rnor_6.0), and 178,671 transcripts from the D15 and D120 libraries were assembled.

### Identification of lncRNAs in the rat anterior pituitary

**Figure 2:**
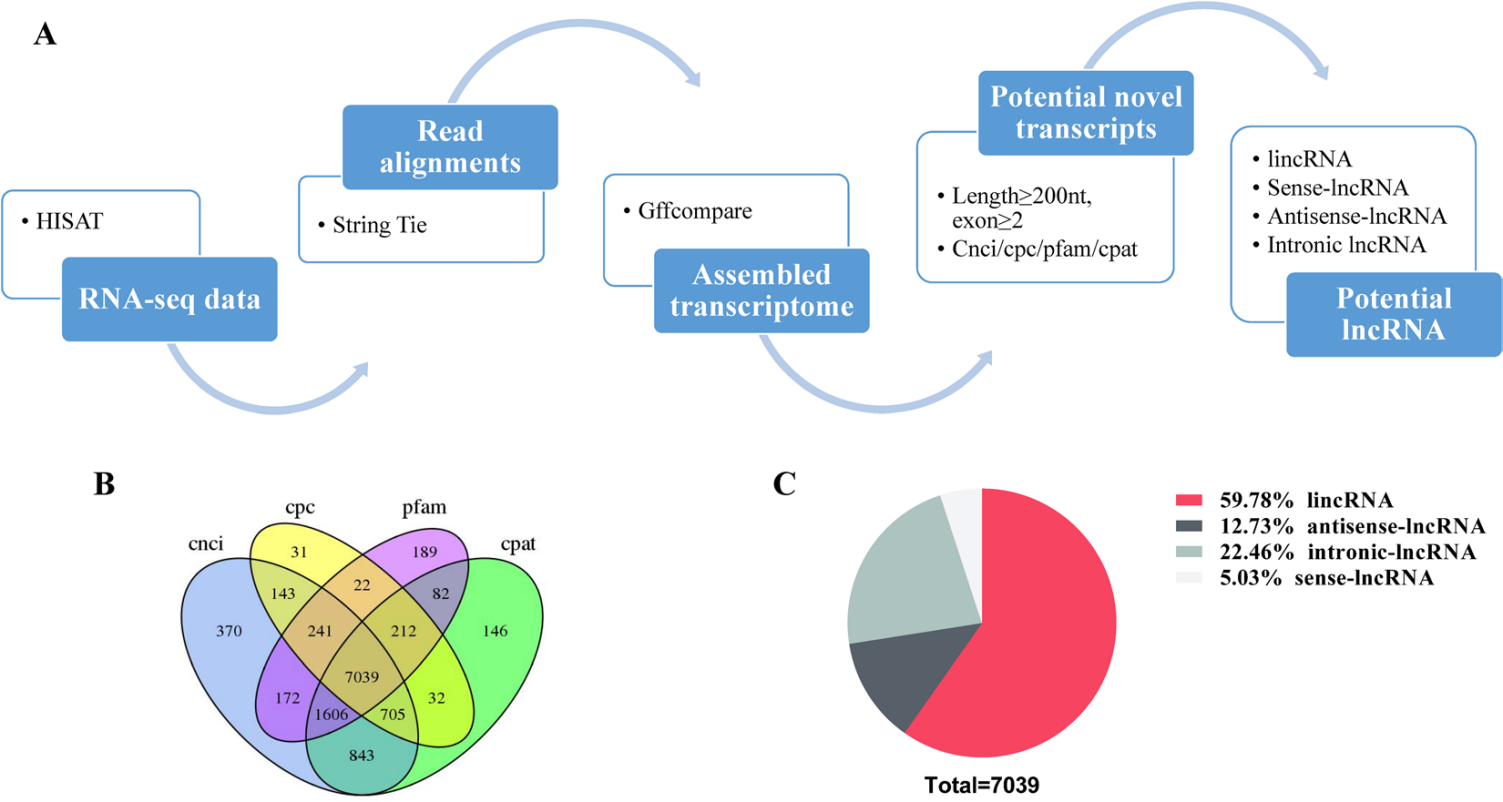
Identification of lncRNAs. (**A**) Pipeline used for the identification of lncRNAs. (**B**) The lncRNAs were identified from the intersection of the analysis results obtained from CNCI, CPC, Pfam and CPAT. (**C**) Classification of 7039 lncRNAs, including lincRNAs, antisense-lncRNAs, intronic-lncRNAs and sense-lncRNAs.

**Figure 3:**
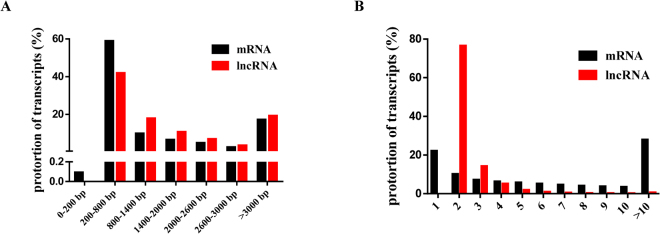
Comparison of features of lncRNAs and protein-coding genes. Distributions of the sequence length (**A**) and exon number (**B**) of lncRNAs and protein-coding genes.

**Figure 4:**
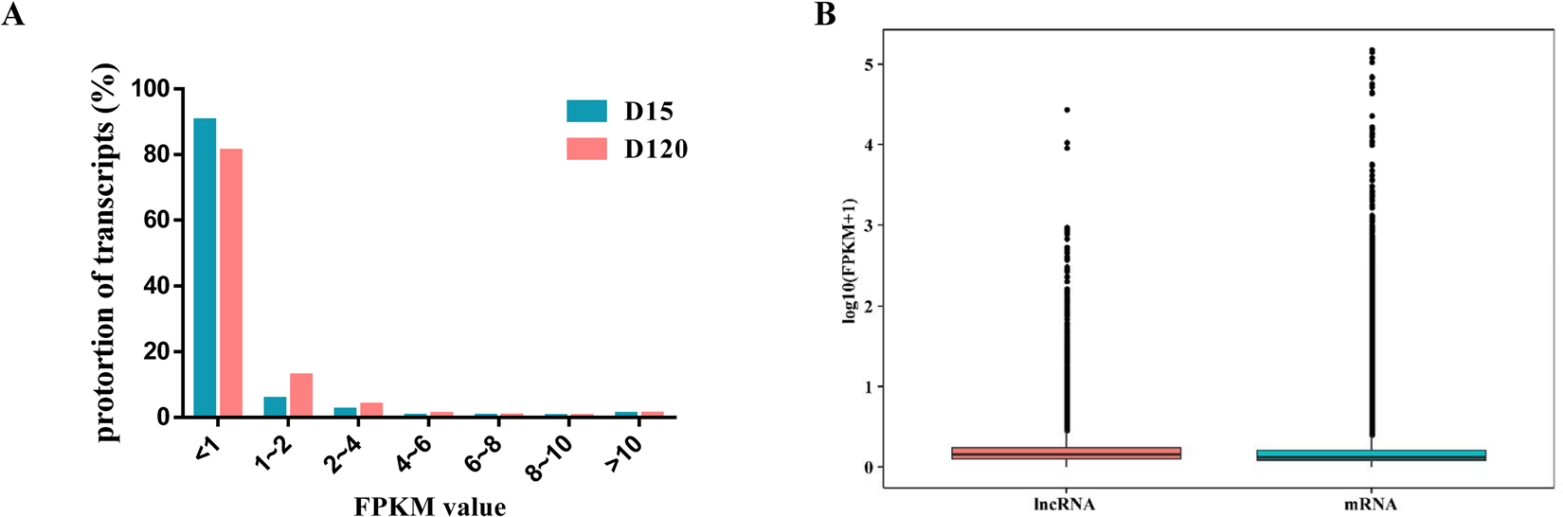
Expression levels of lncRNAs and protein genes. (**A**) The FPKM distributions of lncRNAs and protein-coding genes. (**B**) Boxplots of the expression level (log 10 FPKM) of lncRNAs and protein-coding genes.

We developed a stringent filtering pipeline to discard transcripts without all lncRNA characteristics and maximize the accurate identification of lncRNAs (Fig. 2A). We identified 7039 lncRNAs, including 4208 lincRNAs (58.9%), 896 antisense lncRNAs (12.7%), 1581 intronic lncRNAs (22.5%) and 354 sense lncRNAs (5%), through an intersection analysis of the Coding-Non-Coding-Index (CNCI), Coding Potential Calculator (CPC), Pfam and Coding Potential Assessment Tool (CPAT) (Fig. 2B–C). These transcripts corresponded to 4442 lncRNA genes and an average of 1.6 transcripts per lncRNA locus. In addition, the lncRNA transcripts were distributed in all chromosomes, whereas only a few transcripts were distributed in chromosome Y. Furthermore, 171,632 protein-coding transcripts were identified, and these had an average length of 1728 bp, which was shorter than that of the lncRNA genes (average length of 2052 bp). The analysis of sequence length revealed that protein-coding transcripts were mostly 200–800 bp in length, and lncRNA transcripts were mainly in the range of 200 bp to > 3000 bp (Fig. 3A). The average number of exons in protein-coding transcripts was 4.1, which was greater than the average number in lncRNA transcripts (2.5). We found that the protein transcripts contained an average of two to ten exons in the protein-coding genes and that the proportion of lncRNA transcripts with two exons was 76.5% in all lncRNAs genes (Fig. 3B). We used fragments per kilobase of exon per million fragments mapped (FPKM) to estimate the expression level of the lncRNA transcripts. Almost all lncRNA transcripts were expressed at a level lower than 1 FPKM, and in total, the number of lncRNA transcripts decreased with increasing FPKM (Fig. 4A). Furthermore, we found that the expression level of lncRNA transcripts was slightly higher than that of the protein-coding genes (Fig. 4B).

### Identification of differentially expressed lncRNAs

**Figure 5:**
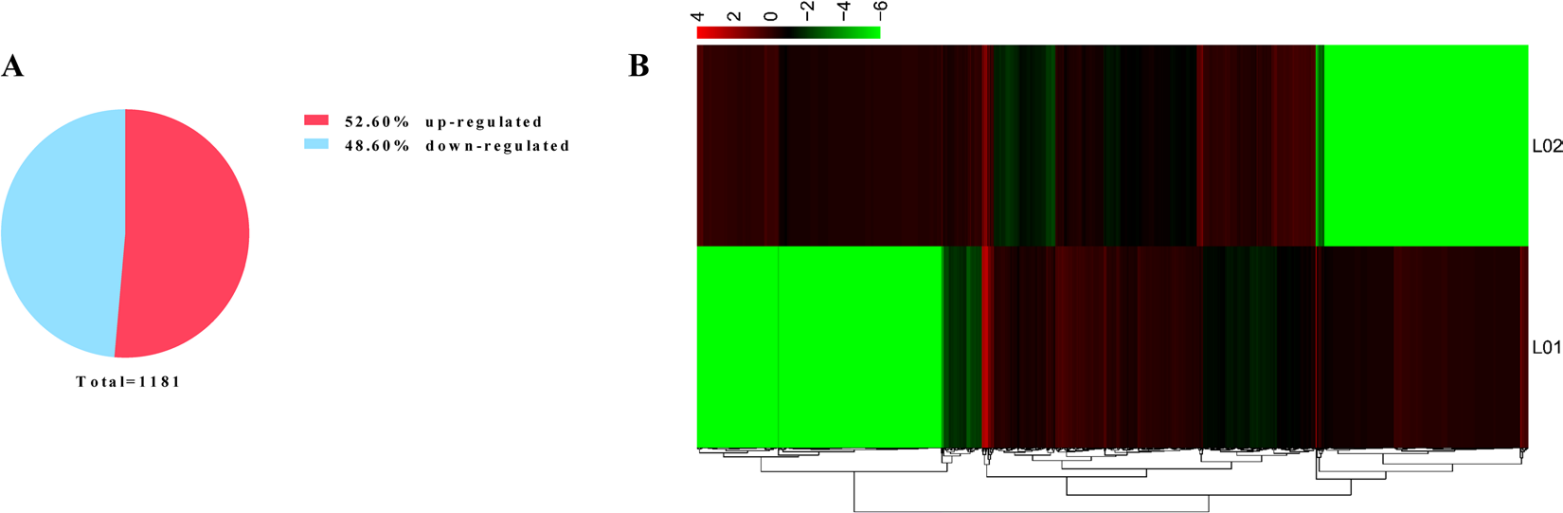
Differentially expressed lncRNAs in the immature and mature rat anterior pituitary. (**A**) Proportion of up-regulated and down-regulated lncRNAs in the rat anterior pituitary at different developmental stages. (**B**) Analysis of the expression pattern of differentially expressed lncRNAs. The highest to lowest fold changes are marked from red to green, respectively.

StringTie software was used for transcript assembly and detection of lncRNA transcript expression, and FPKM was used as the metric for the expression levels of lncRNAs. We screened the differentially expressed lncRNAs using the following criteria: fold change ≥ 2 and false discovery rate (FDR) < 0.05. Therefore, we identified 1181 differentially expressed lncRNA transcripts in the immature and mature rat anterior pituitary (Supplemental Table S1). Of these differentially expressed lncRNAs, 607 were up-regulated, and this number was higher than the number of down-regulated lncRNAs (574; Fig. 5A). We measured the expression patterns of differentially expressed lncRNAs through a systematic cluster analysis to explore similarities and compare the relationships between the different libraries (Fig. 5B).

### Enrichment analysis of nearest neighbor genes of lncRNAs

We investigated the possible functions of the lncRNAs by searching for protein-coding genes 100 kb upstream and downstream of all identified lncRNAs to predict the potential cis-regulatory targets of lncRNAs. A total of 6839 protein-coding genes were closest to the 4074 lncRNAs. Gene Ontology (GO) and pathway enrichment analyses were executed to explore their functions. In addition, 516 GO terms were significantly enriched (P < 0.01; Supplemental Table S2), and these were mainly associated with cellular process (GO: 0009987), cell part (GO: 0044464), organelle (GO: 0043226) and other 16 GO terms, whereas the other GO terms included a small number of genes. In addition, we found that 13 pathways, such as the antigen processing and presentation pathway, autoimmune thyroid disease pathway and type I diabetes mellitus pathway, were significantly enriched (P < 0.05).

### Validation of highly expressed lncRNAs and follicle-stimulating hormone (FSH)-associated lncRNAs

**Figure 6:**
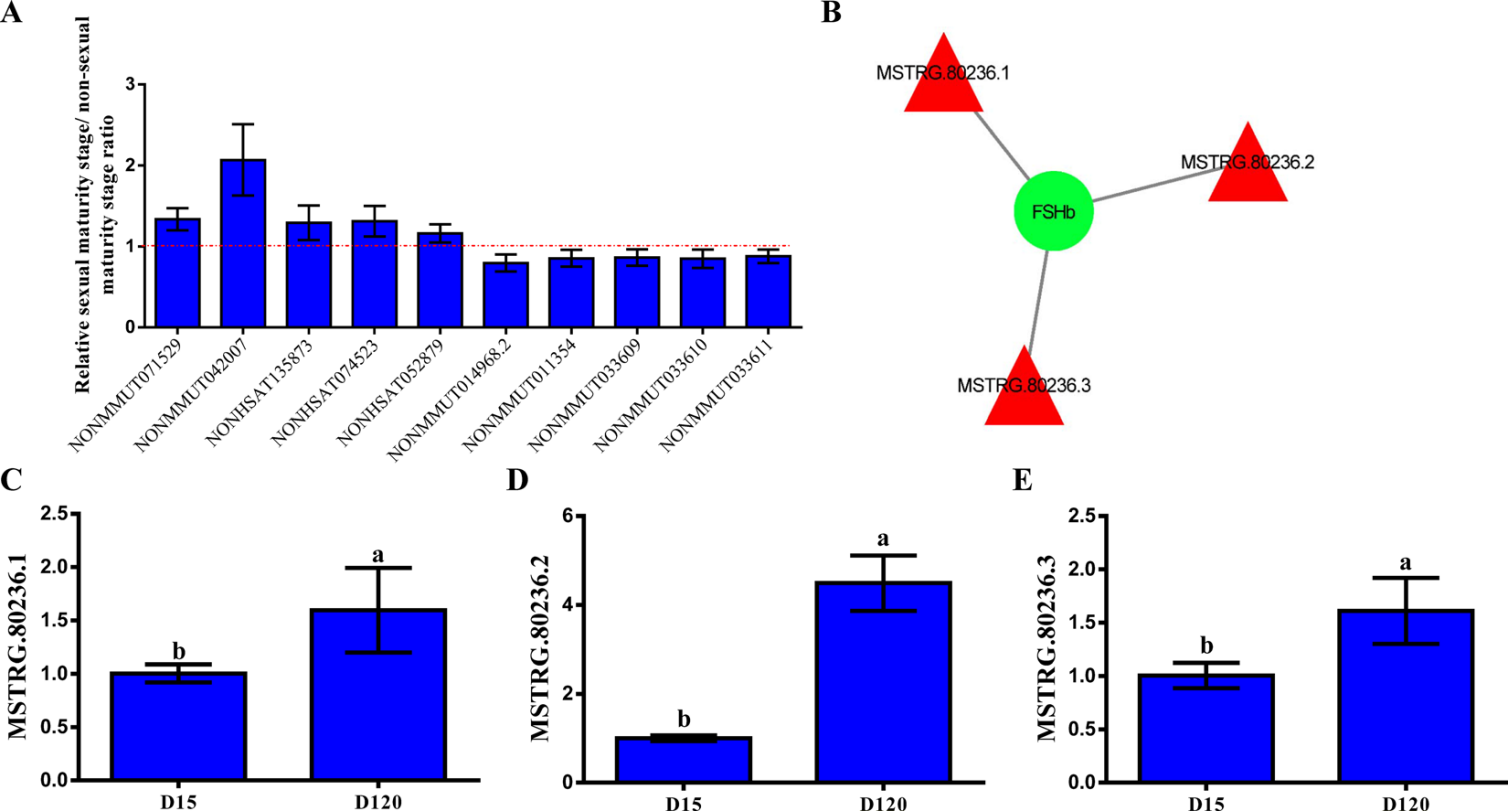
qPCR validation of ten highly expressed lncRNAs and three lncRNAs that interacted with the FSHb gene. (**A**) Expression of up- and down-regulated lncRNAs in the mature and immature rat anterior pituitary. (**B**) Interaction network of lncRNAs and the FSHb gene. The green cycle represents FSHb genes, and the red triangle represents novel lncRNAs. (**C**–**E**) Expression level of three novel lncRNAs, MSTRG.80236.1, MSTRG.80236.2 and MSTRG.80236.3, in the mature and immature rat anterior pituitary. All experiments were repeated at least three times. The data are shown as the means ± SD. Statistical significance was analyzed by one-way ANOVA, p < 0.05 was considered significant, and differences are marked with the letters a and b.

Ten highly expressed lncRNA transcripts were randomly selected to validate their relative expression in the immature and mature rat anterior pituitary through quantitative PCR (qPCR). The results were highly consistent with the RNA-Seq results (Fig. 6A). Furthermore, we further identified the lncRNAs associated with reproduction by constructing an interaction network of lncRNAs and the FSHb gene using the lncRNATargets platform based on complementary sequences in trans (Fig. 6B), and we simultaneously detected the expression level of all three novel lncRNAs (MSTRG.80236.1, MSTRG.80236.2 and MSTRG.80236.3) by qPCR. The results of our previous study demonstrated that the expression level of FSHb was increased in the sexual maturity period compared with that detected in the non-sexual maturity period^24^. The results showed that the expression of these lncRNAs showed the same trend as the expression of FSHb mRNA (Fig. 6C–E).

## Discussion

Recent studies have shown that lncRNAs are involved in pituitary tumorigenesis, mostly in non-functioning pituitary adenomas (NFAs) and occasionally in somatotroph and corticotroph adenomas, prolactinomas and FSHoma^25,26^; however, studies on lncRNAs in the normal pituitary are limited. In rat pituitary, miRNAs and genes have been more of a focus than lncRNAs^27–30^. In this study, we identified 7039 lncRNA transcripts from the immature (D15) and mature (D120) rat anterior pituitary using the Illumina HiSeq Xten platform. It is known that the pituitary secretes several types of hormones and plays a key role during the developmental stages. Moreover, in the immature pituitary, the hormone content should be in an appropriate range for affecting body development and growth. In the mature pituitary, hormones mainly affect the reproductive capacity. To the best of our knowledge, this study provides the first identification of lncRNAs in the rat anterior pituitary at differential developmental stages.

The development of high-throughput sequencing has yielded extensive knowledge on the transcription of eukaryotic genomes and has helped improve our understanding of RNA-mediated gene regulation^31,32^. RNA are classified into two main classes based on their coding ability: ncRNAs and protein-coding RNAs. In this study, we maximized the removal of protein-coding transcripts by developing a highly stringent pipeline for the selection of lncRNA transcripts, and this pipeline included an integrated analysis of CPAT, CNCI, CPC and Pfam to predict the potential coding capability of lncRNAs. Therefore, we identified 7039 putative lncRNAs in the immature and mature rat anterior pituitary. However, only information on lncRNA expression levels in NFAs and the normal pituitary in human^13,33^ is available from recent studies; therefore, we could not compare the characteristics of lncRNAs in the anterior pituitary of rats to those of other mammals. However, Feng Wang *et al*. reported information on lncRNAs in the renal cortex, renal outer medulla, liver, cardiac left ventricle, adrenal gland and hypothalamus in Brown Norway rat and alterations in Dahl salt-sensitive rat^34^. Notably, the putative lncRNAs had similar characteristics, namely, fewer exon numbers and shorter transcript lengths than protein-coding transcripts. Three lncRNAs associated with FSH and ten highly expressed lncRNAs were randomly selected to validate their expression levels in the rat anterior pituitary at the differential developmental stages, and we obtained results that were consistent with the RNA-Seq data. Therefore, all of the results yielded powerful, high-quality evidence that confirmed the identified lncRNAs.

lncRNAs, as a heterogeneous type of ncRNAs that includes large numbers of different species^35^, have been shown to display powerful biological functions in some biological processes, including epigenetic modification^36,37^, cell differentiation^38^, transcriptional regulation^39,40^, development and diseases^41,42^. In this study, 1181 lncRNAs transcripts were found to be significantly differentially expressed in the rat anterior pituitary at different developmental stages. Furthermore, we performed a microscopic analysis to determine the morphology of the immature and mature rat anterior pituitary, and the results showed several differences between these two time points, including the obvious differences in the degree of boundaries and the quantity of capillaries. Therefore, we inferred that some factors might lead to these differences in the morphology and functions of the rat pituitary and that one class of these factors might be lncRNAs. It is well known that the pituitary is the one of the most important endocrine glands and that it plays a crucial role in reproduction and development, producing secreting FSH, growth hormone (GH), prolactin (PRL) and four other hormones. Recent studies investigated some of the lncRNAs that are involved in reproduction and development. For example, Jeannie *et al*. (2015) indicated that lncRNAs are directly involved in X chromosome inactivation in mammals^23^. In addition, two highly overexpressed lncRNAs, PRNCR1 (prostate cancer-associated non-coding RNA 1) and PCGEM1 (prostate-specific transcript), can bind to the androgen receptor (AR), and the knockdown of two lncRNAs leads to reduced transcription of many canonical AR-targeted genes^43^. Therefore, differentially expressed lncRNAs might be potential reproduction- and development-related regulators.

It is well known that unlike miRNAs, siRNAs and others, the functions exerted by lncRNAs cannot be inferred from their sequences or structures^35,44^. Therefore, we explored putative protein-coding genes close (<100 kb) to all identified lncRNAs, and 6839 protein-coding genes were collected as the nearest genes of the 4074 lncRNAs. GO and KEGG enrichment analyses of these protein-coding genes identified 516 significant GO terms and 13 significant KEGG pathways, respectively, and the highly enriched pathways were associated with the antigen processing and presentation pathway, the autoimmune thyroid disease pathway and the type I diabetes mellitus pathway. Moreover, several GO terms, such as reproduction, reproductive process growth and hormone secretion, is strongly associated with development and reproduction. These results might have identified the potential roles of lncRNAs in the transcriptional regulation of associated genes. Interestingly, we found that some of the cis target protein-coding genes, such as FSHb, Cenpi, Crhr2 and Arrb2, were involved in pituitary development and reproduction (Supplemental Table S3), which implies that the corresponding lncRNAs play regulatory roles in pituitary development and reproduction. A few recent studies have indicated that lncRNAs are involved in cis-regulation of reproduction and development. For instance, Monnier *et al*. (2013) indicated that lncRNA *H19* binds the methylated DNA-binding protein MBD1 (methy1-CpG-binding domain protein 1) to regulate some imprinted genes in cis and trans^45^. Other studies have reported that lncRNAs could provide signals for the deposition of DNA methylation in cis^46,47^. The transcription of lncRNA Malat1 (metastasis-associated lung adenocarcinoma transcript 1) plays a cis-regulatory role in development in adults^48^.

Unlike small ncRNAs, such as miRNAs, siRNAs and Piwi-interacting RNA (piRNA), lncRNAs can fold into complex secondary and higher-order structures and thus do not have a fixed regulatory mode^49,50^. Recently, lncRNAs were categorized into four functions, namely, signal, decoy, guide and scaffold; additionally, each lncRNA might perform one or more than one of these themes^51,52^. lncRNAs are known to be involved in reproduction and development in mammals. FSH is an important reproductive hormone in the rat anterior pituitary. Therefore, it was interesting to explore the lncRNAs associated with FSH. In our study, as shown in Fig. 6B–E, we constructed a regulatory network with lncRNAs and FSH and detected and identified three lncRNAs. The competing endogenous RNA (ceRNA) hypothesis was first proposed by Salmena *et al*., who described a complex post-transcriptional regulatory network mediated by miRNAs^53^. Additional evidence also supported this hypothesis; for example, the Xia Tian group focused on a gastric cancer-associated ceRNA network that included eight lncRNAs and nine miRNAs^54^. The lncRNA linc-MD1 competes with miR-133 and miR-135 to regulate myoblast differentiation^55^. Notably, our previous study reported several miRNAs that directly regulate FSHb, the specific gene of FSH^18^. As a next step, we plan to investigate the regulatory mechanism of ceRNAs around FSH.

Overall, this study provided a catalog of rat anterior pituitary lncRNAs to further understand their regulatory roles in rat reproduction and development. In addition, our future studies will focus on investigating the regulatory mechanisms of lncRNAs associated with reproduction and development, particularly in relation to FSH, at a molecular level.

## Materials and Methods

### Ethics statement

The experiments were strictly performed according to the guidelines of the Guide for the Care and Use of Laboratory Animals of Jilin University. In addition, all experimental protocols were approved by the Institutional Animal Care and Use Committee of Jilin University (Permit Number: 20160312).

### Animal and tissue preparation

Wistar rats were provided by the School of Medical Science of Jilin University. Anterior pituitary samples were obtained from each of the D15 and D120 rats, and all of the samples were immediately snap-frozen in liquid nitrogen and stored at −80 °C until RNA extraction.

### Hematoxylin-eosin staining

Immature and mature rat anterior pituitary samples (removing the neurohypophysis) that had been maintained in 4% paraformaldehyde for 72 h at 4 °C were processed using routine histological methods. Sections were then subjected to hematoxylin-eosin staining. The morphology of the pituitary tissues was determined through fluorescence microscopy (OLYMPUS, Japan).

### RNA isolation, library construction and sequencing

Six samples of total RNA from the anterior pituitary, including three immature pituitary samples and three mature pituitary samples, were extracted using the TRIzol reagent (Tiangen, Beijing, China) according to the manufacturer’s recommended protocol, and the RNA quantity was assessed with a NanoDrop ND-2000 spectrophotometer (NanoDrop Technologies). After testing, the three immature and the three mature total RNA samples of the anterior pituitary were combined into their respective groups to construct the immature and mature anterior pituitary libraries, and the main processes for constructing the two libraries were as follows. First, the epicenter Ribo-Zero^TM^ kit was used to remove rRNAs from the sample, and fragmentation buffer was then added to randomize the rRNA-depleted RNAs. In addition, we used the rRNA-depleted RNAs as a template, and the first cDNA strand was synthesized by random hexamers. We then added the buffer, dATP, dUTP, dCTP, dGTP, RNase H and DNA polymerase 1 cDNA chains to purify the cDNA using AMPure XP beads. The purified double-stranded cDNA was then repaired at the end (plus A and connected sequencing joints), and AMPure XP beads were used for fragment size selection. After degradation of the U-containing chain, we obtained the cDNA library by PCR enrichment. In addition, we used Qubit2.0, Agilent 2100 and Q-PCR to assess the quality of the cDNA library. The libraries were sequenced by BioMarker Technologies (Beijing, China) using an Illumina HiSeq Xten platform.

### Transcriptome assembly

Clean data were obtained from the raw data by removing reads containing adapters, reads containing over 10% poly-N, and low-quality reads. The reference genome and gene model annotation files were downloaded from the Ensembl genome browser (Rnor_6.0, ftp://ftp.ensembl.org/pub/release-85/fasta/rattus_norvegicus/). Efficient comparison between the reads and reference genome was performed with HISAT2^56^, and the mapped reads of the two samples were assembled using StringTie^57,58^.

### lncRNA identification

We filtered the assembled novel transcripts from the D15 and D120 libraries to obtain putative lncRNAs through the following steps. (1) Single exons of transcripts and transcripts less than 200 bp were removed. (2) StringTie (1.3.1) was used to calculate the FPKMs of each transcript, and FPKM values of 0.01 were removed. (3) The remaining transcripts that overlapped ( > 1 bp) with *Rattus norvegicus* protein-coding genes were removed. (3) Four types of lncRNAs were identified from the remaining transcripts using class_code information annotated as “i”, “x” and “u”, representing novel intronic, antisense, and intergenic, respectively. (4) The CPC^59^, CNCI^60^, CPAT^61^ and Pfam^62^ tools were used to assess the coding potential of the remaining transcripts, and positive lncRNAs that were not common among the four predictive methods were removed.

### Expression analysis

StringTie (1.3.1) was used to calculate the FPKMs of lncRNAs and coding genes in each sample. Prior to differential gene expression analysis, the read counts were adjusted using the edgeR program package with one scaling normalized factor for each sequenced library. Differential expression analysis of two samples was performed using the EBseq (2010) R package. The resulting FDR was adjusted using the posterior probability of being differentially expressed (PPDE). FDR < 0.05 and |log2 (Fold Change) | ≥ 1 were set as the threshold for significant differential gene expression.

### Target gene prediction and functional enrichment analysis

We searched for coding genes 100 kb upstream and downstream of all identified lncRNAs and then predicted their functional roles as follows. The names of the neighbor genes were used to form a gene list that was inputted into R-project for GO analysis. We used KOBAS software^63^ to test the statistical enrichment of differential gene expression in KEGG pathways. GO terms and KEGG pathways with P < 0.05 were considered significantly enriched.

### Quantitative polymerase chain reaction

Primers were designed through Primer3 Input (http://bioinfo.ut.ee/primer3-0.4.0/) and synthesized by Changchun Kumei Bio (Supplemental Table S4). Total RNA was converted into cDNA using an InRcute lncRNA First-Strand cDNA Synthesis Kit (with gDNase) and a FastQuant RT kit (with gDNase) (Tiangen, China), and quantitative RT-PCR was performed on a Mastercycler ep Realplex2 system (Eppendorf, Germany) with an InRcute lncRNA qPCR Detection Kit (SYBR Green) and SuperReal PreMix Plus (SYBR Green) (Tiangen, China) according to the manufacturer’s instructions.

### Statistical analysis

The data are presented as the means ± SDs from three independent experiments. The data were analyzed using SPSS 19.0. The significance of the differences was determined through one-way ANOVA, and p < 0.05 was considered significant.

## Electronic supplementary material

Supplementary Information

Supplementary Datasets
