# Supplementary material for: Identification of long non-coding RNAs in the immature and mature rat anterior pituitary
Source: Sci Rep. 2017 Dec 19;7:17780. doi: 10.1038/s41598-017-17996-6 (PMC5736705; doi:10.1038/s41598-017-17996-6)
Supplement: Supplementary file 1 — Supplementary Information [file 41598_2017_17996_MOESM1_ESM.pdf]

Identification of long non-coding RNAs in the immature and mature rat anterior pituitary

Dong-Xu Han, Xu-Lei Sun, Yao Fu, Chang-Jiang Wang, Jian-Bo Liu, Hao Jiang, Yan Gao, Cheng-Zhen Chen, Bao Yuan*, Jia-Bao Zhang*

Department of Laboratory Animals, College of Animal Sciences, Jilin University, Changchun 130062, Jilin, P.R. China

*Correspondence:
Jia-Bao Zhang
E-mail: zjb515@126.com
Tel.: +86-431-8783-6551

Bao Yuan
E-mail: yuan_bao@jlu.edu.cn
Tel.: +86-431-8783-6536

Table S1. Differentially expressed lncRNAs (1181).

#ID	target gene	D15 FPKM	D120 FPKM	FDR	log2 FC	regulated	sequence
MSTRG.100158.1	--	0.026	0.298	0.002	3.246	up	CTCCTCAAACCTCTTCCTTCCTCTGCCTTTGTTTACCTTCAGAGCCATCCCCTCATTCCAATCTCCATCTTGATCTCAGACACAAGACTTTACCTCCAGAGGCCTCCCCAGCGCCTTTACCTGTTTATCCAGCAGGTACTTCGCAAAAAGCACATCTGAAGTTGAGCTTTGGCGAGGCCCCCTGACCATACCCACAGGGTTTCACCCATAGTCTCTGTGCCAGGAAGTGGGCCTCTTTTCCCAGTTACTCCCATCAGCACAACCCAGCCAGACTCTTAACTCCTCTTTTTCTGGCACCTTCTTTCCAGTTTATCAGCAAGCTCTATGGCCCACACCCTTCAGATAGACCCTGAGCATTCACAGCTCATATTCTCTTCCCAGCTCCCCACTCTAAAGCCTGCCATCTCCCCTGGAGTCACTGTGGTAGCCTGAGCTCCCGAGTTCTACTCTTGCCATTTTCATCTCTCCTCCAAACGACAGGCAGAGAGGCTTCATAATGAGGTGAAGTGTCCAGCTCCGTGGTGAAGCCCTTGCTTCTCACTATGAGGCACTGGTTCAAATCCAGCCAATCTAGCTCTAGAAGTTTCCAGTGAGTCCTTCCAGCTCTTCGCCAGCTGATTTTGTGTGCCTGGTGCGGGTCTTCCCAAGTAGCCTCTATCCATGCTTTCTCACTGGGCTGTAGGCTTCTCTGAACAGGACACTCAGCTCTTTGCCTCTCAGATTGCTTCTCCCTGGGAGGAGCCTGGGTATTAGGTAGTCATGACAATGAGTGAATGAAGAGACAGTTCAAGGACTCAAGCTCTGGTCCCAGGCACTCGTGTCACATACAGGC

							CTGAAGGAACCCCATCCAGGCAGGCAGAAGCCTCATCTTATTATTGTAGCTTCTGACAAGAAGCCTAGC<br>CTATGTTAGGAGAGGATCTAGGTAAAGCAGACTGGGTGTGGATTCCATCAAGAAAGTTCCAAAGTCAC<br>ATGTGAGCTCCAGTATGGGTCCTGAAAATGGTTCTGTTGTGTAGGTCTAGCATCCCAGCCTTCTGAAAA<br>CTCAAGAAGGCTTTCTGGAGGAAGAGGAGTGTGACAGCATGGTGACCATGGCCACAGAGCGTGAGTT<br>GAAGGGCCATCCATGCTTACACTGAAACAGTGATACTTTTCGCCCTGCTTCCTAATCCTTG
MSTRG.100182.1	--	0.011	0.969	0.000	7.083	up	CTAAAATTACAGATATGGAGGTCAGTTAAGACCCCACCAGCCGGGTAACTTTTCATGTAAGGAAGTCAG<br>CCTACAAGTTTCTGGTGGACCAACCCGGGTAAAGAACAGGCCCTAAAAGACCCCCTCTGAGCCAGCAG<br>CTTGCTGTGGAGGGTGTGCTGCTGCTGGGGGCATCTAGAGCTGTGAAAATCCTCTCACGCACAGAACA<br>AACCCAGTGCCTGCAACTGAGAAGCTTCCCATCATGGAAGCTCATTCCAAACACTGAATTCTAGGCTAT<br>GTCAGTGGTGCTAACTCCCAAAGTCTGTGGTTCCTGCCCCCTGATGCAGAAGGGATGACTTTGGACAA<br>CGCTGCCTTCTAGGCAACCCTACCTTCTAGGCTTCTGTCAATAAATAACACATCACATGGAGTGTAGCAT<br>TTTTTCCTTTGTAATCAACCTTTATTTATTCCTTCCTTTATTTCCTCCAACATCCACTTACATATTCTCAAAT<br>ATTTGTGTACATATTCACCTTTGTTTTGTTTGCTTATTCCTTCCGGCCCTCATTTCTCCGCGTGCTGATTCA<br>CTAATTTTCATTCCTTGTGACGTTGATCTCATAAGTAAATGGGGCAGAGTGGACAGTTTCTATGCAAGGC<br>TTAATCTTAGCTCAAAGATGCAGGCACGGGACAGACACATACATTACAGCTGTCACATCAGCACCCACC<br>CATACATCCTAAGGCTAGCCTATAGACCATACTCCTTACATAGATTCTCACAACTACTTATTCGCTGAAAA<br>CACAGTCATTGTTTTTATGGCATTTGTCAAAACTCTATCAGTGCAAAAACCTTTGCCTGATATGTTTCAG<br>AAGATAAGGAATGGGGGAAAGATAAAAAGATAAATTATGCCAGCCCCTGTCATCCTTCCACCCATCTATC<br>CATCCATCTATCCATCCATCTATCCATCTATCTATCCATCCATCGGTCCATCTGTCCATCCGTCCAACTGTT<br>CATCCATCCATCCATCTGTCCGTCTGTCTGTCTGTCTGTCCATCCATCTATCCATCCATTCATCATTCAATC<br>ACTCTGATAAATCTCTACTGCTGTTGCTCAGCACCAACTCCTGAGCTGCCAATTTTCTACCTAGAGTGAC<br>TGGGGAAGGCTATGTTCAACTGTAGCCTCAAGGGCTGAATATGATTTCAGAACTTGTAAAGAAGAAATG<br>CAGTCCATCCAGACAACTGGATGACATGGATGGATGTGGGGTGTAGGGTCTAAAACACCACATCACAG<br>CTGGGTAAGGAAATTGCCCTGTGCAATATCTAAACACACTGCTTTGAGAGAGCTTAAGGCAAATCTCAG<br>CAGGATTGATTGCCAAGCCAAAGAGTTTTCTGTGTCCCCAAGGAAGACCTGGCTTGGCCTCTCATCGTC<br>AATTGTAAGGAGGGTATGGAGCACGCAGGCACCCAAGTGTCAGGTTGGTTGTTCCTGACACAGTCTGA<br>TTTCCTGCTGCTGTCTCCTCCACCCAGAAGGAAATGCTCCAAAAAGCTGGTTAAGATTCCCCACTGGCC<br>CTAAATGCCTCATGGTTATGACAGAGCCTTGTGATATAGCTTTCATGCCAAATGAATAAAATATGTAGATG<br>AAAGTTTTTCTCCCCAAAGGAAAGGTGCTGGAAGAATTGACAGAAAAGTATTTAGAGGCAGGGTACTA<br>GCTATGTTGACCCCTGGTAGAGCATCCCTGGTCCTCCTGGGCAGAGATACAAAACTGCAGAGAGATAGA<br>AGCCAAGTGTGGGCTCTCAGGAATATGGCTTCACGTGTCATAGCATGGAGCTGGCTAATCCCACAGGCC<br>CCAGTTCTAGCCACAAGCTGAGACTAGCAAGGATAGAGGGGCTTTGAGCCTAACTCTGTGACTCCTTGT<br>GTCCTCTTGCCCTGAATGGCCCTATGTGAGCTCCTGAGCCTCCTCTTAGCTCTTTGGAGCTGGTAGAACT<br>TGTCCCTAAGTCTCAGGTCTCCAGAGGACAGCTGATCCTTCTGTCTAGGCAGGAAGCATAACAAACATC<br>AAATAAGAACAAAAACTATAGGAACCACTCAACACGCACTTGCTCAGATGTAAACACATCTGGCTAATA<br>AAGTGCGGTCACCATCAGGGTCTCATTTCATCTTCAGAAAAACTGAAGGGTTAAGCTAATGTGTTCAGG<br>GCGTAGAGTATAATCCCCACTTCACGTACATTCAAGCCAGTACAAGCCAATGGCAGCTTTACTAAATGTT<br>CACAGGGGCCCATTGCATCAGACTCCCAAACCAGTGTGCTCCAGCTTTGTTTTCCCCAGGAAGATGGTG<br>TTTTGAATGCAAAAACAGTGAGTGGTCCCATGGGTGGATGTGCTATCTGAAGCTGTGGTTCCTCCTCC<br>ATCCACGAGCACCTGTGGAGAGGGTAAGGTGGACCCAACCTCAGCCGAGCGGGGCCTCCTGACTTCA<br>GCCCAGGTTGTTTCCACATTACCCACCCTAACCTTGACCTTGAGCATGGCACTCCCCATTCTTCCAGCCT

							CCCCATGGGTGGTGGGAACATGTGGGACAGGTGGCTTTGAAGGCCACTTCCATATTCAAGCTGTTGCTG GAACAGATCTAAGGACAGGATGTGAAGGTAAGAGATCGGTAACTCCCAGAACTCCATGGGTGGAGTAG TCTAACTAAGGGCAGGGGCAGAGGGCACGGAAGCAGCCCTTGACCGACTTCCCAGGGAAGGCCCACT GCCCTCCGCTTCCTCCTCCAGCCAGGGGGTCTCCTCCGCTTCCTCCTCCAGCCAGGGGGTCTCCTCCGC TTCCTCCTCCAGCCAGGGGGTCTCCTCCGCTTCCAGGTCTTATTGACTTTGAGTTTACAAGGTGTGTGG GTTACTAAGGCTTGGGATGATTGCCCTGGGGCCAGACCCGCCTTGGGAAAGCATCCTCCTCACCCCGGC CCCACAGAAGTGGGTGGGGCCACAAGCTGGGGTCTCCTAGAGCTGCTTAGAAGTTACAGTGGAGAAC CATCAAATGGAAAACAGGAGAGAGAAGTGGAAGGCTGGCTCAATCTGGAAAGGCGGAGGAGAAGCC ATGGACAGCTCCACTTTTACACCCTGAAGCCCTGAGTTCCTAACGCAGCCTGGCTCTGCTGGTGGGTGA AAGCTCAGGTGGGTGAGGAGCTTTATGGTATTTACTTGGTTTATTGGCTCTGGGGAAGAGGATATGAGT GAGGCCCAAAGTAGTTTTCTGAAGATAGGACCCTGCAGAAAAGGCAAGATTTATGACTTCCGGAAAGC CTCAAGGTTACTGGGCTGGGCATGGTCAGCTCTCCTTGGTTCTGTGAGCTTTTCAGGGAGGGGACCCTA GGAAAAGAAGTCCATTCCCCCAAATTCTCTTTGAATATCCCCCAGAGACAGCTCCACAGGGTGGAGTTG CTGTGATTCCTCCATCATTTTAAGGAGGCGGAAACCGAGGTCAGAAGAACCTTCTGCTCTGTAGGATCC ATGTCCATTCAGGTTCCCACACTTTGTCTCTGGGAAGCTGTCACTAAAGTGACCCTGGAGTGGGTAAAT ACTGAGTCACCCTCATTAGGGTGGTGGCCTATAGTCACCACCTGTGTGCCACCCCAGTCAGGCCTAGAA CAACAGCTACCTATCCTGTCCAGTGAGGTGGCCAGGCAGCCTCTAGTGCAGAACCACACAGCAAGGGT GCTTGCTCTCCAGGAAATACTGACTCCATGCCTGTGGACTCCGGCCCTGACCAAGCAGATCTGGAAAC AAACGTTTCGCTGACAACCCTACTTCACAGGCACAAGAGGCTGGTTCCTGACACAGCCGGGGCCTCCC GGTCTTCAGTGGCCCCTGTTAGCAGGATAGATTGTGTTTCTGGGCTTAATTAAGGGACAGACCATAAATA TTCATGCAGCCAAATGAGTCTGGCTCACAAGTGCAGCTGCCTCCCGCTTGGGGAAAATTAACATTGTTT TCCAGAGGAGAGGAATGAGGCTGCTCACTGCAGAGAAGGCTTTTCAACAACCTCTCTAGGACAGGAC AGAAAGGGACAGCCACACTCAGAGGCCCTGCACCCTGGGGAATTGTCCTGGCCTCTGGGACCCCTCTG CTTCCCCAGTGAGGCTCAGTGGGGCTCCGGAAGGGGGGCTTAGCAGGGTTTTTAGCAGAGCAGAAGG CTCCTAACTGTTTAGAGAACTGGTTTTAGCAAAACGTTGCTAAGGGCTAAATCCACCTGCCACCTGTATT TGTCAGGAAGCTTTCCTGGGGCCTCATCACATATGATTCATAGTAACTAAAGAGCTGAGTCATGTGGTG GACCATTTTAACCTGTAAAGCCTGAAGTGCTGACCACTGGGCCTTGTATGGAGAGTCTGCTGGCTCCTA GAACTGTCTAGAACCACCTCCTGTTGGACATCTGCTATTATTATTAAAAGTCTCAGAACTTGAGCTGGCA AGGTGACTCAGTGGGTAAAGGCCCTTTGCCACAGAAGGCTGATGACGTGAGTTTGGTCCTCAGAACCC AAGGTGGAAGAAGAGAATCAACTTGGGAAAGCTATCCTCACATGCTGCAGCATCAGCACCACCACCAT CACCATCACCATCACCATCACCATCACCATCACCATCACCATCACCATCACCTTCATAAATAACATTAAG AATAAATAAAAGTCCTGTCATTTCAACATCCATGGTGTCCTAGAAGTTGAGAGTGCTTTTGCCTTGTGGA CTTAGAATCTTCTCTGCAGACTAAAACCTGATTTCCACGTCTACTCTGCCCCTTCCTTACCTGTGACTGT CAGCAAGTCACCCGCCTTCCTCCTGCTGGGACTCCCTGAGACCCCAAAATAAGTTGGACTGTGATTTAC TGGCAACACTTACAAATTACATATTGCAATGATATATCATTCATATGTAATATGATTATAAACTACACGTTT AAAAAGTGGGACCTTGAAACATTAGGAGCCTTACCTGTGGTCTGGTCACTAGGAGTGGACTGCAAAGC CAAAACCCGATCCTGTCTCTCAGGCAGCCCCACTTGCACCATGTCCTCAGCCTCTCTGAACACCTGCTG TAAGGGGGGGGCTCGCTATCCCCATCCCCACCCGCTCCCACCCTGAGCATTCACATAGGAATCTGCCCT ATATTTGTAGGAATCTATATTTGCCACACTTTGGCTCCTGGTTTGAATAGCTTCTTCCCATCTCTGTAAAG TCCAGAAAGAGAATGCACACATTCTTGAGGCTGTCTCCCAACTTTCTAGGAGCCAGGAGCAGGCTGAG CCTAATGCATTTGCAGCAAGCAAGACCTGGGCAGAAATTTCCTGTTTCTGTTATATTCCCCTACAGGAAG

							CCCTCCCTGATGCCTAAAGCTCCCAGTATTCCTGCCTGGACCGCTTCTCGTTGAGACAGGTCAGTAACA<br>ACCCCAGGACTCTCTAACCCTTGCATTCTTTCTGCTGGTTTTCAACGTGCCCTGATTGTCTGCTCTCTTC<br>CTACGATTGTGTGGCAATAATGAATCCCAGCACATAAATGCCTTGCATTGAAGTTATCCTCACGACCAAT<br>CCCACTTGTAAGATGCTGGACCTGGGACCCATGGGGAGGAGAAACAGACAGTAAGAGGCCTGCAGAG<br>AAGCCCTGTGTGTCCCTTACGGGGAAACCCTCCTGCCCGCTTCTCCTCACATAGGTACCGAGACGTCAT<br>CTGCGGCTGCTGCAGATACTGACTAGTTGGGGGTGACACAGACAATCGAGAAGCTGTTGATAAAGCAA<br>ATCCCGTGTGCCAACCAGTTTCTAAGCACACTGCCTTAGTCGAGCCCCAGGGCAGGTATTTCTATTCCTG<br>AGCAGGTTGGATGTCCTGGGGAATCAGGCTCGACCCAGGACTCTGGTGGGTAAGGCTTTTAGCTCACC<br>CCCAGCAGCCCTAAGAGGCCAGATCATATCCTTTCAGGGAGTGGAAAAGGAAACACTGAGCCCTTTTA<br>CAGGGTTTCTTGTGTGTGCCTAGCAGCCTCTGAGGATGGCCGAGCTGCCACGGATGGAAGAAACAGTT<br>TATTTTTCCTGTGAGCCGCTCTGGAGCCTTTCAAAGGTGTGAAGTTGCAGAGCCTCCAAATGCTGACTC<br>CATGGGGCTGCACAGGGCCAGCCCTGCCTGGGCCTCAGACCCCTCCACCCACCCACCCCCAGTCACAA<br>GCTCCAGCTCTTCATGTGCTGTGGGAGCTCCCCTAGGCCCATCTGGCCTCTCCTGTGCTGAAAGGCTGC<br>AGAGTGACAGGCCAGAAACCCGATTCAGTGTTTATTCACTAAACACTGAGAGAGCCCTCCTGAGTCTC<br>CTCCCCACCCAAAGCCTCTGCTACTTGGGGGGGTGAGGGGGGGCACTGTCCATCTGCCATGCTCAGCAT<br>ACCTGGTGTACTTGCCCCAGGAGATCAGACACAAGGAGGAGCATCCGGACTGGCCATGAAGCAAGAA<br>AAAATCTGGTAAGGCGGAAGAAAGAACTGCCACCCGGTGGCATCAGTCGAAAGAGGAGGAACCCCAA<br>GTCCTGTGGATTCTGATACTTCTGTTAGATGATGGGAATAAGAAGAGTGGTCAGGAAGAAACTTCTAGA<br>TTGCCCTTTGATACAAATATAAACCAACATGGCAGCACCAAGGATGAGCATCCCGTAGCTCACTGCAGC<br>AAGAACCCTGGCAGCAGCCTTCCAGACCCCAGACACCTCCCTGCCCAGCTCTCAGCTGTTGCTTCCAC<br>CATTGCTTCCAGAAGAGTAAGCAAGCCGTCCTTCTGGTACCTCTGTTGCTCAGAGACACAGAGATGGA<br>GTCAAGGAGGACCAGACAGCGGTTCAAATGTGAGAAGCCCAGGAGATTGTCATTCCAAAATAGAGTCA<br>CCCCCCACTGTGAGCCTGGCCTTCATGTTCCAGTGACAGGTTTAAAGTAGAAAACCAATTCTTGGGGGA<br>ACACAAAATCATCACTCTGAAGAACATGGAGTAACTCAGCAAACAGAACCAGCCCACTGATGTGGAGG<br>GCAATTGCCTGGCACTCTATGCTATATCCATCCCCCAGCGAGGCAGGGCAAGAAGGCCCAGGGACCAG<br>GCAAAAGCTGAGTTCTGGCGAACCATCGCTCACACACCTACATATAACTCATGCCCACCCTCAGGTCTG<br>GGTCACTTGGTACCCATGGTTTCCAATGTTCTAGAAGGATCAGGACTAGGTGCGAGTATTTTGTGGGTAT<br>GTCCTCCATGGTGAAAACATTTATTAAAACCCCCAAGGTAGGGCTTGAGGTGATAGTTCAGTCAGTATA<br>GTGCTTACCTTGCAAGTATGATGACATAAGGTCCACCCTTAATCACAACACTGGAAAAGCAGCGATAGG<br>TGGATACTTGGAGCCCAGTAGCCAGTCAGATTAGCCTAGCTGGCAAGTTGCAAGTCAACAAGACACAG<br>TATCTCAAGTAAACAAGGTGAACAGTGCCTGAGGAGCCACACCTGAGGCTATTTTCTGGCCTCCGCATA<br>CATCTGCTCGAACATGCATGCACACAAATATTCATGTGCACCTGTGCACACACATGTACATGCATGTGAA<br>TACATATGTACATGCATGCAAACACACATGTACACACACAGGTAGATAAATCTTCAAGGGGCCGTGCATT<br>CACACGCAGAACCTCCAGCTTTAATTCACCTGTTTAGCAATTGAACTTCATGGAGGCAAGCAAATGTCT<br>AACCTGCCTTGGCATTATTACAGCATAAGGGAAAGGCTTGACACTCTGGACACTTGATGGAATGAACGA<br>ATGGATGGATGGATGGATGGATGGATGGATGGATGGATGGATGGATGGATGAATGGGCAAATGCCATATT<br>CCTTCTAGAGCTTAGAAAATGATGTATATAAAACAAGAAATAGAGCATAAGGCAGAAAGTGGTGACAAA<br>TAAAAGAAATGGGACAATAACGGAATTGGGGAGACAAGACAAAGCCAAATCCTGACACCAGGTGAAA<br>CTAAAGGTAGGAACAATACTAGATGCAGTGGGCAGAGAGCTGGGAAGGCATTCAGGAAACAGACTTTT<br>TCTAAAAGATACTTTTCATTTTTCTTTTCTTTCTTTATCCCTTCCTTTCTTTTTTTAATGTTTTTTATTTTTAA<br>GAAAGCTGTGGTTTTGCATCCAGGGCTAATGAGTTTTCCTTTCTTTTCCCATGTAAGCTGTCCAGTTTGG

							CTAGAGGAAAGGATACATTCACGGCAGGGTTTAGAGACAGAGGCTGGACCCTGAGCAGGAGGGGTTG<br>GGGGAGCAGAGGAGACTCAGAGGCTACTCTCAGTAGCTGTTCTCTGAAGCAGAGGAGTGAGTGTTCTT<br>CCAGAGCGGAGACAGGCTGTAGGGTCTGGACTGGCCTGTGCCAGCCAAGGGTTGCTGCAGAGCTCCAT<br>CTGAGGCTGAGAGGCCAGTCTTGAGAGAAACCAGGCTACAATGGACAGGGTCTCCAGCAACTGCTCA<br>CTGACCTAGAGTGAACTGAGTGAGGGAAATGATTTCCTCAAAGAGTCGTGCGCTTTCCTTGGGTCTGA<br>ACTGTTTGTGGCAAATGTAAAAGATGAGATTAAACAGTTACAGTGATATATAAAGCAAAATTTCAAACAT<br>GAGCAGGTAAGACACAGACTGGGCATAGGATGGGAGAATTTTACTTCCTGTGCTCTGTAGTTGTTTTCT<br>AAATCTCCAGCTCTTTCCCTGAGGCTTGCTCCTCCTCTGCTTCCAGATATGATCCCCGAGGATCCTGCCT<br>TGAACCCCTGCTTCCTATATTTCTCACAGACTCCTTGCAGCATGAAGCTGCCTTCCTGGGTGGTGACAGC<br>CATGCCAACCGGATGTTGGATGTTGGATGCTGCCCCAGTCAAAGTCATCATTATTACAGTTAATACCTGT<br>GGCCCTGCATCCAGCCTCGGCCTTCCCTTCTGATTGCACTTTTACCTGGGCCTCAGGGGCTGTGACAGG<br>TGTGACTGGGAGTCAGTCACTGCTGCAATGGCATCTAATCCCAATAAACTTCCCACAGAGATTCTCAAG<br>GGAGAGAGGCATGGGTCTAACCTAGGCAATAGAGAAGCTAAGATTCTGTCTGTGCCAGACGTTTACAA<br>CCCTGCCTCTGGACTCCGTCCTGTGAAGATCATCTCTCTCCCTCCCGTGCTGTGGTGGATGTTCTTCTCC<br>AGTGGAGTTCATCACCGTAACGACGTTCAAACTGAAAGTGGAGCTGACAGAGACGTGCTGTTCTAGCC<br>TTGCTGTACTCTTACAGATACATATTCTTTAAACTGCTAGAGGGAGACTAGGGTGTAACTAAAGCCTTTG<br>CTGGTCTGAGCAGAGCCTGGGCTGGCTCTCTAGCATTACAATAATAATATGGACCCGACAGTGGGAACA<br>GAAGAATTGTTGAATGGAGCAGCGGGAGGGAACATCTCTCTGTGGTCACAGCTGTCCCCTGTACTCCA<br>CAGTGACCTGTGGAGCCCAGTCTCTGCCATCACAGCCAGTCTGCAAACATGTTCTTGCAGAGTGGCAAT<br>GAGCATGGGTTTCTCAGACCCACAGAAGAATGCAGGTATGTCCCACACAGCAGTGACAAAGATTGGTG<br>TAGGAGAGACAAGTAAGACCTTCTCAAGAGAGGCAACCAGGGACCTCGGGGCCACAGGGAAACCATG<br>ATGTTAACCACAAGAGAATCCACTAGAGAAACCCTGCCCAGAGATGATGTTTCTCTTTCTATGCTGGCT<br>CAGCACCTTTCCAAGCTGTGAGGGTAAAATGGAGTCTACCTTCCAAATCTGTGCTGGTCTGTGTGAGGA<br>GTCAGCTGAACCTTACTTGTTCTCTAAGCCCCAGGGAAATGCCTCCCCACAGAGTCCCCAGGGACTGT<br>GGGTTCATGGAGCTCTCTTTTGGGGACTTATGTATTTTTCTGCCTTTTGTTTTATGTGTTTAATTATTTTAA<br>AAAAGTGTTAACTTGATATTATCTCGCAAGCCAAGGGTTGTGCTTGGGCCTGGGAATGTAACCAGAAAT<br>GAGCGATTGTTAGAGGGAAAATTGTGGCCCCAAAAGATAAGTTGAAATTCCAGCCCTAAGTACCTCAA<br>GATGTGGCCTTATTTGAAAAGAGTCGCCACAGGGGTAATTAGTAATGTTGCTGTGGTCAGAGTAAAGTG<br>GCCTAGTCCTAATCCAATATCCCTGGTGTCTTTCCAAAAGACACAAACAGGACACCCAGTCATGGAGAT<br>GGGGGTTGGGGGTTACACTGCCACCCTGCAGTGAGGGATCAGGGCCACCAGGAGTTGAGAATACAGG<br>AAGGACTTCTCCCTGGAAAGCTAAGAAGCATGGCCCTACCACATTTGGTCTCAGCCCTTCAGACTCTAG<br>CCTTGCGGCTACATCCCCTTCTCTGTTTACCTGCCTGATTTGTAGTACTCTGCTGTGGCAGCCCTGGGAA<br>GCTGAAGCAGAAATTGTCTTTATTCGTAGAAAAAAACATAGATGGACAATCACAAAAGTGTAGAATCCA<br>CCTATAAGAAACAGTCATGAGTCATTCAGTCTCAGAAAAGGATTAGGACAGGCTTGTACAGTAAGCACA<br>GTTGAGGGTGTGTATATGTGCGTGTGTGTGAGTGTGTGTGAGTGTGTGTGCATGTGTGTGTGTGTGT<br>GCGCGCGCGCACACGTGCAGGTTCACATGCACATGTGGGTGTGTGCACGTGGCACGTGGAGGACAGA<br>GGTTAGCACCAGGTATCTTACTCAATTGCCCTTAACCTTACAGAGAATCTCTCACTATACCTCACCAATG<br>GCTGGCAAGCAAGGCCCAGGAATCCTTCTGTGTCTGCCTTCCCAGCAGGCGCTCAAACAGGTGCTCAC<br>CACTGTGCCCAGCTTCTTACTCACATCCTGGAACTCTGAACTCATGCCCTCAAGCTTGTACAACAAGCA<br>CTTTACCCACTGCACTGGCTTCCCAGTCACCCTGCGGTTGAGCACTGAAGGACGGGGAGGAGGTCTCT<br>GGAAGAAGGCTATTGGAGAAGGAACAGCACCCCTGTGTCTTCACAGTCTCTCAGGAGGAACTGTGCTC

							TCAGAACCGTGTTCTGCCCCGTGACAGATGCTCAGCACCCATCCTTTGGCCTTGCAGAAGAACCAGACTTTAGGCAAGGGAAGAAGAAATCAGGAGGGAGTGTTCCTGGCTGCCACTGAGATGTACTACTTCTGCAACTGAGAGCAGAGACAGAGCCCAGCAGAGAGGCCCCAAGGAGATTTAGAACAGGGCTGGCTTTTAAAAGAAAACAAAAAAAGCAAACTCGGGCCGTCCTCTAGGGCTCCAGCTCGGCAGGACAGGAAAGCAATTTATGGTCAAAGGAAGTAGCCTGACAGTAAACAAAAATACAGTGACCCCGAGTTCCCACTGGACAGAAGACAGATGGGGCCGTCGTAAAAATGAGATTAATTTTAATAAGGGCTTTCACCAAGCTGCAGACCCTGGTGGCCAAATCCTGTCCAAGTGCCAAGGACAGGCAACACTGTTGATGGATGGGGCCAAGCTCAGAGAGCTGAAGACGTGGGGACGATCGGAAGTTTGGGACTGGCCCCTGCCTGTGATGTGGCAGCTTCTCCCTATCCACTACCAGGGTGGGAATTCCCTGTCCCCTCCTCTTGGTGGAGACCACGGGCTCTGTGCTTGTATAACTCTAGGAATGGGGAGCTCATTATCTAAGCAAGCTGTGGGCTAGCTCTCCACATACAGACACTGGGAAAACTCTGTGTCATCAGTCATGGGAATATCTGAACTGGCAGTTAGCGCACACTGCTGGAGAAGTCACCGTGTAATAGGTTATGGGAAGAGTGGCATTTAGGATTGGTTCATGCTTCCAGAAGCAAAGACAGGCAACACCCTTATCCTAGCTGTTGACTGAGCCCTAATCCTGATCACAGGCCAAACCCAGATCTCTTGTAGACTCAGCCCTAATCTCAGATGGCAAACTGAGCCTTGAAACTAAGTCCTAACTTAGACTGGGTCTTGTTCCACTCTGAATCTTAACCCTTTATTGACTGAGCCCTGATTCTTCTGACTGAAAGGGAACCTGATTCTTACCCACAGCCTCATGCATAATTATGTGGTGTCTGTACCAGAGCAGAGGGAGGTACAAATGTGAACATACACAGCTCACTGCAGCTCATCATGAGCCTAGAGCAATGCCCATCTGCAGAGTTATAGCCTTGAAGCACCGGGCTGAGTGGAAAGCATTATAAATGTTGCATCCCCAAGCTCTCACAGCTCCTCCTCCCCCCACCCCTCCCCACATGGAGTCAGCATGTCACTGCTCACACACTGCACAGGCTTCTGCTCTCCGGACAAACAGAGTCTATATTGCTCCCTGGCCCGCAACCCCCACCATCCCCACTCCTAGACACTAGGTCCTGACCTGCTCAGGATTACAGAGCTGTATAGCTTCATAGAGTGATGGGTATCAGCAAAGGCCTGGTTAGAGCTTTAGTCACTTTCTTTATCAGCATGCTAACTGGAGTGATGGCCTCGCAGAAAAACAACACCCTGTGGGGTTTCAAGAGCCAAGTACCTGCCAGCTGTAGAAAACCTGCCCTGGTGATTTCCCAGCTCTTGACCCCACTGGCATCTGATAGCCCAATGTCTTGTCAGCTGTTCTCCCAATTATGTATCAATAAATGTCACACAATCGAGTCTTGGCTCCCTAGAGAGTGTTTGAAAGTCAGAGATATTTGCCTAATGCAAGCCGATGTCATGTCAGGAATTAGTTTGACTAATAGGCCTTTAATGAGATCCTTGTGAGGTTTGGGCTCCAAGCAAAGGCTTTCTGAAGTTTTCCT
MSTRG.100182.12	--	0.442	0.000	0.000	-5.855	down	ACTGTCCATCTGCCATGCTCAGCATACCTGGTGTACTTGCCCCAGGAGATCAGACACAAGGAGGAGCATCCGGACTGGCCATGAAGCAAGAAAAAATCTGGTAAGGCGGAAGAAAGAACTGCCACCCGGTGGCATCAGTCGAAAGAGGAGGAACCCCAAGTCCTGTGGATTCTGATACTTCTGTTAGATGATGGGAATAAGAAGAGTGGTCAGGAAGAAACTTCTAGATTGCCCTTTGATACAAATATAAACCAACATGGCAGCACCAAGGATGAGCATCCCGTAGCTCACTGCAGCAAGAACCCTGGCAGCAGCCTTCCAGACCCCAGACACCTCCCTGCCCAGCTCTCAGCTGTTGCTTCCACCATTGCTTCCAGAAGAAGAATCTCTCACTATACCTCACCAATGGCTGGCAAGCAAGGCCCAGGAATCCTTCTGTGTCTGCCTTCCCAGCAGGCGCTCAAACAGGTGCTCACCACTGTGCCCAGCTTCTTACTCACATCCTGGAACTCTGAACTCATGCCCTCAAGCTTGTACAACAAGCACTTTACCCACTGCACTGGCTTCCCAGTCACCCTGCGGTTGAGCACTGAAGGACGGGGAGGAGGTCTCTGGAAGAAGGCTATTGGAGAAGGAACAGCACCCCTGTGTCTTCACAGTCTCTCAGGAGGAACTGTGCTCTCAGAACCGTGTTCTGCCCCGTGACAGATGCTCAGCACCCATCCTTTGGCCTTGCAGAAGAACCAGACTTTAGGCAAGGGAAGAAGAAATCAGGAGGGAGTGTTCCTGGCTGCCACTGAGATGTACTACTTCTGCAACTGAGAGCAGAGACAGAGCCCAGCAGAGAGGCCCCAAGGAGATTTAGAACAGGGCTGGCTTTTAAAAGAAAACAAAAAAAGCAAACTCGGGCCGTCCTCTAGGGCTCCAGCTCGGCAGGACAGGAAAGCAATTTATGGTCAAAGGAAGTAGCCTGACAGTAAACAAAAATACAGTGACCCCGAGTTCCCACTGGACAGAAGACA

							GATGGGGCCGTCGTAAAAATGAGATTAATTTTAATAAGGGCTTTCACCAAGCTGCAGACCCTGGTGGCC<br>AAATCCTGTCCAAGTGCCAAGGACAGGCAACACTGTTGATGGATGGGGCCAAGCTCAGAGAGCTGAA<br>GACGTGGGGACGATCGGAAGTTTGGGACTGGCCCCTGCCTGTGATGTGGCAGCTTCTCCCTATCCACTA<br>CCAGGGTGGGAATTCCCTGTCCCCTCCTCTTGGTGGAGACCACGGGCTCTGTGCTTGTATAACTCTAGG<br>AATGGGGAGCTCATTATCTAAGCAAGCTGTGGGCTAGCTCTCCACATACAGACACTGGGAAAACTCTGT<br>GTCATCAGTCATGGGAATATCTGAACTGGCAGTTAGCGCACACTGCTGGAGAAGTCACCGTGTAATAGG<br>TTATGGGAAGAGTGGCATTTAGGATTGGTTCATGCTTCCAGAAGCAAAGACAGGCAACACCCTTATCCT<br>AGCTGTTGACTGAGCCCTAATCCTGATCACAGGCCAAACCCAGATCTCTTGTAGACTCAGCCCTAATCT<br>CAGATGGCAAACTGAGCCTTGAAACTAAGTCCTAACTTAGACTGGGTCTTGTTCCACTCTGAATCTTAA<br>CCCTTTATTGACTGAGCCCTGATTCTTCTGACTGAAAGGGAACCTGATTCTTACCCACAGCCTCATGCAT<br>AATTATGTGGTGTCTGTACCAGAGCAGAGGGAGGTACAAATGTGAACATACACAGCTCACTGCAGCTC<br>ATCATGAGCCTAGAGCAATGCCCATCTGCAGAGTTATAGCCTTGAAGCACCGGGCTGAGTGGAAAGCAT<br>TATAAATGTTGCATCCCCAAGCTCTCACAGCTCCTCCTCCCCCCACCCCTCCCCACATGGAGTCAGCATG<br>TCACTGCTCACACACTGCACAGGCTTCTGCTCTCCGGACAAACAGAGTCTATATTGCTCCCTGGCCCGC<br>AACCCCCACCATCCCCACTCCTAGACACTAGGTCCTGACCTGCTCAGGATTACAGAGCTGTATAGCTTC<br>ATAGAGTGATGGGTATCAGCAAAGGCCTGGTTAGAGCTTTAGTCACTTTCTTTATCAGCATGCTAACTGG<br>AGTGATGGCCTCGCAGAAAAACAACACCCTGTGGGGTTTCAAGAGCCAAGTACCTGCCAGCTGTAGAA<br>AACCTGCCCTGGTGATTTCCCAGCTCTTGACCCCACTGGCATCTGATAGCCCAATGTCTTGTCAGCTGTT<br>CTCCCAATTATGTATCAATAAATGTCACACAATCGAGTCTTGGCTCCCTAGAGAGTGTTTGAAAGTCAGA<br>GATATTTGCCTAATGCAAGCCGATGTCATGTCAGGAATTAGTTTGACTAATAGGCCTTTAATGAGATCCTT<br>GTGAGGTTTGGGCTCCAAGCAAAGGCTTTCTGAAGTTTTCCT
MSTR<br>G.100<br>182.3	ENSRNO<br>G0000005<br>8886	0.000	0.222	0.000	7.984	up	GCCGGGTAACTTTTCATGTAAGGAAGTCAGCCTACAAGTTTCTGGTGGACCAACCCGGGTAAAGAACA<br>GGCCCTAAAAGACCCCCTCTGAGCCAGCAGCTTGCTGTGGAGGGTGTGCTGCTGCTGGGGGCATCTAG<br>AGCTGTGAAAATCCTCTCACGCACAGAACAAACCCAGTGCCTGCAACTGAGAAGCTTCCCATCATGGA<br>AGCTCATTCCAAACACTGAATTCTAGGCTATGTCAGTGGTGCTAACTCCCAAAGTCTGTGGTTCCTGCC<br>CCCTGATGCAGAAGGGATGACTTTGGACAACGCTGCCTTCTAGGCAACCCTACCTTCTAGGCTTCTGTC<br>AATAAATAACACATCACATGGAGTGTAGCATTTTTTCCTTTGTAATCAACCTTTATTTATTCCTTCCTTTAT<br>TTCCTCCAACATCCACTTACATATTCTCAAATATTTGTGTACATATTCACCTTTGTTTTGTTTGCTTATTCCT<br>TCCGGCCCTCATTTCTCCGCGTGCTGATTCACTAATTTTCATTCCTTGTGACGTTGATCTCATAAGTAAAT<br>GGGGCAGAGTGGACAGTTTCTATGCAAGGCTTAATCTTAGCTCAAAGATGCAGGCACGGGACAGACAC<br>ATACATTACAGCTGTCACATCAGCACCCACCCATACATCCTAAGGCTAGCCTATAGACCATACTCCTTACA<br>TAGATTCTCACAACTACTTATTCGCTGAAAACACAGTCATTGTTTTTATGGCATTTGTCAAAACTCTATCA<br>GTGCAAAAACCTTTGCCTGATATGTTTCAGAAGATAAGGAATGGGGGAAAGATAAAAAGATAAATTATG<br>CCAGCCCCTGTCATCCTTCCACCCATCTATCCATCCATCTATCCATCCATCTATCCATCTATCTATCCATCCA<br>TCGGTCCATCTGTCCATCCGTCCAACTGTTCATCCATCCATCCATCTGTCCGTCTGTCTGTCTGTCTGTCC<br>ATCCATCTATCCATCCATTCATCATTCAATCACTCTGATAAATCTCTACTGCTGTTGCTCAGCACCAACTC<br>CTGAGCTGCCAATTTTCTACCTAGAGTGACTGGGGAAGGCTATGTTCAACTGTAGCCTCAAGGGCTGAA<br>TATGATTTCAGAACTTGTAAAGAAGAAATGCAGTCCATCCAGACAACTGGATGACATGGATGGATGTGG<br>GGTGTAGGGTCTAAAACACCACATCACAGCTGGGTAAGGAAATTGCCCTGTGCAATATCTAAACACACT<br>GCTTTGAGAGAGCTTAAGGCAAATCTCAGCAGGATTGATTGCCAAGCCAAAGAGTTTTCTGTGTCCCCA<br>AGGAAGACCTGGCTTGGCCTCTCATCGTCAATTGTAAGGAGGGTATGGAGCACGCAGGCACCCAAGTG

							TCAGGTTGGTTGTTCCTGACACAGTCTGATTTCCTGCTGCTGTCTCCTCCACCCAGAAGGAAATGCTCC<br>AAAAAGCTGGTTAAGATTCCCCACTGGCCCTAAATGCCTCATGGTTATGACAGAGCCTTGTGATATAGCT<br>TTCATGCCAAATGAATAAAATATGTAGATGAAAGTTTTTCTCCCCAAAGGAAAGGTGCTGGAAGAATTG<br>ACAGAAAAGTATTTAGAGGCAGGGTACTAGCTATGTTGACCCCTGGTAGAGCATCCCTGGTCCTCCTGG<br>GCAGAGATACAAAACTGCAGAGAGATAGAAGCCAAGTGTGGGCTCTCAGGAATATGGCTTCACGTGTC<br>ATAGCATGGAGCTGGCTAATCCCACAGGCCCCAGTTCTAGCCACAAGCTGAGACTAGCAAGGATAGAG<br>GGGCTTTGAGCCTAACTCTGTGACTCCTTGTGTCCTCTTGCCCTGAATGGCCCTATGTGAGCTCCTGAGC<br>CTCCTCTTAGCTCTTTGGAGCTGGTAGAACTTGTCCCTAAGTCTCAGGTCTCCAGAGGACAGCTGATCC<br>TTCTGTCTAGGCAGGAAGCATAACAAACATCAAATAAGAACAAAAACTATAGGAACCACTCAACACGC<br>ACTTGCTCAGATGTAAACACATCTGGCTAATAAAGTGCGGTCACCATCAGGGTCTCATTTCATCTTCAGA<br>AAAACTGAAGGGTTAAGCTAATGTGTTCAGGGCGTAGAGTATAATCCCCACTTCACGTACATTCAAGCC<br>AGTACAAGCCAATGGCAGCTTTACTAAATGTTCACAGGGGCCCATTGCATCAGACTCCCAAACCAGTGT<br>GCTCCAGCTTTGTTTTCCCCAGGAAGATGGTGTTTTGAATGCAAAAACAGTGAGTGGTCCCATGGGTGG<br>GATGTGCTATCTGAAGCTGTGGTTCCTCCTCCATCCACGAGCACCTGTGGAGAGGGTAAGGTGGACCCA<br>ACCTCAGCCGAGCGGGGCCTCCTGACTTCAGCCCAGGTTGTTTCCACATTACCCACCCTAACCTTGACC<br>TTGAGCATGGCACTCCCCATTCTTCCAGCCTCCCCATGGGTGGTGGGAACATGTGGGACAGGTGGCTTT<br>GAAGGCCACTTCCATATTCAAGCTGTTGCTGGAACAGATCTAAGGACAGGATGTGAAGGTAAGAGATC<br>GGTAACTCCCAGAACTCCATGGGTGGAGTAGTCTAACTAAGGGCAGGGGCAGAGGGCACGGAAGCAG<br>CCCTTGACCGACTTCCCAGGGAAGGCCCACTGCCCTCCGCTTCCTCCTCCAGCCAGGGGGTCTCCTCCG<br>CTTCCTCCTCCAGCCAGGGGGTCTCCTCCGCTTCCTCCTCCAGCCAGGGGGTCTCCTCCGCTTCCAGGT<br>CTTATTGACTTTGAGTTTACAAGGTGTGTGGGTTACTAAGGCTTGGGATGATTGCCCTGGGGCCAGACC<br>CGCCTTGGGAAAGCATCCTCCTCACCCCGGCCCCACAGAAGTGGGTGGGGCCACAAGCTGGGGTCTCC<br>TAGAGCTGCTTAGAAGTTACAGTGGAGAACCATCAAATGGAAAACAGGAGAGAGAAGTGGAAGGCTG<br>GCTCAATCTGGAAAGGCGGAGGAGAAGCCATGGACAGCTCCACTTTTACACCCTGAAGCCCTGAGTTC<br>CTAACGCAGCCTGGCTCTGCTGGTGGGTGAAAGCTCAGGTGGGTGAGGAGCTTTATGGTATTTACTTGG<br>TTTATTGGCTCTGGGGAAGAGGATATGAGTGAGGCCCAAAGTAGTTTTCTGAAGATAGGACCCTGCAGA<br>AAAGGCAAGATTTATGACTTCCGGAAAGCCTCAAGGTTACTGGGCTGGGCATGGTCAGCTCTCCTTGGT<br>TCTGTGAGCTTTTCAGGGAGGGGACCCTAGGAAAAGAAGTCCATTCCCCCAAATTCTCTTTGAATATCC<br>CCCAGAGACAGCTCCACAGGGTGGAGTTGCTGTGATTCCTCCATCATTTTAAGGAGGCGGAAACCGAG<br>GTCAGAAGAACCTTCTGCTCTGTAGGATCCATGTCCATTCAGGTTCCCACACTTTGTCTCTGGGAAGCT<br>GTCACTAAAGTGACCCTGGAGTGGGTAAATACTGAGTCACCCTCATTAGGGTGGTGGCCTATAGTCACC<br>ACCTGTGTGCCACCCCAGTCAGGCCTAGAACAACAGCTACCTATCCTGTCCAGTGAGGTGGCCAGGCA<br>GCCTCTAGTGCAGAACCACACAGCAAGGGTGCTTGCTCTCCAGGAAATACTGACTCCATGCCTGTGGA<br>CTCCGGCCCTGACCAAGCAGATCTGGAAACAAACGTTTCGCTGACAACCCTACTTCACAGGCACAAGA<br>GGCTGGTTCCTGACACAGCCGGGGCCTCCCGGTCTTCAGTGGCCCCTGTTAGCAGGATAGATTGTGTTT<br>CTGGGCTTAATTAAGGGACAGACCATAAATATTCATGCAGCCAAATGAGTCTGGCTCACAAGTGCAGCT<br>GCCTCCCGCTTGGGGAAAATTAACATTGTTTTCCAGAGGAGAGGAATGAGGCTGCTCACTGCAGAGAA<br>GGCTTTTCAACAACCTCTCTAGGACAGGACAGAAAGGGACAGCCACACTCAGAGGCCCTGCACCCTG<br>GGGAATTGTCCTGGCCTCTGGGACCCCTCTGCTTCCCCAGTGAGGCTCAGTGGGGCTCCGGAAGGGGG<br>GCTTAGCAGGGTTTTTAGCAGAGCAGAAGGCTCCTAACTGTTTAGAGAACTGGTTTTAGCAAAACGTTG<br>CTAAGGGCTAAATCCACCTGCCACCTGTATTTGTCAGGAAGCTTTCCTGGGGCCTCATCACATATGATTC

							ATAGTAACTAAAGAGCTGAGTCATGTGGTGGACCATTTTAACCTGTAAAGCCTGAAGTGCTGACCACTG GGCCTTGTATGGAGAGTCTGCTGGCTCCTAGAACTGTCTAGAACCACCTCCTGTTGGACATCTGCTATTA TTATTAAAAGTCTCAGAACTTGAGCTGGCAAGGTGACTCAGTGGGTAAAGGCCCTTTGCCACAGAAGG CTGATGACGTGAGTTTGGTCCTCAGAACCCAAGGTGGAAGAAGAGAATCAACTTGGGAAAGCTATCCT CACATGCTGCAGCATCAGCACCACCACCATCACCATCACCATCACCATCACCATCACCATCACCATCACC ATCACCATCACCTTCATAAATAACATTAAGAATAAATAAAAGTCCTGTCATTTCAACATCCATGGTGTCCT AGAAGTTGAGAGTGCTTTTGCCTTGTGGACTTAGAATCTTCTCTGCAGACTAAAACCTGATTTCCACGT CTACTCTGCCCCTTCCTTACCTGTGACTGTCAGCAAGTCACCCGCCTTCCTCCTGCTGGGACTCCCTGA GACCCCAAAATAAGTTGGACTGTGATTTACTGGCAACACTTACAAATTACATATTGCAATGATATATCATT CATATGTAATATGATTATAAACTACACGTTTAAAAAGTGGGACCTTGAAACATTAGGAGCCTTACCTGTG GTCTGGTCACTAGGAGTGGACTGCAAAGCCAAAACCCGATCCTGTCTCTCAGGCAGCCCCACTTGCAC CATGTCCTCAGCCTCTCTGAACACCTGCTGTAAGGGGGGGGCTCGCTATCCCCATCCCCACCCGCTCCC ACCCTGAGCATTCACATAGGAATCTGCCCTATATTTGTAGGAATCTATATTTGCCACACTTTGGCTCCTGG TTTGAATAGCTTCTTCCCATCTCTGTAAAGTCCAGAAAGAGAATGCACACATTCTTGAGGCTGTCTCCCA ACTTTCTAGGAGCCAGGAGCAGGCTGAGCCTAATGCATTTGCAGCAAGCAAGACCTGGGCAGAAATTT CCTGTTTCTGTTATATTCCCCTACAGGAAGCCCTCCCTGATGCCTAAAGCTCCCAGTATTCCTGCCTGGA CCGCTTCTCGTTGAGACAGGAGATCAGACACAAGGAGGAGCATCCGGACTGGCCATGAAGCAAGAAA AAATCTGGTAAGGCGGAAGAAAGAACTGCCACCCGGTGGCATCAGTCGAAAGAGGAGGAACCCCAAG TCCTGTGGATTCTGGATGGAGCTGAGAAGGTCCCTTGACTGAAAGCGCGGCACACCACTGCACTCTGC ACAGCCCCAGGTGCTCCGTGTAGCCCACCCTCAGTCTTCTCTGCTGGAGGTCTCCAGATCTGTGGCTCT CCCAGTGTCGCTGGCTCTCAGGCCTCCCCTCTCAGCACCAAATCCAGCTGTGCAAACCCCCTTCTGGCC CAGGAGGACCTCAGCCTTGGACACGGGGACTACAGGGCAGCCCGTGGGACACACAGAAAGGAGTGA GCTACAACAGACACCATTCGGGCAGAAAGACGCCAGATGCCAGCATTTGAAGACCTGGTTCCCCAGTC GGTGTTGCTGTTTGGGGACATTACGGAACCTTTAGGAGCTGCGGCCTTGCTGGAAGAGTATGTCACCAG AGGGAGCCTTTGAAGGTTTATAGCCACTTCCAGTTTGCTTTCTCGGCTTCCTGTACAATAGAATGTCTTC AGTCAGCTTCCCGCCGAAGCTGTGCCAAGCCTGCCTCACTAATAGGGGTGTCCTTCGGAATCCTAAGCC AAAATAAACTCTTTCCCCAATGAGTTGCTTCGGGTCATTGTTTGTTTGTCTGTTTGTTTGTTTGCACAGC CACAAAAGTCCCGACTACATTGTAACAAATAACTGGGAAAAGCAACTTCAGAAAGGAAGGGTTTATTT TGGCTCCTGTCCTGGGCGTATAGGTCATCATGGCTTGGAAAGCAACTCCAACCCTGGCAGAGGAGTGG AGGAGCGGGGAGATAGATGATGGGGCACAACTCATTTTCTCTTTTTTGACTTTTTTTCTTTCATCCGGGA CCTCAGACCATCAGAGGATGCTTCCCAAAGTTAAGCGTGGTCTTTTTCCTCAGGTGACCCTCTCTGTCA AGATGAAGATTAACTATCACTTGTACTGAGCCTCTACCAAAAGCCAGGCAAGCCTCTGTTCTGAGAGTG CCACTGCAAACAAGCAGCCTGGACTGTGCCTTGCCCAACTTGTGTCTAAAATCAAAGACAAAGACAAG AAGCAAAGTAAGACGTGTGCATGACACCTTGGGTACAGATACTTCTGTTAGATGATGGGAATAAGAAGA GTGGTCAGGAAGAAACTTCTAGATTGCCCTTTGATACAAATATAAACCAACATGGCAGCACCAAGGATG AGCATCCCGTAGCTCACTGCAGCAAGAACCCTGGCAGCAGCCTTCCAGACCCCAGACACCTCCCTGCC CAGCTCTCAGCTGTTGCTTCCACCATTGCTTCCAGAAGAGTAAGCAAGCCGTCCTTCTGGTACCTCTGT TGCTCAGAGACACAGAGATGGAGTCAAGGAGGACCAGACAGCGGTTCAAATGTGAGAAGCCCAGGAG ATTGTCATTCCAAAATAGAGTCACCCCCCACTGTGAGCCTGGCCTTCATGTTCCAGTGACAGGTTTAAA GTAGAAAACCAATTCTTGGGGGAACACAAAATCATCACTCTGAAGAACATGGAGTAACTCAGCAAACA GAACCAGCCCACTGATGTGGAGGGCAATTGCCTGGCACTCTATGCTATATCCATCCCCCAGCGAGGCAG

							GGCAAGAAGGCCCAGGGACCAGGCAAAAGCTGAGTTCTGGCGAACCATCGCTCACACACCTACATATA<br>ACTCATGCCCACCCTCAGGTCTGGGTCACTTGGTACCCATGGTTTCCAATGTTCTAGAAGGATCAGGAC<br>TAGGTGCGAGTATTTTGTGGGTATGTCCTCCATGGTGAAAACATTTATTAAAACCCCCAAGGTAGGGCTT<br>GAGGTGATAGTTCAGTCAGTATAGTGCTTACCTTGCAAGTATGATGACATAAGGTCCACCCTTAATCACA<br>ACACTGGAAAAGCAGCGATAGGTGGATACTTGGAGCCCAGTAGCCAGTCAGATTAGCCTAGCTGGCAA<br>GTTGCAAGTCAACAAGACACAGTATCTCAAGTAAACAAGGTGAACAGTGCCTGAGGAGCCACACCTG<br>AGGCTATTTTCTGGCCTCCGCATACATCTGCTCGAACATGCATGCACACAAATATTCATGTGCACCTGTG<br>CACACACATGTACATGCATGTGAATACATATGTACATGCATGCAAACACACATGTACACACACAGGTAGA<br>TAAATCTTCAAGGGGCCGTGCATTCACACGCAGAACCTCCAGCTTTAATTCACCTGTTTAGCAATTGAA<br>CTTCATGGAGGCAAGCAAATGTCTAACCTGCCTTGGCATTATTACAGCATAAGGGAAAGGCTTGACACT<br>CTGGACACTTGATGGAATGAACGAATGGATGGATGGATGGATGGATGGATGGATGGATGGATGGATGGA<br>TGGATGAATGGGCAAATGCCATATTCCTTCTAGAGCTTAGAAAATGATGTATATAAAACAAGAAATAGAG<br>CATAAGGCAGAAAGTGGTGACAAATAAAAGAAATGGGACAATAACGGAATTGGGGAGACAAGACAAA<br>GCCAAATCCTGACACCAGGTGAAACTAAAGGTAGGAACAATACTAGATGCAGTGGGCAGAGAGCTGG<br>GAAGGCATTCAGGAAACAGACTTTTTCTAAAAGATACTTTTCATTTTTCTTTTCTTTCTTTATCCCTTCCT<br>TTCTTTTTTTAATGTTTTTTATTTTTAAGAAAGCTGTGGTTTTGCATCCAGGGCTAATGAGTTTTCCTTTCT<br>TTTCCCATGTAAGCTGTCCAGTTTGGCTAGAGGAAAGGATACATTCACGGCAGGGTTTAGAGACAGAG<br>GCTGGACCCTGAGCAGGAGGGGTTGGGGGAGCAGAGGAGACTCAGAGGCTACTCTCAGTAGCTGTTC<br>TCTGAAGCAGAGGAGTGAGTGTTCTTCCAGAGCGGAGACAGGCTGTAGGGTCTGGACTGGCCTGTGCC<br>AGCCAAGGGTTGCTGCAGAGCTCCATCTGAGGCTGAGAGGCCAGTCTTGAGAGAAACCAGGCTACAAT<br>GGACAGGGTCTCCAGCAACTGCTCACTGACCTAGAGTGAACTGAGTGAGGGAAATGATTTCCTCAAAG<br>AGTCGTGCGCTTTCCTTGGGTCTGAACTGTTTGTGGCAAATGTAAAAGATGAGATTAAACAGTTACAGT<br>GATATATAAAGCAAAATTTCAAACATGAGCAGGTAAGACACAGACTGGGCATAGGATGGGAGAATTTTA<br>CTTCCTGTGCTCTGTAGTTGTTTTCTAAATCTCCAGCTCTTTCCCTGAGGCTTGCTCCTCCTCTGCTTCCA<br>GATATGATCCCCGAGGATCCTGCCTTGAACCCCTGCTTCCTATATTTCTCACAGACTCCTTGCAGCATGA<br>AGCTGCCTTCCTGGGTGGTGACAGCCATGCCAACCGGATGTTGGATGTTGGATGCTGCCCCAGTCAAAG<br>TCATCATTATTACAGTTAATACCTGTGGCCCTGCATCCAGCCTCGGCCTTCCCTTCTGATTGCACTTTTAC<br>CTGGGCCTCAGGGGCTGTGACAGGTGTGACTGGGAGTCAGTCACTGCTGCAATGGCATCTAATCCCAAT<br>AAACTTCCCACAGAGATTCTCAAGGGAGAGAGGCATGGGTCTAACCTAGGCAATAGAGAAGCTAAGAT<br>TCTGTCTGTGCCAGACGTTTACAACCCTGCCTCTGGACTCCGTCCTGTGAAGATCATCTCTCTCCCTCCC<br>GTGCTGTGGTGGATGTTCTTCTCCAGTGGAGTTCATCACCGTAACGACGTTCAAACTGAAAGTGGAGCT<br>GACAGAGACGTGCTGTTCTAGCCTTGCTGTACTCTTACAGATACATATTCTTTAAACTGCTAGAGGGAGA<br>CTAGGGTGTAACTAAAGCCTTTGCTGGTCTGAGCAGAGCCTGGGCTGGCTCTCTAGCATTACAATAATA<br>ATATGGACCCGACAGTGGGAACAGAAGAATTGTTGAATGGAGCAGCGGGAGGGAACATCTCTCTGTGG<br>TCACAGCTGTCCCCTGTACTCCACAGTGACCTGTGGAGCCCAGTCTCTGCCATCACAGCCAGTCTGCAA<br>ACATGTTCTTGCAGAGTGGCAATGAGCATGGGTTTCTCAGACCCACAGAAGAATGCAGGTATGTCCCAC<br>ACAGCAGTGACAAAGATTGGTGTAGGAGAGACAAGTAAGACCTTCTCAAGAGAGGCAACCAGGGACC<br>TCGGGGCCACAGGGAAACCATGATGTTAACCACAAGAGAATCCACTAGAGAAACCCTGCCCAGAGATG<br>ATGTTTCTCTTTCTATGCTGGCTCAGCACCTTTCCAAGCTGTGAGGGTAAAATGGAGTCTACCTTCCAAA<br>TCTGTGCTGGTCTGTGTGAGGAGTCAGCTGAACCTTACTTGTTCTCTAAGCCCCAGGGAAATGCCTCCC<br>CACAGAGTCCCCAGGGACTGTGGGTTCATGGAGCTCTCTTTTGGGGACTTATGTATTTTTCTGCCTTTTG

							TTTTATGTGTTTAATTATTTTAAAAAAGTGTTAACTTGATATTATCTCGCAAGCCAAGGGTTGTGCTTGGG<br>CCTGGGAATGTAACCAGAAATGAGCGATTGTTAGAGGGAAAATTGTGGCCCCAAAAGATAAGTTGAAA<br>TTCCAGCCCTAAGTACCTCAAGATGTGGCCTTATTTGAAAAGAGTCGCCACAGGGGTAATTAGTAATGT<br>TGCTGTGGTCAGAGTAAAGTGGCCTAGTCCTAATCCAATATCCCTGGTGTCTTTCCAAAAGACACAAAC<br>AGGACACCCAGTCATGGAGATGGGGGTTGGGGGTTACACTGCCACCCTGCAGTGAGGGATCAGGGCCA<br>CCAGGAGTTGAGAATACAGGAAGGACTTCTCCCTGGAAAGCTAAGAAGCATGGCCCTACCACATTTGG<br>TCTCAGCCCTTCAGACTCTAGCCTTGCGGCTACATCCCCTTCTCTGTTTACCTGCCTGATTTGTAGTACTC<br>TGCTGTGGCAGCCCTGGGAAGCTGAAGCAGAAATTGTCTTTATTCGTAGAAAAAAACATAGATGGACA<br>ATCACAAAAGTGTAGAATCCACCTATAAGAAACAGTCATGAGTCATTCAGTCTCAGAAAAGGATTAGGA<br>CAGGCTTGTACAGTAAGCACAGTTGAGGGTGTGTATATGTGCGTGTGTGTGAGTGTGTGTGAGTGTGTG<br>TGCATGTGTGTGTGTGTGTGTGCGCGCGCGCACACGTGCAGGTTCACATGCACATGTGGGTGTGTGCAC<br>GTGGCACGTGGAGGACAGAGGTTAGCACCAGGTATCTTACTCAATTGCCCTTAACCTTACAGAGAATCT<br>CTCACTATACCTCACCAATGGCTGGCAAGCAAGGCCCAGGAATCCTTCTGTGTCTGCCTTCCCAGCAGG<br>CGCTCAAACAGGTGCTCACCACTGTGCCCAGCTTCTTACTCACATCCTGGAACTCTGAACTCATGCCCT<br>CAAGCTTGTACAACAAGCACTTTACCCACTGCACTGGCTTCCCAGTCACCCTGCGGTTGAGCACTGAA<br>GGACGGGGAGGAGGTCTCTGGAAGAAGGCTATTGGAGAAGGAACAGCACCCCTGTGTCTTCACAGTC<br>TCTCAGGAGGAACTGTGCTCTCAGAACCGTGTTCTGCCCCGTGACAGATGCTCAGCACCCATCCTTTGG<br>CCTTGCAGAAGAACCAGACTTTAGGCAAGGGAAGAAGAAATCAGGAGGGAGTGTTCCTGGCTGCCAC<br>TGAGATGTACTACTTCTGCAACTGAGAGCAGAGACAGAGCCCAGCAGAGAGGCCCCAAGGAGATTTAG<br>AACAGGGCTGGCTTTTAAAAGAAAACAAAAAAAGCAAACTCGGGCCGTCCTCTAGGGCTCCAGCTCG<br>GCAGGACAGGAAAGCAATTTATGGTCAAAGGAAGTAGCCTGACAGTAAACAAAAATACAGTGACCCC<br>GAGTTCCCACTGGACAGAAGACAGATGGGGCCGTCGTAAAAATGAGATTAATTTTAATAAGGGCTTTCA<br>CCAAGCTGCAGACCCTGGTGGCCAAATCCTGTCCAAGTGCCAAGGACAGGCAACACTGTTGATGGATG<br>GGGCCAAGCTCAGAGAGCTGAAGACGTGGGGACGATCGGAAGTTTGGGACTGGCCCCTGCCTGTGAT<br>GTGGCAGCTTCTCCCTATCCACTACCAGGGTGGGAATTCCCTGTCCCCTCCTCTTGGTGGAGACCACGG<br>GCTCTGTGCTTGTATAACTCTAGGAATGGGGAGCTCATTATCTAAGCAAGCTGTGGGCTAGCTCTCCACA<br>TACAGACACTGGGAAAACTCTGTGTCATCAGTCATGGGAATATCTGAACTGGCAGTTAGCGCACACTGC<br>TGGAGAAGTCACCGTGTAATAGGTTATGGGAAGAGTGGCATTTAGGATTGGTTCATGCTTCCAGAAGCA<br>AAGACAGGCAACACCCTTATCCTAGCTGTTGACTGAGCCCTAATCCTGATCACAGGCCAAACCCAGATC<br>TCTTGTAGACTCAGCCCTAATCTCAGATGGCAAACTGAGCCTTGAAACTAAGTCCTAACTTAGACTGGG<br>TCTTGTTCCACTCTGAATCTTAACCCTTTATTGACTGAGCCCTGATTCTTCTGACTGAAAGGGAACCTGA<br>TTCTTACCCACAGCCTCATGCATAATTATGTGGTGTCTGTACCAGAGCAGAGGGAGGTACAAATGTGAA<br>CATACACAGCTCACTGCAGCTCATCATGAGCCTAGAGCAATGCCCATCTGCAGAGTTATAGCCTTGAAG<br>CACCGGGCTGAGTGGAAAGCATTATAAATGTTGCATCCCCAAGCTCTCACAGCTCCTCCTCCCCCCACC<br>CCTCCCCACATGGAGTCAGCATGTCACTGCTCACACACTGCACAGGCTTCTGCTCTCCGGACAAACAG<br>AGTCTATATTGCTCCCTGGCCCGCAACCCCCACCATCCCCACTCCTAGACACTAGGTCCTGACCTGCTCA<br>GGATTACAGAGCTGTATAGCTTCATAGAGTGATGGGTATCAGCAAAGGCCTGGTTAGAGCTTTAGTCACT<br>TTCTTTATCAGCATGCTAACTGGAGTGATGGCCTCGCAGAAAAACAACACCCTGTGGGGTTTCAAGAGC<br>CAAGTACCTGCCAGCTGTAGAAAACCTGCCCTGGTGATTTCCCAGCTCTTGACCCCACTGGCATCTGAT<br>AGCCCAATGTCTTGTCAGCTGTTCTCCCAATTATGTATCAATAAATGTCACACAATCGAGTCTTGGCTCC<br>CTAGAGAGTGTTTGAAAGTCAGAGATATTTGCCTAATGCAAGCCGATGTCATGTCAGGAATTAGTTTGA

							CTAATAGGCCTTTAATGAGATCCTTGTGAGGTTTGGGCTCCAAGCAAAGGCTTTCTGAAGTTTTCCT
MSTRG.100427.1	ENSRNOG00000019842	1.066	0.059	0.001	-3.184	down	ATTTGAATTGTTCCCCTAAAACAGCCTTCATGTAAGACAATAACTTAATGCCATGACAGCTCCGCTTAGG<br>CATCAAGACGAACCTTTTCTCCTTAACGGTAGTCCGTCATTAAGTCTGTCTGTCTGTCTGCACACATGTC<br>CCCCTTATGCTCTCTCTGGTGCTAGGGATCCAGCCAGCAGCCTCCAGTGCACTTGGTGTGCGCTCACTC<br>TACAGCACAGTTGCTGTCCCTTCTGACTCTGGTTCTGCTAACCTTGTTACTATAGTAACAATGTCCTCTG<br>CAGGGAGTAAGGGAGCGCCTGGGCTGGCTGTCCTTGGGTTGTCCTCAGTTTCTTCCTCCAGCTACAGGT<br>GGCGGGTGTCAACCCCCTCTACACTGGCTGCAGCTATTTGCTCTCCGGAGTCTCTGGTACAGACATGGA<br>TTTCTCTATAGCTTTGGTTGGTGATCCTGTTTAATCGGAGTTCTAAGCCGACATTACTGTTGTAACCTCAT<br>CTGGGGGCTTGGCTATTGTCTTCACTGGCTGGGAGGATGCTCAGTAGCCTGCTCTGTGTGTTGGCTTGT<br>GGTGTACAAACAGCCCTCACAGAAGCCCCGCCAGGGGCCTGCTGTGACGGCTCCGGCTTTGTTTCCAC<br>ACTTCAATTCTTTAAAAATATAAACACACCCTCTTTCTGGGTGGTAGTGAAAAGAGCAAGGGAATTAAC<br>ACATTGAGCGTAAGCCAAGTGCCCGGCAGCAGATCTATGATCTAATAGCCCTGTGTACACCGCTAAGGC<br>AGCACTGCCTTCTGCCCAGGAGGGTGGGGCTGCAAGAGTTCAGGAGATGCAGTTGGGAGATGTGCTAC<br>TCTCTCTGTTCAAGCTCGAAATCTCAGATGATGACCTTAGCACAGCCGGAAAGTCCATTTCTACTCATAC<br>TGTCCCTGTGTGTTCTGGGCGACAGCGGCATTTGTGGTCGTCATTAGGTGCGGTCACACCTGTGAGAGT<br>CACCGCAGGAAGGATGTCACTCACTGAAGCAAATGTCCTGTACTAAGTCCATAAAACAGGCTCTGGAA<br>AGAATCGCCACACAGCAACTACTTCCTGCTGCAGGGTGGGTGACTTTTCCTGAATGCTGTCCGGGACA<br>ACAAAGACCAGGAAGGGCTGGTTTCTGAACCTGAAGAACCTGGTGAGGACTAGATAGTAAAAAACAC<br>ATTAAGCCTCTACCTACCATCTAGAGATACTACAAAATATGTGTATGTGTGTGTGCGGCTCCAGCAATTG<br>GTTTCTTCAAACAGCTGGGGCTTTGAATGCACCTAGAGCAGCCCTACTCCAAGGGTTCCACTAAGTAAC<br>ACATGGCCGGCCTGAGGCCTTGCTATTATAGACCAGGCTAACTCCGATATCAGAGATCTGCTTCTATGTC<br>CAGAGTACTGGGATTGAAGCCATGCACCACCACACCTGCTTTCAGGACCACAGCTCTGAATGCATCGA<br>AAGTTCCATAAGACTGGATGCTCTAATTCTGGATTCGCCAGTCAAAGTGATTGTGGGTTTCTGTTACTTC<br>AAAACTATCAAGAACATCACTGGGCTTATACACGCAGAACATTAAGGGGACAGAAG
MSTRG.100427.2	ENSRNOG00000019842	0.060	1.061	0.000	4.469	up	GTTTAATCGGAGTTCTAAGCCGACATTACTGTTGTAACCTCATCTGGGGGCTTGGCTATTGTCTTCACTG<br>GCTGGGAGGATGCTCAGTAGCCTGCTCTGTGTGTTGGCTTGTGGTGTACAAACAGCCCTCACAGAAGC<br>CCCGCCAGGGGCCTGCTGTGACGGCTCCGGCTTTGTTTCCACACTTCAATTCTTTAAAAATATAAACAC<br>ACCCTCTTTCTGGGTGGTAGTGAAAAGAGCAAGGGAATTAACACATTGAGCGTAAGCCAAGTGCCCGG<br>CAGCAGATCTATGATCTAATAGCCCTGTGTACACCGCTAAGGCAGCACTGCCTTCTGCCCAGGAGGGTG<br>GGGCTGCAAGAGTTCAGGAGATGCAGTTGGGAGATGTGCTACTCTCTCTGTTCAAGCTCGAAATCTCA<br>GATGATGACCTTAGCACAGCCGGAAAGTCCATTTCTACTCATACTGTCCCTGTGTGTTCTGGGCGACAG<br>CGGCATTTGTGGTCGTCATTAGGTGCGGTCACACCTGTGAGAGTCACCGCAGGAAGGATGTCACTCACT<br>GAAGCAAATGTCCTGTACTAAGTCCATAAAACAGGCTCTGGAAAGAATCGCCACACAGCAACTACTTC<br>CTGCTGCAGGGTGGGTGACTTTTCCTGAATGCTGTCCGGGACAACAAAGACCAGGAAGGGCTGGTTTC<br>TGAACCTGAAGAACCTGGTGAGGACTAGATAGTAAAAAACACATTAAGCCTCTACCTACCATCTAGAGA<br>TACTACAAAATATGTGTATGTGTGTGTGCGGCTCCAGCAATTGGTTTCTTCAAACAGCTGGGGCTTTGAA<br>TGCACCTAGAGCAGCCCTACTCCAAGTTTAGAGTCCAGCTTCAGGAGGAAACCCAGCCTATCATACTCT<br>GAAAAGGAAAAGCAGAAAACGTCACTGGAGTCAAGTCACTGCTGCCACAGTGGGCTGCATTCCCAGA<br>TCTACACCATGGAGACAGGCAGTGCCTGCAAAAGAATACATGCTTCAAACGCTTCCACGAACAGCAGC<br>AAACATACGCCTTAATAAATGGTTCCACTAAGTAACACATGGCCGGCCTGAGGCCTTGCTATTATAGACC<br>AGGCTAACTCCGATATCAGAGATCTGCTTCTATGTCCAGAGTACTGGGATTGAAGCCATGCACCACCAC

							ACCTGCTTTCAGGACCACAGCTCTGAATGCATCGAAAGTTCCATAAGACTGGATGCTCTAATTCTGGATTCGCCAGTCAAAGTGATTGTGGGTTTCTGTTACTTCAAAACTATCAAGAACATCACTGGGCTTATACACGCAGAACATTAAGGGGACAGAAGG
MSTRG.100433.1	--	1.823	0.000	0.000	-4.880	down	GGCAAAGGAGAAAGTGAAGCCCAGGGGCTGGCTGAAGCTAGTCCAGATCAAGCACACTCCCACACACACACTCATGCACTCATGCACACAGGCACTCGCGCACACACACACACACGTGCTTGCACACCCACACATTCTCAAGCACACAGGTACACACCTGTACATGCGTGCACTCACACACTCATACATAAATACAAAAGGGGATTGGGTTGAGCACCAGCACCTCTCTGCCTTGCTGGTGCAGGTGGGTGGTAGGAAGCTAGTCCTAGGCTAAGAAACAACAATCCGCCCAGC
MSTRG.100721.2	ENSRNOG00000026592;ENSRNOG00000026646	0.366	0.000	0.000	-6.432	down	GCTCACTGGTAGGTCAAGGTCAGCCTGGGCTACTCAGTAAGTTCCAATGCCTGAGTGAGACCCTGTCTCAAAACAAACAAATATTCTGGAGGCAGGAATGGTCTCAAAACCAAAACACTTCTGAGCTCAGCCTAGCCTAGTGATGCCAATGTGATCCCAGCCTCTCTGGAGGCCAAGGCAAGCCTAAAGTGGAGAGCAAATTGAAAGCCAGCCTAAGCAGCTCAGCAAGAGGTGGTTCACACCTCTAATCCCAGCATTTGGGAGGCAAGGACAGGTGGATCTCTGTAGTTCAAAGCCAGCCTTGTCTACAAAGTGAGCTCCAAGACAGCCAGCGCTGTTACACAGAAAAACTCTGTCTCAAACAAACAAACAAACAAAAAAGAAAAAAGTTAAGGCTGAGGAAACAGCTCAGTGATAAAGCACTGGCTTCTCATGCAATCTATGTTCAATATCTAGTATAGGAAAAACAAAGAAAGGTGATATTATAACACACCATGTTTGCTAGGAGTCAAATGATTGAGTCAGTCATCACCAACAGAAGTTGCTAACAAGCAGGAAACACCAATTAGCAACTAACTATAAAACTACTCCTGGAGACAAGCGTCTAAGGAGGATGTGGGTTTCAAGTTTTTATATACAGTGACATAACACTGTGGTCCTTTCCCAGATAATATAAAGCACCCCAATAACAAAAGCAGAGACTGCATCCACAGTGATGAATGCCTAGAATTCTAATACTGGGAGGTGGGGATGGGAGCAGGAAGATCACTGCAAAGTTCAAGGCCGTCCTGGCCTATACAACAAGTTCCGACCAGCTACATAGGTCTATCTAAAAACCAGGAAATTATATTCTCCAATTCAAACTGTTCCAGTGTATACTCTGCTTTGTAACCATATTTGTCAGGGATGGAAGGTGGTGATAATGTCTCTTTGTTTGCTATGAGCAATTTTAGGAAGTCATTTGAACAGGTAATGAGAATCAAATGACTGGATTTGGTCACAATGATAGTATGATTTGGTAGTTGTACATCTAGGTAAACCAACTCTGCCTAAATGCTAGAACACAGTAACATTTTCCTTTAACAAGATCATGAAATTCAGGCTCTAGATGCCTGCAGATAGGAAAGTTCTTTTTTTTCTTAAGTCCTACTCCTCAAGCGCACACCTGCCAGATTCATTCAATACAAGGATGTAAGCAACTGCCAGGAAACAAGAAAGGACAAGTATGAGGGCAGAAGGCTCTGAGCCACTCCACTTGCCTCCTGAGCAGCTAATGAACAATGGACACACACACTTCTAGGCTGGTAGGATCCACTACCAAGTCCAAATGCTAACATAGAGTCTAAGATTCCTCTCTTGCAAGAGGAAAACAGTTGTGATTTTCTTCTGAAGACCATCTAAAAAAGGAACAGTGTTTATTTTTAACAATGCAAATTTCTTTATAATACATATGATACTCTTAATCCTTGATTCTCATTATTATTTAGCTTTTAGCTTCCAGGTTATTTATCAGGCTAAATTTCTCCCTAAAATTAATGTTTCTGGGGGCTGGAGAAATGGTTCAGTGGTTAAGAACATTGTCTGCTCTCCCAAAGGACCCAAGTTCAATTCCCAGCACCCACATGACAGCTCATAATTGTCTGTAGCTCCAGCTCCAAGGAATCTGACAGTCTCATAACACTGTACATTAAATAATTTTTAAAAAATTAATGTTTCTGAGCTACTGTAAGATGAAACTAAGAGCCCTTCAGGACTCAGCCTCACGAGCCGGGTGGAGGAGCACACTTCAGGAGGGAGAGGGAGGAGCACACTTCAGGAGGCAGAGGGAGGAGCACACTTCAGGAGGCAGAGGGAGGAGCACACTTCAGGAGGGAGAGGGAGGAGCACACTTCAGGAGGGAGAGGGAGGAGCACACTTCAGGAGGCAGAGGCCGGTGCAGTTTTGTGAGTTCTTGACCAACTAGGCTTCCTCAAAAGATTTTGTTTCAATCCCCTCCCTCCCCCCCAGAGTCCTAAGGCAGGCATGGTAACAGGAGTCCATAATCCCACTCAGTATACAGGAGGTAGAGGTAGGAAGAACAGGAGCTCAAACTCATGCATACATACATAGCAATAACAAGCGGGATGCTACTCAAATAAAGACAGACTGACTCTTAATCAAACACTTGCCCCAAAACCAGAGCTCAGGAGGTTCAGAGGTCAAGGTCATCCTTAGCTGTCTATTGAATGCTTTTAAATCCAGCTTGGGTTACATGTCTTTCCTAAGCCACCCACAA

							AAAAAGGGTGGAAGAAGAGAAGGAAACTTTTAAACATCCCATTGTTAATTCATTTTCCCCTTAGATGACATCATTGTTATTTGGTGTTCTGAGATAGCATCTGGATAAGCCCTGAGTAGCTTGGAATTCATTACATAGACCAGGCTGGCCCTGAGCTTGTAGCACTCACTCCAGCTTCTGCTTCTCAAGTGCTGAGACTACAGTATGGGATGCCAAACCCGCTTAGTCTATTTGTAGAGCAACTAGCAACATCTCTACTTTATCCAGATGACATGTTCTACTTAAGCTACAGCATTAAGTAATATATTAATACTTCAGGTTTCTTTCATGCCTGACAGAACCAAAATTCAATATGCATTAAGAAACAAGGTTTTGGGGTGTATTTAATAAATTAAAAAAGTTAAAAAACGTAACAGTTCTGGGCATTTAGTTTCCCCAGAGCAAAGAGAGGTTCTGGTTTTCAAAGCCAGCATCTCCAACAACAAAGAAGACCTCATTACAAGACTCTGCCATAGTCAGTCTTCTAACGCATTTTTTTTTTTTTTTTGGTTCTTTTTTTCGGAGCTGGGGACCGAACCCAGGGCCTTGCGCTTCCTAGGTAAGCGCTCTACCACTGAGCTAAATCCCCAGCCCCCTAACGCATTTTAAAATGATCATTAAACAACTAAGCTCGGCTGGAAGTGAAGAAATAAAATTTTATCCAAACAGACATCTATGGCTCTGCCACTTTGAAATGGGGTCTTCTTATGTGGCTCTGGCTGGTGTAGAATTTTAGCTCTCTTGATCTTCCTGTCTCAGCCCTGCCTGCCCCTGCTTTTAGGGCCCTCATAAAAACAAACAAGCAAAATTAAAAAAAAACCTTCCAAGTGTTCAGTCATTCTGTGCGTAAGACTATATATAACATTAAGTCATCAGGGAATTTAAATGGAAAAGATTGCATTGTGGGTCAGACTAGGTAATGGATGTAAGACTCTATTTAGAAATGATTCTTGGAATCAGCCATGATGGGTTACAAGTCTGTAATTCCAGCCACTTGTGAGGCTGGGGCAGAAGGACTGCTGAGTTCAAGACAGGCCTGGGCCACACTGTGGGTTCCAGGTCAGTCTACAGAGTGAGACACTGTCACAAATCAGCGAAGGAAAAAGACTATTGAGATTAACAAACTCAAGGAAAAGATACAGAGAGCAGGTCTAGTGGAAAGAGTGGAGCCGCAGTTTAAAGGAAACGTGTGGTTAAGAAGTGAACACAGGTATAAAGATAAGACTTGAGAGGTCTGCTGGTGGCAGCAGTATCCTTGGGTGGCTAGGCAGAGGAAGGAGGAAATGAAAACTAGAGGCACTAGAGAATGAAAGATGTAAGGGGGGGAAATGATTTAAAGGGATTGCAGGTTTTCCTCATGCTTGAGGAACAAGGAGGAAAAAATCCCACAAAGAAGCAACTTGCCCAAATCCTTTTGAGGGATTATTTCTGGGATCCCTTTAAAATTGCTGAATGTGAGCGCTGAGGCACTTTCCCTCCTCCTACTGCAATATGATAGGCATCTTTACTGTCACATGAGTCCTTCCTTTTGCCTTGGCTAAAATGAAGTGCTTTACTCAGGTCGAGTCACTGTATTAGCTGTTTGAATTTTGAGTACAGAACCTCTGTGTACTTAAGGATTCTCAAATACTAGCTAACCAACTACCCACTTCAAGCCATACCTGAAACAAGACAATAGCCATACAATAAAACAAGGTCAGTCCCTCTAAGAAATAGTTTGGTTGAATCGTATTCCATGACTATTGGGATGGTTTCTGCTCCCTAAAGAATTCTAGAAATCAAGTCTCCCTAAAAGCTTCAGAACCGTTCGGCCGCTACATACACTCACGCCCCTTCCCCTCTAATCCGTCTCCCGCCCCACTCACGGCTGGGTTCGTGAGCAGAATCCAATTTTCCATGGCCCACTGAATCTCCGGGGAGGTTCCGCACTAGCGGAGACAGGAGGGGCTTCAGGGACACCAAGAGTCTTCGGTGGCCTGGGGTATCCCCACCTCATCTCGGTGTCCACCCCCAAGACTCTCCAGCTCCAGGAAACCCGAAAACCGACACTGTGGGGCCACTACCCTTCCCGGATGTCTCTCGTCCCCGGAAGCAACCGGGCCTAGCAGCACCCCAGTTCCTTTTTCTTCCAAGGCCGATTTGGGGTGCTG
MSTRG.100727.3	ENSRNOG00000017166;ENSRNOG00000017426;ENSRNOG0000002	0.000	0.111	0.001	4.225	up	TTTTCTTTCTTTTTTTGGTAGGGGACAGTCTCATGTACCAAGGGCTGGCCTTTAATTCCTGCCTCCAACTCACGAATGCTGGGGTTATAGGCACTCCCACTCCATCCGGCCCTTTCCCTTTTTCCTGGCAAGAATCACAGTCAGAGGTGTTGTCGATTACTTGCCTTCTTCCCTGACAGGCACATAAGCCCTTCTATACCACATCTTCTAGCCTTGTCTTTAAACTTTGATCATAGCACACATCCAGTCAAAGCTGGTTTAACCAGTCAAGGTACTGGCACCTGCTGGAAACTCTGACCAAGCCATTTACCAAGGCATTGCAATCAGTCACATTGGCAGGACTGTGGCAATGGCCTGCATTGTTTCTGGAAGGTGGCCAGATACTGAAAGAACATGTAGCTGGGCAGATTCTCTTTGTTTACCAATTTAGGTGCCTTCTGGAATGGATATCACTGGGCTAAGTGCTGGTGAGACACATGCAAACATCGACAAGGTGGTTAGGATGTGCAAAGAATTGTAGGCATAATGAAACAGGGCCTCATGAGAATTTGGTAGAGAGGGGACTTAGCTTCTTAGTGGTTTGTATGGCCATTATTTTGTTGATACTGATAACTACATTTTGTTTT

	6592;ENSRNOG00000037368						CCGGCTACTAGTCTTCCCCACTCCTCTTGTAGTACTATTCACTCATTCATTCATTCATCTCACTGTAAATATTATGTAGCTGTGATTGGCCTGGAACTATGTAGCTGATGGTAGCCTCAAACTCAAGAGTCTCATTCCTCTGCTTCCCAAGTGCTGGGATTAAAGCTGTGTACTTCCCTGCCGGTTTAATGATATGTTTATGTCAGTAGGTCTGTGACTGTCTTGAACCATTCTGTTGCACATTAGTTGCAGTGTGTGGGCTAGATTACCACCTGTAGGTGGAACTGACTTGAGCCAGCCACTGATACTGAAGAATTCAAATTCTGGGCCTGGAGAGATGGCTCAGTGCTTAAGTGCACTGACTGCTCTTCCAGAGGTCCTGAGTTCAATTCCCAGCACCCACATGGTGGCCCACAACCGCCTATAGTGGTTATGCCCTCTTCTGACATGCAGATATACAGATAGAACACTCATACATTTAAAAAGTCAATCAATAAAGAATAAATACAACCAATATATCTTAGAAACAAAGATTTTGAGGTCACTGGGCATGGTTAGCCACCAAGTTCGGGTCCTCTCTAGGACCACTGAACCCCTCTTCCAACATTTCTTCCTGTCAAGGACTTGAATGAAGAGCGTGTACACGATGCTGAGTGGAACAGGAAATGTAAGCCATCAGTTCAGTTCCCGCTGCACTGAAGCTGCTTCATCCAGACGCCTAGAAGCATCTTCCCTGCGGTACACTCGGAGCCCGGAAGGAGGCGGGGAGCAGCCCAGCACGCGCAGGGAAGGCAGAGGTGCGTGCGGGCTGCCCCCAGGGCGAAAGCCCGGAGACCGACGAGAGGCGCGGCCCTGCCATCTCCTCGTGACTCCTCCCCCCCGCGCCGCCCCCACTTCAGCCGCTGCTAGTCAGGTGATTCCGGGCACGTGATAAGCACCACTTACCCTGCTGCCCTGGGAGTGGCAGCCTGCCCAGCCAATAAGAAGACGAAGTTTGGCGGTAGCCCAATTGGAGCTGGGTTTGGGGGCGGGCCTTACGGCGGCTGCTGCCGGGGGCGG
MSTRG.100849.1	--	0.705	0.000	0.006	-4.200	down	CAGCCCAGCACCTGCGCCTGACTTAGTTCTGGTGTACCCAGTATGGGCAGGGCCTCTGGGCCTGTGGTCTCCAGCCACTGTGGGTCTCTGGATCAAGGAAAGCCCTGGTTAGGGTATGATTGCCTGGCTAATGAATGCAAACTCTCACTCCTGCTGGAATTGGGTCACAGCTTCCCGGAGCTCTTGAAACCCTGGCATAGAACTGATTTTTTACTGAGGAGAAGACTGGCCCTTAGTCCCTTGTGCCAGCAGCATCCTCCCTGTGGCCCTCAGCCCTACCACTGTGTCTGCCATCTGAGCGTGTCATCCTCTTGTCCTTGGAGCAACACTTTTGTCCTGAGCTGTGTGAGACTCTGAGAGGCAGGCTTGCGTGAGGTCAATTTTGTGGAGCACAGGCAATATTTCAAGGAAGCTCATTACTGAAATAAAGGCTTATCTACGACACATGGCAGTATTAGG
MSTRG.101043.1	ENSRNOG00000005935;ENSRNOG00000006004;ENSRNOG00000043364	0.596	0.000	0.000	-7.097	down	GTGGGGGTGGGGGGAGGGGCGGGCTCAGGTAATACTTAGCAGTCTCTGTATACCCATCGGTTCATTCACTCGTACGTTCAAAACTGCGCACCAAGGCCATCAGACTGACCCAGTCAATGCCCCCTGGAAGCCGGAACTCTAATGTGGCTTCCCACGGTCATTCACAGGAAGGTGTAGGAACTCTACACGTGTAAAGAAGAAACGGTACCCTAGCAGTAACAGCTACCTGACCTCCATGGGTGACAAGGGTTCTAGGGAGGAATTCTAGGGGAGAGAGCAGAGGCTCTTCAGGATGTGGGCAGAGGTCAGGTAAGGCTGGGCTTCCCCACAACAACTGTGGGATCCTAACCAGACCCAGCAGTGCACCCACCTGGCACCAGCCACCCCTCGATTCCCCAGAGAGAAACTCCCTGACTTCTCCATGCCTACACGTCCTCAGCTGCAAAAGAGGGTAACAAGGACAAATGTTTATCTCCACGGGCACAAGGCCTCAGAGAACAAGGTCTGTCACAAAGTAAGCACCCTCTGCTGCTCCCTAAACACACGGTGGCGATTCATGACAGGGTCACGGTCAAAGTGTCAAAGGTTCTGGGTGGAAAAGCTGCTTGGGTTTGTACCCATTCTGAGCCTGAGTCTGTGATTTAATCATCTCATTAATGCCTAACAACAGCCCCAAGATTCCCAGGGGAAGGTGACAGACCTCGATGTAAGATCATCTCCCAGGCAATGCCAGGACACCGTGGCTGCCACCACACCTGGCTCCACAGCCCGTCCCCACCGCAGGGCCAGGAGCCCTGGCCACACCTCACTGGAAGCTTGAGGGGAGCTTAAGCTTCCCTAGAACTTAAACATGTCCCAGGGCAGACGGTGAGAAGTACAAACAAACAGGAGAATGAGAAGGTCTCAGAAAATACGAAGAAAACACGAAGGGGCTCACGTGACAGTGGGAAACTATTTACAGCAAATGGTCAGCAAAGGCCCCTTTGAGGAGGTGGTGTTCGATGGTGCCACCAGCTATGCAAGGATTTGGGGAAAAACATTCCAGGCCAGGGAAGGTGCAAAAGAAGCTAGCCTGACTAGAGGTGGTAAGATAAGGGGGAGGCAGGGCAGGGCAGGGCTGGTGTAGGAAAGCAGAGGTGAAAGACCAAAATTGGAGCTACAGAAGCCCCCAAAGGCCCAGCATTAGGCTACTCCTTCTTCTTCTAAATGTTCAGGAAGCAGG

							GGAACTTTCAGCAGGGGAAGAGAGAGGCAGGATGGGACTGTCGGGGTCCACAGATCATTTGGCATTCT<br>GAAAACTCCCGTCATGGTTTACAAAGTCAAGGCAGGGCCTAGAAAAGTCAGCGACAATCCTTATGGGG<br>CAAGGAACATGTCCAGAGATCCCAGCACATGCCCGGCTGGGGGTGGGGGACATCCAAGCCACCGTTGT<br>TTCTTCCGCCTAGGACGGATGAGACCAAGGAGAGCATTGTACAGGTGCTCACTCTGAAGCTCACACAG<br>GTACGGAGCATGCTCAGAGCTGACCCAGATGGGGCTCAATCTTAGGACCCAGGCTCCCTGCTAAAGAG<br>ACTCTGACACTCTCAATCCCTAGGAACGTACCTGAAGCCCTGGCTTTTCTCCCTGTTGGCATTAGAACAT<br>GACAGGGCTGCATTTGCCTCTCATATCTGTCCAACCTGGGCTGGGGGAGCTGGATTGATCGGATGGATG<br>GATGGATGGATGGATGGATGGATGGATGGATGGATGAAGGGACGGACAAGCAGACTACTGAACTGACT<br>AAACCATAAACTAAAGAGTGGACAGTGGAAGAACACATGAATGGGCTAGCCACAAATGAGAGACTATA<br>CGAACAGTAAGAACATGGAGCAAGAAGGGGTGGGGGTTGGGGAACCACTGCCTAGGGAAAGGCAGG<br>GCTTAGCTAGCTTCAGACCAAGGCCACAGCTCCCTCACACACCAGCCTTCTCGCCTACAACCAAGCTG<br>GCCTCCATTTCCCAAGGTCACAGGGGGACGACTCTTGAGCCCCCAGGACCGACCTCTTTAGTGTGGCC<br>ATTATCCCCCCCAGCCCCTGCACCAGCTAGCACCTCGACACTGGGCCCAGTGGTTCACTATCTCCAAGC<br>ATCTCATAGGAAAAGGAGGCAGGAGAACAGGGCTCCATCCAGAACTAGCCGGGGCCAGACTTTCAGC<br>CAGTGCCTTCCTCTCTTGAGTCCAGATGACCTCCCAGGGCCTTTCCATCTCTGACATTCTTCCGGGTGGG<br>GATGAGCCTCTGGATTTTTTACACAGCCACTGGGGTCAGCACAAGAATTTCAGACCAAAAACTAGAAG<br>TTGGGAAAGGTCCTGCCCACAGCACCACCCACTTAGGAAAGGCCCACACAAGGAGTCTGCAGGCCCA<br>AGTTAAAGCCCTCCTCCGGTCACTGCCCAACCTCTCCAAGCCTCAGTTTCTTCTATCTTAAAATGAGGTC<br>AACTGTCCCCCACCTGAACATCTGACCTCAACTCAGTTTTGGGAACCGTCTACCATGGCTGCAGCCACG<br>TATGACACTGTACGCCCCGCAAAATTCTGGTTCCTGAAAAATTTTGATAATGGGGTCCAAGGGAAAATG<br>AGGAGGGGGGTCACACTGTCCTACTCCTGTTCAGTTCTGTCCACGACAAACTATGTGGGTGACAGCTCT<br>GCAGAACAGTACAGCAGACAGAACCCAGCAGTGGATCCCGGCCTCAGCTCTCCTCTACTGGGCAAAG<br>AAGGGTGACTCTTGGGCCTCAGTTTCCCCATCTAAGACCTTGTTGGTTCATAACCTGGTGACCTAGGCA<br>TGCCTACTTCTGCAGCTCAACATTCCCAGACCTTGTCACCACTGACTGCCCTGAGGGAGCTCACAGAGC<br>CTGGTGAATCGAGGATACAGATCTGAAGGGAGACTCCTAGTGTTTTCCCTCCAGGAAATGGGGGTGGG<br>GCTCGGGTGGGGGCCCTAGATAAGCACAAAGGCTCTATGCTTATACAGAGCAGGGTCCTCCCAGGACA<br>GATTCTCCCAAAAAGGGGGAAAGACCAGGTTCAGATTTTATGGGCCTGGCCTCACACCACTCTGTTGCC<br>TGCACAGTCACCAGCAAGTCCAGTCAGACACTTCAGAAACTCAATTGATGTCCAATCAGGAGACTTTA<br>AAGTGGCAGGCCGGGTGGCAGAAAGGAGTTAACACAAACAGAGGAGAGGCCCTGTATCACAAGGGCC<br>AGGCAGCCAGCTACTCCTATGTAGACCGGGACCCACAGGAGCTGCAGGCCTGCTCCCAGCTAAAAACC<br>CACCACTAAGGATGTTCATCAACAGAAGCCATCTCTGGAGGCTAAGAAGACCACCCATAACTGAAGGG<br>GGTTCTTCGACTGCAATCAATCAGACTTGCCCTAAGAGGGGGCTACCAAAACTTCCTGTCCACCAAGG<br>GGCTCTGGCCACCCATGAAGGGACAAGCCTCCTTCCACCTCCAACACAAACAGCACCTAAAGGGTCAC<br>ACTGCTGGAGGACAGGGTGACGAGAAGGGTCTTCATGACAGGGATCTTAGGTCCAAACCCTGGGCCCA<br>CTCTAAGCCACTAGAGCCAGCCACCTCCCCCCAAATCTGGGGCTACACTACAGTTCCCTACAGTACCAC<br>TTTGATACAGAGCCTCGTCCAAGCGACTCCTCCCCAAAACTCTCACCTCAGCCACACACACACACACA<br>CACACACACACACACACACACACACCATCCCCACCCACGCAGCTCTGGGGAAACCGGCAGTTAACTTT<br>TCCTAAAAAACCCTGCAAAGGCGACTAGCTGGGGGTAAGAAACAGAGAAATGGGCCCCCATATCCTTT<br>CGCGGCATCTCAGGGCCCTCGGGGGAAGAAAAAAAAAAATCTTAAAAAAAAAAAAAGCAAGAAAGAAA<br>AGAAAACTTTCAGAGTTAACTTTAAATTCCCAGCCCCCTCCCCCCAAATCACCCGGCAGCTTCTCTCCC<br>CCTTATTTTTCCTTTAAAGTGTAATTGTATTGTGTCTCACCTCCCCGAAAGGCTCCCGGCTCTCTCCAAA

							GAAAAGGAAAAAAGCCAACCCTTCGGGTGCCCCCTCTGCTTCCAGCCGCCTCTCCCACATTCTCAGAC AGTTTTATTAAAATCTTCAGACAAAAGAAAAACAAAAATGGCAGCCTGGCTTATTTTTAAAACAGGACA GGGACGCCATATTCTGCAGATCTGCAAAAAAACGGAGAAATCCGGCACTGGCCGCTCCACCCGGCCGG CTGCAAAGTGCGCGAACCGCGCCGTTTGCAATCCGTTTATTTGCATTAAGCCCCATGAATCATTGTTTAC CGGAGAATTGCAGCAGGTTTGAATAGCCCGGCTTAGGAAAACACACACGCACACACTCGG
MSTRG.101209.1	--	0.328	0.000	0.028	-3.770	down	AGCAAAGACTTCTTGACTCTCTGAGAAGAAATCGCTGATGGAGGTGTCTGCCTTTATCCCTGGTAAATT CTACCACCACAGAGGGTTTCAAACTGCCAATGAGCTGTCACTGGGCAGGGGCTTGGAGGAGAGTCCCA CCTCAGGCAGGGCCCCATGAGGTGATGTCAACTGCTTTCCTTCTCTCCTGCTGGGAAAGAGATATCTAC TTGGTGCATGAAACCATCATGGACTCTCTGAGGCAGAAACTGTGGACTCCCAGATTCTACCAGATGCTG AGGAGTGGGTGGAGTCTTCCTCACTGGAGACACATCTCATAGACAGCAGGTGACCTGGGAGGGACAG CAAGAACCAGAGGAGCAGTGGAGTAGCCTAGGAGGATGACCAACAGGACGCATCAAGGCAGACAAG GGTAAGCCTCACCTCTGCTCCATCTCTACCCAGATTTCCCACCCATCAAATGCGTCGTAAACAGCGCACT GGAAAGCACTCTCCCAGCCATAAAGAATTCATGGTGGAGTTGTCAGGAGCCCAGATGGGTAGAGCAGC TGACCTTTGGTCACACAGCAAAGAAGCAGAATCCAGGAGGAAAAAGCAGCCCAGAGAGGTTCAATGT CTCTCTCAAGGTCACACAGCCATTATGGGGCTAAGCCAGTACTTGAACCCATCTCTGTCTCTCAGTCTCT GCTCATCCCTGAAGGCTCCGTTGCCTTGACTTGG
MSTRG.101235.1	ENSRNOG00000013515	0.565	0.051	0.014	-2.575	down	GGTAACGGGCACTGAGAAGCTGTTTCCAGTTGGGAGGAAGGACCTAGAGACAGTGTGCTCCTAATAAT TACTCTGGAGCAGAATGGAGGGGGAAATTTGAAGGCAGCCCAGCAGTGTGTGGACCTGGAGCAGGAA AGACACCCAGCCAAGGACCATGTAGGCCACAGAGGGTCAGGACAGGCCACAGGTCAGGGAAAGAAG TGACCAGACGGCCAGGGATCAAGACCGCAGGTGACTGAGCATAAGGAGGAGCCACGGGAGACAGGTC CAGGTTGTCCACTAGGCCATCTCACTACCCATTTGGGGGTTACTGAAAGAGAAAACCAGTGACTGTGA GATGACCCAGTGATACTGGCACTAGCCACCAAGCCTGAGGACCTGAGTTTGATCCCTGGACCCACATG GATGGCAGAAGGAAGAAAATGACTCCTGCTAATTGTCCTCGGACCCATGTGCACATGCCCTCCCCCACC ACTCCAAACAGTGGCAAGGATTTCACAGCTCTTCCCTGAAGACCAAGCCAACTTGAAGAGGGACAGCT TCTGGTTCTTCTGCTGCTGCTTCTGCAACTCCATACTGGAGTCCTGCCCCAGGACAGACATGGTGATGA GGTCTTTACAAGGTGATTCCTAAGGAAGAAGTACCCCCGGTTTGCAGATGAGAAAAGGTCTCCATGGC AGCAGCATCCGCCCACAGTAAGTGGGGCCACGCCATTCATCTCAGGCCCTTCAGAGCACTAAGGAAGA GGCAGAACTCATGGATTCAGGGCTCCAAACCCAGGGCTGCCTCTGACCCAGCATGTGTGAACCTGGAA GGTCACGTGTTTCCCACCCATGGCGTCCATTCCCTCATCTATAAGGGCACTCTCAAAAGCCCAGATGCTC AGCTTGAAGCTAAAATGTGCCCAGGATGTTCCCTGCCCCAAGGCTTTCAAAGCAGGAGGTGGCACACA AGCCATACAGCTGCTTGACCCGTCGGCCACTGTTCCACATCTCAGAAAAACCTAATTCCTCTTGCTGGC CGCTGGGCCCAGGTGCCGGGCTAAATGAATGGATTTGCCGGAATCCTAGTTCCCTGTGGCTGCTCTCAC ATCCAGAGCAGCATGTGTCAGTCCTGAGCACTTCAGGACAAGTCCCCAACAAGTCCACTGGGGACAAA CCTGTTCCCCAAAGCAGGATCTGCTAGAAGGCAGTGTTGCATTACATTGCTTGATGGTACATTTTATTCC ATCCCATAACCTTTATTGAGCAGAAGTTGGAATCTCCTGCAGAGGCCATTTCCCTTTTTGGGAATGTTTC CTGGCAGGAGACATCCTGGGCAGGCAGCATCCCAAAGGACAGAGAGACAGTTTCAGGACCCCGGGAC AGGCAGAGCAGTAGTGATGTCAAAGGTAAGGCAGAGCTGAGGGGACCACATCGTGGACTGCTTGCTA GGCAAGGCCCATCTACCCAGCATGGGTGCAAGATGAGCTGAAACATCTTTTGGTCCTGAGACAAGAGC TCTGGAGCCAGCTGTGCACCTGAGAGGAGACACAGGACCACGGTGAGAAAGCAGGAGGGCCAGCCA GCATCTACATAGCTACAAGGAGCATTGATGTCTGGTCTTCTGGGGCCCTCTCAGTCACCACGTTAGGGT GCATTTAGCAACCAACTTGAGGCATTAACTCTTCCTGGACTGTCCATACAAACAGGTCCCTTATGCTGG

							AGAAAGAGGGTAAGAAATGTGGGCATTTGTGGGGGTCAGGGAGAGGCATCCTGACACTGGGCACTGCTCTCAGCCATAGCACTCAGAGAGAGGAGGAGACGCGGGGACACTGGCAGCACCTTCGTGGTTTCTGCAAAGGCTGCTGTTTGAGCTAGTGGGCGGTGTGAGGAGTCTTGCTGGCTGTGCAAACACAGTGCTAATCCAGCATGGCGGGACAAAAGAACTGAAAGCTCTC
MSTRG.101347.2	ENSRNOG00000009505;ENSRNOG00000009540;ENSRNOG00000009805	0.324	0.000	0.001	-4.580	down	GTCCTGTTGACTCTGCTTACACGACAGTATCTGCCTTTTTGAAGTCTTAAGCTTCAGGGAGGGGGAAAGACTTGGAAATACACACCAAAGCAGTCTTTGATGAATGCTAGGAACACGTGGACCATATCTCGGGTGGTACTTTTCCACAAGAATATTGGTCTAGCTGGTGGGTAGCCGGAGACTCAAGTTCACAGTGACCAGAGTGAAAGAAACAGTTACCCAGCATCCTTAGGACCATCCTAGCCTGCTAACCGGATGAGAAAAACCTATTTTCAGGTGACAAGATGTCACAGAGTAAGAAATACAGGAACAGATGCTAAGTGGGGTAGGTTTGAGTTCTGTGGTCATTTCCAATTGTGGGGAAATTTTATCATGTACTCAGGGTGTTGTGTGTGTGTGTGTGTGTGTGTGTGTGTGTGTGTGTGTGTGTGTGTGTGTGTGTGTGTGCATGTTGCGTGTGAGGGTGCAGGCCAGTGTGCATGCTGTAGGTGAGGGTTTAACCTGTGCCAGCCAGAGAATCACCTCAGGTACTGTTCTTCAGGCAACTTCTACCTTGGCTTTTTTTTTTGGTTCTTTTTTTCGGAGCTGGGGACCGAATCCAGGGCCTTGCGCTTCCTAGGCAAGCGCTCTACCACTGAGCTAAATCCCCAACCCCTCTACCTTGGCTTTTGTAACAGGCTCTCTCATTGGCCTGGACTGCAGGAGCAGACTAGGCTGGCTGGGCCTGTGAGCTCTAGAGCTCTTCCTGTCTCTGCCTCCCCAGCTCTAGTATTACAGTCGGCCTGTTTTGTTTCGAATCTGAGTTCTCCAACTCAGGTCCTCATGCTTTCAAAGTAGTACTTTACTGACTGAGCCGTGTCGTGTCGTGTCCCCCACCTGACTCCCTCACACCGTGCACCCTTTCTAAAAGTAAGTTTAGTAGTACAGTGCCAAGAGCGCGGCAGACGTGTCCGTAACAGAGCCTGCCTAAGATCTATTCTTCCGTAAGAGGCTAATACCTGCTCTATAGAGATGGCTCTGGTATATTAAAGGTGCTAAACTGGATTTGTCACATTGTCACTCAGCTACAGCATTTTGCATTCAATAAGCCACTTGGGCGTGTATTCTCATACTTCTCACCTTCATGGACTGGGAGATCTACTGCACCTACTTTATTTTGTAGAACAAAGTGAGAACTAGGTAGGCAGAAGTGGTTTGAAGTTGCAGAGCAAAGTGACAGGTGTTTGGCTCACCCGACAGTGGCCTCTCAGAATTAGTTCCCAAACTCAGGATGCAAGCTTCACTCAAAGACCTATCAGTCGGGGTGAGCTGAAGCCTATGACTCAC
MSTRG.101375.2	ENSRNOG00000007097;ENSRNOG00000007229;ENSRNOG00000051720;ENSRNOG00000055933;ENSRNOG00000056153	0.000	1.850	0.000	8.498	up	GCCTCTCGTTATTTAGCTTTATTCTTCCTGCGCGTATATGGTTGGGAACTGAGTAGATAAACAGCCACTTTACAGAAGCCTATGTTCCGGGCGGTGTGTGAGTAAATGTGCCTCCTTCCCTCTATCAGGATACACGGAGAAGTGGATGTCCTTTGTGCGTGTAACTCGACTTTGGAAGTTTGATGTTGCCACCACCTCATCCTGAGAAAAGGGTCCCCCTAGGAGGCAGCAGCCGATTGTCTGCAAGAAGCTACAGTCATGTCCTCACTCACAGGGGGAGGGCAAGGACAGTGGGTAAGGAGAAATGGGGGTGTCCTACAGCAGTGATTTGCCTCAAAGAGGCCTTCTCGAGCTGCAGGTCAGAGAAGGGATCCTGGGGACCTCAGCTACACTGTGACTTGATGATGGGGACTCCGGTTACACTGTGACTTGTGATGATGCGGACTTCAGCTACACTGTGGCTTGTTTAAGAACGAGTCTATCTTTAAGAGGAAATTCTCCTCTTGGTCACACTAGCAATGTTGGCTGCAGTCTCAGCTAATCTGAGAGCATCTCGGGTCTCTGTATAAGCAAAGTCAAATACAGTGTATGGAAAGCAATTCGGAGTGTAGCAAGTTCCCAGGACCCAGTCTCTTGCCACTGTAAGGGCTCCTGAGAGGAGGGCGCTGAGATTGGGTACAGTCAGTCCTGGAAGTCTTCCTGGAGGCGGGGTATCCAGAGAAGGCGCGGAATGCAGAAGTCAGTCCAGACCCATAGAGTGGAGGCCTGAGGAGCTCTGGGGGCTCCAACACTTCTGTCCTCCAAGGGCACCTTAGTGTCACTTTCAGGACCCCCATGGACCCCCGCTGTCTCCTCCGCGTAGATGACGCCGTCCATCTAGCCAGCACCTGCATCCGTCCGTGCGTCCGTCCAGGGGTCCGGGGTACTGAGGTCAGCGCTGGGGGGAGGCGGGGCCGCGGGGCGGGCTGCGGCGGAAGGGCGAGTCGGGCTCCGGGCCCTGGCCGCGCCGCAGCCGCCCTGCGCCCCAGGGCACGCAGCTCCCCAGGCCGAGGGCGGACGCCAGCAGCGCAGGGGGACGACCCCGGCGACCGCGGCGCGCACCCTTCTTGAGACGCGACCCGTGCGCTGCCAGGTAGAGCTCGGCCTGGGCCACGCCGCCAC

							TCAAGCGGCTCAGGCTCTCGGCGCGATGCGCCAGGCGCAGCTCGGCAGCCAAAAAGACTCGGTGCAGCTGCAGCACCCGGCTCTCCAGCTCCGACACTAGGCGCCGGCGGCCCTCCACTTCCAGCAGTCGCCGCCGGGCTTCGGCCAGCTCCAGCGCGCGGCCCCCTGCCCCTGCCGCCGCGCCTGCGCCCCCGATCCCCGCCGGGCTCGCGCCCCCAGACCCCCCACTAGCACCCTGGCGCCAGCGCTGCAGCTCCTGCCCCTCCACGGGTGTCTCGGCCGCTTCGGGTAACTGCGGTGACTGAGTGGAGGTCGGAGTCGAGGTCGGCGATGAGGAAGGGGGCTGGAGGGACGGCAGGGGCTCCCGGGGTGGCGGCGGGGGCGACCCTGGTTCTGCGGTCCCCTCCTGGGCCCCTGGGCCGCAGGCGCTGATGCGGCAGCCCGGCATCCCCCGCCCCGCGGCGACCCAGGGCTGTCGGTGCGTGGGGAGGAGAGATGCTGGGCACGAGACTAGTGAGAGGCGGCCGCAGAGCCCACACCCAGGCCGCACGCGCCGCCCAGCATCTTATAGCACACGGGGCGGAGCGATGGCAGCACAGCCAATCCGAGACTTGCGTGCCCTGAATCTGCCCTGCCTCCCGCCAGGGACCGTGTCCCAAAGTACTTCAAGTTCCCTGTCCGTCTCCCAGATTGGCCCTATTTGGGCATCACCCCAAATTTTGGGTGGATAAGTGAGGGTCACTTTCCTCGCCTGGAGTCACCTCAGTAAACTTCGATGGTAATGCTTAAAATTCATAGATTTCAGTTTCCACCCAGAAGAGGGTTTCGGCTGGGGGTCACCTCTGCTCCCCTGCCGGGCCAGAGGGACAGTAAAAGCCCACCCTAGAACTTAGCTCTCCGGGTCTCTTAGGCCACAAGGTTTGCTAGGACGCCCTCCCCCATCTCTCTGGGTTTGTGGGGTGGCCATCTCTCTGGGTTTGTGGGGTGGCTAAGGCAGACGAACGACTTGGTGTCTACTGGAAGATGGGC
MSTRG.101483.2	ENSRNOG00000016810;ENSRNOG00000016937;ENSRNOG00000017078;ENSRNOG00000022030;ENSRNOG00000022054	0.439	0.000	0.017	-3.928	down	GGCAGACACAGTACCTGTTTCAGAGGGTTGTTTGGATCGAGAGAGGTTGCCATAGCAACCGTTTGGCTCACATGCCAAGAACATTAAGTATAAATTGTGGATATTAAAACAAGTTAAGTGGGGGACAGGATGTGGTGGTGTATACTGGTGACCTCAGCGCTTAGGAGGTGGAACCAGGAGGATCAGGAACTTAAAGCCAGCCTTTGCTTCATTTGCGACTTCCAGGCAGAAGCTGCATGAGACTCTGTTCTATAGAGCAGGCTGGCCGGGAACTCAGAGATCCTCTGCCTCTGCCTCTGGCTCCTGAGTGCTGGGATCAAAGGCGTGTACCACCGCTGTCTGCAGTCTTTCACTTTTATTCATCCTTTTTTGCTGCGCCCACCCCTGGAAGAGGTGACCACGGGGAGTGGTTGTTTTGATGGAGATCTAGACAAACCAGGACAAGGAAAATGTCAGAGATCATGGGATGAGCCTTTGATAAGCCAGATAGCCCAGCTTGGGACGCAGTTCCTCAGGCAGAGTGGCATCTGGATGACAGTAGCAGAAGGATGACAGGTATGTGCTGAGCTTGAGCTGGTCTCAGAGCACTTGTTAGGACACTCCTCCGGTTGTCTTAAAA
MSTRG.101565.2	ENSRNOG00000037165;ENSRNOG00000048139	0.000	0.318	0.000	5.534	up	TTTTTCTTTTTCTTTTTTTCGGAGCTGGGGACCGAACTCAGGGCCTTGCGCTTGCTAGGCAAGCGCTCTACCACTGAGCTAAATCCCCAACCCCTGGGTTTGCTTTTTTTAAGGCAGAGTTTCTCTGTGTAGCCCTGGCTGTCCTGAACTCACTGTAGACCAACCTCACAGATCTGCTCTCTCTGCCTCCAGAGTCTGGGATGAAAGGCGTGTACCACAGGGCTTTCATTTCCAACTTTATGGTAATTGTTATGTTCTTGGAGTGCTGACTAAAACTGTCTCTCAAACTGTCTGTGATCTCCCAGCAGGTGGACTGGAAGTGGCCACAGGTCCTGGACTCTTGATATCGTGAAGTGAATGAAAGAACAAATTATAGACAGATGGACAGAGAAACATAGCAGTGATGACGGCAGTGGTCTGGGCTCTAAGTGACCCTCAGGCTCGACATAGTAGATAGAGGCTTGCTCCCTGAGAGGTCAAACTGGGAGCCTTTGGGAGTCAGGGAGGCCTTTAGTCATGGAGACACACCCTAAGGGACAGTGGGACCCTAGCC

							CCTTACTCTTCCTGTTTCCCATGAATGAGGTGAAGAGCTTTGCTCTGCCCCCACTGCTGCTCCACTGCAGGCCCAGTCGCATGCAGACTGAAACCTCCAAACCACGAGCCAAGACCTCTGTCCTTTACACTTGACTCTCAGGTGTTGTTACAGTGGGCGCACTGGCTAGTGCATGTCAACCTACTGCTAGCTTTGGTCATCAGGAGACGGGGACCTCAGCTGAGGAAATGCCTCCAGGAGATCTGACTGCAGGCAGGCCTGTACAACATTCTCTTAGTGACTGCTGTGGGAGGGCCCTGCCCACTGTGATGATACCAACCTGGGTGCTGGTCCTGGGGGCTGTAAGAAAGCAGGATGAGCAAGCCATGGGGAGCAAACCAGTAAGCAGCACCCTCCATGGCCTCTGCATCAGCTCCCGCCTCCAGGTCCCTGCATCAGCTCCCGCCTCCAGGTCCCTGCATCAGCTCCCGCCTCCAGGTTCCTGTCCAGGTTGAGTTCTTGCCTTGGCTTCCCACAATGAATGGCGATTCAAGATACATGAACTACATAAACTTTTTGTCTCCTGGGTGCTTTTGGTCACGGAGTTTCATCACAGTAATGGGGACCCTAACTAGTTCACTGGGAATGGCTGACTAACACACCCACCGCCAAGCTCCTGTCTCAGAAGAGAGAAAATTGTGTATGTGGTTGTCAGAGGACGCAGATAGGTTCTTTCTTTCCACCATGGATGAGTTTCAGGGATCAACTCAGGCCCTCAGGTTTGGTGGCAAGCACCTTTACCCTCTGGGCCATCTTGATGGCCCTTGAAGGGTTTGTTCTCTGCTTTCCCATGGATGTCACACCCTCAGTGCCATTTTAGATGCACAGAATGTGTGACCCACCCACCTCCAAGTTCAGCCCTTCTCTGCAGAGTCCTAGCTGAGCAAAACTGCTAGAAAAAAGAATTCCTTAGCTATTAGTCTCAGAGAAACAAAGACAGAAGGCGTAGTATGCTCCTAAAACAAAATTACATACATGTAAAGTAAAAAAA
MSTRG.101694.5	ENSRNOG00000013602;ENSRNOG00000033503	0.080	0.880	0.000	4.027	up	CCCAGCTCCAAAAAAAAAAAGAAAAGAAAAAAAAAAAAAAAAGGAAAGAGAAGAAACAGAACCAAGGGGCTGGAAAGATGGCTCAGCGGTTAAGAGCATTAGAGCATCAGAGGTCCTGAGTTCAAGTCCCAGCAATCACAGGGTGGCTCACAACCATCTGTAATGAGATCCAATGGCCTCTTCTGGTGTGTCTGAGGACAGCTACGGTGTACTCAGATAAATAAATAAATCTTTAAAAAAAAAAGAAAGAAAGAAAGAGAGAAAGAAAGAGAGAGAGCGAGAGATAGAACCAAGAAACAAATCTGAAAATGCTGTCAACAGAGTTGCAGAGAGGCCTGGGGTGAGCTGGCAGCCAGAGAGGGCCCAGAGAGGACCCCGCCTTGCCTAAGTGAGTTTTAGGGGAGAGTCCTAAGCTGAGGCCTTCTGCGAACCTGACAGGTTGGCCCAGGAGAGAATAGCGAGTGTGTGAGGGGTATGTACCAGCCTGGGTTCAATGGTGAGGTATGGGTAAAGTTTGAGTGTGAACAGGTGTCCTGTGTGAACCTCGTCCAAGTGTACCTTGAGAGAAGGAGGCAGGATTTGTAGATCTAGTTCATTCCTCACTGGTCCCCCAACCATGTCCCCATCACCCACTGTTTCCCAATATTCCCCTTTCCTGTCCTCTCCTGCTCTATCCCTCCTCGCCTCCCCCTAGTCATTAGCCCCAACCCACCCAGGCGGAAAGAGGTGACCTCGCCCAAAAAGTCCACCCGGGGTTGGCGGCCCCTGGTGACCAGGCGCAAGCCAAGCCAGGGCCCTGAGGACATGAATGGGGCTGGGATGGTCAGGCCACAGATCGGGGGTCCCAGGGCCCGGGGCGCCTCCTGCGGGCCGTCGTAGAATTGCAAGTAGGAGCCCGGTGCGCACGGGTCGCGGGAGGATGCTTTGGCGGTTGGTGTGGCCACAGTGGTCTGGGTGGCTGCGGTAGTCCGGGTGGCCGAGGACAGGCTGTAAACCAGAAAGAAGCGAAACTGGAAGCGCATCCTGTCTTCAGGGGCCGCAGCGCGCAGCCACAGCGTGCAGTCAGTGTCTGGAGTCACGAAGTAGAACTTGCGCGAAGTCGAGTGCGAGCGCAGCTGCAGCCCGTCCCCCTGCCACGTCTGTCCGCACAGGTCCGTCAGATCCGCTGCGGGAAGAGTGCAGGGACCGGGTCATGCCGGATCACCCTGCCATCCCGGACCCGCCCTCTCTGGGTGATCCAGGAAGTCGCGCATCCTCTATAAGCCGGTCCCTAAGGCAGACACGGTCAAGGCAGTAGTGCCCAGCAGGAGCAGCCTTTGGGGCAGCAGCCGAAGAAAAGCCATCCTGGATCTGTGTCCAGCAGGGGGCAGACCTGCGCAACCTCCTTCGGGGTCTTTGCGGAGACAGGGGCGCGGGCCCTGACAGCTCCAGGAAGCGAACAGGGCACAAGCGGGCAGAGAGGCTCCTCAGGTCTCCACACTGTGTTCTGGGCTCACCCTCTCAGGTC
MSTRG.101	ENSRNOG0000003	0.814	0.097	0.024	-2.275	down	CAGAAGAGGGCATCAAATCTCATTACAGATGGTTGTGAGCCACCATGTGGTTGCTGGGACTTGAACTCAGGACCTCAGTGCTCTTAACCATTGAGCCATCTTTCCAGCCCTACCCGTGTCAAATCTTATTTTTTTAATTTAATTAATTTTTTTTACTTATTCACTTTACATCCCCCTCACTGCCCCCTCCCAGTCACCCCCTCCCCAGTCC

704.1	4226;ENSRNOG00000042133;ENSRNOG00000047234;ENSRNOG00000059588;ENSRNOG00000060573						TTTCCCCCTCCCCTACTCCACTGAGTATTCTCCCCACCCTGGCACTTCAAGTTTCTGTGAGGCTAGGCACATCCTCTCCCACCGAGGCCAGACAAGGCAGCCCAGCTTGTAGACCATATCCCCTGTGCAGGCCACAGCTTTTGGGATAGCCCCTCACACATGAACGTCATGCTGCACATCTGCTACATATATGTGAGGAGGCCTAGGTCCCGCCCGTGTATGTTCTTTGGTCGTTGGTTCAGACTCCAGGTTAGTTGACTCTGTTGGTCTTCCTGTGGAGTTCCTGTCCCCTTTGGGGACACACAATCCTTCCTCCTATTCTTCCGTAAGAATCCCCGAGCTCCATCCACTGTTTGGTTGTGGGTGTCTGTACAGCGTCAACTCTTAACACCCGACAGCTCTTCGAGGCCTGGTTCCCATCAGCTTTGGTCACCACCAAGTGTCTCTGAGGGACCTCTCCTGGTGCCTGTTCTAGAATCTGCTCTTTGTTAAGCTGAGTTGTGTCCTGTTGGGTAGATAAACGTGTAAATATCTGTGACTAATGTTTGGGCTGCTCTCAGTTTGGGGCCAGCATGACATTCTTGATCGTGTCCATACGTGTCTGACTTGTGTATCTGCCACACCGTCCTCTGACGTGGGTGTTCCGTTTCAGAGCATACCATTCCCCTCCCTGTCACCAGCCCAGTGCTTGGCTTAGAACCTGCTTCCAGGGCCTGTTTGTCTCAGGCCCTGGCTCCTCCTCCACAGCCCCGCCCCTACCCTCCAGGCCTGGCCAACCCTGAGCTGCATTCTATCAGATTTAGATTCCGCTCTACCTGGTGTTCCTCAGGTTAGGGAAGGGGTCCTGGGCCTGGAGGGGACCCCTCACCCTTGGGCATATCCTGCTACTGGCCGTATGGCTTAGCCTCATACCTTCCCCTCCCTGACCCTGGGTTTCCACTCTCTGCCAAGCATCACGTTGAAGGTCTCCATCATTGGTCTCCCTCATTCAGGGATCTATTTGCTTCCATCCATAGGGCTTTCCTACCTCCAGGGCTGGGCCAAGATAACCTCAATCCCAGCTGCCCTAAACTTCAACAGAAGGCCCTAGGAGAGAAGATGGGCTCTTCTCCCTTGATCCCCTTCCTGATGGCCTCCTCCTGCATCTGCCCCACCCGAGCAGATTTAGAAGAGACAGCCTTGGCATCAGGCCAGCCTGGGTGTGTGTTCTACAACTCCCTGGCTCCGGCCTTGGGCAGTCCAGTCGGTCTTCACCTACAGAGTTATAAGACCTCATCCAAAGATCTCTGTGGTCATCAACGCCCAGCCTGCCAGGAGCTCAGGTCCAGTGAGGGGGAAATCTGACACCATGTGCAAAGCCAGCAGCCAGAGACGTCACGTCAGAGGGAAGAAAGCAAAATGGGGGACTTGGGAGAGTGGCCGAGATGTGGAGGCTCTAGTTGGTGGCCAGAGGAGGACGGTTGGGAGGAGGGCATCAGGGTAAAGATGTGGAGGAGAAATAGGCGCACAGAATCTGCCGGGGCCAGAGACTTGGCATGGTACAGTGGAGTTGCAGTGGAGGGAGGCTAACATGGTTGAGTGTCAGCATTGAGGGCGGGAATAGGAAGAGATGGACAGGAAGGGGGCTGGTCAGGGGAGCCCTTATCTGGACAGGAAGTGAAGAATTGGCATTTCATTCTAAGAGTGATACCAGGTCCCCAGAGATCTTTAGTGTAGTGACTCCTTTGGGTTTGTTTCAGAAAAGCCCTGGCTGTTATGTGTCAGATAGATTGTAGGGGGCAGGAGAGAGGCAGAGGAACCCCATCCAGTAAGAGACGTGTCCTGACCTAAGGGTGTGGCTGTGCTCGTGCCCCTCTGAGGCTGGGTTTTAGCTGCTGTAGGGGTGGAGAAGGGTAGAGGTGGCCATACAGAATGCTCCTAGACCACCATTCCTGGTAGGAAGGCTTGGTGTCGTCACTGGCTGGACAAACCCACCTCAAACCAACCCTTGAGCTCAGCCTTGCCATTCTCCCAGTGACCCAGTTCTCACCACCCAGCTCCCAGGAACCCAGAGGACCCGAGCCTACGTCCCCTGTCTACTGAGAAATGAGGTCAAGGGACACCAGCTGTGTGCCCTCGACTACAGCCCTACTGGAGGCAGTTCTGTGGCTTTGTGGTTCCTGGCTGGCACTTCTTCCTCCAGGCTTCTAGGAACAGTGGGCAGGCTGAGGGAAACTAAGAGGGCAAGAGAGTTCAACAGAAGTCTCTGGAAATGGAATGTAGGCGCCCTCTTGCCTTACTTCCTCTTGGGGAGACAAAGAATAGAAAGGGAGTTGGGGGAAATAACTCGGTAGATAAGAGTGCTTGCTGTTCAAGCATGTGGGACTGACTGCAGATCCTCAACACCATGTAAAAAAGATGGGCGTGGCCACACTCCTAACTGTGAGGGTCAGAGATAAGAGGGTCACAGGGGTTTGCTGGCTACCAGCCTGGCTCCAGGTACAGTGAGAGATCCTGCCCCAAGGGGGAAGTTGGAGCCTGATAAAGTAGGACACTCGATGTCCTTCTCTGACCTCCACACATACATGGGAGCATGTACGTATATTACACACATACCACACACGTGTGCACCATCAATATCACACAGGTGTACACCAAACTTTTAAACAATCCCTGATCCTGTGTTATTTCTATTCAGAGTGTCGCTTCTGGGGCCCACAGGCAAGGGTTTCACCTCTGTCACAGAGTCCCAGCCAGGGTCCTTTGGTGAATGGGAATCTCTGGCCTAGGATGAGTGCTAGCACCTTCCACTGTGTGGCATGGCTGCAGACGGATGACAGGT

							TCCCAGGCCTGGAGGTGGTGCACAGGACGAGTCTTTAATAATGGGAACTATTGGGGTTGGGGATTTAGC<br>TCAGTGGTAGAGCACTTGCCTAGCAAGTGCAAGGCCCTGGGTTCGGTCCCCAGCTCCGAAAAAAAAGA<br>AAAGAAAAAATAATAATGGGAACTATTTGCTGCTGCGGTGGTTCTGTCCTAAGTATTGTACATGTGTGAT<br>ACATGTGAGTGTGTACGTGTGTGCACATCAGCTAGTACCCCTGGTCATCCAGTCCAAGGGATCTGCCTG<br>TCTCTGACTTCCTGGTTTGGGCATTACATGTATGCATCACCATGCCCAGTTTTTCTTTAACTAAAGACAG<br>GTCTGTCTGTCTGTCTGTCTGTCTGTCTGTCTGTCTGTCTTCTATGTGCCTACATGAGTCTGTGTATTCCA<br>TTGCATGCAGTGCCCACAGAAGCCAGAAGAGGACGCTGAATCCCCTAGCGCTGCAGTTCCAAGTGGTC<br>CTAAGCCACCTGGTAGGGGTGTCAGAAGCCAAACCCCAGTCCTCTGCAAGAGTCTTCAGTCAACCCCG<br>CCCACAGCTTTTCTTGTGTGGGTTCTGAGGTAGAACCCAGTTCTTCTGGCTCGTAAGGCAAACTCTATT<br>CCCAAGGCCCTGTCTTAAGTCTTTTGAAGAGCCAGAAGGGGCTGCAGTCGCTCTCGTGCCGTTCTCCA<br>GTCCTGAGGGATTGTGGGTAGGTGGGGTGGGGAGATTCCTGAGGTCGGCCCGTCTTAGAGAATCTAAG<br>TTTCTATATTCTAGGTAGGCCCTATGAATGACATAAAAGTCTGGCATTTTATTCATACGCTGTCAGCCTAG<br>GCTGGACAGAGTTTAGAGCTATTCTAACCTACATCCCAGCCAAGTGTCCCTTGCTGTTTCTCCTGGCCAC<br>GCGCCCATGGTCCATCTCCTCTTATGGCCGCTCCTCCTCCCTTCCCCTCCTTTCTCCTTCTTGCTCAGTCT<br>CCTCTGAGTGGAAACAAACCCTTCTCTCCTAGATTGCTTTTCATCAGGATGTTTTATCACAACGGCAGAA<br>AGGATATGAGGACAGCCAGGCCGAGTCACTCCAGAGAAGGCTTGGGGCTTGGTTGGAGGCACTGAGA<br>GGTTGCCCAGCGACTCTCCCTCAGGGCCCTGTTCTTTCTGTGGGGGTCACCCACCTTCACAGTTCTGGG<br>ATAATATGGATTCTGTTGAGGGGTTGCAGGGTGCCAGGCACAGGACTAAGCATGCTGTGGTAGACATTC<br>CTGGCTCCTCCCATCAGCCTATAGAAGACACTTATTATCGTTTCGATTTTGTAGTTGGGGAAACTGAGAC<br>ACAGAGGGCTACTGGTCTCTCATAGCCAGAAGGCGATCAAACTGGATTTAAAATATTTTATCTATTTTTAT<br>CTTATGTATATGAATGTTTAGCCTGCATGTAGGTATGTATATAATATGTATATATGCACATATATGTGTATATA<br>TAAGTAGGTATATACACCAGGTATGTGCAGGTCCAAGAAGGCCAGAAAAGGGCATTGAATCCCCGGGG<br>ACTGGAGCTACAGACAGTTGGGAGCTGCCATGTGGGTGCTGGGAGCTGAACCTGGATCCTCTGCAAGA<br>GCAGTCGGTGCTCTTAACCACTGAGCCATCTCTCTAGTTCCGGGAACTGGATTTGAACTTGGGTGTTTT<br>GGCTCCAGAGTCCAAGTGCTTTGTGCGTGTGCCTGTGTGTGGAGGCTGAGCCTTCTCATGGTCTCCAGT<br>TGAAGTTCTTAAGCACCATGTGTTTTAGTTATTACTCTCCTTCAGTTCACACACACACACACACACACAC<br>ACACACACACACACACACTACACACCACACACACACACACTATACAACATACACACCATACACACCAT<br>GCACACACACACATACCATACACACCATACACACCATACACACACACACCCCCCACATACCATACACAT<br>CGTATACACACACACACACACACACACACACACACACACACACACACACACTCATCCTCCTGAGGCAACT<br>CTGTGCTTCATCTGCCCCGGGAGAAAAGTCACAGTAGGTCCCCCGAAGTCTTTCTTCAGCACAGCTCTG<br>GGATGAGCAAACTGAGACCCTCAGGAAAACAAATCCAAAAGTTTGAACCCTTACAGATTCGGGGGCA<br>GCATCAGAGGGAGGAATGGGAATTGGGACCGATGTAGTTAGGAGGGGGAGGAAGGGGACTCAGAAGG<br>CAGACAATGGTGGGATTCTAACAGAACTAACATCTGGTGCCGGGCATCTGAGCTCTCCAGGAAGCACA<br>AGCGAGGCCTGTGGGAGAGCTAGACACTCCAGCAGAGAAGATGGTGCATAGTCTTGTGTGGGCGTGTG<br>GGCAGGAACCTTTAAAGAACCAGAGGAATGGGGCAGCTAACTGGACCCGGGGACTTGCTGTTGGAGC<br>TGCTCCTGAAGTGTACCAGTCGGGCAGAGCTGGGAGTGGCAGAGCCGGAAGTGAACCCGGACAGCCT<br>GGCTTCAGAGTCTGAGCTGTCACCCACATGGCATGTCAGCTCCACTGCCCTGCACCTCTGCTCCTGGAG<br>ATGTGACAGCTTATGTCCTGGCCTCTCCACAGAGCTGTAGACAGTTGCTTTCTCTTGGTAAACTTCTGCC<br>CTCCACCAGGGAGTGTCTCTGCCTCTGTAGATGCCTAGAAAACTTGGGATGTCTCCAGGACGCACGACT<br>CCATCCTCAGGGTGCCTGTTGCTACTGAGGTGATGCAGGGAGGCCTGCAGAGAAGCATTTCCTGTTCTG<br>TTTTCTGGGGGACCATCTGCCCCACAACCAGGGCCTTCTCTGCGCATAGCAACAACTCTGAGGTCATTA

							GCTGCCATGAGACCCAGGGAAGGCTGCTCTTAGTGGATGTTTAACCTCACAGGCTAAGCTAGTGGAAGGACTGGAATCTGCACACGGACAGGTCCAACTCTGAAGATTGTGTCCTGCCTTTAAGCTGGTAGGAACAGCCCGGGATGTGACAAGCCCTACACACCTTTCTAGTGACAGTGTGTCAGCCTATGCCACCCCACCCTGAGATTTTAGGGCCATGGAAACAGACTGTATTGCATTGCTCTCCAGGTTCTCGGGTGTGTGGGAGAGCCACCAAGAGGGATACTGGCTACCAGGGCAATCCTTACTTGCTTTCCAGAGATCCTGACTCATTAGGTTTTGAGTGGGGTTCAGAAACCTGCATTTCCAGCTGACTCCTTGAGTTTCACCTTGCGAGGGTGGCAGATATTCACTTGGGAAATTCCACCTGGTACAGCTGTTTGTGGGTGACCCAGCACTGTCCCCTGGCATTCCGTTTTGTCATTTCCACCATCAGAATATCATTCCGTTGGGGAAATCAATGTAAAGAGGTTGGGGAATCTTGAGATTCTCTTTCAAACAGTCACGGCAGCCTTGGGAATCCTCAATCAGAGGAGGAAATGGTGTCTCTGGGAGTTTGGGCAGCGTGCCCAAAGCCAGCTTAAAGTGTCTGCAATACTTAATACTGACTGTCAACGTGAGAAGACGTAGAATTACCTAGCAGGCTAACCTCTGGGCACATCTGTTAGAGAGTTTCTACCCTGGATTAGCTGAGGTGAGAAGGCCTGACCTGACCGTGGACAGCATTATTCATTCCAAGGCCTGGAGGTTCCAGACTGAACCAGAAAGAGAAAGCTGAGCCAGTCTTCAGACCCTGAATACAGTGTGACTAGACGCCATGTGTCCCTCGACACCTGCCTCCTGCCTTCCCCACCATGGTGTGTGTGTGTGTGTGTGTGTGTGTGTGTGTGTGACTGCATCAGGTACCCACAGAGGCCAGAGATGTTGATTCACCCTGGAGCTGGAGTTACAGATGTCTGCCTGACATGGGTGCTAGGAACAGAACTCGGGTCCTTTGCAAGATCAATGCATGCTCTTAACCACTGAGCCTTCTGAGTCCTGTATCCCATCCTGAACTGTAAGCCTCCCTTACGTTGCTTCCCCTGCACCCCCGGCAGTTGATCTCAACATCAGGAAATGTACCTAGTAGAGTGGCTGACGTGAGATTAGAACCTGCATCCCTGAGCTGCAGAGGCTACGTTCTTCCCAGCACCGTATTGCCTCCTTGCATAGAAGCTCTGGATGAGAGGCTCCAAGTTGCCAACTGCTGTTAGGGAGACCAGTGGTGTCAGACAAGGGCAGGCAAGATGGCTCAGTGGGCAAAGGCGCTTGTCACCACACCAGTGTGAGCTCTATGCCTGGGATACAGGCGGTATAAGGAGAGAACTGAGTCCTTTACACAGACATACAAACGCATGCAGATAAATAATAAATAAATAAATCACGACAGCGATGGTGCCTACCGTTTATTGAGCACACATTACGAGCTAGACATATGCTGTCTGATTAAGAGCAGCGTGTGCTATAAGTCTAAAGGCTGAGGAGCCATGCTGTCCCCAGGTGGAAGAGTTGTTCCCAGAGGGGAGAAGCCTGCTTGGAGAAGTAGGGTGAATGAGGTAAGTGTCAACAAGAGATGGCTCACAGAGAAGTCTTGGCCAAGAGAATACATGTGTGTAAAAGCCGTGAGGTTGGATGACCTGCGTAGTATCGTTGTATGGAATCAACAGCAAGGGTGAGAAAACAGGAGGTGGCCAGGGGTTGGGGCCCTGCGGAGCCACCAAATCCAGGTGAGAGCTTTGACCCTAATCCCCCAGAGCAATGAATGTGGAGCCGCAGGACTGTGTGTGTACATTCAAGCCGAACTTTATTTCTTTCATGCCAAAAGTCGTACGTAAGCCCTGAAAGGATGAGGCAACATTGGTAGCTGACAGAGTGGAGTTGCCCTTGAGTTAGTGCCAAGATCTCTCTTACCTCCTGTCAGGCCCATTCA
MSTRG.102085.1	ENSRNOG00000015264;ENSRNOG00000027328	0.000	0.534	0.000	4.725	up	CTGGCACACTCCTGTAGCATCCTCCTTCTCCAGCGACGCACGAGCGATGCGGCCTGAAGAGTCCTTGATTCCCGCTGCAGCTCTCTTATCTCCATCGTCCTGTAGTCGGCCTTGCTTCGGGGTCCCTGTGTTCGGGCGCCACTTGTCCCCGCCAGCGGGGACCCAGTTATTCAGGGTCCCGAAGAGGCTTTCTACCTGTGGCCAAAATGGGGGTGAAGAGAAGAAGGAGACCAAGCAAAAGTTCTGTTGTCAAGGTCTCGTTTATTGGGATGAACATTGCAGCTTTTAAGGCTTTCAGGTAGGGGGAGTGACCTTTGTGAAATATGAAGGAGGGGGAGATGAGGGTATAGTCAGTGATAGCTGGAGGCAAAGAAGTCAGGCGATGAGGTCAGGTGGTGGAGACACTGGGTGTTGACTGAGGAGATAACTGGTATCTGCTCGAGCTGAGGCATCCCTTGAATGTTATACTCCTGTGCCGTAAATTCATGGGAGAGGCTTTATCCAACAATCTTAAGATTTAT
MSTRG.102	--	3.493	0.057	0.000	-4.682	down	AGGAAGAAGAAATTGTGCAGCATATATTGGTGACATAAAAACTGACAGTGTATTCTGTGGTGATACCTGGATCCTTTGTTAGAAGTCTCTTTGGCCTTTACCGTCCAGGGCTCTTAGTTTTGATTTACAATTATGGAGATTGGCTGAGCATATAGAGACCTGAAAATGGGAAAATATTGTTGAGAAAGAACCTGGAACTTTTCATGAGA

212.1							CAAACATGAAAGATATGAACAAGTAGCAAAAGGATAAGTATGAAGGAAAGAGAAGATATCATGAAAATGCCTCTTTCTCTGAGGATTTCAGTTTTCCCTATGAGATGTAACAGGTAGTCACACAGTTAACATTCATGCACAGTTCTTTTCCAAGCAGCACTGAGGAAGAGAAAGAAACACTGCAAGAAAACTATTGTGTCCCACAGAATCCAGACTGTTGAACTCCTCCAACCAGAGGGCTCTGAAAAAAGCCGAGTGCAGGGTCTAAGCATTCCCACACCGGCAAGAAGTGAACGAACATTGCAGAATTCAACAAACCATGAATGTGCATGGTCAGAGAGCTTTTCATGAACCAAGGAGACATTTAACTTAAGTGGGTAATTCAAAAGGTGCTGTCTAATTCTGTCTCAATCTAGAGTAAGATTTCCATTTATAAATATACCTATGAAGGCTATGAAGTCACACTTCTGAATGCTGTGTTTACATCACAGCCTGCATATACCTCAGTGTAAAATTAATGGAGACTATAACTTAACTCAGGAAATG
MSTRG.102233.1	ENSRNOG00000008141;ENSRNOG00000008176;ENSRNOG00000008345;ENSRNOG00000008553;ENSRNOG00000008619;ENSRNOG00000008911;ENSRNOG00000009134;ENSRNOG00000009217;ENSRNOG00000031641	0.000	0.140	0.000	5.464	up	CAAGCACCAAGGCAGCCTCTAGGGGGTGACCTTTGTTCTGGGGTCTATGTGAGACAGGCAGAGGCACTTGTACTAAAGGAGGTAGGAGGAGATGTGCCAAGGTGGGTACAGAAACCTCCCCCTGCTCAGCGGACCAGGCCAAAGAGTGCAGGAGGTCTGTCTCTTCCATGGGGCTCAGAAGGTTGTCTAGTCTGTGGCTGGGAAGAAAGTGCCCCTTAAGGGTTAAAGGCATTCTGTCCTCAGTGGAGGCATCCTCTTGGCCTTGTTTTATGTCCTCTTAATGAGATTCACCTGGGCAGGGCTCCCCCACTCTGCCTGTGGCGGTGTGCAGCCCACTCCCTGCCTTCTGAGCAGACTCCTGCCAGCTGCTGCCTTCTCTGTTTGGCCTAGACTAGGAGCTGTCCCTGGCACTCCTCCCCAGTCAGCCTTCAGGCCTCGCTGCCTGGGGCAGTGACTGGCAGGTACAGATGGCTGCTTCTGAGGCGGGGCTCCCAGCCTGCTGGTCTTTTGAGTCAGGGAAGGGGAGGAGGCTGTCCTTAGGTAGAGCAGGGGCTCGACCCTCCCTCCCCCTGTCTCCCAGGCCTACACAACTGCAGTGCTGAGCCGACTCCTCCAACTGTGGGCCTGTTCCCTCCCTCGGAAGGCCAGGCCTGGTTCTGCTTATTCCTCAGGAACATTTCATAGCTGCTGTTGGGCCCTACTCACCGCACACCTGTGCCGTCTGGCCCGGCCACTGCCAACTGTCTCCAGGGGCACAGGCTTGTGGACTCTCTGGTGCTGCTTTTCAGAGGCCTGGAAGCTGGGCAGAACAAGAAATGCCACTTGACTCTGGCTCCCCCAGGGCCTGTACTGGGCCTGTCACCTCAGCCACGTTCTGTGTGTCCCTGGCAGCCAGCCCACCCTGTCTCTACCCAAAAATCCAGGCTCAGAATAGACTGGACTTGTGACCTGGGAAGCTATGCCCAGTGACCTGCCAATAAGACTCGTGTGCTGGGGGTTGGGGATTTAGCTCAGTGGTAGAGCGCTTGCCTAGGAAGCGCAAGGCCCTGGGTTCAGTCCCCAGCTCCGAAAAAAAGAACCAAAAAAAAAAAAAGTAAGACTCGTGTGCTGGACCTGGGCCTACAGGGACCAGCATGGTGAGCCACCGCCACAAACAGCCTGGTTCTCAGACTGAGTGAAATTGACACTGGGCAGGGCTGAAGGCCATAGTCAAGGGCTCTCATGTCCTGTACTCTGTAACCAGTAGCCAACTCAACTGCATGAGGAAACCCCACAGTGGTGAGCAGGGACACCCCACCCCTCCCAGGACAGCCTGCAGGTGACACTGGTTTTGGAAGACTGTCTGGGTGACACGAGATAAAGTCACCTGGAGAAAGACACAGCTGCCTTCTCTTTGGAGTAGCAGCTGTTGAGCCACCCAGAACTGAAGGACCTTTTCCCACCATGTCCTCTACGCCAGTTCTGGCTGCCATTGTCTAAGCTACAGAGAGTGGATAGAAAAGACCCTCTACCATCCTGTCAGCCTGGCCCTCTACTGCCACTCCAGGGGCCATCGCTAAGAACCGTTAACAGATGAGGAGGCCTGGCCCATCCCTGAGGCTTTGGGCATGAGATGCAGTTAGCGTCTCCAGTTAGCCAAGGTTAGGTACAATGAATGGGAAGTTGCTCCCAGGGCCGCTGCTTGGTAGAGAGGAACTGAGCCTACCGACCTGACCAAGACAGCTGCTCCACCCAGTGAACATCCACCCAGCCCTGCCATGCCCTTGCCAGGCTCCAGGCCTGCTGGGAACCCAGCGAGGGTAAAGAGCAAGAGCCCCAGGGCCTTCCTGCCAGAGTTGAAAGCCAGTGGCCTGAGTGGACTGACCACCCCTCCCCATGCACAGGATCGTGACCCTGCCCCAGTCACCTTTGAAGAGCCTATGCAGGCAGTGGCTGCCAGAACTGGAGGGTTGGGCAGAGGGGTTTCTCTGTGTCACCTGGAAAGGAGAATTGGGTCAGGAGAGGATGATGCCTTGATTTAGGAGAACAGAAGCTCCCCTCAAGTCTCCAGGCTGTGGGCTGGCCCAAGGGACTAATAGGAAAAGAAGGGACTTCTCCAAACAGCTGGGGCATTCGAGAAGAGATGGACAGGGCATTGTTTCACAGGGTTTGACCTGAGGGCTGAGACCCAAGGACCCAGCTTCTTATTCAAAGCCCAGGATGGGTCTTCTGTAGGCCTCTTCCACACGCCCATGTCAGGCTGGGCAGCACATT

							CTTTACAACAGGTGACCAGTAGCTGTGAGTCTGAAGGATCAACTGCAGTGGGCTTGAGCCTACTCACCAAGAGGGAGTCCTGCCTGCCTCCTGCCAGAGCAGGCTCCCACCCGTGTGCATAATGGGGTGCTTCACCTGATACTGAGGAGTCCCAAGACCCCCATATGTGGCACTACTCTCCCTGACTCCCCCTCCCCCAGTTGACTGAAGGCATTAAGCAGAGGCTGGCCCAGTAAGAGCCGCCCTGTGGTGGAGCTCAGGCCAGCCCTGAGACTGATGTTGTGCTCCTGAGTTAAGCTTTAGGATGTCGCTGAAGCCTTGCCTCTCACATAGGGCCTGACCCCAAAGTCTGGGCTCCCAACTAATGCAACAGACGGAGGGGACTCCCAGCCGCCACAGAATGGCTCACCACCTGCACATGTTGCCAGGGAGAGGTTACCTGGGCGTGTGTGTGCCTGCGATGAAGAGCAGGACCCTGGGTACCATTCTCAGTGTCTGCTGTGTTTCTAGAAACCTCAGTTTATCAGCGTATCAGCCTTGCCTTCACTAAGAAGAAGAGAGTGAAAGTATATATACTGTTAAAAGGTGGTAGTGTACTAGTGAAATTTTCACAAGGTTGAGGCAGGAGGACCAGAGGCTGACTCATGAGCTTGAAGCCACCTGGGCTATACAAGATGGGTCAAAGCCTGGAACATCAAGTCAATCCACAAGTCTACAGGGGGATTCTGAAACCCAGCAATGCTAGTCTTGCGATTCCATTTCAAAGGCAAAGCCACTGTGATGAGTTTTTATGCTCTTTCCAAAGTCTGTTCCTTCTGGCAGTCTGAAGCAAGACTCTTGGTGACGCCAAATTGTCCTTAGGAACACTGTGTAGCCTGTCAGGATCTCGGGTCCTCTGGTTACGTCTCCAGACGTCCAGGTGGTACTGTTTCCTGCACCTGGCATTGTGATTCTCTTCCCTCCTATAGGCCATCAGCCTCTCACTGTTGTGTCAAAGCTAAGGACTTCCGGTCTGAAGGTGACATTTTTGTACAAAAAAAGCCAAAACAAAACCCTACTTTCTACTTTATTTCTGTAATGAGCACATTTCATTTTTCCAAAATAAAAAATTAAAAAATAAA
MSTRG.102296.2	ENSRNOG00000010719;ENSRNOG00000011078;ENSRNOG00000011258;ENSRNOG00000012455	0.392	1.037	0.024	2.125	up	GGGGCATTCAGGACTCTGGCTTCCCTTCAAAGGCCCAGGTTGTGGTTCCTGAGCCTTGGGACGTAGCCCAGCAAAGCAGACAACTAGAGGCATGGGATAGGACCGTTCTTTGTGCGTCCTTGTAATGTTGACTGGGAGCCACCTTCCCCCAGTCTTTTGAGCAGTGAGGCCCTTCACTTGGTCGGAGACAGGGCCTCTCACTTGTAGTAACTCCGTGTGTCAGGCCTTCAAACTTCTGGGGAGTCTCTGGGCTCTACATCCTACCTTGCTGGAGGAATGTTGAGATTACAGATCCCAGCTACTGCGTCTGGCTCTATGTGGGTTCTGGCGATTCAATTCAGGTCATTTTGTAAATACCGCACGGCCAAACAGGATGGTGGATTTAGATCAGGGTTTGATGGCGCACTTCTGGAACACCACACTCGGGAGGCTAAGGCAAGAGGATTGTACTTTTGAACTCAGTATGGGGTACAGGGAAAGAGCCTTCCCGAAAGTGGGTAAACCCGAGCTTGGTTCTTGGAACCTCCATTTAAGAAGCATCCGGATACACGTGCCAAATGCCAGGCTCTCCGCTGGTGCCAAATGAGCAGATCTGAATGGACGTTAACCTTGTCTGCAGAGGGGGTGTACCGTTCCACTCTCATCTGCTCTGGGCTTTGAGGTGAGAGTGCTGAGAGGCAGTAAGAGCTGAGCAGAGGGGCTGGGGATTTAGCTCAGTGGTAGAGCGCTTGCCTAGGAAGCGCAAGGCCCTGGGTTCGGTCCCCAGGAAAAAAGAACCAAAAAAACAACCAAAAAAAAAAAAAAAAAAAAAAAAGAGCTGAGCAGAGGCTGGCACCCCGAATCTGTCCCTGCTCCCTGCAATCTTGTCTCTGGACATCTCGGCTGCTCACCTTTAAGAGGCAGTGCCTGAAGCAGAGGGGTGTTTAGGTCCCACTGTGGTCATGTCACTCAGGGCGTTGCTGTTCTTAAAAGTCACAGGCCTAGGGGTTGGGGATTTAGCTCAGTGGTAGAGCACTTGCCTAGAAAGAGCAAGGCCCTGGGTTCAGTCCTCAGTTCCGGAAGAAAAAAAAAAAAAAGATTAAAAAAAAAAAAAAGTCACAGGCCTTATCTTAAAGATCCCTCAGCATAGTCCGGGGCTGGTCGTTTCTTTTTACACTGGCCTGGAATTCTAGGAGCGGCCACCAGGGGGACAAGGTTGCCCGTGTCGTGCAACAGTGGACAGAAACCTAAGGAAGGCAGAGCTAGCAGCCAGCCAAACACCCCCTTTGTCCCTTTTGACCTGCCCAGATCCTCAGCGTCCTTCCCCAGTGTAGACGTTGGAGCCTACAGGACTCTCATCCCCCCTTGGGTAGAGTCGCGGAGAACCCGCTGCACCGGACCAATGGGTCTCTGGTGCCTCGGGCTCCTCCGTTTCCCCTCCCCCGTCGTAGAGCCTCACCACCTCATCGAATTGACTGATTCATTCCTGGTGACTCCTGCAGCCGCTTCCATGGCTGCACCTTGAACTTGCATTAAGTTTTAATTAAAGCCGTTACACCAGCCTTGCCCTTGGCTAAATGCAGCCGTGGGCACAGGGCACATAGATATTTAATACTCTGCCTCTCCCTGCTGATGGCACAGGGTGCCCAAGGGTGTACCCGAG

MSTRG.102333.1	--	0.000	0.211	0.011	3.719	up	CCCCTTCTTCCCTTCTTCTCTTTCTCAGACAGAGTCTCACTATGTAAACCCGGCTAACTAGAACTCGACACCCACTATGTAGACCAGGCTTGCTTCTGCCTCACAAGAGCTGGAATTAAAGTTTACTTTGAGACAAAGTCTCATGTAGACCAGGATGGCCTCAAACATGCTATGAAGCCGAAGATGACCTCAAATTCAAATTCTGATCCTCCCGTCTCTCTCCCTTCCCCCCAACCCCCAATGCTACGGCTACAGGCTTGTGCCACTGTGCCCAGTTTCCTCCGAAGTGCTGGAGTTGAACCCAAGACTTAGTGCATGGGAGGAAGGCGTTCTTCCACAAGCGAAGGATGGCTTTGAACCTTTAAACTTCCTGACCCTGCTTTCCGAGGGCTGGGATTCCGTGGAGGCGCCACCACAGCCAGTTTGTGTGGTACTCAGAATGGAACCCGGGGCCTCCCGTGCGCCAGGCAACGCTTTACCAACTGAGCTACAGCCCGGTTTGGTGGAATCCACAAAGAAGGAAAAGAATGTGCTATAAGAGATGTCACAGACCACCTTCCCCGTACTCACCCGACCCATGCACTCAGGTCTGCG
MSTRG.102341.2	ENSRNOG00000015850;ENSRNOG00000037027	0.560	1.423	0.030	2.079	up	CCCCCCCCCCCCACATCCACAATTAATTAAAAGAAAAAAAAAAACCGGGTATGGTGGCCCACATATTTAATACCAACACTAGGGAGGCAGAGGCCAGCCTGATCTACATACTGAGTTCCAGGCCAGCCAGAGCTACAAAGTGAGACCTTATCTCCAACCCCCTACTCCCAGAAAGGATTTGTGTGTGTATACTAATATTAATTTTTTAAAATTGTTTTTGTTTGATTGCTTTTATTTATTTATTTCTTTTTTGAAAGATCAGATCTCTCCATTCCTACTAGGCAGGAATAAATAAATAAATAGAAATGAATTAAAACCAAGGACCAAGTGTCCTCTGCCTCCCCTCCGGTGTCCGCGTGTGGAACTGCAGGTCCCCTCCCTAACCACAGACTTCCCATTGTTCTCCATCCTGCTCTCCTGGAATCCAGGAGTTGAAATTCTTGATCCTCCCAGGAAAGCTGGCTTGGTTCAGATACCTCGCAGAAGCGTCGTCGCGAGAAGCTTCCTCTTTTATTCCATAAAATCTGCCGTTTCTCCGTGTCATTACACCTTTGCACGGAGAACCCAGCCCTTGCTTTGGGGAACGACAGAGCCTCCTCGCTACCCCGCGGCTCCACGCAGTCCTCATCCGACACGTGGCAGCCCTTCTCAGCTCTGCACAGCGAGGTCCTGCAGCCTCCCCCTGGTGGACGCTTGGAGTTGAAACTCAAAGTGGCCATTCGTTCCAGTCTGAGCCCAGTGAGCCCCGCCAGCTGGCGCCAGCCTGCAGAGAAGGAGCGGGAGCCATGGGAGGGAAGTTGCATCCAGCGCCTGGTGCAGCTATCACGTCCCTGCCTTCACGGAAGTCCTTCCTTTGGCTCCTAAAAGTATTCGGTTATTTCCGGGCATAAACTGTGGCGCTTTGGCCTCAGACACTTAAGTCCTAGTCTGTCCGATGTTCTTTTACTGAGTCCCACTGAGGCACGATCGGGAAAATCATCCAGTTAGTTATCTTGAACTGCGGATTCCTCATCTTCAATGGATGCTTCTGCTCCAGACGGAGTCCTAGCGTGTTAAGGAGACTTGCCCAGGGCTCTCTACCGGGACTTTCTTATCTGCATCTCCCTCCAAGATCCTCCCCCATCAACAGTCCTATCTCCTCTCTGATCTTGCAGGTACCCTACTGCCTACATTCCTTTCTACTGTTATGGAAAAATACCCTGAGGATAGCAAGGTAAAGAAGGAAGGCTTTGTGCTGACTCACAGTCCCAGGTTATTGCCCTCCAAAGCCTGGGCGTCTCGCAGCAGGGCTCAAGGACCTTTTGTTCAGTCCAAGCGATGGTGCCTCCCAGTTAGGGCCGGTCTCCTCACCTCGATTAATTACATCCAGATATTACCTCAAATACTTGCCCAGAGCTTGTCTCTGGCTAGTCTAGCTTCTGTCAAACCAACCAACACACTTTCTCAACCTACACACTTTCTTAGTGTGACCTTCCTCTTACACAGCGGGCAAGTCTAACCAGTGCGAGCGTGAGTGCGTGCGTGCGTGCGTGCGTGCGTGCGTGTACATATTGCACATGTGGACATTAGAGAAGAACTTGTGGAGAATCAATTCTCTCCTTCGTCAAGTGGGGGTGGAATTCATATTGTCAGAGGAGGGCAAGGACCTTTACCCACTGTTATCTTGCCGGCCCCCGATTTTTATTTATGTGTATAGACATTTTGCC
MSTRG.102425.1	ENSRNOG00000017523;ENSRNOG00000017528	0.394	0.000	0.037	-3.684	down	GGGGTACTCGTGCATAGAGATCAGGAGATGAATGGCCGGAGTCAGTTCTAAGTCTTCTATCCAGTGGCTTCTAGGGATTGAACTTAAGTAGTCAGGTTTGGTGGTGAGCACTTTAGGCCAGTTCCCTGGCCCTGTTTAATTCACTGATGGTGCAAAATCATTACATTTTCTACAGATTGGAATTCCCCTCCCCCATACCACACCCCGAACCAGTCCCCTTGGGACAGGGTTTCATACTGTGTCATCGGCTGCCTGGCTTTGAACTCGAAGCAATCCTCAGGATTTCAAAAGCATGCCTCCCGAGTGCTGGAATTACAGCTTCCAGTGACCCAAAGTGGTTTCTGTCCCAACACGTTCCCTCACGGTGAGAGAGACTCAGCTTCAGCCGGAAGCCGGGTGACGGAGATGTGGCCACACCGACCATCTAAAGCGACCCCCAACTCCGATGAAGCCTGGCTGGAAAGACGCGGCTTCCATCCTG

							GGAGCCTGGAGGAGAATCGAGCAGTGGCTATTGCCTGGGCTTGCCTGGGCTTGTTCAGAC
MSTRG.102677.1	--	0.775	0.000	0.000	-6.910	down	TTTGAAAGATTCCATCTCTTGTGACAAACGAAAGCTACTGCCGGGATACTGGACAGGCAGCACCACCAT<br>GTGACTTGGAAGCCTTGGCGCCCACACAAGGGCTCTGGCTAGGATCACAATGGCCTCCCTCTGGCAGT<br>CAGGGCTGGCGCTGGAGCCAGCAAGCTGCAGGCCCAGGTAGCATCAGTCCTTCAATCCAGTCCTAGGA<br>GACAAGCCATCACCTAACAGGGGACCCATCAGCACACCTATTACCCTGCAACCAAGGACCTTCCCACC<br>AAAGATATTTCTATTGATTTACTGCCTCTCGGTGAGCTGTGAAGTATGAGGGCTGCAGAAGAGGGCGGG<br>GAGACCGAGGCAGGGCGCACCTGGCCTTGGAGTCCTGATGGATGCTCCCCACAGGGTTTTGATTGCTG<br>CAGATGAGAACCCCATGGACTTTTTCACATTACCCAGCACCTAGGAAGGGAACTTGTCACTGGCCACAT<br>CTGCCTCTTTGGATTAATTTGTATTCTGCAGGTGACGACTAGTTTCCGAGGGGTTCAGAATTGATCTCAC<br>ATTCAGTTTGTCAACAAGATACATTCTTTGATTGGGATTCTTGCTGCCCCCAGCGGGGGCTGCAGCACG<br>TACTCGGTGACATTTCTTATCGATCCAGCTGCCTCTGCGCCCGCCACCTGGGGTTCATGTTCCCCCTGCA<br>CAGGGAGGTGGACTGAGGAAGGCTTCGAGGTCCCCAAGAGCTGAGGCGCTGTGGGAATGGAGGGTAG<br>GTGTCTTGTGGCAATATCGGGGTTTCTGAAGGCCAGACAGGTCACAGGAGAGGAGTGTCCCTTAGTGT<br>AGAGTGACTACCCTCCTCCTGATCAAGGATTGTTGGACCCAGCATGCTCTGCTCCAGGCAGCTTTGCTC<br>ATTCAGTTCTCAGTAGAACCCAGCTGACAGGGAGCATTTATACGTTAAAGACCAACGGAAGGAAAAGC<br>TGGTTCCAGAACAAGGTCCCTCGGCACACCAAGGTCTGGAAGCCACAGCTAGGTAAGTACCCGACACT<br>AATCCTCCCCCCAAGGCAAGGACACTTCCCTGTACCAACCAAAGGGGGAGGAAAAAAATCCTCATTT<br>AAAGCTTAATTAAACCCCAAGGGCTCTTAAGGAAATTTTATGCAGCCCAAGCTGGCCTCCAACCAATTG<br>GGTAGTTGAAGATGACCTTGAACTTCTGTTCCTCCTGCTGCTGTCTCTCCAGTGCTGGGATGACAGGTT<br>TTAAATGGTGTGCTTGGTTTCTGTATGCACTGCTGGGAATCCAACTCAGGGCTTTGTGCATGCCGGGCA<br>AGCACTCTGCCATCTCAGTCACAGCCCCAATCCAGTCAGGTTCTTCCATGCGTGTTTTAATGAGCCTCTA<br>GTGCACCAAGTCCAGGGGACAAGGCACAGAACGATCAGATAGTTTGCCCAGATCACACAGCTGGCAC<br>ATCTGCCTGAGAGGAGGAGCCAGGCTGTCTTAACCTCCTTGGCTAGTGCTCGTAGTGAACGCTCTACGC<br>TGCGCTCTCACTGCCCGGCTCTGGGACAGGCACTACTGATGCTGCTTGATGAGTCTGACAGGCTGTGCG<br>AAGGTCAGCTGGAAGTGGGGTGGAGCGGCTCCTTTACCATTCAGTTCTCTTGGTTCTGTTCCCCTGGGA<br>CTGAGTGGCCCCCTTTGTCTGACACAACCTAGGTGATGTTCTTTTGAGCTTTCCACTCTGTGGCCGCAG<br>CAGCGCACGCCCCGTCCTTCGTTTAGCCTAATGAGTTAAAGTACCCGGGTGCAATGTCCCTGAGCTTCT<br>TCCTTGGTCCTTGGTGGAACCTTCCACCTGCCCAGTGCAAATTGGGTGGCAGAGGTGGGCAGAGGTGC<br>CAGAGAAACTGGATAGCCCTTCTGTTTTAATAATCCAGCTTTCCGGCACCCAGAGACTCCAGTTTCAGA<br>GTTTCTCCTCAACCTCTAAGAGCCTTGTGGGAAGGGAAAGCCGGGAACCATGAGGCTGGGCAGTGGGC<br>CCTGCTGGGCTTGCGGTGGAGTCTGTAAAGGAGAGGCCTGTGTGGCTGGAGGGAAGGAGAGGCTCAG<br>TTCACTGATAGTGAGGATGCGGCTCAGGCAGTGAGAGCCTGCGGGCCTGCACTGGAGAGAGTCTTCAG<br>TCCTGCAGCACACAGGAAAGGAGTTCCCCATCTCCATATCCACTGGTGGAAACAGAGAGCAATAGAAG<br>CTGCAGGGACAAGCTGCCTTCTGCCCTCTGTGCACAGTGGTTCTTGATTTATGGGGTTGGCCATTCTGTA<br>GGCACTAAGGCTGATGCAAGGGAACAGTGGGCGCAGAAACACCAATATCTCCCATCCCTTGAACAGGA<br>GCGTGTTAGGAACTACAGGTCATAACCACGATAGCCCCCAAAAGATGATGCCACTTGACAACGGAGGG<br>ACTGCCACCCACTCCACTGCAGCCCTGCCATCTCGGTCCATGCCTAGGTGCCAGGACTGTGCGTGTTCA<br>GTGGCTCTGATGAATGCTGGCCATGAAGACTGCTCAAGGTCTGTGAAGTACCCGGCTCACCAAGACAG<br>ACTTGAGTGGAATCCTGAATCCACATTTGTTTGCCTCCCCAGGACAGGTTCACGCAATGCTCAGGAACA<br>ACCTCCCATCGAGTAAGAGGAAGCTGGGAAGGAGAACTTGGTGAGGTCTCGTAGCCTCTACTACAGCA<br>GTCAGCCTGGAAGCAAACGGAAGACGAACAAAGACAGGAGAAGGACCAGGGTGGGAGATGTTGAC

							ATACAAGAAGATGCCACCATGCTGGGATCTCATTCAAGTCCCAATACCCAGGACTCTTGCTCCCCCGCCCCCCAGTCCAAGTGACTCCCACTGTAGATGTTTGGGGTCAAAAAAATACAATTAGTCTCAAAGTCACTGTCAACCCCTGCCCCACATCAGTCCCCAGAGTCACGCAGCGGGTCCCCAGAGGTAAAGCTG
MSTRG.10287.1	ENSRNOG00000023863	0.026	0.282	0.049	2.676	up	GTGTCCTCCAATTCTAAACATGTTACCGGAACCTTCTAAAGCAAAGCCTCATCATGACATCATTTCACCGACAAGCATTATTCTCCAGAGCTTGTGCCCTAAAGGCCATGCTGAAAACTACTGGCCACTGGCCACTGCATCCTGTCTCCTTTTCTAGTAAGCACACTCTGAATAGCTGCTTAATGAATTGAAAGGAGTGAATGGTCACCAAGGAAGAAACAGCTGTTCTGGGAGGATGCTTGAATTGTCAAGATGAGGAGAATGAGACTTGGCAAGGCTAAGCCACAGGTAATTTTTCTAAACTGCTCATTGTTAAAGAGATTTTATCCTATCATCAATGACTGCTCGAAGACCTTTGGTTTGATTTGCTATGTGTTCACTCAGACAAATCACTATAAGAAGACTGACTCTAGGAGGGCAGATCTTTCGGTTGATTTTGTTGGCTGTTGGATGTTCATGGGAGTGAATGCCTAATCAGCAGCAGTCCCCTTTAGCAGATGAACAACTGGAGCTGGGAGAGGTTAAAGGACCTATGTGCGCATCAAGTGTCCAGAAGGAGAGAAGCTGAATTCTGTACACAG
MSTRG.102876.2	ENSRNOG00000018647;ENSRNOG00000018691;ENSRNOG00000019244;ENSRNOG00000019423;ENSRNOG00000019589;ENSRNOG00000019749;ENSRNOG00000019864;ENSRNOG00000019979;ENSRNOG0000002	0.000	1.180	0.000	5.390	up	GTGCTTGCTAGGCTCTACCACTGAGCTAAATCCCCAACTCCTATGTTTTTGTCTTAATTCCAGGTATGGTGATCTAGGGCTGCTTTGGACTGTCTGCAGCAGCTGACTATGATTTGCCTCATGCTGTAGCAGAGGAATGTTTTTGCCAGCTGTAGATAGTTTCTGCGATTGTGTGACATTTGTAACTCTGGGAACGTCAATCAGAAGTTCAAGAAGTTGAGAGGCAGGGTTGGTGGTTGGTGGTCATTCAGGTGGTTGGTTGTTGTTAGTTGTGCTCTCCCTGTCCTGGGCTCAGCAGGCCTTCCCTTCCGAGGCACTTGCTGCCGCTGACTCTGCTTCCGTCTTGTGATGCTGCAGCTGGAGGTAAAGATGCAAGTGGAGTACATGTC

	2307;ENSRNOG00000022354;ENSRNOG00000022455;ENSRNOG00000043331						
MSTRG.102876.3	ENSRNOG00000018647;ENSRNOG00000018691;ENSRNOG00000019244;ENSRNOG00000019423;ENSRNOG00000019589;ENSRNOG00000019749;ENSRNOG00000019864;ENSRNOG00000019979;ENSRNOG00000022307;ENS	0.000	0.477	0.001	4.308	up	GTGCTTGCTAGGCTCTACCACTGAGCTAAATCCCCAACTCCTATGTTTTTGTCTTAATTCCAGGTATGGTGATCTAGGGCTGCTTTGGACTGTCTGCAGCAGCTGACTATGATTTGCCTCATGCTGTAGCAGAGGAATGTTTTTGCCAGCTGTAGATAGTTTCTGCGATTGTGTGACATTTGTAACTCTGGGAACGTCAATCAGAAGTTCAAGAAGTTGAGAGGCAGGGTTGGTGGTTGGTGGTCATTCAGGTGGTTGGTTGTTGTTAGTTGTGCTCTTTGGGAGTCTAGGCTACAGGCTCATCCCCCTGTTGTCTCCTCCGAGTATGCTGCCATGACGTTTGACAGGCCATATAGCGGAGCTCGTGTCCAATTCTGTAGTCATGGACAGTCCATCATCCCCACTCCTACCTCTGCTCTAG

	RNOG00000022354;ENSRNOG00000022455;ENSRNOG00000043331						
MSTRG.102946.2	ENSRNOG00000004577	0.391	0.000	0.000	-5.732	down	CTCGGTTCTCAGGAAAGAACTGTCCTTGGCACACCTCTATTGAAAGCACCTAGTGGCACTGTCCCTGGGCTTGTGTTTCCTTACGGCTGTTGAGACTGCGAGGCCTGCTTATTTAGTGAAATTACCTTTGAGCAGAGTAGCACTTATCTAGTCAAGTGTTCCTCCTTTTTAAACGAATTATGCAAGCAACCCGGGCGCGTGTTCCAGGAGTTACAATGCCAAGCAGGATCAGCGAGCTCAAATCCCCTCAAACCCATGAAGCCGTCGTCATTTCTGAGAACCAGGTTTTAAAAAGATTCCACGGAGCTTTGCGCCAGATGACTTTAATCGGGGTGAGGGCTCCACAGCCATTGAACCGGGAGCGAAAGCATAATTCTTTGAGTGAGATGTCCCGATACCTAGTCATAAAGATACCAGTTGAGAGTCCTCTCACTGTCGCCATAGCTTGAGATCACTGGCCTGCTGAAACCGAGATCAAAAGACGGCTCTTAAACCTGTGTTTTCTTTCAACATCTTCTTTTGACTTGAGAATTACAGAACTATTTGCGAAAATACCGAAACGGCTTAAGCGGAAACTGCTGAGTTCGACCTTGTCCCTGCAGATCTGTGAAGGCCTCCATCACGAGAGGAAGAGCAAGGCCAGACAGACACATCACATTGTTTCGGCTCACCCCAGATGAGCTGACGACCAAGAGATTTCTGTCCTCTTGGAGACAGCACAGATGCTAGTCAGACTGTGGAACAATGACCTCAGTCTGCCACTCAGCGAAGGTGAAAGGCGCCAGTGGCTGGTCACTAACGTGGTCCAAAGCTTACCAGACCATATGAAAGGACATCACAGAACCAGCCACATCCCAGATGCCTTTCTTGCTATCTGTAGGGATAGAAAGTGATGCTTACAGTACAATGGGCAGGGGCGTGTGTGAAACCAGCTGCCGTACCTCTTACCAGCAGCGCCTCTGAGGCCTGTCTGATTAGTTTGTCTGTGGCCAGATCAATCAGCATTGACCCTGCCTCAGTTCACACCATCGCAGCAACAAGTGCTACAGCACACGGTGTGACTCCCGTGTGCTGTGCTGTCTTGCTTTGTGGCCTGGCAGAAGTATCCTGAGACAGGGGTACACGCCTATTCCCATTTCACAGAAGCCCAAGGAGATCCATGTCACACAGTCCCTGACTCCAAATTGGTGACTTGAGACTCATGCTGTAGAAGAGGGGGCGTGTTCTGACCTCTGGGGCTTGGCTTTCTATGTCCGGTCCTTTTCCTGAGCCTCCGCCCAGAGTTCTGACCTTGCTGACTGAACCGTTGGAAAACTGCGGTTGCTTCCTCTTGTTTGGTGGTGGCTTCCTTCTTCTGTTCCTCTACCCATGAAAATAGCTCTTGGCCCTAAAGCTTGCCTGCTTCCCTTAGCTCTCACTTTGTTCCCTTCCAAACTTCAGAACACCTAAAATAATCTGGTCTGAACTCTGCTTTTTCTCCCTCTGACTCGAGGTTGTCTAGCCTCAACACAAATCATCGACGTGCTGAGCAGACAGCAAAGACACCCCTACTTCCTCCTGTACCCTGAGAGTTTCCATCAGGGCTACTGTTGGGCATCATCAAGAAAAGGAGTGGTCCCAGGAGCGAATTCACCCAACAACTGACATTTGGGGGCTTCGACTTTGTGTTAAGAACGTGGGCAACTGCCCTTCAGCAGTGCTCCCCGTGTCCTTCTGTGCCTTCTGCAACCGAAGGTAGACATGGTCATTGCAGGAACCCAAGGGCCCTCCCTGGGTATCTGATGATCTGGGCGGTTTCCATGGGTTTTCAGTGGTACAAGTCTACGGAAGCCATGGCAGTGCTCGGGTCTACCTCCCTGCGTCAGTGGCTCTCGTCTATTAAGTACCAATCATGTATGTGCACCAGATGAGTATCCCAAGGGATTTGCCGCCCAGTGCTTCTGTAAGCAGAGACTGGGTTGGCCAGGGACATTATCAAGCTTGAGCTCTTCTTTGTCAGATGCTACACCCCGACTTAAGTTGAGACCGTCAGGATTCTGACTATCTAACCCACTTGCCGGAATGTATTCCCTCTCTTAAAGCGTATGCACGCGTCACACGCACTGCGGTTTATTGTCACTCGAGGGGCGCCAAGATCCATTCTCTCTGAGAACACACAACGGAAAGGCTATCAGACCCCTGTAAAAGGTCTAGATTGTTCTTGGAAC

							CCGGCAATGGTATTTTCAGTTATTTCAACAACGTTAACGGAAATGGTTCCCCCGCTCTGTATAGAAGTTTCTTTTTAATTTGTCTTTTGTGGTCATTTCATAATAATCTTATAATCATACTACTGTCTTCTCCTATTAAATATGAGGTTGGTAGAGGAAGAAAAATACGTCCAGTGGCCAAGATTCTTTGGAGAAAGCATGCAAAGTTAATATGGGTCTAAAGTAAATATATAGCCGTTTCTCTCTCTGACCACAAAATAATACAGATCTTGGGAACATTTTCACACTATTCATTTTAAAATAAAACAGAGAAAGTGCTCGCGGATGAGTGCCACAGAAGGCC
MSTRG.102970.1	ENSRNOG00000005087;ENSRNOG00000005279;ENSRNOG00000025317;ENSRNOG00000040303;ENSRNOG00000048315	0.000	0.411	0.003	4.043	up	GGGATGCCACAGTCCTGGTGTCTCTTCATAGCAATAAAACACTGACTAAAACAATTGTCTTAAAAGAAAACAAAACAAAAAATCGCTGTAGGCATGATGCTTTTGTGCACCGTGTAAAGGCGATCCTTATTATTCAAATGCTGATTTCTTTGCCCCCAAATCTGATTGCCCTAGGACATGCCACTGTAACGCTTATTCTGAGCATCTCTTGGTTCAAGTTTGGACCCATTGAAATGTGTGCCGATGACATGGGCTCAACAACATGAGAAAATAGTTATGATGCATTAAGGAACTGAAACAGGACACAAAGCTGTCCCCAGAGGTCATCAACTATACCCAACAACCACAACAAAGGCTGGACAGAAACACACCAAGAAGTAAAACCTGGCTGGGG
MSTRG.103041.1	ENSRNOG00000027332;ENSRNOG00000027343;ENSRNOG00000027360;ENSRNOG00000030319	0.686	0.000	0.028	-3.770	down	TGGGCACGGACAAGTGGTCTTCCTTATGTAAGACAGCCACCCTACTCAGGATCTACAGTCTGGTTTATTGTAGGACTGTGGGGTAACTAGTGCCGTGATGATTTAGGTCCATCAGACACCACACACATTTTCTTTAGCCATTTTCTTCTCATCGAAGCACAAGAAAAATCACATGGGAAAATGAATCCATTATTGGTGATTCTGCATGCTTGTTTGACTTGCACATTCCTCACGCTGGCCATGGAACCATATACAATGTTCCTTTAAAGAGTACCTTGGCAGGAACCATTAACAGCTGTGCTTTCACCACAAAAGAATACCTCTTTTCTTTCATCTTCCCAGAATCGAT
MSTRG.103101.1	--	1.145	0.128	0.017	-2.368	down	GGAGCTTTCCCCAAATGTTTCCTTGGATCATCCTAAAATGCTAGTACCAGAAGCAAGAACCCGAGAAGCAGAAAGCAGAGATAGAGCACTGGGACTAAAATAAGATACCAGAGACAGAAGTGAAATCAGTGGGCATCTAGAGGTGTAAATACTGTTTGTTCGCCCCTAGGTATCTGGCCTGATAACGAAGGCTCTTGGACATTTGACCTCTGAAATAAAGATACTGAGGGCCACAGTCAAAACAGGAAGGAGCCTTGATAACATCACATGAGGAAGGTCCTCACTCGTGGGATTTTCAGTTAACTTACAGCCCATAGCCTACTGGCTCAGAGGGTGAACTAACTTCATGTTTCAAAAGATTCTAGGGAGACCATCTACAGTTTGTGATTATAATAGATTAGCAGGACTTTGAA

							GCAAGATGCCATGATCACACATGATTTCAAGAGCCAATAGGACAATTAAGAAGGTGTGTAAGCTTGAAG<br>CCACAATTTAAGGGAGGAAAAGGCCCTTTAGTGACTCTTAAAAGTGTTCTTGATGTGAAGTGAAAATCA<br>GAGAGGGGCAAATTTGAAATGCAAATGACTCTCCTGCTGCAGCATCCATTCACACTCTCCCAGAGAAC<br>ACAGAATAATGAGAGCATTTGTTGCCTTTTTGGGTGATCACATGAGGAGTTGGGCTCTGACTTTCAGAA<br>CTTCCTGCTCTGCCAGATTTCTTCCTGGCTCTGGTTCCCCTTCAGTTTACTTCTTGGAATGCCTATTGACC<br>TTTCCAAGTCTTCTTTCTTGCATGGTTTTGGTCTGATTATAATTCTGCCCTTTTCTTTGTGCACAGTTAATT<br>TCCTAGTCTGGTTTGGTGTATATGATAACAGTTCAAGCCATGTGAAGTTATAAGCAGATGGCAAGGTATG<br>GACCCAAGACAGGACCCTGTTGGATTTTTCTGAGTCCATCCTTTACGATAGTGTTTTATCTTGTTTAATAG<br>AAGTGATATAAATATATTGGGCACAAAATTATATAGAGAATTTTTCCTGAAAGAATTAAAGGAAGTGATG<br>CATAAATAAAATATGGCATGACACTGTCACTTAGTATACACATAATACTATAAAACTTAATCAAAGTTTTA<br>TGGAAAAAAAGGCATCAGAAATAAAAGTAATTTTCATAACATATTATTCAACAAATGTATTGTAGTGGTT<br>TGAATAAGAATGGTCCACATAGACTCATATATTTGAATGCTTGGTCATCAGGAATTGGCACTATTTGAAA<br>GGATTAAAAGAATCAGGAGGTGTTGTTTAGTTGGATTGGGTTTGGCTTTGTTGGATAAAGTGTGTCAAT<br>GGGGATGGTCTTTCAGGTTTCTAAAGCCCAAATCAAGGCCCTCTTACCTTTAGGACTAAAATAAGATAC<br>CAGAGACAGAAGTGAAATCAGTGGGCATCTAAGTGTGAAAAGACTTTTCACTCCTAGGTACCCGGCCT<br>GATGACTAAAGCTGCCTACGGGTCAGGTGCAGCTCTCAGCTACTGCTCAGGTGAAGTGTGATGTGTGCT<br>ACCATGGCCTTCTTCATAATGAAAATGAGCATAAGCCTCTGAAATGGTAAGCAAGGCCCCAGTTCAATG<br>CTTTCTTTTGGTCTTTCCTCCATCTCCTCTTCCTCCTCCTATTCTTCTTCCTCTTCTTTCTCTTCCTCTTCTT<br>TTTTTGAGAAAGGGTTTCTCAGGCTGATTGGTCTTGAACTCACAGATTTGCTTGCTTCTGTCTGTTGAAT<br>TCTGGGATTAAAGATGTGTGTTACCACTGCCAGGATTTAAGTGCTTTCCTTTATAAGAGTTTCTGTGGTC<br>ATGGTGTCTCTTCACAGCAATGGAACAGCAACCAAGGTACTTACTCTTGCTTTTTAAATATGTAAAGGTT<br>TTCTACACTTAAGTACATATTAGAAGTGGGAGGAGAGGAAGTGAAAGAAAGTATTTCAATACGGCTCAG<br>AGGCAGATAAAGAAGGGGAAAAGAACATCGGCAGTTTGAGGAAAGACATGTGATAATAGCTCCCTATT<br>TTGGTGTTTCTCTGATATTCATTGCTCAACACAATAAAGGAAGCATAGACTGTGAATGGGTATAGAGATT<br>TGCTTATTCTCTGTCGCTTAGAGAACATTTCAAAATTTCACAATCTCAGACTAAGTCACGTGAATTTAAA<br>GCCCATCTTCATGCAACAATACTCTTTTATAATTAACTGCATGTAGAGTTGGTTAGAGTTACTTAAGTATG<br>GGATATAGGACATGATTTCTAGACAATAAGTGGTATGTTGGAGACAGTGGTTGGCCAAGAGCTGTAAGG<br>TTAAGTTAGGGACAAGTGCTACATACAAGAAAGCCAGTAGAATGACCACATCTTATCTGTACAAGTGAG<br>CTCAAATGTAAACCAATAGGAACCAGAATTTGTGTCACGGAAGATGTTTTATACCTTTGCTACATGAAGA<br>TGACTAATCTCTCTCGCCTCTACAGGCTTTTCCCTCGAACTGGATGATTTGAGAAGGAATGGTATATTTTT<br>TGTATCAGTTTTCCAGGTCTCAAATTCTGACTCCCTTTGACATCAGCATCCATAGGTAATTTACTGTGAAC<br>ATCAAAGAGTGTCTCAAGGAATAAAAGCTGACAATAAAGAAGAATCTTTGGACGTAGATACACTTCCTT<br>CTCATCTCTACACATCCCCACAGTTCCCAGTTAAGGGAAATGGTCTCGTTGAAACTAGACATGTTTGAC<br>CAGTAGATGGAGTAAGTGTTTCCCAAGATTGAGCAAGTGATTCGGGGTAACTTGTACCTGATAACCAAC<br>AGCTATAGCCTTTCAGCAAATTTTACTTAAAAAAAAATAGACAAAGTGATTGCCAATAGCTAAATGTTGA<br>GTTTGTATCTGATGATTATAATGGATGACTTGGGTGGCTTTTCATTTAAAAAAGAGGTAGAAATTCATGA<br>CTATCTGGAGCAGGAGTATTTGAAGTGGATAGATATTGGGGTGCAGAGGCAATTTGGGGTACTATGGGT<br>CCCTTCGTTTATTCATAATGTTTTCTATTGTCCAGGAAACTCTCCTGCCCATCCTCCCTCGCACATCATTG<br>ATTTTCCAAACACAGCCATTTCATTCCATAGTTTGGCTGTAGTTGCATGTGAGAGAGGGCAGAAGTCTA<br>CTTTGAAGGCAGAACTAAGGAGAACGGCTTCAGATCATTCAGACTGTCAAACCTTTTCTCTGGCAGAA<br>GCCCTGGGGATAGTACACTGGGAATCGTTTCTCTTGCTTGCTTAGGGTGCTCCAAATTTGCCAGCGATG

							GCCAATTTCCAGAGATAGCAGAGAGAAAACAAACGAAACAAACAAACAAACAAAAAACCCCAAACC<br>CTACTTTCCTTAGAGAAAGCCAGTAACAACAGCACCCTTCAATGAAATAAATAGGCTATAGAAGGTAAA<br>CAGCCACAGAAATCAATGCTAAGTAAAGATTCTCAGAAATTCCAAGTGTTTTAGCCAGAGGTAGGGTTG<br>GGGGTTGTGTGTCCTAAGAAGCATTATCTGATGGGTATGACATGTGCACCTGAATTATCACTCTCAGTAC<br>ATTTTCACATTTAAATATGTATCTACATGTACCCACAGAGCCCCATATATTCGAGAACAATGGATGTAATC<br>TCTGGAAGCTGACAGAAAGAGCCATACATTTTTGAGGGGCCTTAGAATTAGGATAAGTTTCCTTATAAC<br>CCCCAGTGTTTGGTTTTCCCAAACATCTTCCTCAATCTCCAGAGTCATCCAGTATATCTCTTTGATCATTT<br>AAAGGGAAGGAAGACTACAGACTTCCCTCTGGATCAATGAGTCAAATCGGGGCATTGCCTTTAATGCTT<br>TGACTCTTCCCACATGTGTCTTTACACTTCCAGGGCCAGCGTTCAGAAGCTGTTCAGATGATTTCTTAGT<br>CACACATGGCCACAGGCCTCATGATGGGCATGTTTACATGTCTAGAGGCTGAGAGGAGTATCTGCAGTG<br>GGCATCTTGGTGGTAAAGAGGCTGGTCTACTTGGGCGACTCAAGATCACGTCATCTGCATAATCTCACA<br>TGATCTCAAGCACTTGTTTATCCTAGACTCTAGGCTAGGCCCACAGGGCATGTCTCCGACTTCACGAAA<br>GAAAACCAGTTGTGTAAGATTGGCTCCTTAGATGAACTCAAAGGCCAGCTAAGTTAGGTGTTTGTCTCG<br>TGTCCCTCCTTAGACAGGTGGGCCTATTCCAACCACACTCCATGGTCCTCACCTCCAAAACCAATGGAA<br>AGTCTGGTCTTTTAGGATCCTATCAAACTCCTTGGCTCTGATATTTGAGAGTTTCCTGAATGTAGGTCTTA<br>AAGGATGGCAATGGGTTGACATGCAAACCTTTCAGACACACTTTATTTCTTCACCTAAACTTTTAGTGA<br>GCTGTCATGTCAATTAATCTTTTCTAGTTTCTTAGATATTTCTCTTCTTATTGTTCTTCACATCTAGGGAGT<br>GAGAGGTTATCTTTGGAGATTTTTTTTCTAGTGCTATTCTTGGTGTTATTGTGGGCACAGAGAATTTCCTG<br>CTTTACAGTAAACTATAACCCCAAACCTCTACCCTAGACATGCACCACATTAAATAGAAGATAAGGCTTT<br>ATCTAACTCACCTCGCTCATTGGCCTGTGAAAACTCTACTTTGAATATTATAACCACAATATTGACACATA<br>ATTCTAATTTCAACTACTTATATAATATAACTGGTAACCCTATCTAGATTTTATTTGCCTGGCCACAGAACT<br>AGGGTTAAAAGGTGAAAAATACCATTAAGTCACCATGTGATAAACTTGGATCGTATTCAAGGCCAACAT<br>TTCTGTCTTCTAAAATAAAATTATAGTTAATATTGCTACAGTTTGGAAAACACATTGTAGAAAGGTCTGTC<br>TTTGCATCCAGGTCTCCAAGACCAATATTAATTAATTAATTAATTAGTCAATCTCAGTTAGGTACTGAAGA<br>GGAAAAAAACCTTCAGAATGCCATGGGGAACTGCAGTTAACCTGAAGATCTATTTAATGCTGACATGTT<br>ATTAATGCAGGTTTCAATTAAGAACCACTTTGGATCTAAGTTTCAATTATCAATATTTCTTCTCTCTGCTG<br>CTGATTAGAAGACCTGAGTTGGTTGGCTTACATAAAAACATCCTATCTCCCGTTGCTGGCAGTGTTTTGC<br>TGAGATAAACTCATTACAAATTGTGTGTAATTACCGCATTGTGAACAAGGGAATGGCAATAAATTAGGTA<br>ATCAAAACAGCATTTTCAAATAAGCCATAAGCAGCTAAAAAGAGCGTTTCTGAATGACAAATTTGGCAT<br>GACCAGGTAGGTTAAAATTACCATTATACATTATTATCTGTTGGATTTTATGTATTTAGAGCAAAGAGGAA<br>GCCTGCCGTAGGCCTTAGCCTATGTGCTTCTCTGATTGTGGATGGGTACATTTATTTAGGGACTGTCATGC<br>TTTATAAAGGATGAAAACACGCTTTAGGGAAGGAAAACTAATTAACTTCCTCTCTCAGAGGTTGGTAGG<br>CCTAGCTCTTGCACTTTAGATTTCAATAGGACATCCCCATCTTTGGATGAAAAAATGCTGACTCTCCTGT<br>TCTGGGAATCTTAGCTCACCCTAAGGCCACTCAGCACCGAGCCCTGGCTGCGTGCCCGGCTTTTGTGGC<br>AGGGAGTAACCACGAGCGCTTCTTTCAACTTACGCTGAAGTTTTAACTGAGACAGCACCATGTGCTAGC<br>GTATCACAGGAAATGGGGCTTCCCTCTAAAACCGCCTAAATCAAATCTCATTTCCATTGGCAACCCTTGA<br>CTGGCCCCCATTTTTCCAATCCCTACACCTGCAGAAGATCTCCCTGGAGCAGTCAAGAACGGCAGGGTG<br>CATGTAAATCTCTTATAACTTGGAGGTGCCCAAGGGTAGACCCCTTTCATCAGGAATAAGGCAAAGGGC<br>TGTGCTGTGCAAGACTCTTGAGGGATGTGTGGATGAAAAAAAAATAGAGGAACAGTAAATAACAAGAG<br>AAAGAAAAAGCAGAGCGAAGATGCTCTCCCCTTTCCTGAAGGGAACCGAGGTCCAACCTTCTAAGGC<br>ACGGAGCAAGTGGGCAGCGGCGTCACCCAGTCCGCATACAGGGAAGCCTGAGTTGAGCAGGGTGTTC

							TTTGAAGACATTTCAAAATATTGGGAACGTTCTCCATAGTTCCTCACCATCCCATAAGAGCCGAGTGAGGGTATTAGCTATTTTCTCTTGCAATCAATTGCAAAACAATTGCAAAGAAACAGAAGCTGGACTGAAGTCTGGACGCAACCTATTGTCTCCATCAGGGATACCCGTCTGATATGTAAGAAAAAAATCCCATTTTCAAAAAGGTCCCAACCCCACAATCATTACAAGGTTTTATATATATATTTACCCTTCTGTCTATCATGATTAGCCTCTCCAACAAAGTAAAGGTCTGCTAAAGTAATTTAATGGGTGTCCACTGTGTTCAAATTTTAAGGTTTGAAAATTGCCATAAAGGAGTCAAATCACCTCCTTAATTAGCATCTCTGAGGTTATTAGGTCTTTATCATTCAATTCGATATCCACAAGGTAGCTTGAAAACTGTCACTTAGTGGCAACAGAGGTTGAACAATTGAAAAAAGAAAGCATGATGTATCTTTGTGTCAAAGTCTAGTTAGAAAGGGCTTTTATTGAATTATCACAGTAAGATTATTTCTCTTGTTTGAAATAGATCTCCTTGGGGCTTTGTTGTTTTGTATTTATGTAAGTTTATTCTCAGGCGTCTGAGCCCACTGTTTTGTAAGTTCATCTCACAGACTGGAACCTCTGGTCTTGAAGTTGCCTAAGGAGGAGGCAAAGGGGTTTATATACTTAGCCTTGAAGGAAAAAAAAAAGAGGAGAAGGCAAAAGTCAAAGAAGAGGAGGAAAAGAAGGAAGCCAGATGAAGGGAAGAATGGAGAGCAAAGAAGAAAATCCAGAGAGAAGACAGTGTGGTTACAGAGAAGAAGGGATAAATTTAGCGGTCTGGCTGTTGGGGCTTCCAAGGGAATGCCAATTATGACAGCTGAGACACCTCTGACGGCTCTGTCACCTCTGAGCACGGAGACAGCAGCACTATTTTTAACAAAGGTCATTAGGGAGTCATGCCTAGACATTACCATTTGGTGCTCCTTGGGATGGTTCCAACCCGAGCAGCATTTGGGCAGAGATCTGAGTGTTGCTTGCCACTCAGTAGCCAGCTAGCCAGCTGACCTGGGGCCTGGCAAAGTCTCGTTTTCCTGACAAAGCAGCACTTCCATTAAGAGAAGGAAGGGCACTCCGGCTATTAATTTCTGCTAACCCTTAACCTGGCCAATAAAACACATGCATGTGCAACTTTGTGTCTATTTCAGGGCAAGGAGGCCCTTGGAGATCTCTGGTTGGCTTTGAGCTCTGTGTATCCATGCCATTCTCTTGGTTCTGACTTCTTTGTTCATTCCTTTTCCTGACAGGCTGCCTGGTGGGAACACAGCTGGTCTAGGATTCTAACAGGCTGAGACTAGACAAAGTAGGGAAAGAACTCATAGAAGCTTCTTTCTTCTTAATAGTTAAAATCTTTCCTTACAGAGAATGTCTACATGCTTTTAAATGTTAAACAGTGCTGTTTGATTGCTTTGACTTTCTGAGAAAAAATTTAACTCCTCATTGCTCCTTGATAACTTCTTGTCTTCTGAAGGTTTCAGAAATCACTAGGGAAAGAAAACATCCATTTATAGCAGGTTTCTGGTGCCCTGTTAATATCAGGAAAGTAAGCAGCAAGCAATATATTACTACCAATGGTCAAAAATTTGTTGATTTATTTCAAAGTATGATTTTTGTCACATTTAATGCAGTTTCTTTAATCTGTTGGGTTGTCTCTTGAGTGGAAGCATTTATTTATTGTTTGT
MSTRG.10311.2	ENSRNOG00000015557;ENSRNOG00000015716	0.000	0.300	0.000	4.948	up	AGAACAATGGAAGGTTGAGAAAAGGACCCTTGTGCTCTGCTCTTCGCCATTAGTGATGCCTATCAGACGGGATGCCGCTATCAAGGCTTGCAAAGATCTTCCAAGACACAAGAAACATCAGCCACAAACTCCCACTGCCAGCCATATACAGAGCTGCTTCACCATTCCTCCTCTGCAGTGACAGATGGGACCATCTGAAACCATGAGCCAAAATAAACTCCTCCTCCTGTCAGTTGCTCCAGCGAGGTATTCATTCAAGGAGAGAAGAAGGAAACGGATGCAGAGATCTTGCTCACTGGGCTACTGTGAATGTGAAATGCTATGGAGTCAACCAATGTCGTCTGTCACTATTGGCAGTGACTGGGCTCCTCAGCATGCCCACAGGAATGCATTCAGAGACAGTAGTGTGACAAATTGTCCCTTGGTCCAGAAATCCCACAAAGGGGTCGGAGATGGCTCCTTCAGGTACATCCCAGGCCTTCTTGTCCTCTACAGACCTACCCCAATTAAAGGATTCTATATAAGCTTGCAGGATTCCTAGGATTCCACTTCTGTATCCAGAATGTCCCCTTGTCTACTTTTTAGGAGGGGCCAGTGGAATACTAAGGACAGCTGACAGGCCTTCGAAGCCTCCAGCTGCATAGGGAGCGACTGATCTTTGGCTCCACTCCAATTGTTCCTCCTCCACTGTTTCTCCTCCACGTTACCCTCCAGGGAGGTGGCCGGCTGTGCTGAAGATTGGCGAATTCTCTCTGCATCTGCTGAACTGAAACAGCATATTGATGATGGCCTGCAAATGTGAATTAGATGGGAGCTGCTTTGCACTTCAATAGAATAACAGGTGTTAGTTCTTTGTGGATGGGAGAGAGGGTGCTTTTCTGCTGTTGGGATGCAGGGGAGGGGAAGGGAGTTGCCTCCTCACCTGTGTCACTGATGATTTCTGAGCACTTGTTTTGGATCTACTGTCTGCCTGCCATTTTAGTCAAGGTATCCTGTGATTCCTTTCCATAGACACACCCTCCAGGAAAAATTTTGTT

							ATCTGAAGTGCAACAGTCAACTTTCTGTTACCACAATAAAAATGTCTGGCCCTGG
MSTRG.103182.1	--	0.701	0.046	0.001	-3.012	down	AAGAAATCCATGTTCAAAGGACAGCACAGCAGGGACCTCGGCCTTCTATTTCAACTGGTCGTTCTATTT<br>CACTAAAATCGAGTCTGGAGTTCTTCACAACCAAACTTCTCGTTTTTACTTCGTCAGCCTCACAGACCA<br>AACACTGCAGGACTGTACACTGGAGTCCACGTCAATGTTCTTCCTTATTCATTCCTGTAACAGAATCTGG<br>AGTTCTCCACGTCAGCAATGACCGTGCCGCAGAGGAAATAGCACGTACAGTGGCACCCATGTGCCAGG<br>AAAGCAGAAACAGAAGCCACCTGATGGGCCTCTGGGAGGAGTGGTTATTCATTTCCATTTTATCTTCTT<br>CTTACCCAGGAATAAGGACAAAATGCCTGGAGGAGCAATGGACATCTTGTAACGAGAAAGAGGGGAA<br>GTTGCTCAACAGATGAGAGAAAATTACAATAAAAAGATTCTAGCTACCTAGTGGTGTCTATAAAGACTT<br>GTACTAACTCTGATCACTTACCTCTGGCCTCCCTACTTCAAACCAACTCCTGGAGCCAGGATTTAACCAG<br>CATCCATCCATCAGACTTTTACATCCCTAACCTCGGAAAGCAGAGAGCAGCTAAGAGTCCGTTTCAGCC<br>ATCATTGGCCCAGAATGTAATCAATCCTACTTACAAAATGAAAGTCATTTAAACAACCAGAAACTTAAA<br>CAGAGAGGTCCAGCGGATTTCAGAATTGGCAAGCACATCCATTTGCTCAGTCACTCTAAACTCAGGAC<br>ACCAGAGACTGGGACATGGACACTGGGCCAGGACAAGGAATGGACAGCATAAGAAAGGAGTCTAACG<br>GACGAAAGCCAGCTCTACAAAGAACCTCCAAGAGCCTTCCAGCCACAGAAATTCCATTTAGCAACAGT<br>CATTCCCACGGCCCTGAGCCTGGAAGGAGGTGGTGATAGGGCGGCCTGTGTCTGTGCTGCTGAGGGGG<br>GAAGAAGCCATTGGGTCAGCCACACACACCAGACTCCTCCACATTCTGGAAAACAGAATGTGAACAGT<br>TCTGAGCGCCAAGAATCCTCACGCAGAGGAGGGGCAGGTCCCTGCACTGAGGACCCTCCAGTCCCGTG<br>AGAAGACATGACTCAAGCGTTTCCGAGTTTGCAGCAAATGCTATAGATCCAGCTGTCTTCAAAACGGGA<br>GGAGTCGTGGCTCCTGGGGCATCGTACTTGCTGTCTCACCTACACAGACAGCAACAGCAGGCTTGGGG<br>AACCTGCTCCATCTGTAATGAACAGAGACATAGCAGTGCTGTCAACAAGATCTCAGGGACCATTCCCAC<br>AGCCTGGCTCTGGGGCCAAATCTCTCCGTCTCCCCCCCATCATGATGCCTGCCCATTGGAATGAATCTGA<br>ACCCTGAGCTTAGTTCTGACCCAGGAATGATGGCCTCTCAGGGGCCGCCCTCCTTCCTTCCAAATATGTT<br>TCTGTTCCTTTTCCCACTCTGAGTGACCCTCCCCCACCTGCTGTTTCCCAAGCTGGTCCTGGGAACGTTA<br>TGCCCATGGCCCACTGGTGATCTTGTTTTACATGATACATTTCCAAAAAAGACCAAAAAGTCCTTTCCAG<br>GAAAATGCCATTTTAAAAATTCAGCCCCAGACACCGTGGCAACATAAGGAAAACAAAGGACTTGGCAG<br>AAAGGTTTCTCTGAACTGGGACTTTCTCTCTAAAATACCTTTTCCAAACTGTGTGCAGTAGGACTGGTG<br>CCAGGGGTCAGTTCTGGAACAGGCAAACAATCCCAGACACCACGGTTGCTGTCTACAACATCCCCTCT<br>CCTTAGCTGGACTACTCTGGGATGCTTAGACTTAATCCAAGAGACCTTCAAAGGGTCTCCAAAGAGAG<br>AAGCAATCACAGAAAGGTTGGAAAATAGTTTCTACCTGCCTTTGGAAGGGACTCCTTGCTACCACCACC<br>AAGTGGAGGGCCAGCAAGTCATACCATGTTTAGAAGAGAGCTGGTCTACTCATTAGTCAAGTGAGAGG<br>AGACGAGATCTCCCATGGAGCAAGCAGTGAGGCCCTGGTTGCTCTTGGGTAGGCTCTAAGACTCTAAG<br>CAGTATGACTTGTTCCCTTTAAGAAGAAAGGCGGGGTTGGGGATTTAGCTCAGTGGTAGAGCACTTGCC<br>TAGCAAGCGCAAGGCCCTGGGTTCGGTCCCCAGCTCCGAAAAAAAGAAAAGGAAAAAAAAAGAAGA<br>AGAAGAAAGGCGGATACAGACACACACACACACACACACACACACACACACACACACACACACACA<br>CACGAAAGAACACAGAGAAAGCGGCCTTCTGGAGACCCAGAAGAATCCAACTCTGCCAAGCACCTTG<br>CCTCAGACTTCCAGCTTAAGAAAATCATGTTGTTTGAGTTACCCAGAACATGGCGTTTGTCATGGCAAC<br>CTGGTCATTATTACCACCTCATGGGGGACAACCAGTCTCAACAGGCTGTGGAAGTTGCCAGCAGAAGT<br>AAAGCCTGAGATACAACCGTATGACTTCAGAGCACGAGCGCACAGAACTGTCCAACGTTTCTCAGTGC<br>TCTGCTATGTGCATGTGTCTGTTCCACTTCGCTGCCGGGTAACAAAAGGCCCCATGACCTCACAGCTGA<br>AGTGACAGCATTTACAGGGCTCCTGTCAGCAATTAGACAGAGCTCACGGGGAATAGCTTGCTCCCCCAC<br>CACTTGTCAGCTGGAACAAGAGGCTGAAGGTTGGGCCAAATCACCTGGAGTTTCATTCAGTTTTCTGG

							CAGCTGACATTGGCTGTGAGCAAGAAAGTCAGCTGGGGCTGTTGTCCGAAGCTCCATGTGGTCTCTCT<br>ATGCTCTCTGAAGCCTAATCCCAACATGGCGGCTGGTTCTCCTGAGAGAGCGACCTGAGGAGACTCTG<br>CTCGCCCCTTCCCAGCAGCTTGGGAAGATGCCTTTCTCTACCACCTCTTCAAGAGGAGTGAGTCACTGT<br>GGCCATTCCACGTTTGAGATTAGACCACTTTTAATGGCAAGAATGTCTAAATGGCCTCAAAAGCCCCAC<br>AGCACCTGGGGCGTGAGCATGGTCGTATATTTATTAGTAACGCAATACTATCGAAAGGGCCTCATTCAAG<br>AAGTCAACCCTTTGAACACGGGGTGGAATTCCAAAACTCATCTAGGAAGAGGCTAGTGACTACTTCTC<br>CACCCTGGTCTCTAGAAGATGAAGGTCACTTACCCCTCAGGTCTATAAGACAATGCTGCTCTCAGTCAG<br>GATACTTCTCCTCAGACACTGCTTGCCTAAGGGTCAGTGATGTGTCCTCAATGCAGCCTTGTCCCATCTC<br>AGAACTTCTGTAGAAGCGACTAACTCTGCACAGAACACCCCCTCCTCATCCAATGTTGCTTCTTGTATA<br>ACTCATTTTTATAAAAAGCAACACCCAGGCTGGAGAGATGGCTCAGCCGTTAAAGGCTAGGCTCACAA<br>CCAAAAATAAAGAAGTAACACCCTTGTGTCTATTTTGCAGACAAACGAGGCTCCCAAGAGATACAGTG<br>CTCTCCCGAACTCTGCAGCCCACTCTCTCCGGTTAGTAATGGGCAGAACTAGCACCATCTGCCCAGCCC<br>ATCTCAGGCATTTGTGCCTGGCCCAGTTCAAAC
MSTRG.103334.1	ENSRNOG00000030629	0.394	1.202	0.013	2.302	up	GCACAGTCTTTTTATTATTGTTGTTGTTGTTATTATTATTATTATTTCTTTTATTTCATAAAAGCAGTGTTTAT<br>TTTATTATAGCAGGACCTCATAACCCAGACAGATTGCTAATATAGCTGTGTGTTTGAATCAATGCCTTTGA<br>GTCCTTTGGGCCCCATCCTCCTCTGTGAGGCCAACAGGGGATGTATCAGTTGCATTGAGAGGAGATGAG<br>GTTTTGGTAACATGTTCAAGGGCGCACCTCATGGAACCCAGATGAAAAATCCTGATTTATAATCTGGAA<br>ATCGTGTAAAACACCAGACTGACATCTGTACACTTAACGCGGCGATCAGGCGCGGTGGTGGAGCCCAG<br>GACGCCGCGGTGGTGGAGCCCAGGACGCCGGCGTCCCTCTGTGAAGACGCCCGCAGGGGCGTGCGGT<br>CCGAGGTTGGGGAGGGAGTGTTTGAGGGGGGCCGCGGGCCCGGCACCCGGCGTGCCAAGGGTTAAGT<br>GCTCCGATCGCCGCTCTCGGCTCCCCTCCCCTGTGTCCTGATTTTCAGGCCCACCGAGCCGCCCGAGAA<br>AGTTTTCAATTCGACGAGGCTGGCGCGTTTCAGGGGCTGAGCTCCTTAGAAAGCTTCGGGGGCCCCCC<br>AAGGGCCGACGTGCAAGGGAGGGGGCGACTTGCCCTGTCCTGGACACGCTAATTACAAGGATTCGGTT<br>TCCCCGCTCTGAAGGAATGCATTACCTGCAGCAAAGCAGGCCCGGCCTCTCAGGAAGGCCCCAGAGGA<br>AGCC
MSTRG.103349.3	ENSRNOG00000057031;ENSRNOG00000058827	0.437	0.000	0.000	-6.234	down	CAGGGAATAGCTTCAGCAGTACAGCCCTTCCCGACTTCAGCATCTGGCCCATGGCAGCCATGACGATAT<br>AGTTTTTAGTAATACCATTCTTATTCTTGTATTTCAAGTTCTCTCTCCCCTACTCTTTGACCTTTAAACATT<br>GACTACACAATCAATATTAATTGATTATGGTGCATTCATTAGGATACAATATAGACATGCACAGATCAATC<br>AACTCTTCCAACTACAGGGATGTCTTAGTCCATTCGGGCTGCTATAACAAAATGTCAGAAACTAAGGGT<br>TAGGATGTGGTTCAGATGGTATAGAATGCTTACCTAGTATGCTCAGTGCCACGGGTTCAATCCCCAGCAC<br>TCCCTAACCTGGGCACGGTGGCATGCATTTGAAATTCCCATGATGGAGAGGTAGAAGGTAGAAGAACC<br>AGAAACCCAAGGTTATTCTTGGCTACACTGAAAGTTTGGAGCCAGTCTAGTCTTCAAGAGCCCCTTTCT<br>CAAAGAATAAAATAAATTAAAATTAATTAAAATGAAACCTTATAAGGAACAGAATTTTATTCTGAACAGT<br>TCTAGAAGCTGGGAGTTTGAAGGTCATGGGACTGGCAGAGTCAGTGCTTGTGAGATCCTACTTCCTAGT<br>TTACATTGTGGAAGGGGCAAGATGTCTCTCTGAAGTTCACTCATAAGATTCCATCTGTGGGGCTGGAGA<br>GGTGGCTCAGCGGTTAAGAACACCGACTGCTCTTCCAGAGGTCCTGAGTTCAAATCCCAGCAACCACA<br>TGGTGACTCACAAACATCTGTAATGGGATCCGATGCCCTCTTCTGCTGTGTCTGAAGACAGCCACAGTG<br>TATAAATAAAATTTATATGAGCCAAACTCATATAAATAAAATACAATAATAATGATAATAATGATGGTGATA<br>ATTGCACCTGAGTCAGCCAATAGCATCCCAGAAGACCCACCCATCACAGTGACACTGGGGGGGGGGTG<br>GGGGGTGGGGGGTGAACTGAGAAGCAGGGCAGGGGAGTGTGGGCAAATTTGCTTACTGAGAAAAGCC<br>ACTGAGCCCATTCAAAGGGGGTAAAATGAGACCCAGCAAGCCCTATGCAAGGCTCAGTGGGAGTCTGC

							AGGCCACCATTGTGAATGTGGCATGGCTCCCACCAGGTGTGGGCTAGGGGAGGAAGATGCAGGCTTATTCATTGGTGAGGCTCCAATGGCCACGCTCTCTGATGAGCAATGACTAGGTGGTATCAGGCACCTCCCAATGACGCCCCCAATGACGCCCTTCTGGCCAACCCTAAGGGACCAGGCTTCTGGTTCAGAGTCAGATAGCTTGGAGGGGAAGATGAGAAGGGCGATGGGGAAAGCTGAGTGTGTAGCGACTAAGCCTGCCTGGAGAGCTAGTTGAAGGCTGTCACTGGATTAATTAGAGTAACACATTGCTCATACCCAGCGTACCCTCCAAGTAGGAAAGGAGGCAGCAAAAGTTGAATGGGAATAATAATAATAATGATGATGGTAATAAAGAATTAACTGATACCGAATGTTTTCTCCTTGGTCTCTGCTTGCCCAGGTTCTGACGCGTACAGCTTGTAATTAGTCTCTACCTGCAGATGTGTGGTTCCAATGACTTGTGGATTAAGGAGAAACTCTAGAATTTCTCAGTACCTGACAAGTTGTAACGTGAAAAGCTACACTTAGTCCATTAAAGATTTTTCACAGTGACATTTGAGTGCAGCTTGAGAAATACACCGCCGTGCCCTTCATCGTTTTCAGGGCATGGGTGTTGGGAAGGAGGTTGCTACGAAGAACAAAACAAAATGGATTGCATGCGCGGAGGTGAGCTAGTGAATGCTGATAAATGTACTTCACTGAATGTCAGCAAATGTCACCCGAGAAGCCATTTGCAGCTACCATAACACTCCCCCCCCCCCCCGAACCAACAAAGAGGAGCAGAAACAGGCGAAGTGAACTGGGCGTCTTGCTTCCACATGAATAAGCAATCAGTCCTCTCAGAAGTTACACCAGAAAGTTGTTAAACCTCACAGAAGAAGCTTTTCAGCCACACTGACACATGTCTACCTTGCTGCAAAGGGTTGGGGACCGTCTGACGGCACTGGGGTTGTGACTCCAGGTTTGACTTAATAGCCCCCCCAGCCCTGTGGGTGCCTGCCTTTTGTATGTGGACAGACTGACTGTCTCAGAGGCACGCTTTCTGGGTTCTTCCTACCCAGCATGCTGCTTCTGCAGATTCCAAGAATCCCAGCTCCTTCTAATCTAACTCTCCTTGCAGGACAGGACTCCATAATGCAGCTTCCATGGCAACCACTGGTTGGCCTAGCCCTTTAGTCCCTCGATCAGAAACAGGACTTGAGTGGTGGAGTAAGTTTCTGTTTCATTCCTGGAGATTGACCAATACTTAAAGCCTGTGTGACTTGGGTTCGGAGCGTCTAGAAATTCTTTGAGAATTCTGCTTGGGTCTACATATTCCTCTCTTCCTCCACAGCTTGGTGATTCTCCACAGATCACGTAGCATATAAACACATTCTGCAGAGAAATCATCGAGAGAGGTGAACATTTCTTGTCGCACTAGGAATATTCTCTTTTCTGCTTTCCGTTTTCCACTTGGGGCTTTTCTTTTTTTCAGACATGGTCTCATGTAGTCCAGACTGGCCTTAAACTTGCTATGTAGACAAGGCTGACCGTGGACTGCTGACGTACCACAAGTCTATGCCACCACAGCCAGAGTAAGTTTATACTTCAGAGTTCAAACCTCAGAGCACTTATTCCGGCTTCGGGAGGCAGCATAGTGTTTCCAGTCTAAGCTGCAGAAGCGCTGGCAGTGTTGTGGCCTGGAGGTGGACTCAGAGGGCGGCGCAGAGCCTTACTTGCGGAGAGGCAAGTCCCGGCTGCTGCGGGCCGAAGCGGGTAGGGGCGCGCTCAGAGCTGCGCGCCTTGCCTGAGCCCTCCTCCCGGCTTTAGCGCTCACAAGGCGGCGCCGCCAGCGAACCGCGGAGCCCGGTGGGCCCACGGGCGACCAGGCACTGGCTGGAGTACAGGGCTGTTCCCGAGCTGCATCTGTAATCCTTGTGCCTCAATTCTTCCTGAGTGGCGAGGGTTTTCGATTTCAGCGCTTTCGGCACAGCCCTCGGCACCTGCCCAGCCTTCCTCCAGGCCTGGGTCCCCCCCCCACACACGGAGGCGGGGGGTTGCGGGGATTGGGAGGGACTGGCCGGGTTCACC
MSTRG.10342.1	ENSRNOG00000014673;ENSRNOG00000014726;ENSRNOG0000001	0.000	0.803	0.000	7.087	up	CAGCAAAGCGTTTATTACATCTTGAAGAGGAGAGACCGAGCCCCAGAATGGCGCTGCTTATATACACCCTAATGTGGTGTGTCCACACCTGATTGGTTGCTTACCCATGATCTCATTAGGCACGCCCCGGAGTGGGCAAAGACCTGGCACGAAGGCACTCTTGCACATGCGCACACAGTTAACTTCCTGAAAGGAAGTCGGCTGGTGCGGCGGAGGCCAGTGCCATCTTGTAATGGCGAAAGCTATCCCAGCTTTCCACGTGGGGCACAGTGGAAGCCAGCACCATCTTGTGGTGGCGAATGTTATTGCGGCCCTCTACATCTCCCCCTTATTATTTTATTAATTAATAACAATAAATTATTGTCTAGGCTGGTCTAGGTCCCTGCAGTTACGTCCATCTGCTGTGGCTCCTGTCTTAGGTCGTTCCTCTATGTCAGAGCCTTATCGAAGGATAACCCTATCACCACCGGGCCCCGTGTACTGTGCATGAGGAAAGTTGCCCGTCTCTGGATACCACAGTGCTGTGTGACAGTAACTGAATGACCTAGGGCGAACTCTAAAAAGAGGCCAGCCAGATGATGAGTTAGTGGGGGAATCATGATCACCCTATCACATGCAATGAGGAAACCTCAGCGATGTGGAAGGGCTGATCAAAGAATCATTGAGTCTCTCTCATCCCAGTGCAATGGATCC

	4946;ENSRNOG00000032246						ACAGATCCATGGGGTGCAAGAGCAGAGAACTCAGATACTGACTAATTTTTGAAGATAGATCACCAAATTTTAGGGGAGGCCCCTTGTTCAATGGCCACAAGTGCTTGAGTGATAACGACCTTGTCACGTTTCTGTTGAGATCTGAGTTTGCAAACCAACCATAGCATGAACACTAATCCACAGCATAGGGCTGCACCAAACAGAGCCACTCCCGATAAGTAGTGGGCACCTCATTTGGTTCACCTGAGCTGTTACCACCAAGGGCCGTAGTCAAGCCGCTCCTATAATAGAATTTAGAATTCCCAATTGTTAGCAACTACTCCAGAAAGTTCACCCACTGTGCTTGCCTAACAATAGATTGGGGGAGGGTAACTCTAGACACTGCAGACACAATGGCTGCAGCAGTAATGGCTGCTACAATAGCAGCGAAAGTGTCAAGGTCTTTTCCAGGACAGTTCAGGATCAGTCATTTTTCTCTGGAACAACCAGCAGACAATGGAACCATTTCTGAGATCTGTACAACCAGTGCAGAGTTCCCCAGTCCACAGCAGCTTTACGCAGGTGGCATTCCTGTGAAGCCACAGTCTGTAAAATGACCAGTTAAGGCAGCAAGAGTCACCAGGGAAATGAGGGGGGCAGCAACGGCTGGAGTCTAGTCTTCTCCAGCAAGCAGCAGTGCACAGAGGGTCAGAGAGCGGGCCATAGTGGGACGGGACACATGCAGCGGTAACACTGACTGCAGCAACGGCAAAATCTCTGTGATGACAAAGGATGAGGGGGAATCTTCCATCTTCCTCTCAGGGCAGAGGAATGACTCCAGGGACCCGAACCACGAGAGCTGAATCTTCATGCAACCAGGATCAGCAAGTCTCACCAACCACCTCTGGCAGCTGGCACACTCTTGTAGCATCCTGTAGTGTTGGGAGCAGCCTTGAGATGGCGAAAGCAGCCTTCAAGTTAAAAAGCAATTATGTTCTAACCTTATGCTGGAATAATAAGCAATATTCTTCAGGTTAAAAAGCAATTATATTTTAGCCTTATGATAGAACAAAAAACAATAGCCTTAGGTTAGATCAAAGCAGCACCTGCAGCTCTACATAAATTCAATAGATAGCATAAATCTAGGTGTCAGATCACTGAGTACTGGTGGTCAGGCACTGACGGTCAGGTCACTAAGCAACCCACCCTGCTGTTCC
MSTRG.103447.1	ENSRNOG00000015415;ENSRNOG00000015566	0.874	0.000	0.000	-4.918	down	GGTGATGGAGCAGCTACAGCAGCAGCAGCAGGCTTTGCCTCGCCAGGTAGCGGAGCGGGCTCTGGGCTGCGGCTATGCCGGCTTGGGTATGCCAGGTCTGGCCGAGAGGTGGCGAGCACCACGTGGCTGCCTTTAAAGAGACCGTTCACTTCTTGACCCGAATGCAGCCGAGTCCATTCATCACTTCTCGCTCTTCTTGGGATGTGATGGATTAGCTCTGCTTCCCAGCTCTGAGTCACCCTCGATTCTCAGAAGCTTGTCATGATCTAAGCTAGACCTTCCTCTCCAGTCATGGGCTCGGCTGGGTTCTGTGACGCTCAGTGAAACAGAAGACCGTCTTGGGACCACACTGTCAGGACATATCAGCCTGCCATTTGTGTCCACAGTGACCTCTCATCTTCCAACCAGTTCAATGCATCCATCCCCACTTAAGGCTGTGTGTGATAGGCACTTATAACTTGCAAATGGTGACACTGGCATTTCTCATTAAAGTTTCATCGGTGGAAATGCTTGCTGTGGGTCAGTTCTCAGGATCGTTTATTTGATTCGGGTTCCTGGCATGAGACCAAGGCAAAGACTAGGGGAGCAAATTGTCACAGGCCCATCTATTCCCCTGTCTCAGACAGAG
MSTRG.103483.2	ENSRNOG00000015059;ENSRNOG00000028504	0.557	0.000	0.002	-4.531	down	CTACACACACACACACACACACACACACACACACACACACACACACACACACACACACACACTGCACAGCACTGCTGCCTTTCATATCCATATGTAGGCTAAGTAATTCAGTACCTCAAAACCATGTTTTAGAGGGAAGTTATTGTGTGATGCCATGGTAAAAATTAAGTCCCTCAAATACTCCTTTAGAAGCAATTAAAACAAGCTTAAAAATAAATAAAATGAATAAAATCATGATTTCAATGTAGAACTATCAATAAGCAAAACCAAAAATAATTAAATGTGACAAGCTCCAGACTGCCACACCCAAAAAATAATCATCTTGTTCTTTTACTTATATGGCTATTTAGTTTTTACACAGGTCACCAGAGACTAAGTTTCAAGTCCACATCAGAAGGAGTATTTGTTAAAACAAAACTAAACCCATGATTTAACAGAAAATGTGACAGACTCCAGCTTCAAAGGAGAAAGACAGTAGTCACCCCAGGAATACAGACTACTACCATGGTTTCTCATGGCTCTGTAAGATAGGGAAATGGGACAGGGCTTGGAGATGAGCCTTCGGTCTCAAACTGCATACTAAACATGGGATGGAGTAAGGCTGATGGCTAGATAACCACCTTAGAGAAATGAACTAGAAAAGACTTCTAATCTACCCACAAGCAGCTAATGCTGACTTCCATTTAGAGTAGTCATTGCTTCCCCCATGGCGGCCCATTGGGAAGCTGATATTGCAACATTACAGTT
MSTR	ENSRNO	0.356	0.000	0.017	-	do	CTACACACACACACACACACACACACACACACACACACACACACACACACACACACACACTGCACAGCACTGC

G.103483.3	G00000015059;ENSRNOG00000028504				3.928	wn	TGCCTTTCATATCCATATGTAGGCTAAGTAATTCAGTACCTCAAAACCATGTTTTAGAGGGAAGTTATTGTGTGATGCCATGGTAAAAATTAAGTCCCTCAAATACTCCTTTAGAAGCAATTAAAACAAGCTTAAAAATAAATAAAATGAATAAAATCATGATTTCAATGTAGAACTATCAATAAGCAAAACCAAAAATAATTAAATGTGACAAGCTCCAGACTGCCACACCCAAAAAATAATCATCTTGTTCTTTTACTTATATGGCTATTTAGTTTTTACACAGGTCACCAGAGACTAAGTTTCAAGTCCACATCAGAAGGAGTATTTGTTAAAACAAAACTAAACCCATGATTTAACAGAAAATGTGACAGACTCCAGCTTCAAAGGAGAAAGACAGTAGTCACCCCAGGAATACAGACTACTACCATGGTTTCTCATGGCTCTGTAAGATAGGGAAATGGGACAGGGCTTGGAGATGAGCCTTCGGTCTCAAACTGCATACTAAACATGGGATGGAGTAAGGCTGATGGCTAGATAACCACCTTAGAGAAATGAACTAGAAAAGACTTCTAATCTACCCACAAGCAGCTAATGCTGACTTCCATTTAGAGTAGTCATTGCTTCCCCCATGGCGGCCCATTGGGAAGCTGATATTGCAACATTACAGTT
MSTRG.103489.3	--	0.000	2.267	0.000	6.636	up	TTAGGACGTGATGCTGTATGCCTGGGGAACAGCATACTGACCACATCTGCCCAGAGCAGGACCCAACTGGGAAGATACGCGGCTTTTGGCTCTCCCTCCACCGTCACTGAAGTCTCCACTTGGCAAAGCAGACTGTCTTTACTTTTCAGGTAGCATGAAATAACATCAAGAAGTTAGAGTGGCATCTAGAGTCAGGTAGCATGAAATAACATCAAGAAGTTAGAGTGGCATCTAGAGTCAGGTTCCCGGGAGCTTGAGATACCCCACCACCCTTCGGAAGGTTCGAGCTCTTGTCCAACAGGTTTAAACACACATTGACACTGCCCCACTTCTCTCCGTATTCCTTGTGGCTTCCCCCTACCCCAGTCTAACAGACTGCCACAGGCTTTACCAGCGCTGCAGCAGTAGACAGAAATAAGCTGCTTCTCATTACCAGAATGTCCATCACATTAGCAAGGGTCACGTGTGCATACAGGAAAGGGCC
MSTRG.104018.1	--	0.096	0.591	0.001	2.990	up	GCTTGGCATGTGTGAGGCTCTGGGTTCAAACCCCAGCGCTACAAAATCAAAATTAAATCAAATCAAGTAAACACAGAAGTAGCAAAACTTAAAATAGGAATATGCTTGGAATTTAAATTCAGCACAACGCAGAGAGGGCTTTCCTACTCTTTGGCTTTTCTTCCTATCTATAAATAACATAAGGAATTCAAACAACTCCATCTTGAGAAAACAAACAGCCCAATTAAAAGGAGCAAAGGGCCTGGGATGCAGCCTAGTGGTAGAGCCACTTGCCTGGCAGGCCCAGTCCCAGCAATGTACAGAGAAGAAAAGCCACTCAAAAGATGTGAATAGCATATCTCAAAGGAAGACATGTGACCGCTAACACCAGTGCTCCGCTCATCAGTAATCGCTGGTGGTGGGAGGGGGGGTGGAGATGGTAAAACCCCACTGAGACATCACTTCACACCCGATTCCTGGAACCCACCTTTTCCTGTGGTCTTGTCTTGTCCTCTGGTGGTCTTTGGAGAGGAGATCGGATCTTGAAGGTTCTGCTGCTCTGAGTGGCTTCATCCTCTCTGTGACAGGTTCCGAATCCAAAGAGATTGCTTGGAGACACTGGAACTGTGAAAGCGGCTTTAGCTTTTCCCAGACAGTCACCAGGAGTGCCAAGATACCAAGTCAAGCCGCCAGTCACTTGTGCATGTGAGTGGGACCAAGTCCCTTTCCATTGCAGAGGAGACACTGAGTAGCGTTTCGTGTACTTCCTCTCACACCAGGTCACAGTGGCCCAGGCCTTGGAGCCTGAAAACTGTTACTTCATCCAAGAAAAGGCTGACCAGTCTCTATTAGGTTCCCCGAAGACCACCGATGAGTTCTGATTTTTGGTTTCTTGCTCCTCTACCCTACCAGCTTTGGCAGGAATCTGCAGTGAAGTAGGCTGATCTGGGGCGCGGCTCCTTTAAAGGAGCC
MSTRG.104026.1	--	0.386	0.012	0.001	-3.738	down	ATTTTAGCTTATGAAAACCACACTGTAGTTACCCTTAATAACACAATTATGGGATTGCAAGGAAGGAGCCCATAATTGCATGTAAAAATTAAAGTTTAAAGGATTTAGTTTCATTTCTCTGTTAGAATCATTCATAGGTCTTACAAGTTGCGGGGTGAGTCCCTGGCTCATTCTAGAGAGCTCCCACTGCCCACTAGTAGCGGGTAGCCAGACTTTAGGTATGCACATTTAAAAGTCACTGTCCTGATTCGTAAAATGGAGATAATTTTACATACATAACAGGGTCATTCTGGGATGCAGAAGACACTGTGCTATAGAATGTCTAATGCCTTAACTGGTAAAAAGAAAGTGGTCCATGGGTGGCATTATTAGCACAGTGACCATTATCATTGAGCATAGATGGGTTATGTAGTGACTTGCTGGTACTAAAGGGCAAGCTCGGGTCTCAGGAGCCTGAAGGATGCAGGTAGGAGGGTGATCATATCTCTCCTATTTGTTCCTCACTGTTTTCTCATTCACTGTGTCACAGTGGCAGATGGACATCTGTTGCTGGAGGAC

							GTAGGACACTCTGGGGAAGGATATTTCTGTTTTTTTTAAATCCTGTTTATTTAAAACAATATTTCTATTATTTTGTTGAGCATTTCATACACTCTATTTTGATCCTATTCCCCTCTCATATTCTCCAAGATCCACCTCTATCTCCTCCTCATTCGATTCCACAGCTCCTTTTCTGTCCTTTGTACTGAGTCCAGTGTGTGCTTGAGTCCAGTGCGTGCTGCCCACACCCACTTGGGTGTAGGGCCATCTCCTGCAACATGATTGACTAACCAGAGGCTACGCCCTTTATGAAAACTGACTCTCCCTCTCCTGGCAGTATCAGCTTCTAATGGCTCCACAGTCGGGGTAACAGAAAGGACAGAGAAGGGGGTTCACTCAGCTCACACGGAGGCACACTTGTGTACACGCGCGCATGCGCACACATACACACATGCTTCTCAGCTACCGCATCTCCTCCTTTCCAGATTGTCCATCTGTAGAACTTGACACTATTGTCACTATTATGTATCCCAGAAAGCACCACTCAATTTTCTCACTTGTCACTTTGGAGGACACTTTGGGGTTTCTTTCCTTTCTTCTCTCCCTTAGTTGCCCACATTTTAAGAGGAAGCCAAACAAGAGAAGGCTCGACCACGCTGATCTGAGAAACAGAAAGTGCAAGATACAAGAGCTCTGCCTGCTGCTCAGTGGCAAGCTTGACTGGCTGCTAAAACTCTCTTGGGAACAAGGAAAATGCCACAGGAGAGGATTTCTAGAGTTCTGTGGCATCTTTAACAAGTCTGATGCAGATAGAAGCAACCTGAAATACTCTCGAGGGACACTTGGAGCATTTAGTAAATAATGATGAAGGAAGAGGAGCATCTGTGTGTGCACATATATGTGCATATGTGTTAGATTGCATTTAACTGTACCCATCGACGTGCAACTAGTGGCTGCCTTATTGGACAGCACCAAGGAAAAGACTGCTAATGGTGTGAAATGCCACAGAACAAGGAAGAACATGACAGAATTTTCATGGGACTCATCAGACTGAGGAGGATGGCTTCGGAAGGATGCAGACGAATCGTTGCCTCCCACAGTTCCCTCTCAACTTTGCCCTGCTGGGCTCTCTCCATTCAATTATGTTTCTCCAGCTTCCAATTTTTTTTCTACCACCAACCCACTCAGTCATTTTCTGCCAATGGCAGGCTCTGGAAAGCAGTTCCCTCAGGCCTCCCAGTGACAGGTGCCTGGGCTGTTCTAGTGCTGTTTACAGGAAGTACTTGAACTATTCTAGCCACAAAGGGTGAGCTCTTTGCATTTTTTCCCAAGTCAGCTAAGGAACAGGAAGCCATGTTCAATTGTTGCTTGCCCAAGAGTGATGCTCATAGCCCTCTCCTGGTTGGTGTTTTTCCGAACAATGGAAATAAATCTGATGTAATGTAGCAGAACACGACAAAGGACGGCTTTCCCCTTAACAAATCCTCATTTGAAATGGTGCCCAAACCCTGCACGAGGAGCGATCTATTTCCC
MSTRG.10413.1	ENSRNOG00000016292;ENSRNOG00000036742;ENSRNOG00000055297;ENSRNOG00000061899	0.019	0.286	0.002	3.620	up	ACCTAACTACCACACCTACCTACTACACCTACCTACTACACCTACCTACTACACCTAACCACTACACCTACCTACTACACCTACCTACTACACCTAACCACTACACCTACCTACTACACCTAACCACTACACCTAACCACTACACCTACCTACTACACCTAACCACTACACCTACCTACTACACCTAACCACTACACCTAACCACTACACCTACCTACTACACCTAACCACTACACCTACCTACTACACCTACCTACTACACCTAACCACTACACCTAACCACTACATCTACCTATTACACCTAACTACTACACCTAACTCGAGGTTTTTTTGTTTTTTGTTTTGTTTTAAGTTCCACACTTCTTTATGTTTTTAGAAACAGGGTCTCATGTAGCCTTGCTTGATCTTAGACTTAACTATAGCTAAGCTTGACTTTAAACTCTTATTTACAAACCTCACCCACGAAACAAGTTTCTCTGTGTAGCACTGGCTGTCTTGAAACTCAGCTCTGTAGACCAGGCTGGCCTGGAACTCAGAGATCCACCTGCTTCTGTCTCCAGTGCTGGGATTAAAGGCGTGCACCACCACTGCCCAGTTCTTAACAAATTCTTTTAAAAAAATTTTTTAAATTATGTGTATTGTAGGAATGTACATGAGTGCAGGTGCCTGTCAAGATCAAAGGTTCGAGATCCCTCTGAAACTGGAGTTCATGGTGGTATAAGACACCCAACATGGGCGCTTGAATTCTATGGCAGCATGGGGAAAAGACAAAGACTTGTCAGAAAGC
MSTRG.104229.2	--	0.000	0.352	0.000	5.898	up	GCAGGAATAAAAACTATTCTCTAGATTTCCCCATCTCCTTTGTGTAGTTCCTTTGCAGATCACTCTGTCCTCACACATAGACCTCAGACCCCTTAAGAGCATACATAGGCAGAGCTGTCACAGACATACATTGATATACACACACACACACTGGGCACTATGGAGGCTTCTTGACCACAGTTACCTATGGTGAGGCACAGAAAGTAAAAGCAAAGAGATCAGAAAGACAGGCACAGGACCAAGACAGGAGAAACTCAAAAGGTTTATCTTTACTTCATCTTTTTCTACTGTGCTTGGTACTATGTTCTTCCGTCAACTACAGTTGGCATGGTCTGCCTACCTAGCCTTACTGTAAGGTTACAAAAGGGAAGCCTGTCTTTCTTTCCAGTGCCTTATCCATAAGAACTACTAAATAG

							TCCTGTGTCAGAATACTATAGAAAGACTGATCATGAGGATTCTATTCTCTCCTTCCCTCAGACAGGGCCTCGTCCTGTACCCAAGCCGTCGTTAGAATGCAGCTTGATGTGAGGCGTCCTCTCGCCTCTGCCGTCCTAGCGCTAGGTTTACTTGGATGTGCCACAGCGCCTAGCTCTAAAGTTCTTTCCCCTCCTCCACCTTGGGGTTTATGTTTTATATTTGTTTTTGTTGTGACATGATCTCATGTATCCCAGTCTGTCATTTAACTCACTGTATGGCCAAGATAGCCTTGAACTCCTTGTCCTCCAGTCTCAATCTCCCTAGTGCCAGAATTACAGTCATGAGCTACCATGCCTGCTGTATGTCATGCAGGAGATCAAACCAAGGGCTTCACGCAACGAGGCAAGCACCCTGCCAACCAATTTATATCCCGGCCCCTGGAGTTCTTTTCTGAAAGGCCTCAGGTCCTGAAGTGATTTCCGGTTCCCTGAGGAAAGGGCATGTATGTGCAGCCCTGAAGGAAACCACAAGCAGCTTGAAAGCAAGTGGACTGGGGTGGCGTCAGCTTGGTTACTGGTGAAAGAGACCGGAAAGAAGAAAAGCACCGTGGGCAGGCGGAGCCATTAGGATGCTAAAACAGTGGGTCTCAAACAGACCCCTTGGGATGCATTTTGGGTGTCCCCACTCCCACCAAAGCGTTGAAATGTGGATTTCAGGTGGGGCCTAAAATGTCCAGCACTGACAAGTTGCCAGGTGATACTGATGTTGATTTGATGGTCCACATTCCACACCTCAGGAACAGCTGCCATAGGAATACAGACCTAAACATACGGGAATACTGGGAACGAGCTAGAATGATAATTCGGGTTTGGTTAAGCTTTCTCTTTTGGTTTACCTTAAGTAGCGGTAGGCTGGGCAGGCCCTCCAGGCAAGCCTCTTGACCTATATTGAAGAAGTAGAAGATGAACTAAATGGTCTCTAAGGTTCTCTGGAAGTCATCAGTATGTCAATATGTGGTTGGGACCGAGGACTGCCATTTACTGTATCCACTGGGTCTCTTCTGGAGAATCAGCTCATTGATCTCCAGACCTATGCTTGGCCAGGTTTGCCCGCTTTCTCCTGTCCTCTCTTCCCAGTGTATGTGGTGACGGACACTAATGTCTTTTTCTCTTCTGCCTCTTCCCATCTGCACACCTCTGTTCCATATGGAAGAGATAAAAAGAAGTGCTATTTATGGAGTCCCTACTGTGTGCCAGCACCACACTGGGGATGCCTAAAGACAGCCAAGAGCTTGCCCTGGTAGTTTTAACTAAAGACAGTGCTCTTTTCTCTCTGCAGCTGGTCTTCTGGCCCGTCTGTCTGCCTCTGTCTGAGTTCTCTTTGCTCTGGGGAGGGATTGCTGTCCTCAGCTCACCTTGTCTGGCATTCTCTGTACCTGTTTTGGC
MSTRG.104229.3	--	1.288	0.052	0.000	-3.806	down	CTCCGTGGCAGGGTGCAGTGGCCCAAGTCTGGGTCCTGTTTACCTCGGGGAGCATGTTCTGACTGTAAACCCAAGCTTTTTTCTTTTTTTTTAAATCTTTGCTTGTTTAAATATGCATGATTAGCCAGACTATCATTGCATTTTCATGTTCTTTTATTTTCTCCTGGTTTTGTGTCTTAAGTGACCTTAATTGCCTGAAACAACTTTCTAGGTGGTGGGGGTGACCTCAGGTCCAAGTAATGTGTTCTGAATGACTTTGCATGAAAGCGGAGAGTTCCTTGGATAACTGTAGATATCCTATGGTATCGAACAGACTCTGTCTCCACCACCCTTCCTGACAAAATTAGTGATTTTTCTCTGTTCCGAGCCCTTGGGGGCTTGGAAAGCAGGTGCCTCAGGGTGTTTTGAATGGCGTGCGTATTAGTTAATTAATGATGTTTAGGGACACTTAGTGTTTGTTTTGCTTCATAGCTGTTTGCCTACATAAGAAAATCAAAGACTTGTCCTTAATAATAACCTTGGGTAAACTAAGTTCAAGAGAGAAATATAGGGGTTGGGGATTTAGCTCAGTGGTAGAGCGCTTGCCTAGAAAGCGCAAGGCCCTGGGTTCGGTCCCCAGCTCTGAAAAAAAGAACCAAAAAAAAAAAAAAAAGAGAGAGAAATATAAAATGCCAATTTTATAAGGACGTATAAAACCTTTAATTTAAAAATGACTCGTCTTTGGGCCTAGAGAAATAGCTCAGCAGTTAAGAACACTGGCCACTCTTCCCGAGGACTGAGGTTTGATCCCTGACATGCACATAGCGCCCTCATCAGTTTGTAACCCCAGTCCCGGGGATTCCAATGCCCTCTGGCTACCCAGAGTACTGCATGCACATGGAGCACACACATGCGGGCTGTATACGCCTATATGCATAAAGCATTAGAAACATTTACCCACCTTTCTCAGAAATATGGATTAATGTATTAGATTTTATTGATGACTTAATTAATAAAATTAAGGTGTTAAAGATTTTATAATATGAAGAGCTTCAGCTGGGAGAGCACCAGTACTGAGCGGCAGAGGCAAAAGCAATGCTGAGAGACTGTCGGTGGGAGAAGAAGCCTGGCTAACAGCCTGGGAGTCACATGGACTGTAATGTAGGAAGTGTGCTTGGGTTTGTTCTTGCTTTGTTTGGTTGGCCTGTTTCCCTGACTCATAGAGAGTTTTTTTTTTTTTAATTGAATGTCACTTCCCCATCTTAGGCACATTTACCTGAGCAAAACAGGAAGACGGAAAATCAAACCCAGCTCTCAAAACATTTTTACCAGGAAGTGACAACTTAGTCACTTCCATTCCCTTCATTGATCAAAGCGAGTCGTATGGCCACAGTTGACTGAAAGAGGAAG

							AGTGAGTGACTGGGTTCCTACCCACATCTCTGACAGTCTCTTTCCGTCATGCATCGTGGCCAGCTACTG<br>AGACAGGTGTCATTTCATCTGTAGGCACCACTGACCGCCAGTGCCCTCCTCCTCTGCGTTCCTGTTCCC<br>GGAAGCTCACCGCAGAGTGACAGCACTTTCTAGGAGTTCAGTCCTAAGCCTCCTCACTAGCACTTGCT<br>GATGTCTCTGAGTGTGGCTGGGATCCATGATTTGGAGGGACCCTCCTCCCTGGCAGGGAAGAGGGCAG<br>AGGGCAGCAGCAGAACTTATGTGAATGTTACATTCTCTTCAAATGTTATGAAATAACATGAAATCTGTGA<br>AATAGGAACTGCCAGCCTATGAGACTAATTCAATGCTGTCCTAGCTGTGGTGGACATGACTCTACCACTT<br>GGGAAGTAGAAGAAGAAAGATCAGGAGTTCGAGGTCATCCTAGGTTAAGTGGAAAGTTCAAGGACAG<br>CATGGGCTATATGAGATGCTGGCTCAAACACTTAACTGTCTGAATAAACAGCTATCCTTGAGCAGACATG<br>ACTCAAGCACAACTTACTTGGCCAGTGACCTTTCATTCTATGTGAGATCACCATCTCTTGAGATCGAAAA<br>ATGAATTTTCCACGGCCAGCGGCCTGAGCACACGTCCTGGTCCTTACAGTCTGACAGGATTGTGTCTAT<br>CTAGATGGGGAAACCTCCTGTCCACCCCACAGGGTTACGCTGGACTCGCCCACGGTCACATTTGTAAGA<br>TGAAAAGATTGCAAGATTGTAAGTCCTTAGACCCAAACCTTCTGTAGCCCGTGCCAGTGTTGAGATGAA<br>GACAGAACCTTTCAAATGAACAGTTACTTTGGGGTGAGCGGTGGGACAGAGAGGCCAAAAGATGTCA<br>GGTCCCCGGAACTGGAGTCACAGGTTAGTTGTGACCCACCCGATGTGTCCTCTGGAAGAACAGCTCTT<br>AACCACTGAGCCATCTCTCTAACCCCTACAAGGGTTAATGTAACTACTATTAAGATTCTGCTCAGAGAAC<br>ACTCCAGACACTAAATATGTGGGGGTTTGCCCCAAACAGTTTTGCAGTTCACTCCAGACACCAGGTGCT<br>GACATTTAACTGTTTATGACACTAACTGGATGTAACACCAAAGCTTGGGTTAAGGGCTCACCTCACAAG<br>ACTGACCCTGACTTAAGATACCAATTGTAAGTACTAGGGTTCCAGTTGCCGGCACTTCTGTCTAATTTGA<br>CTGAAAATTATTTCATCTTCCTCAAGTTTGATCATTTGCAATAATGGCTTGCACCACTGAGAGAACCTCT<br>TCTTCGTTCTTACCAATTTAAATTTTTTTTTATTTATTATATCTAAGTACACTGTAGCTGTCTTCAGACAC<br>ACCAGAAGAGGGCATCAGATCCCATTACAGATGGTTGTGAGCCACCATGTGGTTGCTGGGATTTGAACT<br>CAGGACCTCAGGAATAGCAGTCGGTGCTCTAACCACTGAGCCATCTCTCCAGCCCCGTTCTTACCAATT<br>TAATCTGAAGGATGTCGTAAAAGATGCAAACGTATGGGTAGATGAAGAGGTGCGTAAGACAAGCTCTA<br>GAAGGTGCCCCGTACAGGAGTTCTCTCCCTGCACTGGGGACACCGCCCCCTCCCCCGCCTCCAGCACA<br>TAAATGTGTTCACTCACCGCTCCATTCAGGATGATTAGGTACTGCTTCACAGGGACAATTGATCCAGTCA<br>CTGGCCATTGGTAATTAACTTCATCTCTATTCCTGTTCCTCAGAGGTCTGGAGAGGGCAGTGCGGCCTAA<br>CACTGAATCATGCCATGGGCTTTCTGGCAAACAGCTCCTTTTCTGAAAGCAGCCTTAGCATACATATACT<br>CTTAATTTTCTGGTGAGTCTAAAGGTTTTTAGAAGGTCCTGTGACTGAACCTAGGACAGAAGACCAAAC<br>TCATCCTTGTTATATCACAGTATTATAGTTATTTTGACTTTTCAAGAACTTGAGAGTAATGGGTAGTAGTA<br>CTGTTATTTCCAACTTTTTATGTGGGAGGTCAGAGGTGTGGCCATCCTTAACATATATGAGGCCTTGTGTT<br>CCATTCCAAGCACTGAAAATAAGTGAGTAAATGGATATGCACATCTCGCTGAGGCTAGGTCTTGCCTCA<br>GCCTCAGCCACTCTTCTCAGCACCTTGACTGATCGTCCCTAAGAGTTCTCTGTCTCTCGTGCCCAACTCC<br>CTCTCCTTCTCTTTCCTCAGATACAAAGGTTCTAATTAAGGGAGAACCGCAAGGCTTCTTCAGATCAGA<br>CTGCTCACCCTACGGTTTACCCTGTAGTTTAAGAAGCTCATTTGGCTGGGTCGGCCAATTTAGGGTTAAT<br>CGTGCCCAGAATCACCATGAGTCTGTAATTAAAGGACCTTGCCAGCTCTCATCACTGTTGTATTTCCAGG<br>CTCTGGCCCAGCCTGCTGCACTGCAGATACTCAATAAATATTTGCTGAGGGAAAACCACCATTAACCAG<br>AAGACTGCACAAGTTAATCATTGTATTAATTGTGAGGAACATGACCAGTTCAAGTGCCTGTAGCAGCTA<br>CTCTCTGGTCCAGCCACTCTCAAGTAATAGGCAGATCCATTCAGGAGACTAAGTTGTGACTCAGAAATC<br>CTCTTTACAACAAGGAATCAGAAAAACGAATTTCAGCTTTGTGTGGTGACACACACCTTTAATCCCAGC<br>ACTCAGGAGGCAGAGGCAGGTGGAGCTCCATGAGTTAGAGGCCAGCCTGGTCTACAGAGCAAGTTTC<br>AGGACACCCAGGAGTATATAGAGAGACTCTATAAATAGACAGATAGGTAGGTGGATAGATAGATGGATG

							GATGAAGGGTGGGTGGATGGATAGATAGATAGATAGATAGATAGATAGATAGATAGATAGATAGCAGTGA CTATGTTCACCTAGACACTTGACTCCAGAACTGCCACAGTCCAGATCATTCCTAGTCTGGAAAGACTTA ACATCTCAGTTCCCTTAGGAGACATCTCAGACCTGCGTTTCTTCCCCCCCCCCACCCCCCCCCACTGACT CAGCATATCACATCCTATCACTTATGGGTCTCGTAAGGCTCAATAAGATAAGCTGTCCATGACCAGGGGT CCAGCTGTCCATGAAGGATTTCTATGCTCCGCTCATACAGCTCCCATGTTTCTCTCCCCATAACAGAAAC CTGGCTCTACTTCCAACACAGCCAGGAGTTACACTGACTCACAGAGCTCTGCTGCCCGCAGCAGAGGT GATGGGCTCAGTCTCTGAGAGGATAAAAAAGAAGCCAACACAAATTCCATTCTCAAATAGTAAGGACG TCATTCTCTGGTTAATAGGAATGTAACTTTGTTTGACACAATGCCTAAAATATGGAAATACAAGCCAGAT CGTTGTCATCCTGAGCCTTAACTACACTCCTGCCCTCCTAAGTCTGCAAAGGCATAGCTTTTGGTGAATT GATGAGTTGATGTACTTCAAGACAACCCACAGCCTTTACCCTGGGTCCCAGCCGCTGCACACATGCACA GACTGTCGGCTGGATCCAGTGGCCTGCAGATGGCGTGTGGAAGTCTACCCAGGGTGCTTATCAGTCTTG CTCCCATACCCACTGCAGGACCCACGGCAGTTCCTTGTTTTGGTTTTGGTTTCATTTCGTAAAAAGAGG AAATATAATTATGTTCTCATAGAGCTGCTTGAACACCAGCAAGAAGTTTTCGGTGAGAGTGATTAGGAA GGAGAAATAGTTCTTGGACCTGAGGGGCGTTCCCTTGTTTTCATCACTCCATAAGAGACAAGCTAAGAG GCTTTCTTCTATCTTATTTCCATCCTAGCCTGTATTTCTGTATTTTGTCCTCCCGACCGTATTGTATGTGATA TGAAAACTGTCCCCCAGGCTCTCCAGGAAACTCTTCACAAGCCAGACTAGAGAAGTCCTCCCTACCTC CGTGGGAAGAGCGTTGAATTCTAGGGCACACTCTCTCCAGAATGCTACCTACCATCCAGATGTCATTTTT AAAAATCTAAAGGCAGAGTGAGCCATGGCTTCTAGGTTTAGAACCCCAGAGACTGGGAAAAATAAAGG TCCCTGCCAAGAGAACAGTGGAGACAGAGTATTAGGTGTAATCTGGTTGTCATGTTCTTTTTCATGTCAC TAACCAAGCAGTCAAAGACCTACTAGGTACCTAGAACTGTGTGCAACTAAAACTCCAGTGGGAAAGCT GGGCTATCCAGGTAAACTTGGATGTGGAATTACCACCGGCTCTAAGGTGCAGAGAGCACCGTGCTCTG GTCCCCCAGAAGACAGAAGAAGGGCGTATGCTCTCAGCCACAAAGTCATTCTTCATCCCGCAGGAGGA GGGTTATTGAACAAGGTATTGTCTTCCAAATGGAATCTCAGTCTAGAGTATGGTCAGTAAGCCTGAGCTC CCACCCACCCTCTCACTGACCTAGCTTTGCAATACAACCACGTCCTCCAGTCCTATCCAGTTTTGCTTCT TGTGTGCAACTTGAGGATCTGGTACTGTCCACAGGACAGTTTTCTCTTAGTGAGGTTTCTGACCATTTTT CTGAGAATTGGTTTTGAAATCCTACACTGGGGGACCCAGTAGAGAGACCATCTTACATAGGGGTAGCCA ACCCAGGGAAAGACTGGAGGAATTCTAGCCTTAGAATGAATGCATGAACTTTCTCAACTAAAAATTTAA ATACCAATTTAAATATCTTTTGTTCTTTGAGTTTATTGTAAGTCATAGTAAGTTTGTGCTTTGTTCCAGAA ACTCAATATGCTTTCTTCCGTCCACCAAGAAAGTTAAGTAAACACCTTGCCTTCTACCCTTTTGAATCCA GAAGTCAGTCCTAGCTTCTACGAACCAGGGTGTGATTCAGACAGAGACAAAATCGTGATGATTGAAAG CCAAATACGCACTATTACCGCCTGCCAACACTATTGATTCACTTACAGAATATCGGCAGTCCCAGAAGAC CCCACCAGCCTTCGTAGTGAGAGACTGGTCATATACCAGTTCTGAGTTTCCATTCCCATCATGGCGCAGG AACAGACAGGGGTCAGTTCTCTGCAGAGGGACCACATGCAGACCTGTTGGGAGCGTTATCCTTCCATC GGATATGCATAGATAGATACATCTTCTCAGAGCAGAGCAACATCATAGAAAGTCTCAGTACTCTAGGTGT CTGGGAGCCAGGTTTGTCTTTTGAAGGTCATGAGGGCCTGTTTTTGCAAATGGACGCTTGCTGAAGCAG GGCGTTCCTGCGTCTCCTTCCCTGCTTCAGTTCAGCCCAGGGGGAGTCTCTCCAGCTGCCTCTCCTACT GAGGCTGGTCCTTCCTCGCCCCTCTCCCTTGTTATGCATGCAGTGTGGCTCCCTTGCCCAGCCCTGACTC CCAGATGAGGTGGTTATTTTCTCCTCCTGCGGCAGGGCCCACCCTCTCTCCTTGGTTAGTGGTTAGTGGC GCAGCTGCAGCCTCCCCGCACAGCGCCAGCACCAGCTCAGAAGGGCTTGCGATGTTTTTACCTGTCTCT GAGGGGGAATGATCCGGGCCCAGTCTCCATACAACTACAGCTGCCTTGCCTACATTTCAGACTTACAGC TCCTTTCTCCCTGGATTTTGTTTTTGTTTTTCTGACAAGGCACCTGACAACCGGCAGCAAAGAGGCCAG

							TTTGTTTAATTTAAATACACTGCGGCAAAGTGAAGGTAATCGTCCACACAGGCCATCTGCTGCCTGGCAGGTATCCATGAGTCAGGAAAGAGATGGAGACTGAAATGTCCTCCGTCCTGTTGCCATGATAACAGCCTGGCAGGGAGGTGGGGGATGTGGGGAGGCAGGGCTTGCTGTCAACATTACACTTTCTACCACAGACCTCTTTGGACTTGACTACTTGATATGTACCAAAAAGATTTAAAAAAAAAATCTTACCTGTTCCATATGGAAGAGATAAAAAGAAGTGCTATTTATGGAGTCCCTACTGTGTGCCAGCACCACACTGGGGATGCCTAAAGACAGCCAAGAGCTTGCCCTGGTAGTTTTAACTAAAGACAGTGCTCTTTTCTCTCTGCAGCTGGTCTTCTGGCCCGTCTGTCTGCCTCTGTCTGAGTTCTCTTTGCTCTGGGGAGGGATTGCTGTCCTCAGCTCACCTTGTCTGGCATTCTCTGTACCTGTTTTGGCTCCAGTGTTTGATCTGTGTTCTAGATAAGGGGCTAAGTCATCATCTGCATTCTCCTCTCCTTACATTCCTTGTGCCTGCTGGAAATGAAATTGATTCTCCTATACAAAAATCACAGTACTGGCCGAGATTTTGGCACAATGGTAGGGGGCCTAGATTCCACTCCTACGAAAGGGAACCAGGGGAAACCAAAGTTAGCTTCAGTCCCGTGAAGGCTTGACTGAGTCACGTTCCTTCTGACCTCAGGACTGTGGTAATAAGATCCCACTAACTCTTCTACTTTTTCTGAGGATTTTTGCTTTGCTCTCGGATTCCCCGGTGCCTCCAGCTACCCACTGTGCTGCTGCTGACTCTGGACCAGTTGCTCTTAACGGAGAGGCAGGCAGTCCCTGTGGAGAGGAATTTCCTTGACCATGTCCTGCTGCTGAGACAAGGTGCTAGGATACTGTACTGCAGTCCCACAGCTGGGTTCTCCTGGCTTCAGCCTGCTAGTACCTGCAAGGAGAGTGCTATACAGCAGGTCGAGGGGGTCACTCACTGTCCCACTGTGCACCACGCACCCAGTGTGCATGCACATGATCAGAAGGCCCTTTCCTCCGACTACCTTTCCAAAGCCAGTCCTGTACCATGCAGGGCAGTCTGTTCTCTGTGCTTCGCTTCTTCAGGAC
MSTRG.104229.4	--	0.528	0.000	0.000	-7.785	down	CTCCGTGGCAGGGTGCAGTGGCCCAAGTCTGGGTCCTGTTTACCTCGGGGAGCATGTTCTGACTGTAAACCCAAGCTTTTTCTTTTTTTTAAATCTTTGCTTGTTTAAATATGCATGATTAGCCAGACTATCATTGCATTTTCATGTTCTTTTATTTTCTCCTGGTTTTGTGTCTTAAGTGACCTTAATTGCCTGAAACAACTTTCTAGGTGGTGGGGGTGACCTCAGGTCCAAGTAATGTGTTCTGAATGACTTTGCATGAAAGCGGAGAGTTCCTTGGATAACTGTAGATATCCTATGGTATCGAACAGACTCTGTCTCCACCACCCTTCCTGACAAAATTAGTGATTTTTCTCTGTTCCGAGCCCTTGGGGGCTTGGAAAGCAGGTGCCTCAGGGTGTTTTGAATGGCGTGCGTATTAGTTAATTAATGATGTTTAGGGACACTTAGTGTTTGTTTTGCTTCATAGCTGTTTGCCTACATAAGAAAATCAAAGACTTGTCCTTAATAATAACCTTGGGTAAACTAAGTTCAAGAGAGAAATATAGGGGTTGGGGATTTAGCTCAGTGGTAGAGCGCTTGCCTAGAAAGCGCAAGGCCCTGGGTTCGGTCCCCAGCTCTGAAAAAAAGAACCAAAAAAAAAAAAAAAAGAGAGAGAAATATAAAATGCCAATTTTATAAGGACGTATAAAACCTTTAATTTAAAAATGACTCGTCTTTGGGCCTAGAGAAATAGCTCAGCAGTTAAGAACACTGGCCACTCTTCCCGAGGACTGAGGTTTGATCCCTGACATGCACATAGCGCCCTCATCAGTTTGTAACCCCAGTCCCGGGGATTCCAATGCCCTCTGGCTACCCAGAGTACTGCATGCACATGGAGCACACACATGCGGGCTGTATACGCCTATATGCATAAAGCATTAGAAACATTTACCCACCTTTCTCAGAAATATGGATTAATGTATTAGATTTTATTGATGACTTAATTAATAAAATTAAGGTGTTAAAGATTTTATAATATGAAGAGCTTCAGCTGGGAGAGCACCAGTACTGAGCGGCAGAGGCAAAAGCAATGCTGAGAGACTGTCGGTGGGAGAAGAAGCCTGGCTAACAGCCTGGGAGTCACATGGACTGTAATGTAGGAAGTGTGCTTGGGTTTGTTCTTGCTTTGTTTGGTTGGCCTGTTTCCCTGACTCATAGAGAGTTTTTTTTTTTTTAATTGAATGTCACTTCCCCATCTTAGGCACATTTACCTGAGCAAAACAGGAAGACGGAAAATCAAACCCAGCTCTCAAAACATTTTTACCAGGAAGTGACAACTTAGTCACTTCCATTCCCTTCATTGATCAAAGCGAGTCGTATGGCCACAGTTGACTGAAAGAGGAAGAGTGAGTGACTGGGTTCCTACCCACATCTCTGACAGTCTCTTTCCGTCATGCATCGTGGCCAGCTACTGAGACAGGTGTCATTTCATCTGTAGGCACCACTGACCGCCAGTGCCCTCCTCCTCTGCGTTCCTGTTCCCGGAAGCTCACCGCAGAGTGACAGCACTTTCTAGGAGTTCAGTCCTAAGCCTCCTCACTAGCACTTGCTGATGTCTCTGAGTGTGGCTGGGATCCATGATTTGGAGGGACCCTCCTCCCTGGCAGGGAAGAGGGCAG

							AGGGCAGCAGCAGAACTTATGTGAATGTTACATTCTCTTCAAATGTTATGAAATAACATGAAATCTGTGA<br>AATAGGAACTGCCAGCCTATGAGACTAATTCAATGCTGTCCTAGCTGTGGTGGACATGACTCTACCACTT<br>GGGAAGTAGAAGAAGAAAGATCAGGAGTTCGAGGTCATCCTAGGTTAAGTGGAAAGTTCAAGGACAG<br>CATGGGCTATATGAGATGCTGGCTCAAACACTTAACTGTCTGAATAAACAGCTATCCTTGAGCAGACATG<br>ACTCAAGCACAACTTACTTGGCCAGTGACCTTTCATTCTATGTGAGATCACCATCTCTTGAGATCGAAAA<br>ATGAATTTTCCACGGCCAGCGGCCTGAGCACACGTCCTGGTCCTTACAGTCTGACAGGATTGTGTCTAT<br>CTAGATGGGGAAACCTCCTGTCCACCCCACAGGGTTACGCTGGACTCGCCCACGGTCACATTTGTAAGA<br>TGAAAAGATTGCAAGATTGTAAGTCCTTAGACCCAAACCTTCTGTAGCCCGTGCCAGTGTTGAGATGAA<br>GACAGAACCTTTCAAATGAACAGTTACTTTGGGGTGAGCGGTGGGACAGAGAGGCCAAAAGATGTCA<br>GGTCCCCGGAACTGGAGTCACAGGTTAGTTGTGACCCACCCGATGTGTCCTCTGGAAGAACAGCTCTT<br>AACCACTGAGCCATCTCTCTAACCCCTACAAGGGTTAATGTAACTACTATTAAGATTCTGCTCAGAGAAC<br>ACTCCAGACACTAAATATGTGGGGGTTTGCCCCAAACAGTTTTGCAGTTCACTCCAGACACCAGGTGCT<br>GACATTTAACTGTTTATGACACTAACTGGATGTAACACCAAAGCTTGGGTTAAGGGCTCACCTCACAAG<br>ACTGACCCTGACTTAAGATACCAATTGTAAGTACTAGGGTTCCAGTTGCCGGCACTTCTGTCTAATTTGA<br>CTGAAAATTATTTCATCTTCCTCAAGTTTGATCATTTGCAATAATGGCTTGCACCACTGAGAGAACCTCT<br>TCTTCGTTCTTACCAATTTAAATTTTTTTTTATTTATTATATCTAAGTACACTGTAGCTGTCTTCAGACAC<br>ACCAGAAGAGGGCATCAGATCCCATTACAGATGGTTGTGAGCCACCATGTGGTTGCTGGGATTTGAACT<br>CAGGACCTCAGGAATAGCAGTCGGTGCTCTAACCACTGAGCCATCTCTCCAGCCCCGTTCTTACCAATT<br>TAATCTGAAGGATGTCGTAAAAGATGCAAACGTATGGGTAGATGAAGAGGTGCGTAAGACAAGCTCTA<br>GAAGGTGCCCCGTACAGGAGTTCTCTCCCTGCACTGGGGACACCGCCCCCTCCCCCGCCTCCAGCACA<br>TAAATGTGTTCACTCACCGCTCCATTCAGGATGATTAGGTACTGCTTCACAGGGACAATTGATCCAGTCA<br>CTGGCCATTGGTAATTAACTTCATCTCTATTCCTGTTCCTCAGAGGTCTGGAGAGGGCAGTGCGGCCTAA<br>CACTGAATCATGCCATGGGCTTTCTGGCAAACAGCTCCTTTTCTGAAAGCAGCCTTAGCATACATATACT<br>CTTAATTTTCTGGTGAGTCTAAAGGTTTTTAGAAGGTCCTGTGACTGAACCTAGGACAGAAGACCAAAC<br>TCATCCTTGTTATATCACAGTATTATAGTTATTTTGACTTTTCAAGAACTTGAGAGTAATGGGTAGTAGTA<br>CTGTTATTTCCAACTTTTTATGTGGGAGGTCAGAGGTGTGGCCATCCTTAACATATATGAGGCCTTGTGTT<br>CCATTCCAAGCACTGAAAATAAGTGAGTAAATGGATATGCACATCTCGCTGAGGCTAGGTCTTGCCTCA<br>GCCTCAGCCACTCTTCTCAGCACCTTGACTGATCGTCCCTAAGAGTTCTCTGTCTCTCGTGCCCAACTCC<br>CTCTCCTTCTCTTTCCTCAGATACAAAGGTTCTAATTAAGGGAGAACCGCAAGGCTTCTTCAGATCAGA<br>CTGCTCACCCTACGGTTTACCCTGTAGTTTAAGAAGCTCATTTGGCTGGGTCGGCCAATTTAGGGTTAAT<br>CGTGCCCAGAATCACCATGAGTCTGTAATTAAAGGACCTTGCCAGCTCTCATCACTGTTGTATTTCCAGG<br>CTCTGGCCCAGCCTGCTGCACTGCAGATACTCAATAAATATTTGCTGAGGGAAAACCACCATTAACCAG<br>AAGACTGCACAAGTTAATCATTGTATTAATTGTGAGGAACATGACCAGTTCAAGTGCCTGTAGCAGCTA<br>CTCTCTGGTCCAGCCACTCTCAAGTAATAGGCAGATCCATTCAGGAGACTAAGTTGTGACTCAGAAATC<br>CTCTTTACAACAAGGAATCAGAAAAACGAATTTCAGCTTTGTGTGGTGACACACACCTTTAATCCCAGC<br>ACTCAGGAGGCAGAGGCAGGTGGAGCTCCATGAGTTAGAGGCCAGCCTGGTCTACAGAGCAAGTTTC<br>AGGACACCCAGGAGTATATAGAGAGACTCTATAAATAGACAGATAGGTAGGTGGATAGATAGATGGATG<br>GATGAAGGGTGGGTGGATGGATAGATAGATAGATAGATAGATAGATAGATAGATAGATAGATAGCAGTGA<br>CTATGTTCACCTAGACACTTGACTCCAGAACTGCCACAGTCCAGATCATTCCTAGTCTGGAAAGACTTA<br>ACATCTCAGTTCCCTTAGGAGACATCTCAGACCTGCGTTTCTTCCCCCCCCCCACCCCCCCCCACTGACT<br>CAGCATATCACATCCTATCACTTATGGGTCTCGTAAGGCTCAATAAGATAAGCTGTCCATGACCAGGGGT

							CCAGCTGTCCATGAAGGATTTCTATGCTCCGCTCATACAGCTCCCATGTTTCTCTCCCCATAACAGAAACCTGGCTCTACTTCCAACACAGCCAGGAGTTACACTGACTCACAGAGCTCTGCTGCCCGCAGCAGAGGTGATGGGCTCAGTCTCTGAGAGGATAAAAAAGAAGCCAACACAAATTCCATTCTCAAATAGTAAGGACGTCATTCTCTGGTTAATAGGAATGTAACTTTGTTTGACACAATGCCTAAAATATGGAAATACAAGCCAGATCGTTGTCATCCTGAGCCTTAACTACACTCCTGCCCTCCTAAGTCTGCAAAGGCATAGCTTTTGGTGAATTGATGAGTTGATGTACTTCAAGACAACCCACAGCCTTTACCCTGGGTCCCAGCCGCTGCACACATGCACAGACTGTCGGCTGGATCCAGTGGCCTGCAGATGGCGTGTGGAAGTCTACCCAGGGTGCTTATCAGTCTTGCTCCCATACCCACTGCAGGACCCACGGCAGTTCCTTGTTTTGGTTTTGGTTTCATTTCGTAAAAAGAGGAAATATAATTATGTTCTCATAGAGCTGCTTGAACACCAGCAAGAAGTTTTCGGTGAGAGTGATTAGGAAGGAGAAATAGTTCTTGGACCTGAGGGGCGTTCCCTTGTTTTCATCACTCCATAAGAGACAAGCTAAGAGGCTTTCTTCTATCTTATTTCCATCCTAGCCTGTATTTCTGTATTTTGTCCTCCCGACCGTATTGTATGTGATATGAAAACTGTCCCCCAGGCTCTCCAGGAAACTCTTCACAAGCCAGACTAGAGAAGTCCTCCCTACCTCCGTGGGAAGAGCGTTGAATTCTAGGGCACACTCTCTCCAGAATGCTACCTACCATCCAGATGTCATTTTTAAAAATCTAAAGGCAGAGTGAGCCATGGCTTCTAGGTTTAGAACCCCAGAGACTGGGAAAAATAAAGGTCCCTGCCAAGAGAACAGTGGAGACAGAGTATTAGGTGTAATCTGGTTGTCATGTTCTTTTTCATGTCACTAACCAAGCAGTCAAAGACCTACTAGGTACCTAGAACTGTGTGCAACTAAAACTCCAGTGGGAAAGCTGGGCTATCCAGGTAAACTTGGATGTGGAATTACCACCGGCTCTAAGGTGCAGAGAGCACCGTGCTCTGGTCCCCCAGAAGACAGAAGAAGGGCGTATGCTCTCAGCCACAAAGTCATTCTTCATCCCGCAGGAGGAGGGTTATTGAACAAGGTATTGTCTTCCAAATGGAATCTCAGTCTAGAGTATGGTCAGTAAGCCTGAGCTCCCACCCACCCTCTCACTGACCTAGCTTTGCAATACAACCACGTCCTCCAGTCCTATCCAGTTTTGCTTCTTGTGTGCAACTTGAGGATCTGGTACTGTCCACAGGACAGTTTTCTCTTAGTGAGGTTTCTGACCATTTTTCTGAGAATTGGTTTTGAAATCCTACACTGGGGGACCCAGTAGAGAGACCATCTTACATAGGGGTAGCCAACCCAGGGAAAGACTGGAGGAATTCTAGCCTTAGAATGAATGCATGAACTTTCTCAACTAAAAATTTAAATACCAATTTAAATATCTTTTGTTCTTTGAGTTTATTGTAAGTCATAGTAAGTTTGTGCTTTGTTCCAGAAACTCAATATGCTTTCTTCCGTCCACCAAGAAAGTTAAGTAAACACCTTGCCTTCTACCCTTTTGAATCCAGAAGTCAGTCCTAGCTTCTACGAACCAGGGTGTGATTCAGACAGAGACAAAATCGTGATGATTGAAAGCCAAATACGCACTATTACCGCCTGCCAACACTATTGATTCACTTACAGAATATCGGCAGTCCCAGAAGACCCCACCAGCCTTCGTAGTGAGAGACTGGTCATATACCAGTTCTGAGTTTCCATTCCCATCATGGCGCAGGAACAGACAGGGGTCAGTTCTCTGCAGAGGGACCACATGCAGACCTGTTGGGAGCGTTATCCTTCCATCGGATATGCATAGATAGATACATCTTCTCAGAGCAGAGCAACATCATAGAAAGTCTCAGTACTCTAGGTGTCTGGGAGCCAGGTTTGTCTTTTGAAGGTCATGAGGGCCTGTCTCTGAGGGGGAATGATCCGGGCCCAGTCTCCATACAACTACAGCTGCCTTGCCTACATTTCAGACTTACAGCTCCTTTCTCCCTGGATTTTGTTTTTGTTTTTCTGACAAGGCACCTGACAACCGGCAGCAAAGAGGCCAGTTTGTTTAATTTAAATACACTGCGGCAAAGTGAAGGTAATCGTCCACACAGGCCATCTGCTGCCTGGCAGGTATCCATGAGTCAGGAAAGAGATGGAGACTGAAATGTCCTCCGTCCTGTTGCCATGATAACAGCCTGGCAGGGAGGTGGGGGATGTGGGGAGGCAGGGCTTGCTGTCAACATTACACTTTCTACCACAGACCTCTTTGGACTTGACTACTTGATATGTACCAAAAAGATTTAAAAAAAAAAATCTTACCTGTTCCATATGGAAGAGATAAAAAGAAGTGCTATTTATGGAGTCCCTACTGTGTGCCAGCACCACACTGGGGATGCCTAAAGACAGCCAAGAGCTTGCCCTGGTAGTTTTAACTAAAGACAGTGCTCTTTTCTCTCTGCAGCTGGTCTTCTGGCCCGTCTGTCTGCCTCTGTCTGAGTTCTCTTTGCTCTGGGGAGGGATTGCTGTCCTCAGCTCACCTTGTCTGGCATTCTCTGTACCTGTTTTGGCTCCAGTGTTTGATCTGTGTTCTAGATAAGGGGCTAAGTCATCATCTGCATTCTCCTCTCCTTACATTCCTTGT

							GCCTGCTGGAAATGAAATTGATTCTCCTATACAAAAATCACAGTACTGGCCGAGATTTTGGCACAATGG<br>TAGGGGGCCTAGATTCCACTCCTACGAAAGGGAACCAGGGGAAACCAAAGTTAGCTTCAGTCCCGTGA<br>AGGCTTGACTGAGTCACGTTCCTTCTGACCTCAGGACTGTGGTAATAAGATCCCACTAACTCTTCTACTT<br>TTTCTGAGGATTTTTGCTTTGCTCTCGGATTCCCCGGTGCCTCCAGCTACCCACTGTGCTGCTGCTGACT<br>CTGGACCAGTTGCTCTTAACGGAGAGGCAGGCAGTCCCTGTGGAGAGGAATTTCCTTGACCATGTCCT<br>GCTGCTGAGACAAGGTGCTAGGATACTGTACTGCAGTCCCACAGCTGGGTTCTCCTGGCTTCAGCCTGC<br>TAGTACCTGCAAGGAGAGTGCTATACAGCAGGTCGAGGGGGTCACTCACTGTCCCACTGTGCACCACG<br>CACCCAGTGTGCATGCACATGATCAGAAGGCCCTTTCCTCCGACTACCTTTCCAAAGCCAGTCCTGTAC<br>CATGCAGGGCAGTCTGTTCTCTGTGCTTCGCTTCTTCAGGAC
MSTRG.104416.1<br><br>NONCODE_ID：NONMMUT025494	ENSRNOG00000060896	6.794	18.914	0.017	2.236	up	CGCCCAGGCGGAACGATACGGCAGCGCCGAAGGAGCCTCGGTTGGCCCCGGATAGCCGGCTCCCCGTC<br>CGTCCCCGTCCGGCGGGTCCCCGCCTCGTCGCCCCCCCGGGTGCGGGGCGGGTCCCCCCGCCGGGCGT<br>CGGGACCGGGGTCCGGTGCGGAGAGCCATTCGTCCCGGGAAACGGGGTGCGGCCGGAAAGGGGGCCG<br>CCCTCTCGCCCGTCACGCTTAACGCACGTTCGTGTGGAACCTGGCGCTAAACCATTCGTAGACGACCTG<br>CTTCTGGGTCGGGGTTTCGTACGTAGCAGAGCAGCTCCCTCGCTGCGATCTATTGAAAGTCAGCCCTCG<br>ACACAAGGGTTTGGTCTCCGCGGGGGGG
MSTRG.104416.2<br><br>NONCODE_ID：NONMMUT025494	ENSRNOG00000060896	0.000	3.317	0.000	6.788	up	CGCCCAGGCGGAACGATACGGCAGCGCCGAAGGAGCCTCGGTTGGCCCCGGATAGCCGGCTCCCCGTC<br>CGTCCCCGTCCGGCGGGTCCCCGCCTCGTCGCCCCCCCGGGTGCGGGGCGGGTCCCCCCGCCGGGCGT<br>CGGGACCGGGGTCCGGTGCGGAGAGCCATTCGTCCCGGGAAACGGGGTGCGGCCGGAAAGGGGGCCG<br>CCCTCTCGCCCGTCACGCTTAACGCACGTTCGTGTGGAACCTGGCGCTAAACCATTCGTAGACGACCTG<br>CTTCTGGGTCGGGGTTTCGTACGTAGCAGAGCAGCTCCCTCGCTGCGATCTATTGAAAGTCAGCCCTCG<br>ACACAAGGGTTTGGTCTCCGCGGGGGGG
MSTRG.104	ENSRNOG0000000	0.057	0.420	0.002	3.082	up	TAACACGAGGGGCTGTAGTAAAAGGTCACAGCATTAGGAAGGTTGAAAACCACTGTTCTAGAAGTCCA<br>CAATCCTTTCCTGCCTCTAGTGAGGGCTTCCCTTCTAATCTTCATACAGAAAAACTAAGCAAGGACCAG<br>GGACCAAGAGCAAATTCACCCCATAAAGGAGCCATTCCAGTATGCATAGGGCCTTCAGAGGTCCAAGA

524.1	6046;ENSRNOG00000006062						GGGAGAGAAACATCTTCAGTGCTGAGATGGGGTCATAGACACAAGCTGCTGTCCCCAACCAAGTTTCTCCAAGGGACTCTCACCGAGTATACAAAGCGTGCTGAAGGGCATCTCCTAACACCCAGCTGGCAGGGTCGGTGTGCAGATCCAGCAATGGACCCTGTACAACATCTACCACATGGTAGACACAGTGACGTGTCTTGTGTAGTGCAGCTGCTGTGCATGGCAGATCTTTGAACTGAAGTTTCCAGAGAAAAGCCACACCTACTGTAAAAGACTTCAGGACTGAATGACTCCCGCTTCCCGCTTGTGGAGACCTGCAGGGGTCTGCAGTAATATTGGACTATCTGTAAGTGCATTTAGTAGCCATTCATTGTCTACTTCTGGTTTGATCCAACATCCCTTCAACTACCAGATAAAACCTTTGGAACGTGTCCATTTAACAGATCCCCGTCCTCCACCAGCCTAAAAGCTAGGAATGCCGTCGCGGAACATCGGAACCCTGTGCTGGAGACTCCCCCGCTTCGCTGTGACATACCTGTCTGCAGTCCTCATAACTGTCTTTGGCAACACAGCCTGCTGTCCTTCTGTGACTATGAATGCTCGTGTAACTTCTTCCACTGTGGGACACACTATTTGGGGCCACCTGAATCTACATTCCTAGGCCATAGTCACTCATGTTTGACTGAGAATAAATAATCTCATTCCCTTAAAGTGGTAGCTGTAATTTTCTCCACACACCCTAGTGACCCAGAACTGTCCCAGCAAGCCAGAGAGCATATGGCCACTCAGTGAAAGGCCCAGCACAGTACGTAGCCCTTTGACAAGTCCTCAGTGATGATACTCATGGAATGGTATGTCC
MSTRG.10471.1	ENSRNOG00000049016;ENSRNOG00000049451	0.000	0.506	0.031	3.453	up	GAGAGAGAGAGAGAGAGAGAGAGAGAGAGAGAGAGAGAATGTTGTGTGTGGGTGGAGATCAGAGGAGGGCCTCATGTATCATTCCTTATCCTCCACCTTGTTTGAGGCAGAGTCTTTCTGTTTCTCTGCTGCAGGTACCAAGCTAGCTGGTCAGTAGCTTCCACAGAACTCCTATCTCTGCACAATTTGCATAGGAACGTTGGTATTTAGAGGCATATAGA
MSTRG.104865.1	--	0.000	0.296	0.001	4.225	up	TGTTGAAACCCAGTGAAAGAAGAGGAGCCTGGTGATATGAAGGGAGCTTGAGCAAAATTTGGATAGGCTGATCATGTGACTTGCTCACAAGAGCAGGAGGCAGCAAATGGAGACCTTTAAAGAAAACCATCACTGAATACAATGCAGAGATCAATGAGTCACGAGGAGTACAGCCCCACGGATACATCTATATCACAGGCTCATGGAACATTGCAGAAAAGTGGGTGGAAATAGAATAAGAGCAAGAACACCAGTGAGGTCGCTGTGAAACCGTCTCTCCTAGGAAGGGCTGCATAAACAACACTGGAAATAATAGCAATATCAAAGGGCATGTTAACACGGAAGGCTGAAATTTCACGTGGTCCTTCTCCTAGACAAAGTACTACAGACATTAAAACGATTGCTCAGAAGCAGAGAACGATTTCCAGGATGCAGAGTTGGTAAATCTAGTGCCCAAAACAACACTGACAGACATTACAGATCCAAATGAAATGCGCTCCAGTTGTAACTACATTTCATCAAGGGTCATTTGTGTTCCACACTTGTACCTTTGCAGAGAAAAACAATCAAAATGGGTGGAAAATAAAACCGATTAGGTCTGAGATGCATCGCTGTCAGATTGTTATTCCTGAACCTGTGCTCATTGCACTGGGCTCTCACTGAGCAC
MSTRG.104973.1	ENSRNOG00000004110	0.000	0.346	0.011	3.719	up	CAGGGCCCATTTCTTCTGGTACATCACAGATGGGTGTTGTATAAATAGCATCATTGCCCACTGTTCTCGCTGTCCAAATCTCTTCGTCCAAGCAAGGTCTTCTGTGTTTCTCAACACAATTGGCTATGGCCTCTGGTCACCCTTCACCATGTCCCTACTTCAGGGATCCAAGAACTCCTGCAACTCAGGGCTCCAGGCTGCCACCACCTCGGCAGTGTGACAGTGTGTTCAATCAAGAGAGCAGCCTCAGGATCCGGAAACACGCTGAGCAGATCCAAGTCTGCAGCAGATCCAAGTGTACAGGCGCGTCTCCATCTGGCCAAGTGTCTTCTGTCTGGGACACGACCACCCACGCCATTCTTCCTG
MSTRG.105071.1	ENSRNOG00000005424;ENSRNOG000	0.040	0.231	0.035	2.769	up	GTTTTCAGATTAGACTCCATATATGGAGAGGACTCAAAAGGTCTGGCCTTTGGCTCACGCTCTGTGAGTTTTAACATGACCGCTGTTGCCATCGTTGGATGACTCAGGCTCCCACTGTAAAATCCAGATAAGCTGGCACGGGATGACGCCTTCCATGTGGCACAGCAGCAAGTTTCAGAGGAGCGGCATTGCCCCACTAGCCTTCCAGTGTCTTCTGCTGGTGGCAGTGGCGTGGGGCTGGGGCCTCTTCTGCCTTATTGGACTTCCCATCGCTACCACTGCAGTAAACATCAGAGCCTGTCTCTTAACCCACTGTTGACTCTCCCTGTCTCCTGTTAGAGAGT

	00032523						TCAGAGCCCAAGTGACAAACAGACAGCAGGCCCGCCCAGCCTTGCATCTGGGTCCAGAACTCTATCCT<br>CTGGTTATGCCAGTCACTACAAACAGCCTGGTCGGGGAGGGGGGACTTTCTCTGTCATTCCCTGGTGTT<br>AAGGGGCATCCAGAACACCTCTTGCTCCCTTTAGGACCTCCCTGTGACACTTGCTACCGTCCAGAATGC<br>TGGCCCATCTGCTGTTATCCCAGCGAGACTGTTCATTTCCTCCTTGCCTGGAACCTGGAGTTTGTTTCCG<br>GGGCTGAGCAGGTGGCTGGAGACCTGAACACAAACAGATCATGATTTGGGAATTGACTTGCTTACCGT<br>GGAAATAAATGTTCCTCCTCCTGAGGAGGAGTTGCTTGGCCAGGTTTCTGAGTAATCCTTGTATTAGCT
MSTRG.105587.2	--	0.000	0.219	0.000	5.141	up	GGGAGCAGCACACTGGCCATGCCCCTGGAGGGGGGCCCTGTTCTTGAAGTCATTGCAGGTTCATGTTG<br>TCTCTGCTGACAGCTCTGTCAGTCATGCCACACTTTCTGCTGGTGTTGAGCTCTGGGGCCTTTGCACAA<br>ACAACACCACACTCCACGTGGGTCCCTTTGCTGACTTAGGAAGACAGTTTTGTCCGCGTTCAATCTCAA<br>CAATGTATTTTATTCACAATTGCCAGCAGGACCGAAGTGGCTTTAGACCAAGGCTTCTACTTCATCTGCA<br>AGGATAGCCCTGGGGCTACTATAACAAATTATCACAGATCTGGAGGCATAAAGCAACTTAGACTTACTCT<br>CTAACAGTTCTGGGAAGTCAGAAGTCTAACAATTTTCTTTTAACTAGGCCAAATTCAAGGCCACATGCC<br>CAGTACAGTTTGTATACAAGAAGCCGGTCCTTGCCCCTGCAGATGCTTCTGCCGGTCAGTGCTTCTGTG<br>CTTGTGGCTGCCTCCTCCTCACACACAACTTCAAGTCTTTTTGTACACCATTTTTTCATCACCCTGGCCT<br>TGTGCATGTGCCATTATAAAATCCCCCTCAGTCTTTCTCTTCTGTTGTAACACAGGATAACATTTACTGTC<br>TCAGGAGCCAACCCGGGGGACCCATAGTTGATCATCTCTGCAAAGGCTTTGCTGTGCAACCGCAACAG<br>TCAACATTCACAGGGTTAATGGATTGCGCCTGATGTCTTCAGGCCACCAGAGATGTCTAGCAGCTCTGG<br>AAACCCCTTTGCAGGGTGGAGACATGTGTTTTGAACTTCTGATTTCCCCTCTGGCAGGGTCAGCCTTAA<br>AGAAGAGCTCAGCGAATGCTTTGTGACTGAGTGTGGATGGCAGGTGCTGCTATCTGCCTTCCCTCCCAG<br>CTCCAACCTCAAAGAGAAAAAAATGTCAGGGCTCCAGTTCCTTCCATGTAAGTGGCTTCCTATTCTCTA<br>CCCCCTCAGAAACCACAGAAAGCTCCTCATTAGAGGCTGCCACTCCACATGCTGCCGGGCACCTCCCA<br>GGGTCAGCTTTTAGGGTTATCCCCCCCGCTGCAGAACATGAAGTTCACTCTTGTTGTCTGGGAGTCAGA<br>GCAGGCTGGGCTGCAGTGTGATGACTCCCTCCTCGTCCATAATTTCAAATAGGAGGACAGGGTGAGGTG<br>AGGGGGTAGGTTGGTGGGCTGGGTGGGTTGGGTAGAGAACTGACATTAGCCTATTTCTTTCAAAGGAG<br>ATTGGGCCAGAGCATAGGAAGTCCTTCTGTCCTGGCTATATCCACAGTTGCTACAGAACCTGGCTCCCT<br>GCTAGCAGAAAAAGAAAAGTGAGTCTTAAGCTCCTCCCTTGCCCCCATAATCCCTCATCCAGCCATGTC<br>CAGAGTATCCTGGTCCCTGGTCCTAGTCTTGGGGTTCCCTGAGGCTAGGACAGGTATTTTGCATCTCAG<br>ACTTGCAGTCTCTGCCCCTTCCCTAGGATCACTCTGCCCAGATCTGAATGGCCTGGGTTTTATACCTGTT<br>CTGCTTGGTAGCTGAGGTCTCCACTCCCTCCCCATGCAGGAACAAGGGGGCTGATAGAATGGGATACTA<br>CGGGGATTTGTAGGTGGAGGAGGGTTCTCTTCTTTCTCTGTGCCTATTGTAGCCAGGGAGGTCCAAAGA<br>GCAAGCAGGATTATCATGTCAAACTCTGAGAGCAAAGACAAGGTCTTAGAGGCCCATACTTGGTTTACC<br>CCAGAGTCCAGAGCAAGTTTGAATCTTATC
MSTRG.105589.1	--	0.401	0.000	0.004	-4.319	down	CGGAGTGCATCTTCTGTGTCCTCAGACTTTACTGCCCATTGAGTAAGCCCTGACCAAGATTTTACACAAT<br>GAGAGAGAGGCTAGCGTTACGGTTTGGGATCTAAAAGCACAGGTTGCTTTCGCAGTAACACATGAAGT<br>AAAATAAGTAAATCCAAAATGTAAAACATAGTAAGAAAAGTGAGATGGGAAGAGAACAGTGAGAGAG<br>AGAGAAAGAGAGAGAAAGAGAGAGAGAGAGAGAGAGAGAGAGAGAGAGAGAGAGAGAGAGAGGAGA<br>AATGCTGGGAAGAGAGACAAGAAAAGCTATTCAAGACCTTTCCAGATACTGAAAGCCAGGGTTACTCT<br>TTTGTATACAAATGTACACAGGAGGCCGCAACTCAAGCTTTCTCTTGTAAAGTAGTCGAGTGTCAACAG<br>TTTGAAAATGCCAGTTGGAGGGCCCAAAGACAGTCTGACAGCGAGAAATGCCTCAATCCCAACTTAAA<br>AAGGGTTCAAAGTGCAGTGTCCCTCGGTTTCCTTGAGGATTGATTACTGGATGGCCGCCATCCTGTGAC<br>CGAGATCCTCAGGTGCTCCGGGACAAGAGTACAGTAAGGGGCTGGATAGCTCAGACACGGAGTGTTTA

							GCTGGTGTGTGCATAAGGGTGTGGACTTGATCCCCTGCATAGGTAACAGCTGTTGGGATGGCTCCTACTATTCCAGTTGTTCGGTACCCACTGGGAGACCAAGCTGCACATCTGCTAGATTTGTGTGGGGAGGCCTAGGTTCAGCCTGTGGGTATGTCGGTTGGTGGTTCAGACTCTGAGAGCCCCAAGAGTCCAGGTTAATTAACTCTGCTGGTCTTCCTGTAGAGTTCCTAGGCCCTTCAGGGCTTACAATCCATCCCCCTGTTCTTCCAGAAGAATCCCC
MSTRG.105618.1	ENSRNOG00000047234;ENSRNOG00000058463;ENSRNOG00000059588	0.000	0.636	0.000	8.412	up	TGCACACACACACACAACCACACACACATGCATAATATAAAAGACATGGTCCTCACAGTTATAAGAAGGTGACCACACATTGTGATCTTATTGTTAGAAACAATTATTATATGTAAAAGACTCCAGAGTACTAACCATCATACTTATAAGGAATCTCCATTAATATACCATTTTACATCTTCTTTCCACCTGTCAGTACACTCTAACTTTTCAAAATTAATTCACCATCTTTTCATTAAGAATTAAGTAAGAAAGTGCATTTTTGGTTAAGAATTTAAGAGAAAAAAAGGTATAAAAATCCTGTCTAAAAGAACTGCAGGAAAAAAATGGAGAAGAGCCTGAGGGAAAAATGGCCCAGTGACTGGCCCAAATCGGGATCCATCTCAAGGGGAGCCTCCAAGACCTGACACTATTACTGATGCTATGGTGTGCTTACCAACAGGAGCCAAGCATGGCTTCCTCTGAGAGGCCCAACAAGCAAGTGACTGAGACAAATTCAGATACTTATACCCAATCATTGGATTGAAGTCGGGGACCTCTGTGTTTGAATTAGGGAAAGGCTGGAGGAAACTGAGGCGAAGGGTTACCCCAAAGGGAGACCAGCAGTCTCAACGAACCCAGACCCCTGAGATCTCTCAGATACTAAGCCACCAACCAGGCAGCATACAGGAAAATTTCTCCAAGGTCCTGGACACATCTCCAAGCCTCCGGCAGGGGACTGCCTGGAGTGGCCTCAGTGGGAGATGTGCCTAACCCTTGAGAGATTTGAGGCTCCTGGGATGGGGAGGCCTGGCAGGGGTCAGTGGGTGCATGGACATCCACTCAAAGACAGGGGAGAAGAGGGATGAGATGAGGAACTGTGAGAGGGTGGGCTGGTAAGGGAGGAATGCCTGGACTTAAAAAAAAGTAATAAAAATAAATTAAAATAGATAAGTAAATAATTTACTTTAACACAATTAGAAAGGACCACTGCCAAAATGGAAAAACCAACATTACTATCTAACAGCCATCTACGCTCTGTTTCAAACACAGGTGAAAGACTAGTGAGGCTCCTGGCCAATAAGAATCAGGTCCAAGGACTGACATCCATTGCTTGTGTCGCATAACTCATTCTTTTATCTACTTTATCAGGCAACCAAAGGCTACTCGTGAAGCATCTCAGTGTAAGTAATTAATAAAAGCAACACTCAAACAATTGTGTGTTGACTTTAAAACAAGAGAACTAAAAGAACTGAAAAAATAAAATTCCCTGTAAGTTAACAGCAACGGTAGCAATAATTATTCTTGTCATGATGATGATGATGATGATGGTGGTGATGATGATGAGGATGATGATGATAAGGTTGGCCTTAACAAAGAAGATGCTTTGTTAATTTAATTACTTTTTTCCTACTTACTAATTTAATATCAGAAGCTGAGTCACGTTTGCATTCAATAAAATTGATTTATTAATTTCCTCTAATCTTAGAAATTACTGAATAAAGCCCCTCATATAAACCACTCTTATCCTTCACTCTACAAATTCTAGGATGACTTTATTTCTATTAAAAGCATTTCCCCCAAATTGGAATTTTAATTATAAATGTGGCAAAAAAGAAAAAATGACCATCAAAGGTACCAGGCTTTGCATCATAAAAAAGCCTACTTCTTTGAAATTCACACAGCACAGGGACATTTAATACACACAACCAACAGACACTTCAGATTTTTTCATGATTACTGATTTGTTCAGTTTGGAAATGTATACACTTGGAAAAATGGATTTTGGAAAATAAAGATAATCACATGGAATAATGGTATAACGTTGGGGACGGTGCTTTTATGGGTTTTCTCAATGATTATACCCAGTCAAGAGTTTGCTTACTGACATGTATTCAGATTCAAAGATGGCCATTGGAATAAAATCTCGCTAGCTTACTGTGTGTGTACGTAGGTGAATGTATATGCACTGAGTGTGTGTGTACTCCCCAGGAAACATAACACAGAAGAGTCAACTTCGATGGCAATCTATATATGAAAATTCTGAAAGATTATCACTTTTACCTATCAACTGAGGCTAAGATGTTGACGACAAACTTGGGAAACATACAGTTGATGTAGGATCAAATGGTATACCTCTCCTAGGACTAAAATTGTAGAACTATACAAACTCTATGAGGGAGTTGATACTCTTTGAAGGTGTCTCCATTTCTAAAAAAAAAAAAAGAAAAAAAAGTTGTGGCAAGGGACTCACCAGGTAAATGTCATACTGTTAGTTCAGTGTGTCACTGAACATCTACAAGTAACTTCTATGTTGTCTCTAAGGCTGATATTAAAAAAATAAATAATGTCACTGTTCTGTACGAACACCAGATTCTGTGCATCATCCCACTAAAATCACCTAATAAGATAGCATGTGTTTGTAGTGCACTGTTAAGGACATCAGAGGCTGCAGAAGTACATGTGTGGTACTGTCCACATCTCGGCGTACAAGCATGCGCGTGTCACACACATATAAACTC

							CAACCCCCCCTCCCCCAGATATTTCATAGGACACAACCAAAAAGACAGCTTGAAGCCCTTTGACTTCTG<br>AAATTCTATTCATTTTTAAGTTTCACTTTGTGAGTTTCTATCTTTTCCCTGAATAAAAACAAACCTGCCTG<br>ATGACCCAGAAGGTATTGAGTGAAGTCTATCTCATATCCATATGGAAATAACTCTCTTATACCCATCTTGT<br>TACTAGTGTATCTTATTACTTTTAATTATGGGATGCTAAAGATCACATCCAGTATACTATAAATAAGTAATA<br>AAAACTTCTTGTTGAACAATAGCAGGAATGAGTTTAGTGTTTAAATTAAATGAGGCAGAAAAGTTTACC<br>AACAGATCTTATAATATGAGAAATTTGAAAATCATCACTCATTTAACTATCACATTAACATTGGGGCTAAG<br>TTTTCTTAAAGGTACTAATGATGAGGGGACTAATTCACTGCAGTATTAAGTCTTTAACCAGGATTTCATTT<br>ATAACAAATGGAGGGTCCAAGGTGATTACCACTCATGCTGATGACACTGTCAGTGTCTAACACTACACA<br>ATTCGATTTCTTCTGGAAGCCAGATAGTTCATCCATGATGGTCTTTGAGGTACAACCTATACAACAAGGG<br>TAGGGACAAAGCAGGCCCCTCTGTCCTGTAGCAGCATGGGGTGAATCTGTGAGATGGGACCGGAGCTG<br>GTGGGAACGACCAGAGACAGCACAAAATCAATGTCCAGCTGAGCCCATGCTTTCTGAACTAAAAGTCA<br>AGACAAGTGATCCAAAGCCAAGAAATGGAGTAAGTCTCGGTGTCTGCCCTCTGTGTCATTGTGTAATGA<br>AGGCAAACTCCTTGTGTGCACGCACCAGTCCATGCTTAATTCAGCAACCCCTATGGTATTTTTTAGCTCT<br>CATACTTCCCAATTATTTGACTCTAAAGGGAAAGGTATTTTTTCCACGTGGAAAGGTTTTCATGAGAGAA<br>TGAGACAAGGGGGAGGGACGAGAGAGAGAGGAAGAGTAGAAGAGGAGAGGAGTAAAGGCCAGCCAT<br>GAGCACGTGGAGGGAAGTGGAGAGGAGAATAGGGAGAGAAGTGAAAAGGGGAAGAGGGGAAGATG<br>GAGAGCAGGAGAGGAAAGGATAAGTTTTAATTACACTAATCATATGCAGCAATAATATCCAATTATGCAA<br>AAACCCATACAAGAGCAACCATGAAACTAGTCTCGCCAAATGGCTCTATACATCAGAAGTCACTGGAG<br>ACAGGTTTTATACTTCCCTGGCTTATTCTTTTCCCTTTACCTTTTTGTTGTTTGTCAATGGTGATACTTGGT<br>TTTGTTTTAAGACAGGGCCTTTCTATGAAACTCAACTGTTCTCCCCATTCCTCTGCCCACAAGTGTTGTG<br>TTTATGCACAAACTACCATGTCCACCTCCAGCATCCCTCTATTTAGGCACACTCATGCACTGCTTATGAG<br>AATGTAGGTCATGCCAGTCAGATAGGTTGGTTAACTAGTGACAAGAAGTTTAACTGAAGCAGGCAGTGT<br>AACTTTCCCTCATTTCTCTGGAAAAGGGGGCTTTTATGATTGTTTAATTTCTTAACCCTACAATGTTTTGG<br>GTACCATTTAAGGCTCTTCCTAGAAAATGATCCACAATGTACTATCACCCACTAAAAAGAGTCAGGGATA<br>TTACTAAAATTTCTTTTTTTAATTTACTAAAATGCATTAAATAAGATTAGTTATACAATGAGACTTATACTA<br>TAATGGTCAAACATTAATAAATTCAGCTATAATGATCAATATCAAACAACGTCTTTTTTATTAAATATACCC<br>AGAGTTTCATGTGTGTGTGTGCATGTGTTCAATCATGTGTGTGCTCACATGTGTGTGTGTGTGTGTGT<br>GTGTGTGTGTGTGTGCGCGCGCGCACGTGCTCAGGTGTGCCTGTGTGTGCTCAGGTATGTGTGGTTGCC<br>TGTGACAGTATGTATGTGTTTCTTTAAATATTTTTCATGGGAGCTGTGAGCTCATTCTGCCCCCACTTTCT<br>TACAAGCATTACTATATAGTTTTTAAAATCCTTCTAAGATCCATAATCCTATTGAATCAGGAATAAGAACA<br>TGTATTTAGTTTCAGGCAAACAATTGTTCGACAGAAGCCCCTTCAATTTTTAATTATTCCCTCTTTCCTCT<br>CCAGCTTCACTGTTATTCTCTGATTTCTAATCTTGAAAGCCAGTCAGGGAACATTATTTATGTTACATTCA<br>ATGAGGAGGGAAAGAAGAAAAGGCATTTTTATTCAGGACACTAACACTAACCTTGAAATGTTATTTTCT<br>GAACATTTGTTCTGCTCACTGAGCAAATTTTAGTCTTCAAGTCAGATGTCTAAATACAGTGCTTAAGCTC<br>TGCTTAGAGGCTAAAAATACAGACTTCTGCACAAGGTGATGGTTACAAAGGAAGGTGTTTAGAATATGG<br>TTCAATGGGAGCTAAGGTAATTGAACCTCAACTACATAGTTCAAGCCAGAAGAATTCCTCATCTACGCT<br>GAAGCTTTGGAGTCAAATCTTAGCTATTATTCTCTGAAATGTGTTTTACTTAAAAGGAGAATCAATTAAA<br>GAAGAGGTTTCCTGAAACTCTTCACTGGGTCCTCAGTTTGATCACCCTAAGTCATGGGGTCCATTGTCA<br>ATGTCTCTCTCTAAAAGAGAATAGCTACAGAAGCAGGCTCGGAAGACATACTCAGTCTACAAGCCCTGC<br>TGGCTACAAAGCCCATGTGAAAATTGTCTCCCCTTAAGAAAAATCTATGAGCACAAGTCAAAGAAGTTT<br>GGACGACCAGCATCACCAAATAAAGCCAATCTGTCACATAGAGAACATGGCCCATGCAGGAAACACAT

							ATCCCCTGACTGTGTCAGACTACTGTTCAGTGTCTTAGGCATAACCAGCCCAAAAGCAGAAAACCAAGAAAGCAGACATGAGAACAGCACAAAGCTGCCTGCAATGAGGAATCCTGACGTTCTATAAATGGAAAAGATTTCAATGAGTGAAAGTGGAATGGAGGTGTGCTCTTGAGTGAATTGTGCCCAGCACTATAAAGACAAGTGATGACAGTAATTGTTTCTAATCCAAATAAATGCTCACGGAACATGTCTCCTCATGCTGCCCTGCCTTGAAGGCTTTATTTAAGTGCCAGGAAGAGTAACTGACGGGGGTACTTCCTTCAAAATTATGTGCTGAATGGGGAGCTCCTTTCTCACTTCCCCTTTCATATGATTTTTAACTTATCAATGATTTTAAGGGGTGTCTACATTATTTCCTTCAATCTCTAAGAATTCCCCTTTCAACGCTCAGAATTCCCCCTGTTAAGAGAGTACAAAGGAGATAAGTGAGAAGAGAACCACGAGGCACTAAGTTAATCTCACAAAACAGTATTCGAATAATTTATACAAATCACATATGTTAGCCATTGTGAAACAAGTTTCTAGAAAATATTTGTTCTTGTGAAATGGGCAAATATGAG
MSTRG.105939.2	--	0.527	0.000	0.000	-6.074	down	GTCTGTTATTGTTTCTGTTTATCTTAAGTGAAAATTAGTCTTTAACTATTTTAGCCTTTGCTAATAGTTATTATTCAGCATAGTAATAGGTTTATATTCATAGTTTATCAAATATGGCCACTATGTTATTTTAATATATATTTTCATATTTACTTTCATTCTGGAATACATTCTATTTATCAGGAAGGAATATCTTAACTATTTGGTTTAATTTATATAGTTTTTTTGAGATTCTTATGCACATATTTATAAATGAAATGTCATTCATTTTTGGATATGACTTAATCTGTATTCTACCTTTTATTCTAAACAGGATCAGCTCTTACAACTGTAAGTTAGTGAGTACACCTCTGCTCTCAGAACTTCTGAACACTGCCCTAGGAATCATGTCAGTAGCAATATCTGAAATTCAAATGTTTCCAGAAGGGGTAAAAAAGAGGGGGGGATAAGTTTATATGTAAAAAGCAGTCTCCAAGAGATATAATTTATGGAATATTAGATATTATATATCATATAAGTTGTATGATATATATAACTATGAATATTATGGAATTCCAGTTGTTAACGATACCATTTTATATATTAAATTGCATCTTTTTATGTCTAAAGAGACTTTTGCTTATTTTTCATAATATTTGTGTTATTTTAATACACACCTTTTTTTTGCAGAAATGTAGGTTGAAGCTTATAGCCATTATAAATTATATAAGGAGAAAAATAGCACAGTAACGCAGAGTCTTGTAAGGTGAGTTTCACACAGGTCGCTCCAATCCTGATGTGAAGCAAGCATTTGAGTTAATGCTATGTGAGATAAAATAGATTCAAGACGTTAAAGTGAAACTCACAAGATAACAGTGCATGGATTTTTTATTTATTTATGTGAAATAATACATTAGGAAGAAATTAATAAATGAGTCGATATCTTCCTAACTGCAAGCATTGCTTTGACTAGAGGCGATTAGCAAGATTCTATAGACAAGATGACCATTATCCGACTTGCTTTCCCTCTGCAGATTGAGGCCTGGTGAATATGATGGTAGCCATGGTGAGCAGAGCTTTGTCTTTCACCAATGAAATCCAAAGAGTTTTAGATCCATTTATGTCACTTGGGTCCTGAACCTGGATTGGAAGATGAAGAACAAACTATAAAGACTGGAATCTAATTTGATGGGTGAAGGCTTTTGTGTATAGTTTTCAAATTTTCTAAGTTAAATTTTCTTCATTGATAAAATTAGAATAGTGTTGAATTTATTGGAATATGTCGAGTAGTCACAGTGTGCATGCCGGTTGTGAGGTACTTTTCATATGAAGTACTTGATATAGGTGGATAATTGCATAGATGAACAAGTAAGTTTTAGTTCTGTTACAGATAACACAAGGGACATCTGTAGGAAAGGGCACACAGATTGCTTTTTTTCTTCCTGTCTGAAGAAGTTTGTTCAAGAAATTATGAAAGGCTTGAGCAGATCATTGTTTTTCTTCGTTGAAATTAAAATGAGTGCACTTTTATGAATATTAGTTACTTTTCTTATTGTGACAAAAGACTCAAAGAAAGCAGTGTCAGGAAGGGATTGTTCATTATGGCTCAGTGTAATGGTACAGTCAATCATGGCAGGCTCACAGTGTAACGGTACAGCCCATCATGGAAGGCTCACAGTGTAATGAAACAGCCCATCATGGAAGGCTCACAGTGTAACGGTACAGCCTATCATGGAAGGCTCACAGTGTAATGAAACAGCCCATCATGGCAGGCTCACAGTGTAATGAAACAGTCCCTCATAGAAGGCTCACAGTGTAATGAAACAGTCCATCATGGCAGGCTCACAGTGTAATGAAACAGCCCATCATGGCAGGCTCACAGTGTAATGAAACAGTCCCTCATAGAAGGCTCATGCTGTAATGGAGGGCAGTAATGGACGGTGTGCTCACCTGAGTTCTGCTGCTTCCTTTGGCATGAGGCTGACATAGCTGTGACCCTGCATCTTTAGTCAGGAAGGGGACAGATGGAAGTTACTCTTCTGCTAGCTTTCTTCTTCTTTTGGTCCAGGATTCTAGCTCTTGGGCTGTTGCAACCCTCATTCAGGATGGGTACTCTGTATCAGTTAGGTGTTTCTGGAAATATTCACAGGCTCTCTCAGGGGTTCATTTCTTAGGTGATTTTGAATGTAGTCAAGTTGACAAGATTACCTATCCCAGATACAAATATGTAGCAAGCCTATCTTTTCCCAGCAGCTTTGAGCTAATCAAGTATT

							ATGTGTTCCCAGTGTCATAATTTCTGCATTCTGTTATAATCTTTTTTTTGTTAGTCTACCTGTCTACATAGCCACCTTTGTATTTTATGCATAAGATTTTTAAATAACAGTTTTCACCCCGACCTGGCAAGC
MSTRG.106038.1	--	0.000	0.256	0.031	3.453	up	CAGAAGTATGAGCACCTGAGCAGGAGGTCACTGCAAGCTCCAGGTCCCTGCCTGCTTGAGCTTCTCTCCTGACTTTCTTTGCCGATGAAAAGTTCTGTGGAAGCAGAAGCTGAATAAATCCTTTCCTCCCTGAGTTGCTTTGGGCTCAGTGCTTTACCACAGCAAGAATAATCCTAACTAAGACAGAAAGACTATGGAAATGGTGAGTAAGAACTTAAAGGTCTTTGTTTATATTACTAGCTGTGATGATTAACCTTGGTTGTCAAATCCTGGGAATGTCTGTCTGCAAGGGAGTTTCAAGATTAGTGTAATGGAAGTTGAAAGGCCCATGCTGAATGCGGGCAGTAGTCCCAGACTACATAAAATAAAAGCTGGGAGGAATGAAGGGAGGAGAAATTGTGGTTGGGTATATAATAC
MSTRG.106143.1	--	0.370	0.000	0.049	-3.592	down	CTGTGCAGCACAGCCAATACTGTGTAAAATGTGAGCCCAGCCTGGATGATCTCCTTAGAGGGAGGGCCCATACCAATGTTCAGCATTTTAGTTATGTCTTCAATTTCCTTCAAGACAAGTATCCTCATTTTCTATTTTCCTTTCAGACTCCAAATTCCATCTCTTCTCTTCTGAAATCCTCCTCCAAGGTCCTTACTAATTTGTTGATCTCTGTCAGCAACGGGGGCCTGGGTACCTGGAAGAAAATGGGGGACAAGAGACACAAAGAAGGACTCCAAGACAAGAGTCTGATCAAGGCGACAATTTTATTTTTCCCCAATGCTGAATTTATACCATAACAGGGGTAGCAGAGGGTAGGGGGAAGTAAGAAACATTTACGCAGGCCAAGGACACAATATTCGTGTGGTCAAGGAATTGGCTGATGTTGACAGGATGTCAGAAAGGTCACAGGTTTGTCATATCTATTAGTCAGCAGGATGTTGGTTTTTCAGAAAGGTTACAGGTCATCACATTGGGTATCTTAACGTCAGATGTTATCATATGATTACTCATCTTGACCACCATATTTG
MSTRG.106187.2	ENSRNOG00000058463;ENSRNOG00000059588	0.420	0.000	0.000	-7.507	down	CAAGAGAAAGTTTTTATTAGTTGACCAGTGACTACACTTGGTTTGGGGGACTCAAGTACAGACTCAAGTCTATGTCCTGGTGGTCTTTTAAGCATAAACACCACATCCTGGGCTGAGACATACTCAGTGAGCAAGAACAATTAGCCAGAAGCACAATTACAGAAGCCAAAAAGCAAGGTTAGTACATTTAGGGACATTCCCAAAACTATGGATTTGATGGATTCCGTCTCTGTTTTTGTTTTTGTAGTATGCTGAGCTGCAGGGTGTGAACAACATTTTCATCATGCATTAGTTGTGCTAAGGTCCTGGGTACTGTTACAGTTTTGTTTTGCTGAAAGTATTTCCTTTACCAAATGCTTTAGTATTTTTTTCCTTTGTCCAAAAAAAAAAACAAATGCTCTCCAGTTGTGAATTGATCTCTTTTGTATCTGCATCATTGATTTTTACGTTAATTCTACTGTTGGTACTAGTCTTGGTTATTACTATAACATTATGATAAATTTTAAAAATCATAATATCTAAATCTGACCCAGTGAAATGACTCAGTGTGTAAGGGTACTTGCTTCCTGAGGATCTAAATTTTGTCTCCTGTACCTATATGAAAAAGGAAAGAGCAAACTCAAACGTGTTGTCTTCTGTGATATGCATGCATACACATCACACACTTACTTGTATGTATGTACACATAAAATAGTATAAATATGTGTGTAAGTGCAAACAGATATAAATATGTATTAATTTTTTAAAAGACAGCCAACCTTTTTTTTAATTACATATTTCTGTGGTCATTTGAGATTGTTTGCATCTCCATATCAACATTAAAAAAGTTTGTACATTCCTAAATAAAACACTACATGATTGGTTGACATTACAATGGATTAGGGATAGCACTTTTATTTTTTAATGCAGTTGTATGATTTCTAAAGGGCCACCCCAACCAGCCTTTTCTCCTGTTTGAAGAGTCCTTTCAATTCTCTGTGGAAGGTTATTTGAATTAAGTTGTTTACAGTGTGTTTCCTCTGTTACAAATGGTAGCCCGTGTTTTAAATACATTAAGAGTTTTATATGTATAATCTTCTTTATTGTACAGACAAACACTGAACCTCTTGAGTGTAAAATTATTATTATTATTAGTAGTAGTAGTAGTAGTAGTAGTAGTAGCAGCAGTAGTAGTAGTAGTATTACAAATTACATATGAGATGCCCAAGGCCCAAGTAGTTAAATCATTTCCTCAAGGTCAGGCAGCTAGGAAAAAAAAATGAATTCAGTTGCATCTTACTAAGTAGTTTAAATGTCCCAGGAAACTGAATTTTATTATCCTTTATTGCCCATTATCCTATTAATGTGGGGTTTTGTATGAGCTGGTATCTAAGAGCTGAAGGACGGGTAGTTGAAAGAAGGTCGTTTTGCAGTGGTGAAAAAGCACAGACCTTGAAACAGAGAAAGTTGATGGCTTTGTCTTGAGGTACTATTCAATGCATATGCCTGCTGCATGGGAACCGGAAGTGACCACATGGAGACTTCGGGCAAAAAGCCTCTTTCCCTGTTG

							GGTGAGGCACAGCCCTTTCTATGGGCTAGCCATCTATAAACAGCGTGCCTAAGATTTTTCCTTTCTTGAG<br>TAATTCACATGTGATACATTGGCAATAGCTACAAGCCCTGACTCCCTCCTCCCTCAGATGCTCTCGGGGC<br>AGCAGAGAGAACACAGAGATTCACAAATGAGCTTTCTTTGTAGAGCACTGTCTGCTTTGAAATTTTTCA<br>TTAAATCTTTTTCACAAATAAATTGCCCAGTGTCCATGGGGAATATTTCCATCTTTTAATCGTGTGCAGCT<br>TGAATTGACTCTATGATTTTTGAAATGCTTGGCCTGGCTGGTATGGAGGGCACTGGCAAGGGGGAGGGG<br>AAACACCTTCTGAGGCCCATCCCTTGAAATCTATTTTGAATGTAAGTAACAGAAGGAAGCCCCAAAGGG<br>AGCAATTGGAGTGAGCTCATATTTTGTGATGTATTAGCACCCGCTGGGACTGGAATTCTAAAATGCAGA<br>AAGTTAATTATGCTCCTTACCTATTGAGTTAGTATTGGTTCCCGAAGTGAAATCTTGGGGTCCAACCTTC<br>CTCTCTAGGCTGCAGAGTCGTTCTAGATTGATGGTTTAAATAAAATGTCAAGTATCTGATTGCCATGGAA<br>ACATTATTACAAAGTGGGCCTAGTCTCAGTTCCTGCTGTAGAATTCCAATGATGTGAATTAGCATGGTCT<br>CAGGACACCTTCTAAAATGGCTGGAGTTGCATCTCTGCAAAATAAGTGAGGCATGCATGCACGAAACG<br>AGAAACAGTGTGACATGCTCATGTCTCGATGCACACAGCTCTGAGTTGGACACATTCCAATCTCTTTGA<br>AAAGCCAGGAGCAGGTGATGCCGATAGACATTGGACTGGAATGGTCCTCCCTTCTTGTAAACCAGCCA<br>GTGAGAATTGTTTGTAATCCACAGAGGAAGAATGTCAAGGCCAGTGCTGGTCACCAAGAGGTAAAGCC<br>CTGGAAGGTGCAGCATGCACTTTGGGTCCCGCTCCAGGACCTCTTCATGGAACACATGTTCCAACTGCC<br>TTCTCTAGCCTGATTAGCAGAGCACTGCTGATTATGTTATGCAAATGATGGGAGATGGATTTCACATTTCC<br>CCAAGCCAGGCTTTGCACAGCTTTGCATTTATTATCCCCCTTTAAAAATACAGAGTCCTTAGAGGATGGG<br>GATGGCTCGTTTTTATTTGTTTGCCAGTAGAGTTGGCACAGACCTGTCAGGCAATCATCTTGGGCTGTGA<br>TCTGCAAACTTGCCCTTTGTTGTTCTATCCCCCAACCCCTGATTGTTTACAAGACATACAGAGACATCTA<br>CACACCTCAAAACAAGCTATTTCCTGATCCTGCAAAATTTGTCAGTCTGTGTGATAAAAATGCTACATCA<br>TTGGCTGACAAGCTATCAGATCAACCTAGAGTTCAGTCCCCGGCATCTTGTCCTCATTCTCAGCTCAGA<br>ATGAGAAATACTTAGCAGGTCCTGGCTCTCGATTGTACAGTCTACCACCTGCCCCTTGGCACTTATGATC<br>CTGCTTTCCATTTACTGATGTCTATCTGCCACCAACAATCTGCCATTCCACTTCTTTGGAAATAGAAGGAT<br>TGAACTTGTGTTTGTTGCATTACCAATAAGATCTGAATGTCAAAGTTGGCTCTCTCAGTTTTACATAAAA<br>ATTTTAAGAACCTGTCCTCATTTCAAATGGTTCTAAACATGGTGGGGACATGGTTCCTGCTCTTCCGACT<br>CCAGCTGTGAAAGTCAAGGAAAGAGTACAACTAGGAGCACTTTCTAGTGCTCCCATCTTCTTCTTCTTT<br>TAGATTTTACTCAAAGGAGGAGCTAGTATGAATTACATAAAATGAGTGACCAGAAATTAATAAGAAAGT<br>AATATCAGCTGACATAACCTTTCCATAGGGGAAGGGCTGCTGGTAAAAACATGGGAAGAGATATACCTT<br>TGCCAAATGAAGACAGAATTCTACAGCTCTAAAAAGGATGAGGAATTTATAATCCTGCACATAGCCTATT<br>GCATTGTATTTTTTTTATGAATCATGACTTCTAGACATGTTTTATCATATACAAAAATAATGTATACACATAC<br>ACATCATATGTGCATATTTGTATTCATTTTAACAATTGTTTCAGATCAGTGTGGCATACTTGAGATGTACTA<br>AGATAATTGAATATATAAAAATGTAAAACATTAGGCCTTTTAAGTTGATTGAGATTGATCACAACTGCTAA<br>TAGCAGCTAACATTTGCTGAGCCCTTTGTGGGCCAGGAGCACTGCAGTCTTTTTCCGCTGGTAGACCGA<br>TGCTAGTAAACAGAAGCTGCCTCCAATTTTCAGATAGGAAAATGAAGGCAGAATTAATGACCAATTGCT<br>CTATAATGTAATGCCGTCAGGACTCTCAAAAGTCTTCAGGCTCAAAGCATGAGCTACACATCGGTAGGG<br>AGTCTTGTAAGTTCCAGCAGCAGCATGCCAGTGCTAGAGTGCTCACATAAGAGAATTATTGTTTTACAA<br>ATCCAAAAACTCAAAAATTGGCTTGCAAACTGGTTCTGCTGTTTGAATGATAGGCCCATTCCAATTTTTC<br>CAGTCTTATATTTTATAAAGGGGACATATTCTTAGATCTCTTATTGTGGCAAAGCATATCTTTCTTCTTCCT<br>TCTCTAGATATCCAAATACTTTTCTAGTGTTAGTGCAAAGTAAGTAACATGCCAACATGCCAACTGGTTA<br>AAAGAAGAATTCATTTTCTTTTAACCCCTAAGAAAAACAGAAGGCTTGTCTTTCTTTGACTCTTGCAAA<br>ATGTCTTGCGTTAGAATAAAGTCTGATAAAATTATATTCTAAGTGGCTTTTTATTTTTATTACATATTTTACA

							ATCATTCAGTGTGTATATGTTTGTGATTGTGCAACCTAGTGTGCACATAAGTGCCATAGTGAACACTTAG<br>AGGTCAGAAGACAACTTGGAAAAGTCAATTTACTTTTGCTACCGTGTGTGGGGCCTGGGGATCTCAAG<br>CACTTCAGTTTAGCAGTAGCTGGCTTATCTGCTGAGCCATCCTGCCAGCTCACATTGTCTGTCCTAAACC<br>AACTCCTTAGGCGAGGGAAATGCTTGAGTCACAGACCCAAGAATAACCCTCCATACTCACCTGGTAAC<br>AGGAGAGGGGAGAGAATGACAAACACTTCTCGTTCACACATCTATAAAGAAATATGTAGTATAGGAAGT<br>GACAGAAAACTTACCTATGCAATGATCTGCAGAACACACATAAGTAACATTCAAACACAGACAATAAGA<br>GTGGAGAAGACAATTTAAGGAATTATGGTAGCTAAGAAATAAAAAGGGGCCATGCTAGTTTGGTGTGA<br>ACTGACGCAAGCTAGAGTTATCTGAAAGAAAACAACCTCATTTGAGTAAATACCTCCAAAAGATCCAG<br>AGGGATTGATGGGGGAGAGCCCAATCCATTGAGGGTAGTATCATCCCTGGGCTGGGGGTTCTAGGTCCT<br>ACAAGAAAGCAGGCTAAGCAAACCATTGGGAACAAGCCAGTAAGCAGCTTCCTCCAGGGACTCTGCA<br>TCAGCTCATGCCTCCAGGTTTCTGCCCTATTTTAGTTCCTGTCCTTACTTCCTTCACTGATGGACTACTAC<br>GATGTTACAGTATAATCCAAGCAAATCCTTTCCTCCCAACCTAGCTGTTCAGTATGGTGTTTCATCATAGC<br>GATAGTAACCCTAACAAGCACAAGGACGGAGTTTAAAATGGGAAGTTTAATCATGTTTTACTGCTATATT<br>TATATTGCTTTCTTTTAGCACAAATAGTTAAGAGTTGTAATAGAAAACCTGGAATGCAAAAAATGGAGCA<br>GTCACTCACCTGGTGGATGGAGTGTGTGTGTATGGGGGGGAGTGCATATAGGTTCTTTGGGCAGCTACA<br>GTTGGAAAAACGGTTGTAAGGAAGCTGAGGAGGGTCCAAGGCTTTCTAAGACTATTGCAGTTGTACTG<br>AAACACTATGAGCTGAAAATGAGCATGCTGACTAATTTATTTGTATTACTTGAGCAAGATGATAAGTTTC<br>CCAGAGTATGGAACACAGCACTCAACACTATGTATCTTAGAAATATGGTCCCTGCTGTGGGGCTGTGGT<br>GGAGTGGGGGAGGATGAGGCCTACCTGGATGTCTTTCAGTCCTTGGAGAACTGACCTTCCAAAATGAT<br>GAAGGTAGTTCTCATGGCATCCCTGAGCTCTCATGAAATCAAGGTATTTTATGACACCTTGAATCATTCC<br>CTGTCTTCCTGTGTGACAATGTTGTCTATTGCTCAAAAAAGGAGAAAGTAGGAGAATGAAGGAGAAAT<br>GAGGCAATTAACGCCTGGTCAGAGAGGAGTGGTCTTCTGTACAACAAGCTACCTTGATAGGTTTTCTGA<br>TTCCAAGTAGTCAGTCCTAAACACTTATGCATGTGAACAACATTAGATGACTCAGCAGATTGTGTGTGT<br>GTGTGTGTGTGTGTGTGTGTGTGTGTGTGCGCGCGCGCGCAGAACAATTAATTAAAGAAGGAAATACGTAT<br>TTGAGAGAAAATGTGGGATCATGGGAGATGTTGGAGAGCTAAGAGGGAGGAATAGAAACGATGTAAAT<br>ATAGTAGTCATATAATAAATTTTCAAAATGCAGGAATTATACATACATACAAGAAAGAAAAATTGAGGAG<br>GATACTCAGTTTCAGCATCAATTACCAACCTCCACATAGATGTGCACACAAAAACACACACCTAATAAC<br>AGTTGGCAATGAAAGGAGAAAAGTGGGAAAAAATAGACTAATGCTTACAATTCAAATTAACAGGAGAA<br>TGTGATAGGTTAGGGGTTATATAATAAAATTAAGATTGCATGTATTTTTTTCTTCCTCTGTCCTTATCTATG<br>GTCCATGGCAATTCCTGATATAATTTGAGGTTTTGCCTAGAGAAATGAGAGAATTGCAAAAACTGAAAC<br>ACTGACTTGGCACTATGTAAGAAGAGAACGGTCAATTGATAACAAGATTATTACTTGGGATTCTGCCAA<br>GACATTAACATATGAACCTTAGAAGGGCAATGTGAGATGACAAACTGTTGTGTTGACCTTGTAAAATAC<br>AGCATTGTTATAGAAGCAATTTTAGACACTAGTGGTTTCAAGAGAGAGTTTTCCCAATTGTGATTGAGA<br>AATGTCACCCCATCAAACATTACGCAGAATGACTCTGAAGTAAGGAGCAGGAGATGAGCTACAGTTTTG<br>CTTGGGCCTGGCATGTGTCATTTCTTGCTGTGGCACTTCCTGTTTCCTGTGCTGCCTCGGCTAACCACTC<br>ATTGTAAGGTAATACTAAGAGCCATGAACAGTGTTTTGTGTCACTGGTCACGTCCCTAACAACACAGTG<br>CTCAGGAATAGCCAGGTCACACTTGTGCTTTATGGCTGGCTTTGTTAGTCTACCTTCCCTCTCCTTATGC<br>ATTTTTAAATTTTTTTTTATTTTATTGCTTCTTTGTGAATTACACATTATGTACCCAACTCCCACTCATTTCA<br>CAGTTTTGTCACCCTTCCCTTGACAAGAAGAACCCAAAGAAACAACCAAAAAAAAAAAGTCTCACCATG<br>GAAAATGCAGTATGTCATGATGTGTCACACAGCATACCATTTGTCAAAACAGTATATTTTTGCACGTACT<br>CATTTTAATGAGTCATTGGTCTGGTTGGAGTTCTCTAGGTTCTGCAACACTATCAATACTGCACACTGAA

							GACTCCTCTTGGATATCCTGCTGTTTCACTGAGTCAAGGAGATCCTGCAGCCTTGGTTCTGCAGTCCATTCACGACTCCAGCAGCTCATAGGCAGGGTAGATGTTGGAGAGGGGTAGCTCAAAATCCAGGGTTTGGGCAGTAGCTGATTGATCAGCCATCCAGCTCTTCTGCCCCAACACTCACAGGGCTATCTCTCCATTACCCATGCCCCAAGTCAGCTTTCCCATTAAGGACCAGCTATCCTGAGTGCTGTTGCCAGTGAGTGGTAAGGCCAGCTCTCCTTCATCCATATCACCAAAGCCAGCTCTCCTATGCTGCCCAGGCAAGGGGTAGGTACAGCTCTCCTGCACCCATGTATTTTCATTTACTCATTTGAATTTTTCTTTCAATAAAAATGTTATTTATTTTACAAAAGTTTCACACTTCTAAAATTTTTCATGCAATGTACTTTGACTCATCAAGGGTGAGGCTTCCAAGCCTTGGAATTATGACAGGTTTTATCTCTTGAAGATAAAGCAGAGTTGCTGTGAGTTAACCTGTGTAATGCCCATACTTTGAGAAATTGGTATGGTTAGTCTTATCTCCAGACACCTTGTAATACCACAACCATGATTCATTCCTTGAGCTCCTCAAGGTTTCCAACTGAATCCAAGCCTTTTGCGCCATCATAGCGCCATGAGTCATCCCTCAGCAAGTCTTCAGATAGATCATCTTCAAGTTCCTTCCTTGAGTTTCTGGCCTCAGTTCCCTGGTCTATAAGCTGCAAGATGAAATATGCTCTTTCTTCCCAGTGTTGCTCTTGGTCATGGTGCTTTGTCACCATAATAGAGATCAAACTAAGACAGAGGTCATGCAAATTCTTCTGATAGAGCCCAAGCTTTGAAACTCCAAGAATTTAAATAACTGAATGACTGTAACATGGCTGGTCTTGGGCTGGACTGAGGCAGGGCCACAGGCATCATGACTCCCCGTGACAG
MSTRG.106192.1	--	1.182	0.000	0.000	-5.643	down	CCTCCCTTTGCCTCTTGTGACTATTTTGTTCCCCTTTCTAAGTGAGATTTAAGCATCCTAAGTTGTTTAACTGCTTAGGTTCTGTGGGGTGTATCATAGGTATCCTGTAATTTATGGCTAGTATCCACTTATCAGTGAGTACATACCATGCATATCCTTTTAGGTCTGGGTTACCTCAGATATCAGGATAACATTTTCATGTTCCATAGTCTCCATGTGGGCCCTTGAACAAATCTTAAAAGCTGTCATCTGGCTGTGGGATCCCTTCACCAAAAGGGCTGCCTTGTCTGGCCTCAGTAAGAGTATGGGCCTACCCTGAAGAAACTTGAAAGTGGGGGGGGGGCACACTCTTTCTTAGGAGAGGGGAGGGGAATGCAGGAGGATCTCTTGTGAGGCTGAGAGGGGAGACAGCATTTGATGTAAATAAATACATTTTTAAAAATTTGAGACTTACTTTCCAAATGTACCTGGTCTGCTCAGTACCTTTTCCCCAGATATAAAAATATATTTAAGTGGAGTATCTTATGTAGTTATATGCTCTTAATAATTGTTATTTCATAGAGCCAAGTCTGATTGTGTATACTAGTAATCGCACTTTTAGGAAAGCTGAGGCAGGAAGATTATAAATTCAGGGCTAATCAGTGATATAGCATGAGAAGCTCTTTTAAAACATACATACATAAACACACACACACACACACACACACACACACACACACACACACACACACACACATATCCACATGCATGAAGGCAGGACACAATATCCCAATATATATGTTATGTAGACT
MSTRG.106192.2	--	0.000	0.236	0.004	3.943	up	GTCTCCATGTGGGCCCTTGAACAAATCTTAAAAGCTGTCATCTGGCTGTGGGATCCCTTCACCAAAAGGGCTGCCTTGTCTGGCCTCAGTAAGAGTATGGGCCTACCCTGAAGAAACTTGAAAGTGGGGGGGGGGCACACTCTTTCTTAGGAGAGGGGAGGGGAATGCAGGAGGATCTCTTGTGAGGCTGAGAGGGGAGACAGCATTTGATGTAAATAAATACATTTTTAAAAATTTGAGACTTACTTTCCAAATGTACCTGGTCTGCTCAGTACCTTTTCCCCAGATATAAAAATATATTTAAGTGGAGTATCTTATGTAGTTATATGCTCTTAATAATTGTTATTTCATAGAGCCAAGTCTGATTGTGTATACTAGTAATCGCACTTTTAGGAAAGCTGAGGCAGGAAGATTATAAATTCAGGGCTAATCAGTGATATAGCATGAGAAGCTCTTTTAAAACATACATAAACACACACACACACACACACACACACACACACACACACACACACACACACACACATATATATATATATATACCCACATGCATGAAGGCAGGACACAATATCCCAATATATATGTTATGTAGACTTTTGAAAGGGTAAGAGATTGAGACTCTCATAAACCCACACCATCATGACACCATATACCATGATCTTGTATGCACAGACAGCA
MSTRG.10626.3	ENSRNOG00000019206	3.595	0.033	0.000	-5.545	down	TTAGTGTACTCAAAATAGATCAGGTTTGGTGAGCGAACAGAATTCAAGACATTCACATCCTTTACCACGAACCAATGGGCCTATTCTCCAATCACAGGAGTAGCCACAGGCAGTGAGCCTGAACCAGGTCCCAAAGGATCACGATTCAAGGTTGGGAACTCCTGCGAGCCCCATCAACACCTGAGCACTTTGAGTTCCTATGGAAA

							TTCAAGTGTTTCTTACCTTTCACTTCAGGTATCCTCAGGCAAGAGAGCTTCAACTGATGATTCAGGGCAGGCAATGTTAGGCTTCACCAAACACAAGCATAACCTGTAGTCACTTTCCCTTCAGGTATCCTCAAGCAAGAGATCTTGAACTGATGACTTCAGGGCAGGCAATGTTAGGCTTCTTCCAACTTCATCTGATGTTCCATACTCAGTTCCTCTGCAAGCCGTCCTTGTCCGGTATGAGACAGGGACTCTGTCCCTTTCACTTCAGGTATCCTCAGGCAAGAGAGCTTCAACTGAGGACTTCAGGGCAGGCGATGTTAGGCTTCTTCCAACTTCATCTGATGTTCCATACTCAGTTCCTCTGCAAGCCGTCCTTTTCCGGTATGAGACAGGGACTCTGTCCCTTTCACTTCAGGTATCCTCAGGCAAGAGAGCTCAACTGAGGACTTCAGGGCAGGCAATGTTAGGCTTCTTCCAACTTCATCTGATGTTCCATACTCAGTTCCTCTGCAAGCCGTCCTTGTCCGTTATGAGACAGGGACTCTGTCCCTGAAAGTAAAGCAAATAGCACATTCCCCCACTTTATTGGATTCCTTGCAAATCATGGAGACTGTAATGCATTGTGGTTAGTTCATAATTTATCGAACACTTGGGCTCTGTCAGCACTCCTTCCGTCCTCTACAAATTTGCCCTCACAATCCATATCAGAGATATTTTTCCACCAAGGTACTGTTTGGGGACATGAGTTTCCCAATTCTATCACCGAATTCCTGGATGTTACCATGCAACAAAGTTCTTAAAATGGGACGAAGCCAATGTCTGATCCCGAAAACTACAGTAGTATATACATCACTTGAGA
MSTRG.10626.5	ENSRNOG00000019206	2.829	0.123	0.000	-3.505	down	TCTCCATGCTCATATATCTCCTATACAGGTTTTGACTGGAACGAAATTCCTGTGAGTGCTATTGCTGTGGTATCTGATATTGATCACCACAGAGAAAGTGTCTGTTCAAACCCTCTCAGGTCACCAAACACAAGCATAACCTGTAGTCACTGAGATATATGTTATTATCAACAGCATGATCCTAAGCATACAGCTCACTTTCATAATCAAATGGAAACACAATTTCTTGTTGATATTTGCTTGGAGAAAAGAACAATGTCTAAGATAACAGGACTACCATCAGCCCCAAAACACATAGCCAAAATGCCAAGTTAACCTTCAGCAAGGCCACATCAAGACCATAAGGAGAGATAATCCCGCAGACACTCCATGCCTATCTTCTACGGCCATACCAAAATATAAAATTCCTAGAAGGAGTGTGTGTAAGATTCAAACTTGCATGCTCACATTCTTAGATCCTAGAAGAGGAGGAGATGTATGTCCAGACCATTTTGATACAGTCAATCCATAAGAACTGCTGGCAAACTACCCGGATAGACTGACAAAAAATGTCCCAACAATAAACATAGAAACTAGGATTAAAATGGGTGTTCTCCATACATCCATGAAGGTGGTATGAATCCTGGGACATTTGAAAGCATGCACTTCTAAATCCACAGACAAAAAGGCTACATGCCAACCTTTTAAATTCCAAGGCTCTCTTTCCAACCTAATTGCCATGGCCACAATCAGTCTTTCAAGAAAACAGAGCTTTGTTTTCACACAGGAAGAGTCCACATTCCCATAGTTCCTGAGAATTGCTACTGCCTCATTCCACATGTCGCCTGTAACCTTCCCGTTATGTATCTCTGTTTTATGAAACAGAAGGGTTATCATTCATGTGAAGGCTTCAGACAGAGACAGAAACACATTTCCT
MSTRG.10626.7	ENSRNOG00000019206	0.965	0.000	0.000	-6.833	down	TCTCCATGCTCATATATCTCCTATACAGGTTTTGACTGGAACGAAATTCCTGTGAGTGCTATTGCTGTGGTATCTGATATTGATCACCACAGAGAAAGTGTCTGTTCAAACCCTCTCAGGTCACCAAACACAAGCATAACCTGTAGTCACTGAGATATATGTTATTATCAACAGCATGATCCTAAGCATACAGCTCACTTTCATAATCAAATGGAAACACAATTTCTTGTTGATATTTGCTTGGAGAAAAGAACAATGTCTAAGATAACAGGACTACCATCAGCCCCAAAACACATAGCCAAAATGCCAAGTTAACCTTCAGCAAGGCCACATCAAGACCATAAGGAGAGATAATCCCGCAGACACTCCATGCCTATCTTCTACGGCCATACCAAAATATAAAATTCCTAGAAGGAGTGTGTGTAAGATTCAAACTTGCATGCTCACATTCTTAGATCCTAGAAGAGGAGGAGATGTATGTCCAGACCATTTTGATACAGTCAATCCATAAGAACTGCTGGCAAACTACCCGGATAGACTGACAAAAAATGTCCCAACAATAAACATAGAAACTAGGATTAAAATGGGTGTTCTCCATACATCCATGAAGGTGGTATGAATCCTGGGACATTTGAAAGCATGCACTTCTAAATCCACAGACAAAAAGGCTACATGCCAACCTTTTAAATTCCAAGGCTCTCTTTCCAACCTAATTGCCATGGCCACAATCAGTCTTTCAAGAAAACAGAGCTTTGTTTTCACACAGGAAGAGTCCACATTCCCATAGTTCCTGAGAATTGCTACTGCCTCATTCCACATGTCGCCTGTAACCTTCCCGTTATGTATCTCTGTTTTATGAAACATAAGGGTTATCATTCATGCGAAGGCTTCAGACAGGGACAGAAACACATTTCCTGACATTCCAGTGTACATTCTTGAACTATGCTGCCCCCGCTAAACTTACATTTGCCTCA

							CAACCTACTGCACATTATTGCTGATGGAGTTGATAGGGCTAACTTCCAAGATACTGACAATGATCTCCTC<br>AACCGGAAAGCATGTTATAACTACAATAAAAGGTGAAGCCTTGATATAGGCCACGATTTCTGGTGATTTA<br>GGCAAGGAAAGAACATTCTAGGCTCTCTGTCTCCAAATTTCGAAAGAAGAATGGAGGCCATGTGAGTC<br>CCTGGTGACATTTGTGATCCCAGGTCCTGGACCTCAGTCATGATGATAGTGGCGGTATTGAGTTTTCACA<br>CATTTTGTTCAGTAATCCAAGGAATATTTCATTGGAAATCATCATCAATCCAAGCAAGCTGGTGTACCCC<br>CTGCCATGACACTTAGTGCATTCAAAATTGATCTGGTTTGGCGAGCGAACAGAATTCAAGACATTCACA<br>TCCTTTACCATGAACCAATGGGCCTATTCTCCAATCACAGGAGTAGCAATAGGCAGTGAGCCTGGAGTT<br>CCTTTGGAAATTCAAGTGTTTCATACCTTTCACTTCAGGTATCCTCAGGCAAGAGATGTTACATACTCAG<br>TTCCTCTGCAAGCCATCCTTGTCCGTTATGAGACAGGGACTCTGTCCCTGGAAGTAAAGCAAATACCAC<br>ATTCCCCCACTTTATTGGATTCCTTCCCAAATCACGGACACTGTTATGCATTGTGTTTAGTACATAATTTAT<br>CGAATGCTTGGGCTCTCTCTGCACTCCTTCCTTCCTCTAGATCTTTGCCCTCACAATCCATATTAGAGATA<br>TTTTTCCACCAAGGAACTGTTTGCGGACATAAGTTTCCCAATGCTATCACCGAATTCCTGCAGGTTACCA<br>TGCAACAAATATCTTAAAATGGGACAAAGCCAACACCTGATCACGAAAACTACAGTAGAATGTACATCA<br>CTTGAAAACCATCCAAAGAAATTGACACAAGGTACACAACAGAATGAGACCACTAATATGGCTTAATG<br>GAAGTTGTCTACAAAAATAAGTAAATCTCAGTTCAGCTGGATATGACAGAGAAAATCAGGCCTTACTCT<br>TCCCATGGAAACCACCAGAATCTTAATAGCTTTGGCCACAATCGGAGCACCCTCCATACTCATGACATAC<br>TACACCTATTAGTCCTACTCTGCCTTCGATGAGACCCGATTCAAATAGCATATAAGGTTCTCATCCACATT<br>AGATGAACGGACTATTTAAGTACTCTGTCTCTATCTCTCTTCTTCTCTTTCTCTCTGAAATTGTCTTGC
MSTR<br>G.106<br>27.1	ENSRNO<br>G0000001<br>9206	2.326	0.288	0.037	-<br>2.159	do<br>wn	GCCTATCTTGTATGGCTATAACAAAATAAAAATTAGTAGAAGGACTCTGTGTAAGAAACAAAGTTGCAT<br>GCTCACTTTCTTAGATCCTAGAAGAGGATGAGATGTATGTCCAGACCAAATTTGGTTCAGTCAATCCATA<br>AGAATTGCTGGCAAACTAGCTGGATAGACTGAGAAAAAATGTCCCAACAATAAACATAGAAACTAGGA<br>TTAAAATGGGTGTCCTACATAAATCCATGAAGGTGATATGAATCCTGGGACATTTGAAAGCAGGCACTTT<br>TAGAAACCACCGACATGAAGGTTACATACCAACCCTTTAAATTTCAAGGGTCTCTTTCCAACCTATTTGC<br>CATGGCCACAATCAGTCTTTCAAGAAGACAGAGCTTTGGGGCTGGGGATTTAGCTCAGTGGTAGAGCG<br>CTTACCTAGGAAGCACAAGGCCCTGGGTTCGGTCCCCAGCTCCGAAAAAAAAAGACCCAAAAAAAAA<br>AAAAAAAAAAAAAAAAAGAAGCCAGAGCTTTGTTTTCACACAGGAAGTGTACACATTCCCATAGTTCT<br>GGAGAATTGCTACTGCCTCATTCCACATGTCGCCTGTAACCTTCCTGTTATGTATCTCTGTTTTATGAAAC<br>AGAAGGGTTATCATTCATGCGAAGGCTTCAGACAGGGACAGAAACACCTTTCCTGACATTCCAGTGTAC<br>ATTGTTGAACTATGGTGCCCCCGCTAAACTTACATTTTCCTCACAACCTACTATACATTATTGCTGATGGA<br>GTTGGTAGGGCTAACTTCCAAGATACTGACAATGATCTCCTCAACCGGAAAGCATGTTATCATTAGAGC<br>AAAAGGTGAAGCCTTGATTTAGGCCATGATTTCTGGTGATTTAGGCAAGCAAAGAACATTCTGAAGGCT<br>CTCTCACTGGATCCAAGTCTCGCAAGATGAATGGAGGCCATCTGAGTCCCTCAGTGACATTTGTGATCC<br>CAGGTGCTGGACCTCAGTCACGATGATAGTGGCGGTATTGAGTTTTCACTCATTTTGTTCAGCTTTCCAA<br>GGAATGTTTTATTGGAAATCATCATCGATCCAAGCACGGTAG
MSTR<br>G.106<br>27.2	ENSRNO<br>G0000001<br>9206	2.571	0.245	0.008	-<br>2.523	do<br>wn	AGGCCTGTGTGTAAGAAACAAACGTGCATGCTCACATTCATATATCCTAGAAGAGAACGAGATGTATGT<br>CTAGACAATCTTTGGTACAGTAAATCCATAAGAACTGCTGAAACACTAGCTGGATAGACTGACAAAAAA<br>TGTACCAACAATAAACATAGAAACTAGGATTAAATGAGTGTTCTCCATACATCCATGAAGTTGGTATGAA<br>TGCTGGGACATTTGAAAGCATGCACTTCTAGAAGCCACTGACAAGAAGGTTACATGCCAACCCTTTAAA<br>TTCCAAGGCTCTCTTTCCAACCTAATTGCCATGGCCACAATCAGTCTTTAAAGAAAACAGACCTTTGTTT<br>TCACACAGGAAGAGTCCACATTCCCATAGTTCCTGAGAATTGCTACTGACTCATTCCACATGTCACCTGT<br>AACCTTCCTGTTATGTATCTCTGTTTTATGAAACAGAAGGGTTATCATTCATGCGAAGGCTTCAGACAGG

							GACAGAAACACCTTTCCTGACATTCCAGTGTACATTGTTGAACTATGGTGCCCCCGCTAAACTTACATTTTCCTCACAACCTACTATACATTATTGCTGATGGAGTTGGTAGGGCTAACTTCCAAGATACTGACAATGATCTCCTCAACCGGAAAGCATGTTATCATTAGAGCAAAAGGTGAAGCCTTGATTTAGGCCATGATTTCTGGTGATTTAGGCAAGCAAAGAACATTCTGAAGGCTCTCTCACTGGATCCAAGTCTCGCAAGATGAATGGAGGCCATCTGAGTCCCTCAGTGACATTTGTGATCCCAGGTGCTGGACCTCAGTCACGATGATAGTGGCGGTATTGAGTTTTCACTCATTTTGTTCAGCTTTCCAAGGAATGTTTTATTGGAAATCATCATCGATCCAAGCACGGTAGTGTACCCCCTGCCATGACACTTAGTGTACTCAAAATAGATCAGGTTTGGTGAGCGAACAGAATTCAAGA
MSTRG.10628.1	ENSRNOG00000019206	1.172	0.072	0.000	-3.180	down	AGGCCTGTGTGTAAGAAACAAACGTGCATGCTCACATTCATATATCCTAGAAGAGAACGAGATGTATGTCTAGACAATCTTTGGTACAGTAAATCCATAAGAACTGCTGAAACACTAGCTGGATAGACTGACAAAAAATGTACCAACAATAAACATAGAAACTAGGATTAAATGAGTGTTCTCCATACATCCATGAAGTTGGTATGAATGCTGGGACATTTGAAAGCATGCACTTCTAGAAGCCACTGACAAGAAGGTTACATGCCAACCCTTTAAATTCCAAGGCTCTCTTTCCAACCTAATTGCCATGGCCACAATCAGTCTTTAAAGAAAACAGACCTTTGTTTTCACACAGGAAGAGTCCACATTCCCATAGTTCCTGAGAATTGCTACTGACTCATTCCACATGTCACCTGTAACCTTCCTGTTACTTATCTCTGTTTTATGAAAAAGAAGGGTTATCATTCATGCGAAAGCTTCAGACAGGGACAGAAACACCTTTCCTGACATTCCACTGTACATTGTCGAACCTTGCTGCCCCTGCTAACCTTACATTTGCCTCACAGCCTACTGTACATTATTGCTGGTTGGAGTTGGTAGGGCTAACTTCCAAGATACTGACAATGATATCCTCAATGGGAAAGCATGTTATGACTACAGAAAAAGGTGAAGCCTTGATATAGGCCATGATTTCTGGTGATGTAGGCAAGCAAAGAACATTCTGAAGGCTCTCTCACTGGATCCAAGTCTTGCAAGTTGAATGGAGGCCATGTGAGTCCCTCAGTGACATTTGTGACCCCAGGTTCTGGACCTCAGTCACGATGATAGTGGCAGTATTGAGTTTTCACTCATTTTGTTCAGCTTTCCAAGGAATGTTTTATTGGAAATCATCATTGATCCAAGCACGCTGGTGTAACCCCTGACATGAGAATTAGTGTACTCAAAATAGATCAGGTTTGGTGAGCGAACAGAATTCAAGACATTCACATCCTTTACCACGAACCAATGGGCCTATTCTCCAATCACAGGAGTAGCCACAGGCAGTGAGCCTGAACCAGGTCCCAAAGGATCACGATTCAAGTTTGGGAACTCCTGCGAGCTCCATCAACACCTGAGCACTTTGAGTTCCTTTGGAAAGTCAAGTGTTTCTTACCTTTCCCTTCAGGTATCCTCAAGCAAGAGATCTTGAACTGATGACTTCAGGGCAGGCAATGTTAGGCTTCTTCCAACTTCATCTGATGTTCCATACTCAGTTCCTCTGCAAGCCGTCCTTGTCCGGTATGAGACAGGGACTCTGTCCCTGAAAGTAAAGCAAATAGAACATTCCCCCACTTTATTGGATTCCTTGCAAATCATGGAGACTGTGATGCATTGTTGTTAGTTCTTAATTTATCGAACACAGGGGCTCTCTCAGCACTCCATCCGTCCTCTAGAAATTTGCCCTCACAATCCATATAAGAGATATTTTTCCCCAAGGAACTGTTTGGGACATAATTTTCCCAATTCTATCACCGAATTCCTGGAGGTAACCATGCAACAAAGTTCTTAAGATGGGACGAAGCCAATATCTGATCCCGATAACTACAGTAGTATGTACATCACTTGAGAACCATCCAAAGAAATTGGCACGAGGTACACAACAGAATGAGACCACTAACATGGCTTAATGGAAGTTGTCTACAAAAATAAGTAAATCTCAGTTCAGCCGGATATGACAGAGAAAATCAGGCCTTACTCTTCCCATAGAAACCACCAGAATCTTAAATGCTTTGGCCACAATCGGAGCACCCTCCATAATCATAAATTTCTACACCTATTAGTCCTACTCTGCATTCGATGAGAACCGATTCAAATATCATATAAGATTCTCATCCACATTAGATGAATGGACTTCTTATGTACTCTTTCTCTATCTCGCTTCTTCACTTTCTCTCTGAAATTGACTAGCAGTGACTCTCCTAGCTTCTCAGTTTCTCACTCACATTCTCTCTATTTGGTTTCCTTATTTCTGTCAATCAAGATATCTGCTTTAATATCACAGTGTCCTCTTTTCCACTTCACCCCCCTTTCTCCCCCCCCTCTCTCTTTCTGTCTCTCACTGTCTTCCTCTGTCTCACACATGCTTAGCCTCTTCAACAATTGTTCTTTCTCTCTTTCTCTGTCCATGCTCATATATCTCCTATTCAGGTTTTGACTGGTACTAAATTCATGTGAGTGCCATTGCTGTGGTATCTGACATTGATCACCACAGAGAAAGTGTCAGTTCAAACCGTCTCAGGTCACCAAACACAAGCATAACCTG

							TAGTCACTGAAATATATGTTATTATCAACAGCATGATCCTAAGCATACAGCTCACTTTCATAATCAAATGG<br>AAACACAATTTCTTGTTGATATTTGCTTGGAGAAAAGAACAATGTCTAAGATAACAGGACTACCATCAG<br>CCCCAAAACACATAGCCAAAATGCCAAGTTAACCTTCAGCAAAGCCACATCAAGACCATAAGGAAAGA<br>TAATCCCGCAGACACACCATGCCTATCTGCTACGGCCATACCAAATTATAAAATTCCTAGAAGGACTGTG<br>TGTAAGGAACAAACTTGCATGCTCACATTCTTAGATCCTAGAAGAGGAAGAGATGTTTGTCCAGACCAT<br>TTTGGTACAGTCTATCCATAAGAACTGCTGGCAAAATACCCGTATAGAATGACAAAAATGTCCCGACAAT<br>AAACATAGAAAGTAGGATTAAAATGGTTGTTCTTCATACATCCATGAAGGTGGTATGAATCCTGGGATAT<br>TTGAAAGCATGCACTTCTAGAAGCCACAGACAAGAAGGCTACATACCAACCATTTACATTCCAAGGCTC<br>TCTTTCCAACCTAAAGGCCATGGCCACAATCAGTCTTTCAAGAAAACAGAGCTTTATTTTCACACAGGA<br>AGAGTCCACATTCCCATATTTCCTGAGAATTGCTACTGCCCTAATCCACATGTCGCCTGTAACCTTCCCG<br>TTATGTATCTCTGTTTTATGAAACATAAGGGTTATCATTCATGCGAAGGCTTCAGACAGGGACAGAAACA<br>CATTTCCTGACATTCCAGTGTACATTCTTGAACTATGCTGCCCCCGCTAAACTTACATTTGCCTCACAAC<br>CTACTGCACATTATTGCTGATGGAGTTGATAGGGCTAACTTCCAAGATACTGACAATGATCTCCTCAACC<br>GGAAAGCATGTTATAACTACAATAAAAGGTGAAGCCTTGATATAGGCCACGATTTCTGGTGATTTAGGCA<br>AGGAAAGAACATTCTAGGCTCTCTGTCTCCAAATTTCGAAAGAAGAATGGAGGCCATGTGAGTCCCTG<br>GTGACATTTGTGATCCCAGGTCCTGGACCTCAGTCATGATGATAGTGGCGGTATTGAGTTTTCACACATT<br>TTGTTCAGTAATCCAAGGAATATTTCATTGGAAATCATCATCAATCCAAGCAAGCTGGTGTACCCCCTGC<br>CATGACACTTAGTGCATTCAAAATTGATCTGGTTTGGCGAGCGAACAGAATTCAAGACATTCACATCCT<br>TTACCATGAACCAATGGGCCTATTCTCCAATCACAGGAGTAGCAATAGGCAGTGAGCCTGGAGTTCCTT<br>TGGAAATTCAAGTGTTTCATACCTTTCACTTCAGGTATCCTCAGGCAAGAGATGTTACATACTCAGTTCC<br>TCTGCAAGCCATCCTTGTCCGTTATGAGACAGGGACTCTGTCCCTGGAAGTAAAGCAAATACCACATTC<br>CCCCACTTTATTGGATTCCTTCCCAAATCACGGACACTGTTATGCATTGTGTTTAGTACATAATTTATCGA<br>ATGCTTGGGCTCTCTCTGCACTCCTTCCTTCCTCTAGATCTTTGCCCTCACAATCCATATTAGAGATATTTT<br>TCCACCAAGGAACTGTTTGCGGACATAAGTTTCCCAATGCTATCACCGAATTCCTGCAGGTTACCATGC<br>AACAAATATCTTAAAATGGGACAAAGCCAACACCTGATCACGAAAACTACAGTAGAATGTACATCACTT<br>GAAAACCATCCAAAGAAATTGACACAAGGTACACAACAGAATGAGACCACTAATATGGCTTAATGGAA<br>GTTGTCTACAAAAATAAGTAAATCTCAGTTCAGCTGGATATGACAGAGAAAATCAGGCCTTACTCTTCC<br>CATGGAAACCACCAGAATCTTAATAGCTTTGGCCACAATCGGAGCACCCTCCATACTCATGACATACTAC<br>ACCTATTAGTCCTACTCTGCCTTCGATGAGACCCGATTCAAATAGCATATAAGGTTCTCATCCACATTAGA<br>TGAACGGACTATTTAAGTACTCTGTCTCTATCTCTCTTCTTCTCTTTCTCTCTGAAATTGTCTTGC
MSTRG.10628.10	ENSRNOG00000019206	0.674	0.000	0.001	-4.054	down	CTTCAGACAGGGACAGAAACACCTTTCCTGACATTCCAGTGTACATTGTTGAACTATGGTGCCCCCGCT<br>AAACTTACATTTTCCTCACAACCTACTATACATTATTGCTGATGGAGTTGGTAGGGCTAACTTCCAAGATA<br>CTGACAATGATCTCCTCAACCGGAAAGCATGTTATCATTAGAGCAAAAGGTGAAGCCTTGATTTAGGCC<br>ATGATTTCTGGTGATTTAGGCAAGCAAAGAACATTCTGAAGGCTCTCTCACTGGATCCAAGTCTCGCAA<br>GATGAATGGAGGCCATCTGAGTCCCTCAGTGACATTTGTGATCCCAGGTGCTGGACCTCAGTCACGATG<br>ATAGTGGCGGTATTGAGTTTTCACTCATTTTGTTCAGCTTACCAAGGAATGTTTTATTGAAAATCATCATC<br>GATCCAAGCACGCTGCTGTACCCCCTGCCATGACACTTAGTGTACTCAAAATAGATCAGGTTTGGTGAG<br>CGAACAGAATTCAAGACATTCACATCCTTTACCACGAACCAATGGGCCTATTCTCCAATCACAGGAGTA<br>GCCACAGGCAGTGAGCCTGAACCAGGTCCCAAAGGATCATGATTCAAGGTTGGGAACTCCTGCGAGCT<br>CCATCAACACCTGAGCACTTTGAGTTCCTTTGGAAATTCAAGTGTTTCTTAACTTTCACTTCAGGTATCC<br>TCAGGCAAGAGAGCTTCAACTGATGACATCAGGGCAGGCGATGTTAGGCTTCTTCCAACTTCATCTGAT

							GTTCCATACTCAGTTCCTCTGCAAGCCGTCCTTGTCCGTTATGAGACAGGGACTCTGTCCCTGAAAGTAAAGCAAATAGCACATTCCCCCACTTTATTGGATTTCTTGCAAATCATGGAGACTGTGATGCATTGTGGTTAGTTCATAATTTATCGAACACTTGGGCTCTCTCAGCACTCCTTC
MSTRG.10628.15	ENSRNOG00000019206	1.205	0.099	0.033	-2.435	down	CCATCTTTGGTTCAGTCAATCCATAAGAACTGCTTAAAAACTAGCTGGTTAGACTGACAAAAAATGTCCCAACAATAAACATAGAAACTAGGATTAAAATGGGTGTCCTCCATACATCCATGAAGGTGGTATGAATCCTGGGACATTTGAAAGCATGCACTTCTAGAAGCCACCGACAAGAAGGCTACATGCCAACCTTTTAAATTCCAAGGCTCTCTTTCCAACCTAATTGCCATGGCCACAATCAGTCTTTCAAGAAAACAGAGCTTTGTTTTCACACAGGAAGAGTCCACATTCCCATAGTTCCTGAGAATTGCTACTGCCTCATTCCACATGTCGCCTGTAACCTTCCCGTTATGTATCTCTGTTTTATGAAACAGAAGTGTCATCATTCATGCGAAGGCTTCAGACACGGACAGAAACACATTTCCTGACATTCCAGTGTACATTGTTGAACTATGCTGCCCCCGGTAAACTTACATTTGCCTCACAACCTACTATACATTATTGCTGATGGAGTTGGTAGGGCTAACTTCCAAGATACTGACAATGATCTCCTCAACCGGAAAGCATGTTATCATTAGAGCAAAAGGGGAAGCCTTGATTTAGGCCATGATTTCTGGTGATTTATGCAAGC
MSTRG.10628.2	ENSRNOG00000019206	2.229	0.083	0.000	-3.910	down	AGGCCTGTGTGTAAGAAACAAACGTGCATGCTCACATTCATATATCCTAGAAGAGAACGAGATGTATGTCTAGACAATCTTTGGTACAGTAAATCCATAAGAACTGCTGAAACACTAGCTGGATAGACTGACAAAAAATGTACCAACAATAAACATAGAAACTAGGATTAAATGAGTGTTCTCCATACATCCATGAAGTTGGTATGAATGCTGGGACATTTGAAAGCATGCACTTCTAGAAGCCACTGACAAGAAGGTTACATGCCAACCCTTTAAATTCCAAGGCTCTCTTTCCAACCTAATTGCCATGGCCACAATCAGTCTTTAAAGAAAACAGACCTTTGTTTTCACACAGGAAGAGTCCACATTCCCATAGTTCCTGAGAATTGCTACTGACTCATTCCACATGTCACCTGTAACCTTCCTGTTACTTATCTCTGTTTTATGAAAAAGAAGGGTTATCATTCATGCGAAAGCTTCAGACAGGGACAGAAACACCTTTCCTGACATTCCACTGTACATTGTCGAACCTTGCTGCCCCTGCTAACCTTACATTTGCCTCACAGCCTACTGTACATTATTGCTGGTTGGAGTTGGTAGGGCTAACTTCCAAGATACTGACAATGATATCCTCAATGGGAAAGCATGTTATGACTACAGAAAAAGGTGAAGCCTTGATATAGGCCATGATTTCTGGTGATGTAGGCAAGCAAAGAACATTCTGAAGGCTCTCTCACTGGATCCAAGTCTTGCAAGTTGAATGGAGGCCATGTGAGTCCCTCAGTGACATTTGTGACCCCAGGTTCTGGACCTCAGTCACGATGATAGTGGCAGTATTGAGTTTTCACTCATTTTGTTCAGCTTTCCAAGGAATGTTTTATTGGAAATCATCATTGATCCAAGCACGCTGGTGTAACCCCTGACATGAGAATTAGTGTACTCAAAATAGATCAGGTTTGGTGAGCGAACAGAATTCAAGACATTCACATCCTTTACCACGAACCAATGGGCCTATTCTCCAATCACAGGAGTAGCCACAGGCAGTGAGCCTGAACCAGGTCCCAAAGGATCACGATTCAAGTTTGGGAACTCCTGCGAGCTCCATCAACACCTGAGCACTTTGAGTTCCTTTGGAAAGTCAAGTGTTTCTTACCTTTCCCTTCAGGTATCCTCAAGCAAGAGATCTTGAACTGATGACTTCAGGGCAGGCAATGTTAGGCTTCTTCCAACTTCATCTGATGTTCCATACTCAGTTCCTCTGCAAGCCGTCCTTGTCCGGTATGAGACAGGGACTCTGTCCCTGAAAGTAAAGCAAATAGAACATTCCCCCACTTTATTGGATTCCTTGCAAATCATGGAGACTGTGATGCATTGTTGTTAGTTCTTAATTTATCGAACACAGGGGCTCTCTCAGCACTCCATCCGTCCTCTAGAAATTTGCCCTCACAATCCATATAAGAGATATTTTTTCCCCAAGGAACTGTTTGGGACATAATTTTCCCAATTCTATCACCGAATTCCTGGAGGTAACCATGCAACAAAGTTCTTAAGATGGGACGAAGCCAATATCTGATCCCGATAACTACAGTAGTATGTACATCACTTGAGAACCATCCAAAGAAATTGGCACGAGGTACACAACAGAATGAGACCACTAACATGGCTTAATGGAAGTTGTCTACAAAAATAAGTAAATCTCAGTTCAGCCGGATATGACAGAGAAAATCAGGCCTTACTCTTCCCATAGAAACCACCAGAATCTTAAATGCTTTGGCCACAATCGGAGCACCCTCCATAATCATAAATTTCTACACCTATTAGTCCTACTCTGCATTCGATGAGAACCGATTCAAATATCATATAAGATTCTCATCCACATTAGATGAATGGACTTCTTATGTACTCTTTCTCTATCTCGCTTCTTCACTTTCTCTCTGAAATTGACTAGCA

							GTGACTCTCCTAGCTTCTCAGTTTCTCACTCACATTCTCTCTATTTGGTTTCCTTATTTCTGTCAATCAAG<br>ATATCTGCTTTAATATCACAGTGTCCTCTTTTCCACTTCACCCCCCTTTCTCCCCCCCCTCTCTCTTTCTGT<br>CTCTCACTGTCTTCCTCTGTCTCACACATGCTTAGCCTCTTCAACAATTGTTCTTTCTCTCTTTCTCTGTC<br>CATGCTCATATATCTCCTATTCAGGTTTTGACTGCTACTAATTTCATGTGAGTGCTATTGCTCTGGTATCTG<br>ACATTGATCACCACAGAGAAAGTGTCTGTTCAAACCCTCTCAGGTCATCAAACACAAGCATAACCTGTA<br>GTCACTGAGATATATGTTATTATCAACAGCATGATCCTAAGCATACAGCTCACTTTCATAATCAAATGGAA<br>ACACAATTTCTTGTTGATATTTGCTTGGAGAAAAGAACAATGTCTAAGATAACAGGACTACCATCAGCC<br>CCAAAACACATAGCCAAAATGCCAAGTTAACCTTCAGCAAGGCCACATCAAGACCATAAGGAGAGATA<br>ATCCCGCAGACACTCCATGCCTATCTTCTACGGCCATACCAAAATATAAAATTCCTAGAAGGAGTGTGTG<br>TAAGATTCAAACTTGCATGCTCACATTCTTAGATCCTAGAAGAGGAGGAGATGTATGTCCAGACCATTTT<br>GATACAGTCAATCCATAAGAACTGCTGGCAAACTACCCGGATAGACTGACAAAAAATGTCCCAACAATA<br>AACATAGAAACTAGGATTAAAATGGGTGTTCTCCATACATCCATGAAGGTGGTATGAATCCTGGGACATT<br>TGAAAGCCTGCACTTCTAGAAGCCACCGACAAGAAGGTTACATGCCAAGCCTTTATACTCCAAGCCTCT<br>CTTTCCAACCTAATTGCCATGGCCACAATCAGTATTTCAAGAAAACAGAGCTTTGTTTTCACACAGAAA<br>GAGTCCACATTCCCATAGTTCCTGAGAATTGCTACTGCCTCATACCACATGTCGCCTGTAACCTTCCCTAT<br>ATGTATCTCTGTTTTCTGAAACAGAAGGGTTATCATTCATGCGAAGGCTTCAGACAGGGACAGAAACAC<br>ATTTCCTGACATTCCAGTGTACATTGTGGAGCTATGATGCCCCTGCTAAACTTACATTTGCCTCACAGCC<br>GAGTGTACATTATTGCTGGATGGAGTTGTTAGGGATAACTTCCAAGATACTGACAATGATCTCTTCAAAC<br>GGAAAGCATGTTATCACTACAATAAAAGGTTAAGACTCGATATAGGCCATTATTGCTGGTGATTTAGGAA<br>AGCAAAGAACATTCTGAAGGCTCTCTCACTGGATCCAAGTCTGGCAAGATGAATGGAGGCCATGTGAG<br>TCCCTCAGTGACATTTGTGATCCCAGGTCCTGGACCTCAGTCATGATGATAGTGGCGGTATTGAGTTTTC<br>ACACATTTTGTTCAGTAATCCAAGGAATATTTCATTGGAAATCATCATCAATCCAAGCAAGCTGGTGTAC<br>CCCCTGCCATGACACTTAGTGCATTCAAAATTGATCTGGTTTGGCGAGCGAACAGAATTCAAGACATTC<br>ACATCCTTTACCATGAACCAATGGGCCTATTCTCCAATCACAGGAGTAGCAATAGGCAGTGAGCCTGGA<br>GTTCCTTTGGAAATTCAAGTGTTTCATACCTTTCACTTCAGGTATCCTCAGGCAAGAGATGTTACATACT<br>CAGTTCCTCTGCAAGCCATCCTTGTCCGTTATGAGACAGGGACTCTGTCCCTGGAAGTAAAGCAAATAC<br>CACATTCCCCCACTTTATTGGATTCCTTCCCAAATCACGGACACTGTTATGCATTGTGTTTAGTACATAAT<br>TTATCGAATGCTTGGGCTCTCTCTGCACTCCTTCCTTCCTCTAGATCTTTGCCCTCACAATCCATATTAGA<br>GATATTTTTCCACCAAGGAACTGTTTGCGGACATAAGTTTCCCAATGCTATCACCGAATTCCTGCAGGTT<br>ACCATGCAACAAATATCTTAAAATGGGACAAAGCCAACACCTGATCACGAAAACTACAGTAGAATGTAC<br>ATCACTTGAAAACCATCCAAAGAAATTGACACAAGGTACACAACAGAATGAGACCACTAATATGGCTTA<br>ATGGAAGTTGTCTACAAAAATAAGTAAATCTCAGTTCAGCTGGATATGACAGAGAAAATCAGGCCTTAC<br>TCTTCCCATGGAAACCACCAGAATCTTAATAGCTTTGGCCACAATCGGAGCACCCTCCATACTCATGACA<br>TACTACACCTATTAGTCCTACTCTGCCTTCGATGAGACCCGATTCAAATAGCATATAAGGTTCTCATCCAC<br>ATTAGATGAACGGACTATTTAAGTACTCTGTCTCTATCTCTCTTCTTCTCTTTCTCTCTGAAATTGTCTTGC
MSTR<br>G.106<br>28.27	--	1.960	0.000	0.000	-<br>7.885	do<br>wn	CTCTCACTGTCTTCCTCTGTCTCACACATGCTTAGCCTCTTCAAAAATTGTTCTTTGTCTCTTTCTCTATC<br>TATGCTCATATGCCTGCTATTCAGGTTTTGACTGCTACTAATTTCATGTGAGTGCTATTGCTCTGGTATCTG<br>ACATTGATCACCACAGAGAAAGTGTCTGTTCAAACCCTCTCAGGTCATCAAACACAAGCATAACCTGTA<br>GTCACTGAGATATATGTTATTATCAACAGCATGATCCTAAGCATACAGCTCACTTTCATAATCAAATGGAA<br>ACACAATTTCTTGTTGATATTTGCTTGGAGAAAAGAACAATGTCTAAGATAACAGGACTACCATCAGCC<br>CCAAAACACATAGCCAAAATGCCAAGTTAACCTTCAGCAAGGCCACATCAAGACCATAAGGAGAGATA

							ATCCCGCAGACACTCCATGCCTATCTTCTACGGCCATACCAAAATATAAAATTCCTAGAAGGAGTGTGTGTAAGATTCAAACTTGCATGCTCACATTCTTAGATCCTAGAAGAGGAGGAGATGTATGTCCAGACCATTTTGATACAGTCAATCCATAAGAACTGCTGGCAAACTACCCGGATAGACTGACAAAAAATGTCCCAACAATAAACATAGAAACTAGGATTAAAATGGGTGTTCTCCATACATCCATGAAGGTGGTATGAATCCTGGGATATTTGAAAGCATGCACTTCTAGAAGCCACAGACAAGAAGGCTACATACCAACCATTTACATTCCAAGGCTCTCTTTCCAACCTAAAGGCCATGGCCACAATCAGTCTTTCAAGAAAACAGAGCTTTATTTTCACACAGGAAGAGTCCACATTCCCATATTTCCTGAGAATTGCTACTGCCCTAATCCACATGTCGCCTGTAACCTTCCCGTTATGTATCTCTGTTTTATGAAACATAAGGGTTATCATTCATGCGAAGGCTTCAGACAGGGACAGAAACACATTTCCTGACATTCCAGTGTACATTCTTGAACTATGCTGCCCCCGCTAAACTTACATTTGCCTCACAACCTACTGCACATTATTGCTGATGGAGTTGATAGGGCTAACTTCCAAGATACTGACAATGATCTCCTCAACCGGAAAGCATGTTATAACTACAATAAAAGGTGAAGCCTTGATATAGGCCACGATTTCTGGTGATTTAGGCAAGGAAAGAACATTCTAGGCTCTCTGTCTCCAAATTTCGAAAGAAGAATGGAGGCCATGTGAGTCCCTGGTGACATTTGTGATCCCAGGTCCTGGACCTCAGTCATGATGATAGTGGCGGTATTGAGTTTTCACACATTTTGTTCAGTAATCCAAGGAATATTTCATTGGAAATCATCATCAATCCAAGCAAGCTGGTGTACCCCCTGCCATGACACTTAGTGCATTCAAAATTGATCTGGTTTGGCGAGCGAACAGAATTCAAGACATTCACATCCTTTACCATGAACCAATGGGCCTATTCTCCAATCACAGGAGTAGCAATAGGCAGTGAGCCTGGAGTTCCTTTGGAAATTCAAGTGTTTCATACCTTTCACTTCAGGTATCCTCAGGCAAGAGATGTTACATACTCAGTTCCTCTGCAAGCCATCCTTGTCCGTTATGAGACAGGGACTCTGTCCCTGGAAGTAAAGCAAATACCACATTCCCCCACTTTATTGGATTCCTTCCCAAATCACGGACACTGTTATGCATTGTGTTTAGTACATAATTTATCGAATGCTTGGGCTCTCTCTGCACTCCTTCCTTCCTCTAGATCTTTGCCCTCACAATCCATATTAGAGATATTTTTCCACCAAGGAACTGTTTGCGGACATAAGTTTCCCAATGCTATCACCGAATTCCTGCAGGTTACCATGCAACAAATATCTTAAAATGGGACAAAGCCAACACCTGATCACGAAAACTACAGTAGAATGTACATCACTTGAAAACCATCCAAAGAAATTGACACAAGGTACACAACAGAATGAGACCACTAATATGGCTTAATGGAAGTTGTCTACAAAAATAAGTAAATCTCAGTTCAGCTGGATATGACAGAGAAAATCAGGCCTTACTCTTCCCATGGAAACCACCAGAATCTTAATAGCTTTGGCCACAATCGGAGCACCCTCCATACTCATGACATACTACACCTATTAGTCCTACTCTGCCTTCGATGAGACCCGATTCAAATAGCATATAAGGTTCTCATCCACATTAGATGAACGGACTATTTAAGTACTCTGTCTCTATCTCTCTTCTTCTCTTTCTCTCTGAAATTGTCTTGC
MSTRG.10628.9	ENSRNOG00000019206	1.192	0.056	0.001	-3.314	down	CTTCAGACAGGGACAGAAACACCTTTCCTGACATTCCAGTGTACATTGTTGAACTATGGTGCCCCCGCTAAACTTACATTTTCCTCACAACCTACTATACATTATTGCTGATGGAGTTGGTAGGGCTAACTTCCAAGATACTGACAATGATCTCCTCAACCGGAAAGCATGTTATCATTAGAGCAAAAGGTGAAGCCTTGATTTAGGCCATGATTTCTGGTGATTTAGGCAAGCAAAGAACATTCTGAAGGCTCTCTCACTGGATCCAAGTCTCGCAAGATGAATGGAGGCCATCTGAGTCCCTCAGTGACATTTGTGATCCCAGGTGCTGGACCTCAGTCACGATGATAGTGGCGGTATTGAGTTTTCACTCATTTTGTTCAGCTTACCAAGGAATGTTTTATTGAAAATCATCATCGATCCAAGCACGCTGCTGTACCCCCTGCCATGACACTTAGTGTACTCAAAATAGATCAGGTTTGGTGAGCGAACAGAATTCAAGACATTCACATCCTTTACCACGAACCAATGGGCCTATTCTCCAATCACAGGAGTAGCCACAGGCAGTGAGCCTGAACCAGGTCCCAAAGGATCATGATTCAAGGTTGGGAACTCCTGCGAGCTCCATCAACACCTGAGCACTTTGAGTTCCTTTGGAAATTCAAGTGTTTCTTAACTTTCACTTCAGGTATCCTCAGGCAAGAGAGCTTCAACTGATGACATCAGGGCAGGCGATGTTAGGCTTCTTCCAACTTCATCTGATGTTCCATACTCAGTTCCTCTGCAAGCCGTCCTTGTCCGTTATGAGACAGGGACTCTGTCCCTGAAAGTAAAGCAAATAGCACA

MSTRG.10640.1	--	0.571	0.012	0.049	-2.996	down	CACGAGTTAAAGAAAAGTATATTCCTCTGGGGACCACTATGTCTTAAAGACTCTTATTAGTCTGCACTTTTACCTCGGTACACTCAGCTTACTGTTTTAATGGCATTCTTGAGTGCGTTAACAACTGGGAATCTGATTCTTGTTTCTGCTCCTTTTCCTTCTGTTTTATTGCTTTGTCCACCCCCAATATTATGTTCTTTGTTTATCTTATTATATTTTTTTTGGAGACAGAAAGGGAAGAGATCTGGAAGCAAAGAAAGGGGAGGTGAAACTGGGAAGAGTTAAAAAAAAGGAGAACTGTGGTCACGATTTAAACAGAAAAATTAAGAATGGAAATGAAAAACTAGGACAATAAATTTTCCTTAAAAATACACCATTTTATAATGAAAACCAAAAGCAATTGCAAAAAGACAAGAAAACATACAAACAAACAGAAACAAAAGAAAAGACATGCTTTTGTGGGTTTAACTGATGGAGCATGGAAAAAATCCCCCCCCAATCTCCCCAAAAAAAACACATCTGCA
MSTRG.10641.1	--	0.791	0.051	0.003	-2.946	down	CTTATTTCCTGTGGTTACTTTCTGTATATTAATCCTTACATCGTTCTCTGTATCTCTAATTTCTATGCATTTACCCTCATATCCCTTTTTTGTACAAATAAAAATCTCTTCCTCTTATGACCCATAATTGACCATTCAAATGCTTGAGTTATTAAAAGAACAGTTCAACGTTCCTACTAATCAGGAATATGTATATTCAAACCCTACTATGATTTTATCATTCACAAATAAGAATGGCCAAAATCAATGAAATAAATGACCGCACATTGTAGAAAGTGTGAAGAGTAATAAGAATCATTATACACTAATAGTGGCAATATACAGTTGTATTTCCTTTTGGAAATCAGTGTGATTTTGCCTTTTAACCCTGGGAATATTACACTGCAGATTAAAAAGAAAAGACCCTATATTCTGTATAGAGGAACTTACTTCCCATTGATGGCTGCTATATTCAAATCAGAAAGAAAGAGGATACAGCCTAGCTGTCCAGAAATGGGGAAATGGTTAATGAAAATATGGTACAATACAAAATGGATTATTACTCAGCTGATAAAAAGCAATAGTACTCATAGTTAAGGAAGGAGAAAAAAAATCATCCTGAGTTACCAGTACCTCAAAACACATATCAGACTATGCCTAAGGCCTTGATCATTCTATACACACTGAGGATGGCCCTTTTCCGGAAAAGGACCCTACATTTCTGTTGAACATAGAGAAGAAAAGCAGGTACCTAACTAAAGCTTTTACATACTGAATAGTGTTTGTGGTACTGAAAAGAATTCTGCCTGGCACCTGAGGGGGCATTTAAACCAACTCAACTGTATATTATTTAATCTACCCTGTCAGCTGCTTACATCATACATTGCTACAATAGTTCCACTTCCCTGGAAAAAAGCTACTACTACATGATGAACTAAGATCTACTCACTGAGACAGAAAACATTTCTGTCCTTGTTCCCATGGCAAAATACCTTAGAATATTGCGAATAGCTTGTACCATTGCCTACTTACAATGTGTATCAATAAATGTATTTCTAATAACATTGTTATACTCATAGTGACATGTATTACTGCACCTTCATCATAGAAGCTAACATACACTTTAAATGGGGCCAGATACAGAGACCCATATCTGGATAGTATGCAGTGTGAAAGACCTTGGGCCATTCTGTCCATAAATCAATATTGCCATCCAAGCTCCTCCTTCATGATTGAGCAAACCTTGTAGAAGCCATTGTAAAAAGATACTAAGGCCAGTGATGCTGGGAGGAATGCCAGCAAGGCAACATACATTTCAGACATAGTAGGGCTGATGCACATATGAACACATACAGAAAGTAACAACATGCACAGGATCTGCATAGGTCAGTCCATACACTGAGAGCAGGAATGGCATGTGATTCCCCACTCTTCAACAAGTAGCTATTTCATATACATTTACAAATAATTTAGAAAATATTGTTTGGATGAAAAATTCTGTTTGTTTGTTTCTTCACCGGTAGATATCTTGTCTTTCTCTAACACACAATGAAAGTATTCCCTATCTGTGTTTGATAAATGCTCTCCAGGATCCATTTCCATGAATGTGCAGTTTA
MSTRG.10643.1	--	1.210	0.000	0.000	-4.991	down	GTGAGATAGAGGTTGAAGCCTTTCTAATGTAATCATCATATAAGGAGGCTTCAGTTGTTGCTTTAACGTGGACCTGATTTGTCCAGTATGCAGGTGAGGAATAAACCTTGGGAGAAATAAACTCTCCTCTTGAGGAACTCTGATGCAGACATTCATTCAGAGGCAAACAAAATCCACTTTTTGGGCTGAATGTTTTTATATCATACAGTGAATCTACACACAGTACAGAGCCAATTTTCTGGCCGTACTGAAAACAAGTCTATTTCTTAGCAGTTATCTGAGAGTAGTAACAGACATAACTGTGAGAATCTGCATCTCCGGCTGGCTGTCAAGAGCACTGCCACATGCTTGGATTCTGAGGAAAATAACATGGTCATTCTCACATGGAAATAATGTTTTTTAAAGTGATTTGTCACCGTTATTTAATTAATGATTTTGTTTACATTTCAAATGATATCACCTTTACTTTTTATCCATTCAC
MSTRG.106	--	0.747	0.066	0.009	-2.566	down	TGATGTTCAAACCCTTTTAATTTTTGGAACTTGTGAGACAATTTTGCATTCTGTAGAGTAGGTTGGCCTAAAATTTCATTTGAAATTTGAATTTGACTGTGGTCATTAATTACTTTTAGATTTTTTTTTTTGGGAAAAGAC

44.1							CACTAATGTTGGCACATGCCCTTATATCTAACAACATAGAGGTGTAGCCAGAGAGTGTACACTGAGTTTACTTACAGCCAGTATTACATTTTAGAATTTTTTTGTAACAACCCCAAAAATATATTAATAAAATTAAAATTGTGGTTGTAAAAGATTATTTCTTCTCTTTGGTATCTTGAGTGGTAAGCCGCAGGTTGCTGAATAGACAGCTTATATCAAAAACCACGGAGCAGAAGCAGAGAGAGCTAATTAAAATATCTTGAGTTTTTTAAAAGATAGAAAACCATTCCTAGGGATATCTTTGTCCAGTAATAATTGGGAGTCAAATATGTGAAAATTAGAAACCAACAGTGGACATATCATTCAAACTACTGCATTCTACTCCCTGTTACCACAGTCTCACAAACAATTCATAATAAAAATGTATAGTATACAAATTTAAAAGACTCCATTAATTTTCACAGCATGAACATTGTTTAAAAGCTTAAAGTGTCTTCTGAGACTCAAGTCAATACTTTCAATGCTCCCTCAGTAAAGTCAAAAGCAAGTCACATATTTCTAGCATACATTAAATAAAATACACATTACTTTTCCTCTGAAATGAACTCAGGCTGTCCTGAGAAATGCTTAGGATGACCTCAAGCATCATATTTTTCTGCTCCACATCCAGGGTGCTGTTGTTCCTCGTGTGTAAAATATAACCGCATGTGATAAATTCAAATTCAAATCTGTTTATGTTAGGCAAGTATACTACCAAACATTGATACCAAGCATATATGTCTGTGAATTTTCAAGCTGCACAGCATTTCCATTTCCAAGTCTTTCACAATCCAAGTTCATCATAAGACTGCAAGTATCATTTGGAAAAAGGTTCCTTTAAAAGTATACTTTCTTACCTCAAGAGAGAAGAAAACGATGATATAAACAGAGAAAAATCAAACCATAATGGTGTTAATGAATTAACATCTTAATAATGGTGATCCATTTAATATTTTCTGGTATTATTGAAGGTCTGGGTAACTTCTCTAGCTCTGCCCTTTGCTGCATATGTGCAGCTGTTCTTCTAGGTTATGGCTCTCTCCACACCATGAATGCTACTGAAATTACTGGTCATCCTATGCTACTTGCATCCACCAAAATGTGGGATGCCTTTCACCAACTGGGCTGCACTTTTACCTGTAGACTCATCTAGCATGTTTCAGGAGACTCATCCCTCCTAGAGAGTGACAAGCCTTAGCTGCTCACTCTCATGTCTTGAAGCCTTCATAACAGTGCCCCTAGGTTGAATCTAGTTCCAAGTGAGTAACAGAGTTATAGTTCATCATTTTTGCAATACAATTTCTGCATGCTGAACCTGAGGCATGACTTTCCAGAATATTCACGTCTATGATGCTAGTCTCTTTTTTATCACAACTGATTGATTGTTTAGACTCAGGTGGCCAGGACTTATTATTCATCCAAAGTATGGTTTCTTCACTTTGTTTGCTCTGGTATTGAAATTTCTTCTTTGAAATTTCCTACAGTACATTCAAGAAACAGGGAAATTGTGGAGGATTCTGGCTAAAAGACTGAGTGTTAAATACAGTGTGCTCCTATTAATTTCTTTTTTTTTATTTAGTGTGATGAAACAGAAATAAGGAGAGAAAGAGAGGGGAGGAGGAGAGATATTAAACAAAAAAACCTCAAGGATAAGTAGATTTTGAAAATGTTAGCCAATAGAGGATATAAATCATTTGAGAAGACATTTCTCACAAGGCATCCAAAACACGATGAAGAGAAAGTAAATTCAAGAACGAGACACGTATGAAATCTGATGGCAGCAGAAATTCTGCTTTTATTTACTAATGTTTGTTAGCATGCTGGATAAAAATACTATTCAATAAAATCTCAGAATTTTCACATTGCTTGTCACCAGGTATACAGTATGGTCATGAAAAAATAAAACAAAAGAAAACAAAGCAAATACTTCATGCTGTTGTTCTGGACAGAGAAAGATGGTCAAACGAAATGAATTATGAAATGCCTTGGAATCACAGTACATCAAATTCAGTGCATTACTAATGGGACATCTATTGAATCCTCTCCTCAGCCAACCAGTGGTATTCTTCTGCAGAAGAAATTCAACCTCCTGAACATGAAGATGTTAAGCT
MSTRG.1068.1	ENSRNOG00000011720;ENSRNOG00000039955	0.037	0.705	0.000	4.427	up	TTTGGTTCCGCTCACCGAGCTAAACTTAACCTTTGTAAATTTTTTGGATGTGGGAGGAGGGTGGGGAGAGGGAGGCTTCGTTATTTATGGATGGGGCAAAAATCAGAACTTTTGGCATTTTGTAAACATTGTCGGAGTCTCTTTCATGTACACTTTCTTTTTTTTTTTTTTTTCAATATTTTCAGAAACCTTTTTATATAACGGAATCTCAACCTAAACCAAATCCTTTGAACCTGGCTGGGTTTAGCCTGGGACTCGTGTCAGTCATGTTCACCCTGTGCCATATTTGGAATTGCAGCATTAGCCAAGTGTAACGTTGTGAGTCGTGATATTGCCTAACTGTGTCAACCTCTGTTGGGACTGAAGAATTGTAAATACTACCTTTCCTTGAATCCATGTTTTTACTTTTGCACGCATCATGAATTGTTAAACAAAGTACTTTTCACCAGCATCTTTACTGTTATTATTACCAAAAAGATGTATATAAAAGAATGGAACTGAAGAGTAAAATATATAAACCTACTTAGATTTGAAGGAGGTAGCGTATCTGGACAGAATTGGTTTGGTACGTCATGGTGCCCATGTTCCCAGCAGTTGGCATTGGAGGATTGTGCAGTTTCATATCCCAAGGGATATTGACGAGGAATTTGATTTGTGAAGTTACTGTGTGCATCTATGTACATGACATGGAAGTGGCTGGT

							GCTTTACTGAAAAGGACTCACTTGTCTGGAAACACTAATCCTTCAAGAGCTCCCATCCGTCCTACATGTCCTCCAGACCCAGAGGATTTGCTCAGAGTGAGCTGAGAAGCAGAGCAAGGGTCTCACTGCACAGACTTGGTGTGACTGTCACATCTGCAGATACAGAATTCTCTTCGCTCACAGATCGAGAAAGTGGTTCCCAAAGCCATTCTTCGGAAACCTCCAATGGTGAAAGATACCTTTTCCTAAAAAAATATTTTGCCTTGATTTCTAAGTATCTGGATGCACTTTAAATGAAAGCCCATGGTTATTTATAAACTCATAGCTGGCAGTGTTTCTTTTGTAAGCGTGCACTTCTAAGGGGAAGGTAAGATATTTTGTCAGTCTTTGGATGTATTTTCTAATCCCACGATTAGACATTTACTCAAGGACTGGTTTAAAAATAGAAAGTTTAGTAGCATGCTGTCGGAACTCTGGGGGAAGGTCCCACCTCTTCCCATGAACTCTTTAATGTTTTTCTGATGTAGGACTTCCTTTGATGTGCCATAGTAGGAGTCCTTTGCATTTGTCATGATTCAAGGTAAACAGGGTCCGTGACATCTGCAATGTCCTCTTCATTTCTTTGCATGAATCTGCTAAATGAGCTTTTTATTGTTACTCCCCCTCCCCCTTTGTTGTGGTCAGTATTTGTACATTAACTTGCAC
MSTRG.107023.1	--	0.036	0.408	0.024	3.103	up	GCTATGACTTCATAAAAACCAATGTTTTTTTTTTTTAATTTTTGATTCATTGGGTAGGAGACCTCCCTCATGAATAAACTGTCAGTATGCAGAGGAATCTGTAAAAACTAACCTCAAAAAAAGAACTATGTTGATGTTGTCATTGGCACAATGATTTACTGATTAGAAGGAACTGGCTGTTCATGCGGCTGGATTGTCAACTCAGTATGGGGTTGGTCTGTCAACGTGGAACACAGAAATTTTCTGAGTAGCCTGTACGCCAGAAGCCATCACCTGTGAAGAGCCACTTCAGCATCCTTGTCACACGTTTTGCCTTTCAAGTAAATAGACATAAAAAGATCACCTGCTTTTTCTGTCTGGATTTCTTTCTGCAACAAGGCATCCC
MSTRG.107080.1	--	1.154	0.102	0.003	-2.693	down	GGGTTCTTTGGAAATTCGTTTGGATAGTGTTTTGCTGAGGAAAACTCATGATGGAATGAATATTCAGCTGTAGCAAACACAGAAGAAAGGATGTTCTGCCAAAGCAAGAACATGAAAGTACACTCGTAGAATTCTTTGTTAACAACATGGGACTCAATAGAAACATCAAACCCCTTCCGGTGGTGTGCTGCAGGTTCTTGGCACTTCCACAGCCTTGTGCTGATTGGCGGGTTGATGTCAGGTGAGGCAGACAGATACACATGCTGTGGCAAGACACATACTGAAGCAAGGCTCATGGAGGACACATGATGTTTAGAGAGTAAAAATAGGACTGAACAGACAGTAACTGAGATTGAGTTAGACTTGCTTATAGAACTAATATGCGACACTTGTACAGGCCTCGTGTTTTAGCCGATTTTGCATTGCTGAGAGAGGCACAGCTACTTCTCCTGGCATTCTAGGCCACGGCCTGGCTGTGTCTGCTGATTCCTGTTTGTACTGGGTTGTTGGTGTAACTTCAAAATGTTTATGAGTGGATGGAGCTGTCATTGCTAACCTGTGAACTGAGCTGCCGATTTCTAGACTACACAGAGAGTTGATCCAAAGGACCTTTCTAAACAGGTCCACTTCCCCCATATTTTTTTCTTTCCCACTACCTCTGGTGGGTGGTGGGCTAAAAAGGAGGTTAAAGCATTTAAAAAACCCTTCATTAAATATAGGTTTTGGAAAAATGTAAGTTTCATCCATTTCCAGTAATCTTCCTGACCGTTCCTGAAAGGAGATCCCCAAGAGGGACGGATGTTATTCAGATATTCCACTCATGATGAACACTATGACGTCTCTTATTTTTCAGCACTGAACACTGGTAACTCTCTGTGCTTATTAATATTCACTGTAAGAAACAAACACCACCAACAGGAAACCCTTCTGATGAGTATTGAGAAGTATGGTGATCTGTGAGCATTACACTAAGTCATTAAGAGTTGTTTTACTGTTATGTAAATTTAACAGAATAATAACAGTAGGTTTTCCTCTAGGACTCTGATGTATTTAGCCACATGATTTTAGCCTTGGTAATAGTGATAGGTGTGGATTCTATCTCATGGATCAGGACTTAAATCCAATCAGAAAGTGTTTGATTGCCCCTACGATATGTGTGCCACTATTGTATCAATGGCCACATCTTTCCCAGCAGGTAGTTATTCACTCTCAAGTTATTGACTCATAATCAATTTTTCCCTAACACTTTCTAGCACTGTAAAATTTAGCTTTAAGCAATGAAGCTTCTATGTCCGTACCAACTTGAATTCTCCACATTTGTTACTTAAGTATGTGATCTCTTTAGCAACAAAGTCCTACTATCAAGTTCTGGAAGGTGGTTAATTGTATTGTCAATAGCACGTGATATTTGGACACCAGAGGACACCACTGGTTAATATCTCAAGAAGCCATAACCCATTACTTATACTGATTTTTGTTTGTTTGTTTGTTTGTATGTTTGTTGTTGTTTGGATGATTTGTTGGTTAGTTTGTTGGTTTTGTTAGCAACTGAAACAAAATTATTGAAACTACTTTTTTTAACCCAAAGTCCTCTTCAATGAAGTTTGATTAAACAAATAGTTATATATTTCTAGGTTTTTTCATTTCTACAAAGTGACAGCCAGATAATTATGTCACCT

							GTCACAGAGGATAACCTGTGCAATAGATAATATGTTTGTCTCCCTACCTTTTCATAGTTTGTAAGAGTCCA<br>TTTATAAGGGGAGACATTATACACTACTGTTCAAATGGCCATCTAGAAAATTTCTATGATAGTGACCTTAA<br>ACATACCATATTTTTTAAAATAAACTTTATAATGTAATAAAAGACATCACAAATGATTTATGGATATAGCAG<br>CAAGTGCTCTATAGATTTTAGTGAAATAACTACAATCAATGTAAAAATTTTATCTCCATGATTGGTGCATT<br>TCATCAATGGCCAGCTAAGACATTAGTTTCCATAAATGTTCTCCTGTCTTCAGGTTCATTTTTCTTATGAT<br>ATGGGGTTTGTTCTCAGAGAAACAAGTAAATTCATTCAAATATTTTTATGCATTTACTGTAGGTCAACAAT<br>ACTAGAAAGAATCTCTACCTTAAGAGAGTTTACGATCTAATGGTTGGGAAATATAATTGATTGTGAAGTA<br>GAGGTGTTAATGTTTCACAAGATTAGACAAAAACAATCTTGGCCTTTAGAACTTCACTTCCATCCCTAG<br>GGAGCACATTCTGGAGGAATATGGCAGTGGTGACAACATGCATTGTTCTGACTTCTAATAACTTCATTTA<br>AAGGAGAGGGTGTTCAGACTCTGTGAGTGGAAATAACTTGCAATATTCCAAGTACATGGTGACTTTTTT<br>TATAGTCTGAAAATAAGCCAAGTCTTTGTTTACTGGAACACAGATGATAATAAGACATGACCAATTTAGG<br>GCAACTTAAACCTACTTGATTCTGATAATTATGCTTTCTTCCTGACATTTGAAATAAATAACAAGGCTCAT<br>GGTTTTCATTTGAACGTGTCTACTTTGTAAACTGTTTTTAAATATGTTGTAGTTTCCTATCACTGTCATAA<br>ATATATTACCAAATAAGTCTCCACAACTATTACTGTTGAAGGTGACACAATAGGCTATTCTAATGGTGCAT<br>CATAAATTATTATAACATTGTCTGCTATGTTTTCAGACAGATATCCTGACTGAACTTTGTCTTTAGTGGTA<br>AGTTTCATTTGTATTATGTTAGCAATACAATTATGGTATAGAATTTTTGTATCTTCCATTCTCCTAATTTTTC<br>ATTTGATACCTGTAAATGTCTTTCAATTTTATTTTTAGTCTATTTGTATTACCTGTAATGTAATGGTTGATAA<br>AGAAAAACTAATAGATTAAAATATGTTAATATTTCTGCCAAACATACAGAAAACTAAAATTGGACTCTAT<br>TTACCTGATAGAAAATATGGAAAATTCATAAATCCAGCTACATATCCATGTTTTGATTAAGGTTTTTCAGT<br>AAGAGCAAACATGATAAAAAAAAAAAAAACTAGCAAGCCTGGGTAGCATGTGAGACCTGGCCCTTGTG<br>GTTGTGAGCATAGGAGAGCTCACCTGAAGGGCATGAGAGAGGAGTAGCTGGCCTTACCCGTGGACTGA<br>GCAGTGCTAAAGAACTGGCCCTTGTGACGTGAGCACAAGAAAGCTTAGCTACATCCAGGCTGATTCAG<br>GACTTAGAATTAATCTACCCCAAAACCTACCCCATCTATGAACTGGTGGAGCACATGAAGGGGCTGATT<br>TGTCCGAATCAAAGCTACAGGTTCTTCATGACTCAGGGCAACAACAGAACATTCAAAAAGAGTCTGGT<br>GGATCCAGTATTGATAGTGGAACATAAGCCAGAAGTCCCAAACCAGAGCAAAGACTCATTTTAGTGAA<br>CATTTGCAGGTAAAGTTGTTTGGCAAAAGGTTTTACCATAACACGCTGTAGCTTCCACAGAGATACGGG<br>TTTTTTTTATATTATTTAATTTATTTGGTTTTAATTTCATTATTTTTTGTTTGTTTTCCTTTCTGGGGAAGTTT<br>ACAAAGGCAGAAACCAGATATCAAGGGAAGGGGAGATGAGTAACATTGGAGTCTATGATGTAAAATTT<br>ACCAAGAATCAATTTAAAAATTAAGAGAATAAATTATTGAGCACCTATACTATAGAAGGCATTGTATCATG<br>TCTATGTTTTCACAGGTTTATGAAATGTATAGTCACATTTAATTTACATAGCAGGACATCCTCTGCCAAAT<br>TCCATCATGATCAAATAAGCAAGAGAACAGGATGCTCAGAAGCCAGTGGACTTAAAAAGCAACCTCAA<br>CTTAAGACAGAGGAAGATCCCTATTACATAAGCTTACTTCACTATATTTCAAAAATCTGTTTCTATAATTC<br>TTTCTTGCATACTACCCATTATTGTTCATTACCTTATGACCAATCAGTTCCCTATTCTAGGTGCTGTGTTTA<br>AGCATGGACTTTCTATAATAAGATGAGCCTTCCTTAAATTAACTTTTGTAAGTGCACTTATTGTCAGTTAG<br>CAAACAACTTCTATCCCACACAGCTATCATACGGAGCACAGTACTTGCCCTAAAGTCCTTAGTCTAGGGT<br>GTGGCATTCAATAAGCACTGACTAAATGTTGACTGTTGGAACATTGTCCATTGGTTACTTGCATTTTACT<br>TACTGCCATATTATCATCCCAGAGGAGGCTAAAAGCGCAGAAAATCAGAAATGAAAGTAGGAGAAGTG<br>GAAAGATATGGACTTACCACTGACAAATAAATTCACATTACTATCACATTTCAGAATCAGAAGTGGGTCT<br>TAACACTTAAAATTTTCACTTCAAAATGTAACAACCACACTCACAATAAATTCATGCTTTAAAAATGTAG<br>TGTTGAGAATGACATTAATACAAGACGAGTTATTTCACACACACAAAAATTTTAAATCTTAATTCAT

MSTRG.107135.1	ENSRNOG00000004216;ENSRNOG00000004234;ENSRNOG00000004242;ENSRNOG00000028155	0.000	10489	0.000	18.006	up	TGTGTGTGTGTGTGTATTGCGCTATGCCGATCGGGTGTCCGCACTAAGTTCGGCATCAATATGGTGACCTCCCGGGAGCGGGGGACCACCAGGTTGCCTAAGGAGGGGTGAACCGGCCCAGGTCGGAAACGGAGCAGGTCAAAACTCCCGTGCTGATCAGTAGTGGGATCGCGCCTGTGAATAGCCACTGCACTCCAGCCTGGGCAACATAGCGAGACCCCGTCTCTTTTGGCCACTTATATTATTCATTAGTTAACTTAATTACCTTTTCTTAAATCAAAT
MSTRG.107135.2<br><br>NONCODE_ID：NONMMUT055409	ENSRNOG00000004216;ENSRNOG00000004234;ENSRNOG00000004242;ENSRNOG00000028155	0.083	224.383	0.000	11.228	up	CTCGGGAGGCTGAGACAGAAGGATCACAAGAGTTCTGGGCTGTAGTGCGCTATGCCGATCGGGTGTCCGCACTAAGTTCGGCATCAATATGGTGACCTCCCGGGAGCGGGGGACCACCAGGTTGCCTAAGGAGGGGTGAACCGGCCCAGGTCGGAAACGGAGCAGGTCAAAACTCCCGTGCTGATCAGTAGTGGGATCGCGCCTGTGAATAGCCACTGCACTCCAGCCTGGGCAACATAGCGAGACCCCGTCTCTTTTGGCCACTTATATTATTCATTAGTTAACTTAATTACCTTTTCTTAAATCAAAT
MSTRG.107212.1	ENSRNOG00000007031	1.691	0.066	0.000	-3.815	down	GGCTCATCGGACGGCGGCCGTGAGAGCTGCGTTCTCATTGGCTTCCCGCCCTGTCCGTCTCCCCTTCTCTGCAGGCCGCAGGGGGCTCAGTGAGTCGGAGCTGGAGCAGCCGCTTGGCTTGCTGAAGCTGGCGCGGGATTTTGGGAGGGGGCTGGAGCAAGGGCCGAGAAGGAGAAGTGGGGCGGGATGATTCATCCCTTTCCACCCCCACCCCCCACACGCTTCCTTCTGAGCTCGAGTGCAGTCACCCGGGAGCAGGGGGCGTGAGAACCCCTCCCAATCGTGAGTGGGCTGCTGTTGCCTGTCGCCGGCGCTTGGGGCTTGCCTTCCAGCTCTAGTCTGCGCGCCGACCCACCCCTTCTCCTTCCGCTGCTGGCATCAAGCTGGCACCTCCCCCGTGCCTTCCTGGGGGCTTTTGGGCTTTTCTCCAGCACTCAGAAGCAGCTGGAGCGCCCGCATCCATCGTGTTTGGGGGAGATGGTTGTGTGTCATGGCTGCAGTGCCCCCTTCTTGCGCTCCCCCCCCTCCCCGGTAGCTTTTTCCCTACTCCGAGGCAAAGGCTCAGTATTCCTCCAAAAGCTGTACCACCATCGCTACATGTTTTAAATGTGTGGAGGCCCCTACGCCTTGGAGTGAAGGTACCTAGGTTCTTCTCACCCTCCCAGCCTCTCGAAGCTCATCCCTCAGCATCTGGCTGGGTTTTAGAGAGGCCCCAGGAACATTAGTTGAGTTGAAGCAGGCAGAGTTTGTGTGTTTCTGAGTGCTATTGTTTGGAAACTAGCTATGTGAACGAATTGCTATGGCATTGCAGATTCCAATGTTTGATTCCAAACGATATTATCCAATGTCACATGCCCTTAGCATCTACCATAAATTCCAGAATAGGGGGCGGGGTG

							TTATTTTTGCTTCTGTGAGGTAATGAAGGAATCTAATGATAGCCCTTGTATTCTAACCGATTTCTCTTTTCC<br>CCAAATCGTGCTAGAGATAATGGATCCTGCCCCTTGCCTGCATAACCCTTAAATGAGGACGACGATAGA<br>ATCTTTGAATCAATATATCAGCCTGTTACTCAATGGGTAGAATCTTTGAATCCAGTGACATTCCAAGGAA<br>GTAATTTATCTTGGGGGCACACATAGTTAATTTTCTGAGTGATTCATAAAACAAGTGACAAAAATGGTTT<br>TCAAGTCCCGTGGGATCTTCCAAATTTTCAACTTCCAGTAGATAACGATTATTCATAGCTTCATTTATAAT<br>TTAAAAGCACTTTTTTTTGTCCAGGGAAAAGAAGTACTTTCTATAATCTTTGATTTTTGGAGAAATAGTA<br>CAAATAAATTGGATTATCTCGCAAATATATTTTTTAGCACAATGACTACAGTAGTTGATCATTGGTTGTGT<br>GGGAGGAAGAATTCACATTGTTATTTAAAACTATGTTAGTCACAAATCCTACAGGGTTTATTTCTAACCC<br>ATATTTTAATTAGTTGAGACCAGAGCTGCTAAGGTATATGCAATCTGTTTACAGGCAAAGAGTTTCTTCA<br>GATTTGATTGGGGGCATTATCTATATTAGTGCAGGATACAAAGACTACATTAGTCCAAAGACACTTTGCT<br>CATGCTAAGTGATTGATTCTTAAGAAATTGGTGGAGAGGGTCATCCACCCTTGCATGTTACAATACTTTC<br>ATGCATTTTGCATTCGCTTGTGCAAGTCAGGGAAACCTTGGCTGGCAAAGTAAGGAGCTGTCATTCTTC<br>TGGAGTCTGGGCTTTGCCTACCACGGTATACCATGAAGGAGGTCTGGGGATTAACTAGGGTATGCCGTG<br>TAAGAGGTCCAGTCATTAGCAGACAGGCTACCCTTGGTTCACAGCAAAGGGTGGGTGGTCTGAGGGAC<br>TGTCCAATGGTAGGATTCAGTGTCAGGTACAAATACCCTCAATACGATGCAGACTTACGGAACAGTTGA<br>TCAATTAGGAAATAGCTTAAGATGAGTTAGCGTTGGGTGTGTTTGGTCAGGAACTAGCACATCAGTCAC<br>TCCTAGAGAGCTCTCCAAATGGCTGCCTTTTGACTCTTCCAGACATCATGACTTGAGTCAGTCCCCGTA<br>ATTAGAACAGTTATTCCCGTTTTTCAGATTAATGAACTGACTTTCACGATCCAGTACTGGTGAGGCTAGT<br>ATTAGAACCCAAGAAAGGGAAGGTTGTAGTTCCTTTTCTTCATTCACCAAGTAAATACTGAGTCCAGAT<br>TTTGTCAGGTCCCCACATAACAAAACCTGAAATATTCAACTTTTGCTATCCCACTTAGCTTCTCCGAGAG<br>TTTACCATTTTTAAAATTAATTTCTCTATTTTTGTTAGGCCTTTGAAGATGTCCTCTTGTGTTGTACTACTT<br>GACCATGATGTACTTTAAAGCCCACCACCCCGGGTTATCTTTAAAGGGGAATTTAAGAAATCTGAATGC<br>GATTGGTGACATTGTAGAATGTCAGCCCGCTGGGAGATTGTTTTCTGTTCCAGCTGGTGGTTGACCGTG<br>CCCTGGAGTGTGGGATTGCTGGTCCTGTAGTTTCATCATCCCTGGTAATGACTGCAGCTGTTCTCGTTTG<br>TGTAAATACCGGATACGATCCCATGAAACTTACCATTCGCCCGAACGATGTTCTTCAGCAATGAGGAGA<br>GCAAATTGGAGGAGCACTGGCAGCTTTTCCACTCTTAAAGGAAAGATGAATGTTTTCTAATGAAAACTA<br>CAATTGCTTTTGTATTTCTTAAACTGTAATGCTTGGTGGCTAATTCTGTATTCTCTCTGAGTTAAAGAACA<br>AACACAAGGAAGGAGGTATTTTCTGGTATCCCTCCCTGTTCTCATTTAGAGCCATTTGTCTTCAGATTTG<br>TGGGTGTGGGGAGGAGGGGGTGGTTCTTCAGCACCATGTCCACCTCTGTGCAGTCATGCTTCCCACCGT<br>GATGATAATGGACTAAACTTCCGTACCTGTGAGCCAGCCGCAGTGAAATGCTGTCCTTTCTAAGAGTCA<br>CCATGGTCATGGCCTCTTCTGCCAATAGAAACCTTAACTAAGACAGGACCCCAAAGTGGAATTAGAACA<br>ATAGCTGCTAGTGCCCAGTTTCTGGATGAGCTCAAACCAGCTTCAGGGCCTTCTTCACTTAGGAGCCGT<br>GCTCAGAATACTCGATGATAGGGGTACTTAAGCCCAGATTCATATTTATGCATGTTACACATCTAGTTATA<br>GCAGGCTGAATTCTGGGTAAAACGGGCTTCTAAGAAAAAAGCATACCCCCCTTTCCTGGAAGTGCTTAC<br>CCAAGGAGAGGCACAAGGAGAAGAGACTGAAAGTCTCCCCTCTGCCAAGGATGAGTCATCGTTGCTC<br>AGTTCAGCTCATGGCCTTTTCTTTTTCTAAAGAGTGTATTCTTGCTTCCTTGCCTGCTCCAATTAATCTTT<br>TGAGATTTGGATCCACTGTTTCCCCTCTGAATTCTTCTCTTTCAGGAGTATGAGGGTCGAGTACTACTGA<br>CATAGAGACCAGAGAAAAATAGGGGTGTTATGCACCTTGGATTAGGAGAGTGCCCATCAATATTGTTAA<br>CTGAGACAAGTAGTTTGCATTGGAATTGTTTTACTTTTGTGTTGCTGGAACAAGAATACCTGAGAACAA<br>CTTAACAGGGAGAAATTATGTCTTTTTCTCACCTCACCGCTTCAGAGGCTTTGGTCTACCCTGGGTAAA<br>AAATGTAGCAGAAAAGAGCACTTACATTATGGCCGACAAGAAGCAAAAAGAGATAGGAAGGGACCAA

							GGACAAGATACCCTCAAGGACCTACTCGTGGCTTGCTTCCTCCAGGGCATGCTTCTTGGAAAAGCCTAATCAAGATAAAGAGTGAGTGAGTTGGGGAGAGAGCAGGTGCGTGTTGAAAAGGGAACTTGGAGTCGTCTGGGGATGCGGAGCAAAAGGAAGTCTCAGTTACTGCTGACGGGGACGCAAAATACAGCGTTTTGCAGAATTTAGCAGTTTCCTACAAGGGATTTGGAAGTCCCACATACCCCTTCCGCCCAGCAGTTGGCCCATGGCTTCTTTACTGATAGATCAAGAACCAACTGGGGAACAGGACCTTAGCATCAGACCCACACCTACATGAGTTTATGTCCGTCTGCTTCCACTGTGGCCTAGAAACTCCAGAGAGTTCATGGTCCCTTCCCTGGAATCTGACGTATTCAGTTTTTTGTTAAGTATTACAATAAATGACAATTTTCAAATAACATGATAGGCATTTTCTCTCCCTTGGTGATGCAGATATAGGCCAGGTTAGACCAGATTAAAAGTAGATAAAGGCAAGTTTATTAGGAGGAAGCTTTTATGGGGTGAGGTGGGGGGTTCACCAATCTCACAGGTTGAGGTCAGCAAAGTCACAAATCCGGGCAACAAGGGGGGTTTATAGTGTTTAGGTATGGATCTGGGATGTTGTCTATACATGGTCAAAAACTGAGTCCCTGAGCAGGCACTCTTGGGTGGGTTGTGTAAACTCAGAGACAAGGAATGATGACATGTTAACCGGGAGTTTTCTCCAAGCAGATGGGGGAGTTTCCTCTGTGTGGG
MSTRG.107212.7	ENSRNOG00000007031	0.000	1.303	0.000	9.061	up	GAAGCTGGCGCGGGATTTTGGGAGGGGGCTGGAGCAAGGGCCGAGAAGGAGAAGTGGGGCGGGATGATTCATCCCTTTCCACCCCCACCCCCCACACGCTTCCTTCTGAGCTCGAGTGCAGTCACCCGGGAGCAGGGGGCGTGAGAACCCCTCCCAATCGTGAGTGGGCTGCTGTTGCCTGTCGCCGGCGCTTGGGGCTTGCCTTCCAGCTCTAGTCTGCGCGCCGACCCACCCCTTCTCCTTCCGCTGCTGGCATCAAGCTGGCACCTCCCCCGTGCCTTCCTGGGGGCTTTTGGGCTTTTCTCCAGCACTCAGAAGCAGCTGGAGCGCCCGCATCCATCGTGTTTGGGGGAGATGGTTGTGTGTCATGGCTGCAGTGCCCCCTTCTTGCGCTCCCCCCCCTCCCCGGTAGCTTTTTCCCTACTCCGAGGCAAAGGCTCAGTATTCCTCCAAAAGCTGTACCACCATCGCTACATGTTTTAAATGTGTGGAGGCCCCTACGCCTTGGAGTGAAGGTACCTAGGTTCTTCTCACCCTCCCAGCCTCTCGAAGCTCATCCCTCAGCATCTGGCTGGGTTTTAGAGAGGCCCCAGGAACATTAGTTGAGTTGAAGCAGGCAGAGTTTGTGTGTTTCTGAGTGCTATTGTTTGGAAACTAGCTATGTGAACGAATTGCTATGGCATTGCAGATTCCAATGTTTGATTCCAAACGATATTATCCAATGTCACATGCCCTTAGCATCTACCATAAATTCCAGAATAGGGGGCGGGGTGTTATTTTTGCTTCTGTGAGGTAATGAAGGAATCTAATGATAGCCCTTGTATTCTAACCGATTTCTCTTTTCCCCAAATCGTGCTAGAGATAATGGATCCTGCCCCTTGCCTGCATAACCCTTAAATGAGGACGACGATAGAATCTTTGAATCAATATATCAGCCTGTTACTCAATGGGTAGAATCTTTGAATCCAGTGACATTCCAAGGAAGTAATTTATCTTGGGGGCACACATAGTTAATTTTCTGAGTGATTCATAAAACAAGTGACAAAAATGGTTTTCAAGTCCCGTGGGATCTTCCAAATTTTCAACTTCCAGTAGATAACGATTATTCATAGCTTCATTTATAATTTAAAAGCACTTTTTTTTGTCCAGGGAAAAGAAGTACTTTCTATAATCTTTGATTTTTGGAGAAATAGTACAAATAAATTGGATTATCTCGCAAATATATTTTTTAGCACAATGACTACAGTAGTTGATCATTGGTTGTGTGGGAGGAAGAATTCACATTGTTATTTAAAACTATGTTAGTCACAAATCCTACAGGGTTTATTTCTAACCCATATTTTAATTAGTTGAGACCAGAGCTGCTAAGGTATATGCAATCTGTTTACAGGCAAAGAGTTTCTTCAGATTTGATTGGGGGCATTATCTATATTAGTGCAGGATACAAAGACTACATTAGTCCAAAGACACTTTGCTCATGCTAAGTGATTGATTCTTAAGAAATTGGTGGAGAGGGTCATCCACCCTTGCATGTTACAATACTTTCATGCATTTTGCATTCGCTTGTGCAAGTCAGGGAAACCTTGGCTGGCAAAGTAAGGAGCTGTCATTCTTCTGGAGTCTGGGCTTTGCCTACCACGGTATACCATGAAGGAGGTCTGGGGATTAACTAGGGTATGCCGTGTAAGAGGTCCAGTCATTAGCAGACAGGCTACCCTTGGTTCACAGCAAAGGGTGGGTGGTCTGAGGGACTGTCCAATGGTAGGATTCAGTGTCAGGTACAAATACCCTCAATACGATGCAGACTTACGGAACAGTTGATCAATTAGGAAATAGCTTAAGATGAGTTAGCGTTGGGTGTGTTTGGTCAGGAACTAGCACATCAGTCACTCCTAGAGAGCTCTCCAAATGGCTGCCTTTTGACTCTTCCAGACATCATGACTTGAGTCAGTCCCCGTAATTAGAACAGTTATTCCCGTTTTTCAGATTAATGAACTGACTTTCACGATCCAGTA

							CTGGTGAGGCTAGTATTAGAACCCAAGAAAGGGAAGGTTGTAGTTCCTTTTCTTCATTCACCAAGTAAA<br>TACTGAGTCCAGATTTTGTCAGGTCCCCACATAACAAAACCTGAAATATTCAACTTTTGCTATCCCACTT<br>AGCTTCTCCGAGAGTTTACCATTTTTAAAATTAATTTCTCTATTTTTGTTAGGCCTTTGAAGATGTCCTCT<br>TGTGTTGTACTACTTGACCATGATGTACTTTAAAGCCCACCACCCCGGGTTATCTTTAAAGGGGAATTTA<br>AGAAATCTGAATGCGATTGGTGACATTGTAGAATGTCAGCCCGCTGGGAGATTGTTTTCTGTTCCAGCT<br>GGTGGTTGACCGTGCCCTGGAGTGTGGGATTGCTGGTCCTGTAGTTTCATCATCCCTGGTAATGACTGC<br>AGCTGTTCTCGTTTGTGTAAATACCGGATACGATCCCATGAAACTTACCATTCGCCCGAACGATGTTCTT<br>CAGCAATGAGGAGAGCAAATTGGAGGAGCACTGGCAGCTTTTCCACTCTTAAAGGAAAGATGAATGTT<br>TTCTAATGAAAACTACAATTGCTTTTGTATTTCTTAAACTGTAATGCTTGGTGGCTAATTCTGTATTCTCTC<br>TGAGTTAAAGAACAAACACAAGGAAGGAGGTATTTTCTGGTATCCCTCCCTGTTCTCATTTAGAGCCAT<br>TTGTCTTCAGATTTGTGGGTGTGGGGAGGAGGGGGTGGTTCTTCAGCACCATGTCCACCTCTGTGCAGT<br>CATGCTTCCCACCGTGATGATAATGGACTAAACTTCCGTACCTGTGAGCCAGCCGCAGTGAAATGCTGT<br>CCTTTCTAAGAGTCACCATGGTCATGGCCTCTTCTGCCAATAGAAACCTTAACTAAGACAGGACCCCAA<br>AGTGGAATTAGAACAATAGCTGCTAGTGCCCAGTTTCTGGATGAGCTCAAACCAGCTTCAGGGCCTTCT<br>TCACTTAGGAGCCGTGCTCAGAATACTCGATGATAGGGGTACTTAAGCCCAGATTCATATTTATGCATGTT<br>ACACATCTAGTTATAGCAGGCTGAATTCTGGGTAAAACGGGCTTCTAAGAAAAAAGCATACCCCCCTTT<br>CCTGGAAGTGCTTACCCAAGGAGAGGCACAAGGAGAAGAGACTGAAAGTCTCCCCTCTGCCAAGGAT<br>GAGTCATCGTTGCTCAGTTCAGCTCATGGCCTTTTCTTTTTCTAAAGAGTGTATTCTTGCTTCCTTGCCTG<br>CTCCAATTAATCTTTTGAGATTTGGATCCACTGTTTCCCCTCTGAATTCTTCTCTTTCAGGAGTATGAGGG<br>TCGAGTACTACTGACATAGAGACCAGAGAAAAATAGGGGTGTTATGCACCTTGGATTAGGAGAGTGCCC<br>ATCAATATTGTTAACTGAGACAAGTAGTTTGCATTGGAATTGTTTTACTTTTGTGTTGCTGGAACAAGAA<br>TACCTGAGAACAACTTAACAGGGAGAAATTATGTCTTTTTCTCACCTCACCGCTTCAGAGGCTTTGGTC<br>TACCCTGGGTAAAAAATGTAGCAGAAAAGAGCACTTACATTATGGCCGACAAGAAGCAAAAAGAGATA<br>GGAAGGGACCAAGGACAAGATACCCTCAAGGACCTACTCGTGGCTTGCTTCCTCCAGGGCATGCTTCT<br>TGGAAAAGCCTAATCAAGATAAAGAGTGAGTGAGTTGGGGAGAGAGCAGGTGCGTGTTGAAAAGGGA<br>ACTTGGAGTCGTCTGGGGATGCGGAGCAAAAGGAAGTCTCAGTTACTGCTGACGGGGACGCAAAATAC<br>AGCGTTTTGCAGAATTTAGCAGTTTCCTACAAGACGTTCAGACTATCAGAACAGTTCTTCAGGGGACAT<br>TCCCATCCGAGTGATTCTAAATTATGTCAGGATAAGTCTAGGACTGTCATTATTACCAGGACACTGACGA<br>TGAACTTGGGGACACCACTGAACCACTATAATATTTCCCTCTTCCAAATGTGGCCACACCAAGGATTTG<br>GAAGTCCCACATACCCCTTCCGCCCAGCAGTTGGCCCATGGCTTCTTTACTGATAGATCAAGAACCAAC<br>TGGGGAACAGGACCTTAGCATCAGACCCACACCTACATGAGTTTATGTCCGTCTGCTTCCACTGTGGCC<br>TAGAAACTCCAGAGAGTTCATGGTCCCTTCCCTGGAATCTGACGTATTCAGTTTTTTGTTAAGTATTACA<br>ATAAATGACAATTTTCAAATAACATGATAGGCATTTTCTCTCCCTTGGTGATGCAGATATAGGCCAGGTTA<br>GACCAGATTAAAAGTAGATAAAGGCAAGTTTATTAGGAGGAAGCTTTTATGGGGTGAGGTGGGGGGTT<br>CACCAATCTCACAGGTTGAGGTCAGCAAAGTCACAAATCCGGGCAACAAGGGGGGTTTATAGTGTTTA<br>GGTATGGATCTGGGATGTTGTCTATACATGGTCAAAAACTGAGTCCCTGAGCAGGCACTCTTGGGTGGG<br>TTGTGTAAACTCAGAGACAAGGAATGATGACATGTTAACCGGGAGTTTTCTCCAAGCAGATGGGGGAG<br>TTTCCTCTGTGTGGG
MSTRG.107	ENSRNOG0000005	0.281	0.000	0.000	-5.368	down	TCCAGGTCATCAACATGAATTTGTGATATAAATGTATCTGTCTAGATGTATAGGTCTATTATGTGACATATA<br>TTCTATAGATCTGTTATATGGACAGATATTTATCTGTGTCTATGTGGATAGAAAGATATTTAGTGTACTTTT<br>AATTATGTTCTTAAATTTATGTGCCATTAATTACATATAGAGGTTTTTATGTACATATTTTACTGTGGAACAT

233.1	7934						TAAATTTTCCAATATGTTGCAAAGACTTGATTTTAATAAAATCTGTAATAAGAAATAGGCGTCTATGATGT<br>GTTCATTTTCTATCAGAGGCAAATCTTCTGAAAAAGTTTTAATGCTAATTCATTATGTATTACCAAACATAT<br>TATTCATGTACTTTAATGTCATCATAATTTACTTTGATGTTAAGAAATTGATGTCAATGTACAAAATTATTA<br>AACATGAATAACAATTGTTTAAAATTTTATTAAATAAAATGGTGGTTTTCAAAAAACAAAGAAAGAAAG<br>GAAGGAAGGAAGGAAGGAAGGAAAGAAGGAAGGACTCTGTATTAGGAATCAGGGACCAAATCAAATA<br>TTACACCAAAACATTTATCCAACATCCTGTCATTTAGGGTGTTATACACCCTTTAGGCATTCTGTACCATC<br>AAAAGGAACACAGAGCACGTGCGCACACACACATACACACACACGCACACGCACACGCACACGCACA<br>CGCACACATGCATGCATGTGTGTGTATTCTTATTTCCCAGTCTGAACCACAGTTATAGCTCACCACTTCC<br>CAACCTTAAGTATCTGATATGACTCTCAAGTACAGATGTCACCTGTGTCCCATCAGAAGTTACTAATAGC<br>AGAGGATCCCTCCTGGATTTCAGACCAGAGCTCCTGAACTAATTGGAACTACAATGTCCTTAAAAGTAC<br>CTACAATTTTGTAAACTTGAGGTTGTTAAGTGGACAGAGATGTCTTTCTGGTGGATGATTTTCTCTGAGC<br>TATGAAATCATTTTTTATGTGCATTAAACACATGGAAGCCTGAACCAGCTCCAATTCCAGGCCCTGGACA<br>CTTGCAACTTATCGTCTCTGGAAAAACACAAGGAGTGTAACTTGCTTCCGGTCATTTCATCACAAAGAG<br>CCCGAGGTGCATTTTTCATCCGAAGCTATTTCATCCATACATCTCCTGTGCCCTTCCTGGATTTTAATAAT<br>CACTGTTTACAGCTGTTTCCTCCATTCTCTCTCATTTATTTCCAGAAGTCACATTTTCTGCAAAAGTGCCC<br>CATAAATCACATCCACCAGCCTAGTTGGGCAGGATGTGCTCTTTACTGATGACAAATGTGGTGGTACGAT<br>TTGTTCTCAATACAAATGGTAGTAAAATTGGCTTTGAGCCACTGCTCACATTTCTATTTTTGTCTCTCTCT<br>CTCTGCCTTTGCAAAAGATACCTCCTTAGAGCACAGCTCAGGGGGCTGGGGATTTAGCTCAGTGGTAGA<br>GCGCTTACCTAGGAAGCGCAAGGCCCTGGGTTCGGTCCCCAGCTCCGAAAAAAAAAAAAAAGAACCA<br>AAAAAAAAAATATTTCCAACACTTGTACAAGTTATTCTATTATATTTTATCACTTATCACACACACACACA<br>CACACACACACACACACACACACACACACACTCCTTTTATCTCGGTCTGTATTGAGAGTTGGGGAGGC<br>AGAGACAAAGGGGGCTGGTTTTGAGCCCACCACAGGAATACATGGAAATGAGACAAGTACATGGACA<br>CAGAGAAAGAGACAAAAAGATTATTGTGACCTGCGAGTGCAAACCCAGGCCTCCCCCAGCCCCAGCT<br>GCCCTCTGCTCACTGAACCATGTGCTCTGCAGCTGGGACACCGCCAGAGGCTGTGCTGAACAATTAAAT<br>TATCTCCACAGATGGCCACTCCTTAGCCAGACGCTGAACCGTCTTCCCGTTGGATGTGTTAGTGATGATC<br>TGTAACCTATTAAAAGTAACCAGTCAAATCTTAAGATAAAAGATGGGTTTTTAAGAGACTTAGGGAGCA<br>GATTATTAAAAGAATCTTCCCATCTTGAAATTTTATTCCTTTCCTTCTGATTATTTGAAGGTATAATAAGTT<br>TGTATATTTGGACAAATTATCCCTGTTGTCATGAATAAAAGAACTAGGTAGCGATCCCTGAACTATTTTTA<br>TCATAAAATACCTTAGGAAGATTGTGGTGATGGAGGCAAAAATTACACCCATAAAAATCCCTTGAACATT<br>GATACTCTTTGCAGGTTAGGAGTTATGAATGACTGTCTGCCACAAGCATGCAATCAAACTGCATCAATTC<br>AGTACTTCTACATCTCAACTGCAGCACAAAATTCAGGTGCCACTGTTGGTTTGAATTAGGGAAAAAAAG<br>GTAACCTGTGATTTAAAATAGAATGCATCTAAGAGCAGGTCTACTAAAACTAAACCTCAAAGTTGTTCAT<br>TAAAGAAATTAGTGAGGGCAATGAGGCCACGAGACAGAGGGAGGTCTAGTAAAACCAGCCAAAGCAG<br>GTCATTAGATCGATTCTTGACAGCAAAGGCAGCTGGATGGCAAGCGACGATGCTCTTAATGAGGCAACC<br>TCAGCAGCAGGGGGCAGAGAAAGAGCTTCCCATGGGAGCAAAGGAGAGTCTAACAAGGCCATCCTGT<br>GTGCATCCTTCACCCAAGAGTGTGCCGATAGCTGCTTGGGATGAGTTTATCTAGAACTCTCTAGATGTTT<br>TTCTAGAACTTTCTAG
MSTR<br>G.107<br>298.1	--	0.000	0.334	0.031	3.453	up	GTTTTATTGCTGTGAAGAGACACCATGACCATGGCAACTCTTACAAAGGCAAACATTTAGTTGCGGCTG<br>GTTTACAGTTCAGAGTTTCAGTCCATTACCATCATGGTGGAATGCATCCGGGTGAATATATGGTGCTGGA<br>AAAGGAGCTGAGAGCTCTATATCTTGATCAGAGAGACCAAGCGTCCGGTGTAAGGAGTATCACAGCGC<br>CCGGGAGGTGGAAGCAGATGAGTACACGGAAACTGACTTCACAACGTGCAGAACAATGATTCCAACG

							TGAGCAGAGGCTGCTGTAGCACCCAGAAGGC
MSTRG.107376.1	ENSRNOG00000060802;ENSRNOG00000060998	0.165	0.564	0.003	2.533	up	TCCCCTTCTGCCTTCCAATCTGGCTGTAGCAATCTCACTTACTTCTCTAAAACCATGTCTGTCTGCCTGCCACCATGCTTTCCACCATGAGGATAATGAACTAAAATTCTGAACTGTAAGCCAATCTCAACTAAATGCCTTCCTTCGTAAAAGTTGCTGTGGTCCTGGTGTCTCTTCACAGCAATAGAAGCCCTAGCTAAAACAGGACTCAATGTTCCTGGGCATTGTCATTGCGATTTAATAAGAGCTGAGCGCAGAGTAAATCACTGTCCTTAAAAAAGAAATAACACAAATGGCCTTGTGGCCCTACTCCTAATGCATAACACCATTAAGAGGGGAATTTTTATGGGGCTTATTTTAAAAGTATAGTATCTCCCTTTTTAAAATACATTTTAATTCCTCCATATCATTTACAGAAAGAAATAAATCAACTCTTATTTTTGAGACTCTGGATGTACAAACATGACCAATAAGAAAAGTTTAGCTTTAAAAGTTGTGCAATTTCTAGGAAATATATCTCCATTCATTTTCATGTTATTTTAGTAAGATTCTTGAAGCTGGGTAAGATATAGAGAAGAAAGATTCATTTGGCCCACAGGTCTGGTGAATGGAAAGCCCAAGTAGCAAGGTGCTCCTTGGGTGAGCACTCCCTAGATGATGATGCTCCTCGGGTGAGCTCTCCCTAGATGATGATGCTCCTTGGGGGAGCACTCCCTAGATGATGGTGCTCCTCGGGTGGGCTCTCCCTAGATGATGGTGCTCCTCGGGTGAGCTCTCCCTGGATGAAGATGCTCCTCGGGTGAGCTCTCCCTAGATGATGATGCTCCTTGGGGGAGCACTCCCTAGATGATGATGCTCCTCGGGTGAGCTCTCCCTAGATGATGATGCTCCTCGGGGGAGCACTTTCTAGATGATGGTGCTCCTCGGGGGAGCACTCCCTAGATGATAGTGCTCCTCGGGGGAGCACTCCCTGGATGATGAAGCTCCTCGGGGGAGCACTCCCTAGATGATGGTGCTCCTCGGGGGAGCACTCCCTGGATGATGGAGCTCCTCGGGGGAGCACTCCCTAGATGATGGTGCTCCTCGGGGGAGCACTCCCCAGATGATGGTGCTCCTCGGGGAGCACTCCCTAGATGATGGTGCTCCTTGGGGGAGCACTCCCTAGATGCATCAGAGCACAAGAGAAAGCTAAATGGAAATAAGCATGAGCATGAACAAAATAGATCCAAAAACACAGTACAATAATGCAGACTTGTTCTTTCTCCCACTCTCATAAAATAAAATAGAAACAGCTCTTTAGGACCATGAATTCAATTTATAAAATAAATAAATAAACTCATATAAGTTTACTCCTGGTAATTCAAACATGCTGGGCTGCAATTTACTCTTCAATTATGTAGAATAAAAAGCCTTAGTAAACAAATTAATGGTGTGATAAGCAGTGGCAACTCCCACCTTTCTATAAGTACGGCCCTTAGAAGCTACCTTCTGGTCAGAGGCAAAGTTAGCTAACTTGCTCTCAAGCAGAATGGGAGCAGGAGGCATCTCATGTTATGGTTAAAGTAGAGCTTTCCAAGTAAGGCAGACATTGTGCAGCTGAGACCTCATTGCCTGCACAGCCCTCCCTCATAGCCTGACATTCCCCTGCTCCCGCACAGCCCTTCCTCATAGCCTGCCATCCTCCTGCTCCCGCACAGCCCTCCCTCATAACCTGCCATCCTCCGGCCATCCGCATTTATCTGTATGTCACCTATTCAACGCCATACCCTGTGTGTGTGGTTAGTGCCTAGCCAAGTGTTTGCTGGACAAACAAATGAATGGTTCAGTAAAATTGGACTGGATTATGAAGCCATGGCTGCTAGGTAAACAGTGATCTACTTGAAAGTGTCTGGGGTCATCGAATTCTAAAGAGTTAGAAAGTTGTTATTTATAACCATAGAAGGCCAGGGGGTGGGGCACAAATATGTGTACAAAGTGGCGTTAGTCTACCCCTGGGAAAAGATGGCAAAGCTCGCATCACATTCTCAACAATCGTTAGAATCACTGATCTAACGTATCTCCTCTCTTCTGACCTTTGTCACAGAACCAACCAACACCGCTCCGATTACATAGCTCCAGTAACAGGCATTTCCTCAGCTGGACAATGTCAACTACTCCTGCACTTTATAATTCTGGTACCATTGAACCCCAGTCCTTTCCCAGGAGCCTCTGAGTAGCTGCAATGGCCATGCTCTGGAGTCACCCAAACACCCTGCCTAGTGCCTAAGCCATCTGACCACACTTTGTGGGTTTTTGACAGCTGTCATAACGTACTCTGTCTTTCTTATATCCAGGCTGTAAGTCTCCAGCTCTTCCTACTGCACTTAAGATTTCTCCTCCTGTGCCTGTGCGGGGGGCTCTGCTGGGCCCTGCATGGCCCTCCTGTGTTTTGGTGTGCACTGAACCTAACACTTCATCCTTTTAAGGGTGCCCTGTGGCCCTACTGACTTAGCATCAGCAGGTAACAAAGTCCTTGTATTTTTTTTCTGTATGAGCGCCATAATTCTGCACCCGTGGCCCTCTTGACTTTATTGAAGCAGATAGTCCGAGGATTCTTAACAGGGCTTTGCTAGTATCTTTATCAGATTCCTGCCGAAATGCAGCTAGTCACTGCTTTGGGCTGAGGCTCGTGTGTAAGTTCTGCTAAGTTCTATCTACCTGGCTTTAGCCACCACCCTCCCCCCAAACATTTCATAATTGCTCTAATTTAGATTTCATGCAGATTAACATTCCACTTAATATCCAACT

							TTTACTGCCCAAATTATAAACTGGATGTTGCACAAGAAAAGTCCAGTAGAGATGCAGGGTGAGGAGGA<br>AGGAAAGAAGAGAGATCCAGAATCTGGGTTTGAGGCGGCCTTGGGTTGCCACTTTCTAGCTATGGGAC<br>TTCAGACATGTCACATAATCTATTGCATTCTCTCCACTCTCCACTTGGTGGTCATTTCTCTCAGATACTGC<br>ACTTGTCAAATGAAACTTTTCCGACTGAGTTATTTCTTATAGGTTTGTAGCTGTTGTTTAATTTCATTTGA<br>TTTGATTTTATTGAAACAGGGTCATCTCATGTTGACCAGACTGGCCCAGTTCATAATCCTGCTGTCTCAA<br>CATTCCCAAGACTACAAAGACAGCCTTTGCCTTCATGACTGATTCTTTATAGCTTTTACTTCTTTGAGCTT<br>TTCTATTTCACTTCATTGTTTTGGGATTACTCATTAAACTATCTTTGTTTCAGTTCCTTCAAAATCCCTGCC<br>AGGCGATCTCTAATTTTTATGTTGGAATAGCTGTTTGTCTTTCTGGCTACTGTTGGTTCTTGAAATAACAA<br>TTGAGCTGCTTTTTTTTCAGATTCATGGTTATTTTTATTTTATGTGCACGAGTGTATATATATGTACCACATA<br>GGTGCCTGTTGTCCAAGGAGGCCACAAGAGGACATTAGATCTCCTGGAATTGGAGCTACAGATAGTTAG<br>AAGCTGCCATGTGAGTGCTGGGACCCAAACCTAAGTTCTCTGTAAGAGCAGTCAATGCTTTTCGCCATT<br>GACGAGTCTCTCCAGCTCCCTAACGGTCATTTTCCAACTGTTAGCTGGACCTGTGAGACTCCAGGCCTG<br>ACTCACATGCTCTGATGCCTGGATGAAGAAGGGCACCTGTCTGGAGGCTTCTGGGAAGATATGGAGTG<br>AGCTTTTCCACTGAGCATCCTTGCTACTGTGCAGTGGAACCCTCCACTCTCCACTTGGCTCTCTGTAATA<br>CTTCACTGACTAGCCACCAGGTTAAGCTTGTCCCCTTATGGGCCTTTAACAGTTTAAGGCAGTGTTCTCA<br>ACTTTCCTAATGTTATGACCCTTTAAAATACAGTTCTTCATGTTGTGGTGACCCCCAACTATAAAATTATT<br>TTTGTTGCTACTTCGTAGCTGCAATTTTGCTACTGTCATGAATTATAATGTAAAAATCTGTGTTTCCTAATG<br>GTCTTAAGTGACCCCTGTGGAAGGATCTTTCTACCCCGAGGGGGTCACAACTGACAGGTTGAGAACCA<br>CTGGCCTAAAGGGTAAAGGCTCAGCTTTTCAGTTGGAATGGGGCCTCCATTTATTTCATGGTGGTTCCCA<br>GACAGCTTGCTGAGTGCTTCTTTCCTGAGAGGAAGGTCTGGCTTCGGGTGGATGTTTTTGTTCATGGTC<br>TTTGGTGTCTTCTTGTTGCTAGTTTTCCCAGGATCTAGTCTAAGATATATTAAGAAAAAGAAAACCTAGA<br>GAGTATGTTGCTATGTCCAACTTCACATCACAAGGCTCCTAGAAAGTCTGACTTCTCTCCACATTTCAGT<br>CTTTTTATATTTATCGTATATATAACACGGAGGGTTTCTTGGCTTTCTGTTATTCAGGAAAGAAAAGAGAA<br>GTCTGTGCTCTCCCTCTTCCAGAGCATTGTCTATACTAGGCTTTAACCTGATAAAACTTGCAGATTCCAC<br>AAGGCCACGGGATGGGCAAGTGTAGGATTCCTTCCACATCACTATTACACAGGCTGCCTCTTCCAAGCC<br>AACCCCTGCATATCCCTGAGACTGCCTCTGTCTGCTCCTGTTCTGTCTGTCCTTCATTCATGACACTGCA<br>CCCTTTAAGGGACCATGTTTTTTGAGCCTGTGCAGTCAATTCTTGATAGACATGAAGTTTGTACTGTTCG<br>TTTAAGTGCTGGTGGTACCCTGCCCTCAGCTTTTCCTTTTGTATGTGTTAGTCATTCCTCCCAGCTAGAG<br>AATAATACTCTCTGGAGGAAATGAGCAAAAAATATAACTCATACCCCTCTGTTCCCTCAATTTCTAGCAC<br>AATACCAGGATTAATGGGGTGGCTCTATACATTGGTATGCTGTTTTGAATGAAAATGGTCCCTATAGGTTC<br>ATATGCTTGAATGCTCCCCCCACTACTTAGTGGAACTGTCTAGGAAGGATTAGAAAGTGGGGCATTGTT<br>GGAGGGGGTGTGGCCCTGTTAGAGGAGGTGTGTCTCTGGGGTGGGGTTTGAGATTCAAAAGACCATAG<br>CAGGCTCAGGGTCACTCCTTTGCCTGCTGCCTTCAGATCAAGATGTTCAGCACCCACCATGCCTGCCTG<br>CCTCCTGCCGTGATCTCCATTATGATCATCATGGACTAACCCTCTGAAACTGCAAACAAATCCCCAATTA<br>AATGCTTCCTTTTGTAGATTGCCTTGCCATGTTTCATCACAACAATAGAACAGTGACTATGATAATTGGTG<br>TTTCTTATAGCTTTAACTTGCATTTGATGATGTTGCTCCCCCAATTGGAGGACCGTGGATACCTACATGTG<br>AGCAAGACATCTGTAGACTTCTTGCAGAAGTACTAACTTTTCAGAGTTAAAAAAATCCATGTCAAAGAT<br>TGAGGTGACCTTGGCTTTTGTTTCTTATTTCTGTCAACTAGATCTGCAATAGCTCAAATGCGATTTCTTAC<br>TGTCCAAGAGTCTGTCTTTAATGGAGTGAGCAAAACAAAGTTCAAAGTACTTTGAAATATATACAATATT<br>CAAAACTCCCCTGCACTGAAGATAAAGTCTTTGGTCTATAACTGATTTTATAATAATTTTATTTAGTGAAC<br>ATATTAGCAAATTCATGTCAGACAGAAAGAGCTGTTCGAGATGAAAATATACATCAGAAGGCAATGAGC

							AAACATGGGCTTATATGAATAAGTAATATGCCACAAATACACACCTAGTGAGACAGGGGGATGAAGAAT<br>AAACCTGATGGGACTTTTAACATCACAGAACTAGTTAATGAATTCCCAAAATATTTTAGAAATTTTACGT<br>TGAGTATTTACGGAGCTAGATCTGGCACTGAAAGAAAAAAGGAAGAGAGTTAAAAATAAATATTAAGA<br>ATATTTGCAGATCATATGTTTAATATGTAGGATACTGACCCAAGATTGGACACGAGGACAAAGTTCTTAA<br>GTGTTACACTGGGGAATTTTCTTATCCATGGAGAAGTAGAAAGGGTTTTTTAAAGCTATGGTTTTGTGGA<br>GAATGCACGATAGATCACTGGTTTTTTTAGGTAAACTATGGTTTTAACAATGAATACTCTCCATGGTTTTA<br>TTAAGTGGCTTCTGAATTTGCTTTCGAGAAACATGTAGAAAGAAACACAGTAAGCACAGTGTCCAGTG<br>ATAAGGACAGGAAGCATCACTCTTCCCGGAGACACGGAGATCAGACATCACAAATTACCTTCCTTTCTG<br>AAGTTCCCTAACAAATTGACGACCTCACTTTTGCTTAAACATCAGTAACTGAGAGAGAACTCTGTCCGG<br>CCTTGTTTAATGACTAGAACACCATTCTTCACTCAGTAGAAGTCCACTTCTCTGAATCATTCATCTCAGT<br>TTGGTCCTGGGAAGTCTGCTGGGCCTTGCCACCTTCTTTCCCTTCATCAAATTGATGTTAAGTATTCACT<br>CTCATGGGATTCCTTCTTCCTGTTAATGAATACCTTCTTCCAGCAATTTCTCAGATGGTCTGTTTTTCAAG<br>AGCATTGTTACTTCACAGTTGTCCTTGCCATGCCTACCTCATCAATGCTCAGAACATGTCACTCACTCTT<br>GCCTATGACAGACAGAGGCCTATTTCCTCTTCCTGTGTCCAACAGGGACTCTCTATGAATACTACTTGGG<br>ATTACAGAAAAGGTTTTCATAACGTCACCGTACCACTAGCTCATACTTATTAATTACAACCCTTGGTGTTC<br>TCAAATTCAATCATGAACTATTTAAGTTTCACTGCCTACGTTCTGCACATATTAAACATATTTTTATGAACC<br>TGTACTCAGGATTTTATATTTATGGTTTCATGGACCACTACTCTTGTCTCCTCCTTAAATCCCATAGACTGG<br>AGCTTAGGACATTATATTCTTTCCTGAAGGTGTCAACTACCGTGTCATTCACTGCCACTCTCAGTGTCAG<br>CCCTGGGTCTGTGTTATTAGCAATCATCTAGGTGCTCCCTCACCTCACCACCATCCTCACCATCCTGGTAT<br>CGGCTCTCCAACAAGCTGTCCTCTCCTCTTTCGGATACCCATCTCCTTCCCAATCTCTATCCTAAACACCT<br>CAGCTCTCCAACTGATGCCTCTTATTCTTTGAGCTCTCTTACTCTAAACTCCTTCCTCCGGCAGAGCCTA<br>AACCCTGTTTGTCACCTTTGTCTGTGCCTTCCCATCAGATCTGATGATAATATATGGATTCACCACCTCGC<br>ACATGACCTCAAATCTACAGCAATAGGTACTGTAAAATAAAACCAGAAATCCCCTAACCATTTACCTACT<br>CTAAACCAAAGTTTGGCTGAATGTGGCTGGGGAAAAAAAAGAGGAGGAAAGATCAAGAAAGGGAGG<br>AGAAGACAAGGAGGTTATAGTGTGTATGGTAACTTTATTACAGTATATATGGTGACTTTATACAGTATATAT<br>GGTGACTTTTCTCACATATGCTTAAGACCACAAATTCCAAGGGAATCCCAAAAGCTGGGGTTTTTTTTAC<br>TCCATTCACCTAGACCATAATATCCTCACTCCCCACTCAATATCCCAGCAACTGTGGAATAAGGCACTTA<br>ACTTGCTGTTAAGACATCTGGATACCCAAGTATGTCAGAAATCCTTGACTGTGAAAACCGACTCCCTTG<br>TGAACTTATCTCTCTGCCCATGACTACCAAGTGTCTACTGCTAGTCTAGCCCATAGTCCATCCCCACTGG<br>ACACTGAAAACTTGACATCTCCACTTGGGCATCTGGTACTTCAAAATCAACAATTCTGAAAGCTAACTC<br>CTTGTTTGACCCTTAAACCTGCTCTCTAAAACCTTCCTAATCCAAGTCAGCAACCCAATTACATTTGCTC<br>AAGTCAAGTTCCATGGCACAACTGAATGTTCTTTTCCTCTCACATACCATCTATAATCCAATGAGATGTC<br>ACCTTCAGAATATACATTATGATTGTATCTAGAATCTGACTACTTTTCGTCTCCTTTGAGGTTATCTCTCTG<br>GTTCATGCCATCATGTTCTTCTAACTGCTCATTCTTCCTCCAAATCTACCACCATCGTTCCTGTGTTAGAT<br>TCTTGACATGATGAGCAAAGTGAGCTGCATAAACTTGGTTCCCTGCTATGGGCAAAGACCTTTGACCTT<br>TTAACTCAGAGTAACAGCCCAAGTGGCGACATGGCGGAAAGACTCAATCTTACCTTTTGGCCTTGGCTT<br>GTCTACTGTTCTTCCCAGGCTCACTTTGCTATCCTTTTCGGGGTTTCTGAATGTTCCAGAAATGCACATA<br>GTAGATGTTGTGCCCAACGTCTGAAATGATCTCTCGGGCATCTCACCTCCTGCAAGTTCTTGGATTGGTG<br>CCACCTTATCAAAGATTTCTCTCTGATTACTTCTCCAGCTCCCATTCCTCAGTTTGGACCTTCTCTGTGGT<br>GCTTTAGAACCCTTCCAGGTATCCTTTCTGGTTGCAGTCTTCTCTTTCTCCTTCCCAACCCCAGCCCTCC<br>AAATATACTTTTGGGCAAACAGATTCTTTTCTCTTTCTGCTTTAGCATATCCCAATGCTTACAACATCTGG

							TATATATCAGGGATTCACTAAACTATAGTAGAATTGCGAATCAAATGCTATCTTATTGAAACGTAAATCAG<br>GAATGTTATTTGTTTTCCTATCTTAAATTATAAAACTCTCTAACACATATTTTCTCCCTATATAAAAATTTAA<br>ATTAAGACAAAAGATATTTTAAGGAAGAGTTCATCTTGCTGTTTTTGGATAAACACTAAAATATATTTTTC<br>CTCCTTTAAAACAGTAAGATTAGTGCAATTAAAGAACATACTTTCTACTCCTTTTACAAGTTGAGTTCTT<br>GGATATAATCAGTGCTGTGTATATGATGGTAATTCCAAAAAGAAATGGTGATTCAAGTGGTAAATCTGAA<br>AACAGAATGATTATATTATGTGGTCTGTATTTTAGGGAGCCATGAATTTCTGGAGATTAACATAATCCTTG<br>TTTTGAAAGGATTGACCAGAATTATAAAGATTTTCCTTCCAGGATTTGAGAATGGATTGCTTTGTTTTTCT<br>GTCTTTGAGGAAGTGTCTATTTTAACTCTCAGGCCTTATTCATCTTCTCAAGAGCTACTTGGAATCAAGT<br>CGAATAAAGTCATTCAGCACTATGAGCTATAAGGCAGGTGCATCTGAGGAACGGGCCTCATCACAGCCA<br>CGGCTGGGTTTTACTTGTGGTGAGTTCCCGGCCCTGTGTGCTTGAGCCTTCCCAGTCTGTTCATTTACAC<br>TGCTCGACAATCATGAAATGGGAAGCTCCCTTTCCTCACACACTAGCATCACAGCATCCTGACAAACAG<br>CAGGCTCTCTAGTTGATGATCTTCTCAGCGTTCTCTTCAACTTCAAATAGTGTTTAAAGAATCAAACACC<br>CAGCTTGGGTTTTTTTCTTTTCTTTTTTTTAAACCATGTTCTTTCTGGTGACTTCTGTGGAGAGTGTTCAT<br>GTATTGGTTCATGTATTGATAACATCAGGCTCCTGAAAACATGCCCTCTATATTCTTTGCCCTCTAAGTTC<br>TCCAGTACATTTTTCTAAGCTCCCTTGGTCACTATTGAAATTTAAATATAGAATCATAAGGCACTGTGCTT<br>GTGTCCAAAAGGTGGAGACAACCAATGACCCTACCCAGCTATGACACTGATGAACCACATCAACACCA<br>GCACGGCATGATAACCATAAGGGTGAAACAGTGGCACATGTATTTTAGCCATAACCAACATCTCTTTAAT<br>TATATTTAACATTTACTCAACATGAGGAAGACCATGGCTGGTACTGGAAACCTACCTAACGCTTCAGTGC<br>TAGTAAAATCATGGTTATTGGAGAACCAACAGCTACCTATTCGCTAAACCAGCACAGTACCTAACAACA<br>ACCTATAAACAATTGTCTTTATACCCACAGATCAGTGTAGTCTTCCCCTCATCAAGGAAACTTTCTTTGC<br>AACAGAAAACCACAACTCACCAAAAAGTAAAATTCTGGAGCCCAGCCCCAATGCATGCATTCATGAAA<br>CACATTCTTTTCCATAGCTATGTATTCAAAGCAAGCTACACTCATATTTATGTATCTTGTGTTGACCTTGCT<br>TCTGTGACGGGGGATAGTGGTGATGGCTTTCCAGTCAAGCCCTCGGCAAAGATTTGAGAATGAAAAAG<br>ATGAAAACTGACACAAAGGAAGTGTTTGGCTAAACTGAACACGGATGTGAGACAAAGTGCAGCATGTT<br>CTAGGAGTCCTTGACCTGTGATCAGCAGAAGCATGTAGTAACTATTCACCAGGGTGAACTGAGAAGTTT<br>CTCACTTTACAGATACAATAATTAATAATCCTTGCTACCTAGGATGGACAGTTACATGGGCAATAATCCAC<br>ACCTTGCCTCCAAAACCTCAGTACCAGAAGTTCATATGCAACCTAGAAAAAGAACAGAAACAGGTCTA<br>TCTGTGGCTGGTGGGGGAGGGGCCAGAGGAACAGCAGAGTGAGTTTCCAGAATGGTGACTTCAAAAA<br>CATCAAGAAAGTCCTAATGATATCCATTTTCAATGTGTTTTTAAAGTATAGAAGTATTTCTAAGTGTTAAT<br>GTAACCTATCCTATTGCCTCTGTAGGAGGCAACCTATCTATTTCAGAGATGTGTATCATATTTAACTGCCA<br>GAAATGTTAGATCAGCAGTTTTCAACCTGTGGATCGAGATCTCTTAGGAGGTCAAACAACCCTTTCTCA<br>GGGGTCACACATCAGATAGCCTACATATCAGATATTTACATCATGATTCTTAACAGTAGCAAAATAACAG<br>TTAAGAAGTAGCAACAAGAGTAATTTTATGGTTGGGGATCACCACAACATGAGGAACTGTATTAAAGGT<br>CTGCAGCATTAGGAAGGTTGAGAACCACTGGGCTAGACTGTGTCTAAAGGCTCTGTCTAAAGAAACAA<br>AGTGCATTAGGTTTCCAGACTATTTTACTCTTTCTGTAGACGGCAAAAGCAGAGTGTAGTGTTGCTTCTT<br>GTTTCAGTATCACTCTCAACCTATGAAAGACTGGAAGAGCACTGTAGTTTTCTAGATGAGGTGCATAAA<br>AAAATTCCTAATAAATCATGATAGATGGAAACAGAAGTATCATATTTGACTTTTTAAAAAATGTACCTATG<br>AACTCCACAGTCCAAGGAATGTCTACTGAAGAAGACTCTTCCTTTTCTTTCGGCAATTATCTAAAAAAC<br>AATGTCAAATTTATCCAGAGAAAGGTACAGCTGAGGCCGTCCATTCTGGCCTGGCTTCTCCTGGCCATT<br>CTCTGTCCTGGCCTCCACATGGCTTTCACACAGCCAACAAAGTGGGTGATGAGTTCACCTCTTACTATTT<br>CAATTTGCTCAGACGTCCTTTCTTTAAAAAAAGAAAAATTCTTTCTGTAAACAAAGGGCCATTCTTCTCT

							CCCTTTGTTTTTGTGTAGTCCTTTTGACGAGTTCCCAGGAATAAAATAACCAATCGTGGAGGGGGAGAT<br>GAGAGAGAGAGAGAGAGAGAGAGAGAGAGAGAGAGAGAGAGAGAGCAGTTGCTCTTAGCAGCCTTT<br>GTATAAAACTGTGAGAAGCTGGCGACGCCCCACGGTTAGCATCCCTGACACTCTGTTGCTTCCACATGG<br>CTCTTTGAATGTTCCTTGGCCAAAGACATCATCATCGCACATGCCCACTAGCAATTTTAGAAGAGAAAG<br>ATTATAGTGAAAGGAGAAACTGTTGTTTTTTGACGTGGTTTGGAAGTTTCCCATCCTACACAAGTGTCA<br>GAAGCAGTGGGGATAGCTGAAAATCCACTGAGTATAACAGGTTTTTACAGCTTGCTTGAATGATTTTATA<br>CACACACACACACACACACACACACACACACACACACACACACACACACATGCATATCCTAGAAGT<br>TGACTGAGAAATATTTTTTGGAACTATTTCTTGGGGGAATTTAATTACCATCAATGTATAAAAATAAACAG<br>CAGAATTTCTGCCCAGATGGCATGATCAATGGTTCTTTTTCAGAAAAAAAATGTTTTTAAAGCATCCATA<br>TCTGTCTATATATGAAAACGAAGCCTCCTGTTTACCCTGCTTCATGCTTAAGTTTCCATTGAAGTTGAGG<br>CCTAAAGCATTCTCTTTCAAAAGAAAAAAAGTTACGAAGTAGCCTTCTTGCTTGGAGGACACAGTGAG<br>AAAACAACTGGGGGCCCGGGAGGCAGAACCACAGCAAACACAGACTCTGAGATGCCTTGAGCTCAAA<br>CTTCCCCCGCCTTTAGGACATCAGGAAAGGTGGTTTCTATTTATAAAGCAAAATATAAAACTGCAAAGTC<br>AAGCAAGGTGCCCCACAGTCTTCCCCCTGGGTTTGTGACCAGTACACTATTTTTTTTTCTTACTCTAAAA<br>AAAAATATATATAGCATATAAGGTTGGTCCTGCTTTTATTGGAATTTTCCAATAAATGTCTACTTACTTGGC<br>TGTCATTTTCCCACACCCATGTCTTATACCTCAGCCTATCTTGTCTATGAGTAGGGAGCTTGGAGATGTAA<br>CACATAAGCCAGACAACAACCATCTGGGAAATCTGTCCTAACAACCAATGACAGCATTTCTATACAAAT<br>ATGTACGCCTGCATCCCTTCCACCTCCTCTCACCCAAACTTAGAACCTGTTTGCTTTTATTAGTTTATTTG<br>ATGCTGAGGAAACCTCTGAATTGTTTGAAATATCTTCATAATAAAGATGCTAGACGTGGAAGATGCTATT<br>TTGTCTAGTACCAGGGCATTGCATCTGTGTAGCTCACAGAGTAGATTTGTAAATGTTTTCACCATTAAAA<br>AGACATGATCACGGTTTGTATTAATCAGCTCGATTTTAATCATTCCACAATATATACTATCGAAACATTGTA<br>TTGCATAATATGTGTGTGCATATTTCATCCATTTGATGCATTTTAGTGCATGCAACCTAACATGAAATTTCT<br>TTTTCTTGCTCGCTTGTCTTCAGTGACCCTAGCAGGAGGTAGGTAAACCTTATATACGAAGAATTTACTC<br>TGAGTATGGCAGATAAAGGTTCTGACCTGTGAGCCGCCCTCAGCAAGGAAGGCTACATGCACAATGGT<br>TTTAGGTTCATCATTTGCGTTTTGCTCTTGCGGTTACGTGTCTTAATTTCTCTTCATTTGGACACAAGAAG<br>TTGTAAGGGCGACCAGGAAACTATTATTTGGTTTAAATCCTATAACCCGTTATAATTAATATATGACAAAT<br>CGGGTTGTCAACCTTTGGGTCTTTTCACATTTAAGTCTTCCCTTCCCTTCCTCCCTCCCCTTCCTTGCCCC<br>AACCTCCTCTTTCCCCTCCTCTTGCCCTGCCCTTTGTTTCTCTTTTTATTTCTTGGACCCTACTTTTTTTCC<br>TCTCAGTATCCAAGACATTCTCCGAATCTCCTCAACTGACTTAAATCATGTTTGTTCTGGAGCAGAGTTT<br>AGACAACCCGAGGCACTCCACACAACCCTCGAGAGGGGTTGGCTTAAGAAAGGGGTTTCCCTAAATCA<br>GCTGCCAGGAACACACATGCCAGTCCCACCCTCGTCCCGTCCTCCAGTAACACCCACTACCCAGGCCT<br>CACACTACATTACCAAAACTATACAACTCCCACTCCAGCACAGCAGAGCCTCTCAGACCACAGCACAA<br>CACACCACCCCCTACAGTATTTCTTCTGAAAACAGCAATAACCAGGTAGCCAACCCCTCCCCCTGGTGC<br>CTGTCTGTCTCTAGCTTCCAACAGCCAGGACAAGCCAAGGCCCTAGGGTTGATTCCACCAGGCAAGGA<br>TTTGCAGTGAAGGGCAAGTCAGGGGGAAGAGAGGCCAAACCACTGGCAGTTTTTCAAATGCAAAGAC<br>GAGTTCAGACTGCTGGGTAAACGTGCCTGGATCTCTGGCCACAACCCCAGCACTCCAAATCTACAGCTA<br>GGACCTTCACATTTATGGATTCCAAGTTTCAATACTCTGATAGATTCAGGTAAAGAGCAGCTAGGACATA<br>AAGAAGGAAGGCTACAACCAACTCCCAACCTCCTAACAATGTTGTCCCTCCGGAATTAAGCCTGGGAC<br>TCATGGGGGTTTGTTTTCTGCGCGGGAGAAAGAGCTGAGCAGGAGAAGAACTGGAAAGGAGTAGGGT<br>GTGGTGATTCCTTGAGGGAAAAGAAAGGGAAAATTAAAGTCACTGCAGTACTCCGCTGCCCTAACCCA<br>GCGGGAGGGTCCTGAGGCGGTGGGCCCACCCGCTGCTCAGTTGTCTACGCCCCTGGAGCACTCTGCGC

							GCAAACACGCCCCAGAAAACCATCACAACCTGTCACTCCCGCCCTGAGGCCCCTGACAAGAAGGAAAAACTTGTTCCGGGGCGCAGCAGAAGCCAGGCTAGCCAACGAGGACAATGTCTATGTCTGAAACTAGAGAAATAATGAAATAACTGGACCAACAGCTTTCTTCCTCCTGGCCGCTGTTTTCCACCAGTACGCTTCCACCCTGCTGTTTTCCACCAGTACGCTAATATTTACAGTCGCCCCAGACGAACCTCTGCTGGGTCCCCCCTCCCCCGCCCCCAGGTAAACAGGCCAGGAAGACATAGAGGAAAGCTGTGGTCTAGGGGACTCACAAAAAACACGTCCCGGCCTCTATTTGTGGGGTCCCGGTGCCGGCCGGCTTACCTGTGGATGCAGTTTCCATGGCGACGGGAGGCGACCGGGCGGGGCCGGAGCACAGTTCCCGCGACGACACTACCCCAGCTCCAGCGCCGGACCCCCAAGCGGGCTCCCCATCCTGCTGCGCTGGTCTGGCCCCCTTCGGCGTCGCTTCTTCGTCTTCCCCCTCCCCCCGGCCCCCGAACAACTGGGACCCGGGGTTAGCCGGAGACAGTGGCTCATCTTGCAGATCCGGCGGTGCGGCCATGACTGGCTATGCCCCAGGCTGGGCGACTGTAGCTTGGCAAGGCCCGGGTGCGGTGGCTAGGGACGCGGACGCTCCCTGCTGCTGTCCCTACCGGGACTCCGGGCTCCAGGAGGCTGCAGAGTCTCCGCGCCACCGTCGCTGCTTTTTCTGCAACTCAGGCCGAGACGCTGCTGCTGTTCTGGGCGCTTCGGCTCCTCCTTCTCCCCCTCCTCTTCCTCCGGGGCCCCGGGGCGGCTGCCGCCTGCGCTCCTCCCCTGCTCCAGAGGCAGCTCGCTGTGCCTGCGCTGCTTCGCTACTCCCAAGCCTGTTATTACGCAGGTGAAAATGGCCTGCTTGGTCCGAGTCCTCCCTAAAGCACGTCAGCCCCGGGCCTGCTAATGGCAGGCTCTGATTAAGGGATGTGCCAAACCCACTTGATTTTACCCTTTATCTGGGGAAGAATACACAGAGAGGACGAGCAGGTACTGGCTGCAGCTTGACTAGATAAGAAGTTAAAGCCAGATTGGAGTCCTGGTACTCTAGGACAGCAAGCGAGACTAATGAATCTCAAGACAACCGTTCTGAGGGTTAGCTGCAGAAAGGACACTGGCCTGAGCCGTAACCTGGTGATCTGCAACGTGGAGGATAAATCCTTTATCCTCAAGTTCCCAGAACCCGTACAAACAAACAAACAAAAACAGTAACAACAAAACAGCGTACTGGAATGGGC
MSTRG.107376.2	ENSRNOG00000060998	0.474	0.000	0.000	-8.534	down	TCCCCTTCTGCCTTCCAATCTGGCTGTAGCAATCTCACTTACTTCTCTAAAACCATGTCTGTCTGCCTGCCACCATGCTTTCCACCATGAGGATAATGAACTAAAATTCTGAACTGTAAGCCAATCTCAACTAAATGCCTTCCTTCGTAAAAGTTGCTGTGGTCCTGGTGTCTCTTCACAGCAATAGAAGCCCTAGCTAAAACAGGACTCAATGTTCCTGGGCATTGTCATTGCGATTTAATAAGAGCTGAGCGCAGAGTAAATCACTGTCCTTAAAAAAGAAATAACACAAATGGCCTTGTGGCCCTACTCCTAATGCATAACACCATTAAGAGGGGAATTTTTATGGGGCTTATTTTAAAAGTATAGTATCTCCCTTTTTAAAATACATTTTAATTCCTCCATATCATTTACAGAAAGAAATAAATCAACTCTTATTTTTGAGACTCTGGATGTACAAACATGACCAATAAGAAAAGTTTAGCTTTAAAAGTTGTGCAATTTCTAGGAAATATATCTCCATTCATTTTCATGTTATTTTAGTAAGATTCTTGAAGCTGGGTAAGATATAGAGAAGAAAGATTCATTTGGCCCACAGGTCTGGTGAATGGAAAGCCCAAGTAGCAAGGTGCTCCTTGGGTGAGCACTCCCTAGATGATGATGCTCCTCGGGTGAGCTCTCCCTAGATGATGATGCTCCTTGGGGGAGCACTCCCTAGATGATGATGCTCCTCGGGTGAGCTCTCCCTAGATGATGATGCTCCTCGGGGGAGCACTTTCTAGATGATGGTGCTCCTCGGGGGAGCACTCCCTAGATGATAGTGCTCCTCGGGGGAGCACTCCCTGGATGATGAAGCTCCTCGGGGGAGCACTCCCTAGATGATGGTGCTCCTCGGGGGAGCACTCCCTGGATGATGGAGCTCCTCGGGGGAGCACTCCCTAGATGATGGTGCTCCTCGGGGGAGCACTCCCCAGATGATGGTGCTCCTCGGGGAGCACTCCCTAGATGATGGTGCTCCTTGGGGGAGCACTCCCTAGATGCATCAGAGCACAAGAGAAAGCTAAATGGAAATAAGCATGAGCATGAACAAAATAGATCCAAAAACACAGTACAATAATGCAGACTTGTTCTTTCTCCCACTCTCATAAAATAAAATAGAAACAGCTCTTTAGGACCATGAATTCAATTTATAAAATAAATAAATAAACTCATATAAGTTTACTCCTGGTAATTCAAACATGCTGGGCTGCAATTTACTCTTCAATTATGTAGAATAAAAAGCCTTAGTAAACAAATTAATGGTGTGATAAGCAGTGGCAACTCCCACCTTTCTATAAGTACGGCCCTTAGAAGCTACCTTCTGGTCAGAGGCAAAGTTAGCTAACTTGCTCTCAAGCAGAATGGGAGCAGGAGGCATCTCATGTTATGGTTAAAGTAGAGCTTTCCAAGTAAGGCAGACAT

							TGTGCAGCTGAGACCTCATTGCCTGCACAGCCCTCCCTCATAGCCTGACATTCCCCTGCTCCCGCACAG<br>CCCTTCCTCATAGCCTGCCATCCTCCTGCTCCCGCACAGCCCTCCCTCATAACCTGCCATCCTCCGGCCA<br>TCCGCATTTATCTGTATGTCACCTATTCAACGCCATACCCTGTGTGTGTGGTTAGTGCCTAGCCAAGTGTT<br>TGCTGGACAAACAAATGAATGGTTCAGTAAAATTGGACTGGATTATGAAGCCATGGCTGCTAGGTAAAC<br>AGTGATCTACTTGAAAGTGTCTGGGGTCATCGAATTCTAAAGAGTTAGAAAGTTGTTATTTATAACCATA<br>GAAGGCCAGGGGGTGGGGCACAAATATGTGTACAAAGTGGCGTTAGTCTACCCCTGGGAAAAGATGGC<br>AAAGCTCGCATCACATTCTCAACAATCGTTAGAATCACTGATCTAACGTATCTCCTCTCTTCTGACCTTT<br>GTCACAGAACCAACCAACACCGCTCCGATTACATAGCTCCAGTAACAGGCATTTCCTCAGCTGGACAAT<br>GTCAACTACTCCTGCACTTTATAATTCTGGTACCATTGAACCCCAGTCCTTTCCCAGGAGCCTCTGAGTA<br>GCTGCAATGGCCATGCTCTGGAGTCACCCAAACACCCTGCCTAGTGCCTAAGCCATCTGACCACACTTT<br>GTGGGTTTTTGACAGCTGTCATAACGTACTCTGTCTTTCTTATATCCAGGCTGTAAGTCTCCAGCTCTTCC<br>TACTGCACTTAAGATTTCTCCTCCTGTGCCTGTGCGGGGGGCTCTGCTGGGCCCTGCATGGCCCTCCTGT<br>GTTTTGGTGTGCACTGAACCTAACACTTCATCCTTTTAAGGGTGCCCTGTGGCCCTACTGACTTAGCATC<br>AGCAGGTAACAAAGTCCTTGTATTTTTTTTCTGTATGAGCGCCATAATTCTGCACCCGTGGCCCTCTTGA<br>CTTTATTGAAGCAGATAGTCCGAGGATTCTTAACAGGGCTTTGCTAGTATCTTTATCAGATTCCTGCCGA<br>AATGCAGCTAGTCACTGCTTTGGGCTGAGGCTCGTGTGTAAGTTCTGCTAAGTTCTATCTACCTGGCTTT<br>AGCCACCACCCTCCCCCCAAACATTTCATAATTGCTCTAATTTAGATTTCATGCAGATTAACATTCCACTT<br>AATATCCAACTTTTACTGCCCAAATTATAAACTGGATGTTGCACAAGAAAAGTCCAGTAGAGATGCAGG<br>GTGAGGAGGAAGGAAAGAAGAGAGATCCAGAATCTGGGTTTGAGGCGGCCTTGGGTTGCCACTTTCTA<br>GCTATGGGACTTCAGACATGTCACATAATCTATTGCATTCTCTCCACTCTCCACTTGGTGGTCATTTCTCT<br>CAGATACTGCACTTGTCAAATGAAACTTTTCCGACTGAGTTATTTCTTATAGGTTTGTAGCTGTTGTTTAA<br>TTTCATTTGATTTGATTTTATTGAAACAGGGTCATCTCATGTTGACCAGACTGGCCCAGTTCATAATCCTG<br>CTGTCTCAACATTCCCAAGACTACAAAGACAGCCTTTGCCTTCATGACTGATTCTTTATAGCTTTTACTT<br>CTTTGAGCTTTTCTATTTCACTTCATTGTTTTGGGATTACTCATTAAACTATCTTTGTTTCAGTTCCTTCAA<br>AATCCCTGCCAGGCGATCTCTAATTTTTATGTTGGAATAGCTGTTTGTCTTTCTGGCTACTGTTGGTTCTT<br>GAAATAACAATTGAGCTGCTTTTTTTTCAGATTCATGGTTATTTTTATTTTATGTGCACGAGTGTATATATA<br>TGTACCACATAGGTGCCTGTTGTCCAAGGAGGCCACAAGAGGACATTAGATCTCCTGGAATTGGAGCTA<br>CAGATAGTTAGAAGCTGCCATGTGAGTGCTGGGACCCAAACCTAAGTTCTCTGTAAGAGCAGTCAATGC<br>TTTTCGCCATTGACGAGTCTCTCCAGCTCCCTAACGGTCATTTTCCAACTGTTAGCTGGACCTGTGAGAC<br>TCCAGGCCTGACTCACATGCTCTGATGCCTGGATGAAGAAGGGCACCTGTCTGGAGGCTTCTGGGAAG<br>ATATGGAGTGAGCTTTTCCACTGAGCATCCTTGCTACTGTGCAGTGGAACCCTCCACTCTCCACTTGGCT<br>CTCTGTAATACTTCACTGACTAGCCACCAGGTTAAGCTTGTCCCCTTATGGGCCTTTAACAGTTTAAGGC<br>AGTGTTCTCAACTTTCCTAATGTTATGACCCTTTAAAATACAGTTCTTCATGTTGTGGTGACCCCCAACTA<br>TAAAATTATTTTTGTTGCTACTTCGTAGCTGCAATTTTGCTACTGTCATGAATTATAATGTAAAAATCTGTG<br>TTTCCTAATGGTCTTAAGTGACCCCTGTGGAAGGATCTTTCTACCCCGAGGGGGTCACAACTGACAGGT<br>TGAGAACCACTGGCCTAAAGGGTAAAGGCTCAGCTTTTCAGTTGGAATGGGGCCTCCATTTATTTCATG<br>GTGGTTCCCAGACAGCTTGCTGAGTGCTTCTTTCCTGAGAGGAAGGTCTGGCTTCGGGTGGATGTTTTT<br>GTTCATGGTCTTTGGTGTCTTCTTGTTGCTAGTTTTCCCAGGATCTAGTCTAAGATATATTAAGAAAAAGA<br>AAACCTAGAGAGTATGTTGCTATGTCCAACTTCACATCACAAGGCTCCTAGAAAGTCTGACTTCTCTCC<br>ACATTTCAGTCTTTTTATATTTATCGTATATATAACACGGAGGGTTTCTTGGCTTTCTGTTATTCAGGAAAG<br>AAAAGAGAAGTCTGTGCTCTCCCTCTTCCAGAGCATTGTCTATACTAGGCTTTAACCTGATAAAACTTGC

							AGATTCCACAAGGCCACGGGATGGGCAAGTGTAGGATTCCTTCCACATCACTATTACACAGGCTGCCTC<br>TTCCAAGCCAACCCCTGCATATCCCTGAGACTGCCTCTGTCTGCTCCTGTTCTGTCTGTCCTTCATTCAT<br>GACACTGCACCCTTTAAGGGACCATGTTTTTTGAGCCTGTGCAGTCAATTCTTGATAGACATGAAGTTTG<br>TACTGTTCGTTTAAGTGCTGGTGGTACCCTGCCCTCAGCTTTTCCTTTTGTATGTGTTAGTCATTCCTCCC<br>AGCTAGAGAATAATACTCTCTGGAGGAAATGAGCAAAAAATATAACTCATACCCCTCTGTTCCCTCAATT<br>TCTAGCACAATACCAGGATTAATGGGGTGGCTCTATACATTGGTATGCTGTTTTGAATGAAAATGGTCCC<br>TATAGGTTCATATGCTTGAATGCTCCCCCCACTACTTAGTGGAACTGTCTAGGAAGGATTAGAAAGTGGG<br>GCATTGTTGGAGGGGGTGTGGCCCTGTTAGAGGAGGTGTGTCTCTGGGGTGGGGTTTGAGATTCAAAA<br>GACCATAGCAGGCTCAGGGTCACTCCTTTGCCTGCTGCCTTCAGATCAAGATGTTCAGCACCCACCATG<br>CCTGCCTGCCTCCTGCCGTGATCTCCATTATGATCATCATGGACTAACCCTCTGAAACTGCAAACAAATC<br>CCCAATTAAATGCTTCCTTTTGTAGATTGCCTTGCCATGTTTCATCACAACAATAGAACAGTGACTATGAT<br>AATTGGTGTTTCTTATAGCTTTAACTTGCATTTGATGATGTTGCTCCCCCAATTGGAGGACCGTGGATACC<br>TACATGTGAGCAAGACATCTGTAGACTTCTTGCAGAAGTACTAACTTTTCAGAGTTAAAAAAATCCATG<br>TCAAAGATTGAGGTGACCTTGGCTTTTGTTTCTTATTTCTGTCAACTAGATCTGCAATAGCTCAAATGCG<br>ATTTCTTACTGTCCAAGAGTCTGTCTTTAATGGAGTGAGCAAAACAAAGTTCAAAGTACTTTGAAATATA<br>TACAATATTCAAAACTCCCCTGCACTGAAGATAAAGTCTTTGGTCTATAACTGATTTTATAATAATTTTATT<br>TAGTGAACATATTAGCAAATTCATGTCAGACAGAAAGAGCTGTTCGAGATGAAAATATACATCAGAAGG<br>CAATGAGCAAACATGGGCTTATATGAATAAGTAATATGCCACAAATACACACCTAGTGAGACAGGGGGA<br>TGAAGAATAAACCTGATGGGACTTTTAACATCACAGAACTAGTTAATGAATTCCCAAAATATTTTAGAAA<br>TTTTACGTTGAGTATTTACGGAGCTAGATCTGGCACTGAAAGAAAAAAGGAAGAGAGTTAAAAATAAAT<br>ATTAAGAATATTTGCAGATCATATGTTTAATATGTAGGATACTGACCCAAGATTGGACACGAGGACAAAG<br>TTCTTAAGTGTTACACTGGGGAATTTTCTTATCCATGGAGAAGTAGAAAGGGTTTTTTAAAGCTATGGTT<br>TTGTGGAGAATGCACGATAGATCACTGGTTTTTTTAGGTAAACTATGGTTTTAACAATGAATACTCTCCAT<br>GGTTTTATTAAGTGGCTTCTGAATTTGCTTTCGAGAAACATGTAGAAAGAAACACAGTAAGCACAGTGT<br>CCAGTGATAAGGACAGGAAGCATCACTCTTCCCGGAGACACGGAGATCAGACATCACAAATTACCTTC<br>CTTTCTGAAGTTCCCTAACAAATTGACGACCTCACTTTTGCTTAAACATCAGTAACTGAGAGAGAACTC<br>TGTCCGGCCTTGTTTAATGACTAGAACACCATTCTTCACTCAGTAGAAGTCCACTTCTCTGAATCATTCA<br>TCTCAGTTTGGTCCTGGGAAGTCTGCTGGGCCTTGCCACCTTCTTTCCCTTCATCAAATTGATGTTAAGT<br>ATTCACTCTCATGGGATTCCTTCTTCCTGTTAATGAATACCTTCTTCCAGCAATTTCTCAGATGGTCTGTT<br>TTTCAAGAGCATTGTTACTTCACAGTTGTCCTTGCCATGCCTACCTCATCAATGCTCAGAACATGTCACT<br>CACTCTTGCCTATGACAGACAGAGGCCTATTTCCTCTTCCTGTGTCCAACAGGGACTCTCTATGAATACT<br>ACTTGGGATTACAGAAAAGGTTTTCATAACGTCACCGTACCACTAGCTCATACTTATTAATTACAACCCT<br>TGGTGTTCTCAAATTCAATCATGAACTATTTAAGTTTCACTGCCTACGTTCTGCACATATTAAACATATTT<br>TTATGAACCTGTACTCAGGATTTTATATTTATGGTTTCATGGACCACTACTCTTGTCTCCTCCTTAAATCCC<br>ATAGACTGGAGCTTAGGACATTATATTCTTTCCTGAAGGTGTCAACTACCGTGTCATTCACTGCCACTCT<br>CAGTGTCAGCCCTGGGTCTGTGTTATTAGCAATCATCTAGGTGCTCCCTCACCTCACCACCATCCTCACC<br>ATCCTGGTATCGGCTCTCCAACAAGCTGTCCTCTCCTCTTTCGGATACCCATCTCCTTCCCAATCTCTATC<br>CTAAACACCTCAGCTCTCCAACTGATGCCTCTTATTCTTTGAGCTCTCTTACTCTAAACTCCTTCCTCCG<br>GCAGAGCCTAAACCCTGTTTGTCACCTTTGTCTGTGCCTTCCCATCAGATCTGATGATAATATATGGATTC<br>ACCACCTCGCACATGACCTCAAATCTACAGCAATAGGTACTGTAAAATAAAACCAGAAATCCCCTAACC<br>ATTTACCTACTCTAAACCAAAGTTTGGCTGAATGTGGCTGGGGAAAAAAAAGAGGAGGAAAGATCAAG

							AAAGGGAGGAGAAGACAAGGAGGTTATAGTGTGTATGGTAACTTTATTACAGTATATATGGTGACTTTAT<br>ACAGTATATATGGTGACTTTTCTCACATATGCTTAAGACCACAAATTCCAAGGGAATCCCAAAAGCTGGG<br>GTTTTTTTTACTCCATTCACCTAGACCATAATATCCTCACTCCCCACTCAATATCCCAGCAACTGTGGAAT<br>AAGGCACTTAACTTGCTGTTAAGACATCTGGATACCCAAGTATGTCAGAAATCCTTGACTGTGAAAACC<br>GACTCCCTTGTGAACTTATCTCTCTGCCCATGACTACCAAGTGTCTACTGCTAGTCTAGCCCATAGTCCA<br>TCCCCACTGGACACTGAAAACTTGACATCTCCACTTGGGCATCTGGTACTTCAAAATCAACAATTCTGA<br>AAGCTAACTCCTTGTTTGACCCTTAAACCTGCTCTCTAAAACCTTCCTAATCCAAGTCAGCAACCCAATT<br>ACATTTGCTCAAGTCAAGTTCCATGGCACAACTGAATGTTCTTTTCCTCTCACATACCATCTATAATCCAA<br>TGAGATGTCACCTTCAGAATATACATTATGATTGTATCTAGAATCTGACTACTTTTCGTCTCCTTTGAGGT<br>TATCTCTCTGGTTCATGCCATCATGTTCTTCTAACTGCTCATTCTTCCTCCAAATCTACCACCATCGTTCCT<br>GTGTTAGATTCTTGACATGATGAGCAAAGTGAGCTGCATAAACTTGGTTCCCTGCTATGGGCAAAGACC<br>TTTGACCTTTTAACTCAGAGTAACAGCCCAAGTGGCGACATGGCGGAAAGACTCAATCTTACCTTTTGG<br>CCTTGGCTTGTCTACTGTTCTTCCCAGGCTCACTTTGCTATCCTTTTCGGGGTTTCTGAATGTTCCAGAA<br>ATGCACATAGTAGATGTTGTGCCCAACGTCTGAAATGATCTCTCGGGCATCTCACCTCCTGCAAGTTCTT<br>GGATTGGTGCCACCTTATCAAAGATTTCTCTCTGATTACTTCTCCAGCTCCCATTCCTCAGTTTGGACCTT<br>CTCTGTGGTGCTTTAGAACCCTTCCAGGTATCCTTTCTGGTTGCAGTCTTCTCTTTCTCCTTCCCAACCCC<br>AGCCCTCCAAATATACTTTTGGGCAAACAGATTCTTTTCTCTTTCTGCTTTAGCATATCCCAATGCTTACA<br>ACATCTGGTATATATCAGGGATTCACTAAACTATAGTAGAATTGCGAATCAAATGCTATCTTATTGAAACG<br>TAAATCAGGAATGTTATTTGTTTTCCTATCTTAAATTATAAAACTCTCTAACACATATTTTCTCCCTATATAA<br>AAATTTAAATTAAGACAAAAGATATTTTAAGGAAGAGTTCATCTTGCTGTTTTTGGATAAACACTAAAAT<br>ATATTTTTCCTCCTTTAAAACAGTAAGATTAGTGCAATTAAAGAACATACTTTCTACTCCTTTTACAAGTT<br>GAGTTCTTGGATATAATCAGTGCTGTGTATATGATGGTAATTCCAAAAAGAAATGGTGATTCAAGTGGTA<br>AATCTGAAAACAGAATGATTATATTATGTGGTCTGTATTTTAGGGAGCCATGAATTTCTGGAGATTAACAT<br>AATCCTTGTTTTGAAAGGATTGACCAGAATTATAAAGATTTTCCTTCCAGGATTTGAGAATGGATTGCTT<br>TGTTTTTCTGTCTTTGAGGAAGTGTCTATTTTAACTCTCAGGCCTTATTCATCTTCTCAAGAGCTACTTGG<br>AATCAAGTCGAATAAAGTCATTCAGCACTATGAGCTATAAGGCAGGTGCATCTGAGGAACGGGCCTCAT<br>CACAGCCACGGCTGGGTTTTACTTGTGGTGAGTTCCCGGCCCTGTGTGCTTGAGCCTTCCCAGTCTGTT<br>CATTTACACTGCTCGACAATCATGAAATGGGAAGCTCCCTTTCCTCACACACTAGCATCACAGCATCCTG<br>ACAAACAGCAGGCTCTCTAGTTGATGATCTTCTCAGCGTTCTCTTCAACTTCAAATAGTGTTTAAAGAAT<br>CAAACACCCAGCTTGGGTTTTTTTCTTTTCTTTTTTTTAAACCATGTTCTTTCTGGTGACTTCTGTGGAG<br>AGTGTTCATGTATTGGTTCATGTATTGATAACATCAGGCTCCTGAAAACATGCCCTCTATATTCTTTGCCC<br>TCTAAGTTCTCCAGTACATTTTTCTAAGCTCCCTTGGTCACTATTGAAATTTAAATATAGAATCATAAGGC<br>ACTGTGCTTGTGTCCAAAAGGTGGAGACAACCAATGACCCTACCCAGCTATGACACTGATGAACCACA<br>TCAACACCAGCACGGCATGATAACCATAAGGGTGAAACAGTGGCACATGTATTTTAGCCATAACCAACA<br>TCTCTTTAATTATATTTAACATTTACTCAACATGAGGAAGACCATGGCTGGTACTGGAAACCTACCTAAC<br>GCTTCAGTGCTAGTAAAATCATGGTTATTGGAGAACCAACAGCTACCTATTCGCTAAACCAGCACAGTA<br>CCTAACAACAACCTATAAACAATTGTCTTTATACCCACAGATCAGTGTAGTCTTCCCCTCATCAAGGAAA<br>CTTTCTTTGCAACAGAAAACCACAACTCACCAAAAAGTAAAATTCTGGAGCCCAGCCCCAATGCATGC<br>ATTCATGAAACACATTCTTTTCCATAGCTATGTATTCAAAGCAAGCTACACTCATATTTATGTATCTTGTGT<br>TGACCTTGCTTCTGTGACGGGGGATAGTGGTGATGGCTTTCCAGTCAAGCCCTCGGCAAAGATTTGAGA<br>ATGAAAAAGATGAAAACTGACACAAAGGAAGTGTTTGGCTAAACTGAACACGGATGTGAGACAAAGT

							GCAGCATGTTCTAGGAGTCCTTGACCTGTGATCAGCAGAAGCATGTAGTAACTATTCACCAGGGTGAAC<br>TGAGAAGTTTCTCACTTTACAGATACAATAATTAATAATCCTTGCTACCTAGGATGGACAGTTACATGGG<br>CAATAATCCACACCTTGCCTCCAAAACCTCAGTACCAGAAGTTCATATGCAACCTAGAAAAAGAACAG<br>AAACAGGTCTATCTGTGGCTGGTGGGGGAGGGGCCAGAGGAACAGCAGAGTGAGTTTCCAGAATGGT<br>GACTTCAAAAACATCAAGAAAGTCCTAATGATATCCATTTTCAATGTGTTTTTAAAGTATAGAAGTATTTC<br>TAAGTGTTAATGTAACCTATCCTATTGCCTCTGTAGGAGGCAACCTATCTATTTCAGAGATGTGTATCATAT<br>TTAACTGCCAGAAATGTTAGATCAGCAGTTTTCAACCTGTGGATCGAGATCTCTTAGGAGGTCAAACAA<br>CCCTTTCTCAGGGGTCACACATCAGATAGCCTACATATCAGATATTTACATCATGATTCTTAACAGTAGCA<br>AAATAACAGTTAAGAAGTAGCAACAAGAGTAATTTTATGGTTGGGGATCACCACAACATGAGGAACTGT<br>ATTAAAGGTCTGCAGCATTAGGAAGGTTGAGAACCACTGGGCTAGACTGTGTCTAAAGGCTCTGTCTAA<br>AGAAACAAAGTGCATTAGGTTTCCAGACTATTTTACTCTTTCTGTAGACGGCAAAAGCAGAGTGTAGTG<br>TTGCTTCTTGTTTCAGTATCACTCTCAACCTATGAAAGACTGGAAGAGCACTGTAGTTTTCTAGATGAGG<br>TGCATAAAAAAATTCCTAATAAATCATGATAGATGGAAACAGAAGTATCATATTTGACTTTTTAAAAAAT<br>GTACCTATGAACTCCACAGTCCAAGGAATGTCTACTGAAGAAGACTCTTCCTTTTCTTTCGGCAATTATC<br>TAAAAAACAATGTCAAATTTATCCAGAGAAAGGTACAGCTGAGGCCGTCCATTCTGGCCTGGCTTCTCC<br>TGGCCATTCTCTGTCCTGGCCTCCACATGGCTTTCACACAGCCAACAAAGTGGGTGATGAGTTCACCTC<br>TTACTATTTCAATTTGCTCAGACGTCCTTTCTTTAAAAAAAGAAAAATTCTTTCTGTAAACAAAGGGCCA<br>TTCTTCTCTCCCTTTGTTTTTGTGTAGTCCTTTTGACGAGTTCCCAGGAATAAAATAACCAATCGTGGAG<br>GGGGAGATGAGAGAGAGAGAGAGAGAGAGAGAGAGAGAGAGAGAGAGAGAGAGAGCAGTTGCTCTTA<br>GCAGCCTTTGTATAAAACTGTGAGAAGCTGGCGACGCCCCACGGTTAGCATCCCTGACACTCTGTTGCT<br>TCCACATGGCTCTTTGAATGTTCCTTGGCCAAAGACATCATCATCGCACATGCCCACTAGCAATTTTAGA<br>AGAGAAAGATTATAGTGAAAGGAGAAACTGTTGTTTTTTGACGTGGTTTGGAAGTTTCCCATCCTACAC<br>AAGTGTCAGAAGCAGTGGGGATAGCTGAAAATCCACTGAGTATAACAGGTTTTTACAGCTTGCTTGAAT<br>GATTTTATACACACACACACACACACACACACACACACACACACACACACACACACACACACACATGCATATC<br>CTAGAAGTTGACTGAGAAATATTTTTTGGAACTATTTCTTGGGGGAATTTAATTACCATCAATGTATAAAA<br>ATAAACAGCAGAATTTCTGCCCAGATGGCATGATCAATGGTTCTTTTTCAGAAAAAAAATGTTTTTAAAG<br>CATCCATATCTGTCTATATATGAAAACGAAGCCTCCTGTTTACCCTGCTTCATGCTTAAGTTTCCATTGAA<br>GTTGAGGCCTAAAGCATTCTCTTTCAAAAGAAAAAAAGTTACGAAGTAGCCTTCTTGCTTGGAGGACA<br>CAGTGAGAAAACAACTGGGGGCCCGGGAGGCAGAACCACAGCAAACACAGACTCTGAGATGCCTTGA<br>GCTCAAACTTCCCCCGCCTTTAGGACATCAGGAAAGGTGGTTTCTATTTATAAAGCAAAATATAAAACTG<br>CAAAGTCAAGCAAGGTGCCCCACAGTCTTCCCCCTGGGTTTGTGACCAGTACACTATTTTTTTTTCTTAC<br>TCTAAAAAAAAATATATATAGCATATAAGGTTGGTCCTGCTTTTATTGGAATTTTCCAATAAATGTCTACTT<br>ACTTGGCTGTCATTTTCCCACACCCATGTCTTATACCTCAGCCTATCTTGTCTATGAGTAGGGAGCTTGGA<br>GATGTAACACATAAGCCAGACAACAACCATCTGGGAAATCTGTCCTAACAACCAATGACAGCATTTCTA<br>TACAAATATGTACGCCTGCATCCCTTCCACCTCCTCTCACCCAAACTTAGAACCTGTTTGCTTTTATTAGT<br>TTATTTGATGCTGAGGAAACCTCTGAATTGTTTGAAATATCTTCATAATAAAGATGCTAGACGTGGAAGA<br>TGCTATTTTGTCTAGTACCAGGGCATTGCATCTGTGTAGCTCACAGAGTAGATTTGTAAATGTTTTCACC<br>ATTAAAAAGACATGATCACGGTTTGTATTAATCAGCTCGATTTTAATCATTCCACAATATATACTATCGAA<br>ACATTGTATTGCATAATATGTGTGTGCATATTTCATCCATTTGATGCATTTTAGTGCATGCAACCTAACATG<br>AAATTTCTTTTTCTTGCTCGCTTGTCTTCAGTGACCCTAGCAGGAGGTAGGTAAACCTTATATACGAAGA<br>ATTTACTCTGAGTATGGCAGATAAAGGTTCTGACCTGTGAGCCGCCCTCAGCAAGGAAGGCTACATGCA

							CAATGGTTTTAGGTTCATCATTTGCGTTTTGCTCTTGCGGTTACGTGTCTTAATTTCTCTTCATTTGGACACAAGAAGTTGTAAGGGCGACCAGGAAACTATTATTTGGTTTAAATCCTATAACCCGTTATAATTAATATATGACAAATCGGGTTGTCAACCTTTGGGTCTTTTCACATTTAAGTCTTCCCTTCCCTTCCTCCCTCCCCTTCCTTGCCCCAACCTCCTCTTTCCCCTCCTCTTGCCCTGCCCTTTGTTTCTCTTTTTATTTCTTGGACCCTACTTTTTTTCCTCTCAGTATCCAAGACATTCTCCGAATCTCCTCAACTGACTTAAATCATGTTTGTTCTGGAGCAGAGTTTAGACAACCCGAGGCACTCCACACAACCCTCGAGAGGGGTTGGCTTAAGAAAGGGGTTTCCCTAAATCAGCTGCCAGGAACACACATGCCAGTCCCACCCTCGTCCCGTCCTCCAGTAACACCCACTACCCAGGCCTCACACTACATTACCAAAACTATACAACTCCCACTCCAGCACAGCAGAGCCTCTCAGACCACAGCACAACACACCACCCCCTACAGTATTTCTTCTGAAAACAGCAATAACCAGGTAGCCAACCCCTCCCCCTGGTGCCTGTCTGTCTCTAGCTTCCAACAGCCAGGACAAGCCAAGGCCCTAGGGTTGATTCCACCAGGCAAGGATTTGCAGTGAAGGGCAAGTCAGGGGGAAGAGAGGCCAAACCACTGGCAGTTTTTCAAATGCAAAGACGAGTTCAGACTGCTGGGTAAACGTGCCTGGATCTCTGGCCACAACCCCAGCACTCCAAATCTACAGCTAGGACCTTCACATTTATGGATTCCAAGTTTCAATACTCTGATAGATTCAGGTAAAGAGCAGCTAGGACATAAAGAAGGAAGGCTACAACCAACTCCCAACCTCCTAACAATGTTGTCCCTCCGGAATTAAGCCTGGGACTCATGGGGGTTTGTTTTCTGCGCGGGAGAAAGAGCTGAGCAGGAGAAGAACTGGAAAGGAGTAGGGTGTGGTGATTCCTTGAGGGAAAAGAAAGGGAAAATTAAAGTCACTGCAGTACTCCGCTGCCCTAACCCAGCGGGAGGGTCCTGAGGCGGTGGGCCCACCCGCTGCTCAGTTGTCTACGCCCCTGGAGCACTCTGCGCGCAAACACGCCCCAGAAAACCATCACAACCTGTCACTCCCGCCCTGAGGCCCCTGACAAGAAGGAAAAACTTGTTCCGGGGCGCAGCAGAAGCCAGGCTAGCCAACGAGGACAATGTCTATGTCTGAAACTAGAGAAATAATGAAATAACTGGACCAACAGCTTTCTTCCTCCTGGCCGCTGTTTTCCACCAGTACGCTTCCACCCTGCTGTTTTCCACCAGTACGCTAATATTTACAGTCGCCCCAGACGAACCTCTGCTGGGTCCCCCCTCCCCCGCCCCCAGGTAAACAGGCCAGGAAGACATAGAGGAAAGCTGTGGTCTAGGGGACTCACAAAAAACACGTCCCGGCCTCTATTTGTGGGGTCCCGGTGCCGGCCGGCTTACCTGTGGATGCAGTTTCCATGGCGACGGGAGGCGACCGGGCGGGGCCGGAGCACAGTTCCCGCGACGACACTACCCCAGCTCCAGCGCCGGACCCCCAAGCGGGCTCCCCATCCTGCTGCGCTGGTCTGGCCCCCTTCGGCGTCGCTTCTTCGTCTTCCCCCTCCCCCCGGCCCCCGAACAACTGGGACCCGGGGTTAGCCGGAGACAGTGGCTCATCTTGCAGATCCGGCGGTGCGGCCATGACTGGCTATGCCCCAGGCTGGGCGACTGTAGCTTGGCAAGGCCCGGGTGCGGTGGCTAGGGACGCGGACGCTCCCTGCTGCTGTCCCTACCGGGACTCCGGGCTCCAGGAGGCTGCAGAGTCTCCGCGCCACCGTCGCTGCTTTTTCTGCAACTCAGGCCGAGACGCTGCTGCTGTTCTGGGCGCTTCGGCTCCTCCTTCTCCCC
MSTRG.107376.3	ENSRNOG00000060998	0.455	0.009	0.000	-4.784	down	TCCCCTTCTGCCTTCCAATCTGGCTGTAGCAATCTCACTTACTTCTCTAAAACCATGTCTGTCTGCCTGCCACCATGCTTTCCACCATGAGGATAATGAACTAAAATTCTGAACTGTAAGCCAATCTCAACTAAATGCCTTCCTTCGTAAAAGTTGCTGTGGTCCTGGTGTCTCTTCACAGCAATAGAAGCCCTAGCTAAAACAGGACTCAATGTTCCTGGGCATTGTCATTGCGATTTAATAAGAGCTGAGCGCAGAGTAAATCACTGTCCTTAAAAAAGAAATAACACAAATGGCCTTGTGGCCCTACTCCTAATGCATAACACCATTAAGAGGGGAATTTTTATGGGGCTTATTTTAAAAGTATAGTATCTCCCTTTTTAAAATACATTTTAATTCCTCCATATCATTTACAGAAAGAAATAAATCAACTCTTATTTTTGAGACTCTGGATGTACAAACATGACCAATAAGAAAAGTTTAGCTTTAAAAGTTGTGCAATTTCTAGGAAATATATCTCCATTCATTTTCATGTTATTTTAGTAAGATTCTTGAAGCTGGGTAAGATATAGAGAAGAAAGATTCATTTGGCCCACAGGTCTGGTGAATGGAAAGCCCAAGTAGCAAGGTGCTCCTTGGGTGAGCACTCCCTAGATGATGGTGCTCCTCGGGTGAGCTCTCCCTAGATGATGGTGCTCCTTGGGGGAGCACTCCCTAGATGATGGTGCTCCTCAGGTGAGCTCTCCCTAGATGATGGTGCTCCTCGGGTG

							AGCTCTCCCTGGATGAAGATGCTCCTCGGGTGAGCTCTCCCTAGATGATGATGCTCCTTGGGGGAGCAC<br>TCCCTAGATGATGGTGCTCCTCGGGTGGGCTCTCCCTAGATGATGGTGCTCCTCGGGTGAGCTCTCCCTG<br>GATGAAGATGCTCCTCGGGTGAGCTCTCCCTAGATGATGATGCTCCTTGGGGGAGCACTCCCTAGATGA<br>TGATGCTCCTCGGGTGAGCTCTCCCTAGATGATGATGCTCCTCGGGGGAGCACTTTCTAGATGATGGTGC<br>TCCTCGGGGGAGCACTCCCTAGATGATAGTGCTCCTCGGGGGAGCACTCCCTGGATGATGAAGCTCCTC<br>GGGGGAGCACTCCCTAGATGATGGTGCTCCTCGGGGGAGCACTCCCTGGATGATGGAGCTCCTCGGGG<br>GAGCACTCCCTAGATGATGGTGCTCCTCGGGGGAGCACTCCCCAGATGATGGTGCTCCTCGGGGAGCA<br>CTCCCTAGATGATGGTGCTCCTTGGGGGAGCACTCCCTAGATGCATCAGAGCACAAGAGAAAGCTAAAT<br>GGAAATAAGCATGAGCATGAACAAAATAGATCCAAAAACACAGTACAATAATGCAGACTTGTTCTTTCT<br>CCCACTCTCATAAAATAAAATAGAAACAGCTCTTTAGGACCATGAATTCAATTTATAAAATAAATAAATA<br>AACTCATATAAGTTTACTCCTGGTAATTCAAACATGCTGGGCTGCAATTTACTCTTCAATTATGTAGAATA<br>AAAAGCCTTAGTAAACAAATTAATGGTGTGATAAGCAGTGGCAACTCCCACCTTTCTATAAGTACGGCC<br>CTTAGAAGCTACCTTCTGGTCAGAGGCAAAGTTAGCTAACTTGCTCTCAAGCAGAATGGGAGCAGGAG<br>GCATCTCATGTTATGGTTAAAGTAGAGCTTTCCAAGTAAGGCAGACATTGTGCAGCTGAGACCTCATTG<br>CCTGCACAGCCCTCCCTCATAGCCTGACATTCCCCTGCTCCCGCACAGCCCTTCCTCATAGCCTGCCATC<br>CTCCTGCTCCCGCACAGCCCTCCCTCATAACCTGCCATCCTCCGGCCATCCGCATTTATCTGTATGTCACC<br>TATTCAACGCCATACCCTGTGTGTGTGGTTAGTGCCTAGCCAAGTGTTTGCTGGACAAACAAATGAATG<br>GTTCAGTAAAATTGGACTGGATTATGAAGCCATGGCTGCTAGGTAAACAGTGATCTACTTGAAAGTGTC<br>TGGGGTCATCGAATTCTAAAGAGTTAGAAAGTTGTTATTTATAACCATAGAAGGCCAGGGGGTGGGGCA<br>CAAATATGTGTACAAAGTGGCGTTAGTCTACCCCTGGGAAAAGATGGCAAAGCTCGCATCACATTCTCA<br>ACAATCGTTAGAATCACTGATCTAACGTATCTCCTCTCTTCTGACCTTTGTCACAGAACCAACCAACACC<br>GCTCCGATTACATAGCTCCAGTAACAGGCATTTCCTCAGCTGGACAATGTCAACTACTCCTGCACTTTAT<br>AATTCTGGTACCATTGAACCCCAGTCCTTTCCCAGGAGCCTCTGAGTAGCTGCAATGGCCATGCTCTGG<br>AGTCACCCAAACACCCTGCCTAGTGCCTAAGCCATCTGACCACACTTTGTGGGTTTTTGACAGCTGTCA<br>TAACGTACTCTGTCTTTCTTATATCCAGGCTGTAAGTCTCCAGCTCTTCCTACTGCACTTAAGATTTCTCC<br>TCCTGTGCCTGTGCGGGGGGCTCTGCTGGGCCCTGCATGGCCCTCCTGTGTTTTGGTGTGCACTGAACC<br>TAACACTTCATCCTTTTAAGGGTGCCCTGTGGCCCTACTGACTTAGCATCAGCAGGTAACAAAGTCCTT<br>GTATTTTTTTTCTGTATGAGCGCCATAATTCTGCACCCGTGGCCCTCTTGACTTTATTGAAGCAGATAGTC<br>CGAGGATTCTTAACAGGGCTTTGCTAGTATCTTTATCAGATTCCTGCCGAAATGCAGCTAGTCACTGCTT<br>TGGGCTGAGGCTCGTGTGTAAGTTCTGCTAAGTTCTATCTACCTGGCTTTAGCCACCACCCTCCCCCCAA<br>ACATTTCATAATTGCTCTAATTTAGATTTCATGCAGATTAACATTCCACTTAATATCCAACTTTTACTGCCC<br>AAATTATAAACTGGATGTTGCACAAGAAAAGTCCAGTAGAGATGCAGGGTGAGGAGGAAGGAAAGAA<br>GAGAGATCCAGAATCTGGGTTTGAGGCGGCCTTGGGTTGCCACTTTCTAGCTATGGGACTTCAGACATG<br>TCACATAATCTATTGCATTCTCTCCACTCTCCACTTGGTGGTCATTTCTCTCAGATACTGCACTTGTCAAA<br>TGAAACTTTTCCGACTGAGTTATTTCTTATAGGTTTGTAGCTGTTGTTTAATTTCATTTGATTTGATTTTAT<br>TGAAACAGGGTCATCTCATGTTGACCAGACTGGCCCAGTTCATAATCCTGCTGTCTCAACATTCCCAAG<br>ACTACAAAGACAGCCTTTGCCTTCATGACTGATTCTTTATAGCTTTTACTTCTTTGAGCTTTTCTATTTCA<br>CTTCATTGTTTTGGGATTACTCATTAAACTATCTTTGTTTCAGTTCCTTCAAAATCCCTGCCAGGCGATCT<br>CTAATTTTTATGTTGGAATAGCTGTTTGTCTTTCTGGCTACTGTTGGTTCTTGAAATAACAATTGAGCTGC<br>TTTTTTTTCAGATTCATGGTTATTTTTATTTTATGTGCACGAGTGTATATATATGTACCACATAGGTGCCTGT<br>TGTCCAAGGAGGCCACAAGAGGACATTAGATCTCCTGGAATTGGAGCTACAGATAGTTAGAAGCTGCC

							ATGTGAGTGCTGGGACCCAAACCTAAGTTCTCTGTAAGAGCAGTCAATGCTTTTCGCCATTGACGAGTC<br>TCTCCAGCTCCCTAACGGTCATTTTCCAACTGTTAGCTGGACCTGTGAGACTCCAGGCCTGACTCACAT<br>GCTCTGATGCCTGGATGAAGAAGGGCACCTGTCTGGAGGCTTCTGGGAAGATATGGAGTGAGCTTTTCC<br>ACTGAGCATCCTTGCTACTGTGCAGTGGAACCCTCCACTCTCCACTTGGCTCTCTGTAATACTTCACTGA<br>CTAGCCACCAGGTTAAGCTTGTCCCCTTATGGGCCTTTAACAGTTTAAGGCAGTGTTCTCAACTTTCCTA<br>ATGTTATGACCCTTTAAAATACAGTTCTTCATGTTGTGGTGACCCCCAACTATAAAATTATTTTTGTTGCT<br>ACTTCGTAGCTGCAATTTTGCTACTGTCATGAATTATAATGTAAAAATCTGTGTTTCCTAATGGTCTTAAG<br>TGACCCCTGTGGAAGGATCTTTCTACCCCGAGGGGGTCACAACTGACAGGTTGAGAACCACTGGCCTA<br>AAGGGTAAAGGCTCAGCTTTTCAGTTGGAATGGGGCCTCCATTTATTTCATGGTGGTTCCCAGACAGCT<br>TGCTGAGTGCTTCTTTCCTGAGAGGAAGGTCTGGCTTCGGGTGGATGTTTTTGTTCATGGTCTTTGGTGT<br>CTTCTTGTTGCTAGTTTTCCCAGGATCTAGTCTAAGATATATTAAGAAAAAGAAAACCTAGAGAGTATGT<br>TGCTATGTCCAACTTCACATCACAAGGCTCCTAGAAAGTCTGACTTCTCTCCACATTTCAGTCTTTTTAT<br>ATTTATCGTATATATAACACGGAGGGTTTCTTGGCTTTCTGTTATTCAGGAAAGAAAAGAGAAGTCTGTG<br>CTCTCCCTCTTCCAGAGCATTGTCTATACTAGGCTTTAACCTGATAAAACTTGCAGATTCCACAAGGCCA<br>CGGGATGGGCAAGTGTAGGATTCCTTCCACATCACTATTACACAGGCTGCCTCTTCCAAGCCAACCCCT<br>GCATATCCCTGAGACTGCCTCTGTCTGCTCCTGTTCTGTCTGTCCTTCATTCATGACACTGCACCCTTTAA<br>GGGACCATGTTTTTTGAGCCTGTGCAGTCAATTCTTGATAGACATGAAGTTTGTACTGTTCGTTTAAGTG<br>CTGGTGGTACCCTGCCCTCAGCTTTTCCTTTTGTATGTGTTAGTCATTCCTCCCAGCTAGAGAATAATACT<br>CTCTGGAGGAAATGAGCAAAAAATATAACTCATACCCCTCTGTTCCCTCAATTTCTAGCACAATACCAGG<br>ATTAATGGGGTGGCTCTATACATTGGTATGCTGTTTTGAATGAAAATGGTCCCTATAGGTTCATATGCTTG<br>AATGCTCCCCCCACTACTTAGTGGAACTGTCTAGGAAGGATTAGAAAGTGGGGCATTGTTGGAGGGGG<br>TGTGGCCCTGTTAGAGGAGGTGTGTCTCTGGGGTGGGGTTTGAGATTCAAAAGACCATAGCAGGCTCA<br>GGGTCACTCCTTTGCCTGCTGCCTTCAGATCAAGATGTTCAGCACCCACCATGCCTGCCTGCCTCCTGC<br>CGTGATCTCCATTATGATCATCATGGACTAACCCTCTGAAACTGCAAACAAATCCCCAATTAAATGCTTC<br>CTTTTGTAGATTGCCTTGCCATGTTTCATCACAACAATAGAACAGTGACTATGATAATTGGTGTTTCTTAT<br>AGCTTTAACTTGCATTTGATGATGTTGCTCCCCCAATTGGAGGACCGTGGATACCTACATGTGAGCAAGA<br>CATCTGTAGACTTCTTGCAGAAGTACTAACTTTTCAGAGTTAAAAAAATCCATGTCAAAGATTGAGGTG<br>ACCTTGGCTTTTGTTTCTTATTTCTGTCAACTAGATCTGCAATAGCTCAAATGCGATTTCTTACTGTCCAA<br>GAGTCTGTCTTTAATGGAGTGAGCAAAACAAAGTTCAAAGTACTTTGAAATATATACAATATTCAAAACT<br>CCCCTGCACTGAAGATAAAGTCTTTGGTCTATAACTGATTTTATAATAATTTTATTTAGTGAACATATTAGC<br>AAATTCATGTCAGACAGAAAGAGCTGTTCGAGATGAAAATATACATCAGAAGGCAATGAGCAAACATG<br>GGCTTATATGAATAAGTAATATGCCACAAATACACACCTAGTGAGACAGGGGGATGAAGAATAAACCTG<br>ATGGGACTTTTAACATCACAGAACTAGTTAATGAATTCCCAAAATATTTTAGAAATTTTACGTTGAGTATT<br>TACGGAGCTAGATCTGGCACTGAAAGAAAAAAGGAAGAGAGTTAAAAATAAATATTAAGAATATTTGCA<br>GATCATATGTTTAATATGTAGGATACTGACCCAAGATTGGACACGAGGACAAAGTTCTTAAGTGTTACAC<br>TGGGGAATTTTCTTATCCATGGAGAAGTAGAAAGGGTTTTTTAAAGCTATGGTTTTGTGGAGAATGCAC<br>GATAGATCACTGGTTTTTTTAGGTAAACTATGGTTTTAACAATGAATACTCTCCATGGTTTTATTAAGTGG<br>CTTCTGAATTTGCTTTCGAGAAACATGTAGAAAGAAACACAGTAAGCACAGTGTCCAGTGATAAGGAC<br>AGGAAGCATCACTCTTCCCGGAGACACGGAGATCAGACATCACAAATTACCTTCCTTTCTGAAGTTCCC<br>TAACAAATTGACGACCTCACTTTTGCTTAAACATCAGTAACTGAGAGAGAACTCTGTCCGGCCTTGTTT<br>AATGACTAGAACACCATTCTTCACTCAGTAGAAGTCCACTTCTCTGAATCATTCATCTCAGTTTGGTCCT

							GGGAAGTCTGCTGGGCCTTGCCACCTTCTTTCCCTTCATCAAATTGATGTTAAGTATTCACTCTCATGGG<br>ATTCCTTCTTCCTGTTAATGAATACCTTCTTCCAGCAATTTCTCAGATGGTCTGTTTTTCAAGAGCATTGT<br>TACTTCACAGTTGTCCTTGCCATGCCTACCTCATCAATGCTCAGAACATGTCACTCACTCTTGCCTATGA<br>CAGACAGAGGCCTATTTCCTCTTCCTGTGTCCAACAGGGACTCTCTATGAATACTACTTGGGATTACAGA<br>AAAGGTTTTCATAACGTCACCGTACCACTAGCTCATACTTATTAATTACAACCCTTGGTGTTCTCAAATTC<br>AATCATGAACTATTTAAGTTTCACTGCCTACGTTCTGCACATATTAAACATATTTTTATGAACCTGTACTC<br>AGGATTTTATATTTATGGTTTCATGGACCACTACTCTTGTCTCCTCCTTAAATCCCATAGACTGGAGCTTA<br>GGACATTATATTCTTTCCTGAAGGTGTCAACTACCGTGTCATTCACTGCCACTCTCAGTGTCAGCCCTGG<br>GTCTGTGTTATTAGCAATCATCTAGGTGCTCCCTCACCTCACCACCATCCTCACCATCCTGGTATCGGCTC<br>TCCAACAAGCTGTCCTCTCCTCTTTCGGATACCCATCTCCTTCCCAATCTCTATCCTAAACACCTCAGCTC<br>TCCAACTGATGCCTCTTATTCTTTGAGCTCTCTTACTCTAAACTCCTTCCTCCGGCAGAGCCTAAACCCT<br>GTTTGTCACCTTTGTCTGTGCCTTCCCATCAGATCTGATGATAATATATGGATTCACCACCTCGCACATGA<br>CCTCAAATCTACAGCAATAGGTACTGTAAAATAAAACCAGAAATCCCCTAACCATTTACCTACTCTAAAC<br>CAAAGTTTGGCTGAATGTGGCTGGGGAAAAAAAAGAGGAGGAAAGATCAAGAAAGGGAGGAGAAGA<br>CAAGGAGGTTATAGTGTGTATGGTAACTTTATTACAGTATATATGGTGACTTTATACAGTATATATGGTGAC<br>TTTTCTCACATATGCTTAAGACCACAAATTCCAAGGGAATCCCAAAAGCTGGGGTTTTTTTACTCCATT<br>CACCTAGACCATAATATCCTCACTCCCCACTCAATATCCCAGCAACTGTGGAATAAGGCACTTAACTTGC<br>TGTTAAGACATCTGGATACCCAAGTATGTCAGAAATCCTTGACTGTGAAAACCGACTCCCTTGTGAACT<br>TATCTCTCTGCCCATGACTACCAAGTGTCTACTGCTAGTCTAGCCCATAGTCCATCCCCACTGGACACTG<br>AAAACTTGACATCTCCACTTGGGCATCTGGTACTTCAAAATCAACAATTCTGAAAGCTAACTCCTTGTTT<br>GACCCTTAAACCTGCTCTCTAAAACCTTCCTAATCCAAGTCAGCAACCCAATTACATTTGCTCAAGTCA<br>AGTTCCATGGCACAACTGAATGTTCTTTTCCTCTCACATACCATCTATAATCCAATGAGATGTCACCTTCA<br>GAATATACATTATGATTGTATCTAGAATCTGACTACTTTTCGTCTCCTTTGAGGTTATCTCTCTGGTTCATG<br>CCATCATGTTCTTCTAACTGCTCATTCTTCCTCCAAATCTACCACCATCGTTCCTGTGTTAGATTCTTGAC<br>ATGATGAGCAAAGTGAGCTGCATAAACTTGGTTCCCTGCTATGGGCAAAGACCTTTGACCTTTTAACTC<br>AGAGTAACAGCCCAAGTGGCGACATGGCGGAAAGACTCAATCTTACCTTTTGGCCTTGGCTTGTCTACT<br>GTTCTTCCCAGGCTCACTTTGCTATCCTTTTCGGGGTTTCTGAATGTTCCAGAAATGCACATAGTAGATG<br>TTGTGCCCAACGTCTGAAATGATCTCTCGGGCATCTCACCTCCTGCAAGTTCTTGGATTGGTGCCACCTT<br>ATCAAAGATTTCTCTCTGATTACTTCTCCAGCTCCCATTCCTCAGTTTGGACCTTCTCTGTGGTGCTTTAG<br>AACCCTTCCAGGTATCCTTTCTGGTTGCAGTCTTCTCTTTCTCCTTCCCAACCCCAGCCCTCCAAATATAC<br>TTTTGGGCAAACAGATTCTTTTCTCTTTCTGCTTTAGCATATCCCAATGCTTACAACATCTGGTATATATCA<br>GGGATTCACTAAACTATAGTAGAATTGCGAATCAAATGCTATCTTATTGAAACGTAAATCAGGAATGTTAT<br>TTGTTTTCCTATCTTAAATTATAAAACTCTCTAACACATATTTTCTCCCTATATAAAAATTTAAATTAAGAC<br>AAAAGATATTTTAAGGAAGAGTTCATCTTGCTGTTTTTGGATAAACACTAAAATATATTTTTCCTCCTTTA<br>AAACAGTAAGATTAGTGCAATTAAAGAACATACTTTCTACTCCTTTTACAAGTTGAGTTCTTGGATATAA<br>TCAGTGCTGTGTATATGATGGTAATTCCAAAAAGAAATGGTGATTCAAGTGGTAAATCTGAAAACAGAA<br>TGATTATATTATGTGGTCTGTATTTTAGGGAGCCATGAATTTCTGGAGATTAACATAATCCTTGTTTTGAAA<br>GGATTGACCAGAATTATAAAGATTTTCCTTCCAGGATTTGAGAATGGATTGCTTTGTTTTTCTGTCTTTGA<br>GGAAGTGTCTATTTTAACTCTCAGGCCTTATTCATCTTCTCAAGAGCTACTTGGAATCAAGTCGAATAAA<br>GTCATTCAGCACTATGAGCTATAAGGCAGGTGCATCTGAGGAACGGGCCTCATCACAGCCACGGCTGGG<br>TTTTACTTGTGGTGAGTTCCCGGCCCTGTGTGCTTGAGCCTTCCCAGTCTGTTCATTTACACTGCTCGAC

							AATCATGAAATGGGAAGCTCCCTTTCCTCACACACTAGCATCACAGCATCCTGACAAACAGCAGGCTCT<br>CTAGTTGATGATCTTCTCAGCGTTCTCTTCAACTTCAAATAGTGTTTAAAGAATCAAACACCCAGCTTGG<br>GTTTTTTTCTTTTCTTTTTTTTAAACCATGTTCTTTCTGGTGACTTCTGTGGAGAGTGTTCATGTATTGGTT<br>CATGTATTGATAACATCAGGCTCCTGAAAACATGCCCTCTATATTCTTTGCCCTCTAAGTTCTCCAGTACA<br>TTTTTCTAAGCTCCCTTGGTCACTATTGAAATTTAAATATAGAATCATAAGGCACTGTGCTTGTGTCCAAA<br>AGGTGGAGACAACCAATGACCCTACCCAGCTATGACACTGATGAACCACATCAACACCAGCACGGCAT<br>GATAACCATAAGGGTGAAACAGTGGCACATGTATTTTAGCCATAACCAACATCTCTTTAATTATATTTAAC<br>ATTTACTCAACATGAGGAAGACCATGGCTGGTACTGGAAACCTACCTAACGCTTCAGTGCTAGTAAAAT<br>CATGGTTATTGGAGAACCAACAGCTACCTATTCGCTAAACCAGCACAGTACCTAACAACAACCTATAAA<br>CAATTGTCTTTATACCCACAGATCAGTGTAGTCTTCCCCTCATCAAGGAAACTTTCTTTGCAACAGAAAA<br>CCACAACTCACCAAAAAGTAAAATTCTGGAGCCCAGCCCCAATGCATGCATTCATGAAACACATTCTTT<br>TCCATAGCTATGTATTCAAAGCAAGCTACACTCATATTTATGTATCTTGTGTTGACCTTGCTTCTGTGACG<br>GGGGATAGTGGTGATGGCTTTCCAGTCAAGCCCTCGGCAAAGATTTGAGAATGAAAAAGATGAAAACT<br>GACACAAAGGAAGTGTTTGGCTAAACTGAACACGGATGTGAGACAAAGTGCAGCATGTTCTAGGAGTC<br>CTTGACCTGTGATCAGCAGAAGCATGTAGTAACTATTCACCAGGGTGAACTGAGAAGTTTCTCACTTTA<br>CAGATACAATAATTAATAATCCTTGCTACCTAGGATGGACAGTTACATGGGCAATAATCCACACCTTGCCT<br>CCAAAACCTCAGTACCAGAAGTTCATATGCAACCTAGAAAAAGAACAGAAACAGGTCTATCTGTGGCT<br>GGTGGGGGAGGGGCCAGAGGAACAGCAGAGTGAGTTTCCAGAATGGTGACTTCAAAAACATCAAGAA<br>AGTCCTAATGATATCCATTTTCAATGTGTTTTTAAAGTATAGAAGTATTTCTAAGTGTTAATGTAACCTATC<br>CTATTGCCTCTGTAGGAGGCAACCTATCTATTTCAGAGATGTGTATCATATTTAACTGCCAGAAATGTTAG<br>ATCAGCAGTTTTCAACCTGTGGATCGAGATCTCTTAGGAGGTCAAACAACCCTTTCTCAGGGGTCACAC<br>ATCAGATAGCCTACATATCAGATATTTACATCATGATTCTTAACAGTAGCAAAATAACAGTTAAGAAGTAG<br>CAACAAGAGTAATTTTATGGTTGGGGATCACCACAACATGAGGAACTGTATTAAAGGTCTGCAGCATTA<br>GGAAGGTTGAGAACCACTGGGCTAGACTGTGTCTAAAGGCTCTGTCTAAAGAAACAAAGTGCATTAGG<br>TTTCCAGACTATTTTACTCTTTCTGTAGACGGCAAAAGCAGAGTGTAGTGTTGCTTCTTGTTTCAGTATC<br>ACTCTCAACCTATGAAAGACTGGAAGAGCACTGTAGTTTTCTAGATGAGGTGCATAAAAAAATTCCTAA<br>TAAATCATGATAGATGGAAACAGAAGTATCATATTTGACTTTTTAAAAAATGTACCTATGAACTCCACAG<br>TCCAAGGAATGTCTACTGAAGAAGACTCTTCCTTTTCTTTCGGCAATTATCTAAAAAACAATGTCAAATT<br>TATCCAGAGAAAGGTACAGCTGAGGCCGTCCATTCTGGCCTGGCTTCTCCTGGCCATTCTCTGTCCTGG<br>CCTCCACATGGCTTTCACACAGCCAACAAAGTGGGTGATGAGTTCACCTCTTACTATTTCAATTTGCTCA<br>GACGTCCTTTCTTTAAAAAAAGAAAAATTCTTTCTGTAAACAAAGGGCCATTCTTCTCTCCCTTTGTTTT<br>TGTGTAGTCCTTTTGACGAGTTCCCAGGAATAAAATAACCAATCGTGGAGGGGGAGATGAGAGAGAGA<br>GAGAGAGAGAGAGAGAGAGAGAGAGAGAGAGAGAGAGCAGTTGCTCTTAGCAGCCTTTGTATAAAACTG<br>TGAGAAGCTGGCGACGCCCCACGGTTAGCATCCCTGACACTCTGTTGCTTCCACATGGCTCTTTGAATG<br>TTCCTTGGCCAAAGACATCATCATCGCACATGCCCACTAGCAATTTTAGAAGAGAAAGATTATAGTGAA<br>AGGAGAAACTGTTGTTTTTTGACGTGGTTTGGAAGTTTCCCATCCTACACAAGTGTCAGAAGCAGTGG<br>GGATAGCTGAAAATCCACTGAGTATAACAGGTTTTTACAGCTTGCTTGAATGATTTTATACACACACACA<br>CACACACACACACACACACACACACACACACACACACACATGCATATCCTAGAAGTTGACTGAGAA<br>ATATTTTTTGGAACTATTTCTTGGGGGAATTTAATTACCATCAATGTATAAAAATAAACAGCAGAATTTCT<br>GCCCAGATGGCATGATCAATGGTTCTTTTTCAGAAAAAAAATGTTTTTAAAGCATCCATATCTGTCTATAT<br>ATGAAAACGAAGCCTCCTGTTTACCCTGCTTCATGCTTAAGTTTCCATTGAAGTTGAGGCCTAAAGCATT

							CTCTTTCAAAAGAAAAAAAGTTACGAAGTAGCCTTCTTGCTTGGAGGACACAGTGAGAAAACAACTGG<br>GGGCCCGGGAGGCAGAACCACAGCAAACACAGACTCTGAGATGCCTTGAGCTCAAACTTCCCCCGCC<br>TTTAGGACATCAGGAAAGGTGGTTTCTATTTATAAAGCAAAATATAAAACTGCAAAGTCAAGCAAGGTG<br>CCCCACAGTCTTCCCCCTGGGTTTGTGACCAGTACACTATTTTTTTTTCTTACTCTAAAAAAAAAATATATA<br>TAGCATATAAGGTTGGTCCTGCTTTTATTGGAATTTTCCAATAAATGTCTACTTACTTGGCTGTCATTTTCC<br>CACACCCATGTCTTATACCTCAGCCTATCTTGTCTATGAGTAGGGAGCTTGGAGATGTAACACATAAGCC<br>AGACAACAACCATCTGGGAAATCTGTCCTAACAACCAATGACAGCATTTCTATACAAATATGTACGCCTG<br>CATCCCTTCCACCTCCTCTCACCCAAACTTAGAACCTGTTTGCTTTTATTAGTTTATTTGATGCTGAGGAA<br>ACCTCTGAATTGTTTGAAATATCTTCATAATAAAGATGCTAGACGTGGAAGATGCTATTTTGTCTAGTACC<br>AGGGCATTGCATCTGTGTAGCTCACAGAGTAGATTTGTAAATGTTTTCACCATTAAAAAGACATGATCAC<br>GGTTTGTATTAATCAGCTCGATTTTAATCATTCCACAATATATACTATCGAAACATTGTATTGCATAATATGT<br>GTGTGCATATTTCATCCATTTGATGCATTTTAGTGCATGCAACCTAACATGAAATTTCTTTTTCTTGCTCGC<br>TTGTCTTCAGTGACCCTAGCAGGAGGTAGGTAAACCTTATATACGAAGAATTTACTCTGAGTATGGCAGA<br>TAAAGGTTCTGACCTGTGAGCCGCCCTCAGCAAGGAAGGCTACATGCACAATGGTTTTAGGTTCATCAT<br>TTGCGTTTTGCTCTTGCGGTTACGTGTCTTAATTTCTCTTCATTTGGACACAAGAAGTTGTAAGGGCGAC<br>CAGGAAACTATTATTTGGTTTAAATCCTATAACCCGTTATAATTAATATATGACAAATCGGGTTGTCAACC<br>TTTGGGTCTTTTCACATTTAAGTCTTCCCTTCCCTTCCTCCCTCCCCTTCCTTGCCCCAACCTCCTCTTTC<br>CCCTCCTCTTGCCCTGCCCTTTGTTTCTCTTTTTATTTCTTGGACCCTACTTTTTTTCCTCTCAGTATCCAA<br>GACATTCTCCGAATCTCCTCAACTGACTTAAATCATGTTTGTTCTGGAGCAGAGTTTAGACAACCCGAG<br>GCACTCCACACAACCCTCGAGAGGGGTTGGCTTAAGAAAGGGGTTTCCCTAAATCAGCTGCCAGGAAC<br>ACACATGCCAGTCCCACCCTCGTCCCGTCCTCCAGTAACACCCACTACCCAGGCCTCACACTACATTAC<br>CAAAACTATACAACTCCCACTCCAGCACAGCAGAGCCTCTCAGACCACAGCACAACACACCACCCCCT<br>ACAGTATTTCTTCTGAAAACAGCAATAACCAGGTAGCCAACCCCTCCCCCTGGTGCCTGTCTGTCTCTA<br>GCTTCCAACAGCCAGGACAAGCCAAGGCCCTAGGGTTGATTCCACCAGGCAAGGATTTGCAGTGAAGG<br>GCAAGTCAGGGGGAAGAGAGGCCAAACCACTGGCAGTTTTTCAAATGCAAAGACGAGTTCAGACTGC<br>TGGGTAAACGTGCCTGGATCTCTGGCCACAACCCCAGCACTCCAAATCTACAGCTAGGACCTTCACATT<br>TATGGATTCCAAGTTTCAATACTCTGATAGATTCAGGTAAAGAGCAGCTAGGACATAAAGAAGGAAGGC<br>TACAACCAACTCCCAACCTCCTAACAATGTTGTCCCTCCGGAATTAAGCCTGGGACTCATGGGGGTTTG<br>TTTTCTGCGCGGGAGAAAGAGCTGAGCAGGAGAAGAACTGGAAAGGAGTAGGGTGTGGTGATTCCTT<br>GAGGGAAAAGAAAGGGAAAATTAAAGTCACTGCAGTACTCCGCTGCCCTAACCCAGCGGGAGGGTCC<br>TGAGGCGGTGGGCCCACCCGCTGCTCAGTTGTCTACGCCCCTGGAGCACTCTGCGCGCAAACACGCCC<br>CAGAAAACCATCACAACCTGTCACTCCCGCCCTGAGGCCCCTGACAAGAAGGAAAAACTTGTTCCGGG<br>GCGCAGCAGAAGCCAGGCTAGCCAACGAGGACAATGTCTATGTCTGAAACTAGAGAAATAATGAAATA<br>ACTGGACCAACAGCTTTCTTCCTCCTGGCCGCTGTTTTCCACCAGTACGCTTCCACCCTGCTGTTTTCCA<br>CCAGTACGCTAATATTTACAGTCGCCCCAGACGAACCTCTGCTGGGTCCCCCCTCCCCCGCCCCCAGGT<br>AAACAGGCCAGGAAGACATAGAGGAAAGCTGTGGTCTAGGGGACTCACAAAAAACACGTCCCGGCCT<br>CTATTTGTGGGGTCCCGGTGCCGGCCGGCTTACCTGTGGATGCAGTTTCCATGGCGACGGGAGGCGACC<br>GGGCGGGGCCGGAGCACAGTTCCCGCGACGACACTACCCCAGCTCCAGCGCCGGACCCCCAAGCGGG<br>CTCCCCATCCTGCTGCGCTGGTCTGGCCCCCTTCGGCGTCGCTTCTTCGTCTTCCCCCTCCCCCCGGCCC<br>CCGAACAACTGGGACCCGGGGTTAGCCGGAGACAGTGGCTCATCTTGCAGATCCGGCGGTGCGGCCAT<br>GACTGGCTATGCCCCAGGCTGGGCGACTGTAGCTTGGCAAGGCCCGGGTGCGGTGGCTAGGGACGCGG

							ACGCTCCCTGCTGCTGTCCCTACCGGGACTCCGGGCTCCAGGAGGCTGCAGAGTCTCCGCGCCACCGTCGCTGCTTTTTCTGCAACTCAGGCCGAGACGCTGCTGCTGTTCTGGGCGCTTCGGCTCCTCCTTCTCCCC
MSTRG.107506.1	--	0.000	0.238	0.011	3.719	up	CCGGTTCCAGATACAGACGAGAAGGTCTCCAGGTGGCTCCCATTCCCTGCTGTTTCCATCATCCCAGGCATTCTATTTTTATGCCTGAGCTTACAAATATTATAAAGCAGGGAATGGCCTGTGCATTATATTATGTCCAAGTTTTTCATCAGAGCAGAAGAGACGTCTAAGATTCAGATGTCATGTCACACAGCAGAACAAACTTTCAGCATCCCCATCTTCACACTTCAGAGAGCTGTGGTGTTCCTACAATATTCTGTGGGAAGTCACATCTTTATGACTCTGTCTGAGGAAATCGTACATCAGAACAATTCCTGAGACTGTTTCACAGTTAATCACACTATTTTCAAAAGAAATGATTAATCTATCACCTTACATTTAGATCATCAGGCTCATAACATAGGACTAAGTCGCTTTATATCTCTCCAACCTCAGGAAGTGTGTCAGGTCAGATCCTGTCTCATCTGTACTAGAAGAAATTCCTCTCAGTAATGGGTAGAAAGGCCAATCTCAGGACAATTTTTC
MSTRG.107634.1	ENSRNOG00000006472;ENSRNOG00000029835;ENSRNOG00000038480;ENSRNOG00000058593	0.067	0.344	0.045	2.555	up	CTCCAGGGAACCCCTCAATTTCCTCTGCCTCCTCTGCATGCTGGACATGATGTGGGAACACGCAGCGTGACCTGAGGCCGCAAAGATGTTCTGACTAGAGTCCAACTTCCTGAGCTCCCAGCGCTTCACAGAGCTTCTGGTGAATTTCTGGTCCACCCCGGGACATTCCGATTTCCCACAGAGAAATAAGAGAACAACACTGCTTCCCTGAGGGCGGTGAGCTCCATCCCTGAGCACTACAATCTCCATTTTTGGAAAACTGTTTACCAGGGTGTAAGACTCCGAAAGGAAATTCATCTGTGAGCCTCCTGGGAAATACTAAGACCTCAACAAGACTGCAGAGGTCTGCTGCGTTCCTCAGCTTCTGGGAGGCTCATTCTGATTAAACACCAGCTTGAAATGAAAGCATTTGTATGCAGAAGTGAAGGGTGTGAGGGAAATAACAGAGAGAAGTGGGCACGAACCACCACTTTTTGGGTTGGCCCAATCCCCAACTTTTCAATGGCTGTCAACGCTTTGTCTGTTCTCCTCCTCACCTCCCTCGGTTTGTTTTGTTTTTTCAATAGAGCTGAAACTGGCAAACCATAAC
MSTRG.107679.1	--	0.000	0.360	0.011	3.719	up	AAATGCTCTAAGTAGGTTCTACACACACTTCACAATCCCACTGTTTTCAGACCTGCCTCTACAATCCCACTTTTTGACAGAACCAGTTTTCCAAGTCTCTGAGATCACTTGATTTGTTCTTTCTATATATTTGTTTTTTTAGCTGTCTTTGAGTATTGTTTTTTCCCTCTTATTTGCTGTGTCTCTGTCTGCTATTCTTTTTATCTTTCTGAGTTTATGCCATTTAAAACTCTTCTGTGTTCGTCTCATAAGAATAAAGATAACCATGAACGTTTAGTCTACTTCGTAATCTTTTCTGGAGTGGGGGAACATTTCTTTCATTTATTTTCTTGTCTCCAGAATGCTGC
MSTRG.107700.1	--	0.742	0.000	0.000	-5.312	down	CCAGCTTTACATATTTTATTGAAACAGTGGAAAGTAGATTAATTCAATTGACTTATTTTTTTTTAATTTTCCTCATTATTCTGCGTAAAGAAAACATGCACGTACACAGCAACAGTAAAAGCAGACGCCTACATCAATATTGGACATGAATGTTTGTAGCCGCCTTGCTCACGGCAGCCAAAATGGGAACATAATACAACTGTTCATAAATTAGAGATGGACAGACAAAATGTTTATCTATATGAAGGGGTGTTATTCACACATTAAAGGGGTGGCACATTGATATGTAAAATAGCACGGAGGTACCAAGACTGCATCCAGAGCTGGCCAATCCCTCCTGGGACCTGGGAGTCAACCCATGTTATTCCTTCACCACAGTCATCTGTGCCCGTGTGTCTTGCCAAAGCTGATGAAAACACTTCATGGTTGCCTTTAAAGCACGGGACTCCTGGAAGCTCCTCAAGCCCCGACCGGATACACTGAGAGAAGTCACGTGGCTGCAAAGACCTCAGCAGGAACAAAGGACAGCTGTTGAAACCACCAGACTCTTCCCCACAGGACAGTTAGGAAACACATTTCTACTCCATGGTGGGTTTCCATGGAGAACGGGGTTCTGAAACTGTGTTTCATCTGCTTAGAAACATGTGTGTTTATGTACTTTGCCTCATTTTTGTCTCTTTGGTCAAGGACATCTCAGGAAGTAAGAACTGGGTGTTTGGTTTCGTACTGTACAACAAACAGCATGTAACCTGGCAGAATCACAACAATACATGTTCATCCCCTCAGCCTTGAATTCTGACAAGGCACCTCAAACATAAAACAGGATCC

							ACTGTGGAGGAGTTAGGGAGAACACACAGCTCTGGTCCTGATCACTTGAACACACGGCACACAACTGCACAGGAGAATGCTAACCCTGTCTGGCTAGCACTTAAGACCTACCTGGTTTGGTGTTTTTGTTTTTGTTTTTGTTTTTATTTTTA
MSTRG.107702.1	--	1.057	0.018	0.000	-4.545	down	CTCCTGTATCCTGTAAATGAGTGCAGTGAAGTCTCCTCGTCAAGCCTGCGCTTGTTAGGATTCCCATTTTACAGAAGAATCATGAAGGACTGAGGAAGAGCCAGCAACTCACCCGTTCATGGGCATCCAGCACCCAGCAAGTGAACTCCAAAAGTGGGTCCTGAGCCCAGGGAGCTGGCACAGATGTTCCTCTTTCAGGAAGAATTCCAGCACCCTAAAACATGGCATCTCGGGAGCAGCCTGAAGAGCTTGGATTTCCTGGATCAGGTTTAGCTTCATTCACAGCTTCACTGCCCTTGGCTATACAGGAGGCTTGATAGGTATGGCCTCCGAGGGAAAGGGGAGCATTGCACGGCTTTGCACAGAGACAATTCCTGCCCAGTTCTGAGTCTTAGAATCATCACACCCAGGCTTCAATGTAAAGAATGGCTTTGAGGACAACAGGGCTGGAGACTAAACACGGAAATGACAGACGCTCGAGGCACAGATGGCCACGCAGGGAGAGAAGTCGACAAAAGGATCTGCTGAGTGGGTGACTGAGGGATGGCAGGAAGGAAGAGTCGAGATGAAGGGCATCGTGTCAGTCAGTTTCCTGTTACTGTAGCAAAATGTCCAAGATGATTCCTGTTCTGGGATTCTGCACTGCCATCGTAACTGATGGCTTCTGTTCAAGAGGAGAAGGTGAGCGGGGCCCAAGCGTTTATCTTCCTCCGCTTCTGGACTGTGGCCGCGATGTGACTACAAGGCTGTCTCACGTTCCTGCCACCATGCCTTCCCCATGTCAGGGATGGTGTTCTTTTTAAACCCTAAGCCAAAAAACCAGACCTCTCATTAAGCTGCTCTTGTCCGATATCTGATCAATGAAAAAAAAAAAGCAAAGCATTGGATCAGGCTGCAAACAGCCGTGATCTGATTATTAAAGGATATGACATTTGCTTTAAGCTTTGGCCTTTGTCTCTAAGGGTCCAGTGCCTACATGCTGACTGCCACGTGAGGTCTGTGTAAATGATCCACCCATAGGGCCATGCCGGGGTTCACCTCATTTCGGACTGCGGGACTATTTTGGATCTCATATTTGGGGACTATCTACTCATCTACTTTTGAAGGTGAGATTCAGCGGTAACGCAGGGATACCAAGCTAGGTCTTCTTAAACCACCAGCTTCCATCTAACCTATATTGCTGGGTCTTACTGACCTGTGGCCTGGAATTCTTTATGCAAAGGGTATCTCTGTGGTCTAGATAAACTCTTGTACTCTGGCATTTCACCACACAGCTGTTTCCTCTGTAACCTTTTAGTTCCTTTGTTCCTCCCCACGTCTAGCAGACCTTTGTCACCTGGACAGGATCTTCCTAGACAGCTTCCAAACCACATCAAGAGATTTTAATTTCCTCCCAGCTTTGTTAGCATTTCACATTGCTACAGGTCAAAGCACAAGGGTGTTTGTGAGGAACTAAGAAATGTCACTAACGTGAGTCCCAAACTCCGTGATTTCCCTCCCAGTAGAGCAATGGGGAGCTTAGCTAGCCATGGTCTGCTGCTTGGTCACAGGTTCCAAAGCAATGGGCCCAAATGATCGTGGTCTGAAACCTCCAAAGTGGTGAGTGAAACCTCTCTGGGGCCTGGGCAGATGGCTCAGTGGTTAACACACTTGCTGTGCGTGAAGACTTAAGTTCAAATCCCAGCACTGGGATGGCATGAACCAGGAATCCCAATGCTGGGGAAGGATGTTCAGAACAGGCAGATTCCTTGGAGCTGGCTGTCACTGGTCTAGCCAATAGTGAGCTCTAGGTTCACTGAGAGACCCTATCTCAAAGAGTAAGGTGAAGATCAATTAATAAAATAAGACATCTGACATCTACCTCTGGCCTCTGCACACGTATGCACACACACATATGTGACTACATACACACACGTGTGTGTGTACACATATGCACATGGCCACCATGTACAGAGGAGCCTTCTCTTACTATGGTGGCTGGAAGGAAAGAAAGAGAAAGAGGGGAGCTGGGGTCTCTAAAAGTCTTCTGAGGACGCCTCCCGTCACCTAAAGTTTTCCCTTAGGCCTGCCTCTTTAAGGTTAAAGTACATCCCACTAACACTATGAACTGGGGAACAAGCTTATGACACGAACCTCTGGGGTGTGCTAAGGGTCCGAAAGGGATGGAGCCAACAGCAAAACCAGGTATGTCCTCTTCATGCCAAACCATGTCCTCTACCTCCACTCACCTCTGCAAAGCCAGCGTCCAGGAGGCACCTTGGGCCTTTCT
MSTRG.107703.1	--	0.767	0.000	0.013	-4.001	down	CGTTCCAGCCAGAACTCCCACTGTGGCTGCTCACCGAGTCTCTTCTCACTGGCTCGCCCTTTGCTCCATCTGCTTCTTCCTTGCAGTGTATTTCAATAAATCCAGTCTTCATCTGTCCGTGCCTTGCTAAATTGGTTTACTGCCTGCTTCACCAGTCTTCTGAATGCTGTGTCTTTCTGATTAGAGTTGGTACAAAGTGGTAGCTCTCTCAAAGTCAGTGTGGGAATGCCCCCTGTTCCTTGCCAGAGATGTTCATACTTGTCTGGCCACAGGCCTTGAAGCAACCCTGTTGGGAAACACTGGCTTGAAACTTTAAGACTACAGAGAAGCCATCTTCTGCAGGAATC

							CCTTCAGCAACCTATAAGCATTTACAT
MSTRG.107703.2	--	0.631	0.000	0.022	-3.851	down	CGTTCCAGCCAGAACTCCCACTGTGGCTGCTCACCGAGTCTCTTCTCACTGGCTCGCCCTTTGCTCCATCTGCTCTGCTGTGACGTCCCCTGAGAGGACCAGCGTGGGCTGGGGAGTCAGCCAGATTGTATGACAGCATCTGCTTCTTCCTTGCAGTGTATTTCAATAAATCCAGTCTTCATCTGTCCGTGCCTTGCTAAATTGGTTTACTGCCTGCTTCACCAGTCTTCTGAATGCTGTGTCTTTCTGATTAGAGTTGGTACAAAGTGGTAGCTCTCTCAAAGTCAGTGTGGGAATGCCCCCTGTTCCTTGCCAGAGATGTTCATACTTGTCTGGCCACAGGCCTTGAAGCAACCCTGTTGGGAAACACTGGCTTGAAACTTTAAGACTAC
MSTRG.107769.1	ENSRNOG00000010646;ENSRNOG00000010650;ENSRNOG00000010914	0.370	0.000	0.017	-3.928	down	TCTCCACCTGTTTATGAGAGATAAAACCACTCATAAACAGGACAGAGCAGTCTGCGGCAACAGGGAACCTAAAAATAGCATCTGTAGCAGTGAGTGGGTCCCCCGGCTTTTCTACCAAAGGATGCTGAGTCCTGTGTGCTGTGGTGTGGGGAGGAACTGGCGACGTGGGCCCAGCTGAAGGGACACGACTGTCCTTGCCATGCAAGCCTGGGGAGCACAGTTTGGATCTCCAGGACGTCACGTAAGTGTGAAGGGCTTGCCTGTAATTTCAGCCCAAGAAGTCTGAGAGAATCTGATCTCCAGAACAAACAGCACGCAAGGCTGGCCAGGTCGGTGAGCTCTGAGTTCAAGTGAGAGACTTTTTGCCTCAGGAAATGAAGTAGAAGAAAATGATTCCTGAAATCAACCTGGGGCTTCCACATCCATAAGCAAATAGGTTCACCCATAAACATGCAAAAACATGCATACCCAAATGACACCACACATACACACATCAAAATTGGAGGGGGTGATGTCTTAATTAGAATTTTTTCGACATACCAGGCCTGAATTTGAACTCAGACTCCACATCCTAGTTGGATGTCTTAAGGCAGCTCATCTTGCTCCAACCATTTCTTCATCTGCAAAGTGGACATAGCAACAATACCCACCCTAGGATGTTATGTGCATAAGATGATACGTTAAAGATGCCTTGTACAGTACTGGGCACAAAGTACCTAATAGCACC
MSTRG.107915.4	ENSRNOG00000058646	0.000	0.833	0.000	9.745	up	CGGGCTCGACTCTCTTCCCCCAACCCCCCTTTCTCTCCTCTCCTCTCTTACTCTTTTTTTCTCTAGCAGGAGCTGAAGCTGGTCCCGAGCGGGCCAGGGCTGCGGTTTCCATCTTGAGCTAGTGTCCCTTGAAACTTCTCCAAGTTCCCGCACGACTTACCGTCTCCTGACTTCCCTGTCCACACCCGGCTACCCATCGCAAAACGAGCCGGGAGAGGAGTTTAGGAATGCGGAGCACCCCAAATGTTCGAGTCAAGAGTTTTATGTTTGTTGTTTCCCACCAATGGGAAAGAGAAGTAGGGGTGACCAGAGAAACTTTTCCTCCCGGAGAGCCCAGCCTGGCGGGGGCGAGAGCAGCAGGATTTTGCGCGCAGCGCTCGTGTACACGCGTGCTGGCCCGCAGCCTGCGCTGGCCCGGTAGGGTGGGGGCACCGTGGACTAGACCACTGCCTGGGCAGAATTCTTTTAGACAGACCTAGTTGGGGCGTCTCGTTCACACAACCGATCTTTAAACTTGCTACTACAGAAAAATCCAGATTTTCATGGGCGCCCCTTGAAAATAAAAGAGCAACAAGTCACTTTCCGCGCGCGCTTTGAATTCGGGCTACCTCCTCCACACATCCCCGAATGTTAAAAACAAGGGGAAAGAAGTTACTTTGGTAAGAAGGAAGGTAGAGGTGGGGGAAGCGACACTTTTAACTTCTTGCTGCTCTCCCGCCCCCACGCGAACGGAACATATGGCTTGTCACTATGCCCTGTTTGGGGGTGCCAAGCAATAAGACCCACGGAACCCAATGGCCTTCAATGAATTATACCGCCCTTTAAAACCGCGAACTATGTGTTTATTTACTATTAGGTTTGGCTGCCTCCCACCGGCACAGGCTCGCGTGCTGCAGTGGAGACCCGGGCGGAAATCGCGGGCCGCGCCGCCCAGGCCTCGCACGTTTCTTCCCAGCATTCGAGGCCGCCGGGTCTGTTATGAAACCCGGTGAGCCGAGTGGCTACGAGCGAGTTCCATTGCGATGTAATTAGGTCACCCCATTACAAGTACGTTTCTCTGGAAAGGACGGAGCGGGCGGAGCGGAGCGTTCGAGGCGCACCACCCCTTACACACACGCACACTTTAAAAAAAAAAACAAAACTTTATTTATCGCCAGCTTCTGGGCAAACCCAGGGAAACCATCCAAATCACAACTGTTTTAGAAACAAGTTTTTTTTCTTTTAACTTTGCGAAACACGACTTTTGCTGCAGCCCACGAGCCTAACATTAATTCCCCGCGGAGTCTCTGCTTGATTCGTCTCCAGTGACTTGAATTGATGGCTCCCCCATTTTTAACACGGCTGCTGCCCCTGGGTTTTGGTGCTGCTGCTGTCCAGCCGCCGGTTGCTTCCCTCCCCCGCTCCCATTTTTCATCTTTTCTTGAAATCTGGCAAGCGGCAAGAAGGGAAATAACTTCCATCCACCTTAATTTAAATCATTCGGGTTTGTAGGTTTCTTTTTAAAGGGATGTTTGCTCG

							GTCCCCTCCCCAGTTCCAAGGGGGTTGATGTGTGCGCGCGGATTTCGTGCCGTTTCTCGGTGATGAGGT AATGAGTTCCAGATTTCGGGACAGAACAGGGTGCGTTAAAACCACAAGGTTAGACTGAGTCCTGATGT GAAGCTATTTCGGCTTTTCATTCCGCACGACCCGCCCGGCTGGTCTAAACTCCCGAGTGTTTGCGGAGC TCAGCTGCGGGAGACTCTGACCGGGGAGGGGCGACCTCGCCCGGTGGTGCTGTCCCTGGGGCGGCGG AGCGGGCTTGGACACTCCGAACTGCCTAAGTAATTTGTTGTTTTTCTTTTTTTTTCTCTCTCTCTCTCTC AAAGAAGAAAGGCGATTCCTCGAATTTCCTACCCCTTCCCAGCTCCCTTGGGCCTCCCCTCCCCCGCTT CCCGGGGCGCGGGCTCCGCGGGCGGCGCGCTCCCGGGTTGTTTACCCGCCCCGCCGACCGCCCGGGTT TGTTCCTGCTCCCAGAGCCCGTCTCTATAATTAAACTCTTTTTTCTAATTGTCGGGTTGCAACGTCATTTA GGGGACTTTCCAGGGAGGTGGGGGGAGGGAGCACTCCTTAACCCTTCGCTAACCCTGCGTCTAGGACA CAAGGCCCGCGCAGAGGCCGGAGACCCGCAGGTAGGCCGCAGGTACCCAGCCAGCCTGAGCCAAGCC GCGAGGCTCCAGAGACTCGAGCCCAGGCCTCCCATGCGCCGGGCCGCACAGGCCGCCGCCTTCACCTG CGCCGCCGCTTCCGCCCGGGGGGCGCCGCTGTCGCCGACTTCCGGTTCGCGCGTCCCCGGCCGCGCAG GTATTTTGGGGGTGGGGGCGGGGCCGCGAGGTTTTGAGACCCGAGAAAGTGAAAGTCCTCGGGAAGT CCGGAGGTGAGGAGACTGTGTGAGTCCGCCAAACCCGAGCCGAACCAGTTGACCTTGCTGAGGGGGA GCTTGCGGCGCGGGGTCGCCTGCCCCCAGAAGGCGGAACCGGTCAGTGGCGCCAGGCGACGCCTCTG AGAGCCTAGAGGGGTTGCGTTTCCTCCTAGGCTCCCGGTCATTTGCGAAATCTCCCAAAGGCTTCGGAT TGAAATCTGATCTTTCCCAGTCCTTGCTACTTGAGCCCTGGGGGTGGCCTCGATTTCCCTGTGTTAAAGG TAAAAAGAACTGGCCCAGTGAAGCCCTGAAGTTACCTGGGTTACCTGTCTGGATTGTGGACCCCAGGC ACAGTGATTCAGATCCTCTAAGGATGGACCCAAGGAACTGGCACTGAGCAGTTGGGGATTCAAGTGTT GGTTCTGTCTTCTAGCTGTGATTTATTTTTATTTATTATTGACACGAGGTCTTGCTATGTTGCCTAGGCTGG CCTCAAATTTGTGTATTCAGGGGAGCCTGGGAAGAGTCAGGTGGGCTAGGAATGTGTCACGAGAACCT GTTGTGTAACCTTTCTGAATATTCTGATTATTACTACAAATGCAAACAGTAGGCATGGGAGTCTTCGAAT AGGGGTAGAAACCAGTCTCCTATGTGTTTAAGTCCTAAGCCCCTCAGAGCACTTAGTATCATTGAGCTG TCACATCCTCCAGAACCAGGCAATGACCCCAAGGACAGGGCAGTCCAGATCCTACTGTAAATGAAGCA TTTGCCCTGGGTTTGAGTCCTTAAGAGGTGAAGATACAAGGCTATCTCCTAGACTACTGAGAGTCCAAC CCTGATCCTCGCTGGGACCTTAATGGGGTATTTAAACCTCCATTCTTAGGTTAATTGTTTCAGATCCCTCA GGAGTAGTTGGAAACTCTTCCCTTGGCTGTACCCTCTTGCCTGGGCCTAAGGAGAACAAACCCCTCTTC TTAAACCACAGCCCTTGACTTTCACTATTACTTCCACACCCCCAGGGCATTCTTTGTGCTCTTATTTAAG GGAAAATAAGTCATCTTCCTCCTACCCCTGTGCCTTTGCACATGCAAATCATGTCTGAAGTCACGGGCC CAAGGGTTTTTTGTTTTGTCTTGTCTTGTTTTAAGACTGGGTCTCTCCTTGTAGGTCTGGTTGTTTTGGA ACTTACTGTATAAACCAGGCTGCCTCAAACTCAGAGACACTCCTGCCCCTGCCTTCCAAGTGTTGGAAT TAAAAGCTTGTACCACCACACTTGGCTAATTTTTTTTTCATTTTTGAGATAGTTTCATGTAGCCCAGACTG GCCTCAAACTCAATTTATAGCTGGGGATGGATGCCCTTTGAACTCCAAACCAGGTGCCAAGATTATGCC CAAGAAAGTTTGTAATCATTGTTTTGTAGCTCAAACATCACTTCCTATGGAAAACATTCTTGGCTGCAGG CCGACCTCAGCCAACACAATGTCTCCTCCTGCTGTGTGCCCATGGAATGTGTGTTTCCTTGAGTAGTTTT GAGTTTGATAGCGCCTGGTCCTTCTTATGGCTGTTTTACTTTTTAATACAATAAAGTTGATCTTTGATATAC GTATTTATGAATATTAACAGATTCACTGAGGAGTCACCACAGAGAGTTCCGTCATCCACAGCAGTTCCAT CAACCCTCAAAGAATACTTGGTAGTGTCCCTTTGTGGTCAGACCTTGCTCGATCCTTAATCCCTGGCAAC CACTGATATGTTCTCAATCGCAATAATACTGTCTTCTCTAGAGTGTCATATAATTAAAGCCAAACAATACA TATTTCACAGCACAATGCCCGTAAGATTTATCCACGTTGCATATATTGATAGCTCATTCCCTGCTATTGAC AACAATAGCCTCCTGGTGTATAACTATATAATAGTTTGCTTACCGTGAACCTATGGGGCATTTGGGCTGGA

							AACAGAGCTGATTACATGTTCATACACAGATTTGTGTGAGAGCATAGCTCTAACATCCCTCGGGTAAATA<br>AAGATTTCTAGGACCATAGAAATTAAAACTACCGAGCCTTTTGGTATTGAGACAGAGCTTCCATATAACC<br>CTGGCTGTCCTGGAACTGACAGCCACCCACCTGTCTCAGCATCCTGGGGGTAGGGATGACGCCACACA<br>CAGCTCTAACAAGCTGTACAAAATGGACTTGACAGTTTTGCATCTCTGACATGAGTCTCCAGTTGCTTT<br>ACAGCCTCAATGCTTAAGTTTCCATGTTTACTTCAGTCATTCTAGTAGATATACAGTCATATCCTCTCATAC<br>ATCTGGTTTGGATTGGATAGAGAAAGGGTCTTGCCATAACCCAGACTGGTCTAGAATTCACTAAAGTCA<br>ACTCCAGCTGGCCTTAGATTCAAGGCATGTCTCCTGCCTCAGCTTCCCAAGTGTAAGATCCCAGGTTTG<br>AGCCTCTATGCCCACCTTTTTTATTACAACTTAGTTCACATGTCTCCTATAGCAATGTTCTGTCTGTTTAGC<br>TGTGACCTGTGCAGTCTCAAGTCCTTAGCATATTTTTAACTGGGTCATTATCTTCTTTCTATTGAGACTTG<br>AGGTTTTTGTTTGGTTGTTTTGTTTTACATATTCTAGATACAGTTCCTTTGTCAGACATATAATTTGCAAGT<br>TTTCTCAAGCCTGCACCTTACCTTTTCATCCTTTCAACAACATGTCTTTCACTGGAGGGAAAATGGTTTC<br>TAACTTCAATGAAGTCTGATTTTTCAATTTTACCTCTTGTAGATCATGCTTTTGGTGTCACAGCTGAGTCC<br>TCCCCAACCCAAGTCATTAAGGTCTTGTTTTTGCCTGACCTGGACACAGGTCTGTAGGCCAGCTAATGT<br>AGAAGCTAAGCTAGGAGGATCAGAACTCAAGGCCTGCCTGGGTAACTTAGCAACACCCTGTCCCAAAC<br>AATGAAAACTCAAGGGCTGGGAACAGACTGCAGCTGTAGAGAGCACCCCTGGCATAGCTTGAAGCCCT<br>CCGTTCAAGCCTCGGTTCTGGGGAAGTCGCTTTCTTCCAAAAATTGTATATTTTTAAATAATTCAATTAAC<br>AAATGTTTGTAATTGTTCTGTTACTGCTGTTTTGAATGTCTGATTAACATATAATATCCTGGTCTCCTGTAA<br>TCTTAGATATGTGAAGGTCAAAGGTACAACTGGCAGGACCATCAAAATGCAAGCCATCTTGTCAGCCTA<br>CCTACAGTCTTTCAAAGTCAGTTTGTGTTTTCATTAGGGTTTCTATTGCAGTGAAGAGACACAATGACCA<br>TGGCAACCCTTATAAGGAAAACGTCTAATTGGGGCTGGCTTACTGTTTCAGAGGTTCAGTCCATCATCAT<br>CATGGCAGCGTGCAGGCAGACAAGGTGTTGGAGAAGGAGCTGAGAGTTTTACTTTGGGATCACAGGG<br>AGCAATGAAAGAACTGACCTGAGCTGCTGAGACCTCAAGGCTCACCACCACGGAGACACAGTTCCTC<br>CAGCAAGGCCACACTTCCTCCAACAAGGCCACATGCTCTTATAGTGCCACTCCCTATGGACTATGGGCT<br>GTCCCTATTCAAACCACCAGTTGGCTCTCTTTTCCATTTATTCTTTTTCTGTTGTTTGTTTTTGGTGCTAG<br>AGATTGAACCCAGAGCCTCACGTGTCTGTAGCCCCTGAGGAATCTTTTTCTGTCTTTCACCTTCAATCTA<br>TTTGTGTCTTTGGATCTAAAGTGAGTCCCTTAAAAGTGACATGTAGTTGAGTTACAGTTTTTCTTACCCA<br>TTTTGCTGACCTGTCTTCTGACTGGAGCGTTTACTCCCTTGCATTTATATATCAGTAGTAAGGGGGCTTTA<br>TTCTGCTACCTTAGTGTTTTTTCTTTCCTCCCACCTTTGCTTCTTGTGCTGGGATGGATCCTAGAGATGCA<br>CACAAGCATACACTGAGCTGTGTTCCCAGCCCTCACTGTATTCTCTAGATGCCTAAGAACTACTATCCTT<br>CATGCCCTGCATTCCTAGAGTAGTTTACCAGCTTGTTAGTAAAAGATGCGTTCTAGGATCCCAGGGTTGG<br>GTGGGCAAGATACCGCCACAGGCTTTCCAGCGCCCTCTTCTGGCCTCTGTAGGCACTTCACACACTTGG<br>TGCACAGACACAAGCGCAGGCAAAACAACCACACACATAAAATCAATCCCCACCCCCACCCCACAAA<br>AGAGACTTACGTTGGAGTTCCTTTTTTTTTTTCTTTTTTCTTTTTTCTTTTCTTTTTTTCGGAGCTGGGGG<br>CCGAACCCAGGGACTTGCGCTTGCTAGGCAAGTGCTCTACCACTGAGCTAAATCCCCAACCCCTTATAT<br>AGATTTCTATCACTGCAAAAAAGAGTCCTACTTGGAAATCAGGGATGGAGTGTCTGCTTTCTGACTTAC<br>TTTCTTTTCTGTAGCTGTGATTTAAAAATAAAAATAAAAATAAAATGACCAAAAAGCAACTTTACAGGG<br>AAAAAAGTGTTTATTTTGGCGTATGGTTACAGAAGGAGAGTCCATGGTGGTGGGAGGCATGGCAGCAG<br>GAGCTGGACCATGAAACTGGCTGATCGAATTTCATCCTACATGAAGCTGGACCGTGAAGGGGGTGAGG<br>CTACAGACCCTCAACGCTCACCCCCAGTGGTGTACTTTCTCTAGCAAAGCAGAGCCTTAGAGCCTCATA<br>ACTGTCCCAACATTGCCACTCATACCTCAGATCAAGCACTCATATATGTGAATGTATGGGGCGAACATTT<br>CTCATTCAAACCACGGCATTTTCAGTACACAGGGTCCTAGAGTTAACCCCCAGAATAGCACAGATTTAA

							AAACATAATAATTTCTAAGATTTTTCTAGCCATAAATACAGCAAATGTATTTATTTAGGTCTTTTTATCGGT<br>GGGAGATAGAGTGGGAGGCTGCCGCGGTTTGAACCTAGCAAGCCAGACATACATGCTACTTCTGAGCT<br>GTCCCTCACACACACTTGATTTATTTAATCGGTGCTTTTATGATTGGTTGGCTGGGGTTGTCTTGAGACA<br>GCATCTTTGTCTGTAGGCCAGCCTGGCCTGGAACTCAATGCTCCTCCAATACTTCCTGAGTTCTAGGATT<br>GCAAGCCTCTACCCCATGACAATGGATTTTGGTATATAGATGCTAATTTTTTTAGCTTTGTACCTAATATTT<br>CCCATTTTTGAACTCTCATAATTAAATCTTTGTTTCTAGTCATTTATTACTAATTTATAGAAATAGAGTTATA<br>TTGTTTGTCTTGTCTCCTATGAGCTTATAAAAATCACTTAGTTATAAGAGGAGCTTTTTGGTAGATTTCTT<br>GGAAGACTTTGTTTTCACTGTAAATAACAATGCTGTCTGCAGTCGGAGGCATCTTCCTTTCCAGTGCATT<br>TTTGCTGTTGAACAGATGTCAGGAGACCCTCATCTTGTTCCTTCCTAGGAAGGAAACTTCCATCCTCCTA<br>CATTAAGTCTGATGTTCCCTGCTAGTTGTCTTTAACCACTTCAAAATTTAACCATCTCTCTTCCCAGCTCT<br>CAACTCAATCCCTAGTGCCCAGCACATAGTAGACCCTTTATAAGTACTCAGTAGGAAAAAGTGAAGATC<br>CCGTTATCGCCTGCTTTGTTGACGTATCCAGCTTACTTTCCTACCTGACAGACAAAAGGGGACTTGGTTG<br>AATATGCTCAGACATCACAAAAAAATAATCTGGCAATAAATACTTGTCAGATTCACCTAGGCACTGGCCC<br>CACAGCAGCCACTATTTTAAGGGGCAGAGCACTTGTAGACCAGCTTTCTAGCAAGCCTTTTACAATGTC<br>TGGGCATCTATCTGGGAGGAATAGGTGACTTCAGTAAAATAAATATTTTAAATCAAAAGCATTCCATCCC<br>CAACAGAATTCACACACAAAGCTCTCAGCTTTAAAAGTGAAGAGTTTCTGGGTGAGACTCCCACTGCA<br>CTGAGACCTTGGGCCCTCAGCTGGGTAGGGGAGTTTCACTACATCTATTTCAGCCACTTATAGAAACAG<br>AAAGGGGAAAGTGGGTGGCTGTGTGCCCAGCAGCCGTGGCTCTGTTGTATTAATATCTACCAGGTCCAA<br>CTTTTATTAGAGTTTTGTTTTACTTTGGGGGACTGAAGAGGCAGACAGAAAAACCTTGCAGCATCTGAG<br>CTGTAGTTTCCCACCCTTCTCTCTAAACATTCCTCTTGGTTACCCTGCTGCCAGCCCACAGCCCGGCCCC<br>ACCTCACAGTGCCTGGAGGGTGGTTAAACTGCTCCCTGAGCACCCAGCACTGTGGTCACACCTGGCAC<br>CTTTGGCATCAGCTTTTCTCCCGTTCCCAAAGGGGTAGGGGAGAAAGATTAAGTTATTTGTTCCTTTTCT<br>GGCACAAGTGTGGGTGTGTGGAGACCTGGCCAGCCTTTGTCCCTGGTCCCCACCCAGTCCAATGCACC<br>CACCTGCAGCCACTGGACTTGAGTCTAAAGTTTGATACATTTGAAAGGCCCTTTTCACTCTAGGTACCT<br>CCTTAGTGTCCCCAAAGCATTTGGAGCCACATTCTCCATAGTCCCTGATAATTTACCAGACCCTGCTCCC<br>ACAACCAGAAAAAGAATTTTAAATGAAACCACCATGTTGATTTTCTAAAACCTGGTTTGTGCATTTGAG<br>AGCAGCTTTGAAATACATGCTCATTACCTCTGACGAAGGAGGAAACTGGGGTGAGAAGGGGAGGGGA<br>AGAGGCAGCAGGTGACCCGTAGCCAAGATGGCTGGGGAAGATCAATATTCAGTCTATGGCTGACCCTC<br>AGAAGCCCCGACTGAACCAGAAAAGGAGCTTGTCTTCTGTGGCTGCCACCACCCAAGGCTGAAAGAG<br>GAAACACTCGTGCACGGGGAAGTTCTGAGCCTGCAGCCCTTGAGGCTTGAACAAGCCCATGAGAATGT<br>GGGGAAGCACCCTGCAGTGGGCCTGCCTGTCAGAACCCAAATGCCAGCCTGATGCTCTAACAAACTCA<br>TGCTCCCTGGGTTACCAAGGAAAATGGGGAAAGAGCCCTCTAATCCCTGCTAGGAACCAGTAGGAGGT<br>AGGATGATCTATGCAAAAACTGTAGGCGGTGGCCAATATGAAGATTTGCCAGCAGGTTCTTGTCCTGAT<br>CTCAGGGTGGTCTTCAGGGCAGATGGAGACCATCCCATAGCCTGTTAGTTGTGAAGTGCTTAAAAGTAA<br>ACGAAGCTACTATTCTGTTCTGGTGTCAGACAGTTGGCTCTGTTACTGTCTAAAAGGCTGGGATGCCAC<br>AGATCCCAACCCTGAGAGCCATGTCTTCAAGAAGCAGGCATGTCTCAAGCCAGACCGGGATCCACAGC<br>TCTTGGCAAACTGCCCATGGACAGCTGGGGTGGAGGGGATGAGGGGGGTCTGGCTGGAGAAGCTACC<br>CAACAGGGAGAGTGACTGTGCTTCAGTCACAGCCCTGTGACCTCTTCAATGTTGCCTCTGCCAACTGC<br>ACTCTCTACCCGGAGGGATCATGGGCTCCCACACCAGGGCCTGCCCTGTCTGACCAAAGGCAACCAGA<br>TCAGCTGCAGCTGCTGGGTGAGAGACCAAGCACTCAGAGGGACCAGAGAAGCAGCTGGAGAAAGATT<br>CCTGGGGTGTAAATATTTATACAACATGGTGCGTGGGAAGAGGCAAGTCGGGGGAGGTGGTAGGAAGT

							GAAGACACAGACAGATGCACAGAGCCTGAACTCGGGCTCAGGCAGGGAGGGCAGGGACTGCTGGGATCCAACATTAGAGGCTGAGTCCTGGCCAGTTCTCCTGCCCCAGTGATGGGCACAGCTTAGAAACTCATCACACAAGTATGTCCCAGTCATTCCCACTGTTGAATCTACACTGTGTGCATTTGATGTGGATACTGACAGGTACAGATAGAGAGGAGGAGGATCTGCCTTGAATCAGAGCAGACAGAAAACAAGTGTACAGGGTCTGAGGATAAAGGCCTGGGGACTTGTGAGGCTTATGCAAGCCAAGATACCTCTCAAGTCCTTCTATAGTAGATGGCCATGATAGTAATGAAGGGACAGGTTCTGAAAAGGATGAAGTAAATGAAGTACGCCCTAAGTGACACTCATCAGGAAGTGTGTGCCCACCATTATGTGGAACCTAGGCAGACAGGGCCATCCGAAGACACCCTTGGCTGCTCTTGGGCACCCCACCCCTGATGCAGCTTAGTTCCAGGTGTGCCTAGGTAGAAGCTGACCCAGGCATCCTTCCCCTGTGCTTTCTGCAGACTTAGCCATGGCCCAGCAGCTCTGGTGTTCCAGGATGGGAAGGGAGCCTGAGCTGGTGGGGACAGCCAGATGGACTCCAGTCAATTTGCCTTGGACAGCTGGGGAGCAGGGCTCAGGCCACTCCCCTGTCACTCCAGACTAGGAGGGGCACGGTTGTAACTTCTAGGATTGCCCTGCTCCCACAACCACCAAGAAGTGCCGCCTGCCTCTCTCAGTGCCCAGGCTGACCCTCAGAACCTCCCACAGACGGATTCTGGGCTGCTCTCAGTGTCCAGTAGTCTTGACCTTCACACTGACAAACAGCGGGGTACTGGGGAGGAGAAGGACAGATCTCCAGTGGGAATATGCTGTCATCTTGGCAGATCAGCCCAAAAGTAAGAACGATAGCAGCATCAGTGGGGAGAGGGCTATCAGAGAGTCCCAGACTCCACCGGAGACAGACCCCTCTGCTTCAAGTCCCTCATCACTTCTCCAAATATCTTCCCAACAGGACACCACCCCTAAGTTCCTACCTCTTCCTGTGAACCCCGCTGACACTCACTCATCCACCCACGGAACTCCCCCAAGCAATGGGGTCCAGGCCCACACACTGAGCAGGAAATGCCATCTGTCCCCACCTCTGGCCAGGGCCTGGCAGACAGCATCAGTTTGTCTTGAAGGGCTCCTCTTCCAGGACATTAGGGCCAGGGGGGAGCATCTTCAGGCACCCAAGACCTCTGGCACCATGGCCCCTGCCGGCTGGTTGCAGTTCTCCATGCCAGCTCTACTCAAACAGATGAGCACCAACAAATGCTGTACAGCCTCAGGAAAGCTGCAGGGGGAAGACCTTCCAGACCCGGACGCAGCAGAGCGGCAGAGTGGCAGTGTGGCTTGCCCGGAGCTTCAGGGCCGCTGCTGGCCCTTCCGCATCTCGGAGCGTCTGCGGCCGGGTCCGCGGATG
MSTRG.107952.1	ENSRNOG00000006599;ENSRNOG00000038308	0.571	0.000	0.028	-3.770	down	GTCTTGCTAAGGAAACACGTCATAGACTTTTCCTGAAGCAGACACAGGTGAAAGGATGTTTGGATATAGCAGACACATGAAAGGGCTCCTGATGAAGGAGCATAAATCTGACCCCACAGACTGTGGGAGATGAGCACTGAGCTTTGGTTTGCTCCACCTCGCCATTCTTCAGTAAAGACACTCCATGCAATTGTTCCCCTTACATAGCGTTGGGTTGAGCTCAACTTGAGATAAAGCTGCCATGGAGAGAAGCTCGCCCCAGAACTGCTCATGAGGTTCCTGCAGCAGCTTGTCACCTCTGTGCCCTGACCTTAGGTCAGTGGCCAGCCTGGTGGTTTCTTTATGATTGAAGTATAGCTGCCTGGTGTCTGCTTCCTGAATGGACTGGACTGTGGCTGCTCGTTCGTG
MSTRG.107966.1	ENSRNOG00000027707;ENSRNOG00000032248;ENSRNOG00000038322;ENS	0.745	0.000	0.005	-4.261	down	AGGTGGAGCAAACCAAAGCTCAGTGCTCATCTCCCACAGTCTGTGGGGTCAGATTTATACTCCTTCATCAGGAGCCTTTTCATGTGTCTGCTATATCCAAACAGCCTTTCACCTGTGTCTGCTTCAGGAAAAGTCTGACGTGTTTGCTTTACTAAGACATCCTTTCACCTCTGTGCCTGAGCAAATCATTTGACAAAATTACCTTTTTAAAGACCTAGAAGTTTCCTCTTCATATGCCCGTTTTTCTTTAAGAACTGTCCTTTTCCCTATCATGAGCAATCTACACACAATGGGAACTATTGTAAAAACCATCACATTTTCAATAAATGCCACATGCATAGTATACTCAAGTACTTAACAAAACCTTACCTTTCTATCAATATACAATACTTAAAAAAAAAAAACACCTCAGGTTTCTATCAATATACAATACTTCAGAACAATTTAAATTTCC

	RNOG00000050329						
MSTRG.108157.1	--	0.520	0.000	0.013	-4.001	down	GTTTTGTGATTAAAAATTGACACAGACTAGGAAGTTCGGGTGGGCATGATCCTGCTAGATCCAGTTGGA<br>GAAGATGTGCAGTGCATAACATATATTCATAGAGACTGCCTTGGTTACGATTAATAGTGATGTGATGTATC<br>ATGACCAAAGCAACTCTATTTTGCTTACACTTCCAGGTAACAGGTCTATCACAGAGGGAGGTCAGGACA<br>GGAACTCAAACAGGGCAGGCACCTAGAGGCAGGAACTGATGCAGAAGCCATGGAGGGGTGCTGCTTA<br>CTGACTTGCTCCATGTGGCTTGCTCAGCCTGCTTTCTTATAGAACCCAGGATCACCAGCCAGGGATGAC<br>ACCACCCACAATGGGCTAGACCCTCTCACATTAACCACTGGTTAAGAAAATGCCCTAAGGCTTGTGAAA<br>TCTGTGGAGACAATTTCTCAGTTAAGAATGAAATTAAGACTTTTTTCCTTCAATGTATGTGTATTTTATAT<br>AGTTGTTAAGTTGCTAGGATACTAATTCATGTTAAGTTGAC
MSTRG.108405.1	ENSRNOG00000008781	0.437	0.000	0.000	-6.740	down	GCTAGTTTGCTCTGCAGTTCGCCTGTGCCATGGCTGTAATGGGATGGTTTATGGCAGTGTAAGAGGGCT<br>CTTTGCAACTGTGTGCAGACATCATAAGCTGAAAGTCAGTCTGTGCATGTTTCTGATTTTAGAAAATTCA<br>TTTTGAAAATGGAAACTACATGAATAAAATAAAGCTGGCCATTTGTGTTTATGTGAGGAAGAAAACAGA<br>AGACGCCCTGTCTTCCCAGAGTTTCTCTGATACCTGTGTGTGGATAGGGGCTCGGCAGGCCACACTCTA<br>CTGTGACAGCTGTGTTTTCTGCTTCTAATTTGACTTGGAGTGAAGTGAGGGGACTAACCTAAACTTCTA<br>GCTGACTGAGCCATGTCTTCCCTTCACCTCTAGGTGGCAATGACAGCTTTTATAAGTTCTGTCATATTCTT<br>AACTAAGATTTATGAGTTGGAGCCCTGAGAACACTGGAGGGAGGCTACTGAAACTCCATAGTCCTGGC<br>CTTTTGTCCCTTGAAAACTCAAGGAGAAAAATAAAACATTTCCCAGTTGTTGAATAAAGCCCTGGACCA<br>GCATCTCACATGAACTATTTGCTCTAATTGGATTGTCATAGGAAGACATTTTCTGTAGCATTTATGGATAT<br>GAGCTTATGTACTTGGAAGTATACATAGAAAGAAGACCAAGATGAATTTTAGAAAGCCTATTTTCCATGG<br>TATATGGAACAGAGAGAAGGTAAAATAGTTGCACTTCCTCTGGGCCTACGGTGCGGTGGCTTGTGCTTT<br>TATACTTGGAGGCCTCTGAGCTTCCTCTATCCTTCCTCCACTTAGCCATACCCCAACTTGTGGCCTTGGTC<br>CTGCTTGGTTTTCTGGAAGAGAGAAGGTGACATTGTTTCTCAGGGGTATCATGCCAGTATATTTTGTGTC<br>ATGTTCTAAAGATGTTTCACCTGCAATATTAGTAGCATAATACTAATAATTACCACTCAGGAAAATTTTATA<br>TAATTTTTGTATCTTTTTGGTGCAAGTACAGAAGGAGAGAGCAATGCTGGCATTCATTACGGTTTGCTAC<br>AGATAAACCAATACTCTGGCTGCATGTGCATTAGATGTCAGAAACCACTAATGTAGTGACAGTTATCTTG<br>CTTCAGCAGGAAGTAGCATGTCCACATGTATATGTACACACATTTATATGGTCAGTGGGCGCTCTTCCTC<br>ACTGATGGGAAAATAGCTTTTTACATACTCAGTCTACATTGATCCAAAAAAGGAAAAGAAACATTTTTA<br>AGTCTTTGGTAGGTTTACCTGAAACCTAGGTGGATGTGAAATCCTTCTCGAAGGCATACACATGGCATTA<br>TCTTGAGCTGTTTGCCCCAGATATACAAGGCACACTGTTCTGTGTGGGTTCTTCAGTGTGCCTAAATAGT<br>CACTGTTTATCTTGGCATTCACGTGCCTTTCAGCCCTCAGAGTGCACTCAGCTGTGTCATTACATCCAGC<br>ACCCGTCTGCAGTCCTCCGCACCTGCAGCTTCTGCTAATCTTGGGTCACGTGTGCTTTGTGTGGACATA<br>ACTGAAGGTAATCAGTTTGGGATGAGATCACCAGGTTGCTTAACTTTGAGACTAGAAGGAAAACCATA<br>ATCTTTCCTAAAGAGACAGACCTAGAGGCAAAAATAATTAGTTTCCTTCTTTAGACTTTTATACCCTGTC<br>ATTTCTGTTGGGAGCTTCAAATTAATGCTAGGAGATTGAAAGTTCACGTTATAAAGTTTACTGCATTGTG<br>AAATAGGTTATTTTGTGTAAATTCCTTTCTCCTTTAAACCTTTTCCAGAAAAAGATCTTAGTTTCATTATA<br>AGTTAATGCTTTCATTCGTCTTTGTTTTATTCACACAAAAAGCAAGCCACTCACTGTTTATTTTGTGTGTG<br>GAAGTGTTTGCCTGCATATATGTAAGTACACTAAATGTGTGCAGTATGTACAGAGGCCAGAGGAGGGTG<br>TCGGGTCCTCTTGAACCTAGAAATGGCCTGAACTGCCATGTGGGTGTTAGGAACAGAACCCAGATCTTT<br>TGCAAAAGCAACAAGTACTGTGAACCACTGAACCATCCTATCAGCTCCTTCTCACTGTTTCTTATATGTA

							AGTAAATTATTGAGGAGTTACTAAAATGAAATTGCCTATGTGGTACTTGAGTCAGCTGTCTCATGGTCCT<br>GGTAGTAGTGCTTCTGCTCCATATGCTGCTGTGGTTTTCTTGTGCAGATTTCTGCTGTAGAGTCCCCATG<br>CAGGTCCCTGCTGTGGAGTCCCCATGCAGGTCCCTGCTGTGAGTCCCCATGCAGGTCCCTGCTGTGGAG<br>CCCCCATGCAGGTCCCTGCTGTGGAGTCCCCATGCAGGTTGCTGTTTTGCCTAGTAGGAAAGACTCCAG<br>AGATAGGGGTATTGTTCTCACCAAAATGGTGACATTTTTTGTGTCAAAAATAGAATCTAAAGAGACTTTG<br>TTCTGGATTCTACAGCTGCTGGAAGATCCAATCTGGAATATAAATGATCTGAGACAGTCCCTTCTGAGAC<br>AAAGTTAATAGACAAATTTGCACTGAAACAAGAATTTATTTTTAATTAAAAAGATATTGAGTTGGAAACC<br>ACGTGTGTTATCAGTGACTCAACAAAAGGTAGCCCCAGCTCATGAAAACGTCCACGGGGAGTTCTGAG<br>GGTTGAAACCTAGGTCAGAGAAGTGGGAAAAGACTCCGAAAAGGCTGCTTCTAGCACAGCCCTCCCTA<br>GCCATATTACCCAGTGGAAATGCAGAAGAGACCTCAGATCATTTTGTAATGCCTTTCTCTCCTCCCCCAC<br>CTCTAGGCCCTTCTTCAGTAGCCTTGCTTGCTAATTAACACTGGCTTCTGGGGGATATTTACCTTTTCTGT<br>GCTAGATTCCTGTGGTTTTAAAAGAATTCAGGTACTTGCTGATTGCCAAGGAATTCAGATTACCTCACTG<br>GCTGCACACTGTCCAGAATCTGCCACAGCAGCAAGTAGGGGGAAGAGATACCAGCGACAGGGAGAAG<br>TTCTTCCTAGCCTTACTGTCACTCTCCCTGGCAGGCCTTTGCTGTTTGTGAAGTTTGAGTCATTTCTGGC<br>TCTGAGGACAGAGGATCAGGCTGGTAATCCCCTCCCAGACGCTCTGCAGGGCGCTGCCCATCACTGCA<br>GCATTCTGGCAGTTTCCCTGCTGCAGCTGGTTGACAGCAGATCAGAGCTCTAGGCCTCCTGTTCTCTTC<br>CATCAGATTTTTCTAGGTCAGGCCAGGATGAAACCCAGTGGCTTTTGGCAAGGATGCCTGGGATGAAGT<br>CTCATTGTCCATGACCTCTCATCAGGAAGCTCTACATGCTAAAGACAACTCAGGTGCTGCTATTGTGATC<br>TCCTTTGTTTGCGCATTTGTTCTGAATGTAGAAGATATATCTGCCAAGCTGAGTACTTAACTCTCCCATAT<br>ATAGGCTCCGTTATGCTGACTAAGTATCCGCCATCAGACTCTTCAGGCCACGCCACTGTTTGTACACAGG<br>AGAAAAGTTAGTAAGATCTCACTGACTGTTGCTTCCCCCTTTGCTTCTACATTGGTAAAGGTCTTGAAA<br>CAAGCAGCTGAGGAGCTGTAGCACCGTTTATAAATCCCCACTGCAGATGCATCTCGATCACCACCGAGT<br>CATGCTCCAGCATGGCTGTTTCTTTCTTACCATCTAGGGGTCTTGGTAGGTTCTTTTGTTTGTTTGTTTGT<br>TTTCCCTTTTCCTCTGTCAGTTAAAGAATTAAAAGGCAGGAAAAACAAACAGAAACCACTTCAAGTATC<br>ATGTGCGGCAAGTAGTAGCAGGGAAAGATCTCAAGACAGACCACAGAATCAGCTGGTTAACAAAAGG<br>CTTTTATTAGGGGCGGAAGCACAAGGCAAGGCACATTCAGAGAAAGACCCTGCAGAGCAGAATGGGG<br>ACCTGGGAAGCAAAGCCTGGTCTTTCCTCAGAGGCTGGGGATTTATGGGTCTTCCTCTCTGAGTTTAGG<br>CTTGGTCAGTATGAATGAAGGGAGACTTATAGAGCCACAATTTTGGAGTAGACGTGTCCTGATCTGGCC<br>TTGAACTTGTAGAATCTTGTCAAACTGGTCCACAGGCTGTGTGGCTCTAGTGGCTGGAGGAAAAGCCT<br>GTAGGCCTCTGTGTGTGTGGTCAGAAATATGAGGAAGAAGCTGGACGTTTGCTCTCTCTGCTTCCTAGT<br>CATGGCTCCTCTTCCCGTTTGCCTGCCTATCAAGATCTGGCTGACACCAGTCACCTCAGTTGAAAATAGC<br>ATCGGTTGAAAGTACATTTTAAAAGAGATTTTATTATTTGTATTTCATGCTCATTGTTTTACTTGTGTATAT<br>TTAAGTGTACTACATACACATATTGCTGAAAGAAGCTAGAAGAAAGATTCTGGGGTCAGAGGTGGTTGT<br>GAGCTTATACTTGGGTGTTGAGAATTAAACCCAGCTTCCCTGGAAGTGCAGCCAGTGTTTTAACCACTG<br>AGCTCCTAATCTCTCAGCCTCTGAAAATGCATTTAACACACCTAACTTGTCAGGAATGTTAGCTTAGCAA<br>TATGGTTTACTATAGTATATGGTTGTTGCCCTCCTGACTGTGGGCAACTGGCTACAGCTTGCTGCACTGCT<br>CAGGGTCACAAGACAGG
MSTRG.108450.1	ENSRNOG00000011026;ENS	0.053	0.329	0.000	3.202	up	AAAAGGCTATCACTTGACTCCTTCAACAACCTGTGACAGGACCCACAAGACAGCTCTGCAGGTAAAAT<br>ATGCTTGCCACCAAGACTGAGTTCAATCCCTAGGACCAACACGGTAAAAGGAGAAATCTAACTCCCAA<br>AGCATCCTCTGACTTCCATGTGCACAAGATGCAAACATGCAATATTTTTTTATTTAAAAATACACATTTTT<br>ATTTTATGTGTATGATTGTTTTGCCTGTTTGCATATATGTATGTACACATGTGTGCCTCGTGCGTGGAGGCC

	RNOG00000052456						AGAAGAGGGCATCAGATTCCCTGGAACTGGAGTTGGGGATATGATGGGGCAGCATATGGGTCCTGAAAGCCAAGCCCTGTCTCTCCGAGAGAGCACACAAGAACAGCAGCAAGTGCCCATACTTACTGAGCCCTGTCTCCTGACCTAATATTTTAAGAGAAAAAAGATGTATAAAGAACCCAAGCCATCACTGGACAGAGAGACAGAGGCAGGTGGATCTCTGTGAATTCTAGTCCAGTCTGGTCTATGTAATGTGTTCCAGGACAACCAGAACTACATAGTGAGACTCTTGTCTCAAAAAACAAAACAAACAAATAAATAAATAAGAATTCAATAAAAAACAAAAAACAAATCTGGTGCCCAAACACATGCTAAGTCCAGTTCAAACCTTGGAAGATTCCATAAGCACTCCAATTTCAACAGGTGAGAAAAATCAAGGCCGCAGAGCCCAGTGTGACTAAGGTCACACACACACACACACACACACACACACACACACACCACACACACACCACACACACACACCACACACACACACACACACACACACACACACACACACACAGGTCAAGCTGTAGGGACTGAGTATACAGAGCTAAAGAAAAGCTTCAGACATATCCCCAACCAAGGCCCCAGCCAAGAACAGAGGAGGAACAAACATTTCCCAGCTTGTGTCAGAAGCAGAGCCCAGCACTTTGCACACCCAAGACCTTGAGGCAGTGTCTGTCCTTTCTTCTGGGGAAACAGAGGCCCTGGGGCTCCCCTGGAGCCCCCTTGGCAAATTAGGTCCAGACTAGGCAAAACTCTGCGACGACTCAACTCCCAGGCGGGCCTTGAAACACCAGACCAGACATTTAAAAAAAAAGAAAAGAAAAGAAAAAAAAAAAACTGGCAGGATTCAAGAGAAGGGATTAGATCATTGCAGCAGGATGAAGGAAATGAGGAACCTAGGGAGGGGAGGAGGGACCGCGCCAAGAATGTCTCAAGACAACCCCAAACGCCTTCAGATTAAAGCAGCCTCATGCACCAGCAGCTGAGGCCACTGCCAGGAAGTCCTGGCCTCATTCAGCCCTTTTAGGGCGGGGTAAAAATAATCTCATCCATACCCAAGGAGCACACTCACAGATCAGCAAACAAATCCTGGGCACTCTGTGTGGACAGACAAAACTCAACCACACAGACACATGTGTGCAGAAGTCTCAGCCTGGCGTGCAAGCCCACGGGAGTGCATGTGTAACCAAAACACCGTGTTCACGAGCCCTGGAATGGGAGCCAAATGGCAAGCATAGAGCGATTGGACACAGGAGCTGCCAGGCCCAAGCAGGCCAGGGAATGAGGACACCTCCTGCCAACAGCCCGGGGGCCAGCGAGGGGCTGTCATACTCCGCTTCCATCTATCAGGGCAAAGGGTCATCGCTCAGGGCTACTGGGAACAGGAGTTCAAGCTGATAGCTTCTGGGGAGGTAGGCTGTGTCACAGCCTGGATAGCTGAGGCTGAGGTTGCTAACTCAGGAGGGGAGGGTGACCGAAGGAACAGAACAGAAGATTCATCATGACATCATTGCCTGAGAGCATCAAGTTCACCTTCACTGCATGCTCAGGAGTGGCAGAACCGTCGTTTACAGACGGGGAGAGGAAGATGCAGGCAGGAGAGGCGTGGGGCCCAAGGTCATCAGCAGATACAAGCCCAGACGCTAGATGAAGGGAAAGCAGCCTACAGCCGACTTGGATTAAGGACGAGCCGCCCTTGCCTGGTTCTCCAGACTCCACCTACCCCTAGTGTGCCAGGTCCATACAGAAACAAACAGAATAAGAACAAAACAAAGGGATAGCGAAATCTCATCCCTGTCCTCAAACAGATTTCAGCGATGAGCCACTGGCAGGCGGTGGTGGCGTCTCTGCAGCCTTGGTGAAGGTTGTCGTGAGCTGGGGCAGTAACAGCCCGG
MSTRG.108457.1	ENSRNOG00000011026;ENSRNOG00000052456	0.048	0.813	0.000	4.174	up	CCCGCCCTGTCCTCTACCACATCTGGCCTGGGTATGCCCACACCACAGTACACAAGGCCCTCGGGTCATTTCTGGGTCTGAAGTGTGTGCTCAGCTCTGGACTGACACATTAGCCACATCAGCAGCCAGAATCCTCCTATTGTCCCCTCAACACCCGGACAACCGAGGTGTTCCAGCTGTGGGAAACGGAGGCTTCGCGCCGCAAGGAAAGAGCCAAAGTCGTCCAGGGCTACTCTGAAAAGAGAGCACCTCCAAAACCATGCTAAAGCCAAGCACAGTGGCAGATGCCTGAAATCTGAGCATTTAGGAAGCAGAGACAGAAGGAACGGAAGTCTAGGGGCAGCCTGTGCTACATACCTGTCAAACTTAAGGCAACGCGGGCCACCTGAGATCATGTCTCACAAAAACAAGAGCAAAAGCTCTCTACCCAAACAACATGTACATCTGTGTACAGTGCCATTGACACAACAGGGTCTGTCCTTGTACACTCTATGATGTAGAACTCTCACCTCCTTCTCCAGCACCATGTCTGCCTGAATGCCGCCATGCTTCCTGCCAAGATGATAATGGACTGAACCTCTGAAACTGTAAGCCAGGTCCAATGAAATGTTTTCCTTATTAAGAGTCGCCATAACATAGAAAAACCTTGTCTCTAAAATAAAAGTTGTCATGGTCATGGTGTCTCTTCGAAGCAAAAGAAACCCTAAGACACTCCCTGTCCTGGGAAAACTTTTTACTAGTTATATGCATATCAACCTTCAGGTCGCCCCTAGAATGATACCTTCTCTTGTGTTTAGTTTAGTTTTGTTTTTGTTTGTTTGCTTG

							TTTTCTTTTGGGGGTTGAAAGCCTGACTTTCAACAGCTGAGCCATCTCTTCCACCCAGCCACCACCTTCTCAAAACCATATCCTTAGGCTAGGTGTGGCGGCCTATACCTTTAGTCCCAGCACTAGGGAGGTAGAGGTAGAAGCAGGAAGATCTCTAAATTAGAGGCAAGCCTGATCTACAGAGTGAGTTCCAG
MSTRG.108565.1	--	0.321	0.020	0.039	-2.797	down	AAAAAGTCTTGCTTTGTCAATCTTATAACAAATACAGTGGAACGAAGAAAAGATACTTGAAATGAAAAGACATATTTGAAAATTTTTCTAGATCCCAGAATGCATAGCATCATTGGGGTGGAGAAAACAGATTGTGTACAATACTCCATTTCAGAAACATCAAGAAGTAAATGTCTACCAAATCTTTGAAAAATCTTTATTCTTGGAAAGGACCAATTATAGTTGAGTGTAATCCAGAAAATTGTGTTTGAGAGCTAGCTCCTGGAACAGGTAGGTTGAGAGTTCCAGGTCTTGTGGGCTGAGAATGCCGTGTTACCCACTTCCAGAGAAATGCCTAGTGAGTCAGAGGCATCTGAAGGGCTATGATTGGCGTCCTAGAGATCGAAGAACATGTAGAAGGCAGGCAGTTACTGCGTTACGAATCCAAATACGAGCACTTGATTACTCCATATGAAATCATTCTCCCCATAGCTCTGAAATCTCTCTGTGCTTCATCTTCCAGTGTTTACTAGTATAGGCTACCTACATTCTTGATGCCTGAGTCTAAACCCATAAAACATGAAAAAAAAAAAGCCCTAGAAATCTATGTAATAGTTGAATGTTAGGTATCTCTGGAGTGGGATGTGGAGAAATGACTCTGAATTTTGGAGCTAGACAGTTCTGATCAAAGGCCATTTTTATAAATTATATGACATGGGTAAGTTACTGAATCTCTCAGAATCTTTACTGTACTTGTTAACAGAGGCATATGAATGGGTCCTCGTGGTGGTACTTTAGAAGTAAATGACCTACACAAAACAGGGAACAAAATGCTGGGTACAGGGATCTCTTCAATAGATATCTTGTTTCTTTATTTCTTTTCTATCTCTTTTTTCCTTCTCCATCATCTTTTTCTCCCCTCTTCAAGTCTTTTCCTGACCACATTTCATCAGGGCTATGCTGTCAGCTCTTCCATGGACCTGACTGACAAAGTCACTAAGTATTGTTTAACCCAAGTGTTATACCCAATTACTTGTTAAAAGCAACTTTTGAAACTTTAAAACTTGTGGCTCACAGACACTGGTGGTCTCCAATGGTTGAATTTCTGACTCTTGGTTCACTGGTTTACTGGGGGAGTTTTGGAGGTAACCTCATTGTAAATTGATGCTTTATAATTTTCAAAGCATTTTCTTTGGTCATTTTCTTCATGGGACCTGCTAGCCTCTTAGAAAACTGTATCATGGAGAATGCCCCAG
MSTRG.109002.1	ENSRNOG00000004004	0.994	0.000	0.037	-3.684	down	ACAGTCCTCTTCAAGTGCCCCAATCCCATGGTGACAGTGACACCCTGGTGGCGAGCCCGAGTGTGATGGTTCACCGCTGTGCACATTGCATTGGAGTGAAGTGAACAAGGCTGTCACCAAATCACACACGTTTTAATAAGAAATGTTTACTTGTGAACCATGACTCGGAGCCAGCAGAGTAGGCTGTGTCTGTGGACTTGAGCACACCATCAACATTGCTGTTCAGGA
MSTRG.109100.1	ENSRNOG00000007089;ENSRNOG00000007699;ENSRNOG00000052549	6.399	0.320	0.001	-3.277	down	TGTTTTTTCTCCCTGTAGGCTTTACTTCACCTCTGGGTCATGATTGTCCTGCTGACCTACTCACCAGAAATGCACCATGACCAACCATATGGCAAGTGAGCTGCCATTGTCCAGAGCTGGGGTCTGCAGGAAGAGCAACACTAAGACTTCTAGGAACAGTGCACAAGATGGTCGCATCATGGAAAGCTTCTAATCTTCTGACTTATCTCTGGTGTCTGACTTTCCCCTTTTCCTTAAGAGTCTCTAAAT
MSTRG.109112.1	ENSRNOG00000052549	0.587	0.000	0.005	-4.261	down	GAAGGAAGGAAGGAAGGAAGGAAGGCAGGCAGGCAGGCAGGCAGGCAGGCAGGCAGGCAGGCAGGCAGGCAGACCCGAACCAGCATGTGGGAGGGCCAGCATCTTGGGTCATGTGGGTAGTGAGCCTAGACATTTCAAGTGACCACATGGTTCTAAGTGGCCTCTTTGTCCCAGCTTACCTTGAACACTGGGGAGGCTAACTCAAAAATTTCTCCTTCCTAAGTAACATCCTTAGAGTCTGGAAATAGACAGCTGCCCTGGAAGGTGTCCACCAAGCCCAAATCCACCGCTTCCTAAGTGAACCTACCCTTCCCAGAGGAACCCATGTCCAGAGG

							GCTGTGAGAACAGATAAGAGAGCCATGGAGTCACTGCTGGGAGCAGCCATTCCTAGCCTGGGTCACCTGCCCCCATCTGAAATGGTCAGGGCTCCGGGATAGCTTCTCTAAGTCACTATGAGAAGAATTTCAGGCACCTCTTGAAACTCAAGATAAAACCAAAGATATCAAAAAAACAAAGGGTAACTAATGTGGGCTTGGGCTCCCAATATCAGCGCCTTTCAAACTAAC
MSTRG.109113.1	ENSRNOG00000008101;ENSRNOG00000033970;ENSRNOG00000052549	0.000	0.675	0.000	6.097	up	AGAACCACCACCACCCACTTCAAGAACTGTGTCACAAGAAATTCTACCACATCCCAGGCGAGAGAGCACCAGCCCCCTCAGCCTTCTGTAGCCAGAACAAACGTGGGCGGAGCCTGCATCCTGGCAGGAGGATGCCACCCAAGAGGCAGCCGAGTCCTGAGGCAGAAGGCAACAAGAGTCCTGGGTGCCAGTTCCACTCTGCCGTCACCACCTCTGTAATCAGGTGAATCTTTCTGAACTTCAGTATCCACAGCCACAAGAGAGCAAGAAGGATGCCTTGTGGCTGAATCTTGAGGAGGGGAGGGGCCTTCCCAGCAAGCAGCCTCAGGAAGGAGGCACCCGTGTTCTGATGGTGCCGGCTGTGTGAGAACCACCAGTACAAACAAGACTGTTCGACAACCATCGTTTCAGATAACCCACCAGTACAAACAACAGAAAACAGCACGCTGTCTCCAAGGGTTGCCTTGCCTTGATTCTCTATAAGCCTTTTACTGAAGTATACACATGTGATAAGAACAGGCCTCTGGGTTCAATCCCCAGTACCACAAGGAAATAAAGTGTGTCTTTTAAAGGAAGGAGGAAAAAAGAAAGCGTGTATGTAAGTGGACACCCAGGACCAAGACTGAGACACAAATATTTGCAGGTACCTTGTGTTCCCATGCAGCGCCCTCTCCCCACGGTGGTCCATTTCTGGCCTGCCTCTCACCTCCAAAGCACAGATCTGAAGGCCTCGACCTTCGCCTACATAGAAGGTACACTGAGTACACTCACTAGCTTAGGTCCTCCATTAGATTCTGTGAGACTCAGCTATGGCAGCAGACAGTTCACCCTCACAGATGCATCTCCTACACTCCTCTGATTGACATAAGCAAGTCTTTCCCTGGAGGCAGAATCACAGGGTCAGAGTGACCAAGCCACTCTCCTAAACCCCAGTCAGACTCTTACCCTGCTGGGCTCCCCTCCACCCCCCAGCCCTATATATCACATGCATTCATTACTGTTTCAGACTCTTGCCCTGTAAGTCATGCATGCTCCACAAAGACAGGGTGCTGTGCTGTGTTCCCTGGATACAGCTTGACCTAGAACAGATGCACCTCTGGCTAGCCAGAGAACTCCTGCC
MSTRG.109113.3	ENSRNOG00000008101;ENSRNOG00000033970;ENSRNOG00000052549	0.000	0.294	0.000	4.999	up	AGAACCACCACCACCCACTTCAAGAACTGTGTCACAAGAAATTCTACCACATCCCAGGCGAGAGAGCACCAGCCCCCTCAGCCTTCTGTAGCCAGAACAAACGTGGGCGGAGCCTGCATCCTGGCAGGAGGATGCCACCCAAGAGGCAGCCGAGTCCTGAGGCAGAAGGCAACAAGAGTCCTGGGTGCCAGTTCCACTCTGCCGTCACCACCTCTGTAATCAGGTAATTCTGGTGTGTGAACCCTGGGATGACATCTGGAGTGTGGACAGGACAATCGGGCTTCAGAGACAACAGAGAGCAAGAAGGATGCCTTGTGGCTGAATCTTGAGGAGGGGAGGGGCCTTCCCAGCAAGCAGCCTCAGGAAGGAGGCACCCGTGTTCTGATGGTGCCGGCTGTGTGAGAACCACCAGTACAAACAAGACTGTTCGACAACCATCGTTTCAGATAACCCACCAGTACAAACAACAGAAAACAGCACGCTGTCTCCAAGGGTTGCCTTGCCTTGATTCTCTATAAGCCTTTTACTGAAGTATACACATGTGATAAGAACAGGCCTCTGGGTTCAATCCCCAGTACCACAAGGAAATAAAGTGTGTCTTTTAAAGGAAGGAGGAAAAAAGAAAGCGTGTATGTAAGTGGACACCCAGGACCAAGACTGAGACACAAATATTTGCAGGTACCTTGTGTTCCCATGCAGCGCCCTCTCCCCACGGTGGTCCATTTCTGGCCTGCCTCTCACCTCCAAAGCACAGATCTGAAGGCCTCGACCTTCGCCTACATAGAAGGTACACTGAGTACACTCACTAGCTTAGGTCCTCCATTAGATTCTGTGAGACTCAGCTATGGCAGCAGACAGTTCACCCTCACAGATGCATCTCCTACACTCCTCTGATTGACATAAGCAAGTCTTTCCCTGGAGGCAGAATCACAGGGTCAGAGTGACCAAGCCACTCTCCTAAACCCCAGTCAGACTCTTACCCTGCTGGGCTCCCCTCCACCCCCCAGCCCTATATATCACATGCATTCATTACTGTTTCAGACTCTTGCCCTGTAAGTCATGCATGCTCCACAAAGACAGGGTGCTGTGCTGTGTTCCCTGGATACAGCTTGACCTAGAACAGATGCACCTCTGGCTAGCCAGAGAACTCCTGCC
MSTRG.109	--	0.000	0.353	0.019	3.592	up	GTCTCTTTGTCTTAGGGGTCACATGGTGCGGTTGCTTGGCTGTCCAAACAAGCATGCAAAGACTCTGTGACCCTGGAGGTGGAAAATACCTGCTCGGGCCAATCAGCGCAAGGATTAAGTGTAAGGCCAAAGAGGAC

213.1							AGTCCTGGACATCACAGGAGGTGTGGCAGCCTCCCTGACCGATACCCACCAGATCACAGACGTTCTAC<br>CCTTCCTACACGTGACCATCAAAACGGTGTGCAGACACTGTCACCTGTCCCCTGGTGCAGATACGAAG<br>CGTCCTGTTCGGAAATGCTCATCTAACCCCGCCTCCAGGCCAGACCCTTGCCCATCTGAAGTTGGAGTG<br>GTACCCCG
MSTRG.109442.1	ENSRNOG00000058463	0.000	0.620	0.000	9.865	up	GTTCTCTGCAGTGCTATCTAAAAGGGCTAAGTTGGGACAGACTGAGTAGCCTCTGGTTGATGTGTGGAT<br>GAGAGAATGTGGTAGATACATGCTTGGGACACTACTACTCATCCAAAAAGAAGTTAACTGTAGCCTCAT<br>GAGAGAGTGAACTCAGTTGTTGTGTTTCCTGATTGATCACTGCCACACTGAGGCCAATTAACAAACGAT<br>AAAGTCTCAAGAAAATAAGATTCTACAGACAGATGTTAACCTGTCAAGCATCACGTTCTTAGAGTTTTG<br>TAAACTGAACTATTTTATTGATTGACTCTAATTAGTAACATAGATTAAAGTAAATTCGAGTTTGAAAACAA<br>GACGCTAAAACATGGACTGGGGGTGATGGCAATTGAGAGTGAGTCTCATTCACTGCTTGAAACATCTG<br>AACTCTAATAGCCCAAGAAATTCATGATAGTTCATTTTCTTGGAGGAAGACATCCCCGAGACCCACAGT<br>TCTCAACATGTGGGTCACAGACCTTTGACCATTACATAGGGGTCACATATCAGATATTTACACTATGATTC<br>ATAATAGTAGCAAAATTATAGTGGTGAAGTTGTGACAAAATAATTTATGGTTGAGGGTCACCACAACATA<br>ATGAGCTGTATTAAAGTGTCACAGCATTGGGCAAGCTGAGAGCCGCTGTGCTAGACATATTTAGGAATC<br>CCTAAGGACTTTGAGCTTGGTACCTGTGTTGATATATGTTAATTTTGTCATTAGAATTAAGAAGTGGAACT<br>ATAATTCAAACAGCAACTGATGTATTAATCAAATTCAAACATATGCAGTGTGTTGGGGGCACTTTGATAT<br>GCAAACAGTGAGGCACAATGGGCAACGGTCATAACTCCCACACTTTCATCCTCAGCTAGTATAGACAGC<br>AGGTGGTGGAGCCTTTCTCAGCAGCTGGGTGTGGGGCTATGTTAAATTGGTTTCTAATTTAAATTGTTCC<br>CATATCTTTTGTTACAGTAAGAGCTTGCACTGTTAAATGAATGAGTAAATAAGTCAGCTGAGAAAGCAA<br>AGCTAAAAACCAAAGGTCTGTGGGAAGGCTCTCCCTGCTTTTCATCTTCTTTATGTGCATCTGCCCTGTT<br>CTGTGCTCTTCCCAGTGCTCTCCCACCGGAGCCTGGGATGGAATTCTCTGTGGTTGGGGCTCAGTGGTC<br>GGGGCTCAATGGTGTGTCTCTCACTGGAACCTGTAAATCAGGGTAAGTAATAGGCACAGATCTTGTTTT<br>GGTTTCCTTGGACAATTTCCTTTCTGTTTTAGACATAGGCAGACGTAGAAGTTCTAGTGCACTCATCTTG<br>GGACTATGATGAATATTTTAACTGAGTAATTGGTATAATGAAAAGCCAAGCTCAGTCAATTAAAGGCAGT<br>GGGGGTTCTATCCCCAAATACTGTATTCTTGTTATCTGTATCTATCTATCTATCTATCTATCTATCTATCTATC<br>TATCTATCTATCTACATAGATAGGTATAACAATACTATAATTATAGATAGATACACTATATAAGACAAGTAAT<br>TTAGCTAGCTAGATGATAGATAGGTAGATAGGTAGATAGATAGATAGATAGATAGATAGATAGATAGATAG<br>ATAGATAGAATCATTGCCTTCCACAGCATTACTAGTAGGTTAATGTAAATAAGAGTGAAGAGATATTGAG<br>CTATCCCAGGATAAGTGATCATAACTGTCAGTGTATGAGACTTAAATAATGATTAACATGTATCTCAAACC<br>CTGAGGCTCAATAAAAGATAAATATGTAAGCATGAACCTCTGGAACAGGGCTCATTTCTTATTCTCTGCT<br>TTGTTCTCTACCAGAATTGCTACTCCTAGGTAGTAGGGTTTCTGCGTATTTCACCCATTCATTCATATATCT<br>CTTGATCTTGGGACAAATATAAATAGTAATTAATTTTTTTACGGTTCTGATGGCACTGAAGATTATCCTTT<br>GCATTAGCGAATAGTGTAAAACCTAACCACATGGTATATTTACAGTTCATATGACGTAGCATATAAAGTCA<br>CGTGTGTGACCTTGCCAAATTTAATGTCAATTTGTTGTTGGAAGCACTAGAGCAATTGTATGATAGTATAT<br>GGCATTGAGGAAAGTCATTGAATAATTCAGTTCTAGCAGGAAAAAAAACAGAGATGTGCATTAGTGGTC<br>CTTGTAGGTGTATGTCACAGCTATACACATGATGGCATTCTTTGAGTAGAGGCTCTTTAGAAAACATGTT<br>AGGGATCTACGATGTTAATGATGACTGTAGTTGACATGGACTCGTGATATCTTATTTAGAGCAGTGATAA<br>GCACACACATAAGTCAATGATCCTTATTTGATTAGATGTATTCTGCAAATAGACTACTCTATGGTAATACG<br>GGTGCTTCTTTAATGGTGGTCACGAATGTCAAGAAAAGCTTTGGCTAAAACAAACAGGTTACCTACACC<br>GGTATTATTACTATACCTCATATGCCTGTGTTGAACTTGCATTCCCAGTTAAATATTAATTAAGGCAAGACT<br>CGGAAATCTTATGTTGCTCTAGTATAAAACAATCAAATCAGTAGAAATAAGTAATGTGGACTCTCCCTGG

							AATGGTTGATGACAGCTGTCTTACATGTACATGTCAGATGACAGGAGCTGGTGTCTGAAAGCTGAGAGC<br>GTTGATGTGGTATAGATAGTTTGCATTATTTAGCAGAGAGAAGTAAGGGGCAGAGAGATGGCTAAGCAG<br>AGAAAGCCTAATTACCCTAGTTGGAGACTTAGAACCACAGATTGGAAGGAAGAATAGGGTCCCCTAAA<br>GTTCTCCTATTTTTTCCACTGCTCTTTCCCTCAACTCAGAGAAATAAGCATCATAAAAATGAATGTGAAA<br>TATGATTGCATTAGGCCAAATGTGACCAATGAAACAAATTAAATTATCACAAACTAGACGATTGAATTGG<br>CCATGCCCGTAAAACCATGATTCATCACTGAAGGAGAATTTGCATGAGCTTTTTTCTCTGTCTGAGGGGC<br>GCTCCCGGCTTGTTTCAGTGTTTGAGAAACACAGTAGGGATTTCCACTGTTATCAGTGAATACGTGAGT<br>CTGAACAGCCAGTAAGGAGAAAAGTGAAGGCAGGAGAGTAGAGATACACCCACTGGATTGGGAACAC<br>AGAGGCTGTAAGTCAAAGAGGACACATGCCCCTGTTCATCTAGCAGCAGGACTGCACCCCATTGTCAC<br>CTCTCTCGTCTTCTAATTCCTTGAATATCAATCAAAGAGTAAATAAATATATAAAAGATGGAGGCGTCCTC<br>CCAGGGAAATAATCAAATGCGAATCTTTTAATTTTGTGTGAACTTGGGAGAGTGTGAATTATAGATAATG<br>TCTATATTCTTGGTTAGGGTGAATTAGTTCATGTTGTAAAACATGATTGTTAAAAGCAAATGCTTTCAAA<br>ATTGAAGATGGAATCTATGATTCATTAACTATACATGGAGTTTGGAGGGTAATTAACATACATTTTAATATA<br>TTGCTGGTCTATGAGTTCACCACATGAATAATTAAAGATTGGATGTAGATGACTGAGAGAAGTGCCAAA<br>AAGAAGGAGATGGTACAATTATACGATATCATTTCAGTGCTGATTGGTGACTACGAGTGAACAGAAGTT<br>GGAGGTCAAGGACACTGAGGACTTTACTGCCAAGTTAGAGATAAGCACGACGCATACAGCCTAAACCT<br>TGTGAATCTTTGGTTCGTGGACTACATAAAGTTATCAAGGAAGATTGAGAGATAAAAATGGACTGGACA<br>TGTGGTACAAGTGTGCTCAAATCCAGAACCCACCTAAATCTTCACTATGACAACCCTGAGACTTGCTGA<br>CATAGAGGCGACATCACGATCTTCCTTGGCCACCAGCTTATAGAATATGACACTGTGATGGCCAAGGTT<br>GCAGTGATGGGATGCCATGGCAACCGTCCCGAGCCCATGGGGACTTGACATCTCCAGCTCTCATCCTCC<br>TGTGTGCGCCTGCTTATAGAAAAGCTGACCCAACAGATGGTACCAGCATGCTGAGATCTGGAATCAACC<br>TTGCTTCCTTGCTGTGACCACCCCAAGCTCTGCTGACTGAGAGGACATCTTAATCTTCCTTGCTCCTGAG<br>GTTATAAATGACCCTGCTGATGGCTGACGTTGCAGTGATGGACAGATCCTGGGATGCCATGGCAACCGT<br>CCAGACCCCATGGGGACTTGACAGCTCCAGCTCGCATCCTCCTGTGTGCGCCTGCTAATAGAAAAGCTG<br>ACCCAACAGATGGTACCAGCATGCTGAGATCTGGAATCAACCTTGCTTCCTTGCTGTGACAACCCCGAG<br>CTCTGCTAACAGAGAGGACATCTTAATCTTCCTCGGTCCCGAGTTTACAAAAGACCCTGTGATGGCCCA<br>GTTTGCAGTGATGTACAGATCCTGGGATGCCAGGGCAACAGTTCCAAGCCCATGGTGACTTGACACCTC<br>CAGCTCTCATCCTCCTGTGGGCACCTGCTGATAGGTATGGAGGGTCCAATCACAACATGGGGGTTCAGC<br>AGAGTATAGACAGAAAGAGTTAAATTAGTGCTATTTTGAGGACCAAGCAGTGTGATGCCACACTTGCCA<br>AGCAATAAGCCAATCCTGGGAGAGAGGAACCCAGCCTTCCCATGCTAGCCTCTGTAGTATGAATAGGCA<br>CAGCAGGATCACTCTTGCTCTCTACACTCCTGAATCTGATCAGTTTTCCTTAACAAGGAAACACGGCTG<br>GCAGACAGACAAAGTTGGGTTAAAGTAGATAGAACCACAAACTTGCATCGGGAGTGACTCACAGCTTG<br>GAAATTGCAAGGAACATATTGAGAGATGGTTGTACTGACATTTGACCCCATGCATGATGCAGTGGCATA<br>GACTGGGCCCACCTCTTTTTCCCTTCAAATAACAAGAAATTAGTCTTTTCTGGGAAGAAAAGGTACAGG<br>TGCAGCCCAGGCGAAGTCTGTGATTCTAAGTTCTCCAACTGTGGAGAGCATGGAGTGGTGATTCACAG<br>TCTCAGCAGTTTCTCTCTCTTCATTGCCCTTTATTCTTCAGAAAGGCTGACCTGGTTGATGCAGAACCAC<br>CATGGCCTCCCTGCCTGTCCCAAATTAGCATTCTGTCTTGTGTGGAGCCTGGTTCAAGGTCTTAACTTTT<br>AATTGTCAGTTGTTGTAAGAATTTACATGTTTGTTGTAAAATTTACATATTTGTTGTAAAATTCACATGTT<br>CATCAATTGTGAAACCAAATCAGTGAAGTGTAATTCGGTACGTGGATTGCCATTGATCAGATTCAGTGTA<br>ACATTTACACTAAGTTCATTCTAAAGGCTCAGTACACCCAGTTCCATGGCATGAAAGTCTGCTTATGAGT<br>CTCGTGAGTCGACGGAATTTTTGGTCTGATAATAGGACTTTTCATCATGTCTTTCCTTTTTTTTCTGCTTC

							TCGTTTTCCCATAAAGCTGATCGGTTTATGTTTGCAGACATTTTATGTTATAGATACCTCCTCTGATGTCCC<br>TTCAACACCACTACAACTAGTATTCACTTTAGTGTCATTTTGTGCAGGGCATGGTAAAACCACGTAGTAA<br>TATGGCAGAGTTCTTCATATGTGGCGTGTGTAAGATTAATAATATAGCATTAGTTTTAACTTCAGAATGCA<br>AAACCAAATGCTTTCACGTAGGTTTTCATTTTTTTTCTATTTCATATTAAATGTATCATTTCCATTTTAGGT<br>TAGTTTTTTTCCCCTGGTACATTTATGTTATTTATGATAATAATCATGTTTAGCATATCACTACCTATGCATAT<br>CATATTTCGTATTAATATTATCATTCCTATTAATAACCACTAAGTTCTTCTAAATGTTAAGCCACACTCAAA<br>TGTTCCCATACCCGACTTAGACTGTCCATGATAACGTTGAGAAAGTGCAACATGTATTCAGGGCCATGA<br>GTCATTCATGATTCTTTCACAGCACACATTCTGCAAATAGTTAGTGCTCATCAATCAGTAAAGTAGCAAC<br>GTCATATGAGTCAGCGCTTCCGCCTGGGGGAAGGGGTAAGGAAAATATGGTGACGTGAGTTCTTACCAC<br>CCTGTCCTGAGTCAGATCCATATAGAACAGTTCCTTACCATTTCTTCTTTTACTAATGATAATGCAAACAT<br>TACATCTTATAGCTGATGTTTATGTTCTGGATAGTAAAGAATTCCTCATTATTTAGTGAAGAATTAGATATA<br>TCACTGTGTAAGTCAAAATTGAAAACCATTGCTCTTTTATCTGCTTTTAAAAGTCATTTTTTCAACTTCTT<br>GTTTGTGTTTTGTCTGTTGCTCCCCACACAGGGTCTGGAGCTGGTTTCAGAATTCCCTGTGTTTAAGGA<br>CCGCATCAAGAATGTGTGCTCCAGGGGTGATTTCTGCATTCTTCATGGTCACCAGTGGAACGTTTGCATC<br>TCTGCGTTGAGCTTGGGGCAGTGTGAGTACTAGTTACTATCCCTGCTTTTTTATGTGGTCTGTTGAAAGT<br>TGGTTACTTCATTATTGAAAAGTATCTAATACAGAGCTGGTAAGGTAGAAAGAGAGAACTGATTCCTAG<br>ACATTATACCCTTTCCTCCATGCACATGCCCTTGGCATGCATGCTTATAAACACAAATGAATATATTCAAT<br>GAAAATAAGTTTTAATTACAGTGTATCAGTGGAAATATAACCAATGACACCTACAAACTTAATGTGAAAT<br>AGGACATTGAGTTGACCATGTTTGATAAACATGGACTCCTCACTGAAGGAGGTAAAGTGGTTCAGTAGA<br>GAGGAGTGGCTTTCAGTCTGTGTCAGTGCTTGGGGAAAGAAATATTCTTTCCATTTTTATTTGTGAATTA<br>ATGATTCAATCAAGACCCAACATAAAAGTGTGTAGAGCCAGAGTGGGTTGATGGTGAATGCAAGGATAT<br>GAATCCAGGATGGTATACGTGTGCCCGTCCCTGGGTCCATCCTGTAGCAGATCTGCTCTTAATTTCTTGA<br>ATAGTGAACAAATATAGAAAATCATGAAGATAAACGATTGAAATTCTATTCACTTATGAACACGAGTATC<br>AAGGCAAAACAAACAAACAAACACACTCCCCTTAGTAACTGAGAATATGGAATCCAAAGAGGAGAAA<br>GTGGCATCCAAGCCCTTTTGTGTATAGAGAAACTAATGTCAATACCAGATGCTATTTTCTTGCTTTGCTG<br>GAAGTTTAAAGATACTCATCTTAGAAGACAATAGTATAAATATTTTTAAATTATGGTGGCTTGCATGAATG<br>ATTCAGATGACAGATAATCATCAAAGAGAAAGCAAGAAATGTTTTTCTAAGTACACTTTCCTCAGAGCT<br>GGTGCTCTCGCGTACTGACATGTTTATCCATCTGTGTAGATAGAGCCTGAGACAGATATGAGTTCCAGTG<br>CTCTGTCCTTTGCTGCCTTCTCTGTAACTATTTCATTATCCTCTTCTCTCCTCTCCTCCTCCCTCCCCTTCC<br>CTCCCTTCCTCTTCTCCTCCCTCTTTTCTGTACTCAAGATGGACCGTGTGATTAGAAGATCGTCCAAGGG<br>TCAAGGGCTCCATTTGCACCCTGTCGAGGAGGCGAGGAAGACGAGTAATGGACTTTCCCAAGAAATCT<br>ATTCATCTTCAATGGCTTCCACCCCATCAGTTGCTTGTGATGATGTGCGATCTGCCCAAACTTCTTCTCAT<br>AGTTTTGATGGTTTGTAGAAGCTCCTCACTGACTCAATGCCCTCCATGGCTAGGAGCTCTGCTGACATTG<br>TGGAAGCTGCTGGGCAGACCACTGAACGAGTCTGCCATCATCAAGGGCTCTGCACTTTACCAAGAAGT<br>TCATGTTGAATGTGAGTTGAAACCAGCATTCTTTTTTTTTTTAAATTGAATATTTTATTTATTTACATATCAA<br>ATGTTATCCCCTTTCCTGGTTTCCCCTCTGAAACCTCCCTGTCCCATCCCCCCTTACCCTGCTTCTTTTTT<br>TTTTTTCTTTATTTTTTTATTAGATATATTTCTTTACTTACATTTCAAATGCTATTCCCTTTTCTGGTTTCCTG<br>ACCATAAGCCCCCATTCCCTCCTCCTCTCCCATACGGGTATTCCCCCATCCATCCCCTTTACTGACCCCCC<br>AAATTCCCTTGCACTGGAGGTCCAACCTTGGCAGACCCAAGGGCTTCCCCTTCCACTGGTGCCCCAAC<br>AAGGCCTTTCTCTGCTACATATGCAGTTGGAGCCCTGGGTCAGTCCATGTATAGTCTTTCAGTAGTGGTT<br>TAGCCCCTGGAAGCTCTGGTTGGTTGGCATTGTTGTTCTTATGAACCCAACATTCTTAGTTTTGTTTAAG

							TGGTTTGCTTACGGTAATCACTGTACTATTACAAAGTATATAATACCCCTAACAAGTGGCCAGCATTTTGC<br>CAGTGGCTTGTAAGGAGCTGATTCAAAATGCTCTACTAATAAGGCAAACATAAAAAGTGGAAGATAGTT<br>GATTAATAGATAAACCAATGCTGTAGTAGCTCTGTATGCAAATTAGGAGTAGAGGGGCACTTCTTCACCT<br>TATGCAAGTCATGGAGCTGACATTGGAAGGGAGATGATAGCTTACTCAACAGACAGATGAATGAAGAA<br>ATAAAGACTAGACTTTGATCAATGATGACTCCGATGTGGGCATCATGAATGAATCCATATGTGGAATCCT<br>CATGTTCAACCCCAAAGATGAGCAGCACATTCTTTGTTTCATATGATCCTCTTGACATTACGTTTTAGAGT<br>TTGTTTTTATGCACTTTACTACTCTTTTATAATTAAACCATTGTAAGAAATAGTAAACAAACTAAAAACAT<br>AAATTTGGCGATGTCAAAATTAAAATGTATGACAAAGCAAAAATACCATCGGTAGCTATTTAAATGAGTA<br>TTGCTGGTATTGTTCGACTATGGTGATGGCAACTGTACAAAATATTGAACTTTTCTACTCTTGTGCTAAG<br>AGGGAGTTCAAAATTTTTCATTTTTTTCTAAGCACTCTCTCAATTAATGATTGTTAATGAAAGGTGAATG<br>CAATAGTCAATGATGAAGAAAACTAGCATCTTCTTGTCATGGATTCTGTTCTTTGCCTCTAAGAGTAGAC<br>TAATGGAAGGAGAATTCCTTATCCACACCTGGAGGCCATGGTACGTATTACACAACATGTTCGTCTTTGT<br>TTGTATAATTTTTTAAAGCTGTTTTCAAATATCCTATCTATGTTGTGATATTATTCTATGTCATTATTTGATGG<br>CAGCAGACCAGTTTCTAATGTGTGATGGACAGGAAAAACACGCAGGCACATATGTGGAAAGATGAGCT<br>CTGTGATAGTGAGAATAAGTCTACAACTGATTCTAGCAAAGGTAGTTATCCAATGACAGACCCGAAGAT<br>CTTAGGTTTCTGTGATCCGTACGACTTAAAAGTTGTCAATGGTGAATTAATGAGTTAATAACTAAACAGG<br>GTAGTAATGAAAAGAGACCAGTGTTCATCCTAGGTTCATCAAGACTCTGTGTCATCTGAGCAACGAATT<br>TGTATACATCTGGAGCTTAGATCCTCCATAGATGAAGTGCGCCATCACCACCTCTCGAGTCATATTGTAG<br>ATAGGGACCAGGGAGAGTGGGTTTCATTTTTGCTCTTGTTAAAATGCATTTTGAAGCTAACGCTACAAG<br>CAGTTCAAGGAAAGAGCCAGTAACCAATTATATAGTTACTAATGGATAGAAATTAACCTTATTCAACTAT<br>AAGAATAACATTTCTAGACAATAAAATCAGTGCTATGCATGAGGTGGAAGACCAGTGACAGAGTCTGTA<br>TTTGTGCCACCACTGATACTGTTTGTTTAAACAAGAGGATCAAATAAAAGCCATCCCAAATTGCCTAAAT<br>GACCGACTAATGAAAGATTGGATGCAGAGACCAATGCAAATGAGGCTGACTCTTGGTAATGATCTCCTT<br>CACTCTGTATTCCTGAAAGAACAGAGTTGTGCTGGCCCATTTTTCTTTGGTCAGACCTGTGGGTGGATC<br>TCATATCCTGAGTGTGAAGAGCTCTGTGAGTAGTGTCAGTTAAGATTGTGGTCTTGGTATTGTTTTTTTAT<br>GTAATTATTTACTTTTAAAATATTTTTGTACACTCAACACACTTGAATTTTTTCTTTCTTTTCCTAACTTTC<br>ACATCTTTGGAGTTGGTTTTTTTTTTTTAAGCCATTCACAGAAATCGATTTTTAAAGTGTGACTTAGAATC<br>ATTGAGAAGACACTTTGACCTACTGTAAAAATTGGCTTAATATAAACCATTGTTACCTCGATCTGTGTCT<br>GTATATAGCACTGAGGTTAAATCAGAGTAGAGCTATTGTTATAATAGGAGTTATGCAGAAAGGTGTTAGT<br>GTCATCCAAGGATCATAAGTTTTTGTGATTCTTATGTGATTGTTTATAGTAGATGAACAATGCAGCCACAT<br>GGTCAATAAAAGCACAAAGACGTGAGTGAAGAGCACAGGAGCAGCCTGAGCTCCTGAGGGTGGTGCT<br>GGTAGTGTAGGAGTTGTAGACTACAAAGGATGTCTGGCTCATGCACTTCTGACACAAGAGGAATCTCA<br>GTGCTCTCTCGGGGGAGAAAATATGGAGAAAGGTCTTGAGAGCTGTGCATGGCTCCCTGGTTTGGGTTT<br>ATTAGAAATGAGTCCATGAGAGATAAGTATGACAGAGGAAGCCACACTCTAACTCCTAGCTCATGTTTC<br>ACTGGATGAACACTGACAGCGTGTGGAAGGGATGATAAGTGGACTATCAGGCTTTGTTCTGTTTCCAGG<br>GGTCCATAGCTACCGATGGCTAGAGGAGAGCATTACTCTGGGCTCTTTGTGAGTTCAGAACTCATTTTAT<br>CATATGGCAAATAGCCCATCATTACCATGAAGGATCACTAAGTGCTCACTAGACACCATCGCAACGTTAC<br>AGAGTCTCTGTCACCTTGGCAGTGAGCAATGTGCCCGTGGTTGTGTTATCTTTGCCAGTGTCTTACATGT<br>GTAAAAATAGTGAGTTTATCATCGTAAAGAGTAAATGAACAAGGATAAAAATAAATAATGAAGAAAAAA<br>AACCCATGGGGACATAATAGTATTTCTTTAAATAAGTTAACAGTTTATCCATTTTTTTTCTCTTTTGGTTTA<br>AAATTCTATTTTAATTTCCTGGGAACCTAATATGTCTTTAAATGTTTTTATTTTCATATATGTGTGCTGTATT

							TACATCAATCCATCCCTGCACTTAGGCATTACCGTATGAAAATGTCCTCTTTCTGTTCTGCAGGAAGCTG<br>TACCACAGTCGATCAGAACTAATCATGAAGGCCTCTGCCCTCTGGAGAGAGAGAATATCATGGAAGTAG<br>AGTGGATGCACACCTTCTCCTCTCATCATCGCTACTTCAAGATGAAGGTCGCAACACTGACTGTATGGC<br>CCTGGCAGTAGAGATCTCAACTGAGTGATCACATAAGACACCGTTCAGCCGTGAGGATTCGTCTCAGTT<br>CCCACCGTTAACCCTCTTCCCACCAACTCAGGTCCCTTGAACACAGAACCTTTAGAGACTTGGTAACCT<br>GATCCAGCTTCCAATGAAGAAAAATGGCCGCGGTACAGTGCCAGTCTTACTGGGAGGACTTCTCAACC<br>AATGTGTGTTCCAAAGTTGGGGAGGATGGATGCCACTGAGAAAACTCTACCCCAGATCACCCAGGTAG<br>GTTGCATCTGCCTTTGTTCTAACAGGTTAGACACAGCCATCCCTACACATATCCAGCGTGCTTTGGAGGC<br>ACAGTGATGGGTTTTCAGTCAGCCAACCCCTAGCACAGGACTGTCTATTGTACAGGACACCCACTGTGT<br>CAGATGGTGCCAAGATCTGTGCCTTTCGTACACCTTTGGTCATGAAGGCTGGTTCTGACATCACCTGAG<br>AGAGACTTTGAAGTTGCTGGGTCTGGTTTCTTGTTTCCAGGCGATGGTCCCGGTTCTTGATGCATCCCTG<br>GCCTTTGAGGAAAGGATGCTAATAGGTAGAGACACTGAGTTCCGAGGAAGACTGAGATCTTGCCACTT<br>CTAACTAGTTTTCTTGGTCAGAAGCAATAGGATGGCAGTATGATGGGCGGTGATCTAATCATCTAATTAG<br>CAGATGCTCAACTCTGTTGGTTCTATCTGGTGAATACCCAGGAAGCATAAGACTGGAGAGGGTGCCTTG<br>TGTCCAGTGTTACCAGGTCTATTCATGCTGCTGGGCTCATACAAATTGGACAACTGGTGACTACGTCATT<br>CTGTGTTCCTTTAGGACAGGAGGGAGGGCATAATGGCACCTGTGGGTAAGAAGAAATCTGGAACTTTC<br>TGTCCCTTCATTTTTGATTCTTCTCTCCTCAGTTCTTACTTGGTTGTCCAGGCTGTTGGAGTGGCAATGAC<br>TTTAGGGACTCCATATCACACACCCTACTGCCTTGTAATGAGAGACTTGGGGGCACCTGCTTTCTAGCA<br>ACAGAAAAGTCAACCTGAGATTGGCACACACATTTGGCCAATGGGTTTTAGCACATGAAATTTGTTCTT<br>ATCCCTTAATTCCACTCAATTCTGTGGACGGCAGTAAAGCTTTCTGTGGGGCTCCAGCTAGGACAGTAA<br>TTCTCAACCTGTAGATCATGAGCCCTCTGGGGAGTCAAACAACCCTTTCACAGAGTTTGCATGTGAGGT<br>ATTTACTTTACAACTTATAACAGTAGCAATATTACAGCTATGAAGTAGCATCAAAAATAATTTTATAGTTG<br>GAGGGGTCACCCAACATAAGTAACTGTATTAGAGGGTCACAGCACTAGGAAGGTTGAGAACCACTAGA<br>CTAGAGGGTGGGTCCTGACACTTCCTGCCTGCTTTTTCTGCTTTACTCACTTCCCTTCCCACTAACTCTA<br>TAATGTGTCTGCTTTTAGAATCTTAACTTCCCTGGAAACTGCCCCCAGACTCAGCAGCCAGAGGGGCTG<br>GCTCACTCCTGACCACCTACCTACCTCTTGGACCCAGAATCTGAGGATCATTCCTGAACACCCACAATG<br>TACTGCTCTTGTTTGAGTTAAGAGCTGCTATTCCCGGGGCATCTCCTGAGGCACAATTTCTGGGGGCCCT<br>GGTTCCCTTTCAGTGAACCCCCCAAAACCTGGAGAGATATGGGAATGCAAACCTTCTGTGCTGGTGGG<br>AAATATTCTCAGGCAATTCTGTTCACTGTCGAATTGTAACGGGGTTTTGGTTGATCTCCAGGGCCCACTG<br>AACCAGACAGTTAAGGCCGATCCTCCTGACCAGCCATCTGGCACTTTGCAGCTGCCTTTGTTTGCTTTC<br>TGACCAGTGCTAATTGATACTTGAAGGAGAGGTTGTCCGTGTTGTCTTCTCTTTATTTATGATGAAACAT<br>ACACGGGAAACCTCTTTTTTAGTATCAAATCCCACCTGGTAGGCACTCCCTGTCCCTGATGAAGCCTTC<br>AGGGAAGGGGACCTGGACTGCTATTCCCTGGACTGTGTGACAAGAAAAGTCATTTGAAGAAGGGCCA<br>GGGTCCCTGTGGTGACTTGAGGCTGCTCCCTGTGGCTTTGAACTGTGAGGTGACTCTTGGTGTGTGATG<br>GCTTTTCAGCAAGGTCTTCCTCACAGTAGCCATAAGGACACGCCAGCAACGTGACTGAAGGGACAGTG<br>GTGCTTACTGCTCTCAGGGTAGCTGTGAGGATGGTAAACCGGTGACGTGCACTTCATTTATGCTGTAGG<br>TCACCCGTTTTACTATCCGCCAGCACCCAGACCGTCTGTGGGATGATACCTTGGTGCACACGGCAGCTA<br>TGAAGAAGATATGGGTAGGGTTGAGGCCATTGTGAATCTGAAATACTTCAAGTGGAGTTAGCTTTTAAG<br>GGGATCTTCTTCTGCTTTTGTGTGTGTGTGTGTGTGTGTTTGTGTGTGTGCGCGCGCGCACGTGCAGGC<br>CAGGTGTGTCATGTGTCTTCCATAACTACTTTCTGTTTTTTTTTTTTTTTTTTTTTTTGAGATGGTCTCTTG<br>TTGAACTTGGAGTGCACTGATTTTTGGCTGGCCAGCAAACCCCAGGGATCTGCTTGTTTCTGCCTCTCA

							GGCTCACAGATTACAGGTATATACATACACAGTGCTTGCCCTTTCGACATTGAACCCGGATCCTCATGCT<br>TGTGTGGCCAGAGCCATCTCTTCACGCCAACTCCTCTTCCTCCTACCTTTCAATCCATAATGTCACATTCT<br>GCCGTAGGTGGGTGTCAGGCTGACCACAGATGGATTTGAGAAGGGTTGCTCCTATCAGGATCCATCGGG<br>AGAGTCTGTCCTCGTCTATGCTAATATAGGGTGTGTGAGGCACCTGCTGTGGTCCACTAAATCCTTGGTT<br>GGGCCCGGGGTGTTGTGTATAAGCCAGCACCTGTGAACCTGATGCCAACATGGCTCTGTAGTACTTAAA<br>GAAAGCTATGTGGTGAGCTTCCCATGGTCGTGGCGAGCATTTTATGATCCATATCTTTACAGCGAAGAAC<br>ACCATCTGGAGGATCTCCTGAATGTGAAGCTCTAACCCAAGCAGGGCTCATTACCCCCCGGGGTGAGTG<br>AGTGCTCTCTCTAGCCCAGCATCAAGCCTACATCTGCCACTTATGGAAGAGGCTTCCTTGTTCTTGCTCA<br>TCTCCATGTCTGCTCAATTTTGCTTTTCTTCTGAGGATTAAACCAATTGGCTGCTTCCCAGCACAAGTTC<br>CTGCCTAGGAGATGTATTCCAGATGTGGCATTTTCCTTAGGGAAGGACACCACAGTCCCCATCGATGGA<br>AAAGGTATGTCAGGGTGTTTTGTCTGAAAGAATATATTGCATTTTCATGTGTGTCCTGCTCTGCTAATGCT<br>AAGTATGCCTTCCATACCTAGGAGGACCTCCTGATGGATGAATACCTACCCTGGGGGTGAAGGTGGCAC<br>CTCCCCCCAGGCTCCTGGCAAGTGGTTCTTACAGGCCTGGCAGAGACACAGAAAGTAAGTAGGAACTC<br>TTAGACTGTGATAGGGGACTATTGTGCTTCTGCTCGTGTTTCATTGGTCAAGCCAATGAAGGGTGGGGA<br>GGCAGCTTATACCCATGATGTCAAGAGGGAGCACCATCTTCCTGGAACACCTAAGAAGTCTAGCTTTAT<br>TCATGTCCTTTTATAGCTCAATATGCCATTCCTGTTCCTCTCTGGTAAGCTGAGGCTCAACAGAATCTGTT<br>CTCAGAACACTAGGAGACATCACTTGGTTCTGGCAAAAACTCAGATCTTTTTGTTTCCCATTTCATGAA<br>GACCTCTTTGGCTCAGGGGCCCCACCATGGTACACAGGTTGGGGTCCTCCCCTTTAGCAGATGGGTGGT<br>GACCTCAACTGAGAGGTCACGGGTCACACCTCCTGGATCTGGAGCTGGATGTTGGGAAGCAAAGTCAG<br>TATTGTTGTATTATCGTTCTCTGATGTCTGAGAAGACTCCAAAGACCCCTGCAAAGGATGGCCACTCCAA<br>AGCCTGTCTACAAGATCCTGATTCTGCCTGCGTGGAGCGGGCACGGCTGTGAGAGCCCCCTAGGTACA<br>GCGGGGCTGCAGCGTGATCGCCTGCTCACGCACAGGAAGTGACGACAGCGGTACACAAGCCACAGGG<br>TTCTATGCTGCTGACGTACGGTGCTTAATGAGAAGTTGCCCGCGTGTTTTTCGCTTTATATGTGACGAAA<br>CATTCGCGGTGCACTTCTTTTTCAGCATCCTATTCTACGTTGAAGACTTCGGGGGTTCAACTACTGACTT<br>CAGGGAAAATGTGTTAGTCTAGGAAGGTAGTCTATGTAGTGAGCCTGGGTTGAGACTTTATCTCCCTTCT<br>TGAATGGTCCTCTGACCTTTCTTATCCAGAATGGGTTTGGGTTGGTTGGCCCTTCCCCCACAACACAACT<br>TCAAAGGAGCAGGGCGATGTTCGCCGAGGCTTCCGGGAGAGGGAGTGGGTCTTCATTTCTGTGAGAGA<br>TGCCTGAGTAGATCCCACTGTTACCTTCAGCCACTTGATGTGGTAATGGATGTGAGTTTGAAGGCCAGC<br>TAGAATTCAAGAAGATAGTACCCAGAGCTGTGTAGACACAGAGGGGGAGGCACCTGTCATGGGGGAAC<br>AGATTGAAGAGGCAGCAAGGAACCAAGGGACATTTGGGTAAGAGTGCAACCTGGGACCTGCATCTGA<br>CACTCCTTGAAGCTCAACACGTCTCTATACCTTTGAGTTATGTTAGCCCGGAGCCTCTTTTCTGTGTAAG<br>TCAGCTTGAGTTGAGCCTTTCCTTACTGTGAGAGGAACGGCTGGCTCCTCATCGAGTTGCCAGTGAGTG<br>TAACTGGCATTCTTTGGATGGTATGTTCTCTTGCTGACTATTCCCACTGTCCTGTCTAAGGTAGGCTGTTG<br>ATACCAGATCACTTTCTTTTGTGGAATTACTGGCTGTGGTCCAGATCCATTGCCGTAGTGTTTGAACCTC<br>CCAATGATGGCAGCACACGCTAATCAATGTGTTTTGTTTTCAGGGAAGGCTCATATGTCCCGTTCAAGAC<br>AGCTTCAGGATGATGGATGGGCGCTGTCCTGGAATCTGCTCTTTCCTCTCGTGCTGGTGCTTCCCAGTGC<br>TCTCTGCAGAAGAGGACCCTGGATCTGAGGAAGGAGATTCTGTCTTGGAGTCCATCACCATCTGATTTC<br>TCCTGAGTTCACTCCAGGAACAGTGGCAAGGATGGTAGCCAACATCTTAGAATCATCAACCTTGATGAC<br>CAAGTTCTACGCACCAGGACTTCCTGGGATCCTGAGGTAGAGGGGGTTGGGGCTGGATTGCTATGGCG<br>ATGATAAGTACTGAGCCATTCCTCTCTGCTCTGGGACGCCCCTACCCTACGCCTGACCTCTCCCAGGCCA<br>AGACAGTATCTCCCTCACCCACCACCCCTCCTGCCTGCCCTTCAGCATCCCTTAACACTCAGACACACT

							GCTGGAACAAGCAAGAGCTCAC
MSTRG.109442.3	ENSRNOG00000058463	0.020	3.845	0.000	8.133	up	GTTGCAGTGATGGACAGATCCTGGGATGCCATGGCAACCGTCCCGAGCCCATGGGGACTTGACAGCTCCAGCTCTCATCCTCCCGTGGGCACCTGCTGATAGAAAAGCTGACCCAACAGATGGTACCAGCATGCTGAGATCTGGAATCAACCTTGCTTCCTTGCTGTGACCACCCCAAGCTCTGCTGACTGAGAGGACATCTTAATCTTCCTCGGTCCTGAGGTTATAAATGACCCTGCTGATGGCTGACGTTGCAGTGATGGACAGATCCTGGGATGCCATGGCAACCGTCCAGACCCCATGGGGACTTGACAGCTCCAGCTCGCATCCTCCTGTGTGCGCCTGCTAATAGAAAAGCTGACCCAACAGATGGTACCAGCATGCTGAGATCTGGAATCAACCTTGCTTCCTTGCTGTGACAACCCCGAGCTCTGCTAACAGAGAGGACATCTTAATCTTCCTCGGTCCCGAGTTTACAAAAGACCCTGTGATGGCCCAGTTTGCAGTGATGTACAGATCCTGGGATGCCAGGGCAACAGTTCCAAGCCCATGGTGACTTGACACCTCCAGCTCTCATCCTCCTGTGGGCACCTGCTGATAGGGTCTGGAGCTGGTTTCAGAATTCCCTGTGTTTAAGGACCGCATCAAGAATGTGTGCTCCAGGGGTGATTTCTGCATTCTTCATGGTCACCAGTGGAACGTTTGCATCTCTGCGTTGAGCTTGGGGCAGTATGGACCGTGTGATTAGAAGATCGTCCAAGGGTCAAGGGCTCCATTTGCACCCTGTCGAGGAGGCGAGGAAGACGAGTAATGGACTTTCCCAAGAAATCTATTCATCTTCAATGGCTTCCACCCCATCAGTTGCTTGTGATGATGTGCGATCTGCCCAAACTTCTTCTCATAGTTTTGATGGTTTGTAGAAGCTCCTCACTGACTCAATGCCCTCCATGGCTAGGAGCTCTGCTGACATTGTGGAAGCTGCTGGGCAGACCACTGAACGAGTCTGCCATCATCAAGGGCTCTGCACTTTACCAAGAAGTTCATGTTGAATGTGAGTTGAAACCAGCATTCTTTTTTTTTTAAATTGAATATTTTATTTATTTACATATCAAATGTTATCCCCTTTCCTGGTTTCCCCTCTGAAACCTCCCTGTCCCATCCCCCCTTACCCTGCTTCTTTTTTTTTTTCTTTATTTTTTTATTAGATATATTTCTTTACTTACATTTCAAATGCTATTCCCTTTTCTGGTTTCCTGACCATAAGCCCCCATTCCCTCCTCCTCTCCCATACGGGTATTCCCCCATCCATCCCCTTTACTGACCCCCCAAATTCCCTTGCACTGGAGGTCCAACCTTGGCAGACCCAAGGGCTTCCCCTTCCACTGGTGCCCCAACAAGGCCTTTCTCTGCTACATATGCAGTTGGAGCCCTGGGTCAGTCCATGTATAGTCTTTCAGTAGTGGTTTAGCCCCTGGAAGCTCTGGTTGGTTGGCATTGTTGTTCTTATGAACCCAACATTCTTAGTTTTGTTTAAGTGGTTTGCTTACGGTAATCACTGTACTATTACAAAGTATATAATACCCCTAACAAGTGGCCAGCATTTTGCCAGTGGCTTGTAAGGAGCTGATTCAAAATGCTCTACTAATAAGGCAAACATAAAAAGTGGAAGATAGTTGATTAATAGATAAACCAATGCTGTAGTAGCTCTGTATGCAAATTAGGAGTAGAGGGGCACTTCTTCACCTTATGCAAGTCATGGAGCTGACATTGGAAGGGAGATGATAGCTTACTCAACAGACAGATGAATGAAGAAATAAAGACTAGACTTTGATCAATGATGACTCCGATGTGGGCATCATGAATGAATCCATATGTGGAATCCTCATGTTCAACCCCAAAGATGAGCAGCACATTCTTTGTTTCATATGATCCTCTTGACATTACGTTTTAGAGTTTGTTTTTATGCACTTTACTACTCTTTTATAATTAAACCATTGTAAGAAATAGTAAACAAACTAAAAACATAAATTTGGCGATGTCAAAATTAAAATGTATGACAAAGCAAAAATACCATCGGTAGCTATTTAAATGAGTATTGCTGGTATTGTTCGACTATGGTGATGGCAACTGTACAAAATATTGAACTTTTCTACTCTTGTGCTAAGAGGGAGTTCAAAATTTTTCATTTTTTTCTAAGCACTCTCTCAATTAATGATTGTTAATGAAAGGTGAATGCAATAGTCAATGATGAAGAAAACTAGCATCTTCTTGTCATGGATTCTGTTCTTTGCCTCTAAGAGTAGACTAATGGAAGGAGAATTCCTTATCCACACCTGGAGGCCATGGTACGTATTACACAACATGTTCGTCTTTGTTTGTATAATTTTTTAAAGCTGTTTTCAAATATCCTATCTATGTTGTGATATTATTCTATGTCATTATTTGATGGCAGCAGACCAGTTTCTAATGTGTGATGGACAGGAAAAACACGCAGGCACATATGTGGAAAGATGAGCTCTGTGATAGTGAGAATAAGTCTACAACTGATTCTAGCAAAGGTAGTTATCCAATGACAGACCCGAAGATCTTAGGTTTCTGTGATCCGTACGACTTAAAAGTTGTCAATGGTGAATTAATGAGTTAATAACTAAACAGGGTAGTAATGAAAAGAGACCAGTGTTCATCCTAGGTTCATCAAGACTCTGTGTCATCTGAGCAACGAATTTGTATACATCTGGAGCTTAGATCCTCCATAGATGAAGTGCGCCATCACCACCTCTCGAGTCATATTGTA

							GATAGGGACCAGGGAGAGTGGGTTTCATTTTTGCTCTTGTTAAAATGCATTTTGAAGCTAACGCTACAA<br>GCAGTTCAAGGAAAGAGCCAGTAACCAATTATATAGTTACTAATGGATAGAAATTAACCTTATTCAACTA<br>TAAGAATAACATTTCTAGACAATAAAATCAGTGCTATGCATGAGGTGGAAGACCAGTGACAGAGTCTGT<br>ATTTGTGCCACCACTGATACTGTTTGTTTAAACAAGAGGATCAAATAAAAGCCATCCCAAATTGCCTAA<br>ATGACCGACTAATGAAAGATTGGATGCAGAGACCAATGCAAATGAGGCTGACTCTTGGTAATGATCTCC<br>TTCACTCTGTATTCCTGAAAGAACAGAGTTGTGCTGGCCCATTTTTCTTTGGTCAGACCTGTGGGTGGAT<br>CTCATATCCTGAGTGTGAAGAGCTCTGTGAGTAGTGTCAGTTAAGATTGTGGTCTTGGTATTGTTTTTTT<br>ATGTAATTATTTACTTTTAAAATATTTTTGTACACTCAACACACTTGAATTTTTTCTTTCTTTTCCTAACTTT<br>CACATCTTTGGAGTTGGTTTTTTTTTTTTAAGCCATTCACAGAAATCGATTTTTAAAGTGTGACTTAGAAT<br>CATTGAGAAGACACTTTGACCTACTGTAAAAATTGGCTTAATATAAACCATTGTTACCTCGATCTGTGTC<br>TGTATATAGCACTGAGGTTAAATCAGAGTAGAGCTATTGTTATAATAGGAGTTATGCAGAAAGGTGTTAG<br>TGTCATCCAAGGATCATAAGTTTTTGTGATTCTTATGTGATTGTTTATAGTAGATGAACAATGCAGCCACA<br>TGGTCAATAAAAGCACAAAGACGTGAGTGAAGAGCACAGGAGCAGCCTGAGCTCCTGAGGGTGGTGC<br>TGGTAGTGTAGGAGTTGTAGACTACAAAGGATGTCTGGCTCATGCACTTCTGACACAAGAGGAATCTCA<br>GTGCTCTCTCGGGGGAGAAAATATGGAGAAAGGTCTTGAGAGCTGTGCATGGCTCCCTGGTTTGGGTTT<br>ATTAGAAATGAGTCCATGAGAGATAAGTATGACAGAGGAAGCCACACTCTAACTCCTAGCTCATGTTTC<br>ACTGGATGAACACTGACAGCGTGTGGAAGGGATGATAAGTGGACTATCAGGCTTTGTTCTGTTTCCAGG<br>GGTCCATAGCTACCGATGGCTAGAGGAGAGCATTACTCTGGGCTCTTTGTGAGTTCAGAACTCATTTTAT<br>CATATGGCAAATAGCCCATCATTACCATGAAGGATCACTAAGTGCTCACTAGACACCATCGCAACGTTAC<br>AGAGTCTCTGTCACCTTGGCAGTGAGCAATGTGCCCGTGGTTGTGTTATCTTTGCCAGTGTCTTACATGT<br>GTAAAAATAGTGAGTTTATCATCGTAAAGAGTAAATGAACAAGGATAAAAATAAATAATGAAGAAAAAA<br>AACCCATGGGGACATAATAGTATTTCTTTAAATAAGTTAACAGTTTATCCATTTTTTTTCTCTTTTGGTTTA<br>AAATTCTATTTTAATTTCCTGGGAACCTAATATGTCTTTAAATGTTTTTATTTTCATATATGTGTGCTGTATT<br>TACATCAATCCATCCCTGCACTTAGGCATTACCGTATGAAAATGTCCTCTTTCTGTTCTGCAGGAAGCTG<br>TACCACAGTCGATCAGAACTAATCATGAAGGCCTCTGCCCTCTGGAGAGAGAGAATATCATGGAAGTAG<br>AGTGGATGCACACCTTCTCCTCTCATCATCGCTACTTCAAGATGAAGGTCGCAACACTGACTGTATGGC<br>CCTGGCAGTAGAGATCTCAACTGAGTGATCACATAAGACACCGTTCAGCCGTGAGGATTCGTCTCAGTT<br>CCCACCGTTAACCCTCTTCCCACCAACTCAGGTCCCTTGAACACAGAACCTTTAGAGACTTGGTAACCT<br>GATCCAGCTTCCAATGAAGAAAAATGGCCGCGGTACAGTGCCAGTCTTACTGGGAGGACTTCTCAACC<br>AATGTGTGTTCCAAAGTTGGGGAGGATGGATGCCACTGAGAAAACTCTACCCCAGATCACCCAGGTAG<br>GTTGCATCTGCCTTTGTTCTAACAGGTTAGACACAGCCATCCCTACACATATCCAGCGTGCTTTGGAGGC<br>ACAGTGATGGGTTTTCAGTCAGCCAACCCCTAGCACAGGACTGTCTATTGTACAGGACACCCACTGTGT<br>CAGATGGTGCCAAGATCTGTGCCTTTCGTACACCTTTGGTCATGAAGGCTGGTTCTGACATCACCTGAG<br>AGAGACTTTGAAGTTGCTGGGTCTGGTTTCTTGTTTCCAGGCGATGGTCCCGGTTCTTGATGCATCCCTG<br>GCCTTTGAGGAAAGGATGCTAATAGGTAGAGACACTGAGTTCCGAGGAAGACTGAGATCTTGCCACTT<br>CTAACTAGTTTTCTTGGTCAGAAGCAATAGGATGGCAGTATGATGGGCGGTGATCTAATCATCTAATTAG<br>CAGATGCTCAACTCTGTTGGTTCTATCTGGTGAATACCCAGGAAGCATAAGACTGGAGAGGGTGCCTTG<br>TGTCCAGTGTTACCAGGTCTATTCATGCTGCTGGGCTCATACAAATTGGACAACTGGTGACTACGTCATT<br>CTGTGTTCCTTTAGGACAGGAGGGAGGGCATAATGGCACCTGTGGGTAAGAAGAAATCTGGAACTTTC<br>TGTCCCTTCATTTTTGATTCTTCTCTCCTCAGTTCTTACTTGGTTGTCCAGGCTGTTGGAGTGGCAATGAC<br>TTTAGGGACTCCATATCACACACCCTACTGCCTTGTAATGAGAGACTTGGGGGCACCTGCTTTCTAGCA

							ACAGAAAAGTCAACCTGAGATTGGCACACACATTTGGCCAATGGGTTTTAGCACATGAAATTTGTTCTT<br>ATCCCTTAATTCCACTCAATTCTGTGGACGGCAGTAAAGCTTTCTGTGGGGCTCCAGCTAGGACAGTAA<br>TTCTCAACCTGTAGATCATGAGCCCTCTGGGGAGTCAAACAACCCTTTCACAGAGTTTGCATGTGAGGT<br>ATTTACTTTACAACTTATAACAGTAGCAATATTACAGCTATGAAGTAGCATCAAAAATAATTTTATAGTTG<br>GAGGGGTCACCCAACATAAGTAACTGTATTAGAGGGTCACAGCACTAGGAAGGTTGAGAACCACTAGA<br>CTAGAGGGTGGGTCCTGACACTTCCTGCCTGCTTTTTCTGCTTTACTCACTTCCCTTCCCACTAACTCTA<br>TAATGTGTCTGCTTTTAGAATCTTAACTTCCCTGGAAACTGCCCCCAGACTCAGCAGCCAGAGGGGCTG<br>GCTCACTCCTGACCACCTACCTACCTCTTGGACCCAGAATCTGAGGATCATTCCTGAACACCCACAATG<br>TACTGCTCTTGTTTGAGTTAAGAGCTGCTATTCCCGGGGCATCTCCTGAGGCACAATTTCTGGGGGCCCT<br>GGTTCCCTTTCAGTGAACCCCCCAAAACCTGGAGAGATATGGGAATGCAAACCTTCTGTGCTGGTGGG<br>AAATATTCTCAGGCAATTCTGTTCACTGTCGAATTGTAACGGGGTTTTGGTTGATCTCCAGGGCCCACTG<br>AACCAGACAGTTAAGGCCGATCCTCCTGACCAGCCATCTGGCACTTTGCAGCTGCCTTTGTTTGCTTTC<br>TGACCAGTGCTAATTGATACTTGAAGGAGAGGTTGTCCGTGTTGTCTTCTCTTTATTTATGATGAAACAT<br>ACACGGGAAACCTCTTTTTTAGTATCAAATCCCACCTGGTAGGCACTCCCTGTCCCTGATGAAGCCTTC<br>AGGGAAGGGGACCTGGACTGCTATTCCCTGGACTGTGTGACAAGAAAAGTCATTTGAAGAAGGGCCA<br>GGGTCCCTGTGGTGACTTGAGGCTGCTCCCTGTGGCTTTGAACTGTGAGGTGACTCTTGGTGTGTGATG<br>GCTTTTCAGCAAGGTCTTCCTCACAGTAGCCATAAGGACACGCCAGCAACGTGACTGAAGGGACAGTG<br>GTGCTTACTGCTCTCAGGGTAGCTGTGAGGATGGTAAACCGGTGACGTGCACTTCATTTATGCTGTAGG<br>TCACCCGTTTTACTATCCGCCAGCACCCAGACCGTCTGTGGGATGATACCTTGGTGCACACGGCAGCTA<br>TGAAGAAGATATGGGTAGGGTTGAGGCCATTGTGAATCTGAAATACTTCAAGTGGAGTTAGCTTTTAAG<br>GGGATCTTCTTCTGCTTTTGTGTGTGTGTGTGTGTGTGTTTGTGTGTGTGCGCGCGCGCACGTGCAGGC<br>CAGGTGTGTCATGTGTCTTCCATAACTACTTTCTGTTTTTTTTTTTTTTTTTTTTTTTGAGATGGTCTCTTG<br>TTGAACTTGGAGTGCACTGATTTTTGGCTGGCCAGCAAACCCCAGGGATCTGCTTGTTTCTGCCTCTCA<br>GGCTCACAGATTACAGGTATATACATACACAGTGCTTGCCCTTTCGACATTGAACCCGGATCCTCATGCT<br>TGTGTGGCCAGAGCCATCTCTTCACGCCAACTCCTCTTCCTCCTACCTTTCAATCCATAATGTCACATTCT<br>GCCGTAGGTGGGTGTCAGGCTGACCACAGATGGATTTGAGAAGGGTTGCTCCTATCAGGATCCATCGGG<br>AGAGTCTGTCCTCGTCTATGCTAATATAGGGTGTGTGAGGCACCTGCTGTGGTCCACTAAATCCTTGGTT<br>GGGCCCGGGGTGTTGTGTATAAGCCAGCACCTGTGAACCTGATGCCAACATGGCTCTGTAGTACTTAAA<br>GAAAGCTATGTGGTGAGCTTCCCATGGTCGTGGCGAGCATTTTATGATCCATATCTTTACAGCGAAGAAC<br>ACCATCTGGAGGATCTCCTGAATGTGAAGCTCTAACCCAAGCAGGGCTCATTACCCCCCGGGGTGAGTG<br>AGTGCTCTCTCTAGCCCAGCATCAAGCCTACATCTGCCACTTATGGAAGAGGCTTCCTTGTTCTTGCTCA<br>TCTCCATGTCTGCTCAATTTTGCTTTTCTTCTGAGGATTAAACCAATTGGCTGCTTCCCAGCACAAGTTC<br>CTGCCTAGGAGATGTATTCCAGATGTGGCATTTTCCTTAGGGAAGGACACCACAGTCCCCATCGATGGA<br>AAAGGTATGTCAGGGTGTTTTGTCTGAAAGAATATATTGCATTTTCATGTGTGTCCTGCTCTGCTAATGCT<br>AAGTATGCCTTCCATACCTAGGAGGACCTCCTGATGGATGAATACCTACCCTGGGGGTGAAGGTGGCAC<br>CTCCCCCCAGGCTCCTGGCAAGTGGTTCTTACAGGCCTGGCAGAGACACAGAAAGGAAGGCTCATATG<br>TCCCGTTCAAGACAGCTTCAGGATGATGGATGGGCGCTGTCCTGGAATCTGCTCTTTCCTCTCGTGCTG<br>GTGCTTCCCAGTGCTCTCTGCAGAAGAGGACCCTGGATCTGAGGAAGGAGATTCTGTCTTGGAGTCCAT<br>CACCATCTGATTTCTCCTGAGTTCACTCCAGGAACAGTGGCAAGGATGGTAGCCAACATCTTAGAATCA<br>TCAACCTTGATGACCAAGTTCTACGCACCAGGACTTCCTGGGATCCTGAGGTAGAGGGGGTTGGGGCT<br>GGATTGCTATGGCGATGATAAGTACTGAGCCATTCCTCTCTGCTCTGGGACGCCCCTACCCTACGCCTGA

							CCTCTCCCAGGCCAAGACAGTATCTCCCTCACCCACCACCCCTCCTGCCTGCCCTTCAGCATCCCTTAA<br>CACTCAGACACACTGCTGGAACAAGCAAGAGCTCAC
MSTRG.109442.5	--	0.167	2.061	0.000	4.369	up	GTTGCAGTGATGGACAGATCCTGGGATGCCATGGCAACCGTCCCGAGCCCATGGGGACTTGACAGCTC<br>CAGCTCTCATCCTCCCGTGGGCACCTGCTGATAGAAAAGCTGACCCAACAGATGGTACCATCATCCTCA<br>GATCTGGAATCAACCTTGCTTCCTTGCTGTGACAACCCCGAGCTCTGCTGACAGAGAGGACATCTTAAT<br>CTTCCTCGGTCCAGAGTTTATAAAAGAAAACCCTGTGATGGCCAAGGTTGTAGTGATGAACAGATCCTG<br>GGATGCCATGGCAACCGTCCAGAGCCCATGGTGACTTGACAGCTCCAGCTCTCATCCTCCTGTGGGCAC<br>CTGCTGATAGAAAAGCTGACCCAACAGATGGTACCAGCATGCTGAGATCTGGAATCAACCTTGCTTCCT<br>TGCTGTGACAACCCCGAGCTCTGCTAACAGAGAGGACATCTTAATCTTCCTCGGTCCCGAGTTTACAAA<br>AGACCCTGTGATGGCCCAGTTTGCAGTGATGTACAGATCCTGGGATGCCAGGGCAACAGTTCCAAGCC<br>CATGGTGACTTGACACCTCCAGCTCTCATCCTCCTGTGGGCACCTGCTGATAGGGTCTGGAGCTGGTTT<br>CAGAATTCCCTGTGTTTAAGGACCGCATCAAGAATGTGTGCTCCAGGGGTGATTTCTGCATTCTTCATGG<br>TCACCAGTGGAACGTTTGCATCTCTGCGTTGAGCTTGGGGCAGTATGGACCGTGTGATTAGAAGATCGT<br>CCAAGGGTCAAGGGCTCCATTTGCACCCTGTCGAGGAGGCGAGGAAGACGAGTAATGGACTTTCCCAA<br>GAAATCTATTCATCTTCAATGGCTTCCACCCCATCAGTTGCTTGTGATGATGTGCGATCTGCCCAAACTT<br>CTTCTCATAGTTTTGATGGTTTGTAGAAGCTCCTCACTGACTCAATGCCCTCCATGGCTAGGAGCTCTGC<br>TGACATTGTGGAAGCTGCTGGGCAGACCACTGTGAGTAATGGCTTGAACTATTTTTTCAGGTTTGGCGG<br>TTTGCTTAGTGGAACTAAATGTGTGACTGCGAAATACAACATGGCATTAAGAATGGGTGCTCCATACCA<br>ATGAGAGCCACAAACACGAATGGAAGTTGTCAAATTATTTATGAAACTCTGAGGATGATTAATTAAGAT<br>CAGAACAATTCCCCAGAGATTAGGAGTGAGGTTTTATAGAATTGTTGGAATAATAGGTGCTCAAATTATT<br>ATTAGCTGATTATTGAACTAATTATCAGACAATAGAAGAGGAGTGATTGGAAAAAGAAAGAAACAGCCA<br>TGATTGAAAATGATCACTGTGAAACATGAGTCACATGGCTGACTACATGTCTGGAACTTTGAGAACTAC<br>TGAAGAAGGAATATAATCCATTCTTTTCATTTCAAGTGTTCTTGTAGAAAGACTACAGGACCATATAATA<br>GGTTCATTTTCCAGTGTTCTTATAAATTCCATTCCATTTTCATAATTGTATCAATACAAGAATGTAGCAATC<br>AGTCTTACAAATAAGTGGAGAGGAAATGGGTAAAGCATTATCCAATTCTCCAAAATATGAAGAGAGCCA<br>AGAAATAGATTAGTGTGCACAGAATACTGTGCCACCTTAAAAAGGGGTTTTTTTTTGCCCATGATGACAT<br>TTATAATGTAAATTACAATGTCTTCATTCTGAGGATGTTTGAAAATTCCATCTCTTTAAGCAATCAGAACA<br>TTAAATATACTCCAAAAATTAGCTGGCCTAGTGGCCAGTGGTAGGTGCGCAGTGTAAGCCATTACAGGG<br>AGAGCCTTGATTTTGTCATCTTCTCTGTTCAGTAGCCCATAGAATGGACACACTGTGTGACGGGAGCATC<br>CTGCATGATTGTGGCCTCTTTCTGCTGCTCTGATTCTGAATCTGAATTGTTTTGCTGAAGTTAAAAAAAA<br>AAGATTCAAATTTTGGTATTGAATGATTTACTTAAGAATACTCAGTGCCTTTTGCAGAAAGGTATATTAGT<br>GCCTGCCTTTAGTACCAGCCTGTTATAGGTAGAGGCCTCTAGAGGCAGATCTCTAAGTTCAAGGCCAGT<br>CTGAGCTCCAAAACAAATTCCAGGACAGATAGAGCTACACAGAGACACCCTGTCTCGAAAAACAAAA<br>GTTGAAACAAACAAACAGTATTCAATACATCATTTAAAAGTACATAGTTAGTGGCAACCAACTGTATCTC<br>GTTGATTGATCATGGATGCATATTAAGTCTTTTATTGATTATACAGTTATGAAAACCTGAATCAATTACTCT<br>CAGTAAAAGAATGCTCTCAATATAAATTAGGAATATCAAGTGTGTTCTTGGCATTTCATAAATCATAGGAC<br>TTTGCACTTTTTTCTATCTCTGAATAGGCAAGGTATAAATCAGTGAAATAGGTGGTCAACCTTGAACCAA<br>TGACTACTGCTCGTAGAGTATGAGTATGTACAACGAACATACTTACATACTCTGATGAACATCACAAAGG<br>TTGCATAGCCCCCTGTCAGTGTTTGAAATGATCGCATAGCTAGTTACCAGAAATGTAGCAGAAAAAGAA<br>CAGTATAGCTCTTTTGGGGTTTTGTGTTTATACATTTTCATGATTGGTCAATGTGGGAATAAATAAACATAT<br>AAATAAAAACAATGACTATAGTTAGGCATTGTCCATTCATAAAAACAGATGAGGTGGTAAGAGAATTATG

							AATATACAGTGAATGTAAAAATCTAAAGAGAAAGTGACTCCTCTTACCTCTGTGGTTAATGATGTATAGA<br>CAATATTATTAAGGGAGTATTCCAAATGTTACAGTAGCTAGTTTGAAATTATTTATCTTCTTATAAAAGACT<br>CAAATTAGTGATTTTCTAAGTCAAAAAGTTACAAAGCTAGAATGAATAGTGAGAATATCATACTTGCCTC<br>ACATTCTGTGTCATATTTGTCTCCGAGTGAAGGAACGAGTCTGCCATCATCAAGGGCTCTGCACTTTACC<br>AAGAAGTTCATGTTGAATGTGAGTTGAAACCAGCATTCTTTTTTTTTTAAATTGAATATTTTATTTATTTAC<br>ATATCAAATGTTATCCCCTTTCCTGGTTTCCCCTCTGAAACCTCCCTGTCCCATCCCCCCTTACCCTGCTT<br>CTTTTTTTTTTTTCTTTATTTTTTTATTAGATATATTTCTTTACTTACATTTCAAATGCTATTCCCTTTTCTGG<br>TTTCCTGACCATAAGCCCCCATTCCCTCCTCCTCTCCCATACGGGTATTCCCCCATCCATCCCCTTTACTG<br>ACCCCCCAAATTCCCTTGCACTGGAGGTCCAACCTTGGCAGACCCAAGGGCTTCCCCTTCCACTGGTGC<br>CCCAACAAGGCCTTTCTCTGCTACATATGCAGTTGGAGCCCTGGGTCAGTCCATGTATAGTCTTTCAGTA<br>GTGGTTTAGCCCCTGGAAGCTCTGGTTGGTTGGCATTGTTGTTCTTATGAACCCAACATTCTTAGTTTTG<br>TTTAAGTGGTTTGCTTACGGTAATCACTGTACTATTACAAAGTATATAATACCCCTAACAAGTGGCCAGCA<br>TTTTGCCAGTGGCTTGTAAGGAGCTGATTCAAAATGCTCTACTAATAAGGCAAACATAAAAAGTGGAAG<br>ATAGTTGATTAATAGATAAACCAATGCTGTAGTAGCTCTGTATGCAAATTAGGAGTAGAGGGGCACTTCT<br>TCACCTTATGCAAGTCATGGAGCTGACATTGGAAGGGAGATGATAGCTTACTCAACAGACAGATGAATG<br>AAGAAATAAAGACTAGACTTTGATCAATGATGACTCCGATGTGGGCATCATGAATGAATCCATATGTGGA<br>ATCCTCATGTTCAACCCCAAAGATGAGCAGCACATTCTTTGTTTCATATGATCCTCTTGACATTACGTTTT<br>AGAGTTTGTTTTTATGCACTTTACTACTCTTTTATAATTAAACCATTGTAAGAAATAGTAAACAAACTAAA<br>AACATAAATTTGGCGATGTCAAAATTAAAATGTATGACAAAGCAAAAATACCATCGGTAGCTATTTAAAT<br>GAGTATTGCTGGTATTGTTCGACTATGGTGATGGCAACTGTACAAAATATTGAACTTTTCTACTCTTGTGC<br>TAAGAGGGAGTTCAAAATTTTTCATTTTTTTCTAAGCACTCTCTCAATTAATGATTGTTAATGAAAGGTG<br>AATGCAATAGTCAATGATGAAGAAAACTAGCATCTTCTTGTCATGGATTCTGTTCTTTGCCTCTAAGAGT<br>AGACTAATGGAAGGAGAATTCCTTATCCACACCTGGAGGCCATGGTACGTATTACACAACATGTTCGTCT<br>TTGTTTGTATAATTTTTTAAAGCTGTTTTCAAATATCCTATCTATGTTGTGATATTATTCTATGTCATTATTTG<br>ATGGCAGCAGACCAGTTTCTAATGTGTGATGGACAGGAAAAACACGCAGGCACATATGTGGAAAGATG<br>AGCTCTGTGATAGTGAGAATAAGTCTACAACTGATTCTAGCAAAGGTAGTTATCCAATGACAGACCCGA<br>AGATCTTAGGTTTCTGTGATCCGTACGACTTAAAAGTTGTCAATGGTGAATTAATGAGTTAATAACTAAA<br>CAGGGTAGTAATGAAAAGAGACCAGTGTTCATCCTAGGTTCATCAAGACTCTGTGTCATCTGAGCAACG<br>AATTTGTATACATCTGGAGCTTAGATCCTCCATAGATGAAGTGCGCCATCACCACCTCTCGAGTCATATTG<br>TAGATAGGGACCAGGGAGAGTGGGTTTCATTTTTGCTCTTGTTAAAATGCATTTTGAAGCTAACGCTACA<br>AGCAGTTCAAGGAAAGAGCCAGTAACCAATTATATAGTTACTAATGGATAGAAATTAACCTTATTCAACT<br>ATAAGAATAACATTTCTAGACAATAAAATCAGTGCTATGCATGAGGTGGAAGACCAGTGACAGAGTCTG<br>TATTTGTGCCACCACTGATACTGTTTGTTTAAACAAGAGGATCAAATAAAAGCCATCCCAAATTGCCTAA<br>ATGACCGACTAATGAAAGATTGGATGCAGAGACCAATGCAAATGAGGCTGACTCTTGGTAATGATCTCC<br>TTCACTCTGTATTCCTGAAAGAACAGAGTTGTGCTGGCCCATTTTTCTTTGGTCAGACCTGTGGGTGGAT<br>CTCATATCCTGAGTGTGAAGAGCTCTGTGAGTAGTGTCAGTTAAGATTGTGGTCTTGGTATTGTTTTTTT<br>ATGTAATTATTTACTTTTAAAATATTTTTGTACACTCAACACACTTGAATTTTTTCTTTCTTTTCCTAACTTT<br>CACATCTTTGGAGTTGGTTTTTTTTTTTTAAGCCATTCACAGAAATCGATTTTTAAAGTGTGACTTAGAAT<br>CATTGAGAAGACACTTTGACCTACTGTAAAAATTGGCTTAATATAAACCATTGTTACCTCGATCTGTGTC<br>TGTATATAGCACTGAGGTTAAATCAGAGTAGAGCTATTGTTATAATAGGAGTTATGCAGAAAGGTGTTAG<br>TGTCATCCAAGGATCATAAGTTTTTGTGATTCTTATGTGATTGTTTATAGTAGATGAACAATGCAGCCACA

							TGGTCAATAAAAGCACAAAGACGTGAGTGAAGAGCACAGGAGCAGCCTGAGCTCCTGAGGGTGGTGC<br>TGGTAGTGTAGGAGTTGTAGACTACAAAGGATGTCTGGCTCATGCACTTCTGACACAAGAGGAATCTCA<br>GTGCTCTCTCGGGGGAGAAAATATGGAGAAAGGTCTTGAGAGCTGTGCATGGCTCCCTGGTTTGGGTTT<br>ATTAGAAATGAGTCCATGAGAGATAAGTATGACAGAGGAAGCCACACTCTAACTCCTAGCTCATGTTTC<br>ACTGGATGAACACTGACAGCGTGTGGAAGGGATGATAAGTGGACTATCAGGCTTTGTTCTGTTTCCAGG<br>GGTCCATAGCTACCGATGGCTAGAGGAGAGCATTACTCTGGGCTCTTTGTGAGTTCAGAACTCATTTTAT<br>CATATGGCAAATAGCCCATCATTACCATGAAGGATCACTAAGTGCTCACTAGACACCATCGCAACGTTAC<br>AGAGTCTCTGTCACCTTGGCAGTGAGCAATGTGCCCGTGGTTGTGTTATCTTTGCCAGTGTCTTACATGT<br>GTAAAAATAGTGAGTTTATCATCGTAAAGAGTAAATGAACAAGGATAAAAATAAATAATGAAGAAAAAA<br>AACCCATGGGGACATAATAGTATTTCTTTAAATAAGTTAACAGTTTATCCATTTTTTTTCTCTTTTGGTTTA<br>AAATTCTATTTTAATTTCCTGGGAACCTAATATGTCTTTAAATGTTTTTATTTTCATATATGTGTGCTGTATT<br>TACATCAATCCATCCCTGCACTTAGGCATTACCGTATGAAAATGTCCTCTTTCTGTTCTGCAGGAAGCTG<br>TACCACAGTCGATCAGAACTAATCATGAAGGCCTCTGCCCTCTGGAGAGAGAGAATATCATGGAAGTAG<br>AGTGGATGCACACCTTCTCCTCTCATCATCGCTACTTCAAGATGAAGGTCGCAACACTGACTGTATGGC<br>CCTGGCAGTAGAGATCTCAGTAAGTAAGAGACCGTGTTTCCTGCCTGTTTTTTTGTTACAGAGATCACT<br>GTCTATTTAAAATTGACATTATGTGTCATATAGTCAAAGACAACTACACATATCCTAGTGCTCAGGGGGTC<br>CAGGGAAGCCAAAGTCAGTCAGTCTGCATACATGAGGCCTACACACAGTGGGTTTGCACTACATTCTG<br>AAACTGAGATCACAGACACACGCTAAGTGAGGCAAAGACTATGTTCATGATAAGAAAGGGACATAGAA<br>GAAAGGGTCCACATAGCACCCATCCTGGAGTTGTCATAAAAAGGAAGATTGGTTTTAATGGGGTCATGA<br>AGTGTGCTCCACTAATGCAGTCTCTAGCAAAATGGAGAAAACCAACTCCCCCTGTTGTGTTTGGCTGCA<br>GAATCTTTTCTGTTGATTTAATTTTTGAAAATGAGTGACTAAATTAATCAGGAAAAAAAAATCCGTGCCC<br>AGAAATGTTAAATGATGGAGATTATCCCCATTGAGAACACTGGCCATGAAAATTAAGATTTATGATCGAC<br>TGTTTTTTTTTTTCTCTTAAACCCATTTAACCAAAAAGATTTAATTGGTTGCATTTGTGTATAAAGAACCC<br>TCCCCAAAACCACCTTCAATACTCATTCTTTTTCATTTATTTTGTACTTTGATGAGTTCCTGAAACCATAG<br>TTATCATCATTATTTAGTAAACCAATCTTTTAAAAAGTAAATTTGAGACAGACCAGGTGTGGGTCGATAA<br>TTTACATGTTTTGTGGAAAGTAGTATGATTACGGTTCTTAGTCAGTGATGTCTACAGAGGGAATTTAGTC<br>CCTCCTGTCTTTGTTTTTATGGAACATGGGGCACAAGGCCACTTTGTATGAACTAATAAGGTGCATTTCT<br>ATTCTCTCGTGGCACTTACGTGACCTATCGTTGTCTATCTCCAGCACAAATTCTTGTTTGCAGGTTGATTA<br>GTAGGCTCAAATACTCCCCGAGTATTATTTCCTGGCGCTCAGTCTCTGAGAATGGTAAAACTGCCAATAG<br>CTGTTGCTTTTCATCCTTGTCAGACCTTGTGTCAAATACGGAGTTCATATAGGAGCCCGACCCCGAAACT<br>CTGGGATTAGATAATCCCAATTTACAAACACTGTAACAGGACAAGTGCATGTGTGGCCTAGAAATAAGG<br>AGTTGGCATTCTCTGCTCACTTTTAAGTTTGGGATGCTTTATGAGGAGGGGCATTACGATGCTGTGAGGT<br>CTTTCACAACTTCAACTCACTGCCAGAAGCACATGCGATGTTTGAGTGAGTCAGGGAATGAACGATGA<br>CCAAAAGAAGGAATAAACGGAATAATGCCTGAAGTAAAGATCCCTTGAGGGGGGACAAAAAAAGGTA<br>TGAAGTCATGAAAACATGGAGGTCTGTGCCAACCGAGGATAATAAAACACTAACTAATGACTAAGTTAC<br>AAAGCAAATGAGTCCATGGCGAGTCTGTTAGGGGATGCGTGCATAAGCGGGCCAGCGTGGCAGAAGGG<br>AGCAATGAAAGAAAAG
MSTR<br>G.109<br>442.7	--	0.240	0.767	0.005	2.430	up	GTTGCAGTGATGGACAGATCCTGGGATGCCATGGCAACCGTCCCGAGCCCATGGGGACTTGACAGCTC<br>CAGCTCTCATCCTCCCGTGGGCACCTGCTGATAGAAAAGCTGACCCAACAGATGGTACCATCATCCTCA<br>GATCTGGAATCAACCTTGCTTCCTTGCTGTGACAACCCCGAGCTCTGCTGACAGAGAGGACATCTTAAT<br>CTTCCTCGGTCCAGAGTTTATAAAATATGACACTGTGATGGCCAAGGTTGCAGTGATGGACAGATCCTG

							GGATGCCATGGCAACCGTCCAGACCCCATGGGGACTTGACAGCTCCAGCTCGCATCCTCCTGTGTGCGC<br>CTGCTAATAGAAAAGCTGACCCAACAGATGGTACCAGCATGCTGAGATCTGGAATCAACCTTGCTTCCT<br>TGCTGTGACAACCCCGAGCTCTGCTAACAGAGAGGACATCTTAATCTTCCTCGGTCCCGAGTTTACAAA<br>AGACCCTGTGATGGCCCAGTTTGCAGTGATGTACAGATCCTGGGATGCCAGGGCAACAGTTCCAAGCC<br>CATGGTGACTTGACACCTCCAGCTCTCATCCTCCTGTGGGCACCTGCTGATAGGTATGGAGGGTCCAAT<br>CACAACATGGGGGTTCAGCAGAGTATAGACAGAAAGAGTTAAATTAGTGCTATTTTGAGGACCAAGCA<br>GTGTGATGCCACACTTGCCAAGCAATAAGCCAATCCTGGGAGAGAGGAACCCAGCCTTCCCATGCTAG<br>CCTCTGTAGTATGAATAGGCACAGCAGGATCACTCTTGCTCTCTACACTCCTGAATCTGATCAGTTTTCC<br>TTAACAAGGAAACACGGCTGGCAGACAGACAAAGTTGGGTTAAAGTAGATAGAACCACAAACTTGCAT<br>CGGGAGTGACTCACAGCTTGGAAATTGCAAGGAACATATTGAGAGATGGTTGTACTGACATTTGACCCC<br>ATGCATGATGCAGTGGCATAGACTGGGCCCACCTCTTTTTCCCTTCAAATAACAAGAAATTAGTCTTTTC<br>TGGGAAGAAAAGGTACAGGTGCAGCCCAGGCGAAGTCTGTGATTCTAAGTTCTCCAACTGTGGAGAGC<br>ATGGAGTGGTGATTCACAGTCTCAGCAGTTTCTCTCTCTTCATTGCCCTTTATTCTTCAGAAAGGCTGAC<br>CTGGTTGATGCAGAACCACCATGGCCTCCCTGCCTGTCCCAAATTAGCATTCTGTCTTGTGTGGAGCCT<br>GGTTCAAGGTCTTAACTTTTAATTGTCAGTTGTTGTAAGAATTTACATGTTTGTTGTAAAATTTACATATT<br>TGTTGTAAAATTCACATGTTCATCAATTGTGAAACCAAATCAGTGAAGTGTAATTCGGTACGTGGATTGC<br>CATTGATCAGATTCAGTGTAACATTTACACTAAGTTCATTCTAAAGGCTCAGTACACCCAGTTCCATGGC<br>ATGAAAGTCTGCTTATGAGTCTCGTGAGTCGACGGAATTTTTGGTCTGATAATAGGACTTTTCATCATGT<br>CTTTCCTTTTTTTTCTGCTTCTCGTTTTCCCATAAAGCTGATCGGTTTATGTTTGCAGACATTTTATGTTAT<br>AGATACCTCCTCTGATGTCCCTTCAACACCACTACAACTAGTATTCACTTTAGTGTCATTTTGTGCAGGG<br>CATGGTAAAACCACGTAGTAATATGGCAGAGTTCTTCATATGTGGCGTGTGTAAGATTAATAATATAGCAT<br>TAGTTTTAACTTCAGAATGCAAAACCAAATGCTTTCACGTAGGTTTTCATTTTTTTTCTATTTCATATTAA<br>ATGTATCATTTCCATTTTAGGTTAGTTTTTTTCCCCTGGTACATTTATGTTATTTATGATAATAATCATGTTTA<br>GCATATCACTACCTATGCATATCATATTTCGTATTAATATTATCATTCCTATTAATAACCACTAAGTTCTTCTA<br>AATGTTAAGCCACACTCAAATGTTCCCATACCCGACTTAGACTGTCCATGATAACGTTGAGAAAGTGCA<br>ACATGTATTCAGGGCCATGAGTCATTCATGATTCTTTCACAGCACACATTCTGCAAATAGTTAGTGCTCAT<br>CAATCAGTAAAGTAGCAACGTCATATGAGTCAGCGCTTCCGCCTGGGGGAAGGGGTAAGGAAAATATG<br>GTGACGTGAGTTCTTACCACCCTGTCCTGAGTCAGATCCATATAGAACAGTTCCTTACCATTTCTTCTTTT<br>ACTAATGATAATGCAAACATTACATCTTATAGCTGATGTTTATGTTCTGGATAGTAAAGAATTCCTCATTAT<br>TTAGTGAAGAATTAGATATATCACTGTGTAAGTCAAAATTGAAAACCATTGCTCTTTTATCTGCTTTTAAA<br>AGTCATTTTTTCAACTTCTTGTTTGTGTTTTGTCTGTTGCTCCCCACACAGGGTCTGGAGCTGGTTTCAG<br>AATTCCCTGTGTTTAAGGACCGCATCAAGAATGTGTGCTCCAGGGGTGATTTCTGCATTCTTCATGGTCA<br>CCAGTGGAACGTTTGCATCTCTGCGTTGAGCTTGGGGCAGTATGGACCGTGTGATTAGAAGATCGTCCA<br>AGGGTCAAGGGCTCCATTTGCACCCTGTCGAGGAGGCGAGGAAGACGAGTGTGAGTTACCGGATTCTC<br>CTTGTCTTGGTTTGAGTGTTTTAATGTAGTGGTTAAGCAGTGTATAGTGTAGGTAGAGATGACCATTTGA<br>GTACCAGTGATAGATAGGGCGACTACATGACATTGTCCATGAGTAATTCAACTATGTTAACTAGAGTGAA<br>TTGTTATATGATGGACATATTATAAAAATCCCATTTCCAGATTACGAATTACTAGACTCAGATTCAGTTTTT<br>TCGTCAGTAGGAGTTACTAATCATAGGGTTTATAACTAAGTAATTAATAGGGAACGTAAAAATAATTAAG<br>GAGACAGAGAGCTAATCTTGGTTGATGATGACAAACGGTGGCATTTGAATCATCCATGATGACTATGAG<br>TCTGAAAACCAAGATTCAATAGAGAAGATAAGTGGCTACATGAAGATTGGTAGCAATGGTGTCATTGCT<br>TTGTCCTCTGCTTTGCTCTGTGTTCCTTAGAATGGACTTTCCCAAGAAATCTATTCATCTTCAATGGCTTC

							CACCCCATCAGTTGCTTGTGATGATGTGCGATCTGCCCAAACTTCTTCTCATAGTTTTGATGGTTTGTAG<br>AAGCTCCTCACTGACTCAATGCCCTCCATGGCTAGGAGCTCTGCTGACATTGTGGAAGCTGCTGGGCAG<br>ACCACTGTGAGTAATGGCTTGAACTATTTTTTCAGGTTTGGCGGTTTGCTTAGTGGAACTAAATGTGTGA<br>CTGCGAAATACAACATGGCATTAAGAATGGGTGCTCCATACCAATGAGAGCCACAAACACGAATGGAA<br>GTTGTCAAATTATTTATGAAACTCTGAGGATGATTAATTAAGATCAGAACAATTCCCCAGAGATTAGGAG<br>TGAGGTTTTATAGAATTGTTGGAATAATAGGTGCTCAAATTATTATTAGCTGATTATTGAACTAATTATCAG<br>ACAATAGAAGAGGAGTGATTGGAAAAAGAAAGAAACAGCCATGATTGAAAATGATCACTGTGAAACAT<br>GAGTCACATGGCTGACTACATGTCTGGAACTTTGAGAACTACTGAAGAAGGAATATAATCCATTCTTTTC<br>ATTTCAAGTGTTCTTGTAGAAAGACTACAGGACCATATAATAGGTTCATTTTCCAGTGTTCTTATAAATTC<br>CATTCCATTTTCATAATTGTATCAATACAAGAATGTAGCAATCAGTCTTACAAATAAGTGGAGAGGAAAT<br>GGGTAAAGCATTATCCAATTCTCCAAAATATGAAGAGAGCCAAGAAATAGATTAGTGTGCACAGAATAC<br>TGTGCCACCTTAAAAAGGGGTTTTTTTTTGCCCATGATGACATTTATAATGTAAATTACAATGTCTTCATT<br>CTGAGGATGTTTGAAAATTCCATCTCTTTAAGCAATCAGAACATTAAATATACTCCAAAAATTAGCTGGC<br>CTAGTGGCCAGTGGTAGGTGCGCAGTGTAAGCCATTACAGGGAGAGCCTTGATTTTGTCATCTTCTCTG<br>TTCAGTAGCCCATAGAATGGACACACTGTGTGACGGGAGCATCCTGCATGATTGTGGCCTCTTTCTGCT<br>GCTCTGATTCTGAATCTGAATTGTTTTGCTGAAGTTAAAAAAAAAAAGATTCAAATTTTGGTATTGAATGA<br>TTTACTTAAGAATACTCAGTGCCTTTTGCAGAAAGGTATATTAGTGCCTGCCTTTAGTACCAGCCTGTTAT<br>AGGTAGAGGCCTCTAGAGGCAGATCTCTAAGTTCAAGGCCAGTCTGAGCTCCAAAACAAATTCCAGGA<br>CAGATAGAGCTACACAGAGACACCCTGTCTCGAAAAACAAAAGTTGAAACAAACAAACAGTATTCAAT<br>ACATCATTTAAAAGTACATAGTTAGTGGCAACCAACTGTATCTCGTTGATTGATCATGGATGCATATTAAG<br>TCTTTTATTGATTATACAGTTATGAAAACCTGAATCAATTACTCTCAGTAAAAGAATGCTCTCAATATAAA<br>TTAGGAATATCAAGTGTGTTCTTGGCATTTCATAAATCATAGGACTTTGCACTTTTTTCTATCTCTGAATA<br>GGCAAGGTATAAATCAGTGAAATAGGTGGTCAACCTTGAACCAATGACTACTGCTCGTAGAGTATGAGT<br>ATGTACAACGAACATACTTACATACTCTGATGAACATCACAAAGGTTGCATAGCCCCCTGTCAGTGTTTG<br>AAATGATCGCATAGCTAGTTACCAGAAATGTAGCAGAAAAAGAACAGTATAGCTCTTTTGGGGTTTTGT<br>GTTTATACATTTTCATGATTGGTCAATGTGGGAATAAATAAACATATAAATAAAAACAATGACTATAGTTA<br>GGCATTGTCCATTCATAAAAACAGATGAGGTGGTAAGAGAATTATGAATATACAGTGAATGTAAAAATCT<br>AAAGAGAAAGTGACTCCTCTTACCTCTGTGGTTAATGATGTATAGACAATATTATTAAGGGAGTATTCCA<br>AATGTTACAGTAGCTAGTTTGAAATTATTTATCTTCTTATAAAAGACTCAAATTAGTGATTTTCTAAGTCA<br>AAAAGTTACAAAGCTAGAATGAATAGTGAGAATATCATACTTGCCTCACATTCTGTGTCATATTTGTCTC<br>CGAGTGAAGGAACGAGTCTGCCATCATCAAGGGCTCTGCACTTTACCAAGAAGTTCATGTTGAATGTG<br>AGTTGAAACCAGCATTCTTTTTTTTTTAAATTGAATATTTTATTTATTTACATATCAAATGTTATCCCCTTTC<br>CTGGTTTCCCCTCTGAAACCTCCCTGTCCCATCCCCCCTTACCCTGCTTCTTTTTTTTTTTTCTTTATTTTT<br>TTATTAGATATATTTCTTTACTTACATTTCAAATGCTATTCCCTTTTCTGGTTTCCTGACCATAAGCCCCCAT<br>TCCCTCCTCCTCTCCCATACGGGTATTCCCCCATCCATCCCCTTTACTGACCCCCCAAATTCCCTTGCACT<br>GGAGGTCCAACCTTGGCAGACCCAAGGGCTTCCCCTTCCACTGGTGCCCCAACAAGGCCTTTCTCTGC<br>TACATATGCAGTTGGAGCCCTGGGTCAGTCCATGTATAGTCTTTCAGTAGTGGTTTAGCCCCTGGAAGCT<br>CTGGTTGGTTGGCATTGTTGTTCTTATGAACCCAACATTCTTAGTTTTGTTTAAGTGGTTTGCTTACGGTA<br>ATCACTGTACTATTACAAAGTATATAATACCCCTAACAAGTGGCCAGCATTTTGCCAGTGGCTTGTAAGG<br>AGCTGATTCAAAATGCTCTACTAATAAGGCAAACATAAAAAGTGGAAGATAGTTGATTAATAGATAAAC<br>CAATGCTGTAGTAGCTCTGTATGCAAATTAGGAGTAGAGGGGCACTTCTTCACCTTATGCAAGTCATGGA

							GCTGACATTGGAAGGGAGATGATAGCTTACTCAACAGACAGATGAATGAAGAAATAAAGACTAGACTT<br>TGATCAATGATGACTCCGATGTGGGCATCATGAATGAATCCATATGTGGAATCCTCATGTTCAACCCCAA<br>AGATGAGCAGCACATTCTTTGTTTCATATGATCCTCTTGACATTACGTTTTAGAGTTTGTTTTTATGCACT<br>TTACTACTCTTTTATAATTAAACCATTGTAAGAAATAGTAAACAAACTAAAAACATAAATTTGGCGATGTC<br>AAAATTAAAATGTATGACAAAGCAAAAATACCATCGGTAGCTATTTAAATGAGTATTGCTGGTATTGTTC<br>GACTATGGTGATGGCAACTGTACAAAATATTGAACTTTTCTACTCTTGTGCTAAGAGGGAGTTCAAAATT<br>TTTCATTTTTTTCTAAGCACTCTCTCAATTAATGATTGTTAATGAAAGGTGAATGCAATAGTCAATGATGA<br>AGAAAACTAGCATCTTCTTGTCATGGATTCTGTTCTTTGCCTCTAAGAGTAGACTAATGGAAGGAGAATT<br>CCTTATCCACACCTGGAGGCCATGGTACGTATTACACAACATGTTCGTCTTTGTTTGTATAATTTTTTAAA<br>GCTGTTTTCAAATATCCTATCTATGTTGTGATATTATTCTATGTCATTATTTGATGGCAGCAGACCAGTTTC<br>TAATGTGTGATGGACAGGAAAAACACGCAGGCACATATGTGGAAAGATGAGCTCTGTGATAGTGAGAA<br>TAAGTCTACAACTGATTCTAGCAAAGGTAGTTATCCAATGACAGACCCGAAGATCTTAGGTTTCTGTGAT<br>CCGTACGACTTAAAAGTTGTCAATGGTGAATTAATGAGTTAATAACTAAACAGGGTAGTAATGAAAAGA<br>GACCAGTGTTCATCCTAGGTTCATCAAGACTCTGTGTCATCTGAGCAACGAATTTGTATACATCTGGAGC<br>TTAGATCCTCCATAGATGAAGTGCGCCATCACCACCTCTCGAGTCATATTGTAGATAGGGACCAGGGAG<br>AGTGGGTTTCATTTTTGCTCTTGTTAAAATGCATTTTGAAGCTAACGCTACAAGCAGTTCAAGGAAAGA<br>GCCAGTAACCAATTATATAGTTACTAATGGATAGAAATTAACCTTATTCAACTATAAGAATAACATTTCTA<br>GACAATAAAATCAGTGCTATGCATGAGGTGGAAGACCAGTGACAGAGTCTGTATTTGTGCCACCACTGA<br>TACTGTTTGTTTAAACAAGAGGATCAAATAAAAGCCATCCCAAATTGCCTAAATGACCGACTAATGAAA<br>GATTGGATGCAGAGACCAATGCAAATGAGGCTGACTCTTGGTAATGATCTCCTTCACTCTGTATTCCTGA<br>AAGAACAGAGTTGTGCTGGCCCATTTTTCTTTGGTCAGACCTGTGGGTGGATCTCATATCCTGAGTGTG<br>AAGAGCTCTGTGAGTAGTGTCAGTTAAGATTGTGGTCTTGGTATTGTTTTTTTATGTAATTATTTACTTTT<br>AAAATATTTTTGTACACTCAACACACTTGAATTTTTTCTTTCTTTTCCTAACTTTCACATCTTTGGAGTTG<br>GTTTTTTTTTTTTAAGCCATTCACAGAAATCGATTTTTAAAGTGTGACTTAGAATCATTGAGAAGACACT<br>TTGACCTACTGTAAAAATTGGCTTAATATAAACCATTGTTACCTCGATCTGTGTCTGTATATAGCACTGAG<br>GTTAAATCAGAGTAGAGCTATTGTTATAATAGGAGTTATGCAGAAAGGTGTTAGTGTCATCCAAGGATCA<br>TAAGTTTTTGTGATTCTTATGTGATTGTTTATAGTAGATGAACAATGCAGCCACATGGTCAATAAAAGCA<br>CAAAGACGTGAGTGAAGAGCACAGGAGCAGCCTGAGCTCCTGAGGGTGGTGCTGGTAGTGTAGGAGT<br>TGTAGACTACAAAGGATGTCTGGCTCATGCACTTCTGACACAAGAGGAATCTCAGTGCTCTCTCGGGGG<br>AGAAAATATGGAGAAAGGTCTTGAGAGCTGTGCATGGCTCCCTGGTTTGGGTTTATTAGAAATGAGTCC<br>ATGAGAGATAAGTATGACAGAGGAAGCCACACTCTAACTCCTAGCTCATGTTTCACTGGATGAACACTG<br>ACAGCGTGTGGAAGGGATGATAAGTGGACTATCAGGCTTTGTTCTGTTTCCAGGGGTCCATAGCTACCG<br>ATGGCTAGAGGAGAGCATTACTCTGGGCTCTTTGTGAGTTCAGAACTCATTTTATCATATGGCAAATAGC<br>CCATCATTACCATGAAGGATCACTAAGTGCTCACTAGACACCATCGCAACGTTACAGAGTCTCTGTCAC<br>CTTGGCAGTGAGCAATGTGCCCGTGGTTGTGTTATCTTTGCCAGTGTCTTACATGTGTAAAAATAGTGAG<br>TTTATCATCGTAAAGAGTAAATGAACAAGGATAAAAATAAATAATGAAGAAAAAAAAACCCATGGGGAC<br>ATAATAGTATTTCTTTAAATAAGTTAACAGTTTATCCATTTTTTTTCTCTTTTGGTTTAAAATTCTATTTTAA<br>TTTCCTGGGAACCTAATATGTCTTTAAATGTTTTTATTTTCATATATGTGTGCTGTATTTACATCAATCCATC<br>CCTGCACTTAGGCATTACCGTATGAAAATGTCCTCTTTCTGTTCTGCAGGAAGCTGTACCACAGTCGATC<br>AGAACTAATCATGAAGGCCTCTGCCCTCTGGAGAGAGAGAATATCATGGAAGTAGAGTGGATGCACAC<br>CTTCTCCTCTCATCATCGCTACTTCAAGATGAAGGTCGCAACACTGACTGTATGGCCCTGGCAGTAGAG

							ATCTCAGTAAGTAAGAGACCGTGTTTCCTGCCTGTTTTTTTGTTACAGAGATCACTGTCTATTTAAAATTGACATTATGTGTCATATAGTCAAAGACAACTACACATATCCTAGTGCTCAGGGGGTCCAGGGAAGCCAAAGTCAGTCAGTCTGCATACATGAGGCCTACACACAGTGGGTTTGCACTACATTCTGAAACTGAGATCACAGACACACGCTAAGTGAGGCAAAGACTATGTTCATGATAAGAAAGGGACATAGAAGAAAGGGTCCACATAGCACCCATCCTGGAGTTGTCATAAAAAGGAAGATTGGTTTTAATGGGGTCATGAAGTGTGCTCCACTAATGCAGTCTCTAGCAAAATGGAGAAAACCAACTCCCCCTGTTGTGTTTGGCTGCAGAATCTTTTCTGTTGATTTAATTTTTGAAAATGAGTGACTAAATTAATCAGGAAAAAAAAAATCCGTGCCCAGAAATGTTAAATGATGGAGATTATCCCCATTGAGAACACTGGCCATGAAAATTAAGATTTATGATCGACTGTTTTTTTTTTCTCTTAAACCCATTTAACCAAAAAGATTTAATTGGTTGCATTTGTGTATAAAGAACCCTCCCCAAAACCACCTTCAATACTCATTCTTTTTCATTTATTTTGTACTTTGATGAGTTCCTGAAACCATAGTTATCATCATTATTTAGTAAACCAATCTTTTAAAAAGTAAATTTGAGACAGACCAGGTGTGGGTCGATAATTTACATGTTTTGTGGAAAGTAGTATGATTACGGTTCTTAGTCAGTGATGTCTACAGAGGGAATTTAGTCCCTCCTGTCTTTGTTTTTATGGAACATGGGGCACAAGGCCACTTTGTATGAACTAATAAGGTGCATTTCTATTCTCTCGTGGCACTTACGTGACCTATCGTTGTCTATCTCCAGCACAAATTCTTGTTTGCAGGTTGATTAGTAGGCTCAAATACTCCCCGAGTATTATTTCCTGGCGCTCAGTCTCTGAGAATGGTAAAACTGCCAATAGCTGTTGCTTTTCATCCTTGTCAGACCTTGTGTCAAATACGGAGTTCATATAGGAGCCCGACCCCGAAACTCTGGGATTAGATAATCCCAATTTACAAACACTGTAACAGGACAAGTGCATGTGTGGCCTAGAAATAAGGAGTTGGCATTCTCTGCTCACTTTTAAGTTTGGGATGCTTTATGAGGAGGGGCATTACGATGCTGTGAGGTCTTTCACAACTTCAACTCACTGCCAGAAGCACATGCGATGTTTGAGTGAGTCAGGGAATGAACGATGACCAAAAGAAGGAATAAACGGAATAATGCCTGAAGTAAAGATCCCTTGAGGGGGGACAAAAAAAGGTATGAAGTCATGAAAACATGGAGGTCTGTGCCAACCGAGGATAATAAAACACTAACTAATGACTAAGTTACAAAGCAAATGAGTCCATGGCGAGTCTGTTAGGGGATGCGTGCATAAGCGGGCCAGCGTGGCAGAAGGGAGCAATGAAAGAAAAG
MSTRG.109442.8	--	0.026	0.678	0.000	5.324	up	GTTGCAGTGATGGACAGATCCTGGGATGCCATGGCAACCGTCCCGAGCCCATGGGGACTTGACAGCTCCAGCTCTCATCCTCCCGTGGGCACCTGCTGATAGAAAAGCTGACCCAACAGATGGTACCAGCATGCTGAGATCTGGAATCAACCTTGCTTCCTTGCTGTGACCACCCCAAGCTCTGCTGACTGAGAGGACATCTTAATCTTCCTCGGTCCTGAGGTTATAAATGACCCTGCTGATGGCTGACGTTGCAGTGATGGACAGATCCTGGGATGCCATGGCAACCGTCCAGACCCCATGGGGACTTGACAGCTCCAGCTCGCATCCTCCTGTGTGCGCCTGCTAATAGAAAAGCTGACCCAACAGATGGTACCAGCATGCTGAGATCTGGAATCAACCTTGCTTCCTTGCTGTGACAACCCCGAGCTCTGCTAACAGAGAGGACATCTTAATCTTCCTCGGTCCCGAGTTTACAAAAGACCCTGTGATGGCCCAGTTTGCAGTGATGTACAGATCCTGGGATGCCAGGGCAACAGTTCCAAGCCCATGGTGACTTGACACCTCCAGCTCTCATCCTCCTGTGGGCACCTGCTGATAGGTATGGAGGGTCCAATCACAACATGGGGGTTCAGCAGAGTATAGACAGAAAGAGTTAAATTAGTGCTATTTTGAGGACCAAGCAGTGTGATGCCACACTTGCCAAGCAATAAGCCAATCCTGGGAGAGAGGAACCCAGCCTTCCCATGCTAGCCTCTGTAGTATGAATAGGCACAGCAGGATCACTCTTGCTCTCTACACTCCTGAATCTGATCAGTTTTCCTTAACAAGGAAACACGGCTGGCAGACAGACAAAGTTGGGTTAAAGTAGATAGAACCACAAACTTGCATCGGGAGTGACTCACAGCTTGGAAATTGCAAGGAACATATTGAGAGATGGTTGTACTGACATTTGACCCCATGCATGATGCAGTGGCATAGACTGGGCCCACCTCTTTTTCCCTTCAAATAACAAGAAATTAGTCTTTTCTGGGAAGAAAAGGTACAGGTGCAGCCCAGGCGAAGTCTGTGATTCTAAGTTCTCCAACTGTGGAGAGCATGGAGTGGTGATTCACAGTCTCAGCAGTTTCTCTCTCTTCATTGCCCTTTATTCTTCAGAAAGGCTGACCTGGTTGATGCAGAACCACCATGGCCTCCCTGCCTGTCCCAAATTAGCATTCTGTCTTGTGTGGAGCCTGG

							TTCAAGGTCTTAACTTTTAATTGTCAGTTGTTGTAAGAATTTACATGTTTGTTGTAAAATTTACATATTTGT<br>TGTAAAATTCACATGTTCATCAATTGTGAAACCAAATCAGTGAAGTGTAATTCGGTACGTGGATTGCCAT<br>TGATCAGATTCAGTGTAACATTTACACTAAGTTCATTCTAAAGGCTCAGTACACCCAGTTCCATGGCATG<br>AAAGTCTGCTTATGAGTCTCGTGAGTCGACGGAATTTTTGGTCTGATAATAGGACTTTTCATCATGTCTT<br>TCCTTTTTTTTCTGCTTCTCGTTTTCCCATAAAGCTGATCGGTTTATGTTTGCAGACATTTTATGTTATAGA<br>TACCTCCTCTGATGTCCCTTCAACACCACTACAACTAGTATTCACTTTAGTGTCATTTTGTGCAGGGCAT<br>GGTAAAACCACGTAGTAATATGGCAGAGTTCTTCATATGTGGCGTGTGTAAGATTAATAATATAGCATTAG<br>TTTTAACTTCAGAATGCAAAACCAAATGCTTTCACGTAGGTTTTCATTTTTTTTCTATTTCATATTAAATGT<br>ATCATTTCCATTTTAGGTTAGTTTTTTTCCCCTGGTACATTTATGTTATTTATGATAATAATCATGTTTAGCA<br>TATCACTACCTATGCATATCATATTTCGTATTAATATTATCATTCCTATTAATAACCACTAAGTTCTTCTAAAT<br>GTTAAGCCACACTCAAATGTTCCCATACCCGACTTAGACTGTCCATGATAACGTTGAGAAAGTGCAACA<br>TGTATTCAGGGCCATGAGTCATTCATGATTCTTTCACAGCACACATTCTGCAAATAGTTAGTGCTCATCA<br>ATCAGTAAAGTAGCAACGTCATATGAGTCAGCGCTTCCGCCTGGGGGAAGGGGTAAGGAAAATATGGT<br>GACGTGAGTTCTTACCACCCTGTCCTGAGTCAGATCCATATAGAACAGTTCCTTACCATTTCTTCTTTTAC<br>TAATGATAATGCAAACATTACATCTTATAGCTGATGTTTATGTTCTGGATAGTAAAGAATTCCTCATTATTT<br>AGTGAAGAATTAGATATATCACTGTGTAAGTCAAAATTGAAAACCATTGCTCTTTTATCTGCTTTTAAAA<br>GTCATTTTTTCAACTTCTTGTTTGTGTTTTGTCTGTTGCTCCCCACACAGGGTCTGGAGCTGGTTTCAGA<br>ATTCCCTGTGTTTAAGGACCGCATCAAGAATGTGTGCTCCAGGGGTGATTTCTGCATTCTTCATGGTCAC<br>CAGTGGAACGTTTGCATCTCTGCGTTGAGCTTGGGGCAGTGTGAGTACTAGTTACTATCCCTGCTTTTTT<br>ATGTGGTCTGTTGAAAGTTGGTTACTTCATTATTGAAAAGTATCTAATACAGAGCTGGTAAGGTAGAAAG<br>AGAGAACTGATTCCTAGACATTATACCCTTTCCTCCATGCACATGCCCTTGGCATGCATGCTTATAAACAC<br>AAATGAATATATTCAATGAAAATAAGTTTTAATTACAGTGTATCAGTGGAAATATAACCAATGACACCTAC<br>AAACTTAATGTGAAATAGGACATTGAGTTGACCATGTTTGATAAACATGGACTCCTCACTGAAGGAGGT<br>AAAGTGGTTCAGTAGAGAGGAGTGGCTTTCAGTCTGTGTCAGTGCTTGGGGAAAGAAATATTCTTTCCA<br>TTTTTATTTGTGAATTAATGATTCAATCAAGACCCAACATAAAAGTGTGTAGAGCCAGAGTGGGTTGATG<br>GTGAATGCAAGGATATGAATCCAGGATGGTATACGTGTGCCCGTCCCTGGGTCCATCCTGTAGCAGATCT<br>GCTCTTAATTTCTTGAATAGTGAACAAATATAGAAAATCATGAAGATAAACGATTGAAATTCTATTCACTT<br>ATGAACACGAGTATCAAGGCAAAACAAACAAACAAACACACTCCCCTTAGTAACTGAGAATATGGAAT<br>CCAAAGAGGAGAAAGTGGCATCCAAGCCCTTTTGTGTATAGAGAAACTAATGTCAATACCAGATGCTAT<br>TTTCTTGCTTTGCTGGAAGTTTAAAGATACTCATCTTAGAAGACAATAGTATAAATATTTTTAAATTATGG<br>TGGCTTGCATGAATGATTCAGATGACAGATAATCATCAAAGAGAAAGCAAGAAATGTTTTTCTAAGTAC<br>ACTTTCCTCAGAGCTGGTGCTCTCGCGTACTGACATGTTTATCCATCTGTGTAGATAGAGCCTGAGACAG<br>ATATGAGTTCCAGTGCTCTGTCCTTTGCTGCCTTCTCTGTAACTATTTCATTATCCTCTTCTCTCCTCTCCT<br>CCTCCCTCCCCTTCCCTCCCTTCCTCTTCTCCTCCCTCTTTTCTGTACTCAAGATGGACCGTGTGATTAGA<br>AGATCGTCCAAGGGTCAAGGGCTCCATTTGCACCCTGTCGAGGAGGCGAGGAAGACGAGTAATGGACT<br>TTCCCAAGAAATCTATTCATCTTCAATGGCTTCCACCCCATCAGTTGCTTGTGATGATGTGCGATCTGCCC<br>AAACTTCTTCTCATAGTTTTGATGGTTTGTAGAAGCTCCTCACTGACTCAATGCCCTCCATGGCTAGGAG<br>CTCTGCTGACATTGTGGAAGCTGCTGGGCAGACCACTGAACGAGTCTGCCATCATCAAGGGCTCTGCA<br>CTTTACCAAGAAGTTCATGTTGAATGTGAGTTGAAACCAGCATTCTTTTTTTTTTTAAATTGAATATTTTAT<br>TTATTTACATATCAAATGTTATCCCCTTTCCTGGTTTCCCCTCTGAAACCTCCCTGTCCCATCCCCCCTTAC<br>CCTGCTTCTTTTTTTTTTTTCTTTATTTTTTTATTAGATATATTTCTTTACTTACATTTCAAATGCTATTCCCT

							TTTCTGGTTTCCTGACCATAAGCCCCCATTCCCTCCTCCTCTCCCATACGGGTATTCCCCCATCCATCCCC<br>TTTACTGACCCCCCAAATTCCCTTGCACTGGAGGTCCAACCTTGGCAGACCCAAGGGCTTCCCCTTCCA<br>CTGGTGCCCCAACAAGGCCTTTCTCTGCTACATATGCAGTTGGAGCCCTGGGTCAGTCCATGTATAGTCT<br>TTCAGTAGTGGTTTAGCCCCTGGAAGCTCTGGTTGGTTGGCATTGTTGTTCTTATGAACCCAACATTCTT<br>AGTTTTGTTTAAGTGGTTTGCTTACGGTAATCACTGTACTATTACAAAGTATATAATACCCCTAACAAGTG<br>GCCAGCATTTTGCCAGTGGCTTGTAAGGAGCTGATTCAAAATGCTCTACTAATAAGGCAAACATAAAAA<br>GTGGAAGATAGTTGATTAATAGATAAACCAATGCTGTAGTAGCTCTGTATGCAAATTAGGAGTAGAGGGG<br>CACTTCTTCACCTTATGCAAGTCATGGAGCTGACATTGGAAGGGAGATGATAGCTTACTCAACAGACAG<br>ATGAATGAAGAAATAAAGACTAGACTTTGATCAATGATGACTCCGATGTGGGCATCATGAATGAATCCAT<br>ATGTGGAATCCTCATGTTCAACCCCAAAGATGAGCAGCACATTCTTTGTTTCATATGATCCTCTTGACATT<br>ACGTTTTAGAGTTTGTTTTTATGCACTTTACTACTCTTTTATAATTAAACCATTGTAAGAAATAGTAAACA<br>AACTAAAAACATAAATTTGGCGATGTCAAAATTAAAATGTATGACAAAGCAAAAATACCATCGGTAGCT<br>ATTTAAATGAGTATTGCTGGTATTGTTCGACTATGGTGATGGCAACTGTACAAAATATTGAACTTTTCTAC<br>TCTTGTGCTAAGAGGGAGTTCAAAATTTTTCATTTTTTTCTAAGCACTCTCTCAATTAATGATTGTTAATG<br>AAAGGTGAATGCAATAGTCAATGATGAAGAAAACTAGCATCTTCTTGTCATGGATTCTGTTCTTTGCCTC<br>TAAGAGTAGACTAATGGAAGGAGAATTCCTTATCCACACCTGGAGGCCATGGTACGTATTACACAACAT<br>GTTCGTCTTTGTTTGTATAATTTTTTAAAGCTGTTTTCAAATATCCTATCTATGTTGTGATATTATTCTATGT<br>CATTATTTGATGGCAGCAGACCAGTTTCTAATGTGTGATGGACAGGAAAAACACGCAGGCACATATGTG<br>GAAAGATGAGCTCTGTGATAGTGAGAATAAGTCTACAACTGATTCTAGCAAAGGTAGTTATCCAATGAC<br>AGACCCGAAGATCTTAGGTTTCTGTGATCCGTACGACTTAAAAGTTGTCAATGGTGAATTAATGAGTTAA<br>TAACTAAACAGGGTAGTAATGAAAAGAGACCAGTGTTCATCCTAGGTTCATCAAGACTCTGTGTCATCT<br>GAGCAACGAATTTGTATACATCTGGAGCTTAGATCCTCCATAGATGAAGTGCGCCATCACCACCTCTCGA<br>GTCATATTGTAGATAGGGACCAGGGAGAGTGGGTTTCATTTTTGCTCTTGTTAAAATGCATTTTGAAGCT<br>AACGCTACAAGCAGTTCAAGGAAAGAGCCAGTAACCAATTATATAGTTACTAATGGATAGAAATTAACC<br>TTATTCAACTATAAGAATAACATTTCTAGACAATAAAATCAGTGCTATGCATGAGGTGGAAGACCAGTGA<br>CAGAGTCTGTATTTGTGCCACCACTGATACTGTTTGTTTAAACAAGAGGATCAAATAAAAGCCATCCCA<br>AATTGCCTAAATGACCGACTAATGAAAGATTGGATGCAGAGACCAATGCAAATGAGGCTGACTCTTGGT<br>AATGATCTCCTTCACTCTGTATTCCTGAAAGAACAGAGTTGTGCTGGCCCATTTTTCTTTGGTCAGACCT<br>GTGGGTGGATCTCATATCCTGAGTGTGAAGAGCTCTGTGAGTAGTGTCAGTTAAGATTGTGGTCTTGGT<br>ATTGTTTTTTTATGTAATTATTTACTTTTAAAATATTTTTGTACACTCAACACACTTGAATTTTTTCTTTCTT<br>TTCCTAACTTTCACATCTTTGGAGTTGGTTTTTTTTTTTTAAGCCATTCACAGAAATCGATTTTTAAAGTG<br>TGACTTAGAATCATTGAGAAGACACTTTGACCTACTGTAAAAATTGGCTTAATATAAACCATTGTTACCT<br>CGATCTGTGTCTGTATATAGCACTGAGGTTAAATCAGAGTAGAGCTATTGTTATAATAGGAGTTATGCAGA<br>AAGGTGTTAGTGTCATCCAAGGATCATAAGTTTTTGTGATTCTTATGTGATTGTTTATAGTAGATGAACAA<br>TGCAGCCACATGGTCAATAAAAGCACAAAGACGTGAGTGAAGAGCACAGGAGCAGCCTGAGCTCCTG<br>AGGGTGGTGCTGGTAGTGTAGGAGTTGTAGACTACAAAGGATGTCTGGCTCATGCACTTCTGACACAA<br>GAGGAATCTCAGTGCTCTCTCGGGGGAGAAAATATGGAGAAAGGTCTTGAGAGCTGTGCATGGCTCCC<br>TGGTTTGGGTTTATTAGAAATGAGTCCATGAGAGATAAGTATGACAGAGGAAGCCACACTCTAACTCCT<br>AGCTCATGTTTCACTGGATGAACACTGACAGCGTGTGGAAGGGATGATAAGTGGACTATCAGGCTTTGT<br>TCTGTTTCCAGGGGTCCATAGCTACCGATGGCTAGAGGAGAGCATTACTCTGGGCTCTTTGTGAGTTCA<br>GAACTCATTTTATCATATGGCAAATAGCCCATCATTACCATGAAGGATCACTAAGTGCTCACTAGACACC

							ATCGCAACGTTACAGAGTCTCTGTCACCTTGGCAGTGAGCAATGTGCCCGTGGTTGTGTTATCTTTGCCAGTGTCTTACATGTGTAAAAATAGTGAGTTTATCATCGTAAAGAGTAAATGAACAAGGATAAAAATAAATAATGAAGAAAAAAAACCCATGGGGACATAATAGTATTTCTTTAAATAAGTTAACAGTTTATCCATTTTTTTTCTCTTTTGGTTTAAAATTCTATTTTAATTTCCTGGGAACCTAATATGTCTTTAAATGTTTTTATTTTCATATATGTGTGCTGTATTTACATCAATCCATCCCTGCACTTAGGCATTACCGTATGAAAATGTCCTCTTTCTGTTCTGCAGGAAGCTGTACCACAGTCGATCAGAACTAATCATGAAGGCCTCTGCCCTCTGGAGAGAGAGAATATCATGGAAGTAGAGTGGATGCACACCTTCTCCTCTCATCATCGCTACTTCAAGATGAAGGTCGCAACACTGACTGTATGGCCCTGGCAGTAGAGATCTCAGTAAGTAAGAGACCGTGTTTCCTGCCTGTTTTTTTGTTACAGAGATCACTGTCTATTTAAAATTGACATTATGTGTCATATAGTCAAAGACAACTACACATATCCTAGTGCTCAGGGGGTCCAGGGAAGCCAAAGTCAGTCAGTCTGCATACATGAGGCCTACACACAGTGGGTTTGCACTACATTCTGAAACTGAGATCACAGACACACGCTAAGTGAGGCAAAGACTATGTTCATGATAAGAAAGGGACATAGAAGAAAGGGTCCACATAGCACCCATCCTGGAGTTGTCATAAAAAGGAAGATTGGTTTTAATGGGGTCATGAAGTGTGCTCCACTAATGCAGTCTCTAGCAAAATGGAGAAAACCAACTCCCCCTGTTGTGTTTGGCTGCAGAATCTTTTCTGTTGATTTAATTTTTGAAAATGAGTGACTAAATTAATCAGGAAAAAAAAATCCGTGCCCAGAAATGTTAAATGATGGAGATTATCCCCATTGAGAACACTGGCCATGAAAATTAAGATTTATGATCGACTGTTTTTTTTTTTCTCTTAAACCCATTTAACCAAAAAGATTTAATTGGTTGCATTTGTGTATAAAGAACCCTCCCCAAAACCACCTTCAATACTCATTCTTTTTCATTTATTTTGTACTTTGATGAGTTCCTGAAACCATAGTTATCATCATTATTTAGTAAACCAATCTTTTAAAAAGTAAATTTGAGACAGACCAGGTGTGGGTCGATAATTTACATGTTTTGTGGAAAGTAGTATGATTACGGTTCTTAGTCAGTGATGTCTACAGAGGGAATTTAGTCCCTCCTGTCTTTGTTTTTATGGAACATGGGGCACAAGGCCACTTTGTATGAACTAATAAGGTGCATTTCTATTCTCTCGTGGCACTTACGTGACCTATCGTTGTCTATCTCCAGCACAAATTCTTGTTTGCAGGTTGATTAGTAGGCTCAAATACTCCCCGAGTATTATTTCCTGGCGCTCAGTCTCTGAGAATGGTAAAACTGCCAATAGCTGTTGCTTTTCATCCTTGTCAGACCTTGTGTCAAATACGGAGTTCATATAGGAGCCCGACCCCGAAACTCTGGGATTAGATAATCCCAATTTACAAACACTGTAACAGGACAAGTGCATGTGTGGCCTAGAAATAAGGAGTTGGCATTCTCTGCTCACTTTTAAGTTTGGGATGCTTTATGAGGAGGGGCATTACGATGCTGTGAGGTCTTTCACAACTTCAACTCACTGCCAGAAGCACATGCGATGTTTGAGTGAGTCAGGGAATGAACGATGACCAAAAGAAGGAATAAACGGAATAATGCCTGAAGTAAAGATCCCTTGAGGGGGGACAAAAAAAGGTATGAAGTCATGAAAACATGGAGGTCTGTGCCAACCGAGGATAATAAAACACTAACTAATGACTAAGTTACAAAGCAAATGAGTCCATGGCGAGTCTGTTAGGGGATGCGTGCATAAGCGGGCCAGCGTGGCAGAAGGGAGCAATGAAAGAAAAG
MSTRG.109626.1	ENSRNOG00000028545;ENSRNOG00000028566;ENSRNOG00000028629;ENS	0.000	1.223	0.000	6.333	up	ATAAGTTTTATGCTAGAATTATTTGCTCATGAATATGCATTTAGCGCCTGGAAGACGGTCAGCCCTTTAAAGCTCTGCACGGTGCTACCAATCGTCTTCTAGCATTTGGACGAGTTGGATTAAGACCTAGAATTCCTTTCACCCTGATCCTACCTCCAGTTCTCTGCTATAACCAGGAAGATTTCCCCACAACCAACCAGTTGTTAGCTTTATATTTCTTTCACCTATTGTGATCTCGTCCTAGCATTTCTATCAGTCATAGGACACCAGGACAAAAAGCAACTTGGAGGGAGACAGTTTATTCTGGCTTACAGCTTCAGATCACAGTCCAGCACTGAAGGAGGTCAGGGGGGCAAGAACTCACACATGGCAGAAACTCATGGCAAGAGCTGACGCAGAGCCCAGAGGAACACTGCTTTAAGGCCATCACGGTTATAAAGCAAGCACTTCACCTTCAGAGCTCTCCATGTAGCATGTAGCATGTAGCATGTAGCATGTCACATTAGGGTTAGGGTTATGTGCTTTCTCCATCATGCATTTATCAAGTTATCAAGTTATCAAGTCTCACGGTGCTGGGCATCTGGGTTGCTTCCAGTTCGGCAACTTCCGGCGCAGCACTCTCCAGAACACCGTGGCGGCGCAGCACTCTCCAGAACACCGTGGCGACTGCAGCAAACTTCCACCTCGCGACCCTAAACACTCGCGGCCTTCTGCACACCATC

	RNOG00000028640;ENSRNOG00000028650;ENSRNOG00000033688;ENSRNOG00000037562;ENSRNOG00000046763						
MSTRG.109663.1	ENSRNOG00000013914;ENSRNOG00000013927;ENSRNOG00000014362;ENSRNOG00000014595;ENSRNOG00000014693	0.863	0.063	0.004	-2.856	down	TGGGGGAACTGAAACGTATGTGACTACGTAGCCAGACAGTCTGAAGAGACTAGACCCTGTGTGATCCCAACACTAGGATATTCTGACAAAGACAGAAAACATCCCCAAGATCAGTGACTTCTGGGGGCTCAGCACAAAGGGTTAAATAGGGGCTCCCTGGGGAGAAGATACAGAAGATACAGGTCCCTGGGAACCTCTACTTCAGCCTGATTTTATTGTAAACCTAAAAGTATCCCCAAAATAAAGTTTACTAATTTAAAGAAAAACGTATAAAATACACTCAGCCTACAGTGTTTACACTCTTGGACATTTGTTCCAGAGCAGTGAGAAAATACCTTCTCACAGACATGTGCACAGTCTAATAGACAGGTGCTTTATTGGTAATTGTCTGCAAGAGGAAATAATTCATGCCCTTTAATAGCTGAACAAACCTCGGTTAGTCCACATAATGGCGTAATACTCAGGAGCAGGGAAGGACATGCTGTCCACGTGTGCAGTAATCTGTGTGATTATTAATGACTCGAGGAAGAGAGCCAGCCTCAAAGGTTATTTACTATATAATTCAACGTATCTCTAAAGTGACAAGATTATAGAAGCAGTCAGTCAGCTTTCTGTCGCTGTGACTTTTAGAAAGAATCTGGCTCAGGACTCCTGAGGTTTCGACCCAGTCATGGTTCCGTTGCTTTTAAGCTGTGACAAAGCAAGGACATGTGGCAGAGGAAAACCACTTCTCTCTCGTGTCAGCCAGGAAGCCGAGGGGGCGGGAAGGTCTCTAAGATGCACACCCCTGTGACTCACGTCCTCCAGCCAAGCCCCAGCTCCTGATGGCTCCTCCCGTTAGGACCTCTTCATTGACTCCATGGTGAAGTTGCTGCCCTGTGAGTCAAGCGGCTCAGAGCTATACCCTCACCTCCTGCTAGAGATGAAGCCAGGGGCTTTGGGGGATACTTTTGACCAATCCATAGCAGATGAAGAATGAGTTATCAGTTGTCAGGGCCTGGTGAAGGGGTGGAGATTGTGACCCGTGACTGTAAGGGACAACAGTGGTGTTCTCCTGTCAGCAGCTGTGATCACATCCTGACACAGGGTTGGCTGTCCGACTGACTCCACACTCATGACGAAATGTCATAGAAACCACAGTAGGTTCCTGGCAACAAGACGGCTCTAGGGCAGGGAGCAGGTACATCTGCAGCTGGGCCACAGTTCATTCCCAAATTTTGCTCTTCTTCGTCTGGGCAGCAGGATTTGTAGATAGAGGAAGAGGAGCAGTGTCTCCAGTCAGACGGTCACCTTGGAGACTGCAGCGTTCCAGGGACGACACGGATTTCTCTTCACTAAGCCAGGGCAAACCCAAGGCCAGTAACAGCCTCAGGGTGACAGTGCAGGCAGACAGCTCAGCATCTAAGTGAGCTTTCCTCTGGTTGGCCTTCCTGAGGGATGAGCCTCTGTGGAGCTGACTGCCTGGCTCTGCAGAGGCGATGGTCTAGTGTAGGAACAGGATGGCCCTGGGTCGAACCCTTGCCTGCGGGAACCCTGGTGTCAGATGAATGGC
MSTR	ENSRNO	0.512	0.000	0.000	-	do	CAGACCAGGGGTGGGATGGGGGCACTCCCTTTGAGGCAGATCAGTATGGAGAATTCTTCCTGGGCTCCAGCCCTCAGGTGGGCTTTAGAGGAATGACGTGTCAAGGCATGCTTGCCGTGAGGATCCTGAAATGGATT

G.109691.2	G00000004711;ENSRNOG00000005041;ENSRNOG00000005153;ENSRNOG00000005174;ENSRNOG00000027990;ENSRNOG00000048402				6.124	wn	TCGTGTCTGTTTCAGACGAGGATTCTGAGACTGTACACAGCATAAAGTGTAACACATCAAAGGCTAAGCTCCCAGAGTGCTTTAGATGTGTGGGCCAAACAGCAACGGAGGCTCAAGAAAAGACGGGAGCTGTGGCTTGGCAGTGGAGGGGAAGTGCTGGAAGTGCCTGTGTGGGCCGGGGGGAGAAAGGCTCCCTGAGACTTGGGCAAAAGAGTCAGGATGCAAAGACAGCACAGGCAGCCAGTTCTTCGGACCCTTTGTAGGGAGTGGAGGAAGAGTAATGGACAATAAGGCCGGGGCCAGGGCTATCCCCAAGTGCTATGTCCTTCCAAGGAGTTGACCCACCTCTGATGTCATCAGGAGCATAGCCGCCCCCTCCAGCTTCTCCTCCAGTGCAACACCACACGTTTCCAGCTTACGTGACAAGGGCTTAATGAAGGATCGGAGAAGCTCCTATCTGGATACACTAACCACCTATGCCCATGTCCCCGAGTGGGACCTACTGTGTGCTCGACCTCTTCTGTCCTGGTAGAGAGTCCCATTACTGTAGGGAGCTTGACCTTGCCAACATCTGAGATCTTAATCAGAGTCCAGACAGGTTCAGCCTGGAGCCTAGCCTGGTGCTCAAGACCCACAAATCAAATCCTCTGCAAGTGCACCAAGCTCTCTCAGCTTTCTCAACTGTTAGGTTTTGAGACGAGGGTCTCATATTGTCACACAGATTGGGTCCAAACTCCTGGGCTCAAGTGCTGTTCCCGCCTCAGTCTCCAGAGTAGCTGACAATATAGGCGCCACCCTCCTGACTTCAGTTTGTCTGTTGGATTGTTTATTGAGATCGGGCCTCACGTACCCTAGGCTGTCCTTGAATGCACTGAGTGGCTGAGGATGATGGCCTTGAACTACTGATCCTCTTGCCCCTACCCTTCCAGAGCTGGGATTATAGACGTGGGCACTATGCTCACCACCCTTTTAAAAGAGCAGTGCTGTACGAAACCCAGGCTCCATGCATGGCCTGCAAGCATTGTACCAACGGAACTACATCTTCAGCTCCCCCTCCCCCCTTAAAGCCCGGCTTTGGTCAGGGGTGATGGCAGCGAGGACTGCTGTCTGCTGTGGTGCGCCAAGCCCCGGGCAGACCGCCAAGATCTCAGTGTTGATGCCGCACTATGTACAGTCGGAGGAGGTGGGCTGTGGTGAGTTGCGTTGGGCGGCCCCTTGCTGACCCTCACTGTCTCGCTTAGGCTGGGCCTGTGGGCAGGAGGAAACAATACCCTCAAGCTATTAGGTGCTACTTGCTGGGCTGGGGAAATGCTGCATCTTGGGCTCAAGAGTCAGTGTTTTCCCTGGCCTCTTGACACAGGGGCAGGCTCTCAAAGTCAAATCCCAGATATGGCCAAGAAAGGGAGCATCACAAGAAGAGGCTGTCCCAGCAGCTAGATGAGATCCTGGGTCCCAAGCGAGGGCTTCTGAGGAGGGAGGGACACTTAGAAATGCTGCTTTGGAGTGACGTTGGAACAGGGGCAGACTGCCTTTGCCAGTCCGGTCCCACCACAGGGGAACGTCTGGACCTGTGTGACCTTTCACCTTCCACTCTAAAGCAAACTCACGTTTACTGTAGGAAAATGGCGTCTGAATGATGAGACCCGAATTCTTCAAAATCAAGCCCACTTAACAGGTGAAAGTTCCTGTGATCTGATTCCTCTGAGCAAAGAAACTAGGAGAGGCACCGCCCTCAGAAAAGCCGTCGACGCTGTCATCTTTTAGGTGGATCCTTTTAGGGGTTCCTTTGCTAATAGCCCAGGATGAAGAAACAAGGAGGGGTTAACTACCCTCATGGTAAGGCATGACAAACTGCACCCGTGAAACAAGACAGGAAATAATTGCAATGGGCTCCTCAACCAGAGGTGGGCAAGGAGCTCTCACCCCCGTGGCTCAATGTGCGTGGGGCAGGTGTGGCTATAGGTAGGTGGCAGTTCTTTGCTGGCAGGACTGTACCTGTACTTGATGTGCTGGGCCCATCAAGAAGCACAGCTTTGGATGGGTGAAGAGGTTGGGGGTCACTGGGGACGAGACAGGATAGAGGGCAGAAGGCAGAGGGTAACCATTGCTGTGATAAACACTGAGTAAAAGCAACTTGAGAACAACCAAAGGGTTTATTAGGCTCATACTTCCTCCTCACCGTTCATCACTGAAGGCATTCCTGACAGGGACTCAAATCAGGGCAGGAACCTGGAGGGAGGAGCTGATGCAGAGGCCATGGAGGGGTGCTGCTTACTGGCTTGCTTCCCTTGGCTTGCTCAGCCTGCTCTCTTATAGAACCCAGGACCATCAGCCTGGGGCTGGCACCACCCACAATGGGCTGAGCCCTCCCCTTTCAATCAGTTATTAGGAAAATACCCTAGAGTCTTGATACAGCC
MSTRG.109795.3	--	0.000	0.471	0.001	4.386	up	CCCTGGATTAACTGAGCAAGGCTCATATGAACTGACCAAAACTGAACCAGCAAACAAAGGGCTTACATGGGTCTGTACTTATATTTATTACAGCTTTCTACTTAGTGGTTTTATGGGGCCCGTGAGTGTGTGGATGAGTGGGTCTCTGATCCTCATGTCTTCTCTTGGTCTCCTCTTCTTCTGTTGGGTTGCCTTGTCTGACTTCTTCAATGTGATGGTTTCCATTTCATTTTATATTTTATGTTGATGTGTTTTGTGATCTGGACTCTTTTCTATGCAGAGACCGAAAGGGATTTGATCCAGATGAGAGGAACTGGGATGAGCAGAGGACATAGAAACTAATCAGGATG

							CATTCTGATGTCAATCAGATCCAGCCGGTTACTTTGGAGGAAGTGTATTGGCTCAGAACACGCCAATCAAGTTCACCGAGCAAAGGGGCGGGCCTTGACAATCTCGCGAGCCCACACCC
MSTRG.109880.1	ENSRNOG00000005826	0.000	0.467	0.004	3.943	up	GCCTGTACTATTGGAGGCCAGCTGTACTTCATGAGTGGCTCTCCGTGACGAGAGCCTACTCAGGGCACCCCCTTTCTCCATGATGTGCAGAAACAGAGGTGGGAAGATCCACATTTAATCTGGGCCACACCTTCTGCTGGCATATCGAAGACGTAGAAGAAACCTCTCCTCTGCCTGCTTGCCCTCACTCTGGCTAGCAAATCCATTCTGGAATTAGAGCCTACCTCTGTGGGATTCTGGCATATGCAGAAGACCAGATGAGACATTTAGCCTTATGGAAAAACTCTGGATGCTTGGACCTTCTGTTGATAGACTGCCAATATTGGACTAGCAGGACCACAGCCT
MSTRG.109893.1	ENSRNOG00000005943	0.000	0.293	0.019	3.592	up	GCTGCATCAGGAAACCAGAATGGTGGTTCATCAGGAAAATTACAACAATTAACATAAGTGCTGCAAAAATCAACACTTTAAATGTCACACTCACTAGTCTGTCCAAAGACTGTAAAAGTACCATCCCCATTGCTGGACTAACAGTAAATATGAAGCAATCACCAGCAAACCTTGGGCTGATGGCTTTACAATTGATGTGACATTCTCTGGGTGATAAAATAGCCTCTCATTTTACAAAACTCCCATCTCTCCCATGTGCAAGATGGAAGAAGAGAACAGACTTCACAGAATTGTTCTACGGATCAGGTGACTCATTGAGGACAGTGGACGTGTAGGGTCCCTGGGATTGAACTCAGAACATCAGGATTTGGTACCTTTACCTGAACAGAGTCTTTATCAATGGCAGGC
MSTRG.109987.3	--	0.484	0.000	0.000	-5.395	down	GAACGCATATGCAAAAACACTTTACCGTTACCTTGTGCCTTTTTTATGACAAGATTTACTGAGGCTAGCCCAAATCATCCTGGCCTTTTCTGGCCCATCTTACCCCAATCCGTCCCTTCTCCTATGCCGGCCGTGCTCCAGGGACGTCCCGTAGGTGGTACCCCCTTTCTTCTCCGATCTCAGTGGGACCTCCATTTGTCGGCGCCAGCACTGACACTGTACCGAGAATCGGGCGAAAGCCTGCTGTTGCTTGGCACTGTGGACACAGATGTTCATTCTGGACTCTTCACAGAACCCCTTTGCCTCCAAATAGGATGCTGGCAGTTCAGACTGCTCCACCAGCAGTTCTCTGTTCTCATTCAGCAAAATCTGTATATGGTTCTTCAATTGAGTTTGTTCACACAGGAGCTGGGAGTGCCTGATGCGGTAGGAACTGTTCACTCATATGAGCTCCTTGTACCTCTCCATAGCATCACTTATTGAGTTGGTTATCATTTGCAAAGCCATCATTCTCATCGCATGTTCAGATTGAAGTATTGGCAATTCAACATCCAGACTGTGTTAGGGCATGGCCCAAGGAACAAATAACCATTTGAACCCATGATTTCAGAATTAGGTGAAATGTAAAAAAAAAAAAAAAAATCCAATAAAAAAATTCAAATTTAAGAAATGAGAGGGGAGGGAAGAATGCGACAAACACAAAGACTGGAGTAAAAACACAACAGTGAGACTCCAACAACAATTCTTTCAAGGAAATATTTCTGCGCAAATCAGCAAACATTTATTTTGAAATAAGGACTCCTAAAAAGGCTATGGAGATGTGGTTCTGCAAAGACCCTCTGTACTGGAATGGGTGGAACCTGTGAAATGCCTGTTTGGGAACAACAAATGTTCAGAATTTGTTACCACAATCACCATTTCTAGGTCCGTTGATAGCTTAAAAAAAAAAAAAAAAAACAATAGAGTCAAAAAAATAATGAAGTTCAAATCAGTCAAAAAAGATTACGTGAAATCAGACTATTGACTGGATTACTTAAACCCAGTTTATACCACATACTTGCTCCTTTGAACTGGGTCCTATTTGTGCTGATTAAACTTATTATCCTGAGCACAGGACAACAGAGCCAGGTTACCAATTCCAAGGAACTGACTTTCAAGAGTTGGCCAGGTAAGCATGAAGGCATTGACTAATTCTGTAAGAAGTTTCCTCCAGTCTGTGTCACAGCCTTGGGTCCAACAGATAATTATGATGAGATTTCTTTTGTTTGAAAACAGGGATTCTCAAACCTTGTTTCCTATTCTTACCTGAATTCCCAGAGGGCATAACTACTTTGACAGGGAAGAGGATTTGTAACCCCTCCTTCCCTCAGGAGAGTCCTATCTACCACCAAAGTAAATAGAAGATGCAAAGAGTTCTGTTGATCCATGTGGGCTTCCACACTATCATGACCTGCAAACTGTCCATCAGACAGATCCTTAGATCCCATCAAACTCCCCACACTCCCTGACCGTAAGCATAAAACCCACAAATACTTTCACATACATATATACCATCCCATATACAAACACAATTAGGATGCCAATTACATCAATGGAG
MSTRG.110233.1	ENSRNOG00000043299;ENS	1.359	0.148	0.048	-2.203	down	TAGGGTTTGGTAGGGAGATGAACAGAGACAATTGGGAAAGAGCTATATAAAATACACTGTAAAAATCTGTATGAGAAAAAGATGGATATAATGTGTTTGAGAACAAAGACTGTATTTTTGTACTCCTCTTATAAACAACATACTCAAAATGACTCATTAGTCCTAGAAAATAAAGATTTACTCACCATTCCGCCATCTTCCTCCTTGTTT

	RNOG00000043334;ENSRNOG00000048675						TGTATGGTATTCTATCCTTGGTTTGTTTATTGCTAACTTATGCACACTTACACACATGCCCTGACCTCACCTGCCCCACCAACACCGACCAAGGTTGTGAAATATCCAGCACTGGAAGTTGACTGGCCAGTTTAATATGTTTATCTAAAAACAGCTATCATCCATGATTTCTGAGTCTAAGAGGAGCACCCTCCCTGGCTACCTATCAGTTTTCTAAAGTAGGAACTATAACCTAGGTTCAGATAACTTGTCTCTGTCCTTGATCTGGGCTTTATCTGTTAAACGGGAATGGCAGTCCTAAAAGGGCTCTTTTGACTTTGTTTTCAGCTGTTCTTGCAATGTAGCCTTAGCCTTCAGTCAGAACTTTTAAGTCTTGTTTCAGTCTCTAGAACGTGAAGGTCAAAGGTAATCTGGCAGCTTTTTGAGTTTCAGTGCAATCCAGTCATCCTCAATTCTAAGGGGAACTAGTTCCAGGACCACCCATGGATTAAAATAATAATACCAATCAATGGATACTAATGTCCCTAAATGGCTGCCTTTGTGTGTATG
MSTRG.110379.5	ENSRNOG00000031216	0.000	0.409	0.001	4.386	up	AATATTGGGTCAATTTCGCCAACTTGAGTCAGGAAACAGTGAGTCTGGCCTTCCTGACACACGTGACTGGACCTCCGCGGCATCTACATGCGACTGTGTTAATAACGCAGACTTTACACCTGCGCAGAGTTAACTGCAGACGGATCCTACACCTCAATGTCAAGGGCCAGGGGAAGAACTTCTAGACGACACCAGAGAAACTAGAGCTCTGCCAGTCAGAAAAACCTGCCTTCCCGGACTATGAAAGCCGCGCATGCGCGCACACTGCAAGATGACGTCAGCAACAAGACAACCACACACACAGCCCCCCAATCCCCTATTCTGCACAGGCGCAACCCAGGGATGGAAGTTATTCAGCGGCTGCCGGGAGCCTGAGTTCCAGGGGTTGCAGGAACCTCACGGTGTGCCACGTCTCTCCTCCAGTTCCCGCAGCCCAGGGCGAACAAGCTCCCGCAGCTCCGCCCGGGTGACGGAAGGCGGCCCCGCTCACCATGGTGCGGATTCCGAGCTCTCCCAGCTCGGCTCACAG
MSTRG.110659.1	ENSRNOG00000007960;ENSRNOG00000008026;ENSRNOG00000008199;ENSRNOG00000008217;ENSRNOG00000008296;ENSRNOG00000008414;ENSRNOG00000030530;ENSRNOG000	0.000	0.331	0.019	3.592	up	GTCTGGCTTGTGTCCCCTTTCTCCTGTGTCCTAATGGTTTGGTGTTTCTGGGTTCTGGTGTCACACTGTACCCCATCGATGCTGCAGTTGGCTGTCACATACCCAATCCTTCCAGGTCCCCGTTCTGTCTGGTGGATGCATGGTGAGAATCCGGATACTGTCATTGTTGGGACTCTCCATGTTGAGGTGTAAGCAGTGTGGCGTTTGGAATTCCAGCCATTGTGGAATACTAACTTCTGTCTGTAGCTCCCCTTACGTCTTCCGTTTAGAATGAATGAGCCCTGTAATATGGCGATAGCAGGGTTGGGTGGCCTTAGCTTACTAGCCTTCATTTGTCT

	00060530						
MSTRG.110737.1	ENSRNOG00000004346;ENSRNOG00000006559;ENSRNOG00000028146;ENSRNOG00000060598	0.368	0.000	0.000	-5.573	down	GTGTGTGTGTGTGTGTGTGTGTGTGTGTGTGTGTGAGAGAGAGAGAGAGAGAGAGAGAGAGATTGAGAGACAGACTGACTGACTGAATGAATGAATGAATGAATGACTGAGGGAGGAGAAAAATACAGAATATGACAACAGTAAGAATATCTGAAAAAGCCATAAGGAATCAATCACAGCATTAACTCATTCAGTTTTTATAACTATACATACATAGCTTGAATCAAAGTTTCTCATTTGGGATGGCAGTGGTCCCTCCTACAGACTAAGACCATCTAACAGAAAACCCAACACCATGCAACTCTTCTGAGTTGTTCACCAGTGTTGTTTAAGAGGCTCTCAAACATGTATGCATGTTATTGCTATGCCTTGGGCTGCTCTAGAGAGGTGGAAGGTAAGTTCCTATTTCTGAAGACATCAAGCACTGCAGATACAGGGCCCAGAGGCCCCTGAACTGGAACTAACTTGAATGCCTCCTTACTCAGAACTAGCTTTTCATGGTACCATAAGGCACCATGCAAGCTTCCAAAGAAGGGAAGCAATCAACAATCCTATCTAGCTACAACTCTGTGAAAGCATCAATGACTGGCAGGCACAGCACACAAATCTTGGTAGTAATAGATAGCTCTATAATTGTATTCAGGAGGAAAATTATGCTTGGTATTAGAAAACTAGCCAAGAACCCAGGGCTAGGTGAAGTTATAGATTTGGGAAGAGAAGCTGACTAAAACAGCCCAATCCCTAACTATATTCTAAACCTTTTTTTAAATCTCTACAGATGAGTGCAGTCCTCACTTCTCATCAAGGAAACTTCTCTTTGCAACACAGATAGTAACAACCAACTGAAATGCCACTCCCAATTGGATCTATCTACAAAACACTCTTGCACTTAAAGCTCATGGGACAGTGTGGAAGAGGTAGCAAAAAGATTCTAAGAGCTAGAGGACCAGGGAGCTGGCTGTTAAGACTGTCTCCTGATAACGTCAGAAGCTATACCTGTAAAGTCTCACCAACATGACAGCCTAAACATGAGCTGAACAAGAACAAAAGACATGCTAACGGGGATGGAGGAGTAGTCATGTAGTTCAATCCTAAACAAAGAACTACAGGCAACTAAGAAATGCTCGGAGTGTTAAAAATAATCTTCTCCAGGGAAAAGGACACCAACTGGTTATCCAATACTCAAATGGTCAACCCTGAAAACATAAGAGTAAATTTACATACATTGAGTAGAATAAATATAAATATATGTGTATTAAATATATGCATCTAAACAATCAATTTTTTTAAAGGCCATTATTGTGAAAGCAAGGAGAGGTACACAGGAGGGACTAGAAGGAGAAAGGCAAAGGAGAAATGATGTCATCATATTATAATATCAAAAAGAAAAGAAAAAACTAAACATATAATTTCTCATTAAAAATCAAGTTGCAGCATCTTACCCTGTCCTAGGAACATCTCTGCTGAAGTACTGGTTACACAACAAGAATGAGAAGGCCCATTCCTCTCCTGGTCGTAGAGCAGTGTCTACTCCACACTCACGACGACAGCACTGTTTAGCTGTGCAGTATGTGTCAGTGGGGAAGCACAAATGGAATTACTGTTCCATCTGACTGGCTCTTGCAGCTGGTGAGAACAGCGCTCATGACAGGAGCAGACTGCACTGCCTGGAGCCAGGCCAGCGGCTATGTGCCAAGGCTGCTCTTAGAAGCCTTAATCCTTTATTGCTCAGCTTGTATATAACCTGCCCCCCACCCCAAACACATGACAAGTTGGGCCCCATTACCTCAGACTCTGCAGTACTGCAGGCTCTGTGTCTTCACTAGAACCAGATGAGTGAGGGTGGCGATAAGCCAGCCTTCTCGCTAAGGGAATGTGAGATACACTGAAGTTCTGTCTCCCAACTGTAGGGAACATGGGCCAACAAAGATCGTGCAGCCAGGAAGAAGACCAGAAGCCAGGCTGGGTCTGGCTGCAGCCCCATCAACTAGAGTTCCAGTGCTTTTCCCTCAAAAAGCAATAGGCAGGCCAGCTACACTGGGAGTGTGAGAAAGCTGAACTGTTTCCCTCTTCTCTGTAAAGGATGCCAGCAACTTAGAGGGACAAGCAGCAAATGTACAAATAAAAACAACAACTGTTACATACAAGTTACATCATAACTGTTAAATCTGGGCAGAAAGATGCATGAACTTAAAATGTTTAAGCAACATACATGAAAATAACGAAAATCAAACCAGACCAAGCACTGGTATGGACACAGTGAAGACTGCTGGGTAACTTTCCCTGTACAGAACCCTAAATAATCTCTCAAAGTAATTCATTCTTCTGTTGTTAACACAGTGGGAAAGCAACCATGGACAAGAAAACAGGTAAGCCCAGTCACAGAGGAATAAAACTCCAGGGCCAGTTTGGAGGCTCTGTTCC
MSTRG.1107	ENSRNOG0000000	0.013	0.472	0.000	5.593	up	TCACCCACTGTCCTGTAACTCAGAGCAGGCACTAAGCCACATAAGACACCATAGCTATGCATATCTTCTAAACCTACCTATATTCGACACCACCAAATAAATCAACAGGTTGGCTAAGGCCATTAGGTTACTTTCTACAGCAAAGGCTCCATGACAATACCAAAACCCAGCAGAGATGTGGAAATCAGAAGTTAGGGCAGAGTTCTGA

43.2	6355;ENSRNOG00000006559;ENSRNOG00000027319						AAAAATGACCTTGGTTCATTTATAGATTATATTATACCACCCTGAAATTCTCCATCCCCGTTAAAGTATACAGGTGATAAAGTGAGGCACAGGGCACCCAGCTGCAGCAAGGCAACACTGTTGACACAGAGGTAACTTGAGGACTCTAGAAAACCCTCCCATGGACGTTTGAAAGGACAACTACGGAGTGCTCAGACTGATTTAGGGTCAAAAGTCTGTAAATCTTTACAAGTGCAGGATGTATATATAAGCCTCCAAAAAATATCTTAAAATAAAAAGAATTTATCACTGCAGAAGTAAACACAGTAAATCCATCCTAAATGTGGGTCTCTGTTTTTAAGGTCCATAGATTACATACTTTTTTTTTCTTAAAGAACCAGAACATTCTTCAAATTCTAAAAGGGCTCTCCAACCGTGCCTAGGCTTACTGATGCTTTAGCAAAAAGTATGACTGGTCTAATTCTTGGCCAATGGTCTTTTAATTTTGACAAATCTTTCAACAGCAAGAACCATAGGGAAATGACTGTTCTAATGGAGCTTATTTTGATAAAGAAATCTAAATAAATTGTAACACTAGAAGAATGGTAAGCAGAAGACTTTACAAAAGCTTGGTTATAGAAAAGTGTAGCCAATCATGAAACTGCAGTTGCTTCTTTCTACAAGCATGGAAGACAGAGTTAAAACATCTTTTAGCACACTGTCAATACTGTAAGTCTACTGAAACTTACCTATCTGGCTTCTCAACAAAAACTTCCTTCCAATTCTTTAACAGTGTATGCCTCTCACCCATGTTTCCTGGGGGTTACTTCCAGCCAAACTGAGAACCCTATATACCAGCAGCCTGAATCCTCCTGCCCACTTTGAGATTCAGCTTAACTCCTCCCTTGAGCAGAGTCTCCAAAAAAACCTCTGTACCCGTATAAAGTACAAACCATGTCTTTCACTTCTGCTACTAACAGACTTTTCTGATATTTTTAATCCCATTATCATCTTCATTTTAATTTTAAATCTCTTTGGGTTTTTCTGGCAGGAGGGGAAGAGGATAATATGGCACACTTGCTATGTAGCCGATGTATGCCGGGCTGTATGTTCATCATATGTTATCATATGTTATTCAGAGGTCCTGAGTTCAATTCCCAGCAACCACATGGTGGTTCACAACCATCTGTAATGGGAGCCGATGCCCACTTCTGGTGTGTCTGACGGCAGCTACAGTGTGCTCATATATGTGAAATAAATAAATCTTAAAAAAAAAAAAGACATGAGGAAAGTACTAAAAATAGAAGACAAGTAGCTTACCACACAGCACAACCCAGCACACATAGAATACATAAGCAGTCTATAAACACTTGCTTCCAAACACTTAGCAGGATCTTTACTACCTGCTTCCTCAGCCCTTACCACACACAAACTCCTACCAAGCACTGAGGACAAAGAACAGAAACTGCGTTTCACCTGCTGCTAGAAAGCATATCCAAGAACACACTTGGGTAAGTGGGAAAACAACAACACTAAGCAACACAAACTAATCAAAAAATAAAAACCAAAACCAGCAGTATGGTGATGCCACCTGGCCTTTAATCACAGCACTCAGAAGGAAGAGGCAGGTGGATATCAACTTCAAGGCCAGCCTGGTTTACAGAGTGAGTTCCAGGACAGCCAAGACTGAACAGAGAATCCAGTCTCCAAAACAACAACAAAGTCCCTGTCTAAAATTAAAAAGGAGAAGAGGGGAGAGAAGATAAGAGACATTTCAATAAATAAACTCAGGCAAAGGTTTTATGAACTAGCCTTAGAATGTATGATATTCTTTGTCCTATCTTAACCCGCTAATCCATTTTATGACCCACTGTTTGAAAAAAGATAAAGAATTACTGTTGGGTGAGGTGACTCAACACTTGTCCCTGCACTCAGAAAGCTACTGAAGCATTGACCAGTTAGATGCAAACCTGGGTTACAAAGATCACACCACAAACAAAAAAACAAACAAACAAACAAACAAACAAACAAACACACAGTCTTTCCCTCAATCTCCAGATTCTACAACCTAATCCTTTCACCCTATTTTATATACAAGAAACTGAGTCCTGGGAGATCACAGTAGCCTAAGGGCAGACAGCCTTATGCCCTGTATGACCACCCCACCCCACCACTGAACCACACGGACAGCCCCTATATTCCATGTGGTTCTCATTTCAACCACACAATGATGATCAGCTGCCAGAAAATACACTCTTCCTCAAAGTGAATGCCAACCATTTCACATAGAAAGTCCTCGAATTTAAGTCTCCTTTAACTAGTCCTCTTCCTGTTTTTTTTTTTTTTTTTTTTTACATCTGTAGTCTTGTCTCATTGCCTGAGGAAGGACAACTGGGGATACTAACCATACTCAAGGACAAAAGAATTACTAATTAGTATTTCTGGAAATTGCATAATACCAAGCCCAGCAATACCTCTCCCTTGGCACTATCTTCACAAGGTGCACAAGTCTCTTGATGTAACAGCAGCTAGAAACAAATAAGCAATTTCGACCTTGAGTGGAAACCAAGAAGAGTGGCCACTGCAAAGTAGAAAAGACCTGGACAAACAGTCCCAGTTCTTTCCAAAACAAATGCTACTGGTTTCCCCCTTTCACACCCAATGGATAGCAATACAGCACAGTAATAATACAGTAAGATGCACCCTCAAATTCAGCCACACATAAGAGTGCCATCCTTTTCCACATCAAGGTTTAGGATTATAAGCCATTTTATAAGTCAGAATTGTGGCTTACAGAGAGAACATGATTTGGACAGGAGTAGGTCTGAACCCTGACAGTGAGCTTGGCTATCTCCTTCTAGTAC

							ACAGTGCAACCCAACAAAGACCACTGTTTCCTGCAGCAAAGTTCCAGGGCCTTTGTACACAGGAAGG<br>ACTAACATGATTTTCTTTATGGAAGGAGGTGGGGGGTAAGACCATTTAGATGATACACCATTAACCCTAG<br>CACTCAGGAGATGCAACATTTACTCAGTTTCTCTTACTTGGTCAATTGTTTTGTTTTTGTTTTGAGACAA<br>AGTCTCAACTATGTAGCCTTGATTGGCCTGCAGACCAGGAAAGTCTTGAACTCAAGAGATCTGTCTCTG<br>CCCACCTCATGAATTAAAGATGTGAACCACTACACCCAGCAATTTCTCTTAATTTGGGGTTAATGTTCAA<br>ATTATCTGGCCTATTTGTATTTTTAAAAATTATACATTTTGTTTGTTCATACAAACAAAATGGAATTTCCTA<br>AGAAACTGGATCTTGTGGTCAAGACCCAAAATCCAAAAGCAAGACCCCAGCATAACATTGAGGAACTA<br>CATGTACCTACTTATTAGCCCATCCAATTGCCAGCTACATCCTACCAATCCCCTGCAGGGAACCAGAATA<br>CTCTCAACCATATCTGGACATCCATACAGTTTAAATGAGAGCCAAGAGGCAATGACGGTCTGGAGAAAC<br>GACCGTGCTGTGCCCTCACACTCCAGCAGCATTTTCTGGCTTTCATAAAGTCACATCACCAGAACTGCT<br>TCTGCTGGATGGATCATTTCCCTAAACTCTAACAAGTATCTCAAAACAAATACCTTTATAAGTAGTACATT<br>CAAGTAACCCCATTGTTCTGCCCTTAATCTCGCTATTGGTAAGATAGACAATTCCCACCTTTATATCATCT<br>CCTACATTAGTGTCTACTGAAATTTGCTAGGTAGGAGGGAGACATAACACCCTTGGAAAGCACTTGGAG<br>AAGAATAATGCTATCTATATCATCTGGTGACTTGCCTAGAAAATCAAGTCCTGCTCATTAGGGTACTTATT<br>CCTAGCTGTGGACATACCCTATGCCTGCCTGGTATCCTCTTCCCACCTTTCTTTATCCTACATAATGCAAG<br>CAGACAGCATATACCTTAAGAGGGTAGTGGAAGGCTGCAGAGATGGCCCAGCAGATAAGAGCACTGAC<br>TGCTCTTCCAGAGGTCCTGAGTTTCATTCCCGGCAATCACATGGTGGCTTAAAACCATCTGTAATGGGAT<br>CTGATGCCCTCTTCTGGAATCTGAAGACACAGTGTACTCATATAAAGTAAATAAATTAATTTAAAAAAAA<br>AACAAAACACTGGATTGGTGACAAGATCTATGCCATACAAGACACTTATTTAATACAAAATAGGCAGA<br>GACAGGCTTCTTGAAGGGCAGGGACAAATGAGGAGAGACTGAAAGGCAGTAGCACCAGAATCAAGAT<br>GACACGCTCTAAAGTCTTAGGAGTAGAACAGCAAAACTGAGCTGTGACTGAATGACCAGAGGCTGGCT<br>GCTTAGATCCCTAGAGTCACCATCCAAAAGCATTCAGATAGCAGGGGCTTCAGGACCAGGAGACTGGA<br>TTGGGGGTAGGGGCTTACTATGGGCAAGTGGGGAGTAAAAAGATCAAATGCCAAGAACATTTTCTTTTC<br>TCAACAGCCTTGAGAACACATCTAAAAAAAGATATTTTGTGTTCACACAACTGTCATTTATGAAGAGAA<br>AATGTTAAAACCTCACTTGTATAAATAAGATTGCTAAATTCTGCCAAAAGAAATGGAAATGAATGCAATC<br>TTTCTAGAAGAGTAACTTACAGAAAAATTTCAAATACACATTTTCTGATCTAGTGGTCTTATTCCTAGAG<br>ATGAAAGAGAAAATCTGAAAACCACATCCAAAAATAGAAATTATGGTAGACTTACATGGTACTTTAAAG<br>TACTTACTTTTTGAAATGCCCACCTGTATAGTAATTTGGTAAAACAAAAACTAGCCAGGCTCTGCTGGTG<br>CATAGCAGTAATCCCAATAATTAGGAGGCAAAAACAGGGTTGATCATGGATTACAAAAGCATCCAGGGT<br>TACTGAGGGCATTTGTCTCAAAAACAAAAATTCAACCAGAGCAGGTACTCAGTAGATCAAAGAGTTTT<br>AGACCAGACAAGAGATTGCCAGAACCTACATAAAGGAGAAGAACCAAATCTAAAAAGTTGTCCTCTGG<br>CTTTCACTTGTGCACCAAAACTCACATATTCTCTCACAATTATAAATAAATACAGGGGTTGGGGATTTAG<br>CTCAGTGGTAGAGCACTTGCCTAGCAAGCACAAGGCCCTGGGTTCGGTCCCCAGCTCCGAAAAAAAG<br>AAAAAATAAATAAATACATACATACTATACATACATACATACAAACTCCACAGAGCGAGGGCTATAGGTC<br>AGTGGTAAAGTACCTGACTAGTGTGTGCAAGTTCTACAGACACCCACTCCTAACAGAGTCCAATTCAAT<br>TATGCTATAAAAGTATAGGTAAAACAACTGAAAAGAAACAAAGATAGGATTAAAAGATAAATTGGGGGG<br>GGGGGGTTTTAGTTTTCTTCAAAACAGGATCTCTGTGTAGCCGTGGCTGTGCTCAAACTCAGAGATCTG<br>CCTATCTAGTACCTTCCAAGTACTAGAACTAAAGGCGTAAGTGTACCATCATCGCCCAGCAAAAGAACT<br>TTCAACCTTAAAAACATCTTAAACTTAACTTTCCAAATTCTTTAAATGACCACATATTACTATTGTTCAGA<br>TAAAATGAAAAATGATGTGGTGTGGTGGTACACACCTTTAATTACAACACTTAGAAGACAGAGGCAAA<br>CAGTAAGTGGCTAGCGGGGTCTGCACAGTGAGACAAATGTTACAAAAAAGTGAAATAAAGGGCAAGT

							CAAAGCTGTAGCAGTTACAGCACTGGAGTAAGCACCTGGCGAGTGTCTGTGAAGCCCTAGATTCCATATTCCCTTGGGCGTGGAGTTGAAGAGAGGTGTATGTCTACACTGTTTTAAACCTAACACTAGACACTCTCTCATCAATTTATTCAGAATAAGATATGTTCTGTGGTGTACCTTGGGAGATGCACATACAGGTTTTATTAAGATTGGCATGCTGTTTAGATTGGGAGAAAAAAAAATCTTCAAAAATAAAAGCTGCCAGGTGTGGCGGCACACACCTTTAATCCTAGCATTCAGGAGGCAGAGGCAGGACTTCAAGGCCAGCCTGGTCTATAAAGGGAATCCAAAACAGCCAGGACTACACAGAGAACTCCTGTCTCAAAACATATGAATGGATGAATGGATGGATGGATGAATAGCTGGGTAGTGATAGCACAACACATTATCCTAGCACTTGGGAGGCCAAGGCAGGCAGACCTCTGTGAGTTTGAAGCAAGCCTGGCCTATGGAGTTGAGTTCCAGGATTGCTACGGCTCTCCAGAGAAATCCTGTCTTAAAAAACTGAAACAGCAAAGGAAAAAGGCCAAACAGAACCGGCTCAGCAGTTAAGAACACTTGTTGACCTTACAGAGGACCAAGGATTAACCAATCCCAGCACCCACACAACCATCCTTACTTAACCCCAGGCCTGAATGTAGTACACAGATATATACAGGTAAAACATACATGCACATAAAATTAGTATACTCTATACATACTTCACACACGCAGGCAGACACACACATATGCCCAAATAAATTTAAGAATACAAAATGTGGGGCTGGAAGTGGTGGTGCTCAAGTTTAATCCCAGCACTCAAAAGCAGAGACAGGCAGATCTCTGTGACTTTAGGCCAGCCTGATGTAAATGAAGAGTTTTCCAGGACAACCAGAACCACCTAGTCTGAAAAAGCAAAACTAAAAAGGGGTAAATTTTGGCTACTTCTTCCAGACTTTCACCTATATACACACGGGTATCACACTGATGTACAAGCACTTACATTCTTGGACTTCCGAATCTCAAATGTTATTTCATTTCAACACTATGATACAAGCACTAGAAAGGCAATGCAGTTGGTCTCTGCAGGGCTGGGAACACAGCTCAGATGGTACGGTGCTTGCTTAACATGAGCAAAGCCTAGTATATGATCCCCAGCACTATATAAATGATGGTGGTGCACACTTATAATTCCAACATTCAGGTAGAGACAGGAGGACCTTGGCTATGTGAGACCCAGTCAGTCTCAACACCCTCCACTGAAAAACAAAAACAAAAAAATAAAAATAAAAAACACCAAAACAGTCTCTGTACATCTTCAAAATAATGAAAAAGATTACCATCTGACCTATCAAATCACTCCTAAAGATCTGGCCCATAAAATTCATCCATATCCACATAAATACTTGTATACAAACTTTTTTGCTTATAAACAGAGCCTCAAAATGTAGCCCAGGCTGGTCACCAATTCTCTACCCTGTCCCTGACTCCGAAGAGCCACCATAACTGACCTTGTATAGAAGTGTT
MSTRG.111024.1	ENSRNOG00000050249	0.629	0.032	0.043	-2.606	down	CCCTATTTCCCTTGCCCAACAACTAGCTTTCCGAGTATAAAGTTAGATGAATAGTCACCCTTACCCATATCCCCCTATTCTACCTCCCTTCTCCAAAGAGGGAGAGAGTGGTCCTCATTTCTCCTTTACCCGGCCTTCAAAATCCTGCCAAAAAGGATAAAAGGCAACTAACTCCTTAGTCTGCAAACTAAAATCCTCCACCATACAACTGTAAGGTCCAAAATGAACCTCTTCTGGGACCCCTTGAATGAGGCTCACAGAAGAAGAGATAGATGCAAAAGCAAGAGGAGCTTCATTGTTCCAATATGCTGAGATTGCTCTATTTCAGGAGAACCCCAAACAGAATCTCACAAGGAGTTATATACCTTTGATAGAGGCAGATTTCAAACAATAAGGGCAGAATTTGAACAATGGTAACTAGGCAGGGTACTATGGGTGGGGCGAAGCGACCTTCAAACTGACTCATGGTAGGTAGAGCATCCAGGACTTTTCGGGGGGGGTCAGTTGAGGGGTCACAGGTGGTTTTGTCAGACGGTTGGCTTACAGTTGTCTCAGGAACTAAGCTGGCTCTTGTCTTGGAAAGGAGTAGTCTTTTGTGCTTAGAGGTTTGTCATAGAAATTTCCCACTGGCCTCAGATTGACCCCAACTTCGGTTCAAGATATTTGCTGCTGCTCCTTGCCTGGTTCAATTGTTTTACAATGGAAGGCTCTTTTCTGGAGCAAATATCTTCTACAGATCAGATGAAATCAGCACCAAGTGATGTCCCT
MSTRG.111027.1	ENSRNOG00000050249;ENSRNOG000	0.694	0.000	0.017	-3.928	down	GGGAGCTTGTGTGTGCTCTATCCACTGTGTCAGCAGCAGCCATTTTTGGAAAGAGTTGATTTCTGCCATGCTGCCTATCCAGAGGCTTGCTAGACAACCTGCATCTAATAGATGAGGTCCATAGTGATGGTCAATTCTGATTGCAAGGGGAGACTATGGAAAGACCATATGAATGGCAATCTCAATCTTGTCCTTCAGATTTGTGGAGAACCTCTGAGAGCCTCGTGTCAACAGTCATCCTAACTGTAACACCTTCACTCATCTTCTGGGAGGTAGGACTATATAGATTTGTTAGTGATGCCTAGAAGACAGAATTGGAAGCGTTACGGTGTTAGCCCTCAGACCCGTTTCCTTCTGTGGACTCCCTTCTACCTTGTTGTCC

	00057162						
MSTRG.111042.1	--	0.372	0.000	0.028	-3.770	down	GAGGTTTAGGAAATAGATAAAAATATGTAACAGGGATTACAAACTGGAGATGGCCACCAGAGGGTCCCACAGACCAGGAAATGAGAGGCTCCTAGGACCCATGAGAATGGCTTTGCCTAAATATGCAGAGAACAGGGACATAGATCCTGAAGAGACCACCTCCAGTATATAGGCACAGTCCCCAATGGAGGGCTGAGCCTGCCCATCTCAAAAGTATTTTACCCAGAAATGTTCCTGTCCAAAGGAAGAACAAGGACAAAATATGGAGCAGAGACTGAAGGAAGGGTCCTCAAGGAACTTTCCTACTTGGGAATCCACACTGTGTGCAGACACCAAACCTGAAACTGTTGTCTTTGTCATGAGGCACTTGCTAACAGGAACCTGGTTTGACTGATTCTTGGGAGGCTTGGTTAGCAACTGAGCAATGCAGATGTGGATGCTTGGAGCTAACCATCAGACTGAACTCAGGGACCCTGGTGGAGCAGCTGTCACAGGGACTGCAGAAGGAGAGGGTTATTGTAACCCAATAGGAAGAAAAACATTGGCCTAACAACCCAGAAGCCCCAGAGTCTTAATCACCGAACACGGTGTGTACAAGGACGAATCCATG
MSTRG.111045.6	ENSRNOG00000051596	3.126	0.369	0.026	-2.257	down	CAGGTTGATCTTTTTCTTTTTCTGTTTCCTCTGCCAGGTCGTTTGACAGTTGTGGAGCCCCTCCCACCAATAAAGCTTTTGTAGGTGAAGAACCTGAAGGATCTGAGGCTGTGCCAGTACATGCTGGAGAAATACTTGACTGGACTCGGGAGTAGGAGCCTAGGCATTGGCCCACAGATGTGCTGTTCTCCTTCTTTGGATACCCGTCGCAACAACACAATCTTCTCTGTCTACTGGAGTTGTCTTGGTTGTAGATTTGGAGATCCTCAATATCCCAGCTTAGTTCTCTAATGTTAAGTCATATCGACTCTTAGCATAGTACTTTATTTGCCACTGTTCTTTCTACCTTTATTTTGCCAGAATTTTCTATGGGTAAGTTATTCCATCTATAACACTTTATGATGTGTAAGCCCACACGAGGGTACCCAGAGCCCATACACAATGTGCTCTTATTTACCTGTGAGAAGTCCAAGCCAGTAACCCCAATCTTAACTCAGTGGTGAGAAGGAACTTAAAACTTAGAACAGGACTTGTTTTCATTCCTTTCTTGTTAGTATCATTCACAACTTCTGTAGAAGTTGGGAGTCCAGAAAGCAAAGGGCGGGATTTTCTCACATGATCTTGACCCATGTTGGGTTTTCTGTCTTCCATCTCCACCTTTAACATTTAATATACCACATCTCTCCAAAGGTCATGCCTATTCTAGTGTGCTGCACTCAGTGAAGAAGTTG
MSTRG.111052.9	ENSRNOG00000051596	1.359	0.000	0.000	-4.880	down	ACCTGATCTCAAAGATGTCAAAATTCTTCTACACTGTTTCTCCCAAGTCACTTTCCTGGGTGATCAGTTCTCTCTGCTTCAGAGTGTCCTGCATTGTTTCCTCTAATCCTTCCTTATTCTTATTCCCCAATCTGGCTTAGCTCGTTCCCAAGGCTGTGTAGAGCAGGCTTGTTTCCCAGCAAAGGATCATATGTTGGTGGTGGATTAGACTCTGTTCTTGGCCACTGTTTCAGGCTTGCCCCCAAAGTGTGCAGCTTCTTCTAATAGCAGCTCTCTTTACCTTAACTTGAATGACTTCGTGCCCAAGGCTATTGGAACAGAATCATAGGAAATCTCCATACCCAGAATACAAATGAACAGTTTTTCTCCCCATAATGTGATTGTGTCACAGATGTTTTGA
MSTRG.111054.1	--	0.499	0.030	0.010	-2.792	down	CTGAGGGTTCCTTCTGGATATGAGTCAACACCAGCATGCAGGAATCCACCAGGATTGGACAGACGATGCATCTGACCGGAAGAGGGGAGTAGTAAGTTTCTGGCATGCACGGAAAGATCCCTCACTCTGTGTAATTACCCAGGAAATGAAATAGTCTTGTCCATGTGGACATTGGTGCCCAAATGAGAAAAATAGTGTATATATCCTGACCACTTAAAAGTCATGCTTTTTCTGAGAGAACCTCTGAGCTCGAAAGACTGTGAAATAGGATGTCTGCCTGTACCAGAATCTTTCCTGAGTGGAGAATTTTATCAGGAAGAAAGAGGCCCAGAGCATGTACATATGTTGAACTCTTGAAGGGCACCCAGTTTGAAAGAGAACATCAAAGGCCTGAATCACTGTGAAACATGCTGTCTGCAAGTACTAGAACACTTCCTGAGAGCAAGGGACACTGCCAAACCACTAGAGGCCAAAAGTTATGATGGATAAAGAGACAGGAGTTCTGGTGACCTGTAACCACATCAGGAGCCCCAAGGGTCACCTCCTGCTAAACCAAACATAACCTTTATCAGTGAAGGTCACTCCTGAGAAACCCTGTTGAGAAACCCTGCAGACAGGAAAGCCAAGGAGGCCTCCTCCTTCCCATTGTCATTGTCTTTTAAGGGAGCTTGAACAGGTTGGCTCCTACAGACTTCTTAAAAAGCCTGGAACACTCTGCTAATTCTGTGTTCCCGTACTCTTCAACTGAGTCAAGAGAAGAAAACAGATATTTGGAACTCTGATCCAATCAAATTAATATTCTGGTGATCAGAAGCCAAGACTGTACAGAGATTTCTGAGGCCTCTCCAGGAATTCTGTCCAGAATCACGCTCTGGAGGACTTCAGTGTGCGAG

							AATGTACAGTACATTGGATGGACTCTGGGGTCTAAGGAATGAAGTTTGTCCTCATTAGGACAAAAAGATACAAGAGGAACGCCCGAGTACATATAGAACACAGGGTGCCATGATGTGGAGCCCGAAGACAAGGGAGGCTGCTGAACACTCTAATGCCTGTATGAGTGAACTTGGACATGAACAGTGTAGCTGGATCTGTTTGCCTTCAGTTCAAGCTGCAATGTTGTCACCATCTCCTCTTTAAGCCCATGTATCCTTGACAAGGTTGTTCTGCCCTTCAAGCTCATATGATGGTTGTGCATGTAGGAGACCTAGCCTTCTGTCACCAAGGAAACAGAAAGCAGAGGAGGAATGTGACCGTGGGAGGCTGCAGGCAAAAGTGATGTGGGGCCACAACTGTACCTGGAAGCTACATGGTACAATCCCTGAGGCCCTGTTCAGACCTTCATTCTATTCTGGTGTCTTCCTAGTGACAGCCCTGCTAGCCAATGACAACAACTTCTCCCTGATCTTCCCCAGCCTTGCTTCCCAGTCCTGAGACTTGAAGAGCACAGAGGAAGGCCTCTTGTAGGTCAGTCACTGGCTCTGGACCATTGACCACTACCACTGCTTTTACTTGGGATGGTGTTCAAATTGTGGGATGTCTGAGAAAGCTGGGTTCCACGTTTTTGCTATGCAACCTGTTCCTCACGTCCTGGACTTTGCCGGCTCCAGTCCTCAGGTACTATCATGGAAGCCATTACTCTCTCCACAGTCCTGTCATTCTTACCCACAA
MSTRG.111055.1	--	1.071	0.118	0.048	-2.203	down	CTAGTAACTGAACTCTACTACCTATTATTGGAAAAGCTTTTGTAGGTGAAGAACCTGAAGGATCTGAGGCTGTGCCAGTACATGCTGGAGAAATACTTGACTGGACTCGGGAGTAGGAGCCTAGGCATTGGCCCACAGATGTGCTGTTCTCCTTCTTTGGATACCCGTCGCAACAACACAATCTTCTCTGTCTACTGGAGTTGTCTTGGTTGTAGATTTGGAGATCCTCAATATCCCAGCTTAGTTCTCTAATGTTAAGTCATATCGACTCTTAGCATAGTACTTTATTTGCCACTGTTCTTTCTACCTTTATTTTGCCAGAATTTTCTATGGGTAAGTTATTCCATCTATAACACTTTATGATGTGTAAGCCCACACGAGGGTACCCAGAGCCCATACACAATGTGCTCTTATTTACCTGTGAGAAGTCCAAGCCAGTAACCCCAATCTTAACTCAGTGGTGAGAAGGAACTTAAAACTTAGAACAGGACTTGTTTTCATTCCTTTCTTGTTAGTATCATTCACAACTTCTGTAGAAGTTGGGAGTCCAGAAAGCAAAGGGCGGGATTTTCTCACATGATCTTGACCCATGTTGGGTTTTCTGTCTTCCATCTCCACCTTTAACATTTAATATACCACATCTCTCCAAAGGTCATGCCTATTCTAGTGTGCTGCACTCAGTGACGAAGTTGGAGCAAGTGTCAAAAGAGTGAAGGCCAAGCTCTGACTGTGACAGAGAGTTTGAGATGGACAAATGATGCTTTGAAAAACACGTGACTTCTCGCTGGGACCTCCATCTACTGTAAATAACCAACAACCTGTTCAAGGATCAATGTGCCTCTGAGATGAGGACAACAAGGTAGAAGGGAGTCCACAGAAGGAAACGGGTCTGAGGGCTAACACCATAACGCTTCCAATTCTGTCTTCTAGGCATCACTAACAAATCTATATAGTCCTACCTCCC
MSTRG.111064.1	--	0.000	0.493	0.031	3.453	up	CTGAGCTGTTGTCATTGGAAGGCACTTTAGTTTTGATGCAGTATTCTGCCAGGGTTGTGGGGACGTTCACTCCATCCTTTTCTGCTTCAGGTTTCATGGATGAGACCTGTTTCCTAATGATCTCAGCGTACTCCTTGTCCTTTCCTTTGCTGTCCCTCCATTTCCTGAACATGACTGAAGCATCCACATTGGCTGGAGAAAAG
MSTRG.111112.2	--	1.646	0.140	0.010	-2.574	down	GGCTACTGGGAACTAACTGAATTCACAAGAAACAGTGTTTCAGTTCGTTAAAACGTTGCTCTATCTTGAATAACAAGCTTATTACATGCGAATCCTATTGGGAACCTACTCAATTCACCATGATACTTGGATTCCATTCCTTAAAATGTTGCTCGATAATGAAAAGCAAACTCATACAAGCATGTCCCATTGGGAACTCACTGAATTCGCCTAGAAATTTTGATTCCATTCGTGATAATTTTTCTATATCCCGAACAGTCCACTTATTACTACTGCGGCCTATTGGGAACTAACCGACTTCACCATGTTACTCAGATTCGGCTCAACAAATTTTTATAAATCTTTAAAAGTACACATATTACAAGAGCAAGCTACTAGGAACTAACTGAATTCACAAAGAAACAGTGTTTCAGTTCGTTAAAACGTTGCTCTATCTTGAATAACAAGCTTATTACATGCGAATCCTATTGGGAACCTACTCTATTCACCATGATACTTGGATTCCGTTCCTTAAAATGTTGCTCGATATTGAAAAGCAAACTCATACAAGCATGTCCCATTGGGAACTCACTGAATTCGCCTAGAAATTTTGATTCCATTCGTGAAAATTTTTCTATATCCCGAACAGTCCGCTTATTACTACTGCGGCCTATTGGGAACTAACCGAATTCACCATGTTACTCAGATTCGGCTCACCAAATTTTTATAAATCTTTAAAAGTACATGTATTACAAGAGCAGGCTACCAGGAACTAACTGAATTCACAAAGA

							AACAGTGTTTCAGTTCGTTAAAACGTTGCTCTATCTTGAATAACAAGCTTATTACATGCGAATCCTATTGGGAACCTACTCAATTCACCATGATACTTGGATTCCGTTCCTTAAAATGTTGCT
MSTRG.111112.5	--	1.019	0.040	0.020	-2.988	down	AACAAGCTTATTACATGCGAATCCTATTGGGAACCTACTCTATTCACCATGATACTTGGATTCCGTTCCTTAAAATGTTGCTCGATATTGAAAAGCAAACTCATACAAGCATGTCCCATTGGGAACTCACTGAATTCGCCTAGAAATTTTGATTCCATTCGTGAAAATTTTTCTATATCCCGAACAGTCCGCTTATTACTACTGCGGCCTATTGGGAACTAACCGAATTCACCATGTTACTCAGATTCGGCTCACCAAATTTTTATAAATCTTTAAAAGTACATGTATTACAAGAGCAGGCTACCAGGAACTAACTGAATTCACAAAGAAACAGTGTTTCAGTTCGTTAAAACGTTGCTCTATCTTGAATAACAAGCTTATTACATGCGAATCCTATTGGGAACCTACTCAATTCACCATGATACTTGGATTCCGTTCCTTAAAATGTTGCT
MSTRG.111146.1	--	1.100	0.000	0.013	-4.001	down	AGCTACCCTACCAGCCCACAAGAACCTTAGCTCCATTAAAGCTTCCTGATGGTTTTCTTGTCTAGAATCCTTTGTCCTCCCTCCTTTCTACACATCCTATTCCTGTGCCACTTCTTCCTCTTCCTCCCTCTTTTGAGACTGAATCTTACTATCTTGGCAAGCAGTCCTAGCTGGATCTCAAATGCCAGGTCCTCCCACCTCAGCTTCCTGGATCGTGGCCGTGTACCAGGTGGCCTGCTCTTTCCAGCTTCG
MSTRG.111161.1	--	0.658	0.000	0.000	-6.188	down	CTCTGGCTCTGGTCCTGGGGTTCCATATAATGTGTAAAACACTCACTGGACAAGCACTGTCGAGTCAGGGGCCAAACTCCTGGCCTATGTCAGCTTTGATAGACTTTTCTTTGTGGTGTAACCCGTGTGTGTCCTCTTGAGCAGCTCTCAGGTCAGTCTTAAGTGCATCTCAGGGTGTGAACTCAGGATGCAGGCTTAGGGTGACTTTGAGGTCCATGCTTAGCACCAGGAAATAGGCAGCAGAAGGGTTGAGTAAGAGGCCTGGCTTAGATGAATCCCAGAGTATCATTCTTGATAAGACTCCCGGACTGACAGGTTTATTTGGCCTCTAAGAGCAGGAAATCCAAGGTTAAATATTTCCCTGATACACTATGGGAATGGTCAGAAGTTCAGGCCTAGGAATCTGGTCTCCTGTCCCTAGTCCAGCACATAGGTAGGAGGAGAATACATTCTTGCATGCCCTGTCTTTAACGTGAACAAAGACCATGTGATTTCGGGAACCAAGGCAGTTGCTTCATGATGCTGTGACTTGCAAGAGAGAGAAGCAGCTAGAGCCAAGGTACAGATGGAGGCTGAGGCAGGCCCTGCTTTGGTTCTGCTTCAGTCAGAGCAAGAAAGCCCTTCAGTCCAGCTGTGTGGCTACATAGGTCATGATACCTGGCACTTGACCTCAGTGTGTGTCCTACTCTTCTGCTAGCTTGTCAGCCATCCTGTGTGGTACTCAACCAATCCTGTATATTCCAGAGACTTCCCCTATGCCCTTGGTGTGCCAGTTCCTATAAGGACAAGCTATCTTGTGGCTATTCTGTTGTGGTGCAAGATGTCACGTAAAGGACTTTCCTCTTGCGAGAATTGATTCATCTGGTCCCGGTCACATTCCTGACCTGAATCCTCCATACAGAGGCAGACGTGCTCTTCCAGACTGATAGCTGACACCTAGGCTGCTTTGGGCCATGAATTCAATCACACACTCCCTCCCGTGTTCCTCTTCCTTTCATTCTAGATGTAGAGGGTGGTGTGATGTCAGTATCTTCTGGAACCACCCACGACACCACCCTAGGGCCACTGTACTGCTCTGCTGCCCTGCACTGTTATTCTACTAATGGTGACCTCCTGTGGAGTTTGACAGAGAGCATGGCAGTCACAGCACTATGGGTCATGGAGGCTATGATATGGAAATAAGAGTCTCCCATGGGTCATTTCTGTGAATGGCAGCTCCCAAATTCTAACGGGGGAATCCCTGAATGATTCTACGTAGGTGTAGTGTTGCTGAGTCCTGGACCTTTAAATAAATCTGTAAGATTCTCATGTCAGACACCTTGTCCTTGGATTCATTTCTGAGTGGTATCTGTACTGCTTTGAGCTGTGGGTTTCTGAATTTTGTAGGGGTGAATCTTGGAAATATCAGGCTCTGAGCTTTGTTTCCAGTCAAACAAGTTTTAAAAGGGCCAATAACCACAAAGGACAGAACTTCAGTTTTAATGGTAACTGTGAGGCCAGTCTATTTCCAGCCCCTTAAAGGGGTAAAAATCTTTCTTGGACTATATATCAAGTGAGATACCATCAGCACCCGACATGTCCAAAGTCAGGACTTGCAAAATTATACCAAGAAATGTATGAAAAAAGGCCTGTGGATACCTGGGCACAAGATTCCTGAACCTGCTATCCTGAACAGACAAAAGTTTCACAATCATAGTCCTGGTTGCAACAGCATCAGCCACAGGCTTCTACTGTGGAGTGGGGCCTTCTTCATAATCTAGGCTTCAGACGTAACCTTTGACCTGAGTCTGTCAGACCCTCTGTATGCTGGAAAGTCTAAGGAGACTTTGGTCCTTGCACCTTTATCCTGTGCACAAATGCTTTCAAATATTT

							CTTAAAGATGTGACTTGGCCTTAGATGGACCAGGCTTCTTGGATGCAGTCCATCTTCTAGGCCTGGGCTCATCAGGTAGAACAATCTCTTCAGTATCAAGAAGCTGATGTCTGAAGAATCAGAAGCTCTGAAATGATAGCCAAGGAGACATGCAAGAGCTGAGGGAGTCAGACAGGGAGCTTGTGTGTGCTCTATCCAGTGTGTCAGCAG
MSTRG.111163.5	ENSRNOG00000026976;ENSRNOG00000060573	0.938	0.000	0.000	-8.629	down	TTGAGGCCAGGATAGTCATGGATTCCTGAGTACAAGGACAGTGGCTCATTGTTGCTAAGGATCCCAACTCAAATTACCAACACTGGACTGAAAACACAAACTAAGCATTGTGACGTGTAAGGCTCTCAGGAGAAGCCAGATCCCAGTGGCATTAGTGTGACAAACAAGCTGTGCCCAAGGAGCTGACACTCTGGCAGAGTCCAGGACATCCAGGAAGACAGAGGTGGTGAGATATCACCAACTATGACTCTGACATACAATGAAGCAGTCGAGGGCCATGAGGCTTTTCTGACTGAGGCAACACAGCCTGGAGCCCAAATCCCGAGGCTGTGCACCAAAGTCCTTCCGTTTGTCACTGGTGAGCAGAGTCTGACCTGAAAGTCTGGACCTGATCTCTCAGTGAAGGCAAAACCACTTGACCTCTGATTTTGAACTGCAATTGTAGCTAATGCTGCTATTGATTTAAAAATCTAGTTCAATATGTGAAAAAATACACTCTTCGATCTCCTGGAACATTCTGTCTCAGAGAGGACCATGTCTACACGTCAAAGGAAAGACCAGATCCTGGTGCTTTCCCACAAGAAGTTGCTCAATGTGGCTACCAGGCAGGCCAAACAAAAGCCTCTGTCTGCAGACAGGAGGGCACCAACAATGTGTCAAGCAACCTCATCTGCATCGTCACAAGCATCTGCAGTGTGGATATCTGGTCAACATGGTATGGCCCCTCCCTTCCCTGGCTGCCACCATCAAGTGTCATATAGGTCATCCATTTCAGTGCCAAGACCTAGAGGAGTTCTGCTGGTTAGGGCTTGGAAAAGGTCCAAGTGCAAATGTGATGGCGTTTTGTGTTATTGGTTATTCCTTAAATGTGGGAAATCTGTGTATCCAGCTGTTACAAACTGCCTTCTTCTCTTGGTTTTCTTAGAATATGAATGGAGATCAGACATTCATAGAGAGTGAAGCCATGTCCACCTGGTCCTACCGAGCAAGAGTTGGTAGCTACAGTTTGATAAATTTGACTAAGAGAACCAACAGATAACTTCCACTGTTGTCTGAGTTGTTGTTTTTTAGTGTCAGTGTGATTATCTGAAACAAAATATTTTTAGTTTCATTTCAATCCAGTGACTTAACACTTCCTGGTATACTGCATTAGCACAGTGTAGCTAAGGCACTACAAAGGGGCTACCCATGTTCTATCCACAGGCAGGAGTGTGAGAAATTAATGCATGCATGTATTCTTGCTTGCTAGTGTTCATCTAAAGGCCTCTGCTCTTATGAAGTCTTTCCCTAAGACTAGGGAGTGGCACTGCTAAAATTCTTGTTACTTCCCATGTCATTTAAGATCATAAAATCGATTCTCTACAGACTTGCTCACAGGAGAAGGATCATCTAGAGGATTCCTTTGTGAAGACTCTCCCTCCAGGTCACATTTCCTGTACTAATTGACAGTTATAATTAAAGATCATGAATTTCTCCCAGTGGCCACCTCGCCGCTCAACATTGAAAACTGTCAGACTTGTGACTAGAGGCTTTAGCTTCTCATTTTACCTAGGGTCAAAGCTGATATCTTGTAGAGTGTCCTTTCTGGCCTTCTCAGTTTCTCCCCATATTATACCTGACAGTGTTTCCATTCAGCCATCAGCTCAACACTATGATGTATGTACTGTATCATTGACTAGTACTTTCTAGGCAGAGATAAAAATTTCACAGCAGACTTGTTGGCCTTCAGGTTGATCTTTTTCTTTTTCTGTTTCCTCTGCCAGGTCTTTTGACAGTTGTGGAGCCCCTCTCACCAATAAAGGTTAGCAACTTGTCGGGCTTTCATGGCATTTATGGCAGTTAGATTGTGCTTCTTAGACTAAGTCCAAATCAGTGCTGCTAGGTATGTATGATGCTTATATACTTTACCCTAATTTAAACAGATGGTGTGGGCACTGAGAACATGTAGCCCTGCAAAGCATTTTAAATTATTTACATATTTTCTCATTTACCTATTCACTCTCCCCTACATTCTGTCTGTAGTCTGTCTCTTCTATCTCTTCTCTTTCTACCTTTATTTTGCCAGAATTTTCTATGGGTAAGTTATTCCATCTATAACACTTTATGATGTGTAAGCCCACACGAAGGTACCCAGAGCCCCTACACAATGTGCTCTTATTTACCTGTGAGAAGTCCAAGCCAGTAACCCCAATCTTAACTCAGTGGTGAGAAGGAATTTAAAACTTAGAACAGGACTTGTTTTCATTCCTTTCTTGTTAGTATCATTCACAACTTCTGTAGAAGTTGGGAGTCCAGAAAGCAAAGGGCGGGATTTTCTCACATGATCTTGACCCATGTTGGGTTTTCTGTCTTCCATCTCCACCTTTAACATTTAATATACCACATCTCTCCAAAGGTCATGCCTATTCTAGTGTGCTGCACTCAGTGAAGAAGTTGGAGGAAGTGTCAAAAGAGTGAAGGCCAAGCTCTGACTGTGACAGAGAGTTTGAGATGGACAAATGATGCCTTGAAAAACACGTGACTTTTCGCTGTGACCTCCATCTACTGTAAATAACCAACAACCTGTTCAAGGATC

							AATGTGCCTCTGAGATGAGGACAACAAGGCATCACTAAGAAATCTATATAGTCCTACCTCCCAGAAGAT<br>GAGTGAAGGTGTTACAGTTAGGATGACTGTTGACATGAGGCTCTCAGAGGTTCACCACAAATCGGAAG<br>GACAAGATTGAGATTGCCATTCTTACGGTCTTTCCATAGTCTCCCCTTGCAATTAGAATTGACCATCACT<br>GGGGACCTCATCTATTAGATGAAGCTTTTTGATACTGAAGAGATTGTTCTACCTGATGAGCCCAGGCCTA<br>GAAGATGGACTACATCCAAGAAGCCTGGCCCATCTAAGGCCAAGTCACATCTTTAAGAAATATTTGAAA<br>GCATTTGTGCACGGGATAAAGGTGCAAGGACCAAAGTCTCCTTAGACATTCCAGCCTACAGAGGGTCT<br>GACAGACTCAGGTCAAAGGTTACGTCTGAAGCCTAGATCATGAAGAAGGCCCCACTCCACAGGAGAA<br>GTCTGTGGCTGATGCTGTTGCAACCAGGACTATGATTGTGAAACTTGTGTCTGTGGATAGCAGATTCAC<br>GAATCTTGTGCCCAGAGGTCAAAATAAACCTGTCAGTCCGGGAGTCTTAACAAGAATGATACTCTGGGA<br>TTCATCTAAGCCAGGCCTCTTACTCAACCCTTCTGCTGCCTATTTCTTGGTTCTAAGCATGGACCTCAAA<br>GTCACCCTAAGCCTGCATCCTGAGTTCACACCCTGAGATGCACTTAAGACTGACCTGAGAGCTGCTCAA<br>GAGGACACACACGGGTTACACCACAAAGAAAACTCTATCAAAGCTGACATAGGCCAGGAATTTGGTTC<br>CTGACTCGACAGTGCTTGTCCAGTGAGTGTTTTACAAATTATATGGATTCCCAGGACCAGAGCCAGAGT<br>CACAAAGTCAGGAGCAGTGTATAGTGTGAAATTAATAGCCAACAGAATCTTCGGTAGCAAGCTAAGAA<br>CCTGCAGAGAAACCTCAAACCCAGCCTGCTGGATGGTGCCAAGATCCAGTAAGGAACACTTCCTAAGA<br>GAAAACTCTTCCTGGGAGAATTGCAGCCCAATAAATGGATGCTCTCATGATTCTCGGTGAAATAACTCAT<br>GGACCTTTTTTCCTTGTGAACAGGGAACTTCAAAACATCATGGGGTGAGGTGAGGTGAGATCCAGGGA<br>AGAAGGCTGCCCTTTGGCTCAATCTGAGAAGTTAGGATGCTTCATTTGCAGGGGGGAGGATATCAGGTG<br>TTGTCCTAAGTGACATCATCATCCTGGTGAAATGTCATGTCACTTGTTCTGAGACCAGTAGGCCTGAGTT<br>GTAACTAATGCCTACACTTTTCAATTAGGAGAATTCCTGGAATTTTTTAAACCTATTTCATCCATGCCAGC<br>TCTTCTGTCTTGTGGCAGACACCACTGACCCACATAAAACCACACTCGTGAGTGCGCATGTGGACACA<br>CACACACACACACACACACACACACACACACCCCAACCCTGTCATCAAATGAATACAGGGGCTACAAA<br>ACTAAAAACATGGTCCTCCAAAGAAGACCCTCAAAGGTAGATACTTAAACAAGTGTTCCCCATCCTCAT<br>CATCCTGGAAGAGGAATTTAAAGTGCTTTATGTGACCTGTGCCCTGTCCACCATGACAGGTAACAAGGA<br>AACCAAGGTCAACAAACGATGGGTGGGACAGGGAGAATCATGGTTGCTTACACACTGATAGTGGGAAG<br>TGAAACTGGGGCCACCCCACTGGCTGCCAGCCATTTTGTCCCTCAGAATGAGTAAAATAGGAAGGCTG<br>TCTGATCCATCGATGTGATGCAGGGATCCTCAGACATGCAGCATCATTGTTGCATTGTTCACTATGGCTC<br>AGAAAAGGAACCAGGCAGAATGACACCTCATTAATGTCCTGTTTACACACACACACACACACACACAC<br>ACACACACACACACACTCACTCTCCATCTGTGTGTGTGTGTGTGCGCGCCGTGTTTGTGCAAGCTTCTG<br>TGAATGCACTTGTGCATGCAATGTGTCTTATTTGCTTTCTCTTACCCATGGCCTGTTTATTTTCCTGACAC<br>TGTTTTGTGGGTCTCCATCCATTCCTCACTCTAACCTTGGGTCATAAACATTAGACACTATGTCTACTGAT<br>TTTGGCCACTATATCTCTTGTCTTCCCCTCTCAGAACTGGTCCACTCCTTACTGCTCTAGTAAGTGTCCAC<br>TGTTTCCTGCATTTAATAAGTGGTTTGGGCTTTATTTGGGGGCATTATTTGATGTTAGGTTCTGTTCAGAT<br>ACTTTCTGGTCATGTTCTTCACAGACGTGGCAAGAATCTTTTCCCAGGAGGAGAATTCAAAGATTAAAA<br>TAAAATACAGATGTCATCAGGATATGGAATGTGAACATTAAATTTGGATGCTCTTTCTTTTGGAGAACAG<br>TAATATCGAGACAAATGTTGTACTGATGCTCCTCTGTGTTTCCAGGGTGGTAGGATTATGCCTGTCAGGT<br>CCCTGCAAGGCCAGGACAGGCTAATGTTACAATGTAAAGCCCAGGGAGCTATGATTACTCCTGAGGTTT<br>GTCCTTCTGAAGAGGCTGGCCATGCAGCGAGGTTGAGTACTTTCCTTGGCCTCTGCTGACAGGTCCAG<br>GGAAAATCCTGACACATAGACTAGAAATTAAACCAAGACTGGTACATAGGAGTCCTTTGGATGCAAAA<br>AACAGCTCCCTGGACAATAGGGATGAAATCCCAACCCAGACTACAGACTTATATACATGGTGTTCCTTTT<br>CCCTTATAACTTCTGCAAAAATGCTCCATGTGAACATGAAATGGACAGTGCCCTCAGCACATCTCCACA

							GTGTTGGTCTAGACTTTTGCCTCAGGGCCTTTTCCTTCCTTCCTGATGATGCTAAGGATCTCTTTAGGGG TTTGGTGCCCATAGAACCATGTGATTCAGTATTTGGTTCCTAGTGGGTAAACCTGTTTCAGAAGGATAAG GCAGTGCTTCTTGGGGAAGAGCTGTGTCCCTAGGGTTCGTTTTATAGATTTCAAAAGCCCAAGTCATTC CCAGTGATTTCCAATCTGCCTCCAGTTTGTGGCCCAGAGAGCTCTCAACTGTTCCTTTGCTGTACCACA ATGTACTAGTTCTGTAAATGTGAGTCTGAATCACAAACTATCTTTTACAAATTTCTTGCCTTGGTCATGAA GTCTTAAGGAGCAAAGGAAAGGAAGATGTAGAACTAGAAGCTCTTGCTTTCATGGATCAAGCAGGCTG ACATTTATTCATTGAGTCAAAGCCTATAGGTACTTCAATGTCCTTTGCTGTTGAGTTTATCATCTCATTTCA GACTAATGAACAGTAAGTAGTTGAAGAGAGAGGCGATGCATAGTCCTAGCCATTTGAGATGCCTATGAG CCAAAACAATGACCAGCATGGCTAATATCCGGTTACCTGAGGCTCCAGAAATACAGAATTTCTAAGGTG AAACCAGAGCTGGGTCAGGCATTTGGACATCAGCAGCCCTTGAGTCACTCCTTCTCATAATTGACTCCA TGTGCATACTTCAGCATGGAGCTCCTGTGAACCCATTGGATGAGTACCCCAGACTTAGGTGGAATAGCT CTGTGGCATGTATCTGCAGTTCAGCCTTATACAGGACAGTGTTCTCAACTAGGGGCAGGGATCCCTGCA ATGTACTTAAGGAACACAGGGCTCTGATCCATTCTTTCTCCCTGCATTTCCTTCAGATTGGAAAAATTAA TTCCGTGTTAATATTTTTGAGATGAGTGGGTGGCCCCATGCCTGAGGCGCAAACCATGCCTAAACTCTG GATATGCTCTCTACAGGATCTTGCAACCCACTGTTGGGTATTTCAACTAATGTGATCCCCATGTGTCCTG GGAGCCTCTTGCTTTGCTAGAATCTCAACTTTCTGGTGGCTATCCTAGTTCCCCACTCCGCATTGCTGCA CACATTCTTGTAATTTTCTGATCCTCTGTATGTCTCCCTGTCTCTCTTCACACCTGATCCTGCTCCCCTTTT CCCCACCCCCTCCCTCTCGCTCCGAAGTGCCTACCTCTCTCTACCTCCTGTGATTATTTTGTTACCCCTTC TATGTAGGAATGAAGAATCCATACTTTAATCCCCCTTCCTTTTGAGCTTCACATGGTCTGGTAGCTGTATC TTTGGTATTCCAAGCTTTTTCTCCTAATATCCACTTACTGAACACACATGTGATCTTTTGTGACTGGTTTA CCTCACATAGGATGATATTTTCTGGTTCCTTCCATTTGCCTGCAACTTTCATGGTCATTGTTTTTAATAGCT GAATAGAACTCCATTGTGTAAATGTACCGCATTTTCTGTATCCATTCCTCTGTTGAGGGACATCTCAGTTG TTTCTAAGATTTCCCACAACAGTCCTGAGCATATTACTACTGTTTTTAGGGAGCTAGCCTTTCTGGCCCTA CTGGAAAGATATCCTTGCCCTCTGAAGGCTGTAACTCAAAGACACCAGAGATCACGTAGTGGGTCTCTG GCTCATAGATATTCTTGGAACATTTCCCCTGTACTTACTCAACAACAAGGTAATCAGAGCAAACTGAACT TAAAAAGGTGAACAAGATTCAGTGGACTAGAAAATTTAGCAGCAGAACAGGCAAAAGCCTCTCACTGC TATACCCTCAGGCACTCATGTGGCATAGAAGATTGGTGGCCACATATACCAAGACTCTGCAGGAAAAGA TACAGCAAGAAAGTTAGATGGCCTGGAGTCATCTCCAGCTTCTTCTGACGAACAGAGGATGGTTTGAA AAAAATGATAGGATACTGAGGTGTCAAGACCTCCATTTCTTAGAAAAGTTTTCTCATTAAGTGGTGTCCT ATGCCCAAGAACAAGATTGTACATATGAATAAATTCTACAAATAAATAAATATAAACATCGTACTATGTTG GAAATTGAATAATAACTGCATTAGCTTTGGCTCGAAGTTTTTCTTGGGCCTAATGACAATTAGTACCCTG CTCCACTGCTCAGGACATGACACCCAGGCCAGCATCGATGCTGTTCTGTCCAGTGCTCTGGAATCCACA ATATCCTAACTGTTGGGTTCTGGGGTTAAGGAGGGACGATAATTGTAAAAGATAATTCTGTTCTGTCATG ATTTATGAATGTTGCCTAAACTTCTGGCCAAAAGAGATAAAACGTGTTTGGGGACAAAAATGATATTTTC TATGTTTTGGAACGTGAATCTATCCTTGCCTACTGAGTTCTCTATAAAGAAAGAAGCAACCAGACTGCC AATCAGTCAGGCTGCAAGATTCCTCCAAACCACCATGACTGACTCACTTCTTTCTCTCTGACTCCCTACC TCCTCTGTGGGACCTGGTCATCATCAGGGCTGGCCTCTGAGAGCTTAGAGTGTATGTGTATGGTCAGAA GTCCAGGATGTTACACAACTGCATAGGACAAAGATCTATATGTCATGTATAAGCATTGGAAACAG
MSTR G.1114	ENSRNO G0000000	0.254	1.159	0.000	2.903	up	TTTGTTTTCAATGTTTTCTGTATGGATGGTATGCCGGAAACTTGTGTAGGCAGTGCCTGTAGAGGCCAGA AGAGGTCGTCAGAGCCCCCTGGGACTGCGATTACAGATGGTTCTGAGCTGCCTCCTGGGTGCTGGTAGT CTAACCTGGATCAATCAGTGCTCTTTCCCGCTGAGCCATCTCTCCAGTGCAGCAAAATATCGTTAATAGT

24.1	5720						GATAATCTTAGAGCTTATTTATTGAATGTATTTCAACTGTTCAGAGGACAAGAGGACATAGTAATGAAAG<br>ATAGCAGGGTAAGTTCAGAACTCATTTCCAGGCAAGACTATGTTTCGAAGCTTTGAGCAAGCTGTCTAT<br>ATTACCCTCACGTTCGAACTGTAAATATGCATCTTGCCCGGCAAGTGAGAAATAAAACTTTATTCAGATC<br>GCCTCTGGGCATGGGCATGGCCATGGGCATGGGCAGCAAGCAAGCCAGAGTGGAAATTCAGTTGTCCA<br>TTCATTCTGCAAGTACTTATTGGAAGGTTGTCATGTGAGGTTCTGCACCAGATGCTGGAGGGAACGGAG<br>GTGAGGAGACCAAAGAGGTGTTAAGACATCGTGCCTGACCATCGACAGGCAGCGATCTAGTTAGGGAA<br>AGGGGTCAGCCATTAATACGTGACTAACTGGGGACAATCGTAAATGACAGAGGCAACGGGTCCAAGAA<br>TAATCAGGAGGAAGAAGGAAGGATGGAAATGAGCCAGATGGTCAGGGGTGAGCTGAAAAAGAACTGA<br>AACCAGAGTTGGACAGTTTAAAAAAGAATTTATGAAGACCCAGAGTAGGAGGAATTGAGCAGAGGAA<br>GGCATTGCTAGTCAAGACTGCAGAGGAGTGCAGCCGGCAATCCCATTGAACCTATGGGCTGTTTTTTTC<br>TTTACAGATGGTTGTGAGCTACCATGTGGTTGCTGGGATTTGAACTCAGGACCTCTGGAAGAGCAGTCA<br>GTGCTCTTAACCACTGAGCCATCTCTCCAGCCCAGGCTGTTTTTTTCTTATTACTCAGCATTTTCTTTGAA<br>ATCTCCCTGTCCTGGAGGCAAATGGGTGGTAGTCAAAGAGGACAGAAAATAAAATGAAACCATTCACC<br>TAGCTTCCTTGTTTAAGAATGAGCATCCCCTTTTATATGAAACTGACTTGCCAAATAGGAAGAGTGACTC<br>TGTAAAACAAAACAAAAACAAGCAAGCCAGGCGGTTTATGAACCTGGGCACATCGCTGTAAGAAGTC<br>ATGCCTTTAACTTATCTTCTGTAAACATAAGAAGAAGGGGAAAAAAATGCTACAAAATCACAAGATTCC<br>CTCATCTGGCCTTACTCTTGTGTTATACTTGTTTTCTGTGAGGAAGTGTCTGGTGACTGGTTGCATTTAGT<br>TTATAAAAATTTTTTAAGCATATAAATAGAGTCTGATGACCTTTGCAGATTGAGGGTCAAGCCCGGGCTT<br>TGTTTAAACCATGACCGCTTGCCTTCTGGCATTACATCATCCCTCTGTTGGTTCAGCCTTACAGGTCGGC<br>CCAAGCACCTTTCCAAATTTGGAAGCAGAGGTACACTCCGTGGGTCCTGTGGAGAATAATGTTACAGTG<br>ACTTCAGCCAGGACATCCAAATCTCCTTGAACACATATCATGGAAAGATTTCTAGGAGTTTGCACTGTCT<br>GCATTCTGTAAGAAAATAGCCTTGAGCCATGGCCACGTAGCCAGGATTAGCATCCGTGGTACTGTCCGT<br>GGGCACTCCTGAAGGTCAGGACCAAGAGCCATGTGCTACCGCTGTGAGTTCATTTACAAGACAGTAAG<br>TTCAGGTTATCTGAAGAGAAGCTAGTATACTAGCAGAAAGGAACATGTTGCTAATGTGTAAGAAACAGT<br>ATCCTGGGAATTAGGAGATGAACAATGACATTAAAAGAACAAATACATAATTAACAGGCTTTGGGCTGT<br>GTTCAGGGAAAAAAAAAGAATCTTACTGTAGTCAGAAAACAATTATTCTGAAATTTAAACTGCATTCTT<br>CAGGACTCAAATAAATGTTAACGCAAAAATGGCGATGCAAGCATATAAGGCGAAAGCATCCATCGTTTC<br>CAAGAAAACTTTTCTCTGTGGAAGGCTGAAAGAGATGCCCACGATGACAATGACTTCAGCCACAGGGA<br>GTCACAGAAATACATGTTGACGACTACTTCTTTTCCAAAACTGTAGCGTCTCCACAAAGTGCGGAACAG<br>GGGTCAGCATCATGCCCATAAGCCAGTTTTCAATTCAGTAAGCAGTTACACGATGTCTGCTTAGTGTTTG<br>AAATGCCAGTGTCTAAAATCAGTGTGCCCCCAGGAGTACATACTCAAGTGTCATGAAGTCTTAACGATT<br>GCTAGTAAAACCTGAACTAGTTCACGTAGGACCCAAACCCTGGCAAACCTTTCTGAAACATCTGGATAA<br>ATCAGCTGATTTTTCTCACCGGAAACAAACCAGGATCAAACGGCTTTTCTCCGATGCTCAATGGAACTC<br>AGAAACGACCAAGTCACCAACTCCTGTGCTAGCCTCTCCCTGACTGCTTTTGACATTGTTTTGTGGTGG<br>TTTTCCTTTGTTTGGAAAGGTGCGTGACAGTCACAAACCAAAGCACCATTTGGTTATGCCCATCGGATC<br>TTGAAAATGAGGAACTGGAAGCCACACAGACAAAGCTGATTCGAGGAAATGTCATGGGTCACACGAC<br>ACCTGGAAGGACCCCTTCCTGTTCTTGCCTTGTTGAATTCAGTCTAGCTCTCCCTCTCT
MSTR<br>G.1115<br>49.1	ENSRNO<br>G0000000<br>8247;ENS	0.000	0.268	0.000	4.599	up	GAGAGAGAGAGAGAGAGAGAGAGAGAGAGAGAGAGAGAGAGAGAGAGAGAGAGAGAGAGAGAGAGAGAGA<br>GAGAGAGAGAGAGAGAGAGAGAGAGAGAGAGAGAGAGAGAGAGAGAGAGAGGGAGAGAGAGAGAGAGA<br>GAGAGAGAGAGAAGGGACCGGGAGCCAAGAGAGCCAAGCCAAGAGGGCACGAAAATGCCTGAGTTA<br>TATAGTGAAGAAGAGCTGGAGGAAGGGAAGCCAAGCTCAGCTTAAACTGTTTTCCCAGTCCTCGGCCT

	RNOG00000008289; ENSRNOG00000008797						GGCAAAGGAGTTGGGGCCAGCACGATTTGAGATTTAAAGAAAAACAAGAGCCGATTCTCCTCGCGCCCTCTGGTGTCCCAACGCAGTATAACCAGATAAAATCCACAAACAAACATCGCGGCCATTTCACAGACCGGAGATGTCCAGAGAGAGGAGAAGAACGCCTGGAATCTTGGATGAGATTGGATACCAACAGAATTTCTAAGTGTGGAGACGGATTAAGACGAGCTACAACAGCAGCAGCAACAACAAAAACAGCAGTGCCTGTGGTGCAGATACTTAGGAGAATTGTAGGAGCTGGGATCCTACGTAGCTTTTACCTTTAGCATGTGCCAAGCAGCTCTGGGTTTGAGCCCCACTAACGTTAAACACAAAACAGAACCCAGAACCTAATAGTTCAACAACTCAGTTCTCAAAGTAAAAGAATAAAGATTTTAAACTATCCCCCACTGACCCTGCTGTGAGGACAAGTGCACCTCACAGCAAAGAATAACATAACATATTTTGGGGATGTAGCCTGGCTATAGCCACCACAAACCATCTGGCCTCGATAAGGCCTGACTCAACAGTCCTGAGAAAAACGAGAATTAGGGTCAGCTTGCCCCAGAAGCAGGGCAGCCTAGAGTTGAACTGAGCCCAAGTTACAAACCAATGAAATTGCTTTGTGTGACTGTTGTCAACTACCTATGTAATTTTCCCTGTAAATCCC
MSTRG.111776.2	ENSRNOG00000006983	0.000	0.341	0.000	6.166	up	TCTAAATGTAAGATTGTGTTCCCTGTCCCCTGATCCCTTCATAAACCACTAAGGACTGCCAACCATGTATTTAACATGGCTCATTCAATGGCATTTGATAAGAACATATTTATAATTTGTTGTAAAACTACTTTAAAAAAATATCACTGAACCCAGGGACTCAATGCAGATACTCTACCCCTGAGCTTGCCCCATTGCTAACATTTATGGACAAATGGTGCCGGGGCAGTCTAGATGTGGAGAACATCTCTCTCCTGGATCCCGGAATGCTGCTCATTACACATGCGTGTGCTGAGCTGGGAGAGGGGACATTCTCAGTGTCCCCAACAGTATTCCTCCTCTGTCTGCAGCAGCTAAAATAGAGGATGGCACCCCACCGCGCCCCCCAATTCCTAAGAGAAGGAAAAGGAACATTGGTCACATTATCCTGTTTCTGAACCTCTTTCTGTTTTCTGATGTAACCAGTCTCGGTCTGTTTTCCCTGACGGCCCTTGTTTTTTCCCCAGTAAGAAGCATGACTCTGAGTACAGACTGATGTTTTTTTAAACGCTGTCCGACATCCCGCTTTGATTTATAACAATGGAATGGCACTGAAGGAAACAACTCAGAAGTATGTTTTTGTCTGCCGGATACGTAGAGGCTATGTAGGAATGGAGGAGCCTCCTATGTGAGAACATAGTGTCATCACCATGTGCGGTTATGCCTTAAATGCCCTTCCGCTGGGCTCATTATAGTCAAAATGCTGGAATTTCCATAAGCAAATCCTACCTCAGTGTACTCCTGGATCCTTAAGGATAGACACCATATATAATCTGTGCGTTTCTAAGGGATCTTCGCAGCTTTAGTTGAATACATTGAAGGCAGAATTCCCAAGGTGTTTTGCCTTGAGTAAGAATCTCACTGTTTTTCCCAAGACAGCTCCTGCTGCCTCCCCCTCAGGGTGTCACCCTGCCTAACTCAGTCAGCTACCACTGACGTAGTCTCCAAAGCCCTTCCAACCCAGGTCATAGGCCCACGTTCCTCACCGTCCTGCAAGCCCACAGTCATGGCAAGACTAGGGCTCAGCTTTCTCCCTGCCTGTTTCCTTCAGGACTCCACCTGGACTCTGGAACCTGACTGAGTCTCTCTTGTCCTTACCGTCTTCATTCTTCTGCCCCTTATGGGTGCCACGCCTTTCCCTCTGAGCCACAGTGGCACACACTTGCTACCATGGATGAGGCCCCGGATTCAAGCCCTTATCCAGGAAAGCAACTAGCTTTTTTTTTTCTTCTTGGTTTTAACCCCCTTTTGTGTAGTCTCCTTTAGACATATCAAAGCAATCTGCGTACAGCAGAATTCTGATTTAGTTGCTATTCCTGTGAGCTTCAGGGCATTCTGAGTGGTTTGGCTGGCTTCAGCCTTTTTCTGTTCCTACCATTCCCATTGTCCTCATATCAGTCAGCCCAGCTCTGCCCATTGTTCTGTGACACTTCCCAGTGATAGGGTCCTGTTCTCCTCTCTGTTAGACTATCCGAGCTCTCTTCCTTGGCTTCCTGTGATCACTAAACAGTGTTGTCCTTCTTACCAAATTCTGTCCTTGAAAACATTTTACCTTGTTCACTGGAGGTCGCCCACATCCAACAACTGTCTTCCGTGCAGTGAATAAATGTTTGCCTGCTGAGTTAAATATCTTCAATGGGTCCCAAAAAAGTATCTTCGGTACCCATGCTCTGGAGTGTACGTCACTTTTAATTTTACTTTCTAAATGCAAATCCATAGTGTTTTCTCTTACAGGAAAAGTCAGTTACTACTGGGAAGGAATTTTAAGAAAGGAAACAGCAAAGACAGCAGTGGTTCTGATGGGAATTTGCCTGCTAGGAAAATAAGTTTAAAGGTGGTGGGAGCTTTTAAAAGACTAAATATCAAAGTATTTTGTTTTTCTCTTTCATGGAGTTATTTGCTGCATGTAAGAACCAATTTCAGAAAAGAAATTAATCACTTTTAATTCTCAAAACACTTTTTTTAAACATAAAGTGATCATGGTTTGTATCTTACTGTTCATAAGAAATTTTATAAATAAAATAGCATTGTAAATAATGTTAATATGTATTATAATTCATA

							CAAAATAAATGCACTGTGATATCTACTGCTGGTGTGGAGTTCGTTATTAGACGTTTGGGGTTGTGCTCATGTGTTGATGCCGTGCAGTCCAGGGTGGCCCTCAGCGTTCAGACTCTCCACCTCATCCTCCTTAGCAACTGTGATTATGGGCATGTGACCCCCAGACCCCCTCTGACAAAGTCTTCCAAGCCTTACCTTTAAGATGAACCTCACTGGCCCCTTATTCTAG
MSTRG.111821.1	ENSRNOG00000008965	0.136	0.495	0.008	2.508	up	CAGGGCTTTACATCTTAACAGAGTGTAGGGCCACGGTGCTTCCTAAATGGCTGTTAAGCCAAACCACGTTAATTTCTAACACTTCGCAGCATAATGAACAGCACTAGTTATGAATCCGTGTCTATCTCTGCTTTCCGCAATAATGGAGTCCTATTTCAACTCCTTGTGCAGTAAGAGATCAGACAGAAAACAGGTCTGATCCCTTACTTACAATGCAGTCCCCTAGGGATTATAGATTTATTGATCTATTTATTTATATTATATAAAAGTAAATGCTAGAGAATAGTGGGCTTCAATTTTGCTTTGCCAGAGTTAGTCCAGCACCCACAACTGCAGAAACCACTTAAGAGACCAAGGACATTATAGACCCTGACCAGCAACGGGAGACAACCTGGAGGAGTTTAAGGATCTTTCTTACGAAGAGAAAAAGTCACTACAGGAGCAGCACCTTCAAGAAAATAAGTATTTGTGTGTCTTTTACGATAAGCCTTACAAGAATTCGGAATGTGCGCCGCAGCACGGCCCTGAGCACTCATGCATGGGACATTTAATGAGCTCAGGTCCTTGGTGTGCTGAGAGGTGGCTGTGTGGTGGCAATGAGGTAAGTAGTGCAGAGATACAGAGACCCAAAGGAGGCGGGCGGGGCTTCTGACACCTTCAGCCCACAGACGCAGGACTCCCGATCCCAACATTGCTCTGCAAAGTCGGGAGGTGGCAGGTGAGGAGGGCGTGTGTGACCATATTTGGCAACGTGTTTCCATTTGGAACTTGTAAAAAGTTCCCCGTCTCGGCAGCTGAAAGCCGTGGTTGTGGCACAAACGGGAAGAGCTACCGGCGGTTTTCACGAGGCCTTCTGATGAAGATGTAGAACTCTCAGTTCCCCCTGCACCACGTCTGCCTGGATGCCACCATGTCCCTGCCTTGATAATAATGGACTGAAACTCTGAACCTGTAGCCAGCTCCAATTAAAAGTTGTCCTTTAAAAGAGTTGCCTTGGTCATGGTGTCTTTTCATAGCAGTAAAACCCTAACTAAGACAGTGTTTCCTCACAGCAATAAAAATCCTAAGACAATAGGTACCCACATAAAATTTTCTGGAATTCATTCAAGCAGGAATTTGTCAAATGAGCCTTGACATCAAATATTAGATAAATTAGTAGATTTTCCCATGAGGCTTTGCATATGATTCTGAAGTATCGAGTACCATTTCTGGTATTAACGTATATTGCAAGAGAGAATATGTCCCAGTTACTGGTTTCCTAGTGATACAGTTAAATGCAGAACAGAAGCTGACCAATTGGATGTCCTTGATCCTCA
MSTRG.111931.1	--	0.016	0.690	0.000	4.208	up	TGAGTTTATTAATTCAAGAACACAAGCACATGTCTGTGTTTGACCATCTTACAGACAAATTTACGCCTGTGGATTGCAGCCTCCTGAGACTCAGAGCCGAGACAAAGGAGAACGGGAATTTCTCTCCCTTAGTCAAATGCTGTAGGTCCAGTTGCCACTGAGAATCTCATGGGAGCAGCAGCAGTCAAACTTAGCTTTTGCAGAAGATAAAAAGGCCCAGAGCTACCTTGCAGACTTTATGCTAGTGATAAGGAGACTCATCATTTGGATGCAAGATGAACCCAATGGTGTGAGGGGTGTCCTGAAGACAGTAGAGACGAGTGGACGCCGATCGACATAGCACAGAATCCCAATTCTGTTACTCACGCTATGTGACGCTTCTGTCTGGAGAGCTGGGGTTTTTCCATGTCGATAGGAAATTGTTATCCAGTGACTTCATGGGCGGCTGCTGCATGTATGGGAGTGCTCAATCTGCACTTGAGTGCTCCTGATTTTTGCCAAGGCGGTCCCCAAGCAGCAGGA
MSTRG.111958.1	--	0.640	0.000	0.013	-4.001	down	GGTGTATCTTGTGCCACTGAGGATAGACCAGGCAGTCCTCTGCTGTATATGTGCCAGGGGCCTCAGACCAGCCCATCTATGCTGCCTGGTTGGTGGCTCAGTTCCTGGGAGCTCCCAGGGATGTGGGTTAATTGAGACCCCTGGTCTTCCTATGGGGTTGCCCTCCTCTTCAGCTTCTTCAATTTATCCCCTAATTTAACCACAGGGATCTCCAACTTCAGTCCAATGGTTAGGTATAAGTATCTGCATCTGTCTCAGTCAGCTGCTGGTAGGGCCTCTCAGAGCACCGCCATGCTAGACTCCTCAAAGCAACAGCAGCAGCGCCACACATTTCACATGTCCAGTGGTTCATGAGTTCTCCACCTGGACGGCCAGTGCCTGCTGACACTGCGAATGCTAAACCAGACCAGTTCTAGCAAGCAACATTATAC
MSTR	ENSRNO	0.000	0.251	0.031	3.453	up	TGAGAGAGTCTGGAGCAGAGACAGTTTTAACTTAGCCATTCCTAATTTATCTCAAGCGTTTTCAATTCAT

G.112110.3	G00000023762						CTTCACTGCTCTGTGCACACAGGAACAGCAGATTGCCATCCCTGGGGAACAAAACGCAACCGGGATCTAGAGAGCTGTCACATTCCAAGAAATATTTAAATTGTGCTGGTTTTCTTCTGGAGAGCTGAGCCCAGGGAATGAAAACTCTCCATCCAGTCGTAGCTTTGCGGGCAATAGATCAGAGGATATTGCCAGGCTCCTAATGGATTATTGTTATCGGGCAGGTTTTCTCTGAAGTGAAATGAGAGATCATAATGCATCATCAGGGGTCTGTCACGAAGGTCATCCAGAAAGAGAAAGTCAAACAAGCGCACATCATATGATTCAGC
MSTRG.112117.1	ENSRNOG00000004149;ENSRNOG00000004179	0.000	0.287	0.031	3.453	up	ATAGGCTACAATCAGCTACCACCAGGAAAAGCTGTAACTGGAGCTCAAGGCAGTGCTCTGGAGACAGGAACTGAGGTGTCCAAGAAGGTAGGCTGCTTGCTGGCTTGCTCTCTGGATTGCTCAGCTAGCTTCCTTAACAGCTCTGTTAAACCTAGGAATGTCGGACACTCCTACATCAATTAGCAATGAAGAAAATACCATACAAGCATACCCGCAAGCCTGTCTGATCAAGACAATATCTTTGGCTGAAGTGACTTCTTCCAAGTCTCTTCACAGATTCACGAAAGTTTGGTTTGTTGTGTGAAACATTTAGTTTAGCCAAGGCTCTTGCTACGGCTCCTGATGTACC
MSTRG.112240.1	--	1.528	0.196	0.043	-2.171	down	CACACACACACACACACACACACACACACACACACATTTTGTCCAAATCTGATGGGTAAATAGTTTTTTGAACTTTACTAACATTGCTAAAAGAAAATGCTCTTATTCTAAATCAAATGTAATGCCTTGCTCTTTTACAGTTGGAAGAGAACAGTCTTAAATTTTCAGTTATGAATAGGAAAAGCTCTCAAAAACTGGAGGAGATCATCGAACAATCATCTGCCTATCCCATGACTTCATGATGGTCTCTGGTTAGCATGCCCCATCTCCAGCACACCAGGAGCAGAGAGGTTCTCCCTCTACTCAGAGAGATTGTCCAAAGGTCAAGAAGTCACTCATTATTTTCCCAAGAAAAGTAAAAAATTGATACTGCTTCCCGCAAGAAACAAAATTGTTGTCAGTGCTACCAGCAAAATACCCAAGGATTGATGATGGAATGGAAATCTGGAAACTGACTATCCATCATCTTCAAATATCCCACACAACAATTTTGATTTTCTTCAGTTATAACAACTGGCATAATTGGTATGTCCTATTTTAAATTTGCTTCTATTTTAAAAGTACCAGATCAGTCTATGGCTTGACAGAATCGTAAGATCATGAGGATTCCTTTTAATTTTTTTTTTATTTTTATGTCTACAGGTATTTGTCCAGGTGGATGGAGAACTGCATGTGACTCCAGAAGACAGAAGAGTTTGTCAGATCCTATGTTGGGAAGAAAAGAAGGAGGGAGAAAATGACACAGTCTGTGAGCCACCATGGGTACTGGGGGTAAAACCCAGGTTCTACAAAGCAACAAGTCCTCTAGCCACAGGGCCATCTCCCCAGTCCCAGTGATGGATTCTTGAGCCCCCAAATCTAAGAATCCAAAATGCGTAAATAATGCTTTCTGAAATCCCAGTTTGCTTGTTTGGCCTCAGTGCCCTGTGCTCAAGCTTGAACAAGACTTTCTATACCCAAGGCATCTTCTATATCCACATCATCATCAATCTTTACAACAAATGCAGGTGTTCTCTGCTATCTCATTTTAAGGACGAGAAAACCAAAATGCAGAGAGAAATTAAGTCATTTTTCCAGGGCCATTCAGCTGTAAAGAAAACCATGTAGCTGACCGTATGGGGTTCTTAAGCTAGAAATGCACACTCAACTGTCCTACAGTAAAGCACAGACAAGGTAAGTTTTCAGAAACTTAGGAATGGTCTTCTAGACTGTGCTTTGGCCTGGTAATGCAGGAACAGCCACATTACACTTTAATGATGAAGCTCTTTCCACAGCCAAGGCTAGGAGGGCTCTTCAAAAAACAGTGGAGCCCCGAACTGAAATAGATAGGAACTGCTACTCTTTAAGCTGTGGTAGAACAGCCCTGCTGAGTAGAACAGGTAAATAATGTCAATTACTCTGGCCTCCAACTTCTCCAGCACCAAATTTTATGAACCAACATAGAATAATCATCGCTTAATTTTTTTTCTTTTTAATTCTGCAGTTCTGTTCCTTTTTTTCACAACAATCAGATGATGAGCAAGTGAATTTCCAGTCATTAATTTATTTATTCATCCAGAATGTGTAGTGAGCATCCACTAGGTGTAGAGCTCTATGGTAGGCCCTAGAAGAAACAAAGAGAAAGAATTTGCTGTGGAAAAAAAAAATCCTGCAGACTTAGAATGAGTTAAGGTGGAAGACATGAGTTGAGTTAAGGTGGAACACATGAGTTGACTTAAGGTGGAACACATGAGTTGACTTAAGGTGGAACACATGAGTTGACTTAAGGTGGAACAAATTCAATGGCAGTCACAGGACGCAGCAGTCTGCTGGTCAAACTCCACTGAGTTTTTTACAGTGGATTTAACTGACACCCCTAAAGAAATGTGGTGGGGACTGGCATTTTTTTTGATCCCTTTGAAGCTAATTTTTTTTTCTTTAAAACTATTCGTCTATTCCACAGCTTAATAAATCATGTATTCTTCAAGCAGCAGAATTACATAAGCTTTCACACTGGTATTTAAATCAGTCTTCCC

							GAACAACTTCAGGGGAAAATTTTGAGAAGCACACAAGAAGTAGTATGTTTCGGGGTGAGAATACTGCT<br>TAAGTCAAAGTCCATTGATTTTTTTCTTTTTTCTGTCTTAAGTCAACTGCCGCTGCATAATTCATCAGGAG<br>ACTCATCATAATGGTAGGAGTGGAGGAGCTGATGAACAGTTTGAAGGATACACAAAGGAAAACGGCCT<br>CCCAGTTATTCCAGAAGCAACCCGCTGAAGACTAACAGGTCAGGCTATCTCGGTCTCTCAATAACCAAA<br>AGGCAGGTCTGCATGCTTGCCTTACGTTTACAGCGTAGTTGTGATGAGGCAAGGCCAAGTCTGAAAGG<br>AAAAGCACCTGGGAAGAGACATGGCATCGCATATAAACAGGGACACACAAGTGTAGTGGGCTGGCCTA<br>ATGCTGCTTGCTTGTAATCTAATGCTAAATGCTGGCCTCCGAGACAGTTGTAGAGAAAGCATGAGATTTT<br>CCCTAAATCCTTAATTTTCCACAAATGCTTAAAACAGTGTGCTTACTTGACTCCTTATTTCCATCTTCTTT<br>TCACACAATCCACTGGGAATGATTTTTGGAGGAGTTTGTTAAATCACCCAGTTTTCCCCTGACTCCACTG<br>TTTTGGCATCACCCATCTATGTCACAGGTCACTGCTTTGCAGCAGTCCTTTCCTTTCCTTCTCTTGTTCTT<br>CTCTCCCTCCCTTTAGCCAACATTCTAAATTCTTAAGAGAAGCCCCATCTAAAATGGCTGAAGAGTCTTT<br>CCTTAAAAATCCCAACACTCTAGAGGCTGAGGCAAGAAGATTGCTAATCTGACACTAGCAGATCCCATC<br>CCAAAACAAATGAATAAATAGACAATCAAAAAAAAAAAATTCTGCTCAAATTTCCAGAACCCTGGACAC<br>ATGATGACCTCTGGATAAGCCCTCCTATAGTGTTAATTCTGTACATTACTGTAAAGCTATGTTCACAGAAA<br>AACAATCTAAGGAGCCCTAGGTGTACACTTGTAACCCAGACCCGGGAGAAGCAGCAAGAATCATTCCT<br>GGCCATCTAAGGAATTCCTGACTAGTGTGGGATATATAACAACCTGTCTCAATAAAATGAATTTTTAAAG<br>CAAGAACCAGCTTCTGAAAATTACAGTTCAGTCCTGTGTTCCAGTCAGGTAAGAGACACAGACCTGGA<br>TGACAGACACTGTACAGCACGTACTCAAATGTTACGCTTTCTTTCCCTCTCTAAGTGTAAGCCCCCTGTG<br>CCCCTGACCAAGCTCTAAGGTTCAGCCCTACCTTCTAACAGTCTGCGCCTTCAACAAACCTGATTCCTT<br>CCTAACCAGTCTCACATCCCAGTATTGACAGGTCATCCAGACTTATTCCCGTAATGTATTCAGCAGGAAG<br>CTGCAGCAAAAGACCTCATAAATTTTAATATTGAAAGTGATCAAAATATAATTTGACGAATTCAGAGGAA<br>CCAACAGAAATGTTTAAAACTTTTCTGAAAGCAAGAGAAAAATGTACACATTTTGAAGTTTCCATTCTT<br>TGTAGTACAAATGCCCATGTTTTCTGATCTATTAAAAAAAAATTATTCAGACTAGAGATACACCTCAGTTG<br>GTAAAGTTTCCCAATACACATGACGTCCTGGGTTTGACTACAAAGGGTACATAAGCCAAGCTCTTGAGA<br>AATGAAAGCAGGAGTATAAGAAACTCAAGGTCATCCTCTGCTACACAGATCTAGGACAGCCTGGGCTA<br>AGTTATCACAAGGGGGAAAAGGGTCCTTACATACAATGTTTCTTCTCTTACTTGTGAGTGGGTAGCATGC<br>GCTTGTCAACTCACCTCACAGCCACCACCACAGATCAGACTGTGAACAAGGAGGTAAAATGTTCTCCT<br>GAAGAGTGACTTTTATTTACCAGAAACAGGAGGCAGAGGGATTGCAGCTACTCAGAAGGTACAGATTA<br>AACCCAATGACTTGTGTGTCCTTTCAAATTAACTCCACTTAGAAAACCTCTCAGCAAATGTAATCTAATT<br>TAAATGCGTATCCAAACTACTTATATTTGTTCCTCCTCGCCTGATGAGCAATTAGTTCTATTCTTGCTTCCT<br>TTCCGGATACACTTTCAGCCAAAACACAGCAGAGGTGAGACACTTTATTAAATCTTCTTGTTAGGAGTT<br>TTCTATACCCAAAGAAATCTTGTGCCACCCCCCCCCCACTTCTGTCTAGCCACTGGGTTTTAGATTTGA<br>CACATGAACCAAAAATTAAAACCTCGTGCTTTTATTCATGACATTTTCTGTTCAGTTTTGGAGAGGAGTT<br>TCCCTGAAATTACTTTGATGGGATGCCCCTGAGTCTGTCCTCTGGTCTAGGACTTCAGTAACAACAAGAT<br>CATTTTGGATAACATGTGTCAATGGCTATTAAGTCACCAAACAACCAGGAGTTCCACTGCTGGGCATAC<br>GCCCTCGAAAAGTTGAAAATGGTTCGACCATTAGTCTCAAGGCATTGCTCACAACCGCCTAAGCCAAA<br>AAGCTCTGTCTAAATGCTTGTCACTAGTGGTAGATGAACTCTGGAAAGTCTACACGAAGGAATTGTATT<br>TGATCATGAGGATAATTAGAAGTGTGGATACAAGCTGTACAGTTAAACCCTGAACATTTTCACCTGTGA<br>AAGAAGCCAATTTGGAAAGACCACTCATTAACTATTTCTTTGTAAAGGAATAGTCCACAAACAATAAGT<br>CTCTCCACAGGTCTGACAGTACCGCTTGATTCAACGTATACTGTAAGGCCTGAAGTAAGTCTTACCACC<br>GGGAGACCCCCTCAAGTGAGACACGAGAACGGAGTTCCATGCAAATTTAAGAAGTTTATTTTTCCGGT

							GCATCGGGGGTCGACCCTCAATCAGCCCCTTGTGGCGAGTGTTTAGAACCCAGAATGACTATAGCAAGCAATTTTTATACTTTTTAGGTCTACAGGTTACATCAGCAAGGTTACAAGGTAAACTTATTGGCTAAGCATATTGACCTTTGCATCTATTGGCTACCAAGTATATGACATGCATTGGAACTCTATATGGAACCAGAGGGTCAGGTGACTGTCAGTACATTCTTTGGATTTTCAAGAATGTTCTGCTCCTCCCAAGAATTGTGGGTGGAGAATGGAACTTAGCCTAGCCTTGACCTTAGGCAGTGGGTGTAAGGCTGTCAAAAGCCCCTAGGGTCCTTCAATACAAGTGAGGGAGGATAGGAAAGAAAAACTAGGGACTACAGGATGGGAAGAGGTCTACAAGAAGCCACTATACAACAGAGAATCGAACTGCCATACCAGGGGCTACGTAAGGGCTCAGAGTTTTAAGATATTTGCAGATCTTTCGGAGAACCTGAGTTTGGTTCTCAGCACCCATTCTGACCTGCTCACAACCACCCTTAACTCAAGCACATTGTGGGATCATGTACCTCTGGCCTGAATGGGCACATGGGCACATGATAGGTACCTGTACTCATGTGCACGCTCCCACATACAAACCCCTACACATAGAATCAATTTTTTTAATGGAATGATCACAACTAACATTAGCTATGAAAGTTACTGACATAGAGACAATATCCCTTCTATATACAGAAGAATATTAAAAGAGTAATGAACTGTGGTCAAGGCCTGAGCATATATTGTTAGATCTCAAAGAAACCCAGCAAAAGTTTCACTTGGTACCTTGGCTCCAGCTAGCGCTTCTCATCCTAT
MSTRG.112246.1	--	0.901	0.000	0.000	-5.795	down	GGAAACCACTATCATAATTCTAATACATTGAAAATTTATAAAAACAAGGGTTAATTCATTCTAAATGGAAATTGTACCTGAAGATTTAAGCCCTTCTTTGTATATTAGAAGAAAATACCACTTATTTGCAAAGAACACTTCTTGGAGTAAGCCAGTGGCTCCTTGGAATGAAGTGTGCTATACAGTGCTGTGGAAAAGCTTGGGACACATGATCAATAACTAGCACAGCGCCATTTGGCAGTGTCCTCAAGGAGTACTGAGGCAGGTATTAGAATAGACTATGAAGGGAAGAATCAAAATGCAGAGTGAAGTTGACAGCAGTCACACCCATAGAAGACTGCAAGTCTTTAAAATGACAGGCCTTTCAAGGGAATTCTGAAATATCTACTCTTTAGGATTAAAATTGTTTGGTTTTGTTATTCATAAGTCTTAAATAACAGAATTGAAAGCTGGAGTGTACTTTCTTTTCATGCCTTTGGTGTTTCTGACATGGATGAAATAATATCTCCAAGTAAAACTACTTTGGCTGCCTTCTTATTTCAAAATGAGTTTATCATTAGGGAAAACTTGAAAACCACGTTGGACAAATAATCCCTTAATGCTTTTGCTGGCTAACAAGTAATTCGTCAAAGCAATCCAATTTCTAAATAGCACACAAATCCTGTAAGACAGTGAGCCAGTGCATCTGTTCCTTCTGTATTTAGACTGTATATGAACATCTTTTATTTGGGGTTGGGGAAGCTAATTTTGAGGTTGAATTCTCAAGTTGGGTAACTTAGCATAAGTGACAAAATATTACTTGCAAAATATTACTCATTTTGGGGCAGGTTCTCTCTATGTAGCCCTGGCTTCTTGGAACTCACTATGTAGACCAGTCTGGCCTTGAACTCATAGAGATCTACTTGTCTCTGCCTCTCCAGTGCTAAGATTTAAAGTGTGTACCACCAGGTCCAGACATAAAGCTCCTATTTCATAACAAACAGCTATATAATCAATATTATTTCAGTTTTTAATCACAATTTTTGTAGTCTGGCAAATATTTTTAACTCAAAAGACACAAAAGGTTTTCTCTTCCATTTTCTCCTAGACATATTTTTAGTTTCCACTGCACATAACATAATTATGCAAAACTGCTTGAGAAGAGATGCAAAGGTAT
MSTRG.112247.1	--	0.658	0.000	0.000	-6.219	down	GTTTTGCTTTATTTTATTCTTAGTTTAACTGAATAAATGTTAAATGAATGTGTGAGACCGTCCTAATCTGGAGATATAATAAACATTTTATGAAAGTGAGCCTTGACAGGTCACTGTGCACATGAGGCAATGACCTGAAGTAAGGTATAGATAAAAGGTTTATTTCAGCTCACAGTGTTGGAGTTCGTAGGCATGATTAAATGGCCCCAGTGCTTTGAGCTTTTGATAAGCAAAATCATTCAGCTTCTGACCACAAAGCAAAAAGCATGAAGAGTGGGCTGGGTTTCCATGATCCTCCAAGGGCACATTCATTCTAAGTCAACCAAAGACTTTATGCCTAGCTTATACCTCTTATAGGTTCAATCACCTGCTGACAGAATCATAACGGGTGCTTGATATCAGTGTAGGCCTGTGAGGAAGAGTCAACTTCCATCTATCACAGAATGTGATGGGGGATATTCTTATTCCTCTAGGGTGGTGGTTTGTGCGAGAGCAGAACATCAGGAAAGACTTGAGGTCCGTTAGCCTGGAGACTTTAAGTAGAGTTGGAAGATTCTGAAATCTCACATGGCTGGTAGGTCTTATCTCCTGTCACTCCTGTGTCCATCCTTCTACCTTGAGTTACCCAGATTCTTTGTGCTCCTCTCAGCGTCTTACACTCTCAGTGTACCATCCCATGCCTAGTGGCTGTTGGTATCTTTATTGATAGATCAAAAACCAATTGGAGACCTTTAGCATCTGGACACTCAGATTCCCGATTGAGTCAAA

							GCTTTCGGACCACTCTCCAACACCTCTCCTCTACCTTGTCTTTCCCCCACCCCCACTCCGCCTCCCCTCAGAAAAGAGCATGTCTCTGAGGGACATCAACCAGATACAGCATAACACGCTACTGTAAGACCAGGCACATACCATCACATCAAGGCTGGATGAGGCAACCCAGTGGGAACAGAAGGGTCCCACAAGCAGGCAAAAAGATAGACCCCAATACTACTGGTAGGAACCCCCCAAGAACACAAGAACACCAAGTTACCCAGTGCGAAGGAAACTTTGAACTAGCCACCTCTTCTATCCAGGCAAGACCTCAAGTGGAGGTTCTGAGATCCCAACAGAGCCCTTAGCTAAAGAAGCCTTCTGGCAAAGTCCTTTTGGCAACATATCCTATATCCTTCTTGAGTCTTCGACTTGAGCCCAATGCTTTCACTGCCATATTAAAGGCTGCATGCGTTTCCTTCCTCCAAAGGTACGAGTGGTTGGAAAACCTGGGTGGTTACGTATAGACCCAACTACTTCTTCAGTCACCCACATAAGAACAGCCCAGCTCGAGCTGAGGAGTCCTTTCCCTCTCTTGATTAAATAAGTCATTAGCTATATTCAAACAAAGATGATTAGTAAGACACTTTTCTGCTTGCAGTGTGATTACAGTACTTTTGTTAGCCCAGAGGCAGCACTGTCTCCTCAGCTAATTAGGGACTAATTGACTCCTCAGCTCTGTTAAAAGCTGTGACAGCCTTCTCTGGCTCCTGTCTGCTGCAATACCTGGGACCGTACCTCTGTGACGGGTCACTTTCCTTTATCCCAATGGAGCAAATTCTAGCTGCCCATTTCTCATTGCCTTGTGATTTATGAGGAAATGACAGGAAGTCTTTGTAATTGAGAATGTCTCTATTTAGTCCAGGACATGTAGCCTTTGCTTAGCTAGCCTGAGGGAAACCAGTGGCTTGTACGTATGTATCTGCTTCTATTTACTGGTAGAACGCTACAGTTTCTGAGGAAGAGGATGCTACCTTTCAAACAAGGCTGGGGGTGAGGGGGGCATTAACATGTAAATGAAATGGAAACTTATATAACACTTCATGACGTAACTTACCTTATATTCTTTTTGGGGGATAGGCTAATTTTTGATTCCCAGAAAGTGTCAAGTGTGTTCATATCCCCTTTCTCTTGAGCACATTCTGAATGCCTTCATGTTTGAGTCTTCCCAGTCTTCTCCTCCAGTAGTCTGTCCTGGTGGGTGTGGGGGGACATCCAATGAAGACCTCCTGGTTTCTCTGTTTCTTCCTCCTTCTCTCTCCCCTTCTTTTCCTCTCTCCTCTCCTCATACTCCCTACCTGCAACACTCCTA
MSTRG.112279.1	--	0.349	0.012	0.004	-3.230	down	CAAAGCTCAGTATGAATTAACCTCATTGGAGCTGGTTACATCAACAAACGTACAGGCTAGGTCGTCTGATACGTCTTGTGTACACATTGCACTCTTTTCTACCTGCATGTACTTTTTTTTAATCTAAACTACTTTTAATGATTAACAGTAATTAACTAATCTTTAGCCTTAACTTCTGAAGGTAACTGAAAACATGTAAACCATGTCGATTAAATGTAGACTTTTGTGTTCAAATAAATACTTGACAAATTATCTGGGCCCTTCAGTGGTATTTTAACCACTGCAAGTTAAAACCGTGTTTCTGCTCTTTGCAAGGGTACTAGAGAATCTGTTAATAGCAACACCATACCTACTCTGTCCAGGAACCACGGCTACGGAGTTACACTACAATCTATCACTTCACAGTTATTCTGGAATCTATAGGATTGACATTTTTATTATATGTGAATAAGATCATGGCACATTTATGCTTACATCTTTCCTGGGTTCCCACTAATAGGAAATTTTCTTAGCTCAATGGTGGTAAAAATTGATTTTAGGTTTTGGTGGTATAGCATGTAAGAAGTAAGAAACTATAAAACTTGAACATCGACATCTTATTGAAGAAAGAAATAGGAATCCAAGAAAAGTTCAAAAACGGTTTATTAGGAGAAATAGTAACAATGAAAATACAGATGAACATAGATGCTTTTCTTTGTTCCTTGTCATCATTTTAGTAGAGACTGAGCATTGGTATCTGGGGCTTTTGATTTAGGCTGGATGGGTTGGACCACAAACCTCTGAGATTCCCTTTTCTCTGTACCTACAGTGCTGGGATCCAAGATATTCATTGACATGTCTGTGTGAGTTTTCTCGAGGGCAAGAGTGGGAGCAGATTGGAATCTAAATTCAGACACACATTTTTGCAAAGTTATCACTTTATCCATGGACTCATTTTTTTTATCTTGCCATTGTTTAATAATACATGGATATAAGTACCTTTCTTGCCTGTTTTTAGTTGAGTTTTTAATTGTTTGACAGTCTTATGCATTTATTTAATGAATTAGTCATTTTCACTCTGTACTTTGCCCCCAAAATCCCACTTATTCACCCACTGGGACTTCTCATTCCAATGAGTTTCCCTCCTACTTTCACGTCTTCTTTTTTGTGTGTGGCCCACTGATAATTAGAGCTCCTTGCCTGAGCGTGGGTGGGAAGTTACTCAGCATAACAAGGGCATTTATCTATGTCTCTTTAGTGCAGGAGTTCTCATTAGTGCAGTGCATTCTCTTTAGTACAGTGTGTTCTCTTTGTTGTCAGAAACCCAATATTCTCTAGAATGGCTAGAATATTTCATGCCTTTGATGAATATTTGATGAACAAATAAGGTAGACCAACAGTCTGTTAATAGTAGATCTTCAGATGACAAGACTTTCATAAAGGCTTTGAACACTTCAATAAATATGGAGCTGAAAATATATGGTGTGAGGGTTCCATAGC

							TTCTAACATAGACCAAAAGATCTATGTTAGACACTTCTGCAAAATTCCAGCCAAATACCAGACAAAAAACTTAAAGCGGAAAAGGTTTATCTCAGATCAGTGTTGCAGAGGCTTCTGAAAGTGTTAACCAGGACAGTAGACTTGGACAGAGCATCACCATGGCAGGGAGAGTGTGCTAGAGGAACTTCTACACATTGTGGTGAATAGTGTAATAGGCAATGATGTAGGTAGCAGCACAGGGATCTGCTTCCTACAGCTACACTCCATCACCTAAAACCACTACCTCCCTAAATAGTGCTACCACCTGACGTTCAATCTTTGAGGACATGAGCTTGTGGGAAACAACATCTAAGTCATAGCAAGCCCTAAATTGTACCAGTGGCTGGAATTACCTCCACAGTGTACAGTTGGAGATTAAGTGTATTCACAACAAGCTTAGTAACTAGTAATCATGCCAAGCTTTGCCAATGTGAGTTATCAGTGATATAAAAGATGACAGGAAAGAATTGTCACCTGGTGAGCAGAGCTTCAGAGGCTTTATGTTCTGTGTGTTCTGTGACACAAGAAAGAGTATGTGTCACCTACGCAATGAGACTGCCCCAAAGTAGTGTCTTGAAGAATGGACAACTATACATCCGGGTTTTGATGAGCAGAAAAGTGTAGGC
MSTRG.112308.2	--	1.445	0.000	0.000	-8.481	down	GTCCTTCAGAGGTCTTTTCTATTTCTTTCTGTAATCTGAATCTTATTTGCCTTTTTAACAGTTTTAAGATGGGCATGGAAATGGCTCATTGATTAAGGTGCTTTAACCAATGAGCCGTCTCCCCAGCTCATCTTAGGTATAAGGCCCAGGATTTGGAACCCCAGTACTCACATAAATACCAGGTAAGCATGGTGACCTCTACTGAGAGGAGGGAGACGAAATCCTGTAAGTAAGATGATTCTCGATTTTAAACTTGGGTGTCTACATCCATAGGCACACAGGTCTATCTGCACACCAAGCTACTGCTACACACACACACACACACACACACACACACACACACACACACGCACACACACTAGAAAAAGGCAAAAAATCAATTTAAGAAAACAAACAGAGGCAATATTGAACAACAGTTAACCACCCCAGGATATAGAACATAATTTCAACATTCTATGCAGGAAATACTACCCAAACCAAAAAAAAAAATCTCTTTTCATGTCAGAGAAAGAAAAGGACATCTGGAACATCTTTGTTCCACAATGGCCATTTCAGGAGACCAGAAGCTTCAGTGTACATTAAGCTACTGATTTATTTTTGCTCTGTGGGGAGAGAAAAGGAAGAAAAAATATCTGGGCCGTAGAAATAGAGTGACTTTATTGCATTGAAACATGTCAAAAGGCTGTCTAGGAATATTCTAGAATGTCCAATAAGTTAACAGTGACAACCGTGCTCTTTAGTATAGCCCACTCGACAGTGTTCATAAGAACAGAAGATGAGTCATGGCACCCTGACAACGCATGCCGAATTTGAAGCAAATTGGAGCTATAATTTATAGTACAATAGAACCCATGACAGAGGCACATGTGGTCCAGGCTCTTTAAATATTCTATAAATAATAGCTGGGAGACGCCCGACTGACACTGAGCAGGGCTGGATGATGAATCATCAGGTAGTTTAAGAAAAACCCTGGCTGATTAAATGCTTTGTTTTCAAACCACCTGCATCGGTGTCTGCAGGAGCAGGACAGATCAAATTGTTCTCGGTTTTATGTTGTTTTGTTTGTCAAAAAAAAAAGCAGACAGACTCATGAATGGTAAGTTTTATTACATTACAAAGCTGATGTCTAACTGAAATCCTGAGGGCTGGGTAAGTCTCATCATCTGTGTTTAGGCCTAGGATCTCGAGCATGGGTTATGACGAGGCAGCGAATTCAATGACAGAACGCCATGGCTTTGGGTTGTATGCTGGTGATTTTATTTTGGTCTTTATAGCATGGATAATTCATTTTCCTACCCTTCACTTCGGGAGTCTTTCCTGTTTATCCCACCAACCAGTTACCAGATGCTCTTTCGCCTAAGCTAATTAGTGGAGTCTCCTTCCAAAGCCAGTGCTTGCTGAGTTTGGTCATTAGAGCAGGCAAAAGGGAGGACACCAGAGACATCTGAGATGACACACATGCTCCGGCAAGCCATCCTCCAGCCAAGTCACGGGGTGCAGTGTGGAGGCTGCAGCCACACAACAGCAAAACATTTGCTCAGGTGGACAGAGATATCAGGTCATTCATCTTTCCCCCATTTACTACAGTGCTCAATGTGGGCCGGAGATAAAGGACTCGGCACTGGCACATGGTGGATGTTAACCATCCTAATTTTATGTTGAGCAGGCATTCTTCTTACAGTTACAGAGGGGACACCCCATAGCAAGTGATACGACTTAGCACTAGTAGATTCCCTCTGGAATGTCTACATCACGAGATCTCAAGTCATTGTTCTGAAAAATGTTAAGTAAAACCACTTGTCACTTGACTTGGGTGTAGGTGAGCGTGTACACCCGTGCAGGGGCATGAGAGTCACAAGCTACAGGTTGTCCCGATTTATCCCTCTCTGCCTTATTTGAGGTAGGACCTCTCAATGCACCTGGAATTAGACAGGCCATCAGCAAGGCCCGACTACCCTCCTCTCCAGACCACTGGGGTTATAGACAAGTGCGTGGCCACATTCAACTTTTCATATGGTGACAGAGGAGCCAATCTCAGGTCCTTATCTGTGTGTCCACAATGTTCACAGACAATGACCCTCAGAACCACCACCCCAGCCACTCGTTGACTTTTATGTCAGATCGCCACATAATACTATCATTTCC

							CCTGATATCTTGTTTTGTATCAGCTACTTGCTCACGTATGTTTCTACCTTCCTAAACTGGTATCTACTACAC<br>GCACATACCAGCTTCATGTGTCACCTCCGTTTGTTTCCCAGAGTTCAGTATGTAATAACTATCTCATATGT<br>TTTCTTTAAAAATTAAGTGAATGATACGGTTGGTACCATAACAGAGTGACACTTAGATAATTAGGAGATG<br>ATGATAATTCTATAGCATAGAGCAAAAATTGTATTTTGTAAAAGCTTAACTTAAGGCTTTACCCTGAAATG<br>ATTTGATTAATGAACCAAACAAAAAGATAACTCATGGAAGAGTCTAGGTTTAAGTACCCAAGGGAAATC<br>AATCATGAGCTTATAATGGGATCAACCAAATTTCTTAGCTAAGTCGTCTAGCTCTATTCTACTATAAAAGC<br>GTATGCTTTGAGGTTCATGGGTGATGCTATTTTGTACTTAAAATTTAAAACTATAGAAACCAAAAGCCCA<br>TTATTTCTCCTCAGAGTATCTGCCAGTTGATCCTTCTACACCTTGATACAATGGCTACTCCATTTAATGTG<br>AAAGGCCAGATCTTCTTTTAAGGACTTTAGGAGAAAAACATAATTATTGTGCCCATAAAAATGTTTACCA<br>AAGGATTGTGGGGTAAGAAAACCAATTTGAGTGAAACCACCTAATAAAGAAGGTAAATCTAATCAAGC<br>GACATTACATATTTTGAAGTACCTGTCTTGTGATTTGTGTACTTGGGATAAGCAAAGAAGCATTTGAATG<br>CAATGATTTCGTGAATTAGATTTGCCACTCGTTTCAGCTGGTGGCTTTTAAAGGACATCTTTAACTTCTCT<br>GAAGCTCGTTGTCTATTGCTATAAAAGGGTGCTCGCAATTCCTAAAACAAATGTCCCTCGGTCTTCGAC<br>AAATAATTCATGAAAAAGTGCCATGAACATTTCACACCCTCTACCAAAAGTTATCGCTTAGGCTTATTGG<br>AGACTTCTGTCAAAGTACAAAGTACAAATGTCTGTACTGCTTCGTCACCTATCACATCTGCATATGGAAA<br>TCTATTCCAGAAAGACATTTGACTGTGTTAAATGAATTATGATGCATACACTCAATGAAACATTATAATGA<br>AAAATATTACTGGACGTTAACATGATGATATGGTGTGTTCAGGCCTTAAGTTACTGAATGCAAAAGTGGC<br>CTGCAGTGAGTACTAGAGAAAACTGCCACCAGGGTGAAATTGTATATTATATTTATAAATTGTCAGGCAC<br>CTGGAATGTTTCATGCCAAACTGGTGACTGAACTGTGTATAACGTTTTGAGACAAAGGTTATCCCTTTCG<br>GTAAAGAAACTGATAATAATTGTCACTTCAGCTACTCTTCCTTTACGGTGGGGGTTGGGAGAGGAAGTA<br>CTGTTGGTCAAAACAGCAAGCATTTTGTCATTGACCTACATTTCCAGACCCTCATCTTTCTATCAAATAA<br>GAAGCAAATTCATATCTCAAGATTATACCTCATATAATCACACATTACATAAGCTTGTCCACCTTCTTGTG<br>TCTCCTGTTTGCCCTTTCAGGCTAGCAAACAGGAGCCCTGTTACTCGTCTCTGTGGATTCTGTTTGTCCC<br>ATTTGTTTCTTTCTCCAGGTGTGGAGTAAGATGGACAGGTAGACAGATTTAAAAGGCTTCCTTATTTTGT<br>CTTTCTCAGAAATGAATTTCTAAGCAACGCTATCGAAAGGAGAACTTGTTTCAACAACAACCCAAGCTG<br>ATATGAATTTCACGACATCTTCAGTTATAATGTTATAACCCAAAATCTCTTGCAGAAGAAAATATGAAATA<br>CCCCTGTCTACAGAAAAAAAAATCAGAAACTCGTTTCTGCGTACTCGTTTCTCATTTCTGTGGTAAACC<br>ATCTTAGGTATTTTCTCCACAGTGACTCAGAAACACACTGTCCATTAGCCTTTAGAGTACAGACACCTGG<br>AAATGAGCTGCAAATGACACGGGGTTCGGTTGAGGTCTCTGAAACGACCAACGTCTGATTTTAAAAGC<br>TGAGCTATACGACTTTGTGAGAACTCCCATCTCCTGCTCTCACTTGGAAGACTCTTTAAACTGATTACAG<br>GAGATATAGTTGCTGGTACTTGGGTGGCAATGGGAACTGAGAACCCCACCCCTCCCTATGCGATCAGCC<br>TTCACTTGGATTGTCTGTTTTATCGCAGTATGCAGCAGTAAGTAAAGAAGACATCAGTGAACTTCCGAC<br>TCCCCGGGGATCACAAAATCATTGTTCATATTGCTAGGAAGCAGGTAAGCCTAACATAACTACCAAGTG<br>CTACAGATGAGGGATGGGCACATGTACTACGGTCTTTATTTACTACGGTCTTTATTAAGTAAACAGGACC<br>ACATAAGATTGCATCTTTTAATCACTTATTGAGTTGTGATTACTAAGGCAGTGGGCCTCAACCTTCCTAAT<br>GTTGTGACCCTTTAACACAGATCCTCATGTTGCAGTGACCCTCAACCATAAGTTTATTTCCTCGCTAATT<br>CATAACTAAAATTTTGCTATTGTTATAAATCATAATGGAAA
MSTRG.112405.2	--	0.461	0.000	0.000	-7.122	down	ATAAGTTGAGAGTCAGACATTCAAACACATGTATTCATAGAGGCAAAACCTATTCAAACCATCATATTCC<br>ACTCTCCGGTTACCATAGACTTGTAGCCACAACATAATGCAAAATGCATTTAGTCCAACTTCAAAAGTCC<br>CCCCAGTCTATCACAGTCACAACAATGTTTAAAAGCCCAAAGTTCAAAGTTTCTTCTGAGATTCATGCA<br>ATCTGTCAACTGTAATCCCCTTTAAAATCAAAATTAAAAAAACAGATCATATACTGATTATACTGGCATCA

							TAGGATATACATTGGCATTCCAAAATGTCATAGTGTGGAAACACTGGACCAAAAATCAGCTGGGTAAAC<br>TCCAAGCTCTGCATCCTCTTGTCTGATGTCCAACTCCTTTCAGCTTTGTTGACTGCAACACAATTCTTCG<br>TCCTGGACTGGTTCCACTCCCTGTTAGCAGCTTTCCTCAGCAGGTATCCCATGACTCAGGCATCTCTAAC<br>ATCTTGGTGTTAAGCACATTGGATATTGTTTTTACCTTCCCCAGGAAATGATTAAAATTTCAAGTTCCAAT<br>GTTAAACACTTAAAATGTTAAATGTTCTTTTTGCCTATTTAAGAAGAGACTAAAGAAACAACTTCAAAA<br>AACAAAGTTAGGGGGCTGGAAAAATGGCTGAGTGATTAAGAGCACTAGTTGTTCTTGCAGAGGCCCTA<br>GGTTTGATTCTCAGCACCCATGACTATCTTTATCTCTAGTTCCAGGGGATCTGATGCCATCTTCTGACCCT<br>CATGGGCATCAGGTATGCACATGGTGCACATTCACACCTACATTCAGACACAAAATACTCATACACATAA<br>ATCAATGCAAAGCTGTTTTAATAAAAACCGAATGTAGAACACAAAGCACAGTTGCATGGTTAAAGTGTT<br>TTTTTAAACATAAGATTTATTTTTATTTCTGTGTATGTATGTATGTCTGTGTACCACGTGGTACACAACTTC<br>AGCTTCTCTCCACAGCTCCTTTAAACCTAGAGCTTTCCCTGTAACTGAGGCTTCATCTCTGCCAATGGCC<br>TTAGCATCTCATATTGCGCCCCTCAGATGCTCTCCATGACCCCTTCAATCAGCAACTTTTATGCTGAGGA<br>AGCCAGTACTACCCAGGAGACACATTGTTAAATTTAGCTGTCAGCTTGAGTTGCATCCTTGGTCCCCAG<br>TTTCTGTGTGCTGATCCTGAAGGAAATACTTCCCGGAAGATTTCACCTGAATGCTATTGGTTGAAGAGA<br>CTCTAGCCACTTCCGGCATGGCAGACAGGGATGTGGCAGGCCTAGCCTTTTTCCCCAGTTCCCTCTGCC<br>TTGCTAAAAACTGTTAGATTGCATTCCTAAAGCTAGCCCTCAAGGTCTATTCCCTTATTTGGTCAATGCCT<br>CCTCCTGAGGCTAACTACCAAGGTTCAGCTATCAAAGTATTTAAGTCCAGCAATCAAAAGTCCCTATTG<br>GATCACTTAATTAACATGTCCAATCAAAATTAAACATCTCATTATAATATGGGGGTTCCCCGTTTATCTTTA<br>TAAACTGCCATTTGCTTATGTGCCATGCTTGACTTTTCTCTGTCCAAAAGCAGTCCTTTGTCCCACTCTG<br>AGACAAATACCTCTTCCCCTTCTCTCTCTCCCCTTTTCTCCCCTCTCCTTGTTCCCTTTACCTTCTCCCTC<br>TTCCCCTATGCCTTGTCTTTGTTCCTTATTCCTGCCCTCTGTCCTTCTGGGCCAAATCAATCTTCTGTGTG<br>CCGAGAACATGGTCTTGGATATCTTCAGCCAGCTCTGAGGTTCTGACATTGGTCACAGGTGATTTTTTTT<br>TTTTCAGCCTCCGTTAATGTTACAGCATCAATTATCATGGTCAAGGTTTTATATTCACAGATTCTTAATTCA<br>AAATATAACAAGAGCTGAGTGGCACATACATTTAATCCAAAACTTGGGAGGCGAGGCAGAAGGATCTC<br>TGAGTTCAAGGCCAACCTGGTTTACAGAATGAGTTCCAGGACTGCCAGGGCTACACTCAGAAACACTA<br>TCTCAAAAAAGCAAAAAACAAACAACCCTCCTCCCAAAACCAACAACAGCAAGCAAAACAACAACA<br>ACAAAACAACAACAAACAACACAAGAACAACAAAACCACCAAACCAAACCAAAAAAAAAAATGACA<br>TGAATGGCCCTGATAGTTGCTTCTGAAACTTCACAAGCCAGGCCTCCATTATGTTGTGCCTCTCTCAACA<br>TTTTTACTTTCCAGGTTCCCACAACAGCTCTGAGTACTCACCAATGATTTTTAGCCCAAAGTTCCAAAAT<br>CCTTATACAATCTTCCTAAAAACAACATGGTTTAATTACAGCAATATCTCACGTTCCTGGTACCAATTTCT<br>GTTCTGATTTGCTTTACAAAGACGCCAACTTGGGGGTAAAAGAGTTTATTTGGCTTACAGCTCCCAGTC<br>CATCATTAAGGGACTGTAAGGCAGGAAGTCAAAGGCAAGAATTGAAGCAGAGCCCATGGAAGAATATC<br>TTCTTTGCTGCATACTAACTTTTCCTTCAGACTTACATACAGCTACTCTCTTACACAACCCAGGGCCATCC<br>AACTAGGTATAGCACCTTCTGCAGTGGCTGGGCCCTTCTATGTCAATTAGCAATCTAGAAATGCCCCAGA<br>GTCCATTGGCTAATTCGATGGAAGCAATTCCTTAATCCACCTTCTCTTTTCCCAGGTATGTCTAGATTTGT<br>GTCAAGTTGACAAAAACTATGATACACTAGACAGGGCTGGATTTGAACTTTCAATCCTCCTGCCTCAGT<br>CTTCTGAATAGCTACCATTACAAGGTTCTGTCATGTTTGGTTATGGAGGATATTTATTCTACTCAGCAGTG<br>ATATGTCAGAACCATTAATTATTCATGACAAATATTAGCATTGAGATATAGAAGTGTTTGAGAAAGCTACA<br>TTTGTTTTCCAACAGCCTCTTTACTTTCAATTACTTGCATTCAATTTAAAAGAAGGTTTCATTTTCAGTTA<br>TGTGTTTGTGAGTACAGGTACCCATGGAGTCCAGACATGGGTATCGGATCTCCTGGAGTTGGCTGTAGC<br>CAATTGTGAGCTATCCAGTATGGGTGCTGGGAACTGGATTCTGGTTCTCTGGAAGAGCAGTAAGAGCTC

							TTAATCACTAATTCGCATCTCTAGCATTTAATTAAAAAAAACATTGTATTTGCACATATGTGTATGTGTGTATGTGTTGGTATAAGACAAGTTGGAGTTGACTCTCCTCTTGTAGGTCCCAGTCATCAAACTCAGGTCTTTAGCCTAGACACTGTGTAACTGAACTCTTGAGAGCCTCCTAGACCACCTCCCAAACTTTTTAAAGTGAGGCTAAGAAGCTACAACTAGTCTGTCAGGAATCTGCATAATCTGTATACTGACAGCTTGTTTCTCAGGGGAGCATCATCTACTGGGGGAGAAAAGATACCACTCCGTAATCAGTATGTAGTCCAAAGCCTCGCTCTTATCACAACTGGCAATGCAGAGTAATTTATCTAAACACACATCTAATTAACTACGTCTCTAATGTAGGTCTCCTTATTTCTCATACCCCATTTTCCAGTGCACAAATGGTCAGATTTTCAAAGGCCAATTGAAGAAATTGGGGCTGAGGCAGAAGAGAATTATGCAAGGCAATTTGCCTCCTTTGTTGCAATCTTTGGTTTTAATTCTCAGGGTAAAAGCAAAGGAAACAGCCACGCAGCCAAGAAGATATATTTTTCTTGTTATCTAGTATTTTAGAAACATTGTTTAAATATATTTGACTGCAAGTCACAGTAAGAAATACAATTTAAATAACAACAGGGGACACATTTTTACCCTGATTATTTATTTTTAAATACAGGGTCTCTCTTTAGGAGTCTCTGTAGTCCTGGAACTTGCTCTGTAGAAAGAACAGTCTGGCTCTGGATTCTACAGAGATGGGCTACTATGGCCGGCTAGTCACAAAGTAACTTTTTTTTTTGGTTCTTTTTTTTTTTCCGGAGCTGGGGACCGAACCCAGGGCCTTGCGCTTCCTAGGTAAGCGCTCTACCACTGAGCTAAATCCCCAGCCCCACAAAGTAACTTTTAAAAAAGATTTACGTGTATGGGAGTTTTGCGTGCACGTGCATGCCCTGTGCCTGAGGTGGTCAGAAGGGGTGGACTCCCTGGAACCACCAGCTACCGAGTGGGTGCTAGGAAGAGAAACAAGGATTTGCAAGAACAACAAGTACTCTTAACTGATGAGCTCATCTCTCCAGCCTCCCACAAACTAATTTTTGCTTGGTTTAGATGTTACGCATTCTTATCTTTTAGATTGTAGTCCATTAAAACTGAATTCATTAAAAAACGAAACTGGCTTTTGTTAGTCAGGATAAAAAGAACGTTGCTTCAGGTAAGTGACAAGTTTCGATCAGCTGCTAAACTGGAATGTTACCATCATCACATAACAGTAAGTTTCCTGCCTTGCACAGCCTCAGCACTGAGGAAGTAGAGTCAGGCAGCTGTCTGTGAGTTTGAGGCCAGCCTGGTCTACAAAGCAAGTTTCAAGACATCTAGGGTTACACAGAGAAACCCTGTTTCGAAAAACCAAAACAAGGAAGAAAACCAACTGAACCACAAAACAGTAAACTTCCCTTATGCTTATATGAAACACAAATATAATTTCCTCTACACCCGGGCATCAAGCCTTCACAGGACCAGGGGCCTCCCCTCCCATTAATGCCGGATAAGGCCCCTTCAGTTCCTTTAGTCCTTCCCCTAACTCCTCCATTCGGGTCCCTAGGTTTCTTAACAGAAGAACGTTTTGGAATCTTTCCCTCATTGGAACAGTCCTTCATGAGAAAATCTAATTGTTCTTCAAATGTTAACATATCTGCATCAAGATGTGTCTTAAAGATCAGTGGCCTGGCATAATTTAACTGGGCAGTAAGTTGCTCTTTCTGTGGGCAACATAATGGCCACGTCTGCAGTTTTTTTTTAGAGCTGCTATGAAGTACATCAGTAATAGGCAGAATCCTGTGGCCTATTTAAGTATCTGGATGATCCGAATGTCATCTGATTTCCTGTCAAGAAATGTAATGGAAATTGTGTAAAGTTAAAAAAGAAAAAACAAACTCGGTTCCAGAGTAGAAGGATAATCGAATAAGGAATTGAATTTTTATCACACAAAACAGCCTTGTTAGAGGCACTCAGTCCTAGTAGCTTTTCTCTTCTTCTGTATTGTAACTGGCTTTCTTCCAGTGATGAATTCTCCTTTCCTCTCAACAGGATGGGGAGGGGTTCCCAAAAGCCTGCTAGGTTCTTCGCATCCCAGTGTGACTGAAAACCACATTGCAAACCTCCTGTGAGGCCAACAGCAAATTAGACACCACAGTGAGCGTGCCATTGGAAATAGGCAGCAGCTGCTCCAGACTGCTCCTCCAGCCGGAATTGCTACTCGCAGCCTCCTCTAGGCTGTTGGGCTGGAAGTGGACCCGGCCGGAGGTCGGGAGGCGGGTCCTGCCCAGTGAGCGCTTTGCGGCCTCCTCAGGTGATGTAGGCCGGGGTCCCACCTTCCACAAGGCCTCACCTCAGGGGACCCGCGGGCGGGCGGGCGGCTCTCTACCTCAGGGGTTCCTGAGGAGGAACCTCGAGGGCGCGGAGGCGAGGTGACAGCTCGCCAGCACGACGAGCTGCCAGCAGTTTCCTCCGCGCGCCCGCCCAGCAGAAGCTACCCG
MSTRG.1128	ENSRNOG0000002	0.000	0.288	0.007	3.835	up	CAACAAGCGCTGCATATTAAGTACACCCCAAATGTACTCTGTTAATACTTATTCAATTGTGGCAGAGTGCTGACTATGGTCTGGACTTTGGTTTTGTTAACTGCGGGACAGACTCAGGTGCAGGAGATTTACCAACCAGAGAGGAAGGAAGATACGGGTGCAAGGACTCAAGACGCCGCTCCCCAAACTACAGTTCTATAATTGATG

25.1	5487						AAAGTGGAGGCTGAGGTACTTGGTCAGAAATCTATCAGGTTCGATAGCAAGAAGCAAGCGGGTAAGTGAAACTCGAGGGTAGGGACCATGGACCCATTGCTGTCCTTTGTGTGTGTGTTCCTGGGAGCGGAACCCACTGTCCCCTGTACGCTAACAAGTGCTCCATCACTGAGCCACACCCCCAGCCTCCATCGGAGCTTCTGATCACGAATGTGGTTTCTTCTTGAATGAGATTTCTACTCCTACTAGGCAAGTTTAAAGTTGGTGC
MSTRG.112828.1	ENSRNOG00000007286;ENSRNOG00000007365	0.000	0.370	0.019	3.592	up	GGATTCACCTGCTTCCTCCCTTCCTTCCACTGCACAGCCCTCTTCCCACCCTGATGATTTCAACCCTTTCTAAAGCTTCTCGAAGTCTACATTCCGTCCATGGGTGTTTCTCATTATCTCTCTGTGAGCTGAAGGGCAAATCCCTTTCCAGAGTCCGGCACCTAACAATGAGCTTCAGAGAGGAAAGTGCGACTCTTGGCATTTACAGAGGAATGTCCTTAGTGAGAGACGCTACTGATGGATGAGGATGCTGGACCGCTCTGCTAGCTGACTGCAGAGTCTGTTCACCAGCTCTGGAGATGGTTACTCGGCCAGATGG
MSTRG.113159.1	ENSRNOG00000004189;ENSRNOG00000004312;ENSRNOG00000004316	0.000	0.290	0.007	3.835	up	CTACATTCTAAATATCCGTCCTTACAGTCACAGGTAAGTGTTGTCCTCACCCCTCCCCAAGGACACTTCTTTTCCCAACAGCTGGAGACCATTACAGAAGAACACAGGCCATCAAAATGCAGGGCCCAGTCCCACTAGATACATCTACGGAACAATTAGTGCTCAGGGGACATGGCGGAAGAGGAGGTGAAAATATTGTAGGAGCCAGAGCATCAGGGAGTTCTCTGTGAGATTGCGTCTCCTAGAAATGTCAGAAGGTACACCCACAGAGTCACGCCAACGTGACTGCTAGACACGAGCTGAACGAAGAAGAGAACGATAGATGTGCATGAGAGGGAACGAGGAGGCCTCAACCTAACAGAACCACAGGCAACCAAAGGAGCTGAGAGTTGGGGAAATAGGAAAAGAAGTTTTTAATATCGATGTCTGGCACCTGAAGACACTTCACAAACCCTCATTCCTGGCACCATGCCCAGCAGCTGCTATGCTGCCCCGTCCTCCTCCTGAAATGGAC
MSTRG.113363.1	--	0.456	0.000	0.004	-4.319	down	AAATTTTATTCGTTAAAATAACAGCCTTGGAAAAAGAGATATTCTCATCAGAAGTCACAGCGGGGATCCAGGAATATCTCGTTAGACTATAAAGGATATAACGTAGTTAAAAACCACGCCAGAACCAAACACCAGCCTCGGCTCAATCTGTGTGTGTGCAGGTGGGAGTCATTCTGACTGGAAACTGGCTTCACTCCAAACACTGGCCCAGTTCCAGGGTCTTCATGGCTTCCTCCAGCTTGAGCATTCCTCTTTCCTGGCGCAGGCGGTACCTCTCAGCCATGGACTGAAGCATCCATTTAGCCCGAAGCTTGTGGTTGCAAGAGCCATAAATTAAGACCTCGGTAAGTCCGGAAGATTCCAGAATCTTCAGTTCAAACATGGCTCGGCTCACCTGCTCGATGTGAGGCATTCGAGATCCCTGATCAAAGGTTCCACTGGGCAGCCGTGAATCTGAGCTCCAATCGATAAGATGAAATAAATCATGTAATACATAACACTTAGTTGTAACGGGGTGATTCTCCCTCTCGTGAGAAATAACACCTTAGCAAAACACACAAAGAAAAGGACGTGTCAGGAAAAAAATATTCTAGAACAGATAGAATAAAACACTTTTAAAAAAATCATCCAAACAGAGCATGACAAACTGCGTGATTTCCATTCTATTTCTTCTGCTATCTACACGTACCTTAAATTACCAAGTGCTCTGGTGCACATAATCCAAATGGGGAACTCTTCAAAGAGCAGAAAATATACATTTCTC
MSTRG.11337.1	ENSRNOG00000047234;ENSRNOG00000058463;ENSRNO	3.242	0.181	0.000	-3.328	down	CAGGAATTTAGAAGGCTTAGCCTGGAGCATTTTGTTCATCTGGTTCTGCCTCTCTTTCTTTAAAATTCTTTATTTCCCTAACCCAAGTTACAACTCGGGCTGTTGGCCACAAATCCGTGTTGCAGATTCTGCGTTCTCTCTGAAGCGGGATTTGTTAGTATTCCACTGCGGGACCAAGCCAAGTCATAATAAAGTCTCTCTGGGGCTCTGCAATGAAGGTCACGGCCAACCTTAGCTCTTTATGTCTCAGTGGCTTCTTTAACCTGCCTTAAAATTGGATGAAGTAAAACCAAAGATGATAAAGGCACCATAAGTTTGATGAGCTTGATCGACGTGTGAAAAAGGAAACAAATGTCCCACCTAGTTTCCTTGTCTGTTGTCAGAGGGAGCCCCAGTTCTGCTCCTGCAAGCTCTGATACACATCTGCAGCCAGAAGGACAAATTACAGGAAACATTCAACTGACCACTGAGGAAAACCACAGTGTGGAACAGACTCACAAAGGATGGTGGTTTTCTCTGTTGCTGGCTTGGCCATCATCCTTCACAGTTTACA

	G00000059588						GCCAAAGAAAGTTGAAAGAAACTCCAAACCACAACAGACACAGTGAACATGGACCCAGAAGAAGGTATTAGGAAGAAGTTAATCAATGGATGTTTTTCCATTCGTCTGTATCTTCAATGACTTCCAACTAATTTGAAAGTTTCAGTGTTCTGAATCATTTTTGGTTCAGTTTTCTAGGATTATGTTGGAGCCACATAGGAATGTCTGGAGAGGGCTGGCCTGGAGTGGGAATGACAAACCAGCAGAAAGACAAATTAATGAAGTAGCAAGAAGAACCTGCCCCCGAGTGTCTCAAGATCACAGCGAAAGCATGGACGGAGGATGGCAGCTAAGCAGACTTAACACAACATTCTGGAGGCCCCATGAGATTCCCCAGAGACTAGGATGCAGGGCCACTGGACCACTGGGTCACTTGGCCCCCTCTGGGGAAATAGTGTGGAGAACCTACAGAGAGGACTGCAGACCCTAAGACATTCCAGGACCTCAGGAATTTCTCCTGCATCTACTCCCACTGCCCCAGCCCCAGAAGCTCCCAACAAAAGATTACAGAAAGTTATCTGTATCGGTCTCTTCTGGAGTTGGCCCAGCAACGGACTTCTGAACATCCAGGGGGTTTGGACTGTCTGAAATGTGGTAACTGAAGACTGGGGCCACCCTAACTAATTTCACTATATTAACCATGCTTACTCTAATGAAAGACACACCCTCAGCTTAATTCTTGTTCCCTCTTGCTGGGGACCTGGAAAACCTTTTGGTTTGGCAAGGTAGTCCAAAGAAACCAAAGCCTCCACCTTTTCTTTTCACCCCATTGGAGGAAGTAGACATTCCCCCTCTGCATGCTCCAGAATAACTGCAGGCCATGCCCAGGGAGGGGGAAATGGTTCACCTGGCTGCACTTGGGAGCATGGTGTGTGTGTGTGTGTGTGTGTGTGTGTGTGTGTGTGTGTGTGTGTAGAGGTCTTCTCTCCCCTCCACACGAACGGAGTGGTTCTGTCTATGGGAGCTCCAGGAAAAATTTCTCTAGAGGCAGCCACTGCCTCAAAGGAGGTACTCCCTGTGGGCTTAAGGCTTGAAGATTCTCCGTCTATTCCCTTCATGGTACATCTCTCTTTCTCAAACAGTGATCTGTATACTCAGGAAACTCAAAACCCACCATCCTCTGAAAAACCTCAGGTTTCCATTAGCCTATTCTAGATGGTCTTCACACTCACCTGCTGATCTGGGATGACTGCCTACAGCTCCTGGCAGTGGAGAAAAGTCACCAA
MSTRG.11337.3	--	0.661	0.000	0.000	-5.549	down	GCCTCCGAAAGGCTTCCTGGGTGATCACCCCCATAGACCCGGACACACCAGAAAACCAGTGAGCAACCAATGTTGTTTTTTTTTTCCCTGTGGCCCCAGATAATGGAAAGAAACTTCAGAAGTCAGAAGGGCTTGAGGGTATGAATAGGTCCCAATTGTTAGAGGTTGCTAGAAGGTTTATAGTAATTGTGACTCTGTAAATGAAAAGCAGAATAAATTATTGCCTTAAGCAACAGTGGCAATGCTCAGGGAGCCCTGTAAGACAGATCACAGAGGGAGAAGCCAGAAACACCAGAAAAAGAAAAAGCTAGAGAAAGACCAATGTGCTTTTCTTTTGTAGGCAAAAAGGGATACTGAAAGAATGAATACCAAATCCGAAGGGGAAAGAAGGAGAATTCATCCTTCATTTGGGTGAGAACTGATGAAAACTGACAGGGATGAGACTCCATGTCATAGATGCCAGGATAACATTAAAGGGTCTGACACCAGCCGTATTTTATAATGAGACCTGTGGAAATCAATCAGCCCCTCCAAAGTGTGTGCCACCCAATCACTCAACACTAGGGTGCCGGCAGAAACAGGTTCATCATGCAGAGGAGGTTGAACATGTCGCAGGGAGGTGACATCCCTGGATGGAGACTCTTTACAAGACAGGAATAATGCAGCCTCATGTCCGTCTTTCATCTCACGATGCCCAACTATTTTTACCGGGCTTCATTAATCTTTGTTGAATACAATGGCATACATCTTTAATGCGGCCGCTGTCAAGTCGTATTTCAGCAGGAGCTGCAAACTGAATGACAGCAAGCAATATTAATAAATTTAACATAGACTATATCAATTTTTAACAGAATGAGTGTGGCAAGCTAAATGTATTATTTTTCATTTAAATCATAGAGTTTATCACCAGTTAAGTAGTAGCGGTGATTTGGGGGTGGTGGTGGGGGGGAGAGGCTTGTGACGTACCACACAAATAGATTTACATCTCATTAATAAGTAAATTAGGAAAATGCCTTTCAAGTTCAGACAAGTACATAAGTGTAATCTCCATATTAAAGGGGTAGTCTTTGATAAACCACGGAGGAATTGTGGCAGCCAAGGATGTGACATGGTGCCAGCACCGGCGCCCGGTCTGCTCTCCCACCTTTAATTATGCTCCTTCATTACTAAAGAGATAAGTGTCTCTTCAGTTAACATTCCATAAACGAACAGCATGCCAGCACCTCCCAAATCATTTTTTGCAGAAACCATGGAAAGGTGGAAACACTTATGAACAAAAACGAATTATCAGCTCCTCTGACTTCTTTACCT
MSTRG.1133	--	0.496	0.052	0.041	-2.297	down	GCCTCCGAAAGGCTTCCTGGGTGATCACCCCCATAGACCCGGACACACCAGAAAACCAGTGAGCAACCAATGTTGTTTTTTTTTTCCCTGTGGCCCCAGATAATGGAAAGAAACTTCAGAAGTCAGAAGGGCTTGAGGGTATGAATAGGTCCCAATTGTTAGAGGTTGCTAGAAGGTTTATAGTAATTGTGACTCTGTAAATGAAAA

7.4							GCAGAATAAATTATTGCCTTAAGCAACAGTGGCAATGCTCAGGGAGCCCTGTAAGACAGATCACAGAGGGAGAAGCCAGAAACACCAGAAAAAGAAAAAGCTAGAGAAAGACCAATGTGCTTTTCTTTTGTAGGCAAAAAGGGATACTGAAAGAATGAATACCAAATCCGAAGGGGAAAGAAGGAGAATTCATCCTTCATTTGGGTGAGAACTGATGAAAACTGACAGGGATGAGACTCCATGTCATAGATGCCAGGATAACATTAAAGTCTTAACCTGGCAGGGTCTGACACCAGCCGTATTTTATAATGAGACCTGTGGAAATCAATCAGCCCCTCCAAAGTGTGTGCCACCCAATCACTCAACACTAGGGTGCCGGCAGAAACAGGTTCATCATGCAGAGGAGGTTGAACATGTCGCAGGGAGGTGACATCCCTGGATGGAGACTCTTTACAAGACAGGAATAATGCAGCCTCATGTCCGTCTTTCATCTCACGATGCCCAAGTAAGTACTTTGAATGTAGAGTGCTAAACTCTCTCATCATTTGGGAAGGTAAGTATTGAGACTTAGAAAGGTTCTGAGGTCGGTAGCACCACTCTGATTTGGCATAGACTGGACATAACCAGCAGTTGAACTTGTAGCCTAAAGCCAGGAAGGAAGATTCCAAGTTCAGTTTTCTTTCATGGAGACGGGAGTTGGTCCTGTGAAATGCCATAGGAAAGGACGCGCCAAAGACCAAATTCTGGAAGACCAGCAGGCCACCAAGCTAGGGCTCATACTGCCCGCTGTGGCCCTCACTGGGCACCCCACATCTGTGTGCAAACCTGGCTCCATTAGGCTTCATCAATAGTTGGGAGATATCCAGACAATATTGAAACATTCTCAAATGAACTTTGCATAGAGACAGTTTCATTCGCTGCCTCCCTCCTCATGCATATTAACAAATGGTATGCGGGGCCTCATTTAATTCACTGATTTTACATTCGGATCACTTTCTCTTTTACAGCTATTTTTACCGGGCTTCATTAATCTTTGTTGAATACAATGGCATACATCTTTAATGCGGCCGCTGTCAAGTCGTATTTCAGCAGGAGCTGCAAACTGAATGACAGCAAGCAATATTAATAAATTTAACATAGACTATATCAATTTTTAACAGAATGAGTGTGGCAAGCTAAATGTATTATTTTTCATTTAAATCATAGAGTTTATCACCAGTTAAGTAGTAGCGGTGATTTGGGGGTGGTGGTGGGGGGGAGAGGCTTGTGACGTACCACACAAATAGATTTACATCTCATTAATAAGTAAATTAGGAAAATGCCTTTCAAGTTCAGACAAGTACATAAGTGTAATCTCCATATTAAAGGGGTAGTCTTTGATAAACCACGGAGGAATTGTGGCAGCCAAGGATGTGACATGGTGCCAGCACCGGCGCCCGGTCTGCTCTCCCACCTTTAATTATGCTCCTTCATTACTAAAGAGATAAGTGTCTCTTCAGTTAACATTCCATAAACGAACAGCATGCCAGCACCTCCCAAATCATTTTTTGCAGAAACCATGGAAAGGTGGAAACACTTATGAACAAAAACGAATTATCAGCTCCTCTGACTTCTTTACCT
MSTRG.11339.1	--	1.102	0.122	0.029	-2.305	down	CAGAACCCTGGGGCCTGGGTCTATGTGGATCCTTAGGGTCCTTTTGGTTTGCTTCTCCCATGTCGCCCAACTGAAAAAGGCCCTACAGATGACTTCTGCCACTGCCACAGCATCTGACCTTTGACCACCTCTCTCCAGAAGTAGCTGGATTCCCCTGTGAGTCACTTTTTCCATCCATCAACACACAACTGCAAAGGTCTATATATGGTGTAGCTCACGTCTCTGGTTAGAAGCCTTCAATATTTTTTTTTCAGTCACCCTTGGGGGAAAGTAAAAAAAGTTCTCACCACACTTAGGATGTCCCTTGGCCTGTTCCCGTTTCCTGCCTCATACCATTTCCTGCCTCTTTCCTTGGAAGTTTAAAATTCTCCCAGGCCCCAGAGCCTGCAGAGTCTTCCTGATACTTGCACTTTTCTGAGTCTCTCTCTGAGAACTTTCCCAGGATGTTAAACTTCCCATTGGTGCCCTCCAGATGTCACCTGCCTCTGTTCCATGTTCTTTTACCAAGCCTTTCAGTTGCTTGATGTTGGAGAAGCACAGATAAAGACAGAAGATACATCTGCCCAGGTGTAAGGGAACTCTGAAGGAGATCAGTAAACTACCTTCTCTGTTCTCTGCTGAACTCCAAGTATTGAGGATAAGACTGGATAGACAGCAGGCATTTTCCAAATGAAATAAAATTGTTCTGCTTCACTGGGGACTAAGTAGATAGGAACTAGTGTGATAGACCCTCTCTCCCTTAGATGGACCAAATGTAGTAGTGTTTGCATTGATGAGAAGTAAGGACCTGAGGTCAGTGCTCAGCATAGAGCACCCAGTTGTCTTTTTTTTTATCTGTGATAAGTATAAGACGAGATGGCCTCGTCTGTCTAAAGCTTCATGGCTTGTATGATATGCAACCTGTTATTTGTCCTCTGCAGAAGCGCCAGTGGGAATGGTCAGTAGTGGAGATTTGCTGAGAGCAGGTTGGCAATGCTTTACTCAGGAGGGCTGAGGGTGGGCTTGGTTCTCTCTCCTGAAATCCTGATTGCAGTGAACATCCTGGTGAACCCCTCTCAGGTGGGAATGAAGGCATCGTTATAAGTGAAATACAATTAGACCACTAATCTCCTGTCTCAGAACTAATGCTAATACCCTCAACAGATGAACATAGAGATTAATATTGTAAAATTTTTAAGA

							CAATGTTGTATGAAAGGAAAGCT
MSTRG.113409.1	ENSRNOG00000004839;ENSRNOG00000005034;ENSRNOG00000005129;ENSRNOG00000005138;ENSRNOG00000025570;ENSRNOG00000025584;ENSRNOG00000025592;ENSRNOG00000025602;ENSRNOG00000029465;ENSRNOG00000033161;ENSRNOG00000046214;ENSRNOG00000046245;ENSRNO	0.000	0.591	0.002	4.137	up	ACAAAATGATGAACAGGAGTCAGTTGCAAGCAGTATTGAGGGATACATCTGCCCACTCGGGGCCTCTCCTGTCAGGGACCCTGGATGCCAGTATGCACTGTGTCTGCCCTCAGCAGAAGGGGAGGGGAAGAGGGAATAAGCCGGTAAGAGCTGTAAAAACCAGGAGCAGCTGGTCCTTAGACCCAACGGGGGCAGTCCTGGGGCTGCTCTTTCCAGACGCTTCCACGATTGTTGCTTTCTCTTCTGCAGCTCTCAAGCCTGGGTCCTATTCCCTTCATGGCCTGCTGATTCTTTTTCTTCTCAGTTCCCTTCC

	G00000047631;ENSRNOG00000047895;ENSRNOG00000052955						
MSTRG.114006.1	--	0.734	0.078	0.014	-2.402	down	CGCTGGTTGCTGCACCCTATGTGAGTCAGAATGGCAGCGAAGAATGCAAAGGAAAGGAAAACTGTCATTGTACTGCCAGCACTCGCCTTCCGTGCCTCCTGCAAAGCTTGCTCCTCTCTTCTGACCTTTAAACTAGTTCTTTATTTCAGGCTTTACAGAGGAGAAGAAAAGAATCAAAGGATCAAATTATCAAGGAAATTACCATAATTTGTCACTCACAGCAACATATCACGTGCATACTAAGTAGTATTTTGAAAGCACCTTTTGGGATATGGATTTTAAATGAAGCTGGTTTCTTGGTCAGAATGCAGCTGTTCACATTCTGACAACCTGTCTCTGAACACCTCCAAAAGGAGGGAGGAAAAAGGCAGTCTCCAAAGGGCTCCACTCAAAGCAGACTGCTTGATGGTACCCAGGAAAGAGCCCCCACAAAGCGACGGGCATCCCCTACACAAGCACAAGGATGAAGCCGACTGGGAAAGTATTGCTTTCCAGCAATGCTTATGTTGAAAAAAAAATCCATACAGAGAAGATTGTCCAAAAACAACGCCTTGACTTCAGTGTTCTTCCTCACTGTTCTATCCCCTTCACCTGGACCAGTGAGGGCCTGGCACACAATAGGTGCTGAATAAATATTTGTTGAATGGATGAAACAACTTGAGAACAACTTAGGGGAGAATGTTCTGCTCTAAGTAGTGAGGCCAGAGAAACAACTGGTAAGGCAAACAAGACAAGGAATCAGTCACATTTGGAAGTAAGAACTGAATCCTCACAGAGAGCACCCAGGGGAGCAATGAGAATATCATTCAGAGCTTGACCACCATGACTTGCTGTCCAACTCATTTCCAGCCAATTCAACCAAATGACTTTATTTGCAAGAACGCCTTTTATGTCTGGACTTGGGGCATATATTATTTCACTCTGTTTCCTGGGAGATAATGTTTGCCTCTGACACAATGACAATGAAAGGAAATGTTTTACTATTAATCTTAAAAAAACCATGCAAGGTGGCAGGACGTAAACACAGCAAAGGAAACCTCATTTTATTTTATCAACTCCCTCCTCCACAAGTCAGTTTTATGCCCCACCACCACCCAAGGCTCTAATTCGATTACGAATTGGACAATTAAATTAGGAAATAAATTCCCGTATTTGAAACTATGATTGCCTTAATCAGATTCCTAGTTCAAATATTGCCCAGCAAACTTTAGACTTTCTTGTGGTGATCTTAATCATCATACCCTTTAATTCTGAAAAGTTACTGCTTCCTTTCCTACCAAGTACATGGTATGGTTCATTTCCCTTGACATTTTTTTAAAATTAGTAATTAAAATTTTTTTAAATTAGTCTTAAAAAGGAGAATTTAGGCAATTGAAGAATGGGTTAAACAGCAACCTGAAATGAGTGGAAATTAGGAAAGATCACCGTGGCCATGTTTTGGTCAATATTATCTTCCATCATTTGTGACACACACACAGAGGGATGCCAAACCTACATATTTTGCAAAAACTTCCCTAAATTTATACTAATCTCATGTTACCTTCCTATTTGCCATCCGTGCTGTCCTGGTGCATTCCTAGCTACTTTGAAATGTAAGCCTTTAGCTTTCTTAGATTTATCCTAAGCAACAGCAGTTGGAAAATGAAACACTTGTATTTTCCCTAGCATATGTTAAAGTAAACACAGAATATTAAAATGAATATCTGTGTACAATAATCTCTCCAACACTGGAATTCCTACTGGAATGAGGGCCACATGTTGGGCAATACTACTCAGTTCTTCTCTTACAAAACAAATGGCATAACGTCTCCTGTCAATATGTCTATGGAAAATAATATTGTCGGGAAGTGTCTTAAGATGAATTTGCATCAGGTCCAGAGTGTTTGAAGATGGTTTTCATTCTGTAGTAACTTCAGCAAAACAAACAATTCTGAAACAGGTTCATCCATTTATCTTGGCAGAGAACAGAGGAAGAACTCCCCTAGGCCTCAGACAGGGAAGCCATTGAAGAAACTGGCGTCAGCAGGTCTTTCTTCAGTATACCAATAGAAATGCCCACCCAAAAAAAAAGGTCTGGAATCAAAACCAATAACAGACCCAATGCCACTCACTCTTCCCCTGAACATGGAAGTGAAAAGGATAGGGTGTTATTTAGCATCTACAATAAGTCTTCACGCCTCTGTGATTTTTCATTTGGAAGAGTGTTAGGAGAATAGTAAATT

							AATTTCTCCTCAAGAGTTTCAGTCTCTTGTGAGAACTGACACTCCTAACTCAACTTCTTCAGAAAGCCA<br>TAAAAATGCATCAGTTCTAAACAGAGGGAAGAAAGTTAATATTTAAAGAACCAGTTATGAACCAATTAG<br>CCTTGGTTTAACAAGAGCTAACATTTACTGAGTACCTAAAGTATATCAAGAACTATGCTATGCAATGTTC<br>CAGCCAAATCTCACCTGTTATAATCATCACAATGCCCCAATAGGGAGGTTATGTTATTGCTGCCTTTTTAC<br>AGATGAAAGAACATTGAAGAATTCCAGACACTATAGAAACTCACTGTTTGCTGCTTCCAGACCAGAATC<br>TTTTGATCACCACATATATACAAACCTTCAGTCAGTCCTGTCATAGCATCTGCTTTTTCAGATTCCCGAAT<br>TAATTCTTCCTGGTTTTAGTAGACATTATCCTATTTGTTACTTCACTAAGAAGCAGAACACTTGACAGAG<br>AATTAAACAAATGCAAAGTTCTAAGACTTGAAAATTAAAACATGATTTCTAGCCTCTCCAAGAGGTAAT<br>AATTATGCTATATTTATTGTATGCCAATCAGTTTATATAAATTACCAAATATAATTAAAAAATATTCACATCT<br>GTGTTTATGTGAGAGGGGGGAGAGAGAGACAGAGAGACAGAGACAGACAGAAACAAACAAAGGCAG<br>ACAGAGAGGTCCCATGGAGTTTAGGTTACAGGTAGCTGTGAGTCTTCTCCCCTCCACCTGCATATGGCT<br>TCTGAACATTGACTTCTAGTTCTTTGTAAGAACATCAAACACACTCACCGGCCAAGCCATTACTCTGAC<br>CCCTCAAAATTATTGAACAAAATAATTTACGTAGTTCCAATTGTCAGATGGAGAAATTGAAACTCAAAA<br>AATAAGGAATTAGACTAAGGCTGTACAGGAGTAATATAGTTTGGCATGAATATCTGACTTAAAGGCCCTG<br>TCATTGTAACTAATATGCTAGACTAAATTGCCTCACACATATAAATAATCATCTTAAAACTACTTGTATGGG<br>CATGTATGATAGATACCTTGGCTGAATGCAAGGGACAAGGTAGATCAATTATTCAAAAATTGTCATCCTG<br>ACTAGCCAGCATCAGGACTATCCGAGAACATATTTGTGATGTAAACTAAACTCCTGGCCCACCTTATTTC<br>TACTGAAATTGAAGACTGCATGGTGGACAGCTTCTCCAAACAAGATTTGCTGAGAGCAAAGGTACTGA<br>TTCATAGTTAATAGCTAGGTACTTACCAGTGGGTTGCTTCAACATCTCTTAAGCCTAGAGACATGTTAGT<br>ATCCCTTGTGATTTTTACATAAATAAAGAAGTCTGGATTTACTCTTTTGATTTTAGTTCAGTAGTTCTGGA<br>TGTAGCCTGAACATACTTGTACACATTAAAGGACATCAGTTTCTAATGTATCGACATGGTTGAAAACTAG<br>TGGTTATAATGAATCCGACTAAGAAATAAATTCTGTAAAGACAATTAAATCTGTCTAACACAAAGAGAG<br>AATAAACCTCTAT
MSTRG.114006.3	--	0.000	0.444	0.000	7.210	up	GAAATATTCTCTGATCCTAACTTTCTAACTCTTAGGACAACCTGTCTCTGAACACCTCCAAAAGGAGGG<br>AGGAAAAAGGCAGTCTCCAAAGGGCTCCACTCAAAGCAGACTGCTTGATGGTACCCAGGAAAGAGGT<br>AATTATCTAGTTTACCTATTTATTTTAAAACAGTGACTCCTGTAGCCAGGACGACCTTGAACTTCTGATAG<br>TCATGCTTCTACCTGCTACTCCTGTACACCCCGAATGTATGTAGTGCTGAGGACTGAACCCAAGGCTTCC<br>TGTAAGGTAGGCAGACGCTCTACCAACTGAACTGTTTTCCCAGCAGAAATATGATTTAATTCTTTCTCTG<br>CAGCCCCCACAAAGCGACGGGCATCCCCTACACAAGCACAAGGATGAAGCCGACTGGGAAAGTATTG<br>CTTTCCAGCAATGCTTATGTTGAAAAAAAAATCCATACAGAGAAGATTGTCCAAAAACAACGCCTTGAC<br>TTCAGTGTTCTTCCTCACTGTTCTATCCCCTTCACCTGGACCAGTGAGGGCCTGGCACACAATAGGTGCT<br>GAATAAATATTTGTTGAATGGATGAAACAACTTGAGAACAACTTAGGGGAGAATGTTCTGCTCTAAGTA<br>GTGAGGCCAGAGAAACAACTGGTAAGGCAAACAAGACAAGGAATCAGTCACATTTGGAAGTAAGAAC<br>TGAATCCTCACAGAGAGCACCCAGGGGAGCAATGAGAATATCATTCAGAGCTTGACCACCATGACTTG<br>CTGTCCAACTCATTTCCAGCCAATTCAACCAAATGACTTTATTTGCAAGAACGCCTTTTATGTCTGGACT<br>TGGGGCATATATTATTTCACTCTGTTTCCTGGGAGATAATGTTTGCCTCTGACACAATGACAATGAAAGG<br>AAATGTTTTACTATTAATCTTAAAAAAACCATGCAAGGTGGCAGGACGTAAACACAGCAAAGGAAACC<br>TCATTTTATTTTATCAACTCCCTCCTCCACAAGTCAGTTTTATGCCCCACCACCACCCAAGGCTCTAATTC<br>GATTACGAATTGGACAATTAAATTAGGAAATAAATTCCCGTATTTGAAACTATGATTGCCTTAATCAGATT<br>CCTAGTTCAAATATTGCCCAGCAAACTTTAGACTTTCTTGTGGTGATCTTAATCATCATACCCTTTAATTC<br>TGAAAAGTTACTGCTTCCTTTCCTACCAAGTACATGGTATGGTTCATTTCCCTTGACATTTTTTTAAAATT

							AGTAATTAAAATTTTTTTAAATTAGTCTTAAAAAGGAGAATTTAGGCAATTGAAGAATGGGTTAAACAGCAACCTGAAATGAGTGGAAATTAGGAAAGATCACCGTGGCCATGTTTTGGTCAATATTATCTTCCATCATTTGTGACACACACACAGAGGGATGCCAAACCTACATATTTTGCAAAAACTTCCCTAAATTTATACTAATCTCATGTTACCTTCCTATTTGCCATCCGTGCTGTCCTGGTGCATTCCTAGCTACTTTGAAATGTAAGCCTTTAGCTTTCTTAGATTTATCCTAAGCAACAGCAGTTGGAAAATGAAACACTTGTATTTTCCCTAGCATATGTTAAAGTAAACACAGAATATTAAAATGAATATCTGTGTACAATAATCTCTCCAACACTGGAATTCCTACTGGAATGAGGGCCACATGTTGGGCAATACTACTCAGTTCTTCTCTTACAAAACAAATGGCATAACGTCTCCTGTCAATATGTCTATGGAAAATAATATTGTCGGGAAGTGTCTTAAGATGAATTTGCATCAGGTCCAGAGTGTTTGAAGATGGTTTTCATTCTGTAGTAACTTCAGCAAAACAAACAATTCTGAAACAGGTTCATCCATTTATCTTGGCAGAGAACAGAGGAAGAACTCCCCTAGGCCTCAGACAGGGAAGCCATTGAAGAAACTGGCGTCAGCAGGTCTTTCTTCAGTATACCAATAGAAATGCCCACCCAAAAAAAAGGTCTGGAATCAAAACCAATAACAGACCCAATGCCACTCACTCTTCCCCTGAACATGGAAGTGAAAAGGATAGGGTGTTATTTAGCATCTACAATAAGTCTTCACGCCTCTGTGATTTTTCATTTGGAAGAGTGTTAGGAGAATAGTAAATTAATTTCTCCTCAAGAGTTTCAGTCTCTTGTGAGAACTGACACTCCTAACTCAACTTCTTCAGAAAGCCATAAAAATGCATCAGTTCTAAACAGAGGGAAGAAAGTTAATATTTAAAGAACCAGTTATGAACCAATTAGCCTTGGTTTAACAAGAGCTAACATTTACTGAGTACCTAAAGTATATCAAGAACTATGCTATGCAATGTTCCAGCCAAATCTCACCTGTTATAATCATCACAATGCCCCAATAGGGAGGTTATGTTATTGCTGCCTTTTTACAGATGAAAGAACATTGAAGAATTCCAGACACTATAGAAACTCACTGTTTGCTGCTTCCAGACCAGAATCTTTTGATCACCACATATATACAAACCTTCAGTCAGTCCTGTCATAGCATCTGCTTTTTCAGATTCCCGAATTAATTCTTCCTGGTTTTAGTAGACATTATCCTATTTGTTACTTCACTAAGAAGCAGAACACTTGACAGAGAATTAAACAAATGCAAAGTTCTAAGACTTGAAAATTAAAACATGATTTCTAGCCTCTCCAAGAGGTAATAATTATGCTATATTTATTGTATGCCAATCAGTTTATATAAATTACCAAATATAATTAAAAAATATTCACATCTGTGTTTATGTGAGAGGGGGGAGAGAGAGACAGAGAGACAGAGACAGACAGAAACAAACAAAGGCAGACAGAGAGGTCCCATGGAGTTTAGGTTACAGGTAGCTGTGAGTCTTCTCCCCTCCACCTGCATATGGCTTCTGAACATTGACTTCTAGTTCTTTGTAAGAACATCAAACACACTCACCGGCCAAGCCATTACTCTGACCCCTCAAAATTATTGAACAAAATAATTTACGTAGTTCCAATTGTCAGATGGAGAAATTGAAACTCAAAAAATAAGGAATTAGACTAAGGCTGTACAGGAGTAATATAGTTTGGCATGAATATCTGACTTAAAGGCCCTGTCATTGTAACTAATATGCTAGACTAAATTGCCTCACACATATAAATAATCATCTTAAAACTACTTGTATGGGCATGTATGATAGATACCTTGGCTGAATGCAAGGGACAAGGTAGATCAATTATTCAAAAATTGTCATCCTGACTAGCCAGCATCAGGACTATCCGAGAACATATTTGTGATGTAAACTAAACTCCTGGCCCACCTTATTTCTACTGAAATTGAAGACTGCATGGTGGACAGCTTCTCCAAACAAGATTTGCTGAGAGCAAAGGTACTGATTCATAGTTAATAGCTAGGTACTTACCAGTGGGTTGCTTCAACATCTCTTAAGCCTAGAGACATGTTAGTATCCCTTGTGATTTTTACATAAATAAAGAAGTCTGGATTTACTCTTTTGATTTTAGTTCAGTAGTTCTGGATGTAGCCTGAACATACTTGTACACATTAAAGGACATCAGTTTCTAATGTATCGACATGGTTGAAAACTAGTGGTTATAATGAATCCGACTAAGAAATAAATTCTGTAAAGACAATTAAATCTGTCTAACACAAAGAGAGAATAAACCTCTATGGATATGTGTG
MSTRG.114551.1	ENSRNOG00000026502;ENS	0.333	0.000	0.000	-5.253	down	CCGGAAGTCTTCTCCGGGCGCCAGGGGGTGGGGTTTGCGCGATCAAGTAAGGGTTTTTCGTTTGGTTCCTATTTTTCTCGTTTGTCTCTAAAATATTTCTAAAATTTAGGAGAGCAGAGGAAAGGGTGAGAAGGAAAGATCAGGAAGGAAGGAAGAGACGTTAAGTGTTGGGGAAATCGTGCTTGTGTAAATGTGGAAGACAAGAAGCCAGGTGTAACCGAGGTAAGCGGTGAGGGGCTAGTAATAGTCCCGAAACCAAGATGTTTGCTGTGTCAAGAACAAACTCACCCATTCGGACTCTGTGATTTAAACTGAAAATGTGTAATTAAACCGCAGGGAACT

	RNOG00000032401; ENSRNOG00000034022						CTCAAACCTCGAAGTCCCAAGGCCTCAGTATTTATCTTATGAATTAACAGATAAAAAAAAATCAAATCATACCCATAAACACAGAAAACTTTATTAAAAAGCACTTTGAAACTTTTCTATGTGCATTTCCTGTTCTGGAGGGTCAAAGACACAAACAAATATCCGTTAATTCTGTTAATTGAGGATTGTCCATTAACGTTTTTTCCACCTTTACTCCTGACAAGGTCATTAGCTGATTTTAAATTAATTTGAACTTTGGTCTAAGCAAAACCAGTTAGGAAAATAAAGTCATAACTTTACTAAACATTCCAAAACTAGAAATTATTTTTCCCAATGAGCATTTATTAAACCCACATAAAGTAAAGGCAGAGCTCATAACTGGCTTACACAGCTATCTTTGTGCAAAATCACTTCCTCATTCAGTCTCCTTTGCTGGGAGCAAACCCAGACTTACACATTTTCTACAATTATATAATACCCACAAGGGTAGCAATAGGCTTGTTCCAAGTTGGAGTCTTCTCAGAAACGCAGAAGAGTGCAGTGATAATCAGTTGGTGTACACTTTGATACACTGAGGTAAACTTGATTTTTTTCCAGGAAGGTACCTGTCTGTACCTTTGCTCAGGAAGCTGGAAAGTAATATAACCATCTGTGGTCTCACCGGCTTCTTCTCGAAAAGTGGATGCAACTGTGTCAGCATCAGTACAGGACATAGAAGTCTGTGTTTTTGAGATACATATATGTGTGATTGACAAGTCTGTGGTCTGGTTGAATCGCTGTTGGTGTCAATCCCCAGGCCAATGGCTCTCAGTTTGTGGATCCTGACCTTGGGGTTTGGGGAGGTATCAAAGGACCCTTTCACAGGGTCATGTAAGATGTACAGATGTGCACATCAGATATTTACATTGCAATTCATAACAGTAGCTAAATTAGTTATGAAGTAGCATGAAAAAAAATTTATGATTGGGGTCACCACAACATGAGGAACTGTATTAATGGGTTGCAACATTAGGGTGAGAACCACTGCTCTAAGCCAACGGTTAGCATGACTCAATTGATTCCCATTGGCCACTGGTTGAGCTTCGTAGGTCAATAGTTGAATCTGGTAGGTCTTCACTGGTTGGGTCTGGTGAGCCATCACTGATTTCTTCTATGTATGGACTGGGGAAAGTCATAGCTGGGGCAGACAAGACAGAAAGTGCCAGGACTTTGCAGAGCATAGCACCTCGTTAAGGAGTGGGAGCAACCAGTGAACAAGGCAGACTTGCCAAAACTAGAAATGATGGATAGTTCTCTAGTGAGTTCTACACAAGCAGGGGAATACTGCTAGTATTCTTTGCACAGCATGCAGGATTAAGTCCACTTTATCACTAGTGCATAGTCAAGTAAACAGAGGCCAGTGTTAAGCCCATTTCAGCAATGTAAACAACTGATACAGATTCCAACTGGTGATGGCTATTCTTGGTTGTCACCTCGATTACATCTGGAATTAACTAAAATCTAAGCCGCTCAGTATACCTAGGAGAGATTTTTCTTAATTAAATGTTTGAAGTGGGAAGCTTTTGCTCTTTACTTGTTTGTTTTCGATCTTGCTGGCAAGTTTATTTCTTCGCTGGCATTAGAGCCTACTTCTTTGGGATTCTGGTGGACACTGAAGACCACCTCAGACATACAGCCTCATAGACTGAACAACTACTGAATTCCTGGACTTTCCATTGGTAGATAGCCATTGTTGGAGTAAGTGGACCACAGCCTGTAAGCCAC
MSTRG.114637.2	ENSRNOG00000004958	0.120	0.485	0.001	2.703	up	TGAGGGGCTAGCGCATGGGATTGCATACCTCACATTTAGGAAGAGTTCTCCAATCTTCATTAAATTTTATATAACCCCCTGTAGACATACCCAGAGATTTATTTCTGTATTTCTAAATTAAATCAATTTGATAATCAAAATCAACCATCACGAGTACATTTCACTAAATAATAATATAGAGATAAAAATTCTTCTATATTTTATATGATTTTAAGATTTTATATTATAAGAGTGTCTTTTTACTCCTGATTTATTAACACCTCTTAATATTAAAATTGTTTCAACAAGGCTTGGTGTGCTAGAGTACACTTTTAACACCAGTACTCTGGAAACAGTGGCAGGGGGACCTCTGTGAGTTTGACCCCAGCCTGGTCTATATTTCAAGGTCCAGGACAGCCAGGATTACAAAGAAAAGCCCTGCCCTATCTCCAAGGGAAATGAATGATTCCATAAAGAACACCGTTTTTAATGAAAGAGATCATAAGCCTGAAACTTCTATCAATAAACTAAAGACAACTTTAGATATTTCAAGCAACTGAATAATAGAAAAGCTTTAATATCTGTGTAATATGTAGATATTGAAGTCAAGCATCTGTTATACAGTGCGGTTCATTAATCCATCACAGTATAATTGAAAACAATAAGTTGAGCCTCACTGTATAACAGGTTTTAGACTTATTTTAACACTTGGAAAATATGTCCTATAAGACAAAAAAAAAAAAGGTCAGTTAAAGTGATTACCAGAAAAAATTTCTAATGTAAGAAACCATTCATCTGCATAATGTCTTACATACTCTTTAAATGTAAAACTAAAAAATTTACTTTGCTTGAATATGAAACATTATTTTATACTTTTTGGCAATAGTTGGTACAACTCGTAGGATAGAATATAACAAATAATATTTGAGACCCATGGAAAAGTACTATGTATTATAACTGTATGGCAATTCAGCACTCAATATGTTTAATTCACTTAGACTGGAACAGCCTCAATTTCTCATAAAGTCTTCAGTTGGTGAGCATTGGGTGTATATGCATGTGTTAGGCATTCATTCTTT

							CTGCATCAGCAGCCCAAAAATTTCAAGCAAGGTTGATTGTAACATCTGATTCCAAAGGTGATACAACAC<br>ACAGGATTCTCAGGCAGCCTAGCAGTTCCTCCTGTAATATCTTGTTTGTATCCCTTCTGTGTTAGTCAGCT<br>TGCTGCTACTTATGTTTTTCACTAAAACTTTCTGTGCACTCTCAACACTGCAGAAGGAAATCAAGTAGC<br>CATGGAGTAGGAGATGGCAGAAGAGCACCAAGCTTGAACACTGGCATCCGGCTCTGAAACTGACTGTG<br>ACCTCAGCTTGTTTCTGTGGTAACGGCATTGAAGAGGCTAAGACAAGAGGACTGAGAGGTTAAGACCA<br>TCCTGTAGACTCTGTCCCGAAGACACAATACAAAAACAGCTTGGTCTTCACATGAAGACCAATGTCAAT<br>ATTATCTAGCATTAGAGAGGAATAAGAGAAATTAATGGGATTTAATAAACCACACTGCAAAATATATATAT<br>ACACACACATATATATATAATTCTATATGTGTTATTCTATACATGCATTCTATATGTAATTGACAATAATAAA<br>AGAAAGGGATTCAAATCCTACCCAGGCTCTGTCAGCCGTTAGTAGTGCATATAACTGTCCAAGTCTCTG<br>GGTGCATCGGTACAAGAGGTCAAATACAATATTCTCCAAGTTTATGATATCTATAGAAATTATGGAAAGA<br>AAGAAGTAGGTGGATAATTTTCTAATTTAGATCCAAAAGATATCTTCCAAATCATCTAGTTAATAGTATAT<br>GCTAAAAAGGTTCTCAGACTAAATCCAGTCTGCAGATATGTGTGCTGTAGAAGTCCCAATACCTAATGAT<br>ACCATAACACTTGTAGGGACATAGGTGGGGGGTGTTGTTCACACTTCTGTGATGATGCTGATGCATATAG<br>ACCTTCTTGGGTCTCAGCATTCTAAAAGTTCCATATACATATATATATATACATATATAGATAATTATTTATAT<br>AGACTACACAGTGTCTGATATCTATAATAAAAGATTTTTACTGGTATGTATTTTTAAAAAAACATGCTCAA<br>AGAATCTAAACAGAAAGTACTTATTGTATGGCTCCATTCATGTAAAGGCCTGAAAAATCACAAAGTAAT<br>CCATAGTGATAAAAAAGTGGGTCAGTGGTCCCCGAGTAAACAGGCAAATGGGACACTCTGGCTTAATA<br>ACAGGGCACATCAGAGTGCTGATTGGCAAACTGTCATAAACTGACAACTGTCTTGCACTGAATTTTGAA<br>AGGATTATTAGAGGATGAAAAGGTGATAGAAAGGTTCTATATCTTGGTATGAGAGATGGCGGCACCAAA<br>GTATACACTTATGAAAACTCATCAAATACTACACTTCAAAAAAGGACATTTTCATAAAGAAAAAATACCT<br>CAAAAGTGCATTTGTACATACACATACACAGGAAC
MSTRG.114676.3	--	0.000	0.577	0.000	6.001	up	GAATGCTTAAAATTAAGGATTTATTACCGAGACGCTCCTATGAGCAAGACTCTGTCTATGCATTTCCTTG<br>CTTACTGCATTTACACTAGCAGGAAGCACGTGGAAAAAGGGCAGTGGGGCTGCCAGGCAGTGGGGGA<br>GCTGGAGACCAGCAGAGGACAAGGACAGATCGTTGTACACACATCCACGGTTACGCACACTTATTCTG<br>GTGTACTAAAAGATGGATTACTTCAGTTGTAGGGATATCCATATTCTGCACCTTTGAGGTTTTGCATTTCA<br>CATCTGAGAAGTGCTGGGGCTTGCTCCTGAAAGCCAAAGAACAGAGTCATGGCAGAAGATATTTCATT<br>CAGCTTCTCCTTCCCATAATTCATTTCGTCTGACAAGGTCACTGTTTGCACCCGTTTCATTAAGCCTGCA<br>AAGCCAGGCTGCTGGTCCAGCCGCAACACTAAGCTTGCTCCCGCGACCAGTCTTTCTCCACCTCTTGCT<br>CCATAGCAGGGCTTCCCCTCAGTGAGGTCAGGCACCAGGGTCTGCGTTCCGAAGTCCCTGACCCTCCAT<br>GGACACTGTGGGCACTGCTTCACACTGCCATCATCTGATTCACCTTGCTGCGGCCCTTGTCACGGTTGT<br>CTGTAGAAACTCCCTCTTCTTGGGCTCAATAGAGAAACCAAGCTCATAACAAGTGCACACGGTCTCCAG<br>CAGGCCCTGAGGAGGAGAATCCAACAGGGGAGTACAGAAGTCACCTCTGAGTAGACGATTACTGCAG<br>GTCTCCAAAGCCCACAGCAGCAAGAATATTAACAGGAACCTCAGAAGTACCAGCTCATTGTTGACTGA<br>GTCCAAACTCTCTTCTGTGTACTTAAACAGATGGTCATGGGATGTCCGGAAGAGAGAACTGCTTGAAAA<br>GTAGAAACATCTGTCTCATTGGAAGAAGAGTCTTGACACCTTCCTCTATTGCAGTACTGGGTTTTGAGA<br>GCTTAGCACTAAGATTCCCAGTGTGAGGGGAACCAGCCACCCTGGCTGATGAGAGCAGACGCTCATGT<br>GCTCTTCTGCATGATCTTGGAGTTCTGAAGCCCACTGCCTCACTGGAAGACAAATTTCTAGGAAGAACT<br>TCAGAAGGAAGATGAGCATAAATGACTTAGTGATGTATTTCTGTGCAGATGACGAGAGCTGAGAAGCTG<br>GAGTAGAGTGTATGCTCAGCCTGTAGATTCTGTGATCC
MSTR	--	0.000	0.249	0.001	4.386	up	GATGGTCTGGATTCATATGTATGAGGTGAAGTGGGAAGATTTTAACATGTGGGCCCCAGAAAGCAAGAG<br>GCAGTCTTGGCACCATGAGAAGATTGCAGCGTCTACCCAGACATCTAAGGAAGCAGGTATCTGCTCACC

G.114676.4							AAGATGCAAAGGCAGTTAGCTGCTGGCATGCAGAGAACAACTCAGGCAGAAGCAAGTAATTCTGTCACCTCTAGTAAAGCCAGGACTCCAGTCAAGTGCCCAGCGAAGATGTGTGCCCATCATTTGTTGACATCAGGGAAATTTCTGATCCATGTGACAGTCCGGAGTTCCCTCTGACCTTCCTTCTCTATGTCTGTATTTCTCCCGTTTCTTCATTTCTTCATCCAAACAGTAAGTCCATGTTCACAACCGGAAGGAAGGAAACAGGATGAACCAGAGGAACTTTCACCTTCAAAGCTGCTGTGATTAGGTAATATACATGCCATCGGCTACAGAGAACACGAACAAGGACGTCAGGAGCCAAAGCAGGCCTCTGGGAAAGGCTTCTCAGAGGAGCTGGCAATCCCCCTGCAGTCAGTCCCTCCCTGGCCTCTACTCTCCACACATCTGATCCTTCAGTGCACGCTGGAACCGTTTGATATTTTCATCCGCCTGTCTGCCTCTTCTGGCTATGTTTCCCTCTGCCCTTTTAGAAATACGCCCCTCATTTTCTCTAGAGCTTGACAGCTACATAGCTCTGGCCAAACCTTTGGTCCACGGTGAGAGAAAGACTGTTCAGGAAGTACCTCTGAGACAAGTCAGTCAGGTCTCGTCCCTGGACTCCACAGCCTCAATTAGAAATGAAGATAGCCCCTCCTCCAGACTAGATGAGTCAGCCTGAACAGCAGATACGGTTCTGACAGACAGACACTGA
MSTRG.114688.2	ENSRNOG00000054951;ENSRNOG00000060998	0.317	0.000	0.000	-5.643	down	CAGGCACACACACATATACAAAGGCACACACACACATATATACACACAGGCACACACATACACACAGGCACACACACACACACACACACATATACACAGGCACACACACACTCACACATCCATCTATATACATTTAAATCTAGATTCCCCAAATGAGAGAAAGCATACAGTATTTACTTTTCATGGCCTGGCTTATTTCATTTAGCACAATGACCTTCAGCACAATTCATTTCCCTGAAAAATGATATAATTGTGTTCTTTGTAACTGGGTAAGATTCCATTGTGTACACATACCACGTTTTCATTATCCAAGATGTGGGCATATAGACTGATTCCACATTTGAATTGTCAATAGTGTAGACTTAAAAGTCAGTGTGTAAGTATGGTTTCTATATTTTGGGTGGGGAATACATTCAGGAGTGGTACAACTGGGTCACATGCTAATTCTGTCTTTAACTTTCTGAGGAGGCACCATACTAATTTGCTTAGTGGCTCCACCAGTTCATGTTCTCGTCAGCAGAATGCAAGGGTTCTGTGTTCCCAACATTGTCACCAAGATATATTGCTTTTTTAATAGACATAGCCATTCTGACTACAGGGAGATAGAATCCCAAAGCAGTTTGCATTTCCCTGATGGATAAGGAGGTTGTGTGTCTGATTTTTGTTTTCTCGAGTATTTATTAGCTATTCGTATTTCTTCTTTCGAGAACTGCCCATTCAGTTCAACAGCCCACTTATTGCCTGAATGATTGGTGTGTTCTTAGTTTTTAATTTTTGGAGATGGAAAAGTCCTAGACAGACCTTAGATATTAATGCCCTGTTGGATATAGAGCTGGCAAAGACTTTCTCCCGTTCTGCATGTAAGTTGTCTCCTCGCTCTTCCATTGCTTCCTTTCCAGAGCTTCCGTTCTCAGGCACCGATTGTTCCTCGAATGACAGACAAGCAGCAAGCCACCTAATGAAGCCTGTGTAGCCCGTGATTCCCTCATCTACAGAGGAGACAAGATGGGAGCTCTCCAAGGTTCCACCCAGCTAGGTCGGTCATCAAGGACCTCTCTTCCTGTCCCTCCTTGTTGGTGGCAAGAACTGAAACCTGCCAACAGCTACAGTGGAGCCAGGATCCCCCGGGCCAGACAAAAGGAGGCTCTAGCAAGAAGAATTCTTCTCTACCTCAGTCTGTGCTGGCCTCATGTGCCCTCCTTGCAAGCTTGGCACAGTTGAACGGTGCTAGGATTAGCACAATAACAGCTTTCATGTCTTTGTCCCCGTTCTGGTAAAGCCAGGGTTCACTTTATCCACAGCTTGGCCTGTAATCTGTGCCCCTGCACTCCCCAAGCGTCACCACCTGGTCTCCTTGGGGAAGGGGTGGGCTCTTCTCCAGGACAGGTCATTCATTCTGTGGGCAGGAAAGAGAGATCTGAATTCCTTAAATCGGAATCAGTCAGACACTGCGGCTCCATGGAGAAGCTTGTGATCCCCTGAGGGGGAATGGTCCAAGACCTGGGAATTTTGACATGACAGAGAAGGGGACACCCACCTCTGTATTCATGCTGCCCTCCGCCCATTCTCAGAGAGTTCTGCCTGAGGTCGACCCCACTTCAGCAGACCACTCTTCCCCAGGCCACTCTTAGTGAGCATGGCCTGCCTGACTCTTCCTTTTACTCCCCCACAAATAAAGTGGTAGAAAGGAATGCAGTTTCCAAAAGCCAGTGGAAATGGTTTGAATCCACTCCACCCCTGTTACTGGCTCCATAGCTGCGGCAGCCAACTGTGGCTTCGCTCATTTCCTCACCTGGAGATGGTCAGAGACTTCCTCCCAGCACTGAGAGAATAATAAAGCCCGTTTTACAGCCTGTGCCCGGGAGTCGCCCATGGTTCTTGTTAAAATGCAACTTCTGAATCCACAGCCATGAGCCCGGCTTCCCGGCAAGGCTCATGTGGTGTGGGTTTGCTCTTTGCTATTCAAAGGAACACGTCTGAGCAGCAAGAGTCCCGCTCAGGTGTCTCCAGAGCAAGAGTGGGGAGTGGGGCAAAGGCTCAATGAACACTAACTGCAGCCATGATGATTCTCTTCACTG

							GGGTCAAAAGGCGTCCCCTGTGAGAACAGAGAAACTTCAGTTCACTTGAGTTCATAAGGAAACAAGAAAAAGATGAGAGAGAGAGAGAGAGAGAGAGAGAGAGAGAGAGAGAGAGAGAGAGAGAGACAGACAGACAGAGACATGTTTGGTTTTGAAGATCCCAGACATGGGGGGCAAAGGAGAGAGTCCCACAGTCATAAGCAGCCCCACATGTGCTCACAAGGTACCCCCATGGGCTGGCTGCAGAGCTCTAGGGCCCAGGCCTCAACTCACCATGCACATGAGCAGAGCTCCCCCCCCAGCAGTCCCCCCAATCCCGCACACGCCCTATTCTGCCAGCTGCAGCTCTCCGGAGGAATTTTCAAAGCACACAGGGTGTGTTTGGCAGGAGTCTCTTCATCCCCGTTGGGATCTTTTGCTTTAAGGACCACATTATCTTTTCTGCTTGAAGCCTGAACACTGAGCGTCCCCCTTTGCTGGTACCATGAAGAGACCCTGCACAACCCTCTGCTGGTAATGAGCAGGGAGGATTTGGGGGGACACTGGGTGAGGTTAGAAAAGCCCAACCCCTTTCCACATCCAAGCGATCTGCCTTAGGTTTCACGCTCCTAAAAATTCTTCTTTGTGCCTCAAACTGCTGACTCATCCAGAGTTGGGGTCCCCTCCCCCCATTTTTTCTTTTTGTTCCTCTTCTTTCTCTGGCTCAGAATTGCCACAAAGCCTGGAGATAGTGAAGTTGTTTTTATGAGTACCTGGGTCCTTACAGGGGTCCATTTTTCCCTTCCCACTCCATCCCAAGTTTCCCAAGGGCCCGGTCCTGCTGGGAAAGGGAGCTGGAGACCTGGTTTGCTCTGCCCTGCTTCTCATACGAGTCTCTC
MSTRG.114725.1	ENSRNOG00000025604	0.276	0.821	0.009	2.318	up	GCAGGCTTAGAAGGCCAGGCTGATGGTTTATTAATGCCCAGAGCCTCACTCACATGACTAGCATAAAGCGAGATGAAAGACAGAGAAAGCCTGGAGGTCTGGAGCCTGCTCCTGCAGGGCGGGCGGCTGAGAGCTGTGACTCATCCTTGGGTAGGAGGTGAGGCAGGGGGCAGCCTTCAGATCAGCCAGAAATGCATCCAAAACACGGGTGCCATGTGACCCCCAGCTCCCCAGAGTGTCAGTCTTAATCTCATCTAAAAGGGCACCAATCACAAGGAAAAAAATAAAATAAAAAAGACTGTGCTGCAGGCCCTGCCTGTACAGCTAAGAAACCTCTGCCACTCTACGTTTAGGGGAAGCAAAGAGCTCTTTGTGCTGAGCTTATCACCTGAGGAGGGAGAGGGACAGAGCAGCAAACTAGCGGGTGACAGGAGGGTGACAGGTGATCTCCTCTCTGTTTCACCATATCTTCAGGGAAAGCTTTCATTGCTAGGCAGCTGAGGGGCCCTGGAACAGGAGGATGGGATCCTCTGTCTGGGTGACCTTTCTATAACTGTCATTCATTTAGCAAGAATCTCCTGTAGCACTGAGGCAGATGACGGGGATGGCGAAGTGCCATCTAGCTTGTTCCTGGGTTTCTAATGCCAATAAAAAGACTCCCTCAGGGAAACACAAAACAACAATAAAAAGACGACTACCTACAGGAAACCCAAACCTGAACGGCAACAGCAAAAACTACCTCAGGATTGTTATGAGAATCAAGCGAGATACAGAGAAAAACAAAACTTGCTGTTAGAGGTCATCCTGGGTCAGAATGGCGATCCAGGTGCTGCACAGAGGGCTGAGAAGGAAATTACCTACCATGGGGAAAAAAAATCTCAGACACTCGCTGAAGGACAAGCAAGCCCCACCTCTACAGGGAAGCTGACTGTCAAGCCTTCAGGAGTATTGGTAAAAATAGCAGCTTCTGTTTCCTGTCAGTTTAAAAGGCCAAATCCAAATGGAGGTCAATCATATACATTCTATCTCTCAAGACATATTGTTAAGACTATATAAGAATGTATTTGTGTTTTTGGCTGGGTGTGGTGGTGTGTGGCTTTAATCCTAGTACTTGGGAAGCAGGGGCAGGTGAATCTCTTGGTTTTGAGGCCAACCTGGTCTACAGAGCCAGTTCCAGGACAGCCAGAGGTACACAGAGAAACCCTATCTCAGAAAATTCAAAAAGACTATATAAGAATGTACTTGTGACTTGGCTAGGTGCGATGGGACGCTCCTCTAATCTCAGCACTCGGAAGGCAGATCAGGTGGATTTCTGTGAGTTCAAGGCCAGCCTGGTTAACATAGAGACCCTGTCTAAAGACAAACAAGCAATCAATAAGAGAAAAGGATGGAGTTGTGTCTACACTCCTTCGTCTCCCGGGAAGAGGAACTCCATACCTTGCATGGAAAATCTGCCAGCATGATCAAGGGCTATGTCTCACGTGCTCAGTGCCCAAGCTGGGTGTCCACTGGAAGGCAGCGTCATTTATAACGAGCCTTCAGTTACACTAGGCAGCGCCACTGCCTGCGCTGAGTACCGTGTACGTCCAAGATCCTTATTTATCCAATGGATAACACAGATCGCATACCTGGAAAGTTTTGCCCTGACTTTGAGCTTGAACACTGGCAGGTTGCAAGAAGAAAAATTTCAGCCACATTAATAATCTCCAGGCATGTCAAGATTTGGGTAAATATTATTTGCAAGTGAAACGGCTCTTGAACTAAGAGAGTTAAAATGGGGAGGAGGGCCAAGTTTATGTGAATTAGTTGTATCCTCAGCCAAGAGAAACCTGTCTCTCCACCTTTCGACTGTCTCAGAACTTACAAAGGGTCCCTGATTTCTGCTGTATATCTCTGAGCAGCCATGGTTACCACTCATTGCCACTGGT

							TACCCCGAGCCTCCCCAATCCTTCCAGCATAGCCTCATTTAGTCTACAATGCCCCACGTCTCCCTATGCTCCCGTGAGGCCGGATGTGATGTCTTCGGCCTCAAGTTTCTACCACTCTCCTTTGCTTCAATCCCAGGTTTCTGCACACACCTTTTGTTCCTGAAAGCCTTCTGCCTGCCTCTCCGTCTGGCCAAATCCTGGCCAAACTTGAAGGAAGCATCTCAGGCACTCCTTCTGTCTAGAAGTTCTGTGTAGCACTCCTGGACAGCACCAAACCCAACCAACCAATTGCTTACATAGTTGTAAAAACACCTGACAGCAGAGGCAAGGTCCCGAGAATTTCTCAAGGGCCGAGGCGCACTGGCACGTGGTGGGTTCAGTACAGAACAGGGCTTCAAAAGTATTCACTTGAAGCATTTTGCCACGTCCAGAATGAAGCAGGGGCATGACGTCAACTCTTTCACCCTTGTCATTGGAGACCTTTCACAGAATCAGGAGGCTTCGAATTCTAAGTCACGTATAGCATCGGTTCCTTCACAAGCATCCTTCTTGGATGGGCACTACACTCGCACTTAAAATTCAGTTCGCCTGTTCATTCTGAGATCCTTCCTGGCTACCAGATGACATCACATAAAGAAGCCTCGCACTCAGAGCCTCTTCTCAAATCCACCTCCTGGTTTAGACGTATTTCCCTGATGCATCATCGCCATCCACTTCTTAGGGATGATGGACTCACACATTATCTGCTTCCACACCAGTTACAGTTCTGAGAGGGTGGGGTGTCTATGACCATCACTCCCATTTTACAGGGGGGATGACAGAGGTTTGCCCAGGAACATGTGTAAACCAAGCAGGCCAACAGCCTCCGCTGATCCTGTCTGCCGGCCACTTTCACCCAGATGGAGTATTCTACTCAGTAAGCTTTGGACAGACAGGAGCTCTGCTTGCCTTCTCCAGGCTCCTTGAGTTTTACTGCTACTCAAAACCCATTCAAACCTCACTTACTCCTTCCTGGTTGTCCTTCTTGGATGAGCTGCTCACAAGGATCCTCCTTCCTGAGACTTAACAGGCTACCCCCTGTGTGCTACCGGCGTGCTCACACTGTCTTGTGAGGACAGCTCTGGTGAGTGCAGTATGTACTTCCCAACTCACCAACTAGATGCACCAACAGAGGGCAAACATTTTTAAAGGACACTAAGTGTTCCAGGCTTCCAGGCCACAAGAACTGGACTCTAACTGGACCCTAATTCAACTGACTTCAGGACATTTACTTTGAGCCCTTGCTTACAAAGAACCCAGGGAAGCAGACACAAGTATGAGCTCTTACGGATGCCCACTTTTCATGGCCACATTTAGTTCCTTCTACAGTCCTGTGACATCGGGTAAGTCAGTTGCCTGGCTGTGCCGTTTGTTTACATGGAAATGGCATAAACAGTCACAATTCACAGGAACACTGAAGAGAGTAAAGGTGACAAATGGTTGCTGCTGTGTTCTACTCTGGACCCTCTGCCTTATGTCTTCTGAAAAGTAAAGTAGGTGGGGAGAAAAGCCACCTAAAACCAGATCATTCTGATGATCGATTTGTAACATGAAATCAGATCCGAGAGTCTCCTTTAACACAGATGATGGAAACTAGTCATATTCTTAGCAACAGAAGTTTCCAGATCTCCCCGGGACCATTTCCAATCATCCAGACTAGTTTGCAGGCATTGCAAGGACTAGGTGACCTCTCTAGACCAGTGGAGTCGTGCTGAATGACAGGGACATTTTGCCCAAGAAGGGTTCTTATTTTTATCTCTCTTGGACTGAAGAGTAGCTCAGCCAGAGAGTGGACAGAACAGGCCATACGTCATGGACTCAGCCCAGGGCTTCCAATCTGCTGCTTGAAGGCCTGGCCTTTCGCCAGGTCACTCTGTCCAGGCAGCCTGCATCTGCCCAGATGACAGGTGAATGAACCAGGCCTCAAGGGGGCTTGAATGTCATCCCTTTGGCTGAGTCTCTTCAGTCATCTGACTGTGTTATGGGCACCGAGAAGCTGCACAACCCAGAGAAAGAAAACAAAAGCTAAACCAAGCAACCCCCACAGGAGAAAATTCCCCTCTCACCTAACTCAAACAGCTGCTTCCTCCCAGGGTGAACCATCGGTAAAAAATATCCAATGGCGTGTGTGCGTGCATGTGTTCGTGAAAAAACGATCACCCAGATAGACTCAGTAGCTTTACAGTCACGTTAAACTTACTCCTGCTACTTCATTAACATTTTCCAAAGGTTTGCAATAGAAAGCAACAGATCACAGAGGAGGTGTCAGCTTAGTGTAGATGGAGACTTAGTATGAGGTGGGAGGCATGGGTGGGCCGCACCGAGGGGCGTTGAGCTCAACTATTTTGTGTCCTAAATGACTGATGTGATCCAGAGTGAGCTGAAGCAGGAGAGCGAGAACAATTAGGTTTCCACATAAGTTTAATTTGGGGTTTATTTCCTTAGGCATGATCCTTCACCGATGTTGCTGCCTTCTACTTAGATGGGGTAGGTGTGAGAGGACCAGGAGTGGATTTTCACTTGGGGTAGCCTTACCCATGGACAGATTCACTGAGAAATTATGGCACACCATGTCTGAGGGGGTCATCTCAGAAATGACAGCACAGCTACCCTCAAGTATTGGGGTTTGGGGAAGTTGTTTTCTGCTTGTCCTGAAACTCTTAGAACTCTTGGGGCCATGAAAATCAATCAATCAATCTCTCCCCACCTCAACCTCCCAGGACA

MSTRG.114741.1	ENSRNOG00000007896	0.041	0.302	0.025	2.708	up	CTCACACATGCCCTGCAAATCACGTTTCTGAGAAACCCGGTCAGTAGCATAATTTCTGCAGCCTTCCAGCTTGTGAGATGAATCCTAATTCTGATGCTTACAAATGGGTTTAGTCAGGTTACTTCTTAGGGTCTTCAACTCATAAATGGAGGTCATAGGAACCTCACAGGGTTATAGATTCAGTGTGGATTCAGTATGGTATTTAGCCATGCCAGGAGCAGATCTCATCTAGGACAGGACAGCTATGATGGCTTGCCCAAGGTGTCTAAGTTAACTTGAGAGTCTCTGAATGGCTCCATGTGACTACAAGACCAGCGTCTTGGAACAGAGATTCTATGATCGCAGAATCAAACCACCTGACAGACAGGGCACTGACGGCAATCCCATCAGCACTGACGGCAATCCCATCGGCACTGCGCTTAGCCTAACTGTATTTGAAGATCGGATGGAAGACTCAAGCAAGCCACAAATCTAGAGAAACAAGAAAACCCAAGCGAACCAGGCGAGGTCAACCTGTGGGAACGATGACAACAAGGAAGGTGACAGCACCCGTGGTACGGTGTTGTAAATGGCGGACACTCTCCTACACCATCGTATTCATTCACTGTGTCTTTGAACAGCCCCTTGGGAGCTAGATTGTCATTATCTTCCATTTTGAGGAAAAGAAAAGCCTACTTCAGCCCCCTGAAAATAGGTGTTTGCAAAGGAGTCATTCTGGGTGGCAGAGCTGTACACAAACAGG
MSTRG.114952.2	ENSRNOG00000004100	0.000	0.403	0.000	6.372	up	CGGGGATCTAGGCTTCTAACTCACATTGCCAGTGTACCAGAGGAGCTGGCAAGTGGTACCATGTGGTAGACATTGTCATAGAGCCAACATCACCGTTTAACCAGGAAGAATTACTTCCTGCCTTCATCCTATTGACTGATAGGTTTTATTCATTGTCTTGGGTTTTCATGTAAAGGCCCTTTTGAATTCTGGGCTATGTCGAGCCTTCACAATCCTGGCCACATTCACGTACAAACTAATAATCCCTAAAGCACTTTCCAATATCTTATAGTTACTTGTTCCCTTGCAGGGAAAGAAGAGCAGGTCTTTCTTATACCCATAGCACAGGTGAGACAATCAAGGTACAAAAAAGAGCAGTTGGCTGCCAGATCAGTACCCGAGAAGCTGGAGGGAAATCCAGTCCAGACTTGGCAGCAAATAAATTCCTGGGACCAAAATCAAGACAACTTCACTCCCTTGTCCTTGTTACGGGCAGGCTCTGCCAGTGGTCCATCTGCCCATTCCTGCACACATTCCTTCAAAGCTTAACTTGCTGCTCTCTTGGGCTATCCACAGCCTATCATCATCTAGGCGGGATTTCTGTGTTGAACTCGGAAATGTGTCATGGAGTTTCCAGTCCTGTCTCTGCCCAAATGTTGTTTTCCCCAACAATCCCCATCGCCAGCAGTCCTAGTGGGTGCCCCTCATCCCACAGGCACTTGCTGAACCCCTCTTTTGTGATAGAACTTGAGCTTGGCGCCAGAGTGGTTATGGTCCGACTCTTCTTTCCTGGAAATGCAGTCCAGCTAAAAGAGTCACGGGAGGTGAGATGTCCTTGCAGCTGGATGCAATTTACACCTGACTGCAGGTGCTGGGAAAATATAGAAGGTGTTTTGCAGGGTGAGTAAGAGCTCATTAGTTTGGCAGAGGAAGAAGAAAGATGCTAGTGGGAGGGAACATTTCAGGCAACTGTGCAGAGGTGTGAATTGGCAGGTGTGTTTGGGGCCCTGCCAGGATCTAGCACAAATAGGACAGAGACCAGGAGATGAAAAAGGCAAGTCTGGAAACAGAAGTGAGACCTGAACGAAGGCCTGCTTTTCTAATGAGATGTGGCTTTCCTCTCTGAGTCCTTCAGGGTTAAGCCAGAGCCCGCTGCCATCAGGTTCAAGTTTCAAGGGTTCCTCTATTGTTGTGTGAAAAAGGAATCGAGGGAGGCAGCTTTCATTTGTCCACAGCACTTAGTAGGTACCCTGACACACAGTAGGTATTTGCTGTTGGCTGAAAGGATGTTTTACAGTTCAGAAGCTCACTGAAGGAAGTTAGTATATAGTCTTGCACTAAGTTGAGGGTTGGTGGAGAAGGTGCTAGACAAGGAAACGGAATTCAGGTCAACAAATTTACCAAGATGGATTTCCTATGGGGAACTAGTTCCAAAAGTATCCAAAGAGCAGAAATCAAAGAACAAAGAATAGTCCTGTGGGTGACTCAGAATTTAGCATCAGGAAGTTGCTGTCACCCTAGGACTGGAGGATAAGATGATGTCACCTGATCCCAGGAGACCAGAATCCTTGGAGTGAGCTGGGTCCCAGCAGAGAGGATCCCATGGACTAGGCTCCCCAGGCAGTATACTCCACTGGAAATGAGAAACAGAATGGAGGATGAGGTGCCCAGCAAGGGAAGGCAGGAAAAGAGCTGGATAGGGCAGGAGGAATAGCTAGGCTTACTGAAATCTAAGGTTTCTGTTCCTCCAGCTGGAAGCTTGGAAACGGGGAATGCAGAGGTAATCCAGGTCCTAGCAAATGGACTGGGCTTTATCCTTCCACCATACAGTCATTGGATGTGGACCTCCCGTGAGGGGCAGCTCGGCCCTTTTCAGTCACAGCAATGATATTCCCTTGCAGCAACCAGGGAGAAAAATCTAGTCTCTAAGAGGAGTGCAGCTGTCCAGACAATCATCACACAGAGAGGTTGCTATCGATGGATGCCCAAGCAGGGAGAAGGATGAATCCTGGTAACTCTCTTCTGCCTCCCACCTGCTCATGGTCTTCCAGAGCCTCCCACTGGTCATACTGGGCAAAAAAGC

							CCAGTGAGTTTGTGAAGGCCATCAGGGAGCTGAGCAGGATAGAAGAGGGTCCCTGTTCAGCTCATGGCAACAGTGCTCTGTGGTGTTGTCTCTCCCAGAAGAGGCAGGGTAAAGGCGTGACCGCTGGGTCGCAGCCAGTTGCCGGTGCGGAGCCTGGAGGTGCGCGCGGACGCCCGGAGCTCGGGATCCATTGTGTTGTGTGAATGATGCGCG
MSTRG.115090.1	--	0.000	0.228	0.011	3.719	up	CACGGATGCTATTTTTCAAGCACTGTTGATCTTATTGTTTTTGTGACAGGTTTCCCATTGGCTTGGAATTTGTCAATTAGTTTTGCTGGCTGGACAGAAAGCCACACGGATCCTCTTGTCTTGGTCTCCTGACATTTGGATTAAAGTCACCTGGAGGTGCAGGCCATCGCTATGTTAAACCTATGAGGTGATGATAAACCACACAACTGGAAACCAGAATGGAAGAACAGAAGCTGGATCCTTGACAGAATTATTGATGGGATAGTCCATCATCTGGGGCTAAGACCACCTCCAGATTTCTTGTTTGGTGAAAATTCCTGATTGTTTACATTTGTTTATTTTGAGCAGTCTCTTACTTGTAGCTAAAAGCATTTCTAATAGATATTCAAATTATCTGCACAGCAAGGGGCAGCCAGAGCTCCAGGGGTTCCAACCTAAAATAATCTATGGAACCGCAGATAAGTGTCTTAGCTTCTTCATGAATTCCTTTGCTCATCTGTGTAATGCTCAATAAATGTAAATTGAACCCTGAATGAGGGACAGTGGCA
MSTRG.115141.1	--	0.377	0.000	0.049	-3.592	down	CTAGAGTCAAAACCCTGTCCCCAGCAGGCCCCAAATTACCCATGATATGAGGAATGGCTGATGAGAACTGGAATAAAACACATCCCCATCTATTGAAGGTACGTCCAGGTGAATGTCCACTCATCGTCAGGGAGCCAGGTTCCTGTAGCATCTTGTGTAAGCATAGTGTTTCCTCGAGCTGACAGCAGATGGCTTGGGGATCTGAGAGAAGGGAAAGTGTTTTCACACAATATGTTCTGTTGGGTTACTGCATAGAGGCCAGAATAAGATGTAGGAGACCCTGTTGTGCTAAGAACCACCTTGGCTTGGAGAGGGATTCTGAGGAAAGATGTTTAGGAGTGGCAAAAACTTGACTCAAAGTCAATTGTGGGTACAAGCTGAAGGCCTTTTGTTCTGCTCACAACAGCTCGTGGCTCAGGCTCCACTGCTTAACTTTGTAGATTGTCTAAAAAGGAAGAAAAAAGAGAAAGAAAATCCGTCACTCAAATGCTACATGAGCAGAGATTGTTAAAATGCTAGAATGAAG
MSTRG.115655.7	--	0.723	0.035	0.000	-3.519	down	CCTAAACCCATTACATAAAAGTAACGAAATTTATTGTAATCATTTTATTAAGGCTGAACAGGAGACAATTTCATGATGCAAAATTGTCCATAGGTTTTGTCACACCTTATTTTGAAATCATTTTAGATTTCAATAGTATGTTATTACAGTTTAATTGTAGATCTCTTGCCCAAAAAGAGCAAGATCCTAATTTTGATCTAGAGTAATAAAAACATGTAAACTTTTGGATCTTAGATAATGTACAATTGTGGATTTTCAAGTTAATAATGTTCTACTGTTATAATCAGTGTCCTCATCTGTTTCCCCTCAGTGATGACATTCTCTCCTCTAAAGTGTTTCTATTGCTCCCTCTCTCTAACGCATAGCCATTGCTTCCAGATAAAACCTTTGGCTCCACTAAGCATGCACATTTGTCCAACACTGTCCTAAATCATTGTTTTGTTACCATTTGCCAGATTTGCTGCAGACTCTGAGTATAGTTAGAGCTGAGACAAGTCTGACCCAGCTTTGAACAATGTCTATCAGGACTTCTTGAACTGGTAAACAGTGTAATACGTATGACAGATACTTAATATTATTATGACAAAGAATATCTAATGTTAATCTGCCTGGATGTTTCTTTATGTGAAAATCAGTAACTATTAGCTATTGGCAGGAGAAGAATGATTATGAGGTACTCTGAAAAAAATACGTCGGTTATGTGTCCAATGAAAGATCTATCTGATAATTGAGGGTGCAAATGAATAAACTGCAAGCCAATGATAAAGCTATAAATTCTGCTGCTGCAAAAAGAGTAGCAGCTTTGAGAAGTCTACGATCTTAGACCACTTAGCAGGCAAATGTGGAGGAGTCCTGTTCCCTCAAAGGTCCTAAAATCTCCATTTCAAAATATGAAAAAACTGAGTCTCAATGCTATACATAAAATAGTATATCACAACAAAAACCAAATAAAATTTTCATATATACACATTAAAAATTTGGTTACTAAGACATGTTACACCCATAAAATGAACCACCATGGAAGCATTCAAATACAGAGTGAAAAATGGACTTATATATAAAGATATTTCTAATATTTCAAAATGGAAAATATTATCAAGAAGTGTCACAATAATTGTACTTTTATTAAAAGATTAAACATATCTAGGGAACAAACTTCAACATAGAAAGTTTTTGTTATGCTGTTATTTTGGGAGATGGGCTTACTTGTAATTCTTTGTATATACTTACACATTCTGAAGTGTATGGTATGTGCATTATTGTTCTGTTTAAGTGAAAATAACAAACACCTCCATGTCCACATAGTATGCATGAAGCTGAGAAAACTTTGGGAAGACTAAAATGACTGAGCACCCGTAAATGACCTGTAAGCTTACCTAAACATCCTTTCACTGACTTCTTCTTCC

							AACTCGCTGGAGTAGGTAGAAACAACGCCAAAGGAGCCCAGAGGTTGATGAGTAATAGGTGCTGGGCT<br>TTGTTTGGAAGAGAGTCCAGCCCTAGCAGGGGTCTCCTTGGGCACAGATGAGAAAGGGAGACATGTGG<br>CAGTGCATGACACAGACAAGTTAACAACTTCCACTGCCTTCAGGCTATCGAGAGTAAAGGCCTCTGTG<br>GAGTTCAAGTGGGACCCAGAGATGGAAGTTCCTGCCTTCAGCTCTTGGTTTCGTGACACTGGGGAGGC<br>AATAGTGTGAGTCACAGTGGGGCACACAGTGCCTGCTTCATGTTTGGCCTCCGGAGAGGCATTATGTCT<br>TCGATTCAACGTCATGGTATCATTGACACTTAATGAACCATTTTGTACACCTGTGGAATGCAAGTGGCCA<br>GCTCCGACAGCTGGTGCACAACTGATTTCAGGCACCCTGTGGTGAGACGCAGCCCTGACTTCTGAGAT<br>GCTGTTGTTGGAAAGCATATGTTTACTCAGGCTCAATTCTTTAGCTTTACCTTTCGCAGTTTCCACTGCT<br>GTTCTATGAAAAGACCTGGTCTTAGGATTTTCAAGCACTGTAGTACATGGTATTTTTAAATTAAGACCTTT<br>GGTTTCAACATGTGGTATGTTCTCAGCCATGGCATCCAGACAACAGGGTCCTTGAAGATCAGGGTGATT<br>CTCGTTGCCTTTATGGATATCCATGTACCCAGGGCCCTGGGGTCTGTCAGAAGCCACAGCTATATCTGCA<br>AGAGAATGTCCTGCCCTAACATCCTGGACCCTAGACCCACTATAAGAAGGAGAACTGTATCTGGACGAA<br>CTGTTGTCCTTGCTAGAAAGGTCCAGGTCTTCAGAAAATCCTGCTGTATTCTTTATGGTGTCTGAATCTT<br>CAGCAGTATCCTCAGGGCTGCTGATCTGGGGTGCAGACACAGACTTGGTTAGCTTGGTAAGTGCCAACT<br>CACGCTCCTCATCTTCAAAAGGCAATGAGCTGATGGAGGAAAGAGAAGCTTGAATGGCACTGGGCAGG<br>CTAGTGTTACTCTCTGTGTGACCACCCTCCAAAGAGTTCCTGTGGTGGGCATGCCTTCGAACCACCTGA<br>TGTAAAATATCGCGATCACTGGGTATCTCCAGGGCTCTGCTTCGAGCCTCCGACCAGGCAACGGAGCTT<br>GGCTCACTTTCGATGCCTGAGTCAGATATTATGGAGGCTGACCTCTTGATAACGCCAGAGAGTACTGAA<br>AGATCTTCCGGCTCTTCCCCAGGGTCAGGCAGGGAAGGCTTGGTGTCCAAAGCTTCCCTCAGACCAGT<br>GCTCAGTGGGTCACAGGGCTCAGCAGGGGTGAGCTTCAGACTTAGCAGTACAGGCTTTCCCTCCTGGC<br>CGACTCCCCTCCCTAGAGTCCTCAACTCAAGAGCAGCTGTTTGGTTTACAGAGATAGAATTATTCTGAC<br>TTATTCCTGCTAAAACTTTGCTTGGCATAGTCTTAACAATACCATAGCTGTCCCTAGAGCCCCTGGGTTC<br>ATGCTGAACATTAAGTCCCAGCACGGGCTCAGCTCTAGGGTAGTTCTTATTGCTAGATTCCACATCAATG<br>TAGGTCAATGCTGGGGTATGCCCTTCTTCGGGGCCTGGACTTCTCCTACATATGTCCACATCTGTTAGTG<br>AATCTTTTACAGCATCGGGCCTTGGGCCAGTCCAGCATTCATCTTCAGGTATGCCTGCTCTGTTTTGAAA<br>TTCACCAATGGATAGGTACACTTGAGATTCTGAACACACATCCATTGGATTCTGTGTGGAAACATTCTCC<br>TCCATCTCTGGACACTGAATAACATCTTCATCAGAGCCCAGCTCAGGTGCTTCTGTAGTAGATAAGGGT<br>GCTTCCTTTCTTGAAGATGACTTTTGATTTATAACACTCTTTTCTTTCCCTTTGAGCTTCATTATTGCAGG<br>ACTTGTCACTGGGACTTCAAAATTAGGATAAACACTCAAGTTATGCCCTATAACAAAAGAGGAAAACTG<br>TATTATGCTTTACAAATCAATTATTATAAAGTATTCAAATGTCTAATGTATGAAAATAATTCAGCCATCCTT<br>TACTAGTATTTTTTGAAGGGGGATGACCTTCAGATTTATGAAAGTTCTTATGTTACAGAAAAAAAAACTT<br>TTCTATTTGGGAATATACAGAACTTTTATGTTAACCATGTGATTCATGTCAAATAGTTTATGTTGATGAGA<br>CAAATAAGGATCTGAACAGAACAGAACATGTGTTAGAGAGTTAGGCGAGGGAATAGAAATTCCCACTA<br>AACCTCTGTGAAGAGATTGCATCTCAGTGTTAGGCCAGTGGTTCACTGAATCTTGTCTACTTAAGAGAA<br>CATCAATTCATAGTCAAGAGGCTACAGCTCAGTAAACCCCTCAGATTGACAGATTGACAGCATACATGG<br>ATACTCCAAGAGAAGGAAAAATTCTGAGAATACAGAAACTTCTAGCTAAGCTTCAACATATGGATCACC<br>CCTCCTCCGTCTCTCATCTGTATCTCTGTCTATCATCTACTTTGCTGTCTCTGTCTTTGCCTCTGTGTGTCT<br>CTGTCTATCCCTCCTGTCCACATCCCACTCATCTACCTCCAGTTCTCATTGTGGGGAGATGGCCACCCTT<br>TATTTCACACAATAAAACTTCAATTATGAGCACAGTTCTTTCTTGAGTTCTATGAGTCATCCAGACAAAT<br>TCTTAGACATGGGAAATTTAACATAAACCAAGAATTTGTATCTAGTGGTCAGAATTGCAGACCACCAGTT<br>TGCCTGAAGAACCTGAAATGAAATTTTGGTGGAGAAAGGTTAGTGCCTCACTTGCAGTGTATAAAACA

							AAAGAGGAGAAAAAGTCCCAAAGAAAAATCAGGTATTTTATTTTGTCATACCACTTATGGTTTATTTATC AGAGTACAATTAGCATCTCTCATTTGCAAATGTGACAAGGTCAATCATAAGCCCCATGGTTACAGAGTAA ATATAAAGTGACTTTGATGCTGGGAAGGTCTGATTGTGAACTCCAACATTACTACTCATGAACACTCTAG TGAATTGAAGAGTTCAGTAATGTCTTTCCACCTCCCTAAACTGTAGGATGGGCAGATCCATAGGAACCT GCAAGCCTTTTGTTCATTCATAAATTATTATGTGAGTTGATGTCATGCAGGGAGAAAAGAGTACTGCTTT GTGCAGAATAAACACAATTATGTATGAACTCCATCATCAAGTATATTGTCTGTATGGTAGTCACACCATAC TGAAGCTTCCAGGATCACGGCCATCTTCAGAGGCAAATAGCAGGGAACCAAAGCCTTTCATCTTGGTTT TTCTTAACTGATTTCTATAAGCCATTCTCCTCCATTCTTTTCTCTCTGGCTTTTAATCCTCTCTTTCTCCTC CTGTCTGCCACAACTAGACAGTGACAAACATTACCCATGTGTGCCCATCATAACCCAAGATCTAAGATAT GTCAGGTGACATTTCCCATATATACCATATAAATAACAGACTCCTTTGTTCGTGTTTCCTTTAACAAATATA AGCCTCCTGTTGACAAACATCTACAGTGGTTCCATTCCCTAACTATTATGAAAAGTGAGTCAATAAACAC ACATCTATGTGGTAGGATTTACCAGTTCTTTGAGTGCACGTCCCTGATTATGACCATATAGGTCTTATAGT TTTGGTTTACAGTTTTATAAGAGATTTTCAATTTAATTCCTGTAGTGGCTGTACCAGTTTATATACCTAGCA ACCATAGAGGTGAACAGAAAGGCAAAGGAAACCAATAGAGAAACAGGGGTGCATGGGAGGGCAGTA GAGGCAAAGGTGGTCAAGCATCCTCCCAGATAGGCTCCCACATCCATTCAAGAGCATCTGAACAACAC AAAAAGACTTGATGCACTTGTATTATTGTTGTCTTTAATAAAGCAGAAGGGGAGATGGGTAGAATACTA GGGAGAATTTGGGGGAAAGTAAATATGATGAAAAAATTGAAACCTTCAAATAATTTATAAAATAATAGGT ATTAAATGTAATAAATAAACACATCACCTTATAGGTTAATATTTTTAAAAATCAAACTAAAATTTAAAAGG CTTCATGTAGTTTTTAAAAAGCTATATCAATAAACTTATCTTAAATATTATGAATTTAAGCCATACATATGC TATTTGCAAACCACATGAGGCTTTGACACATGATTATCTAATAATTTTCAGTGTAATTTG
MSTRG.115656.1	ENSRNOG00000057580	1.168	0.141	0.026	-2.248	down	CGCTTGGTCCTGTGAAGGCTCGATGCCCCACCATAGGGGAATGCTAGGGCAGTGAGGTGGAAGTGGGT GGGTGGGTAGGGGAGCACCCTTATAGAAGCAGGGTGAAGGGGGGATGGGCTAGGGGATTTGCAGAGG GGAAACAAGGAAGGGGGATAACATTTAAAATGTAAATAAATAAAATAACCAATAAAAAAGACAAAAGA AAAAAGGGGGTTCAAATGAGTAAGGCAATATTGTAGTGTAGTAGAGGTTGAACAGCAGCTAATGAGAA GAGAAGAATGACAGGCAGGAGAGATGGCTCAGCGGTTAAAGGCTAGGCTCAAAACCAAAAATATAAG AGAAGAGAGGAGAAGTTTCTGAAGTAACCTGGGGGAAATGACTGAGTACTCAGAAATGGTGTGGCAC CATGGAAAGCCTCATATGACTATGAGGGCTAGCAGGAGTTACAAGCCAGCCACACCTACATGTTAGGGC CTTGGCTAAGTTTGAACCTCCTACTTTATCACGTGTCAGATTAATAGTGCACTTGACCCATGGAGCATCA AAGACTCCTAACATTAACGAAAGTTTGTTTTAAGTGTAAGGCCTTAAAATAATGACTTGGAATAAAAAC TAGTGACTATCCTTGGTAATATAACTGATATCAAAATCACTCAGTCATTAGCATGTGGGATACCAATCTTA ATTTAATTCCCTAGTTTTAATTGTTTTACCATAAATAATTTGAGGATAATTTAAAATGTGTGGGAATAGTAT ATACATAGGCTACAGGTAAACATATTGTTTCATATTATTACCTTGAGCTTCTGGGTACTTGAGGGTGCCCT GCATTAGTGAAGGACAATTGCATACATTCTATAAAACTAATAATATTTGAATTTTTATGGTTTAGCGGTTTT TTGAGACTGGGTTTTATTTATATATGCCAGCTTGGTCGAGTTCCTGGTCCCCGTGCCCCCACTTCCGAAG TGCTGAGGTTACAGGTGTCAACTACCACATCTGCTTTTGGATTTGTATTGGACCAAGAGTTAAAGTAAG TTTACCATTCAGCCAGTAGGAACATTGTAGAATAAAGAAACATGCACATAAAGAATCATGACCCCTTCT GAGATATCTGCCAACAGAGAACTAAAACACTTGTTATTTAATTGTGGCTTTTATTCAAAAATGAGTCCCT GGGAAAGACTCAGTTCTACCTATTTGAAGATTAAAAATTACATTTTCTTTGGTCTTTCTCAGTAAAAGTT AAAAAGTCTTCTGAGGTTAAATTTTGGTTCCACACCTGCTTTATTATTGTTGGATCTCACGGATGCTATAA AGTTTTTTGGTTTTTTTTTTTCTGGAAAAGCTGGTGCTTGCCCTATTTGACAAGTATTTCCCACAAGCTA AAAGGCTTCCCTTTGTTCCAGCTACCTTGTGCCAAGACAGAAATTCTTCTTGCAAAACCCAGAGAGGG

							GAAGGCGGTTCTATTTTTACCCCATCTGTGAAGTTTAGCTCTCCAGCAACTGTATGCACTGTATCCCATCT<br>GGAAAGCTGCTGCTGAAAAACCTGTAAAGTGTGCTCTGATCAAAAGGTTAAACTTTCTGCTTAGATATC<br>CTGGCTCCAAACCCATGGTTAGAAGGTATCAGAGCTCTTAGAAACACTAGAGCCAAAAATTAATGTCAG<br>GTGCTGGGGACCCTTTGGACCTCTCAGTTGAATTGCTCTCTGCACTACTGCCCCTCCTGCCTGAGGAAG<br>CTTGGGGGGGTAAGTCTTGAGGGTTTTTCTCAGGCACAAGTCTTCAGTGCCCCTCATACTCAACTGACT<br>TGCATTCAACCCATGGATACATCATTAATTAAACTGTCAACATCTGACCCCAATTTACCTTCTCAATTCAA<br>TTTTCAGTGTCACACTTTTTTGTCCCTTGGGTGGAGCAAAAAGGAAAAAAATCTGTGTCTCTTCTGTAA<br>AGCTTTTCTTTTAGCCACGAGGAGGGGTGACCCTCCCGATGCAGAAAGCTAAACCTGCCATTGTCAAG<br>AGCTTATTAGTGCTAACAGAGTGGTCCAGAATATGCCAGGTGCTTTACAAATCATCGTCATCATCATCAC<br>CATCACCATCATCATCACCATCATCATCACCATCCATTGGATGCATACATACCCAAGGGCTTCCCATGCAC<br>TGTCACTAAAACCTTCCAGCATTCTGGATGGCTTACAGACTCAAGTCTCTTAAAGCCAGGGGCACGATA<br>CATGCATCTCAGAACTGCTAGTTCCCACATTACTAGCATTGCTTCAAGAACTTTTATTTTTAAATATATTC<br>ATAATGTGAGACAGTCTACATGGTTTTATTGTATACAGCCATAATCTCTGTTGGTTTTTTATCACAAAATTC<br>TTTGTTTTATATATAAATACATGTGTCTGTATGGTGAAACATATATTTAGAGATGTGAGATAAGCTCTTCAA<br>AGATCCCCCAGTAAGTAAGTGACCAAATTAAGCAACCAGATGCTCCTGACTCTGGGACCCAGAACCCC<br>TTAAAATCCCTTCCTTTCCCATCTGTGCTGTTGTCTGGCCTCCAGAGGAGCAGTTTCTGCTGATCTCAGG<br>GCAACACCTGCTATGTCTCACCCTTGGCACACAGATCCTACCCTGAGACATTCATAGAAGCAGTGTTTC<br>CTGATCCGAGGGGATTACCCAGCTGGGGAACTTGCCTTGCAGGAGACCAGTACTCTTCTTTCTAGAGTG<br>GCAGCTTCCCAAGGTCAGGGTTGTTTTCCCCCATTCCTATGTTATAACATTTCTCTAACATTCTGCTATAA<br>CAGCCTATCTAATACAGCATACCCATATCAGAATTCTGGACTCTCCGAAGCAAAGGTCTGTTCTGAGGAC<br>CCTGCATGTCCTTTGCAGACTCACTGTGTATATGTATCCTGATGCCTCTACTGGAGTACAACAGATCCTCC<br>TTATGCAACCATTAGATGCCTGATGTATGCCCTTCTTTTCCTTCAATCTAGGACCTTTAATCTAGGAGCTA<br>GCAAAGGGCATAAGAACATTAGACCCAGCTGGCCATGACCTTTTGACCTTGGCTTTCAGTTCCATGCTC<br>ACAACTGCTGTTCACTGGCTGTCTGCTCCTGAATCCCCCTTTCTTTTCCTCTTTGCTCTGATTTAACTTCC<br>ACTCAGACTTCCCACTGCTAGCATTTCCCCCAACTCTCCTCCCTGAAATCCAGGTTTGCCTTTCCAGAAT<br>CACTTTACCTTGCCCTTCAGCCACATACACTCTGTCTTCGGGTGAACCTTTTCATCCTCGTTACTCGATCC<br>GCATCATGTCAATCAGTCTCACTGAGATTCTGAACTGACTCTGAGAGCAGAGAGCAGTCTTAAGCAGAT<br>GAGTAAACCGGGATCAAGGGTACAGTCATATATCATGTCCAAGAGCAGAGAACTCATTTTCTACTTACA<br>GATCCTAACAGAGATCAGAGCGTTTTTGACATGGACATTGAAATCATCGAGGACCCTACGTTTCTCACAT<br>ACTGGAAAACTGTCAGGAGTGTCTGGAACAGAGAACTGGACAAATCCCCTCTTCGTGCTAAAAGAATT<br>TACCCCAAATTCTACTCATATATATATATATATTCATATGTGTTCTCAGTGTTTTAAAATTATATAAATGAATA<br>TATCCAACCAATCTGGGACATTGTTGAATAGCCCTGCTTAGCCCGCTACAGTAGCTCTTATGATCTCTGC<br>CCCTTCACTTGTACAGTGAAGAAAACCTAAGTTCTCCTCTGCACCTTAGGGATGCTTTAGAGTGAGTCA<br>ACAAAGGGCAAAGGCAAAATATATTCCAAGCAAATGTGCATTTGAGAGAAAAGGAACTAGAGGAAAG<br>AATAGATGTGGGCCTCTGTAATGTACTAGTCCTGTCACGAATGGCCCTCGTTAAACTCAGTGCGTCACA<br>AAATAATGAAAGATGTGAATGTGGTAAAAAAATCCTTGTTGGGGAGAAAGGAGATTGGCAGGTGTGGG<br>AATAGAGAGAAAAAGGAGGGAGTAAGAATAATCAGAATATAAAATATACACATTTGAAGATGTCAAAAT<br>ATAAATAATTTAAGCTTAAATTGAATATTAAATAAGAAAGAGCTAGGAACCTGGAAGATCCTAAAAGAA<br>ATCAAATGAATTGAAAAAACAAAGCAACTTTATATACATATCGGTGTTCCCAATTTTACAGACAGAGATT<br>AGGTAGCTCAGATACACCAGGAGCTTCTCAAGTCACAGCTAGGTAAACATGAGACCAGGGGAAGAAG<br>CCAAGTCCCCAAGGTGTGGGTTACAGTTTATAAGTCACCCATCCCTGGAATCCCCTAATGTCCTCTGGG

							ATCCCAAAGTTCTCTTCAACCTTCTCTCACCTCTTTTGTGGGGCACAGTGAGACCCAAGCCCAAGCCAG<br>CAACCAGAGACAAGCTTCTCCAGTGTGCTTCAGCACACTGTCTCTCAGTGATGTGTGTT
MSTRG.115661.1	--	0.337	0.000	0.000	-7.598	down	GCTGCAAGTATGTATATAGGTATAGGGTCTTCTGAAGCATTAACAGCCTCACTGGAACCTCCTCAACTAG<br>GGATAAAACACCACTAATACCTTCCATGTCTCTTCCATGTATGGTTTTTGTCTAGCTTGACTTATGACGTG<br>CTTGGTTAGGAAGTCATCATTACATGGCTGCTATCTTAATTAGGGTTTCAAGGGCCATGATAAAATACCA<br>CAAGCAAGAGCAATTTGAAGAGGAAAGGGTTTATTTTGTCTTACTGCTTGAGTCCATCATTTGAGAAGT<br>CAGGTTGGGAACTCAAGGAAGGAAGTGATGCAGGGTCGATAGAGGAGTGCTGTTTACTGACTTGCTGC<br>CCATGGTTTACTCAGCCTGTTTTCTTATAACTCCCAGGCCCACCAACCCAGGGATGGCACTGCGCACAG<br>AGAGCAGGGAACTTCCACATCAATCATCAGCCAAGAAAATTGCTCACAGGCCAGTCCAGTAGGGACAT<br>TTTCTAAATTGAAGTTCCTTCTTCCTGAATAACTCTAGCTTGTGTCAAGTTGACATAGATCTAACAATTAC<br>AGTTTCCTTGGCTTCCTGTCCAGAATATTATTTAAGTGCAGAAAAAATGAAGTTTTAAAATGCAGATAGA<br>CCTTACAACATAGTGATAAATGAAAAAAAAACATACAGAATTGTAAATTATGTTGTATTGTGCCATTTGT<br>AAGAAAAATGTGGACTGGGGCAAATCTACATAGAAAAATAAAACAAAACAAAACACACAGTAGAAAC<br>CAAACTCTGGGGAAACAGTGATAGACAGTGACTGCTAATGATATGAGACTTCCTTTTGAGGTTCCAGAA<br>GTTTCTTTGAGGTAGAGAGTTGTGGCACTGCCATGATTAGGAAAATATCTGAACCTGTTGAACTATGTG<br>AATTTTGTCACATACATATTTACTCTTAAAGAAAAAGAAAAGCCCTTCCTTCTGTATTACTCTGAAAGTT<br>CTCTTCTTAGACACCTCAACAACTAGACCCCTGGGAGACCCAGAAAGCAGCTGCTGGGATGATCATGG<br>ATGTAATCTCTTTCACTGCTCTCCTCAGCTGTGGGTTGCCATTCCTCCCTCTCATTGCCACAGATGTACAC<br>AGCTAAATCAACTCTTAGTTCATTTGTTGACCAGTACCATTGCAAGACCCAACATAAATAAATCTAATAA<br>TGCAAATTCAGAGACATATTTGTAGCAAAGTATATTCAATATTCCTGCATGAAAATCAAATTTAAGTGGA<br>CATGGTGCAGTTTATCTCTCCACCCATCATAACCTTATTCCACAAGCAGAGGGTAGGTGCAGAATAAATA<br>TTTATGGAAAACGTCATGGATAAATAAGAACCAGTGCAATATCTTAGCACTCACTAATGAGTAACCAGTC<br>ACTCAGTAAACATTTCAGAATGGCACTCTACTTTTCTGCCATGTGGCCAGACCATGAAGAAGACAAGAT<br>ATAAAGCAGCATATGCCACCAGTGCATTTATTGTGTTTCCCTTTGACCTTCCAAGTGCTTGAGGAGGACA<br>TATGTGGGATAGAAGTTTATTAGTGGCAGTAGACTTCCAAACCTAAGACTAACCCTACTCCCAACTGTTG<br>ATCCACTTTCTCTCCAATCCCTCTCTCTTTGTAGCTCTAGGACCCAAGTCCCATCCTATGCTGGGATTTTT<br>TATTTTTGGTCTTGGACCCATACGGGTTAGTGAAATGTCAGATTATCTCATAGGTACAGTCAATGCACAC<br>TATGTTTCTTACCCTATGTATGAGGATTTTGAAGCCTTCAGGACAAGTTCTCCTCTACCCCCTGCAGCAAT<br>ATTTCCTCCATATTCCCTCCAATTGTCATCTTCTGGCCACCAGCCAGCTCACAAAGTATCTTCTGCTTCCA<br>GCACACTGGAGCTATTTCGTAACCCAGATAAAACTCTTGTCTTCCCGATTCTGGATACAGTCTCAGCTGT<br>TTGAACCTAACATCACTATCATGAAAAGTTCTTTTTCCTCCAGATTCCTTGGTCAGCCTGATCCTTGAGA<br>CTAGGCAGATGTGTTGATAGGCCATCAATTTTGGGACTACCAACCTTTGGTTTACTTGTTCTCATTATAAA<br>TAAAGAACCCATTGCCTGAGGTATATTAAGCACAATTATCTGAACATACCAGCTCTAAAGATGCTTCTATT<br>ATTATGTTACCCTCCAAATGTCTGGAAGCAGCAGATGTTGGCATTATACCTTTTACTGGTGTATAGAAGTT<br>GAGCCATTTGCTTGAGTGTCACTCACTAAATCAGAAGAAAAGCTAGATTGGAGCCTTTGTGTAGTGAGC<br>TCCTTATCAGAGTAGAAACATTCTTTCATGATGAAACGGAGAAATGAGACTTAGGAAATAAGCAAGCGT<br>CACAAATGCTGGAAGCAGAAACTATCAAGATTCATTGGGGCTCCTCTGCAAGGTTATAGAAACAATAAA<br>CAGATTCTAGGGCAAGGAGTAATTCTCTACCCAACCAAATTTCCCAAAAGAGAAGTTTTGGCTTGTGAG<br>GAGCTCTGGGGATACAGTTTTTCTAAGCCATCATCAACACTGACATGGTTTTGTCACTAATGCAGCTGCC<br>TGCAAGTTGTTCATGCTCCTGTAACACCAATCAAGTCATTGCTACCCCAGGCTGGACACTGGCAGACTT<br>GATACTTTTGTCAGATCCCTATCTGAAGTAAATAGTGTTTGCACTAAGTTTCCCAGAAAAAGGCTTACAA

							AGCACAGACCCATGACAGCTTGACTATGACCTTGATTCCTCACAAAGCCAGGAGCCATCTCCAAGTTAG<br>CACTTTTACACCATGCTTCTACACTGGCACCTCTCCAAGAAGAACTCGGCCAAAGGAAATCACTGGAAT<br>TCAATGTTAAATCCTTAACTGTGTTAACATAGAAATAGAGAAAAACACCTAGCATGTTGAAAGTCACAC<br>ATTTAAATCAGGCTTGGAATTTATCCATCTTGTTTGAATAATGACTGCCTTTATAGAACTGCTCATCAATC<br>TCCTGCCCCCTGGCCCAAGAGCCCCTCCCATGAAGTTAAGAGTTCTACCCCTATTTGCTTCTCCAGGCCT<br>GCATTAAGGAACCCTGCAGGCCATAAGCATAAGTGGCACTTTGCTACCTGTGAAGATATTAGCTAATTAG<br>AGTAATTCACACTAAAGTGAGAATGAAGACAATAATTGAACAGGAAGGAGGAAGGTGGCAATTCCGAG<br>CAGTTGCTGAATAAATTGCATGTACTCAGTATCATTGGAGTTCTTTTGAATCATGAGGTAAGCACTGCCA<br>GTCAGACTGCCTAAGTCCGTCACACTTCCTGAAGTCCTCCTGTATGTCACAAAACCTAGCTACTCCTGT<br>GGGGGTATTTCTGAATTGACATGAAGCAAATAATATTTTAGAAATAGGTCCACTCCTCTCCTTCCAGCTC<br>CGTGACCTTGGATAAGTAACCAATGTTTGATGAGCATTTGAGTCCTCATCTGTGGACCATCTGACTATTT<br>GGGTTGAGATGGGTATGTCAGAGTCCTAGAGAGAATAAAGGACAGGGAGATACAGGCTCAGACTAGCC<br>ATGATGTCCTAGAACTCATTGCTACTGACCCCGGTAGATAAACTCCAACATCTAGGAAAGCAGTAGTGC<br>CGGAAGCCAGGACTTTCATTTATACATACAAAGAAGCAGGCAAGTCAGGATATACCTTGAAACATTAAA<br>AGGAAAGGGGGTGGAGAACACATAGCCAATCAAGGCCAATTATTTTACAGATACAATTAGGCCACTTGC<br>TTCCTGCCTCTTGACCTCTGTTTCTAGTCCTTTGCACACTGCTCTTTTTAAGCTAACCCCTGACTGGCCC<br>GGTGGCATGTTGCCAGTACACTGAGTTAATGTTTCACCTACCTCCTTGCAGCTTTAGCCTATAAGGAAAA<br>ATATGTCCTGAACCCAAGAGTGATTTACCTAATGAGATGGCAGTTACATTAATTATATTTAATGTTTCCAC<br>TTACACAATAAATTTGCCTGCACAGCCCTGCCATTATCTCTGCTCTCTAGCCCCTTTCACAGCATGTGCG<br>TCTTTGAGAAATTTTAGCAGCTGTGCCAGCCCCAAAATAGAAGGAACACAGCTCAGAGATGCACCTGC<br>CTGGGTAGCCCGAAGGTCTTCTCCTATTTTGCATTGCAAATGCCCTCATCTCTGAGCCAACATAGAATGT<br>TGGAAAGCCCAAAGAACTCTGGGGAAGGGGGAAATGAGGGTCAGGAATACCTGTCTTACATCTATACT<br>CTGCTACTGACCACCTGGGTACCTTTGTCCAAACTCTTAGAGCTACAGAGGTTAGACCTAAGTTGGATG<br>GATTTGACCATTTCAGGTCCCAGATGAGCATCTCTATGAGTCTGTGTCAGATAGCAAATCTATCTTGAAC<br>ACGTTATATGGACTAAATGAAGTGCTTGGATCTAGGTCTTTACATATGAGGTGAAAAGTTCTACCTACAC<br>TATGAGAAGAGAAAGCTGAAAAGGACTGTACTCCTAAAGTCGTGCTTGAAACATTTCACTGTTGCTTAA<br>CAAATATGATGTCAGGCAGAATAGTAACCATCATGGCCCTTTATTTTGATTACCTGAAAAATAAAACTGA<br>GAGTTGCTTTTTCTATATCTGTGGCAACTATGATATTGTATAAAACAAAAATTCTTTAACACGGTCATGGA<br>TTATAGGCCTTTGTAAGAGTTAGCTTTTTCAACTTGATAATTTAAAATCACCTGGGAAGAATCTCAATGA<br>GGGATTGTCTGAATTGGTGTGGCATTAGGGCATGTCTGTGAGGATTGGTTGAGTTTGTTCATTGATATGG<br>AAAGAGCCCCTAAAAGCAGATAGAATCATTCTCTGGGTATGGTTCCTGAAACATATAAAGAGAATGAGC<br>CAAGACGTGTGAGCGCGCATGCATGCATTTACTTCCCACTCTCGCCAATACATGTGGCTGGTTACCTGTA<br>GTCAGGCCTTGACTTCCCTGCAATGACAGACTATAAGCTGGAGCTGTGAGCTAAGTAACATTAAAGTTT<br>CTGCATTAAAGTTGCTTTTGTCAGGACATTTTTATCACACAGACAGGAAAGGAAACTGGGACATCCGTG<br>TTCAACAAATTCAGGCAATAATTTGTTGATGTCTTTCAAAAGATAATGGAGATGTAATCCTAAACCTTAG<br>AATTCCTTGCAATGAATTATACCATGCTTCCCGTATAGGTGCCTCAGTTTACTGATTCATGCAAGGAAAG<br>GGTTTAGGCTTACTCTGCTTCCTGGCCTTGGCATTAGACTTGGTCACAGTTCTTGCCCTGCTTGAGTTTC<br>TGCCTTGGCTTCCTTCAGTGACTGTGACCTGGGCTATGTAAAATAAATAAACCCGTTCCTCTCTGAGTTG<br>CTTTCAGTCAGTGATTGATCACAGAAATAGTAAAGTAAACAAAGACATCACTTCATGGGTATGAGATTC<br>TATGTCAAACAACGAACATGACTAGGAACTAGGGGACATGAATGCAAACCATGCATCTATACTCAGTGT<br>CAATACATCATGCATTTAGAAATGGTTACTATCATTTTGTGACTTTTCTCTCAATTTAAAATGCAATGTGT

							GTCATCATGTTTGGGTAGTGTCATCATTGTACACTGTAGTGATTAGCAGAACCTAGATTGGCATGGGCAC<br>AATCTGTTGGGAATTGTCTGTTATTTGTGGTGAAAATTCCCAGCCACTATAAAGTGGCCCCTTTTCTGGC<br>TGGAACCCTGCCCTTTGTGAGTGAACCAATAGATTCGAGCAGTAGCCTGCATTTATCAGGTTCTACCTTC<br>TGCCTGTGGATACGATCTGCTGCTTTAAGCTCCTACTGCTTTGACTTTCCCACCGTAATGGAAATGAAGT<br>AGGTAAACCTTTTTCAGTGGGTGGCTTTTGCCTGGGTGCTTTATTAAAGCAGAAGGAAAAACAAAAATA<br>AAACTAACAAGTACTCATACCTCAAAGCCCAGTAAAAATGAATATATCACTGTTAACTCCTCAGAGACC<br>TAGTTACCATCATTTCATAGGACTTCTAGGTTCTGAATTCAGGAATGCCTGGTGTTGTGTTCTTTGACCTG<br>GCTAAGACTCTCCACAAATCAAGCATCTTTGCCATTGACAGAACCCCAGGGGTTTGACATTCAAAGTTA<br>ATATCCATGCATTTTTTCTATGCAGACCAGACATGCATTCTCCAACCTCAGAAAATAAGATTTGGTGATTC<br>TGTACCTTTCCCAACTTGTATGGATTCTTCTGTTTAGCCAGGGCGGCAAGCACACCCTTAGAAAATGAG<br>ATCTATCCGTATCATCTGAAACAGTGATAGCTGCTCCAAGACCCTGAGCACTCCTGGAACCGGGCACTA<br>GAGTTCACCCGGATATGAACTGCAACTGGCTTCTCTCCCTCATCCCCCAGTAATGCAAACTCAGAGGGG<br>AGCAGTTGTTCCACATCTTTACTTTGCTATTCACAGCCCACTGACCCCTGTGACAGTTTGTTTCATGCCT<br>TCTGTGAAATGGGATGTTATACTACAGTGAGCTCATCAGAGGCCAGTAGATCATGGTCCAAGGTGTGAG<br>CTCCAAACATGGCATCTAAAACTTGACAGGTCTCCTTCTGACCCCATTTGAGAAACAACCCTGCAGAGA<br>AAGCAGTGACTGAATGGATTTTCAAGGATATTTATGTGTGTCTGTAGTCAAAAGGATGCTGTTAAAAATA<br>GCAGGGTTTCTATGGATGATTTCATACAAATCTGCTCAATTTAAAAGGCAAAAACTTACCTCCCTTTCCT<br>GCTCTGCAGGAACCAGGAAACTGAGAACAGGTGCACAGTGTTCATACAGGTGAGACACAGATATGCAC<br>CAAGACCATCCAACCTCAGTCACCCCAGGGAAGAGGTTAGCTTTATCCACCTCAACTCAACCTGAGAA<br>TGGAATACAAATTTTGTCCTTGTCATTCTGTTGCTGAGAAACACCATGATCAAAAATAGCCTGGAAGTCT<br>AATGAGAAGGCTCAGCAGTTCCCAGCACTAGCTGCTCTTCCAGAGGTCCTGAGTTCAACTCCCAGCAA<br>CTATCTGGTGGCTCGTGACCATTTATAAGGTGATCTGATGCTGTCTTCTGACATGTGCATATATACGCAGC<br>ACAGCACTCAAACATTAAATAAATAAGTAAATAAAAGCAGCCTGGAAGAGGGATTTATTGCGTCTTACA<br>CTTCCTGGTCACAATCCATCACTGAGCTATATGAGGATAAGAACTCAAGACAAGGACGTGAAAGAGATA<br>TCATGGAGGGAAGATAATTGGGGGATCACTGGTATACTCACAAAGCTTTCGTATAGTGTCTAGGCATATT<br>TGCCTAGTGATGATGCCCCCACAGTGAGCTAGACCTCCCCATATCAGGCACCAGTCAAGATAATCTCTC<br>ACAGACGTGGCTACAGGACAATAAACTCAAAGCAATTCTATGGAGGTTCTGTCATCCCAAGTGAGTTTA<br>GGTAGTAACAAGTTGTCAATAAATTCTAACCAGGACAGATTGGATATGGGGAAAATGTAGCTAGAAGAA<br>TCTTGAGGCAAAGGGGCAGAGGATGTATAAAATACATTCTCCACAATGAGGGACACCCATTTCCTTTAA<br>GGCAGCATGTTAACGCCATGCTTTTAAACTCTGATAATAAGAAACTTGCAGCATCCAAAAACACAGGAG<br>TGACCAGATTGTTAGAGAAGGTAATGTGTCACTTCGCTTTGATTTCTAGAAGTATTTAGCACTCAAACTT<br>ATGTATCTCCAATCTGAGCCTCTCTTTCCTCTACCTTGAAGTTGGTACATTCTCTGACAGCTTGTTGCTCA<br>AAGATTAACTGGAAGAGAGTTGCCAGTTGAACTTGGATAACAGATGTTCAGGATTCTAAATCAAAACGC<br>ATTCCATAGTTTTATGTTGCTTGTAAATATCAACTCCAATCTTTCAACTAATATAATAACAGGTTTTTTTGT<br>TGTAGAGTGTCATTTTAATTGAAGTATATGTCCATATTTCCCTATCACAGTGTCAGTCAAGGTCTAGGTTC<br>CAGCAGACTTGTGCATCCCATTCCCTTTCTGTTTTAGCATTTGGGCACCACTAACACACATGATCCTCTT<br>ACACCCTCCCCTATGAGGAGTACGCTGTTGATGAACTCCTTTGACAAACAAGACAGTAAAAGGCTCTG<br>ACAGGGGATTTAGTTATTACAGCAATAGAGTAGTGTTCAAATCCAATCAATATAAAGTAGGACTAAGCTC<br>CCATCACTCCCCAACCACTCCTTGCAAAACAGGATTTCTAAATCATCCCTTAAGCACCAGGCCAGACAG<br>ATCAACAGCTTCCCCGAGGAAAGTACTTCTCAACCTTTCTAATGCTGCAACCCTTTAATACTGTTCCTCA<br>TGTTATGATGACCCTCCCAACCATAGAATTTACTTCATAACTGTAATGTTGCAAGTTATGAATCATATTGA

							AAATATTTGTGGTCTTTTGATGACCTTAGATAACAAGAATTATTCCAGTCTCAAAGGAAGTGGCCTACTCCCCTAATTTTAAACAAATGACCCTCACAGTCCTGTGTCTTGTTCCAAACATCCACAATGTATGAGAAGTATTAGCATGAGGACCAACTTTCTATTTTCAGTTTATTTTCTACTGATGGTGACTTTATAAGAGCCGCACCAATAACCTCTGACAGAATTCCACAAACAAGTTGATAAATGACTTGGAAGGCCCAAGCAGAGCCCCAAGGATCCCCTAACTGAATGCTTATTTATAATGTTGTAAATATGATACATTGCAGACTAAAATAAAATTTAGCATTTGAAAATAAAAGGATGCTGTGCTCAGCTAGATTTTTTGGGATATTTGATTGAATCGACTTCTGGATTGCAGGTCTTCTCAAAATTTGTGGCATGATATTTTCCTATAAGATACTACAAAGCAGACTTAGAACAGAATTCATTATTGCCTTCTCTGATGCCTTCTCAGATAGTGGAACTCTTATCATGCCGGACTTCGCCTGTCTATAGTACACACTGAGAAAGACTGATCCGACAGCTCTGTCACTCTGAGAAAATCATTAAAACGTTCTAAGTCTTAACTGCCTCCAGCACAAACCTATGCTGTAGCTAATAACAGTAGAAGCATGACCACATAACATAAAAGGCTCACAGTACTTTATTCATCCCCCCACAGATGCTGACAAGCTTAAGAGGACCTGATATGTTGTCTGGCCCAGGCTCTGAGCCAAATGCCAGCCAGTCTCGGGTATGTTTCTCATTTGTTTTTGTTTTTTGTTTTTTGGGTTTTTTTGTTTGTTTTTCTTTTGTTTTGTTTTGTTTTTTTTTACTCTTTAACAACGGAAAGAGAAAACAGCCTTTGGCCTGTATTGACAGGGCCCAGGTCTGGGACACCAAAGGATGGATCTGCTGTTTAATGCTGAGTTTTCCTGTAGAGACAGGAAGCAGCCGTCTCTCCTTTCTCTGGAGCCAGGTGATGAATATAACAGACAAGTAAGAGATGTCACTTGTGGATAATATATCTCAGGGCAGAGCCACCATGTCAGGCTCACCCCATCAATTCTGAGAATCGTGGGACTTAAGTGGTGAATTTCCTCAAGGCAAACATGCATTCTGGGAATTTCTGCTGGGCTGACTTGGAGACAGCCCCCGCCCGCCAAGACCTGCATAATGTACCCAGGATACCCAGTCACTATCAGTTGCCAATTGTATTCTGCACTACTGTCTCCAGGAAAGAGGAGGGAAGTGTAGCATTCTTCTAGCAATAGAACTCTTATGCACAGAAAATAGAAAATAAGAGAAATGAGAAAGAGTTAAGGCTCCACGACTAAATTGCTGGGGTCTTAAATACCTGCAGCCTAATGTACTTGGGATATTCAGATAGAACTAAGCCTCTGTTCCTAGGAACACAAAGATTTGTGGTACTCAACTTGATCTTACAAAGTCAGGGTCCCTACAATAAGGAATGGCTTGTTTTTTCTTGGAAGATGAAATATGTTCAAGTAATAATGATATTGAGCCTGGCCAGGACATTCACCATGGATCTAATCTCATGCTCATCAGCTATACCCTCCGTAAGTCCCCTCCACAAAGGAAGAAAATGTTCTCTATCAAAGATGAGGAAACCAAGACACAAGAAAACTCAGACACTTTTTCTAAACCCTATGGCGGGAAAAGACAGGGCTGATTCTTCACATTTGAAGCCTGATGTCCCAAGATGCTACTCCCAAGTCCTCATTCCACTCTCAATGAAAACCTTAACAGTGCAGAGCTCACATAAGTCTGGAAGTGTAAGCTTCAGCCCAGCTGACATGAAAGTCTGCCCTTAACAGCCTACTGGTGCTATATAGTATTTTATGCTGGTAGTGGGCATGGGTGTCTCTATGGTACAAGGACCATGGCCAGAAGCTCCTGGGTACCACACAGAGCTATCTCAATGTGCATGACAGATAGAGAAAGCAAGCACCAAGCCCTTCTCTGCCTGTGGGGCTCAGATACTATCTGGAAGTGCTCAAGGGCAAAAGAAAAGTGTAAAAGAGCTGATCTTAGTGGCATAAGAGGATCCTGCCCCAGGACTTCAGTGATTACTGAATGCAAAGTGTACTATGATAAGCTGACCCTGAGATGGCTCCAGTGACCCTAACCATGGAGAGAAGCCTTTGTGCAATCCTCTCCTGAGATAAGACAGCATACATGCCTTCTGCTTCTGCTGAGCGTGTTGCTTCTTACCGGTCCTGATATTAAAAGGAAAGAAATCAGTGTCCATTTTGTGAACCATGCTATAGAAAGGAGACAAGGGAAGGTTTGGGATTACAGTGTGCTTTAGCTGAATCCTTCTAACAATTGAGAGAACCTGGATGTGGAACTTCCTCAAGAAGATCACAACCAAAGCAACTCCTTAATGTCAGATTGACTTAACCTCACCAAAAACTAGAGTCTTCAATTAAGG
MSTRG.115664.1	ENSRNOG00000047234;ENS	0.903	0.070	0.001	-2.909	down	CTTTTTCTTTGCTATTTTGAATGCATTTTTCCATTCATTTTTTTCAAAACACAATTCAAATATTCTCTTTTATGAGCAGATTTCCTAAACCTTATATCCACTGTCTTATTCAACCAACTAAGGACCTATTACTATGCAGGAGTCAGATAGAAAGGTCCCAGGACTCAGTGGTGAATAAAGAGTCTTGCTCTTTGCTTTTCTAAAGATTACATTGTAAAGAAGCGCATGAGTCACTTGCAAATGCATTTTCCTGCTGTGGTAAATGCTACAAATGAAGAGGACTTGGGCTTTCAAAAGTATATTGGAGCATTAGAACTAGTTATCGAGGTCAGGGAAGGCTGCCTAGTGCT

	RNOG00000058463; ENSRNOG00000059588						CGGCAGTGGTAATTAGCCTGCCACTGCCTGTGCTCTGTGTTAGAACTTAATGCATTTTATATAGTTTATAT<br>TTCTCTAGTCATGTGTAACACAAGGACAGGACCGTGCTTTATCAACACCTTGATCTTCTGCCCTAGAAA<br>ACTGCCCAGGTGGCAAATTATAGGAAGCTGATAGATCTCCCTTAAAGTGGCTTTTATTAGCATTATATTTA<br>TTCCTGCCATCCTGTGGACTAGAAGTAGTACACCATTACTACGAGAGAGAAATAGCAGAGACGTCTCCA<br>CAAAAGCAAGACCCACATGACAACCTCTGTTCTTGCTGCACCCTGGTACTTCAAGGACAAAAATCAAC<br>CATTTGCTGATGACAAAAGAAATAAGTAAGCTGAGGACCCCTGAGCACTGCAATGCCACTTTGCTCACA<br>GCCTCTCTCTGGTTCTTTGATGGTGATTACACAAGCTGTGTCTTATGCTAATGAGTGCCTGCAAATCCAA<br>TTTGATGATTGTGTTTTGTATTAAGCCTGGTTAAAACAGCAGCTTGCACATAGGAGCAGAGATTAGGGTA<br>TTCCTTGCCAGTCCAGAGCAGAGATCAAATAAGCTTTCTTGGCAAAAGAAATCCATCCTTAGGAGTTTA<br>TGTCTTAAAGCCAATTTTGTGTGTGTGTGTGTGTGTGTGTGTGTGTGTGTGTGTGTGTGTGTGTTTGAAG<br>GAGTAGTGTTTCTGTTTGTGCCATCTGTTCAAGTCCTCCTTCAGAAACCCTTCCAAACCACCACAGTGG<br>AGAAGAAACTGAGACAAACCCCAAGACTGAAAAGATTAAAAGGTGATGCGGGAAGATGGAAACAAA<br>ATTGAAATTCTGGTTCCCAGCATCTCTGTCTCTGTCTCTGACTTTCTGTCTCTGACTTTCTCTCTCTGTCT<br>CTCTCTGTCTCTCTGTGTCTCTCTCTGTGTCTCTCTCTCTGTGTCTCTCTCTGTGTCTCTGTCTGTGTGTG<br>TGTGTGTGTGTGTGTGTGTGCGCGCGCGCGCGTGTGGGTGTGTATATATGCTTGTGTGTCTGGGGGTT<br>GGGGGACAAGACTCCTTGAAACATCCTCAGTGATTTTGATAGGACACAGGCATCATGAGTTTCAAGAC<br>AAGGTGGAATCCTCAGCATTAGAAGGTCCCCCCAGGTTGGTGTGACTTCAGTGTGGATTTAGTGTGGGT<br>TTTCATCCCAGCATGGCACTTGATGCCTTCACTCTTCCAGCCCGTACCACTCTAGGTAACTGGCTCTGCA<br>ATGAGCTGATCCTCTGTCGCTGCTTGACAGCCACCTTCCTGAACTCGACCTACTATTCTGATAACAAAAG<br>TCCCCACTTATTGATCATTTACTATGAGCTACACCCAGAAGGGTCCTTGGCTTACAATTCTTTCAAAAGT<br>CCGAAGAAGATGGTATGGCTACTTAAGATAGTTAGTGCGCGCCCCAGATATCAAATAATGCACACGTTTA<br>CCCTGAAACTTGTCAGATGAAGGTAGGAACTGATTGATCCTTCCCACATTGTGGAAGAATGAAGAAAA<br>CTATTGCCAACTAAAAGCCAAACAGAGAACAAAATTATTAATAACACATCTGGTAACTCAAGATCAGCT<br>TTGGAATGGGAGTGAATGAGTAAAACTCCATCAAATAGGAATACCCCACCCACCCCTTCTCTCTCTGAA<br>GTCATAGTTCCTGATCTCACTGAGAACAAAACCCAGGAGGCAGAACAGATGCTGTGGCTCCTTTACGCC<br>TCTGCCTCGGAAAGTAAACAGCATCCCTCTGGCCATGTTCTGCTCTTTAAGCCATGTGACAGAGCTGGC<br>TCAACTTCCAGGAAATGGAGAAAGACTCTGCCCCTTAGTGGGAGATCACATCACTCTCTGGTGTCCCAC<br>CAAGTGGGACAACACTGCAAATACTATTTCAGACATCTTTAGAAACAACAGCTACTAGATTACATTAGG<br>GAGGACTGTGTTTTCATAACTCTGACATGTCTGTTTTGTCATCAACTATGTATCTGTAGACAGGATGATTT<br>ATTTCTCTGAGCCTCATTCCTTCTCCTAAAGAGGATACAATAGCAGCTACCTCACAGAATCCATTAAGGG<br>ACAACTTGCTCTACACTGACAGACAGCCCAACAAAGAGCTTCTGTCATTATAGTGCTCTTGCAATATGTT<br>ACCCAAACCTGGGTAGCACTGAAAGTGAAGGAATAGAAGACATTATGTGGTGTAATGAGAATGGGATT<br>ACATTCAGATTCATGTTCGCCTTCTGATGATATTTATGTTAGACTTTGAAAACTAAATAGAAACTCAAAG<br>ATTCACTCGGTTACTCAGGCACTTAAATAGGCAAAACAGACACCACCTTGGAACTGCCAGGTTGGTGTC<br>CTGGTTCTTTCCAGTCTAGGGTCCCCCAAATATGTACCTTTCCTGATTCATGAAACCTTTTCTTTCCCAAA<br>CTGTCTTTGGCCTTTCTGATTCTGCTAAGTATCTATTTTTAGGTGAATGAGGATCTGAACTCTCCAAGTTC<br>AATGAGAGGAGAGGTCATACTCACATTTACCATGGCATTATCAAGCACAGAGTATGCATAGTGCAGTACT<br>GTACTACACCCACTGAAGTAATGTGCCGAGTACATACACCCACTGAATTCAGCATGGTAACCAGAGTTA<br>CACTTCTTCAATTTATTCAGGGTGAATAAATTCCTAGAAAACCTTGATATTTCCTGCAATCATTAAACTAT<br>CCAGCAGAAGGATGGATGGCAAAGAGTGATAGTGCATTATGTATCTATAAAGTAATTGGCTCTTAGAAG<br>ATTCTGAGGGGCAATGGACAACGTCAGAGGAAAAGTGAAACATGAAGCAGGATGATGGTGGCCATACA

							TTGCAACAAAAGATGAATGATAAATACATAGAGTGCATAGCAGGCGCCAACAAAATTAGGTCAGATTTT<br>CCAACAATGAAAGGAAACCAAAGAGCAGGTATCAATGGCGATGCTGACTTCATGAAATGTGTGCTGTAT<br>GCATGTGTTTACAATCCTCATAAAATATAAATCGTTGTAAGTTCTTTTTTTGGTGAGCAAATTTATGTTGA<br>GATAGACCGAGTAAAGATGGGGCTGTGAGAAGATGACAATTCCAGGACTCATCCTGTATCTCCTTCATA<br>CTTGGATTGATGACTGTATTATCAGTCTTACAGTGGACCTCTGACCAATAAGTAAGTCCATTCCAGTGAG<br>AAGAGGAAGAATGGGTCTCTTGAAAATGAAGATGGAGACACCTAAGAGAAGCTGTTATGCTGGTTCTG<br>CACAAGTACCAGATGTCGGTAGAGATAAGAACCAAGCAAGTGGAGAAGAGAGATGCTGCAGCTGCCG<br>TAGATGGAGCTAACTTTGGGATGTATGCTATGGGGAAATCACAATCTTTTCTGCTTGTATCACTGTTGCTA<br>AATCAAGGAAGTGCCATACTTGTCCTGGGGATGACAGCCCTGACTCTGGGTACACATTCTGAGCTTAGG<br>CAAGCTATAGTCACAAGGGCGGAATATTATTAGTTTCGCTGTGTTTCAGCTCCAGATTTCTCTGTGAATC<br>ATGTGTGCATGAATCACAGCCACCACTCTTCATATTTTAGAAGCAGATGTATAATTTTTCTTATCCAAGAA<br>AAAAAGTCAATTGCTGCCTAAATATGACTCTCCTGTTGTCTCTGAATCTCTTTGATTTATTCGATTCTGAA<br>CCCTCTTTAGGGGGAAGAGTGAGCATAATTAGCAAAGTTTTAAAAAGAAGGTGACTTTTTATGTATTCC<br>ATCCATGATTCTACTTAGACGTAAGTATTTCCATTTGATAGAACAGGAATTGGAGACTGATTAATTGATAG<br>TTAATTGATCCAGAGTGTCACAAACAGTGGTCATATAGAGCTAGGATTCCCCTTAATCGTGCCACACATT<br>AAATCCTTCGTTGTCATCAGCCCTGCTTGGCTTTCTTGCTTCCAGTGTAAGAAATGACAGTAGACAGAC<br>AAAACCCAAATGAGTAATATTTTTTAAGTCTCATTTAGAAATGGCTTTCTACTCTCCCCACTAGTCATTGT<br>TGTTTGTTGTCTACCAATGTCTAAGGACTCTTGTTTTAATTGCTCTGGTCCTTTGAATCTATGTGCGAAAC<br>GACAAATATCAAGTGGATTGCTCTGAAGGACATGATCCAGGGACATAGGGACCAGTGCTAGTGTTGTTC<br>AGCATAAGTCAGACATGGGCAGAGAAGAACCACCCTTCAGGCACCAGTCACAAACATCGTATGGTATAT<br>CTACTCCTCACCCTTGGTACGGGGTCACTTTCAATCTTCCCAGTGGAATTAAAGCTACATATCAGCTTCT<br>ACTCTGGACAACCCACTATACATTTACGTTTAGAGACAACTGTCTGCCTCTGAATTCCCCAAGCACAGA<br>AGATGCCTAGAGTATTTTCTTCCTCTTGCTGACAAGGAAATCAAAACTCTGAACGCTGCATTGTGCACA<br>GCTATACTACTGAGAGCCCCTTTATTCCTACTGCAGTACAGAAAATCATTTCATTACACTAAACCATATTT<br>CTTATCGATATCATAACAATACTTCTCTAAATATTTTCAAGTCCAAAAATTTTTTTAAAGTCTATAAATACT<br>CTACTGTAGAGTTTTAAAACTCTCTCTATATTTTCAATGATATCATTGAGCCTCAAATTATTTTTAAAAATG<br>TCACAATAAGCTTTTCAAAGGCTGGGGCCATGGTGGCACACACTTTTCATCCCAGCACTAGGAAGGCA<br>GAGGCTGGAAGATCTCTGTGAGTTCCAGACTGCCATGGTCTGCAGAGCAAGTCCAGGACAGAGAAAC<br>CCTGTCTGTAAAATAAAAGTAAATAAATAAATAAATAAGCTTTTCAAAAAATAATAATTATACAAATAGTC<br>TCTGCCCTGCTCTGAATATGATGTTCCTAAAGTATAGCTATTTTTTTTATCACATGCTCATTTTGCTTAAAT<br>ATTTTGTGGAGATTTCACATCTGATTGCTCAATAGAATTGAAAAAATAGTCTGAGACATCAAGCTAAAGA<br>TTAGGGATCTAGATTCAGCATGCGCTGAGAAACTATCTTGCTCTCCTCTCCCTGCTGATCAGGGATGTTC<br>CAGTTTTCTACGATCTTGCGTCCCCTGACTCTTTCCTTTCACCATCTGCGATGCCTGCATGCCCTGGTATG<br>CTGCGGGTTCATAGAGGGTCACTTAAAAATAGTATCATTAAAGACGTGGCTTCTTCCCCTTCATGCAAAT<br>TGGCATTCAGAAAGCACAGTAGACAATAGTATCTTTTGTGGCAGAGGGCAAGCAGAGAAATAAGCAAC<br>ATGAGTGGCAAACCAGTAATAAGAGAAGAACCTGAACTTAGAAAAGGAGGCAACAGTTAAGGAATAA<br>CTTAGTAATTCTAAGGCTCATAAGCGAGTACTAGCATCACACTTTCATGCCTACATATCCCATTTCACTGG<br>AAAACACTCCCAAAAATACTTGAATGTAAAGTATGCTACAACAGTACCATTCTTTCACAGTATAGAAGT<br>GCCCAATTCTATAGGAATATTTGTAAAATTGAATTCATTCTGGAGGACAGCTTCAGTCAACATGCCTGCT<br>TGCTTTGCCAGTGGACAGTCACTATGTCCACAGCACAGCCCCCTGCATGATGCAGTAATAACCTACATC<br>CTGCAGACCTCAACATCAATCCTAGGTATTTTAGGGACATCTGAACATCATAAACAATTCTGTGCACACA

							ACACAAATCACAACGTCAACAGAGTGCTTCTGCCTGGACCTGAATCTGAGCAAGCCTGTCTGTAGCAA<br>AAGTTCTAGACACCCAATACTGGTATTTCTTTTAATGCTCTTGTATGTTGGGGGTTCTGACCATTTGGAA<br>AATCACAATTGTCTCTAAATGTAAATTTGCCAATGGCATGCTCTCTATGAACAGGAATCCTATAGTCGACT<br>TAGCCAAAGGGTCAGGATGTGATATGAAATCTATATATAGTGTCCTGTAAAGGACACATTAGGTGCAATC<br>TAATGTGACCATCAAATCTAAGTAGTGCAGGGCTGTCAAACATTGTGAGAATGGTGAGACAAACCATTG<br>GGGTTTAATGAACTGATGGCATTTTTATGAGCTATACAACCTTACACGGATCCTTTAACCTTCCTGAGTCT<br>TAATTTCAAGGTCCTTGAAAAGAACACGGGGATTTACCACAGAGGGTTGCTAGGAGAATACAATTTGGT<br>AATGTCTTTACTGTAGTGACAGTCACAGTATATCCTTAGAGAGCAAAAATTCCTTCCCTTGCTAATTGGA<br>TCTCTGAGTGCTGCATGAATATTGATAAGCAGGATGACATATGGAGAAAAAGTCCAAATGCCTTGGCTT<br>GTTTGGCAAGCCCCCACCTCCACTCCATGTTCTGACTTCCACCAGCAGTCTGGCTTCATCTCCTCTTCTT<br>ACCATTGTATAACTCCAAGTCTAAATCCTTGAGTCTGCAATGGCAGTCTATTTCTGGTAGGGATATGTTAT<br>ATTGTCCTGGAGCATAGAAATAGAGAAGGAACTTGCAATGCCGTTGGAGGAGAAAAATATGGACAGCT<br>AGCTCAAAAACCTAGACTATCATCCCAGCTCTGGCACTTGCTAGATCCATGAGAGTGATAAGCTATTCAA<br>CTTCCTTAAACCTTGGTTTCCTGAACTGTCAAGTGTAGATAAAACCTATATTGCTATAGCATAAAGAGAA<br>GATAAGACCTGCATTGCAGTGTCCTTATAAGCAAACAATGCACTATTGCATGTAGGGTGCCAAGCACAA<br>CACAGGACTGCCTGCAACCACTGAATATATAGTAGCACAAATAAGACTATGAATGCCTCTACTTATGAAC<br>CACGAAATTATTATCATTATTATTCTGAAGAGCAATGGGATGTGGCAATCTGTTTGCGGTGTAATGTTGAA<br>TTCACTACTTCTTAAAATGTCTCTGATTAGTCAAAGGACTTATTTGTACTTAAATTTGCTCACACAGAGA<br>AACATCCCCCCAAGTATAAAAGCAGTCACCTTCTGTGTCTAGGAAACCTGTTCATTTTAATTAACCTAAA<br>AGGTTTCTAGCAATTCATGTTGATAAAGCACTTTGACACCCTAAGAATAAGAGTCAATTTGAATATGAAG<br>TTCTATTACAACAGCATTTTTACAGTTATTCCTCAAGGAGCTCTTGAGCAATGCAGAGTCTCGAGTTTCT<br>TGCCTCTCATCAGGAGGCTGCCCTGTGCTGCCCACTTAAGGATGAGGTGTCACTTCTTCACAGAAAGAG<br>GAGACAGGGTTAGACATTTTAATAAAAACAGTTAAGATTATGTGGAGATAGAAATTTTACCTTTAAGTAT<br>CAGCTAAATGATGGTTTCTTCAAATCTACAAATCATATTTGGGTACATTTCTAAGAGTTATCAGAAAATAA<br>AAGACCAGAGAAAAATAATAAAGTATTCAAAAATTTTTTTCTGAACCATTGCTGATAGGAGAAAAAAAT<br>ACAACATAAATTGAACTCCATTCTGAGTACGACATGAACAAATGAGGATCTATAGTCTAGGAGCAGAGT<br>CAGAAGAAAGCTATTTACTGAGAATGTCTATCAGGAATAGGGTTCGCCAGATAAACAGACCTAGCAGAC<br>TTCTTGATGAATGCAGGTTATGGAGAACAAAAATAACTGAGAGAAGACAAAAGATGAAAAGCCAGATC<br>TCAAGGGTAAGGCTTTCAGATCATATCCAGCCTGGGACCTTTGGGTCCTGTGTCTGGGAAGCATTAGGT<br>TATCAGCAGCAGGGACTTACCTTCCACCTCTCTTGGGTAAGCAAGAGTAATAGCAATAGACTATAAAGT<br>CTGAGGTGTCCTTCAAATTCAAATCAGGGCTTATCATGCCTGGAGTTGGGGCTATTTTAGATTTTCTATG<br>ACTATTAGGGAGAACATGGGCCGTCAAGTTGTGCAAATATATATAATCAAAGGAAAAGAGGCCATGAAT<br>TTGAGAGAGCAAGGGAGGCATGTGGGAGAACAGAGGAAAGGGAAATTATATAATTGTATTTTAATTATT<br>AAAATAAATTATATCCAGAGTGCTAAACTAGAGCTTGGGTCATTTTAATAAAACTGTATTTTATTAGGGAC<br>AATAATCAAGGGCCAGGAGAAAAATTCCAGATTTTACTGTAGCTTAGCCAAGGAAAAAAATTTGGTAGC<br>TCCACAAACACACAAAATAAATTTGTATATAATTTATACACAAAGTAAAGTCCTAGTGTATTGAAATGTTT<br>CTATGGTCTTGGCTACCATGATGAGCTTCACAGCTCCTCCTTCTCACTCCCACCTTCTCACCATACCAATC<br>CCTTCCTCAACACCGATCCTCCTGCTTCCTACACTAAAAATAAAGCAAGCATTGGAGAAAGAGTGCAAG<br>CCTTAAGTGACTAAATTATTTCATTCATAAATCTTTCTGAGTTCAAGTCTTTATCTCCACAGGATTACCTAT<br>TAATCTATATCTCATGGTCTAAGTGTCCATTTTATTGGCAATGAATCAGATATCAGAGAGGGGCAGTTATT<br>AATATTAACATACCCACAATTGGTTGCCATTAGTGAAAAAAATAAATGTTTCTAAACCATAGTACAATAG

							CTGCAGGTATTGATGACTGTATACTTGAGTCTAGACTGTTTAAAACATTACAGATCACAAGTAGAAGGATTGACACTAATTGAGCTCAAGGATGTCTAGCATTGTTCTATTGAAATTAACTTCCAGTGTGGCTGGGTGAGCTTCTACCAGGCTTACCTTCCTGTGCAAATAGTAAATGACTCAAAACACTCTTTTGAAGGCGAAGCAGTATAGCTGAGAGCAGACAAGTTCTCAGGGAGCACTAACTTTTCAGACTCAGAGAAGTAATAGAGTAAGTTACTGTCTGTGTGGTTTTATCACAAGGCTAGGGACAGTTGCTGGAAAAGACAAAATCTCAAACACTAGAGGGAAGAGTTCAGGACAATTTCAAAGTGAAGGGGAAACATAAAAAAGACTCCAAAAAGAGAGAGGACATGTTCTTTGTACAAATTCTAAATACATCTCTTGAATCCAGAACATGTGTATGGGGGCAAATTCCAATTACATGGCCTAAAGATGAATGATTAGAACTCAGTAGAATCCTGGGACAGGATCTGCTGAGAGGATCTGTTAGGAGATAGCATCCCAATAGCATAGGGAAGGAGCGAGGTTGAAGATGTTGAAGGATGGATTGAAGTAGGTGGATACAAAGTGAAGGTGATACAATGCTTAAAACCATGGGCTAGAAAACCCTTGCCATATATTCACTAGAATACATCCACCTATTATGACAAATGTTCCCAAGAGTTTAGTGACTTCATGATTCATGCAAGGAAGTTTCATTGCTTGTGCAAGAGACAAACAGAGATTATTGTACAGCCAGCAGTCTTTGACGTCAACTCCATTAATTGTCTACTACAACACAGGTCAGAAGTAATACCAGTAAGCAACCACAATAAACTATATCTCTAATTAAATGAGAAAAATAAAGTAGTCTCAAAGCATGATACTCACGAAATCTAGAATACAATTCAAAGTTGCTCAACATGAGAAGAATTTGACCCTGTAAAAAGTGTCAATACAAAATGAGATGACATATAACAGCATTTAATTGCATAATGAAAAATATACTCCTAATACATTCACAATAAATAAAAA
MSTRG.115705.2	--	0.043	2.300	0.000	6.391	up	CACACACACACAGAGCTGTTATCTTCAGCAAGCTAGGGTTTACAAAGACAGCAAGCAGGGATGAGAAAGGAGCAGGAGAGCCCATTTCTCCTAAACTCAGTATGGCTCTCTGGCCTCCAGCCAAAGGAGATCCAGGACATCTCCCTGTCCCTTACTGCAGTGAAGGACCTAACCTCCAAAGAACTTTCCGTGTTCCTCAAGAGGCACACTTGCAACATTTATTTATTGAAGATACAATATAACAAACACAAAGAATACATCACTGGACCAAATGGGGATCCCCTCCACAGGAAGCTTACCTAAAAGCAAGCGGGAAAGAGCACAAGCAAAAGACTGTATATAAGTATCTTGAGTACTGTGAGAAACTCAGGGAACTGGGGCAGGTGCTGGGAGGGAAGAGTGTGTCTACCATGCATGCCTTGTTGCAGCACTGACTACTGCATTATAGTTCAGCAAAACCTGTTGGAGGAGAGGAGGTAGCCTCACAGCTACTGGGAGAGAAACATTTCAAAAAGGTTTCCACAAAGACAATACGGTGGACACATACCCACATATTCCTTCGACCTGTTCAGCTAGGATGAGCTAGGGCTAAATATCAGGGGATGGGAAGGACTGGGATAAATGTGGGAGCAGAGGGAAGGATTTGGGACAGGTCATCAACAGCTCTCCTGCCATCGTAAGGACTGTGGGTTTTATTCTGAATAGCTAGAGAGGCACAGTTGGGGAAGTCTCACTTTGGTTTGAATGGGATTACTATAAACAACAGACTACTGGGGAAACACTAGGAACTAGTCTGGGAGCAAGCTAATTGTCTTGGAAGGGAAGAGTGTCGCTACAATACTTGCTACAGTACTTGCCTTGACGCTGGGTTATCAAGGTTTATAGTTCAACAAAGACCTGGCTGAGCCAAGGAGGCAGCCTTGTAGCTAGCTGAGGAAAAGCAGTTCAGAAGGAAAAATTTTCACAAAGACAGTAAGGTGGACATCTATTCCAAAACCTGTTCAGCTAGGATGGGATATGGACAGTGACAAAGGGAAGGAAGGAGGGGAGGGAAAGGCACAGGGGTGGAAGGAGGGAAGGGAAAGGCACAGGGGTGGAAAGAGGGGAGGGGAAGGCACAGGGGTGGAAAGAGGGGAGGGAAAGGCACAGGGGCTGGGTGGTGGTGAATTCAAAGCTGCTGAATACTGGGAACATTAAAAAGGCAGAGAAAACAGAAGTTACTAGAGTGTTAAGCACAAAGACAAAAGAGAAGAGTTAAACACAACTCCAAGATATCCAAGATTGGAGTTTTCTTTAACGGATCCTGAGAAGACTGGAGTGGAACCTGGTTGAGTTCTGAAGGGACTGGGTACAGGAGAGAATCTGCACACTGGAGAGCAGGCATGTCTGAGCCCCCCCTTGGAGTTCCAGCAATGCAGAATTCCTCTCCTCCAGTCATTACTGAAAGCTGTGAGTATGCCACAAGGTCCTCGCACATGTGGCGTGGCAGAGAAGACTGGGCAACCCCATTGGTTGGAGCCCAGAGAGTACCCCACTGCTCACAAGCAGCAGCTACCACCGCAAACCCAAGCCAACCAAACACCTCCCAGATCCTCCCCATATGATGTGATCAAGACTGCTGTGGTTCTCGGGGTGTGCTGGGAGTCCTGCTGCAATCCAGAGAGGAAAACTGTCCACCTGGGTCCTTGGTGCATTCCCACCAG

							CCATAATCCAATACATGCAGTTGGCAGAGTTTAAAAAATCAACCTGCCTGGGTCTTCAGTAAACACCCC<br>AAAGTATAGCCCTCTTCGCTGGGTAAAGATACCCAACCATTTCCTTGGGAGTGTAGCATCCATCCTAAGT<br>TCCACAGCATGAAGAAGGCCAACTGACTTATGGTTATGGGTCTGCAGGTTCCGTGTGTCACCATCCTCT<br>GCAGACGTTAACCCAGGAACTCAGTGCACAGCCATTGCTGTTCATTGAGGATGCCTTCTCTGCAGATAG<br>CTCCACTCTGCCCCAGCAGCATGGTAAGGATATCCACCAGGTCATCCCCACAAAGCCACAGCCATGAGG<br>GCATGAGGAACCCAGGTCTTCATCAATGTATAGCTCTACGCTTTCACCATCTCTGGGCCAAGGACACCT<br>GCCAAGATGATGGGTGACTCTCACAGCCATCTGCAAGGCAATGACATCAGACCATGCTACTCCCTTGTG<br>AGATAAGGATCGCTGGCCAGATCCTTGATCAACCCTTGACTGCAGGATAAAACCTTCCACTAGCATCCT<br>GGGTGCTCACTCAAAGCGGTGCCGCTGTCACAAAAAAACAGAAATCCAACTACATCTTCAATGGAACA<br>CAACGTGGCTTCCGGAGGGCAAGGAAGCTCTCCTGGGGCCTCCACACGTAGTAGCAACCAGACAGAA<br>ATTTGGCTTGCTTTGTTCAGATCTTCCTGTACCACCATACTAAAGACAAAGATGTCTGCCTAAGCCATCT<br>CGGTACCTATGACAGCCATGCAGCTGCAGGGAGGTGAGACGCCCTTCTGAGTTATCATGAACCAGTGCC<br>ACTGTATAAACCCCAGAATAAAGATGTCTGCCCTTGGTCTTGAGGGTAGATGGCTATGCTAATGCAGCCA<br>CTCGGTAAGTGCAGTAAGCTCCTCAGAAAAGCCATCTACGGTGGAAGAATGCCAAGCCAGGCTTTGGA<br>TGTTTGAGTGCAGCTGTGTTACGGAGCAGGGCTATGGAGCCTGTCTGGGTCCTTAGCATGCCACCCAAC<br>GTGGCTATCCACAGAGTAAAGGCTCCACTGGGTCGGACCACATCCCTCAAGAAAAAGACATTTGCCCA<br>GCTCCTTGCTGTGAGGCATTCTGTGGTTATAAACTGCCCTGATCCTCATGAACAAATACATCTGCGGCAC<br>AGGTTAGAGGGTTTGAAATCCCACCCACATTCTTAGTCCGTAGAGTGTGGACCCAAGAATACACTATTC<br>ACAGAATAAGAGAAATCCAGTGGGTTACCAGGTACGGGGCTCCAAAGTAACACAGCGCCACACAAGC<br>TGACGTCATCAATTCAGCTTCCAGCTGTGCCTCCTCCTACATTCAGAGTAAAGTGATCTGCCCAGATCCT<br>GGGGGAGCAGATGTTGCTATGATGCAGACTTAACAATTCCAGGTCTTAATTTGCAGCCACGACAATGAG<br>GTTACCATCTGGGTGAGGGCGCTCGTTGTGCCTCCATCCCCAAGATAAGGGTATCTGCCTAGATTCCAAT<br>GCAGTGGGACAGTAAAATGATGATTTTCACTGTGCCACCAGCTCCAGGAAAACATCAGCCCGGATCCTT<br>AATGCCCAGCTGCCACAGTGCATATATCCACATGGTAAAGGTGCTCGGTCTTACCATACCCAACCTTTGT<br>GTGCAGCTGTGGCCACAGAGCTGCCCATGGGTTCTAAATACTCCACCTCCATTCCCAGAATAAAATCTT<br>CTCTGATCCTTAGTATGCAGCCACCACAATGCAGCTGCCCACAAAATAAGGAAGGATGATTCACAGTGC<br>CCTTATCCCTAGGGCCAAATAAATAAATGAATAAATAAATAGATAGATAAATAAATAATCAGCCCAGATCT<br>TTAGCGCGCAGCAGTCGAAACATGGTCACACACAATTAAGGAAGCTCAGAAACGCCATCCCTGTGATA<br>GATCCTCTTAGGCCTTTCAAATGCAGCTGTCACGACGCAGTTATCCACAAGTAAGGGTTTCTGTTATAGC<br>AACACCTCAGGTTAAGATCCACTCGGGGATTTGGTACACAGCCAACACCATGCAGGCATCCACCAAGT<br>AGTACGGACTCCGTTAAACCATCATCCCGAAATAAGGATATCCACTTAATTCCTTAGTGCGCAAGCAGCA<br>GCCATATAGGGTGAGGCATTCTGAGGTTGTCTGTGCACAGCTCTGCTGCGCCACCATCCGCGGGAATAA<br>AATTACCATGAAGAACCTTAGTGCGCCACCAAAACCATTTAAGCGATCCACCCTCTAAGGACGCTTCCT<br>TGGGTATCCGTGTGCAGCTCCACTGTGCCTCTATTCCTAGGGTAAGAACATCCGTTCAGTCACTGAAGT<br>GTAGCCACAAATACGTGTAATTCCCCAGGATTATGATAACAAAGAGCTGTGCAGGCGTTGGCTGAGTAA<br>TGGCGTACGCCCAGGTTCCAGCCAAGCGGGGAGACCTCCATGGCTCATCATTGTGCGACGACCACAAT<br>GCGCCTATCCACAGGGTGATAGCAAGCCTCTGTGCCAGCGTCTCCAGGGTAAAAAGATCAGCCAAGCT<br>CCCAGTTGCATCGCAAACTGTAGGGTTCTGTGCTCGAAGGTATCTCAATGCTGCCCTCGCACGAGCCGC<br>ACCATCACAGACATCTGGGTTTTTTCGGCGCCTCCTTTTGCCCATTCCTCGGTCACATTGGTGTAAGAAA<br>GCACCACGAATGAAAAATTCCCAGCTCCGTGGCTGTGTCGCAGCTCTTCTACCCAACCATCTTCAGAGA<br>AAAGGCCGCGGATCTTCTCGGAGAGCCGGAGCTTCCTGAAGAGCCGCGGCAATGATCCTCTACATAGC

							ACGGGCACTTGGATGGGCTATCCACACAGCCCAAAATCACAAAAAGATGGGATAACGATGGCCACCGG GTCCTCTTCGGTTCGTCGTCACAGAACCGTAGTAAAGAGATCCGCTCAGCTCCAAGCCGTGCCGCAGT CTGCAGTCTTTAGAGACGTCTGTGGAAATCTGGTGAGCAGGCAGCTGTGACGCAGTCTGCTGGGCGAG CCTGCTCAGATTTCAGTGCGCAGGTGCGGCAATGCGGCAATCTGCTGGTTATGGACACATTCTGAGGCG CCACCTACCGGGAAAGGACGACAGTCCAGGTCCTCCGTGCAATGAGGCCACACAGTTGATCAGCATGG CTCTATTGTGGCAGCATCTTTAGGATATTGGTGCGCAGCCACACTGGGCAGAGATCTGCAGCCTAACGA TGCTCCACTAAATTGGCGGGGAAGGATATCCACACAAATACTCGGTACACCGCGGCCATAGCAGCAAGG CAGTCAACGGATCTCAGTACGCAGGCGTGGCAATGCGACAGTATGCACTTTAAGAATACATTCTATGGT GCCATCTAGCGGGTAAGAATGACTCTCTAGGTTCTTGGTGTGGGCGCACAGAGAGAAAGCGGGTGCCC ATTGGTGACTGGCACACAACCCCAGTGGTGCAGCATCTTCTTGAGTTACCCATGCCGTCACACAAGATG ACGATCCACAGACTAAGGATGCCCTGCCGGGTCTTCAGGGTGAGAGTGTCCATGCAGTAATCATTTGGC AAAAATGAATGCCTCACTATGTCCCACCTAGGACCAGGCTGCAGCTGCAAAAATGCTCAGTGTTAGGA ACATAGCCGGGTCCACAGGAGAAGCATGGGTAACATCTGGCATTGCTGCGCGAGGCCCTGCCCACAGC TCTCCCTGCTTGGCCCCTCCTGGAGGGGGCGGGGTGGGGGAGAATTCTAGCTGCTTCGCTATTGCACAG ACTATGGGAGACATAGACTACCCTTTTGCCGTGGTAACCTAAGGTGTCCTAGTGACGGAGGCGCAGGGT GACCAGTGTACCCTTACCCGACTGCCGAACCGATACTTTAACCCCGATTCATGAACGGTTTGCTAACAA GGAAGGACTATGTCTTCCAAAATGAGCTTTAAGAAGCTCATTGTGTTGTGTTGCCGGAAAGGGCCGAG CAACGGGGTTTCCGAGAACTCACCAGAGGGCAATTCATGACGCACTAATAATTTCTAACCAAAAGCTAA AAATTAAAAGTCTCACCTTTTTAAATTTCTAAATATCCCAGCAATATGGCATCTTGTAGCAAGGAAGCAA AGTATTTTTCATTTTATCTGTTGATCACGCTGGGGATCAACACACGGAATGCTCCCCCCACCCCAGGGAA GAGCCCCTGTGTCCTGGATGCTCCGGTTCTTTCTTCAGTATTCACAGAGGAGAGCCTTTTGTTAGTGAG ATTCACACAGGAGGCAACAAGACGCTAGGAATCTACCAGAACCTTCCTTAAGAAATTAGACAACATGG GTGAAAATAAAGGAAAGAGATGTATTAGATGTGTGTAAATTTAATTGAAAATCTTAACCTGTGCATCAGT GAAATTGAAAACCCAACCCGTGTATGTGTGAAGTCAGAACCTGCATTCGCATATCCTGAAAATAAACGT GTGTGTTTGTTAAATAAAACACAGGAAGCCAAATAAACTGGGGATTAGATGTT
MSTRG.115705.3	--	3.297	0.000	0.000	-9.841	down	CATTTATTTATTGAAGATACAATATAACAAACACAAAGAATACATCACTGGACCAAATGGGGATCCCCTC CACAGGAAGCTTACCTAAAAGCAAGCGGGAAAGAGCACAAGCAAAAGACTGTATATAAGTATCTTGAG TACTGTGAGAAACTCAGGGAACTGGGGCAGGTGCTGGGAGGGAAGAGTGTGTCTACCATGCATGCCTT GTTGCAGCACTGACTACTGCATTATAGTTCAGCAAAACCTGTTGGAGGAGAGGAGGTAGCCTCACAGC TACTGGGAGAGAAACATTTCAAAAAGGTTTCCACAAAGACAATACGGTGGACACATACCCACATATTCC TTCGACCTGTTCAGCTAGGATGAGCTAGGGCTAAATATCAGGGGATGGGAAGGACTGGGATAAATGTGG GAGCAGAGGGAAGGATTTGGGACAGGTCATCAACAGCTCTCCTGCCATCGTAAGGACTGTGGGTTTTAT TCTGAATAGCTAGAGAGGCACAGTTGGGGAAGTCTCACTTTGGTTTGAATGGGATTACTATAAACAACA GACTACTGGGGAAACACTAGGAACTAGTCTGGGAGCAAGCTAATTGTCTTGGAAGGGAAGAGTGTCGC TACAATACTTGCTACAGTACTTGCCTTGACGCTGGGTTATCAAGGTTTATAGTTCAACAAAGACCTGGCT GAGCCAAGGAGGCAGCCTTGTAGCTAGCTGAGGAAAAGCAGTTCAGAAGGAAAAATTTTCACAAAGA CAGTAAGGTGGACATCTATTCCAAAACCTGTTCAGCTAGGATGGGATATGGACAGTGACAAAGGGAAG GAAGGAGGGGAGGGAAAGGCACAGGGGTGGAAGGAGGGAAGGGAAAGGCACAGGGGTGGAAAGAG GGGAGGGGAAGGCACAGGGGTGGAAAGAGGGGAGGGAAAGGCACAGGGGCTGGGTGGTGGTGAATT CAAAGCTGCTGAATACTGGGAACATTAAAAAGGCAGAGAAAACAGAAGTTACTAGAGTGTTAAGCACA AAGACAAAAGAGAAGAGTTAAACACAACTCCAAGATATCCAAGATTGGAGTTTTCTTTAACGGATCCT

							GAGAAGACTGGAGTGGAACCTGGTTGAGTTCTGAAGGGACTGGGTACAGGAGAGAATCTGCACACTG<br>GAGAGCAGGCATGTCTGAGCCCCCCCTTGGAGTTCCAGCAATGCAGAATTCCTCTCCTCCAGTCATTAC<br>TGAAAGCTGTGAGTATGCCACAAGGTCCTCGCACATGTGGCGTGGCAGAGAAGACTGGGCAACCCCAT<br>TGGTTGGAGCCCAGAGAGTACCCCACTGCTCACAAGCAGCAGCTACCACCGCAAACCCAAGCCAACC<br>AAACACCTCCCAGATCCTCCCCATATGATGTGATCAAGACTGCTGTGGTTCTCGGGGTGTGCTGGGAGT<br>CCTGCTGCAATCCAGAGAGGAAAACTGTCCACCTGGGTCCTTGGTGCATTCCCACCAGCCATAATCCAA<br>TACATGCAGTTGGCAGAGTTTAAAAAATCAACCTGCCTGGGTCTTCAGTAAACACCCCAAAGTATAGCC<br>CTCTTCGCTGGGTAAAGATACCCAACCATTTCCTTGGGAGTGTAGCATCCATCCTAAGTTCCACAGCATG<br>AAGAAGGCCAACTGACTTATGGTTATGGGTCTGCAGGTTCCGTGTGTCACCATCCTCTGCAGACGTTAA<br>CCCAGGAACTCAGTGCACAGCCATTGCTGTTCATTGAGGATGCCTTCTCTGCAGATAGCTCCACTCTGC<br>CCCAGCAGCATGGTAAGGATATCCACCAGGTCATCCCCACAAAGCCACAGCCATGAGGGCATGAGGAA<br>CCCAGGTCTTCATCAATGTATAGCTCTACGCTTTCACCATCTCTGGGCCAAGGACACCTGCCAAGATGAT<br>GGGTGACTCTCACAGCCATCTGCAAGGCAATGACATCAGACCATGCTACTCCCTTGTGAGATAAGGATC<br>GCTGGCCAGATCCTTGATCAACCCTTGACTGCAGGATAAAACCTTCCACTAGCATCCTGGGTGCTCACT<br>CAAAGCGGTGCCGCTGTCACAAAAAAACAGAAATCCAACTACATCTTCAATGGAACACAACGTGGCTT<br>CCGGAGGGCAAGGAAGCTCTCCTGGGGCCTCCACACGTAGTAGCAACCAGACAGAAATTTGGCTTGCT<br>TTGTTCAGATCTTCCTGTACCACCATACTAAAGACAAAGATGTCTGCCTAAGCCATCTCGGTACCTATGA<br>CAGCCATGCAGCTGCAGGGAGGTGAGACGCCCTTCTGAGTTATCATGAACCAGTGCCACTGTATAAACC<br>CCAGAATAAAGATGTCTGCCCTTGGTCTTGAGGGTAGATGGCTATGCTAATGCAGCCACTCGGTAAGTG<br>CAGTAAGCTCCTCAGAAAAGCCATCTACGGTGGAAGAATGCCAAGCCAGGCTTTGGATGTTTGAGTGC<br>AGCTGTGTTACGGAGCAGGGCTATGGAGCCTGTCTGGGTCCTTAGCATGCCACCCAACGTGGCTATCCA<br>CAGAGTAAAGGCTCCACTGGGTCGGACCACATCCCTCAAGAAAAAGACATTTGCCCAGCTCCTTGCTG<br>TGAGGCATTCTGTGGTTATAAACTGCCCTGATCCTCATGAACAAATACATCTGCGGCACAGGTTAGAGG<br>GTTTGAAATCCCACCCACATTCTTAGTCCGTAGAGTGTGGACCCAAGAATACACTATTCACAGAATAAG<br>AGAAATCCAGTGGGTTACCAGGTACGGGGCTCCAAAGTAACACAGCGCCACACAAGCTGACGTCATCA<br>ATTCAGCTTCCAGCTGTGCCTCCTCCTACATTCAGAGTAAAGTGATCTGCCCAGATCCTGGGGGAGCAG<br>ATGTTGCTATGATGCAGACTTAACAATTCCAGGTCTTAATTTGCAGCCACGACAATGAGGTTACCATCTG<br>GGTGAGGGCGCTCGTTGTGCCTCCATCCCCAAGATAAGGGTATCTGCCTAGATTCCAATGCAGTGGGAC<br>AGTAAAATGATGATTTTCACTGTGCCACCAGCTCCAGGAAAACATCAGCCCGGATCCTTAATGCCCAGC<br>TGCCACAGTGCATATATCCACATGGTAAAGGTGCTCGGTCTTACCATACCCAACCTTTGTGTGCAGCTGT<br>GGCCACAGAGCTGCCCATGGGTTCTAAATACTCCACCTCCATTCCCAGAATAAAATCTTCTCTGATCCTT<br>AGTATGCAGCCACCACAATGCAGCTGCCCACAAAATAAGGAAGGATGATTCACAGTGCCCTTATCCCTA<br>GGGCCAAATAAATAAATGAATAAATAAATAGATAGATAAATAAATAATCAGCCCAGATCTTTAGCGCGCA<br>GCAGTCGAAACATGGTCACACACAATTAAGGAAGCTCAGAAACGCCATCCCTGTGATAGATCCTCTTAG<br>GCCTTTCAAATGCAGCTGTCACGACGCAGTTATCCACAAGTAAGGGTTTCTGTTATAGCAACACCTCAG<br>GTTAAGATCCACTCGGGGATTTGGTACACAGCCAACACCATGCAGGCATCCACCAAGTAGTACGGACTC<br>CGTTAAACCATCATCCCGAAATAAGGATATCCACTTAATTCCTTAGTGCGCAAGCAGCAGCCATATAGGG<br>TGAGGCATTCTGAGGTTGTCTGTGCACAGCTCTGCTGCGCCACCATCCGCGGGAATAAAATTACCATGA<br>AGAACCTTAGTGCGCCACCAAAACCATTTAAGCGATCCACCCTCTAAGGACGCTTCCTTGGGTATCCGT<br>GTGCAGCTCCACTGTGCCTCTATTCCTAGGGTAAGAACATCCGTTCAGTCACTGAAGTGTAGCCACAAA<br>TACGTGTAATTCCCCAGGATTATGATAACAAAGAGCTGTGCAGGCGTTGGCTGAGTAATGGCGTACGCC

							CAGGTTCCAGCCAAGCGGGGAGACCTCCATGGCTCATCATTGTGCGACGACCACAATGCGCCTATCCAC<br>AGGGTGATAGCAAGCCTCTGTGCCAGCGTCTCCAGGGTAAAAAGATCAGCCAAGCTCCCAGTTGCATC<br>GCAAACTGTAGGGTTCTGTGCTCGAAGGTATCTCAATGCTGCCCTCGCACGAGCCGCACCATCACAGAC<br>ATCTGGGTTTTTTCGGCGCCTCCTTTTGCCCATTCCTCGGTCACATTGGTGTAAGAAAGCACCACGAATG<br>AAAAATTCCCAGCTCCGTGGCTGTGTCGCAGCTCTTCTACCCAACCATCTTCAGAGAAAAGGCCGCGG<br>ATCTTCTCGGAGAGCCGGAGCTTCCTGAAGAGCCGCGGCAATGATCCTCTACATAGCACGGGCACTTGG<br>ATGGGCTATCCACACAGCCCAAAATCACAAAAAGATGGGATAACGATGGCCACCGGGTCCTCTTCGGTT<br>CGTCGTCACAGAACCGTAGTAAAGAGATCCGCTCAGCTCCAAGCCGTGCCGCAGTCTGCAGTCTTTAG<br>AGACGTCTGTGGAAATCTGGTGAGCAGGCAGCTGTGACGCAGTCTGCTGGGCGAGCCTGCTCAGATTT<br>CAGTGCGCAGGTGCGGCAATGCGGCAATCTGCTGGTTATGGACACATTCTGAGGCGCCACCTACCGGG<br>AAAGGACGACAGTCCAGGTCCTCCGTGCAATGAGGCCACACAGTTGATCAGCATGGCTCTATTGTGGC<br>AGCATCTTTAGGATATTGGTGCGCAGCCACACTGGGCAGAGATCTGCAGCCTAACGATGCTCCACTAAA<br>TTGGCGGGGAAGGATATCCACACAAATACTCGGTACACCGCGGCCATAGCAGCAAGGCAGTCAACGGA<br>TCTCAGTACGCAGGCGTGGCAATGCGACAGTATGCACTTTAAGAATACATTCTATGGTGCCATCTAGCGG<br>GGAAGAGCCCCTGTGTCCTGGATGCTCCGGTTCTTTCTTCAGTATTCACAGAGGAGAGCCTTTTGTTAG<br>TGAGATTCACACAGGAGGCAACAAGACGCTAGGAATCTACCAGAACCTTCCTTAAGAAATTAGGGACT<br>GTTTGATTGACATTCGGCTCCTTTTGTTCCGAGGGGTTTTATATGCTAATTGTTAGCTTGCATCTGGCCTT<br>CAGATCTGTAGAATGTAGTGAAGACTTGCTGAAACGCACGCCTCATACCACCTTGTAAAAGACAAACTA<br>TGTGGTCTCTCAAAGCTTACAGCCCTGTGTTAACTTTAATTTTGAAAGCACTAATGTTTTGTTTTTTTCTA<br>GACAACATGGGTGAAAATAAAGGAAAGAGATGTATTAGATGTGTGTAAATTTAATTGAAAATCTTAACC<br>TGTGCATCAGTGAAATTGAAAACCC
MSTRG.115705.4	--	3.022	0.000	0.000	-9.753	down	CATTTATTTATTGAAGATACAATATAACAAACACAAAGAATACATCACTGGACCAAATGGGGATCCCCTC<br>CACAGGAAGCTTACCTAAAAGCAAGCGGGAAAGAGCACAAGCAAAAGACTGTATATAAGTATCTTGAG<br>TACTGTGAGAAACTCAGGGAACTGGGGCAGGTGCTGGGAGGGAAGAGTGTGTCTACCATGCATGCCTT<br>GTTGCAGCACTGACTACTGCATTATAGTTCAGCAAAACCTGTTGGAGGAGAGGAGGTAGCCTCACAGC<br>TACTGGGAGAGAAACATTTCAAAAAGGTTTCCACAAAGACAATACGGTGGACACATACCCACATATTCC<br>TTCGACCTGTTCAGCTAGGATGAGCTAGGGCTAAATATCAGGGGATGGGAAGGACTGGGATAAATGTGG<br>GAGCAGAGGGAAGGATTTGGGACAGGTCATCAACAGCTCTCCTGCCATCGTAAGGACTGTGGGTTTTAT<br>TCTGAATAGCTAGAGAGGCACAGTTGGGGAAGTCTCACTTTGGTTTGAATGGGATTACTATAAACAACA<br>GACTACTGGGGAAACACTAGGAACTAGTCTGGGAGCAAGCTAATTGTCTTGGAAGGGAAGAGTGTCGC<br>TACAATACTTGCTACAGTACTTGCCTTGACGCTGGGTTATCAAGGTTTATAGTTCAACAAAGACCTGGCT<br>GAGCCAAGGAGGCAGCCTTGTAGCTAGCTGAGGAAAAGCAGTTCAGAAGGAAAAATTTTCACAAAGA<br>CAGTAAGGTGGACATCTATTCCAAAACCTGTTCAGCTAGGATGGGATATGGACAGTGACAAAGGGAAG<br>GAAGGAGGGGAGGGAAAGGCACAGGGGTGGAAGGAGGGAAGGGAAAGGCACAGGGGTGGAAAGAG<br>GGGAGGGGAAGGCACAGGGGTGGAAAGAGGGGAGGGAAAGGCACAGGGGCTGGGTGGTGGTGAATT<br>CAAAGCTGCTGAATACTGGGAACATTAAAAAGGCAGAGAAAACAGAAGTTACTAGAGTGTTAAGCACA<br>AAGACAAAAGAGAAGAGTTAAACACAACTCCAAGATATCCAAGATTGGAGTTTTCTTTAACGGATCCT<br>GAGAAGACTGGAGTGGAACCTGGTTGAGTTCTGAAGGGACTGGGTACAGGAGAGAATCTGCACACTG<br>GAGAGCAGGCATGTCTGAGCCCCCCCTTGGAGTTCCAGCAATGCAGAATTCCTCTCCTCCAGTCATTAC<br>TGAAAGCTGTGAGTATGCCACAAGGTCCTCGCACATGTGGCGTGGCAGAGAAGACTGGGCAACCCCAT<br>TGGTTGGAGCCCAGAGAGTACCCCACTGCTCACAAGCAGCAGCTACCACCGCAAACCCAAGCCAACC

							AAACACCTCCCAGATCCTCCCCATATGATGTGATCAAGACTGCTGTGGTTCTCGGGGTGTGCTGGGAGT<br>CCTGCTGCAATCCAGAGAGGAAAACTGTCCACCTGGGTCCTTGGTGCATTCCCACCAGCCATAATCCAA<br>TACATGCAGTTGGCAGAGTTTAAAAAATCAACCTGCCTGGGTCTTCAGTAAACACCCCAAAGTATAGCC<br>CTCTTCGCTGGGTAAAGATACCCAACCATTTCCTTGGGAGTGTAGCATCCATCCTAAGTTCCACAGCATG<br>AAGAAGGCCAACTGACTTATGGTTATGGGTCTGCAGGTTCCGTGTGTCACCATCCTCTGCAGACGTTAA<br>CCCAGGAACTCAGTGCACAGCCATTGCTGTTCATTGAGGATGCCTTCTCTGCAGATAGCTCCACTCTGC<br>CCCAGCAGCATGGTAAGGATATCCACCAGGTCATCCCCACAAAGCCACAGCCATGAGGGCATGAGGAA<br>CCCAGGTCTTCATCAATGTATAGCTCTACGCTTTCACCATCTCTGGGCCAAGGACACCTGCCAAGATGAT<br>GGGTGACTCTCACAGCCATCTGCAAGGCAATGACATCAGACCATGCTACTCCCTTGTGAGATAAGGATC<br>GCTGGCCAGATCCTTGATCAACCCTTGACTGCAGGATAAAACCTTCCACTAGCATCCTGGGTGCTCACT<br>CAAAGCGGTGCCGCTGTCACAAAAAAACAGAAATCCAACTACATCTTCAATGGAACACAACGTGGCTT<br>CCGGAGGGCAAGGAAGCTCTCCTGGGGCCTCCACACGTAGTAGCAACCAGACAGAAATTTGGCTTGCT<br>TTGTTCAGATCTTCCTGTACCACCATACTAAAGACAAAGATGTCTGCCTAAGCCATCTCGGTACCTATGA<br>CAGCCATGCAGCTGCAGGGAGGTGAGACGCCCTTCTGAGTTATCATGAACCAGTGCCACTGTATAAACC<br>CCAGAATAAAGATGTCTGCCCTTGGTCTTGAGGGTAGATGGCTATGCTAATGCAGCCACTCGGTAAGTG<br>CAGTAAGCTCCTCAGAAAAGCCATCTACGGTGGAAGAATGCCAAGCCAGGCTTTGGATGTTTGAGTGC<br>AGCTGTGTTACGGAGCAGGGCTATGGAGCCTGTCTGGGTCCTTAGCATGCCACCCAACGTGGCTATCCA<br>CAGAGTAAAGGCTCCACTGGGTCGGACCACATCCCTCAAGAAAAAGACATTTGCCCAGCTCCTTGCTG<br>TGAGGCATTCTGTGGTTATAAACTGCCCTGATCCTCATGAACAAATACATCTGCGGCACAGGTTAGAGG<br>GTTTGAAATCCCACCCACATTCTTAGTCCGTAGAGTGTGGACCCAAGAATACACTATTCACAGAATAAG<br>AGAAATCCAGTGGGTTACCAGGTACGGGGCTCCAAAGTAACACAGCGCCACACAAGCTGACGTCATCA<br>ATTCAGCTTCCAGCTGTGCCTCCTCCTACATTCAGAGTAAAGTGATCTGCCCAGATCCTGGGGGAGCAG<br>ATGTTGCTATGATGCAGACTTAACAATTCCAGGTCTTAATTTGCAGCCACGACAATGAGGTTACCATCTG<br>GGTGAGGGCGCTCGTTGTGCCTCCATCCCCAAGATAAGGGTATCTGCCTAGATTCCAATGCAGTGGGAC<br>AGTAAAATGATGATTTTCACTGTGCCACCAGCTCCAGGAAAACATCAGCCCGGATCCTTAATGCCCAGC<br>TGCCACAGTGCATATATCCACATGGTAAAGGTGCTCGGTCTTACCATACCCAACCTTTGTGTGCAGCTGT<br>GGCCACAGAGCTGCCCATGGGTTCTAAATACTCCACCTCCATTCCCAGAATAAAATCTTCTCTGATCCTT<br>AGTATGCAGCCACCACAATGCAGCTGCCCACAAAATAAGGAAGGATGATTCACAGTGCCCTTATCCCTA<br>GGGCCAAATAAATAAATGAATAAATAAATAGATAGATAAATAAATAATCAGCCCAGATCTTTAGCGCGCA<br>GCAGTCGAAACATGGTCACACACAATTAAGGAAGCTCAGAAACGCCATCCCTGTGATAGATCCTCTTAG<br>GCCTTTCAAATGCAGCTGTCACGACGCAGTTATCCACAAGTAAGGGTTTCTGTTATAGCAACACCTCAG<br>GTTAAGATCCACTCGGGGATTTGGTACACAGCCAACACCATGCAGGCATCCACCAAGTAGTACGGACTC<br>CGTTAAACCATCATCCCGAAATAAGGATATCCACTTAATTCCTTAGTGCGCAAGCAGCAGCCATATAGGG<br>TGAGGCATTCTGAGGTTGTCTGTGCACAGCTCTGCTGCGCCACCATCCGCGGGAATAAAATTACCATGA<br>AGAACCTTAGTGCGCCACCAAAACCATTTAAGCGATCCACCCTCTAAGGACGCTTCCTTGGGTATCCGT<br>GTGCAGCTCCACTGTGCCTCTATTCCTAGGGTAAGAACATCCGTTCAGTCACTGAAGTGTAGCCACAAA<br>TACGTGTAATTCCCCAGGATTATGATAACAAAGAGCTGTGCAGGCGTTGGCTGAGTAATGGCGTACGCC<br>CAGGTTCCAGCCAAGCGGGGAGACCTCCATGGCTCATCATTGTGCGACGACCACAATGCGCCTATCCAC<br>AGGGTGATAGCAAGCCTCTGTGCCAGCGTCTCCAGGGTAAAAAGATCAGCCAAGCTCCCAGTTGCATC<br>GCAAACTGTAGGGTTCTGTGCTCGAAGGTATCTCAATGCTGCCCTCGCACGAGCCGCACCATCACAGAC<br>ATCTGGGTTTTTTCGGCGCCTCCTTTTGCCCATTCCTCGGTCACATTGGTGTAAGAAAGCACCACGAATG

							AAAAATTCCCAGCTCCGTGGCTGTGTCGCAGCTCTTCTACCCAACCATCTTCAGAGAAAAGGCCGCGGATCTTCTCGGAGAGCCGGAGCTTCCTGAAGAGCCGCGGCAATGATCCTCTACATAGCACGGGCACTTGGATGGGCTATCCACACAGCCCAAAATCACAAAAAGATGGGATAACGATGGCCACCGGGTCCTCTTCGGTTCGTCGTCACAGAACCGTAGTAAAGAGATCCGCTCAGCTCCAAGCCGTGCCGCAGTCTGCAGTCTTTAGAGACGTCTGTGGAAATCTGGTGAGCAGGCAGCTGTGACGCAGTCTGCTGGGCGAGCCTGCTCAGATTTCAGTGCGCAGGTGCGGCAATGCGGCAATCTGCTGGTTATGGACACATTCTGAGGCGCCACCTACCGGGAAAGGACGACAGTCCAGGTCCTCCGTGCAATGAGGCCACACAGTTGATCAGCATGGCTCTATTGTGGCAGCATCTTTAGGATATTGGTGCGCAGCCACACTGGGCAGAGATCTGCAGCCTAACGATGCTCCACTAAATTGGCGGGGAAGGATATCCACACAAATACTCGGTACACCGCGGCCATAGCAGCAAGGCAGTCAACGGATCTCAGTACGCAGGCGTGGCAATGCGACAGTATGCACTTTAAGAATACATTCTATGGTGCCATCTAGCGGGGAAGAGCCCCTGTGTCCTGGATGCTCCGGTTCTTTCTTCAGTATTCACAGAGGAGAGCCTTTTGTTAGTGAGATTCACACAGGAGGCAACAAGACGCTAGGAATCTACCAGAACCTTCCTTAAGAAATTAGGTAGGAATTTTACCGTTGTGCTGTTTCCTTTTTTGATGTGGTTGGGGGAGGGTAATTTTATAACTGTTCACTGGGTTACCTTCCTGCCTTCCGGTGGTCGTTATGGACCTCAGTAAAATCTTAAATTGCCTCCTGCAGGGACTGTTTGATTGACATTCGGCTCCTTTTGTTCCGAGGGGTTTTATATGCTAATTGTTAGCTTGCATCTGGCCTTCAGATCTGTAGAATGTAGTGAAGACTTGCTGAAACGCACGCCTCATACCACCTTGTAAAAGACAAACTATGTGGTCTCTCAAAGCTTACAGCCCTGTGTTAACTTTAATTTTGAAAGCACTAATGTTTTGTTTTTTTCTAGACAACATGGGTGAAAATAAAGGAAAGAGATGTATTAGATGTGTGTAAATTTAATTGAAAATCTTAACCTGTGCATCAGTGAAATTGAAAACCC
MSTRG.116381.4	ENSRNOG00000004471;ENSRNOG00000005358;ENSRNOG00000005572;ENSRNOG00000005577;ENSRNOG00000024128;ENSRNOG00000024170;ENSRNOG0000002	0.078	0.702	0.002	3.295	up	AGGTTAGGTTACAAAGGTATTTCACTTTTCCAAAATGCTGTTTCTCTTTGTAGACCAATCTGGCCACAAAAGGCTACCTGGCTAAGTATTCGCCAGAAACTTCTATATCCCAGTGTGATCTTCTTGTGGCATTTTTCCAACAAATAATGCAGACCAAACCACAAGATGGCCACCTCACTGGTCACATGGTCCTTAGGTTAATGAGCAGAGGCTGACAGGCTGTCTCCTCACTCTTCCAAGAACCGCCCAAGTGCACACTGGCTCATCTCTGGAGCTCAAGAAATCCTCCTTGGCTACTCCCTGGTCGCTCAGTTCAAGGCCACTGGAATGACAAACGGACCACCAAAATTGGATCCCAGGTTGGGTCACTGGCTCTGTTGGGCTGAGGGTTCTGGAACTAGGATAAAGGGTCCAGAGAAGGCCTGCTCGTTCTGTGGCTCCCTAGGCCCGCCCCTGCCCCCCCAGAGTCACTTCCAGAGCTCACCCAGGCGGCTTGTGACC

	4177;ENSRNOG00000050500						
MSTRG.116412.1	ENSRNOG00000007664;ENSRNOG00000007686;ENSRNOG00000008038;ENSRNOG00000008064;ENSRNOG00000008461;ENSRNOG00000008569;ENSRNOG00000023262;ENSRNOG00000042522;ENSRNOG00000043197;ENSRNOG00000049407;ENSRNOG00000058463;ENS	0.144	0.381	0.033	2.092	up	TTTCTTTGAATTGTTGCTGTTCTATTTTCCAAGACAAGGGTTAGCTCTGGTTGTTCTGGAACTGGATCTGTAGACTAGGTTGGCCTCAAACTCGAGATCTACCTGCCTCTGCTTCCCAAGGGTTGGGATTAGTGTGTGCCACCACCACTCGACGGGGGGAGGGGGGGTGGCTTTTCCAGACAGCTTAGATCTAGGAGAAAACCCCATAAACGTGTAGTTGCCACTTCCTTCTGACCCCATCGGCAACTTATTCCTGCCCTAGTTCATGTGTAATTTATTGACGTGTCATAGTGGCCAGTGGGCTTGGTGTTCAGCAGGGATCCCTGGAGACTGAGACTGCTGGGGATTTTTCTCAGCTGTTTAGGAGTTTTCGTGGAGTCCCACGGGCTGGCGGGGCTTGCCAGGGTAGGTGTGTCTGGGCTCAGCCCAGAGCCAGGGGACAGGGAATAAGAGTGTGCAGGGTCCCGAGTGGGCATGGTTGCAATGTTCAGAATCCAGCTGAGAGGCCAGGACAAGCTTCTCTTTGTTCCTCCCCCACTGTTCTGCCCGAGCTCAAAGGCTGGAAGAGGCCAAAAGAGCATCTTAGAGGGAAGAGTGGAGGCTCTCAGTGCCCTTGAAAAGTGGCCGTCAGGAGTCCGTCCACGACATGGGCATGGTAGTGCACACCTTTGATCCCAGCACTCAGGAGGCAGAGGCAGGTAGATTTCTGAATTAGAGGAAGTGAGTTCCAGTCAGCCAGGGCTACCTAGAGAAACCCTGTCTTGAAAACCACAACAAAGAAACAAGCATACAATGGCCACGGGGCTGGGGAAATACTCCAAAGGTGGAAATGTTGCTTCCCAGCACCCACACAAAAGTCAAGTGTACCTGATTCTTGGTACCAGGGAGGCAGAAATGGGAGGTTCCCTGGAATTCACTGGGCAGGTGCCCTAGCTAAATCAGTGAGCTTCACGTTCATTGAGAGACTCTGTCTCAGAAAACAAGATGGCGAGCGACTGAGGAAGACACCCAGCATTGGCTTCTGACTTCTCTATGCAGGCAGAGATCCATGCATGTACAACATACGTGAACAGGTACACATGGTGGGTCACAGTCTCGGTTAACAATCTTTCCCGAGGACTTAAGTAGGCAGACCTTCTCCCTCTCGCCCATCAGAAATTGAAGTACGGAGATCTGAGAGCCTAGCCCATGTTTTGCTGTGTGACTGAGGCAAGTCCATCTCCCTTTCTGGTTTTGTGGCTTTGAGTAGTTTTCCCCACAGGTTCATGGGAGTTAAAAGAAATCGTGGGGGTAAATAGACAAGAAAATAGAAATTCCAAGGCTGCAACTCTGTATGTGTAAACATGTATGTGTATGCTTGTGTGTGTGTGTGAGTGTGTGTGTGTGTGTGTGTGTGTGTGTGTGTGTGTGTTTATGGCTGATTTTTTTCAGCATCTTCCACATGTTAGATATGCCATCAACCACCAAACTAACCCTAGCTCCAGTTTTCATGGCTCCCACAGAGTGATCATGGTGTACAGTAACAGTGCTGGGTAAGACTTAGAGGCTCCATGAGGTCATTCTTACAGCAACCTGAAAATGTGTCTACACCACTGCTTACCAAATAACCCTGACTCCTCAGCTCTCAGGGTGCCACAGACATTTACCTTCTTGCAATGGGTGGGTGCCTCATGCAGCTAGTTCTGGCCAATGGGTTCTAATTAGGTCAAAGACATTAGTGGCTGGCAAGGGGATTCACTAATCTTCAGTTTGTGACAATCTCTGGCAGTGACCTGGAGGGTCCTCAGCCCAGTGGCCAGAAGCTGCTGAAAGTCTGCCTCACCAGAGACAGCTTGGGTAGGACTACGGATTTCTCTGAGGTCTGGCCTGTGGCTGGCTGTCTTCATGCCCTGAGTCTTAGTACAGGATCTGTTCCAAATCTTCCATTTTATCTCCATGGTGCCTGCTTTGCACTTCACAGCAACCGTGCGAACCAACTTTCTGTATTCTCTATTAAGACCCAAAAGCTTTCAGGTCAAGGAGGTTTGGTCATCCTCAGTCCTGTGCGCCCGGGGTTGATCAACTAGCTATTGCCAATGCATGGGTGGGTAAGGGATGCTCAGGGAAGTTCAAGGTTACAGAGTACAGCATCCAGGCTGGGAGCTGGGTCTGGAACCCAGTTCTCTTGATTCGGAGCTCAGTCAGCAACCGCGCCTGGGCAGAACCGCGGTGAGCCAGCGCAATATCCCAATCACCGCGTGACTCACGCACGCGCACCGTCGCCGGCAGCTGCCACCGGTGTCCTGCTCCCTGGACCCGGTAAGGGGCTGGGGACAGGATCTAGGGAGGCAGACAGAGACTGCGATACGGGCACCTTAC

	RNOG00000059588						
MSTRG.116463.1	ENSRNOG00000046472	0.352	0.964	0.016	2.203	up	CTGGGGGACCTTCAGTGTTAATGCTCCCTATAAAGTCCACACACAGCTCAAAGGATGAGACACTCAGTATTTTGATTTGCATCCTAAATTTGAGGGGTGGGGGGAGACAGTCAATACTCAATATACTCTTGACAAAGCAGGTTAAGTTTCAGCTTTTGACATCCAGATTGTCCCTATCAGGGACTATACAAAAAGTTCAGTCTAGCTACTGAGCTGTACCTCGGCAGATGCTGTGACTACCCTAAGCTTCAGTTTCCTCATCTGTGAAATGGGGACAGTTATTCCTACTTGCTAGGGAACAGGCAATACTCAGAGGTTGAGACATAAGCATGCTGCCTAGCAAAAGACAGGCACTCAGTAAGTGGGCTCACTGGAATGTAGTGATACTCTTGTATGTGATGACAAGCCAACAGAAGGCTTTAGTTCAATGTGACCAACTACAGGAAGCAGTCTCTGTCCTTTAAGTCAACAAAAGGCATCAGACACCCTCAGAGCTGCTTTTTGTTAGTCTGCAATCACTCTCAAACCTACCAGGTAGTCTCTTGTCAGTACTTGGCCTGGAGTTATACAGAGAATTTCCAGTCTTTGGACTTGTCTCTGTTGCTAACAAAAGCAGGGAAGCTTGTTGTCTGGCAACACAGGATGAGCATGGGAGGCACTAGCCTGGAAAGGAACAAGAGGCAGAATAGCCACTCCCAGGAATGTCTGGAAGTTGGACAGTTACTCGGCAAGTACTTGTGGAGTGAGGTCCTTGCTTTTTGCTAAGTAGCAAGAAAACTATTCAAAAGCAATATTATAGTATAAACCAGGGAGAAATGAGCTATGAAGAATGACCAAATGAGGTCATTTTCTGATGACTACTCACAGCAATAGTGTATTCACAATTTATTCCTGCTTCCAAATCTTGAAAGCACACACATTCACAAGGCAGAAATAAAAGCATTTAACACAGCTATGGGAAGTTTTGAGGAAATGAAATGGCTCAGAGCTTTCAGAGAGCCCAGAGTCCTCAGAACAAGAATCTGAAAATGGGAGTCTTCTGCATGAAGACCACTTCCAGCAGCATGGCCATTCATACTTACTCTACTTGTAGGTAGAAGCAGAAGAGCACACTGTACAGTGTACAAACAGGAAAAGCTGGCCAGCTCCAGAGAGCCAAAGGAAGAGTGGGTCTAGTGGACCTCCACTTGAGAAGCTGCAGGTTGAACACGAAGTGGCCAAAGGCTAATGAACATACTGAAGATTAATCACAGGTTCTTGCAGGGTGCTGAACCTGCCATAGGAATGCTTTTAACGTGCATCCGAAAGACCCATCAATTCTTAGATGCCTACATGGAAAGCCCTGTAAATAGTCCCAGAATCTAGCTGCCCTTCATTCCCAAGAAACTCCTGTCACCAAACAAAACTGTCTGGACCCAGGGAAGCAATGGTGATATGGACAGAATACTGGAGTCTGGTGGTTGTGGTCTCTGAATCAGAGCATACCTGTCAGGCAGCTATTTACTTTCACCACATCCTAGGGATTTGCACATCCTGTCCAGAGACTTGCTTATGCTGGACTGTATTGTTCTTAATCTTTCCTTTTACTTCAATGCTCTTCCTGTGGGCAGCCTTGTATAAGAGGCTGGGTAGACAGACTTTAGGAATGTTTAATCCATGCAGCTTTGTGCCTGTAATTTAAGATTCAAAATACAATGAGACAGAAAGTATTCTCATTTTCTTATAAGAAAAGTGAGACACTTGCCCATTTAGACCTTTTCTCTAAGGGGCCTCATATGCTACAGCTGCTGGGGCCAGATCTAGCTAAAAGGTTGCACAGTACAGCCCTCACCACTGACAAAATGAACACAGACATGTAATGAATGAGGAAGGCAGCTATGCTCATAGCTATGCCATCAATGTAGATATATGCTAGAGATCCAAAACACCGAGTGCTGGGATGCCTATCTCTCACCTACATCTACCTGGTTGAGCCTTGCTCCAGTGAGAGGCAGAGCAGATACATGACTTGCCCTGGAGAAGCACAGGGACTGGACCTGAACATGGACAATGTAGGCTCTGAAACTCTTGCTCTCACATTTGAAACCTAGGGAGGAAAGAAAAAGGATAGGGCATGTATGGTGAACAGGGAAAGATGGGAACAATCCTGCTATGTGCCAATTTGAACATAGAATGAGGAAAAGTACAAGAAGTCATAGAAGAGCTTCATTAGAACACATCTCAGAGCAAGGAGCAGGTGTTCAAATAGCCCAGCACTTTGAAGAGAAGCAACTGGGCTTTTTCTGAGAATTTTTCATACTACATTCATAGCACAGCCGTGGAAGCCTGAGCACCTTCCACTCTGCTTGTACTTGGCCTGTGGCTAAGGCCAGAATCAGTACAGCTCATCTCTGATCTGAGCCACCTTTGGGGAGGATGACACATGTAGTCACCAAGAGGGTTGTTATAACAGAATCTGGGTGTCAGACAACAAGGGAAGCTCATCACTCAGTTCTAGAAGACAGAAGTCATAAACCAAGGTGTGGCCACACCCCCCCTGAAGGCTCCAGACAGGTTCTTCCTGCCTCTCCCAGCTTGTGGGGTCTGCCAGAGTCCTTAGCTTCCTGGACATCTGACC

							ACTTCTCTCCAGTCCTCTGGCCATCCATTCTCGAGTATCTAGAGCAGATTTTTCCTGCAGTATAAGGAAATGCTACATTTATATCCATTAGAGTTCAACCTGCTGCATTGAGCCACTCTGGTGAACCTGTTTTAAGTTGTTCTCCTCTAAAAGGCTGCTTTCAGCAAAGCTCACATCCTCAGGCTAGGCCTTCAGCTGTTGGATTTGGAGAAACAGTTCCTTCCTTAAACAGAGGTATTATGTCCACACAGCAGAGAAGGAAACTGAGGCTCAAAAGAAGAGATACCAGGTCCAGCCAAGACCCATGTTCTTGTCCCTGAGAGGCTGGGCTGGGTCTTGTACTTCTGCCCTCTTCCCACCATACTGTCCTTCACACGGGGAGGGAAACCTCCAGCTCCTAAGACAAATGGAGGGGGCACATTGACATTCTGCTCTAATGGCTTCTTCCTCAACACGACCAGATTCCACGAGACTGCACAGCTCTACCCACGATCTCTGATGATGCAGGGCAAGTGTCAGTGTGCCACCTGGTTTGAGGGTGGGTCCCAGGTTCTAGAGGATGGTGTCTGCATCTGTACGTGTGATGGTCAGGAAGTGGCCTTCCTCATCTGTGTGTGAGAAACAGACTTGTGCAGGCTACAGACACTATCACACAGGTTATAGGGCCTGCTTTGGCTTTGAGGAATGGTAGCTTTTATGAGCTTTCTTTCTGTCTTTGATTTAAAGAACCACTTACTGAAAGCTAGAGCCAAGCATGAAAGCTCCTGGTGGCTCCTGCTCAGCCCTGCCTGACTGTAGTCCAGGCAGTAGCAGACCAGCAAGGCTTATGTCCTGGCCCTAACTGCTTCTCTGCACACTGCACACATCACCTGCCAGCCACTCTGGGTGTGAACTGAGTGCTGGAAAGGAGGGTGCAGAAGGCACTTCAGTCTGTAGTTCTGGCTTCCTGGTCCTGTAGCTCTCATTGCTGAAAGCATGTGCTTCTTCACAGTGGTCTAGGTGTCTCATTCTTCTTCCAGTAGTAGTTGTCACTGTCATCCCAGCTACAAAAGTCTCTTAACTTCATGGGGTCATTGATGTACAGATAAAACTCTAGTGAACACCACGTGACTCCAAGCAATTGATGAAGAGACACATCCTGTAGGTTATTCTTGTCCAAGTGTGACTTTTGGCACTGCAGTCAAAACACCCC
MSTRG.116485.1	ENSRNOG00000010539;ENSRNOG00000010549;ENSRNOG00000022623	5.145	0.000	0.000	-6.293	down	GCGGTGGTTCAGGAGGGAGTACTCTAGGTGTCCTGGGAGGCAGAGGGTAGGTGAAGGCTTTCTGAAGCACTGCTTCCACCCAGGAGTGGCAGCCCCACCAAGGGGTCTGTGCTGGGTCTAGGCCTTCCATGGGGGTGGTCCAGCCTCCTTTCATCTCGTGCTGTGGAGGGAAGGCACATTCCCCTCCCACTTGGTTCCCACTGTCCCCACGCAGGACAGCTGGCTGGTTATTTGTAGGGCCGCAGGAACCGAGACACTTTGGAACGCAGAAGTTTGATAAAGGACCG
MSTRG.116517.2	ENSRNOG00000021671;ENSRNOG00000029243;ENSRNOG00000052725	0.022	0.297	0.000	3.774	up	CAGAAACTGAACTGGAAGGGAAAGGCTAATGCTGTGAACCCCGGAGACCCTGCAAAGCCAGAGAAATGCTGTGCTAGGCTGAGCAATTGTCTTCGAGGAAACCCAGGGCCTCTTCCAGGACAGAAGACTTTGCAGGAAGCTGAGTCTTTAGAGCCATGCCCTCTCTTTCACTGAGATGCTGGTGCACGTTGTTACTCAGACAATATGGAGCCCAGGCCTGAGATCCACTTTGATGGTCACGAAAGGCTGCTCTCATGTCCTCCCCATGACACCCCGGGAACCTTTTCTGAACTCAACACTCATTTCTTCCTCACATGTCTTCTGAGCCATGCAGCTCTGCACAGATGAAGCCATAAGGCAGTAAGGCAGATGGAGTAGAGCTACACAAGCAAACCAGAGCACACGCAAAATGGCGCAGTCACAGACACTCATAATCAGTCACCACACTCGAGTCCTAAATCCAACGTCTCCATCACCCTATTCAAGGCTCGGGGAACATTCCAGAAGAGAGAGTGGAAAGAAAGTGAAAGTCAGAATGGACTGTTCCCGAAACACTATCTTCCGGGTGTGAAGTGACCGCTGTGCTCCTGAATTCACAGCAGCTGGGATGACCTGCAAGAGAGCCACATCTCAAGCCACTGACATCCCATCGCATTAGGGAAAAGGCTCACCCAGGCCCCCCAACAGCTAACGGAAGTTGGTGTTTGAATGGGGAGAAGGATGTTTTCTTCAGTTGTACAGTTACTGGCAAGATGTTCTTGAGCCTGTAATGACCCCTTGCCCATGTTCCTACAAGCAACCCTAGTGAAATGCACTGGGTG

							ACAAAAAGAAAAGAATGAATAAAAGAAAATAGAATGGGGTTGAGCTGGGAAGAGAAGTGATCAGCAGGTGGGGGGTGTGGGAATGTAATCAAAGCCCTTTATACACATGTAGGAGATACAGTAAAACCCGTTGCTATGTACGACTCATACTTGCTGATGTGAAGGGCTGGGACCAGTGGAGCCCTTGGGATATTGAGGTTACATCATAGGGAGCTGAGCATCCCTCAGCTGTAACCCAGGGATGCCAGCCCCTGCAGTGCGTCTGGGAGATGCACATGTGTGTACCCACTCAGACCGCAGCAGATGCAGTCAGAGCAACTTCACTCTGGGTTGCTCAAATGGCACTGCCCACCCAGAGCAGATATGAAGTTGGGAGACGAATGCCTTGGGGAATATGGGGGAGAGCTTGAGCAATCCTATTTCGTTGTAGATGGCAAGAAATTCTC
MSTRG.116601.3	ENSRNOG00000021463	0.371	0.000	0.000	-7.390	down	GGCGCCAGCCGTGGACGCGTGACTCGATCCTTCGCGCCCACTCTTTTGCGTGTTGGGTCGTTGGATGTAAGACTGCAGAAACCCGGAGAGCCGTGGGTCCGGGGTTTGGGGCCTCGCCGTGCGCTGGCCAGAGGCCATTCCACCCTTCGAAGAGTTGCGGGAGCGGCTCCGCCACTGCCCTTGGCTGTTCCTGTGCGCCCAGGAGCCGAACGCAAGCTACCCGGACTCCAGCCAGCGGCTGCCGGGCATGCCAGCACAGCGCTAGCGGCGGCCAAGAGGTGGTCTGGCAGGGCGGTGGACCTGTGCGCTGCGGGCATGATCAAGGTTGGAGAGGCTTGTACCCACCGGTGGAGATGATAGAGAACAGAGAAGATAGAAAACACTGATGAAGTCCGCTGCCGCAGCTGGCTGGGAGTGAACCTGGATGCCCCGGAGGGACACCTGCCCCCAGGAAAGTAAGCACCTGTCGTGTGTATCAGAAGACCCCCTACTGCCTCACACACAGTCACTGGCCTCTTCTTGACAACTTCCCCAGGTGACTCTGAGAACTCAAGGACCATGTCCAGCTCAGCCCTGCCCTGGATTCACATTGACAGTCCCTGTCAGAGACCTGAACCCTTGGCCGTGTGAGTTGCATCTCTCCTAAGACCTAACCACACTACAAGGTGGGTTCTAGGATTATAGTGTCATTGCCCCCTGGGCCTCAGACTACCTGCCTGTGAAATGGGATTCTCCTCCACACAGATTTTTGCTTTGCATAGGTCAGTATGAGGCAGCCTGAGGTAGGACCTGTACCACCTCTCCCCACCCCCAGAGCTGTCCTCTCTGCCTCTAGTTTCCAAATGGAGTCTGAATAAGCGAGAGTAAGGATGAGAAGGAACATAAAGGAACCCAACAAACCCTGGATCCAGCCCTCTGTGGGGCTCTGGCTGGTGACTGAGTTCCTACTGCCTTGTGCCTGAAGAGGCTTGATGACCAGCCATGGTGAGCAGCTATGGAACCCCTGGCTGGCCAGAAGATGCAAAGGGGGATATGAATAGATGATCGTGCTGGTTTACGAAGCAGCTGGGGCAGTATGGGAAAGCCTCTAGGGCCTTCCTTCTAGCTGGAACCATAGAAACTCAGGACAGGGCACAGCCATACACTCCTGAAATAAGTCCTCCTGGGATACCATTGATGGCCATTTTGGCAACTGGTCCCTCGACCTATTGGCTCCAAATAGGACTGGTCCACCTGGATCACTCATTCCAGGAGCACAGGTTGGCACTTGAGCCCCCTCTGGCTTGAGGCCTCCCTAACCCAGGCAAATAAGGATATGTCCTGCAGAGAGACTAGGGCAGAGTTATAACCCAGAAGAGCTCAGGCCTAGGAAACCTCACAGCTGTCATCAATGGGTGTAAGAACTTCAGTGCTCTTTGACAGGCACACTGTGGGCCACCCGGAACCCCTTAAAGGGAGGAGTGGAAGTTTGTTAGAAGTCCTGAGTATGCTCGGAGCTTCGGCGGGAGGAAGGGTCTGGAAAGAGCCTGCCTGACACACTTTCCTGACTAGACCACCTCAGGCCTCAGCAGGGAGCAGAGCCTTGATTCTGTTTAGTTTCTTCTTCCTCTTCTCTACCAGGCATCCACTGGTCTTCTGACTTTCATGGACCTTTGTCCTCTAAGGGCGTCACAAAATGGCCGACACAGCTGTCCGCCATTTTGTAGCTGACACCAGCAATAGGCTTGAAGAGAAGGAGACCTCTGCTTCCAGCCTGCCCTGAGTAGCCTGGACAGAGCCCAGGACCTGCCCCCACCATGTTTGGCTTTGACCGTGGCTGCCCTGGGCTTGGCACAGCCAGTTGTTGGCAAGGTATCCTGGGAGTTCTGGCCCCTTACCCAGTTCCACTTATTCCTTGCTACCCTCCAAGGTTTTATCACCAAGCAGACAGAAAGAGAGGTTGTACATCACAGTCCAGAAAGCCAGCTACTTACACTGTACTTTTAGAGGGAGTCTGGAAGGGGTGGGGCCAGCACAAATGGGTCTAGAAATGATGTCCGTACTGCAGCACTAGGTTTCTGAGTGGAGCCATCAGCAAGCATAGAATTGAGTTGATTCCTCTTGGGGAGGAAAGCCTCTGGCCTCCTCCAAATGCCCCTCGTACCTTGACCTGCCCGAGGACCTTGGGCCTCCCTGGCCAAGCATGATATGGAATGCCTTGGCCAGCCAATCTCCTGGCTGGGGTAGCCTATGATATCAAGAAGCAGGACAGGTGCTTGCTAGACTCGGCTGCAGAGTAACAGGGGCAAGCACCAGGCTACCGAGGCTCTGAGGTACATGAAGCTTTT

							CCTGACTATCACAGCACACACACTCTCTCTCTCTCTCCCTATGTAGTGTCTGCCTGGTTCAGGCTCGGCA<br>GGGATCCACCGTAAGCCCCCCAAGAGCTTCCTGTGCCTGTTAACAAGCTCACATTGTTCCTCTGCCTTC<br>CCAGAGATCAGAAGCAGCACATCCCAGAGATCCCATAATGATGCCATCCAGGCCTGCAATACAGCCCAC<br>GCTTTGGTCACTGCCATATTGATAGTACATTCCAGCTCCTCGAGGTCGGAAACTGGCGTGTGTTCTGGG<br>AGCCGGGAGCGGCTGGTTAACTCTGCTACCCCTTAAAAAAGCCGAGGCGGGGGTTCCAAGTAAAGTCG<br>GGAAAGGTGTGGGATAAAATGCCTTTTGAATCTTCTTGGGCAGCATGGGGGTTACTTGGTGGCCCTGCC<br>TGGCCCTATGCTCTCTGCCAGGATAACAAGAAGCTATGTAGAGACGGCAGGGTCAAGCTGCCTCATTTA<br>GGAAGCTAGCTAACTGCCTGCTGGCTCCAGACTTCTTCCCCGCAGCTTCCAGGCAGAAAATTCTGTCAC<br>CCTGAAGTGATAGATCAGGCCACACTCTCCTTCCAGAGACCCAGCAGAAAGTTCAAAATCTACCTTCCC<br>TTTTGCTGGGCTGGATGGAACATCCTGGGACCCGGAAGTGTAAACTTCCTAGAAGAGGAAATGTCTCCT<br>GAAGAAGGGAGAGGCCCAGAGGGAGCTGACTGAGGGCGTCTGTCAGAGAGCAGGCAGAGCATAGTC<br>TCCATGATGTTACGTCATTTCCCGGTCACCTTCCTATGACCTAGATGAGGCTTGATTGACAATATCTATAG<br>ATGAGCCCATGCCTGTGTGGTCAAGGCTGGTGACCTGTAGCCTAAATGTCTAGCCCCAGGCTGAGTCCA<br>CAGAGCAGCCCCCCACCCCCACCCTACCCAACCCCTACACAGTGGCCTTCCCCTACTGTACACCAGGCC<br>ATCTGTAGGCATAAGTCAGCCACTTCCTCAGGGCCCCCAGGACTGTCTGTAACAGACAGCTATCTCTCC<br>AGCTCTCCAGAGAAGGTACGTGGCCAACTGACTTTGCCTCAGACCCTGTTTCTAGATGGGGAGAGCTA<br>GGCTCAACTCTGGGCCTCCTAAGCCAGCTGAGACCCGGAGTCTTCCTTTTTCTCTCATGAGTTTTGCTAC<br>AGGCCATAACTCTAGGGGTGGGCTTGCTACCAGAGCACAACAGACCTCGAGTCTACACAAAGCCCAGT<br>GCAGGTTGGATCCATCTTGGGATCAGCATGAAGTGTGTTAAGGCTGGGAAAGCCAGGTCCAAGACATT<br>TAGATGCTACCCCCCGGGTTTGACCTTGTGTAACTGGTACACCGTCAGAAGAGGGTGCTCAGGCCTGCA<br>CGGCACTCTGCCACACATCTGCCAGAACATTCCAGTCTGAAGCCGGGGAGACTCAGCAAACCCAGGTT<br>CATCTTCACTCAGGTATGATCTTGTAATATCGAGGGGGATTTTGCATTAATCAGGTCTCTAAACCTCTCGA<br>AGAGGTGCTACGTGTCTTGGAGAGTGAGATTTTTTTTGTTCACCCCTAACATGCCTGCCCTAGCTAGTTC<br>CAAGATGGTGCTCAGTGGGCATGGCCTGCCTCTGGACACACCGACCCATGCCTACCCAGTGAGCATAC<br>ATCTTTTTAACTTTATGGGCCGTGTTCACAAGGAGATCCGCCTTCTGCCCAGCCGGATGCCACCCCTCCC<br>GTCGTTCCCAGACTTGCTGTTGGCACGGCCTACTGAGCAGAGACACTGGCCGCTTTATGGAAGAAACC<br>TAATCAGGGAGACCAGGAGAGGGGGGCTATCATGGTGGGGGTCTGGCTCTTGGTGGCCCCAAGCCAAG<br>CTTGAGGTGACTTCAGGGTGACACTGTCTGTCTGCCTCTCTCCTCCAGACATCTGCCTAGCCCTGAAAG<br>GGCCACCTGCCTGCCTAGGCCCTTGGCGCACCTGTCCTGGCCAGCCCAGCTCCTCCCCCAGGCATGCTG<br>CCCACGGGCAGATACTGCAGGACTGAGGCAGAGCTGAGAGGTGGCAGCTGGCAGATGGCAGGGCGTC<br>TGCTGACTGCTCACTCCCTGGCGACCTGTTCAGGAGCGCCATTCATGTGTACTGCCTGCCCTGCCACTG<br>GCCCTCCTCCACAGCCTGCCCCTACTGGGTCCTCTCGGTCTGCTTCAGGACCCCTTCCTTGTCACCCCG<br>TGAGAAAGGAGGCATTTCTGTGGGTTCCAAGCCCCAGGCTTGAGGCCAGTGGTCGAGGCCTAGATGCT<br>TCACAGGTGAGCCTGACACATTGGAACACTTCCCAAATACAGAACTGGGCCTTTTCCTCCTTGTTCATA<br>AATGGCTCTAGAGATGGCCAGAGACCCAGAAAGGGTGACCCTCCCTAAAGTCTGGTAGAACCCGAGCA<br>AACCCCCTGGGTAGGGCAAAGCTGCACTGGAACTGAAGAGACTGTGCTAGCCATGCCTTCCACCAGAG<br>TGTGGAGAGGTACTGTGAGAGGAATGGGTCTAAGACAGGCACCTGTGGGTCAGCCTCACCAGATGCAG<br>AGCCAGGGCCCCTGGAGAGCAGGAGCCTTGTGGAAAGAAACAGGAAAAACAGGTCAGCGGTTCCTG<br>GCAGAGGGACAGACCAGGGCAAAAGCCAGGAGGTGGGAAGGACCTGGGAGATGGCCAAGGAGGGCA<br>GAGGAGGCTTGCCCAGGGCACTTCACTGCTTTGCGGGTTGGGGGCAGGGCTGATGGGCGCAGTGGAA<br>GGATCAGAGAAAGGGTGTCAGCGTGACAGATAGCTTGGCTGTCTTGGGAGCACAGGCGAGGCTGGTA

							GTCAGGGAGGGGGCTTTCCCACCCTTGAGTCCTCACCAGACCCTCAGTAGCCAATGGGCTCTTCTTCCC<br>ACCCTTGAGCTGTTGGGGATAACAAGGCTTGTAACAAAGCACTGCCTTGATGGACAGAACTGGGCAGA<br>TCCTTGTCTCCATATTCTATACCCCCGGCTCAGCTCCAGGGTCCTTTGGCTGGCCAGGCCTCTCCCTCAC<br>CACCACCATCTCCCTGCTTAGCTTTACATCTTACTGTTGGCTCTGCCATGATGTTTGGGACCCAAGGGGA<br>GGTAGGATAGTGACTTGATTCATGGTACAACTCTGTGGTCCAGTGAGCCCCTTCCCTGAGTCTCTGCTCT<br>TGGGAGAGTTCAAACCCCATTTGTCCTTTGTCCTAGGGAAGTTGTACTTCACTGATGACTTCTGAGAGG<br>GGATTAACTCTCTCTCCAGGGCCCACTTAACTACGACAGACCGGTGTGCATGAGACAAGTGTGAGCCT<br>GACCCATAACAGTGCTTCCTCCCTTTCCCTCTGGGCTTCCCAGCATCCAATCATCAGCGAGTCGGGCCA<br>CTGGCCACCCACAAAAACATGCTTGGTCCCATGAAGTTGGAGCATAGTCTCTGACCAGCAAGCCCAGC<br>ACAGGGCTGGGCTTAGAGGCCATTCCATCCAAGGCTTTGCATGCAAAGTGTCGCCAGGCAGAAGGCAG<br>ACTGGATGAAAATGTCATTGTGCTACAGGATATTAAGGTGTGGCCAGGGGAGGCCACCGGAGGCCAGG<br>CTTTCTCCCTTCTGAAGCTAATGATCATTGCTTTTGTTTCTAACCCCAGGGAGCTCAGGACATGGGACTA<br>AGCAGGAACTTCTACACGCCAACACTCCCCTGTGGTGAGTAGTGCTCCACTGTCCCTCCTCTGGAAAG<br>TAAGGAGCCATGGGAGACTTGGGAGTGAGGAAGGCTAGGGTCAGAACGACATCCCTGTCAGTTCTCCA<br>GGACCTGCACTCAGCTGGGATGACTTTACCTGGAGGCCTTGTGCTACCCCATCCCCACCCCTGGCCTGA<br>GTATTGTGCCCCCCACCCCAGCAAACTACCCCAGATGACTCATCTACCCATGATATGATACGGTGACCTT<br>TGACCCCAGATCAGCCTGCTCAGGCTGGACATGGTTTGACAGAGATGCACTTCCATCCATTACTGGCTC<br>CAAGTGGAAGAGATACCCTCCCACCCTGAGGACACACTACAGGCTCACACCTTTCAGGTTACCCAGCC<br>TTGAGAGGCCTCTGGGGACCCCAGCATGAGGTCACCCTCTTTTCTCTGTACCCCAGCACCATCACTCAG<br>TCCCCCAGTGTGATCCTCATACCCCCAGGCCCCCTGGCATTGAAGGTGGACCTGCCAGGAGGTCTTGCC<br>TAGAGTGAGAAGAAAGGCCAGATGAGGGAAACAGAGTCACATCTATTCCCAGGTTGGGCTAAGGCAC<br>AGTGGGTGGTGGCCACAGCCAAGAGGAACTAGGATGCTTGTCTCCTGGTATCTCTCTCCTGCAGCTTGG<br>GCTCTGTAAGGTTTGTGTTGCGATGAACCCCAAATTCAGGGAGGCTTACCGAGTGCAGAATGGGCACTT<br>CTGGGCCCTCATAGCGGCATACCCTGGCCTCCTGCTGATTGCTGAGTAGAATGGTGAAAAGCTGCTGAG<br>AATCCCCAGGGGTCAGCTGAAAGGACCCCATAGAAATGCAAGGTGGTGCCCGGAGTCAGAGGGGGCA<br>GTCTAGACCTTCCAGGCAATCCTGATCATGGTACCTGTGTCCTGCCAGCCAAGGGCAGAACTTGAATGG<br>TTGAGAGACCTGGGGTTGATGTGATGCTCACATGGAGATGGCCAAAGTGGTCTCTAGCCATTCCAGCAC<br>GTCCAGGCACATCCCAGGAACTGACAAGGCTGACCATGGCCAGCCGGCCTACTGAGAGGCTAAGCAGT<br>TTGGGACATCCCTCGGCGTTCTCACTTGGTCCACTCTCATAGATGGGAGCATCAAAGGGGCCCTTAGCT<br>ACTCTTCCCTTGCCTCTTCCTGCCTTGCTGGCTCTGCTTAGAACGTCCTTGAGGATATTTGACCTGCCTG<br>TCAGTGAAGGGGTCCATGCTTGAGACAGGGGGTGGCACCCCGGTAGCTCACATTTCCTTCTGGAGCTT<br>GGGAGACCAGTGTTTCCTATTACCACAGATCCGCACACTGAGAGGCAGGCCACACTAGCACTCTGGGG<br>CCTTGTTCCCAAGCTACTCTGCCTTATAGACTCCAGATCTGGGTACGCAGTGTGGACTTAGACCTAATGG<br>CTTTGTTTTGACCCAAGGGCTCTGTGCTTGTTCCTTGACCCTTGGCAGGGAAGAAGGGAGGTATGGCA<br>GTAGACGCTAGGGTTCATGCAGAGAGCAAAAGCTGCCTGGTGTCTTAGGATTGGGCACAGGGCTTCCC<br>TCTAAGTGCTGGGCAAATCTGTCAGGGGCCCAAAGGAAGTAGGCCCTTCCCTTCTGAGAGCAGAGCTA<br>CCTGAGACCAGAGACTCAGCCTTCTGAGCCAGTTTCTTCAGTGATCCCTGCCTCACAGCTGGAGGATTT<br>GCTGGCTCTGCTTAGGAAGGCTGTGGAAGTAGGGTCTCCAAGTGCATGTTGACACTGAAGAGCTGAGA<br>GCCATAGAAGTCTCTTACAAGGCGGAAGAATGTCCCGGCACACTGCTACTGCTCGGGGACAAGGCTGC<br>AGTTGGCCTTACTTGTAAGACCGGCTGACACTACACTTGTACAAGGTTCCCCCCCAGCAAGCTAGTGGC<br>TATGGGCAGTATGCATAGCTGACTGCTGCCCCAGAGTGGACCTTACCTGTCCCATTATGCAACAAGCAG

							CAAGCTCAACACCAAGCCCTCTGTTCATACCCGGATGTACCCCTGCTAGCCTCACTTAGTCCGAGGCTCTGGGAGCCCCAAACTCAGAGCCAATCACAGAGCCCCTGCTGATCTCAGGACGTGCACGTCGCCAGGGACAGAGTGGCCATGAGTCCAGCCAGGGTCCGCCTGGCTGGCAACCATGGGGAGCGAGACAGGGACAGAACCAGAACCCACAGGACAGGCTCTTCCTGCCTAAGGGGAAGAGGGTGCATGCCCAAGGCTTCTGCCTAGCAGGAGTTCTGGTGGTTCCAACTTCGCTGTGCTAGAGTTGAGTGGGCAGAGGCTGGGCCTCAGCCAGGTGGGCACTGGAAGCTCCCTGAACCTGACTGGCTTTTGACAAATGACACCCTTCCCCCATAGCGGCCACTCCTGTTTAGTGACTGATCAGAGCCGGGTGCTTTCTATCTCTTCTCCACCACTTCCCTTACTCTCCCCTGGCCCCCTTCGAGAGAAACCCAGTCCTCTACTAGCTCATACCCTCACCGCTGAACTGGCCGTGTCAACCGCTCACCATGTTCCCAGCTCACCTTCTTCCTCTAGGAGGGACCTCGAGAAGCCACGGCATCCAGCTCTCCGCCAAGACTGACTGACG
MSTRG.116737.1	ENSRNOG00000006984;ENSRNOG00000007133;ENSRNOG00000008392;ENSRNOG00000008694;ENSRNOG00000026425;ENSRNOG00000029830;ENSRNOG00000029866;ENSRNOG00000030633;ENSRNOG00000031233;ENSRNOG000	0.703	0.008	0.000	-5.277	down	CTGCGCGGAAGCGGGTCCCCGGCGCGCGCGCGCACTCGCGGCCGCCATTTTGGAGCGATCTCGCCCGCGGCCCCGAGCCGGTCCCGAGCGGGTCCCGAGCCTCCCGCCCCGCTGCGCCCGAGTCCGAAGGCGCCGCGCCGCGGGGCCGGGCCCATGTGCGTGCGGCCCGCTCCAGGCGGTCCGCACTTCACCTCTGGGAGCGTCCTGGCCTCCCTCGGGCCTCGGAGGAGTCGCCGCGACCCCCAGCCTGCGGGGCAGGTAAACCAACCAAAACTCTTTGCAGAATGAAAACACCTTTTTTAAAATTGCATGTATACAAAAGAAAGAGCCCAAATGCTTTTTATTTTGCCTGAATACAAGATTTAAACAAGGTATTGCCATAGAAGTCTCTACCACAGCGCGTTCTGTTGGACTCTTTCACGGAACTATAGGATCACATGGGCAGCACTTGTGTTTGATTCTACCCTCTTTCTTCCGGTCGGTTCTGTCACCCTGGGAAATAGAGAAGCAGAACGTACGGCTCCTTTCTTCATTCACTGCTGGATCAATGTCCAGGGCCAAGGCAGTTTTGTTTGTGTGGATTTCTGTTTACTGCTGCATGGTAGAGGCTGCCGAACCCCATAAGGAAACATTGTTGCGGTTTGCACATCCAGTCTGAGAGACTTCTGCATTGGTAAGTTCATCCCACGTGTGCTCACGGCCTACTAGTACTAGTGTCTGTCATTTTATTTAATGTTTTCTACTTCCTCATTCTTTAGTTTTTATTTTTTCTTTCTTATTTTTGTCGACAAGATTATGGGAGATGTTTCATTTTGGCCTTTGCTTTTGTTTGTTTTATTGCTAGTTTCTTAGTTATGTATTCACTTTGTCATGGATGCTAAGAGATTTAGTAGTAGATATAATTCCACTTACATGATTTTTAAACTTGAAATATCTATTCTCTTTTCCTAATAATATAATCCCTTTTCCTTGCCATATCATCAGGAAGAGAGATGTCTACTAAACATAATTTTGGCCAGATGAATGGTTATGTCCAAAGGAATGAATTTTTAGTTGATAGAATAATAAATGCTATGCGTGTGGTAGTACGCATCTACAGCCCTAGACTTTTGTGGAGGCTGAAGCAGAAGAATTTTGAGTTTGAGGCCAACTTTAGCTATATTGTATAAACCAATTTTTTTTAAAAAGTGAGACCAAAACCAAACAAAGTATAGATTAGTTGAGCTGAGCATGGTGGGCCAGGGGTTACTGTTTTGCAGTTTGGTTTTTTTTTTTTTTTTGGAGCTGGGGACCGAACCCAGGGCCTTGCACTTGCTAGGCAAGCGCTCTGAGCTAAATCCCCAACCCCGATTTTGTTATCTTGACCCTCCCCCAACCCCCTTTCTGTGACAGGCTCTTTGTATGAAGCCTCGTGTGGGCTAGAACTGGCCATGAGGATTAGGCTAGTTTTGGACTAACAGAGACCTGCCCTGCCTGTAATCCTGGCTATCCTGCAACTCACTCTGTAGACTCGAACTCAGAGATCCACCTACCTGCCCCTCTCCCGTGTGCCAGGACTAAAGGCATACAGCACCATTGCCAGGCTCAGTCCATAGTTAGTTCATAGAGTCATCACCTTGACATAAGCTAGAGGCCTTTGAGAAGCCAGACTGATTGGCCTGTGGGCATTTTTTTGATTAGTGTGGGAGGCCCAACACACTGTGAGCTGGTGGTGCTGGGTGCTATAAGAACACAGGTTGAGCAAGCCGTGGGGAGCACGCCGCTAAGGAGTGTCCAGCAAAGGCTTCAGCTCCAGTTCCTGCCTTCAGTTCCTGCCTTGACTTCCTTCTATGATGGATTATGATGTGGAAGTATAGTTGAAATAAACCCTTTCCACTCCCATTGCTTATGTCATGGCTTTGTCACAGCAGTAGAAACCCTAACTAAGATGGATTGGTACCAGGCCATGGGGTATTGCAATGACAGTCCTGGCCATGTGTTTTTGAGAGATTCATGGTAGCATCTGAACTTTGGGCTGTGTTCTAGATGGTTTTGTGTGTTAACTTGACACAAGTTAGAGTTGTCAGAGAGGAAGGAACCTCAAGGATGCCTCTATGAGATCCATCTGTTAGGCATTTTCTTAACTAGTGATCAATAGGAAAGGGCCCAGCATGTGGT

	00031915;<br>ENSRNOG00000058371						GGGTGGTGCCATCCCAAGCCTGGTGTCCTGAGTTCTATAAGAAAGCAAGCCATGATGAGCAGGTCAGG<br>TCCCCTGCAAGAACGAGGGCTATTACTTGGTGAGTCATCTCTCTAACCAGAGACTGACAAGTGTACCCA<br>TCAACAAGGAAGTTAACACGGAGACCTCCTCATTTGCCTTGCATTTGAATATTTTAAATTTCACACATCC<br>TATGTCTGTCCTTGAGTCAATTATTTTCAACTCTGACTATATCAACAAGATTTAAGTTTTTTGGGCTGGAG<br>AGATAGATGGCTCAGCGGTTAAGAGCACTGACTGCTCCTCCAGAGGTCCTGAGTTCAATTCCCAGCAAC<br>ACATGGTGGCTCACAACCATCTGTAATGGGATCTGATGCCCTCTTCTGGTGTGTGTGAAGACAGCTACA<br>GTGGACTTATATACATGAAATAAATCTTTAAAAAAAAAAAAAAAAGCAAGCAAGCCATGGGAAACAGGT<br>CAGGAAGCAGCATCTGTCCATGGCCTCTGCACTAGCTCCCTCCTCTAGGATCCAGCCCTGCTTGAGTTC<br>TTGTTCTGACTTCCTCCAGTGAGGACTACAATCTGGAAGGGTAAGCCAAATAAACCCTTTCCTCTCCAG<br>TTTGCTTTTTGGTCATGGTGCTTCACCACAGCAATAGAAACCCTAAGACAGGCTAGAAAAGCCCTTGGG<br>TGCTCCGTGCTCTGTGGGCTGTTCCCTGGGAACTCGGAAGACAATGGAGAGCACTGTGGCGATGAGGG<br>TGGTTTGTGAAGTTTCAGAAGGAAGCAGAGACGCATGGCCATTTATGCGATATTAGGAGTCTGTGGTCT<br>GGTCAGCTGAGACTGAAGAATTGGCTGTAATTAACAAGACCTTATTTTGAAATGAAACCTTTGTTTGAT<br>GGTACAAATTGATGCTGGTTAGCTGGAGCTGAGAAATTAGTGGTGATTAAGGAGAGACCAGTATCTGGG<br>CAGTGGTGCACACCTTTAATCCCAGCACTCAGGAGGCAGAGGCAGGAGGATCTCTGAGTTCAAGGTCA<br>GCCTGGTCTACAGAGCAAGTTTCAGGACAGCCAAGGCTACACAGAGAAACCCTGTCGCAAAAACCAA<br>ACAGAGAAGAAGGAAAAGGGAGAAGAGACCAGTAGCCTTGAGAATTGTGAATCTGGGAGTTTTTCCT<br>GAGGGTCTTCACTTAGACACTCTGGTGTAGATGGAGCTGAGGCTGTGCCTGGGGCACATAGGAAAACC<br>GTGAGCAAGACGGAGTGAGCCAGTGAGCACTGTGTCAACGTGGCCTCCGCATTCATCCCTGCCTTGTA<br>AAGGACCGGGGCATGGAAGTGTAGTCTGAACAGACCCTCCCTTCCAAGGTCACCTGCGATGACCACGC<br>TTTTCACAGCAGGAGAAACCCTAACTAAGACCTGGTTCTGCAAGGTAAACTGTGACAGCCATGCTACC<br>TCATTCAAGACCTGACTCTCAGCCTAGCCCTTATGTTACCGCCCAGTTCCCTTCTGTCACAGTGTGGTTT<br>TCAGTGCCATATCCCTGGCCTGGCACAGCCTCTGCAGCCTCTACTCTCCTTGCCTCAGGTCTGTTTCACA<br>CTTTCTGACTTCCGTATAAATGGAGCTATTGGCCTTCTTGCCCTGACACTCCAGCGTGTTGTGCTTGTTC<br>TATGCAGGGAGGAAGGGTCTGTTATATGGGGAGGTAACTCACCTGTTAACAGGTATTTGAGCTGCTCCC<br>TGTGTGTAACTACTGTGAACCGGGTGGCTGCCCACCTCCTCTCAGTTTAGATTACACATAGGATTTAATT<br>TTTCTTATGTTGGCCCTGAGAGTAGAACTGGTTGAGTACCTGGTGAGAGTGCGCCTAACTTTATAAAGA<br>GTGGTCAGATTCTTTTCAAAGTAGCTGTGCCACATTGTCATTCCCTTCTTGCTCTGGCCCTTCCTTTCTAT<br>CCAATTCCTGCCTCTGTAGTGCTGAGAAACAAACCTAATGACCTTGTATATGGCTTTACCACTGAGCTAT<br>ACTCCCAGCCTCCAAAGCACTGTCCTTTAGGATTTCAAGTTGTAGATTTTATGACTAACCCTGTGACATA<br>TATCATGTTAATTTGGGGTCTGGTGTGAAGTAAGGGTTGAGCTTCAGTTTTGTTTTTTAAATGTATATTTT<br>GTTTTCTGGCATGATATGTTGAAAAAACTACATATACATACACACATACTTTTTTCCGTTTATTTGATTTTG<br>TACACTTATTGAAGATCAGCTGATATACATTGGTGTCAGAGGCTGCTGGGTCAGGTCTTCACAGGTAGCC<br>CAGAGTCACAAACACACATTTAAGAACTGCAGCCAGTCTGTTTTTTCATTTAGGTTTATTTTAGCACCAG<br>GAGAGGTGGCACACATCGTTACTCTCAGCATTTGGGAGGAAGAGGCAGGTGTCCCTCTGTGAGTTTGA<br>GGTCAGTCTGGTCTACATAGTGAGTTTCAGGCCAGCTAGAGCCACACAGTAAAAGCCTGTCTCAAATTA<br>TATATGTTTTTGGCCTATGCATGTCTGTATACCATATGTGTACAGTGGCCACACAGGTCAGAAAATGGCAT<br>TATATCCCCCTGGGACTGGAATTACAGACTGTTGTGAGTGATAGAAACCAATCCCCAGAACCTCTGAAT<br>CAACAGCCAGGACTCTTAACTTCTGAGCCATCTCTTTAACCCCAGTCTTTTAGATTTTTTAAAGTAAAAA<br>TACTATGTAAGATAAGCCCCTCAGATTATTACAGTTTTGTAAACAACTTCATATGGTATTTAATTTAAACC<br>AGTGAGATGGCTTGACCTAAATTCAGGCCATGGGGCCCACATGATAGAAAATAAGAACTGACTCCCACA

							AGTTGTCCTCTGACCTGTGCACGTGTATGCTCTCCTGGGTGAGTGTGTGCATGCATGTGCACTTACACATAAATAAATGTGAAAATAATGTAAAAGACCTGTTCTTTGAAGACAAGCTTTAGCTGTATTTCTTCTTTGTTGTCAGAACAGTAAAAACTGTTATCTGACTTGCTGGCCCACACACTGACAGTGAGCCTCGCCTCATCTAGGATGGACTGATGACACAAGCCGGAAGAAGGGTTGGGGAGGCAGAGCAGGGTGGGGCTGGTGCTGGTCTTCTGATTGCTGCGGTGAGAGTTCATGCACAGTTTGTATACGTGTGAAGGAGAGAGTTGTCCCCTCTATAACCCTCGATGTTACTCAGCAAGATTGCCACAGTAAACTGAGGGGCTAGATGGCAGATGACCACAGCAAACGGCAGGATCAAGCAAAATGTGCTGTCTTTTGTTTGGCTTGTTAGATATAGGCTCTGAGAGTATTCCAGGAATACATGTCCTGTGTTCACTCAGGAGGGATTAGACCATGAGAGGCTGGGTTTCTGGTTTGAATTAACCTTTTCAGGATGGAGAGGCATTCCTGGTGCTGATACTTGGGAGACAGATGTGTGGGCCTCTCAGAGCCATGATCAAAAGGAGAGCCTCTAGAGACTTAAGCACTGTAGGTGGGGGGAAGGTAGGTCACATAAGGGGACCCCAGAGTCACAG
MSTRG.117026.1	ENSRNOG00000022507	0.000	0.377	0.007	3.835	up	CTCCCATTTAATAATTGATTTTGATGCTGAAATTAATTCACAGCCAGTCAGCCGTTCTCCTATTCTTCTTAGCGGCCGTGGGTATTAGCCAAAAGCTGTTCTTACGCCGAGGCAAGGAACTGGTTTTGTTAATTTCATGTCAGGAAAAAACATGTTGTAAGGGCTGGAGCTATGGCTCAGTGGTTAGGCACTCGCTGCTCTTGCAGAGGACCTGGGTTCAGGCGGCTCCCAGTTGTCTGTAACCCCAATTCCAGAGGATCTGAGGACCCCTTCTGACCTCCGTTGATACCTCAGGTGGTTCTGCTTGGCTTCTATCAGTTACCCAGGAGCATTTGCAGTGGAATTTCTTCCCCAAACACGTTCTTCAATCTCTTTCTTATTTTTCTGCCTGG
MSTRG.117106.1	ENSRNOG00000006305	0.000	0.372	0.031	3.453	up	GCACGAGGACACACGTAATTGGATCCCTTAGGAACGTGGTTTTCTGACTTGGAGAATGGACGTCACGCTCTACAGTGGTGGTGTAAGGATGAAAGGGAGCCCTGAGCATGAAACCTTCGAGTATTAATCAAGGAACTGCTATGTATGGCTTTCTATACTCCATGAGAGGACAGTTGTGAGCCTGGGGACAGATCCAGTGACCCGGGATTGCAGCTCCTACCCGTGTCCCTCTGGGGACCATCACCGTGGTCTGAAGGTGTGTGAAGAAGACTTTGTTCCACCAGACGTCAGCTGGC
MSTRG.117291.1	ENSRNOG00000021438;ENSRNOG00000052880;ENSRNOG00000053468;ENSRNOG00000053675;ENSRNOG00000059545;ENSRNOG000	0.559	0.000	0.001	-4.627	down	GACTGAACCCAGGACCTTGCGCTTCCTAGGCAAGCGCTCTTCCACTGAGCTAAATCCCCAACCCCCTATTTACGTTTTTGATTTGTATTTTAAACGGCCTCTCCGATCTGGGTGATTGATCTAACATATCGAAAAACGGATTTCACAGGGAAGAGAGATGTATTTTTTTTCATTCGGTTAAATATATTTCTTAAAAATTAATAGATGAACCAGATTATTGTTGCTTAAAAAGATACATTTGTACAATGTAAAGTTTCAAATCAGTGAAGACTGTCTGTCAAGCTATTATATCAGATTCCAATGCTTTATTCATTTAACTTGGCATCGTGTGGGCATTTAGTCATGAACAAATGTTCTCAGGGTGGACATTTGGGTATCTTACAAAGATAAAATTAAAAATCAGGTGAGGGTGAAGGACTGGGGTGGGGCAAGAGAGAGCAGCCAGGCGGGGACCCACAAGTTCCAGTGGCCTGAGCGATTGTCCATTTAATCCTCACAGCACTTTCAGGAACCTGTTTCTTTCTATTCTCTCAACTCAGTAGATAAGGAAATTAGTTCAACAACACAAAGGAACAGAGTCAGGCCTCCCCCAGTCCGGGCTGGTCCCGTTCCAAGAGCCACACTTGAGAACCTTTCCGATTGACTTAAGAAACATTAAATATTAAAAACCATTGAATTCACGATGAGACGTTAAGCCGAGCCAACATCTATCTGTCTCTATGGACACAGACGGTGGCTTTAGCCTGGTGTGTTCACGCCACTCCCATTACAGCTCGTGGTTAATCTTCT

	00060020;ENSRNOG00000060703;ENSRNOG00000060728;ENSRNOG00000061607						
MSTRG.117351.1	ENSRNOG00000049033;ENSRNOG00000052990;ENSRNOG00000053181;ENSRNOG00000056457;ENSRNOG00000059765;ENSRNOG00000061425;ENSRNOG00000061572	0.779	0.000	0.000	-5.283	down	GTTTTCTGTTTGTTGTTTGTTTTGTGTTGAGACAGGGTTTCTCTGTGTAGCCTCGGCTGTCCTGGAACAGCTCTGTAAACCAGGCTGGCCTCGAACTCACAGAGATCCTCCTACCTCCCAAACGCTAGGATTAAAGGTATGAGCTGCCAGCACCCTGCACTCTGGGTGTTAACCTTTAGATCGGAGCTTGCCTTGAGGGCCACACAACTAAAGAAATTTGGGTGGTAGTCTGGTGTGGTGGTTCCACCTCTAATTCCAGCGCATAGGAGATGGAGCAGCTGTTTTCCATCACACAGTGAGTTTAACGCTAGACCAGGCTATTCGAGACCCTACATACTTGATCAGGTAAAAACACTGATCCACCTGGAAAAGAACAAATTCAGGAATCCTCTGTTCCCTAGGCAGTGGACCAGCAAAGTAATCTAAAGCAACAGATATCCGGGTCTCTTTGGAGAAGATTCAAGGTAAAAGAATTCATTAAAATCTGAAGTTAAAGGTCTGAGGTGAAGGGCTAAGTTCCCGTGCCGGGGAGCCCTTAAGCCATTATTTATAAGATGAGGCCCTGGCTACCAGCCAGGAGACCCGAATCCAGGATGGCTTTTCTCTTATCTCGATTTGTCTTTGCCACGACATTCACCTCCTTTGAGTTCAGCTTTTCCGTTTGTAAAACGAACAAACAGCCTGTTTCCCTCTTTAGCATCCGGAGTCTGATCCGTAGGAAAACAAAAAGCAAACCAACCAGAATGCTTTGGAGAGGTAAACGCACTGGGCCGAGGTGAAGCATCACTCCAGCCGCCTGCGGGGTGGGCTGGGCTCTTTGCAAGGCCTTGACGTCCTCCAACCGCCCGGGGGCGCTCGCTCCCGGCACTTCTTTATCAGCACACCGCCTCTCTATGGTGCTCCCTCCTGGCCC
MSTRG.117376.1	ENSRNOG00000055307;ENSRNOG000	0.456	0.035	0.035	-2.552	down	GTTGCATTGTTTTTGGAAATTGAAGATTCCTGTTGTTTTTTTAGGTTTAATTTTTGTTTGATTTTTTGTTTCGACAGGGTTTCACTGTGTAGCCCTGGCTGTCCTGGGGCTCATTCTGTAGACCAGGCTGACCTTGAACTCAGAAACATCCACCTGCCTCTGCCTTATGCTTGCTGGGTTTACAAGCCTGCATCACCGCCCCTAGCCATTTCAGAGTTGTGCAGACTCCAACTGCACCCAATTGGAAGAAATGCCACCTCTGAGCCACCACCCCTGACTGAGCTCACCAAGCTTCTGTAAGCACTCAGTACTGTGTTGCTGTGGCTTTGCTGTTTGAATTCTTCAGGTAAGTGGGCTCCTGCAGTGCTGTGTGCCCTTCTGCTCAGTCACAGGAGTGAGGTCGGAGCAGAGCAGTTA

	00057943						TGGAGAATTCTTGTATTACTGTGGAGTGTTGTTACTGTGAATTGAAAATGCAGACTTCCCAGATGACTGATACTTAACTGTACTTTAAAGTCGACATGCTAGAGTCGTGGAGGATGCAGGTTTTCCTAACTTGCATGGCGCATCACTCTGATGATCTTGTAGGATGTATGACAGCTATGAAGACTAACTGTGCCGCTGTTGTTCACTGGAAGTGAGGCTTGAAGAGGAGCTGCTCTGGGCATTTTCATGGGCACCTTGGGCCTTCCAGAGCTTTGGTCTCTGAAATCAGTTCACTTGCTTCTCTTCAGAATTTGATTTAAAAACTTCTTAAATTCCTTGTGTACATAGGACAACACATGTTCATTGTAGAAAATGTAAGAGTCATAGATAAAGCAAGGTGACAAGCTCTTCACACACTGAGGTGACAGACAGTTGTTGGCATCCCCTTCTGGTCCTTCCTGGCAAGTGTCACTTGAGCTCCTCAGGAGCTGTGACTGGAGCCACAGGTCACTGTACTGGCTTTGGCATATATTTTGACATCCCTTTTGTCTTTATTTTTTAGGTGAAACATAGTTTCACTGAGTAGGCTAGGTTGGCTCCAAACTCATGATTCTCCTGCCTCAGCCTCCTGATTCTGTATCTTTTAGAGCCATCTGATTCCTTTCCTCATCTGTCTTATAGCACTTCCCATACCTTCTGTGCCACTCATCTGCCTTGGGTAGGCTGTGAGCTCACTGGGCAGTGCTGCCTTTTGTCTTTCTCCATGTGTCCCATGCTGTTACAGCTTGCTTACCTTTGGGAGCCATGTGCATTCTTGATAGCTCTTGTACCAGTGTGTCAGAACCAGCGATTTATTTGCAG
MSTRG.117504.3	ENSRNOG00000009779;ENSRNOG00000010103;ENSRNOG00000010588;ENSRNOG00000010793;ENSRNOG00000010977;ENSRNOG00000047393;ENSRNOG00000053769	0.666	0.072	0.037	-2.239	down	CCAGTCATGCATCGGGGCCGAGCTGAGCCAAGAGTGCTAACAAAAAGGGTCTTGTCCCTGCACCATTGTCTGGAGATCAAGCCTTCCAGCGGCCCAGGCCTGGGCCTGGACAAGGGACAACATCTACAGGAAGAGTGTTGCTCTGCCCAGGGCCCTGGTGACAGACCAGCGGTGTTAATATGAAGGAAGAAACAGGGAGACAGAAGAGAAGATCAACCATCTGAGAGAGCCAGGCCCAGAACCAGATCTTGTCTGGGGAACAGAGGAAGTCTGAAGCTTCGCTTTGGATAGCTGTTCGAACTGTCTCTTCGGACATTTCATTCCAAACCTGCTTATTGTTTTGTCCTCACAGCTTCATCAGCAGTCTCCACTTTACTGATGGCAGTTGAGGTAGTGGCCTGACTCCAGCAGTCCCTACCTCTCTCTGCCCCCTGGTGACCTTCTAGCTGCAAACGCAGCGGCTTCTTTCTAACCAGCAGCCGCGGTGCTGTGCTCTGATTTCCTGGTTTACAGCGCCACCGTTTGGACAGTTTTAACTAAGAGGCGATCGCTCCGTCTCGCGTTCATCGGCAATCTTCCACTGCGAGTATGCTGAATGTGGGTAATGTCAAATGCTGAATGTCAATAGGTTTAGCCCCCACCAGAGCTTCATCCATGCTGGGTTATCATGAAGGGCTGTGCTAGAGATGTGAATAGGAACCAAGGAACTGGCTACGACTCCCAATCTCTGTCATCTCACTTGTGCCCAAACTATCCCAGGTTCTCTGTTCCCTGTCCCCTCCACCACCTCCCACCTTCCTTCAGCACTAGACACATCCTGTATGTCCACCTTGCTACTTCCCGGTCCACCTCAGCCCTCCTACCTCAGCTTCTGACTGCTGCCGAGCCATCGCCACACAAAGAATAAATGTTTATTACATTTTAGTTTTATGGGTAGGAGTGTCTGTGTGAGCCAAGCATGTGCCTGGTACCCGCAGAGTCGTTATAGACAGTGGTAAGCTGCCTTGTGGAGCCCAGGTCCTCTGCAAGAGCAGCTGAGCCACCTTGCCAAACCCTTATTTGTTCTTTATTACAAAATCAGGGGCCAGGACCAAGTGCACACCTTTAACCCCAAGCACTCAAGAAGCAGAGGCAGGAGCATCTCCGTGAGTTTAAGGCCAGCCTGATCTACAGAGTGAGTTCCAAGATAGCCAATACTAAATAGAGAGATGAGAGAGGCTATTTCATACAAACAAAACAAAAAAGCAGAGACAGGGGGATGGCTCGGCTGGCAAAGGCATTGACCACGAAGCCTGATGGCCTAAGTTCAACCCTTAGGACTTACACAACAGAAGGGGAAAACCAACTCCTGAAAGTTGTTCCAGGACTTTCACATGTGCACATAAAAAAGTATTGTTAAGACAGAATCTCACGGGGCTGGAGAGATGGCTCAGCGGTTAAGAGCACTGACTGTTCCAGCAACCAGTTCACAAGGATGTGTAATGAGATCTGATGCCCTCTTCTGGTGTGTCTGAAGACAGCTACAGTGTACTTAACTTATATAAATAAATCTTTTTTAAAAAAGACAAAAGCAGGACATGTTCACCGTGGAAACTCTGAAACACAGATGAGCAGAAATAGAGAACTCAAGGACCTCCAGGGTGCTGTCACCTAGAGAGAAATGTGGCTTCTTCGTTGTGTTTTGTTTTTTCTGACACCAGGTCTCACTATGTAGCTCTGGCTGACCTGGAACTCACTATGTAGACCAGTCTGGCCTTGAACTCACAGAGATCCACCTGTCCCTGCTTTCCAAGTGCTGGGAATAAAATCATACACCACCCCACCTAGCAGGAAGAAGCATTGTTAAGACCGTGCTATCTGGGGGCTGGGGATTTAGCTCAGTGGTTAGAGCGCTTACCTAGGA

MSTRG.117504.4	ENSRNOG00000009779;ENSRNOG00000010103;ENSRNOG00000010588;ENSRNOG00000010793;ENSRNOG00000010977;ENSRNOG00000047393;ENSRNOG00000053769	0.371	0.023	0.007	-2.956	down	CCAGTCATGCATCGGGGCCGAGCTGAGCCAAGAGTGCTAACAAAAAGGGTCTTGTCCCTGCACCATTGTCTGGAGATCAAGCCTTCCAGCGGCCCAGGCCTGGGCCTGGACAAGGGACAACATCTACAGGAAGAGTGTTGCTCTGCCCAGGTGAGCTCTCAAAGCCCTGCTCTCCATGCGGCACTGTCTTCAGCTCAGGCCCAGTCACAGGGCGCTCCAGAGGGCTTTAAAGCCTGCCTTTGGTTGGTCAACCCAACACTGATGGAGAGATGTAAACTTGTGGTTCATGAGCCACAGACAGGCCAAATGACCAGCCCTGAAAGCACCAGCCTACAGGGTAGGGCCCTGGTGACAGACCAGCGGTGTTAATATGAAGGAAGAAACAGGGAGACAGAAGAGAAGATCAACCATCTGAGAGAGCCAGGCCCAGAACCAGATCTTGTCTGGGGAACAGAGGAAGTCTGAAGCTTCGCTTTGGATAGCTGTTCGAACTGTCTCTTCGGACATTTCATTCCAAACCTGCTTATTGTTTTGTCCTCACAGCTTCATCAGCAGTCTCCACTTTACTGATGGCAGTTGAGGTAGTGGCCTGACTCCAGCAGTCCCTACCTCTCTCTGCCCCCTGGTGACCTTCTAGCTGCAAACGCAGCGGCTTCTTTCTAACCAGCAGCCGCGGTGCTGTGCTCTGATTTCCTGGTTTACAGCGCCACCGTTTGGACAGTTTTAACTAAGAGGCGATCGCTCCGTCTCGCGTTCATCGGCAATCTTCCACTGCGAGTATGCTGAATGTGGGTAATGTCAAATGCTGAATGTCAATAGGTTTAGCCCCCACCAGAGCTTCATCCATGCTGGGTTATCATGAAGGGCTGTGCTAGAGATGTGAATAGGAACCAAGGAACTGGCTACGACTCCCAATCTCTGTCATCTCACTTGTGCCCAAACTATCCCAGGTTCTCTGTTCCCTGTCCCCTCCACCACCTCCCACCTTCCTTCAGCACTAGACACATCCTGTATGTCCACCTTGCTACTTCCCGGTCCACCTCAGCCCTCCTACCTCAGCTTCTGACTGCTGCCGAGCCATCGCCACACAAAGAATAAATGTTTATTACATTTTAGTTTTATGGGTAGGAGTGTCTGTGTGAGCCAAGCATGTGCCTGGTACCCGCAGAGTCGTTATAGACAGTGGTAAGCTGCCTTGTGGAGCCCAGGTCCTCTGCAAGAGCAGCTGAGCCACCTTGCCAAACCCTTATTTGTTCTTTATTACAAAATCAGGGGCCAGGACCAAGTGCACACCTTTAACCCCAAGCACTCAAGAAGCAGAGGCAGGAGCATCTCCGTGAGTTTAAGGCCAGCCTGATCTACAGAGTGAGTTCCAAGATAGCCAATACTAAATAGAGAGATGAGAGAGGCTATTTCATACAAACAAAACAAAAAAGCAGAGACAGGGGGATGGCTCGGCTGGCAAAGGCATTGACCACGAAGCCTGATGGCCTAAGTTCAACCCTTAGGACTTACACAACAGAAGGGGAAAACCAACTCCTGAAAGTTGTTCCAGGACTTTCACATGTGCACATAAAAAAGTATTGTTAAGACAGAATCTCACGGGGCTGGAGAGATGGCTCAGCGGTTAAGAGCACTGACTGTTCCAGCAACCAGTTCACAAGGATGTGTAATGAGATCTGATGCCCTCTTCTGGTGTGTCTGAAGACAGCTACAGTGTACTTAACTTATATAAATAAATCTTTTTTAAAAAAGACAAAAGCAGGACATGTTCACCGTGGAAACTCTGAAACACAGATGAGCAGAAATAGAGAACTCAAGGACCTCCAGGGTGCTGTCACCTAGAGAGAAATGTGGCTTCTTCGTTGTGTTTTGTTTTTTCTGACACCAGGTCTCACTATGTAGCTCTGGCTGACCTGGAACTCACTATGTAGACCAGTCTGGCCTTGAACTCACAGAGATCCACCTGTCCCTGCTTTCCAAGTGCTGGGAATAAAATCATACACCACCCCACCTAGCAGGAAGAAGCATTGTTAAGACCGTGCTATCTGGGGGCTGGGGATTTAGCTCAGTGGTTAGAGCGCTTACCTAGGA
MSTRG.117638.1	--	0.374	0.000	0.013	-4.001	down	GTTAAGAGTAAGAAAAGGCTGTTAAGAAGCTAATTTTAATAATATATACTTCCCTCCTTCCTCCTTCTATCTTTCTCTCTCTATTTTTCTGATCCCTGAACCAAACTCTCATGTCACAAATATGCTCCATGAATCTATCCCAATATATTCTTACTGAACATATTTTGAATGCAATTTTATATTAAATTAATTGGAAATTACATCTCTTAATTCCAAACTACTATGGGTAAAAAGTAGTTTTGGATGGTGATGTGTGGGTATCCTAAAATCACATTCAGTATCTGTCCCCACAAAGTTTCCTGTATAGTGATGATTATTTACGAATGGTGTTTCTAAGAACTGTCCAGGCAAACATGGGAAGAATTGCACAGGTATATAAATTGCTTCATGTCCTAAATCTGCAGTATTAACATGTTGATAGAAAAATCTCTCTCTCTCTCTCTCTCTCTCTCTCTCTCTCTCTCTCTCTCTCTCTTTCTCTCTCTCTCTTTCCAACACACGCACGCACACACACACACACACACACACACACACACATGTTTAAGATTGTGTCGTTATATTTCTGTTTTAAATATTAAACCAAATTTCTTATCATGAAGAAAAATTACTCATACTTTAGGATTTATTAAAGATATAGGGGAAATGCTTAAAGGTTCCTAAATTCTCACCAGTAAGGTGAATATTTTATCAGTTAATGGGAAGTTAT

							GCACGCAAAAGAATTGGAATATTTTTTAAATGGAACATTTAAAATTAAAATACTGC
MSTRG.117649.1	--	0.000	0.441	0.031	3.453	up	ATTTAGACTCTCTGTTTATAATGCCTTCAGGATTCTGCACTTCTCCCCCTTGCTGCATGAGGAAGCCTCTCTAACGATGTCTGGACATTGCACCATTTTAGATACCAGAGGTCCAATTTTCTTAGAAACATAAAAAACTGATCTCATTATCATCACAAGTATTGCAGAAAGAATTTTATTCAATACATTCCTTCAAAAATTCAGATGACTCTCTGCTCCTAGACCCG
MSTRG.117719.1	ENSRNOG00000022245;ENSRNOG00000058463	0.000	0.794	0.000	10.443	up	CTGACAAGGGTTATTCTTCAAGGATTACTGTGATTACAGTGAGGCTGTATGCAAGGCATTTGGATATATATATATATATATATATATATATATATATGAATTTTTTATTCTCTCTCTCTCTGTGTGTGTGCGCGCGCACGCACATGCATGCGTGGATCTGGATGCCTGTGCAAGCAAAGCCTAGAGGAGACCATTGGATGTCTTCTTCTGTCACTGTCTTCCTTATTTCTTGAGACAGGGTTACTTACTTATCCTGGATCTTGGCTGGCTCCAACAAGCCCTAATATCCTCCTATACCTGTCCCTCAACAGTGTGAGAGATGACCATGCCAATATATGTGTGTGTGTGTGTGTGTGTGTGTGTGTGTGTGTGTGTGTGTGTGTGTGTGAATTGATTTCAGGTCCTCGTGCTTCAACAAACACTCTTATCTACTCAACCATCTCTTTAGGTCCTTAATTATGTCAATGTTGAATATTTCAAGGTTAATCTTCACCAGTATTTAGTAGTTAGATGTTTTCTTCAACAAAAATGCAAGAAACTTTATGCATTAACTGGTCGATGACTACTGGTGGTTCCTAGAATTTGGAAAGTTGGTAATGCATAAAGACTCAAATATCTGAATTAAACTCTAGAATCACCTGTTTGGAGATTAATTTGTTACTTCTTGTATGCATGTCTCTCAGAAGAAGATATAAAAACAAATTGATAGCATGCTTGACTGCACAGTTGCAGTAGAGAAATTTTGAGTCTGTTATTTGCCTGGAAATGATTTCCAAGGCTCTACATTAATTTTTGATCATTACATAATCATCATAAAAGAGATTTTTAAAAATAGCTGCATGAAAAGTTGATGGAAAATTAAAAAACATTGAAAAATACTTTCTGATGCACTTTAGTAAATTAGAAAATATCAAGGAAGCTATCTCAATTAAATACTGTTAATTTCATTTTATTAGTGGGTCCTCTTTGAGACTTTAAGGTCTACAGTGTCCATCTTCTCTACCTCACTGTTTGTGCTGTTTGCAAGTGCTAGAAAAAGGAGCATTGTATGATGACACAACCAGATGCCTTGTAAGATGTTTCATGAGCTACTTCAAGTTTTGTTGATTTTCAAACTTAGGTCAGTATTAGTCTAAAGTAAAGTTCAAAGAACTACAATCTGATTTCTGTCAATCACTGCATGTGTGGTGCTCTGCAACACAAAACCATGTATACCTAAGCCTTTCTGGTAAAGATGAGCTGTGCAGACTCTTCTCTGTTCTAGACTTCTCATTCTTCTGCCTTCCAGACTCTGCTCCTTATTCTTTCTAGTATGATCTGGAATTATGATTCAGCCCATGAACCACAGATCATATTTTCTTTTACATCCTTGTCTGTTTCCTAAACCTTCCAAGAAGTTCTTTAAGTACTTAGAATCTTGAGAATTTTTTTAGTCAATATGTGGGCTAGTGGTTATCTTTTCTTTCTTCACTTCACAGATTTCATTGTGGCCTAATGACAGTTAGGTGACTTGTGCAGCAAAGCCCAGCTGGATAGGTGTCATCTGCCTTCTAGTCCAGTGCTCTTCTCCAAGTGCTTTGATTTTCTATTCCTTGTCCAAGCCCATCCCTGGTTGATAATGCTGTTTCTATCCTGCTGTGTCTCTCTCAGGAACTTATCCTTGTCCTGAGAAGGTTGCCAGTCTTGGTAGGAATTTTCCCTGACTCACTTCTATCCACACTGCAGGACTGAGTTGGTATCTCTGACGTTACTGACTCTAAAATGCAAAAGGTGGGTCTTGTCAATTCAACATCTACTTTCTAACTATGGACAGAATAGGTGCAGGTAATCTTAAGAAACTCAGTAAACTTTATGGTGGTAATTTCAGTACATGATAACATAGGCCATTTTCCTAAGCTTAGAATAAGATGCATATTTGAATACATACTATGTTCTTGGTGGTCTAATCTTTAGGTTTTAGATCCCTAAGAATATCATCGCACGGAAACCTGATCTTTTATATTAGATCAGAAAATTTACTCCTAAGAAATAGCTGGATAAAACAACATACAATAAAACAAAAAGACCCAGAGTGTAATATTTGAATTTTCACTTCTTTTCATTTTCTTTGTCAACCATCTTTAAGACCTGCTATTCTGAAAAGGGCCCCTGAATTTGTTTCAGTTTTTGTAGTATTGGCTTTTATCACCTAGCTCCAGGTCTATTGGCTGCCTCCCTCTCTCAGTACCCAAAGCCACATAAACTGTTCTCTGTAACAAAATAGCACATCAACAAACAACTTTCAGGGTTTGTACTCAACCTCAAGGTTTTATTGTAACTTTTCTGAAATTCAAGGTCTATGTGTGCCTTTAAAGTATAACGTTTTTATCTTATGGTCATACTGACTCTATTAAGTGAGAAAGGACAGGTCTGAAGCTGAAGGCTACTCACTGGCAATACCTAATCAATGCTAAAGTCCACATTCTACTACTACTACACATTCTAATCTACTACACAAACCAATGCTCTATGCACATTCTCTCTGACTTTAGCCATGGTAGTT

							TCCTCATAGCTCTAAAGGTATTTTGCTTAGAATTTACTACAAATCCAATAGACCCTTACTCAATATTTTTTT<br>TACCCTTTCTCATATTTTGTATATCTTATGTTTGCCCATAATCACTACTTAGTCATTCAACCAATATTCGCTG<br>CTTCTCTGGGCCTCTTTATAAGCCTTCTCTCAGATACGTCTATGTGAAACAGACAATGGATCCATCATAA<br>GTTTGTGTATAATATAGTATGTGTTTAGGTTCCATTTGCTCAGAGAGCAGAAACTATATCTTAGATGTCAA<br>ACAAGACATACATGTGGTACTCAGTAACTGGTACTACACACCCAAGTAAAGCACTGAACTATTAACCTA<br>TCTATAAGAATAATGTGAAATAATTTATCAACAAAATAATCTAAGAAGGTGCTTCATAGGGATTCCAGCTA<br>AAGGGTTCTTTTATGTACTTTGTATCTCCAGTAGGAAAGAACACTGATAAAATCAGATACAATTGATGTT<br>TTAATTTCAAGGACAATATAATATTACCATTTAAACTTTAAAAAGGAAAGATCTAGATAAGGTGCATTTGC<br>AAATAACTTTCCATATTGCTACATTTAGCCTGTAAAACACCTTTAGATAAGAAGATTGTAACTAAATTTAC<br>TATTTTGAATAGTTAATACTGTTCACCAAGAAATTAATTGCCATATCTATCAATAAAGATTAAATGCCAGC<br>TGCACATCTCATTTGATTTCATTAAAATTTATTTCTCTACATATTGTATACATATATTTAGCTTACTAAATGT<br>GTCTAGCTCAGTTTCATTTTCAAGTATTTAGAATTTCCATAAAATGTTTGGGTAAGGACAGTTTCAACTTC<br>TCTCTCTAGGTTAAGATCTTAGGTTCCTTACCTGTAGGCAAGTATTTGGAGCTTATTCATACAAAGTAATA<br>CTTGTGTTAATAGATTTTGTCTCATTTTCTTATTGCCTTTTGGTTAGGCTGGGAGAATCGTAAAAAATAGA<br>AAAAGAAACTTGAGAAGTAAAGTATGAAAGAAAGACAGTGTCTGAATTTTCCACAGAGTTCAGGGCTA<br>TGTAGTAGGCTCAGAAAGGGAAGAATATAAGAATTTCTGGTCAAAGACTTCTAAGTCTATTCCATAGTTC<br>TTAGAGATACTGAGTTTACACTGCTCTACATAAAGGTAATAATATGCTTATCTTAGCTTTTTTATAAATTAA<br>ATATTTTCTGTCTCTGAATGATGTTTGTGTAAGTGTTCGTATATGCAAATACAGGTGTATAGTGGTGCAGT<br>GTGTGTGGAGATAAAGAACAAGTTTGTGTGATGAGCCTCCTCTTCCACCATGCTTGAAACAATGTCTTC<br>TGTTCATAGCTCTGTAAGCAAGGGTAGCTGGCCCTTAAATTAGAAATTCAGTTTTCTTCACAGGTTCTGA<br>GATCCTGAATTCTGCTCATCCGGACATTTTGACAAATATTTGTAAAAGGAATCATTGTCTTTCTGGTCCAT<br>TACCTGCTTATCTTCAAGATAATGGCATTTACAGTACAAATTGTACCCATTAATTATTGTTAATTCAGTTTA<br>AATGACAAAAGTGAAATTCTGTTCTTTGTATTAATTTGTTGTGTTTGTGAGTATATGGTGTTGAGGGGGC<br>AGGGCATGTGTGCCATAGCATGCATGTGAAGGTCAGGAAAACAGCTTTGAATCTTCCCTTGGGAACTCT<br>TGAATTAAAAAAGAACATGAAGAAGACACCAAAGGTGGGGAATATTTGATGACTTCAAAGACAATTGA<br>ATAAATGGATAAAAATGTGAATGTATGATAGAAATAATTGAAGTAAATATCAAGAGGCCTATAGGAAATG<br>GTGAATCAGTATCCAGAAATTGTGCTAAAAAATATGCTCCTTTCCTATAATTTATCTTGCTGTATTTTCCAT<br>ATAGTATATTTTTAAAAAAATCACTGTCTATCTTGCCTCAGCTTGAAAGTTTAGTCTGATTTTATTTCATAA<br>TTAGACATATTAAATTTATAACAACACCATTTTTTATTAAAATTTTGCTTGATAACAATGTATTTTATGAGA<br>AACATTTTGAAGTTTTGTTTAATACTTTTGCATTTTCTTACCCAGTATGGTTTGATTAAAAATAATCATAAA<br>TAGTCATATGCAGAGAAATGAACAGCTAAAGAATTTAAATGTCAATGGAAGACTGGTACAAATATTTAAT<br>GAGGATGGAAAAAATGAATGTAATAGCATACAACAGTTATGACTTGTGTCAATGTTTTTCTTTATTTCTG<br>CTATAAAATTTGTAAGTGGATGTTGAAATAACTACTATGCATTGGTAGCAAGCTGGTTTCCTAATGCTGGT<br>GGGTTAAAAAATGTAAATGTATAAAGAAAAAAGGATTAACAAATAGGTGGGGGAGGGGTTTCCTTCAA<br>ATGGATGGAACCCATTCTTTTGAAAGAAAACTCCCCTCTTACCTTAATCCTGAAGCTGGTTAGTTCTCCT<br>CTGTCCTCCTCCTAGGGGAGAAATACCCAAAGTTAAACAATAATAGAACATGGTCTTGTATTAAAGTCA<br>AGTTCAGTTCTGGCCAATCCTTTCTTCAAGTCTCACATGATTCTACCAACTGTCTGATATGATGGGGCTT<br>CCACAGAGTTGAAATGAAACCCGTTTTAAGTATTAGGCCAAAGGGAGTGGGCAATAACTGTGTAGGAT<br>GTAAACTATGGTGTCTCCTGCTGAATTAGCCTGTCTTGTCAGAGAATCCGAATTAATTTCAGGCTTTCTT<br>AGAGCAGGAAAATTCATGAAATAGCACTACTTGGGCAATTAATCAGTAGAAGTGATAAGGTAAGGACT<br>TCAAGAGTTCCCATAGAGAACTCTTCCCATGTAGGTGTCCTAAAATTCCTCTTGCCTTTGGAAATAAAAT

							TATATTGATATGGGCAACAATGTTTGAGAAGATGTTTAATTTTTATAAAAATTGAAAATAACATGTTATGG<br>GAGGATTAGGAAAAAGTCTCACCTGAAACAAGAAGCCACAGGTGAGATTTAATTTTTTAAAATGCTTTC<br>TTTTATCTACCAAGAAGGCTAAGATCTCAGTTCATGCAAGACCAAGGAACTATAGCCATTATCAGATGGA<br>TTTTTCTTCCTTAAAGTATCTGTGATTGAATAGGACACCTGGTGTCTTTGGAAAGACAGATAGAAAGAA<br>GACTAAATTTGAGTATTCAATACTAAAGGCCCTAATAACACCTCTGTCGCCCAGCAGCTAGATAACATGT<br>GTTTCCATAGAGTCATCTGTCTGGATGGATATGACAGCAATTCCAATTCATTGTTGGGATAAGGTCTGAG<br>AATGTATTCATTTACATGGGCTTTGTATATGACAGCTATTATTAAATTGTTAATTACTTCACATTACATAGTC<br>TCTGACATTTTTTCTTATTTGAATTATTTTTGTTTAGGGAATTGTCTAGTAAACTGATCTTAAACGTCAGTT<br>TACAGCTACCCTTTGGCATAATTTGAGAGAGGGTGGCTGATTGAGTCCCATACAAAATCAATACCAGAC<br>AATTGATTGAATTCTGTGAGACATGAAGGAGAGTAGAGACCAAGAATGAGCCTGAACCCATACAAGAT<br>CATGTGCTATTGTTATTTAACTTTGGGTAACTTATTAAAAAACTTCATCAAGTAAAATGACATTAGGTGAG<br>CAAATGGATGCATGCTGGAAACTCAGTTTGTCCCAGTATGTTACAGAGACAGCAAAATGAGGGACATTG<br>TTTGCAGTACCCTAAAGGTCATTAAAAAATGTGCAGGAGGAGAAAATGTTATGGGAAACTGTGATACAT<br>CTGCAAATCTTGTCTTCCTCCGTCACTCCATCTGGTTTGCATTGGAGTCACCCTATGAACTTTAATGACCT<br>TAAAGTTGCATGACATAAACTTGATATAATAACTTCTTCAGCTGCAGCATAATGTTCTCCTTTATCAGTGG<br>TGAATATTCTAAAAGTTGCTATCTTGACAATTAATACTCTACCATCTAAACATTATAAAATCACCATACTAT<br>TTTTCTGAAACAAATACAGCCAAATATTGTAAACCATTCTTAAAATTTAAAAAATGGCTTATTTTATCTTA<br>TAAAGTAACTATTTTGTTCATGGTTGTTACAGTTTAGAACCAAACTCTTCGTGTTTGGAAATCATGTAGA<br>AGTCAATGAAAGAAACAAACTGTAGATATATTGTCTGGGTTGAGTCAGATTAGAGTGACCACGCAGCAT<br>CTACACTTCAGGGATAAGTGGTGGCTACTCGAGATAAAGAGGGAAATAAAGGAGAAATTAATAGTGTTG<br>ACATTGCTAAGGTTACCATGATGTCCTTAAAAGTCATCTTCTCAATGAAAGTTCACATTATATGGGAGAA<br>ATTATTTTTGTCATATTTGGATATGCCAACATACAATTTGTTCTCAATAATATTCAAGTAAATCCTGCCTGT<br>ACTTTCACACTAATTATACCTTTTATGTATCCCAGTGAATTTTCTCACATATGTGCCTCATCTAATGTGTGA<br>AGTCAGCATTTCCTTTTAATCTCTATTCACATAGATGTGTAAGACTGGTATCTCTATCTGAGAAACATGTT<br>TAAAAACACTTTTCTGTTTCACAGTATAACAGACCACAGTGAAATAATTGGCTTGTTTAATTCCCAAACT<br>ATCATTTCTACTTCTATTGCCTGTAACTGATTCCCCCTTTGCTTACAACTTTAGAGAGTTCTGAGACTGAA<br>ATACATAGGCAGGACATGCTTCAAGTATTTAGAGCTGATTCCTATCACTTGGAATTCTCTAAGAGGCAGT<br>TTTTCTTGATTTAACAATCAGATACTGGAGATTATTAGTTAATTTTCTAGATCATTTTGATAACCAACTTAA<br>ACCATGGCAAAGTCTTTCTTTGGTGAATTTATTTTTTATTTATGTGTAATTTCCTCAGCTAAATAAGAATTT<br>ATTCTATTTTCCATCTAAACTTAAATTTAGGTGCAAACTTGAATCCTAAAAGGATATTTTTAATGTAACTTA<br>GCTTTATTAGTGTTTGCAAAACTGAGACAAGAATAAAATTTTAAAAATCTGCTGTTATTTCTCAGAGTAA<br>ACTATCCATATTTAAAAATAAAATAAAAATGTCTTTTCCCATTAGAGCACTGGAGAAAGGAGTCAGAAG<br>ACATCAGTTGTAACCTCAGCTGGGGAACTCTGGGCAAGTCACTTAACCCTTACTTATAGTGTCATCACTA<br>CAAAAGAGAGTTAGAAGAAGCATACACATTTGAAAAAAGTAATATTATGCTGTTACTCTGAGCATCTCA<br>CTTTAGAAAGATATTAGACAATGATTTTTAGTGTTTTATTTGAGCCAAATTTAAACACCTTTATAAAGTGA<br>CCAAATTAAACAGAAAATTAAGTGTTAGATGAGCATACTTTTTTGTCCAAAGTAATATTTTCATTATCACA<br>TTATTTAATAATAGAAAATAACAAATTGTTGTAGGAGACAGTGTAAGGTCAGCTCCTTTTGCATTCTGTG<br>TTTTTCTGACTACCTTTAAAAGTAAAACAGGTACTGTCTTAGTTCTTTGGGCACTTACATTAAGTTCCTTT<br>GCAGATTTCTACTACAAAATTTTGCTTTTATGTTTAAAACTCTAGCAATAAAAAACTGGGACTGGTGCCA<br>ACATTTTTCTAAATTTTTGAATGTGCTAGTTGCAAATATCTGGAAAGAGAGGATTCTGAGGGCTGGAGCT<br>GAATATATGTCTGCTTATCTTCAGCCAGACTTTGGTCTCTTGGTGTGTCACAAAAAGGCCCAGGTGGAA

							CTAATTCTCAGTGGTTTTCATTTGTTTCTGTGCGGTGAGGCAACATGAAAGAGGAACCCATGCATACTTT CATATACTCTGAAAATATGTAGCTATTAGTGTCTCATTTATTTTACTGAAGCTCCAATTTAATCTATTCCTG TTAATTGCTAAACAGTACTCAAGAAAACATCCCACTTTAGTATCTTCTTTAAAACCTTATCTGTACTCAGT TTCTTCTTTGAGTAACCCAGAATAAAAATGGAACCTAAGTGAAATCTGGGTTATTTAAATATGATAACAT ATGACTTTGTTTGGTCAGTAGGATTTTCAAACCAAGTTTGTTTTTTTAACACATCAGTATTGTAGACTTTC AAAGACAAGACTAAAATGGAAAGGATGGATTCATGCTTGCCATTTGCCTGTGCTGTTGACTTTTGTGCT GTGAAAGTTTATGTTGAAATGGCACTGCAATTTTAAGAAGTGAATATTATTCTTAGATTTTAGATTCCCAG AAATTATGGGATCTGGGCGTGTAAAAGAAATGATGTTCAATTATGAAAATGGATAAGTAATGACCTCCTA CAGCATTTCAATGATTTGCTCACCTACTCCTCTCCTACTAAAATTTTTTAATCAGTAGATTGCTTGATTTTT AAAAGACTCAGGCATCTGGAGATGTGAAAGATGGCTTTCTATTTCCATGTTCCTATGCATATGAAACTGT ATATCATCCTGAAATCAACAGCTAGATTCTGTCTTGGCAGTAAAGAGAAACCTTTAAACTGAATGAACT ACAGTTTTCTTATTCTATATTAAAGCATATTATTATTCTAAATCAGAGTAGTTACTGTTCTCTACACTGTTCT GTAACTTCCAAACCATTTTATCTCCCCTTTGTAAAATATGAAAGCATAAAACCAATTTCACATTGGCTGAT ACTATCTTTAGACACAGAAGATTGATCCCATCTAGAGTGGTACACTGATAATTTCTCATATACTCTGGTGA GGTAGATGGTGAAGTAGATTTTTATGTTTGCAGAAAACCAATATTTACCTCTGATTTTCTAAGTTACCATC AACATAGAACTACATTATAACATCACGGTGAAGAATTTTACACTGAGGATTAGATTACATTTTTATAATGT CATAGACTTTAAAGCAGTGGTTCTGTGGGTGTGACCCCTTTGGGGACTGAATGACCCTTTCCCATGTGT CACCTAAGACCATTAGAAAAAACAGATATTTACATTGTGATTCATAGCAGTAGCAAAATTACCTTATAGG TAAGCTGAGAGCTATTGCTCTAAGGTGTCTTCTCATGTTATGTTAATGTTCACTTTACTATGTATACATCCA AAGCTTTAACTGATACATGGTACATGGGCAACATTTTCTTTACCTTCTAATTACTGCATATATAAAGAGCT GTATTGTACCTTGTCCTCTTCAGTTTCTTCCCAGTCTGTCTCTGTTTTCTCAGGTAAGACTGTTCTGGAA ACATAAGGGAAAGGAATCTATTGCACTATGGCTAGAAGATTTCTTACAAAGAGCTGCTCAAGAACTGGG ATCCTGAGCATCATGAGCTGAGGGGCCCCAGTATGCCCTACCCTGCATGTTTTCATTTAGGGAGATGGCT GAAGAACTGTATCTGCAAACAGTGCCACAGAGGTTACTACTTGATCTGTGTTGGTGCTCTGAGGATTCA CAGAGAGCTACTCATAATAAAGTTTTATGTATTATACAGAAATAGTATTTCTGATTATAAATGATAAGTAG ATTTATAATCTATAAAAGTCACAGTTCTATGCACTGAGATCCTATGTGTAGGAGACATGATCATCACATGG TCTTCATTGAGTTTTTTAAAGCTTTAATAATTACTTTGTAGTCAATTATATGTTATAAAGAATAAAAAATAT TACATTGGAAAATTTTAAATGAGAGAATTGAATAGGAATGTAAAGATAAATTCAATATTAAAAGACAAAT GGAAACACAACTGTGTATCACTTAACTCTATTTTGTGCATTTCAGTATGCAAGAATACAATGTTCTCACA CACTAATTCCATGCATGCAGTAATTTTTGCCTCTGATAGTAATACCTCTTCATAATGATGCCACAGTTGCC CTTTCAGTGTTGGCAGTACAATTATTTTATGCCTGCACACTCTGACTCCTTTCCCTTTTTTTTTTTTAATTT GGAGCTGGGAACCGAACCCAGGGCCTTGCGCTTGCTAGGCAGGCATTCTACCACTGGGCCAAATCCTC AACCCCACACTCTGACTCACTATGGGCAGGTATACTTGAGGATGTTGAAAGCCTGAGATGACTACATTC TCTATTACACAGTGTTTGTTCTGCAGCTAAGATTTAAATAACAGCAATGTGTCCATATATTCTGCCCAAAT TAAGAAATCTAGTTTGCCCAGAAAGCAGAAACAACAGTTTCAGGCCCAGTGGTACATGTTGGTAAGCC TACCTTCTAAGATACTGATTTCATGTATTTCTTCACCTACCCTTCTGTGAGGAAGTTTATTTCCTAATGTTC CACTTCGCCTAGATGTTTGGTACTTTCTCATTTGCATATCTACACTGTTCCAGTTTCTAGTTTTAAGGAAT GCTTTCACTCCAGTCACATTGTGAAGACTTGTAAGAAGGAACTCATATCTGGCTTTTATTCACCATGATA GAAAGAGGTGATTCAAATGCCAATACATATTTGTTCTGTCCTTCTGGATGCCTAAAAAAATTGTGTTTAT TTATTGTTGACTTGTTTATAAGACAGGATCTCATGTATCCCAGGCTAGCCTCATACTCCCTTTTTTCTGAG AATGACTTTGAACTCTTGGTCTTCCTGTTTCCATTTTTCAAGTTCTGAGATTACAGTTACACATTACTATG

							GTTAATATTATTTTCTATATCATCAGGGTATGAATTAGTTTTAAATTTTCCTTGAATATTAGAGATGGTGCCT AGTCTTAGTTTTCTTATTAAAGCTACAAAAGGTTAGACTCTACAATTTCAATTGTAGGAGTTTCATTTTGT TAAATATATCTTTCTATATGGTAGTAAATACAAAACCAACCACAAAAATTCCTTATTCCATCCCAGAAAAG TCACAAAAATTTAACTTATAAGTGTAAGGATAAAAATGAAAAGAACTAAACCCTAAATTTAGTAATATTT TAACTTTCTTTTGAGTGCTCAGATGTCTGCAATGTGTCCCTAGTATAAAAAAACTTACAAATCAGCTGTG AAAAAAATCTCAGTTGAAACCCAGAAACCATCTATAGAGTTATGTATCAGAGGATTTGGGTCAAATAGG AAGAAAAATCAAAATGTATCATGCATGTTATGTCATCCAGGAATGTAAGAGAAGGACCTTTGTTTCCTCT TCAAGGGAGATTCAATTTTAGTCATTTTTAATTATAGAAAAGTTAAATTAAAAACTTTAAATTTCTGCAGT TGCAGAAATTTCCATCTTCTTCTATAGTGCTATATGGGTCCTCGTATGTCATCATTTTTTCCAAGACACATA TGACCTCAATGTGTTTACCACATATCATGCCAGTTTGTAAATATAAAATAATTGGTACTCACAGTAAACAC CACTATTATAGAGAAGTGAGAAGCATTTAATTTTATCACTAGGGGATTTTGCATGCTTCTGTTTGTCTGTG AATGATAAAAAATACAACCGATTTTTGTTCTCCTCATTTAATCCTTAGCTAGATAGACTATATTTATGTTAG TTAGGGTTTCCATTGTTATGATCATAGTCACCATGACCAAGGCAACTTGCATAAAGGAAAACATTTAATA GGGGCTGGCTTATAGTTTCAGAGGTTCAGTCCATTATCATCATGGTAAGAAGCATGTCACTGTGAAGGC AAACTTGGTGCTGGAGGAGCTGAGAGTTCTACATCTTGATCTGAAAGCTACCAGGAAGAAACTTTCCT CTGCACTGGGAGGAGTCTGCACATAGGAAACCTCCAAGCCTACTAACACCCCATAGTGATGCACTTCCT CCAACAAGGCCATACCTCCTAATAGTGCAACTCTATGAGGCAAGCATTCAAACACATTTGCCAAAGGAG GTCAAACCTATTCATGCTACCACATTTCACTCCCTGCCCCACATAGGCTTGTAGCTATAACATATGCAAAA TGCATTTAGTCCAACTTCAAAAGTCCTTAAAGTCCATCACAGTCTCAACAATATTTAAAAGTCCAAAGTT CAGTCTCTTCTGAGACTCATGTGAACTCTTAACTGCAATCCCCAATAAAATAAAAATAATAATAATAAAA ATACAAATGACATGCTTCCAGTATCACTGGATATGTATACACACCAAAACTTCATGTAAGGAAATACTGG ACCAAAGCAACACCAAAACCCAGCTGGGCAAACTCCAAACTCTGCATTTCCAAGCATGATACCCAACT ACTTTCAGTTTTTTGAGTAGAACACAATTCTTTCTTTTGGGTTGGTTTCACTTCCTGTTAGCAACTTTCCT TAGCAGGTATTCCATGGCTCTGGCATCTCTAACAGCATGGAGTCTCTAAGGCAACTTCTTCACAACTGCT TGTTCCAATGTCTAGGATCCACACATGATCTTCTGGGTTTCTCCAAAGGGCTTGAGTCACTTCTGCTCTG GCCTCTATAGCATTCTAGGCTCTGGTTGACTCTATTCCACTGCTGTTGCTGTACTTGGTGGTCATCTCATG GTACCAGCATCTGCAATATGCTGAGATCTTCTGCTGCAACTAGGCTTTCCCAATAACCTCTTTTACAGGC TCTCTTCATGGTGCCAAGTCTCAACTTTTTTGCAGGGCCCCTTCAGTTCTGGGACATCAACAGCAACAG AGACTGCACCTTCACCAATTGCTTTTCCTGGCTTCTCTAAGCCTCAGCTGCTCTCCATGACTTGTTCATG TCTTCAAAACCAGTACCATCTGAGTGATTCTTACATATTACCAAGTCCAGCTACCTGCAGGAGGTCTATC TCTGGAACACAGGTGGTTTATATATATATATATATTTTTTGTGCTCCCAGAAAGCACTTCCCAGAAGATTT CACCTTAGTAATGCTGATCTCTTTTTGAGGACTGTTAATTGCTTAGCTCCAACTAACCAGTATCAATTGTT CCTGTAACCTCTTCTATTCTTGACTCTAAAGTCAGAGACACATGGCCTAAAATATCCCTTGTTTTATTACA TTTCCACCAGTTTCTGTTTTCAATGATTTACTTTATTGCCTAAGCTTGTCTTGGAACTTGCTCTGTAGACT GACTTTGAACTCAGAGATCTACATGCCTCTGTCTCCTGAATGCTGGGAATAAAGGTATACCATCATGCCT GGATCTAAGCTTTTCTTTGCATTTTCACACAATCCTTATAAGGCCTTGATTGTAGTTCATTCTAGATATAGT CAAGCTGACAATTGAGAATAGCCTTCATATTCCTTCCCTTGTCAATAAAACACATAACCATATCTCCTTAT GTCCAATTGAAAACAATAACCTGGTCATAATAACTTCTAACATGATATAACTATCTCTTGTTTAATTGCAA ATGCATAAACAGAAGGCTTTGCAGGTTGGGATCTTGGCCTCAGTAACCCACTCTCTTAATCCATTTAATA TCTTTGAACACAGGATTTAGCTCCATTCCACTTCCTGGTGCCCCTTTAAACTTTGAACCATATATTTTACA TTTTTTTCTTCCTGTGACTATGCTTGATCAAAACACTTCATACAAGTGAACCACATGGCTAGGTTTTTTTT

							TTTTTTTTTGAGACTGCCTTTGTCAATGCAATTCATTTGGATTTCTTCAATTTAGCTTCAGGCTGACTCTT<br>AGGAAAAGGGCAAAAACCAGCTAAATTCTTGATGAAATTATCACACAAACCATCTCTAAGCCACATACT<br>AAAATTCTTCTCCTCTGAACTCTTTAGTTAAGCCCTTACAGTTCAAATCATGCTCAGCATGACTGTCTTC<br>CATGCTTCTACTAGAATGGACCATCAAGCCCCACTTAAAACATCCCAAAGCTTTCCAACGTCCCCAAGT<br>CCACATTCCTCCCAACAAAATTATGATCAGGCCTATCACAGCAATACTCCAGTCTCTGGTACCAATTTCA<br>ATCACAGACTTTCCATTGCTGTGAAGAGATAGCATGATGAAACAACTCTAAGAAAGGAAAACATTTAAT<br>TGGGGCTAGCTTATGGTTTCAGAGGTTCCATTTATCATCATGGTGGGAAGCATGGCAGTATGCAGTCTGA<br>CTTGGTGCACCCAAGAGTTCTACTTCTTGATTGAAGTCAGCCAGTAGGAGACAGTCTTCCTCAGGGATC<br>CAGGAGGAGGGTCTCCTCTGCACTGGATGGAGCCTGAACATAAGAAACCTCAAAGTTCCCCCACCTCC<br>TATAATGACGCACTTCGTCCAAAAGGCCATACCTTTTAATAGTGCCACTCCTTATGGGCCAAGAATTCAA<br>ACACCAGAATCTATGGGGTGGGGTGGGGGATGTCAAACCTATTCACTCACCACAATATTAAAAACAATA<br>TGGGATCACAGCAGTCTTCTTTGCTGCAGTATCTGTTCAAATGTCAACAAGAATATAGAATATCTCACAT<br>GCATTTTTGCCATAATACTTAATATAAAAATTGTGTGCTTAAAGATTAATTAAGCTAATTAAAATTCCAATA<br>TAATGAGAAAGTTTCCCTTGAGGCCTCGCTCTTAGCTAAGGAGCTATATTGGCAGTTGGCAGTTGTTAG<br>AGGAGGAAGAATAGTTTTTCTTTGGGGTTGTGACCACTACTTTAGTAGATGTTTACACACATATGCTCTC<br>AAGGCCATGTTTAGCATTAACAAGATTTAGAGGAATGAAAGAGAATATGAAATTGGAAAAGGGACATGT<br>TGGAGGGTCCAGGAGTTAGTGGTGGATGCAAAGTAGATCTGATCAAAACATATTGTATACATGTATGAA<br>ATTCTGAAAAAATTAAGAAAATATTACATTAAGTATGTATTATTATTTAGAAATCCATAATAGAGCATCTTC<br>AGAAGACATAAATTAATGCTTATTAATTTATGGCCAACTGGAACTCAGTAATATTAATATCTACCCTAATG<br>CTTCTGTACTTTAAAGTTTTTATATTTAATACTAAAAGTTGACTTATTTCTGACATAAAAACAAAATTTTG<br>ATTTTCATACACTGTATTGCAAACTCCATCTCTCTAAATGTCTTAACTTATGATAAAGTATATTTTACAAAT<br>TTTACTTTGTAAAATGTTTTATAGTATAATATATCTTTCACACACTAATTATAATATTATTTCATATAGTTACC<br>CTGGGAAGAAAGTGAAAGAAAGGTAATTTGGTCCAAACATCCCATAAGACCATTGGCTTTGCTACACAT<br>AGAAAGAGAAAGTCTAATAAAAGATGAAGTTTTGATTTCCCATTCTTAGTATTCTATCATTACTACATTCC<br>CCCAACCGCAACATCATGCGGTTGCTGGGAGGGTTTAGTGTCAATTGTAGAGTAATTATGATAGGCCTGT<br>TTTGCAACGACTAGCTGGGCGTCTCCAAGTCTAGCAGGTATTAATTATTTTTTTCAGAATTCACCAAATG<br>ATGCAAGTTCTCTCAGAACTGTACTCTACAAACTTCATCTCTTTTGTTTGTTATCAGAAAAATTGTCTCTT<br>TGAGACACAGTTCTGCTATCATCTTCAGAGAAAATAAATTTTACATGTGACTTATTGATAATCAAGGCATT<br>GGAAACTACAGAATTTTAATTTAGTTTCAGATATTTGGATCAAGTTGAAAGCCAGAAGAAATCTGCACA<br>TGAGAGTCGTTGTTTATTCCTTGGTTAAGTGAAGATGTTGGAGAATATGAATATTTTAAAATGAACATTTC<br>GCCTAAAAGTTGCATAATAGTTTGAATGTCTTGTTAACTGAATCAACCTAAATGACTTCTAATAGTTTATT<br>TATCACATGGCAAAGTTGATCACATTTTCAAAGTAATACATGTAATCTTATTGTAGTATAAATAATTATGGC<br>CAGTTTGTGTAAAAGGAATCTGAACCATGGTAAGTTTGAATGTAAAGGTGATAGTGTTGATGGTTTTGA<br>CATGAAAGTTAAAATAAATAAACTTCATCTTTCATCAGATCTTTATATTTCATTATAATGCCTCCTGAAATA<br>CATATTTCAAAATAACCCTCAAGAATTAGCCAATAGTTACCAGAGTGATTTTCAGTAGCCAGAGTAGTGG<br>AGAAAACATGTGTAAAATGCCATGGAATCCAGTGAACCTTACACAGTAATGTGCATGTACAAAAGAAAT<br>CTAGAAGAATGAAAAAGATCAATATATCTGGTACTGTTGTGCCATGTAAAAATTGGAGAATTTATGTTAA<br>AATGCAATATTAGTTTCAGAGCTGTCTATAACTGAAAGCTTAATGTTTTTAAGATTTGTGTATATCTCCAG<br>TGAATAACATTCTGAAATATCTGTCTGAACAGTAAAAGAGTCTGTTTATCATTGTCTGCCACTTATACCA<br>GGTCACCTGGTATATTTTTATTATCTGGGCTTGCAACTAGGGCCTGTTGTTTGCTATTCAGGCTCTCTACT<br>ACTGAGCAACACACCAGTCCCTACAGTGACATTTTCCAGACTAGTCACAGATGTATTCTCTGCAATGTTT

							TTGTGTGTATTTGTTTCAAAGCTCATAGTAAAGAACAAAGTCATGACTGTCTATTCTGACTATAAGTACC<br>CTATTTCTCTTTTCTTGACATTTATTCTGTAACACTTTGAACAAATAGTAAAGTATGGAAGCTTAAAACTA<br>TAGATGAAAGGTATATGGCAAGCTATTTGCCAATCTTATCTGGTTATGCTTACACAGAAGGAAATATTACT<br>GCTTTATTTCTAAAATTCTAAAATTGCTTAGTGAATTGGAACAGAATTTGTAAGGAAGCCACAGTTAGTA<br>ACAATGAAATACACATGTCTTACCATAAGCAGAACAATTAATGCAAAGGCAGGATTCCTTTAAAAAAAG<br>ATAGTTTCTATGGAAAAACAAACAAGTTTCTGCTGAGTGACTAAGACAGGTTTCATGGACACCTGATGA<br>GTGTGTCTTTTTGATTTTCATTTCATGAATGCAATTAATGTAAACTTGTTTCTACAAACTAAATAATGTATA<br>TAAAAGATGATTATTAGTTCAACTCTGTATTGGTTTATCTCTCTTGAGAGAGAAGTCTTCTAGGGGAAGA<br>AGAATGAGAAGCTTAATTCAATAGACCCCTCTTGCTTATAGGGCTGGGAATGGAGGAGTGGAGTTAGGG<br>GAAAAAGTGAATGCTTTAAGCCTTTAGCATCCTTCCAGTGTTCTTGAAGACAAGGACAGAATGGCTGTG<br>ACTTTTGAGCCAGCTTGCTCAGGAAACTGTGGTATACTGACAACAAAATTCATAAACAAACTCCCCATC<br>CAGGCAGCTGAGTTTAACAGATGCCTTATTAATAAGATCTAGGCAAGGGCAGGAAAAGAAGGGAAGTT<br>AATCTCTTATTTTGATACAGTTTTAGACAGATGAAGCCTTTCAGCATTGCCCCACACAGTGTCTGTAGTG<br>TCGATTTGACTACAAAAGTGGCACATTTAAGGGGAAACTCTTTAGCCATCAATGGGAGGCAAATAGACC<br>TGTATCTTAGGGCTGAATTCAGTTCTAGAAGCTTGATTTTATCAGAAGACACAAAATAGAGCACAAGGA<br>AGCTATATCCACTGAGAATTAATTAAGAGAGACAGCCAGTAACAAATAGCTTGATCATGGATATATATATA<br>TATACACATGTATATATATATACATATATATCAACCACAGTTGCTTGCACCTTTCTATTTCTTATAAGTTCTTT<br>GCAATAACGTGTTCTTGTACACCTACCTCCACAGAAAGGTATCTGATATCTGACTGAATAGAAATAGTAT<br>ACTTTTTATAATACCTCATACAAGATATGTCAGTAAAGTTGGAACATCCTGGCTGCCGTTTAAGTTTCTAA<br>ATGTATAAAATTGTTTTAGGTAGGCCTCTGTCATGGGTAATTTATTAGTGTGAAGTTTCCCTGATTATTTCT<br>CTAGTTTTCTATGGGATGTTTGTTCAGATAAACCAGGGAGCAGAGCTGCCAGAATCCATACTCAAATTTT<br>CCTTTAATTACTGCTTTTTAAAAAATAACTGTTTCCTTTAAAGTTTTCTGTTCACAGCATGCCCTCCCCAC<br>ACAAAACCCTTCCCTTGTACCTTTACATGTTTCTAAAACAGATATCTCACCCTTTGCCCAACCCAGCACA<br>CCCAAGTACATTGCAGTTCCCTTTCTGAAGCCAATGAAAACAGCTACAGAACACTGATGTTGCTTTCAT<br>TGTGGCTGGACAATAGTACAATGCTAATTACCTTCAGTTTTGAACGGCTTACCAGAAACATCACTTCTCA<br>AGAACAGGTTCTTGGTTAAGCCTGGACTGTGATTTCCTCTTGAATCTGTGCTTTGTGCTTCTCTCCTAAG<br>CAGCACTGAGTGATTTACCAGTTATCACTTGCTCTAGCCCCAGTAGGTTGAAAGTAGTCTTTATTACTTA<br>AAGAAAATAGGAAAGTCTGCCTCCTGGGTATAAACATGAATTTTCTATGTCGAGTTAGAAAAGGATGCA<br>AAGGGTAGATTTGATCGGTATTTCTGCAAGAGTCAAAGTCTCTTTAAGCCATAGCAGGTAATGCTTTTCT<br>TGTGAGTCACTTGGCAGAATCTATCTACATATCTTTCTGTATCACACTTGATCTAACTTTACAGATCTTATT<br>CACGCCATACTCCCTTTAAAATAGATTGGAGTGTGACTTGAGTAAGCACCATAAAGCCAGAGCCTATGC<br>TTTCATCTAGTTCTGGTTGGTTTTATACGTGGATGTGATTTGAACAGTACCTATGCAACCTCAACATAGGC<br>TGGGAGAGAATGATGCCATACTAAATGAAGCACTGAAATGGAAACAGATAATCTCTCTGTTGGGAAGA<br>ATTGTGATCCTTCTTACTCAGAGTCTAGCAAGCAGGGATGTTTAAAAACAATGATAAAATGCAGGATCA<br>CTACTGATACAATGACAGCAGGCAGTGTCAATTTTGGTGGGCAAAACAACAGAAAAAGTTGATGAGAT<br>CTAGAAAAAATACAATACCCATGATTTAGGCCCACTGTAGGGAGCTAGTGTTCAACCACTTTGGTTTTTA<br>TATGGCCTTCAAAAGTTAATGTCAACTATGATAATTTTAGAATCAAAATAAAAAAAATTGCTTTTACCTG<br>ATTGTTGATCAAAAGAATGAGATGGAGAAAAAGAAAATAAACCAACAAGCATACGATGTTAGCACAGA<br>AAGTAAAATATTCTCTCAGGGACATCAGGGAAGGTGGAATACTATAACAATGCATGTTCTATTGTTATAC<br>TATTC

MSTRG.118107.1	ENSRNOG00000010480;ENSRNOG00000010887;ENSRNOG00000010921;ENSRNOG00000010999	2.242	0.000	0.000	-5.395	down	TAAGTCACTAGGAATCTGGACATCTAGAGGACAGTAATCTTCTGGTATCTTTTTATCCAAGTCAATGTCTGTATTCTTTATCACTTCAAAGGTACCATGGTGGGGAAAAAGAGAGCCTAATGGGGTATTAAAGATTAAAATAATTAACAAACTACCAAGTATAACACTTTACCCCCACATCACTAAAACATGCATTCTTTCCACTGGAAATGTGCACGCTGAGATGGGGGCACTTGGAGGCCGAGGCGGGAGGGTCAAAAGTTCACAGCCAACATTGCCTACAAGAGACCCAATCCTGACAATCCCCAGACTGACCAGTCTTACTGCAGTAGCCGCTACATTGAAGAAA
MSTRG.118148.1	--	0.274	0.000	0.049	-3.592	down	GTGCGTCTAAGGGTCTGAAAAGCAGATGATCTCTGATCTGATCCTTCTGGCAACTTCCACAGCCCCTGGAGCTGCTGTGTGGTGACAGAGCTCTCCTGGTACAGAAGGAAAATCATCCTCCACCCCTGAACAGAAGGAGAAAGAGAAAATGCGGCTAGGGTGATTAAAACCTGCAGACAAAGTGTTAGTGCCTCATGTTTCTTCCTGATTCAGAAAGATCATACCCTGGAAAACCAACCCATCCAACTCCGGCCAAGGAAGGGCCCTTGGCTTTTTCCCTGGGAATCCGTGCATGTTTCAAGCTGTATGGGAGTACTCCATCCTCCTCCTGGATGGCTATCCTGTGAGAAGCCATGCACTGATGCATCACACTGCAAGAGATGCAGGGTCTGCTACACAGTTTCTAACGAGCTTGGAGCCATCTCCATGACTCACACCTCTGACTGGCTCTGCTCATTAGCTGTGCATAGGAATGAAGACAGCACAGCCTGGATCTGTGCTCATTCCTATCCTTGTTATTAAGTAAATACCATTTTGGTGAATAAAATTAGACTCAACCCACGTAAGCAAAATGACTTCTTTCCCTTCCTGGAAATCTTATTAACATAACACAGCCAGATAATTAATCAGTACCAGGCAATTGTTAAATGACTACTAAAAACAGTGATTAAAATAATAAACTCCAACTTGAGACTATTTCTCATTCAAAGGGGATTCGTGTGTTCTGTTTGGAATCTTCTACCAGTGTGCAGTAGGAAATGCATACAACTACA
MSTRG.11820.1	ENSRNOG00000019861;ENSRNOG00000020021;ENSRNOG00000020044	0.961	0.095	0.008	-2.515	down	GACTTGTGTGCCTATGGGCATGTAGGCCTGTGTGAAGGTGTATGCAGTGGTGTGTGTACGTGTGCACACACCCACGCCCACATATGTATAGGGCTGGTGTGTCTGTGAAGGTCTGAACTCCCATGTCTGCCTGTGTGTGGGATGATAAGAGACTCCCTGGGGTTTCCCAAGGAAGGCATCCCCAACCTTATCACCATGGCAACCCCATTGCCCATCCCTTGGGCACTGGAGGGGTGGGGCCTGGCAGGTGCAAAGCAGGAGATAGCAACTGCAGGACTCAGACTTCTGCCAGCACACGGAAGCATAGGAGTGGTGTCTGTCTAGACCTCAGTTAGGTCCACAGTGGTTCCTCCAGGCCCCTACCTTCCTAGAGCATTATAGACTGCTCCACACTGTAATCCTGTCGTCTCTGCCCTGCCTGTCTCTCAGAAGCCAGACAGCTGCTGGTCAGAGGACTGCCACTCACTGTGTCTCCTTTGCCACTCCATGGTATGCATCCCAACCTCATGGAGGCCAGTGGGAGTTCCTAGTGTTGGATCTCTAGGTGTGGATACCAGCCCCTCTTCTCCTGTCCACCTTTATCCTAAACATGCCTTCCAGAACAGTGCTACTCCATCAGGACCTTCTCCATCTGGGGGTCTGCAGGCCTCTCACTCTGAAAGTGACTCCACCAGTGGCCTGATCGCCTGCTTCCTTTCCCTAGGTCCTGCACAAGGGAGGTAAGACGAGCAAGGCTTCCTTAACTAGGACACCAGCTTAGACAAACGGTTCCCTCAATTGAATTACTGAACAGATAAAGGATTCATCAAAAATACCTCGTTGGGGTAATTTTACAGTATTGAATTGCAGAGACCGGTTAATAGAAATGTAATGTGATCACTTGGCACTACAGGGATTGGAGTGGGGTCCCTACCCTCCTCTATAGATATGGCTCAGTAAAAAGGGAGTTCCCAGAACTGGCTAAGGCTCCAGCATGGATGCTGGGAGTGGGGTACAAGGAGTATCCTGCTAGATTCCTTGTGTGCCCAATAAATTTCAGTTTCATAAAACATGTGACTCCAGATAAGGGAGCTAGAGGAAGGCTGTCCTCCACAGGGAACTG

							TAAGGGTACCCACTGTGCTCCCTAGGGGAGGCTGCGGTCTCTTGTGGGGATCTTGGACAACTCTTCCTG CTGGTGGGCTTCTAGGGATATAAGTGTTTTTATGCCTTGCTGGGACTCTAACTGCAGATGGGGCTTCACT GACATCACATTAAGGTTGTCTTTGAGCTGCCTCTTCAGGGGCTGGGACATCAGCTACCAAACTGACTGA CCAAGAGAAAAGATTGTTTTTTTCACTGTAGGTACTTGAACCCTCTTCCTCCATGAATGGGAGACGTGG AACTCCCTCACAGGATACCTCACAGGATACCTCACTGAGGAGTGTTGATGGCAGGTCTTGGAGTGTACC AGGGACCTGGACCCTTTAGCTGTAGAGTCCATCTCTCAGGATCCTGCTCAGCATTCCCTGGCCTCCCCA AACCCTCTCCTCCTAGGACACCCAACCTGTAGTCTCTCTGTTCCTCTTGTTAGCATGGAGGAACTGTGAT GCCCAGTTCATGGCCTGAGGAGTATAGCCTAGCGTAGCTTCCTCCATAGTGGGGCTTACAGTTTGCTACA GTCTAGTAGCCCTTCCTCTGCCTAGTCCTTGCTCCCCATTTCTGTACACACCTATGGTCTGCCCTTGGGG AAGCTCACCTGCTCATCCTGAATCCTGGCTTTAAGGGTTTGGAATTCAGTGCCTACGCCATGTGCCATTG CTCATACCAGGGGCCTGGGTCAGACTCTAGGGCATAGCTACTGAAACAGCTGTAGAGGACCCATGTCTT CTTGTCCTGTAGCTTGAATACATCCTTCCTGCCAGACCCGTGAGTGTTAAGCCAGTTGGCAAGTTTGGG GTGTTGGGTCTCACCTGGAGACACCCACATCACATCACTAGGTTCATTGGCTCTGAACCTACCTGCAGA CCTTAGCCTGGCCACAGAGTTAGGGCCAAGTCAAACCCCTTCACCTGCCTCGTGATGATCCTGGGAAC AAGTGAGCCTCTGGGAAAGCTGGACTGGTGACCTAGCCCCAGGCGGGTGGCCCTGCCAAGGCTATTGG TATGGAATCAGGTTTTCTTATTCCTCTGTCCCTTTGTCTTCCTTGACATAAAGGCTCCCCCTTCCCAGGAA GTTTGTCTCTCAACCTGAGGAACTGACATGGTCTCGGACCATTCTGAACACTCTAGGTTCCTGTCACGG TTAGGCTGGCAGGTGAAGACAGTTTCTCCAGGATTCCCTGGTCCTTCATCATCACTTGTTCAGTCATCTA TGTCACCTTGGAGAATCACCTGTCGTGCCCCTAGTGAACCAGAGCTCTTTGGATACAACAGGCCTCACA TCCCCATCCACACCCAGCAGTCACTCTGGGAGACACCCACATCACTATCCAATCACACATGGATTGCAG AAGTCACTTTTAAGTCAGGGTCAATGTGGTGTCCCAAGGGCTGGGGTAATGTACCATGTGAAATGAGTT GTGAGCTGGCAGAGGGCAGACCATGACAGTGATGGAAGTGGGCCACGGTTGCAGAGAACACAAGGAC ACTGTGGTGAAGGCTGTGTAGGCTCCCCTGATTGGCTGATAACTTAGTACCTAAGTGCTGAATCCAAGA CCAAATGAATGCTTGAAATTCTTGTAGCTGTTGTAAGCCAAGTCTACTGAGATGGCTATAGGATGCAGCC AAGGTGGGAATGCTAGACAGCAAGTCTCAGGGGGCCCATCGGCAATTCAAAACAGAAGGTAGGAACT GAAGGCCCTAGGGATGGGCCCATGTCCCCTTCCCGACTTGCCACTTGTCATGTGGCCTTTCCATCATGCA GCCTAGTCTCTGAGGCTCAAGTGCCTAGCATTCTGCTCTGTGGGGGGTGAGAACTTCAGAGCGGTGCT GAGAAGACCACAGTACACTGGACAGCAAGTCTCAGGGGGCCCTTGATGTTGGCTGCTCTGGGTCCTGG ACAGTTCTGAGTCCCCAGTATTAAGCAAAGGTGGGTTCTGGATGTCTCCTCTGCTCTTTGGGCCCTTGA AAATTTTGTGGGTAGTAGACACAGAAAGAGGAAGCCATATTCTCTGACTCTAGGGGCTGGTTATCCACA TAGGAATAATCCACGGGCCCCAGCCATGATGTCCCCGGCACCCCTTTTCTGGGCTGTCAGTAGGTGAGA GCACAGCAGGGACACTGGCTCAGTGGCCAGGGCAATGACTGGGGCACAGTGGGGTCAGAGCCCCAAC CGAAGGAGGCAAGAAGGTAAGGGGAGCCTTGGATGCTCCTGCCTTATCTGCTTCTCTCACCATGGCAA CAGATGGAGGTTTGAAAGAAAGAAAGGAGAATGCTGTATGAGGTGACTGGGATTTGGAAGCAGGGAT CCTTCAAGGATCATTCAAGGGGGAGGGATAGCATCAGTCCCTCTCTCTAGGCCATTGACCCCCAAGATT GTGAGTAGGCTCTGGAGTAGGGCTGTCTCCTTTTTAAGACACTTCAACCTGGCTCCTGGGGTATAATGG CAGATAGGATGGGAAGGGTTTCATGTCCCCATCTGGATATCTGATGGGTTGAAGTAAACAACATGAGGG AGAGCTTGACATTGGGCACGGCTCTGTCTCCCCCAACCCTCAGGGAAGGTTGCAGCCTGGAAAAGGC CTCCCTGGGACACCAATCTCACCACGTTGATGATCATGCCCAAGATGTCTCTGAACCCTTTCTCCCCTCT TCCCCAGAGAGCTTCCTGGTTTGACCCGGCAGGTAGAACTAGGCTATGGGAGTCCCACCTGGCTGCAC TCTCAGGCTCAGCCTTCACTCAGGACTCTTGACTTCTTCAAAGGCAGCCTGAGAGTAGGTCTGCAGGTT

							CAGCAACCCTGCGTGGGACATTAAAGCCTGTGGTAGTTCATATTGGTTTATGATGAGATAAGAGAGGGACTTGGCCTTCTTAGTAGGCCTAGGGAAATGCCTGCAAAAACAGTGGAAACTTTCACCACGGGGAGGGGAAGCAGCCCTAGCCAGCTGTTGCATTCCCATGTACTGTGGGAGCTTGGCACTTGAGCTGAGGCCTTGGGCTGGGCACCACCCTCTGAGAGGGAGGTCATCATTGACCATAAGCCTTGGTACCCAGGAGCAACTTGCCAACCTGCCTTTCCTCGGGACTGCAGCCTTTAGTCCCCAGTATACCAGTCTTTCTGCATCTTCCCTGACAGTCAGTGGACTGGGGTATGGGTTCCTTAGATGAGGCCCAGTGGAAGAGAGTAGTCAACTCAGTCAGTACGTAGGCAGATGGGAGGGAGTAAAGAGGAAGGGGCTGGCGTCAGTGGGAAGACCTTTCCTCAGTTGGAGACAGTGGTAAGGATCCCATGGTCCTTTCTACCCCAGGGAAAACACTGATCACCTGCCTAGGCTCTGGGTCCCAGACTACACGGTGATACAGTGTCCTACAGCATTTGCAGAGCCTAGGGCACCACCATGTGGCTCTCATTGTCCAGAGTCAGTACAGGACAGGATCTCCTTGGGCCCCAAACAGAGCAAAGGATAGTATCTAAGATCACCTGGCAGCCACTGCATGGACTCGACTATATGCCCCTTCCTGTCTGCTACAAGCCTGGTGGTCCAGCCAGGCCTCCCAAGGAGGATGGTAAGGTAGGTATTTGTCTCATAGCTACCCCAGTTCACACTAAGGCTGGACCCAGTTGACCTATGGGTACCTCCAGTGGTCCTGTACTCTGTGGACTTCACTGTGGTTGGTGTTCTCTCCCACCTTTAGTCTACTACCTTTGGATGACTTCAGTAGGACATGATTGATGTAATCACATGCCAAGCCCACCATCCCCCCTATTAATCCTGGGAATTTTGTTCTTCCCAGTTCCTGCATTCTGATCTGACCTCCAAGGCCCCTGCAATAATGGTCCCTGTGAACGGGAGAGGGTGCTGAGGGGAGATGGACCAACTCAAGAGGCTGTGAGTTCCTATAGTGCCAGTATAGAAGGTTTGGGGTTCAAGTGGGTCACTTTAGCCTGTGGGGGCCCCTGACACTTGTGTTGGAAGGAAACAGTGACCCCCATCCTTAGATATGTTGGGAAATGAAGGATACTGCCTGCTTCTATGAAAGGTTTTCTTCTCTCTGCCCAGTACACAGAATCTGGGCCAAGCAAAGGGGAGGAAGAGGCAGCTGCCATGGGGCCAGGCTGTTGAGACCCCTCTAACCCCAGGCCCGTGTCTCTTGATTCCCAAGAAGTCACTGCAAGGCACCTGCAAGCATGGACTTCCTGTCCTGGGCAAGCAGTGGGAAATGGCACTGACTTGTCACCAAAGGACGTTTGGGCTGACAGGCTGGGTGGGTTGGGATTACTGCCTCATTGCTGTGGTGGGTGATACCTTATAAGCCCCATGTCCCCTTGCAGGGGGAACCTGGTACTTTGTCTGCTATCAGACTTGAGATCACAGGGGTAACACAGGCTAGCCTCAAAGTTCACCAAGGCCCCTGGCCTGATATGGAGGTTGGCTAGGCTTCCTCAGCTGCCCTCTGATTGGAAAGGGAATGCATGCAGGGGACCTGGGTCCTCTGGTCCTGGTGCAGGAGGAACTCTCTGTTGGAAATAATCCACCATTAGGGACTATTTAGAGAAGTAGGAGAGAGGCAGGTAATCTCTCCCCAATCAAGATGGAGTTCTTGGCCACTTGGTAGGATGTTCCCATTATGTGTATCAACTTAATTTCACTGTTTTCTTGTTGCTAGAAGGCAGGAAAATGAGGCAGGTGGGCTGAGAGATGGCTATGGTCAGCCAGGGTAATGAGGGACCCATGGGAAGGGC
MSTRG.118405.2	ENSRNOG00000008037;ENSRNOG00000009255;ENSRNOG00000022043;ENSRNOG000	0.804	0.000	0.000	-5.192	down	AAGCTGGCCATCTGTCTGGAGACTCGGCAGTAGGGAGGAGTCAAGCCACAGCTTTCTTATGGAACAACTGGCCAAAGGAAGGAGACACCTGGTGTTGTGACCTTCCTCATAGAAGACAATTAGTTGGGGTATCAGCCTGGGAACTTACAAGGCGGAACCTTAACTTGGGGGCGTGGCCGAATCACCCTGGTTTGGAAACAGTTCTCTTTCCTTTAGGCTCAGGAGAGGTTCTTCGCGGGACACTGGCTATCCGACCTCTCCGAGGGAGGGTTCGTAGATGCAGAGCTCCGCCAGTATTTGGCTAATAACCGTCCCTTCTGGGGCGGAGCCAGCCCCAGTTTATAAAGTAGCAGAGCGCCTCCTTCTGGAGAATGCCTCACTCCCATGCGTATTAGGGTAGTACGCAGTACGCTGGCCTAGGGGCGGAGCTCAGCCCTCGAGGAGGCGTTTCCTTTTAGTTCTCAGACTCACGCCCCTCCGCCTTTCTTGAGCTACCCTCACAGTGGCACAAAGCATGGTCGTTCGCGTGCTGCCGGCCTGAGGGCGGGTCTGGCCCCAGAGGCGTGTCCGAGAGGCCAAGGACCGCCTCCTGAAACACCAGGTGGTACAAAGCGCTGTGGTTCACGGGTCGCCAGCCTGGAGATGGGGGTGGGGTGGGGCCACCTGATCATTCTGGGGGCGTGCCCGGAAGGCTAAGCACCGCCTCCTGACGCATGCCCTGCCCAGCCCTCCAGGACTGTTCGCTGTGGCACAAAGCGCCTCTGTTCGCAAAACGCGGCCTCGGGGCGGAGCTTAGCACCCCTAGGGGCGTGTCATAGG

	00031129						ACGAAGCACCGCC
MSTRG.118495.1	--	1.002	0.000	0.022	-3.851	down	TTCAAACCTGCCCTGCAAACCGCTCACCAGGGCGAACAGAGTCTGGGCTCTGGGCTTTTGACCTGGGCCATACCTTAGTACACTTTGGTTCCTTAGTTCTCTGTGCAAAGGGGACGAGATCGCCTCTCCTCAGAGCGTGGTTGTGAATTTCAAATGAGAGGAAGCATGAGGCATGCACAGCAGTCTGTCTGCATGTGGTAGATAATACATTGTCTCGAAGAACCGCGGCTTACAAATCGTTAGGTCATGAAGCAAG
MSTRG.118515.1	--	0.274	0.000	0.001	-4.716	down	TGGAACAATTGTACCATCTTCCCTGTGATAGACTGAAATTGCTCTGAACACATGAACTAACATAAATTTCTCTCTTTTAAATTGTTTTGGGTAGTTGTTTTGCCACAGTGACACAGAAAGCAACTAATTTGATGCAGAATTATTATGCATGTGTGCATGCAACACCAGCATGTGACTCCACTGTGGCCAAGCTGGTCTGCTGTTTCTACTCTGTTTCGCTCCACTTACTTCTCTGCCAAATATAAACTATGACCTACTTAAGAGATCTTGTCATTGCCTTGCTGAAGACCTCTAGTGGCTTTCTTTCTTTTTGGAATGATGCTAAATTCTTTATCAGAATCTGCAAGGAGACCTGGTGCAGAGTGATGCAGTCAACTGGCTTCATTCTTCTGCCACTGTGCTTTTCCCATCATGATGGACTGTGTCTCCTGGAAGTCTAAGCCAACATAAGCCCTTTCCTCTTTAAGTTGCTTTTGTCAGGGATTTTTATTGCAGGAACAGGAAATGAAACTCAAGCATTGCATTGTCCCTGGATGAAAAGCACAAGTTTCACTAAGGAGGAGGGCAGAGACGACTGGTGTCCTAGCTGTCCACCAAACAGTGACTGCAGAGAACACATATGATCTCTGTTTTTGTACTGGAAGATCAAACAAAAAGAATGCAGCAGAGAAGCTCCTGCCCGGAATGCAAAGAAAGCATTTGCTGTCTTCTTACCCTCTGAAGAACTGGTACAGGGAAAGATCTGCCATCAGCCCTGGAGTTTACTTCTTTCTGTCATTACAAATTACATAGCGAGTCATCAACCATATATTAAGCATGAGGTAACTAGGCTCAGAAAGGATAAGACATTGGCTCTGTTCCTTGGCTTAAAAGCATCAAGTCGGGTTGTGCACTCAACTTCATGAATCCATAAACACATGGTATAACCATCTTTCCACAAGCAACAGTCATGGCTGAGGTCTGGTCATCCTCTCTAGCTGCAGAAGGGGGATGGGTAATGGAGCTCAACGGAGCAGCAGAGGGCGCGTGGTGACTTGTTTCTTCAACAGGAAGCAGAACTCTGAGAAGACAGAGAAATTAAAGCGTCCCCCGCACAGAGCAGAAGGCAGTGCATGCCTGAGCCAGGAGCCACCTCTAGTCAGAGATAAGTCAGAGAACGGACCACAGTCACGGAGATGTTTCTGGGGAGAAAAGACATGGCAGAAGTTGTTGCTTAGGGCACAGGATGCCATCCACACCTAAGGAGCTCCTACTGGCTGCACTGATGTGGGATTGTGTCCTAGTGATTCTGACTTCAGCCTCCTATTTCAGCTTCATGAGCAGTGAAGGAGGAATGGCAAAGGCAAACAAATGCCCAGGATTTCACAACAGTCTGCTCAGCTGAATCCAGGAGACGAAGGAGCATCAAGTATGGCCAGTTTCCTGTCCAGGGCCTCTGGAGATTCTGAAGCCTGGACTTCCTGATAAAATGCACTGCATTTCAATGCCTTTGTGCCTTCTCCACTTTCCTCATAAACATTATTTTGTCATATTCTGTCAGCTGTGAGTCCATGTTCTTGTCTTCAAGAAGTTTTTGGTCCAATGGTGCAGGGAGAAATAGACTGTGACACGCACTTCCACACCGAGTTAAACAGTTCAAAGCAAGGGAAAACAGTTCAAAGCACTATGTCTCTGACAGACAACGCTGGCTTGGAGAAAGAGCCCTCTGGCA
MSTRG.118515.2	--	0.194	0.592	0.008	2.335	up	GTTTTGCCACAGTGACACAGAAAGCAACTAATTTGATGCAGAATTATTATGCATGTGTGCATGCAACACCAGCATGTGACTCCACTGTGGCCAAGCTGGTCTGCTGTTTCTACTCTGTTTCGCTCCACTTACTTCTCTGCCAAATATAAACTATGACCTACTTAAGAGATCTTGTCATTGCCTTGCTGAAGACCTCTAGTGGCTTTCTTTCTTTTTGGAATGATGCTAAATTCTTTATCAGAATCTGCAAGGAGACCTGGTGCAGAGTGATGCAGTCAACTGGCTTCATTCTTCTGCCACTGTGCTTTTCCCATCATGATGGACTGTGTCTCCTGGAAGTCTAAGCCAACATAAGCCCTTTCCTCTTTAAGTTGCTTTTGTCAGGGATTTTTATTGCAGGAACAGGAAATGAAACTCAAGCATTGCATTGTCCCTGGATGAAAAGCACAAGTTTCACTAAGGAGGAGGGCAGAGACGACTGGTGTCCTAGCTGTCCACCAAACAGTGACTGCAGAGAACACATATGATCTCTGTTTTTGTACTGGAAGATCAAACAAAAAGAATGCAGCAGAGAAGCTCCTGCCCGGAATGCAAAGAAAGCATTTGCTGTCTTCTTACCCTCTGAAGAACTGGTACAGGGAAAGATCTGCCATCAGCCCTGGAGTTTACTTCTTTCTGTCATTACAAATTACAT

							AGCGAGTCATCAACCATATATTAAGCATGAGGTAACTAGGCTCAGAAAGGATAAGACATTGGCTCTGTT CCTTGGCTTAAAAGCATCAAGTCGGGTTGTGCACTCAACTTCATGAATCCATAAACACATGGTATAACC ATCTTTCCACAAGCAACAGTCATGGCTGAGGTCTGGTCATCCTCTCTAGCTGCAGAAGGGGGATGGGTA ATGGAGCTCAACGGAGCAGCAGAGGGCGCGTGGTGACTTGTTTCTTCAACAGGAAGCAGAACTCTGA GAAGACAGAGAAATTAAAGCGTCCCCCGCACAGAGCAGAAGGCAGTGCATGCCTGAGCCAGGAGCCA CCTCTAGTCAGAGATAAGTCAGAGAACGGACCACAGTCACGGAGATGTTTCTGGGGAGAAAAGACATG GCAGAAGTTGTTGCTTAGGGCACAGGATGCCATCCACACCTAAGGAGCTCCTACTGGCTGCACTGATGT GGGATTGTGTCCTAGTGATTCTGACTTCAGCCTCCTATTTCAGCTTCATGAGCAGTGAAGGAGGAATGG CAAAGGCAAACAAATGCCCAGGATTTCACAGTTGGTATCTTTCCGGGGGTTTTTGGTTAGTTTTTTTGTT TGTTTGTTTTGTGCTTTGTGATCGTTCTGAAAACTGATGGGGACTTTCTTTCTTTCTTTACATCTCCAAAT CCCATGGCTGTCTGATAATGGAGAGGAGGGGATGGCTGCTGGGCAGACTCCTGAGATGCTTTCTCTTTT AGTTCACAGTCAGCAACCTCTGATTTCACCTTCACTCTCTTTCTCCATTAGGCAACAGCCTTCTATGGTT ACAATGTCATTTTGGCAGGGGCTGGGAGGATAGAGAAACAACACTGCCTTGTTTCAAAGGCAGTCTCA TGGGTGAAAAAAGCCCTGCTAGCATGTAGCCTGCCTCTGCTTTTGCTTCTCTTGGGCTCCTGCGTGTTTC TGCAACACATGCAATTTACTACAAGCTAGAATAGATCGAAGCCCCACAGAAAATAAATCACTGTTACTC GCAGAGAACTCTTAGTGTTTAATTAACCCCTGGACCTCAAACAGCTCTCTGTAAACTCTCTCTTGCTGA ACAGCAGAATCTATATGGCAGTCCTTTGTTCTGGGATGTGAGTTCCTTTGGTCAAAAAAGACAAACTCC TTACCTATTCTCAACAGCGACTAAAAAACTAGTCTCCAGATGGTTTAAAGATAAAAATAAGGCAAGTTG TTTCTGTCCGCTGTTAAGATCAAAACCTGATCCAGAGAGAGTGCGTAGCCCTTGTCTCCTAATGATCCTC AGTCTATTATCCGGACTCTCCTCAGGCTACTGTGGTTAGGATGGGAAGGAAACTCTGCTAGGTCAGATAT TCTCTGCACCTCAGGGGCTCTCTCTGGTTAGTGAATTATTTAGTCTCCTTTAGAATTTTTAGGCATCCATC TCTTCAGAAACACAGCATCCTCTGGTGACAGCTTTGAATCTTCAGTCACTTCAGAGAGTACCCTCTGAG GAACCAGAGTCTAAGGCTGCAGGTCTATGACAGAAGGGAAATCTCTTTTGTGAACACTTCAACTCCAG GAGTAGATTCCAAAGACTCTTTCTTCTAAAAGTGAATCCTGGACAGATCCATAGGGGAAGGGAGAATTT AGAGGCCCTAAACCAGCCATCATTCACCTTTTATGAGTCAGAGACTCTTTAAAAAGCTCCCCAGATCTG TGAATCCTCTGTCTAGACAGAGGAATGCCCCAGTGACCCCTTTTTGATTATTCTTACAGCACCGTTGAAG CACAGGCTCATAATATTTGGTTCAATTATTGGTCAAAATGAGGCCACCTATCATGGCTTTAGTGAAGGCA CAGTGAGGAAGGAAGGTTTAAAAAATGGCTTTAGCATCCAAAAGTGAGGTACACAGAGCATGTGGAG AAGGGACACACTCTCCAAGGCCATCCCTGATTTCTGCTCTTAGAACTAGGCTGACACCACCCCTTATCT CCTGGGAACATGGACCCTGGCTACATCTTGCTCTGCTTTCTCTTATGGTCCTGGTGGAGGGCTCAGAAC CTCTTCAGAAACCAAGCCTTTGTGCCCTTTTCTATGGACAAATTCCTGCCACACACGGTTTCTAGGCTAG TGTCATTGTGTTTTATGGCCTTTTGGAGTTCTGGACTCCTCTCTCCAAGATGTGAAAGGACTTTGAATTA TTTTTCTCACAACTCATGTCTACCATTCTTGATCCTAGACAGTCTGCTCAGCTGAATCCAGGAGACGAAG GAGCATCAAGTATGGCCAGTTTCCTGTCCAGGGCCTCTGGAGATTCTGAAGCCTGGACTTCCTGATAAA ATGCACTGCATTTCAATGCCTTTGTGCCTTCTCCACTTTCCTCATAAACATTATTTTGTCATATTCTGTCAG CTGTGAGTCCATGTTCTTGTCTTCAAGAAGTTTTTGGTCCAATGGTGCAGGGAGAAATAGACTGTGACA CGCACTTCCACACCGAGTTAAACAGTTCAAAGCAAGGGAAAACAGTTCAAAGCACTATGTCTCTGACA GACAACGCTGGCTTGGAGAAAGAGCCCTCTGGCAACCCTAATGTAGGATGATGCTCATCACAGAGGAA GCCTCTTAGCATTTTTTTATAGACCATGTAGAATTTGAGGAAATGCAGGCATATAAGGAGAGATGAGGCA GAGGGGGAAGGTGAAAATGAAAGCAGCCCAAAGGAAGACAGAGGAGAGAGAAAAAATAAGAAAGCT TAAAAGGAACTCAGATGCCTCGATAGAATCAGCCTAGAATTCAAAGGTCCTAGATTGCAGATTGCAGAT

							ACAATGTGTATGACACTGATAGGCAAGTTTTCATAATTCATTTGCAATATTTTACCTATGAATTGAAATCAGTTGTATAATAATTCAAGTGTCTACTTTAAAAGTATATAATGCTGTAATACAATTCATCACCCCATGTAGATCCATGCTAAAAATTTTCCAAAGAGATCACATCTTTCTTTGAAAAGAAACAAACAAGCCTGTATCTCATATTAAAATATTTAGACATTACTAAGAGGTACATGCTGCCCAAAGTGTCATTTTGGAAATGGGACAGCTTCTCTGTGTTTTGTTACAATGATGTCCAAACTTTACTGAACAGTTAACATGTGTCCAGCAAGTTGCTATGCATACTGTATACCCCATCAATGCATGAACTCAATGTGTTAAGTTTGACTGTTATTATTGTATGTTATTATTCCCATTTTACAGAAGAGAAAGCTGAGGCTGGAAGATGCAGCTGCCTTTCTAAAAAAC
MSTRG.11873.1<br><br>NONCODE_ID：NONMMUT064278	ENSRNOG00000020332;ENSRNOG00000020354;ENSRNOG00000051534	0.496	0.000	0.000	-5.340	down	TGTAACTGTATTTATTGGTGGACCCAGGACCTCTGGTGAGGCCCTGGTGAGGAGGGACAAAGGATGAAGGAAGGTGCGTTGAACACTTTATGATGGAACTGCTTCCAGACTAGGCAAGGGGAAGGTGAGGCCTCAAGCGCACGGCCACACCCAGTTGCCCTCAGACAGAAGTGGATGACGGGCGGGCACTGGGGCAGCATAGCCAAAGGGGCCTACACACTCGCTGTATACATCCATACGGAGAGACTCAGGCTATCTCCGGGACTCCAAACCAGTGCAATCGACTTAGTGCAAATTCAAAAGGGGTGAGTGGGGGAGCAGAGTCACGGATGCTTTGAGTCTCTCAAGCAGAGAAGGAACAAAAGGAAAGAAATATAGAGTTACAGGAAAGGAGGAAGAAGAAAAAAGACAGGAGGGAGATGATGAAGTCATCCCGGGCTAGAGGCTTGGCTCCAGGATGATGTGGGTGGTGGTCTCCCGGGCCAGGCAGAGTTAGTTGGCCATGAAGATGGAGTCTCAGGGGTGGGTGGGTGCTGTGTGGGTCTGCTCTTTCAAGATGTTGTCACCGTCTGTTCCTTCAGCTTTACCTTCGAGCCGATTCCTGAGGCAGGTAGTGTAGTGGTTCTGAGTGCAGCATATTCTTGATTCAGAACTAGACGGACTTAAAGAAGTCCCCGGATTCAAAGGCCCAGACATGAGCTGGGTAGCACCATTTCTTTCATCTTGAGGGTTCAAGGTAGGGGGAAGAGGATCCAGAGAGCAGCAGAGATGTGTTAGCTCTTTGGGCAAAGGAAAGAACAGACGGCTTCTACGGCAGTGTCCCACGGCACCAGCCCACGGTGTTCAGGAAGGCTGGATGACTGTCCTTCTGTCCTCTCCATCACACCGGACCATGTCATGTCTTTCTATCACGTTGACCACACCTGTCATCCTCGCCTTCAGTGACTGGCAGGCACATCCACTTCTGCTAGAGACCTGGCCTAGTCTCCAGTCCGAGCTCTCCGAGACACCGATCACTGCTCCGTCGCCGCCCGCAAGGCCCTCCTTGGCCACGGACTCTGGAGGCCAGTGCCTCGCTGGCTCACCACCCCAGCTGCTTTACCTCGCTCTACCTGCACCCAACCTCCCTCCCTAGAAAGCTCATTCATTCTGCCCCACCTGCCTCGCCTTGTCTGGCTTCATCCTGTGCCCTTGACTCCCCTGGAAATGTTCTGTCTGTCATGCCAGACCCAGGGACTGAGCGGTTAGGGCATACAGTGCACCAAGTCCACTGTGGGCCCTTTCCGCACCAGTCCCTGGGCCGCAGTGCCTCATGGGAATGGTGTGTCTGCAGGCTGGACCCACTGCAGCCTCCTTGCTGTAAAATCCCTCTGGAGTCCGATACCTGCAGCCCCCGTTCCCTTCCTGTTGACACCCTGCACTGCCCTTCTTTCCCGTTCTCCGGCCATCGAAGCTCTGGCAAAGGCCTCAGAAGGCCCTGCCAGAGCCACTCTTGAACCTGCT
MSTRG.11884.6	ENSRNOG00000020369;ENSRNOG00000020405	1.458	9.413	0.000	3.454	up	AGAATACGTTCTTCAAATTTGGTGTAAACCTCTGAGAAGACAATTACATCAGATGAATAATAGTTTTATTTATAGATATATAAATGTTATGGCCTTGATGCAGGAGGCACAGGTTGGTAGATTGGTGGTTGATGCCATTGGTTGCAGTTTGTTTGTTAAGTAGTCCTCCTCCAGTGCTGGAATTTCAGCGTAGGCGCTCCTCACAGCTGCTGAATCCCATTTTCTCAGGGACAGTGGTGCTGTAATAGGATTGGCCGCTGTTTTCCTCATGGGACTCTGGGTTTTTAAACAATAACCACTGGTCCCTAAAGTGGAAGTTCCTGGTTTCTTTGGAGACTCAGTTATGTCATGTGATGTTTTGCTGAAGCAGACAGGTGAGAGGCTGTTTTGCTGATCCAGAGTTCTAGTGCCTTTCTCTCACTCTCTCTCTTGTTCCATTCAGAACACCGCAGTACTCTCCAAATCTTTTATATGCAGGCACGAGCTAATTGGAGAAGGACCCAAGCCTTCTGAGCCATCTCTGTTTGTTTGCATTGTTGCTGGCTGCATGTGCAGCGATCTGGGGCTGTGTGCAGTGCAAGTCGTCTGTACAGCAGGTCAGCCTGTTCTGTATCCTGTTGAGACATGGGAGATAAAGGAAAGCGAGCACAGGAAGGCTTGCTTTGGTGGAGACACCTGATCAGCTTCCTGTGCCCGTGAGAAGCCTTTGCAAAGTTGGCTACCATTTTTTCACCTCTTGATGCTAAGCTCTGATGTTTCACGGGAAGTTCTGCCCCAGCATCCTTGGACCTTTTAGGCTCAGAGAATCCCAACTATGTTGTGACATTGGTACT

							GTGGCTAAGTCTGAGCTCAGAGATGGGTTACTGTCATGGTCTGAATGGGGCTTAGGAGGGAGGTTCTCA<br>GTAGATCTATAGAAACTTGTAGACCCACTAGGTGTTTCATTTTCACCCCGAGGCAACTATACAGCCCAGC<br>CCTACTTGCCTATAGTGGGGAGGAGTGAATTTTCCCTCAAGTTGCTTGGGTTCTAATCCCCAGATTTGGA<br>GCCAATTTGTTGCTCTAGTTTCCAAAGTCAATGAGACCATATATAGAACATAGAATCCAATGCAATCAGG<br>TCAATCTCAGCTCCTCCTCTACAGGCCACTCTCCTTGCTCTTTTGCCCTTCCCTCTCTCCAGCTGCTACC<br>ACCATCAAACTTCCAGTCTCACCTTTCCCTTCTAGTCTCCAATCACAAGCTCTAGATTTATTGACAGGTT<br>AATATAGGAAGAAGGTTCCCATGAAACCACCTGGGCACTAAATGGTCTGTTCTCTGGGGGAAGAATTAG<br>CATCACAATACAAAAAGCAAGACTGGATAGATAATTTAAATGGGAAAACTACCTATGCTTTAAGACCTC<br>CCTCCAAAGACACAAGGAAGCCTATGGCCCAAGGCCCACAAGGGTTCCCAGGTTACCCCTTTGGCCTA<br>CAGCTTTCCAAAGTCCTATCTGTGGCCTAATTCCTTGCACAGCATCTCGCTAGAGCCTATGCACGGGGTA<br>TCAAGTGGGCCACTATATTCCTCAACTTCTCATGGAATCTAGGAACCCATCCCGAGATTTTCACAGGGAA<br>TGAAGGCTTTCTTCATGTTCGAAATACGACGTACAACATTCCAAAAGATCTCCATGTGGCTAAAGGCCT<br>TCTTCAAAGTGTTATTGAGACCTTCTGTCTTCCCTAGTTTCTTTGTGAAACAGAAAGGATCTTAGAGATT<br>CTTGGTGTGTCGTCAACTTCTGGAAAGATTACACTGGTATGTTAGGCCTTCCTGAGAAACACAAGGTCT<br>CAGGCCTTGACAGAATTCGATCTGTGTCCTCGAATATTGCAAAGTTGAACCTAAGGTTTTGCCTAGATTC<br>CCGGTCAGTCTAAGAATGTCCCAGGATCACACAGCATTTAAAGTCTTCTCCAATACATCCCTGGCATAGA<br>AGAGCTTTTGGTGTTCTTACTGCACCGAAGGCTTTCAAAAGGTATTCACGGAAACCTAAGGCCTCACCA<br>AATCCAAACTGTGGTTTTTCACTGGTTCTAACAGTCTTCCACATTCTCACTGAGGAAGAAATCATTCCCC<br>TCCTTCTCACTCATGGCTATAGCTTACCAGAGATTTTCCTTCAGATCTAAAGTCTGTCTTAGATTTTCATT<br>AAGGCTTAAAGTCTTCCTTAGGACCTCCTTGGACTTGAATGTTTTCATTGGTTCTCTCTTGGACTCAATC<br>CTGCTCCAGTTTCTCACGGGAACCTGTTCCATTTCTATGATGTTTCTAGGGTCCAAAGCTTTTCCCAGGT<br>ATTTTCAGAGGACTAGTACATTCTCTAAATTACCATTGAGGTGTCATGTTGTCAGGAGAATGTATCCGGT<br>ACCTTTTCTTCTGTCAGAGTCTCACTGATGTCTAATAATATCCCAGGTTGAAACTAGGTTCTGAGCAGTC<br>TCCTGTGTCTACCTGGAGCCTCATGCCATCCCAGGATCTCATGGACACCTAAGGCCTACCCAGTAATTCC<br>CTGCCTGTTTTGATCTCGACGGTCGGTATGCACAGTTGCTAGAAGAAAAGATGTAGTTTCTTATGCTGAG<br>TGTCTTCCCATAGGTATATGGCCAAGTCCCTAACTAAGGTCATAAATCTATCTTCCCAAGCTTCTCACCTC<br>TTGATGCTAAGCTCTGATGTTTCACAGGAAGACCTACCCCAGCGTCCTTGGTCCTTTTAGGCTCAACGA<br>ACCCCAACTATGTTGTGACATTGGTAATGGAGCTAAATCTGAGCTCAGAGATGGGTTACTGTCATGGTCT<br>GAATGGGGCATGGGAGGGAGGTTCTCAGTAGATCTATAGAAACTTGTAGACCCACTAGGTGTATCATTT<br>TTCACCCCGAGGCAAGTTTACAGCCCAGCCCTACTTGCCTATAGTGGGGAGGAGTGAATTTTCCCTCAA<br>GTTGCTTGGGTTCTAATCCCCAGGTTTGGAGCCATTTTGTTGCTCTAATTTCCAAAGCAAATGAGACCAT<br>ATATAGAACATACAATCCAATGCAATCAGGTCAATCTCAGCTCCTCCTCTACAGGCCACTCTCCTTGCTC<br>TTTTGCCCTTCTCTCTCTCCTAATGCTATCACTGTCAAACCTCCAGTCTCAACTTTCCCTTCTAGTCTCCA<br>ATCACATGCCCTAGATTTATTGACAGGTTAATATAGGAAGACGGTTCCCATGAGATCACCTGGGTACTTG<br>GTGGTCTGCTGTCTGGGGGCAGAATTAGCATCACAATACAAAAAGCAGGACTGGATGGATAATTTAAAT<br>GGGAACACTACGTATGCTTTAAGACCTCCCTCCAAAGACACAATGAAGCCTATGGCTCAAGACCCACA<br>AGGGTTCCCAGGTTACCCCTGTGGCTTTCCAAGTCGTATCTGTGGCCTAATTCCTTGCACAGCATCTTGC<br>TAGAGACTATACGGGGTATCAAGTGGGCCAATATATTCCTCAATTTCTCATGGAATGTAGGGATGGGAAT<br>GAAGGCTTTCTTCATGTTCGAAATGCGACGTACAACATTCCAAAAGATCTCCATATGGTTAAGGCCTTCT<br>TCAAAGTGTTATTGAGACCTCTGTTTTCCCTAGGTTCTTTGTGAAATAGAAAGGATCTTAGAGACTCTCA<br>GTGTGTCGTGAACTTCTGGCAAGATTACACTGGCATGTTAGGCCTTCCTGAGAAACACAAGGTCTCAGG

							CCTTGACAGGGTTCGATGTGTGTCCTACAATATTGCAAAGTTGAACCTAAGGTTTTGCCTAGATTCTCTGTCAGTCTAAGAAGGTCCCCAGGATCACACAGCATTTAACGTCTTCTGCAATACCTCCCCGGGATAGAAGAGCTTTTGGCGTTCTTACTGCGACCCAAGGCTTTCAAAAGGTATTCACGGAAACCTAAGGCCTCACCAAATCCAAACTGTGTTTTTTCACTGGTTCTAACAGTCTTCCACATTCTCACTGAGGAAGAAATCATTCCCCTCCTTCTCACTCATGGCTATAGCTTACCAGAGATTTTCCTTCGGATCTAAAGTCTGTCTTAGGTTCTCATTAAGTCTTAAAGTCTTCCTTAGGACCTCCTTGGACTCGAATGTTTTCCTTGGTTCTCTCTTGGACTCAATCCTGCTCCAGTTTCTCATTGGAACCTGTTCCATTTCTATGATGTTTCTAGGGTCCAAAGCTTTTCCCAGGTATTTTCAGAGGACTAGTACATTCTCTAAATTACCATTGAGGTGTCATGTTGTCAGGAGAATGTATCCGGTACCTTTTCTTCTGTCAGAGTCTCACTGATGTCTAATAATATCCCAGGTTGAAACTAGGTTCTGAGCAGTCTCCTGTGTCTACCTGGAGCCTCATGCCATCCCAGGATCTCATGGACACCTAAGGCCTACCCAGTAATTCCCTGCCTGTTTTGATCTCGACGGTCGGTATGCACAGTTGCTAGAAGAAAAGATGTAGTTTCTTATGCTGAGTGTCTTCCCATAGGTGTTCGGCCAAGTTCCTAACTAAGGTCAAAAATCTACCTTCCCAAGCATCTCACCTGCCTGAAGAATCTTATCTGAATCTTGGCTCAATTATTTCATGGAAGGAGGCAGTTGAATGGTTAAGATCGCAGGAAACCCTCCATCCGTAGGGAAGACCAACATAGTCCCTTTCAAAGTCTTCAGCAGTCCAGGAAACATCAAGCTGCAGTTTCTGTCCAGTCCATCCATCTGGCAGACACCACCCATGAATCAGCAAAGGGAGCTCCTTCCTGTACAAACAGAGTGGGTCCTCTGGATCCGAGAGAGTTCTTGAAAGTCACAAGAAGCTTATTCCAGCTTTTTGTGCCTTTGTCTCCCTCTAGTGTTCAACTCAGGACACTGTAGTACTCTCCAATTCTTTCACTGGCAGGGAGGAGCTAACTGGAGAAGGACCCACAGCATCTGGGCTTATGAGGCAACTCTTGTGTTTGCAGAGCTGATGGCAGCATGGGCAGCGATCTCCAGCAGCCTGCAGTGTACACAGTCTGTAGAGCAGGTCTGGTTCTTCTCTGTCCTGCCTGAAGAATCTTATCTGAATCTTGGCTCAATTCTTTCAGGGAAGGAGGCAGTTGAATGGTTAAGATCGCAGGAAACCCTCCATCCGTAGGGAAGACCAACATAGTCCCTTTCAAAGTCTTCAGTAGTCCTGAGGAAGTGACCTTCACATTCAATTTCTCATAGGAAGTAAGTTGGACTGTAGAAAGATTTCTCATTTTACACGGAGGTTTGGGTCACCATGAGCCAAGGTAAGATGATGCTAGGTAAACAGCACCTTTCTCTGGAACTCAGTTCAGTTGCTGCGTGAAGGAAAACGCCATACAATCTTAGTTTAGAATCTACAACTAACAGAATCGCTGGGAACCGAAACTAGAATTTCATAATGTATTTCAGTTATAAATCCTCTGGTTGCCCATGTCGGCGGGTCCGCACCTGAACCGGGGACTTCTTGCTACCTACATTGTGTCTCCTCGCTCTCTCC
MSTRG.11885.1	ENSRNOG00000020369;ENSRNOG00000020405;ENSRNOG00000020410	0.613	3.361	0.000	3.158	up	CTTGCTTTGGTGGAGACACCTGATCAGCTTCCTGTGCCCGTGAGAAGCCTTTGCAAAGTTGGCTACCATTTTTTCACCTCTTGATGCTAAGCTCTGATGTTTCACGGGAAGTTCTGCCCCAGCATCCTTGGACCTTTTAGGCTCAGAGAATCCCAACTATGTTGTGACATTGGTACTGTGGCTAAGTCTGAGCTCAGAGATGGGTTACTGTCATGGTCTGAATGGGGCTTAGGAGGGAGGTTCTCAGTAGATCTATAGAGACTTGTAGACCCACTAGGTGTTTCATTTTCACCCCGAGGCAACTTTACAGCCCAGCCCTACTTGCCTATAGTGGGGAGGAGTGAATTTTCCCTCAAGTTGCTTGGGTTCTAATCCCCAGGTTTGGAGCCAATTTGTTGCTCTAATTTCCAAAGCCAATGAGACCATATATAGAACATAGAATCCAATGCAATCAGGTCAATCTCAGCTCCTCCTCTACAGTCCACTCTCCTTGCTCTTTTGCCCTTCCCTCTCTCCAACTGCTACCACCATCAAACCTCCATTCTCATCTTTCCCCTCTACTCTCCGATCACACAATCTAGATTTATTGACAGGTTAATATAGGAAGAAGGTTCCCATGAAATCACCTGGGTATTTATGGTCTGCTCATTTGGGGGCAAACCCTCTTGAGGAAGCAGAATTAGCATCACAATACAAAAAGCAGGACTGGATAATTTAAATGGGAACACTACCTATGCTTTAAGACCTCCCTCCAAAGACACATGATGACTATGGCCCAGGGGCCTCAAGGTTTCACCGGTTATCACTGCAGCCTACAGTTTTCCAAAGTGGATTCTGTGGCTTAAGATTCTGGCCCAACATCTCACTAGAGCCTATGCAGAGAGTATCGATTGGGCCTATATATTCCTTAACTTGCCATGGAATCTAGAAACACATCACCAGATTTCCACTGGGTCTGAAGGCTTTCGTCATGTCCTCAATGTCACCTATATCATTCCACAGGTTCTCCATGTGGC

MSTRG.119168.1	ENSRNOG00000010543;ENSRNOG00000011543;ENSRNOG00000011567;ENSRNOG00000021420;ENSRNOG00000060379	0.698	0.000	0.000	-5.969	down	GCAATGAAGGTGGTCCTGCAATGAAGGTGGTCCTGCAATGAAGGCTGTTGAGAGGAATCCGACCGTGTTGCGTCTTCCTTGCTGGCCGAGGTGGTGTATTGCTATGCTACCCTGGCCTCAAACTCAAAATCTTCCAGCTTCAGGCTTCTAAGCACTGGGGTTACAACTGGGGTTAGAATCTGGATTTAGAACTCTCCATTCCTCCAGCATACTGTCTGCATGCCACCATGCTTCCTACCATGCCAATAATGTACTAGACCTCTGAATTCTAAGCCAACCCCAATGAAATGTTTTCCTTTTTAAGAGTTGGCATGGTCACGGTGTCTCTCCACAGCAATAGATACAAGTTGGTACCAGGAGTGGGGTATTGGTGTGATAAGCCAGACCATGTTTTTGTTTGGAGGAATGTGGACTTTGGTACTTTAGGTTAGGAAAGCAGTAGAATGCTTAATGATCAACCCTAGTAGGAGTATGGAAGACAGTGGTGCTGCTGAAGGTGATTTGAATGGTGGTGGCCTGGCTCAAGAGGTTTCAGAAGAGAGAAATGTAGCCTAGTGATATTGTGACATTTTGGTGAAAAATGTAGCCACTTTCTGCTCTTGTCCAAGAAATCTGCCCGAGGCTAAATTGAAGAATTAAAGAGTTATAGCTTTAACACAAGACATTTCAGAACAGCCTAGTATTGACTATGGTATGTAGCTATTAGGGACCAGTCTTATGCATATCTACAATGAAAAGGAGCACGCTGGACAAGGAAAAAATACAAAATATACAGTTTGAAATGAAAAGGGGCTCCAGGAAGTATAAACAGATTAAAGAAAAGCCCGATGCTATGTAGAATAAAGGGAATGGTGACCTCAGAGCAAGACCCCCTCCAGCTAAGCTTCCAACTTGTGAAAGAGAATTAAAGAAAAGCTTAGGGCCAGGCATAGTGGTACATGTCTTTAATTCCAGTACTGGGAAGGCAGAGGCTGGAGGATTTCTAAGTTCAAGGCCAGCCTGGTCTATAGAGTGAGTTTCAGAATAGCCAATCTTTGGCAATGAAGGAAGCCATCAAAAACAGAAAGCTAATGAAAATGTAATTGAATAAGGGAACCATATTCCAGCCCCAAAGCAGTAGAACTTGGTAGCTTTGGCTGTGAGGTTTTGGCTTTAGAGTCAAGGATACAAGAAAGGGTTATGGAACCTCCCTCTATGACTAAGGTAAGCATCGGAGGCCAGGCATGTATCCCTGCATAAAGGCCTAGAGAGGTCATTGAGAAAAGCTGTGATTGCCTTGGAGACCCCAAATTCTGAAGATGCCAGAGCCATGAGATGCCTGCCAAGGAAAGCTGCTAATAGGGAGTGGAACTGGCCCAAGAGAAAGAAGAGTATTTCAGTCAACAAAGACGAAAGGAGTGGGAGATATGAAGAGAAGATCACGTTGGTATCGGACATGGAAATGCAGAATTTGGAGTTTGTCCTGCTGGTTTTGGTCTTGTTTTGGTCCAGTATTTTTTCACTATGGTCCCTTTCCTGCTTTTTGGAATAGTAATGTATGTTCTGTGTCATTGTATGTTAGAAGTACCTGATCTGGTTTTCAATCTTGATTCTACAGCAGATTGCATTTAAGAGATTGCTAGAGTCCCAAAAAGAGACTTTGAACCTTTAAGCAAGGTTCAGACTGATAGG
MSTRG.119569.1	ENSRNOG00000008305	0.124	0.489	0.009	2.510	up	CACACCTCAATGCTTCTGCACCTGCTGTCCTTCTGCCTGGAAAAGCCTCTCTGTGCCTGTCCCCTGGCTAGCTCCTCCATTTTCAGATCTCACTAGCCACCTTCCTGCAGGCTGAGCCGACTGCCCTACTTTACGGTTTGATCCCTGCCCCAGGGCCACCATCAATTCTGGAACAATGGCATCACTGTTCTTATCCCTGTCTCTCTCGCCAGACCGAGGGCTCCTTGTGGGCAGGCACTGAGCCTTGCCTCTTTCTCTATCTCCCTGTAAATGGGAGCTGCACAGTGCTGGATGGGGAAGTCACCTGTAAATGCAGGAGGGGGTGCTGCTCCTGTCTCTCAGTGGGAAGCAGCCAAAAGCATCTGCCAGCTGTCTTCCAGCCATGCCTCACCCAGGCTTCGAGGTTCTTTAACCGAGATGACAAGATGTGACAGAAGCACTGAGAGACAGATAATTAGCAAAAGCTTCCTTTCCAGAGGCTTGCCTCCCTGTTCACCTTTTAACGTGTAGTATTTTAATATATGCAGAACTGCTTTCCCTGGCTCCAGTAGTCCTCCAGAGCTGGGCGATTGCAGGGACCCTGGTTCCCCTCCTTTTTTTTCTCCCAGGGTACACCGGCAGTCCCTGTACTTGGATCTTGAGCCACGGTGAGCCCCATTGAGGTGCCTGTCAGATTGCCAGCCTCTCTCACCTGCAGCGAGCGCCATCTCACTACATCTGAAAGCCACAGCTCCTCTCTGTCCAGGGCTCTGCACAGCTGGCTCCTTCACCTCGGACCCTCTCTGCAGCCGTGTGGCCATGGCCGTCCTAGGTGACCCTTGTGAAAGGGTTATTCAACCAGCTAAGAGGTTGGGACCCACAAGTTGAGAACCGATGCTCTAAGCCTTCGTGATAGAACTTTCAAGTTCAAGAAAACCTGTCAGCTTCATGATATCCTAAACTCTGCAAGGCTACCCTCAACTTGAAGATCATTATAGTCAAGTTAGAGAACAGACACGCACTCGGAAGACTGCATCTCAGTAACAACCACAGGTGGACCAGGGGTGTGGATGTGAGCGCTCGAGCTTATGGAATCTTCATGCCTTCGTCTGTAAAATGGATTGATA

							ACATCTTCTTCTCTGGGTGGCATCTTGCATTGATTACATGAGATTATGTATGGAAAGGACCTGGCTTATAGCCTGGGAATGGTGTGTGCTTAATACACGATAATTACACTAATAGTTGACGT
MSTRG.119569.2	ENSRNOG00000008305	0.000	0.280	0.004	3.943	up	GGAAAATGTAAAATTATTATGAAGGCAGCGGCAGTGCTGTCGGACAGAGAATTGGGGCACAGACCATAAGCCTGGAGCCTTAGGTGGAAACCACGGCTTCCGAAGATGAGACCAAGTCCTAGGTGACCCTTGTGAAAGGGTTATTCAACCAGCTAAGAGGTTGGGACCCACAAGTTGAGAACCGATGCTCTAAGCCTTCGTGATAGAACTTTCAAGTTCAAGAAAACCTGTCAGCTTCATGATATCCTAAACTCTGCAAGGCTACCCTCAACTTGAAGATCATTATAGTCAAGTTAGAGAACAGACACGCACTCGGAAGACTGCATCTCAGTAACAACCACAGGTGGACCAGGGGTGTGGATGTGAGCGCTCGAGCTTATGGAATCTTCATGCCTTCGTCTGTAAAATGGATTGATAACATCTTCTTCTCTGGGTGGCATCTTGCATTGATTACATGAGATTATGTATGGAAAGGACCTGGCTTATAGCCTGGGAATGGTGTGTGCTTAATACACGATAATTACACTAATAGTTGACGT
MSTRG.119633.1	ENSRNOG00000006604	0.558	0.000	0.001	-4.800	down	CAAGGCTTTTGGGTGAGCCTGGGCCTGGCTCTCCCTGGTCCCTCTGTAGTAATTAACAGTCCTTTGTTCCCGTCCCTGATCTCGTCTGGCAGAACCTTAATCATCCTCTTCCCTGCAATGAGCAGCCTTCTCATTAGGGCCTTAAGAACCTCTATTATAAACATGTAAATGGAACTCAACAAGGAGGGAGATATTTGACTGCTCCCGTTTCCTCTGTGCGGCTGCCAGCTCTGAGCTGCGCACGCCCGTGATTTCATTGCTCAGCAGCCCGGCGCCAGCGCGGGGGCCCCCACCGCCCGTCACCAGCCGCTCCATCTTCTCCTATTGGAATTTAATTACCTTCTATGGGAAATGTTTGAGGAACCTTTCTCCATCAATTGATTCTGAAGTCAGATTTTTATTCCAAGATGCTTAATGGTGCTTGAGCCATCCATGGGAACCTAAGAGCAGGTAGACCTGGGCCACAAGGAACACTTTAACAAGGTTAGGAAGGGTCATTACAGTTATGCCCCTGACTCTTCCCTGGTCAATCTGTCATCCAGCACAGATGTGTGTGAATCTCTTTCTGACGGCCCACAGACAATTAGATTCATGAGTCTCCTGATTTGGACTGGACTGGAAGTCCTTTCTGTTGTCTGCCTGATGTGGAACAAACCTGGGACAGGACTTGGGGATGCCCGGAGGTTGCCTGTTTGCTGACTGAGCACAGTAATGCTTCCGTTTGCACCTGAGTCCTTCGAACGTCTTGTTTCCTTTCCTCTCTGCAGACGTCTTCTGTCATTTCATGAAGACAGGTATCCAGGCAGATCCCTGCTCTGCCAGGGATGGTGTGGAGAACAGGTCAGAGGCCCGAGAAGGCAGCCTGCCATCTGATGACTCTGGGCTGGCCAGCATACCCAGCCC
MSTRG.119843.1	--	0.273	0.000	0.037	-3.684	down	CTCTGTCCCACAGGCCCTGCCTGGATGGGCTCTGGCACCCAGGTGCGTCCCACTATTGTGCTGTGGACTGAACCCAGAGCCGAGATGGAGTGAGGAGAGAGACCCGGGCTCTCACAGAGGATCATTTGTCTTTAAGACGACTGAGCTCCAGGCTCTGGCTGAAATGGCGACACAGCTGCCCCAGGGCTGCTGGCCCCTGAAGCTGCCCGGAGGATGGAGAGATAGGAGAGGACACATGTCCCTGAATGACACAGAGCTCTGCTTCCCGCTAGAGGTTATGGCTCCACCCACTGATCTCTTCAGACTCTCCTGGGAGGAAAGAAGGAGAGATGAGAAAATGAAGCCCGTGTCTTTGGACATGACCACACATTAGCAGGGGCATCCGCTGAGTACGTGTATCTACTTGGGGACGTGTGCTCTGGTCTCTTTAGACTTCTGAAAGTCATCCCGTCTGTCCCCCTGCTTCCAGACAAGTAGTCCTGTACCAATTCTCCCACACAGCAAATGGCTTTCGGATTTGTTTCAGACTCTCTAAGGCAAGATTTCTCACCCTCTCTTAGTCACCTGTTCCGGTATCTGGCTTACCACACCCACCCTCCGGAAGTTCTCCCCGCTCTGCTGAAATCCAATCCTTCAGGCAGCTCTCAGTGCACACATTGTGAGGAGGGTCTGAAGACACTGGACAAGCTGACAGCCTCATGCTTTTATGTGCCTAAGCAGTAACCTAGACGCAGAGCCAGGTACCTACTCTCCAGTTGAGGGGTGGCTATGTAAGCGAAT
MSTRG.120484.1	ENSRNOG00000008312;ENSRNOG000	0.000	0.312	0.007	3.835	up	AAACACTTCCCACCCACCACCCCTGCCCTACGTCTCTGATCTTTAAAGGCACAAGTGTTTAATCAGGTGAGTGCAGTAAAAGTGAGAGAAAAGAGCGATAGAAAGTTGCCAAGATCGAAAACAGTGGGCTCCAGATTCAGAATCTGTCCAGGTCAGGCACAAGCTTTTACCAAGCTGCACTCCTGACTGTAGACAGACGCAGAGGTCAAGAGCTAAGGCCCCTAGACTGTTTTGTGGATCGATCTCCACAAACTTCAACCTTGAGGTCCCAGATGGTCCAGGTGGTGGGAGAGCTGAAACCGAAGGCTCTACGGACTTGCTCCCCTGGCTAGGTCCGTGAA

	00008400;<br>ENSRNOG00000008530;ENSRNOG00000008680;<br>ENSRNOG00000048924;ENSRNOG00000050714						GATCTGGGGTTCAACCTTGGTCCCGCGCGCCCCCTGCTGGATACTGCCTGCAGAGTGCAGAGCAGATT<br>GTGGTGCATGTGGCTGCCACCAGCGTGCCTGCCTGGGAATCGAGACTTGCTGCTACAGCACCGGCA
MSTRG.120549.1	ENSRNOG00000009450	0.244	0.735	0.011	2.287	up	CTCTCACAGAGACAGCAAAGGACAGACACTGCCCCAGGTGTGAAGAGATTCTACCCTGCCCAGTCCCT<br>TTCCCATATGCAGTGTCTCTTCCACATGGAGCACTGCTGGAGGAAAGGATGATCTGCTCCGTCAGTCTC<br>TTGGTGACAAGGGACAAAACCCAGTTCATACAAAACCCTGGTGCAGAACAGGACAGTGTGGGAACAG<br>CTGTCTTGTTTCATTGTTGAGTTTGTGTTTGTGATTTGTGGTGTGAGGACATCAGACCCAGGACCTTATG<br>CATATGAGACCAGAACTCAGTAACTGAGCTGAAGCCTTAGCCCCTGTTTCTGTTATCATTTTTTGAGTCA<br>TGGCCTTACAACATAGTCCATGCAAGCCCTGAATTGGCGATTCTCCTGCTTCAGGATACCGAACAGTGG<br>CTGCCACCACAATCAACTGGAAACAGTCACTGTATTTACTCCATTCTCTGATGACAGTTGAAAAATCCA<br>GACATGAGAGCTTAGTTGCGCCAATTTGACTTCTGTCTTAGGGTTTTTCCTTTCACTTGGTACAGAGTCC<br>ACCTCTCCCAGGAGCTGAGGTTTCAAGGAAGACACAGAGTGTTTGGGAGGCCAACCTTTAGTTGAAGC<br>AAACACAGGAGGAGGAACACAGGTTCTTGGCAGCAGTCAAGAGGCATTGAACATTCATGTGGCCCAG<br>CAGTTACCCTTTGTCCCGAAGGTTGACATCAGGCTCACAGAGACCCTTGTGCATGACAGAGATGCAGCT<br>GTCCTCCATATTGTAAAAAGGAAATGGCATATTCATGTGATGAATGTAACAGCTTAGCATGCACACACTG<br>GTCGTGTTTCGCTTGTTTGCGTCCTCCCTGTCCTGGTCCAGATGCCCCTAGATGAGTATACGAAAGCGTT<br>GGTGCCAGCAGCACCTACCTTGCCTTGGCATTTCAGGATCCGTATGTGCCTAACAAGCAGTCACAGAGG<br>AGAGACATGTGCAAAGGACAAGGAGGGAAGCAGGGGTGTCCCAACTCCAAACAAACCACCTCACCAG<br>AGGGGCCAGCCTGAGCCCTGCAATGACTGACTGCCTTGGCCCCTCCCCCTCTGTAATCTCCGGGCTGGA<br>GCTGAAACAGAAGACAGGTTTTTAGGATGTTAATCCACTGTCTTCTCGGAGTGCCAGCTGTCTGAACAC<br>AGAGCAGTTTTACAGACTGAATGTCTCCCTCTGAAAGTGCTCCTGAGCTCCGGTATTCTACTGTGGACT<br>TCTGCTGGGAGGTGTCTGCCCTGTTCAATGCCTAGCTGCCCACAAACGACAGCCTAGTTCCCCCACATC<br>TACCCACCCTGTCCTGTTCTGCACTAGGGTTCCCTGTGAACTAGGTTTTGTCTCTTGTCACCAAGAGATA<br>GAGCCGATCAGCTCTTCCTCAGCACTGCCTCAGGTGGAGGAGACACTGCATGTTCCAGAAAGGTGAGG<br>CGATGGAGCCAACTGGAAGAGTTCCAGGTGCCTGGAGGTGACACAGTGTCAAGTGTGGACATCTCAG<br>GCTTCAAGCAGTGGTCCCAGAGCTGCAGAGCAGGGTCAGAAGCCAGTCTTCCAGACTGATTCCCAGTG<br>CCCAAACTTGAATAGTTCCAAAGCTACCATTCCTTGGCCCAAGTAGAGATGAATGTTTTCTCTGCTGCCC<br>AGTGACCAGCTTAACCCAGGGCTTAATCCAAGGACAACAAAAACCACCCACAGAGAGGAGGGGCAGA<br>AGATTGTTTCCTATTGGTGATTTGACAAGTTACCACAGACTTGGTAGCCTTAAAGAATATGTATTTTAGAC

							CTGGGACACCTGGCTCATGCTTGTCGGCCACGATTCAAGAAGCTGAATCAGAAGGAGGATTGGTGTGTATTCACAGTCAGCCCATTTGTGAGTTCCAAGGCAGCCTGAGCTACAGAGTGAGACCAAAAGACGAGGGCAGGAGAGACTGCTCAGTGGTTAAGGGCACTTGCTGGTCCTCACTGCTAGAGGACCTGAATTCCAATCCCAGCAGCTCACAACCATCTGCAAGTACAGCTCCAGGGGCTTGGATGTACTCTTCTAGCCTCTTTG
MSTRG.120901.1	ENSRNOG00000009193;ENSRNOG00000009303;ENSRNOG00000010156;ENSRNOG00000010176	0.000	0.664	0.002	4.137	up	TTCGAGTGAAAAAGCTGGAAGCCGCAGCTGCTGCACTGGCAGCCAAATCGGAGAAGATTGTTCCAGAGAAGGGGGCTGGAGACAAAAAACCTGCAGTAGGCAAAAAAGGAAAGAAGCCTGCAGGGAAAAAGGTGGTTACCAAGAAACCAGCAGAGAAGAAACCTACCACAGAAGAAAAAAAGTCTGCTGCATAAACTATTAAAATTTGTTAAATTCCAGAGAGCAAAAAGATACAGCCTATTTGAATAAATCAAAGATGCAGTG
MSTRG.120992.1	ENSRNOG00000011302;ENSRNOG00000054617	0.000	0.261	0.002	4.137	up	TAAGGAACACGGATAAACAAAGTGAACCTCTCCCTGCATGTCAATTATTTTTTGCAAAATGGGTGTGCTGGAACACAAACCAGGCTTGCAACACCCCAGGGCCACATGGCTTCTGCCTTTTATCCCTGGTGTATACCAAGCTGGCCTCAAATTCACAGAGATCCACCTGCCTCTGCCACTCGAATGCTAAGGTTAAAAGTGCGTGCCACCAGGCCCGCCCATGGCTGGGTTTGTTTGGGAACAGGGTTTTTTTTTTTTTTTTACTATGTAGTTAGTCAAATGGGGTAGCTTGAATCTTACCTTATAACCCAGCATTGTGTAGCAATTTCCTGGAGCATCCACCTTAGAAGGAAGTTTAAGACACAGGGTGTTCTATGGAGAGGTAAAACATTCCATTCTGTCAGTTACCGGATATATCTGCATCCAGGAAGAGGACGTGGCCTAGGGCAATCAGCTTTCTTCCTGGAGAGTCCTGTAGAAGGACCTAGATAAAATCTGGTGGCTGTCAGAATTCCTGGCAGCTGAAAGAATGAATGTTCCAGTCCTGGAGAGGGAGCATTTAGATGGCACATCATGAGACTTTGTGTTTATGTGTGTGTGTGTGTGTGTGTGTGTGTGTGTGTATGTGTGTGTGTGTGTTTATGTGTCTGTGCATAGATTAGGGGTGGGTGTG
MSTRG.121020.1	ENSRNOG00000029127;ENSRNOG00000033496	0.412	0.000	0.010	-4.070	down	GCAGAAACTGGACTTTCAGGTCTGGCTGCTGCCCCCAAGGCCACTGGAATGTGGGGGAGTGGAGACTACCAAGAGGCCCTGGGGACTTGGGGTGGGCAGATGGAGACCAGACCACCTAAAAGCAGAGGAGGGGCAGGTGAGGGGCAGACCACCATGAAAGAGAACCAAGTCGTAGCTCTTTCACTGGACTGCATGCTACAGAAAAGGACCCTAAAGTGCTCGGCCCACTGCAACCTGAGTGGTCTTCCTAGTCTCCAAGAAAAACCAGACGTAACTAGAGGGATTACATGCAAGCATCACCACACTAGGGCTTACAGACTTTAAAAACCGAGACTGGAAAGTTCTGATGAGGGCTGCTGACACCCGTGGCCTATCGCAGAGGGCTTCCTTCACGAGTTCCCAAAGCACACTGGATACAGGAGCCTGGCCGTGAGTTGGCATTCAAGTGAAACAGCTAATAATAATTAATAAAAAAAAAATGTAGTCGTGGTCATGGAGGCTACTGCTCTGTAATAAACCCTCTCTGGGCTCATTTACTTAATTCCCACAAGAATTACATTAGTATTTCTAGTACTTGGTTATAGATCAGAAATTGGGGGGATCCAAAATGATTGAGTCGCTTACTAGAAGGTACAGAGGAAGTGGGGAAACTGAGAGTCAAAGCCAGGTTAGCCTGCTACAGTGCCCACTGGAACACAG
MSTRG.121	ENSRNOG0000001	0.341	0.000	0.000	-5.027	down	CCACTTAGAAGCAAGCCCACCACAGCTCTCCAAGGAAGAAATGACAACCTGGCCTGAGGCAGATTAGTGAAAAGTTATGGTTTAGAGGCAGAATTAATAAGACTTGGTGAATGACTTGATGTTCAGGAGGTAAGAGACACCTGGGCTGTTTTTAAGTTTCTAGTGTCATTATCACAAACAGGATGTACAATGAGACAAGAGGACAG

088.2	5953						TAGCACTGAGGACCAATTTGTTACCAGGACAGAATCAACAAACGAGTGTAGTGAGCTGAATGCGTAAGTGAGAAGTGAAGCGACAAGTCAGACCAGAGATAGTGAAAGCTCCACTTGCTCGGGTGAGACAGGCGCAGATACTAATTACACTCAGAACAGAACTCAGGGAAGAGGACAGCAGTCTTCAAAGGAATAAATCCAGAAAGGGAAGACCACAAGAACTCTGAAGATGACAGGAATCACGGTGACTAAGGATGGCCTCGAATCCACGATGCAGCTGAAATCAACTTCTAACTTCTAGTCCTTGTGCCTCCACGAGTGCTGGCCTTACAGGTGTGTGCCACCATGCCTGGTTTATGTGGTGCTGGATGCTGATACTGTGCGCATGCTAGGTAAGCACTCTGCTTTTAAACATGAATATATTGGGTCACATGGAGGGAGGAGAGAAATGGACTCCAAGTTGCTGGGTGGAATACATAGTTGCTAAAGGTTTGGTTTTCATTAGAAAGTGGTAGTACAAATCTAGGAGTGGGTCTGAATGGGAGGAGGGCTTCAGGTCACTGGGAATTGTGCTCTCAAAGGTTTTCTGTTTTCCACTTTTGAACCATTTGAGCAGATACCCTCTGCTACATTCTCTCCACAAGATATATTGTCTCACCAGAGGCTCAAATCAACTAGGTCGAAAGATCATGAACAGAAAGACCTACAGCAATGTGCCAAAGCACACTCTTCCTAAGGCCTGTGTTTTCTTTTTCTCAGGAATGTTACATCAGGAGAAAGCAGAATACAGGAAGTAGAGTAATCAATCAATCAATCAATCAAACAGGGATTCAAATTCACACAACTTGGTTCTAGAACCCAGGAATTAAAACAAACAAACAAAACCACAAACTGATGGGCATCACAGGAGCTGAGAGAAAATAGGGTTTAAAGAGAGCAGAGGCTGGCAGTGTGGCTCACAGGTGGTGGGCCTGACTAGCTGCATGAAGAAGCCCTGAGTTAGTTTCCTAGAACCATGAAGAGGACTGAGCGGACAGACAAAGGTGGAAGAAAAAGAAAAAGAGGAAGAGGAAAAGAAGCACAATAAAAGCAACAATAAGAAAACGGGCCAGTGTTAAATAGAAAGGTTCTAGTTGTGCAAGTTAAGTCCCTAAAGCTAACTGTATCAGGTAAAGAGAATCCTGAGGAGGTATAAGGGGCAATAGATTTTCTGTGTGCAGACTGTGCATGTATATTATCTTATATAGCTTGTGATGCCCTTATGAAATGGGCCACAGTACTGACAGAAAATAGAAAGTTAAACAAGATGTTTGTTGCTCTTCCAGGGGACCCAAATTCAGTTCCCAGCACACACACTGTGTGGCTTACAACTGCCTCAAACTCCATCTTTAGGGGATTCAATGTCCTCTTCTGATCTACATATGGCATAGACACCCACAGACATAGATACTTACACATCAAGAAATGACATGCTTATAAAGAGTTATTAAATGACAAGGCCAATGGATGTCTCGACTCTGAAGCATTTTCTCTTTTTGTTATACCATGCTGTTCTCCAGAAGTCTAG
MSTRG.121309.1	ENSRNOG00000042133;ENSRNOG00000047234;ENSRNOG00000056577;ENSRNOG00000058463;ENSRNOG00000059588;ENSRNOG000	0.595	0.069	0.022	-2.282	down	GAATTCTCTCTCCCAATGTCCCTGAGTTATCAGCCATAATCAGCGGCCTACTAGGTCTGTGCTCATGGTATCTGTAATTATATTCATTTGTATGGATGCCCAGGGAATTCCACTGACTGGCCCCTTAAGTGCTTAACTGCAAACAAGGACATGCTTGAATTACAGCATCATTTGGGGTCTTGCACATTTCAAGGGACTGATCTTAAGTTTATATACTGTTTGAATGTGTGTGTGTGTGTGTGTGTTTGTGTGTGTGTGTGTTAGCACACAAAGCTTAAAAAGTAAACATAATAATAATTCTGTCTTTTAAGCATTTTTCCATAACACACACACTCTGTTTGTGTGTGTGTGTGTGTGTGTGTGTGTGTGTGTGTGTGTGTGTGTGTGCAGGGGAACACAATTATGATCTAGGAGAAATCATGAATCTGTCTAGCTTGAGTGGGAAGATATATAAGCTGACAGCTGAAATTGTATTTCAGTGTGATACCAAAATCATTTTCTCTCCCTTTCCTCTTTCCTGTTTTTCTATTTTTTTTTCTTCTTCCAGATTTAGAATCTTATGGGTAAGCCTCTTCCAGTGGCTTCATAATTAGCTAGATTAATTAGCACCTGTCTGTGGCTGTAAGAAATAGGGGCACACGGTGTGGACTCCTAGTCCACGCCAGACACTTAAACAAAACGGTGGGAGTCCCATGGCTAAGCCCCTCGACATCTGTATAAATTAAAACTATCTGGAGCCAGCCCAAGGCCTGTGTGCTCTCATGAGAGCTGGGCAGGTGCATGGAGCTGCACTGGCGATTTGTCCGGATGACCCTGACACGGTGAGCTTAAGGGTAGCTGTTGGAAAATACTGGAAACCCGAGTTCTGTCTTGGAAAAGTTCTGTCAAATTAACGGAAGTCAATGTAAATGCCAGTCGTGCAGATCTTCTAGGAGGTAACAAGACACAAAGCATCAGGCCATCAGACATGCTGCCCCTTGAAGATGGCTATTTATTTGGTTTGCAGAGCTTGTAAGAAAAGAAAGAGCTTTCAGTTGAGGAGGAGCCTGGCTGTTTTTCTTCCTTTCCGTTTGTTTATTTACAGAGGTGGCTCCTTTAAACTTTAACTCCTTATGCAGCCAAGGTTGAAGCTCTACATTCTGTTTCTCCTACCCCCTCCTCCTGAGTGCCAGGCAGGGTTAAAGGTGTGGACCACATATTTGTGACCTACATCTCCCACCCACAATAGGGGCCTGCCTGTTTCTTTACATAGCAT

	00060573						CTGCTAAACATTCTGAGACAGTCAAAACTTTAAATCCTTTGTACAGAAGACCTGAAAGGCGTCAAGGC<br>TGAGCGGTGACATACCTTGCAACCCCGCGTTCCCTAACCTCACAGGAACTCGTCCCCGAATACCTCCAG<br>CTTCAGTAATATGTTTAATTTGTTTAATAATTTCCAAGAGGTTTTAATATTCCTAGCACATCATCTTCAAAA<br>ATAAATTCATAACAGCCTTATTAAAGTCACTCCATCATGTGACATAAATCGAATCTTTTCCCTCTTGGAGC<br>TAGGCTTCCAAGCGTTGTATAGCACACGTTCAAGGTCAAGGGGCACAAAGCCAGAAGTACACATGGAA<br>ACAGTCTGAAGGCTGTGACACACTGCCTTTCCAAGGAGATCCCCTGCTGAATGGTTCCTGGGTAGGTGT<br>CTAATATAGTAATATATTTATAATTGGATTTTAATTGCTATCAGATTTTAGTGTGTGTCTGTATTTTCAGTTT<br>GCTTCTACCGGAAGGACAATGCTCCAAATGAAATTATACAGTGCCTCTCCCAGGGCAATTTGTTTTCCAC<br>CAAAAATATATTGGAGGAATAAAATGCACGCGGTGATGTTGTTTGCCTTAGTTTAATAACTTAACACACG<br>GAGTCGATGGCGGTTTTTACTTTTTTCTCTCTGTGGCTCACTGCAGCAGCACAGGTCTGAGGGCAGATC<br>ATGGGCAGTGTGCCACGGAGTCCCAGGAAATGCTCCTGTGTCCTGAGGTGTTAATTCTGTCATCTGTAA<br>AATGGGACAGGCAGGCTTACTTATCTGATGGGCTAGTTCATGGCCTCTAGCAGATCTACCTTGAGAGTA<br>GGAAGTGAGACATGAGGAACTTAAATGCCTCCGTTATTGATGGTTTCATAGTATTGGGCTTCACAGTCCT<br>CGTGGTATAGTTATTTGAAAACGACAGAAGGGACGAGGCTTGGTTCGAAGAGGGCTTCACTAGCAGGT<br>AGGAGGGGCTTGGTTTGTAGAGGGCTTCACTAGCAGGTAGGAGGGCTTGGTTTGTAGAGGGCTTCACT<br>AGCAGGTAGGAGGGGCTTGGTTTGTAGAGGGCTTCACTAGCAGGTAGGAGAGGCTTGGTTTTGATCCC<br>CAGGGCTGCAAAATGGTGCGGGCAATTCCAGTATTCAGGAGGTGGCATGGGGTACAAGGTCTCTTTTAC<br>CTCCATAGTGTGCTGGAACTGACTTGGATTACACAGTAACCTGTAGACACACACACTTATCACACACAC<br>TTATGCAGGCATGCAGATTCTCTTTTTGTCTCTCTGCCTCTCTCACACACACTCTCTCACACACACACAT<br>GCACACATGTTATCTCTCCCCCCTCCCTTTCTCTCTCACACACATTCTCTTTCAAAATAAACAGATGCATA<br>GACGCACAGATACATGCATGAATGCTGCCCACTCTCTTGCTCTCTCTCTTCCTCTTTCTCAAACACACCC<br>CCTCTCCTTCCTTCTCAAACTCTAACACACACACACACACACACACACACTCTCTCTCTCTCTCTCTCAC<br>ACACACACACACACACACACACTCACATTCTCTCTGTCTCTTGCTCTTTCTCTGTCTTTCACATACAGGTGC<br>ACACAAACACATAAGACATACACTAAAACACTGAATAAATGATTATAAGAGATAAGAGAGAGTGAGGA<br>AATGAGGTGATGAGAGAAAGGCTTGGGATTTGTTAGGATGAAGGCATTCTAGGCAGCCACTGCACAGC<br>ACAGTGGCTGCAGTTGGTGACAATGTACTTTGAACATGAATATTGCTAGAGTAGATTTTAATGAACTCAC<br>CGCCAAAAAAATTAAGAAAGTAGGGTATGTATGTGTGAAGCTATGGATATGCCAATTAACTGTCTTCAGG<br>CACTTTACAGTGTATACACATGTCAAAAGATCACAGTGTTCACTGGGCATGTGTATTGTTTATGTGTTATC<br>GATCCTTATGTAAAGCCGGAGGAGACTTTGAGAAGACACAGGGCTTCCAGAAGTGGCTGTTGATCGAA<br>GGGTGCTTGTATTAGCCAAGGTCCGTGTCTTAGGTCCCCAGCAGAAGTGCCATCTAAAGACAAGTGACC<br>AGATGTCTTCCCAGCACTGAGTGCACAGCAGCACATCTATAATTTAATGTAATAGGTGTTTCCAAAGAGA<br>TCGCCTCGTTAATCCTCGTGTCAACTGGTGTTATCCTATGGCTTAAAAAGTAGATATTTCCCTACTGTTTA<br>AGTGTTAAGTTAATGTTACGTGTCGTTTTTCAAGATGCGCTCGTGGAACTGAGGTCATTTCAACTCAAAT<br>GCATTTGCTAAGGGCTGGAGAGATTGCTGCAAGGTTCAGAGCACTTGTTGCTTTTGCAAAGGACCAGA<br>GTTCACTTCCCAGTATCCACAGGGTGACTCACTATCTGTAACTCCAGTTCCTGGAGATCTCATGTCCCCT<br>TTTGGACTCCACAGGCATTGCACCCACATGGTATGTACAGACAGATAAAGCACACTCATAAAATAAAAA<br>GAACTTAATAAAATTGCCTATAGGGCCTTTGAGAGAATAGGTGTTTTAGGTTTATTCCTGCCAGCAATTAT<br>ATTTGTGTGACTCATGATGTAATGCACGCATACCAGTGTGTGAAGATGCACGTCAGAGGACAAGTGCGA<br>GTCTTGGAGCTCATCAAGTACTGTATGCTGGCAAGCAGGCTAGCAGCTTGCAGGGATCCTCCTGTCTCC<br>TCGTCCAATCTTGCTATAGGAGCCCTGGGATGACAGTGTGTGTCCCTGCACTCAGCTTTCTTATAGGGCC<br>ATATCCACCTGCTTTTGTGCCAAGCACTTTACCCATGGACCCCATCTCTTTAGCCCTCTGATTGAAAACT

							GGAAGGGTAGGCCTCCTCTCTAGGATGAAATCTCATCCTAAGGAGTCCAGTGCGAGCTGACAGTCACA<br>GTGACTGGATGGGTTTGGTTTGAGCTGAAATCAGCAGGCCTTTAACAGCATTAGGTCACCCATCTGTGT<br>CTACGTGTTAGAACCCTGTGGTGATCCTCCTGGGTCCAGAAGAAACCTGTCCTCACATGAGGGGAATGC<br>ACAGTCTGCAGGCCGTTACCCTCTTATTGCCTGGAATACCAAGGATGATAAACGAAAGCTCACTGAGCT<br>GATGTCAGTGTATGGGAGACTAGTGCCCGAGTTTGTGGTGTCCCGTTCCTCATATAATGCAGATCTCTTG<br>ACACTGTGGTGATTGGCTTTTGATAGGAATATTGTTAATGGATACAAAATATGACATCTAATGGAATTTTA<br>AAACAAGGCACTCTTACGTTTTATAGCCAATTTGTTTTCAGTACTACTGTTACAGGAAGAGTTGGCAATG<br>CTAAAAAAAAAAAAAAAAGGAAAGAAAGAAAAGAACAAAATTCTTTAGTTCCCTCCATGAGTAAACAC<br>CCCTTCTTCCACCCTTCTCCCGCAGGGGTACATGAGGACAGACTGTCTGAGGGGGTGGGGGATACTGA<br>GAGTGTTAGTAGCCTAAAGGCTATAAAAACCAGCTTGAGGAATGTAGTGGGGTAAGAAAGGGCTTCTG<br>TTATAGCCATATGAATTTGGATGGAAGGCTAGCCAACCTCCATGGAACTGGGCCCTCTTGTCAGATGAG<br>GAAGCTGAGGTCCAGAGCCAGGCTTAGTTCTGCTTAGAGTTCATTTCAAGGCCCCTTGGCAGCACAGG<br>ATGGTCTAGAATTGAAGTTCTATTGTGCCCTGTCAAGGTGTGAGAAGGAAAGGAACTAGAGAAAGGTT<br>ATGAGAAAAAGAAAGCAAGAAACATATGGAACCCAGGCGAGACCTGGCTTTTAAACGATTGTTCACCA<br>CAGAAACAATGCGCGTATTCTTTTCTGGAGTATCTCTGCTGCAGAGCCAGCCTCTGGCTCACCATTTGG<br>GCCTTGGGTCTGCCTGAGTTTCCTCCGGAGAGGTCAGGACTATGAGGCTAACTCCACACTCCTTTCAGA<br>GGCTGGCAGAGAAGCTGCTGGAATTGGCATGAATCACAGGCCTGCCCCTGCCTCCTCCGATACCTCCCA<br>GGATATCCATTTTGTTCCTGCCTAGAGGTAGACTGCAGTCAGGGTCCAAATATATAGAAAACAAAACCC<br>AGCAGCATGAATTATTACACGCCGTACCCACCTGAATATAAAAATAGTGTTTCCCCCAATGTCGCATTATC<br>TCCTGGTCCTGATAAGGGAAATACCTTACTGGAGGCCGAGCCAGGTGTAATAGGATGAGAGGTACAGG<br>G
MSTRG.121318.2	--	0.000	0.246	0.000	5.095	up	ATTTTATGAAGAATCAGGGTATAAAATGAGATTGTTATCAGTTTTGTTGAGCATTAGCCACTGGCTGTGA<br>GATACTAGATGTAAGATAAACAGTTTGAGCTTTTTCTTTTATCTTTATCCTATTTGGTAAAATTAACATGGT<br>CATTGAAAACAAGACTGTTTTATGAAGGTTCGGTAAAGCAAGTGGGCATGTACACCACAAAACACAGA<br>CAGGCTGCTGGGCATGCATCTTTACCCATTTTGTGAATTACTTTGTGCAAGATAAATTCCCTGGTATCTTA<br>CACCCATTACCTACGAACTCACCATTTGTTTCATGATGTGTTATATGGGGCAAATTTAAAACAGCAGCTG<br>GGTGTTTCTCGTTATCCCAATTTAATGGAGCTAATATTGGTAATTGGAGGTGGGAGTGGCTCAGCAGAGA<br>ATACTTTGTATTTAAAAATAAACTAAGAAGTATCATCACTATGAAGCTATCCCCCCACCCCCAGTTCTTCT<br>AGTTATTTATCTTAGAGGTGCATTAATTAATTTTCTTTCATTATCTGGTACTTAGTCACAAAATGCTGTCAC<br>TCTCATTATGTCATCCTTCAAGTTGCTCTGTGCATGTTGTGTGGCCGTGCATTCATTCATTCATTCTCCAA<br>GCTGACATTGACTCAGAAATCAAGATACAGAGCACACATGCACACACACACACACACACACACACATG<br>CACACACATGTACACATACATGCACATATGTGCACACACATACACACACAGGAACATACACATGCACAC<br>ACAGGAACATGCACACATAGGAACATACAATGCACACACACACATACAAACACACATGCACACACACA<br>TGCAAATACACATGCACACACACACAGGACTCTGACACACACAGAACCTTTGTGTGCAGAAGTTCACA<br>GGTAGTCAGGGATAATGGTAGAATCAAAGCTGAATGGTGAGCCCCAGATGAAACCGGAGTCAGTGAAA<br>GATCAGACAGGAATCTGTGATAACTTTACTAGTGCTATAAAACTTGAAAACTGAGAAAAGAATGAGGA<br>AAAATGGATGGGACTAGAAAATGTTAATCAAGAAAACCCAAACTCAAAAAGACAAGTGTTATATATAAT<br>CTCTCACATGCAGAGCAGAGCTTCTAAATTATTATGTATTTACATGGAATGAATATAGTGGAGACCATGGT<br>ATTAGCAAGGGACCCATATGATGGGAAGAGGCACTAAGAAAGGGAGCAGGGAAGGTCGTAGAATGCA<br>CATGGCATCTAAGTGGAAAGGGACAGCAAGGAAATGGAGGAGATGGGCCAGAGAAGAGGATCAACCA<br>AAGCTTAGAAATTCCGATGCCTTGTACGCTAATGTAAGTAATCCCAAGCAAACATGGAGGAAAGCATGC

							CTTAGGAATTAAAATAAGTAGGCACTGCCTCAGAGACGGACATAAGAAAGAGCAGGTGAGCCAGCCAG<br>CCATGACTCACCCACACAGGCAGCAGATAATGGAGCTGGCATGGGTAACAG
MSTRG.121321.1	--	0.385	0.003	0.000	-4.894	down	GTGATCCTAGCCAACTTGATGAGGTTCTAACCAATGAGAGAGGCTGTCTCAAACAAAAAATAGGAGGT<br>ACCTCAGGAATAACATCTGAGATTGTTCTCTGGCCTACACACACACACACACACACACACACACACAC<br>ACACACACACACAGAGTCCACTGCACCACTCTGCCATTGTTGCTACACTGCCACAGACCTGCCACCCA<br>CTTCCCAAGCCAAGGTTCTTTGTAGTCACTGTGTTTGTCTACTTGCGTCAATGTCACCCAAAAGAACTT<br>AAACACATGAACTATTTACTTTGGTACACAGTTTAGCTGGAGATCGCCTGACTGCAGACCTCTGGGCTT<br>GTGCTAAGGCAGAGCATCATGGTGGAAGAGTATAGCAGAACAGGAGAGCTGTCCTCATGGCAGCTGGA<br>GATGAGAATGACTACAAAACTGGGCTTCCTCCTCCTTCCCCTTTGGATCTAGCCAGGCCACCAGCCCTT<br>TCAGGAAAGGTCTTTGGCTCTCAGTGAGTTGTCTGTGAGAATGTCATCTCAGATATACCCACAGGCTTG<br>CTTGGCCAATCTCACAGGAGTGTGTCAGTGTGATCAAGCTAAGAGTCAAGATCTCCCCATCGCATCTAT<br>CATGGGCTAGCGTAGGAGCCACCCCCTCAGCTAAAACCCAAAGAGCAAGGGTAATACTGGCCTCTCCA<br>AAATCTCCTTCACTCTCAGATACACTTGCCCCTTCGTTACATGATTAAGTGTCCAATCTTCCCTCTCACAT<br>AGCATAATTAGTTTGTGCACTGAACACGTGTTTACCTCACCTTTCAAAAGTCCCCAGAGTATTTGGCCA<br>GGTACGCTGGACATAGCAGTGTGTAGTAGATGGTTCAGTCACTAGCACCTGGCATCTATAGCTGATTGTA<br>GTCCGTAAAAGAATAAGCCTAACCTTGTCTATTCTCACCTCACGAATAGGAAAGCCTGGTGAGCAGACT<br>GATTGAATCCTGATACCATCATCACAAAGACCACTTCATGGAGATCTTTTCTTTGACCTAATCAACACTG<br>ATTAGCCTTCAACAGCAAGGGAGGGAAACCAGGCCAGCGACCCAGAAAGGCAGAGAATTCCAAATCT<br>CATCCCCTTGACATCGCTCTGAACTTACTGTTCTGCCTAAGCTCCTGGTCAGCTTGCATTCTCTTTCTGG<br>GAGACAAAAGGTAACACCCTAGAGATTCAGAAGGAATCAAAAGCAGAAAGAAACAGTGGTCTGGGCA<br>GAATTATCCTCAGAGTTGCCATTATTTACATAATGTCTCATCTGTTTAATGGTTTTCCAAGGGGAAAAGCC<br>ACCTACGGATGCCACTAGTCACTTTTGATTTCCTGGAAGTCAGAGACGCCTCTTATTTAAAGGCCTTGA<br>GAAAGCCATGACCTGGGAACAGCTGAGGTAACTTGAAACTTTGAACAAATATTTATAGTACTTAATATGT<br>GGCTGGCCACATGCCAGGTACTGCATGATACCTGAGCCGTTCAACCACCTTTTGAGAGACTTTGTAAAC<br>AAGCTTGTCAGTCATGAGCCTAGCAAGTATCAACTGAGCTCGAAGCGTAAGTTGAAAGCATATTATTGA<br>GAGGATCATTTCTCCAATGAGAAGATACCCCAGGATACTCCATATTATTGTCCACATGGGTAACACTCCA<br>GGAATTTTCATGGTAATAAAATCCTATTTCCACAAATTACCCAATTATCTGATGGGTTCTCTATCTTGGAG<br>ACATTTCCCATTTGTTCACTCAACTCTATTCTGGTGTCTGACCATTTTTTTTTAAGACTAAGGGCACTAAA<br>AGAAATTTTATTGCCGGGGCTGGGGATTTAGCTCAGTGGTAGAGCGCTTACCTAGGAAGCGCAAGGCCC<br>TAGGTTCGGTCCCCAGCTCCGAAAAAAGAACAAAAAAAAATTTTTATTGCCATAGTTATTATAGGAACT<br>GTCCTGGTTAGTTTCAACTGTCACCTTGACACAATCTAGAATCACCTGGGAAGATAGTTTTAATGAGGA<br>ATCACCTAGATCAGGTTGGCCGTGGTGGGGTGGGGTGGGCAGGGGATATTTTGACTTTTAAACTGATAA<br>TATCCAAATCATTATGGGCAACACCATTCCCTAGGCTGGTCATTGTAAAAGCGAAGAAAGCGATCAAGG<br>AAGTGAGTAGATAACATTGACGTATTTATTCTCTTTGTTCTTGACTGTGGATATGATCAGACTAGCTGCTT<br>GAGCTTCTGCCTTAACTTCCCTGAAATGACTAACTGTAAGTCAAATGAATCGTGTCTTCTCTTATGTTGT<br>TTTTGTTGTTGTTTTGTTTTGTTTTGTTTTGCTTTGCTTTGCTTTGCTTTGCTTTTCTTTGGTTTGGTTTGG<br>TTTGGTTTGGGTTTGGTCTGGGTATTTTATTACAGTAACAGAAATGAAACTAGAACAGAAATTTACAATT<br>TTATAGTCATTCTAGCCAACATAGGGAGGTTGCGTGTTCTCATTTCCAGACATCCCGTCTTCTGAAGGGC<br>ACCACCTTGGTAGGGGATCCTCAAATGATTCTGTCTGAGGTTGACAGAAAAAAAATCTCTTCATGTGTT<br>CTGTGTACGCTGGTAACCTCCTTGTTTGAAGTCTGCCACCTTCACCTTAATGGGAATAGTCAGGTAGCCA<br>AATTGTGTTTGGCAAGCCACAGTGAGAGTAGCTCTTTCTCACCTATCGACTAGAAGGCAGGAGGTGTGC

							TTAGACGCTTTCTGTATTCTCTTCTTTCTGTGTGTTCCCCCTGGAATATCCTTTACTCTCCTGTGGACAAGGTCTGTGGGAAAGAAGAGAAGAAGAGATAGTATTAGATCCCCTAGCTTCATTCTTCTCACCCTGATGCACTTATGCTATCTCCAATTGGAGGGATTTGGTCTTGTGTGTTGTGTTGCCATAATAAAACACCTGGAACTGAGTGCCATACGGTGATAAAAAATATATTTCTCCTGCCTCTGGAAGTTGAGAAGCCCAAGAAGAGGAGAACCATGTTGCCTCCTCTCACGGCAAAAGAAGACAGTCAAGAGGATGAAAGCACAGGGCAGCAAGGGCAGCAAGGGC
MSTRG.121322.2	ENSRNOG00000060573	0.000	0.226	0.000	7.027	up	TATAAACTTGGCCATTGTGGATTACTCCAACTTGTAACCCCACATCCCTCCATGAGGTCAGAGATGATGCTAGGAGGTCCATCTCTGCATTATTTGGTCTGTTCCCTTAGCGATTAGTGCCTAAGCCCTTGAGACTTTCCAGTAGTCACCTCATTAGCAGGAATCCTGGAGTAGGTAAACTGGGCTCGCCACCATAACTAAGCACCCTCTCCTTTCACCTCTATTACTCTCACCTCTTAGGAAGCTGAGGGTTCTAGAAACTCGATGCCAGGGATGGATAAGGACCAAATATATATGTTATTAGTTACCTAATAGAGTATATCATCTCTTATGTCAATGTACATGTTTATTGTCATCATATCAGTTCCCAGAGTAACATAAAAGATTAATGTTGTCAGGAAGGTTCTTCGGTAGAGCCCTTGGCACTGAGGCAGATACTAAGAGCTACACAGAATGACTGTGGACAACAGGGGTTCCTCTGTGGTCTAGAGAACCAGTGCTTCCCTTTGGCTGAGACCTGAACGGGACCTTTTATGGAACAATGGGCCTTTGAGCTGATCTGGACTGAACCAGCCCTGGAAAAGGCTGGATGAAAGATGTTAGGCAAAACATGGCTGAGATGGTTTCTTTCTTGGGAAGTGTGTTGCGCCTCTGTCCTATGTCTGTGGAGGAGCCACATTCTTGGTACCTAAGAGGAAGCATAATGGCAGGGTGTTCCTGCAATATAGCTGAGGCTCCACAAGCAATTCCATTATCGCTGTTCAGAGCGAGTCTCCTAAGACACATTCAGCTCCAAGCAAAAACAACACCACAGGGCTGTTTTCCTGAACTAATTTCACTCATTTGCCCCAGTTAATATTAATACGCCCAAGGCCATATGCTCCTACTCATCAGAGCACATGGATAATAGACAGCATCGTAGCATCTTCAGGTTGAAATGGACCTTAGGTTCTTTCAGTCCAAGAGACCCACTGGAGAGGCAAGGAAACAGGTGAAAGAGAACTGAGAGTCAGACACACAGAGCTAGAGCCACAGGCAGGTCCTGACTGCCAGTCTGCAGAAGGTTCATCCACAGTCCCTGCTGGCTTCATATGAGCGCCTCCCTAAGTGGTAGGTAGAGAGACTATCGGGTGAGCCAGTGAGACTTAAACTTCACTGGTCTCTCTCCAGTGTCTCTGGCCTCTCATTCACCGAGAGAGCATCCACTTGCCTGTCCAAGTGGATAACTGGTTTTGCTCTATGAAGGAAAAGGCAGGATAGAACAATCTACCACGGTCGCAGTACCGTTCTCCTCCTGAATCAGCTCCAACTTGGACCAGATCCCAGTGATGATGGTGCTCTCTGACTCTGCACATGTTTACAGAACTCAGAGAAGCCACCCCTTACTAGACTGTTGCAACAACTACATGAAACGGACAACAAAGCAGAGCCTCCCTCGACTGTTTCTGAAACACTGACTCCAAGAATCCCACAGATGCCAACATCCGAAGGCTCACAAGCTCTTTTATAAATTAGGGTGTTGTTCGCCAGTAACCTACACATGCTTTCGCCTGTTTATAGTCCCCCATACGATGTGGACGCTATATAACCATATTGCGTCATATTGTTTAAGGAATGATAACGAGGAGGAGAGTGTTCATCGGTATAGGTGATTTTGTTCCTAAACAGTTTTTCACCTTCAGCTCACTGAATCTCCGGGTGCAGAACCCTTGAATACAGAACTGGCAGACGCAGGGTCCTACTGCCTGTAGCTCTAAGCCTGGCCCTGGGAGGACCCAGGATGCTTCGTTCTCCAGTAGTTTGTCCTTGCTGATGCCATGTTGAGTCTGTGTCTTTAAGGGACAGCAGTCACCCAGAATTCCATGTGTTCCACATGTATAACGGTAATTACTTAGCACTACCACCTCCACGGATAAAGTCAGTCAGCCTTTGGAACTTATTTCTACTTCTCTTCAGCAATCTGAAACCTTCATTCTCACCTACATGGAACCTTCTGGTCACAAACAAATAAATCACATTTCCAGTAGTCCCAGGATAGTTTGGCTTGGATGAAGAAAACAGGGTGGGAGTGGCAACATGAAGGACATTTGCTATGTTAGGCTGTCACTAGGTCCCCAGTGGAATCAAGAAAAATTACAGCCAATAAAACAACATTTTTCAAAACATGGCACTACCCTTAAATTTATCCTGAGAGAGAAATAGGTTTGGAAAGGAATATTTGACACTCAATATCACAAATGAATTGCGTTATTAATTTATAATAGAATAATGGTTTTGTTTCAACAGTCATTTTCCTGAGCGTTGACATTAATTTTCTTCTTCTTTTGAATCATCTCAAAATACTGGCATGTACAGCACCCCCTACATGCTACCCAAACCTCTTCACTCCTGCTGTAAAAATACAAAAGCGATTCCGACTGGTTGGCTC

							ATGGGAACTGCTGGCTGTCCTTTATGGTAATGAGAGGAATGAAGACAAAAGCAGCTGATATTGACGGA<br>GGCCGACTCTCTGCCAAACAGGGCACTCAGGAGTTTAGCTGGATTGTTCCATTTAATCTTTCCGACAAA<br>TCCTGTAAGGTGTATGCCTCTGTTAGAGTTATTTTGCATATGAGGAAACTGAGGAAAGAAAGACCAGTT<br>TTCCAGAGCAAAGGCCAGCATTGGGTAAGGTTCACAATTACAGGTTTAATAATTCATCAAAATGCACTG<br>AGGCCAGTCCTTTTATATAACACCAGAAGTAAACATTAACTTTAACTGTACCCATATCTAAGGAATGAAG<br>AATAGAAAAATGAAGTGTAATACCTCTATAATTGAGATTAAATATACACCTTTGGGGGAGAATGTTTTGT<br>ATATGGTACATGTGCCTCTGAGTGTGTATATGTGTGGTGGCATATATACACATGTATGTCAGTGTATGCAG<br>TTTTTCCATGCGAGGATGTCAGATGTCTTTCTCTATCTCTCTCTATTCATCTTCTCCTTGGAAAAGGTGTC<br>TCCCTGAACATAGAACAAGACAGGCAGTGTGCAGCAAGCTCAAGCAAGCCACAGTAATGGGGTCACA<br>GGGCTGTTTGCATGTGGCCGCACCTGGCTTGCCGTGTGAGCACTGGAGATTCAAACTGGAGCCCCCAT<br>GCTTGCAAATCAGGTGATTACCTACCTCTCCAGCCCCATGTTTTATTCTTAATTTTAATCATCAGTCCAGT<br>TAGGCTCCTGGTCCCCCTTATCCTGTTATGTTGATTTTACCACATCATATATGACTGTTCCGAGAGTCCTG<br>CTTCAGAAAGATCAGAATACCTGTTGCCAACCCCAGCTCTGTCACTCTTGTGCTACGTTGAGTCAATCA<br>CGTCATCCTTCAGACAGACTGTTGTAAGACATTGGGCCTGGGGTTGGGGATTTAGCTCAGTGGTAGAGC<br>GCTTGCCTAGCAAGCGCAAGGCCCTGGGTTCGGTCCCCAGCTCCGAAAAAAAAAGAAAAAAGACATT<br>GGGCCTAAGAATCCAGAATTCACAGGAATGGATTGATCCCAGCATAGAGACTAAGCACTGCTAATACAC<br>TGCTCCTGAAGACAGTGCGGAAAAGCAGCTTGTTGAGGCGCCAAAGATCTGATACACAAAGACTTCAT<br>CTTGCTCGCAACCTGTCAGAAGGGAGCCCCAGGTAATTGGACAGCAGAGGGGGATACCAGGCCGTTGA<br>GAAGCATGATGGCACGAGGAGCTCGTTAAGATCTGTGTCCCAGCTATCATCCTCCTAATTGAACAGTTA<br>CACAAATAGACATATGAGATGATCGTGACTCTAATTAAGGCTCCGACCTCAGAATCTAGTAAAAGCCTGT<br>ACAGTCCTCCCTCTCCCTTTAAATAGCTGCTTTGGGAGAGCTCTAGGGAGACATGCTGGCCTTTTAGGA<br>CTCTACTAGAGCAACAGGGAATAGCATGCAATCATCCCTTCATGTTTCTTCTGCAATCTGCATCCCCCTA<br>ACTACGTCTACCCTTTAGTTTCTGGGGTTGAAATGGTTTGGCAAGACGGGTTGTCATGGACTGCCAATG<br>CTACCCTCTGCTGTATTGCTGTAACCCCTTTTCTGCAATGCCCTCCACCATCACCTTAGCCCACAATGCG<br>CTGGGTCTGAGAGTGTCATCTGCTGTTCTGAGTTAAGTTCTCTCCCTGTTGGTTAACTGGCTTCCTTGAT<br>GATTAAATAAATGAAATGAAATGCTTAAGGGCTGAGAAGGTGGCTCTACCACTGAGGTACTTGCTTTGC<br>AAGCACAGGGACCCATGTTTAATACATAGAACCTACGGTGGGGCCATTCCTTGCCACATATACACATAAT<br>TCCTAGCACTAAGAAGGCAGAGACAGGTGGATCCTTGGGGCATTTGGGGCAAGCAGCCTAGCTCATTT<br>GGCTAGTTTCAGGCCAGTGAGAGACTCTGTCACAAGAGAAGCATAGACAGCAACTGAAGAGCAACAC<br>CAGAGATTGTCCTCTGGCCTTTATCCACACGCATGTGCACATGCATATACACAAATCTTCATACACGCAC<br>ATTCTCACACATGCGATTCTGTGCACACAGGAATATGCATGCAAACACTGAGGTATTTTCCTGGCCTATA<br>TACACACACGCATGCATATTGGTGTAATCTCCAAGCCGAGGATTCCGGCAGACAAACAGTTCTGAGTTG<br>CTGTGAGCTTTTTGTCTTTCCCGCTGCCTTGACCTGCACAGAGAAGGGGGATGCTGTTGCCCACAGGAG<br>GTAACCTTGGGAGGAGGAGTGAAAAAATATCAAAGGTTGCCTTTGAATAATGGCGAGTCCACTGGGTT<br>AGAGAAATTCTAAAGAGAGGGGTGAAGGCGGTCAAATATTTGTACACAGCATATAGTCAAAGTGCCTG<br>CTTCCTGTCACGTGCTGGTCCCCTTGATGCTAATGAAAGTCACAATATTCATCAGCAGGAGCCCAGATAA<br>AACCCCCGGTGCATATAATTTGAACTTGAAGTTGACCACCTCTGTGAATTCTTTTTCCTTCTTTCCTAAA<br>CAAAGGGCCCATTTTGAGGGCAGTTTGACACAGCTACCTAGGAGAGCGTGGTGACCTGGAATTCCTTT<br>AGATCCACTCTCCCCTCACATCTGAGTGGCACAGCTTTCCGCAGGGGACACTTAGATGGAAGAGAACC<br>CAGCACACTAGGAGACTGAAAGTCACATTACATAAAGAGAAAGTCAGTTTAGAGAGAGGCCTCCTCCC<br>CTCTTGGAATTTCAAAAAAGTATTTTTAACACTTTAAAAGTGAAAAATGGTGGCCTTTTGTTGGACCCA

							GAGCATGAGACACAGAAAAGTCCTCATCTCAACTCTACATTCCTATCCTGTCCCCTGAAGGGCTCTGACAACCTCCAAAATTACTAAGACCCCAATGGGCAGAACACTGACGAAGGCCAAGTAGGTGGGAGTGCAAACACGCCTAGATCCTGCTGGTGGGATTTGGTTTCCAAAAGTCCCAAGAGCTGTGACTGATATTAAGCCACCTCTGTTATCTTGCTTACTTAATAGATAGGCACATTTTATCGGCTGTGATCTGCATGCGTCTCCAAATCCAATAAAGATGTACATGTGAATGATATCTCTGGGGTCTGGCCCTATTGTTGGCATCCTCAGTAACAGCCAAGAGTCTATTTCGGAGGTGAAGTTGAGACGTGATCCACAAAGGTGCCTCTACAATATACTAAATGACTTCTCTCATCATGCTTCTGGCTCATGTATCTGTCAGTTCCTGCTGGGACCTCGGGGACTCCAGGGGAAGCATTCAGGGACACTGTAGAGGGTGACTATATATTTAAAAAGGTAACGAGGAAAAAATAATAGGAATTCACACCAAATCCTCACTGCCCTAAACTTGCCCTAGCACACAGTAAGTGAATGCACCACGTGCAGTGGCTAAAGAAGTAGGTAGCAAGTATTTTAAGATTTGATTCTTAAGAAACATTAGAAAGACTCGGTAGTTAGGAATGGGTGTTACTCTTACTGAGGACCCAAATTTGGTTCCCAGCACCCACCTAAAGCAGTACCTAACCTTCAGTAACTCCAGCTCTAAGGGATCCAATGCCCTCATCTGACCTCCATGGCAATCTGCACCTATGTGACAGTATAGATACAGACATACACACACACACACACACACACACACACACACTCACACACACACAGAGGCTCGTAAGAAACCTCAAAAGAATGATTTTTAAGCAAAGCATGACAACTGCAATCCCTGTTCTTGGGAGTAGAGGCAGAAAGGACAGGTCTAGTTCAAGTCTAGTCTCAGCAAGACAGCAAGTTCAAGGTCGCACTGGGCTTAGAAAGCTCTACAGGAACTTTTTGCTCAGACAGTACTGTGGCCAATAGCAGACACTTCTTCACACCACAGCTCAGAGAACTTCTAACCCAACATATGAGGTCTGGTAATTGAATGTGGGAATTGTAAAGAAGAGGAGG
MSTRG.121323.1	ENSRNOG00000034226;ENSRNOG00000042133;ENSRNOG00000047234;ENSRNOG00000049016;ENSRNOG00000049451;ENSRNOG00000055008;ENSRNOG00000056577;ENS	0.757	0.092	0.035	-2.267	down	CAGCACCTCCCTAAGGCAAAACCAGATCACCCACAGTTGGCCGGAGGCAGCTGTGGCTGCCAGGGATGGAGCTTGTAGCTTTGACTCCTTAAGGCTTCTCAGTTTGGGAGGTGGGTAAAGGCTCCATGCATTTGCCTAATGGGTTCTGAGGGGCTGCTGGTCACCTTGGAAGATGAGGTGTTTAAGCTGGGTCAGCTATAGAGTGAGACTAGGGGAAACCCCAGGAGTACAGGGGAAATCTGCTTCTCTTGCTTCCTGCTGAACTTGCTGCCGAAGCTGTACTTCACAATGCCTGCTTCTTTGTAGGAAGCCAACATCTCGGTTACTCCCATAGATGCCCGAGACTCATGTTTTTTGGCATCAAGACAAAAATGATGAGATACTGACGTGCTCAGATGGGCCGTGCCTGACTTCAAAGCAAACGGAGACAATGAAGACTACGCCTCCAGCCAGCTATCTAGAGGGGTACACTATCATTGTGGTTAACAGGGAAGCCAAGGGCAAGACCACAGGCCAGGATGAGACATGACATGGGATGGGCACATCTCTCTGTTTGGAGCAGACATTCAGGGAAGGACCAGAAGGAGACGCTGATTCTCTTGGCTTTTGTTTTTCATGTGTTGAACGAGCAGTACCTGCTGCCAAAGCTACCCTGGTATGTACAAGCAGGGGTGAGGCTGGCAGATGTACGGCATATTGATTGATATATGTGCATCCATTTGGCATGGTAATTACCCAGGACCCACCTAGGAAGACGCTTCTGTTTGCCAAGCCCCATCCTATATCTGGAATCTTCTTAATCATAGCCTGCTCTTTTTTTTCCCTCCCCTTGTTTTCTTTCTTTCTTTTTATTTCTGATGATGACAAATTGACAGCTATGAATATGGGATAGGAAAGTAACAAGAATGAGTTCTAGGGATAGGCACTCTGATAGGAAAATGAGAGCAAGGGGAAAGAACCTGTACTGCTCTTCCAGAGAAATAGGTTCTCAGCACCCATATTGGCTGGCCACAGCCACCTGTAAGTCCAGCTCCAGGGATCTGGTGCCCTCTTCTGGCCTCCGTGGACACCTACACTCTGATGCACATACCCACATACAGACCACATACATAGACAGAAATAAAAATAACATCTCAAAAAACGAAGAGGCAGAACAAGAATGCTAAGATCTGCTGCTAACATGCTATGTCTGTGCTGTGTCATGCTATGTCTGTCCTGGACGCCACCTACAGCGCAAACAGGGCAAAGATGAGGCTAGTGGTATCCTTAGAGGTCAAGGCTGGGACTTCGTGCAGGAGCACGTGGACAGAGAACCACTAATGGACTTTTTGATAAGAGGGGCTAAGTCAGATTTTAGAACCATCACTAGCTGCAGTAATCCCTAATGTGTAGCTGTTGCCTTAGCTAACTGAGTCACACAGAATCTGACCCAGGATGGCTTGGGTGGAGAAAGGTTGAAGCTGGTAGGACTGATGGGGAATTCATTAGAACATGAACTTTGGATAACTCCTTAACCAGTAAACACAAACCATGTTCATGTCATATCAAGACAGCCTGTGGAGAGTTTCCTCCCAGATTCCCTGCTTAGATGAAATGGTGTATGTGTGTATCTTGAACTGAGGTAGAGAGTCGGGAGCAAATAGTGTATGCAAAAGC

	RNOG00000058101;ENSRNOG00000058371;ENSRNOG00000058463;ENSRNOG00000059588;ENSRNOG00000059805;ENSRNOG00000060573;ENSRNOG00000061718						GTAATGAGTTTGATTCTTAAAGTGGTACATCTGGAGTCCTCACTGAACTTTTGTGAAGACATATTTAGGGAGACCTTAGAGAATGAGGACAGAACCAGAGGAAAGCTTTGCTCTAGAGAATGTGCTTAGTGCCATAGAAAGCAGGAATTGTGGGAGTCTAGCCTATTGATCCCGGCAGTCAGTGTGGCTGGCCATCACTTCAGGACCACATGGAGAAGAGGAGACCAGGGCACAAGGAATGACCAGATCGGTTAGAAGAGCCAGGCTCACACTTCAGACCTCTTCTCACTCTTCGATAAGGAAGTCCAGGATGAGTTTCCTCTTCCCTTGCTCCATGCAAGAGGTAGTATGCTCCAGTGGGAACCATTCAGTAATCCTTCAAGCCCTGCTGGTGTTCTGGACAGGACAGCCATTTCCCATGGGTGGCATTTACACCAGTGTGTGCTGACTGAAAAGAACATGACAATTGTAGTCAATGTTTCCTGTTGAATGTCCTATCTTTGGAGAAAACAAGCAAGTCATGACCATGGCTGTAGTCATGAAAGATAACCATGAAAAGCAGGTATCCCTGGTGACCTTCAAAAGTCCCATCCCTGGCCATCTTCAAAGAGATCAGTGAGGTGGGAAATAGGTATTTGGTATCATTCTTGGTGTTGCTTCTTGACTCTCATGGAACTTGAGTCTTCCTGGTAAATACAGGCTAGGAAGGGTAGGGTCTCTTACTGACAAAGTGTGCTGAGAAGGCTAGGCTGAGTGACCAGTGAGCTCCAGAGATCTGCCTGTCTTTGCTTCCCTAGCCTTGGAATTACAAGTGCAATGTGGATTTGGGGAAATCAGGCTTAGGTCTTCTTCCACACATGGTAAGCACTTTGCAGACTGATACATCTCCCCAGCCCTTGAAATTTTGAAAGGAAAGATGTTTTCATGACTTTTCCCAGCCTTTAGAGGGGATTCTAATAACACAGGCTGGCCACCTACCAGGTTGGATCTGAGCGTGGTTCAGTCATAGTGACTGAACAGATAATGTAGCTTCAGAGTGTGTACGTAAGCGTCTGAAGTATTTGCAAAGTGGGAATTGGGAGACTTCACCCTCCTCGATCATTAGTCTCAGTCTTTTCAGCTGTAAAACAGGGGTGCTGGTAACCTGAAATCCCTGATTGTCATGACTGTTAACATGTATGTAACATGCATGTAGCAATGGCATTCCCATGTGAAGTGGGTGGTGCACATGTTGTTGGTACCAAAATACCTCATATTCTGGTCTTTCTGAAGTCCTGGAGGCCTCTCGGAGAACCGGTGATGATCTCACAAATTCTGTCTTTGAAACTTTCTCTGGCCTGCGTGGTTTTCACTTACCCTAACTAACACCAAAGATTACACGATTGACTGATAGAATAGCAGCAACCTGGAGAGCTTCCACAGCAGGCCCAGGGCTATACTTTCCGTTTTTATTAAAATTCTTATTAACTTGTAGCCCAAGAGCTTTTAAGCCAAGCCTAATCTCCTTTAGGGCCCCTAAATTTGATCAGAGCAATTTGTGTGCAGGATTCTGGTCCCCCTCTGTGTGGATACTGTGAAGCCAGCTGATGTTATTCTGAAGTTACCTCTGGTCCTCAGCCCCTCTGGTGTGGTTTCCCTTCAGGATACTGTTTCCATCCTGTCTGTGGAGACAGTTGACTTCCACAATTATGCCTCCATTCCCTAGGTTGGAAACTTTTTTTGGAAACTCTAACTTTATGAAAAAAAAAATCAGGTCCCAATCTTTCCTACCTCAAGGATGGTGAGCATATTGGCACTTGTTCCTGGGGGAAAAAGAATTCTGTCAGTGAAATCGTACCCTCATAATGGGAAGGGAACAGAGGCAACAAGGAAGAGGGAACTGAAACATTGGGTGTGGCTCAGAGCATCGCCACGGGGCAGTTATGTAAAAAAAAATATGCTGCTGTTTCCCATAGGCAAATGAAAAGGTTGCCTTCCTCTGTGGCTGGGTGTTACTCTGTAGACTGGGCTCTTGTAGGAATTGTACACCTGTTCCCCCCTGGCCTTTGTTCTGAAAATGGTGCCTTCCCAAGGGCCTTGGGAAATGGCTCGCATGCATCCTTCTGTTGATAGGTGGTCTTAAGTTGAATGTCATCACCTTGGTAATCCGCTAAAATTTAGAGCTCCTGAAAATAGCAAGTCTCTTTGCAGCTTGCTGAGTAGAAGCCATTTCTTTCCTGACTTAAAACATACGTAGAGACTTTGTGATTTCTAGTCCTGTTCCTGAGGAGGTCAGGCACACGCAGAGTCGACACTGAGAAGGATGGTGGCACTGTTCTTAAGGACTGGCCATGCATGTCTTGCATAATGGACAGATCTGATGGCCATATGGTAATTACTACCCTAGATCTGAAAGCCACTCCTAAGAAGAAACTGAAATATGATAGCTTAAGGTGACATAGCCTCTCTAACTTGTACCTTCTTTGCATTATGGAGATGTCCAGACAGTACCAAACTCATTGCAATTTCTCCATATTGGGAGGGGGTGTCATTTTGTCTTTTCTTATTTATTCATTCTACAGAGTCATGGAGCCAACCCTGGCACAGTAGTGATCAAGCTAGGTGTATGGTGAGAGATTAGAGAAAAATATTCCACAAAACCATTATCCTGACACTGGTGTTTAATGACTCATAGCTTCTCTTATGGCATTTTATTATCATAGAATTCCAGAGGAGATATTGTCATTGTTCCAATTTATAAAGGAGGACACCGATAGTTAGGACCACCCAAGCAGCCTATGTTAGAACTTGGACTTGAATGTGCACCCTTTAAGTCCTACACCATGTTCTCTCAACCAGCCTTCCC

							AGAGATTCAAGATTTCAGCACATGATAGATCACACACACACACACACACACACACACACACACA<br>CACACACACACACTTGTAATTAAATGTCAAATGTATTTAGATCATCAGAGTTTAGAAGAGGCAAAACTT<br>ATGATAATCAAACAAGGTAAGGTAAAAGAGGTAGTTTAGAGGTAGTGGTTGGTGGGTTTGGTCAACTC<br>AATGGAAATATGTCTAGAGGATATATATCCTATATGTCTAGAGGAGAGACCTATAGCTAGAGGATAGACCT<br>ATAGCTAGAGGAGAACAAAGAGAAAGACTCAGCTGTGTACAGTAAAAAGATAGCAAGACCAAGAATG<br>GAACAAAAGGCTGAAGGGCAGGAAACAGGGTAGACAAAGTGAGGATCTAGGTAAGGATCTCAACTTT<br>AATCCAATTTTCCCCCTTTGTGCAACCTGGTGAATGATGTCTTTTAGGAGATTTCTGTCCCATGAAGATC<br>AAAATTATGAGAACTCTCCTGCCTGTACTTTTATCTTCAGCAGAGAGATAAATAAATGAAATTAGATATAT<br>TAGGGTTCAGCTATCAGGATATACTGGAAAGCTTAACAGCTAGTCACTTAGGCAAAACAGAAGTTAACG<br>GACAATCTTTGAGCTGCTATGATAGCCATGCAGTATGGGCAGGATGTGGTTCCAGTTTCCTTCTGTCATT<br>CCTGGTCTTTCCCCTCGTCTAGTCAAGATGGCTCATGTTCCAGGAACTAAACTAAAGAAAAAAACAAA<br>GTAAAGAGATAGGTAAACAACATCCAATGTTTGTTCAGAACTAGGGAAATCCCCAGTGTGTGAAACTTT<br>CAATGGAAAAATTAGGAAAATCCATGACACACGAGAGAGGCTGAAAATCAACTTTCACCTGAGTTCTA<br>GGGGAACTGAACAATCATATGTCCTTTTGTTATAACTCGCTTATAAGGTCACATCTAGCTGCAAGGGAGG<br>CTGGAAAATGTAATATAAATATATAATATAATTATATAATATGATATGTAATTTTGACATGTGCCCACCTAAC<br>ATCATTCTTCCTATGGAATAAGGGGACAAAACATATTTGGCCATAATTGTTTTTAATAACATTCAAATCTG<br>GTGAAAGAGAACACCATTTACCATTTATGTCAGGCATAGAATCTCTTTAAATGCATTCCAAAAAGCCAG<br>GCTCTGTGACTTTCCTACTGTACTAGATGCAAGGTCTCTCTTTTCCCAATTCAGTAGGCATATCTGAGAG<br>TGGCATTTGTTGCAGGCAGCCTTGACCACTGTGAGTTTCATTTTGAAAAGTATCAAATAGCCCAGGGAT<br>GTCTTGCAACACCCCAACTCTTGGTTGCAACACACTGTCATGGCCAGACATGGAAATGCAGATGTGCCT<br>TAGCAGATTGCCAGATGGCTTTGAGTTCAAGAGTTATGGAGCTTCATCTGGGGACTGGACTGTCTCTCT<br>CCCTAAATACAAAAACAAAAAGGTAGAAATAAATCACCCAAGCAGGAAATCTATATTTAAATAGTATCA<br>GAGGTATTTGACTATGGGCTCTAGAAAACCCCCCTATAGAAAACTTGAGCTGGGCCAATCTTGCCCTAAT<br>TTAACTAGCTAATGAGAATTCCACAAATCGTTTATAGGGGAAAATCTGATTTGTGTCCTGGTCCATGGGG<br>AGGTAATGTCAACAGTCCAGCTGGCACATCAGAAGGGATGAACCAATAGACACTGGAGGCATAAGGGT<br>ATCTAGCCCTGCCCTGTAGACATGCCATTGAACTGTGGTCCTTGCCAGGGAGATTCTCTATACGAAACCC<br>CGCAAATCGAATGGACCACATCTACCTTGTTGATGTTCTAGTTTCCAGGCAAAGATCAGATCAGGGCAG<br>GATGAGACCCAGGAGGAGGCTAGAATATGGCAATGTTTGGAAACTCATTCTTCACGAGGAAAAAAAAA<br>CCTGAGATATTGTCCTTCGTGCCCAAGTAAATCTTGAATTTTTCTCGAATCAACTAGACACTTGCCATGT<br>GTTTCCCATCACATTTTGTGATGTTTTCTTGTGTACCCTGGAGCTTCCAGCTTTCAGAGAATAAACATTCC<br>AGAGGCATTAGTCTGCTCGTGTGAATTTCAGAGCTCTGAGGAGGGAAAGGATGGGGATGAGAATGCAC<br>TCGTCCAGGCGACAGCTTTACTTGCCTGGAAGGATTTATCAAGTCATGAAGGCTTCCTGTCTCAGCCAC<br>TAAGACAGGCGCAAAAGCCTGGGAGCTTTTGGCTAGGCAGTGGTTGGGGAGGTATTAGAGACAATATA<br>TTTGCACCAATTAAGAAGTAAAGAGCTACAATATAGTATACACAGCCATCGGAATCATGTCTTCGACGTT<br>TACTTAATGACATAATAGACTGTTCTTGATGCAGCTTTAAGTGGAGAAATAAAGATCTGAAACTGCATGG<br>ATAGAATGATCCCAATTTTATCAATTTCTGGATAAAGAAAGAAAAAAAAAGACTAGAAGGAAATCTTCTA<br>AAGTGGTGGCAAAGGGGCTTGAGAGATGGTGGCTTATCAATTCAGAGCATAAGCTACTCTGGCTGAAA<br>ACTCAAGTTTGGATTCCATACCTACATCAGGCAGCTTGCAGACACCTGTGTCTCTAGCTACAGGGAGTC<br>CCACGCCCCCTCCTGGCCTCTGTAGGCATTGCATTCACCTGTGCACATTCTCACGCATAGACATACACCG<br>GATGTAGACAAAATTTAAAATAAAATAAACCTTAAAACCAAAATGTCAGCAGAGTCCCATAGCAGGCAT<br>CTCCAGTGTCCCGGCCCTGCCCTTTTGGTTGACTGCCCCATAAATGACAGCATCTCATGGCAGAACTTA

							CTGCATCTTTCCATGTGCTGTCCTAGGCTGCTTGGGCTTCGTGAGAATCAGATGCCTCTGGAAAGAGTC<br>ATGTTTAGAGACCTTTAGATTTGTGATCTTTCCACTTTCATCCTTATAATCTCAGGGCTCTGAGGAAATCA<br>GAATTCTTTGTTGGTAAAGTGGACCCATGGGGAGGCAGAAGTTCCTATACTCATTTCTTGGTATCTGAGT<br>CATAATGAGAGAGAACACAAATCCCTTCGCACCAGACTCCCTCATATAAAACAGTCCCGTTTGTGTGCA<br>ATCTACACGTGTGTTCCCATGTGCTTCAAATCAACTCTCGATCAAGTCTTACACCTGATGTGATATAAAC<br>GCCATGTAAACAGTTCTACGATATTGTGAGGGGAATAAAGACCCAAGACTAAGTGTGCACACATTCTCT<br>GCAAAGTCATCCATCTAAGGCCTCACTGTGTTTTCCATTCTGGGCTTGATTGCATTGAAAAGCACAGAG<br>CCTAAGGACAGAAAGAGCTGTCTGCACCTGTGAGGTGTGATCTACAGAGCCAGGGATAGCCGCATTTA<br>CTCCCACTGTCCTCTGCTCCCTGACAGATGACATGGCTGGAGAAATCGCTCCCCATATAAGGGTTGAAG<br>GGGGAGGTGAGGGCAGAGGGAGAGAGAGGAGGAGGGAGGGAGGGGAGATGGGAGAGAGAGACACT<br>GAGAAAAGAAAGGGGAAGGGGAGCAGGGAAGAAGGTGAGGAAGACAGACAGACAGATAGACAGAC<br>AGAGACACAGAGGTGCTGACTGATAATCTAGGGCCCCAGAAACAGTGCCCTACTGAATTCCTTTTGCTC<br>TAAGGCATAGATACATACCAGGGGAGATAAAGACTGCATGCTCTAAGTCCCTTGGGGCAGCTCCTCACC<br>CCCACCCTGGCAATAATCTATGTGGGCATCTGTGAAGGTGAGGTCTGGACCAAGTTAGGCCAGAGAAG<br>TGCCATTTTCCTCCTGGGAGGCTCCTAGGTGCTGGGTCTTCCCCTCCATTGCACTGTGTCTCCAGCCCCA<br>CCCCCCACGGAGTTACAGGATTTGGGTGTCAGGCAGGTGGGGGCCTTAATGCCTTCTTTGCAGGACTCC<br>TGGAGACCCTCAAATGAGCTAAATAAACGAGTAACCCTTTGGATATTGCAGGATGAAAGTAAATGACTT<br>GCCTCAAGACCATAATGAATGCAAATGAATTCTGTACCTTTCCACAGGTTAGGGGGAGTCTAGAGCAAC<br>TGCTGGTGGCAAGAGTTGTCAGAACAGGAGAGGGAATCAGAATCAATGGGAATGGGGAAGGGGAGAG<br>GGAGGGGAAACGTAAAGTGGTCCTTAGAGCAGCTATTAAGAGAGACCTCTCTTTCATCACCGCCTTCCT<br>GGGCTTGTAATCAAGTCTCTGATGGTTTCTGCCACTGGCCTCCTGGGAGCCTTCTCTTCCAGGGCCACA<br>GTGCCAAGGGGCAGGGCCCAGAGGAAAGGTTCACCCATGTAGCTCTCTGCAGGGTGTTCAGTGTCCTT<br>CAGCTGCTTAGGGAAGCTACTGGAAGGAAACAGACCAATTACTGTGGCAGGATCTGGCTTTGCCTTCAT<br>CTCCCCTCCCCTGCCCATCGTAGACTCTCAACCTAGAACATCTACATGTCTGTAGCCAAACCAAAGAATT<br>TTTCTGCTTGCAAGTGACCATTAGGTTGGCAGAAGCACTCCCTCTCCTGCTGCCTCTCTACCTACCCACT<br>GCAGACAGATGCCTGGAGTCATAGCTGATGGGACTGCACCTCTCCTGAGCTTGACCCTCCCCCTACAGC<br>ATCCCCTGCACACCCCCTACATGCTACTCAGCAGATGGGCTATGTCAGTCTGACTTCCCTTACCTTCAGA<br>TCTCTTCTTGGAGCTCCCTGCTCATTCTCTACCGTTCCTCGGTACCCCCCATTCCTCACCCTTAAGAACCT<br>GTGCCATGAAATCCCAGATGAAACTTGTATTGGTGAGGCTATAGAGACAACTTGGTGGCCGAGGGCATG<br>GGCTACTCTTTCTCTCCATTAGCTCTGACTCCAGGCAGCTGCCTGCAGTGGGTAGGTAGAGAGGCAGCA<br>GGAGAAAGGGAGCTCCTGCCAACCTCATGGCCACTTGCAAGCAGGGGGAAAGCTTTGGATTCCCAGA<br>ACCCATGTCAGGGGGCTCATGACTGCATGTAACTCCAGCTCCAGGGAACCCAGTGTCCTCTCTTCTGAC<br>CTCTTTGAGCATCAGGTACATATGTGGGGCACATGCACACATGCAAGCAAATGCTCATACACATAAAAA<br>ACAACTAAAATAACTCATACAAATCTTAACAGAAAGACAAAAACAACCTAGTACTATTTTCTGAATGGA<br>CACAAATCTGCCTTAGTTCCTCCAGTATTAAGTGAGCATGTAGTGCGTGCCAGCCACAGGACCTGGGAT<br>TTTGTATCTGGCTGCCAATAAAACACAAACAACAATGATGGTGTCAGTGTTCTGGCTGCTATGGAGGAG<br>TCTTTAAGAATGCTGACTGACTGAGCAGCTATGACAAACAACTGGTTATCCCCTAGGCTCATGGGGGAG<br>CAAACATCCACACCAGGATGTGGCCCGCATCAGTGAACCTGACTCAGTTCCTCAGGACCCTCTTTCTCC<br>CCAGAGGCTGTAAGTCCCCATGCTCCTGCAGCGGGAAGATGGTCTGATAGACGTACATGGTGGTCCTCC<br>CAGAAGCCTAGCCATGCTCAGCCTTTAGAATTAGTTCAAGAGCACAATAGATAAAACAGATTTTTATTTT<br>CAGACTGTGGTCTGGAAACAAAATCATCTTCTGTAATCTTGCACTTCTAAGAGAAAACTTCCAAACATC

							AGGTGGACATTGTCAGACAGAGCCTCTCTTGTGTTCTAGGCTGGACCTCCAACTCAATACATAAGCAAC<br>GATGAACTTCTGACCCTCTCTGTTCCTCCACCCAAGTGCCAGGGTTAAGGTGAGCATCTCAGCTTTCTG<br>ACTCTAGGGGTCAGACCCAGGACTTCCTGAATGCTAGGCAAGCACTTTACCCACTGAGGTGCACCCCC<br>ATCTCTACACAACACGCTTTCCTACAGGCCCCTAAATCCTCCAAACTCCCATTCAACATTTTACTTGTAC<br>AAGCTGAAGCCTTTTATGTCAATAATAGTGTTGGCTTCATATGCTACTTCCCTGAGTAATGTAGAATTAAT<br>TAGTAACAATAATAATTAATATAACACATTATGTATTAATATAAGTCGTGTCCATTACTCATGTATGTCATCA<br>GAGTCTTCTGGTAGATAAATATCTAAACCTTGAACAACAACCAAACGAAAGATAATTCTCTCCAAGAGG<br>GTATCTCAGGTAACCCCATAATAGAAACGTTAGGTACCCGAATGTCTGCACAACCCAGCGTTGTGGTGC<br>ACATGTGTAATCCCAACACTCAGGAAGTGAAGGAGGGAGGATGTAGGGTTCAAGGTCATCTTCCTCTTA<br>GACAAAGTTCTAGGCCAGGCTGAGCCACAGTGAGGCCCTGTCTCAACAAATAAAGAAAAGAAGGAAA<br>GATTTGGGTGGAGGGCAGGCCCGGCACACTGAACTCACACACTTAAGTTCGAGGTTCAATTCATGGGA<br>ATAATACTGTTGGGTTGGTTTTTAATCAGACTCAAAATTACATCCTTACAAAACACTCTCTTTACACTAGT<br>TCATGGGGGGTGGGGGGTGCAGGCCAACTACTCAATTAAGTGCCTGTCTTCCTCCACAGTAGAGTTGA<br>AGCAGGGGGAGATTCTGAGTGGAGATTTCCATCCTGGGCTTGGATAAGTACTTGAAAATTAAAGCTAAG<br>AACAATAAATATCTTAAGTTTGAAGTTTGATTGGGAGCGGGGGGAAAGACTACATCTTTAGGAAAAAAA<br>GAGGGGGGGAGGAGCAGGTAGAATAATGTCCCCAGGCCAGGAAAGTATCAGCCATTTTCAGATAAATT<br>ATACCCTCCTCCTCCATGGGAAAAAAAAAAATCATAGTCAGACTTAACTGTCCCTTTGGCACTTTTGTCAA<br>ATGAGTTTTAGTAGCAGGAGCTGCTAAAAATATAAGCCAATTTCTTTCTTCATTTAAATCAAGCGCTTAA<br>AAAAAGCATAAAGCTTGGAACTGGTGCAGCGGCAGCCTTGCAGCAGGGCTGCCTACATGCAGTCTGGC<br>AACACGGCTGTGGTCTCTCAGTGGCAGCTCAGGGTCCTCTGACTGTCAGAGAGAGGCAGCAGCAGGG<br>TTACAGACCGTCCTCTGGAGCCTTCAGCCCCTGGAACCCAAGTTCAGAAGCTGCCCTTGAGGGTATAGT<br>AACCAGGTTAGAGGTGTTTCTCTATTTAGGAACAATGTTCAGACACATCAGCCCACAGCTACACTGGGA<br>CAGGATTTGTGCAAATAAGTAAGACATGCCCTCTCCGCTTAGGCACTACGGGATTCATAGGATCACATAC<br>CATATCGCCGTTTAGAAAATGAAGTGGCCATTTGGTACTGCCTCCGAGATAAGAGCGAGTGGAGGTTCC<br>AGAACTGTCTGCCTTTCAATAAAACACTCTTAAGAATGTCAAGGTCACTCAGCAGTTAAGAGCATGTGC<br>TATTCTTTCAGGGGACCTGAGCTTGGTTCCTAGCACCCATGTTGAGGAACTTATAACTGCCTGAAACTTC<br>AGCTTCAGAAGGATATGATGCCTGTAGTCTCCTCAGGGACCTGAACTCACATGTGCAACCTACATGTGC<br>GCGCGCTGTTTCTCTATTTAGGAACAATGTTCAGACACATCAGCCCACAGCTACACTGGGACAGGATTT<br>GTGCAAATAAGTAAGACATGCCCTCTCCGCTTAGGCACTACGGGATTCATAGGATCACATACCATATCGC<br>CGTTTAGAAAATGAAGTGGCCATTTGGTACTGCCTCCGAGATAAGAGCGAGTGGAGGTTCCAGAACTG<br>TCTGCCTTTCAATAAAACACTCTTAAGAATGTCAAGGTCACTCAGCAGTTAAGAGCATGTGCTATTCTTT<br>CAGGGGACCTGAGCTTGGTTCCTAGCACCCATGTTGAGGAACTTATAACTGCCTGAAACTTCAGCTTCA<br>GAAGGATATGATGCCTGTAGTCTCCTCAGGGACCTGAACTCACATGTGCAACCTACATGTGCGCGCGCG<br>CGCGCGCACACACACACACACACACACACACACACACACACACACAGAGAGAGAGAGAGAGAGAGAGA<br>GAGAGAGAGAGACAGCAGACAGACAGACAGACAGACACACAGACACAGTTCCAGACACAGACACAC<br>ACAGACACAGAGAGAGAGACAGACAGACAGACACACACACACATAGTCACAGTCACAGTCACAGAC<br>ACAGAGAGAGAGAGAGACAGACAGACAGACAGACACACAGACACAGTTCCAGACACAGACACACAC<br>AGACACAGAGAGAGAGAGACAGACAGACAGACAGACACATACACACAGTCACAGTCACAGACACAG<br>ACACAGACACAGACACAGACAGACACAGACAAACAGACATATGCACTCATGTACACAGAAACACACA<br>CACACATTTTCTTAATTAAAATGAATATTTCTTATAAGAATTCCAAAGGCTTTGAAACCCAGGGAGCTATA<br>TCCATAATGGTAAAATTTCAGGAATACAATAGAGTGAAACGAACCCTTGTGCTATCTTGTGTGGCAGTTG

							TTTGTTTGTTTTGCCCAATAATCACACAGGTTCCGTTTAAATTACACCACCCAAAGATAATCAATGATTCT<br>GAAATCTAGACACTGACAAGCCCTCAGAAACTAGCTAATCCAATTCTCATACTTTCATGCAGAGAAAAT<br>CCTGGAAGTTCACACTGTGGGGTGCTATCTACTGCTCTTCCTGCTTGCCAACCCTGGTCCCTGAGAGCA<br>GCACACACCCCCTTCTTAGTGGAGTCTTAAGACAGGGAGGTAGCCTGCTGCTTAGCGCCCTCAGATCAG<br>GATGGTCTTTCACTCTCAAGCCTCTGGTTCCTTGCTCTGTGCTTCCTAAATGAGGCCACCTTGAATCTAA<br>GCCCCCATGGAACATTGTGATTCAGAGAAACATCCTGAATGAGACTTCTGAGAGAGCTTTGCTGTGGTT<br>CAATCTCAGTCTGTAATGAATCATCAACACAGTTATTAATGTCACTTTGACTTCCACAGCAAAGCTACAC<br>TTTACCCTGTCCCCTCTGTCAGCCACATCAACCCCGTGTCCAAGCATGCGTGGAATGGATATAGAACTAC<br>TTGGGATATTTCCCACAGTGAGGATCCTAACCAGCCATCCCCACAGACTGCTTCCAACTCGGCCTGGTA<br>ACAGTGTGGAAAACTTAACATGTGCCTGACGCCATGCCACTGGAAGGAGATCAGTTTCAAAATAGGTT<br>GACCTCTGAACTTTCCTAAGAATCCAGTCCTAGTCACCCAAGCTAATTAATGCTCAGGTATTAGCACTAA<br>TCACATCCCCAAGTTCCTGGCTACCTAGTGTTCCTTTAGCACCATGTGGACTGCCTTCGTGATATTTTACA<br>CTCCAACTCTGAGGACATTACCGTGACATTTTCCGTAGGCATAAAGATATTTTTCCTCTTCTCAGCTGCA<br>ATTCTGTCCCTTAGCAGTTATAATTTAACCGCAGTATCTTTTGAATAAGAAGCATATACTACTTGGGGTGT<br>GGAGACTTGGCTCAGAGGTTAAGAGAGTGCGCTGCTCTTTCCGCAAACCCAGCCTTGGTTCCCAGCAC<br>CCGTGCCAATCACCTCATAATGGCCTATATCTTTAGCTCTAAGTTTCTGGTCTCCACAGGCTCCTATACCC<br>ATGTGCACATACCCCAACATACACAGATAAACATAATTGATAATAAAAGAATAAACATGCACAGTAGTGA<br>AAATAGTCCTGGTTTCTAGATAAATCTTGCTAACTATGTCGTCTTATGAGGCGTTTGTTATGAAACTTTAC<br>CAGTTCCATGGCTTGATCTCCACGGACCCAATACTATCTATCATGGAGGTGCTCCAAAGGGCTCCATTGC<br>TCAGATTGGGAAATAGAGCAACACTTCATGATGTCATTTGCCAAAGTTCACGGAGCTGTTAAATGGCAG<br>GGTTGGACTAAATCTCACTCATCTGGTCTAATGACTACAGCATATGCTTGCTAGCACACTGAGTGTTTTA<br>ACATGGATTTTATTATTGAATGTAACACCCTGGTTGCTGGCACGGCGGGGTTCTTTTTACAAGGGAAGAT<br>GTCTGTAAAACGAGCCAGTTGGTCTCTGTCAGCAAAGCCGTACTTAGAGTTCACAGAGATAATTACGTC<br>TCCTCATTTGAACCCCCAAAAGATATCCAAGAACAGCGTTTGTCTTGATTTCATAGACGAGGCCTGTTCT<br>TCATACCAGGACTAAGGGAAACTATTGGCTGAATTAACTCCCCAAAGCCTACTTGAGAGAATCCTCTCT<br>CCCCATCCTGATTCTCTGACCTACAACACTCTTTTCCTCTGTGATTTCTTCCTTGGGTATCCTTGGAGCTG<br>ATTTGGCAACCACCGATTGACCCAAGCATTGGACATGTTTGCATATTAGAGACAGCAAGCCCCTCATGT<br>CTTACCTAGCAGACCCTGTGATTCCTTGCTCTGTTTTCCCAATAAATTCAAAAGCTAGAAAACCTTGTCA<br>TAAACTTCATTGTTTATCATACTAATATGTTACCATGCCCCCATGCATTTAGTAAATATTTAGGATTTTGAC<br>CTAAAGAAACAATCATATTTTAGGCAAGTGTACTCTTTCGGGCCAAGTGGTTAACATTTTTTTTTTTGGC<br>CTGTCAGTATCTACTCTTTCTGTTTGCTTGTTAACTCACTTGCTTATTTATTTATTTATTTACTTACTACCAT<br>AAGGTAGACTTTCTTATTTCTGGAGAAGATGAAACATGACTTGTGTATGCTTATATACCATCTCTGTGAG<br>GCTACCACAATCAACTCTGAAAAGTATTTCAATCATCTATCCACCCCTATAATTCAGATGTACAATACATG<br>CGTGCACTGTTCATTTCTTGAATACATCCATTCCATTTCAGGCATTTGGTGCTCCGAGCCTCCGGAAGCA<br>ATAGTCTACATTCTGATGCTCTTCATCTGAGGCTGGAGTGATGGAGGGACTGTCAATGTTTTTGTCAACC<br>TGACACAAACTTAGACATACCTGGGAAGAGGGTATGTCTCCATCAGATTGGCCCATCACATGTCTGTTC<br>CGCATTTGCTTGATTGCTGATTGATGAGAGGGCCTGACCCACTGTAAGCTATCCCATCCCGAGACAGGT<br>AGGCCTGGACTGTTTAAGAAGGGAATCTGGGAGGTTGGAGAGATGGCTCAGCGGTTAAGAGCACTGA<br>ATGCTCTTCCAGAGGTCCTGAGTTCAATTCCCAGCAACCACACGGTGGCTCACAGCCATCTGTAATGGG<br>ATCCGATGCCCTCTTCTGGTGTGTCTGAAGACAGCTACAGTGCACTTATATATAATAATACAGAATGCTAG<br>ACAAGCCAGTAAGCACCAGTCCTCCATGGCTCCTGCCTTTGCTCGAGCGTGAGTTCCTGTCCTGACATC

							TCTCCATGATAGGCTAATGTGGAAGAGAAGCTGAGAAACCTTCCCTCCTCAAGTTGCCTTCCGTCGCCG<br>TATTTATCACCAGCACCAAAAGCAACCCAGACGGAGAGCAAAGGACACCAAAGGCAGAATTTTGTATC<br>TGCCTTTGTCATGGAACTCACAAAAGAGTGCTCTCAAGAGAAACGACCCTGGTAAAGACTTTGGCATT<br>GTCTATGTGAGGACTCTCAAGATGTAGGATGTTGTACTTGCTTGGTGTGTCACAGTCACCTCAGAAATTA<br>AAACGTTGGTGCCTGACCAGAGGCTTAGTTTGTATGGGCCATGTCCGCAGCAATTCTAAGTACTTAATG<br>ATCACAGTGCTGGGAGGGTGGGTATTAAGATAACAACAGATATGGAAGCCAGATATGTCTGGGAGGGCA<br>ATGTGGATCTCTGAGCCCTGCTGTGTACAGACCTAGGGGTTAGGACCCACCCAAGTGCTGATTCACTGC<br>TCAGTAATCAATCTGGTAGGGGGAATCATGCCTTCTTCCCTGGGTGGATCTCCCAAATCCCCAGCTGCA<br>ATGCTATCCTGTAATTCTACCCACCCGTCACATGAGAGCACTGGACCTTGATGGACCTGCTTGCCTTGAA<br>TTTGAGCTCCAGCGAGGAATGCTTCTGCCGAACTTAAATCGCAGAGAGCTAGACTGTGCTCCTAGCTGG<br>GATGCCCAGGGCATAAGGGTCAGGGATTAGCAGACCTGTGCTGGCACAACTGAAGTCTAGAGACCCAT<br>TCTCAGTGGCACCGAGGCAGGGAGGGGTCCAACTCTTAGGGCTGAGCCTCCCTCACAGAGGAAAAGG<br>AGACTGTTTACTGACCGAGAGGTTGGCTGAGCTGCTTTTTTAGGATCACATTCTAGCAATTCCAGTTATG<br>AAAATCAATATGAAAACAATACTCCAGTTTCTTCCTCTGGAGTAAAATAAGACTTGAGATTTCTGAGACT<br>ATTTTCAGCCTCTGGGTTGGTGACTCTGTAGGTCACCCGAGCACTTAGAGAGTAGTTGACACACACAGG<br>GAGCTGTTCCATGAGCCTCCTGTGATCTCATTTAATCCAGACGGTGGATCGACATGATCAATTCTATTGTC<br>GTCATCTCTACTTCGGGAGGTGAGGAAACCAATAGAGAAGATGAAGGACCCGCCCGGCTCACCCGGGC<br>AGTGCGTGGTAGACTTCTGTTTTTAACCTAGGCCACCTCCTCTACTCTGGGACTTACACCAGCATTACTG<br>GTGACTGCTTCTCTCTCCACTCAGTAAACAAGCCCTCGGCTTCCCCAGCACTCAGACCATAACCTATAC<br>CGAGTGCTCTCTGCTTGCTGTACTCCTATAGACATTGCTAACCATTCACTGGTCTGTATTTGTTTTATTTAC<br>TCAGCAAACCTACAATCTTGGGAATCTGACTTCCTGGATTCCTAGCTTTTCCTGATGACTTACTAACTGT<br>GTCATTTCGGTCAAGTTCCCTAAGCCCTGCGCCTTCCTTCTCTGGCCTCCTTTATTCAAATCGAACTGAT<br>AATAGTGTGCATTGTGTAGAGCTCTTATGAGGATAACGTGACATGGTACTTATTGCATAAACATGGCATA<br>CTGCCCCAGCTTGGTGCCTGCCAAACAGTAGGCCCTCCATCAATGTTTCTGCTGGTATCGCCAGGCCCA<br>ACACTGGTTGCTAGGGATGCTAATATGAGTAAGACATAGACCTTGGCGTCACAGAGCTTATAACTTAGG<br>CAGGGTGGAGAGGTGGGTGCAAGTCATTAAATTGGATCTGAACTCACAGAAGTGTGTTCCAAGCCTTT<br>GGGGCATAGAAAGAAGGGAGTAGTTCTTCAAGGGGCAGGCCGTGAAGTGAGGTGAGTTGCCAGTTCC<br>TCAGGTACACTCACAGAGAAGCATCTAGACCACTCAGAGTGGGAAGAGTAAGGAATAGAAGGCTACTA<br>TTTTCATGGGAACCCCACCTTCAAGAAGGCTGCAAAATACCAACACGGAGAACAGAGGCCCCCCATGC<br>AGCCATAGTTGGGCCTATAAAAGGGTAGAAAGCCAGTGTGGTGGCAGGCAAAAGTTGCCATGAAGGGG<br>GAGGGGCTTGGCTGGAAGAACTGCACACAGGTACTAGTGATCTCGGTGCCTGGAGGGCAGAAGGCGG<br>GCCCCTGACATTGCCAATTGGTCTTGCCAACAAGCAGAAGGATCCAAGGCCTGGCTGAGGTCCTAGGC<br>TCTGTAGCCAGTAATAGGTAATGGTGAGGCTTGGTAGAGGTGAGGCACACCCTGCCAGTGGGGCAGCC<br>ACAATAGTAGCTGATACTATCTTCCACAGAGTAGCAGAGCTGGATTGGGGAGGGGGAGGGCTTCAAAG<br>ATTCCCTGGGTGGCATCCTCCCTCCTTCAGGTCAGAGCTTGGGAACCTGCATTCTAGCAAGCTTCCCTG<br>TCACTTCAGTGCCTGCAGTGAGTGGCTTCACCTTGTAAGACTGTGGAAGAGGGAAACTTGCTAAAAAA<br>CAAAGATATGGGAGCTGGGGGTGTGGCTCAGCTGGTAGCACAAAAGACTCAGGTTTGATTCTCAGCTC<br>TTCGTGAAGTAGACACTGTGGTATGCACAAGTAGCCGCATCATTCTGAGGATGCGGGAAGAGGATCAG<br>AAGCTCAAGGTCGTTCTTAGCTATATAATGAGTTCAGGGAAGACTGGGCTCCAGCCTGGGCTTTATAAA<br>GAAAGTAGGAAAGGAGGAAAGAAAGGGGAAGCGTGTGTGTGTGTGTGTGTGTGTGTGTGTGTGTG<br>TGTGTGTGTGTACACGAGCACGCAAGCAAACACATGCACTCGTGTGTAGATTCAGTCATTTAGTAGCCG

							AGATGGAACCTCCAGATCACCAGTGTTCAGAGGTCAGGGGAAATTTGTGTCTATCATCAAAGTTTCTTG<br>TAAGCCTCGTTCACCTACTGCCCCCAACCCCTACCGTCTTAGTCTCAATGTGAAAGTTATAGGTCAACAG<br>GTGAAGATTTGGCTTGAAGAGAAGTCATAAGTTCTTACAGGGAATGGATGTCTTTATTTGACCTCACATC<br>TTCACCCACTTTGGATGACGCAAACTGTGTTCCCATCAGCCCTCTCCCACGTGCTCGAGGTTGAATCAA<br>GTCTCCAGCCATCTGTTTTCTGAAAGCCCCCTGTGTGTGTGTGTGTGTGTGTGTGTATGTGTGTGTGTGC<br>GCGCACGCACGTAATGGGGAGAGAGAGAGATGGAAACAGAGACAGAGAGATGGAGAAATGGAGACA<br>GAGAGACAGAAGAGGGAAGTTCTGTCTTCCTTCTTGCCATGGAAAGCCAATCCCTGGTCCTTTCCAGCT<br>TTTTCCTATGGAAGGGAACCGACAGGGAGCTTTGATTAGCAGTATTCATCTGTCACTCAGTGGGAAAAA<br>AAATAAAAAGGTGACTTTCGAAGCACATGCTAGGACCTGTGAGGAGCGAGAGAAACCTTATAAATTCT<br>GGGAAGTACCCTCCGGTGGCTGGGAGGCGGGGCTGCGTGTGCTGGCTTACAGCATCCTTGTCAGTCTG<br>TCTCAAACTTTTCGTTTCCCAGTGGAAAGTTAGTAAGGAAGGCTTTAAACGTAAGTTGCCCACCAGAA<br>GACCTGCATCTGTGACCCCCCCAGAAAACCCCACCCTCCC
MSTRG.121548.2	--	0.718	0.008	0.000	-4.720	down	TGAGAATTAGTCACTTTTATTCACTATGCATCCTAGGATACCATCTTGTCACACACAGCACAGAACCACA<br>CAGGCCAAACACAGAAGCTAACCATTCAGTGTGTCCAGTACTACTGTCCAGAGATTCTCCCTGTACTCA<br>TCCTCCAGACAGCGCAGATGTGTCTTCAACACACAAGCTTTCTAAAATGCATATATTCCTGACAACAGG<br>ATGACTTTTATCATCCTGAGAAACCCCCACACCGTGGGTTTTCTAAGATGTGTGTGTACTCACTACATTC<br>CCACCATTAGCTCTTAACGATCCCCTTTTCTCCACAGCCTTCCAAACATTTGTTTTCTTCTAATTTTTTAA<br>AGGCTTATTTTTATTTTGTGTATATGGACATTTGTCTGCATGTTCGTATGTGTAACACATATGTGCAGTGCC<br>TGAGGCCAGAAGGAGGGCATCAGATCTCTGGAATTGGACAGCTGCCGTGTGGATATAGAGGATCTACA<br>GGATTATGTTCATGTATCTTTCCAGTCCCCACCTCATATGTACATTATATATGAGATTGCATTTATATATGTG<br>TATGTGTGTGTTTGTACATGTGTGTACATGTATGCTATTTTAATTTTGCTGAGGTGTATTCTCAGTGTGGTT<br>TCACTTATTACCCTGACAGTGATATTTTAACAATTTTTCACATATTTATTGGTTGGTTGTACGTCTTCAAGA<br>AATGTCTATTTAGGTCTTTGTGTGTTGACTGTCCTCTCTCTCCGTGCCGAGTTCATTCATGGTTAGTTTTC<br>TGTATTTTTTGGTGTTTGGTTGGTTGGTTTTTGGTTTTTAAGATAGGATTTCTCTCTGTAGCCCTGGCTGT<br>CCTGAAACTCCCAAAACTCTTCCTGCCTCTGCCTTCCAAATCCTGGGATTACAGGCGTGCACATTTTTAC<br>ACCTATTCCGTATTCTGTTCCCTCTCTGTGTTGTTTCCTTGGCTGTGCAGTGTTGTGATCATTGCTTTTGT<br>GGTCATCTTTACAAAAATAAACAAACACTCTTTCTAGATCTGTAGGCATAAATTTTTTTGCAGCCTACTTT<br>TTTTTTTTCTTTTTCTTTTTTTAGGAGCTGGGGACTGAACTCAGGGCCTTGCGCTTGCTAGGCAAGCGCT<br>CTACCACTGAGCTAAATCCCCAGCCCCTGCAGCTTAATATTTATCAGTGATGAAACCAATTTTGGGCGGT<br>ACCATCTTTTGATGGAACAAGCGTAATTAGTAATATAACGCTGTTTGGTAAGGTAGAAGGGTAACAGCCT<br>TCACAGGCAATGGAATTTAAAAGTTAGGCCTTTAAGAAATCTGAAACAGAAAAACACATAGTCAGGTC<br>ACCTAAAAAGAAAAAAACAGAGACTCACCACCACCTTTTCCCTCTCTCATATTCCATGAAGGATCTGGC<br>CACAGATCACCAGGACTTTGCTATTCTTTCATCACTTTGAGCCCT
MSTRG.121550.1	ENSRNOG00000057867	0.036	0.353	0.009	3.093	up	GTCCGCCAGCCCGCAGCCCAACCACCGGTCAGGAGTCTGGGTCGAACTCACCGGCACTCCAACACTTC<br>GACCGGCCATCTTTACCTGCCCTGGAGCGATGCGGTTGCCATGGTCCCATGACAGAACACCCGTAAGGA<br>CACAGAGACCCTAAACAGCGGGAGCAACCGGCTCTTAAGTCTATCAACATTTATAGAAAACGCATCTGA<br>AAATCACCAGACCATTCCTTCGTCACAAAGCCACTGGGATGAGGTAGGTACACTCTTTAAAAGAATAGA<br>AATTCTGAGCTGCTTGAACAGCGGCTGGATACAACACAAATGTCTCATAGTCCTAGGGGTGAGGTGCTA<br>ATGAAATCCTGGTGTTGGCAGGGACATGTCCTTTCAAGGCCTGCAAACGGGGAGGGACTTTATTTGCCT<br>CCCTCTGGTTTCTACCTGTGACTCACTGATTTGATTTCCTTGGCTCCATCTCTCCACACACTCTCTCTTCT<br>GCCAGAGATGCCTTACTCCATGCCCCAAATCCCCATAGAGTCTTTTGAGTGGAGTCTGTTTTTAGGAAA

							CTAGGGCAGGTGTCCTGCTGCCTGCATTCATGGCCTTCTAACCATTTTCATGCCTGGCACATTAATCGGTTCTCTCTCTTTCTCCCATTTCC
MSTRG.121622.1	ENSRNOG00000034226;ENSRNOG00000042133;ENSRNOG00000047234;ENSRNOG00000059588;ENSRNOG00000060573	0.000	0.277	0.000	7.354	up	GGGAGAATCTTTGCAGCAGAAAGCAATATTTCTAAGTGATAATAGGTGGCTCATCATTTTCAGATGTATCAAGCCCTGTTTGTTTCTAAGAATAGAACAAAATCTACAGGCTTCAATAAACTTAACCTTACTTCATACATTAAAGGTAGCCATGAAATAAAGCCTCCCTCATGACCTGGCATTCTGGTGTGTTTCTTCAAATGGAAAAATGGTGCTAGTAGGGCCGTGATGTAGTTACAGTTGGTAAAGTGCCTGCCTAGCATCTGAAGCTCTGAGTTTGCTTTCTAGTCCTACAAGAATTGGGCATGGTGGCAAAGACTTGTAGCCTTAGGATTTAGAAGACAGAAGCAGGAAGATCAGAAGTTTGAGATCTTCCTTGACTACATAGTAAGTTCAAGGCCAGACTACGCTCCATAGGACTCCGTCATAAAGAGAGAACTAAGGAAGGGAGGATAGAAAGAGGGAGGGAAGGGGAGAGAGGAAGAGAGAGAGAAAGAGAGACAGACAGAGACAGAGAGAGACAGAGACAGAGAACAGAGACAGAAAGACAGAGAGAGGAAGGAAGGGAGGGAGGAAAGGAGGGAAGGAAGGAGAGAGGGAGGGAGTGGGGAGAGAGAGAGAGAGAGAGAGAGAGAGAGAGAGAGAGAGAGAATGAATATACTGGTAGTATTGGGATTGGAGAATCCATTGTGTCTGTGCCTCCATGGGGTGTGTTGGTCTTGATAGAAATGGCCTTGATTATGTCAGTTCTGACAGAAAACACATGTTGAGATATGGACATAATAATGGCCCTGCCCCTGGCACTGTGCACTGTGGGAAGGAGGTCAGTCTGGACAAGAAGTCTTTCTTTGGGCACATTAGTGATGCATGAGATTTCACTTCTCAATGTCAGAAGAAATATGTCTTCCTCAGGCATAAGCTTAGCACCAGACAAAGACAACTCAAGAGAAGGCATGAAAAGTTTCTAAACTGGGAAGCGAGCAAGCGAGAGAGAGAGGGGGGGCACAGAGAGAGAGAAAGAGATTGAGATTGATTGATTGATTGATTGATTGATTGAGAGTCAATGAGTCAATGAAAAAGAACTGTCCTGGTTCTTGGGGAACTCTGGCTTGGGTCATGAGAAGATTTTTGGAAGAATCCTGGGGCTACTGAACAATAAACAGAGCTTGGATGAAAATCACTGGGCAAAAATCTCCCATGTGATGCTTGAGAGCTGCTTCATCATCAGGTCTGAAGGGAAACAAAATCACCACTTGATCCAATAATATTTAATTTACCCAACACCAGCAGCAACTTGACAATGTGTTAGAGACATCAGCTGTAGAGAGTCAGTGGGTCAAGAGACTGGATTCAGTTACCTCCCAACAGCAGTGCCTTCTCTTCTCCAGAACCCTGGGAAAGCAACTCCCTAGCCTTTGAATGAAGCCACAGCAGGGATGAATGGCCCTGGTATACCTATGCATTTGTCCCATGTTCTGCATTCTGGAAAGCAGAACTGATGAACAAATAACACTGTGGACTTCTCAGAACAGTCCACAGGATGTGGACCCAGCTGCAACTGGTCACACTTCTCCTTCCTAACTTTTCCAGAAGGAACTATTGCAAAAGAAAACAAAATCCCATGAGATGCTCAAAAGCCAAAGGACCCTTCCCACTCATCACTGGGTTGTGAAGAAGGGAAAACAAGCAGGTGGGGCAGGCAGAGGAGATGTATTAAAGATGTTTAAATGTCTGCTCTTGCCACTCTGAGGCTAATTCTAGAGTATTCTGTCTGTTTGCTTTATTGTCTTTTTCAAATGCTCGAACACTGAGACAGCTGCTTGGAACAGTGAGTTCTCCACGTGGTTCCTGCTAAGCAGAAACAAAGAGAAGCTTCCTTCTCCCTACAAGGCCAGCACCCCCTACAGTCTATGAGTCAGAAATTGGTCCTGGAAGAGCCGGCTCCATGGAAGCTTGGGCTCCTCTCTATTATCTTCCCCCAGGGACTAGAGAGATCGATGCCAGAGAACGGCCCAGTAGGAGTTCATGGGAGTGAAAAGTGAACTGGAAGCCCAGGACCCTAGGTGCTAGATGGAGTCTAGGAAGGAGGAAAATGTGTGGCTGTGGTATTCTAAAGCCCATCATTTGGTCTAGGGAATGAACTACAGCCAAGGTTCGGGCCTTTGAAGACCCATTAGATCTGCCTTCTTCTCAACGCACCCCATTTTAATGTTAATGTTAAATCTCCTTGTCAAGATCATGTGCCTATGGCTTAGAGCTAGCCACAGCAAAAATTTGCATGAATGCGTGCATGTGTACATGTTTGTATGTGTACATGTATGTGTGTGAGGCTAAAGGTCAACATTAGGTAACCTTTCAAACACTTCTCCACCTTCTTATTTGGGGAAAGGTTTCTTCATGAGCTGGGGCTCACTGATTCTGCTGTGGTATCCCAAGAGATCCCTTTGCCTCTGCTGCCTGGAGCTGGGATTCCAGATGTGTTAGTCTGGTATTGAACGCAAGTCCTCGTGCTTCTGCGGCAAGCATTTTCCCCTACAAAGTCATCTCCCCAACTCCAAAGAGTTGTTTTTATAAAAGAATGTTCCAGCATGTAAGATATGCACAGTGAAGACTCAGCTTTAAGTTGTTTATTTTCTATGAGGAACAGTGAGAGGGGCGAGGCTGTTCATACAAAGGA

							GCTTGGGCAGTGTTGTCAGGACCTCCAGCTCCCTCTAAATCACCCACACTATCATATGATCAATACGTTC<br>CAAGGCAGAGCACCATTTATCCTTTTCCAAGCGCCAGAAAACCAAAAGAAAGAAAAAAAATCCCACAT<br>TGTTACATGGGAACAATAATGGAGTTATTAATTAAGTTAGTACTCAGTATATATTTGGGGACATAAATAAA<br>TGACGAAATCTGAGTACTTTGGCCAAATGGGAATAGAGGGAAATAAAAAACAAACAAACAAACAAAC<br>AAACAAACAAAAAAACACAAAGAACTGACTCCTCCCAAGGCAATAAAATTAGCCCCATTTGGCATGAA<br>TATAGATAAAGTCACATGACACATGCAGCTGAGACAGCTGCGTTCAGGACATGACACCCATAGAGCTGT<br>GTGACCTGGGGAGGTATGTTAGCCCCTCTATGCCTTATCTATAGAGGAGATATCTTCTTATTGTATGCAGC<br>TCTCTGTGTACATGAAGTGATATAGTAACCTTACAATCAAATGGCAGCATTAAAATTTCTAGATAGGATAT<br>TTGTCAGAAACTGCTGCACAATGTATCTGGGTCGTAGGGCATCTCATCCCTGCCTTTAACACACCACTGT<br>TTGCTTGTTACTGTTGTTTATTGTTGTAGTTGTTGTTCAGAATCAAGATGCAAGACATACGTGAGATCAA<br>AGGCCCTGGGAACAGACAGAGGAGACAAATAGCCATCCCTCATGCCTCGCCAGCCTTCCTCATTTGTGC<br>ATAGGTTTAAAAGGCGCCTGCTGATCTTTGCCTCTCATCTACAGTGACAGGTTAATACTTATTGTCAACC<br>TGACAGAATTTAGAATCACCTGGAAGACAGGCTTCTGAAGGTGTCTGTGGGGGCAGCTTCTAGATTAG<br>GCTAACAGGGGTCAAAAGACCCATAGCACCATCCCATGGGTTGGGGTTCTAGATTAACGCAAATGAAA<br>GTGAATTGAGGACCAGCCTTCACTACTCTATGTTTCTGGAAGTTTCTTGGGCTCCTGCTGCCACGACTTC<br>ATGGAGGACTGGGAGCCAAATCTAACCCCTCCTTATTTAAGTTGTTTTTGTCATGTATGTTGTGACACAG<br>CAATGGTCCCAGCTTGCCTAGTGGCTGCCACCCAGTACTAATTAGTGGAAGGGAGGAACGGGAACCAA<br>CACAATTTTCTCCTTTCTATACTAGATCAGTAGAAGCCATTAAATGTAGAGCAGTGGCATCGGAAACTGA<br>GGCAGGATCATGTTCCCTTTCTGAGACAAATTTCAAGCCTAGAGAACAAGAAAAACCAGCTCCTTAAG<br>AACTTACTCATGCCTTTCCAAAGAATTTGCCATGACCACCATCCCTACTAATGATGATCACGTATCAGCC<br>TCAGAGGCCAAAGCTTTGTCTAGAGTGTGGGATGAGAAATTCCAGCTGCACAGATGTGATCCCCAGAG<br>TGCCCAGAGTGACAGCCTGGAGTTCTTTACTACTCCAGATGAAGGGCAAAACCATGTCTTCCTCACTGA<br>TTCATCCTCCCAGTTGGAGATCAGCCAGGATGTGTCTTCAAGCCCCGCCCCAATACTTGGAAGTGGGCT<br>GAGCAATCTGCACTATTCCTCCAGTGAGGTAACTAAAGCTTGGCAAAGACATGTTACTGTTCTTCTTATA<br>TTACGTTTACTTGTTTATTTGGGGTGCTGAGGAGGTGACCCACATATGCCACAACATGAGTGTGAAAAG<br>AGTTGGCTAGCTTTTTTCCACCTGTGGGTCCTAAGAGTCAAATTGTCAGGCTTGGTGGCAAGTGTCTTT<br>ACCTCCCGGGGCATCGTGCCAGTCCCTAGCACTTCACCACGTGTTATTTTTAACCTACCTTATGTCCACA<br>TGGCTATGCAGCTCGCTCCTTTGTGGGGGGTAGTCTGTTTTTATTGAGTCAATAACTCTTGAGAACTTGC<br>TATGGTCCAGCCAGCTCCCAGTTCTTCACAGATATCTTCTCTCTGACTCCAGTTTGCTGATGCGGGGAGC<br>TTCCGAGTCAACCGAAGACTGCGTACTTTGGAGAACAAAGGGATGCAGGCCTGCTATACTCTCTAGCAT<br>TCGGGAACAGAGGTAGGGATGAGCAAGGAGCATGCAAGGGGGAAAGGCACTCAACATTCACGTGTGC<br>GCTGACCCCTGCAGCAGGAAGCCATGGGAAGAGCACGGCCCACTGTAGAAAGAGCTTTCCTCAGAGT<br>CTACCTGAGTCATCCTAGGACCTCAGCGCACGGCAGGTGTAGAGTACCACGCAGAAATAGACAAGGAG<br>GCGCAGCTAAGTACAGCCCAGTACCTATAAATAGGTAAACCAGGGCCTCTGATTCAGGAACCATCTCTT<br>ACCCATTGTTAATTGGAAAATAACCAGCTCTCACAGGTCCTCCCACACAAGATGAAAAGATGACTTAGC<br>GCACGTCATAGCTCCTTGAATGAAACCTAACCCCCCCAGCACCCCCAAGATGCTGCTTGCTTCCTAAGG<br>AAGTGGCTCACTTTTCCTCTTCATTCTTCTCCCTGGCTTAACATCATCAGACATTAAGCCACCGTTTAAC<br>CTGGAAGATAAAAAGCTCACCCTGAAGATGCTCCTCTCCTTTTCTTCCTCATCACTTACAGCGAGCCTGT<br>CTCTTTCCATCAGGGCCCCTTGTCAAAAGCCAGGGGATATTGCTAATTTCACCCACAAATACCTGATCTG<br>AATCCGGAGTAATTAGCAGATCGGGTAACCTGAGAGATACTGATCTTTTTTTATAACCACAAAAAAGCTT<br>GTCACGAAAGATATTTTAGTTCAAGAACATTTCCAATCAGGAAACTTCACATCATGTTCCAAAATACCAT

							CCTATCTCGCCTTGGTACATCTAGGCTGTGTGCCCGATGGAGGTAATGGTGTGTGTTCTTGGGACAGTGC<br>TACATCTGAGCGTGCCCAGACAATGAAGTAAGTATATACTGAGCACGTGCCACCAGCTTGGGCAGCTGT<br>CCACACCGAGGGATCAGCTCTAACCAAACTCTTTTCATCAGGTTTAGCTTTCTGTTGTGATTGAAAAATG<br>GTTCATTGATAGGGAGTGTCTGAGTCTCCTGAGAGTTTTCATTTTGGACGGACCTGAAAATGGAGGAGC<br>TTAAATGATCTAGGACACAATTAACGGAGCTAGGGAGGTGACTGCCTTACTACAAATGTTTCCCATGTAG<br>GCATAAAGGCCTACGTTCAGATTCAAAGACCAACACCCACATAAAACCTAATTGTGGGGTCACACATCC<br>AGTGCTGGGCAGATGAAGACAGAAGCTTCCCTGGAGCCGATGAACCTGGCCAATTTGGAAAGTCCTGG<br>GTTCATTCAGTGAGGGAACTCATTCAGTGACGGACCTCATTCAGTGAGGGACCTCATTCAGTGACGGAC<br>CTCATTCAGTGAGGGACTTCATTCAGTGAGGGACCTCATTCAGTGACGGACCTCATTCAGTAAGGGACT<br>TCATTCAGTGAGGGACCTCATTCAGTGAGGGACCCTATATTATAAATAAGATGGAGAACAAGAGAAGAA<br>AACATCTGACATGGATCTCTGATCTCTACACACACACACACACACACACACACACACACACACACACACA<br>CACACACACACACACACACACACACCCCTATCCGATCTTGAAGATATAGTACTATGGAATAGTTTCTAAGGT<br>AGAAGATGATCCATACGAGTCCCTTCTCTGATCACCAACTGTGTCACTGAGGTTGCCATGTCTATAAAAT<br>GGTAATGCTAATTCATGTTCCTATCAAAGGTTGTTGGAAAAAAATATATATATAGTAATTGTGTGTGTGTG<br>TGTGTGTGTGTGTGTTTGTAAACCCATCAATATTCCTCAGTAACCACTATGTCACCTACAGATATTACTCA<br>CTCAAGAACTGTCAAAAGTGAGCTCAAA
MSTRG.121710.1	ENSRNOG00000010447;ENSRNOG00000010642;ENSRNOG00000010784	0.000	0.471	0.001	4.308	up	CTGGGGTTTAGGGAGCTTGATATAAGTTTTGGTTTGGGAGTGTTGGGGAACTGACACCTACACGATGGC<br>TTCACACTGGACATTGGATTCGAGACTCATTCCCTATAATATGTTTACAACTTAAAGATGCGACGCGGGA<br>GTCAACTACCACAGGTCTGGCACCCCCACACGACTAACACGAGCATTAAAATAAACAACAAAAAACAG<br>GCCAGCAGGTGGCTGAGTCAGAGGATAAAAGCTGGCCTTTTACTGCTCTGGAAACTATGGTGGTGGAC<br>ACAGGAATAGCATGGCGGTAGAGAGACACCAGAGTTTCACGGCATATTCTCTCTTTTGGGATGGGGCAT<br>CTGATCAGTGAGCCCTGTTTCCGCTTTCCCATCCCTGGAGTTAAGGATACCTCCCAACATGTCTGCCGTC<br>TCACTATTTTGGAC
MSTRG.121886.1	ENSRNOG00000009439;ENSRNOG00000037659;ENSRNOG00000046191;ENSRNOG00000053574	0.000	0.707	0.004	3.943	up	TTTTTTTTTTTTTGGTTCTTTTTTTCGGAGCTGGGGACCGAACCCAGGGCCTTGCGCTTCCTAGGCAAGC<br>GCTCTACCACTGAGCTAAATCCCCAACCCCAATAAGTTCTAACGTAGCCAGAAAAACCTGCATGTGAAC<br>ATTTATTCTGACTCACATAGCAGTCATTTTATCGAGGATGTTCTCTGTGATTGACTGGGTATCACGATTCT<br>G
MSTR	--	0.000	0.473	0.000	5.713	up	ATTAGGTTTCTCCTCTGCTGCTCCCCGCACACTGTCAGCGCAGTGACTTTGTTGGGGCGCTGAGCTGCG

G.121929.1							CGTGCTATATTTAACCAGTGTTCTCAGCAGGCCAGCCTGTCAACAGCTGAGAAGTTTGCAGCCGGCCCTGCTCAGTTCCTAGGCAGCTGGGTTATCAGCCCCGCATCCCTGCTCACCCCGGAAAGATCCCGGGAAGCCGGCAGACCCTTTTAAGGAACCTCTGCTCATTTCACCGAGATTGCTATCATTTGGAGAAAAAGCAAGTGAATGAAATAGCTGTCAGCACACTATCTCTAGATCCATTTCTAAAACCAAATCTCCCCCCTTGTTTATTTTAAAAACCATAGTGATTTAGTGATTTTCCATCATAAAGATGTATTACATTCTTCAATACATTTGTGACTGACTGGTATGTTTACATTGGCAACGTACAAGACTAGAGAGGTGTCATTCATGATCAATGTTCCACACAGATATGCTCTGTTGATATTTAGTGCCCAACTCTGAGATTTAAGTTTAAGCCTATCAGTAGCAGAAAATATCTCTTATTCCTTGCTGAATATCCGATAATATCAAGGAATCCACTTTCCATAATACAAAACAAAATAAATCCAAAAGCCCCTTATGGATATTTACAGAAGAAATAGGCCCATTATTCTTTTGCTGGAATGTTCTAGCACATTATTTTTTGGTGGAATGTACAGGCACACTGGGCTGTTTCCACTCTTCTTCGCTGCATTGTGGATTGGAGCCAACCAAAGACCAACTCTACAGCGGTGAAATGACACATTCCCAGAGCTCTCTTTGGTGGTTTTCAACTTGGAACTGTTGTGTGTGGTACGGATGTCAGGAGTGTGTGTATTGTTCAAAGTTATCTCTGCCTTTGTAAAAATGAATGGATGGATGGATGGAAGGATGGAAGGACGAACCAGTTCACTCCTGGTGGTTCTGAAAGGTTTTCTCAAAGAAAAAGGAAAGCTTAACACATACAAAAGTGCCTCCAGGCTCTGTGTACACAACTAGATCATCATTAGCCAGATGCACACGGTGGAAAGAAAGTAAAGTGTTCCTGCACATGACTGTCCTCCCAATACTCGGAAGGCTAAGGCAGTAGGATCAGCAATTCAAAACCAACCTTAGCTACATACTGAGTTTGAGGTCGGCCATGGCTACCTGAGACTTGTCTCAAAGTAACCATGAAATAACAACAATAATAATTATAAATAAAAATGATGAAAAGAGACACTCGGGTTAAAAGTCAAACATTTTTAATGTTTAAATTTCCTGCAAAAATAATAGATTTCCGGATACAAAAAAAAAAAATCTTGGTCACTGTAATGACTATTCCTGGTTGTCAACTTGACTATATCTGGAATGAACTGCAATCCAGAATTGGAGGACTCAACTGTGATCCAGATTTTGAGGCTGGAAGACACAAGTTTCTGACCTAGATCTTGGCATGAAGATCTTGAGGCATAGTGGCCAGGAAAGCTTACGATCGGGCAAGGTAGTACATGCCTCTAATCCCAGGAGACTGAAGCAGAGATCTCTGAATTCAAGGTTATCCTGGGCTAGCAAGACCCAGATCTAGGCATATTGGTACACACTATCTGCTGAAGGCCTACATAAGGACATTAGAAGAAAGAAGGCTCACTCTTCCCCTGCTTGCATTTACTTGCCAGCACATCTGTTGGAATCTACTTCTACAGAAGAGCAGCTGAAACAATCAGCCTCGTGGGACTGAGCAACTACTAGGTTCTTGGACTTCCCATCCACAGCTGCCCATTGTTGGGTTAGTTGGACTTCAGGCTGTAAGTCATCACAATAAGTTCCCTCAATATAGAGAGACATTCTATAAGTTCTGTGACTTTAAAGAACCCTGACCAATACAGTCACCACGATGTTTATATTTAATTGTCGGCATGACACAGTCTAGAGTTACCTGGAAAGAGAATCTCAATGAAGGACTGTCTAGACAAGCTTGACCAGCATGCATGTCTGTGGAGGACTTATGTTAACTAATGTGGGAAGATTCAGCCCTCTGAGGAAGGTGTTACCCCTGGACCAGGTTGTATGCACATTCCTCCATGGTCTCTGTTTCAGTTCCTGTGTCCGTGTTCCTGCCTTGAGTTCTGTGCTGACTTCATGATAGACGATAAGATAATAGCAGAAATAAAC
MSTRG.121935.1	--	0.340	0.000	0.005	-4.261	down	GTTCAAATCTCTGGATAGCACTGGGATCTTTGAACTAAAAGAAGAAATAAAATGATAAAGGCAAGAGACTAAGAAGGACACCTGCCCATCTTGCTCCAGCTCTTTTTGGGGGCGGGATAACACTAGCATCCCTTCTCACGGCCAGGTTTCAGCAAGTTTGGCAAGCACATGAGCACCACCATGTTCCCAATGCAAGACATTGCGGGAAGGAGCTCCTTGTTGGTAAGTTATCAGAACATATGGTAGAAAGGCAAAGAAATGTTGGCTTTCATTTTGATTTGTATTCTCAATTTTATTTATTTTTGAGAATTTCATACATGTATAAAGTGAAATATGATCTGTCCACACCATCTCCCCATCCAATTTCCCCACATCATCCCACCTCTCCCCCTCTACAGTATTGGATTTTTGTCTGTCTGCCCATCTGTTTTCGAGACAGACTTTGTCAATATCTGGTTGACCTTAAACTCTGGATCCTCCTGCAGATACAACATCCGGACAGATGAATATATCTTTCAGATGTTCTGAACCATGTTAGAGCTGTCTCTTCACAGATGATCCTGAGGTACGTCACACGACAGGGCTCCCACCCTCCATCCTTCTGCATTTCCTTGGTGTCTCTGGTCTTTCTGTCCTAAGATTACATTTCTCAGAGAACAGATGATATTGAATAACCTTCCTTGTCTTGGTTCAGCCCT

							TGCTCACTAGGGTACCTGACGTTAAAGACATCTGCAAGAGAAGGTCTATTTCATTTCTGTCATTCCACAGAATGCAAATTGCAGATGGACAGAGGGATTCCGGCAGAGGCAGTCATCTGTCGATGGGCTGCAGAGTGGGAAAGCACAGAACCTTCAGGAAACATAAGGATGGCCCAGCCTTCAAGACTCACTGCAAAAGTCCACTGGGAAATACAGTGCCCATGCCTGTGATCCCAGCACTTGAGGCAAGAGGGTTATAGAAAGCTGAAGGGCAGCCTACAAGGAGTCTATGAATGAGAAGAATTTAAAGGACAGGTG
MSTRG.122099.1	ENSRNOG00000042133;ENSRNOG00000047234;ENSRNOG00000058463;ENSRNOG00000059588;ENSRNOG00000060573	0.102	0.296	0.011	2.271	up	AGCAACCGAACTCCTGCCTCGTGCTCTGTGCATGTTAAACTAAAACAGCCTGTTGACGGCTTAAAATAAACCTGCCTTCTCCCACTTAGCATTTTATCAAATGAATGTAATTTTGTTTGCTGATGAATTTAGTTCGGCTTCAAGGAGATTTTTTTCCCTTCTTCCTGGGGAGGCTGGAGAATCAGAATTGCCCTGCCAGAGGATGAAAAACAAGCCTAGGGAGAAAAGACGGGCTGATGTAGGCTGAAGGCAGATCCTAATTAAATCCCAGAAGCCCCTACCATCTAGAACCCGAGCAGCGTCAACATGCTGGAGAGAAATTCAGTGCCTCTGACCACCCGCCTCACACCCACTACACCCTTCTCTCTGCTGAACAGAGGCTGAGAGGGGCACCTCTACCTTATCTGAAGAAGGGACCTGGACCTTCAACTTCTCTATTTCACCGTGCTGTATTTCTCAATAGAAACCTTAGTGTACTTACACTTAGCAGGGTATTTTGTGCTTAAACTGTTAAATGTGTTTGTTCATCCCTATGGAAGAGCCCCAAACTTCATTCCAAGAGCTTGTTAGAAACTATTGTTCTTTGGGTAAAACTATCTTTTTGTTTTAAAACTCAATAAATATAATACTGATAATCTTTTACTGAGGGCACACCATTGGTAGTTTTGTATTACGGTCTAGAGGGGAACTGATTCTCGAACTGGCACTAGGACCATTTCTTTCTGGCACCCTGCACGGGAATGGAGACAGACACACCGCAGCAGCGTTCAGGGAGAGTAACTGTCTTAATTAGTTAGTAAGGGTGAGTGAGCCTCTCCCAGGCAGGCCCAGTGTAGCATGGGTTGGTGGCTCTGTCTCATCCTCTCCGCTTGAAAAGACCTACCCACCAGGAAGACCGAGTCCACAGAGCAAGTGCCCAGGCTGTCTTCGGAGTGTGTCAGCCTTTGGCCTCCCCAGATAATTACAACCTCATGACCTGGAAGCACTGTTTTCCAGGTTCCATGTTCTTGCGATTGCAAGGACTCTCAGAAACACATTTTCACAGCGAGACAAGTCCTTTGAAAACTCTGGACATAATTGCCAGAAGTACCCAATGATATCAAAATAAAGATTCAGCTTCAATTTAATGACCCGGGGTAACTGAAATTTGGTTGGAGGATTTATGAGTCTAATTTCCAATGTAGAATTGGGAAAGTGAAATATAAAGCCTGGCTAATATGACATCAGAAGTTGTCAGGATAATTTCAGGATGGCTCTTTCTAGAACAGCAAGAGAATTGAACAAGCTAATTGACTGACAATTTGAGGGTGTGCTCTTGTTTCTACACTCTCGAGCATGGGGAATATCTTAGCAAAATGCTTTCCTGTGAGATGTTCTCCATATAGCTAGATTACATCCCACTGAGAGTACCCCTTATAAGCCTGACATGAAACGGACAATACTTTCTTGGATCTACAATAGCATGATTCCTTGAATAGCTAAGGTGTGGCAGCTCCATGCCTTATGAATATTATGAACCCATTCGTGTATGAATATTAAGATTGATAATGATACATATACACACATCCAAGCTTATTGATTCCCTATATAATAACATAAGCCAAAATTCAGTTATTAGGAAAAGATACTCTCCTTAGACAGAATTTAAGGTATACTGTCTAATTCAGTAGTTATGACATGTATTAAATGTAAATATAATTCTGTCGGGTGCTTTTTGTTTTTTTTTAATGTGGGTTCTCCCATATCCCAGGCTAGACCAGAATTCACTGTGGAGCTGAAGGTGACTTTGAACTTCTGATCATCGGTTCCTCGCCTCCCTAGTACTAGGGTTAGGGCGTTCACCACCACAACTCATTTATATGGTATGGGGGATTGGACCCAATGCTTCATGCATACTAAGCAAGCACTCTCTCACGTAAGCTCCAGATAAGACCCCAGTAATCTTAAAGTTGATCATCTGCGTGGTATCTGTATGTTTCTATTAGGATATTAGGTTTCAAATACCTAATTTTGAGGCCTAACAGCCACATAGAGCTAGTGGCTATTAGAAAAATAAAATAGATTTAGAAATCTTTCATCACTACAGAAAATTCTATTGAACAATATTGATTTATAGGTTAAAGAAAATCTTCACATACATTAAATTAGATCTCATTGCTCTCAGTTTAGATTTTTGTAGTCCTGGCTGCCCTGGAACTCGCTATGCAGACTAGGCTAGCCTCTAACTCACAGATCTCTACCTACTTCTGCCTTCCAAGTGCTGAGCTTGGAATACACCATGCCCAGCCCTAGAGCCCAAGACAGGGTCTCTAGGTAGCCTTGGCTGTCCTAGAACTCACTCTGGTAGACCAGGCTGACCTTGAACTCAAAGAAATCCGCCTATCTCTGCCTCCCAAGTGCTGAAATGCTGGGATTAAAAGGCATGTGCCCCCACTGCCTAGCAGAGTTTATTTCCTTTGTCTTTACAATAAACTTTCCCCTAAACCAATTTCTGTTGCACAGTCCTAAA

							AGCAGGCTATTTTATCATGCTATTCACATAGTGGTTCAGTTTCTAATTAGAAGTTGATGGAGTCCTTTCAT<br>GCACCACAGTATCATTTGCGCAACATTTTCCAGCTGCATTAAGAAGTTGAATTATCTCACAGAACATAAC<br>AAAAGACAGGGAATTATTCAACCGTCTAGAGAAGTAAATATATATGAAAAATGTATATATGTGTGTGCAT<br>GTGTGTGAGCATGCCCATGAGTGTGTGTGTGTGTGAGTGTGTGTGTGTGTGTGTGTGTGTGCATGTTTG<br>TATGAACTGCAAACTAATACACCATGAAAAGCTGCATCACTATCTCATCAACAAGAGCAACTAAGCCCA<br>GTGCCCATCACAAACTTCTGACCTCCACAAAGGAAGCTGTAGAGTCATCCTTTAAAGGTGCTGAGTCAT<br>CCTTTAAAGGTGCTGAGTCATCCTTTAAAGCTGCTCCTCTGCCCAGAGCACACTGCTCATGTTTCCTTGG<br>GCAAATAACGTTGCTTGACTAAACCTGCCACCAAGGAAATAGGGAAACATGAAACCTGCCCACAGAGC<br>AGCAAATGCTTAAGGAGAGTAATAAAAACTGTCACAAAGGCCTAACGGTTTCTGTCTCTGTAGGGGTCT<br>CCAAATGGACAGAAGGCATAGAGAAGGTAAATACACAGAAGGAGATGTGTTTAGGGAACTGGCCTGTG<br>TGGTGATAGAGGTTGAAAGGTACCAAAATTCACTGTCTATAAGCTAGAGAGCCAGGGTTTTCAATGCGA<br>TGGCAGGTGCTGGTGTGAGCCCTGGTGTCTGAAGGTCAGAGACTCGGAGCTCTCAGGCAAGCTTCAGA<br>TGGAAATGAGGAAAAAGTTTGATCCTTTATGACTCTCATGTCCTGTTAAGGCCATTGGTGGATTTAATGT<br>TGCTGCCGGGTAGGAAAGGTCCTCTGCTCACTGACTCAGGTGTTAGTTCCTTCTACACAAGTCCCTGAA<br>GCACTCACAGACATGGCCTCATGGGCAATTTTGCAGTCTCTCTCTCTCTCTCCCTCCCCGTCGTCCCTCC<br>CTCTCTCCCTCTGTCTGCCTCTCTCCTTTTCTCCCTACCCCCACAGCCTCACAAGTACACTCTCATACAC<br>AAGCACATACACTCATAAGCACATGTGCACACATATTTACAAGCACACATAGACCCTCACAAGTATAAC<br>AAGTATAATCCCTCTCTCTCTCTCTCTCTCTCTCACTCTCTCTCTCACTCTCTCTCTCTCTCTCTCTGTG<br>TGTGTGTTTGTCTCTGTGTCTGTCTGTCTCAGTGTGTGTGTCTCTCTCTGTCTGTCACACACACACACAC<br>ACACACACACACACTTAAGAACACAAGCACACAGAGACACTCACACATACTTGTAAGCACACATGACC<br>ACAAGCACTTATAAGCACACACAGACCCTACAAGCATAATCTCACACACATACACATGGGAGCATTTTA<br>TAGACACAACCTCATGAGTACCCAAAAGTAGCTTTACTTTAACTCTGTCCCATTGTGACATAAAATTAAC<br>CACTGTGCTCTCTTTTGTGACTCCTGTTACAATGCAGCTGCTTTCTAGATAGTATTTTTATCTCTGACTAG<br>AAAAGGAAGTCTCATTTTAGAACATCTGAGGTACTTGGTTCTCCCACATTATCAGCAAGACGGCTGTGG<br>TTTAGAGCCTACTGCAAGCCTAGGGCTTGCTGCCCTCTTGGTATAGCTAAATCTGTGAATGGATGCACAG<br>AACAAAGACGTGTTTATAACTCTCCCTGTTCTAGTCCCACAGTCCCTCACCAGCAGTAAGAAGTCTGCT<br>TTCTTATAACTTGAATGGTTTTCATTAGAAATTTTCCCAGCCACAGGGCCAGAAAAACAGATGCCGCCG<br>AGAATCTTTATGTAATAGTTGTTCCCGTAATCATTATCAGGGCATCCCAGAAAAAGATTTCCTGGTTACTT<br>TCTTCCACATAGGTAAGTTGAGATGAGGTAATCTTTAAGAAGAAGCTTGTAGTTAGTCTACATGGCACCT<br>AGGGACCACGCCTGTCGCGGGTCCTTTTATTCCTGTGTAAGACAGCACACGAGCAAGACAGTCTCTTTC<br>AGGTCCCACAGTTCTGACAAAGCCAGGGATAAAGTGGGAGCTTGTCTTCTGCATTTGGGCTTTATACAT<br>GAATCTAGCAAGCATCTTCCTTTAGTTATTTCTATAACTAAAACAGCTTAGACGATATGACTATATTTACC<br>AGTCCATCTACCAAAGGCCTTCTGGACACTTGCTCACGTAGCCTTAAGATCGCCCTCTGAAGTAAGTGT<br>TATCATGTGCTTTCCTAGGGCTGAGGAGGCAAAGCATAAAGAGATAAATATGACCTATGGGTTATTTTGC<br>TAAAGAGTGTCCTAGTCAGAATTTGAAACCAAGTCCGACCAACGCGCCTGTTCATACTTCTTATTCTCTG<br>TCTTTCTCTCTTGCCTCCAAATTTCGTGTGCCTTGGAAATGGAGAGACAGTAAACCTTAAGTCCCAGAA<br>AAGGAAACTGAATTACTTGCGAATCAGTGTGTGCATTGGGTAATGGCCTACATATTAAAGTGAGAGTTA<br>GAAATGAAAACCACTTCATCGTAAAATAAAAATGGACTCTTGTCCCTGCAGCTCTATTACGGATTTTGCT<br>TCTACGATCTAATTTTTGTCTTTTAAGCGTGCTTATTTTGAATCTAATCCAGTTGAAGCAAGCTTATAATTA<br>AAGTGGCATTTACTACGATGGACGTTTTTCATACTGATTAGATGGGAATGTCTAGAGTGGAGAAAGTCCC<br>TTGGGATCCTGGTACATCCAGTGTCTTGGTCACGTAGTAGGGATGGGCAGCTGAGCTGCCCCCTGGGGA

							GAAGACATGAGTCACAGATGTTTTCAGACGGAGAGCCACAGATGAAACCAGGGAGCTGGGTTCTCAAT<br>GGGATGTTCGTCCCTGGGAGAATCTGTGTCATAGGCTTTTAATTGGTTGTTCATGCAAAGAAACAAAGG<br>AATGAATGAATGAATGAGCACATGGGAAGTTTCGATGATTCTCACACCACTTGTGCTAAACTGCAAATG<br>CTAGCATTACAGCAATTGACATCAGTTGTTATTTCTAAGCTATTATAGATCAAATCATCTTATGTTTAAGAA<br>CTACAGGACCCCAAATAGCAAAGCTTAAAAATGACGCCCTAATGATATCAACTACTGGAAGTTCATAAA<br>TAATTTCTTATTATTTCAATGAATAACATTTGCCAGGCTGAGTAGCTGATGTTCAAAGAACCACTACCATA<br>CTTAGACATCGTGCCTCAAAAAAGGGGGGAAAAGTATTCTCAATTCCTGTTTTCTTAGCATGGTGCCCA<br>GTATTTGAAATAATAATTCTATGTCTTTTTGCTCATAATAATAAAAAATATGTTCTTGCCATTGCTTGGCTG<br>GAGAAGTGGCAGAAAAACCAATCACTTCTTTGTCAGATAAACAGGTCCACAGACATCTAGCAATCTCA<br>ACGTCAACACCAGAATTTGGTTTTGATGAAGCAGTTGGGGTCTTTGTTCTAGATTTAAAGAAGATATTCT<br>GTTGGCCATGAAGACCTAGGACATGCTATTCAAAAACCAGTTTCCCCAGTATATTCAGAACACTATGTCA<br>ACCCTCCAAAAACTGTAAAAGATTACGTTTAATTTTTTAAAAAGAGAGTGAATATTCACTTTCATTTATTA<br>CTTCAAATTAGAATATCATGCAGGGAATTAAAGGATTTTCTCTAATCCCCGTCCCTCTTTTTATTCCAGTC<br>TTGAAGCCTGATTATAAAACTTGGGATTTGGTGCCTATGGTGAGTCTAAATATGGATTAAACAATTAAGA<br>ATCTCCCGCTTGTAATATACTACTACTTTATATAAAGAATGACTCTGATCATAGATTCTTACAGGTATTTAG<br>AAAAGAATTTAAGACTACAGGGAAACCAGTAATTTTGTCCCACCACAAACAAATCAATCTGTGATTTGT<br>GCCTTGAGACTTCCCCAGGGAGATTTAGGCAATTAAGAACGGGAAGCCCTTAATTAGAGGGGAAAAGA<br>CTTTCAGCTATGGTGGATGGAGAAGTGGATGAGTCCCAGAAGGGTGCTCTGTGCGGTGTAAGAGTTGG<br>GTACTTGATACAATGTAACTTCTAAAACAGCATTGGAACAGCTTACTGCCAAAAACTTCAATCATGCCC<br>ACGTCTTCCTGGAGTACCTTCCTCTTGTTCTCGGATCTCAGTGGCTGGTTTCTTTGGCTGTGGTTTTGAT<br>GGATTCTTTGAGTAAACGGCCCCTCCTTTCCCCCCCGTGGCACTGAAGAAGGCATATCAGATACAACAC<br>CTTCCAGATCTTGCACTGGGTTTGCCAAAAGGATGTCTTTTATAATCCATCACAAGCAAGGAAAAATGA<br>CTTCTACACCTTTCTGCCAGAGTTCATTCCACATGAGCTTTCCCACTTTGACTGAGGGGTTTTATGACAC<br>GTTATCTTTATAGAGTGGGCTCTAAATCCCAAATCCATCCTGTTCTTAAACATGGAGTTTGAACTTTTCCA<br>AACAACTATCCAAATTTTAACCTCCAGTTTAATTTTTTAACTGAGAGTTCCTTGATTGCACTATAAATCTA<br>CAGGTACCGAACACAGAGTCTTCAATCAAGACAGAGTCACTGCGAAGCAGGTATAATTTGTGGTTTGG<br>ATTTTAGGAAGCAAATAAATAAGAAAAGAAATGGAAAAGTAAGACAACACTAGGTATTTCAATGTGAA<br>GACTTCAAATTAAAGACTGTACTTGGGATACTCACGATGTATTTAATTACAAATCTAAACATAAAAGTAA<br>GGTTGAGTATGTATGGAAGAGTTTGTGGCAAAGCAAAAATTGACATTTTTCCTAAATAATCCAGAAAAT<br>AGAATAATCTAGGTATCATAGGCTAAAAACAAAATTCTCTGTGGTCTACTGGAAGATTAAAGGTGAGCT<br>GATACTCGCAGCCTATGTACAGTGTTAACTAACAGCCTTCCTCTACCCCCTGGATGCCCTCTGCAACAGA<br>TTTCTAAAGTGATCAAGAACTGCTAAGGCTCGCCTAAGTTTATTTCCCCGATACTGTTATTTCATGTGTTA<br>CGCTGCAGGCATCTTGCCTACTTTCTTGCCTACAGGGGATAATTTTTACCTAAATGTTAACATTAGTGACT<br>TGAGCATAGTATTTCTTGAATATTTTTATCTCCCTATTTTTAAAGGGCATCCATTACCTTGATGCTGTTTCC<br>CGAACTGGTGTTTGTCATGCTGTTTGCAATAATTTTCAGAAGGCAAAAATCTTAAAGGCGTACAGGGAT<br>GCTTCCTTGAAAAAGAATTTTCAAAGACATTTGTTTCTTGGAGCTGTTGCTGGGGTTTTAAAATGAGAA<br>TGTTCTACCTAGGAAAGCATACAATGTATGAATAGGACATATTTTAACCACTGTGGATGCGTTCACCTTTC<br>CCCCAAATATTTTTGTTTTCTTTTCATTCCTTTTCTTACATTTCATAAGAAATTATTTATGTACTCAGATTTT<br>TTATTTTTTTTATCGTTCCTTGCTGTCAGTGCACATAAATATATGATTTCAGGATCCTTTCTGGTTGGACTC<br>CTGAGAGATTGCAACATCAAACAGGTTTATTTAGGCCTGTGCTAAGCAGAACCACAGGAAGCTAGCAA<br>AGGCTGTTAGCAGAAGCCAAGAGGAAATCTTTAAAAACGCGATGAGCTATGTCACACTCACAGAGGAC

							ACAAAGAAATTGCATTTTTGCACCTCCACAGATAGCCTCAGTAGGTAGCAGAGTGCTGGGAAGCCAAGTGCTTAAGGAGGATCCATGCCCATAGCACAGAGGAGTGAGATGCCTGGGCTGCCCAGCCCGCATCACTGATGTCAGAGCAGGAACACCGCCCCCAGCCTCAAGCTGTTCCGCATCTTGGGAACTCCGCAGCAAAGCTAGTGTGTGTGTGTGTGTGTGTGTGTGTGTGTGTGTGTGTGTGTGTGTGTGTGTGTGTGTGTTTACATTGTAGGGAGATGCCCCCTTTAGTGCATGGGTATTGTAGTCATTAGATCCAAAGACAGACTCGGCTGCTATTAAAACAGAAAAAAGAAAGGAAGGAAGAAAAAAAAAAGGAAAGAAAAAGAGAATTGTTGCAGGGTTTGTCCTTCCCCCTGATGGCTGTTCACATTGATTTGCCGCATAGATTGATTGCTTTCGGCATTGGCACCCCTCCTGAACAACGGGGGAAGAAAATCCAGCGTCCGGGCCAAATTCAAGAATGATGAATGAATGTGAAAGACACATTTGGTGTCATGCTGAGTTGCGATGGATAACAGCACTTAAAAGTAGTAACAGCTGGATTTTAGTTTTTAAAATTAGGTCTGTGTTATTAATGGGGGAATTGGGATGCTGGGAGTCTGCGAGGCAGATTAAACGCTTATCTGAAATGAATGGGCTTTATGCTCTGTCTTGGGAGGGTGGCGGCAGGTGGATGTGAGGGGAGGCTCTGGCCCTTACTGGAGGACTTTTACTCAACTGAACCATGGCTCTCTGTTGAAGCCCACCACTGGGGCACGTTAACTGAAGCAGCGTAAAATGGCTTTTGTATTTATTGTGGGGATTTTGAAAGTGTATTGTCTAATAAATCTCACACTGTCTTCTTCATGAAGATATCAAGGAGAAATTCCACACAGACTTTAGGGACAGAGAAAGCTATATTCTCCAGAGAATTGATGTTATTTCTCCTGTATAGACACTGAATCTTGTGACTGGCAGGGTGTTCTGCCTGCTTAGCTCTAGTGAAGCAATTGGTTGAATGGAAAGTCAGACTAAGACCTCCAGGGTATATTGGTGCTTAATGCTTTTGTCGCTGACTTAATTTGTCATCTTAATTTTTACTTCAAGTTTAGCTTTATGTCATTTTTTATCTGTTAATATATTTTTTCATATGCATATAGATACTATAGAAGCAGGAAGTATTTTCTTTCAGGGGAGAGGCAAAATCTCGGGATCTTCTGTATTCCAAGCTAGTCTCAAATTCTATAAGTGGCTGAGGATAGCCTTGAACTCCTGATCCTCCTGCCTCATCTATTTTCTTTAAGTCATTAATTATTATTCAGAAATTTATAACACAGCAGTTATAAAGGCACATTTTCTTGATTTTTGAGGAGGTGCACATAGAAGCACAGATGGAAAGTTAATGCCGCG
MSTRG.122114.1	ENSRNOG00000009580	0.289	0.000	0.013	-4.001	down	TGCCTCACGGGTGAATTCCACGCACCGAGGGACTCTATCTGGAAGGAGGTGGACTTTACCTCTGAAGATGACACCATAGCTTGTCTTCCAGCTCCAAACGCTTGTATATACTCATGTACATGCAGCCATACACATGGACATAGACCCTGCACACAAGTGTATACACACACACTCACTCACTCACTCACACAAAGAAAAAAGTATCAAATTAGTAAGTTCCAAATTATGAATGAGGAAAAAAATCAAACAATGACAATAAGGTAAGAAACTAATAACTATTGTTAAACAAAATCCAGGAGTGAAAACACTATGTGTGACAAAGAACACTTACACTGGATTTTTTATCATCATCATCACCATCATCGTCGTCGTCGTCGTCGTCGTCATCATCATCATCATCATCATCATCATCATCATCGTTTTGAGACAGAGTTCTCTGATTAGCCTTATTCCTGGCTGACCTGGAATTTAGTCTGTATGTCATACTGGCCTCAGATTCAGAGATCTGTTTGCCTCTGCCTTCTAAGAGTTTGTATTAAAGATGTGTCCCACCACCACCCAGCCCCTGGATTTTGAAAAGAAAAAAAGAAAAAAAAAAGTACAAGTATATGTTCTTTGTAGGAGTCTATCCAAAATAAAAATCTTACAAAAAGTGTGAATCAAAGGATGCACAAAAAATAGACGAGATATGCAAACTAGCCAAGTCAAGGAATCTCAGAGCCAGCACTAAAGCAAATGGGAAGTTTTCGTGTTTGTAAAAATTACACAGTCATGGCAAAGAGAGAGGAAGACCCCCTCTACACGGGAGCTCTTCAGTACAGCGTCCAGAACTGCCTTGTTTGCTCTCCTGGGTATGCCATGGTCAGTGGATCTGGGTGCATCATCCCGGATTGCTCCATTGCCACACTCTTAGTCACCTGTCAGCCAGCTACCGCAGCTCTGGCTTCAGTGGAGCTTCTCTGACGTGTGCT
MSTRG.122131.1	--	0.549	0.000	0.037	-3.684	down	CCCCACCATCCCTGACAGTCAGCCTCAGTGTCAGCCGAAGGCTTTCATGTCCTGGATCTGCAAATGTGGGTTATGTGACAAGAGTGTTTGTCTAAGGTGGTAAAACCCACGAGTCAAGACTTTTATAGGTTTTTCTTCTTTGTAAAAGGGCAGCAGGGAATCTGTACGCCACAAAGAGAAAGACAAGGAAATGACAACAGAAAGAAGTACGGATACCACCAAAGTGATGAGCCAATGAGTTTTATTAGAATTACAAGAACATGGATGAGAAGTTACCTACAAGAGCGGAAATGACTCAAAGACAGCTGACTCACCAAAGCCCACCCCACCGTGGTTAACCAT

							TCACAGAAGCCCAGACACCTGGAATGGACTACACAACCTGCAGGATCCTTCCCAGGAAGTTCAGCTGCAC
MSTRG.122148.1	--	0.204	0.624	0.047	2.158	up	CAGAGGGATGTCCTCTCACTCTGGCGGAGAGTCCAGGGCTTAGAGAGTCTCCTGGAGAGTCGGGAAGACAGCACAAGAAGCTAGTATTTGGAGTTACATGTCTTTATTTGTGTCTGCATGTGGGAGAGCCTGCCACAGCACATTTGTAGAGGTGTGGAACACTTACATGGACACACAGAGGGGCAACGTTACCTGACAGAAAGCCCTGAGCTTACTACTATTACAGGAATGAAAGTTCCTCAAAGGTGGTCCTCGATGAAGGAGACAGTCCTCGCTTGTCCAAACTGCTTTATTCACGGAGGATAAGGATGTGTACGACTTACTCGGGGTGAACCAGAGATTAACTACCATTTGCAAGAAGGGACGCCCAGGAAAAGAAGCTCGTTGGCTAACCACCTGGGGCCTCTCCAGACACCTCGTTAGTATGGAGAACTCTGAAGTCCACGCTACATGATGGGCTGTCACGATCATCTCCCTGGGGTGTCATGGTCCCAACTCCTGTGGTCCTCAATTATGAGTTTTACTGGGGTTCTGAACTTTGCTACTACTAGTTCTTCTGTCAACATGACGTTTGAACTTCAAGTGGGTCCTGAACAACTGGAGCGGGGACCATCCTGAAAGCTGTCGTCTGTCTGTAGGATATGTTCTTCTATCTGGGCTGCCTTGCCTGGCCTCAGTGGGAGAAGGTGTGCC
MSTRG.122172.2	--	0.291	0.000	0.000	-6.966	down	CTTTATTAAAAAAATTTAATCTCTGACAGAAGAACACCTTGACAACATGGTATTGTTATTAGCATGGCTATAGAAAGTTTGTTTTTACTTATAGAAATAAAATATAGAAGAATGAATACAAAATATAGTCATAATTTATTTAACTTTAATTTCCTGCAGATGCCCCTTTTCATCAGACAAGATGTTTACAGCCCATATCTGCAATCTCTCATAGCTGGTGCTTCTTTCTCATAAAGCCCCACTTATTATTATTATTACTATTAACATGATTATAAATAGTCTTACTGTGAACTAATGCTAAATTCACCAGAGAAATGACTACTTTATGATCAGTATATAGAAATCATTCAGATTTATTAGTAGTGTCTTCTAAGATTTCCGTGTTTTAAAGAATGCTGATAAGAGTAACGGAAAACATTTAATCAAACATTTAATCAAATCAATATTGGGTTTATTTTAGAAGTTACGTGGGAGGCAGAGCAAGCTGAGGCCTGGTACCCTTGGGAGAGATGTTGTGTTCTTCCTCCAAAGCGCCGCCGTCTCTCCTCATCTAACTTTACGTTCCCATCTGCTCTTACTTTGGGGTGTGCAAACTTGTTTGCCCTGTCCTCTCATTTGACACGTGATGAATCTTGAAGTCCTTGAGGACTTAAAGGGATAAATTTGAACTCTGCTGTGTGGAAAAAAATATGCAAAGACGATAGATTTCAGGGAATTCAGTGGCCTTTTCTTTGGGTTCCTGGGCCAGGATTTCACTGTTTTCAGAAAAAGAGTTTTGTATGGTGTTTGCGTTCCTCGGCACCATACTCACAGATTTCCAAAAACATGTTCACTGTGCAGCACCCATTAGCTTCCTCCGTTTTAATCTCTCCCAAATTAATTTGTGCTGTATATTTGAGAATCCAGTCTCCAAGTCAGTAATTACGTATTTAAATGTTAAGACCGACCAGCAGAGGCATTTTCACGGCTCTAGATACGTAGCGTGATTCCTGCGAACATTTTAGAAACCAACCTTCATTATTAGCTTCCCGCACAGTCTCGTAATGACAAGTCTCCGACTCTGATATAATGGTGTGATAATAAAAAAATAGGGTTTTCTTTTTCTTTTTTAAATCCCCCTTCTGGATTCAATACTCAATAAGAGCGCCAAAAGTTTCAGGGATTGTGGTTGGCTTAAAGGTTTCCTTCTTTTTTTCTTTCTTTTTTTCTTTCTTTCTTTTTTCAACACATGGAAGCAATAACTCTGAACTAGAGAGGCCAGTTGGAGACGATGGTAACTTTCCATACCATTGTTGTATCCTCTCTGTGCGGTCTTCAAGCTGAGGATAGGAATCGAGAAGTCGCTCATACATACGCAGAGTTTGTAAGGGGATTGGGTCCTGATAGTTTGAGCCTGTGTGAATTATTGTGCAAAAGGAGAGTCTCATAGTGTTTAACAGAAGGAAGTTTGCTTTTCTTACTGTTCTGTGTTCCAAAGACTGATTTGCTTTTTCTGTATAAACCCGTTGCTACCAAGTTATAAATATTGTATTTTAGTGTGTCAGGTTATGAGAAGAGGACAAATCCTAAATAATTTTGAGATTTTTGCCTATAATATCTATCTATCTATCTATCTATCTATCTATCTATCTATCTATCTATCTATCCATCTATCTATCATCTATCTATCTGTCAATCATCTATCATCTATCTATATATGTGAGTTATATATAATATAATGTATATTATATATAATAGTAAATATATCTATCATGTTGATCAATTAAGAGTCATCAGGATACTAATGTGATGAATAATTTCCTACAACTTTGTTATCTTAATTTAAAAAAGTTTTTATCAGCGATATATAATTTGCTAGCTACAGATGAAGCAGGCATCATGGGAAATGTGCCTTTTGGCTAAAACATGGGAGCTAGGGCCTCCTGAGAGCTGGTCAGCAGGAGTGTCTGACTGTGAGCCGGGCGTTGTAAAGAGC

							AGTTTTGCCCAGAAGCCTGTTGAGTATTTCTTGTGAGCAGCTTCTGTGGCCACAGACAGTGCTCATATC<br>CTAGTGGTCTCAGCTTGTGCTGCGGATGGGAGTGCCCATGGGCATCTGGGGTGAAGGCCTTTGTTCTCC<br>CTCCATGTTCCGTGTACTGTCTCCACCACCTGCCTTCTCTCTGCTCACTCTGTATTTTCAGAGGGGTGGA<br>GGTTAGTAGTTTGCATGGCTGTTCTGGAAACCTTTAACTCTGCCCGGCTGCTGTGCTGTAGACAGAAAG<br>TTCCTTTTTCTTGCCAACTTTTGAGATGATACGAATAATAAATGATAATAAATGATTTATGTGGAAGGACG<br>TAAACAAGGAACAGTGAGAATAGAGAGTAATGATCATCTGTCAGCTTATGGAAATGAGCATTCCCAGGT<br>GTGAACAGGAAGCGTTCACACCCGAAGCTTTTTGGGGATGTTTTACTTCTTTGGCTCTAAACTCTCACC<br>CCTTATCACATTCAAGTCTTTAATTTCCAAAACATCACTTTGTCTACCCTCTGCTTGAAAACCTCTAAGG<br>ATGCTCTTTTAATGCAGCAGCAAATTGCTTATGCAAGGCCTTGGTTGCCTAAAACAGTGGTCCAGTCAG<br>GGTTCTTGCACCTTTGCGTGAAGCCCGTGTGCTGGTTACACTGACCATTTCTTCAGCCGTAGTTCATCCC<br>TGTGTTTTTGTTTTTTTTTTTCACTCAGTATTTTAGGCAATGTCTTATACAAGCATTTGGTATGAGAAGTG<br>GAGTCCTTGCTTTAGGCACTCTGAGTTTATTAAAAGGAATGGATGATAAACTGTGACCATTAGGAATATA<br>ATACAACACAGTCAAACAGAGCACTGTGGGACTGTACAATCTTTGACAAAGGAATTGATACGTAGCCA<br>GAGGATGGACATGAGCCTAGAATGAAAATGAATTCAAGCTGGGTAGAAAGGGGTATAGTTCTAGGCATA<br>AGCACGGTTTTGACATGGTTAGAATACTGCATAAAAGGCTGTAGTCTGAGTCAAGATTGACAAGTAGGG<br>ATTAGAACCCAAAGAATCTTGAATGAAATATTACAGAATTTACCTCTGTATGGAAGAAATTCCATAGCCG<br>AGCTGGCTTCCCTTGGTATGACTTCTAAGTTTCCAATAAATATGCATAAAAGAGACTTTATAGGACTCTT<br>GACAAATAAGTTGTAAGTAATCCTTGCTGTTAGCATTTCAATAATTAAAATCATATAATTAAAGGATTTTA<br>AGCAGAGGACTAACAGATAGATTTTCTTGTTAGAAAGAGTAGGCTAAGAGGTTTATTAAAATACTATCTC<br>CATAATTTAATTGAGGGATTTGAGGATCTTCAACAACTAAGAGGTAGGGTTGGCAAGCAATGTGGCTTT<br>TAGAGAATAGGAGAATGAATTTGAGAGAAAGACTTGATTCATGAGAAAGGTATGAGGCAAGGACTGAA<br>GTGGTTGATGGAGTGTCTGGGAGGCAAAAGAAAACACACACTAGGCTTTATTAATATACACATGGCTTC<br>ATGGAGATAAGAATATCATTGGGAAAAGTGAGCATTGTGAATGAAGTCTTGAAAACATGGGCAGATTAC<br>TGTTTCAATGTCGAGAGACATGAAGCACATCACTTCCTCTTGTCTATGGTTTCTACCATTCTGCTTAGATT<br>TAAGCCATGTTTCTTAAATATATAAATATAATAATCATAATAATACCTTCAAGGCTCAGTAGTATCATGTTG<br>AGGATTTTATTACAGGATACAGCCATAATTATTTCACTTTAGGATTAAAATGTATTGTAAATCTCATTGCAG<br>TTTTTGAGTCAAGCTTTGTCAAAGGTATTCACATAGGGGGAAGATAGCATATTATGACTAGGTACAGTTT<br>GTGATTTTAGGCATTCTCTGGAAGACTAGAGCATTCTGTGTGGATAAGGAGGGGTTACTGGGGCTAGAA<br>ATGGGTTTAATCATTACTATGAGGGTGTTTGCTTAGCAGAGAACACCTTTTTCTTGGTTTGAATGCTCCC<br>CGGCAGCCTCCCTGACTTCTCTGCCTTCTCCCCTTTCTTGCCAGCTCATGGAGTCAAACTTCTAATATCT<br>CTCAGATCTGTCTTCCTCTTGTATTCCCCCAATAAATTCAAAATGAGCCCAGAGACTGCAAAGTTTAAAA<br>GAAAAACGTGGAAAATAGCAGTCAGCAAGGTGAACATTTTCTAAGAAAATGCAAATAACTTAACGTGT<br>TTCTCCTGTTCAGTATACTGTTAAGTGTAATTCTATCCTAAGGAGCCTTTATTATGGTGGGGCCTACCCCT<br>TTTAGTCCTAGTATCTACCGGGCTTTATGTTAAGGGCGTGTTGTGTTTTGTCAAAGGCTATTTTTTTTCTG<br>CATCAGTTGTGACAGTATTGTGACACATGTGCTTGGTTCCATGTGCGTTGTATATTATTTTGACCAGTTTG<br>GATATGTGGAACCATCTGTCTATCCTTGGACTGAAGTCAATTTGATCCTAGTTTGTGATATTTGTAATATG<br>CTGTTGAGTTGAGTTTATAGGAGTCAAGCTTCCTGAGTTCAAGAAAGGACCTCTTGTTCCCCTTGGAAG<br>CCAGGTCCCAGAGCCCTGTAGGTCATTAAGCAAAAACTCAGTGTTGTTTAAATGAGTTGTTTGTGTCCT<br>AGTATCACTCTTGATTTATTTGAGGGCGAGCACAATTCTTGCTGTTCTTGTTGTTGGAAGGACCGATAGG<br>CAAACTGCTTGCTTATTTTCACATCACCACTCCGTAGAACAACAAAAACCAATCATTGTAGCTTGTGTTT<br>GTAAACCGGGAAGTAGGGAGTAGCTGTGACCTTCTGTGTTGTGTGCTCACCTGTGACCTTGTCACAGA

							GGGGTTTTGTTTGTTTTTGTTTTAAAATAAACCAGAGGTACTTTTCTTCTCTTTCCCTATTGAGTCTATGA<br>AAGAGATTTGTATTCACATGTTGACCAGTGAAGTTTCATAGGCTAGTAAAATCAGTTATCTGAAGGAAG<br>CCAGTCTTCCGTTTCATTCCATATGATAGCTTGTGTGAATTAGTTTTTGTAAATAGTTCCTTTAACACCCA<br>GAGTTACGTTTTTCACTTAGTGCTATAATCCTTAATCTACTTGTATCCTTGAAGAACATGACAAATGGTGT<br>AAAAGATTTCTGTGTGGGCTAACTTCTTTGCTGTTGTGAACGAAATAATAAAGAGAAGTAAGGTCGAAT<br>AGAAGCAGTGGAGAGCTTTACTCTGGCCCATGGCTCTAGAGCAGTGGTCCTCAACGTTCCCAGTGCTG<br>CTGCCCCTTTATACCGTTCCTCATTTGTGCTGACCCCTGCCATAAAATCATTTTGTTGCAGCTTCCTATCA<br>TTTTGTTACTGTTAAGAATCATAATGTAACTGTGTGACCACGAAGGGGTTGCATCCCGCAAGTTGAGAG<br>CCAGTGTTTTAGAGCATTCGGCTTATGGTTTACTTGGTCCCAAGTAGTTCGGTAGGACATCATGGCGGCA<br>GGAAGGAATGAGGGAGACCCTCCATGTGACGCAGGCCAGAACACTGAGCGGGGGAAAAGGGAAAAG<br>CTGATGCTCTGGGTTTTTTTTTTTTTTTTTTTTTCTTCTTTTTCTCATTTTACTTTGTCCAAGCTCCAGTCA<br>AGTAGATAGCGCGGCTCACCATTGACCTTCCAAGCACAGTTAACCTGTGTGTGTTCCCTCAGATATGCG<br>TGGGTATGTCTCAATATCTCTCAGTTGATTCTCAACTAAACCAAATTGGCACTTGGCATTCACCAGCATA<br>ATTTTACCTCCTTATTTGGTATGGAATAATATCTTACGTTTTTCTATTAATATATATTATATTATCATTATAATA<br>TAATTATACTATATGTAATGTACATGTATATTTATATATTAAGTATAAAATATCACTTACAATATTTCCAAATA<br>AGTAAAGGAGTTGGCTTAGCACTTGTTGCTCTAACAGAAGATTGGGGTTTGGTTCCAAGCACTCATATG<br>ATGCCGCCTCATAACCATCTATTACTCTAATTCCAGGATGCCTTTGTCTGACCTCTGTTGACACCAGGCAT<br>GTATGTTGTGGAATACATGTAGGTAGATAACACGCTCATTTATATAAAACAAAATTTAAAAAATTTCAACC<br>ACAGTAAGTTCATGTAAAATTTTATAACTGTTAAACATCAGAGGGTGTTTTATGCGTGGTCACCTTGCAC<br>AACTTGTTAGGCTAAATGAGGCAGCATGGTAAATTCTCCAAGATAGGCTAGGCACGGGTTGTCAGCCTG<br>ACAGGCATACTTCTTGTTTAGACTCACTATGCTTTCTGAGGAAACAGCTAATTGTCTCAGTTTGTCATGC<br>CTCTTAAGTAGGCAGCAATATTTGTAATTTTGGAAGATCATTTAGCTTGGTTCTTTAGTATAATTCAATTTA<br>AAGCTCAAATTAGTCCATGTATGTTTACAAGGGCCATCCTCTCCTTTCATGAGTGAGTTCATGGTCACAA<br>GAAATATGGTTATGTTTAGAGATTAATTACTGCTAATGAATTCCCTGCTAAACTGTGAACAGTTAAATTAG<br>TGCTTATTGCAAAGAACCCAAACAAACAAAAAAACAGACAGCAGTTGAAAATGAAGACTATAAACAG<br>AAATCAAATGGTTTATTATTGTTTTGATTTTCAGGATTAATGCCAATCTTCCAAGTGCTATTTTAATTTGCA<br>GAGATGTCTGGAAAATTTCATTTGATTAAGAAGTGTTGAATGTTTTGATTATTCTTTTTCAAAATAGGAA<br>GATTTGGTTCTATTTCATTATCCATTTTGAGAGTCCTGCCTTTCTGACTTAAGTCCTTTGGGGAAATCAGG<br>TACCAATATTAACTATTTAAAGTAGTTTTGTGTCCTAGATGGCATAAGATACCTATTTGTTTTCATCAAAAT<br>TGCTAGCTGAAAGTTGTAATGACATGGCTCAGTAGGCAAAGGCACTTGCCATGCACATCTGGTGACCTG<br>ATCATGATCTGAGAACCTGCCAAGGGAAAGAGAGAACTGACGTCATATAGCTTGTCTCTCACCTCTGCA<br>TGGTAGCTGTGATACACCCATGTCCCTTCCCTCACACATGGTAATACATTTAATTTTTTTTAAAGAAAAAT<br>GAAGTTAAAATATCTAACTAAAATATCAACAGTTTTTGAAGGCTATAGATATCTTTATGTGTGACTCACAT<br>GTTCAAACCCATGTCTGAAGCATCCCTAGCTGTAACTAGGCCACTTTTTGAAACAGGCCATGGCTACTC<br>ATGAGTTTGATAGGGTGGGATTGCCCTCAGCCCGGTATTTCTTTCCTGGATCAATTTTAAATCTCCAGCC<br>GATGGTTTTGATAGTACAAAACACATACTTTGAAACAGGCCCGTGGTGTTTTGGTCCTTCCTGAGCTGG<br>CTTCAACTTTCCTATTGTATTGTCCTTAAATCATTTTTTCTCCTTTGTTCTCATACTCTTTACTTTTGATATG<br>AATACCTTTAGTCAGCAGTCAGAGAAACTGAAATGAAGCTTTGTCTTTCATCTCATCTCATCTCATTTCT<br>TGACAATCTCCTGCCTCAGCACTTGACCTCCAAATTTATACTCCATATATTTTTACCCATGTACTTTCCTG<br>GGCATAGGTTCCTAACTTAAGTATACATAGCAGACCATCTTGAATGTATTTTGTGACTAATAATTCTAATAT<br>CACATAATCCTATATATTTTTATTAAAACCACCTTTCATGCAGTAGATCCAAAATCAGTTGAAGTACAGTA

							AAATAGGTACGACACATACGTTGATAAACTACTGTGGAATGTTGAATGTGATGGAGTATTTAATTACGTGGGTAGCTAGTAACTTAAAGCAGTGTTTGTTTCAGCAAGACCTGAATTGTTCAGTAAAATAGGACCCACATTGACCGGTGTTTAAACTGGGTCCTGGGAGCCGCAACAACACAGGTGTTGGCTGCGTTCTGCCATTGAAAGATGCCACACGGCCATCCCCTGGGCTGTTCAGAGAGCCCTTCTCTTCTCTTGGTTTTCCTTCCTCTTTTCCTGTCTTCTTGCCTCTGCAGAGCCCTCTTTCGCTTCTGCCTTTCACGACAGTTTCTCCATCCTTAGCAAACATGCAGATCTTTGTAGAAAACACACAGTTCCTCCGTCGCCGTAAGCAG
MSTRG.122247.1	ENSRNOG00000010240	0.394	0.000	0.000	-5.774	down	TTGTGCATCAGATAAATGACATTTAAGTAACACTGATAATGGAATCACAGTTGTCGGGTTACTCATAAAAATCTAAGCCTACTCAAAACTACCAGAATCCTCAAGTTCAGGACTGAGAGTCTGAAAACTACTGATACTGAAAACAGTTCAGTCTTCTGGGTCACTTAACCCTGCTAAGTTTATACCCAGACAAAAATTTTTGGCATCGAATAGCTAGCGTCTTTTGGAGCTGTTTGAGTCATTTAGATGGGCTGGCTGGGGGTGGGACACTGTCATTTCTTAAGCTGCAATAAGCTTTGGAAAGCTGGTCTGCTCCTGTGTATGCCCCATGCTCCACAGTGTACACAGTTTGGGGTTCAGAGGACACGATCTTTAGCACATTAAACACCAAATAGAGCAGACACAGATCAATAGAAAAAAATAACTTCTTTTTATTTACAAAAACAAAAATCCAAATATTGGATGCTGTAAACAAAATTCACAATCTGTTCCCTCTAGCACTGATACGAACATGTCAGTTTTAAAGAGTCCATTTTCCGTCAACTCCTTTTACCGCCTGTGACACACAATTGTCAAAACATTCCAAGAGTGACCCAGGGTGCAGCTTGCTGTAGTAGGACAACTCAGAGCAGGCAATGGTTGTAGGGAGGTTATCACTTTTATCTCAGCAAGTCATTACCATGACAACACGTGACACGGAGATCCCTCCACAGCACTGAAATGGTGATGCTAAAAGAAATTATTAAAGAAAGAGTATATTCAAATAGATGCTATATATGAAAACAGGATTTTTTTTTTGGCTTTTTTGTTTTGTTTTTTATCTAACACACATTTTTCTTTCACAACAAAAGCTTATTTTTGGACAGCTGCTGACAGCTTTTAACATAATACATTTCATTTACAAAAGAGTTCACAATATTTATTTAACAAGCATTGCATTAAAACAAGGATATGCACAGTAAAGCAACCAACGTCAGCAAACAGAAGGGGTGGGGAAAAAAACCCAGTATGTTCACGTTTCATTTCACTTCCTTCTTGGCTTGAATATGCCGCCCATCCTTGTGGTTTTTTGGACCCAGATCAAAACAGTCTAACTGTACCAAAGAGCGCAAGCGCTCCATTCAACTTTTCAAAGTCCGTCCCCCTGCAAGAGTTCTTGAGGCAGCTGAATTTACTGCGGATGAAGTAAACTTTATAAAAGGACACAGTCACAGGCTAAGTGTTCTCTGACATGCCAGAACCTCCAGAAGGCAAAACTTCTCCCTGTTTCAAAGTAACAGTGCACTAGGCCAGATAAACACAAGAGCAAACAAACCGTGGGCACTGGTTAGATGGAGAGAGAGCTGTTCTTTGCTCTGACATTCAGATTTTTTTTTAGGCCACACATCCACAAAGAGAGACATATATTCCATTGCATGTTCACCTATATATGCACAGGACTATAGTATGCACAAAATAGAAATAAATTATATAAGGGTTAAAGTAAATGCCAAAAGTTGTCTTGAACACAGCCTGTTCTATCTGATTGCCATGTGCTGGCACTTTTGATTAAGCTACCAGCCATAAAGTAAGCAAGCAAGGAATATAAACACTTGACAAGTAAATGAAAGACATATTTTCTAATAAGGCCCCTTTCAAACAAAAACCCCTGCTGAAAATTAACAGCAGGTTGCTACTATGGCAGAGCCCAAGATCTCCAGACTATTCAAAACAGTGGATAACTCATATATGCAGAGTTAGCGGGAGAAGAAACCAGGTCACCAGATGAGCCTCTGCCCCACCAGCAGTCCAGGAGAACTTACAGGCGAGCTAAGCCGGCTAGACTGTGGTTTCTGATAGGTGAGTTCCAGGCTTAATCTGCAAGCTGAACAATGTTTCCTGGGACTTCGAAACTGATCTTTCAAATGCAGGGCAGTGAAGGGGAAAACTGGACCACCATGGTAGGATCAGTTCTCTATAAAAGAGAGTGGCAGTGTTGGAGGCTGTGACAGCTTGGTGAGGATTCACAGGGAGGCTCTAGCATCAAGCCGATCTGGTTCCTGGTAAGGCTTCAAATACAGGTGCACAGAAGGGTGGTCCGGACACATCAACCTGGACTAATCTGGCCATGAGAAGCTGAGAGGTGCTGGGAGATAGCATCATTGCCAACGTGAGAGTGTTAAGTTCTCCCTAGCTTCTCTGTAACTGACTGGCTCTGCAGAGGAATTACCATACTTAGCAGTCATTCATGAAAGTTCAAGGGGACCAGACCCTGTGTGATTGTTTCACAACAAGAAAGGAAGGTTTTTAATGCATTTGCTTTGCTTGTGAAATGTATTTTTTTCAGAGACCAGTGTCTACAGGGCTGGGAAAGGAGTTAAGTTGAGCTGTCACTGAACATGAGAGTATCAGTTCATGTCACAAAGGTATACAAATGGT

							CTCTTGGGAGAGAAAACGAAAGTTGGGTTTGGTCAAATACAAAGTGATTTAATTTAAGGTACAGTACTAAATATTTCTATGCAGTGTGACAACCAGATCAAACATATTTCATATTGGATAAATTTATAAAATGTATATATATATAATATATATCCT
MSTRG.122263.1	ENSRNOG00000029843	0.000	0.630	0.007	3.835	up	GCCTGGTTGCTTCTGTACACCTCAAATGCTGACTTCTGCTTATCATCCACATGGCAATTCACAGCATGCAGACGAGACTCTGGGGAGCCCTTAGAGTCTTCTGACTTTTTGTTCATGATCATTCTTGGTTTGAAGCCATGCGTTAAGAAGTATTCGAATAAGTCAGGATGAACTTTCTCCACCACAAAGAGATGGCACCCTGCAGCATCTTCGTCTTT
MSTRG.12234.2	ENSRNOG00000045568;ENSRNOG00000050741;ENSRNOG00000061795	0.149	0.636	0.001	2.713	up	GGCGTGTTGAGAGGAGCACAGGATGGAGTGGGGGAGTGAAGGAAAAGCTAAGATGGAGCTTAGATTTTTTTCCGGTAAAGAAACTGATGTCAGCTCAAGCTTAGGGACCGGGGGTGGTAAGTGAAGGGAAGTGAGTCTGAATAATTATGCCCTGGGTGAAGGATTTTTCATTTATTATTTTTTTTAGACCAGGTGATCTTCCTGCCGGTTTCTTGGGTGCCGAGATCACATGTGAGCAACACCATGCTCAGCCTAAATAGCAAATTCTAAAGGAGAAAATGAAAATCCCTGTGCCTTAAGCACAGTGCCCAAATTTCTCACTGCCTGGGGAAAGGCTGGGTTGTTTTTTTTTTCTCCCCCTCCTGTTCTTTCCCTGATGCATCTTTCTCTTGCTTTTTAAAGCACCCTAGGTTTTACTGTGACTCTTTTTTTCTCTTCCATGCCAATCCATCCATTTATCCTTTGAGTCAAATAAGATTTTTACTTTGCCAAAATCCCTGTCTGCCCTAATGTATTATTCACCCTCTGGTCATTTATTCAGCTTTGACTTCCAGAAGCTGTTAAGCCCTGCACTTCTTCCTCTAGTTGTCAGTTCAGTCTGATGAGTTCTCGGGCCGACTTCATCTGGCCTGGCTTGGGGCTCACTCCGGTCTGGAGTGAGAAGACGAATTTTATTTAGCTTGGGATAGGGCTTGCACCCATACACAGAAGCCACCCACAGACACACTCACTCCAGACACTTTGTGCCTTCTCAGCTCTCAGAGGTCCCTAAGCAGTCCCAGCTGCTTGCTTGTCATCTTTGGACATTAGTGGCCCTCCCTGGGAATGGAAGTCCCAGGCCCATTCCAGATGTGCCAAACCCGATCTGTTTCAGTGGCCCAGCACTCAGGTCATGTGGTTTGTAGTTGAGACTCCTTGGTCCATGCTAGTGCTTAGTCCTGAAAGTCCTCCAGCTGAGAAGCAGGTCGTTCCTCCTCCCATCTTCATTCTTCCCATGAGCCTCCTCTTCAACCCCAGCAGTACTTGGGTGAGCATAGGTTCCAGCAGACCTTCCCAGCATACATCCCTGGAGGGCCAGCCTAAACACTAGTGGGTTCTGGGAGGAGAAAAGGGGCGTGGTGGGGTCTGCAGAGAAGTTGGCAGCAGTTCCCTACCTAAGGGAATCCACTGGCAACAAACAAACAACAACAACATCATCATCAAAACACCCTGGGGGCTGGCTTCCTTAGGTTTGTAGGAGGTTTTTCCCTATAAAAGGAAAACAATAAAACTAATGAAGAAAACTGGGTGTCTTAATGGGAGTGTCTTTGCTGAAAGGCCTACCTTAGCTGAGCTGAGTGGCCTCTAGTTGGGAGACTTTAACTCCAAACACAATAGGGCTGAGGGCAGTGCTTCTGAGTCAGACTATAGTTTCTTATTCAAAACCCAAAGTATTAGGTGACTGGCTCAGGTCATGCCTGGCTTAGGTGCTGCTTGCTCAGGGTGATTCAACTCTGCCCTACAATTTAATTTTATATAAATATTTACAGAGCAAATCCAGTGACTCTATTGGTAGAAAATGATGGGAAAGGAGTCCTAGGCCTAAGGGAAACTTAAGTTCTGTCTGTAGTGTAGCAAAAGACCCAAATCTAGGGGCTGGAGACCTTTATTCTTTACAACTTGGCCTCCTTAGCTTTAACTGTCAATGTGATAAAGCCTTGTCATCTGACAGGAAAGCCTCCATTAAGGAATTGCCTGGAGCAGACTGGCCTGTGGGCGTGTCTGTGGGGGATTGTCCGGATTATTAATTGGTGTGAGAGGCCTCAGTCCACCGGAGGCCGCACCTCTC
MSTRG.122354.1	ENSRNOG00000010608;ENSRNOG00000058371	1.336	0.110	0.002	-2.809	down	GCCAGTCTCCAAGTTCTTCCCACTCATCCAGCCTCTGTGGTTTTCATAGGCTCCTCTACAGGCTTCTGCAGTTTCCTTCAGTGTATCAGGCTTTTGTAAGTCCTTCTTCCTGGTGAATCCTTACTGTCAGTTCCCCTTTCCAAACACTGTGGGTGAAACAAAGAGTTCCTGTAGGTTCTCTCTCTCCACGAGCCTCTTTTGTGACGGTCAGAATATTGGGTTTTGATTACTTCCTCGCTCCTCTTTCCCTTTTATTAGATTTTGAGGGTAAGATCTGTGAGCTAATCATTGCAATTTTCCTGGCAGCTGTTACAGAGCCGAGGCAAATAATTGCAAATATCGATCCGGAGAAGAGTTCCAAGCAGAGCAGCGAGGAAGGTTGTCTAGACCTGGTGCTTGAGATGGACTTAGAGGGAAA

							GGAAGAAAGCTAGTGACTCTGGGAGAAAGAAGCAGATATAGACCAGTAGTGACAGACAGCTAGAGAT<br>ACCTAGGAGGAGCATCCGTGCTTCCTTATCCTCCTGATGTTGCTTTCTGGAAGGCTGCGGTTTTGGCTTC<br>TGAAACTAGTGGTTAGCACAATGTTCTTGGACTATCCATCTTGTGCACTCTGGCCCTCTAACTCAGGAA<br>AGACCCATGTTCTTGGACTATCCATCTTGTGCACTCTGGCCCTCTAACTCAGGAAAGACCCACTGCTCT<br>CCACAGTTAAGGTGGGTACTGGCCTGTTCAGTAGCTGGCACTCAGCAGTGGCAGTTGTGTCATGACACT<br>CCTCTGTGCAGTGTCACCAGGCTCTGACAGTAGTGGAGGTGTCATTGCTTGGGTGACGGGTCACATCCC<br>CAGAAACAGAATTCTGACGGAGTGGGGGTGGGAGGATAACTAGCTCCTTCAGGAAGGCAGAGGCCAG<br>TCCAAACTGCTTGGAGAGGTCTTGAAGTCCGAAAGGGGCAGTGTGAAGACTGAGTAACCAGCCTCTG<br>GAAGAGTGAAAGCGTTTTAAAGGCACCTCTGCACAGGTGTGAGGGAGGAGACTTTTCTAAGGATGGA<br>AACATCGCCTCCCAGAACAAATGCTTTTAAAAGACAGCTCAGGACTACCCGGGAAAGCATTCCCACAG<br>GGAATTTATGGCTAACTGGGCTGTGGATGAGGGCTTGCTATCTCTGCGTTTGTACAAATGTGAGTGATGA<br>GTCATTAGGGTAATGGTGCTAGCCTAGTAGATAGCAACCAGCATTTTGAAAACATGGATCTCAATAGAAG<br>AGAAAACATCACTACTGAGGAGAGCCAGGGTCACTATCAGCTTGTGCGCGCACTCTGTGTGCATGTGC<br>ATTCCTGTGCGTGTATTTCCATGTTGTTCATCCTGCTGTTTCTACATTCCTAGATTGTAGAAATGCCTAATG<br>CCTCATGCCTAATGCTTGCTCTCTAGGCTGAGCCACCTCTGCTCACACAGGGTCTTCTTTAACTTTGCAA<br>ATGACATCATTTTCACATCCTCCTAGTTTCTATTAGCTGGCAGAAGCTGGGACATTTGAGCACTCAGAAT<br>CCACCTCTGAACGCTTTTGGATTTGAAAGATCTGCAGAGTGACGGAAACTTACTGAGATGCCTCCTCAT<br>CTATGGTCCGTCTCCTTTAGTCTCAGGCCAAACTGCCTGTCATTCCCATGCACCAGAGAACCTACAGATA<br>TATTGGGTAGTCACCAACCTTACCATGGTCGACAATATAAAGTGCACCTAGCTTCATAGAATCTTGGGGG<br>GCAACCTCCTGCTAAATTAAACAACAGGCCAGGTTGGGGGTGGGGTGGAGGCCGGGTTAAAGTGAGTA<br>AGTAGGGCTCTGTCTCTGTGCCTTTTCCTTCTTGGTGCCACTTACTCTGACGAAGATATATGGTTGTGGG<br>AGTCACTGATTAAAGTCGCAATAACTATAGATCTGAGGGAAATGTGTCTGCAAAAGATATGAATCCTAG<br>AATTTTGGGTACGCAGGTTGAAGCTCTACATCAAGGTTCAGAGCAGTTTTAAAATAGAGGACTCAGGCT<br>GGAGAGATGGCTAGGTGGTTAAGCACACTGGCCGCTCTTCCAGAGGACCTGGGTTCAATTCCCAGCAC<br>CCAAACTGCCTATAATGCCAGTCCTAGAGGATTTGATGCTTCTTCTGGCTTCCCTGGGCCCTGCACACAC<br>ATGGTTGGTAGAGTACTTGCCAGGTGAGTGTGGTCCCTGAACTTGGATCCCTAGCACACACATTAAAAA<br>TGCCAGACATGGTGGCTGTAACGCTACAGAGGGAGAGACAGGAGGCTTTGCTGACCCACGAGTCTATG<br>AATCACTGAGCTTCAGATTTGAGAAAGATTCTGTGTCAAAAAGATGGAGAGCAATAGAAGACAGTGCC<br>TTACACCAGTCTCTTGCTTTCCTGCACAAACATGCACACACATGCATATGTACCCCCACACATACACATT<br>TATGTGAACACACATACACATACATACACACACACACACACACATACATGCACACACGCACACACGCAC<br>ACACGCAGTCAGATGAATTGCATAGCTTATAAATTCCATTGCTTTGGAAACTCGAGTACAGGGATATGTG<br>TGAGGCCATCATGGGCCACACAGTGGATCCTTGTTTCAAAGAGTTGGGGGTGGAGGGAGAGATAGAAA<br>GAGAAGGAATGAGATGAGATTACAGGTTAGTTCCTGCCTGGTGAGCCACATTCTGGTGACAGACATCCT<br>GGGGTTTCAGTCGTCTGCCAGCCAAGTGAGGTCTTAGAGACCAGCTTTGCATTTCATTGATAAAGACTT<br>GCCATTCCTTAAGTACATTTGTGTGGAGCTTCTCAGATGTCAGGAAGATAGCAGTGTTGGAAATGTACTC<br>CAGGACGTGGGACCGTAATCATCACTGTGCCCATTTAACAAAGGAAGAGACAGAGACCCAGAGGAGTA<br>AGGTATGGATGCTTAGTTACAGAGTCCATAGGCAGGAGACCTGAGGTTAGAGCCAGGCTCGCCACCAG<br>CATTACCTGTTCCCTCCGTGTGGTTTAGGTTCAGAGGAGCCCAGGGTCTACTCCACTCCATGTTCAATTC<br>TGAGCAGCACCTATTCAGGGGTGAGGGGCCACATCAAAGAATCTGCAGGAAGGTGGCTTGAGACAAA<br>GATCTGGAGGGACAGACAATAACCTGGCAAAGAAGGGCCATAAACAGCCTTTGAGCCAGTCCTGAGAT<br>AGGTGGCTGCATTCCCAAGATTGGTGGACAGGACTGGAGAAGAGGGGTTCCCCATAGGATTAGAGGCA

							AGCTGAATTTTGTCTTCCAATGCTCCCATTCACTTGTTAGAAAGCAAGAAGAAAAACAATCAGAAACTAGCAGGCAGTGTGATTCGTAGATAAAGTGAGGGCACCAAGGTAAGCTTTTTGACTAAGGTACACACTACCCACTCCAAGAATGGGAAGATGGCCGAGTTGGCAAAGTGCCTGCTTGGCAAACATGAGGACCTGAGTCTGGATCCCCAGAATCCATGGAAAAGGCTGGGTGTGACAGTGTGTGCTGTAGCCCTGATTCTCAGGGTAAAGGTGGAAGCAGAGACAGGTGGGCCTCTGACGCTCATTGAGTCCAGCTCAGTCTGTGAGCTTCAGGTTCAATGAAAGACTCAGTCTCGAAAAATAAAGGTGGAGAGGAAGGCACCTGACATGGACCTCTGGCCTCCACACACATAAGCATACATACACAGATCATAGCACCTACAGAATGTGTGCATGCATGCTTGCATACCCATGTGCACACATACAGGCAGAGAGAGAGAGAGAGAGAGAGAGAGAGAGAGAGAGAGAGAGAGAGGTGGATTGACGGTATAGCTCAGTGACAGAACATTTGCCTACCTTAAGTGAGACTCTGTATTACATCCCCACCACCACAAAGACACAAAAGATGACTAATAATATAAGCATTGAGAGAATAGGCTGTTGAAGAGATATCTTCAGAGAGAGCTGCTGAACCTGCATATGTGCCTCCTGAACGTGAAATTCATGAGATGTCATGGGCAGCTCGATCTACCTCCCTTGTTCAAATGTGGTAAGGAAAGGATGCTTGGACATTTTCTGGGACCATGAGGGACCAGCTGGAGAACGACAGCACAGGAGCAGGGAGTTTTGAGTGGAGGAGATGATGAGGAAGAGTGAGCCACCGGAAAGAAGACAGGTGTCACTGAGCAGTTCAGAGACTAAGGCCAGGGTAGTCCTGCCTTCCTGCTTTGAACTTGCGTGGGTGAGAAACTGTCCAGCAGCTCAGCCGATGATGGAGAAAGAGAAAAGTTCTGATCCCAGGCGGGAGCACAGCGAGGTCAGCCTTCATGTGTGGACACTGCAGTTCTTTTGGGTCTCAGAAGTGGCTGTGTGAGCAGAAGGGCAATGCATTCTGTGGTGAAAGACAGAGAAGGAGAGTCCATTTCAAAGGGTCTTGGAAAACAAAAACAGGCAAATACTGGGGCAGGTGCCACCAAATGTGGCTGCTCAGAATATGAGGGTCTTTAATGGCTGGATTAGAATGGGAAAGAGTAGCAATCTGTCTGCCTGATGCTCTACTAACTTCTGATGGCTGAAGAGTTAGATGTGGGAACAGTTAGGAAAAAATACTTCCTTAGCCAGTATTTCAGAGACTCCCATTGGAAAGAGAGGCTCATCAGAA
MSTRG.122470.1	ENSRNOG00000011071	0.284	0.000	0.010	-4.070	down	GCTGGACTCTCCCTGCTCCCTATAGATCCAGGCTCCACAGTCAATGAATTTACTGGAGTTACTTACAGGAACATGGGTTACTCTGAGGCAACTGAATCACCAAGCAGAGGCGATGACTCATGGGTGCTGGAACCCTGGAGATCTCTGGACAACATGGAGTCCTTATTGTTTATACAACCTTAAGGAAGGGGAAGGCTTTATGCATCTGGAAAGCTTGAGGGACCTCCTGAGACTTCTGAGTTGCTCATTATCTGGGACTTAAAGAGCCTCAATTTAGAACCTTTTGAGTCTTAAGGAGCTTCTGTTCCAAGAAAATAGCTACACAGTACAATTCACATGAGTCCTTCTGCCCCAGATCCTTTTCTCCCCATCTTGGGAATTTAACACAGATTCCAAGTCTATTTTCATCTCCTCAACTTGTGATGAGGCTTAAAAATAACTTATAAAAAAAAAAAGAGGTAACCAGATGTGGTGGTACATACCTGCCATCCCAGCACTCAGGAAGAGAAGGAAAAGAGATCAGGAGACCAAGGTCCTTCTTAGCTCCATGTGGAGATTACCCAGGCTACAGAAGGCCATATTGTTTAAGAGAAAATGATTAAAGGAAGGGAGTATTTAGTGGGGATTTTACATCTGTGGTCTACTGATCAAGAAAGCCCTTTCCCGGGGATACCAGATGTGATGTAACATTGCTCTTGTGGGGAATGACATCTTCCAGAAATGCATGTGACAATTGCAGGAAGCTGCTTCTAGCTAGCACAACTTAACAAGAGGTGGGGTGGCAGAGAGAGCTGCAACCCTGCTCCTATGAGCTGGTCCACCATTGCTCATCATGTATTAGGACAGCTCCTCTCTATGTAACGATACCTGGTGGGTTTATCTGCTTTTCCTAGGAGAGCAGGAACTTTGTTCATCATTACCTGCACACTGTTCCCAGAGCATTCCCAGAGTCTGAGGGCTCTGTTGGTACTGCCAGCATAAAGGCAGGAAGGAAACCTCTAATCTGTCCCCATAATCACT
MSTRG.122490.3	ENSRNOG00000033584	0.002	0.240	0.000	5.812	up	CTGGAGAGATGGCACAGTGGTTAATAACACCAACTGCTCTTCCAGAGGTCCTGAATTCAAATCCCAGCAACCACATGGTGGCTCACAACCACCTGTAATGAGATCTGATGCCCTCTTCTGGTGTATCTGAAGACAGCTACATTGTACTCATATATAATAAATAAAATATTTAAAAAAAAAGAAAAATGCCATCCAGTGTATATTAAAAGGTTTGAGTTAAGGCTGGGGAGATGGCTCAGAGGTTAGGAGCACTGACTGTTCTTCCAGAGGTCCCGAGTTCAATTCCCAGCCACCACATGGTCAATTTCAACTAAAAACGATGCCCTGGACTGGTTCCTTGTTCAGG

							TATAAATTCAATGAGGCAGACAGCTCAGTGGTTAAGTGTGCTTACTCCTTTCAGGACTCAAGTTTAGCA<br>CCCAGATCTCTCAAAGCAGCCAAACTCCAGTGTCAGATTCAACATCCCCTTCTGGCCTAAAAGGGCTTG<br>CAGTCATACACATACCCCCAGACCTCATCCTATAGAAACATTCCTCCATCCAGCCTTTATCAATTTCTTTA<br>TCCCACTAACACCAAGATCCCAAATATACCTGAAACCCCAAACTGCTGTATGCAGAGTCCCTTAAGCCT<br>AACTCAGCTCCATATACTTGAACTGGTTTGGACTCAATCTGTTTAATCCTTTGATAGGGAAAGATTCTCT<br>TGCTAGCATGTTGACACCAGCTTGTTATCCCCACAAACTAGGAGGCAGGCTTGCCACCAATTTCTTTTTC<br>TTTTTTTTTTTTTTTTTCGGAGCTGGGGACCGAACTCAGGGCCTTGCGCTTGCTAGCCAAGTGCTCTACC<br>ATTGAGCTAAATCCCCAACCCCTTGCCACCAGTTTCTCAGGCCAGCGTTGTCAGTTATACACAGGTCTA<br>AACAGTGAGGCCCCCTTTTCCTAAGCAGTCAACAGGCATCCTTCCCTGTGTGTTCTCTAAGGTTGCTTTT<br>GGTCTTTCTTAGCATCCTGTACCTCCACCGATGAAGAGAGCAGATATTTTTGGAGGATCCTTGGCGCACT<br>AGCATTATATATTGAGTATGCACAGTAATAATCTGGACTTAAGACCCATCGATCGGAATGCAAGGGTTGG<br>AGAAATGGCTCAGCATTTAAGAGCTGCTCTTCCAGAGGTCCTGAGTTCAATCCCCAGCAGCCACATGGT<br>GGCTCACAACCATCTGTAATGAGATCTGATGCCCTCTTCTGGTGTGTCTGAAGACAGCGACAGTGTACA<br>TAAAGTAAGGTACACTCTAAGTACAGAAAGATGCTTAAAAGCCATTTCACAACACTGCCTTGCTTTTTA<br>CGGTTTGCCAGCTTGAGTAAAAGGTCTACAGGTTTTAAGCTTCTCAGAAAACCCTGTCCACTTGTTTCT<br>CCGTGTAGCTGTCCTGAAACTTGCTCTGTAGACCAGTCTTGGAAATGAGAGACCCACCTGCCTCTGCTG<br>AGGCTAAAGGTTCAGTGCACCAATTGTTTTGAAATAATGAACAGCATCTGACCCTGTTCTGGGCCTGAG<br>ATTCATCAACTAATAATGAGCCTTTGACTGGGCTCAAGGGATTTATCTGCCTGCCTTACTCTTCGGTTACT<br>CTTGATTAGCCAAGGAATCAAGAATGAAATCAAAAGGGAAATATTCACATAGAAAAGACACAACAAAT<br>AACATGTATGCACTGAGTGGGCAAACTTTGAAAACTCAATTTCCATGTAAGTGAAACATATTATACCAGG<br>GCCCTCTGGTTCATCTTCTGACACAAGAACAACTGAATAAATATTTGCAGCTGAATGTTTATTGCAGCAC<br>TCCCAAGTGATCACTGTTGGATGAATAAGGAAACAATTCATAACCAATAAAAATGTTGAACTGCCTTTTT<br>TACAGTAATTGTACACTCATTGTGCTTAGTCTGTAAAGTTGTATCCTCAGCTCACCCATAACCTTCCCAG<br>AATAGAACACTCTGTCATACATTAACATAGAGCCTTCAAAAGGTATACACAAGGCTCAACTCTGCAGGC<br>CATACCAGATGCTGTCCCATCCACTAGACAGTTTAAGAGGGACACAGCAAGGGCCATGCAGACCCCATC<br>TCAAACATCCCAGTACTAATACTCTGTATTTGCTTCTTGTGTCTGCTTTTTCTGAACATCACCACATCCAG<br>TTTTCCTTCGCAAGAAGTCTCCTCTCACTGGCCCTAAAAAAGAAAAACAGGTATCACATCGCTGCCAAC<br>TTTAAAACAGAACACAACAATCCACTTTAACGGTCCATCTCAGAAGCCAGATCCGTAATTATATTTTTTC<br>AACAGGAATTCAGATTCGGGATAGGATCATCATAGATCAGACCGGTGACATTATGTTCTGCAGAAAAAG<br>GAAAGAAAAGGAGCATCGCTCACATGCATTTCTGCTCCAGGAGCTTCCCAGAGGACGGGCATCTCAAT<br>GGGCTGGCATTCACTGCTGGCAGAAAGTATCATGACGTGCCAGGAGTAAGAACTTTAACATGGTCAAG<br>TTTTAAGCAACTTTCCTCCCTTCACGAATTCTCTCGGATGCTTTGAGCTCCCCGCCATCTTTGTTTCCTCT<br>GGAAACTGCTTTGAAGACGCGAAGAACAATGGCGCCATTGTCCCTGTGAATATAATCGTGTGTCTCTTT<br>AGGTGATATATCCGTACCTTCCGAACAGCACAAAGCTCCTAGATTGTTTTTACAAACCCTGCTGTGTTAA<br>GAACCCCCACCCCCAAGCTGGGACTGAGGAGCTAGAGCGCATGCGGCCTTTGGGCAGGCTGCTCGGTC<br>TACCGGGGCCTGGGCTCCAATCCCGAAATATCGTTAGCTGCTGCGCTAGGATAGGAAGTTCACAACGGT<br>ACCCAAAACTCAAGTCTCAGATGCTACTCCAAATAACCGTAACCTCCAAATTCTCTTTGCGCCCCCATAC<br>TTAATGTGATCACCCGTCTGTTTAAGTCACTCACCTACACCACAAACAGCGAACTCAAGCTCCGTCGTA<br>AAAGAAAGAGCCTGGCGCCCCGCAGCTCCTTTTATAGCTCCCTTTGCGCGAGATCGAGGCTGCTCCTCC<br>TTCTTGCACCGATTGGACGCGGTTGCACGTGGGGGCGTGGCTTTTCTTGAAGCAACGCGTGGGAGCCG<br>GCGCCTGGACTCTCTCTTTTCTTCCGGGCCATTCCGGGGCGTGGTGCGCGCCGTCCGCCTGCCGCGTGC

							CTGTCTATTTCTTCGCGCGCTCCCGCTGGCTCGCCTCCACCAGTCTCTTCAGAGTCTGGCGGGCCGCTGGGCTCTACGTGGCGTGGGAGTGGCGCCAGAATTCAAACGAACTGCCGCAGTTGGAGGCGAGGTCACTGCCTGCTTCGAGCCTAGTCGGCACGGACGGTCGGCGGCTGGGAGTGGCCATGACTTCGCCGCGCTGGCGCCTTTGCGCGTGGCTGGTGGAGGAGCTTGGCTGCTCGGGTGACCAGCCCTGTGCTGTGTATTCAGCCCGGCCGCTGAAAGTACCTTGGAGGACAGAACTACGCGGGTGAAAATCGAAAAGCTCTCCGTTTATCTTTCCTACACTCTGGTGGGACCGCTGGTCTGCAGAGGGCCACGCTGTCTCGTGCACATTATCCAAACCTTGCTTGGGCGCCCAGCCAGGTTCAACACCCGACCCCTACCTCTCCTCCTTGGTATCAGACACTAGTGACAGTTATACTGAGACAATCATGAGAAACATTCCGTTTTACAGAGGAGGAAGCAGGTCAAAAGCAGCCAATTGGCCTAAGGTCACATAGCTTCGTTATTTTTAAGTTTTTCCCTCCTAAATTCTCTGCGGATTGACAGCGTCAAACATAAAATTCAAAGTAATTTGAATGTATGGATAGCCATACAACATTTGGATATTTTACCAATTTGGAAATTCAGGTCTTTATTTTGTAGCATTTTTAGCTTATTAAGATTATTTGTGCATTTGCAGTCCATTGACAGTTGTCCTCTGGTCAGCATACTTGTGCTGTGGTATGCATGCCCCCTCCCCATCATCCACACGCTCAATAGTAATGATAATAAATAAATAAAAGTCATCTGGGTAAGAGATTGTGAGGAACATCACATAATGTTTGCCATAGAAAAGAAGGTAGGGAACAGAATCACATATGGTAAAGTTTGTTACAGCGTCTGATTAAGTACTGTGCCTCTGGGCTACATTTCAAGCCTGTGGTATTAATTTGGTTGTTTTTAAATATATATGCTCAGGTACGCACATGCATGTGAATGCTAAAGGACACCCTTAGTAGTTATCCTCATGAACACCATTTATCTCCTTTGAAACAGTGTCTCTTACTGATCTGGAGGTCACAAATTAGGCTCTACTGGCTGAGACTGTATATCCTTCCTTTTCTACCTCTCAGCTCTGAGATTATAGTCATGTTTCACAACTAACATCATCTTTATGTGGAATCAAACTGGGGTCCTTGGGCTCAAAAAGCCAATTCTTTACCTATCAGAGCCATCTCCACAGCCACAGTATTAGGTTTTGAAGGTCAGTAGATGAATATCATCTTCACAATGATTTCGTGTTAGCTTTCAACACTCATAACAAGTGAATTAAGATCAGTAGTTCTCAACCTGTGAGTTGAAACCCTGTTGACAAAC
MSTRG.122499.2	ENSRNOG00000010699;ENSRNOG00000043223	0.556	0.049	0.043	-2.421	down	CTGTGCAGGCAAGCTGGGTGTGTGGAAATCAGAGATCCACCTGCCTCTCCTGAGGCTAAAGGTGTGAACCACACCAGTTTTGTTTTGAAATAATGAACAGCATCTGACCGTGTTCTGGGCCTGAGATTTACCAATTAATGAACCTTTGAGCGGTTTCAAGGGATTGTCTAGCCTTAGACTCCTAGGTTGTATCTCCATTTTTCACTTGACATGGAAATTCAGTTACTCTTGAATAGCCAATGAATCCAGGATGAAATCAAAAGGAAAATTAGTATCTCTTCAAACAAACAAAAAGGAGACACACCAGACAACATGGGATACAGCGGAAGCAGTGCTAGAGTTTATAGGAATCAACATGCACCTAGGGAAAAAAGCTGAGTATCAAACGAAAACCTGTTTATCATCCTAAAGAAATAGGAGTAAGGAAATATGCATGGAACCAAGAGACGTCGATTGTCAGAATAAAAATAGGGTGGAGACCACCTGACTGCTCGAGCTTCAACTGCACCCAAACATGGTACAGAGAACCCAGAAAGAGTTCCACAGTCACAGCCAACTCATTTTTCAAAGTTAGCAAGGTTTCCTGTGAAGAAATTAAGTCCATGTCATCAATGATACCAGGAAAAGTTAACATCAGATGCTGTCCTGTCCACTAGACAGTGTAAGAGGGACACAGCCAGGGCCATGCATTTCAAACATCGCAGTACTAATACTCTGTATTTGCTTCTTGTGTCTGCTTTTTCTGGATATCACCACATCTAGTTTTCCTTTGCAAGAAGTCTCCTCTCATTGGCCATGCATTCCTGCTCCAGGAGCTTCCCAGAGGACGGGCATCTCAATGGGCTGGCATTCACTGCTGGCAGAAAGAAGTACCATGACGTGCCAGGAGTAAGAACTTTTAAAGGAATCAGGTTTTAATCAAGTTATTGTCTGTTACGAATTTTCTTTGAAGCTTTGGGCTCCCCTCCATCTAGTATCTTTTTGCAAGTGCTTTGAGGATGAGAAGAATGACGTTGTCACTGTCCTTGAAAAGGATTCACATGCATGTAATCGATTGTCTCTTGGATGTCACTGGTACCCTTGAGTGCAGCACAGCTTCCGTGGATCGAGATCCAGGCTGCCCCTCCCTCTCGCGCCGATTGGACGGGAGAGCACGTGGAGGCG
MSTRG.122	--	0.127	0.399	0.011	2.326	up	TTTTAATTAGCCAAATTCATGAGTAAATTAGCACTTGGAAGGCAAACTCCTGGCTCTGTGTAGAGGAAGACAAAGGAAGAAGCATGGTTCAAGATGGAGAGCGCCCAGGAGGCTGCTTCCATAACATGGGCAACATATGATACGTTTGGACTGGGAAGTCGGGAAGGAACATGCAAGGAGGGCCAGGGAGCGCTGAGATGAGGT

790.1							CCCAGCTACTGTTCCAGTGTCATGCTTCCTGACTACTGCCGTGCTTCCCTTAATGATGGCCATGGTTATG<br>GACTCTAACCCTCTGAAATGACAGGAGGTGGAGACAGGAAGAAATCATAGACAAATTCATCTTTCCAA<br>GGAGCATGATCCACTTCCTACATGAACCTTACCCTCCAGTTTCAGCACTGCTCATCCAGACCTTGAATCC<br>ATCAGGCGATTCGTCTGACTGCTCCTCCAAAGGCCTGCTCTTATCCCCACTTGTACCAGCACTCAAGTAT<br>CTGGGCAGTACAGGATGTATTGTACTGACCATTTTGGAACATGGATAAGTCCACATGGACTTGCTACTAG<br>TACTAGAGGAAAGAAATAACACTGGGAGGACCAGTTGCACTAATGGCACGCCCACCAGCCATCAGTTT<br>TTCATGCCCTTCCCAAGTGATTCTGTGTTGCTGCAAGTCCACAATCAAAACCATGCAGAGTGCAACCTT<br>GGTAGGAGGACTGCTACATTCCTGCTGCATAAAATTATTTGCTGATAAAGTCAAATGATATACGCCTTCA<br>GGTATGAATTGCTTATGGGGCATAGGAAAAAGAAAATATGGACAGTAGAGAAACTAGAGATTTCTGGCA<br>ATACCTTGTAGCTCGATTTTATTGGACATGTTAGAAGTTACAGATTACACTCAAGAAATACATAGCAGAA<br>GTTATGTTATTTTTAAACAGACAGGCATCAGTGGGGTAAGTTGAAGAACAGAGAATTCTAGGGTAAATT<br>CTACTCAAAATGGGAAGAGATAGAGAGATTGAAAAAGTCACAGAAATAGTATTTTACCTCTCCTTACCA<br>AAAATATGTTAAAAGGAGGCTTTATATTTTTTAAATCCTGAAGTGATCAAAATGCTCTTAGCATCTTATGT<br>TTTGTGTAGGGAGCAAAGGGGGATGTATGTGAGTTTCCTGAGAGTTTGTCAAGCACCCCAGACAAACT<br>GGCATCGTCCTCATTGTAATAGCCATGGCTATTGTCATTTAAATCCAGTCATAGTGAGCCACATGATTTTC<br>TTTTTAAATTATTATTTTTAAATGACTAGGTGAAAATAAATGGGTTGTCTAAATTATTTAATCAAGAATTAA<br>AAATGTAAACAGGAAGAGAAACTTGTTTTCTGTACCACTTTTTTCTTTTGCTTAGTAATTAATATTTTTCT<br>TGACACAGGGTCTCCCACTATAGTCCAAATAGTCTTCCAAATCCCTACCTAGATAAAATGTGTCCTAAAT<br>TAAAGATGCTATGATCTCAGCATCCCGAGCATTTCAGGTTATTCTTTTCTCATGTTGTCAATACATAAAAT<br>GTAAAATGATGTCAAACTGGATTTTAGGACCGTTTTTCTAGATTGCTTCTTTAAGGAATTGTGAATAGAA<br>TTAAGCAAGGATGTGGGTGTCAACTAAGAATATTGAATGCAAATGAGAAGCTTTTGGCTAAGATATAAA<br>TTTGGGATCTTTTGGCCTTTTTAAATTGTCTAGATTTATTAAAAATCGAGATAATCTAATGAGAAAATATAT<br>TAAAAGTCAGAAATAACCCCAGGCATGAACTGAGGCAAACTTGAACATTCTGTGGTTGGGTATACTAAA<br>ATAAGAAAAGAGTTTTCTCTTTGCAAATGTCTGGTCAATAGGAAGAGAGAGAAACTACAAAATTATGAC<br>ACCACAGGGCAAGAGAGGAAAAGCAATAAGCTGCATGGAATATTGTACTGTTTGGGGCACTACAGTGA<br>AAGAAATGTAGTCACAAATTTTGTCAACACACAGGACATTAGTGACTTCAAGGACAGCTGAGTTATATA<br>GCAAGACAAAGTTAAGTGAAGGGGAACCAGAAATGATTAAGACTTCTAATGTTACAAAATGTTACTGA<br>GATCCAGTAAACACAAAATGTACATATAAAATAGGAATTATGAAAGTTAATAATAATAATATTAATTGTGC<br>ATTTTTTTACAAATTATTTCAAATCAAAACCCAGATACCTTATTTTTTTAAAAATGAATAACCAATATAATT<br>CATTAAAATTTCAACATAAAATTATGATGGTAGTTTAAAAAGTATGGTGTATGAGATTGAATTTGAAATGG<br>TGAACTATGATTGTTTCTAAATTCTGCCATCTAAGTTAAAGTCTTGTGTTCTCCTGCATATTTTGATCCTTT<br>AGGATTGAGTCTGAAAATATCTGATAATGACTGGACTCCAGGTTGAATATGAAGATTTGTGTGTCTAACT<br>TGGGGTACACCTTGAATCTGGGAAACTAGAACAGGGTTATTGGAGAGGGGAAAATTTTTAAGGGTGAG<br>GGGGTAGTGAAACACAGGTGATTTCAGGATATTGGTGGTGCCAAGACTCCAGAATATCATTGAAGATAG<br>CTGTCCATAGGTAAGGAGCAGAATCCTAAAGTGGACCTGAATGTATTTCAGGTGGCACAGACGGAGGA<br>TCAGGTCTACCCAAGCTTTCAAAG
MSTRG.122952.1	ENSRNOG00000012386	0.184	0.005	0.009	-3.196	down	AAAAAAAAAAAAAAAAGTCTGTAAAGCATGCAAATGCCAACGTACCTGTACGCACCACTCAGAGAGCT<br>GCTAGCATGACAAGACCCTCGGCCATCTTTAGGCAAGGCGATGGAGACCGACTGATTTCTCCGTCTTCT<br>TGCTCACAAGTTTTAGAGATGCGGTTGTCACACACACCAAAAATTGTACGCTGAACAGCTTGAAAGCC<br>TGTGATCACGAGGACTGAATTTATCAACGGCAAAACAGGGACTGAAAAAACTAAGTTCCTTATCCAAG<br>GATTCTCTGAGGCTGCCACCAAGGAGGTGCTGGGCTGAATTTTTTTTCTCTCTCCCTTACTCCTCTGAG

							AGTTCAAAGCTCAGAGCTGACCACTGCAAAGGCCACTGTCACATTTGCTGAAAATCAAACCACTGAGACCGGCTCTGCGAATACGAACGTGAGAACCGGAGTTGCTTGCACAGGGCGCAGTGCCTGTGAGTTAAAAATAGAGTCCACCTTGCCTCCTACCTGCATAATAAACATGATCCCTTCAAGATGAGTAGAAAGTTAAAGGAAATAGCTTGGCATGGGACACACACAGCCTGTTGAGATAGTCCCATGAATGACATCTGAAGGCTGGACATTCACGTTACAGGTTCACAACCTACTATCAAGATACAGAATCAGTGGTGACTACTGAGCCCAGTGAGTGCCTCCTCAGTCTGGGATAATAAATGACAGTGACAGCAGCGGTCATCCATCCCAGGTTCCCTGGAGACGAGTGAGTGAACAGTGTACATTCCTTTATAGCCTCGGCTCTCAGCCTTCCTGATGCCGTGTGCGACCCTTTTAATTCAGTTCCTCGTGTAGTGGTGACCCTCAACCGTAAATTACTTTTGTTGCTACTTCACAACTGTAATTTTGTTACTGTTATGAATCATAATGTAAATGTCTGCTATGCAGGATATCTGATATGCAGCAGGTTGAGAACCATGGCTTTATAGGGAAGGGTGGAAGCTGTGGTTGGGTCCCACACTTATACTCCTTACAACGAGGTGCCAGGTCTAAGAGTTTCCACACCTCTCTCTTGGGCATCCTTGTTTACTCTTTGTCAAAATGAACAAAAAGACAGATATTGGATGTTATTTTGTGATGTCTCAAACAGTGAGCAAATGATATATTTACCAAGCCTACATGGTTTGAACGAACATGAATTCTAGCTGTAAATATTCTAGGGAATGAGTCTGGGTAGATGCTGTCCACTAATAAATCTTGTTTTATACTGGGCATAGCAAAAGCCATGCTCATGCCTGAATTATTTAAGCTTTATCACGATTACAAGTGGTAAAGATATACATTTGCTTAGGATTTTAAAAGAATGGCAGGCCCCTCACTCATCCTCGGGGAATATGCACAGACATCAGCTTAAATGAGAATCCCTTTTCCTCCGATGAAAACTGTAGGCTCTGACAAGTCAAACACAAGGTTCCCAAAACAATTCGGCAAGGACAAGCTAGAGACCAGAGCAGGGCTGCAGACGTCTTTCTCTGACTTATGCTCTTGCTTGCCCATGGCCTCTGTATTCTGCAGGAGGAGCTAATTTTATCTCCAGGCCTGTGGGTTAAAGTTAAATGGCATGGCTTATGCAAAGTGCATAATTCAAGTCTGAACAGAGCTTGACTCTTCTCATTTAAAAAAAAAAAAAAAAACATCAAGTCAGGGCACTGGGTCCCACAGGAATCCTCGGTTTATAAACAGTTGAATTGGTTTGCATGATATTTCTTGATGCCATTCATGGCTTTAAAACATCGCAGCTGGTTTCATTTTATACCTAATCAAATACAAGTAGGGGGACTCATGATCAGGTTAGCTAGTCGACTGATTTTAAGAATCAATGTTCACCATGCTTAATATTGTTGTTTAAAATTTTTAAATGTAAGAGGCAGAAATTTTTTTTATAAGGTTGATGAAATTCAGACCTGAAAACAGTATGGGAGTTCTTTTTCTTTCTTTCCGTCTTTAATTATTAATTAGTATCTATGTTCTTGAGACAGAATCTCACTGTCTAGCTATGACTGGCCAGGAGCTCACTATGTAAATGAGTCCAGCATCAGACTCACAGATACCTGTCTGCCTCTGCCTCCTGAATGCTGGGATTAAAGGTTTGTGCTGCCTCACCCTCAATAAAGTCATTTATAACAGACTTTAAGTAATTTGGGAGGGGGGCTGGAGACATGGCTCAGCAGTTGAAAACACTGGCTTTTCTTTTAGGGGATCTGGGTTTGATTCCCAGCACCCACATGGGGGCTCAAAACCATCTATAATTCCAGTTCCAGAGAATCCAACGGCCTCTTCTGAACTCTCTGAGCACCAGGCACACTTGTGATACCCAGACATATATGTGGCCAAAACGCTCATACATTAAAAAAAAAAAAGACTCTTAGAAAATAATGGGATGATATTTTAGCTGACATTTTTGATAAAGTCCATCACGGTTCTGATGACTGTATCTTGCCCAGAGATCTGGGCGGGAACTTCTGTATACTGACCAAAGCTGAAAACCATCCTTATTTTAACTATGGTATATCAATAAATTCTAATTTAACAATACTAATTGTATCCTACAGTGTTATTATCATCAGTTTTAAAGATCACTGTTATACTGAGACTAGGATTGGAATCTGAGATTAGACGATGGAAAGCATGATGGAAGGCGTGAAACTGCTTACTGCAAATAACTCCAAAATAATTCTCTCCAAACAACTCCGGAAGTCTTCCCACTGGGATGACATACTGGGGAGAAAGAGTTCTCCTGGAGCACTCTGGTTCCCTGTAGCTGTGGCTACCTCTGTTTAATGTGAACCAGGCTCAGGGAATTGTGGGTACCTAAACTGTCACCAGCCAGAGGCAGCCAGAACCCAGC
MSTRG.122973.1	ENSRNOG00000045885;ENS	0.421	0.000	0.000	-5.894	down	GTTCTGACACCTGCACAGGCAGAGAGAACTACCCATGTCATGGCTTTTAGGTGGTGAAGAAGACCAGAGTGTGCAATGGGATGGCAGAATGGCTCTTGTGGGGCAAGGTGGGCACTGGTACTTTGTAAATATTACCTTCTTAATTCTCAAGATGACCGTGTAAGGTATCATTAATTCATATTTACTGACTGAGATACTGAGGGTCAGAGATGTGACCTTTAGGTTTTATTTTTTTAATTCATAAGACCCTCCTAAGATTTTTCATTCATTCACTTGTTTG

	RNOG00000048975						ATCGTTCATTCAACAAGTACTTACTGTCTTAGTGTGGGTTCCATTCCGTGTGATATATGGAAGTGTTGTTTTACAAGGTACCCCTTGCTTGGGTGCTGGGAGAAGATTCCAGACAGGAAATATTCCTCGGGCTGTTTGTCAGCTACTTGGTATAACACTGTGACTGTTCCTGTAAAGCCCCAGTGGCCCTCATTTTCTCTGTGAATGTTTATGAGCCACTTAGATATGTCAGCAGTTAAGCCAAAGGAATGAGTTTGTTATGGTAAAGTGTCAGGCGTCTTGGCAGGTTAGCAGGGAAGATATTTTTGGATAGGGAAAGATAACTACTTTCCTGCCTTACGCACAAAACTGTCTTCTCCCACGATAATTAAATGGTCTCCTGCTTTTGTATCTTTTTTATATTTCCTCCTCACAGCCCCTAACTGTGATGTCATCTCCTATGAACATCGAGCGCGTGGCCTTCTGAGGACAACTGCTAATCTATACATGCCTCAACAGCAGTCCCAGGCTTAAACACTGGGTGAAGTAGGGCACCACCAGCCTCAGGAAGAGTGCGCTTCATAAGTACCATCCCTTTGAGTCCTACGATAGCTGTTCAAGTGAGCACGGCCCGACACTCACTCCAGTCCTGGGAAAACGGAGGCTTGACAGAGTCAGGCACCCAGCAGGGTTACGGGCTAGGCCGGGCCGTTCTTGCTGCATTGCTCTGCCTACACCACCACAAAGTCTCTAGGAAGGAGTCTTGGACACAGATTCATGTATGTGGGCTGCTGCTCTGTGTGCTGCATCACCGTGTGCCCTGTGGCAGGCAGTGAGGCATGCTCGTGAACTAGCCACCCGGGAACACGTGGTTCTCTATGAGTCTCCAGGCAGGGTCAGGATTTCAACCATTGTCACCTCCCCTTTGGAAATATCTGCTGTCATGACTCCAGGGTCAGCTCTCCCAAGTGCCTCAGCTGAGAACACACTCTCCCCCACAGGTCACCTCTGGGTAGAGATGTCATCTCTCCCTTGTTTTGGGGCTGGCTCACCTGAGGGCTGTCCCCAGGGTCAGCTCTGATGTGCTGTCCTGGTGAGGTGCAAAGCTGGCTCTTCTAGGTGTTGCAGCTGGTGGGAGGCAGGGCCAGCTCTCCTAAGCTCATGACCCTGTGGCAGCTTTCCACGCTGCTGGAGATGGTGACAGTCGGGGGGTGGGAGGGGGCGGGGATGAGGCCTCACCTCTGCACTCATGCCCACCGCACAGCAGATGAGTGGTGGGCTCAGCTCTCCCACACTCACACGCTGTGGTGGTTCACCCACACCTCCACCACCATGACCACAGCTAGCTCCACTGTGCTGCCTGGGTGAGGCTCAGGGCCCTCTGTCCAGAGTGCTGCTGCTAGTGAAAGATGAGGCCGGCTCTCCAGAGTGCTGTAGCCAATGAAGGATGGGGCCAGTTCTGCACAGCCCCTGGATATCCACATGGTCCCATGTTGCTGCCCTTACCGGGGTCATCTCCATGTTCTTCAGTGATAACATGAGCCATGGGCATCAACACTGACCTCTGCTGCTGCATAGCCACAGACTCAGACATGGCCCTCAGAGGCAGCTCAGGATCTCTCCATGGCCCCAGGAGGCAGGGCTGGCCACTCACAGCAGGCCACTCCTCTCCTCTCTGGAGTCTCCAGTTCCATCTCTCTTCACAATGCTCAAGCTGCTCCACTTCTCTTTCTCTCCCATCTGACCACCACATACTCACACATTGTGATGGCTCTGCCTGGAGGCTGGCCACATAGCTGACAGGCCTCTGGGTAACAAAGGTAGCTGGTGCCATGCGCTGGAGGGCAGGTCTGTCTGTGGGTGGCATGGTAGTCTGCAGGTCTCTGTCTTCTTCCTCCTGAGCTGCACTGCCTGGATTTGATTTGATCATTATGAGTCTTAGGCGTGAAACGGCTTTGGCCACCAAGCCAGGCATCAGTCTAGGACGAACAAGGGACAGCCATCTGCTCTGCCCTTGGCGTGTAAGAACAAAACCAACAAGGTGTCTCCCACTGCTGGGGGTTGTGGGGATAACATTCTTAGTAGAATCTTGAGAAATCCCAATCCCGGGCTGGAGAGATGGCTCAGCTGTTAAAGGCTAGGCTTGCAATCAAAAATATGAGAGAAATCCCAATCCCAGTGATGTCATTAAAAATGTGGCTTTCTGCATTTCTGCCTGTCTTCCTGGGAATGGCAAGGTCACATATATTCCTTTGCTAATAGATGTGCTGTGTGGCTGGTGTTGGAGCTGTGGGATCAGCTTGCTTATTTTGCAGGAGACTCTAAGAGTTAGTGTGATTACCAGTGCTTTCTTCTTTGCTGTGGGGAAAGTCACAGAAG
MSTRG.122973.3	ENSRNOG00000045885;ENSRNOG000	0.660	0.053	0.008	-2.657	down	CACACACACACACACACACACACACACACACACACCACCACCACCACCACCACCATCACTGACAATAACAACAAAACAAAGCACAGAGAACATCTCATCTTAGAAGCTGTAGTGTGTCACAGTGTGTCCCACAGTACATCCCTCTGTCCACACATCTTCACTTGCAAATGTTCATTGCAATGAGTCATTGGTCTAGTTCAAGGTCTCTGGTTCCTGGGACACCATCAATATTGGATCCTCACTGGGACACCTTCTGGTTAACCTGTTGTTGCCCTGTGTCACGGTGATCCTGCAGGACTGACCCTTTCACTTATGACAAGCATTCACAGATGATACAGATTTTGATGTGACCAATTCAGAGCCCTGGATCTGGGCCTGGGTGGCAGCAAGGTAGCTGGTCAGTGTACCAGTTCT

	00048975						CCGTTATCTCCATTGCCAGCGTGAACTCTCCAGCATTGCTCTGGCTAGGTCATCTACAGCAGCCATCAGAGGAAGCAGGGTTAACAATCTGGCTCTCATGCCCTTGGGACCAGCTCTCCCAACTACTTAAGGTTGAGGGGGGTGGGGGGAGGGCATCACCCCACACCTACACCATCTCATCCATACTCTCACCCTCAGGGCTGGCTCACCTGCATCCCCTCCACCAGGGCCAGCACTACTCTGAGGTGCAGGGCCTACTCTCCTGTTTGCTGTAGCTGGTGAGGGGCAGAACCAGCTCACCTGCTGTCATGACTCCAGGGTCAGCTCTCCCAAGTGCCTCAGCTGAGAACACACTCTCCCCCACAGGTCACCTCTGGGTAGAGATGTCATCTCTCCCTTGTTTTGGGGCTGGCTCACCTGAGGGCTGTCCCCAGGGTCAGCTCTGATGTGCTGTCCTGGTGAGGTGCAAAGCTGGCTCTTCTAGGTGTTGCAGCTGGTGGGAGGCAGGGCCAGCTCTCCTAAGCTCATGACCCTGTGGCAGCTTTCCACGCTGCTGGAGATGGTGACAGTCGGGGGGTGGGAGGGGGCGGGGATGAGGCCTCACCTCTGCACTCATGCCCACCGCACAGCAGATGAGTGGTGGGCTCAGCTCTCCCACACTCACACGCTGTGGTGGTTCACCCACACCTCCACCACCATGACCACAGCTAGCTCCACTGTGCTGCCTGGGTGAGGCTCAGGGCCCTCTGTCCAGAGTGCTGCTGCTAGTGAAAGATGAGGCCGGCTCTCCAGAGTGCTGTAGCCAATGAAGGATGGGGCCAGTTCTGCACAGCCCCTGGATATCCACATGGTCCCATGTTGCTGCCCTTACCGGGGTCATCTCCATGTTCTTCAGTGATAACATGAGCCATGGGCATCAACACTGACCTCTGCTGCTGCATAGCCACAGACTCAGACATGGCCCTCAGAGGCAGCTCAGGATCTCTCCATGGCCCCAGGAGGCAGGGCTGGCCACTCACAGCAGGCCACTCCTCTCCTCTCTGGAGTCTCCAGTTCCATCTCTCTTCACAATGCTCAAGCTGCTCCACTTCTCTTTCTCTCCCATCTGACCACCACATACTCACACATTGTGATGGCTCTGCCTGGAGGCTGGCCACATAGCTGACAGGCCTCTGGGTAACAAAGGTAGCTGGTGCCATGCGCTGGAGGGCAGGTCTGTCTGTGGGTGGCATGGTAGTCTGCAGGTCTCTGTCTTCTTCCTCCTGAGCTGCACTGCCTGGATTTGATTTGATCATTATGAGTCTTAGGCGTGAAACGGCTTTGGCCACCAAGCCAGGCATCAGTCTAGGACGAACAAGGGACAGCCATCTGCTCTGCCCTTGGCGTGTAAGAACAAAACCAACAAGGTGTCTCCCACTGCTGGGGGTTGTGGGGATAACATTCTTAGTAGAATCTTGAGAAATCCCAATCCCGGGCTGGAGAGATGGCTCAGCTGTTAAAGGCTAGGCTTGCAATCAAAAATATGAGAGAAATCCCAATCCCAGTGATGTCATTAAAAATGTGGCTTTCTGCATTTCTGCCTGTCTTCCTGGGAATGGCAAGGTCACATATATTCCTTTGCTAATAGATGTGCTGTGTGGCTGGTGTTGGAGCTGTGGGATCAGCTTGCTTATTTTGCAGGAGACTCTAAGAGTTAGTGTGATTACCAGTGCTTTCTTCTTTGCTGTGGGGAAAGTCACAGAAG
MSTRG.122986.1	--	0.784	0.098	0.031	-2.203	down	GCTGTGTCAAACTGAAGCTAACTAGGGCATCTTCAAAAGACACACAGTTGCTTTCACTCATTCAATAAAATGTAAAGGCCCGATGAGATTAACTAATTGGAAAGACAGCAATGCACTGAAGGGACCGCTTTGATGTGTTTGGTGATCATAGTAGGAGACCCTGAGAAGTCTGGTTCTGCTGTGATAGGAATTCACACCCAGGTTGAGGAGGGAGGTGGGTAGGTGTCAAGAAAGACTTTCTCAGAACAGCACATTAAAATTGAAACTGATGGTCAATCCAAACCAAGCCTCAGGAGACAGTTCCGGACTGAGCAAAAGCAGGCAAAACCCATTGCTGAGACAATAGCTTTGTTACCAGTGATTCAGGAACAATGGAACCTTTTCTTAGGAAGGATAGATGTGGATTCTATATCTTGTTTTGTACCTGGGAAGTAAAATGTATAAAAAGCCTTCCGATCTGATTGGATAAGCAAGCAGAGAAAGCCTTACAAGGACTTTTGCCGCAAAGATGTTTTTAACAGCAATAGCCCACGATAACTAGAACAGAAAAATAATGACAAGATATGTCAATAGGCTCTTACTATTGAAAACCATTGTAAAAGATACTGTAAACACATTTACTGATGTGGAAAAAAAATTCACATTTCCAAAAGAAATAAGCAAGGCTTGAAGAATACATATAGGATGAGTCTGTGCTGAGCACAAAATAGGAATTCAATAAATATTAGCTGAATGAACAGCAAATGAAGTCGTCTAGGCAGATAAACACCAAATAAAATGTCCACATTCATTATTCTTGCGTGATGGGATTATTGGTGATTTGAAATTCTTTTCTGCATCTGCCTATCTGGGCTATCTGGTATTTTATTTTTTGTTGGATAATGCACATGAATTACTTGCACAATAAAAGGGAATAAATTTTTTAGAATCTATCCAAGGCAAGCAATGACTTAGCAAGGTCAATTTTAAAGATCAACACTTGTAATTAGAAAATGAGACTTTGGAAGAAAGCAGAAGATGATGTGGCCACGTGGGTGCA

							AGGGATAGAAAATAGCTGTCCTGTAAGCACTGAGGGTAGATAGACCTGCCCTCGATGTCATAGAAAAAT<br>AAATGCTTTACACCAGTCCATTTCACATTTATTATTTTCAGCTATCCAGTCCATCCCACATTTATTATTTTC<br>AGCTATCAAGTCCATCCCACATTTATTATTTTTCAGCTATCTGCAACACAACACATTAGTTAAATATTAGC<br>GAAGAACATTTACATTGAGGCAAAAATGAAGCCAATAATCGTATTATGATGCCAAAATTTTTAACCGAAT<br>AAAGATCCTTGTGTGCCAAACATCGAAATATCTCGGAAAAAAAATCTGCAAAACAAATGTCATTTGGAT<br>CAATTTCTGGGGTGACTGATTGCACTGTCCATGTTCCTCTGATGGTCTAGCATAATAGCAACTATGGCCT<br>CCAATCAAACCACTGGCTGGACACAAACACGGCCAACTCTGAGACTGCCATCACTAAAAGCATGTGCT<br>GGGCAAATAACACAGCTGATTCTCTGATGTCCTTTCACAGCCAAATCGTAATCCTCTGATTGTTCATGTC<br>ATTAAGAGAATTCTGTTAACTTTGAAACTCTGGAAATCCTGATGGAGGAAAGCGCTGGCTCTGGTCCAT<br>GCAAATCTGAAGAGAGCAGACAGCAGGTGCTGTGGAGAGCTGGCCAAATGGTGTTAACTGTGTCCAG<br>AGGGACCTCTATATATTCAAAGAACAGCTGCTGTATCCTCCATGCACCCCCATGTCTTTATGAGCAAAGT<br>GGCTCCCACCTTTCTGAGATACCTCTCGAACCAGGATGCAGACTCCTTTCTTGGGGAATGAATACACAA<br>GAGGGTGAAGAGGTCAGTAAGTTAGATGACAAGAGCAGGAGGTGGCTGATAAAGAGGAAAAGAAAA<br>ATACCAGAAGATATAGTTCAATGATAGACTATTTGTCTTGCCTGCACTAGGCCCTTGGCTTAATCCCCAGC<br>AAAACACACACACACACACACACACACACACACACACACACACTGGGAGAGAGGGGGAGGGGAGAA<br>AAGACTGTATTGCCCTAGCTCATGAGGAATTCACACAGTTCTCAGCTGTTCTCTTGAAAAGGAGATTTG<br>ATAATTGTGTGACACACTCAAGAAACTGAGTAAGGAAAAGGCCATCACACACATAACCATATTCCCAAC<br>TCTACTCCCCCTGTACTGGGATCAACTGTGTGCAGGAGGTTAACTTGAATGAATCCAGCCCTTGAAGCT<br>GGGAGTACCTCTTACTAGGCTTATCATGGGCACAATCTCGGATGCAGTGCTCACTTACCGACCATTCAGA<br>ATTAACTCAACTGAATGGTGAAATTAGAAACAAGCGGAATTATAGTCCCACAGGCTAGCTTCACAATCA<br>GCACGCGCTGTGACCATCACATGTGACCCATATGACCAACAGGAAACCCGTAGGCTTCCTAGAAGGTAT<br>AAAAAGTCACCTAACCTTCTCCCCACTCTACAAAGGGAAGGGCTATTGAGTCTTCTGTTGACAGTTAAA<br>GGAAGCAGGCACCTGTCAGGAGCTAACAAACCCACTACAGTCTCACACATGAACACAAAAGTGACAA<br>ACATTACAAGAACCCTTTATAGCTACATGGGAGCAGAGGCCTACTGAGTGGAGTGCCAGGTGACCCTCC<br>CATTTCCCCTTACATGCTCACTCTGTATGGCTGTAATCTCAAGCATCAAACTATTCTTGTTCCTTCCTCTT<br>GGAAAAAGCACAAACAAGTCAACAAGTTAAATGCTTATTTCATGGGACTCAGCTGGGTTTCTGTAATTA<br>GATTACCAAAAACTCAAAATGAAAGAGGCTAAGGAAAGGGAAGGGAGCTGACTCTTCAGATTATAGCC<br>TAGTCAGCAGTCCCCACTGGTAACCTTTAGACTTATGCTCACCACACCAGTGACCAATACCCCGAACTC<br>CCTGCTCCTGTACCCATTTCCACAGGGCCTCCACCCAACTCAAGTAGGGCTTGGCTTCAACTTACACCA<br>TTATTCAATCTGCTGACTCCTGAGCAGACTTGAAATTTCAGAGTCCTATTGCAAGCTCAAGGGCATGGT<br>GAACATGTACCTAAGACAGATGACCTTGACCCTTGACCGTATGAGTCTTTCATACTATGGTGAACTACTA<br>CAGGGAGTATTTCATACTACAGGGAGACCTTGGAGAACCAAAAAGGATAGAGGTGTGGAAAAAGCA<br>CACCAGCATCATTCTGTACCACAGCAGAGCCTAAAGCAGAAAGAAAAAGTAACTTTGATCTTGTCTTGA<br>TTACAAATTTTGATATTTTGTTCAGCATGATGTTGATGGATGTAATGATGTTCTAAAGACTGAATTAAACA<br>ACATTTATCTTGATTTCCAAGATTTGTTGTTGTCACCTTAGATTTCTTTTTCTAGCTGAGAGTCTCACTCA<br>CTCCATCCTGTCTCTACAAAATTACTGTCTGTTGAGTTCTAATAATATATCATGCGCTACTCTTAGAAAATT<br>ACACGTGATCCTTGCAATACTCTTGCCAATAATGAGGAATGTTCTTGTTTTACAAATAGAGAATAGGGAC<br>TCAAAAAGTAAATGGGATAGGATCTGGAGAGGTGTTAAGAGGTCGTAGGGATGGAGACCAGGAGACA<br>GTGGAAAGGAGCAGGAACTAGGTCAACCACTGGGGATCCTCCTTTGGGAAAAAGATATGTGAATGCAT<br>GAAGAGGCATGATGTAGGCAGCCTTAGATTTGGGGAGGGCTCTATGATGCCTGCAGAGCTCTGAGGGAT<br>TCAGAGGAGGCTTTGAAGACAACAGCTTCCTTCCCCATTCACATACGGACCAAGAGAAGCTCCCTTCAT

							ACTAGTGGGTGTTTTAACAATCAAGCTTTACATTTCTTAAGATTACTTTACATTTGTGGGAGGAAAATCATTATAAAACCTGTGGCCAAAAGTGGTTTGGTTGCAATTTAATTGCTCATAAAGAATAAAACAAAGTATTTCACACATGCACACAAAACATCTAATTTGAATTAAAGCCATGGGTCAGCATCTTCGTCTGTCATGAATATCACATTCTCATTAACTCGAGGGGATCCATGGAAACACCACCAAGAAACTGCAGAGCTCCAACAGGGATTGAGCTGAGAGATGGGTTTTTTTTTTCTTTTTCAAAGAATGTGTGTGTTATATTGACTAGCAATACATCAACTCTGGCTCCCGAATGATAATCCTGCTAATATTAGCAGTATTATGAATGACTTGCAATGTTGTTAACTCTTGGGCTACATCAAATATGCAATGTTGATCGGAATAAGCTGTCAACAGTGGAAATTGTTGGGTATCAACATTCTGCTGTGTGTGGGCTGGCACCTTTTCACAACTCTGTTGACTGTCTCGTTTGCCTGGTGGCATTCCATTTACAGTTGATGATGGCATTCACCGAAGTCCAGGAGGAATAGAAGGCATATCCAGGATCTATCCCTGATGCTTCTCCCACTTCAATCCACACAAACTCTCCCATCATCAGAACATCGCCAGCGCCTGCCAGCACCACACCGATTCAACCACGTTGTCCTATCTCATTCAGGACTGAAAGGTTTTGTCTCCTTTGGTAGTACTACCTGCTCAGCAACACAAACTGTGTATTACTTGCTCCCACACACTCATCTAAGAAATAAACACAAACTAGGATGGGATGTAAATGGTTTATTGGGGTCAAACGGAGACAAAGAAAGGCAGACAGGGCTCCAAGTGTAAGTTCTAAAGCACAGAAAATCTGAGAAGACTTGAGTCCAGGTAGGACTATTTGGGACAGTCTATGAAAATGTATCCAGAGGATGGCTCTACACCAAAGTAGTGAGAAACTAAGGTTAACTCCTGGGCTACATCTTGAGAGCAAAAGCAAGCACAAATAGGGAGAGGCTGAGATCAGGGAACATTCAACATCCACTTGAAGGGTCTGTAGCGACTAGACAGACAGCCATGAGTCTGGTCTGTAGCTAAGGACTGAGATCATGCCTGACAGTGAGAACCAATGAACCATGTGACTCTATCCCCCCAGAGCTGCATACAATGAACTTCCACCATCTCTAAGACAACATCAAGTGTGCTCGTCCCTTTATCACTGGGTTCCAGGGATCCAGGGCTTTGCTCCTTGTTTTCTGGCTATGTTGGTATCCAGCACTAGTCACATTCTCTTGCCTGCCATAGGCCATGAAGAAAGTTCCCACCTTCCCCACATATAACTGAACTTGTTTGTGATGCATAAGATTAAGTTCCAACTGATCATTGGAAGAGAGTTATGTGGCTAGCGCGTGGAGAATGCCACAACAGCTCTGGCCACAGGCAGGCAGTACACACCTTTTGGTATGGTTGCTATTGTTGATTTGTTTGTTGTTACTGCTGTTGTTGTTTCACTTTGGTGATGTCAGTGCTCCATCCTGGACTCTATGCTTGCCAGGTAACTCAGAAACAACCCTTATCTCACATGGAATTTCTCTACTTTACTCAGAAGCCAGTGAATTGCAAAGCAATTCACCAGGTTGTTACAAATGTCCAGGAGCAATGAGTAAATGACTACAACATTTCTGTACATTGGAATTCTTTCCCATATCGACTAGAGAGTTGGAAATCAGATCTTTCTGGCTAAAAAATAAAACCCATTTTCAGTTCCCACAACTTCCTCTACCATTTGCTTCCTGACTCAGAACTCTACTGTTTTAGTGGATCAATATAGTCCATGAACAATAAGAAACTAGTTCCAAGAGAGGACCATCACCCAAGCCTTCC
MSTRG.123016.1	ENSRNOG00000023622;ENSRNOG00000030069	0.000	0.285	0.007	3.835	up	GTCTATCGCCTCATTGTTAGGTCCTGAAAGCCGAAGCCACTCGATGTCCAGTGGTCCTTGGTCCTCAGGGCTAAGGGTGAACATGCAGGGCAGATGAACAATTTCCCCTCCAGCTTCCTGAACTATCTGCTCTGGAGTAGTGATGCTCACACTCCCAATGAGATCTGGAGAGAGAGAGAGAGAGAGAGAGAGAGAGAGAGAGAGAGAGAGAGAGAGAGAGAGAATGTGCATAACATTGAGGCATTGAGTATCTATCTTGTTGCTCACTGGCTGACTTCAGGCTGAAGCCATTCAGGAGTCAGGATAGGGGGATGTGGGTGGGACTTGGATGCCCAGGTTAGTTTGTGGCTTCATGAAGTGGAGCGGCTATGGGCATTTTTTCATGCTAAGTGATTGCCTTAGAGCACCACCACCTTGCTGGCATCATGCAGGCATGAGCCTCCACCAGGAAGCTCTTGTTCTTCTCACCAGGCGCAGACAAGTGAACTTGGCTAGAGCTCTGAAGGTCAGCCTTGTCTACA
MSTRG.123128.2	--	0.467	0.048	0.043	-2.320	down	GTGTAACTGTCCTCAGCTGGCCTGAGATCCGCCCTAACTGCCCTAACGGCTGATGTAGCTGCACAGTGGTGGTGAGCCTGAGGTAAGCCTCCAGCTAAGACCGTTTGCACTCAGACTCATTTTCATCTCCAGCTTGCTCTGTAAGTTGGGTGTACCTCAACAATATCGTGGCAAACTACAGATGGGCCAAGCCGGGAGGAAAACCAATGACTCTTAAACCCAGGGTGCCATGGAGACCCTCTATAGTCCAGGACAGCATGCGGTTTGCAGCTTGCCTTATTATAGATGCTCTGTGAATTCTTCCTGGACATCCGGTCCCTTCTCTCACGGGTCCTCAGAACTTCTG

							CCAGATGAGGTTTCTGTCCTAAGTGGGGGAAATCTGCCTCAGGTTATCTGAGGGCAGAGGCCGTGTGGACTCAGGCCTCTGTGCAACCAAGCTCCTCTAGGAGAGCCCATCCTCACCCTGGGCAGAATGCTGGGCTTCATTCATATTCATCGTCACTTGGAGCTGCACCTTTTCTCCAGTCTGTGCAGGGATGTGCATCACCATGGCAACGCTAGAGGTGAAATCACTCAAGTGTTGGCAAGCAGGGTTTCTCTCTGGAGCTTGGAAAAATGGAGAGCAGGCTTGTGGTGTCACAGAAAGGAAGATGCCCTTAGAAGAACCCAAGCATTCTCTATTCCATTTGGGGACTTTCCCTTTAAATAAAAACTGAACAGTAAATGGAAAGAGAAATGTTTTTAGTCTCTGGATCTGCTACCCCAGTGAGCCTACTTGGAACGGACACGCTGATCCTTCTGGCTTTCCTAACCTGCTGTACCTTGTAGACTGTTCTGCCTGGAGTTACTATTTTAAAGTAATGAAGCTGGGGCCCACAAGTGTGATGTTTGTGCCTGTTGGCCAATTTCTGGCTCTTTGTCTAATTTGGGGGACTAGAAGATATTTGCTAAATAACTATTTGACAGGCTACTGGTGGCTAAGGGCAATGAGGACATGCTCACACACCCAAGGGGTCTTGAGATAAGCTGTTTCAGTACCTGCTCGAGGCAGCTTGCCCTGAGACTTCTGAGTACATGCAGCAGCCCCTGGAAATGATTTTGACAGCATTGCCTTTGGGCAGGAATGATGAGGAGGAGAAAGGTATGGGAGGATGGGTGTGGGTGCCAGGTACTGGGAAACTTGGGCTGGTTAGGGGACCTGTGCATACCCAAGGCTTAGAGGAGACCATCAGTGCAGATTGCCTGATAATTCTACATGATTCTTGGCTTGATTTTATTCTACCAGGGACAAAGAGGCACTGATAGTAGTTCTCCTTTTAATCAGAACAGTTTGGAATACCTTGTGAAATAGCCTTGCCCTCTTCCCCATCCCCCTTCTCTCCTCTACACTCCCCTCCCATTCTCCCTTCCCTTCCTTTCTCTCTCTTTTTCTCCCCCCTCCCCTTCCCATATCCACTGAGTACTTGTCAGGCCTCAGGCATTATGAAGGGCTAAGTTACAGCCTTCCTGGAAATAGCCAGCAGTCAGGAAGTTAAAGTGAATATATCATAGATAAAGGCACTTCTTCGCAATTCTAGTTTTCCTGTTTTTAATCAGGGACTTTGATATTAAGTCTAGGTTATTCGATTGGACAGATAGACTGACAGACAGACAGGCTGTGACCCTGTTACCCTGAAATTAAGGTTAAGGTAGGGTTCTAGTCCCTTGATTTAGTCTCAG
MSTRG.123250.1	ENSRNOG00000049016;ENSRNOG00000049451;ENSRNOG00000060802;ENSRNOG00000060998	0.299	0.018	0.001	-3.143	down	CTTGTAAGCCCTCCTGCTTGAAAGATGCCCCAATTCTCTGCTCCTTTTATTTAGTGTTTAAAGTTCTTTACAACCCACAAGCTAGCTTATCTCTTTAGATCCATCTGGCACCAAGTCATGCCTAAGCTCCAGAGAGTTGCCAGGAATGATGAGTGTTCTGTGAATGCTTTCACAGCCTCCCATTATCTTATACACGTGCACGCACGCACGCACGCACGCACGCACGCACGCGCACGTACATACACACACACACACACACACACTCTCTCTCTCTCTCTCTCTCTCTCTCTCTCTCTCTCTCTCTCTCCTGTCCTCTGCATTCCCTGCTGTGAAGCCCTTCTGCTCTCAATGTTTCCTCCTGACCTCTAATTTCATACTTAAGTTGCATTGTAACTGTGTATGTTATAGGAGCATGTTTTATCTTTGTATCGCTCACTGATAAGTACCATAAGGTTTACTGTAAAAAACAGAGAGAGAGAGAGAGAGAGAGAGAGAGAGAGAGATCTACTTAAGGAAATCTAAAATAGATATTTTAAAGCAAAGTGAAAGAAAGAAAGAAGGAAGGAAAAAGGACAAAAAGGAAAAATGATTCCAGAGGGAAAATAACAGTGAGAACATAGTATACAGATAGGTATTTAAAGATGTGCAATTTGTGGCGGAAAAATAAAGACACTAGAGAGATTTACATTGTCCTTGCATTGTAGAGCATGAAAAGTAACATGCCCATTAAACAGAGATGTTTTAAGGTGTGCTCCAGGGGAGCTCAATTTTACATGACTGAGGCATCCCCAACATGACTGGCCCAGAACATCCCTGGTTAGATTTTTCTGCATCGTTTCCCCACAAATTCTAAGCAGATGAAGTCATTAGAACAGTATGTGAGGCTTCCCGCCAGACCCTGGTGCCAGGCCCTGCCTGACATAGTTTAAGACCCACCAGATGCATTCCACCCAGAATGGTCCTAAGCAATACACTACTCCTGGCGACCGGAACTACGGGACCCAGATGGTCTTGCTGCCGTGCAGGAACACACTTCTGTTAGAAAGTTCCAAATAACCTGAACTCTGTCTAAGCCTGGGATGTCCGCCTACCTTTCCGCAGCTTCATAGCAAACTGGAGCTACTCCCCAGATTATTCGAGAAACAGTATGAGTTAAATAGTGAGGGCAGCTTCTAAGGGGTAGGCATAATGCACATAGGCTACCAAGTAGCTGGGAAAGTTAGAATAAGGAGAAAAAACTACCAGTCCAAAAGGCAGCAAGAACATAAATATATACAAAATATTATTTATTAAATTATATAATAAATATATACAATTAAATGTTTTAAGAAGATTTATTTATTCTATATATGGAAGTATTACTCTCATAGGATACTCCAGAAGAGGGCATCGGATCTCATTACAGATGGTTGTGAGCCACCATGTGGTTGCTGGGATTTGAACTCAGGACCTCTGGA

							AGAGCAGTCAGTGTTCTTTATTGCTAAACCATCTCTCCATCCCCCTAGTCAAAACATGTAATGGTAGTAT<br>ACAAAGGTGGGGCTATTACTCCAAGGCAGTGTGCTATCTAACATGTGAGGAGCCTACATTCAATGCCCA<br>GTACTGAAGGAAAGAAACGAAGCTGAGCAGAGAGGAAGATTAGGAGTTCAAAACCAGGCCTGGCCAC<br>ACGGTGAGTTCGAGGCCAAACTGGGCTATGTAAAAGTGTGTCTCAAAATAATGGTGATAGACGATATTG<br>AAATATATTAATTTTAATAAGTGCAAATAGGAACAAACATCGAATAGAATTAAATAACCTTGATGAATAAA<br>AGGTATAGCAGTGTGTTGCATGGTACACTTAAATCACAGAGAGGGTCTAATCATGAACCCATAGGAACC<br>CACCAAGATTCCTTCCAAACACACAAGTCAGAGAGTGGCAAGGAAAAGGAGAAAAAAATACTTTCTC<br>TCTCTTCTCCTCAGTACCTTTGAGAGCCAGGATGGGGGTGGTATTTCCCATTCGAAACAGTCCTCTGATT<br>CTGTGTGAACATCAGCTGAATGTCTTACCGTTCTAGTCAACCCTGATACTGCCTGGAGTCAGCATTTGAT<br>TCTGTAGGTCGAAGGACCCAGTATCAACCAACCACTTCCATTATAAGTGCCAACCACAATTCCCAGGTT<br>TCCCCAGCACCAGGGACAAACTAAGGATCTCCACAATGCTGATACTTTCCCAGAGCTCAGGAAGGCAC<br>ACTCCCTAACATTTACTAGCTCACTGCAAAGGCTACGGTCAGAAAGCATCAAATGCGAGAAATACGTAG<br>GGTAGTAAAAGGGAGTATGCTTGCGTAGATTAGATTTTCTCTGCCACTGTGCATACACCAAATATTGCAA<br>TTCATTCACTAGGATAGAAGCCTCCTGAATCTCGTCTTTAGGGAATATTTTAATGTAGGCATGATATAGGC<br>TTACAGATTTATTAACTGGGTCTCCAACCTTTCTCTTTTCCAGGGTATAAGAGGCAGGGCTGAAAAGTTC<br>CCAGCTTCCTGTTACTCTTCAGTCTTTCTGGTGAACAGCTCTCATCTTGAAACTATCCAGAAACCTACCA<br>AACGCTGCCATTTGATAAAAATACACTCTTATCACAAAAGAGATTCCAAGGAACTCGGGGGCCGTCTGG<br>CAGACAGGCTAGCTATCTGCATCACTCAGGAAATGGGCAGGTCTTAGGAGATTTGTGTCAGTGGCCAGG<br>GTCAAAGGCCAGTAAAAGAAAAGATGTTCCTGACACCCCCTAGCTCAAAAAATTTAAAGCATTTTAGG<br>AACTCTGTGTCAGGAGCAGGAGGCAGCAACCAGATATTTATCTATCATGCCACACACAGACAATCCATC<br>ATCAGAGGGAGAAATTCCAGTACAAATCTCCTGATACCAGCCTCTCAGGTAAGAAACCAGTGAAGGAG<br>GCAGACTTAAATGGCTTAAATAATGTCATACTGTATTTTCATTTGCCTTTTTAAGATCCTTATTTATTTTGT<br>GTGTGTGCACATGCATGCCCATAGAGGCTGGAAAGGGATGTTGGACGTCTTGGAGCTGGACGTACAGG<br>CATTTGTGAGCCACCTGATGTGGATACTGGGGTCTACACTGTGGCTCTCTGATAGCGCAGTAAGCGCTC<br>TTAAGCACTGAGCCTTCTCTCCAGCCCAATACTGTACTTTTACATACACAAGACATGTAAGTAGTGTTGC<br>ATGTAATAATTAGCAAGCTATAAAACAGCTCTGAAGACTGACAGAGCTATAAAGATGGCCTCAGGACAT<br>TCAGAAGCCTGGCATGCACACGTGTTGAATTCCTTTACAATATAATAGAACATGTTTTCTAAGGACAGCT<br>AGGACAGGGAAAGAAAACCTGCTGTGTACTAAACGGATTCTGCAGTGCCTTTAAACAGAAGGCCATCG<br>TGCCATTTGTGATAACGTGGATGAAACTGGAGGCCTTGAAATGAAATGAGCCGGAAGACAAATGCCAC<br>GTGACCTCACAGTGAAACTGTGAAACCTACCTTGGCGCGTGCAGAAGCAGAAAGTCAAACAGGGATT<br>ACTGGGATTCGGGGTGCACACAATGGTATTAAGGTGAGGTAAGGATATGAGCGAGGGAAGGGTGTGTG<br>TCTGTGTGCAGCTTCCCTTCCCTCTCTGGGGACAGCACTCGCCTAGCTTATCAACGTGTTTGCCACTGG<br>GGATTAGCAGGAGCTGGGGTGAGAAACACTAGGGAGATATGGACCAAAGGAAACAGAATACCAGTGA<br>GACATCAGTTCAAGTGGGGCATCAGTTCAAGAGGTTACTACAAAGCTTGGTGCCTAGAGATAACGGCAT<br>GCACTGTATTCCTGAATATCACTACAATTAGGTCGGAAACGTGCCCACCATGAGAAGAGGCATGTGGAG<br>TGAGGTATATGTCAACTAGTGACAAAGGTATACATTCTTGAAAGCGTCAGGATGCGCCACCTACGTATAC<br>ACAATTTTGTCAATTAAGACAAATGACGGTTTTAAAAGAAAACGAGAAAGTTTCCATTTTGAAACAACT<br>CACAACCAGAGAAGAAATTAAAGTAGAACTGAAAATTGTATTCTCATATATATGTCTGCCTGCATGCTGT<br>CTTGCTCCCCCGCCATGATAATTATGGATTAATCCTCTGAAGCTGTAAGCCAGCCCCAGTTAAATGTCTTC<br>CTTTATAGGAGTTTCTGTGGCCATGGTGTCTGTTCACAGCAATAGACCCTCACTAAGACACTAAGGCTTT<br>GAACTTGGGGTCGTCCTACTTTGACCTCCCAACGTCTGGTACCGTGAGCATGAACCACCATACTGGGCT

							TCCTTTCGTCTCTCCATCGATGGATTATTCATAAAAATAATCCTATAAAAATGTAAGGAGTGGAGTGACTC<br>TGATACTTAGAAGGAAGGCTGCAACCTTAGGTGTCTCTGTTAGCAGAGGTCAAAGTTCACAAGGTACA<br>CGGGCAGCCTAAAAAGGGTGGGAAGATAAAATAAAGATAGAAAGACGGAAACAGAAGCAAATGTTAA<br>ATGGGATAATCGATGGAGCTTCTCTGCTGCTGTGATAAAATACCACAATCAAAGATAATTAGGGAGCTGA<br>AGCTCCAGGGGGCTCGATGTTCGCAATGGTGGGAAGGCAGGGTGGCAGGAAGCGGGGAGATCACATC<br>TCAGCTGCTACAGGAAGCAGAGCCTGAACCAGATGCGGAGTGAGGCTAGAAATCCTCAAAAACCCAC<br>CCCATACTTCCTTCAACAGAGCTGTACCTCCTCCTCAAGGCTGCATAACCTTTCCAACAGGGCCACTGG<br>CTGGTGATCGCGTGTTCAAATACTCAAGGTTATGGGAGACATTTCTCATTGAAAACACCACATAGGGTT<br>CTGTAAAAAAACAAACAAAACAAAAACAAAACGAAACCCAACGCAACCCAAACCAGACTGTTAATGG<br>CACCATTGATGGAGAGAAGAAAGGAAGTCCAGTGTGGTGGTTCATGCTCACGGTATCAGAACTTGGGA<br>GGTCAAAGTAGGAAGACCCCAAGTTCAAAGCCTTAGTGTCTTAGTGAGGGTTTTATTGCTGTGAGGAG<br>ACACCATGGCCACGGAAACTCCTATAAAGGAGACATTTAATTGGGGCTGGCTTACAGCTTCAGAGAGTC<br>AGTCCATTATCGTCACGTCAGGAGGCTTGGCGGCACGCAGGCAGACATGGTGCTGGAGAGGGAGCTGA<br>GAGGTCTACATTTGAAGGCAGCAGACAGAGAACTGCCTTGAGCTTCTGAGACATCAAAGCCCACTACC<br>TAGTGATACTCTTCCTCCAAAAAGCCCACGCCTACACCAACAAGGCCACACCTTCTTTTTTTTTTTTCTT<br>TTTTCTTTTTTTCGGAGCTGGGGACCAAACCCAGGGCCTTGCGCTTGGTAGGCAAGCGCTCTACCACTG<br>AGCTAAATCCCCAACCCAAGGCCACACCTTCTAATAGTGCCATTCCTTGGGCCAACCATTCAAACACAT<br>GAATCTATGGGGGCCAAAGTTATTCAAATCACCACACTGGGATACAAAGAAAGACCCTGTCTCGTCTGT<br>TACCTTCCTCTCCCCACCAAACCTAACAAGTAAATCATTAGGTTCAGTGCTTCTTTTAAAAATCAAATTA<br>TATAACACACTAATTGAACAAAAATCCCACCTTACTCTTCCAATATTATAGCCATGAGAAAAAGAAAGAG<br>TATTTCCCAATGCTTGGCATTACCTTGATTCCAAACTGAGACAGACACAAAATGAGAAAGGGTGTCACA<br>GGTGAATCTTATACAGCTGACATAATCTTTAAGCACATCACTAGTGATTCTAACTGTTATGACCTTATTGG<br>TTTTACCACAGGATAGAAAATTTAATAATTAGGAAACTGATTAACACATTGTATCACAATAACAAATTAG<br>AAAAATGATCAAATCATTAGTTCTAGAAAAAAATGTTTGGTCAAATTCATCACGAGTTTATTTTATTCTTG<br>TTGGTTCTGAGCCTTGTGCATGCCAGGCAAGGGCTCTACCAGCGAATGGCACCCCTACCCTTTCCTTTG<br>CAATTCACAATTCTAACAAACTAAAAATTAGTAGTAATTTTCCTGATCTGATAAAGAGAGCGTATGAAAT<br>AATAGCAGAGTGTGCAATAAACATCAGGCACTTAATTATTAGAATATATTTTAGATTCTCCTTTAATATTAG<br>ACATAAGACATGCTGGCTAACACTTACTTGTACTTAACACCCTGCAAGTACAGGTTAGATACAGATATAG<br>TAAAAGAAACAAAAACATTTAAAAAGATTGTACCTATAAGACATTATTTATAGGTATGCTTTTATAGAAA<br>ATAAATGTTACCCAGGTTAACCAAAATATTTGGAACCAGAAGTGTTTTAGACGTCTTTCCTTTTTTTTTTC<br>TTAGATTTTTAAATATTTGCCTGTGTGTAATGAGATGGGGGTAAACAGGACCAAGCACATAATTCATTTAT<br>GTTTCACACATACCTTACTTGGGTTAGGAAGATCCACCAATGCCTAAATACCCCAGGCTTGGTTCTCAGT<br>TCTCAGGAGGCAACAGGACCCTGAAGAGGTGGTACCTAGTGGGAACCTTATGCGACTCAGAGTGAGCC<br>CTTGAAGAAGGGCTCCTGTGTCTCCTCCCTCTCTCCCTCTCTGTACTTGGTCAAGAGGTCAAGGGGTTT<br>CCTCCCCCCACATGCTTGTGCCGTGATGTATGCACTGTGCTCTCAGAGCCCAAAAGCTAGGACTCAACC<br>TGCGAATCTGTGAACCCAAATCAGCCTTCCTCTTGCCGAGCAGATAACTTAGGCGTTGATTATGTTAACA<br>AGAAGCTGCCTAGCACAGGACTTTCCTTGGATAACATCCTCGCACATGTTTACGTCACCTACATTTCCAC<br>TGTGAGCTGCACCATGGGGGCGGATGTGGAATTTTCCACTTGTGGTTTTGAACGACGTTCAGAAATCTG<br>GGTGCTGGACCACATGAGATTTTTTTTTGACTAGGAATGCTCAATTTGTAATTAGTAAAATTAGGCAAAT<br>GTTAACAGTCTTGAAAGTAGTATATACAATATAAATGTCAACTGTGTGTTTCCATTCAGTTTGACTCCCTT<br>AGGCAGGCATGCCAAGGACACTGCCATCAACAGGGGTAAAGTATAAAGAACCTACCAATACATTTTGA

							AAAACGATTGTGATTTTTATGTGAAGTGTTTTTGGGGTTTTGCTGCTGGTATTGTTTTCTCGTTTTGTGTTTTCCTGTTTGTCTGTTTCTGGGGCCAGGGGTTGAGCCTCAGACGCATGGACTCTGCCACTGATTATAATTCTGAAAAGTTTAGATTGAATTGAAAATTATGATATGACTCTTTACTTTTTTTAAAGAAGATGTTATCAAAGAAATGAAACACTGTAAAAGAAAAAAAAAGGAGATAATTTACACAAAAGTTGGGGATAATTACTGAAACGATAACTTGCTTCTGAACCTCTACAATGTGGGTCTAAGGGCCCTCTGTTTTTAATTCCTCAATAGCGTTCATGAATTTTAAACACTGTTTCATATGTATGCCATGCTTTCAAAGCCTAGAACACGATGTTGTGTCCCTGCGACACAGGATTCTTGGGTTTTCCATGTACAGCCTGAAAGAACATGCTGATTTTGTAAACGTTCAGAAACGTGGCCTGAATGATCATTCTGTCTCCAGAGGAGGAGGGAGAAGGTGGCAGGTGCAATTCCACATGGAAGCGAGCTCTTGGGGAACATGCAAGCACTCAGGCTGAGCGACTGCCTTTATTTTTAGTTTTAAATGGAAAGTTTCCTGTGCAAAGCCATCCGTGCAACTATCAGGTATCTATCCCGACCCCCTACACACACATACAAGGTAGCTAGTCCTCCTCTGACCACCATCCAAAAACATTAGTCGGGAAGCAGCTCACAAAGGATGAAAGGGTTTGCTGTAAGCCTTAGTTAGCTCCTCACCTCATAAATCTAATCTGCTGGAGCTTTACAAGTCACTCGGTTACAACAGTAAACAGATTGCCAAGAATTTGCAGACGACTAGCTTTTCAGAAAAGCGTAAATTATAGTGGAATAAGTACGATCCATTTGCATGCCAATATGCTCCCTTGGGTGGAGAGAAAAGCCCAGCGTGGCATCTGGTCCCTGGAATACAGCCTGTGGCAGTCTGAATTTCCTAAAAATAGTTATGGTGAGGCCTATCCAGACACTCTGCCCTGCTCTATCAAGGTGTGGAACTTACTTCCACTCTCCCCAATCTGACTGGGCTTTGAGATGGCCTCAGGGAACAGAGTGCAACAAGTGATACTAGCTGTTTTGGTCACAAAAGATGATACATCCTGGCTTTCTTCCTCTAGGGTCCTGTGTTGAGCCAGTGCAGAAGAAACTGGGTTACTGAAAGCTGTTTTCTCTGGAGAAACAAGAAAGCCCAGCTACTGTAGTCCTCCCAGCCCAGGCTGCACACACAGGGTGGGTTAGCCCATCAAGCCTGCAGTTCAGCCAGCCCAGAGGGTTCCCAGAGCGGAGGTGAGCTATTCCCCCTTAGCCCTGGTCAGACTGCAAGATTCATGAAGCCAGCAGTCATGACTGCTGCTTTGCAACCCTAAGTGCTGGCGTGGTTTGTGACAAACCACTAGATAATGAGAACAGAGAAATGTCGTAAATGGAGAGTACTGAATGCATATATACACACACACCCTCATCCGCCCAAAGGAGAAGAAACTCAGAGCCAATTTCCTGGCCAAGTTTTCCAATAGAAGAAGCGGGAAGACCCAGGGCTTATCTCAGATAAAAGAAGCCATTACAGGGAAGCCACACCCTTCCTCCTGTGTGAGTGACACTCCTGAGGCACCTGAAGGACAGTGCTCCAGTCTACACTGGAAACATCTACAAGAAGAGCCCACAGGACCCCTCTTGCTTTGTTTAATTGTAGCTGAGCCTATGTGAGCCTCGGTCCAGTAATAACTCCCCTTTACTGTCATAGAACTCATTTATGTTTTTAACTTTAATTCTAGGAAATTTCACCCATGTATACGGTGTATCTTAGCAAGTCACCCCTCATCTTTCCCTCCAGCTTACTTCTGGGACTACTATTATATCCTTCTCCCAACTTCGAGTCCTTTTACATTTATTTCTTTATTTTCCCTCTGAGTTCAATTAGAGCCCTCCTTATACACACTGGTGTAGGGTGGGACAGGAGAGAGCCTGGGAACCTGGTCTGAACTCAGTCCTGGCCACACTCCTAAACAAAAGCTTGCTCTCTCTCTCTCTCTCTCTCTCTCTCTCTCTCTCTCTC
MSTRG.12365.1	ENSRNOG00000012736;ENSRNOG00000020813;ENSRNOG0000002	0.476	0.000	0.000	-6.418	down	CTGGGCTTGAGGCAAAGTGGGTCTGTGAAGCCCACGACACCCTCCATATGGAAAGCCGCAGGAGCCCAGTGACTGCATCATTCAAGTCTGAAGATTTCCTCCAGGCATCCCTCGGCTCCACCCACAGTGACACAGTCTCTGTGCAGCACGCATTTCCTGGGATGATGACCACAGTGACACAGTCTCTGCCCAGCACGTACTCCCTGGGATGATGACTTGTTTGTTGACTGACTTCTGTGTTCCCATGTGGGTCAATAGTAACTTCTTGGCTTGCTTGCTTTCTCTGGGGCGGAAGTTGTGTCAGGAAAGCCTCAGGCTCCTGTACAAAAATAAAAAGGAAGTCTGTGCCAAGGGGGACCACAGCCCTCACCAACGGACAAAGGGTAGGGCCCTCCCAACTACATGTCATTGATTCTGCTTGGAGACTCAGCTGCACTACCCTGAGACCAGAGCTCATTCCAGACTAGTCTTCTGGATACTGTGGCCAAGCAGCTGTAAATGCTCACAGATGTCACTCTTCCCCTATAAGCTTCTGGACAGACAGACAAATTCCCAAAGAATGAGCAGCAGCAGGATTTCTGGGGATGGCTCATTCCAGCCTGGCACCTCATGCCCCAGGAAAGCGGTGAGCAGCTGCCAGTAGGAGGAAGGGAGAGCGAAGCAGCTGTCCCCATCCGCCCTGAAC

	0881;ENSRNOG00000020892;ENSRNOG00000023318;ENSRNOG00000023668;ENSRNOG00000024239;ENSRNOG00000030616						CCCAACTCACTGAGCCCTTTCAGCTAAGGACTAAGGACTCTAGACCAGCAGGGCTAAGAAGAAGGTGGGGCTGAGAACTGAATACTGAGAAAGGAAACTGATGGAAATTTCCATGACTGTTGTACTCCAAGCCTTCAGCTGTCAAAGAGAGCCTGGCTAGAAGGCCCAGGTCACAGAGGTCACTCTTCCTCAATCCCTCTTCCTTCTTTACCCCACCATTCAGACGAGCCAAACCCTCCCTCAGCTGTGCCTTCTTAGGGCATCTCTATCCAGCTCTGATGTCAGAGTGCTGGCTACAGTGGGGACCTGTCTGGCCTGCTCTCCCCGACCGCAGAGCTCCCCGACAGAGTGTGACACCAACCCCACCAAATGCATTCTGAGTTAACCTGTGTTTTATTTCGTTTATATCACTGCTTGTGAGCTAACAGTTAAAATGCTCCCTGTCTTCCTTGAGTTCAGTCTCAGCTTGATGATGTAATTCTGCACAAGGCTGCTGGGTCAACCACATAATTATTCCTGGTGTGGCCTTGACTTGGTGCTGGGGACTTTCAAAGGTTCCCTTAGGTTTCAGCCGGTTCCCACAGATCTGCCCAGTGCAAGCCAGCACTGTGGCCTGAAGAAATCAGACAGGAATATATCATTATTCCGGTATGACCAGTTCTGTAGGCTGAACGGTGGGCACGGAGCCCCAGAGAGGCCTGGGGACTGCTCTTCCTTCTACTCAGCCCGAATGTTGAATGTCCCCACCTTCCACACGTGTACTTTGACACTTGTCCACCCCATCTTGGCTGTCACAGAAGGTTTACACACAGAGGCCCCAAGAGTCCCCCACACATGGTCCCAGGCTGGTTCCTCCATGTACATCCTATACTCAGGAAGTTCTGCACTCAAGAGTTCCACGTCTCTGACTCAGTTTATTCATCACTGAAATGGGACGGAGCTATGAAGCTGTGATTCTGGCTCAAGAGATGTGCTCCTGTAAGTGGTGGCTGTCCTACTGTCTCCGGCCACCTGCCTACCCACCCTCTTCCAGCACTAAGCAGAGTGGGGCACACTGGATTTTAGCAGGAGGCTGGCTATTGAGTTGCATTCGGCCCTGGATCACCAATAGTTGCCCAATCCCTCCTAGAACCCTAGAGCTCACTGGAGCCAGGCGCACAACGAGAACGGGTTAAACATTTCAAGTTACTTTATCTCTGTGCTGGAACAGGAGGAGGTGGATGAGAGGTAAGAAGAAAATTCAGGCTAGAGGCTGGATGGAATTTTCCCGAGTTCATAAACAGGGTTGCTAAGGGAGGTCACCATGGAAACGGTCACGTGTGCCTGACGTCTGGTTATTTCCAGCTTGGTGCAGGACCATCGCACAGGCTCCTGGCTCTTTCATTGGCTGTCTCCACTCTCACCACCCAGAGCTGACACAGAATTGGTCCAGGGCTGGGACGTACTGGACGCTGTTGTCCTGAGCTGAGGAAGATGTAGGTTCACTGCCAAAGTCATTGCAACAGCAACCGCTTTTCTCTCAGACCCTGGGGAGTGGGGAGCTGCCTTCTTTGTGCTTGTACTTTTCCTACCCAGAAGATGCTGACTAGTCAGGGCCTGCCATGACTTTGAATGCTAACTTTTTTCCTGTTCCACAGTGGCCAAGGAAAATCCTAGCTTTGATTACCCACCTCAGGAGATATTCATTCCAAGACTCTCACCCTTTTAGGCCTGAGGGTCTGGCTGAGCCAAGCTTACAAATCCAAGTTTTCCAGTTTCTAAACTATAGGCTTGCTCTCCAGTGAACAGCACCGGTGTCAGATTGAGGCAGTATGGCCCTGTGAGGTGGAGAGTGGGCATAACTGATGTTGTTAACAAAGCGCAGAGCCCCAGGAACAGGACCCCCTTAGAAGTCTCCCTCTGGCTCCACACCAAATGCTCTTGGGATATCAGCAGCCTCCACAGCTAACACCGAATCCTCCCCCACAGACGTCCTCAGAGGTCCATCCGGCTCTGACATTCAATAATCCTGAACTAAGGCTGGGTGTGACAGTGGACATCTGTAGCACTAACTGCAAGAGTGGGCAGGAGGGTCGGCAAGAGGTGAAACGTGGCCTCTGTCGCAAACAGAAGGTCAGACATGGTGCCGCACACTTTCAATCTCAACACTGGTGAAACAGAGGCAGGTGGAGTGGACCAGCCTGGTCTACAGAGCAAGTTTCAGGACACAGAAGAGAAACCCTGTCTCAAAGAAAAAACAAATCCCATGCAGATGCTCAGTAGGTTGGTAGATTGAACAACTGATTTATTTTGTCTGTTTTTGCAGACAGTGTCCCTCTGTGTAGCCCTGGCTGTTCTGGAACTCACTGTAGATAGACCAGGGTGGCCTGGAACTCAGAGATTCACCTGACTCTGCCTCCTGAGTGCTGGAAATAAAGGATTGAAGGCCCGACATGACTGGTTGATTTTTGAGACAGGGTCTTGTGTAAAAAGCTGGCCTCCTGTTTTCAATTTTGATCTTGCTCTCCCTCCCCCCAAATGATGAGCTGGAAATGTAACCCAGGGTTTCTAAGCAGGGCTTTATTAGTAAGGGACATCTTCAGCCAATGAACCTAACCCAACCCCCTGCCTGATACCCACAAAGAATCAGTACAGAGCACACAAGTCAACAGTCGTTTGTAGCACACCAAGGGCTTCCCACCGGCACTCACACACACCTCTACCAATTGTTCCCAGCC

MSTRG.123680.2	ENSRNOG00000020981;ENSRNOG00000027799;ENSRNOG00000029649;ENSRNOG00000030073;ENSRNOG00000030688;ENSRNOG00000037167;ENSRNOG00000037171;ENSRNOG00000037172;ENSRNOG00000037175;ENSRNOG00000052063	0.616	0.000	0.000	-4.930	down	CAGAAGGAAGACATGAGGCTGCACCACCTGCCTGGCCACTGGGACGCAAGAGAACTCTGGTGACATGGCCACTGTGGACCAAGTGCGGTGCTAGGGGCCACATGCCCGCCGTGGACTTTGGTGAAACAAGCGCTGAACACCTGCTCCTGAACACCTGCTCCTGAACACCTGCTCCTGAACACCTGCTCCTGAACACCTGCTCCTGAACACCTGCTCCTCACTGCCTTGATGGAGGCAGGCTGTAGCGATGGTTCTCAAAGGAGATGGTGTTCTGCTTCCTACGGGGACACACCTAGCTGCCTCAGTGTGACCCCAGAGTTCATGGCTCTCCTTTGGCCTCTGGTCTGGGCTCTGGTAACCCTTTTCCCAGACTTAGCACCATCTCAGGTGAGGAGCCCTGGCTCCGCTTGACCTCAGCCCTTACTCTGCAGTCATCACTCTCGGGGCTGCATGATGGTCTCCACCGCTCTTCCAGTCCAGTTCACGGCAGCCCTTCCCCGAGATCTGGCTTCGTTCAGCTCTTTGGACTGAAATGGTCCCAAACTGCAGAAGGCTCTGTTTGCTCAGTTCTCTTTCCCGCAAGGGAACCTGTTTCCTCCTCCTCGACTGGGATTCAGGCTTAAGCCAGTCTCTCAGTAGGGCCTGTGGCAGAAACACCTCAGGTCCATGCCAAGCTGCCTCAGTTGCCTCAGGCAGGCTTTGCAAAGGCAAGGGGCGTAAACACCAGCAGCCATTCACCTGGCCTAGCTTGCCAGCAGACATACCTGAAACACTTTCTGACCTCTCTGTGGGAGAGACAGCCAGCATATCCAGAGCTTGCTACCACCCTGACTGACAAACATCTCTGGCTGGGGACCGTGAGTAAGTCTGGTTCCAGTCCTGAGCTCTGGGTCACTGTTCCTGGGTATGCCTCCCACCAATTCAGCCTGTGAAGGACTGAAAGAGATGCTGAGAAAAGTACACACTTAATAAGGTTGTGACAGGCCTGTGGGAACCTCAGGGAGGGGCACCTCCAGAGGCCCTCAGAAATCGTGAAGCCATGTATGAAGAGAGCTGGTGATTCTCCTGCACCGTCAAACCTTAGAAAAGCAAAAAAAGGCTTTCTCTGGCTGGGTCCACCAAATATTTTATCACAAATATAGGACCTGAACTCGAGTTCTACCAGAAACCCATGTCCCAAGAAGATCCTTAGAAGGGTTAAACTGACCAAGGCTCCTCCCTCAAAGACAGCCCTAAGTGCCTGTGACACAGTTCAGCTGTGACAAAACCCTGCCACCCACCTTCCTGCCCCCAACACTAACTGAACTTGGGCCTCTGGTCTTTGTGAGTCCACAGGGTTTCCAGAATGCCCTGGTGGGTTCCAGAAGCAGCCATGGCTGTAGCAGACCCCTCGGACACATGACGGGCATGCAGGAAGGAAGCTGACTGACAGCAGAAGAGAGTACCAGTCAGCCATCTGCATTTGGAGGCAGACGGGATGGGCTCACCTACAGTCAGTGCAAGGCCATCTTCTGTAGATGTCTAGCCTGGAACATCTCCTGATTTTCGACATGTGAGCCATGCAAGAGGGCAGTCTGAGGAGTTCATGGCTGTTATCATCAATTCCCCTAACTGCCTAGATCCAGTAGGTATGGCCAAGAAATAAGAGAATCAGCAGGCCAGATGTCACTCCACCCCACTTCCAGGCTTCCCAGAAGGTGGACCTGGAGTCAGGAGGCAAGACTGAGCTGAGAGACGGCTAGTCTCATACAGCTTTGCTGGGCCTTTTCACTCCCCCCTCCTGCCTCATGATGTTTTTTCTCTCTCTCCTTCTCTGGGGGATGGCCACAACTACAGTCTACATATTTATTCAGCATTTTTAATAAACCCATTTCATCTTTATGTGGGTGCATGTGTGCACATGTGAGTGTTGGCATGTGCATATTTTCTCATACAAAGGCAGTAGCT
MSTRG.12370.2	ENSRNOG00000012736;ENSRNOG00000020813;ENSRNO	0.521	0.000	0.001	-4.716	down	CTGGCTGGTACCCCTTTCTCTCCCCGCCTCCTTTCAGCTCCCCATTGGGTGAAGCGCACTTACTGAAGCAGCAGCAGAGGGAATAGGTACGGTCATCTAAGTCCCAACCTGAAACGAACTCATAGCTTGGCCCTTACAAGCCTGGAAATGGGGGAGTCAGAACACATCCTGTAAGATGAGCAAAGCATTAATTCAACATGGTGGTGACCAGACCCTCAGGTGTGACTGCAGCACCCCAGTTGTTGAGCCACATAAGGAACAAGACCCCTTAGGCAAGGTGTGAAACGGCATCCAGGAATGTACTCAACCCAAAGGGTGTCACATCATCTCAGTATGAGTAACCAGGCCTCACCATATCACATGGAGAACCCCAACCAACCAACACCACAACTGGGAAAAAGAGACTTCAGCTCACTGCAGTCAAGACTGTGTGTGTGAGAGACGTGTGTGAGAGAGGTGTGTGTGTGAGACATGTGTGTGAGAGACATGTGTGTGTCATGTGTGTGTGAGAGACGTGTGTGTGTGAGAGAGACGTGTGTGTCGTG

	G00000020881;ENSRNOG00000020892;ENSRNOG00000020904;ENSRNOG00000023318;ENSRNOG00000023668;ENSRNOG00000024239;ENSRNOG00000030616						TGTGTGAGAGGTGTATGTGTTGAGTGAGGTGTGTGTGTGAGAGAGACGTGAGTGTGTGTGAGACATGTGTGTATGTGTGAGAGAGACGTGTGTCATGTGTGTGAGAGGTGTATGTGTTGAGTGAGGTGTGTGTGTGTGTGTGTGTGTGTGTGTGTGTGTGTGATGTGCACGTAGGCTGTAGTGGACAGGACAGCAACTCAGTCACAGATGCAGTGGTGACAGAAAAATACCGTTCAAATCTTTCATTGCTCAAGGATACAACCATGGATTTATTTATTCCCAGAAAATTGAAGGGTTTATCCAATGTGACAAACGCCTCTCGCCTGCCATGAAACATCTGTAGCCTTTAGTGGCTGTGGCTGTGTATGACAGCTATGCCTG
MSTRG.123818.2	ENSRNOG00000008981	0.026	0.379	0.000	3.674	up	AATTAACTATATTTTTCCCTTTTTTAGTACTTAAAATTAATTTTTAGATCAATATATTCTGCCCTGTGGTGAAATACTCCTTTGTTATGCTTTCTCGTATGAAGTCAGACTCTGGGGCCCTGGGGGTATTGTGATGTACCGTTGAAGGTCCACTGTGTGTGCAGCTCTTCAGTCCAAAGTAGGCAAAGTCTACTTCGTGCAGTCAGCCAGGTATGCAGAAGACAACTTCTGGAGTCAGTTCTGTCAACCATGTGGGTCTCAGGGATGGAACTCAGTTGGTCATGATTGGCAGCAAGCACCTTTACCTGCTGACCCACCTCAGCACTCCTGATGGAGCAGATGTAGATGAACAATATCATCTATGAAACATTCCAAACAAAACTAACTTGAATCCATCAAGCCTTCCCATCAAACACGCAATTTTTTTAATTTGTTTTATTATTTTTTGATGTCTGTGTGTTATGTCTGCATGTATGTCTGTGTGCCATGTTTGTTCCCGGTGCCCCCTGAAGTCAGAAGAAGGAATCAGATCCCCTAGAACTGGAGTTTCAGAAAGATGAGCTGCAGGTGGGGGCTGGGAATTGAACCTGTGTCCTCTGGAAGAGCAGCTGGTGCTCTTAACAGCTGAACACCTCTCCAGTGCCAAACACACCATTTATAAGAAATACAACAGGTGAAATAACAAATTCTGTGCCATTCTGGAGGATAACTGGTGTATAAGCTTCAACAATGTATCATCCTGGAAGAAACAATGGCTGTGTGGGGAAAAAAAAAATCTAAGGGACATTACAGCCTGACCCAGATCCTATTCTGAACAGACAAGCTATAAAACACCTTTCAGCACAATTGAAGGAGGAACGAAAGCATGGAATATTTGATAAGATGAAGTTGTGTTGCCATGCAAGCCTTGGAGGTCATTAAGAAAACGGCAAGTCACAAAATGGTGTGTGTGCAGTAGTATCTCGGTTTTAAAAAGTTAAATGTGTGTGTTTACAGACTATGAAACAGGATTTACCCTGAGGTTGTGCTGATTACAATCTTTGGAAGTAAAATCATACATTTTTTTATCTTATATTTATCTTCATGTAATATTTTTGCTTTATACATTACATTTGTTCATTAACAATGAAATTTATTTTATGAAAGTTTATTTTATTTATTCATTTGCATTTACTTTTTTTTTTTTTCGGAGCTGGGGACCGAACCCAGGGCCTTACGCTTCCTAGACTCATGTGATGCTGAAG

MSTRG.124090.2	ENSRNOG00000013136;ENSRNOG00000013634;ENSRNOG00000014168;ENSRNOG00000032908;ENSRNOG00000058371	0.508	0.000	0.000	-5.620	down	GGCCGGGTGGGGTACGCGGATCACGGCCAACTAGGATAAAAGGTCTTGCGGCCTCCCAAGTGGAGCCGGAAGCTCGACCGGTAATGACATTTTTTTCCTAAGGCGGAAGTACTCTGAGCCGCCTCCCGGATCCGGGTGGGAGCGGGTTCTGTCTTTTCTCAGCTTCAGGTCTAAAGAGCCAATTAGCACCTGCCTCGGAGGCCTGGGTTAGTTGGCTCTGCCCAGACGTCGCCTTGACTGTTCAATGATTGTGTCACGCGACTCGGGGGTTACCTCAAGGCCCCGGCCCTAGCTTTTGTCATCCTCACAAGAAAGAAGTTCAGTTTTCTCGGCCTTAGAAGATCGCGCTTTGGTGAAACAAAACATAGAAACATCAACCTGGATAGGGTCGCTGCGGAGCAATCGACTGAGCTCTCCAGTGGGAAGGCCTTGAGCACATCTGGTCGATAGGTTTGTAGGACATCTCATGAAGAGGTGCCATTTGGGCCATACCTCGCAGGACTCAGATGATTTCGACCAACAGAAAATGGGAGGACAGAATAGTCCAGTCCCAGCAAAGGAGCAAGGTCTTGCTGTAGACTTGTTTCAGCTACTGTTAGTGACGCGTTTACTGCGTGGATACTTTTTTCTTACTGGGGAAGAAGAGTAAAAGTAACATCGAAAAGAAGTGGGAAAGGATTGGAATTCTCAAGCTCAGTCCTGGAAGAGCAATGCTTATGGGATCAAAGCACACAGGTGGCTCCCAAGACTCTCAGAAGGCAAGCTCTGCAACAGGCCAAAGAACATGACCTACCACCATCCATCCCTGGACTGAGAAGCAGAAGAGCATGTTAAGGGCTGGAGAGACAGCTCAGCGGTTAGGAGCACTGATTGCTCTTCCGGAGGTCCTGAGTTCAAATCCCAGCAACCACAGGGTGGCTCACAAACATCTGTAATGAGATCTGATGCCCTCTTCTGGTGTGTCTGAAGACAGCTACAGTGTACTTACACACACACACACACACACACACACACACACACATATACACATCGTTTTTTTAAAAACAAAACCTGTTAGGTCAGTGTTTTGTTGATCAACCACATGAACTGATTGTGGAGGAATGTACGTAAACTAGGGACTGCCAGCTTGGTGAATCCAGATCACAAATGTCCACAGTGACAGACACTTACGACATGCAGGGTAGGTGTGAAGGGGATTGTAGGGAAGAAAGAGTCCATGCCAAAGTGGAATCTCAGCATCGAACTGGGCTCTGAGCTAGGGAGGAGTGCCAGGTTCTTCCAACCTTGCATCACAGCAGCTTAATTAGTTCAGGCTAATTGAACGCAAAATGGGAAATACTTGGTTGAGGTGGCTCGTTCCCAGGTGCAGGTTGCAAACAGCACCTGTTAACAGGTTTAGTAGGCCTTGCAACCTGCCTACTTCACTCCTCCCTCGGGACCCTCCTACCTTCAGGAACTCATTCTCTTGCAACCTATATCCATTGTGTCCAAAAACAAACATATAAACCTACTTCAAAGCTGGGCACAGTGACAGATTCTTGTAATCCCAGAATTTAAGACATGGCCACAAGAGAGCAAGGTCACCCTCAGCTATATAAGTGAATGCGTGATCAACCTGGCTTACATGAGACCATTTCAACTAATTGTAAATCTAACTAAACATTATGGTGTTTATTGTGGCCTCATCCCCATGGCCTCTGCTTCTGTTCCTGCCCCCGGATTCCTGTCTTGAGTCCCTGCATTGGCTTGCCTCTATGACAAATAACTCTGTAAGACAAATAAACCATCTCCT
MSTRG.124159.1	--	0.000	0.424	0.019	3.592	up	CTCACCTGTGCTGCATTAAACATCTGGAGACCAAAGGACAGCTCACTGGATTGATTCCTTCTGTTGTGTGGGTTCTCATTTGTAGTTCATGCCACATCTCAGACACACATCTTCCTCTGGAGTGTCTGTGGGTTCAGAGGAGTGTCCTTACGTACATGAAAAGGAGCCCCGAGTGTTGAAGAGAGGCGTCTCAGCAGTGCCGTGCCCTGTCCCATGTGCGGTGACATTTGGGAGATCCTGCAGCCATTAGGAGTGCTTTCCTAACAGTTTGTTCTGGGTCTGAG
MSTRG.124235.1	ENSRNOG00000019404;ENSRNOG00000019428;ENSRNOG0000001	0.459	0.028	0.009	-2.901	down	GGGTTCTAGAGGAGTTTTGAAGATTCAAGAATGACAAACTGCAAACACGTAAAGTGGACAGGACGAGACAAGCCTAGGTATGCTTCCAAGAAGCCCTGGAGCAGCTGCAGCCTGGAGAGAGGCAGTGCACTGTGAGGCACCTGGGGTGCACTGGGCACGACCACTCAGAAGGTTCCTGGCAGTGTCTGCAGATGCCAACGGAGTCAGCTTCATCAGGAGATGAAGCGAGATCCTGCAGGGTGGATCTTGACTGTACAGTGAGCCCAGGAATGCCCACCCAGCCTACCCAGTCATTTGTTGGAACCTGCCTCTTCCACAGGTCTCCCCTCTTTCACGTCTTTGCAAAGATGACCTGTGCAGCGGACTTTCACGTCTTCCCCAAAGTCCTCATGTGGTGAACTCTATAAAACTTTCTCTCACTTCCAAACTACCTCAAGCACAATCCTCCCTGCCACAGACTGCTTATTGGTTGGCTGTTTTGGCCACAGAGTGAAAGGTCTTGGGTTTGGGGTTCAGGTGGGACACTGAGGCAGATTATCCCATCTGGTAAGAGATTTATTAATTTAACACCATGTACGTGGTGGGGCTCCAATAAGAGTTCAGACCCTCAAGGCA

	9472;ENSRNOG00000019481;ENSRNOG00000019492;ENSRNOG00000025643;ENSRNOG00000025676						AAGTGTGGGTTTCTTTTAGGTCTCAGAGGAAGGGGAGTTGTCCTGAAGTCAGGCTGTCCCTGACTCCC<br>CAGTAAGCAGCAGGAGAGGGGATCTTGGGTTGCCAAGAAGGAATGTGTAACTAGAAGGATGTGGGGG<br>CATGAGGAGGAGGGGTTCGAGTTAAGATGTACCCATTATGTGGGTGCAGGCATGTGTCCCGCTAGACGC<br>CCCAAACTCTGGTAATGAGCCTGCAAACTTCCTTGGCGAAATAAAAATGGCCCTGGAATCCAACCGCTT<br>GGGGAGTGGATTCTATTAGGATGTTACAGACATGCTGCCAGTAGAGGGCGCTGGTCATTGCCTTCCTACT<br>GGCTGATGGGCTGGAGAGGTGGCTCAGCTGTTCAGGACACTTGCTGCTCTTCCAGATCATTTGGATTCA<br>GCTCTTAACACCCAGGTCAAGCGGCTAACAACTCCCTGTGGCTTCTGCCCCAGGGGATCTGAAGCTCTT<br>TCCTGGCTTCTGTGGTTACCTGAACACATGTGCACAAGCATATGTATACATATAAAAATTAAAATTCTAAA<br>AACAAACAAACAAACAAACAGGCTTCTGGAGGGGACCTTGGTCTGAGCTGGAGGGTAGTCTGGATTTA<br>AACAGAACATTTCATCTTCAGGAACATTTGGTTCTGTGGCCTTGTTCTGGTTCACATCTCTGGCATGTTT<br>GAATTGAATTGGTGGTTGTAGGCATTCTAGAAGGCTTGAGCCCACGAGGGTGCCACTGAGGGTTGTAAT<br>CAAGTCTACGAAAGCAGCCAGGAAGAGCAGTTGCATTGAAATATACCTGGCGACAGGTTCTTTGAGGC<br>AGGTTCTCACTGTATAGCCTAGGCTGGTCTTGAACTCTCATTTTCCTGCCTCAGCATCCCGAATGCTGAG<br>ACTATAGGCGTGTTTCGCCACGCCTGGCTTAATCGCCACAACTTTAAGAAACCGTGGCACACCGTATGT<br>TGTGTCTATGCGTATTTGTGTGTGGGCATACACCTATGTCCACGGTAAACCTGGTGTTTGCT
MSTRG.124265.1	--	0.280	0.000	0.049	-3.592	down	ATCAGTTCGGTTTTGTGGTGATCTTGCAGCAGCCCCTTGTTTTTTTTTTTGTTTTTGTGCACACTCTGTTT<br>TTTTTTCTTTTTTTGTCTGTTAATCTTTTGACTGTCTATCTGTGTGACTGAATGCTTTTGTCATTTTCAGCA<br>GACCTCTATTTTCTGGACTCTTATTGTTTTCTCACCATCCCACTTGAGATGCTTGTTGGCAGCTGTTACAG<br>GAAATACAGAATCCTACTAGAGGCCAGAAAAAATGTTCCAGACACAGATGGAAGGCACTCACTTCTGC<br>CTGTTGAGTTCAATAAGCCTAAAGGTAGGGAGCACCAGGTCTACCATCAGACTACAATTGTGGGTCTCT<br>GAGGGGCTGAAAAAATGCCCCACCAATTTGGCTAAAGTAAGTAAGAGATGTGAAGAAAAAGTTGCAG<br>AAACTTGAAGGGGTAAGCTATGTCACTGAGAGAGTTAGTGTAAGTTGCAGAGAAAATAAGGTTGATGT<br>GTGTTTCATAGTCTGTGCACATAAGTGGACAGCATCCAGTAGCTATAGTATCCAGTGTTTTGTGGATACA<br>GGGACCCAACACTATGCTCCTGTGCCCCAGTGGGCCAGTGTCCATCAAAGACCTTGGGTACAGGGAAC<br>TACCGGCAAGAAACAATACAGTGGACCTTGGTGTGGGCTGGGTAACCCACTAGTTCATGGTCATCTCTG<br>ACTGCCCCTATCCCTTGCTCATGAG
MSTRG.124535.2	--	0.000	0.266	0.000	7.039	up	GTGTTGTATATGAATGTGTTCGCATTTGTATGTATATGAATGTGTTGTATATGAATGTGTTTGCATCTGTAT<br>GTATATGAATGTGTTGTATATGAATGTGTTCGCATTTGTATGTATATGAATGTGTTGTATATGAATGTGTTCG<br>CATCTGTATGTATATGAATGTGTTGTATATGAATGTGTTCGCATCTGTATGTATATGAATGTGTTCGCATCT<br>GTATGTATATGAGTGTGTTGTATATGAATGTGTTCGCATCTGTATGTATATGAATGTGTTGTATATGAATGT<br>GTTCGCATCTGTATGTATATGAATGTGTTGTATATGAATGTGTTCGCATCTGTATGTATATGAATGTGTTCG<br>CATCTGTATGTATATGAATGTGTTCGCATCTGTATGTATATGAATGTGTTGCTCTCAGAATCCAGAAGAGG<br>GTGTCAGATCCCCTGGAAATGGGGTTGCAGTGGTTGTGAGCCCCCTGCGATGGATGCTGGGAACTAAA<br>CACCTATACTTTGCAAGAGCAATATGCATTCTCAATTACTGAACCGCCACTTCAGCTCCCAAATAGTGTT<br>TGTAAGAATACTTACTTCATGAAAGACATCTTTGGTCTAGAATTCGGGCCTTACTTTTTGCTATGTTATCA<br>CATGAGACAGGAGCCATTGTTTCTGAAGGAGTTGGGAGATGCTGGCTAATGTGTTTAGAATCTCTACAG<br>AAAACAGTAATATAAACCATTGCCTTATGGACAAGAGTTATTTAGACTGTCAGTTCAATGTCAGATAAGT<br>AAATAGTGTAATTTTAGAAAAACAATCCCACACTGATTAAACAGTAAATTCCTTTCAAAGCAAACTATAC<br>GAACAACAACAACACATGATTTGCTACAGGGTAAACACCATAATTCTGAATAAATGCATTCTAGCTTTCA<br>AAATAAATTTTTTTCCATTTCTTCAGAGCCACATTCATGACATCTAACTTATACTGTACATCATCACAACT<br>TTGAGGCCATGAACTCTGGATGGGAATCCATAGACTCAAGTGGCTCCAGGAATATAAACATATATTAACT

							CTTCCAGAAGGCGAATTACTGTCTTAGCTGGATCTAAAAACAACACAGACATCAAGAAAATAGAGCTTT<br>CAGATGATGTGACGGAAGATTTCCATTATTGTGTTCCTAAGGACACGCCCCAGCACCCTATTAAAATTCT<br>GAGGGAGAATTAATCCCTTTTAGGTAAAAGAAAGATAGCATCCCTTGAAGGCGGAGACCATGGTGGCA<br>CTGATGGGGATGGGTTTTATTCAGTGGAGTGTGCTGGTCCCTGCTGGGCTAGTTGACAGAGTCTGTTCT<br>AAGATACAGAAGACACTAGTCTTGATCAAGCAGAAGAAACAAAGTTTACTTTTGAGGGTGGCAGAGCT<br>CAAGCTGCCATGTGGTGAAAAGATTAAATAGAATTGCCAATGAAAGCTGTTGGGGCTGCTTTCTGTCTA<br>GGACTGTCCAAGTTACAGAAGTTCTGTCTTCGACGTAGAACTTAGCACCAGAGCATTGCGAAGTGAAG<br>CAAGACAGTCATCCTGCCCAAAAAAAAAAGCAAACTTTCCTCCTACCCCAACGGCAACCTAAAATCCTT<br>ATGTGTTTTTGCCATTCATACTAGAAAATATCACCCTTTCTCTCCCTGGCTATTACAGAGTCTTCCCAAGC<br>CCCGCTTCATTCCTATCAGATATTTTGAATACTTTGTACACAGGGAGGCTATCTGGATTTGGACTTCTGAT<br>TCAAGGTAGAGAGAATGGATAAAAGGAGCATTACTTGATATGTGTTCTCCACTTGTATATTAGTTTTCTCT<br>TTGTCCCTTTTCCTTGTCCTCTCTTCTTTCTCCCTCTATAAATAAAAACCCAGGAGTCTGAAAATTCAAG<br>CTATTTGGCCCAATCAGTTCACTACCACCATCTCCATAGCCAAATGCGTACACCTAACATTTCCTCTTGCC<br>CTAAGTCCTGTCAAAAAGCAGGGTGGTGAGGAAAACAGTATCTGTGCTCCAAAGTGACTCACCTCTCT<br>CTTCCAGACACCAGCACACCGAAAGACTCCCTTCTTTTCTTTTTGTGCAAAATCATGCATTAAAGTCTC<br>AGTGCACCCGATGCCAGCAGGTGTGTAGTCAATGCCTCTATTACTCACAAATGTCACTTCTAGTCAGCC<br>ACACACCATGGAACAGACTTGTCAGCAGCGGTAATCTACACTCTGACCTTGACTGGAGGAAACTAGTA<br>ACTTACCTCACTTCCCTATTTCCGCCACCTCCAGGATTCCCTAGAATGCTCTGACACCGTTAGCCTACATT<br>TCTAGAGCGCCTTCTTTCCTGTTGTTGTATTTAACCAGCTCCAACGGCTTTGACAGATGGCTTAAATAAT<br>CTTTTAGATGTAAAACTTTAGTCCTCAGATAATTGGCTTCATGTTAGGATCCCACATGACTGGATTTGATA<br>GTGGATACAAAACCTAAGCTTACTCGAGTATTTCTGCATGGCAGAGAATACTGATCCGGAAGGCAGGCC<br>AGAGGAAAGGGCAATCGCTGTTCTCTATCTAACTCTTCCTCTGCAGCAGCATGGAGAAACAACCCACTG<br>TCAACACTGTTACTATTTAAGAAGCCAGGGTGGGGATTCTGTGTCAGGGATGCGAGATGCAATAGAAGG<br>ATGTACAAGTTGGAGTTGTTCAGAACTGAACAAGGACCTGTGTGGGCTGAGTGCAGGGAGAGCAGGG<br>ACCTTACTGGTTTTTAAGTAGGTCAAGCTTCTGTACAAGTATTGCCACACAGCATACACTCCAAATTTTG<br>TTTTGGTGGCTAAGTAGTTTGCCTGGGAGTAAACTGCATGGAGAATTTCTTAACATGTGCTTCTAATAGT<br>TTTAAAAAACTAAATTTAAATAATCTTTCCTACTGATTTAAACTTATAAGGACAGAAATAATTACCACCAA<br>GCTTTGAGTAGGTAAGAATATTTCATATGCTCTGAATGTATTACCACAGTCCCCCAACATTTAAAGCTGG<br>GAGATTTTGAAATATGTATTTATTATTTAAGAAGTCCATTTGTGGGGCTGATTTTTATTTTCCCCTGTAATA<br>GCAGACACTTTCATATACTATACTGTGGCTTTTGGTGCAAACAGATTTCTTTTTTATTTTGTTTTGTTTTGA<br>TATGTTTATGGCATGTGTTTTGGAAACAGAAATGCAAGTCAAAATACCTCTTTAACAATGCTTTTATTTCC<br>TGTAAAATCGCTCTCCTTATGTATGCTTTGTCTTCCTGAGTCCTGGCTACTACTAAAACCATGAATTCCGA<br>AACTTTACTTGGTAGGCAAAGGAAAAAAAAACATGACTTTCATTTTTATGTGTTAATGAATAGATGGAAAT<br>GCATCTGGTGACGTAAACTTCTTAGTGCAAACTTAGTGCAAAGAGTTCTTGACCTGTCTCTGATTTTGCC<br>AAATGTGTATATTTTCCTGATAGACTAATTTTCTTTTCTTTTTTAAAAAAGATTCTATTTATTTATTATATATA<br>AGTACACTGCAACTATCTTCAGCTACCAGAAGAGGGCATCAGATCTCATTACAGACGGTTGTGAGCCAC<br>CGTGTGGTTGCTGGGATTTGAACCCAGGACCTCTGGAAGAGCAGCCAGTGCTCTAACCACTGAGCCAT<br>CTCTCCAGCCCTAGAACAATTTTCTTAAATCCAGAGGTACTAATTGCATCAAAAGTCTCTGCCCAGCTCA<br>ACCCCCCTTTTACAGGGTCTGATTCAGGGAATTAAATGATCTTTCCCCAAATTCTTTGTCATGTTGTATGT<br>TTGTCATGAGATTTAGATAAGTTCAACCAAACTCCACAAGTAGACAAGATGATAAAGATGATTGACATC<br>ATTTTACCCCTGCATTTGTTGCTTTACAAAATGTCCCAAACAAATGACAGTCTTCTGTTTGTCAGCTAAG

							GTGATAGTTATACTTGGCACTTACAGACATTTCTTCCAGACAAGGGAACATTCTCCTGTAAGGAGCCTTGAACATCTAGTAGGTAAGACTGTGTCATAAGCTCTGAATGTCTCATCAGAGTCTCCCAGCTAGAGAGTCAGTAGCTAGAGACATGAAAGTTGGCCTGTCCCAGGTCACACAGGAATACTACCTACCAAGGGCTTCTCTTTTATAGTTACAGATGGTGCCTGTAACATAGCAGAAGCACCATGAGTCAGGCATTGCCAGCACCGAATAGCCAGTGGCCATTTTCAAAATGGCTACTTCAATGACTTCATTAAATTTCTGCAAACACTGGCCTTTGCATATAAGCTCATCAGTCCAGCCATATGAGGGACATTCATTATTTATCAGTACTCTTATTTTTTCTAATGGCTGAGCATGATTAGTGTCTGGATTGCCATTTGCATGAGAGATCCTTATGTTACTAGCAAACATTAAGCACAGCTACCTCCTTTCAGGCTTTCAGTTGTATACAACATGGACACCAACCTGAACCCAGGGAGAAGCCACCCTGGGGCATTCAGTCTATAGCAGCAGAGGAGTCCTGGGACTTCACTTCTAGCAAGCAAACTCCTGAATTACCCATTTACACTTCAACTTTGCAGTCAGAATGGTAGGTTTCTCCCACCCCTATCTGAGACTTTTAAAATAAGTGCCAAAAGTGTACACTGTTGTGGATTGCCTTCTTGCTGTTTGTATGTTAATGCTAATTCTGCCCACCCTCACCCCCACCCCCTGCCTCCATAAGGAGCTGATCACACACTCAGGTGATTCCACATGAACCTGCTCCCCATTTTAACTGGTCAAATAAAGGCTAGAGCCTATGATTAGGGAGTGGAAGGGAAAGGTGGGGTTGGATGTTTTAGAGAAGGAGAGAGAGGAAGGAGGACGATGAGATGGAGGGATGGAGAGAGAGAGGGGAAGATGAAGGAAGGGGAGGTAGAAGATAGAGCAGAATCGCGTGGCCTGGATGAGCAGGAAGTAGCAAGGTATCTCATGGCTGGAGAATAGAGTAGTAGTGACTGCCCAATCTGGATGTGCAGTTTATAAATATAACTGAGTTTTGTGTTCTTTGCATGAGCATATTAGGGTTGGAGATTTACTGATAAAAATCTGACTGGTATTAAATATTAAGACTGTCTGGTGTTTTCCATTTCCAATGATTTAAGTAGACAAGAGAGAGGGTGAGACCGCAGATATGTGGCTGGTCTGCAGACCAGCAAGAAACCAGGGTGAGATGGCCCCTTTCCAGGATCAGGTCAGGAAGATCGGGCGGCTGCTGGTCCTAGATCCTAGCCGGAGCTAGCTTGGATTTTTAAAATTTACATGCAACAGTACACTACCTGAGTGTGATGGTACATGCCTATATCCCCAGGTACTCTGAAAGCTGAGGCAGGAGGATCATGAGTTTGAGGCTAGCCTCAGTGTCTTAGCAAAATGTATCTTAAAGTAAAAATTAGAAATCTTGGATTTTATCTCACTGACTGCACAGGTCCTGACGACATGTGGAAAGCCTTGGGTCCCATCCTCAGTACTGAA
MSTRG.124787.2	ENSRNOG00000031939;ENSRNOG00000045945	0.151	2.046	0.000	4.049	up	TACTCATTTTGCAGAACAGACTTATACCCTGTGGTTGGCCTTGGAACATAATAGACAAGCAGTTACTGATGGTAATAATCTTTTCAAGTGGAATGTAAGGAGGTCTCTTCTATTAGAGTGGGGTATGAAGGACAAGACCATCCAATACCGAGTATAAAATACATCACAGTCTTTGTCTCAAAGTCTTTCCAATGTTGTAAACTGATCTCCATGAAACCCTTCTGGATGACCCATCAGGACAAGTACAAATTTACACGTGCACAGTCTTTCTGGATGACCAGCAGCTCTTGATTGATGTGCATCAGCACTTCTCTGGGTCCCTCCAAAACTGTACACTTGCTGGAGAGAGGCGAGGTGAAAGTACGGCCTATTAAATCCAAAGAAGTTAAACTAAAGGAGGAAAATGCAGTCCACAATTGCTATGGACATTACAGATGAGATGCAATAGTGGTGTATTTGCTGTGCACACTTTGTTCTCCTCCTGTACCACTTCTCACCAAGGGAGATCTTGTCC
MSTRG.124970.2	--	0.007	0.222	0.011	3.297	up	CATTGCATCACACATGCCCAGCATTGAGGACAACATTTGTAAACTAAAGTAGCTCAGGATCATGGTCCTAGCTCCCTCTGGATGCTTTGCTGTACCCCTCCAAGGAGAAGAACATTTTCCTAGTCCTGGAATCTCTTCCTCCAACAGAGGTAATACATTATTCATGTTTGCAGGCCTCTTCTGAAACATATGTATATTCAGGTAGGGAAGAGAGTAGTTCCTGGGAAGGGATTAGTCCCAGGCAGGCCAGCATGGCTGTTCTGTTTTGTGATACAGTTTTTCTTTCTAGGAGACAAATTCTATCTGTGAGCATTCATAAACGACCTCAGTGTGGAGGGTAAATTAACTGTTTAGATTCCCACCAGGCACTGTGGCAAACGCCAGGATATTGATTTATGTCTCATTAGAAGGCATTTATTTAGCTCCACGCTTGCATGATTTGTATTTTCCTTCTCTTTGAAGCTTTGAAGCTGTCCACTTCCAAACTCAAGAGGTTTCCCAGCTGCAATACCCTGCTGTTGGATGGGCTGGGGCTCCAGGGTCTCTTCTTACTGATTGGTTTCAGTTTTTATAATTACCTTACTGTGTGGGAGTTGGGGGTTGGACACAGCTGTCGGGGAGGTGTCTGGGTCGGAATCCAGTTTGTCAGAGTTTGTGCTCTCCCCCTACCACGTGGATTCTGGGTATAGCTCTAAGC

							TCTTGTAGGTAAGTGGCTTTCCCTGCTAAGTCATTTTTTCAGCCTGGAGTTTTGTCTTCTGTGGTGTGTTTTATTTGCAACCCTAATTTCTATAAGCACCAACTGAGGGCAAGAGTTGAAGCTCTGCTGGTAG
MSTRG.124974.1	--	0.965	0.000	0.028	-3.770	down	AGAGAGAGAGAGAGAGATTGATTCAGGGGGAAAGGCTGTAGCGCATTTGTTAGTCTCAGTAGACTGAGCAGCTGTGAACTTTCCCTTCACTAAGGCCATCCCTAACTCAAGCATCAGGAACTGTTCCCTACAGCTTTTACTCTGGACTGAGCTCACCCTCCCTGTTGCCTGCCCTGATCCTCAGCTTCCACCTGGTTCCCAGCAGCTACACAAACAGACCAGTTCTTTTAGTCTGAGGCAGGG
MSTRG.124975.1	--	0.430	0.000	0.003	-4.375	down	GAGAGATTGATTCAGGGGGAAAGGCTGTAGCGCATTTGTTAGTCTCAGTAGACTGAGCAGCTGTGAACTTTCCCTTCACTAAGGCCATCCCTAACTCAAGCATCAGGAACTGTTCCCTACAGCTTTTACTCTGGACTGAGCTCACCCTCCCTGTTGCCTGCCCTGATCCTCAGCTTCCACCTGGTTCCCAGCAGCTACACAAACAGACCAGTTCTTTTAGTCTGAGGCAGGGATTCTGTTGCAGCTTTCAACCTGGAGTAGCAAGCGCTGCTGAGGTGCTGGGGTCCTCGCAGCTGGGAGAACTGAAGTAGAGGAGGAAGGTGTGGCCAGGTCGAAAGCTCTGGGCCAGGGTGTGGTGGTCTCACAGGTGGCCATTTCTGAGCACCTTCTCCACTGCTGTTTCTCACCCCCTGGCTTCTGATACTCGCAGGCTGCTTCTGACATTCCCATAGTGACGCAGGATGGGGAGGGTACTGTGGGCAACAAGTAGACACACCACCCAATCCGGATCAGGCGACCCAAGGGTGTCTGTTAGCCCCGCCTCCGCCCCCCATGTCCCACCCCCAGACTGCGGCCAAGCTGCTCAAGTGGTTCTAGCTGTTGGTGGGGTCCTGCAGCTGTGATAGGCTCCTCCTCTCCGGCCCTTCCTCCCCGCACACCTCTCAGGCCCTCCTGGGATAGCTGTCCTCCTCTGGGGCCCTGTACTTGCTCCCAGCCATGGTGGCTAGCTCACTGCTCTCCTCCTTTACCTCGTTTTCTCCTTGGCCATCCTGGTCCAGACAGGTAAGGTGAGGGCTGGGGCACCCGGGGACCCTGCCCTGGTGCCTGGGGGTCGTTCTGTGCATGAGCGACTTTGTCTCTACCTGAC
MSTRG.125022.1	ENSRNOG00000047003;ENSRNOG00000049939	0.000	0.774	0.000	4.841	up	AGCCAAGAAAGAAGTGTGACTTGCTTTTTGTGATTTACCAAATCAAGACTCAGTATCATTACTATAGAAGCCCATGGAAAAAATTATGAAGTTTTCAGAAATCACCAGTTGAAGAAATGGTGGTTTTGCTTATATTGAATTCTGAGTTTCAAAGTTATGTTTCATTTTTCTTTATTCCTTAATGAGGCTTTTTATTAAGTTTTTTATTCACCATTACACACTTAGATGCACAGTGCTAGGATCGTAGTAGGTGCTCATTAAATCACTGACCATTGGGGAGGAGGAATGATAAGAAAGTATGTATTTTCTGTAAATTCTCTTAGTTTTGGGAACAGGTGGTTTTAGGTTTACCATCATAGCTTGGTATCTTGTCCCACATAAGACAACTTGACAGAAC
MSTRG.125026.1	ENSRNOG00000047003	0.066	0.612	0.035	2.769	up	AGGTACTCAGCACCCCTCGTTGACAGTATGTACTCATTGATAAGTGGATATTGACCAAAACCTCAAATTACCCAAAATGCAATCAACAGATCTCAGGAAACTCAAGAAGAAGGATGACCAAAGTGCAGATGCTCCTACTCCTTCTTAAAATGGGGAACAAAAATATCCATAGGAGGGGATATGGAGGCCAAATCTAAAGCAGAGACCGAAGGAACAGCCATTCAGAGTCTGCCCCACATGTGGCCCATATATATACAGTCACCAAAACTAGGTAAGACTGATGAAGCTAAAAAGTGCTTAC
MSTRG.125160.1	ENSRNOG00000046601	0.000	0.660	0.007	3.835	up	GGCTGATTGGCAGATCGATATCAGCTGATGTGGAGTTCTCCTAAGACATGCTCTCATGGTGAGGCGAGACTGCCATTCCCCTGCCATGGTGCCGCTGGAGCTGCGGCGGCAGGCCAAGCTCCTGAAGGCTCCATTCTATCTGTGGATCAGCAGGTGCTCCATGGGAACACCAGACTCTGTTCAAACTGAGGTGACCAGCCACACCTCAGAACCAGAACCTGAA
MSTRG.12529.2	ENSRNOG00000021202;ENSRNOG000	0.483	0.000	0.000	-6.617	down	GAGCTACTCCATGCAACTTTTTGTGTGGGTCCCTGGGTTTCATTTGAACTCAAGTCATTAGTCTTTTGAAGCAAGTGCTTTTGCCAGCTGAGCCACCCCCCTGCTCTCTGTCACTGCCCATTTGTGGTGCTTGGGAATTAAACCCAGGGTCTGCTGAATGTTAGGCAAGTGTTCTACCACTAAGCTATCTATCTCGATCCCAGAATAAGTATTTATTTTTGTGTTCCAAAATGAGATCCAAACTAGGGCTTTGAGCAAGGGTAGGCAAGTGTTTTACCACTGAGTTCTGTCAAATCTAGGTGGGTTTCTTAAAAGATGATTTCAAGGCAGGTAGTAGTTTATTATTTTGAAGTTGCTTAGCCATTAGTCCTATAAGAAGGTGCTGAGAGGCCAAGTGCTGTCACATGTCTCATCAACA

	00021203; ENSRNO G0000003 2042						GAGAATGATACTTAGGTCTACCTCACAGGCCTGAGTCTATGGAACACAGGAATGTGTCCAATGCAGCAT TACTGCCTCTCATGGTGCCCAAAACATAACCATTATCCTGTTTTGGGAAAGCTTTGGAATCTGAGTCAGA GTGGATGTCATTTTGAACAATTTGGCTATTTTTATGCTTCATAACATAGTACCTTGAGTATTTATGACTTTG CTTTGCTTCTCCAGAATGAATATAATCACTAGTTCCAGGCTAACCATAAAGCAGTCATCAGGGGCAAGC ACATTTAGTCCAAAACTCTGTCGGCAAGAGAATTAGTTTGTAACTGATGAAACAGATTCAGAGAGATTG CTTAACTTACCTGAGTTTGTGCAGCTTAGTGAACATTAGTGCCAAAATGTAATGCCAGTCAATCAGGCTG CAGATCCAATGGCCTTAAATACTCCAAAGTGTCTACATGTAACCTATCACCTTGTTACACACACACACAC ACACACACACACACACACACACACACACTCATGTGCCTCAGTCCTAAGTGAGAAGACTGAAGGCAGG CTATAATGAGAATCGATATTTTGAACTTTGACTGTTTTTTATTTCTCTTTCAGACAAGGTCTCTCTATGTAG TTCTGGCTGTCCTGGAACTTAATGTGAAGACAAGGCTGGCCTTGAACTCACAGGGATCTGACTGCTTCT GCCTCCCAAGCGAGTGCCGGAATGATAGGTGGGTGCCACTAGGTCAGCAACATTTCATTTTTTCTTCCC ATTATGATGTGAATTCCAAATGGAGAACTTTGTTTTGAATACAATAACCTTAGCATCAGTGCCTGAAACA GGCAGTAAGTAGAAACTACAAAGGTTGTATTAATGAATGAGGGAGGTGTATGTTACACTAACTGCTCGA GAACTTTCTGTTTCTTCTAATATTACATACATAGTTTTTGCTACAGAACACAATACCCTGTTTGATTTTAA GAGCTTCCAATAAAAATGCACTGAAATATATCCTGACAGGAAGTGAACACTAAGTGTTAGTATTGGATCT GTGCAAATAGGACAGTACTGAGGCCAATACAAGTATATTGGCCTTTCTCACACTTGGAAGAAGATTTTG ACTTTTGAAACAACATGGTACAAATTATTGGTAGATTTTAGGATTGGCTTACAAAACAATCCTAGATACA ATCACAGCTTACAAAACAACTTTTGCCAAGGAAAAGCATGTTTGATGGTGTTTTCCCATCAGGATAGCT GAGGATACACAGAGAAACCTTGTTTCAAAAAAACCAATAAATAAACAAATAAATGATTTTTTAAAAAAA AGTAAAAAAAGAAAGAAATGTAAAACGCAAATCTGATGGTAAGAATATACTGGGAGAAAAAGTGTAGG AGCCAGCAAGATGGCTCTGAAGCTAAAAGCACTTGCTCTGTGTGCCTGACAACCCAACTCACATTTTAA AAAAATCTATTATATATACTACCAAAAATATATATGCAATATATAGATATAGTGGGGGCTTTCTCAGCATTC CTGCAGCAAGATGGGAGGTAGAGATAGGGCAGTCTTCTGGAAGCTTTTGTACACACTGAGTGCCAGAC ACAAAAGATCCTGCCTTACCAGTGGAAGGGGAAGCCCTGGGTCCTGCTAAGACTGAACCCCCAGTGAA CTAGACTGTTGGGGGGAGGGCGGCAATGGGGGGATGGTTGGGAGGGGAACACCCATAAGGAAGGGGA GGGGGGAGGGGGATGTTTACCTGGAAACCAAAGAATTATTATTACGTATTAAATAAATTAAAAAAAAAA AAAGAGATCCTGCCTTAAAAACAAGGTGGAGGGGCTAGGAAAGTGGCTTAGCAGTTAAGAGAGCTTG CTGCTCTTGCAGAGGTCCCAAGTTCAGTTCCCAGCATCCACGTGGGGGGGCTCACGAATACATAAAGCT CCAGCTCCAAGAGATCCCATGCCTCTGGCCTCCATGGGGAACTCGCCCTCATGTGCATATACTCACGGAT TTATAACAGTTTAAAGTAAATCTTAGAAAGGTAGGAGAATCAACTGAAAAGTTGCCCTCTGACTTCTAC GTCTGTACTGTGCGGTGTGTGCGCGCGCACACACACACACACACACACACACACACACACACACACA CCCACCCACCTCTACCCCCCAGCCCCCAATTAAAAAGAAAAATACAGAGGATAGACGGGGTTGGGAAC AGGTGGTAGGGAAGAGAAATGACTAAAAATTTAAAACTACCCACACATCTTAGGGTTTAATTCCCTAAA TGGAATTTAAAATGCCCCCAAATTGGGGACATTTTGGGGGTGAGGAAGAAGCCCAGTGCAATGGAAAC CCCCATGTAACTTATGAGAGTAATGTTAGCAAAGACTCCTAGTAATGGGGGACACAGAAGTTGAATTGG CCATCTTCTGTAATTAGTCAAGGCCTCAAGTCGAGTGACGGGGACACCAACCCAGCCACAAAATCTTCC ACTTATAGTTTGTTCTGCCTGCACAGTGTTCTGGACCAGAACCTAAAAAAATCATCAGAGACACCAGAG AGACTTCATCCAGTAACTGATGGGGAACAGATGCAGAGTCCTACAGCCAAACCGGTTGTAAGTGGAGC TCTGGGGGTCCTGTAGCAGGGATGATTCACAAAGAACCCACACCACTGAGGAGTCCTTGGGTCACTTAT GGGAAGCTTCAATAATGGAAGAGTCCTGGTCAACTTGCTCCTGAATATGGACCTTTTCCTCATGAGGGG ATATTAATAGGCCTAGTTCCATGAACCTCTCCTGTGGCTGATTCAAGACAGCAACGGTCACGTTCGTCTT

							AGATCTAATCTGGTTTAAATCTAAAAGCTCATTACCTCAGGCTTCATTGCTCAGGAAGTAGATAGCTGAAAGGCTTGGCAGGGTCTCCATCTGTAGGATTTGTGAGAAAACTATTCAGTCACGGCAAATTGTATTGTCACAATATGTCAAGTATGAATAAAAGCATTTCCCAACAAACTTCCTTTTGAAAAGTAATTTTGAAATAAGGTCCTATGAGCTTAAAGCTCACAAAAGGTGGGTCATGGCTTAGAATAGAGAAACTCTAGCAGAGGTCTCACTAAAAGAAAACAAAACAAAAAAACCCAACAACAACCCTCCCTCCCCCCTTTTGAGAAAGAGACTCGCTATGTAGTTCAGGAAGGTATCGAACACCTGATCTCCTCCCTTGGTCTCAAGTACTGAGATTACCAGCGTGCCACCGGAGTCCGATTTAGTAAGGTCTTTCTTTAGCTTTTAGTAAGACTTGGTCAGTTCAGAGATTAGGGATGATGATGATAATGAAGTCACCATGGTCTGTCCTTGTGAAGTCAAAGTCATCAAAGCGTTCACGTCATCACGTCATCACGTCATTCTTTCAGACC
MSTRG.12529.3	ENSRNOG00000021202;ENSRNOG00000032042	0.272	0.753	0.030	2.146	up	CGGGGTTGGGAACAGGTGGTAGGGAAGAGAAATGACTAAAAATTTAAAACTACCCACACATCTTAGGGTTTAATTCCCTAAATGGAATTTAAAATGCCCCCAAATTGGGGACATTTTGGGGGTGAGGAAGAAGCCCAGTGCAATGGAAACCCCCATGTAACTTATGAGAGTAATGTTAGCAAAGACTCCTAGTAATGGGGGACACAGAAGTTGAATTGGCCATCTTCTGTAATTAGTCAAGGCCTCAAGTCGAGTGACGGGGACACCAACCCAGCCACAAAATCTTCCACTTATAGTTTGTTCTGCCTGCACAGTGTTCTGGACCAGAACCTAAAAAAATCATCAGAGACACCAGAGAGACTTCATCCAGTAACTGATGGGGAACAGATGCAGAGTCCTACAGCCAAACCGGTTGTAAGTGGAGCTCTGGGGGTCCTGTAGCAGGGATGATTCACAAAGAACCCACACCACTGAGGAGTCCTTGGGTCACTTATGGGAAGCTTCAATAATGGAAGAGTCCTGGTCAACTTGCTCCTGAATATGGACCTTTTCCTCATGAGGGGATATTAATAGGCCTAGTTCCATGAACCTCTCCTGTGGCTGATTCAAGACAGCAACGGTCACGTTCGTCTTAGATCTAATCTGGTTTAAATCTAAAAGCTCATTACCTCAGGCTTCATTGCTCAGGAAGTAGATAGCTGAAAGGCTTGGCAGGGTCTGATCTCCTCCCTTGGTCTCAAGTACTGAGATTACCAGCGTGCCACCGGAGTCCGATTTAGTAAGGTCTTTCTTTAGCTTTTAGTAAGACTTGGTCAGTTCAGAGATTAGGGATGATGATGATAATGAAGTCACCATGGTCTGTCCTTGTGAAGTCAAAGTCATCAAAGCGTTCACGTCATCACGTCATCACGTCATTCTTTCAGACCACCACCTGTAGTCCTGGCCAGCGAGGAACACCAGAGCCCACCACACGGG
MSTRG.12529.4	ENSRNOG00000021202;ENSRNOG00000032042	0.126	0.393	0.027	2.233	up	CGGGGTTGGGAACAGGTGGTAGGGAAGAGAAATGACTAAAAATTTAAAACTACCCACACATCTTAGGGTTTAATTCCCTAAATGGAATTTAAAATGCCCCCAAATTGGGGACATTTTGGGGGTGAGGAAGAAGCCCAGTGCAATGGAAACCCCCATGTAACTTATGAGAGTAATGTTAGCAAAGACTCCTAGTAATGGGGGACACAGAAGTTGAATTGGCCATCTTCTGTAATTAGTCAAGGCCTCAAGTCGAGTGACGGGGACACCAACCCAGCCACAAAATCTTCCACTTATAGTTTGTTCTGCCTGCACAGTGTTCTGGACCAGAACTGGGGCTGGAAGAGATCAGAGAGCCTGTCAGGATGTGACCCAGGTCCTGTACATATATGTTCTGGCTGAATAGCTTGATGTCCTGTGGCAGCTGGGGCTGTCATTGACTCTTCTGCCTGTTTGTGGGAACCCTTTCCTCCTACTGGGTTGCCTTGATCCAGCCTTGATGTGATGGTATGTGCCTGGTCTTATGCCATTTGAATTGATGTCCTTGGGAAGCCTGTTCTTTTCTGTAGCAGGGATGATTCACAAAGAACCCACACCACTGAGGAGTCCTTGGGTCACTTATGGGAAGCTTCAATAATGGAAGAGTCCTGGTCAACTTGCTCCTGAATATGGACCTTTTCCTCATGAGGGGATATTAATAGGCCTAGTTCCATGAACCTCTCCTGTGGCTGATTCAAGACAGCAACGGTCACGTTCGTCTTAGATCTAATCTGGTTTAAATCTAAAAGCTCATTACCTCAGGCTTCATTGCTCAGGAAGTAGATAGCTGAAAGGCTTGGCAGGGTCTAGAGGGAAGAAAGTGAACACTTGCTGAATGAATGAAATAATGGAGAGGGCAAATGGTGGTCACGGGGGCGATGCTACCTACCTCCATCTGTAGGATTTGTGAGAAAACTATTCAGTCACGGCAAATTGTATTGTCACAATATGTCAAGTATGAATAAAAGCATTTCCCAACAAACTTCCTTTTGAAAAGTAATTTTGAAATAAGGTCCTATGAGCTTAAAGCTCACAAAAGGTGGGTCATGGCTTAGAATAGAGAAACTCTAGCAGAGGTCTCACTAAAAGAAAACAAAACAAAAAAACCCAACAACAACCCTCCCTCCCCCCTTTTGAGA

							AAGAGACTCGCTATGTAGTTCAGGAAGGTATCGAACACCTGATCTCCTCCCTTGGTCTCAAGTACTGAGATTACCAGCGTGCCACCGGAGTCCGATTTAGTAAGGTCTTTCTTTAGCTTTTAGTAAGACTTGGTCAGTTCAGAGATTAGGGATGATGATGATAATGAAGTCACCATGGTCTGTCCTTGTGAAGTCAAAGTCATCAAAGCGTTCACGTCATCACGTCATCACGTCATTCTTTCAGACCACCACCTGTAGTCCTGGCCAGCGAGGAACACCAGAGCCCACCACACGGG
MSTRG.125297.1	ENSRNOG00000015297;ENSRNOG00000015386;ENSRNOG00000015402;ENSRNOG00000015623;ENSRNOG00000022598	0.065	3.483	0.000	5.491	up	TGCATGGTAGACACAGCTTTAATCCAGCATCTGGGAGGGGGAGGCAGGTAGATCTCTTTGAGTTCCAGAAATAAAAATGAAGGAATGAATGTATAAATAAATAAAAACAAAATAAAATAAAATTAGCGCAAGCTAACAAGAAGTTGGCAGTGCTACACAGGGTTTAAATCTGGTTCTTTTGTTTCGGGTCATACCGAAGTTTGCTAATGTTTGTTCAAATCAGAACACAGGCATAGCCTGGTATAAAGAACAGATACGGTGGTTCTTCCCCAGACCCACACCTGGCTGCCCTGCTGAGGACCCAACAGAGCAAGGGCAGGGGCCCTGGCTCCCCTTGCCGGGGCCTCTCTGTGGGACACACAGGTCTGCTC
MSTRG.125339.1	--	0.662	0.057	0.030	-2.462	down	CATACACAGAAAATAAAAAAATCTTCCAAAGTTGTGGGCTCTGTTTATACGTAAGACCCTACAGGGAGATCTCAGTCAGGAAGCAGAACGAGATGAATTCTGATGCCCCCTCCTTCTCTGTTCTCCCAGCTCAGAAGGAGTCTCATAAAGTCCAAGTTGGCCCTGAACTCATGATGAAACCTGGGGATAATCTTGAACTCTTGATCCTCCTGCCTCCACCGCCAAGTGTCAAGATGGCGTGCCGTCATGCGCAGCTCTCTTTCCTCTCTTCATTCAGCCTCCTGGCCCAGCTCTGGGATGGTACCAGTCACAACCAAGCTGAATCTTCCCTTCTCAGACATACTTCTCAGGAAATGTCTTCACTGGCATTCACACAAGTGTCTCCGACCTGAGTCTACACGGCCTCGAGCTGACCATGGTTAACCACTGGGCATGGAGAGGTGGCTCAGCAGTTAAGAGCAAGGGTTGTTCCAGCAGAGGACCAGAGTTTGGCTTTCAGAACTCACATCACAGCTGCCCAGAACTTCAGCTCTAGAGGACCCAACACCCTCTTCTGGCAGCTACTGGTACTACAATCACACCACACCCACACACACACACACACACACACACACACACACACACACACACACACACACACAAATCTTAAAGATTACCCACCAGATCTAAAATTCCACATTAACACATAGCCCAATCAACACAGTGCCTTCCAAGTGCCCCACTTAGTCCACAAACAAATCTGTTTACTCCTAGCTGTTTATGACTTAGAAAACACAGATGAGACAGGATGCTCTCTGCCCTGCTCCAGATAATCCTCAAATCCCACATGTGTGTTCACTGACCATTTCCTAGAGATGATTTTCCCAAAACCTGTGGGGGTCCAGGGTCACAGCAGCCCTTCTGCTTTTCCTGGGTGTTTCCCACAACAACAGGCATCATGGGTGACCGCAGGTCCCTGCCCCATCAGTCACGAGCTCTGACCATAAGAACCTGAAAGGAGATACCTATTTCAGGATTGAGACCGCGGGTGCCTCCTTCAGGGTCGCCTTGCTCCTGTGTCTTCCTTTGTCACCACCCTCTGGACCCGGAGCCTTGGCTGTTTCGGCAACAAGTGCCTGGGCAATCCGACAAGACGACCAATCTCCACTTGGGGGGTGAGATGGGGGACGAGCCGAGCT

MSTRG.125430.1	ENSRNOG00000018420;ENSRNOG00000018613;ENSRNOG00000018863;ENSRNOG00000018949	0.362	0.000	0.000	-4.918	down	GCTGGCTACTCTTATGTTGACACAAGCTAGAGTCATAAGACGGAACCTCAATTAAGAAAATGTCTCCATATTTTTGAGCTGCATGCAAGCATGTGGAGCATTTTCTTAACTAGTGATTGATGGGGAAGGGCCCAGCCTATGGTGGATGGAGCTGGTGGTCGTGGCTAAGCAGACTAAGCAAGTCATGAGAAGCAAGCCAGTAACACCCTTCCACAGCCTCTACATCAGCTCCTGCCTCCAGGGTCCTGCCCAGACTGAGTTCCTGTCCTGACTTCCTTCAGTGATGGACTACAATGTGGATAGGTAAGCCAAATCCCATCCTTTCCTTTCCAATTTTGCTTTTGGTCATGGTGTTTCAACATAACAGAAATCCTAACTAAGTCACTTGGCTGTTACCTAAAAACCTAATACTTGACAGATTCAATAAAAGAGAAAAAATTTGGGGGACGGGGGTCTTGAGAAATAAAATGAACATGGTGCATTAGAATAGCTGTGAAGGTCTGTGCATATAGAAGGTCTGGTCTATAGATATAAAAGGTCTGTAGATATGGAAGGTATGTAGATGATATGGAAGGTCTCTACATATGGAAGATCCATACATATGGAAGGTGTGTACATATGGAAGGTGTGTACATATGGAAGGTGTGTACATATGGAAGGTGTGTACACATGGAAGGTGTGTACATATGGAAGGTGTGTACACATGGAAGGTGTGTGCACATGGAAGGTGTGTGCACATGGAAGGTCTGTACATATGGAAGGTCTGTACATATGGAAGGTCTGTACATATGGAAGGTGTGTACATATGGAAGGTCTGTACATATGGAAGGCGTGTACATATAGAAGGTCTGTACATATGGAAGGTCTGTACATATGGAAGGCGTGTACATATAGAAGGCGTGTACATATGGAAGGTCTGTACATATAGAAGGTCTGTGAAAAGCTATGTGTACCTGAGTACAGCTGGCCTTGAGGGCCAGAAGTAGACGCTGGATTCCTTGGATCTGGAGTTACAGCTCTGCAGTTGGTTGTGAGTTGTTAGACATGGGTTCTAGAAACTAAACTCCTGTCCTGTGCCAGAGCAGCCAAGTGGTTTTAACCACTAAGTTATGTCTCTGCCTCTGAAAATGATCTGTTCAATTAGCTGAAGGCTAGATCTGCCATCAGGAAACATACTGAATTTCGCTAATGGCTCTACAACCTGATTTTACTGGACAGTAATTATCCATATGAAAAGTAATCACAGCCTAGCAGTATGGTGCACTCTGATCTCTGAGTTTGAGGCAAACCTGGTCTACAGAGTTCCAGGATAGCTAAGGCTACACAGAAAAACCCTGTCCTGAAAAGACAAAGAAACCACAATCATGCAGGTGATAAAAATATAAAAACCAGTCCCACAGCTCCCATGTAGACCCTTATCTCACGTTCTCTAAAACACATGATTCTGGAAACATCTAAACACCTCATAAGGGAAATTCACTTTCTATAACTTAAAAAGAGTTGGTTATCAG
MSTRG.125638.1	ENSRNOG00000061718	0.016	0.394	0.000	4.022	up	GGACCTTGATCTCCACCCTGTGAGAGCAGCCGGCCTCCCGCTTCCCTCCTCCACCAGGCTTCCGGAGTGATCTGAGAGCTGAGAGTATAACTCAGGGGTAGAGCACTTGCTCAGGATGTAAAAGCCCTAGGATCCATCCTCAGTACGAGACACGGGTTCGTTTGTTTCCCTTACTGCCTGTGGCCTGAGACCCAAAAGTAGTTAGCCAACACAAACTGTACTCGATGCAAGGAGGCGAGAGAGGAAGAGAAAGTTGGCTTGCTTATGAAGGTGCCTCCAGTAACTGTGGCAACCGACCCATTCCATTCATCATACAAATCGTTCAAACCATGGAATTAAATCCACTTCCCAGAGAACTGGGGTGGAAAATGAACAAATACTAGTCATGGCTGGCACAAGCTATTAACTTATCCCGTACAGAGTGTCACACTGGCAACAAAGACGACCCTAGCAGGTCCTCAGGTGCTCAGAAGAGGGGAGCAGAAACTAACTGCCCAGCAAAGTGGAGCCCACACCGCACTATTACTCATTAAGCAAATATTTTTCAAAGTGACCCAGGGCTGCAGGAGAGCAACACGCCCATTCCCTATTTATAACGTTTCAACTTGCTGGAGTGTCTTTCCATCTCAGCCGTGGCACCATGGTCCGTCAGAGTCACACCATAGGAAAATGCTCGCCCATGTCTCCGCGCCCTCCCCTTCCAACTCCCACAGGCCATGCTCTCACCGGGAGTTTTGGCAAACCATGCAGGCACCACTGTGAAAGTCAGAAAATAAAACGAGCCGTGCTCGACTCTTGGGTAAAGCCATTCCCTTTAGAGGAAACAAACGTATGGGAAATCCAAGTGAACCATCCGGTACCTAGCCCTTGGCCGAGAGCAAAAACTCTATCAAACCTTATTCTGGAATATACTGTAAAATGGGGCTAAAACTGTGACCTCCGGGACTTGTAAAAATGCATGCTGTAGTTAGTGCTTTAAGAGAAGATATTCAATGCAGTGATAAGTACTCAATAAATGAGGACTGTTTATATAATATATTAATGAGAATATTACATAATACTTTATATAATATATAAATTAAATAATATCCCAATGTGAATGGTTACCAGCCATTTGTTTAGCCCTCCTCTGAATACTCC
MSTR	ENSRNO	0.000	0.250	0.000	5.757	up	TAAGAATTGGTAGCAGTAATTCACTAACTCTGGCCATCTGGCAACATATTTTTTAATCTTTATTTCTGGAATCAGGACTACGTAAAATGAAGAAGCTTCTAACCTCATAGAACCCCCTTTTCCAAACAGCATTACTATATG

G.125 970.17	G0000004 7234;ENS RNOG000 00058463						CAGGACACAGAGGCCTCAAAATGCCTGCAAAGCCTTGGCCCGAAATCCCTAGGAAAGCGAGCACACT GCTTATCACTCAAAAGACTAATGAGATCTGGGTTCTGTTTTGACAATCTCATCCTGTGTTAACATCAATC CTAACTCGTTTTATAGTTAAGGAGGATGAATGAGGGAATGGCAGTCAGTTGGCATAACAAGCCACAAGT AAGAGGAAAATGAGTCAGAATCACTGAACTCTTAGATTTTAAAACAGCAGTTGGGTATAACAGTGGAA ATAACAGTACTATCGTAAGAAGATGAAATTGAATTTTAGGAATATGTTTGACAACTCTTCATCTCTCTCCT CTATCTGTCTGTCTCTGTCTCTGTCTCTTTCTGTGTGTGTGTGTGTGTGTGTGTGTGTGTGTGTGTGTGTG TGTGTGCTTGAAATTACTATAATTTTGATCAAGAGACACCACGGGTTTCTAAGACTCCCTCCTGGTAGGT TCTGCACTTCCTCCAGACAACTTCACAGGCATCCTACAAGGGAAACAGTTACCACTTTTAACTGCTTCA ACTGTTTCACAATGTTTCATAGACATATCCACAAAGGGATCCTGTCTTTGAAACAACAAGCACCTGCAA CTTTTAGTGTGTATGATTCCTTGACACACACAAAAAAAAAAGATCTTACTTTCTTAGCAACCAAATGAC GGCTCTGCATTTTCCCCATGTTTTAATGAATCTTTTCTTTCTATCCAACATATGGAATTATAGGGTTTCCAC CCACTTTTTGTCTTATATGATTAGCCTTCCTGTATATGAACTGTATATATGGTCAGGCTTCTTGTGTATAGA CTGCATATATGATTAGCCCTTTTGTATATAGACTGACCTACGTTACTGGTATCTTTTGAGAAATTTAAAATA ATGGATACACAAACTTGTGAACACGGCAGCTTCAGTGTTAGAAGGGCAGGCACATGAGACTTGTGCCA TTCAATAAAACAGCTGGAAGAATGTGAAAATTCAAATAATGGTGTGTTATAAATAAAATATTCATGGCTT ACCATGGGAAGAGGAATACCAATTATCTTGCTCACAACTGTTACAGACATGTTATAACGGTATCTACTGC TAGGTTGAAGTTTGTCTTGACTTTCTAGCATGGCTACGGAGAAATTTAAAGGCACACACATGGCTTCAA ATGCATTTTGTTTTAAATCAGGGAGCACTTACCTAAACATTTAAAATTTCAGGGTACTGCTACAGTAAGA AAATTTCACTGCCCTCATAATTCCACCCTTGTTTTGTTTAGTATATATTACAAATAAGAATAGTAATGTGAA AAATGTTAAAGTTTATTGATCTTTCTAATTATCTGGGAGGTATATGACAAAAGTAAAACAGATTATTTCAG CTCTGGGTTCAGAACAGTGTACAGTTTTTCTTTTCACTGAAACACTACACTAACACAAGTGATGCAATA TAAATCTATTTCGTTTTTCCGATCATAAATTTTTCACTGATTTATAATTTAAATCTCAAAGGTTTGTAGAAT AATCTTCTGAAGATCTACTGCTTAATTCCAAGAAATAAACACCATGAAGCCTACCAGTTTACTAAGAAG AAAGCTTAAACACTTTCTGAAGAGGGTCACATTAGGACACCCAAATATTTTTCTTCAGGGTTTGCAGAA GTAGTTTATAAATGTCCTTTGAATGACATCTAACTTAAGAAAGACAGCAATTATAATAAACAGGACTCAA AGTTCTGTAATAAGGGAAAAACAATTTCATTTGTTTACAACTAGAAGAAAATATGATACACTATTACTGA CTTATCTAGATTGAAATTAGTGCATATTAATTAATTATATGCTGCTTTACTAAAAATAGCCTGTGTATATGC ATGTTCTAGTTATTTATTTTGGGACATCTCAGGAAATTTTTAATTTTTTTAATAAAATATAATTTAACATAAT TTATGTAATGAGTAGGTTAATTTAAAAGTTTTCATTTTGAGTTTAAGGAAGATTACCGTAAGATTCATAAA TCTTATTTATCGGCACAACAATAGAGAATCAATCTCTCCCCTTCAAATAACTCACTACCCCATGGTCTTAT CAGCATACTGAAGGCTCCTTGAGTCACAGAATCAAGACAACCAAACAGCGACAGCAAAAGAAGCAGC AGCCGGGAGCAGGTACAAGTT
MSTR G.126 191.1	--	0.038	0.526	0.000	3.559	up	CAGGACCAAGGGCTTCCCCTTCCACTGGTGATCTTACTAGAATATTCATTGCTACCTATGAGGTCAGAGT CCAGGGTCAGTCCATGTATAGTCTTTAGGTAGTGGCTTAGTCCCTGGAAGCTCTGTAGGTTTTCCACACG TTGTACTATAAATTCCCTCAGACTTGGTTGAGTTTATCTGCATTGTTTCCATAGCAACCAGAGGGCAGTC TGACACAAACCTGATAAACAGTAACTATGAAGAACAAAAATTATAAAATTGTAATTAGATTTTTTTAATT AAAAAACCCAAAACAACAAAAACAAACAAACAAAACAACATCAGAAGAGTGAGGCAAAACCAAAGT GTTTTTATGGAGCCTAATGCAATATAAAGAAGATGAGAAGGAAGCTGGGGGATGAATGCAGCCTTACTT GCTGTGCTTGCCTGCCCCACTGCTCTCTGCAGGCTGCAGACAGAGTAAAGCACTGCTCATCAAATGTGT TAGACCACAGGGTGTGTTAACCAGTTCTGTAGCGTTATTGACTCCAAAGCTAAAGTCTTAGGTCTCTGA ATTTAAGATGCATACTTCTTATAAAGGGATGAATGTATAACATACGTTTAAAACGTGGGGCTAAGGAGGA

							GGACGCTAAAACGATGAGCTCGATGCCATGGGAGCACCTTGTTAGCTGTTGAAATAAGAATTCACATAGTTCCTCGGTCTCTTAAGACCCACACTGCTTTGGACAGATAACACACAATTTCATTTCATATTGGGAAGTATTTTCTAAATAAAACATCCAATTGTTTAATGATTTTTAGATTTG
MSTRG.126191.2	--	0.644	0.000	0.000	-4.841	down	CAAGGGCTTCCCCTTCCACTGGTGATCTTACTAGAATATTCATTGCTACCTATGAGGTCAGAGTCCAGGGTCAGTCCATGTATAGTCTTTAGGTAGTGGCTTAGTCCCTGGAAGCTCTGTAGGTTTTCCACACGTTGTACTATAAATTCCCTCAGACTTGGTTGAGTTTATCTGCATTGTTTCCATAGCAACCAGAGGGCAGTCTGACACAAACCTGATAAACAGTAACTATGAAGAACAAAAATTATAAAATTGTAATTAGATTTTTTTAATTAAAAAACCCAAAACAACAAAAACAAACAAACAAAACAACATCAGAAGAGTGAGGCAAAACCAAAGTGTTTTTATGGAGCCTAATGCAATATAAAGAAGATGAGAAGGAAGCTGGGGGATGAATGCAGCCTTACTTGCTGTGCTTGCCTGCCCCACTGCTCTCTGCAGGCTGCAGACAGAGTAAAGCACTGCTCATCAAATGTGTTAGACCACAGGGTGTGTTAACCAGTTCTGTAGCGTTATTGACTCCAAAGCTAAAGTCTTAGGTCTCTGAATTTAAGATGCATACTTCTTATAAAGGGATGAATGTATAACATACGTTTAAAACGTGGGGCTAAGGAGGAGGACGCTAAAACGATGAGCTCGATGCCATGGGAGCACCTTGTTAGCTGTTGAAATAAGAATTCACATAGTTCCTCGGTCTCTTAAGACCCACACTGCTTTGGACAGATAACACACAATTTCATTTCATATTGGGAAGTATTTTCTAAATAAAACATCCAATTGTTTAATGATTTTTAGATTTGA
MSTRG.126212.1	--	0.419	0.000	0.037	-3.684	down	GTAAATTTAAATCCTGATGTACAGAAAAATGAGAGCTCTGTTTTAATATTATTTCAATTTAATAAGATTGAAATTATTATTAAGAGATTAAGGGTAGATCAATTAATGTTACTGTTTGTCTATGAGATTAGCACAGAATAAAATGTGTGTATAGTAAGGAACTCTCCCTAAAATTGTCTTATTTCTTCAGAAAATAAAAAGAAACAATCCATAGCTTCTTTTGGCAAACATAGGCATAAGAATAGATAAATTTTTTTTTAATTAGTGAATTGCTAAGAAAGAATTAAAAATAACCGTTGAAAAATACTGTTCTCTCCTATATTCTGCCATTAATTTTGAAACACTTGTGCTCCTGTGTCTATACACCCTTGATAAGAATAGGGTTTTCATTAGCATAGATCATCCTTTTTTTTTGAATGGATAACCTCCAGTAGACTTCCTGTGCTCCAGTAGATGCTCCCCTCACCCTTATACTTATGCTCTTGTTTGCACTAATTTGGCCTATAC
MSTRG.126429.1	ENSRNOG00000045975	0.000	0.380	0.031	3.453	up	GCTCCCTCCTGGAGACAGCCTGAATCGCAGTGTCCTGCTTGCTTGAGTGAGCAGGTTATGGCAGGAACAAAGAACTGCTGGAGTTGGAAGACCCAGTAGCTGAGCTGCCTGGGGAAGAGTCTCCTCCCTGTCAAGTTGCCTGAAAGTCCTTCAGAGAGCTCCAGGATTACAGCATTGGTAAATGGTCACCCATATTGAGTGGGCTTTTTCAGTGATGTGGCTACCTTGGAGTTATCCATGATTCCATAAGTAACCCATAAACTCCCTGGTTCACCC
MSTRG.126480.2	ENSRNOG00000015886;ENSRNOG00000016032;ENSRNOG00000016151;ENSRNOG00000016254;	1.314	0.163	0.031	-2.205	down	GGAACACTCTGGAACCACGGATGCTGGAAACAACACTTTAATTTGTACAAATGAACATCATATTCAAGATGGGCAGAGAGAGCAGGGTGTGGGAAAGAGAGTAGCCAACAGAGAGGGCTCGGGAGAGAAAAGACAACTGGGCCCTCAAGGGAGGGAGCTCATGGGCAGGATGGTCAGGGACACAAGGACCCTGTGCTGGGTAGTGGCAGTAGTCTAGCTTCCCAGCAGGCCAGGCTGACTCTGGCTCCACTCTTCTCCTCCTACAGTTCTCTTCCAGAGCCTTGGGCCACTTGTCTCTTGTCTCTTTAAATGTCTTTCCAAGGGCTGAAGTACTGGCCCTGGGGCTGACAGTGGGCCAGCAAGTCCTGCCAGCGGATGTAAGTCTGGGTAGCCAGGTCCTTGAGTTTGGTCCGGTGCATGGCCTGGGGGCTCTCCCGGATATGGGTGCTGATGTGGCGGTCCAACTGCATGCACAACAGCTGCAGCTCACCCCACTGATCGGGAGTAATGTCCTGGCTACTGACCACGACCCCAGCCAGTGTCAGGAGGCTGTGGCACAGATAGCAGACCTTGTGGTCTAGCTCGGCACTGGCCTTTGACTGTTGCCCTGGACTGGATGGCAATGTTTCAGAGCCCAGACACTGCCTGAGGGATGATGGCCTCATGAGGCTGAGGAGCTGGCTGAGCAAGGCTAGCTGGGTCTTCTCTCCCCAGAGAGGAAGTGACTCCTGCTGACCCAGCATCCGGGCAATAACCATGAGACTCAGCTGACTTCTAAGCTGCTCAGAACTGAGCCATCTTCTCCTCGGGAATGAAGGCCCCAGTGGCCTTCAGATCCTTGGGTCCTTAAGCACTTGGGGAAGTAGAGACGAAGGAAGAAGAGACCG

	ENSRNOG00000016660;ENSRNOG00000023941;ENSRNOG00000023995						GAGGATGGGGCTGGGAGTTGTCCTGGGATCCTCGAGCCTAGGTGACAGGGCAAACTCCCAAGCAAATACTCTGTATTTAATCACCCAACCTTGCTAGGGAGATTGGACAGTGGCCTTCCAGTATCTCCGGACACTGGTAACCCGGCAGTCGGCCCTGTGCATGGATTATTTATTCTGATAGGGATGATACGGGGGGGGGGGCTTACATATGCCGGGCAGGTGCCAAGCCACAGAATTTCATTCCTATTGTCTTCTGGAGTGACTTCTGTATGGCCACCACACAGTGCTAGGTAACCCACTCAGAACGGAAGCCGGCTGGGTTTCAGATGCTACTGTCTTGAGCCATGCCAGGGAGTAAGACGAGCAGAGGAAGGAGAGCAAAGAAAGGCCTGCATTAAAGCCTGCTGGGAAAGGCACATTAGAGACTGGATGCTGAGAAAAATGCTCGACGGTAACAGCAGTGCTGGAGAGAACCTCCGACAGGTCCCTGAAAAGACACCGCAGGGACCAGATGCAAGGCCCATGGCTCTGGGCAGGCTCAAGTTTCACATGTGCTTGACTACCGAGTCACATGCTCATTGGTCTGTCTGTCCATCTGTACACTGTGGTTCAATAAAGCTATCATCCTAAAAAGAGATTTCCCCTGGAAGAGATTTGTACATGTCAGGTTTAGCAAATTAAAATACAGGATGCCCTGTTAAACACAAGCTTCAGATAAAGAACCTTTCTGCTTTACTGTCTTAAAAGAATGCTATTCTGCATCGTTTGTATTTCATCTGGAAATGGGAAAACTAGCCATGACCCCCACTTATGACAGGGAAGACAGAATTCTGCCAGTCTTTAGTCTTGCCATCCTTCTGCAGAGGCATGGGGCGGGAGATGGGGCTCCAAGAGTTTCCCTGGGGACCCTGAGAACTATAGCGTTGAGCCAAAGAACGGTCAGTAAGAAGAGCCTATCCTGATCTCCAGGTCCTGCCTGAGCCCCAGCCTCCTCCTCCTCCCCTCTTCT
MSTRG.126480.3	ENSRNOG00000015886;ENSRNOG00000016032;ENSRNOG00000016151;ENSRNOG00000016254;ENSRNOG00000016660;ENSRNOG00000023941;ENSRNOG00000023995	0.328	0.000	0.000	-5.549	down	TGCTGGAAACAACACTTTAATTTGTACAAATGAACATCATATTCAAGATGGGCAGAGAGAGCAGGGTGTGGGAAAGAGAGTAGCCAACAGAGAGGGCTCGGGAGAGAAAAGACAACTGGGCCCTCAAGGGAGGGAGCTCATGGGCAGGATGGTCAGGGACACAAGGACCCTGTGCTGGGTAGTGGCAGTAGTCTAGCTTCCCAGCAGGCCAGGCTGACTCTGGCTCCACTCTTCTCCTCCTACAGTTCTCTTCCAGAGCCTTGGGCCACTTGTCTCTTGTCTCTTTAAATGTCTTTCCAAGGGCTGAAGTACTGGCCCTGGGGCTGACAGTGGGCCAGCAAGTCCTGCCAGCGGATGTAAGTCTGGGTAGCCAGGTCCTTGAGTTTGGTCCGGTGCATGGCCTGGGGGCTCTCCCGGATATGGGTGCTGATGTGGCGGTCCAACTGCATGCACAACAGCTGCAGCTCACCCTTGTGGTCTAGCTCGGCACTGGCCTTTGACTGTTGCCCTGGACTGGATGGCAATGTTTCAGAGCCCAGACACTGCCTGAGGGATGATGGCCTCATGAGGCTGAGGAGCTGGCTGAGCAAGGCTAGCTGGGTCTTCTCTCCCCAGAGAGGAAGTGACTCCTGCTGACCCAGCATCCGGGCAATAACCATGAGACTCAGCTGACTTCTAAGCTGCCTGAGAGAGAAGAACAAGGGAATCTGAGAGGGGTAGGCTAGAGGAACAAAGGCTTCTGAATGTTCTGGGAAGCATGGGGTAAGTCAGGTTACCCGAGAGAGGATAATACACTTTTCTTTGGAGCTGTAACCCCCTCAGATCGATATGAATCTGTGGGTCTGGTTGGGTGGAAGCTCTACTCAGAAATGGACACCATTCCTACCTCTGCGTAATAAACATGCACAGATGAAGAGACAGAACCAGGTGAGGCTCAGTGGTAGAGCATAGCATGCAGAAGAGCCTGGGGACTGAACCATGAACAAAGCAATAATCTGACAAATGTAAAGACAGAAGGGGCTGACACTAAGGAGGAAATTACCCCCCTCCTGCCTCATGCACTGACTCTCAAATTTCAAAGGACTTCAAGCAGACCGGGTGACTGGCCTCCTGAGATCAGGCTTTATTACTTGTTCGTGTGTGCATGTGTGTGTGTCTGTGTGTGTGTGTTTTCACCCATAATTTGGAGACCTTTCCTTTGGGATTCCCCTCCTACCATCCTGAGCTTCAGAGGAACTCACTCTGAAGTAGCTTTTGGGTCTTCATTGGACAGTTAAGAGAGTATAACTTCCCTGTGGGACAGCTAATGTGACAAGGCCTTATAGTGCTGTTTGTCCTCCTGTCTGCCAGCAGGACAGACTTTAGTGGGGTCAGCAAAGAGAACAAGAGAGGCAGAGCACCAGGAATGCCCATGAAGCCACCAGCCCCAGCTCCTAGACCATTTGCTCCCCGAGCTACTTGTGAGGTGGCACAGGCCTCTTGCTTCATTCAGCCGAGCTGGACGTCTGAAGCAGCCATGTGGCAATGCCTAGTCAGTACTTACTCAGAACTGAGCCATCTTCTCCTCGGGAATGAAGGCCCCAGTGGCCTTCAGATCCTTGGGTCCTTAAGCACTTGGGGAAGTAGAGACGAAGGAAGAAGAGACCGGAGGATGGGGCTGGGAGTTGTCCTGGGATCCTCGAGCCTAGGTGACAGGGCAAACTCCCAAGCAAATACTCTGTATTTAATCACCCAACCTTGCTAGGGAGATTGGACAGTGGCCTTCCAGTATCTCCGGACAC

							TGGTAACCCGGCAGTCGGCCCTGTGCATGGATTATTTATTCTGATAGGGATGATACGGGGGGGGGGGC TTACATATGCCGGGCAGGTGCCAAGCCACAGAATTTCATTCCTATTGTCTTCTGGAGTGACTTCTGTATG GCCACCACACAGTGCTAGGTAACCCACTCAGAACGGAAGCCGGCTGGGTTTCAGATGCTACTGTCTTG AGCCATGCCAGGGAGTAAGACGAGCAGAGGAAGGAGAGCAAAGAAAGGCCTGCATTAAAGCCTGCTG GGAAAGGCACATTAGAGACTGGATGCTGAGAAAAATGCTCGACGGTAACAGCAGTGCTGGAGAGAAC CTCCGACAGGTCCCTGAAAAGACACCGCAGGGACCAGATGCAAGGCCCATGGCTCTGGGCAGGCTCA AGTTTCACATGTGCTTGACTACCGAGTCACATGCTCATTGGTCTGTCTGTCCATCTGTACACTGTGGTTC AATAAAGCTATCATCCTAAAAAGAGATTTCCCCTGGAAGAGATTTGTACATGTCAGGTTTAGCAAATTAA AATACAGGATGCCCTGTTAAACACAAGCTTCAGATAAAGAACCTTTCTGCTTTACTGTCTTAAAAGAAT GCTATTCTGCATCGTTTGTATTTCATCTGGAAATGGGAAAACTAGCCATGACCCCCACTTATGACAGGGA AGACAGAATTCTGCCAGTCTTTAGTCTTGCCATCCTTCTGCAGAGGCATGGGGCGGGAGATGGGGCTCC AAGAGTTTCCCTGGGGACCCTGAGAACTATAGCGTTGAGCCAAAGAACGGTCAGTAAGAAGAGCCTAT CCTGATCTCCAGGTCCTGCCTGAGCCCCAGCCTCCTCCTCCT
MSTRG.126568.1	ENSRNOG00000018623	0.143	0.449	0.038	2.269	up	CTTCTATTAAAATCCAGCAGGTTTCACCTCAGTGTACCTGTTGCTCTTTTTCCTTTGGTGGGAACCATAG CTGCTAGAATCTGTCTCAAGAGGGAACGGTCTCTGTTTCTCCTCAAGTTTTCCCAGCGGGTTCTAACTG ACCTTCACTGAGGGTGAGGATGGAGGTGGGGGCTGCTTCTGGACATGCTGGATGATGAAGTGTGGAAG ATGGCAGTTTAGATATCAAAGATCCTAACAGTTGCTACAAATATGCAGGATCCATGAGTGTGTGGTGGAG AAGTAAGAGCCAATGTCCTACAGATAGGCAGATGCCACAGGTGGAATCACACAGGCCCCTCAAAGCAG GCTAAAGCATCTGTGAAAGCAACACACGGCCATAGTCGGCCATCCCCTGCCCTGGGTCTTATTCCTGAT TAAATAGGCTGGACTGATCAAACTGTCTGTGGAATTAGTCCTTTGCCATGAATGATTTGAAGGAAACTT GAATTCCAGAAAGGGTTTTTTTAAAGAGAATTTTCCTTTTGAAGTATTTAAAGGATCAGGTTTTGTCTG CTTGTTTCTTTTTGTCATTCCTAACAGCGTCAATGAAGGGATTACAGAGTCACAGATCCACTGGTGTCAG AGCAGCAGCCGCAGTCTGGGTCAGGAGACCAGGGGTATCTTTCTCTGGAACCCAGTTGAGTCACCGTG TGAAGGAAAACACCACACAATCTAAGTTTAGAATCAACAGGTAACAGTCGCTGGCGCAGAATCTAGCA TCCTAATTTTACAAACTATTCTACCTTAGAAATCCTCCTGCCTTCTCTCTTCCGCTTCCAAACCCGGAAGT CCCGC
MSTRG.126732.1	--	0.769	0.000	0.003	-4.375	down	CCTAAAGCAGTCATTCTGAAACCCAATCTGGGGACAGTGCAGACTAAATAAAATTTCATTTTGATTTGG CCTCCCGCTGCGAACGGGACTGCTTTGTCTCAGCAGACAGAACCAGCAAGCCAGAAAGAGTTTCAAG AATATCCTGGAGGCAGAAGCGTCTTAAATATGAAAATCAACGCAGACACTTCAGAAGGAGGTGCAGCT TCAGCACGAGTGCGAAGAGTTTCCTGAGGACTGACCACAGAGGAGACACCACCTAAGACACAGGCAA CAACTCTATTAAACGCGATCTCTGAGAGACTCGTCTGTATCCCGGTCATCCGTATCCATTTGTCTGTCTTC CTTCCTTCCTTATACTCCCTTAATCCTGTTGGCTTTGTCTGCCTTGTTTTCCATTCCCTTTCTCAGGCATCC ACTTCCAGATAACTTTACTGAGCTACCCTCCTGCAGCAACACTACTTAGAGAAAGGCAATCAGGG
MSTRG.126828.1	--	1.085	0.098	0.005	-2.617	down	AAACTTATCTTTTACCTTTTAATCATAGCTATAATCATAAATTTTTATTTTTTACATTTTTAATGTTTTTGAG ATTTCTTACCAAAGTACTAATGCATTGATATCATTTCTACTCCTCCCTCTCTGATTTAACACCTTCTTTCCT TTTCCCACAATCTGTCAAATTAATCTCTCTCTCTCTCTCTCTCTCTCTCTCTCTCTCTCTCTCTCACACAC ACACACACACACACACACACACACACACACACCAAATAAGAAAAATGTACCTACTTCTACTGGTATATT TAGGATGACCACTTGGGCTAAAGTAACCTGGAGGAAACTTTCTTCATCTCTCAAAGTCACTGATTGGCT GTAGCTCTTCATGGAGAGATGGGACCTTGTGAAAGACGCACATCCACGTTGACACATGAGCTAGTATTG TCATTATGTAGATTTTCTTAGGTGACTATGTTGTTGAGATTTCACGAATGCGGTCTCTCTGTCATGTCTGA

							GTTCCCTATCTAGTAGTAGTTGTCCTTGTCCTCTGGTTCACACAATCTTTTTGCCTCTTCTAACACAATTTCCCCTGAGCTTTAGTTGTATGGATTATGCTGTAGATGTATTTGGGGCATTCCATTTAGTTTCAGCACTTTGAGCAATTTAATCTGTTGAAAAAAAAAACCCTTTTGGAAGAAGCATGAGGGCTACTCTTATCAAAGTGGCAAGCGTGAGAGCTGCAAGTCGGGAGCTAGGGCTAGGAGTGTTCAGTGTCTCAGACCTAGGAGTGTGTAATATCTCAGGTTGAGGATTGTACAGTATCTCAGGCTTAGTAGTGTGCAATATCTCAGGCAGAGGAGTGTACAATGTCTCAGGCCTAGGAATGTGAACTGTCTCAGGTATAGGAGTGTTCAATATCTCAGGCTTAGGAGTGTGCAATGTCTCAGGCCTTGGAGTGTGCAATATCTCAGGCTTAGGAGTGTGCAATGTTTCAGGCTTGGAGTGTGCAATGTCTCAGACCTTGGAGTGTTCAATATCTCAGGCTTAGGAGTGTGCAATGACTCAGGCCTTGCAGTGTGCAATGTCTCAGACCTTGGAGTCTGCAGTGTCTAGGGCCAGCTTGGTTTTCAGATTTTCATTGCCGAGGATGTTGAGTAAGATGTTGTTATGTTAAAGACTGGGTTATTTTGTTAAGCAGAAGAGACCAACTCACTAATTTTTGTTTATCTCTACATTAAAAAACCAGTTAATTTTGAGGCCTGGCTTATATTTTTCTTTGTGAAGTGTTAACAAGCATGTTTGCAAAATTGTGTTTATTTAATTGTTTCTCAATTTGCATATTTAGTTTTGGTGCTTTTGAGTTAGTGCCCCATTCTGTATGCCAGAATACCTGGTAACTCACTATTTATGTAGCCAAGGCTGGTGTACACTGGAACAAGAAGCAATCCACCAGCCTCACTTCATCTTTTGAGTTCAGGAATTCCTGGCATGAGCCTCTGTGCTGGCCCACCTTTCTTAGAAGCAGAGTTTGAGAGATTAAAATACATTTGTAATGTAGAAACTAACTAGTGATAGCCACAACAATTTCCATATGGATTCTATGCTAGATTGTGGAGCCACTGATAGGTTGACTCATTCAGTATTTGTTATGTGAGCCTTAGTTTTCTGTCAAGATTTTAGCACTGAACTCAGCTTTGCTGATCTGTATGTGATAAAATTCATTTACTGTCTGTATGGGCATCTATAAGTTGTGAACTTATTAGTTGGTGACACGTGATACTTGTGAAATATTCAATCTTTAGTTTACCTAATAGTATGTTAAGAGTGAAACATTTTGCCTGTTTTTAGGTTCCTTGTGTTATTCATGAAAATAGGTCGATTTGTCAAAGAAAACAGAATAAACATGATAAATGTCTTAAAGACAAAAGCTCTGTGAGATGTTTAGTAATAGTCTTTGTTATTGGATTCCACATTATCACAAATAATGGAGTATCAAAAGCAAGCACAAAGAGACTGAAGTAGTCTAAATTGTTGATATAGTAGTCAGAATATCCATACCTTTGATTTAACTACCAGGTGAATAGTTAAGGATATATTAAGTTAATGTTAATATAGAATGTCTTATTTTGTTTTTTAAAATCTTTTTCCTGGGCTGAAGAGATGGCTCAGTGGTTAAGAACACTGACTGCTCTTCCAGAGGTCCTGAGTTCAAATCCTAGAAACCACATGGTGGCTCACAACCATCTGTATTGAGATCTGATGCCCTCTTCTGGTGTGTCTGAAGACAGCTACAGTATCCTTATACATAATTAATAAATTAAAAAAAATCTTTTCCCCCTCCGTAGAATGGAAATGACGCCTTTTTGCTGTAAGTTTTAGCAGAATGATTTGTTTAGAAGACTGATTTGTTGGTGTTTCTAGTTGCAATAATGTGTGTTGTCTTCTAAAACCTCTAAAGGATAGAGAAAGTTATTTCTTTGAACACAATAATGAATTCACAATAGTTACAGAAACCAATTGCATGGATTCCATGAGCCCTGCTCCCTAAATGTTTAAGAGGCTGGTGGATCTAAAAGTGTTTCTGTTATCGCATTTATAGATCAGGAAGTTTATGTTTGTTCCTATGGGACACTAAATATATTCGTATTGGAGGAAGAAAAGTGGCACGAACTTGTAACATATCAATTGGTCTGCTTGCAAACAGCAGAGATATTAAATTGAATCAACTTTATTTTTTAAAGAGAATAACTGAACATTATTCTTCCTATATAAATCTCTCTGCTTTCAGGGTGCTATTTGCTAATTCTGTGTTTGTTTTATGCTTGGGAAATGGTTTTATCCACTCTTGTGATGC
MSTRG.127177.1	ENSRNOG00000002091;ENSRNOG00000013388	0.955	0.009	0.000	-4.440	down	CTGGATAATAATCCTGTTAACTAAAGTCTTTTGCTTTAGTTTTTTTTTTTTTTTTAGCACTGTGTGAAGGATGGAAGATGCCCTTGAATTTAACAAGTTCTCAGTTGAACACTTGCTACGTGACAGACACTGTGTTAGGGCCTGGAGAACATCAAATGAGTCACCAAACACCAAACAGCTGTCCAGAGACAGGTACCTATGTGTCTGACGGCAAAGAAGCACACTAAGCCAGATGCAGACCTTCTCACATGAACATTTTAGTCTTCACATTCAGCATCAACCCGAGTCCATAAAAAGCTACAGCAAGCCGCTTTGACTGACAATACCGCATCAACGCTGTGCACGTTCTAGTACAATGTTTCTTGTTTGAATCACGCAGAAAATGTTGCCAAATATTCCTTATAATATAAATAGGGAAAGTAGCTATGTCAAATGAAAATATGGGAATTAGAAGGGCTCAGTCTGAGCTATGACAAAGAGAGTA

							GCTTTGTTGGAAGAGTTGGGGGACTCACCCTTTGCTACTGTTCCTTGTTGGGTGACTTGTATTTAAGGTAAAGTTGGTGTAAAGCTGAGACATTCAACATGGGACAGTTTTATTATTATTATTTTTTTTTTTTACCTTTGAACAAAGCATAAAAGCATCTCCCAGATACCTTGCAGCAACCAGAATCTCTTTGTTTTCTATCTCAGCTCTACTTGGAGATCCACTGCCCTCATTCAGTACAAGCCTCGCAGGGGCGGGGTGGATTTTATGCACATGCTAAACATGGGGCTGCATCATCCTTTTAGGAAGAAAAAAGAAAAACAAATCCTGAGCCTGAATCCCTATTACATGTGATACACTGTTCAGTGAACAGGTCTGAATCCCTATTGTCACATACAATATACTATAGTAACAAGGAAACGTATCATTTTAAAATAAAAATGTTGCTTGATAATAGGCAATAAATATAAAGGACAGTCTTCAATAAAATATCTAATTAGGAAAATTATCATTTCATTTCATTTCACGGGGTATTTTTGTAACTCCTTCAAATACAACCTCCCACTCCATAGTTAAAGTCATAAAAGAGGAAAATACTGAAATATGCATGTCGGTATCATATGTTCCAAAGGCCCAGCTGTTTACATGTAAGATCCGGAGATGAAGACCGAATACGTATAAACTGACTAATGTGGAAGGAAAAGCACACGCAAGGCTTCAGCAGTAGTCGAATTTTCTATTGGAAAGCTCTTCTGCTGCTTTAACATGGTCTCCTCTACCCTCTCCACTGTTTACATTGCAAAGTTGAGTACTTCCTGAATGCGTTGTCTCCTGCCCTTAGACTATTCCTTGTGCAAGTATTTATCTTGTTTCTCTGAGGTTTCCTTTGAG
MSTRG.127177.2	ENSRNOG00000002091;ENSRNOG00000013388	0.006	0.541	0.000	5.303	up	AAAGTCTTTTGCTTTAGTTTTTTTTTTTTTTTTTAGCACTGTGTGAAGGATGGAAGATGCCCTTGAATTTAACAAGTTCTCAGTTGAACACTTGCTACGTGACAGACACTGTGTTAGGGCCTGGAGAACATCAAATGAGTCACCAAACACCAAACAGCTGTCCAGAGACAGGTACCTATGTGTCTGACGGCAAAGAAGCACACTAAGCCAGATGCAGACCTTCTCACATGAACATTTTAGTCTTCACATTCAGCATCAACCCGAGTCCATAAAAAGCTACAGCAAGCCGCTTTGACTGACAATACCGCATCAACGCTGTGCACGTTCTAGTACAATGTTTCTTGTTTGAATCACGCAGAAAATGTTGCCAAATATTCCTTATAATATAAATAGGGAAAGTAGCTATGTCAAATGAAAATATGGGAATTAGAAGGGCTCAGTCTGAGCTATGACAAAGAGAGTAGCTTTGTTGGAAGAGTTGGGGGACTCACCCTTTGCTACTGTTCCTTGTTGGGTGACTTGTATTTAAGGTAAAGTTGGTGTAAAGCTGAGACATTCAACATGGGACAGTTTTATTATTATTATTTTTTTTTTTTACCTTTGAACAAAGCATAAAAGCATCTCCCAGATACCTTGCAGCAACCAGAATCTCTTTGTTTTCTATCTCAGCTCTACTTGGAGATCCACTGCCCTCATTCAGTACAAGCCTCGCAGGGGCGGGGTGGATTTTATGCACATGCTAAACATGGGGCTGCATCATCCTTTTAGGAAGAAAAAAGAAAAACAAATCCTGAGCCTGAATCCCTATTACATGTGATACACTGTTCAGTGAACAGGTCTGAATCCCTATTGTCACATACAATATACTATAGTAACAAGGAAACGTATCATTTTAAAATAAAAATGTTGCTTGATAATAGGCAATAAATATAAAGGACAGTCTTCAATAAAATATCTAATTAGGAAAATTATCATTTCATTTCATTTCACGGGGTATTTTTGTAACTCCTTCAAATACAACCTCCCACTCCATAGTTAAAGTCATAAAAGAGGAAAATACTGAAATATGCATGTCGGTATCATATGTTCCAAAGGCCCAGCTGTTTACATGTAAGATCCGGAGATGAAGACCGAATACGTATAAACTGACTAATGTGGAAGGAAAAGCACACGCAAGGCTTCAGCAGTAGTCGAATTTTCTATTGGAAAGCTCTTCTGCTGCTTTAACATGGTCTCTTCCTGAATGCGTTGTCTCCTGCCCTTAGACTATTCCTTGTGCAAGTATTTATCTTGTTTCTCTGAGGTTTCCTTTGAGCCACAAACCGGGAGTGAAACTGTGATGCAGCTGCATCCCACTGAGCATGCGTAACAGCGCTCTTTGTCTTGAGTCGGACCCCTGAACCTCTGCATGAAAAACAGTCAGGGGAAGCTCCC
MSTRG.127391.1	ENSRNOG00000011825;ENSRNOG00000012473	0.000	0.491	0.000	4.895	up	TTTCATTACACCATTTCCATACATGTCTGCAAACACAGTTTAGTCATGTTCATCCCAAATAACTCCTTTTTTACTTTCAAGGCTCCTTGATTCAAAGACTTATTAGAGTCGCTCGCAGGTAAAGAGCTGCTGTCACAAGCCAGGGCAACTTACTAGTGGCTACACCACCAGAGAATACATTTCTCTCCAGCAACCACTAACTCTGAAGATCTCCAGGGAGCAGAGGGGCCTGGTGAGAGCCTCCCCCACTATGATAGAATGTTGACACATAAGATCTTGTGTCGGTCATCATGGCTCCTGTGAGTTCATGACTACAACCATCATGCCGTGCAAAGAGCACCACAATCCACGTCTTCCTCTACTGTTCTGTTCTTTCTACTTCCTCTGCCACAGTGGCCCTTGAACCTTGGAGTGGGTGATGTAGATGTTCCACTTTGTGTTCAGAAAGGACTCTCCTGCAGTAGAGGGGAATGGCAGGATGGAACT

							AGGTCATTCCGAGCCTGGCGAAGCAGGCTGAGGCAAAGACGCTCTAACTGTGCTCAGACTTCGCCTTGTTCTGTGTCAGCATCTCCCTCCAAGCTCCTGACGTTGGCTGAACCAGCTGCTGGCTGTCAGGCTCTGATGTTTTTCTCTCTTTTGACCTTGACGACCTCTTTC
MSTRG.127557.1	--	0.000	0.409	0.031	3.453	up	CCAGGGACTATTCTTCTCTAGGACCTTAGCTCTACAAAGGCAGCGAAGAGGGAAGATCTGAGGACCGAAGGTCTAACATGTGGAGAAAGCAAGAGTTGAGAGTTCAGCAACCCTGTAAGACAAGAAGACAGTTCCAGAACACAGAGGGGAAAGTCGCATGAAATCCAAGAGGAAAATGGAACCAAGAGCCACACGGAAAGAACTTAACAGCACCAGACTGATACAAATGAAGCTGCTGAAGTAGCTTTGGGCCATCAGCCG
MSTRG.127576.1	--	0.461	0.000	0.022	-3.851	down	TTCCATTTTAACCAAATGTTGGTCATGAGCCTGTGGACTGAAGGCTTGCCCAGTTCACAGGGTGTTTGCTGTCTTGAGGCTTTGGGAACATATTAGTAAGAACCATGCAGCCAGCAACTTCTGTTATGCTTTCTAGACCCAGGCATTTTTTAATGTGGCTACATTTGGTAATGTTAATTCAAGGCCAGTGATTTCTCAACTGGAATTTTACCCTAGATGACATATGACAGTGCCTGAGGCATTTCTCGTCACAGCTGGTGCAGGAACAACACTACTGAGGCTACATGGCTGTGGTTAACTACACTGCATAGGACAGTCCACAATACGAGTGTCTGTCATGCCAAGCTTGAGGAGGCCCAAGCTGAGGACTCTGCATGACTGGCACCTAGTAGGTGCTGGGATGCTGGGCTTATTGATGCATAGTGGGGACATTTTAAGTATCCATCACAGCCTAGCCCTGGAAATATTGAGGACTTTGTGTCCAAAGAATTGACATCTACTGACAAGGTATAAGAAATAACAAGGAGG
MSTRG.127587.2	--	0.000	0.336	0.000	5.351	up	GGAAAAAAAGCTAGAGTCTAGCTTTGCTTTTCAGATGTGTGGATTCTTTGCAGAATGGGAGGATACATCTCAGAACTGTAGGGTTGAAGCTAGTGGAGCGAGCCAGCTTGTGGCTGAAATGGAAGCTTTCCCGTGGGCAGTTGATAGTCCCCGCTCTCTCGTGAATCTGTGTGTTTTCTGGGAAAGCAAATCATGTTCTTTAAATTTTTTTTCTTAAAAGATAAAACAAACTGCATGACCCTCGACAGTCTCCTCATGTGAAGTGGTTAGAAAGTTTTGAGTGGAATTTGTTCTCAAACACTGCCAAAAAGGGTGTGTGGACACAGGCTGCAGAGATAAGCACAGCAGGGCCTAGGAGAGGCCAGGGCCAGCTCTTGTGGAGTGCGACAGGGGATACTTCTCTGAAAACAGGTCTCTGGGAATGCGGTTCTTCCACTCTGGAACTGCTTCTGGGGGTGTTTCCCTCCGCTCTAAGACAGTGTCTGGAACCTGAAAAAACTCTTAGCGTTTGCTCCTACAGGTGACTGGGATGGGAAACCATCTACCAAGGAAATGGGGGATAAGAGAAGGAGGGAGGAAAGATAGAGACAAAAACTCTATTACCTCGAGGCACTTGGCGGAGACAGCCAGCCGCGGTGGTGGGTCTCCTTCCTCACAGCCACCCTGGCGCTCCGCACCGTGCTTAGCGGGTCTGCGTCACTTCCAGAGAAGCTCAAAAGCGCGGGGTGCGAAGAAGCTCATCACCCACTAAGAGCCCCGCGGGCAGCCTTGGGCCGGGGGTGGGACCCGCCCGGACTAGATATTATCCGGCTCCCCGAAAAGGTGATCAATCTCCGGTCATGTTTTCAGTCCAAAAACTAATTGGTACCGGGAGACCTAGCCTGGGTGGCTAAGGGCGAGGCCAAGCTAGACCTCTGACTCACCCTATGAGCGCCCGCCAGAAGGTGGGTTGGCCCTGGCACTACGGCGGCGGCGGGAACCTATTCCGAGAAGGAAGTCAGGAAAGAAGGTGGCGGGCACAAATGGGGGCAACTGAGCAAAATACCCGCAACTGAGCTGGTCCGCAGTAGCTGGAAGGCCCTGTGGGCACCATACATCGGCTCCCAGGCGCGTACCCTCCCGCCCCGGTCTAGTGGAGTCTGAGCAGCTGGGCTAGGCCGGCTCGGAGCGCCCCCTCCTACTCAGGGCGCTGTGCTTGGCGCTGCAAGCTCGCCGCAGCCCCTGGAGAACTGGCATGTAGAAGCTGGGGGCCTGATGCCGAAAGATTCCGGTGCTAGCCTGGCCGGGAGGCAGGAAGCCGGGGTGGTATTGCAGC
MSTRG.127645.4	ENSRNOG00000023923	0.000	0.253	0.007	3.835	up	GTTTGGCACAGGATCTCAACTTGATGACTATTTCTTCTTGTTCTCCTTCACCTCACAAGAACCTTCCAAGCCACAATTCTTTGGAAATCGACACAAGGAGCTTTACTTCAAATTACCATCTTTCGCTTAACAAGATCCATCTCTAAGGTGTGTGGTGCTATCAGCACTGGACTCAAGTTTGGATAACCAAGAACATTGACAATAGCCTACAGCGTTTAGATGTCTGTGGGACCACACTGGATGACTCAAAAAGAGAACGCTCATTCCAGAAAAGAAGACTCAAGAAAGAAAAAACACAATGCTGTAGATGTTCAAGAACATGGGGAAATGATGAAAGTCACTGG

							AATACTAGTTAATTAGCTGTCCAGCAAGTTTCCCTGTGATTCACCACCACTGCAGAAATTTGCATCACAGCATCTCCTAAAGCTGACACACCAGTGCCATCAAGAAAGTATTTCCCCACGCATATTTATTAAGTAAATGGATTATCAGAATGATCTTCAATGGATATTCAAACTTCCTGTTCTTTGTTAAATAAAGCGTTTTAGCCAGG
MSTRG.127728.1	--	0.889	0.000	0.000	-6.140	down	GTGGGATTCAATACAGTCAGGGGACCTGGTTCATAAGCATTATACTCTTCAGCGCATTGAACTCCAGCAAGCACTTATGGGATTACTCTAGCACGTGCTATGGTACGAGACTTTAGATGTATGTGTATATATGTTCTATGAGGGGAGATCACCCGTATTGTTTCATTCACTGGGCTTAGTCAAAGTCTCACGATCAATTTCAAAGTCTTTATTGAGTGTCTATTTTATCCCCAGTCTTCAATTTTGCATTCTTCGAGTCTTCAAGTCCTCTTTAGCTTGGCACCAGTGTGTTGATGGAAGGAGTTAAGTAGATATTGTGCCCTAAAGAAACTATACTTCTTCTTGAAGTGGTACCTATTTTAACATAGCCGACATCACTAGCATTCTTTTGGTGTCTGTTCTGGCTAGCTTTATGTCCTTCGGCATGAGCTAGAGTCATTTAGGAAGAGAACCTTAGTGGAGAAAATGACCTCACCCAACCTCACAGACTGGCCTTTGGGCATTCCTGTGGGGAACTGTCTTGATTGATAATTAGTGTGGGAGGGCCCAGCTTCCAGGGTGCTTCAGTTCCTGTCATTTAATCCTGTGTTGATTTAATGATACTGGGATGTGGAAGTACAAGCAAAATCATCCTTTTTTTTTCCCTCCAAGTTTCTTTTGGTCATGGTCAAGCAACGGAAAGCTAAGACAGCATCAAGACTAACAAAGCGTTTAAAGTATCATCTTGTTTTATCCTCTTGAAAATTCTTTCATCCTTTGACTTCCAATGTCAGTCAGACCAGTCCTTTCAGCATTTTCTCCCATATCCTTTCCTGTCTTTCCCAACTTATCTTTATTCTCAGAGCTTACCTCTCTTCTCTTCTCTGTTCCAACTGATATTACAGGATCCATCACGTTCCTCCAAGTTCTCCATTTGCAAATGCTCTTCCCTTTATCTTGAAGGCCTCTTCAGGATCCGGTGATCCACCCACAGCCACTGTCTACATTCAGTTCTGGTATTTCCTCTGGAATTCCTCTCTCTTACCCACACAGGGGAAAAAGGATGCTCACTGATTCCAGGGACCCCTCTTGCAGGCACACTGGGAAAGAACAGACACCTTCTCCTTGTTGGCGTTCAGTGTACAGTCTGGTGTGTTGTGCTTATGATCACAATTCCTTCTTTTGGATAGCTGCTTCTGCTCCACTGGGATCTGGTCTCGCATCTGGAGGCCCTGATTTGATCCATAACATTGCAAAACAAAACACTGATAGATTAAGATACACCTACAGGCCTCGGAAGCCTTTGTCACACTGGCGTCTTGGTGGCAGATGGTCAGTGCGCATCTGGGTAGGAGGAAAGTTTTGGGCCACTCCTCTGAGGACTCAGAACCCAGTGCAGCGAAGCAAACACATCAAGAGTCAAGCCTGGCTGCAGGGATCGCAAGGCATCTGAGCTTCTGGGTTGCGGACTGAGTGACTGCTAAAAGATTCGCCCGGGTCCTCC
MSTRG.127728.2	--	1.172	0.086	0.002	-2.898	down	GTGGGATTCAATACAGTCAGGGGACCTGGTTCATAAGCATTATACTCTTCAGCGCATTGAACTCCAGCAAGCACTTATGGGATTACTCTAGCACGTGCTATGGTACGAGACTTTAGATGTATGTGTATATATGTTCTATGAGGGGAGATCACCCGTATTGTTTCATTCACTGGGCTTAGTCAAAGTCTCACGATCAATTTCAAAGTCTTTATTGAGTGTCTATTTTATCCCCAGTCTTCAATTTTGCATTCTTCGAGTCTTCAAGTCCTCTTTAGCTTGGCACCAGTGTGTTGATGGAAGGAGTTAAGTAGATATTGTGCCCTAAAGAAACTATACTTCTTCTTGAAGTGGTACCTATTTTAACATAGCCGACATCACTAGCATTCTTTTGGTGTCTGTTCTGGCTAGCTTTATGTCCTTCGGCATGAGCTAGAGTCATTTAGGAAGAGAACCTTAGTGGAGAAAATGACCTCACCCAACCTCACAGACTGGCCTTTGGGCATTCCTGTGGGGAACTGTCTTGATTGATAATTAGTGTGGGAGGGCCCAGCTTCCAGGGTGCTTCAGTTCCTGTCATTTAATCCTGTGTTGATTTAATGATACTGGGATGTGGAAGTACAAGCAAAATCATCCTTTTTTTTTCCCTCCAAGTTTCTTTTGGTCATGGTCAAGCAACGGAAAGCTAAGACAGCATCAAGACTAACAAAGCGTTTAAAGTATCATCTTGTTTTATCCTCTTGAAAATTCTTTCATCCTTTGACTTCCAATGTCAGTCAGACCAGTCCTTTCAGCATTTTCTCCCATATCCTTTCCTGTCTTTCCCAACTTATCTTTATTCTCAGAGCTTACCTCTCTTCTCTTCTCTGTTCCAACTGATATTACAGGATCCATCACGTTCCTCCAAGTTCTCCATTTGCAAATGCTCTTCCCTTTATCTTGAAGGCCTCTTCAGGATCCGGTGATCCACCCACAGCCACTGTCTACATTCAGTTCTGGTATTTCCTCTGGAATTCCTCTCTCTTACCCACACAGGGGAAAAAGGATGCTCACTGATTCCAGGGACCCCTCTTGCAGGCACACTGGGAAAGAACAGACACCTTCTCCTTGTTGGCGTTCAGT

							GTACAGTCTGGTGTGTTGTGCTTATGATCACAATTCCTTCTTTTGGATAGCTGCTTCTGCTCCACTGGGAT<br>CTGGTCTCGCATCTGGAGGCCCTGATTTGATCCATAACATTGCAAAACAAAACACTGATAGATTAAGATA<br>CACAGCCTCCATCCCTCCCTACTCTGGGCATATCATTGTAGTGAGGAGAAAATGAATCAGAGAACTTGT<br>GCAGTGAGTAGCTGCAGAGACTGACCCTACAGGCCTCGGAAGCCTTTGTCACACTGGCGTCTTGGTGG<br>CAGATGGTCAGTGCGCATCTGGGTAGGAGGAAAGTTTTGGGCCACTCCTCTGAGGACTCAGAACCCAG<br>TGCAGCGAAGCAAACACATCAAGAGTCAAGCCTGGCTGCAGGGATCGCAAGGCATCTGAGCTTCTGGG<br>TTGCGGACTGAGTGACTGCTAAAAGATTCGCCCGGGTCCTCC
MSTR<br>G.127<br>731.1	--	1.112	0.000	0.000	-<br>6.040	do<br>wn	CTAACTTTCTGTTACCAATGTTACAAATTTTTAATCATACCCAATAACTCATAAGCCAATAATCTATAGCCA<br>AGACATGCAGAACATATTTATACTTTTAAAACCAGTTAAACAAAAGTGAAGTGGCTACACACATCTTATA<br>TATTTAGACCAAACACAATTTTCTTCCGTTTTCTAGCTGTAATTTCAAGAGAGGAGCACCTTGTCACCTG<br>TTGAAGCCAATAAACACAATCACAGAGATTTTTTCTAGGAAAAACAGCTAGCACCTCCCTTACCATAGA<br>AGTCGAGAAGCTGCTGGCCCCTTCAAGAGTGGCTTGTGCAGACAGTCAACCTCAGGCAAGGCTTCTCC<br>AGCTGCACTAGACTCCTATCTACAGGTACAGGGCTCAAAAGGCCAATTGAGGTTTTTTTTTAATGAGTTA<br>AAACTAAATTAAGGGGTGAACAACCAGCAGATAAAATTTTAACCATTTTTTCTTTTGACCATGAAACATA<br>GAACGTTCAAGCAATGAAACAAAAAAACACAGACATAAACTGACAGACATGGGCAGATTGGTACACA<br>ATAGACAGAGAGGGAAGAGAACCAGATGATGTTCCCCCACAGGGATCCAGATATCCTTCCTAAGGGAC<br>CCAGAAGCAGAAATAAGCATTTCTCCAATAGGAGCCAGCAAGGAGGCAGGACCTTCTTCTCCTGACCT<br>CCTTCTGTCTCTAGGAGAACTTCAAAAAAGCACCCATTTTCCTTCTCTTTCACCAAAGCATCCCTGGAG<br>AGTCTAGGAGGACTGCTTTTCACAAACCCGTTTTCTCTTATGTCTAGGGTGTAAACTGTCTCTAGTCAGA<br>ATCCAAAGACTAAAAGCATCTATGAGAATTATCTTTGCTTCTCTAGATTTTAGCATAACTCTAAAGAACA<br>CATACACAAAAGCACTTTACCATTACCTTGTCCCTTTTTACAAGGTTTATTTACCACAGGCACAAATCTT<br>CTTTACTTTCCTTCACATGTGCTTTCGTTTTTTTTTTTTTTTAATTAACTTGAGTATTTCTTATATACATTTC<br>GAGTGTTATTCCCTTTCCCGGTTTCCGGGAAACATCCCCCTCCCCCCTCCCTTTCCCTATGGGTGTTCCC<br>C
MSTR<br>G.128<br>025.1	ENSRNO<br>G0000001<br>7206;ENS<br>RNOG000<br>00017611	1.375	0.142	0.011	-<br>2.436	do<br>wn	GTCATCCTCCCGGAGCAGGAGTGAAGATGCTGTGTGGTAGACTGGGAAAGGGTCCAGAGACAGGACC<br>TAGAGCTGGAGGCCAGATCAAGTGAAGAGATGGAATCTTCCAGACCAGTGGTTCTCAATGTTCCTAATG<br>CTGAGACCCTTCAGTACAGTTCCTCATGTGAAAATCTGAGAGGGAGAGCGATGGCTAATGACTCCTACA<br>CCTACAGATGTTTCAGCAGGCTCTGTTGTCTGGTCCTTCAGATTCATTTGTTTGTTTGCTTTTGGAGACA<br>GGGTTTCTCTTTGGAGTCCTGGCTGTCCTGGAACTTGCTGTGTAGACCAGGCTGGCCTCAAACTTCCTG<br>AATACTTGGATTAAGGGCATGTATCACCATGCCTGGCCTGTCTGATTCGTCTGTATCTGCCTCGTAAGGA<br>CATCAATGGCACCAGCTGCTGAACTCAGGATGCCAAGATACATAACCTGATTTTATTTAGTCATCGGCCA<br>AGTTCTTCTGGAGCTGAGAGGTGCTCAACCGATAGCTGGAGGAGCTCTCCCTCTATTCCCTGAGTCAGA<br>TGCCCTGGTGAGCAGGGACAGTCTCTGAAATGTGTGGAGAATCCAGGCTTTGCAGCGGCTTGTATATGC<br>AGATTTTGTGGATGTATGTGTGTGCACACTTGGTGTGTGTCTAGAGGCAACATTGTGCCATGAACTTGGC<br>TGTACTTCCTCCACGCAGGAGATGGGATCTTGGCCTTGGCAGCGAGTGGGCGTACGTGTGGACTTGAGT<br>CACAGTAGGCTCAGAATCTGGGCTCAAGCATCTGAAGGCCTGTCACCTGCAAGGCTCATTATAGAAGG<br>GGCCAGGTGCTTGGAGTGTGCCCAAAGCCATACTGACTGTGCTGTCATGAGTTTGAATAGTATTCCTGT<br>GAGAGCTATAAATAACAACTCTGAGCCCAAAGCCAGGGTTTTAGAAAGCCATCCTTAAATGCACAGTGC<br>TTTACAGTCCCTGCTGTCCCTTCCCTGGGTGACTCAGCTCTCTAACCCAGTAGGCCCAGCCTGATCCAG<br>TAACCTTCAGGCTTGGCTAAAAAAGGATAGAGCCAAAGCTGGTCTCAAGAACCCCAGTGATCCCATCAT<br>CCAGTTACCATGTGAGGTTTTGCTACTTTTTTTTTTTTTTGCCTCTGTTCCCTAGCGGAGACATGGATGAA

							CAGGTCCTGTACCAAGAGGCAAGGGCACAGACGTTCCTAATTGGTTGATTCTCGGAAGCCTATTCTATG<br>CATTTGGGATCACAAAAGGTTCTCCCATCCTCAGAATGGATTGGCTCAGATCCCATTCCCTCTAATCTCC<br>AGTGTCCTGAAGCCACAGCACTCAGTCTTGGGAGTGGAAATCCCAGGATAAGGTCCTGCTAAAAGTAT<br>GTTTTCCCCCTCACTCTCAGATGTTTGGGCCAGTAGGAGAAATAGAAATGAAGATAATTTTCACTTTGGC<br>TTAAGAAATGACTACTCGAGGTCTGGAGAGATGGCCTAGTGGTTAAAAGTACTAGCTGTTCTTCCAAAG<br>AATCCAGGTTTGATTCTCAGCAGACTACAACAGTGTATAACTACAGTTCCAGGGGATCTGACTCTCTCT<br>GGCCTTCGTGGGTACCAGGCATGTATGTGACTCGCAGACATACAAGCAGACAAAACAAACCATGTGCA<br>TAGAATAAATAATTAAAAAAAAAAGCACCCACGTCTAGTGCCTCAAATGGCTGAATAGAGTATCAGATG<br>GCAATAGGCTTAAACCTGGAGGTAAAGGTATCGTGGGAGGCAGTGAGCTAGACACAGAGCTAGATATT<br>GGATTCTCTCAGCCCCACCCTCCACACCCGTGCCTGCTTTCTTTCTCCAAGTCTTTACTAGGCTCTTAGG<br>ACAACACTGTCTGAAGGAACAGCTGAAGGTTCAGAGGGTGCATGGAAAGGCCAAAGGGTTTTTTTTC<br>ATTTTCTTCTTTTCTTTTTTAAGACTTCCAGGAGTGTGCAGAGTGCTGGCTTGGCCTTTTAGCCACATGC<br>CTTACCCCCGCTTGGAGCTCAGCGGTGTTTTTATGCCAAGGCCCATACACCTGGCTGTTCAAGCAGAAG<br>GAAAAAAAAATCCATCATTCAGGGTTGTTTTAAAAATAGTGTAACTCTTTGATTAAATGGCC
MSTRG.128099.1	--	0.000	0.244	0.000	6.429	up	GTTTAACTTTATCCTTTATAAAGCCATACATGGATGGTGAGAGTAAGCAGCTGTACAGAGGGGACAGCAT<br>CAGTGTACATACATTCATGTCCAGAACACTTAGCATACATCAGGGTTTATACAAGGTGGCAGAGGCTATA<br>GGCACAATGATACAAAATATAAAGTATATTTCCATCTATAAATACACAGCCGGGGGCAGGGGCAGGGCAT<br>GGACTCCGGATATGGGTTTCCCAGCAGAGGGCCTGGGAGGCAGAGTTGGGATGATCAGGATGTGAGGG<br>ACGTCTTACATTTGATTCGGGTGACAGAAGGGAAGAGACTGGGGAGATACCTCATGCGCACCCCAACG<br>TGCTCACACACATACGAGCCAAATGTGGGTCCTCAGGGAGGGCTGGACTGTGAACACTGACACACACT<br>GGGTGTGTCGAACTTCTGTGGCCTTGCTGCTGTGAGGGGCCCAGGCATGGAAAATGACTGAGACGCTC<br>CAAGTTTGATGAGACAGGAGACAAGAAAGTGGCCCGCCAAGCCTCCTCCCCTGGGACTGGCCCCTCTG<br>TCATCAGCACCAAAACATGACCAATCTGCGGCCCATTAGGCCTCACCTCCTAGTTCTTCTCACTACTGGG<br>TCCCTCCACCTCCCACCTCCCACTTCCCACCTCGAAGTTCCCTAGAGATGAGCAGAGCACGCACACAC<br>ACCCACACTTACACACACGCGCACGCACATACATCCTGTCCACCAGTGACCACAGAAACACCAGTCTC<br>AAGATCTTCGTCCAAGGCCTAAGTGCAGGGGTGGGCCCTAGCTTTGGGTTCCAAGCTCTTCAGGGACC<br>CAAGCCATGGAGGCAGGAGAGGGCTGATTCCCTTAGATGGATCTCCCCGAGATCTGAAGCTGGGAAAA<br>GGGGGTCAGATGTCCCGGGAGGTAGGATGAAGAAAACCCAGAGGCAGGAGGATGCGGCCCACTCACT<br>TGTCTCTCCTGCACTTCCCTGCAGCAGGAGGGTTCTCTCTCCTGAAAGCCCCTCATTAGAGGGATGCAC<br>AGCCACAGTGTCCTCCCTACACCCCCAGAGGGGCTATCCCTAGGGACTCTCCAAAGCCAGGGCCTATCA<br>GGAATCCTGGCACTCATGCCTGAGGCTATCTAAGCTGCTAGGAATCGGTAAGGTGAGAAGACTCAAATG<br>CCCCCTTTCAAAACTAAAATTTGGATTCTTTCCTAAAACATGGCAGGGCAAGGTAACCAGGGGTCCACA<br>GTAACAACTTCAGTATGCAACTGTTAATTTTCCCAGTCTTCATGTCCACCCTGTTCCAACCCAGCTCTTC<br>ACACCTGTCCCCACCCCCAGAGCCATCGGGGACTTCCCTAGGCACCAATCCTGGCTCCATCAGTGCCCT<br>TCTCTGCCATCTCACCTCCAACCTCAGATCTTCCGAGTGGCCGGAGGCTGTTTGTACCTGGGCTGTTCCT<br>AAGGGGTCTTGTTAAGGGCATCACTGGCCGCCATGTTCTCCTGTCACCCCTCCCCCTCTTTCATTCCTGT<br>CTAAGAATGGACAACACTCTCCCTGTTGATATAGAGATTCAACAATAAACAACAACAAAACAGATCATT<br>CACAAAAGAGCAGTGGGTAAAAGTGCTCTCGTTTAAAGGAAGTCTTTGGCAAGAATACAGAGTTGCAT<br>ACGGAGTGCTCTGTGTGCATGTGGTCTCTCTGTGTGTATGTGTGTGTGTGTGAGCCCATCTGTGAGAATC<br>ACACTTAGGAAGGGGACAGACAACAGTGTGGTTCCCACTGTGAGAAGTGAGGTCCCCTCCACCATTCT<br>TCCAGAAAAGCCAAACTCTGGTGTCCATTCTTCCTAGAGGCAGGCTGAGAAGGAGGCGCCCGCTTGCT

							AGCATCTGCCTTGGTCATTCACTCGACTCACACGTGGCTTGGGCCTGAGCACAGCATGGGAAGGTGAGACCATCCATGAAGGCTGATCCTAGACTTGAGTCTGGTCCAGGGCAGGATGCAGAGGCTCTGATGCTTCTCCAAGGCTCAAGTGAACAACATTCCAGCTAGGAGGAGACTCCCCAGACTACATGGAGTCTTTCATTTACAAAGAAAGGGATGAGAAGCAGCTCCTTCTATCGTGGGGAGAAGTACAATTCGTTAGGGGGATGAGGAAATGGGAAATCCAGACATTTGCCTTGAGTATCTCAGAGGGAAGGACACTTGGAAGAGCCTTGATCACTCAGCCCATTTATTCTTTGGGAAGTGAGGGCTCCTGATCCTCACCGGCCTTCTTTAGCTCCTTAAACAATGTCCACACAGTCAAAGCTCCCTGGAGGAGGAAAACAATCCAAGAGACGCCACACCATGGCCACACACACCCTGATCTCCAAATACACCCTGGCTCAGGCCTGAGGGGTTGGAAGTGGCTAGCTGTCCACGCATACGGGATGTTGGCAACTGTGCTGCCGTTTGCCAGAACTGGAGCATGCCATCCCACAGATGTAGCCTAGGGCAGCCAAGGCCAGGGCCAAGGCCAAGGCTAGCCCTGGGTTGTTTGGAATATAAAGACCCCCTGCAGGTCAGGCACTACTTACTGTTAGAAAACAGGTAGGAACAGTTTGTGGCCAGGGACAATAAAGCAATATGGGAAAACAATAAATATTTCTTGTCAATTTACCTATTCTTTTTTTAACCTTTTTCTTATATTTTTTATTAGCTTACAGATTCTATCAGTATGTCTCATGTATATATTATATATATTTATATATCAGTACAATGCCACCCCTGAGGACCCCCATGCGCTTAAATGGAGGCCCGAGCAGTGGCAGGAGCCTTCGCAACCCCGCAAATGGACCATAGGACCTTTGGCCTAAATTCATACCTTCTGTCCCCTGTCCCACAGACAGCATGGTTCTGCTGGGGAGACCGAGGCAGAAGGGGAAACAGGGAAGCTTTGGTACTGTGGGAGGACCATGTAGTTCTCCAGCAGCATTCCCAGAGGTGCTCTGATGTCACCCTGAGCAGCCCAGACAGAGAGTGTCTGCAATATCAGACTCCAAAACACTTACAGAGGGGAAAACAGAGGGGAGGGGCTCCCCCAGGTGCCCTAGGCAGGACTTCGGGAAGCAGGCTCCTGGGATAAGCGAACATGAGGACTCTTTAGGACAGGATAGGTCAGAAAGGCACGGCCCCTATCCCCCAGTACGCTTCAAGAAGATGAGCAAGTGCAAGGATAGTGCTGGCCCAGAATTGGGCTGAGGGAAGGGAGTAAGGCAGTCCTGGGCATTGTCAGTGCGCATGCCCAACTCATGAGCCCACAGCACAGTGCGCCTGCCCAATTCACCGTGAGCCCCACATCGCAAAAAAAGATGCCACCCTTCCTAACGGGTATTCTTCCCCAGACTGTGGCCATCACCTCTTCCTCCAAGCCAACTTCAGTCTCCAGGAGACAGAACAGCTCGAATGCTGCTAGCGGCCATGCCCTCTGTACCAGAGTCTCTGCGGAGGACAGGCTGGAGGCCCCCAGAGCCACTAGAGGGCAGGTGCGGAGAGAACTCTGTACCCATAAGCTAGACTCCTGCAGGAGACAGCTTGTCATCTGACCGGGGGGGGGGGTCCTGCCACCCTATATCCCTAGCCTTCTTTGGGCAGTGTGTATATGTGTATCACAGGGGACACAGACAAAGCAGAGCTGTTTGTAATCTGAGTTGAGTGGACACAGGCAGGTATCGATCAGCGTGTGGGAGTACATTTGGACCCTGCAACAAGGAGTCAGAGCAAGCACAGACCAGGGGTGCCTCGGGTGAGCAAAATGTGAAAAAGTGTCACTGCTTGATGTCATAAGCTGGCCACCAGCAACCTTACCTCCAGAGAAGAAGCCTCAGTTCGACTTCTTCTCAAAGCAGGAGCGTTAGGGAGAACGAGCTTTTTTG
MSTRG.128109.2	ENSRNOG00000014399;ENSRNOG00000014797;ENSRNOG00000014806;ENS	1.327	0.171	0.039	-2.145	down	TAGGAAATTGGGCTAAATGTAGGCTAGCAACTAATTATTCAAACTATGAAATACAAATTTAGATTTTAGATTTTGACTGGTTTAGTGTATGTGTATATGTGTATAGTATAACTTCTTTACAGTCTTTTCCCACTTTTGTGTGTGTGGAGGAGAGTATGAATTTAAGGCTCTTCAGCGTTCATGTGGTGAGACAGTTACCTAAAACGGAAAGCAGCCTATTGTAGTGGTTTTGTTTTTGCTTTTTGTTTGTGCTGCCCGTATGTCTAGCCCATGACTGTTCCATGATATGTGACCCGTCTTCCCGGCCACCTTCTCCGTATCAGAGGCCACCTTCTCTGTATCAGAGGCTAGGGTGATTTGCTTTAGTGACCATGATGACCAAGTTAATCACCTAAGATTTTTCCTGAATCCAGAAAAAGGTTTGTTTTGCTTTGCTTTTTCTTTTTCTTTTTCTTTATTCTTTTTTTTCAGAGCTGGGGACCAAACCCAGGGCCTTGCGCTTGCAAGGCAAGTGCTCTACCACTGAGCTAAATCCCCAACCTCCCTTTTTGATTTCCTTAAAACAGACAGCCTCTCACCGTGCAGCCCAGGCTATCCCAGAACTCAAGTGTTTACCTAGCTATTTTCACATTTGGAGCAGTCTGCTCCCCCACCACCCGTGTGCTGGAATTAGAGGGTCTGTCACCACATCTGGCAACTGCTTGTGTTTAGACACACACTCTGTTGCATTGGCCCAATAAAATAAAAGCGTGGGATCTCCATGTCATTTTTA

	RNOG00000033584;ENSRNOG00000048239;ENSRNOG00000058260						CACATGGGCCATGCTGATCCTAGTGCACGTTCCAGTTTTAGTGTATGTGCTACTCACGTGAGCACTTCTGTTTTCTTAGACAGTCTCATATGCAGCCCAGGCTGGCCAGTAACTCCTGGTCCTCCTGCCTTTGTCTCCTGAGCTCTGGAGTCTGTCACTATCTCCTGCTTGAATTACACCCTGATGAAAAAGATGCTCCTTTTTCATAAATAGCTTATCTCCAGAATAAATTATACTTTCCTGAAAGAAAGAAATAGTACCATGGGTTTGACAAGTAAAGTCATTTGCCACCAAACACAGTGGCCTACATTCCATCCCAAGGTCCTACATGGTGGAATGAAGGAACCATCTTCCTAAAGTCGTTCTCTGGTCTCCATGCATGTGCTACAGTACAGAGCAAATAAAATGTAATAAATGTTTTTTAAAAAGAAGTAGAGGGGGGCTGAGGATTTAGCTCAGTGGTAGAGCGCTTACCTAGGAAGCACAAGGCCCTGGGTTCGGTCCCCAGCTCCAAAAAAAAAAAAAAGAAGAAGTAGAGGGCTGGAGAGATGGCTCGGTGGTTAAGAGCACTCCAGAGGACATGAGTTCAAATCCCAGCATTCACATGGTGGCTCACAACCATCTGTAATAGGATCTGATGCCCTCTTCTGGTGTGTCTGAGGACAGCTACAGTGTACTCATATATAATGAAATTTTATAAAAAATAGTGTCTTGGTATTGAGCAGATGTCCAGTCAGAGAGGAGAAAAAAAGCAATCTGATTTCTGTGTAATAGATAATGAAGACGGCATCTGTTTGGAGGGGCTGATTGCTGTTGTTGTTTCAAGGGAAAGCAGGGAAGAAGAGATGACGTGAAGCTGGCTAAAGGCTACTTCCTGCTGCAGAAAGTGTTGATTAAATCTTCTGGGGCAGGGCGGTGTAGAGAAGAAGAGAGACAAGTCTTGTTGTAACCAAGAATGATCTTGATTTCTGTGTGTTTGTTTGCTTTCTATTGATTTTGTTTTGTTCTTTGTGGGCAATATGGTTGAGACAGGGTCCTACTGTATAGTCATGGCTGTCCTGGACCTTGATACATAGACCAGGCTAGTCTCAAACTCAGAGAATCTGCCTGCCCCTCCCTCCTGAGTGCTTTGATTAAAGGCATGCACCACCGTGATCTTGAATTCATAATCTTTTCCCAAGTGTTGACTTTACAGCATATACCCTGTCTCCAAATATCTTTCCTTTAAGGAAAAATGTATGTTGGGAAGGGAGCTAAGCTAACACTATGTCCTTCCCTTCTCCATAACCCAACAGCCAGGCAGTGCTATCTCAGAGGCAGTGTATAAAAGAAGCAAAATAACACATGATCTGTATTTGGCTTGAGATTTGATAATCAGTGTGCAATAGAGTTTCTATTGCTGTGAAGATAAACATTTAATTGGGGCTGGCTTACAGTTCAGAGGTTTAATTCATTGTTGTCATGGTGGGAAGCAAAGTGGCTTGCAAACAGAGAGGAGATAAGAGTTCTGCATCTGGATTGACAGGCAGCAGGAAGACAGAGTGACCACTGACTAGGCCAGGCCTCAGCACCCATCCTATATCATACACTTCCTCCAGTAGTTAGCCACACCTACTCAAACTAGGCCATACTTCCCAATGGTGCCACTCCCTATCAATCTGTGGGGGCCATCTTCATTCAGACCACCACCCAGTGTTCTTCCTACTTGCAATACAAATGGAAGCTTAGTCCTTGCCCCTTCCTGCGCATTGCAGTGGCTCTGTACCGAGGGGGTAGGGATGCTTTCTCTTACTCAGCAGGACTGTGCTAAACAGACTCTTTGAAGGACAGAGAAGAAATAGGGATAAAGCTGGAGCTTTGGCCTAAGGAACTCAGCCTGGCAGGGAAGCCAGTTGCCCTAATGAGTAGTGAAGTACAGAAGGGTAGTGAATGGTAGCTCCCGTTAAGTAGCCAGTTGTCCCTGGCTGTTAGAGGCTGACTGTAGAAGTGCTGTGTGAGCAGCAGCAGTCACAGCAACACTTCTGTCAAATGGCTGCTTGACCGTGTGTGTTCAGTGTCTTAACCCTGAGACTGGCCTTAGCTTCATGCAGCTGCCT
MSTRG.128164.1	ENSRNOG00000017188;ENSRNOG00000017248;ENSRNOG0000001	0.277	0.889	0.023	2.251	up	TCCTAGCTCAGGCAGGTGGGGGTGGTAGTGGAGACTGCCCCACACCCTAGGACATGTCACCAAAGCAGCCCAGCCTGGCAGGCCCACCGCCTGTCAACTCTTCTCCCCTTCTCCAGCAGTGTTGGGCAATGCACTGAAGCTGGTGGGCACTGGGCGAGAAGCATGGTCAAGGCCATCATCCAGGAGCTCCTTCAATCTTTCTCTGAATTAATGCTCCCCCAGCTCGCCTCCGGCAGGGGGCCTTCCACCCTGGAGAGTGGCCAGGCTCCAGTCGCAGCCCCCCTCCTCCCTCCCCCCTCCTCTCCTGTGGTTGCCGGCAGCTGCCTGCGGTCGCCCCATCCCTTGTGCTCAAGGCACGGGGACTTTCCAACAGGGGAAAAAATGACTTAATGAGAGTGGGAGCCGGGGGCGGGAGGGACAGGTCCCTGTAATCACAGCTCCACTGTGGGGCCTGCCTACCTCCCGGTCCTTCCCAGCTTCCTGCTGCCAGCGCCCGCCTTGGGTTCTGCTGGGGGAGGGGGACAGGCTGGCGACCCGGAGATCCTTGTTCAGGTGGTAGGATCTCTGGGGCCCTTGGGCTGCCTTTTCTTTCTGAAAAGGCATGAGTTGCTTGAGGGAAGAACCCGGGACACCCCAGAATCCCTCTCTGGATCCTGTCCAGAATGAATTCGAAGAGTAAGGGGTA

	7409;ENSRNOG00000017856;ENSRNOG00000017996;ENSRNOG00000037793						CCAAAGAGAACCCTTTGCTGTTTCGAG
MSTRG.128172.1	ENSRNOG00000017409;ENSRNOG00000017856;ENSRNOG00000017996;ENSRNOG00000018059;ENSRNOG00000037793;ENSRNOG00000052510;ENSRNOG00000056996	1.510	0.185	0.030	-2.209	down	GAGAGAGTGAAAGAGGCTTCATATTGTTTAGGGAGCTAGAGTTATAGGCAGAGTTGGGTGCTAAGGACTGGTCTGGGGGCTGAGAGCTTAGTATTATGCAAGATCAATACATGGTGTAGATGGGAGATGCTACTCCTAGTTTTGGAGCTACTGACAGTTGTCAGCTGATAGGAAGGGGGGAGACATTGCTCTCTAACAGTATATTGTAGCCTTTGGTAAGTTGGTCACCTTCCAGTGGAAAACCACACATCTAAGAATATTCAGGCAGCACAAACTGGTCTTGAAAAGGGCTTCTTTTTAAAAGGGAAGGCAACAGAGAGGAATGGATCCGGAAAAGAGGAGGAGGAGAGGGATATGGTTAAAATATGTTGCATGAAATTCTTAATGAACTAATAAACTCAAATACATAAACATACACAACACACACACTACACAAGCATACACAACACACACACACTACACAGACATATACAACACACACACTACACAAACATACACACACTACACAAACACACAACACACACACTACACAGACATACAACACACACATTACACAAATATACACAACACACACACAAACATACACAACACAAACACACAAACATACACAACACACACACTACACAAACATACACAACACACACATATGCAACATACACACATTACACAAATATACACAACACACACACACACACACACGCACACGCACACACGCACACACTTAGTTCACCATGAACAAGAGTTATGCATGCTCCTAACTGCTAAGCTGTCTCTCCAGGTCTTCTCTTGTCCTAATGCACTGACTGTCAGTGCTCAGTGGTATCAAGGAGAACAAGGATAAAAAACATGGAAATATCTTTCTTTACTCCAAACTACTTGTGTATATTTTTTTGTTTTTGTTTTGTTTTTAAATGATACATTTAAAATATGTATTAAAAAAAGAACAAAATAAGAACCCCAAAACAGAGCAAAACAAAAGGCAAACAGGGTTGTAGTTCTGCATGGTTTTAATTCCAGCAGACAGATCTCTGAGTTCAAGGCCAGCCTGGTCTACAGAGAGAGTTTCAGGACAGCCAGGGCTAACCCTGTTTCGAAAAAATAAAATAAAACAAAAAACGTATTTATTTAATTTTATTTGTATGCTTGGTTGTCCACATGTTGGGCGTCATGTTCACACAGTGCCTGAGGAGAGCAGAAGGGGCATTAGGTCCCTCCAGAACTGGACTTACAGATGACAGTGGAGTGGAAACTGAACTAGCTCCTTTGCAATAGCAACATGTGCTCTTAACTACTGCGCCTCATCTGTTTGTTTGAGGCAGTCTTGCTATGTTCTCCAAGACAGCCTAGACTTTGCTTTGTAGCCCAGGCCAACCTCAAACTTGTCTTCTCCTACCTTGGCCTCTTACGTGCTGAGGTCGAAGCCATGTGGTACCACACTCAGCATGTTAGCCATAACTTGGGCAATGTTTCTCATATTTCAACAACTGCTGCCTTCTTGTTTGTCTTTTAAATTTGGATGTTTGTTTTCGTGTACATTTGTGAGCAATACATCATCTCGGGTCGTGTTTTAGAAGCCGAGGGAATCTTAATCAGCGTAATCACTCAAGCTTCTCGTTTTGTGCTGAGCCCCAGCTGAGCAGTGTGTGGCGGCACGATCCTGGAGATGTACAAATTGGCAATAGAAGAGGGGCTTGATGGATGTCCTCGCCGGTCCTTTATCTCCCATGACATTTAAAGGCAGCATGATGTCCTGTCCTGAGCAAGACGAGGAGCAAGGGCTGGGTCTCCCGCTGCTGATCCCAGACGGTTACTCGGCACCTGTGGATTTCAGACAAGTTCTCCCATCACCCTGGAGCTTGGCATTTTGGTTAGACAGACCCTCCAGCAAGCCCCCAGGATCCACTGTTTCTACCATCACCCCTGGGGTTGAGTCCAATCCTCTAACAAACACCAAACACGGATGAGCGGCTGCAGTCCAGTCCCCTCGGTCATCACCACTCATGAATCAGCAAGGGCGGTTCTATCAGGAAGAAATCGCCAGGCTCCACCAATCAGCCTGGATCCGAGGAA

							GCATCAAGAAGCCGCCAGAATATTAGAGCTCTTTGTCGAGTTACTCTCTACGAAGTCATGACAGCCAAAGATCAGCAACGAGACATGAGGCAAATCGAGGCAAATCAATACAAGAGTGTCCTCCACAAAGACTAGCGGGACAGAGCAAGGCAAACCAAAGCCAGAGCCTCCTCCTTCTGGCTGTGCGATCAGACTTCTGTTCTCTAAACATCACATGCCCTCTCATGTGTCTGCTTCAGCAAAACATTCTTTCGTGCGTCTGCTTTAACATTATTTTACATGTTCGCTTCAGCAAAACATCCTTTCACCTGTCTGACCTAGAAAAACATCATATGACATAACTGAGTTTCTAAAGAAACCAGAAAATTTCCACTTCAGAAAGGCCCATGTCAGATAAGGTAGAAGGTGGGGATTGATCCCAGAGGTTGTTCTCTGACCACCATATGTTGTGGGTTGCATATGTGCTGGACCCACACACATAAACTCACATATGAACACATG
MSTRG.128202.1	ENSRNOG00000003597;ENSRNOG00000018639;ENSRNOG00000018697;ENSRNOG00000018975;ENSRNOG00000019052;ENSRNOG00000019243;ENSRNOG00000019294;ENSRNOG00000019506;ENSRNOG00000019704;ENSRNOG00000019772	1.528	0.000	0.001	-4.627	down	CTGAAGAGTCCTCTGCCCGGCTGCTGCCCCCTTCCTCTGGCAACCTTGGCGCCCGGGCTCTGCCAGGCACTGGCAACTCTTGGGCCATGTAGAGCAGCCCTGAATCCTGGAAAAGCTGAGCAGGTGAGGGACCTGTAAGGTTGTAGCAATGCAGGTTCATAGGTCAGAGACGGGTGTGGAGGCTGCGGGGGCATTAGCAGATGCTCCAAGAGAGGGGGTAACAGCTCAGCCTGCAGAGAGGACAGTGGGGAGCCCACCCCAGCTCCATTCCCATCCCCTGGAGGCCGA

MSTRG.129081.2 NONCODE_ID：NONMMUT002077	ENSRNOG00000023993;ENSRNOG00000024030;ENSRNOG00000045623;ENSRNOG00000045775;ENSRNOG00000057389;ENSRNOG00000058463	13.472	0.589	0.000	-3.734	down	AGCTAGGGGTACCTTGGCCAAGCATGGCAGCAGACGAACGAGGGGTGTGTGTGTGTGTGTGTGTGTGTGTGTGTGTGTGTCTGTGTGTGTGTGTGTGTGTGTGTTGTGTGTTGAATAAGGTGTTGGTATGAGGCAGAAACAGCCCCTCCCTCTCCAGGAAAGATGATTATTATTCCAAACTAAGTTGAGGTCCCAGCCTGGGCAACATCAAGTATCAACACCAAACTGTCTGTGGAGGTGGTTTTCCGGATCCATCTCACCCTCTGTTCCAAGCAGGAAACACAGACCCCAGCTAGGAAGGCTTGGCCTATCCCTAGTCCCCGTGTCATCCCTCCCCGTCACGGATGTTGGACAAATGACCTTCAGCTGACATGATATACAGGTAATTTGGGGCAAGGAGGGCATGAAGAAATCAGGTTTCCTGAGCTGTAGACGGATCTCTTGGTCCATCAGTTCTGGTTACTAGTTTCTGACTCAGGACCACCCACAAAAGCCGAGAGCCCACCTTGGTTCCCTAGACAATGCCAGGACAACACTGGGCCAAGATACCCCCGAATGGGGCAGGGTGGGCACTACACCTCCCGACCTTTCTACAACCCGACCCAGGCACAGGTGCTCTGTAGGTTGGCTGGCCTCAGACACCCACCTGCTGGGTGGAGCCGGACTCTTCCAAGGCCTCAGCTTCAGGTTGGCTGACCTGAGCGAGCGCGGCAGGCCCGACTGCCTCTCTGCCTGCTGGGGGCTGTGTGGCTCCGTGGCCCTTGGGAACCAAGACATTGAAGCAACTCGCTCCTTCCCTCCTTTGAATAGGCCATGAGCACCAGAGTGAGTTTCTGAACACAGCCGTCTTCTCTCCACTTTGTGGTCTCAGCCTTCTGCCTCCAAGGTCCACCATAGACAGCTCAGGTCACCTGGTTTCTAGTTCGGGCTTAACCTTGATTTGTATTCTTGGAAATAAGTCTTATTCTACACTTCATAGATAGGGTCTCCTGACACTTGATGCTATTGACTCTTACCTCAAGCCCTGGGGGATGACGGTGGCCAGGCATGAGGGTGGCCACCCATATGGCACCTGACCCCAGTGATGGGTGAATTCTCAGTTTCCGCTGGGCTCACACTGGATGGCAGGTGCACACACTACTTCGAGCCCTGCCACTACCCTGAATCTTGATACTTGGATGAAGGACAGGACCCTTGATAACTGTGCCAAGGGACCTTTGAAGCCAGAACCTTCTACGACCATCCAGACTGTAGAGATGCTGTGAGCATTGGTCTCAAGAGTATCCAGGTCATGTCAGTGCCATGTGGAGACAGCTTGAGATGATGGGGAACTTAGACCCATCAGTTGATCTAGAGGGGCGGGGAGGGGCTGACCAAGGTGGAGGGAGAACCTTGGAATAGCTGGATGCTGAGTGTAACTGCTCCTGATAGGCCCTGGGGACTTCAGGTTTGGTTTCTGGCTTTCCAAATGGACATGGCATGAAAATTCCCCAAGCGCAGAAGGTGCAGAAGAGTCCCTTTACCAAGACCATCGCAGGTCATCCTCATACATCATTCACTGAGTTCTCTTGCTGAGATTTACAGGAATGCCACAACTGGGGCAGATCACTGATCACAGAACCTTTCCAGAGCTACATATTCATTCCTCTGCTCATCGACATCGAGGCTACAGTCAGAGACCCCATTCTGAACTCCCATGGTCTCTGTCACCCACAGCCGTGTGTAAACATGGCCGCCTACCCAATAGCCTGGACACAGCTCAGTTGGAGCCAGTCTGCCTTGCGAGGGTTGACCATACACCTCACCTGATCCTCCTTATTCTGAGGTGAAGCTGCCCACACCACCCTTATTGCTCCATTTTGGGAACCGTTCCCTCAGAAGTAGCTCACAATGCTTTCTGAACTGTCTAAGTCTCGGGATTTGCAGAACCCCTCACACACATACTGTACTAGGTTCATTTCAGACAAACAGGCCAGGACAGAGAGCTTCCCTATGCCAGGCCGTGAGGAGCCATGGCTTGTTCACATCCCTCCCTCCCTCTGCCCCTAGTAGACTGGAGATACAGGTGGCAACCAACAGGGCCAGGC
MSTRG.129081.3 NONCODE_ID：	ENSRNOG00000023993;ENSRNOG00000024030;ENSRNOG0000004	0.388	16.296	0.000	6.125	up	GGGTGTGTGTGTGTGTGTGTGTGTGTGTGTGTGTGTGTGTGTGTGTCTGTGTGTGTGTGTGTGTGTGTGTTGTGTGTTGAATAAGGTGTTGGTATGAGGCAGAAACAGCCCCTCCCTCTCCAGGAAAGATGATTATTATTCCAAACTAAGTTGAGGTCCCAGCCTGGGCAACATCAAGTATCAACACCAAACTGTCTGTGGAGGTGGTTTTCCGGATCCATCTCACCCTCTGTTCCAAGCAGGAAACACAGACCCCAGCTAGGAAGGCTTGGCCTATCCCTAGTCCCCGTGTCATCCCTCCCCGTCACGGATGTTGGACAAATGACCTTCAGCTGACATGATATACAGGTAATTTGGGGCAAGGAGGGCATGAAGAAATCAGGTTTCCTGAGCTGTAGACGGATCTCTTGGTCCATCAGTTCTGGTTACTAGTTTCTGACTCAGGACCACCCACAAAAGCCGAGAGCCCACCTTGGTTCCCTAGACAATGCCAGGACAACACTGGGCCAAGATACCCCCGAATGGGGCAGGGTGGGCACTACACCTCCCGACCTTTCTACAACCCGACCCAGGCACAGGTGCTCTGTAGGTTGGCTGGCCTCAGACACCCACCTGCTGGGTGGAGCCGGACTCTTCCAAGGCCTCAGCTTCAGGTTGGCTGACCTGAGCGAGCGCGGCAGGCCCGACT

NONMMUT002077	5623;ENSRNOG00000045775;ENSRNOG00000057389;ENSRNOG00000058463						GCCTCTCTGCCTGCTGGGGGCTGTGTGGCTCCGTGGCCCTTGGGAACCAAGACATTGAAGCAACTCGCTCCTTCCCTCCTTTGAATAGGCCATGAGCACCAGAGTGAGTTTCTGAACACAGCCGTCTTCTCTCCACTTTGTGGTCTCAGCCTTCTGCCTCCAAGGTCCACCATAGACAGCTCAGGTCACCTGGTTTCTAGTTCGGGCTTAACCTTGATTTGTATTCTTGGAAATAAGTCTTATTCTACACTTCATAGATAGGGTCTCCTGACACTTGATGCTATTGACTCTTACCTCAAGCCCTGGGGGATGACGGTGGCCAGGCATGAGGGTGGCCACCCATATGGCACCTGACCCCAGTGATGGGTGAATTCTCAGTTTCCGCTGGGCTCACACTGGATGGCAGGTGCACACACTACTTCGAGCCCTGCCACTACCCTGAATCTTGATACTTGGATGAAGGACAGGACCCTTGATAACTGTGCCAAGGGACCTTTGAAGCCAGAACCTTCTACGACCATCCAGACTGTAGAGATGCTGTGAGCATTGGTCTCAAGAGTATCCAGGTCATGTCAGTGCCATGTGGAGACAGCTTGAGATGATGGGGAACTTAGACCCATCAGTTGATCTAGAGGGGCGGGGAGGGGCTGACCAAGGTGGAGGGAGAACCTTGGAATAGCTGGATGCTGAGTGTAACTGCTCCTGATAGGCCCTGGGGACTTCAGGTTTGGTTTCTGGCTTTCCAAATGGACATGGCATGAAAATTCCCCAAGCGCAGAAGGTGCAGAAGAGTCCCTTTACCAAGACCATCGCAGGTCATCCTCATACATCATTCACTGAGTTCTCTTGCTGAGATTTACAGGAATGCCACAACTGGGGCAGATCACTGATCACAGAACCTTTCCAGAGCTACATATTCATTCCTCTGCTCATCGACATCGAGGCTACAGTCAGAGACCCCATTCTGAACTCCCATGGTCTCTGTCACCCACAGCCGTGTGTAAACATGGCCGCCTACCCAATAGCCTGGACACAGCTCAGTTGGAGCCAGTCTGCCTTGCGAGGGTTGACCATACACCTCACCTGATCCTCCTTATTCTGAGGTGAAGCTGCCCACACCACCCTTATTGCTCCATTTTGGGAACCGTTCCCTCAGAAGTAGCTCACAATGCTTTCTGAACTGTCTAAGTCTCGGGATTTGCAGAACCCCTCACACACATACTGTACTAGGTTCATTTCAGACAAACAGGCCAGGACAGAGAGCTTCCCTATGCCAGGCCGTGAGGAGCCATGGCTTGTTCACATCCCTCCCTCCCTCTGCCCCTAGTAGACTGGAGATACAGGTGGCAACCAACAGGGCCAGGCTGGGGGTGGGG
MSTRG.129251.1	ENSRNOG00000053696	0.000	0.537	0.000	5.500	up	ATCAAAAGGTACTGAATTGAACTTAAAAAAAAAAAAAAAAACACTACAGCCTGGGAACACTACTACTCGGGTTTTCTTCTCTCAGAAAAAAATTAACCGTTTGTTTGTTTGTTTGTTTGTTTGTTTGTTTGTTTGTTTTGGAAAAGGCATACGGAGAGCACAGAAAAATGGCTATCTCAATGACACCTTGTTTCAAAGAGGGTTTTAAGTGCTTGTTTCATTTACCCCAGGGTGAAGAAATTATTTAGGAATGCCTAATGGGAAAGATCAATCCAGTTGTGTGTGTGTGTGTGTGTGTGTGTGTGTGTGTGTGTGTACACATGTGTGTGCACGCCTGAGCTGATAAACTATACCTGTTTCTTGCATCTTTTCATGACATTTCGTTTCTACATAGAATTTCTTATCTCTACCTGAGAACCTAGGGTCAAAGGCTTGCTTCAGAATTTGGGTTGAGAAACGAAGCCCAAACTGGCTTCCTCTACCTGCTTTATGTCTGACTCACCACGGCTGCTTGGTATGCCATGATAGTCCACTGACGTATTTTCCCTACAGATTTTCTTTCTGGGAATTCCCCCGCAGCTTGTGAGCATAGTCTGCTTCATCTTAACTCTTCCTTCCCACTGCAGTTTCTTTCTTGTCTCATTCCAGGGACTTCTTCCACGTTTTCAGTGACCTTCCTTCCAACATCCTCCAATCCTGCCAAACAGAACTCCATAGAACTCCGTAGAGCAGAGCTCCCTGAAAAGACCAGCGCCTGACTGCAGATCCAGGGTAGCTCTGGGCTGCAGCTCGTCCTCCGCCTCTCTCCGCGCAGCTGGAGCGGCCACCGCGCAGGGCTGGCCTGAAAGGGCCTGGGACTCCCCCGCGTCCAAGACTTTCGGGCCCAGGCCTTCTCCACGCTTCCCCGGGACCTCGGGACAGTCG
MSTRG.129408.2	ENSRNOG00000034226;ENSRNOG00000042133;	0.271	0.000	0.000	-7.765	down	CGGTAAATGATAAAACATTAGACGTAAAAAAATGATATAGCACTTTATAGATGGTAAAACTGAAAGGATCAGAAAATTAGACTGCACAGAAAGTTGTCAGTAGAGCAAAGTGTAACAGACACATGCACACACACACACACACACACACACACACACACACACACACACACACTGGTTATGGCTTTGCCTTCTCCAGACAAAAATGGGGCTATCCTGGACAAAAAAACATACCCATTCTGTTCCCCCCCCCACAAAGTTCAACCCTTTAGTACTATCTTTATGACGTGTCAAATATTAAGGCATTGACAATCAACGAACCATTTGGTGGCCTCTAGTAATTAAGAACCTATCCAGGTCATCTGGGAGTGCACGTTTTGGATACGAATAACAAAAGATAATATTATTAGACTCTCACAGTCTATAACGCTGCTAAAAATGTAAATTACACATGGGAGTCAGAAGCTTGGATATAAAAGATGTAA

	ENSRNOG00000047234;ENSRNOG00000049016;ENSRNOG00000049451;ENSRNOG00000055008;ENSRNOG00000059588;ENSRNOG00000060573;ENSRNOG00000060998						AATACCTCAATATTTTCATATTGATTCCATAGTAAAATGACAATATTTTAGAGATGATGAGTTATATAAAAACCAATTTCACCAGTATACAAGCTTTGACTTTAAAATGGGCATTAGAAAATTTTTAAAATGTGCTTTATCTCCTACTGCGCCTGGTTCTGCTGCTGGGCGGGACAGCAGCAAAATAGAGAGGCGTACACTACCAAAGAAACCCGGGAAGGAGGAAAAGATGAAATCTAGCCATCACAGAGACAAGAATCTGAAGAGAAAAGAGATGAGTCTTCAGGGCACCATGCTATCTCAAAGGGAAAACATGTGGGTCGTTCTACACAAGAAAGGAATCTCTCCCCACCATCCTAGGATACCCGGGGCAAATCAGGTCTCCCCTCTTCTCCCTCTGAACGGAACTGGCAGTTCATCACTTTGCATGCTCCTCTGTGGAACTCTGAGCTGACTGCAGTGCAGATGGATGGCTCTTTAGATAGAAGGGAGCTCAAGCCAGTCCTTATGTTAGAATAGCATCCACCCTCGGCGATCACAGTTATTGTGAGCATCCTGAAGTTCTTACTGTGAGATCACAAGACAGGGCTCTCTTGAACCACTGTGTACTTAGGACATATGCGCCTGTGCACATGAACGCACAACGCCATCGCTGACTCCTCTGGTGTAAAGTATTATTTCTACAGCTCTCCTTTAATATTGCTTTAAATTCTAAGTACAGATCGCAACTGTGTTATACAAATTGTAGGCGAACCTTCCAAATGGCCTTAAGTAGCAAGGTCAATGCTACTGTTTTCTCATCCCTTTTGGCCACTGGGATAGACTAACTTTAATGTTTAAATAGATTCTTCTCCAAAGGAAAAATGAATAAATCAAGCTAAAAATCAACAATATTATACTAAAAATAGGCAGTGAATAACAAGCACACAGGAAGTAAGTCTTGCTTTTATAATAATGAGAAAAATCAAACCGGAAGTTAATGCCACTTAAAAGTTGGTCCACAATTAATTTTAACAGTTTAATGTGTTCTTATACATTGTGTTGACATATATATGTATATATCATCATTCAGGATTTTGGCTTACCCATTTCTTCAAAAATTTCATTCAACCATTATTCATTATTTAAATCATTCTTTCATTAATTTTTGTGGGGTTTTTTTGTTTGTTTCTTGTTTTTGTTCTGATTTATTTTTTGGTTACATGTAGCAATTGTTCTGAAATTTATGCTCGGATGTTTACAGGGATATTTTAGGTATTCTGGTTGACTATGTGAGCTTTATGTTTGTAAGTGATATTTGTTACAACTGTTACACTTGTAAATTCATTTATAGGACGACTTTCCTTCAATAGTTAGTTAATAGCCAAGTTCTAAGCCTTGGCAGGCTAGTTTACAATGAAAACTTATCTGAGATTTTTTTATATCCCGGCAAATAAAACGGCAGATGTATTCAGAAACTCATTCAGACTCTGAAACAAATATTTGTTCCTTTAGACTCAGAGTAACCACACTAAAGAACAAAGATGTCAAGCGTATATCTATTAAGAGCACAAACATATAAAATTATTAATGATTTTAAAATGTAGATGGGAAACAAAGAAATATGGTCTTCACAGGGGCATATTTTTTTCCCATGATATGTTGACTATAATAGTCCGAGAATTCACAACAGTCTTGGCAAGGCCCACAGCAGATACTCAATGAATATTCTGAATGAGTTGAATGGCCCAAAACAGACATGCTGAATGTTAATGATGAATGAATGACTAATTCATGGAAATAGTGGAGGGACCACTAATGCTTTACAACATGCTACATGACCCCATTTCTATGCTGTCAGAATACCCCAAATGCCTTACGCTGTAATGGGAATAGGTACTTAATAAGTTTCCTTGAACAAGTAGATGAAGAAAAAAACCATTGGTGCTTTGGAAAAAGAGCCATGAATTGCGGAGATCAGAACCAAACAACCTTTTCTCATACAGCTCTTGGATACAAGTCTGAGCGAACCGGCTGTGACCAGTATCTTCCCATCTATGGAGGCCAGGATGATAGGGTCATCCTCTTGGGACTTGATGAAGACCAGACATCTTTCCTTGATTTACAATGCTGTCAGGCACAAGGAGTAACTCAGAGCCATTATTATGCAACTACTTTCACAGAGGAGGACAGAATTCCTTTCATTTACACTGTAAGGGGTAGACAGAATATCCTGAGGAATGTGGCAGTCTACCTCAGGATCAATCCTTCACCAACTTCAAAAGCACCCTCCTAAAATAAAATTTAAAAAAAAAAAAATCAACCCAATTGCTGCATACGGATGATACACCTTGCTTTTCTTTTAAAAAGTGAAAATGTATAGGAAAGCATATATGCACAAGACAACAACTAATGAAGGGGCCATAAATATATGGGAGGATTTGGAGAGAGGAGAGGGAGACATGTAATTAAACTACAATCTTTTAAATTAATAAAATAAAAAACAAACAAACCAGGAAAAAGAAGTTACCATGACTAATGGGGTGACATCCTAAACAAGTCATGCTCAGACCTCATCCATCCAGAGGGCAAAGGAACCTTTATGGACAGGGCCAACTCACAAAGCATTCCAAAATTTTCACAAGCTTGCCTCACAGTAGAGCTTTACAGGAGTTTCATCTGAAAAGATCGACTTCGCCTAAGAGCTTGCTCACAAAACATCCGAGAGGTGCATTTCGAGAAGACGAAGGAAATGCCAAGATGCTATCTGTGACTGTCGCAACGCTTGCTTTACTTCTTTTCTGTCAGAAGCAGACAGTCACAGCAGATTTCCAATGGTGGGCACATGTAAATGGCATTTGGCTGAAAAGACATAAAGA

							GGCATTCACCAGGGAAAGTCCCACCCCACTGAGTCTGTATGAGGCTCACTCCGGCCCCGTTGTCAATGA<br>AGACAGCGGCTGTCAATCAATTCCCACTCAGCAGTTAAAGAACCCCAAGGCAGCTCCTCAGTGGGGCA<br>TAAATTGCTCCCACAGTTTAGGTGGAAGCAATGTATTTTCAATCAAGGGCAAAGGGTGGAATAACTAGT<br>TCCTTCGTCATATCCTCTTACGAGTGTAGCCATCCCGGCTTCTGCACCTCTGGATTACAGTACTTGCAAA<br>ATCCTCATTCCGGGAATTCATAGGCTCACACCTAAATCCCCTCACCAGGGCCACAAGGCTAAGCGAGCA<br>GCCCACACAGACTTCCAGAGAATACCAACTGAGGCAGAAGGGTTGGAGCAATGACACTTTGCCAGAG<br>CAATGCAGAGCACAGTGCTGTTGTTACCGGACAGAGGACAAGACCTTTACCCTGGGGTGACGGCATCA<br>TCGTGAGACAGTCACCAAGAATGCCATCCAGAATCAACATGGCATCTTACAACTTCAACTTTGGACTGG<br>AGATCGCTTAATCTCTTTTATGCCCCGTATCCTTCATGCTACCTTAATGCGGGCTGCTTATTTAAAGAAAA<br>ATGTGTCAAGAAGAGCAGAGTTCTTTACAGACATCACACTGCTAGAGAGAGAATCAACGAGGCTCCTT<br>GGTGAAGCAGTCTGCAGGCCAGCTAGCGTTCAGTAGCTCTTCTAATGTATGGAATGTTCTACTGTTGCA<br>AAGGGGCTCTATGCCTTCTTGAGAAGGTTGTTACCAGGATGCAGGGGGAGCCGTGGAGTGGAGAACAG<br>GCCTTGGTCTTCACACATATGAAGGGATGGATGGTTTATGCCCCGTGCTTTCGCACCATGCCTCTGTAGC<br>ACTAACTGTGAGGAGAACATGGCCCAACCACAGCTACTGAGCTTACGTCCTGCTACAGTCAGAGGTATA<br>GGACCTCAGCAGAAGAAAGACAGTGTCTTAGAACATGGTAGGTTAGAGAGGGTTTAAGTCAATCACAC<br>AAAACACCTGTCGAGACTTGCTAGGAAGACCTCACTTGGGAGCTCAATTAATAATAAAATATTGGTCCT<br>ATGTGTTTCGTTTCCAATACGCTGTCGCAAATTCGCAACCCAGTCAATCCTCAGAGCATGCCAGAAACA<br>GGTACTGTTCCCACTGCAAAGATTAAGAAATTCGTCAAAATAGCTCCCTTGTAGAACAAGTGGAAGCCA<br>GATGTCAGAAGCCGTATGCTGGGCAGATGTTAGGGATTTAAAATGAGAACTACCTGAATGCCCTGGATG<br>AAAACATGGTGTTCTAAAAATAGAACACTGGCACCCTCCTGGGATCAACTGTTCGTTTCTGTGTTCTAC<br>ATGAGGGGCACAGGGGGGACCAACTTCTTGCAAATCTGCACAACCATGCTCATGGATTCCTAAGGTCCT<br>TCTCAGCAGAAAACAGTCGTGTTCACTAAAAACGGGCTCACCACTAAGACACTGGTCTCCTTGTTGGA<br>GAACTCAGGGAGGGACAGGACAATACTTGGTAGACAGCTAGGGACGCATAGGCAGTGTGGAGGGGAG<br>AAGTGTTACTTGTAAGCTGATCTGCAGGATGCAGTCAGAGCAGAGGATGTTGCAAGGTAAGCCTGGGT<br>CAGCAGCACACAGGTGCTGGCGCTGAAATTTGTTTTCCGAACATCTGTGCCTAACATAGCAACCGCCAG<br>GGCTGTGGAGGCAGAAATAGCAGATCTCATATGGCCTTATCCAAAACAACAAAATAAAGCACAGTATCT<br>GTCACACAAAATGACATCCCTGTCCCCAGGTTTAGCTATGTATTTGGCCACCTCATAAAACCCGAATCAA<br>TCTTCCCTTCTCTCCTGGCACCAACTTACAAGGCATCTCCAATCTCCTGATCTCCCAGACTGAAAGAAA<br>ACAGCCAGGAGGTGAGGCGCCCCCCATCAGGTGTCTACTTTCTTTTCAGTAGGAATTTGAGACTAAATA<br>CAGACACAAGTTCTCTCTTGGGACTTCAAGAGCGCTCCACAGGAAGGAACATACCAAGCACACATCCA<br>GAAAGCACGTGAGCTATGCTTTCGCCCCTAGGAGAACGCACAGAGAAAGCAAGGGCTCCTATTTCATG<br>GGAAGTGATTCCCTCTCATCTGATGGGCAGAGACCACATCGTCCCACCTGCCACGTGAGTCACTTGGGA<br>ACTGCTTGTTTTTAATTCAGTCTTTTCCCACAACCTGAAGAATATGCAGGAGACCCCAAGACGCTGACG<br>TACAAGGGCAGATTTATTTCTCCCATGAGAAGAAAACCACCAGGGCAATGGTACCTTGAAGTTTTTATT<br>GTCGAGCAAATAAATAAAAGCCCCTTATTTTTCAAAAGGAGAGATGAATGCTCAAGACCCTCCAAGGTA<br>TAAGGTCCCACCCAGTTCCTGAAACTTGCCTTTCCCGGCCCAGCCTTCCTTACTACCATTAACCTGCTTC<br>TTTCTCAGACTGCTCCATTCTGAGGACACATACCAGCCTTGATGCATCAAAACTTACTATTGCTTGTGCC<br>TAGAATGGCCTTCCCTCCGGTTCCAGGTCTCCCCTTGAACACCGTCTCGCCAGAGAACTTTCATGACCA<br>TTGGATTCACAGAGCTGCCCCACAACACAGAGTCTAGTTCCTCTCACCTTTCATAGCAACTACACTGAC<br>TCCTGACACATCCCACGATTCGAGAACTCTTACCATGTTCAGTGAGTAGCTACCTTTAATGGAAATGTAC<br>CGTCTATCGAGGAGGAAGCTTCTGAGGTGCTATGCAGTGTAACCCATTGGTCAACTCTTCTGCGGGAAA

							GCTGGACGCGGTTGTGCATGACATATTCAAGAGCTGGATGCCACCGATGAAATCAGTGGACTTTAAACG<br>AGAACTCGGCCTATTGCAATCTCATTACCTCTACCTACACATACTCAAGCGCCCATGGATACCTGCCAGC<br>CTGTCTGTCTGTCTGTCTATCCATCTATCTATGATGGAATACAGACATTATCTGTCTATCTAAGATGAATTA<br>TGTACTCTATGCATCCATCTATGTCTGTCTGCCTGCCTATCTATCTATATACCTACCCACCCACTATCTATCT<br>ATCTATCTATCTATCTATCTATCTATCTATCCATTATCTATCTGAAATCAACTATATATAGGGAAGTCCAGGC<br>TAGAACAGCCGAAAATCATTCTAAAAGTGGTTTTGAACGAACAGAGTTGATTTTGTAATTTAACTTCTT<br>GTATCTTCCACTGGCTGAATATCTTTATGACGTTTGTCCTTACAATTTAAATGGCCGGCCATTGCTGGACT<br>GCAGGGTTTTGTTCCTTAAGCAGCTACGGTGTATAGAAACTAAGATGGTTTAACTCTAAGGAGCCCAAG<br>ATGAAGGCCTGTAGGGAAGGAAGTTCTTCAGTTGCACAGTGCTTCATGGAACTGGTTAGTCTGCTGGTC<br>TGTTCATCAAATGAGCATTGAACTATACTTCCAGAAATGACCAGGTTTTTTTGTTTTTGCCAAACTGTGT<br>GCTTGGCACACGACATACAGAGCTGTAAAGTGACAAGATACTTCATGTAGGAGGGGCATCCACATTTGA<br>GTACTTCATGTCCGTCTGGGAAGCTCATGCTGACAACAATGGCGGCAGGGGTGGTGGGGGCGGCGGCT<br>AGACCTCTGTGGTAAAGCCTTGAGGACATCAGTATGGCTACATAAATGTAGTGTGACAGCTGCAAGCCA<br>TAGCCTTCAACGAGCAAAGCCAGGCTCCACTCTCCTGGAGGTGGGCTTAACGTAACTTGAAAGACAAC<br>AGTCTTACACCCCAGTCCAATGTCTAGAGCTCTAAAAACATCATGCTCACTGAAACAGTCGGAATCAAG<br>ATCATAACATGATTTGATTCCACGAATATCAACTGTCCAAAAGAGTCAAGTATGCAGATGCAAGTAAGA<br>ATGGGAAAAAGTGGCTGACAGAGCACGACGTCCACCCTACCGTGGAGCATTATAAAATTAGATGGCAG<br>AAGAGATTACATAGCCCTCTACGTACACCAAAATTCTTTGAACTCTATATTTTAACTCAGGAATTTATGGT<br>ATGTAAATTACACCTTCATAAAGCTGTTAAAAGCGGGAAGGAGAAAAACACCTAGAGTGTTTAAAATCT<br>TTCAGTTCCTCAGAAGCAGGGAGAGAGATGAGAAGACAAAGATGTTCTATAGAACTCGTCATGTGGAC<br>CCTTTCCTCCTCCTCTTTGCCTGTTCGTGCCCATTCACCAGGAGCTGGGACAGCATTGTCAGGCATGGC<br>GCCTTGGAAAGCACACAGAATAGAACCACTGGCCAGCCCACGAGAGCCAAATCTACAAGAGCCCTTCC<br>TGGCTTCCTACCCTCAGGTAAATCACTTTAAGGCCATCTCTGCGCTCCACATCCCTCACCTCTAACAAAA<br>ACAATGCCAGCGCTCATCCCCCAGGGCTGTTGAGGTCATTAAGTTCATCTGAACAATGATAGAAGGTGA<br>TTGCTGCTGTTATTACCATCCCTTTAGGATCCTCTCGTGCATCCTACAGATATCAGACTTGTCTGTAGGGC<br>AAAATATGAAAAGGGGGCTGGGGGCTGGGCAGCAATGCCCAGCATAAACTACACTGTGTGTAAACCGG<br>GCTTGATGAAAAAAATGTTAGAAGTGGTACCATTCTGTTACTTCATGAGCACGGTAATGTGCAAAAAGC<br>AAGTGGACCAAAGAAAATGATTGTCTCCTAAGTAGGAAGGTTGAAGGTCATCTGAAACACCTTCCTGA<br>GGATCCTTTGAACTTGCACTTTTCATTTTATAATCTACGAAATGCGAACACTACAGAAATACTTTGTCTAC<br>TCAACTGTTGAGGTCAAAGTAGAGCTGGGTTGGGTTGACTGGTGTGTCTTTAAAAAAAAAAAAGTACT<br>GTATTTTCCCACGCAGTGAAGTGTGCCCAGCAGGGATCCAAACTATATACGCGGCAATACAGCCCGATT<br>AAAAGCTTTTTAATGAAGGCACTGTAAACAGCATTCCCCCTTCTGCTTTAGAATATTTCAAATACAGAAT<br>TAGCAAGCCCCAGAGACTGGAATTTCTGTTACAGGAGAGATGAAAGGTTAACTGTCTGAAAAGGAAAG<br>AGTATGTTGGACATATTCTATTTAGAAAATAAAAGGGTTTCCTAATAAAAGCAGAGCCCCCCACTTGATG<br>CGCAGGACGCTCCTTCAGTGTTTGCACACACAACCAAGTAATTAGAAAGCACGCTCAAAAAAAAAAAA<br>AAAAGACTAAAAAGCCATTGCACACATCGGCCCCTTGTTTACCAGGCTTGCTAGAGGCCCCCCGGAGG<br>TCAGTGTGATTGACAGGATTTTGGTGTTGGTAGGAGGGTTGATAATGCACACTCATCCCTTGAGAGGAG<br>AGCTCTGGGTCCTGACCATGCTTTAATAGGAGAGCAGCCACAACGGGGAACAATGCAGCAGGGGGAGC<br>CTGGGCGAGCGGTGGGTGGAAGGATGTTCAAGGATTGCAAATGGCATTCCCAGTCAATTAGCACTCATT<br>GGGAAGCTGATGTATACCATGCCAAGAGAAAAGAAAGGAAGCTAAAAGTTCCCAGGGCGAGATTCTGA<br>GTTTGGCACAGACAAGGCAAGTCCTGCAGTCATTTTCCTTAGGGTCGCGTCGGGAGCCCTAGCTCCCA

							AATGCATCTTTATGGACTGACATTTTATAAACTTTACGAACAAGGCATTCTAGTGAGGCAACCCTGGTTT<br>CGGTAAAGGACAATGATGCTGCTCTCATTAGGCATGTCTGGTGGGCAAAAATAAACAAATAAATTAGTG<br>ATGATGATAGTGATGATGATGCCAAGAGCAACAGCAAATAAAGCTACTGCTTTGAGCCACCAAGTCACC<br>TTAATTTTAATGTCTTTGTTGAACTTGAATTCACAGACACTCACTGAACAGTGAGTATGTTCTGTAACAC<br>TCATGAGTGGGAGGGGTTCGTGTTAACTACTCCCAATGTTCCTGCTCTTCAGGATCCCCATGACTCACTG<br>AAAAGACTATCCAGCCAGTTTCACTTGACATATTTATGTTTGGGGTATATGATCACAGAGATAATGCAGT<br>TATTTCAATCACATGTCATTAAATTGGTTTGATGAATGGGACCTGCAGAAGATCTGGTCCTTAAGGGCGT<br>GCAGTGGTTACTGCAAGGAGATCGGCTGCCGCGGGAGAAAAGCGTTCACCACGAACAAAATGCTCTTT<br>TAAAACCCCGGTGAGCAGAATCATGGTGCAGTTGCAGATTCTGCTTCTCGGAGCGTCAATTAAACCCAC<br>AGGCTGAAGCACAGATTACCCTAAAATGGGATATTTACCCTTACATGACTCCTTTATCTGAAAACAGAAG<br>ATCATTTCTTAGGGCAGAAGAAAAAAAAATAATCTTGTCTTGACAAGGTCAAACTAGGTTTTCCTAGCT<br>TAATCCTTTTGGAAAACGTGTTGTCTCTTAATCCATTTCACCCTGTGGAAGGTCTTGTCCTGAATTTCTAT<br>ATGATGCTGAAACAATAAACAGACGTGCGGGGTGTCAAAGTTCTGAGGGGGAAAATACACAGAGCTTC<br>TTGAAACCGTGTTCTCCCTTCTAAAATAAAACACGCAGGAAACCAGCGTTGTTTCTTTCGGGCCCCCAT<br>GAAATGATCGCTCCCTCCCCTCCGCTCCCGCCCCTGAAAAAGTGCACACTGTGGAGAATATGCAATGAG<br>ACACCTCCCTAAACAGCGAAGCAGCCAGGAAGCAGCCATTCCAAAGGCAACGCAAATCTGAAATAAA<br>CATAACCTTGCTTAAGTGTATTTTCAAGTTTTGATTTAAAAAAAAAGGGGGGGAGAAAGTTAGGTTTCA<br>GAGAAGGCAGACTTGCGAATTCACCTTCACTCTGGGCCCTACAGGACTGAACTGGAATGGGAGAGGG<br>GTTTCCTCCCCACCACACTGCCACCCGCCCCACCTCTGAGGTGTGATCAATAAAGTAAGTGCCAAGGTG<br>ATAAAGCACTGTGACTCACTTTCCCACTCCGTCCTGATTTCTCATTCATAGCTTCGTTCCCCTTCTCTAAA<br>GGGGTGAAACAACCCTCCGTTCTTTGCCACAAGGCCCACAGAACCATTTTGGGGCTCAGGGGGACAGT<br>GTGTCACCAGGCAACTGAATTAAACACAGGAAAAGACAGATGTGAAGGCCTGAATTCTTCAAAGGCA<br>AGGGGGGTTGACACGTCATAAATCACTGTGTCACTTGCTCTAACGAAAACTGGTCTCGCCTACTGTACT<br>GTACCCGAGGCTCGAGTTGGCTAGACAGGGAGAGTGTGTTTGCACAGCACATCTGGCCTATCGCTAGG<br>AGCTTTAGAAATGCAATGAATCAGAGGGGGCATGTCGAGGGTGGGGCTTCCTCTCCACACAATAAAGC<br>CTTTTGATGGGAGATTCAAGAAACCAGTCACTAAAAAATACCAAAAACGTTAAAAATGACTCAGGTATG<br>AAATGAAAAGACTTTAACATGCATACACGCACGGTAGACTTTATGATCTCAGCGATCTGCCTACGGTCCC<br>TGTATTTTCTAGTCTCAAGATGAGAGGGAGAAGCTGTAGACGATTGGCATCTGTATCTCGATCATTCGAG<br>CTCTTACATAGTAGTCAACGGAAAAGTCATATGTCACCCACTGTCGTACTTTCTCATGATCAGCCCATCT<br>AGTACGAGAACTAGATTTGAACCAAAACTTGAGAAGCTTCCTGCCCGCTGGGCACCCCCTGGCAGAGC<br>TGCTGGAATCAGAAGGAACTGACAGGAAGTGTGCATTGATGTGTCTGCCAGGAGTATTTTTGTCCTGAT<br>CGCTCAGGGTGGTCAGTAGTCACTCCTCCCCTGGCTACAGCATCCTGCACAAGAGTGAAACACACCGT<br>AAATACCACATGCACTCATTTTTTGGAGGGCCCGTTCTCTTCAATTTAGTTCAAAAAGACTATCTGATAA<br>GTGTAAAACTGTTTAAAACTTAGGTGGAAAGTACTTTGTGTCATTTTATGATTAGACTTACATATGACTTA<br>ATGCCTTGTCTGGCCAGAACATGCATGTCCTCCTCCCGCAGCAAAGATGGCCACGGCAGTACAACTCAG<br>TTCCGTTCCTCTCCATGGTGGTAATGTCCAACAGTGTCCTTGTGTTAGTCTCACTGAACCCTAAAAGTCA<br>CAAAACTAACACTTCATAGGAGATTACTTTTTCCAATGTAAGGCCAAAATATGCATTTTTTTTAAAGGAA<br>AAGGGTGACATATATTTCTGACTTCTAAAATATAAGGAGTGTGAATATTACTTTGAAGGACTCATGGGTC<br>ACCCAAAGCAGTGTTTCTCCGGGCCGCCAAAACATGTGTCTCGGCCTTTTCTCATTTGGCTGGCTTCAG<br>TTCAGACAGGACTTAGAGAGTTTGCAGCATCTTTAATGACCACTAGAGCTCTCTTTTGTTCAACTGCAC<br>TTGTGGCTGGCCAGTGACTTTGGTTGTTTGTGGAAGTGAGAGAGAGAGAGAGAGAGAGAGAGAGAGA

							GAGAGTGTGTGTGTGTGTGTGTGTGTGTGTGTGTGTGTGTGTGTGTGTTTATTGTTCTGTTTTTCAGCTTGA<br>CACAAACCAGAATCATCTGAGAGGAGAGAGGAGGGATCCTCAATTGAGAAAACACTACCATAAGATGA<br>AGCTAGAGGCAAGCCTGAAGGGCGTTTCTTCATTAATGATTGATGTGGAAAGGGGCCCAGCTTATTGTG<br>CGCAGTGTCACCTTTGAGCAGGTGGCCCTGGGTGCGATAAAACAGAAGGCTGAGGAAGCCAAGTAGC<br>GCTCCTGCATCACCTTTGTATCAGTTCCTGCCCTGCCGGAACTCCTTCCCTGGGTGATGGTGTATACACG<br>ACCTGTTGGCATCTATATTTCAATTATTCTATGAGATAAAAAGCCTTCCCTACCCAGGCTGCTTTTGGTTG<br>TGGTGTTTCACCACAACAGCAGAAACCTCACTGAGAGAGAAATGGGTACCAAACTAAGGAGTGTCGCT<br>ATGACAAACGTGACCCTGTGCTTGGGGCAGTGGTGGAGCTTTGGGCTAGCAAAGCCCTTAAGTGCTTA<br>GAGCCCAGTGGGCTGTGTGTGGGAGTTTGGAAGGTGAGAATCCTGACAGCGATGGGGAAGATGGAGC<br>TTCAGGACGCAACTGAGAGCCCCTCAGAGATGCTCTCCAGTTGTTCTTGTGATATTTGGCTCTGGGTTC<br>CGGTCAGGTGGAGCTGGAATAGTTGGCTAGGCTTATCAAGAGACCAGGACCACCTCAGAGACCTTTGC<br>CTTGCTAGGACAACTAATGCTAGTTATCTGGAGCTGAGAAAGGAGCAGTGGTTAAGAGTAGGCTGGCG<br>TCAGTGTGTGGTAAGACCTGGGAACAGTTTCCTCAGAGTCAAAATATAGAAGCTGTGTTCCGGGGGCA<br>CCGAAGGTAGAACGCTATGGTGGCAGCTGACACTGGTCATGTGTAAGTTTCCCAGGTGGTACTGATCTT<br>TATGGCAGCTTGGGGTTTGGGAGAGCATCTGAAGTTTGGTACTGTGTGGTAAGGCTGAATCCCCAACAA<br>GAGCTCAGGGGGAAATATCGGTGAACTGCAGCCAGCCGCAGAGAACCCAACATTTTGGAAATGCTGGT<br>ACCAAGAGATGATGAGCAAAAGCGGTGGCAGCCTTGGAATGGGGCTAGCCTTGGAATAGGGCTAGCCA<br>TGGAATGGAGCCAGCCTGGGCCCATAAGCCAAGCTGAGTGCTATGATGAGGGCTTTGGAGCCTAGAAG<br>AGACTGTGAAACCTAGAAGTCTGACTCTGTACTTTGCATGGCTGGCCTTCGGCTTTGCTCTGATCTGAAT<br>GTAATCGAGCCTTTGTTCTTCTCTCCTGGAGTAAGAAAGTATTTACTTATCTCGATTTTACAGGAGCCCA<br>CTGTTGAGAGACTCTCTGGAGTTTTGGAGAGACTTTAGACCCACTGCATACTTTAGAAACGCTTGAATT<br>TTAAGGGACTTTGGTTGTTTCAGAGAAATCGAACTTTTACATTGATTGCATTTTTAGAGACTGTGGGGGA<br>TTTTAGAAGTTAGAATGTTTTATGCGGTCATATTGATAGTAATATGAGGTCCTGGGGGTAAGGAAGAAAG<br>GAAAGGTAGTTGAGCCCTGAGATGTTTGTATGCCGAGTTGGCAAGGAGTCAGTTGTGGTGGGTGGTTAT<br>AGGTGTACTTGACAAAACCAGAATCATTTGGGAGGAGTGAAACGCAACAGAGAGACTGCCCCACTAG<br>GTTGACCTGTGAGCAAGCCTGTAGTGAATTTTCCTGATTGCTGATTGCTGTGGGAGGGCCCAGTTCATG<br>GTGAGCGATGCCACTCCTGAACTGGTGGTCTTGGGTGCTATAAGAAAGCAGGTTGAGCAAGCCAGAAG<br>GAGCAATCCAACAGTGAACACTCTTCCGTGCCTTCTGCATTCATCCCTATCTTGAGTTCCTGCCCTGACT<br>TCCCTCATTAATGGGCTATAACCTGGAACTGTAAGGTGAAACCTTTCTCCCCAAGTTGCTTTGGTCATGA<br>TGCCTATCACGGAAATGGACACCCTGAGGTGCTCTATCTTACCTCCGCTTTTTATGAGGCTCATGTTCTAT<br>CTACAACCCCCAGAGTCTCTACTCATCATGAAGGCTATCTGGATCCCCCATCCAAAATGAAGTTCAGAG<br>AGGGTTTGCCCTGCTAACAAACACAAGATCAGGGCCATCATCTAACCAGAGCTCTCACCATTCCCACTG<br>ACCAAGCTGAGAAATCCAACGTCTGAGATGCCCTGAAATCTGAACACAAACGTATTCTGTATATGAGAT<br>ACTTTGGCTCTCCAATGCTCAAAGGTAAAGCCTACACAAATATTAACACCACCCCCTTCAGAATCAGAA<br>ATGCTTCTATTCTTAAAATCTCTGGATGGTGGCATTCGACCTACACAGAGTGTTTAATACATGGAGGCAA<br>TACTGTTAATATTAAGCCCTATTACTACTGCTGCAAAAGAAACCAACAATGGCTGATTCTTGTTACTGTTC<br>GTATCCTTGCTTCTGAAGGACGTAACATAAGACAGAGACTCCTGGAAGGCAGCCATCCTAATGTTATGG<br>AAACCAGGGAACCGTCCCCCATCACACAGAGCTGTAAAAATGGGTCTGTGAGGTACTTAGAAGAGTAG<br>CCTCGGCTCCAATCTGCCTGTCTTCCACTTCTTTAAGCGAGAGCCCCCGAGAGCCCCCTCGGAGTCTGT<br>GTGAGGCACCTGTTGTCCCACTGCTTTGCAGCAAGCTCGATGCCCACACACTGGGCAGAATGCTTTGTG<br>CAAGTGCCCAGGAGGTGCCCTCCAACCCAAAGGCTCACAAAGGAACCCAAAGGTTCACAAGGGGGCG

							AGCAGGTGGAGAACCAAAGGACCGGCCACGAGCCTTCTGCCTGCTAGGAAGCAACACAGATGCATAAAGAGAGGATTATGGTCCCAGGCACAACGTCAGTCTGTTGGAGAGGGCTGCATCGTTAGGAGAGAATGCGATGAGTACAGTTGCTTCCGAACTCTACAAGCTTGCTGCAAGGGCTCCGAAGGTTTAAGTGCACTTATCTGTCAGCCCTTCGCTTTCTTTACTGAAATAAAGCCATTTGTAATAACAAACGAAGGTTTTCTTCTTTTCCTTATGCAAATGAGCAGCACATGGGTCCTTTTCATCTGGCCGGTAGGGGGTAATGAAGGAAGGCAAGCAACAGAGGTGGACTGGGTTACCATTCGCAATTAACACTACACATGCATACATACATACATACATACATACATACATACACACACACACATATATATAGACACATGCCATTCGCAATTAACATTACATATGCATACATACATACATACATATACACACACATGTATACACATATGCCATTCGCAATTAATACTACATAGACATGCATACATACACACACA
MSTRG.129409.1	--	0.000	0.259	0.004	3.943	up	GGGGCGAGCAGGTGGAGAACCAAAGGACCGGCCACGAGCCTTCTGCCTGCTAGGAAGCAACACAGATGCATAAAGAGAGGATTATGGTCCCAGGCACAACGTCAGTCTGTTGGAGAGGGCTGCATCGTTAGGAGAGAATGCGATGAGTACAGTTGCTTCCGAACTCTACAAGCTTGCTGCAAGGGCTCCGAAGGTTTAAGTGCACTTATCTGTCAGCCCTTCGCTTTCTTTACTGAAATAAAGCCATTTGTAATAACAAACGAAGGTTTTCTTCTTTTCCTTATGCAAATGAGCAGCACATGGGTCCTTTTCATCTGGCCGGTAGGGGGTAATGAAGGAAGGCAAGCAACAGAGGTGGACTGGGTTACCATTCGCAATTAACACTACACATGCATACATACATACATACATACATACATACATACACACACACACATATATATAGACACATGCCATTCGCAATTAACATTACATATGCATACATACATACATACATATACACACACATGTATACACATATGCCATTCGCAATTAATACTACATAGACATGCATACATACACACACACACATAATTACACACATACCATTCACAATTAACAAATATACACATATATGG
MSTRG.129412.1	--	1.694	0.102	0.000	-3.278	down	TGCATGTATACCTAACAATAGCCATGAATTTGAAATAGAGCAAGGGAGTACAAGGGACACGGTGAAGGGATGAATGAGAAGGGGGAAATGATGTAATTTTGTGATATTCTCAAAAACTGTTAAAACAAGCAAACAGAAAAGATTAGATTCCTTTTGTATAAACTAATGAAAGTTGAGTGAGCCCAAGCTACAATGTCTCCAAACTAGAAAATGCAGAGTGTTAAAGCTAGCCTTTAGAGTTAGTATGTGACAAACTTAGAAAACTCCTTACTCCCGGACAGATGCAGACTGAATTCCAAACTAACTTCTAAAGACGTCTAGATTGTCTCGAAGTAACGACACTCATATAAGTACATTATTAATATTTTAAACGGTGGCTTTGTACTCCATTCCCAGGGAGAAGAAAAGGAATCCTTGGCTCTGCACAATGGTTTGGTCTGATTAGAGAGCAGATACCGCTTCATGAATTCCCCCTTCACCTCAGCTCTTGCTTGTGCTGTAGGGATGACTGGAGAACGTGTGTTAACCTCAAACCACAGCACCAGGAAGGGCGAAGTAAGGCATTCTCGGTCTCCACCAAGAACTGCTTTGCGATGCACAGTTATTTCAAGACGGTAGAAATAAATGCAGAAATAAAAAAGCAGGGCAAACAAACCAACTGTACAAGAGCAAACAAGGTTGGGAAGTAGCAAAGGAATATGCAAGAGAATTAGTCGAGTAGGAGATAAGACAATGACCTTCTTTTTGTTCTTAAGTAAACCCAGTATATTATTGATATAATACCAATGCTTGGGAGGCTAAGGCAGGTGGATCATGTGTTCCAGGATAGCCTGGGTCACATAGCAAGACTTTGTTTCAGAATCAAATAATCCCCCCACCCAACCCCTGAAAGCAAAACGATCCGAAGCGCACTTGAAACACTATGGGAGACTCGTGCCTTAATGGAGGGGCATTTAAACTAACCAGACCCTGTTCTAGGTGCCGTACAGCAACCTGTGCTTTCTCTTTATGTTCTGCATTGACATTTATATAAGTAAGAATAATGAGATAATTTGGGGGTCTAACTTGGCGTCCCTGTGAGCTTGACTGCACAAGGATGACTGGCTGAGATAGGGACCCGGGTGACAACATGGTCATTAAACACTAGACGTCGTCTCAAACCCCGTGCTTCTGCAGAGAGCAAGTTGAAGCTTTCCAGGGATCAATTATTTTCCCTGTCTAGCCCTAATGCATGTATTTAAATTTTCCTGAAGTTACTGAACACCAATAACGAGCATTACTCCGAATTTAACCCCCTCATTAATCTTGATAAAGAGACGTGAGTTCACATCGGAGGCAGAGAAAGAATAGGGATTTATGATGCTCCGGATATGTGCACATCCCTCATGGGACACAGAGCAAAAGCACGCTCAGTAAGTGTCCTTTCACCATCTCACTCTAACATGAAAGTTCAAGATGGAGAAAAAGACAGTCACTTCCCTTTATCATCCTAGCTGCTGCTCCTTATGACGTCTTCCTAATTCTGAATATTGTTATTATTGACTCAAAGCAGGTCTGTCAATGTCTGTGGATATATATTCCCAGCAAAAAAC

							ATCATGTCAGAAAAAAAATACTCACTGGATCATAGATCAAAAAATGGAAATGACAGTATGTTATCTGCCT<br>TGAAAAGATGCAATGGGATTGGTCATCTTCCCTTCCCCCTGGTCAACACTAAGGAGGCTCAAATAAACA<br>GGAACAAAATAGCACACAATATTTAGGTAAAAGAGAACAGCAGCTCGTTAAGTACAGGCACTTTTCTC<br>AAATGTATAGCAGTGTGATCAGCTGTCCTATCCTCATCAGACACTGCTGGTCCAGTAAGAGTATTATTTC<br>GTAATCGTTAGGTTATTAATCTAGACCCTATCGGCAATCCCCAGCTGGTTCTAGGTGATCCAGAAGAGAG<br>GGAAGGGCTGCTCTGGCAATCTGGAACAGGCTCCCAGGGGTCGAAACTTGGCAGAGGAGAACAGAAG<br>TGGTCACAAGGAAGCAGAGAGAAAAAACTAATTAGAAGATCACCATGCCCAGAGGCTCTGGACTTCTG<br>GGGGGGAAAGAAGCAAAACTCAATGTTGGCAGGAGGCAGGAAGACCAGCCATCTGGGGGAAAATCAT<br>AAAAAAGAAGTCAGTTAAACCTACAGACTCAAAGGCTCCTGAGTGCTTCCCAGGTTTGCCAAAGAGCT<br>GTGTAGCTGGTATTCTTAATGAGGGTCCTGGCTCATTGAGAAAACTCACCATACTCTACCAGACCTGGTC<br>TTGTAAGGACGATGGCATTTCAGAACCATTTGCAGTGAAAGCATTTATAACTGCTTCGTTGTTTTGAGGA<br>TTAAACAGATTTAACCAGAATTTCCTCTTCCTAGAGCCCGCCAGAAGAATTCGTTCCTGGAAGGACAGT<br>TACAGTTTATCTTTGCTTAAAGCAGGCGACATACACAGAATTAATCATAGCTCTTCAAGAACGTATCCTG<br>AAAACTTTTATTTCTTGGTTTCCTACAAACTTTAAGGTCCTTCTAGGTAACATGATAAACTACTCTGATAC<br>AGGAAGAATCCTACAGGAAGATCATACTTTAGGAAAGTAGACCCCTCCTTCCAAAAGACACACAGCCC<br>CAAAGGAAGCGGGTGTTCATTCGCTCCATCAAACAGTACTTCCTTCCCATTGGCATTGTGGCTCCCCTG<br>CTGACAAGTAGCCCCGATACCTATGTATTTAATCACCAGCTTCATTGACATGTTTCCACGCATGTCTCTCC<br>TATTTGGTAAACTGCATGCATTAGCAGCGAAGACCTGCTGGAAGCAGAAGCAAGCTAGACACACGGTT<br>CCCATCCATCTTGTCTCCCTACCTATGAGGAACCGTGACTTCTGTTAGGAAGTGAAAACTGGTGGGCTT<br>GAAATTCCTTTTCCAGGAGCAAGCCAAAGCGACAGCAGAAATTTACTTCCCGGGTCGAGTTTTACGGA<br>GCTGCAGAGTGGTTCCTGCCCGTGGAACGGAGCAAGCTAATCTACTCTGCAAGAGACAGTGACCTTTT<br>AAAGCAGGCTGTGATAGGCTGAGTCCTCACTTTACAAAGACTATCAGTTCGGAGGCAGAATCATCAGA<br>GCAAGGCGATTCTGTAAGGACTAATTCAGCTATTCCTTTTTCAAGGTGAAGGGCAGTCATTTCTCAAAC<br>CTTAGAGGTTATGGACCAATGGTACTGCACATATATGCATAGGAATCCAAACACTCGATAGCCCTTGGCT<br>GATAGATTGAGGGCCACACGCCTAAAAGAGCTGCTCCGTCTCACTAATCCTAGTCTTGATTTTTTTGTTT<br>GTTTGTTTAAACTGAGCCAGTATCATATTTTAAAAGTCACAAGATTAAGATGTCTATGGATTTCATGTCTT<br>CTCGGCAACAGTAACAAACGGCTTCCGAGAAATAGTTTCTGCAAAGAAAATGATACACTTCAGTCTCTT<br>GTCTGCTCTATAGTCAACTTTGGCTTCACTGTATCGAAATGAATGAATGGAGATCTGACTCAATAGCACG<br>AATGGCACCTGCGAGAAAACATTTGGAACTTTAAACTAGAAGGTGGCAGTACACATCTGCTGGGGACA<br>TGGCAGGACACAGATGGGCATCGTCACACTTGCCACACCCAGGACTGTCAAAACAAGGGTCGGATGTA<br>AGTGAAAAGAGCTGGGTGGCGTTTGAAGTCCTGACTCCCTAGACTGTGGGATATGATCCCTGAGGTAA<br>AGCCATTCACTCATCAAAGCATGGCCTTCCATCAGTGGCTGACAGTCTCCTCGCTGGGCCATTTTCACA<br>GAGCACTTAATTGGGGGATACCTTGAAAGTTCTAACGGCATCTCAGGTGTTGTTATTAATCTCTTTCAGG<br>CTACATGTGACTGACATCTGTGTTCTTCCTGCTCACCAACTCTTGCAAACCAGCACCCTAAAGACAGTG<br>ACCTCAACCAGAGTCAAGCTAGTTAATCTCTCCTGTTTGCTCTCAATCATACAGGGCAACAAGGGAAGG<br>GACTGTGGCTGCACCGGGAGGAGAGCATGTGGGACAGATTAGGCGCCCCTGTAAGGAAGGTTTCAAA<br>GATGGGGATTGTGGGGTGGGGTGGGGGTGGGGGCGCTCAGAGCTAACTAGAGGCAGCTGCTAGAAGG<br>TGGTTTCTGTATTTATATCCTCCCTTTCCCCAAAGCCTCTTTGGAGCATTAGGAAGGAAAGCACATGTTA<br>AGACACAGTGGATCAGTTTCTCCTGAACATACGTAAGGCCTGAGACCATAACTCAGCATGTTTGGAGAC<br>AAGGATACCGGTGAGAAGAATGAGGTAAATCGCTGTAAACCTTTATTTGATAATTTTCTTAAACGCTCTT<br>TCATCTAACCACATTAATACAGCAGAAAATTGCACTGTTGGCAGATGACAGTCGTGATAACTGGGAGAA

							GTCTACGGCAACGGTAAACTCAAGCTAACTGGTGGCTTAAAGCTGACCTACCAGCTGCAAATGGAACA<br>TCCTGTTGCCTCTGTGACCCCTGCCCTCCACACACACACCCCCAACCCTGCAAATTCCACCTTCAAACT<br>CTCACCCCAAGGCTTCAGTGTGGCTCGCTGCCAACACCTTCATCCTTTCTCGGGTACAAAAGTAGATCT<br>GCTGTTCTATACATATTTCAGCAGGCTCAGCTCTGGGCTTTAACCGAGAATTAAAGTCACATACTATTTTA<br>AATAACACAGAGAATGTATTGTTCTGACAAGAGTCTCCCTTTCCCTGCCTGCCCTGACCTCTCTTCCCTT<br>CAATATTGTCCTCAATAACGGGCTTTCTTCCCTCCTTGTAAAGTCAGTATGGGGCACTGAGAGGAAGCA<br>GGGGGGGAGGGAAGCGTTTCTATTGCTCTTGGCACTGAATAAGACGATTTTCTAAAGGATGGAATAGCT<br>CTCCCCATTTTAATAAAATTCCTGCTTCCTATACGGTATTACTCAGGTCACTTCAAGGTGAGAAGGTGCT<br>CAGAAACAGATGAGAGCAGGAGACGGCACTCGTGGCTTGGGCTCAACACAGGCCACGGGCTGTAAAT<br>CAATGCAGTATGTCGACCTGCCCCAGCCCCGCCATTGCCCATATACCATAAGCCGTGAGCCAAGCGATTC<br>CATGTGCTTGGACCAACAGCAGCTGCAGATCTCTAAAATAACACATTTTCATTCAAATTTTAAGAAGTTC<br>CTTCTCAAACTCGGGGGTGCTCTGCCTTAACAGGAGGAACACCAGAATTGATTTTCTCTGACTTGGGAG<br>CGGCTTGCACCTGAGCTTCTAAAGCAAAGTAAAGAATGGGGTAACATTGGCCTTCCCCCTTAGAACAG<br>CTCTTCTCCTCTTCCCAGTAGACAACCAGTGGATCCATTTAATTCAGAAACAAGTCCCTCCTTTCTCTTT<br>CCCTGTCATCCACCCATGTATCTACCTGCTCTTCTTTTGATTGCTGCAAAGTTCTAGCTCCTGCTATTCAC<br>ACACACACACACGCGAGCGCGCGCACACACACACACACACACACGCACTCACGCATGCACGCATGAA<br>CACCCACATGCACACATTCATATGCATACAAGTGCACGCACATGCTCTCACAAGGCTACCAACGTGACA<br>CCTGAATTCCCACCTGAACTAATCCTTAGCTAATACCCTTGACAAGTTGCATAACATCTCCAAGTGTCAA<br>TTTCCTCTTCCATAAAGTCAAGATGATAATTGTTGTCTCATGAAGCCACCAAAGAGTGCCGCAGCTGTC<br>AAGTACACTATGAACAGCAGTTAACATCACCTGCCCCTCCTTCAGCAAGCGTGTATCACTGCCCTTTAA<br>ATATATACATTATGAACAGGAAATTTTCCCCCAAAATCTGCCAAAGTTGTAAATAACCGTGCTTTCCTCTC<br>CATTGTCAGTCTCTTATTTAATTTTAAACCTCTTCTTTCATAAGCACATTACTCTATGTTGAGGCTTTTTTT<br>TTATCCAATGGTTCATTTAAAAAAAAAAAGGGGGGGGGTAATATAATGATAAACATTAGTCCGAACTTCTC<br>ATGAGCAATGTTCTAAATTTCCCAATTCCATAAAAGCCAGAATACAATTTATATCCTAACACACAAAAGA<br>AAATCCTTCTGAAACACACTACTGTGAATACTAACAATATTTCAACCCACTGAGTTAAAAAGATGAGAG<br>CAGTCCTCACTCTGTCTTCCCTGTCCAGCAGGAAGCCCATCACACACACTTACAGCAGGGCAGGGGCT<br>GCCCCTCTCCCTTCCGCAGCGAGCCCTGTGCTCCCTTCTCCTTCCCCAAACATCTGCAGTTGAGCCTAA<br>GCGCAGCTAACCATTCCCTTATAAACATCATTTATGACAACGCTGGCCATTCAACGTACGGGCTTTTAAA<br>ACTAATGGTTTTTTTCCCCCAAATGTTCCAGTTTTACTAACCTATCTTACAGACATCGAAAACAATTTGGT<br>GCTATATTTTAAATCATCATGATATTAAATCTGTCGTAGCCACACTTGTATCCGGTAGCCACACTTGAATTT<br>TTCCATGGAAAACTCCTCGTCCCCTCTTCCACCCACCTGGGCCTCTGCTACTTCTTTCTCCACCTCTCAA<br>AAAGTTTATTCTGAGGTGATGTGAAAGAGGTATCAGGTAGGGTGTGGGGGGTGACATTTCATTAAAATA<br>AACACATGTATCCTCAACGTCAAAAGGACCTCACCTTGGCTACTAAATGTGGCCGGTGTCTATCAAAGG<br>ACCTGGACCAGCCACACTCCTAACATGTATGGTTCAGCAACCTCCTCTCCTCCCCTCCTCCCTCCCAAA<br>GCTACAACCTTCACAACAAAATGCATATAAGCTGCCAGCGCCTGTAGTCCAAGCAATTTACTCTCCATCT<br>GCTCAGAAATTTTTTTTTCCTTTTAAATTCATCACTCAAAAAGAGGAAGGCGACGTTGAGCAACGTCGA<br>GACCAATACAGCCCAACTTGTGCTCTGAGCCGGGCTGGCAACGTTACATCATGTCTTCTATGCCGTGCG<br>TGAAGCATGAGCAGTGCTTAAATAATCATGTAACGTTCCCTGAAGCAACGGTTTTCAAAAATTCAATGG<br>CGGATTTAGCTCCTTAGGTGCCTTGGTTGCCACCGAGAATTTCTGTTTCTCAGTCCCCGAATGGCTCTAC<br>ACTTTCTAAAGCCATTTGGTGATTCGTGTCACGTCATCTTAGGCAACTTTATTTTTAAAAACAGAACTTG<br>ATCAAGACACAAAGGCCTGATTCTGGTCCTGAGAATAGCTTCCTTCATACACAGCTTAAAGTGCTAAGT

							CTTATCTCCCGTAGGGATAGACGGTTCAAACACCAGGCTTCCCCACCCCTGGATCATGCAAGCATGCTTT<br>CAAACTTATGTTGCAGGCAGGCAGGACACTTCTGAGGGGCTACTCCAGCTGAATATGCTCTTTGCAAAG<br>GGTTCTTTTTGTTCCCTTAACGGCACTGTGCGGGGAGGCCATGATGAGATGGGTGCCAACTTCCCAGGG<br>CAGAGACACCTTACAGCAGGAAATGTGCATGCAGGACTGTATGACCCATGGCAATGTTGCACGCCCCA<br>CTACACAGGACACTGGCACAATTCCACAGCTGGGCTCACCTCCAGGGAGCAGCAGCACAGTCCTTTAT<br>GCTGTGCCCACAGTCCTTTATCCCCCATGGTGCTAACGATGCCCCCAAGTCAGTTGGTCCCAGTGCTCA<br>GCTTATTCAAAACGATCTATTTCTACTTCATAAATTCTCAGTCAGCGGAATAATTTACTGAGAACAGGGG<br>GTGTTCTAGGCTCTTAATTTAACTCCTAATTACACCACAAACGCACCGCTCCCCTCTTCGTGTGCCTAGT<br>CGGGTTTCTCCCTGTGAGATGTGTATTTTTCTATCCCATACATACCTGCCTACAGCTGCAACACCTTAAAT<br>TAAAAAGTCTTCAGTAGTCAAACGGCAGTTAAACGGAGAAACTAAAATCACGGAATGTAAAAGTGTGG<br>GTCCTCTAAGTCTCTAATAAAAATGTACTCGGTTGCTCTCCTACAGCCTGAGCATAGAACTTAGACCAGC<br>ATCCAAACCCAGAGAAATAAAAGGGATGGAAATTTACTATCAGTCACTATCGGTCATTTCCATTCTCCCT<br>GGCAAGAGCCAAACTCCGCCATTATTAAAATTATCTGTAAAACGGGGTAGTTGAGACTAGTTTCCTGTC<br>ACACCAGCTCTGGCCACCTTCCTGTCCTTCCCAGGACTTGGCCTTCTTCCCTCTTCCTCCTCTGGTTCTT<br>AACATCAGGGGCAGGAGGAGTAGCAGCGGATCCACATCAAAATGGGAAAAATAATTCCTAAGCAGAAG<br>AGGGCTTAAACGCAGCAAGCGCCCCAGAATGCCAATTCACACAGAGACACAAGAAGCCAAACCAGCC<br>CAGAGCCTGGTGCAGTGGGGGCTGAGGTACAGCTTCGGTGCCTAGCATAGTAGCCACTCCACTGAGAG<br>GTCACCCTTGGAATTATCTCTCCCCAAATTCAACCATGATGTACTTGCTCTCCAAGGCCACGGCACTCCA<br>AACACACAAGCCCTCAGCCTTCCCTGCCACTCTGTGAACCACCCTTGGCCTTGTCTGCCGCCAACTAAA<br>ATGAGATGACTGAATGGTGCCTCTTGAAAGGTCGAGATAACAAATCCAATTTACAATTCTCAAGGGGAT<br>GGAACCAAAAGGTGGAGGCGTCCGAGTGAGTGGGAAAAAAAATTGTATTATCGCTTAATTTTATCCATC<br>ACAAAGATCGCCTCTCGTTGAGGACGCTGGGCTAGATAGCAGTGCTCGACAGGATGGGAGGGGGGGG<br>GGATCTCGGGAAGGTGTGGAGGGGAATCCCACCTGCCCTTCAAAGTACAACCCTGAGTTAAATCCTCG<br>TCTATTATTTCCGTTCTTAAAATAGTCTGATAGCAAGATGTTCAAAGGCACACGCAGCAATAGAGTAGAG<br>GAGCCTCCTTTCAAAGGGCCAGGGCAGCCATTCAAAGAGTTATTCAGGTCACAGAGAAATTACACTTC<br>CTTATGCATCTATAAAGAATTCAGAGTTTTCCATTTTTATTAGCTTTATTGTAATGGCAAAGGTTTTCAGG<br>CATATCATGAGAGGAGCATTTACAGAAGCGCTGGGCAAACCAGGCTCACACAGCTCCCGCCTCTCTTTA<br>TGAGGTCCGCAGACCGGCAGACATTTTGAAAGGACTAACAAATGCATAGCATCTCACTTGCAAGATCCC<br>GCTAATTCACCTACATAGCCCAACACTTCATGTAAGAAAAAAAAAAATCCAGAAGGATGGCACGTTCTTT<br>TGTCCACTACCTAGACTCCATACGATGCCTCGTCTTTCTGCAAATAGACTCCTTGGGACCACCCCTTTCC<br>TCCCCCAGAGTCCGCATGCGGTCGTTGAACTGTCACAGAAAATGTCTCACTTTCAAATGTTTTTCTCTA<br>GTCCTGTGATCAGAAAACTGGAATTATTTTCTGGTTCTTTGTTTCATGCTTAGAGTCCCTAGTTTCGGATG<br>CGATTCCATGATCATTCTGTTTTTTTGTTTTTTGGTTTTCTTTTAATTGATCCAATCGTGTCACCGGCTAAT<br>TTCATTCTCATCTCCTGGCTTCTCCCTTGCATCTCCATCGTGGCATTGGACACGTTCAAAAATGGAAGAC<br>AAAAACAGGTCATACAGCGAGCAGAGAATTCTCTGGCGATGGAGGTTTCGATCTTTGCTATTTCAGTGT<br>TACTCCCGCTGAGCCTAGCGTGCCAAGAATACCACTGGCGCTGTTCAGAGACACTCGTGATGGCTCAAT<br>GGAGAATCGGGGAGACCTGGGCACTTAACTATGAGCTTATTCCCAATGATATTGTCTCAAATCACTTCCA<br>TCCTCACAGAAACATGAAAGTGGAGCAATGACAGATAGGAAGCCCAGCATGAGCTGCCTGGAAGAAC<br>TCTGCTTAGCAACACAGCTTCATCACCCCACCAGGCGTGTGAGACCAGAGGCGGGGGTGGGGGAGGG<br>AGTCTAGGAGGGAGAGAATGGCATAAAAAGCCTACTGAAAACGCTACAGTATGAGAAAAGCTATCAAT<br>CGATCGATCAATAAATCAATCAATAAGTAAAATAACCCAGAGAAATGGATGCTTAGAAAAGCTTGATTTC

							AACAGGTAAGTACAAACGAAAATCACTGGATATCTTATTCGCAATGAACCAAACAGTATGTCCCTGTGT<br>CTTTTTTTTTTTAATAGAAAATAATGTGTTACCTACAAAGACCTATGCTCATAAAAATTTCCACGCAAAGG<br>GAAATATGGTAATATGCTTGAGTCTTGACTTATTTTTGGTCCTTGATTTAATAAAGGGGAACTGGTCCTTT<br>CAGGGATCATTCTAACCATCTAGGTCAGTGGTTCTCAACATGAAGATAGCAACCCCCCCCCCAGGGTCA<br>AATGACTCTTTCACTGGGGTCGCCTAAGCTCATTAGGAAACACAGGTTTTCACATTACAATGCGAGACC<br>GTAGCAAACCTCACAGTTATGAAGCAGCTTTATGGTTGGGGACGCCACAACATGAGAAACTGCATTAG<br>ACAGCTGCAGCAGTAGGAAGGTGAGGGGCCGCTGCTTTAGGTAACATCAGACTTCCTTCCGTCGTCTTA<br>TTAACAAAAATGTCTAAAGCTTTCCTCTTCCTTACTCTTTTTTTTTTTGTATATATTTTTTAACCTTTATGA<br>ATCCTAGCTCCTACTTAGATATAGGTTATGACAGAGCGCAGAATATGAGTTGACTTTTCTGGGGGTGGGG<br>GTGGGGATACATATTTGAGCTACCGTAAGCACACCGATCTTCCGATTGGTCTCAACCATAAACAGCGTG<br>AGAGAGCAGATCAGAGCTCCGGCAGACATTTCTAGTGTTGGGAGGGAATCCAATTACCTGGTCTAATGT<br>AGCCCAAACCCGAGTCCTTGAATCGGTTAGTAGCAAAATGCACTATTGTTGGGGTGAGTGAATGAATGT<br>GCACAGAGTAGCTATCTCGAACCACTCTCTCTACTGATTTTCCTACAAGAAACCAAGCTAGTAAATGCTT<br>CTGTCCCAGCTCTGCCGTGATTCTGGCATGAATGGTGACTTAATAAAGCATGGGGTTAATCCCCTTCAGA<br>TGAATCTGCTCCCTTTGGGGCTCATCCAGAAATTCTAACATATGTCCTATATGTCTCCAGTACTAGCAATC<br>AACAGGAAATGTCTACTTATTTGCTTATGGTCCTTACATATAGGCGGATGTCTCAAAACTGTTACAACCT<br>GTCTTACTACTAGAAAAGACTAGTTGTTATTAGAATGGGCAATCATCCAATCATCAATAGCATAAAAAAT<br>GATAGATGATAGACAGATAGATAATAGATAGATAGATAAGTAGGTAGATAGACAGATAGATGATAGCTAG<br>CTAGATGGATGATATATAGACAAATAGATAATAGACAGTTGATAGATAGATGATAGATTGATTGATTGATT<br>GATTGATAGAAGATAGATAGGCAGACAGACAGGCAGACAGATAGATGATAGCTAACTAGCTAGCTAGAT<br>GATATATAGACAGATGATAGGTAGATGATAGATAGATAGATAGATAGATAGATAGATAGATGATAGATAGA<br>TAGTTGATAGATGATAGATAGATAGATAAATAGATAGATGATAGATAGATAGATGATAGATAGATGATAGAT<br>GATAGAAGATAGATAGATAGATAGATAGATAGATAGATAGATAGATAGATAGATGATAAAAGTGCATCCA<br>AAATAACTCCTTTGATTAGACCCCATGCCTAACTAGTAACCAACAAGCACTTATAGAATTAGTTCACAAA<br>AGTTTCAAGGGAAAGCATGCAGCTTGGTTATGGAGAATGCCGGATGCTGGAGAACCCTAGAGCAGTAC<br>AGGCGTAAGTGGGCAAGGCGCGGTGCAGTCTTCCACAGAAAGTCTGGCCTGAGATGTTGCAGTCGGTT<br>CCTGTGTTAGCACAGGGAAGTCTACTTAATGAACTGCCAAGGGCAGAGCACAGCCACCATGAGAATAC<br>ATTTTGTGTTTACCAGACTGTGAAATCTGCAGTTAGATCCCACAATCCTCTTAGATGAATAAGAGTTAAA<br>GGAAGTTTATGAAATAACCTTCCAGACACAAAAACTCAGTAGCAAAATGAGAAATAAAACAGTTTCCA<br>AAAAAGAAAAAAAGAAAAAGAAATTTCTCAAAGAATCTAATTTGGCAAATACCACAAAATTTGCTTAG<br>TCATCTACAAACAGTAACAAAGCATCACACACAATGAAAGATAGTTCCCTAAGCGTCTTCAAGCATATG<br>ACTTCAACAATTTCCTTTTGCGATAAAAACAATAATAATCATAAAACAATAATAATCATAAAAAATAATAA<br>TAAAAAATGATGGAAAAAAACCTCGGGAAACTCTGTGAATTTTTTTTTCATTTTATTAACGAAGCAATAT<br>AAACTTGAATGATGTCATTAGGTCCACTTACTAGTTAGTCATTTCCAAATGTCTTACACCAATAAAAGTA<br>AAATCTCAAGTGTTTTATAAAAGTAATGGGCCTGGAAAAATTGCTGAAAATACTTTTCTATATCATATATG<br>TTTCTTTTTATTTTCATGCCTCTGGGGATAAAGAATGGCATGCAAGTTATTAACTGTAATATGGCCAATGT<br>AATGACCAGATAACAATAAACATGGTCTTCAAGATGTCGCAAGTCCTGGGTCTTCTGTACTTAGAAAGT<br>ACTTAGAAATCTGTTCAAAACTATGATAAGATGACCCAGCTTAACCCTGGCTGTATAAGTTTCTGTCCTC<br>CATCCATTTCTTTCTAATAGGTCACGTGTCCAGAATGAAGTCAATTAAAAACAATTTTCCACAGATTGAT<br>CACTTTTTAAACACAGTGCACCTTGAAGGCGGTCCTTTACGTAACGTATTTCCAGAGTTACATTGCTAAG<br>TTAAAAAAAATCAAAGTAATACCCCAATAAGAGATGCCTGGATCACTAATTTCAAATAAATGGACAGAG

							AATGAAAGGCATTTTTCTTGGTGGATGATAGACTCCGGATTAAATCTTCTAACATGAACGTGGGCCTTGT<br>CGTCCTAACGTCTCTTCGTTTCAGCTAAGGTCACCGTGAAACGAGGGATTATCAAATAAACCCAATCAG<br>AAAGAGACTCAAAGTTCTCAGAGCCTGTGGTTTCATAAGATGTCTTGGTACCCTGATTGTCGGTCTTGT<br>AAGACGTGAAGGTAATTAAACTGACATGGAAATCACTGCCCTGAATAAATCCTAAAAACAGTTTATCTA<br>ACCTTTTGGTCCTGGGAGAACCCAGGGCTGTCCTAGCAGCTGTCCCTCCTTACCGTCCAGTGACCTTCA<br>TAAAAAGATTAATGCTCAACGATCACACCCGGAATAGCTTACACACGTCTTCACATGGACGTCCCCTTC<br>AGGAATGCAGTTGCGCTAACTGTGCAGAAAAGGCTTCTTGAAGGAACCAGGTTCATTCACAGCCGCAC<br>CAGTCTCTCCCTCAAGCATGAGGGAGAGTTAAACCGGTCTTTAAAAAGGACACTTGGTGAGATAAAAT<br>TAAAATTTTGCTTTCAAACTGCAGGGCCCTGAATACTTAACACTTTAGCGCAGTGTTGACAGAGATTAA<br>ATTTCAAGTGATTTGACAAAAAGTCAAGCAGTATTTTAAATCTCAGATCGTATATACAACATTATTACCTG<br>AAGTAAAATGAGCAATTTTAAAAAAATTAACAGGAAAGATATTGATTTTAAAAAAAAAAAAAAAAAGGT<br>GTATGGGAAAACACTGGCCACTGGATTTCTCCCAAGTGGAGGTATTAATTCCAGATATTAATCATGTGTC<br>CACTTAAAATTCTGCTCATTTGAGAGCAATACACACATGCACACACGCTCAATCTATGAGTACCTGCAGC<br>TGCCTCTAGGTAAGGTTCTCCTCTCCAAGTCAACGGTTATTATCCCTAACACAGACACAGTCACATAAAT<br>TTCCTCGTAAGGCTCAGGGCTGTGTAAACATTCATCTCGCTCAAAAAATCCAAAAGGTACCTTTGAATAT<br>TTATATGCTACTAAGGCAAGGAAGGCAAAAATCCAAAAAATAAATATATTTAAATGTTCAAATGCCTTAA<br>CCGCTTTCCTTAAAAAAAAAAAATGCTGTGAAATCACATCTCAGTTCAGATCTTCTGACATGGGATTTTTT<br>TTTCCTCTATCTTACTGCAAGCCATGTAAGAGCCACCATGTTTTGCTGGGAAGGTATCTGCTTGGAAGAA<br>ACCGGGTGCTGATATTTTCATGCAAAAATGCAGAGGATTGCCATGTCCCGCTTCTAAAAAAAAAAAATATT<br>ACCGCAGCCCTCTTACTTCTCCATCCTTTCTGTTCGAATCTTTGAAGTGACGAGGTAATCTTAAATTGGA<br>GTTTTAAGGGGCACCTGACTGGCTTGTCAGACTAAACTGGCATATTAACTACACTCATTGTGTCACCAC<br>GGGAGCCTCAGTTTTTATCATTTTGATATCCCCTCCAGGGTTTCAATCTTCTCTCTGGGAACTCAATCACG<br>CTCTGTGATGCTCTGTTCGTACATGCGGATGAGAGAGTTTCTATTTAAAAATACATGGGGAGGGGGGGT<br>GTCAGGCACTACTGATTGAGAAGGTATTCCATGATGATCCCCATTTTCAGTTAAAATGCGCACTTATAGT<br>TTTGGATTTAACACCCCTGCCTCTGCAGTCAAGACAGTCATTTAGCTGAAGTCAATCAAGCGTATGATTA<br>GAAATAATAACTCACAAGCAATGAATTCAAAAAGATTAGATCTTTGCCGAAATGCACAAGTGCAGCATC<br>CCATTACGCATCCCTTCCTCTCGGGTTTCTAGAGAGCAGACCGGAAAAAGAAAAAAAAAAAAAAGAAA<br>GAAAGAAAAAAAAAAACAAACAGAAAAAAAAAAATCTCTCCTGGGCGCCATAAGCCGCCTTCACTAGGA<br>ATCTGACTGATCACTCTTTTTACTGTGATCAGAAACCTCGGTCCACTGGAGAGATGATCAGACAGATAAT<br>CAGCCCAGGAGTTGGGGGGCAGTGGAATCTCCTCCATCTTTTCTTCCCCTGTGGCAAAAGTGGTGTTTT<br>ACACTGACTAAGTGGATCCGAGGCTGGGTGTAATTTAATCTTATTTACATAGGCTACGAAAGGCAGAGG<br>AACATGGTGGAGGCTCAAGATAAGGTGGGTGTCCAACAGTAAAGAATGGCTTTCTTCTTACATTCTTCA<br>GATTAGGCTTCACGCCATTGTCCGGTATTTCATTGAGGTTGAGGGCGAGAGGTATTTAGGAAAACTGTG<br>TTTGGATTTCATCAAGTATGGTGTTGATACACAGTTTCCATCAGCCTTCCCAGTGTAACACTTTCCACCA<br>CCGGGAGGATTTGGTGTCACTAGTAAAATCTGGAGCACCTGAGGGAGCGGGTACAGGGTGTGGGGGGC<br>TTTACCTCCACCAGAGGGCAAGCAGTGCAATTCGCAGTCAATGATGTTAAGAAATGGGGAAGACTGGG<br>CCAAGGTGGTAAGTATTTTGTTTGAGACTCTAATGTAGAAGGGATGCCTCCCTCTCCCCGGTGGTTTGCC<br>TTCCCCACTCTACTAACTTTAGGCTGCACACAGGACAACACTAACAAAATTCACACACATAAGGGAGA<br>GGAGAGCAGCCTGGGAATTGAATTTTTTTTTTCTGCAGATAAATCCCAAGGCTAAATACACTGCACGTA<br>GGAAACTGAGAGCCAAGACCATATAGAAGCTTCATGTAAATGAAGCCTACGGACATCCTCTTAAGAGAC<br>AGATATATTTTTAAAATGCATACGTGTGTGTGCCGGCGTGTGTGCACACACGTTTCTTTTTAAAGCCCGT

							CTCGATTAGTAAGGGAAGGTATCTTGCCGACTAAGGTACACCTGACCTACAGTCAAGCACTGAAAGTCAGTCATCTCACAAGACCTCAGTTACGCTCATTACAAGTCGTAGGCTAAGAAAATGACGCTGAGGACATTTGGGACATCTATTCAACAACAATGATGACAAACGACACCTTAAAAAGAATCTGGAAGGTGCTTCAAATACTTTTTCACCACATTTCTCAACATTTTCCTTTTCCTTCAACTGCCAATTAGAGAATCCAATTCCATCCCTCTATCATGATGTCACATCTACATGCAGAGGACACTTCAGGGATCTTAGGACAGAAATGCTGGCCCACTTCTAACACATCACACCTCCATGGAAGGACCACCAGGACCCTGTATGAAGTCTCTTTCACACTGTGTCTCTCTTTTCCCTGTGACCCAGAGAACATTCACCCTGATGCAGAAGATACAAGTTTGCATCTTGCCATTAAATTTTAGTCCATAAAGAAACATAGGACACACCTAGGCATGCACGCACTCACACAGGCACATATACACACTCATATACACTCACATACACACATACACACACTCACACACACACTCATACAAAACACAAGCACACAGGCACATATACACACACACTTACACACTCATATACACTCACACACTCACACACACTCATATGCAAAGACACTCATCCACACACATACACACATGCACACATGCACACATACATACACACATGCACACACGCGCACAGGCACACACACACGCACACAGAGTCAAGCTTTCTCAGTACATAAGAGGAAACAGAAGCAAATAGCATGCACTGTGCTTTCAACTGTTACTTTTGGGAACCATTACTGTTATAAAGGAACAAACACAATAACCACTGGCAGATACAGGGAAGTCTGACAATGTGTGCTCACTCCTTCTCAGCTGACATCTACCTGAAGGAATGGGCTTCCCAATGTTTACAGCCAGGTGGTTACTTTACTACAGTTTTAAACCTGGCAGGGAGGAGTAGGGATAGACAGAAAAGACAGACTGAAAAACAAGTCTACCACAAGACTACCTTTGTTTATTCAGAACTAAAGAGGAAAACATCAAGAACCACTCCTCTGAAGTCTTTCCCCTTCTGCTGTGGACTGAGACACGTCAGGTAGCCTATGAACTGAATTACTCCATCAAGTCCCAACCAAACCTTCAAATGGGAACGAGAGAAAAGTACAGTTTAATTTGTATTTATTTATCATTTTGTGTCTGCCTTACGGAAAACTACCCATCGAAAGTAGGTCAAGCCATGAAATATTTAGATTAAAAGCTGTGGGTTTCAAGGAGTTAAGAAAAGAGAGATCCTCCCAGAACACCATCTCCAATATTTTACTGACTCGTTAAAAGCTTTGTGGCATTCCTGTTATGTGACAAACCCATTCCCAGGCTAGAGTCTATGTGCATAATGTGCAGTGTATAAATATGCAGCACACAGACTCCATATTCAGTCCTGATCAGCCACTCTCTCCACTCCTTGGGAGCACGTGACGCCATTGGAAAAAAAAATCTGAAGCTTAGGGCGCTAAGTCAATAATAAGTTCTCGAGGTGTCCCAGTTACTTTGGGCTGGCAGGGAAACGGGTGCGTGTGAGATTCCTTTTAAACAAAATTCATTAAATGTACTTCTGCCTCTCGTGTACTGTCTCTTTTCAACATTCCCAAACACTATCTGACAGCACTTGCACAGTGAGGGAGCTTCTGTCTCAAGGGTGGGTAGCTGCATTCCTGAGGCCAGGACACTGGCAAATCAGAAGATGAGAGGCATCTTCAGCTTGCATCAGGTGACATGTGTCAGAAATTACACTTGGCGTCACCTTTAGAAACACTGGGTTTGGTGACATCCGGAGAGGCGGTTTCCTGACGGTTGCTGCACACCTGAAGTTTCAGAAGCCACATTAGAGCGAGCTGGGGGCGGGGTCTCCACTTTTGTCTGCCATTTTGGAGGAAATGACTATACGCAGCCAAGAAGAAACCTTGGAGGAGCCGCCACCCGCCGGGTCTCTGATGAAGATATTCGAAATGGCTGGCTGCCTCATCAGTGAACCACACAACCAGGAAAAGGTCCAAGAGGATCGCATGCCTCTGCTTCTGAAAACCGTGCTACCTGTGCTTCAAGGCTGTGTTCAACACCATGCCTTTCCCATTCCCAAGAAGCACACGGCTAGCGGGTGCTTGACTAAGGGTGTGTTGGAGGAGAGACTGGGCTGACAGCATCCTACAGCTTCATTGAGAAACCTAAAACTCTAGTGAGTGCGTTACATAGTATGCTACCCCTTGTGCACCCCTGCCAGCCAGCCCTGCTAAATGACTCC
MSTRG.129412.2	--	0.001	0.728	0.000	9.217	up	CTAACAATAGCCATGAATTTGAAATAGAGCAAGGGAGTACAAGGGACACGGTGAAGGGATGAATGAGAAGGGGGAAATGATGTAATTTTGTGATATTCTCAAAAACTGTTAAAACAAGCAAACAGAAAAGATTAGATTCCTTTTGTATAAACTAATGAAAGTTGAGTGAGCCCAAGCTACAATGTCTCCAAACTAGAAAATGCAGAGTGTTAAAGCTAGCCTTTAGAGTTAGTATGTGACAAACTTAGAAAACTCCTTACTCCCGGACAGATGCAGACTGAATTCCAAACTAACTTCTAAAGACGTCTAGATTGTCTCGAAGTAACGACACTCATATAAGTACATTATTAATATTTTAAACGGTGGCTTTGTACTCCATTCCCAGGGAGAAGAAAAGGAATCCTTGGCTCTGCAC

							AATGGTTTGGTCTGATTAGAGAGCAGATACCGCTTCATGAATTCCCCCTTCACCTCAGCTCTTGCTTGTG<br>CTGTAGGGATGACTGGAGAACGTGTGTTAACCTCAAACCACAGCACCAGGAAGGGCGAAGTAAGGCA<br>TTCTCGGTCTCCACCAAGAACTGCTTTGCGATGCACAGTTATTTCAAGACGGTAGAAATAAATGCAGAA<br>ATAAAAAAGCAGGGCAAACAAACCAACTGTACAAGAGCAAACAAGGTTGGGAAGTAGCAAAGGAATA<br>TGCAAGAGAATTAGTCGAGTAGGAGATAAGACAATGACCTTCTTTTTGTTCTTAAGTAAACCCAGTATAT<br>TATTGATATAATACCAATGCTTGGGAGGCTAAGGCAGGTGGATCATGTGTTCCAGGATAGCCTGGGTCAC<br>ATAGCAAGACTTTGTTTCAGAATCAAATAATCCCCCCACCCAACCCCTGAAAGCAAAACGATCCGAAGC<br>GCACTTGAAACACTATGGGAGACTCGTGCCTTAATGGAGGGGCATTTAAACTAACCAGACCCTGTTCTA<br>GGTGCCGTACAGCAACCTGTGCTTTCTCTTTATGTTCTGCATTGACATTTATATAAGTAAGAATAATGAGA<br>TAATTTGGGGGTCTAACTTGGCGTCCCTGTGAGCTTGACTGCACAAGGATGACTGGCTGAGATAGGGAC<br>CCGGGTGACAACATGGTCATTAAACACTAGACGTCGTCTCAAACCCCGTGCTTCTGCAGAGAGCAAGT<br>TGAAGCTTTCCAGGGATCAATTATTTTCCCTGTCTAGCCCTAATGCATGTATTTAAATTTTCCTGAAGTTA<br>CTGAACACCAATAACGAGCATTACTCCGAATTTAACCCCCTCATTAATCTTGATAAAGAGACGTGAGTTC<br>ACATCGGAGGCAGAGAAAGAATAGGGATTTATGATGCTCCGGATATGTGCACATCCCTCATGGGACACA<br>GAGCAAAAGCACGCTCAGTAAGTGTCCTTTCACCATCTCACTCTAACATGAAAGTTCAAGATGGAGAA<br>AAAGACAGTCACTTCCCTTTATCATCCTAGCTGCTGCTCCTTATGACGTCTTCCTAATTCTGAATATTGTT<br>ATTATTGACTCAAAGCAGGTCTGTCAATGTCTGTGGATATATATTCCCAGCAAAAAACATCATGTCAGAA<br>AAAAAATACTCACTGGATCATAGATCAAAAAAATGGAAATGACAGTATGTTATCTGCCTTGAAAAGATGC<br>AATGGGATTGGTCATCTTCCCTTCCCCCTGGTCAACACTAAGGAGGCTCAAATAAACAGGAACAAAATA<br>GCACACAATATTTAGGTAAAAGAGAACAGCAGCTCGTTAAGTACAGGCACTTTTCTCAAATGTATAGCA<br>GTGTGATCAGCTGTCCTATCCTCATCAGACACTGCTGGTCCAGTAAGAGTATTATTTCGTAATCGTTAGG<br>TTATTAATCTAGACCCTATCGGCAATCCCCAGCTGGTTCTAGGTGATCCAGAAGAGAGGGAAGGGCTGC<br>TCTGGCAATCTGGAACAGGCTCCCAGGGGTCGAAACTTGGCAGAGGAGAACAGAAGTGGTCACAAGG<br>AAGCAGAGAGAAAAAACTAATTAGAAGATCACCATGCCCAGAGGCTCTGGACTTCTGGGGGGGAAAG<br>AAGCAAAACTCAATGTTGGCAGGAGGCAGGAAGACCAGCCATCTGGGGGAAAATCATAAAAAAGAAG<br>TCAGTTAAACCTACAGACTCAAAGGCTCCTGAGTGCTTCCCAGGTTTGCCAAAGAGCTGTGTAGCTGG<br>TATTCTTAATGAGGGTCCTGGCTCATTGAGAAAACTCACCATACTCTACCAGACCTGGTCTTGTAAGGAC<br>GATGGCATTTCAGAACCATTTGCAGTGAAAGCATTTATAACTGCTTCGTTGTTTTGAGGATTAAACAGAT<br>TTAACCAGAATTTCCTCTTCCTAGAGCCCGCCAGAAGAATTCGTTCCTGGAAGGACAGTTACAGTTTAT<br>CTTTGCTTAAAGCAGGCGACATACACAGAATTAATCATAGCTCTTCAAGAACGTATCCTGAAAACTTTTA<br>TTTCTTGGTTTCCTACAAACTTTAAGGTCCTTCTAGGTAACATGATAAACTACTCTGATACAGGAAGAAT<br>CCTACAGGAAGATCATACTTTAGGAAAGTAGACCCCTCCTTCCAAAAGACACACAGCCCCAAAGGAAG<br>CGGGTGTTCATTCGCTCCATCAAACAGTACTTCCTTCCCATTGGCATTGTGGCTCCCCTGCTGACAAGTA<br>GCCCCGATACCTATGTATTTAATCACCAGCTTCATTGACATGTTTCCACGCATGTCTCTCCTATTTGGTAA<br>ACTGCATGCATTAGCAGCGAAGACCTGCTGGAAGCAGAAGCAAGCTAGACACACGGTTCCCATCCATC<br>TTGTCTCCCTACCTATGAGGAACCGTGACTTCTGTTAGGAAGTGAAAACTGGTGGGCTTGAAATTCCTT<br>TTCCAGGAGCAAGCCAAAGCGACAGCAGAAATTTACTTCCCGGGTCGAGTTTTACGGAGCTGCAGAGT<br>GGTTCCTGCCCGTGGAACGGAGCAAGCTAATCTACTCTGCAAGAGACAGTGACCTTTTAAAGCAGGCT<br>GTGATAGGCTGAGTCCTCACTTTACAAAGACTATCAGTTCGGAGGCAGAATCATCAGAGCAAGGCGATT<br>CTGTAAGGACTAATTCAGCTATTCCTTTTTCAAGGTGAAGGGCAGTCATTTCTCAAACCTTAGAGGTTAT<br>GGACCAATGGTACTGCACATATATGCATAGGAATCCAAACACTCGATAGCCCTTGGCTGATAGATTGAGG

							GCCACACGCCTAAAAGAGCTGCTCCGTCTCACTAATCCTAGTCTTGATTTTTTTGTTTGTTTGTTTAAAC<br>TGAGCCAGTATCATATTTTAAAAGTCACAAGATTAAGATGTCTATGGATTTCATGTCTTCTCGGCAACAG<br>TAACAAACGGCTTCCGAGAAATAGTTTCTGCAAAGAAAATGATACACTTCAGTCTCTTGTCTGCTCTATA<br>GTCAACTTTGGCTTCACTGTATCGAAATGAATGAATGGAGATCTGACTCAATAGCACGAATGGCACCTG<br>CGAGAAAACATTTGGAACTTTAAACTAGAAGGTGGCAGTACACATCTGCTGGGGACATGGCAGGACAC<br>AGATGGGCATCGTCACACTTGCCACACCCAGGACTGTCAAAACAAGGGTCGGATGTAAGTGAAAAGA<br>GCTGGGTGGCGTTTGAAGTCCTGACTCCCTAGACTGTGGGATATGATCCCTGAGGTAAAGCCATTCACT<br>CATCAAAGCATGGCCTTCCATCAGTGGCTGACAGTCTCCTCGCTGGGCCATTTTCACAGAGCACTTAAT<br>TGGGGGATACCTTGAAAGTTCTAACGGCATCTCAGGTGTTGTTATTAATCTCTTTCAGGCTACATGTGAC<br>TGACATCTGTGTTCTTCCTGCTCACCAACTCTTGCAAACCAGCACCCTAAAGACAGTGACCTCAACCAG<br>AGTCAAGCTAGTTAATCTCTCCTGTTTGCTCTCAATCATACAGGGCAACAAGGGAAGGGACTGTGGCTG<br>CACCGGGAGGAGAGCATGTGGGACAGATTAGGCGCCCCTGTAAGGAAGGTTTCAAAGATGGGGATTGT<br>GGGGTGGGGTGGGGGTGGGGGCGCTCAGAGCTAACTAGAGGCAGCTGCTAGAAGGTGGTTTCTGTATT<br>TATATCCTCCCTTTCCCCAAAGCCTCTTTGGAGCATTAGGAAGGAAAGCACATGTTAAGACACAGTGGA<br>TCAGTTTCTCCTGAACATACGTAAGGCCTGAGACCATAACTCAGCATGTTTGGAGACAAGGATACCGGT<br>GAGAAGAATGAGGTAAATCGCTGTAAACCTTTATTTGATAATTTTCTTAAACGCTCTTTCATCTAACCAC<br>ATTAATACAGCAGAAAATTGCACTGTTGGCAGATGACAGTCGTGATAACTGGGAGAAGTCTACGGCAA<br>CGGTAAACTCAAGCTAACTGGTGGCTTAAAGCTGACCTACCAGCTGCAAATGGAACATCCTGTTGCCTC<br>TGTGACCCCTGCCCTCCACACACACACCCCCAACCCTGCAAATTCCACCTTCAAACTCTCACCCCAAGG<br>CTTCAGTGTGGCTCGCTGCCAACACCTTCATCCTTTCTCGGGTACAAAAGTAGATCTGCTGTTCTATACA<br>TATTTCAGCAGGCTCAGCTCTGGGCTTTAACCGAGAATTAAAGTCACATACTATTTTAAATAACACAGAG<br>AATGTATTGTTCTGACAAGAGTCTCCCTTTCCCTGCCTGCCCTGACCTCTCTTCCCTTCAATATTGTCCTC<br>AATAACGGGCTTTCTTCCCTCCTTGTAAAGTCAGTATGGGGCACTGAGAGGAAGCAGGGGGGGAGGGA<br>AGCGTTTCTATTGCTCTTGGCACTGAATAAGACGATTTTCTAAAGGATGGAATAGCTCTCCCCATTTTAAT<br>AAAATTCCTGCTTCCTATACGGTATTACTCAGGTCACTTCAAGGTGAGAAGGTGCTCAGAAACAGATGA<br>GAGCAGGAGACGGCACTCGTGGCTTGGGCTCAACACAGGCCACGGGCTGTAAATCAATGCAGTATGTC<br>GACCTGCCCCAGCCCCGCCATTGCCCATATACCATAAGCCGTGAGCCAAGCGATTCCATGTGCTTGGAC<br>CAACAGCAGCTGCAGATCTCTAAAATAACACATTTTCATTCAAATTTTAAGAAGTTCCTTCTCAAACTCG<br>GGGGTGCTCTGCCTTAACAGGAGGAACACCAGAATTGATTTTCTCTGACTTGGGAGCGGCTTGCACCT<br>GAGCTTCTAAAGCAAAGTAAAGAATGGGGTAACATTGGCCTTCCCCCTTAGAACAGCTCTTCTCCTCTT<br>CCCAGTAGACAACCAGTGGATCCATTTAATTCAGAAACAAGTCCCTCCTTTCTCTTTCCCTGTCATCCAC<br>CCATGTATCTACCTGCTCTTCTTTTGATTGCTGCAAAGTTCTAGCTCCTGCTATTCACACACACACACAC<br>GCGAGCGCGCGCACACACACACACACACACACGCACTCACGCATGCACGCATGAACACCCACATGCA<br>CACATTCATATGCATACAAGTGCACGCACATGCTCTCACAAGGCTACCAACGTGACACCTGAATTCCCA<br>CCTGAACTAATCCTTAGCTAATACCCTTGACAAGTTGCATAACATCTCCAAGTGTCAATTTCCTCTTCCAT<br>AAAGTCAAGATGATAATTGTTGTCTCATGAAGCCACCAAAGAGTGCCGCAGCTGTCAAGTACACTATGA<br>ACAGCAGTTAACATCACCTGCCCCTCCTTCAGCAAGCGTGTATCACTGCCCTTTAAATATATACATTATGA<br>ACAGGAAATTTTCCCCCAAAATCTGCCAAAGTTGTAAATAACCGTGCTTTCCTCTCCATTGTCAGTCTCT<br>TATTTAATTTTAAACCTCTTCTTTCATAAGCACATTACTCTATGTTGAGGCTTTTTTTTTATCCAATGGTTC<br>ATTTAAAAAAAAAAAGGGGGGGGGTAATATAATGATAAACATTAGTCCGAACTTCTCATGAGCAATGTTC<br>TAAATTTCCCAATTCCATAAAAGCCAGAATACAATTTATATCCTAACACACAAAAGAAAATCCTTCTGAA

							ACACACTACTGTGAATACTAACAATATTTCAACCCACTGAGTTAAAAAGATGAGAGCAGTCCTCACTCT<br>GTCTTCCCTGTCCAGCAGGAAGCCCATCACACACACTTACAGCAGGGCAGGGGCTGCCCCTCTCCCTT<br>CCGCAGCGAGCCCTGTGCTCCCTTCTCCTTCCCCAAACATCTGCAGTTGAGCCTAAGCGCAGCTAACCA<br>TTCCCTTATAAACATCATTTATGACAACGCTGGCCATTCAACGTACGGGCTTTTAAAACTAATGGTTTTTT<br>TCCCCCAAATGTTCCAGTTTTACTAACCTATCTTACAGACATCGAAAACAATTTGGTGCTATATTTTAAAT<br>CATCATGATATTAAATCTGTCGTAGCCACACTTGTATCCGGTAGCCACACTTGAATTTTTCCATGGAAAAC<br>TCCTCGTCCCCTCTTCCACCCACCTGGGCCTCTGCTACTTCTTTCTCCACCTCTCAAAAAGTTTATTCTG<br>AGGTGATGTGAAAGAGGTATCAGGTAGGGTGTGGGGGGTGACATTTCATTAAAATAAACACATGTATCC<br>TCAACGTCAAAAGGACCTCACCTTGGCTACTAAATGTGGCCGGTGTCTATCAAAGGACCTGGACCAGC<br>CACACTCCTAACATGTATGGTTCAGCAACCTCCTCTCCTCCCCTCCTCCCTCCCAAAGCTACAACCTTCA<br>CAACAAAATGCATATAAGCTGCCAGCGCCTGTAGTCCAAGCAATTTACTCTCCATCTGCTCAGAAATTTT<br>TTTTTCCTTTTAAATTCATCACTCAAAAAGAGGAAGGCGACGTTGAGCAACGTCGAGACCAATACAGCC<br>CAACTTGTGCTCTGAGCCGGGCTGGCAACGTTACATCATGTCTTCTATGCCGTGCGTGAAGCATGAGCA<br>GTGCTTAAATAATCATGTAACGTTCCCTGAAGCAACGGTTTTCAAAAATTCAATGGCGGATTTAGCTCCT<br>TAGGTGCCTTGGTTGCCACCGAGAATTTCTGTTTCTCAGTCCCCGAATGGCTCTACACTTTCTAAAGCCA<br>TTTGGTGATTCGTGTCACGTCATCTTAGGCAACTTTATTTTTAAAAACAGAACTTGATCAAGACACAAA<br>GGCCTGATTCTGGTCCTGAGAATAGCTTCCTTCATACACAGCTTAAAGTGCTAAGTCTTATCTCCCGTAG<br>GGATAGACGGTTCAAACACCAGGCTTCCCCACCCCTGGATCATGCAAGCATGCTTTCAAACTTATGTTG<br>CAGGCAGGCAGGACACTTCTGAGGGGCTACTCCAGCTGAATATGCTCTTTGCAAAGGGTTCTTTTTGTT<br>CCCTTAACGGCACTGTGCGGGGAGGCCATGATGAGATGGGTGCCAACTTCCCAGGGCAGAGACACCTT<br>ACAGCAGGAAATGTGCATGCAGGACTGTATGACCCATGGCAATGTTGCACGCCCCACTACACAGGACA<br>CTGGCACAATTCCACAGCTGGGCTCACCTCCAGGGAGCAGCAGCACAGTCCTTTATGCTGTGCCCACA<br>GTCCTTTATCCCCCATGGTGCTAACGATGCCCCCAAGTCAGTTGGTCCCAGTGCTCAGCTTATTCAAAAC<br>GATCTATTTCTACTTCATAAATTCTCAGTCAGCGGAATAATTTACTGAGAACAGGGGGTGTTCTAGGCTC<br>TTAATTTAACTCCTAATTACACCACAAACGCACCGCTCCCCTCTTCGTGTGCCTAGTCGGGTTTCTCCCT<br>GTGAGATGTGTATTTTTCTATCCCATACATACCTGCCTACAGCTGCAACACCTTAAATTAAAAAGTCTTCA<br>GTAGTCAAACGGCAGTTAAACGGAGAAACTAAAATCACGGAATGTAAAAGTGTGGGTCCTCTAAGTCT<br>CTAATAAAAATGTACTCGGTTGCTCTCCTACAGCCTGAGCATAGAACTTAGACCAGCATCCAAACCCAG<br>AGAAATAAAAGGGATGGAAATTTACTATCAGTCACTATCGGTCATTTCCATTCTCCCTGGCAAGAGCCA<br>AACTCCGCCATTATTAAAATTATCTGTAAAACGGGGTAGTTGAGACTAGTTTCCTGTCACACCAGCTCTG<br>GCCACCTTCCTGTCCTTCCCAGGACTTGGCCTTCTTCCCTCTTCCTCCTCTGGTTCTTAACATCAGGGGC<br>AGGAGGAGTAGCAGCGGATCCACATCAAAATGGGAAAAATAATTCCTAAGCAGAAGAGGGCTTAAACG<br>CAGCAAGCGCCCCAGAATGCCAATTCACACAGAGACACAAGAAGCCAAACCAGCCCAGAGCCTGGTG<br>CAGTGGGGGCTGAGGTACAGCTTCGGTGCCTAGCATAGTAGCCACTCCACTGAGAGGTCACCCTTGGA<br>ATTATCTCTCCCCAAATTCAACCATGATGTACTTGCTCTCCAAGGCCACGGCACTCCAAACACACAAGC<br>CCTCAGCCTTCCCTGCCACTCTGTGAACCACCCTTGGCCTTGTCTGCCGCCAACTAAAATGAGATGACT<br>GAATGGTGCCTCTTGAAAGGTCGAGATAACAAATCCAATTTACAATTCTCAAGGGGATGGAACCAAAA<br>GGTGGAGGCGTCCGAGTGAGTGGAAAAAAAATTGTATTATCGCTTAATTTTATCCATCACAAAGATCG<br>CCTCTCGTTGAGGACGCTGGGCTAGATAGCAGTGCTCGACAGGATGGGAGGGGGGGGGATCTCGGGA<br>AGGTGTGGAGGGGAATCCCACCTGCCCTTCAAAGTACAACCCTGAGTTAAATCCTCGTCTATTATTTCC<br>GTTCTTAAAATAGTCTGATAGCAAGATGTTCAAAGGCACACGCAGCAATAGAGTAGAGGAGCCTCCTTT

							CAAAGGGCCAGGGCAGCCATTCAAAGAGTTATTCAGGTCACAGAGAAATTACACTTCCTTATGCATCTA<br>TAAAGAATTCAGAGTTTTCCATTTTTATTAGCTTTATTGTAATGGCAAAGGTTTTCAGGCATATCATGAGA<br>GGAGCATTTACAGAAGCGCTGGGCAAACCAGGCTCACACAGCTCCCGCCTCTCTTTATGAGGTCCGCA<br>GACCGGCAGACATTTTGAAAGGACTAACAAATGCATAGCATCTCACTTGCAAGATCCCGCTAATTCACC<br>TACATAGCCCAACACTTCATGTAAGAAAAAAAAAAATCCAGAAGGATGGCACGTTCTTTTGTCCACTACC<br>TAGACTCCATACGATGCCTCGTCTTTCTGCAAATAGACTCCTTGGGACCACCCCTTTCCTCCCCCAGAGT<br>CCGCATGCGGTCGTTGAACTGTCACAGAAAATGTCTCACTTTCAAATGTTTTTCTCTAGTCCTGTGATCA<br>GAAAACTGGAATTATTTTCTGGTTCTTTGTTTCATGCTTAGAGTCCCTAGTTTCGGATGCGATTCCATGAT<br>CATTCTGTTTTTTTGTTTTTTGGTTTTCTTTTAATTGATCCAATCGTGTCACCGGCTAATTTCATTCTCATC<br>TCCTGGCTTCTCCCTTGCATCTCCATCGTGGCATTGGACACGTTCAAAAATGGAAGACAAAAACAGGTC<br>ATACAGCGAGCAGAGAATTCTCTGGCGATGGAGGTTTCGATCTTTGCTATTTCAGTGTTACTCCCGCTGA<br>GCCTAGCGTGCCAAGAATACCACTGGCGCTGTTCAGAGACACTCGTGATGGCTCAATGGAGAATCGGG<br>GAGACCTGGGCACTTAACTATGAGCTTATTCCCAATGATATTGTCTCAAATCACTTCCATCCTCACAGAA<br>ACATGAAAGTGGAGCAATGACAGATAGGAAGCCCAGCATGAGCTGCCTGGAAGAACTCTGCTTAGCAA<br>CACAGCTTCATCACCCCACCAGGCGTGTGAGACCAGAGGCGGGGGTGGGGGAGGGAGTCTAGGAGGG<br>AGAGAATGGCATAAAAAGCCTACTGAAAACGCTACAGTATGAGAAAAGCTATCAATCGATCGATCAATA<br>AATCAATCAATAAGTAAAATAACCCAGAGAAATGGATGCTTAGAAAAGCTTGATTTCAACAGGTAAGTA<br>CAAACGAAAATCACTGGATATCTTATTCGCAATGAACCAAACAGTATGTCCCTGTGTCTTTTTTTTTTTA<br>ATAGAAAATAATGTGTTACCTACAAAGACCTATGCTCATAAAAATTTCCACGCAAAGGGAAATATGGTAA<br>TATGCTTGAGTCTTGACTTATTTTTGGTCCTTGATTTAATAAAGGGGAACTGGTCCTTTCAGGGATCATTC<br>TAACCATCTAGGTCAGTGGTTCTCAACATGAAGATAGCAACCCCCCCCCCAGGGTCAAATGACTCTTTC<br>ACTGGGGTCGCCTAAGCTCATTAGGAAACACAGGTTTTCACATTACAATGCGAGACCGTAGCAAACCTC<br>ACAGTTATGAAGCAGCTTTATGGTTGGGGACGCCACAACATGAGAAACTGCATTAGACAGCTGCAGCA<br>GTAGGAAGGTGAGGGGCCGCTGCTTTAGGTAACATCAGACTTCCTTCCGTCGTCTTATTAACAAAAATG<br>TCTAAAGCTTTCCTCTTCCTTACTCTTTTTTTTTTTGTATATATTTTTTAACCTTTATGAATCCTAGCTCCTA<br>CTTAGATATAGGTTATGACAGAGCGCAGAATATGAGTTGACTTTTCTGGGGGTGGGGGTGGGGATACATA<br>TTTGAGCTACCGTAAGCACACCGATCTTCCGATTGGTCTCAACCATAAACAGCGTGAGAGAGCAGATCA<br>GAGCTCCGGCAGACATTTCTAGTGTTGGGAGGGAATCCAATTACCTGGTCTAATGTAGCCCAAACCCGA<br>GTCCTTGAATCGGTTAGTAGCAAAATGCACTATTGTTGGGGTGAGTGAATGAATGTGCACAGAGTAGCT<br>ATCTCGAACCACTCTCTCTACTGATTTTCCTACAAGAAACCAAGCTAGTAAATGCTTCTGTCCCAGCTCT<br>GCCGTGATTCTGGCATGAATGGTGACTTAATAAAGCATGGGGTTAATCCCCTTCAGATGAATCTGCTCCC<br>TTTGGGGCTCATCCAGAAATTCTAACATATGTCCTATATGTCTCCAGTACTAGCAATCAACAGGAAATGT<br>CTACTTATTTGCTTATGGTCCTTACATATAGGCGGATGTCTCAAAACTGTTACAACCTGTCTTACTACTAG<br>AAAAGACTAGTTGTTATTAGAATGGGCAATCATCCAATCATCAATAGCATAAAAAATGATAGATGATAGA<br>CAGATAGATAATAGATAGATAGATAAGTAGGTAGATAGACAGATAGATGATAGCTAGCTAGATGGATGATA<br>TATAGACAAATAGATAATAGACAGTTGATAGATAGATGATAGATTGATTGATTGATTGATTGATAGAAGAT<br>AGATAGGCAGACAGACAGGCAGACAGATAGATGATAGCTAACTAGCTAGCTAGATGATATATAGACAGA<br>TGATAGGTAGATGATAGATAGATAGATAGATAGATAGATAGATAGATGATAGATAGATAGTTGATAGATGA<br>TAGATAGATAGATAAATAGATAGATAGATAGATGATAGATAGATAGTTGATAGATGATAGATAGATAGATAA<br>ATAGATAGATGATAGATAGATAGATGATAGATGATAGATGATAGAAGATAGATAGATAGATAGATAGATAG<br>ATAGATAGATAGATAGATAGATGATAAAAGTGCATCCAAAATAACTCCTTTGATTAGACCCCATGCCTAAC

							TAGTAACCAACAAGCACTTATAGAATTAGTTCACAAAAGTTTCAAGGGAAAGCATGCAGCTTGGTTATG GAGAATGCCGGATGCTGGAGAACCCTAGAGCAGTACAGGCGTAAGTGGGCAAGGCGCGGTGCAGTCT TCCACAGAAAGTCTGGCCTGAGATGTTGCAGTCGGTTCCTGTGTTAGCACAGGGAAGTCTACTTAATGA ACTGCCAAGGGCAGAGCACAGCCACCATGAGAATACATTTTGTGTTTACCAGACTGTGAAATCTGCAGT TAGATCCCACAATCCTCTTAGATGAATAAGAGTTAAAGGAAGTTTATGAAATAACCTTCCAGACACAAA AACTCAGTAGCAAAATGAGAAATAAAACAGTTTCCAAAAAAGAAAAAAAGAAAAAGAAATTTCTCAA AGAATCTAATTTGGCAAATACCACAAAATTTGCTTAGTCATCTACAAACAGTAACAAAGCATCACACAC AATGAAAGATAGTTCCCTAAGCGTCTTCAAGCATATGACTTCAACAATTTCCTTTTGCGATAAAAACAAT AATAATCATAAAACAATAATAATCATAAAAAATAATAATAAAAAATGATGGAAAAAAACCTCGGGAAAC TCTGTGAATTTTTTTTCATTTTATTAACGAAGCAATATAAACTTGAATGATGTCATTAGGTCCACTTACTA GTTAGTCATTTCCAAATGTCTTACACCAATAAAAGTAAAATCTCAAGTGTTTTATAAAAGTAATGGGCCT GGAAAAATTGCTGAAAATACTTTTCTATATCATATATGTTTCTTTTTATTTTCATGCCTCTGGGGATAAAGA ATGGCATGCAAGTTATTAACTGTAATATGGCCAATGTAATGACCAGATAACAATAAACATGGTCTTCAAG ATGTCGCAAGTCCTGGGTCTTCTGTACTTAGAAAGTACTTAGAAATCTGTTCAAAACTATGATAAGATGA CCCAGCTTAACCCTGGCTGTATAAGTTTCTGTCCTCCATCCATTTCTTTCTAATAGGTCACGTGTCCAGAA TGAAGTCAATTAAAAACAATTTTCCACAGATTGATCACTTTTTAAACACAGTGCACCTTGAAGGCGGTC CTTTACGTAACGTATTTCCAGAGTTACATTGCTAAGTTAAAAAAAATCAAAGTAATACCCCAATAAGAGA TGCCTGGATCACTAATTTCAAATAAATGGACAGAGAATGAAAGGCATTTTTCTTGGTGGATGATAGACTC CGGATTAAATCTTCTAACATGAACGTGGGCCTTGTCGTCCTAACGTCTCTTCGTTTCAGCTAAGGTCACC GTGAAACGAGGGATTATCAAATAAACCCAATCAGAAAGAGACTCAAAGTTCTCAGAGCCTGTGGTTTC ATAAGATGTCTTGGTACCCTGATTGTCGGTCTTGTAAGACGTGAAGGTAATTAAACTGACATGGAAATCA CTGCCCTGAATAAATCCTAAAAACAGTTTATCTAACCTTTTGGTCCTGGGAGAACCCAGGGCTGTCCTA GCAGCTGTCCCTCCTTACCGTCCAGTGACCTTCATAAAAAGATTAATGCTCAACGATCACACCCGGAAT AGCTTACACACGTCTTCACATGGACGTCCCCTTCAGGAATGCAGTTGCGCTAACTGTGCAGAAAAGGCT TCTTGAAGGAACCAGGTTCATTCACAGCCGCACCAGTCTCTCCCTCAAGCATGAGGGAGAGTTAAACC GGTCTTTAAAAAGGACACTTGGTGAGATAAAATTAAAATTTTGCTTTCAAACTGCAGGGCCCTGAATAC TTAACACTTTAGCGCAGTGTTGACAGAGATTAAATTTCAAGTGATTTGACAAAAAGTCAAGCAGTATTT TAAATCTCAGATCGTATATACAACATTATTACCTGAAGTAAAATGAGCAATTTTAAAAAAATTAACAGGA AAGATATTGATTTTAAAAAAAAAAAAAAAAAGGTGTATGGGAAAACACTGGCCACTGGATTTCTCCCAA GTGGAGGTATTAATTCCAGATATTAATCATGTGTCCACTTAAAATTCTGCTCATTTGAGAGCAATACACAC ATGCACACACGCTCAATCTATGAGTACCTGCAGCTGCCTCTAGGTAAGGTTCTCCTCTCCAAGTCAACG GTTATTATCCCTAACACAGACACAGTCACATAAATTTCCTCGTAAGGCTCAGGGCTGTGTAAACATTCAT CTCGCTCAAAAAATCCAAAAGGTACCTTTGAATATTTATATGCTACTAAGGCAAGGAAGGCAAAAATCC AAAAAATAAATATATTTAAATGTTCAAATGCCTTAACCGCTTTCCTTAAAAAAAAAAAATGCTGTGAAATC ACATCTCAGTTCAGATCTTCTGACATGGGATTTTTTTTTCCTCTATCTTACTGCAAGCCATGTAAGAGCCA CCATGTTTTGCTGGGAAGGTATCTGCTTGGAAGAAACCGGGTGCTGATATTTTCATGCAAAAATGCAGA GGATTGCCATGTCCCGCTTCTAAAAAAAAAAAATATTACCGCAGCCCTCTTACTTCTCCATCCTTTCTGTT CGAATCTTTGAAGTGACGAGGTAATCTTAAATTGGAGTTTTAAGGGGCACCTGACTGGCTTGTCAGACT AAACTGGCATATTAACTACACTCATTGTGTCACCACGGGAGCCTCAGTTTTTATCATTTTGATATCCCCTC CAGGGTTTCAATCTTCTCTCTGGGAACTCAATCACGCTCTGTGATGCTCTGTTCGTACATGCGGATGAGA GAGTTTCTATTTAAAAATACATGGGGAGGGGGGGTGTCAGGCACTACTGATTGAGAAGGTATTCCATGA

							TGATCCCCATTTTCAGTTAAAATGCGCACTTATAGTTTTGGATTTAACACCCCTGCCTCTGCAGTCAAGACAGTCATTTAGCTGAAGTCAATCAAGCGTATGATTAGAAATAATAACTCACAAGCAATGAATTCAAAAAGATTAGATCTTTGCCGAAATGCACAAGTGCAGCATCCCATTACGCATCCCTTCCTCTCGGGTTTCTAGAGAGCAGACCGGAAAAAGAAAAAAAAAAAAAGAAAGAAAGAAAAAAAAAACAAACAGAAAAAAAAAATCTCTCCTGGGCGCCATAAGCCGCCTTCACTAGGAATCTGACTGATCACTCTTTTTACTGTGATCAGAAACCTCGGTCCACTGGAGAGATGATCAGACAGATAATCAGCCCAGGAGTTGGGGGGCAGTGGAATCTCCTCCATCTTTTCTTCCCCTGTGGCAAAAGTGGTGTTTTACACTGACTAAGTGGATCCGAGGCTGGGTGTAATTTAATCTTATTTACATAGGCTACGAAAGGCAGAGGAACATGGTGGAGGCTCAAGATAAGGTGGGTGTCCAACAGTAAAGAATGGCTTTCTTCTTACATTCTTCAGATTAGGCTTCACGCCATTGTCCGGTATTTCATTGAGGTTGAGGGCGAGAGGTATTTAGGAAAACTGTGTTTGGATTTCATCAAGTATGGTGTTGATACACAGTTTCCATCAGCCTTCCCAGTGTAACACTTTCCACCACCGGGAGGATTTGGTGTCACTAGTAAAATCTGGAGCACCTGAGGGAGCGGGTACAGGGTGTGGGGGGCTTTACCTCCACCAGAGGGCAAGCAGTGCAATTCGCAGTCAATGATGTTAAGAAATGGGGAAGACTGGGCCAAGGTGGTAAGTATTTTGTTTGAGACTCTAATGTAGAAGGGATGCCTCCCTCTCCCCGGTGGTTTGCCTTCCCCACTCTACTAACTTTAGGCTGCACACAGGACAACACTAACAAAATTCACACACATAAGGGAGAGGAGAGCAGCCTGGGAATTGAATTTTTTTTTCTGCAGATAAATCCCAAGGCTAAATACACTGCACGTAGGAAACTGAGAGCCAAGACCATATAGAAGCTTCATGTAAATGAAGCCTACGGACATCCTCTTAAGAGACAGATATATTTTTAAAATGCATACGTGTGTGTGCCGGCGTGTGTGCACACACGTTTCTTTTTAAAGCCCGTCTCGATTAGTAAGGGAAGGTATCTTGCCGACTAAGGTACACCTGACCTACAGTCAAGCACTGAAAGTCAGTCATCTCACAAGACCTCAGTTACGCTCATTACAAGTCGTAGGCTAAGAAAATGACGCTGAGGACATTTGGGACATCTATTCAACAACAATGATGACAAACGACACCTTAAAAAGAATCTGGAAGGTGCTTCAAATACTTTTTCACCACATTTCTCAACATTTTCCTTTTCCTTCAACTGCCAATTAGAGAATCCAATTCCATCCCTCTATCATGATGTCACATCTACATGCAGAGGACACTTCAGGGATCTTAGGACAGAAATGCTGGCCCACTTCTAACACATCACACCTCCATGGAAGGACCACCAGGACCCTGTATGAAGTCTCTTTCACACTGTGTCTCTCTTTTCCCTGTGACCCAGAGAACATTCACCCTGATGCAGAAGATACAAGTTTGCATCTTGCCATTAAATTTTAGTCCATAAAGAAACATAGGACACACCTAGGCATGCACGCACTCACACAGGCACATATACACACTCATATACACTCACATACACACATACACACACTCACAC
MSTRG.129435.2	--	0.000	0.262	0.002	4.137	up	AGCTCCGCCCACAGACTGAGGACATTTCCTTTCGGGAGATAACACCCATGATGTTCGGCGTATGGCATGCTGAAAACTCATCGTCCAACAATTAACTTACTGGGGGTTAGGGAGGTGACTTGACACCTTTGCCGTGCGCTTTCCAGATTGGCCTCGCCTCTCGTGTGTTGTCACGCCTCGATGGGAATGCATCCTGAGGATGATGGAACCCACCCAGACGCTTCCTTCTTCTGGATCGTCTCTTCCCATTGATACTCTGCGTTCCTTCCCACAATTCATCACCATGCTTTGAGGATAATAGCTGTCAGCGGGTACTTTCAGCAAATTACCAAACCTCATGGTGATTCGAGGAATCCCCTCTCTTGAAGCTTGCTGTTAGAAATAAATGTGGCTCTGGCCGTGCCTATCCTTCAGACTCTACCATTTAGATCAGTCCCAAGAAGATTTTTTTTTTAAAAATGAACTGTTGCTGTAACCAGGCATGGTGGTACACGCCTTTAATTCCAGCGCGCAGGAGGCAGAGGCAGGTGGGTCTCTGAGTTCAAGGCCAGCCTGGCCAACAAAGTAAGTTCTAGGACAGCCATGGCCACACAGAGAAACCCTGTCTCAAAACAACAAAACAAATAAAACAATAGCCAATCAACTACAAACCCTACAGCTACTACACACACTGTCAAAATATAAG
MSTRG.129435.3	--	1.123	0.000	0.000	-5.422	down	ATTTCCTTTCGGGAGATAACACCCATGATGTTCGGCGTATGGCATGCTGAAAACTCATCGTCCAACAATTAACTTACTGGGGGTTAGGGAGGTGCCATAAAGAAAGAATGATATAAAGATTTCTATCACGAGGAGACACTTGATTGCCTGTTCTCAGCGTTGAATTCTATCGTAAGACAAAAACAGGGTCCCATAACTCCATGTCTTGTTAAGAATATCACATTTGGATCTGGAAATGATGGGCCAGGTAAAACCTGAACTTTGGAGATTGAGCCAAG

							GTCATTGGCCTGTGCGGAGCTATCCTGGTCTACAGAGTGAGGGCTATGTACCAAGAATCCCTTCTCAAGACAACAAAAGAAGAGTACGTACTATTTGCTCAATTTAGAAGATTCGTCGGCTTCTACGGAACCTTAGTTTTCCTCCTAGACCTCTGTTTTCCAGGAGGGATTTATGATGTACCCCATCAGTCACTCCAAATCTTGATTGCTTTCAGCTCCAAAGTGATTCCATAGACATCACCCTCAAAACCCATGACAGTTACTTTAGAATGAAATGCAGCAGGCCTCCTCGTTGTTAAGATCCCAATAAAAATGCAATTGCGCTGCGCCCTACACCGTGTCATCTCTTGTGCGCATGCGCATCACCCTCCTGCCACACCGCATGCCCCCTTGCCTTTTTCAAAATAAATTCTCTGCTTTCCAG
MSTRG.129435.6	--	1.006	0.069	0.013	-2.741	down	TAATAATATGTATTGTTTCAGGTCCCTTGGGAACCTCTCCAAGCAATAATTTTCGGCAGGTGTCCCGTACCTGGCATTGAGGCTTTTGTTTTATACTTTGGGCTTTCTGGGAGAAGGGTTACCCATCCTTCACTCATTCTTGCCGAGTAACTCCATGCAAACTGCCCTATGTAATACTTTTATCTTTTTACATTATCAGTTTGGGTTAACTACCTGTTGACGTATGTTGAACTATCCCTGCATCTCTGGAGGAAAAAAAAATCCAATTTGTTGGTGACTTGACACCTTTGCCGTGCGCTTTCCAGATTGGCCTCGCCTCTCGTGTGTTGTCACGCCTCGATGGGAATGCATCCTGAGGATGATGGAACCCACCCAGACGCTTCCTTCTTCTGGATCGTCTCTTCCCATTGATACTCTGCGTTCCTTCCCACAATTCATCACCATGCTTTGAGGATAATAGCTGTCAGCGGGTACTTTCAGCAAATTACCAAACCTCATGGTGATTCGAGGAATCCCCTCTCTTGAAGCTTGCTGTTAGAAATAAATGTGGCTCTGGCCGTGCCTATCCTTCAGACTCTACCATTTAGATCAGTCCCAAGAAGATTTTTTTTTAAAAATGAACTGTTGCTGTAACCAGGCATGGTGGTACACGCCTTTAATTCCAGCGCGCAGGAGGCAGAGGCAGGTGGGTCTCTGAGTTCAAGGCCAGCCTGGCCAACAAAGTAAGTTCTAGGACAGCCATGGCCACACAGAGAAACCCTGTCTCAAAACAACAAAACAAATAAAACAATAGCCAATCAACTACAAAC
MSTRG.129467.1	--	0.010	0.894	0.000	6.701	up	CGGGGAGCGCCGGGGAGAAGCAGGGCCGCGCGCTGACAGGGCCGCGCGCCCCTCCCGCGGCGAGGTTGCCCGGACGCCTTAGGGCTCACGCCCCTCTCCAGAGGCGGGCGGAGACGCTTCCGAGCCAGCTGTGTGACATCCGTTTCCCCCCGCCGCGCTGACGTCTGGAGGTGTAAGTTCAAGAGCTGCCATTGGCCAGGCGCAGCTTGGCTCTTCCCCCTTCACTTCCCACCCCAGCATCAGCCCAGACTCGGAAACTGAGGCCAATTGGGAACCTGAAGAACCAGTTCCACCTGAGAGGCCTGAGGGAGAGACAGAAGCGCTGCCCTTGGGTGATGGGCGACAATCCAGTAAGGTCCCGTTAGCTGTGTGACAACAGTGATCCTACTCATTTGTCTAGGCACCTGTTGCGTGACGCTGCCTTTGCAGGCAATTGCAGGATGAAAGCATGTTATCAGATCAGGTACACTCGCCGGAAGGGAGTTTCTCGAACACAAGAGTAATCCTGACATTCCAGGCTCTCGAAGTTACTGTTGTTAACACCTTGAAATCCAGTCTGTCGACTCTATGAATATTAGTGCCGTGCCGGTATGACAGACTTTGGTGGCCAAGGTGGGAAAGTAAACTCAATCTTTCTGAAGTCTTAAATTTCAGTGGTGTAACAGGTTCACTGATGTGGAGAGGTCAGCTTTCACAGGAAAGAAGGTAGGGAAAGTTAGTTGTCTTAGACCCTTCTGCTGCTATAACAATACTACAGACATTAAATTGTGAACAAGTTTATCTGGTTCTGGAGGCTTGGGAAAGTGGAATAACATGCAGTGTCAGCTAAGTGGCAGGGTAAAAGCACTGGAAACCGTCTATCAGGTTTTATCAGGAACATACTCTTGTGGTAGCAATTCATTTTTGGTGGCAGAACTCCAGTGTCCTCATCACCTCTTAAAGGACCCCCTCCCTGTAGCGTTGCATTCATACTGTTGCTGTAGCAAATTTCAGTGTAAGTTTTGGAGGACATTTTCAACCATGCTAAGGAACCTAAAACAGGAGAGTTGTGTCTGTATGGTGAGTGGAGGAAAAGAAACAAAAGGAAAAGAGAGGGCGATTTGTTTCTGATGGTTGCTACTCTTCATTAAGAGAAGCCCCACTGCTCCCTGCTGAAGAAAAGTGGAGGGTATATTTAAAAGCTACCGAGGGGATTGAGGATACCACTGGAATTTTGCAAATAACGGTTCTCTTTCAGAAACTGCCCTTTTTAAGTCTTCATATTCAGAAATCCCACTGCCAGGGCTTGATCATAATGAAATGGAGTCTCAAAACAGAGACAAAGATTTGTTGGGTATTCATATACTTGTGCGGCCTGAGCTTCTTTTCCCTACAAATATTTCCCTACCCGCTTGCCCGTTGTTCTGCCCGTAAGTGACTAGAACGCCATCCGTGAAGCGCAGAGTTATGGCCTTACCCATCCTTCACCATCTCAGCCTGGCTGTTTTCTTTTTTAAAGAAATCCCTCCCCACCCCC

							CCAGAGCTGAGGACCCAACCCAGCGCTCTACCACTGAGCTTAATCCCCAACCCAGCCTGGCTGTTTTCT<br>TGAGCAAATGAAGCTAACTAAACTTGTGCAGAAAGTACAGCCCTGTCAGTTTCCTGTAAGTCTAATGAG<br>TGTCTACCCTTTGACACATCAGACCCATACCTTGTGTTCACCAAAGGAAATAAAGCCCTCCATCGGCAC<br>AGAGACTTGAACAGAAATGCTCATCTCAAGTGTACTCATGATGGCAAAATTCTCAAGGAGCCCAGATGT<br>CTGTCAGTGGGAGAATAAGGAAGTTGAAATATTCAGTGCAACACTACTCATGAATTAAAAATAAAATAG<br>AATATGAATGAATCTCATAGATGTGTGTTGAATCAGACTGTGGAGTTAGATCGGAGAATGCCTTCCTGAT<br>TTTAGCAAGTCCTTAAAAGAGAGGCTGGCACCGTGCAAACCCATCAGGGCCACATATGTGCTCAAACT<br>GCAGCTTAATGTTTCTAAAGATGAGGTAGGCAGACAACTGAGACCTGGAGTTTCTGAAATTTACAGTAG<br>CTACTTTATTATAATCCTTAGGCAGTCAGGAGTGAACAGGTTGCCCTTGGTCTTGTGGCATTCCGTCATA<br>CAAGTGACATTTGTGTTGGGTAGTTAAGAAAGGTTTGTGTCTGCTCTGACTGCTCCCTTCACCCACATA<br>GATCTTGAGAAAACAAAATGAAATTCCTTAAGTGTTAACATTATCACTTAACTCTTTCTTCACCACACCT<br>CAGCTCTCCATCTAGGGTTTCATACCTGTAATCCCAGCCCTCAAGAGGCTGAGGCAAGAACTTTGTCAC<br>AAGTTCAGGGCATTCCTGGGATGTAGTTACTCTCTAAAACAGAGATGCAGGGCAGTCAGTTGCTGTTAT<br>TGTCTTCGTGGTTTTAATGATAAATGTCTCCTGTAGTCTCAGACATTCAATACTTGGAGTCCAATTGGTG<br>GTGCTGTTTAGGTAGATTTAGGGGGAGTGACCTTGTTGGAAGTGCTGTGGGTCAGGCTTTGAGGTTGCA<br>AAGCAGGATAGCATTCTCAGGTTACACACTCTGCTTCTAAGACATCGACAACGACAAGGACGAGTTCG<br>GAATGGCAGCCCTTAGCTTCCAGCTTCAGGTGTCATGGCCTGTATCATACCATCAAGGACTCTAGTCCTG<br>AAACTGTGAGCCCGGATAAATGAGCTGCATTGGTCATAGTGTTTTTTGTTTTTTTGTTTTGTTTTGTTTTG<br>TTTTTTTCATCAAAGCAGTGGAGAAATAACTAAAATAACTATTAAAACCATAGGAACTCTTTACCTATAAT<br>GCTGATACATTTTCAAGAAATGAAACTATCTGCAAGAGTTTTGTCAGTTGATTGTTAAAAATTAATAAAC<br>CCCATGATGTTGCGTGGCCAGAGACTAAACTGTGCTTGCAGCTAACCCTTGGTGGGTCTGACAGCATCT<br>GGCCTCGAATTATCAGCTACAGCACATATCCTCTTAAGCATAAACAATCACCAACCATCAGTGTCATTTAT<br>GACCACTGTGGCCGTAGTGGAGAAGGTGGAAGATTCCCCACAGTCATGTCTGAATACATCCGACACAA<br>GAACATCCTCCAGACTTAGAGTGGGCAGAAGAGGCGGATAGAGACTCTTACATTCTGTATTATAAGGGC<br>ACATTTGTACCTGACATTACAGAAAGAATATAGAGTCTGCACACACATGCTATTAATAGCTTTTTAGGCA<br>CTGCTAATGGTTTCTCTCAAGTTAACCCCTGCTGCCAGCCCGGATGCCTGTAAAGAAGTACAAAGTTGA<br>TATCCAACACACAAAAACTAAAGCATTCCAGGAAGTGCCTGAAGCCAATAGAGAAGGACTCGAGGCG<br>GGACAATGAGAGGCACAGAAATCACAAGACTCATAAATATGGCTGACAAGAGGGACGACATAGAGTGA<br>GTTCTAAAATGAATGACTATTCCCAAACCACAGTAGCTCCCTGTGGAATCTGATGTCTGGAGGGGACCT<br>GCAGGCAGCATATCTTTTGAGGTGTTAGTCTGCTGGCCTCTCAGGTTGCTGGCTCTGAACAAAGTTCAA<br>TTTAAATATTTAATTCCTAACTCTCCTCATTGGCCTTTATGGTGACAGTCACAACTCTTACTATTCTGGTTT<br>TCTAAATCACAAAGCTGAGTATTTCAGCTACTGTGTGACTACTGTTTTCTGACCATTTAACAATGACAGC<br>CCTGTTCTATCTCCTAGATAGCGCATGACAAAGTGTCCATAGACTCCTCTGCTTTCTGAGGACTGCTAAC<br>TCACAGAGACCCTTTAGTGCTCAGAATAATCCCTTCCCGAGTCCAGTCCTCTCGTGTCTTCAGTGCTCTC<br>TTGCTAAGACGTTCTGCAGCATTGTGATATTCTGGCTGCCTGTTTGCACCAAGACATCAAACCCAGCTGT<br>CCTCCCCAGGCATGTCTCCAGCCAGGGAGACAGCATTTAGCATCCCATGTCACCAGTGTGCAATGCATT<br>CCTTCCTGTACTGTCCATCCCACCCTACCTCTTCTTGAGTTCTTCTCTTTTTATTATTACTTTTAATGATTCT<br>TTGTCACATATTTGTCTGGAAGTTTGGGGATTGGTTTTTCCAAGGGTTTATCTTTCTGGTGTTTACTCCCC<br>CAATTCTGTCCCACTAGAATGTCAAGTACATGCAAGAGGAAAAACTCATTTTTTTTTTTTTTGATTTTCA<br>GGAAGATAATTTTACAACTATGTGGAAGAACTAGAGAAGGCGAACAGGAAATTGGTAGGAAGACTAGG<br>GATGATTGATCAAAGGATCAGAAGGGAAACTTTGTATTAATAAAGGTATCCTGGGAATAAGGAAGTAAG

							GTGCGACTTTTCAGGTTTCGCTAAATTATTTATTAGTTGAGTAGAAGGAAGAGAATGATGGATAGGAAG<br>GAAATGAACCCCAGAATTACTACTTGCATGTTCAATGTTCCTTATTGTGCAGCCTTTTTTCAAATGGGAG<br>GGATATTCCAACCCAGGATCGACTTCTTACAGCCTGGTAGGATCTGCACTGAAGCCAACATAGGAACTT<br>CACAGTAAACAGTTTGACTAAAAGCCTCACATTGGGCATAGTGACACACACCCGTGATCCCAACACTTG<br>GGAAGCAGAGGCAAGAGTACTGAAGCACAATCAAGGTCAACCTAGTCTATAAAAAGTTTAGATTAGCC<br>AAGGCTAGACAGCGAGAGTGTCAAAAACCAAGAAAACACCTTGTACTTCTTGTCTAAAAATATACAATT<br>TTAAAATTCACATTTGCCTGTAAAGCATTCTGATGTGTAAAAAGCTTCTTAGGGAATGAAGTTGGGTAAT<br>GATTGCAGCACATTGCTCAAACATGTTCCTGATACAAGGGCATATTGCATGTACCGCTAATCCTACCAGT<br>TGTGGTTGAGGTGATGGATGGCCTTTATAGGAGACCAAAGATACTTTGCATCTGAAAATCATAGAACATC<br>CCAGATGTTAAATCATTTAACAGTTTGCCCAGTCATGTTTTTGTGGAAATACTTAAAGCTAGAAATGTAA<br>GAACCAACAAGTTTTAGGAGAAGTCTCTTTAGGAAGACAACATATTCTTTGATTGGGTTCAGACATTAA<br>CTTCCACCTAGACAGTTCTTCCCTTGCCTCTTTGAGGTCCCAGCCCACATGTCACCACCTCTCACAAGA<br>CCTCTTAGTGGCTCTGCTGAACTGCTCTGTAGAACTTCCCAGCCATTACCTGCTGCTGTGGTTGCTGGTT<br>TAGTTAATATCACCCCAGTCAGAATTAATGCTTTGTTTCTAGGTCTTGTTCTTGCAGTAATAGATAATATGT<br>CCAGAGGGTCCTGTGGAATCCAAAACACAGGTGAAAGCAAATAGTTATCCTTTAGTTTTCCAAAAAGTA<br>AGTTTATAGAAGGGAATAACTAAGCAGGATTAAATGATTTGTTTTTCTCCAAACTTTACGTATTACTGGA<br>ATAATTAAGGCAAATTCGGGGGCTCATTGGACTTGCTTAAGATCCATATTTATCATTTTAATGAAACTAAT<br>TCAGCAGAAAGATCTATTACTCCTATTCCCTCCAGTCAGTGTCCAGCTAAGTAAGCCTTATAATTTTTATT<br>TTCTCATTTTCAACTGATGCAAATTAATCTTTCTAGGTCTCCCTAACCAATATCATTCATTTTTCTCTCCTA<br>GGATTTAATAATGTTTGGGTTTCTCGTTACACTGATATTAAATTGCTATCTGTAACCCAAGGCCAGATAAT<br>TAAAACTATTTCAGTGACTCAGAAAGAGCAAGCTACTCTCCTGACACCACATGTTAATAGTTTGTGATAT<br>TATTATAGTAATCAACCAAGTATCTACATTGCGACCCTGCTTGTTCTACCTTGAATGCGGTGACTAGGAG<br>ATTGCCTCCTCTTCACCCATTGTAAATAACTAGAGATAAAACAATCCTAATGAGGACCCACATACTGAAA<br>GAACTGTGTAAAATGTATACTCTTTTCTGTGGTAGGTACAAGCAGGGGACAAGGGAAGGATGCCTGGA<br>CTCACACTCATCACAGACCACTGCCTAAAGCCTTCACCTGATTCTCTGTCTACTCCTGTAGCCACAGGC<br>ATCCACCCGCATTCCAGTTCCCGAATAATGACTCAGAAGCTTATTATGTAGTATTTAAACTAGGTTTATTC<br>ACAGACGAGTTTGTAACCTGTTTATACTATGCTATGTCTGCCAAATGGCTAGTTACCTCTCCTCTACTTCC<br>TCCAAGTGCGGGTGGCAAATCTTCCCACTCACCCATCTTCCAGAGTTCCCCTCTCTACCAGATGTCCCAT<br>CCTCTAATCCTGCCTCAGGTAATAGGCCATCAGATATGTATGTGACACACAGTGCAAAAGAGATTATCCA<br>GATGTGTCTCCCTTTTGAACTAATAAAGAGCTCTTTATCTAGTATCAATAAACTATCCACAGTAAGAACA<br>ATTATGAAAACTATCAAGAATTATATCCACAGTGTCCAGTCACATGCCTTTAAGAACTGTGGGGGAAGTG<br>CTCCATTATCTATCTGGGTAAGCACCAAGTTCTGTACCTAAATCATTTTCTATCGTAATTTGCATTACCAAC<br>CTAAAGACATCATCTTAGACCTTAAATCATTTTCTTAGATCCTAAACAGCTTAAGTTTGATGAGACTATAA<br>CTAGCTAATCTTCAATCCTGTCAGAGACCTGAGAAGGAGAAAATTTACCTCAATAAATAGAAAGTACAG<br>AGGAAGCAGCTTTGTCTCTATAGAAATGGCTGAGGTTCCTGGCTAACTGGGCAGCCACCCAAGGTTCCC<br>TGTGTCCTGTGTTGGAGCATCATCTTCAGCCTTCTGAATCTCTGACGGACATTCTGTGAAGCAGGAGTG<br>GAAAGACTTGCCTACTTTGTCTTTACAGAGCTCAGTGATAGACTCCCCTGCATCCCATTTGTCCAGATTG<br>GACAGCATGCTGGCAGCAGTCAGGGCAGGGTGGTTTGGGGGCTGTTTGTCCCCCCCAGTTGTTGTTGT<br>TGTTGTTGTTGTCCCCCGAACCCCGCCTGGCAGCAGCTTTGCCACACTTGAAGCCAACTCCGTAAGGA<br>GGCTCGTCAGCGTTCAACGTCGTCTTCCAGGTAGAACACGGTGCTGTCAGGAGCAGACACGGCTGTCT<br>TCCTTTCCTGCGACAGTGCACAAACGGCTGTGTAAATTACATGTCTGAGAGAGAACTGGCACAGAATCC

							GACTTAGTCACCATCTCACTCTGCTTCCAGGAAGATTATTGTTGTTGTTGTCGTCGTCGTCGTCGTTGTT<br>AAGAAGGCTTACCTTTACCACTGCTCTCGGAGTCTGTGTGTTGAGTCTGCGCCACCTCACCTCCAACCC<br>TCAAGGGCAGGTAAGGTCAGGCAGCGAGTCCCGGGCACAGGCAACACTTGTCAGCAATAGAAGCCAG<br>GACAGTCTGGCTCTAATGTCAGCAGCCCAACCAGCAGCTGCTGCAACTGGGTAAATTAAC
MSTRG.129467.2	--	0.000	0.613	0.000	8.649	up	CGGGGAGCGCCGGGGAGAAGCAGGGCCGCGCGCTGACAGGGCCGCGCGCCCCTCCCGCGGCGAGGTT<br>GCCCGGACGCCTTAGGGCTCACGCCCCTCTCCAGAGGCGGGCGGAGACGCTTCCGAGCCAGCTGTGTG<br>ACATCCGTTTCCCCCCGCCGCGCTGACGTCTGGAGGTGTAAGTTCAAGAGCTGCCATTGGCCAGGCGC<br>AGCTTGGCTCTTCCCCCTTCACTTCCCACCCCAGCATCAGCCCAGACTCGGAAACTGAGGCCAATTGGG<br>AACCTGAAGAACCAGTTCCACCTGAGAGGCCTGAGGGAGAGACAGAAGCGCTGCCCTTGGGTGATGG<br>GCGACAATCCAGTAAGGTCCCGTTAGCTGTGTGACAACAGTGATCCTACTCATTTGTCTAGGCACCTGT<br>TGCGTGACGCTGCCTTTGCAGGCAATTGCAGGATGAAAGCATGTTATCAGATCAGGTACACTCGCCGGA<br>AGGGAGTTTCTCGAACACAAGAGTAATCCTGACATTCCAGGCTCTCGAAGTTACTGTTGTTAACACCTT<br>GAAATCCAGTCTGTCGACTCTATGAATATTAGTGCCGTGCCGGTATGACAGACTTTGGTGGCCAAGGTG<br>GGAAAGTAAACTCAATCTTTCTGAAGTCTTAAATTTCAGTGGTGTAACAGGTTCACTGATGTGGAGAGG<br>TCAGCTTTCACAGGAAAGAAGGTAGGGAAAGTTAGTTGTCTTAGACCCTTCTGCTGCTATAACAATACT<br>ACAGACATTAAATTGTGAACAAGTTTATCTGGTTCTGGAGGCTTGGGAAAGTGGAATAACATGCAGTGT<br>CAGCTAAGTGGCAGGGTAAAAGCACTGGAAACCGTCTATCAGGTTTTATCAGGAACATACTCTTGTGGT<br>AGCAATTCATTTTTGGTGGCAGAACTCCAGTGTCCTCATCACCTCTTAAAGGACCCCCTCCCTGTAGCGT<br>TGCATTCATACTGTTGCTGTAGCAAATTTCAGTGTAAGTTTTGGAGGACATTTTCAACCATGCTAAGGAA<br>CCTAAAACAGGAGAGTTGTGTCTGTATGGTGAGTGGAGGAAAAGAAACAAAAGGAAAAGAGAGGGC<br>GATTTGTTTCTGATGGTTGCTACTCTTCATTAAGAGAAGCCCCACTGCTCCCTGCTGAAGAAAAGTGGA<br>GGGTATATTTAAAAGCTACCGAGGGGATTGAGGATACCACTGGAATTTTGCAAATAACGGTTCTCTTTCA<br>GAAACTGCCCTTTTTAAGTCTTCATATTCAGAAATCCCACTGCCAGGGCTTGATCATAATGAAATGGAGT<br>CTCAAAACAGAGACAAAGATTTGTTGGGTATTCATATACTTGTGCGGCCTGAGCTTCTTTTCCCTACAAA<br>TATTTCCCTACCCGCTTGCCCGTTGTTCTGCCCGTAAGTGACTAGAACGCCATCCGTGAAGCGCAGAGT<br>TATGGCCTTACCCATCCTTCACCATCTCAGCCTGGCTGTTTTCTTTTTTAAAGAAATCCCTCCCCACCCCC<br>CCAGAGCTGAGGACCCAACCCAGCGCTCTACCACTGAGCTTAATCCCCAACCCAGCCTGGCTGTTTTCT<br>TGAGCAAATGAAGCTAACTAAACTTGTGCAGAAAGTACAGCCCTGTCAGTTTCCTGTAAGTCTAATGAG<br>TGTCTACCCTTTGACACATCAGACCCATACCTTGTGTTCACCAAAGGAAATAAAGCCCTCCATCGGCAC<br>AGAGACTTGAACAGAAATGCTCATCTCAAGTGTACTCATGATGGCAAAATTCTCAAGGAGCCCAGATGT<br>CTGTCAGTGGGAGAATAAGGAAGTTGAAATATTCAGTGCAACACTACTCATGAATTAAAAATAAAATAG<br>AATATGAATGAATCTCATAGATGTGTGTTGAATCAGACTGTGGAGTTAGATCGGAGAATGCCTTCCTGAT<br>TTTAGCAAGTCCTTAAAAGAGAGGCTGGCACCGTGCAAACCCATCAGGGCCACATATGTGCTCAAACT<br>GCAGCTTAATGTTTCTAAAGATGAGGTAGGCAGACAACTGAGACCTGGAGTTTCTGAAATTTACAGTAG<br>CTACTTTATTATAATCCTTAGGCAGTCAGGAGTGAACAGGTTGCCCTTGGTCTTGTGGCATTCCGTCATA<br>CAAGTGACATTTGTGTTGGGTAGTTAAGAAAGGTTTGTGTCTGCTCTGACTGCTCCCTTCACCCACATA<br>GATCTTGAGAAAACAAAATGAAATTCCTTAAGTGTTAACATTATCACTTAACTCTTTCTTCACCACACCT<br>CAGCTCTCCATCTAGGGTTTCATACCTGTAATCCCAGCCCTCAAGAGGCTGAGGCAAGAACTTTGTCAC<br>AAGTTCAGGGCATTCCTGGGATGTAGTTACTCTCTAAAACAGAGATGCAGGGCAGTCAGTTGCTGTTAT<br>TGTCTTCGTGGTTTTAATGATAAATGTCTCCTGTAGTCTCAGACATTCAATACTTGGAGTCCAATTGGTG<br>GTGCTGTTTAGGTAGATTTAGGGGGAGTGACCTTGTTGGAAGTGCTGTGGGTCAGGCTTTGAGGTTGCA

							AAGCAGGATAGCATTCTCAGGTTACACACTCTGCTTCTAAGACATCGACAACGACAAGGACGAGTTCG<br>GAATGGCAGCCCTTAGCTTCCAGCTTCAGGTGTCATGGCCTGTATCATACCATCAAGGACTCTAGTCCTG<br>AAACTGTGAGCCCGGATAAATGAGCTGCATTGGTCATAGTGTTTTTTGTTTTTTTGTTTTGTTTTGTTTTG<br>TTTTTTTCATCAAAGCAGTGGAGAAATAACTAAAATAACTATTAAAACCATAGGAACTCTTTACCTATAAT<br>GCTGATACATTTTCAAGAAATGAAACTATCTGCAAGAGTTTTGTCAGTTGATTGTTAAAAATTAATAAAC<br>CCCATGATGTTGCGTGGCCAGAGACTAAACTGTGCTTGCAGCTAACCCTTGGTGGGTCTGACAGCATCT<br>GGCCTCGAATTATCAGCTACAGCACATATCCTCTTAAGCATAAACAATCACCAACCATCAGTGTCATTTAT<br>GACCACTGTGGCCGTAGTGGAGAAGGTGGAAGATTCCCCACAGTCATGTCTGAATACATCCGACACAA<br>GAACATCCTCCAGACTTAGAGTGGGCAGAAGAGGCGGATAGAGACTCTTACATTCTGTATTATAAGGGC<br>ACATTTGTACCTGACATTACAGAAAGAATATAGAGTCTGCACACACATGCTATTAATAGCTTTTTAGGCA<br>CTGCTAATGGTTTCTCTCAAGTTAACCCCTGCTGCCAGCCCGGATGCCTGTAAAGAAGTACAAAGTTGA<br>TATCCAACACACAAAAACTAAAGCATTCCAGGAAGTGCCTGAAGCCAATAGAGAAGGACTCGAGGCG<br>GGACAATGAGAGGCACAGAAATCACAAGACTCATAAATATGGCTGACAAGAGGGACGACATAGAGTGA<br>GTTCTAAAATGAATGACTATTCCCAAACCACAGTAGCTCCCTGTGGAATCTGATGTCTGGAGGGGACCT<br>GCAGGCAGCATATCTTTTGAGGTGTTAGTCTGCTGGCCTCTCAGGTTGCTGGCTCTGAACAAAGTTCAA<br>TTTAAATATTTAATTCCTAACTCTCCTCATTGGCCTTTATGGTGACAGTCACAACTCTTACTATTCTGGTTT<br>TCTAAATCACAAAGCTGAGTATTTCAGCTACTGTGTGACTACTGTTTTCTGACCATTTAACAATGACAGC<br>CCTGTTCTATCTCCTAGATAGCGCATGACAAAGTGTCCATAGACTCCTCTGCTTTCTGAGGACTGCTAAC<br>TCACAGAGACCCTTTAGTGCTCAGAATAATCCCTTCCCGAGTCCAGTCCTCTCGTGTCTTCAGTGCTCTC<br>TTGCTAAGACGTTCTGCAGCATTGTGATATTCTGGCTGCCTGTTTGCACCAAGACATCAAACCCAGCTGT<br>CCTCCCCAGGCATGTCTCCAGCCAGGGAGACAGCATTTAGCATCCCATGTCACCAGTGTGCAATGCATT<br>CCTTCCTGTACTGTCCATCCCACCCTACCTCTTCTTGAGTTCTTCTCTTTTTATTATTACTTTTAATGATTCT<br>TTGTCACATATTTGTCTGGAAGTTTGGGGATTGGTTTTTCCAAGGGTTTATCTTTCTGGTGTTTACTCCCC<br>CAATTCTGTCCCACTAGAATGTCAAGTACATGCAAGAGGAAAAACTCATTTTTTTTTTTTTTGATTTTCA<br>GGAAGATAATTTTACAACTATGTGGAAGAACTAGAGAAGGCGAACAGGAAATTGGTAGGAAGACTAGG<br>GATGATTGATCAAAGGATCAGAAGGGAAACTTTGTATTAATAAAGGTATCCTGGGAATAAGGAAGTAAG<br>GTGCGACTTTTCAGGTTTCGCTAAATTATTTATTAGTTGAGTAGAAGGAAGAGAATGATGGATAGGAAG<br>GAAATGAACCCCAGAATTACTACTTGCATGTTCAATGTTCCTTATTGTGCAGCCTTTTTTCAAATGGGAG<br>GGATATTCCAACCCAGGATCGACTTCTTACAGCCTGGTAGGATCTGCACTGAAGCCAACATAGGAACTT<br>CACAGTAAACAGTTTGACTAAAAGCCTCACATTGGGCATAGTGACACACACCCGTGATCCCAACACTTG<br>GGAAGCAGAGGCAAGAGTACTGAAGCACAATCAAGGTCAACCTAGTCTATAAAAAGTTTAGATTAGCC<br>AAGGCTAGACAGCGAGAGTGTCAAAAACCAAGAAAACACCTTGTACTTCTTGTCTAAAAATATACAATT<br>TTAAAATTCACATTTGCCTGTAAAGCATTCTGATGTGTAAAAAGCTTCTTAGGGAATGAAGTTGGGTAAT<br>GATTGCAGCACATTGCTCAAACATGTTCCTGATACAAGGGCATATTGCATGTACCGCTAATCCTACCAGT<br>TGTGGTTGAGGTGATGGATGGCCTTTATAGGAGACCAAAGATACTTTGCATCTGAAAATCATAGAACATC<br>CCAGATGTTAAATCATTTAACAGTTTGCCCAGTCATGTTTTTGTGGAAATACTTAAAGCTAGAAATGTAA<br>GAACCAACAAGTTTTAGGAGAAGTCTCTTTAGGAAGACAACATATTCTTTGATTGGGTTCAGACATTAA<br>CTTCCACCTAGACAGTTCTTCCCTTGCCTCTTTGAGGTCCCAGCCCACATGTCACCACCTCTCACAAGA<br>CCTCTTAGTGGCTCTGCTGAACTGCTCTGTAGAACTTCCCAGCCATTACCTGCTGCTGTGGTTGCTGGTT<br>TAGTTAATATCACCCCAGTCAGAATTAATGCTTTGTTTCTAGGTCTTGTTCTTGCAGTAATAGATAATATGT<br>CCAGAGGGTCCTGTGGAATCCAAAACACAGGTGAAAGCAAATAGTTATCCTTTAGTTTTCCAAAAAGTA

							AGTTTATAGAAGGGAATAACTAAGCAGGATTAAATGATTTGTTTTTCTCCAAACTTTACGTATTACTGGAATAATTAAGGCAAATTCGGGGGCTCATTGGACTTGCTTAAGATCCATATTTATCATTTTAATGAAACTAATTCAGCAGAAAGATCTATTACTCCTATTCCCTCCAGTCAGTGTCCAGCTAAGTAAGCCTTATAATTTTTATTTTCTCATTTTCAACTGATGCAAATTAATCTTTCTAGGTCTCCCTAACCAATATCATTCATTTTTCTCTCCTAGGATTTAATAATGTTTGGGTTTCTCGTTACACTGATATTAAATTGCTATCTGTAACCCAAGGCCAGATAATTAAAACTATTTCAGTGACTCAGAAAGAGCAAGCTACTCTCCTGACACCACATGTTAATAGTTTGTGATATTATTATAGTAATCAACCAAGTATCTACATTGCGACCCTGCTTGTTCTACCTTGAATGCGGTGACTAGGAGATTGCCTCCTCTTCACCCATTGTAAATAACTAGAGATAAAACAATCCTAATGAGGACCCACATACTGAAAGAACTGTGTAAAATGTATACTCTTTTCTGTGGTAGGTACAAGCAGGGGACAAGGGAAGGATGCCTGGACTCACACTCATCACAGACCACTGCCTAAAGCCTTCACCTGATTCTCTGTCTACTCCTGTAGCCACAGGCATCCACCCGCATTCCAGTTCCCGAATAATGACTCAGAAGCTTATTATGTAGTATTTAAACTAGGTTTATTCACAGACGAGTTTGTAACCTGTTTATACTATGCTATGTCTGCCAAATGGCTAGTTACCTCTCCTCTACTTCCTCCAAGTGCGGGTGGCAAATCTTCCCACTCACCCATCTTCCAGAGTTCCCCTCTCTACCAGATGTCCCATCCTCTAATCCTGCCTCAGGTAATAGGCCATCAGATATGTATGTGACACACAGTGCAAAAGAGATTATCCAGATGTGTCTCCCTTTTGAACTAATAAAGAGCTCTTTATCTAGTATCAATAAACTATCCACAGTAAGAACAATTATGAAAACTATCAAGAATTATATCCACAGTGTCCAGTCACATGCCTTTAAGAACTGTGGGGGAAGTGCTCCATTATCTATCTGGGTAAGCACCAAGTTCTGTACCTAAATCATTTTCTATCGTAATTTGCATTACCAACCTAAAGACATCATCTTAGACCTTAAATCATTTTCTTAGATCCTAAACAGCTTAAGTTTGATGAGACTATAACTAGCTAATCTTCAATCCTGTCAGAGACCTGAGAAGGAGAAAATTTACCTCAATAAATAGAAAGTACAGAGGAAGCAGCTTTGTCTCTATAGAAATGGCTGAGGTTCCTGGCTAACTGGGCAGCCACCCAAGGTTCCCTGTGTCCTGTGTTGGAGCATCATCTTCAGCCTTCTGAATCTCTGACGGACATTCTGTGAAGCAGGAGTGGAAAGACTTGCCTACTTTGTCTTTACAGAGCTCAGTGATAGACTCCCCTGCATCCCATTTGTCCAGATTGGACAGCATGCTGGCAGCAGTCAGGGCAGGGTGGTTTGGGGGCTGTTTGTCCCCCCCAGTTGTTGTTGTTGTTGTTGTTGTCCCCCGAACCCCGCCTGGCAGCAGCTTTGCCACACTTGAAGCCAACTCCGTAAGGAGGCTCGTCAGCGTTCAACGTCGTCTTCCAGGTAGAACACGGTGCTGTCAGGAGCAGACACGGCTGCTCTCGGAGTCTGTGTGTTGAGTCTGCGCCACCTCACCTCCAACCCTCAAGGGCAGGTAAGGTCAGGCAGCGAGTCCCGGGCACAGGCAACACTTGTCAGCAATAGAAGCCAGGACAGTCTGGCTCTAATGTCAGCAGCCCAACCAGCAGCTGCTGCAACTGGGTAAATTAAC
MSTRG.129552.1	--	0.374	0.030	0.013	-2.625	down	TGTGTGTGTGTGTGTTGTTTAAGCACATTTGTATTTTCTCCAGTTTTCTCTCATTCCCTTTCTCTCCCCTTGCCCTTTGAGGAGAGAAGAGCGTGTTTTGTGTGAGTGTCCCACAGGTCTCTGGGTATTTCTTTCCTGTTTTCTCTTTGTTGTCTGGCTGGGTGACTCTCTTCTGTCCTTAAACTTATTGATAGTGGCTTCTGTTGTCTTCACTATACTGTGGAGCAGGGCTGGGGAGTTTTAAGTTTGCATTTCTTCCTTCTATAATTTCTCTCTGATTATTCTTACAGCTTGTCTTCCTTGGTTAAGTCTTGTATTTTTACTGTTGGCTTTGAGAGTTTATATCTCATTGCTGAAGAGTTTTTCTGTGGCTGTTAGACCTCTTACCTTGGTATGCTGGCATGTGATTCATCTTGGTGTTAGTATTAATTATCCTTAAATTAAAATTAGGATTTTTCTCAGTGACAGGCAATTTCTGATTGTACCCTTTGTAATTTATGTGTTATGTTAGACTTGGGAAACACTTCAGTGTCTTCATAAGTAGGTACCTTGTTAAGTTTTTTAGCATGTAGATCTTAGTCTGCTTTCATGGGGTACTGTTCCAATGACAGTTTAATTTTCCCCATGCCGTTTTGATTTCCTTCATTGATAATCCTGAGCTCCCAGTGGTTCCTACTTGTGCTACCTGAAAAGCTAAAAAATTCTTTCTTAGATTACATCATGGATGTCTATTGAAGGGGGAGAATGTGCTGGGTTTTCTTACCACATCCTGCTCTCCTGGTGTCTGTGGAATGGGGAAGGCATGTGGCTTCTTAGATTGTCCTTTGTGCAAAACCTGGATCCCGCCTGCCTGTTCTCCCTGCCTATTCTGGGATCTGTGAATGAGGAAGGTGTCCCTGAGACCTGTGAGTACAAAG

							AGGTCCTGTGTATCAGGCAGACACGTTAGAGATCGCTGCAGAGGCACCAAATGTGTCATCACCACATTC<br>AGGAAAGAAAGACAGCTTGGTAATTCCCCTGACAGGAACTTGATGCAGGAGTGAACCCAAGTTTGGA<br>CCCTCCATGTCGCTCCATTGCCTTCCATCTGTCTGTAGTTTGTGTTTCATCTTTGTATCTGACCTCTTCCTA<br>ACAAAGGGCTTTGTTCTGTACTTACTGAACAACACAGAAAGCATCACATGGTGAAGCAATGGCATACTT<br>CACAGAAATCCTTAACAGTGTTACAGGGGATGAGGTTGCTGACCCCCTGAGTCAGATAGCAAACATCAT<br>TGTAAGCACACAGTTACCCAGCAGCATCCGCATGCTGCTCCCAACATCCACAATGGATATTTGTTATAGA<br>GCACTGCTTCCATGGGCTGGGGGCTGCTCGGGCAAATGACTGCCATAGCACACGTGCACACATGCACA<br>CATGCACACGTGTACACATGCATTGCCGTGGGTTGGGCATGGAAGAATACATAGTTTATGATTAAAGCTA<br>TGCATTAGCACAGGATTTGAAAGCTATCACATAGAAGATCCCAGACAAGTAAGGTCACATCATTTTCATC<br>CAAGCAGGTTAAATAGGCTGAGTATAGAGTGGGGTATCAGGCTAGGTTTATGTGCTGTCCATGTGTATTA<br>TGAGCTGGGCTGTGAGGCAACAGTTCTAGTCTCTTAGAGTGGAAGAGATAAAACGTACTAGCTCACCTT<br>TGACCGTATTGCTTGCTATAGTTGGGTCTCTAGCAGGTCCCGTCTGCCTGACTTGCTTATCCCAGGTAGA<br>AAAGGTGAAGCGTAGGCAGGGTTGCAGGCTACGTGATCACTGCCCATGGAGTCACTCAGCTTCCTAGG<br>TTTTTGGTCTTTGCTTGTTATTGAAAACACTTACATATATGCGACTTGTCATGAGTGGTACTTACACGTTG<br>TGTATTGATCTGATAATTTTTTTCTTCTTTATTACCTTGGGTATTTCTTAGATCTGATAATTTCTTAAGTACT<br>TATCAGGTCAGGCAGCCCAACAGTTAAACTTGGTTAATTGACTATGGAGTTACCCTGCTTTCAAAAGCA<br>TCATCCTCATCCCTATGTTTATTTCTAGGAGAGGTATGTATATATTCTGGGCTAGCATATTGATAAACATAA<br>ACAAACCTCTTAGGACAGGCTGGAACATTCCCCAGACTCTGCCAGATCATGCTTCCCATTTTCTTTTCCT<br>GAGCTCTACATATTTTTTTGAAGCTTCTGATAGATAGCAGGAATAAAAATATAAAAATGCTTATCAATATG<br>CATTTAGAATAAAGATGGCATTTATGCCACATCAGAACCAAAATAACCACAATCCTATATTGTAGTAGGTT<br>TTGTTATAGTTAAATTCAAAATATTTCTAGCATTTCTTCCCTTGAAAGTATTTCTTATTCTTTGGTTGGACA<br>CAGGCTGAGAAGTATGTTTCCACAGCTGCAGGTAAAAATAGCGGCAGCTGCAGCAACAGCAACAACA<br>ACAACAACAACAACAACAACAACATCAACGACAACATCATCATCAACGACAACTCATTGTCTGTTTTTG<br>TGTGCCACAAATGACTGTCTTTTCTCCATGGGATTGAGCTTTTACGTATTAGAAAGTGTTCTACCACTGA<br>GTTAAACTCCAGAGTCATTGTTTTAAAGTTGCTGTCTGGACCCAGGGGCCTGGAATCATTGTGGTCAGC<br>ACACAGGTGCCTCAGC
MSTR<br>G.129<br>66.2	--	0.000	0.220	0.031	3.453	up	GTTAACGTGATGAGGGTAATTTCAAATGGCATTCAATCAAAAATTCTGAAAGTTTTATTAGTGAGGTTGG<br>GGGCTTTTTCAGTTCAGATGAGGCTTGCTCCAGCTCAACTCGGCATTTTCAACTAAGATTTCAGAGAAT<br>GAAATGTTCTAGGATGCATGTATTCAATATTGCACTGATTTCCATACATACCAAATAGTTGGTTGCCTTCT<br>TAGAATATTGATTTGCGTTCCTCGCATTCATCCAAATAAATATCACATAGGTAATTTAATCACCATCATAGA<br>CAATCTTTAGGGAGCCAATAGTCACTAGCTGCTATTTCTTGGTACAATATATATTTCTGTGACCTCTCTCC<br>TTAGTGATAGGTTTTTCTCCTTTTGAAAGGTTGTAACCAATTGATAAGAGGATTGAACTGTAGTGTCAGC<br>TTTGTGATCTTGTGCTCTGTGACTATTACTGTG
MSTR<br>G.129<br>725.2	--	0.000	0.248	0.000	4.999	up	GCTCGGCCCAGGGAGTGGCACAATTAGAAGGTGTGGCCCTGTTGGAGTAGGTGTGTCACTGTGGGCAT<br>GGGCTTAAGACCCTCATCCTAGGGGCCTAGAAGTCAGTATTCTGCTTGCAGCCTTCAGATGAAGATGTA<br>GAACTCTCAGCTCCTCCTGCACCATGCCTGCCTGGATGCTGCCCTGTTCCTGACTTGATAATGGACTGA<br>ACCTCTGAACCTGTAAGCCAGCCCCAATTAAATGTCCTTTGTAAGACTTGCCTTGGTCATGGTGTCTGTT<br>CACAGCAGCAAACTTCTAACTAAAACACAATCCCCTTGTCACCGCTGTATGACATACTCACGATATGCTG<br>TCCTGTGTCTTGCCTGCTTCTCAGAGGAGATGGTGGCTGGACCAGCTGTCATCCCAAGCCCTCGCTGCT<br>CATTCTCATTAGGGGGAGCTCAGGAAGAGATGGATGAATTAAACTGAACAGAGTTCCTGTGTCCGCAGC<br>TCTAACAGTGGCTGGGTAACTCTGTGGCTACTGTCCCCCAGGTCTGAACACACGCCCGGTGACATTTTG

							ATTGTGGCTTCCACTCTTTTAAATTTTCAATGATCTCAGACTCTATCCGTGCCTGCCTTGGACATAGTAAAAACTGCAGCTGGAGGATAAGGAATTTGCTGGCTCTGCAACACTGGATTTTTACAGCTCAAGTAACTTCCTCCTGAGCTAAGGAAAGAGGGAAGACATCTGTTTTTCTGTTTTCAAATGTGACATTCTTTCCCCCGGGAAGTACTTTCAAATGTGTTTGGGTTCTGCCTCCTGCGTTTGCCTGCCTCCCTGCCTTCATAGCAGAACTATTTCATATTTTTCCAGAACTTTTGGGTTCCCACAGTATAAAGAGGTGCCCTGAAATGAAAAACTCTACAATTTTCTATTAGTAAAATGTGGGGACACTAAGGCTAGCAGATAAAAACAGGATAGTTTGTATGCACATAGTTTGGTAAACACCCCACCACCCAAGTACACAACAGACACTCTCGGCCATGTCTACAGCTTCGGTTGCAGAGCTGAGGGAGAGGTAAAATCTGGAGCATTTGGGTGGGAAGCTTCAGAACGACCTTGCAGAGAGGTTGTGATGGGGCTGGCTGGAGAATGTGTGTGACAGCTGACAGGGTAAGACTTTCCCATAGGAAACACAATGGTGTGGGATTAACTGGGGCTTCCAACTTCTTCCACCCGGTTCTAACCAAGCATTCACATTCCTTCTATTCCCCAGGCTTTCTCTTTGTGAGGTAGAAGCTAGACACTACCAAGGAACAAACTCTCGGGACGACGGGCTGGTGTCGCCGTCTCCCTCTATACTTAAGCTCCCAGAGCATTTCTGCTGGACTCCCTTCTCTTTCACGGTGTTACCT
MSTRG.129773.1	--	0.043	0.431	0.024	3.103	up	GGCCCTTGCAGCTGTCGGCCCCAGTCTTACACATGTCCTCACATGATGAGGGCATTCCTAGGAGGCAGGCATGACATAGATTCCTGCTGCAGCTATGATGAATGAGGATTCCTCTCCAGACTTAAGGCCAGCCAGCTCTCTCCTAGGTTCCAGGACCCACCTCCCATCAGTCACCAGACATACCACCGCTCCTTCACTTTGCTGGAAAGCCACCCTCTCTTCCCAGGGGACACAGCTCTCTCAGTCTTTTCCACCTGGAACTGTGTTACCTGTATGGTGTCAGTAAATCCTAGAGACTCAGAAATAGAGAGAATGAGCAGTGACCAAGGGGCAGGGATCAGCTCAAAC
MSTRG.129859.1	ENSRNOG00000016719;ENSRNOG00000050799;ENSRNOG00000053805	0.091	0.388	0.028	2.427	up	GGGAAGGGAAAGCTTTCAGTAAAACAGTGAAGTCATCCAATGAGGGAAAGATGCACAGGGGACATTGGGTGAGCAAATACCTTCTTTTTTCTTAAATTCTCACATATTTGAGTCAAGGTGTGCATGTAGAGAATGATCCTGAAACTTAGAGAAGAGCCTTCTGGAAGATAGTTGAATGTTCTGTTTTGTTTTTTTCCTTCAGGAATAAGCAAGGCAGAATGAAATGAGGTTTTGCACTACAGTCCTTGTCAGGCTAGGTGGGAGTCAAGTGACAACCTACACTTCTGCCACTGCCAGGCTAAGGCTTGGGTTTCAGTTTTTCCATCAAAATGATGATCACAATTTCTTCCCCTTTTCTAAAATCTCCATAACTAATGCAGCTGCACACTCTTTGGAGTAGGACATCTTGCAAATGATACGTGTCTGAAATCTACCTTAGAGTGTTGGGTTCTGAGGGGACCTGATGAACACGGCTGTTCTTGTTGCCCATAGTTTTGAGACAATTTGATCACACCGACATAGATAGAACATTACCGTTATTCTGGTAAACCTGGTATCAGAAAACAGGGCTTAGAGCTGTACATAAATTGGCTGTGTGGTACTGAACTCCAGAATGAGGCAGAATTACTCACCCGTTGCCAGAAGCACAGAAATCTCAGGCTTTCTCACATTGTCTCTGGAGCATTTCTTATAGGCAGGCAGGGCAGAACACAGACTGAATTGGCAGCCAGCTCCCTTTCTGTCTGGCTTCCCCTTCCGTCCTTCCTGCTCGGGTGGAAGCGGTAGAAGCGCCCTGCTCCTTCGGCGGTGGATCGCCAAGCTGGCGCGGGGACCAAGGATGAGCTGAGTCGGG
MSTRG.130258.1	--	0.000	0.262	0.011	3.719	up	GCAACATCTCCCACTCAGCTGATGACAAGGATCTACCAAGCTGAACAGCTATTTGTGCAACAAACTTCACACATGAGAGAGAGCTAAGGAAACCAGTGAGACTCAACACCTAAAAGACACCAAATTCAAGATTTGGTTGACAACCTAGAACTCACAGACAGCACTTGTGGATCATCTCACAGTAACTGACATCACATGTGAGTATGGCTGGCACAAACTAAAGTTTGGGTCTATCATTCATTTGAGGCCTAGAGCATTCCTGAGAGATGCTTACTCTCTTCCTGATACTTGCAACTACCTCTGGAAGTGAGAATTAGGTGCAACATAGCTCAGTGTCAGCTCTGGTGTCAAGAGACTGTTTCCACGCCTCTGTTCCTTAATCCTGAGCAGCATGATTATTGCCTGGTCAGTGCTGATGCTGTAGAACATCCCAGGTACCAGGCAGCCTTCTAGGCCCAAGAGGAAGGGACTTTACTG
MSTR	--	0.000	0.961	0.000	5.427	up	CCGGATTCATATGAAACTTTACATGCAGGATTTTCCAAGGAGTTTAAGGAGTATGAAATAGACACATCCA

G.130<br>482.1							GAGATTTCTCCTCCTTGGAGTTAACAACAGAACCTACAAACTGGTGTGGCGGGGATTGCCCAAGTGTA<br>AATGACCTCGGGGCAAGAGTGCGCCTCACCTGATCCTCCTGGTTGGTGTGCCGGATGAGGAATCTCAG<br>GAAGTTGAACCTGGAGCATTTCCGGATAAGGATGAGGTCAGAAGACCCATCTCCCAGATGGGCAAATG<br>GGGACAGGCCCCCAGGGCTCCGGGGACAAGCACAGGACATGTTGGTGGAGTTGATAGCCAGGAACTT<br>CCCACATACCACTTGCCACTCTTCCACTTCCTCAGCGTTCTCCAGGCCATACAGGGCTTTCTTCTGTTCC<br>TCTTCCAGCTGTCGCTTGCTCTGCCTGCACACGGAGCACCCAGCCCGGCAGGGTTTCTGGTCTCGTGGA<br>GA
MSTR<br>G.131<br>044.1	--	0.085	0.304	0.041	2.291	up	GCACTCTGGACCCACTGTTGTCAACGGCAACAATATTAAAGAAATAACTAGAAATGTACACAAGAACCT<br>ATGGGTTATTTTTGTTTCTCCAGACAGGGTTTCTCTGTATTCTGTCCTGGATCTCACTTTGTAGACCAGGC<br>TAGCCTAGAACTCACAGATATCTGTTTGCCTTTCACACTCTGCTGGGATTGAAGGTGAGTGACACCACA<br>CCCAGCTCATACAAAAAAAGAAAAAAGAAAAAGTTGTTCCTTTTTAAATTTCCCAAATTTTTAATTTTTT<br>TGGTGAATATATCATTAGAACTATTTGTGTACTTATAGGTGATTCAAAAAAACAAAAAACAAAAAACAA<br>AAAACATGTAAAGTGCCTTAAGTTCCCTCAGGGTCTTTGGTATCTGCACCTTTGTCCCTCATTTTACTAAT<br>CAATTTTCTTAGTGTTGAGGAGAAGAATCAAGGGAATACCTCTTAGCCAATGTCTTAGTTTCCTTTTCAA<br>GCCCAACCCTTCTGTTTATTCAGCTGCAGGGTACCCCTCACTTACCTCTCCAGACCACGCAGCCCCAAG<br>CCTGTTCTCCTCCTAGGTGCCCACTTGCTCCCAGATGGCCAGGAAGAAGTGGGTAGTGGGGAAGCTGG<br>ACCCAGTCAGCAGGAAGCCTGTACACAGGTAGCAAGCAGCCAGACTCAGCACAGTCCAGGAGGTGTC<br>CTCCATACAGGATCCAGCCACTGCTGTGACTCGAGTGGATGTCAGCCAGGAATGGGGCGATGGCTGGG<br>AAGTCAGTAGGAAAATCGTCAATGACATATTAAGTCTCCCTGGGTAAGTCCTGGATGGAGATGATCCCA<br>TTGGTGCCCACATAGAGGTTGCTGAACTGGACACCGTAGAAGCTACTTCTACGTACTGCTAGCTTCACA<br>GCCGCGGAGTTTTCAACGTCGCCCTCCCGCAGCAGCCGGTCTCCCCACGTCTCCCCGTAAGGAAAGAG<br>CTCTTAAGGATGCAGAGCCCCGGCTCCTGACAGCAACACCAGCACCAGGAGCAGCGATAGCGGTGATG<br>GCGGTGCCAGCCGCCTGGCTGTCCGGTCCCAAGACATGCTCACTCCTCCCTGGCTCTGCCCTCCACACC<br>TTGAGTTTCTG
MSTR<br>G.131<br>258.2	--	0.000	0.487	0.000	8.554	up	GCAGCCTTATTTATAATACCCAGAAGCTGGAAAGAACCCAGATGCTCTTCAACAGAGGAATGGATACAG<br>AATATATGGTACATCTGCACAATTTAGAGTCATTAAGAGATATCAAATTAATAACGATGGAGAAAACAAA<br>ACATTACTGGCTCTTTATCTGACATATGGAACATATGTATAAGACACAAAATACCTTATTCATCAGAAATA<br>AAGTAATGCAATTAAATATGCTACAAATTTAATAAATGCTTAAAATAATGGCACAGTAATTATCAAAAAAT<br>ACATGAAAAATACTAAGCCCACAAATCGTTGGTGAAGTAGAAATACAAAAGACACTGTTTGTATAGTGT<br>TGCTGGAACAAAGAACCCTGGTATTTGTTTGTTGCATCTAAAATTTAATATAGCTACTATAGAAATTAATA<br>TAAATCTGAAATTATTTGTGATTCAATAGTACCACTAAAGGAATATGTACCTGAAGGAATCTGTCTATTCC<br>AGTTATAATTCCATGAATATCTTTACCTTCTCACTGTTCACAATATCCATGTTACAAAATTGGAAAAATAAT<br>TTATATAGTCACTAGCCATTTATTTAACCATCAAAAAAGCAAAATTATATCATTGGCATGGATATTGATGAA<br>AGTAGATATCATTGTGCCAGTTGAAAATTCAGAAGTGTGAAGACAATTATCTAAATCTTATGAAAGTGGA<br>CAACTGCTGTTTGTTTGACTGTGAAGCCTCTTCTATAGAAGGAAAGTCAGATGTCACGTTATAAACATG<br>GTCACAGTTTTCTGATGGTTCACACTGCCATAGAGAGGATCCAACTATTCCTGTTTTGCTAAATGGCCAT<br>GTTGTCAAACTGCCTTCCAATTCTTATGGTTTTTGTCCATACATTACTGCTTTTGTGAGCTGTGGTCAGAG<br>AAATGTATAGGTGTACTGTGCAGCAATTACTGTAGATCTGTGTAACAGATATAAGTGCTAAGACAGTGAG<br>AAGAAAGCTGAGCATAAGGATGCGGAAACATTGCAGAAAAAGAGTAGACAGAACTTGAGGAGCGAGA<br>GGATGCAATCATGATATCTAGACATGACCCAGATTTTGGAACTATGGACTCACTGCATCTACAGTCGTAT<br>GCACAAGTCCTGTACACAATTAAGACAATGAATTAGGGAAGATCCCAGCAGGAAGTACTGTGTGAACC

							AATCGATTATAAAGAAAATAAAACAGGAAAATATAAAGAACATGAAGCTGGAAAGGAAATGTGTAAAG<br>AGAGTAGAACCAAGTTAAGGATCGGTGTGATCAAGGTACATTGTATACATTTGTGAAGACGAAATAAAA<br>ATTCCAGTATAATAAAAAAAGTTCTGAAAATATATTAAGGATTTTTATATCCTATGGAAGTCATTTTCTCCC<br>TAAAAGTAGAAAAGAGTATCCTAACTTTTGTAAGTTGCAGAAAAGTCAGTGAAAAAACGTAGTTACATT<br>AGTTCACTGCATGTTGTCTGCATTATGGAAATATCAAACTGAGGCTCATTAATCTGTACAGTTAACTTATA<br>TCACCTGTTCAAATAAGATAAAATGAAACACTGTGTACTGATCCACCAAAATCAAACTGCACACTAGCG<br>ATTAAAATACAAACGGACAAGTGCAATGTTCTTAACCTCAACCGTTTTACTCTTTGAAACAGTGAATAC<br>ATATGAATATAGAAGTTCTAACCTGATACACAGCATCTAGTATGGGGTCTTTGTGACTTATACGGCAAGC<br>ATTTTAGGGTTGCACATTGCCATTAATTATGTCATTCAGTTTTAAAATCCTAGGACAACAAGTTCTAAAAT<br>CCATGGGCTTTGGTCCTGACAGCCAGGGATTGCACTCTGTACATGATCACTTTATATTATTTAGCTAAACT<br>CTGAAAGGCAATTTGCTGCCTCACAAAATTAAATTGATTCATTGCTATGGTTATGATAAGGGTTTCTTGT<br>GAGAATAGATATAAATTTCTCATGACAAAATTTGCTTTCTCTTCTAGTGTTTTGTGGGCTATTGATGAGCT<br>TACAGCTCTTTCTTCCCAAACTAGCACTTACAATAGATTATTTTGTAAAGATCGTTTTTGACTTCTTCTTC<br>ATCATCTTCACCACCACCACAACCACCACCACCAACAACAACACCACCACTACCACCATCATCAACATC<br>ATCATCACCATCCTCATCTAATTTACCTGAAATTGAATTTGGAATTTTGTTAAACCAAAGAATGAAATGA<br>GAAATATTCTGCGTGAAGAAGAGATATTGATGAACAATATCTCCTGTACAGTCACTTCAATGTTTTAAAA<br>ACATTGCCAAACTTGCTTCAGATGATTGATTGAAATGCTAGTGCCCTTATACATCAGGGTTTGAGAGTCT<br>CTTAGTGAGAGAATGGTCTTTTACAGCAAGGCCTTCTTTATCAGACTTTGATTTAACCCATCTACCAACA<br>AAATTAAATTGTCCCAGAAATATTATAGTGCCTCTCCAGAAACATCTTTTATCCAATAATGCACATCTACT<br>TCTCTTTCTTAATTGTCATGATGATATGGGCTTAGGATACAATATATTCTGATTTATTAATGGTATTTTAGTA<br>TTATTTGGACTAATTATGAAAATTTCAAAGGAACACAACTACTCCATTATTTTATCCCATAAAATTTTAATT<br>TGATCTTGAAGAGTTTGATGAAAACTGAAGTATTGAAAATGTATAACTTCCCATTCAAAACTTGTAACAT<br>CCATCATGTGAAACTCCCACTGAACTTCAATGGATTAATGTCTCTTAAAGCATGTAAAGACTCTCCTACT<br>GCATTATTGCAACTCTACAGAAGCTTCACTCCCATAGTAGCAGTTCCATATTGGCTGTAAACAAATCACT<br>TTCTATGTGTTGGGAAGTGTTTAAATCATTTTCATTAAGTTTTGATATGAAACCAGTTAGGATTTTGAGAA<br>AAGGGAGCAGACACGCCCTAGAAAGTTTGTTTTTCAAATTCTTTTCTTAAAATTAGACTTTTTCATGAAA<br>TATTTTTGGATTATGGTTCCACATCCCAATATTTCTCCCCCTTTGTCTATACCACTCCTGCCATCTGTATCTA<br>CAATCCTTCTGACTCTCACTAGTAAACAAGCATTCATCTAATAAAATAAAATTCTTAATAATAACATGTGA<br>TAAAAATAACATAAAAGTTAATGAATTTAGTAGAACAAAACAAGCAGATGGAAAAGAGACAAAAAAAA<br>AAGCCACAAGAATCAGATACAGATACAGAGACCCAGACTTCCATTCACACACTCAGGGATTCCATGGA<br>AACATAAAACCAGGAAAAATATAAACACAAAACATACAGAGTAACAATTAATAAATAAACAAATAACAA<br>AAGGAAGAAGAAATTATGAAGAAAAAGTCCTGACCCATTATGAAACAAGGATCCTCCAAAGATAATGT<br>TTAGTTACCTCAATGCTTTATACGCAGTGTACCCTTAAAATTAGTTTGTGTACACAGTGAGATTCCCTTGG<br>AGAAATGTAAGTTTTCATTCTCAAATGAATACCCATTGGAGATAACTTCTGGTTTAGAAATTAGGTGATG<br>TTCAGGGCTTGGCTTCTTTCAAGGGCAAATTAATAGGAAAGGAGATCTGTGGGGTGCTGCCTTCTGAGC<br>ATCATGCAGTTGTGACACTCAAGAAGTCACAATCTATCTGAAAAGACCCTATATAATATTGATCCTTGTC<br>CCGCGCGTGACTTTCCGCTGGCAAGAAGCACGCGAGACATAGAAATTCTTGCTATGAGTGATACTTTAT<br>TCCTATCTCATGGAGAAGCCCTGCGCGCCAGTCAGGCGCGCCTATTTAACCCTCGGGCACGCATCAACG<br>CCTGATTGGTTGTTTACTCATGATCTCATCAGGCACGCCCCGGAATGGGCAAAGACCTGGCAGGAAGGC<br>ACTCTTGCACATGCGCATAGTTAACTTCCTGATAGGGGGGCCAGCTGGCGCAGGGAAGGCTGGCGCCAT<br>CTTGACTGACTTGGCCTTCCACGTGGGGCGCAGTGGAAGCCAGCGCCATCTAATGGTCGCGCATGTTAT

							TGCGGCCCTCTACATCTCACCCTTATAATTATAATTTTATAGCAGAAATGATCACCCTATCGCGTGCAATG<br>AGGAAACCTCAGCGATGTGCAAGGGCTGATCAAAGGAAATCACTGAGTCTCTCTCAATCCCAGTGCAA<br>TGGATCCACAGATCCATGGGGTGCAGGAGCAGAGAACTCAGATACAGAGAGTATCCCTCTCCTCCCAG<br>TGCAATGGACCCACAGGTCCATGGGGTGCAGGAGCAGGGAACTCAAATACAGAGTAAATCCTGAACAA<br>GATAACCAAATTCCAGGTGAGGCCCTTTGTTCAAATGGCTGCAAGTGCTTGAGTGATAACGGCCTTGTC<br>ATGTTACTGTTGAGATCTGAGTTTGTAAACCAACCATAGTATGAACACTGATCCACAGCATAGGGCTGCA<br>TCAGACAGAGCCACTCCCGATAAGTAGTGGGCACCTCATATGGTCCTCCTCAGCTGTTGCCACCAAGGG<br>CCGTGGTCAAGCCACTCCTATAATGAAATTTAGAATTTCCAATTGTTAGTAACTACTCCAGAAAGTTTAC<br>CCACTGTACTTGTCTAACAATAGATTGGGGGAGGATAACTCCAGACACTGCAGACACAATGTCTGCGGC<br>AGTAATGGCTGCTACAATAGCAGCGAAAGTGTCAAGGTCTTTTCCAGGACAGTTCAGGATCAGTCATTT<br>TTCTCTGGAACAACCAGCAGACAATGGAACCATTTCTGAGATCTGTATAACCAGGACAGAGGTCTCCAG<br>TCACTGTAGCTTTACACAGGTAGCTTTCTGTGAAGCTATAGTCTGTAAAATGACAACACCAGTTACCAGT<br>TAAGGCAGCAAAAGTCACCAGGGAAATGAAAGGACAGGGGTAACAGCTAGAGTTCAGTCTTCTCCAG<br>GAAACAGCAGTGTACAGAGTCTGAGCGTAGGCCACAATGGGACGGAACACGTGGCGGTAACACTGA<br>CTGCAGCAACGGCAAAGTCTCTGTGATGACAAAGGATGAAGGGAAATCTTCTATCTTCCTCTCAGGGC<br>AGAGGAATGACTCCAGGGACCCGAACCACGAGAGCTGAATCTTCATGCTCCCAGGATCAGCATGTCTC<br>ACCAGACACTCAGGCAGCTGGCACACTCCTGTAGCATCCTGTAGAAAAAACACAAACATTCCCTCTTC<br>CCCATATAAAATATCTGGACAGGAACATGTCAATAAGTGGATCTCTCTATTTCACCTAGGCAGAAGTATG<br>CCTAGTTGTAGGGTGCCATAAACTTTGGCATCCAAATTTTTAAAATTGAAATATTTTAAAATTGAAATAAA<br>AGGAGCATTATTTAGCATACAGGGATAACTCCCCCCTTTTATTTATAAAGATATAGTAAAGATATTATTTAT<br>TAAGGTAAGTCCTCGAGGATTAAGTTGAGGACACTGAGCACTTGTATTTATAAATTGACTTGTATACCAA<br>ATTATTGTCTCTAGCATAATTGTATTGAATATATAAAGAATGAGATTTTTTGACTAATTGTTCCTATGTAAG<br>GCCTATAATCTGTCTGGAATCTTGTCCTCCCTTGTTAAGGAGCCCAGAAAATCCAGTTTGAGTTTTTAAA<br>TGTCCTATAAAGGAACAGTATATAGTGCTCTTAACCACTAAGCCATTTTTCTAGCACCTCACAAACTTTAT<br>TTACCTAAACATAAGCATTAATTAGAAATGTCAAATCTTGTAAATATTGTCCAGATTTAGTCTTACTAGAA<br>ATCTCTGAGCTGGCAGGGTGAGGCTGGGAGTTGAAAGTAAACAAATGGAGTCTTCCTCTTTTCGTGAG<br>GGTGTGTTATCAACTCTATTACCATTTTTAAAGAGCAGGGTCTATGGTTTTGTTTAGTTGTCTGAGCCAG<br>AGCACTGAAAGGACAATGAGTTGTCAGACAATTCACATTGAAATCGTCACTCACTGATTTCAAGTAGAA<br>AACCTGTCTCTAATAAAGCATTAAGTAATTGAAATCAATTGTAAACCACAAGCCACACATTGGCTATCAG<br>TATATGAATAGAAAGCTTGATTTAAAAACAGTGGCTACAGGACGTAATCTAATAATCTGTGCTGAAGCAA<br>CAGAAACTCAAGAGAATAAACAAGTGATCCAATCATACATGTAGCCTTTTCATTAGATGAACCACCTATA<br>AATACAGTAAGCGCATTTCCTATGGGTTGTACGTACAAATATACAGGAAATACAAAAACATGTAAAGAAT<br>AAAATTATCAATTTTGTCTGAAAAATTTGTATATGTGATAGGCCAAATATCAGTATTTAGTAGTAACCAAT<br>TAATTGTTGTTTGGAATAAGATATGTTTTACCAGGTTTCTATTTAAAATACTTCCATGACTCTAGTCCACA<br>ACTCTGTACTAACACAGCTGAAGCTTTATCTACAATGTGCATAACAAAGACCTAAGTCCTCTGGTAAGG<br>TGAGGTACTTAGCCAATTAACATATCTTTAGAAGCTTAAAATTAGCTTCAAATATTCTTTTCTGGAGTAGG<br>GTAACAGCTACTCCCCAAATATTAAAGCTCATTTTGAGAGCAAAGGTTTGTAGTAAACATTTCCATATAC<br>ATAGAAAGATTTAAGTTCTAACTTCTTACACTGAAGAAGCAACAAAATTACCTAATATGAGGCTGTTAAC<br>CATTCTTAATGATATCACTTTATGGGCTCTCTGAAACTATAAGCAAGCTCACGAAATGAAAACTCACAAT<br>TGTTATGACGAAAACAAATTGTATAAAATCAATCCTCTAAATCTGTCTTGAAATGGCAGTTGGAGTAAGG<br>AAACTGGCTGTAATGCTCTCACAAGTTCTAAAACCTCATCAATCCTTATAAATCCTGTAACAATCTCTAC

							CTGTTTGATTTTTCCTTAATTAAAAATAAGTTTGAGTTAGTCAATGTAACACTGGCAATCCTAAATCACA ATCTTTAAGTTCTCTTTGTAACACCAGAACACAGCCACAAGGTCACCTGAATTCTGAGTACGTGACTAG GCAGCTGTTCTCCCGGCAGTGGAAATTAACATTAAAATACAGATAACTTCTGCAAAAGCCACAACTTTG GAAAGTAAAACTTATTAGGCAGCTGTTTTTGGGGCAGTGGAATTAGCATTAAAATACAGATAACTTCTG GGCAAGCCACAGCTTTGGAAAGCAAAACTCAACCTCCAACAAACAATCTAGTCTCCAAGGCACCACA GGTCTATCTATTATGTTACAAAGTTTGACTGTCAATTCTTACATTTGGACTCACAAAGGTGTGGCCTCCA ATACCTCAACCAGGGACAATGTCCTAAATTCTTTTAGAAGCCTGGTTTCTAGCATAAGAATTCCATAAAA ACATTACCTCAATTTAGACCTGTTTCCCTAGGTTGGCCCATGTCACATGCTATCTAAAATGACTCCATCCA GATTACTAGCTTTATGTCAACTTTCTGTTACCAATATTATACCCTTTTAACCATATCCAATAACTTGAAAGC CAATAGTCCACAACCAAGGCAAGTAGAGCATATTTATACATATAAAACCAATAAGAAACAAAATTGAAA TGGCTACACCTATCTTTAATATTTAGACCAAACACCATTTTTACCCTTTTGTTATGATTTTAGGAGAAGCA CCTTGTCACCTGTTGAGTCAAGTAATAAGTCAGGGATTTTTTTTTAAGAGAAACAGCTATCAACTCTTGT ATGAAGAAGCTGTTGGCGCTTTTAAGATCAGCTCGTGTAGACGCTTAGCTTCTGGTCAGCTTCTCCAGC TAATCTAGACACCTGGCTCTAGGCACAGGGCTTCAAAAACCTGATTGAAACTGTTTTTTATTCATTTGTT CCTTTTTGAAACGAGTTAAAACCAAGTCAAGGTGTGCACGACCAGCAGATAAAGTTTTTTACCATTTTT TTTCTTTTGACCACGAAACATAAAACATTTAAGCAACGAGAAACAAAAACCACAGACATAAACTGACA GACATGGACAGCCTGGTGCACCAGAGACAAACAATAGACAAAGAGGGGAAAAAGCCAGATGGTGTTC ACAGCGACTATCCATCGAGTGGAGCTGATATGGGCGATGGATTGTCTCAATTCCTGGACTAGTCCACCA ACGTGTTGAAACCCAATTCTGTAAGAAACTGTATTCCATAGCTTCTGAATTTCTTCCGTGAAACGGAGG CCTCTCGTAGCGCCGGCGATGTTTTTGTCCCGGGTTTCTCGGCACCAAATGTCCCGCGCGTGACTTTCC GCCGGCAAGAAGCACGCGAGACATAGAAATTCTTGCTATGAGTGATACTTTATTCCTATCTCATGGAGAA GCCCTGCGCGCCAGTCAGGCGCGCCTATTTAACCCTCGGGCACGCATCAACGCCTGATTGGTTGTTTAC TCATGATCTCATCAGGCACGCCCCGGAATGGGCAAAGACCTGGCAGGAAGGCACTCTTGCACATGCGC ATAGTTAACTTCCTGATAGGGGGGCCAGCTGGCGCAGGGAAGGCTGGCGCCATCTTGACTGACTTGGC CTTCCACGTGGGGCGCAGTGGAAGCCAGCGCCATCTAATGGTCGCGCATGTTATTGCGGCCCTCTACA
MSTRG.131865.3	--	0.000	0.163	0.002	4.137	up	AAAAATTGGAATGGTAAATGTTAGAAAGTATGTCCCAAAAGCTTCATCTTTAAAATTTAATTTGTCATTC TTTGTTCTGAGTTTATTATTTTAAATGTATATCTAAAAGCATTGTTCTGAATTCTCTCCCAGATTTCCTGAA GATACTGGTATTTTAGCATATTTGATCTTTGAGGTTTTGCAAAACAACGTTAAAGGACGCCATTTGAGAG ATATACCTCAATCATTTCTACCCAATAGTATTTGATAAGTGTACTGTATACTTTCAAATCTGTGTGTGTATG TCTGTGTGAGTGTGTCTGTGTGTGAGTGTGTGTGCGTGTGTGTCTGTGTGTGTGTGTGTGTAAGCTTACT TTAAAGGGGGGCCTTAAATCAACAACAGCTATTTGTTAGAACCCAAATAAGCCTTGAAATGTTTTTTTCT CTGAAAACTGAGTAGTTGGTTTGAAATATAGCTTTAAGGTCTCAGTTCATAAATGTCATTCAGACCTCCT GCTATACTCTTAATGAGTAGAAGTATAACAAGAAGGGGGAAAACCATGAGTCATTATAATGATCTCCATT CCTTTTGGGTCTTCTCGACTTGCCTTTGTATACAGGATCACCATGGAAGTGGGAAGCATAGGCCTTGAA GGAAGCCATAATATAATACAAAATCAGTATGTAAAAAATTGGGAGAGATAAGTACCAACATATGGAGTAA TGAGTAAAAGTCAACCTCCATGAGGTTGTGCTCACCCTTTATCCAGTGCTGACCAGCCATGAGAAGAAC ATCTTGAACCAATTATTGCTGACCTGCATCTTTTATCCAATAAGACAAAGAACATTGAACAAAGAAAAG AACATTGAAAAGACACAATTTCAGTGTGAATTGTGAGATCTGCATTCCTAGGAAACACATGTAGATCAG AGAGTAAAGGAGCTCAGAAAGGCCAAGCTGGAACACATGGAGTGACCCATGCCTCCAGCCACATATGT AGAAGAGGATGACATTGTTGGGCATGAGTGGGAAGAGAAGCCCTTGGTCCTGTGAAGGTTCAATGCCC CAGTGTAGGGGAATGCCAGGGCAGGAGGTAGGAG

MSTRG.131912.1	ENSRNOG00000024053	0.000	0.318	0.019	3.592	up	GGATTCCTGGAGAGCTGCTAGTTTTCAGTCGATGTTGGAATTCAGAATCATGGGCTCTTCATTGGTGTCAACACTGATCTCTGCTTAAGACTGCAGCAGTCCCATTCATTTGCAGTCTTCATTTCTGTGTCAATCCCACATTTGCTCCATTTGCTTGCTTTTTCATCTGTTGTGCTTTTGCCTTGTTGAAGCATGGAAAGGCAGGCATGTTTCTAGAAGACAGAGTGATTTTCAAAACCCCAGTCTTGCCTGGTGGAAACAGGTAGAGGTTTCTTGAAGCTCCGGTCTCCAGGTAACGCGGGGGGTCAAACAAGCGGCGGTTGAGGGCATTAACTATCTTCTCTCTCTGGTCGGACCCTTGATCC
MSTRG.132111.1	--	0.436	0.000	0.028	-3.770	down	TGTCTCCTGAGTGCTGGGATTAAAGGCATGTGCCACTACAACCCGGCTTCTCTTAGGTTCGTATATTTAATCTTCTAAGCCTCAAATGTGTTTATATGTTTCAATTTTGTGTTACTCCAGGTCACAGCATTCAGAGAAACTGCCTTAATCTATTTCAGTGTATTTACTTCTTTTCAGCTATATCTAATCTCCTTTGCAATATATCCACTGACTTACTTTTCAGCTCCTGCATTTTAAAACATATTTTTACATTAATTTAGTTTATGTCTATGAGTGTTTTATCTAGTTATCTGTATACCATGTGCATACAAGGTACTTGTAGAGGTCAGAAGATGGCAAAAAATTCTCCTGGAACTGCTGCTGTTGGGTATAAGCTACCATGTGGGTGTGGGAATCCAACTAAGGCCCTCTGTAAGAGCAACACACATTCTTTTTTTTTTTTTTTTTATTAACTTGAGTATTTCTTATATACATTTTGAGTGTTATTCCCTTTCCCGGTATCCAGGCAAACATCCCCCTCCCCC
MSTRG.132300.1	ENSRNOG00000004776;ENSRNOG00000027767	0.000	0.807	0.000	7.913	up	TCCCGGTTGCGGGCCGCCGCCGCGGGCTGTGGGGACGACTTGGTCGGCGTCCGGCCCGGCAGTCGGATGAGAGGGCGCCGGCGGCGACACCAGCTGCAGCAACGAGGCTTCTTCTGGCTCGCACGGTTCCGAGGCTCCTTCGAAAGAAGAATAGTAGACTGGGATCTCTCCATGAAGAAGAGGGGCAGAAGGAAGGCTGAAAAACTGACGTGAAACATGTGCTCCTATGATGTGTGCCACATGCGTGCAGAGCCAGAAGAGGTGAGAAGAGGAAACGACAACTTTAGAAGCAGAGTTAAACACCAGTTCTACGCATTGTACCAGAACCTGGCCCATGTGAAGTAAGTGAGTCACAAATACAGGATTCTGACACCGTGGAGGGAGGGCAGGAGTCCCTGCAGGTTCTGTGGTCGGCAGGGAAATGATTTGGGTGAGGATCTTGCGTTGAGTACCTGCCCTGACTTCACATCACGATGGTCCCTAGACAGCAAAACAAGAAGGCTAGTGAAGAGAAGAACATGATTGGCATTCAGAATCAGCGGCACAACTTTGAAGTCCTACAGATCAAGTGCGAGAACATTGAAAGGAACATTTCTACAGTGAAGAGTTAAGTGACAGCATTCTTATTGTCTGCCGTGTATACTGTGTGGGAATGAGACATCATGTCAGCGGCTGGGAAATACAAAGGGATCAGGAAGAAGTGTGTAGTGATGGCAGTGTCATCTGTAGACTGGGCCAGAAAAATCCACTGAGTTCAGGACAGCAGTTGGCGATCTCTACTGGACAAAGGATGAGAGAAGCCTATCTGTGGTCTTACAAAGACTTCAGGGCTACAGTTGTGATGAAGACCAGGGATGGGACAGTCCTCTCCCTGTGTGCCATTGCATACTAACATGAAAGCTTCACTCTGGGGATGATAAAGAGATCTTTCACCATAAGCCATTGTGTGTGGGTGAACAATAACGTCACCAACGTTAAGTGGAAGTGTAGATCCCTGGAAGAGCTCATGTATCTGGCCTGATAAGGCCCTGTGTTATAGTCCCTGTGTCAAAAACAAAGTAAGCTAGACAGCCTTGTGAACCAGTTCCTCAGTGGAGTCATTACTGGAGCTCCCACCAGCGTTGCAGGTGAACACTGTTATCGATTATCGCTGCATAGGTGAGATCTGCTAACCCAGACCAACTCAGAGCATCTCAACAGGCAGTTGGCACAGGCACAGAACCTGGTTTGGGAATTCAGTGTGAATTTGGGACTGTTAAGGAGAACAGGAGAGGGTAGCACAGCCAGGAGAACTGAATGTGCCTCCAGTTCAGTTCCAAATCTCGGGGAACAGGGCAATGGCCCATTTTCCAAGATGGTATCCTTGGAAGTGACTGAAGCTGAGAAGCCTCGGAAATAATGATGGCAGGAGAGGCCTGTACACAGTAAGCTGGAGAGAGTGGGTCAAGCAGGAGGAATGAGGGCTAGCCATTTATTCTCCATGGAAATAGAGGAGAGCAGTGCAGACTTGCTTTTACTCTCTTCCTAGAAAGATATGATCGTGGTTGGGTTTTGAGTCACAGGAGATAGACACACAGGAGAGGCTCAGAGATCCTTGCCAGTACTAAAAATGTCATTAGGTTCCTTCCAGGAGACAGAGCTCCCTTAACCACTCCTTCTGCTCCCCACCCACACTCCCCTTTATTTTGCCTCCTTCCAAGTGACAGATCCAAATTCTGTAGTAGAAAAATAATCACACTTCGCCTAACCCCACATCTAGCCCTCCGCTCCCATCAAACTCCATGCCCAGTATGGATCGATGTTTTTCAGATAAGAATTCAACTTTCCTCTTTTTGACTTACAATTCTA

							ATTCTTATTAGTAACAAGTATTTTGTGGGATTGGAGGGATTGAATTCAGGTCATCCCATTGAGGTTCTCAAACTTCCTGATGCTTCTACCTTTTAATACAGTTCTTCGTGTTGTGGTGACCCCAATCACAAAATTGTTTTCATTGCTACTTCATAATTGTCATTTTGCTACTGTTATGAGTCGTAATGTAAATATTTGTATTTCCTGATGCTCTTAAGTTACTCCTGTGAAAGGGTAGTAAGACAACCCCCAAGGGGTCACAGCCTGCAGGTTGAGAATTACTGCAGTTCCAAATCTCAGGAAACAGGAGAATGGTTCATTTAGCTATTCCCTGGAACACTAGAACACTCCCGTTCTGAGTCTCACTGTGCTGCTTTTGTGTTTATCAATGGAACCAAAGCCCTGAAAGAAATGACATGAGGAAAGACAGTTATTTTCAGATATTAATATATCAATATCAGGTAATTCGCTCCTTCTTTACTTGACCAGGGCCCTTGGGCAAAACTCAGTAGTAGCAGAGGATGTTCTCTCCTTCCTAGTGGGCAGGGTGCAGATAGAATAGGAGCTCAGGGGTGGTGCAGATGAAATCTGATGGAAATACTGATACCAACCTTTCCTCGGCCCCTTGGAAAGTGGAAATCTGTGAGCTCGAAACCAACCTAAGACTTTCAGACTAGCCAGAGATACATAGTAAGATCCTCTGTGATGGTTTGCCTATGCTTGGTCCAGGGAATGGCACTATGTGGAGGTGTGGCCCTGTTGGAGTAGGCGTGTCACTGTGGGGGTGGGCTTTAAGACCCTCCTCCTAGCTGCCTGGAAGCCAGTCTTCTTCTGTTTGCCTTCAGGTGAAGATACAGGACTCTCAGCTACTCCTGTACCATGCCAGCCTAGACACTGCCATGCTTCTGCCTTGATGATAATGGACTGGACCTCTGAGCCTCTTTTGCTGTGAGACATATAGAAAACTGAGAGGCACACATGGAGAAGACGTGATCACAGAATGGAAAGAGCACAGAAGCACCAATGCATGCGTGCAGCCAGTGCTGGAGCTGCAGTTTGAGTTTGACATGATTCAGGGGCTGCAGGAATGAGCTAGTATTCCTCTCCTTCTTCCTCACCCTCGCCTTCCACAGAATCCACACCAACCTCTTCATAATCCTCCTCTAGGGCAGCCATGTCGTCACGGGCCTCAGAGAACTCCCCCTCCTCCACGCCTTGACCCACACACCAGTGCACAAAGGCACGCTTGGCATACATCAGATCAAACTTACTCCAGCTTCAGGAATCTGCCTGTGACCTCCAAAGGCATTTGCAATCACATGGACATACCCCACAGACACACATACATGTAATTAAAAATAAAATGAATTAATCTTTTAAAAAGACCAATGTCACATGTCTCTCTCATTAAATATTTATACTAGCTTCTAAATGTGTGTAATTAAAGTTGAAATAAGTGTGAGAAGAAGGACATTACCTCCT
MSTRG.132300.10	ENSRNOG00000004776;ENSRNOG00000027767	0.000	1.250	0.000	8.511	up	CGGGCCGCCGCCGCGGGCTGTGGGGACGACTTGGTCGGCGTCCGGCCCGGCAGTCGGATGAGAGGGCGCCGGCGGCGACACCAGCTGCAGCAACGAGGCTTCTTCTGGCTCGCACGGTTCCGAGGCTCCTTCGAAAGAAGAATAGTAGACTGGGATCTCTCCATGAAGAAGAGGGGCAGAAGGAAGGCTGAAAAACTGACGTGAAACATGTGCTCCTATGATGTGTGCCACATGCGTGCAGAGCCAGAAGAGGTGAGAAGAGGAAACGACAACTTTAGAAGCAGAGTTAAACACCAGTTCTACGCATTGTACCAGAACCTGGCCCATGTGAAGTAAGTGAGTCACAAATACAGGATTCTGACACCGTGGAGGGAGGATCTTGCGTTGAGTACCTGCCCTGACTTCACATCACGATGGTCCCTAGACAGCAAAACAAGAAGGCTAGTGAAGAGAAGAACATGATTGGCATTCAGAATCAGCGGCACAACTTTGAAGTCCTACAGATCAAGTGCGAGAACATTGAAAGGAACATTTCTACAGTGAAGAGTTAAGTGACAGCATTCTTATTGTCTGCCGTGTATACTGTGTGGGAATGAGACATCATGTCAGCGGCTGGGAAATACAAAGGGATCAGGAAGAAGTGTGTAGTGATGGCAGTGTCATCTGTAGACTGGGCCAGAAAAATCCACTGAGTTCAGGACAGCAGTTGGCGATCTCTACTGGACAAAGGATGAGAGAAGCCTATCTGTGGTCTTACAAAGACTTCAGGGCTACAGTTGTGATGAAGACCAGGGATGGGACAGTCCTCTCCCTGTGTGCCATTGCATACTAACATGAAAGCTTCACTCTGGGGATGATAAAGAGATCTTTCACCATAAGCCATTGTGTGTGGGTGAACAATAACGTCACCAACGTTAAGTGGAAGTGTAGATCCCTGGAAGAGCTCATGTATCTGGCCTGATAAGGCCCTGTGTTATAGTCCCTGTGTCAAAAACAAAGTAAGCTAGACAGCCTTGTGAACCAGTTCCTCAGTGGAGTCATTACTGGAGCTCCCACCAGCGTTGCAGGTGAACACTGTTATCGATTATCGCTGCATAGGTGAGATCTGCTAACCCAGACCAACTCAGAGCATCTCAACAGGCAGTTGGCACAGGCACAGAACCTGGTTTGGGAATTCAGTGTGAATTTGGGACTGTTAAGGAGAACAGGAGAGGGTAGCACAGCCAGGAGAACTGAATGTGCCTCCAGTTCAGTTCCAAATCTCGGGGAACAGGGCAATGGCCCATTTTCCAAGATGGT

							ATCCTTGGAAGTGACTGAAGCTGAGAAGCCTCGGAAATAATGATGGCAGGAGAGGCCTGTACACAGTAAGCTGGAGAGAGTGGGTCAAGCAGGAGGAATGAGGGCTAGCCATTTATTCTCCATGGAAATAGAGGAGAGCAGTGCAGACTTGCTTTTACTCTCTTCCTAGAAAGATATGATCGTGGTTGGGTTTTGAGTCACAGGAGATAGACACACAGGAGAGGCTCAGAGATCCTTGCCAGTACTAAAAATGTCATTAGGTTCCTTCCAGGAGACAGAGCTCCCTTAACCACTCCTTCTGCTCCCCACCCACACTCCCCTTTATTTGCCTCCTTCCAAGTGACAGATCCAAATTCTGTAGTAGAAAAATAATCACACTTCGCCTAACCCCACATCTAGCCCTCCGCTCCCATCAAACTCCATGCCCAGTATGGATCGATGTTTTTCAGATAAGAATTCAACTTTCCTCTTTTTGACTTACAATTCTAATTCTTATTAGTAACAAGTATTTTGTGGGATTGGAGGGATTGAATTCAGGTCATCCCATTGAGGTTCTCAAACTTCCTGATGCTTCTACCTTTTAATACAGTTCTTCGTGTTGTGGTGACCCCAATCACAAAATTGTTTTCATTGCTACTTCATAATTGTCATTTTGCTACTGTTATGAGTCGTAATGTAAATATTTGTATTTCCTGATGCTCTTAAGTTACTCCTGTGAAAGGGTAGTAAGACAACCCCCAAGGGGTCACAGCCTGCAGGTTGAGAATTACTGCAGTTCCAAATCTCAGGAAACAGGAGAATGGTTCATTTAGCTATTCCCTGGAACACTAGAACACTCCCGTTCTGAGTCTCACTGTGCTGCTTTTGTGTTTATCAATGGAACCAAAGCCCTGAAAGAAATGACATGAGGAAAGACAGTTATTTTCAGATATTAATATATCAATATCAGGTAATTCGCTCCTTCTTTACTTGACCAGGGCCCTTGGGCAAAACTCAGTAGTAGCAGAGGATGTTCTCTCCTTCCTAGTGGGCAGGGTGCAGATAGAATAGGAGCTCAGGGGTGGTGCAGATGAAATCTGATGGAAATACTGATACCAACCTTTCCTCGGCCCCTTGGAAAGTGGAAATCTGTGAGCTCGAAACCAACCTAAGACTTTCAGACTAGCCAGAGATACATAGTAAGATCCTCTGTGATGGTTTGCCTATGCTTGGTCCAGGGAATGGCACTATGTGGAGGTGTGGCCCTGTTGGAGTAGGCGTGTCACTGTGGGGGTGGGCTTTAAGACCCTCCTCCTAGCTGCCTGGAAGCCAGTCTTCTTCTGTTTGCCTTCAGGTGAAGATACAGGACTCTCAGCTACTCCTGTACCATGCCAGCCTAGACACTGCCATGCTTCTGCCTTGATGATAATGGACTGGACCTCTGAGCCTCTTTTGCTGTGAGACATATAGAAAACTGAGAGGCACACATGGAGAAGACGTGATCACAGAATGGAAAGAGCACAGAAGCACCAATGCATGCGTGCAGCCAGTGCTGGAGCTGCAGTTTGAGTTTGACATGATTCAGGGGCTGCAGGAATGAGCTAGTATTCCTCTCCTTCTTCCTCACCCTCGCCTTCCACAGAATCCACACCAACCTCTTCATAATCCTCCTCTAGGGCAGCCATGTCGTCACGGGCCTCAGAGAACTCCCCCTCCTCCACGCCTTGACCCACACACCAGTGCACAAAGGCACGCTTGGCATACATCAGATCAAACTTACTCCAGCTTCAGGAATCTGCCTGTGACCTCCAAAGGCATTTGCAATCACATGGACATACCCCACAGACACACATACATGTAATTAAAAATAAAATGAATTAATCTTTTAAAAAGACCAATGTCACATGTCTCTCTCATTAAATATTTATACTAGCTTCTAAATGTGTGTAATTAAAGTTGAAATAAGTGTGAGAAGAAGGACATTACCTCCT
MSTRG.132300.3	ENSRNOG00000004776;ENSRNOG00000027767	0.569	0.000	0.000	-6.975	down	TCCCGGTTGCGGGCCGCCGCCGCGGGCTGTGGGGACGACTTGGTCGGCGTCCGGCCCGGCAGTCGGATGAGAGGGCGCCGGCGGCGACACCAGCTGCAGCAACGAGGCTTCTTCTGGCTCGCACGGTTCCGAGGCTCCTTCGAAAGAAGAATAGTAGACTGGGATCTCTCCATGAAGAAGAGGGGCAGAAGGAAGGCTGAAAAACTGACGTGAAACATGTGCTCCTATGATGTGTGCCACATGCGTGCAGAGCCAGAAGAGGTGAGAAGAGGAAACGACAACTTTAGAAGCAGAGTTAAACACCAGTTCTACGCATTGTACCAGAACCTGGCCCATGTGAAGTAAGTGAGTCACAAATACAGGATTCTGACACCGTGGAGGGAGACACACCAGAAGAGGGCATCAGATCCCATTACAGATGGCTGTGAGCCACCAAGTGGTTGTTGGGATTTGAACTCAGGACCTCTGGAAGAGCAGTCAGTGCTTTAACCACTGAGCCATCTCTCCAGCCCCTGAGAAGTATTCTTTATTGTGAGTTTTGCCTACATGCTTTCCTTCCACCCAACCTCCAAGGCTCTGCTCCACCAGCAAACCCTCTGCTAGAGCACAGGGGCTGCCTCACCTCGTCTTTGATTTCTTCAGCCAGAGGAAGCAGTCCTCATTATGGCCTTTTCATACGCAGGGCAGGAGTCCCTGCAGGTTCTGTGGTCGGCAGGGAAATGATTTGGGTGAGGATCTTGCGTTGAGTACCTGCCCTGACTTCACATCACGATGGTCCCTAGACAGCAAAACAAGAAGGCTAGTGAAGAGAAGAACAT

							GATTGGCATTCAGAATCAGCGGCACAACTTTGAAGTCCTACAGATCAAGTGCGAGAACATTGAAAGGA<br>ACATTTCTACAGTGAAGAGTTAAGTGACAGCATTCTTATTGTCTGCCGTGTATACTGTGTGGGAATGAGA<br>CATCATGTCAGCGGCTGGGAAATACAAAGGGATCAGGAAGAAGTGTGTAGTGATGGCAGTGTCATCTG<br>TAGACTGGGCCAGAAAAATCCACTGAGTTCAGGACAGCAGTTGGCGATCTCTACTGGACAAAGGATGA<br>GAGAAGCCTATCTGTGGTCTTACAAAGACTTCAGGGCTACAGTTGTGATGAAGACCAGGGATGGGACA<br>GTCCTCTCCCTGTGTGCCATTGCATACTAACATGAAAGCTTCACTCTGGGGATGATAAAGAGATCTTTCA<br>CCATAAGCCATTGTGTGTGGGTGAACAATAACGTCACCAACGTTAAGTGGAAGTGTAGATCCCTGGAAG<br>AGCTCATGTATCTGGCCTGATAAGGCCCTGTGTTATAGTCCCTGTGTCAAAAACAAAGTAAGCTAGACA<br>GCCTTGTGAACCAGTTCCTCAGTGGAGTCATTACTGGAGCTCCCACCAGCGTTGCAGGTGAACACTGTT<br>ATCGATTATCGCTGCATAGGTGAGATCTGCTAACCCAGACCAACTCAGAGCATCTCAACAGGCAGTTGG<br>CACAGGCACAGAACCTGGTTTGGGAATTCAGTGTGAATTTGGGACTGTTAAGGAGAACAGGAGAGGG<br>TAGCACAGCCAGGAGAACTGAATGTGCCTCCAGTTCAGTTCCAAATCTCGGGGAACAGGGCAATGGCC<br>CATTTTCCAAGATGGTATCCTTGGAAGTGACTGAAGCTGAGAAGCCTCGGAAATAATGATGGCAGGAGA<br>GGCCTGTACACAGTAAGCTGGAGAGAGTGGGTCAAGCAGGAGGAATGAGGGCTAGCCATTTATTCTCC<br>ATGGAAATAGAGGAGAGCAGTGCAGACTTGCTTTTACTCTCTTCCTAGAAAGATATGATCGTGGTTGGG<br>TTTTGAGTCACAGGAGATAGACACACAGGAGAGGCTCAGAGATCCTTGCCAGTACTAAAAATGTCATTA<br>GGTTCCTTCCAGGAGACAGAGCTCCCTTAACCACTCCTTCTGCTCCCCACCCACACTCCCCTTTATTTTG<br>CCTCCTTCCAAGTGACAGATCCAAATTCTGTAGTAGAAAAATAATCACACTTCGCCTAACCCCACATCTA<br>GCCCTCCGCTCCCATCAAACTCCATGCCCAGTATGGATCGATGTTTTTCAGATAAGAATTCAACTTTCCT<br>CTTTTTGACTTACAATTCTAATTCTTATTAGTAACAAGTATTTTGTGGGATTGGAGGGATTGAATTCAGGT<br>CATCCCATTGAGGTTCTCAAACTTCCTGATGCTTCTACCTTTTAATACAGTTCTTCGTGTTGTGGTGACCC<br>CAATCACAAAATTGTTTTCATTGCTACTTCATAATTGTCATTTTGCTACTGTTATGAGTCGTAATGTAAATA<br>TTTGTATTTCCTGATGCTCTTAAGTTACTCCTGTGAAAGGGTAGTAAGACAACCCCCAAGGGGTCACAG<br>CCTGCAGGTTGAGAATTACTGCAGTTCCAAATCTCAGGAAACAGGAGAATGGTTCATTTAGCTATTCCC<br>TGGAACACTAGAACACTCCCGTTCTGAGTCTCACTGTGCTGCTTTTGTGTTTATCAATGGAACCAAAGC<br>CCTGAAAGAAATGACATGAGGAAAGACAGTTATTTTCAGATATTAATATATCAATATCAGGTAATTCGCTC<br>CTTCTTTACTTGACCAGGGCCCTTGGGCAAAACTCAGTAGTAGCAGAGGATGTTCTCTCCTTCCTAGTG<br>GGCAGGGTGCAGATAGAATAGGAGCTCAGGGGTGGTGCAGATGAAATCTGATGGAAATACTGATACCA<br>ACCTTTCCTCGGCCCCTTGGAAAGTGGAAATCTGTGAGCTCGAAACCAACCTAAGACTTTCAGACTAG<br>CCAGAGATACATAGTAAGATCCTCTGTGATGGTTTGCCTATGCTTGGTCCAGGGAATGGCACTATGTGGA<br>GGTGTGGCCCTGTTGGAGTAGGCGTGTCACTGTGGGGGTGGGCTTTAAGACCCTCCTCCTAGCTGCCTG<br>GAAGCCAGTCTTCTTCTGTTTGCCTTCAGGTGAAGATACAGGACTCTCAGCTACTCCTGTACCATGCCA<br>GCCTAGACACTGCCATGCTTCTGCCTTGATGATAATGGACTGGACCTCTGAGCCTCTTTTGCTGTGAGAC<br>ATATAGAAAACTGAGAGGCACACATGGAGAAGACGTGATCACAGAATGGAAAGAGCACAGAAGCACC<br>AATGCATGCGTGCAGCCAGTGCTGGAGCTGCAGTTTGAGTTTGACATGATTCAGGGGCTGCAGGAATG<br>AGCTAGTATTCCTCTCCTTCTTCCTCACCCTCGCCTTCCACAGAATCCACACCAACCTCTTCATAATCCTC<br>CTCTAGGGCAGCCATGTCGTCACGGGCCTCAGAGAACTCCCCCTCCTCCACGCCTTGACCCACACACC<br>AGTGCACAAAGGCACGCTTGGCATACATCAGATCAAACTTACTCCAGCTTCAGGAATCTGCCTGTGACC<br>TCCAAAGGCATTTGCAATCACATGGACATACCCCACAGACACACATACATGTAATTAAAAATAAAATGA<br>ATTAATCTTTTAAAAAGACCAATGTCACATGTCTCTCTCATTAAATATTTATACTAGCTTCTAAATGTGTGT<br>AATTAAAGTTGAAATAAGTGTGAGAAGAAGGACATTACCTCCTCAGTGCTGGTCTTCTGTGACATTTATA

							TTGTATTAGATATTATAATTAAAATTGAATAAATATTATGAATTCTTTGAGAGGATACATCGGTTAAATTAGAGGCTGTATCATCTAATTGTGTCTGAGAATACCCTGAAACAATTAGTAATCGCCTGGTCATTTCTGGATCCAGTATAGTTGACAATCAAAATCAACCATCGTATGCACCCCAGGTCCTCTTCATGAATATTATATTTTCAGACAGAGACTCACAAAATTTACACAGGCTGCTGTCAAACCCCTGCCTCAGCCTTCTCAGTGGATGGGATTTCAGATGTACATGGGCCACTGCACACAGTTCAGCAGATTTGGGTTTTCTTGTCTGTTTATTCATTTCTTTTGTTACTTGGATATTTCATTCTTTACCTGAGGGTTTTGTACTTGGTTCTGCTATACAGACAGTGCTACTTTGGACTCCCTATGCAGTGTCAGCTTCCCTGTCAGCTTGCCTGGAACTTGTGATCCTCCTACTTCAGCCTCCAGGCGCTGGGATTAGATGATTATACA
MSTRG.132300.6	ENSRNOG00000004776;ENSRNOG00000027767;ENSRNOG00000047234;ENSRNOG00000058463;ENSRNOG00000059588	0.569	0.000	0.000	-7.443	down	TCCCGGTTGCGGGCCGCCGCCGCGGCTGTGGGGACGACTTGGTCGGCGTCCGGCCCGGCAGTCGGATGAGAGGGCGCCGGCGGCGACACCAGCTGCAGCAACGAGGCTTCTTCTGGCTCGCACGGTTCCGAGGCTCCTTCGAAAGAAGAATAGTAGACTGGGATCTCTCCATGAAGAAGAGGGGCAGAAGGAAGGCTGAAAAACTGACGTGAAACATGTGCTCCTATGATGTGTGCCACATGCGTGCAGAGCCAGAAGAGGTGAGAAGAGGAAACGACAACTTTAGAAGCAGAGTTAAACACCAGTTCTACGCATTGTACCAGAACCTGGCCCATGTGAAGTAAGTGAGTCACAAATACAGGATTCTGACACCGTGGAGGGAGACACACCAGAAGAGGGCATCAGATCCCATTACAGATGGCTGTGAGCCACCAAGTGGTTGTTGGGATTTGAACTCAGGACCTCTGGAAGAGCAGTCAGTGCTTTAACCACTGAGCCATCTCTCCAGCCCCTGAGAAGTATTCTTTATTGTGAGTTTTGCCTACATGCTTTCCTTCCACCCAACCTCCAAGGCTCTGCTCCACCAGCAAACCCTCTGCTAGAGCACAGGGGCTGCCTCACCTCGTCTTTGATTTCTTCAGCCAGAGGAAGCTCTGGGTGGTTCCTGAACGTTTCCAGAATTTTCCCTTCATTAATGTAGAATTCTCAGGTCCTCCAGCTGATATGTCTGCCTGTGTCACACCACACTCCCTGCCATGGTGACAATGAACTGAACTGCAATTGTAAGCCAACCCCCACTCGATGATTGCCTTTATAAGAGTTGCCTTGCCCATGGTGTCTCGTCACAATGACGGAACACTGACTAAGATAGCTTACCAAAAGTCACTTTTATTTTTAGCTTTGTACACTTAAATGCATCTGAGCCATACAGAGTTTTGTTTCATCTCAGACATATATTCAATTTTAAGAAGATACAACAGTATCCCTTTGTATAGTGGGTTGGTCTGCTAGTATTCAAATGCCAAATTCTGTACCCTGAGATCCGGCTGCTCCAAAACAAGGAGATCCTCACATACACGAGCTTTTGTTACATAAACCTTACCCCCCAACTTATCCCTGTTTGGATAATAAAGATGTCTGCAGACTGGACTGCTCAGAAGAGAGGGGGAGGAGCCTGGAATCTCAGGCAGGGACCATGAGAAAAGAAGAAAGAAGAGCACAGGGGCAAGAAGTCACCATGGGGTAGGTGGATCGTGACCACATGGCCATGAGGTTGGCCAGTAGTAATAGGAGTGGCCCAAGTGGGACATGGTAAGTGATATCTCAGAGTTACTGCCAGGGAAGTAGAAAATAACACAGAGAGTTGAGGTCTGCCCAGCTCTGGTGCTTTAAGGCTATATATATATATATATAATGTGTGTGTGTGTGTGTGTGTGTGTGTGTGTGTGTGTGTGTGTGTGTGTTTGTATCTTTTATTTGGGAATGAAATTATAAGATGGGGTAGAAACCCCACAACACCTTTGGTTTCGGAAACCCCATTTGTAGCTAACAAAATGATCTTCCATGCATATCACATTTCTTGGTTTTGCTTTTGTCCTGGGGTTTTGCTTTTATTTCTTAAGGTTGGGGTTGTTTCTTTGTACTTTTTGTCTGGCACTTTCATTGTCGAGGGCTTGTTTTGTTTTGTCAACTCAGTGTCTCACTTTGGCCAGGGTAGCCCTGTACTCACTATGTATTTAAGGATAGCCCTGAACAACCTATTTTCCTGCCTCTACCTCCCAAGTGCTAGGATTACAAGTGTGTACAATTGGACCCACCCATTTGTGCCTCTGCGTATACGCGAGCACGCCCTTGTTGCTAGGGATGGAACCGAAGTCCTCACATGAACTAGGCAAGTACTCTACAACCGAGCAATATTTCTAACTCATGTTTGCCTTTCAGAACTACTAATCCTAGCCGGCCTCCACTAGACCTGAAACAGTGGAGAGGATCTGGTCTTCTTTTCTGTGAGAATGTTTTGATCAATGTTTTGAGTTAGTGTGCAAAATAATGACTTCCACTGTAAAATAATTACAGTTGCATGTTCTTTGTTAAGACTTAGTACATGGGGTTGAGGATTTAGCTCAGTGGTAGGGCGCTTGCCTAGCAAGCACAAGGCCCTGGGTTCCGTCCCCAGCTCCAAAGAAAAAGAAAAAAAGAAAAAAAAAAAGACTTAGTACATGGGGAGATCACACAGCCATGTCACCTGTGTGCAGGTCCAAGGACAACTTGTAGGACTTCAC

							TTTTTTTTCCTTGCAGCAGTCCTCATTATGGCCTTTTCATACGCAGGGCAGGAGTCCCTGCAGGTTCTGT<br>GGTCGGCAGGGAAATGATTTGGGTGAGGATCTTGCGTTGAGTACCTGCCCTGACTTCACATCACGATGG<br>TCCCTAGACAGCAAAACAAGAAGGCTAGTGAAGAGAAGAACATGATTGGCATTCAGAATCAGCGGCAC<br>AACTTTGAAGTCCTACAGATCAAGTGCGAGAACATTGAAAGGAACATTTCTACAGTGAAGAGTTAAGT<br>GACAGCATTCTTATTGTCTGCCGTGTATACTGTGTGGGAATGAGACATCATGTCAGCGGCTGGGAAATAC<br>AAAGGGATCAGGAAGAAGTGTGTAGTGATGGCAGTGTCATCTGTAGACTGGGCCAGAAAAATCCACTG<br>AGTTCAGGACAGCAGTTGGCGATCTCTACTGGACAAAGGATGAGAGAAGCCTATCTGTGGTCTTACAA<br>AGACTTCAGGGCTACAGTTGTGATGAAGACCAGGGATGGGACAGTCCTCTCCCTGTGTGCCATTGCATA<br>CTAACATGAAAGCTTCACTCTGGGGATGATAAAGAGATCTTTCACCATAAGCCATTGTGTGTGGGTGAA<br>CAATAACGTCACCAACGTTAAGTGGAAGTGTAGATCCCTGGAAGAGCTCATGTATCTGGCCTGATAAGG<br>CCCTGTGTTATAGTCCCTGTGTCAAAAACAAAGTAAGCTAGACAGCCTTGTGAACCAGTTCCTCAGTGG<br>AGTCATTACTGGAGCTCCCACCAGCGTTGCAGGTGAACACTGTTATCGATTATCGCTGCATAGGTGAGAT<br>CTGCTAACCCAGACCAACTCAGAGCATCTCAACAGGCAGTTGGCACAGGCACAGAACCTGGTTTGGGA<br>ATTCAGTGTGAATTTGGGACTGTTAAGGAGAACAGGAGAGGGTAGCACAGCCAGGAGAACTGAATGTG<br>CCTCCAGTTCAGTTCCAAATCTCGGGGAACAGGGCAATGGCCCATTTTCCAAGATGGTATCCTTGGAAG<br>TGACTGAAGCTGAGAAGCCTCGGAAATAATGATGGCAGGAGAGGCCTGTACACAGTAAGCTGGAGAG<br>AGTGGGTCAAGCAGGAGGAATGAGGGCTAGCCATTTATTCTCCATGGAAATAGAGGAGAGCAGTGCAG<br>ACTTGCTTTTACTCTCTTCCTAGAAAGATATGATCGTGGTTGGGTTTTGAGTCACAGGAGATAGACACAC<br>AGGAGAGGCTCAGAGATCCTTGCCAGTACTAAAAATGTCATTAGGTTCCTTCCAGGAGACAGAGCTCC<br>CTTAACCACTCCTTCTGCTCCCCACCCACACTCCCCTTTATTTGCCTCCTTCCAAGTGACAGATCCAAA<br>TTCTGTAGTAGAAAAATAATCACACTTCGCCTAACCCCACATCTAGCCCTCCGCTCCCATCAAACTCCAT<br>GCCCAGTATGGATCGATGTTTTTCAGATAAGAATTCAACTTTCCTCTTTTTGACTTACAATTCTAATTCTT<br>ATTAGTAACAAGTATTTTGTGGGATTGGAGGGATTGAATTCAGGTCATCCCATTGAGGTTCTCAAACTTC<br>CTGATGCTTCTACCTTTTAATACAGTTCTTCGTGTTGTGGTGACCCCAATCACAAAATTGTTTTCATTGCT<br>ACTTCATAATTGTCATTTTGCTACTGTTATGAGTCGTAATGTAAATATTTGTATTTCCTGATGCTCTTAAGT<br>TACTCCTGTGAAAGGGTAGTAAGACAACCCCCAAGGGGTCACAGCCTGCAGGTTGAGAATTACTGCAG<br>TTCCAAATCTCAGGAAACAGGAGAATGGTTCATTTAGCTATTCCCTGGAACACTAGAACACTCCCGTTC<br>TGAGTCTCACTGTGCTGCTTTTGTGTTTATCAATGGAACCAAAGCCCTGAAAGAAATGACATGAGGAAA<br>GACAGTTATTTTCAGATATTAATATATCAATATCAGGTAATTCGCTCCTTCTTTACTTGACCAGGGCCCTTG<br>GGCAAAACTCAGTAGTAGCAGAGGATGTTCTCTCCTTCCTAGTGGGCAGGGTGCAGATAGAATAGGAG<br>CTCAGGGGTGGTGCAGATGAAATCTGATGGAAATACTGATACCAACCTTTCCTCGGCCCCTTGGAAAGT<br>GGAAATCTGTGAGCTCGAAACCAACCTAAGACTTTCAGACTAGCCAGAGATACATAGTAAGATCCTCTG<br>TGATGGTTTGCCTATGCTTGGTCCAGGGAATGGCACTATGTGGAGGTGTGGCCCTGTTGGAGTAGGCGT<br>GTCACTGTGGGGGTGGGCTTTAAGACCCTCCTCCTAGCTGCCTGGAAGCCAGTCTTCTTCTGTTTGCCT<br>TCAGGTGAAGATACAGGACTCTCAGCTACTCCTGTACCATGCCAGCCTAGACACTGCCATGCTTCTGCC<br>TTGATGATAATGGACTGGACCTCTGAGCCTCTTTTGCTGTGAGACATATAGAAAACTGAGAGGCACACA<br>TGGAGAAGACGTGATCACAGAATGGAAAGAGCACAGAAGCACCAATGCATGCGTGCAGCCAGTGCTG<br>GAGCTGCAGTTTGAGTTTGACATGATTCAGGGGCTGCAGGAATGAGCTAGTATTCCTCTCCTTCTTCCTC<br>ACCCTCGCCTTCCACAGAATCCACACCAACCTCTTCATAATCCTCCTCTAGGGCAGCCATGTCGTCACG<br>GGCCTCAGAGAACTCCCCCTCCTCCACGCCTTGACCCACACACCAGTGCACAAAGGCACGCTTGGCAT<br>ACATCAGATCAAACTTACTCCAGCTTCAGGAATCTGCCTGTGACCTCCAAAGGCATTTGCAATCACATG

							GACATACCCCACAGACACACATACATGTAATTAAAAATAAAATGAATTAATCTTTTAAAAAGACCAATGTCACATGTCTCTCTCATTAAATATTTATACTAGCTTCTAAATGTGTGTAATTAAAGTTGAAATAAGTGTGAGAAGAAGGACATTACCTCCTCAGTGCTGGTCTTCTGTGACATTTATATTGTATTAGATATTATAATTAAAATTGAATAAATATTATGAATTCTTTGAGAGGATACATCGGTTAAATTAGAGGCTGTATCATCTAATTGTGTCTGAGAATACCCTGAAACAATTAGTAATCGCCTGGTCATTTCTGGATCCAGTATAGTTGACAATCAAAATCAACCATCGTATGCACCCCAGGTCCTCTTCATGAATATTATATTTTCAGACAGAGACTCACAAAATTTACACAGGCTGCTGTCAAACCCCTGCCTCAGCCTTCTCAGTGGATGGGATTTCAGATGTACATGGGCCACTGCACACAGTTCAGCAGATTTGGGTTTTCTTGTCTGTTTATTCATTTCTTTTGTTACTTGGATATTTCATTCTTTACCTGAGGGTTTTGTACTTGGTTCTGCTATACAGACAGTGCTACTTTGGACTCCCTATGCAGTGTCAGCTTCCCTGTCAGCTTGCCTGGAACTTGTGATCCTCCTACTTCAGCCTCCAGGCGCTGGGATTAGATGATTATACA
MSTRG.132300.7	ENSRNOG00000004776;ENSRNOG00000027767;ENSRNOG00000027850;ENSRNOG00000052581	0.314	0.017	0.050	-2.562	down	TCCCGGTTGCGGGCCGCCGCCGCGGGCTGTGGGGACGACTTGGTCGGCGTCCGGCCCGGCAGTCGGATGAGAGGGCGCCGGCGGCGACACCAGCTGCAGCAACGAGGCTTCTTCTGGCTCGCACGGTTCCGAGGCTCCTTCGAAAGAAGGTGGCCCATAAAGACGGCCCTAGGAAGGGGAACAGAAATGAGTGGACGGAGGCCTGAGAGGCAGGCTCCCGTGGCCGGGCAGGCCGCTAGCTGACTGGGCCTCTGGCCGCAGCGAGCTGCTCGGCGCTGCGCACCTGGCACCCTCGACGACCCGGGTGGGCGTCAGCCGCGGGGTACCCAGGCGGCCAGAAACGGGCTACATACTTTGGGCCCGGAAGCCTGGAGAAAGATGGGCCCGAGCTGTGGGGACTCGAGGGAGGAAGCGAGTCGGGTCGCTAGGCCGCTCGGTTCGGTCAGTCAGCGCGGTGGTAGGGGCAATGGCAGTCCTGCTCAGACCACTTTATCTGCAGCTCCGCCAACGGCGCGTGTGCCTGCGACACAACAGGAGCCGCAGGAGCACACCACCCACTCCAAGAGGGGGCGGAGCCGGCCTTGGGGGCGCGCCGGAGGGGGGGTGAAGGAGGGAGGGGGTGCGGGGGAGGAGGGGGGTGGAGAGATGGCTCACCTGGTTGGGTTGCCAGCCTGACAAAAGGCTAGCTACTCGCGCCCAGCCCCCCTGCTCAGAGGTTACTTGGGGGCCTGAAGCCCATTCCTCTCTTCTGAGAATAGTAGACTGGGATCTCTCCATGAAGAAGAGGGGCAGAAGGAAGGCTGAAAAACTGACGTGAAACATGTGCTCCTATGATGTGTGCCACATGCGTGCAGAGCCAGAAGAGGTGAGAAGAGGAAACGACAACTTTAGAAGCAGAGTTAAACACCAGTTCTACGCATTGTACCAGAACCTGGCCCATGTGAAGTAAGTGAGTCACAAATACAGGATTCTGACACCGTGGAGGGAGACACACCAGAAGAGGGCATCAGATCCCATTACAGATGGCTGTGAGCCACCAAGTGGTTGTTGGGATTTGAACTCAGGACCTCTGGAAGAGCAGTCAGTGCTTTAACCACTGAGCCATCTCTCCAGCCCCTGAGAAGTATTCTTTATTGTGAGTTTTGCCTACATGCTTTCCTTCCACCCAACCTCCAAGGCTCTGCTCCACCAGCAAACCCTCTGCTAGAGCACAGGGGCTGCCTCACCTCGTCTTTGATTTCTTCAGCCAGAGGAAGGTATGGCCTATACCTTGAGTGCTAACCTGGGATTTAGAAGCCCATGGCATCCTATCTAGGGAAAGTCTCCAAAGTATCCAGCACTGGAGTAAATGAAGCAGGACATGTTACTTCCAGCTCAATGTTGTACCCCAAAGGACTAGAAGTGGATGATTGGCTAGGGTCCAATACCGTATCGGAAAGATGTGTTCTGGGTGTTGCGAGACATGTCAGAGGAC
MSTRG.132307.5	ENSRNOG00000004776;ENSRNOG00000004819;ENSRNO	0.000	0.313	0.000	5.095	up	CATTATTCATACTATTTGTATGCATATGCAGATGTGCCTTCAAAAACATTCTGGGATGGGCTGAGGATGTAGCTCAGTGGTAGAGTCCTTGCTTACTATGCATAAGACCCTGGGTTCCATTCTCACACACACACACACACACACACACACACACACACAGTCTGTTAGTTGGATTGGTACTACTCTCACCTACAGTCCTAGCTACTCAGGTGCCCAAAATGAGAACCCTCAGCCCAGAACTAGGTCAATGTGGACAGTATAGCTAGGCCCTTTTAAAAAAAAAAACAAAATGTGAAACCAAATGTAGTCCACACCTAGAACCCCAGATTATTGGAGATTGATTGACTTTGAGGTCAGCTTGGGCTACATAGTAAGATACTAGTTCATAAAACAAAGACCAGAAACAAATTCTGTGAAAGTCCACAGATTTAACCAGATTTCCAAAGGATGTCTCAAACGATTTTTGATGTTGTTTTGTTCATTTGGTTGAGAAAGGGTCTCATATAGCCAAGGCTGGCCTCAAACTTACTGTATACCCAAGAGTGACATTGAAATG

	G00000004903;ENSRNOG00000005303;ENSRNOG00000005387;ENSRNOG00000027767						TGGATCCTCCCAAGTACTGACATTAAAGGCAAGTTCCACAATGCCTAATTTTACTGGGCCAAACTTTTTAGGTTGACGGTCTTTTTTTCATACTTAGCTAACATGTACAAGTCCTTGAGTTCTATCCCCAGCACTACCAATATAAACGGGTGTGGGAGGGAGACTTGTTTTCTAAAGTGCCCAAGTCTTCCTAACTGCAATCCTGAGTTTCTACAGCCCAACAGGCAGGAGCTAACATGGACTCCCTAAGCTATAGGGTACCTGTAGGATGATGTCATACAGGTGAAGGCAGGTACAAGATAGAATGAGGCCTGTCATTGGACGAGAAGGAAGGATGGGCGGGAGAAAAGTTTTAGAGAGAGGAGGAGACTGGGGCGAGAGAGGAAGCAGCAGAGAGAACATGGAGGCGGATTTTTTTTCTTTTCTTTTTTTCTGGAGCTGGGGACCGAACCCAGGGCCTTGCGCTTGCTAGGCAAGCGCTCTACCACTGAGCTAAATCCCCAACCCCATGGAGGCGGATGTTAAGATTCCTCTCTGCACATTTACACATTGTTATGAATATTCTTAAGGGATGGATGTGTACAGGGCTTTGTATGTATAGATGAG
MSTRG.132379.1	ENSRNOG00000009605;ENSRNOG00000009687;ENSRNOG00000009749;ENSRNOG00000010348;ENSRNOG00000010846;ENSRNOG00000011702;ENSRNOG00000022417;ENSRNOG00000022939;ENSRNOG0000003	0.455	0.000	0.049	-3.592	down	GCCTCACTCATGCCCAGGCAGTGGAATGGATTCGCACTGGAGGCCCCCGCCTTTGCCTGGTACTCCAGCGGCCTGGCTTCGAGAATTTCGGGTTTAAACAGGGCCTCAGCCACCTTTTTTTCAGCTAAGGAAATTGAGTGACTCTAGTACTTCTTGGCCTTTTGGCTAAGATCAAAAGTAGAAAATCGAGTCACTCCCTTCCAAAGCCAGATTCCCTCCGTAGCTCTTTTGCATTAAAAAAAAAGTGAAGGCTCTACATATTCTTGTCCTGGTACCTGGAGATTCCAAGCACCTTGAAGATAAGACGGTGTGCGGGACCCGTATCGTCTTCGGCCTCAGGGCAAAGGGGGCTTCCGGGGCGGCCTCTACCTGGCCCGGCCCCCTCCGGCCCCGCCTGCTTTGCTAGGCGCGGTGATTCATTCCCTCCTTTGCCCGGGGGCCCC

	9496;ENSRNOG00000057147;ENSRNOG00000059720						
MSTRG.132589.2<br><br>NONCODE_ID：NONMMUT074424	ENSRNOG00000003178;ENSRNOG00000021434;ENSRNOG00000030619	0.530	0.032	0.012	-2.951	down	GTTGCATACCAGAACCAAATTATTTTGGGTTCTTTTTAGTTTCTTTGAATGCTGTTCATTGCCCATTGTCTGACCTTTGTGACTATCAAAAGTTTCAGATGTATTCTGAGCAAACCACTAGCTCCAGCTATACAAAAACCAAGAGTGTCACCAGAAAACCTCTGAGGGCTATAGCAACCTGTAACCTGTCTATTGGACATGCTAACGTATCGTAAGTGTATATTTTAAGTGTATTTAAAGTACAACTTTGTTTTGTTACCATAAAAATCATGAAGCTAACTTATTGGAATATGGCCTTTGGAGTAAAATGAATCTTTGTTTCCAAGTTCATATTTGGCTCCAGGATAAACTATCTCTTATTTCCTTTAAATTGAGAGCTATTTTTTTTAATCAAGAAGACTGAGGTACTACTACTACAAGACTGTCCAACCCACTCTGATAACAGACAGGATTCACATATGGTAGATAAAAGGGCATGTTAGGGTTCTAATACTGTGAAAGAATATCGGGCAAAAAAGGTTTTGTCATTGGGAATCTATGTCTCCTTATGGATCATCATATCTAGAAAAAGGGCACAGAAAAGGGAGTGCTGGTCCTCAGCTGCCTTTCTCCTTTCTCTGTTTTAATTCAGGATAGACTCCCATGGTGACTTACACCTTCAAGGTACACTGTTTCACTTTAGTTAATTCTCCCTAGCAACCCCTTCACACAGGCACATCCAAATGCATGCCTCACTAATGTCCGAGCAACTTTTTAATCCAAACTAGTTGACAAAGAGGAGTGACGATCATACTAGCCAACTAACACAGAGCAAAGTGTAAACTCAGCTGGAATATTGCCCATCATATAGTAGGACTGGAGTTTTTCTTCTCTAGCTGTTGGCCGAAGGTCCTTAATCCTGAGACTTCTTTCCGATTTCTGTGCAGGGTCCTCTCCATCTTCCAAACAGGCATGTTTTTGAATCCTCACGCGATGCATTTCTTTCACTTATTCTTCTTACAAGGTGGAGCTCTTCAACATTCTATCACGGCTGGTCAAGGGTTTGTGAGGCCCCCACCTCTGGCTGAGGAGCTACTGGCAGTTAATGGTTGATAAATGGCAGGGAATGAAATGTAATCTGTTGTGATAGAAAACTGAGAAAT
MSTRG.13265.1	--	1.359	0.117	0.002	-2.747	down	CCTCCCACCTCCACCCAAACAAATAACACTCACTTTTCTTTTTCTTGTCCAACTACATAGAAGAGTTGTTGACAAGATTTTTCTGCCTGCAGTGCAGAAATCCCAGATGACCAAGCCTTTTATTATGGTCTCTTTGAAGATAGGCAAAGAACCCTTCAATTTTCAGAACCAGAGCCGCCCAAAGAATATTATTTGGCAATAGAAAGGTTATTAGAGAATGGGATAAGTATACATTTTGATGTGGAAATACATTTATAATGTATGCTGGAATAAAGCAGAAAAATAAATGAACATAATGTATAATGTATAAAATGTGTTATTTATCCAGAGGCAAGAATTATAGATATACACATAAGTATATATATGTATATATATATATATATACATACTGCATACATACATATGTACATCTATGTATGTATTTATGTATTATTATCTATGTATCTATCATATCTATCATATCTATCTATCATCTATGTATCTAACCATCTTCCTATCATACATAGTGAGTCTGTATCATTGAGGTTTTCTTTTTTGGTGGGTTTTGTTTTGTTTGGCAAGGAGGAAAGGGCTTATTTGGTTACATATCCATGTCATATTTTACCATGTAGGCAAACCACAGAACAAGAACCTGGAGGCAGGAACTTGGAGGGAGTACCTGAAGCATAATGATGAATGCTGCTTACTGCCTTGACTCCCAAGGTTTACTCAGCCTGCTTTCTTATACACTTCAGGACTGGTGGCATTACCCATAGTGTCCTGGGCCCTCCAACATCAACCATTAATTAAAAAATGTGCTATAGACTTTTCTTCAGACCAGTATGATGGGGTAATTTTCTCAACTGAGGTTTCCTCTTTCCAGTTGACATATATGTATAGGCATGGGCAGATGTACATTTATATGTATGCATATAAATTATGCTAGGGGCAAACGTGGCCAAAGGAGATAGTGATGGAGGTAAACCTTTGAATACTTCTGCTTCTGAGGGAAGATGCATTGATAAGGCCTAATCTAAAACAATTTCACATCTTCTGTAGTTTAGTAGTATATATGTGTGTGTGTGTGTGTGTGTGCGTGCATGTGCGTGCGTGTGTGTGTATGTGTAATGATTAATTAAAACAACAAGGAATAT

							AGATGTCTCAAAAAATGATTCCTTTTTTTTTCAAATTTGCAAGTAAACTAGAAGTAAAGTGGTTAGTAGT<br>TTAAATAAAGGTCAGCTATTTGAAGGTCCAGTCACTTTAGCATCTGTCCATCATAGCTCTTTCCCTGAAA<br>CTTAGCTAACAGACACTGTCTACTACTCATTTAAATATCCAGATCTGAAATCCATTTTTTTCCTGATAATT<br>GAGATTTAGAATCCAGAAATCAACAAATTTGCTGGCCAGCTGGGTTAGCGTTCTCTTTCTTCTAATGTAG<br>AGTTTCCAGAGGTTGTTGAAGTCTTTTACCTGCTTGCAGAAATTATTGCTCTCCTAAATCATGCAAACTG<br>GTCAAGCTGGTTCAATTTTTCTCTGCATAAACTTACAGACATTGATTTGCGCGTATTTTAGTTCTTTTCCA<br>AGAAGCTGCAGCCTACCTCATTATAGGAATGTTCAATAGCCCTACAGAGTATCCAAGTCATCAGCTGAGT<br>AGATGCTGTCATGAAGAAGGTTGTACCCAGGCACACTGCCAAAGACCCACACATTCACTCAATGTGTG<br>TTCTTAGCCAATAAAAAACTACAGATTCTGTCGCAGCAAAGAGAAGTGGTAGTATTTCAATATGTCTGTT<br>AGCCATGTAATAAAGTAAGAACCTAACTGATTTTTATGAAGTGTTGTCATTTTTTTAATGAATGGTTTGAG<br>AATGTTGAAGTTATTGCTAAAAATTAGCATTTTGGAGCATTAGCTATAGACCAAGTATATAATGACTGATT<br>CAGTATTATATGATGAACAATATCCTATAGCATTAATTTTATAGGGGATTTCCACTAGTATGTATGGAAATC<br>AAGTTGATTACAGATACCTTTAATATTTAGCTTCCTACCAGATGTATTAGTAACTCAGTAGTATATGGATAC<br>TATAGGACAACCTTTTCTAGAATTACCCCCATGCATCTTCTGGCCTTATAATCCCCTGACTGTATGTTTTGT<br>TTTCGTTCTCTTTTTACAAAATAGCCCAACCACTTTCTACCAGAAATCCAAGGATCATTATTAATTTTATT<br>CTTACCATATTGGTGTCCAAAGCTAATCAATTGCTATTCTGTTATTCTATGCCTTTGTCCTCTGAGAATAAT<br>TTACAAATAACGGGCATCTGCAATTGTCATGGTAGTGACTAATATATTTTATTTTACAAGAAGAACAGTGT<br>TTTATTTGAATGAGTATTGTCATGATTGGTAATCACTGAGGCAAACTAGAGAGTAGCCACATGGAAAGG<br>CAGGACCATTGTTCCCAGGTCTTCTAGTATTCCACAGGAAGTTGGTGATCAGCATTTTCGTCATCTATTTT<br>TTTATCTCATTCTTTCTAAGTGGTGGGTTTATTTTTATGCACAAAGTGGAATGTGGTATAACTGTCTCCAT<br>TGAACTGCCTCCTGGGACTTAACATTTGAAATTTCCTGATAATATTTTTCCAAAAGGAGTAGACTTTAAC<br>GTTGATTGAATTCAATGGATTTTCATTTTGTATGATAGACACTTTATTCGCTAGAGAGTTAATGCCTCTCAT<br>GACTCGATTCTTTTTAAGTGACATGGGATATAATTGTTAATGTTAAACTGTTTAGAGTGGCTAAACAACTT<br>TAAGGATTACCTAGGATTTCAGTCAAGCAAATTAGTCCTAACACTAATGTTTCAGGGGTTGAGGGTGGT<br>GATCATCGTACATTTAGGTGAAAAGTACCAGAATTTTATTTTGTTTTTTTTTAGCATTGTCTGATAGCCCC<br>AAGGTCGTTAGTGAGTGGGAAAATCACAGCTGAAATTTCAGTGGTATCTACGGGAGCATCAAAGAATA<br>GAAAGGCTAGTGACTGGCAGTTTCCATGACAACAAGGGAAATTTTCAGGCTAGAAAGATGTGTAGGTT<br>ACACCATCAAATGTGCCAGAGTTTGGTCTCCATGACGACAGCAGTGTGAATGGGGAAATGTATGCTTGT<br>GAGATTTTGTTTTAAAAGCCTGATGCGTTATTTTAGAAGAAATTCCTTACCAAATCAATATCTTTTCTATT<br>TTATTTTACCTAAAAATTACAGTCAAAATAAGCAATTCTGTTTTTTTCATACTTTGGAGAAAGTGATATTT<br>TGAATATTTTTATCATAAAAGACTTTGCTAATTTAGAAATAACTTCTTTGGCAACATTTCTAATACAGACA<br>ACATGGAGAATACATTTTTAAAGATGCATTTACTTTAGAATTTCAACATTTATCTCAGGGAACAGTTATTT<br>AAACCTTCTGGTGTTTACAGATACAAATCCACTACCAGAAAATATATCAAACCTATATGCCCGTGCCTCA<br>AGAGACAAGAGTTACTATAGTCCCAGGATAGAAGAATACTTAGTGTAATTATCATCTGAGCTAGTTTCCT<br>GCTTTAGTTTGTGTTGAAGCTTTGTGTGAGTTTTTCTTGCTGCTGTTTCAAAATCTCTAAATTGCAGAAA<br>TGAAATCAATCTGAATGCACTATGCTGTGTGTATAACTAAGAACACATCTACTATGTGTTACTGCAGGAC<br>CAACTCAACCATAGAAACTATCACTTGGCCTACACCTAAGGCTGGATCTGTGGCTGTGTTTGTCTTGGTT<br>TCTTGGGCAGCATTGTGTGGGGGGAACTTGATTGGTAAAATAGATTTGTTTGTCCTGTGAGGAAATGTA<br>AAGTTTATAAAACTGTAAGATTTAAGAAAATGAAAGAGATCATAGAACCAGCTGAAGACTAGCAGCAG<br>CTGGGAGGGGTTAGAACACCCTGACTAGAGGGGACAAGAGGAAAGATGACACCCAAAATGAGGAAAG<br>GGGAATTCCCAGAGATGGCCACAAGACCAGAGCTGCAGCCTTTCATTGAGAAACACAGAAACCAGCAT

							CAGTCAGAGAACAGTAGCTAGAAGAGTACCAGTCTCGCACGGCCTTCTCAGTGCCCAGTGGTGCATCTCATCAGTGAACCAGGCAGAAGCTGCCTTACACAGATGGCGGTAGATGTAGTCCATACAGGTTGCTCTCAGGTGAAGAAGAAGGGGGAAAGATATAGGAAAATTTATTGTAGAGTAGGGAAAATATGTAGTGTGTCAGAAATGACATGGATCCCATAGTGTCAAGAATAACAACTATATACATATATGCATGTATGAATGTCTATGTAGGCCAGTAAAAATTAAAAGGTTTTGCATGTAGCTTTTGCTACCCAACCTTAAGCCATGGGTAGACCACACTACCAGCCCATGCCCAGAGAATATTGGATCCATCGATGCAAAACACAGGAACATGTTGCTCAATTTCAACCTGTCTTATGGTTTAATTGCTGGGCACCTCCAATCTACCACAGCTAGTATGCTCTTCCCACCATGCCCAGCCCAGCCTTCTAAATCTGCCCTTAACTTCAGTTGCTCAGCTGCCTCCAGTCTGAGCTCAACACCCGACCTCCTGCCTGGAGAGGTGGCCTTGCTGTTCCTCCCAAGAGCTCACATGGCTGGTTGGCTTTTCTCCTTCCAAAGCACGGTGAAAACTCTTCCCCTCTCCTTCTCTCTCCTCGCCTCCCTGGGGACCCAGAAGTTCCGCCTATTCCTTCTGCCCAGCAATTGCTCCTGGCAACTTTGCTGATAGATCAAGAACCAATTGGAGAACAGGACCTTAGCATCAGACCAACCCTCTACAAAGAGTGTATAAACAATACTGTAAATTTAGAGCAATCATTACTTTTGTGATATATCAAAAATTTGAACATTATTAACATAGAATATTTATTAAGGTCTTTTTAAAAACTAATTTTAAAAAAATTCCCTATACTTGATAAGCACATATCTAATTATCCATTTACAGTGTGCAGTTTGGTGGAGATTTTAGTAAGTAAGCTAAATATTTTTATATTTATTTTTGTATATGTGTGTACTTCTTTTTACATGTATGTGTGGGTATGTTTGCATAGGTCAGAGCCCCACTTTGATTTCTATCTCAGTCAGGCTCCATATTATTATTATCACTATCACCATCACCATCATCACAACCACCACCACCACCACCACCATCATCATCATTGTCATCATTATTATTTGAAACAAGATCTTTCAATGAACCTAGAGCTCACATATTCACCAAAACTGGCTATCCAGCAAGCTACAGGTTTCCTCCTGTCTCTGCATCCCAAAGGCTGGGCTTACAGGTGCATGATGCAGTACCCTGTAAGTATTGAGGATTGAACTCAGTTCTCCATGCTTATTTAATAAGCACATTATTATCAATAACTAGGCAATCTGTGCAGTTCTTACAGTCCTCCTTTCTAGAGATCTGAACTATATAGCCTTGGAACCCTCATTAAAATAGTAATGAACATTATTTTAACATTTTCCCTCTATCTTCAGGCCAGATGTTGCAGAAAGTGAGTGAATTCAAAACTCATGTTGATGCCTGATGTTAGGGGATGAAGGAAGAATTATAAAGCCTGTTGTGGTCTTTGAGTGATTTAGTCGAAAAAGAAACTCCATATAAGTGACTATAAAAATAAGAGGTATGTGATAAAATGTGAATAAAAAGATTTCAACGATTCTAAGTTCAATACTATTTTATTCTTAAAACATCAGAATGCAATTCATTCAAGAGTTCCCCTATACTGGAGGCAGGCAAGAATTTTCTGTTATTTTTGTTCTCTTTTGTTTGGATCTTGAGCTTCCTAATGATGTGACCCTTTAACACAGTTCCTCATGTTACATTGACTCCCAACTATAAAATTATTTTGTTGCTAGTTCACATATGTAATTTTGTTGTTGTGATGAATGGTAATATAGTTATTCGATATGCAGGATCTTAGTTGATGTGCAAGATATCTGATATCCAACCCCCAAAGGGGACAGACCCCACAGGACATGAGTCATCATCTTACAGCTTTATGTGTTAACCATGC
MSTRG.13269.1	--	0.440	0.000	0.000	-5.987	down	TAAAATTACTGTCTAAAATTCTTATGTTTTGGCCAGTGCTATACAGATTCTGTATCCTTTAAGAGTGCTTACTGCTCTTACAGAAATCTTACATCAAAATGACAGAAGATTATACAAATATATGTGGCTGAGTGTTTGGGAGAGTCAGGGACAGTGTTGATGGGATTTTACATGTGCCGCCTTCTCAAATATCAGCTAAAAGTGAAAAGGTGTTCCACATGTGGAAAGGCTGAAGCATACGGAAATAAAAGGATGGTTATAGTCTGGGAATACCATGAACAGTGGAGATCTCAGAGAACTCAGAAGACTCCTGATATAAGAGACAGTGAGATTCAGGGAAACTGGGACAGGGGTTGGACAGAAGGCCAAGCTTCATCACTAACCTTAGCTATTCATCTTTAAAATTAAACATGTCCTCTCATCTTTGTTTTAGTATTCATTAAGTGTTTAGAAACACATATCTTAGAATTGTGTACAGGGCACCATAATAAAAGTAACACATATCTTTGTGGTATAATAATAATCAAATTTATGTGTTCATTCATTTACTTAGCAGTTACTCTTTACTTTGCCAGTAAACCTCAGACATTAAAGATTTCATTGAGCAAGAAATGCCCGTTTGGTCTTCCCTGTTTGTTCTCTGTTGTCAAGAAACTAGGGAGTTGAGAGGTACTGGTAACTGTGGAAGCAGTGCCAAGGTGGTCAAGGATAGATTGCCAGCAGACTAAAGCCGGGAGCATTAAGGTGTAAGTAGAATATAAAGGGACTATGTGAAGAATGAAGGGGCTGTTGAGTAGAGAAAGTGGCGAGAGCATAGGACCAGACCTTTCACTC

							TGGGCCAGTCAGTGTTCAGCTGACTGATTAAGCAGTGGTGGAGATTCTGACTTTTACTCGTGGGATAGAGGCCTGAATATTATTAGCAAATTGGGGAAGATGGCAGGCTTGTGAGCTCTGAGTTTTGACTGGCTTTGACTCTTGGAGACACAGTGGGAGGCAATAGATTCTAAGGACCAATAAACTGGAAATTCTTTGTAATGTTGGGAGGGAGCTAGGAAAAAATGTGATTAGATCTAGGTATAGATGTCAGTCAAGGCAGCTTCTGATATGCTGTGTGTATTTGTGTGCAGACATTGATAGAGGCTAGCTATGCTGGAAGCAGTCTAGAGGACACAGGGTTTATGTTCTGGAGAGGTTGAAGTGCTCCAATCAGGGTCTGGTGCCTGACTTAAAAGAGGATATATTTTGAGGTGATACTTGATGCTCAGTGGGCTGTGATAGCCAATCAGTAAGGGGTGGGAATGCTTACTCACGTGACTTCTTCCACACATGAACCAGACATATCCTTGCAGCCCAGCATTGGGATGCCATCACTAAGATGGTCTCAGTGATACTTTACATCACTTTACTGGTCTCAGTGATAGCTCTATGCCAGTGGTTCTGCACCTTCTTGATGCTGTGACCCTTTAATATAGCTTCTCATGTTGTGGTGACCACCAGCCATAAGATTATTTTTGTTACTGCTTCATAAACTGTAATTTTGCTACTTTTATGAATTGTAATGTAAATATTTGATATGCGGGATATCTGATATAAAACCCCCATGAAAGGGTCATACTCCCATAGGGGTCATGACCCACTGTCTTACTTAGCGTTTGTACTGCTGTGAACAGACCAAGGAAGTCTTATAAGAACAACATTTAATTGGGGCTGTCTTACAGACTCAGAGGTCCATTATTATCAAGGCAGAAGCAAGGCAGTGTCTAGGCAGGCATGGTACAGGAGGAATTGAGAGTTCTATATCTTCATCTGGAGGCCACTAGAAGACTAACTTCTAAGACAGATAGAATGAGGGTCTTAAAGCCTACTCCTACAGTGACACACTTCCTCTAACAAGCCCACACCTTCTCCAACAAGGCCATACCTACCTAACAGAGACACTCCCTGGACCAAGCGTATTAAAACCATCACAACAGCAGATTGAGGACTACTGATCTATGCTGTTCTTTCAGGAGAAACAAGTTTACACTTCAACATTATAAGTGTCTAAATTAGGGGTTGGGGATTTAGCTCAGTGGTAGAGCGCTTGCCTAGCAACCGCAAGGCCCTGGGTTCAGTTCCCAGCTCTGGAAAAAAAAGAAAAAAGAAAAAAAAATAAGTGTCTAAATTATAACCCAGCTGGGTTTTTGTTGGCATATGAATGAGATTTATTAGTTCATGCTTCTTTTACAGTTTGTAGAATTTGTTTTCTATATTATGGCTCTTTTGATACATAAAATAATAAAATAAAAGCTATATGAGTGTCTGTATATGTGCTACTCACTTTAAATACTCTCTGAATACTGTCTCCTCTTTATTTCTCTTTCTTATTTCCTCTTACTGTAATTACTATAATTTATCTTGTTATGTTTTTACCTAAGATGTTGGAAGTATCTATATAATTTACAAATGAATAGTAACTGTGATATTTTAAAATAAATAAATAGTAAGAATATAAATCTTTCCACAGTGTTTTTACTTGCCTACTATTCATTACATTTAGACTTGGCATGCATTTATCAGTACTGCATTGAGTTCTGTTGCTTTATAGTACTTCACTGTCTAAAGAGAATAGGGTTTTTGCATACCCATTCTCCTATTATAGACATTTTAATTAGAAGCCACCTAACCTAAATTACCTTTCCCTAATCATAGATAGAGTTACT
MSTRG.13280.1	--	0.656	0.060	0.005	-2.636	down	CCGAAGTCCAGCAGTTGCCTGTCAAAGGCTCTTGGGCTCTCTCCCCAAGCCACAGATTAGTCTTTCCAGATGGGGCTAAATGTTCATTCTGGGGCTAGCATTCACACACTAGCATCTTGTGTATTTCAATGCCTCAGACATTCTTTGTCCCTTGGATCAACGCTTTCCTCCTTTTCCTGTTACAAAGTAATACAGATTTTGTGTGATGATAGCATAATGACAGACGGAGACCAGACAGGCAGTCATGAAGGCTCACAGGTGAGTCTCAAGGCTTCTAGAAGGGGAGGCCTGGGTACTGAGCCTTCCAGGCCACCATCTTTCTACCTGGTTTTGACACTTGTACACCACACCCACCTGTTACTCTCATATTCTACACTCCTCACTTAGAGAGTTGATCTCGAAAGGATGAGAACTGCATTGCGGTTTCCTTATTCTAATTATTTAATATAAGTAAGTTTTTTTAAGAATAATCTCATGAAGTAACTCATAAATCAGTTATTTATCAAGCACCTAATAAAAGACTACTTGAAAAAGAAAAGTTTCTGAGCTCGGGGTGCACATTAGAGCTAGGACATAAGAGCCCCAGTGTTAAAGAATTTGTCTCACCGTAGAGATGGATGGGAGGAGGAATTGAGTACACACACAGGACTGAAAATGGTGTCTTGGGGAAGCGGAAAGAGGTGACATTTGAGTTTTGAATTAAACATAGGAAACCTGTGGGTGTGGGAAAAGCAACCCCCAGGGGCGTTGATCGTGGAGAGGTTGGCAATCTGGAAAGGAGGAAGCAGGCTGTGGAGCTGTGATCCACACGGGAGGTTGAAGAACGGGACTAGACGGAGTCTGAGAGGCAGGCAGTGAACGGATGATGTACGGCAGGCTCTCAGTTAGCCCTGCTCCAGGTGACACTGGGTGGGATTCACCCCACAGGCTTCTGTGTCTCCTCTCTCCCCTCTGAAGGTCGTGGCTT

							GTGCTTGGGCCTCCACTGCCACTACCGATCCTCAAAAATACAGCTCCCTTAGTCAAGCGATCTCTGTCA<br>GTTAGGAATGTAGTGAAGAAAAAGTGTGCTAATAGTCTAGAGGCTCTGGGCAGAGATTTCCCCTCAGG<br>ATAGGAGATGGACTGTCTGTGAGCTGAGCTTTGCACATGAAGGGCAGATCGCAGCATGATCCTGGCTGC<br>TTCCAACCAACAGATTGCAGGAGCTTTGAATAAATTACACGCCCTCCCTCGATTTGCTTAATGTGAATTA<br>CTATTTCGTACCAGCAACAAGTTGATTAGAAAACTATAAAAATGGGACCCTCGGACGGTCTGGTGACAC<br>CTTACACAGTGCCAATGGCTCTCAGACCTCTCCTCCCTCTGCCATAGGCCAATGGAAGTGCATTAGCATC<br>TGTCAACTCGTCAGATTTGCTCAGAAGAAGGTGTTTGGCCCTTGATCTCCTTGGTCATAGATACCCAGG<br>GAGGTCATAGATACCCTATTTCTCACATCAGGGGAAATAGGATTTAGAGAAAAGTCAGAGGTACACGTG<br>TATTATTTTTGCTGCGGCATGGTTTTCCTTTTGCAGAACGTGCAAGGCACTATTTGAAATTCATGAAGCA<br>AATGGGTTAATTTGCACCTGCAGAGTGATTGTCAAGCCAGCCAGGTGACAGTCCTCAGTGAAACTTGAT<br>TTGGAAACCCCAGCTGGGAAGGCGGGCTAGGGGACTCAGCAGGCTGGAGTTCTATGTCGGGGAGAGA<br>AAAGGGAGAATTGGTGCTTATCATCAGCGAAGAAAGCCCTACCTGGTGCTGGAGCACTTGAGATGGCT<br>TGGACCAAACAGCTTAGGCGTTATTCGATTTCGACTTAACAGCCCCAAGATGAAATGCCTTTCATTCGA<br>CGTTGTTAAACAGGGACAAGCTGGGCCTCGCTTCACTTTTGAGTTTGCCAAGTAAATGGATTGGCGACC<br>GCAGAGCAGTGCAAGACATTTTTTTTCTGCATCACTCTCCCAGTGGCAGCTTTTTAGCTGAGAGCAATA<br>CTGTCTTTACTCCTGAGTATACAGAAAGGGAAAAAAAATTAAAAAAAAAATTTAAAAAAAAAAAAAAA<br>AGGCGGCAACATCTCTTTATGTAATGCATCTGGCGCCCTCTCGTGGCTCATTCTAAGAATGCAAGTCTGG<br>GATTATTTTCCTATGGAAGATGCCTGTCTACCCAGAACTGGTAAAGGAGGGAAGAAAGTACCTATTTAC<br>GTTCATTTTACCAGCACACAATGTGTGGGACCGTTACTGGGTTCCTCCCTATCTCCTACATCTCATGAATC<br>TTGGAAAATCTGCTTCTGCCCATGACTGATTTAAAATATATTGGCATTAAGGAAAGGATAATAAAAATTT<br>GAAAGGTTAAATTTTTGACAGTGAAAGATATTTTGAACAAAATTTTAATATGCGTTCCAAGCCAATACAA<br>TGTTCATGTGATTCTCAATTCTTTCCTAATTAGAAGTTAGTCTGGTTTTTGCATCATATCCCAGAGGGAAT<br>ATTCTCTTCTATTTCTTAGCTGTGGGCACTGTTTAACATATAGCATAGTTAAAATTTTTTATTAAATTTTATT<br>ATTTATTGGGTGCGCACATACTATATGGGTGTGAATTCCTCTGTGTGCATTTTGGTCTGCTTCCTCCATTC<br>TGTGGGTCTTGAGGATCAAACTCAGATCCCCAGGCTTAGCTGCAGTTGCCTTTAACCTCTGAGCCATTTT<br>GTCAGCCCTTTAATATACAAATTATCAATAGCAAAATTAACCAGCACTTGCTTGAGGGCAGCTCTTTTAG<br>GAACAGCATCTTCAGTTCCATTTAGAAATGTGGGCGTTAACTGTAAAAACAGAAGAGACTCGTAACTAG<br>AGACATTTGTGTTAGAATCGAAGCCTGAACACCAGAATGGTAGAGTGCATCTCTATTGGGCAAACAGGT<br>TGGTCAATCTTGAAGTACAAAGTTGTTAATAGACAGGAAGGCATTGAAATTAAGTTTTTGAAATAGAGT<br>AAATAACCAATTTGCCACTTTCTCTTGATTTAAAGGTGTGATTCAGTGATGTTGGTCAGTGGAAGCTCTC<br>TCTGTCTCTCTCTGCCTCTGTCTCTCTGCCTCTCTGTCTCTCTGTGTGTATGCACGTGCGTGTGTGCATGT<br>GTGTGTAAAGAAAAGCTGTTTAACATACACCACAGTGCTTCTCACATAGATAAACTTCCAAATGATGGT<br>AAAGACACACTCTGTCTCAGGGGTCATCTGCTAGACAAGGTCCTCACATGCTTCCTTCTGTCTTCTGTG<br>TCAGCAGAACTCTGCAGAAGCCTGAACTGTGATGACTTGCGTTAGACTCGGTTCTGAGAGCTGAGAGG<br>ATGCATGAGGTGCCTGGACCAGGCTCCCCATCACTGGATAGATGTAGGCTTGAACTGACAACACTTTTT<br>TTCAGACCAACATGGCAGACTTTATAGATAAACTTCAATGAACTGCTTAGAAATATAATCACAGTGAATA<br>AGAACTATTTGCCTACTAACATGAATTAATGTCTTCATAGAGAAATACATTTTAGTTTGATCAGTATTGTT<br>TGTTTTGACTTAAAGTCTAAGTCTATAGCCACTCTTTGCTCTGCTCTTGAGATCCATCAGGGTCTGGCTA<br>ACAGATCATCCTAACACACTTCTATGCATTTTCTACCCTTATGGTAGCCCATTGAGACTGGCATAGTGAC<br>AGATGGGAAAAAATGGAGACCATAGAGAATAAATACATTGTTTGGGGTTATTAGGCTGGGAGGCACCAG<br>ACCAGCACTTATCACCAGCAATCTGGCTCTATGATATATATTCCCTCCCAATCAGTGCTGTTCTGAGCTAG

							CCCACTGGTGACCAAGCATGACAGGGGTTAGTATATCAGGAAAAGGAACACAGAAGTTGTAAGATCAACACATCAGAAGACAATGTCTGAATTCCATAGGTGCCAGTGTGTTAGTGCAGACGCAGCGCAGATCCTGAAGAATGCAGAAGGGAGGAGGGGGAGCCTAGGGTGGATCTGAGAGCATGTTTCAGGGAGCCCTGCACATTCCTTTTCGCATCACTATCTCCACAGTAGCTGAGAGAGGGACGCATTCAAGTGTCTGTTATGGATGAAGGCATTACTGTATGAATGCACAGTAGAATAGCTTTTAAAGGGGAGTGAATGGCGCATGTGCTATCGTGAGGAGGGCATTATGCCAAGTGAAACAAGTCCATCACAGAATGACAAGGACTTCAAGATTCCACATACTGAAGTTTCTAGAATAATCAGATTCATAGAAACAGAAATGAAAAAGATGTTCTTCGGGGGCTGGGGAGAAGAAAGGGAGAGTCCTATTCTAGATTTAAAGTTTCAGAGACTGAACAACAAAGAAAGTACGCTTAGCTACCATAACTGTTGTGATGGGGGATTGTTTGTTACGCAGATTTGTCACAATATAGACACGTATGGAAATAAGTGAAAATTTTTATGAAGGAATTATGGGTAAAAGGAGAATGAGAAAATGAAGAAAGGCAGGCAAGATGACTCTGTGTTAAAGGCACTCTCTGTCAAGCCTTTAGCCCGAGTTCCATCCTCAAG
MSTRG.133224.1	ENSRNOG00000047234;ENSRNOG00000059588	0.293	0.000	0.000	-7.526	down	GGAAAAGCTTAAAGAAGGGGTTAGGGGGCCTTTAATTAGAGCCACCCTTTTATGTATACCCCATGGCATGTTTAGGAATGTGCTCTTGTCTGGTATTAGTAAGCTACACATGTTTTACATTATGGAATAATATGTGACCTTAGTTTGATGCTTCTCTTTCCTTGCCTAAAAGGAAGTGGAGTGGGCAAATTTTATGTAGGAGCCAAGTCTGAGTGAGTCCTGTTGATTTATAGAGCATTTGATATCACTGGAGTGTATTTATGCTGAGATAGCAAGAAACATTCCCCACTGAACATCCAAGTAAAGGAAGCTTCTGGATAGTTGGGATGAAGGAATAAAGTTAGAGTGGAACTGGTCACAGAAATCATGGACCGGAGTAAGTTGGGAAGCCCTCTAGGATATCATTTTTCAGGAACCCCAGTCACTGCCAACTCCAGGTGGACATTCATGAGGTTTTCTCAGTACCTGTGATCAAGACAACTGACTTCTTCCTAATATAATCACAAACACCTATTCTGCTGCTTTGGAGTTTATCAACAGTTCAGAAGATGGAAGTTGGTTGGAAGGCTGTTGAATGAAGAACACACATTTGGAGCAAGGAGACTGTGGCTCTAATTTGTACCATTCTAATGGCGGCTGGGTAATCTTATTGCCATTATTGCCTCTGAGCACATGACTTTCATCATCTATTATATAAATCTGATGTAAAGTTTCCTGTTTAGAATTCTCATGAATATGAATGAACATGGAAACACTTGGAGATGAGCAAAAGTTTATGAAAGCAACAAGAGAATCTCATAAGGTACCCCTGGGCTCCATCACTCAAGCATCATGGCAATTTCAGCTTGTTATCTTTCTATTGGATGTTCATAAAAGAGAAGAAGCAGAGGGGCTGAAGTTGTTTCCATGTCTGGAATAATGATAGGGATTATCTGCCAAGGTGCAATGGAAAGACTTTAAACACAAAATTAGTGTTTATAGAACAGGCTCTGTGTTGGTTTCCATCCGTGCCGAGCTCTGGGCATGGAGATGAGGAAGATTGGTAGGTTTCCTTTTATTCATGGGTCTAATTTAGCCATCGCAGTGCATCTTCTGTGCAATGGGAAGAACATTTATAAAAGTGAATTACAAAACAGAATTCCCCTCTTCCAGTCTTCTCTCTACTATGTCTTAGTTGGTTTTATTGCTGTGAAGAGACACCATGGCTGAGGGAACTCTTATAAAAGAAAACAGTTAATTGGAGCTGGCTTACAGTTTCAAAGCTTTAGTCCATTATCGTCATGGTAGGAGAAACATGGCAGCATCCAGGCAGACATGGTTCTAGAGAAGGAGCTGAGAGTTCTACATCTTGATTCAAAGGCAGCTAGGAGGAGACTATCTTCTGCAGGCAGCTAGGGGAGAAGACTATCATCCAAACTGGGTAGAGCCTGAGCATAGGAGGAGATCTCAAAGCCCACCCCCCAGTGATACTCCCTCCAACAAGGCCACATCTCCTAATAGAGCCATTCTCTGTGGCCACGAATTCAAATACATGAGTCTATGGGCCCAGAGCTATTCAAACCACCACAGCTGGCTTCCCCAGTGTATTAGCTAATTTTTTTCTCATCCTGTGACAAAAATACCTAACTAAAGCAACTGAAGGAAGAGATAGGGGTTTTTTGGCTCACTATTTGAAGGTACAGTCCTTTGTGATGGGGAAAACACTGTAGCAGGAGGCAGATAATCACATTGTTGTCTGTAGTCAGAAAGCAGAAAAAGATGAATGTGGTAACACTGATAATCAGGGTAGCTAATTCCCATCTCAGTTACCTCCTTTTGAAAATGCCCTCATGGGCACAGTCAGTGTTGTGGTCTTGTGGTGACTCTAGTCAAATTGGAAACGAAGTATCTTGCAGCTACTGTGAGCTAGGAACCAAAAGTTTTGGAAGGCTTGAATGTATTTTTTAATGTAGAAAAGGCTTGGAATACTGAATGCAGAAAGGAGTTTCTAAAGACTAATTGGCAGAGCAATACCTCTTCAAGCCCACCAGCATAATCTCTACCACCACATATCTCACAGCTCATACTATGTTGGAGACCTTCA

							GTCACACTTAGACAACTGGCACTCAGACAAAGAAAGGGACTTTTCCTGAAATTCAGCTGGCCAAGTAG<br>ACCCAAAGCTCACCCCCACAACCAGAAAGCCATATGTGATAGCTTTATTATTTGATAAATGTGTGATAAA<br>CATATTAGTAAATGAATTAAAGAAATAGATGTCACAGTGTGACAAGATAAAAAGATTAGGGGTCACAGA<br>GACAGATTGTCTGAGGTATAATTTATTCTTCTGTTGTCTTTCACTGCCCTTCACAAAGCACTGTTGCTGT<br>GTTTTTGTGAAGCATGGCCTGAGAATTTAGCTGCTTCCCTTGATAGAGCAGTGGAACCCTTCCCTAAGT<br>GCTACCATGTTTCTACAAAGGGGTTCTTTGTTTATTTACTTTTTAACCTTTTGAGAAAGTCTGTCTATGTA<br>ACCAGGGTTGACCCAGATCTCTCTATGTAAAACAAGCTGACCTTGAACTTACAGTGATCCTTTTGCTTCT<br>GCCTCCCAAGGGCTAGGATTATAATCATGTGCACAAACCTAGTATAATTCAAGGGTTCTTACATCACAGA<br>CTATATATACAGTCATGTTCTGGTAGTAGCCAGGGCTGACAGATCTGACCTCTTCAAAGAACTTCTGGGA<br>GATATATAGAGGCAAAAAGAGAGCTACTTCCTGAAAAGTTAAGAATGTGGCACTTGTAGAGAAGATTTG<br>AGGTACATGTTGTTGCCTCTTGTCTCAGCCTGCCAGAGACGAACTTTTTTAGTTTTGAGTTGTGCAGGCT<br>GAGAATGTCTTTATTTGTATCGAATACGGGCTCCTGGCCTTGGAAAGATCTGTAGTTAATATAACAATAA<br>AGTCCATGTGTGGCCCCATGAGGATTTGGACTATGGATGTATAGAAATGTGCCCACTCTGAGTAGTCTGA<br>ACCTGCTCAGATAAACAAAGATGGCCAATAACTCTGCAAACCTAAAATCTCAAATGGGACAGGGTAGA<br>GAGGAAGCAGAATTGAACTCCTCCAGTGCTTCTCGGCTCCATATTGAGGATCTGTTGACTGAAACAAGA<br>AGTGAATGCCCCAGAGTAGGGAAGCAGTTGCAGCTGGCAGAAGCAACCTGCTATTGAAACTGATAATG<br>CCATTTTAATTAGACTAAAGCATGAATGAGAGCTCCATTAAGACTTCTCCTTGGCGCTCTGGGTCACTGG<br>GCGAGAGAATGTGTTCCTTTTACGAGCTTTGCCAAAGCCCACCAGCTGCTACCTTGCAGGAGAGTTACA<br>TCTGAGTCATTGGTTTCCAAAAGGGTGACAAGTAGGCTGTGTATGGCTGAATGAGAGGGGCCTGTCTTT<br>CCTCGGTTTCGCCAGCTTTGGCCTGGCCCACAGCCATCACAGCAATGGCCTTGATTTGTAAACCAGTTG<br>GAAGTTTCAAGAAGTTATTACGGGAACAGACTAGGGGAGAAAGTAAAAACCAAACAGCATAGAAGGA<br>GAAAGCAAGAAGGCCTGGCTGGGCTTGTGGTGGGAGGCCCAAGAGATCTGTTTTCCTGATCTTACTTA<br>CCAGCTCAATACTTTATAAGCTTCTGCATCTCATGCCTGGGGGTCAGCTCTTAATGAAGACAGTGATGAC<br>GATGCTAATGACAATAAAAAGAATGAAAGGTTCTTGTCCATTTTTTTCCAGAAAGTGTGAGCCTGGAAG<br>TGTATCACATGGGTGAGGTCACTTAGCCTTCCCTCTAACCTTGTAATAAGGGCTGTTCTATTATTTTCATG<br>TCAGAAGCAAAGATAAGGTAGCATAGAGAAGTAACTTACCTCTAACAGTCAGGAAACTAGAATTCAGA<br>GCCAAAGACTGGGCATTCAAGACAATGACTCTGCCAAGTTACTCACTAAATGTTGGGAGACCATCCTGC<br>ATAAAGAAGCCTCGGACTCTCTACTTTTTAAAGGCAAGAGACACGCATATCATATCCACTGTAGGATCTG<br>GAACAAATCCATTTTAGGCCTGGAGTGGTCTTTGTGCTCTTTAAGTACTTGTTGACACACTGAGGAATTC<br>TGTTGTTTTGAGACAGGGTCTCATGTAGACGAGGCTAGCATTGAACTCACTACTTGGTCAAGGTTGGCT<br>TTGAACTCATGATCCTTCTGTCTCTACCACTTGAGAACCTGAGATTACAGGTATGCGCCACCACACCCA<br>GATTGCTCACTGAGTTTTGAAGAGGCAGCTAACTGTGTAAACTAGATAGGAAACTTTGTTATATATGATT<br>TACTAGACAAATGCTGATCCTCATATGCATGCATTTGTTCATTCATTTGGTGAAGGAGCATCTCCTGTATC<br>AAGCACTGAGCTAGGTCTTAAAAATAAATGAGTGTGATTCCTCCTTCCTCACTTCAGTAGATGTGAGAA<br>TGCCTAGAACACAGTGGTTACACACATACATGTGTGCATTTAGCTACACAGGTCACTTTAGTCCAGCATC<br>TCCTCCCAGTTCTGCTCCTTACTGTCTGGGTACCTCCTGGGGCTAGGACGTAACACATTTCACATCATTT<br>CATCCTGGTGTTCTGTGTGTTTGTGAGGGTCGAGTAATTGATTATCAGCCTCCTGAGGCTGTCTCCGCCA<br>GGATTGTTTTTTTCTTTTTTTGCATTTTAGTGTTTCTCCAGGTGAACCCCATGCATGTTTAAGTTTGCAAA<br>GTACTGCAAAGGCGTGGGACCCAGACTCTGTCATGTGTGAGCGCTCAACAAATAATCTTCGAGTGATTG<br>CATGCATAAATTGCTGACACTGAATGGGGGAAGTGGGCCGCTTTTACTAGGGCTGGGCAGGACTCCAG<br>ATTTTCATTTTGCCAAGTCTGAGGAGACTGCGAGGAGGTGGAGTCATGTTTCTTTCCCTCTCAAAGAAA

							AAAAAACCACTTGGTGGGGTGCATTTAAATGTCCGGAAGGATAGACTCCATCACATTCATGTGGGAAAT<br>GGCGAGCAACTGAGCACATGACCTGATGGGAACTGAATGGATGAGCCAGATCTTAATGAAGGCCAGGA<br>GATAAAACAGGGCGGGGTGATGAGTTCACAGAAGGTCTGAGAACTCCTTGTAGATGGCCTGTGGCAAG<br>ATGTTAAAGCCACAAGTTTACAAGCCTGTGTGCTTGCTCTTGACACTCTGGTTCCAGGGACTTCTTGCA<br>ATTTAAATCCTTCCTTCCTCTTCCCATACCATCAATCATAGGTGGCACAGCCTTGCTATCAGTCGATTTCA<br>AACTAATAGTTTTGCTTTCCTGTCTTACTTTCTTTTCTCCTTCTCCCCTCTCCAGTATTCCCCCTCCTTCTT<br>GACCAGAGGGTTGGTGTCCAGACTTTGATGAGAGCCGGAGCTACCCTGTGGCATGGCATTTGTCAAGG<br>ACAAGGGTATGTGGGCCTTTATGAGTAATCACAGCTGCTTGTTTGGAGTCATGGTTTGAAGGAATCTCC<br>CTCCCTCAGTAGTGTGATCTTCTCCTCCTCCGAACATTCCCTTCCCACCCCTCCTCAAAAAACTTGCATA<br>AGAGCAGTTGGCTTCATATAATAAAATACAGATTGGCATCATGAACTACATAAACAAGAGGCATTTAATA<br>GACTGTGGAGAGTTCTGGCAGTGGGAGGAACAGTAGCAGCCAAGGAGACACATATGGGTGGCTTAGC<br>AGCATCCTGGACTCATAGGAAGAAGATTCCTGCTTTTAATCCCCAGGGTAGGAGAGGAGGTGGGATACA<br>GGGTTAGAAATGAGGGAGGCACAGCATCCCCAGACCACTACTATGCCCAAATATTTCAAGTAAAGTGCT<br>TAGATATTTTTAATAAACATTTACATAGTGCTGACTCTAGGGTAGACACCATTCTCTAAGCCCTTTACAGA<br>TTTCAAACACAGTTGTAGTAAGGTGTGTAATAATTTGCATTCCATGACGCTTATCTGCTGTAAGCAAATAT<br>TCAATGATTTTTGAAAGCAAATTTCCAAAGCTCTTAAACTATTGCTCACATCCATCTACAGCTAATCTCTA<br>CTCCTACTACCAGCCCAGAAAGCCATTCATTTCCTTTTTGTCCCTCTAGGTATGCCTGTTTTGGATGTCTC<br>ACACAAGTGGCAGAAAATATGGATACATTCTTTTGTGAATAGCTTCTTTCACTAAGCACAGTTTGTTTCT<br>TCTTGTTGCCAACTTGTGTTTCAGTCTATGGTTCTAGTGAGCTACATTTCTTGTATCAGTTCATACTCACA<br>GTACCTGAGGACGCTGCTTCCATGACTGGGAGCTATAGATGAGGAAGTGGAGGCATAATTCATAGTCTG<br>AGAGGTACTGATTGAAACCTAGGTAGTATGACCTTGGAATCTATGCTCTCAGAGCTTTTGCTCTGCTGCC<br>TCTCAATGAGCACAAAATAGGTATTTATGCCCATTTGGGGGAGTTTGATATGTATTCACCCCCACTTGGG<br>GGATTTCTTTCATAAAATGGCCACTACACTTCTAAAGAACATATGGCTTAATTTGTGTACTTTGATAGTGT<br>TCTCCCCCCACCTCGGCGTGTATGTGTGTTGTGTATGTGTGCATGTGTTCATGTGTGCAAATGAAGGCAC<br>GCATGTACTATGTGGAGGACAAAGTCTTTGACTTCCAACTTATTTGGGACAGCCTCTTATTGTTTGTTGT<br>TGGATACAACAGACTGGATAGTCTGGGAGCTTCCCGGGATTCTCCTGTCTCTGGCTCCCATCTTGATATA<br>GGAGTGCTGATATTGCAAATCTATACTACTGCATCTGGCTTTATGTTAGTATTGGAGATCTGAACTCAGAC<br>CATCATGCTTCTGCTATTTCCCTAGCTCCTGACAGTGTTTCTTATCAATGCTTATGTGTTGAACACATAGG<br>TAGGCACTGAGAGGAACTGAGAGACGTTCTTACCTTCAAGAAATGTAGTGATTTTGTTTAGTGGAGAA<br>GATTCACATGCATATGATAATGTGGATGCATTAGATCATAATAGCCCAATGCCTAAACACACAATACTGGT<br>ATCAAAACTCAAATGGATGATGTCAGTGGGAAAAGGTTAGAGCTTGCAATGGGAAGTGGCTTTAAAGT<br>AGTCCCAGATCCTTGTCCTAATCAACAACAAATTTTAGGAGTGGAAGCTTGCAGTTATTTTACCATCCCT<br>CTTCTGTCTGTCTGCCTTTCTGTCTGTCTGTCTGTCTGTCTGTCTGTCTCTGTGTGTGTGTGTGTGTGT<br>GTGTGTGTGTCTGTCTGTCTGTCTGTGTGTCTGTGTCTGTGTGCAGTAGCGGCTCATATGTGAAGGCCAA<br>AGTACAACATTCAAAAGTAGAGTCTCTTTCTACCATGGGTTCTTTTGATTACAATCAGATCATCAGGCAT<br>GTGAGACAATTGCTGTTCCTCACTGAGCCACCTCAACAGCTTTCTCTTTTGTATTGTACTGTCCCTTCCA<br>CCCACAAACTTTTTTCTGTAGTAAGTTCCAACTTTTAGATTCCATCTGTTTGTCATACCAAAAAGCTTTTC<br>CTGCTAAGCTGTTTTCAATACAAATTGCTTAAGGAAGGATTGCTCTCTTTGGAAACTTGTCATCGGGAAC<br>AGGAAAGAGAGAGGAAGAGGAGAGGAAAGGAGAGAAAAGAGGGGAGAAGCAGGGAGGAGAGGGG<br>AAGAGAGGGGAGGAGAATACAGGAGTTAATTTTAAATGACTCACAGCATCCGTCTTTTGGCCACATATA<br>CCTGGCAGATAGGTTCAGATATTAAATTGTCTCCAGGGTTTACTTAGCCTTCAGAAGATTACCCTAAACT

							CAATGGCTTAAAACAAAAACCAAGGGCTTGGATCATAATTCTGTAGTCACCTAGGCAGCTTTTCTCTGC<br>GTTGGTCCATTGGTTTATCAAATTTCGATTCGGCTCAGGAATCTGTGGTGAGCAGAGGATTTGGCTTGGG<br>CCTGGATCATGTACCAACTATTGGTAAAATTTTTTGTCAGGTTAGGACTGTTGGCTTATTATGTGAATTTT<br>CTTCCATGTGGCCTGTTAGCCTCTGACAGTGGGAATGCCCACAGTGTAGACAAGTTCCCTACAACAGCA<br>ACATAAGACAACTCCAGTTTCCTAGCGTTTTCCATACCTTGGCTTGTGTCATGTTTGCTATCTATTCCCAG<br>CATGTCAGATGACAAGCTCAGATTCAAGGAGTGGAGGGATAGACTCCACCTCTTGATGGTAGATCCGCA<br>GTGTATCTCAGGATATGAATACAGAGAGAACAAATTTGTGGTCATTTAATCATCCACAAACTCAGTTCAT<br>ACAATAATAAAAAAGTCAGCCAACATGTATTACATGCTTACAGTGTTCATATATGGCTCTGAGTTCTACAT<br>ATATGTTTCCTACAACAGTGCTGAGACCCGAGTAATAGTTTCCTTATTTTCAGAGGAATGTCTTGAGACT<br>CTCCAAGGAATTCATCTAACACAGTCATATAGCCAGTAAATGTGGAGGCAGGGGACCTCAGGCTTCTCA<br>TCTAGGCAGTGTAGCAGGCTCCAGAGCCTGAGGCTTTGTAAGCACTCTGTTGCCCTGTCCATTATAAGC<br>TTGATGAAACTTGCTTCATATGCCACCATATGGCTAACAGCTAATGTACTAATATCAACTGAAGACTTAG<br>GAAATGCCTGGCTAGTTGTTTCAGACACTTGAGTATATAGTGGTGTTTCATTCTCACCTCAGTCTGACCC<br>ATTAAAAACATACGTTTTATCTTCAGCTTACAGATGAGGAATCCAAGGCTCTAGGAAGTAAGTTATTAGT<br>GCAGTCTCACAGATGAGTAAGCCCTTGTGTAATTCATAGCATCCTTTCATTTTAGACTTTGTTTCAGTCTC<br>AAGTTATAGGCGTGGGGGAAGGCACTCTTAGACCTTTCCAGAAGTTAAATAAAGAACACGAATAAACA<br>TAAATGAAGAAAAAAAAAAGGTGCTAGATGTGATGTCTCATGGCAGTAATTCCTGCCCATGGAAGGCTGA<br>GGCAGAGGATCACCATGAGTTATGAGCAACTTGGGCTGATGTACTGTCCCCATGCCATAGAGCAGGAAT<br>CATTGCCCAATACAAGCGACGTAGTGGGGTTTGCAATTCAGTGACTTTAAAATGCTCCTGTACTTGCTTC<br>TTGCTCATTAGGTTCTTAGCCTTATAGAGATTATATGGATTCGTTTTAGACTGGCAGAGATAAAATGTTAG<br>AAAACAGCTTATAAAGTCTCCGTCCCTGGATGATGACCATTTAGAGCAGTGCTTCTCAACTTTCCTAGTG<br>CTGTGAACCTTTTAATACAGTTCCTCATGTTGTGGTGACCCCTAACCACAAAATTATTTTTGTTGCTACTT<br>CACAACTGTAATTTTGCTACTGTCATGAATGATAAATGAACCTGACATGTTGGACATCTGATATGCAACC<br>CCTATGAAAAGGTCCCTTGATCCCTCTCTCCATCCATGGGTTCGTGACCTACAGACTGAGAACTGCTGG<br>TTTAGAAGAACTAAAAGTAAGAGAATTGAGGAATGTTGGTATAGGGACATGGAGTCTGGGAAAATTTCC<br>AGAACTGCCAAGTAATTATTATCTTAATACTTCTACAAGAGTTGATTTTTAATTAAATGTTTGTTTGTTTGT<br>TGCATATACATACAGATGCATGCAGGATGTAGACTTGGCATGTATGTGGAGGTCAGAGGGCAAATTGCA<br>GAAATTGGCTCTCTCCTTTTACCATGTGGATGCTGGGAATTGAACTCAGGATAACAGACTTGACGACAA<br>GCACCTGTATTCAGTGAAAGTAATTGGGTGATAATTAGAAATAGTACCATAGATGATGAGTACATTGATAT<br>TTCGTAGGTGGCTCTCACTTGAGAAAGGACAGATAAATAAGATGCTATTGAAACTGACATTCTAGGGTT<br>ACAATAAACTGTTGAGGCTCCCAACTTTTCAGATTTATGCCTCTTGGATCCCCGAAGAAATAATGTAATT<br>CCAAATGAAGATAACTGAGCAGTCTCAGGAATCAAGATAATGGCCCAGTGGCCACTTAAAACCTGTGC<br>ATGAACACAATAAAAGCTGCCTTTTGACTTAAAAGAGTGGCTAGCACACAAATGAGTGATTTGCATGTG<br>GCATTCAGAGTGGTTTAGGAGAGGAGCCAGTGCCGATCATGAGAAGCCAACACGAGTGAGCCTGTCCT<br>AGAATGGCAGGCCCTAAGAAGAGTCTCCGTCTAACATAACCTTCCATTTATACATAAGTGAAAGCAAAG<br>CCTCCCAGTTCTGTAGCTCTCTTGCATTGAGGCCAAAGAAGGAAAATGAAGATGATGAGAAGGGATGT<br>TTCTGTTGATTTCTAGGGTTAGTGCCAGCAGCCGCAGGCAAAGAATACCATGGACAATGCCAACTAAGT<br>CTTTCACTGGAATAGCACACTGTTGTGCTCAGCGAGAACCATCATCTTGCCTGTCTCTTCCTATTGTCAT<br>CCTTTAAATTATGAGAAATTATAGAAAAAAATACCAGAGCACCATATATCTGGCTCTCACATAGAGTAGT<br>GTCTGCTTTGAGATGAGAGTAGGCCAGGTTATGGTAGTAGTGTTTTGTGGCAAATGCTACAAAGGGGAA<br>GAGCCCACAGCAAGAAAACACCAAAGTCTCTGGTACTTCCGCTAGCAATATATGAACACTTGTGTTGTT

							GGTTTTGTTTGTTTGTTTTGTTTTGTTTGAGACAGGGTTTCTTTGTGTGTAGCCCTGGCTATCCTGGACTCAGTGGGTAGACCAGGCTGGCCTTAAACTCACAGAGGTCTGCCTGCCTCTGCCTCCCAAATGCTGGGATTAAAGGCCTGCACCACCACTGCCCAGCTGAAAATTTGTATTATATCTGCAAAATGTCTTAAATTCCTGGGAGACAAAAAAAAAGTAGAACAGAAAATGAGAGTTCTCAAGCAGAATGTGCTAGGAGGTCAGTGATACACATGCGCATTTTCCTTTGGGCGGGGAGAGCTGTTGAGGGGTCAAAAGAAGCCCCTGTGTCACTTGAATGACTGCAGTTTCATTGACTCTACTCATTGAATGACTTGACTTGTGGACTGTAGTGCTCTGAAGTTGCTGAGCTTAACTAGAGATTAGAATCTTTTGGATGACCTGGGAGAAAGTATGGTTGGTACTGTGTGGTAAATAAATCTTGAATTTGGCTGTTTGGCTCATAAAAGTCCTTATAAATGTTCCCAAGCAACAAACATGGAAGGAGTTAAAGGCACACTTTGGAGGTCTGGATTGCGATTTCTTTTCTGTGTGTCATATATGGACAAGATGGCAGTAGGCTACATAAAGGGAAAGGACTGTCTCTTTGTGTCCTGGGAGCAGGGCTGAGATAGGAGTGGGGTTGTTCTTTGTTAACATGTGCAATGGTGTTTGTAGAGAGAAAGAAATCATAAACTTGCACTTGGCTAATTTTTTCGATTGTTGGAGTAGAAGGTCAAAGAGCAGGATGTTGCATGTACCTCAGCAGAAGCTGCTCTGCTGGGAAAAAAGAAACAAATTCCCTGTGCTCCAAATGGAGAGTATTTTCTTTCTCCTGGAACAGACTGTTTTGGGCTTTTTTCTTGCATTTTTACATTTTTTGTATGTGCAGCAACTGCTGTTTGCCTTGTCTAAATTTTAAATCATCGGATCCATCACTGCGTGAGTCTCCTTTATCATGTGTAATATTCAACATGTCCAGGCTGCTTCATATAGGTTTCCTGAGAGAGTTTCAACCCTGCTGTATGCTTCCCTTGCCATACCCTTCTGGCCCTCTGGAAATTGACATTCTGCAGGTCTGTTCTCTCCTCTATGGACTGCCATCATTATCTGACTACATTTCAATGTCTAGCATATGGTGATTAGCTCCGACACTGGGTGTTTAAGCTTGCCCTGCAGATTGATGTACATCCATGTGCACGTGCCTTCCTCCAAATGCAAACAGAGCACAGGTTATTGTATAGGTGATCATACATTTCCTATGAAACTAAGTAAAGTTTTTTCCTC
MSTRG.133687.2	--	0.407	0.038	0.045	-2.350	down	AAGACTAATGGAGTAACATACCAGGATTAATTTTCACCCCAACTTTCTTAGTGTAAGCTTTGCAAGTACTGTTGAATTCTAAATGTATATAATGAAAAGGTTTCATTGACAAAGACTGTAAATTATCATCTGCCACTGGTCCAATGATCTCGATCACTTGCTTTACTCTAAATACTCCAATTTGATGTGGAACGAATGAGCATATCACATTCTGAATACATCCTTCATCAATTTTGCCCCTTTCAGGATCCATTTTAAAGTGTGCAGTTTTTTGAAAATGGTACATCACAGGAAGTGATATGGACTGATTTTTTACAATGCACAGAATATCTGAATGTTCACCCATATGACAAGGTGCAAACGTAAGTGCTTTTCCTGGATCAAACTGTAATATGACAGGAAGTCCAGACCCTGTCAGTGCTAATTCCACTTTTTGAAGTCGATTACCAGGTTAACTGAAAGATGTGCTGGAGGTCTCACCATTACAGATCTCATGTAGTAGTAGTAGTACAGAGTAGTATCCACATGACACTGGCATAAAAAGATACCTTTTTCAGTGTGATCAAATTGAAGACCCAGACATAAATCTACACACACAGGGAAACTTTTCATGGTGTCGGAGTTATCATCTCTCAAAAATCCTTCTTTACTTCCCACAAAATCAAATCTCACAAAGAGAGCATAGTCTTGCCTATGTGAAGGTCCAACATCCTTTTTAATATCAACTACTAGCTTTGGGCTGAAACAAAATGTAATGACAATCTTTTGATAAGGGAGTAATGACCCTTCATTGGGAACGCAGGACATAAAATCGGTCATATCTATTTTCTTTATTTTGTTTAAGTAGGAAAGATTATTTAAGGCAATATCTGTCCTTTGATGAATATTTGCTCCCAGCTCTTCTCCAACACAATCATCTTGCATTATTGCCACCCAGTTTACAGTTTCAGGGCTATTGTTATACAGAAGAGCATGCTCAATTTTTGATGTTCCAAAGAACACAGAACCAAACCGTATACATTCCAATTTCCTTTCACTATTCATGTGAAACAATTCGATTATCTGTTCAACTACACGCACTTTGATGTTCAAGAATGTTTCCGGACGACCTTGCAAACTCACTCTTGCCACTTCATTTACAGTTCGGGCCTGGTCTGCACAGAAATCCACTTTGATGACCATTGATGATTTAGCCTTTACAATACCACTACTTGGAGAAATGACAATAGGTAACTGACCCTGGTAATCTGTTTTAAACATCCCTGGAGCTTTGCCACGGTTAATAATAGTAATCTCTTTACAATGCACTTTACTATTGGCAACCAATGTGCCAAAATCAACTACTGGCACAATTTCCAGTCGACAGACTGGAATCAGCCCAATTAGAGGAATATCCAGGACTTTATTTCCTACAGAAATAAAGATATGATCAAACGTGTCTTCATTTTTATTAGGATGATATTCCACCATTGCT

							GTCATCTGAAGGCCAGAAGCTAGCTCCTTATCCAGACTGGTCAACAACAATTTGAACTGTGGCTTCGATGGCTCTTGAAACCTGATCTTCTGATTCCAACGGCCCAAATTGTGTACTGTAAGTGGGAGGCGGTACACTTTCCCAGATACTGCATCCTGGAACTTCAGCTCCAACGGGTTTACCCGCAGCTGAACTTCCTTCTTGGCTGTATTATCTACATCCCGAGGGGCTACTGAGCCCCGCTCTAGATCCATTGCAGCCCTCTTGAAAGCCCGCAACCGGATGAGGACTCGACCAACTCCG
MSTRG.133865.1	ENSRNOG00000034116;ENSRNOG00000038299	0.578	0.000	0.037	-3.684	down	GTCAGAGGACTTTTCTGTGTTGGTTCTCTCTTTTCACCTTCATGAGTGTTCCAGGAATTGAACTCAAGTGGTCATTCTCACTGAAGTAGCATTTGAAAAATTTTCTTTCTGGGTACAGTGAACCACATCTGTGTATCCATCAAAGGAGTATCCTTAAGATCTGGTCTGATCCCAGGGATGTAGGCCAAAATGTCCAAGTAGTCATGTAGAGGCCCATTGATGAAGGTCAGGGTCAGGCAGCAGGGCAGCTGAAAAATTATCCTGGGGGCAGTCAAGAAGAGAGGCTGGTATGAGTCAGTCTGGCATCACCTTTAACCGGAAGGTCATTGCTACACTGATGTGTAAACAGGGGCCAGGGACAGCTTCTGG
MSTRG.133874.2	ENSRNOG00000003748;ENSRNOG00000034116;ENSRNOG00000038299	0.000	0.501	0.000	5.427	up	AACCTTAAACATCAATTTTGGTACAAAATTGTCTTTCCTACAATTTCGAACAGTCCTGTCCTCGTTCCCTCCCTTAGACACTCTTTGGGTGAATGAGATGGCTTGGTTTACCTTTGGAGCTCAAGTTCGAGCAGTACAGTGATGGACAACACAGGAAAGGATGGATCAGTGAGGAATAAACCCTGCAGAAAGGGAAAAGGATGCTGAAGGAGCATGGTGACTTCCGGAGTGATTGGTGATACATGCTGATGCTGGGACAAGCAATGATGCTGACTGTGAGCTTGCTTAGTGTCCTGATGGTGGTGACAGAGAAGCTGTATTGATCTATATCCCAGATTGGACAAGCTGCCTTGCCTCAAGCTTTTAGACCTTATGGGACAGAGAACTTGGAGACTGTGGAAACCGCCTTAGAAAAATTAATCAAATGGAGAAGTTCTACTGACCCTTCTCTTTCGTTTATTGTGATGTTATTCTGACTCAATCATGGGCTGTACTGGAAACCATTCAAATATTTGGGAAAAAAGAAATGACAATCATCAGTTGGAGGGCTGGTTCTGGACGTTTTCAGAGACATAAATAGACAACCGCCTTGTATGAAGTTACATTTGCAGTGGATAAAATTGGGATAGGATCCGGACTTAGAACAATGATAGGATCTGGATTTCAGCAAGTGTTACTGCATGTGTTAAGGTTCCTGATCTTTTATTAATTAGTCATGCTAAAATTATTTATTCTACAGTATTTAATGTAAGTTGTGTTGATTGTATAATTTCTAATTGTGTTAATGTATTGAAATCTGGTATTTCTGTTATGGTAGTCTATCAATCAACTTTTGTTTTATTGCGTATGAGTATTACAGGACCTTGGTATTCAGAAAAAAAGCTTGCAAATGCTGGAAGAAGTGAGTATGGCTTTAAGCAGAACAAGAGG
MSTRG.13397.1	ENSRNOG00000061147	0.086	0.410	0.019	2.668	up	CGAAGAATGAGACTCATCCCCAACGGTGCCCAAGCCTGATAATGCCTGCCTAACTACCAGAGACTCAGGGAAGCATTTGCACCAAGTGAGAGGGATCACACACACCAATTGTCGGTGATGGATGGAGAGCCCGTTGATCAGCAGCCAGAAGGTAGATCTGGCTACCTTCTGAAGGTAACCAATGTTACTGGCAATCGAAGAGTAACTTTGTGCATCAGGCAGGGCCCAGGAATCAAGGAATGTGTGCTGCGTGCTTTCCAAATTCAGTTTATTATCACATCGGGAGGTGAGCTCCAGGACGTCCCCCACTTTATTTTTCTGTGCTGGATGTTGCTCTCATGTTCTTATACATGCCAGGCTCTGGTGCTAAGCACTGTTTTCAGCTTCTTCTGTGCTACGTAGAAGAGAAATCAGAGGCCAAAACACCCTAAACTGCCCAGGCCACAGGGTAAGGCCATCAAGGTGTGCCCACTCTCTTCCTTCAAAATCTTTTACCAATTTATGCCGCTATTTACTGAATTTAACAAGTGGAGGCAGCTACGCAGTCTGACAGCTGTAAAGCGAATATATTTCTCAGTGCTAAACGGTCAAGATCTGGCAACATTCAGAAAAGTAAGACGGTAGAAATCTCCACGTTCTCCATTAACTCCATGGTGTTTTCCCAGCTCAGCACTGGTATCTG
MSTRG.134140.1	--	0.000	0.405	0.019	3.592	up	CACCCAGTTTCTGGAATGATGTATGTTGGTGTCTTCCAATTCAGGCACAGCACATAGTGCCAGTGAGACCTTAGGGAGTCTAATTTGAGTTTCCATTTCTTTTCCGATGAAACCTGAAAAGTGGAGAATTTTTGGTACCTCACAGTTCCATTCCAAGATCTTTGTTCTCCCTTCAGCAAATAGCTTCAAGGGCCTAAAGGAGCGTGACTACCATCATGTGACAGTGATAAACAGAGACTCAAGGAAAAGGACAAAATTCCTAACTGTAGCAGTTTCCTGTGCTTAAATAGCTTCAATCTTC

MSTRG.134209.1	ENSRNOG00000057166	0.006	1.814	0.000	5.369	up	CTCTTTCCTGAAGAAGGGCCACATCTCGGGGCTGTGGAGGACCCTGGTTCTGTGAGGCACGAGAGTTGGGGGAGGATCTCAGATTAGGTGAGGGTCGTAAGTTCTGCCAGTCGGGTGGAATTGGCCAGTCGGCAGGGATCCAGTCAGGTGGTAACTGCCAATCTGGAGGGCCTTGCCAATCTTGAGGGCTCTGCCAACTAGGTGGAGCCTGCCAACTGGGTGGGCTCTGCCATCCAGGTGTACTCTGCCAGGCCATTGGGTTTGGCCAGACAATTGGGCCAGGCCAGACAATGGGGTTTGGCCAGATCACTGGATTCTGCCATGCAACGGGGTTCTGCCAAGCTGATGGTGTCTGCCTAGCTGGGGGACTCTGACGTGCTGGTGGGGTCTGCCTGGCTGTCTGGTTTTGCCAGCCTGATGGGTTCTGCCAAGCCAGTGGTGTCTGCCA
MSTRG.134383.1	ENSRNOG00000029935;ENSRNOG00000038132	0.327	0.000	0.022	-3.851	down	ATTCACTGAGACTTAAAATAAATTGTAACAGAAGATTGGTTTCGATATGCTTTTCAAAAAATATTACTAACAGGGTTAAGAAAGAGAAAAGGGAACACACTGGATTCCCCATTGGCTTCCATACAAAATGGGGGAGCATCATTTTCAATATTATCTTCTTGCAGAGTGGAAATATTGGAGGTTACATAATCATGGTCGCACAAAGCTGATATGACATTTGGGGGGTCCTTCAGATCATCCAAAAGTATGTCATAATCTTGAAGATTTTCTCCAAAGTCACATATTGGTGTCCCATCCAAAAGGAAAAGCTCTGGTTTTTCCATGCATTGTACCATATGTTTGTCTTTTCCCCCATTGGGATCAAGCGGACTTTTCTCTTCTTCATTTTGCATCAGATTTAGACTCTCACTGAAATGATCAGAATCATAATCCACTCCCTTCCAAGATAGTATCGAATTTAGCTCTAACAAATTTCCTTCAGTATGATTCACATTCATTTTATCCCTTCAATGAGAAACACAGAATGGGGATTGAATGTAGATGCAAACCAAGGGATTACTACTTCTAAGAAACCATCATTCAGAAAAATCCAACAGACAATAAATTGAAAAGATTATGCCAGGTTCTCAACTAAGAAGCCATAAACCAATATTGCCCTTTTTGCACTTTTCAGTTACAGACTCTATAGTCTACTGCTGTAATATTTCAGAATAGATTACAGCAGTTATAACCCAGAGGAGCCCACTTCACAGTGAAATTCCAG
MSTRG.13448.1	ENSRNOG00000021108	0.000	0.333	0.011	3.719	up	AGTGTGTGTGTGTGTGAGTGTGTATAGTGTGTGTGTGTGTGTGTGTGTGTGTGTGACTCAAAACCCACAGCTCTCAAATTTTATAGTTTCCATGTTGTTAGATAAAATTACTTAAAACCTCCTGAGAGGTATTTTAATTTTTAAAAAAGACACAAACCATGCTCTACCCTAGAATGAAAGAAAAACAATGGCCATGTATAAAAGTCAGAGACTGAACTGAAAAGCCAACAGTGTTTAATCTTGTGAACAAGATTAGCTTGGTACTAACTAGAACCTCTTCAGTAGTATTGAGTCTCTCTCTCTCTCTCTCTCTCTCTCTCTCTCTCTCTCTCTCTCTTCCCCTCTGTCTTTCTCTCTCTCTCTCTCTCTCT
MSTRG.134807.1	--	0.000	0.310	0.000	5.390	up	GCCCATTCATACACTGGAACAGGAGAAAAATCACAATAAATATTTTAAAAGAATATTTAATGACAACGAAAGGAAGTTCAAGAATATCTATACATCTATGTCTATAATCTATATTATATTTATGCATGAAAAAGCAGATATTGAAATTTGTATATATTAAATACACATATATCACATGAAAAACACTGGGCACATAGAGATGAATACAATTCAAGAAGGCATGGGGGTCCTTAGGATTGATTGCTGATAGCATTCTAGCATTCCATTTCTTCTGTGAAATCATCGGCTTAGTAATTTTGTTTTTTCCTGCAACATAGCTGTTTTATTTTTCTGGCAAGAGGGAAAAGGGAAGGAAGCAGGAACGCAGGAAAGGGGTGGTAAACAGCATTCAATTCCAGTGAAACAGCCAAAACAGTGAAAATGCCATTTGCAGCTCACGAGATTGACCAGGCACGTGCGCACGCGCGCGCGCACACACACAGAAAGAGAGAGAGAGAGAGAGAGAGAGAGAGAGAGAGAGAGAGAGAGAGAGAGAGAGAGAGAGACTGAGTCAGGTACAGTGGACCATGAGACTGCAGGGGACATGAGCATACCGTTTTGTTTTGTTTTGTTGTTTTGTTTTGTTTTTTCTTTTTACTAGCTGCACTGGCTGGCTGGCACAAATACAAAGTGGTACAATGCTTATGAAAGGGAGCCTTTTGGCAACACACAGGGAAAGTCTTTGAAACGTCTATATGTGGATCCTGCAAGTCCACAGTTAAGGAGAGAGCATCCAGGATGCTAGCCTAGATTGGCTTGACAAGGAAGGATTCACTCCTCAGGGCACACAGTGTTGGTTTTAAACACAGGGAAAGAACTAGGCACCACCTGAATGTCCAACAATGCAGGAATGATTACAGAAATGTTAAGGTCCATGGATACACAGGAGCACTCTGCAGGCACTGAAGATGACGGTCCATAAGCCTGTTGAATAGCATGGTAAAGAAGTTCAACGCGTTGATTTCTGTTTGGTTTTAGTCGCTGGAAAAAGCAGGTTAAGAAACACCACATACATGAACCACTCTACAAGAATGTTAGAAGGGAAGTGGGGTCTGGGGCG

							AGAGGGAGAGAGCGGGGAGCAGAGAGCCTGGAAAAACAGCACACCAAAGAAGATAGAGCTTCTCTCTGGGTATTAGGATGGTGGGAAGCAATTAGACTCTTGCTTCTTTCTCCCACATCAGACTTCCTTCTCCTTCTCCAGACATTTCTACCATGAGTTGCATCACCTGTGCAGTAAACAAAGAGACAGAAAAAACGCTTTTAAGAAACAGACCGACCACCACCGGGAGAAGAGGCAGCCTTTAACCTCTCCAATGGCACAGATTAGTGGGATTATGCTCTAGCAAGGGGCCCTTGCTGGGGTTGGGGAGAAAGGGAGGGAAGGAGAGGACAGAAGAAGAGAGGAGCTGAGAGCAGAG
MSTRG.134961.2	ENSRNOG00000002660	0.000	0.350	0.007	3.835	up	GAAAAACCTTTCTGTCCACATGGCTCCACCTAACTTCACTTCTAAAAAAGAACAATTGGCATCTGGTTTATCTGGCATTGGCTGGATGATCCTGATGGCAGCAGGACTTCTACAGAATGTGGAGGTAGCTGTGTGCCATACAATGAAGGTCAGGGTACACAACTGTGCTGGTTGGACCTCCGGAAGGCCCTATTCCTGAAACTGTGTTTCTGTCAGCCAAAGGTAGGATTGTGGATGAATCTGACTGGGATGGGATTGGTAGAACAGATTTCCAAGCTGGTTAGTCTTGGGGCCCCCAAAGGTTTCTATGGGGAAGCAGGATGCTTTTGGAATCCCTCAAGAGGCCTCCAGAGTGAAGAGAGAACCCAGAACTTTAAAAAAATTTTATTCTGACACTTTTTCTATTTAAAACATGGATATATAGC
MSTRG.135279.1	--	0.000	0.455	0.031	3.453	up	GGAAAATATCATCCTGAGTGAGATAACCCAATCACAGAAAGACTTACATGGTATGCACTCATTGATAAGTGGCTATTAGCCCAAATTCTTGAATTACCCTAGATCCCTAGAACAAAGGAAACTCAAGACGGATGATCAAAATGTGAATGCTTCACTCCTTCTTTAAATGAGGAAAAAGAATACCCTTGGCAGGGAAGGGAGAGGCAAAGATTAAAACAGA
MSTRG.13565.1	--	0.000	1.207	0.000	5.757	up	GTACTTTCTATTTTTGCCCTTTTTGTTCTGGAGGAGATGTCTTACCCATCGCTTTCAGATGAAGATCTGGAAGTGCAGGAAATCCGAGGACTAGTTCATCAGCTCCCCATCGGTCTGAGCATTGATTCAGAACCATGCAGTGAACAGCAGGCTGCGTATCATCTTTTCTACCACATGGCTGACATCTGGCACACTCAAGCCACAATGCCTCCAAGTATTTGCCTCTTGCTTTGTGCCTTAGCGTCCTCCGTGTGAAAATAAACTTTTATCTACTCTATCTGTAGAAATCCTGTGTTTTGAGGTGACATACAGAAATATTCACTCTGATGAGGAATGGCGAACTTCTTCTAAATACACTCATTGCTGATGGTTGCTCGTTTATTCTGTGTGAGGTGTGAGGGACTGAAGACAAGCACTCAAAATTCCTCTGCAAACCATTTGTTCTCTTGAATTTTCCTCTGCGTCCCATTTTTTTGTCTTTTTTTTTTT
MSTRG.135682.1	ENSRNOG00000060970	0.000	1.311	0.000	5.787	up	GTTGCGTCTTCCTTGCCGGACGAGGTGGGCGCGACAAAGTGGTGGCCCGTACGGGGACGAGGACATCCTCGTGGATCTCAGAACTCTTCAGCTTCAGGATGAGCGACGTCGGAAAAGTTCCCGAATCAAGAAAATCCAATTTGGGTACCTGAGCGCTTGACTCGAAAATTGCCTTCTGCTCTTCCTGAAGATGAGACTACGAACCCTACTATTGCTACTGGGAATGATAATACAGGTTAATTCGATCACCCTGTGGGCTATTGCCAGATCCTGGCCAGTACCCATGCCAGTCCATAGCAATTCTACTGTTTTACCAACCTTTTTCTCTACCTCCTGTTTGCGTGATGTTCCTTGTATAGTGCCAACTGGAGAAATGCCCCAACGGTATGCCGCCACTAACGTTTTTCTGTTGCATACCTTATGTTTTACCATTAAAGACTCTTCCCTGCCCTGTATT
MSTRG.1357.1	--	0.272	2.030	0.000	3.466	up	CAGGCTGGATGAATACTCCCAGGAGACAATGTGGACATCTCAAGACAGGCACTGGACAATGGCTGAAGACTTGTCTGAAGACTGGAAATATTTTGAAAATTCAGAGAGAAAAAAAAGGCCCCTCTGACTACACCGTACTCTAAGGACAGTTTATGCAGGTGATCCTGTCCACCTAATACAGTACATGATGTGGAAGGACAAAAGCACCTGCAGGAGCTGTGGTGATGACTCACAGGATAGAGCATCTGCTGCTCTCTCAGAAGAACCACATGGACCCTAACAATTGGGTGCTTCCTACACAAGAAGTCACATGACCACAGAGTGTCATTTGTCAAAGAACCAAGAACTTGTTCAAAACTCTACAGAGCATCCAATCTATCACGTGGATATGCAATAATGAGAGCCTTCTGAAGATGCCTAGACAATCCTGGGATGAGTAACAGTCTTTTGTGGAACTGAAAACTGAGTCCTAATGACCCTGGCACACTGATGTGTAAACAGCACAAGAGATAGAGTCATAGCTGAAGACCGTCCAATC
MSTR	--	0.061	0.668	0.000	4.053	up	CAGGCTGGATGAATACTCCCAGGAGACAATGTGGACATCTCAAGACAGGCACTGGACAATGGCTGAAG

G.1357.2							ACTTGTCTGAAGACTGGAAATATTTTGAAAATTCAGAGAGAAAAAAAAGGCCCCTCTGACTACACCGTACTCTAAGGACAGTTTATGCAGGTGCTTCCTACACAAGAAGTCACATGACCACAGAGTGTCATTTGTCAAAGAACCAAGAACTTGTTCAAAACTCTACAGAGCATCCAATCTATCACGTGGATATGCAATAATGAGAGCCTTCTGAAGATGCCTAGACAATCCTGGGATGAGTAACAGTCTTTTATGGAACTGAAAACTGAGTCCTAATGACCCTGGCACACTGATGTGTAAACAGCACAAGAGATAGAACCAGAGCTGAAGACCATGCAATCCAGAACAAAGAAAGTCAGAAATGGCCATTCCAGAAGTGATATCCTTTCTGACCAGGACTTACCTAGAGAAGATGCTCCAGTTGCTGTGGCCCATTGCTAGAAAGGTCAGCATAAGCCTGCAGCCCCCCTGGCAGGAAGCTCAAAATACACTGCCCAGAACTTACTCAACATTCCCCAGAAGTGCTGTCTTTGTCCATCATTACTTTGAGACGAAAGGCCAGACTCCTTCACTCTAGTCTCTCCACAATCGACGTTCACCCTCCCAAAACGCTTGCGAAGACGGGAAAACATGTCTTAGCTTGTAACCGCCAGACTCAGACTGAGAGAAACTAGGGCACTGGTTGTCACTACAACACTGGTGACATCACAGAGGATGCCAGAACTCTCTAGGAGACAATAGAATGTCCAAGAAGGGACAGCTGATGTCATGGGGAGCAGACTTTGCCTCCTGCTAATGTTCTCCCTGTACTGAGCATAAGCAGAGGTGCCCTTTCCAGGAGACTTTCCTGGGGCAGAGCCACTGAGCATCTCCTGCTTCCAAAGCCAAATTTTCCTCAAGGCTTGATTATAAAAACCTTAGTAATTCTTATGTATCTAAATGAATGATTCCTTCCATATCTTGCCACAAAATGTCTCATCCTAACTCAAATTGTCCACATTCACCAATGTTAGCCATTAAAACTTCCTGAGATTTCATACCAGCTTGAATATGCAGTCAAAAGTTCTCTTGTCTCATTCTGTACAGGTGCTTTATTTCACATCAAGAAATAGTCTGCATGGTAGGTTACAACATGGGTGTGTGTTAGGGGAACACTCATGAAGTCATGTGCTTAGCAATCGACAATTGCCAGAAAATTCCTTAGTTGAAAGAGTTGAAGTCTTTATGGTGCTAGGAACAGTGTGGAGACCAGTTGGCAGTGGAAAGTGCAATATTGGACTCACCAATGAAGGGAACCTCATGCTGCTTGTTGGTGTCTCATCTCTGCGCCAATGTCGCCAAAGGAGCACTGCTGACAGGCCTTCTATCAGAAGCTGAGCTTCTATCAGAAAAATAAAATAGTCAAGAGATATAGGGGGTGCAGATAAAGGTGTAGGACGAGGCTATACATATCACTACGTGGATAGAGCAAGGACCCAGCACTTGTGCTCTGGGTCATGGGCTTCCCCACAAACACAAAAGCATCTGTCACAGTAAAAAAATGGATACTGTATTGAGTGCACACACTAACACTGATTGATCAGATCCATACCCCAGATTTTGGGGGCAGATCCATGGCCCCAAATATCTTCCTCTCAACTTATACAGCAACAAATAAGAAAGAAAGAAAAAAGTCAAAACAAAAAGCTCATGCTTCTCATATGAGGATTGCAGGAAGTGTGATCTGGTGGTCAGTTCTGATTAGGCCATTTTAGATAGAACAGTGCACAGTGATGCTTAATGTGATGGGTCCTATATAAAGCTTTCTGCAATATTGGAATTTTAACAAGCCTCATCCAGAACATACAAAACAGGAAAGGATGTGATCACCATGTCCTTGCTCTCTATCAGGTTATTTTTCTGTGTCCGTGATTGTCAGGCATAGACAGCAACAATCTGAAATAGCTGGTAGACATGGAACAAAGTGGCTAAAACTGTAGTTGTGGGTTACACACCTCAACTGTCAAAGGCTAATGCTGTCCTCACAGTCCCTGGAGGCCACTCTTGTAAGACTGTCTACTGGAAAGTAGAAGCAGGTTTATCTGAGCTCAAAGTGAACCTGGCCAGCTAGGAAGAGGGCAACATGGGATAGTTGAGACCCTGTCTAAACCAGCACAGAAACCCACACTTCCTTCAAAGCATCATCTATGAATTCTTGATTATCTACCATTCTCTCCCTACCAGTCTTTCAGACTGGAATTGTAGAAAAGAACTGGAAATGATCTTCCTATTCTGAAGACCTTGATCTCTCTTTCCTTAGGTGCACATTAAGAACTCATGCAAATGGGTATGACCATCTGAACATCTTAGTATCATCTACTCAGTATATCCAGCATAATACAGTGCCTATGGCAATCATCAAGCTTCCTTGAGTTTTGCTGACCCAGCAAGAAGTATGAAGTACTGAGGTCAAACCCCAGTGATCCTTTCAAGGGTTTAGAAGAAAGCCATCAGAGAAACTCCAATTCTTTGTCCTTAGATTAAATCAAGCTGATACAATGTGAACCTGGTGGGCTATTTAACCATGCATCTTTCTGCCAACCCCACCTTTCTGAGTTCAGTCCATGTGACCTGCATGCGGAGAATGCATAGAGTGTACATAGACCACAGTGAGTCCTAAAGAGGACAACAAGCCTGGAATCCAGTGTCACTGCAAGGAGTTTGGTCTTCGCTTAAGAGACCTCCTCAGTGGGTTTCAGTTCTCCCTCTACTCTATAGAGACCAAGAGATGTAAGCAGCCAGCTGTTCCCTATGCCTGCTGACCCACGCAATAAATGCAGTAAATTTTAATTTCT

							GTTCAATTGTTCAGTAACTTACATACACTTGCATATTGCTGTGGAAGTGATGGGGTTTCGTGATCAGCATTGCATTTAACAATGCATAACCAATCATCCCTCCTAGAATGATTCTGTATATAGAGAAAAAAAATTTATTAGAATGGCTTATAGGCTGTGGTACAGCTAATTCAACAAGGAATGGAAGGTCTAAAACGCAGTAGTTACCCCTGTTGAAGCCAATCCCCCTAGCATAGCTGTAGCCATTTTGTTCCATGCCGGCTAGATAGTTCATATTCTTGCTGTAATATGTCGCATATCATTGTTGTCTGAAAATAATTTAACTCACAGCAGGGTCAGGAATGATCACATAACTTGTGTATGTTTTTTTTATCGGTTATTTTGTTTATTTGGATTTCAAATGTTAACCCCTTTCTAGTTTCCCCTACAATAACCCTGCATTCCACCCCCTTCCCCAGCCTCCTCGAGGGTGCTCCCCCACCCACCCATTCCTGAACCACTGGGAGGAAGTTGTTTATGTTGTGAGGTTTCTTAATCTTAAATTTCATAATTATAAGCTTCATGTACGACCAAAAAATTAAAGGTCAATATGAGGGCTGCAGAGATGGCTCCATGGTTAAGAGCACTGACTGCTCTTCCAGAGGTCCTGAGTTCAAATCC
MSTRG.1357.3	ENSRNOG00000052999;ENSRNOG00000058047	0.006	0.326	0.000	4.986	up	CTAGGAGACAATAGAATGTCCAAGAAGGGACAGCAGATGTCATGGGGAGCAGACTTTGCCTCCCTGCTAATGTTCTCCCTGTACTGAGCATAAGCAGAGGTGCCCTTTCCAGGAGACTTTCCTGGGGCAGAGCCACTGAGCATCTCCTGCTTCCAAAGCCAAATTTTCCTCAAGGCTTGATTATAAAAACCTTAGTAATTCTTATGTATCTAAATGAATGATTCCTTCCATATCTTGCCACAAAATGTCTCATCCTAACTCAAATTGTCCACATTCACCAATGTTAGCCATTAAAACTTCCTGAGATTTCATACCAGCTTGAATATGCAGTCAAAAGTTCTCTTGTCTCATTCTGTACAGGTGCTTTATTTCACATCAAGAAATAGTCTGCATGGTAGGTTACAACATGGGTGTGTGTTAGGGGAACACTCATGAAGTCATGTGCTTAGCAATCGACAATTGCCAGAAAATTCCTTAGTTGAAAGAGTTGAAGTCTTTATGGTGCTAGGAACAGTGTGGAGACCAGTTGGCAGTGGAAAGTGCAATATTGGACTCACCAATGAAGGGAACCTCATGCTGCTTGTTGGTGTCTCATCTCTGCGCCAATGTCGCCAAAGGAGCACTGCTGACAGGCCTTCTATCAGAAGCTGAGCTTCTATCAGAAAAATAAAATAGTCAAGAGATATAGGGGGTGCAGATAAAGGTGTAGGACGAGGCTATACATATCACTACGTGGATAGAGCAAGGACCCAGCACTTGTGCTCTGGGTCATGGGCTTCCCCACAAACACAAAAGCATCTGTCACAGTAAAAAAATGGATACTGTATTGAGTGCACACACTAACACTGATTGATCAGATCCATACCCCAGATTTTGGGGGCAGATCCATGGCCCCAAATATCTTCCTCTCAACTTATACAGCAACAAATAAGAAAGAAAGAAAAAAGTCAAAACAAAAAGCTCATGCTTCTCATATGAGGATTGCAGGAAGTGTGATCTGGTGGTCAGTTCTGATTAGGCCATTTTAGATAGAACAGTGCACAGTGATGCTTAATGTGATGGGTCCTATATAAAGCTTTCTGCAATATTGGAATTTTAACAAGCCTCATCCAGAACATACAAAACAGGAAAGGATGTGATCACCATGTCCTTGCTCTCTATCAGGTTATTTTTCTGTGTCCGTGATTGTCAGGCATAGACAGCAACAATCTGAAATAGCTGGTAGACATGGAACAAAGTGGCTAAAACTGTAGTTGTGGGTTACACACCTCAACTGTCAAAGGCTAATGCTGTCCTCACAGTCCCTGGAGGCCACTCTTGTAAGACTGTCTACTGGAAAGTAGAAGCAGGTTTATCTGAGCTCAAAGTGAACCTGGCCAGCTAGGAAGAGGGCAACATGGGATAGTTGAGACCCTGTCTAAACCAGCACAGAAACCCACACTTCCTTCAAAGCATCATCTATGAATTCTTGATTATCTACCATTCTCTCCCTACCAGTCTTTCAGACTGGAATTGTAGAAAAGAACTGGAAATGATCTTCCTATTCTGAAGACCTTGATCTCTCTTTCCTTAGGTGCACATTAAGAACTCATGCAAATGGGTATGACCATCTGAACATCTTAGTATCATCTACTCAGTATATCCAGCATAATACAGTGCCTATGGCAATCATCAAGCTTCCTTGAGTTTTGCTGACCCAGCAAGAAGTATGAAGTACTGAGGTCAAACCCCAGTGATCCTTTCAAGGGTTTAGAAGAAAGCCATCAGAGAAACTCCAATTCTTTGTCCTTAGATTAAATCAAGCTGATACAATGTGAACCTGGTGGGCTATTTAACCATGCATCTTTCTGCCAACCCCACCTTTCTGAGTTCAGTCCATGTGACCTGCATGCGGAGAATGCATAGAGTGTACATAGACCACAGTGAGTCCTAAAGAGGACAACAAGCCTGGAATCCAGTGTCACTGCAAGGAGTTTGGTCTTCGCTTAAGAGACCTCCTCAGTGGGTTTCAGTTCTCCCTCTACTCTATAGAGACCAAGAGATGTAAGCAGCCAGCTGTTCCCTATGCCTGCTGACCCACGCAATAAATGCAGTAAATTTTAATTTCTGTTCAATTGTTCAGTAACTTACATACACTTGCATATTGCTGTG

							GAAGTGATGGGGTTTCGTGATCAGCATTGCATTTAACAATGCATAACCAATCATCCCTCCTAGAATGATT<br>CTGTATATAGAGAAAAAAAATTTATTAGAATGGCTTATAGGCTGTGGTACAGCTAATTCAACAAGGAATG<br>GAAGGTCTAAAACGCAGTAGTTACCCCTGTTGAAGCCAATCCCCCTAGCATAGCTGTAGCCATTTTGTTC<br>CATGCCGGCTAGATAGTTCATATTCTTGCTGTAATATGTCGCATATCATTGTTGTCTGAAAATAATTTAACT<br>CACAGCAGGGTCAGGAATGATCACATAACTTGTGTATGTTTTTTTATCGGTTATTTTGTTTATTTGGATTT<br>CAAATGTTAACCCCTTTCTAGTTTCCCCTACAATAACCCTGCATTCCACCCCCTTCCCCAGCCTCCTCGA<br>GGGTGCTCCCCCACCCACCCATTCCTGAACCACTGGGAGGAAGTTGTTTATGTTGTGAGGTTTCTTAAT<br>CTTAAATTTCATAATTATAAGCTTCATGTACGACCAAAAAATTAAAGGTCAATATGAGGGCTGCAGAGAT<br>GGCTCCATGGTTAAGAGCACTGACTGCTCTTCCAGAGGTCCTGAGTTCAAATCC
MSTRG.1357.5	ENSRNOG00000048796;ENSRNOG00000048976;ENSRNOG00000052999;ENSRNOG00000054246	0.064	0.676	0.000	4.001	up	ACAAGGGAACCTGCATGGGGAGAATGCATAGAGTGTACATAGACCACAGTTAGGCCTAAACAGGAGAA<br>CAAGCCTGGAACTCAGTGTCACTGCTAGGCGATTGGTCTATGCTTAAGAGGCGTCCTCAGTGGATCTCA<br>GTTCTCCCACTACTCTATAGAGACTAAGAGATGTAAGCAGCCAGGTGTTCCCTATTCCGGACATCAGGT<br>GCTTACATGGACCACGGAGATGGCTTCTCCAGGATTATCATCAGCTGAACAGGGTACAGCTTGGTTTCA<br>GGACCCTCACCCCTGTGGGTTCGAACTCAGATGATAAATACACCATCCACAAGACTTGAGTGATTGGAG<br>ACACTCAGATGTCCTACCCTGATACTCTAGGCCAACAACTTTGGATAAAAACACATTTGCATGGAGGAC<br>ATGGTCAATAGACTTATCAGTTCCCCTATTTGAAATACCAAATGCCCGAGAAGTTCCAATAGGTTACAGG<br>ATGAATCTTGGTCCACATGTTCCAAATACTTTTGGTATTTTAGAAACACGTTTGTAACACACATCTCTAAT<br>ATATAAAGCTAACGATGATCCTGCCAGTTAGAAGAAATCAGAGGAGGTCTAGAACATAAGGTTATTGCC<br>TGGAGCACAATTCTCTCCCTGTTCCTACTGTCAGATATCCACTGTATTTAAAGAAGCAATGATAGTGAAG<br>TACATTGCTGCCCTGTCTGAACTCAGAAAAGGTGGACCCTCCATGACTCCTGATGGTGTTTTTATATCAA<br>TGTAACATACCAAATGAAGTGAAAAGTGAAGGCCAGAAGTTCACAGTATAATAGCATTGGCCTTACCAC<br>ATCCTCATTCCCCAGAAGTGCTGTCTTTGTCCATCATTACTTTGAGACGAAAGGCCAGACTCCTTCACTC<br>TAGTCTCTCCACAATCGACGTTCCCCCTCCCAAAACGCTTGCGAAGACGGGAAAACATGTCTTAGCTTG<br>TAACCGCCAGACTCAGACTGAGAGAAACTAGGGCACTGGTTGTCACTACAACACTGGTGACATCACAG<br>AGGATGCCAGAACTCTCTAGGAGACAATAGAATGTCCAAGAAGGGACAGCTGATGTCATGGGGAGCAG<br>ACTTTGCCTCCTGCTAATGTTCTCCCTGTACTGAGCATAAGCAGAGGTGCCCTTTCCAGGAGACTTTCCT<br>GGGGCAGAGCCACTGAGCATCTCCTGCTTCCAAAGCCAAATTTTCCTCAAGGCTTGATTATAAAAACCT<br>TAGTAATTCTTATGTATCTAAATGAATGATTCCTTCCATATCTTGCCACAAAATGTCTCATCCTAACTCAAA<br>TTGTCCACATTCACCAATGTTAGCCATTAAAACTTCCTGAGATTTCATACCAGCTTGAATATGCAGTCAA<br>AAGTTCTCTTGTCTCATTCTGTACAGGTGCTTTATTTCACATCAAGAAATAGTCTGCATGGTAGGTTACA<br>ACATGGGTGTGTGTTAGGGGAACACTCATGAAGTCATGTGCTTAGCAATCGACAATTGCCAGAAAATTC<br>CTTAGTTGAAAGAGTTGAAGTCTTTATGGTGCTAGGAACAGTGTGGAGACCAGTTGGCAGTGGAAAGT<br>GCAATATTGGACTCACCAATGAAGGGAACCTCATGCTGCTTGTTGGTGTCTCATCTCTGCGCCAATGTCG<br>CCAAAGGAGCACTGCTGACAGGCCTTCTATCAGAAGCTGAGCTTCTATCAGAAAAATAAAATAGTCAA<br>GAGATATAGGGGGTGCAGATAAAGGTGTAGGACGAGGCTATACATATCACTACGTGGATAGAGCAAGGA<br>CCCAGCACTTGTGCTCTGGGTCATGGGCTTCCCCACAAACACAAAAGCATCTGTCACAGTAAAAAAT<br>GGATACTGTATTGAGTGCACACACTAACACTGATTGATCAGATCCATACCCCAGATTTTGGGGGCAGATC<br>CATGGCCCCAAATATCTTCCTCTCAACTTATACAGCAACAAATAAGAAAGAAAGAAAAAAGTCAAAAC<br>AAAAAGCTCATGCTTCTCATATGAGGATTGCAGGAAGTGTGATCTGGTGGTCAGTTCTGATTAGGCCATT<br>TTAGATAGAACAGTGCACAGTGATGCTTAATGTGATGGGTCCTATATAAAGCTTTCTGCAATATTGGAATT<br>TTAACAAGCCTCATCCAGAACATACAAAACAGGAAAGGATGTGATCACCATGTCCTTGCTCTCTATCAG

							GTTATTTTTCTGTGTCCGTGATTGTCAGGCATAGACAGCAACAATCTGAAATAGCTGGTAGACATGGAACAAAGTGGCTAAAACTGTAGTTGTGGGTTACACACCTCAACTGTCAAAGGCTAATGCTGTCCTCACAGTCCCTGGAGGCCACTCTTGTAAGACTGTCTACTGGAAAGTAGAAGCAGGTTTATCTGAGCTCAAAGTGAACCTGGCCAGCTAGGAAGAGGGCAACATGGGATAGTTGAGACCCTGTCTAAACCAGCACAGAAACCCACACTTCCTTCAAAGCATCATCTATGAATTCTTGATTATCTACCATTCTCTCCCTACCAGTCTTTCAGACTGGAATTGTAGAAAAGAACTGGAAATGATCTTCCTATTCTGAAGACCTTGATCTCTCTTTCCTTAGGTGCACATTAAGAACTCATGCAAATGGGTATGACCATCTGAACATCTTAGTATCATCTACTCAGTATATCCAGCATAATACAGTGCCTATGGCAATCATCAAGCTTCCTTGAGTTTTGCTGACCCAGCAAGAAGTATGAAGTACTGAGGTCAAACCCCAGTGATCCTTTCAAGGGTTTAGAAGAAAGCCATCAGAGAAACTCCAATTCTTTGTCCTTAGATTAAATCAAGCTGATACAATGTGAACCTGGTGGGCTATTTAACCATGCATCTTTCTGCCAACCCCACCTTTCTGAGTTCAGTCCATGTGACCTGCATGCGGAGAATGCATAGAGTGTACATAGACCACAGTGAGTCCTAAAGAGGACAACAAGCCTGGAATCCAGTGTCACTGCAAGGAGTTTGGTCTTCGCTTAAGAGACCTCCTCAGTGGGTTTCAGTTCTCCCTCTACTCTATAGAGACCAAGAGATGTAAGCAGCCAGCTGTTCCCTATGCCTGCTGACCCACGCAATAAATGCAGTAAATTTTAATTTCTGTTCAATTGTTCAGTAACTTACATACACTTGCATATTGCTGTGGAAGTGATGGGGTTTCGTGATCAGCATTGCATTTAACAATGCATAACCAATCATCCCTCCTAGAATGATTCTGTATATAGAGAAAAAAAAATTTATTAGAATGGCTTATAGGCTGTGGTACAGCTAATTCAACAAGGAATGGAAGGTCTAAAACGCAGTAGTTACCCCTGTTGAAGCCAATCCCCCTAGCATAGCTGTAGCCATTTTGTTCCATGCCGGCTAGATAGTTCATATTCTTGCTGTAATATGTCGCATATCATTGTTGTCTGAAAATAATTTAACTCACAGCAGGGTCAGGAATGATCACATAACTTGTGTATGTTTTTTTTATCGGTTATTTTGTTTATTTGGATTTCAAATGTTAACCCCTTTCTAGTTTCCCCTACAATAACCCTGCATTCCACCCCCTTCCCCAGCCTCCTCGAGGGTGCTCCCCCACCCACCCATTCCTGAACCACTGGGAGGAAGTTGTTTATGTTGTGAGGTTTCTTAATCTTAAATTTCATAATTATAAGCTTCATGTACGACCAAAAAATTAAAGGTCAATATGAGGGCTGCAGAGATGGCTCCATGGTTAAGAGCACTGACTGCTCTTCCAGAGGTCCTGAGTTCAAATCC
MSTRG.135743.1	ENSRNOG00000025705;ENSRNOG00000025730;ENSRNOG00000037707;ENSRNOG00000037709;ENSRNOG00000043128	0.067	0.435	0.025	2.857	up	GATGGAATTAATAGCATCAGGTCGTGATTCCCGAGATTCATTGTTCTTTGTGTGCATTACAAAAAAATGTTTAAAGCCAAGAAGACTATGAAGATTCCATAATTTCTAAAAGATGACCTAAGAAAGCATAGGACACCATTAACAACAAACTAGTGACTATTCTTGGACACATGAAAAGGAGGAATTGAATAAGAATACTTGAGCGTTTGGAAGTCTGATCATTAATTCATTCTTCTAAGCCGAAGAGTTGCTATACATTGTCAAGTGTCCCTGGGAAGTAATAATAAGAAACTGCCCTTTTTTGATTCACGAAATAGTCCATCAACTCCAGAAACCTTGTGCACTCTATGAATTAAGACATTTGGTGTGCAGTTATCAGCTGCATGCCAGAAGAGGGCATCAGATCTCATTACAGATGGTTGTGAGCCACCA

MSTRG.135774.1	ENSRNOG00000011729;ENSRNOG00000024135	0.584	0.000	0.049	-3.592	down	GGGTGAGAATTTTTTTAACCATTATTCCTGCTATTTGGTAGACTCAATGTATCCCAGGAGAAAGTCTTCAGTATTTAAAAACGTGTCCAGGAGAATGCAAGCCTCAGGATGATGTCAGTGTCCTCTGTTTCCCTGGACAGCGTTTTTCTCTTGATCATGAGCAGCGTCGAGCACAGTGAAGACACTCAGTACTTCTGCGTAAATGTACTGTTTCCTCTGTCTCAAGTTCAACGGAAACTTTTAGCACAATGGATCCCACGTTCTCTCGCTATGTACCCAGACACTCAAAATCCTGAAATACCGGCTCTATCTCTAACCTGTCAGATATTTTGCTCACAAGCTCCATCTTCC
MSTRG.1359.1	--	0.111	0.397	0.005	2.526	up	TTGCTGGTCTTTATCTTTCTTGTTGAAGTCAACCAAATATTTCTGGTTCAGTGCACACTCAAGATGTCCCTCCTTGCTCTCCTGCTTCCGTGGGACCAACTTCTTCCTCCATGTGTTCTCCATCAGGAGCTGGCTGAGCAGGTTTCTGGAAATACAGTCTGCATAGTAAGATCATGCTGCCCCTTTCTCCCATTCCTACTCCCAAGTCCCACTAACTCTACCAGGTCCCAGATACCCATGATGACTTAATAAAATACATCTCGATACACATAATGCTGCTGCACAAACCACAAAGAGTTAATTCGGGGAAGAATAAATTGTTTGCTTGGCCTGAGTACCAATTAGTGTCCACATCTCTCAAAAAGAACATGGATCTATAACTATCCATGAAAGCCCAATGCATGGGGCCAACATCAATTAGTAGTTGGCAAACACCCGCAAAAGAGGTCAGTAGAACCAAGAGAAGGTCATGCTAACCCTGTGGTCCACGCACTGAGTGTTGTAAAACCTGGTATGACCCCAGAGGCCCAGGTCAGCCTAATAACGTTCTGCTGACGTCAATCAGTGCTGTTCTATCTAGAGCCTTCTGCAGATGCCTAGACAATCCTGGGATGAGTAACAGTCTTTTATGGAACTGAAAACTGAGTCCTGATGACCCTGGCACACTGATGTGTAAACAGCACAAGAGATAGAACCAGAGCTGAAGACCGTCCAATCCAGAACAAAGAAATCAGAAATAGCCATTCCACAGGTGACAGCCTTTCTGACCAGGACTTACCTAGAGAAGGTCTTCATAATATGGATTTGGCCTGAAGTGTGGCCTGGCCCCAAATGATAGAATACAATGAACATCACCATTTAGGAAGATATGAACTCAGGGTGGGTCCTGAACTACACCAGTCTTGGAAGGACACCTTCTGAATTCTACATATTGGACCTTCTTCAGCTTCAGAAACATGTTTTTCTGCGTCCACCACACCAGAATATCGGCATCCAGATGGAGGGTTCCCTGGCTCCCTTTATTCAATCAAGAGGAAATCTGCAGTCCAGCCAGTTCAGCTATCAAGAGCCTAGGCCACACCTCCCCATGCTCCCCCACCCCCAACGCACCCAGAATCCTGTGATTTCCTTTTTCCTGTCCTGATAACAGACCATATATCAACCCAAAATTATAATCTGCACAACGAATACAAATATACAGGCAAACGTGGCCACATAAGCAAAGAACTGTGCTGTTGACAGTGGGGCTCCCAGAACAAAGCATTCACATCACCATGAAAGTGTTACAGGTAAATACTCAGGCCAAGACATAGACTCGTATATTCCAGGTCTGGAGGGAAATATAAACATATAACGGTTGTGCATATGCATTTGGATGTATGCACACACAGACTCACAGACTGAGACACACCCACAAAAAAAAAAAGAGAAATGTAAACAGAGTCAGAGAAAGAGAAAAGCAAATATTGAAACAGAGAGCACTGTGAGAACCAGGAGAAATGCATGCACCATTGCTGTGTCCGAGGGGCTCCCACAGACACACAGATAATAACAAACAGGCCTGAGCCTGACAACTTCTAGTCAAGCATTGAGATGAACATTTTGGGACCAGGCCCCTCAGGCTGCTGCCTGGATCTTCCAGCACTCAGTCCCCTGCCTAGCAAGTACAGAGACTCACCATAAACTCACTGCAATTACAATCTTGCAAAGCCACCAGCTGTCAAGGGCTTTGCAAATGCACACTAGACACTCACTCTCCCTGACTTTATCCCCGAATTCTTCATTCAGACCACAAGTTCTGATTTTCAATAAATGGAAGCAGATGAGTCCATTTTCCATCTCGCTCCTCAGGAAGGGTCAAGGTTCTGAATCTCCTCTATATGGGAGGTGAGGTTTAGCACAGGGTGGTTAGGGAGGCACAGCTTTCTAGAACCCAAAACCTTAATAGGAGAAGATGAAGGTGACCTTGCAGACCCAGGAATGAAACAAGAGCATTTGGAGCGATTCATACCTATTGTTCTTGGATCTCTCTGTCAGAAACC
MSTRG.135900.1	ENSRNOG00000037661;ENS	4.375	0.000	0.000	-8.586	down	ATTAATGTTTATTATACAAAGGCTATTGAGATTTGCTGCTGGGGTAGCGTAAGACATAAACCAGAAATGAACAGAGAACTCATAAACAGACCAACAGAAATGCGCTCACCTGACACTTGGTAGATGGCAGAAGGAAGCATAAAGAGATGAATGCTGGTACTTCTCTCAGTTCCCCTTTTTATTCTGTCTGGGACCCCAGTTCATAGGATGTCACAACATTTAAGGGGAAATTTCCCTTCTATGGTAAATCTGTCTAGATCTGCACTTACATACAAGC

	RNOG00000045618;ENSRNOG00000048011;ENSRNOG00000049849						ACAGAGCTGTCTCCTAGGTGATTCTAAATCCTATTAAATTGGCAATGAAAATTAATCATTATAATTCTACTTCCTGCAAGCCTGACATCAAACATCACCTTAGTTATTCTACCCTGGTTCATAAAGTCTCATTTGGTCTCTCTCTTTAACAGTTTCAGCTCTGCTCAAAAAATCAGTCTCTGGGTTCGGTCCTCAGCTCTGAAAAAAAAATCAGTGTCTTCTGAGACTGCAGACCATCTCTTCATTTGAGCACCTATGACATGAAGACACAAGTTGAACACTTCTAATACACAAAAACAAAGAAACGTTCCCACCCCAGATAGGAGCGAATGGGAGAATAACAAGGAAGGCTGGATTAAAAAGCAAAACAAAACCCCAGTTACAACCAACACCTAATCCTATAGCTCCATAGCTAGCATACAGCACTCATGACGGGATCATCTGAGCTCTAAATTTGGGTAGTACCAGCACTCCAGTTCTGCCACCTACAGCACATGTCACTTTTGTCTTGGGCTTGTTCCACTGTGTTCTTGCAGCTTTCCTCAGTGGATAAGCAGTTTTAGACTCTCCAATATCCTGGTCCTCTGCTGTAACTTGGGCTTCATCTTTCCAGCTCCATGCAGCGGCTTCTCTGGGTCTTTTTTCAGGGAATCCAATCAATTATACCCAGTGCCTGGATTCATGGACTTTCTGTAGCCTTGGAGCAAGTCTTTGTGATTCATTTCCACTTGCTTCTTCTATGCCTGGTAATGACACAGCAGGCACAGCACCGCATGGCTCCCACAGCAGCCTTGGACAGGAACAGAAGTATTCTAAAACCAAGGGAATAAAGAGAAATAGAATCACCACTTGACCTTTTTGTAGTCTTGACAGAAAATCAGATACAGACATAAACATGGCGTCTAGGCTGAACTGAGGCTCCTTTTTTTGGCCTGTGCTGCTTAAATCCATAGACAATTTCGCCCCCTGGTGGCTACTTAGCATTGTTTCATTCAGTGTCTATACATTTTATTCTGAGAACTCTGACCATATTTAGAAAATTCTGGAAGGGCACAAAGCCTTTAAGTGAAATGTTAAATGAGGCATTTAAGTTAATTATATTCTTCTTCATATCTTACAGAGTTTCCTAGGGATCAAAAATAATATTTATAACTGAGGGTGGAAATAGTTTATCTATGGGGTTTAAGGGATGGCTTCCATGGCCACAGACAAAGAAATTATCTGTTAGGAGCAAAGCACATGGTCTTATTTGGTGCCAATGGTATTCATCCAATACCCTGCTCACGAACACAGTTTAGCCTCAGATAATTCTCAAGACATGAGAAGAAACCACACTGTGCATTCTTTATTAGTTTAAATCCTGACTACTGCTAAGAAGTTTGGACTCAACATCCTCA
MSTRG.135900.2	ENSRNOG00000037661;ENSRNOG00000045618;ENSRNOG00000048011;ENSRNOG00000049849	0.041	2.286	0.000	6.195	up	AGGCTATTGAGATTTGCTGCTGGGGTAGCGTAAGACATAAACCAGAAATGAACAGAGAACTCATAAACAGACCAACAGAAATGCGCTCACCTGACACTTGGTAGATGGCAGAAGGAAGCATAAAGAGATGAATGCTGGTACTTCTCTCAGTTCCCCTTTTTATTCTGTCTGGGACCCCAGTTCATAGGATGTCACAACATTTAAGGGGAAATTTCCCTTCTATGGTAAATCTGTCTAGATCTGCACTTACATACAAGCACAGAGCTGTCTCCTAGGTGATTCTAAATCCTATTAAATTGGCAATGAAAATTAATCATTATAATTCTACTTCCTGCAAGCCTGACATCAAACATCACCTTAGTTATTCTACCCTGGTTCATAAAGTCTCATTTGGTCTCTCTCTTTAACAGTTTCAGCTCTGCTCAAAAAATCAGTCTCTGGGTTCGGTCCTCAGCTCTGAAAAAAAAATCAGTGTCTTCTGAGACTGCAGACCATCTCTTCATTTGAGCACCTATGACATGAAGACACAAGTTGAACACTTCTAATACACAAAAACAAAGAAACGTTCCCACCCCAGATAGGAGCGAATGGGAGAATAACAAGGAAGGCTGGATTAAAAAGCAAAACAAAACCCCAGTTACAACCAACACCTAATCCTATAGCTCCATAGCTAGCATACAGCACTCATGACGGGATCATCTGAGCTCTAAATTTGGGTAGTACCAGCACTCCAGTTCTGCCACCTACAGCACATGTCACTTTTGTCTTGGGCTTGTTCCACTGTGTTCTTGCAGCTTTCCTCAGTGGATAAGCAGTTTTAGACTCTCCAATATCCTGGTCCTCTGCTGTAACTTGGGCTTCATCTTTCCAGCTCCATGCAGCGGCTTCTCTGGGTCTTTTTTCAGGGAATCCAATCAATTATACCCAGTGCCTGGATTCATGGACTTTCTGTAGCCTTGGAGCAAGTCTTTGTGATTCATTTCCACTTGCTTCTTCTATGCCTTTCTCTTCATCCTTTAAGTCCACTCCTATGGTGGCCAATCCAGAGGTGATCAGAGACGCGGGAACTAGAATCCTAACAGCGGAAGAGCTAGAAGAACGCAGCATCATTTCTCTCTTCTACCTGGTAATGACACAGCAGGCACAGCACCGCATGGCTCCCACAGCAGCCTTGGACAGGAACAGAAGTATTCTAAAACCAAGGGAATAAAGAGAAATAGAATCACCACTTGACCTTTTTGTAGTCTTGACAGAAAATCAGATACAGACATAAACATGGCGTCTAGGCTGAACTGAGGCTCCTTTTTTTGGCCTGTGCTGCTTAAATCCATAGACAATTTCGCCCCCTGGTGGCTACTTAGCATTGTTTCATTCAGTGTCTATACATTT

							TATTCTGAGAACTCTGACCATATTTAGAAAATTCTGGAAGGGCACAAAGCCTTTAAGTGAAATGTTAAATGAGGCATTTAAGTTAATTATATTCTTCTTCATATCTTACAGAGTTTCCTAGGGATCAAAAATAATATTTATAACTGAGGGTGGAAATAGTTTATCTATGGGGTTTAAGGGATGGCTTCCATGGCCACAGACAAAGAAATTATCTGTTAGGAGCAAAGCACATGGTCTTATTTGGTGCCAATGGTATTCATCCAATACCCTGCTCACGAACACAGTTTAGCCTCAGATAATTCTCAAGACATGAGAAGAAACCACACTGTGCATTCTTTATTAGTTTAAATCCTGACTACTGCTAAGAAGTTTGGACTCAACATCCTCA
MSTRG.135925.1	--	0.000	0.785	0.000	5.871	up	GGAGCGCAGCGCTTACTCCTCGCATTCCTCTCTTTGAGGGCTGGGGGGCCAGTGGCTTCTGCTCACCGGCTGGGGGGGGGGGGCTCTGGGAAGCGCAGGAGACGCGCAGGACGGAGGCGGGCGAGCCAGGTTCATGCCAGGCTGCCTGCACCGCAGCCCGTCTGAGCCTGAGGCTCCGCTGTTTTCTCGTGTCCCGTGAGCGGAGGTAGCACCGCCGACTAGGGTGCCCCGCGTCCCGCGTCCCAGCTCCCAGCCTGGTGCGCACCGTTCGGCTTGAACCAGGTGCGGCTGGAAGGAGGCAGCACCAAAGCTAACTCGCTCCTAAGACTCTTGGAGGCGGGCACCTTCAAGAATCCAAGTCACTGACTGAGCTGAGAGGTGGTACCTGCGGCTCTAACTAAAGGGTCTAATGATTTAGAAACCTTCCAATAACCTGTCCCCTCTCCGGTTCATCCCGAAGTCCACGGAAGCGTCACAGAACTCCAAGAATTTCCTGAACTTCCCAGGTATCCTCCCTGGCTAAGGACTGGAAGTGGAGTGAACCTGGTGGGAGACTTTGCGTCCGGGGGCTTGGGAGACACCTTCCAAACGCCTCCTCCTCGAGCATCCCATTCATCTCTGGGCAGCTTTACTGGCCGCCCCAAAAGCCAAAGCCTGCGCTCTTCTGAACTCTGCTCTCATCTCTCCGTGGACATGGAACTACACGCCTGAAACCTGCTGGACCTTACTGGTTGTTTTGCAAGACTCAGCTGCCAGAGAACGTGAACAATGAAGCTGCCACTTCTTCTGGCCCTGGTGGTCTGTTCTGCAG
MSTRG.13628.1	--	0.408	0.000	0.049	-3.592	down	GAATGCTACCAGATAGCATCTGCGACCTAGAGAAAATCTTCCCCATTTTGCTGTAACTACCTCTAACCAGGTACCCACCAATATATTTTAGATGTACCTCAGTGTATGGCTTTGTTCTGTACAAAACATCATTTAATTGCTTCTACATTGGTGCATGGAACTCAGTTGAGGATAATCTAAATCTGTATTCCTAACCCACAGATACTCTAAATGGCTCCAGAATGAACAACCGCTTATTTCCTTTAAGGTGAGAGCTGGTTTTGTTATTGTTGTTGTTGTTGGAGGTTGTGGTGGTAGTGGGTTTTTTTCTTCACCCTATGAATGTTAAGTCATTTCTGTTTAATAACATATGGACCATAACTTTACGTTTCAAAATTCCTTTGTCCATCCTGCTCTTACGATACTAGAGTTTGTCTCTACAAATGTGACTTCAGCAGCACCTGTAGAGATAGCAAGTCTGGGGCTGGAGAGGTGACTCAGTGGTCTAGATCATTGACTGCTCTTTCACAGGTCCTG
MSTRG.136281.1	ENSRNOG00000049853	0.000	0.465	0.019	3.592	up	TGTCTGATTTATTTCTGGAAAGTAAGGTTCGACGCAGGGACCTGGCAGGCTGTGAAGGAGCTTCAGCTTCAGGCAGGGTGTAGTTCCCACTCCTAGGCTACGATCTTCTTCTGGGTCAGATTATGATAACAGTAGCCTGATCAGTAACCCCTGCAGGGCCTGGTGGACTTCTAGCAGCCCAAAGCATTTCCTTTTGGGGTGGAACTATCCGGACACTGGGATGGAAATCTGGAGATAAGTCTTTGCCGTTACATTTGAAA
MSTRG.136379.1	--	0.413	0.000	0.010	-4.070	down	CTTGTTCTCGCTCGGCTCTCTCTCCCAACCTGAGCCCCTTCAAACACACGCACCCCTTCACGCAGCAAGCCACAAACAATAAGAGCAAGGGGAAGAAGCCAAAGCCGACTCCGGGAAATTGAGGATAGCAGACACCCTGGCAGTTTTACTGGGACCCAGCAACTCTAAAATGCAGCTTTCTATGCAAGAAAGACCTATGTGATAATTAAGAGACTCCTTCCTTAAGAATGATGGTTTCTGCAGTGCTGGGAGTGTGGACTATTCGAAGTAGACTAATTCAAGAAACATGTTTGATCTGAAAAGCCTCATTAATCACTAATTATAAGGTCAGACATATTTATATTTGTAGTGTCCATCTTTTCTAACAGTTGGTAGTTATAACATCTGATTCTCTGATTGCTCTAATTTTGTCATTAAGCAATGAATTAAAATATGAAATCCCTAGGACTGGTGATATAGGTCCATGGTAGACAGCTTGCCTAGAATATACGAAGTCCTGTGTTTACTTAGCAACACTAAAATGAACTAAGGTAAGAGGTAAGATACAAAAGCTCTTCATTAAGATTATTTCCAGGGACCAGGGTAATATTCACTCTAAATGATCATTTAGAGAGACTGAAAAATAAGAATAGCTGTCATCTTTCTATGTCACTAATTAACTGGGGATCCTAACGAACTGAGAAAATCAGCTTCAGT

							AAACTGCTGGAAAACGGATCAAGTCTCAT
MSTRG.1364.1	ENSRNOG00000048796;ENSRNOG00000048976;ENSRNOG00000052999;ENSRNOG00000054246;ENSRNOG00000058047	0.228	1.044	0.010	2.665	up	GTCCAATTCCAGCTCTCTTGCACTTTCTGAGACCTGGGAGAGGAAGGCAGTTATTCAGACACTGATTTGGTGGGGAAATGCAAGGCAGCTGTCGGAAGGCTGCCTTCTACCACCTCAGTTCTATTGGACAGTCAACATGGACCTTTTAACTGATATCATTGTTGCTAACTCTGTGGGCAGGGAAAATCTCCAGCAACCGTCACAGTAATGTGGGCTGCCAATATGGGACCCAGACTTATACCAGTGCAAACCAAGAACAGGGCAATGGAAGGGACCTCATTGGGTTGCAGGAAGAAAGGAGATGTCCATATGTCACCAAGGTTAAAGCCAATGACAAGTACTAATTTGACATTTGCACTTGGCTCTTATCCC
MSTRG.1366.2	ENSRNOG00000048796;ENSRNOG00000048976;ENSRNOG00000052999;ENSRNOG00000054246;ENSRNOG00000058047	0.383	0.974	0.045	2.031	up	AACATGGGTGGGTGTTAGGGGAACACTCATGAAGTCATGTGCTTAGCAATCGACAATTGCCAGAATATTCCTTAGGAGAAAGAGTTGAAGTCTTTATGGTGCTAGGCAAAGTGTGGAGACCAGTTGGCAGAGGAAAGTGCAATATTGAAGCCACCAGTGAAGGGAACCTCATGCTGGTGGTGGGGTTCTTCTCTCTGCACCAATGTCGCCAAAGGAGCACTGCTCACAGGCCTGAGTAAGGTATACCATGAGCTTCTATCAGAAAAATAAAATAGTCAAGAGATATAGGGGGTGCACAGAAAGGTCTAAAATGAGGCTATAAACATCATTAAGTGGATAGAGCAAGGTCCCAGCACCTGTACACTGGGAAATGGCCACCACACCAAACACAAAAGCATCTATCATAGTGAAAAATGAATACTTTACAGAATGCACACACTAACATTGATTGATCACATCCATAGTTCAAATTTTGGGGGCAAATTCATGGCCCTAACTATCTTTTTCTCAACTTATATAGCAAAAAATAAGAGAGACAGAGAAAAGAAAAAGTCAAAACAAAATCCCAAGTTTCTCATATGAGGACTGCAGGAGGTGTTATATGGTAGTCAGTTCTGATTAGACATTTTAGATAGAACAGTACACAGTGACCCTTGATGTGATGGGCCCTATATAAAGCTTTTTTGCAATATTGGAATTTTAACAAACCTCATCCAGAACGTACAGAAGAGGTAAGGATGTGATCACCATGTCCTTGCTCTCTATCAGGTCATTTTTCTGCATCCTTGATTGTCAGGCATAGAACAGCAACAATCTGAAATAGCTGGTAGACATGGGAAATCGTGGC
MSTRG.1369.1	ENSRNOG00000048796;ENSRNOG000	0.000	0.701	0.000	6.209	up	AAGAGGGATGGAGAAATGATCACTTGTGTTAAGAATCAAGGTGTCCTCTGTAATATTCAGGTCTATACTGGCCCTGTGCATCAGCTTCAAGGACCCATTTCTGATTTTCTTTGGGGTTCCCGTGATGATGCACTCAGACATGATTGGAAAGAAACAAAACGGTGGTCAGACTTGATTTATAAAATGCAGGCAACATTGCAAACTTATGTTGCAGGATATTTGATTCATATTATCAGCCGCAAGATAGTGTTATTTACTTTAAAAAAAATTACGTCTGGCAGTTCAGTTCAGCACTAGCACCGATATTTTACCCTAGAGCTTTTTTTGTTTGTTTGTTTGTTTTTTGTTTTTGTTTTTGGATGAACAAGTACTTTCTTATTTTGTGGCCCAGCCCTAGCTCTTACTTCTTTGCACAGTCACAG

	00048976;ENSRNOG00000052999;ENSRNOG00000054246;ENSRNOG00000058047						AGAGACCTCAAACATATACTCAGCCAAAGGCAGGGGAATGTAGAAAAGAGCCTAAGGCAATACCACATAATGACATTGAAAGTGGGTGGCCAAAAGGTGTTATAAAATTCACCTCACTTCAGTGAGTGATGACATACCAACCTGGTGGCCTACGATTCTGGAATATTAATCAGAATCCTTTCGTCATATTAGATCTTAATAGAAACAAGGAAGCAGTCCTTTCCTGAAGTATATATTCATCATACGTAAAGAAGATATTACATCCATATACAACTCAGAATAGAATTATCCCCCAAGACCGGAAAGATCTGGCAACATTAATATTAGAAGCTTCTCCTGTGTTACAGTGGTAAGCACAGAAGAAAGAGGAAGATAGGGCTGAACACAAAAGCATCAGGCCAGAGCACTGTTTCTCAGGATGAGTTGCTAGGTGAAGGTAAATAAACCTTACTAAGTTTAATACAGTATTACATACACTGTTGTAAGTAACTGGAGCCGAGGCTATCCCCAAAGGTTTGCCTGTCTGTGAAATCTGATACCCTAACTCAGGTGTCTTCCCTGGCCTCAGTGGGAGAAGGTCCACCAGCCCTACAAAAACTTGATGTGTCTTGGTGGAGGCACACTCTGAGGGATATTCCTCTACTCTCTCAGAGGAGATGGTGTGAGGACATTGGGGAGGAACTGTGGGAGAGAAGTAACAAGAGAAAGGACAGCAATTGGGATGCAAAGTGAATTTAAGCCT
MSTRG.137062.1	ENSRNOG00000005076;ENSRNOG00000005352	0.626	1.698	0.020	2.192	up	CACTGTGGCTAGGTGCTGGGGATACATCACTGACTAGCACACAGCTCCTGGCCTTGAGGAGCTCACAGTCTATTGGAGGGGGACATACATGAACACAATCAAAGAACTACATACATGTGCTACAATGAGAGGTATATACAAAGTGCTGTGGGAGCCAGGAGGGAATCACATATTAGCCACTGGGAGGTGAGGACCACCACTTGACTCCTGACTCCATTATGCCATCACAAAAGCAGGGGGATTGCTGCTGCCTGACTCTGGTTAAAGTAACTCCGTCTCCTGAGGTTCTCCTGTAAGCCTCGTCCATAACGCTTGTCAGCAAGGGTTAGTTAACCAGTTAGCTCGTTTAACACTGTGTAGGGAAGAAATTGAATAGGAAGGTGTCTTTAGGATCAGAGGATATTGAAAGTAATACAAATCTTGGATGTTAAGACGGGGTGAGGTGGTACCATGGGGGCACTGAGCCATGAATGGCAGGCCCTGGGCAGTGAAGCACCTGGAGTAACTGCCTGAGGAGAGAGGAGGACAGAGGTCAGCTACCTCAGAGTTTGATACCCAGCTCTCATCAGATCAATCAGTAACCTGTCATTGATGCTCACCTGCTTCATAGGTATGCCCAGTTTTGTGGGTGGCGCGGTGGTGCCCACCCCCCAGGAAGTAACACACATGGGACATTAAAGGTACAGATCTGAACTTAAGGTCTAAGTAGCAGTTTGTGATTACAATAGAGCTATCACAGGCTGTTTCTAATCACCTGTCCATCAAGTGGCAGTTACAAGTTTAAGAATTAAAAGTTAGGAACTGACTGTTCCCACCGAATCCCATCTTGAGACACAATTTTTCGTTGTTAGGAAGAAGATAGAACATATTGGTTAAACGGAACCCTTTTGGAGGACGTGGCATGGCATGGGGAATATCAGAGAACCTAATAGTGAAGTGCCACCAACTTGGAGCTCTTTTCCCTGGTGGGATTGATATCAAACTTCAAATCTTTCGAGAATTACTACCCATAGCTCTCCAAGTGCTCCCCAATTCCTTGGACCCTGTCACGATTCAGGAACTAAAGGGTTAATGCTAGCCCTAGGCCCCCTACAAGAGCCTGGGGGTGGTGGTGAGCATTTTAAGTATTGGCCCAATCCTCAAGCCAGGACTCACTGAAGGCTTTTTATTGAGGAAGGCCGAGTAGCCTGCCAGATGTGAAGGGCTAAGGAGTCACATTGAACAGGGCGCTGACATATACTGGGAAGGCGGAAGCTAGGAGTAAGAGCTAGGCTGGAGGCTCAGGGGATGAAGACAGGCCAGCTATCAGGAGATGAGGGTAAGGGGCAGGATCAAGTACATATAGCACACCAGACAGCTCTGAGATTTCCAGTATAGAAGAAGGTCAGGAAAATGGTGGCATCATTAACAGAAGCAGATCACCAGAGCCATGCGCCAGGGATTTAAGAGAAGCAAGTTCTGGATTGACCTTACCTTAAAGAACTGGCCGGTGACAAGAGTGGGACACCTGGAGAACACCCACACACAAAGAGATGAGGAGGAACCCACACAGAAACTAAAAGCACAGTGGTGTAGAAGGGCTCAGCACGTGTGGGATGACAAGGCCAGACAACCATGGAGAAGATGATTTGACTATGAGAGTGTTGACAGCCTTAGGAGAATGGATTTCCTTGGAGTTAGGATGTGTATGCAGAATTCAGGTCCCAGGTTGATGCAGGGCCACCCCAACTCATGCTGTCCCTTTGTGCTCACTAAAACTGGTTGAGCTTGGAAGAGTAAGGGTACTCGTCAAGCCTGCATGAAGTCATGGGTTTGGTCGATAGAAACACACCGTGATAACATAAGTGACAACAAGGGGGTGGGGTGGGCAAGACAACTGGAAGTTCAAGGTCATCTTCACCAACAGAGCAAGTTCAAAACCCAAAAACTAAAACCCAAATACAACAAGAACATCAACACTAACAGCAACAATGGTGACCTTTTCTG

							GAGATGTTTCCTTCCATCCATCAAGAGATTGTCATCCTAAGCCGATTTTATTATAACAGACATTTCAGTGT<br>CAATTGCTGGGAATGTGGCAAATGCACCTGCATAGCCACAGATGGTCCCTTCCCCCCAGGGAAGGAGA<br>GAGAACAGTAGGGGAGAACAATGGCCAGCCAGGGAGGTAGTTAGGTCAAGTGGGTGCTTTGGCACAG<br>AGGACATAGCAAAGGTCCTGGATACTGATTCACATTAACCGAATTCACCAGACTTGCTCCTGGGCCTGG<br>CATCTCTACACATTGCCCCTAAACCTGGTAGGAGAGAGGCAAGTAGATTCAAGGAAGGGAAATGGAGA<br>CCCTTCGCTGAGAGACTAAGAGAGACCAGAAGAACTCTTTCTAGAAAGGGGAAAGAGGCGCAGGGTC<br>AGTTGCTAACAGGGGATGTAGGAACTCATGTGTTCTTGCTCCTGTCTCCTTAGTGAAGCAAGAGTTGGG<br>CTGTGTGTGACGGCTGGAGATGAAAACCAACAGTCCTCCTAAAGTCTTTCAGAGCCACTGGGAGAGGG<br>TGAGGATGGGAGAGAGAGCGAGACTGTATGGCTGGAGCCGGGCTACCACCCCACTAGCCTCAGCAATT<br>GATGACAGGTGAAACTTCTCAGGACAAACAGAACGAAGAGGGAACCCAAGCTAGGGAACAAAGAAC<br>ACTGGGTCAAAATAGGGCTATTCTTGTCAAGCACAGGGCTGATTTTATCCAAACAGCCAAAAGCCCTGG<br>ACAGACATTCTATCCACTTAGGTCCTGCTAGGGACTTTATAACTGGGGCTGACTATAGTTAAGTGCTTTG<br>CCCCACAACTTTCAGCAAAGTACTGAAGTTGCACCTTGATAGCAAGGTAAATCTGACAAGCAAGTGCT<br>CCCAGGGCTAAGTGTGCTCCTTTTCCCTCCCACTATGGTTTTAACGTAAGTGGACTCTTGTCCCTTAGCA<br>ATGTCCTCTCAGCATGTGCACCCATTGAAGCTCTCCTACCAAGACCTGTTCCGGCTCTGTGACATCTCTT<br>TAGACTGGGAGGGGGACATTCATTCAACCAGTACTTTCCAGTCTCAGAATGCCATTGTTGCTTGCAAAA<br>AAAAAAAGTTAGAGTGCTTTCTCTTAAAAATGCCTCATCTCTGTGGCTGACCTAGGAATGGACGGAAAC<br>ACACACACACACACACACACACACAC
MSTRG.137143.10	--	0.000	1.079	0.000	8.040	up	TACTTGTGTTTCAAAGGTGGGTAAATTGGTGGATGAAGAAAGAGAAAGGAAAAACCTTGAAGCCTGGA<br>CGTTGTCTTTAGGATAAAATTAGTAACTGCCGAGGGTTTGTTCGGTGATGTTATTTAAGACAAGGTGAAA<br>CCTACTCCTGCCATCTGGTCATGGTAGCATGAACCTAGAAATTCTGAAATTCTCAGGGGCAAAGACAGA<br>AGTCTCAAGGAAAGACCCAGGCCAGCCTGGTCCATGTAGTTAGCTCACAACAACTAGGGCTTAGCATT<br>GTTCTTTGCTTTTAAACAATACCTCTATCATGTGAAACCTATGAAAAATTAATCATTCCAAAATTTTACGA<br>AGACCAATAAGTATCTGGAATGGGTAAAAATGAGCCATCATAAAGAAAGAAAAAGAAATATTCCAGAG<br>TCACACAAGGAAAAGAATCCCATAAAACAAGAAAAAAAAAAAAAAAACTGGTGAAATTACTCAAAGAT<br>GAGCTGAAGAACTCAGTACAGCCATCTTTGGCGTTTTTTTCAGACAGGAAGGGCGGGAGACAATCACT<br>CACAAGAGCTTATGAGGAGCTCTACTGCCATCATGAGGATGCGGTTTCAAAACTGACAACACCACTGA<br>CCTTACTTCCCATGATCCTCAGCGATGGCGGAAGCTTTTCTGATGACAAGATCAGCTCTGGGTGCCGATT<br>TGGGCTTCCTGGACTTCTCCTGAGGCCTCAACAGAGACTCTGTAGTTTCAGTACTGCTTTCTCATCAGC<br>ACCTGGACAGTGAGAGGCCTAGCTGGCCTGCCTCATTCCTCTTCTCTTGGGAGGAACTCTGTGCAGTCC<br>ACCCTCAAATCCTGCATTGCTCCCCATCTGCACCCAGCAGCATTCTGCCTCCCTGCTTGCCCCTGGAGTC<br>TGATGGATTCCTGGTCTTGAGAGAGATCTCAGGGATCACTTCTAGGGAAAAAAGGACATGTGGCAACC<br>TCAGGGCTGAACAAAAGCAAACCATCAGGAGACCTAGTTCTTACCTACTTCACACAGACTTCCCTGGAT<br>ACCACGCTGTGTGTGATCCAGACAAGGGCTCTAGACAAAAGGCCTTGTGTGTCCTGTGCCAGGCTGAC<br>TCTAATGGCCCTGGATAGAGGATCCAGATACCTGTCAACACTGAATGCTTTACACAACCCCACTTGGAG<br>AAACATGTGCGTTGAAATCAGAGTGGTTTCCAAATGGATCTGTCACTGATTCTCTCTAGGGGATGATTTA<br>GTTCACACTGGAGCAAGGGCACAAGAGTCTTCCGACATTGGAGGCCTATTACTGTTTCCCATGACATAA<br>CTATATATGCCTGTTACTGTCTTCATACTAGATTATATTATCACAGGAGCCTGGTCCATATAGTCAGTTCAC<br>TACAACCAGAGCTTTAGTCCTTTGCTTCTAAATCTCCACAATAACTATGGTGTAAAACCCATGAAAAAAT<br>CAATCATTGTAAAATTTTAAGAAGACCATTAAGTATTGGAAATGGATAAAAAACGAGCTGTCATACAGA<br>AAGAAAAGGAGATATCTTAGGTAACAAAAGGAAAAGGCAAAAAATAAAATAAAATACTGGTGAAATTA

							CTCAAAGTTGAGCTGAAGAAGTCAGGACAGCCACCCTTGGTGGTTTTTTTAGACAGGAAGGGCGGGAGACAATCACTCACAAGCGCTTGTGTGGAGCTCTACTGCCATCATGAGGTTACGGTTTCAAAACTGACAACACCACTGACCTGCCTCCCCATGATTCTTAGTGATGGTAGAAGATTACAGGCAGAAGATCAGCTCTGGTTGCCTATTTGCGCTTCCTGGACTTCTTAGGTCCCAACAGAGACTCTGACTGTAGTGCTTTCTCTTCAGAACCTGCCTCATTCCTCTTCTCTTGGGAGGAACTCTGTGCAGTCCACGCTCAAATCCTGCATTGCTCCCCATCTGCACCCAGCAGCTTTCTGCCTCCCTGCTTGCCCCTGAAGCTGATGGATTCCTGGTCTTGAGAGCGATCTCCGGGATCACTTAGTTCTATGTAAAACCAAAGAAACTGGGAGGATGGAGATCCTGCCCCTTGACTTCCTGTGTCAATGTCTTCTGACAGCTGTTGGTCCACAGGGAGAATACACCAGGGTCCAGATCCATCTGTAGTTGATGCAGGAGCATAGCCTTAACCCATGAATTTCAGCACTACAAATTCAAGCAGACAAGGGAGTTTATAAAGTGCTTGTCTTCAGTTCTCCCGTTTTCTATCACCAACTTAATCAAGAGCATTCTCCAGGACAGCCAGGCCCATCACCAATTTCCCCGGTAACTTCTTCGTCCTCATAGACAGGGATGAAGGCTTGGTAGGAGAGGTGAAACACACCGTACAACAGCCGTGGCTGATGACTTTTTGCCTGCTTCGTCTCCAGTCCTCCCGTCTTGTTTTTTGGTCTCCATTTGTTTGGCGTCCTAGGCTTCCTAGAATCCTGACTTCTCTTGGCGCGATAGTTCTCGGGTTTTGGGGTTTTTTTTTACCCCCATTCCTCTCCAAGTCTCTAAAACAACTAGGACACACCGAGACTTTTAGCAAGAACATGACGTTCTGATCCCAGGCCTGACTATGCTACGTAAAAGGACCCTAGAGCTCGGAGACCCCACGTGTGGATCGTCTCTGTGCCCCGAGGGGGAAGGGAGGGAAGAGGGACCAGGAGAAGCCAGGGTGTGTCTGCAGGTGGCCTCTCTAGTCCCGC
MSTRG.137143.14	--	0.000	0.558	0.000	6.603	up	TACTTGTGTTTCAAAGGTGGGTAAATTGGTGGATGAAGAAAGAGAAAGGAAAAACCTTGAAGCCTGGACGTTGTCTTTAGGATAAAATTAGTAACTGCCGAGGGTTTGTTCGGTGATGTTATTTAAGACAAGGTGAAACCTACTCCTGCCATCTGGTCATGGTAGCATGAACCTAGAAATTCTGAAATTCTCAGGGGCAAAGACAGAAGTCTCAAGGAAAGACCCAGGCCAGCCTGGTCCATGTAGTTAGCTCACAACAACTAGGGCTTAGCATTGTTCTTTGCTTTTAAACAATACCTCTATCATGTGAAACCTATGAAAAATTAATCATTCCAAAATTTTACGAAGACCAATAAGTATCTGGAATGGGTAAAAATGAGCCATCATAAAGAAAGAAAAAGAAATATTCCAGAGTCACACAAGGAAAAGAATCCCATAAAACAAGAAAAAAAAAAAAAAAACTGGTGAAATTACTCAAAGATGAGCTGAAGAACTCAGTACAGCCATCTTTGGCGTTTTTTTCAGACAGGAAGGGCGGGAGACAATCACTCACAAGAGCTTATGAGGAGCTCTACTGCCATCATGAGGATGCGGTTTCAAAACTGACAACACCACTGACCTTACTTCCCATGATCCTCAGCGATGGCGGAAGCTTTTCTGATGACAAGATCAGCTCTGGGTGCCGATTTGGGCTTCCTGGACTTCTCCTGAGGCCTCAACAGAGACTCTGTAGTTTCAGTACTGCTTTCTCATCAGCACCTGGACAGTGAGAGGCCTAGCTGGCCTGCCTCATTCCTCTTCTCTTGGGAGGAACTCTGTGCAGTCCACCCTCAAATCCTGCATTGCTCCCCATCTGCACCCAGCAGCATTCTGCCTCCCTGCTTGCCCCTGGAGTCTGATGGATTCCTGGTCTTGAGAGAGATCTCAGGGATCACTTCTGCCTCCCCATGATTCTTAGTGATGGTAGAAGATTACAGGCAGAAGATCAGCTCTGGTTGCCTATTTGCGCTTCCTGGACTTCTTAGGTCCCAACAGAGACTCTGACTGTAGTGCTTTCTCTTCAGAACCTGCCTCATTCCTCTTCTCTTGGGAGGAACTCTGTGCAGTCCACGCTCAAATCCTGCATTGCTCCCCATCTGCACCCAGCAGCTTTCTGCCTCCCTGCTTGCCCCTGAAGCTGATGGATTCCTGGTCTTGAGAGCGATCTCCGGGATCACTTAGTTCTATGTAAAACCAAAGAAACTGGGAGGATGGAGATCCTGCCCCTTGACTTCCTGTGTCAATGTCTTCTGACAGCTGTTGGTCCACAGGGAGAATACACCAGGGTCCAGATCCATCTGTAGTTGATGCAGGAGCATAGCCTTAACCCATGAATTTCAGCACTACAAATTCAAGCAGACAAGGGAGTTTATAAAGTGCTTGTCTTCAGTTCTCCCGTTTTCTATCACCAACTTAATCAAGAGCATTCTCCAGGACAGCCAGGCCCATCACCAATTTCCCCGGTAACTTCTTCGTCCTCATAGACAGGGATGAAGGCTTGGTAGGAGAGGTGAAACACACCGTACAACAGCCGTGGCTGATGACTTTTTGCCTGCTTCGTCTCCAGTCCTCCCGTCTTGTTTTTTGGTCTCCATTTGTTTGGCGTCCTAGGCTTCCTAGAA

							TCCTGACTTCTCTTGGCGCGATAGTTCTCGGGTTTTGGGGTTTTTTTTTACCCCCATTCCTCTCCAAGTCTCTAAAACAACTAGGACACACCGAGACTTTTAGCAAGAACATGACGTTCTGATCCCAGGCCTGACTATGCTACGTAAAAGGACCCTAGAGCTCGGAGACCCCACGTGTGGATCGTCTCTGTGCCCCGAGGGGGAAGGGAGGGAAGAGGGACCAGGAGAAGCCAGGGTGTGTCTGCAGGTGGCCTCTCTAGTCCCGC
MSTRG.137143.15	--	1.632	0.093	0.000	-3.190	down	TACTTGTGTTTCAAAGGTGGGTAAATTGGTGGATGAAGAAAGAGAAAGGAAAAACCTTGAAGCCTGGACGTTGTCTTTAGGATAAAATTAGTAACTGCCGAGGGTTTGTTCGGTGATGTTATTTAAGACAAGGTGAAACCTACTCCTGCCATCTGGTCATGGTAGCATGAACCTAGAAATTCTGAAATTCTCAGGGGCAAAGACAGAAGTCTCAAGGAAAGACCCAGGCCAGCCTGGTCCATGTAGTTAGCTCACAACAACTAGGGCTTAGCATTGTTCTTTGCTTTTAAACAATACCTCTATCATGTGAAACCTATGAAAAATTAATCATTCCAAAATTTTACGAAGACCAATAAGTATCTGGAATGGGTAAAAATGAGCCATCATAAAGAAAGAAAAAGAAATATTCCAGAGTCACACAAGGAAAAGAATCCCATAAAACAAGAAAAAAAAAAAAAAAACTGGTGAAATTACTCAAAGATGAGCTGAAGAACTCAGTACAGCCATCTTTGGCGTTTTTTTCAGACAGGAAGGGCGGGAGACAATCACTCACAAGAGCTTATGAGGAGCTCTACTGCCATCATGAGGATGCGGTTTCAAAACTGACAACACCACTGACCTTACTTCCCATGATCCTCAGCGATGGCGGAAGCTTTTCTGATGACAAGATCAGCTCTGGGTGCCGATTTGGGCTTCCTGGACTTCTCCTGAGGCCTCAACAGAGACTCTGTAGTTTCAGTACTGCTTTCTCATCAGCACCTGGACAGTGAGAGGCCTAGCTGGCCTCCGCTCAGCTCTGTACCTGGTCCAAGTCCCTCAGTCAAAAGTGTCTGCATATTCTGAAAGGTCTTGCCTGCCTCATTCCTCTTCTCTTGGGAGGAACTCTGTGCAGTCCACCCTCAAATCCTGCATTGCTCCCCATCTGCACCCAGCAGCATTCTGCCTCCCTGCTTGCCCCTGGAGTCTGATGGATTCCTGGTCTTGAGAGAGATCTCAGGGATCACTTCTGCCTCATTCCTCTTCTCTTGGGAGGAACTCTGTGCAGTCCACGCTCAAATCCTGCATTGCTCCCCATCTGCACCCAGCAGCTTTCTGCCTCCCTGCTTGCCCCTGAAGCTGATGGATTCCTGGTCTTGAGAGCGATCTCCGGGATCACTTAGTTCTATGTAAAACCAAAGAAACTGGGAGGATGGAGATCCTGCCCCTTGACTTCCTGTGTCAATGTCTTCTGACAGCTGTTGGTCCACAGGGAGAATACACCAGGGTCCAGATCCATCTGTAGTTGATGCAGGAGCATAGCCTTAACCCATGAATTTCAGCACTACAAATTCAAGCAGACAAGGGAGTTTATAAAGTGCTTGTCTTCAGTTCTCCCGTTTTCTATCACCAACTTAATCAAGAGCATTCTCCAGGACAGCCAGGCCCATCACCAATTTCCCCGGTAACTTCTTCGTCCTCATAGACAGGGATGAAGGCTTGGTAGGAGAGGTGAAACACACCGTACAACAGCCGTGGCTGATGACTTTTTGCCTGCTTCGTCTCCAGTCCTCCCGTCTTGTTTTTGGTCTCCATTTGTTTGGCGTCCTAGGCTTCCTAGAATCCTGACTTCTCTTGGCGCGATAGTTCTCGGGTTTTGGGGTTTTTTTTTACCCCCATTCCTCTCCAAGTCTCTAAAACAACTAGGACACACCGAGACTTTTAGCAAGAACATGACGTTCTGATCCCAGGCCTGACTATGCTACGTAAAAGGACCCTAGAGCTCGGAGACCCCACGTGTGGATCGTCTCTGTGCCCCGAGGGGGAAGGGAGGGAAGAGGGACCAGGAGAAGCCAGGGTGTGTCTGCAGGTGGCCTCTCTAGTCCCGCCC
MSTRG.137143.2	--	1.133	0.000	0.000	-7.383	down	TACTTGTGTTTCAAAGGTGGGTAAATTGGTGGATGAAGAAAGAGAAAGGAAAAACCTTGAAGCCTGGACGTTGTCTTTAGGATAAAATTAGTAACTGCCGAGGGTTTGTTCGGTGATGTTATTTAAGACAAGGTGAAACCTACTCCTGCCATCTGGTCATGGTAGCATGAACCTAGAAATTCTGAAATTCTCAGGGGCAAAGACAGAAGTCTCAAGGAAAGACCCAGGCCAGCCTGGTCCATGTAGTTAGCTCACAACAACTAGGGCTTAGCATTGTTCTTTGCTTTTAAACAATACCTCTATCATGTGAAACCTATGAAAAATTAATCATTCCAAAATTTTACGAAGACCAATAAGTATCTGGAATGGGTAAAAATGAGCCATCATAAAGAAAGAAAAAGAAATATTCCAGAGTCACACAAGGAAAAGAATCCCATAAAACAAGAAAAAAAAAAAAAAAACTGGTGAAATTACTCAAAGATGAGCTGAAGAACTCAGTACAGCCATCTTTGGCGTTTTTTTCAGACAGGAAGGGCGGGAGACAATCACTCACAAGAGCTTATGAGGAGCTCTACTGCCATCATGAGGATGCGGTTTCAAAACTGACAACACCACTGA

							CCTTACTTCCCATGATCCTCAGCGATGGCGGAAGCTTTTCTGATGACAAGATCAGCTCTGGGTGCCGATT<br>TGGGCTTCCTGGACTTCTCCTGAGGCCTCAACAGAGACTCTGTAGTTTCAGTACTGCTTTCTCATCAGC<br>ACCTGGACAGTGAGAGGCCTAGCTGGCCTCCGCTCAGCTCTGTACCTGGTCCAAGTCCCTCAGTCAAA<br>AGTGTCTGCATATTCTGAAAGGTCTTGCCTGCCTCATTCCTCTTCTCTTGGGAGGAACTCTGTGCAGTCC<br>ACCCTCAAATCCTGCATTGCTCCCCATCTGCACCCAGCAGCATTCTGCCTCCCTGCTTGCCCCTGGAGTC<br>TGATGGATTCCTGGTCTTGAGAGAGATCTCAGGGATCACTTCTAGGGAAAAAAGGACATGTGGCAACC<br>TCAGGGCTGAACAAAAGCAAACCATCAGGAGACCTAGTTCTTACCTACTTCACACAGACTTCCCTGGAT<br>ACCACGCTGTGTGTGATCCAGACAAGGGCTCTAGACAAAAGGCCTTGTGTGTCCTGTGCCAGGCTGAC<br>TCTAATGGCCCTGGATAGAGGATCCAGATACCTGTCAACACTGAATGCTTTACACAACCCCACTTGGAG<br>AAACATGTGCGTTGAAATCAGAGTGGTTTCCAAATGGATCTGTCACTGATTCTCTCTAGGGGATGATTTA<br>GTTCACACTGGAGCAAGGGCACAAGAGTCTTCCGACATTGGAGGCCTATTACTGTTTCCCATGACATAA<br>CTATATATGCCTGTTACTGTCTTCATACTAGATTATATTATCACAGGAGCCTGGTCCATATAGTCAGTTCAC<br>TACAACCAGAGCTTTAGTCCTTTGCTTCTAAATCTCCACAATAACTATGGTGTAAAACCCATGAAAAAAT<br>CAATCATTGTAAAATTTTAAGAAGACCATTAAGTATTGGAAATGGATAAAAAACGAGCTGTCATACAGA<br>AAGAAAAGGAGATATCTTAGGTAACAAAAGGAAAAGGCAAAAAATAAAATAAAATACTGGTGAAATTA<br>CTCAAAGTTGAGCTGAAGAAGTCAGGACAGCCACCCTTGGTGGTTTTTTAGACAGGAAGGGCGGGA<br>GACAATCACTCACAAGCGCTTGTGTGGAGCTCTACTGCCATCATGAGGTTACGGTTTCAAAACTGACAA<br>CACCACTGACCTGCCTCCCCATGATTCTTAGTGATGGTAGAAGATTACAGGCAGAAGATCAGCTCTGGT<br>TGCCTATTTGCGCTTCCTGGACTTCTTAGGTCCCAACAGAGACTCTGACTGTAGTGCTTTCTCTTCAGAA<br>CCTGCCTCATTCCTCTTCTCTTGGGAGGAACTCTGTGCAGTCCACGCTCAAATCCTGCATTGCTCCCCAT<br>CTGCACCCAGCAGCTTTCTGCCTCCCTGCTTGCCCCTGAAGCTGATGGATTCCTGGTCTTGAGAGCGAT<br>CTCCGGGATCACTTAGTTCTATGTAAAACCAAAGAAACTGGGAGGATGGAGATCCTGCCCCTTGACTTC<br>CTGTGTCAATGTCTTCTGACAGCTGTTGGTCCACAGGGAGAATACACCAGGGTCCAGATCCATCTGTAG<br>TTGATGCAGGAGCATAGCCTTAACCCATGAATTTCAGCACTACAAATTCAAGCAGACAAGGGAGTTTAT<br>AAAGTGCTTGTCTTCAGTTCTCCCGTTTTCTATCACCAACTTAATCAAGAGCATTCTCCAGGACAGCCAG<br>GCCCATCACCAATTTCCCCGGTAACTTCTTCGTCCTCATAGACAGGGATGAAGGCTTGGTAGGAGAGGT<br>GAAACACACCGTACAACAGCCGTGGCTGATGACTTTTTGCCTGCTTCGTCTCCAGTCCTCCCGTCTTGT<br>TTTTTGGTCTCCATTTGTTTGGCGTCCTAGGCTTCCTAGAATCCTGACTTCTCTTGGCGCGATAGTTCTCG<br>GGTTTTGGGGTTTTTTTTTACCCCCATTCCTCTCCAAGTCTCTAAAACAACTAGGACACACCGAGACTTT<br>TAGCAAGAACATGACGTTCTGATCCCAGGCCTGACTATGCTACGTAAAAGGACCCTAGAGCTCGGAGA<br>CCCCACGTGTGGATCGTCTCTGTGCCCCGAGGGGGAAGGGAGGGAAGAGGGACCAGGAGAAGCCAGG<br>GTGTGTCTGCAGGTGGCCTCTCTAGTCCCGCCC
MSTR<br>G.137<br>143.20	--	0.482	0.000	0.000	-<br>5.643	do<br>wn	CTCTTCTAGATGGAAGCCTAATTCTTTGCCCTGTGTTTTGATAGTTGGGTACACATCAGGCCTTTTCTAAA<br>CTCCCTTGGCACGTGGAATTGGCTTTTCTTGCCGGTCTTCCACTGCTGCAGTTCCTGGATCCAGCGGGT<br>ATAGTAAGTAGGGCTGTAGAACAGTGATGGTGCGTGAAGTTCTGCTTCCAGCATGGGCAAGAGCAATA<br>GGAACACACACCCCTTCTCATAGTGCAGTGCCTCTAACCCATTTTAAAAGTTCATTCTTGAGTTCCTTGA<br>CACAAAATCTTCTCTCTAGCCCATGTTTACCTAGTGCTCACTGTAGTCCTTTCCTACCTTCAGAACACTG<br>GGATTACAAGCAGGAGCCATCTTGATGACGGAGAGTTTGCATTTGTTTTTGAGTTATTTCTACATTTTCA<br>AATCAAAGCTGGGAATAGCACAGTTTCAGTATCCCAGACTACTTGTGGTCCACTTCAGGTCCCACAACC<br>TTCAGGTTCTATGAGGAATTACACCAGCTTCCTGGCATTTCTTGGGGCATTGGAAGACAATGAGCTTCA<br>GTTTGAAGGCTAGCCTTCCTTCTAGCAGGGGCTTATAGGAAAGAAACTGCTGGTGTCCTGTTGAAACTA

							ATTCCTCTTGGGGGCAGCAGAGCTCTAGATAAGTTCAGCAATGCCACCTTACATCCTATTCTCCCCCCAGTTTAGTCTGAAAAAAAGGACAAGAAAAATGCCTGCTTCCTCTTCTGTGCAGATTCTAGTTGTACAAGGCAGCCATTTTACTTTTGCATTTGCAATTCAAACCAAAGATTGCAAACCACCACCCTTCTTCATGAAAACACCTCTCCTTTGTCTAGATAAATTCCCTCAGAGCCCTGTGCATTCAGGCTTGATTTACTTGGTGATACTTGTCCAGTAGGTGAATAAAAAGTAGAAAGAAAGCATGTGAATTCTGAGGGCATGTCTAGTAAATCCTGAATCCAAGTGCTTTTTTACTCATTTCTGTACTTATGAATTTCTGCAGTGTCCTCAGGTTGTGGGAGGATAACCGTTTGTGATACTGGGGTTGCATGTATGCCTGAGGACCTCTGTTCCATCTCCAGCACCCTCTTTAAAGGAGATAACTAACACTTACAAATTGTTCTCAGACCTTGGCAAGCATACTACAGGGGTACACTCGCACCTACAGTAAACATGTATGCTATTAAATTGCTTCACTGCTGATAGTTCGTGTCACCCCCTAGGTTCTACACTAATGTACAGTTAGGGTTCAGTTCTCCAGGTTGTAGAAAGAAAATAATGCCCAATCTCTTCTAGAACACTAAAGGCCAGGGCTGTCATGGGAAGGAAACTGGAGGTAACATGTCTGATGAAACTGTGTCCTCATGGGGGCAGTAGAGCTCTACGAGGAGCCATTGTGGTGCCCTCCTCTTCCACCCATCTTATCTGCCTCATTCCTCTTCTCTTGGGAGGAACTCTGTGCAGTCCACGCTCAAATCCTGCATTGCTCCCCATCTGCACCCAGCAGCATTCAGCCTCCATGCTTGCCCCTGGAGTCTGATGGATTCCTGGTCTTGAGAGCGATCTCTGGGATCACTTAGTTCTATGTAAAACCAAAGAAACTGGGAGGATGGAGATCCTGCCCCTTGACTTCCTGTGTCAATGTCTTCTGACAGCTGTTGGTCCACAGGGAGAATACACCAGGGTCCAGATCCATCTGTAGTTGATGCAGGAGCATAGCCTTAACCCATGAATTTCAGCACTACAAATTCAAGCAGACAAGGGAGTTTATAAAGTGCTTGTCTTCAGTTCTCCCGTTTTCTATCACCAACTTAATCAAGAGCATTCTCCAGGACAGCCAGGCCCATCACCAATTTCCCCGGTAACTTCTTCGTCCTCATAGACAGGGATGAAGGCTTGGTAGGAGAGGTGA
MSTRG.137143.5	--	0.841	0.077	0.011	-2.545	down	TACTTGTGTTTCAAAGGTGGGTAAATTGGTGGATGAAGAAAGAGAAAGGAAAAACCTTGAAGCCTGGACGTTGTCTTTAGGATAAAATTAGTAACTGCCGAGGGTTTGTTCGGTGATGTTATTTAAGACAAGGTGAAACCTACTCCTGCCATCTGGTCATGGTAGCATGAACCTAGAAATTCTGAAATTCTCAGGGGCAAAGACAGAAGTCTCAAGGAAAGACCCAGGCCAGCCTGGTCCATGTAGTTAGCTCACAACAACTAGGGCTTAGCATTGTTCTTTGCTTTTAAACAATACCTCTATCATGTGAAACCTATGAAAAATTAATCATTCCAAAATTTTACGAAGACCAATAAGTATCTGGAATGGGTAAAAATGAGCCATCATAAAGAAAGAAAAAGAAATATTCCAGAGTCACACAAGGAAAAGAATCCCATAAAACAAGAAAAAAAAAAAAAAAACTGGTGAAATTACTCAAAGATGAGCTGAAGAACTCAGTACAGCCATCTTTGGCGTTTTTTTCAGACAGGAAGGGCGGGAGACAATCACTCACAAGAGCTTATGAGGAGCTCTACTGCCATCATGAGGATGCGGTTTCAAAACTGACAACACCACTGACCTTACTTCCCATGATCCTCAGCGATGGCGGAAGCTTTTCTGATGACAAGATCAGCTCTGGGTGCCGATTTGGGCTTCCTGGACTTCTCCTGAGGCCTCAACAGAGACTCTGTAGTTTCAGTACTGCTTTCTCATCAGCACCTGGACAGTGAGAGGCCTAGCTGGCCTCCGCTCAGCTCTGTACCTGGTCCAAGTCCCTCAGTCAAAAGTGTCTGCATATTCTGAAAGGTCTTGCCTGCCTCATTCCTCTTCTCTTGGGAGGAACTCTGTGCAGTCCACCCTCAAATCCTGCATTGCTCCCCATCTGCACCCAGCAGCATTCTGCCTCCCTGCTTGCCCCTGGAGTCTGATGGATTCCTGGTCTTGAGAGAGATCTCAGGGATCACTTAGTTCTATGTAAAACCAAAGAAACTGGGAGGATGGAGATCCTGCCCCTTGACTTCCTGTGTCAATGTCTTCTGACAGCTGTTGGTCCACAGGGAGAATACACCAGGGTCCAGATCCATCTGTAGTTGATGCAGGAGCATAGCCTTAACCCATGAATTTCAGCACTACAAATTCAAGCAGACAAGGGAGTTTATAAAGTGCTTGTCTTCAGTTCTCCCGTTTTCTATCACCAACTTAATCAAGAGCATTCTCCAGGACAGCCAGGCCCATCACCAATTTCCCCGGTAACTTCTTCGTCCTCATAGACAGGGATGAAGGCTTGGTAGGAGAGGTGAAACACACCGTACAACAGCCGTGGCTGATGACTTTTTGCCTGCTTCGTCTCCAGTCCTCCCGTCTTGTTTTTTGGTCTCCATTTGTTTGGCGTCCTAGGCTTCCTAGAATCCTGACTTCTCTTGGCGCGATAGTTCTCGGGTTTTGGGGTTTTTTTTTACCCCCATTCCTCTCCAAGTCTCTA

							AAACAACTAGGACACACCGAGACTTTTAGCAAGAACATGACGTTCTGATCCCAGGCCTGACTATGCTAC<br>GTAAAAGGACCCTAGAGCTCGGAGACCCCACGTGTGGATCGTCTCTGTGCCCCGAGGGGGAAGGGAG<br>GGAAGAGGGACCAGGAGAAGCCAGGGTGTGTCTGCAGGTGGCCTCTCTAGTCCCGCCC
MSTRG.13756.3	--	0.545	0.047	0.003	-2.729	down	AGCCTCTCTTCTGCCTACTGTTCTAGGTACCACCTCCACGCTTGATATTCATAACATCACACACACACAC<br>ACACACACACACACACACACTTCCCTGTTAATGCATTGCCTCTGTTGCAACATAAGAAACATAGTATCACA<br>GTTGTGTGTTGCTTTCTGGGCTGTAACTGTAATCCTTAGAACAGGGCCTAGCACAGAGCATATGCAAAA<br>TTACCAGTTACTGAATCAATAAGTTGTAGGAATAAAATGTGTAAAACCCAACCAGGTTAAGGTGCTATAA<br>GGAGGGCACAGTGGATGGTAGGCCCCTCAAATAAAATAATACTGCTCTACTGGGACTCTCTTTAGGACA<br>GATACATTAGCTGACCCCAAAGGATAGCAGCATTTGAGAGCACGTAATTGGAGCAGAGGAAATACATGC<br>TATCACGGATATAATGAACACAGAAAGATTAGCCAAACTCACAGAGCAGGCCACGCAGAATTGAGTGG<br>AAGCAGGGCAAGTGTTCCCTCCCACATGTCAGGCACAAGACTTGACACTGACAAGAGAGGAGATGGA<br>AAAATGTATCCAGAACTTTCCCCGAAGCTTATATAGCTTCCTACTTGGATCTGAATTCAGGTCCGTGTCT<br>GCCAAGCTGTAAACTCAGCTGACTTCATTTCACCGTAGGGTCTTCTATCCCAAGGGGTTTGCATTGTTAA<br>CCACAGAAGGGCACTGGACACGTGACTCATGGGACGTAATAAAACTAACACATTTGAAAGATATCAAG<br>CTCATGATTATATGATTACGTAGAGAATGCGAAGAAAAAAAATATAAGCTTAAGTTCAGAATGGCTGAGA<br>TGAAATGTTAGGAAATTCAGGCAAGAAGGGATGAAGGCATGAATTAAAGCTGGGTCAGACAGGGACCG<br>TTAAGAGCAGAAGACAGACAGACAGGACAGAGCTTTGTGATTGAATTGCCCCTGGTTTGCACTCTGAG<br>CAGATTTTTACAGAGAAAGGATGATGTCATCTACAAAGTTGAGAAACAAAATAGACAAATCACAAAGA<br>GGATTATTTCCATCATGTCTATCTGGGCACACAGCTGCTTAATGGGTAGATCTGGATTAAACCCCCAGGA<br>TTTATGGCTTTTAGTTCTTTGGTCATTTCTCCACCTGACATCTGAAACCAAATGACTGGAGCTTGTAACA<br>TAAAGCACAAAGAGACAGAGACCTAGTTCAGCAGTTTCGCTGTGTCAAGAAAAGTCTATATTCTGAGC<br>ATGAGAGCAAAATGTGCTGTAGCAGACAGCACTGAACTATGATGTATACACAATATATTATATACACATA<br>GGTCTTTTATCCCTCACTGTGACTTATAAAGCTGAGGACAAAGGGAAAATGCTGTCTTTCAAGAACAAA<br>ACATGGAAGGAGTCACATTTTTTTCCAGCTTATTTCATGAAAAGTGCTTAAGTACTGTGGGGGTTAAATA<br>TAAAAAAAACAGGGAGATGTGCTAACAGATGAAAAGGCTCTTTGCAAAACGTAGCAGGAGAGAGAGG<br>AGCTGAGATTCCAAGTCTTCACTTAGCAACGAGAGTTTTAAGAAAGGCCTCCATCCAACTGATACTTGA<br>CAGATGACGATAACATCTGGATGAAGACTACGAACTCTACAAACTGCAGGGGCCCACGTGTGGAATAAT<br>GCACTTTCTTATTTAATCTGAGACCTTTCATGCTATTGGAAGGGTTACTGTACTGAGTAATCAAAAGACA<br>AAAATGTCAGCTCTAATGAGACTTTGAGTAATAGGTAAAGTATAATGCCACTTCCTTCAAGCACAATGCT<br>GAGGAGATTTTTAAAAAAAAAATAGTGGATTCTTTTATTCCCTCTGAACTAAATGTCCAATTAACAAACAT<br>TTCCGAAACCTGCTACACTAATTCAGGATAATTACGGGGGAACCCAATTCAATTCCCAATTCTTGAACAT<br>TAGAAAGTGTGAAGCAAGATACATCAAGGATTCTTTCAAGCTGTGAAATGAAATGTAATTAGCACAATA<br>ATAACCACTTCAATGGGGGTTACTGGCCCACTCATATCTGCCCAAGCTTTTTGCCCTTAATTTAAATTCCA<br>CAATTGCAAAAGTTAAAAAAAAAAGTTTTCAACTTCTAATCATCAAGCCTTTGCTTAGCACATGTTTGTT<br>TGAAAGACAGTCCTACTGATCCATAGTTTAGGGGAGGGCCTCAATATCTGATGTTCTGAACCTAGAGCC<br>AATGTCAATCTGCTCTGTGAAGCAAACATCAGCTGCTCTTGTAAGCCTTTGGGTTACATGGCTTAAGCA<br>GTGCTTCCTGTAACTGTTAGCCTCTGGACGGCTTTACAATACTAAAACTATTGAAAATTCCCCAAAGTTT<br>TCACTTATGTGATTTGTCTTAATGATGTTTATCCTATCAGAATTTAAGGTAAACTCAACAGGAAAGAAATC<br>ATGCCCAGTACTACAAAATTAGAGAGCTTCCCAGAGCCAGTGAAGTTACGCTTAGAAAAGAGTCTACA<br>GGGGGTTGGGGATTTAGCTCAGTGGTAGAGCGCTTGCCTAGCAAGCGCAAGGCTCTGGGTTCAGTCCC<br>CAGCTCTGAAAGAAAGAAAAAAAGAAAAAGAAAAAAGAGTCTACAACTGCCACCTTGCTAGACCAGC

							ACAATTCCTATATAGACTCTACGTCACACATTTATCCCCACACATAAATGCAGCTGCCACCCCTCATCAA<br>AGAGGTTTCACTATAAAGCAAATGGAGACTATCACAGAAAGCCACAGCAGGTCACAGTATAGAGATCA<br>ACCGATCCTGGGAAGCCCAGAACCAGTGGATGCATCTATATCAGAGCTTCTGCATCTCTGTCTTGGGGA<br>ACATAGAAAAGGAGGCAGGGTTAGAGTCGGAATATAAGAAAGTGTATGAAGCTGTCCCACTTAGAAAT<br>AGCTGCAAAACCAAGATTGAAACAAGAGAAATTTCAACAAACATGTTTACTCAAAATATGGATAATTTT<br>GAGGGGTCCCACCTGTAGACAAAAACATCTAGAGCAATTGATGATGGGTAGAAGAGGGAAAATTAGTC<br>TCTTTCAGGGATGAGACTTCTTATTGGCTACGCAGTAAAAAGTGGTCAACCCTGGGACCATATATATACA<br>AACAGCAAAAAAGACAAAATGATTTATATTTATTTATTTGTACATAACTATATGTGTGTGTATACACATGTA<br>TGTAGATGTAATAACAATCAAAGAAAAGGAGACTATCAATTTAGGAGTTGGGGAAGGATATGGAAAGG<br>ATTGAAGGGAAGAGACCACTGTACACTATCGAGATAATCTCTTGTGCACTGTTTAAAAAGCATTCACCT<br>GTCAATAAAATGCTGATTGGTCAATGAGCTGAAGCAGGACTAGAAGGTGGGGCATCCATCAGTGAGAG<br>AATTCAGGGTTTAAGTTAGGCATGGAAAGATTCATCCCGACACACAGGAAGATGGTAGCATGAAACTG<br>AGGACCAGGTAACCAACCATGTGGCATGACCTTTAAACAGGATAATTGACTTATGAGCTAGTCAGGGAA<br>CAGGCCCAAGCTTATGGCCTACACATTTATTCATACATAGGAAGTCTTAGAATTGTTATTTTGGGGGGTG<br>GGAAAACCTTTAGTTACATAGTCTCACAAGAGAATGAAACAGAAAAGAAAAAAAAATAGCATGCTGGT<br>GTTATGTGTCCTGATCTCAACGAACACTTAGAAGTATTCCAACCCACACTGCAAGAGAGTCTGCACTAA<br>GCGTATCTGTTTTCATAAAATCGGAGTCTTCAGTTCAGTGCATGGATTTTCCACGGATAATCTTTTTTAAT<br>TAGGAACATATATAATTAAGAAATTTATTCAAATATGCAACCTCACCTATTGAATCTGCTTCCCATAAAAC<br>AAGTACTTTAATTATGAGTGAACCATATTCACCAGCCTTTCCTTTTTGCCTGGTTTAGTCAGCAGAACAC<br>TCCGATGCCCTTCTTACCTCTTTACTGAGGACATGGACACTCACTGCCCCTTTCAGCAGCTTCAAGAGG<br>GTACCTAGGGGCACCTTAACCCTCCTCTCACCAGGGCAACTCTCCTGGTTCACTCTCTTAAACATGAAA<br>TCATCTCATTTATTCCCGTATCAGAAAATACCACCATCATTTGCTCCGATATTTGCATTAAACAAGGAAAT<br>CATTCCTAATTTCTCTTTTTATCACAGGCACACCTAATTGATATGTTGCCAAATCATGCCACAACTTTGGT<br>GGACTCCCACCCACGGACTGTCATCTAAGCTACCATTGCGCTTTGCTTTAATTAATGTTGGACACTCCCA<br>GCTGGCATCACTGGACCTCTCCTCGACTCCATAATTCATTCATCATGGCCTGTGAATGTGTGAGCCAAGT<br>CTATCTCAATTCTTAAATACCCAGCTCTGTCTACATCTCTGAGGATGCAGAAGCAAATTGCAACAAGTGT<br>CCTCACCTCTACCACCAATGCAACTTCAATTCGCGCTGTCTCACGTGCCTAACATTCTTCCTGGGGTTTC<br>CTTCGTCTGGAGCGTTCTATCCACACTTTTGTTGACCTGGCTCTTTCTGGCCATTTGATTCTCATCTCCGG<br>TTTTCCTCTCTGAAGGAAGCATCGTCATATCACAGACTGCCAGACTCACCAAGGCTCCGTCGCCACCCT<br>ACATAATTCCAATTATTCCTAACATTTATCACTACCTGAAAGCCTTTTGCTTTTGTCCATCGATGTTTTTTG<br>TCTTTTTCCCATTCATCCATCATCAAAATATATATAATATTCAGTTCTCTATCTCAGTCTGCTAAAACACAG<br>TAATAGTAAATGACTTAAATGCTACACTTCTCGAACACAGAATGAATAGTTAAATAAATATGTAAGTTCAT<br>TTCAATAAAGAAGCTGTCTTGCAAAACAATCCACTAAGTAATTTTTAGGTATTGCCAGTTGTTGAGAATA<br>TAAAAATTCAAACATTCTACACCACCCACTCAAAGGCACACTGAAACATGCCACGAAAAGGGGTCATC<br>TAATCTTTGGGGCTCATTAATCTTCACTATGTACATTTGTGACCTCAAAATCAATAAAAAGAATATTCTAA<br>AAGAGTTTTCCAACTTTTGATTATTTAAACTCTGATTGATAAAATAAATGATTAAAACTCACAGGAATAA<br>ACTCGGGAACTCAAAGCCATCTTAATTTTCTTTCAAGAAAAACAAACAAAATGACTGTTGAAAACCTAC<br>AGATTGATCATTTCTCCTTTGAGAACTAATTTTTACTTCCGTTGAAAGCTTTATCATCAATAATGATAGAA<br>GGAAGATCCCAGAGTGTCTTATATGTTAGAGCAATTCACCTTAGCAAAGTTAATCAGATTAGGCCCCAG<br>AATTGAACTAAATAAAATCACTAATGTAAATGCAGCCTGTAATAATTCCACAATATATTAAATATGCATGA<br>AATATAAATTCCCGGGAATTTAGTTTAATGCCCTTCTAAGTCATGTCTATTAGAATTCTAAATCTCTGTGTG

							ATACTTACAGAGATTTAGGACAAAAATAATTTATTGGAAAGGAGTTTGCAGTATAACCTTAGCATGAACATCAGATGCAACTTTCCAGGCCATATTGTTCAATTCAGAAAAAGCATTTGCCGTTATAAGCTGCGTTTATGAAGTGGGAGGCCATTGTAGAATTCCAAGAACAACTGGGTCTTAATCCAATGGTCTGTACATCGGGGTGGGCTGTTGCCATGGTAGCCACAGTCTGAAGCTTCTCTGCCCTCTCTTGAAGAGTATGGCAAAACCATTACACACTATTCAGGGATGTCTCATCAGATGGATGGATAAGAAAAGCATTAGTCTCACTGGATGCCACAAAAGTCATTGGGAATCTTGTTACAGCCTCCAAGGAAGTACATGGGTGTGTCTTTAAAACTGAATTCGCAGTTGCGGGGTCTTGTTAAATGTTTAGGATGCACACCACCAAACGGTTTGATGTGATGATGGGTGGGGTGAGCTTGACCTCATTATTGAGAAATCAGTGTGCCATTACACTCTGGCCCAGGAAGTGATCATATTTTCTTCTCGCTCTTTCAACTGTGCAGCCCTACTTATTCTTAATTTTCACCCAAATAATACACATTTGAACTTCCAAAGTTGTCATCCCTTCAAAATGAAGACCGTGGAGAGATTGTCATTTGAACAAAGTGAATGAGGATAGAAATGAAATCCAAGATCCATAATTATGTAGAGTGACAGTAAAATGGGGGAAAAAAGGAGACTTGCTCATAAAAAGTGTGACTCAGGAATTGCACCACTGAAATCACCCTGGCTCCTTTCTGAAATCAGTTACCTTCCTGGCTACCATCTCAAAACTCCATGCACGGCAAATTTCGATTATTCACAATTTCCGAAATCTTTAAAACCTAGAAAAGTGCTAATGCCACCTTTTGTAAGGTAGTAAATTTAGCTCGACTACTATGGCTTCGTTTTATGAATTCAGGCAGGGATTGATTAGCTAAACCTGCCAGTCAAAGTCAGTCACGACAGGGCATGGCAATAATCCCAGCATTTGAGAGCCCAGGGCAGGAGGTTCTCAAGCTTGAGATCAGCCTGAGGAACAGAGGGAGACTCCCAGTGTCTACAACACAAATCCACAACAGGAAAAATACCTTTGTTGTACATGTGCCACTTTTCAATAACAGTGCCTGTGGTCAAAACATTGGGTTAGTCCTGCTTGAATTATACAACAAAATGTGTCCCCTTTTAATGATACAGATATATCCTTAGTATATTGTGGGCAAAAAATACATTACTCAGTTTTATCATCACGTCTGTCACTAAACGACCTGTCATGGTTTCCAAATACATAACAACTCTCACAGGAAAAAAATAAAAGAAAGAAAAGGACGAAGAAAGAAACCACTGTGGAACATTCAAAAACACATCTACCATCTACCTCTGAGGGTGATTCACATTCTAGAACATTCTGGAAAACAAGAGAAGATAGAAATGAAATAAAAAAAATATTTTCCAGCTTCATATTTCGAGAAGGAAAATGATTTTGGGATCACACACAGATAGAAATAATTAACTCCTCACATGCCTAACTCAAATGTTAGCCTTTACCTCTGCAGGCATTCACATATGCAGGAGAAATAATACTTTCCAGGATTTGTTAAGGAGACACAAAACCAAAAACATACAAAAATCAGGAGATACACTTCACTGCATTCAAATTAATAACTGCTATCCCTCAGGTGTCAATAAGACATAAACAATACAAGCTCTACCTAAGAGAAAATATTTACGATGCAGATAATTGCCAAAGGTAAGCTCCGAATGTCAAATCAACTCCCAACATAAAGCTGAGCAGACTAACAAAGACTCTTCAGAGGGACAGCAAAGCTCCAAGGGGTAAGAAAAGAAGCTTTACCTCATTAGAGGTTAAAGGGAAAGAAGGTGAAATCGCAGGACAGCACTGTGCATCCATCCAGTGGGTTAGATCACTGTTAGCCCATAAAAGTCACCACGAATAAAAGAAGAAAAGTGGGATGTACACGAACCACAATCAGATATGACCAGGTTAATAAAAATACGTTAGAAAGACATTTGGCATCATTTCACAAAGATGAAGTGCTTTCTGGGAGGGCAAACAATAGAAACAACGTCATTATTGTCGGAAGCTTAAAAACATATGAAATGTAGCATTATGATGAAACAAAATACTGTATAAACCCATCAAATGTCCAAAGAACAGCTCAGAATTATTATAGTCATGGGAAGTCTAGGCTATTCAACGCTACACATGAGCTTTGCATGGTTGGTGTGGTAAAGAGGAGCTCTCATGACCTGTGGAATCTTACAAGGTAACTTGGATAACGCTCATTCACAAATATACGGGCTCGGAATTCTCAAGTTGGGGAAGATAATAGATGAGAGATGATTTAGGCACAGAAGTCCACCTTCCACCCACACAGTTTCTCCTAGAGGGCACTACGTAAGTACCCCTAAAATGCCTTCAGAGATGGGTTCTGGCCACCACATAAAGCTGTTGCTTTCACTTTGGGGACATTTTAATTAGTAGATTATTGATTCTCCAGATTCAATTTAAACGTCTTTGTAGCCTGCGTTTTCCCCCTCTGGTTATCTACACTTATGCAAAGCAATTCTATTTCCATATGAGAGGAGTTCAAGCATTTAAGAAGTTACATTTTCTCCCTAAACTTCCCATATAGAGATGAAATATTGGCAGTTTTTATAAATGCATAATTCCTGAATTTTCTCCAGCATATTAAAAGCTCTTTTGCACGTTGTACCCCAGAGAGCATGATACTTATTCCATGAGCCACATAAATAACTCAGCTCCCTGACTC

							TGACTCCGCTATCATATCTGAAATATTATTTTTCAAAAGGCACACTTGGTACTTAAGATTCTCCTTGTTTAT<br>GGGCTTTCACAAATACATGTTTTGTTTTGCTTTTGGGTAGCTACTTCATAAACATAGTCTTTTGCATAAAT<br>AATGCGAGTGTTTTGGAAATACAAAAATAATCTGACGCAGCTTCTGGGTGAACGTGAAGTCTCCTGAAG<br>TCAAAGGCCACACCCAATGTTAGTGAATAAAATGGCCTGCTTCTCCTGTTGCATTTACTGAAAACTGTTT<br>CATATAAAAATGTATTCAAGCCCCTCATATGCTCAGGCAGCATTTTGAATGCTAACACAGAGCCTTATCA<br>CTAATATCTACTTTTATATAAAAAAAATCCCTTGTTGAACGCCAGCATTCAAAACGTTATTTAATTTCATG<br>CAGAAAATAAGAATTTTTGAAGGGAACTAACAGGTACAGTGGGAAATATGTAAGTTTACAATACAGGAT<br>GTATAGCAAAATAATGGTTCTGAGTGTATGCAACGTGTGCAGACCTGTATCCATGAATTACCAACAATGA<br>AGCAAACCGAGTTTTTGCCCAAAATTCCATCAAACCAAGTTGCTAAGATGTCTCTGTTTTGAGAGCTGC<br>TGCAGATAACCAGGTACTCCTTAAAGACAGTGTGCAATGGTCCATGCCAAGTCTTCTCTCTCCTGAGCT<br>AATGGGCAGCGCTTGAGACTTTTTTTAAATTTAAATGTGAGAAAATAAAATCAATGTTATTGACTTTAGG<br>CAGGAAGAAATGGCATTTCCAACATTCCAGGAGTTTAATCCTCAGCCGAAAGTACACAGTGGGGAGGA<br>GCTAAGAGTTAAGACAGATCGCAGATTCCTTCGATGAGAGAAGATGAACTAAGGAGGGAAGAAATGAA<br>TTGCGGGTTGTCTGAAAGGAGGGTGGCACACAGTTAAGTGTGTGGGGCACGGCACGGAGCCCAGTGG<br>CGGAAGATCTGAAGAGAAAAGTCCCGGATGGCTTTCATCACGGGCTGTTCCTACTCAGCCTTGGCAGC<br>CTTTCCCTTCCTGCAGTATCATTTCTCCTCCTGCACTTTGAGAGTGTGCATCTTTTGTAAGTTTATGAATT<br>GGGACATTCATAGTAAATATGTTCATGCTGTACTTCTAATCTGTCTTTGGAAAGTTAGCATTGCTGAAGA<br>CATGGGTATGGAAAGAGTTGGCTTCATGTTTTAATAGATGTGTGGAATAATATTATCAAAGGATCTAACTT<br>CTCTAAATACTATATACTTCTCTATGTACTAATGCATAGTTAGATGATCTCATGGAATAATTAATAATAAAG<br>ACAAAATGTTAATAATTAGCAAATGACTAAACATTGTGTAGCAAGAAAGTACTTGAATGAGTTATTAGTA<br>AATTAATCATTGGTAAATATCTTATAAAAAATAGCAATGATGATTCCACTGAAAGAGTTAACTTAAATAGG<br>CAGTTCTCAGAATACTCTGTCAGACTTGGGATTCAACAGAGCATGAGGTCTGCTTCCACACAGTTGTGC<br>ACGGCCATCTTCTCTAAGCCAACCAAACACTTCAGCATATTACAATCAGCAACCTCTGGTACCTGAATGT<br>CAGGTGAGAATCCGGAATACTAGGTTTTTTTTGGATTCAATTGCTAAATCAGAAATTTATTTTAAATGAG<br>GGTAAACTGAGGTTTACCTTTCTAATAAAAAGAAGACCATAATATGGTTGAATTAGGGAAAAGTTGGAA<br>GAAGTTGAAGAGAAGGGCCACCCTTGCAAGAGAACCAGCAGTCTCAATTAACCTGGACCCCCGAAGT<br>CTCTCAGACACTGGATCACCAACCAGGCAGCATACATCAGCTGAGATGAGGCCCCCATATACAGCAGAG<br>GACTCCCGGGTCTGGGTTCAGTCAGAGAAGATACACCTAACCCTCAAGGGACTGGAGGCTCCAGGGAG<br>TTAGAGGTCTGCTGGAGTGAGGGGTATCAAAGTGGGGACATCCTCATGGTGACAGGGGGTCAGGGGGA<br>GGTATGTGATGTGGAAGAGTCGAAGGGTGGACAGGAAGGGAATAAAATCTGGAGTGTAGAAAAAGAG<br>ATTAAACTTTAAAGGGGGAAAAAAGGAAGCAGCACGTCATAAAAGAGGCCAATGTCCATTTCTTAGTG<br>GAAATAAGCTATAAAATATGGTAAACAATTTATATATTTGAGATGTCGAAGGATTCTTGGACACGGCAGT<br>TGTTTTTTCTTTAAACCTATCCTATAACCTTAGGAAGCTAATCCTAAGCAATTCTTGATGAAATTTGAAAG<br>TGATACTCTCATGAAGTTGTAATTTTAAGTGTCTGAGAGGATCAGTTCACACTACAGGGAAACTTGAAA<br>ACAGGGCTTGTTTCCTCTGTCGACTCCGTGAGTGTTTTCATGAAGTTTTGACAGTAAGTTTGTGAAAGA<br>TCCTTCAGAGCCAACTCACTTATCGTACTGTGCTATACTCTTTGTAAATATTAATGTTCACAAAAAGAATC<br>TTTCTAAACCAAGAACGTGGCTGGGCAAGCTAGGAGAGAAGATTGACTGTGTTGGTTCTCAACAAAAC<br>TAATTACACAATAAAACATTTCCCCAGCGAAACTTTGTAAAAATTTTAAAACAGAGTTTTTTATTTTAAAT<br>TATTTTTCCATAAAGATAAAAGCAGAAGTTGTATTGAGCTGATTTCAATTCCTGGGTGTAGAAAGGCTGC<br>TACCCATTTATGGAATTATGGTCACTTATTGGATTTATTAAAGATAATCAACAGTATAAATACAGCATGACT<br>ATAAATATTAACGTATCATTTAAAGTCTTATTTTAAAAGGACAATTAAAGCAGAGGTTGTAATGGGTAATT

							AGGATGATATTTTGCTTAGTTAATCTAACAATGAAAAGATACATACAATTTATCCAAATTATACCAAGGCA<br>ATCGCTACTGAGGCAGTTGTGAAAGTCGTTTATTTATCTTCACTAATGGAAGCAATATCGTCTCTGATAA<br>ACTTTTTTTACATATTTTGTGCTGAATATATATGGAAATAATTGTTTTTAATTTCCTTAAACTATGAGGAATT<br>AGCTTTGCCTAGTATTTAAATATTGTTAATTTTACATCATAATATTTAAGTTATATGGTATCATCAAGATAAG<br>GGACCATCACACAGGGGTTTTGATAATTTTCTGAGGAAAGAGTTCATGTATTTTCAGTTGTACAGTGTAG<br>CGCCTCATGTTAGAATGAGTTTGTTGCTCACTGTCTTTTAAGATTAAATATGGTTCTATATTAGATACCGA<br>GTGAGTATGAGATAGACTAAATATATTGTATCCAAGGATACCAGACACATACTTAATTGTTATTTTAGATA<br>CTTCTTTGAAGGCATGCTAGAGGTGAGAGTAATATTTAAGAATAAACTGGGTGAGGTTTATTGTCATACA<br>AAATGTTAATGAAAGCCCTTCCGTCATCTGATGACTTCAATAGGCAAAAGGCTGATTGATCTCTACCTCA<br>GAAATGGGAAGTCTGCAGTAGACGACCTTCAGCAATTGCAGCCCTGAGCATCTTGGACAGCCCCCACA<br>GGTGCACAATCCCATTTCAAGACAAACCTCTTTCTCACTGCTTATATGTATGTATTATACACATATATAATG<br>TATACAATTTGTTCCATTTCTTTATACAACCCTTACATACTCCCATAATTAAGACCTCAAGTTATGAATCAA<br>GATTGCCTGGAGAAAAATTCTAACTACCATGATTGACTTGGAAAATAGCACATCCTTATTGATTCTCAGT<br>CTCCCCTTTTATAAAATGAAATAATACATTTAGCCTTTATGCATGACTATTTTGAAAATTAGGTAAAGTCTA<br>TACATGGAACTGTTTAAAAACCACACTTGCTATAGTGGATGGTCTAGACATTCAATAAGCCATACAGCTA<br>AAAGTAATCTACCCTTTCCCTTTGAAAGATAGGAGATGACAAATGTGCTTTTTATATTTGTAAGGTTTTAT<br>GTTAATCTATTTTTAATAAATATTCATTATGGAGTAAAACTTAAGATGCGAGTTTTGAATATTGACTGTTGC<br>AGTAGAAAACCAATGGTTCCAATTTGAAGACAACCAAGCCAAAAGTTCATTTTGCTTCATACAATGACT<br>GCATTATACATAGTTACCCTATGAGTATAATGACAAATCAAATTATGTGAGATCACTGACATATATAATGTT<br>ATTCCTAATTTTAATCATTCCTAGGAGAATACCTCTAGTTTCATAGTCAATATTTGCCGCCTATTTATGTCTT<br>TCAGATTTTTCTACCATGCCTACTCACTGTCTTACAGTATACCTACCCTGTGAATTCATTCCTATCCTTACA<br>ATTCTCTTCCAGGTACATAATATTTATATTTGTGTTTTGGTTTTATCAGAAGAAATTTTAGCTTATCTTAGC<br>AGAAACTTACAATGTGGAAGCAAAATATGAAAATAATAAAGGAAAAGATGAAGTACATTACGATGTAAA<br>AACTGAATATTACAGAAGACTACAAAGAAAAATTAAAGGCAAGTCACAAATGCACAGATAATTATAACT<br>ATATTTTCAAACACCGAATAAAATAGATAAGACATTATGATTTTATAATGTGTTATTCTTTACATCAAATGT<br>TCATAGAAATCGAAGGTTCTTAACCTTCCAAACTGCCATTCTTTAATACAGTTCCTCGTGTTTGGTGATC<br>CCCAACCATAAAAATATTTTCATTGCTACTTCTTAACTATCATCTTGCTACTGTTAAGAATTATAATGTAAA<br>TACGTGCTATTTTCATTGGTCTTAGGTGACTCCCATGAGAAGGTTGCTCAACTCCCAAATGGGTTGCAAC<br>TTACAAGTTGAGAACCACTTATACAAGTTATCTTATTTAACTTCCTAATAAGCACATAAAAAGACAAAAG<br>CAAAACAGATATTATCAATATTGGTAATGATGATACTTCAGGCCACAAGAACCACTTGAGGTTTTTTCTT<br>GATGGGAAGCTTTCCTAACCTTTACCCGCTAAAAGCCTTCTCAACCTTTTGTATTATGACACCCAACCAT<br>GTCTCCAGAGTTTACAAATTGAGTAGTTGAAACCCAGAGAAAGTAGGATTTGTCCCAGTACACCCTTTC<br>TTGGTGACCCATAAACTAGAAGGAGACTATGGGATTCATGAATGACTCAACAGAAACACTGGTCATGCC<br>TAATGAACTCAACAGCTTATAGCACATGCATTTCTGGACTTGCTTTCTTCCCAGCTAGTTCAGAAATCAC<br>CCCAGGTTCTTTCTCCAAAGGTAAAAAGCTGGTTTTATAAATTCCAAATTAAAAACAATCAATATTAAAA<br>TCTACCTAGGCATAAACAATATTTACGAAGTTAATGGTCAAGTTGTTCATCAAAACATTTCTACATCAGTT<br>CCTGAAGGGTTTTGTGAAACACACGACCCCTCTTATTAATTAAAAAGGTAATATGCCACGAGGTTTACAT<br>TGACTCTGTGATGCATATGCAAGTGACTGAAAACATCACCTTTATCTGGAAGAATCCCAACAGGAAACC<br>TGGTGATTTACTAAATGTTAAGGGTAGAGTTGAATGTACTAATTAATATGAAAGACATGAATTACCATTAA<br>AGCGCCATCAGAATGCCAGCTAGTGTAATTGTGTGCAGTCTCGTGCAGGTAGCAAGTAGTATTTCTTTCA<br>CACCATTGTCATTCCCCAGGTGTGAAAACATGGGATCCTTATATTAATATTCTAAACACCAGCATGTCATA

							AATTACAACCATGGTACGTAGTAGTATTTTAGCTCTTTGTTCAAGTCGTTTCCCAATAAAAGAAGGAAGATAGGAATTGTTCCACAGAGTACATTGCGATCCTTGAGAAACTCAAGATGGAAAGGCAACGAGTGGCAGAGAACAAGTTACTAACCATACCTTCCATGACATACACCAGTGACCCCACATCGCCTTCCTTGATGATGCAGCTGTCCTTCCCATATTCCACGGGGTACATACAATCCACAATCTCCTGGATCTGTGACAGCTCCAAGTTCTTCATGAAGTCATTGTCAAGGATAGCCTCCTTTATGAGATCCTTCGACTGTGGGCTCTTGGGGTAGAAGGGCAGGGTCACGTGGCTGAGATCCTGGATGTCGAAGGCGGTGGGCTCCGCGGAGATCGCCTGGCGCTTGGTGCGCGGTTCACCCTGCAAGGTGCTGGCGCTCTGCTTCTGAGCCTGCTGGGTGGCCGGCCGGATTACCGAGCGGTACTTGTCCAGTTCATTCTGCAGCTTCTGGATCAGTTCGTCCTTCTGATCCAACTCCAGCTCCAGCTCATCGATGAGAGCATCCCGCTGCCTCAGCTCCTCGATCTTCTCCTGGAGCGCGTACTGTAAATCCCGCAGGGTGCCCATGCTCCTCCGGGCTGCCGGGGGCTCCTTCCTGCCGCTGCTCCGCGTCTTGGGGCTTCCTCCCCTCTCGGATCAACTTTCCCTAAAGTCGCCGCTGCCTCGCGAGTGCAAAAGCTTCCCTCGACTTGAGACCAAAGCCAAGCCTGGCTTCTGAGCATGCCCAGTCCGCCGCTCCAGCCACCGAGAGTTTCAGTGTGCAGATTCCACTTCTCCGGGTTTAGGGATGAGCAAAACAGGTCCAGCTCAGGATGAACCTGAAAGGCGGTGGGCGCCGCGGCGCTTGGGGGAGCAAAACCCGGAGGCGCGATGACCCAGGCGCGGGAGGAGGGAGACTGGGCTCCGAGGCGGTGGCTGCTCGGCTAGGGTCACCAGCGCTGGGTGCCAAAGGAAGCCAGCGATGTGTGTGGGGAGCCTGATAAAGCTCGGGGGCGCTGGGGCTGTGCGGGCGGAGCGAGGCTGGAGCGCTACTTCGGAGGTGGAGAGGTGGGAGGGAGAGGACCTGGAGCAAACTGGAGAGGCGTCAGCGGTCTCCGGGCTCCAGAATGTGGGGCTAGGTTCCTGGCGACCAAGGAAGACAGAGCAGACTGCGCGTGCTAACAGCTTTGACTTCTGGAAGGATTGTGACCGCAGCCTCACCGAGGAAAACAGTGTGGCTGAGCTCTCGCGTCCCCCACACCACCTTTCCCACGGGGTGCGCCTCACACAGGTGTCTAGGGCTTGGAAAGAAACTTGCCTCTCCAAAGGTGAGAGCTTACAGTAGTGCCTGGGAGGGATTCCCCAGGCATCCAGGTGCCTTTGGGGAAGGTTAGGGATGGGTATCTGACCCCCGCCCCCAAGCCTTTCCTCTCTCCTAATTTTCCCTAATCTGGGCTGGCCAAACTCAGATCAATGAATTTTTCTAGGGGTGAAAGCCAAAAC
MSTRG.14003.1	ENSRNOG00000018494;ENSRNOG00000056753	0.117	0.635	0.007	2.891	up	CTGGATCTAATGGGACACTTTCCAGGAAATATAGTGGATAGAGAAAAAGTCAACCCCATGGAGAGCAATCGTGAAAATCGAGGCGGCAGAAAAAGGTGTGGGATAAATGATTCATAGGATAGATCGGAGATTAAGGAAACAAACATAGATTGAAAGCAAATTACGTAAAATATAAAATAAGCACATCGTGAACTTTGGAGTCTGATTTGAAAAGCAGCTGTAAGAGTAATTGAGGTTCCTGGTGCTGATGACCTCTTGGCAAGAGTATGTGTGTCAAAGGATGCTTCCCTCCTCTGCAGAAGATGGGGAACACAGGCAGCAGGGGCCCAAAGCCCCAGGTCCCACCTCACTGGTGGGAATGTCAGGATGCTGTGCAACCACGACGACGTCTTCACTCACGCAAATCCTAGCTCCGTGATGACGTCACCACTCTCTGCCCCGCTGCAGGGGGAAGGTGAGGCTTCCAGTATGCTCAGGACTGAAGTATACGTTGGCTCTGCTTTACAGCTTGG
MSTRG.14099.2	--	0.285	0.000	0.037	-3.684	down	AAGGAAGGAAACAACCTCTGTCTGTCTTTGCTGGTGGTGGCAGCACATGCCTTTAATTTCAGCAGCTGAGAGACAGAGGCGGGAGGATCACTGAATTGGAAGCTGGCCTGGCCTACAGAGCTAGTTCCAGACTTGTCTACACTGTATAGTGAGACCCTGTCTCAAAAACAACAACAACAAGAACTCACCATTTTCAGAGCAGAAGTAATTCCAAAGTGAAAAGCCACCAAAGGGAGCTGGATTGCCGCCCTGCACACAACAGCTGAAGAAATGTGGAAGATCCAGGCAGAACGCACCTAAGGCAGATGCTGTCTCAGCACACTGACATAGGCTTTGGCAGCGTTCATTTCTCCACACACCACACACACGGGCACAGCTACGGATCTGCTCCACAAATCCGATCTTGTTTGCTTCTCACTGGCTAGCAATGATGACTCTGAGAGCGATGATATTTTTATGGTGAATGGATGAAGCCTTGGCAGGTGCAGACGGGCTCCAGTGAGGGGCTGGCTTCCTTCCGTTCTTAAGATTTTTCTGACTCACATTTTCCATTAATAATAAAAGCTCTTCCCTGTTTTCATTGAAGGGCTGGTTAAAAACGGAAAAAGAAAAACATCTACGCATTTCCAACTTGCCTCCTAGATGTGTCTCCATAATTTAGAATTCAGAAAAGAACATGAATTGTTT

							CCCACAAGTTATGAATGAGGAATTTGGAGATTTTATGTATGTACACACACACACACACA
MSTRG.14173.5 NONCODE_ID: NONMMUT034707	ENSRNOG00000014551;ENSRNOG00000036592;ENSRNOG00000056787	0.715	0.000	0.000	-5.525	down	AGAAAAGCTGCAGTCCAGCTCAAAGAATAGAATCAGCATGGAAAACACTCAGGAAAATGCAACTAGACATCAGAAGGTAACTCTGAAACCAATCAGACCACACCTCAACCCGTCCTTGAGACTCAGAGGACAGAAATCTAGGGCAGGGCAGACTGCGTGTGGCTGAGTCAGCAAAGAAGTGCCCCACAGTAAGAACAACCTTAGCTGAGACCTGACCAGGACCCCAGAGGGCGGAGGCGCCCCGTCGCACTTGGTGATGGGTCCCTAGCGCCGCGGCGGAGAGTCCTCAGTGCCCCCACCTCTTCCGGGCGTCCGGCCCGCCTTTTCCTGTAAGATGCAGTCGCAATGCCCGCCCAGAGCCTGGACTGCAAAAGGATCAAAGAAAGTCTTGAGTGGGCCGCGCGGCCTCGCCCTGCCAAGGGGGAGCAACAGGTCTGGGAAAGCCTGACAGGTTGACAGGAGTGCGAGAGCACAGTTTGGGAGCCGCCAGGGGACGCCCTGCCTCGCGGCATCTTGTCCGTTGCGTCTAGATAACAGGCTTCGAAAACCCTAATTTGAAAAGCCTTTCGGACAGCCCTTAACTCCACGTCCATTGCGAGCGCTCACAGACGTCGGCCATGACAGTTACATCCGGGTATGATGATGAGCCAAGTCCTGAGTCTCCCGGAAGTGACGCGACGCGGAAGGATACGCGCTCGACCCAGGGATGCTGAGGTGAAGTGGTCTCCGGAAGTGGGTGCAATCTGACTCTGCTGGTCGGACTGGGCAGTAGGAGGAGCGTGGCCCATGACACAAGCCCAGTCACTGTGCTTAAAGCCAACCCTGCTGTACCGTGTTCAACAATCGGAGAGACAGAGGCTGTTGTGTTTATTTCATATATATATGGAGGAACTAAGATTGAGAGAGGGCAAGCAATTTAAGCCCATACGAATAGAATTTGACCTAAGTCAGTATTTCGTGATGACTCCCTTTCTGGAAAATTTAAATTCCTATACCTTGTCACCATAGATTGTTGCCTTTGCAAGCCTTTCTTCAGCATAGTAGCCCAAGAAGTGGTACTTCAGCTCGCCCATGATTTTAATTAAATATCTTTTTTTTTTATTCTGACTTCATTGGGGTTATAGTACCTTCCCGTGTTAGGGAACAAACCTTGGGAAACTGTAGTTTTAAACAATTTATTTCAGACTCGGCAC
MSTRG.14196.1	--	1.116	0.038	0.000	-3.909	down	AGGCCCTGGTGGGTTCGGTCCCCAGCTCCGAAAAAGAAGAACGGGGGGAAAAAAAAAAAAAAACAGAAGAAACCTTTATCTGACTCCCATGTGTGCGTGTTCTGGTGGGATGTCTACTTAACAGTGGCTTGTTGCTGCTTTGGGGGAAACGCATAAACTATACATGGAGTGTTTGCAGATGATTCTTTATGACCTCCACTTTAAACTGTCAGACATTGGTATTTTATCAGTCCACACTGTTAAATAAAGCTAATGTTCTTAGAAAATCAACTTGTACACACTGTTTAAAACCCTCAGGGACAGTCTTCACACTTTCCTCACTCAATTCAGGTACTTTGATGCTATTCTTAAACCTAACAGTGACTTGTATTGTTCTGTTTTGCTTTTATTTCAACGTGCTGGTAGCACATCTTTGATGAATGTCAACTTAAAAACTCAGTTCAGGTATCCAGGTATAACTCAGCCAAACAGATTTTAAAGCTGCATAACTTTCCAGCACACGGTGCCTCACACTCTTATAGTGGCATTCTATAGATTCAGTTATAACTACTGAGCAGATAATATGGGGGTTCCTGTTAACAGTGTATTTTTTTAAACATGCATACATGTATAGCCACCACACACTAGATATATATTTTTCAATGCCACTGATAGCCAGCACAATGAAAATAAGTGACATTCCTAGCTGCTTCTGTAAGCTGACAGATGCAGCTCCTCCTTGATGTGGCTCTTGTTTCTTCATCATAGCTACTACTAAATTCAAGCACTGGCCCTTGGGTGTCTGACCTCTACATTCTATTTATGCAATGTCTTCAGAGAATCTGTGCACTGGCCACTGTGATGGAACCATTGGGCCGTAAGTGCTTTGAGTTTATCAGTAGTGAGTCTGCCAAAGTTGGTATTTTAACATGAAGGATGTAAAATGTCAAACAGTGAGCAGAGGTCTAGGTCTGCATACAGCAGGAAGTCTTATCTCAGTGTACTTTACAGCCTCAGAGACCTCAGTGACAAGCTCCTTCTGATGGTGACTTGTCTTTTTAAACACTTGTTTTCCACAGTGCTACCTAACTGTGGTTAACAAAGTAAGTCACCCCTCTGCTTTCCTATTTGATATCTTACATCCGAAAGAAACTCTAAAACCCATAGCCTTTGAAGACATGGAAAATGGCATTTTTCAGATTTCTAGAAGTTCAGCGTCATGCAACAAAACAGGAACCCCCTTACTCTATGGACCTCATTTCAATATACTGTTTACAGTTTGACGGAATTGTATAATTTAATATTTCTCTTGTACTGTAGTTAATATTTATTTACAGATTTTTTTGTACTGTGTGATTTGAACTTTTTGTTCTTGCTATAATCATTTGTTTATGTAGTAGAGCACTTATGATCACAAATTAAGTTTTTGGGTTTGATTGCACTACATTAAATTTTTTAATGCAGTTCTGATTTTGACTGGACTAAAATAAGCTGTGTCTTAATGTATGTGATGAGTACTTAAATTTTAATTCATGTGGTTCCCCTTTTTTTTAATTCTTTTTTTTTT

							TTTTTTTTTTTTTTTGCATTGTATGTCAGAAGCACTACTTCTTTTGTGCATGTGTAAGACGTAATGGTTCCATTGTATTATTTGACCATGACATTTTGGAGAAACATTCCCAGCTGTAATGTTGTGTATGGTAGTTCTCACTGGATGCTAGACTTTAAAAACCACTATTCTTCTAATAAATTTTGTTGTGAAAACCTGTTTTTAAAGCTTGGTTTGTGCTGCTTCAGATTTTAGTGCTTTTAAACGGTTCACAGAAAAAGGGTGAATAGAGTGCTATGAGATGCACATGGCAGTCAGTCTGTGCCTCTGTCCAGATGAAACCTTTTATTTGGATCACTAGAAATGCTGCTGCAGAATTAGAGGTTCCCATTCTAATGCACACACCACCTTGTCTCTCAAGTGCTCACTGACTGATCCTATCCCTGGGTTATACATTCGTTTTATTGAATTGTTTACTTAAGATTAATCTTTTCAGCAAACCAAGGTTTCACAAGGCCATCTTCTGCCTAAAAGTCAGTTTTTCATAATTAAAGTAAAACTTCTTGGGGTTGGGGATTTAGCTCAGTGGTAGAGCACTTGCCTGGCAAGCACAAGGCCCTGGGTTTGGTCCCCAGCTCCGAAAAAAAAATAAAATAAAATAAAACTCCTTAGTGGATTGTTTGCCAAAACAGAAATCTTT
MSTRG.14198.1	--	0.461	0.000	0.000	-4.918	down	CTAAAATGTCAGTGGTAGGAACTAGCCCTCAAGAGAACGTGAGAGGGGTTGGGGATTTAGCTCAGTGGTAGAGCGCTTGCCTAGGAAGCGCAAGGCCCTGGGTTCGGTCCCCAGCTCCGAAAAAAAAGATCCAAAAAAAAAAAAAAAAAAAAGAGAACGTTGAGAAAAGAGGTTTCATAGAGACGTGGTGAAGGCAGGAGGCCGTACAGTCATTTTTTCGGTATGTCGTCTGGAACCGCTCTTCTCTCACCTTTTGAGTCATTCCTTTGTTGAGATATGATTGATAAATTCGAGATAATCGAGAGTAGATAATGCTTTCCTTGTGGTGCCAGGAAACCTTGTCCTTAGGGAGAGGACACTCAGGCCTGTCACAGCTCAGGGGAGTCTTCCTCAGGCACATGGTTTCTTCAGCCATAAGCACTTGCTCTTGGGAGCAAGGACAACAGCAATAGGATGCATGTTTTGGGAGTCTCCTGGACAGCCGTATCCTGAAAGAGATCTAGATCAGGAGAGAAGAAGTGGGACACGTTCTTCCTGGTAACCGCCTGACAAGTGAACAGCTTGTACACCTTGGTAGGGCACGGGGCATACATTTTCATGTTTTGTTTAAAGAAACAAGCCAAGCTGAATACTCTAGTCCTAGTCTGCGTCGTTTAAGGTCTCCTTTCTCCTCTGTATTTCTCTCTGCTCCTAACATTAGCTCCCCTGTGCTTACTGTTGGTCTTGTTCGGTCAAGAAAAACCTATTTTGGTCATCAGCGATACTGATTTGCCCGCACAGCAGTCTTTGGGTGGCTGCACCCCAACTGCTGGAGAGAGTACCTGACTATTACTCCCAGAGTCTAGAGTCTCACACCTGACATTTAGCTTCCTGCCACATTTGATCTGTTCTTCAGTACACAATAACCTGAGCGGTGACTGGCCACCATTTATATTAGCCACGGGAATGTAAGTATTGCAGTGTTGACCAAATAGGAGAGCTCAGCTATGCCTCACCTCTGGTCTGGAAAAACATCACTGAGTAAATCATTAAGATAGGGTTGTGAAGCAACCAGTTGCTTTTAAACTGTGCCTTTTTTATGAATATTATCTTATCCTTTACCACGCCCTTCTGAACTAGGCTTTGAGCTGAAGGTAGGACGACTGAGTGATCAAGGTCACGTAGGCCATTTCTCACCAAGGAGATAAACCAGGACTGATTCCAAG
MSTRG.14222.1	ENSRNOG00000013840;ENSRNOG00000027156;ENSRNOG00000029501;ENSRNOG00000046530;	0.344	0.032	0.043	-2.320	down	AGTGAATCAAGGATACTTAGGAAGTACATGAAATCTAAATAAAATTAAAAAAAATCCTGCTGTCTTTATTTCCCAGGCTCACCCAGAGAGCACTCACTCTGTGCTTCCCATGCTGTGTGGTAGGCCCTATACCAAATGCAATTCTCCAAAGCCAGACAACATCCTCCATTCATTCATTCATTCATTCATTCACTCACCCCGGCACAGCCAGGCGGGCATCTGAATTTGTATTGAGCTGCCATAGGTGAATCTGACTAGCAGCAGGGTTTTGGGATGCACTGAGTTGGGAGCTGGCATCAGAGTGAGGCTCTTACAATGCTACAAGTTTCTGTTATGCACACTGTATGATCCCAGCGGTGCATGTGAATTGAGAAGAAGAAAATACCTCAGAGTGGCTTTGCTAGCTATTTTCAGGTGATAGAATACAGGTGACTTAACGTTTTTCTCTTTTTGTTTCTATTAGGCAATTTCTCTGTGTGGCATTTGTCATTTTTAAAACTGAGGAGGAAGGACTGGGTGAGGGTTCCTTACTGTATTAGTATTACAGGGTGTGGACTGCGCTTCCTTCTGCAGCTTCTACAGTCCGGGGGCTGAGCGCAGGCATATACAGCCCAGTCTACAACCTGATCCTCTCATCCAGGAAGAATGAGATAGTTCCTAGGTCAAGGGCAGGTTACCTACTGCCCAAGGATCCAAAGAAAGAAGGGGGCATGGCTAATGTGACAGACCCAAGTGTGATGGGATTTTGCTTGAAAATGTACCGGTGATGGGGCTGGAGAGATGGCTGCCAGTTAAGCACATCTGCTGCCCTCATGGAGGATCCAGGTTTGATTCCACCTGGTGGCTCACCACCATCTGTAACTCCAATTCGATGTCCACAGGTACCACACACACACACA

	ENSRNOG00000046769;ENSRNOG00000048708;ENSRNOG00000050585						CACATGATGCACAGACATACATGCAGGCAAAACACTTGTACACATATAAATAAAGAAAAAAAAAGCCTTCACTGAGGCCAGCCTGGAGTACAGTGGGGGTGGAACCTAGGGCTACATGGGGCCTAGACAGCAGAGATGGTGACACTGGGCTCTAGGTAGTTGACTGGCTTAGCAGGCAGTTGGAAGGTCAGCCAAGTGGGATGCCATTATTAGGAGGGTAGGACAGTGGAGAAGGTGCATGAACTGACTGGTTTGCTGGGAGGGAGCCTCCAAGCCAAGGTGCTCTCATCTTTGCTCAGAGCAGCCCAAGCAGGTGAAGCTGGCTGACTTGTTATCTATTGCCACCCACCTCTCCCAGCCTCTTCTGCGTTCTCCCTAATGTGTTCTCATCTTGGCTTTTGCTCTTTCTCCAGCTTTCTGTCCTTGCTTTTGGAGGGGCAAGATGGAGCCTTGCACATCTTTGAGGTCTTCGTGTACCATGTGGTTCCTGGCCCAAGGTTAGAGTCCACTGAGGCAGGGGTGCTTTGCACTTAACCACTTTCCCAGCAGGAAATGTCAAGGCCCAGTGTAACATAGAGGGCTCTCTCTGGCTTTGTGACTGGCGCCTGCCCTGTGCTTCCCTGGGTCACCAAGGCGACTAAATGCTCACTTGCAAGCGAATAATATCCCTGGTCAGGGCCGCCAGCCTTAACCGAGCTGAAACCAAGAGGATGCCTTCCCCACCAGCTCTGAGGCGCGCCCACAGGGCTCTCTGTCCTTTTGGTAGCTCTAGTGAAAACAGATAGCTCCTGCTGCCTGGCACTGTTGGCACCGCCTCGCCCTCCCCTCTCTTTTCACCTCGCTCTACAGCCTAGCTGGCATCTATGGCAGGGAGTAGGTTTGGGACAGGTGATCTAGTGCACAATTAGCAGCTGCAGCCTGTTTGCAGGGGTACCTTCTTCTCAGGCTGTTTTCTCCTGGGGGACTCTTTTCTCCTGGGTTTGATAGAGGGGCTCCTGTCTCCTAAGCCACCCCCTCAAGGGAGGGGCCCAGGGAGGCTGCTCTGGCCAGTGTGGACCAATGGAGCACTCCTGAGAGAATGAGGCTTGACCTGTCTGCCCTGCCTCTGCATAGCCCCACCCATCCCCAACTGACTCAGCTCTCTCTTGACACAGATCCTAAGCCCTTGTCAGACTAGTCTCAGAAAGGACAGGTGATATGGGCTGTGACCAGCAGCCCCAGGCTAGGCAGGCCCTATGGCCTTCTACTCCCTGAGACCTTACAGCCTGGAGCATCTATAACTCCTCTGGCCATCCAGTTGTCAGTCTGCCTTCTGCTAAGAGACTGTTCCACCAAGAGTGGTGGCAGGTATTCTCTGGTATTCCTGTCCCCACCAAGCTCATTGTCACCTTTTCTCAGGAGAGCTGAGCATCACCTCCCCTGTGTGATTCACCTCCGG
MSTRG.14311.1	--	0.467	0.000	0.049	-3.592	down	GGGAGGGAAAGAGGAGGTAAGAAAACAAGGAGAGAGGGGAAGAGAGAAGGGAAGAAGAGGGTGGAGAGAAAGGGAAGGAGACATGGAGGAGAAAAGGAAGGGAGGGAGGGAGAGAGAGAAGGAGAAGGAGAAACAGACAGAGATAGAAAGACAGAGAGGACCAGGATCGTGATGTAGATATTAGATACGCAATCCAGCCCCTAGGGAGAACCTGGGCCCTAGGGTCATCTACTCCTCTGCTCCATGCTGAGCGGGAGGGAAGGAGGACTGCAATAACAGCCAGTACAAACCATTTTCTCTCACCTCACTACTCTTAAGGCTGGTAGAGTATGCCAGGCTCTTGTCAGCACCACAAGACAGCCATTCTTTCAGCAGCCCCAAAGAAACCAAGGCACTTGATGCATAGTCTATTTTCTTTTCTC
MSTRG.14329.2	ENSRNOG00000016302;ENSRNOG00000016356;ENSRNOG00000016656	0.399	0.000	0.037	-3.684	down	TGAGGATGATGCGGGTGGCGGGAGTCCCTAGAGGACAGCCTCCTGACCTTCTGGGTCCTGGAGACCTGGGGCGGTGGCCCGCCCTCGGGAATAGTAGAGAACAGGTCTTTCTGTACCAGCTGAGCAGGTGGCTGTCCCCGCCAGGCTCCGTTCCTGCGCACGTTCCTGCGTTAGCCAGGCCGGGCTGACAGCCAAGTCCCCGACACTGGACTCTAGTGGTCCGCCTGGACACGCTGTACCCGATCGCGGGTGACCTTGACCATCTCCACTCAGTGCTTCCGCAGAAAGAAACCTGGGGGTGTTGTCTACCTTGATCGCTGGCAGGAGAGACCCTGCAGAAGGGAGCAGGGACACTAGGCTCCAGATTGGTGGATGCCACTTTAGTCCTGGAGAGATGTGAGTTACCCACCGAGGTCAGTGATCTGCCCAACCCCTCAGACCTACCGGAAGCCAAGGAGGGATGATGTCGGAGGAAGAATTTGCAGCTTTCTGCTCATGCATGGACTTGAGGGCACAAGGGGAGGCCTGCAGTCAACAATGATAAA
MSTRG.143	ENSRNOG0000001	0.000	0.349	0.004	3.943	up	AACGGGGGTCCTATTCTCAGTTCAGTGGTTCTCAGCTGGCAATTCTTATCGCTTCCTTAATTCTACTCGCTCTTTCTTATGTAGACTTTCCCTGAGAGAATAGATTTCATGAAGAGTCAGCTCTGCCTTTGTTTCCTTGGG

38.1	7230;ENSRNOG00000017298						TGGCAGGGAAGGGAGGGTGTGGGGAGGAAGGGCTGTGGCAGTTCACCAGCCCTCAGCTCTGCATGCACTCTGAGGCCAGCAGGAGAGGTTATGACGCCTGCTTGTGCGAAGACCCCTGCGAAGCGTCTACACGCTCAAGTGTGTTTTCCCTTCTCTACACCAGCTCTAGGTCCAGCAGCCGACGTGGGAAGTCCCTCAAAAACTCCTTGAAGATTAAAAATGTGAATGTCCAAAGCTCTCAGGGATAATGTTGAATGGTGTCTTTGCAGGTCAAGTTCTCCATCTACC
MSTRG.14492.1	ENSRNOG00000019973;ENSRNOG00000049308	0.308	1.419	0.005	2.697	up	GTTTTGTTTTTTAATTTTTTAAAAACCAAGAATGATTTCTTCTACTCAACACCTTCCCAGCCGGATGGAGTTCAAGTCTCAAGCAGCCTTTGGCACCTTCAAGCCTGAGTGGAACCCTACAAAGCCAGGAGCCCCGTGTCCAGGAAAAGCTGGAAGAGCAGGCAGAGGCCTTGTAACCCCGAAGGTGCACTGTCCTTTAGGCAGGCCCCTGAACCCTCAACCTCACCCCTTCTCGGAGGCCACTGTGCCTCCTCTTTCCAGGAAGTGCTGGGAGGCGACAGCCTTTTCCCCACCACCCCACAGATGGGGACAGCAGCTCCTGTGGCCCAGATGTGCAGAGCCCGCGAGGAGGCCACCCGGCAG
MSTRG.14638.1	--	0.406	0.000	0.002	-4.481	down	CTGGGGAGACGAAACAGAAAGGCAGAAATAGAAGCTGTCGACCGTGAGGCAGGTGAGGGTCCGAGAGAAGAAAAAGGAAGAAGAAGGTTGCCATGAGTCAAATCTAAACCAAACCCATAAACATGTGCCACAGTGTATAAGCACATAAAAAAAGCATCAGGTGAAGCCTGTGACACCAGACTTTTTGGAGTCTCAAAAACAACACATAAACATCAACTAACAATTCCAGCCACACCACTGTTAATAAAACAGCATGCAGTAAAAATTAAAGAGTCAAGTGAAGCTTAAAACAACCAACAGTGTTGAAAACTCGACGTCCGATTTGTCTCGGAATGGCAGAAAAGGTTACATGAAGGCCCTGGGTGCTCTTTGTTAAGAAGGTTGACAGAAGACACCATGGATGCTGTGTTTGTTAAGAAGTGTGGCCGAATCTGCTCTGGGTACTTGGAGGAACTTCAAATATTTACAAGTGTAAATAGTTTATTGGAATAGTAGGTTTCTTAAAGCTTCCAAAGTGTGATAAGGGGGCTATAGAGACGTCTCTCTGATTAAGAGAGCGTGCAGCTCTGGCAAAGGAACCAGATTCATCTCCCAGCATTCATATGGTAGTTTACAATCCTCTGTAATTCCAGATCCAACATCCTCTGCTGATGCTGTGTATGCACCAAGCATACACAAATTCACATACCTACATATAGACAAACAGGAATACGCATGGCAATTTTTAAATTAAATTATAAGACCAGTTACACTGATGCCATCTAGAAGTTTTGCATGGGCAGAATTTCACACCCAGCCTCACTGAGGGCTCTGTGTTTTGACATAACAAAAAACAGGGACTGAACACTCAGTTTCATCAGTTTTCAACACATGGCAAATCTTGCTTAATTTGTAACCCTTCCTAATTTTCTCCCTTTAAAACTTATTTTAAAGCAAACTCCATAACCTTGCTGTGCAAATAGTAATTTTTCA
MSTRG.14642.1	--	0.296	0.000	0.028	-3.770	down	CACACACACACACACACGCGCACACACACATACACGCACACACACACACACGCACACGCACACACAAGCACAGAGAGAGAGAGAGAAAGAGAGAGAGAGACTGAGATTCTAGTTTAATATTCTTTCTGTTCTTAGGCTCCAGGAACTCTTAAAACTATCACCAAAATTTAAGACCAAAGTCCAAATACACTTTGGGCAACCCCAAGGGAACAACACACTGAAGAATCAGCATACAGTGAATGAGGTGACTGGACGCAGATGTCTCATAATAAAAGTGAGGTCATGGGAACTTAAATGATCACTCCACAAACAATAGTGAAATTTGTTTATAAAGCACTAGAAACCTGGTTGAAGTCTATCTGGCAACTTGTCTTTTCTGGACAACTTATACAGTGCTTCTTTTAAAGCTCTTAAATAATTCATTTCTTATTAAACACCATCCTCATTAGAGGTTACAATAAGGGACACACACCATGACTATAGGGAAATGTGGGAGAGGAAAAGAAATGCTAAGGGCTCTTTTGAAGAGAAATAGGACAAGAAAAACAAGATCCCAAGTGCATTTTCTCCATTTCCCAGCTCGGGAAACACAGAAGTTCTGTGGCACCCGAAAGTATGTCGCACAATTAAGATAAGTCTTTGAAGATCCTACTTGGCTGAACACTGTGGAAATGTAAACAGAATCCTCTTTTACAGCTATGCAATGCTGTTCTCAAGAGGCGTCAACAATAAGCATTTTGTATTATGCCACAAAAACACAGCACAG
MSTR	--	0.373	0.000	0.002	-	do	CTGGTTGGAGACTTTGACAAGCTCAAGGCCAGGGCTCTGCCTCTAAATCTTGTTGCCAAGGTAATACAGCATAGGCCTGGATCCGCTGAAAGGGATGCCTCAGCCATCTCGTGCCAGCAGGAGCTATCTGATTAGAAG

G.14724.1					4.531	wn	AGAGCTTTCAAAAGGAAGAGAAACAGTACTTTTGCAGAACCCAGAGGAGAATGCAAAGGTGCACATTTTCTGTTTCGGAGTTTTCAGTATGGAACAGGAGAGGACTCTCTGGATTCTAGTCTAAGATCGTTCCAAGGATGTGGTGTTGAGACAGTAGCAGCACGAAGTCTCAAAAGAGCAGCAAGGTGGTCCAGCAGTGTGGAAGTGAGGGAGAGAGAATTCATTACACGGTGCCTCTCCTTTCCCCTAGTGGAAGGGAGACACTGGAGCAGAAGACCAAAGCGTGTGGCTGGAAATGAGCTGAAGTCTTGTGTTTGCTCTTGACGCTTCTCACACAGTTGGCATTCTACTCCCGCCCAAATCGATAGCTTTCAGATAAACGGCATCTCTTTCCCTGTTCCCAGGGGCACACAGATGTCCCCAGGTGCACCATGTTTAGTCCCAAGGCTGTCCTTTCTTCTGGAGCAGTTTCAGAGATGTTAATATTGTCATCTGAGAAATGAGATTAAACTTGATTCTGGTGAACTCTCACTTCTGCTTTCATCTGGGCGTGTTACACACCCAGCTCACTTCCTCCTCCTTCCTTTCTGCTAAAATTCTTCCTAACCCCTACTGTCTTCTCCATCCCAGTTTTGTCTTAGCTACCCTAAATTTCATACTGAAACTCTACTACTCAAGACATTCTTAGAGAAAATAACTGCAAAACCCTTCCAATAACATTTAAGGCCGCCACAGTCCCAGAGGGAAAACTTTTTTTTGTCCACATTGTACTGGCTTCTTGGCCTGTTTCATTTCCACTGTCTACGACAATAAACTCTTGGAGGTTTGTAACTTTGTTTCGTTTAAATCTTGAAAAGCGCTCAAACTAACGCTCTGGTCAATCACGCAGCCTAGAGTCTAGGAATTCACACAGCTCTTTGTGTTAGAAAGAGGTTGAAGACCGAACATATGTTTTGAGTTGCTTCTCTAGAGCTGACTTGTCCACTTCTTA
MSTRG.14774.1	ENSRNOG00000014173	0.095	0.336	0.041	2.291	up	AGATACTAAAATAAAATGGGATGTCCAGAAGTAGACTTAAGTCCACATGGAAATTCATCATTAGTAAACACGACCTCTGAAGTATCGCCAGAGAAGCTGACCCTTTAATAACTGTCCTGGGGGCAACTGGGCAGCCATTCAGGGAAGGTTAAAACTGGATCTGTGCCTCACACCGTACCCTGGAATATCTGCAGACAGGCAGGCATCTTAGCATAAAAATAAAATCACATAGACACTCAAAGGAGGTGTGAGAATTACAAAATATATATAGTTTTAATATGGGGAACTTTCTCTAATTCCAATTCAAGATCCAAATCCAGCAATATTGAAAATCTGATAAGATCTACTACATAAAAATAAGTTAGAGTGGATGTAGAAAAAACAGGAAATGTAAAGTTAAGACATTAATGTCAGGTATAAAGTAGGAACTCTTCCAATTGGGGGTGGGGAGGGTCTAAAGATGGTTTTAAAGGGGCAGAAGAATAGTTTACTGAAAAAAAGAATACGCATGATTCTTTTAAACCTTTGAGTGAAAAGAGAAATATGTGTACATTGGCAAGAGTGAGGAAAAGAGAAATGGGGGCTTAGGTTTTCCTTGGGTTTCGCTTTTTGCATTTTAGATCGGGTCTTATCATATGGCTGGCTTCCCTTTGAACTTGGGATCCTCTTGCCTTGGTCTCCTGCGTGCTGGGATTATAATTATGTTCATGGCCACTTAAGTGAGACACTGTCTCAAAATTAGAAAGTGTTGGTGACATAGCTCAATGGTAGAATGCTTTCCTGGGAGGTAGAAGCAGGGGGATCCAGGAGGATGAGAAGTTTTCAAGACTGTCCTTGGCTGTGTAGCAAGTTCTGGAAAGCCTGGGCTTCATGAGACCCTCGCGCTTTAGAACTTGGATTTCTGGACTCCCAACCTACAGTCACTTTGATTACAACAGAATACCTGCCACCAGCTTCTTAGAGGACAAAGGGCCTGCTGGTAAATTCCCAGCCAATGAGACAGAGGCGAAGC
MSTRG.15055.1	ENSRNOG00000008824;ENSRNOG00000032706	0.766	0.000	0.001	-4.672	down	GTCTGTTAATTTTTCCAGTGTTCCCCAGAGTAGGGGAAAGCGTTTTATTTTTAACCAGGTTTGGTGTTTCTCAGGGGTAACCTGGGAAAACCGAATCTCTTGACACGTGTCTCTTTAGAACACTCAGGTCTGTCACCTAATTAAGAAACTCTATTGACATCCCGAACAATTGTCCCGATGATGGCAAACATTTCAGAAGTTTCCCCCGAAGAAGATCGGCAAAGTTTCCTGGTTTTCCTTTGGTTTGAATTCTCTGGTTTTCCATGGGTTCCCACTGTCGTCTTCTGGAGACGTTGCTGCAAACAGCATTGTCCTGTCTAGTACTATCATTCGCAGAGAAATGTCTCTTCCATGATCTGCTTGAGAATCCAGTTCCAAGTCATCAGGTGCTCTCTTCTGGCTTCCCAGACACCTCCACACATGTGTGGACACATGTTCACACAGGCATACGCACGCCTTTGTATTCTGGGCTGTTCCGAGAAGGCGGCTTATGTAACACTGGGTCTCAGTCCTCTCCCAAGCGAGCATCGACAATTTGATCATCTGAATGAGGCTTCAGTCACAGGCAACTCCAGAGCAGTGATGTAGCTGTAACT
MSTR	ENSRNO	0.514	0.021	0.000	-	do	TAGGCATTTTTGGATTGCTTTAATACACTACAGAGTAAACGTGATATAAAGAGTTGCCATGCTGCTCACT

G.152<br>36.3	G0000005<br>6130				3.673	wn	TAGGTTCTCCCCTCAGTAGAGATGCCATTTAAAACATCATTTCCTCAGTTGAAATGAATTCATAAATACA<br>AGACTAATACCTGTGGAGGAGAAAACAGTCAGTGAGACAGACCAATGCACACTAGGATATCCATCAAG<br>GCCCAGTTCTAGATGGCTTAGAACCAGAGCGCTCATGCTTAACTCATATTGTGTGGGCTAGGGACTCTG<br>CTTGGCAGAGCCACCGCTGTCCAGAGCACTGCTCTGCTGGACAGATAGAACCCTGTCTCATTCTAGCTT<br>TTAATATTTTGTACCAGAAACTGTTGCGTTCTCCCTCTATTCACAATCTTTGAGCAGACCGTGACCTGAC<br>CGAGAGAACCAGAAGTTCCATCATGTGTGCACTGACATAGTGGGGAGCCAAACACCTTTGTTAAGCAG<br>CACCGACGATAGTCAGCTTCCAAGAAGGTGGTCCTGTCAACTTCATTTCAGAGTGGGCCTGAAGACTG<br>GTGTGGTGAGCACCTGTAATATACCTACAATCTTAGTACTTGGGAGGTAGAGGCAAAAAGATCAGGAGC<br>TCTCTGAGGTTGGCCTTGGCTACATGAGACCCTGTTTCAAATAAACAAAACACAACAGAAAGTATGATC<br>ATGAACAGAGAGTAAGAGACCAAGCTGGATTTGTCCTCTGCAGCAAAGCGAGGGAGCTGGTTCAAAG<br>CTACAGATTCTACTTTGCAATGTAATGAGGTTGGGTAGCAATGCCATTAAACACAGTGGATAAAAGCCT<br>GGAATCTGGGCATCCTTCAGCTTCCCAACTTAGTAGTTGTGTGAATTGAGGCAGGAGATCACTTTGCCC<br>ATCAAAGGGCAGGGAGGAACAGGGAGAGGGTATACTTTACAGAATGAAATGTGTGTATCCCAACAGGG<br>TTGATGTGGATAAGGCTGATGGAGAGAACAGATGCATACAGAGCATGCACGGCAGTCAGAGCAGCTTA<br>GGCATGGCTGGGTATGTGTGTGTCTACACCCCAGAAATCATTGCCTTTGTCTCGTACAGTGTGAATGCTT<br>GGGTTGCTGGGGATAGAAACCACATGAGTTGGTTATTAGTTCAGTGATGGGACTGTCCAGGGCTCTGTG<br>GGGCTAGAGCCCTCCCAGCAGTGTGCTGATGTCACCAAGAAGCCAAGGTATTGTGGCTTTTCTGTTCTC<br>TGCCTTGATGCCTCCCATCCCACATAGGCAAAGCACTAGTGAGCACAGAGGCTGCTGACTTCTAAGAGA<br>GGCTCAGTACAGTGGAGAAAGTTGCCTTTGCCCTGCAGTCAACAAGCCAGTCCTCCAAGTCTTCCAGG<br>CTCCAGCTTGCTTTGGTCTCTGTCAGAGTTCTTGAGTAGAAATCCTAGCTGCTTTAAGCGTGGCTGCTAC<br>CACTGCTGTCGTCAGCATCCAATCAGGGCTGCTGCTCTGCTGGCACCAACGTCAACAGATGAGTTTCAT<br>ATAGGGGTACAGCCTTTGTGGGACTAGTAGCTGACAGGAGACATCATGTCCTTTGTCCTGGTAGCAAGG<br>AGGTGGACACTTTGCATCTAAAGGTTGACTATGAGATGCCAGAATTGGTCCTGGGACATTTCCTTGCCC<br>ACAGATGCAATGTCTGTCTATTACAGGTGACCTGTCTTTGCCAATTGCTTTTTAACTGTCAGCCTAAAAT<br>CATCTAAGAAAAGTCTTACTGAGGGATGGCCTGTGGGTTGGTCTGGTGTGTTGTGGGTGTCTTGTCTGT<br>AAATTGAGGTGGGAAGACCCACCCTGAAGGAAGGCAGTAAGTACCATTTCCTGGGACAGACCCTGAG<br>AGATGGAGTGGAGAAATCTAGCTGACAGTGAGCAGGGAGGCAGCATGGGTGTGCTTGCTTCTCTCTGG<br>TACTGACTGTGGAGACGGTGTGATGTTAAGATGCCTGTCCCTAACTTCCTCCACAACAATGGACTCTAA<br>ACCAAATAAACATTCCTCTCTGTGATGCATTTTGTCAGAGTATTTTTATCAGAGCACAGAAATAATCAAA<br>CCACCTTTGATCAAACCCTTAGGCAGAGATCTAGGGCTCCACAACAATAAGATTGGGCTGACCCGACCT<br>ACCTCTGAGGACGTTCCTGATTCAGTCCAGTCTCCTCCTTTCTGGTGTCTCTACATGCTCATGGCAAGTG<br>CAGATCTTTCTGTGGTTGAAGTAGGGTCTGATCTGGCTGTGTAGCTCAAGCTAGCTTAGCACAATTGTGT<br>GTCCTTAGGCAAGTCCACTCATCTTCTAGACTCTTTCTGTATTCATCTGTGAAAAAGAAACAACACCCA<br>CTTCCACTGAGGTGTGTGACAATGTGAGGCAAGCCGGGAACGGATTTGAGTCAGTGGGCTGAGTAAGC<br>CTCAAGAGCTGAAGACAAACTTGTATAGTCCTCTAGAGTCAGAGGCACCAGTCTTCTGGTCTTTACCAG<br>GAGAACTTCAAACATTCTGATTTTCAGACTCTGAGACTTAGGCCAATTCCCTCCCAACCCCTTGCATTTT<br>CTTGGGCCTTCAGCCTAAAACTGAGAGCTATATCCTTAGTAATTCTGGCTCTCAGGTCTTTGGACTTATG<br>TCACAGATTTCCATGGTTGCTAGTCTGCTGGCAGCATATAGTGGGTCTTAGCCTCCATGAAAAATCCTCT<br>CATGCATCTATAGGGTATCTATTGGTTCTTTCCCTGGAGAACCTGCCTCTAGGGGACTCTGGGAAAACTG<br>ACCAAGCTAACAGACAATGCTTGAACCAGTGGCGTCCAGTTGGACGGGATCTCCTTTCTTCACCCTATT<br>TTGTTGAGCTGTGTTGTCACATTCAGCATCCATATGCTCAGTCTCTGTAAGAGGAGTCGTTTCCAGGCAG

							CATAAGTTAGAAAGGTTGATGTGGGCCCACATTTGCTGACCCAAGATTGTCAGGATGGGCTAGCTTAGCAGGCCTCAGTGTAGTTTGCAGGGATGAGCTAGAACCTTCCAGGCTCTTGATTCTGCAGAAGTTGTCACAGAGGGGCCAGCTCACAGGAATATACAAAACAAGACTGAATAAAAATGGCCCCCATAGGCCCACAGGGTGTGGTACTCTTAGGAGGTGTGGCCTTGGAGGAAGTGTGTCATTGAGGTGGGCTTTGAGGTTTCAAAGGCTCAAGCCAGGTTCAGTGGCTTATTCTCTCTTTCTGCTGCTTGAGAATCCAGATGTAGAACTCTTAGCTGTCTTGATAACACCGTGCCTGCCCACACTCTGCCATGCTTCCCATTGTGACTGTACTGGACTAAACCTCTGAACTGTAAGCCAGTTCCAATTAAATGATTTCCTTTATAAGAGTTGCCATGGGCAAGGCCCTGGGTTCGGTCCTCAGCTCTACTCCACAGGCCGGTCTGCTCGTCCACTGTGATCTCCTTCTGGGCTTTCGCTTTTCCGGGTTAGGAGCGCGGGCGGATGCACGGTGAGAAGAGAAGCAGGGTGTAAGCCACCTGGTGTCCGCACGACGGTACCTGGATCTTCCAGGACGGGATCCGCGCCCGCCGCGTT
MSTRG.15398.1	ENSRNOG00000002420;ENSRNOG00000002452;ENSRNOG00000058463;ENSRNOG00000058739;ENSRNOG00000061236	0.658	0.058	0.012	-2.530	down	CTGGGCTGAGCTCACCAAATGCCTTTGCATTTTGATCTTACATAGTCCAGCCTAGTTAGTTTTGGACTCCTGATCCTCCTGCCATCACCTCTCAGGTTGCTGGAGTTAGGATTACAGGAGTGCCCCCAAGCCTGGGTGTACAAGTGAGCTGGGTTTTATTTTCTAGTTGATTATTAGTGGATGTGTGCACACCATGTGTGGGGACACATATGTGCCAGTGTGTATAGGTGACAACTCTGATCAGATCTCTCCTATCACTTTTATGAGGGTCTGTTGACTGAATTCAAGTTACCACTTTTATTTTATTTTTTTTATTGCTGAGGTCTCTCTGTGTAGCTCTGACTGGGCTGGATCTCAAAGAGAGCTACTTGCCTCTCCTTCCTAAGTGCTAAGATTAAAGGTGTACCCCACTACCTAACTGGTCTCATAAATTGTTTTTTAAAACAGTATTTGTATTTGGACAGTGAGTGACAAGCTGCAAACTGGAGGTACATGGAAACAGCTTTTCTCTCTACCCTACTGCCTCAGGATTTGGAGTGATTATGAGGCTTCTCATGGTGTCAAATGATTAGCTGGGAGAGGCCAGCTTCTTTAGACATGACCTGTGGGATCCTCAGAGGCTCTGTATATCCAGCACCCTTAACAGGCCCTTGGGGGTCACTGACAGATCACTGAGGGCTTGTATAAGACGAGACACATAGAAATAGCTATTAAAGAAAAGCTTATCTCCCTGCAGCCCAAATGCTGTCAGAGGCAGGGACCCGCCAGGAGGGAGGAAACTAGCAGGGCAGTGGTTGGTGGGCAAGGTTGCACGGTATGCTTCCTTCGAGGCAGAGGCTTCACAAGGAGCCAGTCCTTTAGTTGCCCAGTCAGAATTACTTCCTCAGAATAAATCTCAAGAGGTGGGATGCTTACGAACCTAGGAAAAAGTTTGGGCAAGCTGCAGATTGTTAAAGGTGAGTAGCATGTGTAGGGGATATGGGGCCACTTCCAGTACCCTGATGATGGTACAGAAAACTAATGATAACTGGCACACGTTAGGACTTGGTTATCTCCACGATGGTTTTTGTTAAAATAATCTTACAATTTTATGTGTACAGCATATATATATATATATATATATATATACACACACCTAGTGCCCATGGAGGTCAGATACCCTAGTACTGGAGCTGTAAACTTCTATGAGCCTCATGTGGGTGCTGGGAATTGAACTCTGGTCTTCTAGAACAGCCAGTGCTCTTTAAATCACTGAGCAATCTCTCCAGCCCACATGGGTGGTTTTAAAATTAACATTTGATCTAGGCTGTTTACCCCTCCCCATCAACTTTACAAAAAGATTTTATTTTTAATTATGTATGACTGTGTGTGTACATGTTCCATTTGGTACCTTGGAGGGTATCAGATTACCTGAAGTGACTGGCCTTGAACTCTATGTAGTCCTGTTACATGTGGTCCTCTCGTTTTCCCTGAGTAGTTAGGGTCACAGTTGTGTGCCACCAAGCCTGGTTAGAAAATTACAAATATAAAACGCATATAAACGTGTGTGCGTGCGTGCGTGTGTGTGTGTGTGTGTGTGTGTGTGTGTGAAGATAATCACTCTGTAGCTTAGGCTGGCTGAGATACTAGGTGTGAAGCAGACAATGCCGTGACTCCATTGCCTTACAAGCTTTAATTACGTCTGCTTTCTGTTTCTGTGGATTTGCCCATCAAACATGGGTCATAGAAATGGGATTTTACTAACGGCTGGGTTCTTTTGTTGGATGTACTTGTCTTATGGTTTCTATTGCTGTGGTAAACACATGAACAAAAGCTACTTGGGGAGGAAAGAGTTCATTTTGGTTACATTTTACCATCATAATCCATCATTGAAGGAAGTCAAGGTAGGATCCTGAAAGCAGAAACTGAAGCCGAAGGCATGCTGCGCTACTACTACATTACACTGGTACAGACTAAGGTGCAGAGCAGACCGGTCTCCGTCTCATTTCTGTGTCCTCCGGTCCTTCTCGCCTCTGCACGAGGCCCCCTTTCTGAAAAGCAGAGATAACAAACTGTAGCTATGTTCTGCAGAGTCTTTCGACCAGAGCACTAGCGACAATCACCGCTCCGAAGTCGAGCAAAGAACGACTAGAGCCCTAACAGTATAGGGA

							GGTTCCG
MSTRG.15430.1	ENSRNOG00000002532;ENSRNOG00000002539;ENSRNOG00000002564;ENSRNOG00000002568;ENSRNOG00000021902	0.000	0.354	0.000	4.663	up	GGCATGTTTATTAAAAAATGAACTTAAAACTGGAACCCCAGAACTGGAGGAAGAGTCCTTGAGCTGAGACTGTCTCAGCAGGTACAGGAACATGTCATCGAGCTGAGTTCAAGGAGAGAAAACCCCTCCTGAGAGTAGTCTTCTGAACACACACACACACACACACACACACACACACACACACACACAGAAGCATGGGTGGGAAGGGGCAGTTCTTCAGGAGTACAGAAGTGTGCGCAGGGCCATGCAGGCAGGATCATCTCAGTATGAGTGGCAGGGTGATCAGAGGTGGCTCTAGTAAAATACAACTTGACCTTTTCTTTCCCAGGCTGGGTACTGAAGCAAAGACCACCAGGACTTGAGGCATCCCAGGCTGATGAGAATGGGGGTCATGTAAGACTGAGGGAGCCCTTGGGATATGCAAGATCAGACCAGATCTGTGAACCTAACCCTCAACCTCCCACCTGCTGAGCTCCTAATCGCTCAAGCCTTTGGTCCAAGGCGCTATCCAATCAGGGTACACTGGCTCACTGCCCACACATTGGCTATTACTTCCTATGGTAGGCCAGGCACTAGGTGGCTGGGTGGAAAAGAATTCTGCAGCTGTCTTGAGGCCTTCAAGCGCAGACCGCAGGCTGATCTTTAACTTCACGATATAACTTCTCTGTCACTACCCTCAGCATCGATGTCACCATGAGGAGGGTACAGTTATTAGGAGAACTTCAAGACCCAGCCACCGCGGCAGCTGCCCCAAGAACAGGAAGACCT
MSTRG.15454.2	ENSRNOG00000002571;ENSRNOG00000002574;ENSRNOG00000025545	0.084	0.307	0.010	2.430	up	TCTGAACAGATGAAGTGGCTCCAACGATTAAAGGCGCTGTTGAGCCAGCTTGCGTTCAAATCCCAGAACTCATATGGATGGAAAGAGAGGATAGATTCTCCATGCACGTATGCACAGTAATACGGTTTTGGGTTTTGTTTTTAAAGTATAGCTTTTGTCGTCAAAGAACGTTATTCTCCATGACCTCGGTGACGATGCAATACTACTAGTAAAAGTACAATCCATGTCAGTATTTATTCAGTTTCAAATAATCTGCCTTTCCTTATATTCTGTAACAGGCTGGGATTGTGGCGCAGTTGGGAGAGTGCTTCATCTATCATGCACGAGAGCCTATATTTAGTTCCCAGCACTGAAACAAAACAAGACAACCAATAAAACACTTCTCCTGCTCTCTTCTCTCCTCTCTTCTGCCCAGGCTGATCCTGAATTAATGATTTTCTTGCCATACTTGGCGGAGAGGTGAAACTTTATAAATGGGCGTAACTCAGTGGTAGAGTACCTGTCTAACAAGTGAAAAGCCGTAAATTCGGTCCCCAGAAGTAAAAGGTCTCATTTGATCCTCCTGCCTCCATCTTTCAGCCGCTGGGATTGCAATGTGCACCACCACTCCCAGCTCCTGGACTTAAAAAACAAAACAACAAGCGCCCCAAACACTCTTAAGATCGCTATGCTTTTGAGAATAATACTTTGTTTTGTTGCTTTCCCCTCAGATTTGTTTGATGTAACCTCTTCAAACACACTCCTGTGTCCCCACTGGGAGCTTTTCCTAGCTTTCTCTCACTTCTCCAGGTCCCCACTGTCCTTGCTGCTACCATTTCTCTGAAAATCTGGCTGTGTCTGTGGAGCAGTGTTTTAGACACCATGAAGACATCCTGTCCATAGTCAGCCGTGGGAGCATGGCTGTATGCAACTTTGGCACAGGATGGTGGTGGTGATTGTTATTGTTTTCATTGAAAGATTCTAAAGATTTTTCACTTTTGAGATTATAATTACATAATTCTCCCTTCCTTTTCCTTCCCATATACCTCTCCTTGCTGTTTTTCAAATTCATGGACTCCTTTATTGTTATTGTAACAGGGCTCCAAACCTTAGTCGGCCCCGAGACCTTAGCACAACTGACTCCATGATGGAGTGCCATTCAGGCTGTAAAACTCAGCTTGCCCATAGCAGCACCACCTGCTAAAATGAAAACAAAGACTTACTCCACCAAAGTCCATGGCTCTGGTTCCCATTGCCTCTGCTTTTGGATGAGGAAACTGTCGTTTAAGGAGGACGTCCAAAACTGTGATTAGTAATATGCTGAGGGGAAACTGAGGCAATGTCGGGAAAGCTGCACCAATCACACTGCGAAAGTCTGGATCAAGCAAGACCAGAAGTCGAGGCAGAAAGGACGAGATCAGCTGCTGGTTCAGCTGGACAGGTCTGGTTAAGGCTTGCTCACCGTCAGCCTCGAGAGATGGCTCAACTGCCACGATCTCTGACTGCCCCTGCAGAGGACCCAGGTCCCTGGAAATACCTCCACACCAATATCAGCTAATGGTGGCAGCCCAGGGCGATAGGCAACTCCACCTCTGGAATCCGCCCCAGCTGCTCGC

							TCAATGCCTGAGTGCATTCATTGGCCCCTGACTCTCGAGAGACACTTCTTAGGGGTGACATCACACTGAGAGGACGCCGTGCTGTAAGTCTGCAGACCGGAGCAGACTAGAGCCTAGTGCTTTTCTTACCCCATCCCATGGAAAGGACATACTGGAATATGTAAAACAGCACTCCACACCATGTGGCTAATAAATGTCATTTCTCTACATTCTGACTGCTCACCTGACCTTATAGGGAACTAGGGAGAAAAATCACCATTAAGTCTCAACCTGTGTGTACTTCAGACATTGAGTGTCCCCAAAAGCCTTTGTCGAAGGCTTGGTTCCAAAGGAGCCACTGTTAGAGATGGTGGAGCCTAGTGAAAGGTATCTCGGGATGGGGAGGAGCTCTACTGGAAAAGGGTTACAGGCTCCTGCTTCCTCTCCTCTCTTTGATTCCCTGTTCATGGTTTAGTACCATTCCTCTACCACACTCTGGTCATGACGTGTGGCCTTCTCAGGGCACAGCTTGTCAGGGGCCCAAAGCAATGAGCTACTTAATCACAGACCGGAAGCTACAGAACTATGAGCCAAAATAAACCTTTTTCT
MSTRG.15544.1	--	0.160	0.914	0.000	3.187	up	TTACACTTCAGAGCCCATTAGTGTCGCGGTGGGAAGCATGGCGGCGCAGGCAGGCAGGCTGCTGGCGAGGTAGCTGAGAGTTCTACATCCAGATCACAGGGCAGCAGCAAGGGATGACTCACATTTCCTCCAACAAGGCCACACCTCCTCATAGTCCAGTCCCTAGGGAGGCCACCTTTATTCAATAGTCTATTAGCCTGTGTCACTGCGTTTATTAACTGCTTTACTAACTTTTGCACATCTAATAACGTCAAAAGGCAAACTTCAAGTATTTTTACTATTTATGTTAAAAACCACAACAAAAAATGCAAGAAATAAACCAGCATTGTCCTAGTTCCCAGCCCCCAAGGCAAGCCAGCTTCACAGCAAAGCTCTGGAGGTCTCAGCTACCACAGACAGTCTCTATTAGCCGTAAGTTCCTTATGAAGTCCTACAGATCTCTAAGGCAATAGGGGCAAGGCTCCGGTTAATGGCCTCCTCCGAGTGTTGGACTAGATGAGAAGGAGAAGAGAGGGAATAGGAAAGGCCACCCAGAACAGCTGCCGCCCACCCACTTCTGACAAATGACTCCTCTTCCTCCCTTTCCTTCTCTCCTCCTTTCTCCTCCTCCTCTCCCTCATAGCATGAAATTTAAAGGGAGCAGTGTTTTCTAAACCTGATAAATCCCAAATGATTAACACCAAGACCTGAAATTTATTCAACAAGCTTTAGGCACAATAACTGAGCAGGTATTCCTAACCCTCTATGCTATCTTCACTACGTCCCGGCCCCATGCCCCAAGTTACTTTTTGTCTCCTTGGCTCACTCTGCTGCAGCTGTCTGTCCTCATGGAGAACTCCTCCTCATGCTCAACCTCATCGTTACCATCTTCTTCTTTCTCCACCTGCCTCTTGGCCCTTCTTCTCCGGAACAGTCATCTGCCTCACGGTAAAGTCCTTCAACCATGCTCTTCTCCTTGGGACCCCAAGCTGGGATCAGAAGTCCAGCCTTTCTGTCTTCTCTGCCCAATCATTGGCTGTTCAGCTTTTTGTTGTCCATCCCAGTGCTTACAGAAGCTCTCTTCCACAAGCACAGTTGGTGAGGTTCCTGGCCATTGTCAACTGGACTCCTCCAGCCCTGCTCACACTCCAGAGGTCCTCTAAGACAGGCATCCTGCAAAAGCAGAGATGGGTGGATCCCCGGGGCTGAACATTATGTCGATGTCCTGAAAGGGTAGATGAAATCAGTCCAAGGAGTTGAATCAGTGAACGCCAAGTTTGATGAGTCCTGTCTCACCAAATGAGGTGGAGAAGCACCCAAGGAAGGCACCCAATGTGGACCTCTGTCTGACCTCTATCTGTTTTTGCTAAAGTGCTTGCTTAGCATGCACTAGGCACAGGAGGATTCAATCCTTAGAACCAAATAAAACCAAGCAGGGTGGCACACAGCTGCAATCCCAGCACTCGGGAGGCACAGTAAGAGGATCAGGTGTAGGGGTTGGGGATTTAGCTCAGTGGTAGAGTGGTTGCCTAGCAAGCGCAAGGCCCTGGGTTCGGTCCCCAGCTCCTAACCAATAAGCATTTCTAGCTCAGACTGAAACTGGTCCTTGAGACGTCCTAGCCCAAGCAAGTGAGAATAGCACACGACAGTCACAGTTCAACACTGGAGAGACCGAGAGATCCATCCAATACAATAAGCCAAGGACACGGAATGTTGTTGACTGGTGGGAGGTGAGTGGGAGGTAGCAAGACTGGGTCTCTGTGAAGCAATCTTCTTCCTGTTCCTACCTAACAACCTCAGTAAGCATTTGTGTCCAACAAACAATCCTTCAGAGACGAGTCTGTAATTTATGGAGAGGAATATAATCCTCTCTTTGCATGTGACGGCCAGGGTTTGGTGGCCCCAGGACATACTTGGGTGGCCACCTGTCAGCTGAGGTCGTAGAAGATGGCCTACAAGGAGGCACTCTTCTGGAGGCTGTCGTGCATGGAGAGCAGAACGCTGTTGCCTTAGCTCCATTCACATTGTCTGACTCTGACCCAATTACCTGGCCTCTGTTATCTGGTGAAGGTTATGCATACTCCAGCAGCACAAGGAACCGTATTAGAGCCTGAAAACACTCTTCATGAAACCGACTCTCTAGAGAAGGGTCTGCTCTGGGCTAATCTTTACTCCCTGGGTATTTACCTGCTGCGTGAGGAGAGAATGCCAGAGAAGCAAGATGGATTA

							GCCAAGGGGCTAAGGAGCTATGTCGACAAAATGGTTTTCCTCTCTCCCCTTGATTAATGAGCAGGAGGC<br>CAGGCTGTGCTTTTTTATCTCCCCTGGATC
MSTRG.15544.2	--	0.000	0.499	0.000	6.603	up	TTACACTTCAGAGCCCATTAGTGTCGCGGTGGGAAGCATGGCGGCGCAGGCAGGCAGGCTGCTGGCGA<br>GGTAGCTGAGAGTTCTACATCCAGATCACAGGGCAGCAGCAAGGGATGACTCACATTTCCTCCAACAA<br>GGCCACACCTCCTCATAGTCCAGTCCCTAGGGAGGCCACCTTTATTCAATAGTCTATTAGCCTGTGTCAC<br>TGCGTTTATTAACTGCTTTACTAACTTTTGCACATCTAATAACGTCAAAAGGCAAACTTCAAGTATTTTTA<br>CTATTTATGTTAAAAACCACAACAAAAAATGCAAGAAATAAACCAGCATTGTCCTAGTTCCCAGCCCCC<br>AAGGCAAGCCAGCTTCACAGCAAAGCTCTGGAGGTCTCAGCTACCACAGACAGTCTCTATTAGCCGTA<br>AGTTCCTTATGAAGTCCTACAGATCTCTAAGGCAATAGGGGCAAGGCTCCGGTTAATGGCCTCCTCCGA<br>GTGTTGGACTAGATGAGAAGGAGAAGAGAGGGAATAGGAAAGGCCACCCAGAACAGCTGCCGCCCAC<br>CCACTTCTGACAAATGACTCCTCTTCCTCCCTTTCCTTCTCTCCTCCTTTCTCCTCCTCCTCTCCCTCATA<br>GCATGAAATTTAAAGGGAGCAGTGTTTTCTAAACCTGATAAATCCCAAATGATTAACACCAAGACCTGA<br>AATTTATTCAACAAGCTTTAGGCACAATAACTGAGCAGGTATTCCTAACCCTCTATGCTATCTTCACTACG<br>TCCCGGCCCCATGCCCCAAGTTACTTTTTGTCTCCTTGGCTCACTCTGCTGCAGCTGTCTGTCCTCATGG<br>AGAACTCCTCCTCATGCTCAACCTCATCGTTACCATCTTCTTCTTTCTCCACCTGCCTCTTGGCCCTTCTT<br>CTCCGGAACAGTCATCTGCCTCACGGTAAAGTCCTTCAACCATGCTCTTCTCCTTGGGACCCCAAGCTG<br>GGATCAGAAGTCCAGCCTTTCTGTCTTCTCTGCCCAATCATTGGCTGTTCAGCTTTTTGTTGTCCATCCC<br>AGTGCTTACAGAAGCTCTCTTCCACAAGCACAGTTGGTGAGGTTCCTGGCCATTGTCAACTGGACTCCT<br>CCAGCCCTGCTCACACTCCAGAGGTCCTCTAAGACAGGCATCCTGCAAAAGCAGAGATGGGTGGATCC<br>CCGGGGCTGAACATTATGTCGATGTCCTGAAAGGGTAGATGAAATCAGTCCAAGGAGTTGAATCAGTGA<br>ACGCCAAGTTTGATGAGTCCTGTCTCACCAAATGAGGTGGAGAAGCACCCAAGGAAGGCACCCAATGT<br>GGACCTCTGTCTGACCTCTATCTGTTTTTGCTAAAGTGCTTGCTTAGCATGCACTAGGCACAGGAGGATT<br>CAATCCTTAGAACCAAATAAAACCAAGCAGGGTGGCACACAGCTGCAATCCCAGCACTCGGGAGGCAC<br>AGTAAGAGGATCAGGTGTAGGGGTTGGGGATTTAGCTCAGTGGTAGAGTGGTTGCCTAGCAAGCGCAA<br>GGCCCTGGGTTCGGTCCCCAGCTCCTAACCAATAAGCATTTCTAGCTCAGACTGAAACTGGTCCTTGAG<br>ACGTCCTAGCCCAAGCAAGTGAGAATAGCACACGACAGTCACAGTTCAACACTGGAGAGACCGAGAG<br>ATCCATCCAATACAATAAGCCAAGGACACGGAATGTTGTTGACTGGTGGGAGGTGAGTGGGAGGTAGC<br>AAGACTGGGTCTCTGTGAAGCAATCTTCTTCCTGTTCCTACCTAACAACCTCAGTAAGCATTTGTGTCCA<br>ACAAACAATCCTTCAGAGACGAGTCTGTAATTTATGGAGAGGAATATAATCCTCTCTTTGCATGTGACGG<br>CCAGGGTTTGGTGGCCCCAGGACATACTTGGGTGGCCACCTGTCAGCTGAGGTCGTAGAAGATGGCCT<br>ACAAGGAGGCACTCTTCTGGAGGCTGTCGTGCATGGAGAGCAGAACGCTGTTGCCTTAGCTCCATTCA<br>CATTGTCTGACTCTGACCCAATTACCTGCTGCGTGAGGAGAGAATGCCAGAGAAGCAAGATGGATTAGC<br>CAAGGGGCTAAGGAGCTATGTCGACAAAATGGTTTTCCTCTCTCCCCTTGATTAATGAGCAGGAGGCCA<br>GGCTGTGCTTTTTTATCTCCCCTGGATC
MSTRG.15544.3	--	0.341	0.000	0.000	-5.223	down	GTAAGTTCCTTATGAAGTCCTACAGATCTCTAAGGCAATAGGGGCAAGGCTCCGGTTAATGGCCTCCTC<br>CGAGTGTTGGACTAGATGAGAAGGAGAAGAGAGGGAATAGGAAAGGCCACCCAGAACAGCTGCCGCC<br>CACCCACTTCTGACAAATGACTCCTCTTCCTCCCTTTCCTTCTCTCCTCCTTTCTCCTCCTCCTCTCCCTC<br>ATAGCATGAAATTTAAAGGGAGCAGTGTTTTCTAAACCTGATAAATCCCAAATGATTAACACCAAGACC<br>TGAAATTTATTCAACAAGCTTTAGGCACAATAACTGAGCAGGTATTCCTAACCCTCTATGCTATCTTCACT<br>ACGTCCCGGCCCCATGCCCCAAGTTACTTTTTGTCTCCTTGGCTCACTCTGCTGCAGCTGTCTGTCCTCA<br>TGGAGAACTCCTCCTCATGCTCAACCTCATCGTTACCATCTTCTTCTTTCTCCACCTGCCTCTTGGCCCTT

							CTTCTCCGGAACAGTCATCTGCCTCACGGTAAAGTCCTTCAACCATGCTCTTCTCCTTGGGACCCCAAG<br>CTGGGATCAGAAGTCCAGCCTTTCTGTCTTCTCTGCCCAATCATTGGCTGTTCAGCTTTTTGTTGTCTTG<br>CTAAAGTGCTTGCTTAGCATGCACTAGGCACAGGAGGATTCAATCCTTAGAACCAAATAAAACCAAGCA<br>GGGTGGCACACAGCTGCAATCCCAGCACTCGGGAGGCACAGTAAGAGGATCAGGTGTAGGGGTTGGG<br>GATTTAGCTCAGTGGTAGAGTGGTTGCCTAGCAAGCGCAAGGCCCTGGGTTCGGTCCCCAGCTTTGGGT<br>GGCCACCTGTCAGCTGAGGTCGTAGAAGATGGCCTACAAGGAGGCACTCTTCTGGAGGCTGTCGTGCA<br>TGGAGAGCTAGAAGAAAACCCATAATGGAGAAGCGTCAGGCAGTGACTTGCTGGCTTACATTCCCCAG<br>AGTGACTGTCAGTGGAGAGCTTTGCTCTGCCCTGACACATGATAACTTCATCAAGTCAGATCTGCTCAG<br>AGGAAGCACTTCTTGCTTGGAGCAACCTTATCTGAAGAAAGCCAGGAGAGCATGGAGGCAGGGCCAC<br>AGGGATTCGCTGACAGCTTCCCTTGCAACATTGTCACACTACAGGCAAAAGGGAAAGGGCTTCAAGGA<br>GCAAGAGTTGCCTATGGGGCAGGTAATAACCTACATCTGCTGTGATCACAGCTTCCATTTATCCAGTAAT<br>GGACATACGTATCGGGGCCAGACATGGGCAGACACTATAGTGTAGCTGCTCTTGCTACTCTGTGGGTTC<br>CTCTTTCTTTTGCCCTTTCAGGCCCATAACACGATAGGAGAGAAAGAAGGATGGAGGGGTCACAGTTCT<br>GCCTGTAGAATCTCAGAAAGGACTCATCCTGAGGCCCCTTCGATGCTATCCGGTGACACCTTTCTGACC<br>TCCAGAACTCTAGGTTTATAGTAGTTGTTATCGTGCCAAGAAGACACTGATAAAATACATACTCAAGATG<br>CAGGGAAGTACAAGAGCCACTCCCAGCTCTCTGTTATGCCAACTGCCAGCGGTGATGTCCCATGCTCAT<br>TAGCCCTCCCTCCGACCCCTGCGGCGTACGCCCAAGGCTTGCTCGCCATCCGTCTGCTGTACTCACCAG<br>AACGCTGTTGCCTTAGCTCCATTCACATTGTCTGACTCTGACCCAATTACCTGATGGAAACAAATGACA<br>GGCGAGAGAATCACTTTGGCTCACGGCTTCAGAGTTTCAAACCATAGTGGCTGGGAACACATGGTGGA<br>GAACATGGTGGGAAGAGTATGGCGGAGACTTCTAAGCACTGCTGCAAACCAGGAAGCCGAGAGGGCT<br>GAGGGCCCAAATGACCTGCATACTCCAGAAAGGTCCTCCCAGCTAAAGTTTCCAGAGTGTCTCAGAAC<br>AGCGCCATCTCCTGCTGCGTGAGGAGAGAATGCCAGAGAAGCAAGATGGATTAGCCAAGGGGCTAAGG<br>AGCTATGTCGACAAAATGGTTTTCCTC
MSTRG.15544.5	--	0.252	0.698	0.025	2.147	up	GTTTCACCCAACACTAAGATAAACATGAGCTGGCAGTGAAAGTGCTACCAAACACGAAAACAGAAAA<br>GATGTCCAAACACTCCGTTGTCTTACTCTGCGTCGACAAAGGTGCATTGTTCAAGAGAACAGAGGAGG<br>TATTCCCAGCCTATGATGCGATCACCCACTTGAACTCGACAGCCATTCCAGAAGAGACAGGCGTCCCTG<br>TAATTCATGTGGGTAAACGGGAGGATAGATTGAGGAGCAGACACTGTTCTTTTAGCTCAATAGACTACT<br>AAAGTGGTAAAAAGCATGGAGTACAGGAGAATTAGGAGTTCAAGGCTATCCTAAACTACATAGTAAGT<br>GCAAAGGCAGCCCAGCCGGGCTCCATGAGATCAGCTCAAACAAAAGACTTCACCACTGAGTGTTCAGC<br>ACTGACAATGAGTGCACCATGCTGCAGAGACGTGATGAGGTCACTGGGCTGTGATTTGGTGAGAATGT<br>GAGGTTCATCTCTGATTTAAAGGTTATGGGGCCAGCGGTTTCACCATTAAGTATGACTGCTGCCAAGCCT<br>GATGACTTGAGCTCAATCCCCATGGGGGAGACCTAGCACCAACTCTAACGAGACGAGCTGCCCTCTGT<br>CCTCCATGGGTTCACAGTGGCATGAGCCACATATAAGTAAACATTATACCCACAAATAGATAAAAGTTAC<br>CGGACAAATAAGTCAGTTTTATCACAGCCATGTTGGACCACAATGGAGGGAGACGGAGTGAGTTCTCC<br>ATCACTAAGACTCTTCAGGTTGAGGCTGGACACTCTTCCCCTACACAAAGATATACTTGGTGCATTTAAT<br>GGGCATATTTCACAGTTAACCTAGGAAATTAAATGTAGACCGGGGATAGAAAGAGGAGAAGTGGAGAC<br>TAGGGAGAGGAGAACAAAACAGGTGGATGCTGTGGTGAATGAATGTGCTTGACCATGGGAAGCAGCA<br>CCATCAGGGGGTGTGGCCTCCTTGGAGGGAGTGTGCCATTCTGCAGATGGAGGGCTCCTAGTGGTCAA<br>GCTTCACCCACTGCTCCTGGCTGCCTTCGGAACAGGATGTAGAGCTCTCAGCCGCTCCAGCTCTGTCTG<br>CCTGCACGCTGCCACACCTCCTGGCCTCTGTTATCTGGTGAAGGTTATGCATACTCCAGCAGCACAAGG<br>AACCGTATTAGAGCCTGAAAACACTCTTCATGAAACCGACTCTCTAGAGAAGGGTCTGCTCTGGGCTAA

							TCTTTACTCCCTGGGTATTTACCTGCTGCGTGAGGAGAGAATGCCAGAGAAGCAAGATGGATTAGCCAAGGGGCTAAGGAGCTATGTCGACAAAATGGTTTTCCTCTCTCCCCTTGATTAATGAGCAGGAGGCCAGGCTGTGCTTTTTTATCTCCCCTGGATC
MSTRG.15548.1	--	0.361	0.023	0.013	-2.814	down	CCGGGCATGAACGAGAAGGCATGAAGGAAGTGTCTTTCATTTGTTGGAGTGATGAAGTACTCTGTCAAGGGAATGAATTTTACAGCAAAGAGAAGAGACTGGGCAGTGAAGTCAGAGGCATGATCACGCTGAACCTGAGCACTGAGGATGAAACCCAGAACTTCAAGAATCCCACTGAGCCACACCCCAGCCCAGCAGCTGAGTTTTCTCTCACTTGAAAGTCAATGCATCCATAAACAGCCAGTGTGTGTAGAAGTGCCCGATATGCACCAGCCCATGGTTTATTTAAAAAAAAAAAAAAGACAAACCTATACTGACCTATACTGAAATCACATGTGGAGGTTCTTTGTAAACCCAGAATTTGTGTAGAAAGGGAAGCAATTACTGGTTGCCCTGAAGACAAATGGAAGCCAAAAGGTTCATCTTTCTTCTGAGCCTCAAAACACAAGTTAATCAAAGTAATGAAAGAGACAAATCAAGGATCCGTTTTGATAGCAATTGATTTTGAGAAGCCGAAGCCAGTTATTCAAAGCGTCCTGCATACTCATTAGTTCAGTTGAAGAGCAACTGTGGGGGTCAGAAGGATTTGCCTACATAGGACTGGCTCAGAGAAAAAAGCTGTTAAGTAGTTTGGTGATACAATTCCATCCCCCTCGAGACCTGGACTGAACAGAGACAAATGAAGCCAGCTCCCGTGGTTGTCCCAGGCAGGGATCATACCCTGTGCAGCTCAGGAAACCTTCATGCTGAATACAGTAAAGAGAATCCCTGTAGGTCTTCCTATTGGTGGCAAAAGCTCCAGGTTACCAATGCTTCATGACCTTGATCCTCTCTGGGCCTCGTTCTGGAGATGAGGACAACACTTACTCTGCCTAGACACAATCATGGAAAGGGTCAGCTGGAAATGGGTGCAAATTCAGTGTTCTGGCTGTTTATGGGAGAGGAAAGAGGGATGCTAGCCCAGAAAGAGTATCACAGGCACCAATGGCATGGACTTTGTACTAAATGTAGACAAAGACTTCTCTTCTTGTGCTGGGGCATCTAACAATCACAGAATACTTAGAGTGGGTGAGGACACTGGATACAGATTAGCAACAGTCACACATGGTCTCTGCCTCATAAACCTTATATTCTAGGAGAAAGAAGAAGCAAACATCAATATTTGTGCAAATAAAAAAGATTATTTCAAGATAGTGTCCTAACATAGTCCATCTCATTAGGGAGGTATGGTTACTGCCCTATCCATGCCTAACCTTGCCCCATCCATCACAGAACCTTATCCTGGCCTTACCTTTGTCCTCTGTGACTTGTGATCTCCTAACCTTCGATGTTATTTCTATGTTGTATAGTCTCCATAATTTCTGTGTGAATGTAGGCTTCATGAAGGCAAAGACCTTAGCTGTTGTTCCTCAGGTACAACCTCCTCATTTTTTGAGACACAGTCTCTCTCTGGCCTGGAACTCACCAAGTAGGCTAAGCTGGCTGTCCAACAAGTCCTTGGCTCTGCAGTCACAGGTGTGCAGCACCAAGATCAGCTTCTCTTTAAACAGGAGTTCTAGGAGTTGAACTCTTATCCTTGTGCTAGCAAGGCAAGCGCTTTGCCAACTGAGCCACCTCTCCAGTCTAATGGCTGTTGTTTTGTTCACTGCTACCCAAGTGTCTAGAACAACAGCTGGTTAATAACTGCTGTTCAGTACATATGTGATGATTTCCAGAGAGAGAGAGAGAGAGAGAGAGAGAGAGAGAGAGAGAGAGGAGTACCATGATCCAGATGTGTGTTCCCATGTGCAGATGCTGCAAACATGGTCTCCAGGGTGATGACATGAGGTGGTGCCATCTGAAAAGGGTGACTACATCCTAACAGTAGTGTCCTCACAAATGGGACCAATGCCTGTAGACTTAGACTTTTCAAAACCTACTTTAATAAGGGTTCTTCAAC
MSTRG.15736.2	ENSRNOG00000003019;ENSRNOG00000003065;ENSRNOG0000000	0.532	1.319	0.046	2.029	up	GCCGGTCCCGCCCGGCCTGCGGTCCTTCCTATCCACCGGGTCGGCGATTCGCGCAACGGCAACTGCTGAGCGTAGCCAGTTCAGATAACCACTTCTGAGAGTACAGTCCAGACGCAGGCTTGGTGGCGTGTGATTACACAAGCAGTGTGTGTGCGCAGCAAGGTCTTAGACCAGGTCTTGAGTATGGACGATCTTTGAAAATGACATTTCACCTGCGAGCAAGCAGGGTGGTGGTGAAAGCTTGTGATTTCAGCACTCCAAAGAGTGCTAGAGGTTCATAGGTTAGAAGCCAGCATAACACTGTCTCAAACAAAAGTCTCAAAACAAAAACTGATAATCTGAACTCTGAAGTGATCTCAGCCAAATTCCCCGGATTTCTGAGTCTGTTTCCTTACACTAACCAGGCTCACGAAGCTGTTGTGGAAATAAACGTATCTCACAAAACGCCTCTCTCACTGCTTTGTGGTTCTTCCTGGATCTGAGTGCCTGTTTCTTGACATTCCTGTTTATCTCCCAACCGGTTGGCTGAGGTGCCTGGGGCCTCAGCCACCTGTCTGCTGCATTGGTGGCTCATAGGATGTGAACTTGAAGATGCC

	3137;ENSRNOG00000003186;ENSRNOG00000003224;ENSRNOG00000028075;ENSRNOG00000042344						
MSTRG.16041.1	ENSRNOG00000016494;ENSRNOG00000016774;ENSRNOG00000024455;ENSRNOG00000029415;ENSRNOG00000030541;ENSRNOG00000054605;ENSRNOG00000060934	0.801	0.054	0.019	-2.633	down	CAACCAGTTCCTCTAGGGTGGTATGTTGAGTAATTGTAGCTCAGTACAAGGGCACCGAGTTTGCCAGCTGTGCAATGTGTGTAAACCCATGGGAGCCTCTGTTAGTCAGACACAGCTCTGAGCCCTCTGCAGCTCCTTCGCTGCTAAGGGAGGTCCAGCACCTGTGCTCCACTATAGCCAGGATTGTGGGGTCCAGCACCTGTGCTCCACTGTAGCCCGGATTGTCCTGCTGTCACACATTACTCTGTCAGCCTGATTTTCTGTTCTGCCTGCTGTTGCTTTTTCATGGATGTGGGTCCCTGCTCACCAAGGATAGTGTATGAACAAGTACTGTCATGTGACCCATAGCTGGCCCTTCTCCTGTCATCCTCTCAAACTGTTGAGGTTCCATTTTCTTAGGCCTTGTGGGTTTGACTCTGGGTACTTACGGTACAGTGAAATAATTGTTGATGGCCTTGGTTTTGTCTAGACCATCTGTGATAGGTGAAACAGCCTGGCCACCTCCCCTTCTCTCTGCATGGCTGGCCTTGGAGCTTCCTCTGTCTTCCACACTTTGTTTTTATCACAGCTACTCTGCTTTGAACAAAGGGCCACACAGCTTTTGCTCAGCTCAGAGGTTGGCCTTCCCCTCACCAGCATTTCTGAAAACACTCAAGAAACAGTTGAAACTGAACATTAAGTACGACCCCCAACTCATCTACCTCCCCGTTACTAGGCAGGCATGGCTTTTTCAGGTTGGAGGCTTACAATCAAGGCAGTGTCCTTAAAAAAGGATGCTAGCAGCCCTACACTTGGGCTGATCTGCAAGGACTTCACAACTCTTATCTACTCAACTCTTCCACACAGCTCTGCTTGGGAGGCCTGTATTAGCATGCTTGAAGCAGATAGAGGCCTGGAGAAGCAGACTAGACATGGCTTTGTAGAGATG
MSTRG.160	ENSRNOG0000001	0.676	0.000	0.001	-4.759	down	CGGCTCTGTTTCCAACAAACAGTCGGGCAGTCTGCAATCTGCCTGGAGGAGCCCAGAAACCCTGGTGAGACGGCCACGCCCACTGCCTCCTCCTCTAACTGAACCCGTAGCTCAATGATTGGCTAAGCTAGCTGTCCAGCAAGCCCCAGGAGTCTTCCTGCATCCATCTTCCCAGCTCTGGGATTAGAGGTATGTGCAGCCACTCC

69.1	8611;ENSRNOG00000018701;ENSRNOG00000018792;ENSRNOG00000018834;ENSRNOG00000033893						CAGCTCTTTACAAAAGTGCTGAACACTCAGACTCGGGTTTTTCCTTGTGTTTGTGTGGCAAGCATTTGACTGACCAAGCTAACCTTAGGGACATTTAAAGAAGTGCTTGTTCTCATGCATCCTCGGCGTTATGGCTGGGAAGCCTCTCACTTCCATGAGGTGGGGACATGGTAAATGCCAGGCTGAGTAGCTCTGGGTCCTTGAGGTGGACACAGCTGTCTGGACAACCACACACAGCTAGGCACCTTCAGGCCACACGTGCTTCATCCCAGAGTTCCTTGTCTGAGTCTTGCTTCCCTCTTGCTCAGGAAGGCAGAGATGCCCTGTGTTCACTGCTTGGGGAAAGGCGAGAGTGAGGTAGGAGTGAGTCATTAGCCCATCAGCTTCCTGGTCCTGCCTTGCCAAGAGGAGAGAGAGACTCATGCCTACCTCAGTTGATGGCCAGGCCCCTGTACTCAGCCGTCTGCCAGCATGTGTCCATGCCACATCTTGCTTTGTGTTGTTCTCTAGGGC
MSTRG.16157.1	ENSRNOG00000020594;ENSRNOG00000020626;ENSRNOG00000020630	0.313	0.000	0.049	-3.592	down	CTGAAGACAGCTACAGTGTACTTATATAATAAATAAATAAATCTTTTAAAAAACAAATAAAAAAAAATAAGACAATCTGGGGTCAGCCCTGAGAGTTTCTTCTGATTACCTTGTCCCAGACAGACTAAGTTTCTATTCAATTAACATCTCACAAGGGGATTAAACTTCTAACAACAGAAACCCATTCTCTTAGTTCTAGAGAAGTCTTAGTTCAGCAAGTCAGTCTCCGTTGGCTGAAATCAGGAGGTCAGCCCCTCACCAACCCCTTCACCTGTTCCAGCCCGCTCTGCTGCTGGCCTTCCTAGGTTTGTGGGTGCCTTCCTCCCACCTTCTGGGCTACGTCTTCAGTCTCTTTCTGTTATCCTCACCCCACCTTTCTTGTTTTGCCTGACAGCTCAGTATTTGTAAGCAAATGCAGTTAGGGTCCACCCAGATTGTCTCTCATCTCAAGACCTTGGAGGCGATGGTGCTCACGTCACCGCGGCCATGTCTTGTGTGTGCTCTGCAGTGCCTTGGACGTGAAATGTGCCCCACGTGTTGAAGGCTGAATGGTTAACCTTAATGGAGGTTTTACGTGTCACTGTCCCAACTGCTCACAATGCCTAAGCGTCATTGTCCTAACAGCTCGCAAAACTTAAGGGCAGAAG
MSTRG.16169.3	ENSRNOG00000027852	1.072	0.107	0.027	-2.382	down	AAAGAAACTACAATAAGGTTGATGTAACTATCAACAAGTAACAGAGATAGGCAGCTAAAATTCCATCATCGAGCAACCACTTCAGTGTAGGCTGGCTTAGGCCATTCCCTAAAGGGGCATGGGGTTCCTAATTTGGAAGATAATTGTGACCAGGCTGTGTTTGTACACACAGAAACACACTTAGACACCAAGCATTCTGGGTCCAAAATGCCTGCCTCTATTCCTCATTGGCTGAGTGCCTCTGGCTACCTTCGTTCTCCACACTCTGACCTCCAGCTTTCTTATCTGCACATCAAGAGAATGCACCCAGTTCTGCAGGTACAGTAAATAAAAAGTGAAACATAATCGGCAAAATCCTGGTGTATTGCAGACTTTCAATCTTGTGGAGTTTGGTACTGTGAGTAATGATGGTTCAAAGGCTGCTGGCTTCCTGTGGCATCCTTCTTCACTAACCCAACGGAGGGTATGAGAAAAATGTCTGTGGTCCCCAGGACAACCCCAACATTCAGTCAGGAGGGACCAGGGAAGATGTGAGCTGAGTCACATGGAGCAAACCTGGGCTGGTAACAGAGGTAGAGGACAGCATGTGAGCAACAGGCAGGAGGTGTGGAAAGGCAGCTTTGGGAGGCCATGAGAGAGGACCTTGGACAGTGAGTGTGCAGGAGGCCTGAGAGCCCTGGGCAGTGAGTGTGCCAGGGGGCTGAGAGCCCTGGGCAGTGAGTGTGCAGAAGGCCTGAGAGCCCTGGGCAGTGAGTATGCAGGGGGCCTGAGGCAGCAGAGCAGAGACCAATAGCAAGGATCATCTTTGAAGAAAGCCTTGCATGCTGAGCTGAGGCCTTCACACGGCTGCCCATAGGAAGGAGGGAGCCATCAACAAGCAAAAATGTACCCTGGAGGTCTACTTTGACTGTGTATATCCTGGATACGACAGAGATGGAAAGTCCAAGGATTAGGAAAGAAATATCCCTTAAGATCAAGGGTCTGGGCCTCCCCAGATACCC
MSTR	ENSRNO	0.009	0.418	0.000	5.425	up	CCTGATACCAATCGGCAACCCCCTGCCTCAGAAACCAGAGCCCTGCCCCACCTCCTCCACAGGCACAA

G.16226.1	G00000003269;ENSRNOG00000003508						GAGTGAGCGAGGCCTATCGCTACTGCTGTGCTGCTCCACATATAAGTATTTGAGAGACCCACGGAGGGG<br>CCAATGAGATGGCTTTTATTTATGAAATAATGCAAGTGCCACGTGGAATTAGGAGATGGTTCCAATTTTT<br>ATTTGTAAATCATCAAAACTGCGAAAGGAGATTTACAGAGTCAGGGGCAAAACAGGTGGACAACGAAA<br>CACCTCAGACACACTTTGCAAAGTTTCAAAGAGGCCGCATGAGGCCTCTCTGGTCAGCCCCTGTGGCA<br>CAACTATGTTCACTCTTGTATTCCCAGTGAGTTCCTTATATCGGCCTTTCTCTTTCCCAACTTTCGGAGAC<br>TTGGCTTTGAGTTCAACAGTTATCTTCTGGTCTTTGTCTGATTCCAGTGAAGTGATAACTGCCTGTTAGG<br>AGACAAAACAGAGTCGTAAGTGGAAGGAATTTTATAATCTCCACAATTCTGAGCAGTGGACCCTCCCTT<br>GGGTTCCTCCTGCCAGCCACTCTCAAGACCCTGGATCTCTTGAGGCCCCCACAGTTCAGTGATGTGATG<br>ACAAGGGACAGAAACTTTGCTTACATCCCTGGCTGGTGAGAAGCAGTTCAGAAAAAAAAAAATCTGG<br>ATTCTCACCAGCTGCTGGGTACCAATGCAGGGACTAAGAAGACGGGATTCTAACCACGCAGTTTACAG<br>ATCTGAAGCTCAGAGAAAGCCTCTCCAGCATGTGAATGAGGGTGAACAGCGCTTCTTAGCTGGGTGGG<br>GAGGCCCGGGAAGAGAGCTCTGCCCTTTCCTCTCGGGATTCTTCCTAGTGAACGAGGGCTACTTTGAAT<br>GCGTGGGCCCTCTTGCCCTCCACTTGAGGGTACAGCATCGTCAAGAATGTCAAGATAGAAGCACCTCTA<br>CAACAGTAGTTCTCAGCCTTCTGAGCGCCGTGACGCTATAATATAATATAGTTATGTTATATTATAACTATA<br>ATACAGTTTCTCATGTAGTGGTGACCCCAGCTATAAAATTATTCCATTGCTACTCTGTAACTGTAAATTTG<br>CTACTGTGATGAATCGTAATGTAAATATCTGATATGGAACCCCTGTGGGATCTTGACCCACAGGATATAA<br>ACTACTGCTCTAATCCCTACTGATGCCGCTAAGCCTGCCTGCCCTCACCCAGCCAGGGATCCCCATTTTG<br>CTGGGTTATTATCTGTCCTTGCTGGTTTTGTGTGTCGACTTGACACAAGCTGGAGTTATCATAAGGAGCC<br>TCCCTTGAGGAGATGCCTCCATGAGACCCAGCTGAAGGCATTTTCTCAATTAGTGATCAAGCGGGGAGG<br>GCCCTGCCCATTGTGGGTGGTGCGTCCCTGGGCTGGTGGTCCTGGGTTCTATTAGAGAGCAAGCTGAAC<br>AAGCCAGGGGAAGCAAGCCAGTAAGTAACATCCCTCCATGGCCTCTGCATCAGCTCCTGCTTCCTGACC<br>TGCTTGAGTTCCAGTCCTGACTTCCTTTGGTGATGAACAGCAGTGTGGAAGTGTAAGCTGAATAAACCC<br>TTTCCTCCCCAACTTGCTCCTTGGTCCTGATGTTTTATGCGGAATAGAAACACTAAGACAATATCCATGT<br>AGACAATTGTACCTCAGCCTTCTTATGCTGTACCCATTTGATGTAGATGTCATACTGGTTTTGGCACACTA<br>GGGCTACCTTGCTGACCTGCTGACTTTACAGTCTCCTCAAACCACCTAGGACAGCACAGCATACGGAG<br>GAAGCTGTTAGGAACACGTGGGCTGAACACATAAACTTCTGAACGACACAGTCTTGGCAGTCAGAGCT<br>AGTTTTTTTTTGTGCTGTACGCTTGTCCTGAGGTTGCCTGATGTCTTATGTTAGACCCCAGCACCACAAC<br>CCCAAAACTACCCCTGTGGCTGGGAGTGAAGTCGGCGGTGGGAACTGTGTTTACGACTCACAGGTCCC<br>AGCACTGCGGAGGTCTACCATGGTAGCGCATGCCCGTAATTCCAGCACTCGGGAAGATCAGAAACCCA<br>GCGACATCCTCAGCTACATGCTCTGTATTATTTAACTTGAGTTTCAGTTTCTTCATACCTCTATCCCCTGA<br>ATGCTGGGAATAAAAGAGTAGATCACCACGCCTGGCAGATGCAGACGACTCTTTTGAGAAGCCGGACT<br>ATGAAATGGGGGAGCAGAGCAGTAGCGGGAAGGATATGTGGAGAAAGGAGAGAATATGAATTTTCCAG<br>CTTGCTTGCCAAGGAGAGCGATTTCCAAGGTTGCTGTGGCCTGAAGGCTCTTGACTTTCTCCTTTAAAG<br>AAGGCAAACATTTTCAGGTTCACGTTGGTTTTCCAAGACAGGGTCTCTCTCTATAGCTCTGGCTGTCCTA<br>GAACTCGATCTGTAGACCAGGTTGGCCTCAAACTTAGGAGATGTATCTGCCTCAGGCTCCTCAGTGCTG<br>TGATTAAAGGCGTATGTATAATGATGTGTATAAGTCTTTGTGTGTCACATGACTGCAGTGTCCATGGGGC<br>CAGGGCATGGAATCCCTCTGCAGCTGGAGTTACAGGCGGTTGTGAGCTGCCGGACATGAGTGCTGGGA<br>GCTAAACTCTGATCCTCTGTAGGAGCAGTGCACACTCAGAACTGCTCAGCCCGGCCTGCCTGTCACTG<br>GCCTTGCTCTTTTCACTTCACTCCAGTTATATTTCCTTACTAACTGACCCCTCAACCATCAGATGTCCAAT<br>ACACAAAGCTCCTCACTTTATCTCACATCATTCCCTAAAACAATAACTCTCAGCTGCGGAAGAAGGGCA<br>AAGGGGACTCAATAATCCTGGCACTGGCTGAGCAGAGGCCAGGGATGCTGTTAGCATCCCTGCATGCA

							AAAGGGTAAAAGGAGCTTCCCACAACCACCCTGCCCAAGAAGCCCTGTGCAGTTGTGAGCTTCCCGG AGGAGGGCTGCCTCAGTCCTGCTCATTGCTATTGTCGGACGAATGCCTGCTGAGTGAACAATAACACTC TTCGGGCAAACAATCTAGTCTCTGCCTATTCTGTGTTATTCCCCATTCCCAGTCCTCATCCTGCCACGCCC CCACTCAGTCACACCCTATGCTCCAGTCCCAGGGAACACGTGGTCTCTGATTATTTTTGTACTTCTGATC TGCAGCTACCAACTCATTTCCCTTGCCACATCTATCTAGTATCATTGCATGGTAAATTCTTACAGATCCCT GCCCACCAATTCTGCTAAACTCAAGAGACACTACAGCACTTTCTCCCTTTAAAGACTGGCTCAATGAGG CATCATGAGCTATAGCTGGCAAACTAAAGGCCAGTGGGCTAAATTGAGAGAGATGTTTTCAGTCCTGGC ATATTGTTCTATGGCCATTATCGAAAACTGAACGCTCCTAGGCAGGACAGGCACAGTAGTGGCTCCGTT CTAATACGTCTGCAGTACTCTGCTGTCTTGATTGAAGTCTTGGGGCATATGACAGGATTTCATGTTAAGG GGGTGAGTGAACTTTATGCTCCTGGGGAACTAGAACTCGAGGACGCTTGGGTGTACCTGGACCTTGTT GTCCTTGCGAATGGGCATGGAACAGACGCACTACTTCTGCCGCAGTTCCTTGGACAGCGGCAAAGACA TAGTCTTTCGGAGCACGTGGGAGGGAGCATTGAAATAAAGTTTGAGGCTTAAGCTGCGGTCCGAGGTC AACAAGGGATGAAACTGCCTGGTGATCGCCTGCAAAAAGCGGGAAAGGAGGTAGTCCAGGGACGGTT CTGGGTCCTAGAGGAACCACTACTATTCACTCATCGGTTCCGGTTCCGG
MSTRG.16226.2	ENSRNOG00000003269;ENSRNOG00000003508	0.008	0.743	0.000	6.212	up	CCTGATACCAATCGGCAACCCCCTGCCTCAGAAACCAGAGCCCTGCCCCACCTCCTCCACAGGCACAA GAGTGAGCGAGGCCTATCGCTACTGCTGTGCTGCTCCACATATAAGTATTTGAGAGACCCACGGAGGGG CCAATGAGATGGCTTTTATTTATGAAATAATGCAAGTGCCACGTGGAATTAGGAGATGGTTCCAATTTTT ATTTGTAAATCATCAAAACTGCGAAAGGAGATTTACAGAGTCAGGGGCAAAACAGGTGGACAACGAAA CACCTCAGACACACTTTGCAAAGTTTCAAAGAGGCCGCATGAGGCCTCTCTGGTCAGCCCCTGTGGCA CAACTATGTTCACTCTTGTATTCCCAGTGAGTTCCTTATATCGGCCTTTCTCTTTCCCAACTTTCGGAGAC TTGGCTTTGAGTTCAACAGTTATCTTCTGGTCTTTGTCTGATTCCAGTGAAGTGATAACTGCAGGTCCCA GCACTGCGGAGGTCTACCATGGTAGCGCATGCCCGTAATTCCAGCACTCGGGAAGATCAGAAACCCAG CGACATCCTCAGCTACATGCTCTGTATTATTTAACTTGAGTTTCAGTTTCTTCATACCTAAAACACAATTC ATATATATATATGACATATGGATATGAATATATTTAAACTCCTTTATATCATACTTTTTATATTCATCTAGTTAA TACATTTTATTTTTTTTCCCTACCCAACAAGTATTGACAGAGCCCTGTTAGGAGCCAGGGCTGAGACCGG CCAACAAACAAGATGGCAGCTCTCTGGCACATGTGAGAGGCGGTAAACAGTTATAGGCGAAGTATAGG CCTCTCTGTCCCTTTTCCTTTGTGTGTCTCTCTCTCTGTGTCTATCTTTTTTTTAAAAAAAAATATTATTTA TTTAATGTATATGAGTACACTGTAGCTGTCTTCAGACACACCAGAATAGGGCATCATCTCATCACAGATG GTTGTGAGCCACCATGTGTTTGCTGGGAATTGAACTCAGGACCTCCAGAAGAACAGTCAGTGCTCTCT AACCACTGAGCAATCTCTCTAGCCCCGTCTGTCTTTTGAAACAAGGTCTCCTTGCTGTCCTGGAACTTG CTTTGTGGACCAGGCTGGCCTGAGAGATCCCCATACCTCTATCCCCTGAATGCTGGGAATAAAAGAGTA GATCACCACGCCTGGCAGATGCAGACGACTCTTTTGAGAAGCCGGACTATGAAATGGGGGAGCAGAGC AGTAGCGGGAAGGATATGTGGAGAAAGGAGAGAATATGAATTTTCCAGCTTGCTTGCCAAGGAGAGCG ATTTCCAAGGTTGCTGTGGCCTGAAGGCTCTTGACTTTCTCCTTTAAAGAAGGCAAACATTTTCAGGTT CACGTTGGTTTTCCAAGACAGGGTCTCTCTCTATAGCTCTGGCTGTCCTAGAACTCGATCTGTAGACCA GGTTGGCCTCAAACTTAGGAGATGTATCTGCCTCAGGCTCCTCAGTGCTGTGATTAAAGGCGTATGTATA ATGATGTGTATAAGTCTTTGTGTGTCACATGACTGCAGTGTCCATGGGGCCAGGGCATGGAATCCCTCTG CAGCTGGAGTTACAGGCGGTTGTGAGCTGCCGGACATGAGTGCTGGGAGCTAAACTCTGATCCTCTGT AGGAGCAGTGCACACTCAGAACTGCTCAGCCCGGCCTGCCTGTCACTGGCCTTGCTCTTTTCACTTCAC TCCAGTTATATTTCCTTACTAACTGACCCCTCAACCATCAGATGTCCAATACACAAAGCTCCTCACTTTAT CTCACATCATTCCCTAAAACAATAACTCTCAGCTGCGGAAGAAGGGCAAAGGGGACTCAATAATCCTGG

							CACTGGCTGAGCAGAGGCCAGGGATGCTGTTAGCATCCCTGCATGCAAAAGGGTAAAAGGAGCTTCCCACAACCACCCTGCCCAAGAAGCCCTGTGCAGTTGTGAGCTTCCCGGAGGAGGGCTGCCTCAGTCCTGCTCATTGCTATTGTCGGACGAATGCCTGCTGAGTGAACAATAACACTCTTCGGGCAAACAATCTAGTCTCTGCCTATTCTGTGTTATTCCCCATTCCCAGTCCTCATCCTGCCACGCCCCCACTCAGTCACACCCTATGCTCCAGTCCCAGGGAACACGTGGTCTCTGATTATTTTTGTACTTCTGATCTGCAGCTACCAACTCATTTCCCTTGCCACATCTATCTAGTATCATTGCATGGTAAATTCTTACAGATCCCTGCCCACCAATTCTGCTAAACTCAAGAGACACTACAGCACTTTCTCCCTTTAAAGACTGGCTCAATGAGGCATCATGAGCTATAGCTGGCAAACTAAAGGCCAGTGGGCTAAATTGAGAGAGATGTTTTCAGTCCTGGCATATTGTTCTATGGCCATTATCGAAAACTGAACGCTCCTAGGCAGGACAGGCACAGTAGTGGCTCCGTTCTAATACGTCTGCAGTACTCTGCTGTCTTGATTGAAGTCTTGGGGCATATGACAGGATTTCATGTTAAGGGGGTGAGTGAACTTTATGCTCCTGGGGAACTAGAACTCGAGGACGCTTGGGTGTACCTGGACCTTGTTGTCCTTGCGAATGGGCATGGAACAGACGCACTACTTCTGCCGCAGTTCCTTGGACAGCGGCAAAGACATAGTCTTTCGGAGCACGTGGGAGGGAGCATTGAAATAAAGTTTGAGGCTTAAGCTGCGGTCCGAGGTCAACAAGGGATGAAACTGCCTGGTGATCGCCTGCAAAAAGCGGGAAAGGAGGTAGTCCAGGGACGGTTCTGGGTCCTAGAGGAACCACTACTATTCACTCATCGGTTCCGGTTCCGG
MSTRG.16226.7	ENSRNOG00000003269;ENSRNOG00000003508	0.155	0.413	0.047	2.074	up	TGGTTCCAATTTTTATTTGTAAATCATCAAAACTGCGAAAGGAGATTTACAGAGTCAGGGGCAAAACAGGTGGACAACGAAACACCTCAGACACACTTTGCAAAGTTTCAAAGAGGCCGCATGAGGCCTCTCTGGTCAGCCCCTGTGGCACAACTATGTTCACTCTTGTATTCCCAGTGAGTTCCTTATATCGGCCTTTCTCTTTCCCAACTTTCGGAGACTTGGCTTTGAGTTCAACAGTTATCTTCTGGTCTTTGTCTGATTCCAGTGAAGTGATAACTGCAGGTCCCAGCACTGCGGAGGTCTACCATGGTAGCGCATGCCCGTAATTCCAGCACTCGGGAAGATCAGAAACCCAGCGACATCCTCAGCTACATGCTCTGTATTATTTAACTTGAGTTTCAGTTTCTTCATACCTAAAACACAATTCATATATATATATGACATATGGATATGAATATATTTAAACTCCTTTATATCATACTTTTTATATTCATCTAGTTAATACATTTTATTTTTTTTCCCTACCCAACAAGTATTGACAGAGCCCTGTTAGGAGCCAGGGCTGAGACCGGCCAACAAACAAGATGGCAGCTCTCTGGCACATGTGAGAGGCGGTAAACAGTTATAGGCGAAGTATAGGCCTCTCTGTCCCTTTTCCTTTGTGTGTCTCTCTCTCTGTGTCTATCTTTTTTTTAAAAAAAAATATTATTTATTTAATGTATATGAGTACACTGTAGCTGTCTTCAGACACACCAGAATAGGGCATCATCTCATCACAGATGGTTGTGAGCCACCATGTGTTTGCTGGGAATTGAACTCAGGACCTCCAGAAGAACAGTCAGTGCTCTCTAACCACTGAGCAATCTCTCTAGCCCCGTCTGTCTTTTGAAACAAGGTCTCCTTGCTGTCCTGGAACTTGCTTTGTGGACCAGGCTGGCCTGAGAGATCCCCATACCTCTATCCCCTGAATGCTGGGAATAAAAGAGTAGATCACCACGCCTGGCAGATGCAGACGACTCTTTTGAGAAGCCGGACTATGAAATGGGGGAGCAGAGCAGTAGCGGGAAGGATATGTGGAGAAAGGAGAGAATATGAATTTTCCAGCTTGCTTGCCAAGGAGAGCGATTTCCAAGGTTGCTGTGGCCTGAAGGCTCTTGACTTTCTCCTGGACCTTGTTGTCCTTGCGAATGGGCATGGAACAGACGCACTACTTCTGCCGCAGTTCCTTGGACAGCGGCAAAGACATAGTCTTTCGGAGCACGTGGGAGGGAGCATTGAAATAAAGTTTGAGGCTTAAGCTGCGGTCCGAGGTCAACAAGGGATGAAACTGCCTGGTGATCGCCTGCAAAAAGCGGGAAAGGAGGTAGTCCAGGGACGGTTCTGGGTCCTAGAGGAACCACTACTATTCACTCATCG
MSTRG.16235.1	ENSRNOG00000003977;ENS	0.000	0.882	0.000	4.784	up	GAGATGGCTCAGCGGTTAAGAGCACTGACTGCTCTTCCAGAGGTCCTGAGTTCAAATCCCAGCAACCACACATGGTGGTTCATAGCCATCTGTAATGGAATCCAATGCCGTCTTCTGGTGTATCTGAAAACAGCTACAGTGTACTCACATACATGAAATAAATAAATAAATCTTAAAAAAAGAAAAACTACAGGAGGGGGTCTGCTGAAGGGAACAGGTCCTTGTGGCCCCTCGTCTGCCTCTTTGCTCCGCTTTGAACTTCCTACCACAATCTTCCTCACCGCATACCCACCATGATGCTTCTCAGTAACCATGGGCTGGCACCTCCACCCC

	RNOG000<br>00037931						
MSTR<br>G.163<br>05.2	--	0.339	0.000	0.000	-<br>6.594	do<br>wn	GTTAGATCATGATGGCTTCTGATAAAACCAAGGCCACAGCTATCATGGCCTTACACGGGAAGTTCTATTT<br>AGAGAGTGTTTAATTAAATGTGTCAGTAGCAGTATCAGCTTTTATTACTGTATAGTTTGTTCAGGTTGGTA<br>TAACTGTCTTTTAAAAACAGTATTAAATTCCTGGAGTCCTGATGATACTGCACAGATATTAGGAAATGAG<br>TCAGAGGGGAATTGATGCCATGGCTGCTTTCTTTCCCAAGGCTTGCCGTGTACAGTTTTTAAATGGTGCT<br>AGGGGTTTGGAAGGAAGAAGTTTCTTTTATGTTGATGTTTGCCATCTATTTGCCAGCCTGTGAGTAGCAT<br>TGGTGTGGTTATTGATTGAAATGACTGTCTTTGGAAAATTGTGCTGGCACTTCAGAACAGCTAGAGGGA<br>GGCTAGATTTGGCACACCGCCAGTTCTTAGGTCTGTGAAGGTAGGTTACAAGTCTTGGATATAGAATTG<br>AGTGTTTCTTTCAGGCTTTCTTGCCTCGAAAGGGCAATGCAGCAGTGATCTCTCTTCTTGGTACCACAG<br>AATGGCCATTTGCATTATAAAAGTGTTTCTTTAAGTGTTCTTGAGGGTTGCCCTGGGTTTGTAGTGGGCT<br>GACCCGGTTTTAGCCAGAATAGGGGAAGACTTGTATTGTAAGTCTAAGTTATTAATCTGAGGTTCTCTTT<br>GCTACATTCAGTTAAAATTACAGGGTAGCGTAAGGCACTAAGCAGAGCGCCCCAGGCTGTGTGTTGGTA<br>CCTACTGTGGGTTCTCTGGAGCTGCTAGGGAGAGGGAGGAAGTGCAGGTTCCTCTTAAAAATGAGGAA<br>ATGCCTTCAAAGTTCTTTGGAGTCACCTTCCTCTGAGGAAAAAAAGATGAAACCTCGCCTTCATCTAAC<br>CTTGTTTCAAAGATTAGGAGCAATATTGCCTTTAAATGCATGTAGGGAACTTTGATTGTTGAGCCTTTAC<br>TGTGACTATCATATAATCGAAGGCAGGTGGCTCAGTGAGATAAACCAAGGGCTTAGCTGACGTTAGCAA<br>ATCGTTATTTTGTTCCCTGTGAAAATGAACCTGGAAGATGTCCTTTCTTTCCACTTACTTGTTCTGTATAA<br>GTGTTATTGTTGTTGCTTATTTTAAACAAAATATGTATCTGGAGTTTAATTCTTACTTTTAAAATATAGTTAT<br>TGTTTCTTAAAAAGTATAATTATCGGGGCTGGGGATTTAGCTCAGTGGTAGAGCACTTGCCTTGTATATTC<br>CTTGTCACTGAGCTGCAGCCTTGGCCTTTCCTTCTCCTTCTCCTGTCTATGTGGGGAGGGCCTCCAGGG<br>CTGTGTTGCAGTATCCAGACGGCTCAACCTTTGTTTTATTACAAAGGTGGCTCTCTTTGACACTGCATTT<br>AAAAAGACTGTAGAAGAGGATAGAGCCTTAGTTGACTTTGCTTGGGCTGTAGAGGTCTGCGGTTGATAT<br>ACTGACCCTGTCATAACCTCGGCTTTCAGTCTGTTGTCCTGTGTGGGTGGCTGTGTACATTTTGAAAATA<br>CAGGAGTAAAGGTAGTAATTGTATTTAGGCTTAATTTAGGTCTTGTAGGGTTTGCTAGCAGAGTAGAGGG<br>TTAGCAGTCCTCTACTTCTAATTGATGGCTCACAGCTGTTAGGTTTATGACAACCTGTGGGATGCAGGAG<br>TCACTGTCCCCGTTTTCCTCATGTGGCAGCTGAGTAGATGATGAGCTCCCAGGCTGAGACGATGTGTGC<br>TGACTGCTTAGGTTTGTGAGACTGTGAGGTGCTGAAGCACGTGTTTGAGATCCATGGCAGCCTTGGTGC<br>AGACTCTCTTCCCCCTATACTGGTTTTTAAATTTCCTTCTTCCTGTTTTTTGTTTTTTGTTTTGTTTTGTTTT<br>AGCATAGCTTCATTTGCGGAAATTGTACTAGTAATAAATAATGTATGGTTTCTTTAGTCATTATTAATTTGA<br>ACCTAAAATAAGAAAAACATGCTAAGTTCAAGTATTCCCTTTGCCAAGAGGAAGGAATACTCCCAATCT<br>TAGAGCTGGGATTAGAGCCTGAGTGGGTTTCCGGGAAGGCACTGCCATAAGGGTGTCCCTGGTATGCA<br>GTCAGGGGCACAGCTTGTTTCCTGCTGTCAGGGAGTTTTGTGAGTGAACCACAGATGAAAATGCAGAC<br>TGCTCTAGTGTGTGCATGCATGCAGTAAAGCTCTGCAGTTAGCTGAATCTCCATAGGCTTTTAGAGTCAT<br>GGTAGCTAGATTAAACTTGGTCACTCTCCATGTCGTAGAACACTTGGCTTGTCTCTGAATCTAGCCGAGC<br>CGAGCCAGTATCCTTTACTTCACAAGCCCAGAGTTGACCACTTGGTTCTTAGATCAGACAATCCAAGGA<br>CCCTGATTTACCAGGACTCTGGCCTGAGGTTAACACTGATGGCATATCTTTATTTCCTGTGTTATGACCTA<br>TGATTCTTGGTTTGGTTTTGGTCCTGAGGATTGAAGCACAGGCCTTACATGTGTAGGCATGCGTCCTCCC<br>AGTCATGCCCCTGCCCTCATTAGGATCCTCTGCAGAAATTACCTGGGCTGCTGCTTTGTGGATGAAGCTA<br>TAGAATTGGATTGACTTGGCTGAGGCTATGTATTTATTGAATAGAAAACTGGGTTATGAACCTAGCCCTG

							CAGAGCTGCCCCAGACTACAGTCACTACAGCCTTCTTTAACTTGGCAATGGTCATTTTAGAATTTAAAA<br>AAATTCCCCTTGTTTTTAATGTCAAAACTGAAAAGGCACGAATGGAGCACACAGGCCCAGTGAAGTGA<br>CTGAAGAGTGACAGTGTTTCTTAGGGGTAGGGTCCCGTGGCTGTATTCATTGACCTCCTCTTGGCTGCTT<br>CTTGCTTTGCTGGGCTGTGAAGAACTCAGCAATGAGCCGAGGGCTGGGAATGCTGCATTACTTCAGGCT<br>GTAGCTACTGACAGGGTGGCCGTGTAGTGGGTGAGGTGGATTGGATGGGGAGTTGGTGTCCAAGCTGG<br>TAAGCCCAGATTCATCTTCTTGCTTGAGGATAGAGTCTGTTCCCATTTTGAGACAGGCAAGTAGTTCTTT<br>ACCATTCAGGGTTTTGGCTAATTAGAGAATAATAACAAATGCCTCATGCTGATGGTCTGCTTCTTAGGGG<br>TGGAAATCCGTTTCTTTCTTTGCTTGCAGTGAGTCCTCTCTAGAAGAAGAGGCTATTTGATTTGTAGTTC<br>TAGGGCAAGTTAAAGAGAAGCTAGAAGACCAGGCAGGTGATGCGGCCTGGCCCAGCAGTGGACTCTC<br>CTGACCACCTGTCCTCATTTCTTCTGAGAGTTTATTACTTCTGATGATTATTTTATTACACTTCAGGTCTAG<br>AAGATATGATGCTTAGGATGACCTTTGGGATTTCTCAGTGTTAGAGCCTTTAATGCTTTATGAACATTAAA<br>AACCATACCTTTCCTTTCTGTAGGAAAATTGGCAAAATACAGTGACCAGAGTGGAGATACAGCTTAGTG<br>GGAGAAACCAGGCCTTGCAAGCCTAGCATGCACAGAGCCCTAGGGTCAGTTCCTAGTAGATGCTTCCC<br>TTCCCATGTCTGCCAGTAACCTCACTTCCAATTACTACCTGGGGGCTTCATTTACCTGCTGCTGGGCAGC<br>TCTGACAACAGTGACAGTGTCCTAACTGTTCTGTGAGCCCAGCTTGATTTGGAGTCAGCAGAGGCATAG<br>AAGAAGCTGGACTTTGTGTTCCTTGAACTCTCCCTTACCCAGAATGCTTCTGAGTTGATCTTTTCTCCAG<br>AGGATTTTATTTGCAAACTTTATTTGAAACCTGGGGTGGAACTTTGTAAGCATTTGCAGACATGAGAAG<br>CACTTACCTTAATCATGAGCAGTCTTCAGACCTCTGACTTTGCTGTTTTCCAGGACATTTTAAAACAGAC<br>TTAAGATGATGTAACTGAAGAAAAAAAAAGCAAGTTTGACAAATCACTGATTTCTGTCAGCATTTAAAG<br>ATTTAAAAATGAAAAGGAACAGTTGGTACTGAAGTCTATAAAAAGGCCCATAGTATTATGGTCTGTCTTT<br>ATAATCCCAGTACTCGGGAGGCTGAGAAGTATAGAAATCTTAGGAAAGTGTGGAAAATCTTGTTTTCCT<br>CCACTGGTGTGAGGATAGGAAGCTTGAGGCTTGCCTGGACTACCTAGACTTTAAACTCAGAAAACCAG<br>AGCAAGATGGCTCTGAGGGTCAAGATGCTTGGTGCCAAGTTTGATGGCCTGAGTTCAGTCCCCGGGAC<br>CCACTTTATTTTGGAAGGAGAGAACTTCTTCAAGTTGTCCTTTGACTTCCCCCCCTCAATTTTACTTGAG<br>GACTTGTCATCTGACATGCTGGTTCCCAATTTACCTTCAGGTGAAGTGCAGACGGCCACACTGTGTACA<br>CTGTTAATGCAGACAGCACACTGTGTACAGTAGCTAGCTCAGTCCCGTCTCTTCATGTCAGTACGTGAG<br>GTGATGAGCCAGGAACACTCCATCTTTTCTCTAGAGCAATATGCATTGTGGATAAGGCTCTACAGTGTGA<br>TTTTAGGTCAAGAAGATAGGTAAATGGATCTTAGACTATATTAAAATGTTTTTTCTTTTTAAATAAAAATT<br>TGCTATTTGCTGTGTTGCTTGTAAAACCTCACTTATTTCTCTTGGTTTGTTTATAGTTGTTTATTTTGTTTAT<br>GAAGTGACAATTTAGATAGTGGGAGTAGGTAAGTCTAAAGAAAAAGTACTGATGTAGCTTCAGCATCAA<br>ATAGCTGTAATATAACATCAGCTTCCTTTTATTTTTCACTCTGTGATGTTTTTACCTTGGCATTTGTGTGAT<br>ATATATATCCCTCTGTATAGTTTTGTTTGTTTTTGAGGCAGGGTCTCACTGAGTATGTAGCCTAGGTTGAC<br>TTTAAGCTCTTGATCCCTTGAACTCAGCACGCTGAGTGCTGGGATTACAGGCGTGACTTTGCAGACCTC<br>CATTTTAAACAACTGTTGGCTTACGTGTCTAGGTTATATAAGTTGATCAGTCTTTTATAGTACTAGAACAC<br>TGCTGTCATTAAACTTTGATATACATTCATGACTAATTCCTAAGGATACAAATTCTCACCGAGAAATTTTG<br>GGTTTTCCTAAAGTTCATACAGCAGGTTTAAAGTTCAGTAGAAAGGTTGTTTGATATGATCGTATAGAAA<br>TAATGCCCAGTGTAAACTACTAGAAAACATAGGAACACATGAAGAATAAATTTAAAGCTACCCTTTGTC<br>GGAGGCTAGAAAGATGGCTCAGTGACTAGAAGCACTTGCTGTTCTTCCAGAAAACCCGAGTTTAGTTT<br>CTAGCACCCAAGGTTGGTGGCTCACAACTCCAGCGCTAGGGGAAGCCTACACACACACAGACACACA<br>CAGACACACACACACACACACACACACACACACAC

MSTRG.16493.1	--	1.332	0.047	0.005	-3.172	down	GGGGAGCACACACTTATTTATGGAGAGCAAGTTCATTACTCCTGCAGAATGATGTGGATAAAATATGATTGGGCTCCTTAATTGACAGAAGCCTATGCCAGATGGCTGAAATGCCACTTTCGTTTTGGGAAGAAAGGAAGCCTTCATGGGAAGGAGGGACTCTTGCGACTACAAGATGAGGACCACCGGGGTTCAGGGAGCTACCATACAGATCAGCCAAGGTCACGCAATCGGTTGCTTTGCCTGGATGTCAACTCTGGCTATTTAACTAAACAGCTCTGGATCCCAGCCACTGTCCTCTGCCACTTGCTGTCTGGTGGAATATTAAGAAGTCTCTAGCATCTTCCTGGCTGCCTTACCCTATCCAAAGATGTCTATACTAAGAAGCCAAAGTAAAGAGATGTAATGTAGACCACAAGCAGTCGCTCAGAAATCCTGACTTGTATCGACTGAGACACCTTCCCAGGAAGAAAGCAGGGATTAAAGATGCGGCTTATCAGTTCCTCTCTGGCTGCTGCACCAGGCAAAGATGAGAG
MSTRG.16494.1	--	0.672	0.040	0.012	-2.843	down	GGGTTAATATGGGTAAAGGGGGGGGGGAACCAGTGGCTATTAGTCAAAGGAAGCTTACAGCTAAAGGAGGTTGCCTTGGATCTCTTTGTCATAAATTCTCATCATGCTCTTTCAGGCTTCTTTTGTTACACTGGTATATCTCTTGGAAGAAACTAGAAGCAAAAGCAGTGCAGGTGTGGTCTTGTTGAACTTTGTATCTTAGTCCTCCAGTGCAGTATCTGTTCAGGAAGAACTGAATGCAAAAATGAATTAAGTCTGTAGAAAGAAATGTAAGAAAATTTAGTAAGAGACAATAAGGATCCCACCTAGAGGGGGGGTAGACATCTGGGGAGCCAGGACAGTGAGAATTCTGTCTGGGACAGACTATATGTAAAAGACCATGGTTGAAATGAAGGAGGAGATGGGGTGTAAGACCCACAGGATGTCACTGACTGACTGTGTGGCTACACTGGGCACAGAAGGGAAATTTACACTATAAAAATGTTCGTTTATACCAGCCAGTGTTACTCTGCGCGTTGCTTACTGCTTTGTTTTATCTTTTCCTTTTAACTAGTTAACTAATATCTAACTATCAAGAGTTGTTTTCATAAAGCTTCAGATGTTGGCATCTCTAAGTATTGGAGTTACAGTTGGCTGACAGTGTTGGGGTCCAGACACAGCTACCCCTTTCAGCTGGACATTTTGTGTTGAGTCTTGAGTGTCTTAACTCCAGCTGCTGATTGTCCCCATGTTTGCACATTTTCTTCTTTAATGCTCAATAAGTACCCAACTCTGTCAGGTCCCAACCAATGAATCACAGAGCACTGAATGAAGGACTGAGGCTTTCAAGTCAGTGGTCCTTGAATTAGGCTCTAGTGGGCTAAACTCCAAGAGATCTTTGCCATATTCACGGCAATGGGAGTTCTCATCCAGGTCTACCAGAAAGCCCAAGTGCTCAGATGACCCGTCCCATCTGAGACTGTTCTTCATCTAAAATAGATTAATTAGGATAAAGGGGATACACAACTTCAGAGATGAATAATCCTCTTGGTACATGGTATCTCTTGTCTTTGTTAGCATAGTTAGTAATAGTATT
MSTRG.1664.1	ENSRNOG00000015465;ENSRNOG00000039776	0.165	0.532	0.004	2.429	up	TGTGGGTATTCACAGAGAACAGATGGGCCTTGAGCCTAGCCTATTCCTGACAGCACAGTATATCCCAAATTGCGCTGGTGCGGAGGGCACTGGGGTCATTCACTGTCTCGGGTGCTCACTGGGAAGACGACTAGCCCCTGGTGTTCTACCAGAGGAAGGCTCGGAGAACAAATGGCCTTTTGCCTGCAGGGTGGTCTGTGAAAGCCACAAAACTATGTTTGGCTGTTTATAACCCAGTCCAATAGCATGGGACCCTGACAGCAATTATGAGAAAGTTGCTTCAATTTAAATACCCCCTCTGTGCGTGCGTGCGTGCCGGCGTGCGTGCCTGCGTGCGTGCCTGCGTGCGTGCCGGCGTGCATACTAGGGCGTGCACATAGAGGTCTGAAGCAAACTTTCAGGAATCATTTCTTTCTTCCACCATGTAGGAACCTCCTCACTCCACACAAACTCAGGTGATCAGTCCTTTTAAAACTCAGGCTGGTGAGCAAGTAAAAGTGCTTGGTGCCAAGCTTAATGGCTTAAGTTCATTTCCCAGGACCCACATAGTGGAAGAAAGAATTGTTTCCTACAATAAATACTCCTTAGCATGCATACATGCTTACATACATAATGTCCAGCTTGGGATGGTGTTTCCATCTATGTGTGACTTTCATCGAATTTTTTGTTGTCTGCCATTATGTACTGTAACCCTCTCAGACACTAGATACCTTGTAAGTGACTCATCTGCTGCACCCATTTCTTAACACCTCTCCATAGAGAGATGCAGATTCTGGCTCATATCCTGTGAAGAGCTTGATACACTGAGGAATTAGGAGCTTGTCCCTGTTCCAGGCTGCCATGAGTGCTTTTCGGATGCTGGGACATATACAGGTAGGAACCAGGAGTCTCCAAGCCCAGTTGTGGGAGGCACTTCATTGATGTGTGGGTTTCTAGGAGCCCTGGAATGCCCCTCCTTGGAGAATATGCCGTCCATCTTCATATGCCCCTCCCTGAAGTCCTGGAACCTTTCTCCCAGGCCAGTATTTCTCTCAGGATTTCTAATTTTAGAGGTTGGAGTCATCTCCATGAACCGACCTCGATGGGGGAAACTACTGGTCTCCTAGATGAGATGAAAAGATGGGGCCGAGAGAGATAGCACTACCTGGGCAAAGCTCGCTTACACACCTCTCCAGCA

							GAGTCTAGGTGTGAGAACAAGAACACGAGCAGTCTGAGTGTGCACATGCAAAATCATCATGTTTACATT<br>CACACGAGTGCCGCACGCACAGGTGTGTGTGCACCCACATGCTCCAGCATAATCATTAATGAGTCTGTG<br>CTATTTGCACACACAGGCACACTTCCTAAGAGAGCTCTGGACTCCTAGATTCACATTTCATTGGCTCCTC<br>CACATCTACTGCTAGCTTTTTCTACCTTTCCTTGAGAAAAAGGCTACACACATTGCGTCCACCCCGCAGT<br>TAGGGCTCCCCTGGCTCCAGGTCCCTTACTTTCAAAGGAGCAATTGTGCTCACTTCACCGTATCTCCTCA<br>GGCAACTGTCTTTTGGACAGCTTCAACTAAGCTGGAGGCTTAGGAGGCCCAGAAGTGACTTAGAAGAA<br>AGGCTTAGCTAATGCTAAGTTCTGTGTGCTCAAGGCTAAGGGACACAAAGCAACCTCAGAACAGTAGA<br>AGAGTCACTAGGGGCCCATATCACCTGAATGAACCTGGTTCGAATCTAAGAACGAAGCCCTCAGCAAC<br>TCCATTTCTTCGAAAAGTCCATAAAAACCGGCCTTTTAAGTTTCCCATTTTGCTTGGGAAACTCCGGGCA<br>GGGAACTCAAATCTAACTGGACTTTGGCACATCGACTCAGCAGGTGGTAAATCCAGCGGGTTTCCTGAC<br>TTGCCCTGGAGGGGAGAGAGGAACGGACTGCTCCACCTCACTGGAAACTTCCCTCTTCAGGGAGAGCT<br>TGACAATCTGACATGAAAAATGATGCTGGTTTCATCTTTATCTAGGGATAGCTCCTGGAGCTGAACTGAT<br>GACACCCCACAAGGCCTTCAAGATATAGAATGTTTAGGGACGAGAGCCTAGCAGGGGCTCCTACCTATC<br>TCCTTCCCAGGGGCTGAGTGTTCTTCACGAGGCCCCAGACAAAGTCCGCACCATTTACCCAACAATGA<br>GCCTCCATCCGTTCCTGAACTTGTCCTCTCTAACCAATGATGCAGCCCCAGTGATCTTGGTTCAGATATT<br>GCCAGAATTTACCCCCATCTGTCCCCACTTCTAGGGTCTCTAGGGCCTAAGCCAGTGTCCAATCCCTTTA<br>CTCTAACTAAGGCTCTCCTTACCGGGTCCAAGATGAGTAACACTGTCAGCATTCCAAGCAACCCTCCAG<br>CCCCTCAGTCTGAACTCTGTCCCCTGTAGCCAGCACTCTGAGAGCTCTCCTTCAGATAAAAGATCCTTC<br>TTAGAGAAATGGCACTCAGTGACAATAAGAGCAGCCAGCTTGAAGCAAAGGCCAGTGTTCACAAGCCT<br>GACAAGACTTCACCTGTCCAGATGCCAGGCTGACACTCAGATGTCACACCCACAGCGCTCTAAGCTTAT<br>GGGAGCACCTTTTGCCTGCCACACCTGAAAATGTGTTGTCACCACAGATGCCATGCACAGGAAACTGG<br>GTGGCAGTTTGGGGAAGAATTTGACTAAGGACTTAGGAATCTCTTCCTTGATCAAAGCAGTGCTTGGAG<br>CTTAAGGTAGGAAGCCACTGGGTTTACAGAATGACATTTAATCGTCCATTCGTGTACATATCTGTGTATCC<br>AACCAGCTATTCACACCCAACCTCACATGCATTCACTCCCTACAGAAGCCTTGGTAAGTACAGACCGGC<br>CAGCCACCAGAGACTCAGTAAGAACAGAACTGCACACTTGCTTGAGTCAAAAGAGCTTGGAGTTAGG<br>GAAGGGCTGGGGTAGCCTCGCAATTTCTAAGCTGGGAAAAGGTGAGGTAGGAGGACGCTGGGTGTGA<br>AAGAAGAAGGCTGGGGATAAGGAAGGAGGAGCCCAAGCTCCAGCTGGAATTGGAAAAGGATTTAGCT<br>GGAACTCAGCTGTCACCCAGCTCAGAGCCATGACACACTCACTGACAGCCCTCCCCTTAGTCCTTCTTG<br>TCCCTCACTAATGACACTCCACTGTACTGGCCACTGACCCCAAGTCCATGACTCCTCAAGGCAGGGCCA<br>AGGTAGGAAGAGCTAGAGAACTAAACTCACCAGCATTTGCCCATCCATACTCCTGACTACAGGCCCAA<br>GGAATGTAAAGCCTTGAGAAGAGCAGAAAGAGAAAGGAGCTGAGAAGCGGGGAGGTTAGGGGGCAT<br>AAGGGACAGCATGGTAGGGGGTAGCTCAGGCTGGCAGTTGGGCCAGCTTAGCCCTCCACAGTAGGGA<br>GGTACCTGGGAGAGTGGACAGGGACTCCCGGCTGCCCACATTGACCAAAGAAGCCACACCCACCTCA<br>GCTTTGGAAGGGCATGTGGGATGTTGTTCGGCCTCAGCACTGACATGCAGTTCCCAAAAACATGGTGCC<br>CTGAGCTCAGCTATGGCACAGTGGCCCCAGGGACAGCAATGCCCTACAACAGAGAAGGTAAAACGCTT<br>TTCTCTAAGGGAAGCTCCAAACCGTGGGGCTGAGTGGAGAAAGAACAGCTGAGCTGACCCTAACCTTG<br>CCAGGGTCATTCAAACACACAGAGGACCCAAGGGGGAGACTCAGGACACACAGGTCCATGGGAACCC<br>TAACTTTAACTCCCCCCCAAACTCAGACATGTGTTCCCAAAGAAACCAAAAGGGAGTCCCAGCACCTG<br>GCGGCTTCTAAGGAGAGGTCAGAGTCCCACAGCCATGGCGTGGCATGAAGTCACCTGAATGGTTAGGA<br>TATCAGGACTTGGAGGTGCACTGTAACAAGCCTGGCTTGTTATGGTGAGCAGTTTTTAGAGGGCTCGTG<br>CTTCACAGTGAAGCTGTCCAGGACAACAGTCCTAGAAGGCCTAACAGCTCTCTCTGGGAAGCAGCAGG

							AGGAAAGGGGTGGGCGAGTCACCTGTGCAAACCGGCAGGGAGGCAGGGAGGCAGGGAGGCAGGGAGGCAGGGAGGCAGGGAGGCAGGGAGGCAGGGCTCATCCCTGCACCGCCACAGCACTACCTAGCCTTTGCTCTCTTCCCTCAGGATGCTTCAAACGCTGCCTAGATTTACAGTTAGAGCAACCGTCCAGGGGCAGCTCCCAAAAATAAGACTTTACAGCTCCAGGGCCTCCTAGCAGATCCTTCAGACTCCCCTTCACTGCGCACTTCTGGAGGGCCTGGAGGATGTCATTTTTGTCCCCTATAATGATATTTTCCTCCCCTACCCTCCCAGTTTCCTTCATCTACCCCTGGTTCCCTCTCTGACTTTAGACGGAAGGTTTGGGTCCTGTGCCTCTGAGGCTCAGTAACTGTGAAATGGGATCCGTGCTAGAGGCTCCCAGCACAATACAAGGACGCAAAAGCTGTTGCTGTTAACCTTGTTGTGCATGGACTCTTCCTCCTGGTAAGTCTGGGCAACCTCCTCACCTCCCCGAAGCCCAGAGGCTCTGATCGTTCTCCCCACAGCCTCTCTGGTCTTACACTCTGTCTTGCAACCAAAGTAGTGCTCTGAGAGGTCAGTCGTGACTGAGGCCCACCGTCTACGCAACTTCAGCAAGGACCAAAGTCCTCTACAGCCTGCAGGTAAATTGGTTTAGTCTTCCCCTAGCATCGCCTTGGCTCTCCCCGTCTCTTTTCATTCTCTCTTTGGACTTTCCATAAGCTCCTCTTGCC
MSTRG.16669.1	--	0.803	0.000	0.004	-4.319	down	TGCTTGGCATTGTTGTACTTTTGGGGTCTCGAGCCCCTTCAAGCTCTTCCAGTTCTTTCTCTGATTCCTTCAATAGGGGACCTATTCTCAGTTCAGTGGTTTGCTGCTGGCATTCGCCTCTGTATTTGCTGTATTCTGGCTGACACAAATATTTTTAAACTGCTGAAGTACTCTTTCCAATACAAGGGACATGGTCAGGGTTAAGAAAATTCATTTCACTTTTTCACTCAGTCTTTAACTTTGCGCTTGTCTTGAGAAATGGAAGTGAGTTGCATTATAATCGCTAAAAACAAGTGTTATTAATCTTCTCACGATGCCTTCCGTGAAAAGCAAAGAAAGCATTTCTTCAATAATGGAAGGCATATCAATGATTCATGGAGCTAGGACACTTGGGAAGTCCAGCTAGTCTTCCAGGACAACATCTCTCCTGATTGTCAGATGTTCGCCTT
MSTRG.16702.1	--	0.486	0.038	0.043	-2.421	down	CTCCTGTATTTCTTTAAGGGAGTTATTTATGACCTTCTTAAAGCCCTCTATCATCATGAGATGTGATTTTAAATCCAAATTTTGCTTTTCTGGTGTCTTTGGTTATCTAGTATTTGCTGTGGTGGGAGAACTGGGTTCTGATGTTGCCAAGCAATCTTGGTTTCTGTTGCTTAGGTTCTTGCACTCGTTTCTTGCCATGTGATTATCTCTGATGTTACTTTATCTTGCTGTCTCCGACTGAAGCCTATCCTTCTTCTGGGCCAATGATCCTGCAAGCCTGTGAGCCTTTGATCTTAGTAGTGAGAGCTCTCCTGGCATAACAGTTCTCTGTGGGCAGGTTTTGAGTTCGGAGGACTGTGGGCCTGCTTTAGCTCTTGTCACAGATAAAGGCAGGAAAGATCCTGTCCTAAGCTGCCCTGTGACTGCTATGACCAGTGGGCTCTTGGTGGGTCTCTCTTTGGATAGTCAGTGGAGAAAAAGTGGGGTCCCACATGTGGGCTTTGGCCTTTGGAGGGTGAGCACTCCTGGCTGACCTATAGGCAGGTTTGGGGTCAGAGACCATCCTGGACTTTTCATATCCTGAAATTTTATTAGTTTCCTTCCAGGTCTTAGTCCAATGATTATGTATTAAGTACTCATTAAATGTGTGGTTTGTTGATTCTTTGACATGCATCCTATTCTGTAGCGTGCTCATATAAGTGGGTTACCTGCAGTCAGGAAGCTACGCTGCTTGTTAGATGGGTAGAAGCCATGTTTGCCATGTATCGTGTCTGAGGCTTGGGTCGTCAGAGTCAGAATGATCACAAGTGAACCTTGATATGTGGTGGGCCTCGGAAAGAGACTGAATGTCTACCTTTCAGTGTCCTTTCTGGGAATGGTGATTAATGACTGAAGGAAGGTCACAGTGTTCCATGGCTCAGATTATTACATACTTGTGAACCCACTGGACGTAATGCCTGGCACATGCTGCCTTAAACATTGCCATTATTATGTTTATTTAATCACTTTTGTAGAAATTTCTTCCTAGTAACCACATTTGAAATACCTTTTCTCTTTTCCCATTCTGTACAATATAATGTTTTTCTTCCCAGTGACCTTTCCTTGCTACTCTAGATCTGATTATTTTTCTCATCTTATTTGTGTGTGTCTATGTCCTTTTCCCTTGTGGTGGCTAGAGTACTTGTTTCTCAGAACACGTTCCCTCACTTGGCTCCTGGCTTGAGAAGTAATTATTTTGGCATTAGCTTCCATTGCCCTGAATTTGCCATCTGTTGCCAACATTACTGAACTGATCCCATTTTCTGAAGTCTGGC
MSTRG.174	ENSRNOG0000000	1.032	0.000	0.000	-6.823	down	GTATTCTTCTACACAAAAAAGTTACGTCCATTGTATAGTGAGATAAGTTATAGTAAATAGAAGGTTTTGGTTTCGTCGTTTACTTATACAAATGTTTTGAAGGTCATGAGCTTCCTAAACCTAAGTTTGGAATTATACAAT

37.6 NONCODE_ID：NONMMUT009751	7462;ENSRNOG00000007515;ENSRNOG00000007624;ENSRNOG00000007652;ENSRNOG00000033065						CCACTCTGAGCTATTTATTGCAAAAGTTTTGAGACTTCCCAAGTTAGGATTAATCAGATTGCTACGATTTTAACAAGTTTGGATAAACACTGATTTATAATTACATTGCCCTGTGTGTCTGTGGGTGGCTCAACAGAGAGGTGCAGCCCTGGTGAGGTGGAGGAAGGCATGGCGGTTCATTTTCAGCTTTCTTGAATTGGAGCACTGAGTATCCTGTATGTACCGGGTGCTGGTGTGAGCATGGCCACTGTGGCAGCATTTTCAAGACATTTTCAAGGTGTGGCCTGCTGTGACTCGGTGAGCCTTGTGTATCGCTTGCAAAGGCTCAAGTGAGCCGAGTGTCTTGGTGGCGTCAGATTTCGCAGCGCAGTGATGTAAGTTTAACTGTTAGTAATTAGAAGACTCTGTGCGCCTTCGTCTTAAAGTCCACGTTTCCATTATTTAGCAGCTCAGCTGCCCCAGAAGGAATAGCTGCTGTAAATTAGGCTAAAGCACGAGAGAAATTAAATCTAGTTAAAGTCCATCACAGTAACAAAAATAACTCCATAAACTAAAAATATTAGAAACTTGACTAGGCATCTTGACTTGGTAATTTTGTTTGCTTGCTTGTTTGTTTTGTTGAAACAGGGTCTCTCTGTGTTAGCCCAGGCTGGCCTGGACTCCTGATCCTTCTGCCTCAGCCTCAGTTGCTGGAATTGTGGATGTATGGCATCATGTCAAGTCACGCACTTATTAAAGCACTGATGTTAAAACTCCAATCAGGCGGACAGTGTTCACTTCTGAAAACCAAACGAAAGTCTGGCCAGGTGGGGAGGTCTGCTGCAGATGGCTAGGACGCCCACTCTGTCACAGCCGTGCTGTGGCTGCTTCTCCTGCACTGTGTCCTCATCTACAGTGGAATAATTTGTATTTTCAGTGTTAATTTCATGAATGGATAGACTTGCTTGCTTAATGTTTAAAATAAATGTACAGATTTTAATGAGTGGAACAGATCTATAAACAGGCTGTTGATGCACCCTTTTTCCCTTGGTTTAAGAGTATGTTTATTTATTTGAAAGGAGCTGTAATAGGATGTTACAGCAGGCTCTGCACTTACATACCCTAACAGTCAGGCCCTCCCCACTCAATGTCCTCCCCACTTCAGGCTTGGCTGAGAGGAAGGCTCTGCAGCCAGTGTGTGTCCCAGAATCCTCTGGTAAGCCGTCTCATTTGTATTCTGGAAAAGGTGATTTTCAAATTTTTGTTTCTGGCAGCCAGAACACCAAGGTTAGTTTGGAAGGAGAGGGGGAATCGTTACTTCATCCGCAGCCTGATTTAGAGTTATGATTATAGTTTCCGCGATTTCATTTCCATTTTAGGTTGCTGTGGCCCTTCCCCCTTTCTCTCTTTGAACTATTAACAAACAAGGAATACCTTTTTACTTTTAGAGCTGTGAATTAAGGAATGGGCAGAGTTTACATTTACACACACCTGTGTCTGCCCAGTTTCTTCCCCTTCCCCCTCTTCCCTTTTGAGATAGAGTCCAATATAGTTCAGGCTGGCCACAAACCCTCTGTGTAGCCCAGGTTAGCCTTTAAACTCCTAATCCTCCTACCTCCACCTGACGAACCACTATGCCCAGCTTCTCCTCCACCCCTTAACATATATAATTTTTTCTCTCTTCCTTTTCCTTAAAATACTAGAACCCATAGTTCTTCCTAGTGCCTTTGAGCTGCCCCTGCAGTCTGACTAATTAGAATCAGTGAAACCCTGAGTAGCGTTACTGTAGTGCTGTTACCGACCAGGATACAAACTGTACAGAAAGTTTGCTGTGTTAATACAAAATCCACATTTGAGTTGTGTTTTATGTATAAATATGTACTTGTCCAAGCTGTATATATCAGTGTGTGTGTACATGCAGCACGGTAAGATGAGAAATAAATGCCTTCCCAAC
MSTRG.17494.1	ENSRNOG00000050794	0.000	0.344	0.001	4.308	up	GCTGGTCCCTCTGCGTGGAGATTGGCTGACTCTTCAGCCATGCCGGGTCTGACCACTTCTGTGTGCAAGCTCTGTGGGGACTCCATGCCTGCAGATGCTTTTCACAGAAGGTATTTGGCGGAAGTATTTCACCCTTCGTCAGCCGTGGTTCCCTCATTCCTCAGTCTGGTCCCTCTGTAGCCCCTAGAGAGACTAGTCTAGAGGGAGTGGGAGGGGCCTGAGAATCACCTTGGGTCAATAGTGTCTTCAAGTTTAACTTGACCGTTTGTTGAGGCCTTGATGGGAACCCAGCTCAGCGTTGGGGATATGGACTGAGAGCTTCTCTGTTAGTACACTGCCCTATTATTGTAGCAGGTGGCGGTGGCACTCAGCATCCGTGTACTGAACAGAGATGATGTAAGGACAGATATACTTTACTGGACACGTGCATTCTTGTCAAGTCCATTCAGCTCCCATTTCTTCTGGTGACAGCCATGGGGGTGCTGCAGATCTAAGGCCAGAGAGTCCCTTTACTGACAGTTTTATGTGCTTTCTTAAATGGACACTGAGAGATGAGACTCAGGAGGACTCTTGGGAGAGG
MSTRG.1753.1	ENSRNOG00000012742	0.074	0.507	0.000	3.442	up	TGGGTTATCCAGATGATGGGAAACTGAGGAATGAGCTGCCAAAGTTTATAGATAAAAATAAAGGCTGTCCTGTCAAACTGTAAGTTTCTGAGCTCACAGAGCATTTCAAAGTCTGTGATAAACACATAGTTACGGGGTTGGGGATTTGGCTCAGTGGTAGAGAGCTTACCTAGGAAGCGCAAGGTCCTGGGTTCGGTCCCCAGCCCCAGAAAAAAAAAAAAAAGAAAAAAAAAACACATAGTTACACATGGATGATCCAAAAGTATCCATTGTACA

							TCTAAGAGTAAATGAAATTACATCCCTATTGCAACTTTGTTGTTTATTGGTCTTTATAGATTCCTGTTTGA<br>ATATTTGTTCTTCCTGTTATTATCCAAGGCTTACTTTGATTACCTGAACTTGTGAAGTGCTGTGAACTTAG<br>GAAGCAGTCTATTTTGTGCTTGAGAAATTGAAAATGAACTCTACTTTTAATCAGAGATTTAATCTTTTAAT<br>CAGAGATTTAATCTTTTAATCAGAGATTTAAATTGGTAACAATGTGTTCTGTGTTTAGTGTTCCTCATTGT<br>AAGTCATGTCCCCTTTATGGCAAGACAGTAATAAAGTGGAAACTGAGGCAGCAGAAAGATGCACCAAA<br>GATTAGCATCTGTGGTGCTAAATTTCCAGTAAGTCTCAAAGCAATGAAAACCTATTTAAAAACCAACCAT<br>ACAATATGCACACTCACTAAATCATCTGATTTTTCACTCCTATCAACAGAATCCTGTGTGTGAAGCCAGA<br>AAAGAGCAGTGTAGGACTGATAGCTGGGAAACGCTGTATAGCACAGAATGATAAGCCCCTGGTATGGT<br>GGGAAGAACCTGTGCTCAGCAGTTGATATTCAAACCCAGAACTCAGAAGTCTTTACTGATGTAGAAGG<br>AATTTAAAAATCTAGCTGTGTTGTGTGTGTCTTTAAGGAAGTAGGAATGGGAACTGCTGGCCTTCAAGA<br>AGTCCCTCCATGTTTGTCAGAGTGTTCGATGTTAACCTGACACAAACCTAGAGTCACCTGAGAAGAGA<br>GAACTGTAGCTGAAGAATTGCCTCTGTCAGATTGGGCTGTGGCCATGTCTATAGCGTATTTTCCTGGTTG<br>CTAATCGATGTAGATGGGCCCAGTCCACTGTGATGGGTGTCATCCTTAGGCAGGTGTTCTGGGCACTAG<br>ATAACAATAGATAAGCTGGAGAGATAGATAGATAGATAGATAGATAGATAGATAGATAGATAGAGAGAAT<br>ATGAGAGAAGTCCAATAAGCAGCTTTTTCCTGGTGGTTTCTGCTTCAAAGTGCTGTCTTGAGCTCTTGT<br>CCTGACTTCTCTCAATGATGAACTGTAACCTATAAGATGAAATAAATCCTTTCCTCCCCAGGTAACTTTT<br>GATCACAGTGTTTTTCACAGCAGCAGAAAGTATTATAGGGCAATGCCCTTCCTCACCCAAACCCTCACC<br>CAGACCCTGACCCTAACCCTAGCCCTGACTCCACCCATTTTCTTGAGGATCATTATTTTCGTCTTTCTGCT<br>CCTTGGCCCCTGTGTTAATCAGTTCTCTGGACTACAAGAAGGCACCTGAAATCCTCCATTGATAAGAAG<br>AAAGGGCAGTTTAGACCCTGTCGTTTCAGAGCTTTCTGTAAATGATTAAGTGACCAGTTACTCTGGCCA<br>TGCTTTGTGGCAGAGAACACATGATGAAGAAACTTCTCCTGTGGCCGCTCACACACAGGAAGTGTTCT<br>CAAATGCATGCCCCACTGAACCAGGGATCTACTACCTTCCAACCACACCAGGTTGACAGCAAGGTTTTA<br>ACATGTGAACCTTGGGGGAGACTCTTCAGATCCAAACCACAACTTCCTCCTCCTATCCAAACTGTCTTG<br>TCACACATCAGACCAGGCCCACTCATTTCTAGGCTTATAGGAGTCCATGTCCTCAGTCCTCCTACCCCAG<br>AGCCTGCACAACAACCTATCATTTAATATTCCCCAAATTCTTTCAAGGCTGCTGTGTTCCCAGCACACAG<br>AGATGCATAGAACAAGGCCTCGAAATGACAGTTCAACTTCTCTCTGGTGGCTGCAGTCAATGTTCTCCA<br>GACTGAGATCTGAACCCGTATCCTAAACACATGCTCTTCCCTTAACTCTTTTGCCCCTCTGCTGGTAGTG<br>GATTTTACACGCTGAGCTATTGTGCCAGCCCTATTATAAGGGAAAATGTATCTGAAAGATCTTGAATAGG<br>ATTCTATTTTTTTGGACCACTCTTGCACACAAAACATGAATTAGGTCAAAACACTCGATTACTGCCAAGT<br>TAGAAGTGTAAGTACAAGGCAATTAAGTCAAAAGCAGACTGGGCAAGGTAGCACATGCCTTTAACCCA<br>GGCACTCAAAAGGCAGAGAGGCAAGTGGACTGCTATCATTCAAGTCCTTCCTGGTCGATATAGAAAGTT<br>CCAGGCTAGTTAGGGATTATATCATAAGACCCTCAACTCCTCATCCCCCTAAAAAGAGAAGAGAAAAAG<br>AAAAGAGAAGAGAAAATAAGAAAAGGGAGAGCAGACAGGATGGCTATGGTATGTAAGTCAGGATTTT<br>GCTTGCTTTCTTTGACCACACATGCAAGCACAATGACTTCATTAGATGTCCCACTAAGCAGGTGACAAA<br>GGAACACATCTTCTGGAACAGCTCCTCAGCTCCTGGCTTTATCACCAGAAACATTTCCCAAGGTTTCAG<br>GACAAAGCAGGCAGTGCTGAAGTCCAAGTGCCCAAGCCCACTCCCTAAAAGCCTGACGTCAACCAAT<br>GCGTAGTCCGGGTCTCACTTTTCCTGTATTCATGGGCATCACTGGTTGTTGGATACTCTTAGTAATATCTC<br>TGGACCAGGGTAGGCAGGTAGACAGCTCACTGTGCAAGTTATGGAACGAAAAAGACCAAATCAACTTG<br>GATTGAGAGCCTCTGAGAGCTCCCTTCCAGGATGGAGGCTGGTGACTGCTCCTTTACTTTGTTTTGCTTT<br>TGAGATAAGATCTCATGTAGCCCAGGCTTCCCCTTGAACCCGCTATATAGCTGAGCATGACCTTGAACAA<br>GGATGATCCTACAGCCTCCACCTCCAGAGTAGCGGGATTACAGGTGTGTGCCATCATTCTCTATTTGAAT

							GAGGGCTGGAGATTCATGGTTTCATGCATGCTAGGCAAGCACCCTATTGAGCTGTGTTGATGGTTGGTC<br>ACCTTGAATATTACGAGGAAGAGAAGCAGACAAGTAAACATGATAAATGGCCCCTGTATGCCCACTGGT<br>GTGGCCAGCAGACTGCCTGCAGGAACTGAGCTGGGAAAAGAGAGGCCATGGTCAGAAGTCTTGAGTA<br>CGATCCTTCCTTTCAAGGTCCTGCCCAGCAGTGCCCCAACCTCAGAGGATCTCTCTTGGAGTTCTACAG<br>GGCAATGTCAAAACACAGGAACATGGACTGGGCAGAAGAGACACCCACTTGGTACAGGCTTAGGCTC<br>AAGTCAAACATTCCAGGACTGACTCCAGGAGCCGGCAGTTGCTGCTAGCTCTTCAATGTTCTCTCAACA<br>CCCAGGGGAAGTCTTGGCTATTATACTTCCCGACCCTGGTAGGTTCTTGACACAGGGTTCCCAACTTCA<br>ATGAACAACAGCTAGACTAAGGGTGACTAGCCACAGAGAAGGGGCATCTTCCTGCCAGGTACTGGAGT<br>ACAGTGCATTGTGCCTGGTCTTCATAGTGTCTTGAGAGAAAGCAGGGCTGTGTAGTTAATGCTTCAATA<br>AATGAATCTGCTTGAAGGTACTAAAGTTAGGAGAACAGAGTTGATTAAGTGTAGCTCATCAGAGGGTCT<br>ATCTCCTGACAATGTCTGTTTACCCACAGCACTGCCTTACCTAACTCCTTCCTTCACAGCTAGTCACACG<br>GAAATAGCAAAAAAAATGTAAAATAACCCTCCCTTGCCAGAGGTCTCAAGTCTGGGACCTCTCTACTTC<br>CACTTCCCTGCCCTCAAGGAGTGGGATGGGTCTTGGTTCCTCCTGTTTATGTCTGGGTCCATTATCAGGA<br>AGGGCACTTTAGTTGAGGGATTGAGTATTATATAATCATTATAAAACAAACCGTTTTTCCACTGTGTGAG<br>AGCCAGGGAGAAGAAACCAAGACCTGCCACCCACTCCTATTTAAACAAACTAATGTCACTCCAGAGGC<br>CAGAAGGAGCCTGCTCTGCCACCCACCCGCACAGAGGCTATCGCTAAGCTGTCAGGCCTGACCTCCAT<br>GTTGGTGGTTGGGGTGGGGGGGAAGAGAAGGGCAGTTCACTACCTGAGGATCTCCAGGGTGTGGTCC<br>CCCCAGGCTACCCCAGGATGAGGAGGATTAACGGGAGAATCTGTGGCAGGTACCACTCCCTCCCCTAA<br>ACCATCGAGTCTACTCAAGAAAGATTACAAAGTACAGCTTTCTGCTGTCTAGATCTAACCTCCACCTCA<br>GTGTCTGGCACACTACACACCAAGACCTTCTGGCACGGAGTGAGGGGCTGCGTCTTACTGTAATTCCTG<br>TACATCTTGAGGACCCTCTCCCCACCCTTGGGGAGGGGGGCTGATACACAGTTTCTGAAGTTGCTGGAA<br>ACTGAACTAGTCACTATGGAGTCTCCAAAGAAAGGCGACTACTAAGAAAAGGTAAACACAGGAACTCA<br>GAACTCCTGGTTAAAATAGCTGGAAGGCACCTGAAGACCTGAACTGCTCTCTGGCGCCCCCATCTCGC<br>CCAATACCAGTTGCAGGGAAGCCTCCTACTGGGTTCCTAAGGAGGAACAAGAGAAGGGTGCTGAATAT<br>TTTTGGGTGACTAAATCTCGCACGGGGCCAGTGGATCCCCAGTGGATCTTTTCATAAGCTTTCTAGAAAT<br>TCCTCAGACCCATACCTGGCAGACAGGCAACGGAGCAAATCACTGGCCGCGCATTCCTGCCCCCTCTA<br>AGGTCCTGGAGTGTGGATCGAGGGTGCCTCCTGAGGACCAGACGCGTATGTGGGGCCCCACGGGTTCT<br>CTCCCAGTTGTGGCTCCCTGCCTTGGACTCCAGCACGCGGCTGCTCTGCCTAGGGCCCCAACCTTCGGA<br>GGATGGAGCAGCGCTGCGTCTCCATAGAATATGGGAAGATCTTGTTCTGTGCACAGAGGACAGGGACAT<br>GTGCTAGGAAGGAATCGAAGAAATGCCAAGAGAAAGCGGGTGTCCGCTGGAACCTGTTCCAGCTGGC<br>GAGTATGCGGCTGTGGGCTTGCAGAAGTTGAGCGCCTTTTCTTTTCACAGCGGCTCGGGATCGCTCTGA<br>ACCGGAGGTGCTTTCGTTTTGCGCGCAGCTTTTGCCCAGCGCTAGTCAGCACAGCACCCTCAAGGGCG<br>GCGAGATCCCGACTTGCTGGGCTAGTTGGCTCCTTCTGGGGAATGAGGAGATTGAGGACTGAGACCCA<br>GGAGGGAGAACACAGAGACCGGAAGGGATGGGGTCCTCCGTGGAGGGAGCCAAAGTAGTCCCGGAAT<br>ATGGCCTTTCCGGGAGCCCGATGGGTCCTGTGCCTGTGTCATCTTCTATGAAATCTTCCACGCCTAATGC<br>TGTAACTGTCTTCCCGGGAAGACATGAGTCCTGACATCTGCGCAGGTTGTGTTGCCCAGCCTGACCCCA<br>TTCCGCAGAGCGTGCAAGCTGACTCTGAGGGTCTGATGGGACTTTTTCCTCCGTGCCCGCCATGTCCAG<br>GTGCCCTCGACCCTGATTTCACCCCACCACTCTGAAAGCCCTGAAACTCTGTCAGGAGGTGACTCTATC<br>ATGCCAG
MSTR	--	0.645	0.000	0.028	-	do	TCTCAGGAAACTTCAATGGAGCTGCAGGCATTTGACAACAAACCTCACCTTTTTAACAACTTAATGTGT<br>TTAATTAAATGTGTTTGTTTTTAATTACAAATAGAGAGGCTGGAAAGTGGGCTCAGCAGTTCAGAGCGC

G.17656.1					3.770	wn	CAGGTTGGAGTCCCAGCGCCGATGGCTGCAGCTCTCGACCGTGTGTAACTTTGTTTCCAAGGGTGATGGCCGTCCTCTTTTGGCAAACATGCTCCAGGTGTACAAACGGTACATAGATCCACATCCTCGGGAAACACCTTCCAGCTCCATGTAGCTGAAAATGACTCCTGATTTCCCGCCTTCACCTCCCAAGTGCTGGGTTACAGGTATGCCATCATGCAGGTCTGCCTCCTTTCC
MSTRG.17730.1	ENSRNOG00000047392;ENSRNOG00000047803;ENSRNOG00000049845;ENSRNOG00000050694;ENSRNOG00000052623;ENSRNOG00000055773;ENSRNOG00000061794	0.691	0.000	0.000	-5.253	down	GTCCACAGTAATCTAGGAGATCTGACATCTCATGAATTTGCATTCTAATTCCCCCCTCAGCCCAAGTAATACTTGGTACATGGTGAAAGGACAATTCTGCTAGGAAGGAAGTAAACAAGATACCATGTTGTTGTCTGACATCTTCTACAAGGCTTATTTTTATTTATAACTGCAGTGCAAAAGATTGAATCAAACCACTACTAAGGAATTGGAGTTGCAAAAATTTAGAAGCTGAAGAACAATCTTCCTTAAAATCAGAACTATCCACTCCATATGAAATTACTGCTATATAGTCTACATGTGCAAGAGAATATAAAGTTAACAAGAAAGTAAATTATTTCAAATATTGTAAGTAGAAGAACCAGGAAGAAACATGCAGGAAGCCCAGTTGAACGATGACTCCAGTTCTGGTCGACCCAGAAACCCGTTTCTCTCATTTGCTGCTATTCTTTTCTTCAACTTCACTGCTTTCTCTCTAATTTAGCTTCAGGTGTGACACTCCTAACTATGGTATAAATTCAACCTGGAGAAAAATAAATTTGTCGCCTTGATCAGACTCTTGTCCTGGCATCCTTCTTAGTGTCTCTTGTCCCCAATTCTCTTCCAGGTACCCGCTGCACCCTCGATGACTGTACTCTTCTGGCCTCTGCTTCTCCTAGTTCATCTCCTCACAGGTTATCCATTTGCTCTAAGCTTCATCAGGAGACTGAGTGTTTAGCAGTCACTGCTGGTTGTCAGGCCAGGGTGTGGCAGTAGATTCTCCAAGGAGTGACAGCCACTTTCTCACTGAGTCTTATGTTTGTCTGCTGACTCAGTTTCTGATGGTGGACACAGTGAAAAAGACAGCGAGGGAGCCCCAGGCTGTGAGAAGCCCTGAACCCTATCTGCTGGGTGAGTGGAGGTCAGGAGGCAGGTGAGGGGCAGGAGGTGTGCTGGGAAAATCAGAGCTCTCTTTTAAATGGGGACCATGATCATCAGTGAGATCAGGAATGTGGTGTGAAGTAGGGAAGGGATTAGCTGCTGTCTTG
MSTRG.17888.2	ENSRNOG00000003348;ENSRNOG00000053961	0.481	0.021	0.018	-2.845	down	CTCCTATGTCACAGCCAGGGGGGCTGGCCCAGGACAAAGAGGGCCAGCTCAGGTAAGCTGAGGCACTGGCCCAGCACCCTTGGCCCACTGCGGTTTCCCTGAATTACCCCAGCCCTCACCTGCTGCCACCTCTCTGCGTCTATCTGGCCCCATGCCGACTGCTCTGAGTATCAGCAGGCAGAAGAGGCATCAGCCTTCTAAGAAGGGACAGCTGAGAAGGTGGACCACGAACTTCTGAGCTTGACTGTGAATGTAGTTTGTGACTTGAAGCAACTAATGACATCATGTAGCTTTTTCACCGGAGGAGAGGAGAATTTCAAGATGGAGTTCCTGTTTTGAGACACTTCAACAACACAGCCTCAATACAGACTATGAACAGGAGACCACAGGGCAGTGGGATTTCCCTTCAAGAATCTCATGCTTGGAGAAGGGGAAAAAATGAAACCTTGCCAGCCACAGGGAGAACTTGTCTTTGGAAAAGAGAAGCCACGTGCTGATAATGTTCACATGCTAATTGATTATGCTGTTGTTGCTGCTGCTGCTGCTGCAGACTCGATCACTGATATCGGAGCCAGGGCTACAGCCGGCCATCATTGACTACAGACAGCAACAGCTCCCCAGGAACCCTCCAGGCTTCCTGTGCTAGAATGGAAGTAACAAGGCTCTTATGGGAATGACCTGAACAACTACTAGGTGTTGGCCTTGCCGACGCGAGACAGCCATTGTGGAGCTCCTCCAGCTGCCCCGATACCCAGACTCAAGGGCGGAGCACTGCCAGGTTCTCAGCCTCTGAGGTGTGAGGCAACTGTTGCTATTAGTTTAAGGCTGTTCCTTAGGAATAAGGACAGGAACTTGCTGTGACTCAGAAGAATCAAAATGGCTGGCTGTGG

							CCGTGATGCACTCTCTCCTGACACTGGGATTTGAAGGCTCTCTCCGTCCAAGACCTCTGTCATCTATACATGATCACTGTGTCTCCTCTGGAGATTAAGGAAGGTTCTCTCTCCCTTCAAGGCCTGGACGATACCTCACTGCTAGTATGATACTTTCTCCCCTCCTAATGCTCTCCCAAATTAAGGCCTTCATTGCTGCCCACTGTCATGAGCCTCTCCTAACAAAGCTCTCCTGAGGGTAACCCCACATCGCCTCCTTCCCACAAACCTATTTCTGAACTTATGACTGATGGACTCGCCA
MSTRG.17888.3	ENSRNOG00000003348;ENSRNOG00000053961	0.016	0.338	0.000	3.728	up	CAGAACTTCTGAGCTTGACTGTGAATGTAGTTTGTGACTTGAAGCAACTAATGACATCATGTAGCTTTTTCACCGGAGGAGAGGAGAATTTCAAGATGGAGTTCCTGTTTTGAGACACTTCAACAACACAGCCTCAATACAGACTATGAACAGGAGACCACAGGGCAGTGGGATTTCCCTTCAAGAATCTCATGCTTGGAGAAGGGGAAAAAATGAAACCTTGCCAGCCACAGGGAGAACTTGTCTTTGGAAAAGAGAAGCCACGTGCTGATAATGTTCACATGCTCTTTTGACAGTGGCGGCCGCCTCCTCCTTACATAACAGCAGCAGGAGTCAGCACTCCTCTCTCCACCCTGGCCAGGACAGCTCTTCCTGGGCCCTTTGTTCCACTGGCTTTATTTCCGTGGTTTTCTGGAGCTCGGCCACTTGACCCCTCGGCCTTACTCCAGTCTACGCTGCCGGTTCTACGCTTGAAGCTGTGCTGTCCTGTTGAGTCCTGTGACTTCTTGGATGTTGGACAGGCTGTGCGTGAACTCTCCTAGAGGGGGGTTACTATTTTTAAAGGACAGAAAGATTTCAAAAAAGACTGTTTATCTTCACTATACTATGTCTTCTATTTTGACTTTTAGAAACCCTGCGGTTTAATTATTTGCCAGTGATTCTTTTAGAAGGGCAGCAGACCCTCATCCCTTCTGACCCGGCATCAGTTAGAATTATCTAGAGTAACGAGGAAGCATCTTAGAGAAAGGAAGCCAGAACTTCCCCATAAGACCTAACTTCACACTGGCTACTTAACGTTCATTAAAACAAGAATCCTCACCTCCTCATTGTGTATGTGGGCCTACAGACTCCCCTCATTGCTGAGTTCCGGAGATCAAATTCCAGTTATCAAACTTGCAAATGTCTTTTTCCTTGATAATATATTTAGATTTTGACCTAGTAAATGACTCAGAAATGTAACTATACCTTGATTTCACATTTGAAAGTGTTTGTAATTTTGCAGATTTCAGTTCAAATTTCAATGTAAATAATTAACTACTTTCTTAAGTTTTCAGTTTTGGTTGCCAAAATTACATGAAT
MSTRG.18000.4	ENSRNOG00000003070;ENSRNOG00000003086;ENSRNOG00000003104;ENSRNOG00000027286;ENSRNOG00000055994	0.023	0.766	0.000	4.783	up	GGGGTAGGGAGAGTGGACCTGTTTAGAAAGGTTCTTTAGAGCAACTCCTGTCTGTGCTGTCTGGAAATCGGCAGTTCAGTTCACAGGTCAGTAGTGGCAGCTCGATCCACTAGCAAACACTTCACAGGTACACCAGCAGTCCAGTTCACCCAATGAGGTCATCACATCAGTCATAAGATCTGCATTCTAGTTCAAGCTGTGCTTCAAAATGTCTGACTCGGGGACATACTCTGCTTGTTGAAACAAGGTCCAACTCATGCAGCCCAATAAAAATAAAATACTATCCAAAGTAGATATTTTCTAAATTTCAATTCACTTCAAATAAAATTCTACCTGCTTCTTATTCTAAAAAAGCAGACTTTGGGCTGGGGAATCAAATTGGGGCTTTTGGTTCATGCTAGGCAAACCAGATATGTTCCTAAGGTTTCCTTCAGTTCTATGATAAAAGTCGTAAACCTTTTTGAAGTGAAATTTATGTAATTTGCTAACATTTCTAGACTGTACAGTACGGAAGTCGGTAGCCTGTGCAAACGAAGCTGTTTTCAAGGTTATTTTCATGGCCACCCTCGTGACTCCGAATGACAAGGCAATCCGAGCAGCTCTGCTCCTTCCGTGTTTATCTCGACGAGGGAAAAACAACCACACCCAAGACCTCTGAACCTAGTCCTTAGGTTCTAGGATTAACTGGCCCTTTCCCTTCCTTTGCACAATAGGCACCCGGATGGGAGAGGTGGGTGAACCCAGGGTACAGAGTCTAATAGGCATTTCTCCTCTTTCCTTCCTTTGTTGGTAGATTGTCGAGAAGGCAGAGAATCTGCAAGGCCCGCGGGGGCTCTGGGCTGGGTCTCTAGCTAAATCCTCAGCTTCCAGCCCCGCTGTCACCCCACTCCCACCCACATCGGGCCTGTGGGCGGCTAGCAACAAGACAAAGCAACTAAAAACAAAGGCTGCGGAAGCATCAGTGTCCACACTGGAGGGCGAGGGCGAAGCTAGCGTCCAGCCAGTGCAAGTAGAGTGGGGTCGCAATTCACCCTGTGACGTAAACCCGCCGGGCGAGGGGTGCGAGAGGACCACAGGGAACTCGGGACATAGGGTTCGGGGACGCGGGAAGAGAAGACGCCCCTCCCCCACCGCAAGCAGGACCCGTCAGTATTTTGCGAAGACCAGAGGCTGCACATGCGCACTCGGTTACTTAGCAACGTCGACGTTAAACTGCCCGAGAGCCTAGCCCAGCAACAATCGAGCGGCCCGCGACCTCCACCAATCAGCGCAGGCCTCATCTTTGTCTCCTCACCAATTAGGGCTGGTGCCGCAAGGAAGTTTCCAGAGCTTTCGAGGGAG

MSTRG.18344.1	ENSRNOG00000003992;ENSRNOG00000004075;ENSRNOG00000037712	0.816	0.000	0.028	-3.770	down	CCCACGTCCGTGCATACAAATCAGCTTCGTCCTGTCTGTGAGCTGCCTGGTATCTCATTAAATTCAAATCACTGAAACCAAACATCCCCTTGAATCTTCATAGTACGTGATCCCAGCATCTGCCTCAGATACCTTTTCAGTTCTCGGAAAGAGGAAGGCACAGACTGTTTGGATGCTTTTCAAAGTAATTTATTTTTTCTTGGAGACACAGCAAAATGGTGGCCAGCCTCTTTTGGTGTAGGAAGCTGTCAGAAGTGAAGGGGTGTAGGCAATGGAACTTCTGTGTGTCC
MSTRG.1848.1	--	0.000	0.279	0.011	3.719	up	GTGCAGTCTAATTGCAGCAGAAGACAGCAGTGTTTTGGAGATGACAGTACCATGAGATGACCACCAAGAGCAGTAGCAGCAGCAGTGGAGTACAGGCAGCTGGAGCCTAGAAAACAAGGTGTGTCTACAATGGGCAGAACTGGAGAAGTACCCCAAGCCCTTGGAGGAGCCCAGAAGATCGTGAGTGGAATCCAGATATTGGATGGTTAGAGTTTGATTGTGACTATGCCCTGATATTTTTCCTTCTTGAGGGATGCCCTGTGAAGTTCAAATCTTCCATGAACCATCTCTCTCCCAAAGGCGTCTGGTCCACAGAAGGAAGCCAGGTCTCCCGGATGACTGCATCGTTGTTCCATGGAGCTTTTAAAATGCAAGTGTCCTGAATGGCAGTGGTGCCAGGTGACAGCTGTGGGCACACCTCATCCTGAAGCGCTGTAGGAACTGAC
MSTRG.18506.1	ENSRNOG00000017352;ENSRNOG00000017417;ENSRNOG00000017657;ENSRNOG00000017915;ENSRNOG00000018114;ENSRNOG00000018526;ENSRNOG00000018693;ENS	0.000	0.248	0.019	3.592	up	AGTGACTCCCCTTGCAGACAAGTCATTGGTGCAGACAAATCCCAGTTTGTCTTATCTATGGCTTGGAGAGCCATAGTAAGTTGCTATTTTGAGATGGCATCTGCTGTTAAAAACTGAGTTACTCCGGAGGCCTAAACTCAGGAGACAGCCACAGGTCCAGGATCTTCAGGAACGGTCACAGCTCCTGGCCCAGATAATGGCTGCCCTACACTTGATCTCAGCCAAAAGGCCGAGAAGCGAGGATGATTGCTGTTCTGTCTGTTGGACTGCCTGAAGCCCCTCATACAAAAGAAGTCAGCTCGTCCTGCTCATCCAAACGAAACTCTGTCCCTTTACAGTGTGAGGACTGAGGTGGATTTTTGTCAGAGAAAGTGGCCGGCTCTTCAAACTGTAGATCTAGCTGGGAGAAGAAAGAGAACGTTTGCATTTTCTTCTTCGACTTTAGAAACCCTGT

	RNOG00000018715;ENSRNOG00000030726						
MSTRG.18799.1	ENSRNOG00000033267;ENSRNOG00000034227;ENSRNOG00000047153;ENSRNOG00000048690;ENSRNOG00000048801;ENSRNOG00000050608;ENSRNOG00000061506	0.248	2.404	0.000	3.975	up	CTGTTGTCAAGGTCTCATTTATTGAGATGAACGTTACAGCTTTTAGGGCTTTCAGGTAGGGGGAGTGAC<br>CTTTGTGAAATATGAAGGAGGGGGGAGATGAGGGTATATAGGCAGTGATAGATGGAGGCAAAGAGGTC<br>AGGCGATGAGGTCAGGTGGTGGAGACACTGGGTGTTGACTGAGGAGATCACTGGTATCTGCTCGAGCT<br>GAGGCATCCCTTGAATGTTATCTTCCTGTGCCGTAAATTCATGGGAGAGGCTTTGTCCAACAATCTTAAG<br>ACTTATGGCAGTCATCTTAGAGGTGAGTCTCTGTGGCCAAACAGCCCAGAATCACTTTGAACCATGCAG<br>TCAAAAAGCGGCTAGGCCCAAATCCAATATGGCTCCGTGCATCTCCCCCATTTTTCTTTTATAAAAGACC<br>ATTAGGTGGAAGTAGTGGTCTGAGAGAGATCAGACATGCCTCACAGAGTGCCCAGCTCGGCCATGGCA<br>AGCCACTCTTGCAGTTAGGACTGCACCCCAATGGCTAGGAATGAACTTATGCTGTAACACACCATTCTG<br>GGCTATACGTGTGTGTTATTTGGGGGAGAAACTGGGCAGAGGAGCATTTGACCGGCCCGTACATTTAAA<br>CGGGGTGTAGCCACCTCTGTCTTAGGATCGACCTCTCGAACCCCAGCCTTATCTGTCATAGGATCACCTT<br>GTCACCCCCAAGGCACCATGTCTTAGATTGGTCAAGGGTCAGAGGAAGTTGCCCATCCCTGAGGATAG<br>CTACATTGGATCATCTTCTCATTCCTCTTACTTGAGCCCAGGGGCGAAAGAGTAGAAGCCAGATGGCATT<br>AATATAAGAGATTGTGGAAATTGAGCCTCTCCCGATAAGTAGTGGGCACCTCATCTGGTTCGTTATCTCT<br>CTGGTTCTCCTCAGCTGCTACCACCAAGTGCCTAGTCAAGCCTCTCCTGTAATAAAATTTAGAATTCCCA<br>ATTGTTAGCAACTACTCCAGAAAGTTCACCCACTGTGCTTGCTTAACAATAGAATGGGGGGAGGGTAAC<br>TCCAGACACTACAGACACAATGGCTGCAGCAGTAATGGCTGCTACAATAGCAGCGAAAGTGCCAAGGT<br>CTTTCCCAGGACACTTCAGGATCTCTGGAACAACCAGCAGACAAGGGAACCATGTCTGAGATCTGCAG<br>AACCAGGGCAGAGTTCCCCAGTCCACAGCAGCTTCCAGCAAGCAGCAGTGCACAGAGGGTCGGAGCG<br>CAGGCCGCGGTGGGACGGGACACAATGACTGCAGCAATGCCAGAATTTCTGTGATGACAAAAGATGAG<br>TGGGAATCCTCCATATTCCTCCAAGGACACAGGAATGACTCCAGGGACCCGAACCAGGAGAGCTGATT<br>CTTCATGCTCCCAGGATCAGCAAGTCTCAGCAACCACCTCTGGCAGCTGGCATACTCTTGTAGCATCCT<br>CCCTCTCCAGTGATGCACGAGCGATGCAGCCTGAGAGTCCTCAATTTCTCCGCAGATCTCCTGTCTCCG<br>TCGTCTGGTAGTTGGCCGTACTTCGGGGTCCCTGTGTTCGGGCGCCACTTGTCCCCGCCAGCGGGGACC<br>CAGTTATTCAGGGTCCCAAAGGGGCGCTACCCTTGGGCCTGCTCTTCCTGTGTCTCAATCGGTGTCTCT<br>GGCTGCATCTCTGTCTGGCCAGAGGTCCCGCCCCCACACTTCCTACCCCAGCTCCAGCTGTCAGCTCTT<br>TATTATATAAAAACCATGTTCCAGTCTGGAC
MSTRG.18799.4	ENSRNOG00000033267;ENSRNOG00000034227;ENSRNO	0.186	0.744	0.001	2.713	up	CTGTTGTCAAGGTCTCATTTATTGAGATGAACGTTACAGCTTTTAGGGCTTTCAGGTAGGGGGAGTGAC<br>CTTTGTGAAATATGAAGGAGGGGGGAGATGAGGGTATATAGGCAGTGATAGATGGAGGCAAAGAGGTC<br>AGGCGATGAGGTCAGGTGGTGGAGACACTGGGTGTTGACTGAGGAGATCACTGGTATCTGCTCGAGCT<br>GAGGCATCCCTTGAATGTTATCTTCCTGTGCCGTAAATTCATGGGAGAGGCTTTGTCCAACAATCTTAAG<br>ACTTATGGCAGTCATCTTAGAGGTGAGTCTCTGTGGCCAAACAGCCCAGAATCACTTTGAACCATGCAG<br>TCAAAAAGCGGCTAGGCCCAAATCCAATATGGCTCCGTGCATCTCCCCCATTTTTCTTTTATAAAAGACC<br>ATTAGGTGGAAGTAGTGGTCTGAGAGAGATCAGACATGCCTCACAGAGTGCCCAGCTCGGCCATGGCA<br>AGCCACTCTTGCAGTTAGGACTGCACCCCAATGGCTAGGAATGAACTTATGCTGTAACACACCATTCTG

	G00000047153;ENSRNOG00000048690;ENSRNOG00000048801;ENSRNOG00000050608;ENSRNOG00000061506						GGCTATACGTGTGTGTTATTTGGGGGAGAAACTGGGCAGAGGAGCATTTGACCGGCCCGTACATTTAAACGGGGTGTAGCCACCTCTGTCTTAGGATCGACCTCTCGAACCCCAGCCTTATCTGTCATAGGATCACCTTGTCACCCCCAAGGCACCATGTCTTAGATTGGTCAAGGGTCAGAGGAAGTTGCCCATCCCTGAGGATAGCTACATTGGATCATCTTCTCATTCCTCTTACTTGAGCCCAGGGGCGAAAGAGTAGAAGCCAGATGGCATTAATATAAGAGATTGTGGAAATTGAGCCTCTCCCGATAAGTAGTGGGCACCTCATCTGGTTCGTTATCTCTCTGGTTCTCCTCAGCTGCTACCACCAAGTGCCTAGTCAAGCCTCTCCTGTAATAAAATTTAGAATTCCCAATTGTTAGCAACTACTCCAGAAAGTTCACCCACTGTGCTTGCTTAACAATAGAATGGGGGGAGGGTAACTCCAGACACTACAGACACAATGGCTGCAGCAGTAATGGCTGCTACAATAGCAGCGAAAGTGCCAAGGTCTTTCCCAGGACACTTCAGGATCTCTGGAACAACCAGCAGACAAGGGAACCATGTCTGAGATCTGCAGAACCAGGGCAGAGTTCCCCAGTCCACAGCAGCTTCCAGCAAGCAGCAGTGCACAGAGGGTCGGAGCGCAGGCCGCGGTGGGACGGGACACAATGACTGCAGCAATGCCAGAATTTCTGTGATGACAAAAGATGAGTGGGAATCCTCCATATTCCTCCAAGGACACAGGAATGACTCCAGGGACCCGAACCAGGAGAGCTGATTCTTCATGCTCCCAGGATCAGCAAGTCTCAGCAACCACCTCTGGCAGCTGGCATACTCTTGTAGCATCCTCCCTCTCCAGTGATGCACGAGCGATGCAGCCTGAGAGTCCTCAATTTCTCCGCAGATCTCCTGTCTCCGTCGTCTGGTAGTTGGCCGTACTTCGGGGTCCCTGTGTTCGGGCGCCACTTGTCCCCGCCAGCGGGGACCCAGTTATTCAGGGTCCCAAAGGGGCGCTACCCTTGGGCCTAAATGGGGGGCGAGAGAAGAAGGAGACCAAGCAAGATTGTATTGTCAAGGTGTCGTTTATTGGGATGAACGTTACAGCTTTTAGGGCTTTCAGGTAGGGGGAGTGACCTTTGTGAAATATGAAGGAGGGGGGAGATGAGGGTATAGGCAGTGATAGCTGGAGGCAAAGAGGTCAGGCGATGAGGTCAGGTGGTGGAGACACTGGGTGTTGACTGAGGAGATCACTGGTATCTGCTCGAGCTGAGGCATCCCTTGAATGTTATCTTGTGTATCTATGTTTCCTGTGCCGTAAATTCATGGGAGAGGCTTTGTCCAACAATCTTAAGACTTATGGCAGTCATCTTAGGGGTGAGTCTCTATGGCCAAACAGCCCAGAGTCACTTTGAACCATGCAGTCAAAAAGCGGCTAGGCCCAAATCCAATATGGCTCCGTGCACAAATCTCCATATCCTATATCAAATTGTATACATAATCAATATTGAAACATTTTGAATCACACTTTTCTGCCGCTATATATGAGGTTGCACCATGCTTGTGGCTCTGCTGTAGCTATTCGGTATATAGATTTTGGTCCCAAGTTTGGTCCATGAGCTGTCAGACATAGGTATCATAGTATCCTATAATTAGTGTCTTTTTTCTTGGTAAACACATTTAGGGGTGTTGTCATCCTTCTTAAAGTATCTTCTGTGTTTTATCTCCTGGGTGCCCATGAAAGGAAAGGAATTATTTAGTGGCATAGAAAAAATGATGCCTTAATTTTGTAATATTTATCAGTTTTCTATGGGCACTGAAAATGGGTTTTATTGGATAATGCAAAAAGAATCTTGACCGTTTTCTCCTCCCAAAATAGAAATAATGATCTTGGGAGTTTTCCTCCAAGTAAGGAGAAGACCTATAATTGTCTCTAGGAAAATCAATCTTTACTCTTTTCCTTGTCATTGAAAAAGTTTTAGAAACAAGGATGATTGTACACATACCTTCAGAAGAAGAGAGCAGTACTGCCACATGAAGTACTGTGGTGATTCCTAGATTTCAAGATTTTGGAGAGATTAGGATGACATCTGTCCCAGTATCTACAATGCCTTCATTTTCAATATTATTTATAAATGCCTTTAGTTTTGACTTCTGATCATATTTCTGGATCACCATGCTGTGCACTGCAGCCACACCCGATTCAAAGGCCAAGACTATGCCTCAGGGATACTGAGTTATGCAGGCTGAATTCAATCACTTTGTATGTCCCAGTTGCGAACAGAAGCACAGCCTCCTGCATGGCATGGTCGTCAAAGGCATGTACCTGCTCTTCCTGTGTCTCAATCGGTGTCTCTGGCTGCATCTCTGTCTGGCCAGAGGTCCCGCCCCCACACTTCCTACCCCAGCTCCAGCTGTCAGCTCTTTATTATATAAAAACCATGTTCCAGTCTGGAC
MSTRG.18799.5	ENSRNOG00000033267;ENS	0.000	1.293	0.000	7.707	up	CTGTTGTCAAGGTCTCATTTATTGAGATGAACGTTACAGCTTTTAGGGCTTTCAGGTAGGGGGAGTGACCTTTGTGAAATATGAAGGAGGGGGGAGATGAGGGTATATAGGCAGTGATAGATGGAGGCAAAGAGGTCAGGCGATGAGGTCAGGTGGTGGAGACACTGGGTGTTGACTGAGGAGATCACTGGTATCTGCTCGAGCTGAGGCATCCCTTGAATGTTATCTTCCTGTGCCGTAAATTCATGGGAGAGGCTTTGTCCAACAATCTTAAG

	RNOG00000034227;ENSRNOG00000047153;ENSRNOG00000048690;ENSRNOG00000048801;ENSRNOG00000050608;ENSRNOG00000061506						ACTTATGGCAGTCATCTTAGAGGTGAGTCTCTGTGGCCAAACAGCCCAGAATCACTTTGAACCATGCAGTCAAAAAGCGGCTAGGCCCAAATCCAATATGGCTCCGTGCATCTCCCCCATTTTTCTTTTATAAAAGACCATTAGGTGGAAGTAGTGGTCTGAGAGAGATCAGACATGCCTCACAGAGTGCCCAGCTCGGCCATGGCAAGCCACTCTTGCAGTTAGGACTGCACCCCAATGGCTAGGAATGAACTTATGCTGTAACACACCATTCTGGGCTATACGTGTGTGTTATTTGGGGGAGAAACTGGGCAGAGGAGCATTTGACCGGCCCGTACATTTAAACGGGGTGTAGCCACCTCTGTCTTAGGATCGACCTCTCGAACCCCAGCCTTATCTGTCATAGGATCACCTTGTCACCCCCAAGGCACCATGTCTTAGATTGGTCAAGGGTCAGAGGAAGTTGCCCATCCCTGAGGATAGCTACATTGGATCATCTTCTCATTCCTCTTACTTGAGCCCAGGGGCGAAAGAGTAGAAGCCAGATGGCATTAATATAAGAGATTGTGGAAATTGAGCCTCTCCCGATAAGTAGTGGGCACCTCATCTGGTTCGTTATCTCTCTGGTTCTCCTCAGCTGCTACCACCAAGTGCCTAGTCAAGCCTCTCCTGTAATAAAATTTAGAATTCCCAATTGTTAGCAACTACTCCAGAAAGTTCACCCACTGTGCTTGCTTAACAATAGAATGGGGGGAGGGTAACTCCAGACACTACAGACACAATGGCTGCAGCAGTAATGGCTGCTACAATAGCAGCGAAAGTGCCAAGGTCTTTCCCAGGACACTTCAGGATCTCTGGAACAACCAGCAGACAAGGGAACCATGTCTGAGATCTGCAGAACCAGGGCAGAGTTCCCCAGTCCACAGCAGCTTCCAGCAAGCAGCAGTGCACAGAGGGTCGGAGCGCAGGCCGCGGTGGGACGGGACACAATGACTGCAGCAATGCCAGAATTTCTGTGATGACAAAAGATGAGTGGGAATCCTCCATATTCCTCCAAGGACACAGGAATGACTCCAGGGACCCGAACCAGGAGAGCTGATTCTTCATGCTCCCAGGATCAGCAAGTCTCAGCAACCACCTCTGGCAGCTGGCATACTCTTGTAGCATCCTCCCTCTCCAGTGATGCACGAGCGATGCAGCCTGAGAGTCCTCAATTTCTCCGCAGATCTCCTGTCTCCGTCGTCTGGTAGTTGGCCGTACTTCGGGGTCCCTGTGTTCGGGCGCCACTTGTCCCCGCCAGCGGGGACCCAGTTATTCAGGGTCCCAAAGGGGCGCTACCCTTGGGCCTTCAGAAGAAGAGAGCAGTACTGCCACATGAAGTACTGTGGTGATTCCTGCTCTTCCTGTGTCTCAATCGGTGTCTCTGGCTGCATCTCTGTCTGGCCAGAGGTCCCGCCCCCACACTTCCTACCCCAGCTCCAGCTGTCAGCTCTTTATTATATAAAAACCATGTTCCAGTCTGGAC
MSTRG.18842.2	ENSRNOG00000003172;ENSRNOG00000003233;ENSRNOG00000003243;ENSRNOG00000037275;ENSRNOG00000047958;ENSRNOG000	0.367	0.000	0.000	-4.880	down	GGGCAATATATTTACCTGGGTCTTCCTGTTGGAATTCCTGGCTTCTAGGACTATGTGTTCTACCTATAGTACTCGAACTGACAAAATGTTGGCATGCTTTATTCGGGAGTGGGCTAAAGGTGAGCGGGTAAAGGCAGGAGGACCCCATCAGTCACCTGACCTAGGAAGGAAGAGAGCAACTGAAGCAATGAAGAGCTGGCCCAGGAACCAGGAGACTGACCCACTCGGCTTGTTTTCCACCGCCTTTCCTTTTTAGGCTTGAGCTCAGTTTCCCTGTATCATCACCAGGAGCTTCTGGAGCACCCACATTCAGCTGGCCTTCATAACAGGCTCCCGACCATGGCAACAGGAAATCTCTCCCGTTCCAATCTCCTCAGAACTCCTCAACTTAAGAGATGATGCAAAAGTCATGAATTCCAGGCCACAAGTTTCTCTTTGGGGTGAGTGATGAGGTGGTCCCAGGCTTGTCCCTTTAAATAAGCAATTCAGCAGCTTTTGCCAAGTCATTGGGTTCATTTCAGTTTTGCCCATCCTCCGCCTTTCAGACTCTGATTGGCCCCTAGGGAAGGAGCCGCCTCTCCATTGGTCTCCACCTTTGAAATCACTTCCCTAAGTAGGCCTGAGTCAGAGAAGCGCTTCGGAGGGCGGGATTGAATGGGTGTTAATCTTAGGATGGGGTTTCTGGCTGCTACTACTTTGGTAAAGCTCATTCCCTAATTTCTGAGAAGACGCTTTCTCTCAAAAAGTGAGGGCTGTGATATAAATCCTTTGTTAGAAAGGTGCCCCTTGGGAGGCCGTGGGCTTTTCGGCGACTAGGACGAGACATTTACTAGAAAAGAATTCACTAAACCCACGAGGGATGGACTAGATCTCCAGTGAAGAATGGGCCTGTGCGGATGACACTTGTCCCTATACCCGGAAGACCTCGGGCTAGCTCTCCAGTGAAGACTGGGGCTGTGCGAGTGACAGTGGTTCCTATACCCCGAAGACCTCGGGAGTTCTGCTGCATCGGTGGCTGGGTTCCATCTTCAGTTCTCCTGTTGCTTGGCGTGCTCCACCTTCGCTTAGCTTAGGCTTCCCGTGAAGGCTGGTGAATCAGTTTCAGGGTTGTCCAAAAGAAGTTCCCAGAAGGAGGGGCTGTAGCCCTTTAAGGACTTCCCCGCTACGGACCCAGAGATCAGTTTAAAAATGCCAACTCACAGAGCGCGCTGCATTCTGGGAAGCCGAGTGTCACCGTCA

	00048257;ENSRNOG00000050809						GAACTTCATTGACCAGGATGCACTGCAAAGTCACACGCCTTCACTTCCTTCTGCTCTTTAAACTGTAGTTTGACGTAGAACTGGTCTAAGCAAGTTGTCTAGGCCTAGGGCTAACCACACCTTTTCAGCTATTGGCCAGTTGGTTAGTTGGTGGGCGTGGCTTAGCGCAGCTCCTCCAGGCAAGGGGGTGGCCTTTTTGCCAATCAAAG
MSTRG.18956.1	ENSRNOG00000005290	0.287	0.853	0.010	2.303	up	CATGCAGGCAGCGCCGCCGCTCGCGGCCCGGCCCGGCCCGGTATGACCCCGGCCCGGGGGTAAGTCAGCGGGTCCCGCGGGGCGCTGCGGGGGCTGCGGGCATCGCCGGTGTGGCCCCCGGACGGCCCTGACGCGGCTGCTGCCTGCTCGGCGGCGGCCGGTGCAAGTGAGTGCGGGGCCGGCAGCAGGGGGCGGGCCCAGCCCGCGTCACCCGGCAGCAACCAAGCGACTCTTTCAAAGGCCCTCTACAAATAAAATAGACTTCCATCCCACCTTGATGGATGCTTATGAATAACGCAGATTCCAGGGCCCCACCTCGCACCCACTGACCCGGAATAGGCACCACAGAGGCTGAGGCTGGGGGATTGCTTGAGCCCAGGAGTTCAAGGGCAGCCTGGGCAATACGGAAAGACCAGTTTTTAGCAACCCCAACGGTACTGGGTGTGCTGGCTCATGCCAGTCATCCCAGCACGAGGGGCGTCGGACAAGAAGACCAGGGAGTTAAAGCCATCCTTGGCTACGGATCACACGGGAGGCCAGCCAGAGTGATGAGACCCTGTTTTTGTTTTTGTTTTTGTTTTGTTTTGTTTAAATTATGAGAACGGGGAATGTCTTAATTAGGATTTTATTGCTGTGAAGAGACACCAAGACCATGGCAACTCTTATAAAGAACAACATTTAACTGGGGCTGGCTTACAGTTGAGAGGATTAGTCCATTATCATCATAGCAAGAAGCATGGTGGGCCTGCAGACAGACATCGTGCTGGAGAAGGAGCTGAGAGTTCTCCATATGGATCCAAAGGCAGCAGGAAGAGAGGCATTGGACCTGGCTTGAGCTGCTGAGACCTCAAAGGAAGGCCATACCTACTCCAACAAGGCTACACCTCTAATACTGCCCCTCCCTATGGGTCTAAGGGGGCCATTCCGTTCGAGTTACCATGGAGCTGTGGTTAAGTGGTACAGAGCTTTAACGTGCTGAAGAAAACAGTTTCACAAATAACCTCGACAGTTGCACAACCTTTGATCCTGGTTAGCGCTGCTGAAATACACACTTATGTTAAAATGGAGGGCTGTGGCAGTGGCCCACTGTCAGAGGGTGGGCCTAGTATTCAGTAGGTAGGTCCTGGGTTGGAACCACAGCAACCCACTGAGGTGCTTAAAATGAAAAAACTCTATATTCTATATTTTAATACTTGAAAATAATTTCAAATGTATTTAAAATCTTGGTATAATATTCTTCTTTCATCCTATTACATTCTCCCAAGGCAAGGGTTGTCTTGGTTTGAGTCTAACCCTCTACACATTTACTTTATATTTATTTATTATGTAAAGAAATAATATCTGGTATTGTTTATGTGGTTCAGAACTTTATACAAGGGCTATCATACTATACATGTCCTCACTCAACATTCTTGGTATGTGCATATATGCGTGCGTATGTGCACATGCATGTGGAAGCCAGAATGCTGGCATTACAGGCATGTTGCACAGTTCAGAGAATAATCCACAATATCTTTGGTTTTGAATAAATGAGTTACTAAGTATATAGCAAGCCAAGGTAAAATGTTCCACAGGAATTATTTACCTGTGTTCTTTGAGACAGAGTCTCTCTCTGGGATGTAGGGTTTGCAAATAGGCTAGGCTGTTGGCCAGTGCGTCCCAGGGACCTGCCTGGCTCTAACTCCCTATCACTGGCATTATAACTGCATATCTCGGACTGGAGAGATGGCTCAGTGGTTAACAGCATCAACTGCTCTTCCAGAGGTCCTGAGTTCAAATCCCAGCAAACACATGATGGCTTACAACCTTCATGTAAATGAGATCCGATGCCCTCCTCTGGTGTGTCTGAAGACACCTACAGTGTACTTATATATGATAAATAAATAAATCTTTTTAAAAAACTTTGAAAAAAAAAAAACCCCAACAACTGCTTATCACTAC
MSTRG.19063.1	ENSRNOG00000009673;ENSRNOG00000009681;	0.051	0.339	0.000	3.260	up	ATCTTTTTAAGACAGAGTCTCCCTGTGTAGTCCTGGTTGGCCTAGTAGCCAGGTTGTCCTGGAATTTACAGTGGTCTAACTAACCTGCCTTTGCTTCAGCAGTGCTGGGATAACAGCCACTATCCCCGGCTTGGTCTCTTTATATTGTGCGTTTTCTGTCAGAGGTGTTGGGGACTGGCTTTACTTTTGTGTTGATTAGCAAAGAGAAGCTATGTCAGAAGAGGTGAACACCAGTGTCTTGACAACACTTTATGTAAATATTAACTAAAGTGATTCATTTATTTTCATATTCCAGGTTTAATTCCCAGTGGCTGTTGTAACTGGGATTCGGTTTTGAAGATGTAATTGATGTTCCGCAGCTGCCTCCCAGTTTTAGCTGGATGAGTAACTTTTCTTGATGATTTATTTTATCTCTAGCCAAT

	ENSRNOG00000012579;ENSRNOG00000012866;ENSRNOG00000013169;ENSRNOG00000023344;ENSRNOG00000023375;ENSRNOG00000023381						CAGGAACAATTGGGTGAGAGTCCAGTATGGAAAATAGGGGCTTGGGACAGAGCTTAGTTGGTAGGGTGCTTGCCTAGTGTCTCGGAGGCTCTGTGGAGGGAAGATGAGAGCTTCAAGGCCAACCTGGGCTACACCACCCTGCATCAAAACAGAACAGTAAACAGAAGGAAGATAGGCATGCTGGGACAGGAAGTCATCTCAACGATGGAAAATATGGTCAGGAAATTGGATTTTTCTCCACATATGTATATGTGCACATGCACATACACAGATCCATATTTTATGCACATAACATTTTAAATATTAGTATTGAGTTTTTTACAAAAAGGAATTTCACATTAATCTTATGTTGGCTTTGAAGTTTTTTTGTTTGTTTGTTTGTTTGTTTTGGCTTGTTTTGTTTTAATGCTTTTATCCCTAAGGGCTCTGAATTATGTTTAAATTGGGAAGGGAGAAGGATCCTCCAGACAAATCTTACTTAACTTGTTACAAAGAATAGTGTTAAAAACACTGATTCAAGCTTTGGACAGCTAGTGTATTGAATAAAATATGAAATATTTATTTTTGTCTGCTTGAGACAGGGTCTTACTATATAGTTCTGGCTAGTCTGGAGTTCAATACATAGACCAGGCTAGTGTCAAACTCAGGATATCCACCTATCTTTGCCTTCCTGCTCTGGGGTAATAGGTGTGACCATGACACCTTTGAAGTGTGTTTAGCCCTCTAGCAAGGTGGGGAAGCAGCAAGAAAAGCACCTGGACACCATGCTGCTTTGGTCTGTTTGCCCTCCTACTCGATACTCTAGATTTTTCTTTTCTCTTTTCCCAGGTGTGAATACTTAACATGTCTTTTTATAAAGTTGTATTATACTTTGCTACCCCCCCCTTTTTTTTGTATGTATGTATGTGTGCATGTATGTGGAGGTTTACTAGTCCCAGTGGAGGCCAGAAGTTGACATAAAGAAATTTTCTTCAAATGGCTGCTTTACTTTAAAAAAAAAAGTCTTAAAAATTTCATTTTATGTATGTAGGTGTTATGTCTGTGAATCATGGGTGTGCCTGGTGTCCCTGAAGACCAGAAGAGGGCATGAGATCCCCTGGAACTTGTGTTATAGACAAATGCTTGTAAGCCTCTGTGTGGGTGCTTGGAATTTAGCCCAGGAAGAGCAGCAGCCAGTGCTGTCTGTTAGCCACTGAGCCATCCCTCTGTCTCCTTTACCTTATTTTCAAATGAGGATTTCTTACTGGACCTGGACCGTGCTGACTCAGACAGCAGGCTCCCACTCTCCCTGGATCTCTGACTCTGGTTATAGATACGCTCGGCCACACCTGCTTTTTTGTGGATGTTAGGGATCCAAATTCAGGTTGTGTGTGTTTGGTTGATTGGTTTTGATTTTAATTTAGTTTTTTTTTGTTTTTTGTTTGTTTGTTTGTTTGTTTGTTTGTTTTTTTAAAACAGGATCTTTATATGGGTCCTAGAACTCAATATGTCAACCAGGCTGTCCTGGAACTCACAGAGGTCCTCTTGCCTCTGCCTCTGCCTCCGCCTCCGCCTCCCAAGTGCTGGGGTCAAAAATGTGTGTCACCACAACTGGCCCCTAAATTCAGGTCTTTATGCTTGTGTAGCAAGGTATTTACCCAGTGAGCCTTACCTCTGGTCTTGAAGTCCTGATTCTCCTGCCTTAGACTACTAAGGGCTGGGGTTAGTTTTTCTTACTATGTTAATTTTTGCAGTGGGCTTTGTGCATGCGGGTAAATCACTCAGCCAACTGAGTTATACCTCCAGATCTAACAGGCCTGTTTTCTTGTCTGGTTGTGCAATCAGTAATTTGCATGGTGCAGTAGTTGGGATTCCCCAAAGAATTTTTCCCAGGAGGCAAGGGGCTATGTATAGAACTATTAATGGTGTTCTGCATTCATCTCCAGGCGTCATTGAAAGGGCTTGGAAACTCAGATATAGCTCTTCAAGGAAACAGTTATGCACATGACCTGTAGTTTGTTAGGTAGAAGAAACAGTGGTGCTGAAGTTAAAGTAACTCGCTGAATTTCCCTGAGCTGTGTTTATCCCTCTGATGTGCAGCTTAGAATGTAAGGAAAATGGCTTTATGTTCTCTTTAAGGGGAGATTCTGGTCTAATCATGTCTGAAGTATGAAATGATCCCTTGAGAAAGTGAAGGAAGCATTGGATACTTAGCTTAGCTTAATTCCTGTAATAATCTCCTTGGGTGTGGTAAGGTTTGGAGCTGGGGAAAAATTACTCAAGCTAAGAAGATATTAGAGGTAAAAAAAAAAGTCTTGGGATGCTGCTTTAAGTCCCCAGCCCGGATTCTCTTTTATATTTATTTATTATGCGTATGTGTGTGAGCTTGCATAAGTGTATGTGCATGCAGGAGGCCTAAGGAGACAAGAAGAGGACATTAGTTCCTGGAACTGGAGAGCTAGCTGTAGGTGGCTGTCAGCTGCTATGTGGGTGCTGGGAATTGAACCCAGGTCCTCTGCGAGAGCAGTAAGCACTTTTAACTGCTGAGCCATCTCTCCAGACCCTTGGCATAATTTCTTACTGAAACATGCTGGTGGCATACAGTTTGGACAGTCCAGAG
MSTRG.194	ENSRNOG0000002	0.529	0.000	0.037	-3.684	down	GGCAGAGGCAGGTGAATCTTCATGAATTCCAGGCCAGCCTGGTCTACAGAGCGAGTTCCAGTACAGTCAGGGCACAGAAAAACCTTTTCTCAAGAAAAAAAAAAAAAAGGCAAAAAGGCCCTAGACTTTCTGTAACCTATGTCTGGCTATGTGGCCTTGACTGTCCAACCCTCCTAGGCCTTCTGTAACCTATGTCTGGCTATGTGG

36.1	8116						CCTTGACTGTCCAACCCTCCTAGGCCTTCTGTAACTTATGTCTGGCTATGTGGCCTTGACTGTCCAACCC<br>TCCTAGGCCTTCTGTAACCTATGTCTGGCTATGTGGCCTTGACTGTCCAACCCTCCTAGGCCTTCTGTAA<br>CCTATGTCTGGCTATGTGGCTTCACCTTTTCTACCCTACTCCTGCCATCCTCTCTCG
MSTRG.19553.1	--	0.068	0.365	0.005	2.878	up	TTTCTAACTGCTGGGATTTTAGGTATATGCTACCATTCCTGGCCTGACTTGATTCTTAAATATTGAGCCTTC<br>TTGAATTTCTCCCTAACAGGGAGCCCTTGATATTCTGGGTGTGCCTTTTTCCCCCCAGCATGAAGCTAGC<br>CCAGTTACATGCTTCCTGTCAGCAATGTTTGTCCGTCTTTAGTCCCCACCCCACCCCCAGACTGTAGGCT<br>CCACTTTTTAAAAGATGTTATTCTTCTGTGACTTGAACCCAGGGCATTCTATATGCTGGACAAGCTTTCA<br>AACGCTGAGCTACAGCCCCAGCCTTCTGTATCCCTGTTCTGACAAATCGTTCTGCAAAGCCTCGGCAGA<br>CCATCACCGTGTGGAGCGTGTCCTTGGAACAGGCTTTGCCTTGACAAGTGGAGCTAGTTTGGGTGCTTG<br>TCTTGCTGTACGCTGACCTTCGTAGGCAGTGGGGTGGGCTGTGCTCCATCCTCAGGGAAACCAGTGGTG<br>TTGGAAGCTCACAGAAGTGGGGAGAAACCTGCACTTAGATCCAGGCAGGCAAAGTTCAGAGCTCTGT<br>GAAGGAATGCGGCAGAAGGACACAGGTAAGCAGAGTTATAGTGCAACCTGGTGGAAGGTAAGGGGCT<br>TTACTCTTGCTTCTTCCTTTCCTGAACAGGCTGAGTATTTCAAAGCTGAATGTTGCCACACAAAAGGCT<br>GCAGGAGAGTCCAACCCTGGCGAGCATACGGAGCAATTCCGGGCCTATGTGTTGACAGCTTGGGTTGT<br>AGCTTACTCTTTCTTGCCTGGTGGTGAAGTTTAGCACCATGTGATGTGGCTTACAGGGAGCCAGCTGCA<br>GTCTGCCAGGGCAACTGTACCTCTCGATGTTTTGTGCACCACGGAAAAGAACTGGATCCATCCACATTC<br>ACCAGAGATTCCTATTTCCTGTAACAGCAGAACTGACAGAGGATCCCAAGGTGATGTGTTTTTTCTCTA<br>ACTGAGTACAAATGAGTCAGATGCTGTCACTGGAGTTGAAGGTAGGCACCCCTGTCTTCCTAGCAGCTT<br>CCTGGGGAGCAGTTCCCTTTCGGGTAATCTGTCAGCCCAGTGTGTTTG
MSTRG.19634.1	ENSRNOG00000008921;ENSRNOG00000009819;ENSRNOG00000050323	0.050	0.390	0.003	3.142	up	TGAAGGACTCCCCAGCTCCAGGTGTCCATTGGCCTCTAACAGAGGCTGACTCCAGAAGGTCACCAGCT<br>CCCAGCTGGCAATGGATGAGTGGAAGGATTTTGAGGTACCTCGAAGGTCAGTGGAAGGGCAGAAAGG<br>GCCTTTCAGTAGGAGCTTTTCACCGGCTCGACGTTGCTACTCTCCAGTACATTAGCATCACAAGATCGA<br>AACAGAAAACTCGGGTATATCCCAAAGGTAGGACAAGTCAGCAGTCAGCAAACTCCAGCAATTCTGGG<br>AAAAGCTTTTCCGTCTCCCAAGTCCATATCTGTTCTCCTTTCCTGAACCGATGGGACTGGAACACTATCT<br>TCCCGAGTGTGACACTTCACTGGAAGGGAAGGGTAGCTTTGCCTTTAAGGCAAGCGTAAATTTCAAAA<br>AAAAAAAAAATACTTCCTCAAAGAGGTAATTCGGCTAAATTTGACATTATTACTTCCATTATGTGCTGTA<br>CTAAACCCATGAGGTGATATTTCGATCCTAGCTCTTCCCCTTGTACATGAATACATTGGGTACCAAGGGA<br>AGGAAGAAAAACTGCTGAGGACTGGGGAAAGGTATGTCTGAAGGGGTCTGGGACAACAGATCTGTCA<br>GTAATGTGCTAGCTTTGCAAGCCTGAGAACCGGTGTCTGACCCCCAGCACCCACATAAGAGGCCTGGC<br>ACAGCAGCCACACGCTGGCGGTACTAGAGCTTTAGACTCAGAGACAGTGGGTTCCTTCCAAACCCTCC<br>TCAGCCAGCTTGTCACAATTAGGTCCATGTCTGGTAAGAACCCTGTACCAAAGAGCAAGCAAGGCAGT<br>TCCT
MSTRG.19672.1	ENSRNOG00000009819;ENSRNOG00000010257;ENSRNO	0.117	0.000	0.001	-4.716	down	CAAATGTGTCACCAATGGATCTTTTAATCCCTAGCTGGCTCCCCTGATTCCATTGCCAGCTGTGTGTGTC<br>AGTGCCTAGAAAAAAGCAAACACTCTGATGTTACTTCAGCAAGGTAAAGCCAGGATGGTTAACCAGCT<br>GCATTCAGTTTACAGCAGTGAGTCTCTGGCTCCTGGCTTTGCTAAAGTCCAGTAGGTTTGGATTCAGTC<br>CTGGGGTCCTGCTTTGCTAGCGAGTTGGTACAGATGTTGCTGGCTGAGGGCTGTAGTGTGAGCAGGGA<br>GGACCTCGGCGGCTTGGGCTGTGGGCGCATAGCCGCTAGCTGAGTGAGCTAAGCAAGAACACAAGGTT<br>TTTGTGCCTCTGAATTGGGAATAAGAAGATACCTGCAGGGGTTGGGGATTTAGCTCAGTGGTAGAGCGC<br>TTGCCTAGCAAGCGCAAGGCCCTGGGTTTGGTCCCCAGCTCCGGAAAAAAAGAAAAAAAGAAAAAGA<br>AAATACTTGCAGCTGGGTGTGGTGGTGCCCACACCTTTATCCCAGCGCTCGGGAGGCAGAGGCTAACC

	G00000010363;ENSRNOG00000058463;ENSRNOG00000059588						TGGTCTACAGACTGAGTTCCAGGATATCCAGGACTACACACAGAGAAACCCTATCTCCAAAAACCGAA<br>AACAACAACAAAAGAGACACCTGCCTTGTGAGTCACTGTGAAAACAAAATGAACCAAGGCCTATAAA<br>GCACTTAGCCCAGAGCCTGGTTCAATAAATATTAGTTATTAATATCATCTAGAGACACGTAGCATAAAGT<br>GGGCCAAGCGAAACAGAAAATGCTTGCTGGTTTCCTCCTGTTCGGGCTCCTTGTGATGACACACACAC<br>CTCTAATGTTCCTTTAAGCTAGTTGCTGTCACCATCTTTAGAAAGTTGACTGTACCTTGTAGGGCATTGT<br>GTTCATCTCTTATGGGGAAACTGAGACCCAGAAACGTTGAGTAACAGCCACATCCATATCCATAGAGGC<br>CTCAGAGCAAGCATATACCCAGGGCTGGTGTTTGCTTTCTGCATAGCCGCTGCCTTTGCCAGACAGCTG<br>TGTCCACACACGGAGCTAGCCATCTTCAGCATGACTTTGGGGGTGGTCGGTGGACTCCAAACTGTTGTC<br>CACCCCACACTTGTTAAACGAACTGGGAAAACCCATGAATCCTGCTCAGACTGAAAACTGTACCGGAA<br>CTTTAAACAAACGTCTTACAGTCTATGTTCTTGATCCAGGTGTGTTTTGGTCCCTAGAGTGGCGGACTCA<br>GCCCAGGAAGGGTTGGGAAGTTGGGAAGTGTATGCAGTAATTCCTAGCTGCCAAACTGGATCCCATGG<br>TGAGACCAGCCAGGCAAAGTCTGACTCCATTTTCACTGCTATTGTTATGTGTATGTACCAGTGCATGTGG<br>GCCAAAGGTTAACCTTGAGTGTCACTCTTATGTGCTATTTGTGTGTGTGTGTGTGTGTGTGTGTGTGTGT<br>GTGTGTGTGTGTGTGTGTGTGTGTTTTGTTTTGTTTTAGGACAGGATCTTTCACTGTGACCTGGGACTCTTG<br>CTGATCAGGGTAGGCTGCCTGGCTATTGGTCTCAGGAGTCCGCTGATTCTCTCAGTACCGGGATTATAGG<br>ACCTCTGTGTATGTGGGGAGTTGGGGGAGACGGGGAGACACAGAGAGAGACAGAGAGAGACAGAGA<br>CAGAGACAGAGAAAGAATGAGAGGATTCAGGGAATTGAACCTGGGTCATCATGCTTACAAGGCAAGC<br>ATTTTACTGACTGAGCCATTTCCCCAGCCCCTCCAATATCTTTATTCAATGAAATTTGTTAGCCAAATGTC<br>CTTGGGTCAGAATCCTTGCTTCCCAAGGCTACCTGAAATAAACAGGTGCTTAGATAAGACACAAATTAA<br>ATACAGCCAGGCACTTTCTCTGGGTCCTAAACACCTACTGCCTCCCTGGGACTCCCACATAGGACAGCA<br>TTCCTTCTAGCTTGTCTCTTTAGAGAGGGGATGGGTGTGGGGGGTGAATGTTGAGGATAGAAGCTTCAA<br>GCTCAGACTGTGTACAGCCGGAAAGACAACAGGTAAAAATGGATACTCTGGAAGCCATGTTTATATGTG<br>TCTGAGCATCCTTGGGAGTTGCCAGGTCCCCTCACAGGTTCCTCAAATATTCCCAGTGGGACAATCATT<br>CAACTCCAGTCCCAGAGATGCCACCTAAGTGTTTGCCCTGCAAGGACTGCTCTGTACCTTCCACTGAGG<br>CCGCTGGGAAGGTTTGCTGAGCGAGCTGTGATTCACTCTACTTACTCACTGCCTGGTTGACACCTTGGA<br>TGTGTGTCACATGCTCATCCCCTCACCCTATAAGGATGTGTGCCCTTGCTACAGAGTGGCAAACTAAGC<br>CCAGAATAGAAACGAATGTGGCCACAAAACAGCCACAGTCACCCTTCCCAAGGAGAGCACCACAGAC<br>ACAGGTGCCTTAGGGTAGGGCTACAGAGCCTGTCCACCACCCCCATCCCCACCCCTGAGTCCTGGCTTT<br>GCCTCCACCTCAGTGTCTGTTCCTTGGCCATTCTTGCCCTTGGTCTCTGTTGAATGAGGCTGATGTGACG<br>GGTAGGAGGCAGGTCATGGTCCACAGGCTCTGAGGCTGGGCAGAGCTGAGTTTGAAGATGGGCTTCGC<br>CTCTTGGCAGGCACATGACACATTCCTCTCCTCCCTCTGCTTGCTTCCCTGTTGGTAAACGGAGAGGATA<br>ATGGGTCCCTCGTGGGGTGGTGAAGAAGATGAGGGAGTCTTAATAGTTCCTTAGTGCCCGGCCCACAC<br>AGGATGCTCAGGGAATATTTTGGTTACTATCCTTGGTTTCCATTCCCCCTTGGATATTTTGCCCTTTTTGCT<br>TATAAACTTGCTACTGAAGTTACTCCCTCCGTCTCTCCGTGTTCTTCAGAAGTTAGTCTTTAGACAGAGA<br>GAAAGCGTCCGCCTGGAGAAAGCCTCCGCTCCCAACCTTTCTGCCTCGTCGTTTCCAGGAATTCCTTTG<br>TCAAGCAAAGAATCTCTTTGTGAAGAGAAGAATCTCTCACTTGGGATTTCTGTTCAGGTTTTTCTCAGG<br>AGTCAGAGCCGCGTAGGCAGTTTGTTTGCCTGGGGGCCCAGCGAGGGCGCACTGAAAGGGCTCCCCAT<br>GCTTCTAGATCTGCCGATCACCTTTCATCCTTCCGCTGTCTTCCTGTCGGGGCACTGGCCCGCCAACATC<br>GATCACGCTGGCCCGGAAGGTGGTGACGGGCTTGTGTTTCTTGCTCCGCGTTTTGTTCCTTTGCTTAGT<br>CAATCGCGTCTCCATGGAAACAGGTGCAGCCTTTGTGTTGCTTTTGCTTTGGGTGAATCAATGCTCCCCC<br>ACCTCCTCTCCTCTTTGGGATCAAAGGGGGAGGTGGACTCATCACAGCAACTGACAGGGTGCCTCGTG

							GTGGCCTGGGGAGCGGGGCAGGGATTAGTCTTCTAGTCAAGCATCCCCACAGCCACAGCGTGGCTGCTGCCCTGTCCCCACAGTGTCCCTTTCGCCTCATGAGACCATCGGGATGCTGTAGGGTATGCAGCGATTCATAAATGTTCTCACCCCAACCCCCATGGCTCGCTACTCTCAGGGGCAGCTGCTGGCCACCTGGGGCGGGTTCCTGAGTAGGGGTCAGCCTGCTGGTGTTAGCTCTGCTTCTTGCGTGTGACCTTGGAGGCAAGGCCTTTGACCTTTCTAGGCCTTGGCTTCTAAGAGATTGGAGAAGCTTAGAACTGGGGAGAAAGTCAGGCTTTTGGTAGCAGGACTGCATTTGCCCTAAAGAAAACCACACATCTGGGTAACAGCAGTTTATGTCCTCTGTTCTGGGGTCAATAGTAAATTCTAGGTTTGGTTCAAACCAATGACATTCAAAACGGGAGCCTATCTCTGAGAAGCATCTTGATTAATAGCAACTGCCCCCTCCTTCTCTCTCCTGCTGGTCCAGGGTGTTCTGTCTTCCCTGCTGATGACAACTTGTCAGAGGCCTTCTGTTTCAGGCCAAAGCTCAATGCTTTTGGATACCTAGGGTCTG
MSTRG.19768.2	ENSRNOG00000023683	0.025	0.691	0.000	4.540	up	AGAAACTATGGTGTGAGGCTCCTGAAGGAGAGATTTTTCTTCCAGGCTCTTTTTTTTTTTCAGGGTATTTCACAGTGGCTGGAGCATGTTGGGACCTAACACAGTCAGGCTGTACCAGGAGCCAGAGCCCAGAGTCTTATAATCTCATGGGAAGATAGGAAGCGATGATTAGTACGCGTTGAATGGCCCCGCTGTGAGTACTCTGAGCTGGACTGCGCCTGGGACAGAATCAAGGGCAAGTTCTAATCTCATTTTCAGGTCTGTGGACAGCCATTCAGATCGTCAGATTAGGCAATCTGAATTGAGGGGGAACACTGGGTGTCCCTAAATGTATACTCCTACCTTTCTGTCACCATCTATCCATATGCTCTCCACTCTAGAAGTACCCTTGTTGGGTAGATACAAATGGCTTTGTCACCTCTCTGAGAATGGACCCCTTCCTCAGGATGAACTATCATAGTTCCAGATGGCACCATGCAAGCTGCCAAGGGAGGGAAGCAAACAATAGTCTCAGCTATGACACCTATGGACATCAACAACGATGATCATGTTACCATAACCCTGAGGGTGAGACAGTGGCTCACACACTGTGGAGGTAACCAACAGTTCTCTAATCGGACTTAAGGCAACAAGAGGGACATGGTTCTATTACTGAAACCTAGCCAATTACCCCGTGCTAGTGAGGAGGAGAATCTACCCCCACTACATCCTAACTACATCCTAAATATGAATCCTCATACCCACAGATGAGTATCGTTCTCACCTCTCACCAAAGAAATTTCTCTTTGCAACAGAGACCGTTACTGAAGACCATAACCAATCAAACCGCAGAAAATAAGAGATTGTGTGGTGACCAGTCCGAACTGATACATCTGTGGTTCAATTCCTGAACCTAAGGTGTAGAGATCATAGTAGAAGAGGGGCAAAAAGATTGTAAGAGCCAGAGGAACAGGAAGTTTGCTGAAAGATTGTGTCTTCTAGAAATGGCAGAGAGGCTATACCCATGACGTATTATCAACATGGCTCCTTAAACAAGGTCCAACACCAATGGACATGGGCATGCTTACATGGAACAAGGAAAACTGACAAAGCTTCAACCTAGACAAAGAACTATGGACAACTAAGGAATTCTGATAGGAAGAGGAATGTCTTCCCCAGGAAAAGTCATTCAGTTGGTTCTCTAGTACCAAGTGTTCATTCCTAAGATCACATAGGTGCAGGTAACATTATATGTCCTGAGCAG
MSTRG.19943.1	ENSRNOG00000002828;ENSRNOG00000002831;ENSRNOG00000045669	0.898	0.000	0.000	-5.312	down	TTTTTTTTCGGAGCTGGGGACCGAACCCAGGGCCTTGCGCTTCCTAGGTAAGTGCTCTACCACTGAGCTAAATCCCCAGCCTCACTGTAACTGATACTTAACCAGTCCCTAGGAGGAAGTGCTTTAGCACTTTATATCTGGGACCGTGGATTGGAGAAATTCAGTAGGGGAGAAATCGGAGAAAGAGACCTGCTCATTTTCTCTAGAGAATTCTTGAAAAGTATACACAAGCTTAACAAAATTATGATTCATCTATTTTTAGTGAGACATTTTAATCTCAGCCAAGCCTAATATGCTCCCTAATTATAGTGATATATTTTCCAAATTACAAATCAAGACACATGGCCCAGCACATGTGTCTGTAATTCTGAGCGTTTGGTACAGTAACATTTGCCTAATGGTAATAAATTATGCTTCTAGGGCACATGAAAGATAAAGTGTTTCTCTTGGTAAGCCATTCCCAAAATATCTTACCTAGATTCTTGCAATTTCTGTGCCTAACTACAAAATTAAAAATGCCTTATTTCTGTGACAAATTAACGTAACGTTCACTCTAGAAAAGCAAAACCGCACAGCTGAAGAAACAATGGAAGAATAATTTATATTAAAATATTTTGCTGCCAGATGCTTCAGTTTTAAACACCATGCTCTCACAATCTAATCCCACTCTGCAACTAAAAAGCCAAATTACTAAAAATAAAACAGGGTTTAAAGCGTTTCTGAGTAAAACTTCTATGTTCTTAAGGAAAAAAAAAAAGCACTACTCAAGTTTTAGGAACCTGTGCAATGAACAAGAAATGGGTCACCTGAC

MSTRG.19996.2	ENSRNOG00000003273;ENSRNOG00000003284;ENSRNOG00000003304;ENSRNOG00000003330;ENSRNOG00000003524;ENSRNOG00000024420;ENSRNOG00000042912	0.491	0.053	0.024	-2.336	down	GCAAATATTTAATATTATAAAATCAAACATATTTTGTATTTATTATGTTTATAATATATTTTTTTAATTATTAAGGGTGCACGACAGCGATTATATAGAGGTCAAAGGTTAAGTTGGAGGAGTTGGCTCTTTCCTTCCATCATGTAAGTTGTGACAGGGAATAGAACTCAGGTCATCAGGCTGGGCAGCAAGTCCCTTTTTCGAAGAAGCCATCTTTTTAAATTACCTTTTAACATTTTTATTAGCTCAAGCTTATAAGCTCAGTAAGGGTCCCTTCCTATGTATAACAAAGCCTAACCACCAGTTTCCCAAAGGGCTGGCTTGCTTGCTTCCTTCTGAGACAGGGTCTCAGTAAAGAGCTCAGGCCGGTTTGGAACTCATGGTAAACCTCCTGCCTTAGCCTCCTGAGCTAGTGAGAGAACAATTGTGTGCCCACCACATCCAGACCCAAAGGTCTCTTTTTTTTTTTTTTTGGAGCTGGGGACCGAACCCAGGGCCTTGCGCTTCCTAGGTAAGCGCTCTACCACTGAGCTAAATCCCCAGCCCCCCAAAGGTCTCTTAATTTAGTGTTATCTGATGTTTCCTCATAAGTAGAAGCCAGGTGTGGTAGTGTACATCTGTAATCCCATTATCTGGGGAGGTGGAGGCAGGAGGATCAGGAGTTCGAAGCTGCCTTATGTTTGAAGTACATAACCAGTTTCAGGCCAGAGTGGATGGTAAGACCTTGTCATAAAAAAGAGACAGAAGCTGGGTGGTGGTGTCCCATGAGGCCTTTACTCCCAGTACTCGGGATGTTAGAGGCAGATGGATCTCTGAGTTCAAGGCCAGCCCAGTTTACAGAGTGAGTTCTAGGACAGCCAGGCCTACACAGAGAAAAACCCTGTCTCGAAAAACCAAACAGGGTAGGGTGGGGATTTAAAGGAAGTGTTTTGAGCAAGAACACCCAGGCGTGCTACTCTAGGACCCTAAAGCCTAGGTGGAGTGCTCCTCCCTAAGCACAGTGGTGAATGGGGGTGGCAGGGGTGGCAATCCAGGATCAGGAAACAGAGGTCAACAGAGGTCAACCTAGAGTTCTCCTGACTCATAAACACACCTTTAGAGGATGGCCAATGGAGGGAACCCCAAACTCCCACAGGAGCTGACAGCTTTTGTCCTGTTTGGCAGCCTTGTCTCCAGCCTCTCCAGTCACACGGTTTCTCTTCCAAACATGCAAAGTTCTTCCACATCCTGTGCCCTTTTCCTCCCACACCCCCTTACCTGGTAAACACCTGCTCTGTCTCACTCATACATCATTTCCTCCAGGAAGGCTTCCAGGATTTCATCAGGCTGGGCTCCCCTCGGTACTCTAAGCCTTCGTAGGTTCCTCTAGAGGTAGCATTTACCATGCCATGGCCCGGCTGCCAGCCTGCTTCATGTCTCCAATCATAGAACAAGCCCCACCCTGTTTGTTTTCCTTCCTTTCTTCCATGTCTAAATAGGTGAAGTGTGCCTGTGTGCATATGTGTTCCTGTGTGTGTGAAGGAGGGAATGAGTACGTGTGTGTTTGTAGAAGCCCAAGGTCGATATTGGGGGTCTTCTTCAATTTTTTATAGATTTATTTATTTATTAAATATACAGTATTTAGCCTGCATACATTCCTGTACCAGATCTCATTACAGATGGTTGTGAGCCACCATATGGTTGCTGGAAATTGAACTCAGGACCTCTGGAAGAGCAGCCAGTGCTCTTAACCACTGAGCCAGGTCTCTAGCCCATCTTCCATAAATTCTTGCTACTTTACTCACTTGGGTCAAAACCACAGTTGATATTGGTTAAGTCTGGCTAGCTAGCATTAAGCAAGGTGAGGGGAGGATTCCTTTTTCTGTGCCTCTAAGTGCTGGGATTCCAGGCAGGCCGCCATACCCAGCCAGCATTTGTGTGGGAGCTGAGGATCCAACCATCAGTTCAACACTTGCCCAGTAAGCGCTTTAACCACTGCCCTCTCCCCATCCACACCTATGGGGCAGGTCCTACGTTAAATGGCTGGACTATAGAAGGTTAAACTAGCTTAGCCTTGTGCTTGTAATTCCAGGAGTTGAAAGGCTGAGGCAGGGAGATTACTATGAGTATGAGGCCAATCTGAGCTACAGTGTGAATTACTGTCTCAAAGTCAGAATAACAACAACAACAACAACTAGTGAAATGCTTGCCTACATGCCTGGGGGTTGATCTCTATTACCGTATAAACTGGGCACAGTGGCACATGCCTGTAACCCTAAAGATGGAGGCAGAAGGGTCAGGAGCTCAAGGTCATCCTTGACTGCATATCAAATCTGAGGCTAGCCTGAGCTATAGGGCGCCCTGCCTCAAAATAACAAAGGTAATAATAATAATAATATTAATATTAATAATGCCAAAGCCTGCCTCAGGAAGTGTATGGGCCAATATATCAAGAGACAAACAGAAAGAAAAGTGTTACTAGACCCTGGTACCCAGAAATGTTTCCTCACTGACGCAACACCACCCTAGGATCTGGGAGCCCTGGGGTAGGGTGGGTCAGATAGGTCACCTGTAAACCAATGGACAACCTGTTCACTCCTGCTGCTCCAAAAGCTGCCAGCCTGGGACCTGGGGCTGAAGTGGGGTTGGCCTCCAACGTGACTTCCGAATCTGCAGGCAGGTAGACTTCCTGTGCCACGGCCTCCAGGATAGCAGCTACAGTGTGGGGACTGGCCAGACTCGGGGTTCCCCCACCAAAGAACACAGACTCCACCGTGCACGTAAAGCGCCGCGCGCGTTCTCGCGGACTCAGGACTCGGAGGCTGTCCCAGGCTATCCACGCGGGGGCGCCTCTG

							GGCCGCCCTGGCCGCTGCTACCCATCTACCGGTCCGGACCCCGGAGGGAACCATGACGTGCAAGGCGGCAGACAGTAGGGATGCGAGACCTCGACAGCGCGCCCAGCAAGGGTGTGCCCCGTGCTGGACCGCGTGGGCCGCCC
MSTRG.2002.1	--	1.094	0.118	0.021	-2.347	down	GTGCAGTTCCCCAAGTGTTTACTGTATCATTACCTTTGAATGGCATTAATTTTTCCAGCCAGAGTTCCTTCATCTGGGATGCTAAGCCAATTTTCATAAAAATCCAAGGTTAGTAAGCTTCCATCGATGGTAATAATAAAATCCTCCTTCTTTGCAGGTCTCAGAATTCCTGGGAGGCCTTTTGTTTTCCTAATGAGAAAGCAGACTGTGGTGGAGCAGTGGATGCACTGACACTGTCTCTCCTCTTGCTGTGCCTTCCATGCAGATGCCTGAAGCCTTGGAGGAGCTGAATGGTAGAGTTTCCTTGTATCTTCCAGAGGAGAAGAAAACACCATCCAGAATCCTTGTGACAAGCACTCGCTTGCGATTGAGCAAGTGTCATGTTGCCTGTCATCTGCTGGCTCATCCTGCTGCATAGGACATCATCTGCACTTGGAACTCCTCGGTCTCCACCAATGGTTCTTCAAGTCTCAAGTCATGTACATTTTCAGATCATTGAGCTCCTCTGCAGGATACTGGTGCTACTAAGGCTGGAGACGGTAAGACATGTGCACATCCAGTGTATCCTCAAAGGCTTCAGTCCTTCATGAGTTCTAGGTTACCTTCATGGTTCTCATCTTTCTTGCTATCTTCTATGACCAGAGATCTACTCAGACTGCCTTTCTAGCAGGTCCTCAATGAGTTCAGGCTCAAAACAAGAACCAAGGATAGTGACTGATGGTGCTGGAGGATAGCAAACATCCAATAGCCAGCAACATAGAAACTTCACAGGACTCTGTGGACTGTGTGGATGACAGTGCACATCCTTTAAATCTAATCTCCAGTTTCAGTGACTTCTGTTTGCTACAGTGAATGGTGTGAGCCTTGTCTAGTTTACTACCAGGCACAGATGGAGTTCTTAGAACTCCCTTTGAAAACATTTAGACATGTGTAGAAATCAGGAAGTAAAGATTGAGTGTTTTGAAAGACACGAGTCCTGCCAATGTAACTATGCAAGCCCAGTGTTAACTTCCTCCATGACGTTCACCTTTCCTTCATGTTTATCTTCGCCTTCCTTTGAAGAATGACCTACAGCAAGTAACTTGCCTTCCAAAGCCTCTGACTTACAGATCCATAAAGAAAACTATGAATTTTAGATTTCAAGTACTAAGTATTAGTCAGAGTTCTTCAAAGAAATAAAAGAAATAGAACCAATTGTATATGTCTCTCTCTGCATGTGTGTTCTGTATACACAGTCATATGATGCACAATGTTTTTTTCAATAGTGTATTTCACACTCAAGCTACAAAACTGCCTAGGAACAGTTTTCTCAGTCTGTATTCCAGCCCTTGACTTACATGTGGCTCTCTATGTGTGTCTACTTGTAAAGTCAGTGTTATGTTTGAAAGAAATCTCAAAACTGTAGAGCTCACAGTTGAGATCTAGTCAGCCTGACAGCCTGAGAATTAAAAATAACACTGACAGATCAATCCCCCAAATCAAGCTAGGAATCAACCTTCACCTATATTCCTCTTTCTATTCAGCCAGCAACTGGCCAGATGTCACAC
MSTRG.2006.1	--	0.518	0.034	0.012	-2.843	down	GCATAGTAATAGGATGTGCAGAAAGTAGGGAGGACAAAGAATTCTAGACAAGAAAACCATCAGGAAGCTTTAATGGAACTAAGGTTCTTATGGGCTGGTGGCGTAGCTCAGAAGCAAGCATGCATTTACTTAGAGGTCCTAGGTTTTCCTCAGAAAAAACAGGGAAAAACAAGGGGGAGGAGAGGAAAGAAAGGAAATCTGGAGTTGGCTTTTTGAGAAAAAAAAACAAAATTCAAAACTTGTTCTAAGGACTCAAAATTATAAATGGGAGAGGAGGCATTGCAACTAATACTAAATAGGTACAAAGGAAACTACAATGGACAATGTATTCCAACAAATCAGACACGCTAGAGGAAATGGATAGAGTTCTAGAAGCACACAAACTATTAAGGTGATTATGAAAAATCAAAATATAAATGGATGGATGATAATTAGAATAATTAATAACCTCCCAACAAAGAAATATCCAGGTAAGAGAAGGTTATTAAAATGAATTCAAAGAAAAGTTAAAGCTAATCCTTCTCAAATCCTTGCAGATCTGAAGAGGAGGAAAGAATTCCAAATTCACTTCATGAGAACAGCATGACCCTCACGCGAAGCCAGAGGAGAATGGCAAAAACAAGCAAAACTACATGAATAGAGGCAAAAATCAAGGAAACATTACATTAGCAATGAAAATGAACAGCATATTTAAAAGATTGCACAAATCAAAACTGGATTTATGCCCGGGACACAGGATTGCTCAAAATAAATTAAATCATTAAACATTAAAAGTATGAAGCATAAAAGCCATGTGATCATGTCAGTAGGTATAGAAAGAGAAATTGACAAAATTTAACATCTTTTTGATGATAATTCCACATATCAGCCATAGAAGCTGGAGTGGTTAGCGCGAGTTGTCAACTCAATGAGTTCTAGAACCTCCTGAGAGAGAGGCCTCCAGCAATATGGTAGGAGATCATCTTGACTCGGTAAACTGTGTTGAAAAGAAGCACCTTTGGCAGATGACATGGTTCTTCGGTCTGAGAT

							CCTAGCCTGTACACAGGTGATGGAGTGAGCTGAGTGTATGCCATCATCACTCAAACTGGGCAAAAATAA<br>CTTTCCCTTAAGCGACTTCCACCCTGGCGCTTTCTTACAGCAACTAGAAAAGTAACGAAGATGAAAGCG<br>ATGTCACCCAACGGAATGAAGACCACACAGGATGAGGCACCAGGGTACATAGTCAAGGGGGAAACAC<br>TGAAAGATTTCCTTCTAACCCGAGGAACAAGACAACCGTGTCCAATCTACCAATTCTATTCAATGCAGT<br>ACCAGCAGTCCTAGCAAGAATGAGGAAAGGATATTGAAAACGGTCTGTAGGTGACAGGACTGTATGCA<br>CAGAAGATCCACAAGAACCAAGTGTGG
MSTRG.20367.1	ENSRNOG00000010665;ENSRNOG00000010676;ENSRNOG00000010839;ENSRNOG00000010970;ENSRNOG00000011196	0.000	0.211	0.000	5.634	up	CTCCGGCTTCGCTTCCCTTTGTCCCGCGCTCGCCCCGCCCCTCGCGCCGGAGCTTCCGCGCTCGCACTT<br>TCCACACGCTCCGGGGAATTTTCTGCGGGCTGCGGCCGGCCCTCCTCCGACGAACAGCCCTTCCAGTG<br>AAGACGCGGCAAGGTTTAGTGCGCCAAAGATTTTTTTTTTCAGGCTCCTCGGATCCCTCAGAAGTCTTT<br>CAATTTGGATCCTGTCCCAGCCGGGCGCCGATTCGTCACGGCGAGGAGGGCGGAGAAGGTTGGGCCGC<br>GTTGCGGGCCCTGGTTCGCGGCCCGGCGGCAGGAACTGCGCAGACTGCGTACCCCGGCTTCACTGAGG<br>CGGTGGGTTCCGCTGCGTGAGGCAAGCGCTGGGGGAAGGCTCGCTGGAGCCTCTCCCGGGAGAGGGC<br>GGGTCGCAGCTCCCGCACACCCCAAACCCTGGCGTTCCCCTTCGCTCTGATCCTCTGTACTCCGCTGAT<br>GGACGTGTGGATGGAGGATGGAGAATGTAGGCGGGGAGGACCCGGATCCCCGCGTTTTGGTCCTTGCT<br>GGCGCTCGGCAGGGCTCCCTGCCTCCACCTAAGCGAAAAGCAACACGGGAGTTTTGTAGTTTTTCCAG<br>ATGCTTCTAGAGGTTGAATGATGGCGACAGGTTTCAGAGCCTAGGGTCCTCATATGTCAGCTCACTCTTT<br>CCAGCTTGCATACCTGTGACAATTCACATCTCTTACCAGGAGGGCTAATTCAGCTCACCCTCGCTTGTCT<br>TTAAATAGTCAGCTCAATGTTTCTTTGTGATGCCCTCCTAGAACTTTTCCAGGGCCTGTTGAAACCCTCT<br>TGATGGGATTGGGCCATCTGCAATCTTGGAGAGTCTTGAAAACCAAATTTCAGGCATCTGGACTGGACA<br>ATTTCATAATTACCAATGATTTCGAAGGGCTAGACTTGTAATTAGGTGCTCAGTAAGTGTGTGTGTAGCC<br>ACCTTTCTGGACTATTCCAGGGCTTTTTTTTTTTTTTTTTTTTTTTTTTTTTTTTACAAATTACAAGTATTTG<br>GCGGACCATAAGAAGTTGGCTAGTGTGGGTCACGTCATGGTGGAGGAAAAAATGAGCAGACCGTCTGT<br>GGTTATCTAAGGACAGTGTTAGCATCTAGAGAAACTGGTTGAATTCTGGGACTACCTCTTGGCCCTTATC<br>CTCGTGAGGTATCTTTAGAAGTCTGCAGAGCCAGGAATAAATGATAATATTGTTCAGTTCTTCCATTGTC<br>CAATATTTTCATTCTGGTGAGATGAAATTAATGAAATAAAAGTGTATTGAAAGGAAAGGCTAAAGGAGA<br>GTTTTCCAGATGAGTGAATAGCATAAGGAAATGTATCTGAAAATGTTTTTTTTTTTTTTTTTAAAACACAA<br>AAAGCATAGAGAAGATGAATGTAGGATCCACACAGTTACGTCAGGGATCCAGTGTCTTCTTCCTCTGTC<br>TGCCCAGTAACTCCAGCCTTGTTACGTCCCTGCTTTAGCAACTGTGGGATTAAGGAATGGTAGGAAAGA<br>GGGCATGTGTGCACATACGTGCCCCAACTGGGCAGTTCTCTGAAGCTCAATGGTGGTAGGACAGGGCT<br>GGCACAAAAGTGGTTTAAAAAACGAGGGGATGGAGTTCTAAGTGGACAGCGGGATGGGTGTTCTTTCT<br>TAGTCTTTTTACTTCTGTGTGGTGCTGGGGTTCGAATCCAGAGTCCTGAACATGGTGAGCCTATGAGTTA<br>CATCCGCAGCCCTCTAGTGTGCCTTTATTCCAAGGGAATCTTGATAGGTCTTCTGACCTAAAATCAGACC<br>TGTTTCCCATCCCTTGCTTCCTCTTGATGGCCTGTGTTTCATTGGTTTGCTTTTTACCCCCTCTGGCTTATA<br>TGTTATCTGTAAATACTTCTTTTATTTCTTCGTTTTGACCTCCTGTTGGAGTGAAAGCTTCTTTGTAATGG<br>GGATGTTTGATATATTTTTAAAATTAATTTTGAAATTAAAATATAATTATATTATTCTCCCCTTTGTCTTCCT<br>TCCAACCCCTCTCATGTTCCTCTCCCTGCTTCCTCTCAAATTCAGGGCCTCCTTTTCTTTGTTTTATATATA<br>TCTCGATATTTAATATATAGCACACACATGTGCATGTGTATATTGTATAACTATATGACTACAACCCATTCA<br>GCCCATTTAATGGTATCTACGTATATGATTTCAGAGCTGAACATTTGGTACTAGGGGGTTCATACCTGGGT<br>GAATAACCAATTAGGGGCTTCATCCCTGGGGAAGACTTTTTCTCCTGTTCTCAGAATTCCCTGGTTGCCT<br>GTAGTTCTCAGTCTAGGGGTCAGGATATTTGATATCTTGAGCAGTGGTTGATATACATACACACTTATCAT<br>CTGTAAAGTTAGAGGATGAGGATCTCAGTATCCGCCTTTGGTTCTTCCTTCACCCGTGAAGATTTCAGCA

							ATGTATAAAGGGGCTGTTAAGATGGCTCAGCTGGAAAAGGTGCCTGCTTTACAGCTTGACCATCTGAGTCTGACCTCAGGACTCCTGTGGTGGAAGAGCAGAACCAAACCCTTCAAGTTGTCCTCTGACATCCACA
MSTRG.20567.1	ENSRNOG00000020658;ENSRNOG00000020674;ENSRNOG00000020678;ENSRNOG00000020684;ENSRNOG00000020698;ENSRNOG00000020730;ENSRNOG00000023628;ENSRNOG00000023768;ENSRNOG00000050347	0.434	0.000	0.000	-4.918	down	AAATTTCTGCCCAAATATTATGCAGGCTGTGGGATAAACCTTACAGAAAGACCTATGCAGAGGAGCAGGACACTGATGTAAGGGCCTTTCATCATCTTTAAGATCATGAGCTGCGAGACAGCTCAGTGATCAAAGCTCTTACTCTGATGTGCACAACATAGTATATATAACATGTGTGAACACATGTGTACATTAGTGTTTGGAGGCCAGATGTCAAAAAATGATTCTCTATGGCTCTTTTTAATTTATTCTTATTTCATGTACATTGGTGTTTTGCCTGCATGTATATCTGTATGAGAATGACAGATCTTCTGGAATAAGGGCTCTACAGTCAGTTGTGAGGTTTCATGTGAGTACTGGGAATTGAACCTGGGTCTTCTGGAAGAACAGCAAGTGCTCTCTATTTCTGAACCATCTCTGCGGCCGCCTCCATGGCTCTTTGACTTTACACATTGAGGGAGGGTCTCGATTAACCCCAGAATTTGCTCTTTGTCAAGTCTCCCTAGCCAACCTGATCTGGGGATCCCTTGTCTCTGCTTTCCTAGGCTGGAATCCCAGGTGGCTGTCAGGTTTACTGAACACTTGTACCTTTTCTGTAAACCTTATTAACCTTAGTGCTTAGGTCTATATATATTAAAAATACCTTTTTGTTTGTTTTTAGGCAGGGTCTTTCCACACAGTCCTGTCCTAGAGCTTGCTATGTATGCCAGGCTGGCCTTAACTCATAGAGATCCTCCTGCACCTGCCTCTCTGAGTGCTGTGATTAAAGGTGTGTGATAACAGGGCCTTTCCTTTAAAAGGGAGAGGAAACTGAGTTTCTCTTACTGCATCAGCCTAAGGTGAGACAGGCTCAAATCTGGAGTTGCTCCTGACAGTCAAGCATTTTAGAAATGAGCCACAAAGAAACATTTTATCTGCAGACATTCAGTGGGAGCCAAAACAAGCAAACAAAAAGCAACCAAAGATAACACAGCCAACTCCCTAGCACGTGTGCCAAAGACGAGGTAGTTTAGGCGGATATTCCAAGCCAGAAACTAACAATTCTCATCTTGTGCAAGAGCACTCAAAGTCCTGTCTATTTTCACAGTAATGACAGCAACAGTACCCAAAGATAACTGTATGGGGGATGGGACTCTGGGCTTCATCATCTGGTGATGAACTAAATGAAGATGTGCTTACAAAGAAAAGTATTTAAGGAGGGAGATTTTGAAGCAGTGTCACAGCAACAGTTTCCAGCTTCCCTTGCATGAGCTACTGTTTCT
MSTRG.20582.2	ENSRNOG00000020730;ENSRNOG00000020751;ENSRNOG0000002	0.000	0.669	0.000	5.602	up	ATCTGATACGTCCTCTATCCGAGGACAATATATTAAATGGATTTTTGGAACTAGGAGTTGGAATAGGAGCTTGCTCCGTCCACTCCACGCATCGACCTGGTATTGCAGTACCTCCAGGAACGGTGCACCCCCTCGGGGGACTTACACTGCTTGTCTGAAGAATAGACCTTCTTGAGTGATTTACTTCTCTTGTCGTCTTTGCTCTCTTTAACTTCTCTACTTCAAGCAGCTCTGCTACACCTGTGATTCCGGGTTCTTCTATACAGCTCTTTTGGGGCTTTTCTTCTTCTAAGCTTCAGATATGTACTTTTAAGTTGTTATGTAAGTTAGGACTTTGACCTTCACACGGCAGATTCGAACACGCCCTAAAACCAGGCCTTCAGAGGTAGAGGCTTCTAGGGCCAGCCAGAGCTATATAATGAGACCGTCTAAATTTCCAAACTGGCTGAAGCTTCTGAGATTTGTTTAGGCATCTGATAGACTTTTTGTTCTGAATGAATGAAAAAAATAAGATGAAAGAAGAGAAAAACCAGATCATTCTTCAAACATTGTTTGTCTTCTTATCGTGCCACAAGTCCTCCTCTGATCCCCTGACCTGCTCCTGCTGGAGCTGGTTTCCAGCAGTAGTA

	0770;ENSRNOG00000020771;ENSRNOG00000023628						AGACAGACTCTTAACCAACTAAAACCAAAACCTTACCATCAAGAAATTGACTTTATTCTAGTATTCTAGAGACTGGAGTCTGCCAGCACTCATATTGCAAATTTATTCTCAGGTGTGTAATACATACAAACTACACACACACACACACACACACACACACA
MSTRG.20606.2	ENSRNOG00000020805;ENSRNOG00000020837;ENSRNOG00000020874;ENSRNOG00000020877;ENSRNOG00000033653;ENSRNOG00000036796;ENSRNOG00000036798;ENSRNOG00000058371;ENSRNOG00000059683	0.435	0.000	0.010	-4.070	down	CCCGGTTTTTGGTTTTTTAAGCAATCAGGTTAGAGGGAACACCAAAGAGAGGAAAAGGGAGGTTTGAGTGACTGAGAGTTCAGAGTGAGGGTGCGGGGCAGAGAGCTGAGTCCCTCCACAGGCAGGTGGTGCAAAGGTAGAGGCCACCGGACTCTTAGCCTGGGAGGCTAAGGTGCGATTCGCCCAGCCAGAGCTAAGATGCTTGGTGCAGAAGCAGCGCAGGAGGAGCTCCTATGCCGGAGGAGTGAAGGAGTAAGAGGTCTATTTGTGCAAGAGAGAGGGTAGGAAGCCTAATCCAGAAAAGATGGATGCCAGCCATCCCGGAAGAGGTGACAGAGACTTTGCCTCCGGCTACAGGTGCTCCTGGCCGCGCATGGCGTTCAGCCCAGGCTCCAAAGGGAACAGGAACAGTTCCAAGAGAGGAAATGGGCTCCATCCCAGAAGGGCATCTCCAGGCGAGGTGAGTCAGAGAAGTCCTCGATCCCATTTACATCTCAGTGAGAAGTGGGTGGCTGTGTAGCTCAGTGGTAGAGCATCTGCCTAGTGTATGCCAGGCCCTCCTCCGGGCTGGAGAGATGGCTCAGTGGTTACGAGCACTGACTGCTCTTCCAAGAGGTCCTGAGTTCAATTCCCAGCAACCGCATGGTGGTTCACAAGCATCTGTAATGGGATCTGATGCCCTTTTC
MSTRG.206	ENSRNOG0000002	0.000	0.238	0.001	4.308	up	CTACAGAGTGAGTTCCAGGACAGCCAGGGTTGCACAGAGAAACCCTGTTCCTTAAAAGAAAAATAAATGAATAAATAAACAAAAGCAGGGTTTCCTTAGCCTAGGCTAGCCTTGAATTGCTTATAAAGGCAAGGGTGACCTTGATTCTGATCCTCCCGAATCCTCTTCAAGAACGTGGGGTTACAAGTATGTGGCTATCACGCCTGG

18.1	0874;ENSRNOG00000020877;ENSRNOG00000020879;ENSRNOG00000020894;ENSRNOG00000020902;ENSRNOG00000020905;ENSRNOG00000054581;ENSRNOG00000059683						TTCATTTTGTTTTGTTTTGTGTTTTTGTTTATTTTAGGTCTGTTGTTGCTGTGTTTCGGGGTTTTTTGAGCAGGATCGTGTCTCTCTATAACATGTAGACCCCCTAGCTGTCCTGGAACTCTCAATGTAGACCAGACTGGCCCTGAACTCGTGAATACTGCCTCTGCCTTAGGAGTACCAGGTTTTATATTTTGAGGCAAGCCCCTCTCCAGCCCCACCGCTATGTAGCCTATGTTCTCCTGGAACTCCCGGCAATCCTCCTGCCCCAACCTCCCAGGAGCTGAGAGTACAAGTATGTCACACCAGGTCCTGCTTGACAACAGGACTTCGTTTGCATCCATATGCTGCAGTGAGGACCACACCCCGTGTTCAACCTTCCCGGACTCTTTCCAACTCAAGTCACTTGCATGACCCTGGCAAATTCGCACAAACATCTGGGATGTCTGAGCGCTTTCTAGAACCAGCATTTTGGTAGCAGATTAAAGAGCTCCACCACTCTAAATCTGACCTTCGTGACCCTCGCTAATGGCTCCAGTACCCCCACACACAAGGACAGAGAAGTCTAGAGGCGCCACCCTCTGGTCTATGACTTGCCACACCCCAAACGCACCCAGAAGCCACTCCGGGCCTCCTA
MSTRG.20749.2	ENSRNOG00000003501;ENSRNOG00000003506;ENSRNOG00000028436;ENSRNOG00000042833	0.115	0.590	0.000	3.031	up	CTGATCCAAAGGCAGTAATGAAACATTGGGTGGAGTTGGAGAATCGTCTTGACCATCAACTGGAGCTGGAAGCACCCACTGCTTATATTCTACACTTGGAGCTACAACCTTTTTAGGTGACTTCGTAATCTGAATTGGGATCTCCTGCACTGGTTTAGATTCTGTAGTTTTGAGAAAAAACAGATCCAGGGGTTCAACTACCATTCTCATGGGAAGTTCTGCATGATTAAGAAAAATCTTCCCTGACATATGTCTTTTAGTCTCTATAGGAGTTGGAGAATGGTGAGTCCCACCAGGAGAATGAGAAGGTGTGATGAGTTCTTCATCCAAAGATTGTAATGACATAGTGGGTGGTATGGGAGACTTGTCTTGACCCTCAAGTGGAGCTGGAAGAACAGCCTCCTTATATTCTACGGTGTGAGCTGCAACCTCTTTAGGAGATTTTTTAATCTGAGTTGGGTTCCCCTGCACAAGTTTAGATTTTGTAGTTTTGAGAAAAAACAGATCAGGAGGTTCAACTACCAGGCCCATGGGAGGGTCTGCAAAATGAAGATAAATTTTGCCTGGTGTATAATTTTTAGTCTCCTTCGAATTTGGAGGGTGGTCAGTCCAACTTGGAGATTGAGAAAATGTGGTAAGTTCCTGAATCAAAGGCTGTAGAGACATATTGGGTGGTGGAGGAGACTCAGCTTGACCTTTGATTGGGACTTGAAGCACCCCCTTCTCACTTTCTACTGTGTGAGCTACAACTTCTTTAGCTGATTTTGGAATTTGAGTTGGGGTCTCCAGTACAGGTCTATTAAGTATTTTAGTTTTGAGAAAAAAAAGATCTGGAGGTTCAACTAACATTCCCTTGGGAGGTTCTGCATAATGCATGTAAATCTTGCCTAGTGTGTGGCCTGTATTTGCATTTGTACTTGAAGGGTGGGAAATCAAAACATGAGGTTGAGAAGATAAGGTGAGTTCCTGATCCAAGGGCTGTAATGATATATTGGGAGGGGATTGAGATTCATCTTGACCCACAACTGCAGCTGTAAGAACACTCTCCTTATATCTAGTACTTGGAGAAATAATCTCTTTAGGTGATTGGGTACTCTGAGTGGCTGTCCCCTGCACAGGCTTAGATTTTGTAGTTTTGAGAAAAAAAAGATCCGGAGGTTCAACAAACGGTCCTGTGGG

							AGGTTCTGCATAATGAAGGTAAATCTTTCCGGCTGTATTTGGAGGGTGGGGAATCCAACCATGAGGTTGAGAAGATAGGGTAAGTTCCTGATCCAAAGTCTGTAATGATGCATGGGGCGATGATGGAGACTCATCTTGAACCTCAACTTTGGTGGGAGGGCCAGCCTCCATACCTTCTGGACTTTGAGAAACAATCTCTTGAGGTGACTTGGTCATATATGTTGGAGTTCCCTGCACAGGCTTAGATTTAGTCGTTTTCAGAAAAAACAGATCAGGAGGTTCAACAAATGGTCCATTGGGAGGTTCTGCATAATGAAGGTAAATCTTGCCTGGTGTATTTGGAGAGTGAGGAATCCAACCATGAGGTTGAGAAAATAGGCTGAGTTCCTGATCCAGAGGTTGTAATGACACATTGGGTGGGGAAGGAGACTTATTGTGTTGCTCAACTGGGGCTCTAAGAACACTCTCCTTATGTTTCTGGGTTTGAGAAACAATCTCTTTAGGTAACTTGGCTATTTCTGCAGAGGTCCACTGCACAGGCTTAGATTTTGTAGTTTTGAGAAAAAACAGATCCGGAGGTTCAACAAATGGTCCGGTGGGAGGTTCTGCATAATGAAGGTAAATCTTGCCTGGTGTATTTGGAGGATGGGGAATCCAACCATAAGGTTGAGAGGACATGGTGAGTTCCTGATCCAGAGGCTGTAATGACATATTAGGTGCGGATCTAGACTCATTTTTACCCTCACCATGACCTGGAAGAAGAGCCTGCTTATATTCCGGACTTCTAGAAACCATCTCTCTAGGTGATTTGGCTATCAGTGTTGGGGACCCCTGCACAGGCTTAGATTTTGTAGTTTTGAGAAAAAACAGATCTGGAGGTTCAACAAATGGTCCTGTCGGAGGTTCCGCATAATGAAGGTAAATATTGCTGGGTGTATTGGGAGGGTGGGTAATCCAACCATGAGGTTGAGAAGACAGGATGAGTTCATGATCAAGAGGCTGTAATGACATATTGGGTGGGAATATGGATTCCTCTAGACCATCACCCTGAGCTGGAAGAACAGCCTCCTTATTTTCCAGACTTAGAGAAACCATCTCTTCAGGTGATTTGGCCATCTGTGTTGCGGTCCCTTGCACAGGCTTAGATTTTGTAGTTTTCAGAAAGAACAGATCTGGAGGTTCAACAAAGGGTCCTGTGGGAGGTTCTGCATAATGAAGGTAAATTTTGCCTGGTGTATTTGGATGGTGGGGAACCCAACCATGAGGTTGAGAAGATAGGGTGAGTTCCTGGTCCAGAGGCTGTAAAGGCATATTGGGTGGTGATCCAGACTCATCTTGACCCTCACCATGAGCTGGAAAAACAGCCTCCTCATATTCGGGACTATGAGGAACCATCTCTTTAAGTAATTTGTCAATCCGTCTTGGGGTCCACTCCGCAGGTTTAGATTTTGTAGTAGTTTTGACCAAAATCAGATCTGTAGGTTTAACAAAGGGTCCTGGTGGAGGTTCTATAGAATGAAGGTAAATCTTGCCTGGTGTCTTTGGAGGGTGGGGAACCCCACTATGAGGTTGAGAAGACATGGCGACTTCCTGATCCAGAGATTTTAGTGACGTATTGGGTGGGGATGGAGACTCATCTTGATCCTCACCTGGACCTGAAAGAACAGCTTCCTTATATTCTGGAGTTTGAGAAACCATCTCTTTAGGTGATTTGGCTATCTCTTTTGGGGACCCCTGCACAGGCTTAGATTTTGTAGTTCTGAGAAAAAACAAATCTGGAGGTTCAACAAATGGTCCCGTGGGAGGTTCTGCATAATGAAGGTAAATTTTGCCTGGTGTATTTGGATGGTGGGGAACCCAACCATGAGGTTGAGAAGATAGGGTGAGTTCCTGGTCCAGAGGCTGTAAAGGCATATTGGGTGGTGATCCAGACTCATCTTGACCCTCACCATGAGCTGGAAAAACAGCCTCCTCATATTCGGGACTATGAGGAACCATCTCTTTAAGTAATTTGTCAATCCGTCTTGGGGTCCACTCCGCAGGTTTAGATTTTGTAGTAGTTTTGACCAAAATCAGATCTGTAGGTTTAACAAAGGGTCCTGGTGGAGGTTCTATAGAATGAAGGTAAATCTTGCCTGGTGTCTTTGGAGGGTGGGGAACCCCACTATGAGGTTGAGAAGACATGGCGACTTCCTGATCCAGAGATTTTAGTGACGTATTGGGTGGGGATGGAGACTCATCTTGATCCTCACCTGGACCTGAAAGAACAGCTTCCTTATATTCTGGAGTTTGAGAAACCATCTCTTTAGGTGATTTGGCCATCTCTGTTGTGGTCCCCTGCACAGGCTTAGATTTTGTAGTTCTGAGAAAAAACAAATCTGGAGGTTCAACAAAGGGTC
MSTRG.20783.1	--	0.144	2.368	0.000	4.406	up	GTCCCGTTGTTGCCATCCTGGGTTTGGCCTCTCCACACACTCGGAATCCCGTTGGACTTTGGTGTCCTTTTACAAAAAGAAAGGAAGGGCTGCCATTCTTTCCATTGTGCCAAAGCTAGCAGCTCACTTGTAGTGCTTTAAAGTCTGAACTCAGGAATCTGGAAGGCCAAGTAACAGAACACAGCTCAGGAAATTATAGGTTTCATATTTCAGGAAAACTGGTGAATTTAGACAGCTCGAGCATCTCCTTCATGTTAGGGAAGACAGCCTTGGCCTCTGAGTTCTGGCTGACAAAGGCTTGCTGCTGAGACGAGCTTTTAGTGCTCAGACTCAGATCAACATGAGTGTGGAGAAGCAATATCGACATCTCGTTTGTCCTTGTCCCAGGGCGATTGCAGAGCCCTCCACCAGTT

							GTGTACAGTGGGATGAAGAGGCAGGTCAGGGCCCTTGTGAGCCCCAGATCTGCTTTCTGGTTTATAGTCTTCTCCTAT
MSTRG.20783.3	ENSRNOG00000004807	0.326	0.975	0.018	2.243	up	GGGCAGCACCATTCCTAGGCTGTGTAAGACAGCGAGCTGAGAGCTAGAGAGATGGCTGTTTCAGAAGACCCAAGTTTGGCTTCTAATTTACATATCACAACTTCCTGTAACCCCATCTATAGGGAATCTGGTGCCCTCTTCTGGTCTCAAATATCAGCTCACCCATGGCAAGTATTCACACAAACATGCACCTAATAAAGTAAAAGTAAGTCTTTAAAGATGTAAATAGAAGAAAGCTATTGAGCGAGCCAGCAGACAGACTTCCCCTCTGGATCCTACCCTGAGGTCCTACTTACGTTTCTGTTCAAGCTTCCATCAAGGATGGACAATGGACTAGGGCTTGGAAGAGCAAGCCGAAGAAATTCCCTTCTTCTGCTTTTGGTTATGGCATTTGTCATAGCAGCAGAAAAGCAAGCAAGAAAAGTGGACAAAGGCGTTGAGTATGAACCACTTCAGAATGGCCACTTCCCATGTGGCTAATTGAACGCTAACCACCAATGTTGACCCAATGGGGCCACTCGCTTTTTTGTTTTTTGACAGTACTTAACTCTGAACTCAGGAATCTGGAAGGCCAAGTAACAGAACACAGCTCAGGAAATTATAGGTTTCATATTTCAGGAAAACTGGTGAATTTAGACAGCTCGAGCATCTCCTTCATGTTAGGGAAGACAGCCTTGGCCTCTGAGTTCTGGCTGACAAAGGCTTGCTGCTGAGACGAGCTCTTACTTTAGTGCTCAGACTCAGATCAACATGAGTGTGGAGAAGCAATATCGACATCTCGTTTGTCCTTGTCCCAGGGCGATTGCAGAGCCCTCCACCAGTTGTGTACAGTGGGATGAAGAGGCAGGTCAGGGCCCTTGTGAGCCCCAGATCTGCTTTCTGGTTTATAGTCTTCTCCTAT
MSTRG.20797.1	ENSRNOG00000005104	0.865	0.000	0.000	-5.253	down	GACAAGCCCAGGTGACTCTAAGCAGGAGCTTGGTTGTGGCAGGAGGTTGCCCTGGCTGTAGACAGGCCCTGCGGATCCCAGCCTTAAGGGGGTCTTTCATTTTTCTGCAAGAGGCACTTACCAGGGAGAACTTTGCGTTAAGTTAAAGGCGGATAGACTCCTGCCTGCCCACCTCTTGCCTGTCTTTAAGCCCCCCCAGAATACACAGCACATGCAAATCTAAACTCCTCTGAGGAAAAAGTTTTATTCAAGGTCCAACAACAGAAAGAAAGTTTGAAATCCACAGGAAGTGAATCTGTAATCACTGATAATGGCACGTCATCTGTGGCCCTTCCACTCCCTGACATCGAGAGAAGGAGGACATCTGTGATCGCTGGTCCTGGTGCTCCTTCTCCTTCCAGCCTGTGGCTCAGTGTTGTTCTCCCAGGAAGAGTGGGTGATGCTGTCCTGGTACCTACGCCTCAGCTGGGCCGCAGAGGACCCAGGGATGTACTTTGAACTTGATGTCTTCCTCTCTGGGACACCAAGGATGTCAGAAGACTTCCAGGCCCATAACTGCCTGTCGCATCGTCTGCAGCCAGCATAAGATGATACGAAGGAATGCTGGGAAGACTCACAGAAGGGCGCCATCTCCAAGACAGTTCTTCCCTCAGAAGGCTTCAAACTGTATAGACTCCGCAGTCTTTGTCCCCTCTGAGTTGTCCCCACTAGTATACACTAGATCCAGGAGCTCCACACACTTCCTCTCCCCGCACATCCCCACTGGAGTTGCTTATGTCTAGTTGTCATCCTCTTGTCTTGTTTTGTGTGCA
MSTRG.20964.4	--	0.014	0.238	0.005	3.231	up	TGAAGAGTAAAAATCCGTTTTATTTAATACAACTCCCCTCAGGGGCCCTCTAGCCGGGTGGCTGAGGGAAGGCAATGGGGGTTAGGTATGAGATTCCATGGCAAAATGGAGGCAGCTCCCATCCGCCCACTCAAGCCACCCTCGGAAACAGCTCAGACCTCTGGAGACTGTTTCTTTAGCAGGGAATAAATAAAGAAAAATTAGTGAGAAAAGTACACGTTTTCTTTTACTATTAAAAAAAAAAAAAAAAAACAAAAAAAAACAGAAGGCCACTGTAGCCAACAGTTTCTCTTCCTTGGGCCTTCAGCACGGAGTGGAAAAGAAAGCAGTTTAAGGATGTCTGCCCTCGCTCCAGTCTCGAGTTTTGCCATTGCCGGCTTGAGGCCTGTCTTCAAATGACCATTCCGCGGGCATTGCTCCCAGTCAGCTGCTGCCCCTTTGTGTGTGTGTGTGGGGGGGGGGGAGGCCATGCAGGGGACCAGGCAAACCTGAGAAGCACTGTACACCTCCAAAGATCAAGGTCAGACCAGAAAGAAAAAGAACAAAGAAACATAACAAAACAAAAAACTAACCCAGTATTTGACGGCAGCAGAGCAGAAAGCGGCTTCTGCGCGGGAGGACCCACCAAGAAGCACTCTGTCGATCCGATCCGAGACACAACATCTGAGCCTCAAAACACTCCAGGGCGGATTCTCCTGGAACCTCCTCTCACCTCCCAAAAATGCAAGGATGTTCCAGTTTAGAAGACAGGCTGGGTGGGCTGGCCATTAAACAGCGCCTCCCATTCTCTGCACACCCAACATTAACAAGGCAGGAG

							GGATTTACAATTGGATGGCTTGGCCTGAAGGAGTGCTACTTTGGGTGGGGACAGGGCTGGTTCATAAATAAGTGTACAGGACTCTTGCAATACAGGAGTCGGCTGCTCTTCTCGGGACACAGGCGACCTTCCGGGTACGTGCATCTGCCTTGGCTGTCTTAAGTTCCATCAAGGGAGCCTTCCGTTCTAGAGAATCTGTTTTCCACGAAATAGAAATCCACCTGCTTCTGCCTCCGGAGTGCTGGGATTAAAGAAGCGTAGCACTACACCCGGCTTCTCC
MSTRG.2105.1	--	0.330	0.000	0.006	-4.200	down	CGGTAACCAAGACAAATGGTTATGTGGTTAGGGCTGTGGGTAAGCTGCCGGAATGTATTCTAAGAAGTTTTCTAAGTGCCTGGGAAGTTCCAGATAATTCCTGTCTTCCCTTGTATCCGTTCCTTACGGTCTTATTACCACCATAGGGTATTTGAAGTAGAATGAGAGGAAAATATCAATGTTATGACTCTGAGATAAATATATTAATATACAGCATCGTAATGATTTGAAAAGGCTACTTTAGGATTTAAAATTTTTTTCTTAGAACACTACTGTTTTCCACTTATTAAAAAACAGAAAAACAAGGTGCTATCCCATAGCTCATAACTTGGTAGTATAATTTATCATTACGCATTAAGGATAGTTTGGGACCCATAAGTTAAGCTTAAAGTGAAAGGAAATTAACAGCATTCAAAATGCCTTCAGGAATCTAGACTGTATTTTAAGGGTGCCCTGTCAGATCACTGCAGGAAGATTACTGTATGGACCAGGTGGGGGCAGCAGAGAGCCAACCGAGGTCTATGGAATGGACCGCCAGCATCTGTCTTTGGAGTACTAGGTGTCTGTGGAGAGGCAGATAACTTTGGAAGAGGAGTCAGGGCTTCAATTCTAGCTGAGAGGGGAGGGAAGAAAAAGAAACGCCATGGAGGTTCCGAATGTATCGGGAGCAGGAGTTGAAGGACTGCACAGTACGCAGTAGTACACAGTATACGGTATACAGATGTAGAAGTACTCTAAGAACGTGGTACAAAAAGCAGAGACAGAAAAGGCCGGCCACTGCTTCACTAACGTTCCTTTTGTACCTTCCCAATATGGTGGGTCAGCTCCTTGTTCAATCCTGCTGGATCTTCCAAAGGCAGTAGCTGCAAATGAACTGAGACTGGAGGAAACCCACCCACCCACTCCTCTTGACCTGGGTTTGCCGCGGAGGATCTGGTTTCCATTAAAAAATACTGAGCCGGGGGCTGGGGAT
MSTRG.211.2	ENSRNOG00000023549	0.094	0.356	0.003	2.586	up	GCACTTGGTCATATTTGGATTATATGATGAATGCTTTGTAGCAAAATGAATGTAATAGAATTAAAAATTTAATGTTGAAGAATTATAATATCTCTTAGTTAATGGTATCGCACATGAATTTTCAAGCAATGTAGAAAGGAACATTGTACAGCTATCACTCAGATAATCATGCAGAAATCCGGCACGGTCATTAAGTGTTGTAGATGGTTACAATGGCATTTTCTAGTATGACAAATAAGGATATTTGAAACGAGCTCACAGGCAGAGGCTCTACTACATCTGATTACAAAGTGGAAGGCATTCATAATAGAAACAGATTGGAATAAATCATGAAATCTACATTAAAAATACACTCCCTCACATTAAGTATTTTCAAGTTCCTTTTACAGTGAACATAAAGTCACGCTGCCGCATCAGATCTGACCTTTCTGCGTGGCTTACCTGAATCAAATGAAAGGAATTTAAAAATAACAATGGGAGATCCCATACCCAAACAGAGTTACAGCTTAGAACCATGACACCTTTGTGTTACTGCTGCCCGAGCTAACTCGATGGAGTTAAACACACTACGTTTGCTTACGCCTCCATTTTCCCTCCCCGTCAGCGGAGTGGTATAAATACGTTGCCCCTGGCCTTTTCAAACCCACGCTGCCATATTTCATTTATGGCATCCAATAAGGTCAAAGGCAAAGAATAAAAACTACCCACAAGGGCTAACTTTGTCAATTTAAAATGTGCTTCACCATCCAGCCCATTGTTAAGCAGGCTGTTCAAATAATGTCTTGGTTCACATGTCTGTGGGGCTCTGGTAGGCTTCAAAGTCTCTGGGAAAGCCTATGCAAGTTTGAAATCTAACCCCTTTCAACCTTTCTATCTTTCTGTCTCCTGTTTTCTGATCTCTTTAATCCAGAATCTTTAATAGAAAAATTCTACTGCAATGCAGGAAACAGCAGGGGCTCAGAATCCCATTATGGGTTATTTAAAGCAACATCTGTAGTCTTTTAAACGTGTCAAGAGCAACTTAGGTGACCTCTGGGGACTCTTCTTCCAACACATCCTATTAAACACACATAAACACAAGTAAAACATATTTTAAGGTCTGATTATAGAAAAATCCTCCGCAGAAACCAAGCTAGCACTAAACGTTTCAATGAAAGTCTGGTCAAGAAAAACAAACAATAGCCCTCAGTCTTATGTAGATCTCAGATTAATTAATTAATGCAAGCACAGGGTTGTTTTGTTAGTTTTGGATGAGGAATATGAAGGAGCAGATCCTAGAAGGCAGCCTTGCAGTGTCTAGTGCTTCAAGGAGCGACTGTGTCTACCAGAACCAACGACCTGAACAAATACTGTAAGAAAGTCACATGTATGTGACTCTCCCTCTACTTAAATTAGAGGATGATGCTGACAGAATTTTCTAGGTCACCTCTAAAAGGTGAAAGACTCAACTATCAGAAACAA

							AGGAAAACCATCCAATGATGTCCAATACATCATTTACATTCTCCTGTGATATGTATGAGTCAAAGCATTCA<br>GTCTTTTTCTTATTTCCTTTCTATTATCATGGTACAGTTGTTCTGTTAATGGAGAACTCTCAGTGTTAACA<br>GCATTACCTAGAATGATGCCAATTCCTATCAGTAGCTTATGGAAAGAAATCCATAGCACAATACATTTATG<br>ATTATAACTAGAGAGATAGGAGCGTACCAGAGTTTGTAACTTAATAGAAATGTTGTAATACAGTACACCT<br>TAATGATGGTAGGTAGTACCTCTAGTCCCCTAATATTGTACAGATAAAGCCATTGTGTGTGGGAGGAGAA<br>GACCAGAGGATGAAAGAGAAAGACAGAGGAACACAGCAACTGTAACAGAACAACACGGCTCACATA<br>AGTAGCAAAATCTTCAACAAAATACCTCCCTCTGGAGTTTTATGATTTTTATAATAATCAGAGAATGAGA<br>GGCATTTGGAAAAGCTCAATGACAGCGGAAAAAAACTAAAATAAATAATAACAAAGAAAATAAAACCC<br>ACATAAAAAGAATGTCTCTAAGGCAAATTTCATCTACACTCAATTGGCCACTGGACCTTCTTTCAGAACT<br>TAGAACAAAGTTCATGACAATGCCAAGGAGTTATTCCTCCCACCACAAACATTCCTCTAAAATAGATTTA<br>ACACCAACATTGCCTGCTACACCAATTTGTGCCTTCCACTTCGGCCTTGTTTCTTACAGGCACTTCCCAT<br>GTAACCTTCAGGTAATACCAGATCTTACAACAAAGCAAACACTTACCGGACGGATCAATACTCCCCCAA<br>GCAATGAAGAGGAAATACCACTTACCGAAATGGCTTCAGCTTCTCTCAGGAGTCTCCAAAGAACTAGC<br>AGAATGGACCCCGATTTTCTTCATATGACCAACGTTTGTAGAGATGCTACTTGGTGATCACTTTCGCCAA<br>TTCTCAGTGAAGACTCTTCCAACTTTTCTTTCCCCAAAGCCAGTTGTTTGGATGGATCCTGCACTGTTCC<br>CTGCTTCCTGATTCTCCTCTTTCTCTTTTGTTTGCAGTTCTGAGGACAGAACCCAGACCTTCACAGCAGA<br>CAGCAAGTGTCCTACCAGAGAGCTACATCCCCCAGTCCTGCTGCTCAATGCTGGAAAGATTCATGTATT<br>TTCATAAATCTACTGTCTGCTCAATAATTCTCCTTAAAGTGGAAACAGATATTGCCAGCGTACACCTTTGT<br>GGCAGAACCAGTTCGATGAGGACTCGTCGTACAAGCTCTACCCCATTCAGCTTCGGAACTCAGAACCC<br>GGCATAATTAAAATAAGTGACATCTGTAAACTTCATTATAATCTCGGTTTGCTTTCAATATCATATTCCTCC<br>AAAACCTACGGCAAGCCATGCTAATAAAGAGAGTGGGGGACGACTGCATCCTCCTTTCTGCTCTTTCCT<br>GACCTCCAATCGAGTCCTAACAAGCAAAGAAATGAACGAGAGCCGAGGGAGGGAGGGG
MSTRG.21291.1	--	0.380	0.000	0.003	-4.429	down	CTGGCAGGTAGTAGAGGTTCAGGAATGACCTCTTCCCCATCCCTTCTGCAGCCCTGGTTCTTAACCATG<br>AAGGAGCGAGCAGCCAGGGTCCCACAGAGCACACAGAAGCTGAGCAGGGACTCCGTACGTTTCAGAA<br>GTCCTTCTTCCTAGGACTTCCTCTGGACCTGAGAAGGGAGAGCTGATCATTGCAGCCGTGGCGGACCA<br>ATTTGTTTGCGTTAATTGCAGCCAATTAAACTTTTATTTGAAAGCTTTCCTAGGTCAGACCCTCTTGGGG<br>AAGTTGATTTCTGGCCAGATGGCAAACCGCTTTTCCGGGCCCTCACCTGCGAAAGCCAGAAGAGGGTG<br>TCAGGTTCTGGAGCTGGAGTTACGGGTGGTTGTGAGCCTCTGGGTGTGGATGCTGGGAACCAATGTTG<br>GGTCCTCAGGAGGAGCAAGAAGTGACCTTAACCACTGAGCCATCTCTTACGCTCTGTGTGCTTCATGTA<br>CTGGTGAAAGGAAGCAAAGGAAAACGAATCAGGGCAAGATATAGCCCCAAGGACACAGCCATTCCCA<br>TGACCCCCTTCCTCCAACTGCTTCCTACTTTCCTCACTTACAGATGATTCCATCACATTGCTGATCCATCA<br>AGGGATGAGGTCAGAGCCCTCGGGATCCTGCAAAGGCCTCCAAAGGCCTGTTAGCTGGCAGGTAAGTC<br>CCTGGTACATGAGGCTATGAGGATCACTTTGCAGCCAGACCATACGTACCACAGAACACCTCACGACAG<br>CCACAGTAGAAAGAAGAAGTATTCGGGGGGCTCTTGTGTGACGGTGTCTTGGGAACACTGGCTTCTAG<br>AGATGCTGAGGTCTCTTATATATGCAAGCTATGCATCCTGAATGTGAAAATCCAGCATCCCAGGTGCTCC<br>CAAACCACGAACTTTTCAAGCGCCTTTGAGACTGCGCAAGTGGAAAATTCCACACCGTGAAACTTTAC<br>TTCCTGCACAAAATCACTAAGCATATTGTATAAAATTACCTTCGGG
MSTRG.21304.1	--	1.496	0.162	0.014	-2.380	down	CTCTCTCTCTCTCTCTCTCTCTCTCTCTCTCTCTCTCTCTCTCTCTCTCTCTGATGACTCTAGCTTGTGTCA<br>AGTTGACACACAAAATCAGCCAGGACAGCACCATCCCTGGCCTCTCTCCACTAAACGCCAGGAACAAA<br>CCAACTATAACAGTCAAGTGTTTCTACAGCTAGGGGGATGGCTCGGCTGACAAACAGCTGAGTTCAATC<br>CCCAGAACTTGGGGATAGTCAGTCACAGTGGTGTACACTCAGAATCCCAGCACCGGGGAGGCAGAGA

							CAGATGCATCTGGGAGGCTTGCTGGCAAGCCGGTCTAGCCAAAACAGCATGCTACTTGTTCTGGTGAG<br>AGACCTTGTCTCAAACACAGGGTGGTAGGCTAGGGAAACGGTTCAGTAACAAAAGTACCTGACCGTGA<br>ATCCCTACCATCTATGAAAAAAGAAAACTAAGGGTGGAGTCTATAGCCCTGGGTGCTGGACGGTGGAG<br>GCATTAGATCCTGGGAGCTTGCTGGCCAGAGTCTAGCTGTAGGTTTATGCTCTAGATTTATTGAGAGAGT<br>GCCTTTCTCAGAAAATAAGGTGGAGGGCCATAGAAGATGCTTTAAGTCAACCCCTGACTGACAAGTGT<br>GCACACATGCATACAGTGCATGCACTACACAGTCACACACACACACACACACACACACACATATACAC<br>ACATACACACTTTTCGCATGCATATGTCTACACATACACAAAACAAGGTCTGAAGAGCAATTGAGGACA<br>CACAATGTTCACACATGCACACACAAATGTGGGCACAAACAACACATACATTCTATATCAGATATCAATA<br>TGTATTCTATGTACAATATATATCATATGCATATACACATTATACACACACACACAAACACATACACACACA<br>CACACACACACACATTATACATATATATTATACTACTCCAGTTACAGGCGCTGTTCAGAGCATGCAGATCC<br>GAAGAGACAGAGCTCAGGAGAGTGGGAAGGAGCAGAGGTTGAGTTAAGGAGTCAATGAGTCAGCTTT<br>GAGGCACCGTCGGAGATGCTGAACGCTACTGGATTATAGATTTTCTATGGCTACTTTCATGTTACAAGAC<br>TTCACTTTGCCTTTTTTAAAAGTCCTTCCATCTCTTAAGTTTCAGTCTCTTCCTCAAGGTCCACACAGAA<br>CACCCAAGGGATTGAAAAACGGGTTCTATTCTCCCCAGAGACACACCCCAGATAAAGGAGAAAATGGA<br>AGGGCCTGCCTCTGCTCTGCCAGTTGAGTGATTGCCACCAAATTGGGTTTTGTGCAGCATAAAGGTCCC<br>TCCAAGGTAAGGGACGTCGTTACCGAGGCACTAAGTGTGACCTTGTTGGTATTCTGAACGCAGCCGGG<br>GCGGTGCTGGACGCAGAGTGAGTGAGCTGAATGAATGTGTGCACATTAAAGGCAAGCCTAGACAGGAT<br>CCTAAACACCGAGCCCCAGACCAGCCATTATCACCAGTATGATGTAAATTAGTAACGAGGCAGCTCTCC<br>TCAGCTTCCAGGGTACCTGACACACCTGCAGGCACCTGCTTGGTCAATCCTCTCTGCAGCCTTGATTGG<br>AACCTCGCTGCAGATGTGCGGCGGGAAACCAGAACTTTCCATAATCTGCTGTGTTCATTGCCCAACACT<br>GGGGAACAAATCATGCCCCCCCCAAACCTGGCAGCGGAAAGCACCATTCTTCTCAGACGGTCTCTGAA<br>GTCTGGGCATGGCTCTACCGGGTCCCATCTCGGGGTGTCTCACAGGCTGCATGCAGATCAGGAGACAG<br>AGTCTTTTCGGAAGGCTCAGCAGAGGACGTCCACACCCAGCTGAAGATGGTTGTTGGCAGGATTCAGT<br>CCTGGAAGGCACGTGATCAGCCGTGCTTCATGAAGACTGAAGTTTACATCCCAGAAGCCATGTAAATCC<br>CAGGTGAGCCCAACAGCCCACCTGCAACTCCAATGCCTAAAGACAGAGACAAAGGGGTCCCGAGGCA<br>GGCTGATGAGCTAAAGTCATTAAACAGTGAGCTCTGGGTTCAAGTGGAAGAGCCTATCTCAGCAAATG<br>GAGTGGCGAGAGAACGCTGAAGATTCCAGACTCATCATCACTGGGCTTCTACACACATGTGCATATGAC<br>TAGACATCCCTACATTCACATGCGTGCGCGTGCACACACACACACACACACACACACACACACACAAG<br>AAAAAATAAAATTAGAAATTAACAAAATTCAAATCCATATTTAGCAAGCTGGGAAGACTCTAGAAGCTA<br>CCTATGAGGTAGGAGTTGTAATCTGACACAGCCTGGTTATAGAAGTGACACAGAGTCACCTTGTATGCC<br>CTGTGAGTTAGAAGCAAGTCACTCAGCCAGCCCATTTTCATAAGAGGGCTGTTACAGTAGGGTATGACC<br>CCCTGATATGGTGACCAAAGGTGTCCCCAGCTCAAAGTCCGCCTATTACGCTTGTTAAGGGACCCCAGC<br>TAGTAGAGAGTCCAGCTGCCTGTGCCCTGGGGGTTCTAAAAAGGGCTCAAGGTCATCTCCTTGGTTTTC<br>ACTTATCCAGGTATTTCAAGGGTGGAAAAATGGTGAAGGAAGAATTCAAGGAAGGAGAAAGCGGGGG<br>AGGGGAGATGCCCAGAGACTGAGACAGAGAGAGGGAACGCCAAGCTCTTCCCAACCATGCACTCTGA<br>TGAAGCTGGTCCTGCCGGGCACAGCTGGATCCCCCGCTGCCTCATGGTGTCTTCATTAGAGGCGTGTGA<br>AAATCGGCACATTTTGTTATTTTGCCT
MSTRG.21307.2	--	2.012	0.048	0.000	-4.295	down	GGAAAATGAAGACACTGAAGTATGTTTCCCATAGCTACGGGGCGAGTTATTAAGTGACAGAACTGGGA<br>AAACACCAACTCTGGAGGCTCCTGTCCACTGGTGCACACAACCAAGCTTTCTAAGGATCTGGTCCTGC<br>CTTTCCTACCTTCCCATCAGCCATGGAGTTCTCCCATCAGCCATCTCTTTCAGCAATGGCTCCAGCCTCT<br>GTGGCAGGTGGGGAGTCAGGGTCTTGGTAGAAAGTCTTAACACGCTCCATCATCATGCTTTGGAGGTCT

							TAATGAAAAGACCATTGGTGATTCTGAAACAAAGCTGGAGATCCAGGGAATGGAGAAGACAGGCAAGGAAGGCTAAGCAAGAAAGGGAAGCCAGTAGCCCCTAGGGCTGAAGGGGAGGCAGGAGATGCTTTGAAACAAGGCAAAGGCTCACTGACCATCACTGCTGCCCACCTCCTGGCAGGGAGAGAGCCAGGATCAAACCCATTGAGCCCTCCTCCCTGTCCCGGTCTTGCTAGGTAGAGCCAAGGTCAAAAGGCTCAGATGTATCCCTCAGAGGCTGGCCCCAGCCAATCATGGAAAAAGGGGAAGTTCAAGGGGATTCAAGAGCCTGAGGGAGGTGAGGGTTACAGGCTCCTGGGTGGCAGACCCTGGTTGACTGAAGTCTTCCCAAGTCCACTGCTGAGGCCCAAGTCACAGCAAGGCATTAGGCGGTGCGTGCGGGTTGTTCTGGAATGATCATCTGAGAGGCTGCCAGCAGTCTCCTCGGCGTTTTTATAATGGGATTTTAATTCATTCCCCCATCGCCCTGCTGAGCTGAGAAGCGCTCCTGTTTGTCTACTTCTTGTTAAGATGCTGCTTGAAACCGGAAACAGGAGGCAAGCCCTCGGGTCCGGTGGCCTCAGCGAGACCCATTCACACTGCCCTCCTTCAGGTACCCCCAGAAGGGGCTCCAAAGGTCCTGATCCTACCTGGGGATGAAGTCTCATCTGAAGAATTATCTAGGTCAGTTTTGTTTGTGGCTTTGTCCGTAGAGGATCATCTTGATTGTTCATTGAGCCAGCCCCACTGAGTGTGGACCCATCCCCTGGTTTAGGGTCCTGGCCAGTGTGAAAAGTACTGAGAGCTAGCTGAGTAGCAAGCAGGCAAAGATTCCTGTGTCTATTCCCCCCCTGCTCAGCCATGGACAGGACGGTTTAAGTTCCTTGACTTCCC
MSTRG.21640.1	ENSRNOG00000003386;ENSRNOG00000027498	0.414	0.027	0.013	-2.814	down	AGGGGGGCGCCCGGCGGCCGAGGCGGCTCTGCGCTCGCTGCGTCCCTCGGTGTGTGCGCGCGCCCTGCCTCCCGGGCGCCCTCCCGGTCTCTCCTCCTCCGGGCCTCCCCTCCCCCGCGTGGCGCTCGTTAGGAGGGAGGCATGGAAAGGAGAGCGGCGCGCCTCTGCCGGGGGCTGCCCCTGTCCGGGGCGGGGAGCCTGGAGAGCGCGCGCGAGAAGATGGAAAGAGGATGGATGCGCTGCGAGAGAGGATGGATGTGGGGAGAGGGGAGCCAGTCACTTTGGAGCCACCGGAGCCCTGTCTGCAAAGAGTGGCAGTGTTGGGCAGAAGCCTTGATTTGCCCCTCACAGCACCACTGCAGCCTGCCTGCATACATCTGCAGCTTTAGAGCCACACGCATTTGGGCCTGCTCCTCTCACACTCTGTTGTCACCAAGACAAGCCACAGACTGATGGCTCAGGTGATGATGGCCCCCACTGCATAACACATGGAGGACTATCAAGAGTGTCAAAAGATGCGTGACTGAGTGTGGCTTCCCTCCAGGCACTGCCTTGGAAGTGACATTGCAAAGGAACGGGTAGACATGTGCAACTTGGATGACCTCTTGTCAGGCATCTTTGACCCCCACACTCACAGCTATAAATCCTGCAATTGACCTTCCCTCTAGAAGAAAGTAGACTGTGGAGACTGCCATGCATAGGAGAGACCAGAGACAGGTGGCTGGTGGCTCACCACACTCTTCCCCCATTGTCTTGCATGTCTCTGGGATGCTTCTGGGGTAGCTCAGGTGGAATGTGGGTTATGGACCTTCTGAGGCCACCCTTATCTTCCGCAAGGAATAGGTCACTGTTTTGATCTTCCAGGCCTGGACAGTGGCAGTATATCCCCAGCACCAAGTACCTCTGGGGCATGATGCTTCAAGTCAGGTTTGTGAGCTGAGACCTTGGAGGCTGCTCTGCCGGTGCTGTACCCTGAGTAGGTTTGGCTATTCAGTACAACATACCCACCTGCCTCCTGTTCCCTCAGCCTGATGTTTTACTCTCCTGTTGTACACCTGTCTGTCCTCTCAGAGTGGCTTTCGGTCAGCACTCTGCCCAGATGCCTTTTCCACAGAGAGAACTTGGGCATCCTGGTAAGGCTCACCTGGAGCCTCCAGACAGACCACCATGCAGTCACTGTGTGTGGCTAGGAGCTGTTGAGACTAAATCGTCTTTCCTGGGTGGAGTGAGCTCACGGTCTAGTGTGGAAACCAAATCTGCAGCCCAATAGCCAAGAGAATCTAGCCAGGGGAGCCCGTCTAGATACTTGCCTCCACCAGTCACCTCGCCATGCACAGGCTCGCTGGGAGACAGTGAAAAAGAATTAAAGGAAACCTCCAGAAAGAGTTTGAATTGTGTGATTGTCTGGCTTATTTCTCTACTTGTCATCTTCTGCAAGTGCCGCGTCACTTCTGAAACAAACCAGTTACTGAATCCCACCAGTCCCATTCTAAGGGAAGTTGCTGCTGAGGCTCTTGGCCTGCCCATACAGGCTGGAATCATTCTCGAGTGCATCCAGGGCCCAACAGAGAGTCACTGTACCAGCCCAGGCCTTTCACTACTCTCCACTCCCCCCAAGGTTCCTGACCACTCTCCCATACTCCCGAGGTCTTGTGAACCAGGATGTGCAGCCTTGAGTAGTGGGTGGGGAAATCCAATCCACCCCAGTCACTTGCT

MSTRG.21640.2	ENSRNOG00000003386;ENSRNOG00000027498	0.316	0.000	0.001	-4.759	down	AGGGGGGCGCCCGGCGGCCGAGGCGGCTCTGCGCTCGCTGCGTCCCTCGGTGTGTGCGCGCGCCCTGCCTCCCGGGCGCCCTCCCGGTCTCTCCTCCTCCGGGCCTCCCCTCCCCCGCGTGGCGCTCGTTAGGAGGGAGGCATGGAAAGGAGAGCGGCGCGCCTCTGCCGGGGGCTGCCCCTGTCCGGGGCGGGGAGCCTGGAGAGCGCGCGCGAGAAGATGGAAAGAGGATGGATGCGCTGCGAGAGAGGATGGATGTGGGGAGAGGGGAGCCAGTCACTTTGGAGCCACCGGAGCCCTGTCTGCAAAGAGTGGCAGTGTTGGGCAGAAGCCTTGATTTGCCCCTCACAGCACCACTGCAGCCTGCCTGCATACATCTGCAGCTTTAGAGCCACACGCATTTGGGCCTGCTCCTCTCACACTCTGTTGTCACCAAGACAAGCCACAGACTGATGGCTCAGGTGATGATGGCCCCCACTGCATAACACATGGAGGACTATCAAGAGTGTCAAAAGATGCGTGACTGAGTGTGGCTTCCCTCCAGGCACTGCCTTGGAAGTGACATTGCAAAGGAACGGGTAGACATGTGCAACTTGGATGACCTCTTGTCAGGCATCTTTGACCCCCACACTCACAGCTATAAATCCTGCAATTGACCTTCCCTCTAGAAGAAAGTAGACTGTGGAGACTGCCATGCATAGGAGAGACCAGAGACAGGCCTGGACAGTGGCAGTATATCCCCAGCACCAAGTACCTCTGGGGCATGATGCTTCAAGTCAGGTTTGTGAGCTGAGACCTTGGAGGCTGCTCTGCCGGTGCTGTACCCTGAGTAGGTTTGGCTATTCAGTACAACATACCCACCTGCCTCCTGTTCCCTCAGCCTGATGTTTTACTCTCCTGTTGTACACCTGTCTGTCCTCTCAGAGTGGCTTTCGGTCAGCACTCTGCCCAGATGCCTTTTCCACAGAGAGAACTTGGGCATCCTGGTAAGGCTCACCTGGAGCCTCCAGACAGACCACCATGCAGTCACTGTGTGTGGCTAGGAGCTGTTGAGACTAAATCGTCTTTCCTGGGTGGAGTGAGCTCACGGTCTAGTGTGGAAACCAAATCTGCAGCCCAATAGCCAAGAGAATCTAGCCAGGGGAGCCCGTCTAGATACTTGCCTCCACCAGTCACCTCGCCATGCACAGGCTCGCTGGGAGACAGTGAAAAAGAATTAAAGGAAACCTCCAGAAAGAGTTTGAATTGTGTGATTGTCTGGCTTATTTCTCTACTTGTCATCTTCTGCAAGTGCCGCGTCACTTCTGAAACAAACCAGTTACTGAATCCCACCAGTCCCATTCTAAGGGAAGTTGCTGCTGAGGCTCTTGGCCTGCCCATACAGGCTGGAATCATTCTCGAGTGCATCCAGGGCCCAACAGAGAGTCACTGTACCAGCCCAGGCCTTTCACTACTCTCCACTCCCCCCAAGGTTCCTGACCACTCTCCCATACTCCCGAGGTCTTGTGAACCAGGATGTGCAGCCTTGAGTAGTGGGTGGGGAAATCCAATCCACCCCAGTCACTTGCT
MSTRG.21678.1	ENSRNOG00000046207;ENSRNOG00000047026;ENSRNOG00000048113;ENSRNOG00000049215	0.000	0.216	0.004	3.943	up	CAGGTCAACGCCCCATTAACATTTCCCCACCCGGGCGCTGCGACCTCAAGAGGACACTGGACATATTGGTGGGGGTGGGGACACGGAAAGTGCCCTTCCAATATTGTCTTCCGTCACCCCCAACCTGGTCTCCAGAGCCTGGCGATAGAGTTCCTAGCTTCTCGATGGATTTCTTCCGGTGACGAAGAGTGGGTGCCATGAACACCCACTCTGGAGACCCTCCTTCCTCCTTCATTGACCCTCTGGGAGTCCCCATCCCCCATTCACCTAGGCTTTCCCGAGTATCCACGACTAGGGACAGTGAAAGATGAAGAAAGAGGGGGTGCCAGAGGCTAGTGTGTGAGGTCTTTCTGCGGGGTAAGACCGGACTGACTCTTCCAGCCAACCCTAACTCCGCCAACGTAAAAGCACAGGCTCCGTGATGCTCCCCAATCTTAGAAGCAGCTCGTCCAACATTGCTGAGCCCAGCTGGCGGGGGCTGGGCACAGGAGCGTGTACGGGGAGCGGCCAGTCTACTAAGCCACAGTTGAAAATTGTTTCCCCAGCCCCAAGCCTGCACTCGTCTCACTCCAAGGGGTCGAAGAGAGAGAACCCTGGGGTGACCTGTCTGTACACTACCCTCGGGGTTCAGTCCTCCCCACCAATGCCTGCCAGCCCGCGTGGGTCCTGATCCTGGCCCGGAAACCAGTCTGAC
MSTRG.21684.1	ENSRNOG00000046207;ENS	0.034	0.268	0.035	2.769	up	GAGAGAGAGAGAGAGAGAGGAGATATGATAATATGGGGGAGTGTATGAGTCTGACTTGACTGGAGGTCCAAGAGGACTTCGTGGGGGTACATGTGAGAACCTCAAAATAAAGAGTAGGGAGGACCAAGTAGAAAGTTGAGGCTGAGGGTTGGTGGATGAGTGGGCCGCGAGAAGGGGAGGGGAACTGGAATGGCCAGGGAGCCACATATTCCTCCTGAATTTCCGTCCCCTGGTCTCTGACAGCCAAGCCCTGCTGACACAGCCTTTGGAGACCTGGAGTGTGAAGTTTAGCACCTCGGAGCCGCCCAGCCCCGCACGTTCAGCTCCAGCGTCCGGCTG

	RNOG00000048113;ENSRNOG00000049215						GGGGAGCTGCTGCGCCAGACGTGGCCTTCCGAGGGCGCCACCGGGGCCCAGATCTGAACCTTAGGGGCCACTGAAAGGAGCTAGGTGCACCGACGCCGCGTTTGCGCAGGAACCACTCGGCAAGCCGGGAAAGAAGAGCTCGTCAGAGGATCTTTTCTGGAGTTGCATATGATATAACCATAGGAACCTCCCGGCTCCCTAACTGCGGAGGGGGCCTCCTGGAATCACCTCAAACACCGATTGCCACCCGCCACTGCCCAGCCCTCCAAAGGCCTCGCCGTGGGCAGCTTTGGAGGTGACTGAAACACAGAAGTGCTCGCCAACGCAGCAAC
MSTRG.21729.1	ENSRNOG00000003699;ENSRNOG00000003741	0.087	0.415	0.003	2.794	up	GTTCCACTGAGCCCCGCTTAGCCCATTCCTCAAGCCCCTGGACTTTGGGGGAGGGGTCCCAGAGGTGGGCAGCGACTTCGCTTCTACAGAGGTTCGCACCTCTTAACCCTTTTCTTACAGACACCTTACCCAGCCATCCGCACACCCACGCCTCCTTTTTTTTCTGGATAGCGGCACTGCCTGGCTGGGATTCCAGTCCAGGTTCCAATGCCTCTGGGGACTCTGAACAAGGGTCTGGGCTGCAGCTCCACGTCGGAGTTAGAACTGGACCTGGGAACTCGCCCAAGATACAATTATGTTGAAAAGAAAACCAAGGCGGAGAGAACCCACATCAGGTTTTAAATGACTGGAAGAGCCCAGCTACAGGCTGGCCAGGTTAGCCCCGTAGACCGCCCCCCACCCCTTGCAGTCAAGCGTTTGGTTGCCCCACAACACCTTTCTTGGGGCGCAGCGATGGGCCAGACTGGGTCCCGACTGTGAGGCGCCAGTCCGGGACCCACCAGCAACCCTCGGACAGAGGATGCCACTCCCGGTGGGCATCGCCAGTGGTGAGAGTAAAAGCCAGATTGACAGGGAAGCTACTATCCAGGTCTTCTCCGACGCCTCTTCCCAGCTCCAGGGGCGCCCCACTGCCTGTGTTTCTCGTCTCTTCATTTAACCATCCCAGGTCCGTGCTGAGAGCTGCCGTGGCCCCTGCACGCCAGGCGAGTCGCTGGGCCGCCGCTTCCGGGCGCCAGAGGGACCCCGCGACCAGGACGCCGCTGTCCTGGGCTTTGGTGCAGGGAGGGAGTGTGGGGCTCCGGCTCCAGCCGGTCCACCGTCCGGTACAGGGCGCAGGAACGCAGCGGCTAGCATCTGGGCGCGCCCTAGTTCCCCCTGAGGCCCCAGGACTAGCCTCCCGACGCTCTGCACCGGACGTGCACTGGCAGAACTAGGAATTACCCACTCCAGGGGCGCTGCGCTGCTGGGCGACGTCTGCCCCCAACTCCCAGCTTGGTGCGGACGGACGCGGGGACCCCGCGCAGGCCTAGCGCTTCGACCCGGGCGGCGCCTCCCCCACGCGTCTAACGCGAAGCAGTTCCCGGCCTGGCCCTGCGGCGAGGCTATCCCCACTCAGAGTACGTGGGCGCAAGAGACTAGCCTGGCCCTTCCCCGGCAGATCTGGCTGGTGCGAAGGGACCCAGGGCCCTGCCAGCTCGCTGCGTGCGTCCTTGCGCATCAATTTTACCATCTGCCAAACACCAGGAGAGAGCACATGAAATATAAAATGAGTCGAGGCATAAAAGTGTTCCGGAATAAAAATGGCGTGTGCCGGCGAGGGATTACAG
MSTRG.21848.1	ENSRNOG00000036677;ENSRNOG00000045636;ENSRNOG00000046698;ENSRNOG00000047905	0.582	0.019	0.003	-3.257	down	GCTAGAACTAAACCCAGGTTCTCTACAAGAGCAACCAGTACTCTTAAACCTGAGCCATTGCTTCAATCCCTGGATTTAGTTTGTCTCACTCCGTAGCACAGGCTGGTCTTGAAGTCAGTGTTCCTGCAGTAGTCTGTCTCCCTTGTGCTAGATTAAAACATTAGCAATGCACCTTAGTTGGTACAGTGCTTGCCTAACATGTATGATCCCCGAGTTTGATCCTCAGCCCCATTGAAAATCGATATGTTAATGCAAGCCTGTAACCCCAGTTCTGGCAGGGGGGCCAGGAGGATCAACTCATTCCTGACTGTACAAGGAATTCTTCTGAGGCCAACCTGAGCTACATGAGACTTTGTCTAAAAACAAAAGCCAGGGTCCTGGGGAGATGGCTCAGGGGTTAAGAGAACTGGCTTCTCTTCCATAGGACCCAGGTTCAAGTCCCAGCACCCACATGGCAGCTCACAACTGTTTGTCTGTAACTCCAGTTCTGGGGAATCTGGCACCTTACACAGACATATGTGTAGACAAAATACCAATGCACAATGTTAGATGTTGTAGAAAGTAAGAGGCAAGAACAGATGCTGGGTTCTGATTTGGGGAAGAGGTAGCATCAGACAGAGGGATGTGTAGGAGGGAAAGGTGGCCTGCAGAAGTATGATCTATTTATGGACCTCTTCCTTCTCCTTTACATTTCCAGTGCTCTACTTCTTGGCTGGTCTTGGCCTGGACACTATCTGTAGTGGGCCTTGGGACCTGAACTTCAAGTTCTCCCAACCACTGAGCTACCTCTCCAGCCTTGTTTGTTTTTAAGATAAGGTCTTCTATAGCTCAGGCTGGCCTGGAGCTTGTGGGGATCCTTCTGCCTTAGCTTCCTGAGTGCTGGGGTTACAGGCAGGGCCTACTATATTCATCTGTCTTGCATTAAAGAACAAACAAACAAACAATCCTGGGACTCAAGAACCACTTGCTT

							CTTGTGGGAAACCAACTGAATATCAGCAGACAATGCTGCACTATTGAGGACACCTACAGTGGACTGGCCTGAGACTGCCCTTCTGCTTGCCTCACAGGGCCTCAACTGCTACACAGCTATTTCTTCCAGATGTGGCCAGAAGAGTGGGTCAAGGGACACCCAGTGCTGAGATGAAGCCCTGCTCTGTGCCTAAGGCATCTGGTTGGCTCCATTCCACCACCAGTGGCCTCTGTCCTCCAGCCTCCCTGGGAAGCCAGCCTGCAGCCCTCCCTCTGGCCCCAGGCTTGTAGCAAGCAAGCCAGAAAGCTTTTTCCCTTGGCCTCCTGTGGCAGAACCATG
MSTRG.2189.1	--	0.557	0.030	0.050	-2.562	down	GGCTGGCTTAAAGGTTCAGCGGTTCAGTCCATTACCATCGTGGAAAACATGGCGGCTTCCAGGCAGGCGTGACACGGCACCGGCAAAACCGAGACTTCTACATCTTGTTGCACGGGCTACATCAGACCCTCTCAGAAGAAGGAATTGGATCCCATGGGACTACAGTTCTAGAGAGCTGTGACCTGCCATTAAGGGCTGGGAATTGAACTCTGGACTCCTGAAAGAGCAGCCAGTAACCGCTGAGCCATCTTTTCAGCCTCTTGTTCTAAGCCAAATTGGTGGTGAGCGGTTATTGTAGAGATCGGCTTTACTGCTGCTCCTTGGTGCCTACCGGGAGTACCATTACAAGAGACTGTGCCGAGCTGGGCTTTGTCAAACAGCTTTCTCCTCTTGCTCGGTAGAGGTCGCTGTTCTCCATGTGCCTGGCGTCTAGGGGAGAAGAACCGATTCGCTTTTCACCCTGTTCTACTGTGTAACCCTCTGAGGTCCCACGTGGCTTTCCCCTAGCCAGACTCTAAGCTTTGACTATTGCCCTCTTTCACTTAGGAGCCCGTCAGAATCCAAAGTTCAGGTTTGTCTGGGATGAAAAAAAAAAAAAAATCAACCGACTCTCAATGCAGACTCAATTTCAGGGTCCCAGGAGACTCGTGGTGTCTCCAGCCTTCAGTCTTTGCTCCAGTGCGTTCAGAGACAACCAGACCGTTTATTTTTTTTTCTTTCAGCATCTGTTGCTTATCAATGAGGCTCCTCGAATTGCAGGAAGCTTATTAAGATTTTGTATTCTGATTCTGTCAAGTCCACTTTTTGGCTGCCTATCTCTTGC
MSTRG.22154.1	--	0.321	0.000	0.037	-3.684	down	GTGAAGCATTCACATTTTGATCATCCGTCTTGAGTTTCGTTTGTTAACTGGATATTTCTATCAGAGAGTACATTATTAGCCAATGTGTAAGTGAAGAAAAGCAAGGAATGACAAACAAGGAGGAGCTGGCCATTGCAAATTGAAACCGTAACCAAAGGTCAATCATGGTAAATGATTGTGATTTTCCCAAATACTGCTAAAATAAGATCTCTGTGTTTGCTCTACCAGGGCACTAATAATTAATTTTCACAGTAGCAGGAGAGTTAAGATGACTTTGACTTTGTTATTCATAGTTTAGTTCCACCAAATCAGGGCAAATTTCAGAATAGGCCTCTAAGGAAAGTTTAAAATGGCAGAAGATATGGTAGCTGGATTCCTCCATTAATCTCAGAAACAGCTTAAGGCTTTGATTCCATTTCTTGCATGGCATGGGCAAGGATATTTCTGAAAGGATTGGGGAAGAACAATTAAAGTATGACCCTTCTCTTGCTGTTAATCTTACAAGCTAATTGATGTGTCCCTTTCCTCCTAAATCCCCAATTTATTTCTAAGTACAAGAGTTTTTGCCTATTGATTTACATATAGGAATCAGCAATGTGTCTTTGTTATTGATTGCCTGCAGCTTTTGCCTTGGGTAGCAACAGGTGCCCCTGATTGCTCCCAG
MSTRG.22195.2	--	0.733	0.000	0.003	-4.375	down	TTGTCTTACCAGCTATTTGCATTCTCTTCCATGCAAGTCTATCCAGGGTGGATTTGTCCTGCTTTTGACAGGCATCCGTAGAACATTGATGACTCCCAGCCCCATGCTCATGGTTGGAATTATCCGTTTACTCATGTTCTCTCACTAACATTGTCTTTAATATTCTCACTAGTACTGAATAATAACGCCAGTATCATACCTTCATGACTATGAGAACACAATTGTGCCAATCAATTTCGGTGAAACACTTTGTCTTGACCTTTGTATCCAACGTTTCAGGACTTTGTACTCTCACTGTATTTCTCTTAAGCTGTGTTACCGTGTGCCAGGTACAACTCCAGAAGAATTACGAGGTCTTTATTCTTACCCTCTATACGTTTTTGTAATTGCAAAAATTTCCTAAATTAAGACGGCAATGCAATAATATTCCAGAAATTTGAACCCTAAAATTTTATTGTCCTCCAAATTTGTTTCCAGGCTTACCTCTGCATTATAACCCTG
MSTRG.22636.1	--	0.835	0.065	0.008	-2.747	down	TTCTAGAAGAGGGAACTTGAGAACCAAGGACAAGAGTGGAGATACTGTTAGGGTTAGAATGTGAAATGTTCCCCATCTTGTTTGAATGAACTTGTATGAATACATAGTTCCCAGCTGGTGGCGCCATTTTGGTATGTTGCCAAGCTTATGTGAAGAGGAGCCTGGCTGATGAAAATGAGTCACTAGAGATGGGATTTTGAAAGTTCTAGTCCTTTGTGATTCCAGCCACTAGTACTAATACAACGGAAATGAACCAAGACTGGCCCATGTCCTACTTAGTAACCAACATACTTATATTAGGTATCAGTGCTCACTAATTAGGACAAGGATGAGAATTTTATGTTTTTAT

							CGCATGGGAGACAAGGTTCTATTGTAAGTCATTTTTTTTCCTCCTTCAGAGATATGCCAAACAGCCCTACACTAAACAGGTCTGAGAAAGATGACTACGATAAGTTCAGAAATGGAATTTTGCCCATGTATACATTAGGTATGAAAGAAGAAAAAAAAATAAGCTTAAGGCATGAAAATGGACCCTTTATTGTAATATCATACGACAACTCATTACAAAAAAAGGCAGGTTTGGCAGTTGTGTGAACTTTTCTGTGTATCTAGAAGGATGTGCAACAGAATGCAAACATTTAAAACAAGCAGAACATGACATGTGTCTACTTAAGACAAGACTGTTCATTTTTCTTTTTCTTTATTTCTGAGCATTTATACCTACACACAATGAAATATGGTCATGCATTTGACAAAGAATTTCTAAATCCTAATTTTAGCAGATGCCTTATCCCCTTGGAAGATCAAACAGTTAAAAACTAGTCATATGTAGATACTAGGTACTCTGTAGAAAGAAAGAAATAGGAAAATGCCTTTTAAAACTGAATAGCTCTAAAGAGAGTGAATGGATGAATAAAAATGAAGGATTGACCAATAAGAGTTGTTACATATTATATGGTCAAATAAAGAGGGCAAAGAAATATTCTTCCTGACAAGCTCTAATAACAGTGTGACCAAGTTGTTTGGATGATTTAAGTATGGCCTTGCTGGGAAGCCGAGATCGGCAAGATAACCTCCCAGTGTCCTTCCAGTCCTGACATGCTTTTCAAAGTTGTTTATTCTCCACAGGCCAGCTGTCTTTGAGGGAACCAGGAATCCCCTCTGTCACAAAGGGAGGTTAACACCTTGACCTTGCCCACTCCCCTGCTGACTGCCAAGGGTGATGAGCATGAGAAGTCCCGGGGTCCATGTCCACAGCCTTCCACAGACACTTCCTTGTCTTCTGGCCATCGCATTTTAAACTCTG
MSTRG.22639.1	--	0.565	0.016	0.002	-3.539	down	TAAGGTAGAATCTTAAGGTCATTTTATTTTGCATTTCTCTGATCACTAATGAATTTGAACATTTCTTTAAGTATTTCTCAGCCATTCGAGATTCCTCTTTTGTGAATTCTCGGTTTAGTTCTATATCCCTTTTTTTTTTATTCAGTTGGTGGTTTTGGGGAGTTCTTTATGTATTTTGGACATTAGCCCTCTGTCTGATGTGGGATTAGGGAAGTTTTTTTCCCAATTTGTATGATGCCGATTTGTCTTTTTGACTATGTCTTTTGCCTTGCCGAAGTTTTCCAGTTTTATAAGGTCCCATTTATTAATTCTTGATCGTAGAGCAAAAGTCATTGGAGTTCTGTTTAGGAATTTTCCCCTGTGCCAATGAGTTCAAGCTTCTTTCCCACTTTCTCTTCTATCAGATTCAGTATATCTGGTTTTATGTCGATGTCCTTGATCCACTTAAACCTGAGCCTTGTGTATGGTGAAAATATGGATCTATTTTCACTTTTTCTATATAGAGACATCCAGTTAGACCAGCACCATTTATTGAAGATGCTTTCTTTCTTCCATTGTATATTGACTTCTTTGTCAAAGATCAAGTGTGGGTAAGTGTATGGCTTTATTTCTGGATCTTCAATTCTATTCCAGTGATCAACCTATCTCTGTACCAATACTACTAATACAATACTAAACAATTACATTTGCTTGCAATTTCAATTTAGAAGAGTTTTTGAATAAATTTCTGAGCTCTCTGTTAATGATGGCTATGAGTTCCTGGCAGACATTAACAAATTTTAGGAGCTCTGGTAATAGAGGGCCTAGTATCCTGCACTCAGGATGTGCTAACAGCAGTCTGACATTTCAGCTCTTGCCTTGAGACAGGCAATAGCAATAGCAGACACTTCCACTGCACCAATAAATAAGGTGGGAAACTCTGGCAATATATATATATATATATATATATATATATATATTCATATTTAGTAAGATACCAGTTAGGGAACTGGAATCACAGAGACAAAGAACCTGTTCAAACAAATCTAAAAGGTTTGTAGATGGACTGCAGTACTGGGGGGCAGCCCCAACTGGGATCTTCTTTCTCGTTGGTTCTTCTTGAGGCAGCAGAGGGGGAAGAATGTTAGTGGAGGGTAATATTATCTTATGAGATATTTAGGGTTGCTGTATAGTAATAAAACGTTTACTTATCTAAGTTACTATGCTACAGTAGCTAACCGATCTTCT
MSTRG.22640.1	--	0.626	0.032	0.000	-3.254	down	GCCTCTTTTTTTCCACCTCTGCAATCTGGGACATTGCTTTCACTTAAATGACGCTGAAACCATTCCATCAAGAACTTTTCATGAGGATTTCCATCTGCCGCATAGCTACTTTCAGTTCCATGTCCTGCTGTCATTCCTAACTAATGTAAAATCAAATGTTAGCATCTCGTTATAAACCACTCCTTTTCTCATTTCTGCCTCTCAATTTTCCTTCTACCCAAGCACTGTTTTGCCCATTCCTTTCCCCCTGGACATATACTTAGATAATAAACTATATCTCCTGTTTCTTTGCACTTATTCAAGATCACGTACCTCCCTATCTTCCTCTTCTTCACCTTTCATTTTCAATTCCTGAGCTCTTGGTTGTATGGTCTGATCTTCTTTCCCATTGCTCATTACATGTAACAGTCATTTGAAATTCCTAAAAGCAAAACCCTATCCGTGTACTCAATGTAAATACATGGAGTATGTAAATTGCATCCTTGATCATTAAATGCTAATCTGACTAATATCTCCCATTCTAGGTCCACTAAGAAAGGTCAAAAACCTATAACTGTGATCAGAAAGGGCCACAGGTCACATGTTCACATATGAACACTTGGCACTTTACTGGCATTGATAGGTCACATATCAACACT

							TAACCCCCCTTTGCTTTCAAAGTTAATGGAAACTGATACATTTAATATTTTCATAAGTTCTAAGAAATGGCTTGAACTTACCTTCATGAAATACACTTTACTACCATGATTGACAAGGTATACTTCCTCTTTATCAACCCACACTAGTGAGTTGGGTACACACTTAAGTCCTTTTACATATAGCCTACTTCTGTCACTCAGGACATCACATTGAATATAGAAGAACCTCTGGTTATGAATGTAATTATTTACAAGTCAGTTTGACAATAGAACTGTTAGAACGCAACCTCAATAACTTCACGTTAGCCTGTGCTTTCAGTAGACACGGGTTTTTACTCTTTATAGCACCAATCCTAATCTTACACTGCATTGCAAGTGTGGAAGAACTGAGGAACTTTTGTTTTTGGCAACTGTAGAAAAAACATAATATTATACTTTTCTGGAAATAGAACACATATGAAAAAGAGAGTTTATTAATTGCACTGTGTATTTTTTTAATTCATTGAGATTATAATTATATCGTACCCCTTCCTGTTCCTTTCTCCAAACCCTGTGTACCTCCTTTGTTCCCTTTCAAACTCCTTGTCTTTTGTTTAATTTTGTTATATGTGTGTGCATGCATGTGTATGTGTCTATGTGCATTTCTAAATATATAAATACTATGTGTTTAGTTTTTATAATAGCAATTGTATGTATGTTTTCAGGATTGATCATGTGGTATGGGATAATGACATGGTATGCTCTTCCCTGGGGAAGATGATTTCTCCACATCACTATTCCTTAATTGTCTTTTGTTCTTTTCCTAGGGTTGAGGCATTGTGTTTTCCCCGCCCACACTAGCAGGTCTATTGATGTCATCCTTGTACAGGGTAGACTCCTATTGGGATTTCTTGTTTGCTCTTCATCTGAAGCTCACAAAATTCTATCACCACTGTTAACTTGGTTAGTCTATGATGGGGTCAAAATGAATATTTAGATCCAGAGAGTACTCCAGAAAGCACTATATCATACAAAACTGACAATCATCCTGACTGTCCATTAATCTACCAATGTGATACAACCACTTTAGTGAATTGTTAGTCAAGTTATTTTTTATCCTGATTTTTGCATTTTACTTTTTGATTTTACATGTTTGGGTTAAATACTTTTTGTTTTTGTTTCTCTCTCTCTCTCTCTCTCTCTCTCTCTCTCTCTCTCTCTCTGTTTTTTTATGTACCATGGGAAACAGAATGGAGGTTGTGCTATACCTTGAAAGCAGAGGCAGAATGTGACCATAATCCCCAGGCTGCATGTCTTCAGAGAAAGTGACATTCATACCCCATAACAAGCCTGCTTTCCTGTGTTCACTGCTGTTGGTGTACAATAGACATACTATGCAAACTAGGACAAGTGTCCAATGATGGATCAATAAATAAATAATGAAAATATTTACCCAGTAAGACAAAACACACCCTACAACAGAAGGAGACATTGCATTATTATTGACATGGAGGAAATATGGAAATGTTTGACAGTGAGTGGCATAAAGGAGAACTAGAAAGGAAAGAAAATGCTCATCCCTATGTAGAGTTCAGAAATATGTCAATTCATGAAGCAAGATAGGTATTACTATGTCAGGAAGGGGAAGGCTATGGAACGAATATCTAATAATAAAAACTTGCATTCCTATAAGATAAGGAGACTAAATTACTAATGTCCCAAAGAAGAGAGAAGTTAGAATAATATATGATACACAAATATTTATGAAGAGAGACTTTATATGCTCTTATATAAATAAGAGTAATTAAGAAAAATACATAGACATGCCAATTTTCTCCCCCTGGTCATAATTTCATACATGTATTTATATAAAAATATCACACTGCATAACTCAAATATGCACAATACAAATGTGGATATGTATACAGGTGTCCTATAACATTCATAACTAACATGTACATTATGCGGGGCTGACAAAATGTCTCAGTGGTTAAGAGTGACTGCTGACAAGCCTGCAGATCTGAGTTTAGTCCTGGAGACCCACATCAGAAAGAGATTGAACAGTCCTGTTCAAGCTGTTCTCTGACTGTAAGACGCATTTTTGTGGTAGGCCCATAATCGCAGGTATGTATACTAGATAAATAAATCTCCGATAATTAAAAGTTAATAACAGATTAATATTTTCAACTCTTAGGAAAAATACAATAAAAGATGGGGAGTTTATAACAGTTTCTAGCCACTAGAGTAGCAAAACTGGAGCTGAGTTGACACAGAAGTTAAGAGCACTTAGAGTTCTTGTTCAAGGCCCACATTCTGGTTCCAAGCATGTCAGGTGATTCACAATTGCCAGCAATTCCAACTCCAGCTCCAGGGAATCAGATATCCATGGATTCTGCAGATATTTACACTCATAGGGGCACCAAACCACGCACAAATACAGGCACACAATTAAAAAAAGAAGTAGACTATAAAACAAGGCAAAGATTCATCATGCAAAGGACAAATTAGATAATCTAATAAATACAAGCTCACTACTAAGTTCC
MSTRG.23221.1	ENSRNOG00000057794	0.699	0.089	0.042	-2.143	down	GTGGTGGTGGTGGGAGCAAAAGCCAGGAGTTGGAGAGCTACCGAATACAAAATAAAGGAGAGAGGCCCAACGCAGACAAGATTTTTGCACAAACAGATTAAAACAAACGAGCAAAGAGAGTAAGGAGGGGGACCGCGCTCCCGGCCCGCAACTGGTGCGCGTTGGCTCCCGCCCCCTCGAAGCCCGGGGCTGGCCGGCTGCAGAGCTTCCAAGCTCCGACCCACTTCCTTTCTTATTCTGAGGGCTTCCTCGGCTCTGCTGGAACCCGAAGGCGGGGAGAAATGAGACGGGAGAAAAGAAAAGAGAGAAAGAAAGAAAACACCTAAAATGTTTAAAA

							TCAAAAGCTAGGAGGCGGCCTGCGGAGAGCCAGCGAGGCGGCGCGTCCTGGTCCCTTCCGTTGCCGCGTTCTCCCGCCCGGGATCATCCCCAGTCGGCGGCTGCTGCGGGCGCGGAGAGCGGCTCAGGCGCTGTGAAGATGCGAGGAGGTGGGACAATGAATTTATAGCGGCGTGCCATCCTGCAATGGCAATTTCAACAATTCGTCACTGCACGCTGCCTCTTAAAGGGAGAGCTTCCCCCACCACTCCCACCCTTCCCGGTCCGCGCCTAATCTGATGGGGGAGGGCGCCGGGAGAGGCGGAGGGTGATCGAGGAAAGGGAGACCCGGAAAGAGGAAGGGACCAGAACCTAGCTAACAAGGTGGAAGGAGAGACAAAGGAGGGAGCAAGGGGGAGAGAGAGATTGAGAGAGAGACAGCATTTGGGCAGAAAACAAGAAAATTCACTTGGCCTCATTTAAAGCCACAGCAACTCAGGGTCTGTTGTGGTATTTCACAACCAAACGGGGTTGGCTAACCTCGGCTTACCTCATCTCCTTAAGGGGTGTTTTGGTGTGTTTTTAAAGTCTCTCCCTAGCTCCTTCCCCTTGCTGTTCTTGCACGGGATATGAAGTTCTTGCCTCATTTTGTGGGCCGGTGCGCTCTGGCCTTGTTGACCTGGTAACTTCACTGGACAGGGCAGCTGCCCGTGCACTCCCAAGTCCATCTTTCTTTTCCCTGGCTCCCTCCAGTCGTCTGGGTAGCTGATCTCCAGACTTTTCATCTTCTAGCTTGTTCAAGCTCCAACACTTGCTGACTTCGGGTTAGGGGTGACTAACTTCTCCCGGCCACCAGCTGAGCAGCCGGGCAGCCACGGGTGCTTCCTGGCGCGTCTCTGGGCATGCAGCACTGCGGTGACCCCAACAGGGCCGGCTGCGGCCTGGGAAGTCCTTGGCTTTGTGTATAAATGGCGGTGCTCCCAGCTTTTTCCAGCGCATAGTTGGTCAGTGCCCACAATCTGTCACTTATCCCAGCCACTGGAACTCTTTGAGCTGTAGGATGTCAGGGTGGGAAAGAGTTAACTCTTGAAGGGAATTCAGCACCCCCACCCCAAACTTTTATATTTGTGCTCACACTTTCTCTCTTTCCTTCTTAAAAGGTAATTAAATGGCAAGCACTCCTTTGTTTGTTTACTCACTACATCTCTCCTAAGTCATTCACTTACTGTCGGTCCCCTACTCTTGCTTCCAGGATCATTTCCAAAGCTCCTATTCCAATGGAAGAATACTTTAGGGGAGGTGATTGGTGGTTATTAGTGGGCAGATGGCATGGCCCCTGCCACTTACTTCCTCCATCCCTTTTCCCCTCTCATTATCAATTCTAAGAAGGTGAACTGGAAAACCCACTGGCTTTCTCAGAGCAAGCTGATGGGTGGCAGGCAGGTCCAATACTGGAATCCGCACACACCTCATATAATTTACACATTTCCATCCTTTTCAGCTTCATTGCTTCAGAGGAGAGCAGGTGCACTTGCATAGCTCCCCCAAATAGTACATTCTCAGGTAAGGTACCAGGCAGTCCAAGAAACAACAGTTAAGGTAAAAGAGGGCATGGGTTTCAGAGAGACAGAACAAGGTGTGTGTGGGGGGGGTGGACATGGGAGAAGTTGAGAAAGACTGGCTGGATCCTGAAACCAATCAGGACCTAGAAGGAGGCTGCTGCTGAAACACTTGCTCCTCAATCCTCAAGATGATCAAGTAGGTTTCCTGTAGTCAGCATTCCAAGGCAGTGGGCAAGCATCAAAGGCAGAGAATGGAGGTACCGGCCATCTTCTCCGTGGCCCAGTAGCCTTCAGGTTTACGACGACCAAAGTAACTCCAGGGAAGATTACCTGGTACCTATTCGTGTAGCAGAGGCCAGCATGAGAACAGCCCCCATAGGTGCATACGTGCGGTCTCCAGTTGGTGGAGAGGTTCGGGAAGAGTTGGGAGGTGCGGCTTTATTGGAGGAGGTACATCACTCAGGAGAAACGCTCTCTGCTTCCAGCGTTGGAATCAAGATGTGAGCTCTCAGCTGACCCTGCCATCATGGACTCAAATCCTCTGAAGCCGTAAGCCCAATTAAACATTTTCAACTTTTATTATTTTATTCTGTCCTGATTTTTTTTTTTTTGTCACCACAATTGGTAAGTAATTAAGATACAGACTTTATTTCTTTTACGCCACTTTCCAAAAAAAAATAATAAAGTACAATATTTGGTATCAAACAAAGTTTCCAAAAACAATTTTTATTTGAAAGGTGCAACTTACTCATCTGTGTCTCACAGAATCATTTAGAAAGCAGGTCTTTCAGGTGACCCTGGGGTAAGGCTGCTAACTGCAATTGTTTTCATTTGTGGACAGGTTCCTTATGGATTTCCCGATGACTTTACCCTCACTGAAGCCT
MSTRG.23383.1	ENSRNOG00000002115	0.000	0.301	0.000	5.271	up	TGAGCTTACAATTACATGCTAGCAGGTCTGGCTTTTTAAATAAATCCTGAGGATTCAACTCAGGTCCCTCCTTGCAAATACTTTACTAACTGAGCTATGTCCTCCGTCCTTATTGTCAATGTTTTGATGGGTAAATGGTCGTACAATTTAATGTCTGCAATACAGTGGCAGGAAGAAAAGGTGAGAGACTTTGGCTGGAACAAGAGGAAGGCCGCTGGGATGGGCCTGTGGAGGCTGCACCTGACCCCTACCCTTGCTCCACTGTGGCATGACATGTCCAAGTGACAAGGATAGCAAAGCACTACAGAAGTGCTGGAAAAGCTGCTCAGTGTAGGCCACTTGCTTC

							ACGTGTAGTACACAAGACCCAGAAGCTTGCCCATGGTGTTCAGGGTCAGAGGGAGGGCTACTGGTTTAATGTGAAATGATGCATGAGATCTCTGGTTTTGAGGTCTCAGCTCTGTGAGCTCAGCAGCGCTCAATAGCCAGCCTTCTGTTGTATACAGCAAGAGTCTACATTCTAAGCACACTCATACAAGCCTCCCTAGGCTTGTTGCCCCCACTGCAACTGCTTCTCAATTTGCTCCCAGGTCTCTAAAAACAATACTGTATTACTTACTGTCCTATGAATTATTAATTATGCTTCCAAATGGAATTATCTAAAAGAGCCCATAATAGTTGTGATTTTTTTCTTTTCCTGCGCTCTATGCCTACCCTTCCTCACATGGACCAACCCTAACTATTTCCTTAAGATCAATTATGCTAAGACCATGACAGCAGCTCTAAGAGAAGCTCTAAGAGTTTACAAGTATAAGGCCAGCATGGGCAACTTGTGAGAGTTTGTCTAAAAATAGAAAGTAAAAAGAAGTGACACATATACTGTTCATCAGTTCTGTATTTATGAATAGCTTCCCTCATTCTAAAGTAAATAATTTACTATTTAGTCCTTCCTCCACTCATCTAGAGCCAAACTGCAATTGAATAATCTTAAACAACCAACCTGCCAAGGCCACCAGTATTGGCTGAGCAATCTCTCAGATTTATAAAGGCCGACCTTGTGACAATCCAAGATTCTGATTACAGTTACCGAGTCTTCGTAATCTTATTATTCGCAGTCAGAACACAAACCCAATAGTTAACCAACTGCCCACTAAGTTTGCCTCTACCACTAGCTAATGCTGTGAGCGATTCATCGGCACAACGGAAATCGTACCAGTCTGCTTGCTTACGGGAAGGTTAAGTGTGGAGTACAAACGTATTGGAGCACCAATGGGGCACCTTGTAGAGTGAGTGTCCGCGGGGTCTCAGATGACCACAGCACCTTGGAAACCGTTCCTAGCAGTGCTTCTCCCAAACTACAAATTCAAGTACCAACGGAAACCTTA
MSTRG.2358.1	ENSRNOG00000018007	0.000	0.458	0.000	6.121	up	CCCTGATATTGTCCTATACACACACACTGTTCTCTCTCTCTCTCTCTCTCAAACACACACACACACACACACACACACACACACACACACACACACACAGGAATATATCATGAAGAGCGGCAAATACTAACACATGCTAGGTACTATGCCATTATTCAGAGTTATGGTGATGGCAGCAGCATGGGCTGCTGAGGAGTACTGTCAATGCTGACGAGACTGTAGGCTTCAGCCCAGCCTTGCCCTCTGACCTTTGACCTGTCAGCTTCCACTCCCTTTGCAGGGCAGGTTTCACATACCACAACTGTCTACTGCATGTTCCTGCTATACTTGCTTCTGACCTGCAAGCAGCCATGTTGGTAATTAGTCTGTGCAAGCCTCCAACATTAAAGGTAAAGGGCTTGGGTTTAATGTAGAGCAGGAAGCCTTTGGAGGGGTTTTAGGCAGGAACAAGAGCAGTTCCCACAAATGTTTGTAAGATCACTCTGGCAGCACCAGAAAGCAACCTCACGCATCTAGGAAAATGTATAAAATCCTTCAAATGTCGTTAGAAACAGAAAAGCTCTAAGGAAAAAAAAGAAATCAAGAGAAACCCTGGGTGCTAGAATTGGAGAGGACATGAAACCCAGGAGATGAGCAAGGACAGTAAGTGCCTGCAGGCCCTTGGCTGTGCTTGGTGAAGATGCCTTCTTAGGTCCCATTGGGCTACATCTGAGATCTCCACCAAAAAGCAAGGAACTGAAATGTCCTTCTAAGAGGGTATGGGAAAAACTGCCATGAAGGGCTAAGCAGAAGACAGACCAGAGTAGTGGAGCTATGAGAAAAGAATCCTATGGACAGCCTCTGTTCTGCACTCCACTTTGTGTAAGACACAGGCCCCAGACTTCAGTGTGAAACCTCAACTTAAAACTAAATGTCTTAACCAGCAGACTGTCAGATCAACCACGGTCCTTGAGCAGTCTCAGAAAGGAATTGGATGTGGTAAGGTCAGCAAGAGGCAGCAATCTCTTGGCAGCTGTGCTCCTGAGGCTCAGAAAGCCTGTTTGTGGCTAAACATTTGACGTCACTGGCAGGCTGTTCCTGAAATGTCAGAAACTAGGGCACCAGGGCATTTCCCCACAAATGTGCACTAACAAAGGGTCTGTGTCCTCCTCTTTGTAGGGTTTTCTAGAGGATTATGGGAATAAATCCTGTAGAAGGGAGGTCTGATCCACTACAATGTACACAGGGAAAGGGTCACTTTACTCTATGAATATGCCACCAATCTTATAAGTCAAAAACTTCTTTAAAATCTGCAACAGAGACCACTCTGCCCATTCCTGCCACACACAGAGACTGTATCTGTCTGCCTTTCTGTCTCTATCTGTCTTTGCTTACTTCAATTTTGCTTATGTTTTATGTGTCCCTCCATGGAATTCCTTCAAGAGACCTTCAAGGAGAAAGAAAACAAAACCTGCTCTGTGGTATGTGGAACTCTCTGGAAAACAATCAGTTTATCCAAAAATCCAAGGAAGGACACAATCACGAGGGAGAGCCAGCACCCTAAAAGGAATCTGGAGAAGATTTAAAGATTACTGAGGAAGTGAAGGAAGAGAAGCCGACTCTGTAGAGAGGATGCAGAAAAAGAGCAGGCAGCTACAGCAGTTCAGGGGGTAAACAGAGTCAAC

MSTRG.2364.1	ENSRNOG00000018158;ENSRNOG00000018207;ENSRNOG00000060573	0.190	0.000	0.000	-5.500	down	ATTTGAGGTATCTTGTGTTCTTGGAGGCAGTGGAGCTTGGCTACAGAATGTTGTACTGAATTTACTTCTC<br>ATGACTAGAATTAAGGGCCTTTGCAAAGAAAACAGAGTGCTTGACCTTGCTCTTCTTCTAGACATAGGG<br>AGTGTTAGTGTTCATTACTCATGTTATCTGACCTCTGTTACCCCTTCCAACATTACCACTCACAAAACAC<br>ACACACACACACACACACACACACACACACACACACACACACACACAAAATTTTTTTTAAACACAAC<br>CAGATCTCTGGAAGATTCAGCTTGTATTGGGTTGGGGAGAACAGCATTTCATTCACAGATGTCCGCTGC<br>TTACAAAGGTACCTGGATGAATGAAGGACACATGCAAATGCCTTGTGCTTGGCTCATGTAAGCACCGTA<br>GATGGGTGCTGTCGCCAGTGTGAATGTGATTTGATAGGCAGTGGGTTACCATGGAGACAGGCTCACATG<br>CAGACAACTGGATGAGCTGGTGGTTGGCTTATGTATGCGAGGGTTATTTCCCAAGGATACTAGTTGGAA<br>AGCACCCTGGTTTCTTGTGTCAAAATTAATCCTGTCTGACTTTGGAAGAAGGTAAGAAACTTCAGTAAG<br>TGGGCAAACTCAGGCTGGATAGAACTAGGCTTATGTCATTGTCCTTAGACTGGCTGGTCACCAGATACC<br>TCCCTCCACTTGTCAAGAAGCAGCCTTCATTCTCCTTACTCCCTTGTTTTAATCTGGAACATTTTACTGTA<br>GACATCTTTCTCTTTAGATCCTATTGCTCTTTCTTTTCTAAAATGTGGGTGAGACTTGATTTGCATCTACA<br>CCTTTCCTTCCTTGGCCACATAAAATTCATTTGATTTGAATGTTTTCCATAAATTATAAGTGTAATAAAAAT<br>ATGTATGCACTTCCCAGGATATTAAAAGAGGGTTGTGGTCAAATCTCTGGGGGAAACTCCCACAATATG<br>CTGTAAGTGTGGAGGCCGTACTAAAAGATGACCCACAGGAAAGCCTTGCTAAGACTGTCTGTGCTCTC<br>CACAATATGCTGTAAGTGTGGAGGCCGTACTAAAAGATGACCCACAGGAAAGCCTATGCTGTAAGTGTG<br>GAGGCCGTACTAAAAGATGACCCACAGGAAAGCCTTGCTAAGACTGTCTGTGCTCTCCACAATATGCTG<br>TAAGTGTGGAGGCCGTACTAAAAGATGACCCACAGGAAAGCCTTGCTAAGACTGTCTGTGCTCTCCAC<br>AATATGCTGTAAGTGTGGAGGCCGTACTAAAAGATGACCCACAGGAAAGCCTTGCTAAGACTGTCTGTG<br>CTCTGTTCATTCTGCCTGTAGCATCTTTGCGAAATTTCTTCCTGTGTGAACACACCCCGGCAAATACCCA<br>GACCTTGTAGTAAGACCCAGTTCTTGCTGTTGCTGCTACCTGTGATGCTTATTCCCAGAGCACAAAAGC<br>TTTGACTTTTGAGTTGGGCTTTATTTGAAAGCTGACCAGTGGGAGATGCTACAGGAAATTCTTTTTGACT<br>TAAACATTGTTTTGGTTTCCTCCTCTTCCTCTTCCTCCTCTTCCCCCTCTTCCTCCTCCCCCTCTTCCTCTT<br>TTTCTTTCTTGTGCTTGATTTAAGAATTAAAAAATAAACTGGTTTCTTTAGCTGAGCAAAATAAAATTACT<br>CATCTTTTTTTTTTTAATTACAAAGAAATACTGACAAGAACATCTCCCAGTTGATATTGGAGTTTTCCATT<br>TTATAATTTAAAATATGGGAGCTGATTCCTAGGTGGGTCAGTTGTCCAAGAAAGAGTTGAGCAGTATATC<br>CAGAGTTACTGTGTTTACGTAAGTTCCTGTCGCTTGGTAAGGTGTCTCAGTGCTTTTCATTAGTTGTGCA<br>ATACTGTTGTAGCCTCTGTTTATGGAATAATTTAAATGCAATTTTCTTTGTTCTGTTGTCTAAGAGATAGTT<br>ATTTATCTTGCTCTTTATAGTATTCTTGGGAACTATTTTGTTACTATGTATTAGTGATTTATGCCCCTTTATTA<br>TTTATTTAGAAGAATAGTACCTTTGTAATGAGTTATTTGGTGGCAATATATTTTGCTGCAAAGAATAAAGA<br>GAATATGACAGAATTTTTTTTTTTGGCTTGGGTGCTTTATAACTTTCTTGAAAGTTGGGATAAAATCTTAT<br>TAATTCCTTAAAAAAATAAACTTACTCTTTATCTCAGTATTGATTGTATGCAAAATAAGCTCTGTCTTTGA<br>GATAGCATACCATAAGATAGTACTTCTTATTTGAAAATGTTAGTTTTAAATGAAATGTGATAGGACACAAG<br>GAAGGATGAGAGGTCTGAGATAAGAGTCTCCAAAGAGGTCCTTAGTAAATAATCTTTAGGTTTGAGGTT<br>TGGTTTGGTTTTTTAGTTAAAATTGGCCTTTTCAAAAATGCAAAATTTATACTTACAACTTTGCTAAATAT<br>GAACAAATATGGAAGAAATCCCTAAAAGATACTTTCTGTCCAGGGAGATGCGGGCTTCTGTCTGGTCTT<br>GAGCAGTGCTTGACCTGTGAGCTGCAGGAGTACCTGCCCCTCACTAAACTCCTCTCCCAGACAGAAGA<br>GTGAGTCTCAGGTCCCAGGTGGACAGTAGGCTGCTGCCTGCAGCAGCTGTGACATAGTATCATTTAAGG<br>TAATGGCTTCTTCCAGTCAACATCACTTGATTAAATTCTCCTCAAATTAAGTAACTGAATTCTAGGTATTT<br>CATGGTTATTAGCAATTTGTGAATGTCACAATGTCTCATTGGATCCATAAATTGGAAACAGTCAACTACA<br>TAGAGGCCTATAGGAAGAGTATATTAACCAACATGAAAGCTAGTGTTAGATTTATTTGGCTCTAGTGTCA

							TCAAAACAAAAGTTGTGGGTGAATATACACTGCCCATTTGAATGTATAGCTTGAGAAAAACAGGGACCACCACATCTAGAAATACTCAGCTCTCATGGGCATACAAAGAAAAGCTTTTGCATGTTGGTGCTTTTCATGTAATGTTCATTCTGTAATCAAAGGCCAGGGGCTCAAAATCAAGGTCGAATTGTTCTGTCACAGGGATAGAGTGGCTTTGGGCAAGCCCCTGGGGTGTCTGGCCTCCTCATGTCCATCTCTTAAATGAAAAGGCATGCCTCACAAGCTCAAACACTACATTCTAACTCCTGTAGTCTGAGAGGTCAAAATTTAGCAGCTCATCTTCCCCTTGCCAGGGTGAAAGTATGTTTCAAAACCTGCTACCTATGTAGAAAACATTTTCATGAGCCTGTAAGCTATCTACTGTCTCCTGAACAGAAACCAGCAGGTTTACCCCTAAGTTTGTTTAGAATAATGTTTATTATACTAAAAATTATAGAAGCATCAAATGCAAGTACAGTGGAAGTCCCCTCTGAGTGTGTTGTACTCGGTGAGCATTCTCAAGAAAAATAAAGCACTACCTGGATGTAAAACTGGGGGTGGGTTCAGAAAGAAGAGTGCTGGCAAAGGCTCGCCTCTTTTGGTGTGCTCATGTGTGACAATGCTACACCAAAGGGAGCCTGTGCTGTCCTGTGGGCTGTGGACAGGTTGACAGCACTAGCTGGTTTGAACTGTCATTTGAGTTCCTGGGAGTTTATGATAGCGTACAGTTCAGAAGTCTACACCACTGTGTATCTCCTTATCTCTGGCTGATAAACTCTTGAGGATTGTGACTTACTTTGTTTCCATTTTCTTCAGCTTTGTGCAGTTCTCTTGTACTTTAACTGTATCTATATTTTCTGTTTACAAGTTAATTTTAAGGGGAAGGGTAGGATTTTAAGATCTTGGTTATTGTAGATAATTTTTTTCTCGTGTTGTAGAAAAGCATGGGTTATACATTTGAGTGAAAAGGCACTGTATTATTTACCAAAGGTGTATTGTTTGCATTTGTTTATAAATGCATTATTTTAGTACTGTAAATTTGGGCATAACTTCTGAGTTTACTACTATTGCTTCAGCTGGCATCTGTCTTTCTCCTTCTCCGTCATCCCCATAAATGTGCAATGTGGGGTGCATAGGTCACAGACCAAACAAGACCACCAGCATGTAATTGTCCACACTTTGGGAACAGCGTGCACTGTTTCGTACACATCAGTCCCTCCAGTGTTGGTTTTTTAATTTTTTTTATTTTGTTTATAAATTCAGCATTTTGCTTTAACCTTCCGGAAAGATCTGTTGAGAGTCCCCATTGCATCTCAATGAGCTCTCTCTTATTTGCCTATCTTTGTATGTATTTTGTATACTAGATTGTGCAGGACGTATTCTAGAAGGGATCAATGGTTTGCATTCAAAATGATGTAGTTTGTCCAAAATATTTTGCCTTTAAAATTGAAATGGGTTAATCTCATTTTTTTCTTGATGATTTGAAATTGTAAGATTTGTCCAGCTATTGCTTAATAAAATTTTGCGGA
MSTRG.23664.2	ENSRNOG00000001684;ENSRNOG00000039850	0.219	0.000	0.000	-5.474	down	GTGGTGAGTGTGGGTGTGCGCGTCGCCGCGGGTCTCGCCGAGCGCCCGCGCGCCGAGGGGGTGTGGCCGCCCTTGCCCCAGTGGGTGTGTTCCCTTTGTCCGGTGTTCCTCGTGCGTCCGTAACGGTAGGAGGGGGCTTTTGGTGCGCATCGCCTGGCGCGCGGGTGACATCCCCAGCCGTGTGTCGGCTGCCTCATCCGGAGCGCTCGGAGACGCTCCTCGGGGTCTGCGGCTCGCCCGCATTGTTCCTATGCTCGCCCTTCCCTGCGGTGGTCGCCCTTCCCTGCGACGGCGAGGGCGTCCCCATCCTCTGAGGGTGGTTTTCCTCCCATCACCTTTGTCACTCCCTTCCCTGTAGTGTGCGCGCGCGCGTCTTCGCGGGTCCCCGAAGGTGCGCCGCGCGGGGGGCCTGCGGCATGCGCGGGGTTGTGCGAGCGAGCGCGCGCGTAAGGCCCCTGCTCTGTGTGTGTGCACGCCTCCCCCTCCCCGCGTTCCTGGGAGGACCCGGGAGGGACGCCGAGCTGGCCCCCACCCACCTGCAGCCTTGTCCTACGCCTGCGCAGGGGCTGCTGCTCAGCAGTGCTGGCGACGTCATTAAGGCCTGGGCGGCCATCCCATAGTGGGGAGGGGACAGGCGTGGGGGGCGCAAAGGACCAAAATGAAGCCGAGGGTGTGCTGGTACCCCTGCACGGTGCCTTTTCCAGAAGCTTGTAGTTTCCAGACATCCTTCAGTTTGTAGATTATTTCCACAGTATGTGCTTATGCCTGTCACCAGGCGATGGCTAACACTGGTTATTGCAGGGTGTTTTATAGCAGCCATAAACAACTCTGGATTCCGGCGGGGGATGAGGATGGTTCAGGGAAACGGTTGAAATCATACAGGTTTTACTGCTCTCTCTGGGGCAGTTTCTCACAACCACAGCTCTAGTTAGAACACTTATATCAAGAATTTTGTATTTTACTTTAAGGAAAATGGGTTCGTTATATGGGAAGACGTCCCTAGGGATTCAGTTGTCACTACTGTGCTTGTCAAGGGTTTGTGCTTTGCGCTCAAGGGCTGCGGATTCTCCTTCTCTCTTGCTCAGTTTCCTTATTGTTGCCTAATTTTCCCTTTTTCTCTTTCAATTTCTGGACTACCTAAGAGATTAAACACAGGTAGACCTAATCAGCCAGGCCATTTTGTTTATTATTTATTCTTTTTTTTTTTTTGCCAAATTTACTTCAGATTCTCCAAAACAAACA

							AACAAAAAAAAATTTTGGAGATGCTGAGAGTTTGACCCCCTCCTTACCTAGAGATAGCCACTGTGTTCAGGTTTGTGTGTATCATTTCCGTGCATAATTTACAACTTGATAATGGTGAATATATAAACAATATATAGTATTGATTTTTAACTAAAGTGATATAAAATTGTGTCTTTGCAACTTGCTTTTTCACTCAGCATCACATTTTGAGATTTATATATGAATAAATCGAATATTTGTACTTTCTTTGAATTATTTTAACTGTTGGTATAGCACTGCATTGTATGAATATACTGTAATTTATATTTATACTCCTTTTGATGGACTTTGGAATTGTTTATAGCCTTAACTCTTTAAAACAGTCCTGCAAAAAATACCTCTGTACCTGTTTGGTGTGCACACTTTTGGAGTTTCTCTAGGAAGTACCCTTAGTCATAATTTTGCTGGGTAGAAGTATATGTACATCTTCCACTTAACCAGATATGATAGAAGCTCTCAAAGTGGTTGTACCAGTTTTAACGGTCACTGGTATTGAGAACACCCATTTCCTCACATGCTTGCTAACAAACTGTTTTTCTCCTTTCTTTACTGACCGTCTTCTTTATGACTTCAGCTTACTGCTAAATTTGCGTACAGACTACACTGGCTTTTGTCTAGCCATGTGACAGCAAATGGGTTAATCTCTCCCAGGCGATTGGTTCGGTCTTTACTGAATTTGGTTCTCGTAACAGTAATTGCAATGAACAGTTATGTTATCTAATGTTGCTTTGTAATGTTATATGTACCTCTCACGTAGCAAGTGAACTGATTGAATTAAAAGCATTTTAAAGGTATGATGTTTTTCTTTAAATCCTTTCCTCGATGAAGTATTTGATATATTCAGAATATATGTGACGTATTTATTTACAAGCTGAGAAGTCAAACAAATGAACACTAACTTACAAGCCTACCAGTGGAATCAAGAAGCAGCCTAACCAGTAATGAAAATGTTCACGTTCAGAGGTGATTATCCTGAATTCATGTTATTCTTGTATTTTAAACATTTTTATCAGATTTTTGTGTACCATTTAACATTGAATTGTGTGTATGGCTCCTATGTTTGAGTAAAACCTATTACTTTGGTTATAGTCTTCTGAAACTTGCTTTCACTAAATGCTATGTTTCTAAGTAACTAATAATTCTTTACTGCTTGTGTAATGCTGGTTGACTATAATGCTGTTTACTTGTGTTTCTGTCGGTGGGCATTTTGACTGTTCTGTTTTCCCACAAGAAATGTAACCAGCACGCCCACTTGGAAGAACTATGGTATAACTGCAAGTAGGAAAGCAAAAGAATAGTTTAAATGCTGGAAAAGCCTCTTTTAATTCCACTGCTCCTCTGTCCACTTGTTTAGGAAATGGTATCATTTGTTTTTTCATCTGTTAATTATTAGTGTTTATAGTTAACTTTTATTCAACTCAATTGTGTGATAAAACTAGCTATAATACCTACAACGACTTGACACCTATTTACTTGTGTGGGTGCATGTGCCATGGCACATGTAGGGTACCCAAGGACACATGTGGATAGGGGATTGGGCTCTGGTTGTCACGCTTGGTGGCAACCCCCGCCCAATGAGCCATCCTGCTGACCTAGTTATACATTTGGAAGAATCATAGTTTTACTTTAAAATGTCTTTATGATACTTTAGAAATTTATCCTTTGCAATGCTGAACTCTTTTCAGACTTCTTTAGTAGCTCAGGGAAATTGCATAAGTTTCAAAGTCTTTTTAGTGCTTTGTGAATAGGTTGGAGTGCCCAGCATCATAAGCTATTGGTTAGGAGTGGAACTGCTGGATTTTAGGGGTATGGATGTTCCACCTTATGAAGTGGTTTCAAATTGGCTCTGAAGTGGTTGTGCCAACTTGTATTTTAGCTAATAGCTTTGTCAATTTATGTACCTTGATCAGCATTATATGAGAGTTATAGGCAACCTGTTCAACACTTGTTATCTTAGATTTTTTTGTATTTGCCAGTAGTATTTGGTGTATCTTTTTTTTGGTTCTTTTTTTTCGGAGCTGGGGACCAAACCCAGGGCCTTGCGCTTCCTAGGTAAGCGCTCTACCACTGAGCTAAATCCCCAGCCCCCTTTTTTGTGGTTTAAATTTGCATTTATTAATGAGATTGAGAATATTTATTTATTCATCAGGTGTTTCCCTTCTGTGGAAAACATGGCCTTGTTTTTTCACCCACTTTTAAAGAGTACTGAATTGTCTTTTAAAGCTACAGACTGGTAGCACTTTTTATTAAATGTGCATTGAAAAAACTCAGAGCATGTGACACAAGTTTTCTACCACTAAACTACATCATACAACCCAATTCTTAAATTTTTTTTCTTTTTTTTCAGAGCTGGGGACTGAACCCAGGGCCTTGCGCTTGCTAGGCAAGCGCTCTACCGCTGAGCTAAATCCCCAACCCCCCAATTTTTAATTTTAATATAATCTAATGGAAGTGTCCCC
MSTRG.23868.1	--	1.710	0.188	0.036	-2.282	down	CAAGATTGTCAGTGTGGTGCACGGCCGACCACTTGTGCTTGCTTGGTGGACTTGTTTGTTTGAGTCACGCTTTACTGAGGAAGTTTCTTTCAGCGCATGCTCCAGATTAAAATCATCAAAGTCTCCATATCCTCTCCAGACCATGCAGCCCCAACCAAGCCTGTCCTCCTCCTAGGCGCCCACTTGCTCCCAGGTGGCCAGGAAGAAGTGGGTAGTGGGGAAGCCTGTGCACAGGTAGCCAGCAGCCAGACTCAGCACAGCTCAGGATGTGTCCTCCATACAGGGTCCGGCCACAGTTGTGACTCGAGTGGATGTCAGCCAGGAATGGGGCGATGGCTGGGAA

							GTCAGTAGGAAAATCATCAACGACATATTAACTCTCCCTGGGTAAGTCCTGGGTGGAGATGATCCCATTGGTGCCCACGTAGAGGTTGCTGAACTGGACACCGTAGAAGCTACTTCTACGTACTGCTAGCTTCACAGCCGCGGAGCTTTCAACGTCGCCCTCCCGCAGCAGCCGGTCTCCCCACGACTCCCCCGTAAGGAAAGAGCTCTTCAGGATGCAGAGCCCCGGCTCGTGACAGCAGCATCAGCACCAGGAGCAGCGATAGCGGTGATGGCGGTGCCAGCCGCCTGGCTGTCCC
MSTRG.23880.1	--	0.665	0.000	0.010	-4.070	down	ATTAGTTACGCTTTCCTTAAATTTCCTCAATTCTAACACATCCATGGGTTGCCATTCAAATTCTTCATAACCCTGTGGATGATCATCGTCTGGAGGCCTTTGTTGTACACTAACTGGATAGACTAAAGTTTGTTTCCTAATAACCTTAGGATGGCTTTCCCTAATTTTATCTTCTTTCTCTACCTGAGTTTTTTCTTTAGTTGTAATTTTAAATTCCTCTCTTAAAGTCTGTACCCATGCTTCATATCTCTCCTTCTGTCTTTCCCCCTGTTCCTTGAGTTCCTGAATTCTCTGTTCAAGGCTTTGCTTCCACACTTTGAATTTTTCTTTATATTGTACTTCTAATAATCTGCTATCCATTATCTTTTGATTGATTAATTTTAAAAAGTTATTCTCCAAGTCTTGCTTCCAAACCTCATTACTTTCTCTCTGC
MSTRG.23909.2	ENSRNOG00000046991	0.000	2.131	0.000	6.026	up	CCTGGCAACCTTTCTTGCCTCAAAGAGGGGTCTGAAGAATGGGAGAGTCATCCTGGGGCACACTTTGGCATTACAGCAGGGAACTCCAAGCAGATGCTTTCTTATGGGCACACTTGGTCTTCATCTGCATAGGCATTTGTCCACATATTCACCACTCTTTCTGGCCAAAGAATGGGGAGTTGACAGTCTTCTTGTAGTCACAGTTCAACAACTGTATTCATTCTCCATTCTTGTTCAAAATGTCTGTTGTCTTATGGGTGGTAGGAGATTGGGATTCATTTTCCCTTCACACACCACAAGACCAGCATACATTCCAGAACGGGAGAGGGGCATGCAG
MSTRG.24214.2	ENSRNOG00000001609;ENSRNOG00000001611;ENSRNOG00000001613;ENSRNOG00000028411;ENSRNOG00000039562;ENSRNOG00000039567	0.698	0.000	0.000	-5.643	down	AGCTTTCTTTCTAAATTATTGGCGGGGAATGGGTTTGAGATATTCATTTAAAGTCTAAGTCGGTTCTTGGCTTGGTTTCTGTCATTTTATTTTCCCCAGGCTAGTGTTCTAGAATTTAAAACCAGCATTTCTCAGGGAATTATGAACAAAAGACAGGGTGCTCTACGTGTCTTACGAAGCCGCAGACGACTTCATTTCGCAGATAGCCACACTTGTAATACCATCACTCAGGAACTAAGGAAGGACTTCCACAAGTTTGAGACCAGCCTTTGTTACAGTGTAAGACCCTGTCTCAAAAACCTGAAGTTACAGGTTATAAAGCTGAACAGGTTACGAGTACATACAACTATGGAAGAGGACCAGAGTTCAGCCACCTGTAACTTAAACTAGCTGCCTGACCCGGAACGCGTGAGTACTAACATGCACATACACAAAAGTATTTTTTTAAAAAAAAGCTGTTAGCCAGCTGACAGAGGCACGCCTTTAATCCCAGCACAGTCAGGCGGGTTGCTAGGAGTTTGAGGCCAGCCTGGCCTACAGGGGAGTTGCAAAACAGCCAAGGCTACATACAGAGAGAGACCTTGTCTCTAAAAACCAAAAATAAAAAAATAAAAAAAGGAAAAGCTATTTTATGGGGCTGGAGAGATGTCTCAGCAGTTAGGAGCACTGGCTGCTCTTCCAAAGGACCCCACTTCAATACTCAGTACCCAGACGGAAACTCAATAATTAACTCCAGTTCCAGGAGATCTGGCACCTTCACAGAGACATACATGCAGGCAAAACACCAATTAATGTAAAAATAAATAAATCTTGAAAAAGAAAAGCTATTTTAGGGCCTGGAAAGATGGCTCAGCTTGTAAGAGCACTTGTTGAATTCGCAGACGACTGGCATTAGACAAAGTCCAGCATGCACATGGCAGCTCACAACCATCTTTATTTCCAGTTCCAGGGAACCAGCTCAGTCTTCTGACCTCCAGTGGGAATACAGTGGTACATGGTGCACATATGAACAGCCAGCCAAAACATTCAGACACAAAGTCAGATGAAAAATTAAACAAAACCACTTTTAATTAAAAAGCTATTTTTAAAGAACGTCTGGATATTTAGCGGACAGAGACTGTCCAGATCCCAGAGTTTGAGGACAGCCTGGTCTACTGAGCAAGTTTCTAGAATTGCCAGGGTTATGTGTAGCCTGTCTCAACAAAACAAAACAACAAAAAACTGGAAATGCAGTCCCCATAGAGAAGCTCAGGTGAGGACAGATGGGAACTTGGGATAGGACATTCTCAGTGTGTGAAAGATATACGACAGGAGCCAGTTGTTTAGTTTTGTGGAAGGCTGCC
MSTRG.24326.2	--	0.000	0.223	0.011	3.719	up	CTTGTCTTTTTGAAACGAGTATGCAAGTGTTATATTTACAAATGAAACACAGAAATAATACCAAGTGTACAACTATTTGTTTAAAGAATGAATAAAGGACCATCATGTATAACCTACAAAATTTTGAGAAATCAAATAAGATCTAAAGTGTATAAGATTTCGGTGTCAAGGGATTGTTTGTGTCTTAAATGGAACACAGGTATTAGCCTT

							AGGGAGGGAGCACCTGCCATCTGACCTCTGGCCGTCTCTCGTAGGCTGTTTCTTGGATGTTTAGCCTTGACATTTCCCGTCTTTAAGTTCCATTTTTTTCAAAGTACTGTTAAAGGAGAGATTGATGTGGATGTTACTTCTAACTCCATGTTAAAACCTCAGTTTCAGAAATATCGGTAGAGCTTGTAAGTTAATGGTGGAAGCTGCGAGGACTCAGGCGATATTTTTTAATGGAGAACTGAGGGATTCTGGGAGTATGCTAACAGATGTCTAGTGACGATCTGTGGACAGTAAACAATCTAGCAAATTACATTACTAGTGAGGAGCAGCTGTTGAATGTGGTCACACGGAAGGCACGAACGGACCCTGG
MSTRG.25245.1	--	0.040	0.537	0.003	3.357	up	ATGGTTAGTATTCTCTAAAAATAATAAGAATTTCATACTGTCAAGAGCAGGAGTACTTCTTGCTTTCCTATTAGCCGCAGGAAGGCCAGAGCTAAGTACCTTTAGAGCTCATGCTGAGGGCCCCAGTCTGCCCACGAAGCTTTACTTAAAGCCTATGGGTACCAACATCTAAGTTTACAGGATGGGTTGCTTTTCATGGACTTTTGAAGTATGGTTTTTATAGATTTGCTTTATTAGTATTATTTTTTACAGCTATGCTTAAAACCTGGCGTAAGGACTCCCGACAGGCAGACCCTCCGGTGTCCTATACTCCACCTCACCCTGGAAGCTGAGTGTGTGCTTCAAGTCATCTTTGTTTCTCCAACGCACGTGACGCTTTTCACAGGAGAAGCTTTACAACATCTGTGAAGCTAAGGGATCCAACCCAGTTCGCTATTCTAAGACCCAGCTCCTTTCTTCCTAACACCTAATATTGCTTTGTGTCATCCTAGTGGCCTGCGTGGAGCGGTAAGACATGGCCTAGCTGG
MSTRG.25256.1	ENSRNOG00000002187	0.810	0.002	0.000	-5.883	down	GTGCTATGCAGCAGACTTTCACAGCAGTGAGTCTTCAAAACCAGTTTTCTTGTGAAAAGTGTATGTGCTGCTGAAAAAGCCAGTCTCAGGCAATGCCTGAGCATGGAGGGCCATGCGACCTAAGACTCACCCGGAAACAAAGATATTCCTATGAAGGTGAAAAGAACATGCTGTCGTCTCTGATCCAACTTTCAGGAACACACAGCTCATAGCCATTGTCCCACTGAAGTCCACGGAAAAGCCCCCAAACCAAGTGAGACCTTCTGGGACAACATCCAAGAAAACTAGAGCAGTCTACCAAGAGCAGAACAATCAACTGGTGGATTTGAGAGTGCCTTCAACAGTTAAACAAGCCCAGAAGCAGTCTGACAATGTGAGGAGTTTGATGTTTACACATGACCTTGAAAATGAATCTGTAAATTCTGATGGGCATTACTGGGGGTATGGAGAGCCCAAGGTTTCATTTTTGGGAAACACTATTAGAAGGTGAATGTGACTCTTAGGACTACGCTCTAGGCCTGACAGGATTCTAAGACAGAACCCGAGGGTTGGGATGATGGGTTAGTCAGGTGTTTGTCACTCAGGCACCCATTTAAAAACCCTGGCACAGCCATGGGCATCTGTGAACCCAGACCAGGAGAGCAAGAGAGACAAGAGGCTCCCCTGAGCTCACCAATCAGCTGGTCAGTCCAGCTGGGAGAGCAAACTCTGGACTGTGAAAAGATGAATCTCAACAAACAAGATGGAAGGCAGAATATCTGTGCTGCACGTGTGAAGCCTTGGGTTCGAGTCCCAGGACATCAATGAGTAAGATGAGGCATGGGGAGGAAGACAGCCAGTGTCAGCTTCTGCCATGTGCACATGCACACAGGATTGGAGAGGCCATTTCTTCCTTTATAGGAGGGGCAGGGTAATCACCGGTCTCTCTACCTTCCTATCTATCGTTTCTTCACCCTATGGACACCTCCAGGATAGGCTGCTGTACCCTTTAGTTTTGAGAGCAGAAAAGTAAGTTTGCTTGATGCTGCCAGTCACTCCCTCCCATAGAGGTCCCCACTCACTCCTTCAATTTCCCTGTCATTCCTTCAGTCCATCCCCGCAACACTTTGGTCACAGAGTTAAAAGTCAGTCAATAATACTGTACTTGTCTTTTTGCTTTGCATAATCTCATTGCTATTAAATACAAAGATGGAGTAAAATGCAAATAAAAATCACCAACTTCTAAAGATTGCTGAACTGTAGGTAACACTTGGCAACGTCTTAGAACAGCATGTAGCACACAGCAAAAACTCAGCAGCGTTGTGTCGCTGAACAGTGGCAAACTGACCAGGAGTAAATACTCCACTTTAATAACTCTTGTCTCAGAAATGTACTTGTAAACATGATCCCTCTGAGATGGTGGAGACCCAGAGCAGTAACAGATCATTTTATCAGATGAGCTGCAAAACCGGCTAATACATGTCCTGTATCAGTTTTTATTAAAAATGAATTCACAATAAAGAAAATAGAACAATATCCTATAAAACTATTAACTCTGAAGTCTAAGATGATACCCAGGAGATCACAGTCACAGGCATAGTTTTGTCCTCTAGGTCTCTCATTTCAGACTTCTCCTGGTTCCTTAAGAATTATCTAAAGGAGGAGTTCTGAGTCCAGGTTCACAGGCCTAAGTTCTCAACCCACTAGTTCACCAGCCTTCTTTCTGCAATATGTATCAAGCTAGATACACAATGTCCTGCCTCGGCCAGCAGATGTCACCGTCCCACATAAATTAACAAACTCCATACAGGATTCTTTCTTAAAAGGCCAGAGAGTAAGCATTTCAGGCTTATGAGCAAACTTTGTCTCTGTCACAACTAC

							TCAGTGCCACCGTAGTACAAGAGCAGGGAAATATATAAACAGTAGGTTCTAACAAAACTTTAGTTATAAAGTAAAACTAGATATCATTTCTTTTTTTTTTTTTGGTTCTTTTTTTCGGAACTGGGGACCGAACCCAGGGCCTTGCGCTTCCTAGGTAAGCGCTCTACCACTGAGCTAAATCCCCAGCCCCTAGATATCATTTCTAAAAACACAACTGGCTGTATGGTACCGACCCTAGTTAAGACTGATGCTGTCCAACAGAAACATAGTATAAGCAATATATGTAATTTAAAATTCTAATTAGTCGCATCAAAAGTTAAAAGGACGACTTACTTCTCATGTTTTATTTACGTGTAGCCAGAGTGGGGCTGCAGCTCAACAGTAGAGCTCTTGTCCAGCACATTGAAAGCCCTGAGCCTGATCCCCAACACCACAAAAATAAATAAGTAAATATAACAACAGTACTAACATTTCACTGTGTAAGGCCAGAACTAAAACACTTTTTGTATTAAATCTTCAAAATCCAGCATATATTTACAGTGTACCCAAAAACAGATGCTAAATTTTTATCAGAAACATTCCATCTCTATTTAGACTAAATATAATTTATAGTTAAAAGAGATTTACACACCGAAAGTACTCCAAAAATCTCGGTTTTATGGTTGGATATCAGGTTTCAAATTTATTAAATTAATTAAAATTTAAGCCTAGGGGTTAGAAAAAATAGCCCAGTCAGTAAAGTACCTGCCGTCTCAAGCACAAGAATCTGAGTTGGATGCCCAGAAGCTGGCAAGGACTGACAGGCAGTATGGTTCCTAACCCAAGCAAAGGGGAGAGAGAACAGACGGATCCGTGGAGTTGACCGGCCGGCTGGCCTAGCTAAATCCATAAGCTTCCGGTTCCGTACGAAAAATAAAGGTAGGGAGTGAATAAGGAGAAACAAAAGTGTATGCACATGGGTACACAGCCTCACTGATGTGAACACACACACACACACACACATTACACAGCCTCACTGATGTGAACACACACACACACACA
MSTRG.25362.2	ENSRNOG00000026991	0.000	0.263	0.001	4.308	up	GCCACATCATCACCAGTCCTGCAAATCACCTCGAAGAGAAGATAATGGTATAAAGCAGGAGGAATGCAGAGACCGTCCGTCCATCGGCTGCGGCATCCCCTCCACTGCCATGGCAGACTTGCATACGGAGAAACTCACAGCCTCCTAGGCTGCAGAGCCACACGTTACTCCCTTTCTGACCTCTGCTCAAGCAGAAGGAAGGACAAGGCCACACAGCACAGTAATCTGGAACTGGAGACCACCCTCCATTTTTCTAAGGCTGCTTGAATGAGCATCAGGACTCTCATCTGTCCACCACTCAGGAGGGGTCTCGGAATGCTTCCTGTGTTTGGGGGACTGCACCGGTGCTAAGAACGATAATCCCACTGAGCATTCTAGACGTTTGCTCTTTGGTTCACAGAACAATGTTATCGAGCGTCTGGTAATCGATGCTAGAAATACATTGACAAATAAGAACGGACAGAACCTACAGTGTGCACTTGGAGGAGTAAAACGAGTGATTCAGTGCGGGAAATGCAGGCTAACCCGGGGATGAGCACAGGATGCTGGAGGAGGATGCAGCCCAGCCCAGGGCTAGCACAGCTTCCCCAAGGTGACATTGAAACTGAGACCTGAAAGGGAGTGGTAGGCAGACGGCTGGAGAGAAAGAAAACAGCCCGGGTAGAGACCTATTGTGAGAAACTGTACTGCATTCAGGGGCCTGCAAATTATTCAGAATCTTAGGAAATCAAAAATTTTACATATATAAGAATTTACTATATGTAACACTATATGCAATAGATAGCTTTAC
MSTRG.25417.1	ENSRNOG00000001752;ENSRNOG00000001754;ENSRNOG00000024960;ENSRNOG00000043345;	0.478	1.251	0.024	2.137	up	GGATGACAGTGGCTTTCTGGAAAGGACAGTGGAGAAGAGATCTATGTGTGGAAAGTAGAACCACGAAGTGAAGTATCATGTGTGCATGCCCAAGAAACATCCTGAAGACACATGAAGTTGAGAACTAGAGAAGCAACTCTACAACAGAGATGGTCATAGTGTTTCCTCTTTCCACTGGGCCAGAAAATCCTCAGGGTTGCAGTTGGTTGAGTGGGCAGTTGACAGACATATGCATGTTGTCACAGAGTCTTCTCTAAGTTAGCAGTGTGTGACTTCCAGCTTAAAGGTGAGCGTGTAGAGACGCTGTAGGAGGGAATCGCAGGAGTTTATGTAGAGTTCATGGAGAAGGCTCGGTCCCATCTTACATCTGCCTGTCCCTCCTCCAGTCCCTTTATTTCTAGCCAGCTTGACTTAGAAAGTATGAAACTGGCTGGGTTTTATTTAATATTTTTAATATATTGAGAAGCATGGTCTGCCTGGACTGCACTTCTCTAGCAATGAGAAATAAAATCGTGCAGCTATTTTAAAAGTTGTATATATAATGTATATAAAACTGTAAAAAAAGGGGGGCAAAGGCATTGCTTTGTTCTACTTCAATTTCTTAAAAATCACTACATGGTACAACATTAGAGTGACATTAGGGGACAGTAGGGTAGGAGAGAGAAGAGGAGACATGTGCTGGCTCATTTGGTAGTGTTTGGGCTCATAGTCCATGGCTAGAAGAGAATTATGATAAAGGTTCCTCAGTTGGTTAAAGGCGGGAAGCAGGTCTACTTACAATTATGCATATGCCCAGGGCTGATTCAGAAGTATAGGAATGTGCCTCCTGGCATCTGGCTTCAGTGTTTGGCCTTCTGAGAACTGGTCTCCACACAGAACAGTCTCAGCTGCTCTTACAG

	ENSRNOG00000048188						TACTCAGTATGAGCACTGTTAGTATAAACCCTAGCAGAACACATGGTCAGCGGTTCTTTTATTTCTTAACTTTATAATGCTGTAGATGTTATGAAGAATTTTTAATTTCATATTGTTTACATTATACAACAGTCGAGGCCTCAAAGTCTCCATTGGGGCTGTCATCAAATGTTTACTCACCCGTCTGAACCAGTGCCGGCCTGCACCAGGTCAGGCCAGAGTGTTCCTAATCACCTAGGTGTTGCCCCCTGGGATTTAATGCTGAAAACTTCAGGTTGGCAGCAATATGGAAGCTTCATTGCAAAGAGGTGAATGTTATTTCCTGTTTTAACCCCTAAAACTTCTCACTAACACTTAGCACATGAGCTGAGGTGGCTGTTGATTTTTTTCTTTTGCTAGAACAGAAACATCTTTCTGTATAAAAGGATAATTAATATGGTTCCGATACATAAGAATTGTTGAAGGCAAGTAAAATCTTTGTACTTTTTACATGATTGCTGTATGTATATTTTGTTTTAAATCTAATAAAATTAGGGACAGAGAGCAGCTTGCTGGGGTTCTTGAGGCCCTTGATAATTAAATTTGTCAATACTGCTATGAAGCAGGAGGCTTGGCCACACAACGGTCTGTATCGCCTCCTGAGAGGGTTACAGGTATTTATTGTACATTTGATCTGTTGCTTGAGAAAGAGAAATTTGTTAAAGATTCTTTTGATAATCTGTATCAAGAAGAAACCTTGTACAAAGTGGAATTATATGTATTAGAGAAGATAGCTGGCCCAGGAATTGTTTTGGTTTTCCTGTTTTTTTGTGTTGTTTCGTTTCGTTTTGTTTTTCTGCTCTAGAACAACTCAATATGGTGGCATTTAAGAATTGATGAAAGCCCTGGTTAAGGATGGAGACTGACTGGTTAAGAGGATGGAGACTGACTAGTTAAGAGGATGGAGACTGACTGACTAGTTAAGAGGATAGAGACTGACTAGTTAGGAGGATGGAGACTGACTAGTTAAGAGGATGGAGAGTGACTAGTTAGGAGGATGGAGACTGACTAGTTAGGAGGATGAAGACTGACTAGTTAGGAGGATGGAGACTGACTAGTTAGGAGGATGGAGACTGACTAGTTAGGAGGGAGACTGACTAGTAGGAGGATGGAGACTGACTAGTTAGGAGGATGGAGACTGACTAGTTAGGAGGATGGAGACTGACTAGTTAGGAGGATGGAGACTGACTAGTTAGGAGGATGGAGACTGACTAGTTAAGAGGATGGAGACTGACTAGTTACAAGGCGCCAGCAGTCCATATATACTTGCTGCCTCTTCAGCTGTGACGGCCCCTCAAGCTTCTCGTGAGTTTGATGCATAACTTCTGTTTTTTGTCTTGTTGTTGGTAAATATTCATTCGCAACAAGTTTTTGAAGGTGCTGACTGGAAAACAAAATCACGTGGTTATTCATTTCTCATTGAACTTAGAGTCGGATAATTGCCTAACAATTTAACACCAAGCTGTTCTCTCTGTGTCTTGTCTTGTTTTTGGTTTTTTAAAAGACTGGTTCTCACTTTGTAGCTCAGGCTATCCGTGATCTCACAGCGGTCTCCCTACGTCAGCCTCCCCAGAACTGGGATTAGAGATATAAGCTACATTCCCAGAACAACAAGCCATTTTAGCTCAAAGATTTGAATGTTCCTAGTTAGGATGGGCATCTAGAGAACAGAGGACCTTCATTTATAACCTGATCTATTTACTAGAAATCTTGTTACTAGAGACCAGTAGTGGCTGCCCAGATCATTAAGGCAAACTGCTCAGTGTTGAAGGAAGTCATGTTCTTGTTGGTGCTTAAAGAGCGTGTTTCCACACACAGTTGTACCTGAGTCAGTGCTGCTCAAGTATGGCTGGATCTCAGAGTGGCTTAAAACATACTAAGCTTGGGTCCGTATCCTGATTCTGGTGTCAGAACTGGGATATGCTCTGACCATAAGGATTTAAAAATGCCCTGGTTTCAGAATGCTACCAAAGGTTGAGAGCAGTTGTGCTCAATTTCCCTTAGAGGTTTCCCATCTACAAAGTAGGAAATTCTTGCTCCCCTCACTACCTCACAAGGATACAGAGGGTGCACGAGAGCGCACTGGGAGTGTTGGCGTTGTGGGTAGAGGACGCTGTAGAAGTGCAAGCATCTGTCACAGTTCTCAATTTCAGCTGCAGCCGCGATCGAAAACGGATAATTGAATTTGTTGTTGTTGTAGGAAGCTCTTAATGTGATTCCGTGAGGAAGCATGTAGGGGTGGAGCAAACGTTGATGACTACTCTTATTCACTATGGTCTTCTAGCCTACTTGAAACTGGCGGTTATATTATCTAAGTTTTCATAGGAAAGAAGAAATATTAAGGCTTAATTCTGTAATGTTCATTGGCTCATAATTCATTAAAAAGTTCATACAAGG
MSTRG.25576.2	ENSRNOG00000001720;ENS	0.529	0.000	0.000	-5.368	down	CCTCTGTCACCCTCTCCCTGAGTTGACTTCAGTGGTTCAGCTTCGATCTAGAACGTCTCTTCTGTCTTGTGCTCTTTCCTCCACAACTCCTAACATCTCAGGACTAGCATTAGCCTTTACCACATGGGTCTTTGGGGACACGTCATAGCCATGTAGCACCGACCTCCACAATACGAGTCAAAACTAAGGAAGCCTCTGGTTTAGGAGGTAAGTAGCTTTGTACCACAGCCTTCAACTGTCGTCTGAGACAAACCCAATTCTCCTAAACTAAAGAAGGAAAAGAGCTACGCCTTCCCTGACAGAAGTGTCCCCGAACTAAAACAAGCGAGTGGCAAACACAAGTA

	RNOG00000047234						CCTATCTGAGGGAGATCAGAAACACAAACTTCGGGGTTTGCTGGGTCCCTACCTGAACTGTCTCTAATCTTCTTCAGTTGAGGTCTAGAAAAGAGAAACCAGCCACCCACACGCACAGTGACACTACCTGCCTCTATTTCCAGCAAACTTAGACAAGTTCTACGGAGTATATTGACCTGAGTAACCAACCCTGCTAAGAATGACCCGCTGGCCAATGAGAAAAACGGATCCAAGACTCCCTGCTGGCCCATCACTCATGACAGCCTGTTCCTCTGGGACTGGAGACCCACTCAGAACCTGGGTGCAAAGGGGGAAAGTTTAAAAACCAGCCAATCAGCAGTCCTGATCTCAGCAGATCCCTGGGCTGGCCTTTGCGCTCTGCGGAAGAATCCACTCAGATCATGAACGGTGGGTGTGGCACAGCCGCCTTGGTCGTGGATCCCCAGGTGAGCAAATAGGAACTGAGGTCCCTTTGTAATGTCAGAAATATTTCAGCTCCTACCTCAAGGAGTGGAAGCAAAGTTGATTTACGATGAGATGTATGGCGTGTGCGTGTGTGCGTGTGTGCGTGTGTGTGTGTGTGTGTGCGTGTGTGCGTGTGTGTGTGTGTGTGTGTGTGTGTGTTTCTGGGTCCAAGTCTTCAATCCACTCCTCCGGCCTGTACTGTAGCTTATAAAACATGCCAATTTTTGGTCTCAAATGGCTACTCTGGGCCAACAGTCTCCCAGCAAAGTTACGACCACCACTTCCTCTTCCCCCCTGGACTCTGATGAAAGAGGCTCTGGCTTGTACTTACCTCCCATCGTCCTCACCTTGGTGCTCTCTGGGTGGCTGAAATCTGTTAGGGAAGTTCGCAGAGCAGCTGGAGGCCGCTCCTTAGACCTGTGCACTCACCGGGGACTTGTAGCCCTGGGCTGTTTTGTGTTTTATCTCCCGGCTCCATGTGGACTTTCTGAGGATAACTCGCTTTCCAACAGAAACCACGATACACGCTTCAAACAGCCAGCCAAATCACTGGTGGGACGGACAGGCAGCTTGGAGGATGGGAGACAGTCAATCCACGGAGGATCTATTGATCTGCCGAGGCTGTGGAGGGAGAACCTGCGGT
MSTRG.25580.1	ENSRNOG00000001720	0.000	0.546	0.000	6.652	up	GTTAATATATGTAATATATGCATGTATATATGTTGGTATATATGATACTTAAATAACTTGCAAACATAATATACATGTTTGGATGCTTGTATTTTTTCTTTATGTGTATTTCTGAAAAGTAAAGTACTTTTGTTCATCTAAAATATTCATGGAATGTTAATATTTTCAAAGAAATAGACGGGAAAGCACTGTATAAGTTGGTCCATGCCCATATTTGACAGAACGGAAGCTGCAACCTGGTTAAATGAATCACAGCTCCGCGGCCAGGAGAAAGCTTTGAAACTGATCCACTCTGCTTTCCTTTCCAGGGAGGAGCTTCTTGCTGCCCACACATTTTGGCTAGCCTTTATTTGCTGGCAGAAAGGGTACAGAGCTTGGTGTGGCCTTGATTTCCTTGCAGTGAGTCCTGGCTCACAGAGCAGAATCATATGCATGGCACCAAGAGTGTCTGAAAAAAATGTGCGCCAGGATATGGGGTGCCCTTCTCCCACTTTGCCCAGCTCAAGGGCACAGGGTACCCAGGACTCCAGAAGAACGCTCAAACAAAGAAACAAAAAGTCCCAGACTTACTTACAAAAGTAAGTAAGATTGGTGGAAAAGATAAACATCTAAGACCTTTCTGGCTCGCTCCTCGTCTGTGTGGGTGGCTTCAGCTGGGGCACACTGGGGACAAGAGGCAGTGCTGTTCGTAGTAGTCAGCGAGACCGGCATAACCTGGGACACATGATTGTTCTTGTTCCTGTGGCCTCTGGCTATACTAGATTGTTCATTCAGCCATCTAGAGGCCTTCCGAAGAGAAATGAGACATGATTCGTGTTTGACACCTGTGGGAGGTGATGCCGTCCATACACGGTCCGTCACGGGATGAGCTCCAGTTTCGTCGCCGTCGTCGTTGTGGTAGGGTCCAAACTGGACTTGGACTTTGGATCCTTCCACCTTCTACTCCTCAACTGGAAGGGTTATAGACGTGTGACCCCCATGTCAAGCTTGAGCCCCAATTTTTTAACCAAGAAGCTTTAATTCAGCACATAGGACAGGAAGAAAGGATGGAGAGGCTCACTGACAAGTGTACTTTCAGTGTGATCCGCTGGACTTGGTTTTGTCTTCGATAGGGCCGTTGTTCTCCGGACATCCCCATCCCCGTGGGTCAGTTTGCTCCCTGGTGTGGCTACAGAGCAGTAATCCTACCTCAGCCGCTTGTATCTGCCAGCCCTGGCTGAGAAGGTACATTTCCCTTTGGTGGGTGAATATTAAGAAGTCAGGTGTTCCGTGCAAATCTCAGGCCATTGTTCCATAGCTGGCAGAGGCCTCCAGGAAGGAAGGCCTCCCACCTGAATATCGGCCCCGTGAGCCTTGAACTCGACAAGTCTCGAATGCTTAGGAAGGTGACGTTATTTTAAGGAACAGGTCAAAGCCTCTGAGTCCTAACTCCCATAAGCCTACTCCAGGACTGCCCGTGGGCAGTCACAGAAGGCAAACCAAGCTGCTGTGGAGGTCGGCCCCCAGGACTGCGTTTGCTCCCACTCAGCACAGCCGGCGGTGGAGTGGGCTACCTGCTCTTTCTATTGCTGTCCTTGGCTCTGACCATGTAGAGGTGCCACACTCGAGTGGGACTGGAACACTGGCTGCCACCCTGCTTGCCTTGGATCTGTGCCTC

							GAAGGGAAACTTGTCGAAACTGCCCAGGTTGGCTGTTGTCTTGTCGCTCCCTCAAAATCGGCCGGCTGGAAGAAAGAACAGATCCCAGCATTCCTTGGGACGGGAGGCAGGTGGGAGAGCAGGTGTGAGGGGACCAGCCGGCTCTGAGGAAGGTTTGAGGGGAGCTTTGTCAGGCCTCCCGCACCTGACAGCCTTCAGCTGCTCACCTGGAAGGCAGCACTCCCCGCCACCTTTCCCGTGGGAATTCAGTACAGCCGTGGCAACCCCTGACCTCTGAGCCGCTGCCAGCAGGTCTCAAGCCTTTCTCCTGTGCCATCACAGTGACATATGTCATTTTTATTGAGGCAAGATTAGATTTAGAAAAAGACTTTTTTTTT
MSTRG.25687.1	ENSRNOG00000038512	0.145	0.000	0.000	-6.107	down	TCCTACTGGTTGGAGCCACCCATACCTTCTGACTGCTTGACCAATATTAAGATACTGCTTCCACCAACACCATAAATTCTAACTTGTGCAGGATGAGTGAGCCCTTGCTGTGGGCATATATTTTAATCCTTTCTAATGGATTCTTATCTCCAAGAAACAGAATCACTGGCTCAGTATATTATGCCATCAAGATAAGTGCATATTAAAATAATAGCAAAAATACCTCAACAAACTGTTCCCAAAGATATTAACACATACATAAATGAAAATAAATTTGTGGACAGTCTACTTAACATTGTGGTAAATTTAAAATATAGACTCGTGTGTGCACATCTCATGATAAAGATGAAGAGAAAGCATGTCTTTCTTAACTCACACTTCCCAGGACTTGACTACACGGACCAGCACAAATCACCTCTAGTAGTACTGAGTCATACACTGGTCTGGCCTGACTTACGACAGCTTTCCGATTAGTTTGGGATAGTCTCAAGAATAGGGCATATGGAGTTGGGTAAACACACTTATCTGAATGATGATTTGCACGAGCATATTTAGAAAGGCGTATCTGAGTAGGGGACTTGGCCTGGGTAAAGTCCGTGAAGGAGAGTTGTTTTACCACACTCCTACAAGAGAGAGAAGTGTAGTGAGTGGCCTGGAGCCTCACTGAGGATGACAGATTACGCCTCTGAGATTCCATCTAGTGAAATGGTATATGTGCATAAACTCAGCTTTCAAAAGAACCAATATTTCCCACAAGTGGGTCCTTCTATTCTATGGAATAAAATTAATTGAAAATATTTCACTTAAAATTCTAACCAAGATGTAGTCAGATGAATAAATAGTCTTACTAAACTGACGGGATGATAAGATTATAACTACTTTAAGTTTAGAAGGAAAGCAATTCATAACAGAAGTCACAATTTAAAGTAACAGCTCTTTCAGGCTCAATTACACAGGTGATGAGAGTTCACTCAGATCCCTGCCTATCACTGTCAATTCCAAGTCTCGTTTCTTAGACACTTTCAAGCCTAAGAAAGGACAGGCCTCCCCAGTATGGTATGCTACACACGAGAAACGATAAAAAATGTTCTAAATATACTGTGCACTGGAAAGTTACCATTATAAAAACCTAAGTTTTGCCACTATAAATAGAATCTGGGAACTACTTTCAAATAGATGAGCCAACCTAGCTGTCCCTTCTCTCTCCATCTCCCTCCTCCTTCCCTTACTCTTTTGGTGTGGTGCTGCAGACGACCTGGATGGGCAGGCGTTCTACTCTGAACAACACCTCCAGTTCCTACACAGTTACCCTGATTCTTATCTTTTCTGCTTCACATCCTCCAGTCAGGGAACACAAAGACTTCCCCAAAGACGACTTTTTAAACAAAATTCAATATAGTAAGAAAAAATGGGAGTGGAGCTCTCACTAGACAGGGTCTAGTAGTTATGCAGTCCTAGCTGTCCTACAACTCATTACGTACACAGACCAGGGTGACCTTCCAGTCACAAAGACCTTCCTTTTTCTGTTTTACAACTACTGTAAACACATAGAGGTGTGCACTGCCACTGAAAGGCAAACTAAAATACTAGCATTGAAGATAAATTTACACTAAGTATGAACTAAAATGTAACTTAATATTTTTGGTAATTGTGTATTATGCAATTTTATAGGAAGAATTTGACATTTAGGGCACATTGCAGTGTATATGGGCTGGAATTTAGAACAGTAAGTACAGTATTTAAGGTTCTGGAAGGTGAGAAGTTAGTCTGTTAACCAAGGAGATGTCACAGACATACACCACAGTGAGCTCCTCTCAGAGTCAGACGCAATTTAACTGTTCTATGGAAAGTCAACCAATCCTCGCTTTAACTTAGCAACTTCATTTCCATCTGCCTTGTCCCACACCCATTCTGCAAATGCTCTTGGACTCAGTGCTGTCTGTCAGCCTGCTGTTTATTAATCGTTAGCTGAAATAGAATCTGGCATATACCACATCTTTAAGAACTCTGTTTTTAACAGCAAGAGATGATTCCAATACCTAATTATTATTTCTTAATGAAATCTCCTGACTGCACCAAGAAGGTTCAAACCCCAACAGGACCTGATAAGTCTAAAGATCTGAAGCTGGCTGAGTATAAAGTGAAAAGCACAATGCAGTAATACAATAATCCTTTATTAGCTATAGTCACTGTTTTTAAACCCTGTGTTAACAGAGCAAACATTTACTAAAACTCCAGGACTAGAGACTTTCAGGACACAAATCTGCTTGAAAACCCCGGCACATAAACAATGCACTAATGGTTAACTGGCCCTTAGAGCAAGCGCACTCAAATGAGGGTCACAGAATCCAACCGTGGCCGTCAATGGAACCCTCAAGTGTCCTCCCTACCCCACAAGTTCCATCTAGCAATAGTGTCACTTTCTCAGAAGCCTAC

							TTACGAGCGCTAGCAACTCTCTCTAGCCTCCACGGTCTCTGTTCATGTCTTCCTAACATGGTACATCAAA<br>TATAAAGATGCTTATGTGCACATCTTTAAATATATCCATGTTCCTCTGAAGCTTAGACACTGACATAAAGC<br>TCAAAGCCACAATATATGTAGCATTCAGTATAGCTTGCTAAACTACAATTTAATTTTTTCTTATAACAAAG<br>TCCTTTCAACATGAAATATGACATAATAGCCAACCAAGTCTAATGAATATGCAACCTATAAACTAGTTTTT<br>CAATCACAATTGAAGTTTGATTTGACCACGGCAGGCTTTCCTCCATACTTCTGTTACAATAATCTTTCCAT<br>CTCTGTAAAACTTAGTAAACATTTACTGAAACTAGTCTGAAGAGTGCTATGCCTACTAAAACTACACAC<br>ATCATGATATCATGCTACTGTTGGAGCCGCAAGGAGAGCCTTCCCTCAGTGTGTCCCAGGCAGCAGAGT<br>GCACCTCTGTCCCGGGATGGATACAGATGTGAGCATCAGAGGAGCCCAGAGTTCTCGTTCCTACATACG<br>ACTAATTTCTTCTCTTTAAACTGTGCTTTTTGACTTTACTTTTTGCATGTGAACATATGGTATAAAAGTATG<br>CATGTGTGTGGTGTGTACTGTGTAATACATGTATGTATAGATTCCAGCAGTTACCTGGTGCTTTTCTCTTG<br>CTTCCTCTCAATTTTATATATTGAAGCCATATTAGTCTCTTGCTAATTTAGAACCATTTCTGGGTAGGGTAG<br>CTATCCAGCTTGTTCCAGAAATTCTCCTTTTCTCCTGAGTATAGGGATTACACGTAGCTGTTAAGGCCAA<br>CTGACTTTTTTATGGATTCAAAAGATTTAAAGTCAGATTTTCATGCCCATGTATGAAGTGCTTCACCCAC<br>AAAGCAACACCCCAGCCCTTACCCTCCTCGTTTCTTTACAGATATTGAGAGCTTGTATATCTTGGCACTC<br>AAAACAACTGTAGAGTTAAGGGTGCATATTCAAACAAATGTTAACTGAAAACAATGATTTTTAAGCTTT<br>AATTACACCATTACTTGTAGCTTTAAAATCTTCCAAGTTCACCTAGAACTTATCAGTTATAGAAAATACCC<br>TGCACTAGCTGTCTCCTACCTTGATGCACCTGATAGGGATGATCTGAATGTTTGACACTACTCTAAAAGA<br>CAGCCTAGGTGATGTCTCACTCTCAGTAGTTATGATGAGACCAAGAAGACACAGTACTTCTAGTCCCAC<br>TGGAACTACACTAGACAAACATATAAGTCAACTACAACCACATCTTCTCAGTTCATTTAAATAAAACAA<br>AATAGCCAAGAGGTTTCAAAGCAGAATGAGAGTATCTGATGCAAGGTTGTGGCAAACAGTGACTAGTA<br>CCTGGGATGGGTACTCTTCCAATGTGGCCCTGGAGAAACCCCTAAGAATGCAGCATGCTCTTACTGGAT<br>AGTACTGGTCTTTCCTTCTTCCTACAGACCTAAGATCAATCAGACACCTTATATAGTGTCTCACTCCAAG<br>ATTCATTTTAAGGACAGAAGGTCAAATTAAATAGAAACAAAGGTTGCTTAAGATGCTTATTAAACTGAC<br>TAGCTTTAGATACATGACTTCCAACAGGGCAATCCATTACCTGCAACATGGTGGTGGCGAAGTTCTTTAT<br>CCTCTGAGAAGGGAGAAGTTCTGGGCCTATGTCTCCTTTCTCTGTGGTTACCTTGCCCAAGGTCCCTCC<br>CATACACAATTTCCTTACAGGGAAGTCCTGTTTTCTGTGAAGACTTGGGTGAAAGTCTGCCATGGTGTC<br>GAGACTGCTTTTCCCAACTCGGAGTTCCAGGGCTACAGTTAGCTTCCCAGTCTTCAGAGCCAGTCAGA<br>AACACCTCCCCACTGATCTTCGGCAGCGGGGACTTCCAGGGCCTCTCCCGACCGCGCTGCTTCCTTTCC<br>TGCTGCTCACAGGCAGTGTGGACATCATCCCTCCCTCTGCCCCAGCTCACAGAAGGCAGGGGTCTCTCT<br>GACGGATGCTGTTCGTCCAGCTCTTCCAAGTTCTCCTGCCGATGCCTCAAGTTTTCCTTCTGCTTTACAG<br>TCTTCCCATCGTTTTGGAGTCTACCAGATTTTGAGTGTCCAGAACCCAGTTCCCTGTCCCTCCTTGACCT<br>TGACTGGTCTGAGTGGAGGTAGAGGGTATAAAAATAAAAATGGATGACTTAATGAGAGTATAAATTCAT<br>TACACAGGGAGTTGGGACCCAATACTAACATATTTTTAGGTGGATAAAATTGCTTTGAAACTTTTACAAA<br>TTGAGATTTGGAGATGAATACTGGAATGGTCCATGCATACATAGCCAACAGGTAAAAAGCAAAACTATA<br>GTGAAACAATTTGTATCTAAAAGAATCTGATCATGATTATGAAGAGCTTAGCATGTTCATATTTGCCAGTA<br>ACAAAATGATGGACAACAATGCTACTGTATAAGGCCAATGAGCCGCTGGAATAGGTGCTAACAGAGGCT<br>GTAAAGTTTTGTAAAACAGTGGTTTTGTTGTGTTTTTAAATCAGATGAGATGACATCATCACTACAGTGA<br>CAGTCAAGAGCAGGCACTGCTGCTTAGTTTAAAATGACTCGCTGATCTCCCCCATGTATCCAGTGCAAG<br>CATGCCCCACACTTGTGGAGCAGACTACATTACAATTCAGTGGAGAAGGAGAACAGAGTGGAGGAAAT<br>TGTTAAGAAGTAAATGACTCATATACTGGGCTAATTTTGTTCCTTATTTTAGTAGAATCTTAGCCAGGAAA<br>AAACTACAAGAGCAATCGCATATGGCACACATGAGCCATACTGTCTTGTATGTCTAAAGTATAAGCTATA

							AATACAAGTTCTAGTTTAAAAATCAAAAAGCCCACCGTGTACATAGGTGCTTTTATAGACTCTTATCAGA<br>CCCAAGTACATTATAAAAACAAGAAAACAAATTAGTAAGTACTCTGTAAGGCCACAGTAATTTTAGCATT<br>CTTAAAGTACTTAAATTAACTACACTTCGGAAATAGTAAAAACAAATTCTTCTTCCAAGAAGAGACGCT<br>CAGGTAACTAAGCTGCTCCCTCCTCGTCAGTCGCTGCTGAAACACTGTGGAAACAGTTTCCGTGAGCA<br>GCAGACCTGAGGAGTGTTTGCTGGGCTGGGTGGTTTGACTCCAATATAAGGAAAAGGATGTAAAAATA<br>CTGCTATGCATATAAGAAGGAAAGCATGCACTAAATTCAACACTTACCAAGTAACTTCCACTCAATATCT<br>CAGTCTCCCTTTACACTTCCTAAAGATCAGGAAATTTCCTTCAGAGGTGGGTCTCAATTAAATCTGTTCC<br>CTTCGTCAGATATTGTTGAACAGATTTTCTCCAGAGTTCATAGTCAAATTTGTACTTGACATAAGAATAA<br>GTGAAATGTAGCAGTTTTTACAATAAAAACATCTGTTCTTTTAGCATTCATTAACTCTGTCTTGACAACA<br>GATAAAGAATTAATTGGCTACTGCAAATAATTAATATAATATTGACCAGCTATGAAATGATGCCCTCATTC<br>AGAGAAGTGGTTTGTAGTGTCTGTTCCAGCCTACTTACGGGGCTATGTGAGTGGTCCATCATTAAGCTC<br>AGAGCAAAAACAACTATTTTTGTTTGAACATGAATTTATGAGACTATGATTGATTTTTTAATTCCTCCTGA<br>ACAGGTAACTGATAGTCATTTTATTTTCTATAGTTTATCTAAGAAGGCATCTTTGAATTGAGAGGAGGAG<br>TGAGCACAGTTGAGAGTTCTACCCTAATCCTTTCCACCCGCTTCTGGTTGGAGAGACACTTAGACGTTC<br>TTGAGACCAAGTTATCTGGCAAATATAATTAATGTCTTCATTGGCAATCTGTGTGTGGTATGAACAGCAC<br>ACACATACATATATAAATGTATCTACATTTTAAGTGTGTGCCTCCATGTTTCTCACTCAAAGGAAGTTTTG<br>GTTTAAACAAAATGCACATCCATATGTATCTATGTATGACTGTATATATGAATTTATGTATGTATGTATATAA<br>AACTTTAAATAAACTTTTAAAATTACAAGTCATTCAACAAAATCACATTATTTGATCTTTTCAAAATTTAT<br>GTAGGGGATATATTTTTATAATTTTACAAATAAGAAAAATGCCAGAAGTCAAACTAGAAATTTTCCTAAG<br>AAACTGAAATGATAGACTTCACAGGTTCCTGGTGGGTGAGAAGCAATAACAAATATGAACATGGTACA<br>AGGCAAGTGGCTCTGAAGAAAAACAAGGATTCACAAATGAATGATTTCTGCTATTTTTGACAAACATCT<br>CACAAATGTTTTGGATAATTTCAAATTTATAATGTATAGTTGACTATTTTTAAGATTAATTGAAGCTTACTA<br>CAGCACAAGCTGAAAGGGCAAGCACATCAAAGCACTCTGATTGTTACATAATTAAGGCAAGCATACTG<br>ACGCACTCTGATTATTACATAATTAAGTCAAAAACCAGATTAAAAAAGACAGAGGCAGAATCGTAAGAA<br>TTGATCTATTGATCGTGTGTTGGGAAAGAACCCTCACATAGAGCAGATACAGGTGGCTTCGTAAAGGTA<br>AGATGAATGGCATACTCTATGAAATCCATCTTGGTGGAACTCAAAGGCATATAAATGGCTCAGAGTAGCC<br>AACATTTCCCTTTCCTTAATACTCTTGATACTTTGAAACAAGCATATATTCCACTCTCTACAATTTTATAAA<br>AGTAACTAAAATAAATATAATTTTAGGGATCCTGAAAAGAAAAACAAAACAAAAAACAAAAAGAACAA<br>AACAAAACAAACCCAAAAAACAAAAACCATAAAATGTTTAAATAGTCTCCTTCTATAAAGACTCACCA<br>ACATACCAGTACTACAATCCAAATACACGGGTTTGATCAAGTTAGAATTAGGGTTTTAATACAAGGACCT<br>AAAATGGACATTTTAGCTGACTACTCCGCTATCCTCATGTTTTTCAGATTTTAAAATAGAGGGGCTTAAA<br>AACGGACTCCAGTGGTAAAGCACACTCCTCCAGAGGACCCAAGTTTGACGCCCAGCACCCACAGGGT<br>GGCACACAACATCTGTGACTCAGTTCCAGGGGCTCTCATACCCTCTTCTGGCCTCCATAGGCACGTGCA<br>GTACCTTGAGTGCAAAACATCCTTACACATAAAATAAAATTTTAAATTTTGAGTTATTTTGTTATATTGAA<br>CATACTTTGAGAGCAAATTTAACAAATAAAAGCCGCCACCATTAAAATCCTGCATTTCTACGATTTGTTT<br>TGACACTAGTGACTAATAAATAATTTTATACCTCCTAGATCCTATTACAAAACATTTAGTAGCAATTTTTTT<br>CTTTGTGTAAGATCCAATATATAATTTCAGAAAAAAGAGAGTATAATAAAGATAGCTATTTAAAACAAAA<br>AATGTTGAGCTTATGAAGGAAGAAATCAAAATCAAGCTACTTTCCAAACAGGCCAGAGAGATGACTGC<br>TAATTACAGTCTTGTTAGGAAAAAACAAATGGGGGGAGGGGCAGCTGGGCAATTTTATTCTCAAACTAC<br>AATAATATCTCTCTCTCTCTCTCTCTCTCCCTCCTTCCCTCCCTCCCTCCCTCCCTCCCTCCCTCTCTCTGC<br>TTACTTATTAAAGGCAAAGGTATTTGTGTGATAGAAATTTTTAAAAGCAGCTGGTTTTCTAAACCCTCTA

							GGATCATCAAACTGAAAATTTTTACTCTCTAGGCTGTCTTTAGTTTTGGTCACTAGTATTTCTGCAAGTATAATGTTCAAATCAAACCTCAAAAATCACTAACATATGGAACTAGAAATTCTCTTCAGTTGACAAATCTCTGGAGGAGATGAACTAAGCTTTGTGTTTGCAAATGTTCTGTTCCCAACACACTTTGAAGAGAATGTGCACCACCACAGCGTCACCACCACCACACTGAACAGCAGGCACTAAAACACGGTCTTCAGAGGGGGTTCTTTCCCTCACCAATCACTCTGTGCAGTAATCAAGCAGCAGAGGAATCAAGACCAAGAGTAAACAGTGAATTTTCCAGTTTGGTACTGCAGGTCATCAGAGGAAAGAAGGGTGAAGAAAGCTCTTTTACCCAATGACTGATGCATGATGCACCTTCTGTACTCGAAAAGCAGTGCACTGCTGAGATTTCTTTGAGGAAAATGATACCTCTAGAGCCACACCCACCATTTTCTCTACCACAGTTCTGTACTTAGGAAGTAGAATAACCCTACAATGGCTTTATCACAAGGAGTGCATCAGTGTCACAAAGCCATGTTCAAGTTCTAAGTCAAAAAGATGACCGTCGTAAATGCGAAATATAGTCATTCATTAAAATAAAATAAAATATAAATTTTAAAATCAAATAGAACATTGCCACGAATCTAAATTGGACTCAGAGCCTGAGGATAAGAGACATGTCTCTGAATCCCTCAGCTGACTTTATGGAGCAAATAAAAAGGGAGTTCTGTCGCTGAGGCAAGGACAGAGTAAGTCACACTGCAGGCATTTGCTGTTACCT
MSTRG.25779.1	--	0.715	0.032	0.000	-3.428	down	CTCAATAAATGGCAGCTATCATGGTAATTAGTTCTATTCCTGTTCCCCATTCCGGCCTGCCACGCAATCAAAGTGCTAATCACACCCTTGGCCTCTGTGAAATCCGAAAGCCCTTCCACGGGATGGCTCATGGAGAGCCTCAGCCAATATCCCATTTTCTTTGCTCCTGACTAAGGCATGTCAGACCAGAAGAAACAGAAGACTGTCTGGAGTTTCCACTAAAAATCTCTGACACAGAAGCAAGCAGAGCCACAGAGAAACAGTGGGGAGAACATGGCTTCCCTTGCCCCTGCCATTCACGGTGCTGTTCGCCACTGCACAAAGGCCAAGGAGGACTCAGGCGCCACAGCAGGTATCTGGGTCAGCTGCCTCAGTCAGCACGGCTGGACCTCCACAGCCATGACTGCCAGGTTACCTACAGCACTTTTCACACAGCAGGCGTGGAATTCTAAAGGCAAGGGCTCTTATGCCTCACCAACGGGGCAGGTTAGCAAGTAGTCTCAGTGTTCCAAACTCTGCTGTAGCCAAGGGCACCTGATCTAGTTCTAGCTTTGCTCTTCAAACCCTTGTGACCTTGGGATACAACAGCCTCTTCCGGTTTCACTCTTCTTACCTGGACAACAGAACACTGACTGAGACTGACAGTCCTTTAGCCAGCTCAGCAGAAATGAGGACCAGGGTTAGAACTGGTTGAGATATTTAAGAACTGCTTTACCGATCCTTACATATCACCTCAAATTAATTGACAGCTTCCAGCATCGTCACACCACCGCCACCATCCTGATGAAAACAGTAGTCACCACTTGCGAGACCAACTGTGAGGATTTCCCAGTGGATTCCTGGATTAGCCTTGGAAAGAATGGGGAAGGGCTATCTGCAAATAAGTAAATGCCCAACAGGGAGGAGGTGAGCCCCAGGGCGCTATTTTGCAAGTACCATGTGCGTATGTAGCGTCTGGAGACATGATGAGGCATGGTTAAGTTGCAGATGCTGGTATGGGGCTGCATTTCTTCCTGGATTAGCCTTGGAAAGAATGGGGAAGGGCTATCTGCAAATAAGTAAATGCCCAACAGGGAGGAGGTGAGCCCCAGGGCGCTATTTTGCAAGTACCATGTGCGTATGTAGCGTCTGGAGACATGATGAGGCATGGTTAAGTTGCAGATGCTGGTATGGGGCTGCATTTCTAACAGGTCCAAGACAATGCTAGTCCACTAGGCTATTCCCAAGAACCGGATCTTGGCAGCAAGCTACCGAACACAAGCATTTGGCAGAGGAAACCTGAAGCATAAGACATCCTTCCTACACAGATTGGCTAGCCATCTTCTAAGCTTCGCTCAGGGAAATGCTAGGCTGGAAAACCAGAGCTAACTGGAAAGAGATAACTCAGTGAGCGCAGGAAGAGTTGCTTGAAGGTCTGTTTCAGAGGTCAAGATACTGTGCCACCCCAGGAGGGAACTATTCATTATATTTGGCCAGCATGAAACAAAAGCACCATGATGACGTAGTTAAATTGCAAAAGAGAGACTACAGAACAGTAGGTACAGAATGACCCTGAGGCAAGAGACAGACAGAGAGGAGCTCATCACCCAGAAGCAGGTGGAAACCTCTAGCCAGTTAAAAGCCAACAATCCCGGCACAATTAACAAAGTCTGCTGTGGTGGTTGTCAGTGTAGGGTCAGTCTTCCTTCCTGCAGAGCTGTCTCCAAGATACTAAAATAGCATGCTTAAGGCCTAAACGTTTATGACCAAACTTGGCCTTTCTCTTTTCACAGCATCTTTTCACTGTGTATTTTCTAGGAGCCGAGTGTGACTATAAACTCCCTTTTCAGTAAAAGACAAGCCTTGACCCCCTTGTGCAACCTTTGTAACCCTTGGTTTCATGTTGTAATCCTACCCAAGGTTCTTTGAGAAACACTCTATGATCT

							CCTTTACCGGAGGGGGTGGAGAGAAGGAAAGAGAAAAAAAGAGGAAGGTAAAGGATAGAAAGAGGGTCTCCTAGAAACCATCGATTATAGTACAGCTGTTCCCAAGATGCTTAAATGGCTGTCTTGAGAAGCCTGCACTTACACTTGAGTGTGTGTTAACACCCATGCTCGAATCTACGTTTAAAATATCTTAATGAAATCTAACTTTGCCTCACAGTGACTCATCCTGAAATTCTTTTGTAGTGCACAGTCAAGCAAAAATCCTGACTTCACCTGAGTGGAGTTCTCTCAAGAACCCCTCAAGATTCTACAGAAGAGAGCACTATACCCAGCCCCCACTCACAGCAAAGCGAACAGCCCCATGTTTAAGAACAAGATCTACAAACGGCATTTATAGATCTATTCCCCAAGAACAGTACATTTATAGAAGAACTGAAAAGAAAGGCCAAGACATGGTGACACATGCTCATTTCATCCCAGAACTGGGGAGGCAAAGACAGGCAGATCTCTTAGAGTCTGAGGCCAGCCTGGTCTACATAGCACATTCTAGGACAGCCAGGGTTACACAGATGCATGGAACAGACAGACAGACAGACAGACAGACAGACAGACATGATATTACCCTACAGAATCTAGTATATATTTTCCTAGAAAGTCTAATATATTTTTCTATCTGGCCATTTATAGACAATATCTTAGGACTCCTTGGGTAAAAAGAGATACACAGATTCAATTAATACCAAATTTCAGATTAGCGACTACAAGTAGCCACAGGTGGTTTTTATTTTCCTCTGGTACATTCCCAGATGTTTCGAACTTTGCCTGGTGACCATAAATTTCTCTTGAAATTAAGGGTCAAGACAGCTCTTGACCTTCATGTGATTAAGTGCACCTCCTAATAATCTAGCTTAGAAAACACACGCCACGGGTAATTAACACCGGAGTTTAGGACAGTGCTGCTCAGTGGTACCAAACAAATCACCATGTTCTCTTATGACGGCAAAATCTGCAGTCATCACACGTATGAAGGCCCCTTCGTGTCACGAGAAGGTACTACTGAGACATAAAACCATGCACTCGGTAAGACTGAAGTCAGTATAACACAGGTGCATAAAATGTAATAAAAACGACTCACGAAAGAGTATTAAAAACATAAACAGTGCTTGTCTTTAAAACATGGATTTATGAGCGATTTTCAC
MSTRG.25976.2	ENSRNOG00000001770	0.142	0.498	0.004	2.515	up	AGGGCCTTGCGCTTGCTAGGACTGAGCCAAATGCCCAACCCCTGGTGTGGACTTTTAAAGCCTTAAAAAAAAAACCAAAATAACACAACACAACACATAACACTCAAAACAAACAAACAAACCCACCCCCAATAGAGCACCTTCTCCAACACCCACTCCAACAAGGCCACACCTTTTAATCCTTCCTAAATAGTCCACCAACTGGGAATCAAACACTCAAATATACAAGCCTATGGGGCCATTCCCATTCAAGTTGCCATAACCCATGTGGTCTCACAGTCCCGACTCACCCTGATAAATACACTTGCTTACCCGGTGCAGAATCATCCTCCTCCCTCAAAATGGAGTTACAACTTGCTTGTACAATGAGCCGCTCGTGGGGCCAAGGGACAGCTTGCAAAACCACTGTTTTTATACCATGTGGAGTCTCTTAGGACAAGATACCACAAGATCTCAACCGGCTTCAAAGGATGTGCTCATTCTGGGGATTGGAAATCTAAAAATCAGATGTTTTGTCTTTCCATACACTCTGAATACTCGGCCATCTGTGTGTGTGTGTGTGTGAGGGGATGGGGGAGAGGATACGTGTGCTTGTTTTGGGGATCAAATTCAGCTCTCTCTACATCTACCCATGAGCAACCTCTCCAACATAGTCATGAACATTTTGATGTGTTCGTCGTGTGCCTCCCCCATCCAATCCAATCCAATGCCAGGTGCTGGGAATAAATACCACCTACTCAACTGCCAGAGGGGGCCACCTATAGGGACAGAAAGTTATGACTTTGCTGAGGAAGCTGGGCACAGTGACACAGTTCTGTACTCCCAGCTACTTTGGAGGCTAAATCAGGAGGATCTGTAAGCCTGAGGGGCTGCCTTGGCCTTTAGAGACAGCCTGTCTCCAAATGGAAGGTACAAGCAGGGCTGTATGGTGAGCACTTGTCTAGCATAACTGAGACCTTAGGTTCAACCTCCAGTCCTGATGATGACGATGGTGACGGCGATGATGCGGGTGACAACGACGATGACGATGATGATGACGATGACGATGATGGCGACAGATAGTGAACTAGATAAAGACTTCAGGCAGTAACTAAACTTGACAAAGATCTGACCAGAGTCAAGCAATCAAGGCAACGGAACAGAATGGGGAGGTGGTACAAGGTCAGGAAGAAAGGAATGGCACACTTGCAGAGATAAAGCAATAAATAAATCCGGTCAGCAGGGCCCTGGAGGCTCCGGAGGAGATTCATTTTGAACCGTCGTTAGAGAGCAATCTCTTCACAGCAGCCTAGAAGGAGGCTCGGAGGAGGATGGCCTGTGGTTGGAAACCTTCGAGCTGCTATAAGAGAAAGGTCTGTGCTGTTCAGTTTCTGTCAACTTGATACAAGCTCAGGTCATCCATCCTTCAGGAAGAGGGTCCTCCACAGAGATAAGGCCTCCATCAGACTGTCCATCAGACCGTCCATCAGACTGGTTTATCTGCAAGGCTGCGTGCGGGAGCATTTTCTTATCGATGAACGAAGTGGGTAGGGCCCTCTGGGGAAAGTGGTCCTACGGCAAGGCTGCGGGACGGATGCTATC

							GGAGTCAGCAGCGAGCTGCTCTGGGACTTCAGCAGGCAGACACCTGGAAGGCAGGTAAAGCAAGACACTTTGTGTTTACCTCGGTTTCAATGTTAGGTTAGCGGGAAGCCAGAATTTGTCACTTAAAAGGGACAGTTGTTTGAAAACAGCCTTTGCCCTCTGAGTTTCCCTCAATCCCTGACGCGTGAAGATTTCACAGAGTCAGACATATGCAAATTAGATTTCTCTTCCTAAGCCAAGGTTAAATAAAAGGGAAAATTAGGAAAGATTGCTTATGGCAGCAAAGAATTTTCAGAGTTTGAGGATGAATTTCGTTTAAGACCAGGATAGATCTCATTGCCAAACAGAAGAGTGTAAAGTGAGGGAAAGGAGGCCTTAGGAAAGCACAGTTCCAGCCCAGCATTTGTGCTGAGGGGTTCTGTTCCCGACCCCTGCAATTACAGGCATTTTCCTGGGCCCAGAGCAGCATTCGGCAAGGAAGCAGAGGGAGGGCGTTCCCTCCTTTCGGGTTTTAGACTGGGTACCGGCCGTAGGGGAAGCCAATTCCCCGCACTGCTCCGGGAGGAAAGATTCCTTTTCTCTTCTCTGGTTCAGCCGGCAGAGATGAGTGCGGCCACGGCGCCCGAGCGCGGCTGGAAGAGCGAGAAAG
MSTRG.26155.1	ENSRNOG00000001892;ENSRNOG00000029912;ENSRNOG00000046981	0.761	0.007	0.002	-3.843	down	GTGGCTCCTATGCACAGTCCTGCCTCCTCTGGCCCATGGTCCTCCACAGCCAGCAGCACTGAGTAGGCAGCTCCTAATGGCGCTGGCGGATCAGGCCGGTGTACCGGGTTAGGCTTGGCTCCGAGCCATCTCAACGGCCAGACACTGCACTTTCAGAAGCAGCCAGCACATCCTGGGCAAATGGTTGTTTTCCAGTCAGCCTGGAATCCGCCTAAACTGGGCCCTGGAAAACCATAGCTGGCTTAGGCCCAAACAATGCCTGGGAGCATGCTCCCACCCCTCACGGCTGTCTGGGTGGCCACACTTGCCCAAACCTAGCATTTTTTCCTTCTGGAAAAAAGAAGCTGGAGTTGGGTGCCAGGTGGAAAGCTCCAGGGTCTGTAGGAGGCTCCAAGCTCACTCAGGGAGGTTCCAAGAGGCCTGGGGAAGCAAGCTAGGAAAGGCACTGGCCCTGGGTCTGACCTCGAGATCCTGCTCTTCTGATGTAGGATGGTTGCTGTGCTTTCTACACTCTCAGTATAGACCCCAGGTCACACTCCTAGACAGTCCTGGGTTTGCTGTTCCTCTTCTTATCCTGAGAACACATTTGAACAGCTCCTACCTAAGGCCCCTTGCCATCCAGCCTCTCCCTACTACCACAGTGGGGTCTTTCTGAGCTCTCATGACTTCCTGCCGTGGCCCGTGGCCTGTGGCCTGTGGCCCGTGCAGGTTGTCCAT
MSTRG.26167.1	ENSRNOG00000001884;ENSRNOG00000001885;ENSRNOG00000001886;ENSRNOG00000021891;ENSRNOG00000030920;ENSRNOG00000042711	2.043	0.082	0.001	-3.487	down	GCCGTCAATGCAGGCATTTATTGTCATTCTGCAGTACTGGGCTCTGTACCTTGCACGTTCTGCGCCGTCTCCCGTTGACCATGCAGTGGTGTGGTCTTGCCATTCCTTTGCTTGGGTGGTGCCCTATCTGGAAGGCTGGTTTCTCTCAGACAGCCCCGAAGACGCCTCCCCTTTCTCCATGGTCCTTGTATGGTCTTGAGCTCCACAGTGTCCTGAGAGGTGAGGGAGTCTAGTAGGAGAGGAGAGCCCTGGGTCCGCAGCGTAGCCGGCTCTGCCTTGAGCAGGAGCTGTTGCTAGTGTTAAATGTCCTACAAATCAGGGAGGGTGTTGGGTCAGGGTGAGGGTCCGTGTGAAGCCCCAGCTTCATGCAGAAGGGCTGAGTCTGCACTGGGTGGGGCCCTGAAATGATGCAGACTCACAGACACACAGAGAGTGTG

MSTRG.26460.5	ENSRNOG00000001108;ENSRNOG00000001111;ENSRNOG00000051294;ENSRNOG00000059322	0.012	0.539	0.000	5.236	up	AAAAAAGATACCTTTATTGACACCTTCCATTGCAAAGGCATTCATTATCTTGAGTGACTTCCAGTCAAAGGGAATCATGTGGTTGGTCATCCAGTAACAAGGATTCCATTGAAACTCCAGGAGATCCCAAAAGCTTGGCTGGGTGAAAAGCAAACTCTGGCGATGTTGGCCCAAAGGACTGACAGTTCTTGCCCAAGTCCTGGCTGTCCTCGGCGGGTCCACACTGCACCGGCTCACAGGCTTCCCACGTCTTCGGATTTTCATCAAATCTCTCATTCGCAAGTTGACGATCAGAAGACACCCTCGGAGAGCAACAGGTACTAGGCTGGCTACTTAAAACTTCTGAGAGAACAAACTCTCCATCAGTTAGCCTGCAAGCCACTCTTTCCTGACATTTGAAAGTCAAAGGTGTTCTTTTGTTCTTAAAAGATACCATGCAAAAGGCAGACAGGTAATGTAGCATGAGAGAAGGGACAGGGTTTTTATAAAAATACACAGCACACCCTAGTCTGTTAAAGGGTAGGCGATAAGAACAGGACTTCCCAGTCACACTTAGAGACTCTTGGAGGAGGGTATCAAGGTAAAACCATGGCAGATGGCGGTCGAAGATCATCACTTCTTGCTTTCCCTCAACTTTATTGATTATATCAGAAATATCTTTCTCCAGAGAGAAGGTGTTCAGCCTGTGACTCTCTGTTAAGAGGTCGCATTTCTCCTCTTCCGGGTTGATATGAGTCTGTCCCAGTAACTTAAAGTCTGGTGGCAAATTGCAAATCTCCCACTGCATCCCTCGTGCTCCCTTTGCTGATTCACAGGGGTGCTCTGGGGAGTTCAGATTGGGGGCATCGATGCTGGAGACATCTGCTCCTGGGCTTCTGGGTAAACCTACAGTTTCAGCTTTGGGAGACAAGTCTATCAGCAGCTTCTTCTCCATTGGTATTTCTGGGCTGCTTCCTGAGACTGCTGCTCCTTCAGAAGTGGAAATCAACTTAACCAATTGTTCCTCCCTGTCATGGCTGCTCAGTCTACGAGTCCTAGCTCTCTTGGGTCTCTGTTCACACACAAACTTATGAGGTCCCTCCGGCCAACTGTCCAAATATGGCTGACTCTCAGACCCGAGCTGCGATTCTTCATCACACAAACGGGCTGTAAAAAAAGCCCAGCAGTCGCTTTGGACTCCTCGTTCCTTTTGCAAGATTTTCTCTGATGTCCTGGTTCCACCCTGATGATTCACGGCAAACTCATCTGGGCGACTTTTATCCATCACCTCTGTCTTCCACATAAATGAACACCGGGTTTTAGCAAATGCGTCCTGGGTGTCTAAGATGGAGTTATGTCTGTTCCTCAGATGCTGTTCCGCCCTCGCGGCCGGGTAGCCGTCCCTCACTCCACGGGAAAGATGAGTGCTTATACGCCAAGAGGCGGATGGTAGCCCTCTTCCCTCATCGTCCTCCCCCTCCTTGGGGAAGCAGTCCTGAGCTGTCTCAACTGTACACACAGTAGTTTGGAAACTGTTGGAGAGCTTTCTGTCTCGCCAACTTTGCTCTCCATGGTCGTTTCCACCACTGGCTGCTTCATTCTGTAGGCCACCAGTTAAGGGATACGCTGGTTCTCTCTTGGCCTCTTCAAAGCCTTCTGCTTCCCTGTGGGACAGACCCTGGCCTCTAGGTGCAAGGCGGCCTATGGTTGAGAGTGAGTTCTCATTACAACCGTTGTTCCTTTTCTTTCCTGTCCTGTTTTTCTTCTGCTTGGTTTTGTCAAGAGTGTATCCACTTTTCCTTAAGACTCTTCCTTCTGTAGCCCAGGCTAAAACTCTCGGCAATCCTCTGCCTCAGCATTCCAGGTGCTGGGTTTAGAGGCGTGAGCGACCACACTTCAAATTCACGAATGCTCTCTCAAGTTACCGTCCTTTTTCTAGAAGCAGTGAGCCAGGCCTTCTGGAAAACAATCATAAATCAGCAACCTTGTTAAGCTGCTGTGAGACAAGAGGTGGTGG
MSTRG.26476.1	ENSRNOG00000001092	0.390	0.000	0.022	-3.851	down	GGGGCTGCAGGCTTACCTGACTTCTGTGGCAATGAACTCGATTGTCCCGACAGAGCTGATGGAGCGCTCTCTTTGCATTTACCCTTTTGTGTCTTTTTTAAATCCTAGGTTTTTATTTGCAGAGGACTGGGGAGCTATACAAGTCTGAGTATCTAAGATACACAGTTCCTCGGTGGCTCCAACTGTTACTTGTTGCCTCCCTGAAGGTGTATGAGACGTGGGCTCTGCTCACCGAGAAGACTCTAATCTAATTAGGAAACATGGACCAACCTGGAAAATGTTGAAGAACAGAGGAGACCGTGCTCATCTGAACATCCCTGCAGCAGACTCCTGAGAACACAGCAGCTTTTTTCTCCCTCTGCCTGGTTTTCAGACGGCTGTGCAGAAACTGGAGACTCAGTACCTGCAAAGGCCAGAAGAGGGCGTCAGATTCCTCTGGATCTGGAGTTACAGATGGTTATAATCTACCATGAGGGTGCTGGGAATTGAACCTTCGTCTCTGCAAAAGCCAGTGCTCTTAACCGCTGAGCTGTCTCCCCAGCTGCACAACTGCCTTTTTTTGAAGAGATGATCCCATGGTGTCTGCATTTTCTTTACCCTCCTGCTGATGGACTGTTATCTGGGCTGTTGTGGAC
MSTR	--	0.618	0.076	0.031	-	do	TTTGTCCTTCATCCTCTCTTGTCTCCCTCCTCCTACCCTTATCAATTCCCCCACCTCACCCCAGCTGTCCCTTTCCCAGATTCATGACTCTTTAGTTTGGTTTTGTGACCCACTTAGCTTAACAAGGGTCATCCTCTGGAG

G.264<br>90.8					2.205	wn	CCTAGCATGGTCACTAGTAGGTCAATGAAGGCAAGAACCCCTCCATTCACCGAATCTATTTGTGGACAA<br>CATTTGACAGCATGGAGTAGAGCCTTCTGGGCCTCAGTTTCATGCCTGGTTGTTGGTAGACCCTCTTCTA<br>TGCAGACCCACTGTGGCCAACAGTTGTTGAGAGCTATTCACTGCAGTGGCTTGCAATGCTGTACCTGGA<br>AGACAGCATTTGGCGCCCTTCTCCATACCTTCCAACTCTCACATTCTAACTGCCCCCTCTTTTGCAGTGT<br>TCCATGAGCCTAGGGAGCGTTGGTATAAATACCTGGTTTAGGGTGGAGCATACAACCATAAGTAGGCTT<br>GGCCTATCTTATTTTCTGAGGCAGAGCCTCTCATTGGACCTGAAGCTCACTGGTGCCACAGCAAGTCCC<br>CTGGACCCTCCTGTCTCTGTCTCCCACATGTTGAGATTGTAACTGTACCATCGGGCCTCCTGGATATTTTA<br>TTTTATTTTATTTGTGGTGTTGGGGATCTAAGCACTTTGACAGTGAGCCATCTCCCCAGCCCTGGAATGT<br>ATTCCTTTATCAACAGTTGCGATTTAAAGGGAACATATAAGGAAGAGCTCATCAGACATCATCCCAGAA<br>ATGTTACAGTGTGCATAACGTTAAGAATCCCACCCCTTCTCTTAGGGTCACACTCCAGATAGCAGCAGA<br>AAGACAAGAGAAAGTGTGTGTGTGCTTAATGCCTCTGTGTATCAGCTTTCTGGAATCTCGTTCTTCACTA<br>ACTACAAACCTCTTAAGGGAGAATACTGTTCTCCTTTTGTTCTTATTATTCAACTGAATTATTTCAGCCTT<br>GGCTTCATTCGTTTATGAATACAGATGGCACCGTTGAACATACCCTCCCCGTCCCCAAACTGTACCAATA<br>TGAAAAGAAGATCAGCAAATGTTTCTTTTGAGACCCTGCTGTGTAGCAGCTGTCCGATGGGCTTGAGG<br>GATAGAACAGCAAACCTCCTTGTGCAAAGGGAGAGAAGAGAGAAGAGGGGAGGGAGAGAGAGAGAA<br>ATGAACAGCAGGCTTTTAAAGCCTGTAGCATTTGAGCGTCATGAGGAGGAGGCCAATCTGTGTTGGGTA<br>AGAGATATTCTGCTACACTTGCAGTCAGGAGCCTGGCCCAGCTGTCAGCAGAGAGGCTTTGCCCCACA<br>AACTGATGGGAGCAGATGCAGAGACCCACAGCCAAACATTAGGCGGAGATTGGGGAATCCTGCAGAA<br>GAGGGGGAGGAAGAATCGTAGGAGGCATGGGGGTCAAGGACACCACAAGAAAATCCGCAGACTTGAC<br>TAGCCTGGATCTAAGGGTGCTCACCCCGGGAAGCCTGCAGAGGAGTGACCTAGGCCCTCCATACATATG<br>TTACAGTTGTATAACTTGGTCTTCACGTGGTACTCCGAACTGTGGGCGCAGGGGCTGTTTCTGACTCTG<br>TGGCCTGCCTTTGGGACCTTTCCCTCCTCTGGAATGCCTCCTCATCTAGCCTCAGCCAGAGATGTGCCTC<br>GTCTTACTGCTGGTTGATATCCTTGGGAGGCCTCCTCTTTCTGAAGGGAAATGGAGGAGTGGATAGTGG<br>GGGAGGGGAAGTGGGGCAAGGACCGGGAGGAGAGGAGGGAGGGAGAAACTTCAGTAAGAATGTAAA<br>ATAAGAAAATAAATTAATAATTTTTTTAAATTGCCCACCTGTGAAGCAGAGGGAACAGCCAGGCATAAG<br>CCCCCGGAGACCCTGATGTTCAATAAATATGTTGTCTTTGCTGAGCGACTTTCTCTGTTTCTCCCTCTCCC<br>CAGGACTCTCGGGAGACTTGTGGAATTCTGTACAGAAGACAGTAGATTCTGACTGCCAAGGGTAGGGG<br>TAGTGGACTAAAAGCACCCTCCTTTCTTCCCCCATCCACATTTAAGTCGGAAGTTTCCCCAGGGCAAGA<br>GTTGAGCACTCGGAACAAACTGACCTGACTCAAACATGGAGAACGTTGTACTCAACTTGACCCAGCAC<br>ACTGTGAGGCCTCGTGTGGCACTTGGATATCAAGACAGATTTCACACTCGTTCTAATCAGATATAAGACC<br>CCTCTGTCACGTTACTTCCTTTGCTGTGGCCCCTTTGGGTCTATCACCACAGAACCACGATTGGCTTTCT<br>TTGCACAGCCACAAGAAAACCAGTTTTGAGAGCTTACTAGGCTGCTGTATTCTAGTAAGCAGTCAGCGC<br>TCTACTGAAAACTTCTGACTTCACCAAAGAAAGCTCTAGGGAAGACTGACCTTAGGCACGCTTGAAAG<br>ACGAATGGTTTATCTTATTTATCTTCTCTGAACAGGACTGGGGACATAGGATGCTCGACCAACACTTAAA<br>GATGTGAGGTGGGGTCGGAATTGTAAGTCGGAGCGAATACAAAGGGGGAGACACTCGGGCTCAGGTG<br>CTGTCACCAATTCTACTGAAGTGGAAACAAAGAGCTCATGCATGCTTCTGTTCGTACTGATCGGACCAT<br>TCACTGCTAGGTTTTCTCTAACAAAAACCACTGCACGACAAGCTTAGAGTGAAGCAAGCTACACACAA<br>TCACTGCGGTACCCAGGCTGAGGTTTTCTGGTTCCCGCGGACGTTAATCACTGCGGTACCCAGCCTGAG<br>GTCTTCCGGTTCCCGCGGACCTTAGGCGCTGTGCTCCTGCTCCCTAGGAGAGGAAACAGTGCATGCTTG<br>TTGACCACTGGGTTCCCTGTGCTTTCTGCTACAGACAGCTCTTGCGTGTGTGGCTCCACTCTCACCCCA<br>GGCTAGAGAAGGAGACCAGAGGAAACCCAGAGGGGGCAGCTTTGAAATATTGGAAAGGATCAGCAGG

							TGTCAAGGCATGGGGACAAACACTGCGTTAGCATCCTGATTATATCCTCCTGTCATGGCTGTCCTCAGGT<br>CTTAGGTAAAACCGGAAGAGCACCAGGAACAAGGAAGGAGGGAGGGTCAGAATAAAATGCACTGACT<br>TCACAGTTTAGAAACAACAAGAAGGGATGCCCATCAAGATTAATGTAGAAATAATTTATGATAAAAGGT<br>AAGCGTCAAGCATGAGTAAAACTTCATTTAAAAAATAAGCTTGGAACCTTGTGTATTCGGGAAGTTTTA<br>GATAGGATTCAGAAACTCTTGTCCACTTAAACATGGGCAAAAATTGCCCTAGACATGGTTCGCCAACAT<br>TTTTACCCCAATATTTATTTATTGGGTTTCTTGCTTAGTCCTCATCCCCCCTTCCAAATGAAACACTAAAC<br>AGGTGCTTGGCTAAGCAGCTAACGGATTTAAACTTTACCAGACTGATCCCCATGCTACCTAAAAGTACA<br>GATGGCTCCTATGTCCCCACCCCCCGGGACTGTTCCCCCACCGCTGGATCGCCCCAAGGATGCTTCACT<br>GGCTAGGAAAGCGCACATGGTTTCCAGTCCCTCTTGAACTGCTTTTTGCTTTCCCAACCACAGACTCTG<br>CACAGCGAAAACAGTCTTCCGCAGCAACAAGCTGAATGTGCTTATAAAAACATCTGTCTAAGTCTCATT<br>AGGAAATTTCCAGAATCGTGTTTAAAAACCTCCGGGCTCCACATTCGGGCGTTTTAGCCTCCAGGCTGC<br>GCATTCGGGTGTCCCAGGGGTAAAGCATCCTTGAACCCACGTCTCCAAGAGAGATGGAACACAGAGCT<br>CTCTGATGCTGATGGGACTTGCTTTTGGGACATCATCCACTTTTAAAAGAGTTTTTCCTGTCTCCTTCTTA<br>GGTAACATGTGGCTGAGGGCGAAAACACAGTTAAAACTCAAACACGAGAGTGTACGCTTGGCAGGAA<br>GGCATTTCACGCCTCTGGAGGAGCAGACCCATTTGGCGGTTTTAGCAGGGCAGCTGCTGTGCCCTGAA<br>GCCCCCGAAGCCCCTCCCTGGTGAGGTTCCAGCCAGTCAGTTTGCCTGACAGTTTAGCATAGAGATTTC<br>CAGGGAGCTGGAAGCAGCGGACCTCCCTTTTTCTTTGTGTGGGGGGGATGATCTAAGACCAAAAATTG<br>CTTGAAATCATGGCTGTACCCAATGATCTCTCTGTATGTTCACGACAGTGATGAAGCTTAGTTTGACAAG<br>AAGACAGTGAGAGATTAATAGTAATGAGTGATGATAAAATACAACAATTACACCAGTATGTTACAGTAA<br>AATTGCTGATATTGCTATATGATGTTTGGCCATTGTGAAGGAAAATGATGGTCCTGTGACCCCAAGGGCT<br>GCAATACCCTCCTGACCATCAAGCTGAGGGTTGAGAAAGCTACTAAGTGACAAACAGGAGGGTGTTGT<br>GTACTGTGGATACAAAGGACAAAAGAACGACCCACAATTTAGCCCAAGTGTAGAGACACACAAGAGAT<br>CCTCTTACAACTCACAATTGTGTATGGTTCAAAACATGCAAACATTTGGTGATGGAATTGCCCACAAAA<br>ATATCGAACTATGACTGACCATAGGTAACTGAAATGTAGGATCACAGGACTATTCGGGAAACAAAGCTC<br>CTGTTACCTCCTTTCAGGGACTTCCAAAGCTCTTTTCTTTCTCTGAAATAATGCCTATTTTCATTTAGCCA<br>GATACTAACAAGAACTGAAGCAGAGATCTTGAGTTTGTGAATATAGCGCTGCAGTGAACAGACACCGT<br>GACCAAGGCAAGTCTTATAAAAGAAAGTGTTTAACTGGAGGCTGGCTTACAGTTCCAGAGGGCTAGTC<br>CATCATCATCGTGGCAGGAAGCATGGCAGCATCCAGGCAGGCACAGTGCTGGAGAAGGTGGTAAGAGT<br>TCTGCATCTTGATCTGAAGACAGGCAGAAGGAGACTGTCTTCCATTCTAAGTGGCTAGAGCTTGAGCAC<br>TGCAGACCTCAAAGCCCGCCTCCACAGTGACACACTTCCTCCAACAAGGCCATGCCTCTTAATAGAGC<br>CACTTCCCGTGGGCCAAGCATATTCAGACCGCCACAAGAGCTAGACTTGGTTCTCAGTAGATGGTACGA<br>AATGGGTATGCAAAAGTGAAAATAACCATGTTTACCAGCTTATTATGCAGAATTATTTGACATGGTTATTT<br>CAAAGTCATATTTCTGTGATAAAATCTCAGAGGGGTTTACTCTCGATACTCAGTCTAGAAACGGTGACCC<br>TGAAGAAAGGGGAAGAAGGTTCGCCGTGGATTCACCGGTGCTATGTTTTTCCATGTGAGAACATAAATG<br>AGCTTGTTAGATCAGGACCAGTGGGGCCAGGCTTTCAGAGTGGTACATCTGTAAGAACTGCCCAAACC<br>AAGAGGAAAGCAAGATCTGCAGGAAGGGTGGGAGAAGCACTGACCAGAGAGGGATGAGCTATCCTTT<br>CACCAAGTCATTAATAAGTCAATAAGCTAAGACAGGATACTTGGATTTACACAGGAGCATTTCCAAAGC<br>AAAGGCCACACCAATGGCTGTTTCCATCCTCATACACGTCTCATTCTTGAGAGGGCTTTTTTTCCCTCTT<br>TGTTTGGATAAATGTTTATACACAATTCTTAATCTATAGTGTGCATTTCAGGATAATGACTAGTTATACAAG<br>GCACTTCCTTTCTGTGTGAAACCAACATTATGAAGTCGGGGTTAACAGAAATCCCAGCAGCATGTGGAC<br>TAGAGGCAGACAACATGAAGTGATCCAGTGTTCAGGATATGGGGCGCTTCCCAATTTCATGTCATCCCAT

							GGCCACCTGCTTTCTACAACAATCCCAACACTCACAGTGGGCTTAGATTACAGCGATTTGGCCAGAGTCAAACAGTAACAATTTGTATTTCAAACAGTAACAATAGAACAACAATAGCATAAACAGGAAAACAGCCCATTAAAGATCCTTACCTTCTCTTTCTTTGCTTTGGCTGTCAGAGAGAACCTTAAGCTTTGGCCGAACTCCAGCTGACATTTGGTTTGGTACACCATGAAGATTCTAGATGATTCCAGAGTTGTTTCTACGAATTCTTTCCTTTGATTTTTTTTAAGTAATATCCTCCACGTTTATTGTACTTTTGTCATCTACGTTTGAGGCTTTGTCAAATATTTAGCGGGATCGGCAGGTTTGGGTTAAGCAAGAAACCCATGTAGGGCGTGGTGGCGCAGTCCTTGCAGTCCCTGCACTCAGGAGGCAGCAAGAGAAGATGGCAAGTTTAAGACCGGGCTAACAACAATAACAAACAAAGCCCATGAAAAGCAACAGAAGCAAATGGTTTGGTATCTCCATTAGCTATGACATTAGTTGATACAGAGTTTTGCTTTAAAGGTTACATTTTAGTTATCTATGCAGTTGACTGAAGCATTTCTCATAGGAAGGGGGCTCGTTAGACTCAGGAGTTAAGGAAAACATAGGTAAAGCTCAAGCAGCCCAGACACGAGTCACACTGTGGTGAACCGCTGATAGGAAAGAAAACTTCAACAGAACAGCCTGCAGGTTGCACAAGGAGCTCCAGGGAAGTAGTCATTACCCACAGGGGTGGAATTTTTAGTGACACAGCAAACAGTCTTTGAGTCGGACATGTTCTTGGAAGTCACCTCAGTCCAGGAGACCAACTGGTTCTCTAAGCTAGACTTGAGTGGAATCCTATCATCGGTGTTATCAGCATGGTGTCTGAAATGAATAGACATGCATTTAAGTTTCTTTAGGAAGAGTCTGGGATTCAGTTCCAGGGAATCTGATGCCTTCTTCTAGCCTCTGAGGACCCTGCATGCACATGTTTTACTACATTCTTATAGGCAAAACACTCAAACACGAAAATACAGATATTAAAAAGATGTTTTGGGGCTAGAGAGATGGATCAGTGGTCGTCAGGCCTGGCTGTTCTTGTAAAGATTTGATTGCCAGCCCCCAATGGTAGCTTCTTATACCCATCTGTAGTTCTGGCTACAGAGGATCTGATGCCGTCTTGTGACCTCTGCGGACACTGCACACACATGCTACACACAATACAGCACTGTCTTATACACTGCCACTCTGCTTTGTGTGTCTGGGAATGTCACCTTTAGGTACCTCATTTAGAGTAGAATCTTCGTTCGTTGGAACTGGCTTGTCCGATTTAATGACGTCAGCCATCATACATCACTAATCACTTGTTA
MSTRG.26506.1	ENSRNOG00000001088	2.041	0.032	0.004	-3.684	down	GGCCAACATCAGCTCTAACCATGATTCTACTTCCACATCTAACAGATTCCATGAATCATAACCTTTTAGTACAGATTTTAACTTCCTACAAAGGAATGGTTCAACCAGAGGAACCTTTACACAGAACAGCTGACTCGGACCTGCCTCTGTAGATCAGCTGGCCTCTTAAATCACAGCTCCACCTGCCTCTAGAGTTCTGGCATTAAAGGTGCGCACCACCACACTAGGCCAAGTCTTGTT
MSTRG.26507.1	ENSRNOG00000001088;ENSRNOG00000061262	0.923	0.000	0.003	-4.429	down	GGCCGCGGATTCTGCAGAGTCTCCCGACTGTGTGCTTCAGCTCAGCCACATCCTAGAGGAGGGGTTCCTCTGACATTTTTAAAGTCTATTTTTAGGGTTGGGGATTTAACTCAGTCGTTGAGCACATGCCTAGCAAGCACAAGGCCCTGGGTTCGATCCTTAGCTCTGGGGAAGGAAAAAAAAAAAAGACAAAATAAAAGTTGTAAAGTCGGGCTGGAAGGATGGCTTAGTGGTTAAGAGCACTGACTGCTCTTCCAGAGGTTCTGAGTTCAAATCCCAGCAACGACATGGTGTCTCACAGCCATCTGTAGTGAGATCTGATGCCCTCTTCTGGTGTGTCTGAAGACAGCTACAGTGTACTTATATATAAGAAATACATCTTTTTAAAAAAGTTGTAAAGTCTATTTCTGTTTGTTGGT
MSTRG.26595.1	--	0.169	0.475	0.034	2.165	up	CATTCGTGAAAATTTTTCTATATCCCGAACAGTCCACTTATTACTACTGCGGCCTATTGGGAACTAACCGAATTCACCATGTTACTCTAATTCGGCTCACCATATTTTGATAAATCTTTAAAAGTACAGAAATTACAAGAGCCTGCTACTGGGAACTAACTGAATTCACAAAGAAACAGTATTTCAGTTCGTTAGAAAGGTGCTCTATCTTGAATAACAAGCTTATTACATTGAATCCTATTGGGAACGTACTGAATTCACCAAGATACTTAGTTACCGTTCCTTAAAATGTTGCTCTTTATTGAAAAGCACACTCATACAAGCATGTCCCTGTGGGAACTCACTTAATTTGCCTAGAAATTTTGATTCCATTCGTGAACATTTTTCTATATCCCGAACAGTCCACTTATTACTACTACGGCCTATTGGGAACTAACTGAATTCACCATGTTACTCAGATTCGGCTCACCATATTTTGATAAATCTTTAAAGTACACATATTACAAGGGCCTGCTACTGGGAACTAACTGAATTCACAAAGATACACTGTTTCAGTTCGTAA

							AAACGTTGCTCTATCTTGAATAACAAGCTTATTACATGCGAATCCTATTGGGAACCTACTGAATTCACCATGATACTTAGATTCCGTTCCATAAAATGTTGCTCTATATTGAAAAGCACACTCATACAAGCATGTCCCATTGGGAACTCACTGAATTGACCAAGAAATTTTGATTCCATTCGTGAAAATTTTTCTATATCCCGAACAGTCCCCTTATTACTACTGCGGCCTATTGGGAACTAACCGAATTCACCATGTTACTCAGATTCGGCTCACCATATTTTGATAAATCTTTAAAAGTACACATATTACAAGGGCCTGCTACTGGGAACTAACTGAATTCACAAAGAAACAGTGTTTCAGTTCGTTAAAACGTTGCTCTATCTTGAATAACAAGCTTATTACATTGAATCCTATTGGGAACCTACTGAATTCACCATGATACTTAGATTCCGTTCCATAAAATGTTCCTCTATATCGAAAAGCACACTCATACAAGCATGTCCCATTGGGAACTCACTTAATTCGCCTAGGAATTTTGATTCCATTCGTTAAAATTTTTCTATATCCCGAACAGTCCACTTATTACTACTGCGGCCTATTGGGAACTAACAGAAATCACCATGTCACTCAGATTCGACTCAGGAAATTTTGATAATTCTTAAAAAGTACACATATTACAAGAAC
MSTRG.26595.2	--	0.162	0.617	0.015	2.479	up	CATTCGTGAAAATTTTTCTATATCCCGAACAGTCCACTTATTACTACTGCGGCCTATTGGGAACTAACCGAATTCACCATGTTACTCTAATTCGGCTCACCATATTTTGATAAATCTTTAAAAGTACAGAAATTACAAGAGCCTGCTACTGGGAACTAACTGAATTCACAAAGAAACAGTATTTCAGTTCGTTAGAAAGGTGCTCTATCTTGAATAACAAGCTTATTACATTGAATCCTATTGGGAACGTACTGAATTCACCAAGATACTTAGTTACCGTTCCTTAAAATGTTGCTCTTTATTGAAAAGCACACTCATACAAGCATGTCCCTGTGGGAACTCACTTAATTTGCCTAGAAATTTTGATTCCATTCGTGAACATTTTTCTATATCCCGAACAGTCCACTTATTACTACTACGGCCTATTGGGAACTAACTGAATTCACCATGTTACTCAGATTCGGCTCACCATATTTTGATAAATCTTTAAAGTACACATATTACAAGGGCCTGCTACTGGGAACTAACTGAATTCACAAAGATACACTGTTTCAGTTCGTAAAAACGTTGCTCTATCTTGAATAACAAGCTTATTACATGCGAATCCTATTGGGAACCTACTGAATTCACCATGATACTTAGGTTCGGTTCCTTAAAATGTTGTTCTACTTTGAAAAGCACACTCATACAAGCATGTCCCATTGGGAACTCACTATATTCGT
MSTRG.26596.1	--	0.179	0.906	0.000	2.985	up	CATTCGTGAAAATTTTTCTATATCCCGAACAGTCCACTTATTACTACTGCGGCCTATTGGGAACTAACCGAATTCACCATGTTACTCTAATTCGGCTCACCATATTTTGATAAATCTTTAAAAGTACAGAAATTACAAGAGCCTGCTACTGGGAACTAACTGAATTCACAAAGAAACAGTATTTCAGTTCGTTAGAAAGGTGCTCTATCTTGAATAACAAGCTTATTACATTGAATCCTATTGGGAACGTACTGAATTCACCAAGATACTTAGTTACCGTTCCTTAAAATGTTGCTCTTTATTGAAAAGCACACTCATACAAGCATGTCCCTGTGGGAACTCACTTAATTTGCCTAGAAATTTTGATTCCATTCGTGAACATTTTTCTATATCCCGAACAGTCCACTTATTACTACTACGGCCTATTGGGAACTAACTGAATTCACCATGTTACTCAGATTCGGCTCACCATATTTTGATAAATCTTTAAAGTACACATATTACAAGGTCCTGCTACTGGGAACTAACTGAATTCACAAAGATACACTGTTTCAATTCGTAAAAACGTTGCTCTATCTTGAATAACAAGTTTATTACATGCGAATCCTATTGGGAACCTACTGAATTCACCATGATACTTAGATTCCGTCCCTTAAAATGTTGCTATATATCGAAAAGCACACTAATACAAGCATGTCCCATTGGGAACTCACTGAATTCGCCTAGAAATTTTGATTCCATTCGTGAAAATTTTTCTATATCCCGACAGTCCACTTATTACTAATGTGGCCTATTGGGAACTAACCGAAATCACCATGTCACTCAGATTCGACTCAGGAAATTTTGATAAATCTTAAAAAGTACACATGTTACAAGAACCTGCTACTGGGAACTAACTGAATTCACAAAGATACACTGTTTCAGTTCGTTAAAACGTTGCTCTATCTTGAATAACAAGCTTATTACATGCGAATCCTATTGGGAACCTACTGAATTCACCATGATACTTAGATTCCGTTCCATAAAATGTTGCTCTATATTGAAAAGCACACTCATACAAGCATGTCCCATTGGGAACTCACTGAATTGACCAAGAAATTTTGATTCCATTCGTGAAAATTTTTCTATATCCCGAACAGTCCCCTTATTACTACTGCGGCCTATTGGGAACTAACCGAATTCACCATGTTACTCAGATTCGGCTCACCATATTTTGATAAATCTTTAAAAGTACACATATTACAAGGGCCTGCTACTGGGAACTAACTGAATTCACAAAGAAACAGTGTTTCAGTTCGTTAAAACGTTGCTCTATCTTGAATAACAAGCTTATTACATTGAATCCTATTGGGAACCTACTGAATTCACCATGATACTTAGATTCCGTTCCATAAAATGTTCCTCTATAT

							CGAAAAGCACACTCATACAAGCATGTCCCATTGGGAACTCACTTAATTCGCCTAGGAATTTTGATTCCATTCGTTAAAATTTTTCTATATCCCGAACAGTCCACTTATTACTACTGCGGCCTATTGGGAACTAACAGAAATCACCATGTCACTCAGATTCGACTCAGGAAATTTTGATAATTCTTAAAAAGTACACATATTACAAGAACCTGATACTGGGAACTAACTGAATTCACAAAGATACACTGTTTCAGTTCGTTAAAACGTTGCTCTATCTTGAATAACAAGCTCATTAC
MSTRG.26596.10	--	0.099	0.631	0.000	3.257	up	CATTCGTGAAAATTTTTCTATATCCCGAACAGTCCACTTATTACTACTGCGGCCTATTGGGAACTAACCGAATTCACCATGTTACTCTAATTCGGCTCACCATATTTTGATAAATCTTTAAAAGTACAGAAATTACAAGAGCCTGCTACTGGGAACTAACTGAATTCACAAAGAAACAGTGTTTCAGTTCGTTAGAAAGATGCTCTATCTTGATTAACAAGCTTATTACAGTGAATCCTATTGGGAACCTACTGAATTCACCAAGATACTTAGTTACCGTCCCTTAAAATGTTGCTATATATCGAAAAGCAAACTCATACAAGCATGTCCCATTGGGAACTCACTATATTCGCCTAGAAATTTGGATTCCATTCGTGAAAATTTTTCTATATCCCGAACAGTCCACTTATTACTACTGCGGCCTATTGGAATCTAACCGAATTCACCATGTTACTCATATTCGGCTCACCATATTTTGAGAAATCTTTAAAAGTACACGTATTACAAGGGCCTTCTACTGGGAACTAACTGAATTCACAATGATACACTGTTTCAGTTCGTAAAAACGTTGCTCTATCTTGAATAACAAGTTTATTACATGCGAATCCTATTGGGAACCTACTCAATTCACCATGATACTTAGATTCCGTCCCTTGAAATGTGGCTATATATCAAAAAGCACACTAATACAAGCATGTCCCATTGGGAACTCACTGAATTCGCCTAGAAATTTTGATTCCATTTGTGATAATTTTTCTATATCCCGACAGTCCACTTATTACTACTGTGGCCTATTGGGAACTAACCGAAATCACCATGTCACTCAGATTCGACTCAGGAAATTTTGATAAATCTTAAAAAGTACACATGTTACAAGAACCTGCTACTGGGAACTAACTGAATTCACAAAGATACACTGTTTCAGTTCGTTAAAACGTTGCTCTATCTTGAATAACAAGCTTATTACATGCGAATCCTATTGGGAACCTACTGAATTCACCATGATACTTAGATTCCATTCCTTAAAATGTTGATCTATATTGAAAAGCACACTCATACAAGCATGTCCCATTGGGAACTCACTGAATTCACCCAGAAATTTTGATTCCATTCGTGAAAATTTTTCTATATCCCGAACTGTCCCCTTATTACTACTGCGGCCTATTGTGAACTAACCGAATTCAACATGTTACTCAGAATCGGCTCACCATATTTTGATAAATCTTTAAAAGTACACATATTACAAGGGCCTGCTACTGGGAACTAACTGAATTCACAAAGAAACAGTGTTTCAGTTCGTTAAAACGTTGCTCTATCGTGAATAACAAGTTAATTACATGCGAATCCTATTGGGAACCTACTGAATTCACCATGATACTTTAATTCGGTTCCTTAAAATGTTGCTCTATATTGAAAAGCACACTCATACAAGCATGTCCCATTGGGAACTCACTGAATTCGCCTAGGAATTTTGATTCCATT
MSTRG.26596.11	--	0.115	0.653	0.001	3.020	up	AGATTCGGCTCACCATATTTTGATAAATCTTTAAAAGTACACAAATTACAAGATCCTGCTACTGGGAACTAACTGAATTCACAAAGAAACAGTGTTTCAGTTCGTTAAAACGTTGCTCTATCTTGAATAACAAGCTTATTACATGGGAATCCTATTGGGAACCTACTGAATTCACCATGATACTTAGATTCCGTTCCATAAAATGTTGCTCTATATTGAAAAGCACACTCATACAAGCATGTCCCATTGGGAACTCACTGAATTGACCAAGAAATTTTGATTCCATTCGTGAAAATTTTTCTATATCCCGAACAGTCCCCTTATTACTACTGCGGCCTATTGGGAACTAACCGAATTCACCATGTTACTCAGATTCGGCTCACCATATTTTGATAAATCTTTAAAAGTACACATATTACAAGGGCCTGCTACTGGGAACTAACTGAATTCACAAAGAAACAGTGTTTCAGTTCGTTAAAACGTTGCTCTATCTTGAATAACAAGCTTATTACATTGAATCCTATTGGGAACCTACTGAATTCACCATGATACTTAGATTCCGTTCCATAAAATGTTCCTCTATATCGAAAAGCACACTCATACAAGCATGTCCCATTGGGAACTCACTTAATTCGCCTAGGAATTTTGATTCCATTCGTTAAAATTTTTCTATATCCCGAACAGTCCACTTATTACTACTGCGGCCTATTGGGAACTAACAGAAATCACCATGTCACTCAGATTCGACTCAGGAAATTTTGATAATTCTTAAAAAGTACACATATTACAAGAACCTGATACTGGGAACTAACTGAATTCACAAAGATACACTGTTTCAGTTCGTTAAAACGTTGCTCTATCTTGAATAACAAGCTCATTAC

MSTRG.26596.2	--	0.011	0.454	0.000	4.728	up	CATTCGTGAAAATTTTTCTATATCCCGAACAGTCCACTTATTACTACTGCGGCCTATTGGGAACTAACCGAATTCACCATGTTACTCTAATTCGGCTCACCATATTTTGATAAATCTTTAAAAGTACAGAAATTACAAGAGCCTGCTACTGGGAACTAACTGAATTCACAAAGAAACAGTATTTCAGTTCGTTAGAAAGGTGCTCTATCTTGAATAACAAGCTTATTACATTGAATCCTATTGGGAACGTACTGAATTCACCAAGATACTTAGTTACCGTTCCTTAAAATGTTGCTCTTTATTGAAAAGCACACTCATACAAGCATGTCCCTGTGGGAACTCACTTAATTTGCCTAGAAATTTTGATTCCATTCGTGAACATTTTTCTATATCCCGAACAGTCCACTTATTACTACTACGGCCTATTGGGAACTAACTGAATTCACCATGTTACTCAGATTCGGCTCACCATATTTTGATAAATCTTTAAAGTACACATATTACAAGGTCCTGCTACTGGGAACTAACTGAATTCACAAAGATACACTGTTTCAATTCGTAAAAACGTTGCTCTATCTTGAATAACAAGTTTATTACATGCGAATCCTATTGGGAACCTACTGAATTCACCATGATACTTAGATTCCGTCCCTTAAAATGTTGCTATATATCGAAAAGCACACTAATACAAGCATGTCCCATTGGGAACTCACTGAATTCGCCTAGGAATTTTGATTCCATTCATGAAAATTTTTCTATATCCCGAACAGTCCACTTATTACTACTGCGGCCGATTGGGAACTAACCGAATTCACCATGTTACTCTAATTCGTCTCACCACATTTTGATAAATCTTTAAAAGTACACAAATTACAAGAGCCTGCTACTGGGAACTAACTGAATTCACAAAGAAACAGTGTTTCAGTTCGTTAGAAAGATGCTCTATCTTGATTAACAAGCTTATTACAGTGAATCCTATTGGGAACCTACTGAATTCACCATGATACTTAGATTCCGTCCCTTAAAATGTTGCTATATATCGAAAAGCACACTAATACAAGCATGTCCCATTGGGAACTCACTGAATTCGCCTAGAAATTTTGATTCCATTCGTGAAAATTTTTCTATATCCC
MSTRG.26596.4	--	0.221	0.594	0.043	2.073	up	CATTCGTGAAAATTTTTCTATATCCCGAACAGTCCACTTATTACTACTGCGGCCTATTGGGAACTAACCGAATTCACCATGTTACTCTAATTCGGCTCACCATATTTTGATAAATCTTTAAAAGTACAGAAATTACAAGAGCCTGCTACTGGGAACTAACTGAATTCACAAAGAAACAGTATTTCAGTTCGTTAGAAAGGTGCTCTATCTTGAATAACAAGCTTATTACATTGAATCCTATTGGGAACGTACTGAATTCACCAAGATACTTAGTTACCGTTCCTTAAAATGTTGCTCTTTATTGAAAAGCACACTCATACAAGCATGTCCCTGTGGGAACTCACTTAATTTGCCTAGAAATTTTGATTCCATTCGTGAACATTTTTCTATATCCCGAACAGTCCACTTATTACTACTACGGCCTATTGGGAACTAATGAATTCACCATGTTACTCAGATTCGGCTCACCATATTTTGATAAATCTTTAAAGTACACATATTACAAGGTCCTGCTACTGGGAACTAACTGAATTCACAAAGATACACTGTTTCAATTCGTAAAAACGTTGCTCTATCATGAATAACAAGTTTATTACATGCGAATCCTATTGGGAACCTACTCAATTCACCATGATACTTAGATTCCGTCCCTTGAAATGTGGCTATATATCAAAAAGCACACTAATACAAGCATGTCCCATTGGGAACTCACTGAATTCGCCTAGAAATTTTGATTCCATTTGTGATAATTTTTCTATATCCCGACAGTCCACTTATTACTACTGTGGCCTATTGGGAACTAACCGAAATCACCATGTCACTCAGATTCGACTCAGGAAATTTTGATAAATCTTAAAAAGTACACATGTTACAAGAACCTGCTACTGGGAACTAACTGAATTCACAAAGATACACTGTTTCAGTTCGTTAAAACGTTGCTCTATCTTGAATAACAAGCTTATTACATGCGAATCCTATTGGGAACCTACTGAATTCACCATGATACTTAGGTTCGGTTCCTTAAAATGTTGTTCTACTTTGAAAAGCACACTCATACAAGCATGTCCCATTGGGAACTCACTATATTCGTCTAGAAATTTTGATTCCGTTCGTGAAAATTTTT
MSTRG.26596.5	--	0.601	0.000	0.000	-5.223	down	ATCCCGAACAGTCCACTTATTACTACTGCGGCCTATTGGGAACTAACCGAATTCACCATGTTACTCTAATTCGGCTCACCATATTTTGATAAATCTTTAAAAGTACAGAAATTACAAGAGCCTGCTACTGGGAACTAACTGAATTCACAAAGAAACAGTATTTCAGTTCGTTAGAAAGGTGCTCTATCTTGAATAACAAGCTTATTACATTGAATCCTATTGGGAACGTACTGAATTCACCAAGATACTTAGTTACCGTTCCTTAAAATGTTGCTCTTTATTGAAAAGCACACTCATACAAGCATGTCCCTGTGGGAACTCACTTAATTTGCCTAGAAATTTTGATTCCATTCGTGAACATTTTTCTATATCCCGAACAGTCCACTTATTACTACTACGGCCTATTGGGAACTAACTGAATTCACCATGTTACTCAGATTCGGCTCACCATATTTTGATAAATCTTTAAAGTACACATATTACAAGGTCCTGCTACTGGGAACTAACTGAATTCACAAAGATACACTGTTTCAATTCGTAAAAACGTTGCTCTATCTTGAATAA

							CAAGTTTATTACATGCGAATCCTATTGGGAACCTACTGAATTCACCATGATACTTAGATTCCGTCCCTTAAAATGTTGCTATATATCGAAAAGCACACTAATACAAGCATGTCCCATTGGGAACTCACTGAATTCGCCTAGAAATTTTGATTCCATTCGTGAAAATTTTTCTATATCCCGACAGTCCACTTATTACTAATGCGGCCTATTGGGAACTAACCGAATTCACCATGTTACTCAGATTCGGCTCACCATATTTTGATAAATCTTTAAAAGTACACATATTACAAGGGCCTGCTACTGGGAACTAACTGAATTCACAAAGAAACAGTGTTTCAGTTCGTTAAAACGTTGCTCTATCTTGAATAACAAGCTTATTACATTGAATCCTATTGGGAACCTACTGAATTCACCATGATACTTAGATTCCGTTCCATAAAATGTTCCTCTATATCGAAAAGCACACTCATACAAGCATGTCCCATTGGGAACTCACTTAATTCGCCTAGGAATTTTGATTCCATTCGTTAAAATTTTTCTATATCCCGAACAGTCCACTTATTACTACTGCGGCCTATT
MSTRG.26596.6	--	0.087	0.774	0.000	3.745	up	CATTCGTGAAAATTTTTCTATATCCCGAACAGTCCACTTATTACTACTGCGGCCTATTGGGAACTAACCGAATTCACCATGTTACTCTAATTCGGCTCACCATATTTTGATAAATCTTTAAAAGTACAGAAATTACAAGAGCCTGCTACTGGGAACTAACTGAATTCACAAAGAAACAGTATTTCAGTTCGTTAGAAAGGTGCTCTATCTTGAATAACAAGCTTATTACATTGAATCCTATTGGGAACGTACTGAATTCACCAAGATACTTAGTTACCGTTCCTTAAAATGTTGCTCTTTATTGAAAAGCACACTCATACAAGCATGTCCCTGTGGGAACTCACTTAATTTGCCTAGAAATTTTGATTCCATTCGTGAACATTTTTCTATATCCCGAACAGTCCACTTATTACTACTACGGCCTATTGGGAACTAACTGAATTCACCATGTTACTCAGATTCGGCTCACCATATTTTGATAAATCTTTAAAGAACACATATTACAAGGTCCTGCTACTGGGAACTAACTGAATTCACAAAGATACACTGTTTCAATTCGTAAAAACGTTGCTCTATCATGAATAACAAGTTTATTACATGCGAATCCTATTGGGAACCTACTCAATTCACCATGATACTTAGATTCCGTCCCTTGAAATGTGGCTATATATCAAAAAGCACACTAATACAAGCATGTCCCATTGGGAACTCACTGAATTCGCCTAGAAATTTTGATTCCATTTGTGATAATTTTTCTATATCCCGACAGTCCACTTATTACTACTGTGGCCTATTGGGATCTAACCGAAATCACCATGTCACTCAGATTCGACTCAGGAAATTTTGATAAATCTTAAAAAGTACACATATTACAAGAACCTGCTACTGGGAACTAACTGAATTCACAAAGATACACTGTTTCAGTTCGTTAAAACGTTGCTCTATCTTGAATAACAAGCTTATTACATGCGAATCCTATTGGGAACCTACTGAATTCACCATGATACTTAGATTCCGTTCCATAAAATGTTGCTCTATATTGAAAAGCACACTCATACAAGCATGTCCCATTGGGAACTCACTGAATTGACCAAGAAATTTTGATTCCATTCGTGAAAATTTTTCTATATCCCGAACAGTCCCCTTATTACTACTGCGGCCTATTGGGAACTAACCGAATTCACCATGTTACTCAGATTCGGCTCACCATATTTTGATAAATCTTTAAAAGTACACATATTACAAGGGCCTGCTACTGGGAACTAACTGAATTCACAAAGAAACAGTGTTTCAGTTCGTTAAAACGTTGCTCTATCTTGAATAACAAGCTTATTACATTGAATCCTATTGGGAACCTACTGAATTCACCATGATACTTAGATTCCGTTCCATAAAATGTTCCTCTATATCGAAAAGCACACTCATACAAGCATGTCCCATTGGGAACTCACTTAATTCGCCTAGGAATTTTGATTCCATTCGTTAAAATTTTTCTATATCCCGAACAGTCCACTTATTACTACTGCGGCCTATTGGGAACTAACAGAAATCACCATGTCACTCAGATTCGACTCAGGAAATTTTGATAATTCTTAAAAAGTACACATATTACAAGAACCTGATACTGGGAACTAACTGAATTCACAAAGATACACTGTTTCAGTTCGTTAAAACGTTGCTCTATCTTGAATAACAAGCTCATTAC
MSTRG.26596.8	--	0.333	1.472	0.000	2.871	up	CATTCGTGAAAATTTTTCTATATCCCGAACAGTCCACTTATTACTACTGCGGCCTATTGGGAACTAACCGAATTCACCATGTTACTCTAATTCGGCTCACCATATTTTGATAAATCTTTAAAAGTACAGAAATTACAAGAGCCTGCTACTGGGAACTAACTGAATTCACAAAGAAACAGTGTTTCAGTTCGTTAGAAAGATGCTCTATCTTGATTAACAAGCTTATTACAGTGAATCCTATTGGGAACCTACTGAATTCACCAAGATACTTAGTTACCGTCCCTTAAAATGTTGCTATATATCGAAAAGCAAACTCATACAAGCATGTCCCATTGGGAACTCACTATATTCGCCTAGAAATTTGGATTCCATTCGTGAAAATTTTTCTATATCCCGAACAGTCCACTTATTACTACTGCGGCCTATTGGAATCTAACCGAATTCACCATGTTACTCATATTCGGCTCACCATATTTTGAGAAATCTTTAAAAGT

							ACACGTATTACAAGGGCCTTCTACTGGGAACTAACTGAATTCACAATGATACACTGTTTCAGTTCGTAAAAACGTTGCTCTATCTTGAATAACAAGTTTATTACATGCGAATCCTATTGGGAACCTACTGAATTCACCATGATACTTAGATTCCGTCCCTTAAAATGTTGCTATATATCGAAAAGCACACTAATACAAGCATGTTCCATTGGAAACTCACTGAATTCGCCTAGAAATTGTGATTCCATTCGTGAAAATTTTTCTATATCCCGACAGTCCACTTATTACTACTGCGACCTATTGGGATCTAACCGAAATCACCATGTCACTCAGATTCGACTCAGGAAATTTTGATAAATCTTAAAAAGTACACATATTACAAGAACCTGCTACTGGGAACTAACTGAATTCACAAAGATACAGTGTTTCAGTTCGTTAAAACGTTGCTCTATCTTGAATAACAAGCTTATTACATGGGTATCCTATTGGGAACCTACTGAATTCACCATGATACTTAGATTCCGTTCCTTAAAATGTTACTCTATATTGAAAAGCACACTCATACAAGCATGTCCCATTGGGAACTCACTGAATTCGCCTAGAAATTTTGATTCCATTCGTGAAAATTTTTCTATATCCCGAACAGTCCACTTATTACTACTGCGGCCTATTGGGATCTAACCGAAATCACCATGTCACTCAGATTCGACTCAGGAAATTTTGATAAATCTTAAAAAGTACACATATTACAAGAACCTGCTACTGGGAACTAACTGAATTCACAAAGATACACTGTTTCAGTTCGTTAAAACGTTGCTCTATCTTGAATAACAAGCTTATTACATGCGAATCCTATTGGGAACCTACTGAATTCACCATGATACTTAGATTCCGTTCCATAAAATGTTGCTCTATATTGAAAAGCACACTCATACAAGCATGTCCCATTGGGAACTCACTGAATTGACCAAGAAATTTTGATTCCATTCGTGAAAATTTTTCTATATCCCGAACAGTCCCCTTATTACTACTGCGGCCTATTGGGAACTAACCGAATTCACCATGTTACTCAGATTCGGCTCACCATATTTTGATAAATCTTTAAAAGTACACATATTACAAGGGCCTGCTACTGGGAACTAACTGAATTCACAAAGAAACAGTGTTTCAGTTCGTTAAAACGTTGCTCTATCTTGAATAACAAGCTTATTACATTGAATCCTATTGGGAACCTACTGAATTCACCATGATACTTAGATTCCGTTCCATAAAATGTTCCTCTATATCGAAAAGCACACTCATACAAGCATGTCCCATTGGGAACTCACTTAATTCGCCTAGGAATTTTGATTCCATTCGTTAAAATTTTTCTATATCCCGAACAGTCCACTTATTACTACTGCGGCCTATTGGGAACTAACAGAAATCACCATGTCACTCAGATTCGACTCAGGAAATTTTGATAATTCTTAAAAAGTACACATATTACAAGAACCTGATACTGGGAACTAACTGAATTCACAAAGATACACTGTTTCAGTTCGTTAAAACGTTGCTCTATCTTGAATAACAAGCTCATTAC
MSTRG.26596.9	--	0.427	1.610	0.001	2.624	up	CATTCGTGAAAATTTTTCTATATCCCGAACAGTCCACTTATTACTACTGCGGCCTATTGGGAACTAACCGAATTCACCATGTTACTCTAATTCGGCTCACCATATTTTGATAAATCTTTAAAAGTACAGAAATTACAAGAGCCTGCTACTGGGAACTAACTGAATTCACAAAGAAACAGTGTTTCAGTTCGTTAGAAAGATGCTCTATCTTGATTAACAAGCTTATTACAGTGAATCCTATTGGGAACCTACTGAATTCACCAAGATACTTAGTTACCGTCCCTTAAAATGTTGCTATATATCGAAAAGCAAACTCATACAAGCATGTCCCATTGGGAACTCACTATATTCGCCTAGAAATTTGGATTCCATTCGTGAAAATTTTTCTATATCCCGAACAGTCCACTTATTACTACTGCGGCCTATTGGAATCTAACCGAATTCACCATGTTACTCATATTCGGCTCACCATATTTTGAGAAATCTTTAAAAGTACACGTATTACAAGGGCCTTCTACTGGGAACTAACTGAATTCACAATGATACACTGTTTCAGTTCGTAAAAACGTTGCTCTATCTTGAATAACAAGTTTATTACATGCGAATCCTATTGGGAACCTACTGAATTCACCATGATACTTAGATTCCGTCCCTTAAAATGTTGCTATATATCGAAAAGCACACTAATACAAGCATGTCCCATTGGAAACTCACTGAATTCGCCTAGAAATTTTGATTCCATTCGTGAAAATTTTTCTATATCCCGACAGTCCTCTTATTACTACTGCGACCTATTGGGATCTAACCGAAATCACCATGTCACTCAGATTCGACTAAGGAAATTTTGATAAATTTTAAAAAGTACACATATTACAAGAATCTGCTACTGGGAACTAACTGAATTCACAAAGATACACTGTTTCAGTTCGTTAAAACGTTGCTCTATCTTGAATAACAAGCTTATTACATGGGAATCCTATTGGGAACCTACTGAATTCACCATGATACTTATATTCCGTTCCTTAAAATGTTGCTCTATATTGAAAAGCACATCATACAAGCATGTCCCATTGGGAACTCACTATATTCGCCTAGAAATTTTGATTTCATTCGTTAAAATTTTTCTATATCCCGAACAGTCCACTTATTACTACTGTGGCCTATTGGGAACTAACCGAATTCACCATGTTACTCAGATTCGGCTCACCATATTTAGTTAAATCTTTAAAAGTACACATATTACAAGGGCCTGCTACTGGGAACTAACTGAA

							TTCACAAAGATACACTGTTTCAGTTCGTAAAAACGTTGCTCTATCTTGAATAACAAGCTTATTACATGCGAATCCTATTGGGAACCTACTGAATTCACCATGATACTTAGGTTCGGTTCCTTAAAATGTTGTTCTACTTTGAAAAGCACACTCATACAAGCATGTCCCATTGGGAACTCACTATATTCGTCTAGAAATTTTGATTCCGTTCGTGAAAATTTTT
MSTRG.26820.1	--	0.094	0.358	0.038	2.417	up	TGAGTCATTTTCTTGATTCGAAATTGATGTGGGAGGAGTTGGCCCACTGTGGGCGGTCCCAACCCTAGGCAGGTGGTCCCAGGCTATGTAAGAAAGCAAACTGAGCAAGCCATGGGGAGCAAGCCAGTGAAGACCATTCCGGTCCTCCTTCTAGGTTTCTGCTTGAGCTCCTGCCTTGATTGGGATCAGGAAGTATAAGCCAGATCAACCCTCTCCTCCCCAAATTGTTTTTGGCCAGTCTTTAATCACATCAACAGAAAAGTAGCTAGGATTAATAGACTCATGTCCCCAACATCCAGCATGGCATCTGACTGAATCAAAGTCACCCTTCACTGGGGTCATTGCTGGTCAAGTGCAAGGTGCAACTGAAGGGCTTTTGAATGTGAGGCCAGGAAGAAGGGCAACTAAGAAGAAAAGCAAGGTGACGTGGAGTCCGGGTGATGAATCCCCTTTGTAACATAAACATCTCCCAGAAGCTGGTTAGAATGTGTTAAACATAGAGATGTAACAGTTTATAAATATCAGAAAGGATATGGCTGCCATGTGGAATAAATCAGTGTCTAAAAAATAGTATAGTGGGGTGTTTGTGTGTGTGTGTGTGTGTGTGTGTGTGTGTGTGTGTGTGTGTGTGTGTTAGCTGTAATGTATTTTCCATGGCAACCATGTCTTTGGTGCATATGAAGGGAACTGGTAGGTTCAATATAAGTGTGCATGGGTAAATAAGCACCTGTTTGGGAAAGAAACCAAAATGACTTTGGGAATGACATTTCCTAGATTAAAATTTAATCAGAGTGGCTG
MSTRG.27199.1	--	0.611	0.000	0.017	-3.928	down	GGGATGGATTCAGCAACATTGCGGCCCAAATCCAGGGCACTAGGTGCCCTCGGGGTAGATACCATAGCCCCTGACCAGGCAAGAGAGGGAGGCTGGTGGGGGAATCTGTTGTTAAGATGAAACAATAGGGGCTGGAGAGATGGTTTAGAGGGTAGAAACATTTGCTTCGCATGGGGAATTGAGTTTGGATCTGCAGTGCACACCTAGAAACTCAGGTATACCTCTATCACTGGGAAAGGGGTCTGTAACTGGCCAGCTAGCCTGGGTCAGTCAGCGAGCACCAGATGCTCTGAGGAACCTTGCTTCAAACACCAAGATGGAGAGAGGTGGAAGAAGACACTTGACATTGACCTTGGCCTCCAGATGCACATGCACACCTGCACACAAACACCTGCAGATGAACACCTGCACACCTGCACATGC
MSTRG.27203.2	--	0.010	0.470	0.000	4.888	up	GGGAGTTTCAACCTGTATGAGACACAATTCCCCAGATATTGAGAAGCACTCAGAGGGCAAAGTGGTATGTAAACCAGGTGTGTGTAAAAGAGGGTGTGAGAGCCTGGTGTGAGAGGCTGGGGTCAGGGAGGAGTTTAAGACTGAGTTTCTTGGTTTACAACTCAAGTACACCATGGAAACCTGTCAAGTTCTGAGGGAGACACGGGTAGGAGAAGTGCTTGGGAGACAAACACCATTCTGTTCATGAGACATTTCTGCATGTATAGCGGAGAGGGGGAAAGCAGCTAACCTCGGATTTATCTCTCTATTTTTCCATACTTTTGACACTGGTAAGAAAATGATTCAAATTTAATAGGATGAGTGTTATGCCCTCGGGAACTGCGAAAATGTATGGTGGCTAACTTTCTTGGTTTTCCCAGAGGGGACTTTGCAGAGCTAAAGCAAGAACAGCCCAACAGTCTGGGGCAAACTGGGATACGTTGGTTACATGTCATGCACACTCTCCTAACCGCAGAGGACAAACAGTATCTCTCTTACTGTTTTCATGGTGGTAGGACACAAACCCTCAGGTTTGGCATATGCCTCCAACAGGGAGGCCCCACAGAACCCTGAGAACCAGCCCCACAGCAAGAAGGTGAGCCACAAACCGGAGCCCCACTTCCTGCTGTTTTGTTGAACTTAGTGCTCTACACTGGGGGGACATTATACACAACACATGTTTACACTTCAGCTCCTTAATGCCCTGCACCATTCCCGTGAAGAAAGGGCCCGTCCACCCTCTGAGCAAGGCATTTTCCTGTTCTTCTAGAGCTTACTCTGAAAGTCAGAGTCACAGCTCTGTTACTCACTTGCTGGGTTAGCCAGGGCCACAGACTTTTACCACGGTAGTGGGTCTGAAGACGTGGAAACAAGATCAACCGATTGGATATTTGTCCCATGTCAAAATCAAAATGAAACATGCACACTGGATGCCTGACACACATCCGTCCACACAGACATTCCATTACCTCGTCTTATAGCTCTAAATCTCCCACACCCCACGGACACTTGGAGGATACACCTGCCTCTGCTGCTGTTCAGTCTGCCTTTGCCCTTCTCCTGCCCTGGAGCCTGGGAACAAGGTGCCCTCTCTCCTACAGCCCGGGAACAAGGTGCCCTCTCGCCTATAG

							CCCGGGAACAAGGTGCCCTCTCTCCTATAGCCTGGGAACAAGGTGCCCTCTCT
MSTRG.27208.1	ENSRNOG00000034226;ENSRNOG00000042133;ENSRNOG00000047234;ENSRNOG00000058463;ENSRNOG00000059588;ENSRNOG00000060573	1.073	0.000	0.000	-9.859	down	GGGTTGAATCATAATGGAATTTAATCTTGTGTGAAAACAACTATTGTTAAAAGTCATCATTAGCTTGTTCTACAAACACCACGTTATCATGCACCGTGCAGCATCCACCGGAGACAATTAACCTTAGAGACTCAGTGGAGTAGCACTGATCCCGATAACTCACACGATAAAGCTTCTGCACCCAAGCTCAGTGGCTCTTACACGCCGCCTTTTAAGAGAACATTTCCACCATTAGACAAACAAAAACCAAAAGAAACTGGGGGAGGGGCTGTGGAAACATCTGTCAACTCTAAGGTTTTAGTCCAAGTCCTGCTACTTTGTTTCAACAAAGTTCAAATAATTTAAAATGATACAGCTCTCGGTTCATAATCTAACTCTAATATTTCCTAATTTTGTGATTGGACTTAAAATGCTTCTGGGAACCTTATGTTTCTCACCCACACAACACAAAGCATAGCCTAATATTAAGGCTTTCAGGGAAATTAGAGATAGAAGACAGACATTTTGTCAAGCCCATAGCAAGTGATCAGCAAGTTAGAGTTATTAACAAAATTTAACATTTATACCAAAGCTATAGAATCTTGGGGAAAAAAAAAACAAACTAAAGACATAAAACCAGTGCAGTCTAAACTGGCTGAAAACTAAGCTAAAAGAAACATAATTTAAAACAACTATCAAAATTTTGCTTCATAACAAAGCAGTATAGCTCCCCCTCTTTCTGTGGTATGTGCAGAAAAACACATACACAGTATACAGAAGAAAAAGGAACATCTAGAAGGGAAGAGGAAGCCACAGAAGTAGAGAGTATCACACATGGCCACCATCCCTGAATTTTCTTTTGGTCTTAAGCCAAATGAGGTGTCTGTGGTGGTATGGACAAGACTGGCCCTGCAAAAGCTCACATATTTGAATGCTTGGTGCCCTGTTGGTAGAACTGTTTAGGAAGGATTAGGAGGTGTGGCCTTGTTGGAGAGGGTACATCAGTAAGAGTACTCTTTGGCTTTGAGGTATCAAAAGCCATTCCTTGTTACCTAGGGCACCCCTCTGTAACAGACTCAGTTTAATTTAATTTTCCTAACTCTCTGCTCGCCCCCTCCAACACACACACACACACACACACACACACACACACCTGCCATTACTACCTAGTGCCATGCCTGCCTGTGTGCTTGCGTGCTGACAGGTTTCCTACATTATGGTTATGGACTCTCATTCTCTGAGACTGTGAGCCCCAAATTAAATGCTTTCTCTTATAATCTGGCTTAGTTATGGTGTTTTGTCAGGGCAACAGGAAAGCAATTAAGTTGGTCCTAAAGAAGCTAGCACTCTAGAAGTTCAAGAGCAGACCAAAAACTCCTAGGGAAAAAAAATGTCATCTTTCCAGCCAAAGCAACAAAAGGGCAAGCTACAAAGACATGAAACTTGCAGACAAAATGAAACAAAATAGCATTCTTCTTTGGCCATCTTATTAGCAAAGCAAAATGGCAGCCCATGCTCCCTCCCTTGGTGGCCGGTATTCCCTCCTTTTCTCTGCTGTATCACTTTGTACTTCTACCCCCTGGAGTGTTGTGAGAGAAAGCAGAGAAGCCTAACTTTCATTACCGCACAGTTATATCACACTTGTACCTCTGTGTCACTCTGTGTCACTGACAGCATTTCCTTCACTGCATCCTTATAGATATGAAGCACATTTCACCCTCCCCATCAGTGGAGCCTTATATGAGAACTTCACCAGGATCCCCCAACAGTGATGTCTCCCTCAGTTAATGAAGGGTATGTGGAGAGTATTGACCTTCCTCTAGCTGTCAACAAAGAAAGAATGGGAGCCACAAACTACCCTTATCACAAGGATACCCTGCTTCACAAAAGTGTGTGTGTGTGTGTGTGTGTGTGTTTGTGTGTGTGTGTGTATGTGTGTGTGTGAAAGAGAATTTCTAATTTCCAATAGGTTTAAATAGCATCTAGCTTTATAATATCTAAAATGTGGATGATTTTCAAAAATTATTTGTTAAACAAAAAAAATCAAATGAGGAAATACAAATACACTAACAGATATAAATAGCAGGTGACCCAGATGTTAGAACCATCCAGAAAACAAGAATAGTACTACACTGTTGTAACCCCAGCACTTAGGGGTAGAGGCAGGAGGAAGGAGTTGTTCTAGGCCACCCCAAGCTACACAAAATAATAGAAAGGATCACAAGGCCATCATTAGGTGGGTAAAAAGCACTTGCCGTGAAAGTCCAATAACATGAATTTGAACTATGGAACTCTGGAACAGTCTCCTTGAAACTGTCCTACCCCAATACACTCTTGTGTGCGCACACGCATATGCACTATAGCAGCGGTATACCGGGGGCACTCTCTGTTACACATAATAATAAAAACTTTTAAAAATTATCCAGTCATTTTTAAATGTTTTACAAATATACTTGAAACACAGATTAAAAGTTTCAACAATAACAAAAAGGGCAATCTTGAGACAATAAACAGAAATTATAGATATGAAAAATAATAAAGAAATAAAATACTCAATGGATGGACTCAACTATGGAGAACAAAGGAAAGAAATCATAAGCCTTAAGATTAGTCCCAGTTTAGAAAACTAAATGAAACTCTAAGAGGGCAAACAAAGAATTCCGTGAAATAACAATTAAAACCAAACAATGGAAAGCTTAAACAAATCAAGTATCTTAGAAGCTCTGAGAGAGAAACGAGACATTATCTAAAGATCTCATCGTTCATGTACAGAGAGATGGCTCAGT

							GGGTGATGTCATCCCTGGGACCCATAGGTAGAAGGAGAAATTCTTACAAATTGTCCTCAGACTGCCACA<br>TGTGCACTGTGTATTCACACACGCACACACAACATACACACATATGCAGGCATGCAAATAAATAAATAA<br>AATGCAACCATGTTTCAAGTTAGCTAAAATCAAAAAAGAACAAGTAGTTCAAAAGGCAGCGAAGCTCT<br>TCATCAGACACAAGAGAGGCAGAAGAGGACATTTCTCCGGTGCTAAAGGAAAGAAACTGTCAGACTA<br>CACACTATATATCCAGCAAAATTATCCTTCGTGCTCCAAGATGAAATCGTCACATCCTCAACTGAGGACA<br>ATGAAAAGAATTTCTCTCTAGCAAACCTACCTTGAAAGAATGCTGAAAGAACGGACGCTCTGAGGATG<br>GACACTCTGAGGATGGGGCGTGGTGAAGGACTCAGAGCACGAGGGAAGGAGGAGGGAAAAAAAACA<br>GACAAAGCCAAAAGCAGATGCAAGCACAGGGCACACTCTTCTCTGAGCTCTGTAAACTGCGTTCAGTG<br>GTTAAGGCAGAACTTGTAGCACTGTCTGATGTGCATGGGAGAAACACTGAAGGCAATTACATTATAAAC<br>AGAAAGGAGCATGTGCTTCTAAGTAAGACAAGGACCACACACTGAACTGACTGGTTCTGCAATCACAA<br>AAGTAGCTGCCACCGTCACAACCAGTGAAGGGCACAAGGGCGTCTCTGTGCCACACCGTGCCTTCCCG<br>ATAGCCTACAAAGACTTCAAAAGATGTCAGGGACATGCTTCACAAAGTCACTCAAGAGAGTAACTAAG<br>CCAGAACAATCTAGTTAGCATCCTAACTTTTCAGAAGAGAGGAAACACAGTTTCCTCCCAAAGCTTAAT<br>TACTTTATCCTAACTGGGCTCAGAAGGAGTAAATTTTCTTCTGCCATTCTGAACACAGGTCTGCTCATTC<br>CCCCTTGGGACCATTTTGCATTGGTCTACTTTGGTAAGCAGAACCTTAACCCTATCAGTTGTTAGCAGAG<br>TTCTGAGTCCTTCACAAAGTCAATAACGTTAACACAAACACAGCTTGTAGACTTCATATAAATTCTTTCA<br>TAAGCTATAAGAATGTAGCAGTATATCTTTTCGGGATGTAAACATGTCAAAATATACAGTAAAGCATTCAC<br>AAATATGCATACTGATTCTAATGACATAAACGTTGCATTTCAAACTCACATTCCAGAGCGCTTTACAACT<br>AGGAACCACGGCTTCACTTTCTTATTTACTCCCCGTTACACACGCCCCATGCGCTGCTGGCTTTACCCTA<br>ACTGTCCGCATGATTTACTGGATATTGTTCTGACTTCCTGTGGCTTGGTCTGTGATATGCTTCTGAGGATA<br>TCCCAACATACAGGTACAGGCTCCCCACTACCCGTGGGCAGCAGCAGATAAAGTCACCGGGCAAACGG<br>GGTGTACTGCAGAAGATCTGAGAGCCTCCTTCCAACCGAGCTAGACCCTGGTTGTGGGAGCCAGACCA<br>GCCAGGGAGAGCAACCCAACAACTGGCTGCTGAGGAGAGACACAGGGCTGGGGAGGGCGGCAGAGA<br>GGAGGATGAATACTGAACAGTAACAGACTGTGTCCAGACCTCCTGGGTAGTCACGTTAGCTACTTTTGT<br>TAACAACCACCTGGAAAAAAAAAAAAAAACTTAAGAGAGTAAGGTTTACTTTAGCACACGCGCAGTCCA<br>TCTCAGTGGGAAAGACACGGAAGCGGGAGCAGAGGGCAACTCCTCACATGGCAGCCATAGTCGAGAA<br>GCAAAGAGAATAACACTGGGGCTCAGCTCGCTTTCTCCTTCCCATTTAGTACAGGGTCTCAGCCTCAAA<br>GTTGGACGGATCTTCACTGCTCAGTCAAACCTCTCTGGAAATGTACTCATAGACACCCTCGTTAGTTTGT<br>CTCCTAGGGGTTTCCAAATCCTAACAGGGTGACAGTCAAGATTACATCACAGTCACCTGATGATTTCAG<br>ACAGTCACTCTAAGAATGACTTTGGAGCTGGGCATGGAGTGCCAGGGCTCTGATCCCAGCATTACGGA<br>GGCTGAGACAGGAAGATCTCCGAGTACTCTGAGGAAAGCCTAGAATAAATAGTGGCGATTTTTCTCAAT<br>AAATAAATAAAAGCACCAATAAATAAAAACAAAAACAACTTTGGAAAACTAGCTTTCCTGTTTTAAAAA<br>AAAAATTCCTTCTTTAGAAAGGCTTTCTGTTATCCTTGAGTCAACTAATCTCCCCACTCTGCAGATGTTC<br>ATTGAACATCGCTCTCACAGGGAGATACAAGAGAGAACGAGGGAACTGTGGAGAGAAATCACAGTTC<br>CCAGGACTAATCAATCACCTCATGATCACTCTGAATAGGGGGATCAAAGGGACTGTGAGGACATGTGCG<br>TTGGAGATGCACAAGTGAAAAGGTGAGGCAGAAAACTGGCTAGCAAGTAAAGAAACACCGGTCAAGA<br>CCTTGGCGGAGTGAGAAGGAAGCCAGAGCTGCTGCGGGAGCTCCATTTGTAACCAGTCCTGTGGGAG<br>GGGGAGGGGCAGGAAAGAGAAGAGCAAAGCTTGCTGGAAAGCGAGCAAGCAAGCGGCATGTTAGAA<br>AGCTGGCACTGGCAGAGAACTGCACATGAATGTCTGCATTTTTTGTTTAATTTTTGTCAGGTTTCCATGC<br>TTATTGGCTAGACAGCTCAGCTCTTCCCCCAAGTGCTGGAGTCCACGGCTCGTCTGGCTCATAGTAGTA<br>CATCTCCCAGTGGGAGCTACAGAGAGCACATAGTGTGACCTCAGCGCTTCTTCTCCTTAACTCTGCCAA

							GATTTCCCTCTGCTCTTTAAAAGAGACCCTAACCTCAGACACTGCAGGCTCTGCTTCCCAGCTACCTCAT<br>CAGAGACCCATGAAACCTGATACGCCTCTTCTGATCCAGCCTCCAGGGCCTTTTCTATGTATCCAGACTG<br>ACCATGTGGGCCCAGCACAAGCTGTGGCCTCCGCCTGCACACCACCCCCCACTCCCTGTTCACCGACT<br>CAGCTTCAGATCACAGTTCCCACCTCCTCACATCCTCGGGAAGCCTGTGCAGACCTCCAGCAGCCGTG<br>GGGACACTAGGTACCTGGCCTGAAGACAGTCACACTTGGTGTGACTACTGTGGTTTCTGCTTCACTGTA<br>GGAGTGTGAGCTGGAACACTTTGACTCGTCAGAGCACTCGGTTTATCATCTAGCCTATAGTAAGCTCGT<br>GGCTGGAACCAAGGAACCAGGAGTGAGCGCTCTTTGACACTTGTGAGTGCATGCATAGAAGCTAAATA<br>CCCCAGACACAACCTCCTGTTTGTGCCTAGAAGGTCAGCTGGCACCTAGATACTAAGGTAGTTAGCACT<br>GACACTTGTAGCAGTCTCTGAGAGGTAGGCAACACTGTAGCTGACGATGTAACTTGTTGGTTACTGACC<br>CCTCCTTTTTTTTTTTTTTTTTGGTTCTTTTTTTCGGAGCTGGGGACCGATGACCCCTCCTTTTTATTAAT<br>GGTGCTCAAGTCCTTGGAAAATGATGTTTCCCTCAGCGTTAATTGAACTTGAAATCACTGGTTTGTACTT<br>TACTGTGGAATTTCCACTTGCCTAACAACATGCAATTTATAAGAAGTGAATCCAAAATAAAAGCCATCGA<br>GCTAAGTGACCAGCAAATAAAAAGAACTAATAAATCTCAAGGTCACTTGGAGCTGAAAACCAACACTG<br>TTCCCCCTTGACAATCACAGATGAAGAGAACAAGCGCGGATCACGCCAGAATCAAGCCTTGAGCCTTC<br>TAGTTGTGCCAGCTGAGTGGGAAATGTTTAAACTCGGTATTCAGAGAGCGCTATGGATTCGTAACTAGT<br>CATCATCTCCTTCATTAATAAGCAGTTAGTGACAAAATCAGATCACATCATCTGTGCAGTTAATTACAAG<br>CTGGGACATAGTCAGGGTATACAAATTTTATTAGCATGGCTGACATCTGTCAACTATTTTGGTTAATTACT<br>CCTGAATTTGGATTCTAGCCTTCCACTATTTCAAATTTTAATATATTTGGCATAGAGGGTTTTCAGGCTGC<br>CATTATCTCTGTCGAGTATTTTTGTCTTATTCCTGTTCTAAAATTGTCAGAAATAAGCACCGTTAGCTTTC<br>GCACTGACAGTTCATCGTGCAGCTAAATAAAATAAATTGTTAATCAGCAGAATACGACCCACCACATGG<br>CTAAAACTCTTTGTCAATGTTTATTAAATGCTCTCTGGGCTGTTATGTAGGACATAAAATGTTTGGAACTT<br>CCTTAATTCCGTGCTAAAGACATAACCCCAAAACTTTTCTTTAGAATAGAGAAATCCATTTGGCCTTTTT<br>AAAAGTAGTTTGGGGGTGGGTGGGCTGTTTGTCCGGTTGTTAAGGAAGCATGACTACATCCATCAGCCG<br>GGAGCACTGACGATGTCTGAGGGTGGGATGCCCGGCAGAAACTAGACTGGAGCTAAGAGAAGTGTAC<br>ACGGTGAAAGAGCAAACACGTGAGCGGAGACAGGACAGGGGAGAGGGGTGAGCGGGGCAAACATGG<br>CGAAGGCAGAGTGAGGTGAGACACCTGACCATACTCAGAGCTCCCCCGAGGCACCAATGCCAACCAG<br>AACACACAGAATGGAAGAAAAAAAAATCCAGGAAAAACAGATTGAGAGCACAAACTGTACCCAGAAC<br>ACTGAACTCAGATAACTTCTCAACTTGAACCTTCCTGTGTTGCTGTTACTGTGTTCTAGACTGTTAAAAA<br>AAAAAAAAAGTCAAGTGAAACCAAATCTGTAAAGCTCACAGCTGCCTGATAATTAGAACCTAGGAAAT<br>CTGACTTTATGACTTCTGCCACCGTCCTAGTTCCAAAGGTACAGTACCCACCTAAGAGAGGCTCCTCAG<br>AGGGTAGAACAGACACTCTCAACTGCAGGGTCTGTTGTGCTCTGTATTTAAGAATTGGGGTTGGGGCTG<br>GAGAGCTCAGTGGTTAAGAACACTGACTGCTCTTCCAGAGGTCCTGAGTTCAATTCCCAGCAACCACC<br>TGGTGGCTCGCAGCCATCTAATATGGGATCTGACACCCTCTTCTGATATTCAGGCATTTATGCAGGCAGA<br>ACCTTGTGTACATAATAAATAAATCTTAAAAAGAAAATAATTGTGGTTAACTGGCAATTATCCAGCACCA<br>TTCTGCATGTGATACTGTAAGTGGCCAAACGAGATTTTGAGTATCAGCCATGTGAGGCACTGCAAATGA<br>GCAAAGGAAAAAGATACTTTCATGTATTCATTACAACTGCTAAAATTAAGAGGACCGACCACATCAAGT<br>GTTGCCATAGATGCACGGAAATTAGAACTACTGTATATCATGGCCCAGTCACATTGGAAATTGATCATTA<br>AAAGACGCCTATGTTATGGGCCCACAGTTCCCATGTTTTAACCAATAAGAAAACTAATCGTGTTGGTGA<br>GGTGGGCCAAGGTGCTTGTTGCCTCATGTCCTGGGTTCGATCCCCAGGACTCACATGTTGGAAGCAGA<br>GAACTAAATCCCACAGATCATCCTCTGATCTCTACACAGGCACATTCCCACATGAAAGAGACATAGGAA<br>GGGAGGGAGGGAGGGAGGGAAGGAGGCAGGCAGGCAGGCAGGCTGCACATCAGATACACATTTCTAC

							AGCAATTCCACATTCCAGCTTCAACACAGTTCAAAACTGGAAATGCCTACCATTGTCATCAGAAGACTA<br>AATAGCAACATGGTGCTTTACCCACACAATGGAACCCTAACCAGGCGATTCACAGGGACAAACTATTGA<br>TGCCTGCTGTACAGATAAAATCTCAGTCATTAGCATGAACAAAAAAAGAACATGAACTCTGTGCCTCCT<br>GCCGCCCGAAATGCCAAACCATGGAAACTAAAACTAACCAGCAAAGCAAAACGTTGATCTTGGTGTTT<br>GATAACTGGAGTACAAGATTTGCCAAACATGGAGTGGATCTTGCAGAGAACTTGGTGTAGGGATTAGA<br>GGTCGTGATTCTGGTTACGATCTCATGATACATTTGAAACATACTAAATGACAGGTAATATATCTGCTATT<br>TATTTTATGTCGATTGCACTTCACAGAGCCATAGCGTTTCAAAAATAAGTCCAGTTTAGGGTAGTTTGGG<br>AATTAACCCGATTGTCCCATTCTGACCTACAAGGTGAACTTATGACTTCGAGAACTGGATGATCTTCCTT<br>CCATAAAACTGACAGCTTTGAATCATAACCAAGTCAAGGGTAATCTAAATTGTTCCTTCTGTTCAATAAA<br>GCAAGCCGTGATGCCCCTTTTCCCCAGGCTCACTGCACCTTAAGAAGCAAAATAGGAAGAAATAAAAA<br>CAGCAGCTTCTCTGAGCCAAGACAAGAGAGGAGTAACAGCCCTTTCGGAACATTCTTGCAATGCTGAT<br>TCATAGCTTCTCCAAGACTCCTGGGACTCCTGGGGATTCCTGGGGACTCCTGGGGACTCCTGGGGACA<br>ACTCTGGACTTGCCTTATGTATCTCAGCACAGCTGACTACTGTCAACTGAATCAGTCTTAGTAGAGGCTC<br>GCCATACCTCAGAACAGTATCACATTTAGTAGCTTTTCAGTTGTCTGTGAGTGGCCCCTCTTGTCATGGC<br>TACATCCAGAGCCTATGCACTCCTAGTTTTGGATCATCGTGGTCTGAGGAACATTAGCTGTCTGTCCACA<br>AGAAGGAGGCACAAACTTTGAATGGGATGTTCACTCAAGGGAAATAAAGGTTGTTTTCCTCGGTTTTG<br>GTTTTGGTTTCGAATGAGTTAGCCCTATGGTGTTTCTAACCAGCCAACTATACTCTCGATGACTGACGTG<br>GACATGGGCCTCTGGCACTGAAGAGTTAGTGGGCAGTAAGCATCAGGATGGGATATGCAAGCTGCTGA<br>AGTGATAGAAGAATTCAAGAAGGGAAAAACTACAAGTTACAGAGAAGAAGAAAAGAACCAGGTAGG<br>GGTCAGCAACAAGGGTAAGAGAAGCCACCTCAGTAAGACAGGCTGGAGTCAGAACTCAGTTGCCCCA<br>TAGTCCAGACAACTCCTCCATCTGAGGCAACAGAACATGGGCAAGACGCCCCACGGTAGGCCGATCAG<br>GAGTCAGGCATGGCTCTACAACTATCCATATTGCACAGACAGGGATGACCACAGACAGAATACTCAACA<br>ACAGAAAATAAACATTTATGAAGTTTCCATCACGGATAAATCTTAGAGTCTAGAATATCATTTTTGTTAAA<br>GAAAACATTTTTACATGCCATAATCATTTTTGCTGTCCCTACACGAAGGCGAAAATCCAGCCAAAAGAC<br>AGGTCACATGAGAGAGTGATACGGCCTAGTTAAATGTTTTATGCAAAAAAAAAAAAAAAAAAAAAAGT<br>CCCATCCATTTGGGTGACGGAATAAGATAGGTCTTGGTAGCAGACATTTAACATCAACCGTAGTGCAGA<br>AAAACAAAGGAACGAAGAGAATGTTTCTTTTTTTAAAGCAATGCATTAAAAGTGCCGTGAGGTGTGAG<br>TGGTGGTTGGTTTGAAAGGTCCCGGGTGAAGGATTACTGTGCATATTAACTACATGTCCTTGGATAACTT<br>AATTACCTCCAGTCCACTTCTTCATCTGTAAAATCTAAGAACAATTCCACTTGCCCTAACAATGTCTCCG<br>GACTGAGAGGATCAAACGAGGGAATGAAAATGAAACTGTCAAGGCTTGCACAGAAGGGAGCACACTC<br>TTCTCCTCGTGTCATGTCTTCAGGGGTACCAGCTACGCTATCGCCTCACTAATAAGTTCCAGTACTACAA<br>AAGGCATGACTCCTATTCAGCTAAAGCTTTTACGGAGATCCTTGGATAATTTTTTATTCTCCCTATTCATAT<br>CTAATTTTGACTGCTGGTATCTAATGGGCAATTACAGGCTTGGATCACTTAAAAAATTATTCTTCGTAGGC<br>TCTAAACATGTATATTATGTTTAAATATTCTCTGTATATAAAAAAACAATCTAGCCTTAGCTTTAAAAATAT<br>CACCTTTTATATCTTCCCCAAACCACTCCTGGAATCAGGGAGTAATAAGACTTTCTTCTTATGAAAGGTC<br>CTATGTTATCATTCAAATTTTAAAGTTTTGGGAGTTGTTTTACATGAGAATGTCTCCCTTCACCCCTGAAA<br>GGCACTGGACAATGAGGCTATAATAAAATGACCCATCTTGATTTTTAAGGGCTGGGTTCACCTTCCTCCA<br>GAGTCCTGAGGATGGCCAGGGCCCTTCCATGACTGCACTTACAAACCAATGAGGTCATCTCTGTTTTAC<br>ATTGTTCTCAGCAGTAGTTAAGCTGTAGTGAGGTGAAGAGACCTCCTTTTTAAGCATCCTGTTGTCTATG<br>TGAGTTCCTGGGGACAGGGGTGAATTTTAAAAGGCAGCCACACATCCTACTGACCCTGAGGATTCCAG<br>ACCAAGGATATCAGGGATGGAGGAGGAGACTGAGGACAACACTGCCTTCATTTAAATATCCTTCCTGAT

							CTAGTGGTAATTACTTCTTTTAAAGTCCAGTCACCTTCAAAATATTACACAAGAGAAAGGAGAGGAGAA<br>AAAAAATGACATGGTTCATGGCCAAGAACATTAATAAAAGTATTTCTAAAGCAGGATCTTATTTTCATTT<br>TCTGTTTAAAATTTTTCATTTTTTATCATCTCCGTTGCCAAATTTCAATCTTCATTGATTAAAAACACAGC<br>AGAACTCCTTGCATACAAATTTATTCAGTTTTGCAAACATTAAATCATCATTAGATTAGAATGACAAGCAT<br>GACAAAATTAATACACCAAAAAAAAAAGGCTCAGGGAATACAAAATCTAAAATGTATTGAATTAGGTTT<br>TAAATTGAACCCACACAGGCTTATGTTCAATGAAAACCAATATTGCATACTTTAAATCAATTATTTCATAA<br>TCATCTCCCTAAAGCCTTTAATTTACCAAGAAACTCTAAAGTTGAGCATAAAAATGCCACAGAAATGCA<br>AATTGGATCTAAACTTCCACTAAATTCATCAAATGTCACTTTTCCTTCAATTTACCACGTCACTTTTCAAG<br>AGTGAATCCTGAAAGTGTCCTCAGTGCCCATTAATGTTACAATTAAGAACCATATTACAGGAAAAATGA<br>AATGACATAATTATTTTGAACAATTATAGATCTAAACTCTTTGAGGTAACTTATTGTGCAAGTCATAGGGT<br>ACCGAGCTCTTTTATATCCTTATGTGTAATTAAGATCTAATTTCCAAGTCCAGCATGCAGTCAAAGGGAA<br>GTCTGACTTTTGAAAGATATTTTTAAAGGAGAGAAAAACCCTCCATTGATTTCTTTATCCCATCCTGTAG<br>TTCTCAAATGATTATGGGGGAGATGCTGAGGCTGGAAATGTACCCTTAGGGTTAAGAAATGATGAGGCT<br>CCAGGACGCAAGCCTAATCAGACAGAGAGCAGCCCGAGATACCTTTGCAAGGCAGGCAGAGAGTGTG<br>GGACCTAGACCTGGGAAACAGCATGGAGAGTTCCTCTGTAAAAGGCGGCCCCTAAGAGGTCAGCTGTC<br>AGAACCTCTGGCTTTGGAGACAGACATTAGGAGAGATTTTTAGCATGTTGTATGCTTGCCAGGGTTTTG<br>TTTTCTCCCAATCACTGCATTCCACAGTAAAGAGAAAAAGGAAAAAGTTCAATGACAACAAAGAAGCA<br>CAGAAACAACTGAATAATAAGAGATCTATAACTCGTTTGAAGCCGAGTCAATGTTATCAACTGATCAGA<br>CTAAGAATTGCCCATTTTTGGTGCTTAACCCTTTGGGATGCAACTTCCTATCTTATTAATGGGGTCAGAAT<br>AGAGATCTGGCAGGTAAGAAAAGGCTCCAGAAACTGATTTTAAACTTCAGTCTATCATCTTTCCCTCAC<br>AAAAAATTACTGGTGAAACAAAGAAAGTAAAATAAGCCTCGTGATGAAGTTGACCACGTCCCATCTTTT<br>AGGAGTCAATAGCTGACCTAGATTTGGTTCTGATTTTTTTTTCTATTGCCTTGGCTGTTACATAGTAGGAC<br>CACAACACAGGATTCTAAAAGCTCCAGATACGAAGTAATCACAATACACTTATTGAAGGCTTCTACCAA<br>TGGGTGGTTCCCAACCCCAGGAGGTGAAGAGATAAAAGATCGGACGTGAACTACGAAGTAAACCGGT<br>AACACAATGGTTCTCAACCTGTGGGTCACAGATGACCCTTACACAAGGGTCTCCTAGGACCATCGGGA<br>AACACAGGCATTTACCTTACGTTTGTAATCTAATACAGAAAAACTACAATTATGAGGTAGCAATGAAATA<br>CTCTTTTGGCTGGAGGTCACCACAGCATAAGGAATTATATTAAAGGGCTGGAGCATTAGGAAGGTTAAA<br>AACTACTGTACTAATGGGCCATTATTTGACGTAATGATTCGATGGTAAGAGTCTGACTGAAGGAAGGAG<br>TCGCTGGAGACCCGTCTTTGACAAGATGGATCATGGCCCTAGCATCTTCTCTGCCTTTCCATTCTCTGTC<br>TGCCATGAGATGGCAACTGCGATTTACCGCCGGGCTGGCATGACGTGTTCCACCGTCAAAGACCAACAT<br>GAGTGCATACGCCAATCTAAGAGGGTGAGCCACAAGAAGACCTTACTTTCTTTGAGTTACTTTTTTCGG<br>GTATTTTTGATAGATCAACAACAACAGAACCACACAAAACTTGTCCAACCCACAGACCTTCAAACAATC<br>CCATAGACAAGGAACCGTCATGAGGCAAAATCTTGCTGAATAAAATTGCCTTGGGCTGGAATCCCAGCT<br>GAACCACCTCGTTGGCAAAGTAACCTTATGTGAGCTACACAGCATCTTCGAGCCAGGTATGACTTCATC<br>CACTAGTTGAAGACGATGCTTATGAAACTGAGAATTGAATGAAGAGACGTGCATGCTTCCAATAACTGG<br>AATGTTAGCTGAGTCATGACGCTCTGTTTTCAAGGGGAGTGAGTGGTACTTCCTCACGGTAAGTGCAGT<br>AAGTGCCCAGGACACTTATCCTTGAACAATTAACACCAAGGACCTTAGTGAAAATTCCAACTTCACACC<br>AAATCAGGACCGGCCAATGAGAAATCCTCTTGCAGGCGCCTGATGAAAGAAACAGCCTGTGGCCTTGC<br>TCACCTCCACTAATGGCTTCAACAGGAGGCCTGAGCCTCTCCCTGCTCACCAGCTCTCTGACCTGGTGG<br>AGTGAAGAGCACAGCGGGCACACTCCTCAGTCAGTCTTCAAAGAGCAGGGAACAAAGACCATCACCA<br>AATAAGCCCAAATTGCTACAAGAACAGGTTATGAGGTAGACAGCTATCTATAGTTTCCGCCTCTGTCCTC

							TTATTATTCCACTATTGAACTTCCTTCAATCGACTGCCTAAATGAAACACGACTTGAAATTGTCTGATGCCTGCGATTAACAATTCTTAACACCTACTAACGTATTGTAGCGTCTGTATGCAAAGCAGCCTGTTTAACAAAGGTTAAGTGAGACATTGTATTTACAGAAGACTTTAACATCAGATTGTGTCTACAACACAGATTCTTGCAGCCAACTGTGGCGAGCAGGCAATTTACAACAATGTCAGGACCCAGTAGTTCTTAGAGACACTTTAAAGATGCGTCTACGGAATTTACTATAGGATTCTTTCCCCACGAAGCAAGGAGTGCTCTTGAAATATTTCTGATTCCGTTGAACTTAGAACGGTGAAAGGACTTCTCATGTGTTTATACTTGATGCTAGTCCTTAAAGACTGACAGACAAAGATTAATAGTTTCTTGGCCAAGGAATCTTCGATGTTGTGGTTTGCTCTGGCACTGTGCTAGGAAATCTCATATAAATGTAAGCAAAAGCAGAGGGAAAAGGGGAAGGAAAAAAGAGAAATGGGGAAAGTTGACACGAAAATCTAGAGGAGATTTTACTAACAATACAAAAGTCTGACAACTTCTTCTATAATAGAAATAAAATCTAACCTGCAGCTTCCCCAAGTATTAAAACACTAACGCAGGCAACTAACATCTATCCACACCCATCCCATACCTACCCAAGGCTTCACATGTTAGGCAGGGTCTCTACCACTGAGCCACGCCCCCAGCCCCTCATTGGGAATTCTAGGCAGGGTCTCTACCACTGAGCCACGCCCCAGTCCCTCACTGGGGGATTCTAGGCAGGGGCTCTACCACTGAACCACGCCCCAGTCCCTCACTGGGGGATTCTAGGCAGGGGCTCTACCACTGAGCCACGCCCCCAGCCCCTCATTGGGGATTCTAGGCAGGGGCTCTACCACTGAGCCACGCCCCCAGCCCCTCATTGGGGATTCTAGGCAGGGGCTCTACCACTGAACCATGCCCCAAGCTTCTCACTGGGGGATTCTTGGTAGGTGCTCTACTACTGAGGGACACCCCAAGTCTCTCACTGGAGGATTCTGGGAAGGTACTGTACCATTGAGCCACACCCTTAGTCCCCAACTTGGTGATTCCAGATAGTTCCTCTACCATTCGGACTTCTAAGAACTGTGAAGTCACACTGGAAGGCAAGTAGTGTCAGCCTGAGTTTAGCTGCCCAGAATCTTTGGGGAGACATTTTAAATCAAGATGAATCCTTGTCTGGGAAAGCCTCCCCATAAATTGAAAGGAAAAACTAGCTTAGATCAGATAGAAACCTCAACACCAATATAAAAGAGCTCTTAAGAATTCTAGTCAGTATTTTCTATAAATGAATATTTACAAGTTAGTAATAACAGGCTATGAATCTGCACAGCGATAAGGCTCAACCACTAAATTCTGGGGAAAGAGGAATAGCAGTATTCAAGTTTACAAATTTTTCACATTGGTCTAGGAAAACCCCTTTTAGGTGGGGAAAAAAAAACATTTACCTGTAGGTTATTAGCAAGCTTTTATTATAAAAGGGGGTAACTTCCTCCTTCAAAAACAACTTCGATGTGATCAACAGTATACTTCACAGCCCAACCCACCACCTCTTATTAAGGTGAAGTCTATACCAGGAGGATTAAAACTCCAGTGGGATGTGTCTCTAGCCATCCTGTGTTTAAACAGCTTTTCTATGTAAAACCTGGCAACATAAATTCCCTCCCTGCAAGAATACACTTTTAAATGAGTAAGACTCTCATACGAGTTCAACATAAACATCTAACAAACTGTCATTTCGTATTGCTACTTATACTTGCTTACATGCTGGCCCCTGAGAACAGCCTGCCCCCGGAGAACTGTCTCGAAATCAAGCATAGGATTGTAATGGCGGAAGTCAATCCTTCAACACCATGGCATCAGAATTCAGCAGCAGCGAACGAAGTAAAACTGCCTACAGTCATCATTGACTGGGGCAGGATGATACAGGCCAGCACTCGCCGACTCAGCCCCCGCATCACCATGTGAAGACAACATGATGATTCTGGTGTGGACACCATAGCCGCGAGCTACAAAGTTAGAGCTAATGCTGCTGCTTTCCTGGAAGAAGTTCACCCTAAAGCCTAGAGGATGGGGTCCTTCCTCACGGTGGCCTAACAAACACTGCTTTGTTAGTCTTTCGTAGACTGAGTTCTTATCGTGAGTCTATGTAGCAGACTCACATGAAAACATTCTCTCTACTGATCTTCTGACTGTCTTCTGGTGTATTATGTGCACATGTATTTATGCATGTACGGAACAGCCATAAGGCAACCAAGTGTGCTTCTTCAGGTACTGGACACCATTTTTTTTTCTTTTTGAGACAGGCTCTCTCCTTGACCTAGAACCCATCCAGTAGGCTAAAACCTAGCCAAT
MSTRG.27208.3	--	0.000	0.462	0.000	6.050	up	GGGTGAGCCACAAGAAGACCTTACTTTCTTTGAGTTACTTTTTTCGGGTATTTTTGATAGATCAACAACAACAGAACCACACAAAACTTGTCCAACCCACAGACCTTCAAACAATCCCATAGACAAGGAACCGTCATGAGGCAAAATCTTGCTGAATAAAATTGCCTTGGGCTGGAATCCCAGCTGAACCACCTCGTTGGCAAAGTAACCTTATGTGAGCTACACAGCATCTTCGAGCCAGGTATGACTTCATCCACTAGTTGAAGACGATGCTTATGAAACTGAGAATTGAATGAAGAGACGTGCATGCTTCCAATAACTGGAATGTTAGCTGAGTCATGACGCT

							CTGTTTTCAAGGGGAGTGAGTGGTACTTCCTCACGGTAAGTGCAGTAAGTGCCCAGGACACTTATCCTTGAACAATTAACACCAAGGACCTTAGTGAAAATTCCAACTTCACACCAAATCAGGACCGGCCAATGAGAAATCCTCTTGCAGGCGCCTGATGAAAGAAACAGCCTGTGGCCTTGCTCACCTCCACTAATGGCTTCAACAGGAGGCCTGAGCCTCTCCCTGCTCACCAGCTCTCTGACCTGGTGGAGTGAAGAGCACAGCGGGCACACTCCTCAGTCAGTCTTCAAAGAGCAGGGAACAAAGACCATCACCAAATAAGCCCAAATTGCTACAAGAACAGGTTATGAGGTAGACAGCTATCTATAGTTTCCGCCTCTGTCCTCTTATTATTCCACTATTGAACTTCCTTCAATCGACTGCCTAAATGAAACACGACTTGAAATTGTCTGATGCCTGCGATTAACAATTCTTAACACCTACTAACGTATTGTAGCGTCTGTATGCAAAGCAGCCTGTTTAACAAAGGTTAAGTGAGACATTGTATTTACAGAAGACTTTAACATCAGATTGTGTCTACAACACAGATTCTTGCAGCCAACTGTGGCGAGCAGGCAATTTACAACAATGTCAGGACCCAGTAGTTCTTAGAGACACTTTAAAGATGCGTCTACGGAATTTACTATAGGATTCTTTCCCCACGAAGCAAGGAGTGCTCTTGAAATATTTCTGATTCCGTTGAACTTAGAACGGTGAAAGGACTTCTCATGTGTTTATACTTGATGCTAGTCCTTAAAGACTGACAGACAAAGATTAATAGTTTCTTGGCCAAGGAATCTTCGATGTTGTGGTTTGCTCTGGCACTGTGCTAGGAAATCTCATATAAATGTAAGCAAAAGCAGAGGGAAAAGGGGAAGGAAAAAAGAGAAATGGGGAAAGTTGACACGAAAATCTAGAGGAGATTTTACTAACAATACAAAAGTCTGACAACTTCTTCTATAATAGAAATAAAATCTAACCTGCAGCTTCCCCAAGTATTAAAACACTAACGCAGGCAACTAACATCTATCCACACCCATCCCATACCTACCCAAGGCTTCACATGTTAGGCAGGGGCTCTACCACTGAGCCACGCCCCCAGCCCCTCATTGGGAATTCTAGGCAGGGTCTCTACCACTGAGCCACGCCCCAGTCCCTCACTGGGGGATTCTAGGCAGGGGCTCTACC
MSTRG.27209.2	--	0.509	0.000	0.000	-7.130	down	CTCAAGCATGCAAACGCTAGGATACTGGCTTATCCCCACTTAGTGCCCCAGCAGGACTGTAACAAAATCAAGTATATTTCCTCCAGTTAAAAAAATAATACAATCTCAGGTTGATGAACATACTGGTACGGACAGAAAAAGAGAGGTGAGATCTGAGAGTCCGAAATTGACAATAAAGTACCAAGGAAGCACAGCAGAAATGGAAAGAGGAATCCAGCAAGGTCTCCTCAGACACATATCAAGAGAGCACGTGCTACGCGTACTTGATACTCTCTGGTGGCGCTGTGACCACCTGAGGTGATCTGACTTAAGAAGACAAGGGTTCCTTTGGCTCACAGTCTTGAAGGTTCCGGAAGGTGGGTTAGTTGACTGGCCTGGATGGTTCTACCATGAGGGCATTAGACACATTGTACAGCATATCATCTCATGGCATCGGAGAAGCAGCTGAGATCCAAATATCTTCTTCAAGATCATGTTTCCAATGGCCTAACTTCCATCCACTACCCTGACGCCCTAATGATTTCCACCAGGTCCCATAGCACCAAGTCCTTCAATAAGGGACTCAGGAGAATACTCCAGACTGAAAGGCCAGCACTATATAAGGACAGATCTAGGGCTCTGTAGTATCTGAAACGTGTTATACAGATATAGATTGGTAATCTTTGCAGCCAACAGTCTGATGTAAAACACACATCCAAATTCCACATCCGGGGAAACTCGCCGTGACAGTAATTGACTTAGCTCACATTTCAACGCATCAGCTTTTAAATGAGAGCAGAAATCATTTGGATTAACCACTCGACTAGCTTGTTTCTAGACTTTGTCAAGAAAATGAAATGCAACAGAACTCTACTGAGGTAATGAGATAAACTGGAAAGGGCTGGTGTGAAGCCCTCTTCAGAGAGGCTGACGATGTTTCCCCAGCCTCTGCTCCAAGCCTGCAAGGCTACAGAAGCAAGGCTATCACACACACACAAGTCACTCCTACTCAACTTCTACCTGTAGGCATACCACCTCAGTGGGGCGTGATCTCAAAGGTGAAAGGGGAGGGAGACAAGAGGAGCAGTCCGAGACAGGAGAGGACAGACTATGGAGGCCACAGAGCATATACACTGGTGATGCTGCATAAATTCAGGCTTTCTGACACTCTGTTTAGTGCAGAGAGTAAAACGGGTGACCAACCCTGAGTGGCATGTTTGCCTCTTGTCCTATGTAAAATCTGTGCTATGACAAGGCTGTGACACGATGGACAAGAGTTTTAGCCCCTAAAATTCTACCTTTCCCCTCCCACCTCAACTCCTATTAATAGCAGAGTCCTTTAGAGATACCCAATTAATACGCTATGCTTATTTCTTTAATGTACTGCCGTACTAAAAACAGTTAGGAATATGGAAGCTAGATGAACCCAGGGTTAGATAAGTCTTCTTTAAAGAGAAACCTGAGGCTTCCCAACACCACAAAGGCTGCTGAGGAAGGTGCCTGTCAGAGCCCACGCTCCCCAAAAGCTCTTTCAGTTCTTGAAATCCAACTGTGTACTTTTTTCCTATAAATCTTTCTCAAAACAGACAATGCCTTTCTCCAAGTCT

							CATTCGAATATTTTCATTAACTTAACACAAACCCCAGTCTTATTACATACATACAGGGCAGAAAATTAAC<br>AAATAACAATGTTTGAAACCATGCGCTTTTGAACACACTCCAAAATAGCTTTTACACTGTTGCCCAGTG<br>GGAGCACATCTGTTAAAATTCCAATCACAGCAGCATTTTGAAGGATCTCATATTGTAGCCCTGCACAGG<br>GCTGGACCATTAACCAATGCGGTTGCTCAGGCTCTGAGCACTTGACCTCGGTCACATAAGAACACTGAC<br>AACAATCAACTGGCTCTCGGCCCAGAGAAGCTTTCAAAACCAGTTTGGAAGCACTTCATAATTCTAAAC<br>ACTTTACAATGAGGGCCCTTTGTACTGACTACTCTTCTACACCGTCCCCCCACAAAACAAACAAACAAG<br>CAAAAGAACCAAAGCCAGCGAGCAGACTACAAGACGATGTGGAGTAAACAAACAAGGTCAACAGACT<br>CCATTAATAATGATGCTTTCCATTCTGGCTTACCTGGGAGCCAATTAATTCCTTGTTTATCCACACGCCCA<br>GGGTTGGATAACTCTTCTTTAAAGAGAAACCCAGGGCTTCTCAAAGCCTCAAAGGCCGCTGGGGAAGA<br>TACATATCAGAGCCCATGCTCCCCAGAAGCTCCTTCAGTTCTTGAATCCCAACTGTGTATTTTTCCCCCC<br>TCTAAATCGTTCGGAAATTTGGACAAGAAAATAATAAGCCACAAAGAAAAAAATGTGAGTTACACACA<br>AATCTGTAACAATACAATGATAAAGATAAAGATATACCCTGGATAATCATCTTTCTGAAGTTAGAAATTCT<br>ATACAGGAATGAGTGAGACACCATCCACAGCCTTTGAATGCAACCTGGAAATGTACTATTCTGGTCTCA<br>AGACAGACATGAAGACATGTATTGCATACACGGACACACATACACTTCACAGGGGTTATAAAGGGGGC<br>ATCCTGCAGGAATTCAGAAAATAGGAACACTGTATCTTAACGGTTATCATACGATTTAAAAAACAATCTG<br>CTAAGGGCTCTGGGGAGAGGGCTCTGGGGAGAGGGCTCAGTGAGCAGAGCTTTGCTGCACAGACATG<br>AGCACCCGAACTGAGATCCCCAGAAACCATGTAAAGGGCCAGATGTCGTCCAAGAACAGGAGAACCC<br>CAGGAGCTCACTGGCTGGACAGCCTAGCCCAATCCATGAACTCCAGTGAGAGACACCGTTTCAAAAAC<br>CAACAAACAAAGACATAAATAAATGCTTTTAGATGGAGAGCAATAGAGAGAGTGACCTGAGATCGAGC<br>TCAGGCCTCCACGCACTCCATCATCAAACCAGAGTCTTTTTGGATCGGATGTACGTTTTCACTCTTGATT<br>CAGACGTTTCTGTCATCTTCCTAATGGCATCCAAGCCGTTAAACTGCTAACGAATTCCTTCTTAATTACA<br>CAGTGGCATTTATAAGTCAACATCACAAATCAGAAAACACCACCGTGAGATCACACGTAAGTAATGTTA<br>CCAACCCCACCCCGTGAAGTGTCCGTGTATGCACATGCACGGCCTTGATGTCTGACTTGAGACTGGAGT<br>AATACATTTCCAGGTTGCATTCGGGTATGTGAACAGAGTCTCGCTCATGAGAATATAGAATTTCCAACTT<br>CAGGAAGGTGGTTTTCCAGGGTACAGTTTTATCTTTATTGCTGTATTGTTGCAGATTTGTAGTAAAGTAC<br>ATTTTCCTTATAAGGTAAGATGGGAAGAACTACCTCCCAAAGGTTTTCTTCTGGGAAGGACACACATAT<br>GTATCTGTGTTTATGGAAGACAGACATCAACCCTGGTGTCATTCCTCAGAAGATGAGCACCTGATTTTTT<br>GAAGCAGGGTCTCTCTCACTATCCTAAGCCTACCAATTAGGCTAGGCTATCTGGCTAGCAAGACTAAGA<br>AATCCTCCAGCCTCCATCTAACAGACCACCACAGCTAACTTTTTAGAATTCGGGTCCTCAGAGTTGTGA<br>GGCAAGTACATTACTGGCTGACCTATCTTATCAGGCCCAGTTTCCAACATTTAACACCATCCGTTGTTCT<br>TAATGTTTCCACAAACTTGCACATATAGCCCAATCCCAAAATTATCCTCGTTCTTCAGATAAAGATAACG<br>AGGCACAAAAGACTGGAGGTCTAAATTTGTTTTAGTAACTGTGAAACAACCACTCATTACATTCATGAG<br>TGTCAAGGGGAAAAAGCCAAATAGAACAGAATAACAAGTTTTGAAAATAAAAGGGTTCTCCTACTGAA<br>TATGCTGATGAGAAACCCATAGAAATGTTAGATTATCCAACTTTTAAACAAGTCAAAGTATGAGTAGCTA<br>AGTCTCTATCAACCAGACATTCTCAAATTTGTATCAGAGCAAAAGAACCATAATAAGTTTCCACGGTTAC<br>AAATGAAATGTATTGTTGAGTTTAGGATAAAGTTTATATTAACTAATGGGCTAGATAATGTTGAAACTTAA<br>GTATGCCAGTTTCTATTTTTAAGCTATACAAATAGATAAGGGAAAATTGCAATCAAGAGGAAAAACAAC<br>ATTGTGATTTATTTAAGCACCTCAAAATGTCATCGTGAAATGGCCCACTCGGGGGGGAAAAACTACTTC<br>AAAGTATTTGCCTTTCACAATGCATATTCCTACTCCACTTGGAAAATTTAATTGTGTTTTGTGTGTGACCT<br>TTAAAGCATCGTTGTCACAGCTTGATGGTAACATCCCAATAATCGCAATAAAGCGTCTACCACAGATGGG<br>AGGTCATAAAATCATAACAGAAACGCTCAAACTTTATAATAGTATTTAGGAGAAATTTCTTTTCTTACACT

							TCGTCTTTCCCTCTTCAAAAATGAATATTGTGTTTCCAAGTGATTTTCCTAGGAAGACACAATAAAAAATGCTGAAGTGCGCCTGCCATTGTTCTCTGAAGGGTTGAACATTACCATCACTCAGCGCTTTAGTCCTCATTAAAGCCTACCCTAAGAAGCGTATTTTAGACACATCCTCCGTGCATGCTACTGAGCCGCACACCGCCATGCCAGGCTTTCTCTTTTTCTGCTCTTTATGCATGTATTTTCTGTGTGTTATTTTCTTAGATGGGGCTGCTTTGGTGCGAGTGATTCACGTGAAAGCTACATCTCATAAACCACACTGAACTCCATGGACGGTGGGGCCACTTGGTGCTTCTGTCCGGCTTCCTGCAGTGGCCGGCTCAGGGGACACCTACTGCAGGTGTTCCTCCCTAATTGGCAGATACGGGAGACAGCACCGTAAATACACACAAGCAGGAAGAGCAGCTCAGGGCAGTGCTTCCACTTCAGGCTACTGATTAACGGTACATTTTAATGGGTGTCCGCTCGTACAAATGGCATTTGCTCATGTTTTGACCAGCATTCAACTGGGTACTTTACGAGTTAAATTTCTGAGGAAATAAGAGATAACTTGCACATTCTAACACTAGTAGGTATTAAAATCACTACACACAGACAGTACCCGCACACAGGTCAGACAGTAAAGCTAAGGTTCAAAAAGCACCTTCACTGCCCATGGTTGGGTAAGAAGGGATCAGCTCTTAGCATCTAGTGAACTATGGACAGTTTGGCCTGGTGG
MSTRG.27269.2	ENSRNOG00000001251;ENSRNOG00000001252;ENSRNOG00000001253	0.220	0.711	0.005	2.405	up	GGTGGGGCACGCCTTTAATGACAGCACTCAGGAGGCAGAAGCAGCAGATCTCTGAATTCGAGGCCAGCCTGGTCTACAGAGTGAGATTCAAGATAAGCAGAGCTACACAAAGAAACCCTGTCTTGAGAAAACAGGAAAAAGGGGGTGGGGGCGCAGACAGAAGCCTTTGGGAATGTGGCAGGTGTTAGGGCGCTGGCCTAGCAAGCCCCAGGTCCTGGGTTCCATCCCCAGCACCACATGACAGGTGTGGTGGCCCATGCCTGTAATCTCAGAAGGTAAGAGAGGAAGGCAGTAGGATCAAGAGTTTAAGGTCATCCTAAGCTACATTGCAAGTTCAAGGCCAGGCTGAGCTACATGAGACCCTGTGCCAAACGAACAAACAAGTCCAAATTCCTAAGCCCCTAAGTCTTAACATGCAGGTTCCAGTAAAAGCACAGCACACATTTCTCACTGGCCTGGCTGTTCCCTCTTGTCTCTCATCATCAGCTCCCACATCAGCATGGCTGGAGGCCCGGGAAGCTTGTATGAGCTCGTAGGGAGAGACGGGCAGCAGGGAAAAAGCAACCTTGCCTTCCCCAGTGCTGGCTTCCCCAGATGACCACTTGCAGACTGTTCAGAGGTTCATAGTATTGGGGACACACTCTGCCATCCTTTCTAAGGCTGCTCAGACCTCCCCCAGGGACCCCCACAGCCTGAGTGTGAGCCATGGGCTGTCAATCATTGGAAAACAGGGAAGTGGAGTGGTCCCAGGAGCATCCTCAGCAACTGTAATGACCCAGGAAAGGCCCAAAGTCAACACGGGGTCTTGGACAGGTCCTTAGTCATGTAAGGTAACCCTCAAGTCCTGAACGACTTCTCCATCTCCAGTCAGGTGTTGGGGTCCACCGGACTCTCGACCTTCCAGATGGCTGTCTCCTGCTGCCATCTACTTAGGACGCCTTGGCCTCTTGGCACTGCCACCTTGGAGCTTGGAAGGATTGTTGGGGGGCAGTGGGCTTTGACGCCGGCCTCTGCTGTCTGTAGACCCTCGGATGTCACGTGGCAGGAGGGGAGGCCGGCTTGAGGCTGTGTTGCTCTGCCACATGGCGCTGGGGAAGGTGAAGCTGACCTCTGGCACTTGGAGCACGTTTTCCTCTGAAGGAGACAGCAGCAATGGGAAGTGTTATTTGTAGGAACTAGCTACCCGGCTGAGAGAGGAAGCCAAGGTGTGGGGAAGAGACAGGCTCCTTCCTCAGTACCTCCCTGTGACTCCACAGGAGGCTGAGACACAGTATAGGGGGAGTAGGGGAGATGGGAGGGGGAGGGGATGAGGAAAGGAGGAGGAGGAAGAGGGGGAGGGGGAGTGAGAGGAACAGGGAGTGATGAAGATCACAGAGGAGGGAGGAGGAAGAGGTAGATTTAGAGAAAGTATTTTGCTAAGTAGCCTGGATTGGCTTCCAACTCTCCATCCTCCTACCTCAGCCTTCCAAGAGTTGCCAGTACAGACAGGCAGCGGTTGCTGGCTGTGGAGACAGGGTCTCAGTATGTAGCCCAGGCTGGCCTGCTACCCCTGATCCTAATATGTGAGCCTCCTGAGTGCTGGGATCACAGGCTTAGAGTTTAATCCTTGAAGGACCACCTGGCGCTAATGTGCTTTGTCATGTGTGTGCATGCACATGTGGGTTTACATGGGTGCAGCGATCAGAGGTTGATACTGGATGCCACTTTCAGGTCATGGCTGGCTTTGGCTAGCTTTGTGCCTCTGTTCCCGCCCATTACTGGGGCTGCAGGTGCACACTACAGCGCTGGACTTTTCCGTGGTTGCTCAAGGCTATGTGGCACCTTACAGACTAATGGTAGAAACCCACGTCTGTAAACAGAACAGTGCTGCCTGTGATCCCAGACCTCAGAAAGCTGAGGCAGGAGGATGGCAATCTGAAGAACGGTCTGGGATACATAGCAAAATGTTTGTCAAAACAAATTAAGAAGAAAAAAAGGAAA

							AGGGTTTCAGTTAGGCACGGTGGTCCATGCTTTAGTCCCAGCACTCGGGAGGCAGAGGCTACTGGATCTCTGTGAGTTCAAGGTCAGCCTGGAATACAAAGCAAGTGCCAGGGTAGCTAGGGTTACACAGTGTCTCACAGCTAGGTTATACCTGTCACAAAAAAAAGCTTCTATGTTTTATTTAAGTCGGGGTCTCACAGAGCCCAGGCTAGCCTCAAATCCACTATATAACTGAGGAAAACCTTGAGCCTCTGATGAGCCTCCTCCACTTCCCGAGTGCTGGGCTGACAGGCATGCACCTCCACACACATCTGTGGTGCTGGAATGGAAACTAGGGCTCCACACATGCTAGGCAGACTCTACCAGTTCAACTCCATCTCAGCAGGTTGTAGGAGACGATCAGAGGGACCTTAACTTTCCAGCTTACCCAACAATCAATTAAGGAAATTAGGAAAAACTAAAGCCAGAACTCAAACCAGAAGCCATACTGTTCACAGACTTCGGCCTTTATGGCTTGAGACAAGGGAAATATCATTTCTCTTTCTCTGATCCTATACCCGTGTGAAAACTGAGGCTGGGTTTCGTCTTGGAGGCCACAGGGACAGGGACGGCAGGGAAAAAGCTCCTCCCTGCTGGCTTCGGTGGCTGGAGGTGCGCTGGGGACAGCTGGCTCTCGGATGGATGTTCCATAGAGGGCTGATGTTTCTCCAGCGCCTGCAACTGGGAAAAGGTACACAGAAAAGCACAGTGAAGACGCGGAGATCATTTTCACTAAACACCTACTGTGTGCCATTG
MSTRG.27645.4	ENSRNOG00000001417;ENSRNOG00000001418;ENSRNOG00000001419;ENSRNOG00000001420;ENSRNOG00000029211;ENSRNOG00000031346	0.010	0.568	0.000	5.952	up	CCCCAGTGAGGGGCTGGGGGCGTGGCTCAGTGGTAGAGCCCCTGCCTAGAATCCCCAGTGAGGGGCTGGGGGCGTGGCTCAGTGGTAGAGCCCTTGCTTAGAGTCCCCCAGTGAGGGGCTGGGGGCGTGGCTCAGTGGTAGAGCCCCTGCCTAGAATCCCCAGTGAGGGGCTGGGGCGTGGCTCAGTGGTAGAGCCCTTGCTTAGAGTCCCCCAGTGAGGGGCTGGTGGCGTGGCTCAGTGGTAGAGCACTTTCCAAAATGTGTGAGGTCCAGAGTTCAATCTACAGTACTGTACATACAAAAATCTGATGATGTTTTGGTAGGGGATGTAATTCTTTCACGAGGTACACATTTAAACGTTTCCTTTTTGCCCACAACCCTAGATTTCATTGCCATGAGTTCCTTTTCTCTCCCTGGAGTCTCATCTCCCTTGTTCTTTTTACAAGCTGAGATTCACTTTGCTGGTCAGCTATTGGCCTTGGCATTTGGGACCCAGCTGCATATACCACGTGGAGCATACAAGGTGTGTACTGAGGGCTGGCGTCAGAATCTGCGTCACAGCTTCTCACAGACTTAGCACATCTGTGGCTGGGTCCTGTGGTTATTCTGAGACACACCAGAGGAAGGGCGGCTCTTTCTAGTTTGCTAATGGGGGAAATGCGTATTTTTCAGCCTCACTTGGGGAGAAGCTTGGCTATTTTTTTAATTTAGAAAACAGAGTTTCCAAGAGAAAGGGAGAGGCCTTGAGTTCAGTGTCTCCAATGATCCTGGACTTATCCTCCAGCCTCCAATTCCCAAGGGTAGGATTGATTACAAGCGTGCACCACCATTCCTAGTTTCTGTGGTGCTGAGGATGGAACCCAGGGTTTTGTACCTGCAAGGCAAGCCACACCCCAGCCTCCTTCGCCCAGTGCTAGGCTGCATGAAAGGAACTTGGTTGTGTGTGCCTCTGGCTCCGGCCTTCTTTTGAACAAAAGGTAGAAGAAAAAAGGGAGGGGGAGGGAAGGGAAAGAAAGAAGATGGCCCACGCCCTACACAGAGAAGGGCAGCAGGTACAATTTAGCTCTACTCTCCTCCCAAGAGGACCATTTTAAAACAACACAGAGGCTGTCTCCTGAGTATGACCCCTTCGCACCTGCTGGCTCCCGCTGCCACAAATTGCTCCAATTCATTGTCCCCCAACAGCCGGCTATCGACACTGGCCACTGCCTTAGAATTTACCCACACACTGCCTAATCTGGCTTCTCACTCTTTGGGCTCTGGATCTTAGGCATTAATATTCCCCCACACCCACCCATGTTTTTTCTTCTTCTAAAGGTTATTCCTCAGCCTGTCTGAGAAGACAGGGACATATGGAAATTTCCAGGATTACATATCTTTCCTGGTCTGAGGGGTTGGAACTAATCACTGGGGCTGTCCTTTCGGCAGTGAAGGATCCCACCTGCCTCTTTTTTCCTGTTCCTGTCATTTCCTCCTCTAAGGAGGGTATCTCCCCCCAACACTTAGGAAGGAAAGCAACCTGGGTTGTTTTCTTCATTTAGAGCCGTAAGAAAGATCCACAGTAGGGGTTGGGGATTTAGCTCAGTGGTAAAGCGTTTGCCTAGCAAGCGCAAGGCCCTGGGTTCGGTCCCCAGCTCCGAAAAAAAGAAAAAAGAAAAAAAAAAAAAGATCCACAGTGCAGCAGTGAGGCAGGGGATTTTATGAAGAGCTTAGGACTGTTGCAAGCCCCCAAGGGAAGGATGAGACCCAGGAGATAGTAGGCTCGCACAGCAATCAGGCTGGAAGGAGATGGTTCCAGTGTAGGCATTGTGACTCCACGGTGCCACCAGGGACCCCAGGCTCCTTTTGCTCATCCCCTGTCCTGCCATCCCCAGCTGGTTGTCTCCCCTTAGTCTTTCTTGTTGCCTCATGGATTCAAGGTGGTCCTAAATATCTCTCCATCACTGTATCAGCGGAATTGAATCACATGACCACTACTATGGCAATCAACAGCAAAAG

							CAAAAAACCCCACAAGTTTGGTGCAGTGACTCACACTGCATCGCTGTGAAGATGGGCTCCATTGAGCT<br>CCAGGGTAGCTTACAGGCCATGACCTACTCAAAGCATTCCCTTCTTTCCCTCTGTTCCAAAGACAGCTTT<br>ATTTTTCCTTCTTCCACAGTAAGTGGATTTTCTCCGTATCATGGCACTCTCCCTAACTTCCTCCTTCATTG<br>TGATTTTGGCCAGAACATTCGACCATCAAAGCCCTACTTATCACTTTGTTACACACTATCAACCTGTCTG<br>ATTTAGGTTCAGATTTCAAGTGGGGAGGGGGGACAGGAGGAGAATCTAACTGGCCCGATTGGATTTCTT<br>TCCATTTTTGACTCATGACTTCAAAGGCCATTCGAAGGCCAGCCTGTATTAGGTTTGGGCCCACAGAATC<br>GTATGCATGCACAGAGGCAGATCTTGTAATATGTGGCGCTGGCATCTAGGAAGCACTGGCTAGAAGTAG<br>AATAGGTAAAGTGGAATGCAGCAGAAAGGGCTATAGATAGTGTGGGCTCTGACGGCCCAGACAGCGTA<br>CGATGAGGGAGGCTGCTCTCCTGAGGAGAGTGGACACGTAAAAGAAGGTAGAGACACAAGGGGCCCG<br>TGCACCAGAGCCACTTTACACATCTGGGCCCCAGATGGCGGACATCACAGGGAGAAGAGGGACCCAG<br>CTGGCAGACACAATGAAGGCTCTCGACACAAAGCCCTACTGGGACCGTGTCCACAGTTTTCAGCCAAT<br>CTAGTGGAGGTCCCAGACGTAGTGGAGCAGAAATAAACCACAACAATGCCTGACTTGCAGAGTTGATG<br>GCATAACTAGAGACTCTTGAATCTGCCTCAATTCAAGAATCCCCTCAATTCCCACAGCAGATATGCTAGT<br>GTCTGCTGGCAGGGAGCTCATGTCTCTCACAGGCACATATGTTCCTGTAAAGCATTAGATAGGTTTGGG<br>CAGGCTGAAGAAGAGAGTGTAGAAGGAGTACCATTTTCAAAAGGCCACAGAGTCCCGAAAGCGGGTT<br>GCCTATCCCAGCAAACGGAAGTGAAATTCCTACAACCCAACACTAGGACTTTTGAGGTCACGGTTGCA<br>GGGAAGATAAAGATTTGAGTGCTGGCTCCTCATGCATTTGAGTGAGCTGAGCAAAGCTCTCTCCATCTA<br>TTCCCCTCAGCTGTAACCAGAGGCCCACAAAAGACTTATTCTAAAGCGTGTTGAGAAGATTCAGTGAG<br>ACACTGCCTGGGTCCCACAGGTCACTGTGAGGAGCACTTACAAATGCAGCATGGGGCCTGCACATTTTT<br>TTTTGTTTGTTTGTTTTGAGAAAGGATTTCAAGTGTGGTGCCCAGGCTATCCTCAAACTCACTTTGTAGC<br>CGAGGATGACTTTGAACTCTTGAGTGCCCTGCCTCGCCTCTGTCTCCTAAGTGCTGGGATTACAGGTGT<br>GCGCTGCCAAGATCAGCAGCGTTCTAGACTAAGGTCTTGAGTAGGCTCCTAGGAGCCCCGTGCGCTCTT<br>TTGGATGGATGTAGAACTACCTTTCAGCCCAGGCCTTTGTTCCTTCAAGGGCCTGTGCATTCTCCCCCTG<br>TCTCCTCTCCTCGTCGGTGCCCTTCCTCGTTCAGTGCGGGTTGGGACAGTCATTACGGGGACAACTGGA<br>GACACTGTCTAGAGACTCAGTACAGACTGCATCCTAGCAACACATTCCCGAGCTAGAGTATGGGGCACT<br>GGAGAAAACAGATTGTGAGGCGGCCTGCCCACAGGTTCCCAAGTCCTGGGATCACAAGAACGCGCAG<br>CCACACCTGGTTTCTGAAGCATCGCAAAAGGGAGCGGAGGGCTCTCTGCATGCTAGGCAAGCACCTTA<br>CCAATTGAGCTACATCTCCATCCCTTAGAGTACTTGCTTTAAAAGACAGTCGGAAGGAGGGCTGTGGCC<br>TTATGGGAACTTGAGGTTTAGAGACATTTGCCATTACCCCTCCTTTTAAACCTGATAAGAAAATGTCATA<br>GCCAACATGGAAGGTGAGTTCTCTCCTTGCTGGAACCCAGAACTCAGCCATGTGTGGAAAGGAAGGGA<br>TGTGACCTTCGCCTGGGGCCCCTCGGGAGACAGTTCAGTGTACTTCTCCATAAACTGTCTGCCTGCTAA<br>GTTTGGAAGTTACCCTTTCCTGGACAGGAATGGGTCTAGGCGCCATGAGAGCTGAGCCTGCAGACCTC<br>CAAGGTCACCCTGTGAGTGGTTAGTGAGCCATCTTCCCCTGCAACCTGGGCGTGGTGAGTAGACACAC<br>CGGTGCCCATTCTTGCTTACTCACCAAGTGCCCCTTCCCAAGGTCACTCCTATGTCCTAGCACAACTCTA<br>ACTAATTAATGACATCTTTTAGCAGAGGGGAGGGAACAGGGGCTCCTGCCCCTGTATGGATTTCTCAGC<br>TAATATGGCTGGGCTGCCTGGTGGTGAGGGTCAGGGCTAGGGTAGAAGATGAAGCAATATGAAATCCCC<br>TACCACCCTGTGTGAAAACCATCTGGAAAAGGAGAACTTCATCCTCAGCGGCAAACGGTCAGAGCCAT<br>CTCCTTGTACCACAGAGTCCAGAGTGACACACAGCTACCAAGGCCTTTCCTCAGTGTTTTGGCAGTGA<br>AGGCTCCGTGGAGAATGCGAAGGGCAAGGCGTGGCTCTCCTTCAAATATTGTCTGGACTGTAGGCTGG<br>GATGATCTGTGTCCTTCAGAGTTCTTCCCTCTTCTTTCCTGCTTGGAGCTAGCTTTCCCTCCAGGAGTTC<br>CCGAGGCCAACGGAAGGTGGATGAACATGCTGAGGTGTTCTTTCTTTGGCATGGGCGCCGCAAGACTT

							CCCCAGAGGGGCTGTGTGAGGTCGCTTCACAGGATTAACTTGCAGACCACTGCCAAGGTGAGGTGAACTCAGGGTTCCAAGAACACATCTGAAGCTGATGATGAAGATCTCGTTTCCATTGTAGAGGTGCTGCACGTCCCAGAAGCTTCCTCAACTGTAGACCATGAGGAATGGAGAGCTTTAGAGCTCCGGCTGAAAATCCTCACCGTAGAAGGCTGATTCGAGCCGCGAACACCTGCGGAGCCACG
MSTRG.27645.5	ENSRNOG00000001417;ENSRNOG00000001418;ENSRNOG00000001419;ENSRNOG00000001420;ENSRNOG00000029211;ENSRNOG00000031346	0.000	0.621	0.000	8.128	up	CCCCAGTGAGGGGCTGGGGGCGTGGCTCAGTGGTAGAGCCCCTGCCTAGAATCCCCAGTGAGGGGCTGGGGGCGTGGCTCAGTGGTAGAGCCCTTGCTTAGAGTCCCCCAGTGAGGGGCTGGGGGCGTGGCTCAGTGGTAGAGCCCCTGCCTAGAATCCCCAGTGAGGGGCTGGGGCGTGGCTCAGTGGTAGAGCCCTTGCTTAGAGTCCCCCAGTGAGGGGCTGGTGGCGTGGCTCAGTGGTAGAGCACTTTCCAAAATGTGTGAGGTCCAGAGTTCAATCTACAGTACTGTACATACAAAAATCTGATGATGTTTTGGTAGGGGATGTAATTCTTTCACGAGGTACACATTTAAACGTTTCCTTTTTGCCCACAACCCTAGATTTCATTGCCATGAGTTCCTTTTCTCTCCCTGGAGTCTCATCTCCCTTGTTCTTTTTACAAGCTGAGATTCACTTTGCTGGTCAGCTATTGGCCTTGGCATTTGGGACCCAGCTGCATATACCACGTGGAGCATACAAGGTGTGTACTGAGGGCTGGCGTCAGAATCTGCGTCACAGCTTCTCACAGACTTAGCACATCTGTGGCTGGGTCCTGTGGTTATTCTGAGACACACCAGAGGAAGGGCGGCTCTTTCTAGTTTGCTAATGGGGGAAATGCGTATTTTTCAGCCTCACTTGGGGAGAAGCTTGGCTATTTTTTTAATTTAGAAAACAGAGTTTCCAAGAGAAAGGGAGAGGCCTTGAGTTCAGTGTCTCCAATGATCCTGGACTTATCCTCCAGCCTCCAATTCCCAAGGGTAGGATTGATTACAAGCGTGCACCACCATTCCTAGTTTCTGTGGTGCTGAGGATGGAACCCAGGGTTTTGTACCTGCAAGGCAAGCCACACCCCAGCCTCCTTCGCCCAGTGCTAGGCTGCATGAAAGGAACTTGGTTGTGTGTGCCTCTGGCTCCGGCCTTCTTTTGAACAAAAGGTAGAAGAAAAAAGGGAGGGGGAGGGAAGGGAAAGAAAGAAGATGGCCCACGCCCTACACAGAGAAGGGCAGCAGGTACAATTTAGCTCTACTCTCCTCCCAAGAGGACCATTTTAAAACAACACAGAGGCTGTCTCCTGAGTATGACCCCTTCGCACCTGCTGGCTCCCGCTGCCACAAATTGCTCCAATTCATTGTCCCCCAACAGCCGGCTATCGACACTGGCCACTGCCTTAGAATTTACCCACACACTGCCTAATCTGGCTTCTCACTCTTTGGGCTCTGGATCTTAGGCATTAATATTCCCCCACACCCACCCATGTTTTTTCTTCTTCTAAAGGTTATTCCTCAGCCTGTCTGAGAAGACAGGGACATATGGAAATTTCCAGGATTACATATCTTTCCTGGTCTGAGGGGTTGGAACTAATCACTGGGGCTGTCCTTTCGGCAGTGAAGGATCCCACCTGCCTCTTTTTTCCTGTTCCTGTCATTTCCTCCTCTAAGGAGGGTATCTCCCCCCAACACTTAGGAAGGAAAGCAACCTGGGTTGTTTTCTTCATTTAGAGCCGTAAGAAAGATCCACAGTAGGGGTTGGGGATTTAGCTCAGTGGTAAAGCGTTTGCCTAGCAAGCGCAAGGCCCTGGGTTCGGTCCCCAGCTCCGAAAAAAAGAAAAAAGAAAAAAAAAAAAAGATCCACAGTGCAGCAGTGAGGCAGGGGATTTTATGAAGAGCTTAGGACTGTTGCAAGCCCCCAAGGGAAGGATGAGACCCAGGAGATAGTAGGCTCGCACAGCAATCAGGCTGGAAGGAGATGGTTCCAGTGTAGGCATTGTGACTCCACGGTGCCACCAGGGACCCCAGGCTCCTTTTGCTCATCCCCTGTCCTGCCATCCCCAGCTGGTTGTCTCCCCTTAGTCTTTCTTGTTGCCTCATGGATTCAAGGTGGTCCTAAATATCTCTCCATCACTGTATCAGCGGAATTGAATCACATGACCACTACTATGGCAATCAACAGCAAAAGCAAAAAACCCCACAAGTTTGGTGCAGTGACTCACACTGCATCGCTGTGAAGATGGGCTCCATTGAGCTCCAGGGTAGCTTACAGGCCATGACCTACTCAAAGCATTCCCTTCTTTCCCTCTGTTCCAAAGACAGCTTTATTTTTCCTTCTTCCACAGTAAGTGGATTTTCTCCGTATCATGGCACTCTCCCTAACTTCCTCCTTCATTGTGATTTTGGCCAGAACATTCGACCATCAAAGCCCTACTTATCACTTTGTTACACACTATCAACCTGTCTGATTTAGGTTCAGATTTCAAGTGGGGAGGGGGGACAGGAGGAGAATCTAACTGGCCCGATTGGATTTCTTTCCATTTTTGACTCATGACTTCAAAGGCCATTCGAAGGCCAGCCTGTATTAGGTTTGGGCCCACAGAATCGTATGCATGCACAGAGGCAGATCTTGTAATATGTGGCGCTGGCATCTAGGAAGCACTGGCTAGAAGTAGAATAGGTAAAGTGGAATGCAGCAGAAAGGGCTATAGATAGTGTGGGCTCTGACGGCCCAGACAGCGTA

							CGATGAGGGAGGCTGCTCTCCTGAGGAGAGTGGACACGTAAAAGAAGGTAGAGACACAAGGGGCCCG<br>TGCACCAGAGCCACTTTACACATCTGGGCCCCAGATGGCGGACATCACAGGGAGAAGAGGGACCCAG<br>CTGGCAGACACAATGAAGGCTCTCGACACAAAGCCCTACTGGGACCGTGTCCACAGTTTTCAGCCAAT<br>CTAGTGGAGGTCCCAGACGTAGTGGAGCAGAAATAAACCACAACAATGCCTGACTTGCAGAGTTGATG<br>GCATAACTAGAGACTCTTGAATCTGCCTCAATTCAAGAATCCCCTCAATTCCCACAGCAGATATGCTAGT<br>GTCTGCTGGCAGGGAGCTCATGTCTCTCACAGGCACATATGTTCCTGTAAAGCATTAGATAGGTTTGGG<br>CAGGCTGAAGAAGAGAGTGTAGAAGGAGTACCATTTTCAAAAGGCCACAGAGTCCCGAAAGCGGGTT<br>GCCTATCCCAGCAAACGGAAGTGAAATTCCTACAACCCAACACTAGGACTTTTGAGGTCACGGTTGCA<br>GGGAAGATAAAGATTTGAGTGCTGGCTCCTCATGCATTTGAGTGAGCTGAGCAAAGCTCTCTCCATCTA<br>TTCCCCTCAGCTGTAACCAGAGGCCCACAAAAGACTTATTCTAAAGCGTGTTGAGAAGATTCAGTGAG<br>ACACTGCCTGGGTCCCACAGGTCACTGTGAGGAGCACTTACAAATGCAGCATGGGGCCTGCACATTTTT<br>TTTTGTTTGTTTGTTTTGAGAAAGGATTTCAAGTGTGGTGCCCAGGCTATCCTCAAACTCACTTTGTAGC<br>CGAGGATGACTTTGAACTCTTGAGTGCCCTGCCTCGCCTCTGTCTCCTAAGTGCTGGGATTACAGGTGT<br>GCGCTGCCAAGATCAGCAGCGTTCTAGACTAAGGTCTTGAGTAGGCTCCTAGGAGCCCCGTGCGCTCTT<br>TTGGATGGATGTAGAACTACCTTTCAGCCCAGGCCTTTGTTCCTTCAAGGGCCTGTGCATTCTCCCCCTG<br>TCTCCTCTCCTCGTCGGTGCCCTTCCTCGTTCAGTGCGGGTTGGGACAGTCATTACGGGGACAACTGGA<br>GACACTGTCTAGAGACTCAGTACAGACTGCATCCTAGCAACACATTCCCGAGCTAGAGTATGGGGCACT<br>GGAGAAAACAGATTGTGAGGCGGCCTGCCCACAGGTTCCCAAGTCCTGGGATCACAAGAACGCGCAG<br>CCACACCTGGTTTCTGAAGCATCGCAAAAGGGAGCGGAGGGCTCTCTGCATGCTAGGCAAGCACCTTA<br>CCAATTGAGCTACATCTCCATCCCTTAGAGTACTTGCTTTAAAAGACAGTCGGAAGGAGGGCTGTGGCC<br>TTATGGGAACTTGAGGTTTAGAGACATTTGCCATTACCCCTCCTTTTAAACCTGATAAGAAAATGTCATA<br>GCCAACATGGAAGGTGAGTTCTCTCCTTGCTGGAACCCAGAACTCAGCCATGTGTGGAAAGGAAGGGA<br>TGTGACCTTCGCCTGGGGCCCCTCGGGAGACAGTTCAGTGTACTTCTCCATAAACTGTCTGCCTGCTAA<br>GTTTGGAAGTTACCCTTTCCTGGACAGGAATGGGTCTAGGCGCCATGAGAGCTGAGCCTGCAGACCTC<br>CAAGGTCACCCTGTGAGTGGTTAGTGAGCCATCTTCCCCTGCAACCTGGGCGTGGTGAGTAGACACAC<br>CGGTGCCCATTCTTGCTTACTCACCAAGTGCCCCTTCCCAAGGTCACTCCTATGTCCTAGCACAACTCTA<br>ACTAATTAATGACATCTTTTAGCAGAGGGGAGGGAACAGGGGCTCCTGCCCCTGTATGGATTTCTCAGC<br>TAATATGGCTGGGCTGCCTGGTGGTGAGGGTCAGGGCTAGGGTAGAAGATGAAGCAATATGAAATCCCC<br>TACCACCCTGTGTGAAAACCATCTGGAAAAGGAGAACTTCATCCTCAGCGGCAAACGGTCAGAGCCAT<br>CTCCTTGTACCACAGAGTCCAGAGTGACACACAGCTACCAAGGCCTTTCCTCAGTGTTTTGGCAGTGA<br>AGGCTCCGTGGAGAATGCGAAGGGCAAGGCGTGGCTCTCCTTCAAATATTGTCTGGACTGTAGGCTGG<br>GATGATCTGTGTCCTTCAGAGTTCTTCCCTCTTCTTTCCTGCTTGGAGCTAGCTTTCCCTCCAGGAGTTC<br>CCGAGGCCAACGGAAGGTGGATGAACATGCTGAGGTGTTCTTTCTTTGGCATGGGCGCCGCAAGACTT<br>CCCCAGAGGGGCTGTGTGAGGTCGCTTCACAGGATTAACTTGCAGACCACAGGGTTCCAAGAACACAT<br>CTGAAGCTGATGATGAAGATCTCGTTTCCATTGTAGAGGTGCTGCACGTCCCAGAAGCTTCCTCAACTG<br>TAGACCATGAGGAATGGGAGAGCTTTAGAGCTCCGGCTGAAAATCCTCACCGTAGAAGGCTGATTCGA<br>GCCGCGAACACCTGCGGAGCCACG
MSTRG.27861.4	--	0.294	0.000	0.000	-5.253	down	CAGATCAAAAACTTTAAAAAGCCTGTTCTGACACCTGCTCGGGGTGTAGCCACCACTTCTGCATACTTG<br>CACCTCTTCAGCGGAAAGACTGTGGGAGTATTTAGGTGGATCATCCGAGTTTCATAGGGAATACTAGGC<br>CTTCAATTTTTTTCTTTCTTCTCCCTTCCTGGTGTCTCTCACCTTCTATTTAAAGAAGAAATCATTTCGCTC<br>CCTTATTTCCTGCTACTACTAATTACTGATTCCAAACACTGGTCATTAAACCTAGAGACTTTTGGGTCTGG

							AAGAGTGGCTCAGTGGAGACAGGGGTTATCTTGGAGTTTTGGAAGCTCTCCAGGTGATTTCGGTGCGCAGCCATGGCCGGGAAGCAGTGCACTAGAGGAACAAAGAGCAGGGCACTAATCAACCCCTTTCGGGTTCCTTAAGTAGTCGAGGTGTGGCTTTGTTACTCCGTAGTTGGGCACGCGGCAGTGGACAGGATTATCTGAAGGAGTCATCGTTGTTGCAGGGCCAACCCTCCGGGTTGAAAGTAGATTGAAATTTATTATTTAACAAGGTGCCCTTACTATTTATAGTTGGACTGTAGGTATGGCGGCGAGAGACAAAATTAACCTTTTCTCTTGCTTTGAAAAAAGACAAACGCCTGTGAAATTAAGCATAAAATGGGGTTTAAAGTGTGTGCCGTTTTGTGCGGCAAGAAAGTGCTGCTGTCCTGAACCATTTTGGGTGTTTGCAGAAATGAGTTTGCCTTTCCTGGGTGGGAAAGTGGTTGCCTGTGCTACTCACATACTGGTTAATTACCCTTAATATATCGACGCAGAGCTACAGAATGTATTGTTAGGTGGAAAATACAGAGGAGACACTACGTTTCTTTTTTTTTTTTTATAAAATATTTTTAAAAACGCTTAGGTTTGTTTTATGTGCGTGCGTGTTCCACCTGCGTGCAAAAAAAGTACACTATGTACGTTCCTGGTGCCCGCTAAACTCAGAAGAGGGCACTGGATCCCCTGGTACTGGAGTTACACTCAACCATGTAGGTACTGGGAATCAGACCCAGGTCATGCAACAAGTGTTCCCTAACTACCCAGTCGTCTCTCTAGCCCTCTTCATCAGGTGATTTTAAAAAGCTCTCTTAACAGTTTGCGAAAATCCCAAATTGTGACACGGATAATTAGATAGTTTGGTTTGATTCTACTAAATATACTTGTTAATACTTATTGTAAAAACAAGCACATTAATCAAGATACTTATAGAATTATTTGACGTCAAGTTTTAGGAACATTTTACGAATCATTTGTTAATCCGCTAATACGAGGCTCAAACCTCAACATACCTTTTTTCCCCATTTTAATGGTTTTTTTTTTTTTTGAAAAGCCATGGTTCTCTTGAATCATTGCAAAAGTAAATGATTCAGAATTGTTTTGATTCTGAAATAATTCAAATTGCGCATGGAAATTAGACAGTAAGACACATTGCTTTTTTTTTTTTTTAAATTATGTTCTTCGGGTGTGTGGGGTTTGTGCTGGCATTCTGGGAGGAGATAGTAGCAGCCCCTTGGCTCTTTACAGATGTTTGTTTTCCTTCTAAGGGAAGGAAGGAAGGGGCTATTTTTGGCATTTTTGCCCTGTTGGCCCTGGGTGAGAATGTATTATTTGCAAACAAAAATACTTTTCAAGTTCATTTCCCCCTGACTGCCTGAATGGAGCCTGGGTCGAATAGTCAGTGTTTGTCCGGGAGAAAGCGAATGTCCATACCTCCTTCTCTCTGCTTCCTTCGTCTCCAGGGGCAAGGTCTGTTTTGTAATGAGGACAGGGCCTTCCCATGGAAGCTTCGGCTCAGGCACCATGTCCTCTGGCAGCTAAACATTGAGGATCGATCCTCCCTTCTTGGTTAGGTCACCCCACCCTTCGGGTTTTGTGAATGACAACTACCCACTTCTTGCCTCTGTGCTATGGATAGAACCCAGGGCTTTGATTGTTCTAGACAAGTGCTCTAACCACTGAGCAATATCCCTAGCCTGGCACCATCATTCCCAAGTGCCTGCCAAGCATGTCTGAGCTGGGCTGCTCTCCCTTCCAAACAGACCCTGGCAAGAGTTTAGAATCCCCCCCACCCCATGTAGGGTATGGGAGTATTTTCAGAAGAGCAACATGGTAATTTGGCTTGTGGTAAAAAAAAAAAAAAAAAACTTGTTCCTCTTTCTTTAAATATCCTGATTTTCCTTACCCACTTGTCTAGATGTGCCTTTGAGAGTCTCCAGCAGAGACCTTGGGCTCCTCACAGAATGTGCACACAGGGAGAATGAAGCCTTTCCTAAATGGCTTTCAGCTTCAGCTGGAGATGAATGCAGAGTGACCGCTGCTCTCCCCCAAGAGCCTGACCT
MSTRG.27873.1	--	0.285	0.000	0.000	-6.594	down	GATGGATGGATGGATGGATGGATGGATGGATGGATGGATGGATGGATGGATAGCATAATAAGTGGCTTACATATCTTGAATCATAGTATGTTGAGGGAAGCCAATAGAGCTCAAGGGAGGCTTGAACTCAAGGCAGGAGCTAAAGCAGAGATCATGTGAGCTAAAGCCTTCATGGTTTGCTCAGTCTTCCTTCTTATAGCATCTAGGACTACTTACCATGGGCTGGATCCTCCCACATCAATCATTGATGAAGAAAATGCCCCACAGATTGTCTACAGGGCAGTCTGATGGAGGTATTTCTCCATTCAGATGCCGTCTTCCTAGGTGCTGTCTAGGTTTGTGTCAAGCTGATAAAAACCAACTAATACACCAACATCCTCCCTCACTGTCCACCTTATTTCCTTGAGATGGAATCTCGCACTGAACCTAGAGCTAGGCTGGCTGCCCTCAAATGCCAGTAATCCTCCTGCATCCACTCTTCCCAGTACTGGGGTTACAGGTCTGTATGTGGCCACATATACTTTGGGGTGGGAGGGTGCTACTGTACATGCAGAGTCAGGGCCTCAGGCTTGAGAAGCAAGTGTTCTTCCCTGCTGAGAAACGTCATATCTCCAGCTGCCTCGGTACCTGTGTACTTCTCGCCTGCTACCTTGCAGATTACAGTCCAGCAGCTCCCAGCAGCCATATCTGTAGTC

							TGTAAAAGTTCAAGGATGCCTATGACCTCGAGGCAGCGGGTCACAGTGTCCTCAGCCCGTACTTTCATC<br>ACCAACCAGGGGCTTCTTTTGAAGGCATCCTAGAAAACACCTGTCCTCCCCAGTAGCTATCCTTACGTG<br>TCACCAGAACTGTATCAGGTGCAGCCATCAAACGTGACTGACAATAGAGTCAGAGAGCCAGGTGCCAA<br>GGGCTGGCAGATGAAGTGTGAATAGATAGGTAGGTGTGCGATTAACTCCTCTAGAATCCCACAGTAAGA<br>TACTGAGGGCATGGCTCAGCAGGAGAGCCCTTGCCTCTCATTTAGTCTAGATTCTGGCCCCTGCAGTCC<br>AGGGGCCAGGACTTGGGGTACTGAAGCTCACAGTTCATGTGCTTTGCTTCACCAGAGATGGGGTCTTTT<br>TTCACCTGCTCTCCAGCACATCTGGACCTTTACCCAGTTGCTGCTCACTAAACGCTCATAAACATATTTT<br>CGCTTGTTTAGTGACCCATGGGATTCTACCCCTGTGGCTTTCTTCCCCAAGGAGAAGCAGAAAGAAACC<br>AGATGAGGGTGGCTTCCTCATCGGGGTCCTTTCACCATTTCTCCTGGAGAAAGTGACGTTCGTCGAAGG<br>GGCATTGATCTTTATGAGAGCAGCTCCAGATGACAGGCCCTGGACAGGTTTCCAAACAGCTGTTGTTCA<br>GCTGAGAGGCGAGCGCTTGATGTATGGCTCCTATCAGAAAGTGTCCAAATGGAAAAGCCTGACTTACTC<br>ACAGCCCCACCTCAATTAAACAACCTGGGCACTTTAAAATGGGGGGTCATAAGTGTCAACAAGATGGA<br>GGGCTTTGCCTCTTGATTACGGCAGGCCGGGGAGTGACCTGGGAAGCTCTCACCAGTACAGATGGCGA<br>GCAGTCCTTGAACCATCGTGCCACCTGGGGTGAGTCTGGCTGTCATCCTGTGGACAGAGCAGGCCAAC<br>TCAGGCAGAGCACAGCTGGAGAACGTTCATCATGGGGTCTGGGATCCTCTCCTCTGAGGGCTCTGGGG<br>AGGAATGCATCTCTAACTTGTCCCTAGGCAGAAACCACGTGCAAGGCAAGCTGAGGCTGGGGCCATAC<br>CACACCCCTATTGTGTTCATTCACGCTCGGTTTTTTCTTGGTTCCTCAAACCCATTAAATAACCCATTAAA<br>GAAGCTCTGACTGTCAATATCAGGCCAGAGCAGCAAAGCCAGCACAAGAGACGTCACTCACCCATCTC<br>AGCCATCCTCTGAAGGCCCTGAATCAAAGGTATGCTCCACCATAGCAGGCTCACAGAGGAAGCCCTGA<br>GTTAATCAGGGTCCCTCAAATCCAAAACTAAGGTGCAGACACTGGGGATTCAGCTCAGGGCATATGGCA<br>TGTGCCTAGCATGCTGCAGGGCCTGGGTTCCAACTCCAATGCCACAAAAGCCAGGTGTGGCAGCATTG<br>ACCCATAAGCCCAGCAGCTACTAGGTTGAGCAAGGAAGATCTGGGGTTCAAGGTCATCCTCCGATACTT<br>CAAAAGTTCCAGGCCATCCTGCTGCACCACACAAGAATCATTCTGTCTCAAAAAAAAAAAAAAAGAAAA<br>GAAAAAGAAAGAGAAAAGAAAAAAAGACGTGTGGGGGTTTGTGGGACGTGGGCATCAGTCATTGTCT<br>CTGGAGAGGCAGGAGTCCTCCAGGCACAATGTAGGGACAGAAAGAGATCACTGGAGGGGCAGAGTGA<br>AGGAGGGAAGGAACGCAGCTCACGTTGCAGTCTTGAGTGCAGTGGTGTCTGAAGTGACAGGCGCCAG<br>ATGTCTCTAGGTAGACACCCTTATGGCAGCTCCAGTGGAGAGAGATCCTAGTGATATTATCAGGGCTGCA<br>ACAAATAAAAGCTGGAAAATAAACATATGGAACATGCCTCATAGTGTATTTTTTATCATCAGTGATCCCG<br>AATGTGCCAGTGAATCTGGCTAACTATAGTCCCGGCCATCTGCCTATGGCTGCCTCTCACTCAAATGCGT<br>TGTGTCAAAGTCCCACTCTCCACCAAGCCTGGCGGACAAGGAGACAGCCTTGCTGTCACACGGAAAC<br>AGGTGAGAGGGCCACACCCTGGACTGAGGTCCAGAGCCATTGGTCAAGTAGCCTGTCAGGTACCTTCT<br>GGGTTTGGGAAGGTGGCCCCTTCCCACGTCAGGTGCAAGTCTCTTGCAGAATGCCTTGTTGCCGCTTCC<br>TGTAGATTAGAAAGAGTCAACAACCATTTCCAGAACAAAAAACCTAAAGGAGTGACGACCTAACAGCC<br>CGCCAAAAGCTCCCTAAGAGACAGACAGCAGTATAGGAAAAAGCATGTGCCAGCACGTATCTACCAGT<br>GGTGTTAATAAGCAGTAGATGGTTAATGACAATCATGCAGTTTCCACTCTCTCACTCACTCTCTCATTTC<br>ATTCATTCATTCATTCATTCATTCATTCATTCATTCTTGGGTTATGTCTAAGTCCTCAGTACAAATTGCCAT<br>CTTTCTGGGCTCCCAGCGGGAGTCTGTGGAGCATTAATGATCATCAATAATGCTGTCAGAGAGAGGGGG<br>AGGCAGGGAGAAGGAGTAGGTGATAGTAGCCATGGAATAGCCCCCATCCCTCTTTCAGAAATGGAAGA<br>CTAATGCAGCTGCTTTGTGCGCGAAGCCCGGTATCTGATGCTTGTTAACGGGTATCTTTAACTGTATAGG<br>TTGCCCTTGGTAAAGGTATTTAATTTCACACACTTAAAATCCATCCACTGGCTTAAGTTCCCTTCTCAACT<br>TCCCTCCCCTCCCCCCCCCCACCTCCCTAACCTCTCAGAGGCCAGTTTCTCAGTCTTGAACTCGGAATA

							GGATACAAGAAAGGGTAGAGAAGGTGTTAACTGTTCCACATACAGCGTGTGCAACCTCCCTCAGACCC<br>TTGCCCGCTTGTAGTGCTTTCTTTCGAGCACTTAACCACACAGTCACTAATCAGGTAGAAACACTTGAA<br>TCACTTACTAACTAGCAGGCTGTCAATGCCGCACAGAGAAGACCAAGTGTTCATTGGCTGGAATTCCAT<br>GCAATTGCTTGCATTTCGGGACTGATATTTTGAGCTAGAAAGATCTTGATTGGAAGTCTTGTTCAGAAGT<br>GAAGCCTTTCCCTCCCCCACCCCAGAAATTGACTTCTCTTTCAATTCCCTGCCTTTACTAGGCGAACTTA<br>CTTTGCTTTCTTATGGCTCTGTATTTTCTCCTATCCATCCTTTTTCCCTGCTCTGGTATGAAAATTCGTGAC<br>ACCTAATCCAATCTGTGATAGTCCCCAAACCATGCTGTCATATGTGTAGGTTATGTGCCTGTTCCTTCACG<br>GTAACAAGCACATTCGACTTTATTCATTATGGAGTTTCTATGATGTCCTAGGCGCTATGCTTGGCAAGAA<br>GAATATCTATCATGGAGAGGAAAACATCTAAGACACCACCACTAACCTCCTGCTAGTGAAAAACAGGCT<br>CAGTTTGTTTGAGAAAATGCCACTTTCCTTGGATTCCTTAGAGAACATCATTTCCATTTGAAATTGTTCC<br>TCTCCGGGCTGGAGGTGCAGCTCCGTTGGTAAAGTACATGCACAAAGCCCTGAGTTCTGTTCTCAGCAC<br>TGTCCAAACTAGGTATGGTCATAGAGGGGTATAATCCCAGCATTTAGGAAGTGGAGGCAGGGCACACAG<br>GAGTTCAAGGTCATCTTTGGCACAGCCTGGTATACATGAGACCCTGCGTGAAAAACAAAGCCAAACAA<br>AAAAACAAACAAACAAACAAACAAAATCCCACCAAGTGTTCATGAAAAGAATTTGGACCTCACCCATT<br>CCCAAGGTATTGTTTGTTTCCGTAGGCCCTGCCGTGCCTTTTTCTCAGGGAGGTTGAACTCTGGGCTTTG<br>TGTCCCCAACTCACCTTGTTCTTACTGACTTCAAAACAATGGGAACCGTATGAGACCATTGGTTGTGTT<br>GTTGTTTGTGTTGTTTCTTTAACTCCCTATTCCAAAATAGGGTCTTGCCCCTGAGCCTCTGGCCTCTGCA<br>GCTTTCTCTCAGCCTGTTAGTACTGGGTAAATCTCTAGACTTCGCCCCTCGAATCTCAGCCATGTGCCTT<br>CCCCAACATCATCTCCCTTATTCCTATAGATTTATAGCTGTTGTTCTTAGCAGCTAAGGAAAATGAATTCT<br>TTTCCTCTACGATACACAGGGCCTTGCTTACTAAACAGGCTAGTGTTAACTGTCAATCTGCCACAATCTG<br>CCACAATCTAAAATTGTGTTGTAAGACCCTTAATGAGGAGCCAGGCATATTAGGTTGCCCAAGTATGTCG<br>TGTGGCATCGTCGTCATTGTTGGTTACAGCAGGAGGACACACCCTGGATGTGGGTGTCACCGTTTCTTC<br>GGCTGGACTGCAATGTTTATGCACAGGTTAAGCCAGCTGAGCACAGAGCGAGGCTGGCAAGCAGGAA<br>GTGTCAATGTTCTGTCCGTCTGCTCTTGACTGTGGATGGGATCTGAATGGGACACTGGAGTATCAACAA<br>CGTGACTTCCCCACAGTGATGGACTGTTTGTTCCCTGGCCGTGTGAGCCACACAAACCCTGACCTCACC<br>TCTTTGCTTTTACATGGCCGTTTTATCACAGCAATAGAAAATGAAGTAGGACCATCATCATATACTCCCAG<br>GAACTGGGAAAGACTTCCTTCCTGAATTTCAATACAGAGCGAGCCCTGCCCAAGGCTTGTCCAGGAGC<br>TCAACAAAACCCAAACTGTTTTCCTAAGAGCAGACGACTTTGCTCAGACCAGGAGAGATGGCCCAGCA<br>GTTAGAAGTATATTACTCTTCTTGCAGAGGAACCGAGTTCAGTCCTCAGCATCCATGACACGTGAGCCA<br>CAACCACTTATGACTCTGGCTTCAGAGACCCTGACACCCTCTTTTGGCCTCTGAAGGCACATCACACAT<br>ACCCCAGTGCATACACATTTTAAAAGTAAATCTTTCCTTGAAAAGAATAAGTTACTCATTTTCTTTTTCAC<br>CTACCTTCATTCTCTAATGGATGTATGACAGAATTTTCCAGAATCTACAGCATGCAACTGAAACAGATTG<br>AATGCAAAAGCAGACAGAAGAACCCAGCTGTCTGCGACTAAAGCAGATATAACAGAAACTCGGTCGCT<br>CCTCTCACAGTTTTGCTTTGTTTTGTAAAATACAGTTATTTCTGGTAAGAATATATACATAATATAAAGTTA<br>GCATCTTGTACGTGTGTTGTCATTTTGTGTGTTCATGTGTGTGAAGGGAAATAAGGGTGTGTGTGGAAAT<br>ATGAGAGACAGCCTTGGGTATTATCTGCTTTGGGTTTTGGTTTCGTTTCATCTTGTTTTTGCTTTGAAACC<br>GTGTCCCTCCCTGACCCCAAATTCACCTAGGATGGCTAACCAGGGGGCTCAGGGATCCTCATTTCCTGC<br>GCCTGGATTACAAAACTAATTTTTCTGATAAACGGTCTGGGGAGCAAACTCTTGCAAGTGCTTTCCCCG<br>ACGCAAGCCACCCGTCTAGTCCTGTTACAGCTACTCTTTCTTTAAGCGAGCTGGCACAAGTATACGTTTT<br>CGTAATTGTCAGTGTGGTAACAGTCAACAGGTATGACGTACCGGAGTAAAAGCTTTTAGAAGTCCTCGG<br>AATCTTTTGAAGTGGTCAGGGAGTATGAGGCCTGATGGTAGAGAGTACAGCTAGCACACAGGGGGTCC

							AGGGTCATGCCCACCACCCC
MSTRG.27878.1	ENSRNOG00000049016;ENSRNOG00000049451;ENSRNOG00000060802;ENSRNOG00000060998	0.394	0.000	0.000	-6.812	down	GGGTATTGGAGGGGAACAAAGACACTGAGGCAGAAAGTGCTCCCGGAAGACACTCTTAGGGTCCCTGCCCCACTGGATCAGGGAATTAACCCTGCTCACCTTTTGTGCATTATTAGCTTAGTTCTTTAGAACGTCGTGTTCATGTCTGCTGGGGCAGCCCCATTGTAGATGGCAAATTGCTCCTCTCCGCTAATCTTTCTATCTCAGCTTAGCCGTTCCTCTGGTATTACATCTCATCATAGTAGGCAAGTCCGCTCTTTCAAGCCTTCACATAGGGGACATCAGCGTAGGTAGGGGATAAAACAAGAGTGTCCTGAGCTGATAAATGACATTTGGAAGTGACAGCATGATACTGTGTCAGATTCTTTTGATTGCCTTTATATTTGCTTAATTTTGTTTTCTTTTGTGTGTACGGGGTGTGTGTGTTCCTGTTTTCGAATTCCTTGTGTATGCGTGGAGGCCAAAGAGCAGTCTTGGGTGCCAGATGCTGTAGGCTAACCGGCTCAGAAGCTTCTGGGAATTCTCCTGTCTCATAATGTACACACCACACATATACATTGAAAGAAAGGGGGAAAGAATGAGGTCATCAGAAACAAGGAAAGTTAGAGAAGCTGCCAAGACATGACATGGAGCATCCAGCACGAGACCCTGCTTCCAACATACAATGTGAAGGACGTTAATAACACTGTAGCAGCGTTGGCCCATTGGGTCAGACTACAGCAAACATGACTGTTACTGCTGGTTCTTGGTAGCAGGGGCCCCTGGGTATGAAGAGTGTGGAAACACTGTTATCTTCTTACTCTCTCTGTGCATATGTCACTCTTCTAAAAATTAACCCTACTTAGGGACTGGAGAGACGGCTCAGTGGCTAAAAGTTCCTCTATGCAAGCATAAAGAGGTAAGTTTAATCTCCAGCACCCACGTAAAAAGCCACACCTGGCCATGCAGGTATTCAGACTCAGACAGTAGGGGAGATACGAGGGTGGCTGGGGCTTGCTGTCCTCTAGCTCAGTCCAGGGGTTGGGGGGACTCCTTTTACAGGGGGATAAGATAGAATGTCATAGAGCAGGACATCCGGTGTGTCTGGTGTGCTCTCTCTCTCTCTCTCTCTCTCTCTCTCTCTCTCTCTCTCTCTCCAAAAAATAGTAGTAGTAGCAGCAGCAACACCTCTAATACAAAGAACACTCATCGTCCAAACCAGGTTTTGATAACCCTCAAAGGTGAGCATCAATCATCCAGAAAATGTATTTGTAAAAGAACTTAGTTCCTTTCCAATGGCGGAGTGCTGTTATCTCTGCCTCCCTGAGAAAGTATGTCTGAATAGAGAAAAAAGAAAGGGGTTGGGGCAAGAGTATGGTTAAGTACAGAGAGAGAGAGAGAGAGAGAGAGAGAGAGAGAGAGAGAGAGAGAGAGAGAGAGAGAGAGAGAGAGAGAGAGAGAGAACACAATGTGAGCGCTTGTGCAGTGTCTGTCTGTCTTCTGTCTGCCTGCACTTAAATAGGAAAAGAATGAATTCAAGCTGTCTCCAGCCCTGTTTCGAAACTTAGTCTAATTCCAACCAGAGAAGAGCAAGGAGTTATTGTTGTTGTTGTTGTTGAGGAAAAAGTAGACTTACACATTATTAAGTTTTTTCCCCTCCTAGAAGCTAAGGAAACCATCTGTGAGAGCCTGTTTGCATGTTTGTTTTCTACAATAAAATGCAAAATACTTCTTCCTGTTAGCGGATTGTACCATATGTCTCTCCTTGTAACAAAATCAGCATGTTTATTAATGAGCCCAATCAGAGGCAAAGATTTAGAAGGACTGCGCGGGAACTGGATTCTTCCATTCAGTATTCCTGCAAAGGAAACTTCTGGTTAAGCGTTGTCGTTGTGCTTGGAAAATAGAAGGCTTGTTTTTGCTCTGAAATCAATTTACTTGGCAGAGGTCAGAGAGAGCAGGAGGAAGAGGCCTGTGACTCTTTTTTTTTTTTTTTTTTTTTGTACAAACTAAAAATCTGCATTTAAACATGGCCCTTCCCTCCTGGCGACTCCCCCAACTGATTTAGTTCCACAATTTATATGTGATTAATATTTAATTCAATCAGTTTCATTTTCCCAGACAGACTAATTAAAGTACTTGATGTACCTACCCCATCTCTGAACACAGGGGCAAGCCTGTGGGTTTCTTCGAGCGACAGGAATCGGACAAAGTCTCAGCTGTGTTAGCATGGCAGGTCATGGTCTCTGTACAAGAAGCCCAGCTCATCACGGTAGTACACACTCCGTGTGTGCACCTTGTCATACGGTAATACCATACATGGGTGCAGTAGCCATGCTGTAATGAGAGAAAGAATTTCCTGCAGTGATACTTGGGGAAGCCAGACAGCAGACCTGGGCTACATGACATCCTCTCTCCATGTCTCAGGTCATGCAAAGGATGGCCTCCAACAGTTCTTGTCAGCGTAGACCTTAAGGGGACACTGAGGATGCTAGACCCTGGACCAGGCAGACTATGTGTGCTTTCTGGTTATCCAGCCTCCATGCAAGTCTTCTTACTCTCTGTTTAACAACAAGGATACGACCCAGCAAATGCCATTTTCTTCGGTCATGTCCTGTGTTTTTCTAGAAATATTGTAAGCGACCAAAATACCCGTAAGCAGCACACTTGCAGTGGAGCAGGGGGCGGATGGGCAATTAGAAAGGCAAGGGCAGGGGCTGGAAACACTGTAGTGCTGTATGAGAGAGACGCTTTCCCTTCCTACTGAAGTCTTTGGATAGGAGTTTTTTTGCT

							GTGGTAATGAGAGGTGAGGAGGAGGGTGGGCAGAGGCAGACTTACAGAAGTCTCTCTGGTAGGTGTC<br>TGTATGTGCAGATCAAAATGTCTAAGTTAGAGTTTATATTGCCGTGATGAAACTTCATAGCCAGAAGTAA<br>CCTGGGGAGGAGCGGCTCACTTGACTTACAGATTACAGTTTATCAGTAAAAGAAATCAGGGTAAGAAC<br>CCAAGACAGGAACCAAGGCAGAGCGATCATGGAGAGAGGCTGCTTGCTGGCTTCCTCAGCTAGCTTTC<br>TTATATACCTTAGGGCCTCCTGATCCGAAGTGGCATTCCAAATGGTGGGATGGGTCCTCTCACATCAATC<br>ATTAACCCAGAAAATACCCCCGTAGACTTTCCTACAGGCCAGTTGATCGAAGTATTATCTCTCAGGGGA<br>GGTTCTTCTTCTCAGATAACTAGCTTATGTCGACTTGACAAAAACAGCAACCGGGACGCCAGAGGTCAC<br>CCTCAGGTGTCAATCATTAGAAGTCATTCACCTCTTTTCTTGGAAACAAGGTTGTTCAAAGGCCCGGAA<br>CCTACTAGGTAGGCCAGATTAGGTGACCAAAAGTCCTGGCGATCTGCCTATCCTACCGTTTCGGCATTGG<br>GATTGGGATGTGCACTCCTGGGTTCTGGGCATTGAACTTCAGGTCCTCACGCTTGCAAGGCCAGCACTG<br>GATGCCTGCGCCATATCCCTGGATTCTCAGACAGATTTTGCAAGTGCAAAATACACTCTAGTGGACAGT<br>ATACAAAGGTTTCTACCTGTTGCAATGAGCTGTATCAAGTGAGAGGGAGAGGAAGATTGAAATGGCTTC<br>AGCCTTTTTTAAAGCATCTTAGGCAGGCGGTACAGTCGGGATTGAATAGATGTGGCTAAACATTCGCTG<br>GCTCGTGGTCTTGTTTTTTAAGTGATTATGGGTTATGAGATGTCTCTGTGGGTAGAGGTGTTTGCCACTG<br>AGCCCGACAACCTGAGTTCAATCCACGGGGGTCGTGTAGTAGAATAGAAGAACCATTTTCCCCCACGTT<br>CCCTATGACTTCTGCACATGAGTTGTGACCATTTTCCCCCACGTTCCCTATAACTTCTGCACATGAGTTGT<br>GGTACCTGTGTGTGCACATCCACAAACACAGAGACATATACATATACCTTATGCACGTACATAAAATTGT<br>TTTCTAGTAAAAAAAAATCTTAAACCCAATTATATTGGGAGAGCTTGGAGAAAAACTTTAGGCACACTT<br>TGGCCTGTTATGCCAATGGGTAATGAAACCCCAAACACACTGCCGTTACAAGCTCGTGCCGTGAAAATT<br>GTCCTCCCTTGACTAGATGACTCTCTTTTACCAGCAGACACAGTCTGAGCTTAAAGGGTAAAATACCTA<br>GCACAAAATCTACTCCCCTGTGTGGCTGACACAGGCAGCCTGCCTACCCACAGAGGTCTGACACCTGG<br>CTCCCAAGTGGCAGCCCAGATGGACAGTGTCCAGCTGTCATGCTGGGGTGGACAGTTGGCCTCCAGCA<br>ATGAGACTATTATGCCAGGAAGGGTAACCATTTCAGCTCTTCTCCACAAATGTCAAATTTTTTTGGATCA<br>GTTCAGCAAGTACAACACAGGGTTCCTGGTCTGTGCCAAGCGGCAGGGTAAGGTCCTGTGAATAAAAT<br>ATCTCCCTGATAGAGAGGCAGTTAATGACCCAATAGAGTCATTGTGTTAGATGCTAGAGTGTTTAGGGCG<br>AACCGTGAAGGCAGACCGGGATGGGCACATGGTAGGAAATATGTGGCTGGAACCTTCCCCTCCAGTCA<br>CAGGCTTCTCAGAGTCCACTGCAGGATATTGTGGGTTCCTGGAAACATCATTCCCTCCACTCTTCCTAGG<br>AGTATACAAAGCCTGTGACTTTAACAGAAGGCTTGCAAACCAGCTGTGCTAGTACACGTTTATAACCCA<br>AGCACTCACACTCAGGATGCTGATGCAGGAAGATCATGCATTCAGGGTCAGCCTGGGCTACTTAGAAA<br>GATCTTTTTGAAACAGAAAACAACAAGAAGGCCTCCACTCTGGAAAGAAATGAGTGAAACCATGTTCG<br>CTGTGTTATTTTCATTAGAAGGCCTTTCACTTTATATGTTCTTATCATTGTTCTTCAGGAGCTAGGGCTGG<br>GGCCCAAGCTCTTGTGGCTTAGCTAGCCTTCCACGCTTGAGGCATATCCATGTTCCCTCACTGGGGGATT<br>CTAGGCAGGGGCTCTACCACTGAGCCACGCCCCCAGCCCCTCACTGGAGGATTCTAGGCAGGGACTCT<br>ACCACTGAGCCACGGCCCCAGCCCCTCCCTGGGGGATTCTAGGCAGGGACTCTATATCTGAGCCACACC<br>CCCAGCCCCTCACTGGGGGATTCTAGGCAGGGGCTCTACCACTGAGCCACGCCCCCAGCCCCTCACTG<br>GGGGATTCTAGGCAGGGACTCTATATCTGAGCCACACCCCCAGCCCCTCACTGGGGGATTCTAGGCAGG<br>GGCTCTACCACTGAGCCACGCCCCCAGCCCCTCACTGGGGGATTCTAGGCAGGGGCTCTACCACTGAG<br>CCACGTCCCCAGCCCCTCACTGGGGGATTCTAGGCAGGAGCTCTACCACTGAACCAAGTCCCCAGCCC<br>CTCACTGGGAGACTCTAGAAGGCATTAC
MSTR	--	0.000	0.398	0.000	5.500	up	CCAAATGAGGCCGCTCTGGCTCTGCAATCAGCCAAGGATTTCTCATACTCCGTGACCGTCATTATTGTGT<br>CTGGGACTTCTACAGCTGCTCGGGTTTTCTGCTTCTTTCTGAGTCAGCTCTGGCCACACATCGCCAAGT

G.280<br>04.1							GAGAGGTGCTAGGGGAACGCCTGAATAGCGCTGAGACAGGAGCCAGAGGAATACATGGGTTGGTGTC<br>CAGCATCCCGAGTTATTCAGGTCCCCAAGCAAGTGACTGAGGCATGTCTGTCAGCAGCTCAGAAAACG<br>AACGAGAGGTGGCAATCCTGCTGAAGTCAGATCACACGTGAACTTTACGCCCCTGGGTCACACACAGG<br>TCAAAGTTAGTTCTCAAATCATCGTGAGTGCCCACGGCTGACTCTGTCCAGCTGGTCTGGGATGCGTTT<br>GAAGTGGAACCTTCTGGTTTCTTTGGAAAGTCAGTTATGTCAAGTGATGTTTTGCTGGGCCCACAGGTG<br>AAAGGGTGTCTTGCTAAGGCAAACACATGAAAGACTGTTTTCCTGAAGCAGACACAGGCGAAAGGAT<br>GTTTGGATGTAGCAGACACGTGGAAGGACCCGGGATGAAGGAGTATAAGTCTGGCCCCACAGACAGTG<br>GGAGACGAGCACTGAGCATTGGTTTGCTCTGCCTCACTATCTTTTGCTTAACAACACCCATGTATTAGTT<br>CAACTTACATAGTGTTGTTGAGCTCAACTTGTGATAACGCCACCATTGAAAGAAACTCGCCAAGGACTG<br>CCTGTGAGGCTCCTGGGGAAGCTGCTTCCGTGGCCTCTCCTCAGGCTGGCGAGCCTCACATTTTCTTCA<br>GGATTGAACTACAGCTGCCGGTTTGAACCTGGTGTCTGCCCGCCCAGAGGACTGGTCTGCAGCAGCTG<br>AATCCTATTTGGTGTTTGCTACGGGACTGAACCGCTGCCAAAGAAGACCAAGCTCGCCTCAAAGAACT<br>ATTGCTGAACAGGTCCACTTCCCCTGTATCCTAATAGCTTTTCTCTTCCACCACCTCTGCTGGGTGGTAG<br>GCAAAGGAGAGGTTGAATCCTTATTAAAAGTAGGTTGCAAAACATTTATGCCTACATGCATTGATGCTGG<br>GTTTCTGTGTCTTGAAGAGAATTGTTCTTCCAGTGACTAAAGTGACATCAAGTGACAGGAGCCGGCTCA<br>TTGCTGGTTCCTGTTCCCTGTGCACTGGATTCTTTTTAAAAACTACTTATCTTTGTGAATTTCATATCAC<br>GAAG
MSTR<br>G.280<br>78.1	--	0.000	0.363	0.031	3.453	up	TGAGAGTGTATACCTGTGTCCCATGTGTGCGTACAGTGCTTGCCAGATGTGCGAGGATCCCCTGGAACT<br>GCAGATGCCTCCCATCGTGAGCCACCAATGTCCACCCCAGGAAGGGGATCTCCTTTGTGTTGTTCCTTG<br>CAACGCCCGTGCGCAGCAACTCTCTGCTCACCCTCCTCGCTCGCTCCGCTCTGAGTTTCATCTTTTGTAT<br>TTTCTGCTTGTCTGTCCTACATTAGGCTAGCGGGGAGCTTGTTTCTTTCTTATCAAAAGAAGGATTTC
MSTR<br>G.281<br>38.1	--	0.429	0.000	0.022	-<br>3.851	do<br>wn	ATCCATAGGTTATTTCTTCACCTTAATTGGTTTACAAATGATACATGCTGTCCTCAGCCACAAAGGTGACC<br>TCAATGCTTATCCTTATTCACACAGGTCCTCTGAAGTGATGAGTCTGTTTTTCATCATAATAATGTAATCC<br>CTGTGACAGGCTCAGGCTCAACGTCCTAGCCCTGGCATTCGCTGAGGACTTTTCGGAGGATGGGGTATT<br>ATTCAGCATTCTCTAGAGCAGCAGAACCAAGAGAATATGTATTATTACACAAGGGATTTAATGGGCTGGT<br>TTACACAATCAGAGAGTAGACAGTCTTGCGGGAGTAAGGTGGAGAGTGATGGACTAGACAGTTAAGGT<br>CCTCTTCTGGCCTCTGTGAACATGCACATGTAGACTCACTGTGTGCTCCTGCACTCATGTACCCATACTC<br>TGACCCTCTCAGCTTTCATTGCAAGCTCCCTGCCTCTTTTCCCACCTCTCCCTCTGTACAACTGTCCATC<br>TTCCCTCATCGCACCTTCGAATTCAAATTAAGGAGGACAACACCATGATATTCATGCTCTATAGTGTCCC<br>AGACATCCT
MSTR<br>G.282<br>49.1	ENSRNO<br>G0000002<br>1691;ENS<br>RNOG000<br>00021705	0.489	0.000	0.013	-<br>4.001	do<br>wn	CCAGGTAGGGAGATTGTGCTCGATATCAAAGGGAAGATTCCCGAAGATGTCTGAGAACTTCTCTGACAG<br>GAGAATGATATCTTCCCAGCCCTGGTCTGACAGCCAGGTGCACGGCTTCTTCCATTTGCTCTTCTCCAGA<br>GAAATGTTCCCTTTTAGAAAGAAGTCTAGCTCATCCTGGGGGACCCTGCCCTCTGCCTGCTCTATCTTGA<br>TGGTCATGTTGAAGGAGAAGAGTAGCTTGTGCTTCTCAAACAGCCCGGTGCAGCCATAGTTGTAGATGT<br>TGAAGGTCAACGTGTCCATGATGTTTTTCAGCCGCTTCAAAAGGATGGAGTCAGGTAGTGACTTCTTCA<br>GTGACAGCCCAAAGACCTCCAGGAAGGCGATGAGGGAATACTGGTACATGGAGTTGACCAGCGCCATC<br>TCGGACAGCACGAAGAACAGGATGGCGCCCCGTCTCGCGGCCGGCCGGTAGCCATCCCGCAGCCGATC<br>AATGTCCAGGGCTGTCTTTTCTGCCAGCTTCAGCTTCTCTGAAACCTCTGTGGCCTTGGACTTGGTCTCT<br>TCCAGGG
MSTR	ENSRNO	0.312	0.000	0.000	-	do	ACTGAGCTAAATCCCCAGCCCCACAGATTTGCTTTTTGATGTTTCAGTTGCGTTTGGTCAGCTGAGCTCC

G.28436.2	G00000001285;ENSRNOG00000001294				5.969	wn	AAAACTTATAAATGAAAAATGTCATAGTAACCCATAGACTTTAAATAGCTTCGTCATAGTATCTGACTTTAAATCCTATTTTATTGGTACTGCCGTTGAGCTTAAATTTGCCCTAGGTATGAATGTATAGAGAGAAAACAGTGTGCAATAAGAAGGCTGATTTGGCACTTCCTTTGGCCTCAAGGACCCACCAGGGGCCCCTCAGCTAGAATGTACAAAAAGGCACAGGAAAATAGAAGGTGTGGCTGCAGGATTAAACAGACCTAAAACACGTTAAGCATCAGTGAATACCCAGGCCTAATAACCAGGTGTTAAATATGCTGGTACTATGGAAGGTCAGCCAAAAGAGGCATCTAGAATATTCCTCCCTAAGCCCCTCCCGCTCTGGGGAGCGCTTTCTCAGTTTCCCTAAATAAATCTATGCTCATAATGCTAAGAAAACAAAACAAACCAGCCAATCATCTGGAACTGGCACAGTGGTTTATGCCTACTGTCCTGCTTGTTGGGAGCTGAAGCAAGACAAGAGGGTTAGGAGTTCAATGCTAGCCTGGGCGGTAATGAGATAAAAGTAGGTGGAGATGTGGTGAAGGGAAGAAGTAAAAAGAAAAATCAAAACACCAGGGATCCAGGGAAGACTAGAAGATGAAAAAAGTCAAGGAGGTTTCCTCAGAGGTGAGAATTGGTGGGCCTGGAGAATGGTCAAGAGAGTGACAGTGGGGACAGCTGAAGATAACACGCACAACGCACTCCTGGGAGGAAAGCATGACGTCTAGGTATCAGTGTGAGCAATGGAACCCACAACAGAAGTGCTTTGTACTGCTTGTTTAGCTAGTGAGCAGAACTACCCCAGATTTCCCGAAAACTACAATATTTCTAGATATCAACATTTACTACAAAGAGGAGAGCTTCACGGGACTTCCAGACACTCACTTTGTGTCTTAGTTTATTTCCTTTATGTCATGTGGAGGGCGCTGCAATCCGTTTTGGTTTAAGATCTCCAAGGGGCCAATTCAGAAATCAAAGGAAGAAAAGCCCTGTGGCTGAGCAATCTTAGGCCCGCCCAATGAAGAGCGACACCTGTGATTGGCTGAGCAATCTTAGGCCCGCCCAGTGAAGAGCGACACCCATGATCCCACCTCTCCTAGAAGCACACGTAGAATCTGACTTGGAGTGGAGCTGCGACTCCATGGGGAGTATGAAACCTCCAGAAGAGCATGCCCTGTGGTACAAGCTGGAGCCTATCCGGGATGAGGGAGAGGCTGCTGGAAACCAGATGGCGAAAGCTCTGTGCTGCAGCTCCTGTGATGAGCGGATCCTAGGCTCTTGTGGTCTTGGTTCTCCAACTGCCCCAGTTTCTTGACGGGCTGTTGATGAAGTGAGAGGCTCTCTGTGTGCAGAAGAACACGTGATCATCGTCTGGGGGCCTAACTGGAGTTGGGATGAGCAGTATAGACAGTCGACCACTTAATTTGTTTATTATATTAAGGGGAAGAGGGTATTTCCACACCAACACGAAGACCCAGCCTTTAAGGAGGTATCATCTAACCTTAAAAAAAAAATTATATGTGACAGTTTTTTATTGTGTGTGTGTGAAAAGATTTGATCATTCTAAAGAATTTAGCCAAAGAACAGAATATCTCAAAGGGATATTCTTGTTGGGAAAGGTTTTTATAAGTTTATATGATTTCCTGTGTTTAAAAAAGGAATCGGAGCTGGTGGTTCACAGTGCTTGATTTCTTTCCCTTGAGAGTCGGGAGGGTTTTCACTGGGACAATCGCAGTGCTGCCAGGGAGAGCCTTAGCCATCAAGAAGACCACTTGCCAGGGACTTGGCCGTGTCAAACAAATGTTCAGCTTCCATTCTGACACTTCTCGAAGAATTTGATTGAAACTATATATGTCTAGATAACACTCAGCAAAGTGGCCCTTCTTATATTTGTTTCAAGATATATTTGGAAACAAGGGAGATGACCTTGGGCTCAGCCACTTTTCCAATTTAGGACTTCTCCCTGGGTCTACTGAGCGATACTCCCCAACACTGCAAATTCGCCGAATGGAAAAACATTCTTGGTACTCGGTGCTGTAAGCCCTGGGGTGGAGGACATTTCCCCCTAGCAATATGCTCTGCCTTTAAAGATGGGCTATATTTACCCACAAACTCTATGTTCTAACCGTCTACTTCAAAATACAGCCAGGGAGCATTTGCTCTTCGTCAGAATACCTTTCTGTAATCTAAATTGAGCTGCCCTTGGTTTTTAAAATATTTTAGCTTCTAAGAAATTTAAAAAATAAAAAAACCAGCCAACCTTAACCCTTTGCATTTGCTTTCTGGCATTGGAAATCTGGTTTTGAAGCCAAGAGTTCATTGCAGCTTGATGTAAGGTCTAAAAGAAGATTTTGAAGGCATCAATGGCAGTCGAAGTCCTACTGGGCCAGCTCCCGACCTAATGCCCAAGTTAGTTTCCAGTATCTGCAGGAGGCCGTGGAGGTGGATGGCTGACCCTCTCTCCACACTGATAGCCTCTCAGATGCTCTTCTGCATATTTTGAAGGCAATTCCATTTTTCATTGTTGCTATGGCCTTTAAAACCTTGGTTTGGATTTGCTTATTTTATTTTACAGGTCTCGGTGTTTGGCCTGCTAGCATGTCTGGTGCCCACAGTGGTTAGATGAGAAGATGGTTGCAGTTCCTGGTCCTAGGAATTGTAAGCCACCATGTGAGTGCTGGGAACTGAACCCAGGACCTCTGGAAGAGCAGTTAGTGCTCTTAACCGCTGAGCCATCTCTCCAGCCCCTAATCATGGTTCTAATTTGAGACACAGATGTCCTCCTTG

							ATAAGATGTATTTACTTTTTTTTTTTTTTTGGTTCTTTTTTTTTTTTTTCGGAGCTGGGGACCGAACCCAGGGCCTTGCGCTTCCTAGGCAAGCGCTCTACCACTGAGCTAAATCCCCAACCCCAGATGTATTTACTTTTAAGCAACTAAGAATTACAGTCTCATTATTGTTTTATCTTACTTAGCATATCATCCAGGATAGTGTTATCTGTTTTCGTATGGTTTCCTAGTGTGTTACAGGGTGACCAGAGCATTTACAGAGGTGGCTATGTTGTGAGCAACCGTCTCCAGCACACATAGCATATTAATTTCCACTAACATGCTGTGCACACCCATACCGCATGTGAAAATAAAGACGCCATATTATAATATTTTAAGGATAGCTTGTGCTCAGGAATTCCCTGTTGTTTGCTCTGGAATCAAGTTTTACGTCAGTTGTTAGCCACAAGGTAAATGGTCTTGACTGGAAATAAAATCAACACAAAAATTCATACCTATGATCCTTTGGAAGATGACCCTCCCCATCTGATGACAGGCTCATCCACAGCGTTTAATTATTACGGACTGCCACATCAGCCTTACTTTGTAATTTCCTAACTTGAGAGCTGAAAACGTTTCTCGTTTAACCTTTCCTCCTTTGCAGAAGCAAGCAGTGCATGGCGGCCGCCCTGGGCATGGCGGCTGCCCTGTGTATCTTTCCATTAGTGCATGGATGTTGAACCAGGGCAGTGGCTGAAGTCACTCCACTGACCCAATTAAGCAAATAAGAGTTTGTGCTTGTGCTGCCTGGGCTGATAGATTTGCACAGGATAAGCAGTAAAGTTTCCTCTTCCTGCTTCGAGA
MSTRG.28588.1	ENSRNOG00000001374;ENSRNOG00000001377;ENSRNOG00000001378;ENSRNOG00000001383;ENSRNOG00000037720;ENSRNOG00000056407;ENSRNOG00000059344	0.384	0.000	0.000	-6.521	down	GTTTTAGGGGACACCCCTTCAGGAGAATATCCGTCACCTGCAAAAGGGAGTGAATGGGGTAGGGTGGCTTGCTTGTGTCTGCCAAGAGGCGGGCCCATTTCTAAATGGCAACCTTGGCTGTCTTTGGGCTCTTCGATCTCTCCAGAGTGCCTGGGCTGGGGGCGGGATGCCAGGAAGTTTCTGATCCTGCAGTCCCATGCCAGGAAGTTTCTGACAGCAACCCTGTTCTGTTCCAGAAGGAGGCCTTCAGCTCGGGGCCACCCGTGAGGCCACCGGGTCTTGCTGAGGTCTGTAGCTGATTCTCGCTTCAGAACAGAAACCTACGTCCCTCGCCACAGAGCACTGTGTTTTCTGCTTAGGGCACTCGTGCCTCAGTTTCCCCTCTCACACCATTACAGGGTGCTTTCTGAAAGCTCCGAGAAGCAGAGGCTATGTCCCCCATAGCTGGCTGAAAGGCAGGAGCTCTCTAAGGACAGGTGTTGCCTTAGTGGTGGTTTCTATTGCTATGGTAAAACACCATAACCTGAAGCAACTTGGGGTGGAAAGGCTTCTTCTTAGATTTCTAGGTAGCACCCATAACAGAAGGAACTTAAGACAGGAACGTGGAGGCAGGAACTGATGCAGGGGCTATGGAGGGGTGTTGCTTACTGGCTTGCTCCCCATGGCTTGCTCAACCTGCTTTCCTAAAGAACCCAGGACCACCAGCCCAGGGATGGCCCCACCCATAATGATCTAGGCCCTCCCATATCAATAATCAATTAAGAAGATATACTGCAGACTTCCTACAGGGCAGTCTGATAGGCAGCATTTTCTCAAGTGAGATTCCCTCTTCTAAAAGAACTCTGGTTTATCTCCAGTTGATATAGGAAACCCGCAGACCTCTGTCCAGGCGTAGGTATTTCAGGCTCTGATTTTCACATTGTCCTGAGGTTCTCTACTGTCTGAGCTGTCAGCATGAACCACACCCAACCTAGCAATTTCCTCCTGTCCTAAAACTGTTTGGGGACTGTCCCCTCCCTACATGGTGGACTCAAGCGGTTTTGCTCAGTGCCTTCTTTCCTGCCTGATCCTGTTCCTGGGGATAGCCAATGACGTCAGCCAGCCTCCAGTCCAACAAGTCTCCAGTGCTGTGTAGAGCACGGGCTTTGGTCCGGCAGTCGGTTTGGAAAACATTCCTGAGCCTTCTGTTTGCTGGAGGATTCCAGTTTGAGCTGAGAATTCTTCCTGTTTACACAGTCCCTTGGGATCTGTAAGCCTCCCTTTAGAGAGTTAATGAATCACGTCCCTGCTGTCTCGAGAGCCGCCTGTAGTAATGATTCAGCCATCTGGTCACCACCGGGCCCTGACTTCCATTCATCTTTGGACCTTAGCTTAGTGCCTACAGGTGCAGGGACTTCTCAGGCCATGGGGAAAAGGAACTGAGCTGAGTTTCTGGGGCCTCGAAGCAGGTAGGTGTGGCTGACAAATGGCCCTTGTCAGACGTATGTGACAACCTGCCTTGCCTAGAGCCAGTGGCTAGAGACTCATCTCTCCCAATAGATACGTCATGGTCCTGGAGGCCCCAGGCCTCAGTCTGTCATGGCTGCAAACCCTGAAGACCTTCATTTTACCCCAGTTACCTTAAAGATACCATCTTGAACCCCAAGTGCTATATTCTGAGGCTCTGGGAGTCAACACTTCAAACACAGGGATTTACTTCATTTACATTTACTTCATTTGATTTCGTGCGCGTGCGCGTGCGCGCGCGCGCTTGCATGCCTGTGTGCGTGTGTATAGACTGTGACACATGTGTGGAGGCCAGAGGATGACCTGCAGGACTCTGTTCTGTCCACTGTGTGCATCCCAGGAATCAAACACTGGTCACTGGACTTGGTGGCCAGGGCCTTTACCCACTGGGTCATTACCACAGCCCGGGAATGTTTTTCAT

							TGTTTTGTTAATGGGGAGAGAGGTATTTGAGCCTGGGTCTGTCTGGGTTGCCAAGGCTGACTCCGGGAACTCCCTGTCTCCCTGGAGTTCCAGGGAGTGGACCACCACACCTCTCTCTCGTGGTCCTTGCGGCCCACAGTGCTTGTAACCTTTCCCCCAGTGGCCCCTGACTTCGTGTGAGGTCTTCAGTTCAGGAAATGCAGGGTTTGGATCTGGTGACGTGCGGACTTTACACAAATCTTTGGCTCCAAGGGCGGCTGGGGGGATGTTAGTGAGTGACTCACAGGCAGAAGGGTGTCAGGCTGATGACAAAGGAGGTAGTTAGGGGTGAGGTATTTAAAACTTGGAAACCAAGCAAGAACTCAGGGAAGGGTCCTGGGGCTGACAGGTAGCAGAGTGGCTAGAGGCCACCAGTAATCTGCATAATCTGCATGTGTTGGGGTAGCGATCTCTGCTGCGTGTGCACACTCCAGTCTATACCATGGATGTGCCAGGCAGCTCCTGGCAATTCACACTGGGACTTGCCCCTTCACACTAGGATTTGCCCCTCCCCCCCTTTTAGCCAACACATGCTTTTGCTGTACCATGGTCTTCTGGCTGCTTTGCTATGACAGTGTTGTAGTTTTGAGGTGACAAGGTGGTCAGGGATAGGGCCTAAGGGGACAGAGCAGAGAGTCTTGACACTCAGGCCTTGGACACTGTGCCATATCTATAGGGAAGGCTGCTGCCCCTACACAGACCTCTGGGTGACACTCAGGCTCCTTCTCTAGAAATCTCATATAGGCTGAGCCAGCTGGTGCCAGGTACCATCTAGATGAGCAGAGCTGCTGACCTCAAGCAGACACGGCCAAGAGGAGCTGGGATGGAGCCCCAGGACTGTTCTGGAAGGGCAGTCAGCTCAACGGTCCCTCTGTGCAGCAGTCCGACTGTATAGCTCTTTCTTAGGCCAAACATCCCAGCATGCTTGTCAAAGAGGCAAGAACCTGCACCCCCCACCCCCAGGGTCCTCCAAGGCTTTTGTCACGTGTAACCTGTGCGGTGGGGTGGCTGTGTGGATTCTGGAGCCAAGCCCCAAGCCTCTGTTCCTAGCTGAAGACATAACTAGCTTCTACAAGTTATAGCATCCCAGCAGTAGAGCAGGGTTCTTACAGTTGCCCAGCCAGGGGGGCAAGGACTTGGTGAAATGTTGCAGATCACATGGTCTGTGCATGACACTATGCACAGCTATGACATAAGTAGGTGATAAGGCCGCAGACTGGGCTCTTCCTTGAGACCTGAACCCTCACTCCCATCCCAGCAGGTGCCTACCAGCAGGCAGGGACACTGAGACAGTGTAGCGGTTGTATCTCTTCACTCTGGTCCCAGCAGGTGCCTATCACCACACAGGGACACTGAGACAATGTAGCGGTTGGAATCCAAGCACAATTAAGTTTCCACAACCTCCCACTTACCCTGGCCACCTGGCCCTGCCAGAGTCATTCCTCTGACTTTTATAGGGCTTTACCAAGGCAGAATGCCGGATGTCACCAGATTGTTGGTGGTACCGATTAAAATGAGGGAGCACCCAGCATGCTGAGACGGATCCTCATAATGGAGACGCAAAGCCTTACGCTTCCTGGCCCTCTGCATCTTCATGGGGGGATCCTGTCTGTTGCCTCGGGGCCCCCTGCCACGAAATGCCATCTCTTCCTCTGCATGGAGAAAGCTACTGCATCTAAATGGCAGGCGCAGCAGCTGGGTGCGGGCCGGCACCTGCTGCCCTGTTCCGCCGATCACCCGAAGGAGGTCTGAGATCATACATGCTCTGGAAGTATTTTATGAGCCAGAACAGTTGACTTCCCAGCCTCCAAGTCAGGGAGGGGAGGGACATGGGAGCAGCTGTAGGATTTACTGTGCTGTTGATTTGGTGGTTTCTGGCCCCAGTGTCTCCTGGGACTGTCTTGAGTAACAGCGGGAGGCCCTAGGAGGAAACCCCATGCATACATACCCTTACAGAAAGGAACCAAGACTGAAGTCAGAAGCACTGGGGTCCTTCATGGTCGGTGATCTGAGGGGTAGGTAACTCTTCCTTCTGGCTGAGTGGATTCCCTGATGGGGGCATTCTCTGGCTCAAACTCCTCTCCCCGCTGCCCAGAGTGAGAAAGTTCACAGCGGCCTGTGGGGCGGCTCCCCCAAGTTGCTTTCTGGCTTCGGTTTTCCAAACTGAAGTATGTGCCGATCCACGGTCCCGTGTGGCATCCAAGGCGGCGTTCTTCATTGTCAGAATGGCCACCCTGGTGGTGTCTTGTTGTAAAATGTCTGTGGGATCGCCCGGTGGCCTGGACAGAGGGCAAGGGAAAACCCCAGCTTCCTGTCGTGAGCCAAGACTTCCTCCTCCATGCACCCTCCCCCACCTGCCTTTAGCACCATCCCTTTACTTGAACCTCTGGGTTCGTTTTGGCTACAAAAATACGATGTGTCAGGAATCAAGATTAAGGGCCAGGAAAGGAAGGACCAGGCAGGATGCGCTTGGAGTGGGGAGATAACCGCAGGCCATGTGGATCCCAGATGTTGGATCCCTGTGGCAGGCGAGC
MSTRG.286	--	0.496	0.000	0.037	-3.684	down	GGATGGAAGGAGGATGTAACACAGCTCCACCCTCAAGTATGCCACCCCAGGTCACAAGTGCTCTAGATCTATGTCTGACTGGAGGGGACAGCAGAGGGACCATGTGGTTTGTGGCAGTTTGGATGAGAACGACCCCAGAGGCTTACATACTCGAATGTTTGGTCCCCAATTTTGGAACTATCTGGACAAGATTAGGAGGTGTGGC

13.1							CTTGCTGGAGGAGGTGTGGCCTTGCTAGAGGAGGTGTGGCCTTGCTGGAGGAGGTGTGGCCTTGCTGGAGGAGGTGTGGCCTTGCTGGAGGAGGTGCATCATGGAGGTTTCAAAAGTCCACACCATTTCCAGTTAGCTCTCTTTCTGCTTACTGATGGAGATGTGTTCTCTTAGCTGCTGTCTACTGGTGAGGTCCCCATCCTGGTGGTCATGGGCCCACCCTCTAAAACTGTAGGCCCCCAAC
MSTRG.28806.2	ENSRNOG00000001151;ENSRNOG00000001153;ENSRNOG00000001156;ENSRNOG00000001160;ENSRNOG00000034226	0.000	0.911	0.000	6.273	up	GGCTCCTCCCCCCGCCGCCCTGCGCGCATAAAGCCCGGCGCGCGCCCGGGCGCCCGCGGAGGCGGCGGCGGCGCCGGGACCCCCCCTCCCTCGGGGTTCCTGTGTGTCCAGGGGGCGAGCGAGGTCCCCTGATTCCTGGTACGGGGCTCGGGCCTGGGGCGCGGCCGCACCCCTCACCCACGGCGGCGCGGGGGCCCAGCCGCTGGGAACGCCCTCCCGGGATGAAGCGCTTCCCGTTTCTGGACCCCACCAGGAATTAATGAGCTGGGATAGTAAGAGCAGGTCACCGGGAGTCAGATGACCCCGTAAGGTTGGTCTCAGGATCCCGGCTGCCTTGGATGGGTCTAGAACTTCACGAGGAAGCCTCCTAAGCTCGGTGTCTTCCCAGGGTTGCTTTGGTGCTGAGATCGTGTCTTTGGAAGCACCCCGAATCTCTGAAGTGTGTGGGAAGAGAAGGGAGCTCTCTGTGCATTCCCTGGTCCAGTTAATCTGGTCCAGTTCCAATTCAGTTACTATCAAGACAGAAGTGGACAGCTCCCAGGAGTCCCAGCATGTACAAGTAGCCCTCTGGGTTCAATCCCCAGCACCGAGGGAAAAGAAGAGAAAGAGAAAGGGACCATGTGTAGCATTATCCTACCTCGAGATCTACAGATCAATAGGAGAGAGCTGACAATTTAGCAGTAAGCTCTTGTGGGCTGCACAGAGGGCTCATCCATTAAAGGCACTCGCTGCTCTTCCAGAGGACCTGAATTCGATTCCCAGCACGACGCACATCTAGAAGCTTATAACCACCTGTAACTTCAGCTCCAGGGATCTATCTGACTTCCCCGGTCTCTGAGGCCACCTCCACTCACATGTACGCATGACACAACACACACACACACACACACACACACACACAGATTAAACACACAGATTAAAATAAATATTGTTTAAAAG
MSTRG.28850.2	ENSRNOG00000001183;ENSRNOG00000001185;ENSRNOG00000021364;ENSRNOG00000037566	1.499	0.075	0.021	-2.808	down	AAAAAAAAAAAAGAAAAAAAGAAAGAAAAAAAAAAAAAAGAAAGAAAGAAAGAAAGAAAATACCCCAGGCTGTGGACAAGACTGTAGGGGATTTCCTTAATTAGAGACTGAGGGGCCTCGCCCACTTGGGTGGTCCCGCCCACCGTGGGTGGAGCCACCCTGGGCTGGCGGTCCTGGATTCTATAAGAAAGCAGGCTGAGCAAGCCATGGGGAGCAAGCCGGCTTAAGATGGTAGCATCAGGGTCGATGGCAAAAGGCTTGTTCTGGCCACCGGGATACCTCACAAAGACCTGGATCTCAGGAGGAAAGGTCTCCATCACCCGGATGTTCACCTTAGAGAAAATTCCATAATCCGACAGGGTTTTCCGG
MSTRG.28885.2	ENSRNOG00000001198;ENSRNOG00000049426;	0.023	0.318	0.005	3.468	up	GTAAAATTTATTTTTGTAAAATTTGACTTTTTACAGTGATTCTATTCTATGTGTGTGCTGATGACTGGGACACCGGACTCCCCCACTTTCGAGTGGCCTCTGTGATCCTAGTAGGGAAACTGAGGCAGAGTGGCGAGTTCAGAGCCAGCCTGAGCAGCAGAACAGACTCAGATCTCCAGTCTTTCATGGTTTCTCACAGCCCCTCCAGCTGGCAGGGAGGCCACCATCTTGATCTGTTCCCCAACCTGTCATGAAGTGCTACGTACGCACCACGAATGGCTGGCTGCATCTTTGCAGTGAACAAGGAAATGTGATTTGCAACCAGAACTTGAGAAGTCAAGCTGCCGCCACTCTGCACGGGCCTGGATAGGGAGGCCCCCTTCAGGTGGGGCCCTATACAGTCTCCAGTAGACCTTACGTGCTCTGACTGGGTGGGCCCCTCACAAGCACCATGTCTTCTGGGCATGTGTCACAATTCTGT

	ENSRNOG00000049604						TCATACACAGGAAGGCAGAATGATGGCAGAAATCTCCTTCACAACAGCTCTACATACAACAGGTGAATTTTACCTATAGACTCTTGGTTTAACCCCTTTGGGGTCTCCATGGCTCCTGCTTTTTGTGCAAATGCTCCCCCAAGA
MSTRG.28960.1	ENSRNOG00000049016;ENSRNOG00000049451	0.327	0.000	0.037	-3.684	down	AAGCAACCGGCCTGCTCCGCTTGGCACCTTCAGCTGGCCCTTTGCAGAGCATCTTCCCAGCTGAGCACGTCACACCCTCTGCACACCTGAGTGTCCCTTAGTAACCAGCAACTCTGCCCACGCATGGCCTCAAGGAGAAGCTACAGTCTATGTACACAGGCCTGAGAACATCAGAATGGGTTGGGGGGCTCCATCTATGTGGCTGCAATAAAGCCCTCACCTCAGCTAGGTCACTTTGGAACAATCACATAGCACACCCTGGCTCACCATGAGACTGTACACTAAGTAAAGTCATTTCAACAGTGTAGAAGCAGACTTCACACCATGGGGGATATTCCAGGAGGTGTCTTCTGTGGCATGCCAGGCCTGGCAACAAAGACTGGATATGTTTTCCAATTAGACTCTTAACTCAATCACCTGGTAAGTTTGGCTCACCATCCTTGCTTTAACTGATACTGGATTCCTGACACTGGCTGGCAGCTGGACGCTGACAGGCCTTTCTCAGGGAGTACAAACTGTAGCATTGCTTACAGTCTGTGTGTGTGTGTGTGTGTGTGTGTGTGTGTGTGTGTGTGTGTGTGTGTGTGTGTGAGAGAGAGAGAGAGAGAGAGAGAGAGAGAGAGAGAGAGAGAAGAGAGATCCATGCTGCTACCCTATGGGTCCTAT
MSTRG.28970.3	ENSRNOG00000051192;ENSRNOG00000058886	0.000	0.314	0.000	7.759	up	AAGAAACAAGTGGGGGATGGTGGGAAGCTTGAGGAATGTGGCAAGTGAAAAGGGGTGTGTGTCACCCAAACCAATGGCTTGTGAACTGCACAGTCTCCTGGGGGCACCTGCCTTTATCTTTGCCAGTGTCTGGGTGTCCATTTTTGTCACAATAGGGATGAAGATGACTCAGTGAGCAGAATAAGCTGTGACAGACATACACCCTGTCACCTGACATCCACCGTGCCTCCCTCCTGTGCTTCTCTGGGGTTGGGTCAGTGTCACATCCCTGTTTCCCCCAGCTAAAGCCCCTCCTCCCCCAGCTCTAAGCGGTTTCCTCCCTGGCCCGCCTTCCCTCCCTGCCACCACCCCTTAATTTTGTTTTGGTTCCCAGAGCAAGCAGGGACTGGCTTCCCGTTGTCATGACAACAGCCATTAAAAAGAGCCAGCCACGTGATCCAGGTTGATAACGGAAGCTAAGTGACTAACCGCCTTGTTTTCCTGCCTGTGGCTGCTGCCAGGAGGCCTCTTAATTGGAGAGGAGAAAGCAGGGGCTGGTGGGCAGCAGCTGAGACCCCTGGAGCGTGTCAGTGCCAGGAGACAGATCCTGCAGAGGAAGCCTTGGCTAGCTCCGGGTGGAGAGGGGGTGTGTGTGGGGGCTGAAGGGGGGACTGTCCAGCAGAGCCACCCTCTCTGTCTAGCGGAGCCCGCCCCATCCCTTCGGCCTCAGCTGAAGTGGAAGACTGGATGAAGGGGGATGTGCTAAGGAGGTTGAAGGAACATCAGGGAACTGAGCCGCGTTGCCATGGAGACGCTCGGGCTTTACTCTAAGTCCGCAGCAGTTCTTAATGTAGGACTGAGGACAGCTTGGCCACCTCCAAAGAGTTGCCAGATAGCACGCAGGTCACCGCGCAAGGCTTGAATCAGACGCGCGGGGGCGGGGGTGTGGGTTAATAGGTTCTTAGGGTACTTGTGCAGCTCTGCCCCAGGGAACCTTCGGTGTGTCTGGGAACATTTTGGTTTGTGACAGCTGTGGGTGCTCCCAGGGTCTGCAGGAAGAGGCCAGGGGTGCCACCACTTAACACCCTGCGGTATAGGAGACACCCCCACATGCCAACATGATTGCCCCATAGCACGCAGGTCACCGCGCAAGGCTTGAATCAGACGCGCGGGGGCGGGGGTGTGGGTTAATAGGTTCTTAGGGTACTTGTGCAGCTCTGCCCCAGGGAACCTTCGGTGTGTCTGGGAACATTTTGGTTTGTGACAGCTGTGGGTGCTCCCAGGGTCTGCAGGAAGAGGCCAGGGGTGCCACCACTTAACACCCTGCGGTATAGGAGACACCCCCACATGCCAACATGATTGCCCCACCCACCACCCCTCACCACCACCACCACCACCACCACCATCACCATCAACACAGCTCTGACCACTGTGATCGACATCAGTATCACCATCATCACCACGGCCCTCATTACCTCCACGGTCACCCCGCTCAACCACCACTACTACCATCACCTCTCTCCCATCACTCCACCGTAACCCGTCCATCAACAGAGACTCCTGCCTGGAACACACAGCAGCAGGCTTAGCTAGGATCTTCAGAACTCCGTGGAAGCACAGTCTGGGGCAGATGTGTGTAAAGGAGGGAGATGAGGGGAGTGCGTGGTTGCCAGTGTGCCCTGTGCCGGCTGCCTACCCGCCCCAGACAGGTAACAAGACCATGCTACAGAGAACACAAGAGCGGGGAGCGCGGGCTCCAAGACTGTTGCACCTGCTCACCATCAACATGGATCCTCCCAGCTCCTAGAAAAGGACTTCTGTTCCTCTCTTCCCCCGCCTTCCCTTTCTTCCATCCACGAATGTT

							TACTGAGCATCCCTGGGGTGTAACCTTGTTCAGGAAGCGGCAGCTTGGGAAATGACGCTCTTGCTGTAC AGTCTAGCAGGGGCACGGATGAGAAGGTGAATCGTGGGGTAAATCGCTTCCTGTTGGTCTGGGTTTAA ATCCCCAGCGCTCGGGTGAGACCCGACAAATGTGTAAATACAGCCCGGGGAGGGCAGAGACAGGATG ATCCCCAGAGCTCTTGGACAGACATTGTAATCCATCAGTGGACTTCAGATCCTGTGTCAAAGAAGAAGA TGGAAAATGACTGAGGAAGATTCCAGACATCAACTTCTGACCTCCATGTGTACATGGACACATACATAC ATACACATACAATATATATATATATACATATACACAATAGATACATGTGCATATATGCATGTGTATATACATGT ATGTGTACATACATACACATACACAATATAAACATATGTATACACACGTGTATGTGTATATACACATATGTA TATATGTATGCATATATATACACGTGTGTATGTGTACAAATACATATATAGGTACAATGTAAACGTGTGTATA TACCTATATATGTGTGTATACATGTGCAAATATACATACACATACATGTTCACATTATAGACATTAGTGTGT AATATACACACGTATGTATATATATGTGTGTATAAATGTGTACCTGTGTCTGCATATACATACACATACATAT ACTCAAGATAATTTTGTGTGTATATATACGTATGTTATATACACATGTGTAAATGTGTGCATATACATACATG TATACATATATAACCATAAACAAATATGGATGGTGCTCAGTGTTATAAGACAAGGCAGGGGAGAGACAG AAGAGAGGGCTGGCCAGAGAGGTCGGGGCCAGCCTCTGTGATGGGGCGATCTTTGGGCAGAGACAGG GGTCGCTATTGATGTCTTCCATCAAGCCTCCAGCATGTACGAGTCCTTGCGTTTGGAGGTCAGTGGGGC TGGAGCAGAGAGAACAAGAGGGAAGATGAACTCCAGAGGAAGACCTGGGAGGCTCATCATGCCTGTG ACCAAGTGAGACTTTCGTTTTAGTCTGAGCTGAGAGCCATGGCGGAGTTTTGGGCCAGAGAGGAAGAT GATGGTATGAGAGAGTAGTGTGGGGCAAAGGGCAAGGACTCTATATGGGAGAGGAAGCTCAGACTCAC CTCCACTCCCCTCCCCCACCTTTCTTTCCTTCTTTCTTGAGAGATGGTCTCACTGTGTAGCCCTAGCTGT CCTGGGACTCACGCTGTGGACCAGGCTGGCCTCAAACTCAAAGAGATCCACCTGCCTTTGCCTCCCAA GTGCTGGGGCTCACAGCATGCACCACTGTGTTCAGCTTTCTGTCTCATTCTTTGCGAGCTCCTGTTCATG AGCTGGGAGCTGCTGTTGCTCCTGTTGTGTGGAGTAGAGGCTCTGAAACTGCCATTGAGTATGTGTGAA TGTGATGTGTGTGTGTGTGTGCATGCCCATGTGAGCATGCATGTATGCCCTGTCTGTGGTCTTTGGAGAG CAGAACTCCTCATGGTTAATACAGTCTCATTAGCCCACTGCCACACCTGATGTGTTTTCAAAGTTCTGAC AAGGTGACTTTCTGGGAGCTGTGGGTAGCACTAGGCATGCAAGGCTGCTCCCGCACCTCCTACACCTGT CCCCCTGCAGGCCTTCCCCTGCAGCAGCTCAGGAGGGGCTCCAGGAGCTGCTAAGAGTATTTGATGCA GTTTAACAGAAAGATCAGGACTGCTTTGTTCCCAGTGCCAGCATTTGCTGGCTACAGAACCCAGGAAA AGGGGGTGGGAGGCTGGAAGGCGAAAATGAATTTCTTAGTTCAGGCGTTTGGGGAGTTTGGAGCAATC GGATTCAGGTATGGTCATATTTAAACGTCCATGCTACCATCAGACCTTGAGTCTGCCTGGTGACATTCGA GGAAGCCTCTGCTGCTGCTCTGTGAGGAGCCGGTTTGCCCCATCTCCTCCTCTTCCCACCTCCCAGGTG AGTGAGTTGGCCAGCCCAAGACCTCCGAGCATCCCAGTTGGCCGGCACCCTGCTCAGAAAGCCCACTC GTGCCTGTTCACGCGGCTCTGGATGGGTCAAGTCTGTGTTTACGATGTAATAAAGACTGCCGACTAGTC TTCCCGGGAACAAGCCACAGCCACTCAGGCAGGGCTGGGCTGAGCCGTTGGATGACATGTGGCATGGA CCTGGCTGACCTCTCTTGGCCCTCCCCAGGCCCCCTGAGGCTGATGAAACACCATGTCTTATGACAGCC ATTAAGGAGGCACGGGATGGCAGGTCACTCACCTTAGCTCATAGAGATGCCCATGCTACAGCCAAGCCC CACTTCCAACTCTGTTCTGCTTTTTATGGGAGCCCAAACCAACAATGCATGAGTCCTTATGAAGCACGA CCTTGGGGAGTGTGTGACCCTCCCCGATGATAGACCCTCGACAGCCTTCTCTCCTTTGTAGTGACACCT CTCTGTCCCTGCTGTCCTGAAGCCTGCCACCCTCCTCTCTTAGGAGTCGAGTCGGAATAGCTGAGCCTT CAGCAGGCTATGTCCCTGTCCTGGGGTAGAGAGCAGGGTCTTTCCTCCAGCTCCATTTTCTCAGTCAGC CCACCGGAGAGGAAGCCCAAGGAAGCCAGACTCACTGGGCATCACCCTCTGGCTGCTGTGTCCCCAG GCCCTGGATCCTAAGAGTTCTGAATGCTGACTGTTATTTCTTTCAATAGTTGGGGAGCTTGGAGAATGGA CTCTGGTTCCTTCTTCACAGGGACAGACTGTGAGCAGGGGCAGATGGATCTCTTGTGAGCTCAGGGCA

							GGCGATGATGGGTGTAGTGAGATCCAGGACATTCAGAGCCGTGTAGAGAGATCCCATCTCAAACACAA<br>AATAGCCAGACGAGACATAGTAAAAATGAACCATATGGAGAACCAGGCATGGATGGCTGCCTGCGATCC<br>CAGCACTAGGGAGGTGGAGGCAGGAGGATCAGGAGTTCAAAATCATCTGCAGTCGCATAGGGAGTTTG<br>AAGCCAGCCCGGACTCCATGATTTGTAATTAGTTAATTATTTAATTCAGGTCTCTGGTAATTTAGAGACCT<br>GATGCTACTCCGGCATTGAATGCATTCCCTTCTGGTTATTCTTGTTTAGTAAATTATGCTTGATTAGTAAA<br>ACTGTAGCAGCCTCCCTTTACTGAACACGAATGCTAACTGCTTTTCCGTTTCTCATTTAATAACAAAGCA<br>GTCCTCCAGTGGACCAGGCAGCTGGTCCCCTCCCGACAAGGAGACTGAAGGCCAGAGAGGTTAATAA<br>ACACAGAGAAGCTCACACAGTCAAAAAGCAGCAGAGCACGGTCTGTCTACTGTTCGTTTCAGCTTCAA<br>TCAGTCCTAATTAAAAACAGCCAGAAAACTAAGTCTACAAAGCGAAGGGGCTAATAGATGTCTCAGTTC<br>AAGTGTCTGGGAGGTTTGGGGTGGGCGGGTTTAGGGACAGCTACATCCCGCGGTTCCGGTGATGTCCT<br>CAGGCCCCTTCCATCGCTGTGGGTTGCATTTCGCCGCGTTCTTTGGCTCTGTAACGTCCTCAGTCACAGC<br>ACTGACGTCACAGGAAATTGGCTTTGGGTCTGGTCCGGCCTAAATGACGCGATCCTTTTGTGCCCACCA<br>CAGTGGGGCAGGTCGCTGTGATTGGTCAAGCCTGCCTGCGCCTGCATCAGGAGGTTGGAGGTCAGGGC<br>AGCCGAGCTCCAGGGAGGCGGACAGACTTCCATTACTGTGCTCACTAAGTAAACGGGAGGGAAAGGG<br>CTCTGTGCCCGGTTCCGAGGACGCAAACCTATCACGAGGCCACGCCCCTGACCTCATCACCTCCCAGA<br>AGCCCCGCCTCCTGCGCCATCATTTGGGGCCTCACGTTTTCAGCCTAGAGATTCTCAGGGGCTGTCAGT<br>CACTCCGGCCGGACAGCGTGACATAAGCTCGAAGACGGTGTGGTTTCCAGTTCTGTAAGGGCCCTTGA<br>AGGGCCTAATTCCAACCTGTGCCAGTGCGCCTGCGTGACTTAGCCACAAGCCCCTACTTTCTGGTCCCC<br>GGTTAAATTTAAAACGCAGCACCAGGGGCTGCAATCCTAGCGCACCTGCAGGAGGTGGGAGGCAGAAT<br>CAGGAAGATCCTCGAACTCGCAGCACAGCTGACATGGCATGAGAAACAGAAAAACAAAGGGCGGAGG<br>AGAGGGCTGGCACCTGAGGATGTCCTCTCACCTCCACACATGCGCAGTGGCGGACACACGTGAACGCG<br>TGCGCGCACGTGAAAATAAGACTATTCCTCACTAATCCCAGCTCCTTTGCAAGGACTCGGTGGAACCAC<br>AGAGCTATTGCTACCGAATGGGAGGACGGGTCTACAGTGCATTTCTAGAAAGTTCTATCACATTTTAGAT<br>AATCTATCAAGTACTTGATATACATTTGCATGTGCAGTACAAACAAATCTCAGCCATCTGAGGGGCGGGC<br>CTCTGTGTTACCTCATTTTGGAGACGTTTAAATGGAGGCACAGAGAAGTGAAGTGAATTGTCCGAGGAC<br>ACACAGCAGGTAGCAGGCGGGCGTCGGGATCTAAAACCATCCCTTAAAAACAACATTCTCACCGCTTT<br>CCATTTTAAGATCTGGGAGGGTAAAGTCAGTTTCTGATTTATCTGAGCGTTGTTTTTGTTTGTTCGTTTGC<br>TTTTTGAGACAGGGTTTCTCTGTGTGGCCCTGGCTGTCCTAGAACTTGCTTTGTAGGCCAGACTAGCCT<br>CGGACTCGCGGTGATCCTCCTGCCTCTGCCTCACCCCACCCCTGTACCCCACGTGCCGAGCGCTGGGAT<br>TAAAGGTGTGTGTCACCACGCCCGGCACTTATTTGAGTGTTTTAAGCAGCTCCCCAAGGCTTTAGTTTTT<br>CACATCCTCCCAAGTTTAGACGTTCTTGGGGAGAGGAGGCATCTGTGCTGGCTTGCTCATCTCCAGGGC<br>TGTGCGTCTGGAGATGGGGCGTTGCCAGGGAGGTCTGTTGGCCGTGCGGTGGGCAGCGCACCTGTGAG<br>GAGGCTGGCGGCTCTCCGGTGTGGGCGGAATGTGAAGCACGCTCGGGGCTCTTTTTTTTTTTTTTCCCC<br>TGCGTGTGGAGGAGACTGAGGCTCCTCCCAACCCTCTTATCCCAGGAAAAGGGAAGGGTTCTGGGTGG<br>GTCTGGGGCCCTGGCGCAGGAGTACAGGGGGCTGGTTAATAGAAACCTCATCTGCTGTCGAGAACACC<br>TTTTCCCGTGGTTGTGGGGGTTTGGGCAGTCCTCAGATGGTCAGGGAACTCCGCCCAGTACCTTTTTAT<br>AGCCATCAAGTAAACCATTAACTGTGGCCCAGTCGCCTGAACCACAGACCTCCATCCGCCAGCCAGCC<br>CCAACACCTATTTAGAGCAGGTGGGTTGGGAGAGGGCAGGGGAAAGATTTCCCAACAACCCTGGCCCC<br>AGGGGCTTCCCGCTCTCTAGTGTCACAGTTCCCTGACCTGCCTTCGAGCTCGCACCCCTCCACGCTGTG<br>GGTCATCTTCCCAGGCCTGCAGTCTCTAGTCCCTTCACCCCCACCCCCATCTCCACCCCACCCCTGCGTG<br>CGCACCTGCTCCAGAATGCCGTAGTCCGCAGTCCGCATTGCCTTTGAGCACCGCCTTCCTCGTACTGGT

							AATTGGCATATGAGAAACGCACGTTCCTTTGCGAGCACGATTCTCCCAGGGAGCCAGTGTGTGCCAGGCGAGCAGGGAGAGGTGACCCACGGAAGCTGAATAAAGGCTGGAGAGCCAGGAACCGTCGGTGACCCTGATGCTGATGTTCAGTTTCAGGGGCCAAGTGAGCAAGCTGCCTGGACCCTGCTGCCG
MSTRG.29072.1	--	0.000	0.280	0.019	3.592	up	AGCTTCCTCTGCTGACTCCCAGCTCAGCTTGGACTCACCAAGTGTTGGTCTCCTAGGGTGAACAGCGCCTCCACCAGGTGCCAGGGTGTGCTACAGCTTTGCTGTCAGAGCCAGTGATGTGCAGAAGACCCTGGAGAGACACTCCACATCCAAGGACAGCTCTCCCCTGAAGGTGAGATGTGCAAACTGTTCTCTGAAGAGATGCTTTCAGCAGTGCGCAGCTCACTTCTGGCTGCACAAGAGAGCTTGGCTCAGTTCCCGCTTCCCATCAGCCTGGGAGCAGGCTGGCTCCCCGAGGAAGCCCTCAGGGGCCCAGTCCCTTCTTTCATCTCTGCAACTGCCCTGGGCCTGTGACGCACAGAATTTCACAGGCAGGGCTGGGGTCAAGCTTTGTATGCCACGGCTCTGGACTTGCACTACCACGG
MSTRG.2919.1	ENSRNOG00000014434	0.000	1.664	0.000	6.652	up	TGGGCACTGGGACTTCTATCACTGGGAGTCAAATTAACCTCTTTCCTTTATAAATTATCCTGTTTCAATTGTTTTCTTACGAACGAAGGTCAGACTAATGCAACATCCACTTATTTGCAGGCTCAGACTCCTCTAGCACTCACAAAATCCCAAGGGTCCTTAGATCCAGTTACCAACGTCCCTCCAGTAGTAGCCTTTCTTGTTCCAATTCGGCACGGCACCCTTCCACCCGTCCTCCAGGGAGGGACCTAGGCAGGCGCTGTTGTAAAAGTTCTTTTATGAACCAGGCATGGGCCACCCACACACGAATCAAGTAGCGTCACGGGATGTCAGCACCTCGAACTCTTTCTTTCACTAGAGCCCCGCCCGGGAAGAGACTTAGCCGCAGGTCGCTCAGGCAAGGATCGCTGCGGGAGGCGACAAGGTTGCCCAATAGTTCCCTAGCTGCCCCGGTGACGCAGCAGCTCCGGCCGGACTCCGGACACCCAGCGCGCGCCGCCCGTCCCCAACGCCCGCAGTCCTCGGCCTCCGTTTTGGATCCCGGCGTATCGGTGCAGTGACCTCAGCGCCCATCTCCGGAAGCCCCAGGCGAAAGCCAGAGGTAGCGCGGGTTAGGAGTGCGCAGGCCGAGGCTCGGCTAGACCGAGTGGCTTCCCCGAGGGGCGGAC
MSTRG.2919.2	ENSRNOG00000014434	2.839	0.105	0.000	-3.695	down	GGCACTGGGACTTCTATCACTGGGAGTCAAATTAACCTCTTTCCTTTATAAATTATCCTGTTTCAATTGTTTTCTTACGAACGAAGGTCAGACTAATGCAACATCCACTTATTTGCAGGCTCAGACTCCTCTAGCACTCACAAAATCCCAAGGGTCATAGATTGCTACATCACCAAGCTCGAAGAGATCTTGTTTCCGACAAATTGAGCCCGCGGATACAGTAATGACAGAAACTGTCAACTAAGCTGCAGCACCTTAGATCCAGTTACCAACGTCCCTCCAGTAGTAGCCTTTCTTGTTCCAATTCGGCACGGCACCCTTCCACCCGTCCTCCAGGGAGGGACCTAGGCAGGCGCTGTTGTAAAAGTTCTTTTATGAACCAGGCATGGGCCACCCACACACGAATCAAGTAGCGTCACGGGATGTCAGCACCTCGAACTCTTTCTTTCACTAGAGCCCCGCCCGGGAAGAGACTTAGCCGCAGGTCGCTCAGGCAAGGATCGCTGCGGGAGGCGACAAGGTTGCCCAATAGTTCCCTAGCTGCCCCGGTGACGCAGCAGCTCCGGCCGGACTCCGGACACCCAGCGCGCGCCGCCCGTCCCCAACGCCCGCAGTCCTCGGCCTCCGTTTTGGATCCCGGCGTATCGGTGCAGTGACCTCAGCGCCCATCTCCGGAAGCCCCAGGCGAAAGCCAGAGGTAGCGCGGGTTAGGAGTGCGCAGGCCGAGGCTCGGCTAGACCGAGTGGCTTCCCCGAG
MSTRG.29686.1	--	0.161	0.547	0.012	2.366	up	CTTGTCTTGGCTTCCTTTTTTGGGTCTCTTGTCCCACATTCTCTTCCAGGTACCCCCTGTACAAATGGAACCCAGCGTGCAGCTGTTTGGAGGTCTCCTGTTCATCCCTCATGAACAGGCAGGGCTGTCTTCCTTGTTTGGCAGAGCCTTTGTTCCCACTCGGAGCTTATGCTCTCCCTGGGAGCCTTTGCTCCCCCCTCTTAGCCTTTGTTCCACTGCTTCTGGCAACCATGGACTGCCGGACCTTGGTTTAGAGGCTTGGTGTGTGTTTCTTCCTGGACGATGCAGATTTCAGTGGTATGATTCTGGAGTCGTGCTTGCATGAAACTGCTAATTCATGGCTAAAGTTAAAAGGCTGATCTCAGAGAACCCTTGCCAAATTCCTGCAGACTCTATACAAACTACTTAGTGACAGGGGAGAATAGCAAAGAGAAGAGGAGAAGAGAGAAGGAGGAAAGCGCAACAGAGGAAGGAAGGGGGAGAAGGCAGAACAGAAAATTTAAATTACCAGACTGCAATGTGCCTCGATATATCATCTAAAATAGTTTACTA

							GCTTTTCCTGAGGAGATGGAGAGGGTGCCCTCTTTTACCCTTCTTTACAATACAACCTTTAAGAAAATATTACAATTACAAATTGTGAACTTTCCAATTGTTGGATAACAGAAACACTGACTTTATTTCAAGAACTATCTACTTCCCTTCCTCCTGGAATGCAAATTCTGTCATAAAAGCTTCTAAGAATTAACAAATAAGGTAGAGGGTCCTCTGTAGCAAGTCTTCTCGGTTTCTTTCTTTTTGTTTTTCTGATACTGTTAATAGGAATTACAAAAGTAAAATAAAATTGTTCAAATCAGGATAACTTTTCAGAGGTTCAGTGAAGGAATGAGGGACCAAATACAGAAACTTACCACAGTAACCCTTGGTAAGCCACGCTTGCAAAGAACACAGATGGGAGTTCCCTTGTACTGCCCGAAGAAGCAACTGAGCAATTGAGCTTTAATCTCTCACAGCTTCAGTGATTTCAGTTTGACACAATGGATTCCATAAATACTTCATGCAGGAGAACAATTTCTAGAAGTCAAGAATTCCTGCCTATAGAATTAAGCTTTCTATGCTTGCAGGAGTCCAAATGTGGTAATCACCATGCAGATTCATTTCTTCACTGGCAGGAGGGTTTTGAGGGTGGAATGTGTACTAGAGCTCCCCCTCCTGTATTACGACAGTCATTGATCTCCTGGGGTAATTACATTTGGCCTGCAATTCTTAGGCCCAAACTCCCAAACCCTTTACAAGGAGGATCCTTAGTTACAAACTGCTCCCA
MSTRG.29804.5	--	0.349	0.000	0.049	-3.592	down	GCGTCGTCTGGGGACTTAACCAGTTTGTGTGGAAAGACGACCTGTTAACAAATCCCCTATGGTCCAAGCAGGCTTCCACAAGAACTAATCTTATGCATAGTGCAGAAAATATCTTAAGTAGTTTAGCTCCAGAAGAAATCATAATCAATTTGGAAGCAATAAATATAAACTAAGCTATCTATAACTTTAACATGCATCATTCATTTCAGTACATGTAAATATGCTCTTAAAAAAACTACGAAACAACAAACATTTTCTCCTTTGTTCCTGTCAGTGTTCTGACCTTTGACCGGTGTCCTTCTAAGAACAACTTTCCGGGGATACCTTTTCACTGTGGGCAATGTGGTTCAGCTCCAGTCATGTGGCCATTGAGAGATGGGATTCCCACAAGTCCAGCTGACCTCTTTATCCAATGTGTTTCCAAGGTCAACGTTGATTTCTCTAAATCTGGTCCTTAACTGGAATTTGTCAGCAACATTCATTCAGGAACTCTGACGTATACCGGCGAGCACCTTGACCGAGCGCGGAAGCAGGACCTGAACGGGGTCAGGTGTGACCTAGAGGCCCGAGCACTGCAGGCTAGATCGGCT
MSTRG.29998.1	--	0.000	0.902	0.000	5.141	up	TTGGGTAGAACCTAGCATTTTATTTAGATCTTCATTAAACTGTTGGAATTGAGAACCAGACATACGTAATAAACCTCCAAAAATAGATCCTGAAAGGCACTTTCTTGCTTAGGGCAAGCAGTCATGGAATAAGCATGTAAACAAGCTGGCTTCTCTGTACCACACCAGCCAAGTCAGCTTTCTCCATGGCCAGCTGCACCAGCTCTGCCCTCCCTTCTGTTAACTCCAGCCAGACCCCCTGTAGGTCTAACCCAAGGTTTTTCTGTAGGACACCTTGGCCTACCTGGGAATGCTGGAAACATGTTAAAGGAATCATGGTGTCAAAGAAAAAAAAATCTTTTAAAAGCTGCCATCTGAGGTGATGGCTTCTCTGTACTTACGCCATACCCCAGAATACAATAAATAAGCAATTAGAAAATGTTCA
MSTRG.30086.5	--	0.465	0.000	0.022	-3.851	down	AAGATAGAGCAACGTTTTAACGAACTGAAACACTGTTTCTTTGTGAATTCAGTTACTTCCTAGTAGCCTGCTCTTGTAATATGTGTACTTTTAAAGATTTATAAAAATTTGGTGAGCCGAATCTGAGTAACGTGGTGAATTCGGTTAGTTCCCAATAGGCCGCAGTAGTAATAAGTGGACTGTTCGGGATATAGAAAAATTTTCACGAATGGAATCAAAATTTCTAGGCGAATTCAGTGAGTTCCCAATGGGACATGCTTGTAAGAGTTTGCTTTTCAATATCGAGCAACATTTTAAGGAACGGAATCCAAGTATCATGGTGAATTGAGTAGGTTCCCAATAGGATTCGCATGTAATAAGCTTGTTATTCAAGATAGAGCAACGTTTTAACGAACTGAAACACTGTTTCTTTGTGAATTCAGTTAGTTCCTAGTAGCCTGCTCTTGTAATATGTGTACTTTTAAAGATTTATAAAAATTTGGTGAGCCGAATCTGAGTAACATGGTGAATTCGGTTAGTTCCCAATAGGCCG
MSTRG.30131.1	ENSRNOG00000002414	0.331	0.995	0.034	2.236	up	AAGAGAGAAGTGCTCTGAAAACAATCAAGCACCAGGAGAGCTCACACGCATGGTCCCTGCGCCCCCAGATTGGGTGGTGTTTGTTTTGGGTTTTTTTGGGGCTTTTTCATGTTACATCCATATCTGTATTTATATCTTATTTGTTTCACCTCCAAGTGTATCATGGCAAATGTACAGATTTTTTTGTTGTTGTTGTTAATAATGTGCTAGGATTTGCTAAAAAAGAAAATTAAAAAAAAAAAAAAAAAGCCGACCTTTTGAGTTTTTCCTAGAATGTGA

							GACAATGCAGTTTTGTGTGTATGGTGCTATGAGAACCCTCAGATGCCCTGCAGGGTCTTGTCAGATGGCCACTGTCCTGCTGTCTTTACAGAGACAGCACACCTTTTTGTGTTACTAAAGGGTCAAACCTGAGAATAGCAAGTGGCCCAGCCAGTAATGACACTTGCTTTCCAGTC
MSTRG.30900.1 NONCODE_ID：NONMMUT002660	ENSRNOG00000002936;ENSRNOG00000002937;ENSRNOG00000047481	0.000	4.270	0.000	7.579	up	CTTCCAGTGGCCAGGGGTTAGGATACAGAAGACACTGCTGGTTGGAGATGCAGCTCAGTGGTAGATGGGCTCTCTCTTTCTAGCAAAGATCCAGGTGTCTCCGAGCGGGACCGTCCCTGCGCGTTTCCATGGACCGACCGAGTAGGGTGGGGTGACCACCCACCGCTCTCCCCACTGTGGCAAACCACCTAGTGATGCTGGCTTTGCGCTCTGGCCTTTCAGCTACAGCCGGGGGGAGTGGGCTGGGTCTTCTTCCCCAACCCTTCCTTGCGAAGAAGGACCTTCCCTGGAAGAAAATGGGTGCAGGGAGCTGGGCGGGTGTCTGATCTCGCTGTCTTCGGAGGGATGGACCCCTAAGAAGTTCGTGCCCCCACCCCACCCCCAGCCTAGGACCGACTCAACAGCAGAGCTGTCTTAAGAAAATGCTTGTCATGGCAACAGCTCCAGCCAACTCCGGCCAGACTGGGGCTCCCCAGCTCTCCCCCAGCAGAGCGGGAG
MSTRG.30925.1	ENSRNOG00000003183;ENSRNOG00000003300;ENSRNOG00000028072	0.224	0.586	0.047	2.074	up	TATTAGTAGAGATTTTTAACATCCCTAGAATTTAGTCTCATCCCTAGCATCTAAACCTCAACAGAAGGCTGACTACATATGAGAAAGCAAAGAGGCCCCTTTATTTTTAATCAGCTATGGTGAGATCTTTCTGCATGTGGCCACTGTGTATCAGGTCTCCGGTCACAACAATACTAGAAGGTAGGTGTCCTTACTAGTATTTTACACAGAGGGAAACGAAGGCTCAGAACGGGTAATTGTTACTCTGGGTGATAATCAGTCAATGAGTGGCAGACCTGGTCACCTATTTCTCAAACCTTTATCTGGACGCTATGGTGCTAAACGCTGAGTTTATGGGTGTGTATGTGTTTTTGTATGATGGGGTTAGGGAACACACAGGATATTGTTGTTGTCCTATCCCAAGTTTACTGAAAACATTACAGCACAACTGAAGATTCCATGTAGTGTTTTGACTTTCATTCCAGATGAGAGACCAGCAGGCAGAACTATCTGCCCAAATCCAAAGCTTCTGTTGAGTCCTTATGGTGACAGGATCTACCTCAGAGGTGGCCTGACTCAAGCAAGACTCCTGGTCTCAGGATCAGCACTACCTCTCCTTCTCCAGCCCAACCCCTGCAGTCTGATGGAATATGGCTCATAGGAGTGTCTGAGCAGGCTTCGTCAGATGTCACACAAAGGCCACTCTGATGCAGAGCTGGGTGTGAGGCTGATGTCCTGGTGTGGAAGTCCAGGCTGTCTAGTGCACCAGAGACGACCTGGTCCACTTTCCTCCTGGTTTGGGAGCAAAGGCAGCAGGTGTTGGTAGGTCCCACGTGTCCAGTCCTGGTTAACTGAACCAGTGTAGAGCATCTTCAAGTGTCAGAGATGCTCCCCCAAACTTCAGAGCTTTATGGTCTATTTCCAAAATAGGGCAGACGCCAACTTGGAAGTTAAGGAGTGACAGGCAGAGGTACAGGAGTGCCTGAGAACTTGGCAGGCAGTCCGATTTTACAAGCAATTCCTTC
MSTRG.30957.5	ENSRNOG00000003594;ENSRNOG00000003973;ENSRNO	0.439	0.000	0.000	-5.643	down	GTTGGAGGAGAGCCCAAGCTTGAGCTTATAATAGACCCTAATGTTGTAACATCAGAAACAGCTCCTAGTTGGTCTTTTAGTATTCACGAATGCTTCCTGCCATGACAGTCTTAACTTGCCAATCAGGATGGCAGTTACCCATGTACATGTCTTTCTGTCAAGTAGGATGTCAATTCCCAAGGAGGTCTTAGGAACTTAAAACGTTATTCAACCCCTACTCAAAATGGAAGTCTTATTCCAAATGGCTTCTTATATTGGTGCAGGTGTCTCTCTTATGTTGAGGTCTATCCCTATCTCTATGTTGAGAGGCAGGTTATCATACGCAGGTGCAGGAAGTGCCGATGGGTGGTTGGTTTGAGTCCAAGCTTACTGTGGCTAACTATACAAAGCAGAGGAATTGTATTCCTTGTATCTTGGTCATCTTGATGAACCCCTAGTGACAGTGACCACCGGACTTGGTTTATTGCTTTGTTCCTTGGCTACCTTGTCTTTGACCTAGAACTGACCCTGTTCTGTGCCTGTCCCTACAATGGTACAAGAGCAGACTGGAAAAAATAA

	G00000004143;ENSRNOG00000004193;ENSRNOG00000004424;ENSRNOG00000027593;ENSRNOG00000027702;ENSRNOG00000039568						ACCTGCTTCAGCCTCAGAACTGGCTGGAGTCGTGTTCTAGTGTTGTCTAGTTGTTTTTCTCTCTTCACTCCCCACTCCCTCCCTGGAGACACTGATGGCTGACTGAGTTGGCTTGGTCACTAGGCCTTAACACAGAGGATAACTTGCAGGAGTCAGTTCTCTTTGTATCAGAGGGGTCTCCGGGATGGAACTCAGGTCATCAGGCTTGATGGCACTCTCCTTCACCTGCTGAGACTACACAGTTTTCTCTCTAGTCCTATACTAGTGGCTTCCCACTCAGGTGCCTTCAGGACACTTTTTTTTTTCCCTGAAGATGAGGGCCAGGATTGGAGCTCTAAATCCACAACATTCTGACTTACACTTCATCTAAGCTCTCAGGTCATCACTGGGTGTGGTAACACACATCTTTAATCCTAACATTTCAAAGGCTGAGGCAGGTAGATCTCTGAGTTTCAGGTTAGTCTGTTCTACAGAGTGAGTACCAAGTCAGCCATGCCTACATAGTGAGAACCTGTCTCAAACAAAAAAAAAAAAAAAATCTCATATCACTTAGAAATTTAAAAATTTCTTGTCTAATTATAGTATCTTTCTTTGTTTTTATAAGAACTTCTAACTGGTAGCAAGAAACTATGGCCTTCTTTCTCCAATTATGTATTTCTTATGCATTAACTTATGCCCTAAATTCCTATTTATTTGTACGTGAACGTTACCATGCAAGCAAATGCTCATCTTTAAAGATTTATTTATTTTAATTTATGTTTGTACACTGTAGCTGTCTTCAGATACACCAGAAGAGGGCATCAGATCTCATTATAGATGGTTCTGAGCCACCATGTGATTTCTGAGAATTGAACTCAGGACCTCTGGAAGGGCAGCCAATGTTTTTAACCTCTGAGCCACTACTCTAATCCTATGTATAATTCTTGCTGAGCCACCACTCCAACCTTATGCAATAATTCTTAATAAAGCCTGCTTCTACCTCCACATGACACGACACCAGTAATCTTCATTGGCATTAGACAGTGAGCTTAGAGCACTGTCCCAGGTTGATCTCTTGTGACTGTAAGTGCCCAGCAAATCAGGTCACTGGGTCATGAAGTTGGCCAGTGGAGGGCGCCATCATTCTCAAACACCTGCATCATTTCTGGAATTCTGTTTTTGTTTTGTTTTTTTTTCCTGACTGAAAATGCATGGCTGTGGGTATAGCTCAGTAGAGGAAACACAGGGTCCTGGATTTAATCCATTGCACTGGCATAAACTGATCCTGGTGGCACATGCTTGTAATTCCAGCACTTCGGAGGAGGTAAACGCAGAAAGATGAGAAGTTTAGGGTCATCCTCAGTTACATGGTGAATTCAAGACCAGCATATACTGTATAACACCCTGCAGAACTTTGAAATCCCGAGGGTCTCCTGAGTGTAATGGGAACACAGCTTACCGCCTGCCATACTGCGTGGGGACTGCCAGCCTCGGCGCGCGGCACTCCCCACTCCACCCATCTAAACATTTTCTTCTCTCTTTTTCTGAGCTCTCAGGTACACAGGACTTCACGGCTCTGCCTCCTAACGTGAC
MSTRG.30957.6	ENSRNOG00000003594;ENSRNOG00000003973;ENSRNOG00000004143;ENSRNOG00000004193;ENSRNOG00000004424;ENSRNOG000	0.307	0.796	0.039	2.073	up	CTCATCTTTAAAGATTTATTTATTTTAATTTATGTTTGTACACTGTAGCTGTCTTCAGATACACCAGAAGAGGGCATCAGATCTCATTATAGATGGTTCTGAGCCACCATGTGATTTCTGAGAATTGAACTCAGGACCTCTGGAAGGGCAGCCAATGTTTTTAACCTCTGAGCCACTACTCTAATCCTATGTATAATTCTTGCTGAGCCACCACTCCAACCTTATGCAATAATTCTTAATAAAGCCTGCTTCTACCTCCACATGACACGACACCAGTAATCTTCATTGGCATTAGACAGTGAGCTTAGAGCACTGTCCCAGGTTGATCTCTTGTGACTGTAAGTGCCCAGCAAATCAGGTCACTGGGTCATGAAGTTGGCCAGTGGAGGGCGCCATCATTCTCAAACACCTGCATCATTTCTGGAATTCTGTTTTTGTTTTGTTTTTTTTTCCTGACTGAAAATGCATGGCTGTGGGTATAGCTCAGTAGAGGAAACACAGGGTCCTGGATTTAATCCATTGCACTGGCATAAACTGATCCTGGTGGCACATGCTTGTAATTCCAGCACTTCGGAGGAGGTAAACGCAGAAAGATGAGAAGTTTAGGGTCATCCTCAGTTACATGGTGAATTCAAGACCAGCATATACTGTATAACACCCTGTCTTGGGCCTTTCACATGCCAGCTACATTTTCAGCTTTAATTGTTACTGTTTTCGGGACAGGGCCTCCCCTTGAACTTGCTGTTTCCTGCTTTAGTCTCCCAAGTGTGGGGTTACACTCTCAAAGAAATTCTTGAAGAAGTTGTGTTTTTCGGTTTATCGGGAAACCACTTGCCGCCTGCCATACTGCGTGGGGACTGCCAGCCTCGGCGCGCGGCACTCCCCACTCCACCCATCTAAACATTTTCTTCTCTCTTTTTCTGAGCTCTCAGGTACACAGGACTTCACGGCTCTGCCTCCTAACGTGACG

	00027593;ENSRNOG00000027702;ENSRNOG00000039568						
MSTRG.3103.3	ENSRNOG00000042347;ENSRNOG00000042547;ENSRNOG00000042826;ENSRNOG00000043341	0.035	1.266	0.000	4.842	up	AAAATAGTTCCGAGTGGGAGGAATTAGAGGAGTGACGTAAACGGAAGCTGGAAACTGGGTGTGGGAGGCTGGAGACCTGAGACAGAGAACCAAAGACAGAAGTATCATCAGCCCGTTCAAGTCCAGAGCTCCAGGCCCTGCACCGCGGGGAATCGCGAGGTACAGACCTGGAGTCGGAGGACATAGGACTTCGTGGGAAGGTAAAACTCCCAGCAGCCGCGGAAAGTCAGCAATCAGTAATCGGCAGCGAAGCGGCGGGAACTGGAGCCAGGCCAGGGCTCACTGATCACCCAGACCTTCGTACCTGTGGAGACCTGGAAGGTGGAGAGGGAGGAGACAGTAGCTAAAGGATTTCAAGTATGTTGGGAGGATTGGTGAAAGGAGGGCAAGGGGTTCAAGTGAATGTGGAAGTGGTACATGAAATCAGTTTTGCCACAGTCTGTTTCACTAGAAATAATCCTGGAGACATCCACTGAGAAAAATCTCCTCAGGGTTATCCTACTGTAGAGATGAGAAGACTTTCTACAGAAGAACTGATACTCGAAGGCAAATAAAATTTTACTGTGCATGTGAGTGATGCCGTCCTTATTCCATGCACATCACTGACCAGGTCTACACACCAGTATTAAATGGTTTTCATTTCAG
MSTRG.31534.1	ENSRNOG00000002525	0.032	0.450	0.000	3.559	up	GCTGATGATTCTTAAGGATGTCCCATAAAAAAGAGCAAATATCTATTAAGATGGTTTTATTTCTATCTCTCTTATTCTATAACTAAGAAAAAAACTCACACCCACATTTATTTATTGTTTATTTATTTTTGAGACAAGATAGCATTTGAACAGGCTGGCCTTGAACTTGTTATGAGTTGTGAATTACTGGACTCCTGATTATCCTGCCTCAACTCCCAAGGGCTTGGATTACACATATGCAGCATCACATCTGGCTGGTATACAGTTTAAAAGCAGCAGCAAGGGAGTGGGTCTGGGTGGTGGCTCTGTAGTCATGAAAGCTTGCTCTTCGTAAAGAAGGGAGTTAGATCCCAGCGTTCAAGTAATAAGTTGGGCACCATGACACCCTTTGCATGATCCTTAAAGCAATGCGGTGGACACTTAGCATTCCGACGTGGAGCTCGGCATCTGTCTCTGGGAAAGGCGAGTGCCTGGGGCTTGCTAGGACTGCGGAGCCTGGAGGACAGTTCGGTGAAAGACTTCACTCTAACTCCACCAATGCAGATGTCACCCTGACAGCAGCCCTCTCATTTCATTAAAAATAGAATAAATTAAATACACCGGTAACTGTGTGCGTGCTCAGAGCAGCAAGCACGTCAGACTGCGCCCCAGTGGGGAGAGGCAAGGGGATTCCCTTAGTTAGGATCTCGGATCCCGGGAGGGGAAGCTGTGACATTCTCTTGCTCCTCCGGCCCCCCAGTGGATGCGGGACTGGGAGGAAACCCGAGACCTCAAAGAGAGCCAGTCTTGGAGCAGGCACAGCGAACCACAGGGCGCCTGGAAGGATGCAGAGGGCGGTGCAGCTCTCTTGGCACCACTTTGGGCAGCCGAGGGAAGCTTCCTGGCTTCTCTGGGCTCATTTGCGTGAGTAAAGCCTGCCCCTATGGGTATTATGCAATTGGAAG
MSTRG.31691.1	--	0.000	0.744	0.001	4.386	up	GTGACATCCTTATGCCAGAGCCTTCCATCGTGGATTAAGGGCCAGCAGCTCTCCAGGGATCCTCTAGGCTTCCAGTGACAGATTGGAACTTGTGAGGCATCCATCCCAGTGGGCTGAGAAAGTCCCAGGTTCTCAGCTTCTCCAGTGTGCCCACAGCCACGTGGGACAACTCAACCCGTATCAAGTAAGCCAGTCTGCGTAAAGAAGAAGGGACCAGTTATGAGCAAGGCAACGTGGGGAAGTGGCCATAATTACTAAGACTTTCAATAAGCCAAGATTAGAAAGGGACCGCGAATGTGG

MSTRG.32277.1<br><br>NONCODE_ID：NONMMUT002669	ENSRNOG00000003071	0.236	0.738	0.011	2.308	up	GTCTTTTCGATTTATTTTATTATGAAAACTGTCAAGAATAACTATTGTTGGAGAGAGAGGTCCTGAAAGCCAAAGCAGAACTTAGGAGACTAGGTCCCAGCTCCAGAGGTAGATAACTCTGACTCCACTGGGCACCCCCGAGATACACCACGAAGCAGAATGAAAACTGGTCACATACAGGACCAGGAGATGGTGGGCCTGCCTTCCTCCATGGACTCACGGTAATCTCCCTGCCAAAACCTCTAAGCGCTGGGATCACACGCACTTCCTTTGTAACATAGGGCGTGGAAGGCACAGTGGAGGGGTTTGGGAGTGCGGCAGACACTCAGCAGATTGCAGTTCCCAGCTCCTGTAGCGAGGGAAATGGTGGATAGAGAGTCAGGCTAACACTCCGCTCATGAACAGAAACAGGTCATGGTCACACAATATAGCCTTCTGCCCATACACCTTTCCTTGCAAATATTCATTGCAATGAGTCATTGGTCTCGTTTAAGATCTCTGGCTTCTGCCTCTCAGATATACTGTTATTACCCTGTGTCATAGAGATCCAGATCTGCAGGACCAGTCCCTTCACGTGCTCCAGCAATTCACAGAAGAGGTAGGTGTTGAGGTGGGCCAGCTCAAAGCCCTGGATCTTGGTCTGGATGGTAGCTAGGTTAGTCAGTCCACCAGAGCCATTGGGTCAGCTTTCAAGCACTGTCCTAGCAAGGGCAAATGGAAGGGCAGGCTTTCCTGTTCTTGTGCCCTAGGGACTAGCTGTCCAGTGCCCACACCATCAGGACCAAATCTATGTTGCCCAGATGAGGTGCAGGTCCTCTCTCCAGGGTGCTGAAGCTTTGAGGGGGGCGGGGCCAGCTATCCTGCCCCGCCAAGGTGAGAGGCAGGGCAGTATCTCCCATGCCTATGCCACAGGACCAGCGCTACTGTGCTGCCTATGTGAGGTCCTCTTAAATAGATGAAATAGATAGAGGGTTGCTCCCACTTTACTATTGGAAAAATCGAGACCCACAAGTGGCGACTGCCCTGCCTACCTAGTAATTTATTAGATATGTTTTTCTCCACTCCACTCTCTTCGCCCTAAGAGACTAACCCACATAGCCAGTATCAACAGCTTACCTATTCTCCAGCTCTAGCTGGGATTCCTACCACTCCTTGCTGACCCCCAGAACTCTATCCTCTCTATCCTTATCTTTGTGAGTGTTCTTTCAACTAAGTACTCTTTAATTGCCCATAATAGATAGACCATCCATCCTGCTGGAATCCCGAGGGCTATATGTAAGTCTTGATCCTACCTTCTAGCTCTTGCCCCATCGTTTGACCTCCAACTCTAAATACTTGCTAGTCCTGATAGCAAATTAGAAGAAAGAGCCATCTTCTCTTTACGCCCACAGCTCCTACTAACTGATACTCATGAGGAACTCCTGTGAGAGCAAAAGCTCCTGGAATCAAGGCTGCTCCCCTCTGCCAGTGAGCTCAGACGTCTGTACCTGCAGACAGCTCCAGCTTTCTCTCAGTAAACAAGTCTGCTCCAGCTGCTGGGACATTAAT
MSTRG.32277.2	ENSRNOG00000003071	0.010	0.486	0.000	4.346	up	GTCTTTTCGATTTATTTTATTATGAAAACTGTCAAGAATAACTATTGTTGGAGAGAGAGGTCCTGAAAGCCAAAGCAGAACTTAGGAGACTAGGTCCCAGCTCCAGAGGTAGATAACTCTGACTCCACTGGGCACCCCCGAGATACACCACGAAGCAGAATGAAAACTGGTCACATACAGGACCAGGAGATGGTGGGCCTGCCTTCCTCCATGGACTCACGGTAATCTCCCTGCCAAAACCTCTAAGCGCTGGGATCACACGCACTTCCTTTGTAACATAGGGCGTGGAAGGCACAGTGGAGGGGTTTGGGAGTGCGGCAGACACTCAGCAGATCTGTTTTCCTGTGGCAAGTGCCTCCCAGTTCCCTTTAAAGCTGAACCAGTTCCTGGGTCAAGTGGAAGAAAATAATGTGCACCTCTGTGGGTTTAAAATCTATCACCAGGCAATCGAGGGCCAGCTGCTCTGGAAGAGAATTTTCTGACTTTTCCCCCATTCAGATCAAGACATCGTGCACCTTTGGGATGCAGTTCCCAGCTCCTGTAGCGAGGGAAATGGTGGATAGAGAGTCAGGCTAACACTCCGCTCATGAACAGAAACAGGTCTGAGGAGACTATCACCATCCTGGTCTTCTCTTTCTTTGGGACGTCCCTTCTGCATCCATGGTATTTCAAGTAACTGTAGAAAACGTCACACAGAAACATCACCAAACTTCAGCTCTGCTACACGTTCTCTCGTCTCAACTAACAGGACAATAAAATACTTTTTTTTTCTCGCTTACTCAGCAAGCATTTCTTGAG
MSTRG.32278.1	ENSRNOG00000003071	0.043	0.445	0.002	3.295	up	TCTACATGGGAGGGAGAGGAAGGAATGATAAGAGACAGTAACACCACATGTTACCACATGCAGAATCCAGATTTAAATACAAACATGACAGCAGAAGTGGACTGCTCTGAGGGACAAGAGGACCCGAAACGAGGGTGGGGGACCAAGGGAGGCAACATACAGCCCTCCAGCATGGACTGAAGACCAGCTGGGGAGATTCCAAGAATCTTCTATTGGGACTGCTGGGGCATTCAGCTTCAAGCACTGAGTTCTCTGCCCAACCCCACACACCCGGTGGACACTCACTACTGAGCTACCCGGCCCCTACTGTTTAAGGTGTGGAAAGTAAGACCGCTACAGACGGTCCACAGCAGCTGACTATGATTTGTCTCCTGCTCTGGCAGGGGTGTGATTCTGCCAGCTGAAAAT

							AGTTTACCTTTGGAATTCTGGGAAATCTCGAGAGGATATAATGCCAGAAGGATATAATCACCCAGAGGTGATAACAGCTGCTGCTCCTCCTGCTACTGCTGGTTCCTGCTGCTGCTGCTTTTTCTGTTTACTAGATTGCTGGTTGCCGATTGCTTTTGCTGTTTTCTGGATTGCCGGTTGGAGATATCCTCCTGACAATGAAGAATGGACTTGCCCCAAGGAACTTGATGCCTCTAATCAGCAGGAAGTATTCTAAAGAGATATACCCTGGAGTAGTTAAAAGCACTGTCTGATCTTCCAGAGGTCCTGAGTTCAATTCCCAGCAACCCACATGGTGGCTCACAACCATCTACACTGGGATCTGACGCCCTCTTCTAGCCTGCAG
MSTRG.32282.2	ENSRNOG00000003071;ENSRNOG00000003221	0.509	0.015	0.049	-2.996	down	CTCCTGGGGAAGCCCCACTTGGGTACCATCAGGCCCTGGGGCATCAGGTTACAGCAGGAAGGACTAAGTGCATCCTCTCCCACTGAGGCCCCATCAGGCAGTGCAGCCAGGGGAAGGGGATCCAAAGGCAGGAAACAGAGTCACAGACGACGGCCACCACTCCAGTTGTTAAGGATCCCACACGAAGACCAGGCTGCACATCTGTTACCAATGTGTAACAGTCCTAGGTCCAGACCCTGCATGCTCTTTGGTTGGTGGTTAGTTGATCTGTAGGTCTTGTGATGTCCTTGACTTCTCCGGCTGTCTGTCTGCTGTTTCTTATACAGCCATAGAGATACTAGGAGTCCAGTCAGACAGGGTAGCACATGGCTTTCAATGTGCCCTACTCATCTCCTGTACAGCCCACGTACTCCTCCATCGGATCTGAACGTCACAACCACTGGGGATTCTCCAGGATAGAAGAGAGGCCGAAGTGATCGACCTGCTCAGTGTCTCCATTGTCTGAACTTGTCCCCTCCGCCAGAGGACAAGACCCGGAGCGTCGAAAGGCTCGATCTCGGTTGCCAGAGCTTGCTCCCGTGCGCCC
MSTRG.32442.1	ENSRNOG00000002964	0.000	0.235	0.004	3.943	up	CTTTCTTTGGCCTTTCTAGGCTTCCGTTTCCTCAGCTTGGTGTGCAGACTCACAGGAGAGTTGGTGACTCCCCTACTGTCACTCAGAGTGTTTGTGGTAGACCACCTTGAAGCTGAGGTTGGGGACAGGGAGTAAGGTGGAAATAGTTTCTATTTTGAGTGGAGGAGAGTTAAAAGCAGAAGTGGGGAAACTCTGACCTGCTCCTTGGCCCCCAACTTTCTCAGCCTCTGCTGCTCACTGAGTCTTACTCATTACACTTTCCAACAGACTGAGGGTCCTCTAGGGGACAGGATGTTTCCCCTGGGCGTGGGACCACCAGCACAGTCATTTCATTCAAAGGCTGAGGATGGGACAACTGCATTCGAGAGAAACCAGGGGTCCAAATGGAAAAGTTGGTCTTTGTTCCATTCGCTTCCACACGAGGGTCACATGAGTCTCTCTTCATGAAGCTTCAGTTCACGGTGGTGGGCTACAGCATCCGATTCAGTTTGTGTTCTAGGTAATGTATTCGATGCACGTCCCTCAGAAAATTGTCCTGTGTTCCTGGTTCTTAATGCTTCCAATCATGTCACTTCTAACTGCTCATACTGACAAGCCGGTTGACAGTTGATTTTTCTCCCTTTCCAGTGCGTGCTGAATAGCATGCTGCGTCTCCTTCATCCC
MSTRG.32524.2	--	0.616	0.007	0.014	-3.354	down	CGGCACGCTTACAGTGACCTCACGCATACAGTGACGGCACGCTTACAGTGACCTCACGCTTACAGTGACGGCACGCTTACAGTGACCGCACGCTTACAGCTACCTAGGAACAGCGAGGAAGAAATGCGGCTCTCCACAGCTGAGACAGCTTACATCCCTTACTTGTTTCCTGCGTATTCAATCAGGAATGGCAAATCCCTCTGAAACTATTTTGTGATTTAAAATGATTATCATTTAATAAAGTCACAAGCGTAAGACACACCATTACCTCTGAATTACACCCAGTATGTCTTCCAACACTATTCTTTCCCTTAATATTTTAATTTGTCCACTATTTCACCAAAATAGCCATGTCTCACTTTCATCCCTTTTTTAAAAATAAATTTTATTACTGTGTGCATATAAACAGTAGAGGTATGCATATGCTGTGACACACGTGTGGAGATGACAGGAAACATTGTAGAGTCAGTTTTCATCTTCCATGTAACAGAATTAAGGCTTGAGCAAAAAGTGCCTCCTTGGGCTGGAGAGATGGCTCAGCGGTTAAGAGCCCTGACTGCTCTTCCAGAGGTCCTGAGTTCAATTCCCAGCAACCACATGGTGGCTCACAGACCATCGGTAAAGAGATCTGATGCCCT
MSTRG.32528.1	ENSRNOG00000003722	0.000	0.481	0.000	5.924	up	GGGGGAAGATGACATTTGAAGTGTAAACAAATAATATCAATAAAAAAATTAAAAAACCAAAACAGTTACAATTACACTTAAAAGTCTTCAAAGATGCTTTTCCAAGAGTGCAGAGCCCCTTGGCCCTCCTTCTGGAAAGTGTACTTCAGTAGGTTTAAGATGTGGCTGGGAAAATGTACAAGGCTTTCCAGGAGACCCAGGGCCACTCCCAGCAGAGAACCACGAGCCTCCAGCAGACGAGAGAGTCAATTCAGTGAAAGCCCCCTGCTCTTGTGGAACTTATCCTGTAGCATCTATTCAAACAAACCAGAGGAGAAGCCCCAGCTAACACAGACCCTGGA

							GAGCAGCGCAGCCATGTTCCCTGCAAAGTGACAGCTTTATTCAGCTCCAGCCAGTGGTCATCACATGGCTGCTCACTGGAATCCACTGGTAGAAGCTTTGGAGTAAATATATACACTCCGGATAGTCCTAAACCCAGACAACCAGGCTCTCCCTGAGTGGAATCCAAAGAGCATATGAGAAGCTCTCCAAAGCTGGTGTAGATAGCCTAGCATTGAGAACTTGCGGAAAGCACTGTGGGACACTGCCATCAATGATGGACTTCACTTGAGCCACGGCCAGGCCTTCCGAGACTCCTCCCTGGGTTTATGCCACACTTGAGGAAGTTTAGGGCTCCAGAAGACCAAAGCAGAAATTGCCAGTAGCCCTAGAAGCCACCTCTGAACCAGTATAGCATCTGTTGATTCAAGAAAATAAGCTTTTCCTGAGTCAAGAGGAGGGGCAACCGACCCTTCCTCTCCACAGTGTCAAAAGAACAAAAGGGATCTGGGAGAGCCATCTAGTCACTGTGAGGACTTTAATCAACCTTACACAGAGACATACATGGAGAGCAGCTAATGGCCCAGACAATGGCTGACGCCAACTAGAAGTCCTGTGACTGAGCCACATTGGGAGGTGACCCTCCAGCCAGAGAGGGAGCCTTTGGATGATGCAGCCTCAACTCAGTCTCATGAGAGACCTTGAACCAGAGCGATACAGTCTGAATTCAGAACCTGCAGGAACTGGGGGATAATATGGGATTGATCTGTCATCTGGCAATGGGTAGCTAACACAGTCGTAGACCCATATGGGGAAGTTATGAGTACGTGGAGAGCTAAGGAAGCCTTGAGAGATTGTATGAAGAGAGGATGAAGTCCTGCACAGCCATGTCAGGGACCAATGTCTTAGACACAGGGAAATGGCGAAGCCTTCTGGTTTGAATAGGGATGTTGGCAGTGTGTGCAAAAACTAGCAAACATAGCTTCCTCCTCCTGGATGACTGCACTCTGAGACTTCTCACCTATAGAGACCTCTTTTGGATATTGTATAAACCCTCC
MSTRG.32532.1	ENSRNOG00000003722	0.273	0.000	0.049	-3.592	down	GGCTTATTAACCGCAGTGACAATAAAGGCCAACCCTATATTTGCAGAGGGGCTGCCTTCCAAGGCTCTTGAAGCACTTTGACTGATCGTATCTGCTTCCCTCGCTCACAGCAGCCCCAGAGAAATCTTATTTCCTTGTAATTAAGCATAACTGCTAATCTCTCCCACAGCTCACCCATAATTGCCTGTTGGACTGACCTCCAGATGGACCACCAGGAGCACCCAGAAGGGCTGACTAGCAGAGTGCAGGTTCAGGTGTCCCCAGGAGGCCAGAAGACATTGGATCCCCTGGAGCTGGAGTTAGAGGCATCTGGTGTGAGCCTCTCAATGTGGCCTCTGGAAACCACATATCTTCCTCAAGAACAAGACACACTGTCTTTTATTGTTGTTGTTGTTGCTTTGTTTCTCTTGTTTTGGTTGAGACAGCATCTGTCTATTACGTAGGTCTGGCTGTCTTGGAATTCTTCCCGTTGACCGGGTTGGCCTTGAACTCACTGAGATCCACCTGCCTCTGCCTCTGCCTCTGCCTCTGCCTCTGCCTCTGCCTCCCAAATGCTGGGATTAAAGTCGAGTGCGCATCACAGTATGTGCTCTAAACTCTTTAGTCACCTCTCCAGGTCCTGAACTGTACATTTTAAGTAGATGAAATATATGTCACTAAAGCTAGAACACTTCCATAATTCTGGTAACTGATCTGATTCAGGGGTCTGTGGCACCCCATTGCTT
MSTRG.32543.1	ENSRNOG00000003816;ENSRNOG00000025151;ENSRNOG00000032058	0.625	0.000	0.000	-5.894	down	GAAACTGAAAGGATCTAGAGAGATGACTCTGTGATTGCAGGTGCTGGCTACTGTACCTGAGAAGCCACACTCATTTTCCAGCACATGGAGCCTCCCCGCTCCAGTTGTAGGGGATCTGATACCCTCTTCTGGCCTCTGCAGGTACTGCACACATGTGGACAGACACGCAGGAAGGCAAAATACACACATAAAAAAGGAAAAAACATTTGAAAAAACACTTGGTAAAATAATCGCATGTGGGACTGGGGATGCCACTCAGTTGGTAGAGCGCTTGATTCGAGTGCACAAAGCCCTGGTTCAATCCCCAGCACTATAAACCAGGGGCCGTGGCACGTGCACGTCATCCCAACATTTGGGAGGTAAAGGCAGGAGAGTTAGAAGTGCAAGGTTACTTTTGGCTGCATAGTGAGTTCAAGGCCAGCCTGGGCTGTATGATTATTTTAAGAACCAAACAGTAAAAACAATTTTTAAAAAACACAAGGATTACTGTCTGCCTGTCAAAGATGCTGTTGAGCTGTGCCTTGGAGAATGGGTGGGTGGATCTGGAGTAAAGATTTGTACTCTGAACATATGCAGAGAGCCTTGTGGTAGATGTGTTTGGGTGGTGTTACACTGGATCGTACCTCTGGTGCACCCCTATCTGCTGGCCAGGAGTATTGTTCGTGTCTGTATTGGTCAGGATTATTTTATTCCAATGTCATGGTGAGGTCCCTCTAACTCCCTTGGGGCTGGCACTGGTGCAGCACCAGCTATGACACTGGAGACTGGCTCATCAGAGTCCAGCAGAAAGGAGACATTTAAAGGGAGAGGGTTGGAAGCCCTCCCCCAGGAGACTCCTTGAGATGTTCCAACTCCCAGAATAGTGATGTGTTGTGACAGGCTGAGATCAGACAACAGGAATTACAGGTAATTTTCTTATTCCCTTGCCATCCCGAATAAAACTTAGCTCGTGAATAGAAAGAA

							AGCCATTGGGGAAAATGGCAAACGTAAATCATTTCTAAAGGGTACAAATGAACGGCTTTGCTAATAGCATAACTTTTATGTTAGGAGCAATAGTGGGTGGAGGAAAAATAGTAAAATATACCCCTTCCAAAATTGATTTAGTTGTGGCCAGAATCTTATGGGAATTTAGAAGTGATGTATACAGGGTAAGGATTATCCCTGGAGATTCTAAGGTCTAAGAAGCAAAATGGTGCCTAGCATTCCCTGCCAGGACCCTACCTCCTCGGCACTTGGGGCAACCAGTGAAGCTCAAAGGCGAGTAAGAGGCTAAGTTGCTTGAAGCCTGAGGTTGGCAGTGATGGGGCATATTTGTTCTTTGCAGCAACCCGTGTGTTTTCCCACCTTACACAATGTGGAGCTGAGGTTAAATATGCCAAGTGACCACTTATTGCCAAATAGCGTTTAGTCTTTAAATGGCCTTCTGACTGTCTAGGGTTCTTCCCAATTCCTGCAGTCATCACAGGCCCAGACCGCCACTTATTCTCTTCACAACCAGGTGGAGAGTGGGGAGAGCCCCGAGTCGATATTCACTCACAGGTCATCATCGAGGAGAAGCTACGAAGATGGGTATTGGCCACTTCATCTATCCACGCTGCCGCTTTATCCAGAGGACAGTGACCTTGGCCATGCAGAAGCTTCTCCATTTCATGAAGCCCCATGGAGTCATTGTTGGTCAGTGTGTGTGCTACTGGTACTCACTCTCTTCAGGAAACCTTTGCTTTTTCTCACACTGCTCAGGTGGCCAAAAACATAAGACTAGAAAGGGAAAACCTAG
MSTRG.32598.2	ENSRNOG00000004537	0.016	0.236	0.002	3.416	up	GTAAATGTGGTGTGAATACCCAGTTTATTCTGAGGTATTCTGATGCTGATCGGATGAGCACAGATCCCACTAAGTCCTGAGCTAGGATCTTTAGGGCTACTGGAAGGAGGAGAAAATGGGCCAGCACCTCAGTTTTCTCAGGACCTCAGTAGCTATTTGTAGAAACCGATCTGTTCTCTGCCCAGAGCCACCGTTCTCTTCTTCCACTCCACAACTTGTATGTCTCGAATGTCTTATTATTCCTAGCTAAAAAAATGAGAAGACTTGGGGTCAGAGAGGTCTAGCTCTTTGGAAACTGGCAGCAATGGCCTCAGAGTGACGATGTGTCGTCTGCTTTCAAAACCCAGCAGCTTTCCTCGTGCATTAGCTGTTTCCCTTGCTGTGAGAACAGACTCCATGTGTAGACGGGGCTCGCCTCAAACTCAGAGATCCATCTGCTTCTGTCTCCCACCTCCTAGGACTACCTAACTCCAACCGATAACCACTCACAAAGGAAGCAGGCTGTGTGTTCTCACCAGAGAAACGAATCTTCAACCTTGGGCACCCCAAAACCATGAGCCGTGGCAAATCTTTCCCCCTCTTGATGTGGCATCTCAGGCTATTACCTCACAGTCACAGAGAACTGACTAGCACACTTGGTGTGGGTCTGTTGGAGAGCAAAGGCAGAATAACGAAAGAACCCAACGTGCGCCTTCTGATGCTTTCAAGGTGTCTCCATCTGTTTTCTGCTGCTATAGTAAAATGCTTGATACTAGGCAATTTGCAAAGGAAAGAGGCTTATTTAGGTTAATGGAGAGTGGGATGCTGGAGAATCTGGTGACAACATCTGTCAAGTTTCTCCAGAGGGTCTTGTTCTGCTTCAACCCATGGCAAAAAGTGGAAGAGCGAGTGGCTGCAGGCTCGAGAGGCAGGACACAGGTGTGGCCTGGCTTCATGTCAGCCTGCTCTTGTGATAGCTGATCTTGTTGTCTGCATTAATCCCTCTTGTGGAACTAGTCACCTCTTAGAGGCTTCATTACCCCACCACCCAGCACATTAGACGAGGAATCGAATTCCAGCGTGCACTTCAGAGGAGACAACCCATATTCAAACCCCAGCAGGAGGAGCTAAAGCAGGAGGCTTTGGTGTCACGCCCACACCACAGAAACAACATTATCCAGGCTGGCGGCACAACTAGCCATATCATCCCTATGCGTAAAATAAAGATATGGGGTCAGG
MSTRG.32720.1	ENSRNOG00000002730;ENSRNOG00000002773	0.562	0.000	0.022	-3.851	down	ATGTCTGTAACGCTAGATCTTTATTGTTCAAAAGAGAAAACTAGAGAGAGGAGAGAGGAGAGAGGAGATCCAGCAGGCAGGAAGATGTGTACTTCGCGTTGCCTGTCAGCAGGCTGAGGGGACAGACCGGTGGGAGAGCAGCCGTCCCTGCTGTTGCTTCTGAGCTTCTGAGAATCTTCCACATTCAGTATCATGGGCTGAATTCTTCGGGCTCCTGAAAGCGCACTATAATCACATCCTGTGATCGTGGTTGAAAATACCCAGATATGCACTACAAAGGGACCGTGTTCACACTGGGGAGTGGTGACTGGAAGGGAGAGGTTTACTTCAGTGTCTCAACAAAACAGCTCTCATCTTAAAGATCACCGGTCGTAGTCCAGTGGCCTGGAATGTGGACATCACTGCTTCTCAATCTTGGACTAGAGGTTCGGGCAGAG
MSTRG.33410.1	ENSRNOG00000002879	0.000	0.367	0.001	4.308	up	TCAGCTGTTACCTGGGTCCACAGAGATGACAGTTAAGAGTTCTTACCACTCTTGCAGAGGACCAGAACTGGGTTCCCAGTACCACATCAAATGATTCGCAGATGCTTGTAACTCTGGCTGTAGGGAATCCGATGTGATCGTGTGGCTTTCACCAGTGCCCACACACGTGCACATACATAGACACAGACACGGGTTCTAATGTTTGGA

							CAACCACAGACAGTGGAGTGTCATGTACCCCAGGCTAGCCTGAAGCTCCCTCTGTAGCCGAGGATGAC<br>CTTGGGTTTCTATCCTATCTGCATCCCCGAGTGTCTGGATCACAGACCAGGCAGGCGTCCTCCCAGGGA<br>GCCAGGGCTCCAAAGTCTCGCCTGCTCGCCTTGTTTTAGAAGAAACAAAATGGGACTGATAGCATGGA<br>GCCCCTTCAGTGGCCGCTCTGACTCTTTCTTCAGGGACAAGAACCACCCGATGTTTAAAGTTTATCCTTC<br>AAGTTCATATTAAATGCCGTCATCCACGAGCCTCGGTACACAGAGACATTTTTTGGCTTTTTTGTTCATTC
MSTRG.33533.1	ENSRNOG00000003211;ENSRNOG00000052574	0.000	0.553	0.007	3.835	up	GAGGGGGTACCGCAGCACCTTGTAGTTATTGTCCTCATGCGTGCTGCCTTCTGTGTACCTCCAGGGCAC<br>ACAAATGATTCTTCACATCGGAGGAAAAGACAATGGGGGCTATCAGTTACATGGTGTCTTTTCATGTGG<br>ACTGCAAATGTTTTCCCGGAAGCTGACCATGGTAGCGTCCAATAGACTCCAGCTACTTAGGTCGGTGAG<br>GCAGGAAGGCCACAGGTGGGTGCTTAGGAGTTTGTGCCAGCCTG
MSTRG.33606.1	ENSRNOG00000052963;ENSRNOG00000058348;ENSRNOG00000058805	0.828	2.418	0.012	2.255	up	CCCACTTCCTGGTTCGTCCAACAGACCAAAGGCTGGAGAGTACCTTCAGACCCTCTGGGATGGCAGGA<br>ACCAACACGTCCCCTGACACTGTGTTTGCAGAGTCTCTGTGGAAGCGAGAAATTACCATCCATTGAAAT<br>CATCATCCCGGCTTATTTATCATCCTGCCACGTCTTCCCAGCCTTGAGCGCTGGGGACTTCTGTGTATTTC<br>TGGCATGCTTCGAGAAGGTTCAGTGTTTTACACAGCTCATTTTGGGAGGCGCCTCCAAGTCTGATCTTA<br>AGATGACCCTGAGCTGGATCCAGTAAGTCTCAGAACAATGGAGTCCTATGTTCAGAACTGGGAGCCAA<br>GAGAAAAGTGCTGGCCAATACTACAGCATGGGAAGGACTAGAATAGTAAAAGCCTTGAAGAACACCAT<br>CAAAACCCAAGACATTGCTGGACTCATTGACTCTCAAAAGCCCTTGAAACACCATTCACTAGCCAATCA<br>TGTAGGCAGAGTGAACCCACCTCCTGTTTCCTAGTCAGCTCATTTTCAATAAAGCTCTTTCTTTCGTCAC<br>AGTATTGGCTTCTTTGCACATTGGTGCATTTGTATCCTTGCTCTGGAACCATCTAGACATGACTGAAGGA<br>TCTTCAGAGTGGTTAAACCCACTCAGTTGACTATACCTTGGTTGAATAAACCTATCATC
MSTRG.3361.3	ENSRNOG00000054417	0.092	0.398	0.002	2.731	up	CCCGAGAACTACAACTCCTAGCATTTTAGGAAGTTTTTTGTTTCTTGGTAGGTAGTGCTTATGCAGTAAC<br>TTTGGCTTTCCAGTATGAGAGAAAATACAGCCAGAGGCATCTGGAAGTGTCCACACTTAAACTGGCCAT<br>GAATGGGGGGAGGGGCTGGGAGTGGGGTGAAGTTAGTGGAGAGGAGGCTCACAAGAGAGGAACCAG<br>GTACCAAGACAACACCTAGCAAAGATATCCACTGTGATATATTAATAAGCACTTCAGTTACTCATTTTTCC<br>TATGTTTCTTTGATATCTCACTATGCCCAGACCAGTATGCATGCTGTGGAAGGGCAATATCAGGCTGCCCT<br>GTTTTTATAGGGAGAAAAGTCACTATAGATTAAATAAAAATTGAAAGCAGTTTTTGAATAGTTATGATTG<br>CTCACTTACATCAGAAACATTTCTCCTTAGTTCAAGAAGAGCACATGTGGGCAAACAGTTATTTCATGGT<br>TTGTTGACACTGAATTCTGTGACTGGATTTCTTGCCTCGACCGTGTTGTCAGGACTTTTTTTTTAAAGAC<br>CTCCTTTTGTAAAAGTACATCAGTCTGGATTCTGTGGGTCGGCATAATTATCTTGCAGTCTTCCTTGCTCC<br>TCCTCAGAGTTACTGGGAAACAACTCTTCATGTGTGTGAACTCTGTTTGATTAAGTTTCACATTTCATGG<br>AGGAAAGTGAAATCCTCAGAGAAAGAGACAATTGCCATGCTATTTTGTCTTTGCTTCATGTTTATCCTTT<br>GGCAGATGTAAGCTTTTTTTCTACGAAAAGTTCCAAGTACTCTCTGAAGTATTTCCTTCCCATATGCAGG<br>TCTCCCAAAGCCAGATCTGCTCACCTGTGAATCAACTGAGGACCCTTTTATTCTCTGAATCTTTGCTCAA<br>TGTGAGACCATCATGTCATCCTGTCACAGAGAAATCCTCAGAGGGAAAAAACATTCATTATGTTCTTCCT<br>GTTCATCCTTTTGGGAGGTATAAAGCTTCCTTCCGTGATAGATTAAGAAGTTCAGAAGTGTTAGTTATTG<br>TCATTCTCATCATTGAGTTCCTTTTCAGCTTCCAAGATTGCTTGATCTGTAATTGGAAGTGGTAGAACTC<br>AAGTCAAAGCTAACTGAAAATTCTTACAGAACATTTTCAATATTCTTCTACTATACAGCTATTGGAGATTC<br>ATTTGTAGATTCCCCTTTCTGTACTGGTACTGTAATGTATTAGAAATTCTAATTAATTCTGAAGAATGCTTT<br>TCTTATATGGTTGTCTAACTGATAAGCTAAAAGCAAAAGCAAAAAATCCTGTAAAGTAGAGAGAAAAAC

							TGTAGCAAAAGACCATGTATCATTTGGAGGAATTGAGGTAAGATTAGTGAGGAGTTCCAGGGAGTGTGAAAATGGGATAAGAGGAGCATTGAAGAGATGCCCCAGTGATGAGTAGTACTTGCTTATCTTAAAGACACTTGTATGCAGTTCTTAGCATCCCTTAGTTGTCTCCAAACTATGTGTCCCTTCAGTTCCAGGAGATCTGATGACTTCTTTGGACCTCTGCAGGAATCCTGCACAAAGAGAGTACATATATACATGATGCAGAGCACTACTGCCCATAAGCTAAAATAAAAACATACTAAAGATTTAAAAGAAATATAAATGATGCATGAGATTGGTTTTATCCACATTCTGATCCTATAGAAATGGTTCTGAACTATTCTACTGTAAATCACGTCATGATTAGCCTGTTTTGCACATTACTGACTTCCTATGTGGTATTATTAGAACAAAATATATTTTATTTCTGCATGTGTGACATCCTTACTCTGTATCATTTTGGGGGTTCATATCAATGTTATATAAATTTCTTCAATGCATTTAAGCTAAGTAGTGGGGTACAGCATCCTCATAGATCAAGC
MSTRG.33856.1	ENSRNOG00000002627	0.000	0.357	0.000	4.531	up	CAGTGGACTGGTGAGTGTGTCTGCCTCTTGTACACACACCTCTGCAGTGCACACCAACTGTGTTGTAAACTGGTTTAACAGTGTTGCTGTGTGGTATCAGGGTAGGAACTGCTGTTGACCTGAGGTCTTTCCAGGTCAGCGTGACTAACGTCACGGCTTATGTTAGGCATAGCGGATTGGCTTCAAAGCTCGTGTTGTGTAAAAACACTGCAATAAAAGAAAGTTGAATCCACACTGGCGCTGAGTTTCCTCTGAGAGTTTACTCCTCATTGGTTGCTTTATTTTCCTCACTATGTAAGTCAGTCATGAAGCTGCTACTCTGTTGCTGTGGCTTGGTCCCCTGCAGATCCCAGTTCCTCTTCAGCTCAGGTACTCAGTTCCATTTGCTCCCAAGGTATCTGATGCCTGATGGCACCATCCCTCAGAACACTTGGCTTGCCCTAAGACTTACTCTCCGTGTCTCAACGAGACACTTAAGCCACACACGTCCATGTGCACTGCTCTGCTCCTGTGGACATGATCCGGAAGCCCAAGGTCATTTCCAGTGACTAGTAGTTCACCTAAGAGCAGACCTCCTGCCATCTTAGTCCCAGGGGCACATGACTGGGAAGGCTCTAAGAGAGCTTCCTTCAGGAACCAGGAAGTTCATAGAGCTATGCTGCTGAGATAGGGCCCACTCCGTTTCTT
MSTRG.33859.1	ENSRNOG00000002627;ENSRNOG00000022144	0.690	0.000	0.000	-4.880	down	TGGGTTAGAGGTCTATGGGTACGAGTAAGTTTCATTGGCTGGTGACTTGATGGAGGGAGGGTTTCTTACACATCGTGTCTATGGTTCTAACTTTGCAAAGAGTAGGTACAGAAAGGCTTATATAATCAACACCAGTGTGTCTTGGTATAGATTTCACCAGTGAAGAAGGGGCAGAGGGGAATCGTGAATCATGCAGGCAGATGGGGGGGAATAAATAGAAGGCATTGCTAACACTTCCCTGCTGGACTTATGCAATATGTGAGTTCATGAATTTTTCATTAATATTGGAGTTCCAAAAAAAAAGGGTTTCATGGAATTTTGACTTCTAAGCAGGCCACCATGACAGAAGAAGACAACTGTTAAAGGTCAACATTCTTGTCAGCAGGGCAAGTCCATGACTCCTGCTTCCTTAATTCTGTGTCCCCTCAAGGAACTCCAGATGCATCAGAGGCACGCATTCGTAAGACAAGGAGGGAACAGACACGGAGATGAACGTACGGACACACAGACGAGTGTATGCTGGTGCTGTGTCTGCACAAAGGACTGATGGTGTGACGGTTTGTGTAGACTCACCCCAGGGAGTGGCAGTGTTGGAAGGTGTGACCCTGTTGGAGTAGGTCCTAGCTGCCTGGAAGCCAGTGTTCTGCTAACAGCCTTCAGATGAAGATGTAGAGCTCTCAGCTCCTCCTGCACCACGCCTGCCTGGACGATGCCACATTCCTGCCTTGATGATAATGGACTGAACCTCTGAACCTGTAAACCAGACCCA
MSTRG.33859.2	ENSRNOG00000002627;ENSRNOG00000022144	0.433	0.000	0.006	-4.200	down	TGGGTTAGAGGTCTATGGGTACGAGTAAGTTTCATTGGCTGGTGACTTGATGGAGGGAGGGTTTCTTACACATCGTGTCTATGGTTCTAACTTTGCAAAGAGTAGGTACAGAAAGGCTTATATAATCAACACCAGTGTGTCTTGGTATAGATTTCACCAGTGAAGAAGGGGCAGAGGGGAATCGTGAATCATGCAGGCAGATGGGGGGGAATAAATAGAAGGCATTGCTAACACTTCCCTGCTGGACTTATGCAATATGTGAGTTCATGAATTTTTCATTAATATTGGAGTTCCAAAAAAAAAGGGTTTCATGGAATTTTGACTTCTAAGCAGGCCACCATGACAGAAGAAGACAACTGTTAAAGGTCAACATTCTTGTCAGCAGGGCAAGTCCATGACTCCTGCTTCCTTAATTCTGTGTCCCCTCAAGGAACTCCAGATGCATCAGAGGCACGCATTCGTAAGACAAGGAGGGAACAGACACGGAGATGAACGTACGGACACACAGACGAGTCACAGGTGCTGTGTCTGCACAAAGGACTGATGGTGTGACGGTTTGTGTAGACTCACCCCAGGGAGTGGCAGTGTTGGAAGGTGTGACCCTGTTGGAGTAGGTCCT

							AGCTGCCTGGAAGCCAGTGTTCTGCTAACAGCCTTCAGATGAAGATGTAGAGCTCTCAGCTCCTCCTGCACCACGCCTGCCTGGACGATGCCACATTCCTGCCTTGATGATAATGGACTGAACCTCTGAACCTGTAAACCAGACCCA
MSTRG.33862.1	ENSRNOG00000002627;ENSRNOG00000022144	0.713	0.000	0.000	-5.987	down	AATGGACACACAGTGCTGCTGTGTTCAGGGCTGGAAGACAAAGGCAGCCCAGCACACCCTTTCCTGCATATCCTGCAGGTGTAGTCCATATCCTGCAGCAATAGGTGAATCCACCGTGACCTGGGTCTTCCTGGAACAGGAGCAGCCACAAGGAAGCACCGTAGATGGTGCTGACCCAAACCGATGTGCTGGGCGTGGGTCCTAAGGAGAAACTCCTGGATCGTTCTTTGATCCAGCAAAGGAGCTGGTGAGGAACAAGGGAGCAGTGAGGCTACAGTGGGCTTCACCCAGCAGATACTCGAGAGGTGCCTCCTACAGTTGGCAGAGAATGAGACACGCGCAGCCAAGACAAGCTTCACTTGAACTCTATCCAAGATAAGAGGCTCCAGCCACCTGCTCACCGCTCATGTCTGACACCACCGTCTGTTTAGAGCCAAACTCTCAGTGGGGCATGGGGTTGGGACAGCTCTGTGTTTGTACCTGTCTGTCCATGGTGACAAAGAAGGAATTAAAGGCAGAAGGCAAGATCTCGAACGATAGTCACCACAGGAGGCAAACTTATGCCAAGTCTCTGGACAGAAAGGAATCTTGATATGGATGGGAGACAAGATTAGCTCAAGTCACCAGTCAGGAGGAGCCTGGGGTCCTCACGTCGGGCAGATCTGTGCTGAAGGGCTGTGCTGGGAGTCCTGGAGTGCCACCCAGATGAATCACTTTCCCTACACAGGGAAGACTGATCTTTCCCTCAGAAGCTGTGTTTTGGGAGGGTGCTGGGGAGAAAAAAGCCCAAAACGCAGATGAGTGGATGAGTCTGGAAGTGGATCCGTGGTGAGGTTTACAATGTCTGGAGTTTGACGTTAACCACTGTACGATCGCTTAGAGATTTAGCGATTCTTATTAAGCCTGCATGTATTCAATGGGGTTTTCTTTCTGGTATTGTTTTTACATGTTTCAGTCCCTCTATACTAAATATACAGAAATAAGCAATGAGCCACGTGTGGTGGTCTAGGGAGGCAGGAGCATCTCTGGGTTCACCATTAGCCTGGGCTATGTTGCAGGTTCTAGTCCAGCCAGCACCATATGGTGAGACCCCGGTCCAAAGTGAAAAAGCAAATAAGTGAATGGATACATAAATAGGTAAAATAAGAAATAACCACCTAAATACCTTACACTACATGGCAGTTGTAAAGGGTAGAAAAGACCCTGAAAACCCGAAGTAGGAAAAAAAAAAACCCAAAGCAAGTAATTATATCTTTTGAGCTTTAGAGAAAAGCTGAAGGTCTCATTCTCTGTCCACACCACACCCTGTGCACATCTGTCACTTTGTATCGGTGTGCAAAGCTAGCAATGTCCGGAGTGTGTGTGTGGGGTGAGGGTGGGGGGCTTGTCTAGCTCTTTCAACTAACTGGGACGAGAGATTTGGGTTTGAACCTCTTCACAGAATTTTGCCATCCTTCCCCAAGCTGCAACCTTGAGTCACATCAAAACCCTGATTATCTTTTAGAACCAATGAGGAGCTGGGCTTGCTGAGAGCCTGGGTGGATGTGTGTGTGTGTGTGTGTGTGTGTGTGTGTGTGTGTGTGTGTGTGTGTGTGTGTATTCATTCCTTAGCGAGTGTGAACAGCCTGTCTCCGCTGTTTGTGTATTATAAACGCCTG
MSTRG.33920.2	--	0.318	0.000	0.000	-4.955	down	GTGCAAGGTAGGGTTTTTATTAATGCAAGTTCTAAATGTGGATTAACCCTCTGGCGAGTCTGTTTCCCAAAGCACTGGAGTGTGCTCAGGTCCCTTTCAAAGAAGCACACCGCTGCCATTATTCTGAATATCAATTGAACAGCATTCTAACTCAGGCCACAAAGCAAGATTTAAAACGATTACAGCTCTCAGCGCTTACATCTAAATAAGTCACCGGAGATTCAAACTGTACAGACAAAATGCTGTAAATCGGTCTTCTCGGGAGAGTGTTGCTCCCTCTCTCAGGCTTTTCAAAAAGTGATTGTTATTTTAGGGCTGAAGAGAGCCTTGGCCACGAGTTGATTCTATTCTTGAGGACATGTTGATGTGAGAGCCGAGCTGCATTTTTGACTCATGGCTTAGGACAGCCGTCAAGGGCATTTTCCTCGGGAGAGAAACGTGTAAGAACACATCTCTTTATTTTATATTTCTATTTTGATGTCTGTCTGTATTTCAGAAATCAAAGAGCTTGCTGAACTTGATCAGGTGAGGTATAACTATGGTTGGAGGTGACTCTCCAAGCTGAGAGGTGCAACGGCTGGGAGCAGCGGGTTACCCCCCAACAGTCAGTTATTTATATCTGTCCATCATCTCTTATCTTCCACCTCTGGTTTGGCAGTAGAAAAAGAATCCCATTTCCCCCACAAGAATCCATAGGGAAGGTTGTCTTCTCATCCAACTCCGGCGATTTTCAGTCACATCCTGTTTTCTTAAATGGATGCTGGGTATTTGGGTGATACCTCTTTGTTTGAAAGGACTCATAAAAATAAGCTACTTTCACGTCTAATTCTAACGAGATAAATGGCACTGTGAGGGGACATTAAAAATTTAAAGTCCTCCTTCCTAAGTGTATTTGTCAGCTGA

							GCACACTGAATCTTGCATAGTGCACACACTTTCCGCTGTCATCGCACGGCTGATGGTACTGAGAGAGTCTCCTCGCTCTTTGGCAAGCCACTTAATTGTATTGTGCCAAACTGGCAGAATCGTACAATATGTTAGTGTAAAGGCCCCACAGTTTAAGGGGGAGATCTAGAAGTGTTAACTGTACACTGTCTAATTATACAACCTTCCCGTCCCAGAATGTGATTTCAGACACCAGCTCTGACATATGGAAAAGGGTAGAACAGCTGGTCATTATGGTTTCCTCAAGACTATTTAGCAACACACTTGAATGTAATTAAAGGGAGACACTAAATACAGCACTTGCGGTACAATGTATTTTTAATTTCTATGGCATGTGGTAAAAGTAAATAGATGAAAGCATTCAAGTTTCTCCACTCGGCATCCAACTTGTCAAACATGCAACGTGCGCTTGGCTGGCGGCAAGCAGGGTAAGGAGCAGGCATCCGTGTGCGGTGTCTAAGAGGCTGCATTGGGCCATGCTCAGAGGAAACATCCAACTGGAACCATGGATTAGAGATGAAATGACAACATTCATCCATTCAGGTGTGTACCCACCCATCCACCCATCAGACAGATGTCTTGTTGGTCTGATAAAGGGCCAGGTGTAGCTCACGGCTTCCTCACTCACTGAAGGCTGTGACTGGGGTGACAAGGTAAGAGTATCAGGTCCACACAGGGGCTGTCCCTTGACAGGAAGTGATGGAGTTGTGAGTGAGTAGTTTGGTGTGATGGCGAAAGAGCCTTCTGTGCAGATGGACAGCTGAAGTGGAGGCTCTCTCAAATCTCTCTCCAAAGGACTCTCGCGGTGACTTCTTTGGGCCCCTCTG
MSTRG.33966.1	ENSRNOG00000003716;ENSRNOG00000042286;ENSRNOG00000046460;ENSRNOG00000049633	0.080	0.438	0.007	2.891	up	TGGGACATTACTGCTCAATGAAGGTATCAGTTTGGTAGGTTTCACCGCATACGAAGTCAGACACAACATTTGCTACAAAGTAACAGCTTCTTTCTGGAAGACAATGGTCCCCTGTGCTCTGAATGTTGAAGTATGAGGCAGTACAACACCTGCCCGCCTGGTCCTCTCCAGGGCGGCTCATGATGCAGCTGGTAGACTTAGTTACTTTCTGTCACTGTGGTAGGGCACTATGGCCAAGGCAACTTATAGAAGGAAGAATTTGACGTACAGTTCCAGAGGGTTGGTGACCATAATGGCAGAGTGGAGGCAGCAGGTAGACATGGCAGCCGGAGCGGGATGCTGAGAGCTCACGTCTTGAACTTGGAGCACAGGCAGAGAGCTCTCCAGGCTCCTGGCCTTTGTGTTCTTCCTCTTCTGGGACATGTCAGGCTGGGTCCCACCTTGGAAACTTGGCTCTTCTCCTAGCTCTCTCTGTCTCTGTCTCTGTCCCTGTCTCTGTCTGTCTGTCTGTCTGTCTGTCTGTCTGTCTCTCTCTCTCTCTCTCTCTGTCTTTTTGTCTGTGTGTCTCTCTGTGCCCCCCTCTCTGTCTCTCTCTGTCTCTCTGTCTCTCCATCTTTCCATCTCTCTCTTTCTCTGTCTGTGTCTCTCTCTGACTCTCTGTCTCTCTCTAAGTCTGTCTGTCTCTCTCCCTCTCTCTGTCTCTCTGTCTTTTTGTCTCTCTGTGTGTCTCTTTGTGTTCTCTCTCTCTCTCTCTC
MSTRG.34044.1	--	3.858	0.393	0.008	-2.501	down	GGCCAGCAAGGCATGGAGCCGGGTCAGCACTGGTGCTGACAACGCACGAGAGATTGGACTCAGCTGCTGTAGTGTGTGTGTGTGGTGAGCATGGCCCTGGGATTGTTTGTGGTCTCCGAGATGGACATGTCCTCTTCCTAATCCCCCTGTGTAGAAAGCATGCTGTCTTTGCTGAGTGCTTGGGCTTTTGATCCTGTGTGCGATTACTACTTGAAATATGAGATGGGACACTATAGATACCATAGATCTTAGGAGAAACAATGTTAGGGAAAGAACATTTAACCTGGAGGAGTTGGAGAGGTTTGCTCAACTGAGAGATGCGGCCTTCATGGCAGTTTGCAGGTGAGGCTTCAGCAGGTATGTGATCTTGCTGCTAACGGAATAGGGCAGGGCCCACAGGGTTCCGTCCATGTCAGAGGTGCGGTGAGCTTATCAGTGTGTGATGCTACAGGGAGAGACCCAGCCTCTGGCCTGAGCCTGAGGAGAAGCCACTCACCAGCATTCCAGTTCCAAGATGCTCCTGGTGCTAGTTAAAATGCAGATTAGACTTGGGAGGGAAGAACAAGGGCTGAAGCCTTTACTCACCTTGGGGAAACTCGTTCTAGCACAGCACCCAGTGAGACTGACAAGGCTTGCACTAGAGACAGGCTGCTGGGAGCCCTGAGAGCTTCCTCCCCACTGTGTGCATGGCCTGTCCCCTTCTGTCTGTATCTGACCTCAATGTCAGTTGATAACATTATTAAGTGCTGTCTGTGCCCTCAAGTATGAGCTGAATGGTCGGAAATGCCACAGAGTCTATATATTTTAAATCACACATGTGGTCCCCAAACATTGAATGTTGGAGAACTGTCTTCAGAAAGTTGGTCTATTTAAGGCTACTGGGGAAAAGGGCGGGTCTCCTTTGCTATCTGTCCACAGCCCCTTTGCTTCAGCAAAGAACAGTGTGCATGCATGCAGTCTGTGTTTGAGAGCCATGCGAGCCTTCACCTCTGTGAGGGCCAGATGGCTGTTTGTCTACAGTTTCCTGAAGGCC

							TCAGCAGAAAGGGATTCCTTTTTCCTAGTAAGTGCTGCTGGCTGAGAGGAGGTCAGAAGTATTTTAATGCCATCTGTTTCATGGCTAAACACGCAGACTCCTTGCATTTTAAAATTCTGTGTCCTTTACTTTTTGGAGCTATACTAGCTTGCTTGGGTGTGTAACTTGCCAGAGTTGACTTTTCTAGTGCTTTATGTAAACTCTGCTGTTGGCACTTGCAGCTGAAGCAGCTGAAAGAGGGGCTGCTCACTGTTGCAGCACTGGGATCCCTGGCAGCTGCAGCGGCAATGCTTAAGGAGCTTTTGCAACTTTGACTTCAGTAGCAGCTTGGGATGATAAAATATGGTGAGTGTCTTGTCTGCTGTGGAGGGCCTGACAATCTGCCAGGTGGGAAGTCTTGTTAGAAACCAGAAACTCAGTGTGGTGGTTATCCCACCTTTTCTTTCTTTCTCCTTTGGTCTATAATGATATTGACATAGCTATTCAGAGTAAAATCTATGTATACAAAAAAATTCTAAGTATGAGGTTTTAAATGTTCTTGAACTTCAGAAAAACTTGATTGGATACAATTGTTTATAATTAACAAGTCATCTTATTTATAAATTTTCCAATATAATATAAATCTATTTTGTG
MSTRG.34044.2	--	0.730	0.014	0.000	-4.339	down	GGCCAGCAAGGCATGGAGCCGGGTCAGCACTGGTGCTGACAACGCACGAGAGATTGGACTCAGCTGCTGTAGTGTGTGTGTGTGGTGAGCATGGCCCTGGGATTGTTTGTGGTCTCCGAGATGGACATGTCCTCTTCCTAATCCCCCTGTGTAGAAAGCATGCTGTCTTTGCTGAGTGCTTGGGCTTTTGATCCTGTGTGCGATTACTACTTGAAATATGAGATGGGACACTATAGATACCATAGATCTTAGGAGAAACAATGTTAGGGAAAGAACATTTAACCTGGAGGAGTTGGAGAGGTTTGCTCAACTGAGAGATGCGGCCTTCATGGCAGTTTGCAGGTGAGGCTTCAGCAGGTATGTGATCTTGCTGCTAACGGAATAGGGCAGGGCCCACAGGGTTCCGTCCATGTCAGAGGTGCGGTGAGCTTATCAGTGTGTGATGCTACAGGGAGAGACCCAGCCTCTGGCCTGAGCCTGAGGAGAAGCCACTCACCAGCATTCCAGTTCCAAGATGCTCCTGGTGCTAGTTAAAATGCAGATTAGACTTGGGAGGGAAGAACAAGGGCTGAAGCCTTTACTCACCTTGGGGAAACTCGTTCTAGCACAGCACCCAGTGAGACTGACAAGGCTTGCACTAGAGACAGGCTGCTGGGAGCCCTGAGAGCTTCCTCCCCACTGTGTGCATGGCCTGTCCCCTTCTGTCTGTATCTGACCTCAATGTCAGTTGATAACATTATTAAGTGCTGTCTGTGCCCTCAAGTATGAGCTGAATGGTCGGAAATGCCACAGAGTCTATATATTTTAAATCACACATGTGGTCCCCAAACATTGAATGTTGGAGAACTGTCTTCAGAAAGTTGGTCTATTTAAGGCTACTGGGGAAAAGGGCGGGTCTCCTTTGCTATCTGTCCACAGCCCCTTTGCTTCAGCAAAGAACAGTGTGCATGCATGCAGTCTGTGTTTGAGAGCCATGCGAGCCTTCACCTCTGTGAGGGCCAGATGGCTGTTTGTCTACAGTTTCCTGAAGGCCTCAGCAGAAAGGGATTCCTTTTTCCTAGTAAGTGCTGCTGGCTGAGAGGAGGTCAGAAGTATTTTAATGCCATCTGTTTCATGGCTAAACACGCAGACTCCTTGCATTTTAAAATTCTGTGTCCTTTACTTTTTGGAGCTATACTAGCTTGCTTGGGTGTGTAACTTGCCAGAGTTGACTTTTCTAGTGCTTTATGTAAACTCTGCTGTTGGCACTTGCAGCTGAAGCAGCTGAAAGAGGGGCTGCTCACTGTTGCAGCACTGGGATCCCTGGCAGCTGCAGCGGCAATGCTTAAGGAGCTTTTGCAACTTTGACTTCAGTAGCAGCTTGGGATGATAAAATATGGTGAGTGTCTTGTCTGCTGTGGAGGGCCTGACAATCTGCCAGGTGGGAAGTCTTGTTAGAAACCAGAAACTCAGTGTGGTGGTTATCCCACCTTTTCTTTCTTTCTCCTTTGGTCTATAATGATATTGACATAGCTATTCAGAGTAAAATCTATGTATACAAAAAAATTCTAAGTATGAGGTTTTAAATGTTCTTGAACTTCAGAAAAACTTGATTGGATACAATTGTTTATAATTAACAAGTCATCTTATTTATAAATTTTCCAATATAATATAAATCTATTTTGTG
MSTRG.3406.1	ENSRNOG00000019453;ENSRNOG000	0.511	0.000	0.000	-5.027	down	CTAATTTGCATTCCCCCTGCTTCAGCCTTTCAAGCGTTGTAATTACAGGCATACTGCATAATGTCTGGCTCAGAGAAAGACTTCCTGCTATAATATGAGCCCCAAATCAAATTAGTACCTGAAACCAGATACTGAATAGAACTTTTGATTTCATTCACCACCAAAAACAGTTTGTCGAGGCTTTATTGATTAGGGTTTACATCATCTGCAAGTAGGAGAGTTATTTACCTCTAATTGTTCCCGTGACACCACCCACTCCTTCACCTACCAGCGACGCCGTGTTCAGAGCAGAATTATCCCTGTGTGCCACAGACTGGATAGAAAACTGTCCGGTCTCTAGAAGTTAAGCACAGCAGTTGCAAGTGGATGACAAGTCCAAGTTGCTCTTTTTCCTCAGTTTTCATTCTCTGTGGTTGAC

	00019623;ENSRNOG00000019626;ENSRNOG00000027436;ENSRNOG00000027459;ENSRNOG00000027487;ENSRNOG00000027514;ENSRNOG00000031113;ENSRNOG00000031459;ENSRNOG00000038445;ENSRNOG00000043328;ENSRNOG00000060429						ATCTTCTCTTCAGTTCTAACATCACAGCATTAGCTACAAAATTCAGTGTCAGCCATCTCTTAGCAAAAGGAAGCACAGAAGCAAAACCCTTTTTGGTCATAGCTTCATTCCAACTATCCTGGTTTCCCTTTTCCCGCTCTGTAGTGTGGAAGCAGAGATTCCTATGGAAGACCAAGTGGTTTGTGAGAAGTCAACCCCAGTTCAAGGTGATTCATCCCATGACAGACCATTAACAGTGTAGTAAAAGCCACCTCTGCAATTGTGTAGCAGAGAAGCACGGCATCAAGTAAAAACAAACGCTCGGGGAAAGTCTCGGGAGGTCCTAGTCAAAGATCATGTCAGCATCTATTATGTCAGTAGCAAGGAAAAGCGAGCCAGGAAAATGGCCGGGCGAGAGTCGTGACCTGGAGGTCTTTTGAAGCCTCTGTCTATCTCCGTTCGACGGATATCCCCATAGGAGATGGTGCACACGCTCAGTCTTTGGATGGAACCCTCCGGACACGCCACGGTCATCTGGACTCTGGGGAAGGTGATAATCACAGAGGCCTAAAGTTCTTGACTTTTCAGTTCTCACGAACCTCCGGAAGCGCCCTTGCGGCAGTAAACCACCAGGTGACACGGCCCGCTCCACAGACAGCAGGGGAAGGGGCTGGTAAAAAGCCAGAAGTCCCGCCTTCTCTGCGAAGCGGTCCAGAAAATGGCTCACAATCTATAGCATTGGTCCAGTGTCTCTCTGGCCCGCCA
MSTRG.34070.2	ENSRNOG00000046445;ENSRNOG00000058463	0.366	0.044	0.036	-2.207	down	AGAACTATATATACAATTGTTAATGTCAATTACATGTTTAAACATTCTAACCATTGTTATTTTAAATTGAAATCTGTCTTTTAGATCTTACTTTTCTGTGGTGGTTTGAATATACTTGGTCCATGGGAAGTGGTACCATTAGGAGGTCTGGCCTTGTTGGAGGAAGTGAGTCACTGTAGGAGTGGATTTTGAGGTTCTATGCTCAAGCTCCGATAAGTCCAGAAGAGAGGCTCCTTCCGGCTGTGTTTGGATCAAGATGTAGAACCCTCAGCTCCTCCTCCAGCATCATGTCTGCCTGCCTGGATGCTACCATGCTTCCTGCCAAAATGATAATGGACTGAACCTCTGAAACTATAAGCCAGTCCCAACTAAATTCGTCCTTTATAAGAGTTGGCTTGGTCATGGTATCTCCTTACAGAAATGGAAACCCTTACTAAGACATGTTGGTGGAGATGGTGATTAGCCACTAGACTGACTGTGATGAATGGATC

							AAGCAGCAAAAGGACTCTACCTGTGCATGTGCACCTGGCTCCAGTGCGGTTCCAGAGAAGAGTTCAAC<br>TGTGCACACTGAGACCGGCACTGGCCTCTGCATCCCCTCCCAGCCTCCCAACAGCCACTGCGTTTTACT<br>TTTCTTGTGTGTGTGTGTGTGTGTGTGTGTGTGTGTGTGTGTGTGTGCACCCACTGAGGCCAGAAGAGG<br>GTATCGAAGCACCTGGGGCTAGAGTTGTAGGCATCTGTGATTCACCCAACATGGCTTCGAACAGTACCC<br>TGATCCTTCATAAGAACAGTAACCATTAACTGTGGAGCAGGCGTCTCAGACCTGCTCGCTCACTGTGCT<br>TTAAAGTCCCTAGTTTCTTTTTTTTTTTTTGGTTCTTTTTTTTCGGAGCTGGGGACTGAACCCAGGGCCTT<br>GCGCTTCCTAGGTAAGTGCTCTACCACTGAGCTAAATCCCCGGCCCCGCCTAGTTTCTTTCTTCACAAGG<br>AAGGTTTCATATCTTTCTCACCCCTCCAACTTCAGTGCTCACCTGTCCTTAATCTTCATTGGTGATTTTTT<br>GACCTCATTCCACCCTCCCCCTTACCGCCCACCAAAAGGCCATGAGCCCAAACACTCTGCCACCAGAA<br>CAGCTTCTCTCCCCACTCAGAGATCATCTGTGAGGTAATGTGCGAGGTCGGGTCTAATGCCTCCCCTAG<br>CTGTCTACTCTTTCTGCCAGGACATGCTTTCCCACAGTTCATTTCTGTGACCCTGGCCTCTGTACTGTTG<br>TCACTGGGTATGGTGCCCTTCAGGAAGACGTGGGGCTGACTGTCTCTTCAGGATGTATAGTTAGATTGG<br>AGATATACAAGTCTCTTCCAGCAGGCTGCCCCAATTTTAAAACCGGAATCACTGTGTGGGATAGAAAAG<br>GCCATCTCTTGGCTCTGAGTGGGCTTGGAGCTCCCTTGTGGCCACCTTCTTTGCCAGAGGCTTTCTGGC<br>AGCCCTAAGAGCTCGCTTCCCTGCAGGTCAGCGTGGTACCAGCCTGACTGTGCGATCACCCCCAGAGG<br>GCATGTGTGAGAGATAAAACTGACTGTCCTCAAAGGCGCTTTGAGCCAACTTAGGTCCAGCTACAAAA<br>ATTAGGAAACACCAGCCCTCTGTCTGTGCCTTCTCTAGTTATTCCCCACCCAAACTAGAAGTCGAGAGC<br>AGAGACTTACAGCCAAGCTTTCCTGCGTCTGAGGAGGATGTAAAAAATGTAGATGAAAGCACACCATT<br>TTTGCTGGGAAAAAAGGCAAACATAAAGGTCAGAGGGAGAGGCGAGATGGAGATGGTGTGTAAGATG<br>TCTGTCCTGACTGTTAGGAGAGGGAGGAAGGGGCCATCTTGGAGATATTGCACTTGGCACACTTGGTAC<br>TCCAGGCTGATGCCCAACTTGACAAAGGCATCAACCTCTGTCCTCGGGGCTCTTATTGTGAAGTGAGGA<br>TAACCATTTCTCACACTCCTGTGGTAGGAGTGGATTCTACCCCAGCTGTGCTCTGCTGGGCTTCAGAGG<br>ACCTTCATTCAGTGACATTGGTACCACAAGAAGCAGTCAACGCTGTCAGACAAGCTCAGCAAAGCACC<br>CTCATGGTCTCTCCTCACTTCCCGGTCTTGTTTTCTCCATTTCTCCATTTGTTGGCTGTACCTAAAAAGCA<br>AACTTTCTAACTCCAGTCCTTGACCCAGATCATTTCTCATTTGTTCAGTTCCCTTCTGAGTCTCACTGGG<br>CACATTGGTCATGGAAGGTAAGCCATGCCCAGTAGTAGGTGGCCAACAGAAAATGAATTTTTTGGAAAT<br>GCTATTTTTGGAGATATTTTTTGTCTCAAATTGCTTTGACCACTTTTTTGTCTCATTAGTCTTTTGCTTGTA<br>TATTTTGGTTTCCATGTGTGTTTTTCTGGTTTTTGTTGTTGTTGTTGTTTTGTTGTTTTGTTTTTTTAA<br>GAGAGAGAGACAGGTAGGTAGAGAGATGGGAACAATCCGGGAGGGGAGAATGGTCATCAGAATATAC<br>TCTTAAGAAACACAGAGTTGAGTAGATGGAATGAGGGTGGATCTGGAAGAAGTTGAGGAAAGCGACA<br>GAGTATGACCGAAATGCAGTACACAAAATTTTCAAGAATTGTAAATAAAGAATTTACAAAGAGAACCTC<br>AAATTAAGACAACATTGAAAATTGGGTTCTTTGGGAAGGATAGTATCTGAATACTGTTGTATTCATGAGT<br>TCCAGAGTAGCCAGGACAACATAGTAAAGACAAAATTTCAAAAGGAGGAGGAGTGAGTCCTCTGTTGT<br>GATGGATGGATACTCTTCCATGGGCCCTCAGTATGAGGTATTGTTAGGAGGTGGTGAAAGGTCGGAGGC<br>AGGGGCTGGTGGGAGGATTTATTGTGGCGTGGCGTATCTCTGCAATTATATTTCATGGTGGCCACTTTTC<br>GTTTGCTACAACTTTCTCTGCTTCCTGTCAGGCAGGATATGTGAACAGCTGTCTCTACCCATGTCCCTGT<br>TGCCATGGTTTTCTACCCAAGCACATGGGTATAGGATGGTCTGAAACTGTAGGCTAAGACAGCCTCTGT<br>TCCCTGAGTTGTTCTTTCTCACACTGGGTCATAGAAATGAAAAATAACCAATACAAAAGTTTCCGTGTA<br>GCTGGAATCCCTGAGACCCTAGGCCGCTGTTCCTGCCCTCTTACGACTGTGTCTGTATCAGAAAATCTTG<br>AAACCCGAAGCTGGAAGTGTACCTCAGCCCACGTTCAGAATGCTCACCTTGAATGAAGAAGACCTGGG<br>CCGGAAAACCAGTACCACAGAAAGCGGGTGTGGAGTTGTGCTCCTGTAACCTTGCAACCTGATTACAG

							GCACAGTTCTGGTTTACCCTCGACTACACAGTGAAGTTGAGGCCAGTCTGAGCTAAAAAGAATGATATCACAAACCGTTTAATACCATCAGGCTCCATTTTCTCTCCAGCCACTGCCTCAGTCAGTGGTTCTCAGTATATCAGCTTCCTCCCAGTGCAACCAGTACTCTGTACCTCGGTCCCACCTAAGTCGCCACTCACTACCCACTTCAGGTTGGTCCACTATGTTCTCTCCGAACTGGCATTGGTAAATAATAATGATAATAATAATAATAACAATAAGAATAAAAGTAGTAATGGCTTTAGTCATAATTTCACTAATGAATAACTGAAACACAATTTTAAAAAGACTAATTTAATCTCTGATTTTCTATGATTACTGTATTTTTAACTTTTTTGCATTTATGTATGTCTCGTGTTTGTATGCCTTGCGTGTGTATGTGTGCACGCGCGCATATGTGGAGGTCAGAGGACAACTTGTGAGAGTCAGTTCTCTCCTTCCATGAGTCCCGGGGCTGTCAGGCTTCCTGGCAGACACCTCTACCTGCTGAGCCATTTTGTGAGCCTCACATTGATTTTTTGAAAGGATTCTTTTGTTGGTCAGCCTTTTGTCTCCGAGAATCAACCCAAGTCAGCTTATACAGAGGAAGGATTTACTTTGTATCCTAGTGTGAGGAGGAGAGGGCTTATTTCCTCTTACAGCTCAGGATTCCATCTTTGAAAGAAGCCAGGGCAGGAGCTGAAAGCCCGAACCTGGAGTCAGGAACTGATGCAGACACCACTGTTAACTCTGATTACAGGAGTGCCCACTTGGCTCTTGAAAAAATATAACCCAGGACCACCTGTCCAGTGGTGGCACCACTCAGTGGGCAGAACCCTCTTACAGAAATCATTAATCAAGAAAATACCCTACAGACTTTCCTACAGGGAAACCTGATGAAGGAATTTTCTTAGGTTCCTCTTCCCAGATGACTCTAGTCAAGTTGTGCCGAATGGACAGAAAACTGAACACGCTTTGGTCGTGATTTTCCTTGCTCTGGGGCTTGTGGCAATCAGTGTATCCTGGTGGTAGCATGTGGCAGAGGGAGCTGTTTAAGTTATGGTGTCACAAAACAGGGGGGAGAGAGAGAGAGAGAGA
MSTRG.34335.2	ENSRNOG00000000064;ENSRNOG00000000065;ENSRNOG00000023937;ENSRNOG00000048308;ENSRNOG00000049895	0.040	0.344	0.000	3.701	up	GCTATCTATTAGCTATCAATCAATCTATCAGTTACCAATCTATCTATCGGCTATCTATGCAATCCTAACTCACTCCTCCATCCATCTATCCTTCATCAATTTATTCACATGTATATGGCTAAGCACACACACATATGGTTGGACAATATTGATTCTTTACTCACTTAGAGTTATGTATTAGATACAGCTTTGTTACTTCTGATTGACAGTATTTTACAGTGTGCTTTTACCCCAGTTTTTATAGTTATATTGAAGCATAATTGACAAAAATCAAACCTGTATACATTTAGTTTGGTCCCTTGAGCACAAGCATGTCTCTTAAACCCAGGGAAGTTCTTTGAGTCTGCCTGGGCTCCTCGGCTGGCACCATTGCTGGCAACTCTTAAGCTGTTACACGAGGCACTGGAGGCTTTCTTTGATTCTCTGTCACAGTCTTGCCTTGTTTCATGATTAGCATTTGAAAACCATGCTTTCTGTCCTCTGTCCAGCATTTGAGTTATTTCAAGGGAAGGGAGACCCTGCCACCTTTGCTCTAACATGGTTTTGGCAGGGGTCATCCTTCTCTGCTTTCTTGTGACTCCCTGCCTCAGATGAAACTTCAGTGAGCATCCCTGAGCATCTCTGTGAGGGTGTATTTGAAAATCTGAAGTGGTTGTCCAGCCACCAAGCTCTTGTTCGCCCTACAGGATGGTTGTGTCTACTTACGTCTGCTAAGGAAGAAAGGCTCCACCTGGACTGTCCAGCCCTGGTTTTGGAGGTCTAGGAATACAAGGACATAGTGTGGTAGACAGCAGCAACAGCATGATTTCAGACGAGAAAATGAACACAGGAAACACAAGAACTCCATTTGATGTGTTCCACAGGTAGAAGGAAAGTTCACCCCAGTTGGAGTGGTGGGAGGCTGGAGTATGTGGGAGACAGAGCCAGAACCATTTCTGGACTCTTTACTGTCATAAACAGAAGCCAGAGGAGGCTTAGGTGGATTCCCATTAAGTGCTTCATTTAGACTCCAGAGGAGACCAGGTTGAGTAGTGATGAGTTTTCGAGGGATCAGCTGAAAGAGGTATAATATGTGCTTGGGCTATTACAGGAGAATCCACAGGGCCAACGTACGCCACATTTCCAGTCACGCTTCTATTCATGCAGTGAAACTGGTCAGCTGCAGGGTTTCATTACCAGCCATGAGTGAGCCAGGGCTGTGCGCAGCATCGTGGACACGCTCCACAAACATTATTTATGGAACAAAAGAAGCCAGGTAGGGAGAATCATGCTCTCTGACTCTCTGCCCCCAGAAGGCCGCCAGACCTCTGTAGGAACTTGAACAGTAGTTAATGATGTCATTTTCCTGGAAAAGTGGCAAAGTTATCCCTCACCGGTGTGGTTCTTTCTGGAGTTGCTCTCCTTCCTGCCTCTGACCCTCCAGGTCACGTGACTAGACCGCAGCTACCCTGGGTCCCCTCCTTGGCATGCATGGAAGGTTAAGCAGACTCTGCAAGTGGAAGTTGAGGATGGCTCACAACAGAGCATGATGAGCTCTTCCTCGGTGAAAGGATCCTGAAATAAAGCAATTCAATCTGCTCTAAAATCCTACCTGGGTTAGCTTCAATGTCATCCCCGGTTTCGCTGTCGCGGGACCACAGATTGCCCCTCCCACTCTCCGCTA

							ACTCCCAGTCCTTTTAGAAAGACTGCTGAGCAGAAGCTGCAGGGTGATTCCCTGTACCTGGGATTTTACACTGCAAGCAGAGAGAAAGCTCCTTTCGCTCCCCTAGATCCCAGGCAGCAGAGCTCCTTCTCAGCACACGCCAGGATGCTCGCCTGAAATCTCTGTGTTCCTGCCCTCAGAATAAAATTGAATTGCTTCGGCTTAAAGACCCTTCTCAGTGAGTTCCCCTCTCCGTGTTTGCAGGCTCCTGCCACCATACTCTCCAAAGAACTAGATTTTTATTTATTTTTTTTTTTGGCACCACAGACACGTACATACTCAGCTAGCCCCAGAAATAGTTGGAATTCTCCTGACCTGGCCATTTCCAATCCGACCTGATAGAAAAGACCTATTTGTACTCCAGGGAAATCCTGCACCTTGAGGATCCTCTGCAGGGGTTGGAATCAGGAAGGCATCGGGGGTGGGGGAGCATAGCCTGGTCACAGAGTGTGGGTTTCTGTCCCAATTCCAGTCACTAATTTACTAATGAAAAGTCCTACTGGGGATAACAGGGGACCTACCTCACCGGTTGTCCTGAGAAGTGAGTAAAAGAATGTTCCTGAAAGCTTTCAGTACTGAATGCATCGGATCAGACAGGCTCCAGACAAAACTGAGGCAATTAAGAAATATGACAATAAATTTTGAGAAATGAGGAGGCAATATCGGGGGGGGGATCATTGTGGAGTGTGAATATACCCTAACGGGGAAAACATCATGTTCATGGACCCTGTGGTGAGTATAGAAATGGAAAGGTGGTTAGGGGGGTGGCAGTGCCCCTCTAGAGGAGAAACAACCCCAAAGAGTGCTCTGAGGTAGAAGGCATCCCCATAACCCTACCCAAGTCTTCCATCTTAAGACCAGACCTCACCCTTCATGGATGTGGTGGCTTCAACTCCTTCTTCCCCAAGACCAGCCAGCCACAGGGGTCCCATGTGGGTTGCAGAGAGGCAGCTCTGCAACGCACAGCAGCTCCAGATCTCGTTTCCCTGGAAACTGGAAGAACCGTTGGAAGTTTGCACTGAGCTCTGCTCTCAAGCGTGGGCGCCGCGATCCTTACGTACGAGGGCTCGCGGAGCCTCCTGGGCCACGCGCCAAGCTCGCGGAGGTAGGGCTGTGCCTTTGCTTTCTGGCGAGTCATCCTTCACCAGGCGCAGGACCGGTATGCTGCTGTTTCAAATTCTGCTCCTCCTGCTAAAAATACAGAGAGGCAGGCTCGCTCTTTCTGAAGCTATGGCCCGAAGTGAAACCCACGCAAACCTCCGGGAGTCTTTGGAAAGCCTCCATACACCCACTAGCAATCGGCTTCCCGGAAGGGTGGGCAAGAACCCCAGAGCTGCCTCGGACACATAGGCGCGGCTTCCCCAGGCAATACAGCGCTCAGTTTTGGAAGGGTCTCTGACTTTAAAGGGCAAAAAATAGGAAAGTCCCTAGGCCTTTTCCTGACTTGAACCTGCTGTGGGAAAGACTCGTGCGCAGGCGCATTCTCTCCACCTCTCCCTCTTCCCGACTCTCGGACCCGGCTTCTCCCAGCCCCAGCTAAAGGCGCAGCCAAGGCAACCCGCGGTTCCGAAGCTGCTGCGCAGGCGGCCTGAATCACCCACACCCCGGACTGCAGGCCAGGGCGGGGTTGCCAGAGGGAGGCCTCCAGTGGTCCAAGTCCAGATCTGGGCTACTACAACGTTCTCCCTTCTCGTGCACTCGAGCACAGGCCGAATTTATTTACCTGAGTGTGAGGGATTTGTCTGCCAGATCTGAAAACGGAAATGGACTCAGAGCTGAACTCACCCGAGGTTCTCCCCAAGACTTCATCCTGGCCTTCTCCATGGCTGGTCCTCTGAGAGAAATCTCTAGCCAGTTCTCCCGCATACCCTATTCATAACCCCCCAGCACCTACAATACTCTACCCACACCTCAAGGCGACACCTTTCCTGCACATTCACCACATAACACACAGACACACACTTGCACACCACACGCCTGGACATTGCACGTACACTGTGTTCACCCTGCGCACATCTCGGAATGCACACCGCTCCCCGCCACAGGACACATACGTACACACCACCCTCACGTACCCCATACTCTACATTCTGCGTACATCCTTCACACAGGTACACAGCACACACCCCTACATGCCAGCACTTCCCAAAGAAACACACTTGGTCCTCCATCCCCGAATACCCTGCTCGCTGTCCTCCATTCCTTCTGAACATTTCCCCTTTCCCCATTTCCTCCTCCAGGCTCTCCCTCATACCGATTCCTACTGGTGAACCCCCACTAACTTCTTCGGTTTCCCGGGTCCAGCCAGGTGGGCGTGGAGAAGAGCTGCCCGGTGGATGGGAAAAGCAAACTCGG
MSTRG.34383.2	ENSRNOG00000023446;ENSRNOG000	0.000	0.210	0.000	5.464	up	GGTAGGTACCAGCTGACGAGCTAGGCCCTTTTCCTTGCCTGACGAGACAGCTGAGGAAACAACCTATGGAGAGAGCTTATTTGGGCCTGGTGTTTCCAGAGGTGCAGTCTGGCCTGCCGGCTCCTGTGCTGTGAACACGGGGAAGGTAATGGACAGAGCTGTCGCCTCTCTCAAGGACGCCATTATAATATGACTCAGTCAAGGGGTAATCCATTGACCGGCTTGAGCCTCAGGACTTAACCAATCACTTCAACAAGTCTGTCAGCTGCACCCGGTCTTTAACACATGAGCTTTTAGGTGTGTGGCTGATCAAAGTCTATTAGTGCCATGTAATCATTTAAAGGGACAGCATTTGTACAGTGTCTGTAAACCAGAAATGAGCTTTTCAATATTATGGCCTCGTGTACTATGCAA

	00047876						GTAGCAGTGTGTAAGTTTCCGTCTGATTGGAGAACTGTGACCTGAGGCAGTCTTGAGGGTAACTGAGG TTGATGTTGCTCCTGCTGGAGCTGCTGGAGCCTGACAGAACTCAGATGTCTCCATTAGTTACAGCTGAC TGAGGCACCAGAGGCCAGCTGTCACCTTGACAGGTTTGTCACTTCAAGTTACTCTTAGATCTTTCTAGT AGAGTTTCTAGGCAACAAGTTAAAGACTAGACAACTTTAGGCTACATAATAAATGCAGGCTTCATAAAG CTGTACAGAGTTTGTGTGGGAAGTCTACTGTCCTAATATTAGGAGGTAGGCCTGTTCTAATAACACCGAC ATAACTAATGCTAAATTTAGCTGCAGATCTTAGCCTGGTTTACTCTGTTCTAGGGACTAAGAGTGAGTTAT GGACTGATTTAAGCAACAGTGAAATGCTGAACGGTCTTTGGCTGTGCCCACTACTAGCAACCTTGAGCC CATCCTTTTCTTCAGCTGATAATACAGTTAGACCTAGCTCACAGTCTCAGCCCTGAGATCTTAGACCATA GTCAGAGCTAGCTCACAGTCTCAGCCCTGAGATCTTACACCAGAGTCAGAGCTAGCTCACAGTCTCAG CCCTGAGATCTTACACCAGAGTCAGAGCTAGCTCACAGTCTCAGCCCTGAGATCTTACACCAGAGTCAG AGCTAGCTCACAGTCTCAGCCCTGAGATCTTACACCATAGTCAGAGCTAGCTCACAGTCTCAGCCCTGA GATCTTACACCATAGTCAGAGCTAGCTCACAGTCTCAACCCTGAGATCTTAGACCATAGTCAGACCTAG CTCACAGTCTCAACCCTGAGATCTTACACCAGAGTCAGAGCTAGCTCACAGTCTCAGCCCTGAGATCTT AGACCATAGTCAGACCTAGCTTACAGTCTCAGCCCTGAGATCTTAGACCAAGTTTCTTATTTTGTTCTAC TAGTACTCTACCAAGAGATCCCTTTTTCTTAGACTTTCTTGGTAGCTTTCGCCTCATGGCTTCAGTCTGCG AGCTTGCACTGTTGCTTTGGGACAGACTATCATGGTAATGTGTAGGTAGCAGAAGGGCTGCCTCAGTTC ACACAGGCCAGAAACACAGACATGGGTTGGCAGAAAGGGTTAGTGTCCCAACACTTTATTCAAGCACA TGTCCCAAGTGACCTAGCTCAGGGTTAGGGCTAGGATTAGGGAGGCCATGACACCTAAAGTGCTCACA CCAGCTGAGAACCAAGATTTCAGCACATGAGCCTTTAGGGGACACTGGAGAGCCGAGCCATTAGCACT CCCCAGGTCTCATTAAGCAGCCTTGGCCTCTTTTGCACAGAACAGTTTTGAAAGTTTTTAGGGTAAACT AGTGCTCATTCTTGCCATGTTATCGTGACCATATGTATAAAAGAGACTTGAATTTGGTTTAATAGAAATTT CTTGTTGGAGGGCTAAGGAGACAGCTCAGTGAGTAGGAACCCCTTCATAAGCATGGACCTGTGTTCTG ATCTCCACAATCAACATGAAACCCTAGGCACGATCACATGCACCTGTAGCCCTGGCTGGAGGGACAGG AACAAGAGGATTGCTGGGGCTCAGAGTGCCAGCCTAGCTCGGGTTCAGTGAGGGACCATCTCAGAACA ATAAGGAAACTGAGAGAACAGGACCCCCACTGCTCTCTGGCCTCTGTGCATACACATGCACACACTAA AAAAAAGAAAAAGATCCAGTTATGCATAAAATTTACCAGTTAGTAAAAGCAAAACCTGTTAAGATGACC TTGTAGTTGCCTGGCCACATTGGTAATTGTCTTTGCTGTGAGTTTTATACTCTTAAAAGTTATATAAAGTC CAATTGAATTGCATAATCTTATGTGTTAAAA
MSTRG.34492.4	--	0.007	1.192	0.000	6.991	up	TGTATTTAAAGTGGAGAAACCCACACTCAGTAAGTCTGCATGAGAGTCAAACAGCATCCCTGATGCCCC CTGTTCTCTTTGCAGGTCCTGTGAATGGAGGAGCTCTCACAGCAACAGGCGACCATACCCGCAGATGTG AAGTACTCCAGAGCATCCCGTGCAGGTCCATGAAACATGAGTTTTCCTGAAGCCATTAAGGTGAGGCTA TCAAAGAATCTGAAGATGGAGTACTGAGGCTGATTGATGGAGAAGATGATTGTCTTTCCCTTCATCGAC ATCCTTCTCAGGACTGATATGACATAAGTCGTAGTTGTAAAGTCTAAGTAAGTGGTGGGATTATCCAAGA ACAAAATGGGATGGTCAGCGATCAGCTCCACTGCTATACTGGTCCTTTTCCTGAGTTCTTTGGATCTAGG CTTGGTATTTTGTTTTTCATCCAGATGAAGAAGTTCAAGCACCTCATTGATCCTCCTTCTTTTTTCATCTC TTGTTATAGTCATTGGAAGACAGAGAGCTGCTGAGAACTCTAAATTGTCTCTCACGGTCACTGTGTGCA TGACCACATCATTCTTAAGGGCAAATGGAATTCTAATTAGATAGAGTTACCACTAAGACTCTAACAAATG TGACCTAGGCCACACCAACAACACACCCAGTGTAATGCTGCACTTAAACTTTGAATTTCACTAATCCCA AGCTCAGAGCCTGGACCAAATGTTTTCTTCCCCCTCCCATTTGCCTCCCAAGAAGAGGACAGGAATTTG GTAACTGCTGCCTGCAGTTATTTGCAGAAGACATCTTCTAGGAGTGTAGAAAGCTGGAGGAAGAACAC AGGTAGACAACAGTGGCCCTCTAGACTAAGTCATGGCTGTTCTTACTAACAGTGTGCCACAGATGTCAG

							ACTCAGGTTAATGAATGACTATAAAGTGTTGGCATGGAAAAAGTGGGGCCTATGAGTATGTTATAGACTTAACTGGAAAGACACAAGTCTATAAGCTGAGCACCTGCCCATACCCAAGCAGAACAGGTCTAAAATTGTAACCCACCAACACTTACATGTGGTACATAACCAGATGTACATTTGAAATTAGCAGGTCGAGGTTTACCGTTTACCAAAATATCTCCTGATAATCCACTTGGATGTCTCCTTGCAGCTAAGACATCTAATAACCTACAAAGGGTTAATTATAGTGTGTAGTTTATTTTCTTCAATGAAAGCCACAGCATTCTTTAGAGGCTCAAAATTAAAACTCTGCATGTCAAGCAAAACGGAAACATAATGTAAAGAACAAGTCAACAGTTTAACTGAAGTCCCACATGTGCACTTTTCTCTTCATCAAGGGCTGTAATTCAGCTCAGAATTGTGAATGCAAGACACGATTTACATAACTGCCCCTCCCTGCTATCTGGGCTTAGAGACTGACCCAGGTAAGGTGCTGCTGTGTTTCGAGAGTTAGGCCTGCAGATGTTCTCTTACCCTCCCAAAACGTCTGCTACATCTTTCTATTTCAGGCCTATCCCCCCACCTCATGAAATAATAACAGGTATCAACACCATGTGCCAATCACTGTACCATTTCCTATTCCTAAAGTTCATTGTGGAAAATGAGACACATCCCTCCTGCACATCACAGCACATAGGACCAGCCTAGCTGCAGTGACAGCACTTAAATAACACCCATGAAGGTGACTGGTTTAGAGCACGTGAACTGCACACAGAGGGCTTGCTGTGCTTTCAGTTATACTCACAGAGATCTGCTCCCATCCTGAGGTCCCATTATGGCATTGAGGCCTGGTCCCATGATCCCACTGATGCTCGACAGTCTTTCCATCACACGTGTCTTCTGACAAAGTGGAAAGCCGCTCTGCACTGTTTCTATGTAGCTGATGTTGTGAAAACTCAACACAACAGCCTCTCCAGTCAGTGTCTCCGGGTCACTGGTGTTTGTTTCGGGAAGGTCACTGTGACGTCTTTCTGTCATTGGTATTACAGTGGGGTCATTACTGGAAGACATCTTGAGAGGTTCTTCCTTCCCAGCGGCTCAAGGACCCGAAGCCTCCGACAGAGTCCCTTGCTTCTTCCTAGAGGCTGCCAGGGAAGGAAAGTGAAGACCTGCCTGGGCGGAGCAGGAAG
MSTRG.34596.2	--	0.002	0.876	0.000	6.349	up	GGACCCTAGTTCAATTCCCAGCACCCACATGGTGGCTCACAATTCCCAGCAGCCGGTGGCTGCTCAGTTCCAGGGGATCTGACTCCATCACACAGACATACATGTAAACAAAACACAAGTGCACATGAAAGTGATAAAAATAAAATCGTTAAAGAAGTCAAGTAAGATAGGCCACGACAGTGAGGGCTCTGCTTAACTTTTCGCTCATGTTTTACTCACGGTTTACAACTCAGTGATATAATAGGAGTCCCACATATTATGGAACACCGAAAAGCTGTATTATGAAAACAGGATACCAGGATGCCTATCGTTTTGATAAAATTATTTCTCATGCTCATGCAATCAAAAGATATTCACTAAACATTTACACAACCAATTGCACTTAATATATCAAAAGGTTTTCCCTATATCCAGAAAGCTAAAACTACATAATTTCTTATTTAAAAACACATAAGTTACTAGTTTGGATATGCCTACAGAGTAGACATACATTCAATTCAGTCTCTAGTTGTTACTTCTGAAATGATCATATAACAGTTATTGTCTGAAACACTAAAATTCTAAGCCACCAAAAGTAATTAAATATATCACATTAACACTTCACAGCCTTTAAGACTTCATACACTAAACGTGGCATGGCAGGGTGATTCCCCCCCGAAATAGATTCTCGATGGATCACTAGAACAAAAATATCACGTCACTCTTGTGAGGTCTTGTAAGAACCTAGACCATTTAAAAGAATTAATCATTAATATATAAATTTATAATTATTAATTGTGCTGGAGTGGATAGTGGGACACTCCGGGAGGGCTGGGGACAGATCAGGACACACAAGGGAAGAGGTGTAGAAGACAGGAGTGAGGGGACCACGGGGGAGAACATGCAGTGGGGGAGGGGGAGAGTGGGAAGCGTAGCGGAAAGACAGGAAAGAAAGTTGCCAATCTCATGTCTCATTATTCAAATGCTCAGTTTGAGTTCTGGAAAAATGGGCTACTGTAGATTGCTACTGACATTCATAAATAATTTCCCATGATTATTCTGTTCTAAAAAGGAAAGTGATTGAAACTGATGGATTCTTTGCACCATGAGCATACCTTGTCAGAGTTTAAATCCTGGTACAGTACAAAGAGAAGCAGACATTTATCCATAGTGTGTCACATACCCAAGGGACATGAGGACTTACAAGAAGGTATTAAAAATAACTGGTATAAAAACATTCTAAGAACTAACTCAGACGAACCTTACATTGTACTTTTATCCACATATAAATAATATATACTGGGAAAGAACCCTCTAGTATCTTGAAATGAAAACTAAGATACTTTTTCAGAAACAGTATAATCTTTATTTAAAACAGGTAAAGCAAAACACCAAAATGGCCAGCTAACCAACCAGAGCACAGCAGAATACCCACAAAACACACGGACATCTAACCAACCAGAGCACAGCAGAGTCCCCACAAAACACACGGACATCTAATCAATGAGAGCACAGCAGTGTCCCCACAAAACACACGGACATCTAACCAACTAGAGCACAGCAGAGTCCCCACAAAACACACGGACATCTAATCAATGAGAGCACAGCACAGAG

							TCCCCACAAAACATGGATATCTAACCAATCAGAGCACAGCAGAATCCCCACAAAACACATGGACATCTAACCAATGAGGGCACCACACAGAATCCTCATAAAACACATGCACTGCTCTTCTTGACTTCAGGAGAAACAGAACCCTGGTTAAGAATATATTCTCTTTGCTTCAGAGGTTTCTGAACTCTGCAACAGTGCAGATAATCACCAGTCTACGCTTCAGTTTTACACGAAGGGTTTCTCACGGGTTTCC
MSTRG.34599.1	ENSRNOG00000034289	0.512	1.658	0.011	2.358	up	GGAGGACTGAGAGGAAAGAAGGGAAGGGAAAGCTGCAGTCTGGTTGCAAACTGGGGACCGAACCCACCTAAATTCTCTCTAGGAGAGAAGAGCTCAGATCCAGTCAGAGACCAGCTGACTACCCTCATATTAGAGTGGCAGCCCTGCACCATGGGCACTTGTTCCTGGCAGAAACAATGTCCTTGTCCCTGCAGTGTGAAGGTTTCCAGCCGTGTAATGATAGAGCTGGTAACCTGTCATTACCTCAGAGGCTGCTTTTTGTTCCACGGTTGGAGCCGCTCTCCACAAGGCCGGATCTCCAGGACTTTGTGTCTGAACTGCTTCATTATGTTGCAAGTTGGAACCTGACGGGTGAGATGTCAATCAAGACAAGAATTTCTTTTTTGTGTGTACACTCTTCTCCACGAACATTTCCCCTTTCTTGCATTTATCCCAAATATTTGTGGAGGGATAAGTCTTATAAACTGTATTTTTAATAAACCCTCCTGAG
MSTRG.34755.1	ENSRNOG00000002165;ENSRNOG00000002178;ENSRNOG00000002188	0.083	0.436	0.041	2.475	up	TGTGTAACTTACATAACCAATGTGTAACTTTCGTGACCAGTGTGTAACTTACGTGACCGGTGTGTAACTTGCGTGACCAGTGTGTAACTTACATGACCAGTGTGTAACTTACATAACCAATGTGTAACTTACGTGACCAGTGTGTAACTTACGTGACCAGTGTGTTACTTACGTGACCAGTGTGTAACTTACGTGACCAGTGTGTAACTTACATAACCAATGTGTAACTTACGTGACCAGTGTGTAACTTACGTGACCAGTGTGTTACTTACGTGACCAGTGTGTAACTTACGTGACCAGTGTGTAACTTACATAACCAATGTGTAACTTTCGTGACCAGTGTGTAACTTACGTGACCGGTGTGTAACTTACGTGACCGGTGTGTAACTTACGTAACCAATGTGTAACTTTCGTGACCAGTGTGTAACTTACGTGACCGGTGTGTAACTTGCGTGACCAGTGTGTAACTTACATGACCAGTGTGTAACTTACGTGACCAGTGTGTTACTTACGTGACCAGTGTGTTACTTACGTGACCGGTGTGTAACTTTCCTAAGTAGATCGACT
MSTRG.34834.1	ENSRNOG00000060904	0.447	0.045	0.041	-2.379	down	CAAAGGGACGGACACAGTCCACCTCTGTGAAACCATCCCAGACCAGTGCAGATGCCTTTAACAATACAGAAAGACATTTGCATCGGAGGATTCTCGGCAGTGACTTTGTGTAGGAGAGCCGAAACAGCACCACATTGCTCTCTACCCTTGGCTCTGTTAGCAGGGATTTCCGGGCTTCTGTCACTTCAGAGGAAGGTCTTGAACCCATGTCCTCAGGTGCAAAGCGGTGTTTTTGCCTGAATTTCCATCTGCGAGGAACGCCCGTCATCAACTTAGGCAGAACAGGACCATTTGGAAGGAAAGCTGCTTGTGCGATTGTAGGGGTCTTGAGATCCTGGCCGGCACTAGGCAGGAAGTAAGAACAGGAGTTTTTACTTCCTCTGACGGGCATACCCACTGTCTACATAAACAGAAACATTCAGGCTGAAAAGGAGTCTTTGTGATCACTGGGGGTCCTCCTCTCCGAGACCCTGATTTTTCTGTTTTTGCTTTTTAATATAGAGCTTTGGACGAATGACATCGATATTGAATTTCTTTTAAATGGTGGGCAGGTCCGGCCTTTCCTGTTGCAACTTTTCACTGGAATTAAATGCATTTGCTCAGCGGAATTCTGATTTGCCTAAAGCTTGAAGCATTTCATAAAATTGAAATTTTATCACCTAATGGTATAAAAAAAAGGTGGGGAAGGGTCTAGAGCCCAGCGTGCTTGACATTTAAATCTAACTTTGCCAATCCATTCACGAGACTAGAAAAGAATTCCCCATGCGTTCTCAGTCTGCCTCGCGGTGCTCTTAGAGGACAGTAATGAGAATCCTCTGTGGGAGCGAAGTGAATATTTGAAGTTGCTCCTTTATTTTCTATCTCACTGAAATACATTTTTTTTAGGTCAGGCTCTTTTCTCTCTGTTGGCCATACATCCAGGCACAGTTTCCATAGCATCACACTTTTGGAAAAATAAGTACAGATGTACCACTGCCAAATATATCCTTCTTGGTTATTCTGAAAGTCGCTTGAGAAAAGTACTGATTTTAAAAGCAGAAACTCAGAGTCGTCCTTCAGCTCAGCACATTTTACCGATCCTTTTAGGCCGGTCTTATCTTTCCCAACAAGTTTAGGAAATAGAGGTTTGTCTGTGTGTCTCCTCGTCTGGCACCACAAGCAGCCCTGGCCTCACAGTTTTCAGTGATGGCGAAGAGTTATGGGAAATGGCTATTGTTGTGCCCACTGGGTTTCCTGGTTAGGGTAACC

							CACAGGCACAGGCTGCCATACATGGTTAGAATGATAGTGAAGAATGTGGTAGGATAACCATTTGTACCCCCAATTCTGTGCCACACTTTCTGCAGAGCTTGCCCACTACTGATTAGCAGCTGTACAGCAGGGGCTCCCAGCCCTTTCTGGAGCTACACATCTCCATGTAACTTTCTCACTACTAGGATGGGGGTGAATGTTAGTGCTGGTACCATAGAGCCACATGGCATCTGTGGGATATTTTTATCTAAGTCAGCAAAGCGTCTCACCTGCTAAACTCCATCCCATCTCACACTGCCAATGGCCTCTCTCTGAGCTTGGCAGCAATCTCTCTACCCATCTAGTTTCCAAGGCAGGTTGCCACCATGCCAGACATACACATCCC
MSTRG.35088.1	ENSRNOG00000002506;ENSRNOG00000058724;ENSRNOG00000059582	0.619	0.007	0.000	-4.910	down	CATACATACATACATACACACACACACACACATATACATATATATATGTATATGTATGTATACATATGTATGTATGTATATACATACACATACACATACACACACACACACACACACACACACACACACACACACACACACACACACACTGTTGTGGCAACAAAAGACAGATGTGAAATGTAACAGAATTGGAGCTAAGAGCCATTCAGCTTCAGCCTTTCCATACCACAAACAAGACAGAGCATCACATGGAGAAGTTTACGTGGTGGCTGCTCACCTGCAGTACTGTGGACCTTTAACATTTGTCCCCTTTTCAGTGAAATACTAAAAAGTACTCATCTACCATTACTGTTTTTGCTGATTTGATTTTATTTTGATGGTAATATTCTATCCTTATGACACTATTGCAACCAAATTGGCTTTACCATCTTGGCTTTAGTAGGTATAGAGGAAAGCGGATTACCATCTTTATTTTGTAATGTGTTAAGCATTATATGCTGGTAGAATCTAGTTTAATTGTTTCAGGTGGAAAGTATTCTTTTTGAGTTTCCATTTTGAATGTGTTTGGACTAAAACAATAAACTACTGATGTCTGCAGCATTTATCTATGTCCCTAATTTAATCTGAAGTTATATTTTCCCAAATGCTTTCTCCCAGTTGCATTTCATAACGGCCTGAATGATACAGCTCAAATCAGCTGCTTCTTGTTGAGGTATATAAAAAAGCAAAACACTGAACATTGTCAGTGCTGGGCATCTAGTACTGATCTTGGTACCTAGTATAGAAATAAAAGAAAATAGCTGTTGGGAGGGAAGACCCGGCTACCCTGACATTGGGAGGGTTAGAGGCAGAAGGATCTGGAGTTCTGCCTCATACACAGCCAGATGCCAGCCTGGGCTACATTCAGTACTGAATGTGTGCAGAAGTAAGTTGTAATAATCTAAAAATGTCTATAGACTGTCTTGAAAATAGGCAGGAATTTGCAGGAACAAGTTACTTGGACTGGAATTTTTAATTTTGCAGCAGGATCTGGAGGCTTTAATCAAGCTGCTCTCGTCTGTCTCAGAGTTATGGTGTCCAGTTGACCCTATTGCCTTGGCCTCCCAAATGCAGGGGCTGCTGGCAGCATGCAGATGTGTATGACACATGGCCTGGCTGGTACCTAATTTTTCTTGCTAGGCAAGTACTTTTGTACAATATATTCAACTTGGCTTAGGAAATTCTTTGATCAACAAAGATTTTTAAAAAGAACCCCCTCCCTCCTCAAAAAAGAAACCGCTCTGTTCATAATACTTCTGACAAGATTCCATTACGTTTTCCTGGAAAATAATTACTTAAAATGAGGTAGGGTGGCACACAGCTGCCAATCTTGGCACTGGGGATGTGGAGGCAGCCTCACCTACTGTGTGTTTTAGGGGTTGGCTGAAACACTTTGACCTACCTGCTCTCAAAACCCCTGACTCAGAAAATAATGTTTCTAAATTAATGATTCCAAGTTGCCTTTGACAAATGGAAAATGGCTAAAATCTCCAAATCTTTTTATTGTGTACATGAATCTGTTATGTTTAGTATCCTTCCAAATTATAAAAGTCTAGTTTTGGCATTGCCAATACTTGGAAAATATTGACGCATTAACTATGTGAGTTGTTCTCTAGAGTTTGGCTTAAGCTGGTCACTCCTTGGAGTACTTTTGTGACTACTCGGTTTTCCATTCTGATGTCACGCCATTAAGGGGGCCCTCCTTTGGTTTCATTAGTAGCTGGGTGCAGCGCCACAGACTGCATCAGTAACTGCCCACAAATGTCGCCTTCTGGGCTGATTTCCTGCCTTAGGGTCTGGTCAGTATAGTAAGCCCTCCATACTGTTGCCTGTGTCAGGCATATCTTTTAAAATAAAATTACATTTTCTAAGATTCCCTCTGTGTAAGCTTGGTTGTCCAAGAATTCTGTAGAACTCAGATCTGCCTACCTTTGCCTCCCAAGTGCTGCAATTAAAGATGTGTTCATGCCAACACTATTGCCCAGCTTTTATTTTCAATATATATATATATATATATATTTTTCCACGTTTCCTTGGAAATATTAGGTAATGAACCTTTTTGTACTTTATTGTGCATACATCTCTAATAACGAATATCATCTAATCAGCCTCTCAGTTTGGAGTCCCATCTCCACTGAGCATTTTCCTGTTATGCTCCATTGTATCTCAGGTTGCCAAGTCTATCCGTGTTCTGACTAACTCCCAGGAGTCCATCCTAACTAAATCACCTAGTTTTGGATGGCATGTTTTATTAATTCATGATCAGTGAGAGCTGAAACTCTGAGGAGATTGATCTGGTAGGCATTGATAGGGGAAATCCCCTTTTCCTGCAGGTATTGCCACGATTGTGTTAGGAAACAAATGATCCAAAGTGTTTGATAATGCCGCAAGGGTCCTATTACCTTGGCATCTGG

							TTTTGTTTTTCTTGATTACTTGGAGTCATTGTTATAGTGAACTTAAGTCATTGAAAAATGTTCATCAAATGTTTGCCAAGTACGTCTGTGACCTGAGTCCTCTTTGAGTGGGTCATGTTAGCTAGCCCTGGCTGTTATGTGCTTGGACAAACCTGCATATATTTAGGAAGTACCCGAGTAAAGAGGTCATGTAGAGACTTGGAACAGAATCCAGGAGCTGTTAACATCCTGTGAGGTGAGACTTCAGGAAGGACTCTTGTGTCTTAGGCCTTTTTTAATCTCACGAGCCTGTTAAGTTTTGATCTAAGAAATGAACTATATCTTGGAGAGGCTCGAAATTAAAACAATCCCTTGTCTAGTGTGGTAACAAAAATTGTGGCTTTTTGAAGGGAAAATCTAAGTATTGATTTGCTTGCTATCAGGAATGACTTGGCATGTTGTTTTAAAGCTGGCACAAATAGACTTGGTATGTGCCAAAGAACTCAG
MSTRG.35453.2	ENSRNOG00000002011;ENSRNOG00000002014;ENSRNOG00000021931;ENSRNOG00000033910;ENSRNOG00000034226;ENSRNOG00000042133;ENSRNOG00000047234;ENSRNOG00000054937;ENSRNOG00000055008;ENSRNOG00000058371;ENSRNO	0.000	0.195	0.000	7.189	up	AGAAAGAAAAGAAAAAAAGAAAACACTGATAGAGTTCTGCTATGATTTGGTCTAAGCTCCGCCTACAGTTACCTGGTAACAGCCAGGTGTGCTGACTCACTGTAAAAGGGGCTGCTGGCTCCCTCCCCCTCCGATCTCCAAGCTCTCCTCCAGTCTTGTTCTTGGCTCTGATCGCTTGCCCCTGATTTCTCTCCCCTTACCCCTCTCTCCCCTTACCCCTCTCTCCCCATTCCCCTCCCTCCTCTCTCTACATGCCCATAGCCGGCCTCTACTCCTCTACTCTCTCCCACTCTCTCCCTTTCTGCCTTTCTCTGTCTCTACCCCCTCAACTTCCCTCCCCATGCCCTGAATAAACTCTATGCTATACTATATGGCGTGTGTGTGTGTGTGTGTGTGTGTGTGTGTGTGTGTGTGTGTGTGTGTCTGTGTGTGTGTGTGTGTGTATGTGTGTGTCCCCAGGGGGAAGGGATGCCTCAGCATGGGCCCAGGGAGGCACACCCTTCCCCCACACCAGACTACACATCCACCAAACATATTCCTTCTCTCATTATCTTTTTATAAAACACAACAAGTTCTATTCTTCAATTGCATGGAACTCTAGGTTTCCCCTGTGCTGAAAGAAATGCTCCAAGTTTACATGGGCACTTATACCAAGATTGTATAGGACACAGGACTTAGCTACGTCACAACAATATCAATCTAGCTTCATAACATACGTACTCCTAAAATGATAAAAAAACAAAATCTTACTCCTGGATTTAAAGCGACAGTAATATATACGGACAAGGTTATCCTAAGGGCTTCATCACATCTGTTTAAAGTTTATGTCTCTCATATGGTTTCTTATGTGTTCAAGAAACTGCTCTATTCAGGCATTGCCTTGGGCATTTTAAAGATATATTTGCTATGGGGTATCTTAATACTGAGGTCCTTAACTTAAGTAACCCATGAATAGAATTATATGTCAATAAATTTTTGTTTTGGTCCTCTTTGTATTTTTGGGTTTTGTTGGTATAAATAGAGTTTATTCAGGGCGTAGGGAGGGGAGTTAAGAGGGTAGTAGGGCAGAGAAAGAGAGAAGTGGAGAGAGAGAAGTAGAGTAGAAGCTGGCCATGAGCACATGGAGTGAAGTGGGGAGGGGAAATGAGAAGCGGAGGGACAAAGGGAGAAGAGGGTGAGAGCAAGAGGGAGCAAGAGGAAGACTGCAAGAGCAGTCCTCTTAGTATTAGTTTAGACATTTACTATTATTATTCAAAGAAAGAATTTACCAACTAAACCAGACTGTCAAAAAAGTTACAAAAAGGTTTAATAATACTATCTTGATTTTATATATATGGAAATTATTAAATAAAATGTATCTAACTCTATTTGTTTGTTCATATATTTGTTCACACAATGAATGCAAATGTTAACGTTGAAATTGTCAGTTCAGGACTGGAGAGATGGCTCAGTGGTTAAGAGCACTGGCTGCTCTCCCAGAGGTCCTGAGTTCAAGTCCCAGCCACCACATGGCAGCTTACAACCATCTGTAATGAGATCTGATGCCCTCTTCTGGTGTGTCTGAGGACAGCTATAGTGTGCTTATATATAATAAATAATTCTGTTTAATAAGAAAGAGAGAAAGAAAAAAAGAAAGAGAGAGAAAGAAAGAAACTGTCAGTACAGTATGCCTAGTTCTTCTTTTACACATCCCAGCTTGTTTAACATTCAGGATGTTTATGAAAATGTGGTAGAATTTATTTGCTCCTGACCAAGGACTTCTATCCTACCTTAGACCATTTAGTTCCTGGAAACACACACACACACACACACACACACACACACACACACACACCTTTTATATTTACAATAAGCCTTAAACAGCACATGAATTGGGCAGATATGCACCCGCCATGCTTCTAGAATCTTCTTTCCCATGATAAACCCAAGTTATTCCTTACGGTGTTTTATCTTGGCTGCTCTCAACTCTAATTGGCCAGCCCCCAGAGCCATGTTTCAAATATGGGATAGCCACACCGTGATTGCTGACTTTAGTTTATAGATCAGCACCTTCCTGACCTTAAGGTTGCCATCTTTTATCAGACTCGTTCTGCTATTCTGGAAGAGACTGCCAACAGAGAGGAGGTATGCATAGCTGAGCCCTGGGTCTACCTAGATCACATGTTTGCTGCTCCAGGAGGGTACTTGTCCACTGTGAATAGGAGTGAGAAATTTTTAACGGGAGTATTTAAAATTTTACACTTGTTATTATGTAAAAACATAGCTCCCCCTCAAAGAGAATAAGAGGTGCTTTGTTCTGGAGCCAAATATGAATGACCAAGCTCAGAGAATGTGTATTTACATTACTCTTAAAAGGATGATCCAATGT

	G00000058463;ENSRNOG00000059588;ENSRNOG00000060573;ENSRNOG00000061718						GGTAACAGTTTTCAAGAAGCTTTTATAGTTATAGAACAAAAAAAAAAAAAAATGAAAGCCCTTTAAAAGTACATTGCTGGGACCATCAAGCAGTTGAGTTGCAGGAAAGCAGGAAAATTCCAGCCCTACAGCTTAGATGTTATCTGGCGACATTCTCACCCTTTGACTGAGTTGAAGCTAGGAGTCTACCAGTGCATTCCAGAACATTGCCTCTGAGGGAACAAGAATGCTAGGTCCAGGGGTTGGGGATTTAGCTCCGTGGTAGAGCACTTGCCTAGCAAGCGCAAGGCCCTGGGTTCGGTCCTCAGCTCCGGGGAAAAAAAAAAAAAAAAGAATGCTAGGTCCAACACAAAAGGAAACAATGATCAGCTAGTAAGAGGGCTACAACTGGCTGACCCAAGAGAATTTGGCTCTAAACCTGAGACACTTCAACTGCTCACATTCCCACAAATTCTAAACCATCAATCTACCCTCCAGAGTTTTTAATGCAGGTGAGCCTATTTTCGGGGAACTTTTGTTGACACATGCACTAGAGTAAGAATATGAAGAATTCATTGCGGGTTCAAGAGAAGAGGAGGGGAAGAGGAGGGAACAAGAGAAGTATTATTGTCTATCCCGTCCTTTGCCATATCTAGTTTGTAGCTGTCTTTAATCACCACTTGTCTGTTTAGATACCTCTTAAAATTAGTGTGAACGCACACATGCTCACATACAGGCAAACAGCACAGTGTCTGTATGGACATCAGAGAAGAACTTTTAAGAGTTGGCTCTTTCCTTCCACCATGTGTGGCCCAGGGATGGAGCTCAGGTCCTCAGATTTGTCAGCAAGCGACCTTACCTCCTGAGCGTCTTTCTGGCCCGTCTTTACTCTTGGGTCTGATCATTCATATGTAACTTTCTTTAGCTTTCGGATTTTCTAGAGTTTGAAACACATATTTATAATAAAATATCTTGGAGATAGGACCCCAGGCTGAACATGGTATTCATTTGCTTTGTATAACCTTATAATAGTCTTGTTCTGGGCCAGAAGTGTACTTGATTGTCTTCTGGTAGAGTTGACTTTTCTTAATTTATTTTCCTACTATCTTTCTTTTCCATTTGCACAAGTTAAGTGAGCCCCACTGCGGGGAAGCTTCACTTGAGAGTTGACCAAAAGAGGTCCCTGGTCATAGGACTGACTAGACGGACTGGCCCTCCCCCATCGCCTCTCTGGAGCTTCTTCCCTAAGTCCTGTGAAAGCCTGGAGTCTAGAAGCCTGTCAGTGCCCATTGTCTGGGACCCAGTCCTGCTACACAGGCCCTCTCCTGCGTTTTTTCTCTTGAGGCCCCACCTATAATACCATCTTCTTTTCCAAAGTCATTTTCATGCTGTCTGTAAAATTCCCTCTAGTTTGAGACAAAGATGCTAAACGGCACTGCCTAGGGTAGCCGTGGTGGTCCTTGGTTTCATTTTTCCAGCACCTTGAGCTCTGTGGTCTACAATTACCTGCGGCCTCCGCTGCTCCAGCGACAACTGTATTTTCTGTGTGAGTTTAAACTGCAGTTGAGTCTATTAGGGAATAGTGACTGTGGCTCAAGTCTCAATCTCTCAAATAGTGACTAGGAATTGTTTCATGGGGGTGCTCAACTGCTTCGGTTCCTCAGTCAGAACAGATCGAGATGAAAGTACAGCATTTACCCGGGGTGGGGGAGGGGTGAGTGAAAATTTGAAGGGCCATTTAGAGATTTTTAGTGTGTTTTTTTTTGTTTTGTTTCTTTGGGGGGGGGGGGGTTTGTCCTTTCTAGTCAAAAGTTAAAATGACATCGTAATTGTTTACCGTCACTGTTGTCTACACAATTAAACAATTAAAATGTCCTGGAGGGTCTGGAAAGTGGCTCATTGTTTGCAAGCACTTACTCTTTTTACAGGAGATATGGGTTTAGTTCCAAACATCTGCATCCACAGAGTAGCTCACAGACATCAATAATTCCACTCTCAAGGTATCTGATACAGCAGGAACACATGTGGCATTCATTCATACATACATACATACATACATACATACATGCATACAGGAGGAATATACACACAAACTAGAAACAAAATTGTTTAGAATGTCCTTGAAACTGGTATTTAAATTGCAAGCGTATGATGATTTCTCTTCTTTTAGTCTATAAACAACTTTCAAAAATATTTCAAAAAGTGGGATTTCACGTTGCTGTCGTCTGTGTAGGCTGTTACATTAACCCACCTTTCTCAAGACTACAAAGTACCCGCAGATTTTGTATCTACTGTAAGGGCCCACTCTGGTTCTGGGAAAACCATCTCTTACTTTGCCCGTATATGGCCTGAAGGAGGACAGGGATCTCTAAGCTTCCATCTCTGAACAATGTAATCTTCAAATTCCATCACAGTGGGGATTCAGTTTCAACATCTGAATCTGGGTGACGTAAGCACTTGTGGTCTGTGACCTACGTATTCCAATCCACAGCCCAAGTCAGTAGATAACTATTAAGATTTGTTTTTTTTTCCTGGAGCTGAGGACTGAACCCAGGGCCTTCTGCTTGCTAGGCAAGCACTAAACCACTGAGCTAAATCCCCAACCCCAACTATTAAGATTTTAATAAAGACTTTTCCTTTACAAAAAAGTAACCTCTGTGTTAAATAGTTAGCTTCCAACCAGTTCAAGTTTTAGAACAACCTTGGGCAAAACTCTTGCTCAAAATTCAGGAAGCTATCTATTAATGTGCTGACTTGACATCAGCAATCAATTTTCAGTCGTGAAATTGGTGAGACAGTCTCTGAAATGTAGGCATAATATACCAGAAGAACACCAAGCTAGGAAGAACACTGAATTCTAGTCCTCGGTTGTATC

							AATAAAATGATATGAACTTATTTGAGTCATTTAATCTCTGAATCGTCTTCTGTAAATCAAAGAAATCGTAC<br>ATTGAACATCTTTACTGTGCTATTGTTTAATCTTTCAGAAGTATGAAATACGAGAATAAACTTGGATATGT<br>TCTGTCATTACAAAACCAATATGATTGTTGGCTATATAATTTACAATGTAAATATTTCAATTTGTAGAATTT<br>TCACATGCATATATGTTTCAGGAAAATATACATCACAGTACTCACCTACAAAATTTCCTAAGATCAGGTCA<br>TCGTAAATGCATCCCAGTATCCTGGAATGGATTCTGAGGGGAATGGTTAGCAGAAAAACCTGCGATATC<br>TTAGTAAAATGTTGTGGTTTTAATAGTATTGCATGAATATTGATTTCTAAGTCATGAAAAATGCACCTAAG<br>AAACACGAAATGGCCAAGTTATTTGAAGAATTTAGATAAATTCTCTATTATCTTTCTAAATTTTCTGTAAG<br>TCTGACATTATTCAAAAGCAAATGTTTGCAGGTAAAACTATTTTACTGCAATATTAGAATCCAGCCATCC<br>ATTCCCATATGCATACATACACACAGACTCACAGCACAGAGACAGAGAAAAGAAGACAAAGAGACAGT<br>GAACCTCAGGGTGGAGAATGAATGAGTATAGACATGATAGGCATAGAGTCACATTTATTATTACTATTGT<br>TGTTGTTGTTGTTATTATTATTATTATTATTATTATTATATCTTTCTTTACAGTCCAGTCATTAATCCCCTCAA<br>GGTCCTCCCTCTGGCAGTTCCTCTTTCCATTCTTCCTCCTTCCCTGCTTTCCCAGAGGATGTCCCCACCC<br>CAACCCCACCCTACTCATTCCCTGGGGCCTCAAGTCTCTCACTGAGGCCAGACCAGGCAGTCCTCTGCC<br>TTATTTGTGTCAGGGGCCTCATATCCGCTAGCATATACTGCCTGGTTGTGAGCCACATTTTTTTTTAAATC<br>AAGCCATACAACAGGGACACAAAGCTAGTAAAAAAAAAAAATTAAAAACTTGCTTCAACATATAGCAAA<br>GTCCTCTTCGTTGAGTAGCTACTGACTTGACTCACAGAAAACCTTTTTGTCTAAAGTCATAGATTTTCAA<br>AAACTAAAATGTTTGAATTATGATGAATCTGTCATTTGTTGTTAAAAATATCCAAATCATGTTGTTAAAGA<br>GAAATAGCTTCCATCTCCAGAAAGTTGCAGAACCCATAGCAAAGAAATTTGAAAATTTGTTTCACATCT<br>ATATTTCATAATTTCCAAGAAAACAGTGTCCTGGGTCCATATAGTAGTTTAGAATTAATGTAGACCTTTTC<br>ATTAGAGCAGGATGGGGGTAGGGTGCTTGTTTATTTGTTTTTCAGCCTATCAGAATGCTGCTGCATATTAC<br>GTTCCTAAACTTATCCTCCCTAACAACAAATATATCCTAATCCTTTGTTTGAAATGCAATCCATGGCTCCT<br>CTGGGAAGTTCATTCCCTCCTTGTAACAAGCTTCTCAGACTCTCTCTTGCAATTCAGGTCTTTCTTTCAG<br>ATGGATTTGTTCTTACTGAGCTCAAGTTCCTGCCAAGGACAAAAAATTAACAGACAAGTGAGCAACGG<br>AGTATCAAGTTCAAATGAAAAAAAATAAAAAGAAAGTGGAATGATGAGTATTCCATCATCACAGACAAT<br>AAAACATTGCTGAAAGAGGTGCCTTAAACTCAGAATTGACAATATAGAACTTGTGAGAATTAATAAACA<br>AAAATGAAGTAGGATGAATTCAAAAGCTAACAAACAATGAACTATAATTCCACCCCAAATTTGAGCCTG<br>CTCCCAAGGGGAACTTTGGATCCCTCTGTTGCTTTGGCTGTCAAAATATTCTCTAATATTGACTCATGAA<br>GGTTTCATGAAGAGGATAGTCTTATTAATTGTTGGTATGAAGTGTTGATAACAGTTTGTATCATTTGAACA<br>TGAGAGTTGTTTTTCTGAGTACAAAATAATCAATTTAGTTGTTTTTTTCTTCAATATTCTAAATATACTGCT<br>ATATTTTCCTGTGGCATAAAACCTCACTGCCAAAATGTCTGATGATAAATTAATTTTTCCTAATATATAAAC<br>ATGGCAAAACATGAAAAACTGTTAATATCTGAATCATCATTTTATCGTATGTTTGAAATAATTTATACCAC<br>ATTTCATTAAATGTAAGATGCTACTACTGTGGTTTGGATCTTAAATACTATTGAAGGCCAATGAGTTAAAG<br>GCTTTGTCTAAAGGCTGGGAATAGCATAGGGGGATAGTAGAAACATGAAGACAGTGTTCTCAACCTTCA<br>TAATGCTGTGACCCTTCTTTCAAGTTTCTCATGTTGTGGTGACCACCAACCATAAAATCATTTTGTGATTT<br>CAACACAATTTTGAAGAAAAAACTGTTATACTTTACAGAACGCTGTACTGTATAGTCAAAGTAAACACC<br>ACTTTTTTAAAGTTGGGCTAGTGTTTTTTCAAATACATCACCAATTAGAAAGATTCTTAATTGCATTATGT<br>GTACACAATTATCTCCTAAAAATGGTAAAATGAGAGTGAATGAAAAAAAGAGTCAGATGAGAAATTGG<br>AAATCTAAGAACTGGTAATCCGAGTTATTTGCTGTATGTCTTCGACCATATCATTAGCTGTAAGTTCCTTT<br>TTTTCTCTCCCTTTTTTAAAAAGATTTATTTATTTATTTCATGTACACTGTAGCTGTCTTCAGACACACCAG<br>AAGAGGGCATCGGATCCTAATAGATGATCAAAACAATTTAAGTAGAATCTTTAAGTGGAATCCCAGCCT<br>GGAGATGTCCTCTCTTTTCGGAAACCGATCTCCACTAGGTGCTAGAACACTTCTGGATTTGCAAGCAAC

							AGCACCTGGCTGCTTACGAGCCCCCCTGGGGTTTAGCTCTCTTAGCCGGAAGTGACGTGTTTTGCTTCCGCATTTGCCGTCAGCTCTCAGTCGCCTTCTCCTAGGCACTCTCCTCACCTGCAAGGAAGCGCGCACAGTTGCCCCTCCCCAACCGGAAGTCAGGTGTCCCAGGGGCTGACTGGAAACCCTGTCCTGGGCGTACTTCCCCAGCTCCTCCCCTGGTGACGTCGCCGCC
MSTRG.35576.1	--	0.051	0.278	0.009	2.839	up	CGACATTTCATTCTCAGAGAGAGTACTCTAACATACCTTGGAATTACTTTTATTCACAACAGTTTTGATATTTTTCTCTTTGTACACTTGTTTTGTATGTTACTGTAGATGCTGCTTCCTTTAATGTCCCCTTTATCACTCTCTATTTTTTTTCTGGTGTATAATATACATGTCCTTCTGTAAATGTGTCAACCAGTTTTTCTTGAGAAGACAATTATTTCTTTCTCCTCTTACCTATAAGTGTGAAATGTGGATAGTCAGCACTGAATAATCAATGAAATATGAATTCAATGAGCAATAACTAAATTGAGGTAAATTTAAATATTTTAATGCAAGGAACCATGGTTTACAATTACAGAAAACCATGTTACAGATTTAATGTGGCCCAGAAAACTTTATGTAGAAACTCATGTTTCAATGTCACTAGATTTGGAAGTATACTATTTTAGGACATTTAAGAGATATTATGAGAATGATGTCCTATAGATAAGATTTTTTTAAAAGCTACTTTATTTAGTGTTCTTAATCACTTCAACATCCTTCTAACCCACCTTGCAAAGGTAAGGGAACAAAGAAGGTTAATAGGAAAAGGGTGTTGTGGACTTGCTTAGAAGTAGTTCCTTGGGGTAATTCCACTCTTCCTTGTTAGGATACCAGCAGTCAAGTTCAATAGCAAACACAGAACATGAATAGCAGCTGGGCATAATCCAGCAGAAACAGCAAGACAGAATTGTGCTGAACCTGCAGAAGCATCCAGAATCAGCTGGAATACCAGTGGAAGTTCTTCAGCGCATTTCTCTATGCTCGACTGTAATGCCAGCATCAAATCATTACACTAATGTGATTGGAGATGACTTCAAAGGCACCTGAAAAACACGCGAAAATCTTACATGCTACAAATGGCTTGACATGCTTAAAATTCTGCCAGTTGCACTTTATGTGAAATGCAGACTAGGCTTCAGCATGATGTAATCCAATGAGTACTTGTATCACTTGAAAACTAAAACCTCCCCTCATTAAGCAGTTGCTGGAGATAATCAGTAATTAGGGAACGAATAGAGTTGTATAGCTTCCAGTTCTTTCTACAAATTCATACTCTGATTGATTGCTTTGTGTTCTTT
MSTRG.35715.1	--	0.359	0.000	0.005	-4.261	down	GTCATCCCTTCAGAAGTTAGCATTTCACAGTTCTCTCCTCCTCTGCTGGTTGTGACATTCTTTATTCTCCCTCTTCCAGAATGTTCCCAGAGCCATCTCAAATCTTCAAGTTTCCATTAACACATACATGTGTATGTGATCATTACTAAGTATGTGCCTTGTGTCATTTGTGTACTTCCCAGGCATTGCTGTGAGTGAGCCTCACAAGAGGCATTTCACAGATAAGAAAATAAAACACAAACAGGTCTGTTGACTCCCCTAAGGTCAAACAGCTAACAGGAGGCAAAGACATGGAATTATGACCTGGAAGCTTGAGCACTATCCTGGGTTGCCTGTGTGGTAACCACGGTGCAGTGAAACAAGATGAGGTGTCCTTAGGAAGAAAACGGGGAGCACACATTCCTAAAGTATAAATCTCTGCTTTCAAATCATTTCCCATGACACGGAGCTTCCTGGGGTTTTCTGCAAATCACTTTTTATAATATCGCATCCTTCGCAGAACCACGGAATTTGTAAATAAGGAGTTATGATCCTTTATGAATCATATTTTTGTGATTGCTTTTGTGCGTTGACATGGCTGTGCCATGAAGAGCTCGGGTATTTGATCAAACAGTATTCTGAGTGTATTTGTAAGGGTTTCTCCAAGTGAAACTAAGATTTTGAAATTAACCTCCTTGATGTGGGACATGTCCAATGTATTGAAAGTCTGACTAGGACAAAACAACTGGGTATCCCTCATGTCAGAGCACTTCTTGCTAGACGAATTTGAGTTGAGCTAAGACATTAGTCTTTGCTTCCTTTAAGGCTCAAGCAAGAACCTCAGCTCTTCTTGAATCTCCAGGGGACCATGGCTAGTCTTAGGAGACTCGTGTGGCAAAATATGCGCAGAACATCACCAATGTCAACAATAGTACAAATAGCAATACCAACAACAACAACAC
MSTRG.35801.2	--	0.550	0.000	0.010	-4.070	down	CAAAATAATAACAGCTGTCGCTTCCAACACCTGGATGGGAGACGGGCTAGGGAAACTGCCAGTGAGGGACACGGAGGTCACCAAAAATCCAGGGGACGGAGTCTGAGCTCTGGAAAAGGCATCTTCAGACTTGGCGTTTCCACTGGGACTCGTTCAAGGGCAAGCAGACAGGAAGACAGCACAGAAGCCAATATGCCGAGTGTGTACTGAGCTGAAAACATCACGGCTCTTCCGGCCTCCTCCAGTGGATCCTACCGAGAGACCTGATTGAAATCATCACTGTGGAATGACAACTCCGAGAACCCAAACATCTGAGGAAGAACACAAAAGTCACAAGAAAGACCCCAAGGAACACTGCAAAGGCACCCGCCCGAGGCAACTCTCACTTACATGAGCTTCCATAATG

							CATGAAGTCACTGAAGATCTTTGCAGTGGCCCAGAGGAGACCTAGTTACATGGATAGTATTGCATATGCT<br>AACCACATTTTGAGTTAGACAGAGTGGTGTTTGATGTTAAATAAATAAATAAAATTGCCC
MSTRG.36035.1	--	0.260	0.000	0.001	-4.759	down	CAGAAAACATCCCGTGCCAGAAGAGCCATTGCCTTTTCAACACATTTACCTAGGACCTAGTTCAAAGTG<br>GATTTCAGATCCCATAATTTATTTATTAATGTTCTAATTAGAATGAAAGTTGAGATCCTATGTCACACTTCG<br>GGAAACCTTTGATTTAGATATTACATAGAGAACTATTTCTACGGATGCCTCAGGATGATCAGAGATTAATA<br>TTGTCACTCAAATATGTGAAACAGCTAAACCTCTGGCCTGGTTGCTTTAGGAGAGTTGAACCCCTGCTA<br>GTATAATTGGAAGCTTGACACACATAAGGAGAGAGCTAGAGATGTGAAAAAAGCATTTAATTACTATGT<br>AGAATATCTATAGCCATGTACATATTACAAGCATGACAAGCTCAAGTAACTATGAGTCACCATGACAGGA<br>TGTTAATTATGCTTAAATAACTATTGGAATGTCACAGTCATTATATTTTTAGCTTCAGAGTTTATTTGAAAG<br>TAGTAACTGAACCTTACAGTTCACTTCAATCATTGGAAACTACCTAACATTAAAAAAAATAAAATAAATA<br>GGAAGATATATGGACTTAGTCCTTCCAGGTGTGTGCTGTCAGCTGAAATTTTCTGGTGCTGGAGAATGG<br>AAGAAAGGCGCCATCTTGGCAGCAACAGAGCAACGTTCACTCGTATCAGATTAGAAAGCTGTGCCAAA<br>AACTCCAAGCAATGAGAATTCATAGGTGTCCCAGGTACGGATGAGCACTTATATGATTCTCTTCTTTCCT<br>GTTTACTGCAAATTCTGCCTTAAGGCTTCTTCTCATCAGGGCTCAGAAGCCGCTCATTTTAAAGGAAAG<br>GGCAGTTGGTTGGCACACTTTTCTGTCTGGAAAGCAGCTTTCTTTTTCACACAAGAGTTAATGTAAATC<br>CGCTTTTGTAAATCGCAAACTTTCAATTTCATTGACTCGAATTTACAGCAGCTTTGTATACCAGAAGAGG<br>TTTTGGTAAGAGTTGAACATTTTTAAACAATGACTTTAAGACATCAACAGATTGTGGATTATATCACACA<br>CACACACACACACACACACACACACACACACACTCACCCCACGTAGCTAAATTAGGAAACATGATTTT<br>CAATTTTATAAGGTTTCTGATTGTTAATGGGTAAAACTCATGTTTCTTTTAATATATCAAATGATATTATGTA<br>TGTCCCTAAAATATGTCCCTGAAATAAGCTGAACTGTGTTTCTGTCAGACTAAATTTACCTATGACTGATA<br>TGTATATTAGCAGTTATTAGTTGACTTTTAAAATTAAACGCAACTTCACGATGATGTTTTTGTAGAAATTA<br>AGTAGTCAAGCCGTGGCATTTCTTGCAATTTTGTACAGTATTTGAGTATAGTGCAAATCAGGTCTGTTCA<br>CAAAATCAATAAAAGGGGCATCTTCTGGAAAATAAGCAAACTCTCAACACCAACCAAACACCCTGCTG<br>TTCCTTTGTCTCATTCGACTTTTCCCGTTTTATTTCCTAGTGGGATTTTACTATCTGCTTTGCATGATTGTG<br>TTCATACCAGGGTCACAGCTTCATCTTACTAACAGAATTCGCTATGAAGACAGCCTTGACATTAAAACCT<br>TCCTCACAAAAACAAAGAGTAATCCATAAACCTTAATAAAAATATTACATGAGCCAATAAATGTATTTTG<br>TTAATTCCCTTTCAGTAACATCTTCTCTATCATCTAAACAAGTTTTAGAGTGTGAATATTGCCCATTTGAG<br>TGAAATAAAATATCCCCTGTTTCTTAAATACTTACGTACAAACAAGGCTCTTGTGGTTAGGTCTGGCATG<br>GGTGCAGCAGTGAGAAGTGGTATGACACTCGATTGCTTTCTATGGTATATGTTGCCATACCTTAGG
MSTRG.3606.12	ENSRNOG00000014791;ENSRNOG00000015071;ENSRNOG00000048353	0.000	0.769	0.000	6.990	up	TTTATTGAGGACTGTCTCGATAGATATAAGGACCTTGACAGTGTTTTTTCGTCTTCATCTTCTTAAATTAC<br>ATTAGGTTATTTATTTTGCAGGAAGGGGGATGTGCACTTGTGGAGATCAAAAGACAACTTGCAGGAGTC<br>AGCCCTCTCCTTCCCGTGACGGAAAGAATGCCCAGTATCAGGGACCAAATTCAAGTCATCATGCTTGGC<br>CACCATCACCCTTACCCAGCTGAGCCATGTCACCAGCCTTGGGTTTTCCTATGAAGATTAAGGGGTGAA<br>GATACAGTCTAATTCCAAACACAGTGTGGGCAAGCTGTTGTCTATCGGCAAGAATCAGGATCAGGTCCA<br>TGGGAAGGAAAGGTTCCCATGGAAACATAAGGATGAGCTGAATTCTCTCCACTCTGACCCAGCAGTCT<br>TGCTGGACACAGGGCAGGGAGTTGAGACGTCACTGGGTAGGATAAGGAAGGTGATCAGATATGTCAGG<br>TGTGGGGGTAGTAGGTTCTTGCTAATAAAAGATTTTGCACGGAAGTCCATGGACCAGCCCAGAGGTTTC<br>TGGAACTCCATGAAAGTGTGATAAAACCTGGAATGTTTGGCAATATCCTAGCATGCACAAGGCCCTGGG<br>TTCAATCCTCAGTATAGATGGTCAGGCGAATGCTGAGGTCTTCAGCCTAATGGGCACTGGCCAGTTTCG<br>AGCGAGGAGGTGATGCCCTCCAATTTTCCTCTTGAGAAGATCCCTGTGGCTGCAAATGCTGAGTTGATC<br>AGAGGCCACGTGGGTCGGGGAGTGAGGGAGACACGGGGTGACCAGGAACACAGGTACAGCCATGGA

							GATATGGAGATGAAGGGGCATGGGAGAGACCTGTACAGGAAACAGCTGAGTTACTGTGTAGGACGTAGGGCTGATGGCAGGATGGGAAGGTGCGACATGAAACACCGTCTTCTGGACATGGCATGCAATCATGGACACACAGCAGATGTGGCTACCTGGTAGCATCGTTTTACATCTCTGCAAATGTCATCGGTGTCAGACTTGATAGGGAGCAGTTGGAATTCTCATCTGCTTGAGCCTTCAGCCTGTTATGGTGTCACAGGTCATATAGCCTCAGGAAAATGGCACTGTACACATTTCGTTTCTACAAACTTCAGCAGCGGGCTTTATTTCACTGGATCTATTACGATTCAGGTCCTTTATGGCAGCGATACCCCCGTTATTTTTCCTGGTGACAGGGATGTCTTCTATCACAGGCGGGCTTGGTAATCTACCCGCTTACTCCCCTTGCAATCTCTTGCTATTCTATGCAGTGGATTCATGCAATGGTGGGTGACCACATGCGATTCAGCGCGCTGCCATAAACCGCCTTCACCCAGTATCTCAGGTTGGCGTTTCGCTATGCAGACAGTCAGGTCATGATTCTTCCAGCCATTTGTATTGTCCTGTGACTTGCAGTCTCTTTTGCCCCTTTTTGGTTTGCTTTTGTCTTTCCCTTGCTGGTTTCCAATAGCTCTTCGTTTATTAAGGAAATTAGCTTAGTACTTGGGTGTTGCAGGCAGAAAAGTCCTTTCTTCCACCTTTATCGTTCGTGGTTAGACCTCAACGTATGTGTTTCTGCCTCGATTTTTCATGGTTTTTCCTTTTTCCCGATTTTAGACAAATATATGCTTGTCCTTTTCCCCTCTCTCTTCTCAGGTTTTGCATTGGGGCATCATTAGTAGGTATTTCTGATTCACCCCGATTTCTTTTGGGGGGGCTGCATCCCTTCACACCCACATCCCATCCCCCTTTTCCAGACTCTGCTTGGGGGCCCCTAACACGGACTCCGTTGACGTCAACTCCGTG
MSTRG.3613.1	ENSRNOG00000014791;ENSRNOG00000048353	0.180	0.000	0.000	-5.027	down	AACTGTTATGTACCTAACTTTCTGTTGAGAAAACCAACACTTTTTGTTCTCCTCTTTTTCTTCCTCTTCTTCCTCCTCCTCCTTTCTCTTCTTCTTTGATTATTTCTATTCTTTTTCTTTTATTCTTATTCTCCCCAAAGGACTCCTACCTCCATTGTGCCTTGGGCAGAATGATCAGTAGGTTATTAACAGTTATCACTGATACATTTGAGTGTTTATTGATGTCTTTCTACCTATCAAATATGGTGCTGGAATCTTTACGTGAATTATATCAGTTAATCAATCCTACCAGTCATATGAAATAAGTATTATGTACCTATTTTTTTTTTGAGGCAGAGTCTCACTATGTAGCCTAGACGAGCCTCCAACTTGTGACATTCTTCCTGCCCCAGCTTTTCCAGAGCTGGGATGACAGGTGTGAACCACCATGCCCGGCAAGCTGCTTTGTGAGGATGGCTTATTTACAATTTTGCACTTAAATAAGGCTGCATTGCTGTTTTTATATTGCACTCTTTCAAATAGATAAGATTTTATTATATATTTGCAGAAAATGTGCCTTAAATTTTTGTAATATATATGTTCATATGTTCACATAGATATGAACTTGTATTTCTGTTGTCTCCTGACGGCAGAAAGCAGGAGCTGAAACAGAGCTCAGTAAATCATGGACTGTACAGACCTAGGTGAATCTAGCCACAACCTGCCGGAAAAAGTTTTGGTACACATCACGTACACTTGTTTATGGATTGTCTATCCAGTGGATGCTTTGAGGCTTACGAAGGCAGTATCGAGGCATTGGAACAGACAGCCAGCATCTCATGAAGCTGAGAGGAGTGTATGGCCAGTGGAAAGTTGGCTAGTCACCTCTTAATGGCTAGGTAACTGGAACGAATTACAGAGGAAGTGTTTTGGAAAAAAGCGAAGGCTGACTCTAAGGTGGCCTCACCAGAACTCTTGTGCCATCTCTCTGGGGGTTGATGATATATGGTATTGCAATCCAATACAGATCATCTGTGTGTTTGTGGGCATGGCCATGTCTCTGCATATTAGAAGACAAGTTTTGGAGCCGTGGAGATGGTTCAGTGGTTAAAGCTCTTGCCAGGAAAGTGTGAAGACCCAAGTTCAAGTTCATGTCTCCAGAACCCATGCCAGGTGGGTATAGCTCCAGGCTCCAAAGAAGAAGGAGATGAGCTTGCTGAGCGGGCTAGCTCGGGAGACTAGCCACAGCAGGGTTCTGGATTTGATTGAGAGGCAGGCCCTGCTCCAGTGAACAAGGCGGAAGAGTAACAGAGGATGATTCCTGACATCAATGTTGAACCTCTGTTTGCACTCTCTCCTACACGTATATGTGCATGTACATATGTAGACACACGTGCACACGTATACACATGTGCATGAAGATGGAAAAAGCCCAAATTAAATAAAAAGGACAAGTTCCACGAACAGCACTTGCCTGTATTAAATGTAAGTTCTAACCTGTTCCCACGTAAAAGTGCCAGCGACTGTATTCTCAGCTGGTTTTGTTCAGATGGCGGAACCTCAGCGCCCACAGTTCACGAAGCTCCGGGAGGCAATAACACCAGAATCCTCTACTGACTTCTTTACCAGACCCTCAGCCATGCTTGAAGGGTTTATTAGAAGACAGATATGTGGTGGTTTGCCCCACGATGCCAGCCAAGCCTATGACCTTCTGTGTGTTCCAAATGCAACCAATGGAGTTGGGGGCAAAGGTATAGTATGTTCCTAGACCCTCTCGCTTGTCTTTGTATCTTATTCAGGATAATTTCTTTCTACCCTTTGCTCTGCAAAGATT

							GGCGAGCACACAGATGTCTTCAGATGAGAGTAGAGAAGCCACCGATGAAGAAATCCCAGGACTCTGGT<br>TGGAGCTATCTCTGGTCATGTGGCTACCTGACTCTTAAGAGCATCACAGGAGTGGGACCATGAAGTGTT<br>ATGTGGGGAGATGACTCCACGTGGTTTGTTATTTAACAGTGGTTGTTTTATGACTGTGAAATATCTGTATG<br>TGAGTGGAACTGTATGGGGTTAGCTTCCCCTCATAAGAGAAGAAAGATACTTTTTGTTTCCTGAGATCCT<br>TTTGGTTGCAAACAAGAATCCAGCTCACACTAGTTGAAATGAAGAGTCAAGCAGCAAAAGGAAATTGT<br>GGACATGTGTGTCTGGGAGGGCTCCTTGCATGAAGGGTTCATGATAGGGAACCTCGATAGGAATCCAGA<br>ACTTGGTATAGCCAAGACATCTCTGGAGCTCTGTGTATTTCTCTGCATTGTCCTTCTTTCGATAGACTGG<br>AACATTCTCCTTAATGAGGAAAGATGCCTACACAATGCAGGTATGATGTTCAAACTTAGAGGAAAGAGA<br>ATGTCTCTCTCCCAGGTATGTCTCAAAGAAGATCCCAGCTAGACTGCTGCAGTTGGGAAGTGAGCTGAG<br>AACTGGTTGCTCCTTTCTTCTCCTTAAGTAACTGCAAGGAGAACCTAGCACTTTTGTAATCCTTTGAAGG<br>TGGTATCTCCAGAGAGATGCTAGGGAGACAGGACCCATCTCTCGGCAGGAGCATGGACAGTGACCAGA<br>GCCATTGAACAGCAACTTGGTTGAAGCTTTGCTAGCAATAGCATCCTCTGTCTTAAACACATTCTAATTC<br>ACAGCGTGTTTTTTCGGGGGGGGGAGGGGGGTTGGCTTATTTTTGCTTTGTTTTGTTTTTCATGATAAAA<br>ATATTGTATTCACAGGCTGGCAAAATAACTAAGCCAGTAAAGGTGCTTATCACTAAGCCTGACAACATG<br>GGTTCAACCCCAGGGTTTCACACAATAGAAGGAGAGAATAGACTCTGGCAGGCTGTCTTCTAACCTCT<br>GTCTGGTTGGGCACCCTGACTTTCACACAAGCACATTCATATACACACAATAAATAAATGTGATAAGCAT<br>GTATATTCTTCAATTTGTAATACATCTCTGGATATTATTCAAAGTACAGTCAAAAGGTATATAAAAAATTTG<br>TTCTGACATTTATCTCAACAAAAATGTATATACCACAAATATTCAACAATGAAATGTGTGCTATACATTTT<br>TTATTTTTATTTTTCCCTTTCTTTCTTTCTTTCTTTCTTTCTTTCTTTCTTTCTTTCTTTTTTTTTTTTGAGAC<br>AGGGTCTCTCTATGTAGTTCTGGCTGTCTTGGAATGTAGACCAGGCTGAGATCCTACCCTCTCTGCCTCC<br>TAAGTACTGGACTTAAAAGCATGTACCACCACACCCAACTATATATGAATTATAATGCATGCAC
MSTRG.3613.2	ENSRNOG00000014791;ENSRNOG00000048353	0.105	0.000	0.005	-4.261	down	AACTGTTATGTACCTAACTTTCTGTTGAGAAAACCAACACTTTTTGTTCTCCTCTTTTTCTTCCTCTTCTT<br>CCTCCTCCTCCTTTCTCTTCTTCTTTGATTATTTCTATTCTTTTTCTTTTATTCTTATTCTCCCCAAAGGACT<br>CCTACCTCCATTGTGCCTTGGGCAGAATGATCAGTAGGTTATTAACAGTTATCACTGATACATTTGAGTGT<br>TTATTGATGTCTTTCTACCTATCAAATATGGTGCTGGAATCTTTACGTGAATTATATCAGTTAATCAATCCT<br>ACCAGTCATATGAAATAAGTATTATGTACCTATTTTTTTTTTGAGGCAGAGTCTCACTATGTAGCCTAGAC<br>GAGCCTCCAACTTGTGACATTCTTCCTGCCCCAGCTTTTCCAGAGCTGGGATGACAGGTGTGAACCACC<br>ATGCCCGGCAAGCTGCTTTGTGAGGATGGCTTATTTACAATTTTGCACTTAAATAAGGCTGCATTGCTGT<br>TTTTATATTGCACTCTTTCAAATAGATAAGATTTTATTATATATTTGCAGAAAATGTGCCTTAAATTTTTGTA<br>ATATATATGTTCATATGTTCACATAGATATGAACTTGTATTTCTGTTGTCTCCTGACGGCAGAAAGCAGGA<br>GCTGAAACAGAGCTCAGTAAATCATGGACTGTACAGACCTAGGTGAATCTAGCCACAACCTGCCGGAA<br>AAAGTTTTGGTACACATCACGTACACTTGTTTATGGATTGTCTATCCAGTGGATGCTTTGAGGCTTACGA<br>AGGCAGTATCGAGGCATTGGAACAGACAGCCAGCATCTCATGAAGCTGAGAGGAGTGTATGGCCAGTG<br>GAAAGTTGGCTAGTCACCTCTTAATGGCTAGGTAACTGGAACGAATTACAGAGGAAGTGTTTTGGAAA<br>AAAGCGAAGGCTGACTCTAAGGTGGCCTCACCAGAACTCTTGTGCCATCTCTCTGGGGGTTGATGATAT<br>ATGGTATTGCAATCCAATACAGATCATCTGTGTGTTTGTGGGCATGGCCATGTCTCTGCATATTAGAAGAC<br>AAGTTTTGGAGCCGTGGAGATGGTTCAGTGGTTAAAGCTCTTGCCAGGAAAGTGTGAAGACCCAAGTT<br>CAAGTTCATGTCTCCAGAACCCATGCCAGGTGGGTATAGCTCCAGGCTCCAAAGAAGAAGGAGATGAG<br>CTTGCTGAGCGGGCTAGCTCGGGAGACTAGCCACAGCAGGGTTCTGGATTTGATTGAGAGGCAGGCCC<br>TGCTCCAGTGAACAAGGCGGAAGAGTAACAGAGGATGATTCCTGACATCAATGTTGAACCTCTGTTTG<br>CACTCTCTCCTACACGTATATGTGCATGTACATATGTAGACACACGTGCACACGTATACACATGTGCATGA

							AGATGGAAAAAGCCCAAATTAAATAAAAAGGACAAGTTCCACGAACAGCACTTGCCTGTATTAAATGTAAGTTCTAACCTGTTCCCACGTAAAAGTGCCAGCGACTGTATTCTCAGCTGGTTTTGTTCAGATGGCGGAACCTCAGCGCCCACAGTTCACGAAGCTCCGGGAGGCAATAACACCAGAATCCTCTACTGACTTCTTTACCAGACCCTCAGCCATGCTTGAAGGGTTTATTAGAAGACAGATATGTGGTGGTTTGCCCCACGATGCCAGCCAAGCCTATGACCTTCTGTGTGTTCCAAATGCAACCAATGGAGTTGGGGGCAAAGGTATAGTATGTTCCTAGACCCTCTCGCTTGTCTTTGTATCTTATTCAGGATAATTTCTTTCTACCCTTTGCTCTGCAAAGATTGGCGAGCACACAGATGTCTTCAGATGAGAGTAGAGAAGCCACCGATGAAGAAATCCCAGGACTCTGGTTGGAGCTATCTCTGGTCATGTGGCTACCTGACTCTTAAGAGCATCACAGGAGTGGGACCATGAAGTGTTATGTGGGGAGATGACTCCACGTGGTTTGTTATTTAACAGTGGTTGTTTTATGACTGTGAAATATCTGTATGTGAGTGGAACTGTATGGGGTTAGCTTCCCCTCATAAGAGAAGAAAGATACTTTTTGTTTCCTGAGATCCTTTTGGTTGCAAACAAGAATCCAGCTCACACTAGTTGAAATGAAGAGTCAAGCAGCAAAAGGAAATTGTGGACATGTGTGTCTGGGAGGGCTCCTTGCATGAAGGGTTCATGATAGGGAACCTCGATAGGAATCCAGAACTTGGTATAGCCAAGACATCTCTGGAGCTCTGTGTATTTCTCTGCATTGTCCTTCTTTCGATAGACTGGAACATTCTCCTTAATGAGGAAAGATGCCTACACAATGCAGGTATGATGTTCAAACTTAGAGGAAAGAGAATGTCTCTCTCCCAGGTATGTCTCAAAGAAGATCCCAGCTAGACTGCTGCAGTTGGGAAGTGAGCTGAGAACTGGTTGCTCCTTTCTTCTCCTTAAGTAACTGCAAGGAGAACCTAGCACTTTTGTAATCCTTTGAAGGTGGTATCTCCAGAGAGATGCTAGGGAGACAGGACCCATCTCTCGGCAGGAGCATGGACAGTGACCAGAGCCATTGAACAGCAACTTGGTTGAAGCTTTGCTAGCAATAGCATCCTCTGTCTTAAACACATTCTAATTCACAGCGTGTTTTTTCGGGGGGGGGAGGGGGGTTGGCTTATTTTTGCTTTGTTTTGTTTTTCATGATAAAAATATTGTATTCACAGGCTGGCAAAATAACTAAGCCAGTAAAGGTGCTTATCACTAAGCCTGACAACATGGGTTCAACCCCAGGGTTTCACACAATAGAAGGAGAGAATAGACTCTGGCAGGCTGTCTTCTAACCTCTGTCTGGTTGGGCACCCTGACTTTCACACAAGCACATTCATATACACACAATAAATAAATGTGATAAGCATGTATATTCTTCAATTTGTAATACATCTCTGGATATTATTCAAAGTACAGTCAAAAGGTATATAAAAAATTTGTTCTGACATTTATCTCAACAAAAATGTATATACCACAAATATTCAACAATGAAATGTGTGCTATACATTTTTTATTTTTATTTTTCCCTTTCTTTCTTTCTTTCTTTCTTTCTTTCTTTCTTTCTTTCTTTTTTTTTTTGAGACAGGGTCTCTCTATGTAGTTCTGGCTGTCTTGGAATGTAGACCAGGCTGAGATCCTACCCTCTCTGCCTCCTAAGTACTGGACTTAAAAGCATGTACCACCACACCCAACTATATATGAATTATAATGCATGCAC
MSTRG.36323.1	ENSRNOG00000046600;ENSRNOG00000048268;ENSRNOG00000055956;ENSRNOG00000056747;	5.269	22.865	0.000	2.881	up	ATGTAAATCTCGCGCCGGGCCGTACCCATATCCGCAGCAGGTCTCCAAGGTGAACAGCCTCTGGCATGTTGGAACAATGTAGGTAAGGGAAGTCGGCAAGCCGGATCCGTAACTTCGGGATAAGGATTGGCTCTAAGGGCTGGGTCGGTCGGGCTGGGGCGCGAAGCGGGGCTGGGCGCGCGCCGCGGCTGGACGAGGCGCCGCCGCCCCCCTCCCACGTCCGGGGAGACCCCCTCCTTTCCGCCCGGGCCCGCCCTCCCCTCTCCCCGCGGGGCCCCGCCGTCCCCCGCGTCGTCGCCGTGGTCCCCCCCTCCTTTCCGCCCGGGCCCGCCCTCCCCTCTCCCCGCGGGGCCCCGCCGTCCCCCGCGTCGTCGCCGTGGTCCCCTCCTCCCTCCCTTCTTCCCCGTCCGCGGGGGGGACGGGGCGGGTGCGGGGGGGCGCGCGCGCGCCGGGCCCAGGGGCGGCGGGTCCAACCCCGCGCGGGCCGGAGCGGGGGGAACCGCGGGCCCCGGTGGGGGGGGGCCGGACACCCGGGGGGGGCCGGCGGCGGCGGCGACTCTGGACGCGAGCCGGGCCCTTCCCGTGGATCGCCCCAGCTGCGGCGGGCGTCGCGGCCGCTCCCGGGGAGCCCGGCGGGTCGCCGGCGCGGGGTTTTCCTCCGGCCTCGTCCTCCCCCTTCCCCCTCCGCGGGGTCGGGGGTTCCCGGGGTTCGGGGTTCTCCTCCGCGTCGGCGGTTCCCCCGCCGGGTGCGCCCCCCGGGCCGCGGTTTCCCGCGCGGCGCCTCGCCTCGGCCGGCGCCTAGCAGCCGACTTAGAACTGGTGCGGACCAGGGGAATCCGACTGTTTAATTAAAACAAAGCATCGCGAAGGCCCGCGGCGGGTGTTGACGCGATGTGATTTCTGCCCAGTGCTCTGAATGTCAAAGTGAAGAAATTCAATGAAGCGCGGGTA

	ENSRNOG00000059586;ENSRNOG00000060896						AACGGCGGGAGTAACTATGACTCTCTTAAGGTAGCCAAATGCCTCGTCATCTAATTAGTGACGCGCATGAATGGATGAACGAGATTCCCACTGTCCCTACCTACTATCCAGCGAAACCACAGCCAAGGGAACGGGCTTGGCGGAATCAGCGGGGAAAGAAGACCCTGTTGAGCTTGACTCTAGTCTGGCACGG
MSTRG.36324.1<br><br>NONCODE_ID：NONMMUT071529	ENSRNOG00000054945;ENSRNOG00000055956;ENSRNOG00000056747;ENSRNOG00000059504;ENSRNOG00000059586	9.954	138.633	0.000	4.562	up	ATTGTGAAGCAGAATTCACCAAGCGTTGGATTGTTCACCCACTAATAGGGAACGTGAGCTGGGTTTAGACCGTCGTGAGACAGATTAGTTTTACCCTACTGATGATGTGTTGTTGCCATGGTAATCCTGCTCAGTACGAGAGGAACCGCAGGTTCAGACATTTGGTGTATGTGCTTGGCTGAGGAGCCAATGGGGCGAAGCTACCATCTGTGGGATTATGACTGAACGCCTCTAAGTCAGAATCCCGCCCAGGCGGAACGATACGGCAGCGCCGAAGGAGCCTCGGTTGGCCCCGGATAGCCGGCTCCCCGTCCGTCCCCGTCCGGCGGGTCCCCGCCTCGTCGCCCCCCCGGGTGCGGGGCGGGTCCCCCCGCCGGGCGTCGGGACCGGGGTCCGGTGCGGAGAGCCATTCGTCCCGGGAAACGGGGTGCGGCCGGAAAGGGGGCCGCCCTCTCGCCCGTCACGCTTAACGCACGTTCGTGTGGAACCTGGCGCTAAACCATTCGTAGACGACCTGCTTCTGGGTCGGGGTTTCGTACGTAGCAGAGCAGCTCCCTCGCTGCGATCTATTGAAAGTCAGCCCTCGACACAAGGGTTTGTC
MSTRG.36329.2	ENSRNOG00000046600;ENSRNOG00000048268;ENSRNOG00000054945;ENSRNOG00000055956;ENSRNOG00000059504;ENS	14.926	228.695	0.000	4.705	up	TCGGGGGAGGAAGCGGCGCGTCGGTCGTGGGACCGGCGCGGCGGTCGCGGTGCTCTCTCGTGCGACGGGGTGCCTCTGCGCGCGCGCGGACGGGTGCCGGACCCCTCGTTTGGGGGGGTTCCCGGCCGCTCTCGCCTCCCGTTGGCGCGTCCCGCTCGTCCTCGTTCGCCCTCGCCTCCCGCCCGTGCACCGCCCGCTCCGTTCCCCGGGGGCCCGCCGCTCACCCGCGCCCCTACCCTCCCTCCCTCCCTCCCTCCCTCTCGCTCTCTCTCTCTCTCTCTCTCCCGCCTCCCGCCGCGTCTCGGCTTCGCTCGCGCTCCTTACCTGGTTGATCCTGCCAGTAGCATATGCTTGTCTCAAAGATTAAGCCATGCATGTCTAAGTACGCACGGCCGGTACAGTGAAACTGCGAATGGCTCATTAAATCAGTTATGGTTCCTTTGGTCGCTCGCTCCTCTCCTACTTGGATAACTGTGGTAATTCTAGAGCTAATACATGCCGACGGGCGCTGACCCCCCTTCCCGTGGGGGGGACGCGTGCATTTATCAGATCAAAACCAACCCGGTCAGCCCCCTCCCGGCTCCGGCCGTGGGTCGGGCGCCGGCGGCTTTGGTGACTCTAGATAACCTCGGGCCGATCGCACGCCCTCCGTGGCGGCGACGACCCATTCGAACGTCTGCCCTATCAACTTTCGATGGTAGTCGCCGTGCCTACCATGGTGACCACGGGTGACGGGGAATCAGGGTTCGATTCCGGAGAGGGAGCCTGAGAAACGGCTACCACATCCAAGGAAGGCAGCAGGCGCGCAAATTACCCACTCCCGACCCGGGGAGGTAGTGACGAAAAATAACAATACAGGACTCTTTCGAGGCCCTGTAATTGGAATGAGTCCACTTTAAATCCTTTATCTCTCTCTCCCGCCTCCCGCCGCGTCTCGGCTTCGCTCGCGCTCCTTACCTGGTTGATCCTGCCAGTAGCATATGCTTGTCTCAAAGATTAAGCCATGCATGTCTAAGTACGCACGGCCGGTACAGTGAAACTGCGAATGGCTCATTAAATCAGTTATGGTTCCTTTGGTCGCTCGCTCCTCTCCTACTTGGATAACTGTGGTAATTCTAGAGCTAATACATGCCGACGGGCGCTGACCCCCCTTCCCGTGGGGGGGACGCGTGC

	RNOG00000059586						ATTTATCAGATCAAAACCAACCCGGTCAGCCCCCTCCCGGCTCCGGCCGGGGGTCGGGCGCCGGCGGCTTTGGTGACTCTAGATAACCTCGGGCCGATCGCACGCCCTCCGTGGCGGCGACGACCCATTCGAACGTCTGCCCTATCAACTTTCGATGGTAGTCGCCGTGCCTACCATGGTGACCACGGGTGACGGGGAATCAGGGTTCGATTCCGGAGAGGGAGCCTGAGAAACGGCTACCACATCCAAGGAAGGCAGCAGGCGCGCAAATTACCCACTCCCGACCCGGGGAGGTAGTGACGAAAAATAACAATACAGGACTCTTTCGAGGCCCTGTAATTGGAATGAGTCCACTTTAAATCCTTTAACGAGGATCCATTGGAGGGCAAGTCTGGTGCCAGCAGCCGCGGTAATTCCAGCTCCAATAGCGTATATTAAAGTTGCTGCAGTTAAAAAGCTCGTAGTTGGATCTTGGGAGCGGGCGGGCGGTCCGCCGCGAGGCGAGTCACCGCCCGTCCCCGCCCCTTGCCTCTCGGCGCCCCCTCGATGCTCTTAGCTGAGTGTCCCGCGGGGCCCGAAGCGTTTACTTTGAAAAAATTAGAGTGTTCAAAGCAGGCCCGAGCCGCCTGGATACCGCAGCTAGGAATAATGGAATAGGACCGCGGTTCTATTTTGTTGGTTTTCGGAACTGAGGCCATGATTAAGAGGGACGGCCGGGGGCATTCGTATTGCGCCGCTAGAGGTGAAATTCTTGGACCGGCGCAAGACGGACC
MSTRG.36329.4<br><br>NONCODE_ID:NONMMUT029654	ENSRNOG00000046600;ENSRNOG00000048268;ENSRNOG00000054945;ENSRNOG00000055956;ENSRNOG00000059504;ENSRNOG00000059586	38.502	0.970	0.000	-4.512	down	GCTCCTCTCCTACTTGGATAACTGTGGTAATTCTAGAGCTAATACATGCCGACGGGCGCTGACCCCCCTTCCCGTGGGGGGGACGCGTGCATTTATCAGATCAAAACCAACCCGGTCAGCCCCCTCCCGGCTCCGGCCGTGGGTCGGGCGCCGGCGGCTTTGGTGACTCTAGATAACCTCGGGCCGATCGCACGCCCTCCGTGGCGGCGACGACCCATTCGAACGTCTGCCCTATCAACTTTCGATGGTAGTCGCCGTGCCTACCATGGTGACCACGGGTGACGGGGAATCAGGGTTCGATTCCGGAGAGGGAGCCTGAGAAACGGCTACCACATCCAAGGAAGGCAGCAGGCGCGCAAATTACCCACTCCCGACCCGGGGAGGTAGTGACGAAAAATAACAATACAGGACTCTTTCGAGGCCCTGTAATTGGAATGAGTCCATTTAAATCCTTTAACGAGGATCCATTGGAGGGCAAGTCTGGTGCCAGCAGCCGCGGTAATTCCAGCTCCAATAGCGTATATTAAAGTTGCTGCAGTTAAAAAGCTCGTAGTTGGATCTTGGGAGCGGGCGGGCGGTCCGCCGCGAGGCGAGTCACCGCCCGTCCCCGCCCCTTGCCTCTCGGCGCCCCCTCGATGCTCTTAGCTGAGTGTCCCGCGGGGCCCGAAGCGTTTACTTTGAAAAAATTAGAGTGTTCAAAG
MSTRG.36330.1	ENSRNOG00000046600;ENSRNOG00000048268;ENSRNOG0000005	14.763	147.596	0.000	4.088	up	TCGGGGGAGGAAGCGGCGCGTCGGTCGTGGGACCGGCGCGGCGGTCGCGGTGCTCTCTCGTGCGACGGGGTGCCTCTGCGCGCGCGCGGACGGGTGCCGGACCCCTCGTTTGGGGGGGTTCCCGGCCGCTCTCGCCTCCCGTTGGCGCGTCCCGCTCGTCCTCGTTCGCCCTCGCCTCCCGCCCGTGCACCGCCCGCTCCGTTCCCCGGGGGCCCGCCGCTCACCCGCGCCCCTACCCTCCCTCCCTCCCTCCCTCCCTCTCGCTCTCTCTCTCTCTCTCTCTCCCGCCTCCCGCCGCGTCTCGGCTTCGCTCGCGCTCCTTACCTGGTTGATCCTGCCAGTAGCATATGCTTGTCTCAAAGATTAAGCCATGCATGTCTAAGTACGCACGGCCGGTACAGTGAAACTGCGAATGGCTCATTAAATCAGTTATGGTTCCTTTGGTCGCTCGCTCCTCTCCTACTTGGATAACTGTGGTAATTCTAGAGCTAATACATGCCGACGGGCGCTGACCCCCCTTCCCGTGGGGGGGACGCGTGCATTTATCAGATCAAAACCAACCCGGTCAGCCCCCTCCCGGCTCCGGCCGTGGGTCGGGCGCCGGCGGCTTTGGTGACTCTA

	4945;ENSRNOG00000055956;ENSRNOG00000059504;ENSRNOG00000059586						GATAACCTCGGGCCGATCGCACGCCCTCCGTGGCGGCGACGACCCATTCGAACGTCTGCCCTATCAACTTTCGATGGTAGTCGCCGTGCCTACCATGGTGACCACGGGTGACGGGGAATCAGGGTTCGATTCCGGAGAGGGAGCCTGAGAAACGGCTACCACATCCAAGGAAGGCAGCAGGCGCGCAAATTACCCACTCCCGACCCGGGGAGGTAGTGACGAAAAATAACAATACAGGACTCTTTCGAGGCCCTGTAATTGGAATGAGTCCACTTTAAATCCTTTAACGAGGATCCATTGGAGGGCAAGTCTGGTGCCAGCAGCCGCGGTAATTCCAGCTCCAATAGCGTATATTAAAGTTGCTGCAGTTAAAAAGCTCGTAGTTGGATCTTGGGAGCGGGCGGGCGGTCCGCCGCGAGGCGAGTCACCGCCCGTCCCCGCCCCTTGCCTCTCGGCGCCCCCTCGATGCTCTTAGCTGAGTGTCCCGCGGGGCCCGAAGCGTTTACTTTGAAAAAATTAGAGTGTTCAAAGCAGGCCCGAGCCGCCTGGATACCGCAGCTAGGAATAATGGAATAGGACCGCGGTTCTATTTTGTTGGTTTTCGGAACTGAGGCCATGATTAAGAGGGACGGCCGGGGGCATTCGTATTGCGCCGCTAGAGGTGAAATTCTTGGACCGGCGCAAGACGGACC
MSTRG.36330.2<br><br>NONCODE_ID：NONBTAT015716	ENSRNOG00000046600;ENSRNOG00000048268;ENSRNOG00000054945;ENSRNOG00000055956;ENSRNOG00000059504;ENSRNOG00000059586	39.295	525.274	0.000	4.508	up	TTGATCCTGCCAGTAGCATATGCTTGTCTCAAAGATTAAGCCATGCATGTCTAAGTACGCACGGCCGGTACAGTGAAACTGCGAATGGCTCATTAAATCAGTTATGGTTCCTTTGGTCGCTCGCTCCTCTCCTACTTGGATAACTGTGGTAATTCTAGAGCTAATACATGCCGACGGGCGCTGACCCCCCTTCCCGTGGGGGGACGCGTGCATTTATCAGATCAAAACCAACCCGGTCAGCCCCCTCCCGGCTCCGGCCGGGGGTCGGGCGCCGGCGGCTTTGGTGACTCTAGATAACCTCGGGCCGATCGCACGCCCTCCGTGGCGGCGACGACCCATTCGAACGTCTGCCCTATCAACTTTCGATGGTAGTCGCCGTGCCTACCATGGTGACCACGGGTGACGGGGAATCAGGGTTCGATTCCGGAGAGGGAGCCTGAGAAACGGCTACCACATCCAAGGAAGGCAGCAGGCGCGCAAATTACCCACTCCCGACCCGGGGAGGTAGTGACGAAAAATAACAATACAGGACTCTTTCGAGGCCCTGTAATTGGAATGAGTCCACTTTAAATCCTTTAACGAGGATCCATTGGAGGGCAAGTCTGGTGCCAGCAGCCGCGGTAATTCCAGCTCCAATAGCGTATATTAAAGTTGCTGCAGTTAAAAAGCTCGTAGTTGGATCTTGGGAGCGGGCGGGCGGTCCGCCGCGAGGCGAGTCACCGCCCGTCCCCGCCCCTTGCCTCTCGGCGCCCCCTCGATGCTCTTAGCTGAGTGTCCCGCGGGGCCCGAAGCGTTTACTTTGAAAAAATTAGAGTGTTCAAAGCAGGCCCGAGCCGCCTGGATACCGCAGCTAGGAATAATGGAATAGGACCGCGGTTCTATTTTGTTGGTTTTCGGAACTGAGGCCATGATTAAGAGGGACGGCCGGGGGCATTCGTATTGCGCCGCTAGAGGTGAAATTCTTGGACCGGCGCAAGACGGACC
MSTRG.36333.1<br><br>NONCODE_ID:NONMM	ENSRNOG00000046600;ENSRNOG00000048268;ENSRNOG00000055956;ENS	42.804	496.982	0.000	4.304	up	CTGTCCTAAGGCGAGCTCAGGGAGGACAGAAACCTCCCGTGGAGCAGAAGGGCAAAAGCTCGCTTGATCTTGATTTTCAGTACGAATACAGACCGTGAAAGCGGGGCCTCACGATCCTTCTGACCTTTTGGGTTTTAAGCAGGAGGTGTCAGAAAAGTTACCACAGGGATAACTGGCTTGTGGCGGCCAAGCGTTCATAGCGACGTCGCTTTTTGATCCTTCGATGTCGGCTCTTCCTATCATTGTGAAGCAGAATTCACCAAGCGTTGGATTGTTCACCCACTAATAGGGTAACGTCAGATGGGGTTTAGACCGTCGTGAGACAGGTTAGTTTTACCCTACTGATGATGTGTTGTTGCCATGGTAATCCTGCTCAGTACGAGAGGAACCGCAGGTTCAGACATTTGGTGTATGTGCTTGGCTGAGGAGCCAATGGGGCGAAGCTACCATCTGTGGGATTATGACTGAACGCCTCTAAGTCAGAATCCCGCCCAGGCGGAACGATACGGCAGCGCCGAAGGAGCCTCGGTTGGCCCCGGATAGCCGGCTCCCCGTCCGTCCCCGTCCGGCGGGTCCCCGCCTCGTCGCCCCCCCGGGTGCGGGGCGGGTCCCCCCGCCGGGCGTCGGGACCGGGGTCCGGTGCGGAGAGCCATTCGTCCCGGGAAACGGGGTGCGGCCGGAAAGG

UT042007	RNOG00000059504						GGGCCGCCCTCTCGCCCGTCACGCTTAACGCACGTTCGTGTGGAACCTGGCGCTAAACCATTCGTAGACGACCTGCTTCTGGGTCGGGGTTTCGTACGTAGCAGAGCAGCTCCCTCGCTGCGATCTATTGAAAGTCAGC
MSTRG.36434.1	--	0.000	0.348	0.003	4.043	up	CCTGGGGTTGTTAACTGTAGACTTTCTCTGGGAAGGAATGAAGGATATGGTCATGGAGTTTTCATCTGTTTATGCAACCACTTCTCCTAACTGTGATCTCATTCCATGTGGCTTTGGGGTTCCTGTTGGGAGCTTCATCTCATCTGGTGTATGCTGCCTGGTTGGTGATCAGTGTCTAAAAGATCTCAGGGGTCCAGATTAATTAAGACTGCAGGTCCTCCTTCTTCCTAAGATTCCACCAGCTTTTCCCTAATTCATCTAGAGGAGTCAGCAGCTTCTGTTCATTGGTTGGGTGCACATATCTGCATCTGACTCAGCTGCTTGTTTGGTATTTTGGAGAGCAGTCATGGTGGGTCCCTTTTTGTGAACGCCCCATAACTTCATTAATGGTGCCAGGTCTTGGGGCCTCCCCTTTAGCTGGAACCCAGTTTTGGCCTGTTGGTGGACTTTCTTTTCCTCAGGTTCTT
MSTRG.36871.1	--	0.369	0.000	0.000	-7.592	down	CACACACACACAGCACACACACACACAGCACACACACACATACACACACACAGCATACACACACACATACACACACACAGCACACACATACACACACACAGCATACACACACACATACACACACAGCACACACATACACAGCACACACACACAGCAGACATGCATAGCATTTAAAGAATTTCTGTGTTAGTTATTATTTGCATTATAGTGTAGTTTCAATATGGGTATTAGTGTCTCATGCGATCATACTTATTCTACAGATGATACAATCAAGGCTCAGAAAAGAGAATAACTTCCCACAGTCATTTGTAGGAGTATAGTCAGTCCCCAGTCTTGAGCTACTAGGCTCTTGGGTCTTTGCCCATTTAGACTTAGCCCAGAGAGTAATTTTGATTTTACTGCCACTTTTTTCATATTGATTGAGATAGAAAAAATAATGGTATTGTAGGAATAGAGTCAGGCAGAATGCCATAGTCTGTAGGGAGAGCTGGCAACTTGAAGGAGTATGCGTAAGAATCGGCTGCCCTAAAACCAGGAGGCAAAAATGTAGTTAGCTCGATTTCTACATAACATGCTCTGAGCATCCTGGTAAACAAAAGTCCATTAAACTCCCCACTTTCGGTGTCCTCTGTTCCAAGCAGAGGGTAAGCATGCATATTTTAAATTAGAAAATGAATTTCAATAAATTAAGTAGAAATCTGTGAGGATAAATGAACCAGCCTATTACAGAGGTCATAGACCTGTGTGACTTGCCACCATCTCTAGATTGTTTCTCTGCTCTCAATAAATAGCACTGATCTGGTTATATATTTACATCCTCAGCCACTGAAAATTCAATCTGAATGCTTTGTCTGTAGTCACATATGATTAGCTTCCTTGAAATTCCTTATTTTGTTAGTTTGTAATAAGAAATTTAAATATATGTATATGTATGTGTACATATATAACTTTTTATTAAAGAATAAGTCACATTATGGTTTTGAGAATTATCAGAAATGGTTTATACATCTATATGAATGGAAACATTGACTTTTATTGACCTAAGCTAAGCTATCCTTTAGAGATTTCAGAAAATAAATGAATCACTCTTTGACATATTGTCCAGAATAAAATAAAATGAAAAAACATAGCCATAGCCTGAAGCCAAACATGGAGTCTAGAAAAAAATTAAAAAGCTCAAAGTTCCATTAAATAAGAAGTTTGGTGGTTGCTTCTCCCAGATCTATTAAGCTGAAATAATTAAAAGCAATGTTTGTAAAAGGCAAATAAGAAGAAATTATAGATTTTATTTTAAAGGATGCTGATCGAGTAATTTCTAAATGTTTCCTAAGAATCTCACACAATTTTACTCTGAGATGATAAACAAGTTTGTCTGTACTTTCACAGAATTCAGATTGGAAGCACTGGTCCCTTACTCTACTTTAAGCTTAGGTGATGAAGCTACCTTCTCAGGACAAGGTCAATAGAAAGACTCTGAGAGATTCAGAAACTCTGGGATGATGCCAAGGGACCATATCTGGAGCCACTTGAAGTGGGACGATGGGTGTGTCAGGACCAGTGGTCTCTGAATGAAGGAAGAGGATCAATGGATAGGAGTAATGGCAACATGAACTCGGCCCCATGGTGTGCGTGCTTGAAAATGCATGAAAAAGCATGTTCTTTTGTATACTTACTAGATATATCACAAAGTTCTCTCCAAGCATCCCTAAATTCTTAGATTAATTCAACTGTAATTTTAAGGTAACAAAGTTTTGAGCAGAAAGTTCTTCACATTGTAATAGGATTATGAAGGTAAGGTAGAGTAAATAATTTGAAAAGGATACTAAGGCCTAGGATCATGAAGTGTGGACCTGAGCAGTTGCATGTGTGTCTTCATGTGCAGGCATGCACATACATGTGTATGAGCACATGTGTTATCCAATTAGATATACAGCTTTTTACCTGGAAATAAGGATTCAGGATTTTTTTTGGCTTAATATTGAAAACTGGCCTTTTAATAATCCAAAAGAGAGAGGGCAAAGTCCTACTTCTAGTCTGCACTAAATAACACATAAAGGTGCAATCACATAAAGGACGCAGCCATCAAAGAAATCTTAGCAACATGGTCAACCAAGCAGAACCTGCATCCTGACAGCATCAGATGGCATGCCAGCATGGAGAG

							AAGACATTACAATTCCCACTCCTAGGTAAAGCGATGCAGGCTGCTCGGAGTCACTGAAAAATGAAAAC<br>ACAGTTCTCCAGGGATGATTCTCCTGAGAAGTGATTCTGAGTGCTCATCCCTAAACACAGAAACATATA<br>AGCATGAATGGACTCAGCAGACTATACAACCATAATAAAGATGAGCTCATGTATCTGAGAGGAGGAGGT<br>TGGGAAGTGTTGGAGGACCAAGGAGAGAGAGTGCCAATGATGTAGGTATGCACGGTACTTGTGCATGA<br>GAGCCCACTAATTAATTAATTAATTAATTAATTAAGAAAAGTGGCAAAGGATGGTGCCACTTTCTGGAAA<br>AGTTAAAGTGGTACATGGTGACAATGGTACTGATGATGGTACTGTAAGAAACTCCATGCTGCCTAATATA<br>GATTCATTGCCACGTGAATGAATGAATGTGGGTGAAGAGCTGAGGAGTTTACATCATAAATGAAGATGA<br>GCTGGAAGAAAATGTATTCTCAAATCCTGGGAGGGGAGTATTGACTAACTCCTTTATATAATGCTTAACT<br>GTAGAAGACCTTGTATAATTGTCATGCTTTCACATTGAGCTATTGTGCTCAGCTCTGGAATGGACTCTCT<br>GAGAGGAAAAAGCAAGTAAAGAGGAAAAAGGTAGGTAAAGAAAGAGACAGCTGAGGAGAAGGGAG<br>AAACCATCAACAGATAAATCTCTCTCAGCACACTGAAATGACAAAGACCTAGGATTCCTCCCATGTGGA<br>AGACAAAAGCAGATCCAAAAGATGTAGTGACCGAGGGAGTTTTCTCTCAAGATTGAGTGGCATTTCAA<br>TTTTAAGATAAATAAAGCATTATTTTAAAGATATAAATCAATATCCTGCCATGCGCTATTCACACTTCTGAA<br>GCTGTGCCAGAAACATCATATTTTTGCAATCCTGGCAAATCAGTGTATTATGATATTAAAGTCTCATATTT<br>GTTTATACTTACAGAGTACCACCTGGTGGCACCCAAGGTAATTTCAGGAAGAGAAAAACAACAACAAC<br>AACAACAACAACAACAACAACAAAAACAAAAGCAAAAACAAAAACAATGAATCCTCAGCCTTTCAAC<br>TTTTCAGTTCGAACCTGAGATTTAGGCAAAGCTATGAAGGTCTGGAAACATTGTTAGGATGAGGTCGTG<br>TGTGGTGGGAGGAAAGTTAGCACCCAAATAAGTCACACAGTACCCCTAGCCACTCCACTGTTCATTAGG<br>AATATCCCATTAGGTAAGTTTCCCTCACCCTTAGCCGATGACTTTGTTTATTTTCTTGTTCTGTGTCTCCC<br>AGACACAGTGCTTAATGTGGCTGGCATACTAGCAAAAATGCAAGCGAAGAGATCAGAGCAGAGACAA<br>ACCTAAGTGGAGCACAGAAGGTGCCAAACTCTGTCACCTTGGCCTCATTAATATTAGGTTACAGCTAGC<br>TGTGCTAGCAGCACTTCCCACCCCGTCTTTCTCATCATTATATAATAGCAAAGTACTATTCATCACTGACT<br>TAAATTGCCTTTCCAGATCTAGTCTTTTGTATATCCTTTATCAACGTGTAAATTGGGCTTCTCCGTGTTTCT<br>TTCCCCCTTTCTCTCCAATAAAGCCCAAATCAGTTGATTTTCCATATTTCTGCAAGTTTTCTGCTTGATAC<br>AAGAAAGACCTCATATAACTAGATGGTTCGGGGTATTGCTGTTATCCATAAAACAGAAGCAGTAGTAATT<br>AATAATTAATTAAGTGAGTCTCTAATAGGCAGAGTTGATGGGGTTTTGTTTTGGTTGCTTCATTCAACTCT<br>CAAAATTAATGCTTTGGGAGGTGAAACCTATTTTCATCTTGATTTTACTAATGATGAGACTGAAGCACTG<br>GGTGGCCCATGGTAACCCAGACAATAGGTAATAGAGAAGAGCCTCAGCTCATAGCAACCCCAGAAACT<br>GTTCCTTCCTAGTCACTCTCCGCAAACAAGAAAAGAAATAGCAGCATAGAATCAAAATGGGGAAAAGA<br>ATATTCTGCCATAGCCAAATGAGCTCCAAGGAGCAAAATCTATAGCAAAGCTTGTGATAACCATCAGCAT<br>AAGATAAGAGACAGGATGAAAAAGGGCTACTTTAAACTGAATATCCAGAGATGTCTCTGTGGAAGGAG<br>ATGACAGATCTGACTGATAAAAATAATCCCTTCTGCAAATAGCAGGTAGGGAGAGTCTCTCACAGAGAA<br>GGCAGACACAAAGGCAAGGGGATGTTGGTAAGGTTTCAAGAACTGAGAGAGAGCTACAGCATTGAGG<br>GAACAAGGGTGTCACCCGGAGCCTGAGAAATGCCTCTAGCAAACAAATAAATGATAAGAAGTTCCAGT<br>GAGGCTGTAAAGAGAACTCTTGCATGCTCTGTGAGGAGCTGGTTCTCAAAAAGCAAAAATAGAATGAC<br>TTTATACTCCACTCGCCCCATTATTATTATGTCTGAAGCAAAGGAAGTCAGAATGTGGAATCTATGTTTAC<br>AACGGCAACATTTACAATACCAAGATGCAGAATCAACAGGTGACCTTCAGTGGATCAAGAGATAGTGG<br>AGATGTGGGATGTGTACTCACAAAATAAATAAATAAATAATAAAAATAATATTCAGCTTCAGAAGAGGAA<br>ATTCTGTCACTTGGAACAACAAAGATGAGCCCATAGGAAATAAAGTCACATAAATATGACAAGATTTAC<br>ATACTGAGTCTATAATATCTTATAAAAACAATTCATAATGGTGTTCATCAGGGATTTTGGCTGAATATGCA<br>ATATTCGTTTGACAAGAAAACAATGTAAAGAATCTTTTGTACCACATGATGGTCTGTAACCAACAGAGC

							ACTATAGTAGAAAAACTTGTTAAAAGATTGGATTTTAAATACTTTTTACCATACAGTAAAGAAGGCAAAC<br>ATCTGAGGAAGTACATGTTCTTTATCTCTACGGTGCCCATCTAAAATGTACACCTAAGAATACACATCTA<br>AATTTCTAACCTAAGAATACAAAATGATGTCAGTTCCTCTGAGAGAATCCTGCCTTTCAGTGCAAAGGA<br>AACCATTGGCAGCTTTGTGTAGCAGAATTATAATCTTTTCAGCATCCCATAGGTGGCTTCTGATAAAATAT<br>TGATCTCTATTCATAGTTTAGACACCACAGTTAGGATATTGTGCCACATGTATAGATGACCAATGTCAAGT<br>GTTCTGATGCCTTTGACTAATTCCCTGTTTAAAGGCCAACAGTCCCTGTTTTTGTAACTGAATTATCCAA<br>AAACATTCTTCTGTATAATTGATCTGAATATTTACTGATAACAGACACTATTAATGAGCATGTTGTCATAAT<br>ATGTCTTCACTAACAAGGGCTGTCACACCAGTGTACTAACAGTACTAAAAGCAGATAAAAAATCATGCT<br>TAATTGCTAGGAACTGAAGACTTGTGGACATTGAGCAGAGCAACTGTTTGACCCAAGAGCTCGACTGT<br>ACCATGTGAGTCAGGGAGCAGCTCTGTGTATGCAAGATTTTGGTCTTTGGTAAATAATGTGCTCAGAAG<br>GAATGTCAAAGGCTCAATAGGAAGCATCATGACATCTGTAATAAGGTCCACCCTGAATGCAATGATGTG<br>AACAGACAAACTGTGCTTCCTAGGAGACTGATTGTACACTATTTTACAGATAATGTCTGTGGGCTTTCAA<br>AGCAATCTCCACAAACCTTATTTCTAGACCTAGAACTACTCAATTAAAGTTTTACAGAGAGAAGTATTCT<br>AGCTTTGTTGACATAGCATCGTGCATGACAGTTTCCAGTTTATAGGTTGTGTGGTTGATGTAGGTAGGTG<br>AGTGCAGGAAGGGATAGATGGATAATGGATGGATGGATAATGGATGGATGGATGGATGGATGGATGGAT<br>GGGTGCATGGATGGATGAGTGCCTGGATGGATGCAGAGATGTGTGCATGAATGGATAGATCAAAAATTT<br>AGGGATGAATGAGAAATCCTATGTTCTGTGTTTCAGAGTGCAATCCTGGAGATATACTCTGCTAGTCAGG<br>AATCTTCTAGGTAATCAAGCCAGAAACCATGATGGGATCAGGAATTGAACCACTGTTGAAAATCAGCCT<br>CTAACATTATTTCTCCTTCCAGGCCTAGAGAACACTCAAGCTGTATAGACAAAAAGACATGATAATTTAA<br>TCAAACAATATAAATGAATGCCAAAGTGTGTAACCCTACCAGGATCTCCTGCTGGAGGTGCTGACTTCG<br>TAGATCTCAGGGCAAGTTTTATATCACTTGCTTCAGAAACACATGCATCTCAAATGCATCTCTGTCAGCC<br>CTTTTGCCCTGTTGTCTCAAGTTATAGCTTCAGTATTCAAAGCCAAATATGCTCCTACATTGATAAGAATA<br>TTCTTCTCATTGACCTAGGCCCTTCTTTTTCTGGAGTCTAATTTTAAAATGTCAACAAATATTATTTTGGG<br>AACATACATCTAACTCTCCCCTGAGATAAGGAAATTGTATTGTTTCTGTACACCAGTAATGTTCCTTCTTT<br>TCTTCTCTGAATTTTCAGATATTTGCACTCTTACAATGAGTACAAGCATCTTGAGGATATGACTGCTTTCC<br>CACATTTTTTCTGCCTTATGTTGATTATGTTCACCTCTTACATATGAATTTTACACCTTCTATTTGACATATC<br>TCAATGACATATCTCAAACATCATTGCTATGGTGCTTTGAAGTAAAACATTTCAAGCATGGTAATAGTATG<br>ATATGAGGGAAATTGTCCTGATTTCTGAGGTGGCTACTCAGTGTGTACTGGGTAGATAGCATATAGGGCA<br>TGGATACATGAGAATAGAAGTCCTATGCTCTTGATAGGTGGAAAGGGATGATGAGAGATCTAATTGATAA<br>TGCCTCAGAATAGAACACAATTTAAAGTGTGTGGATTGTTTATCTTTGGAATTTTCCGTTTACTGTTTGTG<br>TACCAGAGCTGACTGTTGGTAACAGAGACTATATAAACAAAACCATTTATAAGGGAGACCTCTGTACTG<br>TCATTTCACGGCAAACACTCTATTTGCTGATTTAGGACAATATAAATCTGTTATAGACATTTTTTTGTTTTG<br>CTCTGTAAAGTGAGGTCTAGCAAAATCATTATTTCAAAGACTATATTTCCCCCAAGTCTTCAGAGTCTGG<br>CAGAAGGGATAAAGGAGGAGGGTTCATGAGAAAGATATTGAAACTAGACAACAGTGAATTTTGTTCAT<br>CTGTTTAACTCATATGATAAAATATTTTTAAAAGTAGACATTTCAAAGCATCAGTAAATATTGGACTGCTG<br>AGATACATTCTAAAGTGATCAATTTTATACTGAGCATAGTGGCTTATGCCTATATTCCCAGAATTTTGGAG<br>GCTAAAGTAGGAGTAGTAAATGAATTAAAGAAAATTTGTATTACATGGTACATTCAAAAACAGTCTTAGC<br>TATAAAGGGCAAAGGCCTGAACCCATATAAATAAATAAATAAGATTGATAACTTGTATTCATTGTATGTGA<br>ATGTATGATCTTTTTCCTATATGAGCGAACTTTGCAAGCTTTTAATATCACACATATCAGAAAAAAAGTTT<br>TACATTCTACAGTAGAATTATGAGCATAAAGATCTATCCATTGCCTATACAAATCATTTCTTTCCTTCTTGT<br>TTTCAAGAATCATAACATTTATTGTTAGGATTCTAGTCATAAACTCTGGACTTCAAGTCTGATTTATGTAA

							TTAATCTCCAATTGCATCAAGAACATTGTGGTGTTTGTTTATGGACTTTGCACTGAAGCAGAATTCAGATTTATGAGATGGCGTGATTAAGATTCTAGTGGCTTAGTGAGAATAGCACTATCATAAACATTGCAGTGTATTTGTGGTCTCTGTGGAATTCTTGCTTGACTAGATGGAAGAAAAAGCAGACTTCATTTTTTGGTGGCCTGCTATATACGTTTAATGGGGAAAAATAATCTGTAACTATTCTCCTCAACTAGCACCATTGCTGTAGAGTCAGCTGAATGTGACTGAGCTCTGTCTGAGCTGTGAGGCTCAATGAAATCCTTTTAGATTGAAGAGTAGATGATCGGGGAAAAGTTGGAGGAAGTTGATAGACATGCTGCTGTAATAATAAGTTGCCAAGGCCCATCCTCATCTAGGTCCAGGTTGCTGAGTGGGGAGTATGTTGGAGTGGTGAAAACAAAGAACTCAAAGTCAAAATGTGGGTCCAAGCTGGTGGTCTTAATTCTGCTGAGATGGCTTTTCCAGACTGCTTATGCTCTCTCTCTCTCTCTCTCTCTCTCTCTCTCTCTCTCTCTCTCTCTCTCTCTGCTTTTGTTTTCTTGCTATCCTAAAAACTCTCTGGTTGAGACAGCTATCTCTCCTAGCAAAAATTCTAAAAGCTATCTGAGTACCAAAATCAGAACAGTTGTTTAAAAAATAATTCTTGAATCTTCTGGTCAAGTCACAAATCCAGATAGAAAAATTCTAAAAGCAATTCTTGTGTTTCAGGATCAAAATCAGAAAGCCAGAAAAATTCTGAAAGAGCTGTGTGAGCTCTCTAAGTAATTCTTGTGATTTCTGACCTTATCCTGTTGGAAAAATTCTCAAAGAGCTGTTTTTGCCCTCTAGAGATCTCCAGATAGCTTAGCTCTCACCAGAGAAAAATTCTATAGAACTGCTATCACTTTCTGCTAGTTCATCTCATGCAGACCAAAAGACCAGTTTTTGTTTACATACAACCAGGTTTCCTTATGGTTATCCTATGAATCAGCATCCCATGACCCCGGCCCCTTCATTCTTAGGAAACAGCAAGCATTTCTTCTAGCATTGAAAAAAAAAAAGAGATCTGCCTACCTGTGTTAAGAACAGACAATAAGCTAGCTGGGTGGGATCCTGTACTGAAATTACCATTTCTATGATATTTGAACCTAATCTTGCTATGTCCCAGGCAGATCTTGAACTCAGGACTCTGCCTGCCTTTGTCTCTGCCTGTCTTTTTATCCTGAGAAACTGGCTCATTAATGCAACTGCCTTTGTTCCTTTAGGTTCTTGAAGCTCGTTACATAATTCATGTTGCATTCTTATTTTTTGCATTGGAGAAAAATTAAATATTTTTGAACTCATGTTTTTAGAGTTCTTTCCCACAGCTTAAAAGGCTGTCATGAAGGTCCCAGTGTTTACCATTTTGCTGAGACAATAGTCACAACTTCAACTTCTAGATTCATGTTGTCAGATTATCATGGAGTCATAAATGTCAACAGGAAGGAAAGAAAAATGTAAAATATGCACATTATTATTATTCAAACAAGCCTTATACCCATGGAAGTTCACATCTGCTGGCCCTGTCTCAGAGGCGGGAACCATATTTCTGTCTCCACAAATACCTTGCCAGACCTGACAAAAATAAAGCTATAAAAAGGAGCCATAGAAATGGCTCAGAGGTTAAGTGCACACATTACTCTTGTGAAAGAGACCAGTTGGTCCTCAGAACCCACACCATGACCTTACGTGTGACCTGACACCATGGCTTCCATGGGCAACTGCACTTACACACACAAATACACATATACACACACACATACAACTAAATGTTAAAATATATCCTAACAGAAATCTATAAATGAAATGGTTATTTGGCAAAGGCTTCAAATGTGAAATGTTTTAAGAGGACATTATTTTCTCACATATTTAAAACAACATATTATTTACATAAAATTTAAGAAATTTAGTGACAAAGAAAATTAAGGTTATTTCTATTTTACCAACAAGAAATTCAATATTTCAGTCTTCATGCTGACAGGCATATGCACAC
MSTRG.3723.1	ENSRNOG00000016608;ENSRNOG00000016687;ENSRNOG00000016829;ENS	0.049	0.455	0.013	3.019	up	GGAGAAACACAATGCAGCCCCAGCTGCTTATGAAATCTGCCCACGGGACTGCTAAGGGGGTGGAAGTACCAGGAGGGGGTGGAGCTTCTGGCAAGAGACACGAGGGTGTGGGCAGCATTGAAGAACACTCGCTTATCAATAGGCAGTGCACCGCTGGCCCACGGCTCCCCTTCGCTGTTACTGACTGGAAAGGAGCTCTTTCCCCGAGACATTTCACTTTCCTGTATTGAAATAGGGTTATGATAAATCTTGACTGTCAAGATGTGTCCAGGATTGGGCTAACGGAACTTCCTTGAGCAGTGCACAACCCCACAGCAATCAGAACTTCCCTGGGGAGACATAATGACCTGTCAGACTTATGAAAATGAAGGCAGAGGCCAGGAAATGAGCTGGCAGAGGAGAGGAATGGACCTGAAAGAAGAGGCGTCTCTCTCCGGGGAGTTTGCCAAGATTGAAGCCGCCACCCAACACCTGGCCCTGAC

	RNOG00000016877;ENSRNOG00000016930;ENSRNOG00000038622;ENSRNOG00000038625;ENSRNOG00000042414;ENSRNOG00000042927;ENSRNOG00000050348						
MSTRG.37923.1	ENSRNOG00000013720;ENSRNOG00000014098;ENSRNOG00000014409;ENSRNOG00000014785	0.289	1.072	0.015	2.415	up	CACCTCCCCCCAAAGACAGACTCAAAGTGGGGAGGAAGAGAGAAGCGCAGATCTGGCCCATGGCTGCAGAAGATTCCATTGGGCAGGAGCCAGGTGGGACCTTCACACACTGAGGCTGAGGGGAGTCCACATGGGCTGTGGAGCAGGAGGAACGCCTGCAGGCTCTCCTGCCTGCTTCTCTGTTTTCTCCGAAGCCTCCCTTCTGAACTTCCTGTGTAGCGCTGACCCGCAACCATCAAGTACCGTGGATGGGATAAGTGAACTGCCTAAGTGACCAGGTATTCCCATTTCAAGTGGCTGGCATCTGTCCAGTACAGCTCGTCCTCAGCCGGGAAAGGCTGACTCGACAATTATAGCTTCAGGAAAACCCCATGCAGGGAAGGCAGTCTGAAAAATACCTGTTTCGCTAATTCCAAAAGCTCCTACATGTTCTGACGTAGCCAATGGCAGACATAAAATCAGTGGCAGACCAGGTCTA
MSTRG.38004.3	--	0.368	0.000	0.000	-5.027	down	GTTCAAAGTAGATTTCATTTATGTGCTTTTCAGTCATTACACAATGTACAGACATGATCCACTGAATAGTTTATGCTCCACACAAATGGTTAAACAATGATTTATGAAATACTAAACAAAAAGCTTCTAGAAGCAGAGTATCGTTTCCTGCCCCCTCCCCCAAAAAAATCAGCTTCAGGCATACATTTGTGTTTATGTCTATTCCTTGAGAATGTTACGTTAGCAGTGCATAAAGTTTATTCCATAAAAAGAGCTACAAGAGAATTCGATTTTCAAGAGACTCGATGCATTGTGCTTTCAGATAAAAATCCCAAGAGGAAGATCAGTTGCTGTGACAACAGTTAAACACACAGACGTGAGACGCCAGAGAACACTGTCAAGAAATGGAAGTCATTGTGCTTAGGGGGCTTTTTTTTT

							TTTTGGTAAATTTACTTGAATAAATAAAAACTTAAAAAACGTCCCAGTGACTGATATAAAATAGACACAGGTATTTAGGGCTTTCTCTACTTGGCTTAAGAGTTTGAAAGCGAAAAAAAAAAAGTCATGTCCTCAGAAACTAATTGTCATCTGACATTCCTAACCAGGGTGAGTCACTTACACTCAGGCAAGCAAGGGACTTGGGAGAAAACCAAAAAGTGATTTTCCCCTTAATACATTAAGGATTAACCAGAAGTAAAACCAGGATGAACCAATTAATTTTAAAAATGCCTACCTTTCTTTAGAACTCTCAGGCCAAATAGGGAAAGCAATCTACTTCTGTGAGGGGCCTGTTCTCTGAACGACAGCCTGTCCTGAAGTTCACGAGATAATTTTAAAACATCTTGTCTTCTGTCACGAGTCCAGGGTAAGCATGATCCCTCAGTAAGTACCTTATGTTTCCAACCCCCTCACACACTGACTATAATATATTCGCTTAAGAAACCTGGCAGCAACTCCTTGTAATGCTAGGAGGAAGTCTATGATTTACATAGTAATGTCGGTGCTTGTGTCTAGGCAATGTCACTTTATGTTCAATCAGCTCAGTTGCTCATATAGGCCTTAAACTTTGAAGGACCCATGACCCTTATCAAAGTGACTTCGAAATCAAAAGAAGAACAATAACATTGAACTAAAGTTATTTGCATAATAGGTATAGAGTTTAAAAGAATGTCTTCAAATAATTTCCTAGATATCAATCGCTCAGAAGTATCTTTGTTGTCTGTAACATAAAGTGCTACAGTCCTTCAAATAAAATTATAGACTAATTGAAAATATATATTATACATTTGCAGTTATTTATAATCATTCCATAATTTGTAGTGTGTTAATGGAGTGGAAGAGATAAAAGGCAGTCCACATTCATATTTTAAGATAAATTTACTTCAGAAGTCAGTTTTAATCCTTGTTCATGTTCTAAGTATAGCCATTGTGATAAGACCACTCCCAGGAAGACAGTTAGCAAACTAGGTCCTGAAGAGACTGCTCTGTTTTCTATAATCCAACCAATTGGGAGGCAGAGGCAGGAAGATGGCTTAAAATTCAAGGCCAGGCTGGGCACTACATAGTGAGTTGCAGGCAGTCTGGGTTTCATACCGAGACTCATAGTTAGCCTTGAAATTCCAAGCTGGGCATGGCAGCCCACAGCATTAATTAGACTTGGG
MSTRG.38263.8	ENSRNOG00000046536	0.211	0.963	0.004	2.708	up	GAAGAGGCCGGAATGAACATCTGTGACCCCAGTGCCACGCAACAGCAGGTCTGAAATTCTCCAGCAGAAACTTGAACATGACACAGTCCAGGACAAGATCTCCCTTGGAGAGAAGAACTACAGGAGGAGAACTAAGTGTGCAGAGCAAATACACCACTATAACATCTCATCTGTAATGTGCATAGCAATTGTGGACTGCATTTTCCTCCTTTAGTTTGATTTCTTTGGATTGAATACGCTGTATTTTCACCTTGCCTCTCTCCAGCAAGTGTGCACTTTCTGAGGGACTCAGAGAAGTGCTGATGCACATCAATCAAGAGCTGCTGGTCATCCAGAAAGACCGTGCACATGTAAATTTGTACTTGTCCTGATGGGTCACCCAGAAGAGTTTCATGGAGATCAGTTCACAATATTGGAAAGACTTTGTGACAAAGTCTGTAATATCATTTATACTCAGTGTTGGATGGCTTTTTCCTTAATACCCCCACTCCAACAAAACAGACCTACTTCCACTCCTCTTGAAAAGATTATTACCATCACTAACTGCTTGTCTACTATGTCTCCAAGGCAAGCCACAGGCTATAAGTCTATTCTGCAAAAGGAG
MSTRG.38502.3	--	0.000	0.484	0.000	5.602	up	GGGCTGGGCTGGCAGGGGAGGGCTGAGATTGTCGCCCAGTGCAGGGCCAGGACAGCTGCAAATGAAACACAGGCCTAGAGCACTCAGGGAGGCAGGCATCCATCTTTCCCGGAGCCAAGAAATCCCGGCCAAGAGCTCAGCTCCAGAGCGAGGAAAAGCACCAGGCAATACTTGAAACCAAGCAAGCCCCCTGGAAATGTTGGATTCCTGACAGGTAAAGAGCCAAGCCAAGGGCTTTTTTCCTGCTGGCCCAAGCACAAAAGCCAAGGACTCTGGAGAAGTGGGAGATCAGCCTGGAGGGGACTCGGCAAACAGAAGAAAGCTCTTGAGGCCAGAAAAGATTCGCCCCCCTCAGATCCCAATGCTCATATCCAGTCAGTGTGGAGGAAGGGACACTTGGTGTCACAAACCTTCAAGGCCACCTCCTGGTGGAGGCTCCAGCGACAGGCGCCATGGCAGTGTGATCAGATTGTAGCTCTCCTCTACTACTGCTCCAGCACCATACCTGCCTCCTCGAGGCCAGTGTGCTCCCCACCATGATGATGATGGACTAACCCTCTGAAATTGTAAGCAATGCTCCGATTAAATATTGCCCTTTATAAGAGTCGCCTGGGACATGATGACTCTTCACAATAAGAGGTCATCTTGAGGTGAGATCACCCATGTGATGAGTTCAAGTCCAGCCTGAGCTACATAAGACCCTATCTCAAAATGGAATCAGCTACTCAAACACATGACATACGTAGCAAGTGTCTAGAGTCCAGCCCACAGTAATCTACGAACATACTCGTTTCCTAAATTGTAGTGTTTTGGGGATGAGAACTATCCCCTAGAGGCTCAAGGATTTGAACTTGGTCCCCTGTGGTTTTGGGGTCATTTAAGATACAAAGCCTCCAAAGTTTTGGAGGACGTATGTCACTGGGGGTGGACTTGGATATTACATAGGCTCACCCCATCCGCA

							ACTCACTCTCCTGCTTTGTACTTGTGGTAGAGATATGAGCTTTCAATTTTCTGATCCGGCCACCATGCCTACCTGCTGTCATGCCTCCCAATGATGAATTCTTACTCTCTGGAACAGAAGCCCA
MSTRG.38539.1	ENSRNOG00000005464;ENSRNOG00000042277	0.238	0.850	0.038	2.269	up	AATCTCTCACACCTGAAACCCTGGAGCTTTGTTTACGTCTCGTTTGCGGACCCCAGCCATTTGCTATGTACCACAGAGGGACAGCGGGAGCAGGGATGTTGGAATCTCACGACTGATTTGACTTGAGACCTCCCTCACGTTGGCCCCCAGAACTGAAGAGCCTTCTTATACAAGGACTCTGCTGGGACTGGAGTCTCTGCAGATCTGGAAAATCTACATCAGGGAAAGGAGACAGGGGTCAAAGGGCAGCCCTGCAGATGGCTGGAGGGTGAATGAGTGTGGCTAGGAAAACTTGCTGCATTCCACGGGAAAGTGGGGCTGTGTAACCCAGCAACTCCGTTCCTCTTTAATATCACACAGCACCATCCTCTATGCCTTTTGTCTCTGGCTTACTTTACGTTCTTAAATATGAAAAAGGACCCTAGATAACTCCTGTACGTGAGCCAAGCT
MSTRG.38582.1	--	0.000	0.587	0.001	4.225	up	CTCACGTCTCTGGGCTGTTCACGCCAAACCACGGGGAAGACTTTTCCAGATGGCCACATCTCATTTTCAGCTCAGTAAGTGTTTGGGTTTTCAGCATTCTTATATTGAGAATCTAGGCAGTGTGTAGCGCTTTTGTCCTCCTTCCTGACTCCATGATGGTCCTGAGCGTAGAGACCTTCGAGATCACACAGTTGGCTCAGAAGGTCCTTCTTTGAAAAGTGGGGATGCAGTTCCTCCCAGCCCAGCTGCTTCAGCGCTCTCAGCCAGCTTCCCTACCCCATTCTAAAGACCGGATTTGTCTAGGTAATTTCCATGGATGACTTCAC
MSTRG.38950.2	--	0.025	0.302	0.035	2.995	up	GGTGCTCTCAGGAGGGCGATCATCTTACATGTCTCCAAATAGGGTCGTGGGTGAGTTGAGCTTCAAATCCTTTATTCACTCATCAGAGACCTGTGCTGTCTGTGTACCCCCAGAACCACTGTGGGTCAAGATTGCAGGAAGATTCAGGAAACAAAAATATTCCAAAGCAGCTTCCCACTCTCCAACACCGTGTCTGCCTGTGTTGCCATGATGTTTCCTGCCATGACCATAATGGATTAAACCTCGAAGTGTAAGCCTGTTCCAGTGAAATGTTTTCCTTTATAAGAGTTGCTGTAGTCACGGCATCTCTTCACAGCAATAAAATCCTGTAAAGATGCAGCATGGAATCTTAACATCTGCCTCCATGTTGTCCCGTGGCTGGCTACTCTGCTGTCCCCTCTTCTCTCCTCCTCTCCCCTCAAACTTTTCTCCCATCCATCCTTCCTTCTCGTCCAATGACAGGTCTCATTCTATCCTGTACCGGTCGTCACC
MSTRG.39084.2	--	0.412	0.005	0.000	-4.694	down	TTCAAAGGGAAGATAATCGCAGACTCATCCTTGGACCAAAAACAAAAGTGAAAGTACAATGGCTGCCTGGGCAAGCTGACAAAGACAAATACTGTGCTCACCCTCAAACTGAAACTGAAAATACAAGTCAGCAGGATGAACACTCAGAAATGAAAAGAAGACAGAAATGAGGACCATGCGTTCACCCTTGCAGGACCCATAGCATAAAACACTAAGGGGTGAACTCACTGAACAAACAACCGAAGGTGGCAAGACAGCAGAGAAGAGTGGATAAGGTCAAGCCACGAACACAGTCCCTGAAGCAACAGGCAAAAGTGAGCCCCCAGAAGAGGAGGTCAGCAAACAGCCACAAAACAAGGAAGATGAAGTAGTTCACAGACTACGCTGGGTGCCAGATATTGTCTTTGCTCAGGACTGCCTACCTGCCCTTGGAGGTTAGAGATGAGGAGATGTTGAATCAACAGCACAGTCTCCAGGAGCAGGTTTTCAGATATTAATAAACAAAGGAAGAAATATAAGGAGTTTTTCTTTATTAACAACAGATGGCTTGATTCTGCACAAAGGAAAGGTCTGCAATCTGTGTCAGATCAGACTTGGCTGAACGATGAAGTCATTCACAGTTGCCAGCAAACATTAATAATATGGCTCCCAAGAGTGGAATTGCAGTTATCTCTGTGTTCACCTGTGAAAAGGCTTGCAGCTCAGGCATAGAAGATCAGAATTGCTGCAGTGGTGCTCAAAGGATGAGCTCAAAATCGTTTAGAAGGAGACAAAAGAAAAATATTGATTTTTGAGATATTTTTATGTGTTATTACTTGGTAAAACACAATCTTTTTGGACAATCAATAATTGAGATACTAAGAAGTTAAATTAATTTGTTATTCTTCCACATGTCTATCCAAATACATATTTTTCTCTTTGAAGATGACTGATTGGTAATGCAAAGTAAATGTTTAATAAACTTGATTAAATAAACCATTAGATAACATAGAAAGGTACCAAATGTGATGTTTGAAACAACATTTAATGTTAACCTTCAAAATAATTTTGTTTAAAGCCTTGATATAAGCAGTAAAAAATAAGTTGTTTTTCACTTTTCAGGTTATAGAAAGCATGAAAATGAAATTACCAGTTATCCCAGAGTTTTACTTGTAATGAATCATGGATGATATAATTAACCAAAGCTCTGTTGGGTCTTCATAAATCAAATTTTATAAAGATGTGGGAAATCAAAGGGTAGTTT

							GAGACCTGTAAATATTTTCCTCAAAAATACCCGACTTTTACTTAGCCACATAGATGCCTGTATCACAACATACCACAATATGGCAGAAAGAAAACAGAAGTTTTTCTTAATACACATGCTCGTTCCACTTTCTGCCTTCTGTCCACCAGTCCATGCACTTCTGCACAGCCCAGTTACAAAGAAGGATGTGTCAAGGTTGAATGATTACCTAATATTCTTTAGCTCATTCCATTCTACCTTGACTTTTTGTATTCCATTTATCCTTTTTTCATTATTAATCACTCTGTCTTTACTGGTCCCATTTAAGCAGTTATAATTATCTATTCAAAAGAGTTACTAAGAGCCTTTTCCCTAGAGATAGAGTATCCAATAGAATAGTCAAAAACGTAGTGTACAAGGGGGAGTGAAAACTGATATTATATAATGAAACAGATTTAAAATGAATTAAAAATGAGTTCATGTTTTTACATTTAAAATACTTAAAAGTGTTCTTTGATTTCGAGCCAAAACAATTTACTGTATGTATTCATCATTTATGGCACTTTCATATAGAAAGTTTTTATGAAAGTATATAATGTGATTCGAGCATGTTGACCGTAACTTGCTTTTCATCCTTCAGGTCTTCTGTATGCCTCCATTTTTCCCCTAGTAGTAGCTTTTCTTTTACTTCCGTGTCATATAGGCATGCACAATTTCATGTTTAGATAAATTATAGGACACACAAATGACAGAAAATACACAATGCTTACTTCACAGACAGTTTTATATTTTTAATTTTTTAAAAATTTCTAAATTTTATTTGATATAATAATTTCTATTTGCATCAGTTTTCTGCAAAAGCTATAACTTTACTTTATGGCTGAAAAAAAAATTCCATATACTACACTGTCTTTCCCAGTCTTGTGTTTTGGACTCCTTATGTAGTGGAGTAACTTTACTGTTGTGCTTAGAGCTACAGTGAACATGGGTGTCTGTATCTCTATGAAAAACCCAGGAGTGGCACAGCTGACTAATACATTTGCTTGATGTTCAGTTTTTTTAAAGACCCTCCTACTAATTTCTGTCATGCAAGTATAGTTTCCTTTCCCACCAAAGTGTATTTAGAGGGTTCCTGTAATGGACACTGTTGATTGTCAACTTGAAAAGACCTAGAACTAGAAATATAACACAACAAACCTAAGGTCATCCTTGTGAGGGACATTTCTTTTGTTTGTCTGACTGCAAGTTAGTTTTGTTTGTGTAGTTTAATTGAAAAAGGAAAACTCACTCATTGCCCATTTAATAATTGCACTATTTGATTTCTTTTTGTTATATTTGAAGTTTTTGTTTAATACCCTAGATATTACTCCTCAAATAATACATGATTAACAAAGATTTCCTCCCTTCATAGGTTGTCATTCCTTTGGACTAAAGGCTTTCTCTGCTGTGCAGAGGATAATAATTTTATAAGGTTCCAATTTTCAATTGTTATTGCTATCTCCTGAGCTTTAGTCAGAAAGCCCTTCTTTGTGCTGTCTACATCTTAAATATTTTCTCTACTCTGTCTCTGATGTTTTCAGAGTTACAGGTCTTGTATCAAGTCTTGATCAACTTGGGAATTGTGTAGAAGAGAGATATATTTCATTCTTCTATATATGAACATTTTGTTTTTCCAGAACAATTTATTAAGAGGTTTTACTTTAAGGGCCAGATAGATAGATCAGGAATAAAAAGAACTTGCTGTACCTGCAGACAATACAGGTTGAGTTCCTGGCACTGACATGGCACATCTGAGACTCCAATTCTATGGTGCCAAATGCATTCTTTGGTCCTCCATGGCAGCACACACACACACACACACACACACACACACACACACACACACACACACAGAGTACATATTCACGTGCATAAAAAAATCATTGGTATATACAAAAATACATAAATCTATTTTTAGAAATTCTGTAAAAGTCTGTCTCATCTTTGATATATATTTTTGACATCTTTGCAATAATTTCCATAGCTATAACTAAATGGAATTTTATCTGAGTCTTACATTCTATTCCATCATCCACTTGTTTGTTTATTTATTTATTTATTGTTCATACCATGCAGCTTTGTTACTATGGATGTATAGCAAGCTGGAAATCAGGTACAGTCATACATCTAACATTCTTTTCTGGTTGAATGTGCTATCTGGGGTCTCGTATGTTTCTATATACATTTAAGTTTTTCTATGTATGTGAACAGTGGCATGGTCAATATGATAATTATTGCATAGAATCAATATATAGCTTTTAGCTGTACAGTTACTTTAATTGTTTTAATTCTTCTAATTCACAAATATAAAAATCATTTCATGGTCTAGTGTGAAATTCCATTTCTTTCTTTAATGTTTTATAATTTTTTTGTTGAGGTCTTTAACTTCCTTGTTGATGTCTTTGTTCCTAGTTTTGTTTGAAAATAGTAACAGAGAAGAGCTGGAAGGTGAAGTTTTGGGGGCTCTGAAGGACTGTGGAGGTGCAGAGAAGGCAAGACTTTGCCTTGCAAAGACTATAGAAGTTAAGAGGCTGCAAAAATAAGCATAGAGACCCC
MSTRG.39084.3	--	0.537	0.000	0.000	-7.105	down	TTCAAAGGGAAGATAATCGCAGACTCATCCTTGGACCAAAAACAAAAGTGAAAGTACAATGGCTGCCTGGGCAAGCTGACAAAGACAAATACTGTGCTCACCCTCAAACTGAAACTGAAAATACAAGTCAGCAGGATGAACACTCAGAAATGAAAAGAAGACAGAAATGAGGACCATGCGTTCACCCTTGCAGGACCCATAGCATAAAACACTAAGGGGTGAACTCACTGAACAAACAACCGAAGGTGGCAAGACAGCAGAGAAGAGTG

							GATAAGGTCAAGCCACGAACACAGTCCCTGAAGCAACAGGCAAAAGTGAGCCCCCAGAAGAGGAGGT<br>CAGCAAACAGCCACAAAACAAGGAAGATGAAGTAGTTCACAGACTACGCTGGGTGCCAGATATTGTCT<br>TTGCTCAGGACTGCCTACCTGCCCTTGGAGGTTAGAGATGAGGAGATGTTGAATCAACAGCACAGTCTC<br>CAGGAGCAGGTGAGAACTCTGCAGCAAGCTCTATGAACCTGGTGCAAGCTCCTTTAATACCCTCCACCC<br>AAGTCCCACTGGTGGGCTAGCAAGCATTTCTTTCAAACAGCTGTTCTTGATTTTGGCTCTGTCTGTGTCA<br>GCAATTCTTGGTTTCTCAAACAATCTTCAATTAGTTCCTCCTGAAAGAGTTGCCTTTGTAGCTGCTCAGC<br>CCCCAGTGCTTATGCAGAGCTAGTCACCTCCATTCCTCCTACAAGTACCAGCAACAAACAGCCACAAAA<br>CAAGGAAGATGAAGTAGTTCACAGACCCTGTACCCAGGACTGTCTGCTGACTTACCCTACTTGCCCAA<br>ACCTCAGTTTTCTGTAAGGTCAGAAAGAGCTCTTAACTGCCAAGCCACCTCTTCAGACATAGGGTCTTC<br>CAAATAGCCCTTATTGATGAAAACACTCAACTGAATAGGATGCATCTCATTATTGCAGCATCCTGTGGTT<br>AAAAAGAGCCGTAGGATCAGGAAGGAACATATCTAAGATGATGAATGTATATGCTAGTTTCTGGATGTAT<br>GTCTAATGTAGATCATCACCTACAGAGATATTTTGACTAGTTATATTGCCAAGCCTGTGACTCAGTATACA<br>ACAAGTCTTCAGAGTGACACACTTGTGGAAATGGCTTTGATTTTTCAGCATCTGTAGCAGATTTTATCCT<br>TGTCTCATCTTTGTTCCATACCTGGCCAATTACTTAACAAGTCAATTTGCCTAATGCATATCATGTGAAAT<br>GTCATTGTGTGGATAAATAGTATAATGTAATGTGTTTTAAAATTGTTTCTTATAATGCATCACCATAGATTA<br>TAGTTAAATTTTAGTCTCTAAGTAAAGGGAATTATTTCCTGTTAGCTAGTATATTTTCTGTTCCAACAAATT<br>GCTAACTCAGATTAGTAAGAAGTGCATTGTATTGTATGTTACAGGAATAGATTCTTTCTATGCTCTTCTTA<br>AATAAGTTACATTTCTTGGTGTAAGCAAAAAAAGCAAGTTAACAATAAAAAACATTTGGTTTTAGGTTT<br>TCAGATATTAATAAACAAAGGAAGAAATATAAGGAGTTTTTCTTTATTAACAACAGATGGCTTGATTCTG<br>CACAAAGGAAAGGTCTGCAATCTGTGTCAGATCAGACTTGGCTGAACGATGAAGTCATTCACAGTTGC<br>CAGCAAACATTAATAATATGGCTCCCAAGAGTGGAATTGCAGTTATCTCTGTGTTCACCTGTGAAAAGG<br>CTTGCAGCTCAGGCATAGAAGATCAGAATTGCTGCAGTGGTGCTCAAAGGATGAGCTCAAAATCGTTTA<br>GAAGGAGACAAAAGAAAAATATTGATTTTTGAGATATTTTTATGTGTTATTACTTGGTAAAACACAATCT<br>TTTTGGACAATCAATAATTGAGATACTAAGAAGTTAAATTAATTTGTTATTCTTCCACATGTCTATCCAAA<br>TACATATTTTTCTCTTTGAAGATGACTGATTGGTAATGCAAAGTAAATGTTTAATAAACTTGATTAAATAA<br>ACCATTAGATAACATAGAAAGGTACCAAATGTGATGTTTGAAACAACATTTAATGTTAACCTTCAAAATA<br>ATTTTGTTTAAAGCCTTGATATAAGCAGTAAAAAATAAGTTGTTTTTCACTTTTCAGGTTATAGAAAGCAT<br>GAAAATGAAATTACCAGTTATCCCAGAGTTTTACTTGTAATGAATCATGGATGATATAATTAACCAAAGCT<br>CTGTTGGGTCTTCATAAATCAAATTTTATAAAGATGTGGGAAATCAAAGGGTAGTTTGAGACCTGTAAAT<br>ATTTTCCTCAAAAATACCCGACTTTTACTTAGCCACATAGATGCCTGTATCACAACATACCACAATATGGC<br>AGAAAGAAAACAGAAGTTTTTCTTAATACACATGCTCGTTCCACTTTCTGCCTTCTGTCCACCAGTCCAT<br>GCACTTCTGCACAGCCCAGTTACAAAGAAGGATGTGTCAAGGTTGAATGATTACCTAATATTCTTTAGCT<br>CATTCCATTCTACCTTGACTTTTTGTATTCCATTTATCCTTTTTTCATTATTAATCACTCTGTCTTTACTGGT<br>CCCATTTAAGCAGTTATAATTATCTATTCAAAAGAGTTACTAAGAGCCTTTTCCCTAGAGATAGAGTATCC<br>AATAGAATAGTCAAAAACGTAGTGTACAAGGGGGAGTGAAAACTGATATTATATAATGAAACAGATTTA<br>AAATGAATTAAAAATGAGTTCATGTTTTTACATTTAAAATACTTAAAAGTGTTCTTTGATTTCGAGCCAA<br>AACAATTTACTGTATGTATTCATCATTTATGGCACTTTCATATAGAAAGTTTTTATGAAAGTATATAATGTG<br>ATTCGAGCATGTTGACCGTAACTTGCTTTTCATCCTTCAGGTCTTCTGTATGCCTCCATTTTTCCCCTAGT<br>AGTAGCTTTTCTTTTACTTCCGTGTCATATAGGCATGCACAATTTCATGTTTAGATAAATTATAGGACACA<br>CAAATGACAGAAAATACACAATGCTTACTTCACAGACAGTTTTATATTTTTAATTTTTTAAAAATTTCTAA<br>ATTTTATTTGATATAATAATTTCTATTTGCATCAGTTTTCTGCAAAAGCTATAACTTTACTTTATGGCTGAA

							AAAAAAATTCCATATACTACACTGTCTTTCCCAGTCTTGTGTTTTGGACTCCTTATGTAGTGGAGTAACTT<br>TACTGTTGTGCTTAGAGCTACAGTGAACATGGGTGTCTGTATCTCTATGAAAAACCCAGGAGTGGCACA<br>GCTGACTAATACATTTGCTTGATGTTCAGTTTTTTTAAAGACCCTCCTACTAATTTCTGTCATGCAAGTAT<br>AGTTTCCTTTCCCACCAAAGTGTATTTAGAGGGTTCCTGTAATGGACACTGTTGATTGTCAACTTGAAAA<br>GACCTAGAACTAGAAATATAACACAACAAACCTAAGGTCATCCTTGTGAGGGACATTTCTTTTGTTTGTC<br>TGACTGCAAGTTAGTTTTGTTTGTGTAGTTTAATTGAAAAAGGAAAACTCACTCATTGCCCATTTAATAA<br>TTGCACTATTTGATTTCTTTTTGTTATATTTGAAGTTTTTGTTTAATACCCTAGATATTACTCCTCAAATAAT<br>ACATGATTAACAAAGATTTCCTCCCTTCATAGGTTGTCATTCCTTTGGACTAAAGGCTTTCTCTGCTGTG<br>CAGAGGATAATAATTTTATAAGGTTCCAATTTTCAATTGTTATTGCTATCTCCTGAGCTTTAGTCAGAAAG<br>CCCTTCTTTGTGCTGTCTACATCTTAAATATTTTCTCTACTCTGTCTCTGATGTTTTCAGAGTTACAGGTCT<br>TGTATCAAGTCTTGATCAACTTGGGAATTGTGTAGAAGAGAGATATATTTCATTCTTCTATATATGAACAT<br>TTTGTTTTTCCAGAACAATTTATTAAGAGGTTTTACTTTAAGGGCCAGATAGATAGATCAGGAATAAAAA<br>GAACTTGCTGTACCTGCAGACAATACAGGTTGAGTTCCTGGCACTGACATGGCACATCTGAGACTCCAA<br>TTCTATGGTGCCAAATGCATTCTTTGGTCCTCCATGGCAGCACACACACACACACACACACACACACAC<br>ACACACACACACACACACACAGAGTACATATTCACGTGCATAAAAAAATCATTGGTATATACAAAAATA<br>CATAAATCTATTTTTAGAAATTCTGTAAAAGTCTGTCTCATCTTTGATATATATTTTTGACATCTTTGCAAT<br>AATTTCCATAGCTATAACTAAATGGAATTTTATCTGAGTCTTACATTCTATTCCATCATCCACTTGTTTGTT<br>TATTTATTTATTTATTGTTCATACCATGCAGCTTTGTTACTATGGATGTATAGCAAGCTGGAAATCAGGTAC<br>AGTCATACATCTAACATTCTTTTCTGGTTGAATGTGCTATCTGGGGTCTCGTATGTTTCTATATACATTTAA<br>GTTTTTCTATGTATGTGAACAGTGGCATGGTCAATATGATAATTATTGCATAGAATCAATATATAGCTTTTA<br>GCTGTACAGTTACTTTAATTGTTTTAATTCTTCTAATTCACAAATATAAAAATCATTTCATGGTCTAGTGTG<br>AAATTCCATTTCTTTCTTTAATGTTTTATAATTTTTTTGTTGAGGTCTTTAACTTCCTTGTTGATGTCTTTGT<br>TCCTAGTTTTGTTTGAAAATAGTAACAGAGAAGAGCTGGAAGGTGAAGTTTTGGGGGCTCTGAAGGAC<br>TGTGGAGGTGCAGAGAAGGCAAGACTTTGCCTTGCAAAGACTATAGAAGTTAAGAGGCTGCAAAAATA<br>AGCATAGAGACCCC
MSTRG.39133.4	ENSRNOG00000003901	0.315	0.821	0.027	2.098	up	AGATATTAAAGATTTGTTTTTTAAATACAGATTTATTTTCAAATTTCAGTGGTAAGATCTTGAAAAACTCA<br>GTTATTCTCATAGTTCCTCAGCTTTATCTTAAATTTATAATGCTAGATCATGAGGTGATGGTCACATTATCT<br>TCTAGAAGTATAGTTAACACTTTGCTTGAGTAGGCACCTAGGAAAGAATTAATGTACAATGTTCCTCTCT<br>CATTTGCATCAGAATCTTAAGGTGCTAGTCCTATAGCCTGTCATTTGAAAATCTAAAAATACTCTTTAAAA<br>TTCCTGAGTCAAAAAATGAAGCAGCAGCCAGGCCTTGTGGCACACATATTTTTAATCCCAACACCCAGG<br>AGACAAAGGCAGGAGGTTCTCTGATTCTTCCATGTTGGCTTGGTCTACATTCCAGAATAATCAGGGTTTT<br>GATGGAGTTACAATTTTTATTGTGTTGCTTTTTCTTCTAGTGGCAAAGAGGGAAATATTGCAGACAATGA<br>GCAAAACGATTTCAAAACCTCGAGCATCTTGTCTTGTAGTCCTCTATCAGTGAATAGAAGTTGTTCATAA<br>TCTTTAGTCTTACAGTAGCGGGACAAGATTCAATGTATGTAATGTCCATAGTTTGGCAAACAGCAGTCAA<br>AACTTAGAAATGGGCTGGAGAGATGGCTCAGCAGTTAGGACTGGGTTCCCAGCACCCATGTGGTGGCT<br>TACAGTGGTAGTTTATGGCAACTACCAGTAAACTGCAGTTCCAAGGGAACCGGCAACCTCTTCTGTCCT<br>CAGCAGGATCCATAGGCTGGCCTGGTCCTCAAAGAGATGCTCCTGTCTGTTTTTCTATGTGCAGTCTTTA<br>CAGATGAATGGCTGCAGACCAGGCTAATTTTCTTGATCCTTTATACTTTCTTGATCCGTGGCCATGGAAC<br>ACTTTCCCGGATGAAGTCTCGCAGATTCTATAACGGATTTTAATGGGTGAGGGGCTCGACTTCACATAAG<br>ACTCAAACACCCTCAACCCCCGCCCCCAGCCCTTTTCGGGCTGACAGAATGAGTCCCTACACTTGGCTT<br>CGCCCTAGACCTGTCCACGCATTCGGACTTCCCAGCCTGGCTCTGGTCAGTTGACAGCAAGTCCAGAG

							TTGACAACCCGCCATTCAACATCCGCCCTACAAATCCGGAAAACAGTTCAGCAACAAACACCACTGCT<br>CCATCAACCTTCTACAGACAATTTCTCGCGGAGCAGGGAGGAAAGCTCGCGAGACTTCCCCGCCCAAC<br>AGAGATTCCCGAGCCAGGAGGCGGGGCCGAAAGAGGAGGAAGTCCCTCCTAGGCCGGCTCACCTCGG<br>CGCCGCACTCCTCGCGAGATCGCTGAGGTCTGACTCTCCGGAAGCGTGTTTGGCCGTAGGTACTATGCA<br>TTGGACTAATCGGCCGCCGTAGCCCCTGCTAGGACAGCCCGTGCGAACCTGGTAGAGGAGCTCAAAGG<br>GATGTCGCTGCTGGTTCGAGCAGAGTCCGACGGTAAGGTTATTGGAACGGTGGGATGGGGACCAGTGC<br>ATCGGTTGCGACACAACCTGAGGTTTAAGCTCTGGGACTCCAGTATTCTGACTAGCACCACTAGGGACA<br>GCACATTGGTCTTGAGCCTGCCCTTCCTTACGATAAAAACAAACTACTGGGCCTGGATTTACTCTCCGTC<br>AGACGTGGACTCGAGGTTCCCCGCCTCTCTCCCTGTTAAGGATTTGACTCCTTTCCCCTCCAATCCCCGC<br>TCGGTTGTTGAAATTTAGAATGTGATGAGTGAGGAGTCAGTAGAAGACCACATAGTAGAAGACCAGATA<br>GGAATCTCGCCCATGAGATCTGACACGGATCCCCGACCCGCTTTAGGTACAGTCTTGCCGGTTGAATGT<br>GTCTACTATAAAAGGTTTAGTGAAACTTTTCTGAGTGCCTCAAAAACAGGTTTCAGTTGTTCCAATCGA<br>CAGGTGCTGGGGAAAACTAAAACGGACTTTTTCTTGAAGTTAGACTATTGCCACGAAACACACAGGCA<br>CATACATCTTTATTTTCTGATCTCTCCAAGGCTGCCATTGTAGGGAGTGAAAAAGCGGGTTCCACAATCT<br>CAGCAAGTTAGAATACTAAAGCAGACTGTAAAGTTGTATTTGCTGATTGCAGAATTCATAGAAACCATTT<br>TAATATTATTAAAGCTGACAGACATAATAAAGTGGCTACTACTACCTGTACTCAAGAGCTGCTCAGTTGG<br>AGCAACTAATACATGCAGGTAATATAAGAATAACAATGTTAGATTTATTTTTGGCAAATCTTAAACTAGTG<br>CAGAGTCCCTTTTAGTAGACAGCAAGTCCAGAGTTCTTTCTACATTATGTGGGGGGGTTGTTCATTGTCA<br>AAGGAAAAGGATAAGTATAATTTGTATGGTAAGTCTCTCCAAGGCCCTTGTAATTTGTTTTGTTAGACAG<br>GAAACAGCTTTTGTTAAAGTTAGTCTTGAATAAACCAAAATACATTTGTATTCAGATGTTCAGTCAAG
MSTRG.39150.2	ENSRNOG00000004057;ENSRNOG00000004145;ENSRNOG00000004426;ENSRNOG00000004456;ENSRNOG00000052764	0.004	0.613	0.000	5.281	up	GGAACGGACTGAGTGACCCACAGTTCTCCTGGGTCCGGAAGGCGACCTCGCCTGGGAACTGAGTGAG<br>TGGGGTTTCTACGCTAAGATGGTCGCGAGTGCCAGTGTCACTTGCCGCGGCTTCAGTGCCCACGAGCA<br>CCCGGGTGCGCTTCCCAGAGGCCCGTGTGGCGGCGGCGGCGGTGTTTTTCACTTCTTTTTTCCTTTCTA<br>AAACCTCTTATTGGTCCATTCAGACATTTCCGTTAATGCAGCTACGCATTTGCAGCGTACGCACCCTCTC<br>CCCGCTTTGCGAATGTCACCCTGAGTTCCCGTTCCTGGTTGCCGCTAACCTCATGGCAGTCGGGAATGA<br>CCGCTATGGATCCCACGTCCTCTGTAAAGTGACAGGCGTTGGGATATTTAAGAACCAAGGTTTCTTTCC<br>AGGTTTGAGATTTGTCTGAATTCTCGATTACATATATCTATGTTGGGTTGTCCCAGAGTCTTTCCTGTTCC<br>ATTAATCTGTGCCCTTCTAAATCCCCAACCCCATGAATGTTTTTAACAACATTCATGCTAGTGAATGTTCA<br>AAGTATGTAAGTCATCAATGAGGAAACAATACATTTATCGTCTCACTTTTTTTCTTTGTAACCTTGTTATA<br>GAAAGGCTATAGAGAAGTCTTTCTTTTTTACAGAGTGAGGCCCATTGGACAAGAGTGACTTGTTCCTCC<br>CTGACAATGCTGGCTGATCTGTGAGAGCAGGTGCCCATTCAAGGCTTTTGTTACTGCGTCAGGTCCTTC<br>CTCTATGACTTTGCTGTGCCATTTTCCTTTTATTGGTATTTTTTCTCTGTGTGTATGTATGATGGTGTTAATG<br>TGCCACAGGTAGAAGTTTCTCTATGACTTTGCTGTGCCATTTTCCTTTTATTGGTATTTTTTCTCTGTGTGT<br>ATGTATGATGGTGTTAATGTGCCACAGGTAGAAGTTTCTCTATGACTTTGCTGTGCCATTTTCCTTTTATT<br>GGTATTTTTTCTCTGTGTGTATGTATGATGGTGTTAATGTGCCACAGGTAGAAGTTTCTCTATGACTTTGC<br>TGTGCCATTTTCCTTTTATTGGTATTTTTTCTCTGTGTGTATGTATGATGGTGTTAATGTGCCACAGGTAGA<br>AGTTTCTCTATGACTTTGCTGTGCCATTTTCCTTTTATTGGTATTTTTTCTCTGTGTGTATGTATGATGGTG<br>TTAATGTGCCACAGGTAGAAGTTTCTCTATGACTTTGCTGTGCCATTTTC
MSTRG.394	ENSRNOG0000004	0.871	0.009	0.000	-5.166	down	GTAAAAGCATTTGCTGCTAAAAAATGTCAACCTGAATTGGACCACTACCTGAAAAGAGAACCGACTCT<br>CACAAGCTGTCCTCTGACTTTCCCACAAGCACTGTGGTATACGTGTCCATATTCACATACGTACCCACAA<br>GGAAGAACATGCAAGATAAATACATTTTAAAAACATAAACAATAGACCTTGACTTAATATTTTATTAATCA

09.1	8980						TGAAAGTAATTCTTGGGGAGCGTTAAAAATTTGGAAATTGTAACATAAATTATGAGTAACGAGAGGAAG<br>GATAGCTCTCATTTATTAAGTTCAAGTAATTTGTTATTTATTGGTCCAAGTAATTGATGTAGCAGGGGCCC<br>AATTTTACAGTGGATGAAGGAAGTTAGAAAGTCTTTATTTGTTTTTAATACCACAGCCCAAAGATGAATG<br>ACCAAGGACTTTAGCTAAAGATCATTCGGGGACCCGGTTCTCCCGCCAGGTTTTCCTTCTTGTGATCTG<br>AGGTGGTTTGTTACAAAGACCCAGCAGGAAGGGAGAGGGAGAGAGGTGGGCCCGCAGCTTACATGAA<br>CTACCTTCGCAGTTCTCCTATCCAACTCTGTGGTATGGCTGCACACCTAACAGCAAGGGAGGCTGGGAA<br>AACGAGCTTCATGTACAGGCGTGTAGTCTTTCTCAAAGTACCCTAGTCTTGTGCCTGCAGGACAAAACA<br>GAGGACATTAAATGATAAAGATAGTTTAACATAGCGCTAGGCTCCTATTTAATCAATGACTGACTGCAAT<br>GCTTTGCCACAAGACGTGTGAGAAATGGCCCTAGCAAGTGACGAAGAGGCAGGCAGCATACACAGTAT<br>GGATACTTTGGACAACGGGATGATTTGTATCCTGGGCAGGATCGAGGGGATCAGAGTGGCATAATTTAA<br>AACTTATGAATTATTCATTCCTGGAATTTTCCATTTTCCATGTATTAACATTTTCAGACTGCAAAGTAACTA<br>AGACCTAGAAGAACCAAATTTCAAATGGGATTGCGAGATGGGCAAAGTTCTACGTTAGGAAGCTAGCT<br>GATATTCCACAGCAGATCACATTCTGCGTTTGTGTCTCTTGACTGTACCAGTCTGATCTGCCAGGGAGCC<br>TCCGGCCTTGAACACTGACCAGGTAAGAATTCTAAAATCTTTCCTTTTAGGATCGTCATGAACCTAAGTA<br>GGACTCCAGAAGGGTCCACGAAAGTCGCAGTGGTCTTATAGACTAGAGTGTTTAGGTTTGGTGTTCTTA<br>CAGAAAACAGCTACCTATAGTTTTTAACAACATTGGTCTGGCTCTGGGTTTAAAGAAAACATGGATCTTT<br>TTAGTGATAGTTAAAATTGGATTTTCGCCCACTTTTACCCTTATCTTCTTAAAAAAACAAAACAAAACAA<br>AAAAAACTGAGTCGAAAAGAAAAATATGAGCTGGAGAACATTTGAAGTTGTTACCTTAATAATGGTTGT<br>CCCTGTGGTGCCAAAAGTGATTAAGGGATTTCAGACATTACGATAACCTGTGCAAGACAGAGCCGCAG<br>AGCAATTAGTTTTGAGAAGTAAGCCTTGATCTTAATGGATTGGTTATAGAGGTGCAAGACTTTTCCCTTA<br>GCAGTAAGCAGAACAAAGGGTCAGCTTAGGACCGGTGGGTTTCTTCTGGAAGATACTTAATTAACTTTG<br>GAGATGAGTTAAAGCCCGTCAGAACTGGCTGCAGACTTGAGAGGCCCAACTACCCCAGATGTGGAGTT<br>CCCATTAGACACTGACTTGGGATTTTTCACTCTACATTAAAGTCCAACCTCATTATGAAAAATTTTAAGT<br>ATATGTCAAAGATATAATGGTCACAAGAAGTCCCCAGGTCCTTATCATCCAGTGTCAGTTACCATCAACT<br>CACAGCTCAGGTATGCTTTTTCACTCTCAGCCTTCTCGCCCTTCTCCGTTGGGCTGAACGACATGGCCA<br>CTGGAAGTCAATGGCTGGACTTTCCATTACTTATTTATGTTCCCATCTAATGGTGATAGCAAATGCCCATT<br>AGAACAGAAAGCCTCATAGCCTGGCTTAATCGGTTTCTAGAATTTGTCATCCATTTGTTTCTTCACACCA<br>CAAATGCATACTTAGCAGTTAGCAGGCCCAAGACCCTCCTTTGGATAAGAGGGTCGTTTTTCTTTTCAA<br>CTCACAACAATGAGGTCCTTTAAGATCGTCAAATGAAGTTATCTATCAGAGACACATTGACAGAATGAA<br>AAAATGGCATTCACCATACAGCCTTAACAGTCAAAGGTAAGCATTTGACGCATACCACAGTTTTAGAGA<br>CTCGGTGGGAGCTTTCTTTCTTAACCTATTAATATGGTGGAATTGTAAAGACCACACACAAAACAAAAC<br>TCCCAACTCTTCTGTTTGATTGTGAATGTTCTTCCAGTGATCTGACCCCTTTCAAGAAAAGCTCAGGGA<br>GAGAGAGAGAGAGAGAGAGAGAGAGAGAGAGAGAGAGAGAGAGAGCAGGTGTGGGTTCCAACTTAAC<br>AATTTTGCCTCCAACCCGTAGCCAGTGTGCCTGGCGTTTGTTATATATTTAATAGAAGTGTTGAATTGAAT<br>GAACAAAGGAATGAATGCCAGACACATGAGCCTGAGGATGAAACGACATGGGCCACAGTGCTGCCAG<br>TTGCATGGGGAAGTAATAGAGGCCCCACGGGTGAGCTGTGAGCAAAGAGTCACCAACAGGTGGAGGA<br>GGGCGCTGGCCTGGTGGCCCTGTGGAGAACAGCTTTGAGTGGTGTTGAGATGTCTTTGGACTGAAAGG<br>GAGAGAAAATTCAGCTCAGGTATAAGGAATCACATATCGAAATACGCACAGACCCAAAAAGGAATGGT<br>GCAGTCTGAAAACCTTCAGAGACTTGGAGTAGCCAGAGAGTAGCTGACTGAAGAGGTGGGACTTCAG<br>TAGCAGGCCAGCCAAGCATGGCTGAAGAGAATATACAACGAGAACTTTTCCTGGAAAAGATGATTCTA<br>CAGGCAGAAGGGAGAATGGAAGGCAGGCCACTGGGAAGACTTGGGTCTAGGAATAAGGGGACAGAA

							CTGATGACTAAGTGAGGAGAGAAGAGACATCTAGAATAACTTGAAAACCTCTCTGTGGGATGAGGAGCTGGGGAGCAGTCAGCAAGCTTCAGATGCAAGAAGAGAGACTAGAATTCTGATTTCAGGATGTGTTCCTCCATGCAGGTCTGCCTATAGCAGGCGGAGTTGGGGTTGACCATCAGGACTTACAGAAAAACATGGAGACATATAGCTACAGAGAGACCACCCAGACCAGACCAGTTGAATCAGAAGACTTAGGGGAGAGGCCTGGATATCTGTGGCTTCTCAGCGTTCTAAAGTGCAGTAGGGTTGAGGATTTCTAGTTGAGAACTTATTTGAAGAGGGGTGG
MSTRG.39454.1	--	0.321	1.093	0.050	2.241	up	TGGAGAAAGGTTAAATTGGTTTGACTTGGTTTCTGCTTAGATATCAGGAAAACCTGCCCTTGATGCTTCATTCATCCTACCTGAGGCTGCTTGGTACACGGATTTTCATACTGGCTTCCCCACAGAACCTTGTTGTTTCCTTAATGGACTCTTCCAGTGCAATCTGATCCCATAGAAGCTCCCTGTTCTCATTCCTTGCTCTCTGGGCATTGTTCTTCAGCTCAAAAAACTCAGTTAGGAGGGGGCAGAAGCTGTGGCTGAGACACAAAGAAAAAATGGTGAATCCCAGCCAGTCTCCAACTTCC
MSTRG.39473.1	--	0.499	0.000	0.017	-3.928	down	AAACGTCCACGGTAGGGGCTGGAGAGATGGCTCATCATTTGAGAATTTGTTTCTCAAAAGACATGCAGGGCACCTAACAATCCTCTGTCACTTAAGTTCCAGGTGAAACACAGTGACCTCTTCTGGCCTTTGGGGAACAGCATGCACAGACCTACAGAAGGCAAAACACTCATAAATGTAAAATAAATCAACAACAACAACAAACAAAAACAAAAGCACCCAAAGGAGGGAAAACCAAAGGAGAAAACTCAAGGGCACCTTTGCCTGGATATTTTGTCCAGCTTTTAAAGAAATCTAGAAACTGAATTGTAAACTATGTGGTCTGCTTGAGTTGAAATCCCAAATGGAACATAGTACATTGTCAAGAACAAAAGTCATCATCCTAATCAAGAACAAGAAAACAACCAACCATAAAAACCCTATCCCCTGAAAGGTCTTGCAATTTATTATCTCTTCTGTGCAGTTATAATTGATATCATGTTTGAAGGAAACCAAAAGCAAGCAATTCTGTTTCATGACTGCTTCTATACTCC
MSTRG.39512.6	--	0.061	0.339	0.009	2.740	up	TCCATGTTACCTATACAATACTAAAGAATCAAAGTTAGAGACTCATATCAGAGAGAATATGAGGTATTGGAACTTTGGAGCTTGTATTACTCCTAATAACAGTAATATTTTCCAATTTCACTCATTTCTCTGATATTTTCATAATTTGCTTTTTCCTTACAGAAAAATAATTTTCTATTTTCTACATGTACCACATTTCATTACCATTCATCGCCAGACAGGCATCTAGGCTGCATCCATTTTTTTCACTTTTCAGGGCAGCAATGGACCTGGATGTGCAGGCACTCTGAATACAGGGTTTCTCTATATACTTATTATTTAAAATATGTTAACCCTCAGTGATAGTTGATTGCCTCTAGTTCAGGGTCTAGGATTTGGATCCACAGGATTGCCCCTTAACAGTTCAACTTCTATTTCCTTTACTTGATTGCATTGAGCTCCATGGTGCAGACCTATCATTCCACTCTCTGCATGAGGTCAGACAGAACCAGGTATCTCATAATTCCATCTGCCACTCTTGGACACATTCACTCAGGACTTCTTCTCCTCTAATGTGTCTTCTAGATGCTGCTGACAGGAGGCCAGTCAAGGAGCAGCTTCATAAGTACATAGGTGGCTCTAGGTGACATTCCTTTCTGGAGCTATGCTGCACTGGCACAGGAATAGCCAAGCTATCTGGAAGAATAAAGAATTAGTTTGTATTTCTTCTCTTCGAGGTTAATTTAATAACTGACGCTTTCTTTATTTTAACCGGGAGTTCAGTTTTCCTCATTGATTGGTTAGAGTGCTGCTGTTATCGGAGAGTATTCCTATAAACATAATGTCCACAGTGTGTACTGAGCCACTTGTATTTCACATTTTCCAGTCAAGAAGATTCTTGGATATTTTGTTGGTCGATTCTAAATCACTGTGATGAAGCACCTTAAATGCACAAATTTAATGGGGTGGGGAGGGAGATATTTTAACTCATGGTTTCAGAAAATCAGTGCATGGTGGCTTGGATTTATGGGATGGCTTGCCTCATAGTAAAGAAAGCAATGGAGCAAG
MSTRG.39756.8	--	0.000	8.742	0.000	8.359	up	AGCCAACTGAAATGTAGGCTGTAGCAAGTAGTTTTACAAAACATACTTCACAATTAAGTTTTACTTTTGTTTAAATGTGTCCCACTTGTTTTGCTAGTACTAAGCAGTGGCTTTCACATCGTTTTCTTGACACAGACGTCCTAATGCACAGAGCAAAGGCCACTATTTAAAGTTTACAACTTAAGTGCCTCTGCCTGTGTTCAGACTTCTGTGTAAAGACCACTGCACAAGCTTTGCGCTCTGCTCTGCTCTGCTCTGCTCTGCTCTGCTCTGCGTTTGACATAACATAGAAAACAATATGCTAAAAGGATTGGTCCATGGGTAATTGCTGTCACTTTAAAAATAAAGTACATTTTGAAAGAATTAGTTTAAAAGTAATAAGGTTAAAATAGCCCCACTTTTAAGTGATCCATTTTAAA

							A
MSTRG.39759.1	--	1.261	0.129	0.014	-2.445	down	TCCAGCCATGTTTTCGTATCCTAGTGACCATATTACTTTATGTGGTAGTTTGAATGAGAATAGCCCCCATAGGCACATGAAGGATTAGGAGGTGTGGCCTTGTTGGAGAAGGTGTGTTATACGCTTTGAGGTTTCAAAAGACTCAAGCAATCCTCAGTTAACTCTCTCTGCCTCATAGATGTGTCTCAAGATATACGTTCTCGGCTACTGTTCTAGTGCCATGTCTGCCTATCTGGTGCCCCCCTCCATATCATAATGGTCATAGATGCACCCTCTGAAACTGTATGCCCCAAAATACATTCTTTCTTCTATGATTTGCCTTGTTTTATCACAGCAATAGGAAAGTAACTTAGATACCTTGTAAGGAGGTGTGAGTGGAGACCATAGAGAAAAAAGGAATTGTATACTGTTCACGAAAACATAATAATCTGCTCATTGACATAAAAACATATTTGTTGCTCTTTCATTTAAAATAATGACACTATAGAAGGACGGGTTTTTAAAACCAACTCTTGTCATTGAAAATGCCCTATATGTTACAAGGACTATACAGTTAAGAATAAAGAAACAAAGGTTGGGGATTTAGCTCAGCGGTAGAGCGCTTGTTTAGCGAGCGCAAGGCCCTGGGTTCGGTCCCCAGCTCCGAAAAAAAGAAAAAAAAAAAAAGAATAAAAAAAACAAGGTCTGGGATTCAACTGGCATAGAAATATAGTAGTTCTGAGGCGAATGCCAGCAGCAAACCACTGAACTGAGAATAGGACCCCCGTTGAAGGAATCAGAGAAAGAACTGGAAGAGCTTGAAGGGGCTCGAGACCCCATATGTACAACAATGCCAAGCAACCAGAGCTTCCAGGGACTAAGCCACTACCTAAAGACTATACATGGACTGACCCTGGACTCTGACCTCATAGGTAGCAATGAATATCCTAGTAAGAGCACCAGTGGAAGGGGAAGCCCTGGGTCCTGCTAAGACTGAACCCCCAGTGAACTAGTCTATGGGGGGAGGGCGGCAATGGGGGGAGGGTTGGAGGGGAACACCCATAAGGAAGGGGAGGGGGGAGGGGGATGTTTGCTCGGAAACCGGGAAAGGGAATAACACTCGAAATGTGTATAAGAAATACTCAAGTTAATAAAAAAAAAAAAGAAAGAAAGAAAAGAAATATAGTAGTTCTGAAAGCCAAATAAAGAAGACAGACCATCAGTGATGTTCCTCCAAGCAGCAGTTGGAAACCTGAGAGCAAAGGTCAGATGAGGAAAAATCTCTAGCTTAAATGTTAAGTTGTGCTTCCTAATCAGGTGACTCTTCAGCCTGTGCTGTTCCCCATGTGCCTCCCACACCAGGTTTTAACTGAGCAGGTGACAGAATAGTCTGTGTGTTGATATCTCTCCCGTCTACGGTGCTGCAATTCCTTCACAGAGGAGTATGCTGACATACAC
MSTRG.39796.1	--	0.484	0.000	0.006	-4.200	down	AAAAAGGCAGAGTGGGTAGTTAAGAAGGTGATAAAAGACATAAGAAAAAGTTTAATGGAAATGATTTATTACCCTTCCTGTTGGTAACTACAGTATCTTCACATATTGAAAGCTAAAGAGAGGCAAATCTATTCCTAACAGCCACTACAGAAAAACTTCAGACAGGAGAATCTCAACAACCTGGCCAGATGGTCTTGATGCAAGTCAGACAACCAAAGACGTGGGACCTACGGAGTATCTTTCCAGAAGACATGGATTTGTGCTACATTGGGTCAATTGTATGTAAGTAAAGGATCTCACTGTATCTTGCTTCCTGGGGAGTTTCTGAGGTACACAGATGACATGATCCAACCAGGCCAACAGTGCTGATTGAACTTGGTCAGTGTGCCAGGCATGTGCCAGGCGTGTGCCAGGCATGGGCAGAAATAAGGATCTGTCTTTCAGACTTAGCAGGTTTGACCTCCAACCAGGCTTCTAAGTTCAACTGCTCTCTGAAGAACTGCTGTGGAGGAAACAGCTCTGCCTTTCAATTACACAGACGTTAAAAAAATTAAAAAAAAAAAAAGGCAAGAAATACTGAACGCACATTGAGTGTATACTGGGTAATGAGTATTTATTTCAACACATCAGTGCATTTAGATCTCAACACTGTAAGAATCAG
MSTRG.39818.4	--	0.000	0.382	0.000	5.728	up	CCTGCTGTGGGACTTTTCCCGTCAGCTCCCTGATCCACCCCAGTGGGGACACGCTCAGGCGACACTCCTGTCCCGTGCTCCCGCCCTCTTGCCTTTCATTCCAGCCGACAAGACCTTGGAAGCTTTGAAGCCCAGCAAAGAAGGGAAATCCACTGAGAAGAGTCTGGAAAGGAATGGACCGGGCTGCCACTGCTGATTTGTGTTTGGTGTTTTGGTAATAGACTGGACTGCTGACAAGGAAGATTCAATTCCCACGATCTGCAATAGAAAACAAAACGGAAGAAAGAAGAAAAGAAAAGCTAGGGGGGAGGCAATGTGACTAAATACCAGCACATGGAGGTGGAGGCAAGAAGAATCCTTGCTCACAATTCTGGGAACCCAAGGTCTCAGGGAGACACCTTGTTCCACAAAACAACATGTGTGGATGGCTCCTAAGGGAAATAATAACACCCAGATCTGACCTGAGACTTTCACCTGTACACACATAAGCAGATACCCCTCCTAAGAAGACCTCCTGGGATTTGTCAGCTATTTTTTGTATTATCTTTTC

							ATTTTGTCACTCATGCTTAAATAACTTACCCAGTAGTGAGTGTTGCCATTTTATTTCTGTGAATACTTTTATCTATCCCAGGACTTAGCAAACTTTCTTTGACAAAGACCAGAAAATAACTACTAAATGTTCTGTGGTCCTAAGGAGTCTTTTACATTTGCTTAATTCCGACTTGCTAAGTGAAAGTAACTTTTGATAATAGGCAAAAAATACTTCTCTCCCCATGACACTTTATTTCTGAGGGTTCAGATTTGAATTTTTTGTAACTTTCGTGTCTTATAAACTAATATTCCTCTTTAAAAGTTTTCAAGAACCTTCAAAAGTGTAATGACGAGTTTTAGATTGTGGCTGCCAGGAGGCAGCTGTCTAGATGTAGCCTATGTGCCATTGTGTGCCTACTGTTGCTATACAGAATAAGCAGGATACAGCTGTAGGATCTTTTTAGGTTTTATGCTGAGGTCTCTCAGTTTCCATCTGAGGTATCTAGGGCAAACAAACACCGTTGACTAAAGCTTTGTTATCTTTGTAGTAAAATGTAAATGATACAAAAAATAGAGTTGGTTTGTGAGCTAAAGCTTATTTGAGCCATAAAAAATCCACAGGTTAACTATTTCATTTCCTCCTCTGGTCCTTTCTACCACTTGGGATTTTATTTATTTCAATTTCTATATATGCAGGGTTAGGGCAGTATCAGTTTTACTTTCTTATGAATTCGATTTTTATAGAAAGCATTCAACTTCTTAATTTATATTAAATCTAATATATACAATGCTTAATAGGAACAGAAACAAAATATATTGAGAAGGTGTCATAAAAAACTCAGAGTTTCAAAGACTATACATGGACTGACCCTGGGCTCCAACTGCATATGTAGCATTGAATATCCTAGTAAGAGCATCAGTGGAAGGGGAAGCCCTGGGTCCTGCTAAGACTGAACCCCCAGTGAACGTGATTGTTGG
MSTRG.39818.5	--	1.164	0.005	0.000	-5.554	down	GACAAGTTTTGTCAATGGCATATCTAGCTAGCACAACCTACCAGTTGAAAAACATGGACAGGGAACATGGCGCTTTACTTTCAAATGCCTTAATCTTTTGTTTTTATCTCCTTGCAGCTTTGAAGCCCAGCAAAGAAGGGAAATCCACTGAGAAGAGTCTGGAAAGGCACATGAGAGGATGTTTTGCTTCAAACAGACTTGTTGGGTTTTTCTGGAAGTTGCCTGGTGTGATGTTTTGCTGGAGCAGAGAGACTTACAATGTTTAAAAAGGAATGGACCGGGCTGCCACTGCTGATTTGTGTTTGGTGTTTTGGTAATAGACTGGACTGCTGACAAGGAAGATTCAATTCCCACGATCTGCAATAGAAAACAAAACGGAAGAAAGAAGAAAAGAAAAGCTAGGGGGGAGGCAATGTGACTAAATACCAGCACATGGAGGTGGAGGCAAGAAGAATCCTTGCTCACAATTCTGGGAACCCAAGGTCTCAGGGAGACACCTTGTTCCACAAAACAACATGTGTGGATGGCTCCTAAGGGAAATAATAACACCCAGATCTGACCTGAGACTTTCACCTGTACACACATAAGCAGATACCCCTCCTAAGAAGACCTCCTGGGATTTGTCAGCTATTTTTTGTATTATCTTTTCATTTTGTCACTCATGCTTAAATAACTTACCCAGTAGTGAGTGTTGCCATTTTATTTCTGTGAATACTTTTATCTATCCCAGGACTTAGCAAACTTTCTTTGACAAAGACCAGAAAATAACTACTAAATGTTCTGTGGTCCTAAGGAGTCTTTTACATTTGCTTAATTCCGACTTGCTAAGTGAAAGTAACTTTTGATAATAGGCAAAAAATACTTCTCTCCCCATGACACTTTATTTCTGAGGGTTCAGATTTGAATTTTTTGTAACTTTCGTGTCTTATAAACTAATATTCCTCTTTAAAAGTTTTCAAGAACCTTCAAAAGTGTAATGACGAGTTTTAGATTGTGGCTGCCAGGAGGCAGCTGTCTAGATGTAGCCTATGTGCCATTGTGTGCCTACTGTTGCTATACAGAATAAGCAGGATACAGCTGTAGGATCTTTTTAGGTTTTATGCTGAGGTCTCTCAGTTTCCATCTGAGGTATCTAGGGCAAACAAACACCGTTGACTAAAGCTTTGTTATCTTTGTAGTAAAATGTAAATGATACAAAAAATAGAGTTGGTTTGTGAGCTAAAGCTTATTTGAGCCATAAAAAATCCACAGGTTAACTATTTCATTTCCTCCTCTGGTCCTTTCTACCACTTGGGATTTTATTTATTTCAATTTCTATATATGCAGGGTTAGGGCAGTATCAGTTTTACTTTCTTATGAATTCGATTTTTATAGAAAGCATTCAACTTCTTAATTTATATTAAATCTAATATATACAATGCTTAATAGGAACAGAAACAAAATATATTGAGAAGGTGTCATAAAAAACTCAGAGTTTCAAAGACTATACATGGACTGACCCTGGGCTCCAACTGCATATGTAGCATTGAATATCCTAGTAAGAGCATCAGTGGAAGGGGAAGCCCTGGGTCCTGCTAAGACTGAACCCCCAGTGAACGTG
MSTRG.40029.1	ENSRNOG00000007378;ENS	0.436	0.000	0.049	-3.592	down	GGGTTTTACTGCTGTGAACAGACACCGTGACCAAGGCAACTCGGATAAAGACAGCACTAAATTGGGGCTGGCTTACAGGTTCAGAGGTTCAGTCCATTGTCATCGAGGCAAGAGCATGGCAGCATCCAAGCAGGCATGGTGCAGGAGGAGCTGAAAGTTTCGCATCTTGTTCTGCCTGGAACAACAAAGATGTCTTCCTCCAGACACCTCATCTGACTGAAAACTCCAGAGACACACGCTGAGATCCACAGATGACAGCGGAGACCCACAG

	RNOG00000007817;ENSRNOG00000007895;ENSRNOG00000008024						AGGGCAGACTTTGGAACGTCCAGCTACAGATGAACTCTCCTGCCAAAGTCAAATGGCTACCGAACCTGGGGAAAGTTACACGATAGGAAGGAAAACAGGATGTCTAGCTGGAGTTAATCAAATGCTGTTTAAAACCGATGTTAATACCCTCGCAAATTGCACAACCCGCGTATTGTGTTTATAAAAAGGTAAGGGG
MSTRG.40059.1	ENSRNOG00000024504;ENSRNOG00000057837	0.409	0.027	0.007	-2.785	down	ATCTAAACTTAAAATTAAGTAGAAATTTTATTTATATGAGTTTTGTTTTCTTAAACCAATCCTCATTAAGTCTAGTCTCTAAAATCTTACTTAAGGCATGTCTTCCTAATATTTATCATGTTCATAACAATAACTTTTAATCATTCTATAAATATCATTCGACTCATTTCAGAGGCAGATGGGATTGGTAAAGCACAAGACTGGAGAAGACTCTGCTCCAGTTGATCCTTGGTCCTTTGAAGCTTCTCCCCTGTGATCTACTGATATATTAATTCAAATCTGTATCAAGAAGAATAAAAAAATTAGAAAACGTAAAGAAAAATATTTCTTTCTGAAAGATAATACTTTGTTTTTGAACTGTTTGTGTATTATTTTAATATTTTCCAATTATAATGTACTGAGCGAAAGTCATAAATTTTAAGCAATTGAATGATGGCTTATCTGATCTTTCTCCTTTTTGAAAAGTTTATGCTACTCTTCCTCACATTAACTGTCCCATAGTCCCTCTCTGAGTGTTGGGGCCTGGTGATCCCTCCCCAAGGTTGATGAAACGGTGCCCCATCTTCTATAGGATTTCTGCAGGAAGCCACCATGTCTTGCTACAAGACTCCATTTCACAGCACTTCTCCCCAAACTTTGACTTTTACTTTCTTTCTGCCTCCTTTCAGAGCCGTACTTGAGTCTTTAGGAGCGTCGGGCTGGTTGGTTAGACATAACCAGGGAAGAGGAAATCTCAATAGAGGCATCGTATCCACCAGATTATCTGGTAGGAAGTCTGTGGGGGCATTTTCTTGATTGATGATCGATGTGGGAAGGCTCAGCTTACTGTAGGTGATGACGCTTCTGAGCAGGTGGTCTTAAGTTGTATAACAAAGCAGGCCAAACGAGCCATGGGGAGCTAACCAATGAGCAGAGCAGTGGTTTCTCTCTCTCACTCTGTGTGTTTGAAGTATATGGTGCATGTGCATGTGCATGTAAGGCACACATGTAGAGTCCAGAAGACAACTTTTTGGGAGTTAGTTTTTTTTCCCCATGGATTCTAGGGCTGGAGCTCAGGTATGAGGCTTGTATGGAGAATGATCTTAGGTGCTGAGTCATCCCAGTGTCCCCCTCCCTCCTCAATTTCTTCTCATTAATATTTTTCTTATAAATATCCCAGAGAAGTCTTTTTTTTATATTCCACTTCCTCATTAAATACAGTAAAACCTTGGGAAATGCTAACTATTAAGGAAAATACGTGGCTATGTATGTAGCTCAGGGGTAGAGTGACCTTGCCTCAGATATACAAGGCTCCTTAACACTAAAAAAAGCATGGCATTTTAAGGGAACTAAATATGTTTCAATACAGTCAACAACTAATCGTATAGTACACAACTGCACAAGTTCCTGTTTTCACATTCATTTGTGTTCAGAGGAACCCAACATGTTTTTACCCCTCTCTGAAATATTTCCATTTATTGTATACAAAAAGATAGACTCATAATAACCTTGTATTGAGACCATACAAAGATCTTTTATAACTCAACACTGAAAGGACAGTCCAGTAACACAGAGGGCAACGGATCTCACGAGACATTTCTCCAAAGACGACATACAAATGTTCCACTGGCACATTTGAAGATGCCCAACACTATCAGCCCTCAGACAATATGAATCAAAGCCCAGTGAGATACTGCTGAATACAATGAAGGTGTTAAGAAAGAAAGATGAGTCAGTTATGTTGGCACAACCCTCTAATCCCAGCACTTGGTAGATGGAGAAAGGCATATATGAGTCTGAAGCAGGAGAGTAAGATCTGTTCTGAAAATTACAAAAGCAAGCATTGCAGGCCGAGGATACAGCTCAGTGGTAGAGCACTCATCTAGCGTGTAAGTGCTGGGGAGCAGGTGAGAGCACGCAATGCAGGAATCGAAGAGGAACCCGCTGTGTTATTGATGGAGCTGTAAATATGAAATGCTGTAGCCCCAGCACCTTATTGTGCTCAGCATGATTTTTAATAGACAAAAAGGAGAAAACTGAAATACCTATTGCTAGATGGATGGGTAAGCAAGGTGTGGTGTGTTAACACTGTGGACTAATATACAGCCACAAACAGGGAGAGTACCGATGCATCCCATAACATGAATAAACCTTAGAGACCTTAGAACGCACTCTTTCATTTCTGATAAGATTCCCAGCTGGCCTTGAGCTTACGATAATCTTCGTGCCTTTGTGTCTTGAGTGTTGGAGATATA

							AGTGTGCACCGCTACACCTGCCAATGGCGATTATCCCATCATCAATTCACCCACCAGTGTGTTGCTCATTCCTACTGTTTTGTTGTCTTAGATTTTGTTATTTTGATATTTTAAACTGAGGGAATTTAAATGAAGTTGAATATAGAGAAGGATATTAAGTTAAGGAAAAGGGGTACATAGGTACTAAATAAATAAGTAAATAAATAAGCTCATGCCTTTGATCTCAATACTTGGGAGTCAAAGACAGAAAAGTTCCTTGTGAGATTGAGGCCAGCCTAATCTACAGAACAAATCCAAGATTTCCTGGCCTACACAGAGAAACCCTGTCTTAATAAATAATTGTTTTAAATTATCAAAGATAATCTGCAAAGAAGGGAGAAAGAATTGGGAATTTTATAAATGTTTTCAAGGTTACATAGATAATAAATAAATCCTGGCCTTTGATCTCAATACTTGGGAGATAAAGGCATGGACAGTAGATTCCTTGTGAGTTTGAGGCTAGTCTAATATACAGAGTTAATCCAAATTAACCCCAGG
MSTRG.40291.1	ENSRNOG00000013353	0.770	0.009	0.000	-4.499	down	AAAGATATCCAAGTCATAAAATCTGCTAGAGTCACAAAGACAGCACAACTCCAATCTCAGCTGGCTCAGAGAAAGTGTCCATTAACAAGGGATTAACACAAGGGAAATACACAACCAGTCAATGGAAATGAAAACATTTCACACAGCCTGGTCCATTTGAGCCCGCCCGAGGAGTGAGGAGGGGGCTCTGACTACAGAAAGGGGTCTCAGAAACCACCAGCCATGTTTCGAGTTAAAGGGACACACTGTGGGCCTGAAAGTTTGGAAACTTCAGCCAAGCAGATGAGAGAAAGTACTGACTAGAGGCCTCCTTTAATCCGGTCCTTGTGTTTTTCAGGTCCTTCCGGGAAAAGCCACACAGGCTCACCACAGAAGCTTTTTAAGTGTGGAGATTAGTCCAAGTCCTTGCATGTCAGAGCTTTTGGGGTGACGGAATGGCAACCAGTCCTGACAAGAAGCAACTCGAGGGAGAAGAAGCTATTTCCAATTACATTTAAGGAGACTTGGTGCGTCTGTGGTAGGAAAGTCTTAAGGCAGGTGCTGATAGAGTGTCTGCAGTCCTCAGTTGGCGTAATTGAGAAACTCCCTCATAGGCACTCCACGTGTCTCCTAAGTGGCTTCCGAAACCATCAAGGGGACAAGACAAAACAGCACAGAGACCTCTCCTCCAACCGTCATAGTATCTCTATCACGAGCAGGACACTGAGAAAGGACATCTTCCTATTCCCATGGATACACAAGCCTTAGAAAGCCACGTCCCTTCCGTTCGATGGTATTGAATTTGCATATAACCTCTGAATGCACTTTGGGAAGCTGCTGGACTCCAGCCTGGGTCTTCACAACCTTGACCTGGGCCTAGGAGTGAAGGAGCTTAGCCAGCCCTGGGATTGAGCATTTCTCCATAATGCTGTCCGATTCCAGAGTCCCAAAAGTGATCATCCACCTTTTTCTGCTTGTCCTTTGCATGACAGCCTCCACCAAACACACCCTTTTTTGCTTTGCACAGAGCACGGTTGCAATCTTCCCTCCACTTCTTAAAACAAGAAACCAGGAGAAGCAGGGAGCTGGGCTCCTGTTCATCACTGAGTCTTTTGTAAGGCTCTTTCTGTGGCCCCAGGTGACCTGGCACAGCTGCTGGTATTTAGTTCCGT
MSTRG.40291.2	ENSRNOG00000013353	0.000	0.820	0.000	5.871	up	AAAGATATCCAAGTCATAAAATCTGCTAGAGTCACAAAGACAGCACAACTCCAATCTCAGCTGGCTCAGAGAAAGTGTCCATTAACAAGGGATTAACACAAGGGAAATACACAACCAGTCAATGGAAATGAAAACATTTCACACAGCCTGGTCCATTTGAGCCCGCCCGAGGAGTGAGGAGGGGGCTCTGACTACAGAAAGGGGTCTCAGAAACCACCAGCCATGTTTCGAGTTAAAGGGACACACTGTGGGCCTGAAAGTTTGGAAACTTCAGCCAAGCAGATGAGAGAAAGTACTGACTAGAGGCCTCCTTTAATCCGGTCCTTGTGTTTTTCAGGTCCTTCCGGGAAAAGCCACACAGGCTCACCACAGAAGCTTTTTAAGTGTGGAGATTAGTCCAAGTCCTTGCATGTCAGAGCTTTTGGGGTGACGGAATGGCAACCAGTCCTGACAAGAAGCAACTCGAGGGAGAAGAAGCTATTTCCAATTACATTTAAGGAGACTTGGTGCGTCTGTGGTAGGAAAGTCTTAAGGCAGGTGCTGATAGAGTGTCTGCAGTCCTCTGAATGCACTTTGGGAAGCTGCTGGACTCCAGCCTGGGTCTTCACAACCTTGACCTGGGCCTAGGAGTGAAGGAGCTTAGCCAGCCCTGGGATTGAGCATTTCTCCATAATGCTGTCCGATTCCAGAGTCCCAAAAGTGATCATCCACCTTTTTCTGCTGGTGGGGGGTGGGGAGAATGACCTTTTCAAGAAGCTATACAGACAGCTGAGCTCTCCC
MSTRG.40291.3	ENSRNOG00000013353	0.000	0.402	0.000	4.725	up	AAAGATATCCAAGTCATAAAATCTGCTAGAGTCACAAAGACAGCACAACTCCAATCTCAGCTGGCTCAGAGAAAGTGTCCATTAACAAGGGATTAACACAAGGGAAATACACAACCAGTCAATGGAAATGAAAACATTTCACACAGCCTGGTCCATTTGAGCCCGCCCGAGGAGTGAGGAGGGGGCTCTGACTACAGAAAGGGG

							TCTCAGAAACCACCAGCCATGTTTCGAGTTAAAGGGACACACTGTGGGCCTGAAAGTTTGGAAACTTCAGCCAAGCAGATGAGAGAAAGTACTGACTAGAGGCCTCCTTTAATCCGGTCCTTGTGTTTTTCAGGTCCTTCCGGGAAAAGCCACACAGGCTCACCACAGAAGCTTTTTAAGTGTGGAGATTAGTCCAAGTCCTTGCATGTCAGAGCTTTTGGGGTGACGGAATGGCAACCAGTCCTGACAAGAAGCAACTCGAGGGAGAAGAAGCTATTTCCAATTACATTTAAGGAGACTTGGTGCGTCTGTGGTAGGAAAGTCTTAAGGCAGGTGCTGATAGAGTGTCTGCAGTCCTTGTCCTTTGCATGACAGCCTCCACCAAACACACCCTTTTTTGCTTTGCACAGAGCACGGTTGCAATCTTCCCTCCACTTCTTAAAACAAGAAACCAGGAGAAGCAGGGAGCTGGGCTCCTG
MSTRG.40291.5	ENSRNOG00000013353	0.411	0.000	0.006	-4.200	down	AGTCATAAAATCTGCTAGAGTCACAAAGACAGCACAACTCCAATCTCAGCTGGCTCAGAGAAAGTGTCCATTAACAAGGGATTAACACAAGGGAAATACACAACCAGTCAATGGAAATGAAAACATTTCACACAGCCTGGTCCATTTGAGCCCGCCCGAGGAGTGAGGAGGGGGCTCTGACTACAGAAAGGGGTCTCAGAAACCACCAGCCATGTTTCGAGTTAAAGGGACACACTGTGGGCCTGAAAGTTTGGAAACTTCAGCCAAGCAGATGAGAGAAAGTACTGACTAGAGGCCTCCTTTAATCCGGTCCTTGTGTTTTTCAGGTCCTTCCGGGAAAAGCCACACAGGCTCACCACAGAAGCTTTTTAAGTGTGGAGATTAGTCCAAGTCCTTGCATGTCAGAGCTTTTGGGGTGACGGAATGGCAACCAGTCCTCTGAATGCACTTTGGGAAGCTGCTGGACTCCAGCCTGGGTCTTCACAACCTTGACCTGGGCCTAGGAGTGAAGGAGCTTAGCCAGCCCTGGGATTGAGCATTTCTCCATAATGCTGTCCGATTCCAGAGTCCCAAAAGTGATCATCCACCTTTTTCTGCTTGTCCTTTGCATGACAGCCTCCACCAAACACACCCTTTTTTGCTTTGCACAGAGCACGGTTGCAATCTTCCCTCCACTTCTTAAAACAAGAAACCAGGAGAAGCAGGGAGCTGGGCTCCTGTTCATCACTGAGTCTTTTGTAAGGCTCTTTCTGTGGCCCCAGGTGACCTGGCACAGCTGCTGGTATTTAGTTCCGT
MSTRG.40291.6	ENSRNOG00000013353	0.494	0.055	0.022	-2.334	down	AGTCATAAAATCTGCTAGAGTCACAAAGACAGCACAACTCCAATCTCAGCTGGCTCAGAGAAAGTGTCCATTAACAAGGGATTAACACAAGGGAAATACACAACCAGTCAATGGAAATGAAAACATTTCACACAGCCTGGTCCATTTGAGCCCGCCCGAGGAGTGAGGAGGGGGCTCTGACTACAGAAAGGGGTCTCAGAAACCACCAGCCATGTTTCGAGTTAAAGGGACACACTGTGGGCCTGAAAGTTTGGAAACTTCAGCCAAGCAGATGAGAGAAAGTACTGACTAGAGGCCTCCTTTAATCCGGTCCTTGTGTTTTTCAGGTCCTTCCGGGAAAAGCCACACAGGCTCACCACAGAAGCTTTTTAAGTGTGGAGATTAGTCCAAGTCCTTGCATGTCAGAGCTTTTGGGGTGACGGAATGGCAACCAGTCCTGACAAGAAGCAACTCGAGGGAGAAGAAGCTATTTCCAATTACATTTAAGGAGACTTGGTGCGTCTGTGGTAGGAAAGTCTTAAGGCAGGTGCTGATAGAGTGTCTGCAGTCCTCAGTTGGCGTAATTGAGAAACTCCCTCATAGGCACTCCACGTGTCTCCTAAGTGGCTTCCGAAACCATCAAGGGGACAAGACAAAACAGCACAGAGACCTCTCCTCCAACCGTCATAGTATCTCTATCACGAGCAGGACACTGAGAAAGGACATCTTCCTATTCCCATGGATACACAAGCCTTAGAAAGCCACGTCCCTTCCGTTCGATGGTATTGAATTTGCATATAACCTATAGAATCATTCCACATGTTTTAGTATTCAACAGGATGTAAATGTTACGTAAATGATCCTTATGCTATATGGCTTAGGGAATAAGGAAAAGTCTGTGTATGCTTCAGCAAAGGTTCTGTTTGGGTTTTTTTTTTTAAATTGTTTGCCTCTGTTTGAATCTGTGGAACCCTTGACTGTGAAAAGCCACCTATATTCCTGGAAGTGGCCTGAATATGGAAGATATTGTGATCTGATTAAAGAGCTACTAATGTCATAGTTTGCTTTAGCAACTGCCCAGCAAAAACTGTTTGTCAACAGCTCTGAGACAGTAATTTGAGATTCCACTCCAGCACCGGCAAGGCCTGTGGTCTGAGCTGCTTGTTCTCAGAAGGCAGCTGATGCTATTTCCGTGCCTCAGCATTCTTACCAGTGTAACCAGGACACCGTGGGCGGAGCCCCGTTCCCTGCAACAGTCCTCTGTGTGCACTGCTCCAAGCAACTGCGGCAGAAGGGCAGCCTCACCCTTTGCACCCTCCACGTTTCCATGGTTATATTAGTAAAGGATGACGTTTCAGGGTTAGAAGTGTAGCTCTGTGGCAGAGCATTTGTTTGCTTAGCATGGGCAAAGCCGTGGCTTTGCTCCCTACCTGTAACACAGCAGCAGCAACAACAACAACAGTTACA

							TCTGAATGATACCCGGGCAAAGGATTCCTCTTGTTGCCGCCTCCCAGACTCTCAACGCAGGACCCAGGAGTAACGCTCTACACTGGTGGGGGAAGGGGGGCAATGCAAGGCTCTGCCAGTGTGAGCGGTTCTCTCCACGTAGGGAACTCAGGCGTGGACTAAGTTATTCCTGAAGCTCCAGGAGAGCTGCTATCACCCCTGTATTTTCATTTAGCCCGCTAATATGGAGGCCCTTTGGTCTTTACTCAGACCTCTTCTCCAGTTCCATCTAACTCAGCCTGCCCCTTAGAGTATAACTCACCTCTGAATGCACTTTGGGAAGCTGCTGGACTCCAGCCTGGGTCTTCACAACCTTGACCTGGGCCTAGGAGTGAAGGAGCTTAGCCAGCCCTGGGATTGAGCATTTCTCCATAATGCTGTCCGATTCCAGAGTCCCAAAAGTGATCATCCACCTTTTTCTGCTGGTGGGGGGTGGGGAGAATGACCTTTTCAAGAAGCTATACAGACAGCTGAGCTCTCCCTCTCTTCTCTCCCCTCTCTCCCTCTCTCCCTCTTCTCTCGCCCTCTCCCTTCTCTACTTCTCCCTCTCTCTCCCTCTCCCTCTCCCCTCTCTCCCTTCCCCCCTCTGTTCCAATGCTTTTCTTTTCCAACACTGTTATACTTTTTTAAAACTTTTAATTATAAAAACTTTTATTTATAGGGATGTCTGCGAGTCTGTACATGAGCGCTTGCAGAAGCTAGAAGAGGGCATCAAGTTATGAGCCACCTGATGTGGGATAGGAACTGAACTTGGTCCTCTGGAAGAACAGCGAACACTCTTAGCTGCTGAGCCATCTCTCCAACCCCGCAGAGACGTAATCTTTAGACATCTTTTTCTGTCCTTTCCCAAAGATCCCGAGTGTTCTTTTTGTACCAGTATATTTTATACCACCCCTACCAACTGCCAAGTAGACTCTACTCTCCCACAGTAATTAGCCCTAACCGATATTTAAGTGCTTAATTTTTTTCAAGTTCTGTTTGTTTTGCCCATGACACTAATTTATGGTTAGGTTATAGTGACGGCAACCACCATCATTTAAATTTCTAAGAAGTGCGTAAGCTTCTAAGGGACCTCATTTTGGGGAGGGGTGGGGAGAAGACTTAAGGTGTTGTTTATTTTATTATTATTATTATTACTTTAAAATGATCCCAGGATTCTTCTTTCCATGCTTAACTCCATCTAGGGTCTTTAGGGATAATCAGCAGTGAGCCTGTGTGGGTAAAACGGCCCTGAGCTATTCTTTAAGTTAATTATCACTTCTTTTGGTGCCGTGAATGGCTGTTCAGTGACACCGTCAGCAAGTGGAAGGAACCAAATTACTTTAATTATGGAGTAATTGCACGGAGTGATGGTAATTAAATCTTGATCAGTTTAAGTGAGTGAATTAATTGCTCTAACTTGGAATGCACACCACCACAAGAGGGGTTGTTTTGCCGTTTAATTAACACCATCCACTGAAGTCTTATCATATCGGAAATGTAGACAATTAATGAGTCCGATATTACTCTCACCTTCACAGGAACCTGAGCCGAATGCCCTGGCAATGGCGGCAGCATGCTTGGTACGCCTGTCACTTGTGTAGACAAACCGGCTGTGATCTCTGTGCCTCTTGGGAGAAATGTTCAGACAGAAATGCTTCCATGTATTAGCTTCTGACCCCATTCCGTTTCACTATTCTCTGACCTTGTCCTTTGCATGACAGCCTCCACCAAACACACCCTTTTTTGCTTTGCACAGAGCACGGTTGCAATCTTCCCTCCACTTCTTAAAACAAGAAACCAGGAGAAGCAGGGAGCTGGGCTCCTGTTCATCACTGAGTCTTTTGTAAGGCTCTTTCTGTGGCCCCAGGTGACCTGGCACAGCTGCTGGTATTTAGTTCCGT
MSTRG.40293.1	--	0.000	0.345	0.001	4.225	up	CCTGGGGACCGGTGGAACCTTCTTGGGCTTGAAAACTGGGAGCTGCCTTTGTCCATCCTTCCTGTTTCAGTGCTGGAAGGAGAGGGGATGGAGCCCTGCTGATTTGGATCTGGGTTCTTTCTGGGGCCTTCTCATCAGGCCCCTCAGATGGCTGCTGCTGGCTTCCTCCCAGAGCCTGGCCCATTGCAGCTGAATGTAACTGTGTCTTCCCCAGAGTGACCTGTTCCACCACAGCCCTAGGACACCAGGCAGATGGGAAGGCAGCAAGTTAAATAACTCCCCTCAAATCTTAGAGGCAGAAAGGAGTGAGGGGGAAGTAAGATCTCCACTCCCATTGTCTTCTTTCAAAGTTACCCAAGCCTTTTACTTTCAAATCTCCAAATACCGTGCTCCCTCTCTCCCTCCCACCCACCTTTTTTTTTTTCTTCTTCCTCTTAACAACCACAAATTGGAAGAGAGCTATTGTTTAAGGCATTTCTGTTTCTAACTTTTGTAGGCTCTAAGGTTTCTAACAGTTATTCAAATCTGATTAATTTAATTCCAGCCAGTATTTATTATGTGTTAAC
MSTRG.40470.1	ENSRNOG00000012488;ENS	0.254	0.000	0.000	-6.140	down	AAAATGGAAAGGGTCAGTACTACCAATGTTCACACTCCTCGGGGTTTGATTCTCCACATTCCTTGTACACACATCTTTCACTTCACACAGGTTCTCTGACCAGCATTAGTGATGGATCACCTTTCACGAACACATTAGCTTTTGCTGTTTTGCCAGAATAATCCTGGATTTTACTGAGAACTACTCACCGTTTCTCTGTTGGGGTAGCAATCTTTTCCTGAGTCTCTGCCCCCACTGCTTCAAAGGAAGTGAAGTAGCACGGTGCACTCACAGACCTC

	RNOG00000012844;<br>ENSRNOG00000013287;ENSRNOG00000013292;<br>ENSRNOG00000048857;ENSRNOG00000050975						TGCCACCTTCTCCTTCGCTCAAATGTCACAATAAATCCACACTTTAACTTAAAATTTCAAAAAATGAGTA<br>CTTTCAATAATCAAGACTGGGATCTGATTCTGTTTTCCAGGGATAGATTATGAATTTTTAATAAAGCATGG<br>CCACTGGCCATGGAGAACATGTTTCACACTTATAAACATCAGACCCTTGGAATATCTGGCTAAAGGCCA<br>GATCAACAAAGGATGGTTCCAGCAGATTTCCACTGTCTGCTCTCACGCCACTAACTGTACACATGGAAA<br>GGTCATGGAAGCACAGTAAAGGTTTGGATGATTTAGCATAACTGCCTTAGCTAAAAGTATGCAAGGAAT<br>ATAAGTAATAATTAGTACATAGTATACTTACAGCAGTGTAAGAGAAATACTTGCAGTTAGCAGGACTGAG<br>CTGCTGGTTACTAGACGCTTAGAATTCGCAGATGCTACAAGTCTAATAAACGTGAACTAAAACAGTCAC<br>AGCACTAGGAGCAAGTCCTGAGCTTTCTTACTCAGGTTAATTTGTTTGAAGTGCCAAAAATTACTTTCA<br>ATGAGCAAACATGCACAATAAGAGCCATTGTAACAAGGAAATAAACTATTTTTAAATAGGAATTCAGATT<br>CTATCAACAGTTAAAGTATTTTCTTGACTCAAAGTATGCCAAAGGGGTAATATATAAGTTGTGGAAGCCG<br>GGCCTATAAGCCTAGCTTTTTGGGAGGCTAAGGCAGGAAGGATCACAGAAGCGTTGGGCTACAGAGTA<br>GTTTATGCCTAAATCTAGACAGTTTAGCAAACCTTTCTCAAACTATAAATTTAGAAAGGCAGCTGGGCAT<br>AGTCGCACATTGCCTTTAATTCCAGTATCCATGCTGGGGCAGGCAGACAGCTCTGAGTTTGAGGCCAGC<br>TTGGTGTACACAGCTAGTTCCAGGCCAGCCAGAGTTACATGGTGAGGCACTGTCTATAAACAAACAAAT<br>AAATAGCAAAAACCAGTAAAAATAAAAATAAATAAAAATAATTAAGGGCTGGCTTATAGCTCAGTGGTA<br>GACTGCTTGCTCACAGGTAGAACCTCTAGAAATAATCAATCAATCAAAACCAATTAGATATATAAATAAA<br>AATAACTAAGGGCTAGCATATGCCTAAATAGTATAGTGAATTGCCAGCTCTTTGTAAAAACCCTAGACAT<br>AGTATCAGTGAATCCCCTCCCCAGCCCACCCCAAAAATGGGGACTTTTTGATAGGGACTATAAAAATATG<br>GGTATGTTTTTATGTAGCTCTGGCCATCTTGGAACTCACTATGTAGTCCAGAGATCTGCCTGCCTCTGCC<br>TCCCAAGTGTGGAATCAGTGCTGGAAAAGCATACACCACCATGACTATCCTAATACAGGTAAGTTTAAA<br>TCAAAGCATATCACTACTTGGACATGAAGTGATTTTTCACAGGGCCTTACTATTTAGTCCAGGCTGACCT<br>GGAACGAACCTACTATGTAGCCCAGGGTATCCTCGAACTCTGCTGCCTTCGCCTCCCAAGTACCAAGAT<br>TACAGGTCTGGGCCATCATATCTGGCCTTGTCTGCCTGAGACAGGATATACTTAGTTCATGCTAACCTAA<br>AATTTTCTAACCTCCTGCTGGGGTTATAGGCATGTAATGCCAGGTCTAGCTTACTAGGTAGATTTTGAAAT<br>GTTTTTAAGTCAAGTCATATATTAACCATTTCTCCATTTATACTCAAATTATGTGCCCAGGGCTGAAGAGA<br>TGGCTTGATGGTTAAAGTGTTTGGTGTTCTTACAGAGGACCTGGGGTTTGGTTTCCAGAACCTATATGAT<br>GGCTCACAACTATTTGTAAATGCAGTTCCATGGGATCCAAAGACCTCTTCTGGCATTGAAGGGTACTGG<br>GAACACACATGGTGCACATAATTACACGAAGTCAAAATGCTTATATACATAAAAAATACAAACAAATGTA<br>TGCCAAGCACTATATAATACAATCCTGTTTCTACAAATGTTTATTTTGCTAATCAAGATTCAAGGCCTCTC<br>TAGGCAATTCTTTTTTGTTGTTATTGTTTTGTTTTTCAAGACAAGGTCCCTCTGTGTAGCCCTGGCCATCC<br>TAGAACTGGTTCTGTAGAGCAGGCTGGCCTTGAACTCCGCCTCCTGAGAGCAAACTCAGAGTTATGTGT<br>GTGCCCTAGCACCTGGCGTGGTGATGTTTATTAGACATCAATTTTGTAGGCTTCTGTCTAGGCCAATTTA<br>ATGAGCACACTTTGGGGAAGCTGTCCATGATCCAAACAAATGTAAATAAATAAATAGTACATTTTTTTTT<br>CTCGGAGCTGAGAACCGAACCCAGGGCCTTGTGCTTACTAGGCAAGCGTTCTACCACTGAGCTACATC<br>CCCAACCCTAAATAGTACATTCTTAAGCATTTTTTATGATTTCAAACTTCTAACTAAAGCTCCAGAAACAT<br>CCTTTTAAATGTAATCTAGTCAAAATTCTGTGTGGAAACATTACTTCTGAAGCCACAAATATAGTTAAAA<br>CTCTTATTTTACATAAATGCACTGATACCTAATATTACATATGTTTAAATAAACTTTCTGATATTAGATTTCA<br>AACAATCTATCAACTCAAATAAGCAATCTGACGGTTGTGCCCATTATATTTCTGTGATTAAGATGTTATTT<br>CTCTTCATTCCTTTTATATTTAAGACAGAGTCTTACTTTGGAGCTCTGGCTATCCCGTAACCCATTATGTAC<br>ACCATGCTGGCTTCAAACTCTGTCTGCTTCTGTTTCCAGAGTGGTGGGCTTAAAGGCATGTGCTACCAC<br>AGTCCACCTTTTTGGTTTTGAGGCAAGTTCTTTTCCAGTACACAGGCTGGCTATATCCCTGAGGATGACC

							TTGAAGTCAAGAGATTCTTGTCTAAGGACAAGATGACTTGTATGCCTCTAGCCCCAACCAGACTGACAATTTTCAACATATTTTTTTACATAAAGCAAGCTTTTTCGGTTACTAAAAGGTTTTTCTTTTTTTTTTTTTTTTTCTTTTTTTCAGAGCTGGGGATCGAACCCAGGGCCTTGCGCTCGCTAGGCAAGCGCTCTACCACTGAGCTAAATCCCCAACCCCAGGTTTTTCTTTTTCATTTGTGCATGAGTGTTACAGTGGCAGGCCCCACTACTCTACCTTATTTGCTTGAGACAGGGTGAGTCCCTTACTGAATTTGGACAGCTAGGTTGGTAGCCAGTGAGCCCCCCCATGATCCTTCTTGTCTCTGCAGCCGACAGCACTTGGAGACCTCTGTGCTAGAGATTTGAACTCTGATCCTCACGCTTGTACAGCAAATGTTCTTACCTGCTGCACTGTCTCCCTCCAGCCCCTAAACGACTGTTTTCAAAAAGGCAGTAAGATACTTAAGTCTAGTAACTGGAGACTTTGGTAAATAGATTAAAAGCCAACTTAGTCGTTTGTCTATGACAGATATGTAATTATGTTTTCTACCTAAGACTGATAAACAAAAAGAAACAGGAGCTGGAGAAACATCTAGGCAGTTGAGAGCCCTGACTGCACTTGTTGAAGACCTAGTTTTGATTCCTAGAGATTCCCAGTACCCACATGGCGGCTAACAACTTTCTAAAACTCCAGCTGCAGAGGACTGTGAGGTGCAAGGCATCTTTTTCTGGCCCCCAGGGGCACCAGGTGTGTGGTGCACAGACGTCACAGACATACATGCAGACAAAGCACCCATAATCCATACAAATAAAATAAACAAACAGGGGTTACTTATACGAAAACCAAACTTACCAGGCACAGATGTTTGTTTGGTGAGGAAAAAAACCCTCCAAATCTTGGTTTCTAATTATTGTCTTTTCCTATGTTTTGCACTTTGTGTTCCATAATGCCCTGTTATCTTTTTAAAATTATGTAATCTGATAAATCTTGTATTGGCTAATGCAATTTAAAAATCAAGGACAAATTTAACTGGCAACTCCAGTAGGCTGGCCCCACTGAGGATACCCCGTTTCTGTTCTTTAGTCATTGTACTGACCATGGACTCATTCATTTATCATCATATTTTGTTGTTTTGTTTTGAGAGAAGGTCCCTCTGTGTACTCCTGACTGTCCTGGAACTCACAACTCACCATGTATACCAGGCTGGTCTCAAACTCATAGACTCTTGTCTATGCCTCCAAAGCGCTGGGATTAAAGGTATGGGCCACCATGCTGGGTTTTCTCCCTGGCTTTTTCTTCTAATTTTCTTTGATTTTCACCTAGATTTTAAGCTCCTTTAAGGTAAGAGCAGTGTCATCTTTTTTAAGAATCTTGCCAGAGCACCCAGATCCAGAAAGTGACCACGTGAACTTAATTTCCCCAAGGTCTGCTGATGGGAAGTGCCTTCAAGTTTTCTAGCTTTATCACCCAAGGGGGAGGGCGTGTTTAAAGACCAAAACCAAGTGTTTTCAGATTCTTGCTACGTTTGTTAACAGTTGCATTACTCATTCAGCACCACTAGTGAGGACCTTGTAGTGGCTGCTAAACTGGGCACTGGGGATATACAGAAGCAGCCCTTGCTCCCCGCGTACAATGAGAAAGAAAAGACAGTGATAACGCAAACGTTACAACTAAGGTAAACCAGGCACAGAAGCAACAAAAAGGGCTTTGCTGCCTCTCGGAATAGGGTACTGAGAGGCTTCACAGACGCAACGTTCTCTTAGGACTGGAGTGGGGCACAAGGAAAGGTGTGGGCTCCATGTTGGTGTGGTGTTACGCTTGAAAAACAGGCGCCCTGGAGGAGGAGGCACTGCAAGCTTTCCCGCAGGTGGACACACGGTTCAGCTCCTCAGGGGGAAAACGGTCTAACCAACAGCCCAGGAGTCCGATAAAGGGTTGCTCCGGAAAGGGCGCAGAGCCCGGGGCTGTCTTTCTCTCCAGGAGTCGCTCGCCCCGCCCCGGC
MSTRG.40708.2	ENSRNOG00000014887;ENSRNOG00000014904;ENSRNOG00000016414;ENS	0.000	3.722	0.000	6.570	up	AGGAAGGTGAGCTCACTTAAAAGATCGGTGTCTCTACTAGATGAATGGGATTTAGGGAACCTAGAACTCACCTCCCTTTGGAATGGAAGCTTGGTGACTGGATGAAATGGCTGTGGCTGTGGGCTGCATGGGCACACCTGTGCATGGGCACACCTGTGCATGGGCACATGCAGGCATGCTCATGTGCATGCTGGGCTGTTTGTTGACTCTGATGATGGGGTACTTAGAGAAAACATTCCTTCTTGCTGTGGCAAGAACAAGGGCCAGTTCACTGCCCTTCTC

	RNOG00000016804;ENSRNOG00000016828;ENSRNOG00000025757;ENSRNOG00000042029;ENSRNOG00000042195;ENSRNOG00000046229;ENSRNOG00000048644;ENSRNOG00000052228						
MSTRG.40771.1	ENSRNOG00000018239;ENSRNOG00000018399;ENSRNOG00000018528;ENSRNOG00000018536;ENSRNO	0.000	0.326	0.031	3.453	up	GGAATTCCTAGGTCACGGTCTTAGCTACAGAGCAGTTTTGAAGGCAGCCTAGGCTGCATGAGAGCCAGTCTCAAGACAAAACAAAACACTTCCAACTTGCCTGCCCTTTGGCTCTTTGGCCCAGTGCCCTTTATTTTCTGATGATCAACTTCTGCCCAGAGCTCTCTGGTCAGTACAGGCTGCTCTTTGGGAATATATCTGGGTTCAATTCTCAGCACCCACACAGAATCACAACCGCTTGTAACTCTTGTCCTTAGAGATGTACAGCCTCTTCTAGCAAGTACAAGGCCCTGGGTTCAGTCCCCAGCTCATGCTTGGTTGGCAGCAGTGGTCATCTT

	G00000018825;ENSRNOG00000019041;ENSRNOG00000019162;ENSRNOG00000019246;ENSRNOG00000019438;ENSRNOG00000019478;ENSRNOG00000019503;ENSRNOG00000019553;ENSRNOG00000019710;ENSRNOG00000019789;ENSRNOG00000019840;ENSRNOG00000025518						
MSTRG.4116	ENSRNOG0000001	0.000	0.292	0.000	5.534	up	CTAAGTGGTGAGGATTAAATGTTCTTGGTATAGCACACCAATGTATAGTTCTTTGTGTCTGCCACAAAGAACTACCATTTGAATGCCTATTTCATGTCCTAGTTATACTGACATAGGCTTGGACATGAAATGTTGTAAACAAAACCCGACAAAATGGCAACTGTCCTTTTCATGTATCCACAAGCCACTTGTCACCTTCTAGTAAGCAGC

7.2	4659;ENSRNOG00000021843						AAGTAGTTGCTGTGGATCTATTACATGCAGAAGTCCTCACTAGTTATCATTTAACAAACAGGACACAGAACTCAAACGAATCATGACAAAAACTTACACCAACATCCAGAAAAGGAAAGAATGTACAAGCAATAGAGTATCCACGTAAACTATTCAGGTTTTGCTTACAGTAATAAGAGACAACCTCAAGAACAATTACTGGTGGGACAAAGTGTGGTGGCCCACACCTGTAATCCCAGCACGTGGGAGACTGAGGCAGGATTATTCTCTCTGCTTAACCAGCCTGCCCTGTAGTTAAAGCCTTGTCTCAAAAACAAAACAAATGAAAAGCAAGAGAGACCACACACACGCCACATACACATACACATACACATACACACACACACACACACACACACACACTCTTACCAAAAAAACAGACTGATGGATGTTCCATTTGCTAGGTTTCACTTTTGTTTGGAATTCTCATAAAGAAACTTAAATGTGATCAGGGCAGAGATAGAAAACCTATAGTCTCACTCAAATGGGAATTCCACAGATATTCCCCCAGGTTCTTTCCAGTTCATAATTAATTTTGTAGCTCTGAAAAATGGTCCAGGTTCGTCTGCTTCTCAACCAAACCAAACCCTGTATTTACTAACTAGCCTCTTACCAAAGACTACTAGACTATACAAATCATATTACCTACTATTAGAATAGAGAAGAGGATATGGGCTCAAAACTATTTCCCCCATCAAATCCAGAGGTTGGGTCCCAGTGAAGTTTGTGCTATTACACTTCATTGGTTTTAGTAGGAGCAGAGAGACCTCTAGCGCTACCTCAATAAGCAGAGTTTGCAAATAGTAGCTGCTAATGTCACCTTAATGAGACAATGGCAACATGCTGAGTAGCAGCAAACTGAGTGACCACAATGAATGTTTACAGTTTTGCAACCACTTGGAAAATCTACTAGCTTGGTGGCATGTGGTATACACAAGGACGTAGGAATCTATGTATCCAATTTATTTCTGCAAAACAGGTCAAAGAAATTATTTTCCTATTATTCACACTTCCACTCCAAAACGTGCTTTAGAAAAAAAAAAAATCCCAAGATCAGAGGTCTGCACAACCTCCTTCCCAAAGCTTCAACACAATAGACGGTAAACTATTGTCCACCTTTAGCTCGCTCCAAATGCCAGCAGTCACAGGGGTATGAGCTGCAACAGCTCGGGAAGACTGGTCCAAAGGACAGCACTGGAATATAAGAAAATGGTCTTGGCGCATCCTACCCTCCCCCATCCACCACCCTCAAACAAGTCAGTTACCCGGCTGGTCCCCACCCCCCTCTGAGGGCTGCCACCGCCCGCCTGGGCCCGGCTCGCACAAGGTAAGCGCGGGACCCAGGCTCCATGTGCGGCAGCGCGGGAGGCTGCACCGGCTGCGGACACGAGCGGCGGCCCGGCCCCGGCGGTGCGGTCGGCACGCGGGGCGGCAGCATCCCGGTCCGTCCTCCCGCGCGGCCCG
MSTRG.41189.3	ENSRNOG00000009075;ENSRNOG00000015095;ENSRNOG00000021831	0.000	0.616	0.000	7.373	up	GGAAAAAAGCAAAATACATAAAATCCATGTTTGAAAAACCCATCACTGGTATGTGCATAATTTTATAAGGTTTCAGCAGAATAAAATATATTTCTATATCATAAATTTAATAAAAGCCTACTGCAAACACAGTGCTATAACTTATATAGTAATCATGAAAGGAGAAAAGCAAGTCATTTTTCCATGACAACTTAATAAGAATCTGATTATTAAATTAGCCAAACTTCTGAATGTATTTAACATATAAAGCAGTTATAAAAGTTGGGTTTTACTTTAAGAGCAGCACACCCTTCAAAGCCAAATTCATAGGATGAAGCTTTATGTTAAAGGTCAAACATAAAACATATAAGAGCTCAGTTTCAAGAAATGTATACTAGCAAAATACTTAAGACACACACATGAAATATATACCCCCCCCAATCTACACATGAACACTCAAGAATTACTTCCATATTGGAATAGTCATTTTGAGTTCAGTGTCCTTTATCACCCATTATCCAGAACAACATCTTAGGATGACAGGTCAAGTTGGTTTCCTGAAAGGATACAGAACAGGAATCCTGTAGCTCAGGCCTAAGTAACCATTTGTATGGTTCCCATTACAAAGGCTGTTCCTTTAAAGGTATATCATTTTATATAATAGAACAATTTTATACAAATAAATTTGCTTTCTGTTCTCATTATTTATAGGATGCACAGTTGTTATTCTGTTCATGAGCACAGTCATATTCCATTTAAGTTTTGTTTTTTCTCTATTATTAAAATGTATGTAGATAATTTATGAATAACCATGGTTTCACCCATTTCTCTATTATTGCCTGCAAGTTTTAATACAATTTTTAACATCTATTTAAGATACATCAACACATACTTGAATTTACAGTGTGCTAATTTGTTTCCCATTGTTGTGAAAAACATCATGATCAAAAGCGACCAGGGAAGAAAACAGTTTTTCATTTTACAACCGAATGTCCATCACAAAGGGCAGGAATTTAAAATCAGGAAACAGGAGGCAGGAACTCAAGCACAGGCTGTAGAGGAATGTTGCATACTGGCTTGCACTGCTTGCTCTCTTATACACAGGACCGTCTGCTCAGGGGTAATGCTACGCAGTGGGTTGGGTCCTCCCACATCAAGTATTAACCAAGAAAATGCCTCCTAGATATATGTCCATAGGACAAGCTGATGTAAGCAATTCCTCAGTTGAGATTCATTCCTTCTTTCTAGATGTCTGTAAGTTTCTAAACCAACCAGCACAGTTACATGAAAATTTAGGTTCTCTTCAAACCTACTGTCTAGTTAAATTTTAGACATGTTTTAAAACATACACGATTATT

							TTCACTTAAATATTTTACTGAATATCTACTCTATGTAACCACTCTCCCACTTGTTATACTATGGTTGCTACAGTTATCTTGAGTTTGAATTTCTACTTCCCTAGTATTAAGTTAGCTTAGCATTGTTATTATTGGTAATCATGTATCTTCTTTAGAAGAATGTCTATTTCAAGACTTTTTTTGGTTTTTGCCTATTTACTGATGGTGAGTTTTAAAAAAAAAATTTCTGGCTTAACAGTTAATACTACCTACCAAAAACTAGATATGCAAATATTGTCTTCCATTTTGTCTACCTTTGTGTTTTGTTTACAGTTATTAATGCACAAGTTTTAGATTTTGAGGAAGTTTATAAAATGTTATTACCTCTATTTTTGGTATTAAATACTTGTCAAATCCCCTGTCTTGAAGTTTTACCTTAAGTTTTCTTAAGGGGTTTTCACTTTTAGACATTTTTGTTTAGTCTCTGACTATCTGAAACAAAATTTTCACTACATAATAAGGTCTAACTTCATGTTTTTGTGTGTGTAAATATCTAGGTTTTCTCACACTATTAAAAGACAGTCTTTTCCCTTTAGTGGTGGACTGAGCCCATCTCTAGATAAAGAACTGTTTTTACTATTGCATTTCATAGCGGATTGTCCATTCAAACTAGACTGTGTGGCAGTCATTTCCTAGTACAGCCCAGGCCATTGCTCCATTCCTATGCACCTTAGGGCACCTGAGATACCACACTGTCTTTACATCTTCAAATATTTGGTTTCAAGTGTGCCAGCTTAAAAAAAAGTATGAAAATAATTAAAGGTAAACACTATTAAAAATGATTTTGTCCTTTATCTTTATGTAATGGTTATAGTTTCAAATTCAGTGGTCTTTAGAGACAAGGTTTCTTTGTGTAGCTCTAGTTGTCCTGTAACTCACTCTGTGGACCACGCTGGTCTGGAACCTAGAGGTCTATCTGCTGGTCTTCCTCCTGTATGCTAGGGTTAGAAGTGTGCACCCCCATGCCTGGTTCCAAACTCAGTGGTTTATATTCTAGCCTAAGGACCAAAGTAATTTTCTTTGTTGGGTTTTGATATAGTTCAAGTTTTTACTGATGTTCTTAAAAGAAAAAAAAAGGCATTATGAAAGGTGAGATAAAAATAGGTTTAGCTCAGTGGTAGGCCCTGGGTTCGGTCCCCAGCTCATTATGCCACAACTTCTAGGACATCCATATGCACAGAAAAGTGCTCGTCTTGGACCTTGTATGGTTCTGGTTCTCATCTCTAGCAAAAATAATAATTGTTGGGCTACTTAGAGGGCTGTTTTAGTAATATCTTCATTTTATCATTGTTAAACCTTAATCTCCTATCATATTCCAATGTATTACGTGGGTATTCGATCAAAAAACTGGTTGTTTTCCACAACTGGCTCTCTTCTAAAGGTTAAAATCGATGGCGCCGGGTCTGGCGAAGCCCCACCGACTGTCCTTTGGTCTAAGTTATTTTTTTGATGATTTTTGGGGGATTTTCCGGCGCGATCCTAGCGTCGCGCTACTCTCGGACAGGTTATCCCTTCTCTCCCGCTAGATTGATGAGATCAGGCATCACTCGAAAATGGCGCCGAAAGCGCTGAGGCTCCTCATTCAAATTCGTTAGAGCCAACCCCGCCGCCCCG
MSTRG.41236.1	ENSRNOG00000009449;ENSRNOG00000053334	0.556	0.000	0.022	-3.851	down	CTTGGAGAATTTCAAGTTTGCTCTCTGCTTTGTACCTGTGGTTCAAGATGTGTGCTCCCAGCTTCCTGCTACCAGTGCTCCCATCAAGGTGCACACTTAACATTCTGGAGACTCAATCGTCTGGAAGCTGCTTCGGCTGTGGTGTTTTATCACGGCAACAGAAAATCTGCTAGCAGTCTTCCGCAATGGGAGCTGGTGAAGGGATTATGCAGCACAGGGTCAAAACCAGGGAACTGACACTGCGTAAGGAGCAGGAGCAGCCCTTGCTCTTTATCATTGGTGTCGGCTTTCACACAGTCACCACAGTCAAGATGCAGAGTTGCGGGACTCCATGGAGATTTATTTTCCCACCACCATCATTCCCAATCCCTGCTACCTGCCGCTCTTTTCCCTTCCCACAATCCTATCATTTCGTGGCTGTCTTGTAAATAGAATCATCCG
MSTRG.41494.2	ENSRNOG00000034226;ENSRNOG00000042133;ENSRNOG0000004	0.543	0.006	0.000	-5.257	down	GGGAGAGAATGAGAATGAGAGACTATACTGGGAACCCACAGGGGCCAGAAGAGGGTGTCAGATCCCCTGGGGGATGGAGTTACTGGTGATGTAGCTGCTGGGTGTGGTTAGCTCTGGTCCTCTGAAGTGGTTGCAGGTGCTCTTAGCCTCTGGGCCATCTGTGCAGCCCCAGGACTGTATTTTAAAGGGAAGGATTTATTTAACCTGCAGTAGTGAGGTCTAGCACTCAGTGCTGGCCTGTGCTCAGCTGGTGAGCACCAACTGTGGTGCTCCACCATGATGGAAATGTAGAAAGGAATGAGTGCATTCGAGGAGCACAGCGGGGCTGGACTGCTTCACAGCCACCCACTCTCAATGCCTGGTACCTTTCTTCACAGATAGTGATCCTTTCCTAAGTGTGGAGCCCCTCAGACTCCATATGAGTCTAGTGGAGACTGAGTGTTCAGAAGATGTACTGGAGATCTTGGGACACAAGGAGTCTCCCTTTTAAAAAAAGGTTGAAAACAGAAATTCTAATGGACATGGTCTGGCCATTGGGAGTCTATCTAGAGCTAATCCACTCCCTTGTGCTGATGCAGAGAGAAGCTGGGTCCCAAATGATCTTTAACTAACATTTTCCAGGGGACTTAATGAATAACACAGCAGATGAGTCTGCCTTCATGTGAATCCATTAACACAGCCATATAGTCA

	7234;ENSRNOG00000049016;ENSRNOG00000049451;ENSRNOG00000059588;ENSRNOG00000060573						GTGGACCAATTTCTAGGGACTGCTCTGAAGTGGTTCTTCAGGGAAAGGGTGGCCACTTGATGGCATGG<br>AAGTCCAGCACTGGGTAGTTGGAGAAGACTAGATTCATGTCCTTTGTAACTGGGAGAAGCAGGGTTTG<br>CCATTCTGATTGCACAGCAGATGCCTTTTGAGGTTAAATCTTAGAAGGAGGGTCGTCAGCCTCAACTGC<br>AGGGCTCTGCTTCCAGTCAGGAATGAGTATGGAACATGGACCCTCCTTTCCCCAGGCCTTAATTACCGC<br>AGTATCTGTTCTCTGAGTAGTCTGTGCTTTTTAACATTTCTCAGGGGCCAGAAGAGGGTGTCAGATCCCC<br>TGGGGGATGGAGTTACTGGTGATGTAGCTGCTGGGTGTGGTTAGCTCTGGTCCTCTGAAGTGGTTGCAG<br>GTGCTCTTAGCCTCTGGGCCATCTGTGCAGCCCCAGGACTGTATTTTAAAGGGAAGGATTTATTTAACCT<br>GCAGTAGTGAGGTCTAGCACTCAGTGCTGGCCTGTGCTCAGCTGGTGAGCACCAACTGTGGTGCTCCA<br>CCATGATGGAAATGTAGAAAGGAATGAGTGCATTCGAGGAGCACAGCGGGGCTGGACTGCTTCACAGC<br>CACCCACTCTCAATGCCTGGTACCTTTCTTCACAGATAGTGATCCTTTCCTAAGTGTGGAGCCCCTCAGA<br>CTCCATATGAGTCTAGTGGAGACTGAGTGTTCAGAAGATGTACTGGAGATCTTGGGACACAAGGAGTCT<br>CCCTTTTAAAAAAAGGTTGAAAACAGAAATTCTAATGGACATGGTCTGGCCATTGGGAGTCTATCTAGA<br>GCTAATCCACTCCCTTGTGCTGATGCAGAGAGAAGCTGGGTCCCAAATGATCTTTAACTAACATTTTCCA<br>GGGGACTTAATGAATAACACAGCAGATGAGTCTGCCTTCATGTGAATCCATTAACACAGCCATATAGTCA<br>GTGGACCAATTTCTAGGGACTGCTCTGAAGTGGTTCTTCAGGGAAAGGGTGGCCACTTGATGGCATGG<br>AAGTCCAGCACTGGGTAGTTGGAGAAGACTAGATTCATGTCCTTTGTAACTGGGAGAAGCAGGGTTTG<br>CCATTCTGATTGCACAGCAGATGCCTTTTGAGGTTAAATCTTAGAAGGAGGGTCGTCAGCCTCAACTGC<br>AGGGCTCTGCTTCCAGTCAGGAATGAGTATGGAACATGGACCCTCCTTTCCCCAGGCCTTAATTACCGC<br>AGTATCTGTTCTCTGAGTAGTCTGTGCTTTTTAACATTTCTAGAGAGGGGGTGGTACTTTATAAAACTTTT<br>CGTTTTGAGGATACACTGGTTTAGGGAGATGGGTTTCATGGAATTAGATGTCAAAGATGGAAAAGATAT<br>CATGGAATTAGATTGATTGTAGTAACTTAATAGTATTTATCACTTTGTTGTTCCTCTCAGAAAACGGGTCG<br>TTTGCTCAAGTTTTTCTAGTAAAGAGTGCTTCAGGGATACAGCGACATGATACATTCAGGGATACAGTG<br>ACGTGATCAACTTTGGAAGTGATAGTGGTGTAGGCATTTGTTTGGGACTCCCGTGCAGTTTGAATTGAG<br>AGTACTCTCTTTATTATGTTGAAGTCAAATGAGCAATTTCAGGAGCCATGAGAGGAACATGAAAGTAGG<br>TAGGTGTGTCTGCAGGGGATTTGTGAATTGTTCTTCCTTCTGATGGTAAATGTCTGTCCAGGTCCATCCT<br>TCCACCCTTCCCATTGTCACTGGTCATTTTTCTAATGACTCTACATCTCTTAGCTGTGTGCTTTTTTATTAA<br>GCCATGCCTGATCCCAAGAAGTTGGTTAATAGGGGACAGTGTGTCTAGCATTTCCTCTCTCTTGTTCTCA<br>GGAAGCTGTATTGATCTTTCCTGGGGAGCGACAGACTCCAGAGCATGGTCATTATGACTCTGCAGTGAA<br>GCTTATCAGTTCTCTTTCCTGCCTGGGAAGCACCATCTGCTCTTCAGTAATTATTCTCATATGCAACCATG<br>GAATTGTGTTTTGGAGACCTTAATGTGCTACGTTCCCCAGCCCCCTTATTTAAACACTATAACCACCACC<br>ATCCCTAGAAATGTTGGTTTTGATACACCCTGAAAATTGTGCCCTGTTGCCATGCCTCTGCCAAGGCCCT<br>TGCTCACATGGGTGGCTTGCAGGTCTGTCCTCCCTCCACTGTGCTGACTCAGCTCCACTTTGGTGCTGA<br>CGCCTTGAGGATCGTCACTCAGAGCACCCGCTATTTCATTATGCCAGACCCCAGGACCAGGACAGCCAC<br>CCTAAGGTAGCTTTAGCCATTGCCCTGCTGCTGTCCTTGCCCTGTGGCTTCCAGTGCTGCTCTCTCCCTC<br>TGCTTTTCATTCTTTTCTCACCTCAGCAAACCCTAAAGGGTTCTTGTGTGGTGGGAACTGGGTTTCTGTC<br>ATTGCTTGACCAGTTGAACAATCAATCGCAGGACATTCTTGAGCCTTAATGTTCATATTCTCATTCTGTTG<br>AGGAGCCAGCGTGTGGTTGTAAATATCAGTGGGGCTCAGTTTGTTGTCTCTACATGTATCTAGCACGTCT<br>TGCTCAAAATCGGATGACACAAATAGTTTTGCTATTCTATGAAGCATGGACCTAAATTTTTCATCATACTT<br>ATTGATTGATTGATTGTATGAATGGGCACATGTGCGTGGCATACGCTTGATGGTCAGACGGCAACTTGCA<br>GAAGTCTGTTTCCCTCCTCCTGTCATGTGAGTTCCAGGATTGAACTCAGGTCATCAGTCTTGGTGGCAA<br>GCACCTCTACCCATTGAGCCATCTTGACAGCCCAACTTTCTTTTTTAATTATCAAATATTGTTTCCTTATGC

							TATTTTGATAACATGTGTAATCACTTCAATTATCTTTCCTTGCACCTACTCTATGCTGATCCCAAATACTCC<br>CCTTCTACCTTTATGTCTTGTTTTGAATTTAGATTCCACATCTAAGATAATGCTCATCTGTTTTTCTGAATG<br>TGACTTATATCATTTAAATAGCGGTTTATAGTCCCATCTATTTCTTTTAGATAACAAAATTCCATTTTTTATG<br>ACTGAATAAAATTCTATTGTGTGTATATTTGTTCATTTAACTATTGGCTGATGCCTGGTCTGATTCTATAAC<br>TTGGGAATAATGCTGCAATAACTCTGTGTATGTGTGCGTGTGCGCGTGTGTGTGTGTGTCTGTCTGTGTC<br>TGTTTAAATTCCTGAATCTTCATACAGCACTTGAGTAACTTGCCTATTTTATAGCTATAAAGAACATACCT<br>GCGTGTTTACTTTTCTGCCTGATGTTTCTAGGGATGCTAAACATTATTGGTGTTGTGTGTGGTTTCAGACA<br>GTTGGTGTCTGTGATTCCACGGGGGCTGTGGGAGCTGCTTGCTGATGCAGACTGTGCCCCTCTAGGTTG<br>CTGGCATTCAAAACTTTCCCAAAAATTTCATTTTTTGAGGTCTGGCTGCTATTGTTTGGTTGTTTTGTTAT<br>TTGGAAGTAATTTTTGTATTTATTGTGTTTAGACTTGAGCTATTTAATAGTGACATAGTCACATTTGTAACC<br>TTTACTGGTGAAGATACAGCTGGTCTAGTCTAGACTCCTGGGGAGCATCAAGTGTCATCTCACTTTACAT<br>ACATTTTCATATGTGTCTTGTAAAAGCCCTGCCTATTGGTGTACTTGGCTGTGTCTTATAGTGCGTCAGCT<br>GTCAGTTTAAGCCTCTGATTGGGAGAAAACATTTACACAGATGTATAGGAGAAATAACTGCCTCAGGTA<br>CTCGCCAACAGAGTAGGGTGCTCAGTGGTCGGTACATTTGTAGGATTGAATAGAATGGCATACACGAAC<br>GCTATTTGTAGACTCTGTGTTTTAGTCAGGTGCTCACTGCCTTACAAATGCTTGTTTTTCATCCTGTGCAC<br>TGTTGGCAGAAAGTGGTATGGCTGTTGAGGGAAACAGTGTGGTTGTTCGTCAAACAAAGGAATGGAAT<br>TACTGTACAACCCAGAAGTTTGACTTCTCGGTATACACTGAAAAATAGAAAGAAAAACTTGGACAGATA<br>CTCAGGGACTGTGCTCACAGTAGCTTTGGCTGAGGTTGCCTGCAGGTGCAGCCAGCTGCAGAGCCCTT<br>GGGTGACTCAGTGGATATGATCCAGCTTTGGAAAGCAGTTCTGCTGTACGTGCTGTACAGCACACTGAT<br>GAACCCTCAGGACATTATACTAAGGGGAATAGTAAGCAGGCCACATAAGTATGGGATGTTTCCATTTTGG<br>AAGCAGCCATCAGGTCAGAAAGGAGGGAAGGTTGATGGGAGACATGTTTAGTGGTGTGGCTTGTGCTT<br>TGCAAGGTGAAAAACTTGGTGGAGATGGTCTCATAATACTCAGTACCACCAGGCTGTGTGCCTACGAG<br>AGGGTTAAGATGGGAAATCAAGCAACAGCTCACACAGTTGCAGAGTGTTCTTTGGTACAAATGGACCA<br>TGGTGCTGGGAGGTAGAAGAGTTTCTGGGAAGCAGATGAAACGAGGATGGCAGACTGTGGATAAAAC<br>TGTGTTAAAACTGGGCAAGGGTTCAGGGCCCACTGTGCTGCTCTCTTTATTTTAAAGTTCCTGTTTGCCA<br>TTTAAGAAGACTACTTACCTTTTCCATGACCAATAATGCCTCAAGAGAGAGAGAGGAGTGAGCTGGTGT<br>GAGCTGGAGCTGGTCTGCTCTTTAGTGCAGGAGGCTGAGGGGAGAGCAACCGTGGAGCGAGGCAGGG<br>CAGGCTTTTCTGCAGACAGCTAATTTTGTTGTGCCCAAGTGGAACCACTCCCTCAGATTGTCCTCTGAC<br>TTCCACATGTGCACCGTGACATAGGTGTGAGCCTATACACTAAAGTGTGTGTGTGTGTGTGTGTGTGTG<br>TGTGTGTGAGAGAGAGAGAGAGAGAGAGAGAGAGAGAGAGAGAGAGAGAGAGAGAGAGAGTGTGTTG<br>GCAAAGAGGGCTGGAAAGATGGCTCAGCAGTTAAGGGCAAGTGCTGTTCTTACAGAGGACTTGAGTTT<br>AGTTCCCACCACCCACATTGGACATCTCATAACCCTCTGTAGTTCCAGCTTTCTGGGATCAGTTACCTCT<br>GGCCCCAGTGGCATTTGCATTCAGGTGTGCATAACCCCCATGTAGATAATTAAAAATAAAGTGAACGTTT<br>AAAAGGCTAGCACACAAAATAATATTGGCAGCTTGCTTCACTAGGGGAATAATATTATTTTCTAAAATTA<br>CTTTTGTATTTCCTTCTAAGTTTGTAACCATAAATCTAGTGAAATATGAGTCTATCCTGTCTTGTCTAACA<br>GAAAGAGGTGAGATTTCTTTTCTTTTCACTTAGTATTTCTTTTCACTTAATAGACTATATTAATCACAGTTC<br>CTTTGCTATAATGTACAAATTGTATTGGCGAGAAACTTTTAGCATCTACTAAAATGTCAAAATGGAAAGA<br>AGAGAGTATTCTGTGAAGTTTGACAATGGACCACACATGACCCAAGGGAATGGTTCACCAATGAGTGT<br>CAGGTGTGACAGCTGGTAGCAGAGTGACCCTGGGCAGTTTTAACACGAGGAACAACACTCACATGCAG<br>GGACTGGCCAGCAGGGAGGACAGGGACACAAGGAGCAGTAGCATGAATGGTGCACTTGCTCACACAC<br>AGTCAGGCTGGAGATGGAGATTTGTCTAAAGGAGCTTCTTGTAGGAATTCCTGTTTGTTCCGTGAGAGC

							CTGAGGAAACACCTCAGCAGGACTACATTCTTGTGTGGCTACTTCTCTTCAGTAGTTTGCTTCTCAGGC<br>TGATTCGTCCATTGTACAAATCTGAACACTTCCTGTGTACAGAAGCTGTGCTAAATCCTGAGAGAAGCT<br>GAGGGGCAAGGCCAGCAGGAGCCTATGCTTCAAGCTCAGGTCAGGAGTCACTTCCAAAGTGACAAGA<br>GCATTGATACCAGAGCAGATCCCATGCCTGGGAACACTCAGCTGGCCCTAGTGCAGCAGTCCTGTGGGT<br>CTTATGTTACAGTTTATAACAGTAGCAAAATCACAGGAAGTAGCAGTGAAAATAATTTTATGGCTGGGGT<br>CACCACATGCGGATCTGTATTAAAGAGTCTAGCATTAGGAAGGTTGAGAACCACTGCCTTAGGGGATAG<br>AGGGAAACCAGAGCTAGAGATGAAGGCACTGAATTCAGTTCCAGGATTGCTGGTGACAGCCCAAGTTG<br>CCCACTCCTGTTACCTGGGTTAGGGCCTGTTTTCTTCAAAATTGTCTTTACATCACCCAGCCTGATGTGT<br>GGATGATGTAAGAAAAGATACCACATTTTGGTCTCAGGTTCCTACCTATGTTACTCTACATACTCCACTTA<br>GCCACCTCCTGTCCAGCCCTTATGTCTCAACTCAGACACCCTTCTTCCCAGTCTTCAGTCAGGACTGTG<br>TGCACTCCGCAAGGTGCAGAAACACTGGTCCCAGAGGGGTCCAGAGAGGCAGGACAGAAGGCCAGT<br>GGGGTCTAGACGAAGTCCTTCAGGCATACCCTTCAGCCCTGGTACTTTGCTCCATGCAAAGTAACATTT<br>CAAACCAGTGAGTGTACCTGATGGTTCGTGCAATGAGAGCAGAAATCCCTATCGGTCTCCTGTTATAGT<br>CAGTTTCCAGCAAACAATTAATGAGTGATTGGATGCCATTGGAATGTATCAGTCAAGAAAGTGGTGAGA<br>GAAGGGGATTGTGGGTTGTTCCAGTGTTGGCAGCATGTTGTACTTATGGGTTAGGAGGGAAATGAAAA<br>GGGGAGGAAGGGTTGGTTGATGTGTACAGGAGAGTTGGCCAGAGAGCCTGAGAAGGGAGTGCTTTCT<br>GTCGCCTTGAGATGAGGGAGAGGCTTTTCAGTAGCAGCCTGCCATGGACATACACTGTTCCCGTGGGTG<br>ATGGAGCCTCAGACAGGTGCCTGTGGGTTTCTCTAAGCCTCGAATGTATTTGTGGTCAGAAGCAGTGCT<br>GCATAGCTGTAGTTAAGCTCGTCACTTGGGGTTGGATTCTCGTAACATCAATGTGCCTGTGCTTCCTGCC<br>GTCCTGTTCAGGTCTCATCAGTACTGATTTGGGGCAGCTATGAGGACTGCAACGTCCACAAGGGAGCTT<br>TCTGCAGCATACTGTGCCAGACTCTACTAGAGGAAGATTTTCTTCACTGGTATTTTACCGGAGGTTTGAC<br>TCCAGTTCCGTATTGCATTTGTAAGGAAGGCTAGATGTCTCAGGATACAAGAGGGGACGTACTTGCAGA<br>GCTCTAAGCAAAAGAACATGAGGCTGAATTGAGGTGGCTTTGTGGTCATCCGATTTCTAGACGTGTGCG<br>GTCACCTGGTCCTGAAAACCCTCCACACAGAATGGTTTGCAATTTTTTTTGGTGATGGCATGTTCAGAAT<br>CTTGGATCCCTAAATTGAATGTTGGAAATATGTAGGAGCCTTGGAAATTACGTTGTTTTCATATATTAATG<br>TTTTGGTAGACTAATAAATGTATGTAAAGGAACTCCATACTTGTAAGGTGACAGGTATGTCTCAGGGCTG<br>TCACCTGTGTGAGTGCTGTGCTCTGGAGACAGCTCTGCTGTGGAGATTCTCTGTGGACCTCTTGCTGTC<br>CTGGGCAGACTGTGAGATTCCTCCCATGATGCTTTGGTTAGCTGGTTTTCTTTCCTACCTACATTGCAGT<br>GGGAGCTCCTTAGATCAGGAGTTATGAAGAGAAGAAAAAGCATCAAGTAACAATCTTAAATGCAGTAA<br>CCACAGAATTTCTACCTGGGAATCATCTTACAAGGTCTGTATTAGAATATATAATTTTTTAAATCAAATATA<br>TGTGTAGCGAAATGCAGCCAGTTTTTAAAGTCAGGCATTATTGTAGATCAGAGGTGTTCACATTGAGGG<br>AATAAGGATGCTCGCTTTCACCTTCTTCCTGTCTCCTCCTTGTCAGTTGATGAGTTAGTAGGAAGGGGTT<br>AGTGGTCTATGTAAAGAGGAGGAGGAGAGGCTTAAATTTCCTGAAAGGATTAATTTCCCTTAAAATAAA<br>ACCAAAAACAAAAAACCCAACAAACCAAAAACTTAGAAGTTGGTTCCCTTCCCTGTGTGGGCTGCTCT<br>CATGTAGAATGTGGGTGGGAGAGTCTCCATCCATGGACAGATGATGGTGGTATCGGGTGCAGTGCAGGT<br>TAGAGTCGCTGTAGTTACCCTTGATGGTGTGTGGTCCTTCACTGCCTCCTGCACCTGCTGGAACTCAGA<br>GACTTAAGGAAGCTCAGTTACTCCGTTGTCAGTGTGAATGCCGTTCTCCTCTTACATCCTCACTGGGGA<br>GTACTGATTGCAGGGACCACGGTTAGAGCAGCCTGGAACGTGAAGGGAGTGCAGGAAGCTGCAGAAA<br>CTGAACCAGAGCTCAGGAGAGCCTCCGTTGAGTTTTATTATTGGTGGGTTGTCCATTGTTCCTGGAGAC<br>CTTGCTTACCTGCAGTCACCATTATCCTTAGATTTCTCTTGGAGTTTTTCAGAAACTGAAGGGCTGTAAT<br>CCTGCCAGTGGCGCCCCTTCTAGTTAAAATCTAATTATATCATTTCCCTTCTTGCCTTTCTTCCCTCCAAC

							CTTCTCTTCCCTTTTCAGTGGCAGAAATGATACTTATTAGATGATACTTATTTGCTGGGAAACACTACTTA<br>GATTTTAATTGTGACATACCAGTGTCAGTGTCAGTTGCTTCCTGATTATAACTGGCTTTGAAAAACTCTC<br>CTCCTGTGGCACTGCTGTCAACAGAGGTTTCCTTTTAGCTGGTTCTAGCCACAGTATCGATTGGGCCTG<br>GGGATCTGATATACCCAAAAACAAATTAGCCAGCCAGAAAAAAATCTTTGTCCCCACCGGAATAAATAT<br>TTTAAAGACCTTCTTAGATTCTGAAAACCATGAGTACAAATGAAAGAGACCCATGAAAGAGAAAACCT<br>TAGGAGCTTGACAAAACAAAGTGCTGGTGGGTGCGACTGACCTTCACAGAGCAGCTCTAAGGGAACA<br>GTGCCTCCTGGAGGGGAAGAGAAGGCTGGAGTGTCCTGGACACGAGGCATTTCCTGTCGCCACAGCTA<br>GTTTTCACAAGCAGGCCATGGAATTTAAGAATAGTGAATTATTGTGGTATGTTCTAAATGTGTTTGCAGC<br>TCAATGGTTAATTTAACTTCAAATAAATGTCTTTACGTATTAAAGAAAATACATTATCAATAAAATTCTTTT<br>GTTCCTGTTGTATAACTTCACAAAATTCTGTAGAATATTCTGTATAGTGGTAAATGGAGGGCACAGAAGC<br>TTCTAGATAAAACTTTTAGGACTGTACTTAATCTTGTTTTGCTTGGACTACTGATTGGAGAGCATTTTAGT<br>GTCGCTCATGGGAAGTCTGTCCCTTCCTGGGTCACTGTGCTTCACAGCCTCTACTAGTAACACAGGTTAT<br>TTAACAGAAGGACCTAGCGAGTGAGTGGAGCAGTAGCCTGCCACTTTGAGGAAATGGTAGAGAGACA<br>CCAGCACCCCCACATAGGCAAAGTGCAATTGCTACACAGAAGGCACATTGGCATATGAGGATACCCAAT<br>CTTTGGGGTCCTCGAGGCTCACTCTGACTTTGATCTGCTCCCTTAGTGGTTCTCTGCCTCCAAAGGAAG<br>GGCCTCCAACTTGACGGCTGCCTACTGCTCTTCACAGAGGCCTGCAGCCACAAGCGGGAAGCCTCCTA<br>AAGAAAGGGGACAGACTGCAGCATCCACTCCTCAGGACAAAGGAGAGAGGAGCGCTCGAAGCATAGG<br>ATATGGCAGTGTCTATTACTGACGAGTAAAGATTCCATAATAAGTCGAATAGTAATGCCTTTTAAACGTG<br>AATATATATACTTTCCCCTGAATTATTACAAAGGGTTTCTCAAGTGTGACTTTCTTATGTCAGCATATTAAA<br>TTATTCTTAAATTATGTAATAGTGACTGGAATTAGGGATTCTGTTGTGACTTGTAAATTTGAAGTGCATTT<br>TCAGGAAAGTTGTCAAGATTCCCAGGTGGCTCATTTTTATTCCTTTCAGCCGTTCTGGTATGATCCTTCCT<br>GAGTTTGTCAGTTGGGAGGAGACACCCTTTGCCTTCTGTTCTGACTCGGTGGCTGCCTCTGGCTGCACT<br>ACTCAAAATCTTTATTTGTTAGAAGGTCCATTTGGACAGCTCTTGTGGTTTCACATCCACAAAAAAAGA<br>GGGTCCTTCGTGCTTCAAATACATGCTTCCTGTGGTGACCATGAACACTGAGTACAAAGGGTTCCAGAA<br>TCTGCTGGAATTCTGCTACATTGCCCTGCTCATCCTTCCTGGGTAATTCCATCATGGCTCCTTCCAGCTCT<br>TCAAAATGGTCCTGTTTAGCTCCTGAGTTCACAAAGAAGTGTCACACATAGTTCACCCCTCTTCTTTCCC<br>ATTGTCAGGTTGCATTCTGCTGAGAACAGGAGCTTCACATCACTTACTGAGAGACTCATCCTTCTCCTG<br>AAGAGAAAGCAGTGTTTTATATATAAGTGCCTTGGCTGTCCAAGGATTGTTTCTAGAAAGTTCACAGCA<br>CTGTATTCTAAATGTGGCTTCTCAGTGTGCTGGTCACTGCTGTTGTGGTTTTAGCCTGAGAAGCCGAGTG<br>TACCTCAGGCATGGGAAGACCCAGAGCTGCCTTATGAGTATGGAAGCTTGCTAGGACCTGAAGAGAGC<br>TAGAGATGCTCCTGCGTTTCCCTTGGTGGCTTTAAAACCACACACACACACACACACACACACACACACA<br>CACACACACACACACACACACACACACACACACATATTATACAGAGAAAGTATCTGGCAGCAAGATTT<br>GTCCAGACCATAGACCAACTTTTAAAGCTACAAAACCTACATGGACTATCTAGGTGCATGTTCGTAAGC<br>CATCATTTAATCTTTTGAAAACTTGGTTACATTGACATAAAAGTTTTTTACATATAAGTTTCAGAAGGAAT<br>ACAGATGTGTTTTGTGGAAAGGCTATTATATACTGCTGTAATAAATTTCTGACTCTTGCTTAATAAAAATT<br>AAATGGTCCAATTATGAAAAATCCAATACTTGGAATTTTTTTGGTGGGGTGATGGTAAGTGCATGGC
MSTR<br>G.414<br>97.1	--	0.314	0.000	0.022	-<br>3.851	do<br>wn	ATGAACCCCAACACCCCCCACCCAGCTGTGTGACGTCGGACAAGTGACTTACCGTCTCTGTGCATCTGC<br>TTTCTTCTGTGCAGAATGGCATGGCAAGAGAAGCTGCCTGGTGGGACAAATGTGAGGGTTGGACAGGG<br>TGGCATGTGAGTCATGTCTAGCAAGTCGCCTGCCTCTGAGAGCGCTCTGCAGTCGCTCCTGGGGTGACC<br>TTGGGAGGGAAGAGCTTGGCCAGAGAGTCAAGGGTCCTCAGTTCTTGCCAGCGGTATCACGTGTGGTT<br>GCCAGCTCAGGTTTTTTCCTAATGTGCTTGTTCTAGGAGATGTTCCCTTGTAGGTCCTACAAATGTATTTG

							TAAACTGTTAGCTCTTCAGTCGAAGCCTCTACCAGTTAGAGATGGGTTTACTTCCTCTGTGGTCTGTTCATGTCTTCTCTTTATACTTCTTTGTCTTGAGCTGACGAACTGGCTGCTCCTTCCAGCATGGGGTGGAATGAGGGTGACAGCAGACACCATTGCTTTGCCCCCGGTCCTCAGATAAAAACATTCAACATTTACCATTATATGTAATGTTAATTGTGTGGGTTTATTGGTATCCTGCATCTTCGTAAGATTCTGGAATTTTAGTTCTGGGTTTGGTTTGTTGAGTGTTCTTATTATGAATGGTTGTTGGATTCTGTCAAAGATGTTTTCTACCTGGGTTAATGTGCTGTGTGGGTTTAGCTAATGACAGAGTTTGCATTCATTGAATTTGAAAAATGGAACCAGCCTTCCACGTTAGGATAAAC
MSTRG.41498.4	ENSRNOG00000012012;ENSRNOG00000012049	0.109	0.479	0.000	2.875	up	TTCTATAATTATAAGATTTCATTGGAAATAACTAAATGATTGAAAGGGGTTTGCTTTAAAAATTTGTTCCTTCCTCCTTCCCTTCTTTCCTTCTTGCCTTTTCCCCTCCTCCCTTCCTTTCACACTAGAAAGCACAATTTCCTTTATTACTTACCCTTTTGGTTCCATAGCTATAGTATAGTCCAAGGAGGAAACTGGAATTCCTTCCTTGTCTCTGCCTCCTGCTCTTCATTTCACCCAACACCAAGAGAAAGCAGAAATGACTGCCCATGCCAAATCTCCTATACATGTGCTCTGTTCCCCAAGTTTTTCCATGCTTAAGCTTCCATTCCAGGAGAACCTTCCAGAGTTTGAATACCAGTCTCCCTTTTCTTTCCATGCAGGTTCTAGGTCACACCCACCATTGGAGCGGTCCTCCCTAGATGCCTACACACGTTGTACAAAGGTACAGACAGCAGGAAGTTGGGGGCCACTATAAGCCCTCCTGCATTTGGTAGAGACATCACATGACTTAGTAGCAAAGAACACAACCAACGTTCAGAATGTGTTTATTTTTTTCAAAGTTTTTTTTTTGTTTGTTTTTTTAAAGGAAGGTCCAATGGGTAGGTGATGCTAATGGTCAACCAAGAAGGAGACATGGGTGGGACTTATGAACATAAAGGTCAGTTTTGCCCATGAAGTGACCTCTCTGCTTCCTTGTGGGCATGCTTCTTAGAGGGGGGAGCAGACAGACAGACAGACAGACTCCAGGCAAGTGAGGGTAGCTTGGAAGTGAGGAATAATAGCCACCTTCAGCTCACTATGAAAGGCCTCTTTTTTCTAGACAGCTCAGTTCTGCAGGATGTGCTGGTCCTTCTCTTGTCTGTCCACTGTTAGTTTTATTTGTGAGGGGGGTATTTCTGATGATGTGTTCTCATTATTTCTAAGTTAAACACTCATTGATCCCTAGGAAGGGAGAAGACACTGTTCCAAAGAGAAATCATTCTAGAAAGAGGCTTTTCATCCTAGCTGTTATTTTCTCTTGATATAGCTCCCTCTATAATTCACTCCCAACTCCCCCATCCATTGCTAGTTTCTGTGTGCCCTACTTCTGGATAAGAATAACGTCTAGGCCTAAGGGACTTGTAGATCACTGAACCATAAGCAGCAGTATACTTCAATGCCAGTGACAAATGATACCAGTATTTCATTACCTCAAATTCAAGGTTCTGTACAGCCACGGTAGAAATTTTACACTTTGGGCTACTCACGGCCACTGTGTCTGCTATGCTGTGACCTTCTTTGCAAGGGCTGGTTGTCAGACAATGAATCTATTATACCTCAAAGGACTTCCCTGCAAAAACAATATTACCAATGAAAATTCAGCCAATTGACCACCAAGAGTATTTTCTATACCAAGCACAGAGCTAGAACCATATGGGATCAAAATATAAAACATATTTCAAGGCATTTAATAAAAGGAGTAAGAATTGCACACAAATTCTATCACAACATAGGAAACAGCACAAGAGAAGTGTACAGTGCAGTGGGCATCTGCCGAAAGATGGTATTTCCAAGAAGAAGTATATGCAAAGATTTGGAAATGGTAGCCTCTAGATCAGATTTTGAAGGGTTTAAGGGATGCTTGGAGATGAAACACTAGGGTTAGCTATAACTTCAGTCAGGTATCATGTGTCTACTTAAGATTGTTTTCTTCCTGATAAGAATGCATTCTGATCTCCAGCATGCATAAGGTGTGGCTTTCTTCCCAGAACTGGGATTGCCAATTCCAAGTGTAGCCTAGCATTGAAGATGGACCTTGGCCTGAGCATAGCCTGTGAGATGCAGAGGATGGCACGTTTAGTGCTTGAACTAGAGCAACAGCCATCAAATTTCCTCCATTTCCCTTAGTCTTATTTCTTATTGTTATTATCTCTTTAATCTTCCTATAGTAGCAATTGTTACCAATCCACAATCCAGGGTTTTTTTCAGCAGATCAGAACTCAGCCCTATCAGCCTAGCATCCATCCAGAAATGATGGACTACAACTTCATCTGAGTGTTTTAAGTCCTGATCTTCAGGAGATTAAAGTTGAGCATCCCCCTTATTCTGCACCTGCTAATGGCACTCGACCTTTGCTCACCCTCACAGACATCAGACATCATGACAGGTTGTCTTCTGCTGAGTGTGTGGGTCAAAGTGTCCCTCACTTAGATCTCACTCATGTTCGGGAAAATCCAATTCAAAGGGGTTGAGTGGAGTGGTACTCTCTGGGATTCAAAAGACAAGAAGATGGTCTAACACCACTGAGTTTCAAAAACAGAGGTGGTGACATGACTTTCAGAAGCTCTGTGCCTCCCCTGTCTGTTGAGCATCCCCCTGTTTTAAA

							GTACCTCCTGAATTTAGGAATTATGAACATTGATGAGGAAACAAACACAGTGTACTTCATCTTCTTCCCT<br>GCTCCATTCCTTGAAATTCTCCAGTACTGAATGCCAATGCTTGTCTCTCTTGACCATTTGTAGAGGATTGT<br>AAGGGCCCAAAGAGCCCTCCTCCAAAGCAATGCTCACTTCGGAAAAATGTCTCAACCAGGAAAAAAG<br>ACAAAGAGAGTGGACAGTGATAACATGTTCCTTTTTCCCCAAGCTTCTCCAAGACAGAGGATGTTTCTT<br>GCACCCAAATTATGCTAATATTGATCTCAAGCTGGAAGAATAAGCCCCTCTATTCCAGCACGTCCAGGAG<br>AAATGAGACTGTCACTATGCCTTGTTAGCTATCATTCTTGACCACCATGTCTTTACATCCACAGACCATTG<br>GCTTCTGTCCTGAGCTGTAGGGACTTAGAGAGGTCATGTGAATTTATATTCTATGACAGGGTTATGGTTA<br>CTGAGAATAAGGGAGAAGAATATTCTCTGAGGACTAGAGAAAGTAGCCTTGCATTACTTGGCCAACTTT<br>CAAAGCTCCCTGTGAGCTCAGAAGCCATAGGCCAGTCCCCCAGCATCCTTTTGGGCACAAACTGATTGT<br>CACTGACTCCTTCTTTGCCACTGTTCCATCCTGTTGTCATGGCTGATTCTGAGATCTCTGCTGGAATACTT<br>TATCAACAGTGTTCCTAAGGCAGTATGTATCATTTTCGTTATATTAATTACTCACCAGATGCCTATTTCCAG<br>AGCAGTGTGTATTTATCTCCCAGTTAGTCCATCGTGGCCATTGATGGCGATATTTTAAAGTTCTCACTGCT<br>TAACAGTGATGAGACATGGCATTGATCTTTCTTGAAACCTTCTAGAAAGAGTTACCATTTCCTAGTTTGA<br>CTTGGCAAACTGGAGCATATGAGTTGGCCGGGCATGTAGCCAGTGGTTTTTTTCCAAGGGGATTGAGAA<br>TAAAGTTCTATAAAGAGAAATCTGGGATTGAGGAAGCTATGTTGGGACATCCTAAGCACAGCTCCTATC<br>AAGGAGAATCTAGTTTCACCACAATCCAAGTAGGAGGGTGGCATACTGGATGCAATGTCAGCCTAATAC<br>CCTAGCCCTAATGCAGTTTCCTCCTTGGAAAATACAGACAATACTCTTTAATGTTCTTTGCTAGCCTGGA<br>ATTCTGTTGTCAGCACACCCACTCATCTAACAAAGGAACAGTTCAGTTCCCCCTTTCCTAACTCACCCTA<br>ATGTTTATAAGTCTAAAAATCCAAGGAGATAATGGTGGTCCACATAGAAAGAACCTGTCTCAAAACAAA<br>CATTCAAAGTGTGAAAGGAGAAGATCTGACTGTCCTCAGACAAGGTGCTAAGCTGGCAGCCAGACATC<br>TTAGATGTTCAAAAATTAACCACTGAATCAAATAATGCCAAGCACAAAAGAACCCCACCCATGCTATTG<br>TCCTTTAGAAGACATCTACAGCAGATGAGTGTGTCCCTAATCCTGTGCTCTGGATAGGGAAAGTCATAAT<br>GCTCCACAGAATGGAAAGGTGACCTGTGTTTAAGAGAAACAAAGAGGCTTCCCACGGCATTTCCTGAA<br>TGAGTGCTGTGTGCTTTGCCTCTGATCACTATGAATTAAGTGGTTTCACACCCAACCGGAGGAGGCTGG<br>AGGTCAGAGAAGCTGGCCTGCCTGAGGCACACGACTACAATGAAGCAAGAAACCACCCTAGACTCCA<br>GTGTCAGTTTGGTGATATGCAAACCACGCCAAGCGTTAGGAATGCAGAAGCCACCTTGCCACCATGCC<br>AGTGCCAACAGCATCAGTTGGTGAAGTCTTTCAGCCAGGGGCAGCTCCATAATGGCACCAAGTACCAA<br>GAGTGACTCATGCAGATGTGTCCTGGACAAGGACTTTTAGCTTTTAGTTTGCTTTTTCTGTCATCCCGTC<br>CCCTAATTTCATGCTGTCCCTTTCCTCACATGAACAACATCTGTTATCACAAATCTTTAAATGATGCATCA<br>TCTGGAGAGATGAGGCCTGGCTATTTATGCAGAACTCTGGGGATTTATTTGAAGACTATAGCATTTGTAG<br>AGGCTATTAAGGCAGACCAGGTTTTTAAAGTCATCCATTGACAGGAAGATCCATCCCCAGCAAAGCCTG<br>TTTGGACATTAGCTGTCTTGAAGGTGCCGGTCACTTTAGAGGTGCCCAGCGCAATGTGCAAAGGACCA<br>CACTGTGGGCTATCCTGTGGGTCAGACTGGGCCAGGAACAAAGAGCATTTCCAAGGTGATTACTTTAAG<br>AGAGTGGGCTGTGGCCTGGTGACTAACTCTGGGGAAACTTGTTCAGAGAGATGTGGAGGTAGGGTCTC<br>TTCTGGCCAAAGAGAGGAGTGGAAGCCCCTTTTTGCCAAAATCAAGGAACGTACTTCATTTTAGAGGT<br>AGCCCATGCTTCCTCTAATGGCCAGATTTTAACCAACCACGGAGTACTTCCTGGACTCACACCACCACT<br>TCCCACGCAGTGTCAAGCAGGGAGACTTCCTGATAGCGCAGAGGACTCAGAGGTTCTTACTAGGCTGG<br>AGCTGTGAGTAGATGATCTACCCATTTACACAGGTCACTAGGAGAAAGCCAATGGATCATGCTACCAGC<br>CACAGCTCAGAGATGAAACACTGCTTTCCACATCCTACACACTTCTAGCTAATGGTGCTCCCAAAGCAA<br>CTCTTGTGCCAAAACGGCCTCTTTTGAAATCGGCCCCAGGTTCACCATCACTTAGGGCAGAGTGTGGCT<br>TTCTTGGACTTGGACTCCAGAGAGCTTCATGAGGAAGGCCAAAGACCAACAGGCAGGGTTTGTGGCTT

							CTCTCAGCTTCTGGTCTGCTGTTGTCTGTGTCCAGTACCAAATGTTTGCCCCCACCCCTAACCTTAGTCA<br>AAGAAAGGGTGACCAAGTGCCTCTCCAGTACCTCACGGACCTCCTAAAACACCCCATATTCCTGATGCT<br>GCAGGTCTCAAGAGGCACGTCTTCCACGGGCTCCCTTGTTCTTCTCATATCCCAGACGTGGACCTGGTC<br>AGTCCTTTTAGCCAGCTTACTCTTTTCCAGCCTATTGCCCCTAGGTGAGGCTCAGGAAGTGGGTCACCC<br>ATTCTTAGATGTCCTAGATTCTTGATGTTTTTGTTGTTGTTGTTGTTGTTTTGATTTGTTTTTCCCCTTCCT<br>TCCAGTACATGAGACTGTGAGAGGCACTGTCTCCTTGGCAACGGCCGTCCATTTACCAAATGGAGGTGG<br>GTCTACCCTCCTCACCCTCTCTGCTTGCTTCTGTTGCTTTCTTTCTGAGCAGTAGGTGGCGCCACCCTCT<br>CTCCCAACAGCCTGGAAGAGTGACTGAAGCACAATGCATTGTGGCCAGGAGGCTCAGGCATTGTTTTC<br>TAAGTACAATGAATAGGGAAAGCTCAGTGATGCGGGACTCCCTTGGGATCCTCCATTCTTCAACTCCAA<br>TTTTCTAGTTATTTTCTCGTTGAAATTGTTACCAGTTTTGAAGGAGTAGACACCGTGTGCTGGCGAATTG<br>TGCAAACCACCACCCTCTCTCCTTCCTAGCGTTGGGAGGCACGACCCGCTAAGCACCCTGTCTCTGTTA<br>AGACCGCCATCTGTTTTTCTGAGTTGATTTCGATTGGTGCATTCTCACTTATCAGGGACCTCTGATTCTAG<br>AACAAAATCAGCTACTTATTCTAGTTACTGTAAGCAGACACATAACTTTTACTGTAATTTGAAAACCCTT<br>GCACTGTAATGGTATTTAAACAAAAACAACAAAAGCGAATCCTAATTGAAACTGATTTTGTATTTTTGAT<br>TACATCTGGATTTTCACATTTTTGTATCTAATCATAACCACAGCAGAGCTTCCATAGCTTGTGTTCAAGGC<br>CTTGTTTTCTTTCCAGGTTGAAGTCTTAAGATTGTGTTGACACCAAAAGTTTAAGGGGGCAGGATTGGG<br>GCCTGACTAGCGGCACTTGTCATGGAGACATCCCTGCCCTGGTGCCAGGGAGACCTTTCCTAGAAGAG<br>CAAGCCTCGTCGCATCCCATTGTGCTGTGAGTTCCCACAACAGCTCACATCTCCAACAAATTCAAGGCC<br>AACGGTTTGGGTCCAACTTTTGCCAGGGACTCTCTCGACTGAGCAGCCCCCACCCCTGCGTCACCCAG<br>CATCCTGGGCAAGGGAGGACGTTTTTAAAAACACTAAGCTTTTGGAGTTGATTTGTGATTATGCTTTTTT<br>TCCTTCCCGCTGAAAACGTGCTGGGCAAAAGACAGCATGATGTTACCGCCCCTCTGCTTCTCCAAACCG<br>ATACACCTGTTGTGCTCTTTTGTTTCTTAGTGAAGAACATTCTAGACACAGACCTAAATATGAACTCTTT<br>GCCTCCTGTATTTCCAGTTAGCAGTTCTCCCCGTGTATCCCTAACTGTTGATGCGTTCTCCTTCTTAGCAT<br>TGCCTCTCACATTTTACTTTATTTTTTTTTTACTGAACAGCAACAAACCCACAAAGGAACAAATTCTCTC<br>TCTCTCTCTCTCTCTCTCTCTCTCTCTCTCTCTCTCTCTCTCTCTCTGATGTGTAATGTTGTGTTGTCCAGA<br>TTCTGTATAAAAATATGTATATGATGTATCTGATATCTGGCAGCCACTGGCTGTTACTTTAACCAATATAAT<br>TAACAAAAATGCATAATGTTGGTGTTTGATTTTTTTTACAAAAAAAAAAAAAACCTAGTTAAGATGTTGTG<br>TATTTTTGCATGGTTAAAATGTACTTAGTGCATTAAGAAGTGTCGTGGAGGTGCGCAGTGAACAGGCCA<br>GTTTACTTTTTAATCGTAACGGTGACTGGTTGTCAACCAGACTTAGTCCCAATGATGTCTGTCTGCTTTC<br>CTTTAGCATAATTCTATTTATAACTTGTGTTTGGTACTCAAGGTTCAACATAGCATATCTATCTATCTCTATT<br>GTAGTTACTGTTCCTTAGAACCAGGTTGTGGAAGTTGACTCATCAGGTACAGCTGTGTCCATTGCTGATT<br>TCTGTTGACTTACATTCCAATGAACAGCTGAGCAGTCCAGCTCTAAAGCACCAGGCCCTGGAGACAAG<br>GGCGGGCATTTCTCCTTCCCTCGATCTCTCCCTCTGAGCAGTACTGTGGTTTTCCCTGGTCATGGAACAT<br>TATTTTTTCAGTCCTAGCTATATAAAGGAGAAGAAAGGACAAGAAGGTAAGTGGGAGTCTAAGAATTAT<br>TTATATTTTATACATAAGAGTACGTATAATATATTATAGATAGAAAGGTAGATAGATGGCAAATGTTGAGTT<br>AGGCACACAATGGGAGACGTCCAAGGATTCAGAAAGAGATGCAGAGTTTTCCTTGCTTGGAATGGCAC<br>CAGATCCACAGGAAAGACTGAAGAAAAATTGATGGCCTTTTTGTGCCTCAGTTTCTTCATCTTCGGCTG<br>CAGCGTATCCCTTCAGCCTCTCCACCAAGCTCTTTAAGTGAAAGGCAGTGGCTGTCTTCCCTTGGACAA<br>TAATGAATGTATCTACTGTAAGTGCAGACCATGTAGAGAAGTGGCTGGGTTCAGAAAAAAAAAGAGGGT<br>TTTGTAAAGCCTGTTTATTTTTTTAAGTCAGTTTTGAGCATTTCTATTTTATCACCCTTCACGTGGTTTTGG<br>GGAACCCAAATTGTATCAAGGTCTCATGCCAAAAGAAGCCAACAGTTGTTTTCTTCACCTTTTTCCTCC

							ATGCACCATGATTTCAGAGATTGTTTTGGTGTCGTTTTAAATGTTTTCTTGTGACATGTACTGACGATAAA<br>AGTCATCTTGACAAAAAAAAAAAGATTTATACATGAATCAGAAAGTATTTATTTCAATTTTGTACCTTTCC<br>ATTTTAATACATTATAATGTATTGACTCAACTGAGATAATATAAACAGTTCATTTTAATAACACGCGTGTGT<br>GCCTTTTCCTTTTATGTATAGTTGGGGTGGGATAAACACTCCTGGGGTGGAAGAAAGGAGTGTTGACTG<br>GCTTTAGTGTAAGCGGAGGGAGACCTCCCCTGCACCAAAAGCTTCTGAGTTAGAAGCTGGTGCCTTTG<br>GGACAGGAAAAGATGTTGAAGCAAGGAAGTGTGGAGGTAAGTGGTGGCCTCCTGGGTAACTCTGACC<br>CAACTGACCTTGCAAGGGAAACCCAGAGACACTAAACTGAGAAGTTGGAGAGGAAGACTCCGTATTA<br>AAAATAAGGGTAAGTGGGGTAGGGGTGGGGACTAAAAAGTTCTGGATTGTGTTTTCTGGACATAAACT<br>GTTCTTTCCTAATGTTAAAAATCCTGCAGGTGACTCGTTGAAGTAAAATGCCTTCAGACCAACCCAGGG<br>GCACGCAGCGAAAAGGGCTCTTATGCTTCTGTTAGGACGCCTTGTTGAGTGTATGCCCATATTCATGTTG<br>ACTGACTTTGGAGAACAACCTCAAATGTTGTTCCTCTGGTGTCTTCTACCTTTTGTTCGAGGGAGGTCT<br>GTCATTGACCTAGACCCTCACCAAATATGCTAAGCTAGTGGCTAGTAAACTCTAGGGATCTGGCTGTCTC<br>CACATTCTATCTTGCTGTGGATGGGATCATAAGCATGTACAACTATACCCACCTTTCATGTGGGTTCTGGA<br>GGTTTGAACTCAGATCCTCATACTTGAAAGGCAAATACTTTACTCTCTGAGCCATCTCCCAATCCCTAAT<br>CTAGCATTTTATTGTGATGTCAGGCAAAACACAAAATGGGATGTAATCATACTCCATTTTTACGTCTTTTG<br>CTGGAAGGTGGAGGTGGGTGGAGTAAGCAAGGAAGCAGGGCAGGAGGGGAGAAAAGATGTATCCATG<br>GGACTGCAACTGTTTTTAAAGCATGTGCAGCAAACAGGTGGCAGTAACAAGAATCATGGACCTAATTCT<br>CTACTCTCCCTTGCATGCACGAACATGGTTCATTTTCAAACTGTGCAGTCTCGGGAAGGAGGGAAGCAC<br>ATAATTATAGAGAAATCCACGTCAAAGTCAGGGTGTCATCCTCTCCTCGGAGAAGAGGAAGAGTGAGC<br>TAGAGACACAGAGAACGGTGTAAAATGAGTGCTTTGATCAGACCCAGCAGTCCAGGACTCGGAGAGC<br>CGTACACAAGTCTCAACAATTGGTCCATATCCCTTGATATCCTATTTTCCCGGGTACCCACAACTTTCTAC<br>ACATACAACCTTTGAAAAAGCTTCATTTTTTGGTTATTTTTAAAGAGTGCAGGTGCTTACATAAGTCAGG<br>AGAGGGCGCCTGCTGGGTTTCTGGAGCTGGAGTTACAGGCAATTGTGAGCTGCCTTACATGAGTGCCA<br>GGAATTGAAAACTGGGTCTTCTGCAAAAGCAAGGATTGTTCTTAACTACTGAGCCAGCTCCAGCCCTAT<br>CCTTATGTCTGTCTCTCTATGCTCCCCTTCCCACATCCCTGTCTTTTCTAGGCGGAACACTAAAAGAAGA<br>AGCCTGCCTTTGCTTTTGAATCTCTTACCCATCTTTTGTATTAAACAAAACAAAAACAATAAAACAAAA<br>AACAAAAATATGTGACCCTTGTCCAGGGGAGCTGATGACTAAACTGTTTTATGGTTCAAGTCAATAATTT<br>AGAAATGAATCAGTTGTCAATAATATAAAATACATATTAGTCCTATAAAGGGGCAAATTTGAAGACATATG<br>TACTAGAAAATTCACAGGGATTCCCTCTGGTACAGTTTTCTAAAATTCTTCTTCACTGGTGGCAATTTTT<br>GACTTACAGCATTTTCAGATGTTTAGAGAGGGGCTGGGTTCTGAAACAGATGTGAGAGGTCTGTACTGG<br>TGTCCCTAACTAAAATTCACCCTCAAAAACATCTGCATCCAAGGAAATGGCAGGGGATACTTTCCTCAG<br>TTATTGCTCTTTGGAAATATGGGTCTGGAATTTAAGACTGTTACTCTGTGTCAGTGTGTAATTTTGCCTGT<br>CTTAGTCCATTTGAGCTGTTGTAACAAAATACTTAAAGTGGCTTAAAATAACAGAAAATTATCCTTTAAA<br>GTCCTAAAGGCTAAGTCCAAGGTCGAATGGCTGCCAGATTTTGTTTCTATGATTCCTGGTTCCCAAAGA<br>CTGTCATGTTGCATTCCCAGAGCATGTGAGAGAGAAGGACATCTGGATTCTCTCTCTGAAAAGCACCAA<br>TCCCATCCTGAGGCTCCTCTTTCATGATCATGCACAGCCCCTCCCCCTTAAAGGCACCACCTCCTCATGC<br>AATCTCCTTGGGGGTGAAGGTTTCAGCACAGGGGTCAATCTCATTATAGTGGGTCTGAGCTTCCTTGTC<br>CCTGCCAGTTGAAGTTGTGGGAGGGACTACTGCCAGCAATTTCTGCAGCTACCAAGGTGAACTGCTGC<br>CACACACCCCACCCCTAGGAGGTTACTGAGCTCTTGTCAGCACCTGGTCGTTTTGTGTAATGAAGAAGC<br>ATACTTTTCTGATGACAAGACAAGCAGTGATTGAGCCATTCTTTCCCTTTTCTGTCCTCCCTCTCCCTTC<br>ATCCATCTTTCTTTTTCTGTTTTGAACTGTGACCTCTGATGTTTCAAAAAAAGCCACCTCGATTCGGGAG

							TTCCGCTCAGCACAGTCTTTCTTCCTGGGAACAGCACACCCGGGCTAGTTACAGGAAAGAATAAGGCAACCCCTTGCTCAGTCAAGTTCAGGCTACACATGGATGAGTTCCGAGCTCCATTAGCCAATATTAATAATGACAGAGGAATGACAACGTAAGGCACAAATGAATCATGTTAACCTGCTCTCTTCCTGGAGAATCAATCAGGATTTAGCAGAGAGCAGGAGTTTCACTCTCACAGCAACAGAATATTTTTACATAATGCATGCTAAACTTTCTCACAGACTGTCTCAGGAACTATTCCTGTTACTTCTTACCTCTACAATGATTAATTAAATTATTAGCTTACTTTTTTTTAATTACCAAGAATAGTTTCTGCTCTAGCCAAAGCCAGAATAGAAAGGAGTTCCCTACATGCCCTAATAAACTTGCTTCAGACTCCAGGGCTGCATGGCTTCCATACTTTCTTTTCTGTCCTCTCCATACTTGCTCCTCCAACTCTTCTCAAACTGCAGTTAATGGACAGTAGAAGTTTACAATGCTCCCCAAAGACGTCTTCAGACAAATGACAAAGACTTTCAAAGGTAGCTGATGCTGTGATCCAAGAACACTCGCTGGGAATGCTCTCATTGGTCACTCCTCCTTGTCCTGTAAAGAACTTTTAGAGGAGTGAGGTCAAAGCTGGTATCCTCACTGAATGTGAGAAAATGCTCCTGTGTGCCCTTCTGGCTGCTGTACCTGGCCTTAGTGATCCAGATTAGGAAACGTGGCCCAGGGAAACGGAAATCTGGAAAGGATTTGGGGCAAAGCTCAGATGTCCTAAGCGGGTGATTTGGTCAGACTCAGCCTCATCACCACACAGTCACATTACTGCCTCTTCTTTCTTGTTAATAATTCGTGGTCAAGCACACAATCATTCTTGGCTGGGTGCTCTCATCTTACAGGTAGGTAAGTGACAGTCATGCCTGATTCTCAGGTCTGTCCACTCCCAACCCCGAGCTCTTCCTCCAACACAGCCATATTTTACCAGCACACCGCATCCTAGAAGGGTTGCACTGACTGCCATGGGGGTCTACATGGAATCTTGTTGGGTCTGGTGAAAAGTGGATGTTACCTTCCATAAAAAGACAAAACGAGGAAGAAGAGCCTCTGGAAGGTCAGGGCACTGGCCAGTGCTGAAGGCACAGCTACAACAAAGATTTGAGGAAAGCTAAGGTCTAGATTGGAAGCCAAAGCCTTGACAAAGAAAGGGACGTTGAAGGAAGAGGCTGGGGCCGGGGGTTGGGGGGAGGCAAGTAAACAGCTTGGGAAATTGCTGAGGTCACACAAATGGAGGAGAAAGGTCAGCAAAAAGAGGGCAACAGAATGTGTCAGGATGCATTCAGCAAATGCCAGGTATAGCATGTTGAGCGATGGAATTGGGAAAGGCCTTTGGGATTTAAGTCTGTCACAGCGAACACCACGGAAGAGTTAACATTTCTCGACCGTTAAGAACAAAAATCTGGTGTTTCGTTTGACTTTGACAGCTACGTGGCTGTTTCTGCCTTCTCTCCCTCCCTCCCTCCCTCCTCTCTCAGCATCCCCAATGAGAAGAGGTAGAGTGTGAGGAAAGTGGGCCCCCGCTCCACAGTGCCAATACCTTCTTCTTTGGCCATCTCGCCAGCTCTTTGAAGGTACCTATGGTTCACAGAACCCCGAACAACTTTAAGGTTGCTTGCATATCCGCTTTCTGCGTTAAACTGCGAACTGCTTGAGTCCAACGAGAGCTCTGCCCCAGCCCTAGAATGAATTTGTGTACCAGTCCCGCAAGGTGGGTCGGCCCCTCGGCTTTACGTAGTCCGTGGCGAGTCGCGCTTGGTGACG
MSTRG.41501.2	ENSRNOG00000012049;ENSRNOG00000012402	0.103	0.287	0.024	2.177	up	TAATACCAGCAGCCAGTTCCTGCTATTAACCTATTGAGAACAGGAATTCTGAGGTAATAAGCCAAAGGACAGGGAGCCAGGGTTATACGACCTGAACACAAGGCCCAACCTCAAACCTGGCACCCAGCCACCAATGAGACCTCTGCTACATTAGTGGGCCACCTTCCCAGTGCCCGTGTCTCTGCATGGCTATGGGTCACAAATGAAGAACTGTGTTAAAGAGTCGAAGCATTAGGAAGGTTGAGAAGCACTGGTCTCTAAGGAGACTAGAAGGTCAGTGTTAGGTGTCTTCTTCAGTAGCTCTCCACAGTCATTTTTTGAACCAGGGTCTCTCATTGGACGTGGAGCTCGTTGATTGGGTGGTCGGCCTGTTTTGACTTCCCCATCTGGAATTCCAGGTGCACACAATTGGCCTGCTGTGCTCTGAGCTAAGGTCTTCCTGCTTGTGCAGCAACCGAGTTGATTTCCTAGCCACCCTCGAGAGTATTTAAACTTATCCACCATTTATCTCATTAGTTCTGAATTTTATTGTTTTAAATCTCGAAGTTAACTGCATGGATTTGGTTTTGAGCCATGGGCTGGAAAGTGTACAGCCAGTGTCGATTTGATATGCCCTGGAGTTGATGCCCTTTGATGGAACTCTGAGCTGCAGTGGAACTCAAAGCCCAGTCGTCATTCAGTAGGACGTTGATTCTGCAGATGAGATCTGTCTGGCCTTCTCCCGCACAGTTCTCTGTGATCCCCTTGTTCTAGGTTCTGCAGCTCAAGTTCATCGTGAGTGGGTTCAAGTGGCTCCTCCTGGCTGCTCTTTGCCTGGGTTACTCCACCAGATAAAAATGCAGCAATGCTGCATCCTTCATTGCTTTCCTCGGCAGCCTCATTCGGTTCCCAGAAGAAG

							AGCAGCCCTCTGACAGGATGCCCCCGTGTTGTCTTGCAGCAACCCTTTCTGTGGATTTTAATAGACAATTATCACTAAGCACAGTCTTATGTGCTGGGCCCTATTCCAAGCACTTTAAATAAATCAACGTGTTAAATTGTCATTCATCCCTGTGAGGTAACCTCATTCCTCTCCCCTTCACAGAAGGGCCTTTTGTGATGGCTGGAGATTTATGCTACCCTAAAGCCCCATGATGCCCCAGCGCCCTCTCCACACCACCCTGGGCCCCCTTGACTCTCAATTCCAGCTTCACCTGCCTTGTAGGCCCTTGGGTTGCATCGCCCCATAGTTTGATTACTTACCTGCACAGGGAAGCAGCTGGCAAGCTCTACAGCTAACCCACGTTGGTCTTACCTGTACACGAACGTGCCCTTGGGTGAGTTGGAAATCCCCGAAGGGGCCTCCTCTCCACCTTGGCCCTGCAGTTGTTGGGAACTTGACTAACTCAGCTTCTTACAACCTGTGTTGGAGACTGGTTAAAAGAATCTTCTGTTTTGTAGTAAATATCGATGCTTTTGGAAGATAAAACCCAAACTAGCATTTGTTTTCTTACACAGGCCATGCCTCTGTTTCTCTCTTCACCTGTCCCCTGAGTGGCAGGAGCAGGCCGAGACCTGTTTGCTCAGAAGAATCAGCACCAGAGAAGTTTCCTTGCAATCTCTCCCAGTGATCTCAGCTCTTGGATCAGCAAGCAGCGGCTGTATTTAGATTTGAGTAAGAACTTCCTCCCCACCCGCCACCCTGTTTATCTCTCTCAGCTTCTGCAGCCGCTGCAGTTTAGAGCCTGTTGTGCTGGCATGGAGTGGAAGAGGTCAGATCGGGTGCCCGGTGTGTGCTTTTATGGTGTGCGATTATTTGTGAGCAAATTAGTACCATGTCCTAATAAAGGAGGTGACTAGCCTTGCACACAAGCAGTCTGTTTAGGAAATACTGAATATATTTAAATGACTCGCACAAACCCTTCTGGATCCAGCAAAAGGAGGGAAAGGCAGACAGAGACATTGTGTAGAGAAGGGACTAGAGAAAGCGGGCCACTCCAGAGTTCATGTAAAGGTGATACTTGATACTTGCAGGGAATGGACTGAGAGCCTGAGTTAAATTTTCGTGTAAAGTTAACAGTCACCGTGTAAGGAAGCCTGCAACGAGTTCTTTAACTGAGGGAAGGACTTTGTAACTAATTACATCTGGAATTTTCCACACTGGATTTCCTTGGGAAAGTGAACATAGATGCCAAAAAGTTATTGTAAAAACATGTCTCGATCACCAATCTTAGGCTTCACGGGGGGAAGAGCTGACCCATGAACATATACCCACGTTCCCGGTTTGTCTTTGCACGAGCCCAGGCTCAAGCCCCCTCCCCTCCACCCAATCACTATTTCATCTTGAGAACATCCTTGTCAGTGCTCCTGTTGGGCCAGAAGCTTCCCAAAATTCTTTGCCAGCCCATTATTAAACCTGATCCTGGAGTGAATTTGTCACACTCCCCATCAACAGCATTTCTCGCCGATTAACTATCGATGATTGATCGTTCTCTACGTGTTCCTTCACACATTTTGCCATAGGTGTGGGTTTTGCCCACCCTGGAGACCATGTGAATGCCAGAGCCAGCTACCCTCCAGTGACTGTGCCATAGCAGGTCTGCCGAGGGGACAAAGACAGAAGGAGCTGGGGAGACAGCCTACTCCACTCAGTGCCAGAGATAACCCTTTAGCCCGGAAGGGACCAAGTCAACCACTGAACGGTCTGTTAAAATGTCCAACTCGGCTTTAAAATGTGATGCAGTTAGGTGGCGATTAGTCTGGGAGCTCATGACCAAGGAGCGTGACAAACGTGTCTACTCTCCTTCACCTCCTCTCCTCCAGCCATGCCATAATGTATGTCCTCCCCAGTCTCCTCTCCACTCCTTGGCTCTTCCTGCCCTCCACCTTGTGCTCTTCTTCCCTCTCCCTCTGTAGAGATGCTCCTGACCTATGTTTCTGCTGCATCCTGGATAGTTCCCAGGTCTGTCTGCACCTCCATGATACCAAGCTTTTAACTGCCTCTCCAGGGGGCTTTCTCAAATCCTAGGTGTCATCAGGGGGAGCTCTAGCTCATCTTCCCTGGCTGCCCTCTGGACCTGCTGTTGTCA
MSTRG.41510.2	ENSRNOG00000012049;ENSRNOG00000012402;ENSRNOG0000004	0.000	0.434	0.001	4.386	up	CTGCTGGAAGCTGCTGAGGTGGTGAGTCTCCATGTGAGAGGGCGAGCGGCTGGGCAGAGTTCTAAAAGCCACAGAAGACCATCCGGAGACATCCAGAGGGTCCATCAAAAGGCCAGTGGGAGAAAGTCAGCGTCCACCTCAGCCTGACCTTGATTAGCTGCTTCCTGGGCTCAGCGAGCAGAGCCATGCCCAAGTGCTCCTTGGAGGACGGATCTTGTGACTCCACAGTGAATGAATCAGAGGGTCCTAAGGAAGAGAACAAACCAAGCCTTTCATCCCTGTCTGGGCACAGGAACAGGCAGCCCTGTGAGGAGGCAGGGGCAGGGACTAGAGATGGCACATTCACAAACCACACAGTCACACAGAGTATAGCAAGAGTCACAAACTCTCAGCTATACACAGGCAGGTTCTGGGCAGCCAAGTGGGGAGGAGCTCAGAGGGCCACAACTTCTTAGCTGTCTTTATCATATAGCTCTGTAGAGGGAGGGCAATGAGCAGGGAAGCAAAGGGAG

	6326;ENSRNOG00000047258						
MSTRG.41770.1	--	0.751	0.000	0.005	-4.261	down	ACTTGATAGCCCAAAGGAGCCATTCTTGCTATAACGAGAATGGTAAAGACAATGACTAATGTGATTGATTGCTTATTCTCTGACAGGCAGTATGAGAAGAATTCTGCACATAGTAATTTATTTAAGCTTTATAAGTACCCTGGGGTAGGCACTCTACCCTCCCTGTTTCTGAAAATGGGAAAACTCAGAAGTAGATTAAAGAACCAGCCACGTCACAGGAACTTTCCAGATTTTCAAGATTGGGACTGTTGAGACACTAAGCCTTGTGAACTGAGCAACTGCCTGGTTGTCAACCTCCCCAGTGAGAAACAGCCACCATAAGACTATTCCAACTACAGAGGCTCCCAGCCTAAAGGACTAAGCAACTGCTGACTTCTCAGCACTCAGTGTGATTCAGCTCCTGTTGCTATTTTAATGTATGAAGTCTCTCTCTCCCTCCGTCCTTCTCCC
MSTRG.41797.1	--	0.062	0.438	0.018	2.940	up	GGGGGATCCACTGGAAAGGTTTGTATCTGAGGGAAGGCAGAGGAAGCTACTTTCAGTTGGCACTTCAAGGATGCTTGCAGAACTGTGGTAAAACAGTTGTGCAAGAGTCATGCAGCTGGCATACTCCTGTCTTCCTGGATCCTGTCCAGATGGTGAGGGGTGGGCTTCAAGTCCTCTCCTCTACAGGTGAACATTCGAGAGCACCTTAAGAAGTTAGCTGCTCAGACTCATTTATCACAAGCTCCAGGTCCAAACGCTGTTGTGATCCGGGTAAGTCACCCCAGCAGTAAAGACAAACTAAACAATTAAAAAGAGAGAGCTGGAAGTGCCGTGCTTCCTTGTGGACTCTTCTCTAGGGCCAGCTTCTCTCTAGCTGTTCAGAGTGACTTGCTTGTTTGTACGGTGAAGAACCCTTCTATGAAGGTTGGTCTGTCCATCCAGGTTTTGCTCTTCTTAGACACTGTGCCTTCATCCT
MSTRG.41815.1	--	0.000	1.635	0.000	5.728	up	CAGAGTCTGGTTGTTATGATACATGTCTGCGGGTCATGGATGAGACGCCTGGCTAAAGCTCTAAAGAAGATGTGTCCCTGTGTGGGTCTGTGTTCTACCCTGCTGCAGGGCATGGTGGCTTGGGAGGCAAAAGGAGGCTGTATAAAGAGGCAGATTCCTGGATAAAGTCGAATTATGCATGACGATCTATACCAGGACCCAAATCTCAGGTAATTGGTTTCTGCACACAGACGGGCACCAACTGTCGAATAAGACCTCGGAGCTCCTGCTTCCTGACCTGCTTGAGTTCCAGTCCTGACTTCCTTTGGTGATGAACAGCAATGTGGAAGTGTAAGCTGAATAAACCCTTTCCTCCC
MSTRG.41815.2	--	0.000	0.444	0.007	3.835	up	CAGAGTCTGGTTGTTATGATACATGTCTGCGGGTCATGGATGAGACGCCTGGCTAAAGCTCTAAAGAAGATGTGTCCCTGTGTGGGTCTGTGTTCTACCCTGCTGCAGGGCATGGTGGCTTGGGAGGCAAAAGGAGGCTGTATAAAGAGGCAGATTCCTGGATAAAGTCGAATTATGCATGACGATCTATACCAGGACCCAAATCTCAGACGGGCACCAACTGTCGAATAAGACCTCGGAGCTCCTGCTTCCTGACCTGCTTGAGTTCCAGTCCTGACTTCCTTTGGTGATGAACAGCAATGTGGAAGTGTAAGCTGAATAAACCCTTTCCTCCC
MSTRG.41887.2	--	0.422	1.058	0.037	2.035	up	GCTTGCTAGGCAAGTGCTCTACCACTGAGCTAAATCCCCAACCCCTCTTGCCTTGTTCTTAAGGCTTACCTTCTCTTGCTGTATTCTGCTCATAGCATTGCTTCTTTCTTATGTAGCCTAAAGCTCAGCCCTGGGCTTTCCAGAGCTCTGGAGAAGAAAGCTTGGTGGTTTAATGATTAAGTCATCCAGTGGGGCTCACCTGCTCTGATTCCCAAAGAGCCCTGCAGACTTCCCCTGCACAGCTTGAGCTGTTTTGTTTGCCTCTCCTGTGGTGGTGAGGAGAGGGAAGAAAAGTAGACTTCCTCTTCCTGGTAGCTGACTATGTTACTTCAGGGCCCCTAAGGAGACAGAAACTGTTTCAGATTTTCACTGTCCTAAGACAAAGAATCCTGCATTTTGTGAAGCAAATAGATAAAGAAACTAAGGAACAAACTAACAGAATTCAGTGCTGAACCATGGCATCCTGGAAATCATCTGAGCAAAGGGCAATGTTAAGACCACAATGTCAAGACCATTATCTTAGAGATTCTTCTTTTAAAAATTAAAGCATTAAGTGTTCTCTCTGTACTTCGTATGGGATTCTCCAACTGAACATAGACTACTTTAACAGAAGCTAAGCCGATGCCTGTGTGGAACACGTTTAAATACTGGCTGCTAAGTGAGAGCCTGAAATGCTGAGGACACCTGAAGCTAAGTGAGCACAGTGGACTGTCTCAGGATGCACCCGAGACGGCTTTCTTCTCTCTTTGTTTGCCCTATTTT

							GAGAATGATGATTTGGACAGGCAAATGTACTGCAAAGAACTTAGAAAATGCTTGAAACCGAGTCAAGAGACCGCAGGAAAACAGCTACCAAAGCAGCCAAATTTACTTCCCTAACATTGGTGTTCAGACGCTAACAGTCCAATTCACATCTGGTTTATTTGGGGGGAGTGGATGCAGAATGTTACACAAATCATTTGCTGACCATTGCACATGGAAATCCTAGAATTAAAATTTAATAATAATAATAATAATAATAATAATAATAATAAATGGACATTCTGTTTTTTATTTATTGTTTGGTGAGTTTAACCCACACTCCCGGCATCCAATTGTCTCTAGCAACACCGCCAGAACCAACCTTCTGGCTGTAAAATTGAGGTTTCAGCTCTAAATGAGATGACCGAGGCCATTGTCATGCCCCGAGACCCCAGCGGCTCTCGTACTAGACAGCAACACTGTCTGAACTTTCTCTTGCTGCAGTGCACTTGATTGATACCCTGTTTTGAGAAGACAGTGTCAGCGACCTTAATGTTCTGAGCCGAGCTCAGCTGGTGGCATGGTCTGACAACCTG
MSTRG.42060.1	ENSRNOG00000022462;ENSRNOG00000047413	0.080	0.444	0.004	2.921	up	ATATAGCCCACTGTAGCCGATATCTGTGTCCCTCTCGTGATGAAATGCCACCTTTCAGCTGGATGTTATGGAGGCCTCTTCTTAATCGGCGTTTCCGACTTTCAGATAACTCTGGCTTGTGTCAAGGTGACATAAGACTGGCCAGCAAAGGCCATCAGCACGAATGAACATTCTTGTCCCTCCATAGCCTGAGGACACTGCTGGGTAGTAACTGGCCAGGGACTACATTCTCCAGCCCCTGTGAACTAGGCTGAGGCCATGAGACGTGCCCGTAGAGATGGCAGATGCCACCACAGTGCCACCTGTACAGAGAGAAATGAGGAACCGAAGACGATGCTCCTCTCTGCGGTGTCTGCAGTCCACTTGAGACAGGACACACCCCTCGCTCCTGTGAGTCTTTGAACATCATACACTTCGCAAGACTTGGTGCAGCTGCCTCCCTAGCCTCAGCCTGCAATAGGTCACCCCAGAAACCAGATGACGTATATGTGTTACTTTTGCCTTAGCCTCACCTGACAGAAACAACTAAGGGAGGGGAAATTCATGTGGGCTCATAACTTGAGGGGCTTTCAGTCCATCATCAAGGCTCAATGGAGCAGCTCAATTGATGGTAGCAGGAGTGTGGGATGGTGGTGGCTCACATCGTAGCAGACCAGGAAGCAGGAAGTGAGGCAGAAAGCAAGGGCTGAGGAGACTCTTCAGAGGCTCACGCCCTACTTCCACCAGCTGGCCCCACTCCTTTCAGGTCCCATAGTCCCCTAAAACAGCATTATAGGAGATAGGGAATAAGTGTTCAAAAGATCAAGTTTAAACCAAAAAGCACTTGGTGTCTATAGAACTCCTATAACATGCCAACATTTTCCCATGACAAAATCTTTTCAATGCTGAAGACCAAATCCAG
MSTRG.42130.2	ENSRNOG00000001033;ENSRNOG00000001037	0.190	0.000	0.001	-4.672	down	CGGTCATAATTGTGCTGCGTCTGGCAGTCTGGGAGAAATCAGCCACCACGCTGACCAGGTGGAAGTCAACCAAAGTTGCCACATCCAGGTGCAAATGCCCTGGGGGCCTAGCTCATGCTGCCTTGAGGGGACAGCACATAGACAGATGGCTGCCAGGGAAGCCACACTGAAGGGGCGATCTTACCTTGGCCAGGCCTGTTTTGAGTTGGGTTTGAGAGCACTAAGGTGGCTTTGAAGGAGAAGATGAGTAGACCAGTCGCTGTTGAGGGTTTGAAAGGTCGTCTTTGACTCTCAGGGGAATCCTGATCCTCATAGTTTCCAGATGGTTCTTCTAGGGCTACCCCTGAATGAGATTGACTCCGTGGAAAAGCTAAGGGCTAAACTAGTCCTTTCTGGCCTCAGCTGCACAGAGTCAGCTTTCATCTGGCTTAAATGGACTTTGTAAAAATGGGAAAGAGTCCAAGGTTTGGGTTTTAAGTCGACTTGCGCATAGTGAACTTCGGGTGATGCCCTAACCTGTATCGTGACCTCTTTGAATCCCAACCAAAGAGCACTGGGAGGAAGCAGTGAAGGAAGCGGCATGTTGCCGGGGCAATTGAGACCAACCTTAAACAAACACAAGCTAAGATGAAAGGATGGGAGGGCGCGGTAGGTGGCAAAAGGTCCAAGAAGCAGGGATCTGTGTTAGTGTCAGAAACGGCTCAGGTCCAGAATGGGCAAAGCCTTGTGAAAGAGCAACTGCGGGGAGAATAAAGCCGCAAAGTCTGAGAAGCAACTTCGCGGAAGTGGTCTCATGCATCCCCACGATGACCCTGGGAGTGGGGATGGGAGTCATTATCCTCTGAGCTTGTAGGGGCTTCCCAGTGGCAAAGCTGGGATTGGAGTCTAGATCATCTCCGACCACGTGTTAGGCTCTTCCTACGGCGGTGCGTTATGTTCTGTGCCACGTAGCTCAAAGGCTCTTTTTGACGCTGGGTGAGAAGCATGAGAGGGAGGATCTGACCCAGGAGGAGTGGGATGCTCCCATCTTTCCCTTCAGCGTTCATCAGGAAGTAAAACTATCGCGTATGCGTATTTGTCTGTGCGTATCTGTGTACCATGTGCATGCAGTGCCTACGGAGGCCTCAAGGGGGCATAAGATCTCCTGGCAATGGAGTTGTAGACAGTTGTAAGATGTCATGAGGGAGCTGGGAATTAAGCCTGGGTCCTCTAGAGGAGATGCCAGTGCTCTTA

							ACCACGAGGCCATCTTTCTAGCCCCACATCCCTTTGCCCCCATCCCTCCTCAGCAAGTCTTTTGAGGAGTGTTGAGCACTCTGAGATCCAAGGCCTGGCTTTCTCCCTCTGTGACTTCCATAGCTCCATCCATCCAGAACTTACTTGCTTGGTGTCTCTGTTACTCACCAGCCTTACGTTTCTTGGATAGAGGAGCCATGGTCCCTCCTCCACCCACAGCACAGACTGACCAGGAATGTGCCAGCAATCATGGCTGTGACATGGCAGCAATGCCACAAGAAACAGTGACGTTGTCCTGACTTAGCACACACAGACAGAAGGTAGAGTCTGACTCCACGTGCTGATATCAGTCCCAGAGGTATTCAGAGGTTCTCCTAAAGTCCAGAGATGCAGAGTGAATTCCACAACTCCTCTCATCTATGGTACAAATCTGGCAGAAACTCACAGCGGCTGTTGGACTTTAGGCTGTGATGAAAGCCCAGAGAAAGAGGATGGGGGCAATGCCCCCTTTCAGATTCCCAGGCCTGCTTCTTAGGTAAGTTGTTACATTAAACATTGGGCATCTTAAGAGAAGTTTTACACTGGGTGCTGTGGGGAAGGGTGTTCCCTAGCAACGGAGAAGCGCACATTCAACTCTGTATAGTTCAGCATGGCGGCCAGTCCATACTCACTGTCGTGTTTGCCTGAATTGTGTTGTTAGAGTATTGGCTGAGGACTTTAAGTGATGCAGGGATGTGGGCATGAACCTTGCTTTCCAGAAAGTTCTTCAGCTCCTCTCTTCTTGTTGGGAAGTTGAAGGCAGCCTTTTCGTGTCTGTATGTATTCTTGAGGGTCTCCTCAAGCCAAGTCATCTCAGTGTCTCCACCAATTATGTCTCCATCGATATGACAAGTGCCAAGGCCTGTCCTCACCTGGCTGAGCCTTGGTATTTCTCTAGGACTCTGAGGAGCAGAGCAGGAAGCCACTGCAGTCTTACATGGACTCCAATAATCGGCCGCAGATTGCTGTGTCATGGTGACGCCCACTGTCGGGTGTGCTTCCACTTCTAACCGGGTTCTGGTTACACTGTTCCTCACTGGGGCCTCACTACCCGGCCCCTCCCCCTGCCCAGATCCCTGTCCACTCTGCAATGAAGACATTGAGGGCCATCACTAAT
MSTRG.42134.1	--	0.040	0.414	0.035	2.995	up	CCAATACCTCATGGAGCAATTGGACTCAAGTTCAAAGCCAAAAACAGACAGCTGAGGAGCAGGGACCTCAATCTGAGCATCTGGGTTTTCAGCAGCTCCACCTTGCCACAGTGAACCGGAGCACTAGGAACATCAAACACGATGAGTAATCAAGAAAGGCCAAGTCCAAGGCTGCCAATGGAACCAGCTGCAAGAGAAAAAGAATACCAGGAGAGGCAAGCACGTGGGTACCTACTGAGGCAGAGACAGGAAGTACCAAAGCCATGAGGAACTGAGAACATTCCTGGAGCAGTCAAGAGAAGAAGGGGGCCAGTACGGCAGAAAGAGCAGAAGAGCATGAAGAAGAGAGAAC
MSTRG.42140.1	--	0.361	0.000	0.049	-3.592	down	CTCATTAAACCTCTCCACATGGAACCATGTTGGTTGGGTGTGTTCTGTCTGGATGCGAGCTGAGATTTAAACTCCAACTGTAAAGTCTGATCTCTTCTTATGGTCATTATCTAGAGAATATTCCCCTCTGAAAGGAGGGAGGTTTACCTTCTGACAAGCTCAGCTCCTGGATGCAGTGAGGAGCATAAAAGTCAACCAAGCACAGCTTTTGATGCCATGCGGTGCCCGGGGACGCAGGGGTGACAGCGCCTCGCTCTCTAAACACGCTCAGGCTATCCATTCGCTGCCTTATGGTTTTGGTCAGCAGTATCTCTCTCTCCCTATGCTTTTCTGTCCAGGGAGTGTTTGTTTTAGTTTCTTTTACTCCTTTAGACTTAATCTGTTGTCTTTAGATTTCCTTTCTGTTCCTTTCTGTGAAACCTTGGGTTTTTCTCTCCACCTTTTCCTGTTGTTCTCAGTGCTTTGGGCTGCTCAGTTCTCATCGTCTGTGTTCTTTCATCTCAGCCGACGTGGAATCCATTACAGTTCGATTAATCCCCAAGACAGTCATGTGTTCTGTTCCTCTGCATCAGACCTCATCCACCTC
MSTRG.42345.1	ENSRNOG00000012594;ENSRNOG00000012821;ENSRNO	0.030	0.541	0.000	4.605	up	CTTACATGAGCTCCTATTCATAGCCATTTACCACCCCACCCCAACCCCCTTTTTAGAATAACTATAGCACAGGCTGAGAAAGTATTCGGATTGTTGTAGACAGAATTGCTTATTGGGGTGGAAGCTTGAACTAGATGTTTCTTGCTAGGTCTAACTCTGCCCACAGACCCTTCAAACGCCACACCCAGGCTCTCTGTGTTTGCGACCCTTGACCCTTTCACTTCTCCTGGCAGATGTGATGCATTATGTAAGGACGGTCACACCGGGATGGATGCACCAGGCTCTGAAGTCAGGGAGTCCTGGACTGAATCCCAGTGTCTCCCTTCAGTGAGCTTTGCGACCATGGATGAGCAACTTTTTATCTCTCTCAACCCTGGTTTAATCATCTGTCAAGCTGGGTCACCAAAACCCACCCCCGCAGGTTGTAGAGAGCACGGAAATGAACCGGCACATCCAGATCCCTGGACAGAGTGGCAGGCTTGGAGGAGGTACTGATTAAGTGGAAACGTTCAGCGGCAGTTCACAAAGCAAAGCTGGTGGACTTTCACGCG

	G00000029808;ENSRNOG00000033141						TGCACGCACAGGAAAAGTGACTTTCAAGTAAGGGCTGGAGGAGCAGAGCAAGCACACTAGAGACTGAAATGTGGCTCCCAATGCCTGCCGCCCTATAATGGAGCGGCTGGGAGTTACTGCCAGTTTGCAGATGGTAAGGTGCAGAGATGAGTCATTGGCTTAAACATGAAGTCTACTGACACTTTAGGACAGGCGAAGGCCCTGAAGACAGCATCCCACAAAGACAGTCATCTATGAGGAAGAGACTGAAGTAGAAGAGATCATCTCCTGAGACCCTTGGCTTCTCTTCAAGCAATAAGGACTTTTTCAGACCACCTTGTGGGAGTGGAGACAATGATGGGTAGAACCAGATCTTGACCAGCACTGACTCATTCATATCTCTTGTCCCTTACCCATATCCTTCCGCTGTGTAAACCTAACCCTGTATCTGAAGATGAATTTGGTATGTATATAGTTGGCATTCTTGTGAAAAAAGATGGCAACTCCAAAGAGCTGGTATGAGAAACAAAAAGGGCAGGAAACTTTCTCTGCCCATCCAAAGCTTAGCTCTTATCGCAAAAGGGAATTGAAGAGTTTATTCTCGAAACAACTGTGAGTGGCCATGGCCTGGGAACATAGATTTATGCTGTTCCAGACACCATGTTCCAATGTGGTAACTGTTTCATGAAATTATTCTTTTAAAGGTTTATTTATTTATTTTATATAAGTATACTGCAGCTGTCTCCAGACACACCATTACAGATAGCTGTGAGCTGCCTCGGGGTTGCTGGGAGTTGAACTCAGGACCTCTGAAAGAACAGTCAGTGCTATTAACTGCTGAACCATCTCTCTACCCCCTCATGAAGTTCTTATAATTAGTTATAGGAAGATTTTAGAAGACAAGTCTAAATCAAGGCACTTTTCAAATATGTGCCTAGGAACCTCTGGGAGGTGGGCCAGGAAATCTCTGTGGTAAGCCTCAGATGCTATCTATCCGATGACACCCTCAGCTTTTGGGTTTGGTGGGACCTAATAGAATAGGTTAATACATTCCAAAAATTACCCCCATTCCAGTATGTCACAAGGATGTTAGGTCAGAAACAGAAGTGGCCAATGAATGGCTTTTAAGGGAAGTAAATATATGCCAAGATCATTTGGACTCCCAAGTTTCAATATTTGTATTCTTCATGATCATGAGGTCCTAACTGGTCAAATCAGTCGTGTAGCATATTTAATGAAGGGGTGGAACCCCTTAGCTCCCCACATTACAGAAGCTTCAGTTTACTTTCACATTCAGCCTGTAGCACTGCCCTTAGTTCTGTTTTCAGAGATAATTGCCCTGCTTTCACCTTTACCTAAGTCCCAGGGTCATGTTTCTGGCTGTTGATCCCCTGCAGAGGACACACCTCTATTCTGCTTTAGTGGGAAGGGTCTTTACCTGCCACAGGGAAAGAACTTCCTTCTGGCACCAACTGCTTCCTAAGAAGGCTTTGACTAACCTCTCTCAGACCTCTTCTGCCATCAGAAATCTTATTATCTGGATACTGACATCACTCAATGAATAAAAGCACTCTGTCCCAAGCCTGAAGACCCTAGTTCAATCCCCTGAGTCCCATGGTAGAAGAAGAGAATCGACTGCCAGGGTTGTCCCCTGAAACCCCACACCACGGCACACGCATGCCCCTATACATGCACAGACCCTTTTTTTTTTTTAAATGAAGGCATTTTGCTAACCAATGGCAGCTTCAATCTTAAGAGTGGATTGTATGGTTCACGCTAGAGAACCTCATGCAAGAAGAGACAAGGAGAAGAAATGTACTGTGGCCTATGTCCTTGGTCCTAGGCTGGTTAGTGCCTGGGTAGAATCATGCTTTCCCTGTAGCCACTGGGATTCTGTTCTTGGTTTTGTTGTGGATGGAGTTTCTGTGTTACATGTTGTCCCA
MSTRG.42863.2	--	1.039	0.000	0.008	-4.137	down	AATGAGATGAGGATAAACATGCACAGTTGTTCATTGGCCTCCAGACATGCACCGTGATCTCTAAGACTGCACCTCACCTGTATTTACACACACGAACACACACACACACACACAATGTATACATACACACACAGAGACATGCAAACACCAACCACCAAAGCCCGAGAAAAGTTGCTTTCTCTACCATCTGAGGTTCCATCCAGGTTGAAGAACCTGAGAGATCATCTCTCTTCTCTACTTTGAATACTTGATAAAGTAAATGAGTGGTCTTCTAAAGGTGTTATTCTCTCT
MSTRG.42872.1	ENSRNOG00000038041	0.000	0.619	0.002	4.137	up	GGGAGAGAGTGAAAAGACAGAAACTACACTGAAAAGTCACTCTACACAAGCTCTACGACTGTGTCCTAGCTGTGTGACAAGAGCACTGGCTTCTCTTCCAGAGGACCTTCATCTGATTCCTAGATCCCACAGGCTGGCTCACAACCCTCTCAACCTGCAGTCTCAGGGGATCACACACCCTCTTCTTACCTGCTCAGGCACAAGGTGAAAACATTGAAATTCATGCTTATTGACTGGGAAGACTATGACATGCTGTTATTAAGTGTGTTCCCAACACGACTAA
MSTRG.429	--	0.000	0.431	0.000	7.612	up	CGGCGGGGTGCTGGGACAGAGCTGGCCCCGGGCGGTCGTTGAGGGTCCGGGGACTGTGGGTACCACTTTCTTGAAACGGAGGTGAGCTCCTTGGGCCTGACCTGACCACCTTTACCCGGTGCCTCCGAGCCCACC

56.1							CTGCGTGGGAAACCGCCCTGAAGGTGGGAGCTCAAAGGGGAAGTTTGTGTTCGAGAAAAATTTGCAG<br>TTCTGCGGAGTGGAATGGGGTGGGAAGAGAAACCGTGGGGAGGCCGAGAAACAGGAAATGAGAGCG<br>AAGGAGCAAATTGGAGCCAGGAAAAGCCAGCTTCTACATCTGTGTGGCAGCGTGGCATTTGCTGAGCA<br>CTTAAGGCCCAGGTGCGCAGTTAATGAAAACAAAGGCGGGGCACCGGCTGGCCCAGCTCTGCCCTTGC<br>CACGTTTTGGAAAGGAAGCAAATGGGAAAGGTGGTCCAGCCAGGAGAGTACTGTCGTGGCCCTGTCCT<br>GGTCAGGACAGGATGAATCAGCGGCCGGAATCCTCTTCTCAGTGAAACAGGATTGACGGAGACAGTGT<br>GTCCCCGAGTTAAGGTTTCGAAGAAGAACAAATGAGATAAGTGTGAAACTGTCTGAAATGTGTGCGGC<br>ACGCTCTTCCAGAGAAGTAGCTCTAAATGAGACAACAGTGGGAGCAGGGAGGTGTGGAACTCAGGCC<br>CCCAGGAAGGAAATGGGACCCAGAGGCTGAAGGGTTCACTGATAGAATGGGGGGAGGCGGGGTGTCT<br>CTGTTCCAAGAATTGGCAGCCGCAAACCTCAACCTGATCTGATCTTGGGAAGAAACCCCTCTCCACTCC<br>GGTGCACCCAGACAGAAAGAACGTTGCAAACTCAAGTTTGAAATATTGCAGAGTATCAGCAAATGTGC<br>ATTATTTAAAGGAAAATCCTCCACTGGAATCACTAAAATTTCCCCGCAGGTGGGGCTAGGATAGGCAGG<br>GAGTGAATTTAACTAGGGATGGGAGGATGCTCTGAGAAATTATGTCATCCTGGCAGTTCTTGTGCTAAC<br>AGCTTTCTCAGCTTGAGGGCCGAAGCGGATCATCAATACATGGTGAATACTTCAATGCGCGGGAAAACA<br>AAATGCCTCCTCCCTGTAGCAACAGCCATGGGCGTGCTAGGCAAATGATGACGTTCGAAACTAATCTGC<br>AAGAATTCATTCCAGAGGGAGAAGGAAATTAAGGGCAGGTCAAGGTTAATATCTAGAGTCTGAATAAA<br>GCATGTGGTTGAAAAAGGGAAATACATCAAGTATTAGGGCAAAGAAGTGCTAGCAGCATTTTGAAAAA<br>AAAACCGAATACTTTTAAATGGGGATTAGAAATGTTAAAATATGGCGACTCCCTTGCCATAAAAGAAGT<br>TGGTGAAAAGAAAAACACAGGTTTGGAAGGGAGAATACTGCAAACCAGGGGAGGGAATGGAAAAGT<br>AAACACAAAGAATTTTAAAGGGATGAATATTAGTGAGATTTACACGTCTGCAGTGTACATGATGTGTGT<br>GTGAGTGATATGTGTGCGTGGGAGTGTGTGTGCATGTCATGTGTGTGTTTTGTGGGTGCGAGTGTGTCG<br>TATGTATGTGCATGTTGGGGGCTGGGGGCGCTAGCATTTGTTTCTACTGGAGTTTATCATTCTGGCTCTGA<br>ACCAAAGTTAAACTGTGGAGAGAAGGAAGTAAGGCAAGGAAAGATGGCCTCCCTTAGAAGGATGAGT<br>GTAGTTTTTTGCTGTTTAGACTTTTCTACTCCACTCCATGTGTGTTCGGGGATAACGGAAAATCAGATCG<br>AGTAAAAAGAGGAAAGTTATTTTTATCGGGCATGTAGAGTTCTAATAATGTAAAAGAATAATAAGAGAG<br>GAATTACTGAATCTTAGAACTTCAGGACTGAAAGTATTTATGAAATTTTATTCATATTTCAATACTATTTAA<br>ACATAAATGCCTTGGGACACGGATGTGGTTCCTGCTTACTTCTTAGTTGGACTTTAACCTGAAGAAAAA<br>CAAATTGTCTAAAAGGATAAAAGGTGTTATTTTGGTCTACACATGGCATACACACTGAATTAATTTTACAT<br>AGCATAATGTCCAGAAAAGAAGAGGAAGCAGATAATGTGACTCAATTCCAGCTACACAGCAAAACTAG<br>CAAGTGTTTTATTTAATAATTCGAAAGCACGTTCCAACTCACAAGCCCTGCCCCCCCTTTCAGATTTCAT<br>ACTTAACTAGTGGTAATTTAATGAAAAATTAATATCACTGTGAGAAATCTTTTATGCTATAAAAAAAGGTT<br>TAAATGTGCACCACACCCCAATTCCTATATGGTAAAAATTCTCTCCAATGTGTATTAAAAGTAGTTAAAC<br>CTTTGGATTAAAGAGGGCAAAATCGTATGGGTGATTTGACATTAATTCTGTGTGGAACTTGGGGAATTG<br>AGTCCATTTCAGAAATTAAGGCATCATAGAAAGATTAGAATGAACTGGAAGTGAAAATAAAATGATTCG<br>CCCAGCTCTAAATAAAGTTTAAAACAAAAAACAAAAAACAATGTACTCTTCCCCAGGGCTCAACACGC<br>AGAAGAACAGAGAGAAATTCGCTGCCTATATTTTTCACATGTCATATTTTGGCACTAATGTGCAGCAAAG<br>GCGAATTCTGTTTAAGCACGAGAAAGGCACGTTTAGAATCTTCAGTACAAATGGAAACAGCTGGTGATA<br>TTATGTGACGCTGTACTAAACAGAATGTTTTGATTTGGATGGTGAGCTGAGAATTGGGAGGGAAACGCT<br>ATTTGATAATTTTGTCACAGTCCCCACAGGTTTGGGGTTTTCTAGTTTCAATATCACATATTAGACACCCA<br>CGTTTCCATGTCCCTCTCAAGCTTTCTTCAGACCCCTCATCCTTTGTACCACTAACACCGGACCCTGTCT<br>ACCTTCCCAGCTCTGGACTTCTGTTTGATCCATCCTTCCCTTCCCTAGACAAATCCTGGGTTATGTTTATA

							TACCTGTATACACAAAGCCAAATTGGAGTTGCCACAATGGTTTCAGTGTACCACCTTAGCATTAAAAACACTGTCTGGACTTGTCTTATAGTCTCTCTTACAACCCAAGGAGTTTGGGCACCACCGTCCAGCCCATTAGAGGCCATTACCAAGTGGTTTTTTTCTTTTCTCCTATTGAATCCTCCCAACAAGGTTGCATGACACCTCACATATATGTGGGTATAGCCAATCTAGACATCAAAAACATTCTTTACTCCTCAGAACTTTTTGGGTGGGGGTATAGTGCAAAGATTATCTCATTCACTAGTGTGTAAGCCCCTCCCCATGTCTGTCTGCCATGTCTGTCTGTCTGTTTCTCTTTCTCTCTCTCCCTCTGTGTGTGTGTGTGTGTGTGTGTGCGCGCGCACGTGCACGCCCGTGTTTAGGGGAAACAGTTTTTCACTTCCTTAAGTTTGCAACCAATGCGAGGCATACTAGGTCAGACAGACACAATAGCTGTTAATTTCTTACTGTATTCTGTTACAGGAGCATGGCTGTTTCCACAAACTCTAGTGTTTTGTCACTATCACATAAAATACATTGAGAATAAGACCCATGACTTAACTCTCAACCACTAATTTGACTTCGTTTTTACTCCTACACTGATACGCAAATAGTAAGTGATTAATGAATGTTTGTGGAATGGAAGAAATTACAGCCAATAAAATTTTAATTGTGTGCCTTAAAATACAATTTCACAGAAAGCTTCCATTTCTGTTCATGTGTGTACTAAAAGTAACAAGAACAACAAAGAAACTTCTGGACTGTGTTAGCAAGAACCTTTGAACATTAATCCTCACAGTGTTTTTCAGGTAACATTAAGTTCAATGTTTTTACATTAAGATATACTAACGTATCTATTTTTACTTAATTTTGCTGTCCCTTTCACATTTAGTTCTTTGAAAAGCACTTACCAAATCATACTACTTTTCATTCTTTTTACTAGAATTACATGACATTATTCTGTATTAGGTAAAAGTTCAGTTTTTCAATTCCAAAGAGAAATTCTTAGTAAATTTGATTTTTTTGTAACCCTTTTCAGCCTGTTGGGTTGTAAACATTGGAGCTCTTCTATCCTAGAGATAAACATTATTTTTAAATATAATCAATTGGGGCATACGGTATTGAAAGAGTCACCCTCACTTAAAAACAATTCCGTGTAAACAATGTACAAACAGCGGAATTTCACACTTTAAGACTCTTCAGAAGATAGGTATTTTTTTCTTAACATGACATTTGTCATGCGTGTAAAATAATGTTCGTTATATTGTTTTATGGTCTGCCTGTCTAGATACTATTTAGCTTGAGGTACAGAATTGCTTCCAACCTTCTTTAAGATAGTTTCTTGTATCCATCTCCTGTCTCTACAATAATTACCATCCATTCTATGTCTATACAACTTTTATTAATGTTTGGATCTTGCCTCCAAGTCTTTATTGTCCTTTCTAATACTAATGTATCTCTACAACATCAGAATGTTTTCCCAAATGTTCGCAGTTACATTTACATATAGGGAGCATTTTAGCTTCTCTGGTGAAAGGGGAGAAAATAAAGCTGGTGAATGGTTTTCCCTGTTATATTGTGGAAGTTGTTTTTAATTTTATTCTTACTAATTAAATTTTGACAATTTCACACATTTGTATAATGCAGACTGGCCACCGTCACCTCCCACCTTCTGCTACCCACCCTGCTACACCTGGCTATGCCCTGTCTTGTAAATCTCTTCCTCTGTAATGTCTGTTCCAGGGCCTTCCAAATTTAAGCAGAGCCATCTCTGTGACCATGGGTTGGAGTTATCCATTAGAGTCTGATGGGCTCATTAGAGGGTACAAAACTGAAGACATCTAGGCACTATTTTATTAGTCAAGAATGGAGTCTCTCCCCACTTTGACTTTGGTACATGTATTTGAGTGGAATGGAAGGGAAGAGAACAAACATCCAGTGTTGCTTCCAAAACATTGCTTCCCCATCACAATGTAAGTACCTATATTTTACGATCAGCTATTCTTTCTGACGTATCGTAGACAATATTCTATAAACACATGGAAGTTTTCATTCTG
MSTRG.42967.2	--	0.503	0.000	0.010	-4.070	down	GGGAAAGGAGAGGGAAGAAAAGGCACCCTACTCCCAACAAACTGAGGAGAACTAGGCAGCTTTCTGATCAGAGTTCAGATGAAGAAGGTCACAGTAATGTGTGGCCGCTGACTAAAATAACCCCGTGGATGGGAGAGTTAACCACATTAGAAGCAAACTAAAAAGATCAGGAAATATTACAGAAGTTGTGGAAAGATCTCAAGAACCAGAAGATGCTGACATCTGCTTTGCTGTTGTGTCTTTTATGTATGTCTGGAAGCTGCACCTATGAGATCATACAGGTAGACTTGCCTGAATAAGATGTGTGTAATGGAGAACACCAGTAGATATTCAAGCAAAGATGGGGAACTCTTACAAACTCCTCCCTTAGCTGAAGAGCTCCTCATGACTCCTGAGAGAGGAAACTTCAGTCTTCTCGAGGGACAGGCCTCTGGATAGGGTATCCAATTCTAAGTGTCAGTCCTAAACATATACCAAGGGATTGCAGACTGTATTCACACACACACCACCACCACCACCACCGACACCATCAGCAGCAGCAGCAGCCGTAAAAACAACAACAACAACAACAACAACAACAACAACAACA
MSTR	--	0.356	0.000	0.028	-	do	GCTACATATTCAGCTGGAGCCACGGGTTCTCCCATGTGCAATCTTTGTTTAGTGTTTTAGATCCTGGTGTTTTATTAAGGCAAAACAAATGTGTCTGAGAAAAGAAGTTGGTGAGAAAAGGGAGTTTAATGGAGAAAAT

G.44056.1					3.770	wn	GCCTCCATCAGATTGACCTAATGTATAGAGAAGTCTAAGGGATATTGTCCTGATTAATCTTGGAATTTGTAGGGCCCAGATCTCTAGGATGTTGCCACCCTTGAGCTATTAGTCAGAAAATGAAAGGTATGAGGTACAAGGCAAGAGGCAGAACTCCTACTCAGCTTGTACTTCAGTTCTTGAATCCAGGTTCCTGCCCTGCTTCAGTTTAGGCCCTGATATTTCTCAGGAGTTAACTGGTAACTCCAAGTATATCATGGAGTGTAACCTGTCTTCCATAAGTTGCTTTTTATCAGTGTTTTGTCAGAGCAATCATAGGAAATTATAACACTTCCTAAAGTGAGCAACACTGATAAGTTACTTTTCTTCTGAGATGATGGCTTAGGTTAAGTCAACTCTACTCATCACATATTTCTTACCAGCATGCAGCTAAGTTTATGTTTGAGCAGTCCTGCACAAGATAGTCATATTTTATATGCAGACTGCACAGAATAGTTTTGCTTGTTTGTTTTCATGCTTGACCATACAAAAGAAT
MSTRG.44150.2	--	0.000	0.415	0.001	4.386	up	GCTTAAGAAAGAAAGAAAGAGAGAGAGGAAGAAAGAGAGCCAGCACAAAGCCCAGAATTGGACAGCTGACATCCAGGAGGAAGGCTGCGTAAGATGGTCCCGAATAGCTGTCTGGGATGGTGACTCTCTGCTGCCTGGCACCTAGTAGGTGCTTAATAAATGTCTGAAAAGGCTCAGTCATGTTTCCTGCCAAAGAGAAGACCAAAAGAGGGAGATTTTGTAACCAACAAATAATTGACCTTTTGATATCAAAGGTGATTTGCTCATGAAGAAAATAGCAGTGCTAATTTCAAAATGATGAAGAAACTGAGATCCGCAATGACTGTAAAGTTTGGAAGTAGAGGTGCCAAGATGTAAGCTCCGTCCTTGGTCTTTTGTTTGCCTTACTGAGCCCTAAGTTCAGGTCATCAGCTCAGGCTGCATGACTCCCCTGCAAGATCATGAGCTACTTAAAAGCAAGGACCACGACTTCTCTTTGGCAAGGCCAGTTTCTGGCCCAGTGCAATGGCACATAATAGCCC
MSTRG.44150.3	--	0.000	0.369	0.003	4.043	up	GCTTAAGAAAGAAAGAAAGAGAGAGAGGAAGAAAGAGAGCCAGCACAAAGCCCAGAATTGGACAGCTGACATCCAGGAGGAAGGCTGCGTAAGATGGTCCCGAATAGCTGTCTGGGATGGTGACTCTCTGCTGCCTGGCACCTAGTAGGTGCTTAATAAATGTCTGAAAAGGCTCAGTCATGTTTCCTGCCAAAGAGAAGACCAAAAGAGGGAGCTTTTGATATCAAAGATGAAGAAACTGAGATCCGCAATGACTGTAAAGTTTGGAAGTAGAGGTGCCAAGATGTAAGCTCCGTCCTTGGTCTTTTGTTTGCCTTACTGAGCCCTAAGTTCAGGTCATCAGCTCAGGCTGCATGACTCCCCTGCAAGATCATGAGCTACTTAAAAGCAAGGACCACGACTTCTCTTTGGCAAGGCCAGTTTCTGGCCCAGTGCAATGGCACATAATAGCCC
MSTRG.44182.2	--	0.000	0.338	0.001	4.225	up	GCGGCCCAGGCATCCCAGGCCCGCCTGCCCGAGGCTCTCTGTTCCCTATCCCGCCGCCACCTCCGGGCGACCTGGCGGGAGCAGCCGTAATTGGATCTTCGTCGCCTCCTCCCCTCCCTGCCCCTCATCGGGGAGCCGGTTAAAGGCTGGGTACCTCGGCACCACCGGCGGATCCCAACCTGTGATTTGGCTGCCGGGCTTCCCCCGGGCGGCTCGGCGACACCCTGCAGGCTGGGCCCTCCCCCACCGCTGGCCCTGCGACATAAAGAAGCTGGACCTAGAGTCCCATGGATGGTGGAGGCAGCCTAGCATGTCAGCAACACTTCCAAGCCCTAGACTGAGACATCAGGACAGGAGGACGGTGACAACCACCTAAGGGGCTCAAGCCAGGTGAGATCTTTCATCTCCTCTGCCCTTTATTTTTGTCCCAGCCTCATCATCCATCTGGTACCCTTCTTATCGTGCTTGGCGTTTTGTCTGCCTGTACCCTTACATTCGGTCAAGAATGCAAGCTCCGTCGCTTACCTAGGAAGCGCAAGGCCCTGGGTTCGGTCCCCAGC
MSTRG.44231.1	ENSRNOG00000011636;ENSRNOG00000059442	0.818	0.000	0.037	-3.684	down	TAAAAATGTAGCATTCCATTTGTATTTATTTCCCCCTTGCCAAAAAGATTTTTCTAATAATGCTTGTACTGGCCAGGGATGAGCTCCACAACACTATTCAGTTTTCTTGTACTGAGGATTCCCCCACCTCCCTCACATTATTGACTCCTGACTCTTTGCCCAGCATATGATAGTTCTGCTGGTATTTTACTGTTTACTGATGAACATATCTTCATTAAGAATTTTTTGAAAACTCATCAAATTCAATGAATAAGTTTTC
MSTR	--	0.149	0.458	0.007	2.346	up	CTCTTTGGCCCTCATGCAGATGAACTATCCACTTGAGGGACAGAGCAAATGTCCAGGCATTTCTAAGATTCTGCCTCAGCCTACAGTTTCCTCAGCTGCAACCTGGTGTTTTGATGCCTGTCTCTAAATATTTTTTGTTT

G.442<br>59.3							TTGTTTGTTTTGGTTGGGTTCAGTGTTTTTGTTTTATTTTGTTTTGTTTTGCTTTGTTTTTTACCTGAGAAC<br>TGCTCATCTGATCGGAAAGGATTACTGGCAGTTTTAAGTTTGATTTAGTTTTTGTCTTGATGGGCATTAAT<br>GGCCCTCCCTCCTTACTCAGCCCACCTAGGTCTCCCTCCTTACTCAGCCCACCTAGGTCTCAGACAATG<br>AATAATTTCTAGATAATGATTTCCCCCGTGCAATTACAGATGTTTCCTCTTTTCTTGCTAACTCGTGTATCA<br>TGTTTTGTCATAGTAATGAGATTTTCTAGGAAAAGAAATAAACTACCCCATTTCACTAATTCACTCCCTAA<br>GGGTTTTAGTTCTGGTCAGGCTTATTTCTGATTTGCTTGGCTTTTTATCTTGGCAAGTGGGTCTCCAGAA<br>CCTAGTCCTCTACTTGTTACCACAGAGTGTATAACAATTGTACAAGTTAGAGACCCAGAGGCTGTTGTA<br>GGGACTTCCACTTCCATGAAGTTGCTGTGTTGCAAGTTCCACCAACCCTTAGAGTCACAGAGAGCTGA<br>CAGGAAGTGTGGAAGAACCAGGAGAAGTGAGAAGGAGTTTCTCACTTCAAACCTGGAGCCTACCCTT<br>GCTGAATGAAGCAGTGGTTGGAACCACCCCTGAGGAGACGGTTCACCACAAACACAACCATATCTAAA<br>AATCTGAACCCCGTGAGTTAGTTTTTCTGATAAGGAAGTGGTCTTTATTGGTTGGTCAGTTTAGACTTGA<br>AGGAACAATGTCTTTGTGCTTTTAAGTGGCCATGTCCAGGAGAGTCCTTGCAGCAACGGTCCTGAAAA<br>AGTGGTGTTTCTGCTGTTGCTATTTCACAATCTTGTCCCAAACCGGCCAATAACCCGAAGACTCAAGCA<br>ATCCCCCTGCCTCTGCAGCAGGGACTGAGGCATGTGTCAGAACCGGCTAAAGAGTTGTTTTGTGCCTAA<br>TGCTCTGTGTAAGTGGAAAGTCTGAGCTATAAAGCAAGATCTTAAATCACACGCGATTCCTTGGCATCT<br>GTAGATCCCGGCCAAGCCCCTTGGTTGAAAATGCCTTTTACAGGGGTCACTAGAAGGTCTTGAATCCCA<br>GGGAAGGATGACTTGCCCACAAAGCATTGTTATCTCGTTGGGACTAGAACGTCTTCATAGTTGACAAAA<br>TAATTGGGGTCTATTTTTCCCTCACCACTTGAACTGATCCATCACCTGATCCCCCAGCTTCCGGAGAGGA<br>TAGAACAGAAACTATTTCAGAAATGAAAAAATAAGGCAGTGTATATTTTTGAGAACAATTTGCAGAGTG<br>GAGAAAGCTCCTGGCAGCCGCCTCCAAATCTCTCACGTCAGAGGATATGAGTAGGCTGGTGGTGGGGA<br>GATAGCTTCTCCCACTGCAATTTGCTGGTTTAAATCCTGATGGCTGAGAGGTGCCTGAGTGGGGAGCTT<br>GGTGAGTCCTTGCTGTCCTAAGAAAGCCAGCTGTGCCCCAAAAATGGCAATGGTGCCACCGAGGAAAT<br>ATTGTTTGAGCAGTAGAGCATTTGCAAGCTTGACGTGCGATCGGGTTTGATATTCGTTGCTGCCTTCTAT<br>GTCCACAAAGTTACTATGTCCATGTGTTCCCAAATGCTCTGTGTATATATTTTAAGCCAGTCCTTTAGGTT<br>TCTGTAAGCATGTATGATTTTTTTTTTAATTCCCATTTCCTTATTTCTCTCTCTCTCTCTCTCTCTCTCTC<br>TCTCTCTCTCTCTCTCTCTCTCTGTGTGTGTGTGTGTGTTTACACGTGTGCCGTGGAATGTGTGTGGAGG<br>TCAAAGGACAGCTTACAGGAGTCAGTTCTCTTCTTCATCCCCGTGAGTCCTGGGGATTGGGCCCTGGTC<br>ATCAGGCTTGATAAGTGAATGCTTCTCTTGCTGAGGTATCCTGTTGACCCATAAGTACAGCCAGTTTTAA<br>AGATTGGGATCATGACACACATTTATTTAGTTGGGCCCTTTTCACTTACTATTTAAGAGTACATCAGTGTG<br>TCTCACGATATTCTTTAATAACGTGGTTTTTAAATAGCTGCATCGTCAAATGAAGGCAACAGATTCATTCA<br>TCCCATCCTTTCTTAGGGATTTATGTAAGCACAGACTTCCTCTGTTATAAATAATACTGATGGGCATCCAA<br>GAATACGCCTGTGTAATCCCTTTACTCAGCAGGATCAAGTCCAAGATATGGAATTCCTGGCTCACTGGCT<br>GTATCCCCTGAGCAGCCATATGTTCCTCCTGTCCGTGGGAGGTGGGAGCCATGCTAGAGATGCTGAGTC<br>TTCTATAGAACCCGGCGGGAGGGACTCATTCTCTGCTGCCTGTCCTGTCCCTTTCTCTCCTCAAGCACCA<br>CCTTCCGGTTGCCGAGTCTGTGAGCCTCGTCCCTCCTCTTCCGAGGGCCCTCTTGGCTCGACTGTGAAC<br>TGGTGCAGATACATTGTTGCTTTGGTCCTCTTGGCCTCTGTGGAAAGCAGCATCTGGACCTGAGGCTGG<br>GGGAGGAGCAGGTTAGAACTCCTTGCCAGTTTGTAGAGATTGAGATAAACTCCGGCAAGGACTACTTG<br>GGGCAGTGTATCCTGCCTCATGCATTTGAGTTCCTTCTGCAGTGACAATACGTCTTCTGTCATCCCCGTG<br>AGGCATCTTGTTCTTTCTCTCCCCTGTGCCTTCCAGTTACTTGTGGTGCTGCCTAGACACTCAGCTTGTG<br>GGCCGGTATAAAGAGGGGAGAAAGTTCTCCTGTCTCCATGGAGCAAAGCCGTGTTCACTGTTAGAATA<br>CATCCAACCAGACACAGAGCAGCAGAAAGACTATTTATCTCTTCTCAGGAGTCCAGATAATTAAAAAA

							GATAACATTTCTGCAGGTTTGTTTCTTGTTGCCCATTCTGGAACTTGCTACCTAGGGAGATTAGCCTTTCCCCAGCCAGCAGGAGCATTGATGAATTATGTGGCAGGCTTTAATAACGCTCTTATCACCCTGGAGACCAAGCGGGCGGTGAGGGCCGGCCTTCGTTAGCACGTGCTCATCCGTAATCCGTGGACATTCTCATTTGGGATGCACTGTTGTTCATGCTGCCTGTAGGCTCCTTCGGGCATTAGGTTGCAGGGACCCCACTTCCAGAAGGTTGTTCTGAACCTGCCGCCTGACGTCAAGTTTGACCAGTGTTAGCAGAAGCACATTGCTTCTTTCTTTTGGCCTAGTCAGTTATTGTGATTATTAGGCCAAGGCACTGCACAGAGCTTTTCCAGGACAAAGAAACCCCAGGTCTTTCGAGACCTTCTCAGATGCTAATGACTTCAGTAAGTGCAGGTCTACATAGCTGTCTTTCGGGGCTCCTGGTGAGGTGAGGTGATCTCAGTGAAGGCCAGGTTAAGTGAGAGTTCTTGCGGTCTGTAATGTCTGTTTAGATTTATGATCAGAATTGGGTGCAGAATGTGGCTCCATGCCTCAGTTTCTTCCGTGCCTGTAAAACTGAGATCATAGCAGCCTCCTTCCTTAAATCCGTGAGGATGGAAATGAGTAAGTTCACCGAAGAATTTAGCCTCTCTCTGTAGTAAGTACAGAACTATAAGTACAGAACTACCAGCACAGTGTTTTGAATAACACCTTTAATCCCAGCACTCCTGAGGCAGAGGCAGGTGGATCTCTGTGAGTTCCAGGCCATCTAAGGCTGGCTTGAAACCTGCCTTGAAAAAACAAAACAAAACAAGACTGCCTCCCCCGCACCCCATAGACTCATATATTTGAATACTTAGTCATTAGTCACCTGGATGTGGAAAGTCTTTGAAAGGATTAAAGGATTAAGAGGTGTGGCCTGGATAGGGGAAGTGTCACTAGGGGTGGGATTTGAGGTTTTAAAAACCTATGCCAATCTCTGTCTCTCTCTGCC
MSTRG.44322.1	ENSRNOG00000049723	0.693	0.000	0.000	-4.955	down	CTGAGTTCTCACAGTCTTTCGTTGCCAGTGACCATGGAAATATGGGAAGAGCTAGGATGGGAGAGAGGATGGGCCAACAAGCTACCACATCCATGGATTCTTCTCACTTCTGTGCGCTTCGAAGTTGAGTCACAATAGGCAGGCCATCAGCTTATTGCCAACCACAACGGGAGTGCTCCCCTGGAAAAGGATGTCAGCTTACAGTACATGGGGAAACTAAGAACACTATCTCTGAAGACCCGGACGCCTCTGTGAATCACAGTGCTGAATGAGAGCTGGAAACGTCTTGAAGGGAAACCTAGACTCGCCCTTGCAAGGAAATGGCTTATTCCTGAGTAAAACGCCGCCGTCTCTTCTTCCCAGTGACCCTGAGATAAAGCCATTAAGCAGGGAATATAGTTTATAAGCAAACATTTCAATAGCATCAGCATGTTGTCTTTGACCGGGAACACATCTATAAATAAATCTGTGGTCAAAAGAACACTACTGATTAACATCTGATTAGCATATCTGAACGCTGTCTTGGAGACTGCTTTGTTTGTTTATTAGGAGTGACCTGCTTATTTCCAGAGTGCCCAGGAATGAGCACAATGGGAGTGATCACGGAGCACTGGATTTGGGCCAGTATTCATGATGAGCCTGACACACAACCGACACAAACCCCCACCCCACGGTTGTGGCCAAACATTCATAATTTCACAAACGTGGTTTAGACACCTCCCAAATAGATGCCTGAAAATACAGCCAACAGTTGGAGCTTCGGGGCCCAGGGACAAAAACCTTCCCCTCGTTTAAAGACCCCCTGAAACGTGGCTTCCTCCA
MSTRG.44371.1	ENSRNOG00000016064;ENSRNOG00000016346	0.100	0.413	0.048	2.355	up	AAGAGCAGACCGTGCTCTTTTTTTTTGTTCTTTTTTTCGGAGCTGGGGACCGAACCCAGGGCCTTGCGCTTCCTAGGTAAGCGCTCTACCACTGAGCTAAATCCCCAGCCCCCAGACCGTGCTCTTAACCACTGAGCCACCTCTCCAGCCCCCTGGGGTGAGACTCTTATGTCTTTTCTTGCATGTCCCACTCTCCTAGACTAGAACCCGGGCTCTGCTGTCGCTCGAGTGATCTACCATGGGGCCATAACCTAAAACCCAGATCTTAATACGTGAGACGTCACTAATGGAGGCTCCTCGGACTCTCAGGGCCACGACCCCTACTGCACCAGAGAAGCCTCTAGCACAGGACCTGCCTCTAGAAACTTTTCAAGAAACACTGTAACAACCTGTGCCTCCAGGCTTTGGATGCTACTAACCCGGCATCTGGAGGCCTCTAGGCTGCCCCGGGGTGCTGCTCTTGGAACAGAGCAGACAGGGGCCACACCACTGGGTCTCTCAAAGCTGCCACTTGCAGTAGGAGTTTGGTGACAGTGACGGCTGCTACCTTTCATATCACTTTCTTTTAAATTGTGCCAAGAATTGGGACATTTGACATTCAAATTCTCTTTCTCATAATTTAAAGCCGCATGGG
MSTRG.445	--	0.990	0.004	0.000	-6.095	down	AGCCTTCAATACTCACTTGAGATCTAGCTAAGGAGGATAAGAGGAAAGGAAGACTTCCGGAGGGAGCAGCCAGGTAATGCAGACAACAGGTTGCACCGTGCAGAAATCGGATCCAGCAACTAGAGAGCGAACACT

48.1							CCTACCTCCAGCGGAAGGAACACTTACAATGTCCATCCAGTGGAATTTCAAGTGCAACTAATAAATCAA<br>CATCTGTATTTTTCAGTTGGACTGTTTTGGCTACCCTAGCCCCACCTGACTATTCTGAGAAGGGAGGGGA<br>GATAAATGTTTCTGGGCCCAAAGGATCAATGTCTGAATCTGATACAGACATTAAGGAAGATGGGACAGA<br>GAACCATAGTATGTCAGACAACCATAGGCTATGGCAGGCCTGGTTCTTACCAACACTCATAGGCTTGCC<br>CTCCTCACATTTTCCTTCTCTTCTATAACTAGGCAGGTCTTGATGGCTAGCTCTGCTGATGCACTACGTAG<br>GGGTGATGTGTGTCACCTCCAGGCCAGCACATGAGCCAGTGTCTTACCATAAAGCTCTCTTCTGCTGAC<br>TCAGTGAGAACTGGTTAGAGGGAAGTCTCTCTCAGCACAGAGTTTTAAATGCCTATAGGGACATCTAAC<br>TGCAGGGCCTGCAGGGAACACGTTTAGATGGTTTCTTAAACCAAGTGAGTAATCACTGAATGACACAG<br>TTAGGGTGGACCTGGAATGGAAGGTGGAGGTGCTATGAGGACTAAATGAAAATTGACACCTACAGTTAT<br>ATCAAAATTTTGGTAGTTGCTTCAGAATTTAAAGACAGCAAGAAAATATCCGACAAGAATAGCCAGAAT<br>CTAGTATCCTAAAACAATGGGAAGAGTGATCCAAGGAGTGGCTTCAGTAGGCTCTGGGATGTCTGTTCA<br>GACCCTGGGCTCAGAGTAAATGCTCTTCCACTGCCCAGGAGGGATGGCCGCTGGTGGCCTTCAGGAAA<br>CTCCTCTAGGAAAGACCTGTTTTCAGGTCAGAGGTTAAATAGGAGCAACCAGCTGAGGCTTTTGAAATA<br>AATGCAGCTAACGTAATTTCTCCCTTTGTCCACTTTTACTTTGTAATAGATACTGATCTTAAAGGAACTCT<br>CTAATAAGCTTCTTACACGTACAGCTCCACCCTAGTTCCGCACACTGTCCAGCCTAGGATGCAGTCAGC<br>GCACACTGTCCAGCCTGGGATGCAGTCATCAGTGCACACTGTCCAGCCTGGGATGCAGTCATCAGTGC<br>ACACTGTCCAGCCTAGGATGCAGTCATCACCACACACTGTCCAGCCTAGGATGCAGTCATCAGTGCACA<br>CTGTCCAGCCTAGGATGCAGTCATCAGTGCACACTGTCCAGCCTAGGATGCAGTCATCAGTGCACACTG<br>TCCAGCCTGGGATGCAGTCATCAGTGCACACTGTCCAGCCTGGGATGCAGTCATCACACCAACAGACC<br>TCTCGTAGATCTACTGCTAGCTCAACTTTATTAGATTAAGAACATTTCTTCAGCCATACTTTATTTTTCCTA<br>ACATCTTCTAAAATGAGTAAAAGGAGTTAAGTAACAAGAAAGGAAGGACTGCACCTTAGCTTGCACTG<br>ACACTAGTAATATCTTGATACCAAAGTATGATTAAAACTATCTTCACAAGAGAACTACAAACTAAATCAT<br>TAATGAATCCAGGGGTAAAAAAAAAAAATGCAACAGAAATACTAGCAAATCAGTTCCAATAGTATACTGT<br>AAAGATTATATATACCATGTCCAAATGGGATCTATCCCAGAAATAAAAGAGGACTCAAAGACAAAACAA<br>AAAACCAACCTACCAAAAAAGAATCAATCTTCTATAGAAGGAGGTCTGGCCTTTGTCCTAAACTACCAG<br>TGATGGCGACACCTAGAATATGCTGCTTGAAAGAACTGTCCTGTAGTGTGGAGGTTTTGACCATGACAG<br>TCTAACAACATGACCAGTTCCAACCTTTGGAGGAAATGGAGATTTAATGTACTAGCATGAATCAGAGAA<br>TGATCCCTGGCAACTAAGGAGAGTGGTATGTCATCACTCCTGGATGAGTCTGGGAGAGCTTCCCTTGCT<br>AGTGACACTGTATTGTTATACTCCATAGCCAACCAAACAAGTAAAACCTCTATGTTGGAGTCAGACTTCC<br>CCACAGGCCTCTTTGGCTGGTCAGGACCATGGTAGAACTGGTGACTTGTAAGCAGATGGTCTAAACTCA<br>GGGTAGTTCTGGGGCCCTTGAGCCTGTGTGTGGCTTGCTTTAGGTATTTGCTCCAACAGTGGGAAACAA<br>ACTAATCAATGGAGAGAGACAGAAAGCTTTCCCTTATTCTGACCAGCAAGACAAAGACACCTATTCCCA<br>CTACTGTTCTCTAACACCTTACTATGAGTTCTCCCTAGGGCAGCCGGGGCGTGAGGAAGCGCCCAAATG<br>GAATACAAGTGAAGCTATGGTGCAGATGGCATGATCTTAAAAAAAAAAAAAAAAAAAATCACCGTTTATG<br>AACACATAAGTGCAGGCCAGAAACAAAGAACATAGTGACAGTTTAGGACAAAGGAAAGACCATGTGA<br>AGCTACAAGAGAGTGCGTATGGATCTCACTAAACCCATAAAGTTTTGGGCACTGAGAACTGTGAGAAA<br>AAAAAACTCCTGCTGTTTCAGCTCCCCAACGTAAAGTACTACAGACTAACACAGCTTAGGGAATGGAA<br>AGATCTTATGCTTGCAAAGCCCTCAAGACAAACAAGTTAAGAGTCAGGTAGGAAGTGATGAAACCAC
MSTRG.445	--	0.361	0.000	0.000	-5.666	down	AGCCTTCAATACTCACTTGAGATCTAGCTAAGGAGGATAAGAGGAAAGGAAGACTTCCGGAGGGAGCA<br>GCCAGGTAATGCAGACAACAGGTTGCACCGTGCAGAAATCGGATCCAGCAACTAGAGAGCGAACACT<br>CCTACCTCCAGCGGAAGGAACACTTACAATGTCCATCCAGTGGAATTTCAAGTGCAACTAATAAATCAA

48.3							CATCTGTATTTTTCAGTTGGACTGTTTTGGCTACCCTAGCCCCACCTGACTATTCTGAGAAGGGAGGGGAGATAAATGTTTCTGGGCCCAAAGGATCAATGTCTGAATCTGATACAGACATTAAGGAAGATGGGACAGAGAACCATAGTATGTCAGACAACCATAGGCTATGGCAGGCCTGGTTCTTACCAACACTCATAGGCTTGCCCTCCTCACATTTTCCTTCTCTTCTATAACTAGGCAGGTCTTGATGGCTAGCTCTGCTGATGCACTACGTAGGGGTGATGTGTGTCACCTCCAGGCCAGCACATGAGCCAGTGTCTTACCATAAAGCTCTCTTCTGCTGACTCAGTGAGAACTGGTTAGAGGGAAGTCTCTCTCAGCACAGAGTTTTAAATGCCTATAGGGACATCTAACTGCAGGGCCTGCAGGGAACACGTTTAGATGGTTTCTTAAACCAAGTGAGTAATCACTGAATGACACAGTTAGGGTGGACCTGGAATGGAAGGTGGAGGTGCTATGAGGACTAAATGAAAATTGACACCTACAGTTATATCAAAATTTTGGTAGTTGCTTCAGAATTTAAAGACAGCAAGAAAATATCCGACAAGAATAGCCAGAATCTAGTATCCTAAAACAATGGGAAGAGTGATCCAAGGAGTGGCTTCAGTAGGCTCTGGGATGTCTGTTCAGACCCTGGGCTCAGAGTAAATGCTCTTCCACTGCCCAGGAGGGATGGCCGCTGGTGGCCTTCAGGAAACTCCTCTAGGAAAGACCTGTTTTCAGGTCAGAGGTTAAATAGGAGCAACCAGCTGAGGCTTTTGAAATAAATGCAGCTAACGTAATTTCTCCCTTTGTCCACTTTTACTTTGTAATAGATACTGATCTTAAAGGAACTCTCTAATAAGCTTCTTACACGTACAGCTCCACCCTAGTTCCGCACACTGTCCAGCCTAGGATGCAGTCAGCGCACACTGTCCAGCCTGGGATGCAGTCATCAGTGCACACTGTCCAGCCTAGGATGCAGTCATCAGTGCACACTGTCCAGCCTGGGATGCAGTCATCAGTGCACACTGTCCAGCCTGGGATGCAGTCATCACACCAACAGACCTCTCGTAGATCTACTGCTAGCTCAACTTTATTAGATTAAGAACATTTCTTCAGCCATACTTTATTTTTCCTAACATCTTCTAAAATGAGTAAAAGGAGTTAAGTAACAAGAAAGGAAGGACTGCACCTTAGCTTGCACTGACACTAGTAATATCTTGATACCAAAGTATGATTAAAACTATCTTCACAAGAGAACTACAAACTAAATCATTAATGAATCCAGGGGTAAAAAAAAAAAATGCAACAGAAATACTAGCAAATCAGTTCCAATAGTATACTGTAAAGATTATATATACCATGTCCAAATGGGATCTATCCCAGAAATAAAAGAGGACTCAAAGACAAAACAAAAAAACCAACCTACCAAAAAAGAATCAATCTTCTATAGAAGGAGGTCTGGCCTTTGTCCTAAACTACCAGTGATGGCGACACCTAGAATATGCTGCTTGAAAGAACTGTCCTGTAGTGTGGAGGTTTTGACCATGACAGTCTAACAACATGACCAGTTCCAACCTTTGGAGGAAATGGAGATTTAATGTACTAGCATGAATCAGAGAATGATCCCTGGCAACTAAGGAGAGTGGTATGTCATCACTCCTGGATGAGTCTGGGAGAGCTTCCCTTGCTAGTGACACTGTATTGTTATACTCCATAGCCAACCAAACAAGTAAAACCTCTATGTTGGAGTCAGACTTCCCCACAGGCCTCTTTGGCTGGTCAGGACCATGGTAGAACTGGTGACTTGTAAGCAGATGGTCTAAACTCAGGGTAGTTCTGGGGCCCTTGAGCCTGTGTGTGGCTTGCTTTAGGTATTTGCTCCAACAGTGGGAAACAAACTAATCAATGGAGAGAGACAGAAAGCTTTCCCTTATTCTGACCAGCAAGACAAAGACACCTATTCCCACTACTGTTCTCTAACACCTTACTATGAGTTCTCCCTAGGGCAGCCGGGGCGTGAGGAAGCGCCCAAATGGAATACAAGTGAAGCTATGGTGCAGATGGCATGATCTTAAAAAAAAAAAAAAAAAAATCACCGTTTATGAACACATAAGTGCAGGCCAGAAACAAAGAACATAGTGACAGTTTAGGACAAAGGAAAGACCATGTGAAGCTACAAGAGAGTGCGTATGGATCTCACTAAACCCATAAAGTTTTGGGCACTGAGAACTGTGAGAAAAAAAAAACTCCTGCTGTTTCAGCTCCCCAACGTAAAGTACTACAGACTAACACAGCTTAGGGAATGGAAAGATCTTATGCTTGCAAAGCCCTCAAGACAAACAAGTTAAGAGTCAGGTAGGAAGTGATGAAACCAC
MSTRG.44640.1	--	0.377	0.000	0.003	-4.375	down	TGCCTGGGGCTAGCAGATGACCAAACTCTGGTCTTGATGATCTCCGGGACAGTGGGTTGCCAGAGACTGAGGCTTTGGTGACTGCCTGGGTCCTGTGGGTTCATGGACCCACTCCCACTGCTGTTCACCACTCAGTCCTGGTTCCTCGTTCTCACCTCTCAGATCTCTTAAGTCAGGCTCACCAGCCACGATGTTAACATACTGTCTTTAAAAGGTAATGAGAGGGCTAAGTATGTGGCTAGGTGTAGAATAGTTGGCTGGCATGTGCAAAGATGCTATCCCTAAGACAACAGTAGAATAGAAACAAAAACAAAGTAAAGGCGGCGAGAAGGCCCCACTCTGG

							AGCAGCCGAGCTTGGAGGAAAAGCATCCCCTGGAGCAGCTGAGCTTTGAGGGGAAGCCTCCTGATCCTGGAGTAGCTGAGCTTGGAAGGATAACGTTCTGGTATTCTCATCCCAGGCCTGTGATTACCACATAGATGCCTTGGGGCTTGCTAAACACGTTCTTCCTCCAGCAGGACTTGCTGTGTTCAGTTCTTGGCCTTGGAGGGAGTGTGGCTCCTTTCAGTCATCTTAACTTGAGAGAAGGTTGGGTTCCTTCTCTTTCCAGTGAGGCTTTGGCACTGGCTGTTGAATGGCTTTAGTAAGTTTACCTTTGATGTTATCTTCACTCAAACCTGTCAGCTGGTTCAAGAGGTGTGGAAGTGGGTGTGGGCCTTTTTATCTCACTCCACAGCCTGGGCAACTCAAGTAATGAAGTCAGAAGCTGCTGAGAGGGACTAAGATCAGGGCCTTGCCCTGGGAATCCTGCTCACCAACAGCTTCTGGAGTGGTCTTCTACTCACACCGTCCCATCTGTCACGCTTCCAGGTGTACTGAGCCCCAGTACTGTGACTTGGTGTTCCTGCTTCCATGGGGGCTTTCTTCTTGGTCAGGAAAGATGGAGATATGAACAGCTCCTGTTAT
MSTRG.44824.1	--	0.277	0.004	0.040	-3.062	down	GGCAAAATAGAAAAGGGAAATGGATTTATTTACAGTTTGTCCCGACCCCTGGATGAGAAAGCTTTCAGTTTCTTCTCTCACTTTTATTATAGCAGTGGCATAATATTATAGGGAAGACAAAACAAAAGCCAAAGAACATTATTATATTCATTTTACCTGTAGGAACTCTTGGATGTTTCTCCTGGCAATAAAGTTCTTCTCTGCTTGGCACCAGGGTTTTAGATTTTTCTTTTCCCTGACATGAGATTTCTAGGAAAGTACTAATTTTAGGGATCCACAATTTCAGTAATATGAAGGCCAAAGGAAAGCCCAAGTGTTTGTAGTTAGACTGTCCCTCTTTTGCTATATTAGTTTTAAGAGGATGGCTGAAAAACTACTCTGCATGAACTTTGGCTGGACCGGGAAGACAGGTCCTTAGAAGACTTTCCTACCTTCCAAGCAGTATAGTACAATTCACATCATATGAAACGATAACCAGAATGGGCCTATCAATTTCTGGGCCAAATAGCCAAGAGGTTATGGTACCTCATTATACTACAGACACTTCTAGAAATCTCATCTATAATCATCCTACAAATTGTAAGTAATCCATGCAAGTTGTATCCCCTTAGATGCTGGGAGAAGGCCAAAGCATCATGGGAGGGGGCTACACTTCTGGTTTCCATATAAAGATTCACTGGTCCTTGCCTCTAGCCCAGAAGCGCAACAGTTTACAGCGATGCTGAAAGCAATGTCTGATTTGATCAACCACGAGTTTAGGTTCTCTCAGTTCCTCATTTAGGAAGTCATCACCTAAACTGTGCTGTACTGCTGCTCTTCTATTGCAGCCCCTCTTTTTATACTCAGAACTGTGAACAGGAGTATTGAAACCATTCCACTATGTTTCACTTAGTCCATTACTGCTGGAGTAGGGATTCATATTGCACAGGCTTTTTGGTATTTCCTGGTGAGTGGAAGTTCACACTTATCTGTTTTATGAGAGCAGAAACTTCCTATTTTCTGGTCTTCCAAGTCTCCAATTAAGTCTTACACTTGGATCCGATAAGATGGAGTCAGTTTTCGGCAGAAATTGATCACAACGGACTCCTTGGTAGCCTTC
MSTRG.44976.1	ENSRNOG00000051423	0.651	0.067	0.035	-2.372	down	GGGACATTGGAGTGGGCAGGGTCTTCCATAACGCAGAAAACAAAATTCAACAAAATTGTCTTCAACTCTCTTATATTTAGAAGCCCCATTAGCTATATTCTACTTTTACAACATATATTAAATATAATTTAATGAACATACTTATACTCAACATACAGAGATGGCATGCCATGCCTTACTTGCACTTACATGATTCTAGGAATAATCTGCTAGATTTCCAGCCTTTAACTCTTTTCTCTGATCAGTTCCTTCTGTGTTTACTAACAGAGGCATGTCTCTTTAAAAAAAATTAGGGATCTGAGAGGGAGAGATGACGGGGAGGGAAAGTGGGGGTAGTTTATACATGGGAGGAGATGGGGGAGAGTTACAGAGGGCCAGGAATTTGAAAGTAGATTTGTAGCAGTGGGGGAGGAGTTATTGGAGGTAGCCACTAGAAAGTCCCAGATGCCAGGGACCCCAAGAGGTTCCCAGGAACCAACAGGGAAGGCATTAGTCAAAATGGCCAACAAAGGGGAGAGAGAACCTGTAGAGACCATATCCAGTGGATAGGCACTGCCCCCGTTTGAGGGATGGGACACCCACCCACCTCAAAAATATTCAGAATTCCTCCTGTCAAAAGGAAATTCAGGGACAAAAAGTGGAGCAGAGACTGAAGAAAAGGCCATCTAGAGACTGCCCCACTTAGGATCCATCCCATCGGCAGACAACAAACGCAGACACAATTACTGATGCCAAGAAGTGCTTCCTGACAGGAGCCTGGGATTGTTGTCAGAGGCTATGCCAGAGCCTCACCAATAAAAATTTGAATGTACACAGGCCACACAAGGACTGAGTGAGGGGACCCCAGTGGAGAAGTAAGGGAAGTAATGTAGGAGCTGAAGGGGTTTACAACCTCATAGGAAAAACAACAATACCAACCAACCAGAACCCCCAGAGTTCCCAGGAGCTAAACACCAACCAAAGGGTACACATGGAGGAAGCCAGGGATCCAGCTGGATATTTAGCAGAGGAATGCCTTACCTGTCACCACAGGGAGGGGAGTTCCTTGGTCCTGTGGAGGCTTGATGACCCAGGGAACGCTAGGGAGCTAAGGCA

							GGTGTGGGTGGGCGGTCGGGGAGCACCCTCACAGACTCACAGGAAGGGGGGAGGGAATAAGGAGCTTGTGGAAGGGAAACTGGGAAGGGGGATAACACTCCAAATCTAATGTAACTGAATAAAATAACTAATAAAAATGAAAAAAAATATTTGTGAGATTATGATACAATCGCATCATTCTATTTCATTTGGTAAAAC
MSTRG.45046.6	ENSRNOG00000011912;ENSRNOG00000042037;ENSRNOG00000048622	0.613	0.000	0.005	-4.261	down	GGAGGGAGACATAGCAGGGTCACAAATTCAACATTGTCGTCAGCTACACAATGAGTTCAAGTCCAGCCTGGGCTAAATGAGACCTCATCTCTCGAACGCCAAGCCGTGCTTCTGGCAGAAGCTCTGAACCTCTGGGTAGGCTGTGCACTGCAGGGCTTCTCGCTCTGTGTCCATATCCTCAGCTCCTACAGCTCTCCGCGTTCAATGCCTTCTCCCTGGGGAGTGTGGGTCCCATAGGTGCATACGTCCTGGGAACCTGGGTCCGCATGCCCAGCGCACAGTAGGACTCACCGACAAATCTGAGCTCAGTGGGTGCTTGGGGAGCTGCCTAGTTTTGGCTCCTGTATATTACTGCAAACACCTCAGCAAGCGTGGGGTTTCTTCCACACATTCAGGGCCCTTTGCAGCACCAGCTCCCGGCTCTCTTCGGGACGCCAGCTTCCCTCTCACTGCCATGGAAGGCATCATTCGGAAGTCTCTCAGAGATGCGGGTGAAAGAAGAGAGTTCCCTGGGCTCAGAGCCACAGTGGAAACCAGGCTCTGCCTCTCCTGGAAACTTTTG
MSTRG.45092.1	ENSRNOG00000014454;ENSRNOG00000014600;ENSRNOG00000014621;ENSRNOG00000023614;ENSRNOG00000039528	1.013	0.000	0.000	-5.573	down	AAAAATTTATTTGACCAAAATGTAGAAAAAGTGATACCATTACGTATGATACAATCGCAAGAATCTAAAGAACAGAGTAGATTTTATCAATTGCACAGTTTGCTAAAAGTTCATCTCCCTGGTAGTGTGATGCTTAATAAATAGGAGTGAGGGGCAGGGTTCAGATAAGCTAGAAGCAGGTGACTCTCCGTCAGCACTGTAAACTCGAGGTGGCCCCACACTGCTGGGGAATGTGGAATGTTTCAGTGAGATGTTAACTGAAAAAGCAAAACTACAAAGCCAAAAAATATGTGCAGCCTCTAGAGCGCTCCACGTCCAGTTCAGTCAGGAGTTCTCGGTCTTTATTAGTGTCATTCCCAAAGGAATTCAAGTCTCAGCAAACTCAAGCTCCCATTTCTTGTTCCCTGAACAATGGAGATGAAGTTAAGCCAATTTCTTTTTCTATCACTACTTTGCGGCTGGAGAGACCCTTTGTTGCCTAACCCTTGGCATCAATGTTCTGATGGCTGGCAACCTTTATATTATTTGAGACAGAGTCTCGCTGTGTAGCGCTGGTACCTGGCTTTGCTAGCAGTCTTGATCCAGACAGGACTGATCTGAAAAGGGTTGGACTCTGCCATATTCCCTGCTGAGCGTATGGTTAGACCACAGCAGAGAAGTCCTGGAATAAGACACTTGCTCCTCAAAGAACAATTCTGGAGTGAAGGGAATGTGTACACTTGAGCCTGTGTTTCAAGGTGTGGGGCTGACTAATTCCCGAGAACCAAATGCCCTCAGCCTTAAGCAGCTATACGCAGTGTGAAATTCCCTGCCCATTTTAGGAATAAATGCAATTTAAGAATAAGAATAGCCAAAGAAGCTGGTGCCATTTGAA
MSTRG.45379.1	ENSRNOG00000011826	0.000	0.399	0.031	3.453	up	CCCCGTCTATCATATTCATCCACATGATGTCTCCTGGAGTGGATCATATTCTCTGTTGTCACCAGGTCCCCAACACTTGCCGCTCGTTGTAATCGACCCACCGGGTCATACCCGATCCGATAGCGGTGAAATTGCCTTGATCTCTGATGACACAGGCAACCATACTGTGGCTTCCAAGCTCGCACGCACCCTTCACAGCTCCTGGAACGTGCAAAGAACCACTGGCACTTACTTCCCCAGCTTCAGGACGG
MSTRG.45390.1	--	0.466	3.745	0.000	3.580	up	CAACAGCACAGCTCAGGCCAAGTCAGCATAAGAAGAGTTTCAAGGTGAGGAAGCCAGCGCAGAAACAGGGATGTATTGGGGATGCTACAGAAGTGTGCCAGCTGCCTGCGTGTCTGGTGAGACATGCTGATCCTGGGAGCATGAAGATTCAGCTCTCCTGGTTCGGGACCGTGGAGTCATTCCTGTGCCCTTGAAAGAAGATGGAGACTTTTTAGGATTTACCCTCACCGAGCACAGGAAGATAACATTCAAGGGATGCCTCAGCTTGAGCAGATACCAGTTATCTCCTGAGTCAACACCCGGTGTCTCCACCGCATGACCTCATCGTCTACCGCCTGACCTCTTTGCCTCCTGCTATCACTGACTATATCCTCATCCCTCCATCCTTTGTGTTTCACAAAGGACACTCCCCCT

							ACCTGAAAGCCTTAAAAGCTGGAACGTTCACCCCAATAAATGAG
MSTRG.45508.2	--	0.695	0.074	0.029	-2.321	down	TGGGCTAACTCTTGTTTCAGAGAGGCCTACCTTTCTTCAAGGTGTTATATCAACTTGGGAAGAGAATTATTAGAATATAGACTATTGTAACTTGGTAGCATCTTTGTTACACCTAACAAGGTTAAGATTCAGTCAGTAACTAGAGGTAGCTTTCCCATCAGAGTCGAAGGTAGGGCCAGGGTGCTGAATTAAAGCAGTGAGGCACAGTACATCATGGAGGTCGGGAGCTCACTGAATTGTCTATCCATTTGTCCCAGAGTGCATGTTCAGTGGATATATGTAATACCACCATGGAAAGGGTATAGTCATTGCTGAAATTATACCAAAGGTGCTACTAAATGAATAATCAAATAAAAATAGATGTAAATGAAGTCTGATTTTCTTAAGCCAAAGACACCAAAGACTTTACTATTGCTTTAAAGTGTTTCAAAGCATGCCAGTAGTTTCTAAAATGATGGCTGACCCATTACAGTAGAAATTCTATATACTGAGAAAGGATTCTTGATACAGTAATTTCTGTTGTAGTTTGAGACAGGATATCCTGTGACACATGATATCTAGGAACTACCGCTATGGTTGAGGATGGCCTTGAACTCTTGATCTTCCTATTTGTACCTGCTGCATGCTGGGATTACAAGCTTAGGTTAGCGTACTTACCCAATAGTCTTTAAATATTTCATTTGTAAGTCTTCTTAAAGAATGCTGAAAATATGACATTATCTCCAGAATGGCAAAGCAAGGACCCCAAGGACCTTGGCAGACCTGCTGTAATATAACACTGATAGAGGCAGTCACCATTTTCAGAACTCCTGAAATTAACCGCAGACTTGTAGCAGTCTAGAGTGTAGTCAAGTGAAAACATCTAATGTCGACAGGCGTTGCACTGTGCTTTTAGTTACCTTGTTACATCTTCTCTTCAGTCCTTTGAGAAGCAACCTTCAAAACCAACTGCCCTACAAATCATTGTAGCTGTGAACAGAAGAGAAACGTCTGCTGGTACAGGAACACAGAGTGATCCCAAGCCTCCTTCCCCGAGGATTGTCAGTGAGCTGTCTGCCTGAGCACTCCTGGGATTTGCCCTTCTTTGCCCTGGCTCTTCCCTTACTTGGTGCCAGCTGTTGAATATTTTTATTGCTTCAGAGGGTGACAAGTTGTGACAAATAAGATTTAAAAATATGTAGAATGGAAACTAGGGGCTGTGGTACACAAATGGAGCTTTGGTAAATAAATTCTGGTATGTTTCAGCTTAGAAGAACTTAACATGACCGAGAAAGACAGACCTGAGAGCATTTAAGCTTGCAACTTCTGCCTGAACTGAAGATGCTCCATAGCTAGGAAGGCCAGCAGGAGCCACAGAGAAAACTCTCTGCTGGAACATTACAATCATGTCTCAGCCCCATGTCATGAGAGGGAGCCTACCTGTTAATGATATTCTACATTACAATCATGTCTCAGCCCCATGTCATGAGAGGGAGTCCACCTGTTAATGATATTCTACATTACAATCATGTCTCAGCCCCATGTCATGAGAGGGAGCCTACCTGTTAATGATATTCTACATACAATCATGTCTCAGCCCCATGTCATGAGAGGGAGTCCACCTGTTAATGATATTCTACATTACAATCATGTCTCAGCCCCATGTCATGAGAGGGAGCCTACCTGTTAATGATATTCTACATTACAATCATGTCTCAGCCCCATGTCATGAGAGGGAGTCCACCTGTTAATGATATTCTACATTACAATCATGTCTCAGCCCCATGTCATGAGAG
MSTRG.45563.1	--	0.000	0.616	0.003	4.043	up	CTCTTACTAGGATATTCATTGCTACCTATGAGGTCAGAGTCCAGGGTCAGTCCATGTATAGTCTTTAGGTAGTGGCTTAGTCCCTGGAAGCTCTGGTTGCTTGACATTGTTGTACTTTTGGGGTCACGAGCCCCTTCAAGCTCTTCCAGTTCTTTCTCTGATTCCTTCAACGGGGGACCTATTCTCAGCTCAGTGGTTTGCTGCTGGCATTCGCCTCTGTATTTGCTGTATTCTGGCTGTGTCTCTCAGGAGCGATCTACATCCGGCTC
MSTRG.45651.1	ENSRNOG00000032550	0.517	0.000	0.017	-3.928	down	GTTATATGTTAGTGTAAATTATAAGATTTATAACTAGGAAATACTGTTGATGGCAATCATTTTTATTCTGTATATTTAATAATTTAACTGAATATGGCACAAGCCATTTCTTTTCTGTGGATGTTATATGTACCTCTTGTGTTGGTGTCTCTTTGCTTAGATTTGGAAAAAATTTCTTCTTTTATGTTATTAAAATATTTTCTATGTTTTCTAATGAAATTCTTCTCATTGCATACTTTTTATGGAGTTAGAGATCCTACATATTTGGTTCATACAATTGAAAAACATCAATGAGTGAGAATAAATTATATACTTTCTCTATTTTGTCTTCAAACCCTGATAGTCTGTCTTCCACATGATTCATTACTTTGGGAGACTTTCCATTGAACTCCTTACTTGATGTCTTCTATTTTTTCATTTCCATCATTATTTCTATTTGGGTTTTATTCATTTCTTCTATCTCTTTATTAAATTCTATTTTCACACTACAGAATGACC
MSTRG.457	--	0.051	0.330	0.010	2.910	up	TTGGGTATCAAGGAACATAAAATCTGTCTTCAAACATACATATATGCAAGACCCTTGTATTTTTTTCATTCAGTTCTCAATAATTTGGAGAAATAAGTGTTTTTTGTCAACGTTTTGAGGAAAACACCAAGTATGTTGGC

08.2							CAAGGGTGCTTAGAGGCAAACATAAGAAGTTTGATGGCAACTCTGTTTGTTCATTCCATTTCATCATGTA<br>TGATTGCTTGATCAAGACATAAGAGTAAAAATATGTAATTTACAAAGCTTCTGTAGTGATAGTTTAAGCG<br>GTTTACATAGTTTTTCTTTTCACACTAATTCAAACTTCTCATGAAAAGACTTAGACAATTGTTCAAATGCT<br>AAAACAACGTTGTCTTCTTCATTTTCACACAGTTCACGCAAGGAGACACTGATAGATTTAATGGTAAGG<br>TAAAAATCTTTCAACAGATTATATGCATCCCCTCGATGTATCCTATTGTCCTGGTCTAATCCAGCAAGGGA<br>TCCATACTTATAGAAATCAAGTGCATAGGCATCAAGTGGCATTTTTCTTCCTTGACTGTCCAATCTGTGTG<br>GCCACAGCACTGGGGCTTGTGCATGATTGATACAAATTGTGTGTAGAGTGACATGATTTGGAACTCCCG<br>GACTAAGCAAATCCACATCAAAGTTTCCAGACAAACAAACGAAGGGCGAAACCGCCATCCTTCCTTCC<br>TTGCAGCTCATCAAGTGAGACACAAGTGGTGAATCCTCACATTCTTTACCTGCGAACTCTGAGCAGAAT<br>GAGTTGACATACAACCACTTTTATTTTAATTCTTGCTATTTTAAT
MSTR<br>G.458<br>81.2	--	0.000	0.395	0.000	6.447	up	CGGGCCTGAGCAAACTCAAGCTGGACAAAGTCCCAGCACAGAGAAAGGAAGCAGACACAAAGTCCA<br>ACCCCTTCGCATGATGTTATTTGCCAGTGATAGCTCCTGGGAGAGGGGGAAATCAGGCCTTCTTCATGA<br>AGGAATACTGGGTATATCAACCACACTCTAGGGCCAGCAGAAAAAGCAATTCCATGGTTGTGTATATGT<br>GCTTTTTTGTTGGAGGTGGGTATATCAAAATACATTGTATGAAAAATGAGATTGAAAAGAAGCACAGAA<br>AACCAGATTCGCTATTAACGGAATGGTTGTTTTTAAGAGTGTTCTGTCAAGGTGGCAGGCGTTTTAACC<br>ACAGAGAGCATGAGGGTCAGTCATGGCGCTGACTCCTTAAATCCTGGTGTGGGTATGGGTAGAGAGAG<br>AAGTTCCTCTTCAAGAAATCAGGAGAACAGTTTTCCTCTCTCACCTTGGGAGTCTCCACAGCCTTTCAC<br>AGAACACTTGGGCTTTCCATTCCGGTGTCAGCTGTCTGCTCACTGATGAAGGCGTTTATGTTACCGGGTT<br>TACAGTAATACACTGACAAAGTTCCGTGGTGATGACTTCTGACCATTTAACCATTCTGCTGAGACCCGA<br>GTTTGCTGGGAATCTGCAAAAGGGCATTAGAAATTTAAGTCTCGTCAAACCACCATCTCTAAATGGCAG<br>GGTGGGTTCTCTAGCCCTTTCACTGAGAGTTTCTTTCCATTTCAACCATTTCATTAAAAACCATGATGAT<br>GACTGGAATCAGTAAGACAGGCTACTACTTCTCATTTTGTAGGTATCAGGTTCTCAATCAAAGACCACA<br>GGATTCTGAACAATGTATGTTCACTCCAGCAAACAGACGGTTAGTTTGATAGAACCATCAGTTAATCTAG<br>GAGTGAGAGGACACGTGCTGGCTGGATGAGGTACAGAACAATGTGCCCTTCCTTCCCAACTCTTTAAG<br>AGAAAAATTGACTTACATAACGGTTTTATGCAAGCACTCAGGTTTAATTAGTCTTTAAAGATATTAATTTT<br>ACAGATGTGTCCAAAAATTTTAATGCTTTACCAGTAAGTGCATGAGATATACTTTCTCCCACCTACTCTCT<br>CTCTCACGCTGAGTCTGTCTTGCACTGTCAACATCCTAGGAACACAATACTAAATGACAGCCCAGTCCT<br>TGCCCTTAAAAATGTTGGCTATTGAGGTGACAGTTGAAGAAATACTAAGGCTGAGCTCTTCCAGGTCAT<br>GCTTCCATGAAGCAGAAACTTCTAACCTGGCAAGGGAGGGGAGGGCCAGTGAGGACAAAGTTTCTGA<br>AAAAACCTGACACACAAACTGAAATTTGAATGGTTACAGTTTATGGGTGCAGCTGATGTAACAACTACC<br>AAAGCCTGTTCTAGAAAAAGGAGTAATGTGCTCAAAGGTCAACAAGGGAGAAGGAACTTGAGCTGTT<br>TCAGAACAGAAAATGGCATGGCTATAATTTAAGCTGGAAAAGTGGTAGTGACTAAGTAGGCACAAAGC<br>CACACATGCTCACAGAAGTCATCGGTGACAAACCAAGGTTAATTCAGGATTAATTCTAAGAGGGAGAG<br>AGAGAGCTGGTGATGAGGTTTTGAAAAAGACAAAATGCGTTTCAATTACCTTTTTAAAAGTGACTAAGT<br>CAGAAGAGTCCTCTATATCATAGGTGCTCTAAACATTACCCAGCTGTGAAGTGGTAGCATTCTACAATAT<br>TAAATCAGAGAGAGCTTTCAATGAACATTTTAGCCTTCAAAACACTGTGGGAGACCATTAATCCCATCA<br>AATGGCAGCCTTTGACAAATATTTCATTCCGGATTGAGTTCAACATAGGAATTATCTATTAAGCTTATGGT<br>AGAAATAGTAATCTTATGTTAGGGGGATATCGGATTTAGGTTGAAACTGTGACTCTTTCGGTTTGAGTAG<br>CTACAGTAGACCATCAACAAAGTCGGTGGCAAACGACAGCTTACCAACCGGTAGTGACTACTGGAAAA<br>CAATCACTGATCTAATGAAATGAATCCAATATTTTGTTCTTGCGAAAACGGCCTCCAGGAAGAGGACAA<br>TCGAGGTGATGAGCTCCTAAGCCCTCTTCAGAGCTTCATTTCCCCTTTGTATTTTCAGTCCAACTATTTAG

							CCAGTGTTCCACCCAAAATATTAAAGCCTTTCTACACATCTGACACAGGAATACAATACACTCCATAGACAAAGTCCCCAGTGTCGTGTAACTTTTTACTCTCAAGAGGAATTAAGCTGTCGCAACATAGTGATAATTATTAGGGAAGAGATGGGGTGGAAGGTGGGGTACCTAGACAATTATCTTGGTTTAGAGGGACAGAATCGAGTAGAAAAGGCTTTTGTAACTGACTGTGAAGCTTAGTGCTAAGGTGTCAGAAGATATTGTAGGTTTGAAAAGGAGATTCCGGGGGTTGGGGATTTAGC
MSTRG.46049.2	--	0.254	0.000	0.003	-4.375	down	AAATTCTTTGGGAATAGCAAGGTTTCTCTTCCATCTACTTCACCTGAGCATAATTACAGGGCTGTTCATCAGACCACATTCTCAATGGCATCAGAAACAAGAGTTGGTCAGAGTGGAGTTTGTGATAGAACTGAGTTTCCTTTGGTCATTGGTATTCCAATACAATGAAAATTTTGGAATGGAATGTCATAGATATGTGAAGTTACATACATTTAGTTTTAAGGACCTTGAGGTCATTAAGAGAACACCGACTGTCAATATATTGTAAATTTTCATCTCTGAATATCAAACAAAAAAATTAACTAAGAGAACAAAGTCTTTCGTGTAGAAACTAATGCATTAGTTTTGAAACTTAAATATTTCATTTTAAATAATAATTTTGTATAGATACAATTTTTTATCATATTATACCTCATTCTTCTGAGATCTTTAGGATTTTCCAAAAGCCAATGTATTCAAAGTGAAACACTTTAGTAGTAGGGTCTGGTGGGATCCATCAGGTAGCTCCGGAGGGTACTTTCGAAGGAGGTGGTGGAACTCTAATCTTCTTCTGCCCCCTATTTTGTTGCTAGTAGTTAAGTGAATGGTTTTGATTCATCATTAATGATGTTACTGTATATTACCTTTACATAGCACCCAAAACTGCATGGTCAACCAAAATATGGATGGTACCTCCAATATTATGAGCTCAGATTTTTTTTTAATCTGAGGTTTTTTCATATCTCAGGTGTAAGAGCAGAAAGGTGATGGACAAAAATTGTATCTGTTAATTTCTCTGGAAGACATTTTAAAAATGTTTTGATTATAAAATTACTAATCAAGGCTTAATAGATCCTATAATACAAACAGCCCACAAGCCAATGAAGGAACTGTCCAGCAGAATTTATCTGCATACTTGCTTTACTTTTTCAATTATTTCTTTTCTTTTTTAGATTATTTCAACCCTACACAAAGAACTAAGGAAGCTAAGGACTGCCCAGAGCGGGAGAAATAGTCTTCCTTAAGGCCCATTCGGGTTGGAGAGGTTACTCCAACATGTGTTCCTGAAGGAAGGAGCCTCAGGCTGTAACACCTACAGGTGATGCAGATCAATGACACACACAATTTCACACATGAACCTCACATGGTTGTCATCTGAGAGGCTCTATCAGCAGATGCATAAGGCAGATGCAAATACTTATAGCCAATCACTAGACTGAAATCAGAGACTATTATGGAAAAGTTGGAAGAAGTATTGAAGGAACTAAAGAGGATGTCAACCCCATAAGAAGACCAACAGAATCAACTAACCAGACTCTTGGGAGCTCTAAGAGACTAAGCCACCAAGCAAATAGCATCCATGGGTTGGTCCATGGCTCTGGCATATATATAGTAGAGGACTGCCCTCTCTGACCTTAGTGGGAGAAGAAGTGCCTAATCCTGTAGAGATTTGATGCCC
MSTRG.46248.1	ENSRNOG00000013480;ENSRNOG00000022736	0.373	0.000	0.008	-4.137	down	GGCCCAGCAGTTAAGAGCAACTGGCTATTCTTCCAGAGGACCAAGATTCAGTTCCCAGCACCCACATGGCAGCTTATAACTGTAACTCTAGCTTCAGGATCAGTACTCAGACACATAAAATAAAGTTATTTTTTCAACATATTGTCACTCATATGACATTAGTTGAACAAAGATATGCTGCTGAGGATGTAAAGAGAATAATAGAATCCAAACACCATATGAATTCCTGAAAAAGCCAAACATCCCCTTTTGTAAGCACTTACAGGACAATTGCTAAAGGAGCAGTAATTGTGTTGACAACAGTCTTACTGCAGTGCCGCTAACTTAGAAGTAAACTGTCTAGCGGTAGAATCTTACTAAATTCCCAGACGGCCTCCAGTCCAATTCCTAGTTCTACCTCACCTTCCCGAGTGGCAGGAATTATAGACATGGGCCACTCAGCTCAACAACTATAAGAAATCTTAAATCTTTAAGAACTCTGGAATTAAAGACAGACTACATACTAGCTAATAAAACATCTGGATGAATAAGAAGTAGTTCTAGTGATGCTTAAATACAAAGTCAAACTAAAGAGAAGAGAAAAATGTAAGGAAAACAGAACTGGGAGTTGGTGAAATATAACCCATTTTAAAACAAATGAGTTGAATTAACAGCCTTGATTTTAAAACAGTAATGTAATGAGTTAGGCTGGGTAGTGGTAGCACAGGCCTTAATCCCATCACTTAGCAGGCAGAGGCAGGCGGATCCCTATGAGTTTGATGCCAGCCTGGTCTACAAAACAAGTTTCTGAACAGCCAGGCAATATAGAGAGACCC
MSTRG.462	--	0.279	0.749	0.020	2.156	up	CCCACATTTTACACAATGTGATGGTTTGGCCCAGGGAGTGGCACTACTGGAAGGTGTGACCTTGTTGGAAGAAGTGTGTCACTGAGGGGTGGGCTTGGAGACCCTCCTCATAGCTTCTCCTAGGATACTAAATCTGTT

87.1							CCTGACTTCCTTCCGGTGAAGATGTAGAACTCTCAGCTCCTCCTGCACCATGCCTGCCTGGATGCTGCC<br>ATGTTCCTGCCTTGATGATAATGGACTGAACCTCTGAACCTTGGCCAAGTGCATGTGGAGGTCAGAGGT<br>CAACTTGCAGAAGCTGGTTCTCTCCTATCACCATTGCTAGAGATTGAACCCAGAGCCTCACACATGCTA<br>GGCAAGTTGTCGGCCACTGAACCATCTCTCCGGCTGTTTTAACTTCACACTCACCGCATGGCCCTTGGA<br>CCAGCACAGCAGCATCTTCTGCAAGCTGAGTGAAATGAACCTCTCTCAGCTCCAGCTAAACTCAGGAC<br>CTACCCGAAGGGGAGCAGGGTCTCAAACATTCCATAACAGATCCTCCCAGGATGTTCACTGTGCAAGC<br>CTGCTGTATGTTAGTACAGAGCCAGTCTTCAGATCACTTCCCTGACAAACAAACCAAAAGGAAAAGGC<br>ATGATTGGTTCAGAGTGCATGAAGATTAATCTCGATGACTGGAGAACCGGTCTGTGACTTCCATGTCAAT<br>ATTGTTTGTATTCTCTGCGTAGCAGGCTGGGAGCCGAGGAAGCTTTGAGCTTTGAATTGAGAGCTAGGC<br>TTGTCCGTGCTATTAAGAACGTTAAAGCACCTAACCCACGGCACGATTTCACATAATTTTTCTGAAGTCC<br>TGACAGCTGTCTTCTGAGGTAAGAATTACTGTCTGAATAATTCGGAAGAGGAAACCGGTTTAAAACCGA<br>TTTACGCAAAGCCCAAGTAACCCGTCTGCAGAGGGAAAAGCTGTGATTTACACCGAGAGAGGTGATCT<br>CAGAAGGAAGAGAGGGCCTGCTTAAGCTTCATTTTCAAAACAGCCATGCCTTGGCTTGGCTGGGGACA<br>TTTTAAAATTCAGAGAAAGGGCTTGGTAAAGCTGCTGTTTGTAGAATGGGGTGCATGTGGCAGAGGAA<br>GCACTGACTTTAGCACCTCCAAGTCCTGTGTTCCAATCCCCAGTATCACGGAAGGAGGAGGGAAAATA<br>ACCTGAGAACTGGTTCTGAGGACTCGCACTTTCTTCATTTTGTTTTGTTTTCGGGTTGGTCCTTACTTTC<br>GGAACGGCTGTCTTTTAGACCTTCAATTCTGGTTCTGCACACTGAAAAGGAAGAGCAGCTATAAAAGA<br>AGAAATATTTGTCACGTCCTAAGGGCCAATTATTCTATCGTCATGGTTATTTCCTATACTTAGTTCAGGGT<br>GTTGGTTCCTGATTTTGATTTTGAATTGCAGGCAGGAGCAAATTGAGGACATAGGTATGAACGCTGGCA<br>CTGAAACGAAACCAGACTGACTCTTTAAAAAGCGGGCTCCTGGCATACCTTTTAGGGGAGCGCAGGAA<br>AACAACACACAAAAGGGTCCCTCAATAGACGACCTCTTCAATTTAGTATTTAACATTTCAAAGGATTCT<br>GTCTTCTCAAAAGTCAGGGAGATTTTTAAACCATTGTCTTCATTAAACTTCAGAGCAAAAAACCTTGGG<br>TTGTGTTTCATGCACTTTTTTTTTAAATCCTAAATTTGTTTTGAGATTAGAGACACAAAAGACAGCCCTG<br>GGGGTGGGGGGTGGGGGGTAAACTGTGTTCTGTTTTCCTTTCCCCCAGAAGAGTTACAGTTCAAGCTT<br>GTTCAGTGGAATTGTCCTTTACCCATTAGTAGATTTTTATTTTTTTAAGAAGATGAAATCTGCAGTAATGG<br>GAGATTAGTTGGCAGCTACAGATCAACTTCCTGTGCCCACAGCCCATTTCTATAAAACATAGAGACATCA<br>GCTCACCAGATCATGGTACATGGTTCATTATTTTAGCGTTGGACTGTGCGAGGTCATTACAGCAATCCAT<br>CCGCCCCATACATCCACCCACCCCAACATCAAAATAATTACAAACTGTGGTTGCTCAAAGGCACAGGAA<br>CAAGAACAAGGGCAAGAAGAAGCTCTCCCTGATGTGCCAGGGTGGGCTGGTACGGGGGGGGCTCCCC<br>ATTCTTAGAGAAGATGGGGGGAGAGTCTGTATGAGAGGGTCTGTGATTGGGATACAACAAACAAATAA<br>ATAAATAAATAAATAAATAAATAAATAAATAAATAAATAAGAAAAAGTCTCTCTCTAGTCAATGTGGATGT<br>AAAGTTTTTCACGATAAAGCCTGCAATTGGTTGATATGGAACCTGTGGGTAGAAGGAGCAGCCAAGTGT<br>TGGTAACCTACTCCTTTAATCCCAGGATTTAGGAGGCAGAGAGATGCAAATCTCTGAGGTCAAGGCCGG<br>CGTGGTCTATACAGCAAGTTCCAGGACAGCCAGAGCTACCCAGAGACAGGGTTTGTCTTGAAAAAAAA<br>AATCCAGCCAACAAAAACACAGTAACAGTAGGAGCAGGGGCAACTGTAATTTTCTTTTCTAATGTCCAC<br>CAAGGTCAGACACATCTCAGCTGCATCTTCAGTCACAGTGATGTGGGGGTCTCTTGTGTGGTAGCTTCA<br>TCTACTTCTCAGCAGGTAGCCCTTTCTACACTTTTTAGAAGAAATCTGGAGTGCAGGTTTGGCTGCCTGT<br>GGTGGCATCATTGATTGGCTGGTGACTTATTGAAGGAAAGCTTTTGAGACGGAAGCCATTGTAGTGGTG<br>AGTTTTTAGCTTTGCTGCTTTGGCTCCTGGTGGATACACACACCCAGGAACTATTTGGATTCAAGAATGG<br>AACGCACGCACGCACACGTCAGTTAATTTTAATGTCTTGGCTACCTCAAAGGCTGGGCACTTCTAATCC<br>TCTGTCTGCTACCCTGGGTCCAATCTGGGAAGTGGCCAGTGGAAGCTTTATTTCCTGCAGCTCCTATATC

							CATCTGTATCCTGCTTCGTGGAGATTTCTCCATTTCCTCCTCACTGCATCCTAGCCCGTGCAACTCTAAGG<br>TCCCGCCCCATTTCCCTTTGCCCAGCCATTGGCCACTGGCATCTTTATTGCTCGATCAACAACCAATTGG<br>GGACAAGGACATGCAGATTCCCGACTGAATCAAAGCATTACAACCAATCTCCAACAAGCCAGGTATCTC<br>GAACAAGCTCAAGGGAATAGGAGTGAAGTGCACTTGAGAGTAGTGGGAGATGATTATGGGGATGAAGA<br>CCGCGCCAACGTGATGGAACGGCACAGTGGAGGCAGCACCTGTTTAGCCTGGAGACACCAAGGGGGC<br>TGGACCACTTGTGCTGCTTCCTAGCCCTATTGCTTGGGAAAAGCCATTCCCTGCCTCAGGTTTTTTTTTT<br>TCATCATGCAGTGTGAGTTTAGGGGCAGGTAGATTTAAAAGTTTTTTTTTTTCTTTGCAGTTCTCATAGG<br>ATTCGTTTCTAACATACTGCTAG
MSTRG.46388.2	ENSRNOG00000010830;ENSRNOG00000032192	0.593	0.036	0.001	-3.086	down	GGGGCGATCGCGGAACGGAGTCTCTGCCGAAGATGGTCCTGGCAGCGCTCCTGTCGTCCATAGTTCCA<br>AGAGACCCCGTCACAGTGATGAGGCCTGCACTGTAGGGGCGACAGCTTCTCGGCAGGGCCTCGAGAA<br>GACTTGGGGGCTCGTGGAAAGGAGACCGCCAGGGCCTCCCTGCTGTCCAGGGCATCACCTGCTCGTAC<br>AGCCACGACGCTGCGGTAGGTTTAGACATCTGGGATGGTTTCCTACAAAGATGCTGTAATCTTGTAATTC<br>TGTAACAAGACGACAATAATAAAAATGAAGGGCGACTGTGTGTTCGTTCGATTTTATTTCAGCAAAACG<br>CTGTTCACATAAACCAGTCCTGGTGGCGGATGTCTTTAATCTCAGCACTTAGGAGTAGAGGGAGAGGGA<br>TCTCTGTGTTCAAGGTCAGCCTGTTCTACATAGTAACTTCCAGGCTAGCCAGGGCTGTATCGTGAGGTGT<br>GTGTCAAAGGGGAAAAAAAAGTTCCAATTCAAGCATAGTTAATGTGCATCAGGGGCAGGTAACCTATG<br>AAGGATGGCCCTTATGATGATGGAGATCCACTTTAAGCAAACTAAAAAATGATCTCTACAGACTGTTAA<br>GTGCCCTAATTTCTTGTTGACACATCAAAGGGTCAGGAATGCCACCACACTCCTGATCTCTATCCCCTAA<br>TCACCAGAGTACACGGATATAGTACTTTATATGGCACGGGATTTAGCTGATGCAATTAAATTTACATTATC<br>CAGGAGCTCACTGTGTAATCAATCACTAGGGCCCTTATAAAAGGGAGAGGCAAAAGCTTCCAAGTCAG<br>TTAGTTGTAGGAGCTGTAACACAAAATGGTTGAGTGATGGGTGGAAGAGTCAATGAGCCAAAGAGTGC<br>ATGCAGCTCTGGAGAGCTGAAAAGAGGGAAGAAATAGATTCTATCCAGAAAGAACTCAAATCCACCTT<br>GAATTTAGATTTCTGACCTCCACAACTCCCTAATCCAAATAAATGAGTTGCTTTCTACATCTAACTTTGTG<br>GGCGTTTGTTACAGCCATGGTAAGTTCTCACCACTTACATAAAAGTACAAAGCAAGCAAGCACGGACTT<br>AATTAACATTGTAGCCCAGGGTCTACCACTCACTGTACTGTTTTGTCAGAGCCATGACAGAATATCTAAC<br>ACAATGAGGAAAGATGTATTTGACTCACATTTTTAGAGGGTTCAGTCCTTCATGGCACTACAGAGCAGC<br>AGTTCATGTAATTGTAAGGTTGGAGAATCTGGCCTACATGAAGAGGCCAGGCCAACCTCTATAGCGATA<br>CCTCTAGTGACCTTCTTCCACCTAGGCCCCACCTCCTGCCTTTCACTGCCTGCTAATAATGCCAGCATATT<br>ATGAATCCATCAAGGATCACCCCATTGATGAGATCAGAGCCCACATGATGTCTCTGGAAATGCCCTCAC<br>GGACACACCCAGAGGTGAGCTTCATTATGTGTCTAGGTGTTTCTAATCAAATGTGTGGGTATGATCACAA<br>CCACAGCTATCAGACTTAAGAAAGAACTCTTATTTGGAACAGTATTTTTTTTTTTTTGGTTTTGATTATT<br>AACAATTTTCCATTTTGTTGGGTCAAAGAAAGTATTTCACTTAATTTTAACTAATGAAAATATTTTGTCAC<br>TGATTCCCAGGGTAGATATAAAGTATTTGAGTAGACATTTCTCCAAAGAAGATACAAATAGCCAGCAGCT<br>CAAGAAAACACTGAGTATTATGGTTTGAATGAAAATGGCCTCTAAATGCCTGTATATTTGAATACTTCGT<br>CCCCAGTTGGTGGAACTGTTTGGGAAGGATTAGGAGGTGTGGCCTTGTTTGGAGGAGGTGTATCACTG<br>GCTCATATGTTTCAGTACTTGGTCTCTAGCTAGTGGAACTGTTTGGGAAGGATTAGGAGGTGTGGCCTTG<br>TTTGGAGGAGGTGTATCACTGGCTCATATGTTTCAGTACTTGGTCTCTAGCTGGTGGAACTGTTTGGGAA<br>GATTAGGAGGTGTGGCCTTGTTGGAGGCATGACACTAGCTTTGGAAAAGGTCTCAAAAGACTTGTACC<br>TCTCTGCCTTCTGCTTTTGGATTAAGACAGGAGTTGTTATCTGTTCCTGTTGCCATGCCTTTGCTCTGCCA<br>TCATGGACTCTGAAACTGGGAATCCAACTAAATGCTTTACTTCATACGGTGCCTTGGTTGTGGTGCTTTG<br>TCACAGCATAAACAAAGTCACTAAGACACCACTCATTAAGGAAATGCACATCAAAATCAGTGAAGGCC

							TGTGAGATGATCATTTCTGGTAGGTAAATGTGCTTGCCAGATGACTAGAATTAGATCCCAGAACACAAAAAGATATCCAGGGCCAGGGAGACAGCTCAACCGGTACAAAGTACTTCCAAGTCAAACCTGACAACCTCAATTTCTAGATTTCATGGTGGAAGGAAAGAACTGGCTCCTTAAAGGTACCCTATAACCTCACAACTTTTCTGTGACATATCTGTGCTTACAAAGTTGCCCTAGAACCACAACTTCACTGGTACCCCTGTATCCACACATCAAGAGTACAAAGATCAAACAATGCGGTATGCTTTAGCCCCACTATTTTAGAAATACTCTTGCAAAAACTAGAAATAGACAACAAGATCAAGATGGACAAGACTCAAAGAAATTGGGAGTCTTTTACACAGTTGATATGTAAAACAGCAGGATAGGAAATTCTATGCTAAATATAATTATATGAACTAACAATCCCATTTCAGAATATATGCCCAAAAAAATTCAGAGCAGTGTCTGGGACATTTGTACATCCAGATTCATAACATTATTCTTCATAATACTCAAACTATAGAAACAACCCAAGTCTATAAACAGAAAATGTTAATACAAACACTTGCATACATGCAGTGGAATTTTACACAGCCACAAAAAGGAAGGAAATCCTGATGCATGCTATGACATGAAGTTTAAGAGAGGATGCTAAATGAAATAAGCTAGTCAGAAAAGACAAAGACATGATTCCATTTCTAAGAGGCTGAGGATAGTGTAATTCATGGACACAAATCAGAGTGAGTGTGAGGCGGTTCTGGGGAGGAGGGGGTGGTGAGTTGTTTGACGGCAGAGTTTTTGTACAGCTGCCTGGTAGAGAAAGTGTCAGACATGGATAGTTACAATATGAATGTATTTACTGCTACACTGTGAAGATGGTTACCATGGTAAAATTTCTGCTTTGTGTATTTTACAATAAAAGCAATTGATTTTTAATAACGATTCTCTTGGAGACTGAATTTTTTTTTTTTAAATCTTGGTTCATTATGTGTCCTTCAGGCATTACAACAAATCCTATAGTGTATCTTCTCTCTCACAGTTCTGGAGAAAGGAAAGTCAGAGTTCAAGGTGCCAGTAGATAAGGCACCTTGATTCCTGGTTCAGAGATGGCTTCTCTGTGTCCTCTCACTTGGTTTGAGAGTCATGGCAAGTAGGTGTCTTGTCTTTGTAACAATTATTTTTCAAACGTTATTTTTAATGATGGTTTTTAAAACGCTTTAACCTCCTTTTAGCCCACCACCCACAAGAGGTAGTGGAGAAGAAAGAATGTGGGGAAGTGGACCTGTTAAAAGGTTCTGTGGAACAAATCCCGTCTGTGTTGTCTGGAAATCAGCAGTTCAGATCACAGGTTAGTTAGCAGCAGCAACAGCAGCAGCTCGATTTACTTGCAAACACCTCACAGAAATACCAGGTCAGCAACAGTGGTGTCATTACTTAGCAGAGAGAGAGCCAGGCCTCAGCCTAGGCATGTCAGCAGGAGGGACCAGCAGGAACACCAAGAGAAGTTTTGGGCTATGCCTCAGCAGAGTGAAGATCAGCGAAGATGTGAGACCCACAAGCATTGCACAGCTAGTTCTAAGCAAGCTAAACTCAGCCTCTGTCACTGTCCGTCGAGTCCTATTCATACCCTCCAGACATCACCCGTCCTCCACTGGTCTTTTGTCAGCATGTGTCTTTTTTTTTTTTTTTTTTTTTTTTTGGTTCTTTTTTTTCGGAGCTGGGGACCGAACCCAGGGCCTTGCGCTTCCTAGGTAAGCGCTCTACCACTGAGCTAAATCCCCAGCCCCATCAGCATGTGTCTTAACACATATATCTGTCTCAGCTGACACCAGTCTGCCAATCAGCT
MSTRG.46388.3	ENSRNOG00000010830;ENSRNOG00000032192	0.529	0.035	0.001	-2.997	down	GGGGCGATCGCGGAACGGAGTCTCTGCCGAAGATGGTCCTGGCAGCGCTCCTGTCGTCCATAGTTCCAAGAGACCCCGTCACAGTGATGAGGCCTGCACTGTAGGGGCGACAGCTTCTCGGCAGGGCCTCGAGAAGACTTGGGGGCTCGTGGAAAGGAGACCGCCAGGGCCTCCCTGCTGTCCAGGGCATCACCTGCTCGTACAGCCACGACGCTGCGGTAGGTTTAGACATCTGGGATGGTTTCCTACAAAGATGCTGTAATCTTGTAATTCTGTAACAAGACGACAATAATAAAAATGAAGGGCGACTGTGTGTTCGTTCGATTTTATTTCAGCAAAACGCTGTTCACATAAACCAGTCCTGGTGGCGGATGTCTTTAATCTCAGCACTTAGGAGTAGAGGGAGAGGGATCTCTGTGTTCAAGGTCAGCCTGTTCTACATAGTAACTTCCAGGCTAGCCAGGGCTGTATCGTGAGGTGTGTGTCAAAGGGGAAAAAAAAGTTCCAATTCAAGCATAGTTAATGTGCATCAGGGGCAGGTAACCTATGAAGGATGGCCCTTATGATGATGGAGATCCACTTTAAGCAAACTAAAAAATGATCTCTACAGACTGTTAAGTGCCCTAATTTCTTGTTGACACATCAAAGGGTCAGGAATGCCACCACACTCCTGATCTCTATCCCCTAATCACCAGAGTACACGGATATAGTACTTTATATGGCACGGGATTTAGCTGATGCAATTAAATTTACATTATCCAGGAGCTCACTGTGTAATCAATCACTAGGGCCCTTATAAAGGGAGAGGCAAAAGCTTCCAAGTCAGTTAGTTGTAGGAGCTGTAACACAAAATGGTTGAGTGATGGGTGGAAGAGTCAATGAGCCAAAGAGTGC

							ATGCAGCTCTGGAGAGCTGAAAAGAGGGAAGAAATAGATTCTATCCAGAAAGAACTCAAATCCACCTT<br>GAATTTAGATTTCTGACCTCCACAACTCCCTAATCCAAATAAATGAGTTGCTTTCTACATCTAACTTTGTG<br>GGCGTTTGTTACAGCCATGGTAAGTTCTCACCACTTACATAAAAGTACAAAGCAAGCAAGCACGGACTT<br>AATTAACATTGTAGCCCAGGGTCTACCACTCACTGTACTGTTTTGTCAGAGCCATGACAGAATATCTAAC<br>ACAATGAGGAAAGATGTATTTGACTCACATTTTTAGAGGGTTCAGTCCTTCATGGCACTACAGAGCAGC<br>AGTTCATGTAATTGTAAGGTTGGAGAATCTGGCCTACATGAAGAGGCCAGGCCAACCTCTATAGCGATA<br>CCTCTAGTGACCTTCTTCCACCTAGGCCCCACCTCCTGCCTTTCACTGCCTGCTAATAATGCCAGCATATT<br>ATGAATCCATCAAGGATCACCCCATTGATGAGATCAGAGCCCACATGATGTCTCTGGAAATGCCCTCAC<br>GGACACACCCAGAGGTGAGCTTCATTATGTGTCTAGGTGTTTCTAATCAAATGTGTGGGTATGATCACAA<br>CCACAGCTATCAGACTTAAGAAAGAACTCTTATTTGGAACAGTATTTTTTTTTTTTTTGGTTTTGATTATT<br>AACAATTTTCCATTTTGTTGGGTCAAAGAAAGTATTTCACTTAATTTTAACTAATGAAAATATTTTGTCAC<br>TGATTCCCAGGGTAGATATAAAGTATTTGAGTAGACATTTCTCCAAAGAAGATACAAATAGCCAGCAGCT<br>CAAGAAAACACTGAGTATTATGGTTTGAATGAAAATGGCCTCTAAATGCCTGTATATTTGAATACTTCGT<br>CCCCAGTTGGTGGAACTGTTTGGGAAGGATTAGGAGGTGTGGCCTTGTTTGGAGGAGGTGTATCACTG<br>GCTCATATGTTTCAGTACTTGGTCTCTAGCTGGTGGAACTGTTTGGGAAGATTAGGAGGTGTGGCCTTGT<br>TGGAGGCATGACACTAGCTTTGGAAAAGGTCTCAAAAGACTTGTACCTCTCTGCCTTCTGCTTTTGGAT<br>TAAGACAGGAGTTGTTATCTGTTCCTGTTGCCATGCCTTTGCTCTGCCATCATGGACTCTGAAACTGGGA<br>ATCCAACTAAATGCTTTACTTCATACGGTGCCTTGGTTGTGGTGCTTTGTCACAGCATAAACAAAGTCAC<br>TAAGACACCACTCATTAAGGAAATGCACATCAAAATCAGTGAAGGCCTGTGAGATGATCATTTCTGGTA<br>GGTAAATGTGCTTGCCAGATGACTAGAATTAGATCCCAGAACACAAAAAGATATCCAGGGCCAGGGAG<br>ACAGCTCAACCGGTACAAAGTACTTCCAAGTCAAACCTGACAACCTCAATTTCTAGATTTCATGGTGGA<br>AGGAAAGAACTGGCTCCTTAAAGGTACCCTATAACCTCACAACTTTTCTGTGACATATCTGTGCTTACAA<br>AGTTGCCCTAGAACCACAACTTCACTGGTACCCCTGTATCCACACATCAAGAGTACAAAGATCAAACAA<br>TGCGGTATGCTTTAGCCCCACTATTTTAGAAATACTCTTGCAAAAACTAGAAATAGACAACAAGATCAA<br>GATGGACAAGACTCAAAGAAATTGGGAGTCTTTTACACAGTTGATATGTAAAACAGCAGGATAGGAAA<br>TTCTATGCTAAATATAATTATATGAACTAACAATCCCATTTCAGAATATATGCCCAAAAAAATTCAGAGCA<br>GTGTCTGGGACATTTGTACATCCAGATTCATAACATTATTCTTCATAATACTCAAACTATAGAAACAACCC<br>AAGTCTATAAACAGAAAATGTTAATACAAACACTTGCATACATGCAGTGGAATTTTACACAGCCACAAA<br>AAGGAAGGAAATCCTGATGCATGCTATGACATGAAGTTTAAGAGAGGATGCTAAATGAAATAAGCTAGT<br>CAGAAAAGACAAAGACATGATTCCATTTCTAAGAGGCTGAGGATAGTGTAATTCATGGACACAAATCAG<br>AGTGAGTGTGAGGCGGTTCTGGGGAGGAGGGGGTGGTGAGTTGTTTGACGGCAGAGTTTTTGTACAGC<br>TGCCTGGTAGAGAAAGTGTCAGACATGGATAGTTACAATATGAATGTATTTACTGCTACACTGTGAAGAT<br>GGTTACCATGGTAAAATTTCTGCTTTGTGTATTTTACAATAAAAGCAATTGATTTTTAATAACGATTCTCTT<br>GGAGACTGAATTTTTTTTTTTTTAAATCTTGGTTCATTATGTGTCCTTCAGGCATTACAACAAATCCTATAG<br>TGTATCTTCTCTCTCACAGTTCTGGAGAAAGGAAAGTCAGAGTTCAAGGTGCCAGTAGATAAGGCACCT<br>TGATTCCTGGTTCAGAGATGGCTTCTCTGTGTCCTCTCACTTGGTTTGAGAGTCATGGCAAGTAGGTGTC<br>TTGTCTTTGTAACAATTATTTTTCAAACGTTATTTTTAATGATGGTTTTTAAAACGCTTTAACCTCCTTTTA<br>GCCCACCACCCACAAGAGGTAGTGGAGAAGAAAGAATGTGGGGAAGTGGACCTGTTAAAAGGTTCTG<br>TGGAACAAATCCCGTCTGTGTTGTCTGGAAATCAGCAGTTCAGATCACAGGTTAGTTAGCAGCAGCAAC<br>AGCAGCAGCTCGATTTACTTGCAAACACCTCACAGAAATACCAGGTCAGCAACAGTGGTGTCATTACTT<br>AGCAGAGAGAGAGCCAGGCCTCAGCCTAGGCATGTCAGCAGGAGGGACCAGCAGGAACACCAAGAG

							AAGTTTTGGGCTATGCCTCAGCAGAGTGAAGATCAGCGAAGATGTGAGACCCACAAGCATTGCACAGCTAGTTCTAAGCAAGCTAAACTCAGCCTCTGTCACTGTCCGTCGAGTCCTATTCATACCCTCCAGACATCACCCGTCCTCCACTGGTCTTTTGTCAGCATGTGTCTTTTTTTTTTTTTTTTTTTTTTTGGTTCTTTTTTTTCGGAGCTGGGGACCGAACCCAGGGCCTTGCGCTTCCTAGGTAAGCGCTCTACCACTGAGCTAAATCCCCAGCCCCATCAGCATGTGTCTTAACACATATATCTGTCTCAGCTGACACCAGTCTGCCAATCAGCT
MSTRG.46563.1	ENSRNOG00000009846	0.000	0.853	0.000	4.599	up	CTTTAAGGGACGCCATGTAGATAGAAATTGCCAGTTCATTTCACTTCGACACTGAGGAAGTGGTAACCTGACCTCTATGGAACCCATCAAGGTTCTCTGATGGTGTTTAATCCAGTGCCCTGTGTGATTAATGTAAAAGTCATTCACCTTTGCTGTTAGTGACAGCAGTGCAGTATCACTATGCTTGGCTGGAGTTCCTCAGATGGATATTTGACACATATTTTAATGGGCAATCAATAGACCTCTGACTCTGGAAATGGTATTTTGGAGGAGTATAAAATAACCATTATAA
MSTRG.46912.1	--	0.567	0.000	0.017	-3.928	down	GAGACTGTGCTTTCCATACTAATACCCTTGTGTATAGGAACCCTGAGAGATGATTGAAGAAATCCCTTCTCATGGGGCTATGAATTAAGATCAAAAAACATTTGTCATCTATCTAGGAAAGATGAAAAATGATCTACACAAACTGACAACTGGGATCTTCAAACTAGGAATAATTGGATAAAAGCCAAACTCAAAGAAAGAATAAAGGGTGACAGAGGATAAAAGTCCAAGAACCAAAATGGAGTCATCCTGGGAAAGCAAATAATGTTTAAGACGAAGGCGATTTCATTCAGACAGAGCATCTCACAGCACACCTACGCTGTCGTTTTTAGGAAGGCAATCTACAATCTGACTGAAGCAAACCAGAGCCATAAATTACAATGCCTAAGCTTCTGAGAGTCTTAAAAATACATTACCAACCAAAGATTGAGACACAGGGTTCACAAATTCTGTTTGCAACATGGGAGAAGTAGTCC
MSTRG.46949.2	--	0.025	0.363	0.000	3.881	up	AGCCACTTTTTATTCAGTCAGTGAAATTGAACACCTTTGAAGGTTTGGATGGTAATTCCATATACAGGATGGTGGGGATAGAAGGAAGGGGTAGCTGTCACACCGCTCCGCCATTATTGTTCAGTAAGTCACTATATATGGGCCACGTTCTTGTTTTACATGTAAAGTTCCCTACAGCCCTGCACATATGCCCTTCCTTCTTTCAGAGCTAAGCTCTTTACATCTTGAAGCAGCCCAGAGAGTGTTATTATCAAACACCAAAGCAATAAGAATCCTGGGCTTTGGGGTTGGGGATTTAGCTCATTGGTAAAGCGCTTGCCTAGCAAGCCCAAGGCCCTGGGTTCAGTCCCCAGCTCTGGAAAAAAAAAAAAAGAATCCTGGGCTTTGTAAGTTGAGGAAGTGGCTGTTAGGCCCTCGTTAACATTAAAGAGCATGATAGGAAAAGGACTAAGTGCCTCAGAGTAAAAGAAAACGGTGACAGGACCAAAGATACTCAGACATGGTGGAAAAACAACACCAGGAGCATCTTCTTCACAATTTTGTCACCATAGAAACAGCCCGGTGATACTGAGTAAACGTTCCACAAATCCAAACATGCTCTGGGTTCTTTAGGGAGGCCACAGGCTTCAGGTTCCCCTTGATGGAACCGATGTTCCCCACTCCAGTTTGATCTAAGTGCAGCCAACATTAGTGCTCTGTTCTCTTCAGCTCTCACTTCCTCTGCCTTGCACTTGAATGATGTGATAAGGGGAACGGCGCTGGGTGCAAGTGATGTTGGTATGCGCTGCCTTCCTGCTTTGCTTGTTTAGTTGTCTCAGGATTTCCTCTCTCTAGGTCTTGAGAGCGCGTCCCTTCAGTGCGAAAGCAGCCTGCTGAGCAAGATAAGTGGCGAGCACTAGGCTGTAAAATTCTCAGGATACACAGAACATTTTAGCTGAGGGAGCTGGAGTTGTCCGAAGAAGCTGGAGTGGATATGGGGACAGGAGCCTTGAAACTGGAAAAGAAGATCTGAGGCATTGCTAAGCAAAGAAAGTGTTGGCAAGGACAGGGCTGCTGCGGGAGCGCGCATGGCTCGATGATGCAGTCATGTAGCTTGCCAGCTGGAGCCCAGAGCCAGAATAAACCACCGACTGTAAAGGAACCCCTTGGAGAGGAAATGCACGAGCAGGTAACTGTTATATTTTAAAAACTCGAGCTCCCGTTTAAGAAACGGAACACCCATGTCTCTTCTGCCGCATAACCTCAACTTAAAAGTGAGTGTCCGTTAGAATCCACCCGATCTAAAACCTGTGGTCAAAAAGACGCTGTGCTGATGGGAGAGAACCTGCATGACAGCGCTACCTGCTCGGCTCTCTGCTTCATCTCCGGGAAGGAGCGGCTGGGCTCCCATCTCCAGGACGCGGCGCCGCACAGCGAGCGAGGTGATTGGGTTACACCGGGCGCATCCCGATAGCGGGCGCGGGAGCCGCTGGCCCTAAGCCTTTCC
MSTR	--	0.099	0.384	0.018	2.527	up	CTTCCAGGCAGCTGCACACTACCAAGCAAGAAGCAGTCCATGACCATCCTGCAAAAATGAAGCCTTGA

G.469<br>62.1							AGTTTAGCCTCCCAGGCCACTCTGCATTCCCACAGGATGAGGGCACGGAGGAGGGCCAGAGCAGTGGC<br>TGGGAGGCTACCAGGAAGATGGCCTGCAGCAGACAGTAAGAGTGGACCATCAGTGCAGGCCATTGGG<br>CAGAAGAGAAGACACCCTGTGGGGGTCAGCGAGCGAGCGAAAGGACCTAAGGATGGACCTAAGGATG<br>GACCTAAGGATAGGCGATGACAAGGTCACATGGTAATCTTTGGAGCCCAGTCCTCCACTCGATTGACTT<br>CTTGGAATTTGGATCAACCAAAGGAAACGAAGTAACACACAAAGCGAGAGAACAGGAAGAAGTGTCT<br>GTGTGGTGATGTTTACTTGGTGCCACCTGGAGCTGCGTCATAAACTTCCAGTGATTCAGCTAGAGACGC<br>TGCCCTGCCAAGGCAAGGCCTTTAATGTGCTTGGAATATAAGCTGTGTTTAATTTTAAATGAAACTGATT<br>AGAAGTGTATATGTTTTATTTACCAACAGGGCCAAATTGCTTACTCCAGTGGGCTGTTGCTGTACGGGTT<br>TTGCAGGTGCCAAAGTCTATATAAAAACAGCTTCGATTCTAAAGAGAAAATAGGAATTTTCCTACTGGC<br>AGCTCTCATCTTGGTTAATTAGATGGTATTAGGAAGGAAAAGGTTGGCCATGTTATCTAGAAGAAAGTGT<br>TATTAGAGACAGTGTTGTATCCTGAAAATGCTTAATGTCTCACGTTCTGGTCTCTAATTGCCATCTGGAG<br>CAGATACTTGGGAAAATTTTTTAAGCAAACTAATAGAAATATTCTAAAGATGCTTCAGTCAATTCATCAA<br>TAAAGGTGGAAGCAG
MSTR<br>G.469<br>9.2	ENSRNO<br>G0000005<br>5519	0.750	0.002	0.001	-<br>4.117	do<br>wn	GCTCAGTCTCTGGTTTGAGATCTGATGTCCCAGCGCCTTCATATCACCACCCATAGCACTTTGGTGCCAC<br>GGCGGCAGCGCTGAGAGGCCCGGGTCACCGCTGCTGCCGGGCCAGCGCACTCGCAATCCACCTTCGG<br>GGTCCGTCACGGGGGTGGTCCGGTGCTTCTGCGTCCTACGCTCTTCTCCGGGACCTTTTCTGAGGATCC<br>CTGGGACTCGGGTGGCGGTTCGTGGGTGCGGTTTACGTGCTACACCTAAAAACCCGGCCTGGTTGAGG<br>AGACCGAGAAGGCGGGGGCCGCGATTTGCATCTTGGAGATACCCAGTGCAGAACCTCAGATTCCCCGC<br>AGACCTGAGGAGCTGAAAGCCGGGGACACATCTGACCTAGAGGCCGGGCAGCCCCCAGAGTGGTAAC<br>CCGGGGAAGCAGGGTGGCCGAAGCAGCAAACTCGGGCACGATGACCGTGGGCTGCTGCAGTCTGTCT<br>CGCTTGCGGGGATACATACCAGTGGGGTCAGCCGGGACCCCTGACGCGTGGAATGGGGTTCTGATCAA<br>GTTCAGAAAAGCATAGGGGTGAAGCCAGAGAGCGTGGTGGCACACACCTGTCCTCTCAGGATTTGGAG<br>GTGGAGGCAGGAGGAGAAGGCGCTCTGCTGCCTAACATTAGGTTGCAAGAGACCCCTCCGAAAGAAA<br>AACAAACAAGGCCTGGAGTGTCTTCAATTCACTTTGCCTCTTCAGAAGAGGCAGCCACGACTTCTGTTT<br>ATAAGCGAGTTATGTATACACCAGAAGAGGGCATCAGATCTCTTTACAGATGGTCGTGAGCCACCATGT<br>GGTTGCTGGGAATTGAACTCAGGACCTCTGGAAGAGTAGTTGGGTGCTCTTAACCGCTGAGCCATCTCT<br>CCAGCC
MSTR<br>G.470<br>11.1	--	0.531	0.000	0.000	-<br>5.835	do<br>wn	CTCCAACCTATGGCTCTAATTACTGTCTTTGTGCACGCAGGACCAGAGCTGTCTCCAGAGCAATTACAAT<br>TTAGAGGAGATTGAGAGACCAGGAAATGAATCCAAAGGCCAGAAAATCAAAGCGAAAAGGCAATGAC<br>ATTTCCAGGAAGGACAACGATACAAGAGGAAGATTTGGAATATCCCATGCAAAGATCTCAGTACTCTGG<br>TTGCCTGTTGCTAGTGTTTGGATGGGGTTTATGCCCCGATGATTTATTCATCGACTGCTTGGTTGTTAGTG<br>TGACAATACTAAGAAGCGACAGACCCTTAGGAGACAGAGCCCGCAGCAAGGTAATTAGGCCATCAGGG<br>AACCGATGCTGTTCTCATGGAGTGAGTCCTTTCCAGGGAGGTTTGCTTTCTACCCTGCCCTGGGATTTCT<br>TCCTCTTGCACGTCCTTCTGCCGTGATATCATCTACCATAACGTGATGCTGTAGGAGCTCTTGCCACAATT<br>AATGCTGTGCTATGTGGGCCAGGTCAAGCATGGAGAGAGAAAGAATTCAGTGCCAAAAGTTTGATGAG<br>CAGGGTTGACTACCAGTGGGAAAACGTATCCAGGAACCAAGTCTCCAGAGGCAGAGGTACTGTGAAG<br>TAAGGTTGCAGGACCTCGTTGGTAGAGACGCTTTCCAAAGACCTATGCCATACACTGATGATGTACCCA<br>TATGGCGACATAGTAGACAACATAGTTCCCCTTGACGTCTTAACTCAGGCGGTTAAATTTCGTGCTGTCC<br>TTCCAACTTCCCCTCCTTCCTCCTTGCCCGTGTTTCAGGAGGCCCCGAGCATCCACAGACCTTTGAGGT<br>ATCTAGCTGAGGGGAATCTGAGTTGGGGATACATTTAGACTGACTAAACGGGATATTTTCTTAAAGTTCT<br>GACTAGAGTACATGCATAGCCTATCCCTTTGTAAGGGTGCCCTTGGGGGTATGCATGCTGACTGCTGATT

							TGGCCAGCTTCCTGACAAGGATTCAGGTCATAGCAAGATAAGAGTGAATTCTTGGGACTGGAGAGATTGCTCAGTGGGTAAAGCGCTTCCCGTACAAGCCTAAGGACCTATACTCAGATCCCCAGAACCCACATAGGGCTCAATATGGTGCATGTGTCTCTAAATGTCAGCTCTCCACCAGAGACAGGAGAAGCCCTGGAAACTTGAGGGCCACCTACGCCGGAGCGGTCAGCAATAAACGCCAAGAAAGCGAACGAGGTGGCCGACGAGAACCAACATCCAAGGCTGACCTCCGACCTCTTCCACATGCTTGTAGTAGGAAATGCTCATCTGCGTATCTGTGTGCTCAGGCACACGCACACAGATGTCTGAACAAACAAAAACAGGGAGAGAGAATTTTCCAGAACAGTGGAGAAGAAATAAGTTTTGACATGCTGGGATCCAGCCTCATTTAGGAACATTCTATATTACTCGAGGAAAACTAGAGGATGCAGTTCAGTCCAGAATTAAAGTTGTATCCCATCAGGAATGAATATGCTAAATGACAACATATTCAGGTTTAGTTCTCCTTGCGTGTTTTTTTTTCCTTTGGTGGCAATTGTATGAAACGAAATTCCTGTGTCATAGGCGCCATCCTGAAGCAGGACTGAAAACAGCATGTCAGTGTGTGAAGTTGGGAGTTTTTCCTTCAGCCTAATTACCCACGATAGGAAACACGTTCTAACACTGTGTGCTAGGAGAAGCATTAAACTGTCTCCTTAATCTACCCACAGAGATATTATAAAGCTGTTGCTGTATGAAGACGGGATCAAAGTAGGCAGTCCATTTTTTTTTAAATGTATTATACAAAAGTGGGTGGGAAAAAGGATTTTAAGTAAAAATATTGTCACTTTTTATAGCTTTGGGGATATTTATAGCATTAGTCACTTTAAAAAATTTATAGTCTCAAGTTCTTCTTACATGCCGAATACATATTCATGTTGCATAAAGCTTCAAGTTTGTCTTCTTACAAGACATTCCTTAGAGAAAGCTCACAAGGCC
MSTRG.47055.1	ENSRNOG00000012601;ENSRNOG00000013254;ENSRNOG00000030102;ENSRNOG00000031959;ENSRNOG00000050067	0.431	0.000	0.049	-3.592	down	TTGGGGTATGGAGACAAAATCGGAACAGTGTTTTTCACCCCTGCTCTGAGAACTGTGTTCTGCTTGTCCTCCAGAAGGCTAGATGGCTAGAGTAACGCATTGGTAACATGGCTATGACTCCAACTATTGGCTTAAAGAAGATACAACCTGTTTATTTCTCAAACAAGAGAGCAGAGAAGAGTGGTCCAGGCTGCTGGCACTGTTGAGCTCCTTTGGGTCATTTAGGGACGTGGCTCCTTCCCATCTTTTGTTGCATCATAGTTCCTAGGATGTTGTTCTTGGCTGCGCAGTCAAAGCTAGGTCATGACCACCTGAACCACCATGGGCAGTGCAGAAAGCTAAATTGGTCAGGAGTAGGTTGTTTTTTAAACAACCAAAGGCAGTGCAGGTGTGCAGAGGGAACCAGAATGAGTCAGAAAGGCCTCTCTGGATTCCATCCGGGTCTCCAGTTCCAGGATGCAGTTCC
MSTRG.47128.1	--	0.315	0.000	0.000	-6.022	down	CAGGGCTGTGATCAGGATGTAAAGTGAATAAATATATTAATTGAGAGAGAGAGAGAGAGAGAGAGAGAGAGAGAGAGAGAGGAGAGAGAGAGAGGGAGGGAGGCTAGAACTTACATTTCATGCAGCATTTATGTAATGAAACTTCAGACTTGAAAGCTACTGAGGACTTCCATTTCTGTAGTCCTTCCTTTTCACCTGCCATTTCTGTGATCAAGCAGCCATCGTTAAGTTTGAGCCCTGCTTGAATTAATGGCAGGTGACCAAAAACATCCACGGTAAGCAAAACTAGAGTTTTAGGATTCCATAGGGAATCCGTGATGCAAATACTTGATGCACCTGTACAAGAAAAAAAATAGCATGGATGGATTGATGAGCAGATGATGGCCAGAGGCATGCTTAGATCCATTTCAATGCTACCATCAAAAAGGGCAACGGTGAAATCTAGGTACTAGAACATGGGTAATGATTCTGAAATTGCTTAAACCAGGAAATGGTTGTGAGTTAATCATGTTATTAATGTTGGGAGATGGGAGATAAGCTACTACATATCAAAGATAAGATAGAGATGGGTCGGTAAGAACTACAGCACACCAAATTATTTCTCTGGCTCATTGAAGGAT

							AAATGGAAGTAAAGAAGTAGCTAGGGCAGACCCACATACCCTCTTTTTTCTCCACTGGCTTCTGACTCT<br>CTCAAGGGAATCTGTCCTCATTCAAAGAAAAGGAGTAACTCCCCTCAACCAGCAATACACCCATAGATT<br>TTTTCTATGCATTTTTTTCTGTATATGTTTCAGAAAATAGAGCGGTTTCTGCCTTCAGAACAATTACAACA<br>CATGGCGAACATGTTATTCAAACATGGCACTCAAGTGTGGGGCTTCAGCAAGAACGCACTTTTGAAAG<br>TTTCGCATTAGTACGTGGAAAGGGTATTCATTAGGTGACGTGATAAAATCCCAGAAGGCCTGAACCTTT<br>GGGCCTTTGAAGACACAGGTACTGGCTGCTGTGATTTGCCAGGGGTTGCTTACCTTCCTTTTGCTCTGAT<br>AATTTTCCTCTGGAACAGCTGAGCAACAATTCGCTATTATCTGGGGAAGAACAGACATTAATGTATTATT<br>CACTGTGATTTTAATGTACACGGGGATATAAAGTCATATAAAACACTATATGACATTGCTTGACTTGTTCC<br>TTCTCTTACTCCTCCTTGCTGCCCAAACACAGTTTTAACCATTGCAATGGAGAATAGTCCTACCATGCAG<br>TTGGACTGATGCTCCATTAAACCCTCCATTCCATATGAACACAGTGAAGTGACTTGGGAGAAGAGACTG<br>TCTCCCATGGTACATCTATTTTATGACAACATGAGCAACAGGTAGGCTAATAAAGTTTTACTGAACAGCG<br>AGGTTAAAGGAAGGTGATATACCGTTGTAATGTGGATTTTATAATTTTCAAGGGTAGACTAGATATGCTTA<br>AAATGTTTTGTGCTGTTAGAAAAATGCATAATTCTCATTCAAAGAAAAGGAGTAGCTTCACTCAACCAG<br>CAACATATACGTATACATAGATTTTTTTCCATGCATTATTTTTCTGTATATGTTTTATAAAGTAGACTAGTTT<br>CTGACTTCATAGTGATTATGAGTAAAACAAAGGGCTTCCCAGGAAACCTTTGTGAGGTTTAATATTTTAC<br>ATTCTTCCTGCTTAACCTATTCTGTGGGAGTGGCTGTCCCTTGCTACGTGTATCACTGACCATTGCATAGC<br>TTTCATTCTTTCCACTACATCTTAATATCTACATTCTATAGTATTTCTACCTCCTAACTGCACACAGGGATT<br>ACAAAGATCCCTTTCTTTGTGAAAATGGACAACTGTCCCAACAACTTGTTTGCACTTTAGCTGACGTGT<br>GGGACGTGGCCTGATTTCCTTGATAAATCGGATGCCAATTTACAACCAGGAACAGAAGTCTGAAGTGTT<br>CTGAGACCTGGAGCTCGTGGTCTACACTGACTTTGAATCAGTCCTTTGGCTGGACAGTTCCCATCAAAG<br>CAAAACTGGAAATTTTTTTCCTTATGTTAAGGCATTATTTTGCCTATCATAGAAGGGAAATGAAAGAAAT<br>AAGGAGTATAAAGGGCTAGGGAGAGGGGTAGGAAGGCTGAGGGGTAGGTTGTAAATGGGATTTTGCTG<br>GAGGAAATGTTAAATTAATGGTTTCTAGATGATATTATACTCTAAGAAAAATGTCACAAAATGAGAGGCT<br>AGGCAAATTCTAACGAAACATATCAGCTAAAACTTGGTGGAAGAAATGTATTCACACTCAAGCCATGTT<br>AAGATACACTGAAGACTGTTTCTAATATGTGGATTGGGTCTTCATTTGTCTCCGATAAGCAGCTGCTATA<br>AGCAGAAGAGTCAATTCAAAAATCCCAAAATCATAGACAAGAAAAAAAAATCCACAGCACTGTTTGTT<br>ACAGAGAAGAATGGGAGAGAAAAAGTCAGAATTGTGTTGTAGGTGGCAACATATTGAATTCTGCTCTC<br>TTAAGCCCAGTGTACAAAAAGTATTGATTAACAGGTTAGGTCTTGTGAGAGGAGGGGCCGGAGGCACC<br>ATGCTTGAGTAATCCAGGCTGCCCTTGCTTTGTCTGTCAGCACTCCTGCTAGCTCCAGGGAGCAAGTGA<br>TTCTCTCCTTCCTTGCTGTTCCTTCTCAGCCTCAGCAAGTCAAGATGCAGGCAGCTCTTAGTGAGGATTA<br>GCAAATTTAAGAGCTCAGCCTTGGAAATGACTGTAATTTGATTCTATGCAAGATCCATCATTTCTCTGAC<br>TGCGAATGTCAAATAATTTCATACAACCTGCAGAACTGCTGTGGTGAACTGTCACTAGCATTTTTCCAAT<br>CCCGGCATTTTAGAAGAGATTTGGTGTAAGGTTTGGGGGTGCCGTTCAGTGTTAAAGCATGTGCTTAGC<br>ATGTGCAAAACTCTGTTTCTATCCTGAACATAAAAAAAAAGAATCATTATTGATAAGCATGGGGTATAAA<br>TGTGACTGTGTAGGCACTGGATTACACACTGGATTGAACCACAGAAGAGGAGGGAGGAGCTAACTTCT<br>AATAAGAGCCTTCTATTGGCCAGACCCTGGCCCTGCATTCTGTGTGTACTACCCTTCATAATTAGTCCATG<br>CAATGTGCTGACAAGATGTTGACTCCTGGACTGAGAAGATGAATCAGCACTTAAGAACATGTGCTATTC<br>TTGCACTAATATCAGATCACCTCTTCTATTCTCCTTGGGCACACGTACTTATATTACACATACACCCATGT<br>GCTTATGCACCTGTGCATGCACAAGCACGTGCACACATGCACACACACATATATATACACACGCAGACTT<br>ATATGCACATACCCCCACATGGATACAAACATACATACACTCATTAGCAAATACTCCTGTGTACACACAC<br>ACACACACACACACACACACACACACACACACACATACACCACAATTAAACATAAACCTTTACCAAAG

							ATAATGTCTTTCTTGAAATCCGGAGAACTAAGATTGTGAACTCTGCCCCATGTGACTAAGTTTGAGTGATTTTTTTTCTGTTCCTAGAGCGGTGTAGCATCTGGTCCATGACTTAGGAACCATAAGGTGAATACAAGAAGGAAAATAACCTGAAATCCAAACCAGTTTTCTTCTTCCTGTATTAGCTGTTCTGACTGAAACTATTGACTAAATGAGATTTGTGAGAATAGGAATGAATTCTGATTATCTAACTCCCTGCTAAGAATTAAAAGAGTCATATTATTTCTTCTGTGGGTGAGAAGAGGAGTGAGAGATCTGAAATAGTACCTATGGAGTATAAAAAGACGAATTTGGATACTGAAAGCAATAGTTGTCAAAGCTGTCAAAAC
MSTRG.47158.1	--	0.508	0.042	0.009	-2.603	down	CTCCCTTCCCTCTCTTCTCCTCCATTTTTGAACTTACAGAGCCCTCTGACATTTTGAGCAGAAGGCTGCAAATGTTTGCCACAATGTCTGGTCCTATCCCAGGAAATGCGCCTTGCCTGGCCACATCCTGGGAACATTTCCCCCTGCACTTCAACATCAATAAGCACAGACACACACACACACACACACACACACACACACACACACACACACACACACACACTTCAGGATTCTCAGGAGGATGAATGAATATTCTTGGGCCAAATACTGTGTGAGGGTTTGGGCAACATTTGGCAGGTTGAACAAGGCTTATATTTATGGCTTTTTGGCAAGATCATTGCTGATTATCCTGTACTTGGTGAAGAGCATTGTTTCTAATCAAGATAACTGAGCTATCCTTAGGCTTAGACCTGACACAGCAATTGCTGGAAAATGTCATGTTTTCATAATGGATCTTTGTCCACCATCTTCAAATGGAGAAGAAGGTGAACGAATGGCGAACAAAACAAAACAAAAACAAAAACAAAACAAATAAACAAGAAACACTGCACACCTTTTTTCTTCTGGTTTTACAGGTGTTACACCACTTGCTGTTTAAATGCATTTTTTTGAGGTCATTTTTGAAACTAAGACTCTAAACTAGTTAAGTAGGTGAAAATTAATTCATGGAAGAGGTCTCTTATAATTAAATTAAGCCTTTGTCATTTCCCTTAAACTATATTCCTTTCCCTAAAAAAAGGAGACATTTGAAAAGGAAGAAACTGACGGCAGTTGACAATACTTCCAAATGTTGTACATATATAACCTAGTTTTCTCTCCTGAGATAGAATTATGTCACCTAGGCTGCTGATCTTGAACTCACAATCTCTCTGCCTCAGTATAGGAAAAAATCTTAGTTCATGATTTAAATGTCCTCTCTCTCTCTTCCATCCCTCAATACTTACCTTTAGTTTTTAACTGTAAGAACTAGGATAGTTTTATTCATTGCAAATCCAGGCTGTGAACTGCACTACCCATTCCTTTTACCCTGAGCAACCCTTCTTTTTTCTCGGAAGGTAACGATTACTTCAGATTTTTAATTTCGTCTGGGTTTCCTCTATATACCTATTGGCAATGCCCTCTTATTGGTGTTCTTTGTCAATAAAGTGAACCAGGTTTTATTTGTTTTTCCTTGGCTTTGTCTTTCCTCTTTGTCTCCACAGAGACTCTTCATCCTCCACTTTTATGCCTACAGGAAGCTCTCAGGCTTGGTGCCAGCCTGTAGTCTGGTACTTCCCTTGTTGATTAGCCTGAAGTTTGATTCCTCAGCGTCTTCCTTTGCAGATCGCTCCACCTTCCACCTTCTCATTTCACTGCCCATGCAGCACACTCTGCCATGGTTCCTAAACAAGCACACATGGAAGAAATGTATTTTTAGACCTTGCTTGTCTGAAATTGCCTTTCATACATGGTGGATATTTGAACTAAATATAAGACAAAGGTCGAACATCATCTCCCCTCAGACCTGGAAAGCTCTTCGTTGCTGACATGTTTGCCGGCTTAGCTGTTGGTTCTGTTTTACTTCCACTGATGTCTCTAAGAAGTCTGGCGGCATGCACTCGCGCATGCCTAATGCTTTCTGTTTCCTTACGTTTTCAGAAACATTCTAGATGTTCTTTAACAACTAACTGCTCTGAAATTTAATACCTGTACTCCTTTTCCTCTTTCCATCAGTAGAAGCTCTACGCACATGCCCTGCAATATTTCTTTGGTAATTTCCCCATTCCGTTTTCTCTAATACTTCTCTCTTCTTGAGACTTCTGTGAGCCTTGGCTTTGGGTTTTTGAACGACTCTATTTTTCTAAAATGACTTTTCAGATTAAATCCTAGAGACGATATCGGTTTTGTGTTTTCCTGCTCCTCTTGGTGTTTTAGCTTGAGATATTTAATCTCAAAGGCCACTTAATAGCATTCTTTTTTTTCCCCGCTTTTTATAATGTACTCGTTTCAGAAATCTTGTTTCCTTTTCAACTCTGAGTTTGCTGTCTGTGTGGTGCTGACCTCCGTCTGCCACCTTGCTCACCTAACTTCCTCTCAGTTTTGTTTTGGGTTTGCTTTAGTTTCACTCTCACGTTCATCACAGGGACTTCTCTCCGATCGCTGATGGTCTCAGTTCTGTGTGAGGTCTGGGGAGCGTCCTTTTAATGTTAGCAGCCACAGTTTTTTCTCTGAAACCACGCCCTGTGCCAATTAGCTGTGTGTCTCACCACAGCCACACTAAAACCCCAAAGTGTTCTCCATAATGAATGCCAATGCCTGTCGAGATGATGCCGTTTCCATTCCGTTCCCACAACACCCTGCTGTAGCTGTGGGATAAGCATTGCCTCCACTGTCTCCTGGGAGACGAGGCTCCTGGCGGTAAAGCAAGTGAGAACCACATAGGCAACTGCACCCTGTGCTGGTTTCAACA

							CCCTCACGCAGCTCCCCAAAGCATAACTTCAGGCACCTATCACCCTTGAATGGTATGTTTTGGGTGTTCT<br>CAGGCCAATTTGCCCACATTCTGTGCACTTCCTATTCCTCTCTATCTTCTGCTGCCCACCTCGAGGTGGT<br>CTCCATTCCAGCGTATAAACTGCCAGGTGCAGACTTTTCATTTCAATCTTCCAAAATTGGTTTGAATTGT<br>TTTTACCTTATGGTGAAGTATTCCGTTTTTCATTAATTTTTTCTCTTTTTCCTAATCGTTTTAGTCTTTTATT<br>GTCCTTCCAGGAGTGGTCATCAATAGAGGAATGTGGCCAATACACAATATTTAAGCAGACTCCAGACTG<br>AATTACTGGTAGCTTACCATGAGTTCCAGATCGTGCCACTATAGGTCTGGATAGTAAAAGATTCTCCTCT<br>TACAAAAATAACAACAAAACAAACAAAAGCCAAAAGGAGACATGATACAGTTCCTAACTGTTATCTTC<br>CCCAAATAACATGGAAACCAC
MSTRG.47279.2	--	0.514	0.000	0.000	-8.550	down	TTTCTGTTGAAATAAATTATTGCTGTATTTTCCTTATAAAAAATTAGCCTTTTCTATTAAATGCTAGCTCAA<br>ACTTGGAATAATATTTAAACATGTATATGCTTGTTATACATATACAGGCACATACATACACATACACACACA<br>TATGAAGACTCTTTTATCCTCTTCTATCTCATAGTTTCATAGATCATAGACATAAACCATGTGAGTGTTTTC<br>TTCAGACTAGAAGGGCATCATTTAAGAAATTATTCTTTCCTGGGCATAGACAATCTGTAAACCTATAGAC<br>ACAGTACATCAGCTATAGTAGGTTTTTAGGGGCGAGATCATTCTTCTTATCCTTAATATCAGGACTGAATG<br>AAGCTCTCATTTTATAAGTTACATCGTCAGTTACATGGTCTGCAGTACTGCTGCTGAAGTCCTTAGCTTTC<br>CTACCTGCTGCTTCTATGGCTGCCAAACCGTCTTGAGAAATACAGAACAGCTATCAGTTAGCTGCCTTTA<br>GGGAAATAGTGGTAGCCCTGTTTTGGTATAAGAAGCCACTTTATTTAGATATGTAAATGAAGCTCTAGCT<br>TCTACCAAGTCTGGAAAACGTTTGCTCCTCAAATTAAGAACTCCCATTGAAAAAAAAAGAAAAATCCC<br>CAAATACCTAAATAGCTTCTTGGAAGGCGCACAGACTGTCGTCAGAGATGTGGGGTTGAGTTTTGGCTC<br>TGCTTTTTTCCCATCTGTATGAAGTTGGCAAACTCCTTCGCGTCTCTGAAACACTGTTTTTCTCCTGATA<br>AGTTGGGGGGAAATAATAGAGAACTTGGGGTTATTGTGATAACTCCGTGAGCTCATGCTGAAATCACTC<br>GATAAATGGGATTTCCTTTCTTCTGACTATTCAGAGGCGACGGGAATATGTAGGTAAAGCTTCACCATGG<br>TACCAGACTAGGATGTACTCAACCACAAGTGCTTGGAGAAGCAGGCTACCGGAAAGCTCACACAGCAG<br>CATTTATAATGCAATTCAACAGCTCTAACTATAGTATGACCCAGGGCAGCCCTTGACACATATCCTATAAA<br>GTAATTTAAGTTATTGCCTTTTGTTTTTATTGGGAACAGAAAATATAAGGCTTGACCAGGATGAAGCTTC<br>CAGTTTTGCCTTCAAAGCTTCTCTCAAGGACATTAAACACTCTTCCTAATTACTTTCTATTGATTTCTTTT<br>TTGGACATTTCTGCTCAAAATTGACTCCTTATTTTTAGGAAACGAAAAGGATGCTAAGCAACAACTGTT<br>AATGGTGTATTTGGAAGAATAGACTTCAATATTTTAGTCTTGTTTCTTACAGGCATTTGCAGCTAAAAGA<br>AACCATATAACCGTCTTGCTATGTGTCTCAGCGTCTCTTACTATATAAATAACATTTCCTTCCCAGCCTCA<br>ACTGTGAATAATTTTGTGACTTACCCTGCACAGTCAACATTCTCAGGAAAGATGATGTTTATCATAGTCA<br>AGTAAAAAAATAGCTTTTACTCAGTTTCTACCAAGTAGAGATTAGAAATAAAAAATTAGGCACATAACTGTT<br>TTGTGTATTATTTTTTTTATATATTCTGGGTATTTCCTATTATTCTTTCTCTGATTTATAGGACTAACATGTAA<br>ACAAGTCTGGTTAGATGACCCTTCCTTGTAACCCAACCAAACGAAAGGTTATAAACTGGAGCATTTAGA<br>ACGCAGTTCAACGACACTAACTGTAGCATGACCCAGGGTCTCGATGTTAGGTCTGGAAATAGCCAGGTC<br>TTTTTTTTTTCTTTTCTGTTATTGTTGTTTATGGTTTGTTTGTTTGTAGAAATTATTTATTTAAATTTAGAGG<br>GAAACCAAAGCTTAACTTTCATTTGAGAGGATCACCCATTTGAAGATAGGTGATGCACTTTTCAAATCC<br>CACAGTGATGGGGCAGTGAGTAGGGCTGAGGGTGGTGGGTACACACAGACTTCTGTGACTATGAGACT<br>AGTCAGGGTTACCCTGTCTCAAAACCAGCCAACAATACAAACTTACTACATTATATAGAAAGATGTTTTG<br>AAGGTTAAAAGAAAAATATCATCCAGCCAGCAAAAGTAGCCTGACTGCTACCAAATATTCCTAGAAATA<br>AATTTTCGCCTTCTCTAACTGAGCTGCCTGGCTTGCAGTTTTTCTTTGCCTTTTCTCCTTCAGCTCTTGGT<br>GTGTACCGTCAGCTTTTGGAGGAGTTGGTGGTATTAATTTTACATGGAGGTCAGTCAGATCTTATGCAAA<br>TATTAAATACTGCACAGACGTCGTAACTAAATTTAGCCCTTCGTTTGGTACAGTTCCTGATGTCCCGAGT

							CCCCACAGTGCCTATAGCTCAGTTTATTTCTATTCATTCACCCATACACTCACAGGTACATGCATTTCTATA<br>ACTGTGGGTTTGTAAAGGAATAATAAGGGTAATACCTTTCTACCCTTGAAATGGTCACTAAATGAAAATT<br>GAATTTTCTTAAAAGCGTTTTTAAAAGGAAGATGAGGATAATTTGATTGGAGGCCGAATAGAATCCATTA<br>TAGCATTCCAGCCGGTCGGTCACTTTCCTAATTTAAACTCAGCTAGTCAGGATCAGTTAAAGAAAGCTT<br>AACAGAAAAGCCTGAGCATTCTGTTCGGAAGGAGCAGAGTGGCCCATAATGGGACCCAAGCAGTTTCC<br>CCGAGTTAGGACTAAACTAGCATGGATTTGACAGGCATTAGCTTCTTGCTTTCTACTCAGAGTGTGAAA<br>CCTAGAAGTAATAGACCTGACGTCTCCTTCTCTGAAAATATGGTTTAGAAACAAAGGGAATGTTAATGT<br>GAATTCCCGGCTGGAAGATAGAAGGACATGATCTCCACAGTCATGGGGGCAGGGGTGGGGGCGGGCTA<br>CACTTTTTCTGTTGTTTGCTTGTTTTTACTACAAGGTCACGCGTATAGTTATGAAATAACCATCATGAGCC<br>AGAGTACATTCATGGATACATTATGAGCCTTGGCATCAACTGCCTTGGATGGGATTCATCTTCTTTGTTGT<br>TTGTTGGTTTCTACTCACTTTGTTTTGTTTGAGATGAGGCCCTGCTCTGGAGCACAGGCTGGCCTTGGA<br>CACACTCTGGTACAATCTGGTTTCAAACTGAAAAACACAGAACTTCTCCCTCCTGAGTGCGGTGAGCC<br>CATGTGTGTGCCATTGTATCCTGCTGGAGAGGCTTCTTTTCTTTTCTTTTCTTTTCTTTTCTTTTCTTTTCT<br>TTTCTTTTCTTTTCTACGCTGAGAATAAGCATAATACCCTCTAATTCTCCTCCTCCCCAGTTTCCTCAGCT<br>GTGAAAAATCAGGGAACATTCTTTTTTGCTATGGTCTATTGTGAGGATTTGTTATTTAAACCACTACACTA<br>CCTGGCACAGAATGGTTATTAATGAGTCTTGGTTTTTTGGGGTCTTTTTGTTTTTGTTTTGTTTTGCTTTG<br>TTTGTTTATCTTGGCGAGAAGCGTTGTGGTCTTTACTGTACAGCTGCCTCTCATTTGCCTGTTTCTTCTAT<br>GGTCGGGAGGCAGAGGAGCAGGAAGAGAGTCAGACAGAGCTGTACCTTCGCATCCAGCTGGGGCGAG<br>CAGGCTGGATGACTACGGAACGGGACGCTTACAACCTGAAGCTAAGCTACAGTGCTGGGCCCTAAAGT<br>TTTTGTGTCATAGAAAGGAACGTCTAAAACGAAAGATGATCCAGAGTACTCCGTCCTCCAGCTCTGCAG<br>GCACAAAGTACCTTCCATATTTACTTCCAATTTCCCATCCTCTTGGTCTGGCCGTAGGAATGAGTACCTTA<br>GTGCTAGTATGTGGCAAACAGCTTGCCCATTAAAACAGGGCACTCGTGAGCATTCTTAAAGGGCTTTGC<br>TCACCTCGGAACATAGTCTGTGGTTGTTTCTGTCTTAGAAAGGTGACTATTGGGATGAGTTATGCTCTTC<br>TGTCTTAAGAGGGGACCTCAGGAGAGTTTAGAGCATAGGGAACACCTCACTCCTGTTTGAATGCCGGAT<br>GCCTTAGAGAGTGAGCCTCCTGTGTGTCTTTCCCTCCTGTTTCTCTTCCAGTGTCCATGCTGACACTGAC<br>TGTTGAAACCTTCTTTGCTTTGGAGAAACCACATCCCAGGCGGTGTGTTTTCTAGACTTCATTTGTAGCA<br>TGTGACGGAATCAAGACTTCTCGCTGGTACTCATGCTCCTTGAGCAGGAGAATGTCCATCACAGGGTGT<br>CATAGCCTCCAATCCAAAGTCACCGTGACAGTCTCTCACTGTAAAATTAATTTCCACATTGGATGTTATC<br>ATTAGTGGAAATCATAGCCTTTGACAGAATGGCAGAGAGGATTAGTTGTGTTCTTACTAGGGAGAAAGA<br>GAACAGTGGAGAGAACTGAGCACACCTTCTCCCCATTTGGCAGCAACTGTGCCTGTTATCCAATTTTTG<br>AATATTTATTACTAATAAGTCTTTTTTAATAAGTCACGCCTGGTGAGTTACAAAGTCTACATATAAGTTCTT<br>ACCTTGAGTAACAAAAAGGGTCGTGTCAGCCTCGTTAAGGTGATCATTTGTCTCCTCTTGTCAACCACA<br>TTGTTCTTGCCTCCCTCATGGAAGTCCTCAGTTAGCTAAGCATCTGAGGTCTGTTCTCTTTGTTATTTGAA<br>TTATAAAACATTCCTGACCTGCCAGGGTAGCCATGGGTAAGCACTCAATGTATTAACTCATGAGAGCAA<br>CAGATTCCTTCTGTCTGGACCTCTGCTCTTGTGGTTCTGTGATCTAAGCACAGCTGGAACTTGGCTGTG<br>ACCTCATGCGATGTCTAGTCACAGAAAAATAGAAAATTTAATTGGCTTGGACGTATCTCCTGTCTTAAGA<br>ATTAGATCATTCACGACTTCTTTCTCAGGGTTACTAGAATGGTCACCATTGTCGTCTGGAACCAGTCTCA<br>CTATTCCGAGGCTTCAGGGGTAATTCCTGCTCACACATCTTCTGTCTTTTGTTTGACCTTTGCATCCATGT<br>GTAGCTGGGTTACCTGTCTCTAGACAGCAGCGTAGATCACATTGCTAGTTAATGGATGAAATCTCATCTG<br>ACATTTTCAACTCTACTGTAGCCCTTGCTGTTATGTGCTGTTTAGTACAAAAGTTTCAATCTCATATCAAG<br>ACTTGAAGCTATGTATGATTTCCCACAAATTGTAGTAGTTCCAAATCTAGCAGCCCTCTGCAGACTATATA

							GTCATTTCTCAAGAGGCAATGAGCTGAAGAAAGTAGCCTCCTCTGGGTACCAAGAGCCCTTCTTATTCAGCTTCTCTCTCCTCTATGTCTAACCTTAGATCAGGAAAAGGTAACAGTCTGACTTGCCTTACACATTCTGACTTGAACTTCACGTTTGCTGTCTATAAGAAGTACCATTTCACACAGTCACCATCCTTCTGGTTTTCTCATTTTTTCACCCATGGCCAGCAACAGGAGGGATTCTCGCTCTTTCACTTCTCCCTCATTTGTAAGAATGTTCTTAAATGTACACGACCTGTGGCAATTTCTGTGAGTGTGTCAAATATACTACCTAGGTTTCTCTAGCTAAAAATTATTTTGGATCAGTGATGAATTATTATCAACTGACTAGAATTGAACAAAAAAATATTCAGAAAAAAAAACCTTGGAAGGTTCTTGGATGTCCTTGGTGTTATCCTACTTGCAGGACAATCACTTGTTCTAGGTTTGATGAGTTATAGTTAAGAATAAGGTAGCTTGTGTCTAAATTACCCAATAAATGCATCGGTCCTTGTCATTTCTTGCCCAAAAATGAACGGATGAATGAGTGAGTGAATTAATGAATGAATCGACTTTCAGACAAAGGGTAGCAGACTATTTTGAATTTTGAAAGGAAAGGCATACTCAAGCACACACACACACACACACACACACACTTGCATGCACACATAACTATTTACATATGAAAGCACTAACAAATGGGGAATTCATTTCCCCAAACTCGAGCCACATAAGCCTATGTACACACCTAATAAAGTCTGATGACCTGGTCTAATAGAAAAGGGTCTGAAAGAGCAAGGAAGGAGTTCTGGGAGTTCTCTTCACACTGTCTGATAACTGTTCTTTAATTTCACTAAGTGATCTACCTGCTTCTGCCTCCTAAGTGTTGGAATTAAAGGCATGCTCCACCACTGGCCAGCAGAGGTAAGGATTCTTCATGGAGATGTTACACAGAAATCAGGATAGAGACAGAGGTCTAACTTCTTGTTTATAGTCTTGTTTTCCTGGTAAGACTTTCTCTTACACTTAGAAACAATACCAAAGCTTACATATAGTCCATGTTCTTCTTTTGAGGGCTTCGTAGGATACATTATTCCCAAGTCTCCTGCTTATGGTGGTTACTGTAGTCTGGATCCCTTTCCCCACTACCAAATCTGAACTTTGCTGTAAATAAACACTGGTCACAAGAGGTTTTTCCAGAACCTATGTGAGCTGAACAAGAAATGCAGGTTCTACTAGATATTGACATACTATTCATTGTTATTTACAGTGTTTTTGAATAGATGGCCAACTTGGGCTAAACATAACCTGACTTTTCAAAAACCGTTTAGGAAGTAAGGAGTAAGCTGGGAAAGGAAGAGACCAGGGTTTGAAGTCAAGTAGCTTTAAAGAGCATGCTTCCCTTAGTGTGCTGGACCGTCCTGATCCATTCCTGGACAGTGCTAGGGTGTGAAGCCCTTTCGACATGGATAAAAAGAATGACTGCAGCATCTGCCCACCACCCCAAATGGTTTATGTCCTACAATAAAGGGATGAGCTCAGCTACTTAGAATTACTGGAGCTTTGGGAGTAGGAAGTAGTTTATTAGCCCAAGTAAATGGAGTGCTACCAGGAAAAAAAAACAATAAATTATAACAAGAAGCTAATATATTGAGTGTCACATCCGTTTCTGGCCTAGCAGGGATGTAGTCAGGCAAACGGAGACAGGCCTAGCTTCTACCAGCCCTTATAGAAGGTGTTTGCCATGCTTGTTTTGGCTCCCCTGGATATTTTCCAGCAACCAGTTGATAACAGTAAGATGCAAAACATATAGGTCCAGTTCATCAAAAATAGTGTATGAATTAGGAAAGAAGTTTGATCGCATAGATTTCTCTCTGGGGGCCTAAAACTACAGCCATGCCTTGAAACACCTCTTTATTCTCCTGCACTCAACAGCCTTTACTACCATTGGTGGGAAAACAGGTGACTGGTAGCTTTGAGAGTGGAGGCTGTGAAAAATCAAAGCCTGTACCACATGTATCTGTGTGCATACACACCCCTCCCTGCACAGTTTCCCAGCTGTGGCCTATGTGTAGTACACTGAATTTAAGCTTATCTCCAACTAGCTTGCAAATAAATGAGGCTTGCACTATGGTTAGTTTATGTAGCCTTAACAATTACTGGGGGTAAACCCTAATCTGCTTTTCTAACTGATGGCCTGGCTACTTCCCCAGCAGTGTGCCCCCAGATACTTGGTGTCCATCTATCTCCCCACATGGGGGCTTCCTCTCTCTTCCCTTCTTCCATCTTCTCTTCTCTCTCCCCCAACCAGGTCACTCGAAACCCACCTACCTCTAATCGCTCCAGTAATTGGCTGTAGCCAATTTTATAAGACCAATAATTTTATTCAATCAAGGTTTGCACAACAAAAACACATAAACATGAGAAGTCAGTCGTAGGCCTAGACCTCCAGGTAGAGAATTTAGCATTACGATACATAACAGCAGAAGAAACCTCAACACCTATGTTTTAAGTATAATTCTTGTAGACATTATGCATTCGGAGTCTATTATTTTGTCCCTGCCCCTGTAACAACACAAGTATTACCTACAATATTACCTTGTCACTGCACCATCTCGGACCTTGTAGCTTTGGAGAAATAAGACAGAGTATGAACTAAACCATTGGGACATTTGCCGGGAGTGGGATTGGATTTGGGAAGCAGAGGAAAAGGGAAATCGTTTTCTTGAAGTGAAACATTTAATATTACGCATGTTTTCTTGAAACCTCGTGCTCAGGATGATCACACATGAAGCACTGACATAGTCTTGAAAA

							AGATCAACTTCTGTGTTGTTTGGTGAGGTAGATTTTATACCAGGAGCATGAATATTATAGATATCCCTCTA<br>ATAGCACATTCTATTACACGCTGTTAGGAATGAGGAATTCCTGGAAATTTCAGGGAAATATATAGCCTGA<br>AGTTAGTGTGACCAAGTAGGACACACCATTTACTGCTCCTTGCCCTGTAAGCATTAACCTTTGACCTTCT<br>TGAAGAAAATGTACCTATAGAAATAGACATTGGCTGCTTATTTCCACAGCCATCTTCCACACACAGAATT<br>GCAATTGTTTGGCTAACTGGCCTGTAGTAGCGTATATTCAGTGTTTTAACGAGACATACTGTGTGCTGTA<br>AAGACAAGTATTCTAACAATATTATATTGGATTCTCCCCTGAGATGGCTATCAGTCCTAGAGATGTCTAAC<br>CTCAAAGTTAAAACACTTGGAGCATTATGTCTGTCTTTTCAGAACTGTTCTTGAGTTATTTTCTTGGTCG<br>CTCCTTGACACAAAGCACAAGAACTTAAGATTATTTAAAACACTCATCAGAGTTAATTTTCTGTTGTTGA<br>AAACATTACTGCCTGAGTCCTTTATGTAATGAATGATTTCAGATGAAATTGTTACTTTCTTAAACTAGAGG<br>GTGGTTTGCATGTATATGTGTGAGGATTGATCCCAGAATCTTGTGCATGCCCCACATGTATTCTATCAGTG<br>AGCTGCAACACCAGCCCCACTCCACCAGTGCGATCTGTTGTTAATGGAGTCTTTTATGCCAGTGGACTG<br>GTGGTAATGGACATTAGAGTTCTACCAATGGATATCAAATATCAAGCAAAGCCCAAGTACCTTTCTCTAC<br>TATCATGGAGAGGAGGGCATTTCATTGATGTATAAGGAGTAAGGAAAAAATGTGTTAGGCATTTGAATG<br>CCTCAGAAAGAGGAAGAATCGAGCTCAAAATATGAGTGAATTCTGGTATTTAGCTCCTAACTTTAACAT<br>ACACAAATACAAAGGGTATATGTGACGTCTTTGGATTATATTGCCAATACTGCAATAAATAAATGCAGAG<br>TGTTTCTTGGTTGACTTATGTTCTTCCTGGCCCTTACCTGTTCATTTGAACTCTTTCATAGCTGGACCTTT<br>GGAATGGCTCTGTCACTCGCTGGTCTTCTCATCTCCTTTTGCTCCTTCATCAACCATTAAAGGATATACCA<br>CCAATTTTCTTAATCATGACTAAACTCAAGGTCTCTCTATGATCTGCCTACTGAATATTGATTCCAGCCAG<br>GAGTGTCAGTATGTCTCAGATATGGATGCTTGGTTCTGCCACAGCATCCTTACCTTCTTCACTGTATTGG<br>AATCGTTATGCCTTCAGTCAACACAACCCTTCCTTCCCACCTCCACAGATGTGATTTCTTATCTGTTCAA<br>CAGTGTTTCTTTTTCACACAGCCACAGGCCCTCAATGCCCCTAACATATCATCCTAATCTTGGTCTCTGT<br>GTATGTCATTAAATTCATATGACATAATTTATTATTTTTTGACTACAATGAATATTCCTTTATTTCACTGGGG<br>TTTCTTGTTAACCAAGATTAGTTTTCATGCATGCATGTTGTCAAATAATGATTTTTCATTCTCTTTCTCTAA<br>ATCTCTGAGAGAAATTTAAGCTTCTGCCTATTGCATTGAATAGAATTCTTGCATAAATATCAGCTAAGAAT<br>ACTTAAAGCCATAACCTTACTTGTTCTTCCAAACTATGGAACTATCCTTCTTCCAAGAGTTACAGTGTTA<br>CATGCATTTTAGGTTGTTGGCCAAAAGTAAATACTTTCACATGTTGCGGAGGATTACTTTATATATATATAT<br>GTGGGTTTGGGAAAGCTAAAATCACTTCTTTGTAATTTTTTATTTTAAAGATACAATAAGCACATATTTAA<br>AACCCACTCTATAGGACTTGGATGTATGTCCGTGTTATGAGTACTGGCCTTTCATGTGTGAAATCTTGAG<br>TTTAATCACCAATACTGAAAATAACTGTATAGTATAGAATTTAGTATAAACATGGATATGAATGGCTCATG<br>AAAATACAGAAAATGATATAATAAATGTTCATGTGCCATAATCGACCATAACATTTATTATCTTAGTTTCCT<br>TATTGCCCTTTCTTCATACTACCTCAGAGTAGATCTCAGATTTCTGACCATCTAATATATGTATATTTTAATA<br>TATATTACTGAAAGATAAGGAATATGCCATTTTCAAACATAGCCATGAGTCATTTGACCCCCCCCCCACG<br>CACACAAAAAAATAGCGGTAATGCTAAGTATCATTTGTTATTTAGATAGTGATTGGACCTTCTCTCAGAAA<br>CCTCACATTTATGACAGTTTTGTGGAGTTAGTTTCCAAGTGAGGGTCACACACTGTGCTTGGCTGATGC<br>CTCTTGACCCTTTTATGGGCTTAGTGTTAGCTTTTTCTCTTCTTTCATTCTTAGCCTATAGCTAACACTCTC<br>CTGTTCATGCCACCTTTTATGTTCTTCAAGAGCTCTGGTGCTCCACGCTGCAGAGCTCTGTGCTGTTGGG<br>CTTTCACGGTCATGCACAGTCCTGGTAGCTCTGAGTGCGCTCCTTTTATCTAATTCCCTTCCAAGTCATTT<br>TTACCTTCATGGGAATATCACATTAAGATAAAATTTGTAGATGAATGCTTGACGGTTTTACAGACACTTTG<br>TTAATTAAATGCTGATTCAGTATCTATTAAAATGACCACATGGCTTTTATTTTTGTTGGTTATGCCATGGTC<br>TTTGCTTAAAATATTGGCTATAAAGCTGAATCGACCTCACGGTTTCTCTCTACTGACCTTTCATCTGCTTT<br>TGTTTCCAAACACATCTTAGAGGATGAATAAATTAACGGAGGTCATTAAATGCTACATGGTAATTCTAGC

							GACAACTGAATAACTGACTCTCCTATCATAGTTGGGAGTTCATTTCTTTTGGTTTATTCTGTCACTGCTAA<br>AATTTTTGGCTAACCTGGACAATATTCTCTATGTCGGCATGACCTTCTCCCAAACAAATTGATCGTGGTA<br>GAAAATGCAGACTAAAGAATGGAGACCTTCAACATTTAACACGGTAAGATCTAGAAATATACCAAAGTG<br>AGCCAGGCAGATGGGCTGCTGATTTTTGCTTTGTAGGCAGCTCGTCTAAGACTGTACTCTTTTAGAATAT<br>CCTTTAATGGGAAAAGGATCAAAGGACAGCTTACACTTAGACTCATAAAATGTCAGTAGTTTCTCAGAA<br>AATATATAGAAGAACTATGTTTCTGGAATTGCTTTATAACTAAAAAAGATAGATAGATAGATAGATAGATA<br>GATAGATAGATAGATAGATAGATAGATAGATAGATAGATAGATAGAAAGGTAGGTAGGTAGATAGATAGATAGAT<br>AGATAGATAGATAGATAGATAGATAGATAGATAGAAAGAGCTATTGCCTTTGCAAAGAGGATAGTGTACATTTC<br>CTGTTGTCATTCTACCAGCTAGTTACCCAAAGATATTCTGTTACTGTTTGGAAAAGCTGTGACATCCTCC<br>AAGACATTTATGCAACTCTGATATTGTCTTAAGTTGTAGAATGGCATGACTAGACCATCGTGCATAAAAT<br>GTCCTGTCAGGGAAAAATGACATCTTGTACATTTAAAAAATAACCTTGCCGACAATCTTATTTTCCTAAG<br>GATAACATTGTCTAATAAGACCAAGTAATTTTCTTAGCAACTCCTGGGAACTGAAAATGGCCTTTCTGTT<br>TAACTACAGATGACCTTGGTCTTCCAACAACAACAACAACAAAAAAAATCATAATTTTGTTTTATGAGGG<br>AAAACATCCAAAGGTTACTTTTCATTCTGCTGGAAAGGAAGAAATAATATTAGGGGCTTTTCATGGCTTT<br>TAAGGGAGAGAATTATCTAAAAGTTGTCTTAATACCTGCCCCCCCCCCGGATCCCTGAAGGAGGTTAGC<br>TGAAATAAAACACACACTGTTCCTCATACATTTACGTGACCTTGGGAAGTCTGCCTCTGTGTACATTTGT<br>GAATTCTTAACTGGTGTATACTATTAAAGACTGAAGAACCATCTCATCTTTTGAGATGACTTATGTCCTCT<br>TTTAAAATACATAGGATGTTGTAAGATATACTATTGTCATTTCCGGTCCATCCATTCTACAGGAGTCTAAAT<br>GATAGCCAAACACAAGTTCAGCAATTAAGCTAGTTAATCTCCATTGGACTTTTGTGTCAACTTGTTCTTC<br>AGATAAATGACCACCTTATAGGGTGTCTGCAAACTCTTAAATTTCCCAAGTTTATTAAGTAACTCTTGAG<br>TCCCTGCAGTCAGATAACCTAGTCTGCTAGTGAAACTTCTGGTTACATGTTGCAGGGTCTTGTGGCATTT<br>TCTTTGCTAGCCCTATTGAAGACTTCCTCCCTTCCTTGTCTGTCTGTCTGTATGTCCCTTCTTCCCTCCCC<br>CCTCACTTCCTCCTACTCTACTCTGCACTCCTCTTTCTTTCCTCATTGTATCTCCTCCTTTCTCCTGTGTGG<br>ACCCATCTTATCTTTTTCAGGGCCGGGGATGGAACCCAGGGTTATGCACATTCTAGGCAAGGACTATACC<br>AATGAGTGATGCTTACTTCTGTCCTTTCCTGTGGTTTCTTTAGCAAAGTCCAGTGAAGGAGCAGTCAAA<br>TACATGGTCATCATAAGGGCAAGGACGGCATAAGCTATCTGTGCTTTGTTTGCGCGTAGCCTTACAGTCT<br>TTGAATTTGTGGCTACAACGACTTTAGTTGCGACTATTACTTTCTACGTTAATGTTATTGCCAGAAAAGG<br>TTATTTTTCAAAAATACAAGTTGCTGTTTTTCCCTCATGGCCCATTTTACTCATGAGAGAGTCTAACCATT<br>CTGTTCTATAGCTACTTAGACAATGTCTCAGTGACACAGATGTCTTGAAGAATGTCACAGCTTTTCCAAG<br>GAAAGGGAACTGTCTTTGGGGAACTGGTGCATGCTAATCCTTTTCTACTCAGCACCAGCTTGTTTAAGC<br>TGGACTTCTTAGGAGATGGGTGTTCCTCTGAGGTGTGGTCTGTCCTCTCTTGATCTCCTAGATCCAGAAA<br>AGTGTCTGCCAGGCACTATTCCCCCTTTACAGCACCTATATGCTATTCAGCAAAGGATTGAATTACTATGC<br>AGGGGTGGGGGAAAATGCCAACCCAGAATGACAGTGCATCTGTGATTCCGGTGTGGCTCTGACTTTTC<br>CTGTGGACAGCTAATGACTGCTCCCATCATCCACTTACTGTGGAGAGAAGCCAGGTGGGCAGGACAGC<br>ACAGTTTTTCACCTCCGAAAGTGCTTCACGCACAAGAACCAGCTAAGCAGTTTCCAGCAGTGCAGTGT<br>GCTTCCAGCGCAAGGAGTCCTCTTGCATTCTGGGGGAGAATAATAATTTGATGGAGTTTGGGCTTAAAC<br>AAGCCCAACATAAAAAACAAACAACCAAAAATACAGGGTATGGAAGAGCAAGAAACCTTGCAGATTC<br>CTAAATACATACGCATTGAAGATACAGGTAAGGTTAACAACTTTCCTTTTGTAGTTTGGCTGCTTCATATA<br>GGAGTGTTAAGAGGTTTTTTTTTTTGTTTGTTTTTGTTTTTTTGTTGTTGTTTTTGTATATATAGTTTGTTG<br>AATTAACCTCATATTTACCTAATTTTCCGCAAGTAAACCGGTAAAAAGTGTCTTGAACACATGGTCATTC<br>AGGAAGGATGGACTCTCCCTATTGCTCTCAGGAATCCAAGATTCCTCCTCCTAATTCCTGCTCATTGTCC

							ACAGAAACTGGTCTGGCTTCTTAGCAGAAGTATTTGTGTTATGGTCCCAGTCTGTGGGTCTCTCTGCCCGCTGTACTAGTAAGGAAAACGGTGTTTATAGCCTGGGAGAGGG
MSTRG.47542.1	ENSRNOG00000012674;ENSRNOG00000012739;ENSRNOG00000012991;ENSRNOG00000013751;ENSRNOG00000038473;ENSRNOG00000059588	0.000	1.409	0.000	4.948	up	CTGCTCTTTGGAGACAGGATCTCACTGCTTCAGACTTTTCATCTCCCTGCCTCGGCTTTTCAAATTTCCAAATACTGGAATTAGAGGTATGCCTGGTAGATCGATCGATCGGTCTGTCTGTCTGTCAGTACAAACACACACACAGAGCCCCTTTTTCACTAATAGAGGGAATTCCATTGCTGGTCACCTCATTCTAGAGTTAACATGTTGTTCATGTTTCTTCTTCCTGGT
MSTRG.47838.3	ENSRNOG00000018650;ENSRNOG00000019018;ENSRNOG00000054572	0.478	0.004	0.014	-3.354	down	CCGGGGGCCGGCTTTATTTCCCGCCTTTGCGGTGGACTAGAAAGGAGTCACACCCGGGAATTCACAGTAGAAGCCGCAAGTTTCTTTTTAGCCAGGATGCTTGGGTCCGGAGAGCCTTGTCCCGGCAGGCTCAGCAGCGCCACCTTCCGTGGCCAAGAGCGCACTGCTCAGTCCGCCGCTGCGGAAAAAGCTCGGTGGAACTTCCCACAGTCGCTGTTCTAGTGGCGTTCACATTTGAACGGCTAGAGCTTTCTTGGCTTTAATCCCAGCAGCAGGAAACCATTCAATGCTGTCCTGGTCTACATAGTGACTTTCAGGCCAGCCAGAATCACCCAGCGAAACTTTCCGGTCACTTTCTAGCTCCCCGGACGACCTTTGTGACTGGATGACCGACTGCCTTCATCCTCCAATCAGGGTTTGGAGTCTCAGGGCTACACCAATGGAAGGTTACCGGACCTTCAAGAAAGGCCAATCGGATGCTAGAGCCTAGCAAGCGAACGGCGATCAATCAGAGCGCAGGAGCGGCGGCCGGGGAGCGGCTGGCCAATCAACGTGCCAGCGGTGAGGAGGGGGCGGAGCCTTCGCGCAGGGAGCTGTACGCAGCGCGATTGCGCTGCCGCGGAGCCGGAACGGAGCCTGAGACCTGGTCGAAGCTGGTAGGGCTGGGCGCGCGGTTGGGAAGGGCTGCGTTGGGCGTCACATGGTCCTTGAGCATGCTTTTTGTGGATGGCAGTGTCCTGTCACAATATCGAAAAGTTGGTTGTGCCCACGGGAGGAGGACTTGAGCCTGATC
MSTRG.47889.10	ENSRNOG00000012757;ENSRNOG00000012841;	0.298	0.000	0.000	-5.855	down	CAGGCTGCATTTATATATTTATTTGTATACATATGTGGCAATAATCATGAAAGAAAAAGAGACCATGAGTTCCAGAGGTAGGAGGGTGGGAGGGGAAAGAGGAAGTGGAATGATGCAAAAACACTTTAATTGAATCAAAGAAATCACTTGCCACATTTAGATAGTTTCGTATCAATAGCTCTACCTCACAATTAGATATAAATATATTATACAAAGAGTATGTACCATACACTTCACAGTTGCAGTATCCCTGTAAGCAGGAATGTGAACCCCCACTGCTTGGACCACTTAACAACCACTCAGAAGCCTCCTAGAAACAGATGAGCCTCAACATTTCACAGAGTACCTACCTGAAATCTAGTAAAAAATAAATATCTCTAAAACTTATTAGGAAGGCTGGAAAAATATGTGAATCATATACTCTTAATACATAATTTTGAAAAGTTAGTGTAAAAGTGTTTTTAAGAAATTGAGGATCATTTCTTCTT

	ENSRNOG00000012878;ENSRNOG00000048769						TCACCACACCCCCTATTTATGTTCATCCCAATTCCTATAATCTTTAGTCCTATTACTGTATGTGGATGGGTAGGCTGTGTGGGCTATCGACAAATTTTACCTCAAAGACTGGTCTTAACATGACAGAGTAGGCCAAGAGTCAGAATACCAGGCCATAATCCTGATTCTGTCATTAAACAGCCAATGAAGATCTACTCAGTTCTTTAAATGGTTTTTGTCTGTAAAGTTGAAAATAAAATAGAGAATGTGTAAGAGTCTGTCTCGCTGGGGACTGTCTGGTTAGCTGAGTTCCCATTGTAGTGAATCAACAATGTAACTGGGAGTAGATAATGAAGCTAGCTAGATCCAAACAAAGCAAAACAACTAACAACCAAAACCAACCAACTGGCTGGAAGAGACAGTGTGACTCCTTTGAATGCCTCAACACAAATGTCAAGCTGTGTGGGCAATTCATTTAATTACAAGGCACGATGTATGAACAGAACAGCACATGCTGCCCTCACAGTCCTATCTCTACACACATTCATGTGTTGCTGTGCTGTGGCTCAAGCTTGCTCATGTATTACAGCCAGCTTCTCCAAGTCACACCCTCAGCCCCCACAGCTGCCAGTTATGCAGAATGGAGGCCAGGAGAAATTTATACCGATTTTCACCTACTGGCTAAGAAACCAAGAAACTACAGAGTTGTAAACACATCTGTAATAATGGGTACAGAAAACCTTTACTTCTGGTTGTTAATTTTTGTAAAAAAAAAAAAGTTTTAAGAATGGTTTTTACAGTGCTAAATTACTTGGTAAACAGCCAGCAGTCCTCTATATCACTCACAGTAAGATCTCCAGAAGTCTAGTAAAGTCCTTGGTGAAATGCATTGTCACTTAAAGAGAGAAAATGTGTAAGTTGGCATTAGTTTGTAGGTCATACTTGAGGAACTGTACACTTTAAGAGTCTAGACAATGTTTAGATTGTTGTTAGGAATCAGGCTGGTTTAGTAAATAGTGGCTGTTCTGTAGCATTGAATAGTTTAGCTAGGTTCCCCCTCTAGTTGAGCAGGTCGTGTGTCTAATTGAAGAGCTGTTGGTTACTGTCAAGTGTGTGTGCTGTGCTGGTTTTCAATGTGGTTTACAGGTGGGAGAACTGGGTCGGACCGTTGGTTGCCCCTCTCCTTTGGAAGGTTGCCTGGCACCTTCATAACATGAAAGTTAGTCCTGGGGCAGCAACAGCTCTCTCTGTGTGCATTTTCAGGTCAGTTCCAGCTCAGGGGTCTTTTGGCTTGTTTCTAAAGTACATTGTGTTTTCAGCAGTTTGGACTTATCTTTCACCTCTGGGGTAAGCAAGGATAATAGGCTGTATGCTTTGGGGAGTCTTTTGGATAGCCCTGGCCAAAAACTCAAAAGACACTTCTCATGTCTGGTGTTGGGTTTTGTGTGTGTGTGTGTGTGTGTTGGTCTTTGGGTCATCTGTCATTCCAGTAGAAGAGAAAAGCTCATTAAAACTACATGTGTACTTATACACAGAAACACACACACACACACACACACAGTTTGTTGCTGTTATTGGTAAATACTTAATAGAATGATTCCTTATGGCTTTTCAGGTGTCCTTATTTTTATTTTTCTGACCTCCCTCCTTTATCTATCTCCTTCCCTAAAAGTGTCCCCAGCCCCCACACCTTTTCACATTTCTATCAGATCACTGAATCTTTGGTTACTTTTAACTAAATCAAAGTTTAAAATCTTCAGGGAGGCTTCATATACTTCACATCTGGTTACCATAATGCTGAAGGTATTAATAAATACTTTGACTTCAGAAGAAAACCACCCCAAAGGTTGAGGCATAGCTTTCAGTGTTAGCAGGTCAGCTATTTGTAACTGAAGTGAGGCAGCCCAGTATGAAAACTACCACTTGGCACTTTCTTTCAAAATGCCATTCATGGTCTTTCACAGTATGTGGGCAGGATCATACAACATGGTCAGTTTCAGAAGAGCAACTCAGCTTGATGTTCTCTTGAATGAACTGAGGCTGACTTTTAGCCTTCAGTGGTCTGTTAAGAGATGAGAAACCCATACAGATGAACTGGGGTGGGTGCTAAACAGAACCCAATTTCCACAGAAACCTCGTTCTGGGAGAGATTGCTGCCTGTTTTGACTTAAACATATCGTATGCACCCATTGAAGAAATCAGCCCACCCCATCTGATACTTGAGGAATAGGATTAGAGATGCCTGTTTGAAGTTTCTTTTTCCTTTTAAGAAAATTAGTAGGTATACTTAAAAGGGTTACCCATGCTATTATTTTCGGACTCCTGGGGTTGAGGTAAAGTAGCTTATGGGTTAGAACACTTGTGTGCCACGAGTATGTCTCTAGGTTCAATCTCTAACCCTGAACCCACCTGTCAAACAGAATCTAATCCAAAAATTTACAAATATATTTTAAAGTGCCAGTTGATAACCTGCTGTTTCTCTCAGCACTTCCTTTTCCTCTGGAAGGAAGTCTGAGATCAGCTCAGGGCACTCTGAGGTGTTAGCATTCACGGCCCTGTTCTTCCTTACAGTTTGAGGTGCTACATGGTCCTCCATCATCATAAAACAAACTAGAACCCCAGGAGTGTTATTTTATGTTTTCTAAAGCAAAAGTGGTTTGAAAAGCAGGACTTGGGGTTCAGGATGCAGCTCAGTTGGTAGAGTGCTTGTCTAGACTGAGGCCCTGGGCTCCACCATCAGTTCTGCAAGTTCTGCATACACTGGTGCATTCTGGGAGGTGGAGGCAGGAGCATTCAAGGATTAAGACAAGGAAAGGAAAATATTAAATTAAAAAGATGCATGAGTATATGAAAATTTGATTGGTAGTTTTCT

							TTGAGATCTGTACTGTTCAGACTTTGAGACCAAACAGTAAGTAAAATCTGTACATTAAGCTACATGAAGCTGGGTGCAGTGGCACACCTTTAGTTCCAGCATTTGGGAGACAAAGGCGGGCGGATCTCTCTGAGTTCAAGGCCAGCCTAGTCTACAGAGTGAGTTCTAGAACAGCCAAGGCTATACAGAGAAGCCCTGTCTCAAAAAAGCAAAAGAGAAAATACTCAAGATTACGTTTTCCATATAGATTAGTTCTGCTGTTTTTGTTAACTACTGCCCTGTCTA
MSTRG.47980.1	--	1.265	0.101	0.002	-2.820	down	TTCATGTTGATTTTTGTCTCATTGAAGAGCAAAGCTGACACGGATTTGACTTTGGTTAGGAATCACCGTTGACTTTTCAAGCCTTCCAAAATGGCATCTTAGAGGAAGTTGTAAGATACCTTTGTGAAGGATGAGTCCTGCTTGCGTTCCCAGTCAGAGCATCCGTGTCTAGTAGCTGCTACTGATGTATTTATTTTGTTCTGTGTTTATTAACCTCTCTTCTTAACTACAGAAACTGGTCCTCGAGTCTTTGGAAGAGAGGATGGATGATGCCAATGACCCAACAGCTGATGAAAGCCAGTCCTAGAATGAAGAGATGTTTTCTGATCTCTGATGCCCGTACCATCCACACCTAAATGCCATTCCTTGGTAGGGCATAAGATATAGAACCCCCAGGAACAGTGTAGGACATTCCTTGAATAATAGCAAGCTTAAAGAGCAAGCTGTATGGTAGGAATCGAGTGGGCCTACTTTTGTAATGTGTGATGCAGGTTCCGCAGAGTATACCTTCTCATATGCAGTTACTACTTCAATAAATCTTTCACCACTTGCAAATGCCATGCAGATGATGCTGTTTTATGAAAGAACCAAAAGGAATGTTTTAGAGTGTCAGAACTATGATCAAGGAGGCCCTTTAGACATAGGTCACCCACACAAAGATTTTTCAATAGAAACATGTACTGTTTTTGGCCAGAAGCCATTTGAAATATTGAAGTAAATAATAGTAATGACAATAATAATAAACTCACAGGAAGGTGGCATCTGTGTACTTCTGACTGAAGGGGTTCTGGTTAGTTTAGGAGGACCACAAGGAGGCATTGTTATCGAAGAGCTACCATGGAAGTTCAGTGCTAGAAAGAATGATTAAACATCTTGGCAGTATTCTGTTTTACATTTTCATACTGTACTCTTCATACTGTTTTAAATACAATGCCCACACCAGAAGCACATGTGTATAAACAAGTGCCACTCCAACATCAGATATAGTTATCTTTAAGTTCGCCTGCCTTTTGGCTCACTCCAGTCATGTAGTACTTAGCCACCATACTAGAGCGAAATTGCCTAAGAGAGTGATCTTTGGAAAACTTACTGAGAGAATATTGAAGGGCTAATATACATAGGATAATTAATTTAATATACATTTCCTAAAACATAATCATTATTTGGCAAGTACTTTTGCATGCTTCTATGGAGTCAGGTGCTGTTTAGCTAATTGTAAGTTCTGTATCCTAGAGTGACATACTGTGATTAAATCAGAGCTGTACCAGTAACAGGGACACAAAACTATGTTAGCATTTCAGAGGATAGTCAGCATTGTTATCTTATAAAAGGAAACTAAGGAAGATTAATAAAGAGAAAAGCTTTCCAATTATAATGAGGGAGGTCATCTCAGTAAGAAATTGTTTCCCCTACCCTCAGAGGGTAGGCACTTAGAACATTCTATTAGTAACAAACAACAGTAAAGTAAGCAAGTGGTATCCAGGGTGATGTATAGGATAAGGTAGTCCTTAGGATTTTGTCTTTTAGTGAACTGAAGTAATCAGAGGATTTGAATTAAGGTAGATGCAATGATCACTTTTAATTGTCAACTTGACGGATCTAGCATTATCTGGGACAGAAAACTCAAAGGCCTCCCTAGATGAGATCAAACTGTGAGCGTCACAGTGAAGGTTGTATTGATTAGTGTGAAGACCCACCCATGGTGGGTGGCATTATTCATTGATCCTGTTTCATGTAAGAGCATGAAAAGCAGGCTGAGCCAAGTGTTCACGTGTGAGTCCATGGCTGTCTGCACTTAACCTCTGGATGCAATGTTCTCTCATACTCCTCACACTGAGACCTCACTGGCATGCTTGACTTTAACCTGGAACTGTCAGCCAGAGAGACTATTTCATCATTTTTGTTGAGATGAAATAAAGACCACAAATAAGATGGCCAAGGAGTTAAAAGTAGATAAACATCTAGAGGGAGGGGCCTGGAACCCCAAGAAGCCAGAGAGGGGCCCATTGCAGCCAGAACAGTAAGACTACTTTGTTCAGAGAGAGCACATCAAAGGTGCTGAAAATTGATCAGAGTCTGCACACATTTAGAGATACAAGAAACAGGATATCCCGAGAAAGTACCATCCTCTGTTGGCTGCATAGCAGGGTCCACAGAGTTCTGCAGAGGCCTGGAGAGGTCCAGGGAGGTCCAAAGAGGTCCTGCCATTAATATACTCAGGATGTTTTTATGCAGTGTTTCCAGAATCCATGGCCTCTGAGTTCTTAAAAATACAAAACAGTATTGTTTCGTGGCAATGATGTGGAACACATTGTCATATAACATTTAAAAACTATATTAAAAGAATGCAGAGTGGCTGTAGGTGAAGCCTCCCTCCATGCAATGTGATATCTGCCATACAGCTACAAGAGGGCCTTAGCTAGAAAAATCAGTCAAATTTTAAGTGTCTATAAGACTCAAATTTTTAATTTGTTAATTCTCACAAGACACAAAGAAACAG

							CGGGAAGTACTTTATATAGTTCCCACTCCCCTAAAAATATTATTCTCTTTGCTGCATACATAAGACATCAA<br>TAAATTATCTACTTCCTTTGATTTAAAGGACATGCATTTTCAGACATTTGTCAGCAACACGGGATATGTTT<br>TCTACGCGAAAGCTTTGCTTTTTCTCTTTGCACTTTAAAATGGGAAATTGGTGATGGCTTGTTTAAAAAA<br>AAGCTTCATTTATTTATTGAGTCACTAAATCTAATTAGGTCTATTATTCCTAAGAAACTCCATGGTAACTC<br>ACCCAGAAGACTGATTCACATAGATCCTGTGCTTGGCTAGATTATTAGTCCTGCAGCGGAGCTCAGAGC<br>TCTGCCTCGCTTAAAAAGCTGTCAGAATTTCTATTCGTTTCAGGAGTTTGGAAGTTAACTGTGTGAATAT<br>TTCTGGCTGAACTTTGAAGGGCCTGAATGCAAAAGTCTTGATATCTTCAGTGCCTGCTACACCTTACCTT<br>GAATACCTTTAACCTCACTCTACCTTCATGGAAGGTTTGATGAGAGCCATGTGCGTTTGTCAGACGACCT<br>CTTTGTGGCGGTTAAAAGCAGGTGCATAGGAAACAATGACATCCAACTTTCACTTGATTGATTTTCACA<br>ACTAGTAATGCGCCTTAAATATTAAAGCAAAGTTGGATGTATTAGGCAGGAGGAGTTCTCGTCAATTGAG<br>CTTTCTAATGTTGCATCCTACTTGCACAGCATTTGTGAAGACTCTATGTTCTGTAAATCACATGACCTACA<br>ACTAATAGAGAAAATCATTGCTTATAGAATAGAAGCTTATAGAATATAAGTACGGATTCATAAATGCCCCC<br>TCCCCCAGAGCCCAGGAGAAGCAAGTTGCTGATCATGAGCTATCAAGTACTCTGTGCTCTGTAATATCA<br>AATGAGTACTGAAAATCTTTCTCTCTCGACTTCGTGACTGGTACCATCTGTTGACATCACACAATTATGG<br>GGAAAAAAATAACATCTTACTGAGGAGAGAGGAGCAGAAGGCGTACCCAAGTAAGTGATTCAAATGCT<br>CCTTGGTATAGGCAGTGGTTTTACCAATTTGCACTGTTGTGAGGGCTGGCATGGTAGTGGCACAGAACA<br>AGTGATTCTGATGATACTAGTGATTACATAGCATATGCTTGCTCTCTGAGACTACTTCTGAGTTTAAAGAC<br>AGGGATAAGGAACCCTGGTGGAAGAGAGGGAGAGGACACATGACAAAGAGTGACTGCCAGATAGCAA<br>CTATTTCCCTTTGCCACATTTCACTCTAGAAGGATTAATATAACTGGCTACCGTTCTTCCTAGCATTTTATT<br>TCATGAGTACGTTTTGATAAGTTATTTTAAGTATCTTACAAAGATACTGAAATGACAATCATTTTCTCATA<br>CAAAACACTTCACAAAAATTGGCAAAAGTAGTCACATCCATTCTAATGTATTTCCCTTTCTTAAATTATTA<br>AAGTCCAACAAAGGAACAGAAGGGATCCATTTAATGAAGTTTCTGATCTTGATGACCAGGGATTTGCAT<br>TTAGTTGCTTATTAAAAATAAAGTTTTTAACAAGTTCCTATTTTTTCTAAAGATAACCTTAATTCTTATTGT<br>TTGTTAGTGTTATTAGTTCATCCCTTGAGGGTTACAAGTCTGACTCTTTTATTCCTGAAGGCTTCACAGTG<br>ACACGTATGGGAAGGACCAACATAGAGAGACAACTGACTCCTGAAATGCATCTACACAGCTTTACCTTC<br>AGGGCCACAGATCAGTGCCTCTCAGTTGTCAGAAAAGATATTTCCTCTCACTACCAGATTGCACTTTCCT<br>GGGATTTCCTTGGTGGTTGTTTGATATCCATTTTACAGTTGCACTTCTGGTTCATTTAGCTAACGACAAA<br>GCTGATGTAGTTCTTCCCTCTCAAAGAGCGGGATCATTTTGTTTCCTTGTATTTTTCCTCAGATTCTTTCA<br>AATCTCTACCTGGAACTGGGATCCAGCACTGGCTGAGACCAGAACAGAGAGCTAGGTTCATGTGTTCA<br>CAGATTAGTGTCTACCTCTTCAATAAATCTGTAAGAATGGGAGACCATGTTCCGCCTCAGGGAATGTCTT<br>GACTTTTGGGGGATTTCCCAAGATTCCATGCAGAGCAGCAAGTGGGCAGAGAGATATCTCTGTACCTAG<br>CAGTGAAATTGTCACCCACACAAGCATCTTGTTCTGGACTCCTTTAAGGAGTGCTCAGACAAGCTTTGT<br>TGGCATATGAGACCTTTATACTGTGAGTGAAAGGGACTCACTGTGTTCCCTGTCTCCAAGTCCTCATGTA<br>ATATCCTAGGCTTTTCGCTAATCATTGCCATTTACCTATTTGACTTCAAGTATAACAATAACAGTCTATGTC<br>TGAAATTACTTATATTTGTCTTTGTTTTGGTTGATTACTAAGATACATAATGCTTTTTCTTTACACTGAAAA<br>GGTGAATCTACTAGATATTCATGAAGACTCACTTTGGAGAAGAGCCTGATGATTAGAGAACCCCTTGCT<br>GCTTTCAACAAGTAACCTTTGATTGGTTTCCCAG
MSTR<br>G.479<br>81.1	ENSRNO<br>G0000004<br>2133;ENS	1.131	0.111	0.008	-<br>2.571	do<br>wn	CGCATTTCCAATTTCATGCCCATGGCCTCCATCTTCACTGCTCGTTACCTGGCTGAACTAAGAGTGGGCT<br>TGACTTGCTGGTTCTTCCTACCCAGTACAGCCTGATCCAGATTCAGGGTCATTTCAAAAGTGCAGCTCC<br>ATGTGATACCCACAGACCTCAGTGGTTAATTTGATATTTGGCTAAAGGACCGTCATCACAGATTGATGGG<br>CATCTCAGCATCCTGGACCTAGTAGCTAAGGTCCTGAAGTCCACATGAGCATTTTATACTCTAGGATACT

	RNOG000<br>00047234;<br>ENSRNO<br>G0000004<br>9016;ENS<br>RNOG000<br>00049451;<br>ENSRNO<br>G0000005<br>9588						GACAGTTCTCTGTGCTCACGTTAGGGTGTACTAAGTCCTGCCTTTAAAGCACATGGTATGGTTCCATTTG<br>AGGTGTCACCAAAAGGTGTAATGAGTGTGCATTTCCTGGTACAATAGGAAGGAAGTTTCACAGTCTATT<br>GCTGGACATTTTCTTTGTTTTCAAAACTTTGTTTAGGGTCCACATGGATGGTTTATAAGTCAATTTAAAC<br>AAGTACTTGTTCAGTATGTCAAAAACAGAAAAAAAGGGTAAAAAACCCCCAAAACTACTGATAACAAC<br>ATATTTAAATAGATTTGTTCTTTCTAGAGAACAATACTAGGCAAAATTCATGGATCAGTAGGAAGTAGAC<br>AACACAGATGGAGGGATAAATGTTCTGAGGATAAAATAAGGCTCAGTGTAGACTGCAAAATCCCCATTG<br>GTTGTCATTGATAACTAAGAGAAAATAAACAAACAGAACAAAAAAATCAACAATAACAACACACACAC<br>ACATACACACACACACACACACACATACACGGTGCCTGATGTACATATGATTTCACAAGGGATTTAAGG<br>ATTGGGAATCATATCTTCATAATTTTACATTTTCACGTTTTCCTCTAACATCTTCGACACTCTTTGCTATTAT<br>TTTCTTTTTTGTTTTCAGTATGAGTATGTGTTTGTGTATTTTTTTTGTATTTGTATGTCAGAGCAAGCTCAT<br>GTGTGTGTACAGAGAGGCAAAGAGAAAGGTGTGTGATTCCCCATCCTCTAGATGTGGAGTTAGAGGTG<br>TTTGTGACACTCACAACTAGGATGCTGGGAATCGAAATCTTGGGTGTCCTAACCACTGAACCATCTCTAT<br>AGCCCAGGTATCACATCTTTACCAAAGTAAATCATTTAACAGATACAAAGAAAGTGACTAGAGCTGATG<br>CCTCAAATATTATATGCAGTGTTGAGACTCCCCAAGTTCCTAGGAGATCTGGATGAGATATTAAGGTGAG<br>AGAATTGCCTAGTCAAATGTGTAGATTTTTCAAATGGAGAAAATACTGTGGAGATAAGGAACTTATGAC<br>AGAGTTTGAGGAAAGGATATTACAACAAATGATATGTGTTTTATTACCCCTTTACTCAAGGCCATAAAAC<br>ATTCATGTTTACATCTGATACATCCATTTATTCATTTTTCCACAAGAAAACAAAACATGTGATAGCAGTTT<br>GTTTTCTTATCCTGCAATTTAAATATTTCAAACTTGTTGTGCTAGGGCGTCTTCTGGAGTTCTGACATGAT<br>AGTCTCATGAATATCAGCATTATGTCATAAAGCATCATTTAAGGAAAAATCATGAGCTGAACTTCCTTCC<br>AAAACAGGCATTTGTTTGCTTGCTTGCTTGTTTATTTAGATACATTTATCAGCTCTTTTGCTTGTGTTTGA<br>GAGATCTTATCTGGAAATAGTAAACAGCTAGAAATATGCTAGCATACAGTATGAACCTTTAGCTGTCAAT<br>TCATAAATATAAGGAAATAAAGTATGTATATACATATACTACACAGGATGCAACAGGTCTCCCTTCCCCAT<br>AGGGAGCACAAGACTAGAAGTCACTGATAATAACACTGTAGATGATTTGTAAATTATTGAAATTAATTTC<br>TATGATATATATATACATGTGTGCATGTATATTTATATCTCTATTCTATATCAATTGTGTGTGTGTGTGTGT<br>GTGTGTGTGTGTGTGTGGTGGTGTAGCCAGGATCCCTGAGCTCCTGGTGATTCTCCTGCTTCTACTTTCC<br>GAAGTCTGTGGTTACACACACAAGTACCACCATTTCTGTTTTGCGCGGTGCTAGGTACCAAGTCCAAGG<br>ATTCGTGTAATCACTTTCTCAGCTGAGATCCACCCGCAGCCCAGAGCAATATAATTTAAATGTTTACCAC<br>TCTGCCTAAGTATTAATCCAATTAAAAATGGAATATCATCATCAACACAAGACATATAACTATGAATATGT<br>CTGAACCAGTTATGCATGGCATGCTCCCAGGTGGAAATTAAGGTTTACTTTGACATGAGTCATTCTCACA<br>ATAAAAGTCAAGTCACGCTGAATTATACATCTCAAACTGTCAGAGGTCACGTGTTTTCATTCTCTGAGG<br>AAGCCATCTTGGAGCTTTTCCTCATGATCCAAAGCTCAGGGATTAAACAACTGCAAACTGGCAAGTTAG<br>TATGCTCTGTTCTACTTAGGCGGAAACTCAGGATCATGGTTTAGAAACATGAGGTTCTGCAGTCTGAAA<br>ATCAGCCAATGCACTCATTTCATTCATTATGCAAAGATGTTTAAAGGATTTTTTTTCTTTTTAACATTCCC<br>AGTGTGGTAGGTGTAAAGACCATTGAATTCCATGATGCAGTAGGGGTGTGATTTTCAGAGAACTGGGTT<br>CAGTTCTTTGTTCTGTCTGTTGCTGTGGGCAAAAACATATGAGAAATTATATTCCTCAATTTTTTTATGGG<br>TTCCGGTGTAGGGAAGATGATTTCAGCTAATAAAATGCTTGCTACACATATTTCACACCTTGCGGTCACA<br>TTCTCAGCACCACCTGAAAAGCCACGCATGGGGACCTGTGCCTGCAACCCCAGCACCCAGAAGAGGA<br>GACAAGTAGGCACCAGCAGCTCACTGAAGAGTCATCCTACCTGCAAAGACGAGATTTAGGATCATTGA<br>GAATCTTGACAAATATGGCGGACACTGGTGGAGGCAGACACTTGATGACACTGACTTCTCACTTCCACA<br>TTCTTTCATGTGTGCACACAAGTGCAACATACATACCAATCCTAAAAATGGTGAACATAGTAGAAAATG<br>AGTGATTCTCAGATAACATTCCATTCATGGAAGCTCCATGTGCATTTTCCATTTTTGACATGCTTGTTGAG

							AAGTCCTTTATTCAAGACTCCATTGAGTCTGATCTCTAGCCAGACAAAAATGGAGTGGGAACTTCTTAA<br>ATCATTAATGGAAAGCTCTGTTTACACAAGAGTGCTAAGATTTTTAGATGAGATGAGATATTTTGATTAA<br>ATTACCTTATAAAGTGTTTGCAATTTTCTTAGTCGTATAAAGATAAATAAAACACGTAAATCAGAAAAGA<br>AATTATGTCTCATATTTGCATTAATGGAGCTTGTCAAATCCCTGCTAGGCCTAGAAACTGCTGCACACTTT<br>ATCCTGTATGAGACTGGCAACTAGCTCAGCTCAGATGCACCAGGACAAAAGTGGCTGGCTCCTTTTAGG<br>AACACACCACATCTCCTGTAGTGATGTTCATTTCCAGTGAACATTGGCATAGAAAGAAAAAGAAAACA<br>AAGTATTTTCAAAACATCTCTGAAATTATCATTGCTTTCAGGTTCAATGTGGCTTGCTACCCGCTAAGCTT<br>CTAAACCTGAGGGACCTGTGCAAGGTAATAGTTTGTATGCAACAGGGTTCATGCCTGGCACTGTTGCTC<br>TATCCAACTGACTATTACAAATGTCCACTGCCATAGTTGTTGTCTTCCTAACAGCTTCTGAGAGATATGTC<br>TTAGGACTGTGTGATGGCTTCTCCCACAGGCAGCCAACTGAGTTCCCGAGATGGTCTGAACCCCAATCA<br>CTGATGGTCCTATGGAAGCTTTTTCTTAAAGGGGACATCTGGGTGTGGCAGCTTTACAGAATGAAAGCC<br>CACAGTCCATAGCCTTATACTGCCCACAACTACATTTAAAGGGGATTCTTTCTGCCTACCAACCTTTTATG<br>AACTAATTATATAAGAAGGTTCATTTGTTAATGGGCTATATAATATCTTTCTGCAATATGCATATGCTATTAA<br>GTCACAAGATTGTGTACTGCAAGGAGAAAATATGTGACCAATTAAACTTGTTGCTAAATAGGAGGGGGT<br>GAGTGTGGTAGTAGATAGAAAGAAATAGGATGAGGAAGAAAAGGAGAAAGAAAGGGAGAAGAGGAA<br>AAATGGGAGGAAGCTTAAGAATGAGCTTAAAGAAGATGAAGAGGATGAGAAGGAAGATGAAAATGAT<br>GATGATGAGGAGGAAAGACAATGTAGAGTTTCCATGCACAGCTAAAACCCATTTTGAAACACTAATCCC<br>AATCCATGCCAACCTGACTGATATTTTCAAATGCGGTTCCATTTGAGATATTCTCCCAAGTTCTAAACTTC<br>CTATTGTGCCTCCACAGAAGCTCTCCATTTAACTCTAAGAAATTGGAAGGAAGAATTACGGATGCAACT<br>GCAATTTGGTAACAGGAAGTGTTTGTTCCCACATAGAGAAGTACTTAGGATAAGAGGAAGATTTAAGGA<br>AGGGAGCCTCTAGCTGCAGGGAGGACTTGAGTAGCTAACAGTTCACATTGGTGCAATATTGAGGAAAA<br>ACTTCCTGGAGAGTCCTTGCTGATGGTTGGCAGCCTTCTAGAGATGCTAGAACAAGCAGACAATAACTC<br>TAGGCTCTCATGCTGCACATAACTGTTATTTAAGGGTTTGTAATGTGTTTCTGCAATGATGTTCCCTGTAT<br>TCTGCAAGATTTGGTGACGAGAACCCTTCCTATGAGCACACACATTTATACAAATACTATATATTTAGGCA<br>ACAGTATACAGAAATCCATGTGGCTTTTAAAGAATGAGCTTAAAGAAGATAAAAGTACTAGAAAGTCAC<br>ATTCTGCATCACATTTTATGCAAAATAATCACTACTTTTGAGGTAGTTTCAGAAGACTGAACTAGATCTT<br>GAAGTATAAAGTTTTCCCCAGCACTGCTACATAATAGGCCACTGTGTTCTACTGTGGAGTGGGAGAAGT<br>TAGCACATTTCATTTGGCCATTCCCAGAAGCCCCCGTATACACAAGACATAGAACTAGATCATTTCCTCA<br>GAAGCTTGAAGGCTCTGAGCCTAGGCCACAAACACAGTGTCTGAGAGGCAGTTGTAAGTAACTCCATC<br>CATGAACATTGAGTGAAATGATCTCATGGAAGAGTGATACTCCTCTATATAGAATAATTTTATCAGACTTA<br>TTTTTAAGAAACAGTATATTTTATAGATGTGTAAGTTAGCCTTTCTTTTTTTTTTACTGGCTTAAACATTTT<br>TATTAGCAGTATTTTAAAAATACTTCTTGAAATGTAAGTTACTATGCCTTTCTAGTGTGCATTTTATTTCCT<br>CTTTAAGACTTCATTATTCACATTCCAATTTTCTCAGTGAAGAAGTACAAAATCTATCACTTGGGGAAGT<br>TGGTTGAAACAACCCATGAGAATATTTTGCTACAACAGATTTATTAGAGAAAGACATGGATCACTTTTCT<br>AAAAGGTTGTGCTTCATTAACATCAGAAAATAACCCATCCTCCCTTACAAACACTGTATATATGTGTGGC<br>TACCTGCATGGTGAACAACCATCAGTCAGTTCAATAGGTTCTGATTTATTATTCCCTTTAGTTTCCTTTAC<br>TGTGTTTATTATTCATAGAATGCATTAGAATGTCGGGGAGAGTGCTCAGCCATGAATGTTCTTGCTCTGTT<br>AGCCTGAACACCTGAGTCTGAACCCCCAGCACCATAGGAAAAATCAAGAATGGAGTCATGTGCCTGTC<br>ACCCCACAACTAGGGACATGGAGACACTATGTGGTCAAGAGCTATGATGTTGATAACAAGAGATGGAC<br>ATCTCCAGGAGTAAAATGCCTTCAGAGTTCTGGTGAGTATATGACACACTTTCAATATTAATTTAGAGCT<br>GTGGCTCTCAACCTGTGATTTGTGACCTTTCTGGGGGGTGGCTATCAAGTGACCCTTTCACAGGAGTCA

							CATTTCAGATAGCCTGCACATCTGATATTTACATTGCAACTCACAGAAGTAGCAACAAAAATAATTTTATT<br>GTTCAGGGTCACCATAGCATGAGGAACTGTATTAAAGCAACAGAGCATTAGGAAAGTTGAGACCACTG<br>CTCAAGAGATGAGTTACAACCAGGTTCTGGTAAGTCTGTCACTTTATATAATGTCTCAGCACTAGCATGG<br>AAAGTGTGCTTAAAACCAATTCTAGCTAGATGCCTATCTGGTTCTAACATCACCCTTAAAGACACTTCTC<br>AGACCTCTTATGCAAAATATTTGCCATGGAGGTGGTGACTGATGAAGTTTCAAAGCAGGAGAATCTGTG<br>TTAGTTCAGCTTCTCCAGTTCCTGTTCTGCTGCGAAGGAAAGTATAGATTCCCACATGCATTAAAAAGTA<br>TTGCCTTACAAAGAAATTTTCATTGGTGTTTTTCCTTTTTTTTGTTTTTAACAATACTGCTTTTTGTCATTT<br>TTCTCCTGCTGTTCCCCACCCCTCTCCCCATCTTGCCCTTTCCTCTCCAGGAGTTCCCCATTCACTTTCAT<br>TTCATATAGGTCGACAGGACAGTCTCTACTCCCCTTCCCACACCTCTTTCCTCTTGTGGTCTCCTCTCGAT<br>GTTGATAGACTATAGAGTCTCCTGCAGCCCTTCTTTTCACATTTACAAATGTCAAAAGTCAGGCTCCACC<br>AATGAGAGGAAATATGTGAATTATGTTCTTCTATGATGGCAACACCGCTCTGTGTCCCACCCCACAGACA<br>CCCCTATCACTGTTCATTGTTCATAACAGAAAGAAAGTGGAAGTAACCTAGGTGACAGCCAACAGAAG<br>AAGGGGTAAAGAAAATGTGGCCTATATACATGATACATGACAGGATCTGAGTCAGCACTTAGGGAGAAT<br>GGAATTATGAAAATTTTCAGGCCACATGAGCATTTGCCATTCCTAAACAACTGCCGTCAGCCTTCGGATT<br>TCACTGCAAAAACAGGAACGCGAGCTCTGTGATTGAACAAAACCATCTTCACCCGCACGCAATTGACC<br>TGAAGACTGGACAGTGCATTTGGGATTCCAATGCAGGCAGTTCTGTCACCGGGACACGCTTTACAAAG<br>ACCGTGCCTTATTTTTAAGTCACCCCTGAGCACTGCCAACAGTATCTGCTGATGCTGAGAACAAGTCCT<br>TAATTCGGAATTAGCCCATGTTCTTGCAACCCAAATGTGCTAACATTCGATTTAGCTCCATAATTGCGGCC<br>ATAGACAGAATGCCCTCAAAGTGTTCCTGTGGAGAGTACTCTACTTTCTTTTATAAAAACATTTTTATGC<br>CCTGAGTTATCTTCCTGACTTCATCAGATGCCTGCTGAACACCACCTGTCTTTTTGGCATTGGCACATCC<br>TTTGAAGTGCTGCCTTCGCCTTTCCAGGGCAAATGGAGCTCATTCTGTATACCAGTGGTCACAGGTGTG<br>CTTCATTTAAGATTGGGGCTAGGATAGGTAGAAGGAGAGAGGAGCCACCCATGGAAAGGACCATTCTG<br>GACATTCTATATTGACTCCCTGGGATGATCCACTCCCACATGATACCTAGAATTTGCTGAAAAGATTCAA<br>GCAACTTTGAAATCGGCAGGATGCCATTGTGACAGATGGGTCTCCATGTGGCCACGGTGCCAAGTCTTC<br>TATGCATCAAGTGTCTCTGCTGAAAGTTTGAGCCTCCGCCAGTCATATCCAAATGGATGTCTGTCTCCAG<br>ACCCGGAGAGGGTGTGTGAGGCTCCATGATCTTCCCAGTCCCGCAGCCCAAGGCTCTAATCCACCCCC<br>ACCCCCTTGGGTCTACTCAGGAAGCCTGGGAGCTCATAATTGAGAGTTAAATTGATGCAGTCATCATCCT<br>TGATTTCTGCTTCACCTTCTGTTGCAGACAGCCCGGGAAACCTCAGCAATGTCTCCTCTGCATGTGCTA<br>GTTCATGGAAACTCTAGGAAGAAAACACATGCTGGGAGCAGGCATGAGCGAGGAAGATAGCACGCAG<br>ATTCTCCTAAATTGGATGTTTCAGGGCAAACACTTCAGTGTTTAAATGTACATCAGAATCTGTGGTTGTT<br>AGTCTACATGTAAATGTTTCCTTAAGACACACAGCTGCTTAACAAACATAATAAATCCATGGAGGAATCT<br>CTGTTACTAGGGTGTGAGTGGAGTGTGTTAATAAATAGCAGTGTATTGCATTTTCTGTCATGTGTGCATAT<br>GCATTAAATATAAATGTATATGCATATATAACATATGGATGTGTGTATGCTTAGATGGATATATTATATATTTT<br>ATATGGATAGTAACACACCTCATATGTGTGATGGTGATCTTTTTGATCTCACGCCCTTCATTTGGCAGTCA<br>CAATCTGTAAGACCTAATTTAGCGTGACCTTATTAGACAAAGGAGCTAAATAGCCTTTAGGATGTTTCCG<br>AAAATGAATTACAGTGGCTCCTGCCCAGCATTTTGACATCAGAGAGAAAGTATCCCACTTCTTTGCAGA<br>GACTACCTTCTATCTGTCTGGAGGACTATCCCTTGTCTTAATCGGGAGGTCAGTCAAGGCCTGTGGAATA<br>ACAGTGGGTCAAATTGTGAATGTTCATGGGTCTGTCTTGTAGAATTTCCCTTTTGCTACTTGGACTTCTT<br>ACCCATGAACATTTTAACTTGTAATATAAGCTGAGAAAATCCACAGTGCTCTTGAAGATGAGAGCATCTA<br>ATTTTTATTACATTCAGCCTCTCCAGGCTTATTGGTACAGATCGGATCCTAGCCGAAAATCTGGGAACCTT<br>ACATATGAGGGGAAGGCTCCACCCCTTTTATCATATAGTGTGTTAAGATAAAAGGCCTCTGTGCACCCTA

							CAGCATTGCTCACCTCACTTGCATTAAGTCTCCCTACCTCCTCCTTTTCCTATCTGTAAGTGTCACAATGT TACATTTATCTTTTTTGCCTTCCACATGTTTGAAGAAGAAACCATTCTCAGGATTGAACTTGAAATTCAAT GGGTACATTTTATATTGCATATTTTTAGTGTCCATTTTATATGTGCATATATGTAGATGTGCATATGTATGCAT TACATGTTTACATAGTTGTAAATAATTCTCATATGTATGATTGTAAGCTTTCAGAACCCACGACCTTCATTT ACAGGGGACAATCTTTAAGAACTAATTTAATGTCCATACACTCAAGATCTTTCTTTCTGCTGTTTTAGGT GCCAAATTGCTTTTTGCCTCCTACAGAAGTATTTCTCATGAATTGCAATATCATACCTAATACAAGAAATT ATAATCACCGGATTTATAATTTTAAATCTTAATTGGATACAATTAATTAGAGCCCTATTTTTATAGATTCCTT TGATAATTATATTAATTAAGAAGGATGCACCATAATTGGGTTCTGTCCAAACAGTAATATCCCTGGCAGCT GCTGTCTAGAGAAGCCTTCCTCATTATGATGCCCTGAAGAATATGGTAGAAGGGTTAGTGTGTGACTTG GCACTACGTGCACAGTTCCAGAAACACGTGGACAATTCCCTTGCTTAGTGGGTTGTTAGTAGCTCTTCC TAAGATTAATTATAGTCAGGAAAAGTCTGAGGATGGAATCCTAATCTACAGTGTGCATCTGGTGAGAAA GAACTTCTTCTGGTTTCTGAAACCATGTAGGTATGACCCATAGACATATGTTGGAGACTTGCCCATGTAG TATCCTCTGTTGGCATAGGTTTCTGTTGGAGATAAAACTCTGGGCAATGTCATTCTCTAGTGATGCTCTTT GGTGATTTTTTTTCTTCCTTATCAGTGACATGGTATTCATGCAACCTCTTTCCAGGCATTGCCAAGGTTTC TGGAGGGGATGGAGGTGAAGAGCCATTCAGAGCCACCAAACAATCTATAGTAAGAAGAGCTGTGTAGT GGTAAATGCTGGTGCTGATCTTGGTGCTTTATGACAGGCCCAGCTCGAGGGCCTTCAGGTTCTGGATGT GGAAAGAAGGAAATAGCTGGAGTTGCTTCTCATACAAACGCATTTTATAAAATTTACTTCCAAGTCCTGT CTGAAACTCTAGTTCTTTTCCTGTCTCCCACATTTCTCCTACAGAAGCTGCTAAGCAAATCTTGGGCCTG CAAATCTTTGGAACTATTTGAAATCTGCTGGCAGTTATTCTGCTTATCTTGAAAGGAAACAAAAGTCTCT AGAAAGTAAGAGAATTGTGGTAGTAGGCCTAAATTTCATCAGCATTTTCAGCAGGAAGCAAAAGGAAA TTGAGCTAGCACAGAATGCGTAGCTGGGAAATGCAGGGTTGGGATGCTGAAAATGGGACTGAGACATG CTCTGTCACTTGGCATGATGGTTGTGAGATTGAGCTCATTGGATGAGTGTCTATGACTGTCTTTCACCTT GCCATTAACTCCAAACTAATGGTCTTCCTTTCCTTCAACCTAACGTCATTTTAGTTCAGTCAGCTGCCTT CACGTGTTTTCCTCAGGGAAGTCAGTGTTAGCTTGGAAGGCAGGCCTCCCCCTTACTTGCTGGGAGAAT AAGCTAATTTTAATCTCTCTCTTTTTCTCCTCCAAGGCTGTGTGAAGTACTAGAAAAGCACAGGCTTTGT GATTGTACAGTGCTGGGATTCAGTCTTCTCTTACTAATTATCTCCCTTTTGGCAAGCTTCATTACTTTTTT GGAAGCTCGATTTCCTCATCTAGGATCTTCAGAGATTAAGCTATTTTTGCATATTGGATGGGAGCTAGAG ATCTCAGAGTCTGTGGCTAAATGGAGAGACCACAGCACTATAGAAGTTCAACAGCGGTATGTCTTGTTC CTACTGGCCAAGGGGATCATTGCTCCTTCCTCTTACACATTGTAGGTTAAGGGACCATTGTTATCATTTGT ACAATGGGGGTAAAGACAGTCATCAGATACCTGTTGCAGTTATAATTGCCTCAATATAGAATCACTTGGC CTATAAGGCTTATTAATGCATTTAGGAAGAATGTCACTAACATCTCAGTAAGGGACAAGTATCTGGCATT ATGTAGATCTGTATAATATATAAATATATATTACCCATCAATGACAGTTGACAGTCATACAAGTAAAATGTA ACAGTTATCACTTGTCCTGATTTATTTGTACTTTTCCTCAAAGTAAAAATCTGTGTTGTGCTCAAAACAC TGCAAAAACACTTCTTTTAGAGTAAAAGCTAAATTAAGAAACTGAATCAAGAACAATAAATTAGTCCTG CTATTATCAAATTGGACTAGATGGAGAAGAATTCATGTATGTAGATACCTCAGAGTCACTGCTTTCTAAA CACCAACTGCTTTCAGACGTACTTTGCACAATAGTGCTGACTGAGGGGTGATGTTAGTAAAGGTGAGTC TTCAAATTCGAATTGAATCCCATCTGTATGTTGCTGTCTATCTATAGAGATTTTCATGTATATAGTTTTTGC TCAGTAGAAGAGTTGATTCAGGACTTGATTAAAAATCACACCATACTCCCTTTCCAGGGTTGACTCGAC TTCAAGGTGGTTTGGGGATTTGCCAGCTCATCCCCCTTCTGTAGGCTTTGAAGGTTTATTTATTCTAAAA ATTATATAATCGCTTCCCCACAGGAGCTCAAAATGCTTCTGTCAGGAGGCCCCTGCCATCCCTGGAGGC AGCCGAGGGCAGGTGTTGGTCATAGAATTTTACTGACTTGATAGTAAGGAAAATTATTATGGACTTCTGG

							TAACAGTGACATAGGCCTAAATATTAATCTTGTAAAATCATGGAGGACTATTTCTGGGAAACAGATACTT<br>ATGCTCTGTCTGAAGTCATCAAATTATCTAAAAATCCTGCAGAGCAGCCAGTAGGAGTCTCCCTGGTGA<br>CTTTCAGTAATAGCTGCCATCCACCTGCAGGTCTGCGCAGTCACTCCCAGCACCTGGGACCTTATCCAG<br>ACATTAACAGAGGACGGTTGACAGGAGCAGTTGGAAATGATTTAAAGACGGAGCTTGACAATAATCAA<br>GTACCTTTAATTATCAATCAGATAACTGAGTTATTAACCAAATGGTTGGATTTTCCCGTTTTATAAAGAAT<br>GAATTTATGAACACCCCCAACATCACATTTCCAATTAATCATCAGAAAACCAAAAACGTAGGTGCATTTC<br>CAAACCCACATGTGGAAATCTAGATTCCAAGGTAACAAATTTGGGAGAATACTGAGAAAGTGTTAGGAT<br>GCAACCTCCATTCATTGAAAGCATGTTCTGAAGGCTGAGAACCTTTCCACCATCCGATGGTGTGGAAAG<br>AACAGACTATCTATATTGAAAATGGCCTGCCTTAGTCACAGAGCTCCCGGCATCCTGAGCTTGGACTTGT<br>AGTAACTTCTGGAACTAAGATATTACTGTGTGTTGTGTTTAAGTGACCTGATAATAAAAGTTAAGTTTTT<br>ATAAAAACCTGTATAGATTAATGGATAATATATAAACCTCATAATATTTTGTAAATCATACTGTAAGTGGTG<br>AATCCTAACTCTTGCCAGCTATTCCATATAACACAATATAACTTAAAATGCATTTCAAAAACTGAGCCAA<br>ACTCTGTCCAATCAAATATGGAGAATATTTATCTATATGGAATGCCCTGTAACATTACAGTTAACATTGAA<br>ACATGGGAATTTGAGAATTCTGTATTCAAAACTAGCTCTTAACCGATACCTGAATTTGTTAATCCTAGAA<br>ATTGTTAGTTACTTTCAATTGTAAATATTTTTAAAATTCTTAATTTTCAGAAGAGAATGTAGTCATCAAATT<br>AAAAGCAACAATTACAATCAAGATGTCATTGTAGTTTGACAATAAAAAGCAAAATGAAGTAAAAATAAA<br>TCCATGGCCTCTCATCATCTATCAAAGAAATGGGTTCTTGGGTAAAAATGACACCTGGAAAAGAAGAAA<br>GACATACATGTTCCATAAATCATAGCCATGATGAGCTTACCAGGAGAATCAGCCACAAACCACATGTGG<br>AGGGGACATTAAAATAAGGACTTTTCATTAATTGCTTAGAATATTAAGAATGCATTAGAAGACCTTAAAG<br>ACACGCAACTATTACTTTTTCTTTTAGGCATCCCTGCAGGTGTTAAATAGGGAAATTTTACAGAGATCCC<br>CTTCTTTTAAACATTTTAATGAATTATTTTATTTATTTAAATTCAAAATGTTGCTTCCCTCCTGGTCCCTCTT<br>CCCAGAGTTCTTCACCCTTATCCCCTGTCCCATTCTGAGACTAAGCCACCATCAAAGGAGATCTCCTTAT<br>CTTTGTCATGTCAAGTACATGACTGACCAGTGTGAAGTGTTCCAGATCTCTCTCAATACGAAAAACATTT<br>TCCTCAGGTATATTTACTTGATGGAAGGAATTCAAGGGGAAGAGGTGTTCCTCAAGGGGAAAGCTCTTA<br>ACGGCTGTTCTGTCTCGTAAGTGGAAAAGCCATAGGAAATAAATGTTAGGACTATAGATAATAACGACTA<br>ATAGAATAATAAACAGATGCAGAATACGGACATTACAACTGAGAATATATTAGGGAAAAAAGAGAACTG<br>ATAACTAGAAAAATAAATGATCTCAGGCAGATGAGAGAGACAGGGTCCCTAACACACTATCATATTGGA<br>CATATCCAATATGATATCAAATCTGCTATCTTTCATCCCTCACCAAGAGGCTCCTGTGCACTGTGCATGGA<br>CCACATGTGAAAATCTTAATATGGCTTTCTCTGGGAGGATGGAGCCTAAACACTGTCACCTGTGATTAAA<br>GATATCTTCAAACATTGCCCTATCCTTTACCAGATGAACAAAGGTCTCTATACCTGGGTCTTACTTCCTTC<br>AGTTCTTTTTAATTAGTACAGAATAATCAAGGAATAACACAACAAAACAAACAATAAGAAAACAACCA<br>AAAAAACAAAGAAGCTGAAGAAACAATTTAACAATGTCTCCCACTTTGTCAGACATTGGCCCTGCAAG<br>AGTGGGACTTTAGAATGACTGATTTACTAGACAGGACACTTTAGAGTAGTCATGGTTGATGTGTTAATAG<br>TGTAAATAGAAGAGGTAAACAATGTAGAAGACAAACTGGTTGACATATCAGAGTGATGGACTAAAAGA<br>GTCAACTGGAGGGCTAGAATTAAAGTGTTGCAAATGAAATGAAAAACAAATTATTGGGAGTAACCAGT<br>AGATAATGGGGCATGGATGTAAATGAAGGCAGAAACTTGGCATTGTCAGCTGGAAATGTCCAAGAAGA<br>AATACAAACAAATGAAAAGAGGATAAATACAAGCCACACCCCAAATAAACAAACAAAATAACTCAAAA<br>CCACCAAATCCTAATTATTATAGAATGAAGAAAAATTGAGTCATGCTGAATTCTCAACTAAAAGTAGAGA<br>TCAGAAAAGGTCAAGAAAAAAAATAGACATAATGATTTCAAAAGAATTACAAGTCCAGAGGCCAAGAGT<br>CAACCCAAATAAACAATTATATGTGAAAAGTACAAATGAACCACGCACAAGACAGAGAAGCCCACATA<br>AGAAAACGGACTAAGAATGCATCACACACAAGACTATGGCTTGAACAACTATGGGTGAACAACTTGGC

							TGATGGTAGGAAAATGGGGAAAGACACATTGTGCCTCCTTTGGTTATAGAAAGCTGACTAAAGATTAAGTAGAGTCTAGCCATGGATTAGTTAATTCTTTGAGAAAACATAATAAGTATTAAACACAAAGCATCAAATTATGGTGAAATGGGAAATAGATTTATTCATTGTGGAGTGGAGAAGTTGAAAAAGTTGATTATTGGTAGGTCAGTAAGCCCTCAAGGCTAGAGGTTGACCATCACTGTCAGCTCTCTGTGCACTTCTCTCCCTTGTGTATTGTCACTGTCATGTCAGTGTTCTCAATCACCTAAGCTCACAGGGAATATTCATCAGGGAAGGTTCCCATCTAGGCCACAAAACACATATTCAAAACTTACTACTAATTAAAAACTAATGTATAAACAATGTATATTCTGAGACCACGAAAATATTAGAGCAAATAACAAAAAGGCAGCTGGGAAAACAAACAAGAAAACCCACCAAAGCCTCTGAAATGTTGAATTTAGAAACAAAAATTCTAAATGGAAGAAAATAAGTCTCCATAGAGATTAGAAACCACTGAGATATTGAAAATAAAGTCCAAGCAAGCATTTGTAAGATGCAGGAAAACATATTTACAGAGTAACGTATACCTTACGTATACTAAAAATAATCTCAAATCTCTACCTGCCCCCTGAACTTTTGTGTCCAGAGAAGAGGAACTGAGGCTCTACCAAGAATAGTTGAAGTGATCACAATCATGGCAGACCCCACTGGTCCTGCAGAGAAGGCACTGGGACATCTGTGGAGCCCAAAGTGAGAGTCTGAGGAAGGATATAAAACTGGCAAATGGTATTTTTCAAGTGAATGAAAAGGAGAGGATGGAATCGCCACTATCAGACATGCAGGTAAATCACTGTTGCCACCACGTGGGCACTGCCCTAGTCTGTGTCTACACGATTTGATAATGGGCATGAAATGGAACAGTGATTCAAAAGAGTAACACTCACACGTGAAACATATACAGACTGCCTCCCTCTCATGTTACTGCCTACAGAAGGCTTACTGTGCTATGCAACATGTGTGAACTGGCAATGGAAGCATGCGCGCCCTCCTGCTTACATGTGTGAACTGGCAATGGGATGCTGTGGCCCTTCCTGCTCTAGTACCTCATTCATCAAAACCGAACCTAAGAAAAGGATTGAGGGGATCCATTCATCCTTTCACTCATCACAACCTTGAATTATGACTTGGGAGATTTGAAACTCAGTTGGAGGATATATTTTAGCGATATTGTTTCCTTAAGACACTCTGCTGGCTATTCCAGCAATGCCAGGAGAAGATGTATTATTTGATCATATACCCCGCGAGTAAGGAGCTTAATGCATGGAGAGGGAGGTTTATAAAAAAGAGCTAGGCTAGGGTTCATATTCCTGAACACTGCACACACTTTACCTTAGGGCTAACTTAGGGTTCATATACTTGAACACCATACACTCTTTATCTTAGGGCTAGTCTAAGGATTACATACCTGAATACCACACACCCTTTACCTTAGGGTCATAAGTGGCTATTACAAGTTCATGAAGAAAAGCCACTCTTTCAAAGGAGCAATTTTCAGATATCATTCCATTGCTTCCTAAACTCTTGGGCAAATATATGTATCTGTCACCCATGGCATCCTTTACTCAAATTTTCTAGCAAATGTTTTTACCTCACTATTTTGAAACAAGTTCATTGTTATCGTTGGCTATAGATATAATTAAAACCCATGGATACACCCAATATTGTAAGGGTACCAGGAGGGTAGAACTGTTACAATTTTTATTTTACTGCAGAGGATACTGAGAAATGGAG
MSTRG.47984.1	--	0.452	0.040	0.012	-2.569	down	TAACAATTGAGGCTAAGATGTGAATCTTGGGCATTTGGTACAGAGTCAACATTAAGAGTTATTTTTAATGAACCTCAGTCAGACAGGTAAGAAATTGATCCAGAAGGAAGGTCTGGTGTGTGACTGGGTAATAGGATGACTATGGCCAGAAACCTATTATCCTTGTGTTAAATAGCTGGGGATGTGGGTTTTGATGATTTCCACCACATAAATAACTGTGTGAGGTAATAGGAACATTAAATATTAATTGATTATTTTATTATGATACAAAGTAATCCAAGAAACAAAAGAGCGCCACCCCTCCTCCACATCCCTTGACTGATATGTTGGTAGGTAGCAATTTGAAGAGAAAATAAATAATATGTAAATTCGAAATCAATTTTAACAACTAGTTTACTTTCTTTCCCTTTCAGACCTGGAAACCTGTTAGTGAGCATGGACATTGAGGATATAGATAAAATCTTTGTTGTTAAGATGTATCTCACAGAGTTTGTATATATTCACTGAAATTCTTTAAAAATAAAGTATTTCTACAGCCTGCAAGATAGACACAAGGAACCTGTGCAGAATGGCTGGCTTAGTTCAAGTCACCAGATTAACCCCTTATCCACAGGAAAGCACTTCACGCTCACTTGCTCCTTTATTACAGAATCATTTCCCTGTGCAAAACTGTGACATTTTCATCTACTTCTCAACTAACTCAGCTGGAGGAAGGTGTATCTCATTGACATAGCATAGCCCTATGAATCCCAGGATCAATAGGCAGCCTGCTCTGAGGCAGGCTGGGCTTCTTGCACTATGTCTTACCTCCTGACACTGTCTGGCATTCACATCCCTCAAGGGAGGATTTCTTGCCAGTGTCTTCAGGACTGGATTCATAAAACATAGCTACATGGTAGCCCTGCATGAGCCTCACTTCCCCTTTCTTGGGAACTCCAGTGCTTGTGTATATCAGCAGGCAGAGATTATTAAACTCAAC

							CTGAGCTAGGGGAGGACCAGAAGGGATCAGCAGAGGAACTCGGGCCCTTGCCTGAGAATTGTGTGGA<br>GACAATAGCATCTCACTGAGGTCTGTCCATCCTTTTCGGCATGTTCAGATTATGGCAGTAAGAATATGGC<br>TAGGTAAAGCTACCAGCCACTTCTCAGGACTTAGCTACTGGGGGCAGGGAGGGTCACACCAGCTCCTT<br>CTCAGTTCTTGACAGCCCTTGTAGTTCTCTGTAGCAGACGGAGCTGGCCCTTTCCCAGCTTTGGTCACT<br>GCAATTCAGACACCTCTCTTCCTTTGGCAAGAGTGCTCAACCCCAGGCATATTAGGTGCACAAGTCCAA<br>AGTTCTGCTGCAACGTTTGAAATCACCATGTAAGTTCAGAGAGAGAGAGAGAGAGAGAGAGAGAGAG<br>AGAGAGAGAGAGAGAGAGAGAGAGAGAGAGCGAGCGAGCCTCAGAGCCTTAGACGTGTGCTAATCAAC<br>AGAGGAGAGGCTCAATGTTCTACCTCAGTAAACTAATATTTAAAGACAGGTGATTATCGTAATGGTTGGG<br>ATTATAACAACTGAAGAACGCACAGATACAGATGAAGGATGTTTCTTTCTGAAAGTTTAGCTTTACAAC<br>AGCCACATAAGAGCAGAGCAGATAGAGAACAGTCAAACAAAAGAAAGCAGCCTGGGAAGAGGGGCT<br>GCTGGTTATCTGTGTCTCTTGCATTTCTGATGAATGGAGAAAGAGTGAAAAGAGTTTGTGCAGCCCCAT<br>GTTCCCAGAAATATAGAGCCACCACAGAGCTGTGTCCATTCCAACACCAGAGCCTGATCCTGGGCCTGC<br>GGCTGGCCACAGTAGATTTCCATCCTGAGTGGGTGGCCTCACAGCACACAAGCCATGAGTCTGATCATT<br>CACCAGCTGATTCAGATTTTATCAATTCACTCTTTTTAGCCCCTAAAACATTTCTAGCTGCTAGCAACCAC<br>AACTTTTATGCTCGATTTTGTGGGTGTGCAAGATTCTCTGGGAAAATAAAAGACGTCTCTTAGACTTTAT<br>GAAGTTCTAAAAGGGTCTGTATCTAGTGAAAGCCAGCATACTCCATTGATTGATGCACACTCTATGATCT<br>TGTGGCAGTTTAGCTGATCATTGGCATTTATTTTATCTCATTTCTGTGGTTATGATACCCAGTCCAAGTAG<br>GCGTACATAAGACTACAGTGTATTTCATGCATGACACAGTGGGAAAAGTAAGTAGCATTAATATCAGTGG<br>AGTTAGTATGGACCTTTCAGAGTGTAGTTTATTAGTATGAACTTCGACTTCAGACATTTGTCTTATGCACT<br>TACCTGGTGAGGGAGAATCAACTTTGTGAATTAGAAAAATCTACTTTCTGTCTCAATAGAAATAGCCTAC<br>CCTCATTTCTTTAGGAGAAATGCTATTGGCGAGGTCACGCGAGACACTGGTTTTTCTCCTAGCAACCAC<br>CGCACAGATGCACACATGTAAGTGCACATTAGTATGTATTCCTGAATGCATGCACACAAACACATATTGA<br>TACATATGTATACTCCTGTGCATGTACACACACATAATGCCCATACTGCAACTAAACATGCTCATTCTCAC<br>ACAGACACACACGCTCACACGTATACCTATACACAGATACTCACATGTGCTTGCATGTGGGTGCATGCAT<br>TCACACATTCATTCATGCACACAGGTACACATGCTCACACTGCA
MSTR<br>G.479<br>85.1	--	0.824	0.079	0.006	-<br>2.578	do<br>wn	TAGGAAAAATGTTCTTGAATGCTTGCAGAAGCATGAGGGCACACTTGTGTGTGTCTGCACTTGTGCCTA<br>TGGCTGAGAAGGCTTGTCGTGGCTGCGCTGTGGTACGCAGAGCTGCCGGGAGCCTCGGGAGAGTTAGC<br>CGACTGTCTGCACTGTGAATCTCAGTTACCACTGGGCTTCGGATGATCAACGCGAGTCTGGGCTTGTTC<br>TGTCCTGTGCAGAGGCACACCCCTCAGCTCATTACAAGTATTGACAGCGTTAGAAAAGGGAAACCATA<br>AAGCCAGAGATTTCTGAGGATGATGTGAGCATTAATATTCTTGAGTGGACCTCTGGCTCTTATACTGGAA<br>ATTGTATGAAAGTGAGGGCAGGTAATTAAAAGTCCTTTGATACAGTCATTTTCTTTTTTAAATGGTTGAG<br>TTAATAAATTCCCAAACCAGTGGTGTATTTCATTTCTTTTTAATTTAAGAGAAAACAGTGTATGCTCACTA<br>AACCTCTGCTCATAGACAGTTGGAATCTGTTAACCGGAGCAGTAAAAACCAAGATGATTCAGTAGAAA<br>ATTCATCTGCAAAATTGTCACTCAGCGACATACAAGCATGGAAACACATTATGCTCCCAGCCCCAGTTC<br>ATAATTAATTCTTTCATTTCTTAACATAGTTGTCTCGACTTTAATGTAAACTTGAGCCCTTATAAATACTCG<br>TCCCCCATGCTTTTGCCGTTTGCTGGTTTTATTAACTTTCCCCCGACCACCCATTTCTACTGCAGACAAAT<br>AGCTTCTAATTTTTCTTGAACACATGAACAATATTTAACAAAACATAATCACAGGTAGTGTTTGAAAACT<br>AAAATTGGCCATCGTTTAAAATGCACCTTATATGCACCAGAGAAAGGGTGGCCCTCCCTAGTCATTTCTC<br>TCTTGTTTTAATTCTTTGTCCTTAATGGGGATCTCAGTAGCAATTCAATAACCTTCTGCATCTGAACACCA<br>GAAAGCTCTGGCTGTTCTGACACTAGAGACGAAAAATGTTTGGTTTCATTATTTAAATGTGCACGTGAG<br>GGCGAGGTTTGTAATTTGACGTTGGGTTTCATCTCATTTCACATCCCTAGTAGATGTGGCCACTGGGCAA

							GTCACTTAAGCTTTTATTACTTAGTTTCCTTATTTGTGAAATAAATATGTGGAACATGTCTCAGCACATGT<br>ATTACTTAGCTGCTTCTGTCACTTCCGCAGAGGAAGCCTGGAAGATCCTCATTGGATGTAGTGTGGTCA<br>CTTCTAAGGGAGGCCTGTGTGGTACTCCTTAATACTGGCTGTTTTGGTACCGACGTTCTCTTTGGTGTCT<br>ATCATGGAACATTCCATTATCTTCTCTTCCTCTGTGCTTGTGTTTGCTTCACGTGGCTTGACAGCCTGCAC<br>AAACATTTCTAAGTTAGTTCAGAGAGTGCATGTGCCTGCTGACACAGTGAGTGTTTTCCATTCAACCAG<br>CATTGTGTGTATCTTGGCTAAGGTTACTTCACTCTTTCTCGCGAATCTCATTCCATATAGATAATTTTTTCT<br>GCTGCTGTTTTGCTATTGATTTGCCCATTTCTGTCTTCTAAAAATAACAAGAGCATAAGATAAAAGTCAC<br>TGAGCTGGAGTCACTTGATTCCAGCTAGAGATGGTTAGTCGAGGCAATAGTAGAAAAGCAAGAGATTG<br>ACATCTGTGTGAATCAGGCAAGGAGAGGATGTGGACGTTGTTCAACTGGAAAGAAAAAAGAAAACTC<br>AATCATGCTGAAAATAAGTACTGAATATTGGACCAGGATAAAAGCCTGAGTCTCACAGGTCAATTAAGC<br>CCCATGTTACTTTACATGATAATTAAAAGTCTCTTCCCTTTAAAGGGCTGTTGGTGAAGCGTGCTCATAC<br>TAAATTAAATATGATCACCTGATAACCATAGTTTGACATGGACACTGGACATGCTAAATTGTGTTTTAGCT<br>TCCTCTTCTGGTCCTTCGCTAAGTAGCAACTGAAATGTTTCTTTAGGCTATAGAATCAATACTTCAGCCC<br>AAAGTGATTTGGATTTTACTTCTGTTTGTCTACTACTCCCTAGTATGTTTACTAATTTTCTCTATTGATCCT<br>GAGGCCCCAGGGTCAGAGTCACTGTGCTTCACATGTGTGGACTTCAAGCTTGATATTTTGAAATGTGCC<br>CATGCCTGTTCACGCCTGGTCAGATGCCGCCCACCCTCAATTATTTATTTATTTTCTCCTTTTTCTTAAATT<br>TAGTGAGAGAATGGCCACCCATGATCTGATATGACAGTAGGTATGGAGCTGAGTTCCCTGAGGCTGATA<br>CGCTAAATGACAAAAGACTTGGGTTGTTTTGTGCTTTCCACAGACATTGATTCTCTTATAGAACCTAAGT<br>AGGTTTCATGTAGGTTTGTACCATGAGTTCATATGCATACATTTCATCAATGGTGATTACATTTTAATTGTG<br>TATTTCTCATCACAGTCCTTAGAGCTTGGCGGGGCACTGTACATCTCAATAGGAAATCCTGTCAATTGTG<br>TCCAAGCATTGGTCCCAATAGTGAGTACCTGGAGTGAAATTTTACTTCCATAAAAATTATACAATTGTCT<br>TAATTATCCAAATTGTTATCTTAAAAGTTAGCAGTGTCAACTACAGAGAGAACATAATGAGAGCATGCTT<br>CCTTGGCACCTACAAGGTTGTGGGTTCAATTCCATGCACCAGGAGGAATGTGATGGTGGAGAACTAAA<br>GGGATAAGTTAAAACACCCCTATCATGAAATACATAGTATCTAGGACTTCCCAGCCAGTCTAGGCGACGC<br>TTTATGCTTTTCCTAACAGATATTAAGTGTGGGATTTGGATATGACCTTGTACTTCTCTCTTAACCTTGGG<br>CAAAAAGAGGGGAAACCACTGTTGTATGAGCTTCAACATGCAACATGGACTTGTTGGTTCCAGAAAAG<br>GCTTTGGTTTCATGGAAATCAACTACATCCCCAGCTTAGGTTTTGTTTTCCAGTCAGGCATATATTTTCCA<br>GAAACATGTTTTTTAAATATTAGACTCCTGGGATGCAAAACTGCATTTGGGGAGGAGAGTGGGCTAGAG<br>CATGGACAGCTGGTGTGTGGGACATCTGAGAGTCCTGCAGCTCGGTGGGACGTGGGAGGGAAAGAAA<br>CTGGAAGCTGAAGCTGAAGACATTTCAACACTAGGAGTGTTGTCTGCCTTAGTGTGGTGGGGTTTCAA<br>CACTTCCCGTCTGAGGCTTTAGTTTCTGAAGCTTCACACTTTCTCACAGCCCTCCCATATGCCACACTAC<br>AGACATGTGCACCGAAAAGTCTGGAATGGCCCTATTTCAGATGGAATTCTTGGATCCATAAATCAAGGG<br>CAGAGCCTCATGGTATGATGACTGTGGTCACAGAGAATTTGTAATTGTTAGAAGTTTGCTGATTAATTTT<br>GTACATTTTATTAGATACTCCTGAAAAATGAGTGAGACAACTTTTCATTCATGTGATTCTGTATAAAATTA<br>GCACCGTTTGTTCTCAGCTTAATGAGAGTTGCATACAAGCTTTATATGTTGCAGAGTGCCACATTAGAAT<br>GTTACCCAGGTAGGAAGCATTTGCAACATCCCATCAGCTGTACTTTTTGTCACACTTAATGCAGCTGTTT<br>GTCTCCATTTTCAGCTCCTGAACAATTTGCCATCTCACCCATTAAACCACAGGGACTCTAGCACAGCTTT<br>GCAAAACATAGCATCCATGTTAATTCCTGTTTGGTCCTTAAACTATTGCAGCTGGGCATTGATTGTTTCTG<br>AAGCAATGTTTTCCTGTTACTCTGACATCCACCTGTAGAAAGGAGCTAAACTGGTCGTGCCCAGGCTGG<br>GGTTGTAATGGCTTCGTTCCCTACCTCCTCGGGGGTGAGTTCCAGCTGCTGACTCTGTTCCTGCCTGAA<br>AGGAAATCACAGCTGATAGTTAGATATTCCTAGATGAAGAAGGTACCCCTGTAAAACCGTTTGTGTAAA

							GACAAGTGCTGTGGTCACAGGGTTATTCCGACAATTGACATTGCAGTCTTCATTTTTCCAGGAAGCAA<br>GCTGCCCGTCCCTTTCTGTGTTTGACTTCACTCATTTTTAAAAATGTATTTTCCATTGATCCCTTGAGAGT<br>TTTCATGCTTGCTTATGGTACATTTTTATTACACACATTCCCCATTCTCTAGCTCCCAAAGAAGCCTGTCA<br>GCTATAACTGGGGCCTGGAAAGTCAGCTGCCTTTCCATCTGACCTCACTCCTTACCTATTCTGGACAGG<br>AGGGATCTGACCTGAATTTTCAGAGGAGCAATGTCCTCTAACCATCCTGATAGTTCTGCTGTGCCCATGT<br>GGTTAACTTTGTTAGATATCTTCTTTGGACACCAGATGTCTCGGTAGTTTGAATGAATCAACTTTTCTCCT<br>ATTTCACTGAGCTGTAGACACTTCTGGCAAGGCACTGTTCAGAATCCTCCATGAAAGTATGTGGAAGGC<br>AGATTACCAGAACTCCTTATACCCATCTTGAATATCTAAACCCAGGCTCCCAACTGGCTGCCTCTTCTGT<br>TTTCCCTTCAACTGAACATTGACCACCCACAGTTATCACACTGGGCCAGTAAGCTCAAAGTGCTCCTTC<br>AAATTCTTAGGGACATGGGCACTTGTATTTAAATAGTATGTGCCACATAAGCTAGAGGGTCAGAGTTCAG<br>ATCCCTAGAAGCCACAGAAATGGCAGGTATGCAGGGCACCCCACCTGTTACTCCAGCCTCACAAGGTA<br>GAGACAGTGTGTCCCCAGCAAGTCAGTAAAGGAGAGTAGAACTGAGTGAGATCTGAGTTCTATAATAG<br>ACCTACTTCAAAACACAAGCTAGAGAGTAATCCAGGAAGATTCTCCACATTACCTGGAGACTTCCACAG<br>GAGCATGTACAAACATGCATATGTTCCTGCCCACACAGATATACATATAAAGAAAAAGGAAATAAATTTT<br>ACAGACTGATAAAATTCCTGTCTTCTACTGCACCACTTCCCTAGAAAATGGAGAATAAGGATCTATCAAT<br>AGAGTCGTGGTGGCCAGGATGCAATGTTTTGAATTGGGTAGCAGAGTGATCAAGTTTAATAGAATATTTA<br>GTGGTCATTGTATTTCCCTAAATCCTGCACCTTTAAATGTGGTGGATTGACAGCTGTGAGCCCACCTGCG<br>AATACTTTGTATTGCTTTCAAGGGCAGACAGGCTCAGATCCCTGGCATAGCTTACATAGGTCACAGGGC<br>ACATTATAGTCTAAGACCTCATAAAGGGTAAACACCTACTTGGTGTAGCTGCACTGTGATATCTATAGCTA<br>CATTCCACACCATCTGACAGATGTTGGGGTCTTCTGGGAACTGAATTAGTGCTGCCATTTCACTAATATA<br>CAACAAATAAAATTACTAGCATTGTCTCCCACCGGTGGCTCTAGCAGATTCCATCTAACTGCATTCCCCT<br>GTGCCCCTCATGGCTGTGTAGTTACTGGACAGGCGTGGGAACACCTGTTTGCGTATGGTACATTTTTATT<br>ACACACATTCCCCATTCTTTCAGACATCTTTCAGAATACTAATCGAAGCAGCAACATACTGCTCAGGGG<br>AGCACTGCTTAGGAGAGAAGAAAATGGTACTCACATGTGCATAAACATCCTGACTTCTTCCTGAGGTGC<br>AACTGGGGTACATGGGCCACTGTGGCCAGGAGTTTAAAGCATTGAGTGCAAAGGTACCTGAATGTAGT<br>GTTCATGTTTTTGACATGCTATTTCTAAGAGGATTTACACGATAAAACATAGACATACACACTGCTCTGT<br>GGAGTTTATTGATGAGAAAGTGTTTGTATTTTGATCCCATGCATACATGGGACTTACAGATGACTTCTTAT<br>GGGACTTTGATTCACTACTTGTGCCAGATCATCAGGCTCAGTGGCAAAACCATCACCTACTGAACCAAC<br>CATCACCTTGCACTGCCTGGTCCAAGGTAACGCATTCTAAGGCAAAACCATTTTCTTTGACTTCTACCGT<br>GTGAACTGATTTGATCAAATGAAGCACAGGTATTTCACACAACTTAATTTTCCTTTTTAATCCATAAGAA<br>TTAAAATTCTCATGTTGTTAATGAAAGCCATGCTGTGGGCAGCTGGGTCATGTGCACATAACTAGGAAG<br>TCAATTGTGTTAAATTGTTCAACCATTCTTTCTCCCAGGAAATTGAGAAGCCAAACATATCACGAGTAAA<br>GCTTGGTGGTGGGCTAGTCCTTGGATGTCATATGACTTCCTCAGAATGCAGGGCATGCTGACCCTAAGC<br>AAGTACCTTAGGCAAAAGGACCTTGGGCTGTTTTTCCTCTTTCTGTAGAAGACCAGAGAGGAAGTAGA<br>ACCCACATTCTCTGTAAATGTTTTACATAAATGTGTTCTATTCAAGAACTTGCTCCGAAATTAAATAAATG<br>TTAAAGTGAAAATACAATGATTAGAAAATATCAGGGCCTTCACAATCTTCTTGATTGTTAAGACAGTGAA<br>GCTGAAGAGGGCTCTGTACCATTTGACTATTCCAGACCCAATGGATGTCAGAGAGGCCTTGTGAACCAT<br>GTCGCTCTATCCTGTTATTTTAAGATAGCCCCTATGTTTCTTCTTCAAAATGTGTGTCCTCTTGTGAGGCT<br>CCCATGATATGATTCCAAGGTGGAGACATCTCACTGTCTAAGCCTTTGTTCCTATAGGCACAGAGGTCAT<br>TGAACTGGAAGATATCCTTACCAGTAGGTTTGTCATCACAAGCAGAAATGTGTGGGTCTCAAGTCTTCC<br>TTGATATGAGGGCATTTGCCTTGTCTTATTGTATCTTGTCTTATCCTGTTAAGTTGTCATCTCTTGGGAGCC

							TGATCTTTTCAGGAAAAGTAAT
MSTRG.48242.1	--	0.440	0.018	0.004	-3.378	down	GTTTACTAAAGTAGAGGAACTTTTCGTAGTTTAAGTTGGTAAGGTAATTTGATTTGTTTTTATGGTATCATTTTAGTAGTGATATTTTGGGAGCTTTATCTTCAGTACACCAGAGCGAGGTGGGAAGGGGACACTGTCAAATTAGTTCAAGTTCCTATGTCATTCTGGTGCTTAAAGGCCTGATAACTCTTGTTTTCCAGTAACAAATCTTTATGAAGCGAAAGGGTCTGTCATAAGCCCGTTTGTTCTTGAGGCTCCTCTGAAAACGAAGACCTAGGAGTTCTATTGTAAAGGTTGTTTGTTGGTATTTGCTCTCAATCTCTTTTGCTTCCGTGTACTTTCTGTGAGCATATGGCTCTGTCAGCGTTAACAGCTTTTCATGAACATTCCATTTTGAAATACTCTTTCTGAATTAATCTCACTCCCCATATCTCTAAATCAGCAGCTGCTGTGTAACAAACCTTTTCCTGGGGAGACGTAACACCCATGTACTCACCCCAGATAAGGAACCCATGACAGACCAAAGTGTGGATACCACCGAAGTCCAACTTGGTGAACCAATGAGCTTTACTAACGTTACTTACAGGTGTGTGAGTGAGGGGTCACTTACAAGAGCAGAAGCGACTCCTAGACAGCTGCATCGCCAGAGCCACCCCAGCATGGGTGACAACTCGAAAAGTTGGGAGCCCAAAGCACACTGCAGAACCTGCAGGCAGTTCACCAGACTGGAGAGTATCCTTCATAGGTGCCTCAGTTTCTCCAAACCTCTTCCCCATAGCTCTGCTGGTTTCTGCATCTTCCGAGCAGCTGGTCTGGTCTCAGTCTTCTCAGCAGCTTGCTTCTCCTTACTGTCCATCTCAGTCTGGTCCTGGCACCTGCTTCTTCATCTGGGTCCAGCTGGTGACCCTCTCGCCTCTGGTTCCTACCTGAGAACCCTCTCTTTTGGCCCGGAAGTCTGGCCTTAACTCTCCAGCCCTGCTATTGGCTGGTCAGCTCTTTACTTGACCAATCAGAAGGCGATGGAGAACAATGTTTACAAAATACTTAGGCAGGATATGCTTCATAATTATGACAATACCAAAGTCCGCCAGTACCAAACTCAGCAACTGAATGATGCAAAGTCAGTCTTTACACCACCGGGCTCCCGATGCTCCCCCTGAGCTCACAGTCTGGCAAGGAAGGCGCGGCAGTGATAACCGGCTAGCTGCAGCAGCAAGAGCGTGGGATAGCTGGGAAGGACACAGCTGTACTTCGGAGCTTAGCGACGTGGCTGTTGTTGCCTAGTTCCCTTTCTCTTTTTTCCTTTAGTCTGGGATCCCAGCCCATAGGATGCTACCGTCCACATCCGGGGTGCCCTCCCTCCTCAGTTATAACTCTCAGAAAAGCCTTTCACAGAGAAACCCAAC
MSTRG.48331.1	--	0.761	0.040	0.000	-3.333	down	GCTCTTCAAACCTGTGACCACCCGGCGCCAGCTGCGGGACAATAGGTCCTTGTGTCCAGCTGAGGGAAGAGAAGTTGTCCTGGGGCAGAGTCTCTCCTCTGTACACACACCCACTTTGCTCCGTCCCGACTGATGGCTGCCATCTCCCTGGTCTCCCCTGGTGAGGCACTCGTCACTAACCGCGCAGCGGCATCCAGGGGAGCCGGGAACTGCGCACTCGGACCTGGCTGGCTACTTTCAGAGACGGCAAACTTCACTTCTGACTCCCCGGTGGAGGCTCCCCGCTTCCCTGCAGCCCGGTACACATGTCTCAGCCGGTCCCCGTCCCCGCGTGGGCCCGCTCCGCGGCAGCTGAGCGCAACCTCCGAGGATGAATGTCCCTCGACCCCGGGATCGACCCGGCTACAACTGCCTTCCGGGTGGGCCCGGGCGAGCGCAGATGACTGTAGAACCAGAGACTAGGGATCTGCAGTGGCCGATCAGGAGTACCAGACCCGGGGATTAGGGATGTATTTGAGACCATTGTGACAGGGATTGGAGAAGATCTGGCATTGCTACGGTTTGGATTAGACACGGATGACACCAGCGCCAGTGACTAAACAGACCTCCTGAGACACCACGGTCTTGCTGTCCACCTGAGTTCTACCCAAGTGTCTCACCATGTAACACCAGCTACAGACACCCAGCCAACCTCAGGGCAAAGTGTTTTCTCTCTGGCCTTCCTGGACTTGCTGTGTGAGCCTGGTTAGGTTGATGGCCGGCTCTCAGTGATACTCCTAGCTGTGGTGTAGATTGTTGAGGCCTCTGTGTCTAGGGTCTAGCAGCTGGCCTCTGCGTTGTGGCCATAGTCCTGTGGCCCTCACTGTGCTGTTCATGCATGTGTCTCTGGTGATGCTATCTCTTTCCTACGGTGACTCCGTACTGCTGGGCTAAATTGTAAGCTTCTAGTGGCCAATTACAATTACAGTGGGTATTCCTGGCACAGACCTGTCTCGGGATAAAGCCTTCCTGTCATTAGAGAATGTTCTACTCGGAGGCATGTTTTATTCTTTCCTTCCTTTTTCCTTGTTTGGATTTTTGGGACAGAATCTTCATCCTAGGCTGTCCTCAAACTCCATGCAAACTTCCTGCCTCATCCTCTGAAGTGTTGGGAGTACAGTGAGCCCTCATGTCTATCTTATTCCTCATACAGAGCAACGCACTTTCCTAGTGGTGCTGCAGGAGTGAGGGACTTCATCCTCTGAGCCTTCACCCTTTGTGGCTCCCTCCAGCTGGCACTATTCATTCTAGCGAGTGAGTGTCTGACTTGCTA

							ATGAATACTGAGAGGAACATGCTGGATGTTTAGTGAAGGTTTCCTTGCTCTTAAGAAAGGACGTGGCTT<br>CCCTTTTAAGAATGGATGCCGTTGTCTGGAGGGGATGCCTACTGCTTTGCTCCCTTCAGACACCAGAGG<br>ACCAGAGGTGGTAGCCAGAGGAAAGTGTTTATGCTGGGGACAGAAGCATGATTCTGGTGAGAAGATCC<br>TGAAGTGTCATAGACCCATTAACCCACCCTAAACAAAGACTTGGTAAACAGTCAATGCAATACCAAGTT<br>GAGTTGGGTTTTCTATTACCAACAGACGTCAGCATTTTAACTTGGAACCCACGAAAAAGAAGTTAGGAC<br>GTGGTTAAAATAACAGAAACTCCAACTCATTCTGGGAACCTGTTAGTCATTGCTGTGTAACAAGCCACT<br>ACCCATGCAGGAGGTGCTGAAGCAGGGGAGGGGGGGGAAGCCAGTGCTGGCCAGTTCTCCCGGGCT<br>TGGCAGGTTGTTCTGCCCTCCCTGGTGTTGGCTGGGAATCTCAAAGCTACTGTATTTTTTTGGAGGCTCT<br>GCCAAGGTAGGAATGTCACCATAGCTCCAGTCACATGTGCAGTGTCCCTGAGATGGGAACTGGGGTTG<br>GGAGAGCAGGCTATTCTTTTCAGATGGCTTCTTACAAGTCAGTGAGCTAGCCTGGGCTTCTTCACACAG<br>CAGTTGGGTTTCTCAAGAGACAAAATGAACATTACAGAACCACTAAAGGCTGCGTTTAGAGGCCCCAA<br>AGTGTTCCCTCTGCCACACTGTTCTGGAGAGAGATCATATGCCCTCTGCCTCCCTCCACAAGGAGCACG<br>AATAAGCCATATTTCCACGAGAGCCTCTGGCCAACACTGCTGCTCTGCTCCAACCAACAGGAGGACATC<br>TTGTTTCCTGTGCCCCGTACAATCCCTTCCAACTCCCTGACCGTTTGTCCGAATTCTTCTTCTTCAGAATA<br>AACTCTCATTTGATCATCTTTCTTTGGTATGTCCCCCTCCCCTCCACCTTTTCAGTCTCTTTTAGTTGCAG<br>GTAATGGCTTCTCCCCTTATAGTCACTCACGTTTAGTGGCACCAGTGAGACCATGGACTCGTTTGCTCAC<br>AGCAGCTTGTCTAACCCTCATAGATGGTGATCATGTGGGGAAGTATTTTTTTAGTGATGTTTGCCCTGGA<br>CGGCCGCTCTCCGTCCTGAGCACTTTCCGCCAAGCTTTTGAAAGCTGGCAGTTACAGCTACTGAGCCCT<br>GAGCTGGTGAGGAAGGTTTCACTGGGGATGGCTACCGTCATCGATCTTCCACTCTTTGGCACAGCTAGG<br>GATCTTGTATGTACAGGTTCTGGACAAATTGTGAAAAGATTAAATTACACATAGACTGCCACTTCAGCCA<br>CTGAGTTTCAGCAGGAAAAGCAAAGGGAGACCGGGAAGCCTTGCCAATGAGAAGAAAAGTTTACAGT<br>CTACAGCAGTCCACTGGGGGCCCTGCGGCTGTGTAACATCAGTGTGGAGCGAGCGCCCAGACTCTGGA<br>TCATGCTAGCCAGCCAGGCTCTGCAGCACTTCCCTGGGATGTACCCAGGAGGGAGTAGTGGGTGATACC<br>AGAGTTTATTCATTTATTCTGAGACAGGGTTTTGCCACGTAGCTCAGGGCCTTGCTTAGGGTTTCTACAG<br>ATAGGACTAAGCACTCTGACCAAAAATCCAAAAATAACTTGGGGAGGAAAGGGTTCATACTTTCACAG<br>CTCTCAGCTCACACTCTATCATGGATGGAAGCTATAGTCAGGGACTGAAGCAGAGGCCATGGAGGAAC<br>ACTGCTTACTGGCCTTTTCTTCATGCTTTGCTCAGCCTGCTTTTCTGTACCACACAGTATGGCCTGACCA<br>GAGGTGGCACTGCCCTCCACCCCTCTCCCCCTGCTCCCTAATGGGCTGGGCCCTCCCATCAAGGATCAA<br>TCAGGAAAATGCCTGACAGACATGCTCACAGGTCAGTCTCTCAGTTGAGATTCCCTCTTCCCAAGTACC<br>TTTAGATTTGTGTCAAATTGACAAAACAATAAATGAATAAATAGATTAATAAATAAATAAATTAATTAAAA<br>AGCCTAGCACATCGAGGCTAGCCCTAAATTCATGACCCACCTGCCTCTTCCTCCCAAATCTTCCAAGTGT<br>TGAGATTAAAAGTGTACACCATAGCTGGCCATGAAGGTTTATAGTTTTACAAAACCGAGTCATAAATCCA<br>GACAAATTTGAAACACATCTGTAGGCACATCAGAAAGATGGTTACAGCAAGATGGGGGAGCTTTTCTCT<br>ATGCCTCAGATGCTATCTGATGTTATACTTAGCTTGGGGGTTGGTGGAAGCTGGTGGTCTGCTAAGTCAA<br>TACACTCTGAAAGGTGTTTATTAATCACAGGACATGGCTACAGAAGGCTATTCAGGGGACTAAAGGGCC<br>CTCCACATTCCAACCTCTCCCGATTGCCAGGTCTCATCAGCTGATCCCTGCAGATACAGTCAATCCCTGC<br>AGATATGGCCCACCCAGGAATTCTCCTTGTTGGGCCTCTGAAGTTTTGCCTCTGCTGTATGATGGGTCTT<br>TGCTTTCTCAAGGATCTGAGGGGTCTATGGTGCCTTCTGAGGCTCTGTTTACCCAAGTGGTGGCCTGTC<br>CCCCAAGGGAGACTTTTGGGGGCTTAGTTTGCTTCTCTGCATCCTATTTTCCTCTTATTTTTCAGAGACTA<br>TGTAAAAAACATTTTTAAAAAAGGTATGCTAGGAATTGTTTAATTTTTCTGCACGCCCCTATCTTAGCTTC<br>TAAAGGATCAATGATTTTTAATCCCAGTTTTCTTAAAAAGAAAAACAAAACCAAGTTCTGTGGTGTGTA

							A
MSTRG.48331.2	--	0.022	0.708	0.000	5.418	up	CCCTGTTGCTTCCGCCTCCCTGCGGGGCGGGAGGGCGCTGTCATCGCCCCCGCGTGGCTTGTCCGGTGC<br>CGGTTCTCCCCGCAGCTGATTCCTCCCCGTTCACTCCTGTTCTAAAAAGGAAGCCTGGCTGTGGGCCTA<br>GGAGGCGATGGCGGGTGCGGTGGGCCTGGGTCCCTGGGCCTGTGGGTCACACCTCCTGCCTGGGGGTG<br>GACAGAAGACACAGGATCCTCAGGTGGCTACAGGATGACTGTAGAACCAGAGACTAGGGATCTGCAGT<br>GGCCGATCAGGAGTACCAGACCCGGGGATTAGGGATGTATTTGAGACCATTGTGACAGGGATTGGAGA<br>AGATCTGGCATTGCTACGGTTTGGATTAGACACGGATGACACCAGCGCCAGTGACTAAACAGACCTCCT<br>GAGACACCACGGTCTTGCTGTCCACCTGAGTTCTACCCAAGTGTCTCACCATGTAACACCAGCTACAGA<br>CACCCAGCCAACCTCAGGGCAAAGTGTTTTCTCTCTGGCCTTCCTGGACTTGCTGTGTGAGCCTGGTTA<br>GGTTGATGGCCGGCTCTCAGTGATACTCCTAGCTGTGGTGTAGATTGTTGAGGCCTCTGTGTCTAGGGTC<br>TAGCAGCTGGCCTCTGCGTTGTGGCCATAGTCCTGTGGCCCTCACTGTGCTGTTCATGCATGTGTCTCTG<br>GTGATGCTATCTCTTTCCTACGGTGACTCCGTACTGCTGGGCTAAATTGTAAGCTTCTAGTGGCCAATTA<br>CAATTACAGTGGGTATTCCTGGCACAGACCTGTCTCGGGATAAAGCCTTCCTGTCATTAGAGAATGTTCT<br>ACTCGGAGGCATGTTTTATTCTTTCCTTCCTTTTTCCTTGTTTGGATTTTTGGGACAGAATCTTCATCCTA<br>GGCTGTCCTCAAACTCCATGCAAACTTCCTGCCTCATCCTCTGAAGTGTTGGGAGTACAGTGAGCCCTC<br>ATGTCTATCTTATTCCTCATACAGAGCAACGCACTTTCCTAGTGGTGCTGCAGGAGTGAGGGACTTCATC<br>CTCTGAGCCTTCACCCTTTGTGGCTCCCTCCAGCTGGCACTATTCATTCTAGCGAGTGAGTGTCTGACTT<br>GCTAATGAATACTGAGAGGAACATGCTGGATGTTTAGTGAAGGTTTCCTTGCTCTTAAGAAAGGACGTG<br>GCTTCCCTTTTAAGAATGGATGCCGTTGTCTGGAGGGGATGCCTACTGCTTTGCTCCCTTCAGACACCA<br>GAGGACCAGAGGTGGTAGCCAGAGGAAAGTGTTTATGCTGGGGACAGAAGCATGATTCTGGTGAGAA<br>GATCCTGAAGTGTCATAGACCCATTAACCCACCCTAAACAAAGACTTGGTAAACAGTCAATGCAATACC<br>AAGTTGAGTTGGGTTTTCTATTACCAACAGACGTCAGCATTTTAACTTGGAACCCACGAAAAAGAAGTT<br>AGGACGTGGTTAAAATAACAGAAACTCCAACTCATTCTGGGAACCTGTTAGTCATTGCTGTGTAACAAG<br>CCACTACCCATGCAGGAGGTGCTGAAGCAGGGGAGGGGGGGGGAAGCCAGTGCTGGCCAGTTCTCCC<br>GGGCTTGGCAGGTTGTTCTGCCCTCCCTGGTGTTGGCTGGGAATCTCAAAGCTACTGTATTTTTTTGGAG<br>GCTCTGCCAAGGTAGGAATGTCACCATAGCTCCAGTCACATGTGCAGTGTCCCTGAGATGGGAACTGGG<br>GTTGGGAGAGCAGGCTATTCTTTTCAGATGGCTTCTTACAAGTCAGTGAGCTAGCCTGGGCTTCTTCAC<br>ACAGCAGTTGGGTTTCTCAAGAGACAAAATGAACATTACAGAACCACTAAAGGCTGCGTTTAGAGGCC<br>CCAAAGTGTTCCCTCTGCCACACTGTTCTGGAGAGAGATCATATGCCCTCTGCCTCCCTCCACAAGGAG<br>CACGAATAAGCCATATTTCCACGAGAGCCTCTGGCCAACACTGCTGCTCTGCTCCAACCAACAGGAGG<br>ACATCTTGTTTCCTGTGCCCCGTACAATCCCTTCCAACTCCCTGACCGTTTGTCCGAATTCTTCTTCTTCA<br>GAATAAACTCTCATTTGATCATCTTTCTTTGGTATGTCCCCCTCCCCTCCACCTTTTCAGTCTCTTTTAGTT<br>GCAGGTAATGGCTTCTCCCCTTATAGTCACTCACGTTTAGTGGCACCAGTGAGACCATGGACTCGTTTG<br>CTCACAGCAGCTTGTCTAACCCTCATAGATGGTGATCATGTGGGGAAGTATTTTTTTAGTGATGTTTGCC<br>CTGGACGGCCGCTCTCCGTCCTGAGCACTTTCCGCCAAGCTTTTGAAAGCTGGCAGTTACAGCTACTGA<br>GCCCTGAGCTGGTGAGGAAGGTTTCACTGGGGATGGCTACCGTCATCGATCTTCCACTCTTTGGCACAG<br>CTAGGGATCTTGTATGTACAGGTTCTGGACAAATTGTGAAAAGATTAAATTACACATAGACTGCCACTTC<br>AGCCACTGAGTTTCAGCAGGAAAAGCAAAGGGAGACCGGGAAGCCTTGCCAATGAGAAGAAAAGTTT<br>ACAGTCTACAGCAGTCCACTGGGGGCCCTGCGGCTGTGTAACATCAGTGTGGAGCGAGCGCCCAGACT<br>CTGGATCATGCTAGCCAGCCAGGCTCTGCAGCACTTCCCTGGGATGTACCCAGGAGGGAGTAGTGGGT<br>GATACCAGAGTTTATTCATTTATTCTGAGACAGGGTTTTGCCACGTAGCTCAGGGCCTTGCTTAGGGTTT

							CTACAGATAGGACTAAGCACTCTGACCAAAAATCCAAAAATAACTTGGGGAGGAAAGGGTTCATACTTTCACAGCTCTCAGCTCACACTCTATCATGGATGGAAGCTATAGTCAGGGACTGAAGCAGAGGCCATGGAGGAACACTGCTTACTGGCCTTTTCTTCATGCTTTGCTCAGCCTGCTTTTCTGTACCACACAGTATGGCCTGACCAGAGGTGGCACTGCCCTCCACCCCTCTCCCCCTGCTCCCTAATGGGCTGGGCCCTCCCATCAAGGATCAATCAGGAAAATGCCTGACAGACATGCTCACAGGTCAGTCTCTCAGTTGAGATTCCCTCTTCCCAAGTACCTTTAGATTTGTGTCAAATTGACAAAACAATAAATGAATAAATAGATTAATAAATAAATAAATTAATTAAAAAGCCTAGCACATCGAGGCTAGCCCTAAATTCATGACCCACCTGCCTCTTCCTCCCAAATCTTCCAAGTGTTGAGATTAAAAGTGTACACCATAGCTGGCCATGAAGGTTTATAGTTTTACAAAACCGAGTCATAAATCCAGACAAATTTGAAACACATCTGTAGGCACATCAGAAAGATGGTTACAGCAAGATGGGGGAGCTTTTCTCTATGCCTCAGATGCTATCTGATGTTATACTTAGCTTGGGGGTTGGTGGAAGCTGGTGGTCTGCTAAGTCAATACACTCTGAAAGGTGTTTATTAATCACAGGACATGGCTACAGAAGGCTATTCAGGGGACTAAAGGGCCCTCCACATTCCAACCTCTCCCGATTGCCAGGTCTCATCAGCTGATCCCTGCAGATACAGTCAATCCCTGCAGATATGGCCCACCCAGGAATTCTCCTTGTTGGGCCTCTGAAGTTTTGCCTCTGCTGTATGATGGGTCTTTGCTTTCTCAAGGATCTGAGGGGTCTATGGTGCCTTCTGAGGCTCTGTTTACCCAAGTGGTGGCCTGTCCCCCAAGGGAGACTTTTGGGGGCTTAGTTTGCTTCTCTGCATCCTATTTTCCTCTTATTTTTCAGAGACTATGTAAAAAACATTTTTAAAAAAGGTATGCTAGGAATTGTTTAATTTTTCTGCACGCCCCT
MSTRG.48437.1	ENSRNOG00000023509	0.433	0.000	0.004	-4.319	down	CGCAAACCGTGACAAGCTCACGGCTCACGTCGTGTCAGCTCGAAGTGCAGCTGGGCATAATGAGGAAATCAAGGCAGGAGGAGCAGCTTGTAGCAGCTGGGCACACTGTAGCCACCATCAAGAAGGAGAATGAGCCCAGGCTTCCTTCCCCTCAGCTCCCATTCCGTGTTTTATACAACCCATATCCCGTGCCCCGGGAATGAATGGTCCTGCCTACGGTTGATGGGTCCTCCCACATCAGTGGGCAAGACGATGACAGCGAAGGGAATCCCCCACAGGTATGCTTAGAGACCCATCCTCGGGTGATTTCAGATTTTCCCAAGCTGATAGTTAACACACCGTCACACACAGACACCCCTCTGCCAGCCAGCTAAAGCCTTAAGGTCAGCGAAGAGTCATCACAGCCCATAGTCTACACAGGCAACAAGACAGGCGTTGACTCACTGCCTCCTTGGACCTGCACCAGTGATCTGTGCAGGCAGCCGAGGCATGCTGTCAGAGGAAGATGTGGAGATGATAGGCCAGGCAGTGGCCTCAGCTGCCCGGGTCACTTTCTTCTGGGCCCAGGCTCCCTTGGTTCCTGTGTATCTCTCTTTTTTTTTTTCCCAGAGGGAGGGAGGTGGCACGTTTTCAAATGGAGTCTGAGTAGGAGCTCAGTGCCTTGCACATTCAAGAGGACCAAGAGAACAGAGTTTTACAGACTGAAAACGAGAAGCAAACAGAACAGCGCCTCATTCTTCAGAGTAAACAGAGCAAAGGCCAGCCGGTGGCAGATGCAGCCTAGAGCTGGCTCTGTGCAGACAAAGCGC
MSTRG.48470.1	ENSRNOG00000033703	0.598	0.000	0.049	-3.592	down	GGTGGTAGCTCACACGGTGTGTCGAGTGGCAGATGGAGATGCTCTGTGAGCCTGAGAGTGGGAGCTTCCTTCTAAGGAACAAACGCCTCACTGGAAAAGCCCAGTGGTGCCTGGTGCCCACGAGCAAGGTCAATGTCCACAGCAGTAACTCTCAGCCACAGCTCTGTGGCACAGGCAGTGGAAACAGAACCTTCCAACTAGCCTGTCGTCCACTGTTCACCAAGAGAGCCAAGCGGAGCCCAGATAAATAGGTTCCACGAGGCTAGGGGCCAGCAGCTCTCCAAAGGGAAGTTAGGAGATCTGGGCGGCCTCTGCAGAACAGATAGCACAGATCCCACTGCCATCCAATGTCT
MSTRG.48751.9	ENSRNOG00000027229;ENSRNOG00000027234	0.002	0.317	0.024	3.103	up	GAGAAAGGTTGAATCCCCGGGGAACCCTAAAAGGGACAGCAGGCAAACCTGTAGGAAGGTTTGAGACAGACCAAGTCACATAGAGCAATGCCCCAAGCCCCTCCTCCACAGAATTAAAGGTGTGCAAGAATCACTCCATCGTCTGAGAGCTTCTGTCATAAGAGCTGTAACACCGCTGCTTGGGGAAGAGATCTGCTCTCCCAAAATCACCTCCAGAAGCCCTCCTGCACCCCTCGCTGGTTAGTCAGTCTCCTGCTGGCTCAGCCTGGCCTGAAATGGAGGCGGAACAACACCTGCCCGAGTTCTCCTCCTTTCTCCTGGACCTGCCAACCTAGCCGGACCAAAGATCTCTGTGGAAAACCTCCCGCACAGAGTCCCACAGCCACCCTCTAGGTTTTAACCATCCACTA

							AAGCAGCAACTTGATGGACCCCCCACTACTAAATCCCCAGGCAAGGTGGCTGCTAAGGCAGGACAGCCACCTTGCTCCACGCTGCTCCTGG
MSTRG.48848.1	ENSRNOG00000031461	0.045	0.330	0.001	3.156	up	TATTTGGAGAGTTGTGTAAGGAGCAAAGGCTTCAACACACTGAATAACTTCAGAGGGCTTCTTTTCTGAATCGATCCTTTCATTCCTTTGAGCATGACTATGAGCATGTAAAGCAAAGGGTTTTCCACAGTGAGTATATTCGGAGGGAATCTCTGCACTCTGACTTCTTTCATGCCTGAGAAGATAATATGCACATGTGAAAGCTTTACCATACTCATGATCACTTTCATCTGGATGATTTAATGTTACTGTTTCTCTACATATAAAGAGGCATCTGGCCTTAAATTTTTTCACTTCGTTCACATTCCAGCTTTCCCTTGACTATGGGCTGTTTTGCATCTTCCAAGATCTCTGAGATGGCAGAGCATTTATTACATAGCTCAGATTTACATATCCTTTTTCTATAAAACGTTTTTCCCAAATTTAAAGAAAAGCAAAAGAGCTCAGAGCTTTAACACACTAAGTGTATTCCAATATTTACTGTAGTGTTGTTCATACTATATTTGGGAAGTAATCCAGGAGAAATAGAAGCATTTCAGCATTCCTAGTATTCAAGAGAATTTATTACAAATTGAGGTCCCATATGTATTGCCAATGAAGTTGGAAAAATCATTAATTGTAAAACATTTATCATGTTGAAACAGTCTAGTATAACAGCCATCCACTATATATCTTCTCATTGTTATGGGAGAGAGAGTGTCAGAAAAAGATTGAGATACATAGATAGAAAGATAGGTAGAAAGATAAATAGATGGAAGGATGGAAGGATGGATGGATATGATAGATAAAGATAGATACATAGATGGATAGACAGACAGACAGAGAGAGAGGTACATTGTTTCCTTCAAAATCCCTTTTATATACATGGTTTGTGTTTTTATGATATATGACATATCTATGAAAGGAAGATGAACACATTGCATTCACATAATTTAACACTTTGTTCTTCATAGACAAGTGATATAGGGATGGAGGTTAATGCACAACATCCTTCTTACAACAAATAATCTGCCAATTTCACTAAATCTAACAATATCACTACTAATATCTGAATAAGCTAATTAGAAGCTTTTCGACATGTATTTGTCAACCATTCAACATGTGGAGCTTTTACAAAATTAGCAGAAATGTATTGGCAGTTGTACAGTGTAGTTGTAATAGGGCTGTCTTTCAAATGGTGATGTACACTAAGGCATTGTTTCCAAGTCCACTTTCTTAAAAAATTGCATTGCAAAAACAAATCAGATAAAAGCACACGAAATACCATGGTTTTGTTAGAATGTATCAATCACCAATATATTTAAAGCAGTTCTTATTTACACTGTTGCTTTACTTTCAAACTTTCAGCACACATTAAAATTTCTTCAGAGGCATATTTGTATGAGCTTGCATATAAGAAGTACCTTCTCTGTCTTCTAGAACTTTGACAATGTTCCTCAATATTTT
MSTRG.48960.2	ENSRNOG00000034226;ENSRNOG00000050485;ENSRNOG00000060573	0.729	0.064	0.004	-2.656	down	CTTGTGTCTATTATCTACACCAGGGAGGTGACAAAATGCAGACATGGCCACATCGACTGCCGTTTGGTCCAGTGAGGACCGGTTTCCTGGCACACCACCGTGCCCTGTTGTTTATAATGGGCTCCAGGAGTCAGGCAGATGAAGTATATAGTCTACATGGCGGGTAAAGCCTAAAATCTCAGCCTTTTCTGCTCTGTACAGAAAAAGTGTGCTAGCCCCTGACCTAGGCGCATGGAAACAATCATGCTTAAGGAATGACAACCGAGGAGTGACTTACAGCTCCCACAGCCGGGCCTCCCTTGCTGATGGAATAACGACACTTCTTTCATTTGGCACTGGTCGTGCATGTGGCTTCCTGGCTGGGAGAGGTCATGTAAATGAGCAATTTTACCCCTGAAATGGTTAAGTGGGAACTTCCCAAACCTTGACTGCATCGTCTCCTCTCTGAACACCGAGACAATAAACAAAGAGGTGCTCTCTGCGACGCGGTGCTATGCTGGCCGGCTCAGGCAGCGCAGAGACAAGGTGGGTTTGCGTCTACGGTTTGTTCACTCTATTACTTCTGGGCCTGAGCCGTGGTGTGTATCACAGCGGGGTAAGTGTGGTGGAGAGCAACAGCTCACTTGGGGCCAGAAGAGAAAAGGGAGGAAGGAGACAGTGTGCCTCCACAACCCCCTTCATGCTGATCTAAAACCACAGCCGGGCCTTCCGTCTATGGGGCCAGGTCTCCCGGTTGGGTTTGTGCCCTTAGTAGCACCATATGGTGGAACTAACCCTTCAGCTACGTTTCTTGCTCTCCAGACCCAGCCTGGGCACTGCCCCTGGGTAGCTGCATACTAGCCTGGGACGTTGTCATGGTGACCCCTTTTCTGAAGCAGTTCACTGTGATTTCCTAGAACTCGCTTCCTTTGATATAAATCAAACAAACATGACTGGAAAATAGAGAAAATGAATTATACTCGAATCCTTGAGAGCCAGGGGGTTGTGATTCTTGTTCTGGGCAGGACAGGCAAAGGACCCTGAGACCCAAGGAGCCCTTGGGATATGAGGAGTATGAGTAGATTGAATGAGAACTGTCCCTCGTGGGCTCATGTATTTCACACATGAGGGGAAACATTGATTGAACGACTTCCCATATATTTGCAGCGATGGGTGGTAAAAGATGTCTTACTTGAAGGGAAGACTAATAGTGAGATCTGGACGCTAATGGCTGCACTAATTAAATCCTCCTCCAGAACTGA

							GATATGAAGACATTTTTCCCAGAGCAGTGTCGGCCTATCTCTACCACTTCAGGAACGCGTCATATCAAAC<br>ACCAGAAGAAACATGGGTTCCACCCAGTTGCTCACATTATTCATTAAGCAGTGTCAACATTGAAACAAG<br>TTAGCCTTCATTCAACTCTTGAGCCCAGAGTAAGGGGCAGGATCCTTTCTTACCAATGCTGCCCGCACAT<br>GCTGGAGACTGATTGGTGGTGCCTTCTGTCCCCTTGTCCAGAGAAGTCATGGTTTATTGACAACTAAGA<br>CTGCTCATGATTCATTCTGCAGTGGCCTGGGATGTTCAAACTCAGCTTAGAGATGGTGAATCATGTGTAG<br>GCACAAAATGTGTTTGGCAGCTTCCACTTCTGCCCTTGGCTGGGCTGCACCGGGCAGCTAGTGCTGAA<br>CTGGAGAGGCAGAGCCTGGGCTCTGGGGAGCTTCCCAGCAGCAACTCTATCCCACGGCATCCGCATTG<br>ATGGACTCCAATTGTTGCCCGACCTCCACTGCAGTGCTCACTGGAGTTCTAGAGATATCGGTAACCATA<br>AGTTCAGGCTTTGGGCTGCAGGAGCTTACAGGAGTCCTGCAGTTTTGCTACGGGATTCTTCGGCTCTGG<br>TCCTGAGACTAAAGGTCTGCGGAGGGTACAGAACAGACCTCAGCAGCCATTTTGGGCTCCACCATCAC<br>GTGTTTGTGTGGCCTGTAACACGTAACATGACTACGCTTTGAAAAACTGAAAAGCTGAGTTGCTTCCCC<br>TACCTAAAAAAGGCATTTTTTTTGTGAACAATAGAACGTTTGATTCGTGATAATAACACCTGGCAATCCA<br>GACAGACAAATGGGTAGACCTGGGAAGAGGGCCCACAGGCAGGCTCATGAAAGGGTGCCAAGGTCAC<br>TCAGTATAGAAATGCACCTCCTGTCTTCGCCGAATGGCAGTGGAACGTTCCATATGGGAAGGAACTGAG<br>CACCCAGCATACCTTCTGTCAAATTGGAGAATTTACTTCAGAACCGATGCCTGAGCATGCAACCAAAAG<br>TCACGAAGTTCCCAGAAGTAAACACAAGGGTGTAACGTTGCAACCTTACAGGAGGGACGGGAAGACC<br>AGGAGCAGAAGCAGGGAAAACTCAAGGCAGATCTCCAAAAGCCTAGTTCATCGTGGGTGATGCATGG<br>ACGCTGAGCCCCTGGAGCTCTGCACGGGGTTAGAGCAGTCCTCTTCTGTAACCTTGGGAGGGGCCGGT<br>GAATCCTGTTCTATTCAGCTTTCCTGTTCTTGTGACTTTCGTTTACTTCCTGGGTTACCTCTCCCCAGGAG<br>AGAATGCACAAGCTCACAGGAAGTTGATGCACCTTCCAGGGAAGTTGTAGCAATGGTTAGAATTCTAC<br>ACAGCAGTCCACAGGAGCAAAGCACACCTTCACAGCAATGAGGGAAAAAAGACCCAGACCATGAACA<br>GCATGTCCATGGTGACTCCATCTATGCGGAATTCCATATTTCACCCTTTAGTGGTGGCAGAGAGCAATCC<br>ATGGGTCCTGAGAAGAGGCATGGTTAGAACACAACCTAAAATTGTATGACAGACTGTTTGGAGGGATA<br>GCACTCTCCCCATTGTCTTAGGGTTTCCATTGCTATGAAGAGACCATGACCACAGCAAGTCTTACAAAG<br>GACAATGTTTAATTGAGGCTGGCTTACAGATTCAGAGGTTCAGTCATGAACAACATGGCAGGCAGACAC<br>CGTGCTGGAGAAGGAGCGGAGAGTTCTGCATCTTGATCTGAAGGTCACTAGGAGAAGACTAACTTCCA<br>GGCATCTAGGAGGAGGGTGTCCAAGCTCACCCCCACAGTGACACACTTCCTCTAACAAGGCCACACCT<br>ACTCCAACAAGAAGGCCATACCTTCTAGTAATGCTACTCCCTGGGACAAGCATGCTCAAACGGCCACAT<br>TCTACTCCTTGGTCCCCATAAGCTTATTCAAATATAAGTGTCTATGAGGGCCATACCTAGTCATAGCACAG<br>TGCAAAATACATTTAGTCCAACTTCAAACGTCCTGTAGTCTAAAGCAGTCTCAACAGCATTAAAACTCC<br>AAAGTTCAAAGTCTTTTCCCAGATTCATCCAGTCACTTAACCGTAATCCCCTATAAAGTCAAAATCAAAA<br>GGCAAATCACAATACTGCCGACATCACACGATATACATTACCATTCCAAAATGGGGGAACACCGGACCA<br>AAGCAAGACCAAAAACCAGCTGGGCAAACTCCAGACTCTGCATCTCCGTGTCTGATGTAAAAGTGGTC<br>TTCAGATCTCCAACTCCTTTCATGTTTGTGGACTGCAGCAAACTTCTTTCTCTTGGACTGGTTCCACTCC<br>CTGTTAGCAGCTTTCCTTTGGGGTTAAAGGTGCACATCACCAGGCCTGGACCTAGGCTTTTCTCTCGTG<br>GGAACCTGCTCTGTACTCAAGTGGTCTTGAACTCAGGGAACTGCTTGCCTCTGTCTTCTGGAATTAAAA<br>GCTTGTACTATCGTGCCTGGACCTAAGCCTTTCATGGTCACTGTGCCTCAAGATTTGGATCAAAAGCAA<br>GCATCTTCCATATCTGGATTGTAGTTCATTCCAGATTAAAAGTCCAAGTGAAAACAATAACCAAGTAATA<br>ATGATGTCTAACATGATATAACTGTCCTTGTTCAATGGTGGACACATAACACTAGGCTTAGCTGGGTGGG<br>ATCTTGTCCCCAAGTCACCATTTCCTTATGTTTGTCTCCCTTTGTTTCTCTCCTTGGACACAGGATTAGGC<br>TCTCCTTCCCTTCCTGTGTTCCTTCTACTACTTGCACCATACATTTTTGTTTTTGTTTTCGTTTTCTTTTTA

							AGCTTGCTACACTTGATCATGAGAGTGAGCCAGAGGACGAGATCTATGCTGGGCTTTTCTGAGACTTGC TTTATCAGTGCAATTAATTGAAGTTTCAGACAAGGGCAAAAAGCAGCCACATTTTCACCAAAATATTAC AATAGTCTCTACCACATACTAAAATTCTTCTCCCCTGAAACCTCTACAGCCAGGATACAGTTCAAATCAC CCTCAGCACCACTGTCTCCTATATTCCTACAAGGATGGCCCATTAGGCCTCCACTTAAAGCATCCCACTG CTTTCCAAATCCAAAGTCCCCAAATCCACATTCCTCCAAACAGAAACACTGTCAGGGCTATCGCATTAA CACCACTGTCCCTGGTACTAACTTCTGTCTTAGGGTTTCCATTGCCAAGAAAAGACACCATGACCAAGG CAACTCTTATAAGGGACAACATTTAATTGGGGCTGGCTTACAGGTTCAGTCTATCGTCATCATAGCAGGA AGCACGGCAGTGTCCAGGCAGACACTGTGCTAGAGAAGGAGCTGAGAGTTCTGCATCTTGATCTGAAG GCCACTAGGAGAAAATTGACTTCCCGGCAGCAAGGAGGGGGGTGTCCATGCCCATCTCCACAGAGACA GACTTTCTCCAACAAGGCCATACCTCCTAATAGTGCCACTCCCTGGGCCAAGCATATTCAAATCACCATA CCTGTTCAGTATTGGACACACACACACACACACACACACACACACACACACACACACACAATCAAATGAG AGTGAATAAATCGAAATATCGTAAAGTGCATGCCATCAGGCAAAATATTTAGAATGTGAATAGTTAAAAC CTACAGTGTTTACGCTTTAATGGATACAGATGCTGAGTGCAGACACCAGTCATGTCCTAACCACAGGAC CCTGAGCAGGTTTGCAACGTTGCAGAAGTAAGACCTTCCTCACAAAGCTTCAGAGACAGACATCAGTG TTTAGAGCTGCACCGAACGGGCACTGAGCATTTGCAGCTAGGGTGTGATGTCACGGCTGAGGGCCACA TCAGTTTCACCTGTCCCTCTACTCACACACCGCAAGTGGAAAACACTGTGGGACCGTGAGGAGGAAAC ACATTCTGAGCAGCTGAGCAGCGAAGGGTCCGAGAGCTACATATGCTCTCCCTCATGGCTGACTGGGTG CTGCAGCTCCTTGCTAACTGGCCAGCACAGCACCGGGAGACAAGGCTAGACTGCACAGCACTGACCCC AGAAGAGGCCCAAAATCAAAGCATGCTTTCTAATGAGCACTGTGGTGAGACTGTGCGTGTGTAGGCTG GAGCACTGGGAACTGGGGATCATCCCTGGTCTTTTCTGGCTTCTGATGACCATGTCACATTCAGTCTCCC ATAATTAGGAACGGAGGGAATGGACATAAACAGAGGCACGGCCATATTCATCTTACCTTAACCAAGTAC CTGCATTCCCGGGACTTCAGCCCTACCTCTGGATCCCCATGTTCATGAACATTGGTTGCTTCACCCTTTC CACTGCTGACACCCAACTCTTAGCAGGAATTGTTCTTTTATTTAGCAGATGACAGTGCAGTGTTTTAAGC AGAAAATCAAGACAGCAACTAAGAGCAGAACGACTTATGTGGATGGCATGATTGTGTGGATGGCATGAT TGTGTGGATGGTGTGTGGATGGTGTGATCATGTGGATGGCGTGATCATGTGGATGGTGTAATCATATGGA TGGTGTGATCGTGTGGATAGTGTGATCCTATAGATGGTGTGTGGATGGTGTGATCATATGGATGGAGTGA TCGTGTGGATAGTGTGATCCTATAGATGGTGTGTGGATGGTGTGATCATGTGGATGGTGTAAACATATGG ATGGAGTGATCATGTGGATGGTGTGATCCTATAGATGGTGTGTGGATGGTGTGATC
MSTRG.49026.7	--	0.208	0.618	0.025	2.199	up	CGGAGAACCAAGTTAACAGAAGGAGAAGATCAAGGCATAGTAAAGAAGTGAGGACATGGAATGGTAG TTGAGATCACTCGCTTTTCCCAGGCTCTCCCTGAGACTTTACATGCCTGTAAGAAGATAACCTCCTCTTC TCTTCTTAAACCATCTGAGCTTGAGGTTTTAGTCACGGGTAACTCGGTCCCAACAAACATAAGTCTTTTA CATATCCCCTACTTGATGCTCCTTATAGAGAAAGGAAACAGCAGGACTCACTCACCTTTCAGATAGAAG AAAATCAAGGGGGTGGAAAGACGGCTCAGAAGTTCAGTGTTCACCGTTCTTGCAGAGCAAGAGTTCA GTTCCCAGCACACACTAGGAGGCTCTCCAGTGCCTGGAAATCCAGAGGATATGATGCCCTCTTCTGGTG TCCTCTTGGTACTGCACCCACACACATTTAAAAAAAAAAGTCTTTCTAAAAAAAACCAGTGATCTCTGT GCTCCGGGAGTCAGAGTTAGGTCCTAAGGCAGCTCTGGCCTTCATAACACACACAGCTTGGCTCATTAG CCCAGGCTGGCTCCTCTCCATACCTGCTGCGGCCATGATAGACATCCCACAGTTCCAGTACCCCCAAGA CCTTGGGGTCTCTACTGCAACTGAGATGCTCCTTCTCCAATATCCTCTCCTGCCTTCCTCCGGGACTTTG ACCCTGCCATGCGGCCCCCAAGCCTCAGCTTTTCTCCATAACTTCAGTCCTGTGGTTTCCTTGACAGCC GGGGCTATGCTCCACGACTAGTCTCTCAGCATACCAACCAGCTGCTCTCTATGACACCTTTAATCCCTTC AAAACCAGCATCGCAACAGGAGACTCTCCCACATCGTTTAAGTTCTGCTGATTAAGACACAGCCTTGGC

							CCCACGCCCCCATCTCCCAGACCACAGCTTCTGTATGCTGACTCTGAAGAAACTGTTCCCAAAAGATTTTAACTCAACCTTGCTGGCTTCGTATCAAGACTTTATCTTTTCTGTTCCAGCTGAGCGACGTCGACTGTACAAGTAAAACAGAGGTTTCACTTCCGCAGATTCAAAGATCCAAAATAGCACAAGAACTTCACGTGAATTGCTTGGTGCAGCCTTTGCTTCACCGCGAGACTCCGCAAGCCCGGCCTCCACCCCTGAATCCG
MSTRG.49035.2	--	0.960	0.000	0.000	-6.570	down	GGGGAGGGGGAGGGGAGGGAGAGAGAGAGCGTAATTGTTGAGCTGGCTCAGGGGTTAAGAGCGCTCACTGCTCTTCCAGATCTGACGCCCTCTGCTGGCGTCCTCGGGTACTGCATGCATGTGATCCCAGACGTACGCAGAACTCACGAACCAGCGACTTTATTTCTTTTAACTAGTTCTTGAAAAACCATAAGAAACATTTGAAAGTCTACTTTTCTAAAATAAACAAATGAAAACTTAAAAGTATGTATTTAGTTTCTCCTCCTTTTCTAATTCACTCGGGAAAACATTCGTTCCGTTCCTAAAACTTTCCATTTGTATTTCCAGACACGGCACAACTCCTTTTCTCCTCCTAGTCTGTGACTAGCGGCTTACCATAGCACCAAGCCAGGCCCATGCGAAACCAGTCCCAGCCAGTCTTTAAGTGGGGCTGGTGGCACTCAACCTGATGATGTTGTACCTGTGAGGTGTTTGAGTTCCTGGGCAGAGGGGCGCTGTGTCTCAGGTCAAGTGTCATTTCCAGTGTGACATTGCCAGTGTGTTTTTCAGGAGCTCATATAAAGGGGGTACCACTGCATATAGGCAATTACTCCACAGGTACAAAATGATCTGCACGCCGAAGGTACCTCAGCTGGAACTTTCTGGAAAGCCTTGCTAGGACCATGATCGTGGTGAGTCCCCCCCCCCCCCCGGAGCTGGGGACCGAACCCAGGGCGTTGCGCTCGCTAGGCAAGCGCTCTTCCGCTGAGCTAAATCCCCAGCCCCCGTGGTGAGTTTTTAACTACAGGTCACTTTGGCACGGAAGCATCACCCAGGGTTCCACGGACAGACAGGAGCAGGAGACATGAGCGTATCTATGTTTACATTTGTACCTGAAACAGAAGGGGCGGGCCTGGGTCCTGAGCTATATGGGTCTGCCCAGGCAAGTCTGAAACCTGCATGATAGAGAAGCAGGCCAGAAAGTGAGCCAGGACTTCGAGCTTACAAACCCACAGGTGAGCCTCGTCCTTTCTGAGGGAAATCTTGGGTTTTGTCCTTAAGATTCTTGAGCTGATTGGATGAGGCCCACCCGCACGCAAAGGCATTTTTGTCTTAGTTCAATTCAGCTGACTGTAAATATTAACTGGGGCTAAATGCCTTTGGGAGAAGACCTAGATTAGCATCTGAAGCAAACACGCAGAAGTAACCACTGTGAGGCTCCTGGGGGCTGCTCCTGTGTTCACCATCCACAGAGTCTCTCTTCTCTCTGACAGGGGCTGCCTCCTACCGGAGACTCCACATGGGACCAACGATCAGCACATATCATGTGAATCTGTGGGATCTAGACAAGAGAGAAAGAAGCCTAGGCAAGGAAGCAGGTCCAGAAAGAGAAAACTTGTATTACATTCCTGCTCTAGGACAAGCATGATATGCCAAACGACTCCCGCACACGTCCCATCGGTCCTCACCGTGTCCTAGGAAGAGCGATAAAGCACTGTATTAAGCAACTGCAGTCCTCATTTTAACCAACGAGAAAACAGTCATCTAGAAATGAAGTCACTTTCGCAAAGCGACTTTACAGTGCCGGTAGTGGGCATGGATCTTAGACGAGTTGTCTGACGCTCGAACCTCTGTGTCTCCTGTCTTTGGATATCATGCAGAAGACACAAAGAAGACACAAGCAAAGGAGGGCAGAGTGTGACAGGCTTGGTGGGAAACCTGGCTTGGCTCGGGGCTCAGCTCAGACGCCAGCCTTCTGTAAAAGGAATGCAACACACAAACTTCCCCCCGAAACCATGCAGAACCAGAGAGACGCTATAGATACTGGATAAGTGTTTACTGAATGGATAAACAACTAATTAAATAAATAATAACAACAGATACG
MSTRG.49084.2	--	0.000	0.368	0.003	4.043	up	CTGAGATGTTTCCTGAGCTTCTGTGGGGATGGGGGGTGCGGACTCCCTGGACCGAGAGTGAAGTACAGACGGGTAATGTGAAAGCAGAGATCGGAGCAGGAGATGTGAGCTTTCAGTTCGTCCAACAGCTTTGGGGTACCTCACTGGGGTACCTCCTCCTGCTCTCTCGGGTGCTGCAGCAGATCTGCCACGAACTGTTGCGAGAATTCAGGAATTCCAAGTATCTAGGCAGGCGGTTGGCACAGAGCCCCATAGCATGGATGCCCAGTAGGAGTTGGTTTTCCATAATGATGCTGCTTGCAGTTTGCCAAGCCGTGTAGCAAGTGCACTGTCTAGTGCCAGCCTACAGACACTGTAAGAGAAAGGACCTGGGTTTCAGACAGTTCAGGTGCTGAAGTTAGACCCGGGGAGGTTGGAAGTCTTGCCTGTGCTCAGCAGCT
MSTR	--	0.000	0.399	0.000	5.602	up	CTGGTTCCTCTTGAATCTGCCAGTGCCGCTGGCGGCCAGGAACTTGCTTTGCTTAGAGTCTCAGGGTGG

G.49084.3							TCACAGCGGTGCCAGGGAGCTTCAAGCATAGGTGGGATCTGCCCCCTCCTCTTCCGGTTCTGTCCCACGCTGCCCTGCGTGTGCTGTCACGGCTTACGCATCATCATCTGTTCCCACGTTCCGAGTCGCCCAGCGCAGCAAATATTGAGTGCCCTGAAGTGTCTGAGGGGAGGTGTCCTAGGGTGGCTTAGATGGACTGTGCCAGCCCTGGGTTCCAGCCACCTGAAGGCTCATCTAGGGAAAACCTTACTTACGAGACTGCTAGCAGACTTCAGTATTTTAACTTGGGCAAGGTACCTTGCTTCTCCAGTGAGGGACTCTAACACATGAAAGCGAGAGGCACACTCGAGGCAGACCTCCATCCTATCCCCTTTGCTATGTTCTCTGACACACACAATCACCGAGGTAGCACACACATGAAAGGTGGGGAATTAATTCCATGCCTGAGTGGGAGGATCAGATGTCTGCCCTGCCTCTGAAGTGCAGCCAGAGGGTGGCTCGGTGGAGATTTAAGCTGAAGGCGAGAGCCTGAGAGTTGAGCTCTGAACTGTTCTATAGTATGGCCTCCGAATGCCACAGAAGCCTTTAATCTCAGTTCTCCATGGGGAGACACCCGAGTCCTGACTTCTAGGACGTCTCTCTGAGCTGGCAAGAGGAGTTTTGAATGGCCAAGGGAGGAAGCAGAGGTCCGTGGGGTACATGGAGGAACCAAGAGGAATCCCAAGGGCAGACTGGTTTGGGCAGTCACAGTTTCATTCAAGGCAGGCTTTCAGATGCACCGAATGTGGAGAAACCGAGGAAGGGAGAGAGACTAGCACTGTCTCAGCATTTCTTAGCAGTAGTCGCTGCTACTGCGAGCAAAGTCCTTTCCCTGAATAGAGCAGGCAGACTTCCCTGAGATAGCTTGAGAGGGCCACAGGAATGTGTTCCCGAGCTGGGGACAGGCAGATCATCCTGACACACTCCAGCAAGAACAGCTAATACCGGCTTGGCTCCCACACCTTTGGCACCCAGCACTCTCCCTGGGGCTGGGTTGCTATGGACAACCAGAGGCTGTCTCTCAATCTCTTACATCACCCAAGCTCCACAGATGGCAGCAGGGAAAAAGAGTCAGGCTCCAGGAGGCGCAAAGGAGGCCTTCAGGAGTGTACGGTGAGGACGGTGCTGTCCCGAGACTCCAGCTCAGTGAGCATCTCTGTCCACCCCTTCAGAAACGATGCAAAAGCCATTTAGGATGGGCATAAGCAGGCATAGCAGC
MSTRG.49333.1	ENSRNOG00000011374;ENSRNOG00000011423	0.055	0.373	0.002	3.044	up	GCTGCCTGGACTGTGGACACACTGTGTGCTTGTCCTGTGCCGTCTGACTTCCTCGGTGTCACAGAAGCACCACGCAGGCAGGGCTTCTCTTGCAGCTGCTGCATCCCTGGTGCCTAGCAACAGTGTCTAGTACTTAGTACCTGGGAGGGATGGAGAGGTAGAGGGATGGAGAGGAGGAGTAGATGGAGTGGTGGACAGATGGACTGCAAGATGTCTCCTAAGTTCATGCTACCCTTTTGTTATCCAAGAAGTATTCGTTTTTCTGCTGACACCTGCCCCGTGTTTTGTACTAACACATCTTAAGAGGGAAAGTTCTAGATAGCCAACCTTAAAGGAAGCCATATGTCAGTCACATGCCAGCCTCAAAATCCTGAGGGTCCCCAGTGTGGCAACTCTCTCCCCACGCACAGCTATGAGGTTGTCCGTCAATAAATGTGTCCTGGCTGTGCCTTCGGGGTCGAGCCTGGTCTCTTTGGGTTTTCTCATCAGGACATTCGGAACAGATGCCCTCGGCGTACCCAGCCAGCTGACGTCCCGTCAGTGGGGCCTGGCACACGAACCAGGTTTTTCTGTCCGTTGTATCCACAAAGTGCACGCTGCTCACGCAGAGCCACGCTAAGTCGCCATCTTGGGCATCAGACCAACTGCCACGCGTGGTGTCCCGTGTCAAGTACAGGAGTAACGGTGAGGACCTTCACATCTCCCAAATGGCATGGAAGAGATTCCAAGGCCTGGTGGGAAAAGCAGATGCAGGAGACATGGCACGCCGTGGTTGGAGAAGAACCTGGGAGATCACGCTGCCGAAGGCGTGATGCTGAGGTTGGGAGGGCTGATTAGGTGTTTACCAGGAGGATCTCGTCCAGTCTGTAGCCGATATTCTTGAGGAGACTCACCCCTCCTTGGGATGTGGGAAATCACTGGTCCCCACTTCTCATCTCTTCTAGCACCTGGGAGAGGTCGCCAGGGTTGGGTGCCTGGAGCATCTCCCTGCCATCTCTGCACCTAACAGAAGCAGCAGAAAGACGCAGCAACGTCTGGGCTGAACCGGGGAGACAGCAGACATTTATAAATGACCCTCTTAGCTGTTTACAAGACACCTTCTGAGAGAAAGCAGAGGAGGAGAAATAAACTTGAAGTAGGGAACCCTGGCGGGAGTCTGCGATGTGC
MSTRG.49333.3	ENSRNOG00000011374;ENS	0.077	0.500	0.001	3.145	up	CCAATAATCATGACTTCACTTCCGCTGAGCGCTCCGCTAGGGCTCCAGTACCAGGCATGGGGTCCCTCTTGTGGAGCAGGTCTTAAATCCAATTAGAGATCTGTTGGCTACTGCCAAAGTATGTGTCTCTACTGTATGGTTCTTGTGCCGTGCTGGTTGTTGATGTGGTTCAGGTTCCAAGGTTCAGTCCATTATCATCGAGGCAGGAACATGGCAGCATCCAGGCAGGCATGGTGCTGGAGGAGCTGAGAGTTCTACATCTTCATCTGAAGGCTG

	RNOG00000011423						CAAGGAGAAGACTGACTTCCAGGCAGCTAGGATGAGGGTCTCAAAGCCCATGCCCACAGTGACAACATCTATTGTGATGGCAAGCCCAGTCCAGTCCCATGAGCAGAAAGAAGGGCCAGGTTTCAAGCCAGGCTGCCCCATTCTAGGGTCTCCAGCAGTAGACCCTGTACCAACGGCACTCAGGGTCTCATCGATGGTTCTAATACCTGGCACACGAACCAGGTTTTTCTGTCCGTTGTATCCACAAAGTGCACGCTGCTCACGCAGAGCCACGCTAAGTCGCCATCTTGGGCATCAGACCAACTGCCACGCGTGGTGTCCCGTGTCAAGTACAGGAGTAACGGTGAGGACCTTCACATCTCCCAAATGGCATGGAAGAGATTCCAAGGCCTGGTGGGAAAAGCAGATGCAGGAGACATGGCACGCCGTGGTTGGAGAAGAACCTGGGAGATCACGCTGCCGAAGGCGTGATGCTGAGGTTGGGAGGGCTGATTAGGTGTTTACCAGGAGGATCTCGTCCAGTCTGTAGCCGATATTCTTGAGGAGACTCACCCCTCCTTGGGATGTGGGAAATCACTGGTCCCCACTTCTCATCTCTTCTAGCACCTGGGAGAGGTCGCCAGGGTTGGGTGCCTGGAGCATCTCCCTGCCATCTCTGCACCTAACAGAAGCAGCAGAAAGACGCAGCAACGTCTGGGCTGAACCGGGGAGACAGCAGACATTTATAAATGACCCTCTTAGCTGTTTACAAGACACCTTCTGAGAGAAAGCAGAGGAGGAGAAATAAACTTGAAGTAGGGAACCCTGGCGGGAGTCTGCGATGTGC
MSTRG.49445.1	ENSRNOG00000015139;ENSRNOG00000015262;ENSRNOG00000015616;ENSRNOG00000016161;ENSRNOG00000016410;ENSRNOG00000016539;ENSRNOG00000047752	0.753	0.000	0.000	-5.666	down	GGGAAGGGGGGCGATGTCATTGACAGAGAGTTTCCTTCTGATTAGGAGCATCCAGTTCCTTGCAAGTTTGGTCTTTGCCTTCAAGATACCTAACTTGTTCTCCTGTTTCTTCTTTGTTCACGACTCCTTAACAAATCATGACCAGAAGCAATCAGTGACCAGCCACCAGTCCTGTCTCAGGAACCCTCTTCCTATATCAGGGCATGTATTTACCCTCTGAAAACTCCCCAGGATTCCAAAGGTCACAAAATCGCAGGAACTATCTGCAGCTGGCAAAATCACACTCCTGCTAGAGCACGAGGCAAATCATAGTCAACTGCTGTGGACGGTCAGAAGCAGCTCCATATCCCACACCCCTTGGAATGGAAGGAAGCTAAGGCCAGCAGTCTGCAAGGTGGATGGGTGTTTGAGAGCGGCCCTGCGTTCTCCCCTTTCTGCGGTCGGTTCGGTTTAGACATCCACCTCCACGTCTTGTTGGTGTTGCTCCTCAAAATGTTTTCAGTCCTTCACTGAGGGAAGACAGGGCAGGACCGCGGAGGAACGATGCTCACTGGCTTGCCATGAAGACATGGCCCGCTCAGCTTACACAGCCCAGGCCCACGTGCTTAGGGACGGAACCAGGCGCAGGCCAATCTGAAATCCTGGCATTTGGGAGTGGGAAGGAATATCAGGAAGTCGCCATCTTTGGTTACATAGCAAGTTTGAAGCGAGATTGTTGCAAATGAGATCCTGTGTCAATTCCTCCTCTCTCTCTTCCAAGGGGAATTACATCCCGAAGTCACGTGAGCATTAGGGGTCATCCCCCTGTTCTGTGCCTGGGCGGATCTTCCGGTGTTTCTCTCCATAGCTACAGTGCCTTTGTTTCAGTCTACAAACTGTTACACAGTAACTGCAACTCTTGTCCAGCGTCCTGGCCCCAAGACAGGCGCGTGCGCATCGCCATCTCTCACCGCTCGCCCCCGCCCTCCTCGTCCGGGTTTTCAAATTTGAAACCTGCGGCTTTTCCCGCCCGAGCGGGGCCCCCGCGGCGGGAGAGGGCGGGACCGCGCGCCAGCCAACCCAGTCTCTGAGGCTCGTGTCCCGCCCCGAGGATGTGGCCGCGCCCCGCGCACGTGCAGCCCAGGCCCGCGCCTGCAAGGACCCGCGCGACTGCTTGTCGCGGGAGCCGGGCAGCTCGCAGCACCCAATGTCCAGGCTGCTCGGCCCGGCGGAGGGCTCGTCCCCAGAGCGCCGAGCTGCAGCCCGGGTCAGAC
MSTRG.49525.2	ENSRNOG00000000104	0.792	0.007	0.000	-5.035	down	GCTGCTGTACAAGGTCTTTTTTCTTTGTCGTCATGGTTGGTTTTGTACATCATCTTAGCATTTGCATCATACAAAGTGGGGAGCACGGCCATGGCTTTTGGTCAGGTTCAAGGGAGTGCTTGGGGGGGTGGCCCTCCCCTCCCCCAGAGACTGACACATTTAAAGGAAGCAGAGCAACAAGGACAGAGCACAGTTGTGGGAAAGAGGTTCCCTACATGAGCAGGATGGTCCATCTCCCCTGGGTCTCAGTAGGACTTCCCACCCCCACCCTGGGG

							CCAGCAGGAGCACCTCGGGTCTCTCCCTGGTGAGAAGGATAGGACAACAGGAATGAGGACTAGAGCAGAGACATTTGCTAGGAGACATCCCCCACGGGAGCCAGTCTGATCCCCAGGCCTCCTGGTGAGGTCAAGAGCCGGCCAAACCATAAAAAGATGGAGGGGGAGTTGTGGGTGGGCGACCCTGCTAGTTTCATAGCTGGCATTCTTGGGGCTGGAAACCCCATGGCACAAGACTTTAGGATGGCTGGTTTGCTCATCCACTGTGCCGTGTGTGAGGGGTCTCTGGGCTTCTGTCTCTTTCCTGCTCTCATTTACTGGGATGACCTGTACAGCTCTTTCTTCCATGGAGGAAGTATTGTGAAGCTTCGCTGCTAAGGACTCACGTTTGGGTGCTTTGGAGATGAAATGGAGGACATGTACTTTGGATATCCCCCTGTCATCTCCATAGAGGCCCAGAGAGGCCCCCTTTCTTCTCGATGGTGGTGATTTCCAGGTGCAGGAGCCTGATGGCTTTTCTAAAGAGCGCACATGCCTGTCAGTGATGTGCCTGCCTCATTCTGTCTCCAGCCATCTTCCATGCTGTGTGTGTGTGCTTGTGTATATATATGTGTGTTTATGTGTATGCATGCATATGCACATACATGCATATGCACATATGTGTGTATGTATGTGTGTGCATGTATATGCATGTATGCATATGTGCATGTGTGTATGCATGTGAGTGTGTGTACATATGTGTTATGTGTGTGTATGCATGTATATGCATGGTTATACGTGTGTTTGCACGTGTGTCTATGCATGTCTATGCAGTATGTGTGGCCTTTTTTCAGCATGCCATTTGCACATTGCATTTCTGCATGATATATTCATCTGCCCATTCAGTGGACCACACACACCTCCTGCTCTAGGCACGTCGAGTGACGGAACGAACATCCTTTCTGATGCGCAAGCACCCAAGTTCTGTCCTTCCATGAATGTGTTGTATGCACATAGCATCCTTCAAGGGTCACCCACTGTTTTCTCCCTATAGTCCAGGTTGTGTCAGAAATATCACTTATTATAACCCCTGGGCTGCATGTGGGTAGAACCAGGTATGTACCACGTAGAAATTCTCTTGCCTCCACCGTCAAAGAACCTCAAAATCTGCTGCCTGGGGATAGAAAGGAGACATAAATCTAGACTAAGCCAGTGCAGCCTGAAGGGCACTAGGTGGGGCTCTGGGCTCAGGGGAGCCGTGGAAACTACCAAGGGCTTCAGTTGAACGGTCTGGAGTCTCTTGGGCCCACGTGGGTGGTTGCATGGCTGGGTTTCCTTGCTTGTTTGTGGCTCCCCGGGTCTTGGTCTCTACGATGCATCTGGTTCTCTGTACTGCGCTTCTGCCTTTAAGGTCTAAGAGTCCCTGTCGAGGGTTAGGCCTTTCTTCAACTGTTGTCCTTACCTGCCTTTAAGAGTGTGTGGGGGTCACACTCAGGGTCTAGGGAGGCCTGGGGCCCCTGGACTGGTCCCTGCCTGTCTTCGGCCCAGGACCCAGGGGCTCTTGAGAATGACCGTCCTGCCCAGGATAAAAAACAGAAACAAGCAGACAATAGGGGTGCAAGATGGCTCCCCACCACAGACATAGACACAAACACTGGCCCTCTGTGGGATGACACAGGGACCCAAGAGGGTGGGGGAGGGTAAGTCCCGGGAGCTAGAGCTACCGAAGCCCTGCTCCTTAGAGAAGGGAGAGACCAGGGGACTTTTCTGGTTCCCTACACATAAGGATTGTTTAGTCTCATCTTTAAAGTATTGCGGTCTAACTGATATGGCTTTAACTATTGGAAACGGACAGGGAAGACGTCTGTCTTCTCCGAAATAAAAGCGTTAAGTCCACTAAAATGGAAAACTTGCAGAATGCAGAGTGGGGCCAGCGGGACAGGGGCTTCAGGGGTGAGCAAGTCGCCCCCTCCCGGGCCCCTGCTTTTGGGATAGGCCCTGGTAGGGGCCCAGCATGGCTTCCTGGGGCACTGCCTGGGTCTGTCCCAGTAGCATTCTGAGGACCTCAGGTAGAAAACCCCCAAGGGTGGGAGGACTCTAGGCCAAGGTAAGGTCTCCTGTCGGATGTGGCCATCTGTGACTAGCAATCTCAGGGTTGGGAGGAAGCATCTTATTCCTGACTTCTTTACCTACAACTGCCTGCTTTAGACCCACACACATGAACACCAGTCTTAGCCCACATGACAGTTCTCTCTC
MSTRG.49572.1	ENSRNOG00000018355;ENSRNOG00000047234	0.780	0.078	0.012	-2.442	down	CCCCAACCCCAAAAAGTAAGTCTTAAAAAAACATAAGATCACCCTCTTTCCCAGGGCCCACTAGCTTACACTGCAGGTGTAGAATATGTTTGTGAGGACCTGGAGTAACAATTCTTTTCCACTTCTGTAGCCTGAAATCCATTCGCTTTTGTAACATTTACAGCAAAGTAAAAATCTTGGGTTTTAAAGAAATCATCAGGTAGAATCTACATTCCAGCCATATTTCTCATGTCTCTCTGTCCCTTCCTCAAGAATCCAAGCTACATCACCTGTGTAAGACTGTTAGATGGCCTCAACTTAGCAGGTGTTTTCCTCTCCTTTCTTTATATCTTCGTCCCTGGTATTTACTTACTGTGAAATTTGAAATGTTTCTGAGGCCATAAACAAAGAAGGGGGAAAATGCACACTGAGCAGGAGGGGGTTGGGACAAATAATGAGGCCGCATGGTGGCACCTCTCATAAGGGATCATTTCCTGTACTGTTGCAGATACTGTTTCTAAAGTCTGCTGTTACCCCACTAGCTCTCTTGGGAGTCCGTCAGTTACCATCCCCAGTTCA

							CAGAGCAGGAACCAAGGCTTCAAGAGTAAATTGTCCCAAGATGCATTGCCAGAAGAAGAGTAGGAAA<br>CCCTTGCAGTCCTCACAGGTCGACAAGTCAAGTGTGAGTGGAGGGTAGCAAGACTGCAGAGGGTGTG<br>TGTGTGTGTGTGTGTGTGTGTGTGTGTGTGTGTACCCAATAGTGATGTGCAAGCCCATTGTGTACAGGTGG<br>TATGGTGAGCACTCCATTTTCTCTGTCACATTTAAGCCTCCCAGAATCCCCTGTCTCACCAAAGGTCACA<br>GAATGAAAAAAATAACTGAGAGGACCCTGAGCCAAGCTCTGAACATTCTACTGTTTCGGGGCCATATTC<br>CAAATGGGAGCCCCATTTTTTGCAAGAATTTGAGCAGTATGGCCTCCCCTCCTGGTAATTAGTGGTTGTG<br>CCCTCTTTCTCCTCGACACTCTATCAAGATTCCCAGTAAGATCCGGCCCCACCCAGCCTGAAACCACAT<br>CGGGAGAATGTCAGCCTAACATAGCCCAAGGTTCAAGCAAAGGTTTGCAGAATCTCTAAAAGGTGACA<br>GACTCTTTAGGCTTGGAGAGTGAGTCATAGAAGTGCCCTGAGGAATCTGTCAGCCCTGACATCAGATAA<br>CAAGGACTCATGAAATTGGAAGAGTACAGAGAACACGCCATGAGAGGAGGCACCAAAGAGCCTAGGT<br>TCTGGGCTTGGACTTTCCGTGTTAGAATCTGGCCCAGTGTCCTGCAGGGAAGGAGCCTTGGACAGGAA<br>ATTTTCTACCACTTTGTAACTTGAGGATCTCCCAGAAGCACTGCAGCCTTAAGTCTGGGCACCTGTAAA<br>GCCTCTCTCGTATCATGAGCCTAAATGGCAGCTCTGTTGTATCTTACAGGCTGCATGGATTTGCTCTGTG<br>AAAAGCAAGTGGGGGCGGGGCGCAGGATGAGCTCAGCTAAGTGTGAGAGCCGAGGGTAGCTAAGTTT<br>GGAGCAAAAATTCCAAGTGACATCAGTCCAGCGGCAATAGCAGCAGCAGCAGTGGCAGCAACACAGT<br>GCAAGCAAAATATGGCAAAGCCACATCCTGGAAACATTTAGAACATTTCCAATGAGCCGCGCTTAACAC<br>ACAGCTCAGGTTCTGCCAAACTTGAGCCTCTGTGAGTTACCATCACCAAGGTCACGATTTCCACCGCCA<br>TCTGCCTCCTCTCCTTTAAAGTAACTGTCACCGCCTGGTTTCTTTTTTAAACACATAGACAAATATGTGTT<br>TCTGCATGAAACTCTTTGGTTCTTGTTAATCGGGAACGTGAAATTGAGGAACCAGGGTAGATGTCAGGC<br>GTTGCGCATAAGCTTCGCTTGCACCAGGCGCTGGTCCACCCACATCACTACCACCAGCAGCAAAGCAA<br>AAATATCAACAAACCAACACGAGTGTGGTTGTAGGTGAACGCCGGAGTAAAGTGTTGACGTCTCACTC<br>TGGTCATTTTGAGAGGTTCGGGGAGGAAACAAATAAACTAATGACAACCCGGCTTCCTCCCCAGCGAC<br>TCAGAGAGACCAGCACTAGAGGGGCGGGGTTAACAACACCCCCCAAACTGCAAGAATACAGTAGGGC<br>CGTTTTAAGGGATCTGATTAAGACCTAAGGTTGAGAAGAAGGAGAAGGAGGGAAGTGAGCAAAAATC<br>CACCCACCTCCTCAAATAGCCAAGCTCAGGATGCCCCTGACCCGTCCGTCCCCTGCCTCATGGTTACAC<br>ATCCCTGCCCCCTGCTTCCCCAGATCAGACTATCTAATTCTGCAAGTTATTCAACATGGGCGCAGTTCTT<br>TTTTCTGGTTCTGATTTGATCTATGTTCCAGGAGCCGTTATCTCTACCTCTTTAATAGCTTTACAAGCAAA<br>ATATTTGACTGGGTCAGAGCACAAGGGATGGGCAATCCCTGGCACTGATTCATAACACATCCTAGGCAA<br>ACCCCAAAACATGTTGTGAGCAAGTCAAGCACCCCCACCCCCTTTTCTGCTCTCCAGAGAAGCAGCTG<br>GAAGGACCCAGTGGCCCAGGCCCTTCCTGGCACACTGCATTTTTTAATCCATGCACTCACTCTTCCAAT<br>GCGTTTCCTCACCGGGGCCCTTCTATGCCAACTCTTGCTCTGGTGTCTGCTCGGGACCAAGAGCAACAA<br>GCACTTTGCTCTTCTTGGGGATGACAGGCACTCAACTCTGATGCGAGCACCTCACTGGATCGTGACGAT<br>CTCTTAGTGTTTGAAGACGGCGGCTTGATATAGGAGACAGTAACAACGTTAGACTCACGCCTCTGTGGA<br>GTGACATGTGAGCTGAGTGGCCAGAAAGAACCATCTCATAGAGTTCCAGAAGCAGCATCTTCTTGCAG<br>GTCCACTCACAGCAGGTAGTGACAAAGGAATCAAGTTTCAGTTCCTTGCTGGGCCCTCCTGCCATTCTG<br>ATACCATCACTTTCTGTAAACAGTAGAAAAATGTTTCTGAAAGCCAGGACAAGGCCAAGGTTACCAGG<br>TCAGGCCTGGGTCTTAGTCCTAGCTGCCTCCCAAGTCCCTAGGTCTGGACAAGTTCTCAAACTCAGTTC<br>TCTGCGGCACAGACACAGTGGCGATCCCTATGTTGCAAACAGGAGGAAAAGCATAATTTAGACGCCGT<br>TTGTGGAACAACGTGTGCTTCATGGTAGATCCATCTGTGCAAAAGAAAGGAACCAGGCTGATTAATTGA<br>CCCCCAGAAGGAAAGAAAGCAGAGAGAGAGATGGGCGAGAGAGGGAGAAGAGGCCTAAAAATCATG<br>ACTGGGTTTAGAGCAATTGGCTTTGGGTCCTCCCCCACT

MSTRG.49597.1	ENSRNOG00000010882;ENSRNOG00000028997	0.025	0.675	0.000	5.035	up	GCTGGGGACAGATTTGAAAGATGTTGTGGGCAGATAGTGTTTGAGACAGAGAAGGGACTACAGGGAGAAGAGTGGAGTACATACATATACACACATGCATACATATTACACACACATACATACACATACATACATATACACAGGCATACATATTACACACACATACACACACACATATACATACACACCCACACATGCATATACCAAACATAAAATCCACTTTAATCCAATTCAGTTTAGACTCCGGGTAAAGAATGGGTTTCTGGACTCCATCTGGGAATGGCTAGAACATGAGCTCACAGGACTGGAGAAGGGCTGGAGAAGGGGACAGGGATGAAGGGTTTAGCTCAGGGGACTCTCTCTTGCTCAGCCCTGACCCCTCTTCTTTTGAGCCCTCAGGTCTCTGCCCACTGAGCCAGCAAGAAGGTAACTTAGGCTACATCCTTATTTGTCTCTTTCCTTGGGCAGGTCTGACTGCCTAGACTGTGTCCTTCCAGGTGGGTCCATTTCTTTTTAACCAATCCAGAGCCTCGCCACCAGAGAGATGAAGGGCGAGCTGGAATGCGCTGTCTTAGGAAGCACTTCTCAGAGTCCCTTGGTGCACACTCCAAGAAAGTAAGGCTGAGACAGGCCACTTGACTCACATGGGAACACCAAGGGCAGAATCGGCAGATAGGGAGGTCAAGCCCAAAGGGTTCAGCCGAGGAACATTCATTGTGCCAAGGTGAAATGTGCCAAGGTTTGCAATCTTTTCATTAAATGTGGGCTGTTGTTGTGGAGGAAGAAGGGAGCTGAAATCCTCAGCTTATATTGGAGTCCTTTTGTCTTATCAGTAGTGGGCATGTTTGCTCAGGCTAAGCCTAAGTGCTTTCCCCATCTTCAGTGACTCAAACAAAGTCATCTAAGGCAAGCCATGAGCCATCACTACTGTGGGTTGTGTTGTTTCACCAGGAAGGCATCTTCTCGGAGACAATTCTGGGCTCATGCTCTGAGGTGTTTTAAGGCTCCCAGCCTTTCTGTTCCCATTCAACACAGAGATTTCCTGCCGATGGTAGCTGCTGTTGGTTACTACTGGCAGCTGCTGGCTGCTTCCCGTTCGTGGAGCTTTCCCCTGTCAGCCAAAACATCTGCGGGGACTCCTACATATTGTCCTGGAAGACGGTTTCATAAGCAGCTCCATTTTGAGAGTAGAGAATGATTGTACCCCGGGGCAGACAACTAAACAAGGGCATTTGTTCAGCACCGCTCACACGGCTGACCAATGACTTATTCCTGGGGAAGCCAGAAAAGGGCATCCGATACCCTGGAACTGGAGTTACAAACAATTAAAGGCTGCCACGTGGATGCTGGGAATCAAACCCAGGTCCTCTGCAAGAGCAGCACATACTCTAAACCACTGAGCCATCTCCAGCGACGTGGTTCACTTCTGAAGCCTCTTGAGCTCTGTTGGGGTCTCTGGTTTATATGTGTTCTGTAAACAGCTTTTATGACTACGTAAACAAACTTCCTGTGTGCGTGGTGCTCAGGATATTGGTGGGTGAATGGCTGGTTTTGTGTGTCAACTTGACACAAACTGGAGTTATCACAGAGAAAGGAGTCTCCCTTGAGGAAATGCTTCGATGAGTCCCAGCTGGGTGGGAGTGGATAAGAGGTCATTTCCAGAGGAGTTTATGTGAGACAGGAGGAGCCACCATGAGTGCTGGCAACACCATTCTGTGAGCTAGGATCCTGAACTCAAGGGTTCCGAACTTCCGTCTTCACGAGCCGGTCCACGCGCAACGAACAAGCGCGAGGAGGCCCGGCC
MSTRG.49597.2	ENSRNOG00000010882;ENSRNOG00000028997	0.982	0.000	0.000	-6.140	down	TCCACTTTAATCCAATTCAGTTTAGACTCCGGGTAAAGAATGGGTTTCTGGACTCCATCTGGGAATGGCTAGAACATGAGCTCACAGGACTGGAGAAGGGCTGGAGAAGGGGACAGGGATGAAGGGTTTAGCTCAGGGGACTCTCTCTTGCTCAGCCCTGACCCCTCTTCTTTTGAGCCCTCAGGTCTCTGCCCACTGAGCCAGCAAGAAGGTAACTTAGGCTACATCCTTATTTGTCTCTTTCCTTGGGCAGGTCTGACTGCCTAGACTGTGTCCTTCCAGGTGGGTCCATTTCTTTTTAACCAATCCAGAGCCTCGCCACCAGAGAGATGAAGGGCGAGCTGGAATGCGCTGTCTTAGGAAGCACTTCTCAGAGTCCCTTGGTGCACACTCCAAGAAAGTAAGGCTGAGACAGGCCACTTGACTCACATGGGAACACCAAGGGCAGAATCGGCAGATAGGGAGGTCAAGCCCAAAGGGTTCAGCCGAGGAACATTCATTGTGCCAAGGTGAAATGTGCCAAGGTTTGCAATCTTTTCATTAAATGTGGGCTGTTGTTGTGGAGGAAGAAGGGAGCTGAAATCCTCAGCTTATATTGGAGTCCTTTTGTCTTATCAGTAGTGGGCATGTTTGCTCAGGCTAAGCCTAAGTGCTTTCCCCATCTTCAGTGACTCAAACAAAGTCATCTAAGGCAAGCCATGAGCCATCACTACTGTGGGTTGTGTTGTTTCACCAGGAAGGCATCTTCTCGGAGACAATTCTGGGCTCATGCTCTGAGGTGTTTTAAGGCTCCCAGCCTTTCTGTTCCCATTCAACACAGAGATTTCCTGCCGATGGTAGCTGCTGTTGGTTACTACTGGCAGCTGCTGGCTGCTTCCCGTTCGTGGAGCTTTCCCCTGTCAGCCAAAACATCTGCGGGGACTCCTACATATTGTCCTGGAAGACGGTTTCATAAGCAGCTCCA

							TTTTGAGAGTAGAGAATGATTGTACCCCGGGGCAGACAACTAAACAAGGGCATTTGTTCAGCACCGCTCACACGGCTGACCAATGACTTATTCCGTGGTGCTCAGGATATTGGTGGGTGAATGGCTGGTTTTGTGTGTCAACTTGACACAAACTGGAGTTATCACAGAGAAAGGAGTCTCCCTTGAGGAAATGCTTCGATGAGTCCCAGCTGGGTGGGAGTGGATAAGAGGTCATTTCCAGAGGAGTTTATGTGAGACAGGAGGAGCCACCATGAGTGCTGGCAACACCATTCTGTGAGCTAGGATCCTGAACTCAAGGGTTCCGAACTTCCGTCTTCACGAGCCGGTCCACGCGCAACGAACAAGCGCGAGGAGGCCCGGCC
MSTRG.49639.1	ENSRNOG00000032328	0.468	0.000	0.049	-3.592	down	GGTTGTGGAGAGAAACTGTGATTTTTGTAAACTTGAGGGTTGGCCAAGGGTAAGCTTGCACAGTCCCGAGTTGTAAGAGCAAACTGGAAGAGGAGGATTGAACTGCCCTTGCTGGCTTGTCTGTGGTGTCTTTGGAGAGCACCGGATGGCAGCTGTTTATTTGAACTTGGTGTTCGCTACTGGATTGAACTGCTGATATCCTGACAACGAAGATTGGATTTGCGCCCAAAGAACTATTTCTAAACATGTCTACTTTCCCTATGCCCTGATAACTTTTCTTTTCCACTACCTTTGGTGGGTGGTGGGCTAGAGGGGAGGTTGAATGTTTATTAAAGTAGGCTGAGAAAAATTTATGCCTACAGTCAGTCAATTCTTGGGACATATCTTGAAACAGTGGGAACAACGTATGATGAATAATTCACAACATTATAGAGGAAGG
MSTRG.49686.2	ENSRNOG00000013773;ENSRNOG00000014130	0.093	0.481	0.003	2.827	up	CTCAAAAGTGGCTGGTGTGGCTGGGTATGGTGACACACACCTGAAATCTCAGCACTGGGGAGGCTGAGGCAGGAGAATATTGAGTTTGAAGCTAGTATGGGCTATATAGCAACAGCCTGACCAAAATTAGTTTTTTTAAATTTTATTTTATTTTAAAATAAAATAAAGGGAATTGGTGTTTTGCCTGTATACATGTCGATGTGAGGTTGTCAGATCTCCCAGAACTTGAACTATAGACACTTGCGAGCCACCATGTGAGCGCTGGGAATTGAACTCGTGTCCTCTGGAAGAACAGCCAGTAGCGGAACCTCTCTGCACCACCCCCCTAGCATTTGTTTGGTTGGTTTTTTTTTAGAGGAGAAGGAAAAAGAAAGAAAGAAGTGGTTGGTTGTGGTGTGCGCGGTGAGGTGAGCACCAGCTGACCCAGGCTGTTCTTCCCTTTTGCGGGTTCCCCAGCACCTCACTTCCTTTGCAGCATACTAGCCCTGAAGGAACCAGAGATGTCTTCCTGAAGCTTCCCTTCTAGAGGTGCCAGGAACAAACCCCAGAGACTCAACACGGCACAGGTCGGAAGGGAACAACGCCTTTGGCATGAGGTTGTAAAAGTGGCCTTTGCTAACCTGACATGTGGGGACTCAAGAACCCCACTCCCTATGGGCTCCAGTCTGTGCTGCTGGCCCCAGCTCTGGGGCTCTGAGAGGTTCCTGCTGCTACACCACAGCAGCCCACTGCCATTCATGTGTGTCTGTTGCTGAAGGCCTCGGTGGCCCCTGCCCATGAAGATGTGTGCCCCCGTCAGGGGGCTGTTCTTGGCCCTGGTGGCTGTGTGGAGGACCGCGGTGGCATTCGCCCCTGTGCCTCGGATCACCTGGGAGCACGGAGGTGAGTGTGAAAGCCGTAAGTGCCCGCAGCTCTGGAGAGTGTGGGGGTCCCTGCTTTAGATGTTGTGTGTGTGTGTGTAGGGGGGGGTGTCTGTCAGGGCAGGTTTAAGCTGCTGAGATGTCAGGAGGATACTGTGTGAGGAGGAAGCCCTGGTGGCTGCTTCAGGCCCC
MSTRG.4982.1	ENSRNOG00000010938;ENSRNOG00000011451;ENSRNOG00000011584;ENSRNOG000	0.000	0.389	0.003	4.043	up	GCCGGCTTACAGAGCAGAGGGTTAGAAAGAAGCAAGACAAGCAGCATTCCAGGAAAGCAAATACTGCAGATCGAAATTGGACACAAGTTCCTGGAAAGACAAATAACAAAAACCCACACCTTCCACTAAGCCTTCCGCCAGCTCATTAGCTGATGAGAACTCTAAGGACTTGGAGAGGTTGGAATGTTGCAAGTCCAGAGCCAGATCTTCACAAGCCGCCTTAGCACCTCTGATAGCCCTTCATGACCCACCTCTGACGTCATCTGCTGCCTCTGTGGCTGTGAGAAGCTTGACAAAAACTGTTACCGCGGTGGAGCACGGAATTTGGCGTAACCTGGTTCTTTATTTGTCTGCTGCGGTCACTCATCTTTGACTTCAGAATAAACTATCCCTTCTCACCTTTGAGGTGAGAGTTACATTTGTGTACGTGTGCT

	00032232						
MSTRG.49831.3	ENSRNOG00000017092;ENSRNOG00000026564	0.000	0.221	0.000	7.502	up	TGGAAAAGTCTGCATTCGTGGATCTAGTAGGAAACCAAGAACTAACTGCACTGTTTTAAAGTATAACTTGCCTTAAAAGCATTTGCTTATTAATGCTGTGCATGTCTTTTATGACTTTTTAATTTAACAAGTAGATGATGGGAATGGGCAGTTTTTACTTACTGTGTATTCTATGTCAGCATAAATGAGGATTTAATTTTGATTGATGTTGATAATTTCGTGTTAACCTTTCTTGAAAGAGACTGATGTTTATTCCAGAAGAAAAAAATTGCTCTATCAGAGTGAGGTAGTAGATTGTATAGTTGTGGGGTAGTGATTTTACCCTGTTTAGGAGATAACTATACAATCTATTGCCTTCCCTGAGGAGTAGACTTGCTGCATTGTTTTATCTAGAGGATATTATAAACTTAGGTGAATTCTGACCCTTTGTTTTACAGTTAACTTCTTCAGTGCAATAAACATTGTTATGTGACATTTTTAGTATTCATGCTTAAAGAATTTAGGCAAATATCTGAACGGTAAAAGTATCTAGGACCAGGCCTTTTGGGGTTATTAATAGATTCATTGTTCTACATTTCTGGTACACATGTCCAGAGAAAGATGCCTTAGGTAAATGGACATACCAACTGGAAGTGTACTGCTGGACCAAATTGAAATCTGGTTATTTTATTATACTTAAAATGCTGTAACTATTACTATTACTTCACTGGTATATTGATTTTGACAGACAAAGGAAAGGGGCTCTGACTTTTGTCTTGGTTCTTTTCAGACCTCAATTTTACCCACAAAACAAAGGAGTCACCCAGAACTCAGGTGACAAAATCCCTAATAGGTAGCTTTAAAGAAAATATGGGAGGGGTATTAAAAAAGGTGACTTGAATGTTAGCACTGTTGATGGGCTCATGTTTTAGCTTTTTCTAGTGCTCAAACTATCTGTTGGACCTTAGGCAAGGCCAGAAGCTCTGGTTTTTAAGTGTTTGCTGTTTAACTGGAGTAAGAGTGGGAAAGGCAGAATGTGGAAGCAGGGGCAGTTAATCTAACTAATTACAGTGTATTGGTATAGTCAGTATGATCTTTTCATGAAACAAAACTCCTTTGGAAAGGCTCAGTTCAAGTGATTTATCCTGAACCAAATTTAAAATTTTTATTCAATATCTAGTGTAGGTGGACACTTTGGAAAACTTGGTCTTTGTTCTAAATTTGGCATATAAAAGATTGAGGAAGGGGCGGGGATATATATGTCTAAGATTCTTTTTGGCTTAATCTTTCCTTTTGGCTTTTCCTTAAGTAGACTGTATAACCTGCCTGTTGGATGTGTTGCAAGTAGCCGAGAAATTCTGTGGTTTTCCGCCCCTTATTCCTCTAGCATGGATATCTACCGGTTGCAGTTTTGAAACAATCTTTACTGAGGCCTGGAGATGATTATTTATATTTTTGTTATTAATTGCACGAGGACTAACATCCTTATTACACCAAGTGGATGGAATTTGGAAAACTGGTATATTTTAACTACTGAAGTGACTATGGAAAGTGATAGAATCACTTAAAGCTGATCTTTGCCAGTGATAAAAGTCATGAACCGAATTTGCTTCTGTTTTAATTTAGGCATCAAAAAGTGAAATAATTTCTGGGTCAATGGAGATGTTAGCTATAAGTGAGAAGTATGCGGCCTGTAAATAGCGAAAGCTTTAGGTTTCAATTGATAAGTAATTTTTTGGTTACCAAGAGGCCATTTTATGAGACTTGATATCTGAAGTTTTGGTAATTGTTTTAAATAAAGTATGCAGTAGGCTGAGTGGTAGACTGTTAGTTTCTTTTTTTAATCCTCTGATGTAATTGGATTAATTTTTAAAGTAACTTTAAAATTGACTTTTAGTTGATCACATTGTTGGCTTTTGCCAAGAACATGTGGCCAAGAACTTTTAAGAATATCAGTCAAAGGATTCTAAGTGGTTGAATTTTAAATAACTCAAAGGCCTGCCTTAACAGGTATGTGAAGTGCGTCCGCCAAGTAGAAGACCAGCAAGAATACAGTGGGCTCCTAGGAAGAGGTAGTAGGTTGCATAGTTTTAGGGCAGAGATTTTGCCCACAAGGAGTTAACTATACGACCTGCTGCCTTTCTTAGGGCCTTATCATACCAGCATACAGTTCCATGCGTGTAAGCAAGAGTTCTTCGTGCAGGTGGTTCTCGTAAGAATGGGAACATTGCAGACCTTTTGAAGACAGTGTGTGCACTGGTTAGTACACAGCAGCACTGCACTGCGTCAGCGACAGGCATCTAGTAAGAATCTGAGGTTCCAAAGTTAAACCCTAGTTGCTCCTGTGTCTCACATTTGGGGGTTACTGGGTAGTTTGGGAAAGCAGAATTTTCATATTAATTTTTTACTTAATTTTCCTTGTCTTTGACATGGGGAGCATCAGCTATTTCCATATTATGTTTGAAAGAAAACATTTAACAAAACCTTTGGGATATTTATTTGGCTTTAACACCAATTATGAAAATTCATGTGTTTTCTGAGGTGTGGGAGGTAATTGTGTTAAGTGACTCACTATAGCATGCAGCTTGACAACACAAATGAAAGTGGTTGCTGGAATAGTAGACTTAACATTGACAATAAAAATATGACATTTAAAAACCTTGTGCGTTTCTATTTTGTGCTGAGGCTTCCTCACCCAATAGTATAACTTAGGCTGGCCTGGAACCTGAGATCCTTCTATCCCACTTCTCAA

							ATGCTGGTTACAAGTGTGTACCACTGTACCTGACACCAAATTAGAGATCTATTGACTTCACTTTCTATTA<br>GTGACTACTATCATTATTCACAGATGTGTTATAAATGTGGCTACATTTGCATACACCCTTGAGTCTTTGAA<br>CTTGCACAAGTAAATACTACCTTACAAATAATCCATTTCCTCAGTCTGGTTCAAACATTTACCAAATGAG<br>GAAAGCAGGTAACTTTACTGATATAGTAATGTGTTCTATGAAAACTTGGGTAAGGAGGGTGAATGGTGT<br>CTTTGATTGCTTTCCATTGCTGTAATAAAGACCCTGAGGAGGGAAGGGTTTTTGTTAAAATCAGGGCAG<br>GAACCTGAAAGCAAGACCTGCAAAGGAGCTGTAGAACACTGCTGGCTTGCTCACCATGGTTTGCTCAA<br>CCTGCTTTTTATACAACCCAGGACCAGTACCAACTCAGGGGTGGTACCTCCTGAAATGGGCTGTGCTGT<br>GCCGAGCCCTTCCAACTCAGTTTAATATTTCAGACTTCCTGCAGACCGATCTGATAGCATTTTCTCATTG<br>AAGTTCCCTTTACCCTGATACCTTGTATCAAGATGAGAAAACTAGAACAATTGGAAATTTGGGGACCAT<br>TGAGATGGAATTGTGAGGGTCTTATATTTCAGGAGGAGAATTTGTACTATACACTACTGGTGTTGGTGAT<br>TATAGCTATTTTGTGATACAGAGGTGTAAGTTGGTCAGTAATTTTTAACTAGCATTTGCTGTTAGGTCTGT<br>GACAGTCTTGTGTCCTTGGCTGTCTTGGAACTTACTATGGAAGCCAGGCTGGCCTCTGAACTTAAGAGA<br>TCTGTTTGCCTGCTTCTGCCAGTTCTAAAATTAAAGACATGGGCCATCATGTCATTAATTCCAAATCTTTT<br>GTGTGAGAAAGAAATGCATTACTTTACAATAAGTTGTTTCTTGCAGAGGGAGTTCATTGTATTAAGTTTT<br>TTAGGGGAGTCCTGAAAGTTTCTTAACCCTGAGGGAATAGTGAAGACAGGAACAGGCTTTTTTGCATA<br>GCCAAAAAGAGAATAGGTCCCTTGGGTGACAGTTCTATGAGCATGAGCACAACGGCATTGCAGTAGGG<br>GAAAGGCTGATACCCAAAACTTCTCGATCTTGGGATTTGGGAACCAAACTAGGATCAAACTCAGTGAG<br>CATCACACACATCCTGATGCTTTCTTTGGACTATGGAAATTTGGGTCACCTTAAATTGGTTCTTGATGTGC<br>TTTAAAAGCAAGATAATCAGGTAAGTTTGAGTACTAGTGCACTCAGAAAAGGGTAGATGGGAGCAGAG<br>CAGGTGGGAGCCAGCTGAAAACAAGGTATTCCAGAAACCACTCCCCATAGGAACATACGTTAGTCATA<br>CAACAAAACACCATCAGAAGAGATAAACCAGGTCAAATCTTAGCACATACTAGCATCCTATGTCCTCAG<br>CAGGCGTCCCCAGATTTTACAGTGTGTAGAGAATTGCTTGAGGGAAGGAGTAAGGTCTAGACCTCAAA<br>CTTAACCCACTGCCCTGTCTACAGAGGACAATACCACAAATGACTTCATATAAGATGGCTTTACCTTGAA<br>ACAAGGTACCCCAGATACCAATCTTTTACAGACAGCTTCCTGTTTTAACAGCTAGGCAGACTGCAGTTA<br>TGGGCTCATCTGACCCTGTGTTAACTGTAGCGTGAGCCTGATGTGTACTGTGGCCATGACCATTATGCTT<br>CACTGTGGTGCTGTCATTGGGAGTCAAGATGCTTAATGTCCATAACCTGTTCCCAACAAACAGGATTGT<br>TGAGGCTTAAATGTATTGCAAAAGGTAGGTCATGGAAGTGAACACAAGATGGAGCCCAAGTGACCCAA<br>AGGCCAACCAGATGGATTTGTATTATGGCAGAACCACTGGCTCTGTCATTCTTAAGGCATGAGTATGCCA<br>CAGACTTAGGCATTAATGTTTGAATTAGTGTTGGAATTCATGCTGTGTTGACTGGGCATTGGGCTCAGGC<br>TGAGCATTGTAGAGTTTGTGGCCATGGGTTGGTTTGGTTTCACGTCGTAGACAATCCCTAGCAACAGAA<br>GATGGACTAGTTTGGAGAAAAACAGGCCTTTTGAGTTGGGGGCTAATCCCTCCACATAAAGCCCAGAG<br>AAAGGGTATCAGAGTAGACGGTGTCAGTAAGTGGGTTCCCTGCAGTCATTCAGTTCTGAATGATCCATA<br>ACCACATATTGGAACAGGAGCTTCAGATGCTTTCTAGTGGGAAATTAACAGATCAGAGACTGTTGTAAA<br>CTACTGAAGGTGCCAGCTAATGCTGAGACCGCAGAAGTGAACACAAGTTCAGGACAACAGGTTAAAA<br>AAAAAAACAGGCACAAATAAAAACATTGTTAAAATGAGAATAAAAGCTTTAAGTGCTTTCACATGTGA<br>CATGCCCACATCAGGGACTTTCTGGAATCCTGCTCTGATAAGGAATAAAATAGTAAGCAGCAGAAAAGC<br>CAGTTTTTAAGGGGAATAAGTGAGCAAGGAAATGTATTTCATTAAAGAAACAATAAAAGGCATCAATCA<br>GAAAAAGTAAAACTGAAGAAACAGGTCAAGATAGGCTGTTTTAATGTGTGTGGTGATGGGAGTGCTGT<br>AGAGGGAGCTTAGTAAAGAGCACTGACTGCTCTTGCAGAGAAACTTGGTTCAGTTCACAGCACCCACA<br>TGGCAGCTCATAACTGTCTTTAACTCCACAGTTTCAGGGGAGATCTGGCACCCTCAGACATATGACGGC<br>AAAACACATCAATGCTCATAAAGATTACAAGTGGAAGGGATGGTCAAAGTTCAGTACTTACTGGGTTCA

							ACTACACCTACCCCTAAGTCATGTTAAAAAGTCAAACTGCCAAATGAAAACATACCAAGACATCAGGA<br>AGGAAGTGACATCTAAAAGGGGTTGCACAGACTTGGGTTACAGGCTTTTTTTTTTTAATTGTATAGTTTT<br>TTCACTTTACATCTTGCTCACTGCCCACCTGGTTCCCCCTCCCACAATCTGTCCCCCCTTCCTTTCCCCCT<br>CTTGAGTAGGTGGGGACCCCTGGGTATTTCTCCCACTTCGTCTCTGAGATGCTAGGTGGTGCTTCTTCCA<br>CTGAGGCCAGATAGAAGTCCAGCAGGTAGAATATATTCCACATACAGGCAACAGGTTTTAGGATAGCCC<br>CAGTTCCAGTTGTTCAGGTCACACGTGAAGTTCAACTTCACATCTGCTACATATGAATGGGGAAGTCTA<br>GGTCCAGTCGTGTATGGTCTTTAGTTGGTAGTTCAGACTCAGAGCCCTGAAGGGTCCTTTGTTAGGTGA<br>CTGTTGTTGTTCATGTGGAGTTCTATCCCCTTCAGGGTGTGTAATCTTTCCTCTTCCATAAGAGTCCCCAA<br>GCTCTATTCACTGTTTGGCCATGGGTGTCTGTATCTGTCTTAGCTGCTGGGTGGAGCCTCTCAGGACAAT<br>ATACCCCTGTCTGCAAGCATAACTATTAGGGATTGGTGCTTGCCCAAGGGGTGGGTCTTAGGTTGGGCC<br>AGATATTGATTGGCTAGTCCCCCCAGTCTCTTCTCCCGTGACTGCATTTCTTATAGACAGGATGAGTTTTG<br>TGTTAAAAGTTATATGAGTGGGTTGGTGTCTCTGTTGCTCCGTGGGAGTTCCTATCTGGCTACAGGAGGT<br>GACCTCTTCAGGTTCCAATCCATATCCCCAGTGTGGTGAGTCACAGTTAAGATCACTCTATTGATTCTTG<br>GGCACCTCCCTTGTTCCAGGTCTCTCTCATGTCCTGGAGATGTCTCCTCACCCATGTTAGTTGCAGCTTT<br>CCATTCATTTTCAAGACCATCTAGCCATCTCTCCTGTCCTTCTGCACTCCTGATTCTGAATCCTCCATTCC<br>TCTCCCCATACCCCTCAGTTCCCTGCCCCCATCTGCCTCTTATGACTATTCCCCCTTCTAAATGAGATTCA<br>AACTTCCTTGCTTGGGCCTTCCTTCTTGTTTAGTTCCTTTGAGACTGTAGAGTGTAACATGGGTATCAGT<br>ATTTTATGGCTAATATCCACTTATAAGTGAGTACTTACCATGCTTGTCCTTTGGGACTGGTGTACCTCACT<br>CAGGATGATATTCTTAAGTTCCAGTCATTTGTCTGCAAAATCCATGTCTGATTAAGGACTATGCTTAAGA<br>ATACTAATCCTAGCAAAGCTCTCTTATAAATGAAAGGAAAATAATCCCAGGTACAAATTAGAAAGTTTGT<br>TACCACCATTCCTAGCCCATCCCCCAAAGAAGTTCTCTATCCTGAAAGAGTAAGAAGAGGACCCAAACC<br>TCACCGAGAAGGGTCTGTCAGACATTCAAAATGGTCTAGTCGGGCAGCATATAAACCATAGTTACAGTA<br>TGGAGGCAGAAGATCATACATTAACACTTAATAGGTAATAAGCATAAAACGAGAAGAAAAGTGCCCCCA<br>CACATGGAATACAGGAGATCAAAAAAATTAAGAATTTTGTGTGAGTGCATGGATAATATGTTGGGGTCA<br>GAGCAACTTGTCTTGGGAATTGAACTCAGGTCATCAGGCTTGGCAGCAAGTGCCTTTACCCATTGAGCC<br>ATCATGATGGCTAGGAACTTTTAAGATGCTATCTTTCTATATTTTTATAATTAATATTATACTGCTATAATTTC<br>AGTGTAGGTTATAATTTTAAAAGTTCTATCAACCACATCCATAAAATAACAATATGGTACACACACACAC<br>ACACACACACACACACACACACACACACACACACACACCAAGCAAGGAATCTAAAATAATTTGCTTAGTA<br>ATATCACTTCACAAGGAAGGCTATAGAAAGATGGGAGGAAGGGAAATAGAAACTATTATCAAACTGGA<br>AAACAAAATACCAATAGTAGCGTCTGACTTTTTGATAATTACCTCAGTGTAAGGTAATTGAGGGTAATGT<br>AAATTTCTCCCATTAAAATAGAGCAACTAAATAGTTAAAGCGAGATTCAACTATATACTGCTTAAGGACC<br>CTGGAAAGGGAGGGGTGAAAAAAATATCCCATATGAATAGAATCATAAAGGCAAGAGTAGAACTACTT<br>CATCAGATAGAAACCACAACAATTAAGAAAGCCATGAAGGGTTCTGGTGGCCCATATCTTTAGTGCCAG<br>TACTAGGGAGGCAGAGCAGGTGGAATACTGAGTTTGGGGACAGCCTGGTCTACAGAGAAAGTTGTAGG<br>ATAGCCAGCTCTACACAGAGACACCCTGTCTTGAAAAACAAAACCTAACTAACCAACCAACCAAACCT<br>CATAAGTGATAAAAGGATCAATTTGGCAAGAGAAAATAACCTTGAATATGTCTTTGAATATACATGAATT<br>CAGTTTCAGAACACCCAGATATGTATTAGAGAAACATTGACTAAAATGATAGAACTTTTAATAATCTAGA<br>TCACCCAGACCAAAACTTATCAAAGAGCAGTGTTAAACCATTCCAAAGCAGACAACACATTTACAGAA<br>TGTTTCATCCAATGGCTGCTGAGTACACATTCTCATTAAAACGTGCAACATTCTCCAGAGTAGTTACATG<br>GTACACCACAAGACAGATTGCCAACAAATTTTCTTTAAAAGTTGAAATCTAGGGGTTGGGGATTTAGTT<br>CAGTGGTAAAGCACTTGCCTAGAAAGCACAAGGCCCTGGGTTCAGTCCTCAGCTCCTGGACAGTGCAG

							CTTTGTGTCACGTGCTGTCACGTGTTCCGTGGATGCGTGAGGAAGAGGAGCCACTGCCCATCTCTGGGTTTCTCTTTTCTCTGTACTGTCCTTTGAACTGGAGCTGCCTTGGTTACCCCGGACTTCATCTCAGGTTGACCTCCAGGTCCTGCTCAGGTTTCCCTTCTCTGGGTGTCCTGCAGTCAAGGAAAGAATCCCGCCGTGGTAGGGAACCCCTCCTGTAGTCTGAAGCTTCGGGTTGCAGCCACTAAGTCAGCAAGGGGCGGCCTGCAGCACGCAGCATTCTGGGTATTGTAGTTCCCCCATCTTTAGCGCTGCGTGGGTAGTGAAGTGCGCGCGCGTCATCCCGGTCGGTGTACTTCCGCATCCTGCAGTCGTCACCTGTGCCTGTGGACGATCCTCCCGCGGTTCTGGTGAGCAAAAGTGCAAAGACCCGGGCTCCTGCCTATCCCTCCAGGCGGAAGAGTCCGGAGAGCTGGCGTGGGCCACCCACAGGGTTCAGGTGTCAGCCGCTACTCCATCCTCGATGCGTGGAGTCTTTGGGAGTGGAATCAGCTGTTCTGCCCGTCTTAGAGGAGGGTCTCAGTGAAGACCCTCGGGTGGGGAGGGGGCCATGTGAGAATGGGATTTGGGGTCCAGTGCCTCGTCCTAGACAAGCTACAGGTCGGTCTGGCCTGCTTCCCAGGCTGTGACGCTGCAGGTGTGTCCCCTGTGTTAGTGCTAG
MSTRG.5.1	--	0.419	0.000	0.005	-4.261	down	CTGGTTATGAATTTGCCTTTCCTGCACATAATGCTTCTGCCAAAACCAGCATCCATGGACTTATAGAATGGCTTATTTAGTGGCATGGTATTCCACACGGTATTGCTTTTGACCAAGGAAATCTTTTCAAGGCACTGGTGTACTTAAGAAATAAAGTATTTCCTGTGTAAATGTTTGTCTAAGTGGCTACACTTGTGGTCCCTAGTCCATATTTAAATTGTTCAAAACTTAACCTGGAGCCTGAACTTAAAAGAGACTTAAGGGTGACAGCATAGGTGGAAAATAAAATCTACTCTCAGCCAGCTCTTATTTGAGTATTAGCCAGAGCTGAATGTATGCTGTCATTAAAGAGATTTCTCTTAATCTTTTAATAGTGTGTGCAGTGATTTAAAAGATCTTACCAAATATTTTATAGTCATAGATTCCCCTCTGTATGAGTTCTTTCAGGACTTCAAAGACTACTTTGAGATATACAAAGGCTTTAACAGACTGATCATACACTAAAGGTTTTACCCCTGTATGAAGATCCTAGGAAACGTTACCACATTGACTGTCTTTATGGAATTTCTCTGCAGTTTGATTTTTCTCCTATTTTGTAAGACTACTGCTACATGCAAAGGCTTTACTACATTGTTGACATTTATTTATTGTCTCTCTGGATGTATTAGTTTATGTATTTGTAGATAATTGTGCTGTGAGAAGTGCTGGAGTCCATCTCTGGTGGGGTTCTGGAACCTGAGGATAGATGTATGGTCTTGGTAAAAGTGAAGACTCTTGACTGCCTGTTGACTTTCAACCAAGTGGCACTGAATAGCT
MSTRG.50168.1	--	0.000	0.578	0.000	5.427	up	AAGCTGCGGATTCTGTTGGCCAGGTTTGGGAACAAGGTGGTCGTATTGGCTGTGCAGGTGAGAGACACGCTGAGCTGCCACTTGCTCACTCTTCTGAGTGGAGGCCACAGGTACTGGGTATGAGGTACACGGAGAACACTGCGATTCGGCAACCAACTCTGGAGACGCAGGATTGGGGGCATCTAAGGAGCTGTTCTTTAACTTAGAAGGGGATCTGACAGTTCTATTTGGTCTCGGTTTCTCTGTCTTAACTGCACAAGACGAGCCCTTTTGTGACAAGGTGCTCAATTCTGGCTCCATTGCTTTCAGTAAGACATCGAACGCGGTGCTTGTGTAGGAGGGGGGTGTGGTGCTACAGCACGGAGAGCTACATTCACTATTGTCAGTCCTGGTTTTACTGGAGGAGGAGCTGGAGTCCCGCTGTCCATCAAGGCCGCTGAGCCTGGCTGGGTCTGAGCCCTCAGATCTCCATCTCCGCAGTTCTGACCTGTGGTGGGGAGATGAGGCTTTTTTGGGGGAGACCGAATCTCCAGATTCTGAGGCCTCAACAGTCATCCCCGGTGTTTCTCCATTTTGCTTGGTAAATTGCCTCCCCTGTTTAACAGGGACACAGTTGTGTTTCTTGTGAGACTCCGGTGCGCTCTGGAGAGCACTGAACAACGGCTCTTCTGTGTTCTGCTTGTGTTTTGTCTGAAGAGGATTTCTCAGGGGGGATTTCGGTATTTTTTTCAGGTGATTCTCGGCAACAATCTTTTTTCGTTTCACACCTTTAAGAGACTCAGAAGCTCCTTTAACACCAGCC
MSTRG.50170.8	ENSRNOG00000014460	0.000	0.512	0.000	5.500	up	AGGTGGTCGTATTGGCTGTGCAGGTGAGAGACACGCTGAGCTGCCACTTGCTCACTCTTCTGAGTGGAGGCCACAGGTACTGGGTATGAGGTACACGGAGAACACTGCGATTCGGCAACCAACTCTGGAGACGCAGGATTGGGGGCATCTAAGGAGCTGTTCTTTAACTTAGAAGGGGATCTGACAGTTCTATTTGGTCTCGGTTTCTCTGTCTTAACTGCACAAGACGAGCCCTTTTGTGACAAGGTGCTCAATTCTGGCTCCATTGCTTTCAGTAAGACATCGAACGCGGTGCTTGTGTAGGAGGGGGGTGTGGTGCTACAGCACGGAGAGCTACATTCAC

							TATTGTCAGTCCTGGTTTTACTGGAGGAGGAGCTGGAGTCCCGCTGTCCATCAAGGCCGCTGAGCCTGGCTGGGTCTGAGCCCTCAGATCTCCATCTCCGCAGTTCTGACCTGTGGTGGGGAGATGAGGCTTTTTTGGGGGAGACCGAATCTCCAGATTCTGAGGCCTCAACAGTCATCCCCGGTGTTTCTCCATTTTGCTTGGTAAATTGCCTCCCCTGTTTAACAGGGACACAGTTGTGTTTCTTGTGAGACTCCGGTGCGCTCTGGAGAGCACTGAACAACGGCTCTTCTGTGTTCTGCTTGTGTTTTGTCTGAAGAGGATTTCTCAGGGGGGATTTCGGTATTTTTTTCAGGTGATTCTCGGCAACAATCTTTTTTCGTTTCACACCTTTAAGAGACTCAGAAGCTCCTTTAACACCAGCCTCTAAGATTTCTTTTTTGGAAACTTCTGCCCCATTAAGCTCTTTTTGTGCTTCTTCAATTTTGTCTTGGCAGGGCAGCCATCCTCATTATCTGTGTGCTGACACACCTGAGCTCCCAGCCCTTCAAGTCACTCTTAGAAGCAGCTCTGTGGCTCACTGGCTGGCAGGCTGCAGTCCTGGGAGTCCTCCCCCACTACC
MSTRG.5023.1	ENSRNOG00000021573	0.000	0.543	0.011	3.719	up	GCATTCATTGGTTCCAGCAGCTGTTCGTCGACGCAAGGCCCCGCCCCCGCCTCCCGCGCTGCCCCGCCCCGTCTCGTGCGGAGCACACCGGAAGGAAGAGGGAGTGAAGGACCACGCCCTACATTGAGTCTGTGATGAATCCCTATCAACACAAGGAAAAGGTTTATGAAGCCCATTCCTCACTCCATTGCTCTCTGGAGACAGGGAAGTTACCCTCCCTCTCCCTACTGCCCATCCTGGCAACAGTGG
MSTRG.50380.1	--	0.333	0.000	0.010	-4.070	down	CTTTGATTTATTTATTTTTCTTTTGTACTCAACAGATTGTTGAGCTCACAATAGACAACTTGAGGACCAGGAGTTCCGCCTCCTCTTCGAGTGTCTGGATGTGAAATATTAGAAGACCAAAAGGCTAATCTGTGCATCCGTCAGCCAGGGGTGGGAGCCCCATTTCACACGAAGTAATCAATTCATTGACTCTCCTCTCTCTTGTCTTCTTTTCCATCATTGGAAAATCAATGCTGGTCTTCATATAGGGCAAGCCAGTTCTGTAACTCTGAGCTGCAGCTCCAGATCCTCAGAAAATTCATAGAGATGCAAGGTTACTCCAGGTGTCCCAGGGGCTGAAGAGTGGGAAATGAACTTTTATTGGTAAATGGATTAGGGAAGTCTCCCTGGAAATGAGGAAGAACTTCCAGCCATGCAGACTGATGGCTTCACAACAACATGTGTGTAGCTAATGCCACTGGGTTCTGTACTTGAGCTGAAGCCAACTTCCTATCGCCTGCATTTTCTCACAGTTAGGAAGATTGAAGTACTGTCACCATCATGGCTTCTCCTTTGCTCTGCAAAGGTTCATTATGACAATTAGAGGACAATGCTGCTTCCCACTCTCCATATGCTTTTACTTCACTTAGCTTGGAGTTTGTTCTTCAGCCCTTCCCAAGAAATCAAAAGGACATCCCCACCCACCAGATGACAAAGAATCTAGAGAAAATATACAGGGCAATAAGAGGCTAATATTTAAGAAGAAAGATTTTTACTTTTTAGAATCTGAATATATATTTCAAATGCAATTATTTTGGGGCACTGAGTAATTTTTGCATGCTTTATAAACATAAGGCAAAATCATAAAATGTCTGCAAAAGATAGATAC
MSTRG.50404.2	--	0.000	0.339	0.031	3.453	up	GTGGGAGACATAAATAAAGAGCATGTTGACATGACTAAGAACTTGAACCTTAGGCAGCCATGCTGTCCTTGGATGTAGTAGAATTTGAGACATGGATCCAAGCTGGCAGTAGATTACCAGGGAGGCTAAAGAAGAGCTTCATCCCAGATCAAGCACAAGGATGGACAATAGGAACAAGAGAAGAGTCACAGAGCCAAAAAATGATCAAGGATTTCTCTCCGGAATGACCACAGCAGGACTCTTTCGTGTTCTCTTGATGCTCTACTTTCTGCCACTGGCACTTGCAAAGTAATGA
MSTRG.50425.1	--	0.306	0.000	0.037	-3.684	down	GGGATTTGAACTCAGGACCTCTGGAAGAGCAGTCAGTGCTCTTAACCACTGAGCCATCTCTCCAGCCCCCTTCATCCCTTCACATCACTTTCTTCATAGTTCTGTATTCAGCATCCTGCCGCAGCAACATCTGACAGAGGGTCTTCATATCTGAACATCAGACTACAGAGCTGTACTCCCACAGAGTACATAGTACTCCTACAGAACTGTGTTCCTCAGGACAAACAGAAGGGCTTTGCAGCATCACTGGAAATGTTGGGATTGTTTTGTGTAGAGTTGTAAATGTCTAGTTAAGCATCTGTTTTTATAATCCTTTTCAACACATTCCAAGACAATGGAGTGGTTTGGAAATGGACCCCACACTTTATTAATGTCAGGCGGTAGAAGGAAGTGTGACTATAATGAGACACTTTCCTGCATGATGTGGCTAAGCCCTGTCTCCTCTCCATTCCCTAATGCAAAGCAAAGATTCTCCCTTTTTCCTTTTCTATAGAGTAAAGTAGGTCCAAGGCACCAAGGAGCTATACATCCTTACTTTCCTAAAGGATAAGTCTGTGTAGCTCTCACGTTACAGTTTTACATTTATTTTGATCTGAGGTCTGGTTGAAGAAAAAAACATTTATTTGAA

							ACAGGAGAGATAGGCCTGCCATTGAAAGCATTCCTGCTCTTGCAGAGAAACTGGGGTACAGTTCTCAG
MSTRG.50531.1	ENSRNOG00000050767	0.000	0.360	0.031	3.453	up	GGAGTGGGCTCTGGCCAAGGCCCTCTCTCACTTCTCAGTATCACAGAAACGTCTGGTATGCTTTGCTCTAATATGGAGCCAGCAGACTGTGACCTCTAGGGCGAGCTGGTGTGCACCTGCCTTTACATGGCCCATGAACTGTGAACAGAGTTTCCACTTTAAAATGCCCTCTGTCTGATTTGGCTGCAAATCCAAGGCTCTCACAATCCTTTTGGGATCCATCCTTTGCTAGAAGTCTTCACAGAGGTCACAGAAATGCTTTCCTGACTCACCCAACC
MSTRG.50691.2	ENSRNOG00000017820;ENSRNOG00000049124	0.001	1.597	0.000	7.242	up	GATGGCCACAATCTGGTATCTCTCTTGGGATCTAATGTGTCAATAGCTGCTTCCATGGAAGAATAATCGTGATTCTGAGCATGCTGGCAGGTGCTTGTCAAGTGTCTGGGTTCTAATTTTACTACATGTGCCTCATTCTCAAACATACTTGCCCAGCTGGGCCACGGTGGCACTCGCCTTTAATCCCTGCAGAGCAAGGCCTACCACTGAGTTCAAGGATAACCTGGTTCCATACTTGCTCACAAATGAAAGAGCTGCCAACCAGTGATGACGTGAACAGACTGTGACTGGGAATGGCGGGATAATTGTGTCTGAAGAGACAGGATCCAGGCAAGCTGAGTAAAGAGACCTAACCAAGTTTATCCTTAGGTTAAATGTCAGATTGCATCTCTGTGCATTCTGCTTGAAAATTGGTATTTGGCATATTTCTGTGAGCTTAAAATGGAGTTGCAAATATATTAAAATATTAATTACTTTTCCTGGAAATCTTGAAATTGGTTTTCAAATTCTGGTTCCAGATCGAAAAACTGGGCTACATATTTTCACTTTTCACAGGGCATGTTTAGCCAACATGTTGAAATGCATACAGTTGTTCACTTAAATTTGAGTTTGGAGTGGCATAATCTCAGCTTTATTTGGAGTGATGCTACAATTTGAAACTTAAGTACCTTGCAACTGATGCACCTTGAAGGTGTATGATAAACTCATCTAAATGAATATTTGATTAAAATACTAAAACTCTAAATCTATGTCAATATTTTAGATTTATTTTGTCATATGTGTATGAATATTTTGCCTACATATGAGTATACATCTCGTGTATGCTTGTGCCCAAGGAAGTCAGAAGAGTGTGACAGATTCCTTGGAACTGGAATTACAGACAACTGTGAGCTTCCATGTGGGCACCGGGAATGAAATCCAGACAGGCCCTTTGTAAGAACAGCAAGTGCTCTTAACTGCTGAATCATCTCTCCAGCGCTGCTTCTCAATTTTAATGATGGTCTCGGCATCTGTCCTTGGCCAATGGTTACTTCTTTTGCTGGACTTATATCCCAATATGGAGAAGCAGAAGAGAGTCCAGGCATACACTTGGATCTGGCCATGGGGCACCTTCAGCTCTTTCTTCTGGCTGGCTCACCAGGTCTTAAAGACGTGGATCTGAAGTCCCAAGCCACAGGCCAAAGTATGCAGATTCTTGTGTACTCCAGCGAAGAATTAGACAGGGAAATGTCATAACGTATTTCACCTGCTTACACATCCATACTGAGACCAGAGACTGTATCTTCTCCAATTCTGTCTCCAAGTAGCTCATGGTCTATTCAGTAGGATAATTCTGGCATCCTCATGAATCTCCCTTCAGCAATGACCCAAGACCTCATGGCCAGGTAACTGTACACTCTGTCCAGCATAGGAAAATCCCCACTTCATTTGCATCTTGAAACAGAGTACACAGTGATACTTGAGTAGATTAGATGAGCTTTTTTGGTATCCTGGTGTTCAATATTCATTATTCTTTATGGGAAGATGTTCAGAAAATGGTAGTGTCGGGGTGGTCTAGGACACAGTTTTCAGGGTTTTGAAGCCAAAAGTCGCCTACCAGAAGTCACTGAGCTGCATCCCCTTCTTGCCTTCTGCTCTTCTATATCTTGTCATATCAGCGTTGCCTTCATTCATCTATCCATCCCCGCCCTGCACTAATATGCAGAGGCTTCCAGTACAGATGCCATTTGCTATGGGTGGAGACTCAGGCTCTGATAGTCCCTGGCCTTACTCTCGGCCCTTTCCTACTCCGGGGCGGCAGCGCCCTGGCCTTTAGACTCCGCCCGACTTCCCGCCCCACAGGTATTTAAGGCCTCGGGCGCGGCTGGGTCCCTCCCATCTCTTCTCTGCCTGCCA
MSTRG.50691.4	ENSRNOG00000017820	1.238	0.000	0.000	-5.395	down	GTTGGCACAAGCTAGAGTCACCTGGGAAAAGTGTCTCCATGATGGATTGTCCAGTGGGTTGGCCTGTGGACACATCTGGCATCCTCATGAATCTCCCTTCAGCAATGACCCAAGACCTCATGGCCAGGTAACTGTACACTCTGTCCAGCATAGGAAAATCCCCACTTCATTTGCATCTTGAAACAGAGTACACAGTGATACTTGAGTAGATTAGATGAGCTTTTTTGGTATCCTGGTGTTCAATATTCATTATTCTTTATGGGAAGATGTTCAGAAAATGGTAGTGTCGGGGTGGTCTAGGACACAGTTTTCAGGGTTTTGAAGCCAAAAGTCGCCTACCAGAAGTCACTGAGCTGCATCCCCTTCTTGCCTTCTGCTCTTCTATATCTTGTCATATCAGCGTTGCCTTCATTCATC

							TATCCATCCCCGCCCTGCACTAATATGCAGAGGCTTCCAGTACAGATGCCATTTGCTATGGGTGGAGACT<br>CAGGCTCTGATAGTCCCTGGCCTTACTCTCGGCCCTTTCCTACTCCGGGGCGGCAGCGCCCTGGCCTTT<br>AGACTCCGCCCGACTTCCCGCCCCACAGGTATTTAAGGCCTCGGGCGCGGCTGGGTCCCTCCCATCTCT<br>TCTCTGCCTG
MSTRG.50781.1	ENSRNOG00000017800	0.359	0.000	0.028	-3.770	down	GTTTTATTCTCTTCGTTCGGAATTTAGCCTTGGAAGCCCATGCGTGTGCATCGAGGGGGTCAGAACTAGT<br>TGTACCCGAGACATACAACCCTACACGGCTCCCCAGGGGGCTGGGGTCAGTATAGTTGGCTTACCCCGC<br>TGGAAGACGAAGTGGGCCGTCGACAACTGTCCCAGCAGCGTGTATGGGAGGCTACCACGACAGATGA<br>GTAGGGCAAAGTCCGTTTGGATGTGTCTTTCAACTATTTAGAGAATTCCCTCAGTTCTCCATTTGGCCCT<br>TTTTTCCTCTCCTGAAGACCGCGGGAGGGGAGGATATCCCAGGGTCAAAGCCCATCAGGCAGCACGTA<br>GAGTTTGTCATCAGTGTGACTTTTCACCAGGAGGAGGAGGTACGCGGTGGGGATGGGGGTGGGCAAAC<br>ACCTGGGTGGCTGCGAGTTCCTGCTTAGGAGGCTTTACAGCAGAATGTTCATAACGAGATTCTTCTAATT<br>CAACACCTTCCTTGAACGACTTCAATGCAGAGGCGCGCCAGTATTTACATACGCTGCAGACATAGACAC<br>CGGCTCAGTGCTGGCACCAAGCCAAACTCCCCGAGGAGCGCCCCAGGTCCTTGTTGACTGCTGTGACA<br>GTCTGGGACTGTCCTTCTCTTCAAG
MSTRG.51025.4	ENSRNOG00000033196	0.000	0.639	0.000	7.803	up	GGCTAGCAAAAAAAGACCTGCTCTCTCGCTTCTCTCAGGGCCTTGTGGAGAGGAGCATATGAAGTGAG<br>GGGCAGTGACTGAATTGTTATGGATGAAACCTTCTCCAGGAGGATGAAGATGAATCCTGCTCCATCTCG<br>CCTCACCCTTATGCTTCTCTACCTCAGCCCAGAGGCCTGGATACATTCTCTTCACTGCTCTGCAACATTG<br>TTCTTTCATCCTGTGGGTAGCAGTTGAAGCCTACATTTCTTGCTCTGCAGTTAGTCAAGCTTCCGTTTAG<br>CCAAGGAGATACATGGCAGCGCACACACTATCAAGAAGCCTAGAATCCAAGCCAGAGATCTTATATTCT<br>ACCATGAAAGCCGGAGGACGGTTTTTTCTGGTATGAAAGACAAAGCTTTCCAACATTAACACTGACTCC<br>AGTAATACCTGGGAGAAAATACAAAATATATACCCAGAAGTCATATTGCTAAGGAGAAGAATCGGTTGG<br>CCAGCAGAAGGCAGAACAGAGCCTGGAAGAAGAGGATTCTCAACTAAGATTAAGCTTGTGGGGAGGA<br>AGGGGGACTGGAGAAATTTCTTAAATCCCTGCTCAGAATTCCAATGAATTTACCAAGTTAGTGGATCCT<br>CCCTGAGTCAAGATGCTGCTGGGTGAAGAGTTGGCTATTCTGGGTCCCTTCTGAGCATCCTTGATGTTG<br>AGCAGATGAATTTTTAAGAATGAAGCAGAGCTGGTTCAGTTGGTAGAGTGCTTGCCTAGCACACATGAA<br>GCCCTAGGTCCAATCCCCAGCACCGTATAAACAGAGCATGTATGTCTGAGATCCCAGCACTGGGGAAAC<br>AAGGCAGGAGTAGATCAAGACTATAGCAAATTTGAGGCCAGCCTGGATTTCATGAGTCTCTGAAGGAG<br>GAGGAAGAAGAGGAGGAAGAAGAGGAGGAAGAGGAGGAGGACAGAGGGAAATTTTCAGGTAGTCTG<br>TCTTCACATGTAATCTCTTTCAGCTCTTCTCATATGCAGTGCTTTGTCACGCTGAAAGTAGGAATAGTGA<br>AAGCCCTTCCCCCACAAGCCCTCTGTAAGGATGGGTGATTTGCCATTAACTTGCACTTAGCAACAAACA<br>GAAGCAGCCAATGAGAGGCCCTTTTCGACCAGCTCTTAATCGCTTAATGTGCCTTTTGGAATGGAAATG<br>TCTGGAGAAAGAAGACAGACAATGGCTAAAGACTGTGCCTTGTGGAGTGGGCATGTGGAAAGAATCAT<br>CATCCCTCAGAGGGAAATGACATACTGAAGGTGTCAGCAGAAAATATGGCTCTCTGTGGCCAGTGTCTG<br>ACATTCAATTAGCTGGGACACTTAGAAGCTGAGAAGCAAGATGTTTTTGACACTTATCAGGATACATCA<br>CCTGAACAATCTATTTATAGGGGACCTTCTTCAGAAGGACTTGACTGGTGAAGGATGTCACTATAGTGG<br>ACTCCATGGAAATTGCCTGAGACTCCTAGCCTTCTGGCTACTGAGGTGGTGTTTAAATGGATCAAATGG<br>ACCAAACTCCCATCCTCCTGATAGCCAACTATGAGGTTAAACATAATGGTGAATTTCTGGAGGGCTGGA<br>GAGATGGCTCAACAGTTAAGAGAACTGTCTGCTCTTCTAGAAGGCCCACTTTCAATCCCCGGCACCCAC<br>AGGGCAGCTCACACCTGTCTGTAACTCCAGCTTCAGGGCTCCTGACACCTTCGCACAATCATACATGCA<br>GAAACAAGACCCAGAGACAGCAAACCAGTAGCATCTTGAAGTCACACTGCTCATTTGCAAAGCTGAGT<br>GAGACCTGCCCTTCTCAGATCTGAATGCCACACTTTTTGCCTAATATCCTGCGGCCACCCTTCTGGCAGG

							CGACCTTATCACAAAGATCCATCCTGGCTACCGAGTGACATCTTTTCTGATACCACGATTCATGAATAACTGTGAAAGTATTGCTAATTTTGGCTTGTTTGCTCCTTGTACATCTAATGAAATGTAGGCAGAATGCTTGGAAGGAAAGTGAGATGAGGGGTCCTGTCCCCTCCTTCCACACTGTCGGATAACAAGTGAAGTCGCTCAGTAGATGATGCTTGATTCTGAGGTGGAGGCTCAGTGGTAGAGTGTTTGTCTCTCATGGAAGAGCCTTGGCTTTCATCCCAAAACTGAGAAATAAAACAGAACTAGAGTGACAGTGACTGAGCACCAAAGATGACCACGGGGGACAAGGGCTGATAGATTCAACAAATGTGCACGCTCCTTAACTTCAGGGATTGTTCCTATAGTAGAGCAATTCAGTTACTCTGCTAATCGTCACAAGTGGCCCCGAGAAATGTGTCCAGCTCAGACGGAAGGGTTTACAAAGAAAGCCACTATCTTCTTACCAACCCTGGTCTTCCAGGACAAGGACTAAATAGAAGCAGGGGAGAGCAATTCTTGGTACTTCTCTAAGAGAAGAATCTAGCATGACAAGAATGGAGCCATCACTCCAGACAGCAGAGAAGCCTAGAAAGGTAGGAAAAAGACGACTGTGGGAGCCAAGAGTAGGACAAGACCGTAGCATCTGCTGGACCTACCTGTGGTGAGGGAACCAGGTGATAGAATGCTGGAGAGGAAGACAGGAGGCTAGCTAGATGTTGGGCTCTATGTTCCTGAAGAGTTCACAGGCTCACGGGGCACCGGGAATGTTTCTAGAAGGTTCCAAGCTTAGATGATTTCATTTCTTCCAGAACCCCTCTGAGATGAGTTTCAATATTATCCATTGCTTAGAAAGGAAGAATTATAGGCTCACAGAAGCCAAGGCACTTTGCCAAGTTCACACACCTGACCACTGAGTACAATATGGGCTTGTGTCCTAAAGCTACTAAGAAGTCACCAAGAGGTGGGAGCTGGGTTTTGAGATGAGACTCGCCTCCATTTATTCTAGTACCTATCTTCACCCTTCTAGAGGATTCCCTGACAATATGCGTAACTGCTAAATATTCGTCTTTCTCCTGTGGAAATTTATATTGTTTGTTTACACACTTAATGATTACCTCCTCAGCGTATCAATAAAGCAATCAAATAATTGAAATCCTCTGGGATGAAATAGATTATAAATGGAAATTTGTACTTAATGGACAATTATACTTAATGATCAATTAGCTTGGCTAATTTTGCTCTTCCACCTTTCCCTAATAACATTTTGTCAGAGATAAATAGAAGTACTGTAGACTTGAGAAATACTAAAACAGAGAATGATAAACACACAAAAAACCTTGGGTCTTAAGTTCATTTTGTTTGCAGGAAATTATCCTAAGGAAACAATCTCACGCAGGTTTGGTACCCAGTCTGTTTCTACTTTGAGTTTTTGTCTTCCATTCCTCTGAGTTATGGAAAACATCCCACAGGTAAGATGGTTCCCCGTCGGCTTCTATGCACATATGGTCACAGCCAACGCCTCTGTTGATCTCACATGTCCTTACTCTATGGGGTGCAGGGTCTTTACCAGGTATCCTCAGTCTCCCACCTTGTCTTATCTCTGTAGGCTTTGTTATCTCAGAAAATTCTCTTTCACTATCTCCAAACATCCCAGACCCTGTATAAGTTTGAAAGTATCCATCGTCTCCACCATGCCTGAGTTCCCATTGGCTCCACTTCCTATCATTCACCTTCTCAGGGACACGCTTTTGATAGCAAGAGCCCATTGCCTTGTCTTAGGGACTAAGGCATCCAGAAGGTGGATCTAAATTTACACTGGAGCCCTTGGAAAGTACAGGGAAGAAAGGCATCTGTGGTTTTAAAATAAATCTACAACTTACAAATCATGCAATTTCATCCCAAAATATTTTTGGTCTCATACACTAATATTTGAATAAGTCACCAGGCTACATCTATTAATAGTGAGCACTAGAGAAACTTTTAATCTCCTACTAGAGAGAGTATCCAC
MSTRG.51025.5	ENSRNOG00000033196;ENSRNOG00000040249	0.098	0.682	0.000	3.491	up	GGCTAGCAAAAAAAGACCTGCTCTCTCGCTTCTCTCAGGGCCTTGTGGAGAGGAGCATATGAAGTGAGGGGCAGTGACTGAATTGTTATGGATGAAACCTTCTCCAGGAGGATGAAGATGAATCCTGCTCCATCTCGCCTCACCCTTATGCTTCTCTACCTCAGCCCAGAGGCCTGGATACATTCTCTTCACTGCTCTGCAACATTGTTCTTTCATCCTGTGGGTAGCAGTTGAAGCCTACATTTCTTGCTCTGCAGTTAGTCAAGCTTCCGTTTAGCCAAGGAGATACATGGCAGCGCACACACTATCAAGAAGCCTAGAATCCAAGCCAGAGATCTTATATTCTACCATGAAAGCCGGAGGACGGTTTTTTCTGGTATGAAAGACAAAGCTTTCCAACATTAACACTGACTCCAGTAATACCTGGGAGAAAATACAAAATATATACCCAGAAGTCATATTGCTAAGGAGAAGAATCGGTTGGCCAGCAGAAGGCAGAACAGAGCCTGGAAGAAGAGGATTCTCAACTAAGATTAAGCTTGTGGGGAGGAAGGGGGACTGGAGAAATTTCTTAAATCCCTGCTCAGAATTCCAATGAATTTACCAAGTTAGTGGATCCTCCCTGAGTCAAGATGCTGCTGGGTGAAGAGTTGGCTATTCTGGGTCCCTTCTGAGCATCCTTGATGTTGAGCAGATGAATTTTTAAGAATGAAGCAGAGCTGGTTCAGTTGGTAGAGTGCTTGCCTAGCACACATGAA

							GCCCTAGGTCCAATCCCCAGCACCGTATAAACAGAGCATGTATGTCTGAGATCCCAGCACTGGGGAAACAAGGCAGGAGTAGATCAAGACTATAGCAAATTTGAGGCCAGCCTGGATTTCATGAGTCTCTGAAGGAGGAGGAAGAAGAGGAGGAAGAAGAGGAGGAAGAGGAGGAGGACAGAGGGAAATTTTCAGGTAGTCTGTCTTCACATGTAATCTCTTTCAGCTCTTCTCATATGCAGTGCTTTGTCACGCTGAAAGTAGGAATAGTGAAAGCCCTTCCCCCACAAGCCCTCTGTAAGGATGGGTGATTTGCCATTAACTTGCACTTAGCAACAAACAGAAGCAGCCAATGAGAGGCCCTTTTCGACCAGCTCTTAATCGCTTAATGTGCCTTTTGGAATGGAAATGTCTGGAGAAAGAAGACAGACAATGGCTAAAGACTGTGCCTTGTGGAGTGGGCATGTGGAAAGAATCATCATCCCTCAGAGGGAAATGACATACTGAAGGTGTCAGCAGAAAATATGGCTCTCTGTGGCCAGTGTCTGACATTCAATTAGCTGGGACACTTAGAAGCTGAGAAGCAAGATGTTTTTGACACTTATCAGGATACATCACCTGAACAATCTATTTATAGGGGACCTTCTTCAGAAGGACTTGACTGGTGAAGGATGTCACTATAGTGGACTCCATGGAAATTGCCTGAGACTCCTAGCCTTCTGGCTACTGAGGTGGTGTTTAAATGGATCAAATGGACCAAACTCCCATCCTCCTGATAGCCAACTATGAGGTTAAACATAATGGTGAATTTCTGGAGTGCCTCCAAGTTAAAAGTAGAGATTCCTGGGCAAGGTGACACAGCGCTGAAGAAGAGCACTTACTCAACAGCTGTGAGGACCTAGTGTGATGCCACTGCCTCTGAAGGGGAAAAACATTGAAATTCTTTGACTAAGGCTTCTGTTTATGGAAGAGTGCATCAGCCCCATTTGGATTTGGATCAGCACCATCTGGGTCTAGAACATTAGGCTTTAAGCTCTATCCAGACTCCCTGAATCTAGAGTCCTGTGAATGACACAGATGCTCACTCAAGTCTGAGAGCTACAAGACTGTCCTCTATTATGAGGGATCCTGACTTTGGCATCAAGGCTGGTCCCCACAGCAACAACCAAGGCAATAGAGAACCCCGGCTCTTTCTGTGTTCCTCTCAGGGCTGGAGAGATGGCTCAACAGTTAAGAGAACTGTCTGCTCTTCTAGAAGGCCCACTTTCAATCCCCGGCACCCACAGGGCAGCTCACACCTGTCTGTAACTCCAGCTTCAGGGCTCCTGACACCTTCGCACAATCATACATGCAGAAACAAGACCCAGAGACAGCAAACCAGTAGCATCTTGAAGTCACACTGCTCATTTGCAAAGCTGAGTGAGACCTGCCCTTCTCAGATCTGAATGCCACACTTTTTGCCTAATATCCTGCGGCCACCCTTCTGGCAGGCGACCTTATCACAAAGATCCATCCTGGCTACCGAGTGACATCTTTTCTGATACCACGATTCATGAATAACTGTGAAAGTATTGCTAATTTTGGCTTGTTTGCTCCTTGTACATCTAATGAAATGTAGGCAGAATGCTTGGAAGGAAAGTGAGATGAGGGGTCCTGTCCCCTCCTTCCACACTGTCGGATAACAAGTGAAGTCGCTCAGTAGATGATGCTTGATTCTGAGGTGGAGGCTCAGTGGTAGAGTGTTTGTCTCTCATGGAAGAGCCTTGGCTTTCATCCCAAAACTGAGAAATAAAACAGAACTAGAGTGACAGTGACTGAGCACCAAAGATGACCACGGGGGACAAGGGCTGATAGATTCAACAAATGTGCACGCTCCTTAACTTCAGGGATTGTTCCTATAGTAGAGCAATTCAGTTACTCTGCTAATCGTCACAAGTGGCCCCGAGAAATGTGTCCAGCTCAGACGGAAGGGTTTACAAAGAAAGCCACTATCTTCTTACCAACCCTGGTCTTCCAGGACAAGGACTAAATAGAAGCAGGGGAGAGCAATTCTTGGTACTTCTCTAAGAGAAGAATCTAGCATGACAAGAATGGAGCCATCACTCCAGACAGCAGAGAAGCCTAGAAAGGAAATTATCCTAAGGAAACAATCTCACGCAGGTTTGGTACCCAGTCTGTTTCTACTTTGAGTTTTTGTCTTCCATTCCTCTGAGTTATGGAAAACATCCCACAGGTAAGATGGTTCCCCGTCGGCTTCTATGCACATATGGTCACAGCCAACGCCTCTGTTGATCTCACATGTCCTTACTCTATGGGGTGCAGGGTCTTTACCAGGTATCCTCAGTCTCCCACCTTGTCTTATCTCTGTAGGCTTTGTTATCTCAGAAAATTCTCTTTCACTATCTCCAAACATCCCAGACCCTGTATAAGTTTGAAAGTATCCATCGTCTCCACCATGCCTGAGTTCCCATTGGCTCCACTTCCTATCATTCACCTTCTCAGGGACACGCTTTTGATAGCAAGAGCCCATTGCCTTGTCTTAGGGACTAAGGCATCCAGAAGGTGGATCTAAATTTACACTGGAGCCCTTGGAAAGTACAGGGAAGAAAGGCATCTGTGGTTTTAAAATAAATCTACAACTTACAAATCATGCAATTTCATCCCAAAATATTTTTGGTCTCATACACTAATATTTGAATAAGTCACCAGGCTACATCTATTAATAGTGAGCACTAGAGAAACTTTTAATCTCCTACTAGAGAGAGTATCCAC

MSTRG.51025.6	ENSRNOG00000033196;ENSRNOG00000040249;ENSRNOG00000051678	0.029	0.431	0.000	4.263	up	GGCTAGCAAAAAAAGACCTGCTCTCTCGCTTCTCTCAGGGCCTTGTGGAGAGGAGCATATGAAGTGAGGGGCAGTGACTGAATTGTTATGGATGAAACCTTCTCCAGGAGGATGAAGATGAATCCTGCTCCATCTCGCCTCACCCTTATGCTTCTCTACCTCAGCCCAGAGGCCTGGATACATTCTCTTCACTGCTCTGCAACATTGTTCTTTCATCCTGTGGGTAGCAGTTGAAGCCTACATTTCTTGCTCTGCAGTTAGTCAAGCTTCCGTTTAGCCAAGGAGATACATGGCAGCGCACACACTATCAAGAAGCCTAGAATCCAAGCCAGAGATCTTATATTCTACCATGAAAGCCGGAGGACGGTTTTTTCTGGTATGAAAGACAAAGCTTTCCAACATTAACACTGACTCCAGTAATACCTGGGAGAAAATACAAAATATATACCCAGAAGTCATATTGCTAAGGAGAAGAATCGGTTGGCCAGCAGAAGGCAGAACAGAGCCTGGAAGAAGAGGATTCTCAACTAAGATTAAGCTTGTGGGGAGGAAGGGGGACTGGAGAAATTTCTTAAATCCCTGCTCAGAATTCCAATGAATTTACCAAGTTAGTGGATCCTCCCTGAGTCAAGATGCTGCTGGGTGAAGAGTTGGCTATTCTGGGTCCCTTCTGAGCATCCTTGATGTTGAGCAGATGAATTTTTAAGAATGAAGCAGAGCTGGTTCAGTTGGTAGAGTGCTTGCCTAGCACACATGAAGCCCTAGGTCCAATCCCCAGCACCGTATAAACAGAGCATGTATGTCTGAGATCCCAGCACTGGGGAAACAAGGCAGGAGTAGATCAAGACTATAGCAAATTTGAGGCCAGCCTGGATTTCATGAGTCTCTGAAGGAGGAGGAAGAAGAGGAGGAAGAAGAGGAGGAAGAGGAGGAGGACAGAGGGAAATTTTCAGGTAGTCTGTCTTCACATGTAATCTCTTTCAGCTCTTCTCATATGCAGTGCTTTGTCACGCTGAAAGTAGGAATAGTGAAAGCCCTTCCCCCACAAGCCCTCTGTAAGGATGGGTGATTTGCCATTAACTTGCACTTAGCAACAAACAGAAGCAGCCAATGAGAGGCCCTTTTCGACCAGCTCTTAATCGCTTAATGTGCCTTTTGGAATGGAAATGTCTGGAGAAAGAAGACAGACAATGGCTAAAGACTGTGCCTTGTGGAGTGGGCATGTGGAAAGAATCATCATCCCTCAGAGGGAAATGACATACTGAAGGTGTCAGCAGAAAATATGGCTCTCTGTGGCCAGTGTCTGACATTCAATTAGCTGGGACACTTAGAAGCTGAGAAGCAAGATGTTTTTGACACTTATCAGGATACATCACCTGAACAATCTATTTATAGGGGACCTTCTTCAGAAGGACTTGACTGGTGAAGGATGTCACTATAGTGGACTCCATGGAAATTGCCTGAGACTCCTAGCCTTCTGGCTACTGAGGTGGTGTTTAAATGGATCAAATGGACCAAACTCCCATCCTCCTGATAGCCAACTATGAGGTTAAACATAATGGTGAATTTCTGGAGACTGCTGTTGCTCCAGACAGAAACCTTGATCCTCCATCATTGCCTTCTACTCAAAGGCTACCTGCCTTACCAGTAAGGATGCCTTGGTCTCATTGAATAAGGCTGCAAGCACACCCTTCTTCTCCACACTCTGCCTTCCTTTGGCTAATCTTCTCCAAGGCGGCCATCACTTCCACTGCTCTCTGACTTACTGTCTCTCCTCAACCAAGGCCTGTGAGGCTGATGAGGGCAAGACTTCATCTTTCTTGCTGTTCTCCTCACAGAGTCTGTACCAATGTCTGTTGATACATTTTAATGCTTGGCACTCAAATGTACACTATATGGTACTTTTAAGTTTTATGTGCATGTTGCTTTCCATAGGAAACATTCCATGATCCTGTAAGTCTAGATAAACATTTCTCCCATGATGCTATGTTCCTTTTCTGGTACTTCTCAAGCTGCATGATGAGAACAATACAATGCTGGGTCCAGTCACTTTCTTCACTGGTGAACCCTCCATACTTGCAAGGCACCATGTTGCTGAAACTCAAATGTTTGTTTTGTTGAATATTTTTCTATTCAGTCAAAGGCGTAGTTATACATGGTACCTTGTGAACTTTATTTAGGATATGTACTGTATGAAACAGGATCAGTCAGTTGTGATCTTTAAATTTCCATGTGTATATTACCATATTACTGCTACCTCTCAATAGTGAACCCAAATTATTTTCTATTCGGTGAGCATTTGGGGGGATTCTTTTTCCAGGGGGGAATTGTTCCGTTTGGTCTGCCCAGTGTTTACTGGGATGAAAGCATCAGTGGTAAGACTGAAATTGGGGGCTGATAACCATGCACCAGACCATGAACTAAGTGTGACC
MSTRG.51167.1	ENSRNOG00000017444	0.141	0.618	0.001	2.760	up	ATTGCAGCCTGTGGACTCGGTCTGGTCATTGGGGCTTGAGGATCTCCCTCCTGAGAGAAGATTCTCTCTGAGAGTCTTACAGGTGAACATGATGATTGCCAAGGTAGAGAAGGAAACAGAGGCAAAGTGTCCCAACTTTCCAGGACCAGAGATGACAACACCTTTCAACATCATAAAAGAAAAAGAAACACAAGTTCTCTCTGAAGACAGCCCTAGAGCTCCCATCTCTGCTTCTGCCCAGCTTCTCCCTGATACAGATACAGAAGAAGACAGAATTCAGTAAATGTTCCCCACCACTTCCTGAGATGAAGAGTTTAGAGAGCAAAATAGAACTCCACTCTG

							AAGCAGTTACTTGCACAACTTGAGCATCAGGTTCTGAGTTGGAAGCCTTAGCACTCAGTACCAGGACACCAGCATTTTGAGTACATGTTGCCCAGATCTGCCCATATCATATCTGACTATTTAGTTTCTCACACTCTGAGTAGGCACTTGATGTGTATGAAAAGTTGAGAAAAGTCTGGGGTTGTTATCACTGGCGACATCCTCGTGACATTCAGGTTTAGGGCTGATTCCCAGCCCACTGAGAAAAACAAGTGCTCTCAGTCATTGTATTAGCATCTCACCTGTGAGCTATATCTTCAAGTGCAAAGATTTCAGCTATCTAAAATGCAGAGGTATTCTATCTAACTCCCCCTATCCTGAACAGCATTCCCAACCTGACAACATGCCGCTGGGGGAAACTGAAGAGCTGAATCAATGAACACGGAGAGACCAGCTGGGCTCCATGAGGACTGCAAAGGCAACGTGGAGATGTGTGCAGATTTGGTGCTCGCTTTGGAATAGAAGGCATTTCTGTACTTCATGGCAGAGGCATTGGCAGTGAAAGTACAGAAATTATCATGCTTGTTTTTATTTCTGTTCCCATGGAAACTAAAATCGTCTCCTACCAAGTTGAATGAGGTATTTGGAAATAATCAGTTTCAATTTTTCATGATAGCTAAGTATTGCTTTCCTGTTCTCGGCTTACTGGCTTACTTTCATGGAAGAGGGGGACTGACATTGTCTGAGGGCACAAAGACCACAGAAATGATGATTATCTGAAGAGGCATGATCCCAAGGCCCAGATGAGGGTCGAGGCTGAGGCTGCCACTGGCCAGTGCTCACTCACCTCGGCCCTGGAGAGCTCTGAGAGCTCCTGGAGCCATATTAAAGTGTAGTTTACTGGAAGTAGCTGGGTAGTGTGGAATTATGAAGTCATTATTGCTGGGAGAATATAGTAAGTTTTCTCATTTTAAAAGGAAATGTTGGTTTTCTTTCATTTTCACGGAATCCCCTACAGGCTACACTTTAATGTTTAACACCACTTCTCTCAATTAAATGTAAATCCACAAACACGAATTCACTAAGAGTCCAGTGTCTGGTGAGCCCACGGAAGGTCAAGGGTCTTGCTTGTCCTCATTGTGATGCTATGTCTGGGATGCTCACAGCACCAGCAGGAACTTGATCCACCACACACCTGTGGCTGGAATACATCTAATGCCAATGAACTCGTGGTTCTAATTCCTTTCATTCTCAAAGGCTGAGTGGCCCTGTGCTCAGGCAGGAGACTGGGCCAATTATGGAAAAATCAGAAGCTCCCAATCACTCCACTGCCCTTCATGAAGAGTTCTAAGACAGCCATAAACCAGAAGCCAGCGCCTCTGACTCCTCCTGGGCCAACAGTGTCAGCAGCATGTGGTGTTTCTGTGGAAGAATGACTCTCTCTAATGAGAATTTTGTTTCTTCTGTAACAAAATAAAAGATTGAGTCCAGGTTCCCTTTGGTTCTAATGTTTTACATGTAGTAAATGCACTTGATTGAAGAGGAGTTCAGGTTATGTTACTGCATATGAAAACTGGATATTTGTGGTAAAAAAATTTAAAAAGCCGTACAACTGGTTCCCGCTTGGACGGCTCCAAGCTCCCTCTGACCTGGGCGGTCTGCGAGCCGTTCCAGCTGGGCACCCTGCCCTCATTCACAGCTCCATGCCATTTCACACTGCCA
MSTRG.5149.2	ENSRNOG00000034226;ENSRNOG00000042133;ENSRNOG00000047234;ENSRNOG00000058463;ENSRNOG0000005	0.591	0.000	0.000	-7.919	down	CCAGCAACAAAACTCCATTTCATAATGTACACACGACATTCTCACTACCAAAATACTGCTTTACGTGTACAGTCTTTCTGGATTCCACATTTAGAACACCAGACACACTGCACAAACAGAAGTAGGAGCCTGGCAATCAGGAAATTTTTCAAAGGAGGCAGATGTGGAAATGCATGGATAACTGGGTAGTGAGTCTCTTATGGCCAAGTTTAAATATCTGTAAGCATCTTTTTAAAATATGTATATGCACATATATGGACATAGTATGTGTGTGTGTGTGTGTGTGTGTGTGTGTGTGTGTGTGTGTGTGTGAGAGAGAGAGAGAGAGAGAGAGAGAGAGATAAAATGTTATATTTTGAGATGTTTTTAATTTAACTACCAAGGGAAAAAAAATCTAAACAAGAGAAAATCCATATTCAAAGCTGAATATACCTAGCTGAGTTTTAAAAACTGACTTTTCTGTAAAGGTAACACCAACTTAATTTCTTCTGCCAGGGAACTCACGCTAATTTCTTATAATAGTGAATGAATAAGAATAAGACAGGGACTTGATTATGTAGGCGAGTCTCCAAAAACAAGCAAACAATAAAAGGTATTTTCATCATTTTGTTGACCTCCACAAGAGTAAGGTCCCTATGAAAGAACTGATGCCAGCTTTGTCTTGTTTTGTTAAGTATTAAAGTGTGCAAACTTATACCTAAAAAGTCACTGTAATATTTTTATAAAAAGTTACTTGGTAGAACAGTACTAAAATAATTAACCTAGGAAATTCTTTAAGAACTACACATTTATAAATGTTTACACAATAATACAGCAATAATGCCACAGTAAAGAAAAGGGAGGCTGACCTGACCCACCAGTTTAAACAAATTATCATTAACAACCCAATCTGTACACTTAGGTACAGAACTAAGATTTATAACAAGATGTCATATGAGAGTCCACTCAAACACTCCAATGACCTCTTTCTGAGGAAAGGAAGCCTGCTCTCTGAGATGAAGTAACTCATCCTGGAAGTGACCACATGAATCAGCTTTAAGCTACAACTACCTGACTCTAAGCGCACCATCCCTTGGACAAGCCTGCCTCCAACCTGGCACAGTGTAATG

	9588;ENSRNOG00000060573						GTGGCAAGAAGGGCTATTGCACCTGCAGTCACTACTGCCACTTCTCTGCGTCCTCCTCAGCATTGTACTCACACATTCTGTCATAAAAAGGTCAAAAATTATGCGTGATGAATTGTAAGGTCATGAGACTGTGCCAAAAAAAAAAAAATGGCTTTCCTCAGAAACTACTTTTTAAATGTTAGTCTGTAAAGAATGAAGGTTTAACATGCAGTGGGTGAGCGCTCCTGTGCTTATTTGCGTTTGTGCTATTTATTTTGATAATGGACCACAAGAGGGCATCGACCCAGTCAATCTCCAAGCGCAGAAAAGTAGCCAGACAAAGTTGGACTGATAGAGTGCACCTACAAACTTATCTGCACTGCTAACCTCTGATCCTTCTCTCAGGAAGGCTTTGCTTCACTTCTAAAACTGTTTATGCTACCCGTGACCCCACAGTCTTCCTACTCGGGTTACTGTCTCAAGAGCAGTTTGTTTAAATTCATCAGGTCAGCTTCTCCTCCTGGACTGCAAGCTTCAAGACGAACCTTCCAAACAGGCTGCTCAAAGTTACCGCTGCACTGGAGATGTCTCAGCTGCTCCTCCAGAAAACCTGGGTTCTAATTCCAGCACTGACAAGACAGTTCACAACTGTCTGTAATTCCAGTCTGTAATGCCCTCAAACATACATAAATATAAACCAAAAAAGAAAATGTAGATGAAATAAAAATCATTTTAAAAAAGCCAACTCCACTTTAGACATGTACCCGAAGTGAAGCTACCACAACTCTCTAAATCAGTTACACCACAGCACTTAAACAGTTCAATGCTATTTTTAGCTCAGATAAAGAAAAAGGGAGTAAAGGTTAACCCCAATTTTTGCACAGTTCTCTAACCCAGGCATGAAGAGTTCTCCCTCTCCTTTCTCTCTGATGTCTGCCTCACTCGCTCAGTTCTTTACTCGTGTTCCCTAACACACACCTTCTATGGGAAACTCGAGAATGTTACATGTACAATCTAAGCATTTCTAAGAATAAAGGCCTGTGTTCCTAGGGTTATTTAAATCAGGTTTATAGGAAACAACTACTAGCTGAGAGCTCCAGGGGGGAGAGGTCACATCCACAGAGGCTGTGTGGTGCAACAGTTCTTTTAAAAGAAGTATGGGGGCTGGGGATTTAGCTCGGTGGTAGAGCGCTTGCCTAGGAAGCGCAAGGCCCTGGGTTCGGTCCCCAGCTCCGGAAAAAAAAGAACAAAAAAAAAAAAAAAAAAAAAGAAGTATGGCTCTCCTTGTAGTGGTGGCCTCCCAGCACTCTGGAGGCAGAGGCAGGATTTCTGAGTTTGAGACCAGCCTGGACTATGGAGCGAGTTCCAGGACAGCCAGGGCTACACAGGGAAATTTTGTCTCAAAAACAAAAAGCCAAAAGCTTCAGAAATAGATTTTATTCCAACTTAAGAGTCTGATGTGTTTTAGGGTATACAGATCTAACTTGTTAAAAACAGTTGCCGCGCATGAATGGGGCCCAAGATGCAATCCCCAGCCCAACAATGAGGACACACACCTATAATCCCAGCACTGGGAAGAACAAAATCAAAAATTCAGGGTCATCTTCAGCTATAACTAATCTTTCATACATAAGACCCTGTCTCCAAAACAGAAAAGAATTTGATACATTTTAGCACTCAGTACATATACCCTGTTATGGAATACCCCTATAAATATAATATTTGCAATGAAACCTGATTACTGGGTGGCCCATTATGTCAATGTGTGGTCATTTCACAAGTAACTTTGTTCTCAGTACTTTACAGGCTTTATGACTACAGAGGGTTAAGCTATATTTCTGGATCGGAGGGCTGCCTGGACAGCTTGCAGACCTGTTTGACTAGGAGCACTGATCATTTACTGTCCACTTGCATATCTCTGGGAACACTACTCTGTTTCCACTAAGAAGAGACCTTACAGAACTCCTTTTGAGCAGCTACAGCCCCCAGAGTGGAGAAGGCCCATGCCTGCTGCTACCCAACCCAACTGATCCATTTTGGATATAAGGACCAAGGTACTCAGAAGAGATGAGGAGCTCCAAGATAGGAGCCACCTGAGACAGCTGGAAACAAAATAAACATGCAAGCAAGCAAAACTAACACACACACACACACACACACACACACACACACACACACCAAGTCAATTCTTGTAGAAATTCCTTTCAAGCATTTCTGTGGCTTACAATTGACTTAAGAAAGACACTGTGGTGAAACTTGTCACATGGATTAGGATAACATGGCTGTGACATTCAAAACAATTCATTAAACAGTGAATACAAATAGTTAAAACAAAGGTGGAGGGTAGCTCACTAGTAATACAAAAAAACAAAACTAAATCTGAAATTCCATTCTGCACTTAAAAGATACACCATTGTCAATCGAACTGTACAAAAATTAAGTTAGCTATACAAGTTCAAAAATCACTGAAAACTGGAAAAACCAGGTAAGTTTAAAAAGAAAAAAAGGAATCCGACTTTTAAAGGCAGCCAAGAGTTGTCAAAGTAACAGAAAAAAGACTAGGAATTTAAAAGGAACTAGGAAAAGAGAGCTGATCTCCAAAGCTGTAGTTTCTCAGGCAATTCTGTACCTAGTCTGAGCATCGCTGCAGGCCCTAAGCAGACAACCACAGCGGAGGAAAGAAGCAGGTAGGAGTGTCTGACAAAGAAGAAAACAAAACGAGAGTGTATAAGTATATGGCTAGCTTCTATCAAAGACACCTGTCAAATCCTAAAGCTAAAAAGGCCAAAAAGCTAGACCAAAACTTTGGGAAGAGGTTGGAATAAAACAAAAACTAAAGAGT

							CCAGAAGCTTCCAAAAACTGTAAACAAGCAGTGCTCTCAGACAAAGTCCAGAAAGAACCAAAAATAG<br>CAGAGCTCACTAAAGGAGGCACTTGGCCCTTACTCCATTCAGCACCTGCCAGCAGTAGGCTAACCTTG<br>GCCAGCACACACCCTGCCTATCTAAAGGCAAGTGAGGCCTCTCCCAGAAGACACCTTCTTCAAACACA<br>TCTACTATCCCTGCATTTAACCCCAAAAGACTACAATGCCACAAAACAGAAGCACATGACCAAACACA<br>GGTAAAAAGAAAAGAAACAAAACAAAAAGGTTATACAATCTGGACACTGGTGTCAATAAACTAGGCCA<br>TTAAAATTACAATGCACATGTATCAGAGGATACAAGAAAAGATATGAAAAGACAGACAATTTTCACTGG<br>AAACCTCTTTTTAAAAAAGAGATTTTTCAAATAAAAATGCTCTGGCTGTGACTCTAGCTCAGTGGCAGA<br>ACACCTGCCAAGCAAGTGCAAAGCCCTAAAAATCCATTCACCAGACAGAAAAAGAAGGGAGGAAGGG<br>ATAGAGACAGGCAGTCAAAAAGAAGAAAAAAACCACACAACTGAGGTTTCTAAAATTACCACAACCA<br>AGGCTAGACGACACTGAGCACAGCATAAGTCCGTATTCTTACCATCTGGGCACAGATGCAGGTAGCAGT<br>TTAGGTGTAACTGGAGGTGAGAGGCAGAAAGATGGCACGCAGTCCTCAAAGAAGCAACCAAAGTTAC<br>AGAGCTAACGTCAGCACAGAATGCCACACACTGAGACAACGGAGTAAAACCTTAAAGTCCTGAAACA<br>AATAACTGAGGAATCATTAGTATATCCACAGCAAACGCAGCCTCCACCATGAGAGAAAAACAAAGATTT<br>TCAACAGATGACCGAGAATCCCACACCAGATTTTCATTCTTCATTAAAGAAATAGACAGGCTTTCTTCA<br>GCAGCACACATGCAGAGAGAACTGCAGAGCAGCTGGGACAGCGCAGGTCACAGGGGTGGGTGTCAA<br>GATGAGTACTAGCCATGTACAACAACTAAATACAGCCTTACAAAATGGCCTGAAGCAATAATCAAGATG<br>TGGGTGGGAGCCGAGGAAGCCTTGCACCTTTAATACCAGCACTAAGAAGGCAGAAGCAGGTGGATATC<br>TCTGAGTTCTAGGCAAGCCAGAAGTATATATATATAGTAAACCCAAATCTCATCAATCACTGAGGAGGAG<br>GAGGAGGAAAGAAAGAAAAAGAATGAATGCATGCTGAACCTGGATGATACAAGCCTAGTCTAAATTCG<br>TAACACACTGAGTATTAAGGTACACACAGTGTAGTACACATGTAGTTCAATATCCCATCATAAAGATGTT<br>AATAGGCAGAAGTAACAATAATGTTATGTTTGATTAATCCAGAGATGGACAGAAAGGGGGGAATCGGCA<br>GAGAAACAATAAGAAAGTAGGTAAAACAGAAGGACAGCGAGGTAGCATCTAACACACATCAGGCTGC<br>CCATCATTTAAACGGAAAATAACAAGTATTATTTGTACACAGAGAAGCTGGAAGCACTGGGAACTGCTG<br>CTGGGAAGGTAAAAGTGTGGAAAGTCATGCAGCAGTTCCTCAAACACTAAAAAGTACTTACAAATTGA<br>TCCAGATTTTAACCTGACTACACCCAAAGCAGTCAAAGCAAACAGCACAAAAGTTTGCACACATATATA<br>TATCTACAGCAGCATTATTCATAACTTGAAAATGAAGGCAGTGTGTCCACTTACAGATAAATACTGTATA<br>AAACAATTTTAATACACTGTTGAATACAATAAAATATTGCCCTTAAAAAGGAGTAATTCTAGACCTGGGT<br>GGCAGTGATATGCTGAGGCAGGAGCATGAGTCCTAAGCTATTCTAAGACATACAGACAAACATGCCTGG<br>AGATTGGGGGACTGCCTCCCCAGATCCTGTTTTAGAATCCAGACAGGACTGGAATGATGGTTCAATGGA<br>TAAAAGCACTTGCCATATAATCATGAGAACCTGAACTTGAATGCCTAGAATCCACATAAAATACACACAC<br>ACACACACACACACACACACACACACACACACACACACACATGCAATCCTAGGTTCTTACGAGAAACA<br>TGGCAGACAGGAGAATCCCTAGAAACCCAATGGCCAGATAGATAATGTATGCAGCAGCCCATAACAGAC<br>AGACACTGTCTCAAACAAGCAGATGGCAAGGGCTGACATTTGAAGTTGTCCTGACCTCCACACCATGG<br>CATATGCAAAGCCATATTCATAAAACACATAGATGCATAGAAGTGCACACACAAGCATAGTGCACACAC<br>ATATATCACACACACACAATAAACAGGGGAATATGATAGATGTTACAACATGAATGCTTTGAACATATTA<br>CACTAAGTCAAACAAGCCAGTAAAAACAGGGCAAAACTATATGACTGCTTACATGAGCTACCTGGATTA<br>GTCAAATTCAGAGAAAAAGCAGAAGGTGTTTGTTGATTGATAGGTAGGATAGAAAACAGAGCTTTTGT<br>TTAATACCACAGTCTAGCTTTGCAAGATAAAAAAAAGTCTTAAAGACAGGGTTATGTCTTTTTTACAACA<br>AATTTTAAAATATAAAGTAAAATCAAATAGAAAAGTGATAAATTTCTATAGTATTTAGACATAACTGCTCT<br>AAAAGAAGGTTCAACTGAAAATTAAAACCACAACTACATGATATTTATAAGAAACATACTTCAAACATA<br>AATACTAAAGTTAAAAATGACAGATCATAAAATGCATACTAATATCTAGTAAAGGTGACTTGAGGACAAG

							AATATCTTCAAAGATGATAAAAATAAAAATCCAAAATGGTAAAGAAGTCAACTGACCAGAAGTTATCTTAAGCTGTACATAGTGAACATCACAGCCTTATACCCATACTGAAATCTAATACAGATCTCTTGGCAGCCCGAGTACTAAAAAAGGATGGCCATAAAGTACTTGAACAACACATTCAACCAAATGGAGCTAGCAGAAGAAATGTTGAACTCAAGAACTAAAGATTAAATTTATTTGCAAAAAAAGAAAAAATGAAATATTTACTAATAAATATCAGTCATAAAGCAAGAATCAAATGTCCATTTTCCAATTACATGTTCTTTGATTACAGTAAAATTTAACATGGGTATACGCAACAGAAAATACTCAATATTTTTAAGTTAAAGAATATATGTCTAAATAACTCACAGGTCAATAAACACCTCACAACAGATATTAAAAAACAGTTTTGGGGCTGGAGAGATGGCTCAGTGGTTAAGAGCACTGACTGCTCTTCCAGAAGTCCTGAGTTCAATTCCTAGCAACCACATGGTGGCTCACAACCATCTGTAATTTGATATAGTGCCCTCTTCTGGTGTGTCTGAAGACAGCTACAGTATACATTGATTATTAATTAATTAATTAGTTTTAACTGACTATAAAAACAACCTAAAATATATGTAAGGTACCTAGTACAGCAATGATAAGAAAAACTCAAAATGCAAAATACATAAGAAAAAAATGAATGTTAGTAATCTTTTAAAACAAGGGGAAAAAACTTAAAGGGGGAAAAAAAAACAAAGCAGGATCAATGAAGAAAAAAATCATATTTACAAAAGAGAAAAATCAACCAAGCCAAAAGTTGATCCTTGAGACAATTTCATTGCCACATAAACTCCTAGCTAGGAAGACAAAGCAAATTATCAATAACAGCATTTTTAAAAAACTGTTTTCAAACTACTGGTCTTAAATTATATATATTATATATATAATTATATAAATGTGAATATATTTTATATGCATACACACACATATATATACATATATATGTATGTATATATATATATA
MSTRG.51824.5	--	1.219	0.041	0.000	-3.763	down	CGTATTTACAGGTTTGAGTGTAGGAAAGTGTGTGGTGGCAGGAGACGCTGGGCGGCCGCGGAGTTGGGATGCGTTTCTGCCAAGATATCTAGCAGATATCTTTGAGCACCGCGGTGCGGACCTAGCAAGTTCCTAAACTTTTACTTCTGTACCCAGTTGATAACTGTTCTGGCAGCTTTCCATTCAGCTGACAGACACCATTCCTGAATCAGCTGCGGATGACCGAAGGCTGCACGTCCTGGATTTGCTGAAAGCTGAGGAGAGCCAGTTCAGCCAATGAGATTGGTCGCTGCTTCTTGAAGAGAAAAAGTAAACTCTCCATGAGTGTCTCACAGTTCCTGGCCACCACGTCAGGAGAAGGCCCTGAACTGAAAACTAAAGCAAGAAACCCTGACTGGTTCCCATTCTCACTCTAAGGTTCCTCTTAACCAGTAACTGCTGTCAGCCATTGCAGGGCCCTTAGTTAGCTCTCCTACTCCTGCTGATTATGGTTGGCCCCTGCGTCATTAATGCCTTGACCAGATATATACATTCCTGATTGGGAGCTACTAAGTAGATGGTAATTTAAGTTCCAATATGAATAGGCACCCACCACAGAGTCAAAGGTTTGACTCAGGATACAGCTTGTCTTAGTCAGGGTTTCTACTGCTACAATGAAACACCGTGACTGAAAAGCAGATCGCAGCACAGGGCTTATGCAGCCTACACTTTCTCAGTATTGTTCATCATTAAAGGAAGTCAGGACAGGAACTGAAACAGGACAGAAACCTGGAAGAAGGAGCTAATGCAGAAGTATGGAGGGGGTGCTGCTTACTGGCTTGCTTCCCTGGCTTGAACAGTTTACTTTCTTAAAGAACCCAGGACCACCAGTCCAGGGGTGGAAACACCCACAGTGGGCTGGATCCTCCCCATTAATCACTAAGAAAATTGCCTTATAGCTTGATCTTATGGAGCCATTTATCTCAGTTGAGGTTCCCTCCTTTTAGATAACTGGGATGCCAAGTTCATATAAGACTAGCCAACACCCAACTCAAGGGGGAAAGTCATGGCTAACATGTGGATGGGTAACTTTATATTTAAAAGTTTCAGTTTACTATGTTTAAGGTAACTATAAAGCAAAACTGGATTTTCGAATGTTAAGGGCTGTTCAGGATTAATTTTGTAACTGTCTGGTGGACTCTTACAGCTTTAATAAAACCACTTTCTACTTCTACAGAAAAGTTTGCTTTGCAGGAGCTGGCCTCTGCTGGTAGTCAGTGTGTAACTCTGTGTTCTTTGCTTTTAAAAGTCCCTGAGGTCAGGCTGGGTGCTGTGTAGACCAGGAGCCAGTATAGGAGGAATCCCCAGTCCCTGTGTAGACCAGGAGCCAGTATAGGAGGAAAACCTCTTCTTTGTTAAAATTTATCAATGGTCAGACTGGTGAGTCTTTGAAAGACCCCAGACTCATAACAATGCTGTGACCACTGACCACACAACAGTCATTTCTTGTGAACATCAGCTTCGTCCCAGCTTGGACCAAGGCAATGGCTCTTTCAAAGAAGACTCCATCCTTACATCTAATTACAAAAGTCCGCAAAGCCCTTCCGCATCATTTCTGAACTCTGC

MSTRG.51979.2	--	0.000	0.484	0.007	3.835	up	ATCCAGTGTTGAGCTCATTTTCCCTCTTACACAACCCAAGACTCAAACCCAGTGAATGGTGTGGCAAGGCCACCTTCCACAGACATGCCCACAGGCCAGCCAGATGGCCACCTTCCACAGACATGCCCACAGGCCAGCCAGATGGCCACCTTCCACAGACATGCCCACAGGCCAGCCAGATGGCCACCTTCCACAGACATGCCCACAGGCCAGCCAGATATGGACAGTATTTCACCGGGACTCTCTCCACAGGTGGATCTAGTTTGTGTCAAGTTGACAATTAAAACTCAC
MSTRG.52138.1	ENSRNOG00000018972;ENSRNOG00000026376	0.000	0.336	0.002	4.137	up	CAGGTTAATTTTATTTGTTTATTAATTTTATTTGGCAGTTCTCAGCCAGCCAGTGAGCAAAGGCAGCAGCTCCTCTTATCGTAGTCTTCCTCCAATTTTTCAAGTGTTTGTGAGTTCTAATTCCAAGATCAACGACCACCGTGGGTTGTTTCTAATTACCCTTGAATTTCTTTTTCTTCATTATTCCATAAACAGAATTCAGATTTTTGTATTGCTGATTGCACCATCATCACGGAATGTTGACTTGGGGAGAACACGGGTAATGCTCTCGAGCTGGACGTCACTGTTGGGGACCCTCCGGTGTTTCTCCTCCATGCTCAGCATTGACAGCACAGCTCTCCAGTTCTTCATCAACATCACCTTGCTCACAATTCCATGCTATTCCTGCACTAGTTCAGGGACGTCAAAGCAGAAATGTAAACACTTCTGTCTTAGTCAGTGTTCATCACCAAGCTCAAGAAACAAAATTCCAGCAGTCTTCACCCTAGAAATCTCCACTGTGGGGCTTTCCTCCACAGGTGAGTAGTGG
MSTRG.52152.1	ENSRNOG00000018972	0.203	0.725	0.035	2.374	up	CTAAAGTACGGGGTCACAGGCTTCTTTGGAACAGGAAGTGTGGTGTCAGGTACATGGACATGTCCTTCTAAGAATTATTTCAGAATGAAGGAAGAATGGGACACAGTCCCAGGGCAGCAAGACTAGCAAGAATGGACCTGGAAACAGGTTCCAGCCAGCTGGCTTACCTCAGGGCACTATTAGCTCAGTACACAGATGACGGCAGTTAGGGCGTCTGAGTACAGGAGAAGCTGGTAAGGTCCCAGATCTCTTCTGATGTGTCTCTGCCAATGCTTCTTGATGCACTGGGATTACAGGTCCATGCTATAACACTCAGGAGGCATCACTGGGGCAAGAGATGCGATGTGCTACTGGGCTGACTGGCTAAAGGTCTCCATCTAAGACTGCCTCAAAGGACTAACGTTTGTCCACCGCAACAGCTGCCTAGAGG
MSTRG.52167.2	--	0.816	7.273	0.000	3.911	up	ATAAATTACATGTCAGCCAAGCTCTCTCTCTCGGCTGCTGGCTTGGCATTGTTCAAGGTTCTTTTGAGGGACAAGGGACTCTGATTTCCAGATAAAACAACAGCAACACAAGAAAAATAATAAATTCAAAATGATTGCAATTGGAGACATTGCCCAGAATATGCTAATTACCTATTTTTCATGTGTGCATAGGCCAGACTCCAAGCTTTGTTACTTTACAGAAATTATGGGATAGTATCCTCTCATTGGTTGTGTTCATAATACAGAACAAATTAGCTGAATTTTCCCCCAAAGGACAGAAAAAAATAAAACATGACAATTTCAAATAAAATACAACCCAGAGTTCACTTATCTAATTGCAACATGTTAAAACAAGAACATTTTTATTAAAATATTTAATACCAGAATAACTTATAATAGTATGAAAAGGAGTGAAGTATTCATTCTTGTTTCTCACTGCCTCGCATACATCTGGGCAGGCTTGTTTTGTAGTGACTCCCACTTCGCATCCCAGTATCAAGGATGCACCAGAAGAAGCCAATACTTCATCCACTCATACAGTCAGCACTTACACATCTCACCTAGGGCTTACTGCAACTGCCAACGGCACTTAAGAAAACAAAGTAAAAATATACATACAAAATATTTTCATATCAAATTATCTTCATTACACTAGATAACCCCCACCCCACTATGCTATGATACAGGAGAAAGTGTCCTACGATGGCCATCGAGGCCGTCGTGTCTGCTGAGCAACCAGATTAACCTTCAGGGTAAAAAGCGTTTTGTAAAATGAAGAAAAAAATGTCAGTGTGTGCGGTGAAACCAGATGTCATTCACACTTGCAAAAATGTTTTCCAGCCTAAACCAATTACAAAATATATCACATATCCAGAAAAAATGCCCTTCCCCCAAGAGCCTAGTTTGCCGTTAAACCTTTGCTCTCCTACATTAAGGCATCTCCAGATTTCCTTTTCTATTGGGCGTCTCCACATCCCTTCCGTTCGCAGTCTTCCGAAGCTGGGGAGGACGTTATTGTTTGATGGAAAAGACACTCTTCTTCCTTGATATCCATTACTCTCACTCTGTGGTAGTGACATTTAGAAATAAGACTTTCTTCCTTTCGACTCAAGATTTCACTTAATTTTTACTTGAATGTCCTGCTTGGCAAAAATCAGAGACCATTCATGTTTTGTTACCCATACGCTTTAAATTTTAAACATTAATCCTTCTGCATGTATATGTATATCCACACACACAGATTATTTTCTTCGTTGTCTTGTTCTCATTGCAATACAAATTGTCCATCTGAACTGGGATTTGAAATGAACCAAATAAGGGGCTGCGGATTTAGCTCAGTGGTAGAGCGCTTACCTAGGAAGCGCAAGGCCCTGGGTTC

							GGTCCCCAGCTCCGAAAAAAAAAAAAAAAATCCGTTAAAAAAAGAAATGAACCAAATAAAACGCTTTTAGTTACTCCATATGAAAGCTGCTTCCATCACAAAGGAACCCTGAGCTCTAGTGCAAACAAGATTATAATTTAAAACTAAACTTTAGGTTTAGTTTTCCTCAGGAAAAAATTCAAAGATTTATATAATAAGGTTCTTTTTAAAACCTCAGGAAAAAAAATCCCAATTAGTTGTAAAGGGCTTTGGGATACTTTCCAACAGTGCTTGGGAAAACAAATCCTCACGACAATATACTCTGGCAATGTTAAACCGATTTGAAAACATCCCTTCTTAAACATACGCATCACGTGGCCCTGCTGGGCCTTCCATTTTGGATGCGCTAGCCTATGCAAACAAAACCCACACATAAAACTCTCACCATCGCCCTGGAAAACACAAAGAACAGTTTCCAGTCCTGAAGGCATTAAAAGGCAAATAAAAGATCACCTCATCTGGGCTGAACAGATCTCCAAGTTTGAACGTGTGAATGACTGAGTTTGTTTCGGGAGCTTTCCGCGATTGGCTTCAGTCCTGGGCCAGTCTGGAGTGATGTAGTCTAGCAAGTATTGGCGTTGACAGTTTGAGGCACAGCGCTCCACTGTGCCCACGTTGGAGCTTACCTCAGGGAAGCAAGGCACTTACTGTAATGGCAATGCAGACGCTTTATGCATAGTTTCAGTAACATAAAATAAGTTAATATTTTTGACTAAAATGAATTTTTATAACTTATACATCTTTTTATAGAAGCAACGGATATATTTAGGATAGTTGAACTCAGGAGGTTTAAATGGGAGTCCTCTTCACCCCTGCTTTGGCTCTTAGGATGAGGATTACGGTAAAAAACAAACAACAAACAACAAACAACAAACAAACAAACAAACCCACTGACAAGCCCAGCACTCCCTGGCCACTCTAGAAGTGCTGCACCAGTGGCAGCTTGACTGTCTTTACTTCTGCTATGTGGGAAGACTTCTGGATGATGCAACTGGTGGTCGTCCCCTGCGCTTCGTCTTTTCCCTGGGCGAGGTTTGTTAAGGCCATGGCTGCGTTGATCTCCTTCCAGTGTGTGTCATCCTTGCTCGGCCCCCTTCTGTTAGCAGTGCTGTCACCCTTATCACCCTTAGTAGATCCAATGTCTGGGGTGTCTGT
MSTRG.52565.1	ENSRNOG00000042133;ENSRNOG00000047234;ENSRNOG00000059588;ENSRNOG00000060573	0.124	0.379	0.034	2.236	up	GTTGCCATTTCTGTTTGTGTGTGTGTGTGTGTGTGTGTGTGCTTGTTTGTTTGTCTGTATGTATGTTTCTCCATATATACACATGTACACACACACAAAACATACACACATGCAAATACAAACCCCAGGCTGATTTCTAATTCATGATGGAGTGAAACATGACTTTGAACTTGTGATTCCACTGACTCTGTCTCCCAAGTGTTTGTCATTCCATTGTACATGAGCTGGGGTTTGTGTATGTGTGTATGTATAAGTTTCGTCATATAAACTTTTATACATACACACAAAACATAAACACATGCACATATATGGGGGCAATCACATGAATCACAGGCTGATTTCAACGTCATCATGAATTGAAGCAAGAGTTTGAATTACTGATTCCACTGGCATTATCTCCTAAGAGTTTGCCTTTCAAATAGGTGTGGAGTTATTTGTGTGCATACAACCACACACACACACACACACACACACACATACACATACACACACACATCTATAAATGGACACACAAACATAGACATAAAAAAGCAAACACATGCACATACATACGGGTATCACATGAAAGCCAGGATATCAATCATGAATTGAAGCAAGAGTATGAACTACAATCCTCATGACGCTAACCCTCAAATGCTGGTCATTCGTGTTTGTATGTGCTTCTGTCTGTGTGTATGTAGGTATGTACATATATACTCACATACACACATAACATACACACAGACATATACACACATGTGCATATTTAGGGAAATCACATGAATCCCAGGCTTATCTCTAACTCATCCTGAAGTCAAGCATGAGTTAGATCACCTGATCCTCCTGTTTCTGTCTCCAAAGTGTTGGGCACTTGTGTGTGAGTGTTCTTGTTTGTGTGAGTATGTGTTTATGTATATGTATATTCATATACACAAAAGAACATACACACATATAGTCATATGCACATAACTAAGTCTTCCCATGAATCACAAAAGCCTTTGTCTGGACTCAAAATCCTCCCAACACTGACTCCCACATATTTGTCATTCATGTGTGTGTGTTTGCATGGTTGTGTGTGTGTGTATATATATGTATATTCATATAGACACATGTAGATCCCCACAACATACACACAAGCACATAAATATGGGCATCACATGAAAACCAGGCTGATTTGAAACTCAACATGAAGTGAAGCATGA
MSTRG.52720.3	--	1.355	3.361	0.043	2.006	up	TTTTTTTTTATTTATTTTATGTATGTGAGTACACTGTAGCTGTCTTCAGACACACCAGAAGGAGGCATCGGATCCCATTACAGATGGTGTGAGCCACCCACCGTCATTTTTAAAACCCTGTTGTTGGGGCTGGAGAGAGGGCTCAGCGGTTGAGAGTGGCTTGTTGCTCTCATACAGGAGTTCAGTTCCCAGCATCTTAGAGCCACCTCTTCCCCCAGGTCCAGAGAATCCAACACCTTTTCCTGGATTGTGTAGGTATCCATGTGCCAGAGCAAATGCACACAAACACACATGTCCTTCCATTTCCTCCTGGTGAGCAGATCTCTTTTCCCCCAGACCTGAAATTTCCTTTCCACTTGCTATCTTCATCAGCAATTAAACAAAGGATACC

MSTRG.52778.3	ENSRNOG00000018396;ENSRNOG00000018500	0.734	0.000	0.000	-6.948	down	GGGCGGGGAGCCGAGGGGAGGGAGGCTCGGAGGCTCGGAGCCCGGAACGCGGCGGCGCTCAGGAGGGAGCGGTAGCCATCTGTGTACGCGGAGGGCTGAGCAGCGCTTCCTCCCCTCGAGTGCTGAAATGGGAAGGCGTTCAAATTCCCAGCCTCCCTTCGGGACTCCACTTCCTGCCGCTAAAGTTGCTACCTTCATCCGGGCTCTACAAAGCATCTGCCTCAATACTCAGCAGCTTCAGCTAGAGACTGAGAGACAACAAATGCCATGGTTGAGAATGGGCCGGGTGACAGGAGCTTGCATGGGGAATAAAGGGTTAAGAGGCCGCCTAACATGGTGGCTCTTGCTTGTAAGCCCATAATGCAGAAGGCTGCAGCAAGAGGATCAGCCTCAGATCCATATCAAGACCCTGTCTCTAATAGTAAGGATTGCAGAAGTAGTAGTGAATTATGAGATCTCCAAAATTTAGAGAAACCATCAGGAAGGACTTTTACTAAAATGGTGTTTGCTGCCATCATTGGAACCCTAAGGGTTCCGGATAAGGGAGCCTTTGACCTTGCCTTCTTATGGTCTCAGGTCCTCTACTGTCCCATTCTGGTCCCAAGTGCCCTCATGTATAAAACCAAGAAGGGAAATGTTTAACCACAGAGAGAGCAAAGTGTCTAATAACTATTGTTTTTGTGCCCCGAATGTGACAGGGTGCTCTGTGAAACCGTAATGAGCAGGGGGGCCTCCTGGGCCCTGGCTCCTATTAGCCTACTAGGTCCACACATCCTGTGCGTGGCTGAGGCTGGGGCTGAGGAAGAGGCCTCGGCTCCACTGGACTTCCTCTCCAACCAGCACTTACAAGCGCTGTGGGATGGCATGTAGGGTAGCAGCAGGCAGGCTGTCAGGAGGGCGTAGAAATAACGACTTGAGAGGAAGCTCGGCCTAATGGCTCTGATTCTTGACCAGTACTTTAGGGAGCCTTGTCTTCCTCCTGTCCCCGAGGCTGAGTCATAGAGCCCCTAAATGACCTCCCTCTGAGTAACTTCCTGCTTCATCCAGAGGGACCATTATACAGGATCCAGTCAGCCATCTGGGTCTCTTCCTCCTTCCCCAGAGGAAGGTGCTCTGATGAAGAGAGACCAAGAGAGGTGCCAGAGGTTAAGCGTGTGATTGATGGTGGGCCCCGGGCTTGTGACTTCTTGTCCTGGTCTTTTTCTATTGTGCCACAGAGCCCCAATGACAAGAGCCCCAAGAGCAAGAACAAAGACACTTCAGGCCCTTCAGGAGACCCGTTCTGGCCATCCCACGGGACACGATGCTGTACATATTTCCAAACATGCGAACGTTCTCAAACAGAATGGCACAGACAGGAATAGTCTCTAAGCCCAGAACAGAACAGACAGACAGACAAACAAACAAACGAAAATTCCACTTCCTAATTTCACTTCTGCTTTTTCCTGAGACACTACCACACACAATTCCCTGGGTTTTATCTTTACGTTGGGCACATAGAAATGAAGTGTCAAATGGAGCTGCCACAGAAAAGTCCCAATGCTGTGCATGCAGGGGATTTGATCACGTGCATCCTGGGAAGAGTTATGGGGACAACAGAAACAGACATTTAAATGAGGAAGAACAGTTGGGACCTTGTGTCCATTTTAAATGCTTCCGTTAGTGTCCGCTATGTTTTAGTTTATTTATAAAGAGAAGCACAGGTGTTGTGGTTTAGGTCGGCCACAGATCGCCATCTATCTAATCTAAATTCGTTCATCCATTCACATTTGTGGATGTCCCCTTGGTACTGGCTGTACTGCTAGGCAAGAAGAATATGATATCTAACATGTATTTCTGCAGGTACCCCCCTCCACCCCACCATGTGTGCATCTGCTTTTCTTTCTGCCCTGGGATTGAAAATATTTGAAAAAAAATTATATGTACTGAATACAGACTGTTTTCTATCTTGTCATGATTCCCAAATCAATATCATACCATAACTACTTCCACAGCATTTCCAGTGTATTAGGCATTGCAAATCTGAGAGACGTGAGTTAGGGTTTTTGGGAACCTGAGCATGTTGGCTTACGTCTCTAATCCTAGCACTCAAGGGGCTGAGGCAGAAGGATTGCTGCCAGTTTGAATCCAGATTAGGATGCATAGTGTAGTGAGTCCCGGCCAGTCTGGGTTAAACAGTGAAACTCTGCTCCCAGAACTTAAGGAAATGGGAGTATAGAGTCTGTCATAATGGATCATAGGCAAATTCTGCATCATTTTATGTACGGGGTTTACCGACCCAAGGCTCTGGAACTAACCGATCTCCATGGACAATGAAGGCCGGCTTCCATTGCTACTCTCGGGTCACACTCCATCAGCAAAGGAATTCAGGGTAGGAACTCAAAGCAGGAATCTTGAGTAGAAGCCATGGGAGAATGCTGTCTACTGGTTCCCCACTGCCTTCCCCCACTAACAGGACCACGTTCCCGGGGGTAGGGGAGAGCACTGCCCATAAGGATCTAGGCTCTTCCCCATCCACCACTGATCAAGAAAATGCTCCACAGGTCACCTTGTACACTAGTCTGACAGAGGTATTACTTTGCCGAGGTTCCCTATTCTCGGATGATTCTAGCTTAAGTCACGTGGTCAAACAGCTAACCAACACAGAGACCAGTAGCCTCGGCCTGGTGTGGTCAAAGGTTCTCCCTTCAGGCCCTGAACTTGCAAAGCCTTCTAAATTCAAATCTCCAAAATGAGATACCCATTTATTCTGTGCATACGTAGGTCAGAGCGGGCACTGCAACTTCAAAGACCGTCTCAGTGCCGTTCTCCTGGATTCTTAAAATGGAG

							CATCTCGTACTGAATACTTCCTACTAATCGGTAATACAATGTTCATTAACAACAACAAAAAAAGCAAACACAAAAGCAAAACAAAAAGATAGACATTAGAAGCACAGGATCCAAAGCCATAGTCTCTACCAAGTATTTGCTGGCCTTGTATCTTTAGAAACTTAATTTCCTCATGTCTTTATTCGTGTTTAGAACTAACACTATGAATAGCAACACTTGCCTATAGGGCTGCTGACTTAATGCATGAATACATCCAGAGCTCTTAACTTGATGGCTGGAATCGCCCTCAGCATCCAGCAAGCACTCACTATTCTGATGTCTGTCCCTCGTCGCTCGTTACCCGTACCATCCC
MSTRG.52794.1	--	0.480	0.000	0.028	-3.770	down	CATTCATACATACACACATTCATTCATACATACACACATTCATACATACATACATACATACATTTATGCAAGCATGCATGCATACATTCATCCATACATACATATGTACATACAAACAAATTCCCCACATGGCAGGCTATGAGAGACAATGTTTAGGTGACTCAGAGCAGAGTTTAGAACGCCCTGATGGGCTACAATACTATCTAACTCTTTGCAGTCTGCATGTCAGTGAGAGAAAGCCTTGGCCCTGGCAAACACTAGCACATCTATGCGAGAGCTGAGCAAGGCGACCAGACAAACAGCCCACACGCAGATGTGGAAACACATAGCACAGTGTGATGAAGACAGAAGCCAGAGCCAAGTACCTGCTTAGTACCACCCGGGGTCCTAACAACAGTCTGGGTTCCAACCCCAAGAACCCATTGGGATTAGGCCAGCAAATGGCTGAATCTGAGGACTCTAGAGAAAATATCATGAACGGAAAAGCCTTAGCAAACGTCCT
MSTRG.52951.4	ENSRNOG00000047234;ENSRNOG00000058463	0.361	0.004	0.000	-5.159	down	GAATTTTAGAATCTGTCAAGCATATTTGTGGTAGCTGAAGGGGAAATATGCATTTCTCTTTCTTGGTCATTCCAATCAGAGAGGAAATTAGAATAACCACTTGGAAGTATTTTATGCTGTGGTCATCATGGATTTGCTCACGACATATTATATCTGAACAACATGCCTTTTTCCATCCAAAGACCAATATTTTCTTTCCTATATGCAGGTTGTATTTCTTTCCGTCCGTAATGAGTAAAATGAATGGGTCAAATATATTTTCGGAAAAAAGGATGAGCCGTGGTGAATTTTAATCAGCGGATGGTCTTGGACTTGCTTTCCCGTCATTACCTTTCTGATTAACATAATGGACTTCTGCCTTTATACAGTGTTCCTTCTGCAGTGGGAAGGTAGTGATGCTCCCTTGTTGCCCTGATTTCTCTTGGGGACAACCAGAAGTCTGGGTAGAGAGGGTTTGAGCCAGAGTAGGAAAGGGAGATGAATGGAAGCTTTGTTGGCTTATTTTCCTCGAATACACTCTTTGTAATGAGTGATATGGAGAAATTTTAAAGACATACACTGAGCCACAAACCCAAAGACAAAGCAGAGGGAGCCAGTTGGGGACCTGCGAGAATTTTAATGCTTAAATGTTTTACTTCTTTGTGTTGCAAAGTGGGGACATGGCTTCGGGTCAATTTTGAGAGACTCAAAGGAAAAAGGATTGAAGCAGATTTTGAAATTCTTTGCAAGTTTCCCTGAGCTAGGCAGAAAAAGGAGGGCATTGGATTGAAATGACTTTCATTGCAGGACTGAACTCCTTCAGATGTGATTGTATTACAAGATAAGAGGGGATAATTAGGATGGCTCAGTTTTTATCACACTCAAGTGTAGCATGTGTTACAGCAGCATAAGCTGCAGCATAAAGGCTGTCCTCCAGGTATCCAACTTATGACTCAAAGACTGCATGGAGCCAAAGGGAGTTATGGATGCAACCCTCTATAAAATATAAGCTTACCCAAAACGTCGACGTTTTCCTGTTTTGATGGGTGTGTGTGTGTGTGTGTGTGTGTGTGTGTGTGTGTGTGTGTGTGTGTCAGTTACTTGGTTCTCAAGTAAGAGCTTTGTAAATTAGAATGTTGAGTCAGTTTCAAACATTGGAAGTGGCCAAATCTTACTGAAGCCACGTGCCCATTCCATGGCTACCAGTAGTTTGGGAAGCATGACTTCATCTCCCCTAACTACCTCCTTATTCTGCTGACTTCTACTGTTATCTGTGTGCAATCTTATTACTGAAAAATACCATTATTGATCTGAAAAAAAACAAACACCTAGAAGATCAAGGAATACAGAAAGTCTTGGCAAAAAAAAGAAAAAAGAAAATCTGAAAAAGAATGATACATATTTCCATTGAGGCTGAGTAAAATAGAGTTGTCCTTTTTGGTGGCTACAGAGGTAAAGATTAAGGCTGAAGAGATGGCTTAGATGGTAAAGTGCTTGTTAGGCAAGTATGAGGACCTGACTTCAGGTTTTTTGCACCCAGTAAAAAGCCAAGGGTGGTGATACATACCTGGAATCCTAATGCTTGGGAGGCTATGGCAGTAGGATTCCTGGGGTTCGCTGGCCACCCAGTTTAGTCAAATTGGTGAGCTGCAGGTTTAATGGAAGACCCTGATTCAAAATATCAAGGTGGAAGAAAATTGCAGAAGATACCCAATATTGGCCTCTGTCCTTCATGTACTACTGTACACACATGCTCACGTATCCATGAACACACCAGAGGAGGGAGGGTGGGGGAGCAAAAGGAGGAGGGGAAGGAGAAGAAAAGCAAGAAGAGAGAATAAGCAGAAGAGAGAGTACAAAGATTAAGAAAGATACACGGGGCTTGGTAAGAGTCC

							TGCTTGGAATTCTAATGTGAGATAGAATGGTCAGAACAAGTCAAGGGGCAGAGGAGAGCTGACCTGCC<br>CATGAAGGCATATCACATTGTCCCAACAGACTGAACCACAATCGTGGATGGAAATGGGCAGAGACAAG<br>GAGATAGAAAAGTAACCATCTTTGACACTTACACTGAAGCTTTTTTCACTCTGTTGTTTCTGATCATACT<br>TTCCTGTTCTAATCCTCTCAGCGCATCTGCTTCTCTCACCTCTCTCTCCCCATGTCCTCCAGAAGTAACA<br>CCATCATCTTTGCCTTAGATGTTCCAACGTTGTTTCCTCTGTGCTCTCTGCTTTCCCTTCAGCTACAACCT<br>CACTACCAACGTGTATGTTAAAAATGCTGTCTGTCTGCTCATGCCTGTGGCTGTCTGAATGAAACCATCC<br>CACTGCTCATAGTACAAAGATAAAACTCCCGGCACAGAGCTCGTGACATGCTTACCACCTGCCTTCTAA<br>ACCTGCTTTGGTCCTTCTCCCTCTCTTACTTGATCCTTTGGCTACTTAACCATCCCTTCCTAGACAGAGAC<br>ATTTGTAGAGGCTTCTTCCTGTGCTCAAACCCCTGTATTTTTAACTCCTTCCCTCTTGGCAACCTTAGAG<br>CAAAGAGCACTGCCCCAAAAGACTTTTCTTCCTTTAGTAATGGCAAATGACTATCTACATGCTCTATAGT<br>TTTGTAACATTCTTTACTTTCATACACTAACATCCTACTTTGTAAATATATATATATATATATATATTGTCCCC<br>CCTGTATCTTGACCTCCCTCTATTTAAAAATCTTGGGTGTTATATTTCCTATCTTGTGAGACCAGTACCTG<br>CAGATTCATGTGCATGCCCAGTAGCACTCAGGCGACATTGACTCAACAGACAGATAATTGTTGAGGATA<br>ATTGTTGGGTTTTTTTTTAATGTTGCTGGGTCTTTTTTTGAATTATTGTTTTGCATTTATGAATGTGTATAG<br>CTGTAACACTGGACTATGATGGGTGGGTGAGAAGTGAGCACAGAGGCTATCAGGACCCTGAGGCTGTG<br>TGAAACGTCAGCCACTGAAAGAGCTGTAACACCCAAACAGAGTCACTCCTGATAAGGGATCTCTGTTC<br>TACTACAGAAAGAATCTCAAGGTTGTAGTGTTGTTGGGGCATCTTTAAATTAAACTAGTTGTAGCTGATT<br>ATAAAATTATGTATGACATGTAAAAATGACGTATAGAATTATAGAAACTGTTACTGGTAAGATAAGTTAGG<br>AGGTCAAGTTTTTTACCACTAGGCAGGATGACCTAAGTTCCATTGCCAGGATCCACATCAGGAAGGTCA<br>GATGTGAGTCCTATAAGCTGTCCTCTGGCTTCTGCACATGATCTGTGGCATATGTGTTCCCATATATGTAC<br>AAAAATAAATGAGTGTAACAAATATGTGGACATGTATACCTGTTATACTGGCAGATCTTTTGAAACGTGA<br>AACTTGTATGATAATTGGATTAGGTTAGCTTTATCTGTCTCCTTGGCTATTTTTCATTTGTTTTGGGTAAAA<br>ACATCCAAAGTGCTACATTTTTGAAATATGCTATAACAGTTATCACCTTGCTGTGCAGTCGCCTGCTCGA<br>ATCTCTGTGTGTGCCTATGTGTGGTATATGCATGCCTATACAGATGCGTGCATGGGCGTGCTCACCCGCCT<br>ATGCTCATATATGCTAACCGGTGCATGTGTATGTTGAGGCCAGAGGTCAACATTAGATGTATTTCCCCATC<br>TTTTCCCTTGTATTTGGGTGAAACAGGGGTTCTCACTGAATCGAGTGCTTATGAGTTTGGCTAGACTGGC<br>TAGACAGAAAACCCCTAGGATCCGCCTCTCTCTGCTTCTGCTTATTCTACACTACTGTTAGAGTGGGCGT<br>CACCATGGCAACACATGATGTGAGGAGTAAGAATCCAAACTCAGGTGTTCGTGCCATCTCCCTGTCCTC<br>CACGCCAGGACTTCTTGCCCCTCTGGATAGCTAACACTGCGGTGTTCAATCTCCCCTTCTGCTTCCTCTC<br>CAGTCTCTCTCGCATGCTGGGAGATCACCTTTTGCAGCTGCTATTATCCCTCTAGTGTCATTCTTCTGAGG<br>AAGTGCACCGTCTCATTTGCTGTGTGCTTTCTCTCTTGTCACTAGGAGTGACACACAATGTGAAACAAC<br>CAAACAATCCTGATTTTTCGTAAGCAACACTTACGGGTGAACCTCTTAGATTTTAAAATTCGTGCCATTG<br>ATGTCATATGTATATACACATGATGTCATATGTATCTGTGATTCTTCATTATTACTTCAATCGCTCTTCGTTA<br>CTGCATTACAATATTTTAATGTAAATACACCATATATTATTACAAAATACATCAGTGAATCATTCATTAGCTT<br>TGTGGTGAGCTCCGTAAATCACAATCTATTTGATTAAGTTCATTTGATTGGATTACTTCTTAGCTCCTTAC<br>TAATTGCCAATACGTAAGCTTAGCAGTTTCAGATTTCTTACTGTAGAATCTGTTTCTGTTTGGGGAAAATA<br>GTCCAGTATTTTTAGACCAAATTATCTAGAGAGGATTGTTTTTCCTACTTGTTAATAAAAATAGGCAGTTA<br>TTTTCACATTTAACAGCTCCCGTCGTTCTCCTGCTCTGCTGGATAAAATCTGAACTCCGCTGTGGAATTA<br>GGTACCTCTCCCTGCTTCGCTGTTCTGCCCCACCGGCCCCTTCCTCTGCCTATGCTCCTTGGACACTATG<br>AATTCATTATTCACTTTAACAGTAACACAAAGAAGTATTTTTAAAATACAGGCTTCATTTCTTTAACTGGG<br>GTCAAAGTAGGAGTATCTTAGACAATATGGGGGCATGTTTCTCTTCATAATGTCACGCCATGACAAGCCA

							CCTCCAAAGGGAACAGCGGCTTCCAATGAGAGACTGGAGACCTGCTGCTGTGTCCCCGAAATGTGTGG<br>CAGGTTTTTCTGTATCGCTTCAGCATTACATCTACCTTAGGGCAACAGGAAAGAGGAACGTACAAGAGG<br>AAGGCAAGGTCCCGCTCCCCTAAGGACAGGCATGGGATTGATACATGTCAGTTTTGCTTCTTCTCATTG<br>GTCAGAAGCTAGTCACGTGCACTGCCCCAATGCCCCACCCCCAGTTGCTAGGGAAGCTGAACTGTTTG<br>CCTTAGCACAAACTCCCTAGGTTTATTCTGGTTGCTATGGATGAAATGATAAATGAATAACAAAGACGCA<br>AGTGTGAATTTATGTCTCCTGGTCTATGCTCAACGCTAAAACTGTACACGTAAGGAAGGACCGAATTTCT<br>GGGAGGATGCAATTTAGTTTGAATACTTTGGACCGGACGCGGTGATGTAGACCTGTAACCCCAGAACTT<br>GGGAGGTGGAGGCAAGGAGGATTAGGGATGCAATGGCATCCTTGTCCCCATAGCTCATAATAGATCTGA<br>GGATATCCCCGTGTGATGGAAACCGTCTCAAAAAAAAGCCCAAGGGCTGGAGAGGCACATGCATGTGC<br>TTACACATAGAACAACCCAAGATAAGAAATAAAAATGCGGGGCTGGGGATTTAGCTCAGTGGTAGAGC<br>GCTTACCTAGGAAGCGCAAGGCCCTGGGTTCGGTCCCCAGCTCCGAAAAAAAGAACCAAAAAAAAAA<br>AAAAAAAAAAAGAAATAAAAATGCAAGTCCCCGAAAACGTACTTTAAGTGTAACTATGCTGTCTTAGT<br>GTATCAGGAGTTTGTAGGCCACACACATTTTTTGCTTGTATATTTTACAAGAGCAGATGTGGCACATATCT<br>AGTCTGCGTGTGAACTTTTGTGCTTCGTCTACTGTCTAGTTAACTGGCATTCTTTCTCTACTTATTGTCCA<br>CTGGGTGCAGCACTGCCTGGCTTGTAAAAGGATCCCACATAGCCCTGACTTAACAGAGCCCTGAGCGT<br>ACCTGGGAGAGATAGTTAGGAGCTGTTCCCATCATTTCCCCTGGCATTCCTGTTGCCCCTTGTCACTTTC<br>TTCTCTTCTCTCTGCTGTGTTGTAAGACCTAGACTTGCTTTTGGTGTGGTGGCACCTGCCTTGTGTCAAT<br>CCTTAAAGGAAGATGGTGGCATGGACTCCCAGTGAATCAAGGATGCTCTTGCGAGGCTCTCTGCGCTGT<br>GCGTAGCGAGGAGGGCGATGTAACAGCCAAACAGCAGCAGGAGCAGCGTCGTTCTCAGAAACAGTCA<br>CATACACAGCGGCCAGAGTAGGCCTCCTCCCTTCGGAGGCAGCATACTGCAGCCTGGTGAAATGCAAG<br>GCTCCGAGGTTTGAAAATCTCTGGGGTTGAGCATGGCTACTGTAAAAGCCTCGCTCGGAAACCCTGAC<br>GCTCTGAACAGGCAAAACCCCATGTTAGCAACAGCTCGGTCAGGAAGAGAAATGCTACCTTTTCTAGA<br>TGTTTGCCGGGAGGCAGCCCCCTGAAAGGAGGTCTCAAATTACCTGGGAGTGGAAGGTGGGAAGAAA<br>TGGACAGTGGCCGTGACGAGAAGCTGAGGCACGGTGCAGCTTGGTGAAGCCACGCTCTGACTGCGTC<br>CTGCCACCTCTTCATGCAGTGCTGCGGGCTGGTACATCGCCGGCGAGTACGGGTGTCCTATGTAAGTTT<br>CTATTGCTCTACAGTGGTGGCCGGGAGCTGAAAATTGTAACTTGTGTCTGTCGGGTTCATGCTGCCGCT<br>CCTGGGTGCTTTAATCCCTGTGCTTTTGTTCGGGGTTACTTTCACAGCGTTGGAAATAACTTGTAGAAAG<br>AGGGACTGGTTTGATGTGTGTATGTGTGTAGTTGGGGAGTAGCTATTGTCTCAGATATCTGTATCAGTTC<br>AAACCAGTTGATATACTCTTTTGCTCGGAGTTTTCGTATTTGAATCGTGGCGTGGGCAAAGAAATTGTTT<br>TCTGGAGACGAATTACCATGGAGTTTGTCAAATTGCAGACAGCCGCGGTGGCTAATATGAAATCCTATTT<br>GGAAGAGCTTCAGGAAAATTGTACATGATAATCGTTTGATGGATGACACAGCTGTTGCAATATTGCCAG<br>CCTTCCCAATGGCTTTATCGTCCCCTGTCTGTTTCTCAGGAACAGCCCTTATTTCGTGGACCTGCCGTGT<br>TTTTGCAAAATGGCTGTCTGAGCATCAAATTCTAAAAGGCATGCTCTCGCCGATGCCGAAGTATTATTGT<br>TGGAGAAGGTCGGATGTTAATTGAAATACACGGCACCGCTGTGCAAGCAGACAGGCTCGGAGGAAGCC<br>CTGGCTTTATTTTCCTACGCCGTCTGCGTGCAGCCGCTTTATTTTCACAGAACGATGTGAAAGGGAGCG<br>GGCAATTTATGCAGGCTGGCCAGACTGCAGTGCTGAATGGCTAAACCCCCGACTTGAGATTCCTGGATG<br>CAAAACAAACGGAAAACCGACAATGAACACTGATCAGTTCTTAAGGGTGTCACTAAGGAAATCTGTGA<br>CCTTGGGAGTTTGGCCAGCAGAAGGGATTCACCTGAGACCTCAAGACAAACAAGGTCAAAATCTGCTA<br>TGTTCTAAAGCACGAAGTTGAAGATCAGGGATGGGAGGGGACAATGGGATCGTTTGCATTCCTGTAAG<br>ATTTGGAAAGATCTCCCACTATTAAATAAATAAGAGATTTAAACGCTCCCTTAGTTGAACGGGTTTTAGG<br>ATTATTATCAATGTTGTTTGTGTTCTGAATGCAACAGCTGACAGGGCAGGAGTAAATGTAGTTGTGAGGT

							AGCCAGCCAGGATTTTTTTTTAAGGACGACATCTGCAGAGATGGGTGGATTTTTCCCAATAGAAAAATTAATTGATTTTAAAATAACATTTGGAAACAAGTGCCCCAAACTTTTTAGTGTCTCAAACTGCCTAAAAATGAAAACCTTAAAATGATATCAAGTTAAAAATCCTCTATTTTCATAAATTAATGAGGACTCTGACTTCTGTGGCTTTATGTCTTTCTATTTGTCCTGTTCTTTGGGAATTAGAAGAAATCTATTGCTGTATCTGCCTAGTGTGTCTGGGGTTTCTGAAAAATACTCTCAAGACTGTGGTTTTTATTTGGCTTGTTTTTGCCCCCTGTGTTACACCTGTAAGTACATAAAAAGAACAGATGCTACAATCTGTTTTCGTTTTTTTGTTTTTGTCTTTGATTGGAGTCATGACGAAAAAGTGTGACTTTGTTTCTTTATAATTATCAAACTTTTTACCCTGTACTTTGAATAAGGAGCCATAACAATATATAGCATGTGTATGTTTTAAAGTCTGGCATATGAATATTTTTAGAAT
MSTRG.53391.4	ENSRNOG00000024066;ENSRNOG00000024071;ENSRNOG00000031197;ENSRNOG00000056435	0.177	0.750	0.001	2.766	up	TGACCCACTTGAAGCTCTGTTACCATTAGGCTCCTTCCTTCCTCCCCTTCCCTCTCTGAGGGTAAAACGGGAAACACGCGGATCGCACTGACATGCATTAAACACTCCCCACATGTCTAACATATGTTCACATACATTGACCCACGTGATCTTCCCACCTAAAGGGGACGCATGCGCGGCACGTGTAAGTCAGGTGAGCACATACATATATACAGACACACACACATACATACACACATACACATACACACGTCAGGGCCACAGGCCGGTTTCGTTTACCATATATGTAAATTATATGTAAATTACCAGTCTTTAGCCCATGACTATGCAGTCTATACACACTGCATATGCAAAGTCTTAGTTAACATATAGCGAGCGCATCAGGACCTCGCCATACACGTTAATATTAATCTATACGTAAACGCTTTAGCCCGTCATGAGTTATCCATAAAGACGCGCCCTAACCATTAGGCCCTCGTGTCCCAGGTGACCTCGTAAATCCTAAGGAAGGTTTGTGTGTTTATCCCTCAGTTCATCGGCGTGTGTAAAAGTAAAACTAGTTTTACATACGCTATATAAGTTCAGTTTTATATATGCTATGTTTACCATGAGGTCAGTTTTATATACGCTATGTTTACCATGAGGTCAGTTTTATATACGTTATCTTTTAACATGAGGTCGGTTTTATATACGTTATTTTTAACATGAGGTCGGTTTTATATACGTTATCTTTTAACACGAGGTCGGTTTTATATACGTTATCTTTAACACGAGGTCAGTTTTATATACGTTATTCTTAATGTAAGGTCAGTTTTATATACGTTATCTTTAGCATAAGGTCAGTTTTATATACGTTATATTTTAAAGTCTTCCCACGCGAACGGATAGATGTCCAAAGCCTTCTGCACAGCGCAGCGGCCGCGGTCGCTGTTTCCGCCGGGGTTACTCACAGGAAGACGACGTTTCCGGAAGTGATTCCTCCGCCTCCGGCGCAGCGCGGCTAAAACTTCCGGGTCGCACGACCGCCGCCGCCCCCGCCCCACACCGGACACTCAAGCAACGAGTGCGCAGGGATCCCGGGACGAACCGCGGAGTAAAACCGGACGCGGGGGCCGTAGGCCGGAAGCCGGAAGTGCCCCGAGGTCCGTGACGTAGAAAGGAAACCGATCACGAACCGTTTCCGGGCTGACGTTTAAAACGGTGCCCCTTTCTCGCGGCGACGTCGCCTCCGACGGCGTGATGCACATACGCCGCGCCGGCGAGGTTGCTGTCCGTGACGCAATACTCCGCGCCGAGTTCTCACGAGCGAGAGGGCCGGCGGGCGCGCGAAACGGACGTCAGCACGGGGAAGGACTCGCCCCAGGCGGAAGTGAGAGGGGAGAAGATGGCGCGGGTGGTGCAGGCTGTCTATTCCGTCCATCTTGAGTTGGGCGCCTTCCTGGTTTGTCTGAACCACGCCCGTGAGCACCGAGCAGGATGAGGGGCTGGAAGGACAGCTCAGTGGTTAGCGCCAGCTCCGCTTCCTGAATGTATGGCCCGGTGTCCATGTGACCCCACGTTCCAGTGACTCAGTGAGGGCGACGCTGACGTTGTCTTCTCTTCCCCGCTGCGACTGAGCGACAACATCAGGACAGTGGAGCCGGACCCCAGCTCACAGCACATATGGAAATTAGCTACCAACGAGCCCGGGGCGTAAACATGCAAGACGAGGGCGCAAACGCTTGGGAGAAGAGTGGGTCCTTGTGATGTTGGCGAATCAGAATGCAATGCACCGGAAATGGCAGGGTGCCCAGACTGCCTTGGGGGAATCAGAATGGAGGCCAGAACCGATCTTTTTCTTGAAGCACTGCTTACCAGCCAATTTGGAGGAAAAGATGGCCCTCCCTCCCTCCCTTTTGACCCATTTTCCCTCTTCCCCGATACACTC
MSTRG.5377.1	--	0.052	0.528	0.049	2.676	up	GAAGGATTCATTCAAAGCTGTGAAGTGGTGTTACCCAGGGGATTTGGTCACAAAATGTGTTATCAAATTCCATAAAGCTAGCTTAGATAGGATGCCCGGAACACTCGTTGATTTAAATAGATAAATTCAGGAAGTTGGGCTTGTGAATGGGTTTGATGCTGCATTGTAAATGGGAAGCCTCATTGACTCATACTGCATAAGAAATAAGATCTTCACATGTTTGTTCTTCCTTACACCTCAGGGAAACCCATAACATGGCAGTCTACTTTTGACTTTCAGTGCCAGGTTTGAGGATGATTTACGTTTCTTA

MSTRG.53879.1	--	0.000	0.218	0.011	3.719	up	CCTGGTCATCTGCCTCATCCTGGGCCACAGACAAAGGACCTCAGACTAGAAGACAAAATGCATAATTTTGTGTTGTTTATGCCAGGCGTTTTGTTACAGAAATAAAGAATAATGCCTCATGAACAAAACAAGCTTCTAAATTGGGAGTTTACAGACATCTTCTGCAAACAGCCAGGAAGAAAAACACCTGACTGCCTGATCCAGAAGAAGTGACAACATCCAGAAGCATGGACCAGAGCACTGGCACCCAGCTCCACCCACCTGATACCCTTACTGTGGAGAATATAAATTACCTTCGCACCAACCTGTGCTCACTGCCGCTCTATCTAAAGTTATCAGCTATAAAACCAGCCTGCATGCCCATCAGTGGGTGAACGGGAAAAATGGAGTTTTCATTCAACTGTAAAGAAGAATGGAGTAGTGTCATCTGCATTATATAAGTATACACACACGTATATGATGTGCATGTAAATGGGGGACTATTTCAGGATTGGGAGGGGATCATCTATAAGAGAGGGAGAGAAATGGGAGCGAGTAGAAAGAAGTATGATCAAATAAAAGTCTCACTACAGGG
MSTRG.53915.1	--	0.457	0.051	0.047	-2.194	down	GTAGAGGTGAGGGGAAGAGGAAAAGAGATACGAGAACAATAGGAACAGAGAGAAAGGAGACATTTATTTAACTTTATGCACACACCGTCTATACTTAAATGGAATGCTTCTGATTCTATACCGTTGAAGTAGATTTACAGTATGTCCAGCCTAATTTTCTGCTGTATAATAAGCAAGTGCTTCAGTTCGCTTTCTGTTTCTGTGATGAAACAAACAAACAAAAGCCATGTTCAAAAGCATCTTGGAGAGGAAAGTTTATTTGACTTACACTAACATGTCACAGTCCAACGCAGAGGGAAGGCAGGGCAGGAACTCGAGCAGGGCCCTGGAGGCAGGCACAGAAGATGGAGGAGCACTGCTTACTGGCTTGCTCCCTTCAGCTTACTCAGTGTGCTTTCCTGTACACCCAGGACCACCAGACGAGGATGGCACCACTCAGACTGGGCTGGGCCCACCCACATCCATCATCAGTTAAGAAAATGTCCCCCCAGTCTTACCTGGAGCAACCATACATGGGCATTTTCTCAGTTGATGTTCCCTCCTCTCACGTGGGTCCACCGAGTGTCAAGTTGGCAGAAAACTGGTGCCCCAGAGTTTAAATTCTGCTTTGCTGTTGATTAGCAGATGGTACCCTGAGCATGTCCTCAAATCTGTAAGGTGGCATTCTCGAGAAAACACCTCATAAGGGTCCTGTGCGGTGTCAGGATGATGGAAACCCATGTGCAGTCACTAACAGATTGTATAGGCATGTATGGGTGTTTTCTATCTTATATTTCTGGTATAATATATCCTCCCATGGCTGTGTTGTTAAATGCCCGCCCCCTTTGACCTGAAATCCTCCATGTGAAAATGCCCATTTACTCTGGTGCTCACTCTGCCCTTGGAGGGGATTGGGGAACAAGAGGTTTTGCCACACGTCTCATCCTCACAGATAAACAGTGTCAATCAGATCCCCACAGCTTCTGCATCTAACTGTTCCTTGTTGACCCTGCCTTCCTCCTTTGATGCCTCCTATGATGTTGGAATTCATGTCCCCAGCTATGTCATGGTCATGGCTGAGACCATCTGAAGGCTTCAGGGCTGGATGTAGCCCAGCACTATGGAGAAGCAGGACTGTTTCCTTTATTTCAAGCCGTCTCTAAACTCTCGTCGCCTTTCCTAACGCGAGCACATTGTGCCGATGGCTTCTGAGGGCAGCACATTACAACCAGCCTCTTGGTGGACATAGCATCCTCCCCTTGGGTTGCACTGATTCATCTTGCATTTAGTCTTTCAATGTTTATCTTTTTACGTTCTCTGAAGACTTGGTATGATACTTGTTTAGTCTTTATAAAAGGAAAACACCAAATCTTAAAAGAACACCTAGCAGCTCTTGTACTGTCTCACTGGGGATTTTGGTAACATTTCCATTTGCCATTTTCCCAGCCTCTGAGCTTCATTCATTATCCAAATAATTACTGGAATCTTCTGGTTTCAAATGCTATATTAAATGCTTAGGTACAAAAGGGACAAGGGCCAGGGGCAAACGTGCTCTAACACTACATCCTGTAGAAGGTGAAGCAAGAACTAACAGAGACATACATAGGACATAAGATATGACCAGTGCGAGGTCACAAGGAGGGAAGTAGGGCTACCTCAGATGGCGCCAGGAAGGAAGACCTCTCTGAGACAGTGCCTTCTGCTCTCTGGTTGAAGGAAACAGAGCATAAGGTTCTAGGTGGCCCCAGGGTGTACAGGTCCTGATGCAGAACCAAAGGGCATGTCCCAGGAAAAGCTGAGAAGCCTGCAGTGAAAGGAGCGTCCTAGGGGAAGCCTCGGAGTGAGGTAGAGCTGTGTCCTCTGGGCATGGTGCCCTGGGGAAGGAATGTGGGTCTTACAGTAGCTTCATAAGAAGCCACCTGGCGCTCCTACAGTGTCATCGAGGTTGAGTTGTAGAAACCGTGGCAAACTAGAATTCAAGGAAAACAGACCGTGTCATATCCGGGCTAAGGAAATAACCCAGATCTATGTACCTGAGAGGGAACCGTCGATGGCTGACTAAGACTTCCCAGCAGAGGAAGTGGTGTTTGACTGTTCTAGTTAGGTGCCATCTATTTGTTTTGGGCTTTTGTTTTGTTTTGTTTTGTTTTGTTTGTTCGGTTTGTTGTTTTTTTTTTTTCCTGTGTAGCCTTGGCTATTCTGGAATTAGCTCTGTAGACTAGGCTGGCCTCAGACCC

							AGAGACCCGATCTGCCTACCTCTGCCCTCAGGGCACTGTGATTAAAGATGTGTGCCAACTGGCCCCGCTACATAGATGAAACAGTGCTTTTTTTCTAATAAAGGGAGAAAATTCAGGGTCTCAAGAATGGGGGCCAAATGCCGTTATGAGGTTGAGTAGAATGACGGACGAGAGAGCGGTTGATTCTGTAATGGGTGTACTGCGGACAACTCTGACCAGAATGTAAGACTGGAGGACAGTTGCTAGCCGTGGAGTAGAAC
MSTRG.54010.1	ENSRNOG00000015546;ENSRNOG00000030439	0.000	0.379	0.031	3.453	up	GGTAGCACCACGGTGTGAGCACAAGGTTCTCTGCCGGTGAAGAGCAGCACATCAGAGCCTCAGTGAAAGGAAGAAGTCTGCCGAAGTCTCAGGTGATCCGGGTCCGCAGCGCTCAGCACAGACAGGCAGCAAGCGGCTGCAGGATGACAATATGTTCTACACCAAAAGCAGCAGCGGCTACCTTGAGTGCTTAGAAATTGTTACAGCCAGGATTATAACCTCTAAATCATGCGACTCCGAGAGATAAAGCATATTGCCTTCCTT
MSTRG.54283.1	--	0.560	0.040	0.001	-2.994	down	ATGGGAGAGTAGAATGAGGACCAGTGTTCAAAGCAGTAAAAGACACTTTCTCCTAACTGTTCAGGAAAGCAGGACATGATGGTGTTGTTTGGATGAGTCCCAGGGAACTGGTCCTAGCAGTAAGTGGAAGGAATATGCCCCAGTGATAGGCTGAGAGTCAGATCATGGGAGGACTCTATCACAGGGCTACAGTGAGAGCAGTGATTAGGCATGGGGAAAAAAGGGAGTGACAAGTTTGATGAGCCTTGAGAATGGATACATTTCAGATTCTTTCTAACCCAATTACTTATCCAGGATTGTTTCCAGTATCTCTGGAGGGCCTGCAGTAGACAAGACAAAGACATCTCCAGCCAGGGCTGATGTATGGTCTCTTTGAGACAGGTTTAACCATCTTGAGTGACAGTATGTATGGTGCTCCTTCTGAAGTAGAAGAGAGCTCAGCAAATCTCTATTAATAAGCTTGCTCAAAGATAAGAATCTGGATCCCCTAACTTACCATGCTCTCACATCATTTACTTTGGAGTAGTATCTGTGACTTTCTGTGTCAGGGATCTTACATCCACCTCAATTCTCTTCATTCTCCAGCCTCTGCCTTCATCCTTTTTGGACTCCTTGTCTTGGTCATTAGCACCATCTTGGATATATGGTACAGGAGTGGGTTTCTCAACTTTGGATCACCAAGTCTGTATGTTCCTGTTCAAGATGCATGAAAACACTGCGATTCTGCTCAGACATCCTTGTCTCAGGGCTTGGGAGATGTAAGAACCAACACAAACAGTGCTTTGTTATAAAGAATGTTGATGCTATAAAGTACCTTCACTTATCATCAAAGACCAATTATCTAGAAATGGATAAGAATAAAAACAGGAAGCCTGTAAAAGTTGGCTCAGTGATGACTGTGAAAGGAACAGAATGTATAATATTAGGATGGGTTATAAAGAACAGTTATGAAAGTCAAGAAATAGGCTGAAGGCATTGATCATTATAGACTCACACATATTCCTAGGGATAGTCAATTTATTACATACAATGTTATATATACAATAACAAAACTTACATACAAAGGAAAACATATTACACAGAATAATTACAGTACTCAATTTTTAAAAGATTTTCATATCCAGTGATTCACTTTTGCTTTCTAGTTACATTAACATATCACTGATTATAACACATGCATTCTACTGCTTAGACCCTCAGGTATCGAATCATGCCATTATTGGAACATGAGTAACAGCTTGGGATTTTAATTTTTCTTGTTCTATCTTTAAAATATGACCAACACACAAAAACATTTCATTGGAATGTTAAGGAGAGTTCTCGCTGCTGAGAAATTGAGTACAGATTCTAAGCAGACATAAAATAGTCTTGAACCTGTAGCCCCAAGACTGGAGATTAGGGAGCAGCACTAGGAGGATTGCTGGTCTCCAGTCTAGCTCCAGGTTCAGTGAGCGACATTGCCTCAAAGAAATAAGGAGAAGAGTATTTGACCAGAGTACACTGAACCCTCCTTTGGTTTCTGCAAATGTGAGTGAGTATATGACCATGTACATATGCATGTGGATACATTACATATATAAACACTCCCCTCCAAATAGCACATGGTGATTAAACATCTTACCATAGTTCAGTTAATCTTCATGGTGAAGAAATATTAGATGCTTTAACTAGATAGATGAGATTTGCATTCACACTTTTATTTTATTTTGAAGATTATCGTGTAAACATTCCATACCTCCTTTACATTTCCTCATTCCAAATTATTGAATGTATACCTTCACATTTCCCTTCAAATTCATATCCCTCTTTTTTTCTACTAGTTGTCATTGTGTGCATATGTGCACACACACATATACATATATGTATACATACACACATATTATATATAGATTATGTTCTTAGGTATAGTCATTATGTACAATGTCACCTGTTTGTACATCTATATTTTCAGGTCTGATCATTTGGTAGTAGACAATTAATCAGTATTTCTTCCCTGAAGATGACTATCTCTTCTATTCCCAGTTTTCTTGAGATGCCTATAGCATAATCTATAAACTTCTATCCTGGTACTCACAAATAAGTGTGGTCTTCACCTCTCATCAAGGA

							ATCTTTGCTTTGCAACAGATGGAGACCATTACAGACACCCACAACCAATCAAAGTGTTGTGGAATCAAG<br>TTCCAAGAGATACAACTGCCTTACAATTCCACACCTAAAACCCAAGAGAAATTGTAAAAAGATGTGGC<br>AGAAAAAATTATAAGAGCCAGAGGATAGATCAGGAACTTTTTGTGAGGTTGCGTCTTTTAGTAACAAAA<br>GAAACTGCACACATTAATCTCAGTCACACTAATGCCTAAATGTGAGCTGCACAAGGATGACACCAAGA<br>AACATGTCAAAGAGGACAAAGCAAATCCTATGACGTTCAACTCTACATCTGTTTACTTTTACGCAACCA<br>ATGTGAACTCACACTACTTGCAGCTAAACTAATTTCAGAATGTGTATCCATTCTCAAAAGCTTCTTTCCA<br>TGTCTCAGTCATGTGTTTTTTAAATGTGTACACAGTGCAAGCACAATACTATATTTTCTCTTCATAGCTCA<br>TCAATCTTCAACTTTTATAAATACAGGTATAAGAATGCTGATCCCTATACCCACAACTAATTACATTCCCA<br>GGATCCTACATATTACCCTTTTTCCTTACAGTTAAATGTGCTTTTAATTTTCTGTAGGCGTTAACATAGACA<br>TACTAGTGGAATTAAATTCTCAAGAACAACAAAAATAAAAAAGCAATGAAAAACACCCATTCTCACTAC<br>ATAGAGTCACAGAACCTCTTGAGGTTAAAAGAAAATCCCCTCATTTTTTTTTCTCTTTGGTATTGTGCAT<br>GCCCATGCTTTGCAATGAAGCACCCCTACAGTGTACTCTAGTGGGATTTCATTTAGGGAAAGGGGATGT<br>TACAGAGAACCAATGGCACATCTCAAGCCTGTTCCTGAGCCATTTTAAAGGAAGTAAAACACACAGGA<br>AAATGCTGAACGGATTTTATAGCAAAGCATCATGGTGCTGTTATCAGCTTCCTTGCTGGAAATGGTAATG<br>TGTATTCATATAAACACAATGGGGTTGAACATCACATCCCAAGCATTCAGATGCCTCCCAGCGCCCAGCA<br>ACTCACACAAATGTCTATTCAATCCCCTTACAGCTCTCAGCAACTGAGGCTCTCCAACCCTTGCAGACC<br>CTGCAGCTGCCCAGCAGCTCAATGAGCCATTGAGTCTGATGGGAGCGTATCTGTTTCCCTCCTATCAGC<br>AAGCAGAACCAGGAATTTGCTGTTTTATCTTTCCTTAGCATACGGGTGTAAGAATGATAGTGAGAGAGC<br>AGGGTCATCTTCTAGGTTTACGTAGTCTGATTTATCTCATAAACACAGAAGCACGATTACTATTTACTTTT<br>CAACCAAAATGACAGAAAAAGCAAAACAAGTAGAAAACGGAAACAATACAAGGAAAATCAAGAAAG<br>AAACAAGTGCCCTACCTCAAACACGGTTGTGAAAAAAAGACAGACTTGGAGGTTCCCTAAAACTCAG<br>AAGTAGTGATTTTCTGAGTATGGTAACCACTTTACAAACGCTGAAATTTGGCTGGGTATTCTTCATCTGT<br>GAATGCATGAAGATTGCTGCCTGTCAACAGCACTTGCTAAATCATATCTCCACCTTTGAGGAAGTCTCCT<br>CTATCGGTAAATTAATCTGAGTGTGAAGCAGGGAGTGAGTCCATGGAGACTGAAATTGACCACACTGAT<br>GAAATAAAGCACTGTATCTGGCCAAAGCTGTATTTGACTTCATATCAGCAGTGATTTTGCAGAAAGTCAT<br>ATTAAATAGATTGAAGATTATATGGCAGTGTACGTATGCTACACATGTGGTTTATCTAGAGTCAGAGGGA<br>GTTGGCTCAGAGTACATGTGTTTTCAGTGTCAGAGCACCCACATTGGTATGGCTACCGAAGGAAGACGA<br>TATGGTGTGTTGGGCTTGAGCTTGAGAACGTCACAGAATTCCATAACAACTCTCTGACTCTTTTGTTGAT<br>CAATGAATATTTTAAGTGTAGTAATTTGAGGCCATTAAAAATAGCAAAGTCCTTAAAGCCAAGGACTGG<br>ACATGCCTCTTCGTCAATTAATTATATAATTTATTAGGCATGCATTTATCTATTACAATTTTTTTGGCAATGA<br>TGTTTACAGATGGAACCTTTAATGACTGGAAATGTCATTAGAGAAGAAAGAAAAGCACGCAAGTTGTTT<br>GATAATGACTGGTAACTCTTTCAGGTATATTTCCCAAATGGACAATAGAAAATAGTTTGAAACTATTCATC<br>ACCTGTTGAACATCCTCAAGCTCCACTCTGGAATTTTGTTCTTGTTTCTTCAAATGAGGACCAAGTCTGT<br>TTTGAGACACAATGCCTGATGCACGTATAATAACCCGTTCTTTGCTTTTCTTTCTTCATCGCATCATTGAC<br>AGAATGAAAAGAAAGGGAAAGGAAAAAGAGGAATGTTGCCTCATAGGTTGAGCATCACAGCAAAGCA<br>ACAAAAGTGTTGTATTCATGGTAAGAATAAGTAAATATATTTCAATGTTTCCCTCTTTTGCTAGCTTGCAT<br>CTCTATCCAGAAAATCCCTAGTAAGAAATGGAAATCTTAGAGCCAGTCACCACAGCCTAATTATGACAG<br>TTTTCCCAGTATCTATATAAGTAAAGATTTGTTTAATTTTTATGTAGATAAAAAGTCAAACAGGGAAGAG<br>AGTACAACCAAGATAATCTGACAATTACTAGAGAGGAATGTGATAGCATTTTCCTCAAGCTAGAGAAAT<br>GAGGAAAGGATTCAAAAGATGGTTTTATGAATGTAAGTTTCAGATCTTTGAAACTCCCTGGTTATATTGT<br>AAACCTCTGCCATGGACACTTTTCTTGAGACAGGGGTTATTCTCTATTCAGAAAAGAAATCAGAGCTTT

							GATTGTTCATCAAACATCATTGTATTTCACTAGAAAACTGATAAGCTCTTTTTCTGCATAATGAAATACACCCAAAGATACGATGTGTTAATTTTCTGAGGAACAAAGGGATTAATTGAAATATCGGTGGTATTAATGATGTCAATGCTGACTATAATCATTTAGCATTTAACCTTTATAGCTGCCTTTTGCAAAGGAACATGGGGGGATGGGGCCTGATATTTTCTACTGTGTAAAATGAAAAACACTAAGAAGGCTTGTTGACTTTGAACATAGTGTTGGGTATATGGGGAAAGAGAGAAGGTGAGAGAGACAGAGAAGGAGGAACAAAAGAAATAGGAGGAAGGAAATAAAGAAGAAAGGAAGGACATAGGAAGTAGTAGATGGAAGGAAAAGTAAAAACAAAAACAAAAACAAAAACAAGAATGTCTTAGAAGGGTCTTTGTATACAGTAACCTGATACAATTAAGACTTACCACAGAAGGGAACACAGTGACAGACCAGAAATCATGATTCAAAGAAGACACCAAAGAAACCGCCTTGGAAACTTATCACAGATATGGGAAGACAAACATTCTTAGATGAAAGAAGCCAAGAGAGTCCGAACAGTTCCAGAGCGAGTGTCTTCCTTGTTAAGGCAAGTACTTTTATTGTCATGCACACAAAGCCCAATAGCAAACATTCCCAACCACTCAGCTTCTTAAAGTTAAAATAAACCTACTTTTCATTAATTCTTGGAAGAGTTAATAATAATCACTTTCATAATTTGTTGCATAATTTTGTAGCTCCGAGTCCACATTCAGACATGCGATCCCAAGCTAAGGATCCTGGTATCTTAATCTTTACTCATTCATCATCATGTAAAATTGAAATGGAGAGCATATCTAGAAATCCAGATTCTCTGGAGTTGTGGCAAGACAAGACTTCATGATGAAAGCCTGGAACTCTTCTCTTTCCACGGATAGTAACACACATGATACCCAGCTGTTTCAGTAGATTTTACTGCTCTGAAGACAACAGCATGCTTGGTAAAACTAATAAACATTCTTTACTTTTGGAGGTTCAGAGGTTAAGTCCATTATCATCAAGGCAGGAGTATGGCAGCATCCAGGCAGGTATGGTGCAGGAGGAGTCCAGTTCTACCTCTTCATCCAAAGGAAGCCAGGAACAAACTGAGCATCCCTAGGTAGATAGGAGGAGGGCCTCCAAGGCCACACCCACATTGACACATTCCTCCAACAAGGCCACACCTTCTAATAGTACTGCTCCCTGGGCCAAGCATATGCAAACCACTGCAGATATATAGGGCTGTTAGTCGTCTCCTTCCTTGTGAAGCTTGCATGGTACCTTCAGGTACCACGAAGAAGTGTCCTCAAAAAGAGAACTTGGATCCATGTTAGCCTGGGACTAGAAGTCAATAGGCCCTCTTTGTGATGTGCATTGTATCTTCAGCATTAGGGACTTTCCATCCACCTGAGAACTGCCTGGTCATCCTATTGCTGTCCTTCCAAGGTTCCTCTGTCCTTTCCTGCTGCCTTCTGTAGCAGTTTGATGTCCCTGTTCTCTCTTGCTCTATTCTCTCATTCAGAGGACCAAT
MSTRG.54283.2	--	0.438	0.000	0.000	-7.011	down	ATGGGAGAGTAGAATGAGGACCAGTGTTCAAAGCAGTAAAAGACACTTTCTCCTAACTGTTCAGGAAAGCAGGACATGATGGTGTTGTTTGGATGAGTCCCAGGGAACTGGTCCTAGCAGTAAGTGGAAGGAATATGCCCCAGTGATAGGCTGAGAGTCAGATCATGGGAGGACTCTATCACAGGGCTACAGTGAGAGCAGTGATTAGGCATGGGGAAAAAAGGGAGTGACAAGTTTGATGAGCCTTGAGAATGGATACATTTCAGATTCTTTCTAACCCAATTACTTATCCAGGATTGTTTCCAGTATCTCTGGAGGGCCTGCAGTAGACAAGACAAAGACATCTCCAGCCAGGGCTGATGTATGGTCTCTTTGAGACAGGTTTAACCATCTTGAGTGACAGTATGTATGGTGCTCCTTCTGAAGTAGAAGAGAGCTCAGCAAATCTCTATTAATAAGCTTGCTCAAAGATAAGAATCTGGATCCCCTAACTTACCATGCTCTCACATCATTTACTTTGGAGTAGTATCTGTGACTTTCTGTGTCAGGGATCTTACATCCACCTCAATTCTCTTCATTCTCCAGCCTCTGCCTTCATCCTTTTTGGACTCCTTGTCTTGGTCATTAGCACCATCTTGGATATATGGTACAGGAGTGGGTTTCTCAACTTTGGATCACCAAGTCTGTATGTTCCTGTTCAAGATGCATGAAAACACTGCGATTCTGCTCAGACATCCTTGTCTCAGGGAAATAAGTGTGGTCTTCACCTCTCATCAAGGAATCTTTGCTTTGCAACAGATGGAGACCATTACAGACACCCACAACCAATCAAAGTGTTGTGGAATCAAGTTCCAAGAGATACAACTGCCTTACAATTCCACACCTAAAACCCAAGAGAAATTGTAAAAAGATGTGGCAGAAAAAATTATAAGAGCCAGAGGATAGATCAGGAACTTTTTGTGAGGTTGCGTCTTTTAGTAACAAAAGAAACTGCACACATTAATCTCAGTCACACTAATGCCTAAATGTGAGCTGCACAAGGATGACACCAAGAAACATGTCAAAGAGGACAAAGCAAATCCTATGACGTTCAACTCTACATCTGTTTACTTTTACGCAACCAATGTGAACTCACACTACTTGCAGCTAAACTAATTTCAGAATGTGTATCCATT

							CTCAAAAGCTTCTTTCCATGTCTCAGTCATGTGTTTTTTAAATGTGTACACAGTGCAAGCACAATACTAT<br>ATTTTCTCTTCATAGCTCATCAATCTTCAACTTTTATAAATACAGGTATAAGAATGCTGATCCCTATACCCA<br>CAACTAATTACATTCCCAGGATCCTACATATTACCCTTTTTCCTTACAGTTAAATGTGCTTTTAATTTTCTG<br>TAGGCGTTAACATAGACATACTAGTGGAATTAAATTCTCAAGAACAACAAAAATAAAAAAGCAATGAAA<br>AACACCCATTCTCACTACATAGAGTCACAGAACCTCTTGAGGTTAAAAGAAAATCCCCTCATTTTTTTTT<br>CTCTTTGGTATTGTGCATGCCCATGCTTTGCAATGAAGCACCCCTACAGTGTACTCTAGTGGGATTTCATT<br>TAGGGAAAGGGGATGTTACAGAGAACCAATGGCACATCTCAAGCCTGTTCCTGAGCCATTTTAAAGGA<br>AGTAAAACACACAGGAAAATGCTGAACGGATTTTATAGCAAAGCATCATGGTGCTGTTATCAGCTTCCT<br>TGCTGGAAATGGTAATGTGTATTCATATAAACACAATGGGGTTGAACATCACATCCCAAGCATTCAGATG<br>CCTCCCAGCGCCCAGCAACTCACACAAATGTCTATTCAATCCCCTTACAGCTCTCAGCAACTGAGGCTC<br>TCCAACCCTTGCAGACCCTGCAGCTGCCCAGCAGCTCAATGAGCCATTGAGTCTGATGGGAGCGTATCT<br>GTTTCCCTCCTATCAGCAAGCAGAACCAGGAATTTGCTGTTTTATCTTTCCTTAGCATACGGGTGTAAGA<br>ATGATAGTGAGAGAGCAGGGTCATCTTCTAGGTTTACGTAGTCTGATTTATCTCATAAACACAGAAGCAC<br>GATTACTATTTACTTTTCAACCAAAATGACAGAAAAAGCAAAACAAGTAGAAAACGGAAACAATACAA<br>GGAAAATCAAGAAAGAAACAAGTGCCCTACCTCAAACACGGTTGTGAAAAAAAGACAGACTTGGAGG<br>TTCCCTAAAACTCAGAAGTAGTGATTTTCTGAGTATGGTAACCACTTTACAAACGCTGAAATTTGGCTG<br>GGTATTCTTCATCTGTGAATGCATGAAGATTGCTGCCTGTCAACAGCACTTGCTAAATCATATCTCCACCT<br>TTGAGGAAGTCTCCTCTATCGGTAAATTAATCTGAGTGTGAAGCAGGGAGTGAGTCCATGGAGACTGAA<br>ATTGACCACACTGATGAAATAAAGCACTGTATCTGGCCAAAGCTGTATTTGACTTCATATCAGCAGTGAT<br>TTTGCAGAAAGTCATATTAAATAGATTGAAGATTATATGGCAGTGTACGTATGCTACACATGTGGTTTATC<br>TAGAGTCAGAGGGAGTTGGCTCAGAGTACATGTGTTTTCAGTGTCAGAGCACCCACATTGGTATGGCTA<br>CCGAAGGAAGACGATATGGTGTGTTGGGCTTGAGCTTGAGAACGTCACAGAATTCCATAACAACTCTCT<br>GACTCTTTTGTTGATCAATGAATATTTTAAGTGTAGTAATTTGAGGCCATTAAAAATAGCAAAGTCCTTAA<br>AGCCAAGGACTGGACATGCCTCTTCGTCAATTAATTATATAATTTATTAGGCATGCATTTATCTATTACAAT<br>TTTTTTGGCAATGATGTTTACAGATGGAACCTTTAATGACTGGAAATGTCATTAGAGAAGAAAGAAAAG<br>CACGCAAGTTGTTTGATAATGACTGGTAACTCTTTCAGGTATATTTCCCAAATGGACAATAGAAAATAGT<br>TTGAAACTATTCATCACCTGTTGAACATCCTCAAGCTCCACTCTGGAATTTTGTTCTTGTTTCTTCAAATG<br>AGGACCAAGTCTGTTTTGAGACACAATGCCTGATGCACGTATAATAACCCGTTCTTTGCTTTTCTTTCTT<br>CATCGCATCATTGACAGAATGAAAAGAAAGGGAAAGGAAAAAGAGGAATGTTGCCTCATAGGTTGAGC<br>ATCACAGCAAAGCAACAAAAGTGTTGTATTCATGGTAAGAATAAGTAAATATATTTCAATGTTTCCCTCT<br>TTTGCTAGCTTGCATCTCTATCCAGAAAATCCCTAGTAAGAAATGGAAATCTTAGAGCCAGTCACCACA<br>GCCTAATTATGACAGTTTTCCCAGTATCTATATAAGTAAAGATTTGTTTAATTTTTATGTAGATAAAAAGTC<br>AAACAGGGAAGAGAGTACAACCAAGATAATCTGACAATTACTAGAGAGGAATGTGATAGCATTTTCCTC<br>AAGCTAGAGAAATGAGGAAAGGATTCAAAAGATGGTTTTATGAATGTAAGTTTCAGATCTTTGAAACTC<br>CCTGGTTATATTGTAAACCTCTGCCATGGACACTTTTCTTGAGACAGGGGTTATTCTCTATTCAGAAAAG<br>AAATCAGAGCTTTGATTGTTCATCAAACATCATTGTATTTCACTAGAAAACTGATAAGCTCTTTTTCTGC<br>ATAATGAAATACACCCAAAGATACGATGTGTTAATTTTCTGAGGAACAAAGGGATTAATTGAAATATCGG<br>TGGTATTAATGATGTCAATGCTGACTATAATCATTTAGCATTTAACCTTTATAGCTGCCTTTTGCAAAGGA<br>ACATGGGGGGATGGGGCCTGATATTTTCTACTGTGTAAAATGAAAAACACTAAGAAGGCTTGTTGACTT<br>TGAACATAGTGTTGGGTATATGGGGAAAGAGAGAAGGTGAGAGAGACAGAGAAGGAGGAACAAAAG<br>AAATAGGAGGAAGGAAATAAAGAAGAAAGGAAGGACATAGGAAGTAGTAGATGGAAGGAAAAGTAA

							AAACAAAAACAAAAACAAAAACAAGAATGTCTTAGAAGGGTCTTTGTATACAGTAACCTGATACAATTAAGACTTACCACAGAAGGGAACACAGTGACAGACCAGAAATCATGATTCAAAGAAGACACCAAAGAAACCGCCTTGGAAACTTATCACAGATATGGGAAGACAAACATTCTTAGATGAAAGAAGCCAAGAGAGTCCGAACAGTTCCAGAGCGAGTGTCTTCCTTGTTAAGGCAAGTACTTTTATTGTCATGCACACAAAGCCCAATAGCAAACATTCCCAACCACTCAGCTTCTTAAAGTTAAAATAAACCTACTTTTCATTAATTCTTGGAAGAGTTAATAATAATCACTTTCATAATTTGTTGCATAATTTTGTAGCTCCGAGTCCACATTCAGACATGCGATCCCAAGCTAAGGATCCTGGTATCTTGAATGAAAAAAAAAGACAAGGGTTTCCATCACAACAGACTTTCTGTCAATTTGGACTTTCAATGTTCAGAATTTCCTAGTGTTTGTTATGGGCTTGATTTCTTTCTATATTACTGTTTTGGTCAACATGGGAGTGACAGGATGTTGTCCTAAGTAGTTTTAGAAAATTGACCATGCAAGACTAGTACAAACCTTAATCTTTACTCATTCATCATCATGTAAAATTGAAATGGAGAGCATATCTAGAAATCCAGATTCTCTGGAGTTGTGGCAAGACAAGACTTCATGATGAAAGCCTGGAACTCTTCTCTTTCCACGGATAGTAACACACATGATACCCAGCTGTTTCAGTAGATTTTACTGCTCTGAAGACAACAGCATGCTTGGTAAAACTAATAAACATTCTTTACTTTTGGCTGTGGAGTTAAGAAGAAAAGCAAAATTGCAATAATTAACTGGATGTCTGAGAAATTTATAACTCATGTGAATATGTGTACATCTGGTCTAAGATACAGTAAAATTTGTATCATAATGAATAAATTTAACTCAGTGCAACTAGCTATAAAGCTTGTCTGCTTCTTACTACGGCATAATTTCAACAAGTGTTCTGTGTATTTGTCTTTAATATCTTGTCATAAACCTCATCCTTCCAATAGCTTCATACTTCCTTTGTCTTTCTGAAGACTACACTGACTTCTTTCTACTATATTTATGTTTTCCATTTTGATGCCTACAAGAAGCCAGATTTTTATAACATGCAAATTTTAGGCAAACTACACACGTGAACTTGTTAGTCCTTTTAAAAGCCAGAGAAATGGCACAGATGTTAGGAGAATGTACTATTCTTACAAAAGACCCAGGTTCACTTCTCAGCCCCTATATGCTTCCTCACAAACATCTCTAAGTCTAGTTCAAGATCCGACTTTTTACTCTGGTATCCATTGGCACTGCATTCATGAGAATGATACTGAGAGTTTATACAAAAGCACATATACACACATAAATACATCTTTAGAAAGTTTTTCTGGCCTGAAATCTTAAGTACCAACTTTCACATTATCCAGTGGCTGAAGATGATCTATGTTTCAATAACTTGCAAAACAAAATAATCAACATTTTTC
MSTRG.54514.1	ENSRNOG00000020468	0.000	0.235	0.001	4.308	up	TGAGTTTTGTGAAGTGACTCGAGTACAGACAAATCCAGTGACTGAAAGTTACAAACAAACGGGCTATCAGAGTGATGTTTTAAGTTAATAATCATACCAGTTTGTATTCTAATTATTTTTTATGTATCTCTAGTCATTAAAATACATATAAGCTCTAGGTTTCTTTGATATGATAAATACCATAACCAAAAACAATGTGGAAGAGGAAAAGGTCTATTTTATGTCAGTGGCTCCAATCTGTCCTGAGGAAAGGCCACAGCAAGAACAGAGGACAGGCACCTGGAGGCAGAATGTGGTACAGAGCGCACAGGGAACACTTTCCTGGCTTGCTTCTCCAGTGTGCTCAGCTCTCACAGTCCAGGCCCCCTGCCTATGGATGGTACTGCCCACAGTGGGCTGAACCCTCACACCAATTAGTAATCAAGGAAACGCCAAACAGCAAAACTCAAAGGTCAGTCTGATCAAGACCGTCTCTAATCGAGGCTCCCTCATACCATTTGGGGCTATGCCAAGCTGACATCTGACGCCAACTAGGACATATAAAACTACATACATGTAAAGAGTTTTCCCCACAGGGTTCTCCAAATAACTAAATTTAAATCTCAAAAAAGAAAAAGTTTTTAGGACTCAGGCAAACTATAGCTAAAAGAGCAAATCTTAATTAAGGACTTAGATTACTATCATAATTAACTCACAATTTTTTATTGTAATGGTCAGACTCATTTTTTTTGGTTTGGTTTTTGTTTTTGTTTTTTGTTGTTTGTCTATTTGCAAGCTACCAGAATAGCACTGAATATTTTCAATAGAGACAATATAATCTATATCACCTAAAATGTCTATCTGGCCCTTTATAGAGAAAACAAAACTCAGATTTTCA
MSTRG.54580.1	ENSRNOG00000020157;ENS	0.187	0.000	0.006	-4.200	down	AAAAAAAAAAAAAAAAAAAAAAAAAAGAAAAAAAAAAAAAAAAAGGAAAACTTAATTCACCTGTAGTTCCCCAGAAAAATGAATCCCTATTTAAGAGTCATATTGCCACAATCTCTACCTCTTTAGCCAGAAAACAAAGCAAATGTTACTGATGCCCACGATGGGAAATTCTCCTCAAACAGATCTGTGCTAATTTAAAACACATAAACCGAAGTGCCTCTTTAAGTGGTCATAAAAGTGGTGAGCACTTGGAAGGCAAGCCCTGATTTAAACACTGAAGTTACCTCTTTATATGGAAGTACTTACCCTCAGTGCCTGAGAAATAGACTGAAGTCCCTCAAGGCTC

	RNOG00000020167;ENSRNOG00000020204						AAGGCCATCTTTTTTTTTTGGGGGGGAGGGGCTGCAGCAAGGTGGGGGGGGGGGTTAGGAATTGAGACAGGGCTTCTCTGCATTGCTCCTGGCTGTCCTGGAACAAGCTCTGTAGACCAGGCTGCACTCTTTTCCACCTACTCCTGCCTCACAAGTGCTGGGACTAAAGGTATGTACCCCCACCACCAGCCTTCTTCAACATCTTCTTTTACTTAGGCTGGGAGCAAATGGTAGGGCAAGTTAGATTCACTCTCAAAGGCTTACTCTGAAAGATACAACTTTCTAAATCTGAAAAGTGTGAGTAGTATTAACCATACAAAGTGTAATCCACCTAGCCAAAGAACTCTGGATCTACAACAAGTTTGCTAACCTAGACACGAGTTGAAGAGCAAACAGTTTCCCAAAAAGACTGGGTCCTCACTAAATAGATACTTATTGTTCTACCATATCCCGATGTCATCTTTCTGGGGATGCCTGTTAATCTTATATCTTCAGCCCATAGCTCCAAAACTCCCTGGGCTGGAAGAGGATCAGTTCTGCCTCCTGAAAGGAAGCACAAGGAACTGCTGTAAGCAGGAAAACTGTGGACCTTCAGAGTATGAGGACACTCCAGAGACACGGCTCCAAAGAATTTACTTACCCTCCAACAACAGGAATCTGCTCAAAAGACAGCAGCATTAACAAGACATTACCTGACCAACAGGCTATCAGCAACCAGTAGTGCAGTATCAGACAGAGCCCTCCTTACCGAGGATCACCTCTGCTAAGACTATGAAGATACAAGTTTTGGCAGTTTCTGAAAGAATGATGCAAAGGGTAACAAGACCCAAGTGACCAGGAAAAACTTTATCTACTGACTGAAACACTCTGTAAAAGCTTTTCCTCAAAGGGCCTGTCTCTGAGAAGACACAACATTTGGTGGAGCTCACTGTCCCAGACATGTTAACATGGATGCGGGTGAGCAAGCACCAGATGGAAAGACTGTCCTTGGACTCGGTGAGGACGAGCAGCAGGGACCAAAACCATCCCATGCTTACCCACCAAAGCAAGTTTTCTATCAATTTTTGATTAGCTGGCCATAGTAGTACTCAGACAATCAACTCACTGATGGGTCTGATTACAGTATCAATAAATTCTAATTGTTCTATAAATCAAAGTTTTTGTTATCTCTAAAAAAGCTGTGTTCTCTCTGACTATGTTCATACCTAATGTTTGTCTGTGGCTAGGAAACTCTAAATATTTATGGTTAGTAATTTATCTTAATCTACCATTAAAATTAAAGAAGCCTGATTTTCTTTTTACCAAATCCTGTCTTATAATGGATAGATTCACCCCAAGCTTTCAACCTCTGTGAAGAGGTTGTTCTAATTCTCAAAACAGTTTATCAGCAACAAAAAAAAGGCTTACC
MSTRG.5467.1	ENSRNOG00000019318;ENSRNOG00000019418;ENSRNOG00000019424;ENSRNOG00000019442;ENSRNOG00000019532;ENSRNOG00000019627;	1.693	0.179	0.038	-2.320	down	GGCTGTATGATTTATGATTTGAAGAGGCACAGCCTGTCATGACGTCTAACATCTAACAGTCCATCTAACAGCAGTGACAACAGCTCTTTGATGCTTCTAAGGAAGCCCAAGTTCATACCACCCCAGTTGCCCAAAGACCATCTCCTAGGCCTTTAAATAGAAGCTTCAGAGGCTAGCGAGATGGCTCAGCAGTTAAGAGCACGGCTGCTTTGACGAGGATCCGGGTTCAATTACAGCATCTGCATGGCAGCTCACTACAACTCCAGAGAATCTGGCACCTTCTTCTGGCTTCCATGGGCAAGGAGGACTTGCTTGGCTCCCAGTTTGCAGGTCCAGTCCGTCATGGAAGGGAAGTCAAGGAGGTGACTATGTTTCATGGCTGTGCTTGCAGAGCGTGAGGCAGCCTGCTCACATGACTGTAGCTCTTCACATCTTGGCAGAACAGGAAGCAGAGAGAACAGCATCAGTATTCCGCTCGCGTCCGCGGGACTCTCGGCTTCTCTCGGCGGCTGCCGCACCGTCCGAGCGGGGGCCGGGCC

	ENSRNOG00000031739;ENSRNOG00000037298						
MSTRG.54866.1	ENSRNOG00000020072;ENSRNOG00000020073;ENSRNOG00000020084;ENSRNOG00000020095;ENSRNOG00000027469;ENSRNOG00000029244;ENSRNOG00000039476;ENSRNOG00000046264	0.556	0.000	0.002	-4.481	down	TAAAGCCAGATGCGTTATATTGAACCAATCTCAAATAAAGGAAAAAAGAAAACATTTGAAAAATGAGAATATTCTAAGCACCAATGGCCAAAGAACATCAGAGTTGATGGAAATCGAATTAGAAAATGAAAAATGGCGGAAGTGTAAATCAGCCTAAGCAAAGGTTATCAAACTGCAATAATCAAGAAAACTGACAAAATCTACCCAGATTAACCCAGGGAAAAAAGAAAATTCAATTACCAAATATGGCATAATAGAGGGGTATTGTAACTTATATAAAACAGAGAGATTATAAGAGAATCCTCATATGTTTCTTTAAGAGTGTCTTCAAAACTTCTCAAATAAATACTAGGATACTGATCTATTGGCATATAAGGAGATTACACATCATGGCGAAGAAAGATTACCCTAGGACTAAAACATCTGTTCATATTCAAATTAAATCAATGCAATTTACCTTATTAAAATAATGGATAAAACCACACAACTATGTCGATTAATGGAGAAAAGCTTTTAAGAAAATGCAACACTTTTCCATGGTAATAAAACACACCAAGAGGTGGGAGCAGAATGAAAACTCTAAAGACGCAGCTACAGAACCCACCCATAACATCCTATTTAGCAGTGAAAACATACAGGATAAGCATGTTTGATTTCATCGCTACTATTTAACATTGTACTGGAAGTTCTAGCCACTGCAAATAGAAAAGTAAA
MSTRG.55087.1	ENSRNOG00000013851;ENSRNOG00000047234;ENSRNO	0.036	0.417	0.000	3.782	up	GGAGCATATACCTTTACCCAAAGGAAAAGAGGGAGAGCAAAACAGTCGTCCATTTTTCATCTGCTTTTCAGTAAGAACGACTTCCTAACTCCTGTCTGCACTGACACCTACAAATAAATAGCCAGGGACTAAAGCTATTCCATGAGCCAAAAGCTGCACATTTTGTGAATCTTGAACTGAGCCCAAGTACTCCTTTTTTTTCTTTTAACCTAAAACTCTGTGTGTGTGTGTGTGTGTGTGTGTGTGTGTGTGTGTGTGTGTGTGTGTGTAAAATTTGTGTTTTATATACATAAATAAACCTTAGGGATAAAGTTCACAGCGAAGCCAGATGTCTATTTATCCCATGACGGGCGAACCCAAACATGAAACTATTCCCCTAACCAACAACCCCCCCCCCCACACACACACACACAAGTCTGACAACAATATTCTAAATGTGGAGGAAGAAGTGAGATTCTGATGAAGTAAGGAATCTAGCTTCTGGTGAGAATCGAAGGACATGGGCCCAGAGGATCTTGCAAAGCCTTGGATAACATCCACAGGTAGACTTTATCTGA

	G00000058463;ENSRNOG00000059588						ATCTACGACAGTCTCCGTTGCCCACAGGGAATGTGGTCCCTTCCTTCGTTGATCACCTGCTGACACAGACTGCTCTTCGGTTGGTGATTCAGTCTCTGAGAGCTCCCGAGGTCCCAGGTTAGCTGACTCGGTTGGATACTGTGGAGTCCCTGTGCTCTTCAGGTTCCTCAGTCTTTCTCCCAACTCTTCCATAAAACTTCCTGGGTTGCATCTAACGTTTGGGTGTGAGTCCCTGTATCTCTTTCCATTGGTTGCTAGGTGGAGCGTCTCTGAGAACGGCGATGCCAGGTTCCTGTCCAGTTTCCTGTGAAGAGCGTTTGGCTCAGGCCTCTTTTTGATGTTGTAATGGCCATGGACAATGTTAAGAAGCGCTAAAGGCAGTCTCCAGCAGTCACCAGGGCCATGTTGCTGTACACCGCTCAAGGCAATCCCCTTATTCCACAGGCAAGGCTCAGTCAGGCTGCTGCTGGGAGACTGCCTGCTCCTTGTTACATGGAAATGAAACACCAGCCTGTTCAGGGACTGCCCAGCACATTACGAGGAGGAAGCTATAAAAGCTGCTAACATGGCTTACTTTAAGCATTTTTCCTCCTCAGACACTTGGTGGGGGGAGTAAGGTGTTGACTTCCATTTTTTTTTTTTT
MSTRG.55354.1	ENSRNOG00000011225	0.833	0.062	0.035	-2.552	down	GCATCTCCTTGATGAATCCTGGAACCCAACTGGCATCCCTGATGCAGAAACAGGGTGTTCTGTACCCTCTTTTTAAGAGGGTGTGGGAAAACATACAGGGAGAATACACTGTGACAGGTACCTGAATCCAAATCGACTTATACCTAGCCCATAATTATACTGAGATTGAATTAATCAGAGCCAGCCATTTGGGTTCATTCTGGTTAAATGCAGTTAAATAAATAATAAACTTCTATTACACCGAAGTCAACTGAAAGAAAAATCCACAGACAAGAAGAAAGACAACCTGGGAGGAGGGTCAAAGGTCAATGACTTAGTACTACCCACCAGTTTTACTACCAGGCTGCTGAGCCTTGAGAGGTTTTCAGCCTCGTCTCTCTTTTCCAATGCAATGGTCCTTTCTGGGAGGGCACAGCCATTTCTTGATCCTCTGCAGCTTCCAGCAGCTAACAAAGCCTTTCCATATCAAAGATGCTATGTATTTTCAGGAAGCAATCCTGAAATAATACGTGGTAGCCAGACTGAACAGCGACAATCAAGAGACATCTTTGCCATTCAGAGAATTTATGAAAATTCTTTGTTTCTGGAAGGCTCTCAAACTCCACATGCCCTAGCGGGTGAGCTCCCTGCAGCAGCTGGAAGCTGATCTATAAATAGCACAGGGCGGGAAGCGCCCCAGGAGCTCTGCGGGCG
MSTRG.55428.1	ENSRNOG00000003578;ENSRNOG00000031421;ENSRNOG00000042070	0.000	0.995	0.000	4.725	up	TGAAAAGCAACTGCTGTGTTTTTTAACTTTTAAAAAAGCCTTCTTGCCTATACAAATTTCCATCATCATGTACTGTATTCTGAATACTTAGATCAAAATCTCATTTTAATCAGCTTAATCCACAGAAGAAACAAGACAAAATGAAGACAGATAAAAATGAACTCCTCAGTGCATCACATCATGAGGAGAAGACATATGGCACTTATGTGACACTAGATTGTAACTGCTGACCTAAGCTGGGAATGACTGGACCAAGAACTAGTTCAACAGAATGCACACATGAC
MSTRG.55962.1	ENSRNOG00000013774	0.346	0.000	0.005	-4.261	down	GCAGGGCCTTAGTCCCCTCCTCCTTTTTAAAAGAGCATTTCTCACATTATTCATTTAATTCTTTCAGTCATCGATTTTATGGTTTTAGTAATTCTGCTATATTGACCCCTGGGGTACTTATTTTTAAGTTATAGGTACATTAGTTGACTTCCTAATATTAGATGGTTTGCCTCTTGGTTTAGGTTTGTAAACACCTTTTAGGCAGTAATGACTGTTTTGTTAGAAATCCTAGTTTTCTCTTGATGATATTAGACCTGCGCCATATTCCTGACTACACCCTAGCTGCTTCTGTTTAATCTTAGAGTGGGAGGGAGGGCTTGTCCACGACAGTTTGTCATACGTCTCTGTGCATAGATACACATAAGCCTATTTTCGAGGTGTGAATATCCTAAGTCTTGTGCCTATTGACTTAGGCACCACTCTCAGGTTGAAGCTTAGCACCTCACATGTGGGAACTTGTGCTTCAGAAGGCTCTGTGTGAGGGGCGTCAGTGGTCCAGTGTTAGGAGTCCCTGAAGCAAGGGTTTGATATTTTCGCAGCAAAGCCTAGAATTCTGACTGGCGCTGTCCTGCCGCCCGAAGCAACAGTATCAATGGAGGTGTGATTTATTAGAATTCTCCTAGTTTTGTTTG

							TCTGCCTTCTGAAAGGATGATCAGCCAAATTGAATGAGATAGGAAACTTTTTAGGGAAAATAGGAAAATTTAGAGAAGCCATATTCACATTGTTGGAGGATCAGAGTTCCACTTTAAAGTGTGTGGAAGGGACAGCAAATTGACGGGAGTGTGGTTCTGGGAAGAGAAGCTGGGCTGGCTGGATCAGAGAATGGAAGGAACACAGAGGAGTCAGCGTTGCCAGTTCGCCCTGGAGACCTGCTCAGGCCCAGGGTGTGAAGTCCGCCCACTTGCGACCTCCTCACTTCCACAGTGATGCTTTCTCTTTCACTTTCAGCTGCTCAGTAAATCTCG
MSTRG.56092.1	ENSRNOG00000047935	1.018	0.000	0.000	-6.957	down	ATGGCTCAATGTGGTTTAAAGCATTTCAGAGTAGTCGCTAGTACTGTTAGATGTTTTGAGGAAGGAAAATGCCATAAAACAGTGCCAATTTTACAGAGCCATTGTGGGGAATAAAAAGGGTCAATAAATTGAAGGTCTTTAGAATCTTGCCCAGCACACAACCAGCCATGTGTTCATGTTAATTCTTTCTAATATCCAAAATGGCAGTGTTTATATGCCTTATGCCCCATAGGTAATTACCCCAAGAGGATAATGTCAGTTGGAAAGGTCAGTTATAATGGGTCTTTGAAAGGTCTGCTCTGCTATAGTAGGGGATTCAGTTTATTTATTGATTGATTGATCAATTGATTGATTTTTGTATTCATCTGTGTGAGTGTAGATGCCAGAAGAAAGCATCAGATCCCCTGGAGCTAGACTTGTAGGTGATTTATATAAACCACCCGACATGACAGGAGACAGATTTGGGTCTATTGTAAGAGCAGTATGTAATCCTAACTGATGCACTCTCTCTCTAGCCCCGGGGTAGTGGTTCTTTATTTCTACTGCACGGTGGGATTCTCTGAGGGACTTTGAAAATAGTTATGATATTGGAACCAATAGCTGGTCCAATATATCAGAGTCTCTTACAGGGGATGCCTGCAGCATGAGTATCATTTGAAATAGCCTGGGTAACTAAGGTGGATACGGTGTTGACACCAAAGTACCCACTAGAATATATGCACAGCACACATTCCTGTTTGTTCTGGGGTGGAGCCCATGCCTTCAGGTTTGCACAGAAGTACTTTACCAAGGGAGGTATCTTGCCAGTTCTCTAGTGTATTGATTTTAAGTTAACTCTCATATTTAAATATCCAGCCAACATTGAGTTATTCTCCATTTTCTTCCCTTTTCCTGCAATCGTAGAGACTAACAAAAACTTCCATATAATTGTCATCCATTTGTTTAAAACAGCAAAGTCCCCAGACGTTAAGACAATAATATAAAAATATCACTTACAGCCTCATGGCATCTCATCTAATGACTTGCTCAAATATTGCTTTAAAAAGAACACCAAAGTGGATTTTCAGGCGTTCTTAGAACTATTTTCACACATTCAGTACTTGAAGAGTTATAGCTCTTCCTAATGACTGTATCTGAGTGGTATATAATAACCGAGTTCTAAAACACTTGAATGCTGATTCCCATTATTTTGCTGCTGTTATATTGACTTATTGCATTGGACTTTGGTGCTTTAAAGACAAAAGGCATTAGAGCGACTTGTATGATAAATTGCAGAAATGGTAACTTTATAGATTTATAGCTCTTTCGATGTAAATTTGATGTCATCTGCTTGTCACTAACAAACATTCCTTTGCTCAACACTGTACTGACTTCTCTGGAGGTGGAGACTACGTGATACTTTATTTTAAATAAAAGAAGGAAATGCTCCATCGAACTGGAAAATGCTGTGCATCCCTCAAATCTTTAGTACCATACAGGACACTTGGCAGATAGGAGAGAGCATTAAAGGCTGAGCAGTATCAAATTGTGTGTTTGCATTAAGCATATCTAAGGTACAAAAAGTGTGAATTTGAAGTTGTAGCAGAATCGCGTTATAAAGCATTTATACATCAAACTCAGAAGTTAAGATGGGTCTGTAGACGATGTGTGAGCAAGTCTTAGAATTTCGTGGAACAGTGTGGACTGGAAAAAAAGCGTAAGATTGTGAGAAATCCTTCTGGCTGATGTTAATGAGCAGCCGGTATAGAAAATCAGTCCGCTGGGTCATAGAAAGCTTTGATGAGCTCTGAGTGGAGAGTATCACACAGATGTTTTTCTTAATGGCTCTAGTGCAGGAAGATGCCTTAGCGGCCCAATGCTTGAGGACAGGGTCTTATGGGATTTTGTTATAGCTGCTGATCATGTGAGATGAATCTTAATGAAAGGGCCAAAGAAGAAACGAAGAGGATGAACTTAAGATCGAGAAGACAGAGTTGGTCACAGAAGTGACAATGTAGGCATGCAGTAGTAGATTGGGGAAGGTGAACGTGTCACCTGGGTGTTAGGTTTGAAGAGGAGAGTATTCCCAGAGGTTAGGTACTTGAGATGAGCTAGTCTGAGTGACGAATGCTTCAGGGATTACCAGAACACAGGAGGATGTCTACTCAGGGCACACCAAGTTCTACTCCTTATTCATGTACTCAGCAAACAGTCAGGAGCATCGCACGCCCAGCCGTTCTTCCACCAGTTGCTGTTTCAGCAAATGTTAGAACCACATCCCGACCTTATTTTCTAGATTATGAACGAGGTAAATAAGCTTCAAAAGACCCAGG
MSTR	--	0.000	0.228	0.031	3.453	up	GCTTTGTACAGTGCAATAAAAATGAATTGATTTACATTCTTCAACGTGCTGACCTCCAGTTGAACAAGCATCATTTGTTGAAAATGCTATCCTTGGCAGCAGCTCTCTGCCCCCAGACCATTAACCAAGGATTTAGGAG

G.56122.1							TCAGGCAAAGGTCTCTAATGAAGATTCTTCTTCTTTTTTAAATAGGCTTCTCCAAAAGATGAATTCTATTTAATCCCCTTGCTGTAGTTATTTTCATATACGGCTTTTATGAGAGGTGATCCTGATTCCTTTGAACTTGACTAATTGCTTCAGATTTTAGTTTCTTCTTCTTTGAGGTATGGAGACAGCACAAACAACTTCTGGGATCACTCCAGCGCTGCAATTCCTGGAATCTTGGATATAAACCACTAAATTTCCTGAAGGATCTTCTACCAGCATTACAGTTGGGTTCAGGTATCA
MSTRG.56208.1	ENSRNOG00000018735;ENSRNOG00000018774;ENSRNOG00000026060;ENSRNOG00000026108	0.000	0.336	0.004	3.943	up	CTCAAGTTGCCTTCAGCCGAGGGTAGCATGAATGTAAAATGAGACAGTGGATGAAAGAGTTGTCGTTTATCAGCACGTTCAGCTCCCTCGTTTTTCAAATGTGGAGAAGGAGCCTTGAGGAGGGGGACTGTCATCTCCACCACCATCATTTGAGCCTCTTGTCCTCTGCTGCCGGGGCAATGGGTCACTAGCAGTGACTCTGGCTTCTAAATCTGGATCCATTCGGCTACACGGAAGGGCCACTTTATTGTCTACGGTTACTTTGGAATGCAGCTTCATGGTTAGGGTCATCTAGGGTAAGCTGGAGCTCCGGAAAGCTGGGGCCATGTCTGGTTGCTGCCATTTCTCATAGGCTCACCTCTGTGCTTTGCAAATACATATGAACCAAGAACGAAAAGGCAAATGAATGACACCCCCTCCCCATTCTTTGTGTTTAAATATTTAGAATAGTAAAGCCTTCAAGGGCCC
MSTRG.56417.1	ENSRNOG00000023960	0.729	0.008	0.000	-4.374	down	GTGAACTCAAGGATTTAAGGACATGAGTCATTAAAATGGTTTAAAAAAAAAAAAAAAAAGCACAGCTACATGCGATCCATTATAAGAACCAATATACATGAATCCTTTCCATCGGCCGCTTTAGTAGAATTTTATTGGGTTTGTTTTATTTCTGTTTGTGAGGTGCTGTGGAGAAGTACAAAGTCCACAATGTGTGTGTGTGTTTTTTTTTTAATCCCTTAAAGAACATAAATAGTAAGAGGAAAAAAAAATGCACACATTAGAGAAAAAAAAAAAAACTTCAAAACACCACCTCGATTATCAATCAGGTGGCAAAAGATTAATCACAAAATAAATCCTGCAAATAACTTAGAGGAAGAGAAAACAGAGGGGAACATTAAGGGAGAAGTTGAGAGCGGTGTGTCCAGTGTGGGAAAAGAAATCAAAGGACACTGCACAAAGAGCTGGTTATTTTGAGAAACCACCTGGCCAGTGTTGTAAAAGTTTCTTGGAGTCCCACAAAATCTGTTTTGAGACTGCAGCTTCAGGCTAGAAACCTCAGAACTGCTCTGGCACCATTTTCCTCCCAGCAGAAGACATTAAGCCGGAAACGGGAGAGACTCTGCTGTGTTCCATTTGTCTGCGCCGTATACACAAGTATGTGTACACGCCAAGCTGTGGCAGGACGAAGGCTCCGACAGGGGAATCTGCTGTGGGGAACCGCACCCAGCTAGAAAAGTCCTTGTGATTAAAGACTATGAACAAAGTGACAGGAAGCAGGGACAGTGCAGTAGGGAGGCCCTGGGATCTTGGGAAACCTAGGATGCGACTCTTCTGCTGAGTCCTAGGGAAGCCTTTGAGTGAGGTGAGGAAAAGCCCCCTTCAGTAAGCAAGGCTGGAACTGGTCTGGAGGTCAGAAGAGGACATCAAAGGGACCAACTCTAAATGCTTTATGGTGAGTAGCTTGGAGTCAGGCAAACAACCAAGGAAAGCAGTGTGGCTGCTGCCCACTGGATACACCAGGTGTTGAGAAACTTCCGTCCTTCTCTTCACATTTCAGTAAGCCTCTCTGTTCCTCTGAGAAGCTCGTCCCTTTCTTTCTTTCTTTCTTTCTTTCTTTCTTT
MSTRG.56421.2	ENSRNOG00000017181	0.000	0.334	0.007	3.835	up	CCCACTGATCCTCTCTAACAGGCACAAACCTATGATGACTTAGTCCTTTGGTGTGACATATTGTGATTTGACTCTTTCCTACATCCTGGTCTCTGCCCTTTGTCTTGTGTGCCTCCAGCCCTTCTGAACCAAGCACAGCTCAACAACTTTCCTGTGATAATTCGCTTTCTTGTGCTTTTGCCTTGGTTTTCTTCCTACTCAACTTCACAGAGCACTTCTTTCTCCATCCATGTGGCTTGCAAGTGCTTGTCTCTGTCTTTCATTGTCTTTGACTAGACCATCTCACAACACGGAGAAGCAGAAGGTAACTTGGAAACCTGGCAGCAATGTAAGCAATTCGTGCTCGTTAGTAAATTCTGTAAAGTGGCAGACATACTGATTCCCTCTTTCTCCATCCCTTCCTGCTCCGCCC

MSTRG.5645.2	ENSRNOG00000021128;ENSRNOG00000021149	0.181	0.587	0.021	2.325	up	CTCAGCGATCTCACACACGAGGACGAAGAGCAGTATGAAGGTCAGGATCCAGCGCAGGTTGTGCCCCGGGAAATGGAGCCAGGTGCTGTGGTGAATGTGCACCTTGGAGCTCTGGCTGCCCCATCCTGCAGGGAGAGATGCAGAAGAGCTGATTGGCCTGAGAGTTCAAGTTCGTGGGAGAGGAGGGGACCAGAGTGAAGAGTATTGTCTCCAGACTGGGCGCTCAACTTCGTAGACCACTTCCTGGCAAGCGCGCTGGGAGTTGAAGGGGTTTGCTAAATCTGATCTGAGTTGACTGGCAAGCGCGCTGGGAGTTGAAGGGGTTTGCTAAATCTGATCTGAGTTGACTCACACGGTCTTGGAGAAGTTACATGAGGAAGACTCGCGCCTTGTGGGGTGGTGGGGTACCGTCACCCAAACTCTATCTCCTCGCAGCGTGGAAAGGTCCCCAGGCCGGGGAAAGGTTCTGCCTGAGGCCGGGCAGCTGGGACAGCGCGAGGCTCGCTGGCCTCCACCATCAACCCCGCTGGCTGCAGCCTGCGCTCCAACCCCAGTCCAGCGCAGCGGTGACAGCATGGGCCTGGGAAAAGGATGGCCCGGCTCCCGCCGCAGCGCCACTCAGGGGTGAGGCTGAGTCCCTCGCGACCTTTTTTTTGCTCGTGCGGAGCCCCATTCCGGATCAGGCTCTGCGGTAGCAAAACTTGGGGATCCCTAAGATCGCTAGTGCCCATCGTGTCTGGCGCTGGTTCAGGGATGGGGGAGGGTGCGAAGGAGGAGACCCACAGCAGGAGACCCAGGAAGACCCGCCAGCCGGTGGTGCGGGACCGCGCGCCACCCCCACCGGCCACTCGTCCTTCCCCCCGCGCGTCCTCCCGCCCTCCGTCGCGGCGCGCGCGCTCAC
MSTRG.5646.1	ENSRNOG00000021128;ENSRNOG00000021130;ENSRNOG00000021149	0.021	0.139	0.005	2.942	up	TGGAACTTGGATCCCCTGGAGCAGGAATTATTGGTGGTGGTTGAGAGCTGCCTGGCATGGGTGCTACGGAGAACAGAACTCTGTCTCCTCAAGAGAACTCACAGGAGTACCATTAATCACGGAGACAACTCCCTAGTGCCTGATAGTTTTGAATAGTCCACTTGTCACAACCTAGAATCAATTAAGGACGTCAATTGAGCAATTGCCTAAATCAGGCTGGCCCGTGGGCATGTCTGTGGGTAATTGTCTTGACAATTAATTGGTGTGGGAAGACCTAGGCACTGTGGGCAATACTATTTCATGAACAGAGCCCTGGACTGTGTAAGAGTGGAGAGAACTAGATGAGCAATAGGAGAGCAAGCAAGGATCAGAGAGCAAGCAAGGGAGGACCAGAGAGCTAGCAAGGGAGGACCACAGAGCTAGCAAGGGAGGACCAGAGAGCTAGCAAGGGAGGACCAGAGAGCTAGCAAGAGAGGATCAGAGAGCTAACAAGGGAGGACCAGAGAGCTAGCAAGGGAGGACCAGAGAACTAGCAAGGGAGGACCAGAGAACTAGCAAGGGAGGACCAGAGCGCTAGCAAGGGAGGACCAGAGAGCTAGCAAGGGAGGACCAGAGAGCTAGCAAGGGAGGACCAGAGAACTAGCAAGGGAGGACCAGAGAACTAGCAAGGGAGGACCAGAGAACTAGCAAGGGAGGACCAGAGAACTAGCAAGGGAGGACCAGAGAGCTAGCAAGGGAGGACCAGAGAACTAGCAAGGAAGGACCAGAGAGCTAGCAAAGGAGGACCAGAGCGCAAGCAAGTGTGATCCAGATGTGTATTCTTTCTGATTTTGCTTGTGGATGTGATACTATAAGCACCTGTATGTATCCACTCCCAATGCCCTGAATTCACTGTGTGGCCCAGACTAGCCTCAAACTCACAACCTTCCTCCCTTAGACTGGAGTTTTCTATTATTCTATGTTTCCTCTACATTTTCTACATCATTCTTTGTGCAGTTACATGCTGGGCCACTGTCGTCTCTTTCAGTAGTTCAGTCATGGATGCATAAACTTTCAGGCAACTGAGGACTAACGGGGGGGGGTGGCGGTGGTTGGGGGCACATACTAGTCTAGATTGCCTACTGCTGTCATCAGCTCTCAGGTGCCATCAGTCACCAGAGGTTCTGCTGCTGTGTGCACAGTGGCGCCACCTAGTGACAGTTCTCAGAGCACATTCCCATCGTGAGGCAGCACAGGACCAGACGGTATTCCCTGTTCTGGCTCTGCCACAACTTCTTGTGTGGTATGGGGCCAGTCACCTTTCTGAGCTTCAATTTCCTCATTTCTGAATGGGAGGAGGAAGTCATGTAAGTTGGGATGAGATCAGGGGACCAAAGGGGTTTAGCACACAGGGGAGGCAGGCTCTCTATGCTCATTCGAGTGTCTACAACTTCGTCCTATAACACCAAGACCCTACACTTGCCTCACCTAGGTCCTTACAGCAGACACAAGGTGTGGCTAAGTGTAAAGTTCTAGTGAAAAGAGCTGCAAGTGGATGGAGAATGTTTGGTCTGCAGTCTCAGGGACCCATTTACCTCAGGGACACATGTGGCCGCCAGGGAAGTCCCTTCTCAGCTGGATCTAAAGTGAGACCACACGGCCCCCTGCCTGACACATGGGTTCTCTTGGACAGCATGGTATCGGCTGCCCAAAGAGGTGCTCTGTAGCTGCCATGGGTGTGTGATAGGATTGCTTTGTACCTGGGTTTAAAATCACCTCAAATGCCCATCAGTACAGGGCCCCTGAAAACATAAGGAACCTGCCTGGTGATGTCCTTCCCGGGGCTTCAGTGTGGCAAGCAC

							AGAGAACATGGACAGGGAAACGTCTCGCAGACACAGGCATGGGGGAGCATGTGCTATGTGATATCCTTTATGGACTGACTTGTCCGTGTCCATACAGTGAAGGGCGGGCCTCTGGGCTTGCTAACCCTGGAGATCCTGCCACTTCTCTCGCAGCAGGCCCTGGGAGAGTGCTCTAAGCAAAGGTACCCCACTTTGACAATTCTCAACCGTCCTGTGGCGGAACAATAACTGACCTGAATGTAAGAGTCTAGATTCTCACTCCCTGGCAGGCAGGGTGGGCGTGGCTCTTCTGGGGCTGATGTTGCTCAGGCCTTTGTGTGATTAACTGGGTAACTAGTGACCGCATCAGAGGACATCTTCATGATGAGAAAGTCAGCAACGAAATAGGAGGCAATTTGGGCGATGACCCTTTCCAACAACCCCAACCAACGGGCTGGTTAAAAACAAAAGCAAATGAACAACAATACCAAGCAACCAGAGCTTCCAGGGACTAAGCCACTACCTAGAGACTATACATGGACTGACCCTGGACTCTGACCTCATAGGTAGCAATGAATAGCCTAGTAAGAGCACCAGTGGAAGGGGAAGCCCTTGGTCCTGCTAAGACTGAACCCCCAGTGAAC
MSTRG.56536.1	--	0.464	0.011	0.000	-3.664	down	GTCCAACAGGCTGTTCCATTGAGTAACTGGTTAGTGAACGCATGTGCTGAGACACGCGCGTTAATGCCTCTGAGTGAGCTAGGGTAATCGAGATACCACACTACTTTAAGAAACCTGGAAACGTTAGCCAAGGGAGCGTAACAAATAGAATAATCAGGCACTGGTGGTTTCTAAAAGCTTGACACAAGTTCTGTAACCGTCACTGATTTATGTGACATCTGACCTTTCAGGACTGAACAGATGAGCGAACCATCTAAGGGACACGCTCCATGTTAAATTAGCAGTTTTATACAGCAGTGATAGGAAAACAACATGTCCTAGGAAATACTGTGGCCACGTGGATGAAACACACGACCAGACCCAGGCAAGTAGCCCTGCAGCCAGTACTAAGCCATTTTACCAGCTGGATGACCTTGCTCACCCCGTGCACCTCTAAGTCTCCATCTGCCAGTTGAACATGTCCCTCACGTTTAGACTACCATTCAACTCTAACCATCACGTGCAGGGGCAGGGAGCCCTCATCTGGAGCTCGCATAGGTGAAGCAGCTAATGTCAATCCATCCTAGCTTCCAGTGGCTGCTCTAAAAGCACACTTCAACTAACAGTGCTTCCATAAATCCCACTGATCTGCTCTGGAAGCAACTCACACCCGAGCCTTGGGAGTAAGGAAGAAAGTTTCCAGTTTCCTATACAGAAAGCGTTGTGATTCGCTGGTTGGCCGCAGCATAGTGAAGACACACTGGGATTTGTTTTACTTTGTAGCGCCCAAGTAGCTTCTCAGTCCGCTCTAAGTGCCTTAACCCGATGCCTTGCACAGCATCTTAAAAACTCAGACAAGCAACAGACATTTAGGGATATGTGTGCCACCTCAATTCTGTGGGAGAACGGAGCAGAAGTTCGGAATTCAATCATTCAAAAATCAATTGTTAGCTCCCAAAAGGGGGAAAACAACGGAGCATCGTCCTCTCCTTTGGGGATAGGATCCACCTGTTTTAACTCCGGGCTTGCACAAGGAATCACCTGGGCCTGTGCATGTTTAATTTTAACAGTGGCTGCCACTCTCCCAGGTAAACCCGTGATCTTTTTTTTTTTTTTTAAAGACCATGAGTTTCAACAATCTTGACGTTTGTTTTCACTCAAAGCAACCACCCCATCTACCCACAATCCTCCCGGTTACAACTTTGCCTAGAGAGACCGTGACAGTGGTACACTCTGTCCCAACACAAGGTCAAAAGCAAGCCAAGTGCCAAGTATTTCCTTGTTGCTGACAATATTTTCAAAACAAAACGCAAATGAGCAGCAAGGCATTCCTCTTCGCCAAGTTTACACCCACAGTTCAAGCCGCTCTCATCAACTTGCAAAGTTTTTCACTCCAACCTCTACCTTGCAAGCTTTTCGCAGAACCAAAACAGTACAGGTTCTCAAATCTCAGGCACTTACTGGCTGCAAACTCAGTTGCTCAGCTTTGCTCCCAGCCCAGGAGCACAACTGTTCGCACTGAGGGAGCGCACCACCCCAGTACCACCCTGACACGGGCACACACAGCCCTGGCTCCATTAGCGCCCTTCCAAAGCCCTGCTTGACAGCGACGCCTGTCCCTCTAGACTTCTCAGAGCCCCAACTTTACCTGAAGCACGTGGTGAGCTCGCCTGAAGACTTCAGACACGGGTATTTGGTCGTGGTGATCTTGTTTTCCGAGCACACTTCTCTGTCTCGGTCCTCGGGTTCCTCTCGGAAGGTCCACGTGTGTCCCAGCGCCAGGAGTAGCAGCAGCGGCCCCAGGCTCTTGCCCAGCTGCCCCTTGACTGCTCCTCCCCGGCTCAGAGGAAGCGGCGGCACCATCGGGGAAGCTCGCCGGCTTCTTCTCGGCACCCGAGCTCTCTCCCTGCGCTGTGCCCGGGGAGGCTCCGCGGCCGCAGCCGCCTTCCCCTCTCGCCAGCAGAGGGCGCCGGAGAGCTGGGGCGCTTGCACCACCTGCGGCCCCCTTCAAACTTGGGGTGAGAGGGGGTGAACCAAGCTTGTCAAAGGGGTGTCTGCCTTGCGCGGCCAGAGGGAGGATGCCGGAGTCCCGGGAAATCTCTTCTGGACAATCAGATTCGAGTAACAAACAGGCTGAACTTGGCTTC

							AGGCTGCGCCCTCCTCTCTTCCACCCCTGGGAGAGAAGCGCGGAGTTGAGGCAGCCAGGCGCTCCTTTGCATTTTT
MSTRG.5660.1	--	0.000	0.227	0.011	3.719	up	CTGGAATTAAAGAGTTTGATCTGGATACTTAGCTGTCTGGGACCTTTTGGCACCACCCACTTCCCCTTGTCCAGTGAGGAGGAAATGCAAGTCCTCAGTCACCTGCACTGCTGAGCATCAAGCCTAGCATGAGGGCTCTGGGAGATCCACACAGAGAGGCCTCACATCGCCAAGCAGGAAGGGCCCCAGGCTCGTTCAAATAGAGCTACCCTGAACCTGATAGTGCAGCTCGTGTTATTTCTACGTTTTCCTCCCCTTTCCAGATCAGTGGAGAAGCTGGGCAGTGGGGACTGCCTATCTGTAGCACCTTGCAGGCTGAAGAGGAAAGGTCACAGGTTCAAGGCCTGGACTACAAACCAAGACCCTGTCTCAAACAAAAGAGAACCACAGACACAGACTGGAACCATCTGTAGGATCCCTCCTATGCCTGATGTTCAGCGACAACTGCTCCTCCACACAAGCTCGGCTCTCAGGCCATAGCACCAGCTCTGCTGGAAGCATGACAGAGATCCAGTGTAGACCAATCAGACCTGCAGAACCAGAACCCAG
MSTRG.56611.3	--	0.051	0.509	0.002	3.295	up	GGGACCCCTGCCCGGTGTGCCGTCTGGGCACGACTGGGGGCGCGCCCTCATGCGTGGCTCTACGTTGGCAGCACCAGACCCCAGTCTCCTGTTGGACTGTTTTTAAGAACAGATCCCAGGCTTTGCCTGGTTCAGAGGTTAAATACGAAACTGTGTTCCTTCCCCGCCAACGGGAGCCATGGAAGAACGGCCCGCGCTCGCCGCACAACTGTATGCTGCTCCAGCATTCCTGGGCGCTCTCAAGCACAGACGTTTGATTCTGAGCAAGAACAAAAGCAGAGCCGAGAACAGCAGAGCCGAGCAGACTGAGAGCCCTTGCTGAGGCCGAGCCTGCTCACTGAGAGGAAGATGTTGGCTGCTGGGAGGCTGAGGAGCCCTTCGTTTTGAGGTGGGAAAGCGGGTTTCAGCAGGACAGCTTCTCCGTCCCTGACCTCACCATGGCTTCAACTCTCTGGCTGGATGGTTGATGTGACTATGTGTCCCATATGCCGTCGGACTGTGTGACATCCAGGTAGCCAGTGGGCATCGGATGCTCCCAAAAGCTGGACCAGCAGAGCGATGGACTTGGTCAGGCTAAAATGGAAGTGACCTGAGGCCTCGCCCAGGCGGTTTCTTCACAACAGGACAGCTTAAGAATTGAAACACAGGCTCGTCTGGGGAGCAGTATGCCGGAAGCTGGTTTTCAGGCCACAAACGCGTTCACAG
MSTRG.56665.1	--	1.779	0.046	0.000	-4.378	down	CTTCCATTGGGCAGATGGTGGACCATTGACTTCCACAAGATTTATTTGACCAGAGAGGCCTGAGCCTCTTCCAGGCAGGAAGATTTGGTGGCTGGCTGTTCACTATTAAGTTTCTTGAGTGGCAAGCAATTTAAAGTAAAGAAGGAAAATGAAGTAAGAAAAAGGCTTTTCTTTAAAAGAAGACATGATAATTCCGAAGAGCCAACCCCAGGATAAATTAGAAGTAATGCTCTCCAGCATCGGTTGGCAAGCTTTGAAGGAAATCTTATCCTTAAGGTCACATGGAGGCCACTTCTAGTTCGACTTTTTATCTTAAAGTCCTCAAAATCAGTCTAGAGAAGAACAACAGGAGACTGAACTAAGACTACAGGAGAGAGTAAAATGGTGGACCGTGCTATCATTGGAAGTGTATATACCATTGAGAGAGTGGAGGGGGACAGCTCACCAAAGAGAGGCAGCTTTCCAGTCCATCCTGTGGTCTTGACAAGAGCGTCAACCCATTCATATGGACCAATGCATGGAGGGTGAAATATTTTTGTCATTGCCGAGAGGTTTGGAGGGAAAACTGCTGATAAAACTATAAATGGAAGGAGCTGTGTAGGCAGAGTGGAGTCATTTGAGTGAGAGGGAGTTTCATCTATCATCGTGTGAGCCCAACAGAGAAGACATAGTGGTATAAATGTGCTAATTAAGTATTTGGAGTTGAGCCTTAGAGAAACAAAATGCAAGCAGTTAAAGAGTTTGCCTCTGGGGACTGAGAAGCAAAGGGAGGGAGGCAAAGAAACCAGAATTGATGGTTTTCTCCAGGAGCCCTTATGTTGTTTTTGGTTCCCAAGCTATCCGTATTTGTTACTTTGAATAAAAATAACAATGAAATAAAAACTAAAGTATAATAGTGGAAATGTTGTAAAAGGAGAACGTTTATTAGCAATGTACTAAAACTTAACGAAAAGCTATCCAGGAAAAAACTTAAAACACAATTGGAAGATTCAACTTAAGTTGAGTATTGGGAGCTGAGATCCTGTAGGACTAGTTTGGAAAAGATAATAAAATTTTAAGATAATTTTAATTAAAGAAAATAATTTACTTTTAATTTAACACAAATTTAATTAAAAATAACGATATTAAAAGTACCCGAGGATCAAAATGGTACAAAGTAGCAGACCAGAAGGAAGCAAATAGTCAGGGATGGCCCCTCTCTGCTTTCACTGGAACATTTATATGGAGCTTTGTGATAACTTGA

							GAACCAGTATCGAAAACTAATTAAAACATGAAAGCATCTAAGAATTAGTGGCACTGACAGAAGATCATC<br>GATAAAAGGGGGATAATGCAGTAGGGGAAGTGTTTTGTAGGTTTTTATTGAAGATGGGATCAAAGGGAT<br>TCACACACTTTAATTGTCTGTTGAAGCAATTAGATAAAAGAAAAGAATCATCGTAGGAGAGGTCGTTCA<br>TATTAAAGATGGCAAGAACTTGACAGGAGAGCAATCAGCCTCTTCCCCCAGTCACCACCTGGAGATCA<br>CCTTCAACACTTCCTCTGTGCTCCAGGACAGCAGCTAGTATTCTTGTGATATGAAAAATATGCTATAGATA<br>AATCCAAGTAGGATTGGGTTGTGAATAAATAAATCCCTTAGCTCCTGTTGAACTTACCTTGAAATTTGAG<br>CAAACCATATATAGGTTTAAGGCAAAGAAGTTTTCTATTTTATTCTGTGGAACACATGGAAAGTATCTCC<br>TAAAGCTAGCTGGAAGAGCCCAGAGAATTGGATACACAGGCCCTACAGCCATAGACATTGTCCAGCAA<br>CCACAGTTCACCAAGTTTTCAGACTGTAATTAGCGGTTTGGTTTGAGTCAAACTTGAGACACAGGAAA<br>AGACATTAGCAACTCATTATACAGAATAAACAAATTGAGGTTAAATGTTAAGTGGCACACTCAAGATTA<br>GAAATCAAATTAATAGGAAATCTGAACCTAGATGTTAAGTGGCTGGTGTTGCAGTCAAGGGCCCTCTCA<br>GTAAATAATAACTTTCACATCACAACACATTAAATTCATTCATCACCCTGAGACCAAGGCTTAGAGTTAA<br>GGAACAGATTGTCAGCATTGTAAATGTCTACATTCAGGTATGGCTATTCTTAGTACATCTTGGTCCTTCAA<br>AAGGTTCGCAGAAAGGTATAGTATCAGATACTAGATACTACAGAATTAGAAGGTATGGCAGAAACAGGG<br>GGCCTCTGCCAGTCAACATCCCAATCTCTGGATGAATCATGCCCTGTGCCTCTTCCCACAGCTTAGTTAA<br>TATTAGCATCCTTTTACTTTTTAAATAGTATCCTATACTGGAAAATAATTACCCATGTGCCCCTTGTTTATCT<br>GTACATGCTGTGATTAGGCAATAAATATTCCCTCTTCTCTCTTTTATTCTTCCTTACTTTTCTCCTCGCTCA<br>TTAGTTTTCGAGAACAGGTCTTGTTATATAGTTCAGGCATTTCTCAAACGTATTAGCCTTCTGCCTCAGCC<br>TCCAAAGGACCATGATTACAAGTGTGTACCATGATGCTTGGCTCCTGGATTTTCTTTCCAAAGATGCTTA<br>TTTAACAAACAACTTTGAAGACTAGACACAATCCTTCACTCTAGAGAAAAGTCTAAGCATGTTCTTGTC<br>ACAATAGAAAAATTGAGTTCATTAAATTGAAAGTTTCTCTCCCGTAGAACAACCAACTCTAACAGAGTC<br>TATGTGCATGCAGGCATATATCTGCTGTGCTTTTCGGGTGTTCATAGGAGGCAAGTTTCTCTGACATACA<br>AATGGTCTGCAGCTTAGTGAGGAATATACTTTTCATGCTGACCCCAGGGGTCCTGGGTCTTGCTAGTATT<br>GAGGAATTTGTGTTAAGCAAACTTGTTTTCTAGCAAGCTAGGTAAAAACAGAGTCTTAAGCACTCTTAA<br>TAAGCATCTCTTTATCTTTTAAAAATGAGTAGCTGACTTGGGGCTGGAACCCTGCTGTAGACTACATTTC<br>CCACATGCCTTTGCATGAGGACTCTGGTAGAGACTACATTTCCCATATGCCTTTCAACGAGCATGTGATC<br>CCTTTCTGGCCAAGAAGCCAGACAATACTTTGAGATCACAATCATAAAGGCAATGACTACACATGACCC<br>TTGACCTTCTCTTTATCTTAGAGAGAGCTAGCCAAATGGTGGCATTCAGAGCCACGTATTTTAGCCTTAG<br>GATAGAAATAAAATGTTGTTGAGAGTGACAGAGCCTCTCTGTTAGCCAGGTGTCCTATAGTATGGGAAG<br>GGGCTAGGAGCTTCTACTTGAATATATGTGGTTTTGGCTGCTTTAATGGAAGCTTGATGTACACAAGGAG<br>ATGAAGACATGACCTTTGGGAGTCTGTGCTACACTTATTTTTAATAGATGAGAAAATGGAGACCTGGGA<br>TCAATAAAGGACTCGCACATAACTGCAGAGTGAGCAAAACTAGAGCTAAAATTCCTAATTAAACGACCT<br>CTCCAACTCCCGTTTTTCATATAGCTCATGGCCAATGTACCCAATAATATTGAATAAATAAGCTCAGAGGT<br>CCAGAGAGGTAGGAGTGCGGGGAAGTCTTTACAGTGAGAGAAATTGCAAGTGTAACAACTGATATCAA<br>TTATCGCGCCCCAGATGAGGAGATGAATATGGAGACATAATTTGAGCTTCATGAGCTGACGATCGAGGT<br>AAGACTGAGAAATTGCTGGGGTAAAAAGTACTCAGCGGAGGATGAATGATTTACACCACAGTGACGTG<br>GTTGGTGGTTTCTTTCAATTTTCATTGTTATGTCCCAGAAGCCCAATTCACTGCCTTTTTGGAACAGCTT<br>CATTAAGCAAGTGAAATTTGAAGAGGGATCTGAGAGAGTCGAAATAGCTTTCATGGCTAATGGGCGAA<br>GGAAAACTGGCTAAGAACTGACTTCATAAATCATGCAGTATCTTGTTTAAGACATTTCCTAAAATGCCTG<br>TGGGTGTGGTGAAGATGTAGCGTTTTTTTAAGGCAATGTTTGTGATCTGAAGAACATATCGTTTTGTGGA<br>GTGGCAAGAGCAACATTAAAAACATGGATGCGATTTTATACCCAGGATGGAGGTAGACAATTTCCCAGA

							GAATATTCAATTTGAAAGATGCTGACGTGTTTAAGAAATATCCTTTAGGAAGGTGAAGTCCTGTAGACAATGGCCAAATGATTCCTCCTTTAGTTGGAAAGTATAAATGAAGCAAGTTTTATTATTACAGTTGTTTTAAAGTTTTGAAGGGATTATTGGAGCTCTCCTTGAAGTCTTATGGTTTTATAGATTAGTGTGGGAGTAGGCTTGGGTTATAAAACTACCTAAAGATGAAATGAAAACTTTTATAGAGTGGTTGCTTTTGGATAGACATTGAAAATTCCTAGGTGTCAGATATAAAACTCAAGTGGGGAAGTTGCTTTGGGCTGGGACTCATCAAATGTGTTCTCCTAGAGCAAAGGAGGATGAACTCAGGGCTCTTAAAGGAGCTGTGAATCAATGTGGCCCAAAGAAATCCATGGGTGACATTCAGAAACTTTTTTCTATGTCTGCTACCAAAATTTTACGTTTGGAAAAAATTAATTGTGGCAAAACACTCTGCATTACACTGACTAGTGTAATGTTCATTGTGCGGTGAAAGCAATTTTTCTGTACTTTTGCATTCTCTGAAATTACTCTGTTCCCATTAAATTCTATTTGTTCCAATCTACATTGCCTGGAAAGCTCTCTAGGATCTTTTTTGTTAGATTGACTCAAATATTCTAGATATGTCATGTAAATGGAAAGACACAGTATGTATCTTTTAAAAATATTTATTTTTATTCTTGAGATTATAAATTAATAACAACGTCTTCTTGCCCCTTTCTTCCCTCCAAATTTTCCCACTTACTGTTTCCCTCTCTTCAAATTCAGA
MSTRG.56734.1	ENSRNOG00000028712	0.504	0.000	0.049	-3.592	down	TAAGAAGGCAGAGGCATCGTGTGGATCTCTGAGTTCTAGGCCAGTCTGGTCTATGAAGTGAGTTCCAGGACAGACAGGGCTACACAGAGAAACCCTGTCTCAAAGAAAACAAAACAACAAAACAAAACAGTAACAACAAGAACAAACAAAAACAACGCATGCACACAAGTACAAAATTATATGTGTGCAAATATTTACCTTGAGGGGAGGGGTGACATAAGTCCCACATTTTGGTGCTCTCCCTGCCCAGTTGAGCTGCAGGATTCGGGTTTCTTTCACACAGTGTTTCCAGGTAACCTTGGTCTTCTAGATGATGTTCCATTGCCTTCCAGTCAGCTACGTTCCCATGAGAATCCATTCTCCTTGCATTCGTTGCTCTTCCACAGTTGATTGGACCACTTGCTTCAGCTGGCGTCAAC
MSTRG.56784.1	ENSRNOG00000000113;ENSRNOG00000015582;ENSRNOG00000051965	0.000	0.436	0.000	4.599	up	GAAGTCTTCTCTGAGGCTCACCCTCCTTGTTCAGCAGTCAAGGCAAGTAGCTCTTTGCCTGAGAGTTGCGAAGTTCACCTGAACTTGCTTGAACTTGTCCAAAGCTATACCTGTGAGTTAGAATGTGGTCCAGACTCCAGTTAACCAGGGTCTACTACAGCTCTTTGGAAAATGTTCTCTCCTTACTGCTGTCATTCTAGTGCGATTCTACACACGATAACTGTGTAGCGGAGGGAGGCACTCGGGGTAATCAAGCTGCTTTTTTGTTTCCTTCAGGAGATCACGATGTGCAAAGAGAAGTCAAATGCAGTTTCGCACGAGAAGATCGGGAGGCTCTCTGAGAAACTGACCTATAAGCAGAGACCATTTGACTATATATAAATGATACAAGAAAGAGGGCACTGGAGAGATGGCTCAGTGGTTAAGAGCGCTGGGTGCTCTTCTAGAGGACCCAGGTTCAATTCCCAGCACTCACAAGCAGCTCACAGTTCACAGTGTGTAACTCCAGTTCCAGGGGATCCGACACCCTCTTCTGCCCTCCAGACAGGCGCACAGGTGGTCCACAGACTTCCA
MSTRG.56825.2	ENSRNOG00000013766	0.890	2.158	0.045	2.037	up	GTTGTGAGCCACCATGTGGTTACTGGGATTTGAACTCAGGACCTTTGGAAGAGCAGTCAGTTCTCTTAACCACTGAGCCATCTCTCCAGCCCCTATAGGTTTATTCTTTATCCTTGTCTAGCAACCCGAGCTTCCTGGAGAGTGTATATTTGTTGTAAGTAAAAACTAAGCTACTATTTGCTTTATAACTGAAGTGCTCACCGGATCGCATGTACACAGTCTCCCACTTGCACTGACACAGGGTCTTCTTATCTAGTCCATGCTGACGTGAACTCACCATCTTCTTCAACCTCTCTCAAGTGCTATGACTATAGGCATGGGCCACATTCTCAGCATCATACACTTGTCCTAAAACTATTGCAGCTCCTGTGATGTCACCTGCTCTCCTCAGTTGTCAGTACTGTAATTTCTAGCAATGTTGAATATTATAGAAGGGGAGGGAGGGCGGTTAACCATGGAAAAGTGGCTCAGAACGTGTAGGAGAAAGGACAGAGGAGCTGGACCATTCTGAGACTCTGATAAACTCTTAGACAGTAGAATTTAGATTTACACTTGACCAATTTCCAGTTCTATATTAACACATCTTCCCTTCCAATATTAACTACAGAAAGAGATAACACGGCCTTTCTGAAAACACTGGAGGCTAGCCCAGTCAAGAACAGAACAAAAAGACTAGCTCAGAAATTCCAAGTCTAGCCCCCCTAAGTTATATTTTGAAAGGCGTAGTATTACAAGGTGAACCTACCTAAAAACTACATAACTAC

							TTTAGAGTCTACTTTAAGTAATTTTGTAGCTTAGTTGAAGGATACTGTGTGTGTGTGTGAATGTCAATCTCCAGAACAAATAAACAGAACAGACCATAACACACTTTTCCATTCTTCAGTAACATTGTTGCTGTTTTCCTTCTTTTTTTTTTTTTTTTTCTTTTTTTTTCAGAGCTGGGGACCGAACCCAGGGCCTTGTGCTTGCTAGGCAAGCACTCTACCACTGAGCTAAATCCCCAACCCCGCTGTTTTCCTTCTTGCTACTGTCTCTATCCATTTTTGTCCTGCATGACTTTAGTTGCAGACACTGAATCTTCACCCATAAATATTTCTGTGCCTAACTACAAACAAAACGAAAACCTCCCAAAGCCAGTCTCCTACATGTCAGTTAATGTTGGGCTTGTCTTGACGTGATTAGATTCAGGGCATTTAGGGGAGGAGTCCTACATAAGAGAGTTGTGTTCTCCGTGCACCAGGGAGTCCATGGCTTCCTGACTGGTGACTCTTAAGTTTGGTCATTTGGTTAAGGAAACGCCTCAGTTCCTTTTCTGAGACTGAAACTGCTGCTATTCTTACACATGCCCCTTTGAGTGTGTTACCTTTCACCCCGATTTTATCAGGACCATTCACATAATTACAGCTAAGAGCAAACATGGAAGTCCCTCCTCGTAGTGACTTGGAGGGCAAATGAAACAGAGGAACCCAATGCAAGAGCAGAGACATCCATTCCTACAGCTTGAGCTTTGCACAGGACTGCATGAAAATTCCTCTCTAAGGGACCTGTAAAGAGACGAGTGTGATGACGTGTTCACGGGGTGAAGAAAAACATGAGATTTTTTAAATAAAGTTTTAATGAGTAGATGTGAAACAGCAAACAGTATGTTCCCCCATACGAACCTTTTCACTTGTCTGACGTTTTTCGCAAAAAAAAAAAAAAAAAAGGGTAGGTTGAACCCAGGTGTGGTGAAGCACGCCTTTAATCCCCTTTAATCCCAGTATTTAGGCGAATCACATAATTCTTAGCCTAGTCTACAGGAGTTTCAGAACAGCAGGTAACCCTGTTACGGAGAAACAAAACAGGAGAGAAAAGATTAAAAAGGCAGTCCCCACTCCAAGGAAGCTAACGAAACACACACAAAGATAGATTAAGAATAGGCAGTGATTGACGAGTTAGTAGAAAGCAATAGCACCACCCACGCCCTGTTCCCACGCTCAGCCTAGGCCACTGGCCACCCCGGGGCCCTGAGCTCTTATCCCCAGCACCCTCTCTCTGCTTAGGCCGCCTCCAGCTCGCCTGGCCTCAGTGTTCGCAGCACAATCCATCACCTGGTTCCCAGAACTCAGATTGCGCAGTGGTCATGTCATCATCGGCAGGGCTCACAGCCGGAGGCGTGGGTACCCCTAAACCTGCCACGTTTTCATAATCCTCTCAGGCTCCAGACCAGCAGCGCCGGCTAGGAGAGGCCTGATCATATAAGGAAAATACCTCGGGCTCACTAGGGGCTGCCTCGGGGACCCAGGCGCTCCCTGCTCTACCATACATCCCCCTCCCTTCTCTAGACGCCCCAGCTACCCGCCGCTGCACCCCTTAGGCCAAGTACAAGCCTCCACCCAAGGGCTGCGCAGG
MSTRG.56825.3	ENSRNOG00000013766	0.000	0.922	0.000	6.447	up	CGCTGTTTTCCTTCTTGCTACTGTCTCTATCCATTTTTGTCCTGCATGACTTTAGTTGCAGACACTGAATCTTCACCCATAAATATTTCTGTGCCTAACTACAAACAAAACGAAAACCTCCCAAAGCCAGTCTCCTACATGTCAGTTAATGTTGGGCTTGTCTTGACGTGATTAGATTCAGGGCATTTAGGGGAGGAGTCCTACATAAGAGAGTTGTGTTCTCCGTGCACCAGGGAGTCCATGGCTTCCTGACTGGTGACTCTTAAGTTTGGTCATTTGGTTAAGGAAACGCCTCAGTTCCTTTTCTGAGACTGAAACTGCTGCTATTCTTACACATGCCCCTTTGAGTGTGTTACCTTTCACCCCGATTTTATCAGGACCATTCACATAATTACAGCTAAGAGCAAACATGGAAGTCCCTCCTCGTAGTGACTTGGAGGGCAAATGAAACAGAGGAACCCAATGCAAGAGCAGAGACATCCATTCCTACAGCTTGAGCTTTGCACAGGACTGCATGAAAATTCCTCTCTAAGGGACCTGTAAAGAGACGAGTGTGATGACGTGTTCACGGGGTGAAGAAAAACATGAGATTTTTTAAATAAAGTTTTAATGAGTAGATGTGAAACAGCAAACAGTATGTTCCCCCATACGAACCTTTTCACTTGTCTGACGTTTTTCGCAAAAAAAAAAAAAAAAAAGGGTAGGTTGAACCCAGGTGTGGTGAAGCACGCCTTTAATCCCCTTTAATCCCAGTATTTAGGCGAATCACATAATTCTTAGCCTAGTCTACAGGAGTTTCAGAACAGCAGGTAACCCTGCCACGTTTTCATAATCCTCTCAGGCTCCAGACCAGCAGCGCCGGCTAGGAGAGGCCTGATCATATAAGGAAAATACCTCGGGCTCACTAGGGGCTGCCTCGGGGACCCAGGCGCTCCCTGCTCTACCATACATCCCCCTCCCTTCTCTAGACGCCCCAGCTACCCGCCGCTGCACCCCTTAGGCCAAGTACAAGCCTCCACCCAAGGGCTGCGCAGGCGCAAGGGCAGC

MSTRG.5696.1	ENSRNOG00000013663;ENSRNOG00000013981;ENSRNOG00000047591;ENSRNOG00000050257;ENSRNOG00000050806;ENSRNOG00000058252	0.000	0.299	0.003	4.043	up	CCTGCTTCCGGAGCAGAAGCGCGTGTGCACCACATTGTTTGGAATGTGCGGCCAGAAGCACAGGGGACTGTCACTTCTGGGACAAGGTATAAATCCTTATTAAACGAGGAACCAAGGCTTGTGACCTCTAGGGACAGATACAGTTTCTGCAAAGGGCCGATTAAAGAATTGTTTGGCATTCTGACTCTGTTTCCTCATTTGCGACTGAGGGTAAGTCTTCTAGCTGGCTTCTGTAAGACTGTAGTTCTCCATCTGTGAAGTGAAGTAGTACTAGTCCCCGTGTCATAAAAATATTGGGAAGACTCAAGGGAAAATGTAAGCTGGCCGACGTGTAACAAACACCATGAACTATTTGCTTCAGGAAGTAAAGCTTGCAGAAAATTAATTGAAAGGAGAATGTAATTTTTACCTTTTGAAACTTAAAAAAAATGATTCATTTATCTTTATCATACGAGCATCGCTGTTTTGCCTGCATGCATATCAGTGTGAGGGAACTGGAGTTACAGACAGTTGTGAACTGCCACATGAGTGCTGGGACTTGAAC
MSTRG.56960.2	ENSRNOG00000048824	0.000	0.283	0.000	5.898	up	GGGGGCTTGGGGGATTTCTTCAGGAAAAAAAGTCTGGTGCTTTTTCTTCACCATTGAAAAAACTGTGCTTGTTCGGTAGGCGTAGTTAGGAATGCTCGCACGGAGGCTGGGGGCCTTTGCGGAGAGGAGCTTGAGGCCTGTCACAGGTGGTGGCGAGGGTGACCAGGGAGCCTTGGCAGGTGGAGGAAGGTGCAGCCCCAGCTGCCCTCTGCCTGTGCCCAGCTGGATTCGTGGCGTTGCGTTGATTGGTGTCCTAGGTGGACCTGCTGCGCCATGGCTGCGGTTAGGAGGCCCAACTTGTCAAAGGCACTCATGCGTCTCAGTCAGAAAGGGTCTGAGTCCCTTGAGTTGAGCAGGAGAATTGCATTATGTCTCTCCTGGAGATGGCGGATGTGAGATGCTGAGGCCTGGCCCTACCTCATTTCCACTCTTCTGGCTTCCTGACCAGTCTGAGGACTGAGCAGAGGCTTTTTCTCCACCCAATCGGCTGCGCCGAAACGGGAGGTGGGAGGGCTCTAAGTGCTGCTCCAGGGCACAAGGACCCCAGGTGGGTGGGAAGGGGAGGGGATGGAGGGTCAACCCCTTCCCTGCCCAAGTCCTAATGTTAGCCTGGGGTTCAAAGAGCCCGCACAAATGGAGCAAGACGAAACCATCCCGCCCTCTCCTGCCTGTGTGCTGCGGCCTCAGAAGCGGGTCTGCTGAGAGCAGCTCTACAACTTGAACGATGTGTTGATGTTAGGCAGAATCACAGGATGTGTTTTGGGAGAAATTTAGGGAACATCCAATCAACTTCCTTATGTCACAGCTGAGGAAGCTGAGGACATCTTGGTTTGGGCACAGCCAAAAGGAAGCAGCAGGGAAGAAGTCACAAGTTCTTGTGGCTTTTGGTCAGCAGCGCTCTGACATTCTGACTTTCTTTCCCCAGCCAGCCTTTATGCTGGAGGCGGTGTTTACCACGGACAGCAACCAGAGCTGACCAAGGCCCACACTCAGTACTCCTGACCTTCACCATAAGCACTGTTGGCTGTTCCTCCATGCCTCAGAGTTCCAACCAGGCCAAGATATCGCCTGAGCAATCATCTTCCACATCACTTGCCCCCTTCCTGGCCATCGGCAGTCCACAGGGATTCTCTGAGACACCAGAGTAGACAGGCAGCCATTCCTTCATGATGTTTGTCCTGGAAGGATCTCTCTCAAGTCCAATCAGCCATTGTCATTTTTTGGTCACGATTCATCCTCGGCTCACTTAACTTGGTGGAATTTCTCCTTTTATCTCTTAGCATACGAGGGCACTGAGATTGGCTAGGGTTGAGGTCAGGGTAGATTCTGAGAGCAGTGCAAATGATGGGGTGGGAGGAGGGGCGGAGGGAAGAGGGGAGGAGGAAGGGGAAAGGGGGAGAGGGAGGAGGGAAAAAAGGAGGAGGAAGAGGAGGGGG

							ACCTGGCATTTTCTGCACACGATTAAGAGCTACAGTCGGGTTAGAATTTGGCAGATTTCCGCCACCTCCGCCATTCTGTCCTCCCATCTCTCTCAGTGGTACAGAAAGCAAACACTAATACTGTGTAACTTGGGACTTTTCATCTCCTTTTTCACTAAACACTCAGAGGCTCCCATGAGAGAATAGAGAGTACAGAAGGGGCTGGATGGTGTTAACTCTCAGATCTAGGTGAAGTAGGAGGAGTGGGGGAGATGGGAACACACGGTTGTTTCTCCCCAATGGAAGGAGGCGGAAACTGAGGCACAGAGAAAGAAAAGGAATTTCCCAAGATTTCAGTAGAATCAGAGGATCACGAAGCTAGGGGCACCGGCCTCCCAGTCCAGCCGAGGAGCCTCGCAACAACGGCCATCTCTGAGGGTCTGTCTAATCAAGCAGGATCCTGGAGGAGGCTCAAGTTACTCCCAAGGTTGTAGACTTGACTTGAGCCCTAAGTGCTTACTGAGAGCACTTTAGCCAAGGCCGAGTCTACACTGCTGAAGCTATTCTGTCTGTATCATCTGGTTAATTTTTATTTTTTTGCATAGGGAGGCAGCGGCTACATTGCCTCTTTGTGGCAAGGTGGGAGCTCAGGATAGAGAGTTAGGAACTAGGGCGAGCAGGAGCTGCTGGCTCCCAGGAACGTGGGGTAGGCTGTAGCTCGAGGCATTATCCTTCCTGGTTCCGTGTGGAACCTAGGAAAGCTGCTGGGGCTAAGCCACAGGCCCGAAGACTGGGGAACCCTTGCAAGATGGGGAACCCGTTCACTGAACCTCCTCCCCTTGCCTGAAAGCCTCAGAAACCCAGTGAGACCGGC
MSTRG.56978.1	ENSRNOG00000048824	0.044	0.325	0.025	2.708	up	GGGAGCACTGCCCGGGAGCACTTCCTGGGAGCACTGCCTGGGAGCATTGCCTGTGGACACTGACTGTGAGCACTGCCCAGGAGCACTGCCTGGGAGCATTACCTGGGAGCACTGCCTATGAGCATTGCCTGGGAGCACAGCCCAGGAGCACTACCCTGGCACCAAGAGTCTATTCTCTCATCTCAGTTCCAACCCTGCTGTGTGACCCTGAGCTGACCCTCCTCCCGGACATTTGCTTCTCCTCAGCTAACAGTGGGCACAGCTGGGCCATCCCACAGGGTGCCAAGTGAGTTCTAAGGAGCATTCCACAGTTGAAAGCATTTTTGTCACTCATGAAACTCAATGCAGTTCCTCGGGACTTAAGACTGTTTGCTGCACACCGTCGTGTCCCATGTGAATTCTCGTCATCGGAATCTCTAGGACTACATCCAGGTCTGCCCTCTACCAGATGCTCTTGGGGGCTTTTTGACATTGTAAAATTTCCTAGTCACTGCCCTACGTGTGAGTCCTAGATGGTGGTTAGAGGCACGTCTCCTTTGAGATGTAGATCAGAGGAGACAGAACAGAAGCCAGAGAGACAGTGGTCTCCATAAAATCCCCAGAAGCTAAGCAGCAGGCTCTCGATTCAGTAGCAGGTGATTGACAGTAGCCACTCCCTACCAAGTCCTACCAACCTTGAGATGCCAGTTGAAC
MSTRG.57131.1	--	0.238	1.858	0.000	3.358	up	GAGGATTTGGTTTATTGTCGGGATACACAGGCACAGGCACCTGCAGGCGCTTCAGTCACTCGGGGGACTGGCGCGCCCCACGGAACCAAGGATGGGCTTTTATAGGATTTTGGGGAGCAGAAGCAAGCATACAGAAGCAGATGCATGGTTACAGGGATATAATTGGTGGATTCTAATATGGCAAGGGTTTAGCCCGGGGGCAAGTTTCCTAGCTACCTTCCTGAAACATAACGGCTGACTCGGCCCCCCAAGGGTTCAGCCCGGGGGCAAGTTTCTTAGCTACCTTCTTGGAACATAA
MSTRG.57241.1	--	0.000	0.300	0.019	3.592	up	AAGGGATCTCCTTTAACAGAAAAATACTAAAAGTCCTGTGCTCGGCAGATTCAATGAATCAGCGAATTGTTTCTGCAACCATCTGAGCCAAGATGTGAGCAGCCCTCCTGACTTTCTGTGGCATTCCTGTGGCATTCCCAAGCCACAGCCAGGAACTACTCCCATCCTGTTGCATCGAATCTAACAAAGCGTGAGCCAGAGTAAATCCTTCCTTCTCCAAGTTGCTTCTCTTCAGGTCTGACTGAAGAGAAAGTTACAAACGGTAAACACAGGCACCAACAGACTAACACCTCGGTCACTCTGGAGTGAGGACCACGGAGGGAGAAAAGCTGGGGACTTTCTTACCTCACCCAAGCTGTTTCATGAGCAGTGTCTGCCTCCAGGCACAATTGAAGGCTGTGTGGGTCTTCC
MSTRG.57294.1	--	0.000	0.718	0.002	4.137	up	GCGGCATCTGGGAGCGCATATCCCAATCCTTGCATCATTGAGCTGCACTTGTCAGGACTCCATAGAGAGAAATGCACCAAAAAAACTTCTGGTGGTCTTCTCTGTTGCTTCCTCGGACTCCGGCTGATTGGCAGAGTGATGTCAGCTGAGACAGACAGGCATGCTGAGGCAAGACCCGTGGATGATGCTGAAACCCAGGATGGTGTCTGTAGATCTCAAACCAGGTGAGGCTTTTCCTGTCTTT
MSTR	ENSRNO	0.334	0.000	0.003	-	do	GTCTTAACCACTGAGCCATCCCTCCAGCCCCAGTATATTGTATTTTCAATGTTGTTTGTTTTATTTCTTTTC

G.57646.1	G00000011330;ENSRNOG00000011635;ENSRNOG00000026262;ENSRNOG00000045837;ENSRNOG00000046212;ENSRNOG00000050034;ENSRNOG00000061644				4.375	wn	TCACCTATGTCTGTATGGCGTTTCTGTGAGTACAGGTGCTCATGTGCTTGTGTAAAGGTCAGGGACAACCTGAGCACAGGTCCTCACTTTCTACCTCTCATCCATGGCTGTTTATGCTAGGCTAGCCCTTCCCCCTAATCCTAGAGACTGTCCACTTTAGGCTCCTATCTCCCTGTGGGAAGGGCTGGATGACAGACACTACTGCATCCAGGTTTATGTGGCTTCTAGGGACCTAAACTGTTGTTGGGCTTGTTCCGCATGAAAATAAACACCATTTCACAGCCCTGGCTGGGCCAGGCAGTGCACGGGTCTTTGCTGCTCTTGCAGAAGACTTGAGTTCAGTCTCCAGCACCAGGGTCAGGTGGCTCACAACCACCTGAGACTCCAGGTCCAGGGACTTCCAAGCTCTTCTGGTTAAACCTACATGGCAAAAGCAGGGCTGGGAGCTGGAGGCTTCTGGTGTTGGCTTCTCCCTGCAGTAGGGGGCAAATCATGGGCATGTGAAACATGGATCCCTGTAATTCTACAAAGTGCAGAAGCTCCTGATGTCCCTCATGGTGCCCCTGACCCTGCCCACCACTCATTCAGGCTTCAGTAACATAACCTTGTCCTGACCTGTGTGCACTCTGCATCCTCACTCTCTACCCCCTGCTTTTCCGGGTTGGAAGTCTCAGGTGTAGGAGGTGCCTACCATAGGCCTCTTCTCTCAGCATTTCAGCATTTTACATTTTATGTAGTTTGTGGTGTTCCTGTGCCATGATATGCACATGGAGAGCAGAGGACACAATGTGGACTCAGTCCTCTACTTTCACCAGATGATCAAACTGAGGCCATGAGGCTGGGTGGCAGAGCCTTTACCTGCTGATCTGTACCCACTTGACCTTCACCAAGCATCCTTTGCTGTACACAGATATATCAGCCTGTGACCCTCATCCTCCAGACTCTTATAAAGCAAACCCCATAGGTGTGGAGCTCTCATGTTAGCCAGGCCTAAAGGGGAAGGCAGCAAGCCCTTCAGGAA
MSTRG.57652.4	ENSRNOG00000045837;ENSRNOG00000046212;ENSRNOG00000049312;ENSRNOG00000050034;ENSRNOG00000051666;ENSRNOG000	0.169	1.274	0.000	3.520	up	CTCTTCCTCGTTGGCCTGCACCTTGTTTAGGACATTTTCAGGGGATGACGTCTGTCTGCAGGCCTCTCATTATTGCATATCCATGTGATAGATTGGATCCTATGCAGAGTTTTGAACAACTTCGTGGTCCTTTGACAAATGACACTCTGTGGTCATGTGACTTCTTGTGTAGGAAGCACCTGCATAAACTGTCCTTAGAGTACGGTGTAGTCAGAGGGGCCTTTTTTTTCTCTCTGAATTTTCAAAATATTTCAAGTCTTCAGACAAGTCTTCAGCCACTGTCCAGTGCCTGTCTTGAGATGTCCACATTGTCTCCTGGGAGTATTCATCCAGCCTGCTCTGTAATTTGGTAGAAGATTAATAGGGTTCCAGCCCTGAATAAACTGTCCTTAGAGTACAGTGTAGTCGGAGGGGCCTTCTTTTCCTCTGAGATAGCAAAACATTTCCAGTCTTCATACACGTCTGTCAGCCATTGTCCAGTGCCTGTCTTGAGATGTCCACATTGTCAGCTGAGAGTCTTCATCCAGCCTGCCCTGTAATTCAGAATCAGATGGCTTTTAAGATTGTTCCCATCAGGTGCGATCTCTTTGTCCATGGGCCTTGCCCAAAGCTGCAGGGCCAACCTCTACTGCTGCCAGAAAGCTGTCCAGACTGTGCAGAAGTTGATCATGGGGAAGTCTGTGTCCCAGTTTTTGGAAAAAAAAATAGGATAGTCCTTCAAGTGACCTGCTTCAAAGGATTGTTGTCAGAGCTCCTGGCCCAGTGAGTTGAAGGTGGATGCCACAGTGACACAGGAATATTTGCTGACTGACTCCCAGGGTAGCCAACCATTTCTGTTATTTCTTAGGTTTGGAAGCTGTTTGCTCTGAATTTCCTTTTTTTCAGGCAATATTTCTCCTCCTCAGATCTTGGATGGGGTTGAAGATTAGATAGTTTAGTCTTAACCTTTTCTTGTTATGAGAATCAGAAAAACAAACTTGTTCACCATCGTGCAGAAATGTGACAGCCAGGAACCTGGACAGGAACAGGGTGGACATGGGAGGCTCAGAAAGCAGGGCTCTATGGACACTCCACAGCAGTATCAAAACTCCTGTGATAACCGCTCTGGCACCATAAGTCCTGAGAGAGGATGGGGATGCCAGGAGAGTGTGCGTGGTCTGCCCACTGCCTGGGACTTTTAAAAGCTTGTACCGGGGGTTGGGGATTTAGCTCAGTGGTAGAGCGCTTGCCTAGGAAGCGCAAGGCCCTGGGTTCGGTCCCCAGCTCCGAAAAAAAAAAACAAAAAAAAAAAAAAACAAAACAAATGAAAGCTTG

	00055757; ENSRNOG00000060419						TACCACCCCCGAGCACATAGAAGAGCTTGAGCAGAAAGAGGCCGATGCCCAGAAGCCGCTGCTGTGGTAGGAGGCAGTTATGGCGGTTGGAGGTGGGGGTAGAGGTGGAGGAGGGATGGGAGAAGACCGAGGAGTTTCCCCAAAAGACAAGAACAGACACCATCCTTACTACTCCAGAACCCACGCCAATCCCACCCCACCCCTAAAATGCCCAGTCC
MSTRG.57655.1	ENSRNOG00000026262;ENSRNOG00000046212; ENSRNOG00000050034;ENSRNOG00000061644	0.092	0.624	0.002	3.044	up	GGAAGCTTGAGATTGCCATAGGCACAGTATTATGCTAGATATACTGAGTAGATGATACTAAGATGTTCAGATGGTCATACCCATTTGCATGAGTTCTTAATGTGCACCTGTAACACATTCATGGTGATGTGAATGCTTTGTTCTGGGAGCCCCACTGTCAACAGCACAGTTCTTTGCCTATGTGGCCACGTTTGTCTGTTTATTTGTATTCCTTGTGCAGATTATAATTTTGTGTTGATATAAGGTCCGTTATCAAAACAGGAAAAAAGAAATCACAGGTTTCTGGGTGTGTTGTGTGGGGGAGCATGGAGAGGTGTGGCCTAGGCTCTTGATAGCTCAACTGGCTTGACTGCAGATTTCCTCTTGATTGAACAAAGGGAGCCAGGGAAGCCTCCATCTGGATGCTGAGATTCTGATGTCCTGGACGCAGAAAAACACGTTTCTGAAGCTGAAGAAGATCCAATATGTAGAATTCAGAAGGTGTCCTTCCAGGGCTGGGGTAGTACAGGACCCACCCTGAGTTCATATATTCCTAAATGCTGATGTTCATTGGATTCTATCATTTGGGGCCAGGCCACACTTCAGGCCAAATCCATATTATGAAGACCTTCTCTAGGTAAGTCCTGGTCAGAAAGGCTATCACTTGTGGAATGGCCATTTCTGACTTTCTTTGTTCTGGATTGGACGGTCTTCAGCTCTGG
MSTRG.57659.1	ENSRNOG00000045837;ENSRNOG00000050134; ENSRNOG00000051666;ENSRNOG00000060419	0.029	0.606	0.000	4.520	up	CCTCAGTATCAGCAGGATGGGGCCTGGGCACCGTAGGTTCCCCAGGACCTAAAGCTATGTCCTTGAACTCAGTGCCATGCTCAGCCCTTCCAGCCTCTTTCAACCCTTCTGGATGCTCAACTGCTCCTGCAGCAAGGCATCAGTGTGCACATTCTGTTATGTCATTTGTGTGTGTCTACAGGTGGTCAGAGCCTGTCCCCTCTGTTCCTTTCTTTTGCTGTATTTGCCTTTCTGAGGAGGCAGGACTCACAGGTATGGGAGGCAGCCACATGCATATTCTGCAGCCATGTCTGACTCCTCTGAGGCTCAGTCCCACTTACACACCTACCCTCCAACCTCCCCCCTCCATGTCACCCCTGGAGCTCTGTAAACCTTCCTGCTTTCTGTCTTTATATAACTGGTGACATCTGCAGCCCTCAGTGGTGTCATACAGTGTTAGTTTGTCTTTGCACTGCCTGGAGGTCTGACTTAGGAGAATGGCTACCATTCTCATTCCTGTTCAAGGGTCAGAATCTGAATCCTATGTAAGGCTGAATAATATTCCAGCACATGGCTGGGCCATGTTGAATTTACACAGTCACCTATGGATCTTATATTTTGGCTTGTAAACACACACATATAAGACTCAAGTGACACCCAAATGGAGAATTGCTGGAGTCCTCCATGGCTTTCTGAACCGTCTGAGAGACCATCCTGTCCACAGTGTATAGGGCACCATCTACTCATAGCTCACACAGCATGAGAAGATTAGGGAGGGTTAACCTAGCTTCTCTGTCTCCTACTTTCTGCCAGACTAGCCCTAGCATCTTTCTGTATTGCAGTGAATGTTTAGAACCCAGGAGGATTCCAGCTTCCTTGCTCAAATATAGCAAGTTCCTTCTTGCCTGCTTTGAGACTGAGTCTCATGCAGCCCAGGCTGGCTTTGATCTCACCATTAGTTACGATGGCCATGAACTCCTGGTTCTCATGCCTCAGACGTCTCTGAGTGCTGGGTGACAGGTATGCACCACTCTTAAGTCCTGCCACTGGGACAAGAGTGAGCAGAGCCAGCTCTGAAGCACACACTTTGCTGGTGTCAGTGATGACTGACTTATGATCCTTTCTTCCTTGCCCATGGCCCTTCTCCTTGGTGCATGCCATCCTTCCTCCCTGTGACTCATGGGGGACACCAAGCCCGAGGATGTCAGGAAGAAGTTTACTTACTTCACTGGGTTGGAGGCTGTTAGGTTCCTGCGTCAGTAGGTGAGATTCAAGCTAGAAGGTTTGTGGCATGAGAAACGGGAACAGAACTGGAATGGCAGGAACAATCGTTGCCTGGCTCTGAGAAGACCAGGTGGGCAGAAACTCATCGTTGGGCAGGTAGTGAGCTCAAGACCAGCCTGGGCATTGCTCAGCACATGTGAAC
MSTR	--	0.000	0.265	0.000	6.858	up	ATTGCTAACTTGCTTTCAAGTCCACTGTCATCCGGTGTCAGGAGTGGTCCTCATTACATAGCAGGGACA

G.57776.11							GACCGATCAGCTTGCTCTTCTTCTTCCCTTTCACTGCTGCAGAAGATACCAGTGTCAGGAGTGGGCATATCCAAGTAAATGGACATGGGCAGGAAGTGACCTCTCAAGTGGACAGAAATATCACATAAGATGAAAAAAAGGAGCTTTGCTAAATGAAATCTCAGGGATTGAATCCAGACTGCTCACATTATGGTCCTTGATGGTGAGTATCGAGTCTTGTTTTTGTTAAGTCACTTGTAGTTTTGAGTCATCATCTACTCTTTATTTCAATTGTTTCTGTGGTTCCAGGAAAACAAACATTGTTTTCCTCAATGCTCCTTGAGCAATTACTACACTGAGGCAATAACTGCTCACATCCAGTAGCTAACGAATGTCTAATTGTTTATTTCCATGGAATTTTGTATATGTGAAATTGTTTATTATTACCAATTGCTTCCACACTGGATCTCAGACAACTAGAAGTCAAGTTCTTGATGCAATCTAAACCACTATAAATGACTCTTGATCAGTGTGTTAACCTCAGTTTTTTCTATCAGTGCCTTGGAGGATTTTCATCAGAGCAGAATTTGGGGAGCATCATAGGCATTCTGTATATATGTCATAGGGTATCTACAGTGTTGGAAGGAGACTTGCTGTCCTGCCCTTCCTGCACTTTGTCATCTCCATGTAGTCTCCTCACCATATGCTTCCTATTGTGTATAGGAGTAGGATTTGTCTACAAATCCTCTTTACTGGAGGAAGCTCTAGTACCCCTTCCTTATCTTGACATCCCTCTGTGAACTCAGCACCTCAAACTGGACTTGACTTGGAAGATATTTGAAGAACAGTCAGTCCACCAACCCATCAATAAACAAGAGAAAATCAAATAGAGAGGTTTAAGTGCAGCTGAAGTAACAGATGTCACACACCATGGAAAGGGATGTTAATTAACAGAGGTAGTTCAGAACCTCAGCCAAGACAACACCTAAAGTGGACAGAGAAAAGAAAGAGAAGAAAGCAGGATCACAATTCTTAATCCCTTGTCCCCTTACAGAATAATATTCATGTCTAGAATCCAATTGATCACGAGTCCCACACTTAATCTTTGCCCCAACAACATAGACCATTACCATATGTGAGAGTTAAAGATTGAGAAGATCAGAACCAGACTTGGAATCCTGTATGTTCTTTTGATGAACTGAGTGGTCTGGACAAGTTTTTATCACATAGTCCAATGACTGAGTGAGAGCAGCGCCATGATGTACACAGCTTGGGCGCTTGTTCCTCCTTAGCCTGCAAGAACTATTAGCTAGCATTGTTATTAGGGTGATGGTATTCAGAAAATTATAAGGAATCCCCAACCCTTGAATTCAGGCTCTCTTCTACTTCCCACATTTTGCCCAGCTTTTATTTCCATGGTAAGAGGATCTTTTTGTGTTTCTTTTTACTTGCCACCACAGGCTGATTTGCTGATAATCATCCATTTATATTCATGCCTTAGGCTTCCTGCTTTAATTTGGTCTTTACCTTATTTCAAGTTCAGGTAGTTATGTTCTCAGCTCTGGGTTTTCTTTCCCCCAAATGTCTTGTGAACTGTATGTGTGGAGGGGAAATGATTCCATAGTCCCAGTCTCTGTGCACAATAGTAATATGAACACAAGCTTGTTTAGATTTATTTTTTTTTGATGACCTATTCTTGCCCCTTGACACTCTGAGATTTTTGTCCTTGTATAGGTCTGCCCTCACCATCAATTTTAAAACCTGGGTGATCACCCATTTGAAAGAGCCCAACATTTGAAGAGCCTACCAGATGGTGGCTTTGTATGTTTTGCCCAAGTTTTTGCATTGGGAAAATGCCCTCAGCCATGAATCAAGAGGCGACAGAGAAAAGCCAGACAGACTATCTACCAGAAATGAGAAAAGATCCATCTAAAGGTCACAGAATGGCTTAGTAGCCAAAGCAGGCTTCAACGCTGGGAGTTCAATATTTTTGGTGTTGTATGACAACCTGATGTTTTCTAGGCTAGTTCAAACAGAGAACCAAGGCAAAGAGAAAAGATGAAAGATGCATGCTCACCCAACAGCCTATTTACTGGCTTCCATTTAACTTTCTGACTCTTAGTACTTTTATCTGAAAGGGAAAAGTTATAATTTCTTCTGAATTGGATTGTGGTAAGAATTAATTACTGCTGAGGTTTAAATGAGATTATATTTTTGCCTTATCATAGAGTTACCACAGACTTTCCTTTAGTGTTGATGATGACCACTAATATTAATAACAATGCAAGTAGGTACTCACATAATTATGAGCATCTCCAGAGGCTTCTAAGGCTGTAGTTCATTACAAATTGTGTTCAGCTTGGTTGGAATTCTAATCAGCCACTCAGCAATGGAAAGATGGTGAACAAGTTGTATGCCCATTCTTCCCAATTCCTCTCAGGTAGAAACTAGGATTCTTGTTTTGTTTCTAGAATTGTAGGAACATCCAAGAAAAATTGCAGTTATTAAATTACCCTCTGTTCTTCCAAGCTTTCCTTAAAAGCACATGCTAGTCAGGCACTAATATCCCTGAGTTCAGAATGATCCTTCTCTGATAGTGGTACTAGATAAGGCTGTGTTCACGTGGGTGGTAAAGAGGAAAACTCTGCCCCTGTTATGTATGTTAAACCACCAGAATAGAACAGGTATTTTTCATTGTCAGAAACAGTGGAGAGGAAGAAAAAAAACAAAAAGAAATGTGTTAGTACTCCTTGGATAATAGCATCTTCCAAAGGGTTGACAAAGGTGACTTTTATTAACATTGGTCTTATTGCTTCTGCAGGGTTGCCTGCCCCATTTGAATCTCTTCAGGGGCATACTGTGGGCTCAAGTTCATTCTAGCT

							CATGGCCCTCCATTATTACTAATGGGAAGAACTGACTGGGGCAACCCACACGGGCATCAAATCCATCCAGTACATTTACCCTTCCACATTGGCCATCTTATTTGATAAAGTGGCTGAAAATCTGGGCACTATAGAATGAAAAATAAGAAGAAGTGTTGACTTTGCCATGCCGGGGCAGCATTTCTTTGAAATGGTTCTAAAATGGCATTTGGCAGTAGAATCATGTACATTTTAATGTGGTGAAAATTACGTACACACTGGGATGTTCTTATGTGTGCATATACTCATGTGCCTATATGTGTGCACATGGGGGTGCTTGCGTGTGTGTGTGTTTGTGTGCATATAAAACCTCAATGTGTTCTTTTACGTTCTCTACAATGTAAAAAAAATCAATAAAGTGACCTTGATAGTGGCTGAATATGGCATCTCACCAGTTCATCCATTACTGAGTTAACACATGTCATTACTTACCACTCTTAGGGACAGTGTGGTATCTGTGGTCACCGCCCAGAAGCAACATGCAGCTGAGCCTTTTTCTTTATTTCTTTTACTTATTTAAGGTAAGGTATTTTGTTTCCATTATGGTCAGGGAACAACTGCAGCGTATCTGCCACCACATTTCTTTAGAAGGACAGCTGGCAGCTCAATGTCTTCCTTTTGAAAAAGAAAAACAATGTGTCTCTTTCTCCCAAGTTTCAGAAAGAAGCCACTGCACCATCAGGAAATCATGTCACATATTTTATAGTGCTAAACTCTGCTGCAGGAGTTGAGAAACAAAAATGAAAGAAAGTCAATATACTATAGGAATTGGTACCAATGAACAGAAAATGATGGCTGGACATAAATCTCTGCATGTGTAAAGGGTAAGGGAATCACAGTTCCCTTTGACATTCAGGACCTGAGCATTAGAACCACTTCAGATACCCAGAGAAACAATCAGCCTCAGTGGAATGTTTTTGATGATGGTGTGATCTAATGAGGTTCATGGTGTGTTGCAGGGGGACTACAGTATGAAAGAGAATGAAGTCAGTTGAACAGAGTAGGTTGGAAAGAAGAGTGGAAATAATATTACAGTGTCCTGAGAAACGAGAGTCAGGTGTGACACTTTCTCTATGGTCAGAGGTACGAATGCAACAGACCACACCTGACTGTAACACATGTGGGGTGAAGGAACTCTGATCCCTGGTTTTAAAAGTAAAATCATGAGCAGAATAATTAGTGAAAGAAGATTAATGCCACCTCCATTTAAAGAATCTGAAAATAATTAACAAAGATTATGAAAACCACTGTAATGATACAGTAAGAAAAGGCAAATACTGGGCATTACAGACAGGCTAAATGTTGCTGGTATTGTCTACCAGCTTTAGTGGGGGTAACACAGAGTGCTAGGGAACATCTGTGAGCTGTCATTTTCAATGAAAAAAAATGGGAACTTGAACGCTACACTGGAAATGGTCAATGTTTACATTACTTAAGGTAGCAATTTTTTGTAGCTGCCTTTTAGAAAACAGTATGTTTGTCAACATACATAAATGTATCATCTTTCTTGGTTTTGATACTCCTAAAGGCAGTAAGTGGAAATGTTAATTGTATTCCTGTTCAAGGCATGGGTTTGGAAGGCTTCTTAATGTCCAACACAGGACAACTTATACAAATGCTGACACAGTAACTGCATAGGTGATTTGCTGGACCATACAATTGCAATGGCAACTACATGGCAACATGAAAATTAGGATATCCAATTAGGTAGGAAATATTGAAAGACTATTTAATAGGAACTATAATATGATTACCACTGCTTAAGAAAGGATAATTAAGATTTATGCAGTAGTCTTATTGCTGGGTAATTTGTTAAGTATTTTCCATTGGCTATTTTCTTGCATCTTTATCATAGATCCTCAAGAGTACTGGAGGAGGATATATA
MSTRG.57776.12	--	0.049	0.395	0.000	3.429	up	ATTGCTAACTTGCTTTCAAGTCCACTGTCATCCGGTGTCAGGAGTGGTCCTCATTACATAGCAGGGACAGACCGATCAGCTTGCTCTTCTTCTTCCCTTTCACTGCTGCAGAAGATACCAGTGTCAGGAGTGGGCATATCCAAGTAAATGGACATGGGCAGGAAGTGACCTCTCAAGTGGACAGAAATATCACATAAGATGAAAAAAAGGAGCTTTGCTAAATGAAATCTCAGGGATTGAATCCAGACTGCTCACATTATGGTCCTTGATGGGTTGCCTGCCCCATTTGAATCTCTTCAGGGGCATACTGTGGGCTCAAGTTCATTCTAGCTCATGGCCCTCCATTATTACTAATGGGAAGAACTGACTGGGGCAAACACAAGATCTGACAATAGAAAAATGATTCAAAAGCATTTATTGATGAGGCAGATGGAGACTTCTGAGCTGCACACAGTGCTGCAGACCTGGTATAAACACGTTAACCCTGAAATCTCAATGCTCTAACTCTGTTATCATCCCCTGACACACCAAGCATAGGCAACCAGATAGCTGGCAATTAGGTGACTCAAGAAAAATCAAGAATATTCTTGGCCAGAGCACAGGGTTCTCTACTTCTTCATGATGTATCGTGACTTCTTCAGTGAACCACCTAATCTTATTCTCTTGGTTGTTGGACAAAGGAAAAACAGTGTCCTAAATCAAGAAGTGGCTGTCTATCTACTGAGCAAGAGAGAATACTGTCTACCAGAGAGATTGCAACATGTGTATCATTTGTTTAAATTCAATTTTTCTCACTCAAGCAATAACAGTTCATCTTCAGTTTAGAGCCTCCTGACCTTCAGGTCCTCTCAGAATCCAAAATGGTGGCTGTAGTTCCGTCTATCTTTGACCATAGGAAACTA

							CTGGATTGTAACTTTTCCTGTAGAAAACCTCACTGAGGAAATGATCATTCAGCAATTTGAAAGGATTCACCAACATGTCATCAAGGCAGAACCATATATGGAACCTCCTCTCTTCAACAAGCCTGTGATCTTCCGGCTGTGATCTTCTGCTTTATTGAATGGTACACATGTGTCAACCAAGAGAGCATCAACAAGTAATCAAAATCAGGAGGCACTGTGACAGCTGTGCAGTGAGGAGGAGCACTGGTGACAGATGTTAGCAAAGGCAGTCATGACTGAGCCTGGATCCCATTTGCAAAATAGGAGAAAAATAGAAAGAATATAATCAACAAAATACTTAAGTATAACAGTTTTTCAACCTCAAAGTGAACAAAGAAAAGTTCAGTCTGCCCTATTGGCTTGATCAGGAGAATGAGAGCCAGAAGCAAATGGCAGCAACTTTGACCTTCAACAAAGGAATCTGGGGCATGAATATGTGAGAAGAAGGGAGACAGAAGGCTGGCTTTATGGATCTGAATGTCCTGTGTGTGGAAAGAACAGGATCTGAGAACATCTCAAGACAGAGGAGAGACTCATAACCCTAAGAAAGTGGGCATAGGAGAGAGGGAGGTTTGATTCCTTGTGATGTCAACATACATTAGGACCAGATGGTCTGACAGCCACAGGAAAGTCGGCGTTGCAGATCCTTCTTCTAAACTGAAAAGCTCAGCATCCTGTTAATGACACCCCATTTCTGTCTGTCATTTGGGGTTGCAATTATTTAAGAAGCAAAAATGGAACTCATCATGTTTCCTGATTTGTGATATTTT
MSTRG.57776.15	--	0.529	0.005	0.000	-4.148	down	GACAGGCCTTGGGCAGCCTATGTAGTCAGAGGATGGGCAGCATGTCACCAAGTCTGAAATCCTGTGATTGAAGGGGCTGCTATTCAGTATTTAGGTTTCACATGGGACTGGCAGTACTAACCTGCAGCTACAAGCAATTAACATGAACAATCTTGAGAGAATTGCTGAGAAATCTGGCCAAATTACTCTGTGATTTCTCCCTCTAGCATTTGTTTTGTAAAGGGAATAATTTCTGTTGTGTTTCAGGTTTGATTCCTTGTGATGTCAACATACATTAGGACCAGATGGTCTGACAGCCACAGGAAAGTCGGCGTTGCAGATCCTTCTTCTAAACTGAAAAGCTCAGCATCCTGTTAATGACACCCCATTTCTGTCTGTCATTTGGGGTTGCAATTATTTAAGAAGCAAAAATGGAAAAACCTAATTGGGATTCACTTTCAATTTCAAGAGAATAGATGGAAGAAACTGAAGTTACCAGCAGCCTAATTCACGAATGCATGGTCCTTCAGTGTACCTATCCCAGATCCTCTGGCTGAGCAGGTAACTTGTAATTACTGTTTGAATTCTTATCTTAATGAATGCATACATATCACAAATGAGTGGGCATTAGACTTGTATATTCATTTATCAAATACAAAAGGGACAGTCACGAAGATTTCTAAGCTAATATGTATTCCAAGAATTTAGTGCAGGAAAAACAAATCCTGATCAGTCTGCTTTTCTTTTTTTCCTTAACAACCACACTCTAATATCTTCTTCTCACCATGAGAGAGAAGGAAGATAAGAGAGTGTTCTGGATATGTTCGGATAATTATATTTTCCACATTGCTTAGCATTTAGGATTGACAACTACTAATTATCTGAGGCACTGTGTGTCCTTAAAAATAATTCACCACACAAAAGAAATAATTGGATATAATTGCAAAACTAGTTGATATGGTCATAACACACTTTTGTGTTTGTGGACCTCGGTTTCTCTCTCAGTCAAGTAAAGGAAGTAGAATACATTACAATTTTTCAGGATTTGTTCCCCTAAGAACATATCAGAGCTCATCCAAGGTTTTTTATTGAGGGAAAACATTTGAAGAATGCTTACAGGAAGAGTAAAAACATCTCTTTTAGAAAAGAGGTAGATATTTTCAGTGTCAATGAAAATATATGTATATTTTCTGTACTTGGTACAGGAGGCATTTTGACAGAGTGTAGTGTATAGATTTGAGAAGGTCAGAGGTGGACTCAGAAGTAAGTTAGAGAGTGAAAAATAATCTTCTTGGACTATGGGCTTATATTAAGTAGGATTCTCTAG
MSTRG.57776.7	--	0.000	1.363	0.000	8.625	up	ATTGCTAACTTGCTTTCAAGTCCACTGTCATCCGGTGTCAGGAGTGGTCCTCATTACATAGCAGGGACAGACCGATCAGCTTGCTCTTCTTCTTCCCTTTCACTGCTGCAGAAGGTAGGCCACAAGTCTTTCTTCTTGGGAACTGATGACAGCAATTTGTCTTTCTGGGGCTTCCACCCATGCTAGAGCAGTAGCAGCTGGGACCGAGAAGCCCTCCTGATGTTAGCGAGTCAGCAGATTGCTTCTGGGCTGGCCATGCTTCAGTACGTTCCTGTTAAATACCATATTGATTTTAGGGCCTTCTTACATGTGGTCCAGGACCTAACTGGATGAGGATTGGAAAATGAGGAGGAACTGTGTAATGAGAAGAGCTACCCAGCAGGTTTACACACACAGCCTTAAGCTCTCTGATGCTGCTGGTTTACTTTTATTTATTTAACAGATGTAATAAAACATCTATGCTTCTGGGATGACAGAGCAGACAGTGGGAAGAGAGACCTAGCGATGAGTCAGTGGAGATGAGGATTATATTGATGGCAGACAGCAAAAGAAGTTGGTCTAACTCTGAACAACAAGGGAATTAAAATTATGACAAAATAAAAGATGGTGTTACTTTAAAGATCAAGTAAGTATCAGCCTGCCTAGTCAAATTTCACTCCTAAGGCCACCTGCCCTTGCTTTTGGCTTTCACCC

							CTCTCACCTGGAAAGCTGAGGTCATTTCCTCACACTTCTTCTGTCCCTCTTAATTAACTTCCCATGCAACTGTGAGCTGTCTTTGTCTTCTACCATAGTAAAGAACATATGATAGAATATTTAACAGCTCAACCATCTTAAGTTACATAATTCAACAGTATTGAATACATTGACAATATTTTGCAAAATCACTACTAATTTCATTACTTGGATTTTATTCTCTTATTTATCACCATAGAAGTATAATTAATCAGTCACCCTTCCCAACCCTTTTCTTATAGCCTTAAGCAACCACTAATCTGTTTTCTGTCTCTATGGATTTGCCTAGTTCAAATATTTCACACAAATTGAATAGTACATAATATGTGGTCCTGTGTCTGGCATTTTCACTTAGTGTAAACATGATCAAAATTTATTAATATTGAGGTATGTAGATAATTAGAAATTAGACACATCACATATTGGTCACTGGAATCTAAAATGGCATAGTCACTGAAATTTGGCTTTTTCTTAACAAGCTAAACATATAGTTAACATGAAACCCCAGAATTTCACTGTATATGCCTGACAGATGAAGACACATCGGCAAACAAAAGCTTGGACACGAATTCTATGTCAGCACCACCCACAGTAGATAACATATAGAGCCGACATCAACATTTCCCATGTGATGACTGGGTAAGACAGGCCCCAGATCCATGAAATGGGACAATTTTTTAACTCATCTATTCCTCTTCTAACCTTCAGCAGATTCCCGTTGAATTTCACATTAATTTGAGTGCATCATAAATTATCCGGGTATGTTGACTTGCCACTCATCCTATGATAAGGATCATCATGAATAAGAGTCGGTGGTGGAACTGCTTCACGGCAGCTGAGGAGTGGCTTGGAGATTCTACTAACTTTAAAATCACACCCCCCAACTCTAACAACTTCATTTCTTCCTCTAGGCCCCACCTTCTATGAACCACTTTCCAGGAATGTCATTATCTAAGACGAAGCTTCCAAGACAGGGGTCTTTGATAGGGGTACACTTCTAAACTATAGAATAGGGTATATGGTTTAGCATCTTCTTTTAACTTATTATTAAACTATTCATCCATCCTCTCACTATTTTCATCTATGCTTACTATTTTTGTTACTTTTTGGAACATTCTGACTCAGGGACCCTTGGGACATAGTGTGCCTGGCTTTCTGATTTCTGAGACTGAGCTAAACCATGGCTTCTGTTATTCTCAGTTTCCTTGCCATGTTTGTTTCTCTCCAAAGCGCTTCATAATATTAATTCATTTTGAAGGAAATTATTCATTACTGTTTTGAACTCACTATGTAACCCAGGTTGACTTCAAACTCATGGAGATCTTCTCTATATCTCGTAAGTTCTCTTTCTGTCTTCCACTCTCTCTGTCTCTCTGTCTCCCTGTCTCTCTCCTCTTCATGTTTGTCTTCCTAAGCTTGATATGAGTGAGTATAAAATGATGTAGATTTTTCCATCATAAGTACACACTGTTTGAGACAGTGCTATGCAAACTACTACATGCTCAACATATACAAATATAGTGTGAGTGCAAATTTTTATGTAAGTTTTAATGTGTGGATAGTTTGTCAGGTCCTAGCATTTTGTTTACATGCACACATCTACAAAGTTAAATTTAGGGCATGTTAGGAGTTGTGAATTATCTTGAAGAGAGGATCAACTTTAATGTACAGAAGCAAGGATAGTTCAATCCACAGGAAATATAAAAAGCCACCATCTTTTAACCACCTGTGTTTGCTACAGATACCAGTGTCAGGAGTGGGCATATCCAAGTAAATGGACATGGGCAGGAAGTGACCTCTCAAGTGGACAGAAATATCACATAAGATGAAAAAAAGGAGCTTTGCTAAATGAAATCTCAGGGATTGAATCCAGACTGCTCACATTATGGTCCTTGATGGGTTGCCTGCCCCATTTGAATCTCTTCAGGGGCATACTGTGGGCTCAAGTTCATTCTAGCTCATGGCCCTCCATTATTACTAATGGGAAGAACTGACTGGGGGGACAGTGTGGTATCTGTGGTCACCGCCCAGAAGCAACATGCAGCTGAGCCTTTTTCTTTATTTCTTTTACTTATTTAAGTCTGCAGACCACCTTTAAACATTATGAAAAGTACCAAGGAGGGAGATACCTTCCATGTCAGTACCAGCTTGATTTCTCTACATCTTCTGGACAACAGGTGGGTTGTCTTCAGTAATAGGAACTTACCATCAAGTTCTAGTGGACATTAAGGGTGGTAGTACTATCTAGGCTCCTCTTGATCAACAACTTCAAGGAAGATATATTTCTCAGGGACAAGGGTTTATGTTTGATAACATGTAGCTTTTTGGAGGAAAATTTCCCCCCTGCCCTGTTGTAGGTCACTCCAATTTAAAAGTGGGTATGCGTGAAGGAATGGCAAATAGTGAAAAGGATATTTAATTTGAAGACACACAGGAAGGTAAATTAGAGTAGGGAGTGGGAGGTGAAGGATAATTAACACTAGGATTCTTGATAAAACCATATGGAGGTCTAC
MSTRG.57776.9	--	0.021	0.324	0.000	4.140	up	ATTGCTAACTTGCTTTCAAGTCCACTGTCATCCGGTGTCAGGAGTGGTCCTCATTACATAGCAGGGACAGACCGATCAGCTTGCTCTTCTTCTTCCCTTTCACTGCTGCAGAAGGTAGGCCACAAGTCTTTCTTCTTGGGAACTGATGACAGCAATTTGTCTTTCTGGGGCTTCCACCCATGCTAGAGCAGTAGCAGCTGGGACCGAGAAGCCCTCCTGATGTTAGCGAGTCAGCAGATTGCTTCTGGGCTGGCCATGCTTCAGTACGTTCCTGTTA

							AATACCATATTGATTTTAGGGCCTTCTTACATGTGGTCCAGGACCTAACTGGATGAGGATTGGAAAATGA<br>GGAGGAACTGTGTAATGAGAAGAGCTACCCAGCAGGTTTACACACACAGCCTTAAGCTCTCTGATGCT<br>GCTGGTTTACTTTTATTTATTTAACAGATGTAATAAAACATCTATGCTTCTGGGATGACAGAGCAGACAGT<br>GGGAAGAGAGACCTAGCGATGAGTCAGTGGAGATGAGGATTATATTGATGGCAGACAGCAAAAGAAGT<br>TGGTCTAACTCTGAACAACAAGGGAATTAAAATTATGACAAAATAAAAGATGGTGTTACTTTAAAGATC<br>AAGTAAGTATCAGCCTGCCTAGTCAAATTTCACTCCTAAGGCCACCTGCCCTTGCTTTTGGCTTTCACCC<br>CTCTCACCTGGAAAGCTGAGGTCATTTCCTCACACTTCTTCTGTCCCTCTTAATTAACTTCCCATGCAAC<br>TGTGAGCTGTCTTTGTCTTCTACCATAGTAAAGAACATATGATAGAATATTTAACAGCTCAACCATCTTAA<br>GTTACATAATTCAACAGTATTGAATACATTGACAATATTTTGCAAAATCACTACTAATTTCATTACTTGGAT<br>TTTATTCTCTTATTTATCACCATAGAAGTATAATTAATCAGTCACCCTTCCCAACCCTTTTCTTATAGCCTT<br>AAGCAACCACTAATCTGTTTTCTGTCTCTATGGATTTGCCTAGTTCAAATATTTCACACAAATTGAATAGT<br>ACATAATATGTGGTCCTGTGTCTGGCATTTTCACTTAGTGTAAACATGATCAAAATTTATTAATATTGAGG<br>TATGTAGATAATTAGAAATTAGACACATCACATATTGGTCACTGGAATCTAAAATGGCATAGTCACTGAA<br>ATTTGGCTTTTTCTTAACAAGCTAAACATATAGTTAACATGAAACCCCAGAATTTCACTGTATATGCCTGA<br>CAGATGAAGACACATCGGCAAACAAAAGCTTGGACACGAATTCTATGTCAGCACCACCCACAGTAGAT<br>AACATATAGAGCCGACATCAACATTTCCCATGTGATGACTGGGTAAGACAGGCCCCAGATCCATGAAAT<br>GGGACAATTTTTTAACTCATCTATTCCTCTTCTAACCTTCAGCAGATTCCCGTTGAATTTCACATTAATTT<br>GAGTGCATCATAAATTATCCGGGTATGTTGACTTGCCACTCATCCTATGATAAGGATCATCATGAATAAGA<br>GTCGGTGGTGGAACTGCTTCACGGCAGCTGAGGAGTGGCTTGGAGATTCTACTAACTTTAAAATCACAC<br>CCCCCAACTCTAACAACTTCATTTCTTCCTCTAGGCCCCACCTTCTATGAACCACTTTCCAGGAATGTCA<br>TTATCTAAGACGAAGCTTCCAAGACAGGGGTCTTTGATAGGGGTACACTTCTAAACTATAGAATAGGGT<br>ATATGGTTTAGCATCTTCTTTTAACTTATTATTAAACTATTCATCCATCCTCTCACTATTTTCATCTATGCTT<br>ACTATTTTTGTTACTTTTTGGAACATTCTGACTCAGGGACCCTTGGGACATAGTGTGCCTGGCTTTCTGA<br>TTTCTGAGACTGAGCTAAACCATGGCTTCTGTTATTCTCAGTTTCCTTGCCATGTTTGTTTCTCTCCAAAG<br>CGCTTCATAATATTAATTCATTTTGAAGGAAATTATTCATTACTGTTTTGAACTCACTATGTAACCCAGGTT<br>GACTTCAAACTCATGGAGATCTTCTCTATATCTCGTAAGTTCTCTTTCTGTCTTCCACTCTCTCTGTCTCT<br>CTGTCTCCCTGTCTCTCTCCTCTTCATGTTTGTCTTCCTAAGCTTGATATGAGTGAGTATAAAATGATGTA<br>GATTTTTCCATCATAAGTACACACTGTTTGAGACAGTGCTATGCAAACTACTACATGCTCAACATATACA<br>AATATAGTGTGAGTGCAAATTTTTATGTAAGTTTTAATGTGTGGATAGTTTGTCAGGTCCTAGCATTTTGT<br>TTACATGCACACATCTACAAAGTTAAATTTAGGGCATGTTAGGAGTTGTGAATTATCTTGAAGAGAGGAT<br>CAACTTTAATGTACAGAAGCAAGGATAGTTCAATCCACAGGAAATATAAAAAGCCACCATCTTTTAACC<br>ACCTGTGTTTGCTACAGATACCAGTGTCAGGAGTGGGCATATCCAAGTAAATGGACATGGGCAGGAAGT<br>GACCTCTCAAGTGGACAGAAATATCACATAAGATGAAAAAAAGGAGCTTTGCTAAATGAAATCTCAGG<br>GATTGAATCCAGACTGCTCACATTATGGTCCTTGATGGGTTGCCTGCCCCATTTGAATCTCTTCAGGGGC<br>ATACTGTGGGCTCAAGTTCATTCTAGCTCATGGCCCTCCATTATTACTAATGGGAAGAACTGACTGGGGA<br>TACACTGTACAGGTTTAAGAATTTTCTCCAAAGCAAACCTTGCCTCCACTTCATCTTGAATTCAATTTTA<br>AGAAATGCTCTACTGACAAACATAAAAGAAGGGGAACTGGTATTCTCCAAGATGTGAGTCCTGATCTTA<br>TCACGGTTGGTTAAGAGTCTTTATCGTCTCCTCACCGTTACTAGATGATGCTGCTATGCTTTGCCATTCAG<br>GTGCCCTCCAATGCTAACAGCTCCTGTGGC
MSTR	--	0.502	0.000	0.049	-	do	GACAATTTTTAGCTGGCAGCAGATCTGATTACTGAGGCAAGTCTGAAATTAACTTTTCCTCTTGCCATGT<br>CGATTTTCCAAGTCATGTAGATTTCCCTGTGTTATATCACCCATTAACATCTTCACCACCGGTAACTGTGC

G.57850.1					3.592	wn	TGAAAGGTAATCCATTTTTCACGGAAACACCCATTCATCCATGTCCTATTACCCCTGGTGATATCAGTTCATACCCAGAAACCTCAATTTAAGTTTTCCAATCCAGTAGCTGGAAGGAGAGAAGCAACTCCACGAAGTGATTTTCTGACCATGGGCATGGGCATCCTGTCTCCAAATGTTTCCTCAGTAATTACTGCTGGTGTTATCAGATCCAGACTTCTTTCCTCCTGCTTGCATTTCAACTGTTTAGAAGAGAGAGCCCGAGTTAGCCATTTTCAC
MSTRG.57893.1	ENSRNOG00000060024	2.018	0.098	0.001	-3.317	down	GATTCATGACTGCAGCTTCTTTATATTATTAAGTAGCTTATGAATAGTTTTGGGCTTATTAATAAACCAAAATGCCACCAGGAGTGAGAGAGTGACAATAATAATAAAGATGTAACAAGGCCATTCTTCAATAATAAAGTTTTCAGAAATGTACATGATATAATTGAACCCATACGAAAGCAACGGTATATTAATATTTCTGAGGGCATGGAGATTACAACATTAATAAAATGATTTTAATAAAATTAAATATGCAATTATATAAAAAAAACAAACAGAAAAGTCAGTAACATGGAGTTTATACTGTCAGTGCCTGTCTGTTTAGAAATCACCAAATTATCTGCGAGATTTAAAGCTAAGAGTGAGTTGTGAACTCTCTTCACAATCCAGTGTTCAACATTCTAAATGTCACACTATTGAGTATCAATGTGTCGTTAAGAGCACTTTCCGAAAGTGAACCATTAGACTGTAAAGCATGCTGCACAGAAGGCTCGATCCACATTATCCAATAATATCAGCATATTCATCTTGATTCCAGAGTTCTATTCTCAGTGATAGCCATGACTACTTCCTCTATATACCTCTTGTCGTTCTCTCACTTGAAGGGGACTGAACTGGATTTAACTAAGCAGAACCTTTTTAGCCTCTTTATGTTGTTCGGCTCAATCTATTTTCTGAGAATTAATTGAAAGGAATCACCCACGTTCTAACTGGATCTCACATTAACTCCTGTAGTAGCCTGGTGCTCTTCTCGTAGTTCCTGCTATTTTTCTTTGTTCCTGCCGAGCTCATCACCTGTTATGCACACAGCGTTCT
MSTRG.58056.1	ENSRNOG00000014087;ENSRNOG00000014626;ENSRNOG00000025860	0.048	0.273	0.045	2.555	up	GTGGCACATAGCTTTAATCCCAGCACTTGGGAAGCAGAGGCAGGTGGATCTCCATTCGAGGTCAGTTGGAGTGAGTTCCAGGACAGCCTCAACAGCAATAACTCGTCACGCGAGGTCACTCATACCTGCCATTTAGTACTTGGCAGGAGGATCAAGATTTTAAGGCCATACTTAACTACCAAAATAAGTTTGAGGTCAATTTGTGGGGTACATGAGACCCTATCTAAAACAAACAAACAAAAAAAACCCAAACCGGTAGTAAATGGGTTGAGGGGCAGAGCTCAGTGTTAGAGCTTGGGTAGAGTGGTAAGGTTCTGGTTCAAGTCCCGGTATCCCAGATAAAACAAAGCAATGATTCCTTTCATCAGGACAAATACTTCATGGTGTCTGAACCTCTGTCTTTACGGGGTTCGCAGAGAAGAAAAGACCTTTAAAAAAGTGATGTGGTCAGCCAGGTGGTGGTGGTGGTGGCACACGCCTTTCATCCCGACACTTGGGAGGTAGGCAGATCTCTGTGAGTTTGGGGCCACCTAGTCTGCACAGTGTGTCCCAGGACATCCAGTTAGAGACCCTGTCTCAAAACAAAACAATAACATAACAGGGATGTCGTAGCCAGGTGTGTTAGCACTGACTTGTAATCCCAGAGTTCAGGAAGCTGAGGGCGCAGTATTGCCAGTTCTAGGCAGCCTGTGAGCAAGGAAAGGCACACTGGTTGGAAGCATAGTCCCGG
MSTRG.58286.3	ENSRNOG00000014093;ENSRNOG00000030174;ENSRNOG00000053115	0.000	0.250	0.019	3.592	up	GGGACCATGTAGCCAGCTGGCATCCTAGGTAACACCTTCCCATAGCCCGACTGCTGTCATGGCCGAGCTTCTCACAGGAGTTCTGGAGGCTCTGTCCCCATCTCAGCTCAGAGCAGTGCTGAGATGTAATCTGAGAAATGGAGACAGGAACAGGAATATGTGGGAACAGGTAAAGGAACTTGCCACAGTATGTTCAGTTCATTCTCTGTCTCAAAGGAGCACAAACCCAGGTCCTTGCCTCCTCTTTGGCCACAAACGAGTGGCTCCTGGAGCAGGGAGACATCAAATAGAAATTTCGAGTCCGACTTGGACAGGAGGCTATCGTCCTTAGGGCTTGGTCCCCAAGACTAAAGGCTAAGATGCTAATATTTTCGTTATCTTGCGTAACCTGACCCAGGTCTTTCCTTGGCCAGAGACCCAGTTTCCGGATGTGAAGACTTCTAGGCTCCACTGCGGCGCACATAGAAGTTTCGGGGGCGTGGCTTAGGA
MSTRG.583	ENSRNOG0000001	0.481	0.000	0.037	-3.684	down	CAGAGACCTCAGACACTCACCACTGCCTCCTCCCCAGGAACCACGGACAGTGAGTATTTTCCAGTTTCAAGCTACTGAGACATTTCTGTGCAGCCACAGAGACCTCAGACACTCACCACTGCCTCCTAAGAGTCAACTGTCCTCCCCTTCCCCTTCTGCTTCCTTCCCTGAGGCCGGAGCAGGACTTACAAGCTCAAGTTCCAGG

04.1	4117;ENSRNOG00000014336;ENSRNOG00000014761;ENSRNOG00000047379						CTGCTTGTTGGCTGAGGAGTTGACCTTGAATATCATTCTGCCATCTCTGGGTTCATATTTATGAACGGTCTACTGAAAAGCAATGTTCTCCCTGATGCCCAGCTCCTTACGGTGGGCGAAACCTTTCCCCGTGTTGTTCTGCCTTCATCTCCTGACCACTCTGAGAGGCACCGAGGACAAGTCAAGTTGCAGATGAAGAGTCTGAGGTCCCAAGATGAGAAATGATGTGACAGGGCACTTGTGCTGGGGTCCCC
MSTRG.58316.1	ENSRNOG00000014761;ENSRNOG00000047234	0.388	0.000	0.000	-5.913	down	CACTGGGGGTTCAGTCTTGGCAGGACCAAGGGCTTCCCCTTCCACTGGTGCCCTTACTAGGCTATTCATTGCTACCTATGAGGTCAGAGTCCAGGGTCAGTCCATGTATAGTCTTTGGGTAGTAGCTTAGTCCCTGGAAGCTCTGGTTGGTTGGTATTGTTGTTCATATGGGGTCTCAAGCCCCTTCAAGCTCTTCCAGTCCTCTCTTTGTTCTATTTAGTTTTAGTCTCAAGCTCTCTTTCCTTGACTAGTGTCTGTTGACACCGCCCCTCCCCCCAGCATAGCTGTAGCCATTTTGTTCCATGCCCACCAGCTATTTCATTGTTGCTGTAACATGCCTCGCAGTCAATGATGTAGAAAAATAGTTTGGCTTAGAGTAGGGTCATGCTGACTGCATAACCTGTTATGTTCTGTATGTTATGAGGTTTGTTAATCTTAAGAAATTCCACAACTGCATGCTTCACATAGGACCCATCAAATTAAAAGTCAATATGAACTGTTTGATCCAAAATGGCTTGAGTCAGGGGCAACACCAGGCAGCACTTCCAGCATGGGTATATGAGCTCATTGTGTGTGTGTGTGTGTGTGTGTGTGTGTTGCAGTTTTAGCCTTTATAAGCTGGACTGAGAAATGTCCAGTGAGCAGCTTTGAGCCTGAACTGTTGCCCTGGTCGATCGTTCTTTAGATGTGTGTTCAATAAACCATCCCTGTCTGACTGAGTTGGTGTCCCCATGGTTTGCGTGGTGGACTCCTGGACACTGTCTATCTGCCATCCACCTTCTGCTCTACCTGCGTTACTGACAGTGAGCTCCACTTTCCCAGTCTTCCATTCTTAAGTTTGTGCTGATAGGACACTCTAGGAAGGTGGTTCCTCCCAGTGTTAATGCAGTCCATTTACATCACAAATAACCCCAGGGCAGGATGGGATTATCCATCTCCCCTTGCCTTCTTGTCTTCTTGACTGAGGTAGTCTTCCCTTTCTGCCAGCCTTGTGCTAATTACAATTTCTTTCCCTTCACTACAGGAGTCCTCTTGCTTCTAATCTTTTCCCCAACGTCCATAAGAATCTATGGTCCTGTCATTTCATTTCCTTAACTGTTCCCAAACTCATTTAGGCTTTTGGCCAACATAATGTAGAGGTTTAATGAGAGAAGAGTAGAAGAGTGAACTGTGGGCATGGAGGGGGTAGGATGGGGAAGCGGGCAAGAGCAGCGAAGAGCAAAGAGTAAGAGAGAATTAAGAGAAAGTAAGAGAGGGCAAGCAGCTCCTTTTATAGTGTCAGGCACAGCTGGCTGTTGCCAGGCAACTGTGGGGCAGAGCATACCTGGCTATTCCCAGGTAGCTGTGGGGGACAGTTAGGTCACAAATCTCGGGCACTCTAGGTTCCCTCCTTCCAATTCCCATTAGTCTGTGGTACCATTCCTGTGGCAGGTTCATACCTTGTCACCTTCCTTTCAGCACACACGATACCACCTTTCAGACTTCAAGATCATCTGGCATCCATTTAGTAACATCCAGGACCAAAGACACAGGGAAGGAGTGCGTGCGTGCGTGCGTGCGTGCGTGCGTGTGCGTGCGCGCGCGCGTGTATTTCTATGTGCCCAGTGTGTGTGTGTCTATGTTAATATGCCAACTAAGACCACATCCTGTTTGTCCTTGTCCATTTGGGGAGCAAATAATAACACCATCCTGCACTCCCAACCATTCCACTCACATAAAGCAGCCGATTTTTCAGCCGCACAAAACAGTTTGAGGACAATTGGCCCAGCAGGGGTCAACAAAGAACTTGAGCGCCGAAGGTGGTTGTGGGAGCGCAGTGGTAGCTCCGCGGGCTCATGGGAAATGTAGTGCGATTATCTGCAGCTTCGCGGAGCCCAGGTGAGAGAGCGCGAGAGCACTTGCCTAGCTCTCTGGGTTGTTTCAGGTCAGGCTGGTAGAGAGAGCAGCTGGGAGGGGAAAACTTCTCATGTGGCGCGCCCTGTTTTTGACAAGGACAGAAGGTATATTGCCTGCATGTGTGTCTGGGCACCACCTGCATCTGCGGTGTCCAAAGAGGACAGAAGAGGAAGCCAGATCTCCTG

							GAGCTATAGTTAATGAATGGTTGTGTGTTACCCTGTGGGTGCCAGGACTTGAACCCAGGTCCCCTGGAAGAACATCAGTGCTTCTAACCACTGAGCCATCTCTCAGCCCCAGGGCTCTCTTTTCTAACTCAGGCATGGACACAGCACGATGGCCACTTGTGGTCCTATTCAACATGACGTATTGAGAGTTAGCCAGTAGCAGAGCGTGTATATCTATCTCCATGCTCCCCTTTCCAAGACAACGTGCTTAGGCTGGCCTTAACGGGGTACTGCCAGGGCCGCCATCCCAGTAAATAGGGACCTTACTAATGTCACATGACAGGTTCATCCAATCAGGTGTACTGATTCCTCTCTTCTTCACGTCCATCCCTCCCGATGCCACTTGAACTTTCTTTAACCCTTTCCTGCCTGTTTCTTTACCTTGTATGCCTCCAGTGTAAATCAGGTCAGTATTTCTGCTTGCCACGGGTGGATCACAGGGCTTCTCATTATTCACCATGCTGCCCTGCTGTCCGACCATTCTGCCTCGCGTGTCTCCAAACGTGCTCTGGGTGCTAGCCAACTGAACTATTGCCATTTTTCTTCTATCCTTATATTATGACTTACTAGTCATAATTTGAGTTTTGACGGTGCCCCAATTATTTCGAAATACAATTTTATTTAAGTTATTTCACTTGCTAAATTTGAAGTATGCAGCATTGGACTGGAGGGTCAGTAAACACTTACTGCAATAAAAAACTTGTGCCAGCCGGGCATGGTGGTGCACATTTTGATCCCAGCGCACAAAGGCCGAGGCAGGATAAATGTTGTAAGTCTGAGGTGAGCGAG
MSTRG.5852.1	--	0.327	0.014	0.014	-2.916	down	CCACTTGAGTATCTCTAGAACAAATAAATGTATGACACTATTCTTTTTTCATTCTCTGTATCTTTGCCATGATGACTGGTACACAAAAGATATGCAATACACATTTTTTTAAATTAATGAAACAGTTGACACTGGTTTGATGGCGTGCTCACATCATAACATTACTGAAAAAGCAAAGAGAAGATGGCCATTGCATAGCACCCTAGTAGGTTTCCAAAAATCACCATCTTTAATATTTCCCATCACATACCATGGTACACTATGATTGTGAAGGGAATCAGATTATTGTAAAAGGTGTTCAGTTTTCCTCTAACACAACTTCTGGCCACAAGAGCAAGCTGTCATCATTAAGTTCCCTGATTCTCTTCCTTATTGTTTGTTTCTTTTTTGCATGTGTGTATCTGGTATGAATTTGTGTGTATGTATGTTTCTGCACATGTGTGGGTACAGATGTATATGAAAATGCATGTGTGTGCATTGGAAGCTTCCCAGTATCTTTTACTCAAAAGTATCCTGTGTTAACTTGTATCTAGCTATAGTTTACTTTTCTTTTTTCCTATTAACTGCAATGTACCTGATATGTTCTTGAAACAATAGAATCATGTCACGCTCTTATTCAAGACTCCACATTCAGTGGTTTCCCTTTACAATGCAATATGCCCTAGGCTAGAAGAATGAGGAATCCATCACTGAAGAGTCAGGACCTAGGACAAGAGCAGGCTCTTCATTCACACTCATTCTAAGTATTTATGCAAGAAAATGGAGTGCATGAAAGGCTCCAGTCACAGACAAGTGTTGGCAACTAGAAGAAAGAATTTACTGCAAGACGCTTGTGTCTGGCTTTCATCTACAAACCTGTTCAAGACTATTCTTTTCTAAAGAATTCAGGAGCCCGTAGGGCAGTGAAACAAGTGCCAAGTTTGTTAAGCTTGCTTACCTCACTTGAGCATCACATGTGGCATCAGGTTAGTGCTTCCTCTAGCTATGGCTCCTATGGTCCTATATCAGCTGTGGTATGAGTGTTGGAATCCAGATGTGGTTCCACATCCTTTGCTTCCTCCCTCCCTGTTAGTCCAGATGCTTCTGCACACCAGATTGGAAGGATGAATGGGTGGAGTCTGCAGGATCCCAGTCTGGTTGGAAGTGATTGCTTGATAGGAGAGGGAAGGCCTTCTCCAAAGATAATTTTAGTGTTAACAGCTCTTTTACAAGATGACCTGCTCTGAGGAGGATCTTAGTGTTACAGCTCTCTTTGATGATCTTAGCTTGTGACCATACCTTTATTCAGGATGCTTGTATGCATTCACTTTATTCAGGGTAGAATAAGGCTGTATGTGAACTTTGGAGAGTGAGGAGGAGTTTTCAGAGTGAATGTAAAATCATTAGCTAGAGTTTCAAGTACTGCGAATACCTCTTTTATATGGAAAGGCATTCCAGGAATCCCAGGTACTTGCTGGATGAATTTTCATGGGAAAAGAGGAAGTTGAGCTTGTAGGTCTTCCAAGAACTACTTGACCTTTCTGAAGTAGGGGATCGAGGGTTAGAGATTGCCAGAAAAAGTCAGAGAAGGGAAGCCCTGCTGGTAAAGGAAGACCTCCATTTTGCACTGAGCTCAGAGGAGCTGTCCTGTCATGGCAGACTCCATGTTAAATAGCAGGTTGTGCCCACACTCACTTTTCTGCACTCATTCTGACATCTCATTTTTGTCTTTACCAACCTCTCTGCTTTGCTTTTATTTCTGGAATATAAAGGATAGCACCATGCTGTGGCCTTTCTCTACCTGACCTCATTCTCACCAGCTATCTGTTTTGACTAGTGG
MSTR	ENSRNO	2.808	0.000	0.000	-	do	GGCATAAATCTTTTATTTAAAAAAGAGGCAAAGTCATTCAAATGCAATCAGCAGTGACAGATGCAGACAGAGGCCGACTCTGATAGGACAGGCACTCGAGGATTGGCTCCCCCTCGGTGACACATCGCAGAGGGTGA

G.586 22.2	G0000000 0010;ENS RNOG000 00024182				7.988	wn	CCTGAAGGACAGTCAGTGAACGTCAGCAAACAGTGGGGCCTCTGTCTGTTTCCATGTTGCTTGTTGGC CGGAGGTGCTGAGACTGACAGTGTCTTCAGGAGGATGTGGGAAGACGCTGCTGGGTAGGCAGTCAGG GCAGACGCTCAGAGATGGTCCACACTTCCGCTCTTCTATCTTGGAAGCAGAAGACACGCAGTTGCTATG GATGGTGATGAAGCTCTCCCAGATTTGTTTATAAGTTGTGTTCGCTTTTCTTTTCATTCTCTCTCACCTGG AATGCCAACTCACCATTCCTTGATATTTTTCACTCACATGGCTTAAAGAAATATGCAGACTGACCAGGAG CTGCTGGAGGTTGGAAGCCTCTCTTGGAGCCGCCTGGTGTGTGGAGAAGCAGACCCCAGGCCAATAGC AGGAGTTGGCACACTCTAACAGGAGTGTCTTTTTATTGACAAAAGCACTTTAACAAAATTCATCTCAGG GGTAGAATATCCATCTTCATATTGACTTAAAATACAGATAATGCAGGGTGGGCCTCCCGTTCATGTGCCAT GGACCTCTGCATGTGGCTGTCAATAAATAATCAATATTTTATCATCAAATTATGAGCTAGGTTTCAATGTT TTGCAAGAAGAAACTGGAGATGCATAGCTAAACACAATGATGCTTTTGATATGCAAAGTGTGTATTTTAC ATTTCAATAGAGTCCATCCTGAGCAGGCGGCATCCCCTCTGGTCCTAGGAAGGATGGTGAAAGGGACA GCAGGTCTGGAGGCAGCCCTGGCTTTGCTGTCTGTTGGATACTGTGCTGCCGGGCACTTTCTTGACTGA GAATTGGAGGGTCCATCCCACTGTGGGTAGTATCACCCATGGGAAAGTGGTCCTTGTCTGTGATTTGAT GTGGTCAGCTTCCTCTGGTGTAACAAGAGGGCCTCTTGTCTGTCCCACTCAGTGTGTTGGGGGATGATT ATGCTCAGCTAAGACCTGAGGAAAACACAGCTAGCAAGGCTGGCTCTTGAGGGTGGGGAGTATCCCAT GAGAGGAGAAAGAGAACAGCTGGGGCCAGACGAAGGCAAGCTGTACAGGCCACACACACCTAAGAC GTCAGTGAATGTGGAGATTCCAGGGGTCTGTGGAGCATCAGCCTCGGCAGAGACACTGCCACAGATGG CCAGACGAGGGCTTCTGCCCACCCCACCAAAGGAGATACAGCAGGTGAGAGGGGTCACTGGGGCTAT CCATGCTCAGAAAGCATACACTCACAGCGGGCTGGCATACCATGCCAAGTTCCTGGTCGTTCCGTCCTC CAATACGACCTGGGTATCCTTCCTGGCAGTGGCTGGTTAGGGCTGACTCTAGAAATGTGATTCGGGGGT TTCTAGCCACTCTAGAGACAAAGTGGACTCCAGAAGCCCTCGAGCGAAAGAGTCCACGCGTGACTCCG GGTGGTTCGGACGTCCTCCCCAGAAGAGCCCCCGATGTCCACTCCACTTCCCGCGACACGTGCGCCCC AGGCCGCCACGCAGGCCCCGCCCCGCACGTACGTGCCCTTAACCTGCCCGTGGCTGTCGCTGCCCCGC GCTTTC
MSTR G.588 56.1	--	1.506	0.127	0.003	- 2.742	do wn	GAAGGGAAAGGGTTTCAGCACGCTCCAAAGTAACCTGCCACAGAGGTTGAGGGGTCCTTGTCGGTGTT TCTCAGAGCAGCACTGAGCAGCTGTGGCAAACGGACTTAGAGAATGAAGGGGTTGGGCACAGAGTAC AATGGCTTTCCTGTCTGGCCAGTCATAGCCATATTTTGCTGCAGCCAGGGAAATCCCCAAAAGGACAGA GCACACCCCTCTCCCAGTCACATCTTCAGGCCACCATGACTGGGAAGAACCCGACTTCCCAATCTCTCC CCAAGATTCCAGAGGCTTGGTCCCTCGCCAAGCCCAGGGCCAGTTTGGGAATGAGCAGGAGCTGGGGT GTGGTGCCTAGTGCTGCCCTTGACCATGCTCAGATGAGGTGAACGATTAATGTGACCACTACATACCAG ACTAGCGTTCACAACCCTCACAGGGATTACCTAAGACCACGGGAAAACACTGAGATTTACACTACAAC TCATAACAGTAGTTATGAAGTTAGTTATGAAGTAGCCATGAAATAATTGGTTGAGGGTCTACAGGACATG AGGAACTGTATTAAAGGGTCACCGGCATTAGGAAGGTTGGGAACTACTGTGCTAGACTTAAGTGAAATA TTGTGCCCAAGGCAAATTAGAGCCACGTCACATATAAAAGGACAGGGATGGTAAATCACTGAGTTCCGT AGTCGTTTGTGTAGTAAGCAGGCCCCCACAGTTTGAAGAAGCGCCTTGAACTAGAAACAAAACCAAAG CAATGTGTGGTGGCACAGGCCTGTGACCCTGGTACTGAGAAGGTAGAGGCTGGAGCCTGAGAGTCCAA GGTCACCTCGGGCTACATGGAAGATCTCTCACGAGAACAGAGCAAGACCACAGAAAAGGAATCTGA GTTCTTCTTTTTTTTCCCTCTTCTCTGTCAATCCAAACGGCAGAGAGTTGCTTCTGCATCAGGGAACTTT GGCTGACCCTCGGCAGCAGCATTGTAACCGGTTTTCAGGGTGCCCCAACCACCCCAGGAAGAGCAGAA ACTCCCTTGTGTATTCAGTGAGGACATTGGTTGGGAAACACTAGCGGGAAGGCATCCATGCGTACAATT ACCTTTTAAGAACGTCCCACGATATTTATTTTATGTGACTCTCCCTCCCACTGTCACTGTATTTCATTAA

							ATCCAAAAGTAGACTGAGACCAAGTGTTAAGTCCCTCCTCCTCCAGACCTGGTTTGTGGGGAAATTCTTTAAATGAGCAAAGTAGTACCAATGTCCCTCGTCATCTGTGGGCTCCAACCAACCATAGGTGGTTGTTGGAAAGTGTTGAGGAAAAGGTAATTTTTATAACAAACAGGTCACATGTTTCCTCTCAGAGTCACTGCCTACAAGGCTACTGTAGAGCTCCTTATGCGGCATTGCACCTCATTGGCTGACGGAAACGCGATGTCTTGAAGCACCACGTGGGAGGAACTTCACAGGAGATATCATAACACTGTGCCAGGAGTCCAGGCAGCTTTGGTGTCCTTGGGGAATTCTAGACTCCATGTGTGTATTTTTGTGCAGTTTCCTCCATAAGATGAATAATATTAGTGTTGTAGATGTAATGTGAGGTTTGAAAACAACCAAACGCGGTTTTCATAACTTTAAAATTCCTCATTCATACATAAAACAGTAACACAAGAAATAATCCCTCTCCCTTCATCTTTGCTCACCGTTTCCTCACTGAAGACGTAAGGGATGGGACAATTGCTGCTGTCACAACCGTTGCTGTAAGATCAGATCGCAGCATGTCCGTGGGACAGGTTGACAGCCAGCAGCACAGGCAGACACCACTTTCTCAGAAGTGAGTTTTGAAAAGAAACGCTTCTCCAGCCCAGCACACTGCTTTCCCACACAGTGACCCCGAGTTCTGCAAACATGGGCCTATTTCAGAGGAATAGCTCACGTGGTGTTTCTATGCAAGCCTGGACGTCTTAGGAAATCCCACTGGCGTCCAGTGTGTTCTCTAAGGAACCCCACTACGCGTACCGGTCACTTTCAGATACTGAAAGATACTGATGTTACCCTAGACGCCCTTGAGAAGAGAAGGGTGGGTGCTGGTTACAGACCCCAGCTCACAACCTCCTTCCGTTCACGCCATTTGTTGGTGCCCTGGTCTTCCAGTCCATTTTCTGTGTTGAAACTCAGTGTAGGCCTTTGATGGAGATTGTGCTCACGTTTTCTTTTCTCGGAGAAGCATCCCTTCACTTGACACTACCCAGAGCCAGAAGTCTGCCTGTAAACAAGATTAGAATCAGCACGCCTTCCTGCTGTGCGAATGGAAGCCGCTGGAGTCGAATCAAACAGCGGCTGTCCGCTCACCGCCGTTGGCTTGTAGCAGCGTGCCTTTTATCCGACTTCAGTCCTGTCATCTCATTGGTCTGTGCTGGAGCAGAATTAATCCCAATTGAAAGGCAGAGGACAGCAAGCTCCCTTGTGACATGCTGAGAGCACCAGTTGGGGTCAGGGATGCTTCCTGGAGAAACTTCTGGGCAGAGGTACCTGCACTGTGTTTTAATGTCCTAATGGCGCCATCTTTGAAAAGGAAATTGTCTCTAACAAAGACAATTCTTTG
MSTRG.58924.2	ENSRNOG00000005115;ENSRNOG00000005257;ENSRNOG00000005533;ENSRNOG00000005619;ENSRNOG00000005729;ENSRNOG00000005747;	0.518	0.053	0.012	-2.433	down	TCCCATTACAGATGGTTGTGAGCCACCATGTGGTTGCTGAGATTTGAACTCAGGACCTCAGGAAGAGCAGTCAGTGCTCTTAACCTCTGAGCCAACTCTCTAGCCCCATCATTCTTTCACCTAGATATGCACCCTCAAAACCTTTTGTCATCATTGTCATCATCATCGTGACCATCATCATCATCACCATCACCATCATCACCATCGTCATTGTCACCACATTCATTTATTATGTTTGTGTACAAATGGGGATTAGAGAACAACTTGTAGAATTCAATTTTCTCCTTTAACCAAGTGGGTCAAATTCAGGGTTTCAGACTTGGCAGCCAACACCCTCTCACCCACTAAGCAAGCTCCCTGGACTTTCCATACGTAAGCAGTGGTGAGTGTCCCACTCATGCCTCCTCCATGCTAGGTGACACTCTCTGGATGAGCCATGCCCTAGTCCCTGAATGGAACTCTGTACGGCAGTCAGTGTGGACTGAGAGTATAGCTCAGTGCTAATCACGACTGGCCAGATGGAATCTAGGACTACAGAGAAAGTTCTATGTCATTTTCCTCTCAATCTACAAACTAAGAACAATGTTGCTATGGAGATGACCCTGTTGGTGAAGTGCTTGTGTACAGGCATGAGGTCCTGAGTTCCATCCCGGAAGCCTCTTAAGAGTCAGGTTTAGTCTGTTGCCTGACCCCTAGCAATGGTTCTATGGGGCAGGGAGGGGTGAGAGGTGAACTGGCAGAATCCTGGGCTCAGGAGCCAGCTGAGTGAACGCCATCCAGGTTCGGGGAGAGATCCCACTGTCAAAGAGTAAGATGAAGGAAGGAAGCCACGCACGGGCGTAAGTACACCCACGTGTGCACATGCACACCCACGTGTGCACATGCACATAAACAAAAGGAAATGGTAATAAGACTAAAACTAAATAAACACACGCACGGCGGTCACCACCTACTTCCAGCACTCGGGAAGCTGAGACAGGAGGATTGCCATAAGTTCCAATCAGCCTAGGTTCCGTAGTAAGTTCCCAGCTAGCCACACAACAGTAACAAGGACTTTGCTGACACCACCTGCTTTCTTGGTGTATGAGTGTAGAGGAGTCACTCAACCTCTCTGTGCCCCGATTTCTCATCTCCATAACAGAGAGGCTTGGAGGAACACATCATTGTCACGCTCTGTGGTCAGCGGTGCACCTTTGTCCTTTCTCCAGCAAGGTCTCATGGCCTGTGCCGCTCCCATGGGTGTCTCTCTCACTATCTGGAAAAGGCTGATGAATAATCTGCTCACTGCCCCTTTGCCAGTGCAGCTCACGGGGTGCAACCTTCTGCCATTTTCTCTCCCACATCCTGATGTCTGGTCAGGAGCCTCTGATCCCAGCACCGAGG

	ENSRNOG00000005911;ENSRNOG00000006551; ENSRNOG00000006741;ENSRNOG00000006747; ENSRNOG00000052637						AAGCAGAGACTCCAGAACTCATGGGGCTCACAGGAAAGTCAGTCTAGCCAATCCAGTGGCTGCATGTCCAGTTAGAGTCCCTGTACTCCCCCGAGCTGGAATCTTTCCTGACCCACAGACCCCTCCTCACTGCTTCAGGTCCCAGACGGTCTCCAGGCTGTCCCAGATGGTCTCCCCAGCACTGGGATTGGAACCCAGACCTTTGTTCTCGGTAGCAAATGTCCCACCGTGCCCAGCACCAGCCCAGGTGCATGGCTTTGTAGGTTTCTAAGTCTGTTTGTAGATGCATGGTGTAATTCGGGTCGAGCATTGTGTGATTAAATCCTGCCTGTACCCTTTTAGGTCTGTACTGTGCTGCTTCATTTTGTTCCTTAGAGTGAGCAGCACCAACCCACTTCCTTAGCTCTGTGCCAGCTGAGCTCAGCCTTTCCGCAGACATCGCTGGCACCTGCCGCTGTGCCAGAACCCATGCACCTCCCAGAGTCCAAACCTAGCTTTCCCTTTGTTTGGAGTCTTGATGGTTGCTTTATTATTTATCTTTTAGGTTTCTGACAAAGTCTTCCTGTATTGGCTCAGGCTAACCTTGAATTTTTGATGCCTCTACCTCAGCCTTCCAACTGCTAAAAGTTCAGGGCTAGCACAGTCTAGGAGGTCAGAGGAAGATACCAGGTGGTCTGCTCTGTAGCTCCTTGCCTTAGTCCTGAGGGTGTGTACCACGTGAGACTAAAACAGCCTTTTGTTTCACTTCTCTTTAACATCCTATGTTTTAAAAAGCTTTCTTCCAAGATGCAAGGTTGTAATCTGCTCCCCAACAACTTTCAGGTGCAGGAGTATGTGTGCGTTCAAATGGAGGGCAGCACTGGCTGTCAGTCCCCAGACATGCTTCCCAGTGGCATGGAGCTCACTGACTTGGTGGTGAGCCTTAAGAACCCTCCTTTCTTCACCTTTCCAGGGCTGGCATCATGTTCAGATCCGTGTATTTACAAGGCAAGCTGTGTACCCACTAAGACTTCTCCTCACTCCCCTTTTTTTCATGTTGGAGGTAAACCCAGGCCTTGAGCATGCCAGGCAAGCACTCTGCCACTGAGTCAAGCTCAGTGGCTCCAGCCCAGCGGCTTCTTGTCACTCTTGAATTTGTGTCTCCACCCCTCCACTTCCTGTGAACTGGTGCACCAGCTGTGTGGGCGAGAGGCTTAAACCAGGCCCATGTTTTGGCCAGGTATGTCACCAGAAAGTCACTGTGAACTTCCTTTTTCTGTTGAAGAAATTCTTTCTGGCTTTGGTTCTGTCTATTTCAGAGTTCAGGGTCCTCTGGGAGTCAGGAAATGGTAGGAAGTACAACCAAGTCTTCTAGGTCCCTTTTCACCAGGAGGATTTTTCCTCTAGTCTAGAAACTCTGGTCTTGACAGAAGCAGCAGTGGGGACGAGTGAAGCTCCTCTGCCAGCGGGGGTGTGCTCGCTCCTGTGCCCTCTGGCCTGGGTGGCTTTCAGTCCCTGTGGGGTCCTCAGGATTGGGAACAGCTGCTGCTTCTAGGGAGACGAGTAGCTGTGCTTCCTTCTCCTGAAACTCACTCTTGGGACATGAGAAGGCCTGACCAGGGCACTCGCAGTTGGATGCTGCATCTCCAGGTGCAGATGGGGTAACCTTATTCCTTGGAGCCACCATGTGCTTATCTGTCACCAGGGAGGATGCTTTACTTGCCTGGGATGTTGTGTTTCTGTAGAATTGCCTGGTAACCAGGTGCATCTGCCAGATGTGGGCATCCTCTTTTGTCTCTGCAATTCCCTGTCTCATCTTGTTTTGTGGTATGGAGGTGGAACTCATGGCCCCAATTGTGCCTTCTCCATGCTCAGCCTCTCAGTGTGGTGATCTGAGACTGTGGGACAGCCAAGCCACAGCTAGACTCTGCATGGTGGACTTGAGGCTGGTGCTCTATTGGTGAGCTACATATGTTCTCACCTCTTTTTTTAATTTTAAAAAACATGTTAAGAACAATTACAGTTTTTAAGTGTGTTGGTATTTTGCCTGCATGTATGTCTGTGCAACATGTTCGTGTCAGGTGCCCTCAGAGGCCAGAAAGGGTGTCAGATTCCCTGAAACTGAAGTTACAGGTGGTTGTGAGCCCAAACTGCCTTGTGGGTTCTGGGAGAGAAGCTGGTGCTCTTAACTGTTTGAGATTATCTCCCCCGCCCCCTCTTCTTACTTTTATGTTAACAGGTCTCGTGAAGTTGCGTAGTAGGTTGGCCTCAAATGTTCCATTTTCTTGCCTTGGACTGTAGAGTGGCCCAGGCCCTCCTTCTACTATGTTTTGTGGTGCATATTCCACACCTCCTCATTTTGAGGGGTTCTTTTCAGAGAATAATGGTCACAGAATCTTCTCTCTCCCTTCTCTCTCCTCCTCCACTGCTAGAGCAACTACAGGGTCTGTGTCACACATGACTCTGCTTATTGATGTTCTGATATTATAGATGAGACAGAGGTAGACACTTGTCTGCTTCTACATGTATAATCCAGGACCTTGCATAGGAAAAGTATGAGAAATAGGCTCAGAGGGTGGAGCCACTTTGTCCATAAAACATAGCAGAGCTGGAATAAAGGCCCCAAGTTTTGAGCCTTTCTCCATCCCCAACTCCATAGGCATGGGGTAGCAAAACTTAAGATGTGGTGGTGCGCCCCTGTAATCTCAGCACTAAGAGGTGGTCATCCTCAACCTCAGAGATGGTTTGGAGCCAGCCCTGACTCTAAGACCCTGTCTCAAAATACCAAAACCAAAACAAAACAAAAAACACAAAAACACAAAACCCATTGTTTTGAGCTGGATCTTGCAGTACAGCTCA

							GCTCAAAGCAATCCATCTGCCTCAGTCTGCAAAGTAGTTGTGTCTACCCAACTTGGACATTTTTGTACATTTTTTGAAGTAGACAATAGGCTATTAAACAATTGGTATAGAAAAAATGGTTACTATAAAGGAACTAATTAACACATTCATCATCTCTCTCACAGTTATTCATATTCCCCTGTGGAAAAAGCAGTTAGAATCTCATTTAGAAAAGAATCCCGACTCAATAAACAAAAATATTTGAGCACAAATGTCAACCCTGAGCTTGCAAGATTCACTTTGCCCTAACCTAGGTGCCAGCAGGAATGATTTCATTTAACGTTTGACATTTGGTTCACCGCGGGTCTACTTTTTCTCATTAATTGATATTTTAAAATCCTGCTTTAGAATATTATCTTGATGATTAATTTCTGAGCTGGAAGCATTCACTCCCGTTGGGGCTATTTCTGTTTCACCTTCTCCAGCGGCAGCTGACCTATCTCTTGGAGAAACTGAGGCCCGGAACGGCAGGGAGCTCTGGGAATAGAATGCCGCTGACCTTTGCTTATCGTCCTCTCATTAAATGTCCGCCTGCCAGAGACCTTTTCACTGCCTGAGTGGCCGAGATAGCGGCGCGGCCAGGCAGTGACCTTTCCAGGGATCCGACTAGCATCGTAGCAGCCGATTCCTCTCCACCCTCTAGAACCGAGAGAACGCGCCTGTGAAGTCATCGAACTCCGAGACTGGACTCCGCCGCTGACGTCACAGAAGGGCAGCACAGAGTCGCAACGTTCCTTGGGGGATATGAGCTCCAGAGGCCAGAGAGTGGCCCAGCAAGGAGGAAGTCTTGAGGTTTTCAGCGGTACGTAGGAGCGAACTACAACTCCCGACGCACCATGGACCTGTGCAAGAGAGCGCGACAAACTTTTGGACGAGATTGATATCTCGTTAGTCCAACCAGAGAGCCGATTTAGAGCGGCATCCCACCCCCTTATAATTCTCAGGGCTGCTTCCTCGCGCGAGTGACGGGCTTCGCTTGCCATCTAGGGTTTAGCTTTCTCTGACGGACGTCCGAACTGAGGAATCGGAGCTCGGAGAAGTCGAGAGGGTGCTGAAAAGCGTGGACGGTAGCCAATAGCATGCCGGAAGCATCCGGCACCGTCCAATCGCCGCCTCGAAGGTCCGCCCTGGCGCGTGACGTAGTAGGGTGGGTAGCAACAGTTGCCCCGGTGAGGGAAACGGAGGCGCC
MSTRG.58924.3	ENSRNOG00000005115;ENSRNOG00000005257;ENSRNOG00000005533;ENSRNOG00000005619;ENSRNOG00000005729;ENSRNOG00000005747;ENSRNOG0000000	0.084	0.676	0.000	3.721	up	CTCAAAACCTTTTGTCATCATTGTCATCATCATCGTGACCATCATCATCATCACCATCACCATCATCACCATCGTCATTGTCACCACATTCATTTATTATGTTTGTGTACAAATGGGGATTAGAGAACAACTTGTAGAATTCAATTTTCTCCTTTAACCAAGTGGGTCAAATTCAGGGTTTCAGACTTGGCAGCCAACACCCTCTCACCCACTAAGCAAGCTCCCTGGACTTTCCATACGTAAGCAGTGGTGAGTGTCCCACTCATGCCTCCTCCATGCTAGGTGACACTCTCTGGATGAGCCATGCCCTAGTCCCTGAATGGAACTCTGTACGGCAGTCAGTGTGGACTGAGAGTATAGCTCAGTGCTAATCACGACTGGCCAGATGGAATCTAGGACTACAGAGAAAGTTCTATGTCATTTTCCTCTCAATCTACAAACTAAGAACAATGTTGCTATGGAGATGACCCTGTTGGTGAAGTGCTTGTGTACAGGCATGAGGTCCTGAGTTCCATCCCGGAAGCCTCTTAAGAGTCAGGTTTAGTCTGTTGCCTGACCCCTAGCAATGGTTCTATGGGGCAGGGAGGGGTGAGAGGTGAACTGGCAGAATCCTGGGCTCAGGAGCCAGCTGAGTGAACGCCATCCAGGTTCGGGGAGAGATCCCACTGTCAAAGAGTAAGATGAAGGAAGGAAGCCACGCACGGGCGTAAGTACACCCACGTGTGCACATGCACACCCACGTGTGCACATGCACATAAACAAAAGGAAATGGTAATAAGACTAAAACTAAATAAACACACGCACGGCGGTCACCACCTACTTCCAGCACTCGGGAAGCTGAGACAGGAGGATTGCCATAAGTTCCAATCAGCCTAGGTTCCGTAGTAAGTTCCCAGCTAGCCACACAACAGTAACAAGGACTTTGCTGACACCACCTGCTTTCTTGGTGTATGAGTGTAGAGGAGTCACTCAACCTCTCTGTGCCCCGATTTCTCATCTCCATAACAGAGAGGCTTGGAGGAACACATCATTGTCACGCTCTGTGGTCAGCGGTGCACCTTTGTCCTTTCTCCAGCAAGGTCTCATGGCCTGTGCCGCTCCCATGGGTGTCTCTCTCACTATCTGGAAAAGGCTGATGAATAATCTGCTCACTGCCCCTTTGCCAGTGCAGCTCACGGGGTGCAACCTTCTGCCATTTTCTCTCCCACATCCTGATGTCTGGTCAGGAGCCTCTGATCCCAGCACCGAGGAAGCAGAGACTCCAGAACTCATGGGGCTCACAGGAAAGTCAGTCTAGCCAATCCAGTGGCTGCATGTCCAGTTAGAGTCCCTGATGTGAGAACTGAACTCTCCATTCTTTATTTTTTCAGTTTGTGATAGATTCTTACCCAGTAACCCATCGTAGCCTTGAGGCACGCTGCCACAGCCTCCAACCGGCTTGATTTCATTTTATTTTTCTTTGTTGTGTTGGGGTGGAAACTAGAGCCAGCCCTGAGCTAGCCTGGGGTCTGCTCTCTCTTGCTTCTATCAGCCATAGGCTAAGCCTACTAAACCACACAGCATAGCTTCAGAAGCTGCTTTTCCCCAGC

	5911;ENSRNOG00000006551;ENSRNOG00000006741;ENSRNOG00000006747;ENSRNOG00000052637						ACCCAAGGTCTGTGGCCCAGGCTAGCCTCGAACCTGCTATGCAGCTGAGGATGAGAGGGAACTCCTGATCCTCCTGACTCATTATACCTGTACTCCCCCGAGCTGGAATCTTTCCTGACCCACAGACCCCTCCTCACTGCTTCAGGTCCCAGACGGTCTCCAGGCTGTCCCAGATGGTCTCCCCAGCACTGGGATTGGAACCCAGACCTTTGTTCTCGGTAGCAAATGTCCCACCGTGCCCAGCACCAGCCCAGGTGCATGGCTTTGTAGGTTTCTAAGTCTGTTTGTAGATGCATGGTGTAATTCGGGTCGAGCATTGTGTGATTAAATCCTGCCTGTACCCTTTTAGGTCTGTACTGTGCTGCTTCATTTTGTTCCTTAGAGTGAGCAGCACCAACCCACTTCCTTAGCTCTGTGCCAGCTGAGCTCAGCCTTTCCGCAGACATCGCTGGCACCTCCCAGAGTCCAAACCTAGCTTTCCCTTTGTTTGGAGTCTTGATGGTTGCTTTATTATTTATCTTTTAGGTTTCTGACAAAGTCTTCCTGTATTGGCTCAGGCTAACCTTGAATTTTTGATGCCTCTACCTCAGCCTTCCAACTGCTAAAAGTTCAGGGCTAGCACAGTCTAGGAGGTCAGAGGAAGATACCAGGTGGTCTGCTCTGTAGCTCCTTGCCTTAGTCCTGAGGGTGTGTACCACGTGAGACTAAAACAGCCTTTTGTTTCACTTCTCTTTAACATCCTATGTTTTAAAAAGCTTTCTTCCAAGATGCAAGGTTGTAATCTGCTCCCCAACAACTTTCAGGTGCAGGAGTATGTGTGCGTTCAAATGGAGGGCAGCACTGGCTGTCAGTCCCCAGACATGCTTCCCAGTGGCATGGAGCTCACTGACTTGGTGGTGAGCCTTAAGAACCCTCCTTTCTTCACCTTTCCAGGGCTGGCATCATGTTCAGATCCGTGTATTTACAAGGCAAGCTGTGTACCCACTAAGACTTCTCCTCACTCCCCTTTTTTTCATGTTGGAGGTAAACCCAGGCCTTGAGCATGCCAGGCAAGCACTCTGCCACTGAGTCAAGCTCAGTGGCTCCAGCCCAGCGGCTTCTTGTCACTCTTGAATTTGTGTCTCCACCCCTCCACTTCCTGTGAACTGGTGCACCAGCTGTGTGGGCGAGAGGCTTAAACCAGGCCCATGTTTTGGCCAGGTATGTCACCAGAAAGTCACTGTGAACTTCCTTTTTCTGTTGAAGAAATTCTTTCTGGCTTTGGTTCTGTCTATTTCAGAGTTCAGGGTCCTCTGGGAGTCAGGAAATGGTAGGAAGTACAACCAAGTCTTCTAGGTCCCTTTTCACCAGGAGGATTTTTCCTCTAGTCTAGAAACTCTGGTCTTGACAGAAGCAGCAGTGGGGACGAGTGAAGCTCCTCTGCCAGCGGGGGTGTGCTCGCTCCTGTGCCCTCTGGCCTGGGTGGCTTTCAGTCCCTGTGGGGTCCTCAGGATTGGGAACAGCTGCTGCTTCTAGGGAGACGAGTAGCTGTGCTTCCTTCTCCTGAAACTCACTCTTGGGACATGAGAAGGCCTGACCAGGGCACTCGCAGTTGGATGCTGCATCTCCAGGTGCAGATGGGGTAACCTTATTCCTTGGAGCCACCATGTGCTTATCTGTCACCAGGGAGGATGCTTTACTTGCCTGGGATGTTGTGTTTCTGTAGAATTGCCTGGTAACCAGGTGCATCTGCCAGATGTGGGCATCCTCTTTTGTCTCTGCAATTCCCTGTCTCATCTTGTTTTGTGGTATGGAGGTGGAACTCATGGCCCCAATTGTGCCTTCTCCATGCTCAGCCTCTCAGTGTGGTGATCTGAGACTGTGGGACAGCCAAGCCACAGCTAGACTCTGCATGGTGGACTTGAGGCTGGTGCTCTATTGGTGAGCTACATATGTTCTCACCTCTTTTTTTAATTTTAAAAAACATGTTAAGAACAATTACAGTTTTTAAGTGTGTTGGTATTTTGCCTGCATGTATGTCTGTGCAACATGTTCGTGTCAGGTGCCCTCAGAGGCCAGAAAGGGTGTCAGATTCCCTGAAACTGAAGTTACAGGTGGTTGTGAGCCCAAACTGCCTTGTGGGTTCTGGGAGAGAAGCTGGTGCTCTTAACTGTTTGAGATTATCTCCCCCGCCCCCTCTTCTTACTTTTATGTTAACAGGTCTCGTGAAGTTGCGTAGTAGGTTGGCCTCAAATGTTCCATTTTCTTGCCTTGGACTGTAGAGTGGCCCAGGCCCTCCTTCTACTATGTTTTGTGGTGCATATTCCACACCTCCTCATTTTGAGGGGTTCTTTTCAGAGAATAATGGTCACAGAATCTTCTCTCTCCCTTCTCTCTCCTCCTCCACTGCTAGAGCAACTACAGGGTCTGTGTCACACATGACTCTGCTTATTGATGTTCTGATATTATAGATGAGACAGAGGTAGACACTTGTCTGCTTCTACATGTATAATCCAGGACCTTGCATAGGAAAAGTATGAGAAATAGGCTCAGAGGGTGGAGCCACTTTGTCCATAAAACATAGCAGAGCTGGAATAAAGGCCCCAAGTTTTGAGCCTTTCTCCATCCCCAACTCCATAGGCATGGGGTAGCAAAACTTAAGATGTGGTGGTGCGCCCCTGTAATCTCAGCACTAAGAGGTGGTCATCCTCAACCTCAGAGATGGTTTGGAGCCAGCCCTGACTCTAAGACCCTGTCTCAAAATACCAAAACCAAAACAAAACAAAAAACACAAAAACACAAAACCCATTGTTTTGAGCTGGATCTTGCAGTACAGCTCAGCTCAAAGCAATCCATCTGCC

							TCAGTCTGCAAAGTAGTTGTGTCTACCCAACTTGGACATTTTTGTACATTTTTTGAAGTAGACAATAGGCTATTAAACAATTGGTATAGAAAAAATGGTTACTATAAAGGAACTAATTAACACATTCATCATCTCTCTCACAGTTATTCATATTCCCCTGTGGAAAAAGCAGTTAGAATCTCATTTAGAAAAGAATCCCGACTCAATAAACAAAAATATTTGAGCACAAATGTCAACCCTGAGCTTGCAAGATTCACTTTGCCCTAACCTAGGTGCCAGCAGGAATGATTTCATTTAACGTTTGACATTTGGTTCACCGCGGGTCTACTTTTTCTCATTAATTGATATTTTAAAATCCTGCTTTAGAATATTATCTTGATGATTAATTTCTGAGCTGGAAGCATTCACTCCCGTTGGGGCTATTTCTGTTTCACCTTCTCCAGCGGCAGCTGACCTATCTCTTGGAGAAACTGAGGCCCGGAACGGCAGGGAGCTCTGGGAATAGAATGCCGCTGACCTTTGCTTATCGTCCTCTCATTAAATGTCCGCCTGCCAGAGACCTTTTCACTGCCTGAGTGGCCGAGATAGCGGCGCGGCCAGGCAGTGACCTTTCCAGGGATCCGACTAGCATCGTAGCAGCCGATTCCTCTCCACCCTCTAGAACCGAGAGAACGCGCCTGTGAAGTCATCGAACTCCGAGACTGGACTCCGCCGCTGACGTCACAGAAGGGCAGCACAGAGTCGCAACGTTCCTTGGGGGATATGAGCTCCAGAGGCCAGAGAGTGGCCCAGCAAGGAGGAAGTCTTGAGGTTTTCAGCGGTACGTAGGAGCGAACTACAACTCCCGACGCACCATGGACCTCCTTAAAGCCTTCCGCGGACACAAGTGCGGAGAAGCCGAGGTAACCGGGGCAGCGCCCGGCAAGCTGGGCGTGCTGGGGCGGGCGGCCGGGCCCGCGCAGCGCCTGTCGCTTGAGTACAGTCCTGAGGGAAGGGGCGGGCGGTCAAAATGGCGGCGGCGACTGTCGGGACTGTGGGGGAGGGGGCCGAGGTTCGGCCGGGGCGACACCACCCGCCCCCGGACCCGCAGCCCGGCCCCCGCGAGGGGCGGGTGCTCGCAGGCGGCGGGCGAGT
MSTRG.58924.4	ENSRNOG00000005115;ENSRNOG00000005257;ENSRNOG00000005533;ENSRNOG00000005619;ENSRNOG00000005729;ENSRNOG00000005747;ENSRNOG00000005911;ENS	0.000	0.628	0.000	6.519	up	CTCAAAACCTTTTGTCATCATTGTCATCATCATCGTGACCATCATCATCATCACCATCACCATCATCACCATCGTCATTGTCACCACATTCATTTATTATGTTTGTGTACAAATGGGGATTAGAGAACAACTTGTAGAATTCAATTTTCTCCTTTAACCAAGTGGGTCAAATTCAGGGTTTCAGACTTGGCAGCCAACACCCTCTCACCCACTAAGCAAGCTCCCTGGACTTTCCATACGTAAGCAGTGGTGAGTGTCCCACTCATGCCTCCTCCATGCTAGGTGACACTCTCTGGATGAGCCATGCCCTAGTCCCTGAATGGAACTCTGTACGGCAGTCAGTGTGGACTGAGAGTATAGCTCAGTGCTAATCACGACTGGCCAGATGGAATCTAGGACTACAGAGAAAGTTCTATGTCATTTTCCTCTCAATCTACAAACTAAGAACAATGTTGCTATGGAGATGACCCTGTTGGTGAAGTGCTTGTGTACAGGCATGAGGTCCTGAGTTCCATCCCGGAAGCCTCTTAAGAGTCAGGTTTAGTCTGTTGCCTGACCCCTAGCAATGGTTCTATGGGGCAGGGAGGGGTGAGAGGTGAACTGGCAGAATCCTGGGCTCAGGAGCCAGCTGAGTGAACGCCATCCAGGTTCGGGGAGAGATCCCACTGTCAAAGAGTAAGATGAAGGAAGGAAGCCACGCACGGGCGTAAGTACACCCACGTGTGCACATGCACACCCACGTGTGCACATGCACATAAACAAAAGGAAATGGTAATAAGACTAAAACTAAATAAACACACGCACGGCGGTCACCACCTACTTCCAGCACTCGGGAAGCTGAGACAGGAGGATTGCCATAAGTTCCAATCAGCCTAGGTTCCGTAGTAAGTTCCCAGCTAGCCACACAACAGTAACAAGGACTTTGCTGACACCACCTGCTTTCTTGGTGTATGAGTGTAGAGGAGTCACTCAACCTCTCTGTGCCCCGATTTCTCATCTCCATAACAGAGAGGCTTGGAGGAACACATCATTGTCACGCTCTGTGGTCAGCGGTGCACCTTTGTCCTTTCTCCAGCAAGGTCTCATGGCCTGTGCCGCTCCCATGGGTGTCTCTCTCACTATCTGGAAAAGGCTGATGAATAATCTGCTCACTGCCCCTTTGCCAGTGCAGCTCACGGGGTGCAACCTTCTGCCATTTTCTCTCCCACATCCTGATGTCTGGTCAGGAGCCTCTGATCCCAGCACCGAGGAAGCAGAGACTCCAGAACTCATGGGGCTCACAGGAAAGTCAGTCTAGCCAATCCAGTGGCTGCATGTCCAGTTAGAGTCCCTCCTTAAAGCCTTCCGCGGACACAAGTGCGGAGAAGCCGAGGTAACCGGGGCAGCGCCCGGCAAGCTGGGCGTGCTGGGGCGGGCGGCCGGGCCCGCGCAGCGCCTGTCGCTTGAGTACAGTCCTGAGGGAAGGGGCGGGCGGTCAAAATGGCGGCGGCGACTGTCGGGACTGTGGGGGAGGGGGCCGAGGTTCGGCCGGGGCGACACCACCCGCCCCCGGACCCGCAGCCCGGCCCCCGCGAGGGGCGGGTGCTCGCAGGCGGCGGGCGAGT

	RNOG00000006551;ENSRNOG00000006741;ENSRNOG00000006747;ENSRNOG00000052637						
MSTRG.58924.5	ENSRNOG00000005115;ENSRNOG00000005257;ENSRNOG00000005533;ENSRNOG00000005619;ENSRNOG00000005729;ENSRNOG00000005747;ENSRNOG00000005911;ENSRNOG00000006049;ENSRNO	0.000	0.419	0.000	7.637	up	CTCAAAACCTTTTGTCATCATTGTCATCATCATCGTGACCATCATCATCATCACCATCACCATCATCACCATCGTCATTGTCACCACATTCATTTATTATGTTTGTGTACAAATGGGGATTAGAGAACAACTTGTAGAATTCAATTTTCTCCTTTAACCAAGTGGGTCAAATTCAGGGTTTCAGACTTGGCAGCCAACACCCTCTCACCCACTAAGCAAGCTCCCTGGACTTTCCATACGTAAGCAGTGGTGAGTGTCCCACTCATGCCTCCTCCATGCTAGGTGACACTCTCTGGATGAGCCATGCCCTAGTCCCTGAATGGAACTCTGTACGGCAGTCAGTGTGGACTGAGAGTATAGCTCAGTGCTAATCACGACTGGCCAGATGGAATCTAGGACTACAGAGAAAGTTCTATGTCATTTTCCTCTCAATCTACAAACTAAGAACAATGTTGCTATGGAGATGACCCTGTTGGTGAAGTGCTTGTGTACAGGCATGAGGTCCTGAGTTCCATCCCGGAAGCCTCTTAAGAGTCAGGTTTAGTCTGTTGCCTGACCCCTAGCAATGGTTCTATGGGGCAGGGAGGGGTGAGAGGTGAACTGGCAGAATCCTGGGCTCAGGAGCCAGCTGAGTGAACGCCATCCAGGTTCGGGGAGAGATCCCACTGTCAAAGAGTAAGATGAAGGAAGGAAGCCACGCACGGGCGTAAGTACACCCACGTGTGCACATGCACACCCACGTGTGCACATGCACATAAACAAAAGGAAATGGTAATAAGACTAAAACTAAATAAACACACGCACGGCGGTCACCACCTACTTCCAGCACTCGGGAAGCTGAGACAGGAGGATTGCCATAAGTTCCAATCAGCCTAGGTTCCGTAGTAAGTTCCCAGCTAGCCACACAACAGTAACAAGGACTTTGCTGACACCACCTGCTTTCTTGGTGTATGAGTGTAGAGGAGTCACTCAACCTCTCTGTGCCCCGATTTCTCATCTCCATAACAGAGAGGCTTGGAGGAACACATCATTGTCACGCTCTGTGGTCAGCGGTGCACCTTTGTCCTTTCTCCAGCAAGGTCTCATGGCCTGTGCCGCTCCCATGGGTGTCTCTCTCACTATCTGGAAAAGGCTGATGAATAATCTGCTCACTGCCCCTTTGCCAGTGCAGCTCACGGGGTGCAACCTTCTGCCATTTTCTCTCCCACATCCTGATGTCTGGTCAGGAGCCTCTGATCCCAGCACCGAGGAAGCAGAGACTCCAGAACTCATGGGGCTCACAGGAAAGTCAGTCTAGCCAATCCAGTGGCTGCATGTCCAGTTAGAGTCCCTGTACTCCCCCGAGCTGGAATCTTTCCTGACCCACAGACCCCTCCTCACTGCTTCAGGTCCCAGACGGTCTCCAGGCTGTCCCAGATGGTCTCCCCAGCACTGGGATTGGAACCCAGACCTTTGTTCTCGGTAGCAAATGTCCCACCGTGCCCAGCACCAGCCCAGGTGCATGGCTTTGTAGGTTTCTAAGTCTGTTTGTAGATGCATGGTGTAATTCGGGTCGAGCATTGTGTGATTAAATCCTGCCTGTACCCTTTTAGGTCTGTACTGTGCTGCTTCATTTTGTTCCTTAGAGTGAGCAGCACCAACCCACTTCCTTAGCTCTGTGCCAGCTGAGCTCAGCCTTTCCGCAGACATCGCTGGCACCTCCCAGAGTCCAAACCTAGCTTTCCCTTTGTTTGGAGTCTTGATGGTTGCTTTATTATTTATCTTTTAGGTTTCTGACAAAGTCTTCCTGTATTGGCTCAGGCTAACCTTGAATTTTTGATGCCTCTACCTCAGCCTTCCAACTGCTAAAAGTTCAGGGCTAGCACAGTCTAGGAGGTCAGAGGAAGATACCAGGTGGTCTGCTCTGTAGCTCCTTGCCTTAGTCCTGAGGGTGTGT

	G00000006551;ENSRNOG00000006741;ENSRNOG00000006747;ENSRNOG00000052637						ACCACGTGAGACTAAAACAGCCTTTTGTTTCACTTCTCTTTAACATCCTATGTTTTAAAAAGCTTTCTTCCAAGATGCAAGGTTGTAATCTGCTCCCCAACAACTTTCAGGTGCAGGAGTATGTGTGCGTTCAAATGGAGGGCAGCACTGGCTGTCAGTCCCCAGACATGCTTCCCAGTGGCATGGAGCTCACTGACTTGGTGGTGAGCCTTAAGAACCCTCCTTTCTTCACCTTTCCAGGGCTGGCATCATGTTCAGATCCGTGTATTTACAAGGCAAGCTGTGTACCCACTAAGACTTCTCCTCACTCCCCTTTTTTTCATGTTGGAGGTAAACCCAGGCCTTGAGCATGCCAGGCAAGCACTCTGCCACTGAGTCAAGCTCAGTGGCTCCAGCCCAGCGGCTTCTTGTCACTCTTGAATTTGTGTCTCCACCCCTCCACTTCCTGTGAACTGGTGCACCAGCTGTGTGGGCGAGAGGCTTAAACCAGGCCCATGTTTTGGCCAGGTATGTCACCAGAAAGTCACTGTGAACTTCCTTTTTCTGTTGAAGAAATTCTTTCTGGCTTTGGTTCTGTCTATTTCAGAGTTCAGGGTCCTCTGGGAGTCAGGAAATGGTAGGAAGTACAACCAAGTCTTCTAGGTCCCTTTTCACCAGGAGGATTTTTCCTCTAGTCTAGAAACTCTGGTCTTGACAGAAGCAGCAGTGGGGACGAGTGAAGCTCCTCTGCCAGCGGGGGTGTGCTCGCTCCTGTGCCCTCTGGCCTGGGTGGCTTTCAGTCCCTGTGGGGTCCTCAGGATTGGGAACAGCTGCTGCTTCTAGGGAGACGAGTAGCTGTGCTTCCTTCTCCTGAAACTCACTCTTGGGACATGAGAAGGCCTGACCAGGGCACTCGCAGTTGGATGCTGCATCTCCAGGTGCAGATGGGGTAACCTTATTCCTTGGAGCCACCATGTGCTTATCTGTCACCAGGGAGGATGCTTTACTTGCCTGGGATGTTGTGTTTCTGTAGAATTGCCTGGTAACCAGGTGCATCTGCCAGATGTGGGCATCCTCTTTTGTCTCTGCAATTCCCTGTCTCATCTTGTTTTGTGGTATGGAGGTGGAACTCATGGCCCCAATTGTGCCTTCTCCATGCTCAGCCTCTCAGTGTGGTGATCTGAGACTGTGGGACAGCCAAGCCACAGCTAGACTCTGCATGGTGGACTTGAGGCTGGTGCTCTATTGGTGAGCTACATATGTTCTCACCTCTTTTTTTAATTTTAAAAAACATGTTAAGAACAATTACAGTTTTTAAGTGTGTTGGTATTTTGCCTGCATGTATGTCTGTGCAACATGTTCGTGTCAGGTGCCCTCAGAGGCCAGAAAGGGTGTCAGATTCCCTGAAACTGAAGTTACAGGTGGTTGTGAGCCCAAACTGCCTTGTGGGTTCTGGGAGAGAAGCTGGTGCTCTTAACTGTTTGAGATTATCTCCCCCGCCCCCTCTTCTTACTTTTATGTTAACAGGTCTCGTGAAGTTGCGTAGTAGGTTGGCCTCAAATGTTCCATTTTCTTGCCTTGGACTGTAGAGTGGCCCAGGCCCTCCTTCTACTATGTTTTGTGGTGCATATTCCACACCTCCTCATTTTGAGGGGTTCTTTTCAGAGAATAATGGTCACAGAATCTTCTCTCTCCCTTCTCTCTCCTCCTCCACTGCTAGAGCAACTACAGGGTCTGTGTCACACATGACTCTGCTTATTGATGTTCTGATATTATAGATGAGACAGAGGTAGACACTTGTCTGCTTCTACATGTATAATCCAGGACCTTGCATAGGAAAAGTATGAGAAATAGGCTCAGAGGGTGGAGCCACTTTGTCCATAAAACATAGCAGAGCTGGAATAAAGGCCCCAAGTTTTGAGCCTTTCTCCATCCCCAACTCCATAGGCATGGGGTAGCAAAACTTAAGATGTGGTGGTGCGCCCCTGTAATCTCAGCACTAAGAGGTGGTCATCCTCAACCTCAGAGATGGTTTGGAGCCAGCCCTGACTCTAAGACCCTGTCTCAAAATACCAAAACCAAAACAAAACAAAAAACACAAAAACACAAAACCCATTGTTTTGAGCTGGATCTTGCAGTACAGCTCAGCTCAAAGCAATCCATCTGCCTCAGTCTGCAAAGTAGTTGTGTCTACCCAACTTGGACATTTTTGTACATTTTTTGAAGTAGACAATAGGCTATTAAACAATTGGTATAGAAAAAATGGTTACTATAAAGGAACTAATTAACACATTCATCATCTCTCTCACAGTTATTCATATTCCCCTGTGGAAAAAGCAGTTAGAATCTCATTTAGAAAAGAATCCCGACTCAATAAACAAAAATATTTGAGCACAAATGTCAACCCTGAGCTTGCAAGATTCACTTTGCCCTAACCTAGGTGCCAGCAGGAATGATTTCATTTAACGTTTGACATTTGGTTCACCGCGGGTCTACTTTTTCTCATTAATTGATATTTTAAAATCCTGCTTTAGAATATTATCTTGATGATTAATTTCTGAGCTGGAAGCATTCACTCCCGTTGGGGCTATTTCTGTTTCACCTTCTCCAGCGGCAGCTGACCTATCTCTTGGAGAAACTGAGGCCCGGAACGGCAGGGAGCTCTGGGAATAGAATGCCGCTGACCTTTGCTTATCGTCCTCTCATTAAATGTCCGCCTGCCAGAGACCTTTTCACTGCCTGAGTGGCCGAGATAGCGGCGCGGCCAGGCAGTGACCTTTCCAGGGATCCGACTAGCATCGTAGCAGCCGATTCCTCTCCACCCTCTAGAACCGAGAGAACGCGCCTGTGAAGTCATCGAACTC

							CGAGACTGGACTCCGCCGCTGACGTCACAGAAGGGCAGCACAGAGTCGCAACGTTCCTTGGGGGATATGAGCTCCAGAGGCCAGAGAGTGGCCCAGCAAGGAGGAAGTCTTGAGGTTTTCAGCGGTACGTAGGAGCGAACTACAACTCCCGACGCACCATGGACCTGTGCAAGAGAGCGCGACAAACTTTTGGACGAGATTGATATCTCGTTAGTCCAACCAGAGAGCCGATTTTAGAGCGGCATCCCACCCCCTTATAATTCTCAGGGCTGCTTCCTCGCGCGAGTGACGGGCTTCGCTTGCCATCTAGGGTTTAGCTTTCTCTGACGGACGTCCGAACTGAGGAATCGGAGCTCGGAGAAGTCGAGAGGGTGCTGAAAAGCGTGGACGGTAGCCAATAGCATGCCGGAAGCATCCGGCACCGTCCAATCGCCGCCTCGAAGGTCCGCCCTGGCGCGTGACGTAGTAGGGTGGGTAGCAACAGTTGCCCCGGTGAGGGAAACGGAGGCG
MSTRG.58967.1	ENSRNOG00000003011;ENSRNOG00000003029;ENSRNOG00000003149;ENSRNOG00000003279;ENSRNOG00000003307;ENSRNOG00000003443;ENSRNOG00000023830;ENSRNOG00000033368	0.959	0.000	0.013	-4.001	down	GATAGGGACAGCTGTGTGGCCCAGGTCTTCTCAGACAGATGGGAATAGGGGCCACTTCAGGAGGGTAGGAAGCTGGGCTGGGTGGGTTGTTTTTTTTGTTTTTTTTAATGGTTTTTGATTTTTCTTCCTCTTAAATAAACATGAATATATATATATGTGAGATATATATATATATATATATATATATATATATATATTTTTCTCTTATAAACTTTTGTGGAGTGGGGTGGGGGCCTTCTAGCCTCCCAGCATCACTCATCATCAAAGTTCTGACTCAGGAGGAAG
MSTRG.59009.8	ENSRNOG00000004212;ENSRNOG00000004219;	0.573	0.000	0.000	-5.395	down	CCTGGTTCTTGCTACATATAGTCATATTCTATATCTTCACAGCACTGTGTGATTTGTTCTGTTAATGCTACCATTGGCATACAATACCTAGGATATTTTGTGCTCACATCTGTGACATGAGACAACAGTTCATGTCATCCCTTACCTGAATAAACTGTCCTTAGAGTACAGTGTAGTCTGAGGGGCCTTCTTTTCCTCTGAGATAGCAAAACATTTCCAGTCTTCATACAAGTCTGTCAGCCATTGTCCAGTGCCTGTCTTGAGATGTCCACATTGTCAGCTGAGAGTCTTCATCCAGCCTGCCCAGAATCAGATGGCTTTTAAGATTGTTCCCATCAAGTGCGATCTCTTTGTCCATGGGCCTTGCCCAAAGTTGCAGGGCCAACCTGTTGTTTTACAATCCAAAATTCCAGACACTAATTTTACCAGTGAAGTTTCAGAGTCAGATGCTGAAGTCAAGTAAAGCTCCCAACTGTCTTTCCTGATTCAC

	ENSRNOG00000004287;ENSRNOG00000023899;ENSRNOG00000023910;ENSRNOG00000023920;ENSRNOG00000033611						TGACCTCCCAGAAGAAGAGCAGTCCTGTTCCCTCTGAACACTTTCTTAAAAATCCTTTAACTCAAAAAC<br>TTTCCATTGGTTTAGTTATCTAGATTTCCTTTCACGTTGTGTCTTGATCTGCTGGACCTAAGTGCACTTTT<br>TACCTTTGCCATAAATGTGTGTGGTAGGTGAGCCATACCGTAATTAAGTCCATGTTTACATGAATAAACA<br>ACTCTTGCTTTAAATGTCTGTGCCATGGCCCTGCTATACCACACACTAGTTTTTCAGTAAATAATACATTC<br>TTGGAGATCACAGTGTGATCAAATATTTTGTAACATGACTTAGTAACGTTCCCTCGATTTTATAAATCAAT<br>TTCTTTGACGAGGATTCTGGGTGTTGTCTGTCAGGTCTGAGTGCATCATCTTGGGAATCCCAAGGAACA<br>TCAAACTTGGGCTTTTGAAACTGTTGCATATGGCCAACCCGGAGCAAAAACCACCCAGGAATCTACTG<br>ATACTTGACACACATTCATTCACCTGCCTTTTTCATATAGTTTATGGCAAGAGGTCACCACTCTTGACCTT<br>GTTTCATTATAAAACGTGGTACTAAATGTAAGAGTTGGGGTCAAGGAGATCACCCCACAAATCACAAAA<br>TACCCATCTCAGATAACCATGTATGGTTTATTGAATACAAAACATCCATGAATTTCAGGACAGAATTAAA<br>AGTCCAAACCTAAGGATAATAGAATAGAAGAGAATTTAGATTTCCAGTTCACAGGTCCAGTAAGCATATT<br>CAGTAAATCATAGCAGAAAACATCCATAAGGTAAAGGAAGAAAAGGCCATGAAGGTACAACAAGCCTA<br>CAGAATGCCAATTTAATCATACCAGAAACAATT
MSTRG.59013.1	ENSRNOG00000023910;ENSRNOG00000023920	0.127	0.875	0.009	2.839	up	CTTGGAGTAAAAGCGGAAATGCTGAGGTGACCTCCCTTTGTACTAAGGTGTGTCAACTTTCAATTGTTA<br>GTTCTGTACTGGCAGTGAGCTAAGTGCTGGCAGGATTTCTCTTGTCATTTCTATGTCCCATCATCAGGTT<br>TGCTATTTTGTGGATGTTTGTCCTTCTCACCAGCTCAACACAAGCTAAGAGATGTATCAGGTGTTGTCTC<br>ACTGCCGATTGGATGTAATAAAGATCTGATGGCCAAGAGCTGCGTCAGGAAGTATGTGGGCAGAACTTC<br>TGGGCATAGAGAGGGACATAGGAAGAGGCAAGCTGAAGCAGGAGATTCAGAGTAGACATGGGCAGGA<br>AGAGATATGGAAC
MSTRG.59014.4	ENSRNOG00000023920;ENSRNOG00000058276	0.000	0.392	0.000	4.663	up	TGGTGTGGTGGCCATTTCCCAGTGTACAGGTGCTGGGACCTTGCTCTATCCACTTAATGATGTTTATAGC<br>CTCATTTTAGACCTTTCTGTGCACCCCCTATATCTCTTGACTATTTTATTTTTCTGATAGAAGCTCATGGTA<br>TATCTTACTCAGGCCTGTGAGCAGTGCTCCTTTGGCGACATTGGTGCAGAGAGGAGAACCCCACCACC<br>AGCATGAGGTTCCCTTCACTGCTGGCTCCAATATTGCATTTTCCTCTGCCAACTGGTCTCCACACTTTTC<br>CTAGCACCATAAAGAATTCAACTCTTTCTCCTAAGGAATTTTCTGGCAATTGTCGATTGCTAAGCACATG<br>ACTTCATGAGTGTTCCCCTAATACACACGCATGTTGTAACCTACCATGCAGACTATTTCTTGATGTGATAT<br>AAAGCACATGTACATAATGAGACAGGAGAACTTTTGACTGCATATTCAAGCTGGTGTGAAATCTCAGGA<br>AGTTTTAATGGCTAACATTGGTGAATTAGGACAATTTGAGTTAGTGAGACATTTTGGGGCAAGATATGGA<br>AGGAAACATTCATTTAGATGCATAGGAATTACTAAGGTTTTTTATAATCAAGCCTTGAGGAAAATTTGGC<br>TTTGGAAGCAGGAGATGATCAGTGGCTCTCCCCAGGAAAGGCTCCTGGAAAGGGCACCTCTGCTTATG
MSTRG.59017.1	ENSRNOG00000023920;ENSRNOG00000058276	0.040	0.540	0.018	2.940	up	TCCTTGGTGCATGCCATCCTTCCTCCCTGTGACTCATGGGGGACACCAAGCCTGAGGATGTCAGGAAGA<br>AGTTTACTTACTTCACTGGGTTGGAGGCTGTTAGGTTCCTGCGTCAGTAGGTGAGATTCAAGCTAGAAG<br>GTTTGTGGCATGAGAAACGGGAACAGAACTGGAATGGCAGGAACAATCGTCGCCTGGCTCTGAGAAG<br>ACCAGGTGGGCAGAAATTCATCGTTGGGCAGGTAGTGAGCTCAAGACCAGGCTGGGCATTGCTCAGCG<br>CATGTGAACAGGTGTGCTTACATGGACATGTGTTTCCCCCACATGGTCACCACTTATCCAGGCACGGTG<br>AATACTCCAAGGAGCTTTTTATGTTTTAACATTGTGTGTGTT

MSTRG.59018.1	ENSRNOG00000023920	0.000	0.762	0.000	5.271	up	ACATCACACTTCTTGATGGGGCAACAACACTCAAGGAAGCTTGAGATTGTCATAGGCACAGTATTATGCTACATATACAGAGTAGATAATACTAAGATGTTCAGATGGTGATATCCATTTGTATGCACTCTTAATGTGGACCTAAGGAAATAGAGATCAATGTCCTTAGAATAGAAAGATCAGTTCCAGTTTTTTTCTACCATTCAAAACTGAAAAACTGGTAGGGAGAGAATGGTAAATAATCGAGAATTCGTAGATGATGCTTGGAAAGAAGTGTGGGTTTCTGTGCTGGTTTTAGACGGGTCTCAAGTATCACAAGTTGCCCTCTACCTAGCTGGCCAGATGCACTTTAAGCTCAGATAAACCTCCTTCCACTTTTCAATACATAGGCTTACAAGAGTGGCCTCCAAGGACTGTGAGTACAGCATTGGCCTTTGACAGTTGAGGTGTGTAACCCACAACTATAGTTTCAGCCACGATTCTCAGTGTCTACCAGCTATTTCAGATTGTTGCTGTTATATGCTTGACAATCAAGGACGCAGAAAAATAT
MSTRG.59032.1	ENSRNOG00000058517	0.365	1.227	0.026	2.357	up	TCAACCCCCAAGTGCTTCTTCCAGGAGAAAGCAACCAGCTCTTCTTATGAAACAGTGTTGGCTCCCACATGACCCAGGTCTGTGATTGGGGAAGATTTTCTGGAGCTTAATTGTCCAGAGTGGGGCTACATCCTTGATCCATGCTCATTTCCACAACAAAAGGTCTTCCTCCACAACCCAGGCCTTGAGGTGAACCCAGGTCTTTCCAGGACCAACAGACCTGAGAGAACAGAGGGCAGTCCAGGGGAGAGAAGAGACCAGAATGATTTTAAACTGGAGAAAACTCCCATACTAGCCATTTAACAACTTTTGTCCAGACCGTGGACTGCAG
MSTRG.59042.2	ENSRNOG00000057212;ENSRNOG00000058517	0.329	1.399	0.002	2.669	up	AACGAACATTTAGAAGCACAGATGTATTGCTGTTTCTCCTCTAGCACACATAATGAAAGGCGGTCCCCCAAGGTGTCTCTTAAGTGGTGATTTCTAAAGAAGAAAGTGAGTAAGACCTCAGTCAGTCAGGCTGGCTTAGTGATATATAAATTTAGGGTCTCTAAGCAATGACATATTAACTATGAGGAAGAATATTCATCCAAAAAATGTTGTTAATAGTCAGGATGTTTGTCAGCAGGGGACTCCTGAGAAAGCAACTTATTCTTCAGGAAGCTCTCTTACTGGGGTGTGTCCAATTCCAGCTCTCTTGCACTTTCTGAGACCAGATATTGCTGGGGTCTGGCGGTTTCTGGTCTTCTCCTATTCATATTTATCGTTCTTGTTCAATTCAATCAAATATTTCTGTATCAGTGCACAATCAATCTGTACCACCTTGCTCTCCTGCATAGATGTGACGAAGTTCTTCATACATGTGTTCTCCATCAGGAGCGGCTGAGCAGGTTACTGGAAATACATTCTGCATAGTAAGATCTTGCTGCCCCTTTCTCCCATTCCTACTTCCAAGTTCCACTAACAACCCTAGAACCCATATACCCATGAAGACGTAGAATACATCTTGATACATATAAGGCTGCTGCAGAAAC
MSTRG.59055.1	ENSRNOG00000057212;ENSRNOG00000058517	0.000	0.339	0.031	3.453	up	CCATCTGGATGCTGAGATTCTGGTGTCCTGGACGCAGAAAAACAGGTTTCTGAAGCTGAAGAAGATCCCAATATGTACAATTCAGAAGGTGTCCTTCCAGGACTGGGGTAGTACAGGACCCACCCTGAGTTCATATATTCCTAAATGCTGATGTTCATAGTATTCTATCATTTGGGGCCAGGCCACACTTCAGGCCAAATCCATATTATGAAGGCCTTCTCTAGGTAAGTCCTGGTCAGAAAGGCTATCACTTGTGGAATGGCCATTTCTGACTTTCTTTGTTCTGGATTGGACAGTCTTCAGCT
MSTRG.59103.2	ENSRNOG00000051410	3.405	0.028	0.000	-6.021	down	AATTAAACCCCTTCGAGGACATTGAGCTACGTTATAGAAAGTGTTACTTTTAAAACAGAAGAACAAAGAAAGAAATTGTGGAGTCACTGAGATACACTTCCCAGACAGGATCAAGGAAAGACGGCTGAGAGATGCAAGCAGGTGCTGGAATGCTGCCCTTGAGAAGAAAATGTTTTTGCTCACTTTACCTTACACTATGTTGTACTATGTCCTATTTTTGTGGAAGCGGCTCTCATGCTCTCTGCGTTATGACAAGTATATTGTGATAATAGGAAGAGGGGTGATTGTCCCGCGCGTGACTTTCCGCCGGCAAGAAGCACGCGAGACATAGAAATTCTTGCTATGAGTGATACTTTATTCCTATCTCATGGAGAAGCCCTGCGCGCCAGCCAGGCGCGCCTATTTAACCCTCGAGCACGCATCCACACCTGATTGGTTGTTTACTCATGATCTCATCAGGCACGCCCCGGAATGGGCAAAGACCTGGCAGGAAGGCACTCTTGCACATGCGCACAGTTAACTACCTGAAAGGGGGGGCCAGCTGGCGCAGCGAAGGCTGGTGCCATCTTGACTGACTTGGCCTTCCATGTGGGGCGCAGTGGAAGACAGCGCCAACGAGTGGTTGCGAATGTTATTGCGGCCCTCTACATCTCACCCTTATAATTATAATTTTATAGCAGAAATGATCACCCTATCGCGGGCAATGAGGAAACCTCAGCGATGTGCAAGGGCTGATCAAAGGAACACTGAGTCTCTCTC

							AATCCCAGTGCAATGGATCCACAGATCCATGGGGTGTAGGAGCAGAGAACTCAGATACAGAGAGTATC<br>CCTCTCCTCCCAGTGCAATGGATGCACAGATCCATGGGGTGCAGGAGCAGGGAACTCAGATACAGAGT<br>AAATCCTGAACAAGATAACCAAATTCCAGGGGAGGCCCCTTGTACAATGGCCACAAGTGCTTGAGTGA<br>TAATGGCCTTATCACGTTACTGTTGGGATCTGAGTTTGTAAACCAACCATGGTATGAATACTGATCCACA<br>GCATAGGGCTGCATCAAACAGAGCCACTCCCGATAAGTAGTGAGCACCTCATTTGGTCCTCCTCAGCTG<br>TTACCACCAAGGGCCGTAGTCAAGCTGCTCTTATAATAAAATTTAGAATTCCCAATTGTTAGTAACTACT<br>CTAGAAAGTTCACCCACTGTACTTGTCTAACAATAGATTGGGGGAGGATAACTCCAGACACTGCAGACA<br>TAATGTCTGCAGCAGTAATGGCTGCTACAATAGCAGCGAAAGTGTCAAGGTCTTTTCCAGGACAGTTCA<br>GGATCAGTCATTCTTCTCTGGAACAACCAGCAGACAATGGAACCATTTCTGAGATCTGTATAACCAGGA<br>CAGAGTTCTCCAGTCACTGTAGCTTTACACAGGTAGCTTTCTGTGAAGCTACAGTCTGTAAAATGACAA<br>CACCAGTTACCAGTTAAGGCAGCAAGAGTCACCAGGGAAATGAAGGGACAGCAACGGCTGGAGTCCA<br>GTCTTCCCCAGCAAGCAGCAGTGTGCAGAGTCTGAGCGTAGGCCACAGTGGGACGGGAACACGCAGC<br>GGTAACACTGACTGCAGCAACGGCAAAATCTCTGTGACGACAAAGGATGAAGGGGAATCTTCTCTCTT<br>CCTCTCAGGGCAGAGGAATGACTCCAGGGACCCGAACCAGGAGAGCTGAATAACCAGGATCAGCAAG<br>TCTCAGCAACCACTCAGGCAGCTGGCACACTCCTGCAGCATCCTGTAGAAAAAACACAAACATTCCCT<br>CTTCCCCATATAAAATATCTGGACAGGAACATGTCAATAAGTGGATCTCTCTATTTCACCTAGGCAGAAG<br>TATGCCTAGTTGTAGGGTGCCATAAACTTTGGCATCCAAATTTTTAAAATTGAAATATTTTTAAATTGAAA<br>TAAAAGGAGCATTATTTAGCATACAGGGATAACTCCCCCTTTTTATTTATTAAGATATAGTAAAGATATTAT<br>TTATTAAGGTAAGTCCTCGAGGATTGAGTTGAGGACACTGAGCACTTGTATTTATAAATTGACTTGTATAT<br>CAAATTATTGTCTCTAGCATAATTGTATTGGATATATAAAGAATGAGATTTTTTTTTTGACTAATTGTTCTT<br>ATGTAAGGCCTATAATCTGTCTGGAATCTTGTCCTCCCTTGTTAAGGAGCCCAGGCAATCCAGTTTGAGT<br>TTTTAAATGTCCTATAAAGGAACAGTATTTAGTGCTCTTAACCACTAAGCCATTTTTCTAGCACCTCACA<br>AACTTTATTTACCTAAACATAAGCATTAATTAGAAATGTCAAATCTTGTAAATATTGTCTAGATTCAGTCTT<br>ACTAGAAATCTCTGAGCTGGCAGGGTGAAGCTGGGAGTTGAAAGTAAGCAAATGGAGTCTTCCTCTTT<br>TCCTGAGGGTGTGTTATCAACTCCATTAACATTTTTAAAGAGCTGGGTCTATGCTTTTGTTTAGTTGTCTG<br>AGCCAGAGCACTGAAGGGACAGTGAGTTGTCAGACATTTATACTGAAATCACTCACTGATTTCATGTAG<br>AAAACTTGCCTCTAATAAAGCATTAAGTAATTGAAATCAATTGTAAATCACAAGCCACACATTGGCTATC<br>AGTATATGAATAGAAAGCTTGATTTAAAAACAGTGGCTACAGGACGTAATCTAATAATCTGTGCTGAAGC<br>AACAGAAACTCAAGAGAATAAACAAGTGATCCAATCATACATGTAGCCTTTTCATTAGATGAACCACCT<br>ATAAATACAGTAAGCGCATTTCCTATGGGTTGTACGTACAAATATACAGGAAATACAAAAACATGTAAAG<br>AATAAAATTATCAATTTTGTCTGAAAAATTTGTATATGTGATAGGCCAAATATCAGTATTTAGTAGTAACC<br>AATTAATTGTTGTTTGGAATAAGATATGTTTTACCAGGTTTCTATTTAAAATACTTCCATGACTCTAGTCCA<br>CAACTCTGTACTAACACAGCTGAAGCTTTATCTCCAATGTGCATAACAAAGACCTAAGTCCTCTGGTAA<br>GGTGAGGTACTTAGCCAATTAACATATCTTTAGAAGCTTAAAATTAGCTTCAAATATTCTTTTCTGGAGTA<br>GGGTAACAGCTACTCCCCAAATATTAAAGCTCATTTTGAGAGCAAAGGTTTGTAGTAAACATTTCCATAT<br>ACATAGAAAGATTTAAGTTCTAACTTCTTACACTGAAGAAGCAACAAAATTACCTAATATGAGGCTGTTA<br>ACCATTCTTAATGATATCACTTTATGGGCTCTCTGAAACTATAAGCAAGCTCACGAAATGAAAACTCACA<br>ATTGTTATGACGAAAACAAATTGTATAAAATCAATCCTCTAAATCTGTCTTGAAATGGCAGTTGGAGTAA<br>GGAAACTGGCTGTAATGCTCTCACAAGTTCTAAAACCTCATCAATCCTTATAAATCCTGTAACAATCTCT<br>ACCTGTTTGATTTTTCCTTAATTAAAAATAAGTTTGAGTTAGTCAATGTAACACTGGCAATCCTAAATCAC<br>AATCTTTAAGTTCTCTTTGTAACACCAGAACACAGCCACAAGGTCACCTGAATTCTGAGTACGTGACTA

							GGCAGCTGTTCTCCCGGCAGTGGAAATTAACATTAAAATACAGATAACTTCTGGGCAAGCCACAACTTTGGAAAGTAAAACTTATTAGGCAGCTGTTTTGGGGGCAGTGGAATTAGCATTAAAATACAGATAACTTCTGGGCAAGCCACAGCTTTGGAAAGCAAAACTCAACCTCCAACAAACAATCTAGTCTCCAAGGCACCACAGGTCTATCTATTATGTTACAAAGTTTGACTGTCAATTCTTACATTTGGACTCACAAAGGTGTGGCCTCCAACACCTCAACCAGGGACAATGTCCTAAATTCTTTTAGAAGCCTGGTTTCTAGCATAAGAATTCCATAAAAACATTACCTCAATTTAGACCTGTTTCCCTAGGTTGGCCCATGTCACACGCTATCTAAAATGACTCCATCCAGATTACTAGCTTTATGTCAACTTTCTGTTACCAATATTATACCCTTTTAACCATATCCAATAACTTGAAAGCCAATAGTCCACAACCAAGGCAAGTAGAGCATATTTATACATATAAAACCAATAAGAAACAAAATTGAAATGGCTACACCTATCTTTAATATTTAGACCAAACACCATTTTTACCCTTTTGTTATGATTTTAGGAGAAGCACCTTGTCACCTGTTGAGTCAAGTAATAAGTCAGGGATTTTTTTTTTAAGAGAAACAGCTATCAACTCTTGTATGAAGAAGCTGTTGGCGCTTTTAAGATCAGCTCGTGTAGACGCTTAGCTTCTGGTCAGCTTCTCCAGCTAATCTAGACACCTGGCTCTAGGCACAGGGCTTCAAAAACCTGATTGAAACTGTTTTTTATTCATTTGTTCCTTTTTGAAACGAGTTAAAACCAAGTCAAGGTGTGCACGACCAGCAGATAAAGTTTTTTACCATTTTTTTTCTTTTGACCACGAAACATAAAACATTTAAGCAACGAGAAACAAAAACCACAGACATAAACTGACAGACATGGACAGCCTGGTGCACCAGAGACAAACAATAGACAAAGAGGGGAAAAAGCCAGATGGTGTTCACGGCGACTATCCATCGAGTGGAGCTGATATGGGCGATGGATTGTCTCAATTCCTGGACTAGTCCACCAACGTGTTGAAACCCAATTCTGTAAGAAACTGTATTCCATAGCTTCTGAATTTCTTCCGTGAAACGGAGGCCTCTCGTAGCGCCGGCGATGTTTTTGTCCCGGGTTTCGGCACCAAATGTCCCGCGCGTGACTTTCCGCCGGCAAGAAGCACGCGAGACATAGAAATTCTTGCTATGAGTGATACTTTATTCCTATCTCATGGAGAAGCCCTGCGCGCCAGCCAGGCGCGCCTATTTAACCCTCGAGCACGCATCCACACCTGATTGGTTGTTTACTCATGATCTCATCAGGCACGCCCCGGAATGGGCAAAGACCTGGCAGGAAGGCACTCTTGCACATGCGCACAGTTAACTACCTGAAAGGGGGGGCCAGCTGGCGCAGCGAAGGCTGGTGCCATCTTGACTGACTTGGCCTTCCATGTGGGGCGCAGTGGAAGACAGCGCCAACGAGTGGTTGCGAATGTTATTGCGGCCCTCTAC
MSTRG.59104.1	ENSRNOG00000055892	0.242	0.897	0.013	2.448	up	ACACTGTACTCTAAGGACAGTTTCTTCAGGTTAGGCATGACATGAACTATTGTCTCATGTCACAGATGTCAGTACAAAGTATCCAAGGTATTGTATGCCAATGGTAGCATTAACAGAACAAAGCACACAGTGTCATGAAGACATAAATCATGAGTTTGTGTAGCAAGACCCAGGCCTCTTCCAGGTTCTTAGATAGTAACTGAGAACCTCAAATACGGTAGTGATGTGATAGTGTGGGGTGAATCTAGTTACAACTAGCTTCTATATTGACACAGATGACACTTTTTGTGTCATGGTCAGCTTATAATGAATGACTAGACCTAAGCATGAGAGTAACCAAATACTACATTGCTTCTCTAGCTGCTGAGCTTTTAAAAGCCCTGGGGTGACAGCAAGCTTTTGTGGACATACTGGATTGGTTCTTCTGTACTTTAACTCTCTGAGTCACCTCCACTCACAGTAAGTTAGTTCAATTCCATAAATTGGAAATCAGTCACCTGGAAAAAATACATTCTCAATACGATCCCAGATTGGCCATGGAAAAAGGGTAGCTTGAACTGTCCCACCTAGGTTCTGGGCTTGCACAGGCC
MSTRG.59114.1	--	0.068	0.750	0.000	3.747	up	TTCATGGAAATTCAATTCCTTTTCAGATGCTCCAGTTGTTGTGGCCCATTGCTAGAAAGGTCAGCTTAAGCCTGCAGCCACCCTGACGGAAGCTCAAAATACACTGCCCAGAACTTACTCCACATTCCCCAGAAGTGCTGTCTTTGTCCATCATTACTTTGAGACGAAAGGCCAGACTCCTTCACTCTAGTCTCTCCACAATCGACGTTCCCCCTCCCAAAACGCTTGCGAAGACGGGAAAACATGTCTTAGCTTGTAACCGCCAGACTCAGACTGAGAGAAACTAGGGCACTGGTTGTCACTACAACACTGGTGACATCACAGAGGATGCCAGAACTCTCTAGGAGACAATAGAATGTCCAAGAAGGGACAGCAGATGTCATGGGGAGCAGACTTTCCCTCCCTGCTAATGTTCTCCCTGTACTGAGCATAAGCAGAGGTGCCCTTTCCAGGAGACTTTCCTGGGGCAGAGCCACTGAGCATCTCCTGCTTCCAAAGCCAAATTTTCCTCAAGGCTTGATTATAAAAACCTTAGTAATTCCTATGCATCTAAATGAATGTTTCCTGTCAAATCTTGCCCAAAAATGTCTCATCCTAACTCAAATTGTCCTCATTCACCAA

							TGTTAGCCATTAAAACTTCCTGAGATTTCACACCAGC
MSTRG.59143.3	ENSRNOG00000033226	0.000	0.959	0.000	5.757	up	GTAACCGCCAGACTCAGACTGAGAGAAACTAGGGCACTGGTTGTCACTATAACACTGGTGACATCACAGAGGATGCCAGAACTCTCTAGGAGACAATAGAATGTCCAAGAAGGGACAGCTGATGTCATGGGGAGCAGACTTTGCCTCCCTGCTAATGTTCTCCCTGTACTGAGCATAAGCAGAGGTGCCCTTTCCAGGAGACTTTCCTGGGGAGAGCCACTGAGCATCTCCTGCTTCCAAAGCCAAATTTTCCTCAAGGCTTGATTATAAAAAACCTTAGTAATTCCTATGCATCTGAATGAATGTTTCCTTCCATATCTTGCCCCAAAATGTCTCATCCTAACTCAAATTGTCCTCATTCACCAATGTTAGCCATTAAACCTTCCTGAGATTTCACACCAGCTTGAATATGCAGTCAAAAGTTCTCCTGTCTCATTATGTACAGGTGCTTTAAAACACATCAAGAAATAGTCTGCATGTAGGTTACAACATGGGTGTGTGTTAGGGGAACACTCATGAAGTCATGTGCTTAGCAATCGACAATTGCCAGAATATTCATTAGGAGAAAGAGTTGAAGTCTTTATGGTGCTAGGCAAAGTGTGGAGACCAGT
MSTRG.59157.1	ENSRNOG00000053751	0.127	0.555	0.010	2.665	up	GGCCCCAATGATAGAATACAATGAACATCAGCATTTAGGAATATATGAACTCAGGGTGGGTCCTGTACTACACCAGTCCTGGAAGGACACCTTCTGAATTCTACATAATGGATCTTCTTCAGCTTCAGAAACGTGTTTTTCTGCGTCCAGGACACCAGAATCTCAGCATCCAGATGGAGGGTTCCCTGGCTCCCTTTGTTCAATCAAGAGGAAATCTGCAGTCAAGCCAGTTGAGCTATCAAGAGCCTAGAGCACACCTCTCCATGCTCCCCCACCCCCAACACACCAAAAATCCTGTGATTTCTTTTTTCCTGTTTTGACAAGAGACCATATATCAACACAAAATTATAATCTGCACAAGGAATACAAATGTACAGACAATCGTGGCCACATAGGCAAAGAACTGTGCTGTTGACAGTGGGGCTCCCAGAACAAAGCATTCACATGACCATGAAAGTGTTACAGGTAAATACTCAGGCCAAGACATAGACCCCTAGATTCCAAGTGTGGAGGGAAATATAAACATATAAAAGTTGTGCATATGCATTTGGATGTATGCACACACAGACTTACAGACTGAGACACACCCACAAAAAAGAGAAAAGCAAACAGAGTCAGAGAAAGAGAAAGACAAATATGGAAACAGAGAGCACTGTGAGAACCAGGAGAAATGCATGCACCATTGCTGTCTCAGAGGGTAAGACAC
MSTRG.59217.1	ENSRNOG00000026880;ENSRNOG00000049551	0.515	0.000	0.003	-4.375	down	GTTTTTAGACAGTTCCCATGGAGCGTCTGTCCTTCATAACTAAGGGGACGCGGGTCAGAACCCTGCGGAGGGACAAAACATCATGGTGGGTTCATCTTGTGACATCTTCATCCGGAAGAGCAACAGCCAACCACTCAGAGGTTGCCTACCCCTGTCACCCACTTCTTCCAGTGAGGCCCAATTCCTGAACATTCCACAGCTTCCAAAACAGTGCCACTGGCCAGGAAGCAAGTGTCCAACACACGAGGATGAGGGGGATACTTGAGATTCAAACCGTAACATTCGTGTCCTGGTTACGTTTCAGTTGCTCTGACACAATCCTGACAGAAGCAAACACAGGTTTATCCTGACTCCCGATTCAAGGGTAAGCACAGCCCATTCTGGTGGGGAAGTCAAGGCAGCAGGCACCTGAGGCAGCCAAACGCATCACATCTCTAACCAGGAGGCAAAGGGCAGCAGATGCCGCGCAGCACCTCAGCTCCTTTCCTCACAGAGTCCACCAGGGAAGGCCACCACCTACAGTGGGCAGGTCTTGTCACTTGAATTAACACACCATAAGCTTCTCACAGGCATTCGCAGAGGTCCAGCTTCCAGAAGATTCGAGATTCTGTGAACTTGATCGTTAATGGTAACCATCACAACGAATAATGCAATCATTGACAGTCACAGGAACCACGTGAACAACTGCTCAGTCAGAGCAATGACTGCC
MSTRG.59391.2	ENSRNOG00000012860;ENSRNOG00000012928	0.530	0.026	0.005	-3.143	down	GGTTTATTTTCCAGCGCGTCAGGGTCGACTTCAATTAGGCTCCTCGTGGGGCTGAGTGACTGGAAGGTGACCTCGAATGGCTATAACAAGCAGTTTTTATACTTTTTTAGGGGTACAGGTCAAGGTTACAGTTTAAACTTATTGGCTAAGCATATTGACCTTTGAATCTATTGGCTAGCAAGCATGACATGTAGGGCGCCCTCCTGTGGTGAACAATGGTACTGCGCATGCACGGGTTTTGCACCTGGAGTCAGTTGGCCGGGACGGTACCATTCTAGTGAGGTGATTCGTTCTCCGCCAATCCCTGGAGGACAAATAGCAGGGGTGATAGTGGGGTGATTCGTGCTCCGCCAATCCCTGAAGGACAATTAGCATAGCCACAGCCTGTTGTGTATATAAGGCGAGCGTTTTTGCCGCTCAAGGTCCCCATATCAGCAAAGAGGTGGTCCTGCAATAAAGGCTGTTGAGAAGAATCCGACTGTGTTGCGTCTTCCTTGCCGGCCGAGGTGGGCGTGACAATGACATGCATTCGAACTCTATCTGGGACCAGAGG

							GTCAGGTGACTATCAGTCAGTACATTCTGTGGATTTTCAAGAATGTCCTGTCCCTGCTTCTCCTGAGAACTAGGAAGCACAAGGCCCTGGGTTCGGTCCCCAGCACGTGACTGGAAAGCTCATCTGAGGAGCGATGTTTTTTATGAGTTAATTTTTTACTGCATAGGTTGTTTACTCCTCCACAGCTAGTCGCCTTCGTTAGTGAAATCTCTTGGGTGGCAGAAGACACTTTATCACTTTTCTTGTGAGGAAACTTGAAACTCCTCTTATGCCCCACGTTGGTGTCATTTGTCCAGGACGGGTGCTGAAGATACCTGGAAGAGAATGGGGGACAAGAGACATGAAAAAGGATGCCAAGACAAGAGTCTGATCAAGGTGACAATTTTATTTTTCCCAAGGCTGAATTTATACCATAACAGGGGTAATAGAGGGAGTGGAGAGGTAAGAAACGTTTACTTAACTAACCATAATAATAACATCAATAATGGAGGGCTTCAAGGGCATCGCTCCCAATGAGGTGCTTTTTCCAAGGCCTAAGATTTCCTGCCCCTGGCTTCCATTTGTTGGGAAACTCTCAGCCCCAGTGGCCCCTCTCCTTGCAATATGCACACTGGTCCTTTTCAAGGGATTTTCTGTCTCTCTTTGGTTAGAGTCTCTCTTCTCTGCTTTCCCCAAGTTTGGCTGTTTGGAGGTCTCCTGGAGGAGAATGTCGTCGTCCCCCGTGAATAGGCAGGCCTGTCTTCCCTGTTTGTCAGATCCTTTGCTCCCCCTTGGAGCCTTTGTTCCC
MSTRG.59391.3	ENSRNOG00000012860;ENSRNOG00000012928	0.000	0.639	0.000	5.351	up	GTCAGGGTCGACTTCAATTAGGCTCCTCGTGGGGCTGAGTGACTGGAAGGTGACCTCGAATGGCTATAACAAGCAGTTTTTATACTTTTTTAGGGGTACAGGTCAAGGTTACAGTTTAAACTTATTGGCTAAGCATATTGACCTTTGAATCTATTGGCTAGCAAGCATGACATGTAGGGCGCCCTCCTGTGGTGAACAATGGTACTGCGCATGCACGGGTTTTGCACCTGGAGTCAGTTGGCCGGGACGGTACCATTCTAGTGAGGTGATTCGTTCTCCGCCAATCCCTGGAGGACAAATAGCAGGGGTGATAGTGGGGTGATTCGTGCTCCGCCAATCCCTGAAGGACAATTAGCATAGCCACAGCCTGTTGTGTATATAAGGCGAGCGTTTTTGCCGCTCAAGGTCCCCATATCAGCAAAGAGGTGGTCCTGCAATAAAGGCTGTTGAGAAGAATCCGACTGTGTTGCGTCTTCCTTGCCGGCCGAGGTGGGCGTGACAATGACATGCATTCGAACTCTATCTGGGACCAGAGGGTCAGGTGACTATCAGTCAGTACATTCTGTGGATTTTCAAGAATGTCCTGTCCCTGCTTCTCCTGAGAACTTGAAACTCCTCTTATGCCCCACGTTGGTGTCATTTGTCCAGGACGGGTGCTGAAGATACCTTCCTCTGTTGCGCTCCTCTGCTTCCCTCGCC
MSTRG.59419.3	--	0.117	1.083	0.000	3.889	up	AGCGAGACTCCCGCCGCCTCCGCCGCTGCCGCCGCTACCAGCAAGTCCAATACTGGCTGATCGGCAATGAGGATGGAGAGGATAGCAATCCCGGCAGTCGCCCTACTGACGGTGGGTAGAGTAATAGAGCGACAGTGCAGATACCCTGTTGGAGGCGGCGAGCCGGCGAGCCGGCGGCATTAAACCGCGCCGAGGGGGCTGTGAGCACCCGCCGGTCCCCAGCAGTGGCGGCCCGGCCTGGGGCCGGCGGGTCTCCGGGGCTGCTGGTTCGCCTCGGGGTGACTGTGGAGGTCCCGGGAGCCCACCAGTCGAGCGGTAAAAGCTTTTCACCATCCGACTGTTCTTTCCTTTGCCACAGTGCCTTGTGGCAATGTAGAAGGTGGAGTCCCTCCTAGCTGCATCTCTGAATGAAGATATTCTGGACCAGTGTCCCCAGCTCCCTGGTTACTGGTGAGACACCTCTGCTTCTAGGAATCCCAACATACAACATGGCTACTGACCTCCTCAGTTGATATCTTCAAATCCAGGGGAAGTGACTTGTTAGACCTGTGAGAGTAAAAACCCAGCTGTCCTGGAGGCCACAGCGATGAAAGGAAGCAACAACAGCTTTAAAAGATACATATCTCCGCATTTGGACTAGTGCTAACTCCTAAAAGTATTAATTAGGTGGTTATTAAGTGGCGTACTAGTTACCCTTCTCATTGTGGTATGAAGGACCTGACAAAACCATTTTATGGAAAGAATGTTTTTCTTGGCTCACAGTTCCAGGCTACAGTCTGTCATGGGGACAGCATGGCAACAGGAGCCCGGGCTCCTGATTCCATTGCTTCTGCAGCTAGGAAGCCAAGAACTGTAGCCCACAGTATGGTGCCCCCATATTTAGAGCGATGCTTTCCACCTCAGTTAACACAATTGTTTTAGCGAGGAGGCTGTCTTAAATTGTCCTTAGTGTACATAGTCTTTATTTATCAAGCTCAGGCACTCTATTTGCTATTTCTCACTATATCAACCTCCCCTCCCCCAAATCAAAGATAAATTTCAGTCTTTCAATTTCCCAGGTCAAACTTCAGCCCATTTCAAGACTCCTGCTCAATGTCCACGACCCACTTGAGACCTCCTTAAAATGTATGCTGTCTTTTTGTGCTTTACTCTCTGTGTGATGGGTAAGGGGATATTCTTTTAGAAAGAACAAAGAATTGTGTGTCCAAAATTAAACATAGAAATTAATTTTTATTTGTATTGG

							GGGGAAAAGGAATGAAAGCTGCCAGATACCACAAATGTTAGAAATTCCATAAAATTACCAGAGTAGTTT<br>TCTTGCTTCACTATCCAATATTTTGCAGCATCCTTTTTCAGCTACATAGGTATTGATGATCTTTTGGTAAGC<br>CAAACAGTATGTAATATTTTCTGTAGAGAGATTAGAAAGACAACCAATTCATTGTTGGTAATGTAATGTA<br>AATTATTCTCAATTTTTTCTTAAATAAACTGCTTTAGACCTACTGTAATATTGGACTAATAGCTTATATTGT<br>ATAAATCTAGGCACTCTGATGAATTTTACTTTCTATAATTCCAAGACAAAAAAGAAACAATAGAATATGA<br>TATATGTATTTTTGAAATTTAATATTATAGTTATAATGTGATTGGTGCATTGTTAAATATATTCCTGACAGAA<br>TCAATGAAAACTGGATTCTGTGCTTTCAGTTTGATATATCTCATCGAGGATTTTCATACAATTTATTCTAGT<br>TCCATGAATTTTAAAAAAACATTTCTCTCCACCTTCAGTCATCTTAGTAGTCCTGGCATGATATGCCCAGT<br>GTACCTGGGCATATGTAAGTTGGCACTAGGGCAAAACAGTATTTGAAAAAAATATTTCTTCTCCATCTCC<br>TCTCCCTCCCCTTCCTCCCCCTCCTCCTCCACTCCCTTTCTTTTCCTTCTTCTATGTGAGCCTTGAACTCG<br>TGGTGATTATTGTTTCAGATTCTCATGCACTTAAGTTATGGGTGTGAGCTACCATAACCAGTTGCAAGTAT<br>TTTTATGTAGATAAAGATGACAATGATTTAACTACATAAGGAAGTGACTGAAAAACTGTATAATCATACC<br>CTCTCTAAACCCAAAATAAAATTTTCTCCAGTTCATATTTCCTTTATAAAGGGGTCTTGAGTGCCCACTTC<br>CATGGCAGAGTGCAATAAAGAAAGTAGAGGGAAGAGGCTGTGATCCTAAGCAATTAATATTTTACTTTT<br>CAGATCTGATTAAAACAGTTATAAATTGAAACTAAAAATATTTTTACCTCTCTAGGCCTTTCCACATGCTG<br>ATGGCCCTGATTGCCAGCTGACAGGCCTTTGGAAGGTCACTGGATCATGATAGTGCTATTGTAAATTTAT<br>TAATTGATAATCAATTAATTGATTAATCTGCTGAAGAATTTCTGCCAGAGAGGGATAGTGGGAGATGGTG<br>CTTGATTCCAAGAAGTAGATCACTGGAGGTATGCTTGTGAAGGGTATATCTTGTCCAAGTTCCTTTGTTT<br>CTATCTCTCTGATTCTTGGATGCTGTAAAATGAATAGCATCCCTCTCCATCCCTTTCTCCCATGCCTTTCGT<br>GTGTGGGTCTAGAAACACCAGAGCCAACTGACTGTAGTCTGCAAGTATGAACCAGGATCCATCTTTTCT<br>ACTTTAAAGCATTCCTCTTAGGAACTTTGTCATGGCAAGAAAACCACAAGATGATGGGAAGGTTTTCCC<br>AGGGTCTTCCTGAGACATTGGCATGGGCCCCTGTAAGCATCATTAGCTCCTGGTAAATTCATCTGTTCTT<br>ACAGCTCCAGCTTTAGGCTGTAGCTCCCTTTGCTCAGACTTGCAGTTGAAAATAACACAGACATAGGGG<br>TTGAGCTTAGCACGTGCCTCACATGTCCAGGTCTTTGATTCCTGCAACACGATGATCCAAACCAGTCAG<br>AGAGCTGGGATGTGAGTGTAGTTGTCTCAGTCCATTCCCCTGCTTGATCTTCAGTACTAGATGTCTATAG<br>CTTACTCTTTGTATGGAAACTGCTCAAAGCTGCTCTTTAACCCTATCCTGTCAGCCAGGCTATTGCAATT<br>CACTCCTCAATCAGGAGCCCTTCTCCCCACTGAACACCTATTATGGTGGATGTCGTTGGGAACACCTGT<br>AATTTTCATTGACACCCACTTTCCTTTAACTCTATCGTGATTGTACACTCTGTAGAGGAAGGGAAAATGG<br>CTGCTGTGGGAGTGTATCTCTGGAAACAGCCAATAAGGCTCCTTACACAATTTAGTACATAAAAATTCAT<br>AATTCTACTGTGATATATAGGGTTCAAAAAGACAAAGGCAAAAAGAGGGGGAAATAGGTTGCATAGTG<br>ATAAAGCTGCAAACCCCGAAGAAGAGGGAAACAGCACCCTTCTTCTCTCCCCCTCTTTTTATACTGCAT<br>TATTAGAGTTATTATCAGGGAGTGCTGCCACTGGAGTTTGGTTTTAAGTTGTTTGTTCACTTAGATTTTTC<br>AAAGCCTACATTAATGTGGGTTATTAAGTGCTTCCATCCCTCTTCTAGCTTGCTGATCAAAGATAGGGAG<br>AAAGATGGTGAAGAGGAAACGGGGAATGTGGACCTGTTTGGAAGTAGTTCTTTGGGGGCGGTTCCAA<br>ATATGAGTACATGAATCAGTAGCAGTGGCACAATTCTGGAAGAAAACATGAGATTCTGGCTGAATTGGC<br>ACATGTCTGTGGAAGCAGCAAGAAACAGCCAGAATACCATAAAAAGTTCTTTGGCGCTTTTCTCTCTAT<br>GAAGTCACAACAAGCAAACAACAGTGAAACCAGCAAAGCTTTGCAAGGCAAACTAATGCAGGAGCAT<br>CATGCCCTGTCTGCCGGAATATACTTATACTTTTCCAACATCACCTGCCTTCTTATGTGCTTGCCTCAGCA<br>AAACATCCGCTCATGTATCTGCTTTGGCAAAATATCCTTTCACATTCTGCTTCGGCAAAATATCCTCTCAT<br>AAGACAGCTTCATAAGAAAAGCACCACATGACACAACTGAGTCTTCCGAGAAACCAGAGATGTCTATC<br>TCAGCTGTTCTCTAATGTGCAGATGTGGCTTGACAAGACCTTGGAAAACAACATCAGAGCACTCTTAGC

							ACTGACTGACACAGGGCTGCTTTCCCCAAAGCAGCACTCGTCGGATGGAGACTGACACCCTGGCAACCTTCACATCTATGACAGAGGACAGGAGAGATGGCTTCCTGGAGACACAAACACTTTACTAGAGAATTTAAACGTGCTGGAATCATCCCGACACTTGTCTTTTTGTAAAGTTTTATGTTTCGCTCTTCATCTCCTTTATTGTTGGAGTCTAGTTTTGGTTAGAAGTTATAAATTCAATTTCATAATTTAATTTTTGATTTCAGGTTTTTAATATATAC
MSTRG.59480.1	ENSRNOG00000060995	0.040	0.299	0.018	2.940	up	GCAGAGAAGGCTGTGCTGTGTCCTAGTCCTCTACCCCACACTGCCACCTCATCCCTACGAACAGAGCAAAAAGAACAGATGAGGAATGTTCCAGGTTTCTTTCCTCTTTGATTTCTTTATTAACCTAGCCCCTGTGCTACATGTAACCTTGTCACATCCTCTGTCCCATCCCTAAGCCCTCATACCTGTCAGATAAGACCGGGATCTTGCTTTGGGGGGAAGCTCTGGTCCCTTTCTGTCCTAAGAGGCTCTGAGCCATCGCTGAAAGAGAGTCCTCTGAGGATTGAGGGAAGTCCATAGCCAGTGCTCTGACTTCCAGGAAGGTTTGAGGAAGGTTATCCTCTTCTTGTGGTGCCTCTAGTAGGAGCAGCCCACACTTGGGCCCAGCTCCCACTATCAGGAAAAGACAGGTGGCTCTGGTCTTTGCACAGCTCCAGCGGAATGAACAGTGCATCTCTCCTCTCTCTCTTGCAGGAAGTCTCAGCCTCTGGTGCAGGTGCTGTTCCCAAGGCAAAGGAAAGGGAAGTGCAGAGTGAGGACTCCCAGGATCCTTTAAAACATTGTCTGCATTGTCTTCATTAATTTTTCTGTTTAATTTTTATTCCATGTGTATTGATCTCATTGTAGGGGTGTGCATTACAAAGCATGAGTGTTGAAGGTAGATGAAAAAGCATGGAAGTCACTTTATCC
MSTRG.59497.4	ENSRNOG00000029323;ENSRNOG00000050237;ENSRNOG00000060995	0.067	0.567	0.003	3.142	up	GAAGTCCTGTCTTTGTCCACCATTACTTTGAGACGAAAGGCCAGACTCCTTCACTCTAGTCTCTCCAGAATCGACGATCCCCCTCCCAAAACGCTTCCACACTGTATTTGTGTATCCACGTACGTTAGTGGAAATCCAGCATTACTGAGGAATTGGAAACCAGCGCTGTGGCCATGCCTTTCTGTGCCTACTCCTGGCTCACAGGCAGCAGGGGGGCAGACCTTGAGTTCCTCTTCAGGGACCAAGTGATGTTGAATGGGCACTTAACAGCGGCAAATTTGTTACACAGCAGCCAGCTCTTTCCTATTGTGCTGTAATACGCCTCCACATCATGGATGCTGAAAACAAGCTGGGTGAAGAGAAGTGAAAGCATAGCAGGAGAAGATTGGAGGGAGTAACTGGGAGTCGGAGAGGAAGCACGCTATCTCTTGCAGCAGAGACCAATTCTTGAAGCTGATCCCAAGTTGGCCACAGCGTGGTAGATGAGCGACCATGCAGCTGAAGCCTGGTGAAAGCAGAAGTGAGGCGCTGCTCAGAGCTGTCTGGCGTCAT
MSTRG.59500.1<br><br>NONCODE_ID：NONMMUT049261	ENSRNOG00000029323;ENSRNOG00000060995	0.000	0.316	0.004	3.943	up	CCTAATGCTGGCTCTAATTCATTTTCTAATAATTACTTTAAAGTTTCTTTGCAAGTCAAGCATTAATCTGGAATCTATTGTGCAGTTATTACATTGTTTCCAAAACAAGAACACATAGCATGCACCTGGGCCATCGGGGCTGTGCTGTGCTGTGCTCCTATGTCTCAATTTCCAATTGTTTAAGAATCATAACATCAAGCAACATCTAGTTTCACAGAATTCACCAGAGCAGAAGGAGAAGGAAGTAGGAAAGTCAGATATAATAGAATAATTGTGCACACCGAGGCCGTCTGAAAGGCAGCCACACAGAAATGAAATCTATACAGCCGTTGTCCAGGGCTAAGGGTAGGAGAAATGGGAACAACCAGTCTTTACAAAGAGCTGCTGTGGGCTACTGAGATGTCTCCATCCTGGCCTGCTGCTGGGAGTCCCTGTCATCTATGTTGTCCCCAGCAATTATTGACCCTATGCACCAC
MSTR	ENSRNO	0.590	0.074	0.031	-	do	CCCCTTTCCCTCCCTCCCACTCCCGGCAGGACTCTCGAAATTCCACGCATCGTTGCCCCACTGATTAGCA

G.5952.2	G00000037137;ENSRNOG00000047234;ENSRNOG00000058463;ENSRNOG00000059588				2.201	wn	CCTTCAGCGGCTTTGCGGAGCCCAGTCCACGCCAGAAGCTCTGCGCAAACGTATTGGGCATGGGTAGGCACCTGCTGAATCCACGCTAGGAAAAGTAGGAACCCTGGCCTCTGAGACTTTGAAACCCGCATTTCAAGGGTGCTGTCTGTTACCGTATTAACATCTTAGTAAGGTGGAGAAGAACCACACTATGGCCGCAGGAGTGCTGACATGGAGCTTTCCTATTCTTGATTCCCACATCAATAGATTCTCTGCACCTTTATTTGCTGAAGAAAAAATTTTATGTTTGACGAAAAAAAGTTTTTGAGACTGGATAGCCATGGATGTCCTGGAACTTACTAAGTGGACAGGTTGGCCTCAAACTCACAGGTATCTTACTGCTGTTGCTTCCCAAGTTCTGATATTAAAGGTATGTACCACTACCCAGCTTACCTCATATGTTTAGAATAACACCGTACCTCACTCTCGAATTCATATGCCATATACATGTCCATATGAATTTTAATATCCCATTCAAATAATGAATTTTACCTCTAATGAGCCACATTTGAAGATGTTAGTGATGATAAACTGACAGAAGTTGGTACTTTCTTGTTGGTTTTTGTTTGTTTTGTCTGTTTTATTTGTTGAATCCTGTAAGGCAACAGCTCACAGATTTAAAATGAAAAGGACACTTTAGAACTGACACACAATTTTAGACATATCATTTACACCTGCTCTAAACTCATGAAAGACACATAGAACTGACTGTTTCTTACAGGAATGTACAGAGATAATTTTAAGGATTTCAGCTGAAATGCTATCACACTGTGAAGGCAAGAGTTGCACCCATGTGTGGGCTTTCCTTAGCACAAACTACACTTCATCAGCGAAGCTTCTTCTGCAGTAAGGACAGCTGCTGGTATAGAGAGAATCTGTGATTTTAGGGTGCATATCCTGAAATGGATGAGAAATCTATACCAAAACATTCACTCAGGGATCTGAGAACTCAGGTACATATTGGATTTCATAAAATATATGGTGTGTAATTATAGAGGAAAAGAAAATAAATTTAAAAATAAAGTGTTTTGGGGGACTATGGCAAGGCATAGGAGGAGTTTCACGGCAGAAATGATATAATTCGTCTCACGTATAAGTTTCTCAAAAAACTAATTTAAAAACTAACAGTATGTGTTTGTGCTCTGACAATGGCACCCTCTTGCTGTAGCTAGCTATCTCATTTAAAAATTGACGTCTGTCTGGTGACATATCCCGGCAGCTTAACTTCTTGTCACACAAACCTGATGATTTTTCCTTGATCCTCAAAACACCAGGTGGAAGAAGCATGAAAGAGTGAATCACACTTTCTAAGTTTTATGTGGTAGTTTTTTTAATATATCTGCAAACACAGTGATGGTAACCACCAATGGAGACAGAGGAGCATTTCTGATGTCTGTGCTTCATCAAACGGTACCAAAATATTTTTATAACTTTCTTGTTTTATACTCACTTCATAAATTATAAACAAACAGAATTTCCCTTAGTTTAATTGTTAATGATTTCTAGACTTAAGAATATTTGATATTATTCAATTTTATTTTTATAAGAAAATACATAATACTTATCATATATCAGAACAATATTTTATTATTTCCCATATTAAGTAAGAATTATGTGGTATAAGATTCAAAGCGTGTTGAGATATGGTCTATTGCTATGTATTATAATGCTAAATTCTATACTGGAAGGTCTAGGCCTACTGGTAACTTCTCATCTTTACAAGACTGTGTTGTGCAAACTTCATTTCCTGATTTAATACTTTTAGTTAAATAAAATTCGCTACAGCCTGTTACTGGATGGATTAGAGATAGTTGGGTTTGGAGTGTCCTGTTTGGAGGAGGAGAGACCAGGAAGAGAAGGAGGAAGAAAGAGAGTGAGGAAGAGAATAGAGAAGAAGCCACCATGAAGGAAGACAGATGATGAGGACATGGCCAGGAGAAACAGCAAGTATCTGGAGTATACTCTTGGGAAGTAGCCAGGTCTGCAGTAGATTGGGGGCTTAACCCCAGTAATTGTCAAATTCAAATAAAATAAATATAGTCTATGTCTCATTTACTTGTAAGCTAGTCAGGGATAAGTTTAAATTGGTTGTACTACAAGGTGAAATGATGTCTTCATGCAAAAAAAATACCTAGTAAAATCACAAGCGAGGTGTGCACATCATGATTTTCTTCTTCTCATCCTCCTTCTCCTCTTTTTTCATTTTAGTGTGTGTGTGTGTGTGTGTGTGTGTGTGTGTGTGTGTGTGCGCTTATAATTAACAAAACACTAATGTAACATGAGTTTTTGTTTCATACTGGAGGCCCACAGGTAGCATTAAGCAGTTAAGTAATCATGACTAATGCTGGAACCAAGCCACATGACACTTTGAGTTTTACCTGCTAAGTTTCAATATGATGGAGATATTGTGACAAGCTATACAATGTCAGACAAAAATCACAACACTTCTGTGCATTTCTCTGACTCTTATAAAACTATTGTTTTGCTCCAAGGATCTACTATTATGAAAGTCTGTTTAATAAAAACTAAATTAAATAATAAAATCCACAAAAATCAACAATTCTCTCTACAGGTACAGATTCATAAAAAGTGTTTATTGTAATGGCAGAAGGTAGATGAGTAAAATCGTACCCCACTTCTCAGAGTGGACTTGAGTTTAAATTCCTTTTGCAGGGAAGAGTGTCTGGAAAGGGAAATTCATTGGCTAAACCTCCAGGTCTTTAGGTACCTCATTATAATGGAGATCTGTCTTGAGGTTCTGTGACCTGAGGCCCTCTACCTGTGAAGGGGCTGGGGTATCACGGGTCTAAAATCTATGGAGGACTA

							CAGGCTAGTGACCAGGCTGGTTTCATGGGAGTCAGGCAAAACGGCTACTGTCCCTCAGGGTCACAGGG<br>ATTTCAAAACCCTTCAACTGGGAACCAAGATCTTTCACTGTCCCACAGATTTGGAATATGTTTTCAGGTT<br>CTTCTTAAGTTTATAGACAAGAGAAAGATTAAAATGTGAAGAGACAAAGAGTTTAAATTAGCAAACTTG<br>ATGTGAAATGGACTTGAAGAAAACAAGAACAGAGACTGGGTTTCATAGATGATGGTTGATTGCTTATTT<br>TAGAGAAAAATAGTGGGAACACAGAAGAGGGCAGTACATGAGTAATGAGTGATGTCACCAAGTAGATG<br>GAGTGTCAGTCTGTTACCCTTGACGTGTGATGGGTAGTAATGAGCAGTAATGATGAGGGTACTGTGAGT<br>CCTTTAGAAATAAATATATTCTCCTTGTTGCTTACACAATACACATGTGAGCCAGGGAATCTGACCATTAG<br>TGTTGACTCAACCCACCTGACTCAAGCTCATATGCTGTCCACATATCCATCCCACTGTGGGGAAGTGAA<br>GACTGTGCCTTGTACAATATAACCTGACTTTTTCAATATTTCAAGCATCTATAGTTACATTAGTCTTCTAAA<br>ATAAATGCAACCATGAGCCTTCAGATGCACTGATTAATAAAGAAATTATTCTATTTATTTTTGAAATGTGA<br>AAGAAGATGGCCATTGGTCTGCACTGTGAACAAAAAAAACCCTTGTTTTCCTTTTGTACTTTATTAGAG<br>AGTATAGAATAATTTATTAGAATTCCAACACTCTTATATTCAGTCTAGAAATTCTTTTTCTTCAAATGTTCT<br>GGTTTCCATATTTATAAATGGTGATGAGAGTCCAATGTGCATGTGCTGTCGGACTGACAAGTCACAGTAA<br>TGGGCTCCCAGTACACATTCCACCTCTGTTCATAGATGGGATGGTAACCTGTTAAATGACTGTGCTTTAT<br>CGAATCTCAGCGAACAAAACTGAAACAGAGCTTGTATGATGCTTTCAGTTTTATTCTCCTGGAGATTCA<br>CATTTTCCATAAAACACACACTAGTAAACAGCCCATTAAAGCTATACAGACTTTTTATTTTGTACACTTCA<br>AAGCATATGATTTAATTTCTTGTCCAGGTGTGAGCTTTGGCTATCAGTCTTAGATGTTATGTGATTCCATC<br>CCTAGCCTTTAGGATGAAAGTAGGTAGCAGATAGTGTAAGCTTCTAAAAGGTTTGTCTCAACAGTGCAA<br>GAATTGTAGGCTATTCGCAGAGGTGAGCAATTGTTAGCTATTGGGGCTGAGGGCTAAGACAGCCCATGA<br>TAATTTGACTCTGGACTTGGAGTAGTCCAAGTCTCCAAATCATTGAGTTGATCAGCATTGTGGGAAGTTT<br>ACCCCAGGTTTGGCTTGTTGTTTTATTAAAAATTCCAGGTTACCATAATTTTGTGTAAATCATCATTTATTT<br>ATGTAATGCAATGAAAAGTAATTTTTTATAAATGGATTTTGCAAGCTTTTTAAGGACTTCTCATCTCCAAT<br>GCTTTAAAACACACAAAAATTCTGATTCCCCACACAAGGAACTTGTTAAAGCCTGTTTTTTTGTCTAAA<br>ATAATTTCCATTATGTCTCCTTGTAAAAGCACTGTTGAGAAAAGTAAGCTTCACTGAGATGCATAAACAT<br>ATAATCCTAAGGGTCTGTGGACCCAGTCTGCTTATATGCCATGGATGTAATCTTCTGTGTATATGTGGTGA<br>TGTTTCAGCAGAGAGGAAACTGAGCTGTCGGCTTTCTGTACTGATAAGGACTTGGGACAGCAACAGGT<br>CAGAAAGTTACCTGTGAGCAGCAGTACAGCTCAGCTATACAGGATGGCACCTCACTCCAGCATCTCTCT<br>ACAGATAGTGAAGTTATTCCATAGACCAAAGATAAGATCACGGCAAAACCACAGCAGGTGTTACAGCAT<br>CTCAGTGATGTTCACCTACCTTCACACTTCAGCATTTAGAAGATCTCTCTCCTGAATGACAGTCATTGAC<br>AATGAGGCCAAATCATTCACGCAGAAATTTTTCTAGATTTAACATTCAGCACTGTTCCAAAAGCATTAGA<br>TGATAAGTAATATAATAGTTTTAGCATTTTCCAGGATCAACTTCTGCCTGACATGTCATTCAAGAAATTTA<br>AAAGACAAAAAGCAATTTTTAATTTCTTTCTAATGCAACTAAGCACGGGTTCATTATTAAAATAAGTGTT<br>GCCTTAGAGACCTGTGCGTTCCCTCTTATGAGGATATGAATTCTATGCTTTGGTGAATGATGGAAATATAC<br>AACCTAAGATAAATAATCCCTCACTCATCAGTATATAGAAGAAGATTGAACATTCTATTATAACATTGTGG<br>TATATAAAATCTGCTCTGACCATATTTAGATCTCTGGTTGACAAACTACAGATTTTTCTTAGACATATAAAT<br>TTTAATTCAGTTCTATGCTTAAAAGATACAGTTCTTATCTCACCCAAAATATTAAATAATTTTTATTCTGTT<br>GAAAAATGAAGTTTGCTAACATATCCTGAAGGCTCTAGATATTCCAGTGGAATAAAATACACACTACCTA<br>ATATCACTGATCTATTAAGTGGAGGATTCCCAGAAAAATAAGGTTACAGTAAGAATGATTTCAGTTAACA<br>TTAGAAACTCTGGAGAGAATGCTAATATCTTCAGTAAACAAGACTGTACCACATTGGTGATTCTTAAGTC<br>AGAGTCCTGTCTTAGTAATCAGGAAATGTGTAAATTTGTTAAAAATAAAAAAAATTATTTAGTAGTATC<br>AGTATTGTTTTCGCTACAAGAATGCATATAGAAAATGATATGAGTTTATTACAACCCTTGGGATTTAGTCC

							TCAATGTAAATCAGAGAATTGAAATACTTATTTTTGTTTGCAAATTTCTCTAAATACCTTTTATGTTATGAACTTGAAATAGCAATGGCTAATATGCCCCTTTCATATCTTATTTAAACAATTTGATGATATCATCTTAAAAGTTCTGATTTTACATTTTCTGTTTCATATTTTTCCTGAGAAGAATCCATAGGCTTCATCAAACTGAAAAAAGTTACCTGCACAGTTCATCCTTGATATAGGTTATTCTCAAAAGTGCTCAAAAGTGTGCCTTTTCTATGTGCCTGTCATCACATTGGCATGCCCTGTCTTCTTGACAGGTGCATGGTTCAATACCCTGAGCCTCTCCTGCTTTCTTACATCAAGCCACAGTGTAATTGACAATGTAGCTCACCCACTAGTCAGAATACTACTTCAATCTGTGCAGGGTTAATGTTGGAGCTGGTGTCAGTCTGTGTCAGCTGGAGGGCCTGATTTATTTCAAGCCCTGAGCACTGAAGAATTTTTTCTATGTTCTCCAGTTAAAGCTATAATTAAATTATATATGCATAGATTAAATTATATCCACATAGAAAAAGTGTGATAATGATAAATGTTATCTCATGTAGTTTAATATTTTAATCAATTATATGATGCCCAACTCTACAAGATGAAATTTTACAATTATGTTTTCAAATAATTGTATGATCTTTTCAAATTTGCATTTTATTGACAGAAACATGAAGATGAATGACTATTTCTTTCGATTCTCTGTGATAAGAAAGCTACAAAATCTTTAGACTGGTGAGGGGACTCTACCTTTTAAAATATGTCCTATTATTGATCCATGTGTATCAGTTCCTAACTGCCTGTAACTTTACCTCCCAGGGAATCCTATGCCCCTGTACTCCTTATGCAC
MSTRG.59566.1	ENSRNOG00000016890;ENSRNOG00000017045;ENSRNOG00000017084;ENSRNOG00000039001	0.000	0.913	0.000	4.460	up	GGCAATTTTATTGTCTCAGAGGGGCAGAGCTGAGGGAGAGGGCTGCAAAGCTGCTTTCCCAATACCCCAAGAATACATATTCTCTATTTTTCCCACAGTCCCGAGGGGGTCATGGAAGCTGGGCAACCAATCCCAGGGCAATGTGCTACACCTCACCAAGTCAGACAAGTGAGCCAGAGGCTTCACCACAGCACACAGACACACACAGTGCACTTGGGAACCTTAAGTCGGAACTCTAGAAGATGGG
MSTRG.59567.1	ENSRNOG00000017084;ENSRNOG00000017235;ENSRNOG00000017746;ENSRNOG00000017867;ENSRNO	0.588	0.000	0.037	-3.684	down	AAACAAACAAAAAAAAACACAAAACGGGTTGGGGATTTAGCTCAGTAGCAAGCGCAAGGCCCTGGATTATTTGGTCCCCAGCTCCGAAAAAAAAGAAAAGAAAAAAAAAGTATTAAAAAAAAACATAACAAGGTTTATTATGTAGCTTATAAAATAAAAGAAGAGGTTAAAAAAAAGAGTGTACTTGCCTTGGCTGGTTGGTTAAGAGCACTTACAGTGGACCTGGGTTTGATTTCTAGCACTTGCAAGGTGGCTCACAGTTGTAATTGTAGTTCCAGGAGATCACTCTCTGTCTTCTGTCAGCACATGGTGCACAGACATGCACCCAGGCAAAATACCCATATATAGAAAATAAAAATACTTTAAG

	G00000024364;ENSRNOG00000024452;ENSRNOG00000038973;ENSRNOG00000039001						
MSTRG.59712.1	--	0.313	0.000	0.000	-8.200	down	AAAGGGAAGGGTCCTTCGAACACTCAAAAGTACCTTCCAGTCGGAACTGCTTTTCAGAGGAAAGAGCTTTGCCTTCCAATGGGCTCAAGACCCCAGAGCGGGTCAAGAATCAACTAGGGAGAACACACTCAGATTCCTTAAGGCACCGGTACTCTTCTACAGTCTGGGACCACATGGCTGCAACACACCAGGTTAACTGTCCCTACCCATAGGCAAATGGTGCATCTGGCACAACCTACACCACACCCAAGAGTAAGGCGCACCAAGGACTGCCAACAAACGGGTTTTAAGTGTTATAACACAAGACCCACTTCAAGCCTCAGGTGACTCCTTTCCTCCTCACCAATTTCAACTGGCCTCACAGGCTGAGCCAAGAGCCAGCTACTGCCCTACCCCCCACCCCATCATGAGCTGAGGGAGAGAAGGCTTTGCCTGCCCCTTCTCATCCCTGGGAACAAGGGCAGTAGGCCTCCCAGAGAAAGCTAGCACGCCCTTCCACAGCCCCAGGGCACTCTGAATTTGCCTAATTGAACATTAACAAGTTGCTACAATAAAGTTCCTCTGTTGTGAGCTGGAAACTTTTTGGGCAACAACAACAGAAGTCTTGTTACAAACTGCTCTCAAAGCCAGCCTTCAGCCCCGACAGGTGTGAACAAGATTTAAACCCCTCCTCCAATACAATGCTGTCCAAACAAAGGGGAAAGTCAGCCTCAACCTCCTGGGGTTTCTCCTTGTCCAACATCATCTTCACTCACTCAGGCTGAGGAAGCCAAAGCAACCTGTAGAGAGGCTCTTGAGGGCAAGCAATAGGGGAACTGGGCAATGCATGGAGCAGGCTTCCTTCCTGGCCTCACATGTCACCATCTTGGATGCCCCTGCCCCCACTTCCCGACATTCCCTCTCTTTCCTTTACAAGGCATCAGATAGGAAGGGGAGGCCTTTCGTCACCCTCTACCTGCTCTTGTACGACCCAGACACCAGCTGAGGCACCCCAAGAGCAAGCTTGAACGTCTTGCACATGAAGGCTAGGAGTCAGAATCCAAGCTGGTCTAATCCAGACACCTCCTGACCCTTCCTCAGAATAACTGAGGAAAGACAGGCCTCGGAGACTGGATTTCCCATCAAAAAATATCCCGTGACACCAGAAGCAGAAACACAAACACAGGTGCCGGGCAAGAGAGAAGGAATTATTTCTTTCTTTTCATTTCTTTTCTTTTCTTTCTCCCCCAACCCCACCATTTTCTTCTGCTCTGGTGTATTAGAAGGAACATGGCATTTATCTGGAGATTTCTTCCCAAAGCCAACCACATAAACAGGACTTGAAAAGGGCTTATCGGACAAACGATTTGCTCACACAGCCATTGCCGGAAGTGGGAATCCCAGCAGGGAACTCGATGAGCCCCAAGATCACTGAGTGGACAATCTCAGGCAGCTCTTCACTTGAGACAAACGAGGGGGAGGCCAGAGACAGGACTGACAGAGGGAGGACCACTGCAAAAGCTGCGCGCACACACACACACAAACACACAAATAATAATTCTCTCACCTTCATTCTTCTGCTCCAATGAGGCTAACGCAAAGCGACCATTCAACCCAGTAAAGGCATGCGGGAGAGGGATGGTTGGGGGCGCAGGTGGGGAGGAGCAGGGGACTAAGTGCCCACATGTGCTCGTGTCTACTGCACGGCCATTCTCAGCATGACTTTCCTATCCTCTTCCACCTACACTTGGGCTAGTTGGAGGTTTCTGTTGCTATCTTTTCTTAACAACCAAGCTTTTGATACAAACTTCTTCGCTTTCCCTGTGTTGAGGATAAAAATTGCATATAAGATTTTTACGCAGCTTTTAAGGTGAAGACAACCACCTTAGGGGTTGGTTTTGCCCAGGGTAACTTTTAATACCAGTTCCCCTTAACCTTCTCTGGCCCATCCATGAGGAAGAGCTGCCGCCGACACGGGACATAAAATGTTCCTGACCCCTGTGATGGAACCT

							GTTCTTCAACAATTCTGACTAATAAACCTTACAGCGCGGAACAGATCCCATAAGGACATACACAGATT<br>TCATTTTCCTCTGCGCACCTCTCTTCTACAGACACAGGAGGCAAGCACCTCCCAGGACCCCACAGAAG<br>GGGCATAGCCCCACCCCTCAAACGTCACCATAAAAGTTCAAAGACTCACATCCTCCTTCCAGGTAGCTG<br>GGAGGATGTAATTGGTGAGGCAGGAGATTAGAAATATTGGCAGCCTCTAAGTAAGACTTTAATTTACTG<br>GCAGATCAATAGAGCCGGGGTTATATTGGCAAAGTGTCTACAGCTCTATCGACTCACGAGATGCTTCTTA<br>TCAATTTATCAGAAATCTGGAAGTCAGAAGATATTGTTTGAAAAGGAAAAGAAAAAGGGGGTGGGGAA<br>AAAAGAGGAGGAAGCAGGAAGAGTCACAGAAGAGTGTGAAACCAGGGTCCGCAGTGCAGAACAGAG<br>TAGGAAGACATTTACTCCTGCCCAAGGATGGTCATGCAATATATTCAGAGTTAACTAGCCACCCTTCCTT<br>GCAGGAGTCCCCAACCTGTTCACGGGATTCCATTTCCCATAAACCTCTAGTCACACGCTGTGGGGGGG<br>GGGGGGTTGGATGTGCTCAGCAGTAGATCTCTGCCTCTGGGGTCAGTGCCACTGGCCAGAGTTGCAGG<br>CAGTCCCACGAGCATCCCTTCTGCTCGATTAGGGGCTAGTTTTATTTTGTCCGTCTTAATTTTTAACTGCC<br>TCTCTGGCTCTACTTCAGCCAGAAATCCTCTTAACATCTTTGCTAATCTATTCTAAAAGAAAAAATAATAA<br>ACTAAAGGAGAGATATGTATCCTTCTGTCCCATGTTGCTAAACGGACACAAAGGCTTTGTGAGACTGAT<br>GCTAGGACACACATGGTCTCGGGAAACACAGGGAGAATAGACTTGGGTTCCATAAATCATCACAAAGG<br>GGTATGTAAAAGTTATCACACACAGTTAATCCTCGTCTGAGAGGGTTTCAAAATACGCTAGGTCACTAA<br>GAAAAAAGGCCAGGGAAGAAATGTGAACGTTAACCCTTCAAACCTAAACTTCATCCATACCAGAGGTT<br>CCCAAGTATTTCTGCCCGATAACTCCAGGGTGCAGGTCAGGGAGGAAAACCAGAATCTGGAGCACTCC<br>GATGATCAGGAGGCCAAAGGGGAGAGCGATTTCCCCTGGTTTAAAGGAACCACAACTGGCTCATGACG<br>ATCCGCTTTGGTCCCTGCCTCATACAACAAATTTCTGCTTAATTGCTGTTTTGCATCTCGTTGAGACATCG<br>AATAAACAGTACTTAAGTATTTCCAGACAAAAACAATAACTCACTAATGACTAAGGCACTGCTAATTAA<br>AATGCCCCATGATGGCATGACTGGACTCAGAGCCACCACAAGGAACAGAAATGCCCAGGCCTCGAACA<br>CATGAACCCAAAACCCAACCATCACACACTGTGGGGGTGGGGTGACCTGGTCCTTCCTCTATAGGTCA<br>AACGGCCCTGTGGTAGAAAGGAAGGATGACCCTTCCAGGACCTGAGAACAGGCTGTGGAGCTCAGCA<br>GGAGCTGAGGTGTGAAGGTTCTTGAAGGATGCCAGACAGAAGAAATACATCCCAGAAGTTTCTGGGCA<br>AGCATGTGGTTTGATGCAGCCCTGGAGACTTTGTGCACTGCTAAGATCCCCTGAAGAGGAGGTAAGTGT<br>GTTGTTAAACAACCAAACAAGATACCCCTTCTCAAAAGCACTAGTCTTGCAGGCACCATAAAGGGTCTT<br>GAAGGCTCCAGAGGTCGGATCCCTTTCAAAAAAACAAAAAAAAAACAAAAAACAAAAAAAAACAAAA<br>AACAACAACAACAAAAAAAAAAAACACCCAACAGACTAGGAAAGAAGGAAACAAGAGTGTGTGGTAC<br>CCTTTGGCACAGCTAAGTGGTCACCTTAACCCAGTTCCCAGCTTTCCAAGTGCACCTGACCAAGTCCTG<br>GGATCTAGCAAACTTCTCTCCCTCCACCACACACGAAAGCACATCAGCCGACTTCCCCTTGTGAGCCGG<br>AGTCTCAGCAAAGCAAAGGAAAAGAAAGGCTAGCCCGAGTCTCCAAGCTTGCAAATGACTTTCTCTTC<br>CTCCTCCCAGGGCAGGTTAAAGACCTGCTAGTTAGCACCGGGAAGGGGACTGAGGGAAGTCATTGAAA<br>CCACAGGGGCCTAATACCCATTTGTATTCACACCCAGCCCCTTTAGGGGTGTAAAATCGGAGAGGTCGA<br>ACTGTTTACATTCACCTAAAGTCCCAGTAGACAAATCCGGCGCTATGTAGGCCCCCTCTATGCAGAAGT<br>GGCCAGTTGGCTTTTCAGGTTCCCAGTGCAAGGCCACCAAGGTTCTCTTCCTACCCCATTCCAGTTTCT<br>CACAGGGCCCAACTCCATTCTCCCCAACAGAGTCATGGGTGCTTCCTGAAAAAGCCCAGATGCCCTCAT<br>TCGACTGTGTGTTCTACACCATCAAAGGACACAGGGGAGCAAGTCCTCCATCAATGCCCATCATCTCCA<br>TGAAAAGGGGGGCTTGGGACACAGACGCTACCCCTTCCCACCTCCCAGGATCCCACAAAGCCCAGGAT<br>GGCCTTGAAGCAACTGGCCAAGGACTTTAGGGTCTGACCTAGGAAGAAATCTCCCCTCCTTTCTGGTCC<br>TCAAGGCTGACTGCTGTTCAAACACTACATCTCAGAGGGATGCCCCCCCTCCTTCCCAACAGCCCCTCC<br>CCACAGCATCATCAGATCTTGGCCAAGGCATCTGAATCTATCAGATGTCCTAATGCAACGCTGGATCGTA

							TCCTAGAGAACGTGAACAGTTTCAACTTGACTAGGGGAAGGGGTTGGGGGTCATGACACTCCTTTTAA<br>ATGCAGATTTGAACTTTTCACCCGGGGTCAGACAGCTGTGCTGCTCTGTCCCAAGCAGAGACCAGCTC<br>CAGGCCCTTTCTACCTCAGACTTCCAGTTTTTTTTTCTTTTCTTTTTCTTTTCTTTTCTTTTTTTTTTTTTTT<br>TTTTTTTTTTTTTTTTTTTTTTTGTGCCTGCCCAGGAGGCCAGCCTGTTAAAGGAATTAGATACCTCCCTG<br>ATTTAAAACACCAACCAACACCCACTTCTTGTTTTACCAAAGGCACAAATTCCACTACCCTTCCAGAGG<br>GGGCCTTGGATGGGCTAAGAGGAGAAACTGGACCAGTGAGCTAGAACTGGTAACACCTGGGTGCAATT<br>GTTCTTATCACGGGCACTGGCTAAGGACTAGAGTGGGGGTGTCAGCAGTGTGAACACAGAAGCATTTA<br>ACTCCACTCTGGACGGAAGCACACTCCTCGGGACACATGCTCCCCACGGCAGTTCTTACAGACAGTTC<br>TCACTGCTTTACCCTACAAAGAACTGCAATGTGGGGTTCGGTGGTTAAGAGCACTTGGTTCTCTTGGAG<br>AGGACCTGGGTTCAATTCCAACATGGTGGCTTACAATGGCCTGTTATGCCAGCTCCAAGGATCAAACAC<br>CCTTTTCTAGCCTCTGCAAGTGCTAGGGCACACAAATGACACACACGTGCATGCAAGCAAAACACACA<br>AACATATGAGGAAGAGGGCACTATTGGGGTGTTGCCTGCCGTGTGCCAGCACTCCTGGGGCAGACCTT<br>GCTGATTCTCTGGGAGTTCATGGCCAGCCTTGTAACTCTATAGTGAGATTCCAGACCAAGGCTACACATT<br>GAGACCCAGTCTTTAGAACCTGTTGCGTGGGGGTTATGGGAAGACTGCCCACAGTGAGCCAGCCTTGG<br>ACTCTACCCTGACTTCTTACTATCAAAGCAGAATGCTCTGTCTGAACTGCCCTCCCCCTAAAATGTCCTG<br>TAGGACACAAAGAAGTGTGTTCTCCTGTCTGTCTGTCTCTGTCTGTCTGTCTGTTTCTGCCTCTCTCTGT<br>CTCTGTCTCCCTCCCTCTCCCCCCCCCACACACACTCCCTCCCTGGACTGGTGTGGGGGAGGCAGAGAC<br>GGCACAGAGAAGGCCCCTTTCTTATTAGGCTGAGTCTATTCTACCACCAAGGATCACAAGGCCAGTCTC<br>ATTTCTCTATGTAAATGAACTTTTTAGCCACCTCCTCAAAGAAGAAAAGCTGCACGCAGGCCCATCCTG<br>GGGCTCACACAATTACAGCCATTTGCAATCGGGCAGCAGGATTAGCACAGGAATGCGCTCTGCAGCTTT<br>GGGGAGGGGGAGTGGTGCTAGGAAGATGGGGTTCTCCTGGGTTGGTCCACCCAAAGCTCTGAGACAC<br>TTCCACGTCTAGGAGAGTCAGAAATTCCAAGTTGCTGAGGCTCAGGACTCTAAAAGGGGTGGGGGGAG<br>GAGGGGGAGAGAATAGGGGCCAGCAGAGGGCGGAGGGAGGAGGGGAACCTCGCATGAGGGGCACTG<br>GCCCTCCTGACGCTTTGATCCCCTCCGTCCCAAAGTCGGTCGCTAGGAGAAACCAACTTCTCTCCTTGG<br>GTTAATCAAAACCAAATTACTCAGGAATGTGGAAGGACAGGTCTGAGCTTCCAGGTCCCTTTGTCTTTG<br>AGGATTTGAAATGAAAAAAAAAATTTTTTTTAAAGCAGATGGCAGTGGTCTACTGAATAGGGAAAGTC<br>GGGGGAGGGGCGGGAGGGGGGTGATGAGGAAGGAGTCCACTCCATCTTCTGTCCTTAGAAAGGCACC<br>CACAAACTTCCTGCCTTAGTTCGTTCTTAATGCAGTGACATTTCTGTAGATTAATTCCAAAGACTGGAGG<br>AAGTTTTCATTGTATCCTTACTGCCAACTAAAAAACCTAACATTGTAACTTCTTAAATACAAATAAGAAA<br>TAACGCAGAGAGGCTGGGGAGCCAGCTCCCAGGTTCGGAGTTGGAGCCCATCCTGAGCAAGACCCAA<br>GCGTGGTCCCCAACACCCATGTCTGGGTGGCTCACAACTGTTCCAGCTGCAACTCCTGCTCGGGAAGA<br>GTCACTACAGTCTTGGGACCTCTGAGGACACCTACACACACTGTCACGCAGGGACAGAGTTTGAAATA<br>ACTTTTTTTTAAAGACGGGATTTACACGACACATGTGACAAACTGATTATTAAAGGCAAAGTAGGCTGG<br>GCCCAGCAGTGGACTGCTTTAATCTCAGGGCTCAACAGGGGTCAAGGGTAGGCAACCGTCTGTAAATT<br>CAAGACCAGCCAGGTCTGCATAGTGAGCTCCAGGTCAGCAGGAGTTACCCTGTCTCAAAAGCAAACAA<br>CAAACCACCACCAACAACAACAACAATAACAATAATAATAATAATTAGGCAAGTCTACATAAACATCTC<br>AAAAGCAAACCAGTCAGTCATCATCATCATAATAGATAAAAAGGCAAAATCTACATAAACTGAGTAAAA<br>CCCTACTTTTTGCAAAAGCTAAGTAGGTGTATTGCTCAGCATAGGGTTTCTCCCGGAGAATGCTGGGGTT<br>GGGGTAAGAAAGCTGGGAGACTGGATGCTGAGTGTCTCCCAGTTATATCAGAAAGATAACTTTTTCTGG<br>GCATCCTCAGATTCCTTGGCTCAACTCTTGTCCAAAAAAACCTCAGGAGCCTGGGAAAGCTCGCTGGG<br>CGTGCTGGCCAGGCCTGCCATGGTGGAAGAAGGTCTAGAGGTGGCAGGCGGCTGACCTGGGGGGTGA

							ACGAATCCTAGGCTGGGGCTATAGAAGGCCACGGCACTCCTCCCCCAGGCCCTGACACTCCATGATGGG<br>CCTGCACCCCAGGCCCCTGGAAACCCCATAATACAGCACCCTAGTGCAGATCATCATCAGGGACCCTGG<br>AGACAGAATCCAGAACAGCTTCACAACAAAAGCTGATCAAAGACCCCATCTGTACCCCACCTCCCCAC<br>AAAAGATAAACAGAACATTACCAGTTAAGCTACAGAGCCTGAGCCCAAAAGATAAGGTGAGACCAGCA<br>CACAGCCATGACTACCAGCTGGCCATAAATCGTGACTGGCCCCAGGCATAGTTATCTGGGAGGTTGCAC<br>TAAAGTAACTTTTAAAATTCTCTTCACCTTATCTCGAGGCCCCGTCAGACTCGCATACATCACCGGCTCA<br>GATCTCAGCCTTTAGTACTGCATTTGGACTTTCCAAAACAAGGCCATCCCAGTAGGGCCCTTCCCCAATT<br>TCCAGGCACAGCTGTGGACTTTCACCCCCTTAACTAACAAAAGGGACCTTGTGAACAAAATGAGTCCA<br>AAGCATTCGTCTGTGTGATGTCAAAAGAACGCTGCTTAGTCCTTAGTGTATTTATCCTCCTTCTAGGCCT<br>GGTCAGGCCAGGGTGCTTGCCTGGGACCTCCCTGGGTGGGGCCCACAAAGATTAGACACCCAGACTTG<br>TCAGCTACTGTCCCATGCACCTGTGCAGTCCTGGTCCCTCCACATAGAGAGAAGGTGGGGGAGGGTAA<br>AGAAAAGGAAGAAGAGAGGGGAAGAAGGGAGGGAGGTATGGAGGTGGAGAGACTCTGACTCCATAA<br>CTGTAGAAAGTGTCCTGTCTTTCTTTACGCTGGAAAGGACCAGAGTCAACTCATGTCCAGCATGCTGTT<br>GGCTCAGGCTGGAAATGCAGCCAGTCCCATGAAGTACGAGGAAGATGTCCCTCTGACACTGCAGGTTT<br>TATAAAATGTGTGTTTTCACTTACTCCCACCTGTTCACACATTCCTCCAGAGGCCACTGGCAAATGTGTC<br>TGCAATGCATGGTGGGCGACTTGTTCTCTTTCTACCCACTAAGTGGCCAGCCACAGGCAACAATGTTAC<br>CTCCTCAAAGTCAGTTCACCACCAGGCCCTGAGAGAAAAGGATACTAGTTTCCCGATGCTAAAAAAGA<br>CTACTGTGGAATTAAGCCCATGGAACCGTCTGATTATCAGACAGGCAGAATGGCAGGCTTGGAGGCCCC<br>TCAGCCAGCACGCTGCCATTTAGACAAAGCACCGATGATGCAGAACTTGGCAGGAGAAGCCAGGGCCT<br>GCCATTCCATCCTGGAGAAACAGCCTCTACTTTGTGGTCCTCAACCAGGGGCAGGTCTACCTCCTCAGG<br>CCATTCCTTGTGACAAGATGGACAGTCTTGTCAACAGGAGAGAGAGAGCGAGCTACTGACATCAAGTG<br>GCCAGAAAGAAGCCACTTGAGTGAAGGATGCCACAAGCTTAGATATTTATATGCAAATAAAGCCAAGAC<br>AAATGCTCACCGTAAGTGCCCCCAAGCTGACATGATCCACCCCTGAATGTCAACAGGGGACTTGGTCTG<br>AGGTCCACTCCTAATTCCCTAGGTTGAGGGAGTCAGTTGGGAGCATCCTTTTGGAATAGCTTCTAGCCA<br>GGGCTCTGAATTCATAAATTGGAGTTGTTTAAATATGGGGAACTAAAACAATTATAAAAACTATCATCCT<br>CACTGAAGACTGAGTATCAAACACTGACAGAGCCAAGTGCCTGGTTTGCGAGGTGGCAGGTAAAACA<br>ATTGAACGCTCAGCACTGTATGAGCTGGGCTCCTCTGCAGGCAACAGGCTGTCTCTCAGAGATGGCTG<br>GAAATGGGCCATGACTAGCACGCCCAGTTTGCCACATTTGACCACATGGTTCCTGTGACAGGCAACAC<br>GGCAGCGAAGGCGCTGTGACCAGGTCAGCTGGTACAGGAAGGGGGACTGACTAGGAGGCAAAGCCA<br>GGAAGAGTTGAAGGCAGAAAGAGATCAGGCCAGACTGGCTGGGAGGCCTTGCAGGAGAGGGGGTTG<br>GGGGGAGGGCACATGATAATTAACTCAGCAAGTTGATAGTATTTGCATATAAATGCCTCTACAAATCACT<br>ACAAACAAGCCAGGACAAGTCAGGCCTATTAGCATATATAAGCCAGCACTGGAAGGCCTCTCTAGGTG<br>GGTCATGGAGTGGGCTGCTCACTTAGGGGGCCTCCTGCCAGGCTTGGGGGTGGGGGGAGGAAGACAC<br>GTGGGGAGGCTGGGAGGGGGAGGACACTGGTTCCAGTGCAGACCCTCAGACTCCAGAACCCTTTTAG<br>GAGGTCAGAAAGAGGTGGCTTAAATCTCAGCCAATAAGGAAAGAGTGGAAGAAACTCAGGACCTCTT<br>AGTCACTCTCTAACTGGGCGTGGTAAAAAGAAGCAGAGGCCGTCTGCAGGGATGAAATAAATGGGTGG<br>GAGCTTACACGTATGTGTTTCCTCCGTATGCTGTATGCCATATGTCGTATGCCGAGTACCTCAGGTGCCAG<br>TATTCTGTGTTACTGAATTCCCACCACAGGTGTGGGACCACACCCTCTGACCAGTGAAATGAGAGATGC<br>TCAAAGAACTTAAGGGATTGGTTCCAAACCCACGGGAAAAGGTCACAGAGCGGAACCAAGAGCTCCA<br>CTTTGGGGTGAGCTCAATGGTGTTGTGAATAAGGCAGCTTTTAGGAAATCAAGGTCAACAGAGTCCAC<br>AGGTGAGGAACTCTGAAGCTCCGCGTCTGTTTTCCTGGCCCATTATTTTGTACCTTCACCAATGAAAAG

							AGACTTCCTGTCCGCCACCGGTGGGTTGGTACCAGGACTTCCTGTTACCCCAGTTCTCCAGCATCTGGG<br>CAATTCTTCTTGTTGCTCCCAGCAAGGACAAGATAAACTGGCACCTGGGACAACTGGTCACCTGATCTT<br>GGCTGTCAATCTGACGACCATATCTGGAGCCAATTAAAACTAGAGATGATGGGCGGGCAATCCTGTATG<br>GGATTTTCTTAATCACATTATTGGAAGCAGGAAGACCCACCCTAAATCTGGGTGGCACCTTCTGGAAGA<br>AGGAAGTTTCGCTTTTTGCTTGCTCTTGGCGGTCACTCTCGCAGGGCAAGCGTGTGAATCCTGTTACTT<br>TGGCATTCCTTCACCAATATTCAAAAAAAAAAAAAATCTTTGGGATGCCAATGCAGACCAAAGACCTCTC<br>CAGGAATCCTGCCAACCTTCAATGCCTTCTTATAACTGCAGAGGCAACTACCAGATTCTCTGTTTCTCCA<br>GCCTCAGACAGCCACTGCTGGGCCGTGCAGACCACATCCTTTAAGCCAGTCTAATAAATTCCCTTTTAC<br>TACATATATCCATTCTATCCGTCCTGTTCCTTTAGAGAACCCTGATCAATACAGCTCTTTCTTTAAAAGAC<br>CTACAAGTTCTCTGCTTTTCCCCAAAGATGCCTTGTAGGTTTAATAACTGTTGAGCAAAAACCAGTTTCC<br>ACTCTCTCAGTGAGAAAAAGGGAAGGGCCAGGCAGGTGGGGCACAACCTACAGCTGGTATCTCTGGC<br>ATTCTGTTTACTTACCTTACATTTTTGGAGTTAGGGTCTGACTACATAGCTTAGACTAGCCTTGAATTCCT<br>GTCCGTCCTTCCTGCCTCAGCCCTCCCAAATAGGCATGTGCTGCATCCAGCTTATTCTGGAACTATACTT<br>TCAAAGTGGGAGAAATGCTATCTTATGGGAAAGCCAGCCACAGGGCTGAGATTGCACATGCTCCTCAA<br>GTCAGATAGCTGGGGCTGTGATCAGACTCAATAAGGCGACAGCATTCAAGAACCCGGTCAAACAGTGG<br>TGGCTTTGAAAAGCCACTTCTATACAGCAGACACCAATTCCCCAACATACTAGGGCCTCTTCCTTATGTG<br>TCCAGAAGTTGACTTGACACTTTCACGAGGATGCAAATATTCCACAGAGAGATGGTAGAGCTGTTAAAA<br>GGAAAGCAAAATCCGCTTCCCCAAACCAGCCAAATGTACATTAAGAACCAAGGCCCCGAGCCAAAGA<br>GCGTTTTAGGAAGCAGGTCTTGAAACAGCCTAAGAAGCTCGCACTGTTTCTCGAGCAGTTAAGGAAAA<br>CTGTTTAAAAAAAAAAAAAGTTGCTGAGGAGCATTAAAAAATAACAATTAAAGAAATAAAAGTCCATG<br>TGTCCTTTAGCCTAACTCCCCTGGACAAACCGTCAGGAAAAGCTGTCTACAGTAGACCACCTCAGATGC<br>TGGAGGTATGGAAGAACACGTGAGAGTGCGTGCGTCCACAGAGACATAGGCTTTTTAGAGACAGGGTG<br>ATGTGGACTCCTGAAAGGGAGCTTTTTAAGACACCAAAAGGACGCTTACAGGACTCGGGAAGGGGTCT<br>GGGACTGAAACCAGGCATCAAGTGAAGACAACCCCCTCCTCCCCACCCTGTCTTCTCTGTACTGGGTTA<br>ATCTGACTTCAGCCCTCTGGTCAGAGGCTTCAGTATGGGACAGGCAGGCACCTTGGAACTGGAATTTTA<br>ACACTGAAATGCAGAGCCAGCCTGTGACTGAGGTATATGCAGGCACCGGCGTATCCAATAGCTTGGCCA<br>ATACTCTCAGAAGAATCTGTCTCCAATCCTGCTGGCTGGAGTCAGGAACAACCAAGAAGCCAGCCAAA<br>TTGCCCTTTGCTCGCTCCTGCAAGTGGCAGGGATCACCACAGTTAAGGCTCCTCACAGATTAAGAGTCA<br>CTGACAGTGTCCACTCCCAATCTGTCAGAACCTAACATGATTATCCTCAGAGTCATCCCCATGGACTTGA<br>AGTCTCAAAAGCCTCCCCCCAGATACACACTAGCCACTGCTTCCAGAAGCATGCAGACAAGGGTCTCT<br>CTTCCAGATCAAAGCTTTGTAGCTGAAGGGTGCAGGACAATGGATAACAGCAGTTGCACGTGGCACAA<br>CTGTGCCAGTTTCCTCCCTTCAACTCAAGGGTTTCCTAGTGATAACCAGGCCTAACTCTTGCCCCCAAA<br>AGGCCCATTCAGATTAGAGTCAAGAAAGTCACTTCAGAGGGCACTTTAAGATCCCAGTATGACTCCCTC<br>AGGCCAGAAAAAGAGACTCAGCCTTACACCATCTCTATAAGGAGTGGCTTTGCCACAGATTCCGTCTCT<br>AACTAGTGGGAACCAGAAGGGGCAGGCAGTAGCAAGGTAATCAAATGTCTGACCTGTCTTGAGTCTCC<br>TTTCTCTTGGAGGCCTCAGTCCTGACCCTAGAGGTGGAGTTGAAAGAGTTTAGTTAAACTCCTTCCCAG<br>AATGCACACATGTATACACACACGCACACACATAGACACAGACAGACAGACACAGACACACACACACA<br>CACACACACACACACAGATTCTCTTGTTGACAAGCCTGTATGATGGATATGAAGGAGGGCTCACTCAAA<br>AGGCAGATAAAGCACCAGGTACTTCACTAAGAGTCCCCAAATTGGGTTCAAGTAACAGAATACAATTCT<br>TGAGGTGCAACAGAGCCAATCTGCCAGCCCGAAACTGTCTTTTTTTTTTTTTAATTTGCATTGTTATCCTT<br>GCAAACAGTGAGAAACTCTAGGAATTAAAAATGAAAGAATTAAGAGTGAAAGAAAGGCAGAAAACAT

							CCAAAGGGGAGAATTAAAAAAACAAAAACAAAACCCAATAACAACAAAATAAAACAACCTTCAACAA<br>ATCAGCAGCAGGGCGACTTAAGGGCAAAGAACTTTCTAGAAACACTCCATTCACTGTTTCACTTGTGGC<br>TTGTCTTCAGTACTCACTACCCTGTCCCCTGACCCAAAGGATGAAAATCAGGAGGGGAAAAAAAGGAA<br>AAAAAAAAAAAAGACAGATGGACAGATGGACAGGCAGAGCACATCCCTCTGTGTCACTGCCTCTGAG<br>ACTATTAACACCATGCTGCTACCTGTCAACTGGGGCTGGGCCTTGCTTCCCTTGAGTCCCTGGTTCCTGT<br>CCCTGTAACACTGATTTGCCACGTCAAAAATATAAAAATAAAACTCCTCTACCATCCGATCATTTCCCAC<br>GTGTTCAAAGAGAGCAGTCTGGCAGTCAACTTTAAATCATACAGAAAACAAATCTGACAGTTGCTGATT<br>GGATAGTTTTGCTCCTTAATCTGGATTTTGAAAGCAGTTTGCCAATGAACCAAGGTTTCTGCCTGAGTCT<br>CTCCCTTGGGACAGAATGAGGAAACGTTAGCTTCTGTGCGTCTGGTTCTGTTTCAATGTCCTCGCGATG<br>CAGGACACTGTGCTCAGGACAGCACCACTATCTTTAGACCTTACTGCGCATGCTCCTACGAAGGAATCC<br>CCTAGCTTGGGTCCCATTAAAACGGCCTACCCAGTTCCCTGACCACACAGTTCCTTAAGTCAGGCAGAA<br>AGGACTGGAGAGCCCCGGCTATGCTGCGGCTTCTAACAATAACCATTACAACCAGATTCTCCACCAGAG<br>CACGTGGACAACCCTAAGAAGTTCCCGATAATGGAATCTGCCCTCCTGTTGTGTTTGGATGGCACCGCC<br>CCCGTACTGGTTAATTACAACAGCGCTGGGAACCAGAGTAGGCCAACAGGCCCAAACATTACAGAAAT<br>AGGCCAGCCTAACTGCAAGCAACCATTTTAATCACAAATGAAAGCATAAACCACAGCACACACTGTAC<br>CCCTTGTCGTTGCTACAGTGAGTATCAGGTGCGTTATTCTTGCAGCGGTGGACAGCCAGGAGGAGGAG<br>CTTATCCGAGCTTCCTCAGACCAGACACCCCACTGCCTCCTGGCAGGATAGACTCTGGGTCCTCGGTTA<br>GGCAGCAAGCTAGGAACTCTCCCAAGGGGAACTATGCAGACAACAAGTGGGAACCCACACATTTTGAT<br>AAAGACTGCTTAGTCACTTAAATATTTACTTTTTAAGACGAAACTTTTGCAACTCCCCCAAAACACTAGT<br>TTCCTAGGTAAGAATTAATTTGCTTTGGGCCCTCAAGTATTCCTAAACACTTATCTCTCGCTTCCGAAATC<br>GAGAAGACCCAGTCTCTAGAAGATACACTAAGAGGAACAACAGTTTGTATACACACACACACACACAC<br>ACACACACACACACAAACACACACCAACAATAAGGACATATTCCCTGAATACCACATGTGATCAAAACT<br>AAAGTTTACCGATTTATTCTAATGCACAGATTTATTCAAATGAACCAACCACCTTTGGGTCACTCAAAAA<br>TGTCGAGGCCACCACTCTTAAGTCCTAATGGTGAAGGCTGAAATCTCACTTATTTTAGCTCCTTGACACT<br>GGGAAGTCCAATTGGCACTGAGTTTACAGAGCAGTTAAACCCAGCCCAAAACAGCAGGAAAGCACTTT<br>GCTGTGCAGCCAGGACAAGCCAAACAAGAGGCCGCCTGGAAGCACACCGTTCTATTTGGGCCCTTTTC<br>CACACTCCCTTAGCATTCGCTGGGCAAAGAAACCCAAATTAAGGTTTCAGGTGGTTGGCGGTTGCAGC<br>CTTGTTGTTTTTGTTGTTCTCAGAGAGCCAGCCAGATCCTTGTCAATCAAAACAGTTTTCAGAACTGGA<br>GAACCAGAGTTCCATCCCCTAGAAAGTGTACGGATTGGACGGAGGTGAAGAGGGAGACGCCTGACCC<br>ACGTCAGAAGGACAGAAGGTCTTCAAGGCACTTAATTCCTGGCCGTTCCAAAAGGACTGTTTTCAAAC<br>TGTACAAGTGCTTGCTTCTGCCACCGACCCCATGTTGTTAAATAACACTTGTGTGTAATTAAATAAAAGA<br>CCCAGTGTGACCCCCAGCCCGTTTGGAGAGCCACTTTAACATGTCTTCTGAAGACCATACTAGAAATCA<br>AATGCAGGCAGTCTCTTTGGTCCTGTGATTTAGAAAAGGGGGGAGGGGTGCTCCCGTCCTTCATGGCAT<br>GCACACTCTCTGTAAGGGAGCCTTCAGGAACCCCTCAAAGCAAGTGAACACAAATACAGGTGTGCAAA<br>CATCCAGGTATTCACGTCCCTCCAACCACCCCCTTTTCAGAATCCTTCTGTGCTGGGGACTCTGTCTCCA<br>GACTCCTCTTAGCCCACCTCTGCTCCCTGTTTTCCACGTCTGGATTTAGAATCTCAGGCTGCCGGTGGAT<br>GCTGTGATAACAAGAAACAGGCTCCCAGCTCTCTCTACTGCAGCCTTTCTCGATCAGGTTACCCCTGAA<br>GGTCTATGTTTCCCTATTTAATGCAGTCTGGGCCACAGCACTCCAGTCCAGCCCACTGGAACTCGGGATT<br>TTTAGGGTTTCCCTTGAAACCTATCCAGAAACCACAGCCATTAAAAGAACATGAAGGTTTGACACCTCC<br>TGAATTTAGGAATGAGGGGACTCCGGGGGCCAACTCTACAAACTGGCACCTGCTGCTAGATGGCAAAG<br>AAACAGTTACCCCCAGCTACGTGTGAGCTTCAAATTATGCTCTTATTTATTTAGTGACTCGTCTCCAAGG

							ATCTCAAAGCATGAAATGAAGTGAGGTGAAGATGACGTGTAACTTCCTCACTATCTCTCTTCCAGGCTTAAGTCTGTTTTCCTTGCTGGGTCTTTGATGCCTTCCACCATGCAGGCCCTGGCCTTCGTTGCCTCCAGATGAATGTATACTCTCTGTGGTTCTCAGTCGGTGGTTCCAAGAGAGTCTTCAAAGGCTGATAAATAGCAGGGGGGGGGGGGCTTGCCCTCCTAGACTCACCTGCTCTCCGGCCACCTTTAAACCAGTTGAGCACACTGTGAACCACTAAGGCTGGCTGCTGAAACTCCCTTTGAGTGTGCCATGTGGGCTGTGGGCTTTCCCCTGAGGGCAAGATTCCCCATCATCATCATTACATAGGTATGTACAGGACAGAGAGGGCTGTTTGCCAGAAAGCCTGTTTCTCTCTAAGCCCACAAAATGACTTTGGGTTGGCTGGGGGTTTTTTGTTTGGTTGGTTTTTGTTTTTGTTTTGTTTTGTTGTTGTTGTTATTTTTCTTGTTGCTACCTTAACTTGGATATCTCTCCTTTTTAACTGGATTAAAAAAAAAATCTTACCTTTTCCTTTGCAACGTCCCCCCCCCCCAAATAAATGCTGTATGGGTTCTGGCAGAAGAACAAACCGAAATTAGAGGGCAGCCAGGCCAAGATGATCTCCGTAGGTCCCCTCCAGCTCTACACTGATTCTCTCTTTAATTACTCCCCAAACAGGCACATTCCTCACATTGCTGCAAACTGACCAGCGACTTCTAACTCTCTCTCTCTCTCTCTCTCTCTCTCTCTCTCTCTCTCTCTTCTCTGTCTCTCCCTCTCTCTGGCTGAAGAGGGTGTGTTTAGAACCCAAAGTTCTCAGTGGCACCAGTACCCCCAAGTATTAACAAGGTATTAACTAACCCAAAAACAGAATGAGGAGGAGGAGGTTGGGAAGGAAAATCAAGAGGGGATAGGAGGAAGAACGTACTCTGGTGCCCAGCAGACTCTAGCCACTCTCCCCAGCTGCAGAAACTTACGAAAACATCCCCTACTCTCCATTGAGAGTTTAAGGACAGTTTCTGAGGAGAGAAGAGGAAAGAAGAGGAGAGGAAGAAACGACTAGAAAAGACCAAGCAGTTTGCAGCCATCCGCTGTCAGGAAGAAGACTGTCCTTAGGAGCCACAGAGCATTTCAGACAGGACAAGAAAAGGTGCCCCAGCTCAAGATGACTACCACCAGGATCAAACCAAACAGGGCTACCTCGGCTGGTCAAAGCAACTGCTGGACACTTCCAGGTCCCCCTAAGTTCACCTTTTGCGTGCTGTTATGTAAAGGCAGGCCCCCTTGAAATCTCACCGGAAGTCTCCAGTCCTCTCAGACAGAATTCCACCCTCCTCCTAAATCTAGTCTTCATTCCCAGCATAGAAGACACCTGCTTTTTCTCTTTCTGCAAACCAGCCACCCAGGCTGCCCCGGCAGCCGCCGAGTCTTTTCCTACTGCCCAATAATGAACCATGCAAGTGTTAGAAATATAATAAGCTCACCAGGCGAGGAGCAGAATGTATGGAATCGTATTTGCATTAAACACACACTAAAAGGGAGGAAAGGGGGATGCTTGCTTGGTGAAAACAACAGTGTCCCTGTGTAGAAAGCATTTTTCGGTACAAAAGAATTCAGTTTCAAATCGATGTGAATACCTAACCTGTTGGGTGCAGACTAACCCCCTGTCTATATTTTAGCACCAAATTTAAAAAAAAAAAAAAAAAAAGATTACAGTTTTTCCCCCCAAGCTAGGGCCTTGGGCGGAAAAAAAAAAACTGCCTCTGGG
MSTRG.59749.1	ENSRNOG00000017500;ENSRNOG00000017602	0.037	1.208	0.000	5.286	up	GTGCTTGGGGACAGGCAGAGAGGGGGTGGAAGGGAACATAAATAGGGTCGGTGAAGGCCTCCTATGCTAACCTGGACATGGGTCCATGTCTCTGCCTATCAGTAAGAAGCTCTTCATGGGGGGCAAGGAGCCAGTTAGTGGCAGGAGGAACACAGAACTGGCTGCAGTGCAGCAATGTCCTTGTCAAGCAAGCTGCCTGCATCGCCTTCCTGAATAACCTTTGGAGTTTTCTGATGATGACAGCAGGGAGTGGGGTGAAAGGCTACAGCTGTCACAGTGGACGGCTACAGATGCTGCATCCTTAAAAACCTGTCATTATCACTGCGCAGACTCTCAACAGACCCCTATAACCTCTGAACCCTGCTACCTTTAATCCCAAGACCTCTCTACACAACCCTGCCATCCTGAATCCCAAGACTTTTCTAGACAACATCCTCCCAGACAACTGCACCGGGGAGCCTTGAGAGAAAAGCCAGCTTAAGGGCAGGCTGCATTCAACTTTCTGCTGTTTGTTCTGGGTCCTGCTGAAGGCAGCCTGGCTGCCCTGGTCCTGAGTGACTTTGGGATGGCAAACAGTCTCTGCTCCCTGGCCCATAGGGGCCATTTCGAAGAATTGTGTCAAATTCTTACAGATCAAGTAAGAATTTAAGATCAACTGGGGCTGATCTTAGAAGAGGTGGCTGAAGACACAGCTGTAGCAGCAGCCCCTGTCTCCACATCCTCACCCACAGGAGATAAATTAAGTGGTGGCTGTCATGCCTGGCTACCTAACCCACACTCCCAGAGGCACATGAACAGGAATCCAGCAAACAGTAAGCTTTTCCTGGGTGTCTATCTTTGCATTGTCTGGATCTCTGTCTCCTTCCTGCTTCTCTATGTATACTCCAGGGTCATCCCACTTCCAGGCCTAAGGTCACCAGGCTTCGAAGTACTTAAAGGACAAAAGGACCAATGAATAGAGTAA

							CCATCAATAAGTCCTCCCTGGCCTAAGGAGCTGGGTACTCTGGGAACACTGGAGTTCCAGCCTCACAGAGACATGGAAATCAAGTGCAAGCATGGTGTTTGACCTTAGTTTCTTAATGTAAGTAGGGGCAGTAAGAGTGCCCAATAACTGAGACAATACACATAGTGCTGGGTCATGCATGCCACACGTTGTGCTCAATAAATGGGGTGAGTGTGACGTCAGCAAATATCTCTCGTCCTGGCA
MSTRG.59770.12	ENSRNOG00000016953;ENSRNOG00000016971;ENSRNOG00000016977;ENSRNOG00000038741	0.234	1.231	0.001	2.981	up	ATGAACCCTCTGGTTTTTGTGGAGGTCTCAGCAGGCTCACTGGCTTCCATGGCCTAAGCTAACCTTGAGAAAGCTGGGCCAAAAGTGAGGTATCAGTCTGCCATTCAGTTCTCAACCTGAACTAGCCACTTTTGGATCACAGACAGGCCAAGTGGAGGCTTTCTGCAGTTCTCCTAGGCAGAGACCAGATTTACAAAGCTGTAACCAACCTTCCCAAGGACTTATACCATATATACCTCATTCCTGCAGTCCGGCTTCTCCCATATTCTGAGTGAGATGCCCGCTGCGGCAGACTGAAGCACCTGCAGGACCACAGCGGGTCAATGGACTTGCGATGTCACCTCGAGCCGCTTCGCCCCCTCACGCAGTCACCCTCTGTGAAGTGACACAGCTTCCCCGATCCGCCACCTGCTAAGCTCTGTGGGCCTGACGGTAGTTGCCACTCAAGCGTCACCGCTGTGGGCACCGAGACGTGGTGGGCCGCGAGGCTGGCTGCGAGGGACCCTGCGTGCTGACCGCCAGGACACCGCGAACCGCAACGAGTCCGCACTCACCTGCGCAGCACGGTCCGCGGC
MSTRG.59773.1	ENSRNOG00000016348;ENSRNOG00000016953;ENSRNOG00000016971;ENSRNOG00000038741	0.467	0.000	0.022	-3.851	down	CGTAAATAAAACTGCCTCTTGTTGATTACATCAAGACCGGCTTCTCGTGTTTCCTGGGGGTACTTCAACCCGTGACTGGAGCGAGAGTCTCCCCAAAATCTGGGGGTCTTTCATTGGGGGCTCGTCTGGGATCGGAGCGTAACCCCCGGAGATCCGAATGCCTCTTGGAGGTACGTTAGTGTGAGTGCTGGAAAGCGGCCGCTGTGTGAGTGTGAGAGTAGTGTGTGTGTGAATTAAGTCCTGTAGTCTGTGTTTTTCAGTACCGGTTTAAGGTCTTGGTGCACTGTCTGGGCAGAAGAAGATTCCCCACTCCGTTGTTTTTGAGTGCACTCGAGTGGATGGGCTTCCCACTCTGTTGTTTTTGAGTGTACTCGGGTGGATGGGCTTCCCACCCCGTTGTTTTTGAGTGCACTCGAGTGGATGGGCTTCCCACTCTGTGTTGTTTTTGAGTGCACTCGAGTGGATGGGCTTCCCACTCTGTGTTGTTTTTGAGCGCACTCGGGTGGATGGGCTTCCCACCCCGTTGTTTT
MSTRG.59884.1	ENSRNOG00000017932;ENSRNOG00000022593	2.087	0.000	0.000	-8.066	down	TTCGGAGCTGGGGACCGAACCCAGGGCCTTGCGCTTCCTAGGCAAGCGCTCTACCACTGAGCTAAATCCCCAGCCCCTGTTTGGATTTTTTGAGACAGGGTTTCTCTGTGTATCCCTTGCTGCCCTGGAATTTGCTCTGTAGTAGACCAGGCTGGCTTTGAACTCCAGGATCCACCTGCCTCTGACTCCTGAGTGCTGGGATTAAAGGTGTGCACTTCCACATCCTGCAAGATACAGTTTTTCCTGGTGTTAATTATGAATTATTCATGTTACTAAGACAGGAATGATTGTAAGAAGGAAGCAGCATTTTGTGGTTGTTGGTGTATTAAGTAGTTGACATTGCCTCAGGCTAGTATCAGTCGTTTAGTTGGTACTGTTCTAGCACGAGCAACCAGGGCTCTCTACAGACCAGGATTCTGTTCACTCGCCAGAGGAACTCACACACACACCTCACCCCACTTTAGTAAAACAGGATGATGTTGGTCACTGCCTATAAAGTCAGTCTATGGATGCATCTTCCAGGAGTGATGGTATATACCAGTCAGGCCCAGCATTAGCAAGCATGATAGTCCGTATCTGTAAATCCCAGCATTAGCAAAGCTGAGGCTAGAGGGATTGGAGTTTAAAGTAAGCCTAGGCTACATAGGTCCTGTCTCAAAAAACAAAACAAAACAAAAAATAGTCTGAGCATGGTGCTTCATGCCCATAATCCTGGCATTTGTGATCTGAAAGCAGGAAGTTCAGGAGTTCAGAGCTAACCT

							AGGCTCCAAGAAGCTGGCTCAGGGCATTCCACCTGGAGGCTGAGGCAGAATTGCTGAGTTCAAAGCCAGCCTGGGTTACTTAGAAATAGGCCAGCCTTAGCTCTGTACACTGAGTCAAGTTGACAAGGGTGGAAGAGAGATTCTGGGAAAACAGAGCTCAGGTGGCCATGAGTGGGAGAGAAAAGTCAGGGTCACAGGATAAAATGCTGCAGCTGTTTTAGTTTTTGTTTGGGATAGGGTCTCAGTGTTCAACACAGGCTGGCCTGGAAATCAAAGCAATTCTACCTCAGCCTCCTGAGTGCTGGGATTCTAGGCAGGAGCCATCAAGACCTGGTGACTAAATTTTGGCTGCATCATAGTCCAGTGTCCTGCTGTGACAGACTAGCCAGGGCTGGGCAGTCTGAGGACAGGTGAGCATGCGGGCGTGGAGGCGGAGACGTCAACATCAGCATTAGCAGCAGGATGAGGAATTCTGAAATCAGTGTAGCAAAACTGGCTTTCTGTTGTTGGAGGGGGGAACTGGACTGCCTGCCTCCTTTGCCCACACTCAGGTAATGACTGTTTCTCTTGGTCCTCATTAGAGACACCAGATTCTTGAGGACTCCTTGCAAGTTTACATGGCTTTGCTTCTCTATGAGAATAAATACCTGTGTTCTGTTGCCTGGCTGTTAAGGTGTCTTCTATAAAGGACCCTGGGGCATGAAATTAACCCAAGAAACTTACGCTGAGGCACTTACACCGCTTTTCTTGTCCTTAGCTGAGATAGAACTTTCCCAGAACAACTTGAAACCCCGTGTGAAGATGCAGACTCATGCTTGTCTTGGCTTCCAGACTGTCTTGGCTTTAGAATCCGAAGGGGCAGGGAGGCGGCTGCTCTGTCATAGTTTCAGTCATGAAGTGGGGAATGACTAGAAGGTGGAGGAAGGTATGGTGGCCCTGCAGATGTCATCTTAACACTTGCAGGGCTGATGGTTGAAACGTTTGAGTACTGCTAGGGCTACAGAGATCTGGACTGTGTCGTGAGATCATGATCAGGTTAGGTCAAGCTTTACAGTGATGCCTGACATGCCCGCTGTCCATGAACCATGTCTCCCCTCTGTCTTAGCTACAGTTAATGGGCAGAGAGAGAGACAAGGGTGAGAGAGCAGACCTTCTGTGTCTTTTTGAGCCCAGAATTGTGGCTAGCTTTTGCCAAGGGAAGAAATAGGTTTCCCTAGAAGACTACTGTTGTTCAATGTGTTTTCCCCATTCCAGTTCTTCAACCTAAAAGCAGCCTACTGAGTGTAGGTTGTTGTAATTATTCCCTGAGGGCAAGCCTCCCTCCCCATTCACACTTGAAGGAACAAGTCAGGTTATGTGGCTAAGTAAGCCTGTCACACTTGAAGGAACAAGTCAGGTTATGTGGCTAAGTAAGCCTGTCACACTTGAAGGAACAAGTCAGGTTATGTGGCTAAGTAAGCCTGGTTTTTTGGGTTTTGGTTTTATGTGTGTGTGTGTTCAGCTCATTTTGCTATCTGACAGCAGTGGCTGCTGAATATGCGTAGAAATTATCAAGCTGTGGCTAGTGCATGACTCTGGTTATTACTGATCCAGGTCCTAAAGGTCAGAGGGCACTGGCTGCCTCAGGGAGTTGTCATTAGTGGGGCACTGGTGCCTAGCCTCTCACTAATTTTATTTTTTAAGACTTGCT
MSTRG.60357.1	ENSRNOG00000014415;ENSRNOG00000014522	0.283	0.000	0.005	-4.261	down	GCCATCTTGGTGACCCTATGCCGGTAGGATTGTGCAGTGCCATGTAAGGTCTCGGTGTGATGCAGGAGGCTGCTCTCACATATACGGTTATTATTAAATGGGTACTGACCCCCGATGCTGGTCACAAGAGAGATGAGACCTTGGGCACTGTTCCTCAGATACCATCCATCTTCTTTGAGACATTGTGTCCCTCTGTCCAGGGTCTCACAGACTAGGCTAGGTTGGCCGGCTTTTGAGTCCCAGGATCCTCCTATCTCCGTGTCCCTAGAGCTTATATGACCCAGACATTCTGGAAAGGTTGAGAAGGCCTTCTGTATCTTCAGAGCACAGACACGCCAGGGAAAGACCAGCAAACACAGGATTACTGCCAGAACTTAGGAAGGGATGAATAGTAACCGTGAAGGAAGGCACTGAGCCGGAGAGAGAGCGTTCAGGAATGAATCAAATGCAGTCCTGTAGGGAGGTATGTAAGTGACAGGCTAGAGCAGTTACAGTGGGCAAAGGCTGAGTCCTGGGGTGATAGACACGCTGCTCAGGTCACCTTCAAGAAGGTCTGGTGTTAGCCCCCAGGGTCTCCTGTGGCCCGAGAACTGCCATGAGCAGTTTCTCTAGAGAGGTCTAGACTCCCAGTGGAGTGTCCATGTCTGGTTGCTGTGCCCAGGGTACCAGGTCCTGTTCAGGTTCTGCCAGGCTGGTCCCCTACTCCCTCCCTGGGAGGCAGACCTGCCTCACCTCCCTCTTGGAAACTGCCAAGGTTTAGCCCCCGACCCCACCTCATTTTCTTAAGACAGAGTCCCTCACTGAAACTGGATCTCACCGAGTCAGCAAGACTAGCTGACTCACAAGCCCCAGGAACCATCCTGTCTCTCCTCTCCCTGTATCCACCTTGCCTGGATTCTCACATAGGTCCAGGAGATATGAACTCAAGCCCTCATACTTGCTTGGCAAGCGCTTCACCAACAGGTCCATCTCCCTGGCCCCAGAGACAGGGTCCTGAGACAGAGCCTTAGGCTCCAATAGAGCCAGCTGTCCCTGCCTCACACCAGAGCTATGTGAATGTGGAAAGATAAATATCCCATTTGGGTCTTCCTCATGGCTGGCG

							ATATGGAGCAGGAAGCTCGTATCATCTCTGGAGTCCTTCTGTGGTCATCAAACATGTCCTGCGTTCCATCGATTTCT
MSTRG.60439.2	ENSRNOG00000017342;ENSRNOG00000034226	0.620	0.000	0.000	-7.841	down	AAACTTTCCCAAGAAGTCTTAGTGGGACAGATCTTTCCATCTGTCGTCAGCAAAGGGAAGGTGCCTGGTTACCACCAACAGGATCATCTTCCTGATTGGCAGAACTCCATTCCCCCTGGCTAACCCTCACGACTTCAGAAAAATCCCAGAAGTTCAACCTTGGTTTTCTTTAGTTTTTCTTTGCCTCCCTGGCTCTGGGCATGGGAGCCAGGACCTGAGCTCCACCCCCAGCCTCTTTGTGTTTCTTTATTGAAGAGATGTGGGAGAAGCTGAATGGCTGGACGTTGTGCCTACTCCTATTTATGAAAAGAGCCAAATCTACTGGCCTCTTAAAGACACGCCTTTCGGAAGACAAACGGGACTTAGCCTGCCACTCGCGCCTTCAAAGGCCGGTCGGTCCACGGGATCTTCGCTGGGACTCTGCTACCTTAGGCAAGACCAACTTTGAGGGAAAATTTCTCTCCCAAACCACTCCTGCGCGCAAAAAGGCCGCGCCCTGGTCATCCTCTGCTGGAACTGTTCTGGAGGGAACCCGCGCGGCTGTGGGGAGACCCAGGGACGAAGGGGCGCTCCTGCGCCTGCGTACAGGAGCCGAGCGAAGAGGGGCGCTCCCAGTAAAAGTGACAGCTCAAAGACCCGGCTGGTCGCACATCACGTGCCGCTAAAGGGGCGGGGCGAGCCCCGCTGACTGACAGCTCGCTCCGCCGCGCCTCAGAAGGACCCGGCACGGCTCACCGCGCCCGGCGCATCCGCGGCAGCTGCGGACCCGACGGCGTCCGCCCCCTGCCCGGTCCCGCGGGCTGCCGCGGCGAGAGGGCCTCGGGCTGCGGTGCTGCACTCGCGAGGGGCTGTGCGGCGTGGCTGAGCGGGGACCGGTGAGGAGGGGTCGCGGGCGGCGTGGGCGGGTCAGCTGGACGGCCGGTGCAGCCCGCAGGACTGTCACTCCGCCGGCCACAGTGGAGGGCCTCGAACCGGTCAGAGGCCACGACCCGCGCCGGGGCCACCGGGGAAGGCTGGGACACCGAGGCCGCTTCTAGGATTTGGGAAGACCAGTCTTGCACTGGTCTGCGCTCTCCCGAGTTGCGGGTTGATGAGACGTGGGAAGGTGACAAGGGGAGGAGGAAGCAGGCTTGGCGGTCCATTACCGCACACGGGTGCGACCTACGGAGTGAGCGGGAGCGCGGCTCTGCGGGCGCAGGTGCGCTGGCACGGGCTGGGCCCCCGACGTGGAGCTGTCCCGGTGGTGAGTCACAGGTAGAGGTGAGCTGGCAGCTGTCAGGCCGGGCTACCCTCGCCCTGGCTGGGGATCCAGCCTGTAACCCGATGCTGAGGTTCTGATTCATTCCCTACGATCTGCCTCCTCCCTAGTCCTGAAAGCAGGACAGCGACATAGCATGAACCCCAGGACAGGAGTTGAGTGAGCCACATCCCTTTCCTCTTTCCCACACTGTGGTCAGAAGTAAGGATCTTTAGGCCTTTGAGGACCAAGATGGCAGATAAGCTGTTTCGACGGGCTTCCGTGGAAAAAGTTGACACCCGAGATTTTCTTTTCCCCTCAAACACTGGTGATTTCTAGTTGGCGATGATGGGCTATGTCAGTCCCTGTTGCAATACAGAAATTTCTTGGGATGGTTGTCTTTTGGATGGTCTGTGTCAGTTCTTGAGAATGTCACAAATATAGCCATATCACACGCCTTAAAGAAATTTAGCATCTCCACGGATGCTAAATGCAGCTCAATCTTCCTGACAATATTTGTTTCTTCAAAGGTAAGTGGTTTTATAATAACCCCAAATGGCGCTGAGGGAGCTTCTTTTAGGACAGCTCCCCAACCCCCTGATCCCCTAGGGAGCTATAGAGTGGGCAAGTCATGTGAATACCTGGCTTCCTTTCCTTTTCTCCCTTTCACAAAAGGAAAATCATTGGGTTACAAGAAACATCAAGGGTCTGGAGTGAAGGTTCAGCAGTTAAGAGCACTGATCTTTGAGAAGACCAGAGTTCAGTTCCCATCACCCACCTTGGGGGACTCCCAAACACCTGTAACCCTAGCTTCACAGAATCTGATGCTCTCTTCTTGCCTCCTCAAGCACCTATTCACAGGCATACAAAAACAGTTAAAAAATTAAAACAACTAAAAATGAAATTTCAAACATCAGATGAAATGGATTAGGCTTGCACTGTTAGTACTCCAGAAGCTGAGGCAGGAGCATCATGGGTTCAAGGCCATCCTGGGCTGTATAACAAGACCAAATCTAAAGAGAGGAATCAAAAGCTGAATGACAGCCCACTGCAGTAAGGAACAGCAACAGGAGGATGACTTCCACTGTTATGGTGTTCTCATGCTTGGTAGCTTACAGAGTACTTTCAACAGGATCTCCTTGTTCATCTTCACCATTAATTTGGGAGTGGCAGGTGACATTCCTCCTGTGACACAGATGAAGAAACCAAGACTCACTTGTCTAGGGCCACTTAACTATTGGAGGCAAAGAAAGCGCCACAATAATTTGGTTGCTAAAAGTGCTGGGTATAATAGATCATCACCCAAGAGGACATGAGATGGGTGTGAACAGATTACGCCTCCTGCTGGGCTGGAGGGCTGAGTGGAGCATCCTTCTCTGCTCTCCCCGCCTCACTGACAGCCTGGTTGGCCAGCCAGCATCACCATACTG

							TTACCTCTCCCTACACCCTCTGTGGCAACTCAGATTTGTCTCCAGGCTTTGAGAAAGCCAGTAGGAAGT<br>GCATTGCAGAGCATTTTTGCTCAAGTGCTTAGTTTCCAAACAAGTTGGGTGTCCTCAGCTATGATGCAA<br>GACAGCATGGCAGTTCTAGGGAGACGCGCCCCAGTGACTTTTTTTTTTAATCTCCCAACTGGAAGAGAA<br>GTGTGGTAGAATCCATGGAGCTGCTGCCGGCTCTGCTGCGTTCACCTAGATTTTGTACGTGGCATTGTTT<br>GAGGCCTTTTTTTCCCCCTATTTTTAGCACACACAGCTGTTCTTGTTAGGGAAGAACAAGCTTTTTATCC<br>CATCCTCCAAATCCGGGCAGCACCACAGGACTGCAGGGAGTTACCTTATGGCTGTTTCTGCAGGTCAGT<br>TCAGTGGTTCTGGCCAGACTGAAAGCCAAAACAGTCCCGGGTGCATGTATCAAAGCCAAGGTCTGACC<br>TGAAAGCTTTCATTTACTGCATGTCTCAGGGACTGTGTTCTTTTCTCGATAGCACATTTAATGAAGGTCA<br>CAGAAAGCCATTCAGAGTACACGTTGTAAGTTGCTACAGAGGTGCAGGAATTTGAGCTGGTAAGCCAG<br>GAACATCTTGATGACCAGAATTACAGAACATTCAGGGCTGTTACTGGTAAGATCCCAAGAAGCAAAGG<br>ACACGCAGTACAGTTAGAAAGGCTTGTTTGAAGAATGCCATAGTCTCTCAGGATGCAGCTCAGACTTAT<br>CTTAGGCTGACGGTTAGTGACACAGGTACTGACTGATCTCGCCTGTATCTCTAGTGTCAGCAGCTACCC<br>CAGTGATTCCCAGAGTCCCTCCTTACGCATACCTAGTTTTCTGTGCCACAGGGCCTGTTCACTGGTTGTT<br>CTTCCTTCCAGATTTCCTTGTAGGCACCCGCCTATCATTCAGGTAACCACTCCTATGTCACATGTTAAAG<br>GAGACCTTCCCTGTTAGTCAAAATGATCTTCCTGTTTCCTCTGGTCCCCAGCCCACTCCCTTCAGCATAT<br>TGCAGTTTTCTTTATCTGCATTAGAAATAACCTTGTTTCTTTGAGAACATTTTTTTATTATCTGTCCACTGT<br>TTGCTGCTGTATCAACACGCCCAGAACAGTGGCTGGCTTGTAGAAGTGCTCAGTGCATGCTTACTGTAG<br>GACTGTTTATATCAGAGCCTTACAGGATGGGAGGAGGTGCTACTGAGGTGGACACTCGATTGGCTTCTC<br>TTATGGGAGATAGCAGGCTAGCAGCTGACCAATGGGCAGAAGAGTTCAGGCATGGTGGATGGGAAGAG<br>AAGCCAAGTGACGGTCTGTGTCAATCTCCTGGAGGAGCGAGCTATAACAACGTTCTATAAACTGGGTAT<br>TTCAAAACAATAAAAATGGATTCTCTCAGAGGTTTACAGACCAGAAGGACCGTTTCCTTTTCATGACTC<br>AGGTTCCATGTATCTTTCAGCTGCTACTACTGGTTGCTGGTCAGCCCAGGGATCCTTCCTTTATAGCTGA<br>AGCACTCTAGCTTCTGCATTTGGGGGTCTACTATAGTCTATCGTGGTCTCAACAAGTCACATCCATAACC<br>ATCCTTTTCCAAACATATTAACACATATCGGGGGATAGAACTTCAATCGACATTTTTGTGGGCATAATTCA<br>GCTTAATGTCTTCCAACTAAAAGATTCTGTGATCTTTAGTTGAATGCAGTGGTACTCACCTAACGCTTGG<br>GAGGCTGAGGCAGGAGGATTGCAGCAAGTTTGAGATCAGTATCATATTAAGTTTGAGGTCAGGAATGGC<br>TGTGGAGTAAGACCCTATTCTAAAACTCTAACAAGCAAATAAACTAAAATTTTTAAAAAAAGCCCACAA<br>AATATAGTGACATTTTAACAGAACAAATTGGTAGAGGTTGCTTGGCAATGTTTAGGAACAATTACAGTG<br>GTTTAGATGCATTCTTGGAACGCGTCCATGATGCTCCCATCTTCAGGGGATGGATGCACACTGTGCTTCA<br>TGGTGAGCCCTGGGACAGGATTTCCTTCCTTGACTTTTTGCTGGATTCCGTCCAATGGAGTTGAAATAG<br>CTTGTCAGTGTCTAGCATCCCTGTTCTCTTCACCGTGGACTTCACACCTCTGTGTGGAGGAGGTGCTGA<br>CATCAGGGAAGCTATGCCCTCCCTATATCATTGCCAAAGACGGCTACTGAGCCTGTTGGGACATGCAGG<br>CTCTGACCTTCACCCTTGACTACGTATGAACTCTTCAGGAGCTCCTGCCAGGCTGATTTGTGGTTTGTGG<br>TAAAAGAACCTTGCCCTTCTCTCACTAGCTCTCTAAATCCTTATCTTGTTTAGCTTTCAGATGGGATCTTG<br>TTGTGTAGCCCAGGCTAGCCTATTGCTCACAGTCCTCCCTCAATCTTCTAAGTGCTGGGATAACAAATCC<br>ATGCCACAATGTCCAGGTTGCTGGGGTTTTCCAGTTTTGTTTTTGATTGGATAACCCATGGCTGGTATCA<br>AATTTCAAATGACATACCAAGATGCTAGTGACAAGTTGCTGTGATCTTTCCACTGTCCTCCCCATCTCCA<br>CGGGGACCATTACCGTCAGCATCTTGGGGCTCCTTCCAGAGACCTGATGCTCTTTTTATGCTTTCATTCC<br>CAGTTACCACAGGGCTGTGGTTGTCTCTCCTTCCAGTCTTCTTCCCTCCTGCCTCTTCTGTGCCCAGTGG<br>ACTGGCTTGTTCCTATGTGTTCAGAAAGGATGACAAGATCCCTGCCAGGAGGTCTATATTGGGAAGGAG<br>CGTGCTAGAAGAAATGATTCAGTGGTGCCACATTTAAGACAAATGGGAGGAAGTGCTTCTCTGGAGGG

							GTGGCTTCCAGCATCCTGTTGCTTCAACCTCATTATCTGGGCTGGTGCTGACAGGGGTGAGGAACGGCCCTGGGAACAGGGACAGCTGATCTTAGGTTCCAAGGGTGTTCTGAACCTTATTATAGCTGGTGATTATTATGGGGTGTATCTGCCGTGACAAGTACCATGCCCAGTTATAGGGTTATCATGTCAGTCCCCACAAAAACAGTCTGAATAGCCCCTGGTAATATCCCAATTTACAGCTGAGACTCCGGGTATAGAGGAGTCAAGAAATTTGCAAATCCATGTTCCCTGGCTTTGAATCTGTCTCCTCAGCTTGTCTGATCTGCTGCTGCCCTGGGCAAAGCCAAGGTCACCTGTGAGACCCCCTGTCAGTGCTTCTGACAGGTACTGAACACTACACTGGTCCTCTGCCCTCTGTGCCTGGGCGTTCCTGGTGCCCTGCCAGCCCTGGGATTTAGTCTCAAGGCCTGATTGACCCCTCCCCATCAAGAGCACTGTCTCAACAGCCATGTGTCTGCTCAGACCGGTCTTCACTCACCCTACTCACTCTTTTGCCTGCCCTCAGCACTTCCTAGCACTTTGGGGGTGCAGAAGTGCTGAGAGTGGAAGGCTACAGATGGCTTGAAAGCCCTGGCCTCTCAGAGCCTGGCTGCCATCGATTCCTTCTGTGAAGATGCTTAGCTCTCGGCTTCAGTCCTAAGAGTTCACTCACTCCTCACCCTCTCTTTGCATAGACTTTAAATAGCTGCCTCCCTCCCCTCTTTCTTCTCTGTTTCCTTCTTTCTTTAGGAGCTTGTAGAAGTGCATTTTACACATTATAGAATCTACCCATTTCACGAGTCAGTTATTTTTAGTAACGCTACTGAGTGGTAAGACCATCGTCATAGTCTAATTTTAGACCATTTCCATTGCCCCCAAAAAGCAAGTTACTAACGATAGCAGTTCCTCTTTGTCCTGTCCCCCACCCAGGAGCCACCATTTTGCATCCTCGCCATGGTTCTGCCTATTCTGGGCACTGGGCAGTAGGTGGTCCTTAGTGATAAGTGTCATTTTCTAGCTTGGAGCATGGTGAAGCATGGACCAGTCTTTGCTCCCATTCAGCCATTATTTTGTCCTGAGAAAGTGTGGTGCTTTCTGTTCCTCTGTCTTCCAGCATCATCTGCTGTCTCGAAGATATGCCTCTGCAGGGGAGCGGGGAGGGGCAGGTGAATGAAGGTGCCCCTAATGTTCTCGCTGACTCAGCTGGGGACTTTAATTTGGGGATGAGTCTAACTTCTAGGTATCTCTGGCTGGTTTGAACCCACGGTCCTATCGCCTCAGCCTCCCCAGTGCTGGAATTACAGGCAGGTGGCATTAGCCTGGCTCAGACCTTGCTTGAATTGCATGAGTTTGTCCATGAAAGTCTTCTTTCTGTCCCAGGATCCTAGTCAGGCTCTCTGTTGCATTTCTGTCATAGTCCCCTATGCTGGAACCCCTCTCTGGATGACACACACACACACACACACACACACACACACACACACACACAC
MSTRG.60459.2	ENSRNOG00000017905;ENSRNOG00000018226	0.000	0.271	0.000	4.663	up	GTAGGTACCTGCAATCACATGTGCGGGCACACACACACACACACACACACACACACATGCACACATAAAAATAATAATAAATCTTTTCTATGCTAGGTGTGGCGGCTCACACCTATAACACCAATATTTGGCAGACTTGGTTCTTGAGCCTTTAATTTCTCAAAATTGGAATTCTGGTGGTGTTGGCGAGATGATTTACTCTGTACTCATGGCTCTGGAGTCCCACCAAACTGTGCAGATCTGTAGCAGTGGAAGTGGGGGCTAGAAGAGACCCCTTCAAGCTTCCCTCACCACTTTCTACACTGGCAAGGGGACTCTATACATTTCTGTTCACAACTCTGAGTGTGTCCCAAGCTCCAGTTCCGCTAGCATAGCCAGAGCACCCCTTTGAAATCTCTAGTTTCACAAAAGTCTGTGATCCTGGCCTTAGCAATGACCTACCCCGACCTCCCACAATTCCCCCAAGATCAGCACAAGCTCAGCCAGGCAATGGAGAGCACTTCTGGAGTGTCACAAGTGCACACACGAAGCATCGGGTGAACACCAGCCAGCTTGGCAGGTAAAAGAAAGCCCTTGTTGCACAGGCCAACAACCTGGGCTCCATCACCTGAACCCATAGAGGAGATTGAGTGAGCCTTTTACAGGGGATATCAAAGACCACTGGAAAACACAGATATTTACCTTACAATTCATAACAGTAGCAAAGTTACAGTTATGAAGTAGCAACAAAATAATTAATTGTATGGTTGGGATCAGCACAACATGTATTAAAGGGTTGTAGCATTAGGAAGTTTGAGGATCACTGCCATAGAAGAAGCAGATGTCTTCTAACCTCCACAGACATGCATGCCCCCCCCCCCCCCGCACGCACACATATACACACACACAAGTCACACAGGTCACAGGGCAGTGCTCAGGGAGAAACTGAAACAAACTTCACCTATCCTGTGCGACTAAAGGTTACTTACTACTGAGAATCCTCACAAGAGAAAGTAAGACAAAAG
MSTRG.604	ENSRNOG0000001	0.074	0.487	0.005	2.942	up	GGGGGTCACCACAACATGAGGAACTTATATTAGAGGGTCGCAGCATAAGAAAGGCTGAGAACCACTGGGCTGGGGTGTAGGCGGGCTGCACTTTTAAATGTGTTTCTCCTTCAGCGGGAACCGTTTCTGAGTATTGCTGACCTTGTCTAGTGTGCAGGAAGTGTGCACACCTCCCTGGGCTTAGAGTCCCTTCCATTCACGGCTGT

73.1	7431;ENSRNOG00000021714						CCATTCCACAAGCCTCGCTGTCAACAGAAGACGTGGACTGGATATGCAGGAAAACACCAGCAGCTTTTGCCTCAGATGGAAACTCCTCAACCTGCCAAGGGTCAAGTTCAGCCAGCCACTGTGCTGGAGGTTCTGCTGGAGAGGCTGAGAAGCCAAGAGGTATGGGAGCCGAAGGCCTGGAGGATTATGGGATGTCCCTGATCTTCAGTCCATGCTGGAGGCTGATGAAGTCGGAGTTGATGTCAGGGAAGGATGGTGACAGTGTGATGGCGGTGACAGCAGCAATGGGGTCAGAGGCACAGCCTGGCACTAGCGCGTCCTGCTTGACCTTTCCTTAAAGATCAGCGGCAGCGGCTGATGGGTTTCGTGGGTCCCAAAGGAGACCCTTCACACAGCCCGAGCTACGGGCTGCAGAGTCCCTAATTGGCGCAATGAATCACCGCGGGCTCCTGCTGGACACAGAAGAGACAGAGGAGCTGAGGCCTATTGTGC
MSTRG.60678.1	ENSRNOG00000026964;ENSRNOG00000053560	0.781	0.000	0.037	-3.684	down	CCCGTCTGCTTATGAACATACCAGAAAGCATGGCTTCCACAGTATCAGAGGATGGGCAGGATGCTCCTATCAGCTCACACAGGAAGCGTAGGTTCCTGAAGAGCCACGTGGTCATGGCTTCCGTGTCAGGATGGCTTTGCAGTAACTCTTGTAGGCTCACGACATGGTGTGTGTGAAGACGCCACACAGCTTCCAGCAACAAAGAGTTCTGTGCTTTCCATAGTTCTTGACAGAAGGTGAGTCGGGAGAACATGTTGTGGGCCAACAAGCGCTGAGCGCTC
MSTRG.60904.2	--	0.288	0.000	0.000	-5.223	down	GGAAAAACGGTGGGTGACTGAGTTAAAAAGCACAGAACACCCGCTGTCAACACTCCGTGACCTTCCTTGGCTCATCAGTTCTCCTAAGCCCAGGATCTGGTCTGAATCCCCTGGTCCTACAGATGAATTGCACAGTCGGGCTTGAAGAATAGCACTGAATGCCTACGATCAAGCATGAGGGCTTGAGGACAGCTCCTGTTGGTGCAGGACAAGAACTTTCCGGACTGTGTTTTCTGGTTCTCATCCTGGCTATCCGGGTGTTGTTGCCCGACACCTTTGGTAAAGGAAAGGCAATGCCCAGGTCACCTTCGCTGGAAGCAGCCTCAGCACCCAGACGTTCTCTTCAAGGGGGTCTCTTATTCCAAGGCCTTTGTGGATCCAAAGAAACACCTTTTTTCCTCAGAGGCTAGACATTTCCTTTTATCCCTCGACTGATTCTTCATTTCAGCCATGTTAGGAAGGCTTCCTTGTCTCAGTTGGAGACTAGGAGCCGCTTCAAAGACAAACAGCTAAAGAACAAAAGCAGGAGATGGGCCAGCTGGGTGTGCGAGAGTCCACTCACAGGGGGGGGGGTTGTTAATTTGATGGGGGCAATGTTAATGCATTGTTTGTTCTTATTTGGGTTTCACTGCTGAAGGTTTGAAAGCCAGCCTCTTGAATGCATTTGCATGTGGGAACATCTCAGAGCAGACCTCTGGTTTCACCAACCTCCCAGAACTGTGTATTTTGACTTGTGTGGCACTCCTTAAGATACTCTGCACCCTTCTAGCTCAAAGGAAGCCTGCATAACTGTTTCCCAAGTCCTGCTGGCTCAGCCTAGCTCCAATCTTCTTGAAGCTCAGTGCTGTCTGGGGAAGGAGTTCTGGCTAATGGAGGGTGTCTGGCCAAGATGCATCCCATTGATGACCTACTGAGCTTCCTGGACAAATCTTCAATGTCCCTGCTGGGTATGAGCACCCGGGACAATCTTAGAAGGCACATACAGAAGATGCTTCCGGAACCTCCATCAGCCTGGATCCCAAGGGATGAATAGAGCCCCTCCTTCAGTGCCAGGCCACAAACACAGCAGCATTGACAAGACAACAGCGTTTGCTGCCATGGTAATGAATACTTCTGACACAGGCCAGGGATCTGATTCTGAGCAGAAGCTCAACTACTTACAAAAAGTCAACCGCAGAACAGACCCAGTGGCTATTAAATGTCACAAAAATGTGTAGCAACTGGCATGACCTTAACAGTCAGTCAGGAGAATATATCTTCTTCAAGCCGAGATCACGAAAGCTCGTGATTGCCCACACACTCAGTAAAATGAAACCGGAGGCACAGAGGTAACCCCCCCAACCCCAGGTTCTACCTTATTAGTAGCCTGGGATGAGATCAGCTTCTGAAGTCCTCTTCCCGGATACTAGAGGTTGGGGGTGACAGTTAATACCGTCTTGAGGCTGTGGCCCACTGATTGGTCTCAGCGGCTTATACTGAGCACTCCGAAACCAAGTCAGCTTAGCTGGGAGGAGCATCAGGACCAATGATCTTCTCTGTCAAGTGCACGGGTGTTGGGGGAGGGGGCCCTCCCTGAACTTAGCCCAGCATGTTCCAGCATGTGTTTAGCACGTGTGGAAAACGTCTCTCAGCCTCTCTTGGGATGCTGGCTGGTCATCCAGTCTCCTCCGTCTCTTTACTGGCAAGGTCGAGCTAAACTCTCCAGGTGAGAACCCTGAAAATCTGCAGATTCTGCCATTTTTTTTTTCATGATCCGCCTTCGGGGATCATCCAGGAGGGTGCAGAGTG

							ACAGAGAGTATGAGGTCTGATGATCATACCCCCTGGGACTGGTGGCTAGCATAAGCCACCTGGCTTTAGCGTCTTGTGTGCAAAGTGGGTGTCACAATAACACCTTATTCACAGGGAGGGCAGGGAGACTGGACCCAATAAGGCTGGACACAGTGAGCACTGGTATGTGTCTGTCTGCGGGAATGTGATAATATGACCTGTGGGAATTCAGAGGACAACATCAGGTGCCAACCAAAACTTTTTTGACGCAGGATCTTTTGTCTACCATGCATAGGTCAGGCTAACTGTCTTGTTGAGTTTCTGGAGCTTTCTGGACAGATCTCTACTTCCACCTCGCACTAGGACCTCTGGTTTTGCAGAAGGGTGTGCCGCTGTGTCCCGATATATGTGCCTCTGGGGATACAAATTTGAGTCCTCAGGCTTCCGTGGAAAGCACCTACCAAAACTAACCGATGAGCGGCACTCCTCTGTGGCCTCTGCTTCAGATCCCCTCCTCCAGTTTCCCTGTCCTGAGTTCCTTTCCTGACCTTTCTCAGTGATGGGTTGTGATGTGGAAGTGGAAGCGTAATAAACCCTTTATTCCCTACC
MSTRG.60909.1	--	0.000	0.566	0.004	3.943	up	GCAGAAGCAAAAGATAGGAAGCAGAGCAAACACCTATGGGTGTGTGATGGGTAAGGTCGATTTACTGTGTGGCCATAAATAAGATTTGATACTGCTGTAAAGATGGCAAGACAGGCAGCTCGGTGAACAGAACACTGGCTGACGTCAGCCTAACACGGGTGATTTCACTGCGTCCTCTGGGCCATCTTCCATCATCCTAAGAGACTGTGAGTCTGTCCGTTTCTGGTCCATATGGCTTTGAGGTAGCTGTGACCAGAGACAAATAAACAACTCCCAC
MSTRG.61053.2	--	0.000	0.271	0.031	3.453	up	AAGAGAGACAGAGAGTGACAGAGAAACAGTGACAGAAAGGGAAAATTGTAGGTTATTGTTCCATCGCTTCGATCACTGTCTGTTGTTAGCCATCTTGGCAACCATGCCCCGAAACCCTACCTTGGGTAAGATCAAGCATAATGGCAGCAACCATATAATTCCTGCTTAGTTGCACTGAACACCCCAGTCATCACTACCTGTGGAGAATTGTAAGGGGACTCCTCTGATCTAGAGAGTAGTACCCTTGAATGAAATGAGAAGCACAACAAGCATTATTTGTTTTGAAATATATGATGTGGAAAGAATTATATGTGTGGATTTACAGACTGAAAGACAGAGAGACAGACAGACAGGTCGGACAAGACAGAAC
MSTRG.61378.1	ENSRNOG00000014795	2.303	0.004	0.000	-7.054	down	TTTCAACTTTAATATTTTTTCAGCTATCAGGTTTAGCTTTCAACTCTTAGGAATTTCCCTAATATATATCTTCATAATGATAGATAAATTTCATTGTTCCTATTAATAGCCAATGATTTCAGATCATCATAATTAGTAAAGCACACTTCTCACCATCCCTGCTAGGTATCCATGAGATTTGGGGCTTACTGCACCTGCTGGCATTACAAAAGGAGAACACGGTGTCCAGCAACACTAGGACATGGTGTGGAAAACTGAGAACTCTTACTCTGTTTATTTCTTTCATCCAACAAATATGGCGATCGAGCCACTATAATGTGTGAGAGCTCAGTGAGACACTGATAACAAGGGTGAGATTGCTAAACCACCTGTTCTAGGAGCTAAAAGTAAAGCCTTGAAAACTAATGCTCATCTCTAGTAACTTTGCTGAGAAATCTGTTTTAAAAAATCATGTGAAATTAACCACAAATGTGAACTGGGAACTAGAGTCAGAAAAGGTCTTACCTTTTTTTTCCTTCTTTCTTTTATTGGGTTTTTTTTAAATTTACATTTCAAATGTTATTCCCTTTCCCAGTTTCCCAGGCATAAGCCCCCTATCCCATCCCCCTCCCCTTCTTCTATAAGGGTGTTCCCCTACCCACTCGTCCCCTTCCCTCCCTCCCAACCGTCCCCTACAGTGGGGGATCCAGCAGCACGTTCTAGTCTCCTTCTCAGTCTGTTCTCCTTCTCGGTCTGTTCTCCTCCTTTGTTGTTCCTTCTCCTTTCTGGGGAATCCTTCCTGTGGTCTGCTCACTCACCGTCACGAGCTCACTTCGTGGAAGAGCTTCCCGTCCATCTTCAAGTTCTTCGCGGAAGCCTCAGAAGCCAAGAGCAGCTCGCAGAAGCCGACCCTGACGCGGCGCTCGCACCGGATCAAGCACGAGGAGCTGTGAGCGCGCAGCCGCCCGCCGCGCCCGCACGCTGCCCGCAGCCTCCCGGCCCCCATTAGATTGCTTTCTTCCTCCAGCGCAGGGTCGTGATGTGTCCGTCTAAATAGCGTTTTGCATTATTTCTAGATGAGTGCAACTGTCAAAGCAATATGGCTTCGGTGGCCGCGCTGCCAGTGCCAGAGCGGGCCTCGCGCTGCAGGGCCCGGGGCCGACCCAGGCAGACGACCGAGGCAGACAGCGCCGGGTGCGCCAGGGCCTGGCGCTCCTATTGCCAGGCTGATCCGCGGGGCGCGGCTGGCCGAGCGGCGTCTGCGCGGTCCTATCGCCCGCTGCAGCGCGGCCGCTGTTGCCCAGCCTCAATCGTGCCCATTTTTATTCCCCTTTAAACCGAGCAAGCTTTTTTATTCTAAACCTCAAACTTCGATTTTTAACCGGGTTTTTCCCTCGGGTCATCTTATGAGC

							CCCTGTCTTTTATACAAAGCCACAAACCCAGGCACTAGAAATGCCAACTTTAAATTCAAAACATGATGGACTGTGTAGTTTGGAGAGTACAATTGTTCTCTCGTTGTAACGACTTATTTTCACTTCAAGACCCAAAGAAAAACTTTGCATAAAGTACCAAATCCGAATCCTCTTTTCTGCACAGTTGAATTCCAGTCTTTATATCTCTGATTTCAGGAGAGAATGTCCAAATGAGAAACATAAATAGATCTTTTAGATGTAAGTATAAGCCCCCATTTTCAGTGAATTTCCAAGTTACTTATAAGAGCTGACCTTACTATTAAAATATATAACGTGATGAATGACCTTGTAATTTAGATGATACACTTAAGGTTATGTGCTCCGGGGCAGAGCCGGGGTGGTAGGTTGTGATCCTTACTAACTGTACTCTAACCTTTCTTATAGTGTGTCCTTTCAGAAAATGACACTGCATACCTCTTCCTTCCAAAAGGCCAAGGAGGGTTAAAATGGTTATTTCCCTCTACAGTAGGAAAAGAGAAGCCTAGTGTGAATGAGTTTCTCTTGTCCAAAATGTTTAGTGTTCTCTTCCTGTGATAGGGTTCTGCCCTGTAATTCCAACAGTTAGGTCCTTTAACCCAACACTGTTAACAACTGGCCAACACTGTGAAGTACAAAGGATAAAATGTGTTGACATTTTACTGGCTTTCAAATAAAAATATGAAGTATTGAATTTTTTAATTAGTGAAGATTATATCAGGTGGATGAGATTGTGATTTAATACATCCATCCTTTTTAAAATGAATTCAACAAGTAATGTGTCTGCCTTGTTTGAGACCATGAGACAGTATAATCAAAATGAGTCTGAGTTACTAACACCAGGGAAGGAAATCTGAGCTCAGGGGGAATATTGTCATATGAACTGATTTTAGCAGGTTTCAAACTGGTAACATCTCATTCTGTTTACGAATACATACATTCAGTAGTTCTGTAGTGACAGTAAAGTCCTCAGTGACCATGGCTGGGCATCTTCTGATATTCCTTAGGCAGAAACACCTTAGGAAAAATCAAAACAGCATTTTATACAGCTTCTTCCAAAAATGTCAAGCACATTGTACTGGCATAGTGACCCTTTGCATTTCTGATTTTAGAAGGATTGATGAACTCTGAAAATAATGATGTCTTCCCAGTGACTGGGCTTAAAAAATTGGTGTGTGTTGAGTATATCTTACCAAATGTTATAAACTGTCTGCTGTTCTCAGCATGTGAAAATTTTCTTAAGAAACTGATTTTGCAACATTAAAGGGGGGAAACCTATGCAGTTTAAATTTGCTAAACTTCTGCAGATAAACTAAAGCCAAGTAACTATGCTCGGCTCATGGACCAAATAGACATCTCCTGTTTCTCCCTTCCAGGTTCCTCTGTGCAGCTCTGAAATCTTGTTTAGAAAAAGTCTCTGTTTCCTTGGGATGAGAACTTTATAATCACTGAATTGTTCGCTTGGCATCTGTCTAGTAAAGAGCTATTTACAAGATTACATTTTGTAAATGTCTGTTACTACTTGTGTGTCCCTGCTATAGCAACCCAGAAGGAGCCAGGCACAGCATCCCAGATCACCAGCTGATGAGTGACAAGTGATTCCTTACAATGTAGGATTTCTTGACCTGTTGAGTGGGTTGCTGTTGGTATATCTGTACTGATGTTATTCACTCGGCTTTCTTTGGATAACAACAATCAGTTGGCAGTACCCTGCCTGCATGGAGGCCAGAGCTGTTCTGGTCTCTTCCTATTTTTATTTTCTTAAAGAGATTTTAGGCACCTAGTGGTAGAAAGGTAGGGTGTGAGTTTTTTTTTTTTTTCTTTAACTTCTTATACTTCGTTCTTAAAGTAAATAGATGGAGAAAGACATAAATTGGAAATTCAAAAGAGTCTGATTCAGCGAGGCAGTTGCTTTGGAGCTTTTTAGGGTACATAACACACAGTCCTTTGGATGTTTGCTAGACGTCGACTTGTAATAATTGTCCTTTTCTCACATTACTGCCTTGTGCTTTCTGAAACGCATTTTTGTTTGTTTATACATCAAAAATAATAAAGTGCGGAGCCTCCTGTAATCTTGCTTACCATTTTAAAATAAAAATTTCCAACA
MSTRG.6164.3	ENSRNOG00000012690;ENSRNOG00000040249;ENSRNOG0000004	0.152	0.584	0.010	2.481	up	GTCTTCTAAGATGGTCTTTGCTCACCAACTGATAAAAAAAATCAAGTCCTGATTTAAAAAAAAAATCAAGCCCTGTGAGCTCCTTATTTAAAATCCGAATTTCTAGGAGTCTTCCTGTGAGACCTTTGGGAATGAAGGCGTGGGTTTGACCGCAAAGGGCGGAAGTGGGCAGGGCAAGAAGATAGGTTTCCAGGAAAGAGGCGGTAAAGCTTGGGGAAGACCTTGTGATTCGGACTACATAGAGAATCCCTGTCTCTAGAAAAAAAGAGAAGGAAGAGGAGGCCGAGGAGGAGGAGGAAGAGGAAGAGTTGTTCCTGCTTGTCCTGTGCAGGTTAAGGTTTCTGTTTGCAGTTTAAAAATAAGTTCCTGTTTGTGCTTGTGTTAAGTCCTTACAATTTAAGGTTTCTATTTGTAATTAAAAATAAGTTCCTCTTTCTTAGACCTGATATAAACAGCTTTGCAACCCCATCAAATGGGGTACATCCTTCCCCTTGTTCCTTCCTTTTTATGTATTTTTTTAATGATTTATTTTATGTCATATGCATTGGTATATTGCTTGCACGTAAGACTGTATGAAGGTGTCAAATCACTTGGAGCTGGATTACAGACAGTTGTGAGTTTGTCATGTAGGGGCTGAGGATTGAACCTGGATCCTCTGGAAGAACAGCCAATGTTCTAAACAACTGAGCCATCT

	2702						CTCCAGTCTCTCAATCTTTCTTGGGCTGTACAACAGCCAACTTTATTGCCCCTGTGAACATCTTATCTCTC CTATAAAAGGGACTTCAAGTGGCCAACAGGCACTGCTCTCTTAAAGCCCAGTTTAGGGTAAGACAGCT GGCTGGCTAATCACAGGTGCTTATCAAAATATAGCTTACTTTATTGGACAGTCATGGATTTGGTCCTTTCT CCTCATGACTCAAAGGTTTACCAGTAATCAATCTAGCAGTGTTGTCCACATGGTACTGACTGGTCCGGA GACATGAAAAGTAAAAAAAGAAAAAGAAGAAG
MSTRG.61930.2	--	0.061	0.379	0.002	2.943	up	CTGAACTCCACGAGGTATAACTTGAACAGAAATCAAGGTCTTTCAAGTTCCAGGGATGCTCTTCTCAGT GTGTGGAGTAGCATTTCTCACAAAGGGGTCGTCTAAACCTTAGTCACATTTTCTTCTCTTTTCTTTTTTA TTTTTTAATTTTTATTGGTTCTTTGTGAATTTCACATCTTATACCCCAACCCTACTCACTTCCATATCCACC CTCTGACCTTGCAACCTCCCCCACAAAAGAAATGAAAACAAAAGAAAGCAAAAAACAGACAGACAAG ACATTTCACAGTGGAGGCTGTGCGATGTGTCATAGGGTGCCACATAGTACACCCTTTTGCCCAAACAAA TTATTTGCAAATATTCATTGCAATGAGTCATTGGTCTGGTTCAAGGCCTTTGGCTTCTGCTATACTGTCCA TCCCAGATCCTCATTGAGACTTCCAGGATGTCCTATTGTTGCTCTGTGTCATGGAGATCCAGCAGCTTTG GTTCTGCACGACTGGCCCCCTCACAATGCTTCCAAAGCTCAAAGATGGGAGAGATATTAGGGTGAGCC ACCTCATAGCCCCGGATCTGGTTCTGGGTGGTAGCTGAGTAGATCAGGCTGCCAGCTCTCTCATGCTCTA GACACCATGGCCAGCTCTCTGGCTTTGCCTACTTGAGGGGCAGGGCCAGCTCTCCCTCACCGTAGGAG ATGCATGATAAATGAGAAATTTCGAATGGCCACAGCATGGGGCTGCTTCTGACTTCTAATAGGCAGAGG CTCCACTGGTTCTACGCAGAAGGATACACACACCCTGTTCGCACCCTCAGCTCATCCACAGTGGCTGGA ATCAGAGATGATGCTTCAATGGATTGGATTTTGTCTGCCCAGTACCATCTATTTCATTGGAAGTCCGCTGT AGTCACTGAACAGGTGGCCCTATTTCTGAAGCATTTGTAAGAAGCTTTGTAATGGTTTCTGACTATTGGA AAGGTCTGTGGTAACTTGGTTAGCGGTTTGCAGAAGCTTTTTTCCTCCTCACCTCTGGGTATCAGGCAG CAACCTGGTAGCAAACATTGATTTCCTGATGCATGGCCTTAAAGCAATTTCAGCAAAGGTTCCAGATGA ACCTTCTGTGGCAAGCATTGCCGTTTCGTGGGCGCAGAGTGGTAGGGCTTCTGAATCAAGCATCAGACC GGGAGGCAGGCTCAGGCCAGCAGGCAGGCCTGTTTTCCAGGCTGGTAGCTAAGCTGTTTCTCTTCTGC ATCAAATACCAGAAGTAAGCCTTTCCC
MSTRG.62035.1	ENSRNOG00000050343	0.263	0.000	0.000	-6.156	down	CTGTGTTGTTACATTGCATGTACAATACGAAAGGTGAGTGCAACACTGGGTGAGCAAGAACTTAACTCT GAAGTCAGGAGTGTGAGAGGTAAGAAACGCGGCAACTACGATGCAGAAAGGCTGCACTAAGGAGCAG AAGGCGGCGGTCAACAGTGATGGAGATTCATAGTATTGCTATAAATGCCTGTTTAGGAGTCTGCTTATAC TGCTAACACTTGAGTTAGCTGAGCGTATTTGGGTATATAAATTTGCAAGAGATGGCATTTATAGTATGCTG TGGACAATCCACTTAATGTCGTGGTGGGAAAGCCATCTGTGAAGCCAAGCACAGTGCATAGTGTTCTGC CTTTATAACATTGTACAAAGCTAGTAATGGTGAGTCAGTCTGCCACTGTAATTCTCACTTACCCAAAACT GGTGAAAAGAACGTGTCGGTTCCTTTAGCCCCAGTGGGTTTTACTCATGCAGCCTTCCCTTTCTCAATA AACTATGTATTATGGTTTCACACTTGGCAGTGCATACAGTACAGAGAGAGATTCAACAAGGCAGTGGGG ATTGTGTCAAAGACATTGTAAGAAACTGCTAGAGGATGCAACCCTTGTTATATCACACAAATGAAGGGG AAACCACACAGAAGGGAGATGCCACCAGTAATTGCAAAGACTTACAGGAAAGGCAGTGGAATGTGCA CGGAGAACCTAAAATACCACATCATACCCCTTACATACTTACATACAACATGCAAGGAAAAGTGTTTCCT CCTGTTAAAACTCAACATCCACACCTTCTGTTTTTAAACATTTCAAGATCTTTCCTACATACTTCCAATGC CAAACTGAGCCACTGTTCACTGTAACTGAATCTGCAAAGACACAGATGAAATGAGACTCCCCTCCTCTC AGAACAAACGGCTGGGAGTCAGCGTGAGGCAGCAGTCGTGAGGACTGTAGCATGCAGCCTCAGGCAT GTTCTGTGCATGCGCCTTACAGAGGCCCAGTGAGTGTGCTCTGAAGACACAGTGCACTCATAAACTTGG AACCCAATCCATTAAATCATCATACACATGCCAAATTGTACATTTACTGTGGGCAAAACAAAAAAGTTAA AATAAGTACATCTTTAATTCGGGGAACATTTAGTGGAGTCCTCTCTCTCTCTCTCTCTCTTCTCTCTCT

							CTCTCCTTCTCTCCTGCCTCCTACTTTTAAGTTTCTGTTTACACACAGCATAGTTTAGATAACAATATAAA<br>CTCATTTTCTAGGAAAACCTTTTAAATTACATTATTTTATAGGATTAAAAAATACCATTGCCTCATATTTCG<br>AAGCAATTAGGCAGGATTTGAATGTGCTTTAACCAACAGGAAGGCTCGGGCCTGACTACTCCAGTAGTA<br>GGACATGAGCGTAGCGTGCTCAGCACTAAGTGCTTCTGCCTTCTCCCCCTCTGCCGTCTCAAGTCTTGG<br>TTCACTGGCAACACCAAGCACAAAGAAAATGTTCAATACGCACATTTACCTTTCTATAATCATTTATAGT<br>CAGTGCAGAAACCTTAAACGTGTCTGCTCCCCGTCACTGCATCTTAAGCTGTCTGCACTGTTTGCAATG<br>TCCTGTCAATCTTTAGCTATTTTACCTGGTGAAGTGTAAGAAACTCGAAACTCCCTGACAGGCCCAACG<br>AAGACCCAAAGTCACTTTGTAGGTTGGGGTCTGGAGGTGAGTGTATTACAAAGCTGGGTGAAAAGCAC<br>TGGTTTAAACAAATATCACATTCTGTGAATGAATTCCAAAAAGAAGTCTCCTGGGGGTGGGGTGTCCAG<br>AGAATGCAAGTGCATCAACACCCCATGCCATGTGGGTGCTGGCTGCCATGCTGTGTCCTCCTGGACTCT<br>AATTGTTAGTGTGTGGTGTCTGCATAACTCACCACAGTCTAGGACTGTGCACCAAGAATGATTCTACATT<br>CGTGTTTATAGAAGAGAACCCTCTGAACACACGCACTGTAGAGTCTGTGTTCACTGGAGGAGAGTCCA<br>CACCACACCAGTATCTTTTTAAAAATGGATTTTGCGACAGGTGTAGTGGTACAAGCCTTTAGTCCCAGC<br>ACTCAGGAGGCAGGGACAGGCAAATCCCTGAGTTTGAGGTGTAAAGAGGGAGTTCCAAGACAACTAG<br>GGCTACACCTATCTCAGGAAGGAAAGGAAAGATTTTTGCAAAAAAGTTTTAAGTGCACAATGAACAAG<br>TAAGGAATGATTGTTTTCAGGCTGTCTCATGAACTTCATACCCCATATAAAGCACTCTTTAAACAGGGTC<br>ATTGCTTAGCCCCTAAAAGTCCTCATTCTCCAGCAACTCTTACTGCTAAAAAAGCAATGAGGACTTTGA<br>ATCGAGAGATGCCTTTTCTATACATAGTACACTGGCTGCTGTACATAATTGGGGTTGAATCAGGGAAATA<br>TTGAAACTGACATTCAAGTATGAAGAATAACACTAGATTGAGCATCCCAAGTCATCTCCTGCTTTCCCAT<br>CTCCAGTATAAAATATGCAGGTACACGAGGAACTGGACACACTGGCTGACGCCATGACAACTGAGGAC<br>GTGAATGAACTGTGAGGCCAGAGCTCATCACTTCTGGTCTCCCGGTTGCATCTAAACTGAAGCAGCAG<br>CTCTGTGTTTTATTAAGTGTTTAGGAAACTAAGATGGGAGGAAAAACACACCATGCTTACTGTCATTTTC<br>CGGGGCCCTAAAGCCACTTTAAATTAATTAATGAGAACTTGTCTGGTGATTTGATAATGTATACGAATTAT<br>TTTAAATTTTATTATGATGAATAACTCCAGTGTGTGTGATTTTGTGAAGTAAAAATCTTTCCTTGAGGATT<br>AAGAAGCAATGCTAGAGGTGGAACATGAGGTCTTGCACTCCAGTTTGAGAACAAGAATCCGAGGCAG<br>AGAATGGCATGTTGGGAGGAAATCACTTCCTCGAGCACAGGCTGAAGGCTCCGTGCTGTGTCTTGGAT<br>GTCAGGATGTGCTCAGAGGTGCTCCCTGTCCTCATCCATCCCCACCTACAAAGCCTAGGCGTCTGCTGC<br>TAAAACTGATTACAACACAATGGCTTTTGTTAAATTATACTCCCGATTTTTCTGAAGTTTAAAAAAATGG<br>GACTGGAGAGATGGATCTAGGATTAAGAACATGCACTATGACTTCAGAAGACCCAAGGTTGGTTCCCA<br>GTAACCAAGCCAGAGCCTCTGTATACATAATTCAAAGTAATTTTAAAAATGAATCTTTAAAAAAAAAAA<br>ATCCAGCCCCCCCCCCCCCACACGCCTGTAACAGAGGCAGAGAGCAGAGACGAGAGAAGAGGGCTG<br>TGGCAAACCTGGGCGTCCCAGGCTGGCCAGGGCTACACAGCAGGGCCAGGTCTCAGAAGCAAGTAAC<br>TGAGCTCCTGGGTATACAATGCACGGACAGACGGACAGGTAGGTCTGTAAATGTTACAGTGCATGGATG<br>ATAGGTGACTTTTAGAAAGTTTCTAAGTTTTGAAGCTTTTCAAATATTTTTATCTAGCCAATGGCTTTTGG<br>GTTAGACACATTCTGGTACTTACGGGGTATAAAGGGCACAGAGTTCCTCTAGGAATGAGGTTTGTCTGT<br>CAGAGCTGCTTTAATTGTTGTGGACAAGATGTGGGAATTATGCTAGGACTAAAACCAATACATTAAGTAA<br>ATGCTACTAGTCTCATCAATAAACATGAATGTTTTAAGTGCAAAGATTTGGTTTTCTATAACCCGTTACAA<br>GAATATTTTTCATAATTAATTTAAAGAGAAACATCTCTTAAAAGTGACAAGAAGGGAGCCATGTTCCTGG<br>AGGCAGACACTGCGAGGCTTTCAGGTGGGCAAAGCTGGGGAAGAATGTTCTAAGAATGAAGTTCTTTT<br>TGGTATTTTAATTCCTCCTCCATCATTAGCACACACTATAATGTGCTGAAACGGGTCCTGCATATCTGCCT<br>ACAGGCATGTCCGCACTGACAAGCAGCACCAGCTCCACTCATAAATGGACCCTTACAGACACAGCCAA

							GCCAGCACTGGATCTGCCCATGTAAGTTTAAAGACTTAGTCACAGCAACAAACACTCTCCTAAAGTTCTAGCTGCGGAATAAAAAGACGGTGAAATAACGGCTGGAGGCTCTCGGGACCGCCCGCTGCCTTCCAGACCTTTACTCAACACATGCTCCCCACCAGGAGCACAGCAATAAACCTTAAACTGGTGCATGATACAAAGAGTTGTAATTTGTTTGTATCCCTACTGTTGAATCCTGATTTTTGATTCTCAATAGGAAAGTCATGAGTTATAGTAATCAAGCAGTAGTTCAAACAAAAAGACTAAAAGTAACATTATCAAATTACAAAGCATTGCTTCTCATAAACAGGACGCACATAAAATTCAAACTATTCTAAATACAGAATTATGAAACGCTATTTTAAAAGCATGGAAATTTTAACTTCAAACTCTTACAAAATGTAAGACTGTGGCCTCTTAAAAATATGCCCTGTTAGAATGTGCCATGTTTCAGTGCACACAATAGGCATCTATCTCCACTGGCTTCTCAGGTACTGAACATCTCCTTTGGAGATGAGTGAGTGACGTGGACATTTTAAGTGCATTTCGTGTTGTTTTCTTTTAATTCCTGACCATAACTACCTTGATAAACATCTTCGTCAGCCTGTCACTCTGCTTCTATAACCTGGTGTAGAAGCAGCTGTGCTTCAAGCAAGCATCTTCTCAGTTCCCTGGAAGATGAATCCCAAGCAGCCTGCACAGCAGAAGGACGCCAACCCCATGCTCTCCCAATTCACAGGGACACGACTGGCCACGGTGAGACTATGAGTGGGCAGACGGGGAAAAGAATGTGCAAGAACAGCCGTCCCCCGGCGCTTGTTCTCACTGCAGTCAGGAACATTGCACACAAAGCTGACGTCAGCACTATCGAGGAGAGTGACTGAACAGTAATAACCTCCTGTCTCCTTCCTTTTGTGTGTCATACCATGTTGTCAATAGACTCGGGATCTAATGGAATCCAACTGTGGGACCAAGAGCTTAAACTTTCCCCCAAAAAATCAGAGCCAGTCTTCATCCATGGGTATTTTTCTTCTCTCTG
MSTRG.62192.1	ENSRNOG00000047234;ENSRNOG00000058463;ENSRNOG00000059588;ENSRNOG00000061718	0.034	1.276	0.000	5.879	up	GCACACAACACACACTACACACACACCACATACACACCACACACATACCACACACACACAACATATACCACACATAGCACACACAAATACCATACACACATACACACACAAACACACAAACACACACATATGCATGCACTCACACACACGCCACTCAAAATAATAATCATAATAACAATCACAATACATGAAAGGTAGAGGCAGGAAAACCAGGAGCTCAAAGCTAGCCCCTGTGAAATGGAAACTTAAGGGTTCTTTGGAAAGTCGGTTGAATGGTGTTTTCCTGGAGCAAACACATGATGAAAGGTCTAGCTGAAGCAGACTCAGGAGAAAGGATGTTCTGCTAAAGCAAGCATGTGAAAGGACACGTGATGAAGGATTCTTCACTAACGACACATGTGTACTGTCCCGCCTTACACTGCACAGTTGACCTGCATTTGTTGGGACTCCATAGAAACACACCAAAATCTTCTGGTGATGTGCTGCAGTTTCTTGCAGCTTCTGGGGGCTCTGGCCAATTTTCAGAGGGATGTCAGCTGAGATACGTGTGCTAAGGTTAGACCTGTGGAGGACACGTGATGTTTGGTCTTCTCTGATCGCTTCACTGAGAGAGGCACAGCAAAAGTTCTGGCCACCACTGCTGATTCCTTTTTGCTATCCCAACTCTACCGAACTGGACTGCTGGTGTATCTGTGAGGTGTTGTTGAGTGGATCGAGCTGCTGCTGCTGACCTGTGAACTGAACCGCCAATTTCCAGACAACACAGACAGGAGTTGCTCCAAAGAACCTTTCTAAACAGGTCCTCTTCCCCCATATCCTTTCTTTTCCACCACCTCTGGTGGGTGGTGGGCCACAAGGGAGATTAAAGCATTTAAGAACCATCATTAAAAATAAGGTTTGGAAAAAAATTAAAGTTATACCTTTGCTTCATAAAGACTTGTCTCAAAATAAAACACTTCACAATGGAAGCTTAGAAAGGCTGAGTCTTCTAGGAAAGTTCCAGAACAACTGGATCAGACATAGCCCAGAGACCTCCAACCTAACAGGCCCTGGCTCCACTCACCCTCCCATGTATTAACATGGGGTACACAAGAGATTTTATCGAATGGGTTGTCAGTTGTCTGTGGTAGGTCGTATCGTCTGGCTGTGGTAGGTAATGTTACAGCACAAGGCCTTCCACCTCAGACATGAAGCGACTTTAAGCTTTTCTCATCCTGACATCTGTGTCAATAAATTCTGGATCTTCCTGCTCCCTTCAGCCATGATCGCTGTCCTTGTTAAGACTGAAGAATCCTTCCGGCCTCTCCTCCTGTCTCCAGAGTAGTCACCATACAGTGTTTCTCAACCTGTGGGGTTGGGGTCAGGTTGCCTGAGACCATCGGAAAATCAGGTGTCTAGATTACAATTCACAACAGAAGCAATTTTACAGCTATGAAGTAGTAATGAAAATACATTTTTATGGTTGGGGATCGGGTCCAGAACCACTGCTCTAGCAGGAACCACTGCAATCAGTGTAAGCCAGACAGCGCTGGATTCAAGCCTCTGACAATGTCCAGCTTTGAGCTTTTAGCTTTCACTCTTACCCTTCACTTTGGCTGCTTCTCCTAAGGACCACTGGTCTTTTCCTTGTCGTCTTCTGCTTTCCCGACTCCTTTTCTTTTTATTGCTACAGGAGGTTGAACAGAGGATGCTCTTGCTCTGCTCTGGTCTCTTCCTTGCTTTTCCTT

							GAGTAAACAGTACAAAAGAAATCAGCACCCGAGGACCTTAGTAGAGATCCCATCCCCATATCTTGCGCT CAGTTCTCCATAGTCTCTGATAACATGCCATCTTGTCATCAAAACCATTGATGACCTCCAATTACTCCCTG CCCGCCCACACGGCTTAGTCTTCTAGACACCATTGTTCATCACCACCTGACAAGTTGCATAGACTCTTG ACCATTTGAGTTTTGTCAGCCTCTCTCTATATGTGGAGACTCTTCCACCATTAGAGTGCAGCGGTGTCTG AAACGAGTCCTGGCCCATAGCTCGCACTCACTAAATATTTATTGAATGGATAAATGAATTCCTGAGTCCT AATGTTCTGTAGCCTCAGTTGCCTGGAGAACCCAGCCAGGCTTCAGGTGATGGGGGTGGGCCATGGGG GTAGGGGCATGAATAGGAAGAGGCTATTGAGATGGTCCAGAGTTCTTGGGGAGTAAACAAATATGGAA GAAATATGATTCAAGAGTGAAGTAAGAGGAAAATAATCAGAAAGACCACGAAAGGCTCTGGCTAGCGC TCCCGTTGGTGCTGAACCAAGGCCAGGCCTGACTTGCTCGCTTCAGCTGTTGAAGTCTGGTTCTTGTCA TACAGCAACCCCGACATCTATTTTGATATCCATCTTATGTGTCTCTTGGCATGCAGAGGACTCCTATCATT TAGCAGATTTATAGAAATTAGGGAAAGCGGTGTGAAATGCCTCCTGCCGTATGGGAAAGGCAGAGAAA CAGCTGACATTTGGCTTCTTGCCAACTTCACTGAAGTTTCGAGTTTCCTACTCCTGTCCTGTCTTGGGTG GAGTAGCCCTCTTATCAAACGTCTATTCTCTGGGTAGCCATAAAGACACATCCAGGCATCTGGTGGGGTT TGAATATTCAAAGACTCATATGGTAAACATGCATAAAACCCAGCATTTCGGTGCAGCTGTTTTAAGAGCC AAGGTTTTCTTTGTCCTAAGCATGGTCTGTTCCCCCTGCTTCTCTAGCCCTCACGGGCTGGAGCTGACAT ATAGGATGAGGCATGTGCCAAGCCCAGCTCCAGCCGAGGCATCAGGGGCACAGCAGTCTGTGCTGCCA CCACTCCCGTGTCATGTGACTTTTGCAGAACTGACTGCTTGTTCATTATGGGTATGGTCCTCTGGGATTT TTTATTGATAATTGAAAGGTCCTTCCCTCTGGGTATACTTCTGCCCTGAGCCTTATTTTTGAATTATTCCAA ACTGCATATTATGCACAGTTTAAGTTGGTGCTTGGCTCTCAGGCCAGCCTGGCTCACATTCTGTGGAAAC GTACCTCAGCGCCACGTGATTGTCCTGGATGACACAATGCTGCCTTTGGTGTCCCTGAAGCTAAAACAG ATTTATTTCCAAATTATGTGATAAATAATGCTGTGAGGTCAGCTTGACAGAAATAATCAAATCTCCCTCCA TCTAGATACAGCTCACCCTCAGCCCCCAAGCTACTGCTTACGTATTCGTTCATAGGCCGTGTTCTTAGTT CTCGAGAAATTTATACTTAAAGAGGTGATGGTGATGGTGATGGTGATGGTGATGGTGATGGTGATGGTG ATGATGATGAAGGGGTGGTGGTGATGATGATGGTATGCGTGTGTGTGTGTGTGTGTGTGTGTGTGTGTG TGTGTGTGTCTGTGTGTCTGTGTGTAGGAGTGTGTGTCTGGGTGTCTGAAGGGGGCACGTGCATGCCAT GGTAGGAGTGTGGAGTCAGAGGACAACCTCAGGCACTGTTCCTCCCTCCCCCTCATCCTTATTTGTACA GAACATCTTGGTTATTCTTGTTCAGTTTTGTGTAAGTCAGATTAGCCAGCCCACAAACGTCTGGAGCTTC TCCTGTCTCTGCTGTCCGTCTTCCGTCTGGGATTACAGATGTGTGCTCCGTCTCTCAGCTTTACAAATTCT GGGCACTCCAACTCCGGTCTTCATGTGCAGTGAGCAATTTACCCACCAAGTCATGTCTGGAACCCGAAA GAACATTTTCTAAAAGGGTACTGAGCACGTAGGATGCACAAAGCTCCCTGTTGCGAGGATCCATGTTCA TACAGTGTCACGTGAAAACCTCGGTGGACCGCCGATCGACTCTGCCCATGGGAACCGCACACGTGTTT CTCATTCCTCTGCTTATCTTTCCTCATGTGCTGTTTACATCTGTTTACTCTCTGGTGGATGCCTCCACAATT CTTCTGTTTGCAGTGCTAACTGTGTGCAGAAGGAAAATGTCCTTGTTAACATATAACACCGGCCTGTAAT TTCTCCAGAAAAGAATTTTGTTAGTGTTCAGATGAGTGTTACAGCTAATTAGGAACACACACTCATTGG TAAAGTAAATTGCCTGGGGTTGGCAGTAGGTAATTGCTCATGAAGTGTAATGAAGTCTTGCTTTCTCCAT CTAGATAATATCAGTAGACGATAAGCACTTTCTGACTATGTCCAGTAAATTATGCTCAGACTCTGGCTATG AAATATTCATCTTCACCACTTTGGATAGAAGCATCCTCCTAGCGTGCTTCATATTTCTTGTCACCCTCATT TAAAAGAATGAACTATTTCAACCTTCACGGTGACTCAGCCACTGCTGTGAGCATGGTTAATTCTACTCC GCTAAGGATACACGATGACCCCAGAGCCTGTTGAGTTATGAGCTGTGCTGGGAAAATTCCTAGGTAAAT GGTCTCAGGGTCTTAGATTTCTTGAGCCTTTGTGAGTCTTCTGATTCTTCGCACCATGTCATGTCCATACT TCCTGCCTTCCCTGCAGCCTTCCGTTCAGGGTGTACTTCTGGAGCATTCACTGTGTTGGGCTTTCTTCCA

							TGTATAGAGAATAGAGTTCCATTGGAGACACAGTTTTGATGCTTTCCTTGTATGGGGTGCACCTAGAACT<br>GATTACTGTGGATTGCAATTGCTGATTGCAGATCTCCGAGATGTGGAGTGAAAGGAAAAATGACTCGGA<br>AAGAGAAGGCCCTGGAAGTCCGATCGTCATCACACAGGCTTCCGCTGCCAGCCGATGGGGCAGATGCA<br>GAGACCTACAACCAAACCTTAGGCGGATAGAGGCCCCAAACTGAAGATCTCCCTCAGGCTCCTCCTCTT<br>GGGGCTCAGGGAACCCCCGCAGAAGAGGGGAAGGAAGGATTGCAGGAGCCAGAGGGTTGAGGATAC<br>CAGGAGAACAAGGCCCACAGAATCAACTAAGCAGGGCTCGTAGCAGCTCACAGAGACTGAAGCAGCG<br>ATCATGGAGCCTGCATGGGTCTGTGCTAGGTCCTCAGCATATATGACAATGGTTTTTAGCTAGGTGTTTTT<br>GTGGGATTCCTAGCAGTGGATGTAGGATTATCTCAATTCTTTCATCAGCTCCTGGGACTCTTTTCCTTCTA<br>TGCCTTGCTTCCCCCAGCCCTGATAGGAGGCTTTGTGCCTAGTCTTACGGTACCTTGTTACATAGTGTTC<br>AGCCCATATCCCTGGGAGACTGCTGTTTTCTGGAGGGAAATGAAGGAAGAGTGAATCTGGGGAAGAGG<br>GAAGGTTGGGGGAGGCTGGGAGGAGTCGAAGGATAGTAAATTGGGAACTGCGGTTGGAAAACAAAAA<br>ATAAATTAAAAAGAGAGACATTCTGATGACAACGCCATAGCATCACTGCAGGACTGTCCCCTGCCTCCT<br>TAGTGTAGCTCCACACTGCCAACTGTCCCTGGGGACCTGGCAGTGTCTGGCTCCCTCTGTAAGGGGCAC<br>TCACGATCGAATGTGCCCAGATCTCAGACAGTGCATTCTCTCCCATTGATTCCAAACTCCAGAACTCCCT<br>CCTATTTGGCTTCTTGATTCTGGGGATCAAGTTCGTAGTTTACTCATAGTATACATGATGTGAAGTGGGTT<br>TTGTTAAAATTGTGACAGGCTCCTTAAAACAAAACAACAAAACAGAACAGAACAGAACAAACAAAAA<br>CAGCGTTGAGCCTCTCAATTTCCTTCTCTGAGAAAGGAAGGGAAAGCACACAAAGCCGAATTCTATGC<br>CAACCGGTGAGGATGATGTGCCTATGGTCGCGTTACAATTTAGCTGATAAATGAGATTCCCGCAATGCAG<br>GAGGGGAGGCGTAATATGGCACGATCATCTCTAAAAGAGAGTAGGACAAAGTAAGCTTGACTTATGAA<br>ACCTTCCAGGATATGTGAAAATATTCACATTATAAATGTCCTATTCCCTAGGCAAGATGGCACATGTCTGT<br>GTCTCATAACTCTTGGAGGTCGAAGCAAGGTGGGGTGGGGTGGGGGTCGCTCCTGCAGTTGAGGCCAG<br>TCTGAGCTCCACCCTGAAAGCCTGTCTCACAACAAAGTTTTAAACAAACAAACAAACCAGACTGCTAT<br>CACGTCGTGGTATGTGATATGCTCCACCAAGGGAGAAGAAAGAGGGAAAAAGAGAAATTTAGGGGAA<br>AATGAAAGAACAGGGGGGGCTTTGCTAATGTATACCAAGGTAAGCTAAGCATGATGGAGCTGGGTGTAT<br>GATTTTTTTTTCTCTTTCCCATTTCTTCCTGCCTGATTTTGCTGAAGTAGTCATGGCTACTTACAAGTCAG<br>AAAGCAAAATTTTAATGTTCTTAGGTTGGAAAGAAATATATACAAATTTTCCAAAATATATACAAATATAT<br>ATACAAATACATATACAAATATATGTACAAATTTTCCAAAACTAATTAGGGACACCAATTAGGGAAAGTTA<br>TCTTATTAAATTGCAACACAAATGACTTCTGACAGGTGGTGTTTGATCATAAACCCATTGATAGAAGATG<br>ATACTGGTGAGGCTGTGTCCTAATAAACCCATGACATGTTGAATATACCAAACTCAGGAGTGCATCGAAT<br>TGCTGGGTATGGTGGCCCACATCTGTAATCTCAGCACTTTGGACACTGTGGCAATAAAATCGCCACTTG<br>AAGGCCAGCTTTGGATGCCTAAGACTTCATCTCTAAAGAACCAACAAACAAAAAAGTGCCATTACAGA<br>GTGATGACAGCATAGAGACACAGCGTACCTCGGAGGCTCCCTTGTCCCCCTGCCCCATTACTGAGCTGG<br>CTCAGAACTGTAGCTCACCAGTGCTGCCTGTCACAAAGGGAAGGACCCAAGGATGCATTGCTGCTGGC<br>CAGGGAAGAGATCAGAATTCAAAATCCCAGTTGCCCAGTGCCTGTTGCTGTCACAGCAGAGTGACGAG<br>GAAATGTGGAAAGTGGACCCACCGTGGGTCAGGAACTGCCTGTGGTTTGAAATATGTGGCTCCGCGTC<br>AGGTGGTGAGGGCTGCGATTCCATGATTAGCCTCCCAGAGGCTTCAGTTCATCATGCTTCTCCCACATTG<br>TTGGGTATCACCAGGCTCCCTGGCTCTCCTGACTCCGTTCCCCTTTTCTGAGCTTGGTTGAGTCTGTAAT<br>TGGATATTTTCTGAGGACCAACGGCCTTTACACTGCCCTTTGCCCATGTGGCTCTCGTAAGTTGTTCTGT<br>AACCGAAAATATGCAAAGAGATGCTCACTCGGGCTGCCCACACACACCCGAGCCAGCCCCAAATTGTT<br>CTCCGCAGAGAAAACGCCTGAACTTGAGGTGGCAGGTTTACCTCAAAGTATGATCCCTAGCCTCAAGAT<br>CTCTCTCTTCCAACTGTGTCAGAAATTCGAGAGATGTGAGCCAATGTTCCTAGTTGTCTCAAATTATAGC

							ATCGTCCTCCCTGACTCAGAGCAGGCAGTACATGAATTCCCAGAATATTTGGATAGTATCTCAAGGCATGAGCTGACCCAGGTATCTACCCATCGGACTTGTTAATACAGACAGCAGGTATCCACGGGCTTTCCAGAACTCAGGGATTTTCTCTGTTGACTCTGAGAGTCTGGGAATGACATCCCTCTAGGTTCATCTGAAGCTAGGCAGAGGAGGCCGTTGCTGAGTCCCAGTTACACAGCAATTTATGTGTGTGGCTTGGCCAGAGACTTTTTTAAAAGACAGGGTCATACTATGTAGTCCAGGCTAGCCCTGAACCTACGAACTTGCTGCCTCTGCTTCCTGAGTGCTGGGATTGAAGGCATGCATACTTTCTCATTCTCTCTCTCTCCTCTGTGTGTGTGTGTGTGTGTGTGTGTGTGTGTGTGTGTGTGTGTGTGTGTGTGTGTGTGTCTGTGTCTGTGTCTGTGTCTGTGTCTGTGTCTGTATTTGTGTATGTAGGCATATATACTTACATCTAATAGACCCCAAAGGAAACTTTTACCCCACATCATCTGAAGGATGGATAAGTACATGGCTATTTCTACTCAAGGGAATACTTTTCTTTTCTCATCAGAGCACTGGGGGGTTGAACCTAGAGCCTTACAAGGTCCTAACCTAATATTCTAAGCCAACACTCTACCACTGAGCTATATTCCCAGACCCTTTCTTTTAAACTTTAATTTTGACATGGGGTCTCACTAAGGTGTCCTGGCTGACCTTGGACTTGGACTTGTAATCATTCTTCCTCAGATTCCCAAGCAGCTAAGCTATAGGTCTAAGCAAGTGAGCTGAGGTAAGGGTACATGACAGGAAAGAGCAACCACGATCAGTCCCATGATGGGTGGATCTCCCAGATCTGATGCTAGGTAGGTGTGACAGATGTTGAAGCATGTATACATCATCTACGTGTGAACACTTACATCATGATTATAAAGAACAAAAGCAATCAATAGACCTACAGTGGTAGAATGCATGTTCTTCATCTTGGTGGATGAAGGGACTTCTAGGGGTTACAGATGGCGTCTCTGAAGAGATACTAGTTCATATTTAAAGAGCCTTCAAAGCCATATCCTCAGGCCACGTGCACTTGGGTGGACATGTCTAAATTTGCTCAATATGCCTCAATAAAAATTACATATAATAAAAACATATGGACTCGGCCTATGTGTACTTGATAAATGACTATGTAATACCCATATCCATAACATTTAATACTTTAATAAAAGGTTATTTGAAACCAAATGTGAA
MSTRG.62192.4	ENSRNOG00000047234;ENSRNOG00000058463;ENSRNOG00000059588;ENSRNOG00000061718	2.207	0.199	0.004	-2.685	down	ACACAACATATACCACACATAGCACACACAAATACCATACACACATACACACACAAACACACAAACACACACATATGCATGCACTCACACACACGCCACTCAAAATAATAATCATAATAACAATCACAATACATGAAAGGTAGAGGCAGGAAAACCAGGAGCTCAAAGCTAGCCCCTGTGAAATGGAAACTTAAGGGTTCTTTGGAAAGTCGGTTGAATGGTGTTTTCCTGGAGCAAACACATGATGAAAGGTCTAGCTGAAGCAGACTCAGGAGAAAGGATGTTCTGCTAAAGCAAGCATGTGAAAGGACACGTGATGAAGGATTCTTCACTAACGACACATGTGTACTGTCCCGCCTTACACTGCACAGTTGACCTGCATTTGTTGGGACTCCATAGAAACACACCAAAATCTTCTGGTGATGTGCTGCAGTTTCTTGCAGCTTCTGGGGGCTCTGGCCAATTTTCAGAGGGATGTCAGCTGAGATACGTGTGCTAAGGTTAGACCTGTGGAGGACACGTGATGTTTGGTCTTCTCTGATCGCTTCACTGAGAGAGGCACAGCAAAAGTTCTGGCCACCACTGCTGATTCCTTTTTGCTATCCCAACTCTACCGAACTGGACTGCTGGTGTATCTGTGAGGTGTTGTTGAGTGGATCGAGCTGCTGCTGCTGACCTGTGAACTGAACCGCCAATTTCCAGACAACACAGACAGGAGTTGCTCCAAAGAACCTTTCTAAACAGGTCCTCTTCCCCCATATCCTTTCTTTTCCACCACCTCTGGTGGGTGGTGGGCCACAAGGGAGATTAAAGCATTTAAGAACCATCATTAAAAATAAGGTTTGGAAAAAAATTAAAGTTATACCTTTGCTTCATAAAGACTTGTCTCAAAATAAAACACTTCACAATGGAAGCTTAGAAAGGCTGAGTCTTCTAGGAAAGTTCCAGAACAACTGGATCAGACATAGCCCAGAGACCTCCAACCTAACAGGCCCTGGCTCCACTCACCCTCCCATGTATTAACATGGGGTACACAAGAGATTTTATCGAATGGGTTGTCAGTTGTCTGTGGTAGGTCGTATCGTCTGGCTGTGGTAGGTAATGTTACAGCACAAGGCCTTCCACCTCAGACATGAAGCGACTTTAAGCTTTTCTCATCCTGACATCTGTGTCAATAAATTCTGGATCTTCCTGCTCCCTTCAGCCATGATCGCTGTCCTTGTTAAGACTGAAGAATCCTTCCGGCCTCTCCTCCTGTCTCCAGAGTAGTCACCATACAGTGTTTCTCAACCTGTGGGGTTGGGGTCAGGTTGCCTGAGACCATCGGAAAATCAGGTGTCTAGATTACAATTCACAACAGAAGCAATTTTACAGCTATGAAGTAGTAATGAAAATACATTTTTATGGTTGGGGATCGGGTCCAGAACCACTGCTCTAGCAGGAACCACTGCAATCAGTGTAAGCCAGACAGCGCTGGATTCAAGCCTCTGACAATGTCCAGCTTTGAGCTTTTAGC

							TTTCACTCTTACCCTTCACTTTGGCTGCTTCTCCTAAGGACCACTGGTCTTTTCCTTGTCGTCTTCTGCTT<br>TCCCGACTCCTTTTCTTTTTATTGCTACAGGAGGTTGAACAGAGGATGCTCTTGCTCTGCTCTGGTCTCT<br>TCCTTGCTTTTCCTTGAGTAAACAGTACAAAAGAAATCAGCACCCGAGGACCTTAGTAGAGATCCCATC<br>CCCATATCTTGCGCTCAGTTCTCCATAGTCTCTGATAACATGCCATCTTGTCATCAAAACCATTGATGACC<br>TCCAATTACTCCCTGCCCGCCCACACGGCTTAGTCTTCTAGACACCATTGTTCATCACCACCTGACAAGT<br>TGCATAGACTCTTGACCATTTGAGTTTTGTCAGCCTCTCTCTATATGTGGAGACTCTTCCACCATTAGAGT<br>GCAGCGGTGTCTGAAACGAGTCCTGGCCCATAGCTCGCACTCACTAAATATTTATTGAATGGATAAATGA<br>ATTCCTGAGTCCTAATGTTCTGTAGCCTCAGTTGCCTGGAGAACCCAGCCAGGCTTCAGGTGATGGGGG<br>TGGGCCATGGGGGTAGGGGCATGAATAGGAAGAGGCTATTGAGATGGTCCAGAGTTCTTGGGGAGTAA<br>ACAAATATGGAAGAAATATGATTCAAGAGTGAAGTAAGAGGAAAATAATCAGAAAGACCACGAAAGGC<br>TCTGGCTAGCGCTCCCGTTGGTGCTGAACCAAGGCCAGGCCTGACTTGCTCGCTTCAGCTGTTGAAGTC<br>TGGTTCTTGTCATACAGCAACCCCGACATCTATTTTGATATCCATCTTATGTGTCTCTTGGCATGCAGAGG<br>ACTCCTATCATTTAGCAGATTTATAGAAATTAGGGAAAGCGGTGTGAAATGCCTCCTGCCGTATGGGAAA<br>GGCAGAGAAACAGCTGACATTTGGCTTCTTGCCAACTTCACTGAAGTTTCGAGTTTCCTACTCCTGTCC<br>TGTCTTGGGTGGAGTAGCCCTCTTATCAAACGTCTATTCTCTGGGTAGCCATAAAGACACATCCAGGCAT<br>CTGGTGGGGTTTGAATATTCAAAGACTCATATGGTAAACATGCATAAAACCCAGCATTTCGGTGCAGCT<br>GTTTTAAGAGCCAAGGTTTTCTTTGTCCTAAGCATGGTCTGTTCCCCCTGCTTCTCTAGCCCTCACGGGC<br>TGGAGCTGACATATAGGATGAGGCATGTGCCAAGCCCAGCTCCAGCCGAGGCATCAGGGGCACAGCAG<br>TCTGTGCTGCCACCACTCCCGTGTCATGTGACTTTTGCAGAACTGACTGCTTGTTCATTATGGGTATGGT<br>CCTCTGGGATTTTTTATTGATAATTGAAAGGTCCTTCCCTCTGGGTATACTTCTGCCCTGAGCCTTATTTTT<br>GAATTATTCCAAACTGCATATTATGCACAGTTTAAGTTGGTGCTTGGCTCTCAGGCCAGCCTGGCTCACA<br>TTCTGTGGAAACGTACCTCAGCGCCACGTGATTGTCCTGGATGACACAATGCTGCCTTTGGTGTCCCTG<br>AAGCTAAAACAGATTTATTTCCAAATTATGTGATAAATAATGCTGTGAGGTCAGCTTGACAGAAATAATC<br>AAATCTCCCTCCATCTAGATACAGCTCACCCTCAGCCCCCAAGCTACTGCTTACGTATTCGTTCATAGGC<br>CGTGTTCTTAGTTCTCGAGAAATTTATACTTAAAGAGGTGATGGTGATGGTGATGGTGATGGTGATGGTG<br>ATGGTGATGGTGATGGTGATGGTGATGGTGATGATGATGATGATGAAGGGGTGGTGGTGATGATGATGG<br>TATGCGTGTGTGTGTGTGTGTGTGTGTGTGTGTGTGTGTGTGTGTGTCTGTGTGTCTGTGTGTAGGAGTGTG<br>TGTCTGGGTGTCTGAAGGGGGCACGTGCATGCCATGGTAGGAGTGTGGAGTCAGAGGACAACCTCAGG<br>CACTGTTCCTCCCTCCCCCTCATCCTTATTTGTACAGAACATCTTGGTTATTCTTGTTCAGTTTTGTGTAA<br>GTCAGATTAGCCAGCCCACAAACGTCTGGAGCTTCTCCTGTCTCTGCTGTCCGTCTTCCGTCTGGGATTA<br>CAGATGTGTGCTCCGTCTCTCAGCTTTACAAATTCTGGGCACTCCAACTCCGGTCTTCATGTGCAGTGA<br>GCAATTTACCCACCAAGTCATGTCTGGAACCCGAAAGAACATTTTCTAAAAGGGTACTGAGCACGTAG<br>GATGCACAAAGCTCCCTGTTGCGAGGATCCATGTTCATACAGTGTCACGTGAAAACCTCGGTGGACCGC<br>CGATCGACTCTGCCCATGGGAACCGCACACGTGTTTCTCATTCCTCTGCTTATCTTTCCTCATGTGCTGTT<br>TACATCTGTTTACTCTCTGGTGGATGCCTCCACAATTCTTCTGTTTGCAGTGCTAACTGTGTGCAGAAGG<br>AAAATGTCCTTGTTAACATATAACACCGGCCTGTAATTTCTCCAGAAAAGAATTTTGTTAGTGTTCAGAT<br>GAGTGTTACAGCTAATTAGGAACACACACTCATTGGTAAAGTAAATTGCCTGGGGTTGGCAGTAGGTAA<br>TTGCTCATGAAGTGTAATGAAGTCTTGCTTTCTCCATCTAGATAATATCAGTAGACGATAAGCACTTTCTG<br>ACTATGTCCAGTAAATTATGCTCAGACTCTGGCTATGAAATATTCATCTTCACCACTTTGGATAGAAGCAT<br>CCTCCTAGCGTGCTTCATATTTCTTGTCACCCTCATTTAAAAGAATGAACTATTTCAACCTTCACGGTGA<br>CTCAGCCACTGCTGTGAGCATGGTTAATTCTACTCCGCTAAGGATACACGATGACCCCAGAGCCTGTTG

							AGTTATGAGCTGTGCTGGGAAAATTCCTAGGTAAATGGTCTCAGGGTCTTAGATTTCTTGAGCCTTTGTG<br>AGTCTTCTGATTCTTCGCACCATGTCATGTCCATACTTCCTGCCTTCCCTGCAGCCTTCCGTTCAGGGTG<br>TACTTCTGGAGCATTCACTGTGTTGGGCTTTCTTCCATGTATAGAGAATAGAGTTCCATTGGAGACACAG<br>TTTTGATGCTTTCCTTGTATGGGGTGCACCTAGAACTGATTACTGTGGATTGCAATTGCTGATTGCAGATC<br>TCCGAGATGTGGAGTGAAAGGAAAAATGACTCGGAAAGAGAAGGCCCTGGAAGTCCGATCGTCATCA<br>CACAGGCTTCCGCTGCCAGCCGATGGGGCAGATGCAGAGACCTACAACCAAACCTTAGGCGGATAGAG<br>GCCCCAAACTGAAGATCTCCCTCAGGCTCCTCCTCTTGGGGCTCAGGGAACCCCCGCAGAAGAGGGGA<br>AGGAAGGATTGCAGGAGCCAGAGGGTTGAGGATACCAGGAGAACAAGGCCCACAGAATCAACTAAGC<br>AGGGCTCGTAGCAGCTCACAGAGACTGAAGCAGCGATCATGGAGCCTGCATGGGTCTGTGCTAGGTCC<br>TCAGCATATATGACAATGGTTTTTAGCTAGGTGTTTTTGTGGGATTCCTAGCAGTGGATGTAGGATTATCT<br>CAATTCTTTCATCAGCTCCTGGGACTCTTTTCCTTCTATGCCTTGCTTCCCCCAGCCCTGATAGGAGGCTT<br>TGTGCCTAGTCTTACGGTACCTTGTTACATAGTGTTCAGCCCATATCCCTGGGAGACTGCTGTTTTCTGG<br>AGGGAAATGAAGGAAGAGTGAATCTGGGGAAGAGGGAAGGTTGGGGGAGGCTGGGAGGAGTCGAAG<br>GATAGTAAATTGGGAACTGCGGTTGGAAAACAAAAAATAAATTAAAAAGAGAGACATTCTGATGACAA<br>CGCCATAGCATCACTGCAGGACTGTCCCCTGCCTCCTTAGTGTAGCTCCACACTGCCAACTGTCCCTGG<br>GGACCTGGCAGTGTCTGGCTCCCTCTGTAAGGGGCACTCACGATCGAATGTGCCCAGATCTCAGACAG<br>TGCATTCTCTCCCATTGATTCCAAACTCCAGAACTCCCTCCTATTTGGCTTCTTGATTCTGGGGATCAAGT<br>TCGTAGTTTACTCATAGTATACATGATGTGAAGTGGGTTTTGTTAAAATTGTGACAGGCTCCTTAAAACA<br>AAACAACAAAACAGAACAGAACAGAACAAACAAAAACAGCGTTGAGCCTCTCAATTTCCTTCTCTGA<br>GAAAGGAAGGGAAAGCACACAAAGCCGAATTCTATGCCAACCGGTGAGGATGATGTGCCTATGGTCGC<br>GTTACAATTTAGCTGATAAATGAGATTCCCGCAATGCAGGAGGGGAGGCGTAATATGGCACGATCATCTC<br>TAAAAGAGAGTAGGACAAAGTAAGCTTGACTTATGAAACCTTCCAGGATATGTGAAAATATTCACATTAT<br>AAATGTCCTATTCCCTAGGCAAGATGGCACATGTCTGTGTCTCATAACTCTTGGAGGTCGAAGCAAGGT<br>GGGGTGGGGTGGGGGTCGCTCCTGCAGTTGAGGCCAGTCTGAGCTCCACCCTGAAAGCCTGTCTCACA<br>ACAAAGTTTTAAACAAACAAACAAACCAGACTGCTATCACGTCGTGGTATGTGATATGCTCCACCAAGG<br>GAGAAGAAAGAGGGAAAAAGAGAAATTTAGGGGAAAATGAAAGAACAGGGGGGGCTTTGCTAATGTA<br>TACCAAGGTAAGCTAAGCATGATGGAGCTGGGTGTATGATTTTTTTTTCTCTTTCCCATTTCTTCCTGCCT<br>GATTTTGCTGAAGTAGTCATGGCTACTTACAAGTCAGAAAGCAAAATTTTAATGTTCTTAGGTTGGAAA<br>GAAATATATACAAATTTTCCAAAATATATACAAATATATATACAAATACATATACAAATATATGTACAAATTT<br>TCCAAAACTAATTAGGGACACCAATTAGGGAAAGTTATCTTATTAAATTGCAACACAAATGACTTCTGAC<br>AGGTGGTGTTTGATCATAAACCCATTGATAGAAGATGATACTGGTGAGGCTGTGTCCTAATAAACCCATG<br>ACATGTTGAATATACCAAACTCAGGAGTGCATCGAATTGCTGGGTATGGTGGCCCACATCTGTAATCTCA<br>GCACTTTGGACACTGTGGCAATAAAATCGCCACTTGAAGGCCAGCTTTGGATGCCTAAGACTTCATCTC<br>TAAAGAACCAACAAACAAAAAAGTGCCATTACAGAGTGATGACAGCATAGAGACACAGCGTACCTCG<br>GAGGCTCCCTTGTCCCCCTGCCCCATTACTGAGCTGGCTCAGAACTGTAGCTCACCAGTGCTGCCTGTC<br>ACAAAGGGAAGGACCCAAGGATGCATTGCTGCTGGCCAGGGAAGAGATCAGAATTCAAAATCCCAGTT<br>GCCCAGTGCCTGTTGCTGTCACAGCAGAGTGACGAGGAAATGTGGAAAGTGGACCCACCGTGGGTCA<br>GGAACTGCCTGTGGTTTGAAATATGTGGCTCCGCGTCAGGTGGTGAGGGCTGCGATTCCATGATTAGCC<br>TCCCAGAGGCTTCAGTTCATCATGCTTCTCCCACATTGTTGGGTATCACCAGGCTCCCTGGCTCTCCTGA<br>CTCCGTTCCCCTTTTCTGAGCTTGGTTGAGTCTGTAATTGGATATTTTCTGAGGACCAACGGCCTTTACA<br>CTGCCCTTTGCCCATGTGGCTCTCGTAAGTTGTTCTGTAACCGAAAATATGCAAAGAGATGCTCACTCG

							GGCTGCCCACACACACCCGAGCCAGCCCCAAATTGTTCTCCGCAGAGAAAACGCCTGAACTTGAGGTGGCAGGTTTACCTCAAAGTATGATCCCTAGCCTCAAGATCTCTCTCTTCCAACTGTGTCAGAAATTCGAGAGATGTGAGCCAATGTTCCTAGTTGTCTCAAATTATAGCATCGTCCTCCCTGACTCAGAGCAGGCAGTACATGAATTCCCAGAATATTTGGATAGTATCTCAAGGCATGAGCTGACCCAGGTATCTACCCATCGGACTTGTTAATACAGACAGCAGGTATCCACGGGCTTTCCAGAACTCAGGGATTTTCTCTGTTGACTCTGAGAGTCTGGGAATGACATCCCTCTAGGTTCATCTGAAGCTAGGCAGAGGAGGCCGTTGCTGAGTCCCAGTTACACAGCAATTTATGTGTGTGGCTTGGCCAGAGACTTTTTTAAAAGACAGGGTCATACTATGTAGTCCAGGCTAGCCCTGAACCTACGAACTTGCTGCCTCTGCTTCCTGAGTGCTGGGATTGAAGGCATGCATACTTTCTCATTCTCTCTCTCTCCTCTGTGTGTGTGTGTGTGTGTGTGTGTGTGTGTGTGTGTGTGTGTGTGTGTGTCTGTGTCTGTGTCTGTGTCTGTGTCTGTGTCTGTATTTGTGTATGTAGGCATATATACTTACATCTAATAGACCCCAAAGGAAACTTTTACCCCACATCATCTGAAGGATGGATAAGTACATGGCTATTTCTACTCAAGGGAATACTTTTCTTTTCTCATCAGAGCACTGGGGGGTTGAACCTAGAGCCTTACAAGGTCCTAACCTAATATTCTAAGCCAACACTCTACCACTGAGCTATATTCCCAGACCCTTTCTTTTAAACTTTAATTTTGACATGGGGTCTCACTAAGGTGTCCTGGCTGACCTTGGACTTGGACTTGTAATCATTCTTCCTCAGATTCCCAAGCAGCTAAGCTATAGGTCTAAGCAAGTGAGCTGAGGTAAGGGTACATGACAGGAAAGAGCAACCACGATCAGTCCCATGATGGGTGGATCTCCCAGATCTGATGCTAGGTAGGTGTGACAGATGTTGAAGCATGTATACATCATCTACGTGTGAACACTTACATCATGATTATAAAGAACAAAAGCAATCAATAGACCTACAGTGGTAGAATGCATGTTCTTCATCTTGGTGGATGAAGGGACTTCTAGGGGTTACAGATGGCGTCTCTGAAGAGATACTAGTTCATATTTAAAGAGCCTTCAAAGCCATATCCTCAGGCCACGTGCACTTGGGTGGACATGTCTAAATTTGCTCAATATGCCTCAATAAAAATTACATATAATAAAAACATATGGACTCGGCCTATGTGTACTTGATAAATGACTATGTAATACCCATA
MSTRG.62192.6	ENSRNOG00000047234;ENSRNOG00000058463;ENSRNOG00000059588	0.218	0.000	0.000	-6.761	down	TGTGTGTGTGGAGGTGCAAGCATATGTGTGTGTGTAGGTGCAAGCATATGTGTGTGTGTAGGTGCAAGCATATGTGTGTGTGTAGGTGCAAGCATATGTGTGTGTGTAGGTGCAAGCATATGTGTGTGTGTGGAGACCAGAGGACAAGCTCAGGTACTATTGGTACTACCCATGCACTTTTTAAGATGAGGTCTTGCTAAGTAGGCTAAGCTGGTGTGATGGTTTGAATAAGAATGGCCTCCATAGGTTCATAGATTTGAATGAGGGAGTGGCATTATTTGAATGGGTTAAGAGGTGTGGGCTTGTCTAAGGAAGTGTCACTGATGGTGGACTTTGAGGTTTCAGAAACCCAAGCCAAGTCCAGTAGCTCTTTCTCCTGCTGTCTTGAGAACCAGATGTAGAACTCTAAGCTACCATGTCTGTCTGCATGCCGCCATGTTCCTCGCCGTGAGGATAATAGATTAAACCTCTGAAAGTGTAAGCCAGCTCCAATAAAATGCTTTCTTTTCTAAGAGTTGCTTTGGTCATGGTGTCTTTTCAAAGCAGAAGGATATAGAGTAAGACAGCTGGCAGACCAGAAAGCCCCAGGGATACCCCTGTCTTCACCTCCCCAGAGTTGAAACTAAAAATTTGCATCACCACACCTGGCTTTTCTTATGTGAGTTCTGGGAGACTGAACTCAGGTCCTCATGCTTGAAAGGCAAGCTCTTTATTCACTGAGTTATGACCCCACTCCTTGTATTAATTTTCGATGACATACAAAATTAATACCCATATTCAGCATTTTTATTCCCTTGGCAATTACTTTTCTCTCCAGTCCCCTTTTGTTCATAAGCCCCCCAAGTCCTGACCTTAAACTTTTTGTTTTCATGGCTCTTGATGGTCCTACTTTCTGCATCTCTCTTATCCATCTCTCATCAGAGTAAGCATTTCTTCCCACATGAATTCCCGTTGGAAGCTCCTGCCTCTAGTCCAATGTAAAGGCTTACTTTTTTGCTCGATGTCTGTTTAAAATTATTTCCTAACTATTTTGTATCCATGGGGAGTTGAGGTATGGAAAATCTGTAAGGACTGTGTCTTTCTGATGGGCAAGCCACACACCTGTAGCAGTTAACCATTCCTGCAGACAAGCCAGGTGTTATTAGAATCAGAGAATGATTTGGACACCTACCCCTTGGGCACCAAATCAGAAATCCTTTTTGAACTATTAAAAGCCAATGGACTCAGCAGAAAACTTTAGTAGAACTGTTCATAGTTTCTTTTGAGTTGATAATAATCAGAAAATAAATGCACCACTAATAAGAACACTATGACTTTGAAATATGCAAGTATTTTGGCAATTGTCATAGAAATAGATGTGATCAGTTTAAGCCATGGCTTGTAATTTAATAGATATT

							GGCTATAAATTTGTCTAACTTTCTCATACATTACCACATTTAGTAAAATTCAGTCTCTTGGCTTCTCCATTC ACATAGTGATTTTCCCAAGATGCTTAGCACTTCCCACTCTAGCCTGCTTCTTGGGCTTTGACTCCTGTGG GAGGCTCCACACCCTCACAAACTACGCACAGTTTGTGTCTGGTTCCTTCCAGACCTGCCTTTGAGGAA ATGAGAAATACCTGAACCATGGAGTAGGAACATGAAGTAGAGATGCTTGTTAACCTCGGCTCACTTCCA AAACGAACACTGTATGAAAGAAGACATGTAAATTACAAGAGCAATCCTCAAAGTAGGGCAATGATTAG AAGAGAGGGGATTAACACCCCTTCGATCTAGCTTAAAGAGAGTTGTACCAAAAACAGTAAACGGGACC CTAAGCCTCATTAGTCACCCTAGGAAATGGGGAGGTGTGTTGAGCTTCACAGTTTCAAGCACAAAGTTC CTTAACAGCGACATGATTTCCCCTGCAATATATGCATGAAGATTGCTGTGACAATACAAACTGAAAGGTG GCTTACACTAATATAGTAATGTAGAAATGGTTTTCTTATAGCCATTTTCTTCTACTGACCTGCAGAAAACA TGAGAGCAAGCTGCTTATTAAATCATAACTCAGCAAAGATATTTCTTTGGGGAATTTAGCTAACGTGGTC CAGACCCCAAATCTTTCTGCTGATTTATCTTGACATACAGAGAGCTCAGAGGGCCTGGAAGAATGGATG AGTCAGCCTGACTCAGAGAGCCCCATTCTCGGGAATCTATTGCTTTGGTTGATATTTAGAATGGGGCCAT GGACTGGTCCACATGTCTGAGAGCAGTGTGAACTATGGGCTGTGATTTCAGGCAGCAATCCTGAAATCA TATCATGAGATGCTATGGTTTGAATGGACTTCAAAAATCGATGAGTTACGTGAGATATGGCAATGTGATA ACATTAAAAGGTGCTTATGTCAGTGAAGGCTACTCCCTCATAAATGGGAGTAAGGTCCACAGTGGGGAG CACTTGCCAAGTCAGGGTGAGGAATGGAGTTTGGATTCCCAGAATCCCTACAAATGCCAACTGGGCAT GGTGTCCTGCCTGTAGGAGATACAGAGCAAGCTGGTTAGGTAGAGTAGCCATGTTGATGAGCTCTGGGT TTAAGGGACACCGTCTCAGTGAAAAAGTAATTGGCAATGATTCCTGACATCAGCCTCTGGCCTCCACAT GCATGTGCACATTACCACACCTGTGGCATTCTGCCATAGCACACGCATGAAGCAAATAATGAGAGAGCC ATTTCTTTATGTACGAGGTAGGTTCCTGTTTCCCAGTTCAAGGCTATTGAAATGTAAACTTTCCCACAGA TTTGGAGTAATGTTAAATTGTTGAAAGTCTGCATTACCCATGGGCTGTGACAGTCAAGAGATTTACCTGT GACACATTCGAAGCTGGCATTTCTAGTTGTTCTTCCTCTGATAGCTACAAAATGAACCCTCACCTATTGG CATGGCATTGTTTGACCTCCTTATCCACAGAGATAGTAAGCAGTCTCCTTGAGAGAACCATTCTACATGG TTCTAGGGACCATTTCAATGTCAAGAAACTCTGCTATGTCACATCCTTTGAAACCATGGCCTAGAAGAA ATGGGTTGTATAGACAAAGAACTTCCTCATTTTAACTCACTGAGATTCTGATCCCCAAGAGGCACTAGG AGATGTTAACCTAACACAGTCTCTATGTCTTGTGCATGGATGGATTGGGTGAGCTTGCCACTGTCCATTA GCAGTCATACCCCAGAAGCCAGTCTCTGGAAAGAGCAATCAATAGTCAGTACTCTTCATCAGAGAGCTG GCCCTGGCCTTGTAACCCAAGGGGCTGAGTGGGAGGTCTTAAATCTGGATCTTTCTTTGCTCAGCCATG AATAAATTAGCAGCTCCTAGCCTAGGGAAGTGGATATATTTGTGAGATCAATAAATAAATCGAAAATGTC ACTGAGCATAGTTCACCTGAAACCCTGAGGGGGAAGCAGCTATCCGAGCTGGATAGCCATGCTGAAGA GAGGCAGAGCATGTGGTAAACCACAGTCCAACAGGGGAACGCAGAACTGAATCGAAACTTTTGGTTC CCAGCACATGAGACTTCAGAGATCCTCATGGTGAAGGGAGAGGGGATAAGACCAAGTCTGTCCTGCTC AGGGGCTTAGGTCAAGGTTGTACTCTGCATTTTGCTACAAGAGGCCATTGATGACGTCCTAATCTCCTTC CTTACCCTGTCCAGGTTTGTGCGTTCCCACCTTCCACTTTCACGGAAACTCAATGGGTAAATGAAGCAA GAGAGATTTACTTCTTGGCGCAGTTGTTCCCAGGGCTTTCAGGATGTAATCGCTTCAGAGGTTCAGGGA GCCTCGGCCACATAGGGTTTTAAGGCTCCTGGGAGATTCTTAAGTGCTCCTGGGGTAGAGTGCTACTCA AAGATCAAGCCATCCTCACCCCAGCTCATCTCCACCTCTTCACCCAAGAGCTAAGGTGTGTGTGCACCA TCTCCTTCCCCTCCTTTGAGAATTCTGGGAGGCTATTTTCTCACTTACTCTATCTACATTTTATTCTTAGAG AAAACTGCTCACCAGGAGCAGCCAAGTTCTCATTTAATTGCTGTGAAATTGTGGTTCTGGTGTTTAGGT AGTTACTTCAAAACCACCATCTTCACAAAGATGTTTTTACGTGATATTTTTTATGCAATTGCAAATCAATA TTTTGAACTAGTTATTACTCCTCCCCTAAGCACAAACATCTGCAAATAACAACTAAAACTCAAAAAGCA

							GCGGGGCAGTGGTGGCTCATGCCTTTAATCCCAGCAATCAGACGGATCTGAGTTCCAGGCCAGCTTGGT<br>CTACAGAGCGAGTTTCAGGACAGGCAGGGCAGCGCAGAGAAACTCTGTCTCAAAAACAAAGCAAACA<br>AACAAACGAAACTTAAAAAGGAAAAACAAAAGGTAATGAAAGTCACCAGTAAGTGTTTTGTGACACC<br>ACCATAGAGTGAAGTCACAGCCCTACTATCCCCACTGACACAGTTTAAATCCCACTTTTCTCAGGATGT<br>GGTATCCCTCAGTTAGTTCAGTGGTGGGATTTTTGTTTTCAAATGCTCATCTCTTTAAAAAACCCTTTTAG<br>AATTGTTCTGTGGTTTGCATGCTTTGGGGATGGGAGGTGGGGGATCAGACTCATAAATCTCAGATTGTTT<br>TTCAAGACAAAGAAAAGATTCTTTCTCCTAAGCAGAATCGATCTCGCAGGTGTGTGCACGTGCAAGCAT<br>GTGTGAGCATGTGTGCTCACCTGCATGGTGTGTGTGATTATATTCCCACACTGTCTTCATTTCCCCAAAG<br>TGAACAGCCCCCAGCCTCAGCGCGTTAAAACCCTCTAGCACACAGTTGTGCCATCCTTGAAGATATTCC<br>CCGAATGTGCCAAAAGATCAGCGGTAGCCCCTCTCAATTAACATTTCCAGACAGTGTGACAAAATCTAA<br>AAATACACTCAAAGCAAGACATGTGCGTGTGTGCCCATGATTGGAGCACAGGCTCCTCCATCCAGGGTA<br>TTTTGAATGGGTCCATTCAGAGGATGTTAGATGGAGAAACCACTCATCTCCATTTACAGCAGCCAGACA<br>TGGCTGCATTCCTGGATGTGGTATTTTTAGATGAGAAAGTTTAGTTCAGAGAAGGAGAGAGCACAACCT<br>CCCTCAAGCACACACAGGAAGAGGTAAGCCCTCTTTGCTTGACACCTACAGGAAGACCGGTACACGGG<br>CAAGACACTGCCCAAGGAGCACTTCCGGAGGCAGCACTAGCATCATCACTGGTTTGTGAGTGAACCTT<br>TTCTTTGTCCTGTTAGTAACAACTGGGGTGTCATTTGCTTCCCTTCATCCGTGTGTGGCAGGGACTGGCA<br>AGCCGGTGACCAAAATTCTTATTTACTTGGTGTGGTCACATGACCTGATTACTGTGGATTGCAATTGCTG<br>ATTGCAGATCTCCGAGATGTGGAGTGAAAGGAAAAATGACTCGGAAAGAGAAGGCCCTGGAAGTCCG<br>ATCGTCATCACACAGGCTTCCGCTGCCAGCCGATGGGGCAGATGCAGAGACCTACAACCAAACCTTAG<br>GCGGATAGAGGCCCCAAACTGAAGATCTCCCTCAGGCTCCTCCTCTTGGGGCTCAGGGAACCCCCGCA<br>GAAGAGGGGAAGGAAGGATTGCAGGAGCCAGAGGGTTGAGGATACCAGGAGAACAAGGCCCACAGA<br>ATCAACTAAGCAGGGCTCGTAGCAGCTCACAGAGACTGAAGCAGCGATCATGGAGCCTGCATGGGTCT<br>GTGCTAGGTCCTCAGCATATATGACAATGGTTTTTAGCTAGGTGTTTTTGTGGGATTCCTAGCAGTGGAT<br>GTAGGATTATCTCAATTCTTTCATCAGCTCCTGGGACTCTTTTCCTTCTATGCCTTGCTTCCCCCAGCCCT<br>GATAGGAGGCTTTGTGCCTAGTCTTACGGTACCTTGTTACATAGTGTTCAGCCCATATCCCTGGGAGACT<br>GCTGTTTTCTGGAGGGAAATGAAGGAAGAGTGAATCTGGGGAAGAGGGAAGGTTGGGGGAGGCTGGG<br>AGGAGTCGAAGGATAGTAAATTGGGAACTGCGGTTGGAAAACAAAAAATAAATTAAAAAGAGAGACA<br>TTCTGATGACAACGCCATAGCATCACTGCAGGACTGTCCCCTGCCTCCTTAGTGTAGCTCCACACTGCC<br>AACTGTCCCTGGGGACCTGGCAGTGTCTGGCTCCCTCTGTAAGGGGCACTCACGATCGAATGTGCCCA<br>GATCTCAGACAGTGCATTCTCTCCCATTGATTCCAAACTCCAGAACTCCCTCCTATTTGGCTTCTTGATTC<br>TGGGGATCAAGTTCGTAGTTTACTCATAGTATACATGATGTGAAGTGGGTTTTGTTAAAATTGTGACAGG<br>CTCCTTAAAACAAAACAACAAAACAGAACAGAACAGAACAAACAAAAACAGCGTTGAGCCTCTCAAT<br>TTCCTTCTCTGAGAAAGGAAGGGAAAGCACACAAAGCCGAATTCTATGCCAACCGGTGAGGATGATGT<br>GCCTATGGTCGCGTTACAATTTAGCTGATAAATGAGATTCCCGCAATGCAGGAGGGGAGGCGTAATATG<br>GCACGATCATCTCTAAAAGAGAGTAGGACAAAGTAAGCTTGACTTATGAAACCTTCCAGGATATGTGAA<br>AATATTCACATTATAAATGTCCTATTCCCTAGGCAAGATGGCACATGTCTGTGTCTCATAACTCTTGGAGG<br>TCGAAGCAAGGTGGGGTGGGGTGGGGGTCGCTCCTGCAGTTGAGGCCAGTCTGAGCTCCACCCTGAA<br>AGCCTGTCTCACAACAAAGTTTTAAACAAACAAACAAACCAGACTGCTATCACGTCGTGGTATGTGATA<br>TGCTCCACCAAGGGAGAAGAAAGAGGGAAAAAGAGAAATTTAGGGGAAAATGAAAGAACAGGGGGG<br>GCTTTGCTAATGTATACCAAGGTAAGCTAAGCATGATGGAGCTGGGTGTATGATTTTTTTTTCTCTTTCCC<br>ATTTCTTCCTGCCTGATTTTGCTGAAGTAGTCATGGCTACTTACAAGTCAGAAAGCAAAATTTTAATGTT

							CTTAGGTTGGAAAGAAATATATACAAATTTTCCAAAATATATACAAATATATATACAAATACATATACAAATATATGTACAAATTTTCCAAAACTAATTAGGGACACCAATTAGGGAAAGTTATCTTATTAAATTGCAACACAAATGACTTCTGACAGGTGGTGTTTGATCATAAACCCATTGATAGAAGATGATACTGGTGAGGCTGTGTCCTAATAAACCCATGACATGTTGAATATACCAAACTCAGGAGTGCATCGAATTGCTGGGTATGGTGGCCCACATCTGTAATCTCAGCACTTTGGACACTGTGGCAATAAAATCGCCACTTGAAGGCCAGCTTTGGATGCCTAAGACTTCATCTCTAAAGAACCAACAAACAAAAAAGTGCCATTACAGAGTGATGACAGCATAGAGACACAGCGTACCTCGGAGGCTCCCTTGTCCCCCTGCCCCATTACTGAGCTGGCTCAGAACTGTAGCTCACCAGTGCTGCCTGTCACAAAGGGAAGGACCCAAGGATGCATTGCTGCTGGCCAGGGAAGAGATCAGAATTCAAAATCCCAGTTGCCCAGTGCCTGTTGCTGTCACAGCAGAGTGACGAGGAAATGTGGAAAGTGGACCCACCGTGGGTCAGGAACTGCCTGTGGTTTGAAATATGTGGCTCCGCGTCAGGTGGTGAGGGCTGCGATTCCATGATTAGCCTCCCAGAGGCTTCAGTTCATCATGCTTCTCCCACATTGTTGGGTATCACCAGGCTCCCTGGCTCTCCTGACTCCGTTCCCCTTTTCTGAGCTTGGTTGAGTCTGTAATTGGATATTTTCTGAGGACCAACGGCCTTTACACTGCCCTTTGCCCATGTGGCTCTCGTAAGTTGTTCTGTAACCGAAAATATGCAAAGAGATGCTCACTCGGGCTGCCCACACACACCCGAGCCAGCCCCAAATTGTTCTCCGCAGAGAAAACGCCTGAACTTGAGGTGGCAGGTTTACCTCAAAGTATGATCCCTAGCCTCAAGATCTCTCTCTTCCAACTGTGTCAGAAATTCGAGAGATGTGAGCCAATGTTCCTAGTTGTCTCAAATTATAGCATCGTCCTCCCTGACTCAGAGCAGGCAGTACATGAATTCCCAGAATATTTGGATAGTATCTCAAGGCATGAGCTGACCCAGGTATCTACCCATCGGACTTGTTAATACAGACAGCAGGTATCCACGGGCTTTCCAGAACTCAGGGATTTTCTCTGTTGACTCTGAGAGTCTGGGAATGACATCCCTCTAGGTTCATCTGAAGCTAGGCAGAGGAGGCCGTTGCTGAGTCCCAGTTACACAGCAATTTATGTGTGTGGCTTGGCCAGAGACTTTTTTAAAAGACAGGGTCATACTATGTAGTCCAGGCTAGCCCTGAACCTACGAACTTGCTGCCTCTGCTTCCTGAGTGCTGGGATTGAAGGCATGCATACTTTCTCATTCTCTCTCTCTCCTCTGTGTGTGTGTGTGTGTGTGTGTGTGTGTGTGTGTGTGTGTGTGTGTGTGTGTGTCTGTGTCTGTGTCTGTGTCTGTGTCTGTGTCTGTATTTGTGTATGTAGGCATATATACTTACATCTAATAGACCCCAAAGGAAACTTTTACCCCACATCATCTGAAGGATGGATAAGTACATGGCTATTTCTACTCAAGGGAATACTTTTCTTTTCTCATCAGAGCACTGGGGGGTTGAACCTAGAGCCTTACAAGGTCCTAACCTAATATTCTAAGCCAACACTCTACCACTGAGCTATATTCCCAGACCCTTTCTTTTAAACTTTAATTTTGACATGGGGTCTCACTAAGGTGTCCTGGCTGACCTTGGACTTGGACTTGTAATCATTCTTCCTCAGATTCCCAAGCAGCTAAGCTATAGGTCTAAGCAAGTGAGCTGAGGTAAGGGTACATGACAGGAAAGAGCAACCACGATCAGTCCCATGATGGGTGGATCTCCCAGATCTGATGCTAGGTAGGTGTGACAGATGTTGAAGCATGTATACATCATCTACGTGTGAACACTTACATCATGATTATAAAGAACAAAAGCAATCAATAGACCTACAGTGGTAGAATGCATGTTCTTCATCTTGGTGGATGAAGGGACTTCTAGGGGTTACAGATGGCGTCTCTGAAGAGATACTAGTTCATATTTAAAGAGCCTTCAAAGCCATATCCTCAGGCCACGTGCACTTGGGTGGACATGTCTAAATTTGCTCAATATGCCTCAATAAAAATTACATATAATAAAAACATATGGACTCGGCCTATGTGTACTTGATAAATGACTATGTAATACCCATA
MSTRG.62294.2	ENSRNOG00000016541;ENSRNOG00000025274;	0.000	0.218	0.000	5.427	up	CGCAGGACCAACCTATCTAACATCCTGAATCAAGCTCAGGCTGTTGTACAAAGCAAAGGAGTGGCCTCCTCTCCTGAGGTCTGACGTCTCGGTATCCGGCCCTGTTTGTTCTAGTCCAGGGCAATCCCGATTCTGTGGAGTTTTCTTCCTGTACATGCACACAACTCACCTTTGCTTCCTGTGAACAAGGCGATAAGAATGACTCTTACCATTGGCCGGAAGACGCCATAAAAATGACATAGTTTGAGTTTCTATGATCATAAATCCACCAACTTGCGAATCACTCTCAGCCTTGAGGAAGGCATTTGAAACCGCCCAACAGTCTGAAGAAGTCTCATACTCTGGCTCGAGCCAATCACTGTGGTCTAAGGGTCATCGGAAATGCACACTTTCCTAAAATGTAAGTTCAGTTCCTGTGTCCTTCAGCGTTTTCTGGTTTGCATAACCCACATAAACAAATCCCATCACCTCGAATTGCTTATCTCCC

	ENSRNOG00000025285						AGTAGCAGATGAAGGGATGTATAGGGTTCCTGAATTCATTTCAGGAGTTTCCAGGCTGCAATTTTGTTTTCTTGTTTTGAAGCACGGGGAAGCGCTAATTAGGCAGAAAAGATACAGCAGGGGAGAACGGCTGGCTGGACTTGGCATTCTTTTAGCACCACTTGCTTAGCCAAGCTCTCTACAAAGGGAAGCAAACTTCACTGTTTGCTTCATTGTGGATGCTGCAATTTCCAGGTGGTAGATGCAATAGACACGATTATCTGTCCTTTGCCAGGTGGCTTCTGGCAAACATCCCCAACCATCCAGCTGACGTATGTACTTATACTGCTTCTAGGCAGCGGCAGCAGTTCAAGGTGGCTTACTGGTTTCAGCAGGCTGTGCTCTCAGGAAGCGCCATGTGGAGCCTCCTGAGCCGGTTTAAACCTTTGTTACGCCTGGCGTTTTAAGAAAAGGAAGACTCGGGGCGCAGCGTTGGGGAAATGGAGAGGACAGGGTAAAGTCGATCTCCCGGGCCACTATTGAGGGTGTGCAGATCTCCAGACAGGCAATTCACATCGCCCAAGACTTTTCCTTGCTCACCTGTACGATAAGGGGTTGGGCCAATGATCTATACAGTTCGTCCCTACGTATCTCAAATGCGGAGTAAGAGCCAACTCAAAAGTGGCGTTTTAGCTCCCAGCCAAATGCCCTGGCACAAAGAGTCAGAAATGAGAGTCATAAACAGTTGTGGCTACATGAATTGATTTTTTTTTCGAATACTCTCGAGGCTTTACGGTGTGTGTGTGTGTGTGTGTGTGTGTGTGTGTGTGTGTGTGTGTGTGTGGTGGGGTACTATTTCTTGACTGAGCTCCAAAATCCACCAACCAACAAAACCCAAGAAAGGCCGAGTCTCGAGGTGACTGAGACGCCTTCCCTACCAGGAGGAGGCAGCGTGACTGCTCCTTGGAGGAGTTTGCTTTGAATCTAGCGGCCCTGGCGATTGGTTGCGAAACACGTGGGCCCGCAGGACCCAGCCGGGGTTGCTGTGGCTCCAGCTCCGAGCGAGGCAGAGTCCTGTCGCGGGTTCCTCACCAATTTGGGGTGGGGAAGGCAGACACCCGGCAAGAGCCTGGAGTAGAAGTCTGGCTTCTTGCCAGCAAGTAACCCAAAGTCCACTTGCTCGACCCGCAGTGTCCCCAAGGTCCAAACCAGGGCTTCTTCCCTTGCTCCAGCGGGAACGAGGCTCTGCCTGCGGTTGGGGGATCTAACCCAATGAGTGGCTGTGCTTGCCGGTGCGTGGGACCCCCGGCCTGGCCTTGACTATTCCACTGAGCATTCTTAGGTTCCCCGCACCCTTGAGCACAGGAAAGCTCCCAGGGCTGCTTTCCCCTGCAGTGGACGCCCCAGCCCCCGACCCAGCCTTGAATAGGGTTGACCCGGAGAGGCGAAGGTCCTCGGAGCCCCTCAAACTTATTTTCTTCGCGGTCCCCAGGGGCTGCAGCTGCCCGTAGCTGAGCTAGGGCGTCCGCTTTGTCTCCGTTCACTCCCCGCCCCACTTCCCTGGTTCCTCTCCGGCAAACTCCCCCCGACAGCCCCCACCTCCAGCCCCCAGCTCCTCCCGCTCCAGCCCGCAGCGTGGACGCGCTTCCTCCCTCCTCCC
MSTRG.62320.3	ENSRNOG00000016591;ENSRNOG00000016912;ENSRNOG00000048118	0.385	0.022	0.021	-2.808	down	TTCAGAATCTGATTGTTGACTACACGTGTGTGTGTGTGTGTGTGTGTGTGTGTGTGTGTGTGTGTGTGTGTGTGGTGTGTTCCTGCCAAAATGGCTAAAGGTGATTTCCAGTTATAGCCTACTTCTTAAATATTAAACCAAAACCCCCTTTTTCATCTCCAGGGCATAACTCCACAAGACAGCACAAATAGATCTTCCCAAACACACACCACGTCTGTGTATCTCCCATCAGTCATCAGGTGTTCCAGAGGGCTCCAGTGTGGGCGTAACCGAGAACTACATGTACCCAGAGTTTTTCTAATTCCCTAACAGCCGTCCAACATGGATATGAACATGTTAAGTCCCAGCCTAAAGCAACCTTTCGTGGTTATCTAATAAAAGTCCTATAGAAGGAGTTGATCCCTGGGACACTGACACCATTTTAATTAGGGTGGCACAACTAGTCACTGATTATACCTGTGGGAAAGGACTGGTGTGCAACTGTCACATGGGCTGTAATTACACTAGGGGCTTCCGGTGGCTGGTGACATGACAGATGTGAGAACGGGGCCCTGGTGCTCTGCACATGATGTCATCTGAACTCTGTTAGAGGCAGGTATGGCTCTGGGCAGCTTATTCTCATTAGAGACGATCCCGGTCTTGTTTGCCCGAATTAGCTTCCCAGCTCCACAGTTAGACACGCAGGAAACAGGTACTGCTCTGTTGTGGTTAATAGGAGATGTCAGCCCAACTAAAGGAAACCCAAATTACATTAGCACAGGTGTGTCTGCAGGGTTCTCTGAGGACATTAGCATTTCAATCAGATGACCTAGTTAGGAGACTGCCTTTCCAGAGCCCTGAAAGAAAAGACACAGGCAAAGCAATTTCTCATTCTTCTCCCTGGGCATTAAACTCCTGGTTCTCCAGCCCCAGATTTGGAGTCCCCTCTACCACATACCCTTTTCCAGATTCAGTCTGGAGTCCACACCAACCTTTCTGGTTATTTCTGAGGCTCCCTAGTCTCATACAATATTGAACTATGAGTTATGTCTCTTTGGAAGCACAAAACAAGTCTCTATCTGGAAGACAGATTTTCCTTTTCGATATGTACCCATTTATATTATTGGGAAATTATTGAGTATATTTCATAAAGGCAGCTCTGTGAGGACTGATCTAGAACCACAGCTGTGTCATTACACTTTTA

							AACAACAAAAACGTCATTATATGTTGTCCTTCTCGGGCACATTGGGGAAAATCCATAGTAGAGAATCTATGTATGGACTAAACATCTCCGGCCTTGCAGAAACATACACACGTAAATACCTGTTTATGGGGGCTGGAGAGATGGCTCAGGGGTTAAGAACACTGACCACTCTTCCAGAGGTCCTGAGTTCAATTCTCAGCAACCACATGGTGGCTCACAACCATCTGTGATGGGATCCG
MSTRG.62452.1	ENSRNOG00000017945;ENSRNOG00000018067	0.326	1.935	0.001	2.990	up	TCCCAGAATCAAAAAACAAAAATGTTGAATAATCTTACCAAATCTTTGAGTACAACACTTACGGTGACTTAGTCTTCAGATCCGCACTTCCTGCTCCTGCACAGCCACTCTCAGCATTATTCACATCACCAGAGACAGCACCTCCAGGTTCTGACACAGGTAAATCCGCAGCACACGGCTCTGAGGACTCACTTGCTAGTGGCATGATGTCTTGGTGATTACTAAGGAAGGGAAAATGTATCAGGAAGAAAAGAATTGCTACAAATAAATTTTCATACCATAAGATACTTT
MSTRG.62499.1	--	0.000	0.406	0.011	3.719	up	GTGTGACCAGGAAGTGGGGCTCGGCCAGAAAGTCTCCAGGTCCACCTTCAGTGACTCGCTCTCTTCAGAAAGGCTCTACCTCCTAAGGAGTCCACAACTTTTTCAATCAGCGCTACCATCAGGAAACTATGTGTTCGAGTACTGGAATCTATGGAGGAACGGATCATATGCAATCCACTACAGATTGATGGGAATGGCATATCAGGATTTGTGGAATATAAAACATCAGATACATGTAGGATGAGCCTTAGAAAGAACTAGTGATACACTGGAAACTTTCGCCAATTTCTGCACTGTAGATATTCCCA
MSTRG.6282.1	ENSRNOG00000034226	0.358	0.005	0.000	-4.722	down	GTTCACATTAAAGTAAAAATGCAATTATGTATATACAAAATGTTTTTTTTTTTCATCCAGTCTATGTCATGTCCACAATTATGTGATGATTTGTTTATGGATGACTGACCATGAGTGTGATAGTTTGCATATATTTGGCCCAGGAAGTGGCACCATTTGTAGATGTGGCCTTGTTGGAGTCAGTGAGTCACTGTGGGCATGGACTTTAAGCCTAGCTGCCTGGAAGTGAGTATTCTGCTAGCAGCCTTCAGATGAAGATGTAGAACTCTCTTTTCCTCCGTTCCTCCTGGACTATGCCTGCCTGGACACTGCCATGTTCCTGCCTTGTTGATAATGACCTGAACTTCTAAACCTGTAAGCCAGTCCCAATTAAATGCTGTCCTTATAAGATTTGCCTTGGTCATGGTGTCTGTTTACAGCAGTAAAACCATAACTAAGACAAATACCCTATAAAATATCCAAGTTTCTGTGTTTAACAGTATCTGTTCTTGTTTCAAAGAGTATGGATATGGGTCCCAATACCAAGACTATGGTTTACAACAATTGGTAACTCTCATTACAGGGATTAAACAACCTCTCCTGAATTCAATGGGCACTAGTCCTGCATGTAGTATATGAACATATGTGTAGTCAAGAAAATTATATATATAAAACAAAAAATATAAATCTAAAAATAATTTAGAAAAAAGTATTTGGGTTAAATATTCTGTGTGCTAATTTTTGAACAGTAGATATCTTTTGTCCTTTGTCTAATACACAATGGGAGTATTTCCTACCCATGTTTGATAAATGCTCTTCAGGATCCATTGCCATGAATGTGCAGTTTAATATTCTTGTTCAACTTTTAATGGTGGAGTTCTACTTAATTTTTACAGTTGTATGGTTATTATAATAATTGTGTTCAGATAGAAATATGAGTACAAACATATATATTTGAAATAAACTCAACATTTCAAAAAAGCAAAATCATAGCACTCAGAAATATACATAATTATATCTGGCATATATAATTATAATAGATATAAAGACAATATCTATGAAAAGACAGCAGCAGAAATCTAATGTAAGTAATGCATTGTCTTTCCAATAGCAGCAATCATAGTCACAAGGGAGAATTTCCTTGAAAATTTCATTCCCCTTCTTCCTTTCTGTCAGCATTATATTAGGCTTGTTTTAAAGAGATAGGATAGTTAATATGAATAAAAGTATACAAGTTTCTTGAACATGATAGATCAAATAATTGGAGTAGCACTATTTAATGATTTGTCTATGAGTTTCTTTTCTTTCTCCAAGATTGTCACTGTTCTAGCACTTTCATCCCAAACTGTAATTTGAGGTAAAATAAAAATACTTAACATATACATGGCTATAGGAATTAGTTTGAACTACATAATCTACCATGAGTTGACATTGAGGATAGATTTGCAAGATTTAGGATTAACTTGTTTTTTCAAAGCTTACATTGGCAAAGAAGACTTTAATAAGATGCCTTCAGACCATCTCAGACTACGGCATCCATATTTAAATTGGCAATGTGTTTCCTCTCCAGCTAGTGAAGACTAGTGCCTCAGAATGCTTGAACTACTTACTAAATGGCCCTTTGCAACTTAAAAAAATGGTGAACTGCTAATTAAAAATTTGTATTGCACTTTTCTATGAAAAGGAGAAATGAGTCCAAAACACTGAGTGCAACGGAGGGCAAAGTTTATCGAATAATTGTGACAGGGTATATTAAGTCAGGATATATAACTGCCACCTCAATA

							CTTGTTTGAAAATGAAGTGTGGAAAATTCTTTGTTGTACAAAATATTTAACCCCAAGCCTAGGGTTTCTA<br>CCTAGCCTTTGGTAATTGAGTTACCCCCTATCCTGAAACACACACACACACACACACACACACACACAC<br>ACACACACACACCACTCACAGCCTTTATATTTACAATAAGACATAATCAGCACAAGAGCTGGGCAGATA<br>CCTAGCCCCCATGATATTAGAATCTACTTTCCTATGATAATCCTGAATTTATATTGTTTTATCTGGGTGGTTC<br>TTAGCTCTTACTAGGCAGACCTCTTGACCATGATCTGTTGACTACTAATCCATGGCCACTTCTCCTTCTTT<br>CTCTACTGCACCTTCATTCCTGTTTTGAATGCTAGAGCTGGATCAATTACCTCAGGCAAAATTGGTCACT<br>ATCTAGAAAAAAAACCTGGTTACAATTGCTAATGTTTCTGAGCTTGAGAAAATTGTATGCCCAGAGGAA<br>CATGGGGCCATGTTATTTTCTGATGACTCCTCTGCAGCCAAGTCCAGTCTGTGAACCAGGATTGTGATGT<br>ACTTTTTGTTGACAGTATTTCCAAGAGGAGTACAACACTGCAATGGGACTTTAGTGAAGGTGTGTGTGT<br>GTGGGGGGGTACTGAAAAGAAGAGGAAGTACTTAGTTATTCATCTTTAAAGTTGGTTGTCAACATTCTG<br>TGGAAACAAACACCTTCCCAACTTTTCTGTGTTCTAAACCCAATCGGGAGAAACATCTAGGAGTAAGAT<br>TCTAAAATAATTTTAACAGCTAAATGTAATATCTACCATAAAGAACCCTGTCAAGATGTAGTCATGGGGG<br>GCTGGAGAGATGGCTCAGCAGTTAAGAGCACTGACTGCTCTTCCAGAGGTCCTGAGTTCAATTCCCAG<br>CAATCACATGGTGGCTCACAACCATCTGGGATCTGATGTACTCTTCTCGTGTGTCTGACAGCTATGGTGT<br>ACTCATATAAAAATAAATAAATCTTTAAAAAAATAAATAGTCATGGGTGGAAGAATAATGTGATATATAAA<br>ATACAACACCTTAATTTTCAATGAAACTTACTTGTTTTGATATTTTGATATAAGTTTAGGACTTTAATAATT<br>TCTGAACGAACTCTGCAATATTAGCATCACTTCAAGGCTGTTCCTTGCAAATTGCTGAAACACTGAATCC<br>TTGCGGCTCTTTGAGTCTGAACAATCAACCATTACATATACCAGGTGGACCTAGGCCTCATCACACATAT<br>TCAGCAGATGTGCAGCTTGGTTGTCATGTGGTTCCCACACAACTGGATCCTTGGCTGAATCCAAAGCTG<br>TTTCCTGTATGTAGGATATATTCTTCTAGTTGGACTGACTTTTCTGCCCTCAGTGGGAAAGAAAGCAGCT<br>AGCCTTTCAGAGACTTGAAATGCGAGGGTGGTGGAATATCTTGGCAGCCCCACCTGCACAGAGGAGGA<br>GAGGAAGAATTGGGGAAGTGTTGTTGGAGGGGTACTGACTTGGGAGGGGGACAGTGAGCAGAATGTA<br>AAGGGAAGAAGTAAAATATAAAATTAATTTCAAAAGTTAAAAATGATTTTCACATATATATGTTCTAGTTA<br>GAATAAATATATGAGTCAAGAAACTAATAACATCATGGGTCTGTATTTCAGGGGAGGGGAGTGTTTAAA<br>AAACAGTGGTATAAATATTAAGGGAAACTAGTGACAGACTTAATGTCTTAGTTAGGGTTTTACTTCAGTG<br>AACAGAAAACATGACCAAGGCAACTCTTATAAGGATGACTGTTAAGACAGAGATGCAGCTCCCAGACA<br>GGGTCAAGTAAGTACTGGTGACAGGTGCAAGTCGGGGCTGGAATGTCACCCTTGAGAAGAAAATGTTT<br>TTCTTTCTTGGCCTGAAACTATTTTCTGCTGTATCCTGTTTCTGTGGAAGTGGTTCTCATGGTCTTTTGGT<br>AATGGCGAGTATATTTTGACAATAAGAAGAGGCGTGATTTGTTATGAGAATATTTTGAGAAGAAACCTTT<br>GTACATTCATATTTCTTTGTTTAGCCTTGCCTCAGTTGTTCCTGGTATCATGGGAATAATGATCTTATGGTA<br>TCTTACCAAAATGGAAATTGTGGTAATGCATATTCATATCTATATAAGGATTAATAAAGCTTGCAACTGCA<br>TGCCATCGGTTCTGCCCTTGCTCTGTGTCCTGGTGCTTTGCGACCACACCTAGGGCCCCGAACAGAATA<br>GACCTTGACAGATGACATTCAATTGGGGCTGCCTTCCAGCTTCAGAGGTTCAGCCAATTATCTTCAAGG<br>TGGAAGCATGGCAGTGTCTAGGCAGACATGGGGCTGCAAGAGTTGAGAGTTCACTCTATTCTGAATGG<br>AGCTAGTAGAAGATTAGATTCCATATAGCTAAGATGTGGTTATTAAAGCACACACACACAGTGACACAC<br>TTACTCCAACAAAGCTATATCTCCTTATGGTGCAAACCCTGGGCCAAACATATATAAACGGTCACACTGA<br>ATTAAATAGTGATGGTAGTGGAAGCTGAGATTTGAGGAGTGAATATGAGGAGGATAAATATCCCGAAAG<br>TCATCTTGAAAAAGGCATATGGAAGCAAACTTGTGTAAGAGAGGCTTCCCTGCACAAATATACATATAA<br>ACATTTTTCAAATAGGATGAACTTATAATAAGAATGATTTCTCCACTGGACACTATAGTCTAACAAGTCC<br>ATAGTCCAGTGCCTGTATTGCATTGTCTTCTTTGAATTTACTATTCAGAGACTTCCCATACAGTTCAGTCC<br>ATTACAACCCTTCTTGGTTACCATACAGAATTTGAAAGTAAGACCCTATTGCTGAAGTCAACACATGGTT

							AAGTCAAAGAGCAGAGATAAATTTATGTAGCTATAAGCTTGATTCTGCTGTGGATCTTAGTGGATAGCTTTCTTAGTACCAGAAAGCTACAGGTATGGAGTAGGCTACTGCTGGAGAAATATAATCACTGCTTTTGTCAAGCTTGGAAATACTTCCTGCCAATGAATTCTAACTGATAAAATGATGAACTGCCTATCATGAGAATAGCCAACATTTTTAATTGGATTGTGTCCCAAAATGAAACCTATATTTGGCACTGCACCTAAACAGGGCCTTCATACTACTTTTAGATCTACCCCTATTACTGTGCTAAGTCATCTTAATTTAAATCAACCCATAATAACTAATAATTATACCTATAAATCAGTATACCCATGAAAAATATTCAGAGACTAGAAAATCACACATACACATGTATAAATGAGTAGGCATAGTTTATTGTTTAAATGAGAAGGCAGTGATTGTTGTTTTTATTATATAAGTTAATATATGCAATTGTCATATTCCTCACATATAGTATTTTCTGTGTAAAACTATGTGGCATAATAATAATGATATACAGAAAATAGTTGATAGATTCCATAAACCTATGTGGATGGTTCTTTCAATGGATTCATGATCATTAAATAATTAAAACAATGCATAGAGAAAAAGTACCATCATGATATACAGCAATGTCTCTTGTGAGCTTACCTGGGGGAAGGATAAGAGCAAAGGCAATGCAAATAATGTATCATGATTGCAAATTGCTAGCACCAATATACTATGTAAATGCTATGATGTTTCCCTTGACCCTTTGTGGGAATTCCTAATAGAGCTCACATTGTTGAAATGCATAAAATCATATGGAATACAAGTAATATTGGAATTAACAAATTTGTTGTACTGCAGTATCTTTATGTGGCATTAAACATTGTCTTAATCCTCTGACAGTGCTCTGCAATAGTAAATATAAGTGGTATAAAAGTTTTTAACAACTCTAAAATAGAACAATTATGATCTTAGGTGTTATATAAATTTCAGTTTAATCTATGAGTTATATTCTTACTAGAGATCAGTCTTAGAAAGTAAATGTTCCAAATTTGAATTTTATAAACAATGCTTTATTAACCACTTGTGATATATGGGTGATTTTGGAAGCAGGCTTATAGTATGAAAAATAATTTCTCTAAGGAAAATAAAACTGTGAGAGATCTAAGGTTGAAAAAGTAAGAGGACTACTTCGAAAAGCAGTCCTGTTTCTAAATAAAAATTAAATTTCCAAGAATATCTATGAGCTTGTGAGATTCAGGAAATCTTATGAGAATATTTGCAATACTTATTCCATCCTCATGTGTTTTCTAAAAAAATTAAAAAGTATTATTTTATATTTGGCCATTGACACCCCTATGAATCTT
MSTRG.62940.1	ENSRNOG00000060573	0.000	0.584	0.000	6.729	up	CACACACACACACACACACACACACACACACACGCACGCACACGCATACACATATCACAATATTTGCCATTTTCCATGACCTCTATTTTCCCTCTTAGTACCTTTTTTAATCCAAACCTCCTTTTCTAAAACAATTTGACATCCAGTGAAGAGCACGTTAAGGAGCTGGTGAAAATCTTGGTGCTAATATTGTGGCTGGAACCGTAGGACATGTCAGTCACAGCAAAACAATGCCATCTAAAACTTCTGTACGGGGGTGCAAATGTAGCTGCATTCTTAACTCCAGCATGCTTGATAGAATATCATTGGTACAGATTCAGGCCTATACGTTAATTTACGGTGGCTGCCACTCAGCACCACAACAGATCATTCATTTTAACTTGATTTGTATTTGAAAAATAGAAATTTATGAAAAGATAGATAACAGTTTAAAACAGTGACTTAGCAAGATTTCGATTACAAATAGAAGTAATTGACTCAGAGCAGCTACACACACACACACACACACAGGTACACGTTCGACAACAACAAAAGAACAGGAAACCCCCATGCACACCAGTTACATAGAAATGTACAAAAACAAATGGACTTTGTTAGATTTAGGAAACTCTGGGAATAAGACAGCTCCTCTGTTTGGTACCATATGGCTAACATTTTCCATGTGTCCCTGTGTTTCTACTCTCTGCTGTCTCCTCTGTAATTGCCACCCACATCTTGGTCATTAACTTTTACTTAACCCTAAACGCAATCTTCCTCTGCATATCACTGTGAGCAGGTTTTCTTCAACCTTGCATTGAAGTCTCAATGTTACATAGGACCCACCATGTTCTAATGATAGACTATTAATATTGTAATCCTTGTTGAACACGATAGTACATGCCTTTAACATCAGCCTTCGGAAGGATCTCTGTGCATTCAAGGCCAGCTTGGTCTACATAGTGAGTACCAGGACAGCCAGTACTATATAGAGAAACCCTATGTTAGGAGTACTATTACTGTGAAAAAAACACTATGACCAAAAAGCAAGTTGGGGAGTAAAGGGTTCAAATGATTTACCACTCCTTATAGCTGCTCAGCATCAAAGGAAGCCAGGACAGGAACTCAAACAGGGCAGGAACCTGGAGGCAGGAGCTGATACAGAGGCCATAGTGGCATGCTACTGACTGCCTTGCTCCACATGGCATTTCTAGCCTGCTTTCCTATACAACCCAGGACCACCAACCCGTGAGTGGCATCACCCGCAATGGGTTAGACCTTTCCTATTGTGGGTGAAGGGCATAGTGGTTTTTATCATAGACTTGCATGGTTTCTTAAAGTACTTAATGAGCTTCAAGTTTACAAAGAACCATTAAATCCTACTTCACCTATTGAAAAGACATGAGACCATCTCCACATGTGCAACACTTGAGATATTGTAAGATGTAATTGCAGACATAGACTGTCAGATGTGGATCAAGCAC

							TAATTCTTTGTTCCCTGCAAGAGCAATAAGTACTCTTGATCGCTGAGTCGTTGCTCCAGCTCCAGATTAT ATGACTTTTGAAACTGGCAGAAGGAGTGACAGAACTGGAAAGAGGTATTAAAATATCCATTATATATATA TATATATATTCAGTGAGGATGAGATAGGTAACAAGCATATGGCCCAACACCTCTCTTCATCCTGCTGCAA ATGTCACTTTCTTTGTCCTGACCCACTGTGCCATGTCATGTGGAGAGTATGTATCTACAACACTGTGTTAT GTAGAGAATATGTGTGTACAACACTGTGCTATGCTGTGTAGAGAGGTTGTGTCTGAAACACACACACACCG TGCTGGGTTGTGTAAGGGTATGTATCTACAACACTGTATTGTATCAAGGGTAATGCATCTACAACACTGT GTGTCGTGTAGCTCCAATGTGTGCAAACATGGATTTCAGCCTGTACACATCCCTTTACTTGTGCTTGAGT GTGGTAGCATGAGGCTGTTTGACCAGTTGCTTCATTTTGCCAGTAGACATGTCTTTACTGGTGA
MSTRG.62960.2	ENSRNOG00000011951	0.727	0.000	0.000	-4.991	down	GACAAGGTAGCCCAGCTAGAAGAACACATCCCACAGACAGGCAACAGCTTTTGAGATAGCCCCAGCTC CAGATGTTCAGGATCCACATGAAGACAAAGCTTCACATCTGCTACATATGAGCAGGGAGGCCTAGATCC AGCCCGTATATCTTCTTTGTTTGGTGGTTCAGACGCTGAGAGCCCCACAGGTCAGTGTAATCGGTCTTTG GACAGATGTATCCTTTTGTCAAAGAGAAAAGTCACATGGAGCAAGTAGACCACGCCAACGGACAGAAG AATTGATAATTGAGTTGAAGTAGATGTGGTCTAGACAAATCCTGGCAGATGCGACGAGGACAGAAGAC TGTGCTTTAAGAACTAATGACCAGAAGAGGACATATTGTACATCATTCTACTCTTGGCTGTCTTTATGAC TGATCTCCAGGAACATCAGGATGTGCGCTCACAGCTAGCTGTTCCTGCCACTGTGCCTTTGCTCTACAAT CATCGGACAGTTGAAATAAGCCCAATGGAGTAATTTCCTTTATAAGTTGTCTTGTGTTGCGGTAAATATTA AAAGTAACCACAATCCCTTGCTAACTGGCATCACTGAGTCATGTGGCATTAGTGGGGTATATTCATCTCA GTGGCAGCCTGTTCTAGAGCACCACACCGCACTACCTCTGGCTAGTCTCTGGTTTCAGCAGCAGCTGCC TCCTGGCTCTCTGCAGCCCAACTCTGTACACATTCCCTACAACTGCCCCCTGCGGTCACTGTCACAACC TACACCAGCCTAACCAAATAAAACTCCAG
MSTRG.6298.2	--	0.126	0.602	0.019	2.668	up	GAAAGGAATGTAAAAATGCTACAGAGAAGTTTAAGTAAAATCCTAAAACTTTTTCTTTAATGTCACCTA GGTGTTTGGTGCTTGTAATGAAGGGGAGGCTCCAATGAGATGAGGATTTCACTTACAGTGAGTTGCAGT GTTTCGGGGGCAGGTACAACATCCTCCAAATATTCTGTCCTCAGCGTGTAATAGGATGCAGAGAGAGAC AAGTTGATTGTCAATAGTTTGATCTTATGACATTAACATTGATGGCTGCTGGATGAGCAGGTGTCACCTT TTTGTTCAGAACTTTGACCACTGTAGAATAATTGTCAAGCTTGTACAAAGCTGGGCTCGCTTAGCACTG ATGACTGCCTGATGCACGAGGGTCACTTTTCCTTAGAAATGTAGCCACAAAAGCAATATACAGTTCCAG TGCAATTCCCCATCAAAACTCCAACTCAGTTCGTCACAAAGATAGAAAGTCCAAA
MSTRG.6320.1	--	0.045	0.643	0.000	3.816	up	CCAGTGCAATTCCCCATCAAAACTCCAACTCAGTTCGTCACAAAGATAGAAAGTCCAAATCTCAACTTC ATTCACAAACTGCAGCCTAAGGTTCCTTCTAATTAGTGTAATCCAAAGTGTCCTCAGAGAAGTACTCCA CGTTTATCTCTCCCAATGTGAAATTACAGGTCACAACATGGTAACTTTCATCTACTTTTTCTTCTTTGAAA AATTTAGTCCCTGGGCTCCCTTAGCACTGATGACTGCCTGATGCACGAGGGTCACTTTTCCTTAGAAATG TAGCCAATATAATCCCACAGTGATATCTGTTAGTTTAATAAAATTTAAAAATTTTAATAAATGATTCTGATC AATGGGAATGGCCGCAGATAAAAGAATGAAATTGCATATTTATTAACTGCCCTCCAATTCTCGACTGCAA ATGTTTCAAAGATTGTAGTGTTCGACCCGATGTCTTGAATTGAATCGTGACTTCTGTAATCCCTCAATTA ATTCTGAATCGTTCACTTCCTTCTTTTAGCCGCATTCCAAGATTAACGGAAAAAATAAAGGCGAGACTTG TATTATCAGTAATTATTGCGAAGACAAGATTCAATCAAAGAAATCCTGAATGTAACAAAAGGGAAAACA AACCACTTTCCAGTGTTCCCACGACCACAGGAATTGAATGAAGTGGGTGTCACTCCCAGGCTTGGCTC CAAGGGAAGTTGAAAACCGAAAGGATTTCTGGTCTTTGTAGTTTCACCATATTTTCTCTTGCCAGAAGC CAGGGTCTGAAAGAGAGGGGCAACACA
MSTR	--	0.912	0.008	0.001	-	do	GTTGGATTTATTGCTCACAGTAACCAAAAAAGCATATAAAAATAATAGGCTAGTAGAATTCTTTCTTCTTC

G.6320.3					4.021	wn	CTCTTAAAAATATGATGACATTGTTGAAGCTATCACATATTTCATCTGCTGGGCTCCCTTAGCACTGATGACTGCCTGATGCACGAGGGTCACTTTTCCTTAGAAATGTAGCCAATATAATCCCACAGTGATATCTGTTAGTTTAATAAAATTTAAAAATTTTAATAAATGATTCTGATCAATGGGAATGGCCGCAGATAAAAGAATGAAATTGCATATTTATTAACTGCCCTCCAATTCTCGACTGCAAATGTTTCAAAGATTGTAGTGTTCGACCCGATGTCTTGAATTGAATCGTGACTTCTGTAATCCCTCAATTAATTCTGAATCGTTCACTTCCTTCTTTTAGCCGCATTCCAAGATTAACGGAAAAAATAAAGGCGAGACTTGTATTATCAGTAATTATTGCGAAGACAAGATTCAATCAAAGAAATCCTGAATGTAACAAAAGGGAAAACAAACCACTTTCCAGTGTTCCCACGACCACAGGAATTGAATGAAGTGGGTGTCACTCCCAGGCTTGGCTCCAAGGGAAGTTGAAAACCGAAAGGATTTCTGGTCTTTGTAGTTTCACCATATTTTCTCTTGCCAGAAGCCAGGGTCTGAAAGAGAGGGGCAACACA
MSTRG.63352.1	--	0.000	0.387	0.007	3.835	up	GTCTTGGCAGGACCCAGGCAAGAACTTTAGATCTTTCAGAGTTTGTATGTGTTGTTGGCGAGGGGACACTTCCATTTGAAGCTCAGGGCTGAGCCTGTATCTCAGGGCTGAGCCTGTATCTCAGTCACCTTGGTTGAGAGTCTCCCCTGTACCTTATCTGTGAAGTAGGGTGACTGCCTCCCTCATAAACACTGCTGTGAAGGTGAAATGCTGCAGACCGTGCAGTCAACATCCAAGTCCTAACGCTGCCCGTGGGCTTTTATCACCTTCCACACTCAGCAAGAACATGCTTATCGAGCACTGCTCTGCAGGGCATTAGTGCTTCCTATACAGACTTCCTGTGTATCAGCCCATTTTGATGTATAACGGTATGAGGCAAG
MSTRG.63507.1	ENSRNOG00000054825	0.185	0.721	0.006	2.594	up	CTGGTCTGGCTTCTGAGTACTTGGGACAGGGCTCCCTGAGGATGACCTCGTGCTGGTCCTGATTGGACCTGGATTTCCAGAGGACTGGAGTTCATCTGCATCCTGTCGGCCACAGTCAGGGAGTGGCTAAAAACACACAGACAAGCCCAGACACCACTGAGCTGCCTCCCTTCTAGCTGTGCTGGCCCTGGTCACCCTGACACCTTAAAACTGCCTGCATTGAGCAAAGGGAATTTCCCCTGCTGGCTCAGAAATCTTGCAGAAACCTGGAGGCCCGGCTAAAGAGGACCTTGTGGCCATTTACTGACAGAAATCTGCCCCACCCAGAAGCAGCTGATCTTGTTCTGAGACCATAGCAGCGAGGAGCCTTTGATCTCCATCCTGGATGAATGTTAATTCCAGATAGAAGCCATCCTTAGATGGGATGGGTGGTGGGTGTGTGGACGGCAGATACAGAAAGGGGATGACAGGAAGACCAACAGCAATCCAGGCAGCAAAGCCTTGTAGTTGTCTGTATGAGTCAGGACTACACATAACGGGGCAATCACACTCCGGGATGCGTCCAGACCTGGAGACATGTGGAACCCTCTGGTCAGGTTATGGCTATCCCTGCTCACCAAGTCTGGCAACTCAACATCCAGCAGTGCCCTTGCACTAGTTAAGAAAAAGCAACTGAATTTGCAAAAGGGAGCTGCAATGTTGGCTGAATGTAAGCAGCAGCAGAATCAATCCCATCACTGCCAAGACACAGTTGCTGATGCAGGATTCTGAACTTCAGAGGAAACAAAGTAATTCTCCCTGTGGAGACATTGAACCTGATCAAAGAAGGTTTGAGGAGAGAAGGAGGCTGAGAGCAGATTTGCACCAGGCCATTCAGAAAGGGC
MSTRG.63523.1	ENSRNOG00000053086	0.418	0.019	0.024	-2.769	down	GAAATTTGAGTCTGCTCCTACTTTCTGCTTAAAATCTGCTTCCTGACTATGGCTGCAATGTGCCTGGCTGCCTCATGCTTCTGAGGCTGTGCTTCCTCCTCTGCTGTGATCAGATGCAGCCCCTTAAACGGATTCCCTAAGAAGCAATTGAAATTGGACCAGTGACTCCACTTGGCCTTATTCAAAAGACTGAGAAGGAAAAGGCTGGCAAGCAAGAATATTAAGTCAATTAATAATTCTTCAGTGGTTTCTACAAATCCAAGAGAAAGCAATAAATATAAAAAGCATGAACAATATGTACAAAGATCAAACCAGCCACAATGTCCTCTTGAAGGCAAAGTTTAGTGCTGACGTGAAGCCTGACTCTCTTTGATCCTTTGAAGGAGGATGATGGTGAGGACGCTACAGAAGGAAAGATGAAGCATAGCGGAGGTGGTTTATGAAGCTTGACGAGAAGGTGCCATCTTTAACATGAGGAGCAGCAAGTGTGAGCACAGAACTTGAGCAAGACATCCAGAAGGTCCCAGATAGCTGATAAAGGTGGCTATATTAAGTAACCAATCTTAAATGTACACAAGACTGCTTTACTTTTAGAGAAGACAAAACCTAGGCCTCCAATAGCTACTGAAGAGAAATCTATGTCCACCTTCACAGCATCAAAGGGCAACATGACTGTTTTGTTAGAGGTAATGTATCCTGTTGCTTCAATTCTCAGAGAGTATGCCTTTGCTATTATGAAGATTCTTTTATAATCACACTAAATTTGCACTTTTTAAAATTCTTTAAGTGGAACAGTATACTGTAGACACTACATCATGTGTTTACAACATGG

							TTTATTACATATTTTATGCTCACTGTGGAGAATTGTTGACCGGAAAAAAATTAAATATATTGCTGTGGTACAGACTTTAAAGAAATATGTTTTTTGTGTGTGTATGGATATTTTGCCCATATGTATGTCTGTGTACCATGTGTGTTCAGTGCCCACAGAATCCCAGGAGGGACTGGTGAAGATTCTCCCTGGCCTAGCTTTTACTCCAGTGTGTTAATAGCTCCTTTTATAACTTGTAAGTATCACCTCTATCGTTCCCATAGAGCCCCTGCTCACCTATAAGAGTTGTACTCAAGTGTATAGTTTATCTCTCTATTTTATAGTTTCAGTGGCTTACCCATGAGAAATCATTTTAATCCTAAGAGGTTCTACCCATATAACAAGGTAAACAAAATACATCTATTAGTTTTCCAAAGAAAGTGGTCTTACATGCTATCAAAGGCCAAATTCTCATTTGTAAATGGTGCC
MSTRG.63764.1	--	0.866	0.000	0.003	-4.375	down	GCCCAGTAATCGTTAGTAGTCATTACCACCATTCTCAGCAACTGAAGGCCTCTCCAGGCCCGTGTGCTTCGATGGGCTCTCACCAGGCCACCGGGGTACCTGTTTCCAAGTGGATTCCGTTCCAGGTATTGCCGCGCAACCAAACCCCTGCCTAGACTCAGGCCTGCGTAATTGCTCCGCACACAGACATTACTCCCAGGGCCCCAGAAGAGGCCTGGCCCAGGAGGCCGCTATATTTGATTTCTGTAGGTCAACAAACAAACATCTGAAATTAAATAAGCATGTTAGTCATGGCAGGTTCAGGTTGCTGTAATTAGCTTCTGTCTGGCCTGCTGGAGCGGCAGCCGGCCTCTTCTTCCCACTGCCTGTGCAACAAGAACACACCGGGCCCAGGGCTCTGGGGCTGCCGCTGTCCAAAGCTG
MSTRG.63877.2	--	0.004	0.536	0.000	6.135	up	GATAGATAGATAGATAGATAGATAGATAGATAGATAGATAGATAGATAGATAGAGGATAGATAGATAGATGATAGACAGATGATAGATAGATAGATAGATAGATAGATAGATAGATAGATGAGAGACAGATGATAGATAGATAGATAGATAGATAGATAGATAGATAGAGATAGATAGATAGACAGACAGACAGATGATAGATACATTTCACTTTTTACACGTGAATACAACTCTGCTATGTCTATGTGCCACATTTGCTCTATCTAGTCATCTGTTGGTGGACACCCACATACCTTTACTACTGTAAATAGTGCTACATGAACAGGAATGTGCAAGCTATCTGTCTGATGTGCTGCCTCAGATTTCTTCAGGTATATACCCAGAAACGGTACAGTTTAGTCATATGGTCATTCATTTGGTTGCTGATGGCCCTCCATGCTGATTTCTATATGGCTCCTCTGGTTTACATGCCCAACAGTAAAGTATGAACATCTCTTCCCCCACACATCTGCTCCAGAATTTACTGTGGTTTTGAGTTTGTTTTCAGCCATTGTGTATACAGTGGGATAGAATCTCAGTTCTGTTTGTTGGTTGGTCTGTTGTTTTGAGATAGGGTCTTCTGTAGCCCAGAATGACTTAATATGCAGTGCATAGACTGTAGACCAGGCTAGCCTCTCCCTTATGCTCATGCATATTTCTCCTAATTGCTATTATCATACATGTATGCAACAAACCTGGCTTCCTTGGAGCTTTCTGAAGTTTTTTACTTTAGATTTATTTATTTTATTTTTATGTGTATGAATGTTTGCCTATATGTATGAACATGTGCATGTGTGTGCCACATATGTGCCTGGTACCTGCAGAGGTCAGAAGAGGTTGTCGCATCTCCTGGAACTGGAGTGGTTGTGAACCACTATGTAGAAGCTGAAAATTATATCTGAGTCTCCCACAAGAGTGTGCATGCTCATAACTGCTAAGCCATCTCCACAGTCTTAATAAATAGTTTTGATTTATATTTTCTGATGGCTAATTCAAAAAATTTTTTGAATTCTTTCTATATTTTAAATGCATAAAGATTTATAATTGACAAGGATTTTCTCTCAAAGATCCTGAAAATCGGCGCTTTCCCTCCAGAGCTGTTGTGCTTAAAGCTTGATAAAAACTGAAGTCCTTTCCTATCAGACACGTGTGAGCAAGCTTTCCCTGAGCACAAGCTATATTACAGGAAGCTATCTGAATCAAAATGTCTTCTCGTGATTTTCATTAAGGAATTGTCCTGTGTTCCCTGTCCCTATAAGTGAGTGCTGATAGCACTCCTCCATACCTTAGCATGTAAGTCAATCATTAACAACGGTTGTACTTTTCACTGGGTTGTAAAGAATGAGAAGTCAGAGCTACAGAAATCTCCTTTCTTGAGATAGGCAGTGGAGAGAAGAGTTCCACTTGCCTGTAAGCTTCGTAACCAATATGTAGCAGGCTGCAGTCCAACACGCAGGGACATGTTCAAACAGAGGACAGTCACAGGTAAGGAGTGCGGGTATGTTTCTGATTCTCTATCCATTCTGATATTTTACCTCTCACCTTTCCAATCTGTGGACTAAAAATGAACAGGGGCATGGTGTGATTACCATACTAGTGGTGGAAAGAAGGTTGTATGGATGGACCTGCATTTTAACAAGAGATGGGCAGTGTAACTGTTTGTTAACTTACAAAACCAAGCCAAGACACTTCTGATTCCTTCAAGACCCAGCGTTCCAGAGAGAAAATGTGTGTCAGTTACAGGATCAAAGATCAGTTCAACAGCACAGTGGTCACATGTCGGACCAGTCCTTAGGGAAGATAAAGATGGATTAGTGTGGGCTTGGGAACACCTCCAGATCCTACTCAAAG

							GCAAAGAGAGGTTAGATGGTAAATTTAAAAGAATCACTACAAACTACACCAAAGGAAGGTCTGGGTGAGCTATGCTCCTTTTTTTATTTATTTTTTTATTTTTTGACAGACTTTGGGTTTTTTATAATAGTCTGTTTCAAAATAAATTTGTGTTCTTCAGAAAGTTTCCAGATACTCTGCCCTCTAAATGGCTTTCTTCTGCCATTACGTGTGACTCTCTTATGACACATTTATGATGATGAACCAACATTAATGTACCACTATCTACTTAAGCTCTTACTTCATTTACATTAGGGTTGAGTATTTGTCTCAGCTATTTTATGGGTTTTAACAAATGCATGATGACACATTACTGACTTGTACAAAATGTTTCCACAATCCTAAAAGTCCTATGTCCTACCTAGTCCTCTTTTCCTACTCTCCTTTCAAACCCCTAACAACTTGATTCTATGGCTTTACTCCATGTCCTAAATTATCACATGGTTAGAATTAAGCAGCATATGGTCTTTTCAGATTGGCTTGAGTTAGTCATATGCTTTTGAGTTTTCTCTGCATCTTTCTGTGGCCCAATAGTATGGCAACTGTATCCATTTGCGCAGATATTTTAGCATGGCAAGAAGTAGAAACAATAAAAGCGGTGAGTTAGAAAGTGACAATAATATAGACTTAATAGAATCAAGAAAAACAGAGATTTTAACAAAGTGGAAAAACTAGCTAGAAAGATGAGCTCAGATGTCTATGCTAGAATAAAGAAGAGCTCTGGTTCTGGAGCCAAAGGTTATAGAGTGGAGCCAGCCCTTGTTTAAGCAGATGGAGTGGGAGACACATTGCCTTCAACCTGAGAGCCCAACACATGGTAGGCATCCCATAATGTTTCCTGAACCAACGAAATGCCAAGACTTTTTCTAAAAAACGAAGCTTCCTCAAGATACCAGCTTATCGACTCAGAAGCCCTATGTGAGCCATCTCAGGCAAGTTGTAGCTGTCTGGTCAGATGAGCTTCTGCATGCCATGAGATAAGAAAATATTCCCTTGATAACCTTTGTTTGACCTTGGCTTATGAAATGCCTGAATATAAGCAAAGAACTCATGTTTCAACCTAATCACAGAAGTATCACAAACAATTTCCATGCAACGAGAAAAAAAAGGCTACGAGAGGAAGATGAGTCAGCTATGCCTTCGAGTGCTTGCCACATTCTAGAGCAGAGAACGTTCAGGCAACAGTATCAGTAGTGCATGCAATGATATAGGAGCCTAGTCTGAGAGGATGCAGCTTACAGCATGGCTGTGTAGCTGTCCAGGGAAACTGATGGGCCAGCTGGGACTTGCAGAACCCAAAGAAACCTGCAAGTAGCAATTTACCAACTCAGTGATGCTAACATCAAAAAAAAAAAAAAACCCACAAAGAAATAAAAAAAACAACAACAGCAAAAAACCTAATTACCCAGAGCAATCAGTGTATTGATTCAAAATCATTGACTTCTTAACACCAGACTTCTTCTCTACCAAAGCATATAGCATTGGTGGTCCTTCATACCAGATTCATTTCTTACTGTATGATGAAAATGGGGAATTGACCAGGCATGAAGGTAGGGCCTTGTGCTTTGGAACTGGTCATGTCCTCTGCTCAGTTGGCTTACACGCTCAGCACAAGCTAGACAACACCAGAGGATTTTCTACACAAGGTAAAAAATCTTGGAACTGCTTTGCCCAAAAGCTATTCTGTAGAATGGAACTCATCCACAGACTTACAATTTATTCCTTGTCTCTATTTATGCGTATACAACAAGAGGACTTGACTTATCACTATAGAAGTTAATAATGGATAGTGGATGTGGAGGCGTTGGAATAAGTAAAGTGTAGTTCTCTACCCCACTCAAAGCTCCT
[truncated: 567,079 more chars]
